# Supplementary material for: Estimating transcriptome complexities across eukaryotes
Source: BMC Genomics. 2023 May 11;24:254. doi: 10.1186/s12864-023-09326-0 (PMC10173493; doi:10.1186/s12864-023-09326-0)
Supplement: Supplementary file 1 — Additional file 1. [file 12864_2023_9326_MOESM1_ESM.zip › Supp. Mat. Estimating Transcriptome Complexities Across Eukaryotes For Proof BMC Genomics_ESM.pdf]

SUPPLEMENTARY INFORMATION

COMPLEXITY DIFFERENCES ACROSS GROUPS

Among all the higher lineages in this study, clades within have many constituents and a variety of metrics within a group. Deuterostomia classes have a broad range of values for each metric (Fig. 1). Observing TpG, ray-finned fish (Actinopterygii) are relatively conserved, with a range across mean TpG from 1.60-2.02 (a difference of 0.417) while Aves has a broad range, respectively compared to other deuterostomes, with a difference of 1.04 mean TpG and a range from 1.98-3.02. Mammals have a relatively high spread across the quantiles compared to other deuterostome classes (difference of 1.04) with outlier *H. sapiens* at 3.97 mean TpG, being the highest TpG complexity metric across every taxon in this study. Among all deuterostomes mean TpG is not significantly different among all deuterostome classes (KW $Chi^2 = 15.35$, $P = 0.082$). Mean EpT is also not significantly different among classes of deuterostomes (KW $Chi^2 = 9.50$, $P = 0.393$). Two taxa pull the width of the plots down in Cephalochardata and Chondroichthes the Florida lancelet (*Branchiostoma floridae*) at 7.10 mean EpT and the whale shark (*Rhincodon typus*) at 7.74 mean EpT. Certain dynamics change across EpT to EpG among different deuterstome classes. Amphibia become narrower from EpT to EpG, while Actinopterygii, Aves, Chondroichthes, and Echinodermata become wider. In total, EpG have lower complexity metrics in EpG than EpT. Among the deuterostome classes EpG are also not significantly different (KW $Chi^2 = 11.02$, $P = 0.274$). Complexity metrics can also be used to understand annotation quality between organisms, as observed in the stark differences between *Branchiostoma flordae* vs *B. belcheri*.

Drosophila complexity metrics between species are all significantly different TpG ($t = 14.26$, $P = 5.82e\text{-}08$), EpT ($V = 66$, $P < 0.05$), and EpG ($V = 66$, $P < 0.05$). The most robust

annotations within Drosophila are from *D. melanogaster*. Like other model systems, it has the highest mean TpG, however, *D. simulans* has the highest mean EpT and EpG. Curiously, *D. ananassae*, *D. erecta*, *D. mojavensis*, *D. virilis*, and *D. yakuba* along with *D. simulans* all have higher a higher mean EpT complexity, while *D. mojavensis* and *D. simulans* have a higher mean EpG complexity value than *D. melanogaster*.

Among plantae orders mean complexity metrics are not significantly different from each other (mean TpG: KW Chi^2 = 27.47, P = 0.094; EpT: KW Chi^2 = 33.34, P = 0.022; EpG: KW Chi^2 = 33.65, P = 0.020). There is an outlier among all Plantae orders, from the bryophyte species *Physcomitrium patens*, spreading earthmoss. *P. patens* has a mean TpG 2.39, mean EpT 8.14, and mean EpG 8.25. These are much higher than the vascular plant clade in Viriplantae. Viriplantae complexity metrics have IRQR ranges between 1.43-1.65, 5.86-6.56, and 5.59-6.26 for mean TpG, EpT, and EpG, respectively. *P. patens* is outside of all complexity metric IRQ values in the group.

Fungi complexities are widely variable between classes among annotated taxa. The fungus lineages are old and highly diverse, as well a relatively unknown broadly. Thre is not a significant difference between mean TpG metrics across classes, KW Chi^2 = 17.84, P = 0.399. There are only a few outliers in this metric, with Chytridiomyces having the highest width. However, across all taxonomic classes the range is narrow being concordant at mean TpG 1.00 with only outliers being above 1.25 mean TpG. Both EpT and EpG mean complexity metrics are nearly identical. However, among the groups within Fungi there is significant difference for each metric among Fungi classes, EpT: KW Chi^2 = 66.49, P = 8.58e-08 and EpG: KW Chi^2 = 66.44, P = 8.76e-08. Patterns are easier to observe by consolidating Fungi classes into their respective phylum. EpT and EpG mean complexity is clearer to see that Ascomycota is lower and has

nonoverlapping IRQ values than Basidiomycota, Chytridiomycota, and Mucoromycota. When Ascomycota is included among all phyla, there is a significant difference among taxonomic groups for mean EpT (KW $Chi^2 = 40.49$, P = 8.39e-09) and mean EpG (KW $Chi^2 = 40.47$, P = 8.47e-09). Without including Ascomycota in the KW-Test, there is not significant difference among the other Fungi phyla for mean EpT (KW $Chi^2 = 5.151$, P = 0.076) or mean EpG (KW $Chi^2 = 5.151$, P = 0.076).

Supp. Table 1: Fisher Combined Probability Test p-values and T statistic for each taxonomic group's complexity metric used in this study.

	TpG		EpT		EpG	
	$\|T\|$	P	$\|T\|$	P	$\|T\|$	P
**Deuterostomes**	10^{-16}	10^{-16}	10^{-16}	10^{-16}	10^{-16}	10^{-16}
Drosophila	397.765	10^{-16}	2637.79	10^{-16}	2332.525	10^{-16}
**Plantae**	3606.728	10^{-16}	10^{-16}	10^{-16}	10^{-16}	10^{-16}
**Fungi**	47.333	0.8403028	10178.283	0	10165.276	0

ABNORMAL COMPLEXITY AND ANNOTATION

There are also potential signs of *B. floridae* having less resolution in its annotation compared to other deuterostomes, given its lack of complexity. Especially when comparing its sister taxon *B. belcheri*. Since formulating this manuscript, *B. floridae* has a new genome assembly (RefSeq assembly accession: GCF_000003815.2) but is not updated on OrthoDB. The previous assembly is from 2009 while the new assembly from 2020 is able to utilize the new

genomic resources in both sequencing and *in silco* techniques. The new annotation has mean complexity metrics more in line with its placement in the deuterostome phylogeny. Receptively, *B. floridae* GCF_000003815.2 has mean metrics TpG: 1.58, EpT: 10.98, and EpG: 8.90; while *B. floridae* GCF_000003815.1 has mean metrics TpG: 1.00, EpT: 7.10, and EpG: 7.10. These metrics further explain the caveat to the metrics are sensitive to the annotation data being supplied.

When plotting complexity metrics across the deuterostome phylogeny, we observed a pattern where high outlier counts for a specific gene were missing in *Gallus gallus*, chicken. Upon further investigation, the gene was identified as Titin. Titin is the largest known described protein that has a significant role in structural, developmental, mechanical, and regulatory roles in cardiac and skeletal muscles (Fürst et al. 1988; Vikhlyantsev et al. 2012). We found that the previous *Gallus gallus* annotation, build 6a (RefSeq assembly accession: GCF_000002315.6) (Bellott et al. 2017), did not have Titin annotated while the current annotation (RefSeq assembly accession: GCF_016699485.2), used in this paper, does have an annotated *TTN* gene has annotated Titin. This is a key example of how TranD can be used to guide and help utilize annotations properly to obtain the most accurate and complete dataset, especially as annotation and assembly progresses rapidly in the face of new data. This result illustrates the power of complexity metrics in identifying patterns in structural phenotypes of transcripts along with TranD being dependent on proper annotations for accurate metrics.

The fruit fly, *D. sechellia*, annotation has lower complexity metrics compared to its other congeners. The *D. sechellia* annotation collapsed many of its transcripts and exons into genes, thus having metrics that are mostly single transcript genes, without much AS, compared to the rest of the *Drosophila* clade. The Drosophila 12 Genomes Consortium; et al. (2007) found there

to be “appreciably higher frequency of masked bases in lower-quality” in the *D. sechellia* assembly and it is observed in our complexity analyses.

Supplementary References:

Bellott, D. W., Skaletsky, H., Cho, T. J., Brown, L., Locke, D., Chen, N., ... & Page, D. C. (2017). Avian W and mammalian Y chromosomes convergently retained dosage-sensitive regulators. *Nature genetics*, *49*(3), 387-394.

Fürst, D. 0., Osborn, M., Nave, R. & Weber, K. (1988). The organization of titin filaments in the half-sarcomere revealed by monoclonal antibodies in immunoelectron microscopy: A map of ten nonrepetitive epitopes starting at the Z line extends close to the M line. *J. Cell Biol*, *106*, 1563-1572.

Vikhlyantsev, I. M., & Podlubnaya, Z. A. (2012). New titin (connectin) isoforms and their functional role in striated muscles of mammals: facts and suppositions. *Biochemistry (Moscow)*, *77*(13), 1515-1535.

TAXONMICS SCALE RESULOUTION COMPLEXITY METRICS

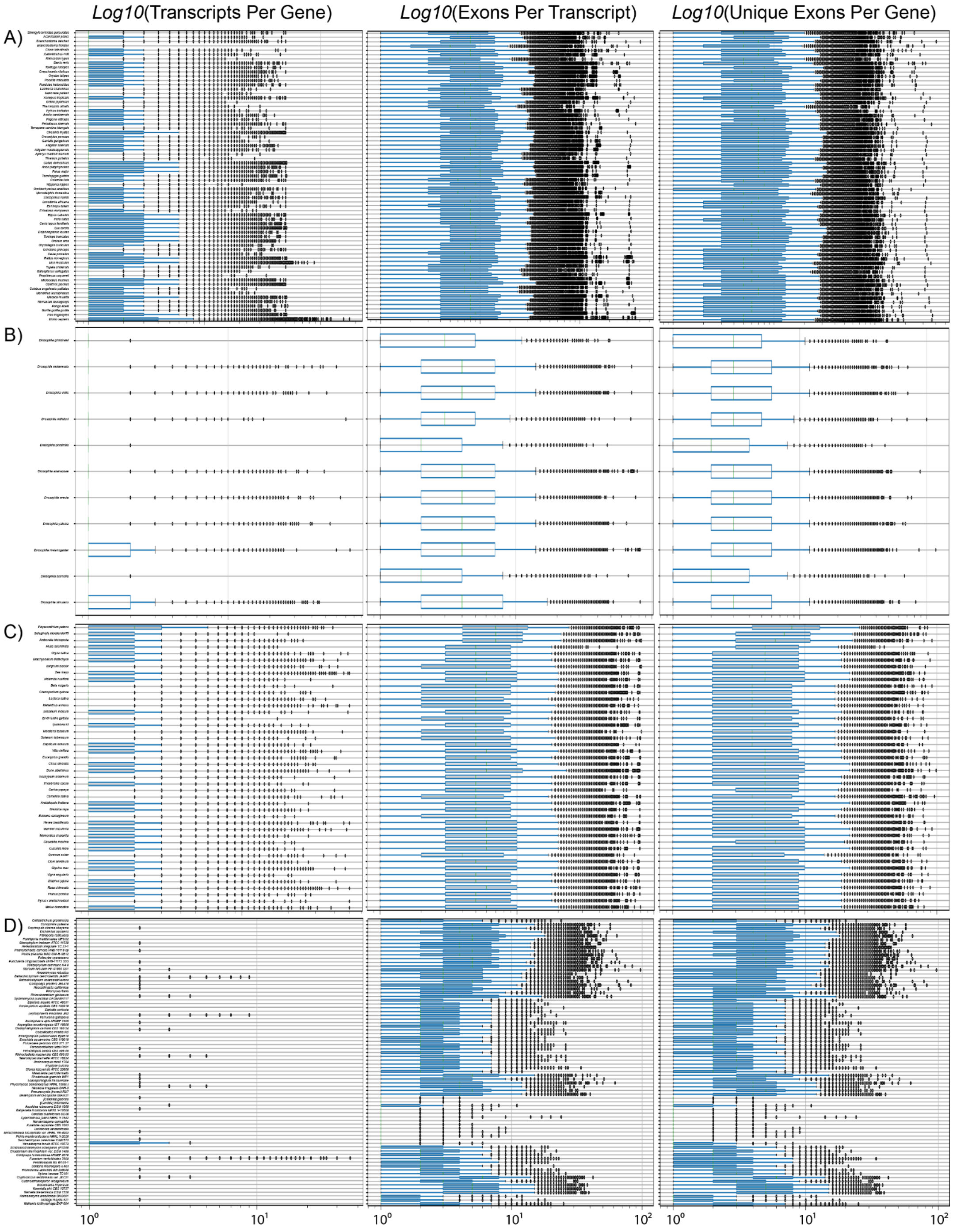

Supp. Figure 1: Box and whisker plots showing complexity metrics for TpG, EpT, and EpG for individual taxonomic groupings in log-scale. Green lines indicate median metrics with the first and third quartiles at the borders of the box. The minimum and maximum range indicated by

black lines. Columns are set to each complexity metric. Taxonomic groups consist of A) Deuterostomia, B) *Drosophila*, C) Plantae, and D) Fungi.

NOVEL GENE PROPORTIONS

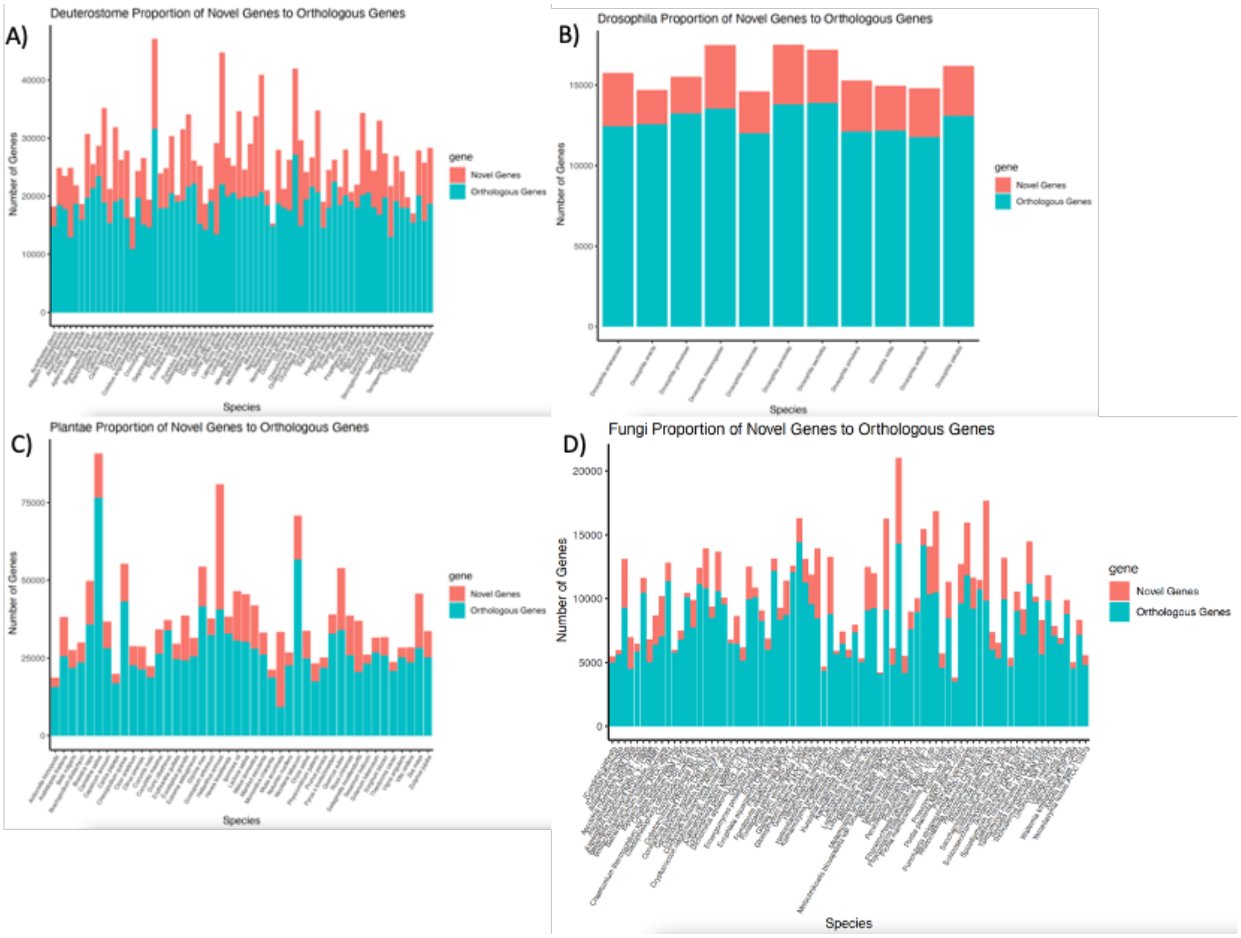

Supp. Figure 2: Proportions of novel genes (Red) to orthogous genes (aqua) for each oraganisms annotations. Panels are broken down by taxonomic groups consist of A) Deuterostomia, B) *Drosophila*, C) Plantae, and D) Fungi.

VARIANCE

Of all our metrics are primarily over-dispersed compared to expectations under the Poisson (Supp. Fig. 2) Both EpT and EpG for all deuterostomes, *Drosophila*, and plants are over-

dispersed compared to expectations under the Poisson Distribution. Fungi have 59/77 taxa over dispersed EpT metrics and 60/77 for EpG. All fungi taxa are under-dispersed for TpG. TpG is have more taxa under-dispersed compared to expectations under the Poisson Distribution than other the other complexity metrics. TpG is over-dispersed for 57/68 Deuterostomes, 7/11 Drosophila, and 33/44 plants taxa. We observe exceptionally high normalized variance in humans across TpG and EpT with variance two orders of magnitude higher than the mean.

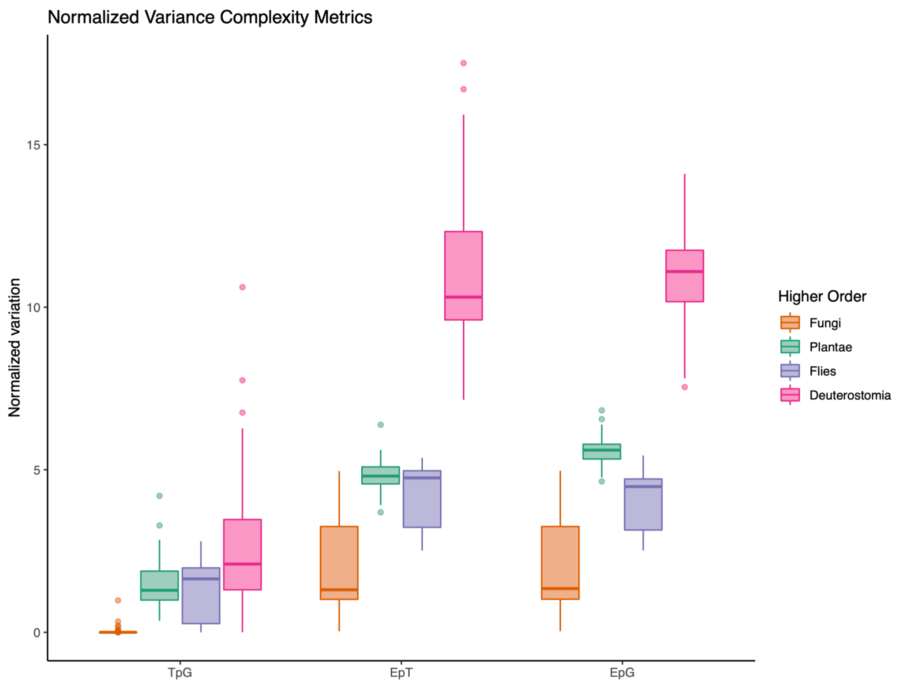

Supp. Figure 3: Normalized Variance Mean Complexity Metrics - Box and whisker plot of normalized variance computed from standard variance squared / mean from each complexity metric - TpG, EpT, and EpG. Higher-order taxonomic groups are colored by lineage. Outlier data are plotted as points above and below whiskers. Whiskers denote the extreme data, the box represents the upper and lower quartile, and box lines represent the median.

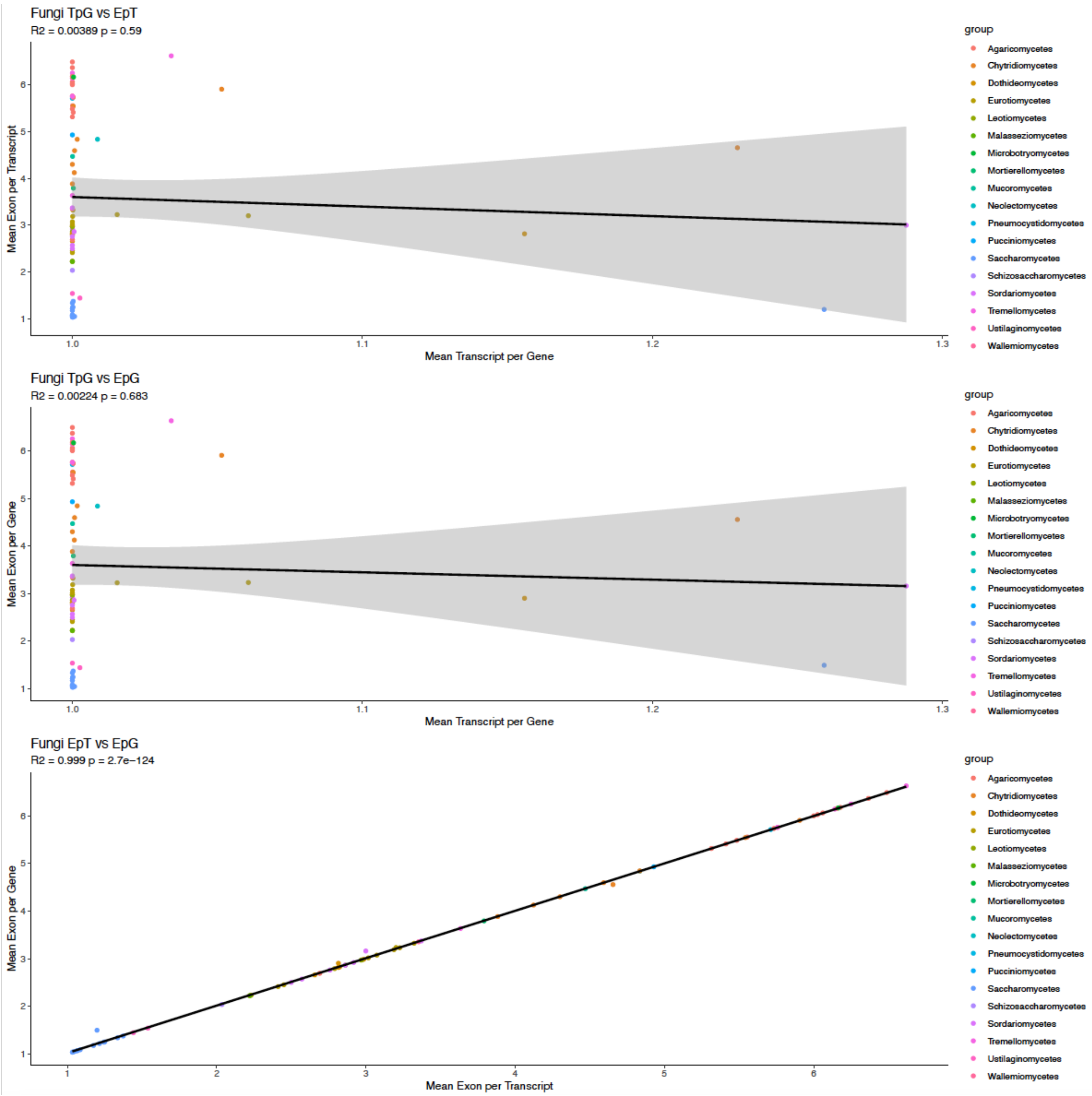

Supp. Figure 4: Fungi pair plots comparing complexity metrics. Metrics are compared among TpG, EpT, and EpG. The p-value and R^2 derived from calculating the Pearson correlation coefficient.

COMPLEXITY CORRELATIONS

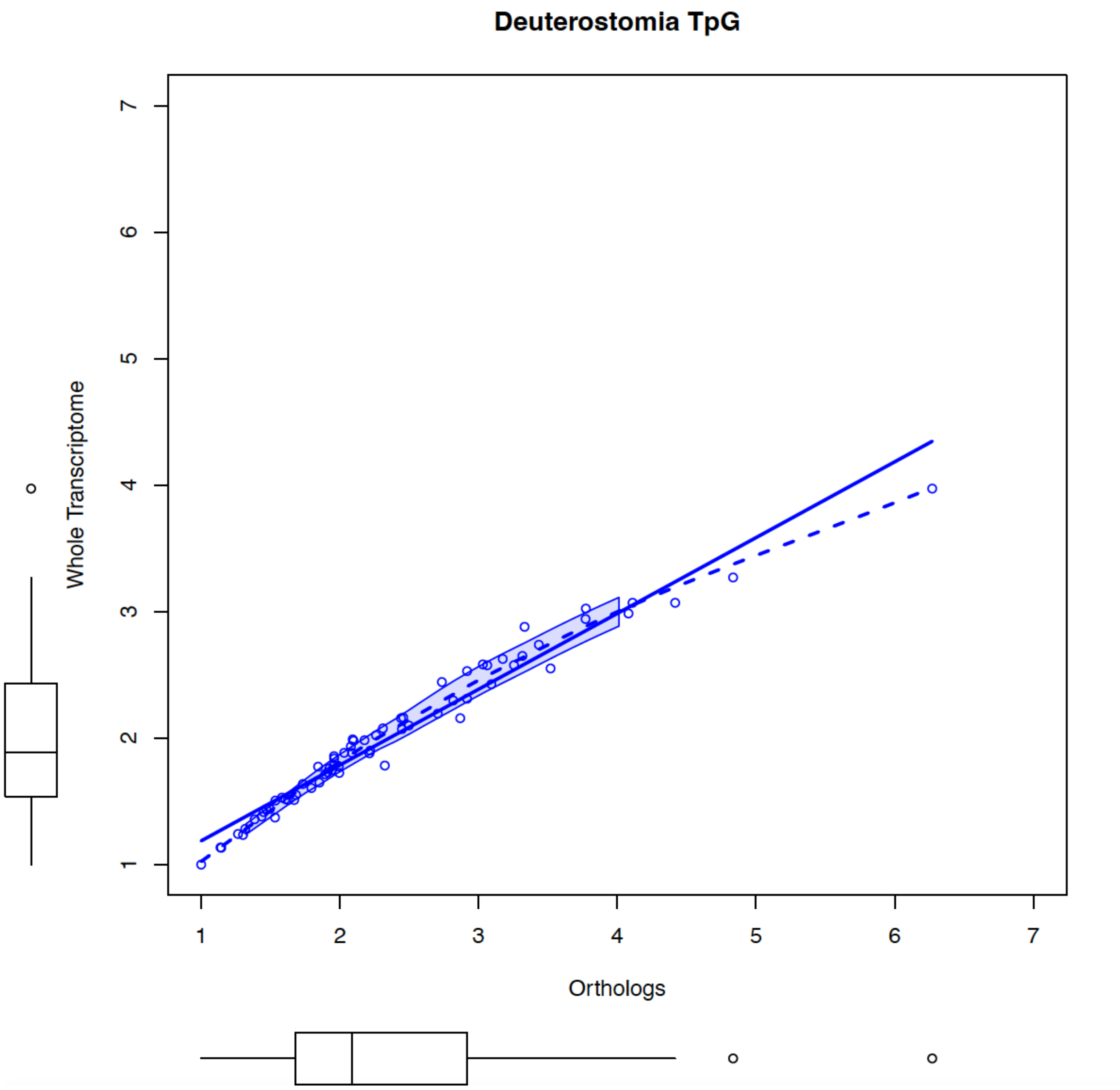

Supp. Figure 5: Deuterostome TpG scatter plot between whole-transcriptome (y-axis) and orthologs (x-axis) for each individual, the regression line (solid blue), the smoothed conditional spread (blue shaded regions) the non-parametric regression smooth (dotted blue line), and box and whisker plots for each dataset at the corresponding axes.

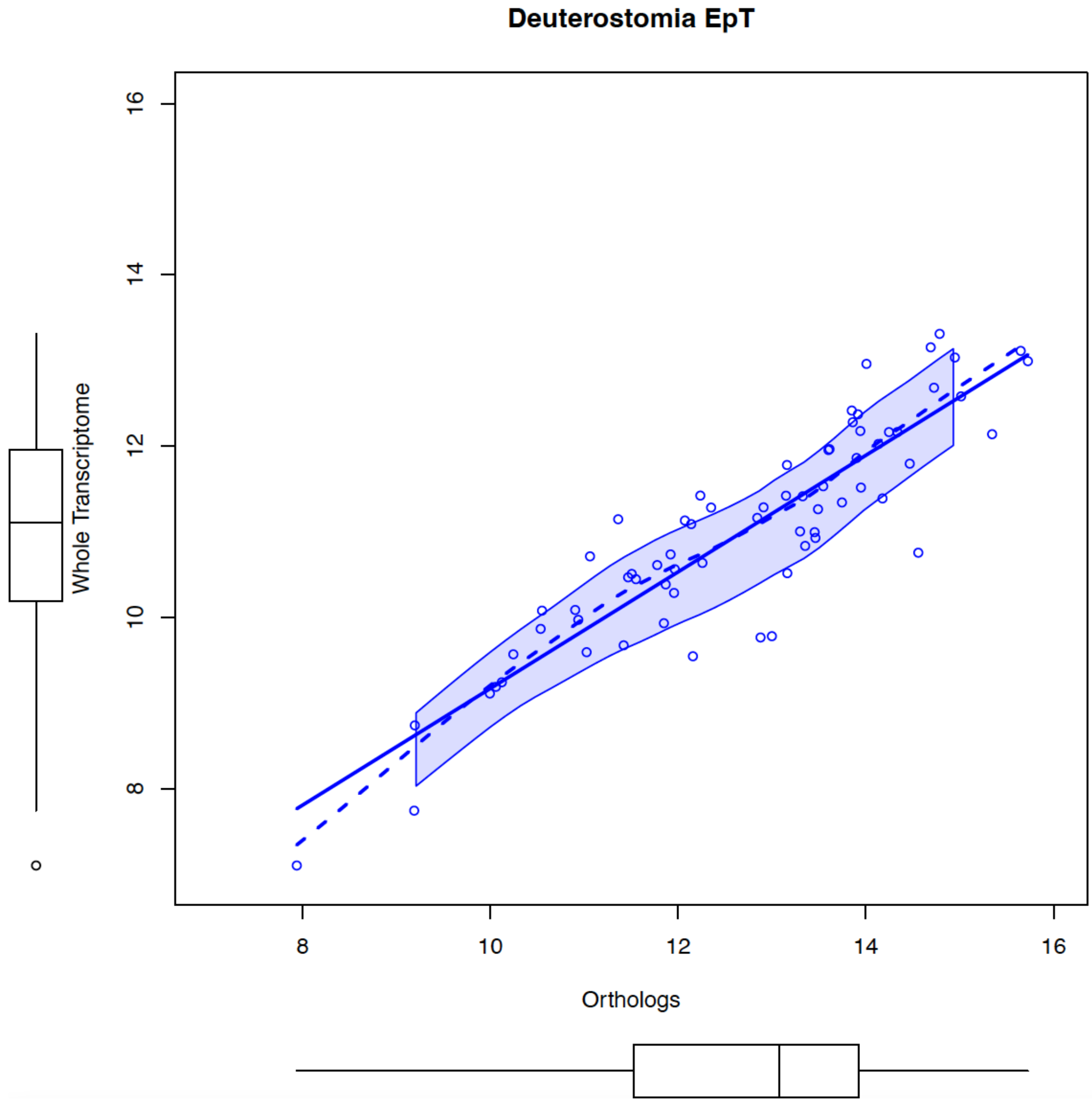

Supp. Figure 6: Deuterostome EpT scatter plot between whole-transcriptome (y-axis) and orthologs (x-axis) for each individual, the regression line (solid blue), the smoothed conditional spread (blue shaded regions) the non-parametric regression smooth (dotted blue line), and box and whisker plots for each dataset at the corresponding axes.

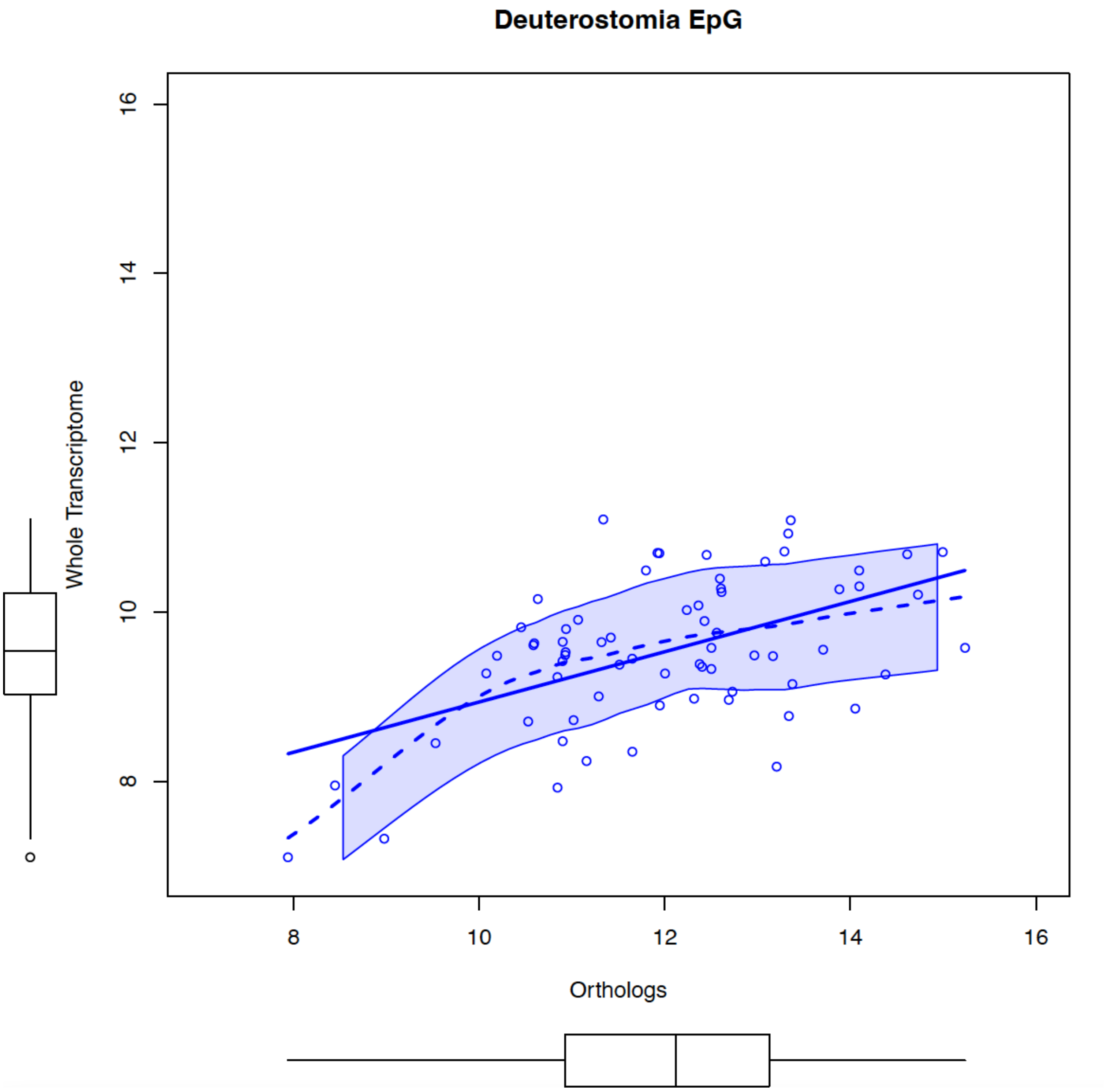

Supp. Figure 7: Deuterostome EpG scatter plot between whole-transcriptome (y-axis) and orthologs (x-axis) for each individual, the regression line (solid blue), the smoothed conditional spread (blue shaded regions) the non-parametric regression smooth (dotted blue line), and box and whisker plots for each dataset at the corresponding axes.

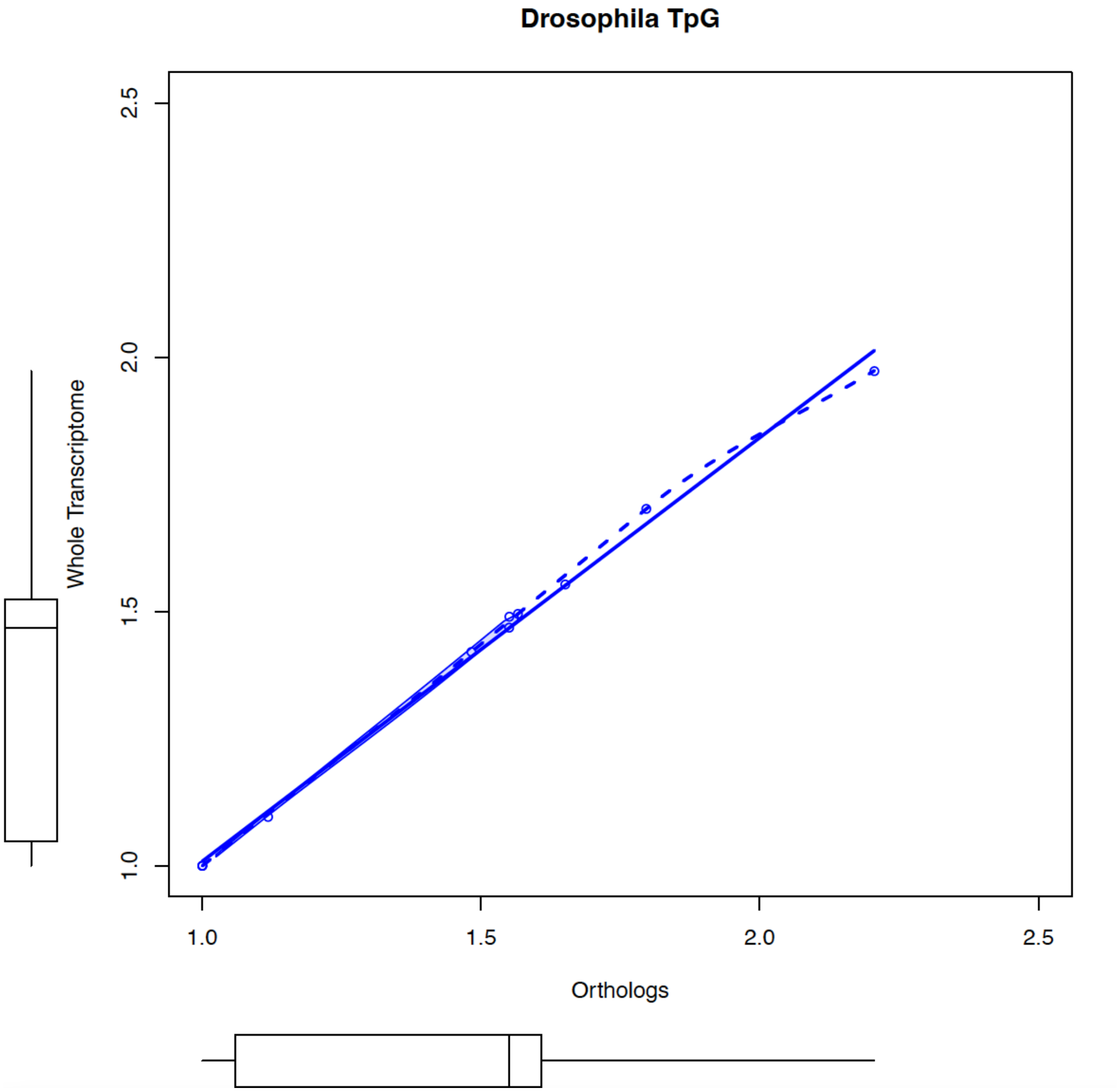

Supp. Figure 8: *Drosophila* TpG scatter plot between whole-transcriptome (y-axis) and orthologs (x-axis) for each individual, the regression line (solid blue), the smoothed conditional spread (blue shaded regions) the non-parametric regression smooth (dotted blue line), and box and whisker plots for each dataset at the corresponding axes.

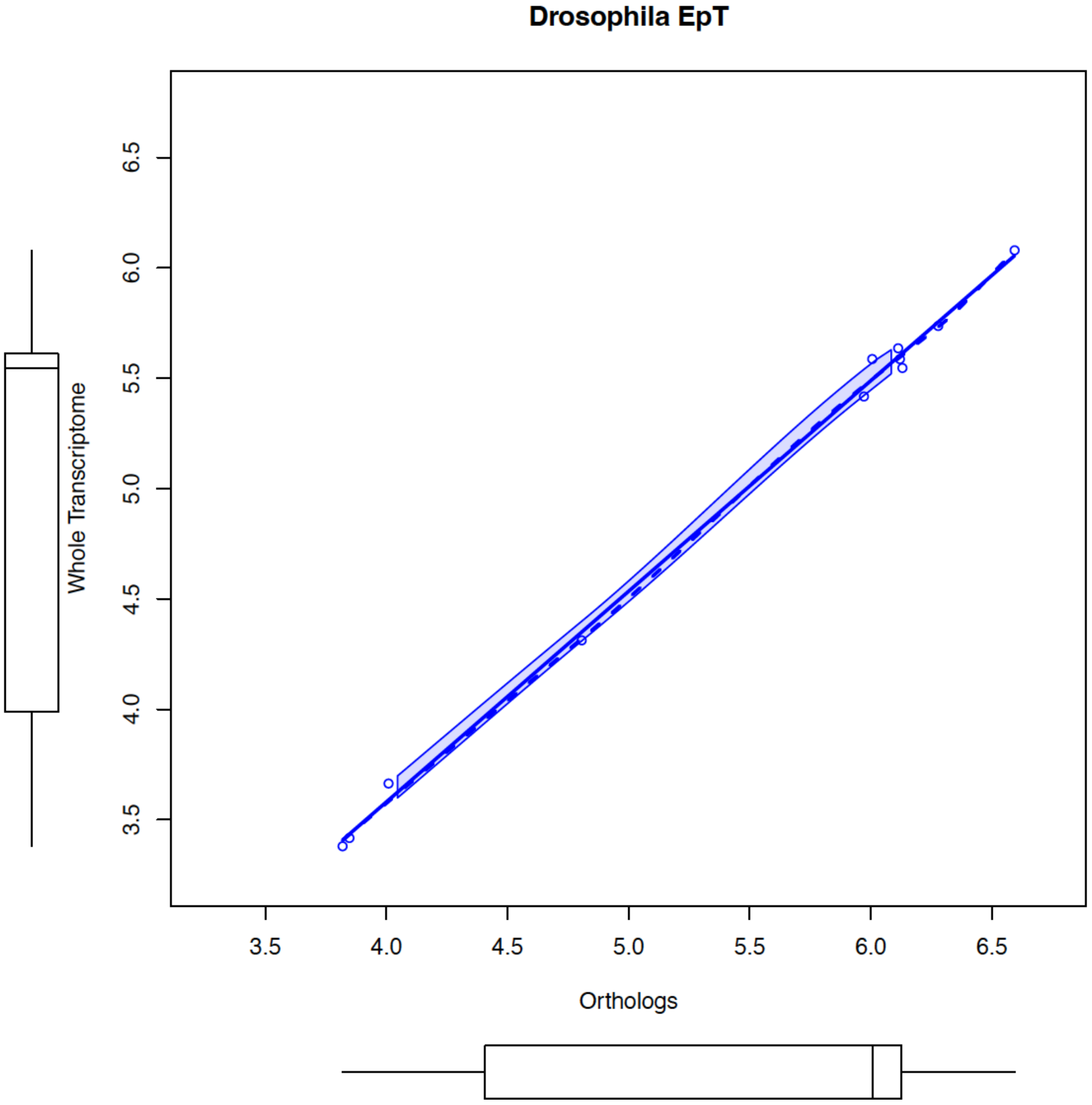

Supp. Figure 9: *Drosophila* EpT scatter plot between whole-transcriptome (y-axis) and orthologs (x-axis) for each individual, the regression line (solid blue), the smoothed conditional spread (blue shaded regions) the non-parametric regression smooth (dotted blue line), and box and whisker plots for each dataset at the corresponding axes.

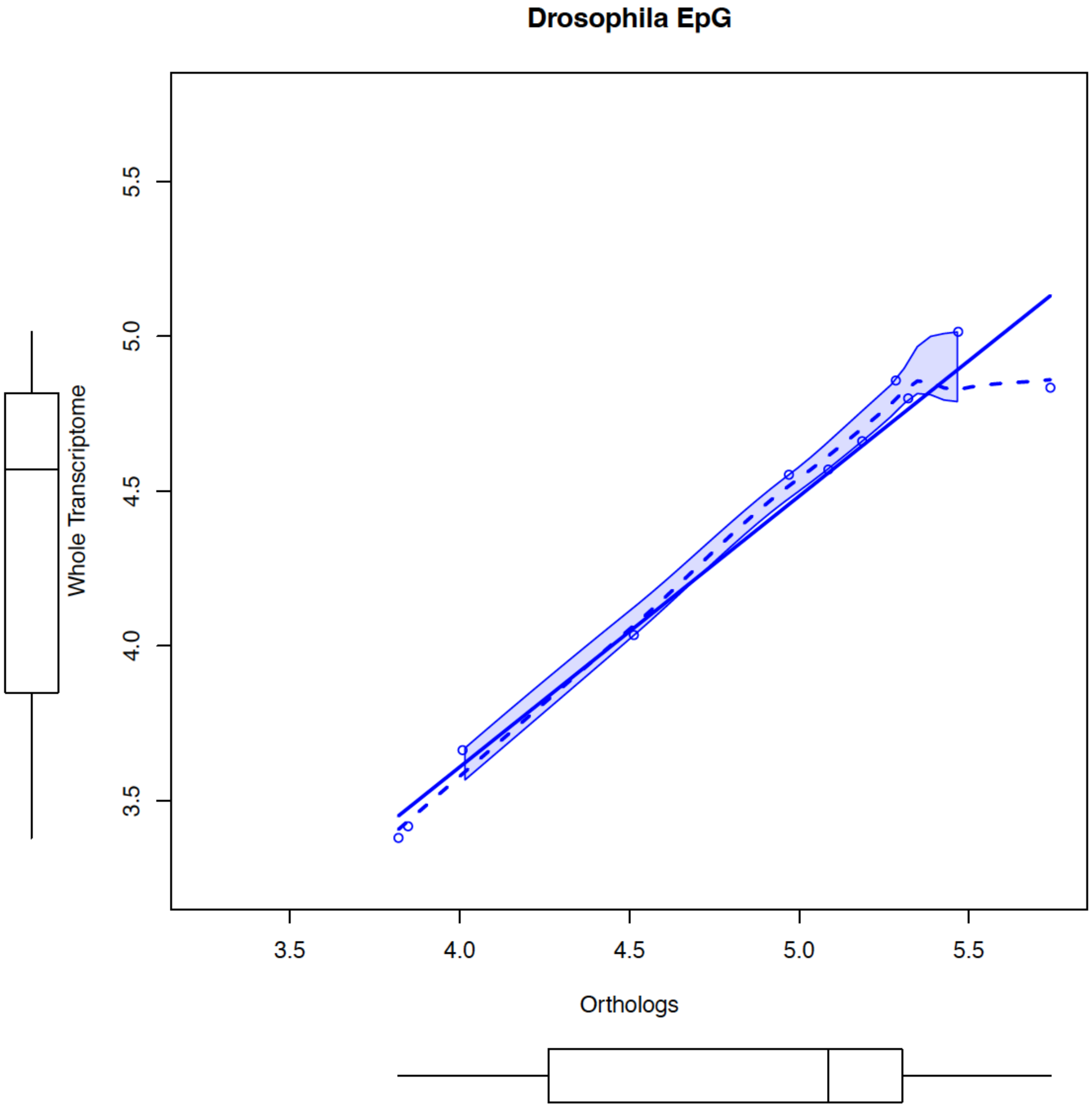

Supp. Figure 10: *Drosophila* EpG scatter plot between whole-transcriptome (y-axis) and orthologs (x-axis) for each individual, the regression line (solid blue), the smoothed conditional spread (blue shaded regions) the non-parametric regression smooth (dotted blue line), and box and whisker plots for each dataset at the corresponding axes.

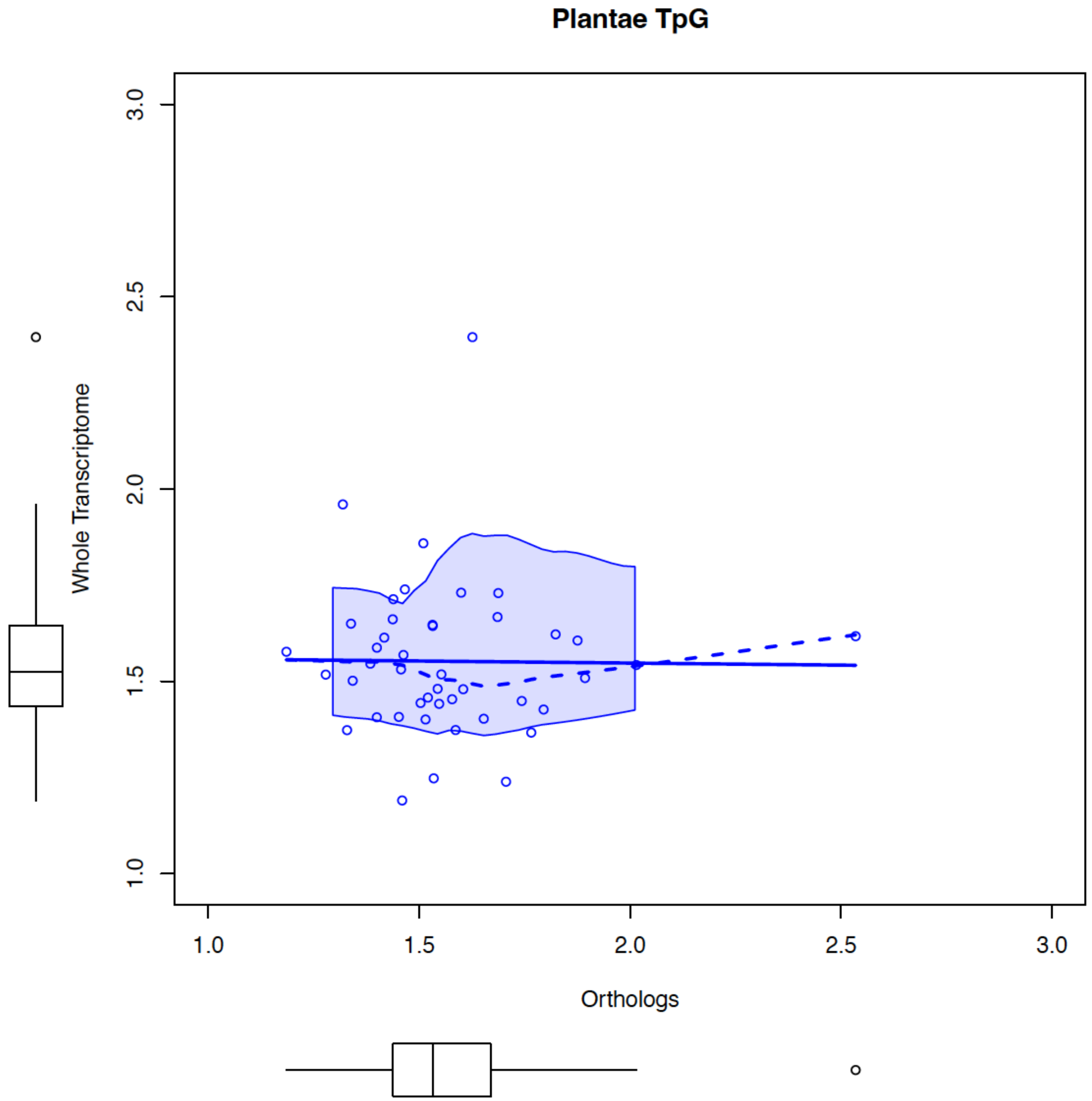

Supp. Figure 11: Plantae TpG scatter plot between whole-transcriptome (y-axis) and orthologs (x-axis) for each individual, the regression line (solid blue), the smoothed conditional spread (blue shaded regions) the non-parametric regression smooth (dotted blue line), and box and whisker plots for each dataset at the corresponding axes.

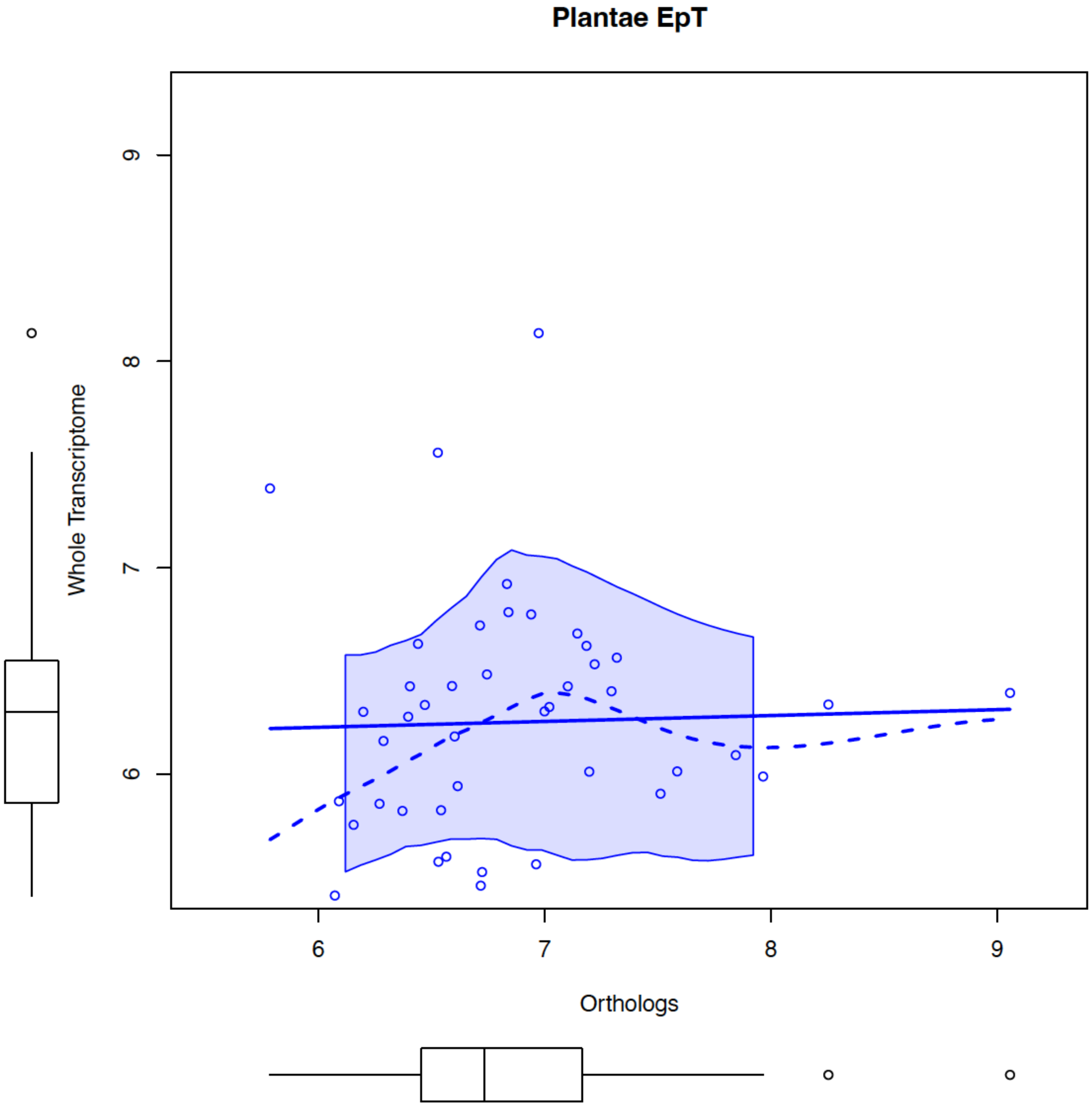

Supp. Figure 12: Plantae EpT scatter plot between whole-transcriptome (y-axis) and orthologs (x-axis) for each individual, the regression line (solid blue), the smoothed conditional spread (blue shaded regions) the non-parametric regression smooth (dotted blue line), and box and whisker plots for each dataset at the corresponding axes.

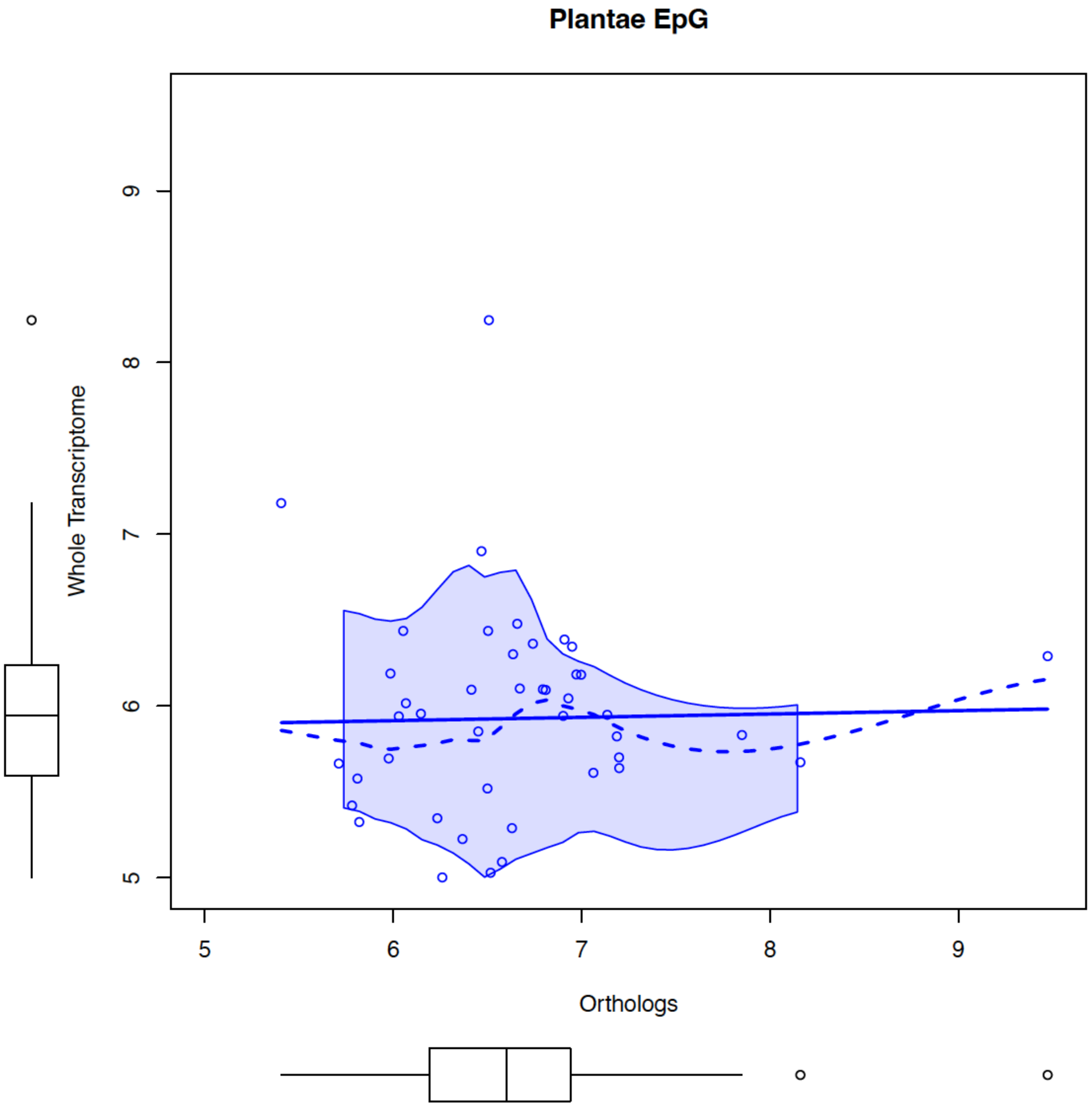

Supp. Figure 13: Plantae EpG scatter plot between whole-transcriptome (y-axis) and orthologs (x-axis) for each individual, the regression line (solid blue), the smoothed conditional spread (blue shaded regions) the non-parametric regression smooth (dotted blue line), and box and whisker plots for each dataset at the corresponding axes.

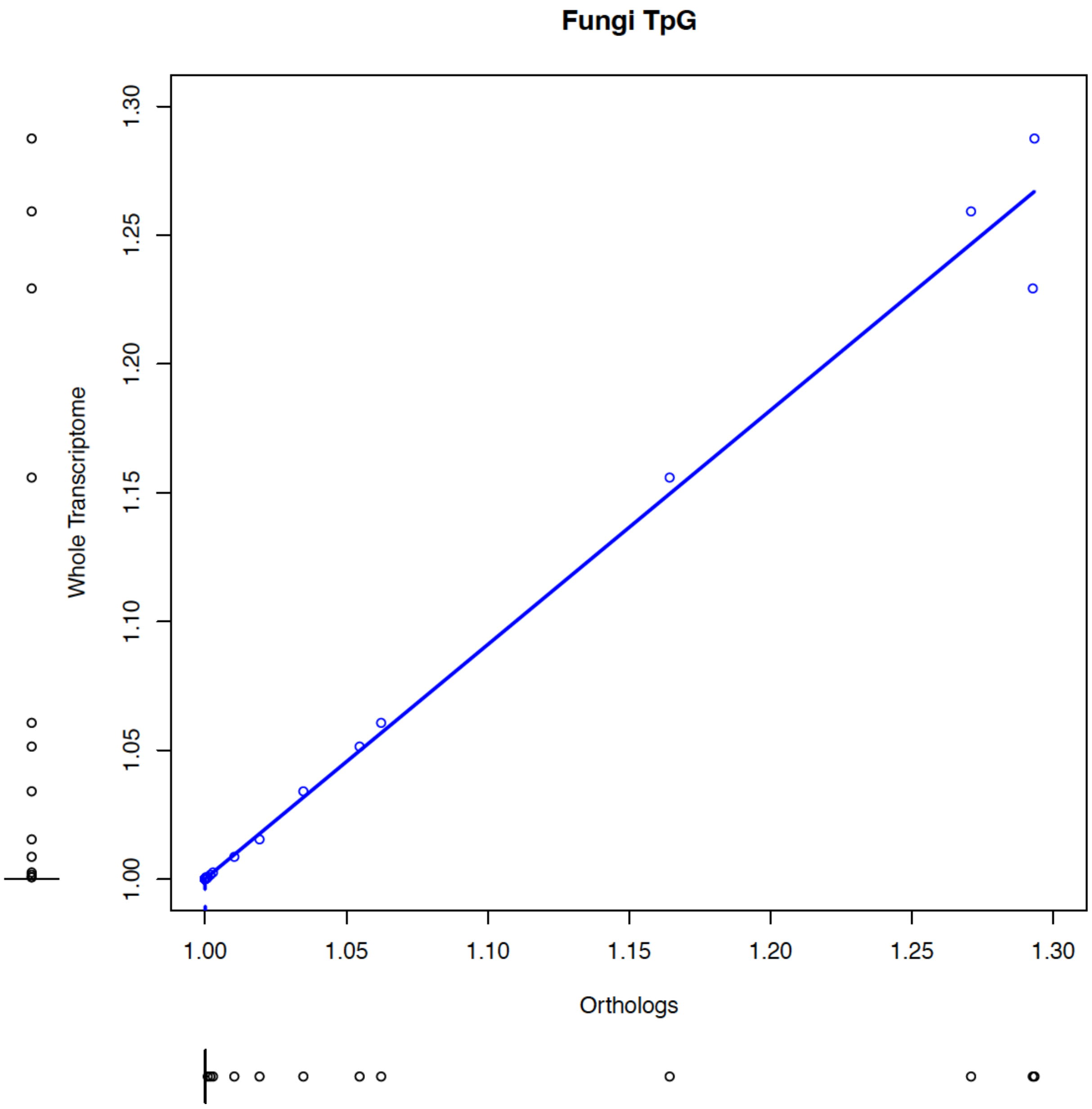

Supp. Figure 14: Fungi TpG scatter plot between whole-transcriptome (y-axis) and orthologs (x-axis) for each individual, the regression line (solid blue), the smoothed conditional spread (blue shaded regions) the non-parametric regression smooth (dotted blue line), and box and whisker plots for each dataset at the corresponding axes.

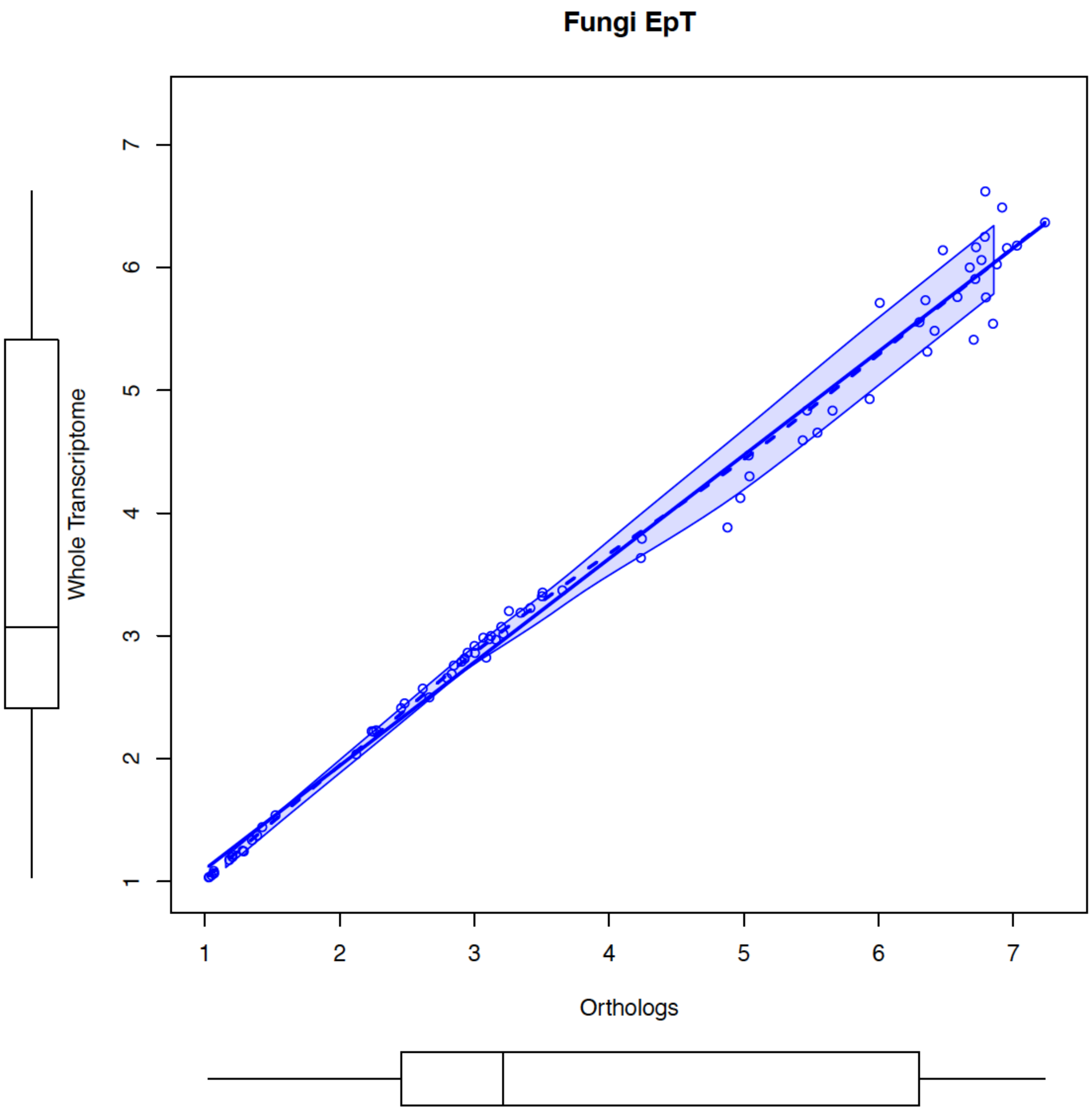

Supp. Figure 15: Fungi EpT scatter plot between whole-transcriptome (y-axis) and orthologs (x-axis) for each individual, the regression line (solid blue), the smoothed conditional spread (blue shaded regions) the non-parametric regression smooth (dotted blue line), and box and whisker plots for each dataset at the corresponding axes.

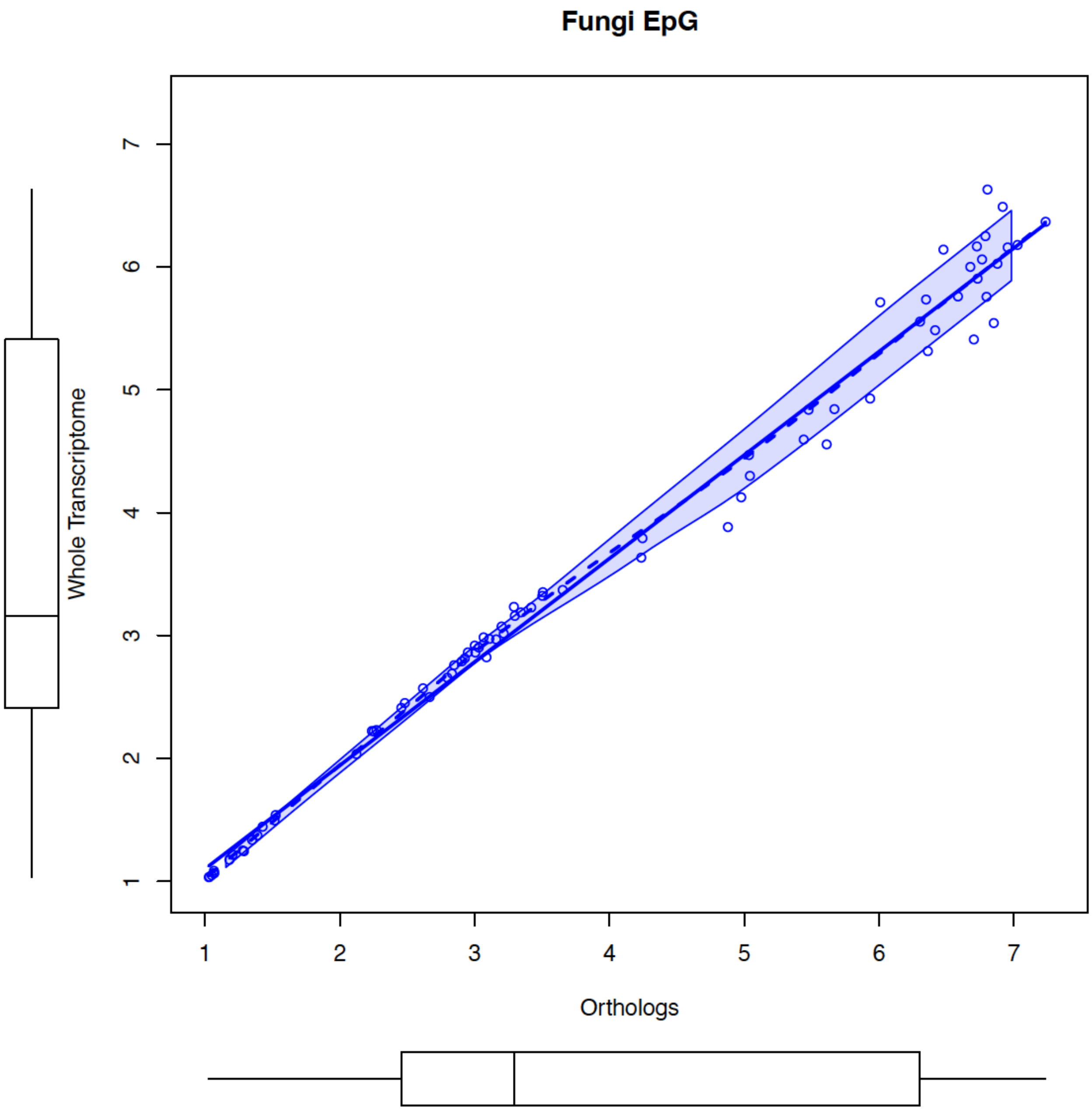

Supp. Figure 16: Fungi EpG scatter plot between whole-transcriptome (y-axis) and orthologs (x-axis) for each individual, the regression line (solid blue), the smoothed conditional spread (blue shaded regions) the non-parametric regression smooth (dotted blue line), and box and whisker plots for each dataset at the corresponding axes.

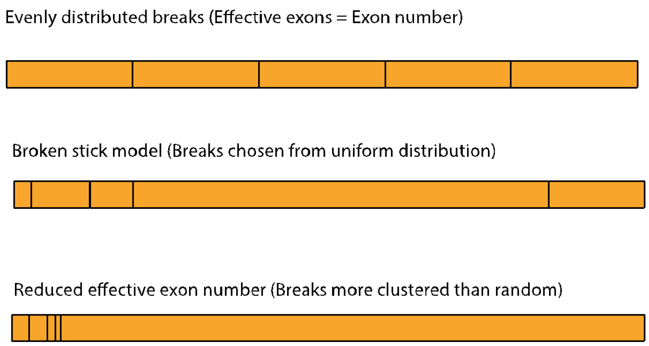

Supp. Figure 17: Effective Exon Number (EEN) captures information about the distribution of exon lengths produced when introns cause breaks in transcripts. When EEN = EpT, exon sizes are evenly distributed, more even than a model of random stochastic intron placement. If intron boundaries are drawn random across the transcript from a uniform distribution, they should follow the EEN distribution from a Broken Stick Model. More tightly clustered intron breakpoints yield lower EEN values compared with the Broken Stick. Patterns of variation in EEN are influenced by parameters of splicing models used during annotation, physical and molecular constraints on intron splicing processes, and constraints from natural selection shaping genetic structures.

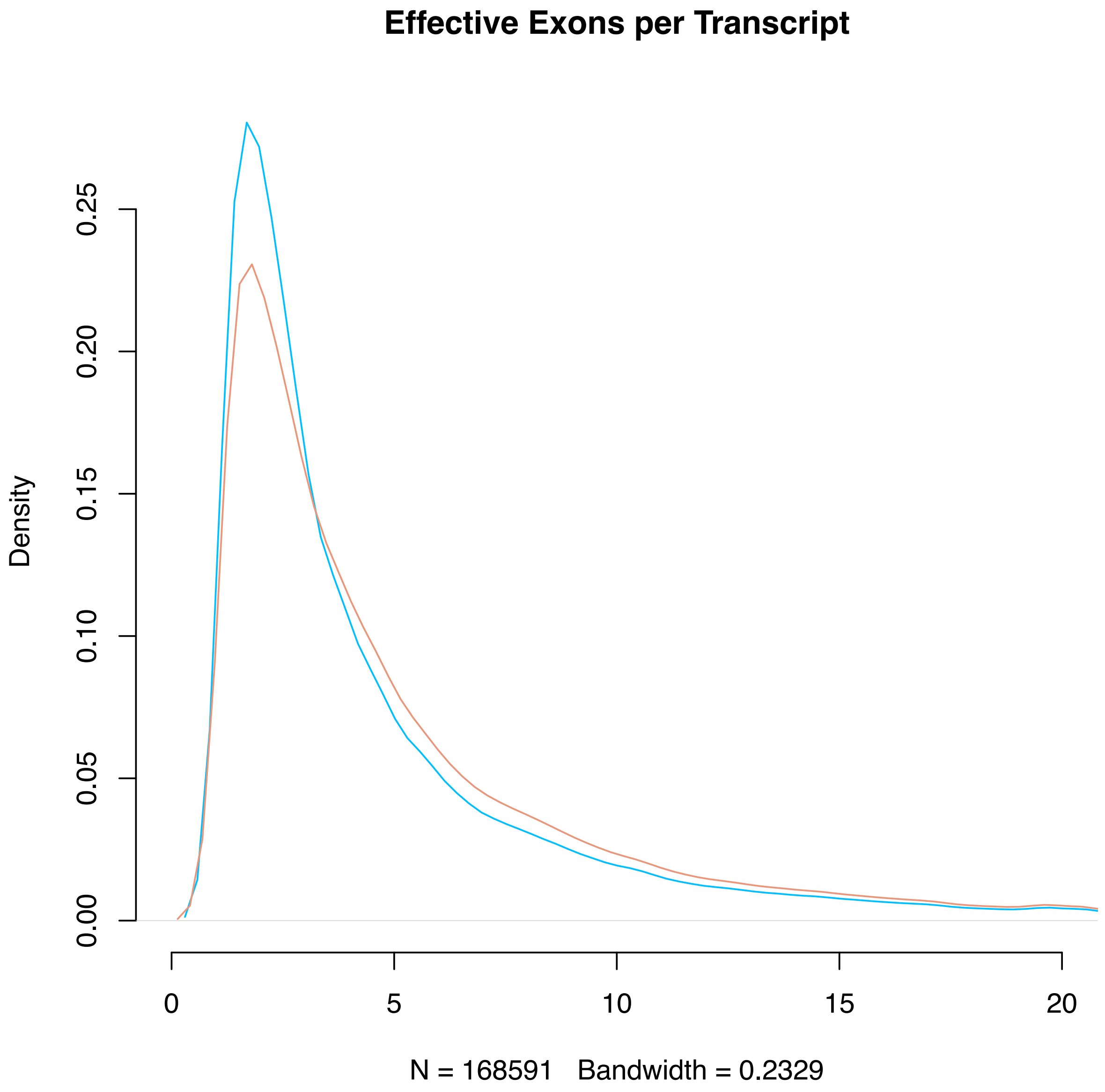

Supp. Figure 18: Effective exon number distribution for all genes in whole transcriptome data and conditioning on orthologs in humans. We observe a significant difference ($P<10^{-16}$) when lineage specific genes are excluded.

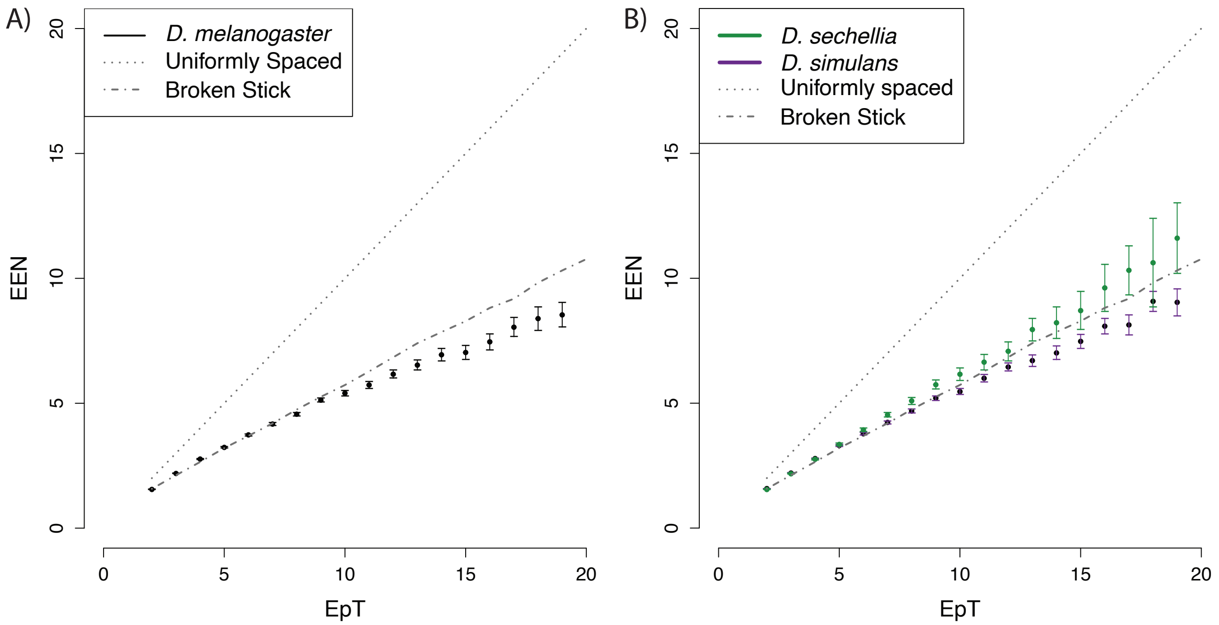

Supp. Figure 19: Mean EEN +/- 2*SE for A) *D. melanogaster* and B) two closely related species *D. simulans* and *D. sechellia.* In *D. melanogaster* EEN is consistent with the stick-breaking problem below 10 EpT. Above 10 EpT EEN is lower than expected, suggesting more tightly clustered introns than chance. *D. simulans* follows a similar pattern to *D. melanogaster.* In contrast, *D. sechellia* shows mean EEN greater than expected, but with wider error bars overlapping with the broken stick model at for higher EpT values. These different patterns for two very closely related species suggest differences in annotation, splicing mechanisms, or selective constraint influencing transcriptome complexity patterns. For some evolutionary comparisons, normalization for differences in complexity may be warranted.

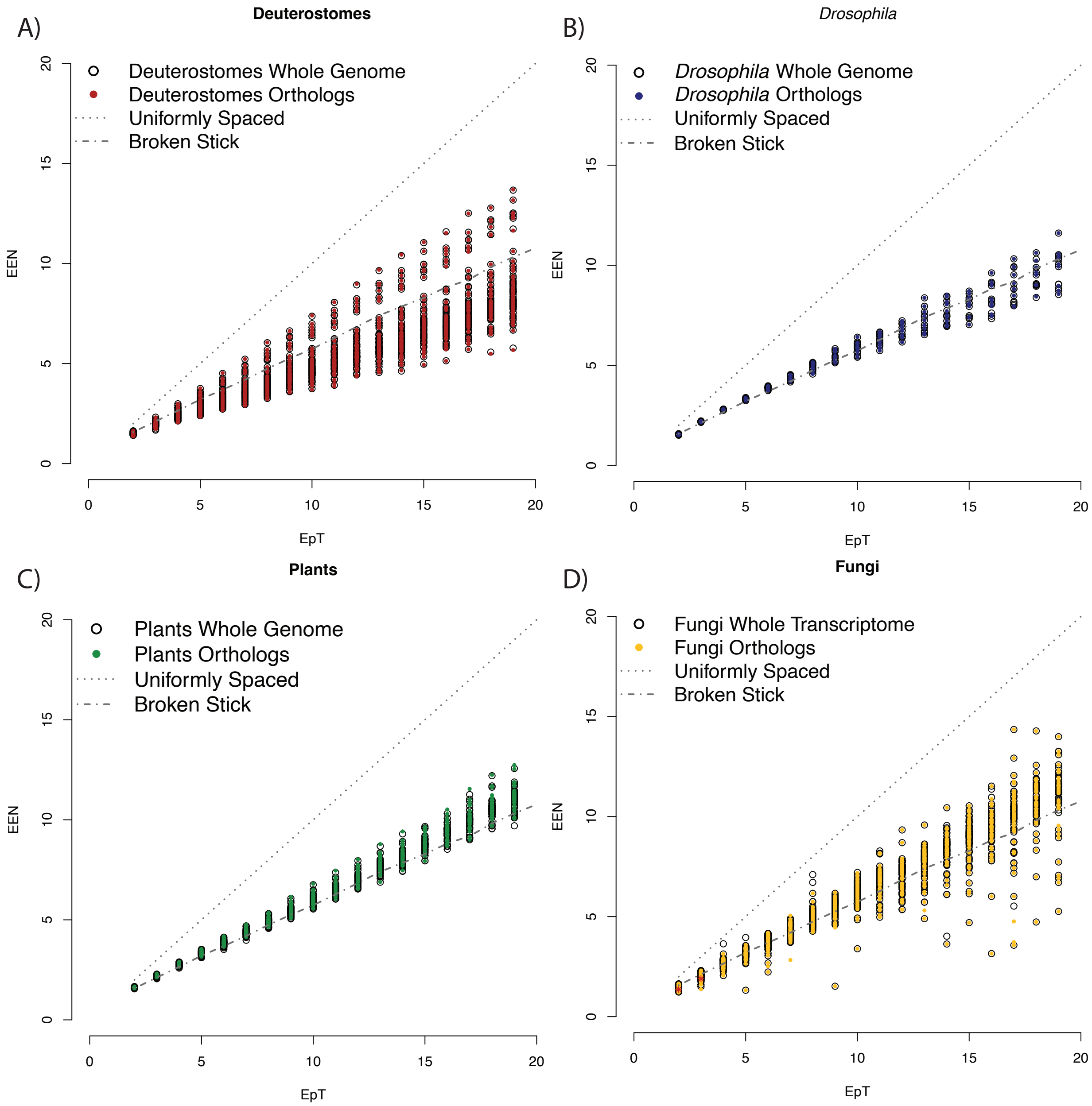

Supp. Figure 20: EEN vs EpT in Whole Transcriptome data (solid circles) and conditioning on Orthologs (open circles) in A) Chordates and Deuterostomes, B) *Drosophila*, C) plants, and D) Fungi. Conditioning on orthologs has only a nominal influence on EEN comparisons to the Broken Stick Model and does not alter inference for any species. The well validated annotations backed by abundant molecular evidence in humans and *D. melanogaster* show departures from the broken stick model. Both have reduced EEN for transcripts with high EpT suggesting more

tightly clustered intron boundaries than expected under stochastic random processes. In chordates and deuterostomes, 62/68 species lie at or below the Broken Stick and 6 species lie above. Plants lie nearly completely above the Broken Stick line, with Fungi having annotations under and over the Broken Stick.

EVOLUTIONARY RATES

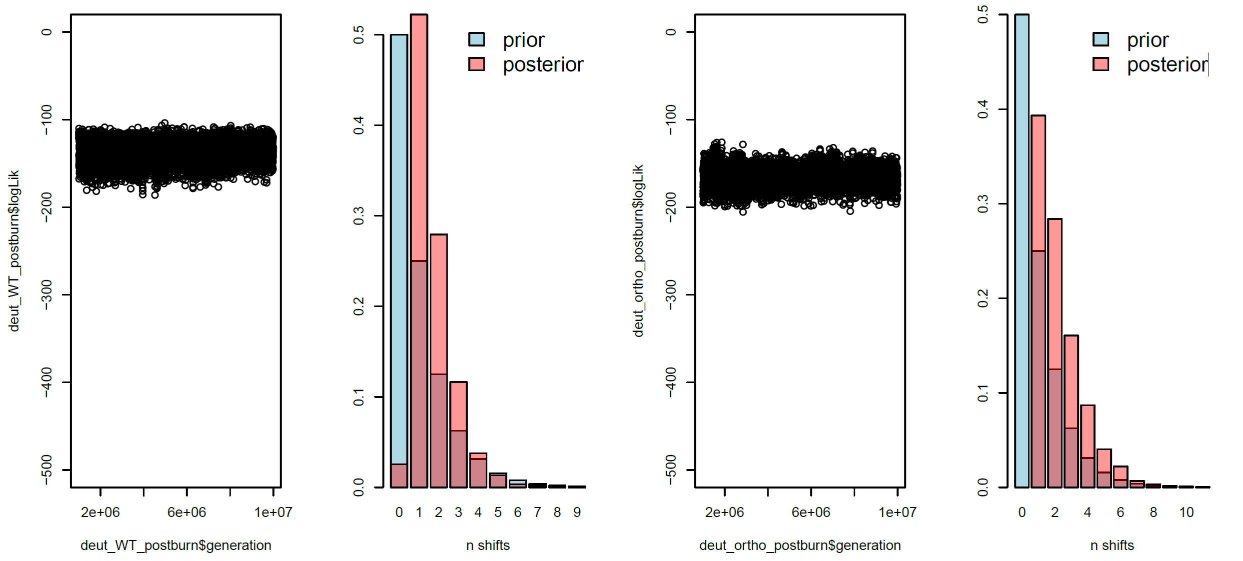

Supp. Figure 21: Deuterostome MCMC convergence post-burnin for whole-transcriptome (left) and ortholog datasets (right) with generation time on the x-axis and log-likelihood on the y-axis. Posterior probabilities for trait shifts for whole-transcriptome (left) and ortholog datasets (right), priors are in blue and posteriors are in red.

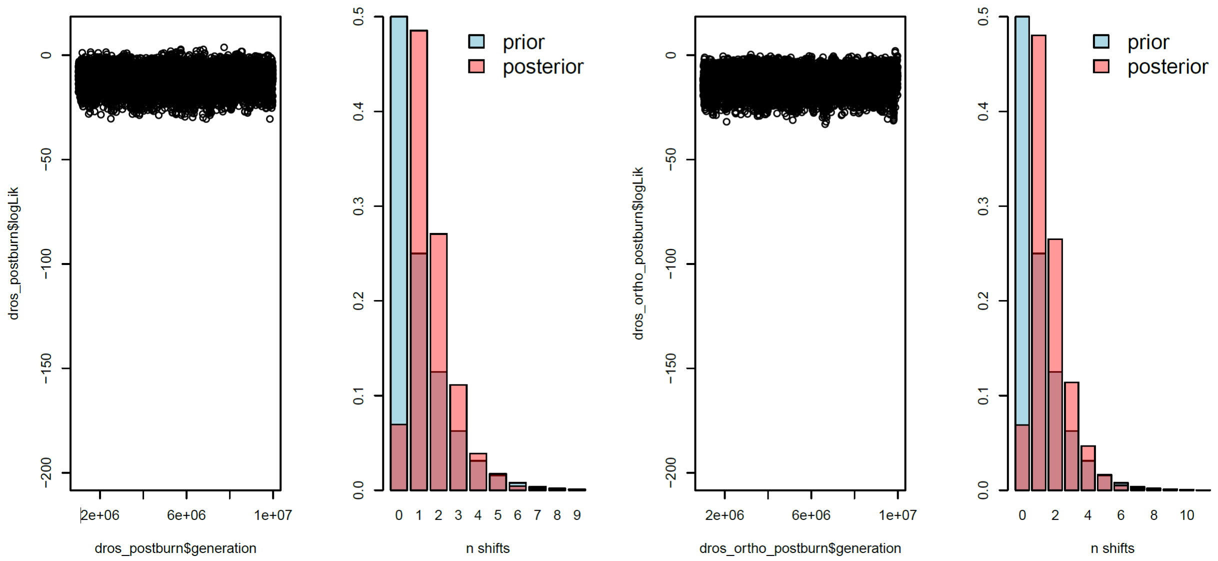

Supp. Figure 22: *Drosophila* MCMC convergence post-burnin for whole-transcriptome (left) and ortholog datasets (right) with generation time on the x-axis and log-likelihood on the y-axis. Posterior probabilities for trait shifts for whole-transcriptome (left) and ortholog datasets (right), priors are in blue and posteriors are in red.

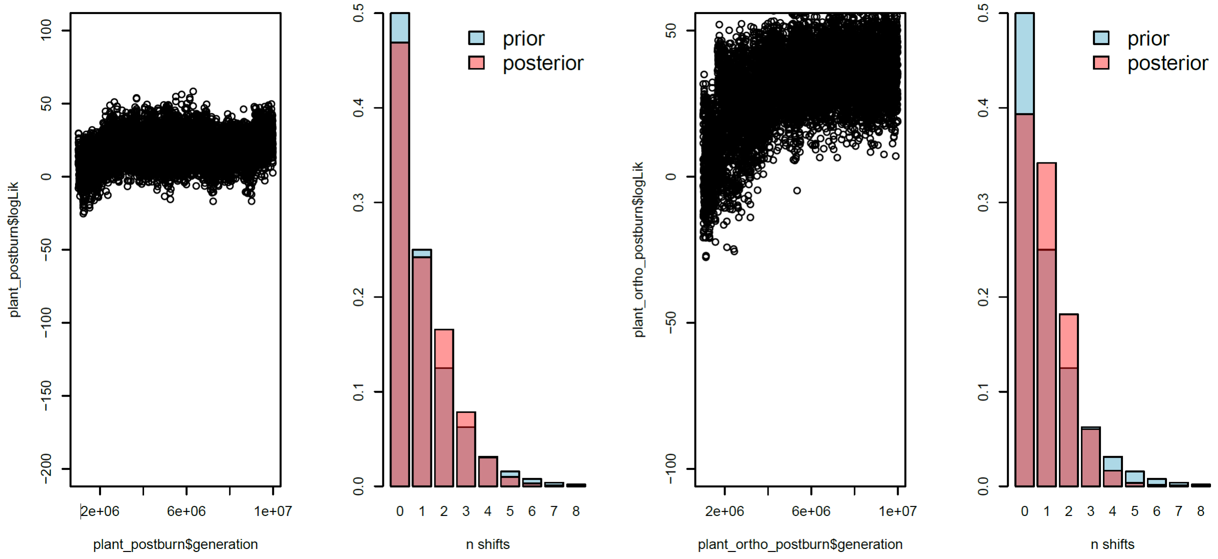

Supp. Figure 23: Plantae MCMC convergence post-burnin for whole-transcriptome (left) and ortholog datasets (right) with generation time on the x-axis and log-likelihood on the y-axis. Posterior probabilities for trait shifts for whole-transcriptome (left) and ortholog datasets (right), priors are in blue and posteriors are in red.

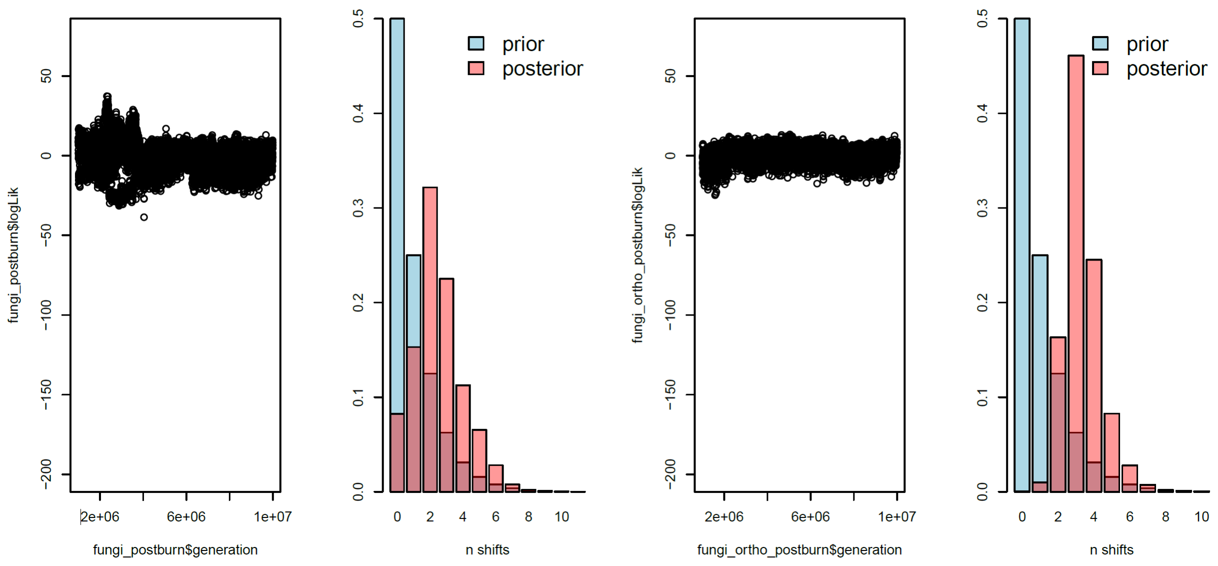

Supp. Figure 24: Fungi MCMC convergence post-burnin for whole-transcriptome (left) and ortholog datasets (right) with generation time on the x-axis and log-likelihood on the y-axis. Posterior probabilities for trait shifts for whole-transcriptome (left) and ortholog datasets (right), priors are in blue and posteriors are in red.

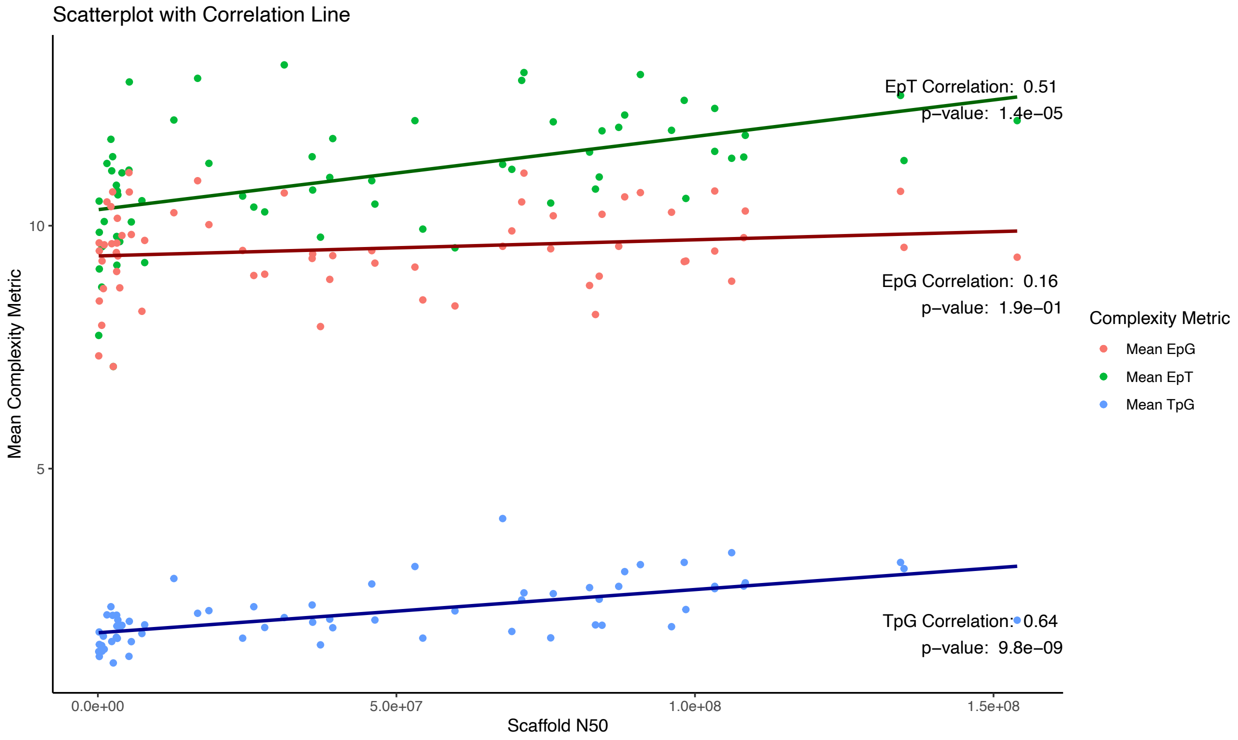

Supp. Figure 25: Pearson correlations plotted for TpG (Blue), EpT (Green), and EpG (Blue) complexity metrics against scaffold N50 for a subset of deuterostomes. Regression lines are plotted in darker corresponding colors. Here we omitted the North Island brown kiwi (GCF_001039765.1_AptMant0) as it is only a contig level assembly with no available scaffold N50. We also omitted the Tasmanian devil (GCF_902635505.1_mSarHar1.11) as it has an N50 of 611.3 Mb, which is almost six times that of our next largest scaffold N50. Note that omitting the Tasmanian devil does not change our overall significance. Scaffold N50 correlates with TpG and EpT, but not with EpG. Even though TpG and EpT are significant, the regression line slopes are slight and likely due to effects sizes. However, it is important to note that contiguity of an assembly does have a correlation to the complexity metrics and care should be taken, even however slight.

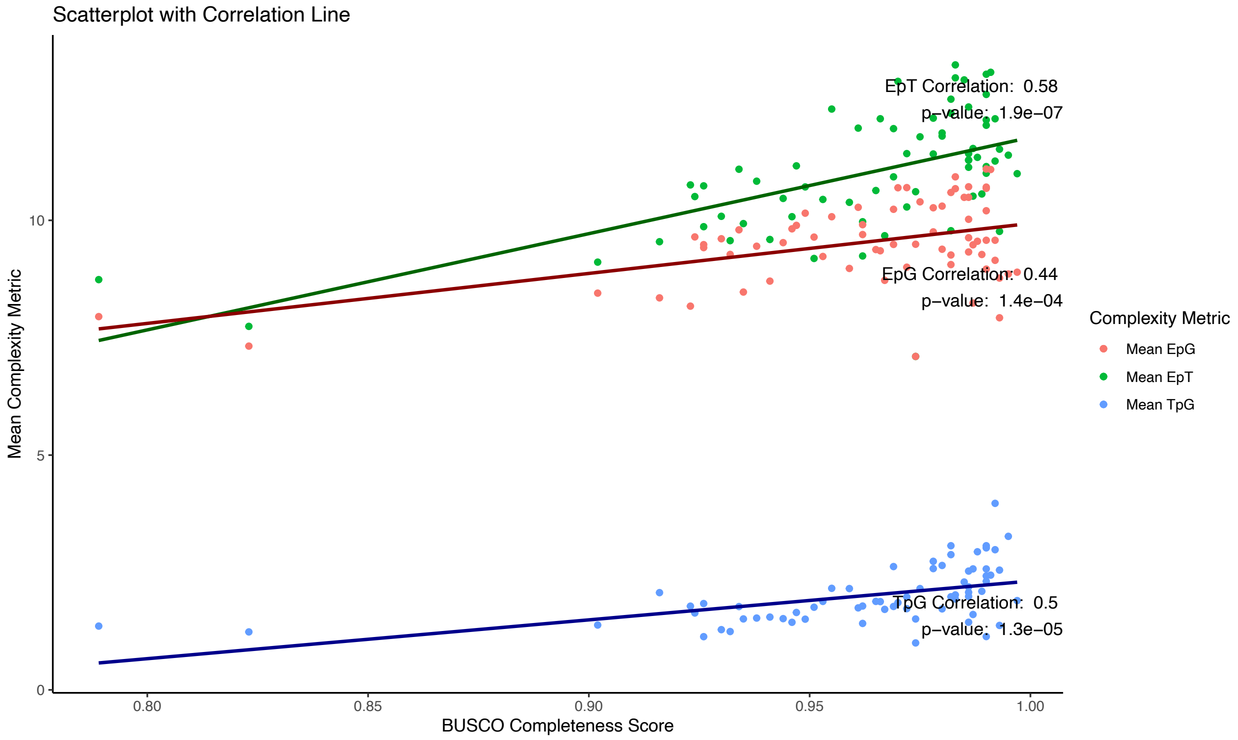

Supp. Figure 26: Pearson correlations plotted for TpG (Blue), EpT (Green), and EpG (Blue) complexity metrics against BUSCO quality analysis completeness for deuterostomes. Regression lines are plotted in darker corresponding colors. BUSCO analysis scores correlate with all complexity metrics.

Supp. Table 2:

Group	Species	Overlapping Transcripts	Total Genes
Deuterostomia	GCF_000001405.39_GRCh38.p13	1088	53285
Deuterostomia	GCF_000001635.27_GRCm39	606	40165
Deuterostomia	GCF_000001905.1_Loxafr3.0	193	25229
Deuterostomia	GCF_000002035.6_GRCz11	900	40167
Deuterostomia	GCF_000002235.5_Spur_5.0	342	32950
Deuterostomia	GCF_000002285.3_CanFam3.1	209	31841
Deuterostomia	GCF_000002295.2_MonDom5	212	33766
Deuterostomia	GCF_000003025.6_Sscrofa11.1	284	27328
Deuterostomia	GCF_000003625.3_OryCun2.0	161	24143

Deuterostomia	GCF_000004195.4_UCB_Xtro_10.0	176	28301
Deuterostomia	GCF_000090745.1_AnoCar2.0	133	21860
Deuterostomia	GCF_000151735.1_Cavpor3.0	125	26253
Deuterostomia	GCF_000165445.2_Mmur_3.0	223	28962
Deuterostomia	GCF_000181335.3_Felis_catus_9.0	240	31499
Deuterostomia	GCF_000186305.1_Python_molurus_bivittatus-5.0.2	66	21995
Deuterostomia	GCF_000224145.3_KH	812	16306
Deuterostomia	GCF_000225785.1_LatCha1	124	26565
Deuterostomia	GCF_000230535.1_PelSin_1.0	196	24504
Deuterostomia	GCF_000281125.3_ASM28112v4	139	24866
Deuterostomia	GCF_000296755.1_EriEur2.0	74	20182
Deuterostomia	GCF_000313985.2_ASM31398v2	135	24789
Deuterostomia	GCF_000331955.2_Oorc_1.1	309	26245
Deuterostomia	GCF_000334495.1_TupChi_1.0	185	27859
Deuterostomia	GCF_000337935.1_Cliv_1.0	163	26539
Deuterostomia	GCF_000455745.1_ASM45574v1	209	23474
Deuterostomia	GCF_000633615.1_Guppy_female_1.0_MT	137	26223
Deuterostomia	GCF_000696425.1_G_variegatus-3.0.2	142	26098
Deuterostomia	GCF_000705375.1_ASM70537v2	70	16999
Deuterostomia	GCF_000708225.1_ASM70822v1	50	15270
Deuterostomia	GCF_000935625.1_ASM93562v1	65	20982
Deuterostomia	GCF_000951035.1_Cang.pa_1.0	326	24314
Deuterostomia	GCF_000951045.1_Mleu.le_1.0	332	24513
Deuterostomia	GCF_000956105.1_Pcoq_1.0	84	20693
Deuterostomia	GCF_001039765.1_AptMant0	233	18636
Deuterostomia	GCF_001077635.1_Thamnophis_sirtalis-6.0	84	19785
Deuterostomia	GCF_001447785.1_Gekko_japonicus_V1.1	70	21187
Deuterostomia	GCF_001522545.3_Parus_major1.1	161	18984
Deuterostomia	GCF_001625305.1_Haploidv18h27	289	25489
Deuterostomia	GCF_001642345.1_ASM164234v2	110	27805
Deuterostomia	GCF_001723895.1_CroPor_comp1	79	19380
Deuterostomia	GCF_001723915.1_GavGan_comp1	85	18700
Deuterostomia	GCF_001858045.2_O_niloticus_UMD_NMBU	283	41929
Deuterostomia	GCF_001949145.1_OKI-Apl_1.0	194	18206
Deuterostomia	GCF_002234675.1_ASM223467v1	162	26669
Deuterostomia	GCF_002263795.1_ARS-UCD1.2	237	30678
Deuterostomia	GCF_002288925.2_ASM228892v3	244	23842
Deuterostomia	GCF_002863925.1_EquCab3.0	229	30303
Deuterostomia	GCF_002880755.1_Clint_PTRv2	539	34738
Deuterostomia	GCF_002880775.1_Susie_PABv2	287	27986

Deuterostomia	GCF_002925995.2_T_m_triunguis-2.0	81	24220
Deuterostomia	GCF_003339765.1_Mmul_10	332	34609
Deuterostomia	GCF_003957565.2_bTaeGut1.4.pri	265	21741
Deuterostomia	GCF_004115215.2_mOrnAna1.pri.v4	166	29644
Deuterostomia	GCF_006542625.1_Asia_NLE_v1	372	27916
Deuterostomia	GCF_008122165.1_Kamilah_GGO_v0	313	29127
Deuterostomia	GCF_009663435.1_Callithrix_jacchus_cj1700_1.1	415	35110
Deuterostomia	GCF_011125445.2_MU-UCD_Fhet_4.1	209	34057
Deuterostomia	GCF_011762595.1_mTurTru1.mat.Y	283	25731
Deuterostomia	GCF_014633375.1_OchPri4.0	42	21256
Deuterostomia	GCF_015227675.2_mRatBN7.2	382	34322
Deuterostomia	GCF_015237465.2_rCheMyd1.pri.v2	198	27777
Deuterostomia	GCF_015476345.1_ZJU1.0	194	24845
Deuterostomia	GCF_016699485.2_bGalGal1.mat.broiler.GRCg7b	539	25275
Deuterostomia	GCF_018977255.1_IMCB_Cmil_1.0	111	21238
Deuterostomia	GCF_900067755.1_pvi1.1	82	21573
Deuterostomia	GCF_901000725.2_fTakRub1.2	180	26907
Deuterostomia	GCF_902635505.1_mSarHar1.11	155	24348
Drosophila	dana-all-r1.04	306	23118
Drosophila	dere-all-r1.04	329	20853
Drosophila	dgri-all-r1.3	6	15507
Drosophila	dmel-all-r6.07	898	34478
Drosophila	dmoj-all-r1.04	249	21766
Drosophila	dper-all-r1.3	6	17502
Drosophila	dpse-all-r3.03	393	27318
Drosophila	dsec-all-r1.3	12	17199
Drosophila	dsim-all-r2.01	647	25981
Drosophila	dvir-all-r1.03	318	22365
Drosophila	dwil-all-r1.04	94	16208
Drosophila	dyak-all-r1.04	453	25139
Plantae	GCF_000001735.4_TAIR10.1	851	43200
Plantae	GCF_000002425.4_Phypa_V3	249	23167
Plantae	GCF_000003195.3_Sorghum_bicolor_NCBIv3	212	31549
Plantae	GCF_000003745.3_12X	293	28320
Plantae	GCF_000004515.6_Glycine_max_v4.0	522	54350
Plantae	GCF_000005505.3_Brachypodium_distachyon_v3.0	246	29919
Plantae	GCF_000143415.4_v1.0	2397	36907
Plantae	GCF_000150535.2_Papaya1.0	168	19816
Plantae	GCF_000208745.1_Criollo_cocoa_genome_V2	184	23672
Plantae	GCF_000226075.1_SolTub_3.0	281	31483

Plantae	GCF_000309985.2_CAAS_Brap_v3.01	1855	49745
Plantae	GCF_000313045.1_ASM31304v1	157	22273
Plantae	GCF_000313855.2_ASM31385v2	207	33319
Plantae	GCF_000315295.1_Pbr_v1.0	258	38968
Plantae	GCF_000317415.1_Csi_valencia_1.0	278	28567
Plantae	GCF_000331145.1_ASM33114v1	213	28783
Plantae	GCF_000346465.2_Prunus_persica_NCBIv2	262	25062
Plantae	GCF_000365185.1_Chinese_Lotus_1.1	104	26711
Plantae	GCF_000471905.2_AMTR1.0	106	18616
Plantae	GCF_000478725.1_Eutsalg1_0	242	31397
Plantae	GCF_000504015.1_Mimgu1_0	318	29503
Plantae	GCF_000511025.2_RefBeet-1.2.2	187	27474
Plantae	GCF_000512975.1_S_indicum_v1.0	162	25978
Plantae	GCF_000612285.1_Gossypium_arboreum_v1.0	168	37626
Plantae	GCF_000633955.1_Cs	1182	90812
Plantae	GCF_000710875.1_Pepper_Zunla_1_Ref_v1.0	248	36672
Plantae	GCF_000715135.1_Ntab-TN90	395	70675
Plantae	GCF_000826755.1_ZizJuj_1.1	482	33504
Plantae	GCF_001190045.1_Vigan1.1	150	28233
Plantae	GCF_001433935.1_IRGSP-1.0	360	33612
Plantae	GCF_001654055.1_ASM165405v1	383	38282
Plantae	GCF_001659605.2_M.esculenta_v8	355	33088
Plantae	GCF_001683475.1_ASM168347v1	682	55201
Plantae	GCF_001879475.1_Asagao_1.1	440	46384
Plantae	GCF_001995035.1_ASM199503v1	122	21160
Plantae	GCF_002114115.1_ASM211411v1	413	41822
Plantae	GCF_002127325.2_HanXRQr2.0-SUNRISE	530	80843
Plantae	GCF_002303985.1_Duzib1.0	503	37161
Plantae	GCF_002738345.1_Cmax_1.0	294	34127
Plantae	GCF_002870075.2_Lsat_Salinas_v7	387	45406
Plantae	GCF_002906115.1_CorkOak1.0	326	53852
Plantae	GCF_002994745.2_RchiOBHm-V2	429	38649
Plantae	GCF_016545825.1_ASM1654582v1	560	38596
Plantae	GCF_902167145.1_Zm-B73-REFERENCE-NAM-5.0	732	45660
Fungi	GCA_000003515.2_ASM351v2	0	7856
Fungi	GCA_000365165.2_Clad_carr_CBS_160_54_V1	0	10428
Fungi	GCA_000978255.2_Sc_YJM1573_v1	24	5858
Fungi	GCA_001574975.1_Ganpr1	574	13912
Fungi	GCA_001636715.1_AAP_1.0	0	6442
Fungi	GCA_001747045.1_ASM174704v1	0	4657

Fungi	GCA_001883825.1_Emmo_past_UAMH9510_V1	0	9078
Fungi	GCA_001929475.1_NeoIirr1.0	0	5536
Fungi	GCA_002006685.1_Batr_sala_BS_V1	123	10186
Fungi	GCA_002104895.1_Anaeromyces_sp._S4_v1.0	136	13083
Fungi	GCA_002104945.1_Piromyces_sp._finnis_v3.0	335	11313
Fungi	GCA_002104975.1_Neocallimastix_sp._G1_v1.0	284	21039
Fungi	GCA_002104985.1_Rhihy1	1019	17656
Fungi	GCA_002918395.1_ASM291839v1	0	6859
Fungi	GCA_002938375.1_Psicy2	0	15936
Fungi	GCA_900106115.1_CBS_141442_assembly	0	5936
Fungi	GCF_000001985.1_JCVI-PMFA1-2.0	0	10134
Fungi	GCF_000002545.3_ASM254v2	0	5458
Fungi	GCF_000026945.1_ASM2694v1	20	5972
Fungi	GCF_000091045.1_ASM9104v1	0	6773
Fungi	GCF_000143185.1_v1.0	32	13189
Fungi	GCF_000149035.1_C_graminicola_M1_001_V1	6	12399
Fungi	GCF_000149335.2_ASM14933v2	90	9905
Fungi	GCF_000149555.1_ASM14955v1	40	16286
Fungi	GCF_000150505.1_SO6	595	5347
Fungi	GCF_000150705.2_Paracocci_br_Pb01_V2	4	8953
Fungi	GCF_000171015.1_TRIAT_v2.0	306	11810
Fungi	GCF_000182565.1_S_punctatus_V1	10	9169
Fungi	GCF_000182805.2_ASM18280v2	0	10548
Fungi	GCF_000182895.1_CC3	10	13657
Fungi	GCF_000203795.1_v1.0	16	8677
Fungi	GCF_000204055.1_v1.0	42	16265
Fungi	GCF_000221225.1_CTHT_3.0	8	7283
Fungi	GCF_000223465.1_Candida_tenuis_v1.0	518	5547
Fungi	GCF_000230375.1_ASM23037v1	0	12469
Fungi	GCF_000264905.1_Stehi1	48	14453
Fungi	GCF_000264995.1_Punctularia_strigosozonata_v1.0	40	11647
Fungi	GCF_000271605.1_Fomme1	36	11411
Fungi	GCF_000271625.1_Conpu1	56	13896
Fungi	GCF_000271645.1_Treme1	12	8292
Fungi	GCF_000275845.1_Dichomitus_squalens_v1.0	40	12497
Fungi	GCF_000281105.1_Coni_apol_CBS100218_V1	0	9367
Fungi	GCF_000300595.1_Phanerochaete_carnosa_HHB-10118-Sp_v1.0	42	14080
Fungi	GCF_000313525.1_ASM31352v1	0	9262
Fungi	GCF_000320585.1_Heterobasidion_irregulare_v2.0	367	13272

Fungi	GCF_000328475.2_Umaydis521_2.0	0	6909
Fungi	GCF_000344685.1_Glotr1_1	100	11885
Fungi	GCF_000354255.1_CocheC4_1	169	12809
Fungi	GCF_000400465.1_Wallemia_ichthyophaga_version_1.0	0	5000
Fungi	GCF_000409485.1_GLAREA	0	13083
Fungi	GCF_000497045.1_PSEUBRA1	2	5889
Fungi	GCF_000512605.1_Cryp_pinu_CBS10737_V1	0	7931
Fungi	GCF_000516985.1_PFICI	0	15413
Fungi	GCF_000576695.1_AUH_PRJEB4427_v1	0	5988
Fungi	GCF_000709125.1_Exop_aqua_CBS_119918_V1	0	13118
Fungi	GCF_000835455.1_Fons_pedr_CBS_271_37_V1	0	12573
Fungi	GCF_000835555.1_Rhin_mack_CBS_650_93_V1	0	11418
Fungi	GCF_000836295.1_O_gall_CBS43764	88	9871
Fungi	GCF_000938715.1_LALA0	0	5319
Fungi	GCF_001027345.1_Triol1	262	8602
Fungi	GCF_001278385.1_MalaPachy	0	4202
Fungi	GCF_001329695.1_Rhoba1_1	34	7376
Fungi	GCF_001477535.1_Pneu_jiro_RU7_V2	8	3811
Fungi	GCF_001619985.1_Xylona_heveae_TC161_v1.0	222	8346
Fungi	GCF_001636725.1_ISF_1.0	0	10061
Fungi	GCF_001638985.1_Phybl2	108	16850
Fungi	GCF_001661235.1_Picme2	270	5701
Fungi	GCF_001661335.1_Babin1	234	6795
Fungi	GCF_001661345.1_Ascru1	277	6962
Fungi	GCF_001661405.1_Cybja1	186	6184
Fungi	GCF_001664035.1_Metbi1	182	6090
Fungi	GCF_001883845.1_ASM188384v1	0	10839
Fungi	GCF_001890105.1_Aspzo1	326	10027
Fungi	GCF_002102565.1_Kocim1	314	7428
Fungi	GCF_002105155.1_Lobtra1	281	11983
Fungi	GCF_002117355.1_PosplRSB12_1	76	12716
Fungi	GCF_002847465.1_Aspnov1	199	11620
