# Supplementary material for: Estimating transcriptome complexities across eukaryotes
Source: BMC Genomics. 2023 May 11;24:254. doi: 10.1186/s12864-023-09326-0 (PMC10173493; doi:10.1186/s12864-023-09326-0)
Supplement: Supplementary file 1 — Additional file 1. [file 12864_2023_9326_MOESM1_ESM.zip › Supp. Mat. Mean Complexity Metrics_ESM.pdf]

MEAN COMPLEXITY METRIC BOXPLOTS

Figures below account for mean metric (TpG, EpT, and EpG) box and whisker plots among a variety of higher-order grouping among Deuterostomes, *Drosophila*, Plantae, and Fungi. Kruskal-Wallis tests were performed, and statistical values are recorded in the sub-header. Y-axes are the metric values, and x-axes have group labels.

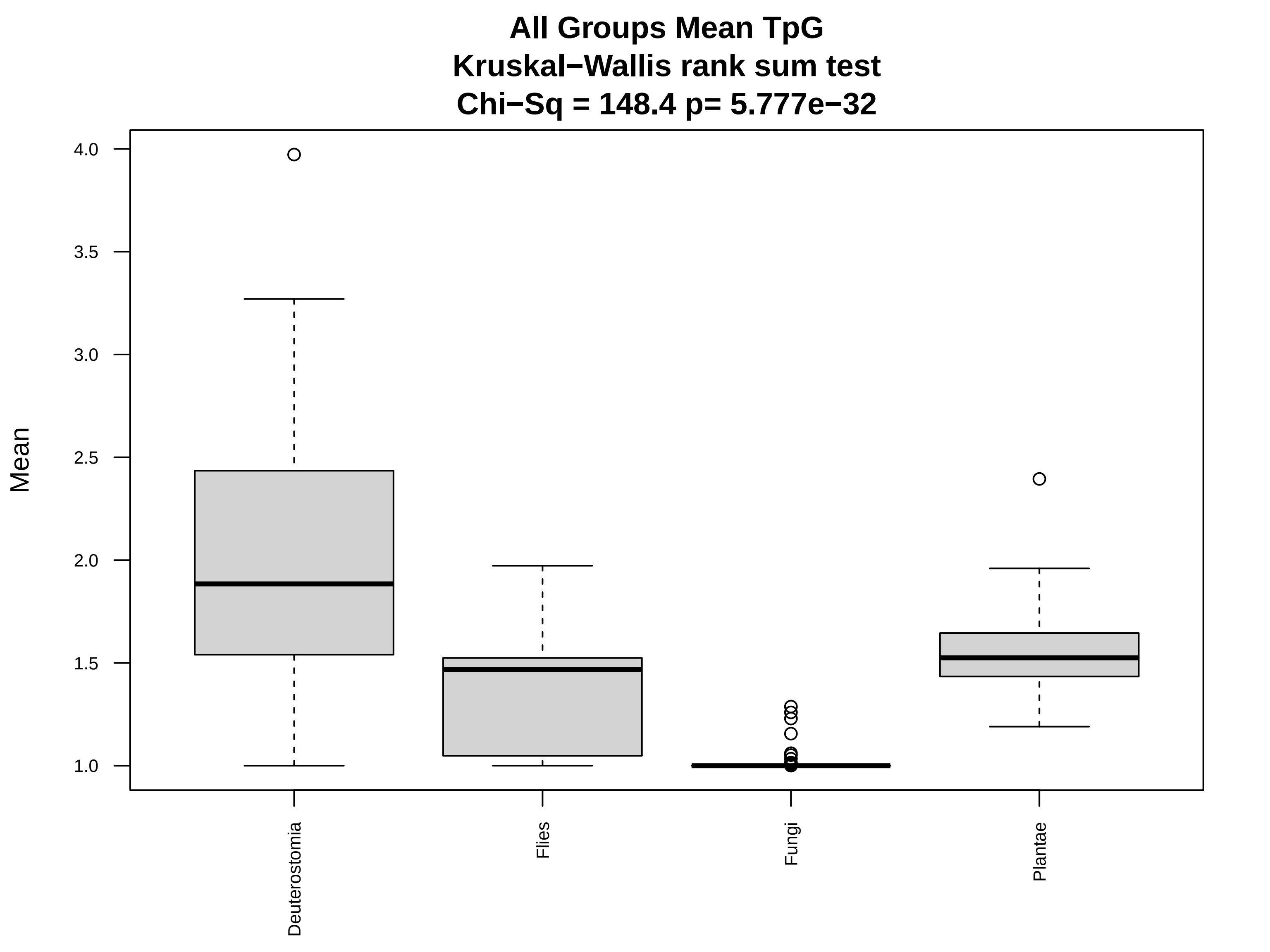
All Groups Mean TpG
Kruskal−Wallis rank sum test
Chi−Sq = 148.4 p= 5.777e−32
Mean
4.0
3.5
3.0
2.5
2.0
1.5
1.0
Deuterostomia
Flies
Fungi
Plantae

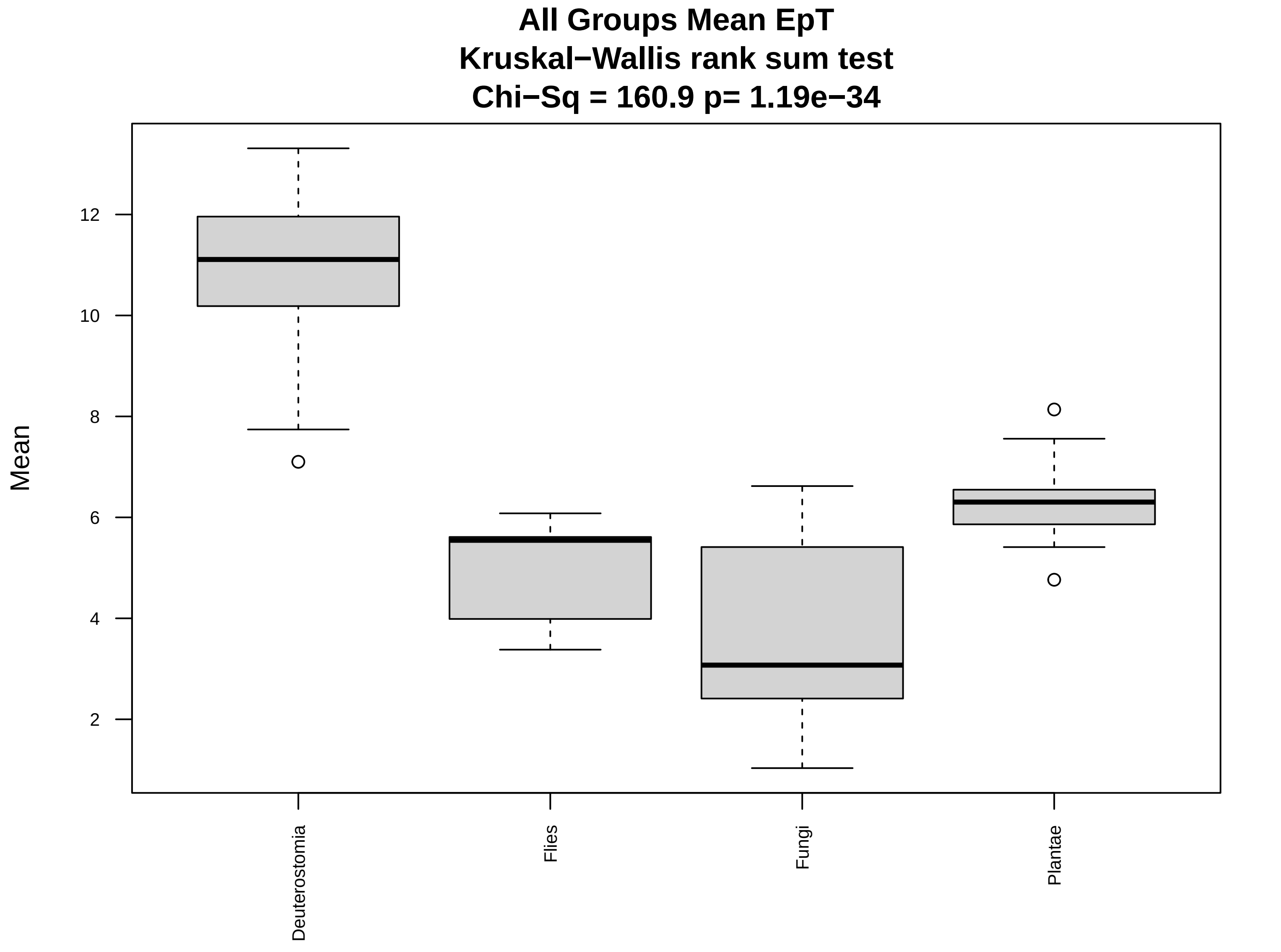
All Groups Mean EpT
Kruskal−Wallis rank sum test
Chi−Sq = 160.9 p= 1.19e−34
Mean
12
10
8
6
4
2
Deuterostomia
Flies
Fungi
Plantae

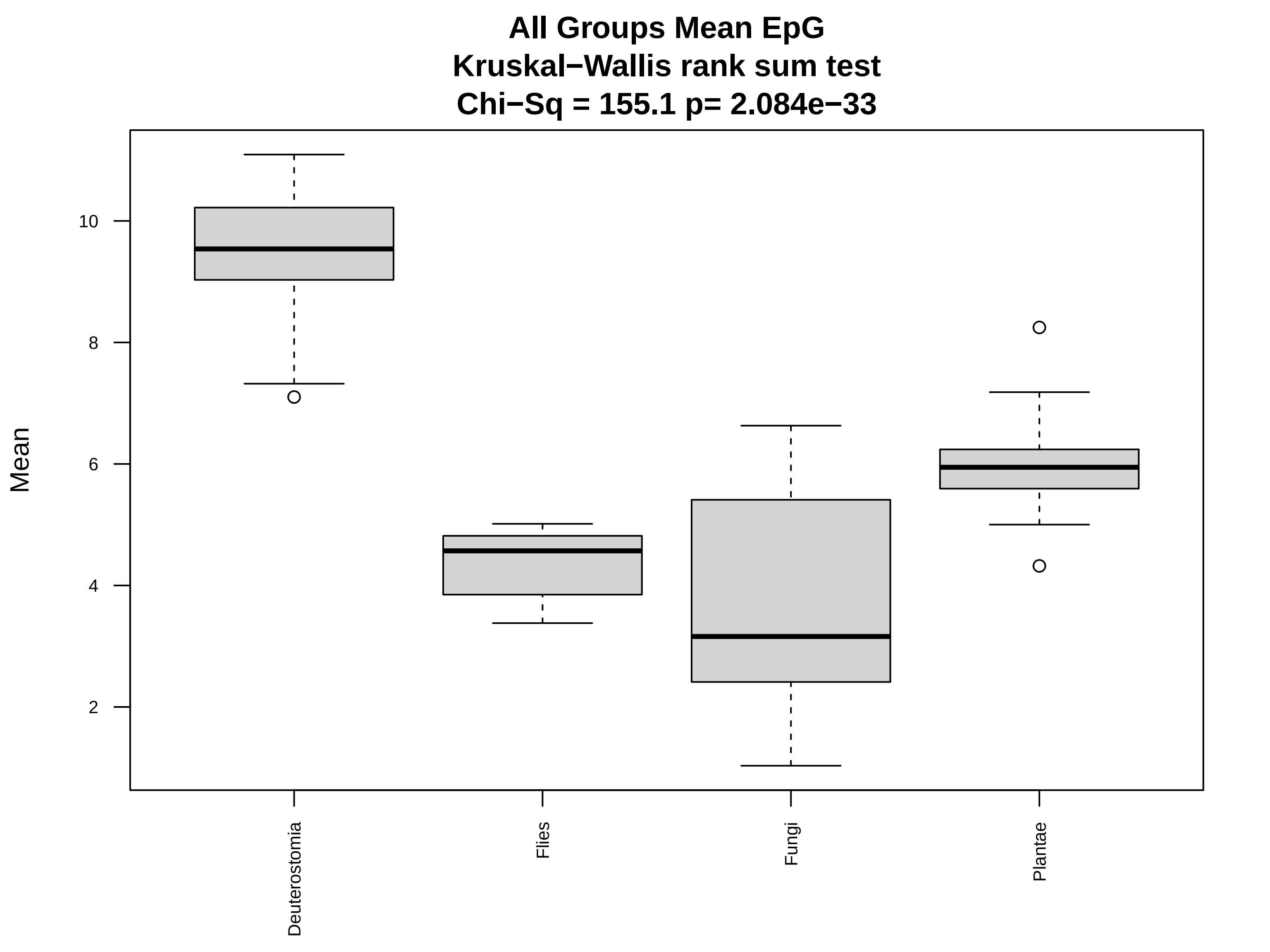
All Groups Mean EpG
Kruskal−Wallis rank sum test
Chi−Sq = 155.1 p= 2.084e−33
Mean
10
8
6
4
2
Deuterostomia
Flies
Fungi
Plantae

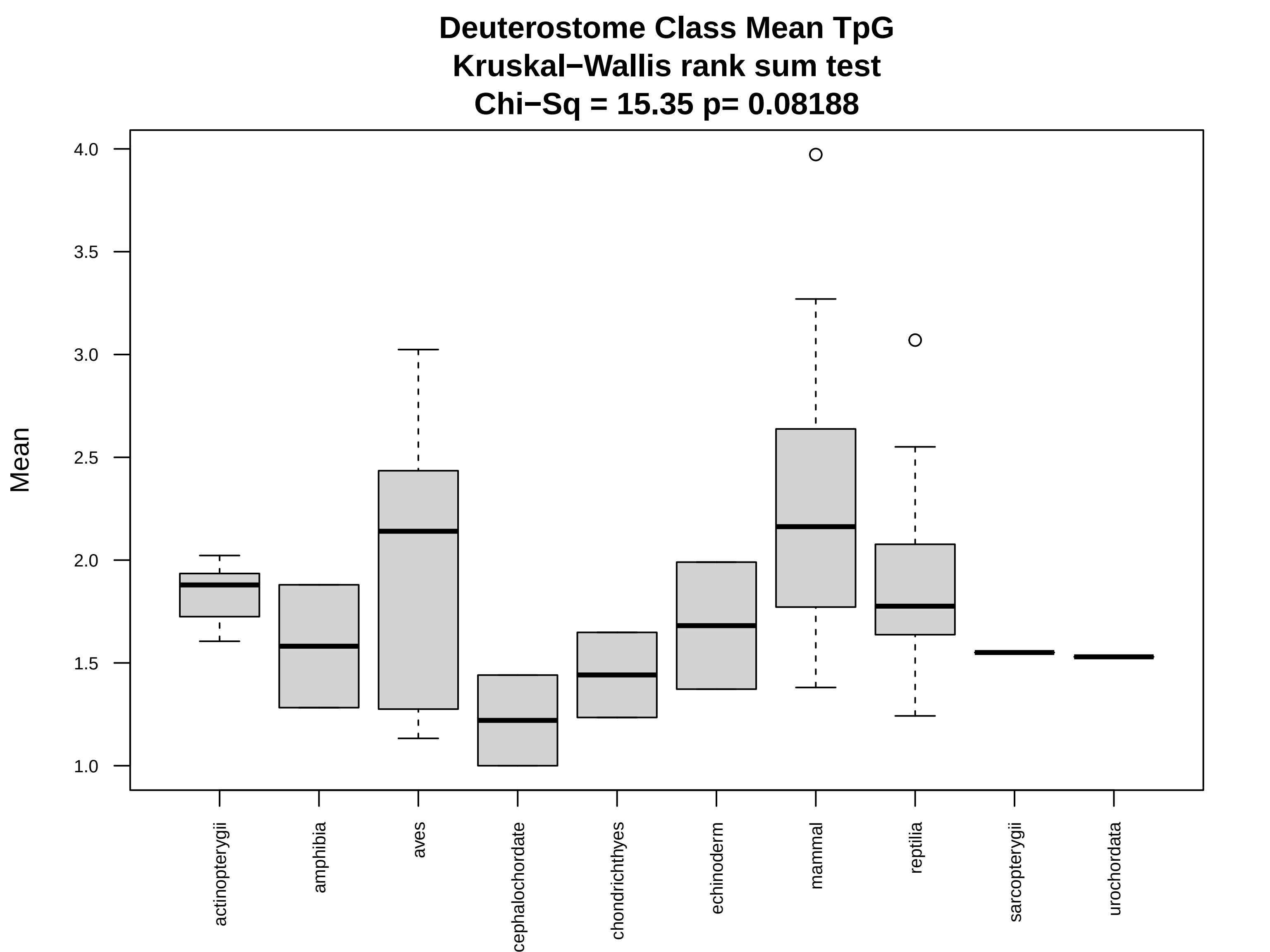
Deuterostome Class Mean TpG
Kruskal−Wallis rank sum test
Chi−Sq = 15.35 p= 0.08188
Mean
4.0
3.5
3.0
2.5
2.0
1.5
1.0
actinopterygii
amphibia
aves
cephalochordate
chondrichthyes
echinoderm
mammal
reptilia
sarcopterygii
urochordata

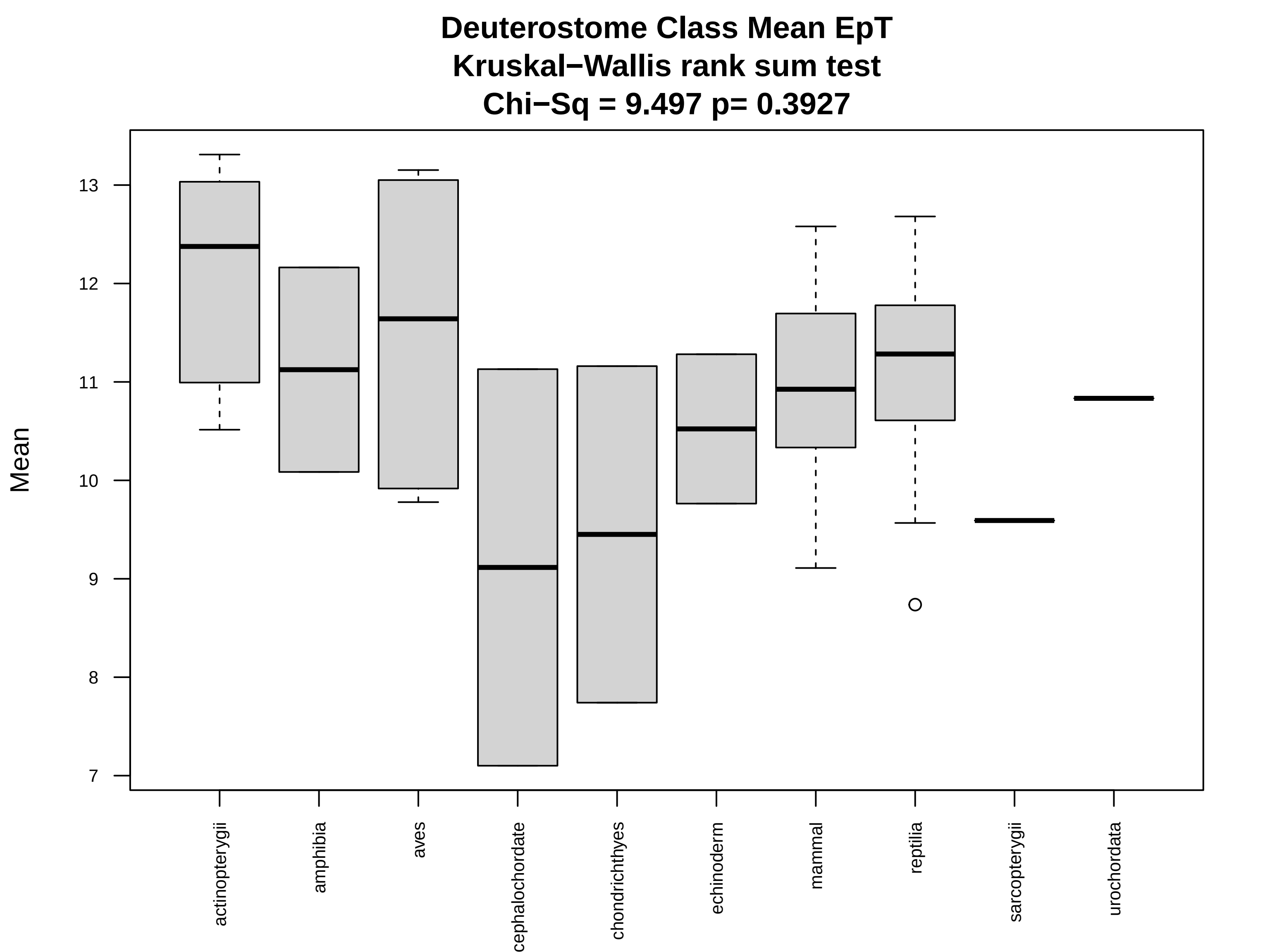

Deuterostome Class Mean EpT
Kruskal−Wallis rank sum test
Chi−Sq = 9.497 p= 0.3927
Mean
13
12
11
10
9
8
7
actinopterygii
amphibia
aves
cephalochordate
chondrichthyes
echinoderm
mammal
reptilia
sarcopterygii
urochordata

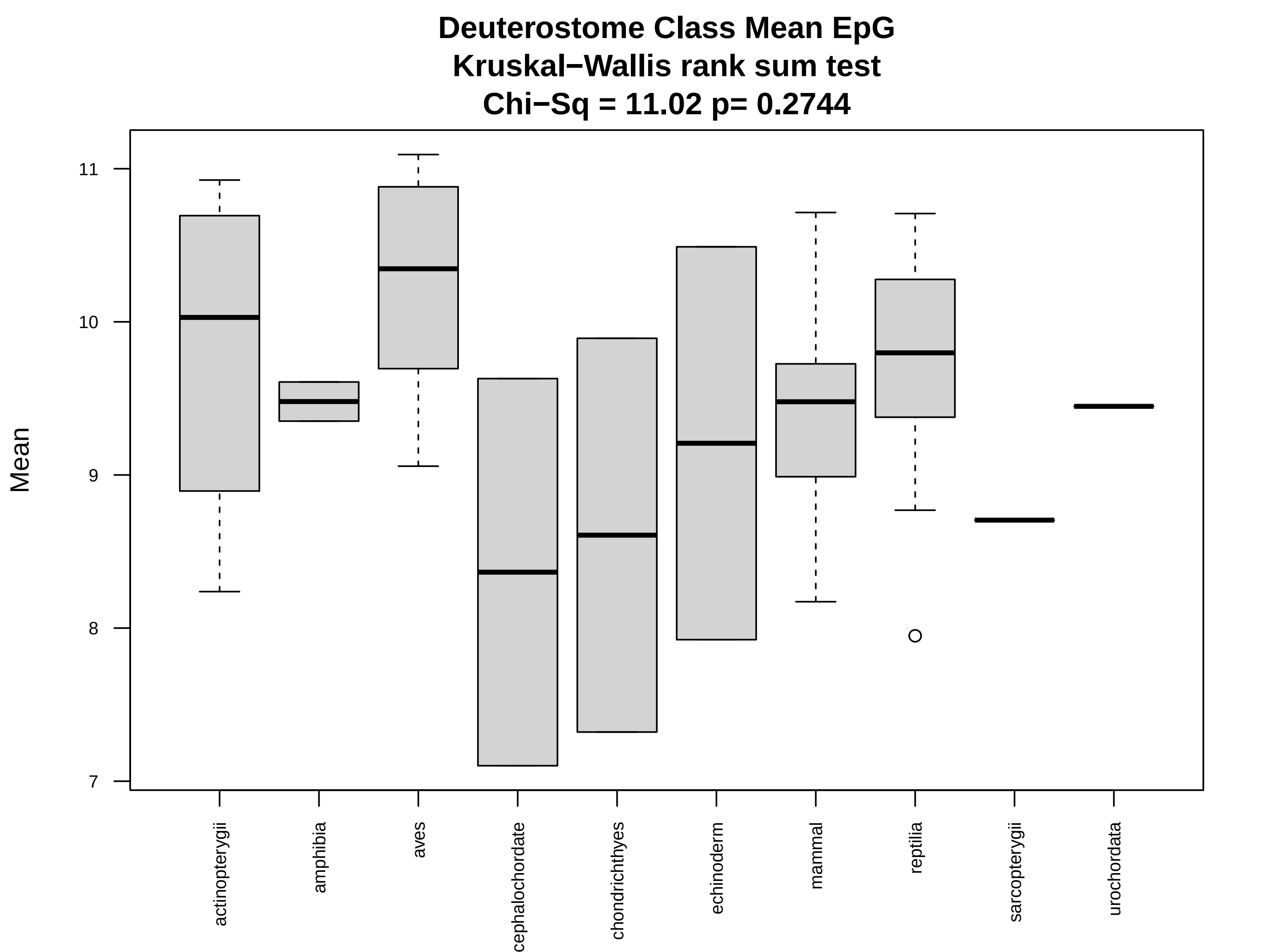
Deuterostome Class Mean EpG
Kruskal−Wallis rank sum test
Chi−Sq = 11.02 p= 0.2744
Mean
11
10
9
8
7
actinopterygii
amphibia
aves
cephalochordate
chondrichthyes
echinoderm
mammal
reptilia
sarcopterygii
urochordata

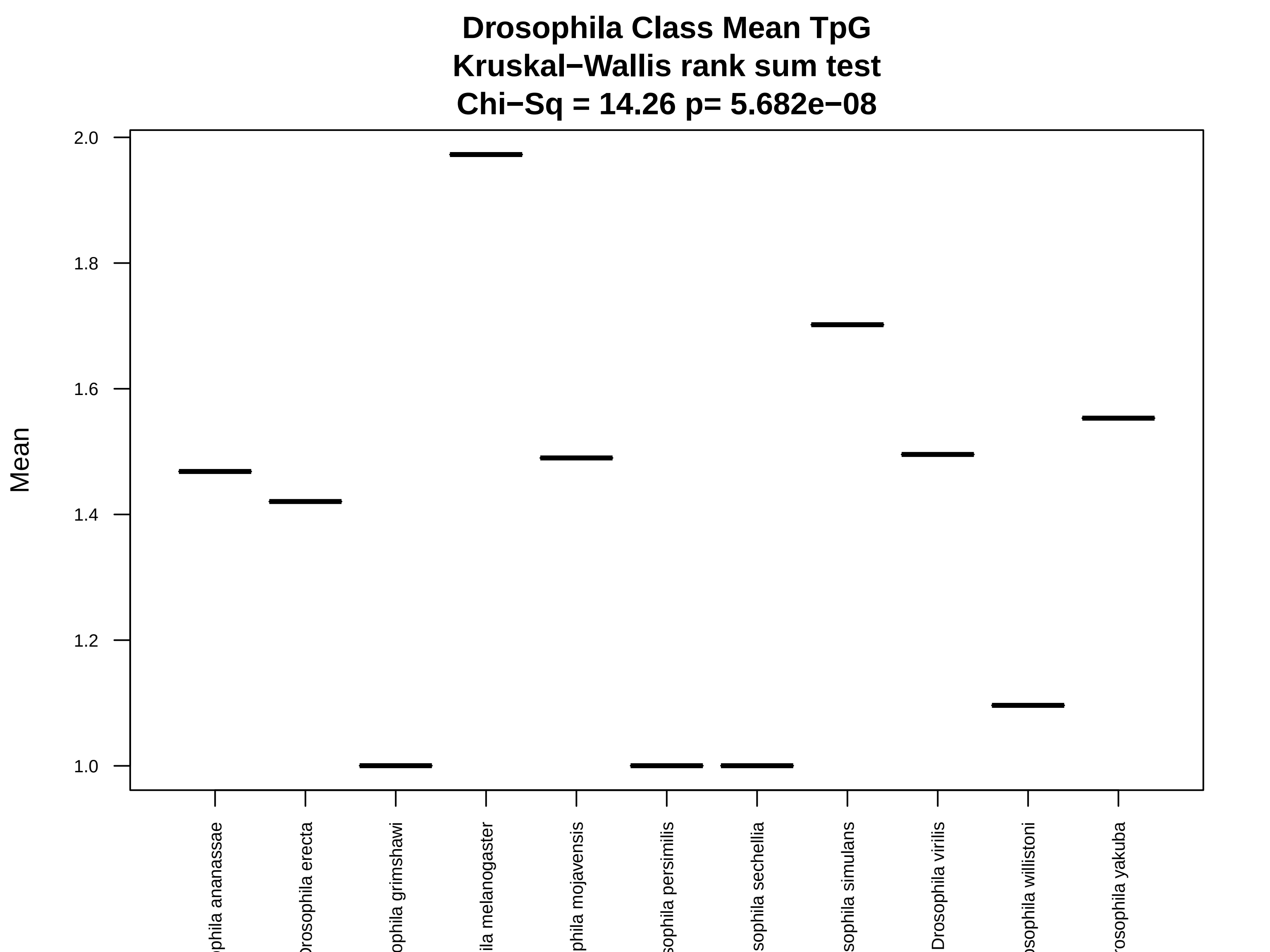
Drosophila Class Mean TpG
Kruskal−Wallis rank sum test
Chi−Sq = 14.26 p= 5.682e−08
Mean
2.0
1.8
1.6
1.4
1.2
1.0
ophila ananassae
Drosophila erecta
ophila grimshawi
ila melanogaster
ophila mojavensis
sophila persimilis
sophila sechellia
sophila simulans
Drosophila virilis
osophila willistoni
rosophila yakuba

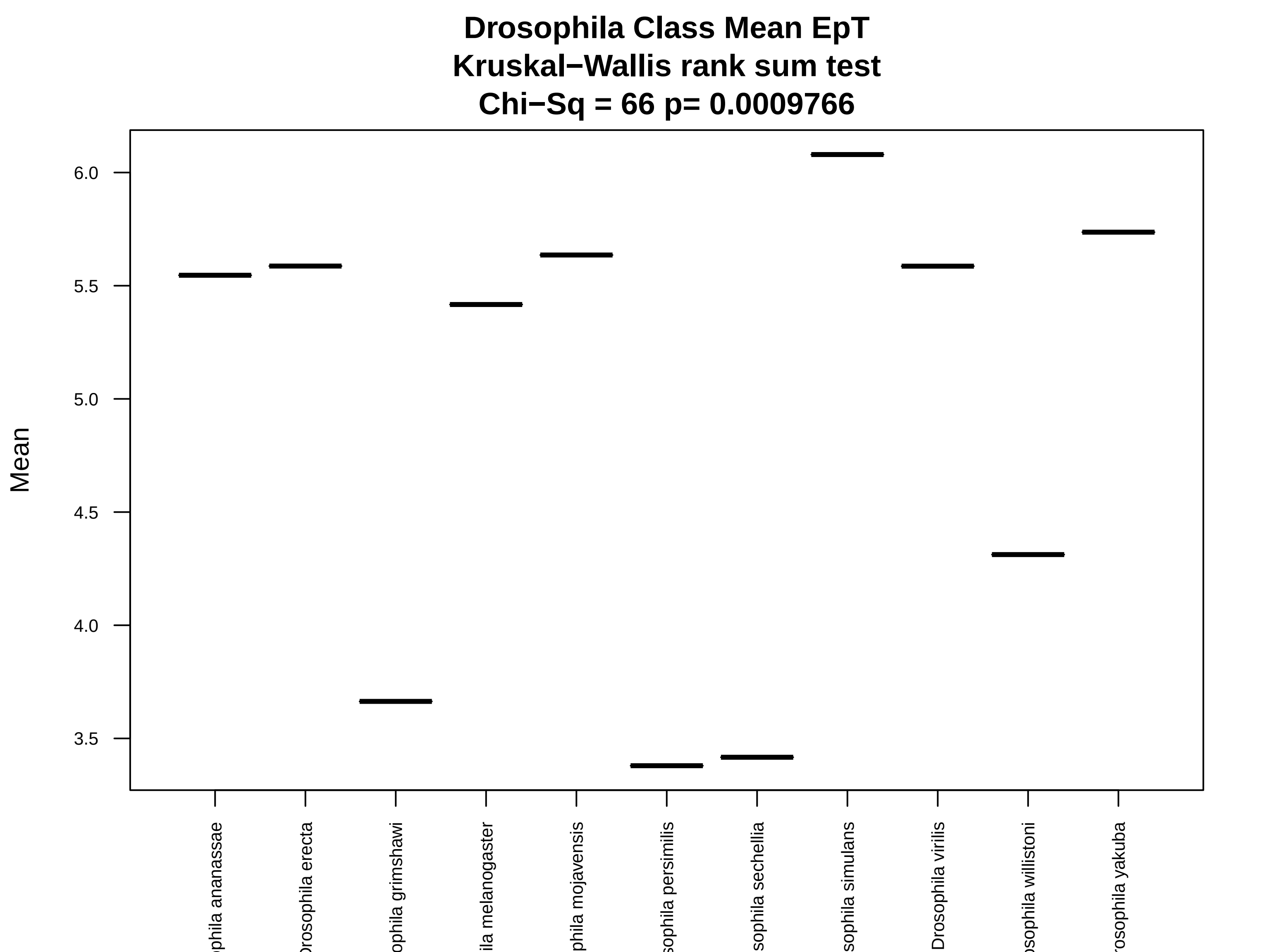

Drosophila Class Mean EpT
Kruskal−Wallis rank sum test
Chi−Sq = 66 p= 0.0009766
Mean
6.0
5.5
5.0
4.5
4.0
3.5
ophila ananassae
Drosophila erecta
ophila grimshawi
ila melanogaster
phila mojavensis
sophila persimilis
sophila sechellia
sophila simulans
Drosophila virilis
osophila willistoni
rosophila yakuba

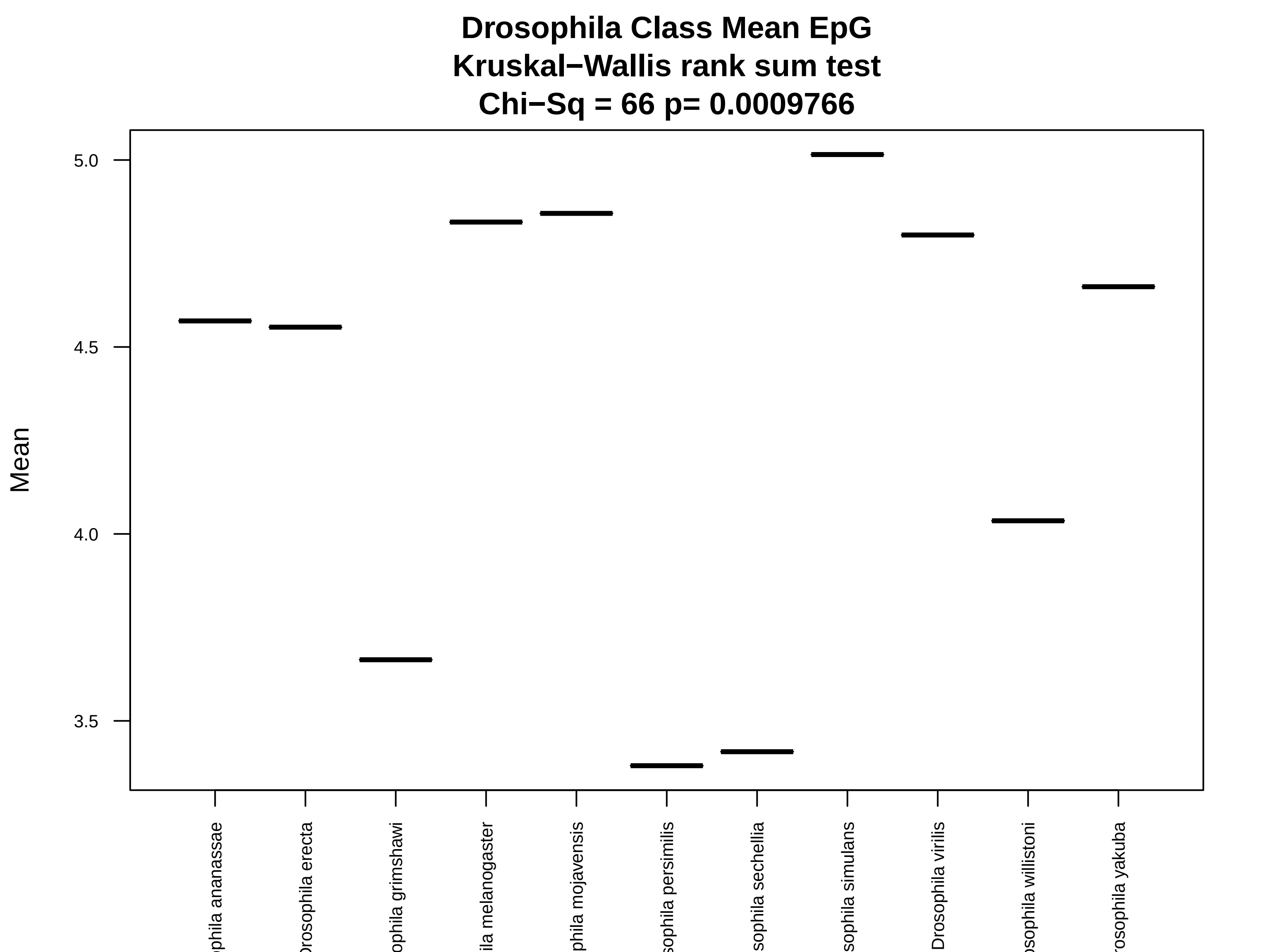

Drosophila Class Mean EpG
Kruskal−Wallis rank sum test
Chi−Sq = 66 p= 0.0009766
Mean
5.0
4.5
4.0
3.5
ophila ananassae
Drosophila erecta
ophila grimshawi
ila melanogaster
phila mojavensis
sophila persimilis
sophila sechellia
sophila simulans
Drosophila virilis
osophila willistoni
rosophila yakuba

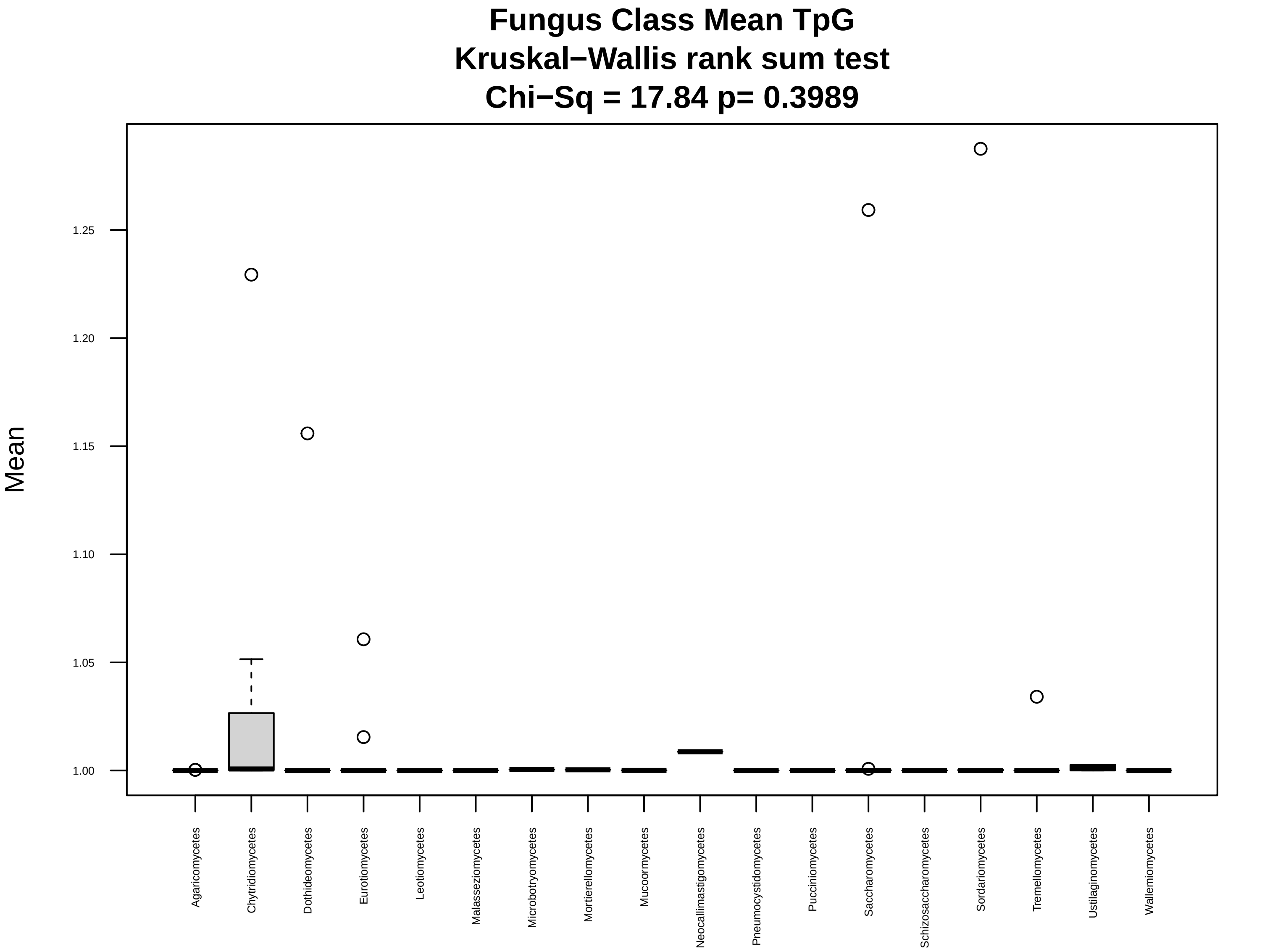
Fungus Class Mean TpG
Kruskal−Wallis rank sum test
Chi−Sq = 17.84 p= 0.3989
Mean
1.00
1.05
1.10
1.15
1.20
1.25
Agaricomycetes
Chytridiomycetes
Dothideomycetes
Eurotiomycetes
Leotiomycetes
Malasseziomycetes
Microbotryomycetes
Mortierellomycetes
Mucoormycetes
Neocallimastigomycetes
Pneumocystidomycetes
Pucciniomycetes
Saccharomycetes
Schizosaccharomycetes
Sordariomycetes
Tremellomycetes
Ustilaginomycetes
Wallemiomycetes

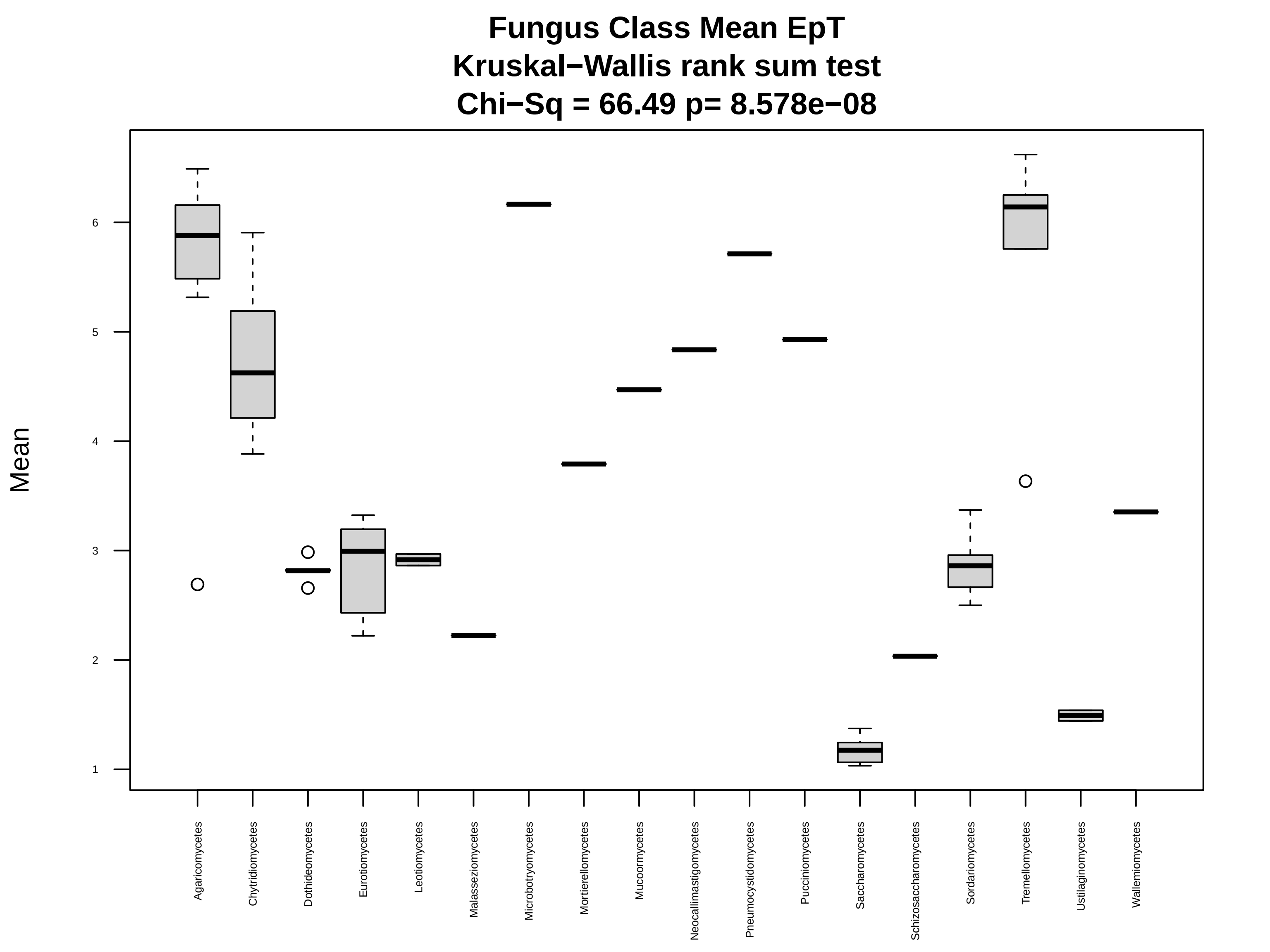
Fungus Class Mean EpT
Kruskal−Wallis rank sum test
Chi−Sq = 66.49 p= 8.578e−08
Mean
1
2
3
4
5
6
Agaricomycetes
Chytridiomycetes
Dothideomycetes
Eurotiomycetes
Leotiomycetes
Malasseziomycetes
Microbotryomycetes
Mortierellomycetes
Mucoormycetes
Neocallimastigomycetes
Pneumocystidomycetes
Pucciniomycetes
Saccharomycetes
Schizosaccharomycetes
Sordariomycetes
Tremellomycetes
Ustilaginomycetes
Wallemiomycetes

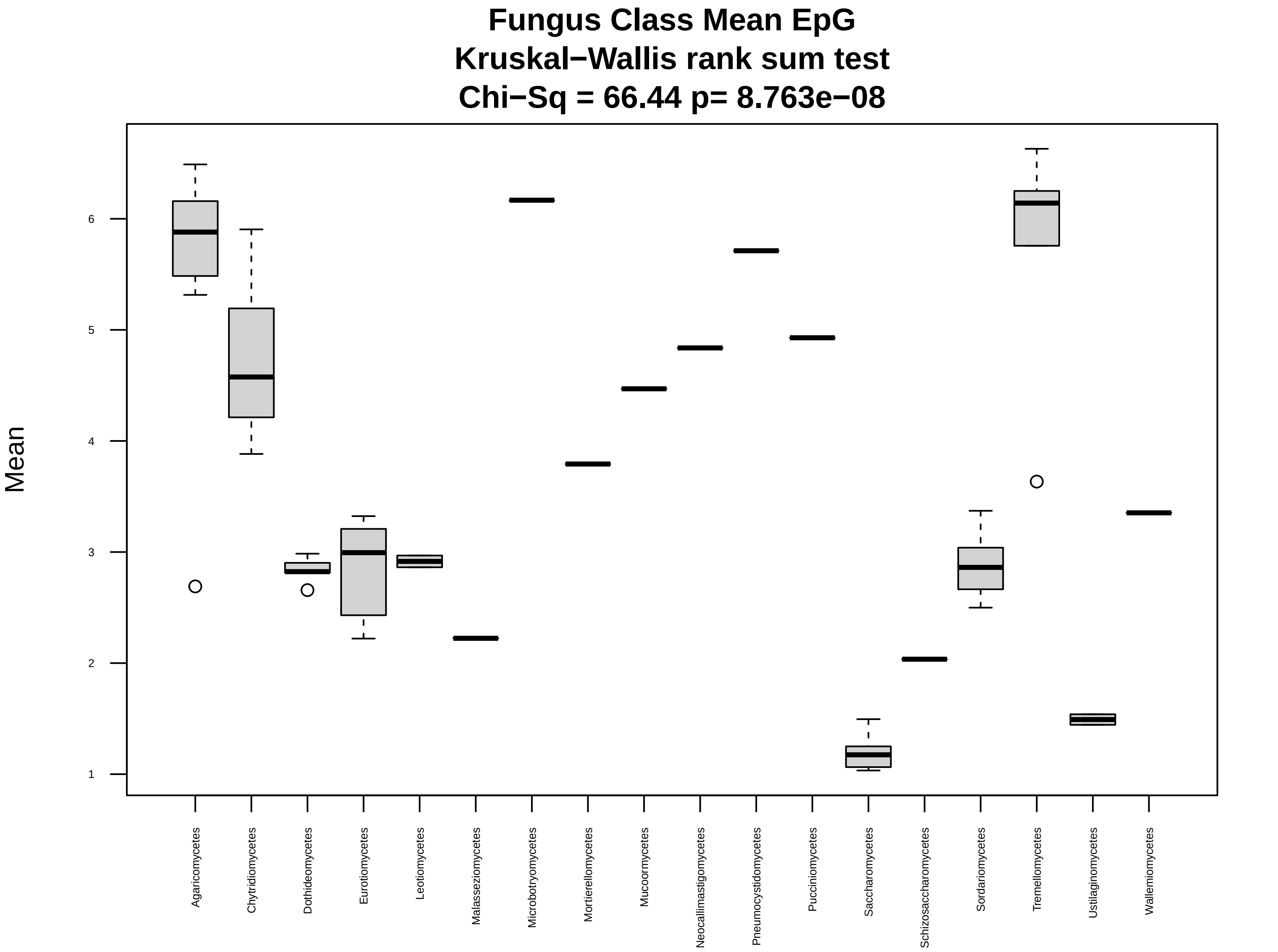
Fungus Class Mean EpG
Kruskal−Wallis rank sum test
Chi−Sq = 66.44 p= 8.763e−08
Mean
1
2
3
4
5
6
Agaricomycetes
Chytridiomycetes
Dothideomycetes
Eurotiomycetes
Leotiomycetes
Malasseziomycetes
Microbotryomycetes
Mortierellomycetes
Mucoormycetes
Neocallimastigomycetes
Pneumocystidomycetes
Pucciniomycetes
Saccharomycetes
Schizosaccharomycetes
Sordariomycetes
Tremellomycetes
Ustilaginomycetes
Wallemiomycetes

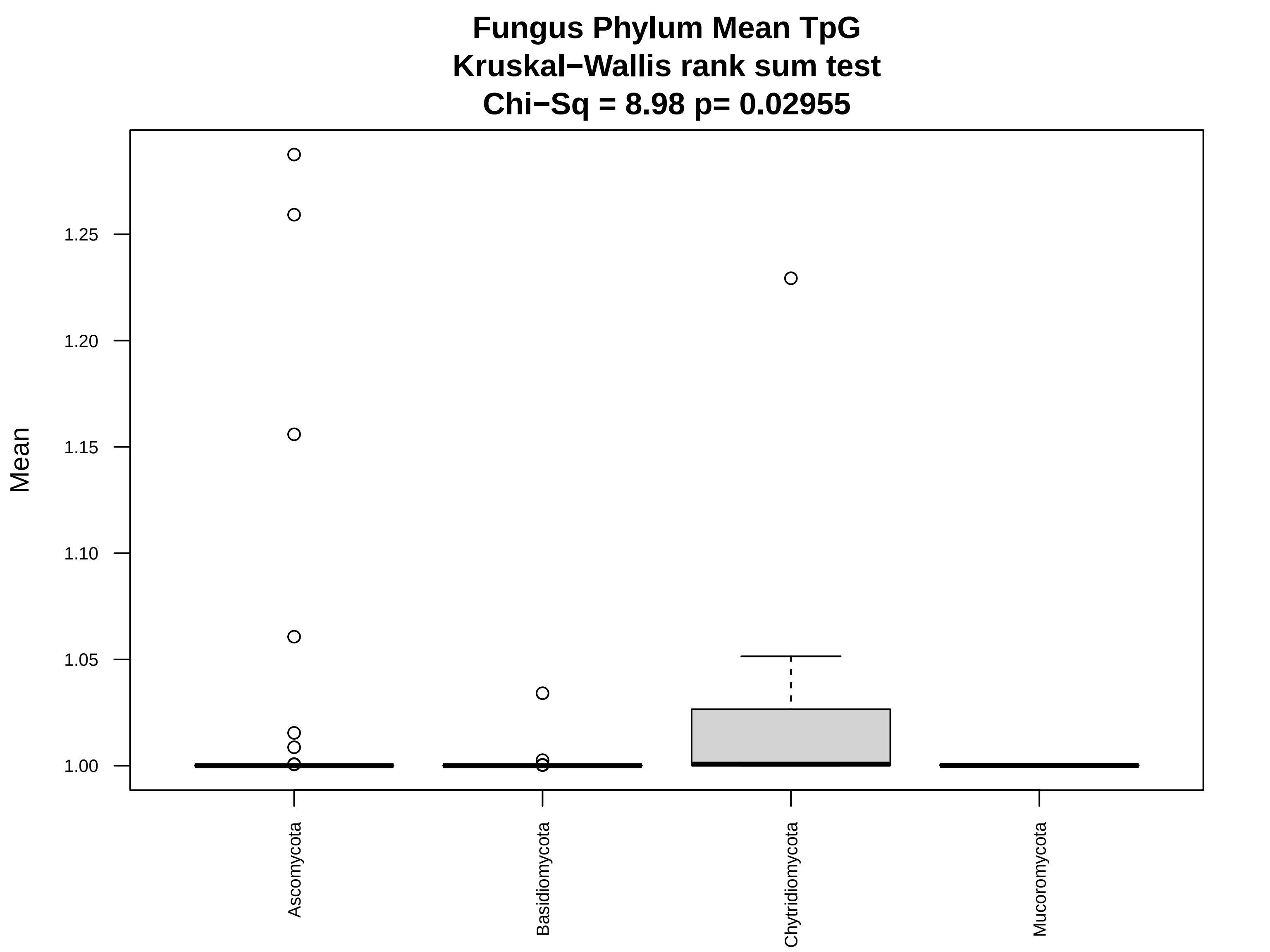
Fungus Phylum Mean TpG
Kruskal−Wallis rank sum test
Chi−Sq = 8.98 p= 0.02955
Mean
1.00
1.05
1.10
1.15
1.20
1.25
Ascomycota
Basidiomycota
Chytridiomycota
Mucoromycota

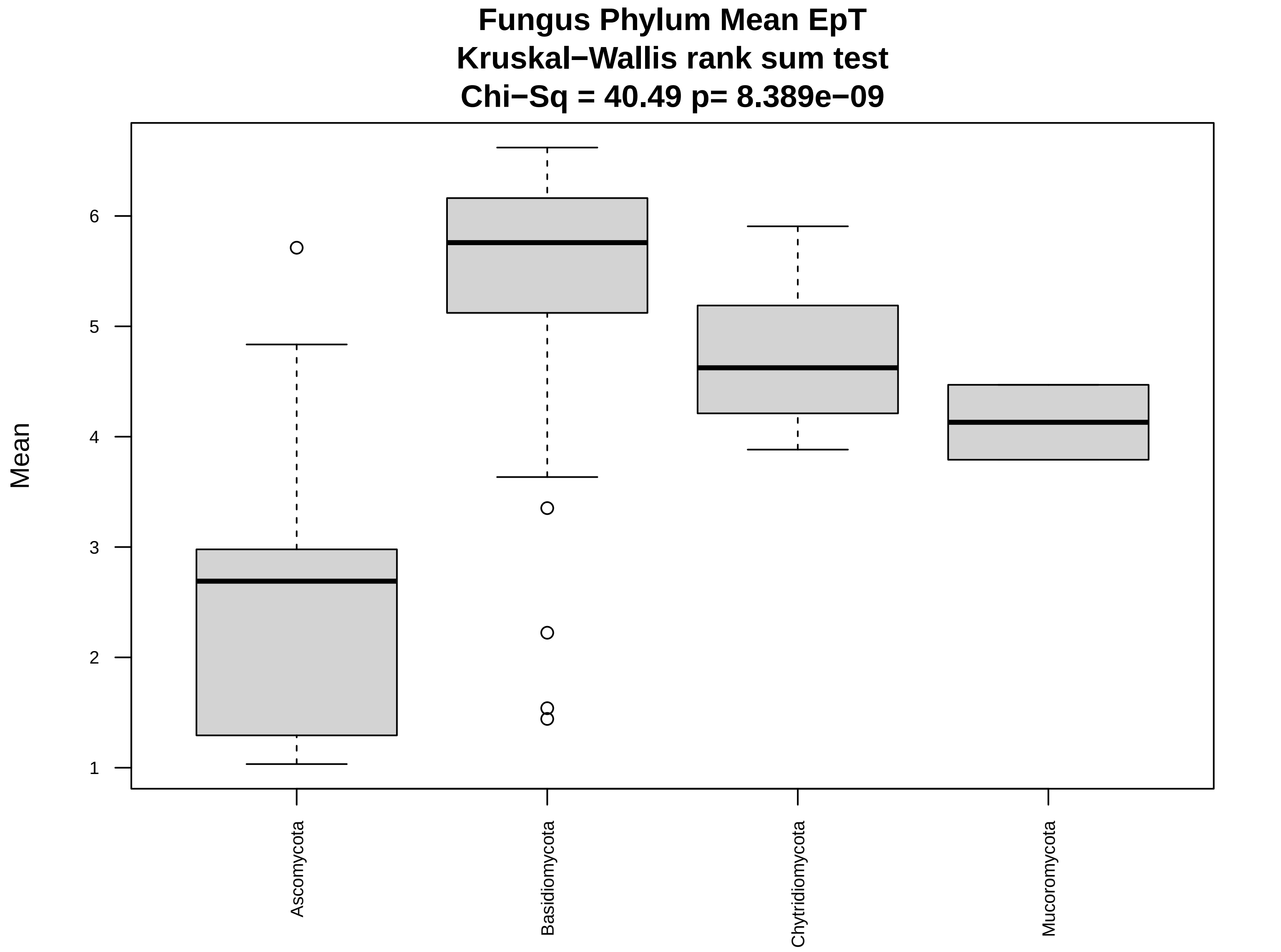
Fungus Phylum Mean EpT
Kruskal−Wallis rank sum test
Chi−Sq = 40.49 p= 8.389e−09
Mean
1
2
3
4
5
6
Ascomycota
Basidiomycota
Chytridiomycota
Mucoromycota

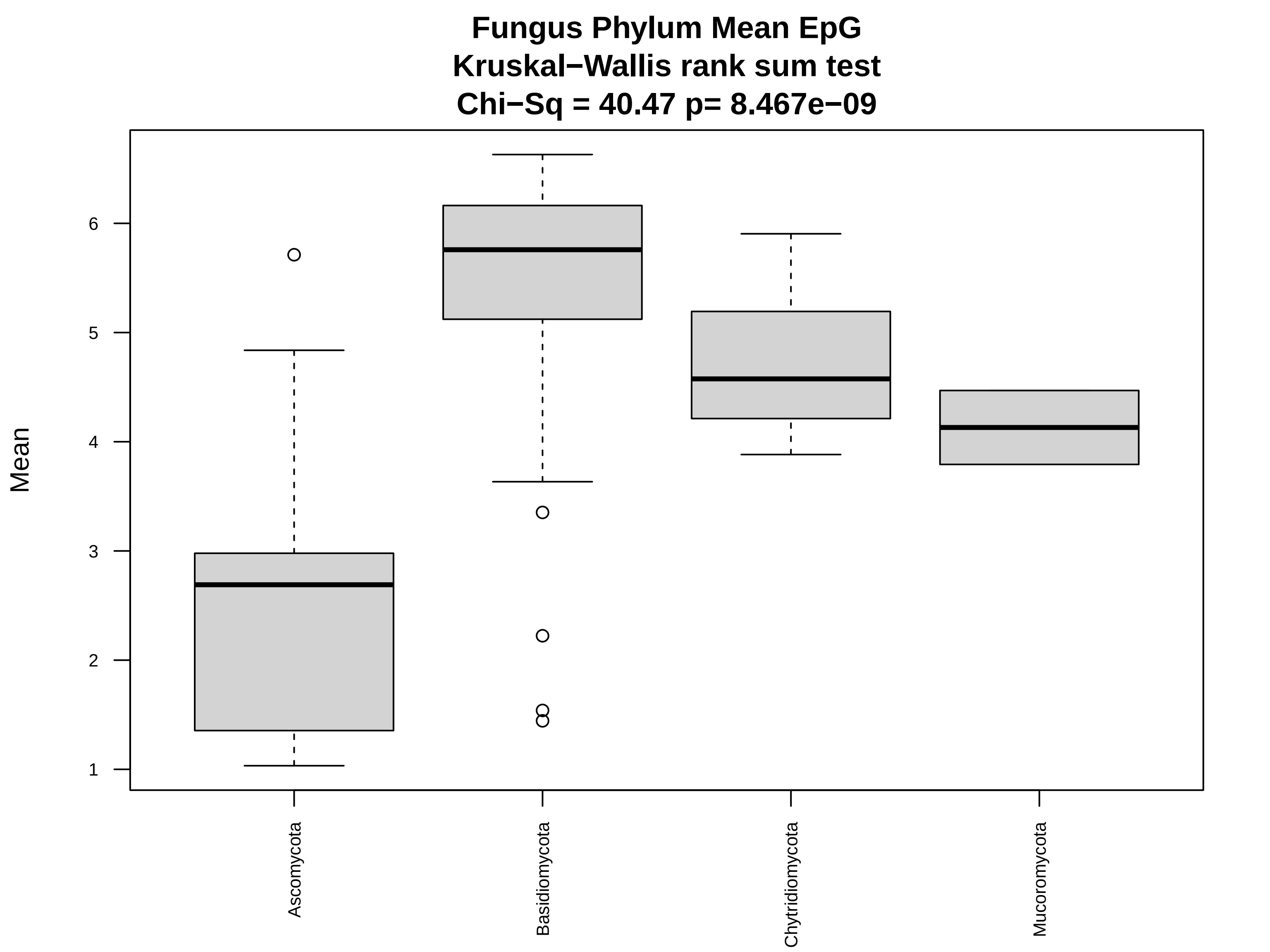
Fungus Phylum Mean EpG
Kruskal−Wallis rank sum test
Chi−Sq = 40.47 p= 8.467e−09
Mean
1
2
3
4
5
6
Ascomycota
Basidiomycota
Chytridiomycota
Mucoromycota

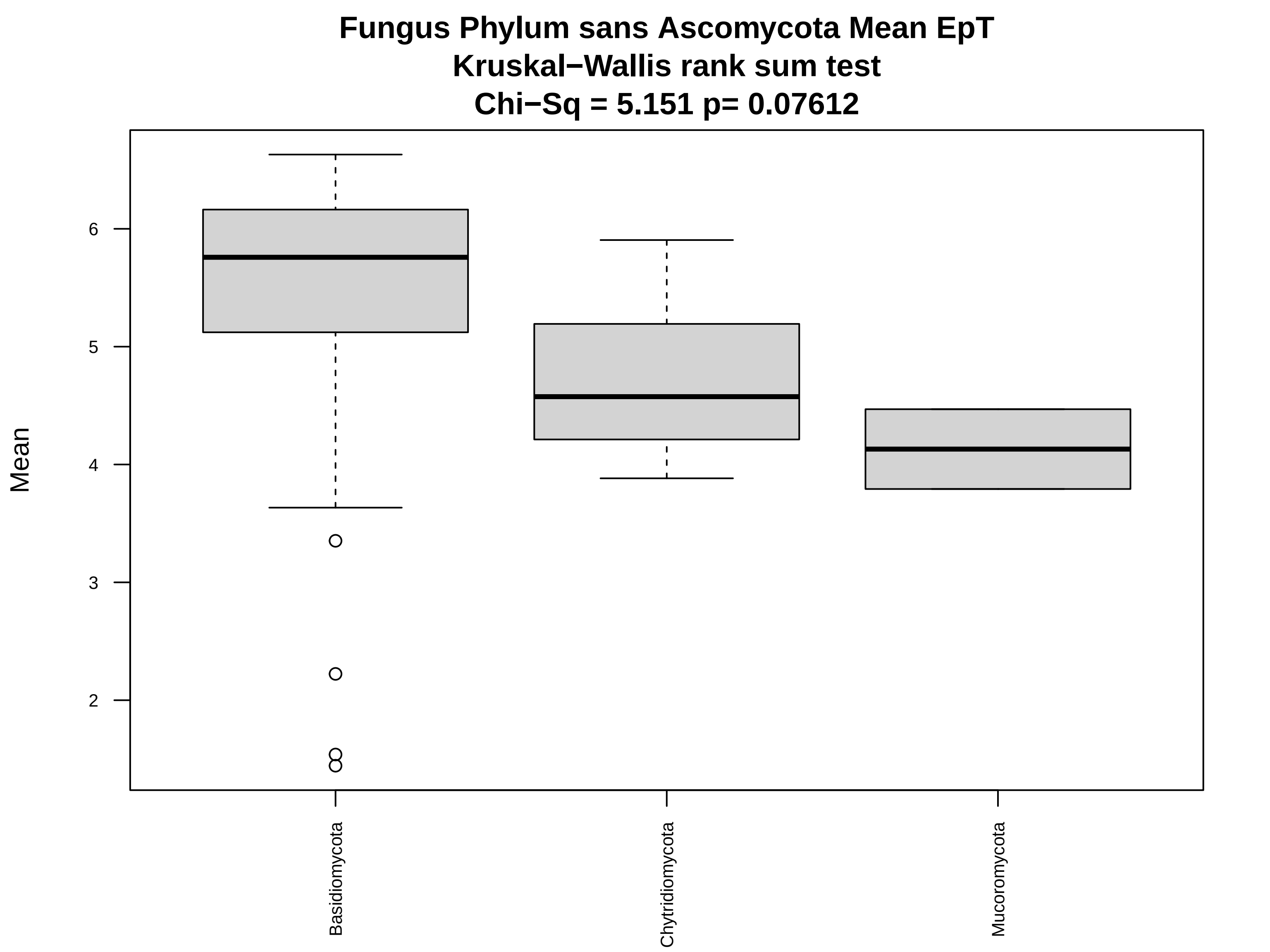

Fungus Phylum sans Ascomycota Mean EpT
Kruskal−Wallis rank sum test
Chi−Sq = 5.151 p= 0.07612
Mean
2
3
4
5
6
Basidiomycota
Chytridiomycota
Mucoromycota

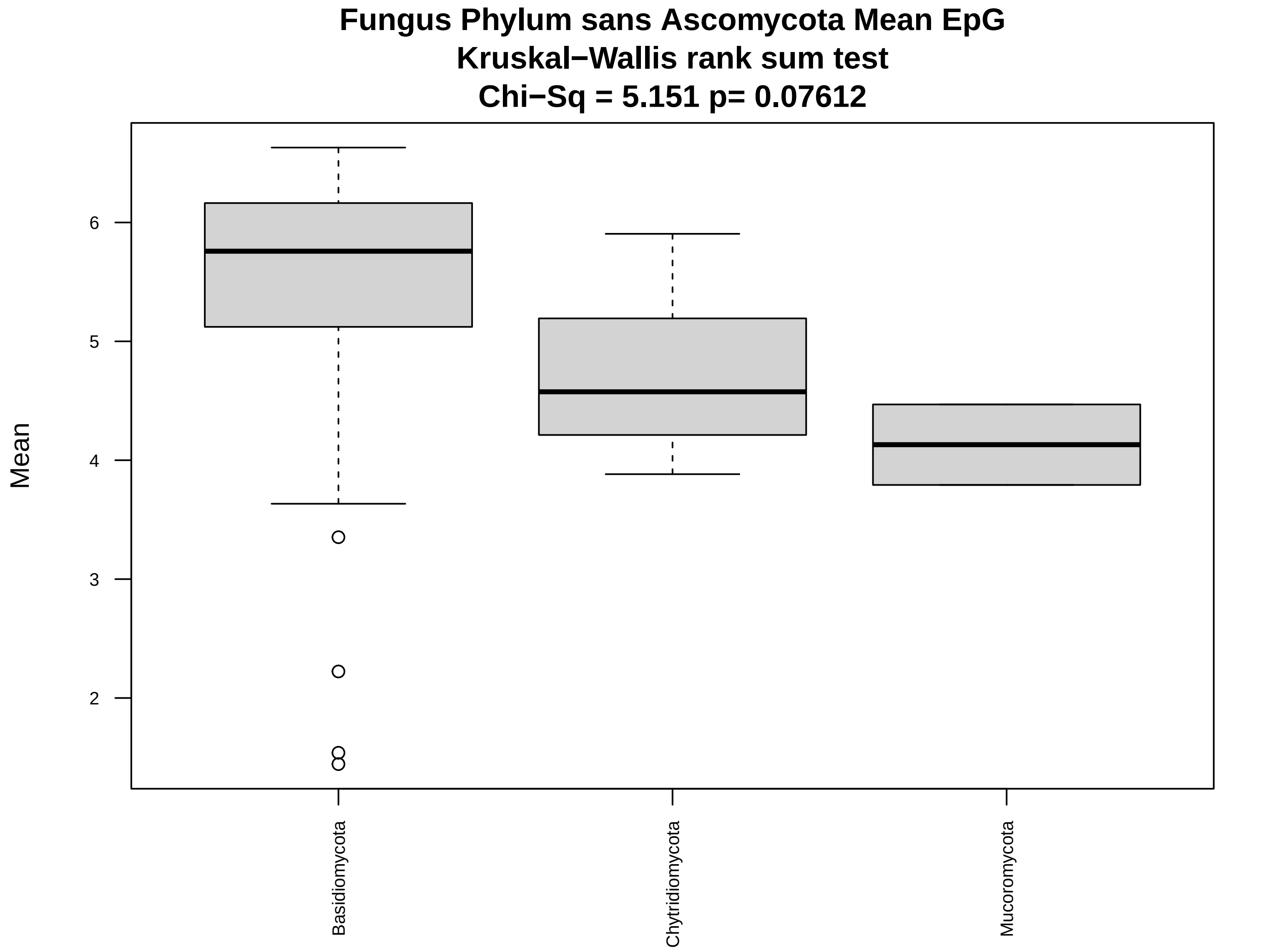
Fungus Phylum sans Ascomycota Mean EpG
Kruskal−Wallis rank sum test
Chi−Sq = 5.151 p= 0.07612
Mean
6
5
4
3
2
Basidiomycota
Chytridiomycota
Mucoromycota

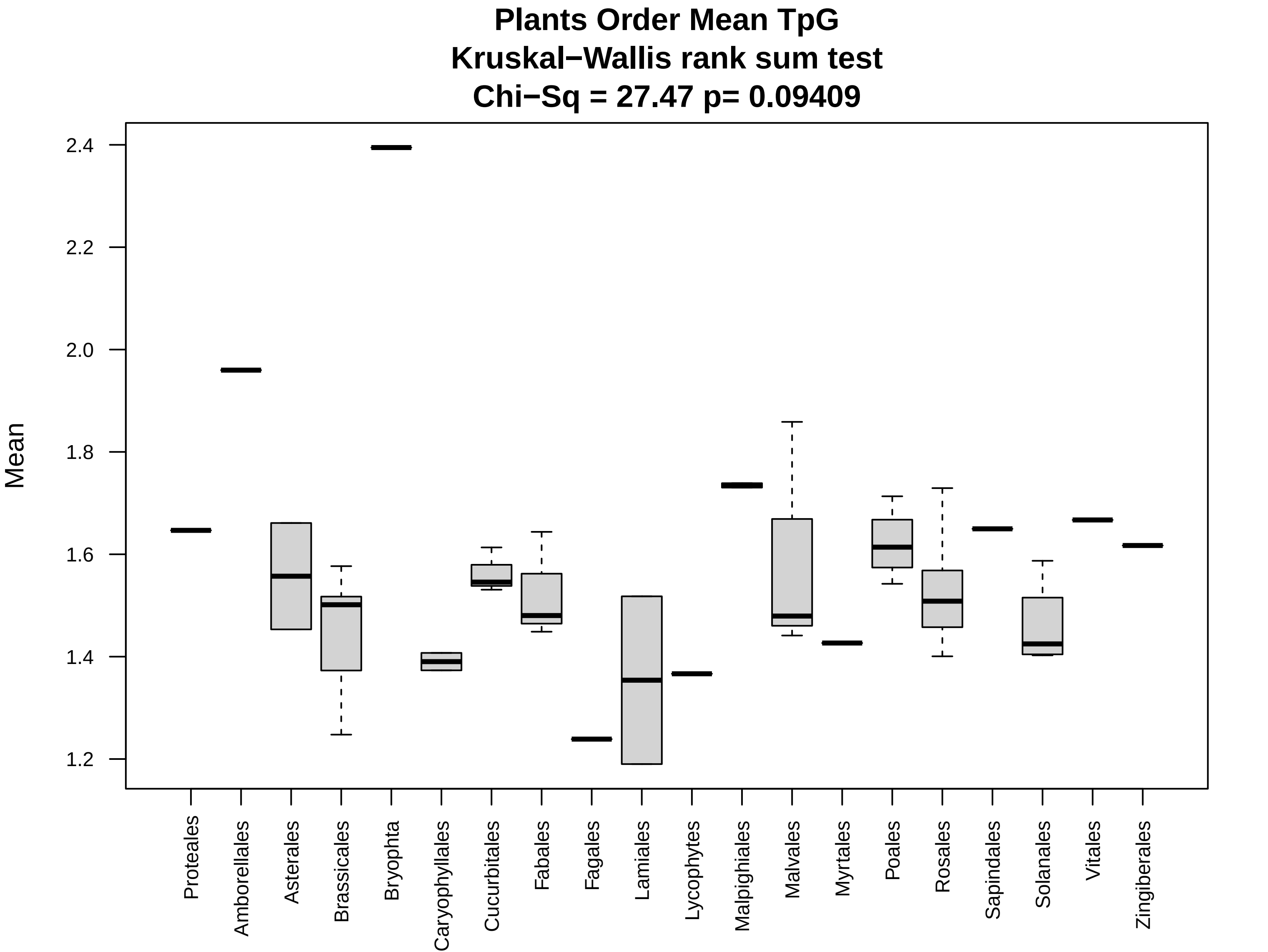

Plants Order Mean TpG
Kruskal−Wallis rank sum test
Chi−Sq = 27.47 p= 0.09409
Mean
2.4
2.2
2.0
1.8
1.6
1.4
1.2
Proteales
Amborellales
Asterales
Brassicales
Bryophta
Caryophyllales
Cucurbitales
Fabales
Fagales
Lamiales
Lycophytes
Malpighiales
Malvales
Myrtales
Poales
Rosales
Sapindales
Solanales
Vitales
Zingiberales

Plants Order Mean EpT
Kruskal−Wallis rank sum test
Chi−Sq = 33.34 p= 0.02195

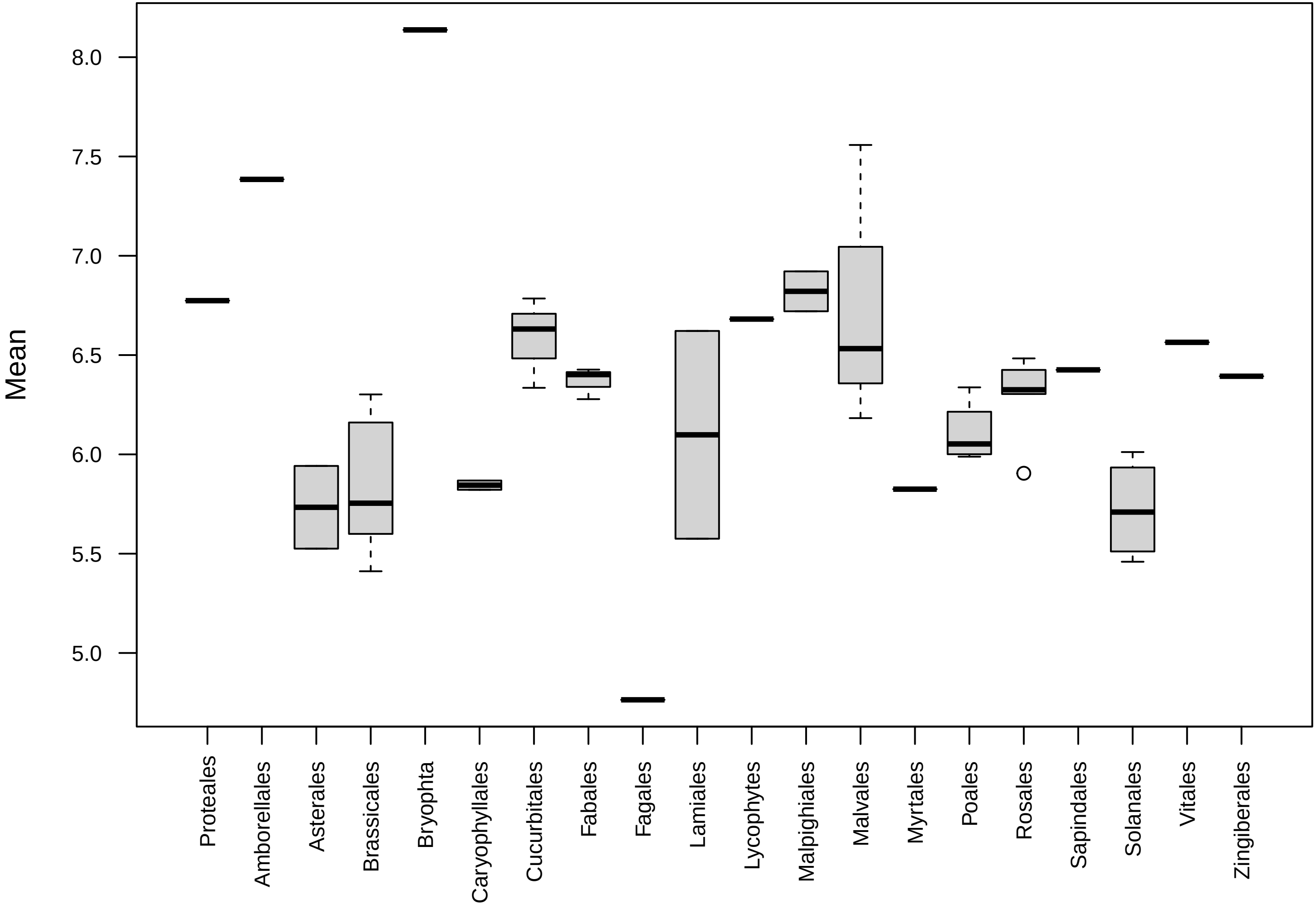

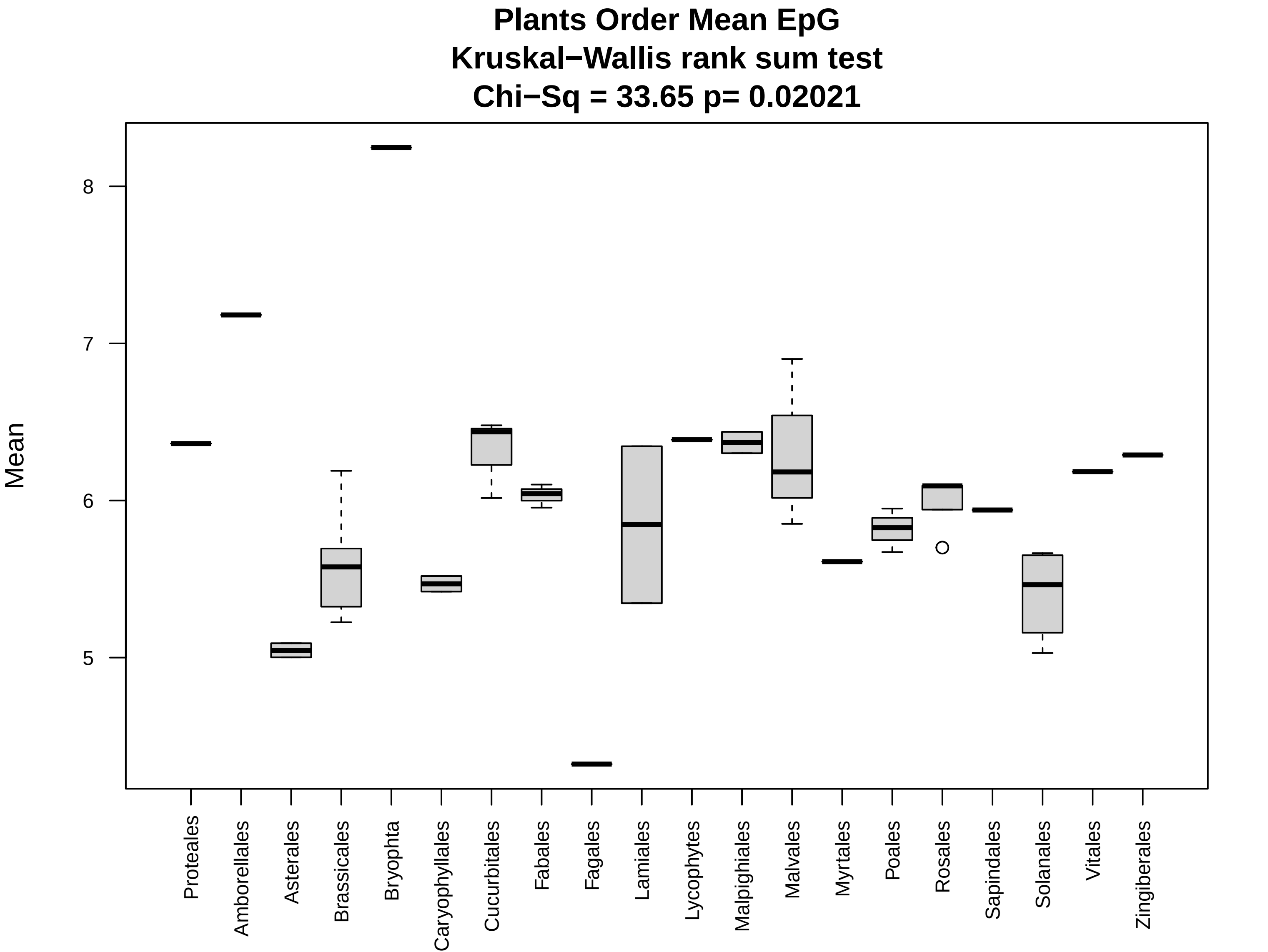
Plants Order Mean EpG
Kruskal−Wallis rank sum test
Chi−Sq = 33.65 p= 0.02021
Mean
8
7
6
5
Proteales
Amborellales
Asterales
Brassicales
Bryophta
Caryophyllales
Cucurbitales
Fabales
Fagales
Lamiales
Lycophytes
Malpighiales
Malvales
Myrtales
Poales
Rosales
Sapindales
Solanales
Vitales
Zingiberales
