# Supplementary material for: Estimating transcriptome complexities across eukaryotes
Source: BMC Genomics. 2023 May 11;24:254. doi: 10.1186/s12864-023-09326-0 (PMC10173493; doi:10.1186/s12864-023-09326-0)

NOVEL GENE DENSITY PLOTS

Figures below are density plots for all novel gene distributions (light green) for complexity metrics (TpG, EpT, and EpG) with densities on the y-axis and genetic element counts on the x-axis. Plots include every organism used in this study.

GCF_000001405.39_GRCh38.p13

Novel Genes

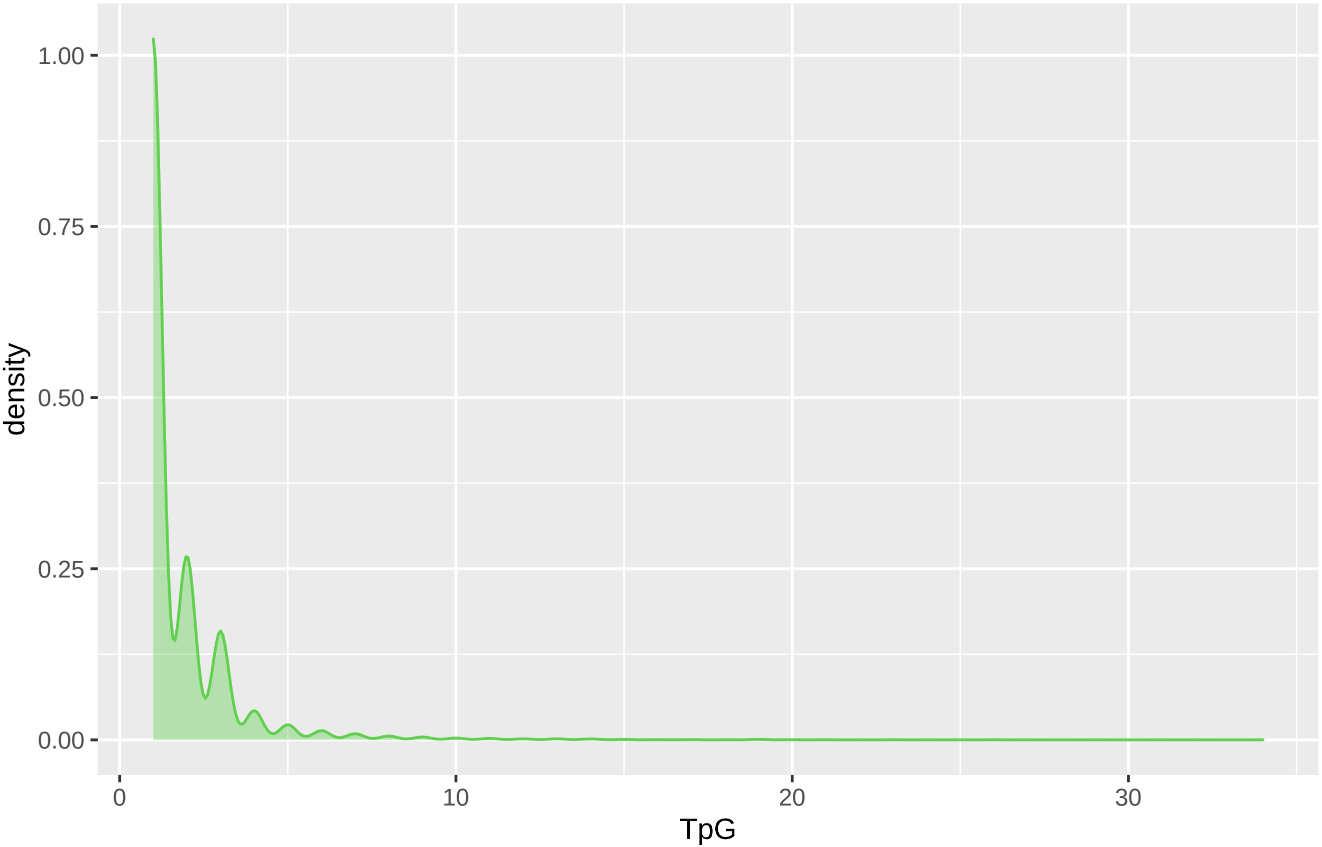

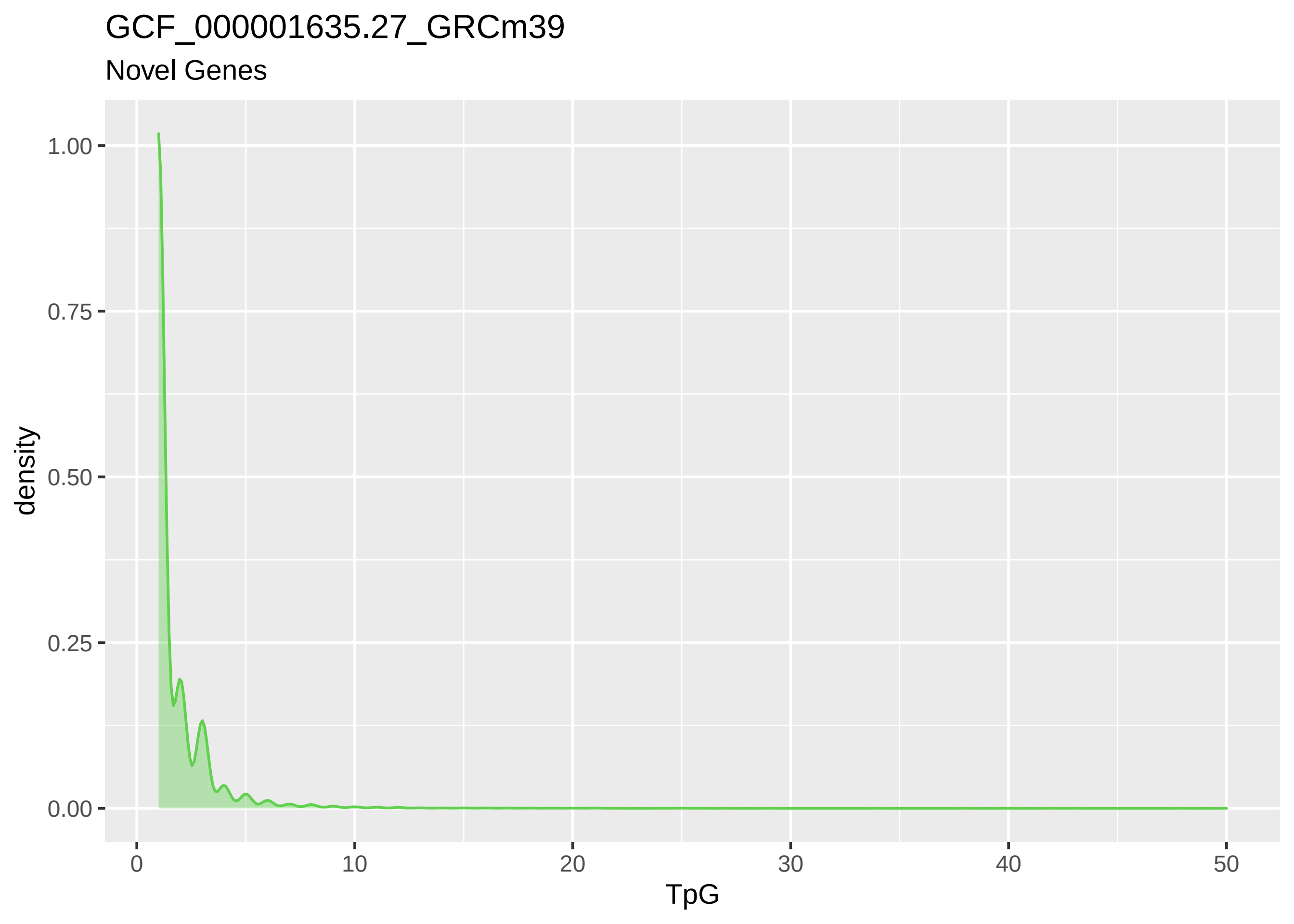
GCF_000001635.27_GRCm39
Novel Genes
density
1.00
0.75
0.50
0.25
0.00
0
10
20
30
40
50
TpG

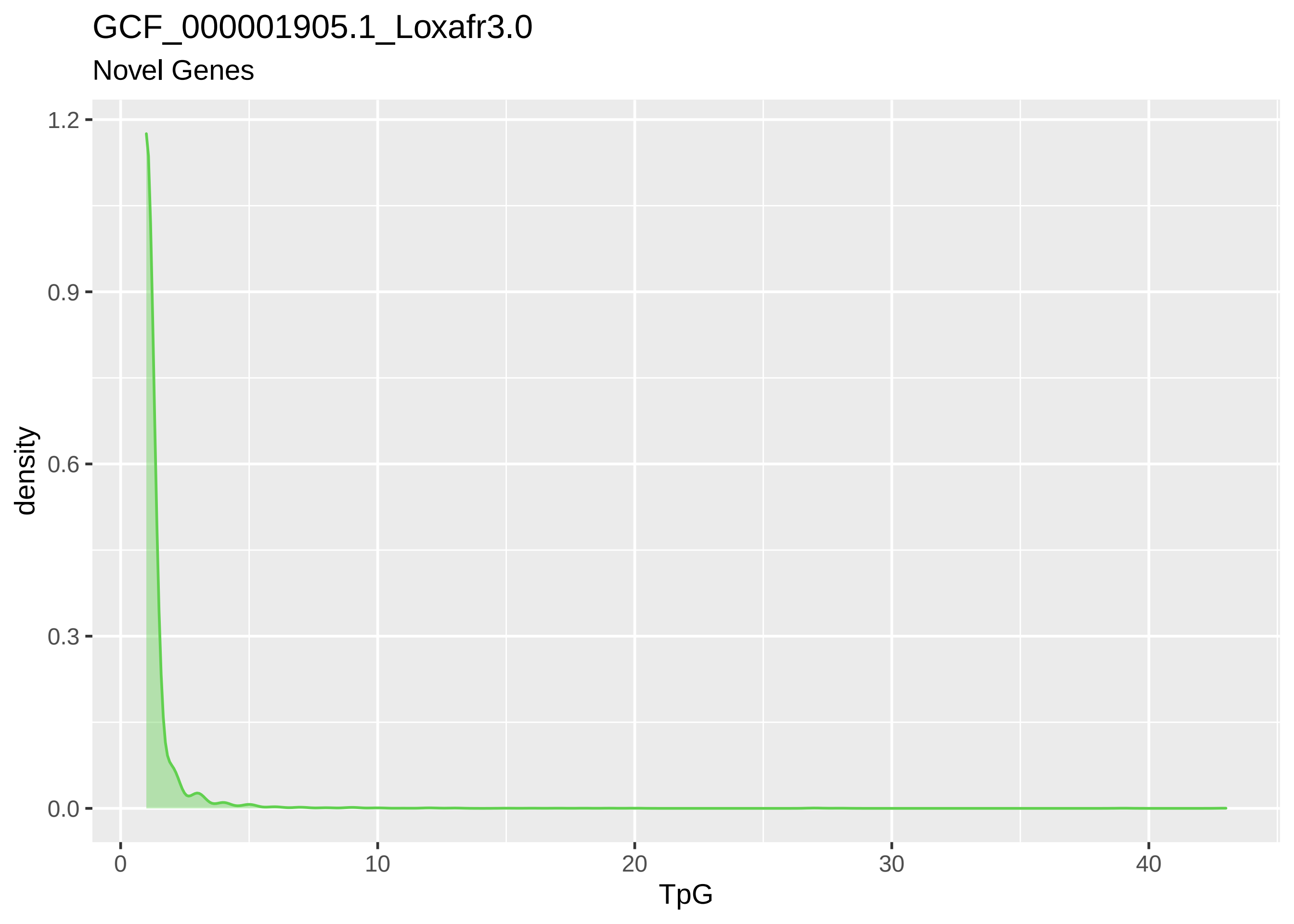

GCF_000001905.1_Loxafr3.0
Novel Genes
density
1.2
0.9
0.6
0.3
0.0
0
10
20
30
40
TpG

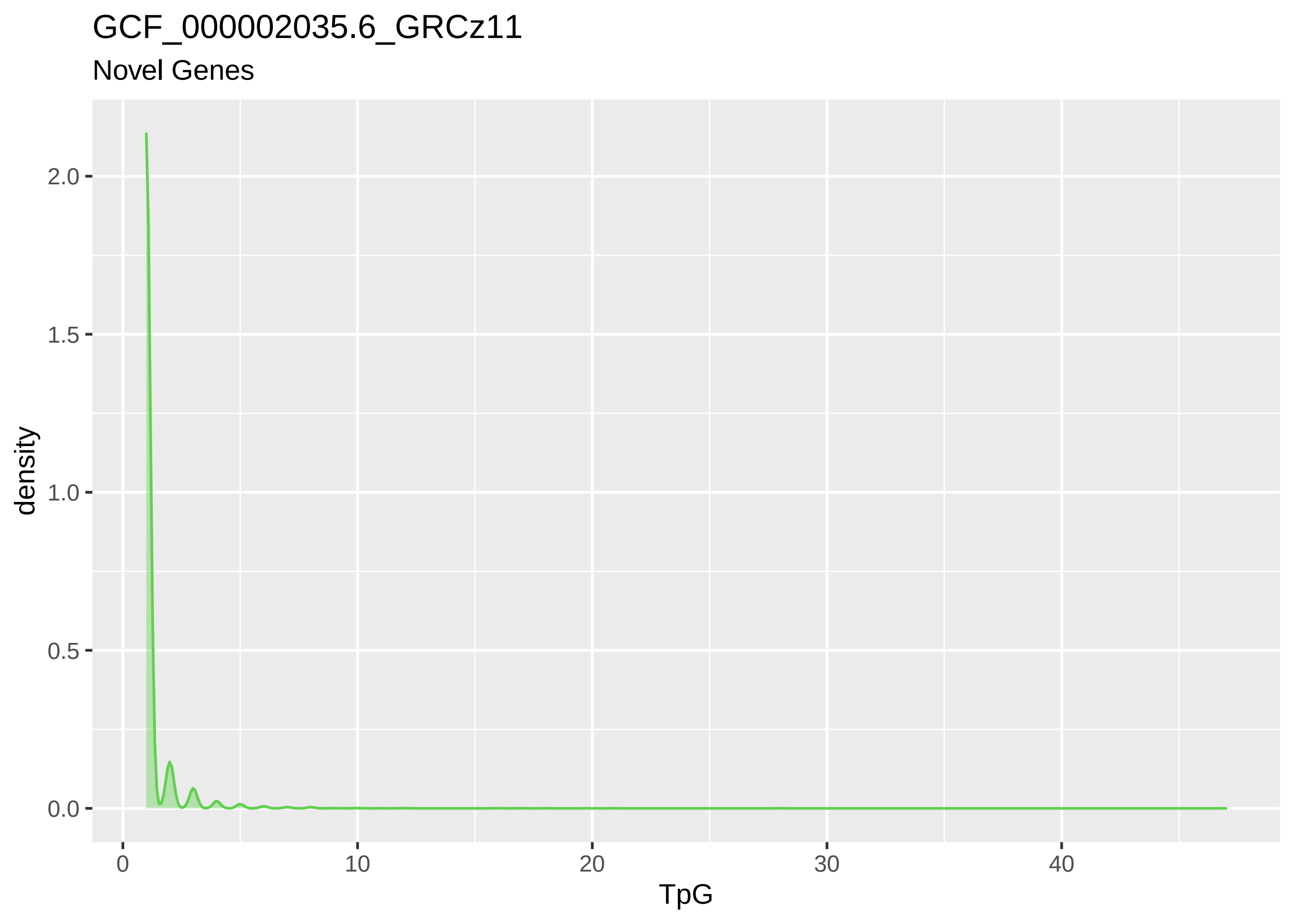
GCF_000002035.6_GRCz11
Novel Genes
density
TpG
2.0
1.5
1.0
0.5
0.0
0
10
20
30
40

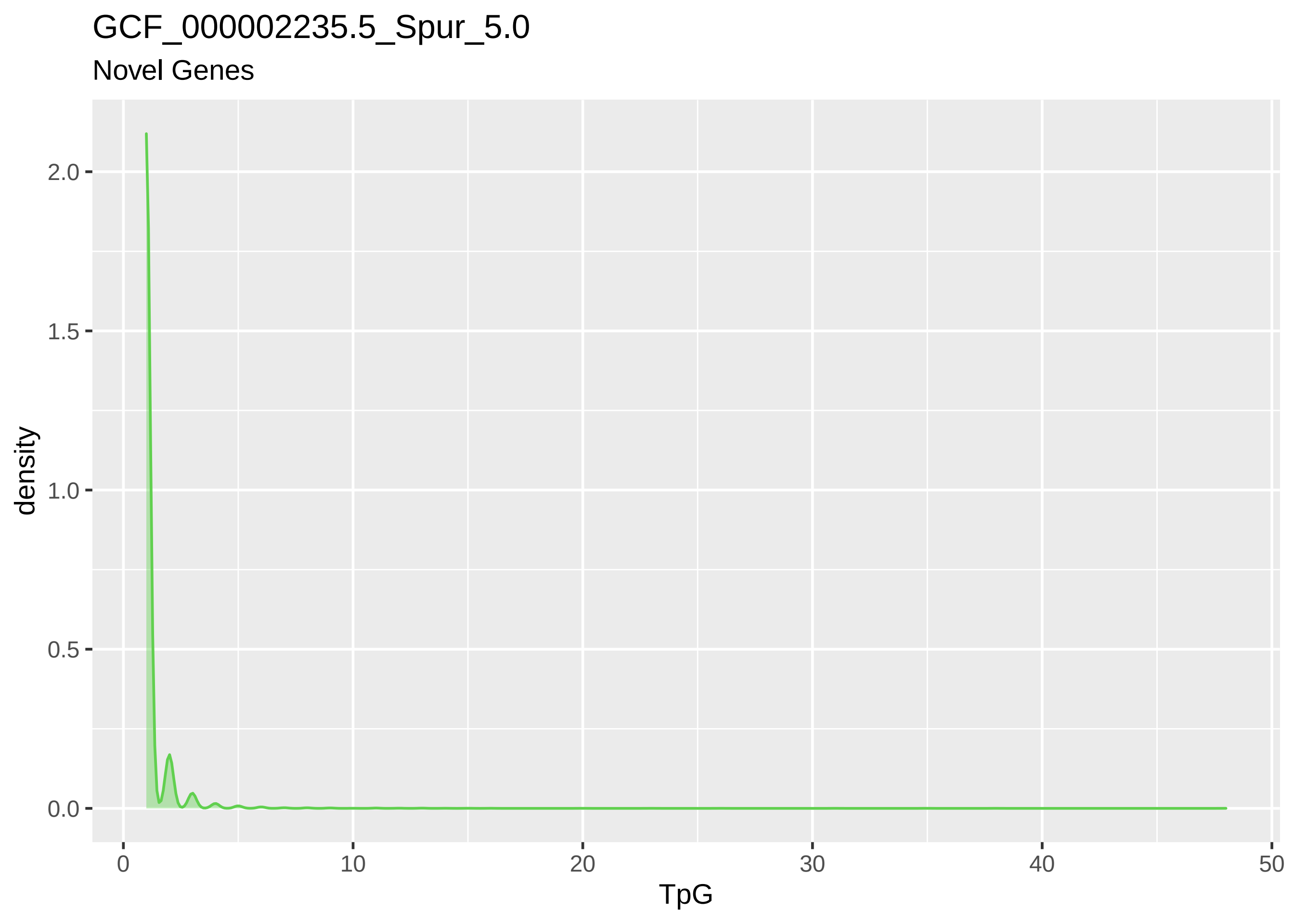

GCF_000002235.5_Spur_5.0
Novel Genes
density
2.0
1.5
1.0
0.5
0.0
0
10
20
30
40
50
TpG

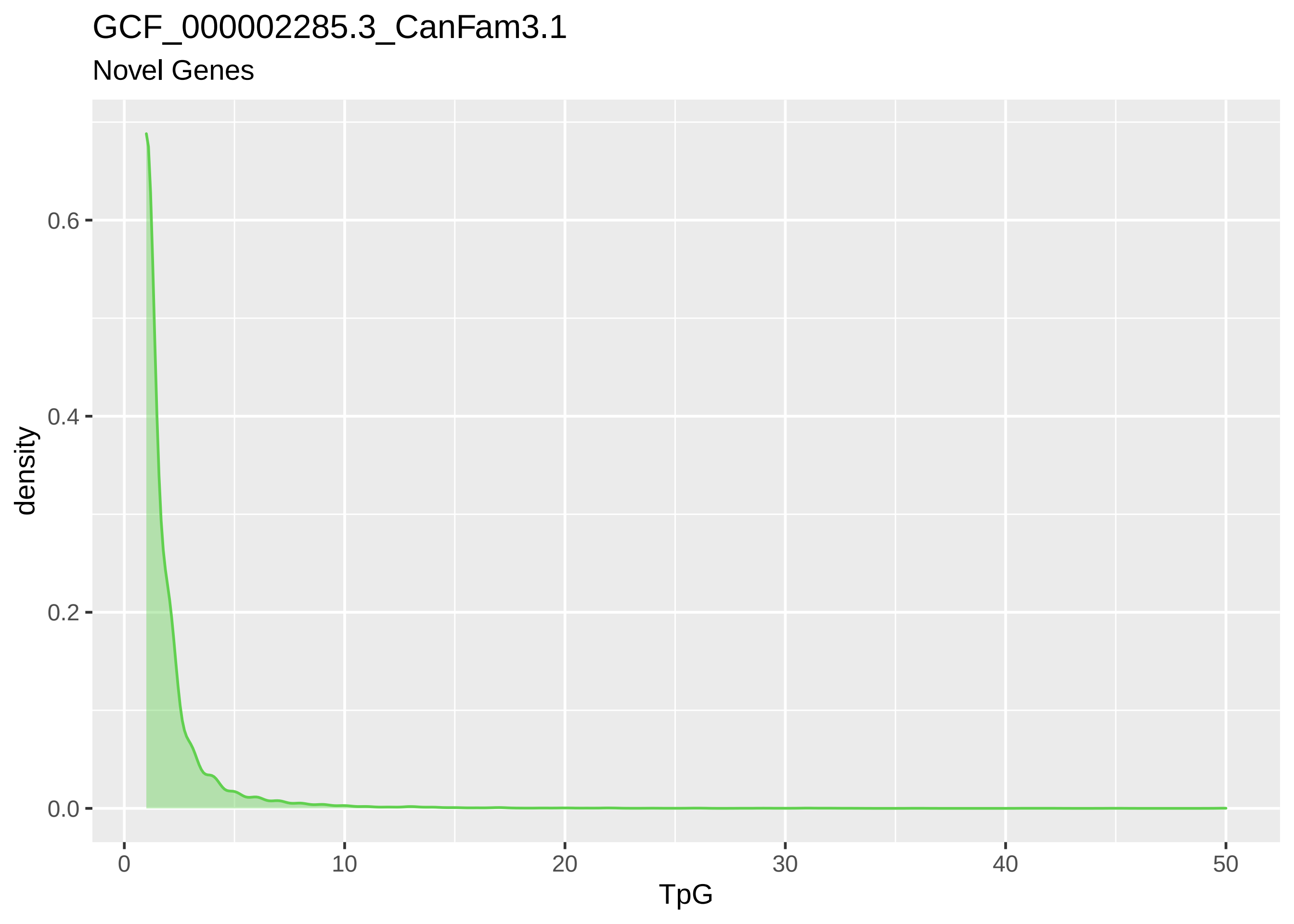
GCF_000002285.3_CanFam3.1
Novel Genes
density
0.0
0.2
0.4
0.6
0
10
20
30
40
50
TpG

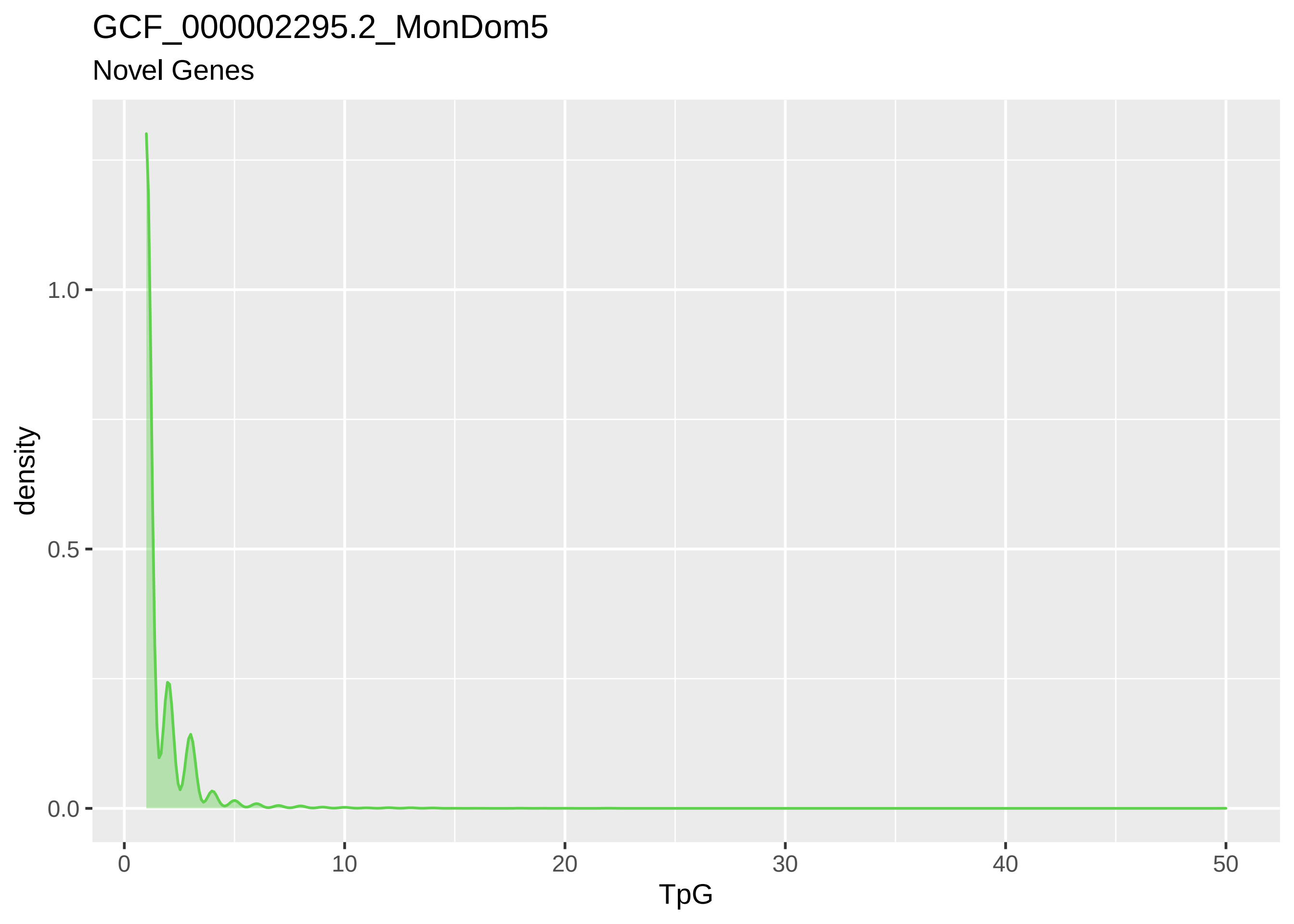
GCF_000002295.2_MonDom5
Novel Genes
density
1.0
0.5
0.0
0
10
20
30
40
50
TpG

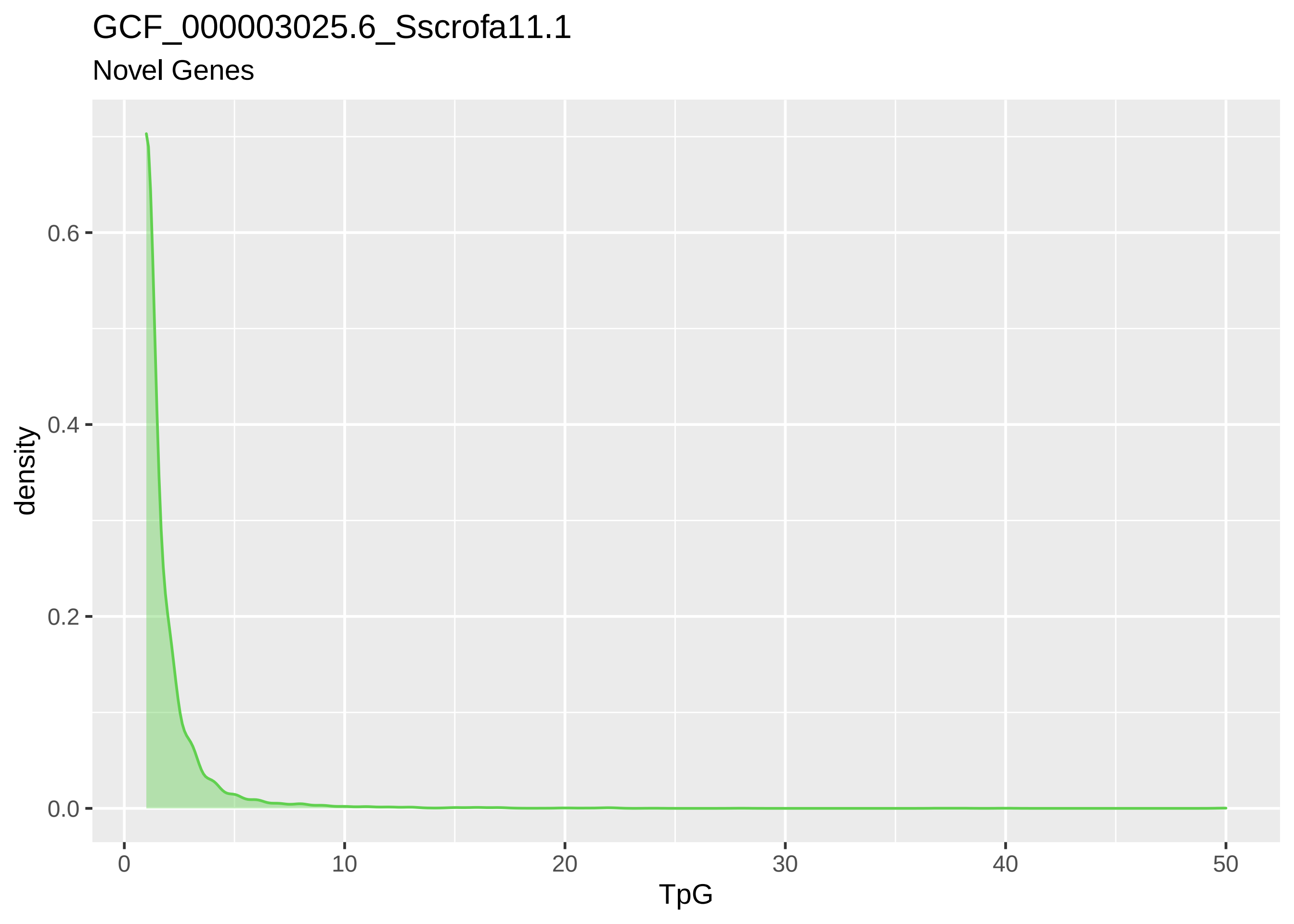
GCF_000003025.6_Sscrofa11.1
Novel Genes
density
0.0
0.2
0.4
0.6
0
10
20
30
40
50
TpG

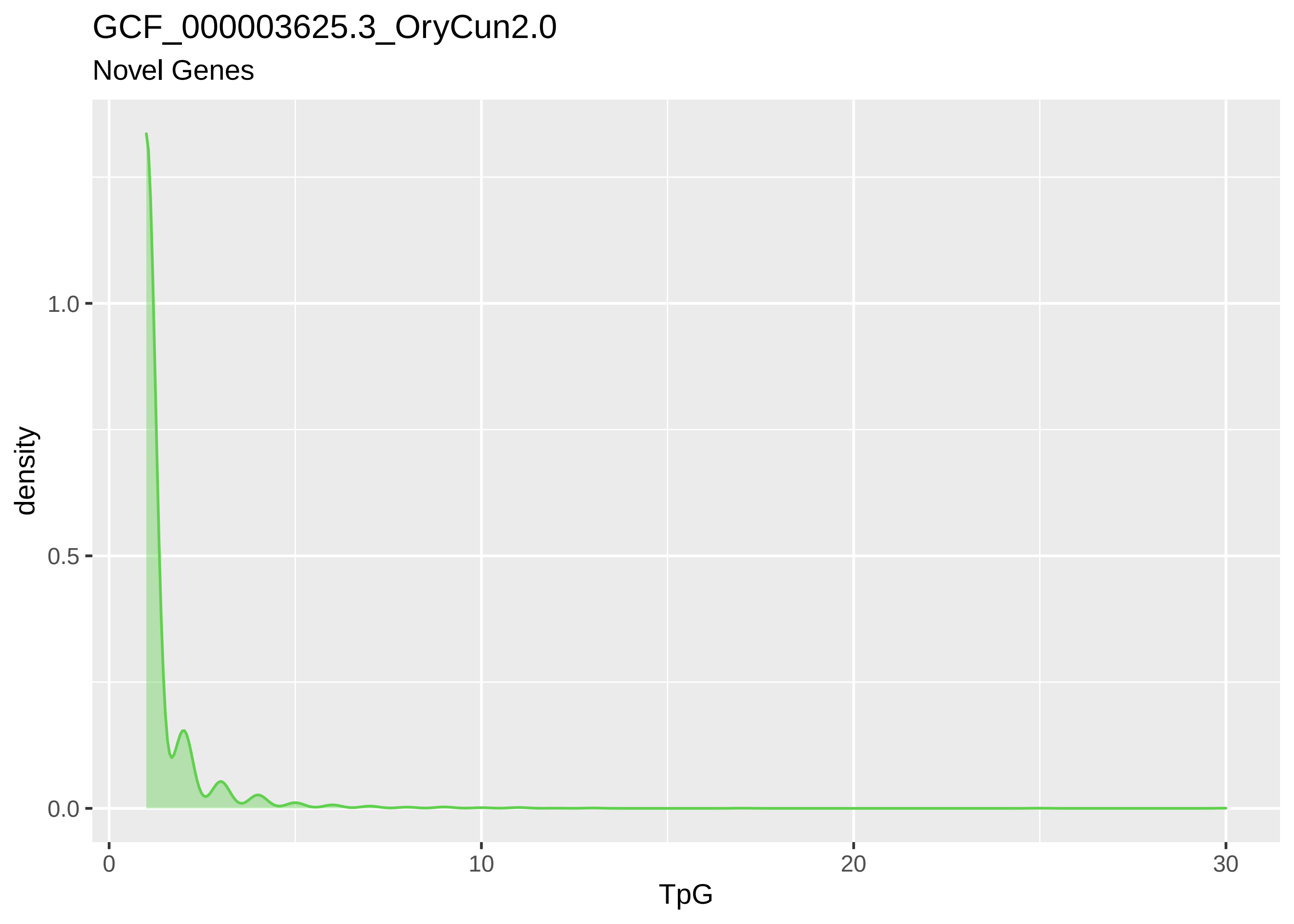
GCF_000003625.3_OryCun2.0
Novel Genes
density
1.0
0.5
0.0
0
10
20
30
TpG

GCF_000003815.1_Version_2

Novel Genes

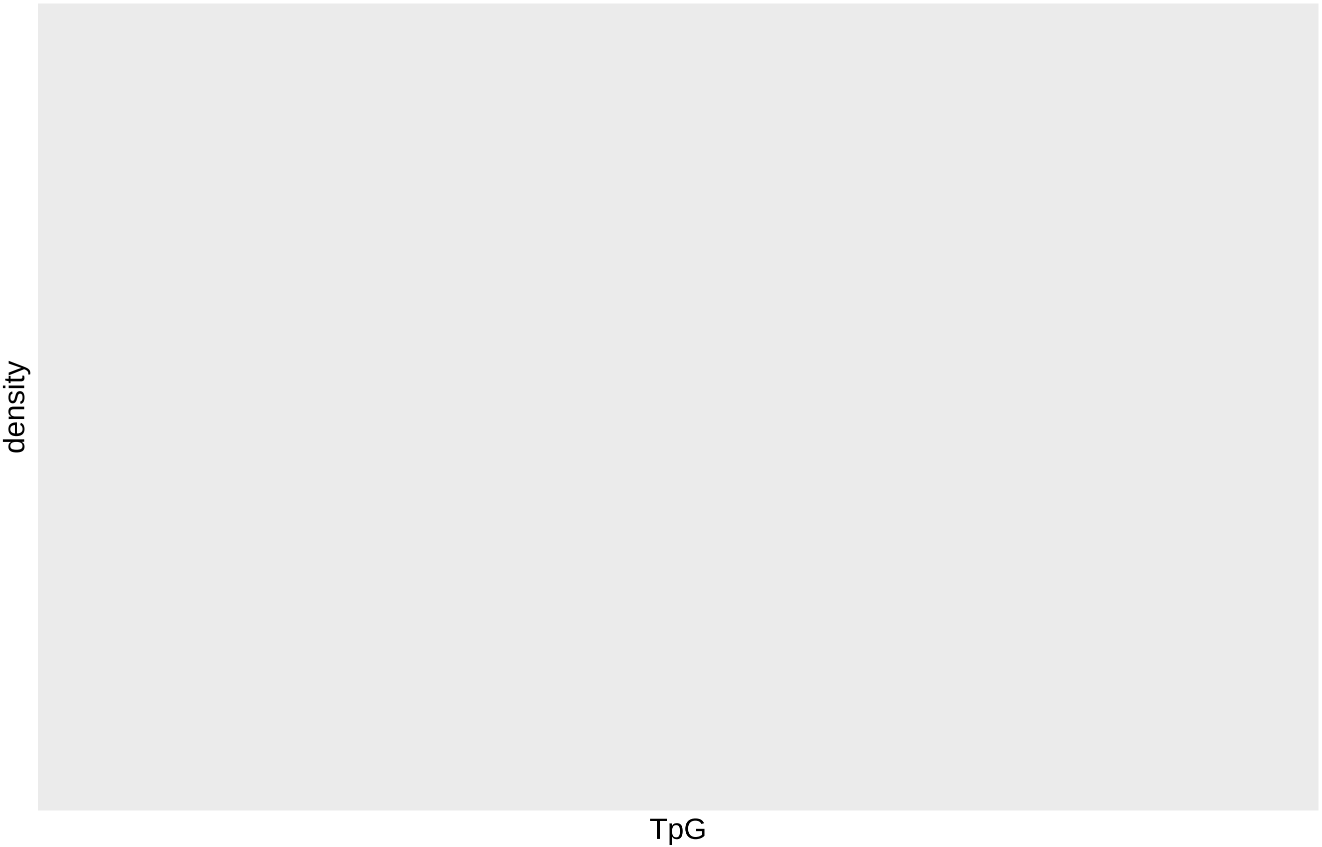

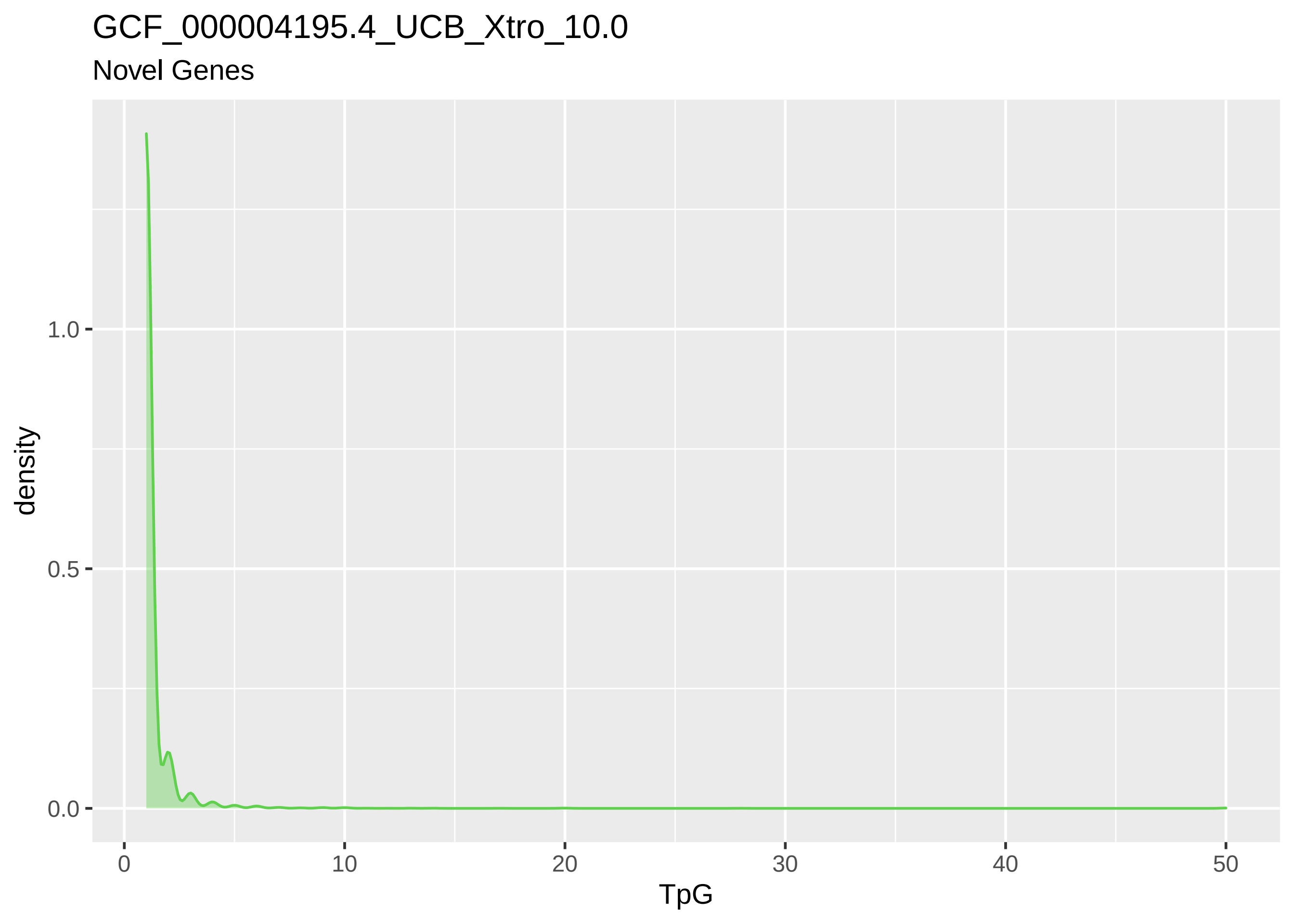
GCF_000004195.4_UCB_Xtro_10.0
Novel Genes
density
1.0
0.5
0.0
0
10
20
30
40
50
TpG

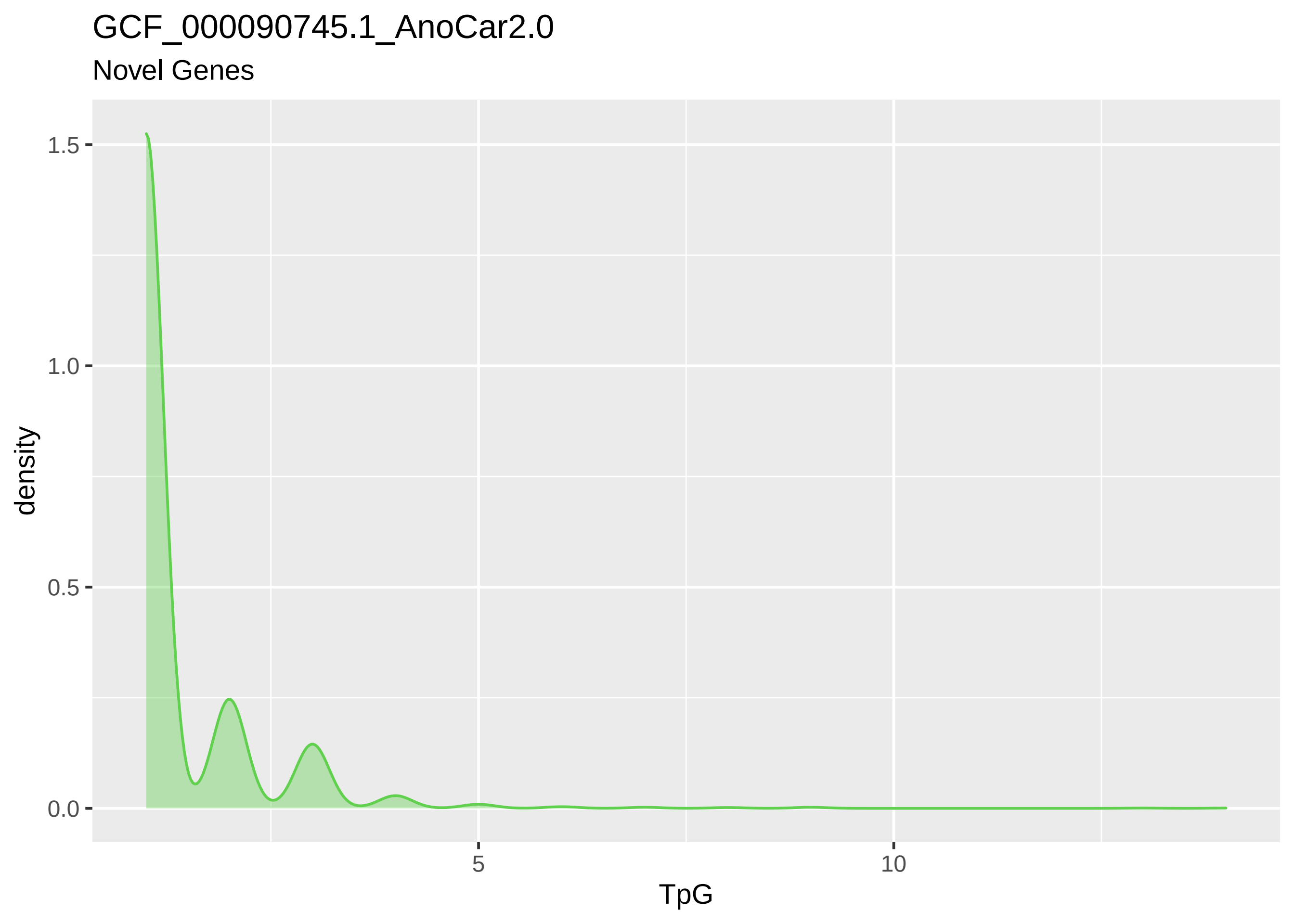
GCF_000090745.1_AnoCar2.0
Novel Genes
density
1.5
1.0
0.5
0.0
5
10
TpG

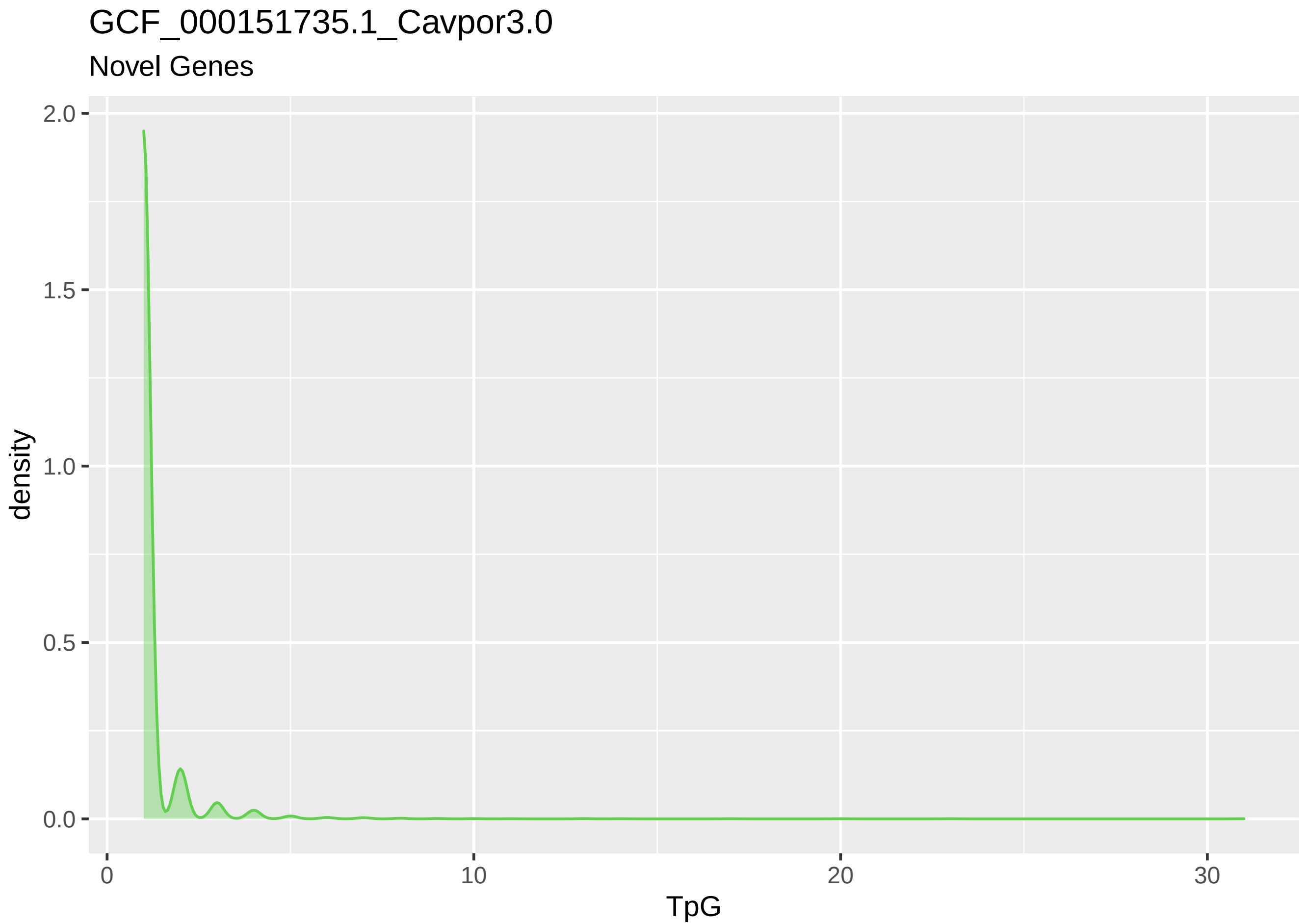

GCF_000151735.1_Cavpor3.0
Novel Genes
density
TpG
2.0
1.5
1.0
0.5
0.0
0
10
20
30

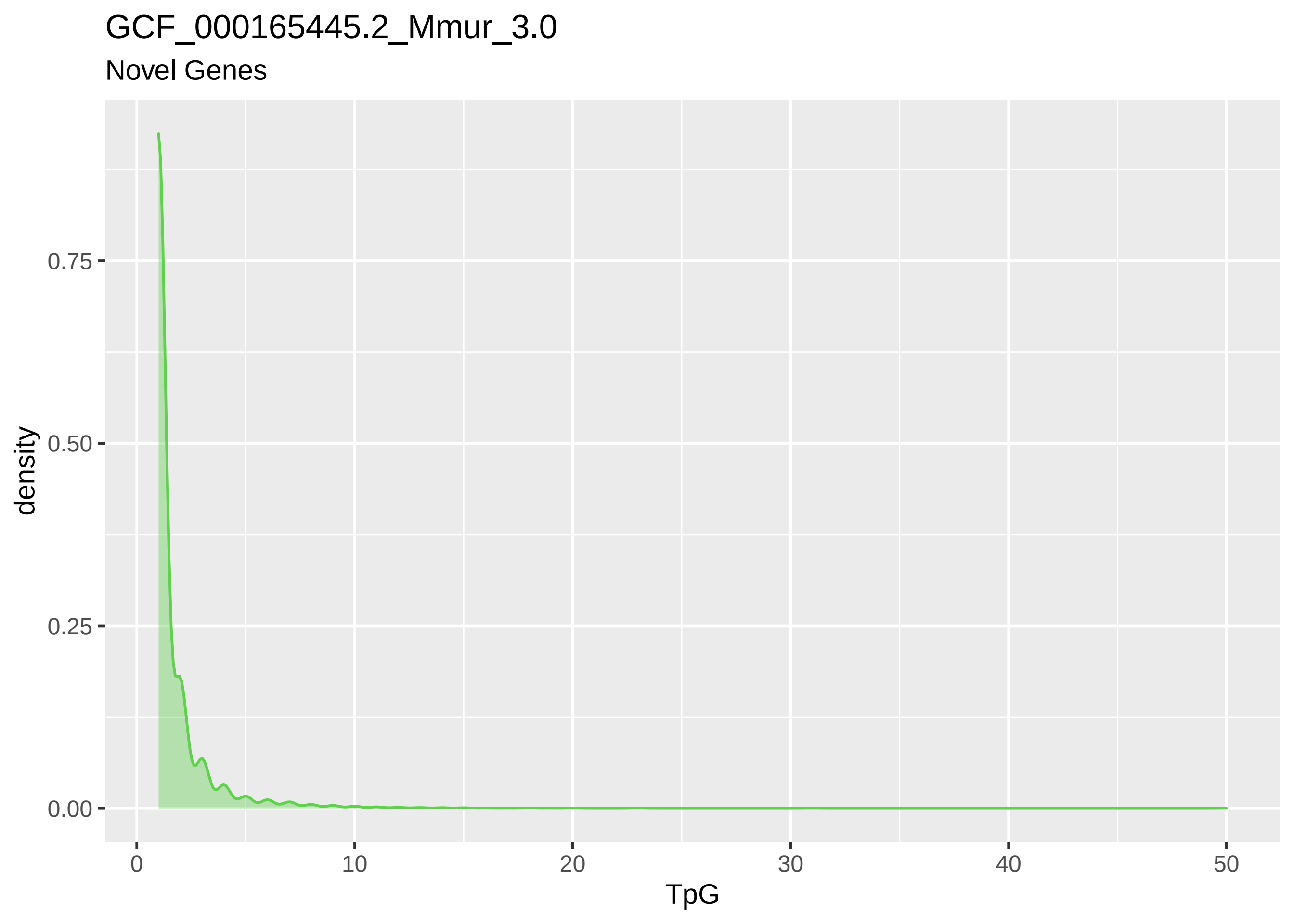
GCF_000165445.2_Mmur_3.0
Novel Genes
density
0.00
0.25
0.50
0.75
0
10
20
30
40
50
TpG

GCF_000181335.3_Felis_catus_9.0

Novel Genes

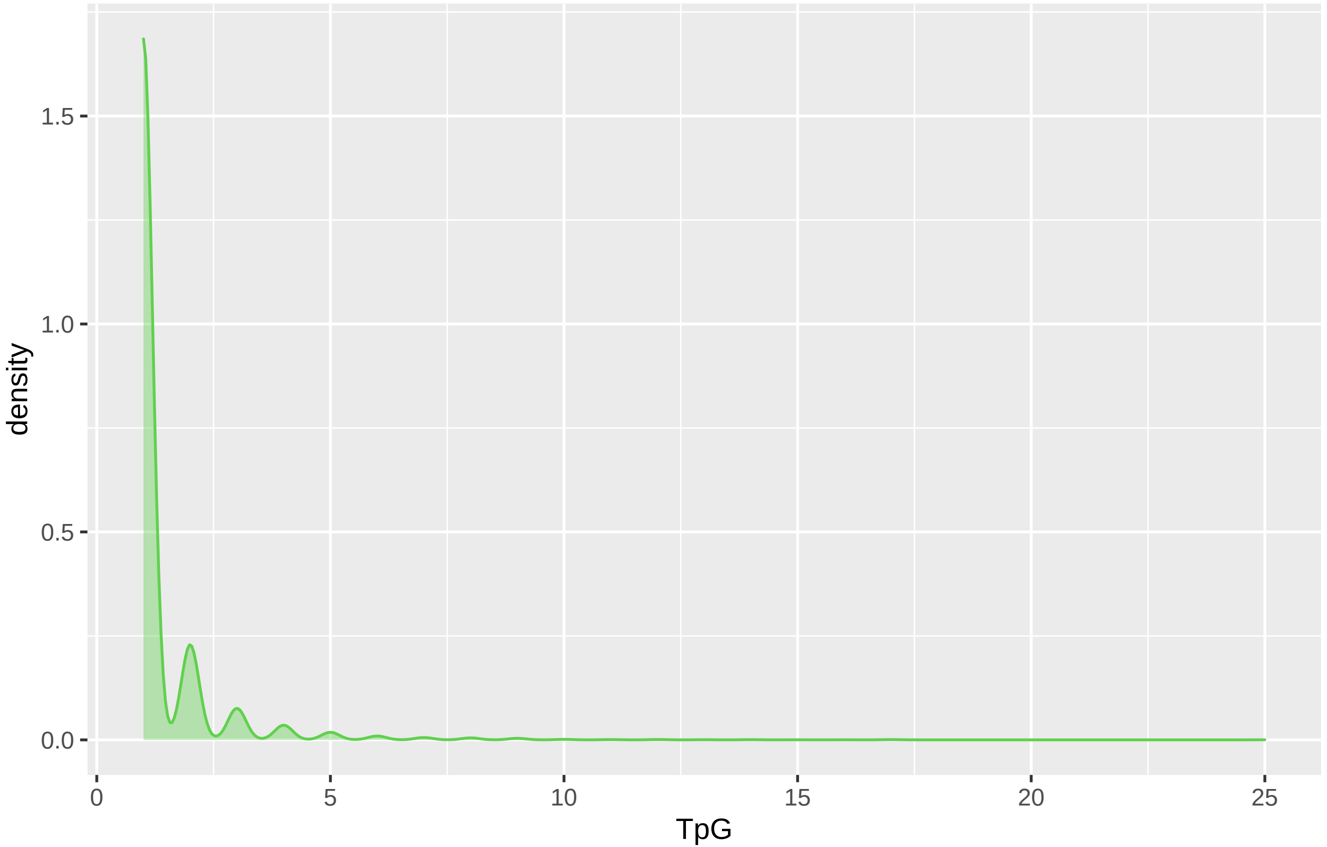

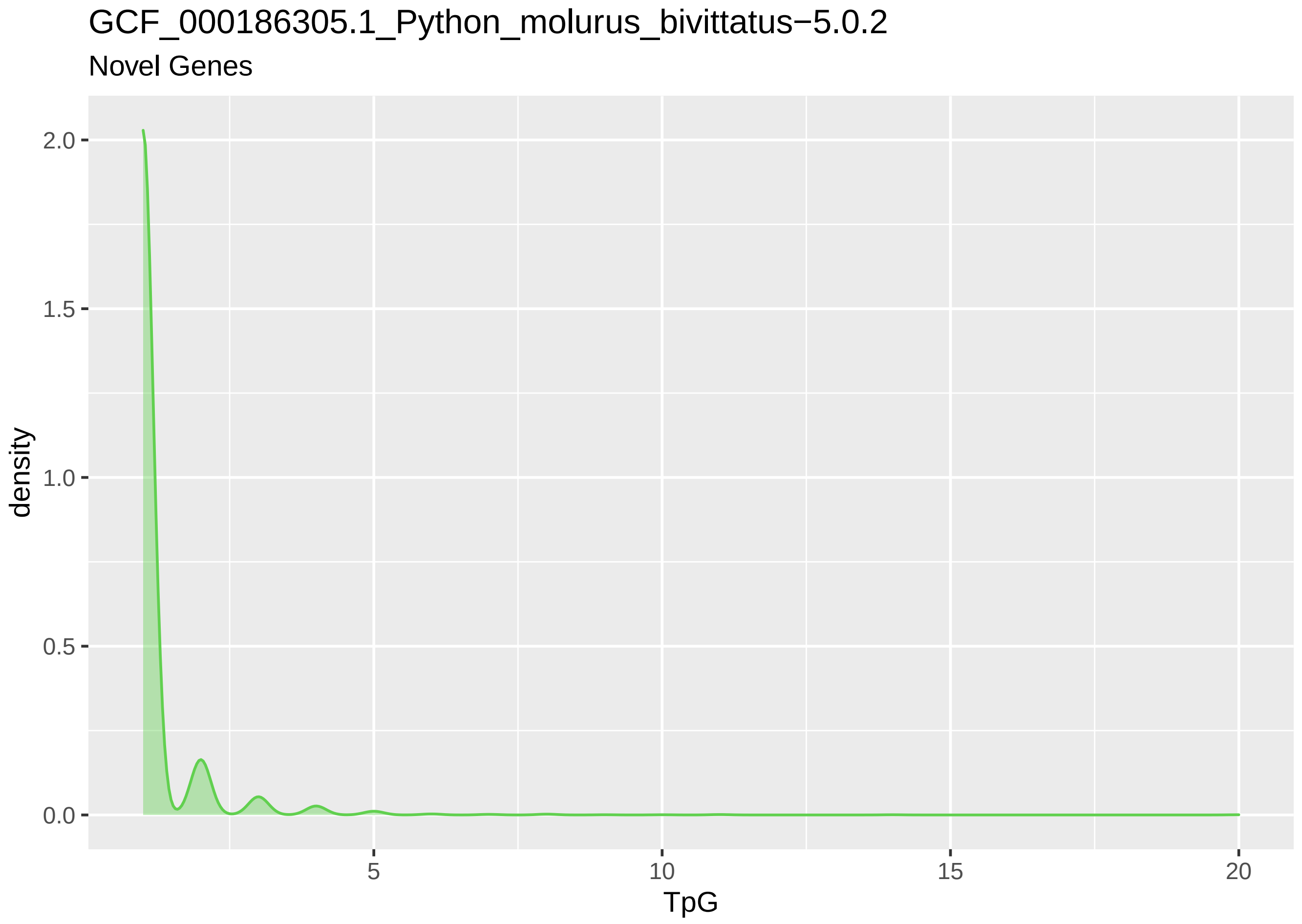
GCF_000186305.1_Python_molurus_bivittatus−5.0.2
Novel Genes
density
2.0
1.5
1.0
0.5
0.0
5
10
15
20
TpG

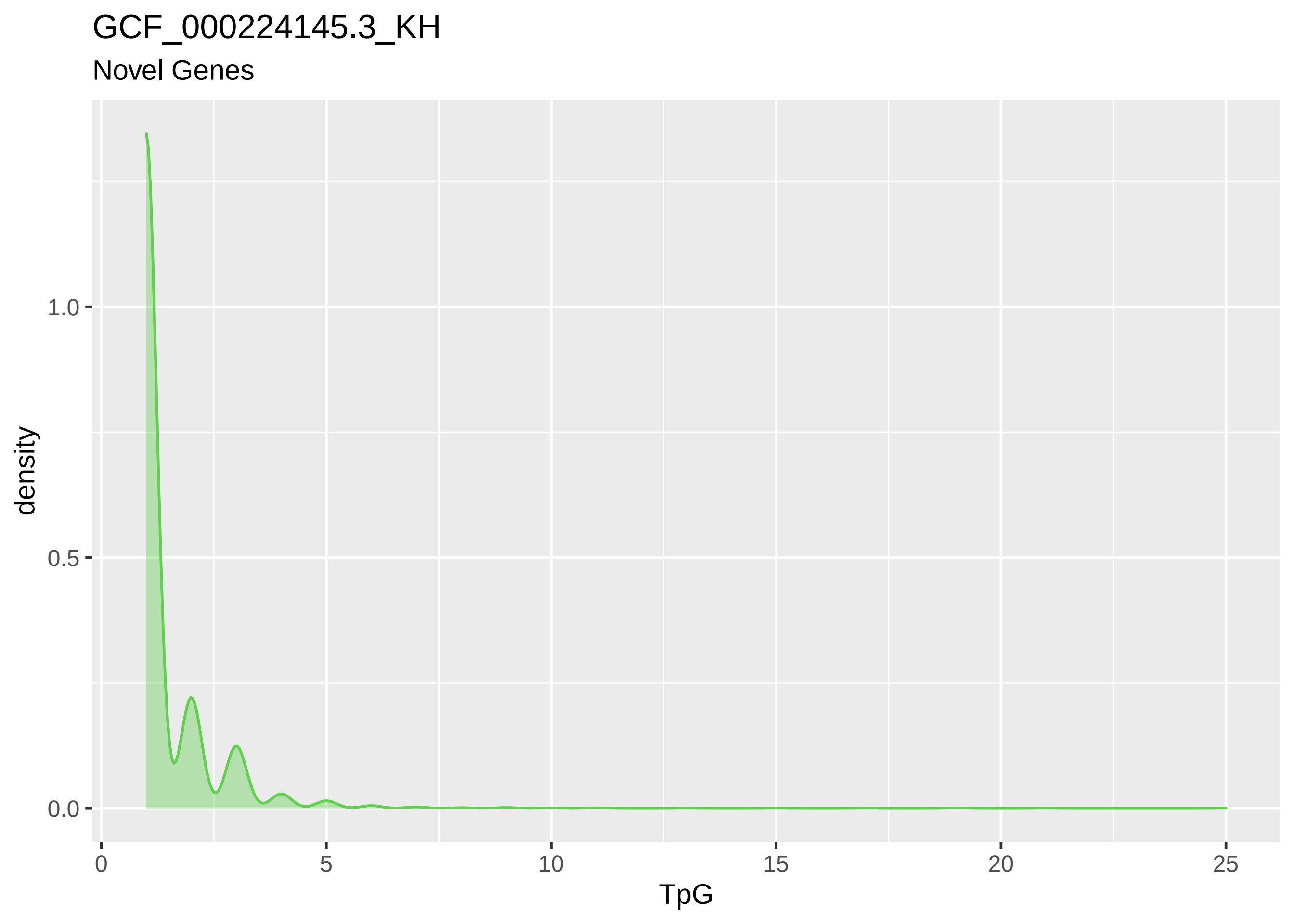
GCF_000224145.3_KH
Novel Genes
density
1.0
0.5
0.0
0
5
10
15
20
25
TpG

GCF_000225785.1_LatCha1

Novel Genes

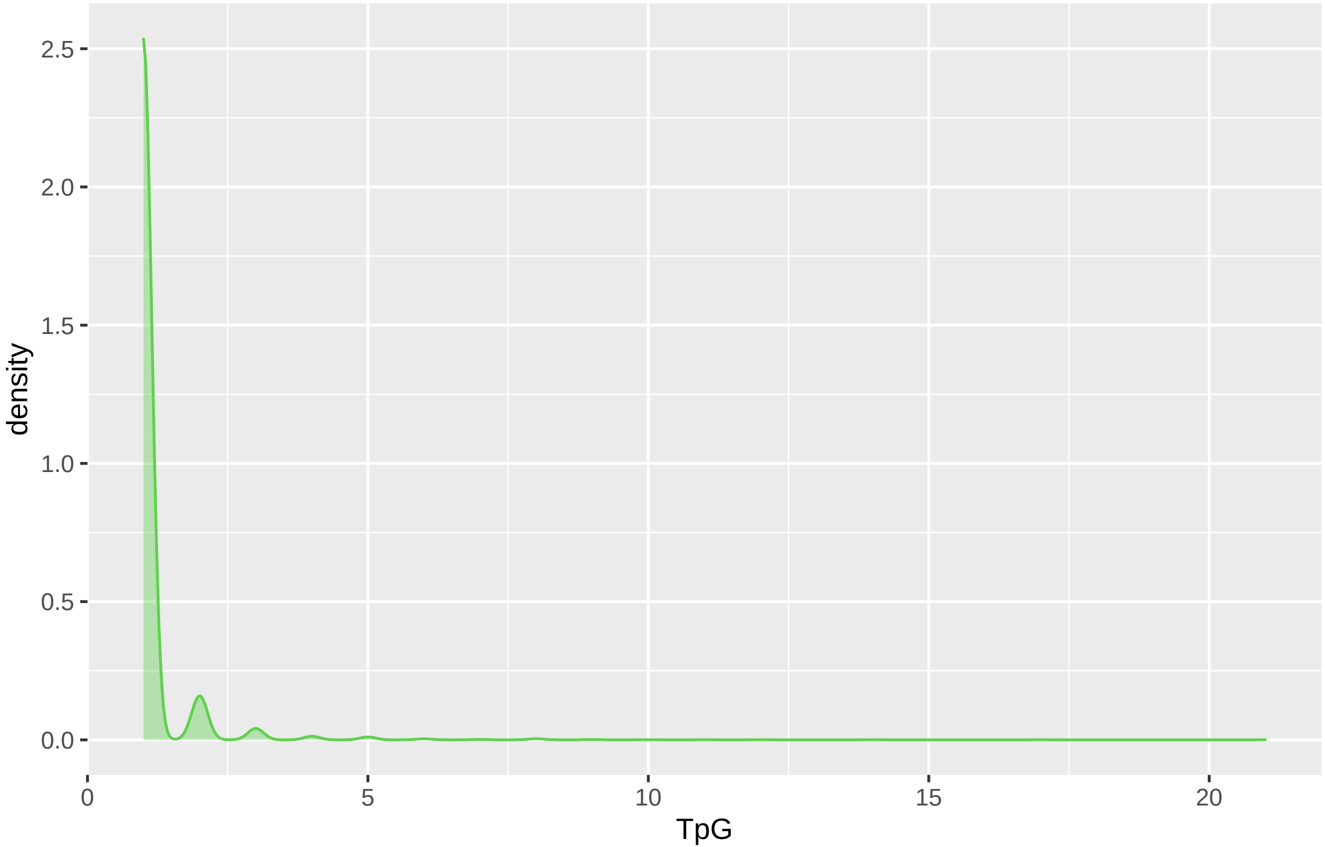

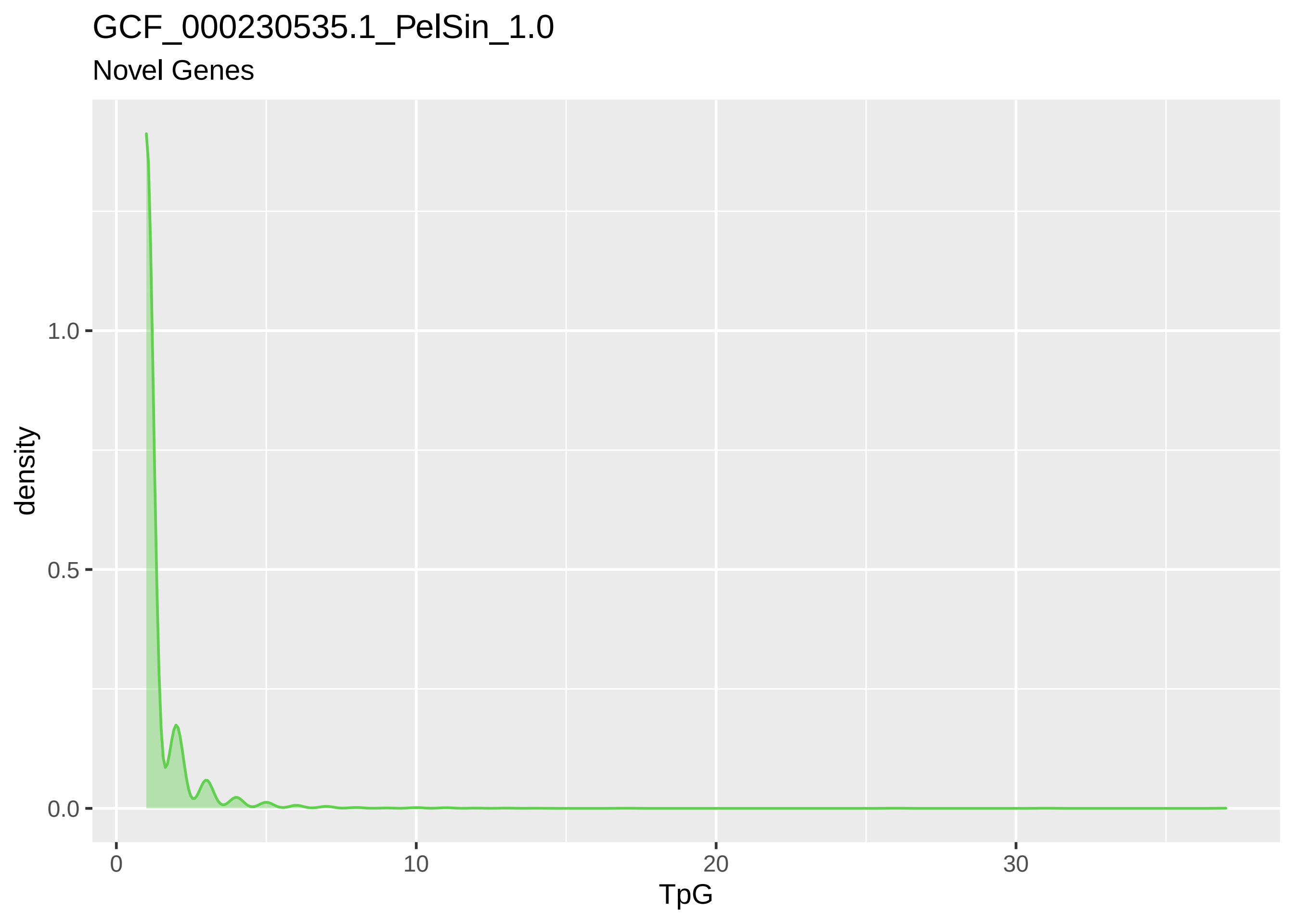
GCF_000230535.1_PelSin_1.0
Novel Genes
density
1.0
0.5
0.0
0
10
20
30
TpG

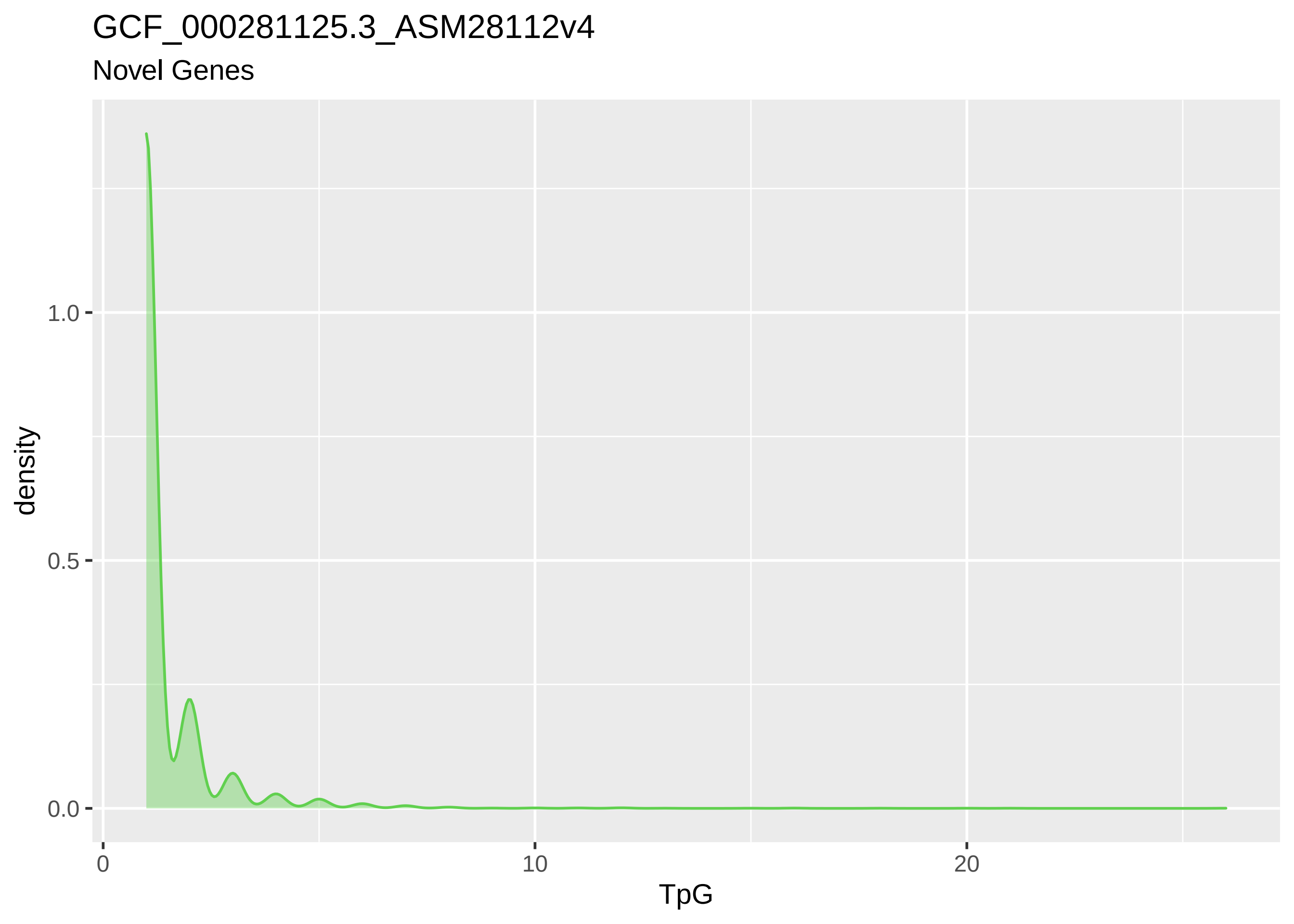
GCF_000281125.3_ASM28112v4
Novel Genes
density
1.0
0.5
0.0
0
10
20
TpG

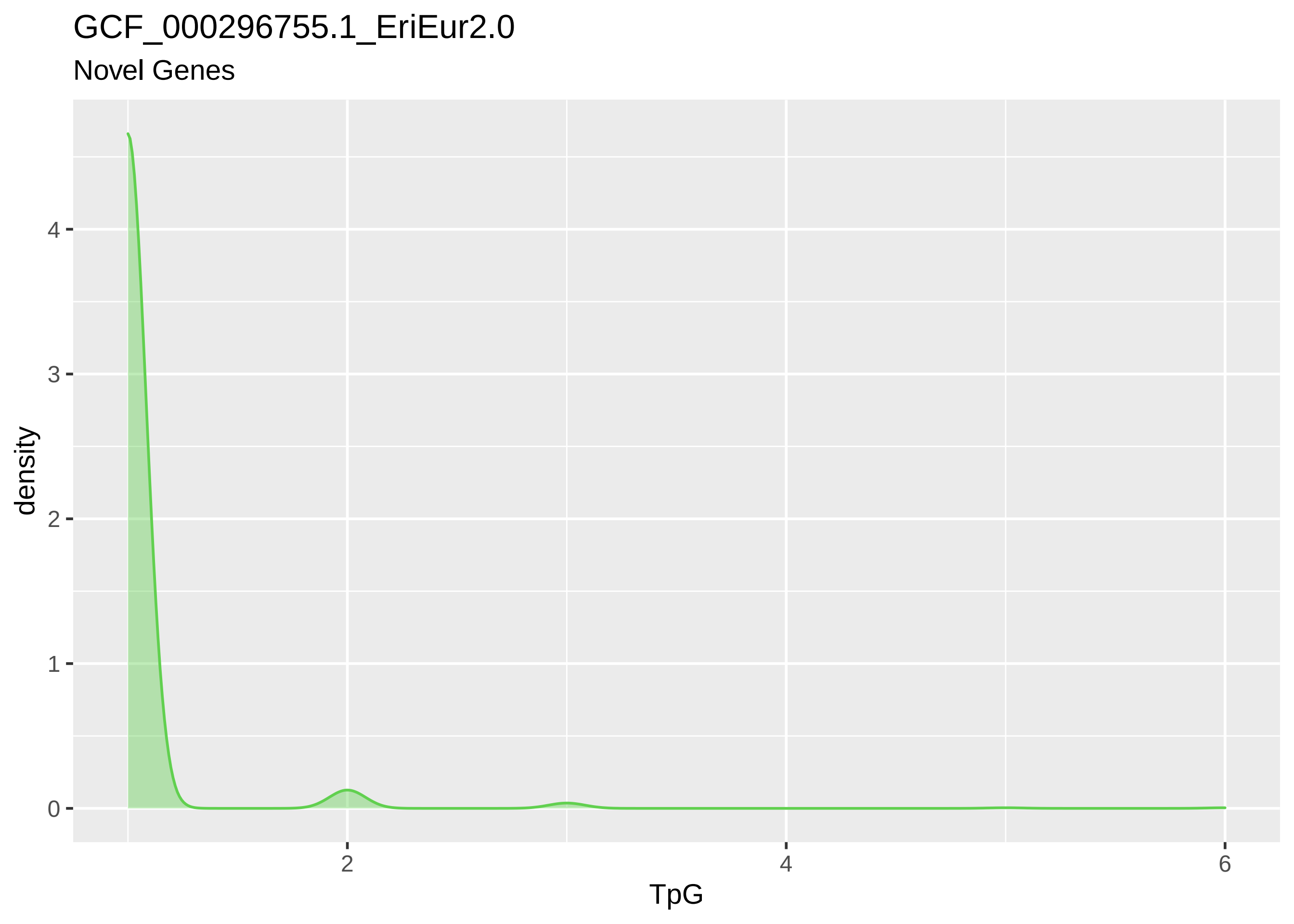
GCF_000296755.1_EriEur2.0
Novel Genes
density
TpG
0
1
2
3
4
2
4
6

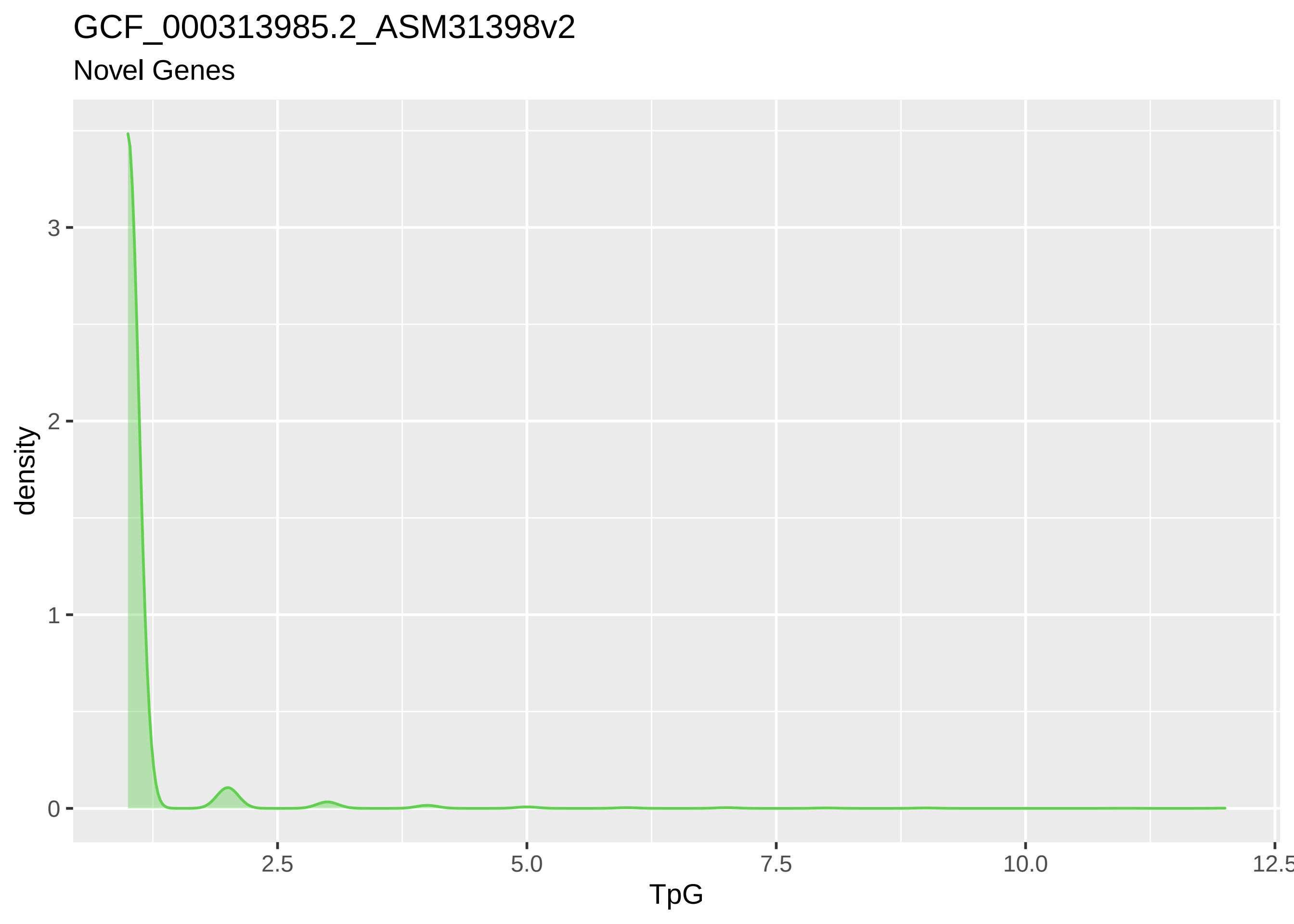
GCF_000313985.2_ASM31398v2
Novel Genes
density
3
2
1
0
2.5
5.0
7.5
10.0
12.5
TpG

GCF_000331955.2_Oorc_1.1

Novel Genes

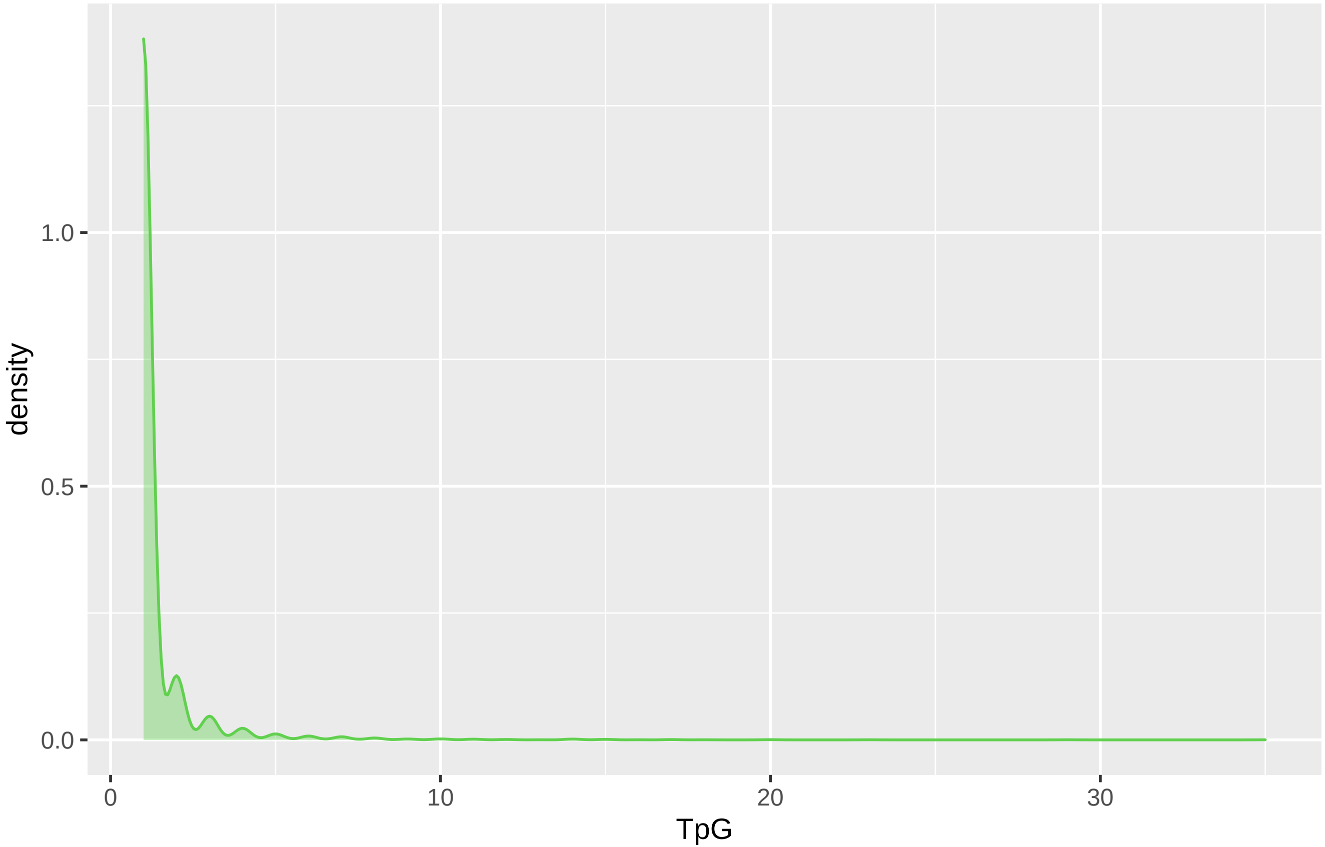

GCF_000334495.1_TupChi_1.0

Novel Genes

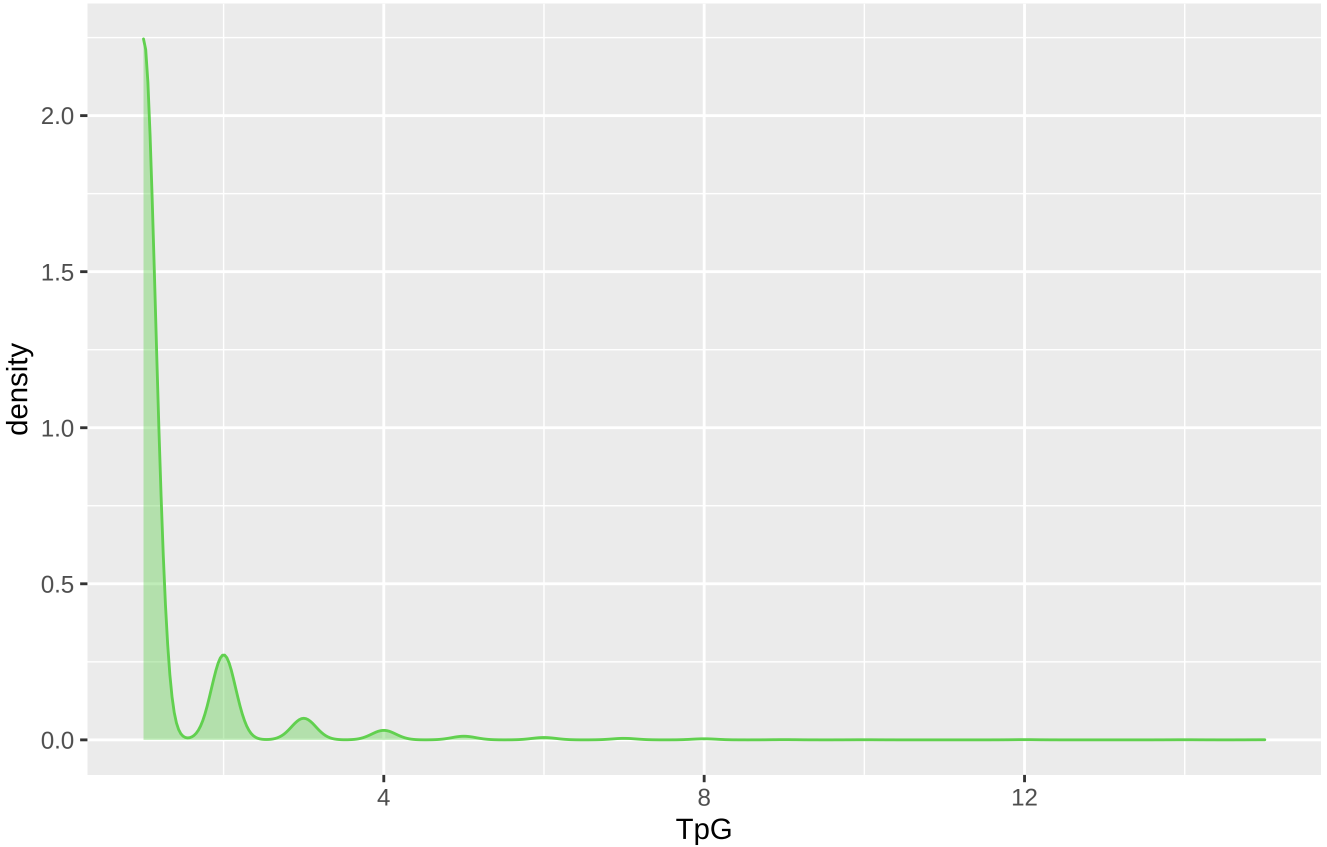

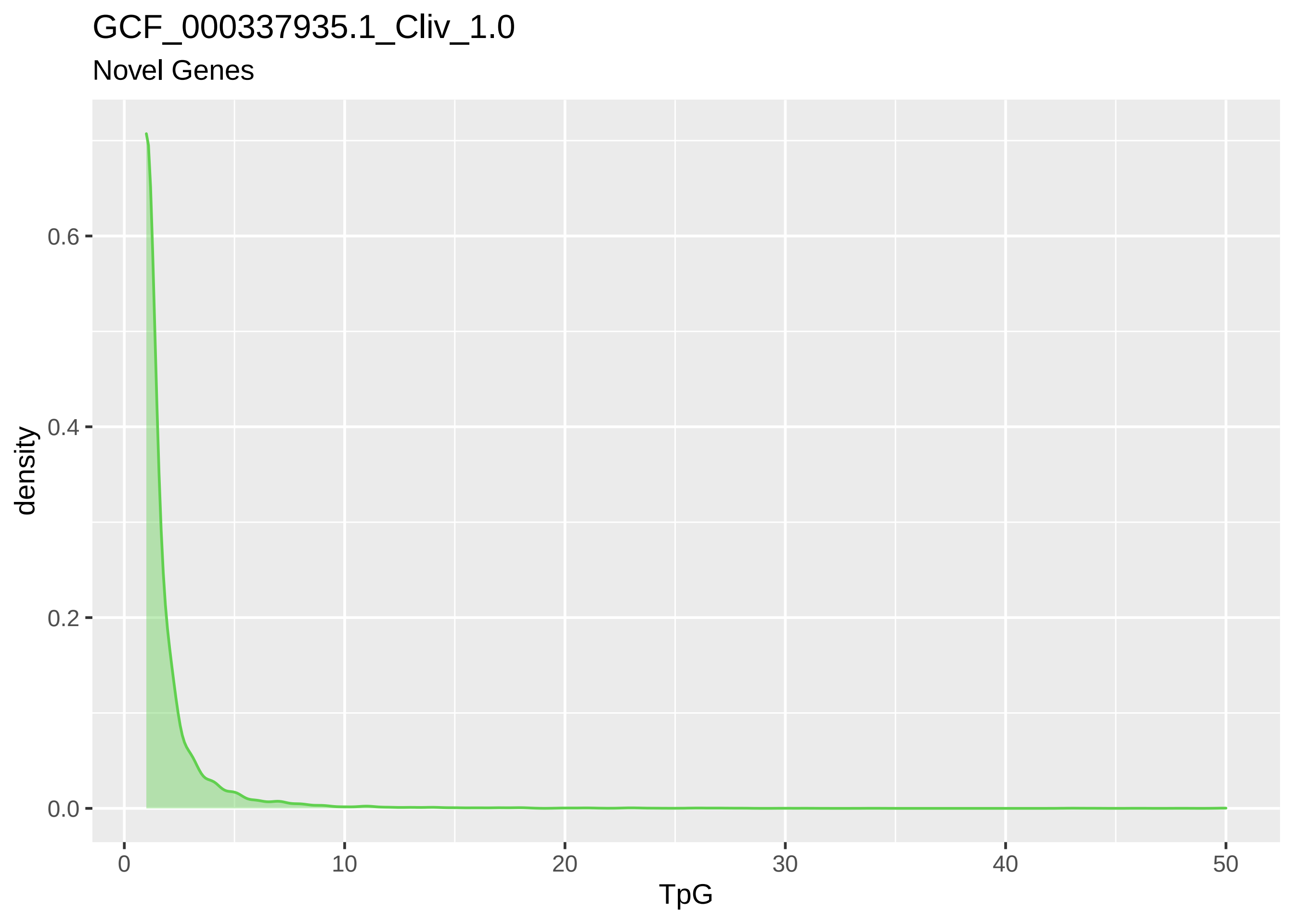
GCF_000337935.1_Cliv_1.0
Novel Genes
density
0.0
0.2
0.4
0.6
0
10
20
30
40
50
TpG

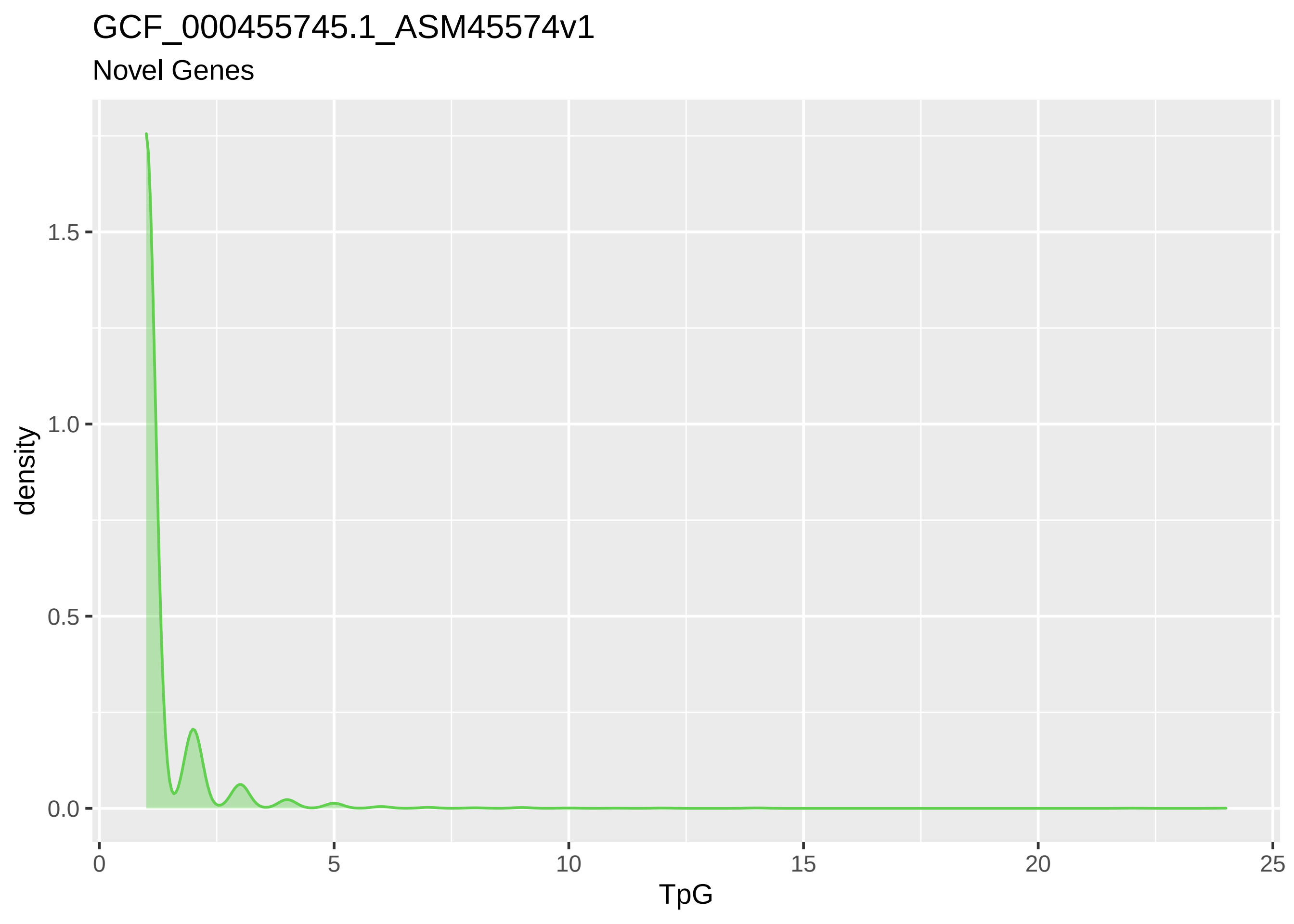
GCF_000455745.1_ASM45574v1
Novel Genes
density
1.5
1.0
0.5
0.0
0
5
10
15
20
25
TpG

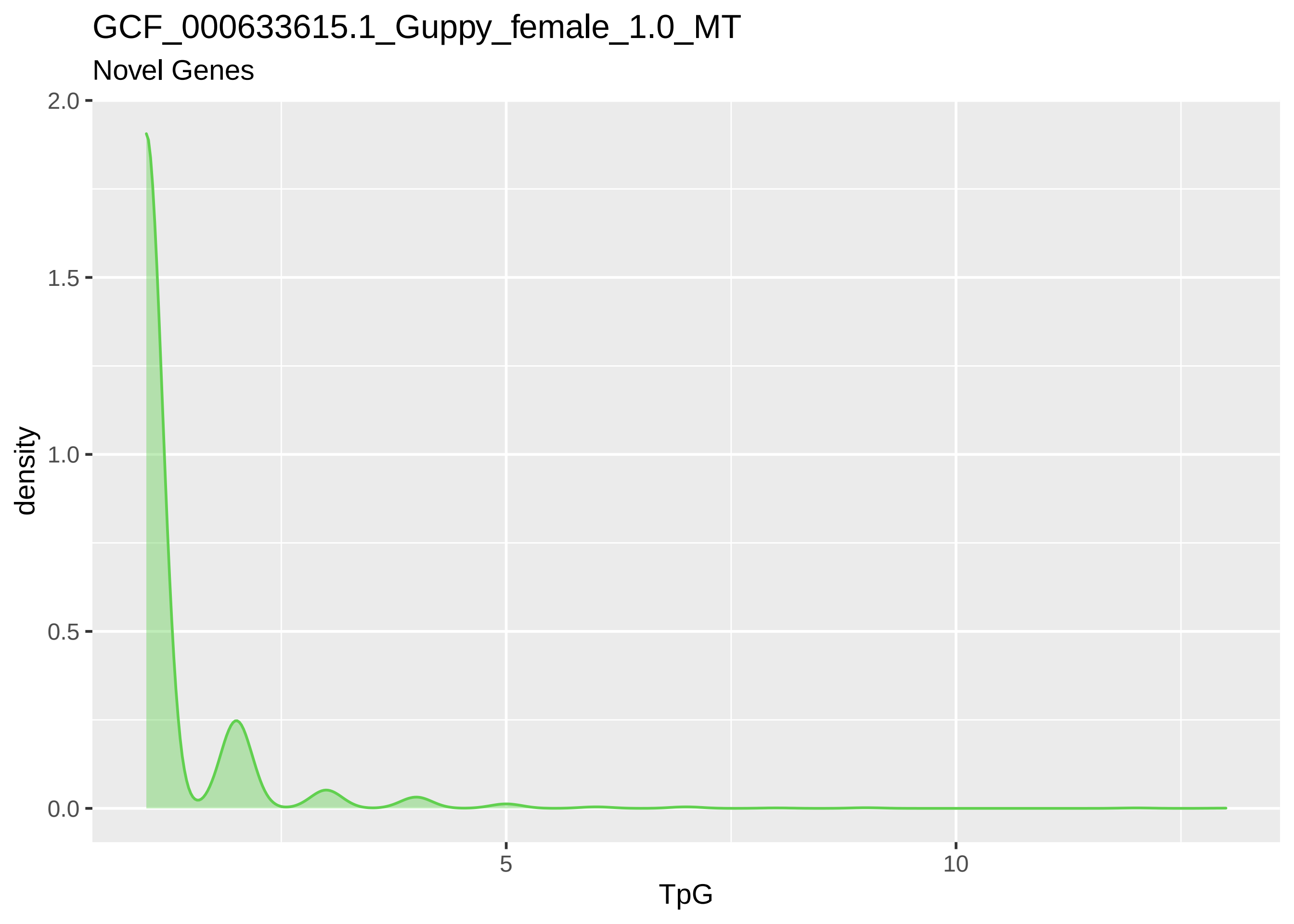

GCF_000633615.1_Guppy_female_1.0_MT
Novel Genes
2.0
1.5
1.0
0.5
0.0
density
5
10
TpG

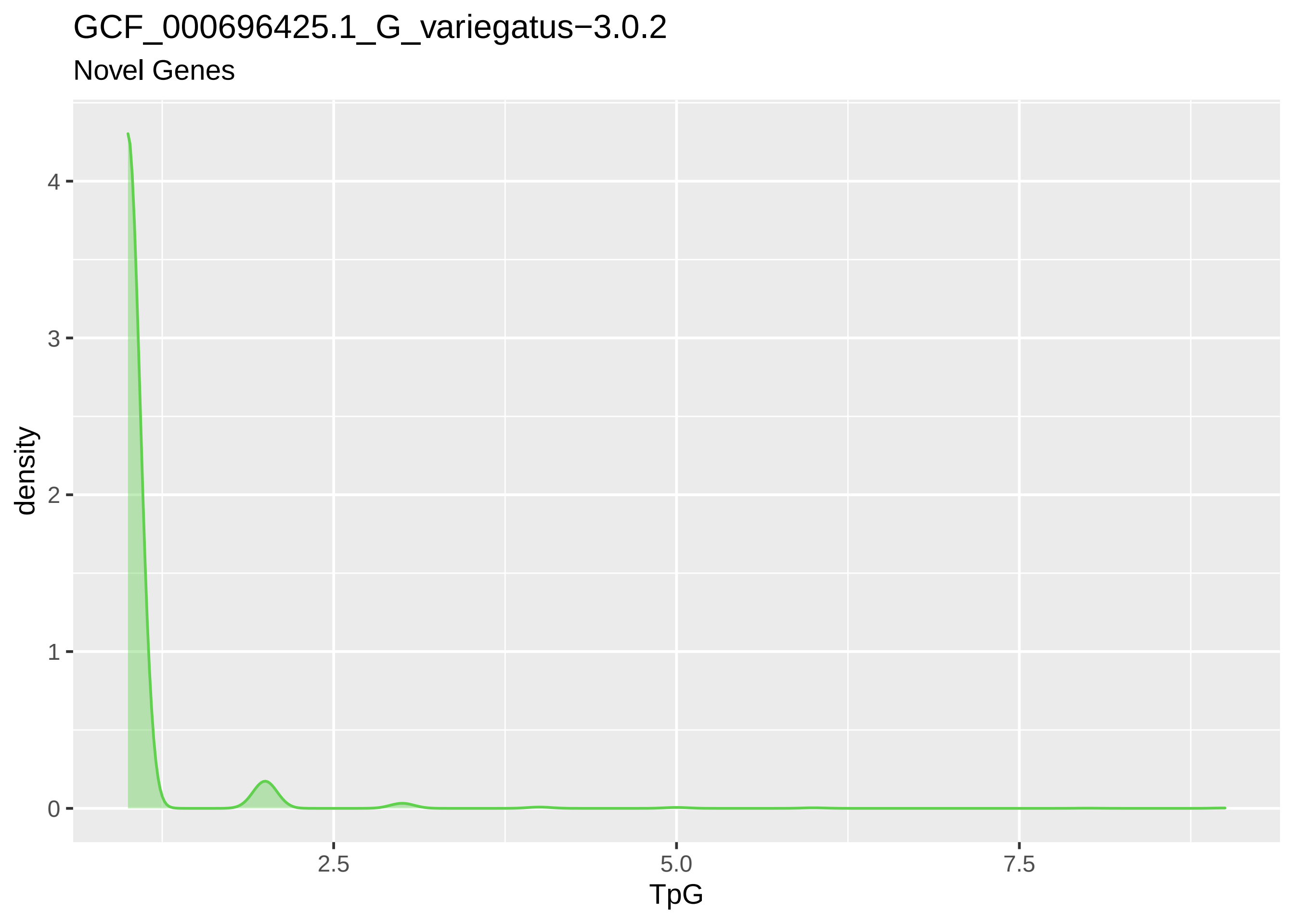
GCF_000696425.1_G_variegatus−3.0.2
Novel Genes
density
TpG
0
1
2
3
4
2.5
5.0
7.5

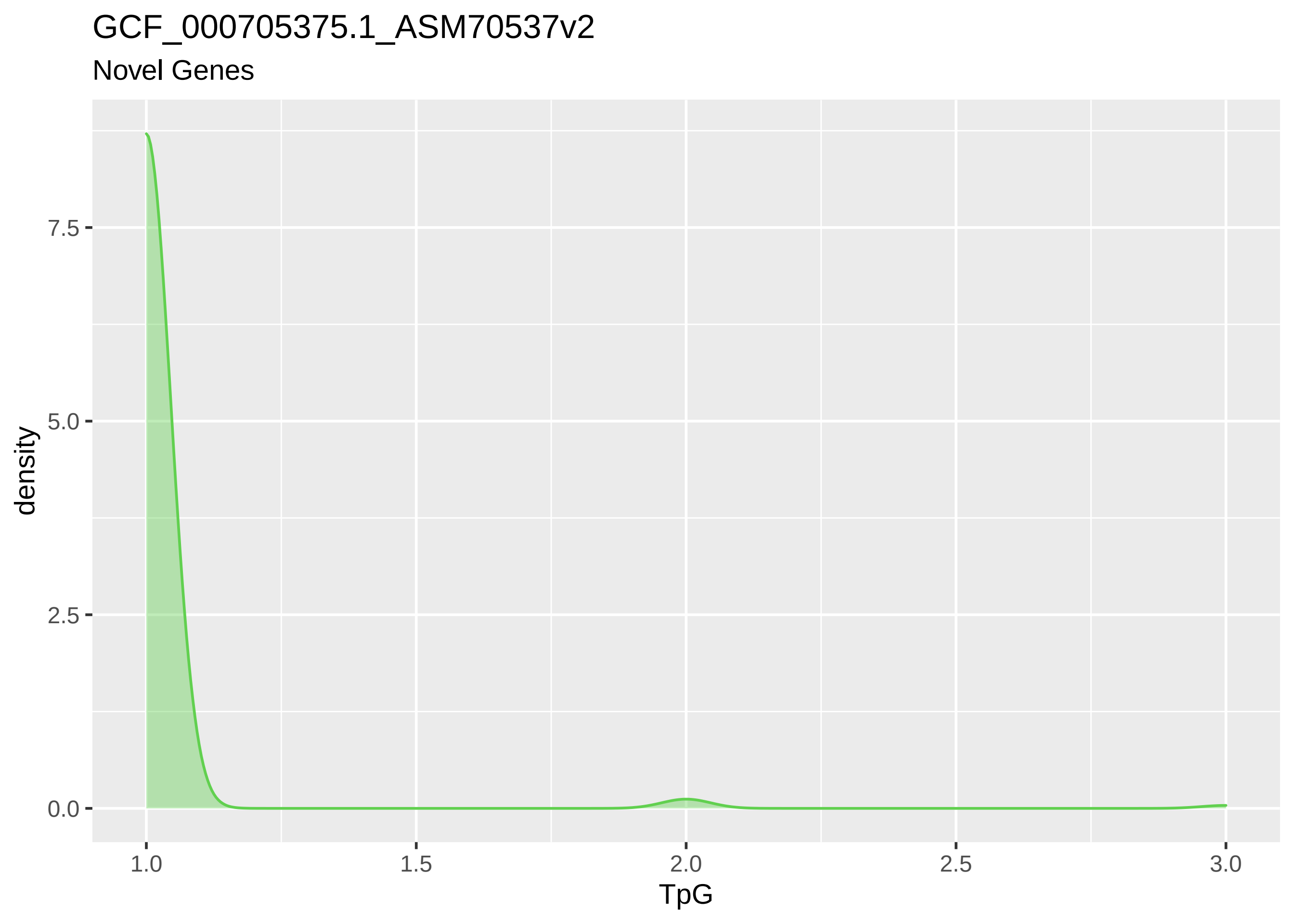
GCF_000705375.1_ASM70537v2
Novel Genes
density
7.5
5.0
2.5
0.0
1.0
1.5
2.0
2.5
3.0
TpG

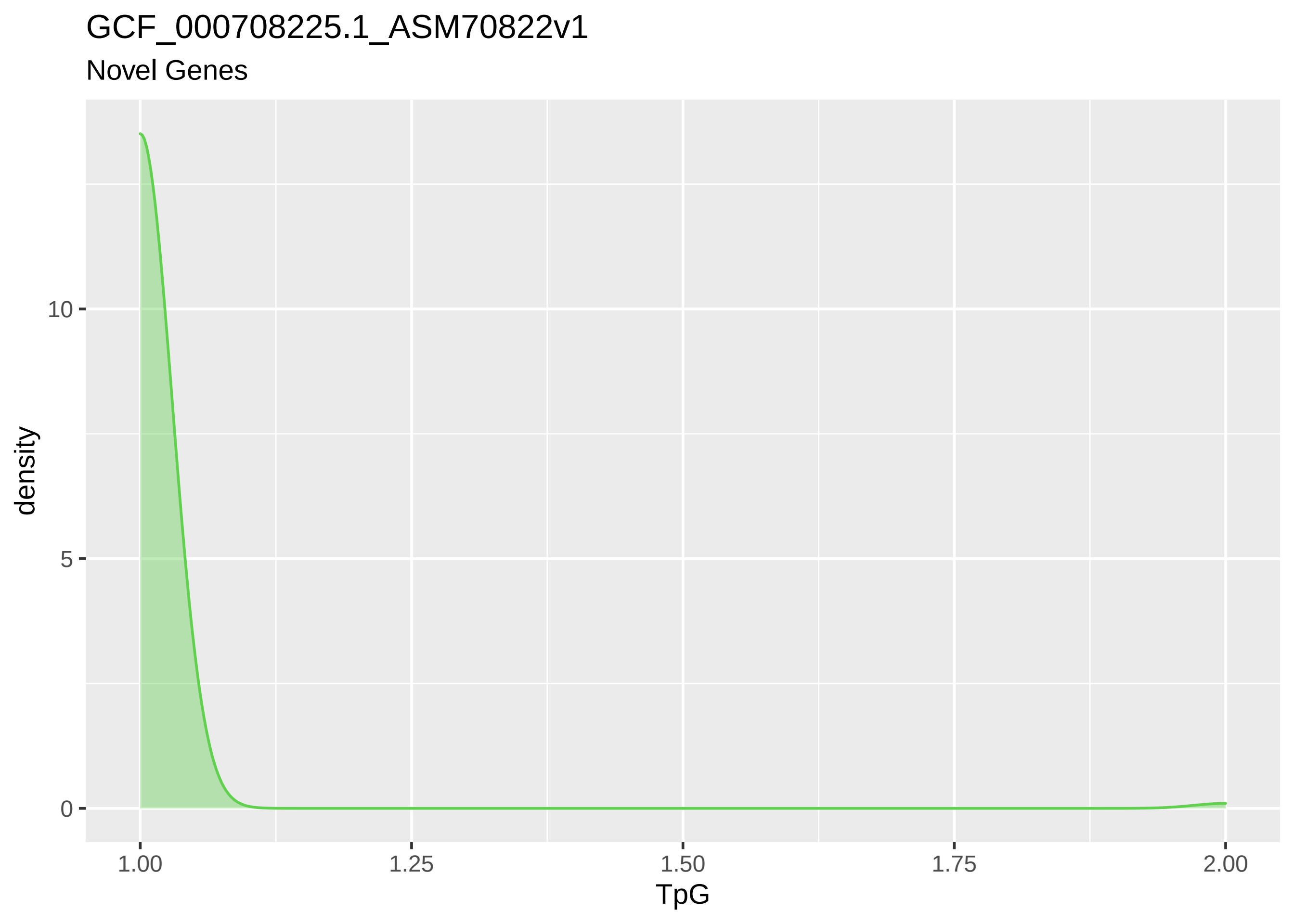

GCF_000708225.1_ASM70822v1
Novel Genes
density
10
5
0
1.00
1.25
1.50
1.75
2.00
TpG

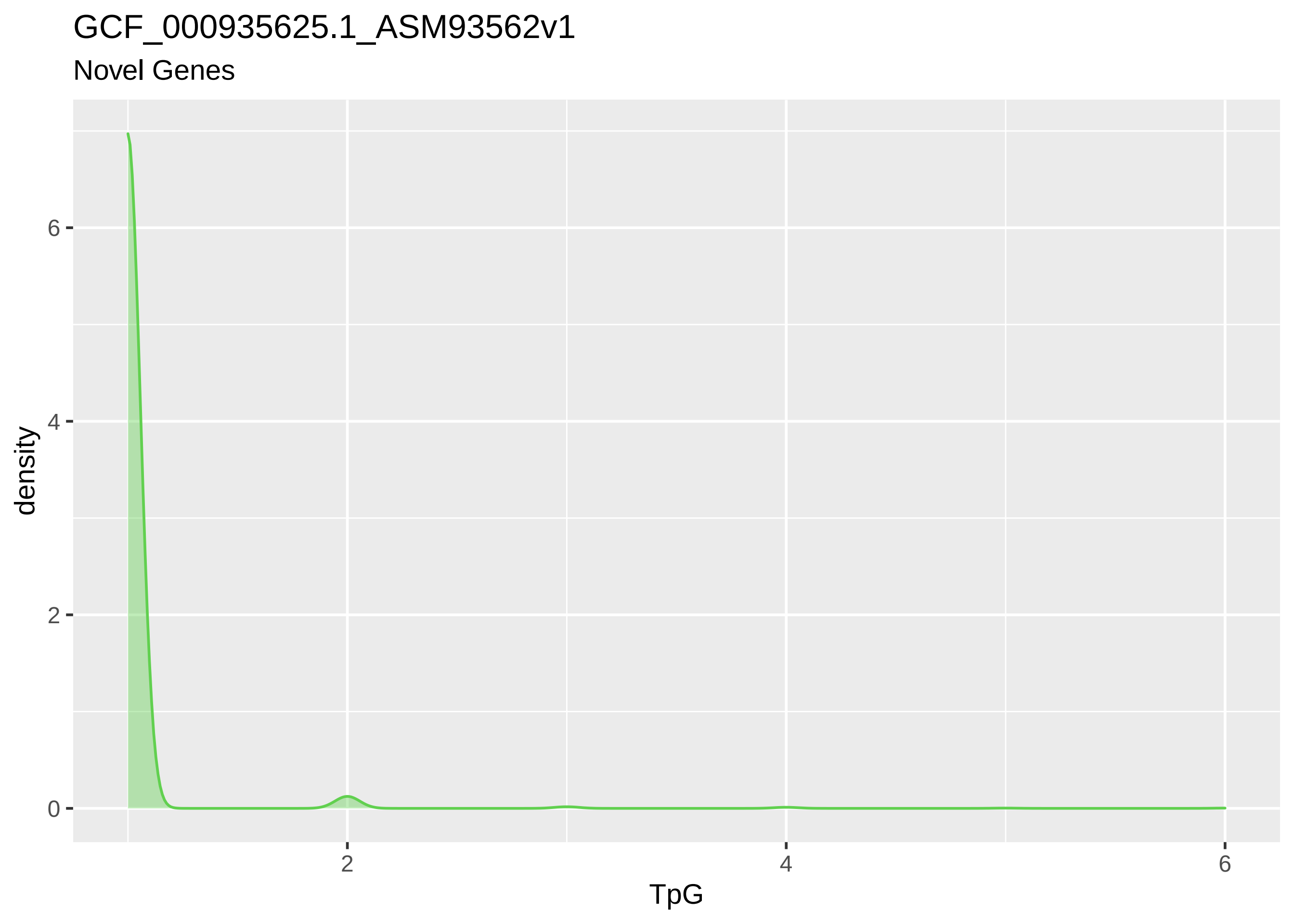
GCF_000935625.1_ASM93562v1
Novel Genes
density
6
4
2
0
2
4
6
TpG

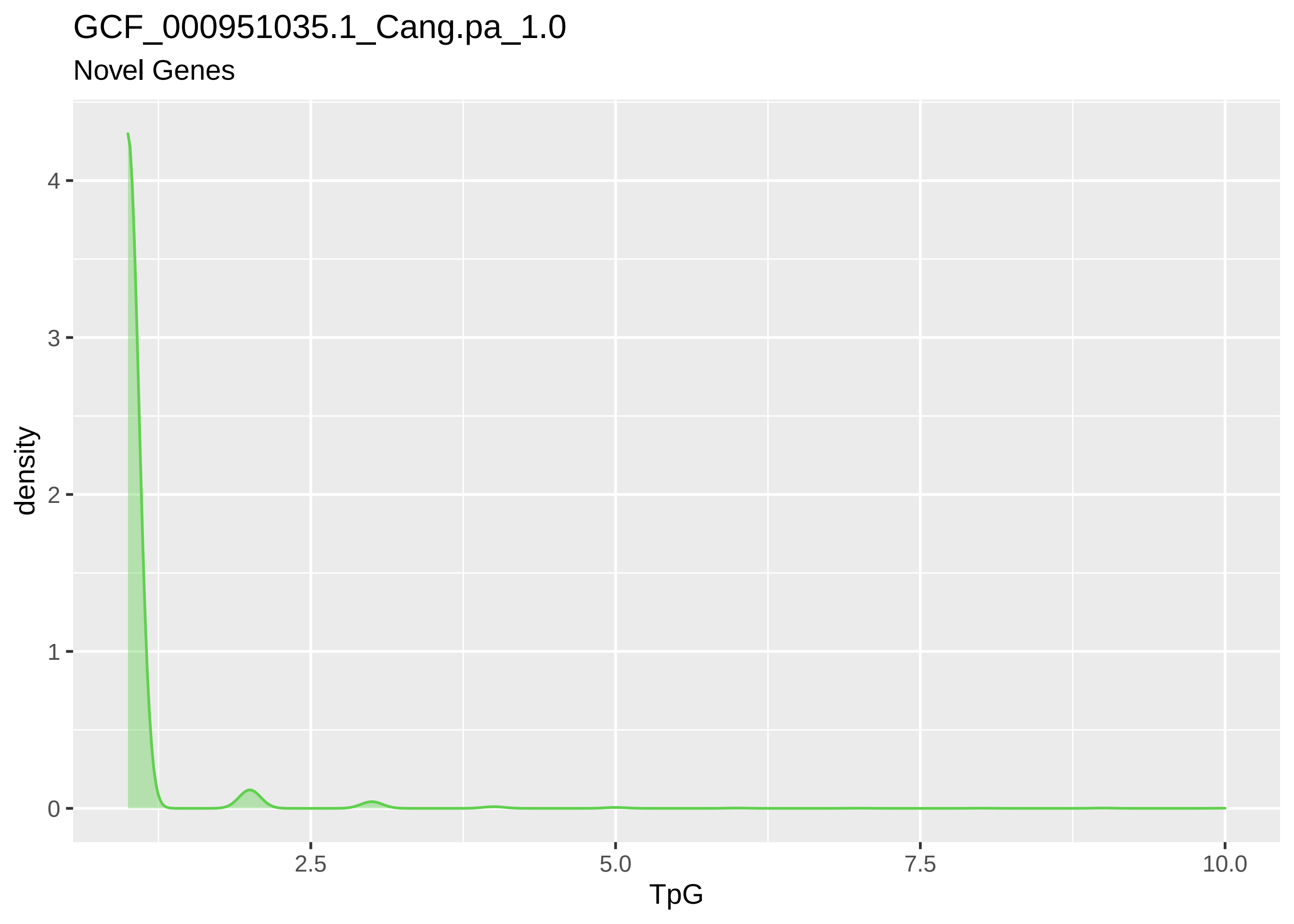
GCF_000951035.1_Cang.pa_1.0
Novel Genes
density
TpG
0
1
2
3
4
2.5
5.0
7.5
10.0

GCF_000951045.1_Mleu.le_1.0

Novel Genes

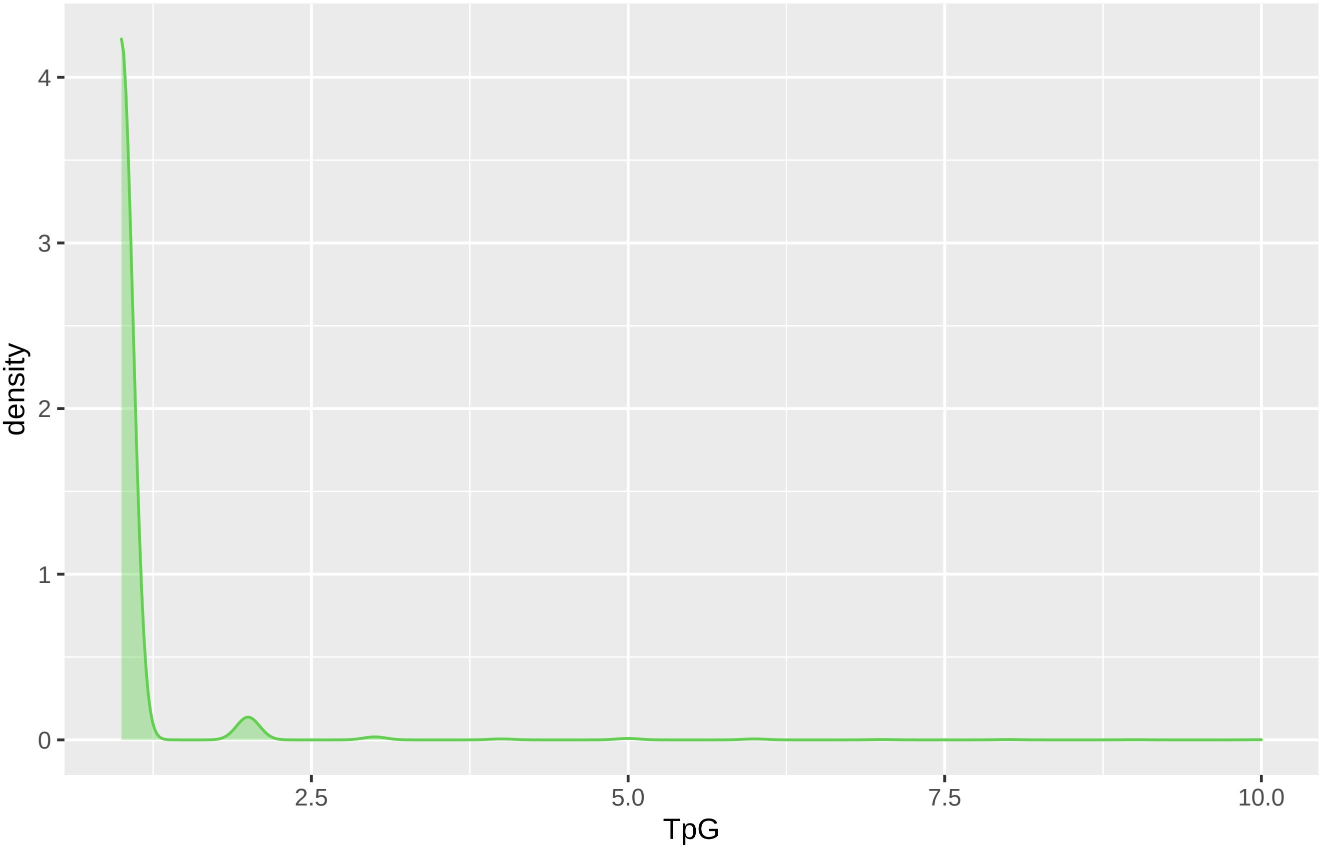

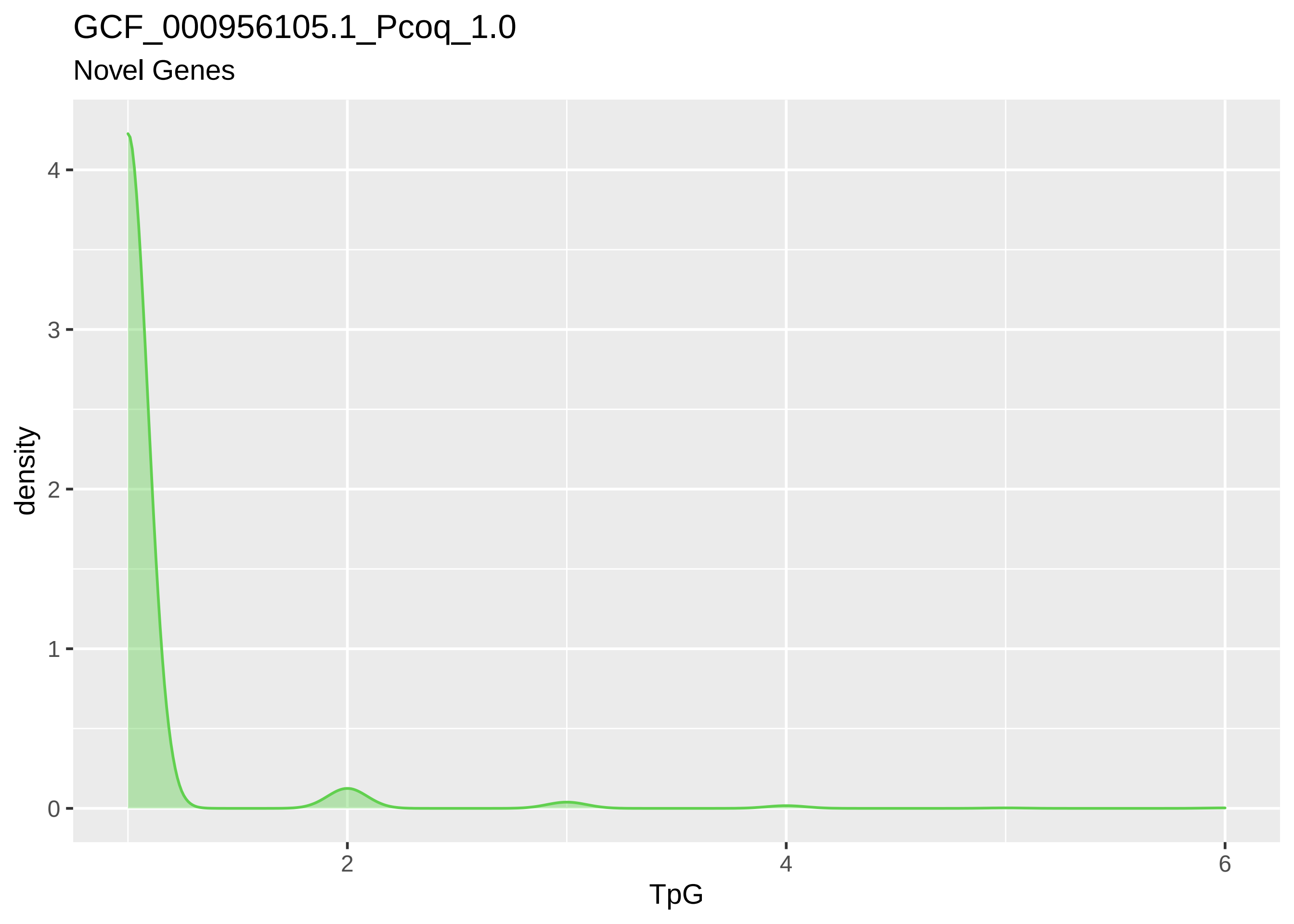
GCF_000956105.1_Pcoq_1.0
Novel Genes
density
4
3
2
1
0
2
4
6
TpG

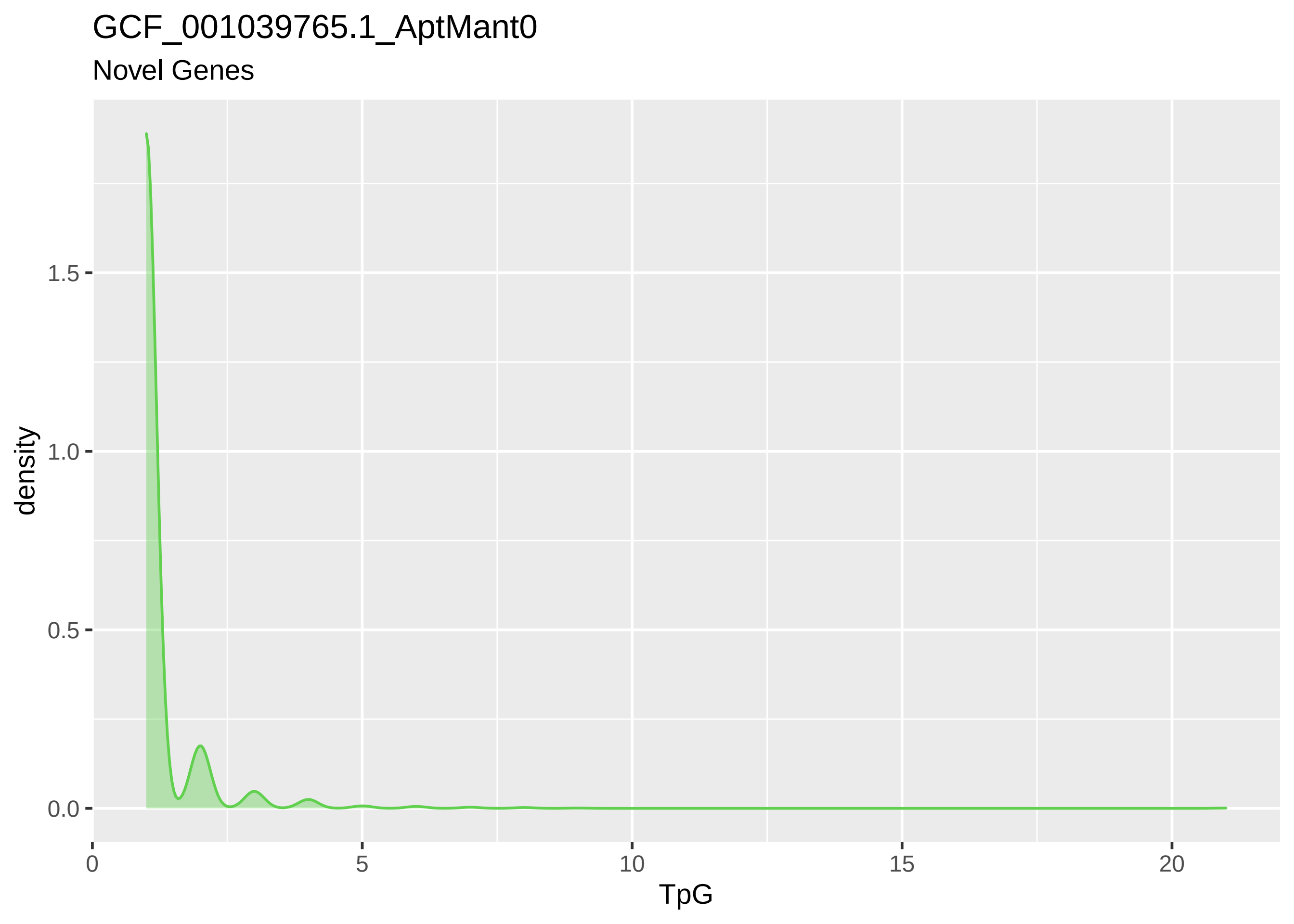
GCF_001039765.1_AptMant0
Novel Genes
density
1.5
1.0
0.5
0.0
0
5
10
15
20
TpG

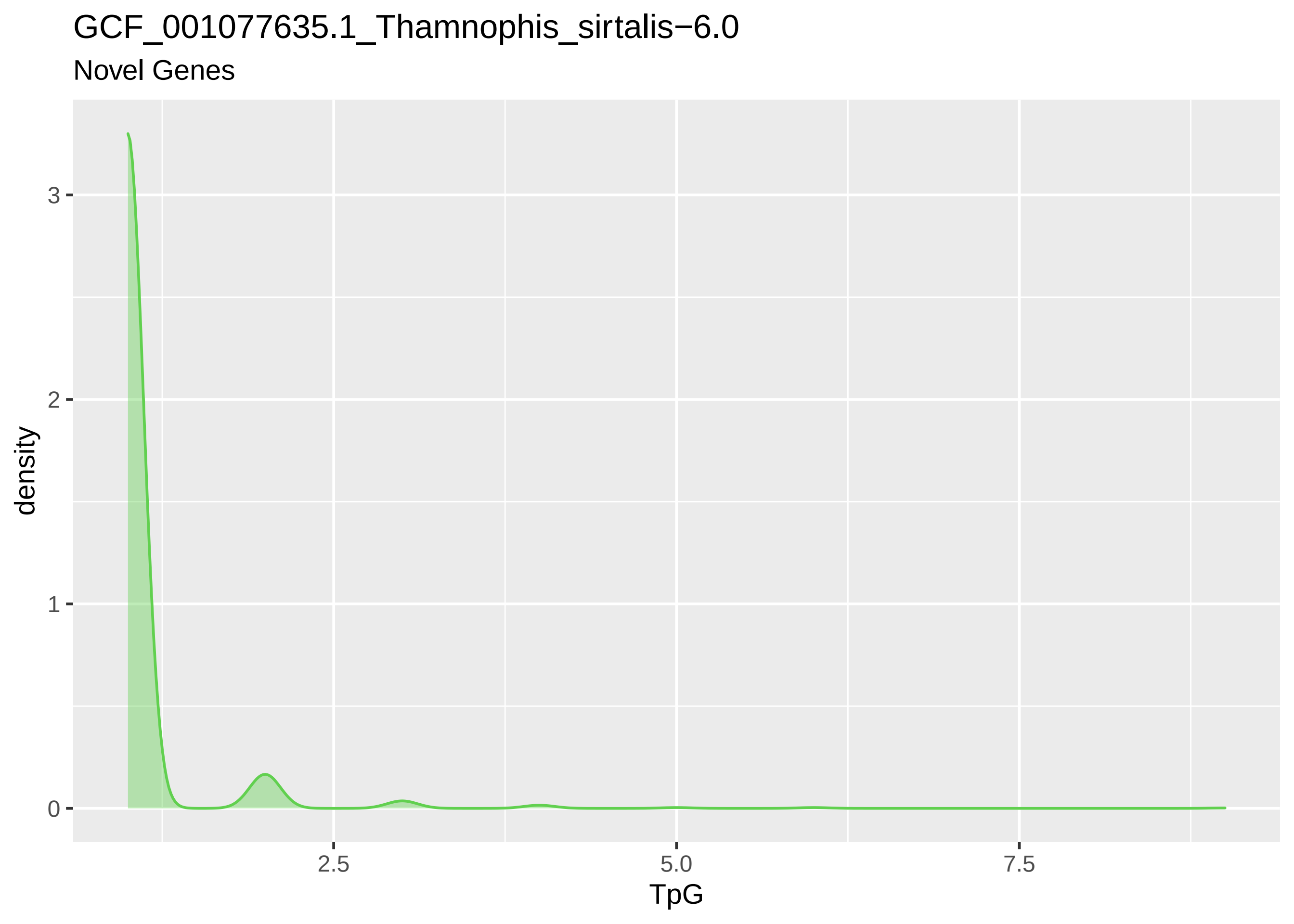

GCF_001077635.1_Thamnophis_sirtalis−6.0
Novel Genes
density
TpG
0
1
2
3
2.5
5.0
7.5

GCF_001447785.1_Gekko_japonicus_V1.1

Novel Genes

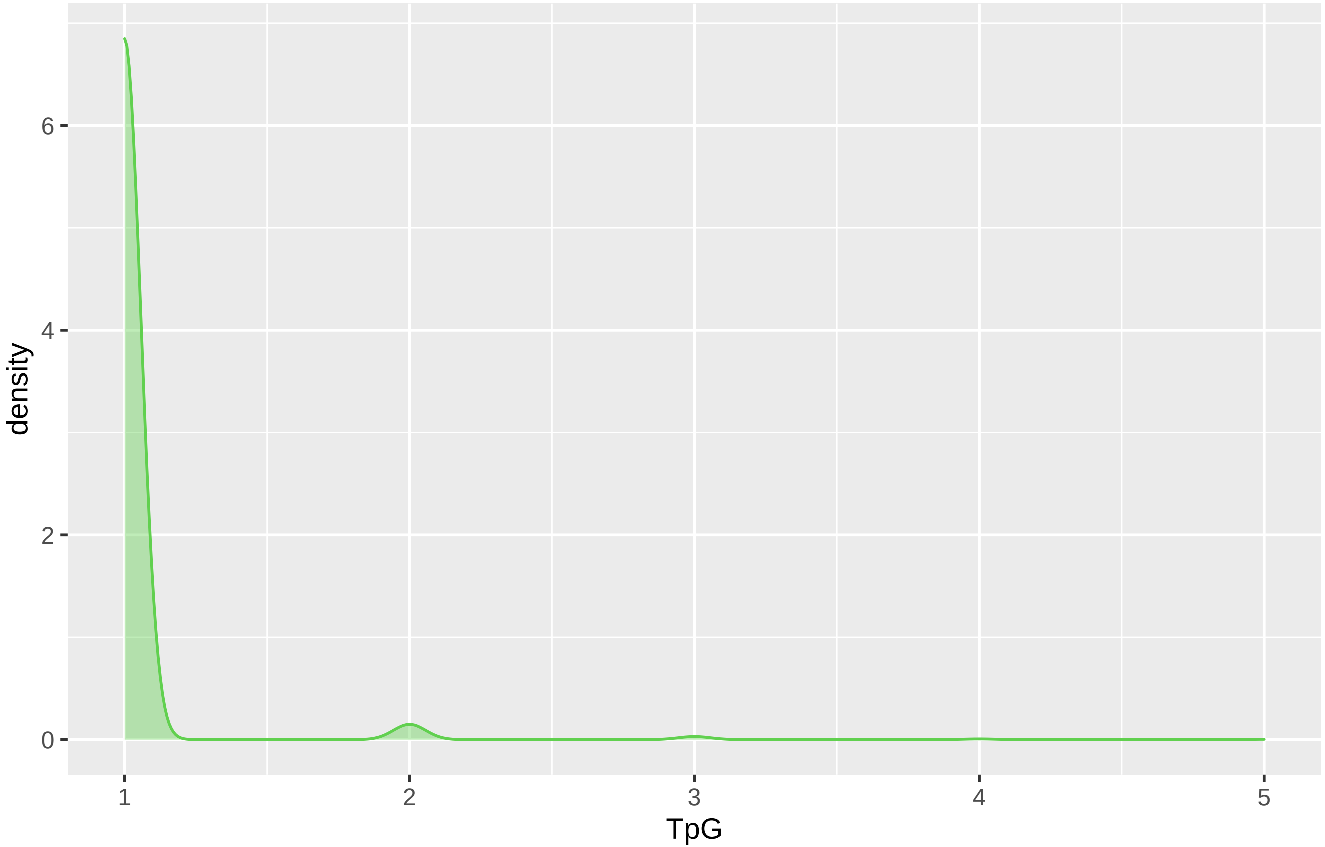

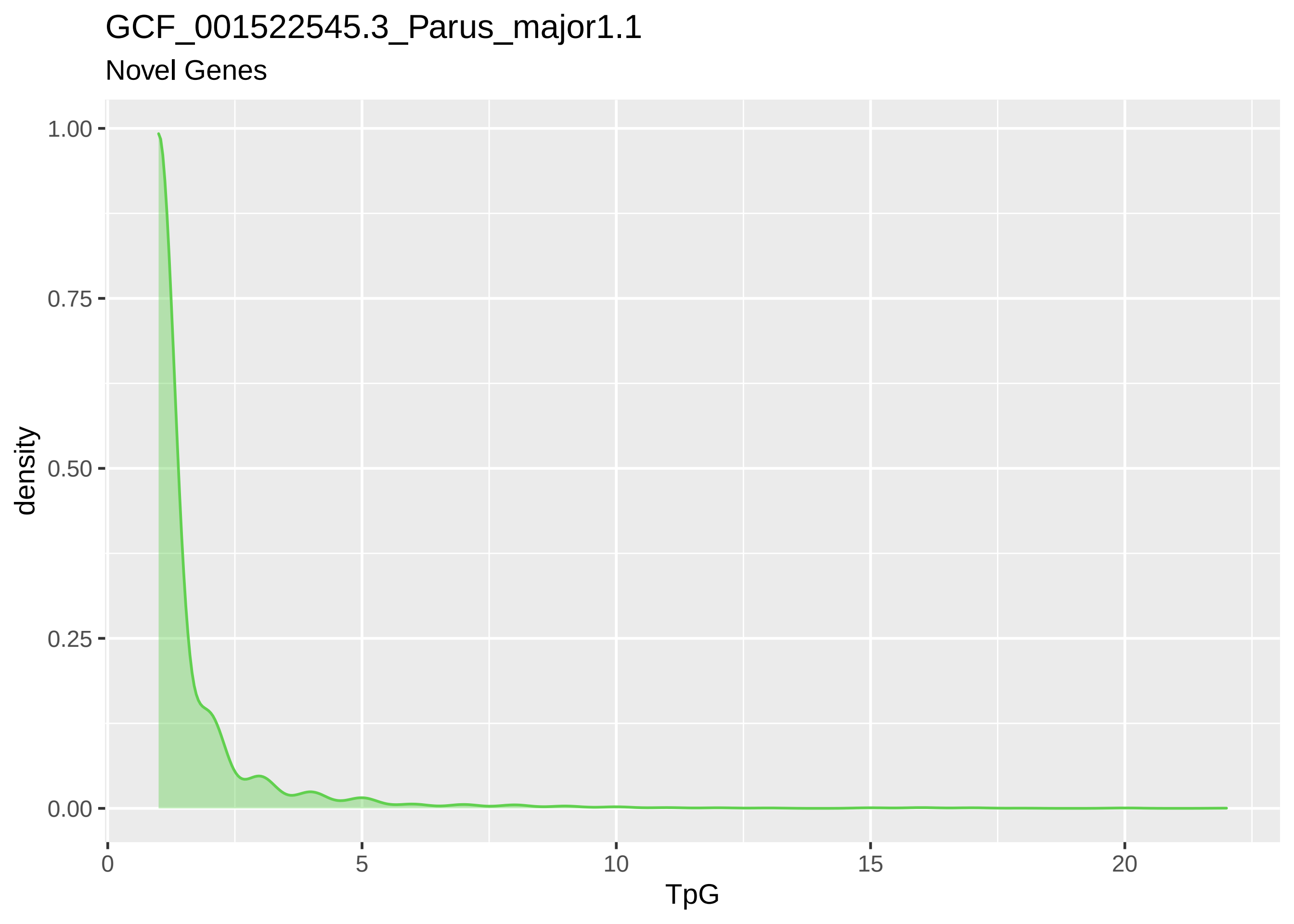
GCF_001522545.3_Parus_major1.1
Novel Genes
density
1.00
0.75
0.50
0.25
0.00
0
5
10
15
20
TpG

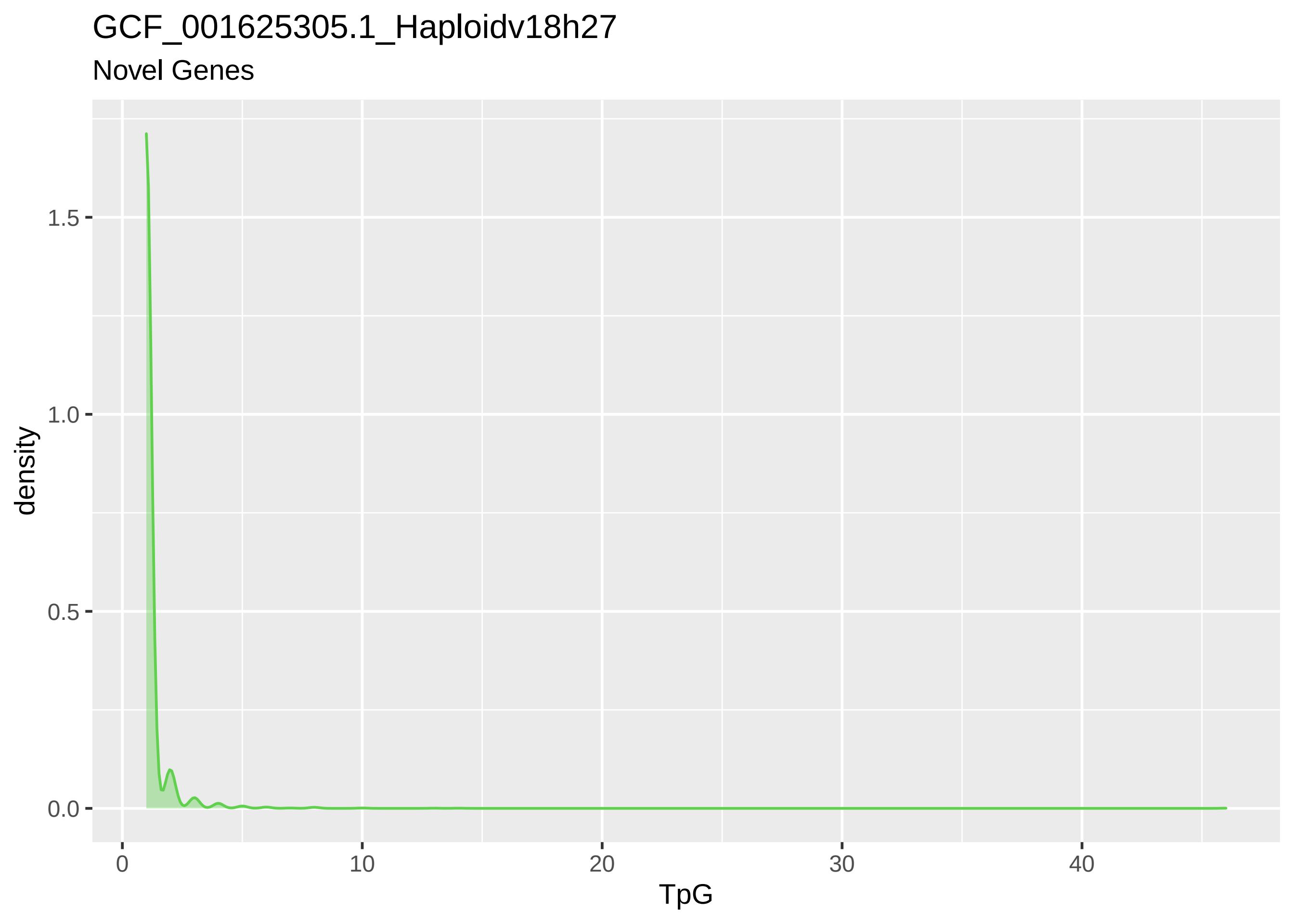
GCF_001625305.1_Haploidv18h27
Novel Genes
density
TpG
1.5
1.0
0.5
0.0
0
10
20
30
40

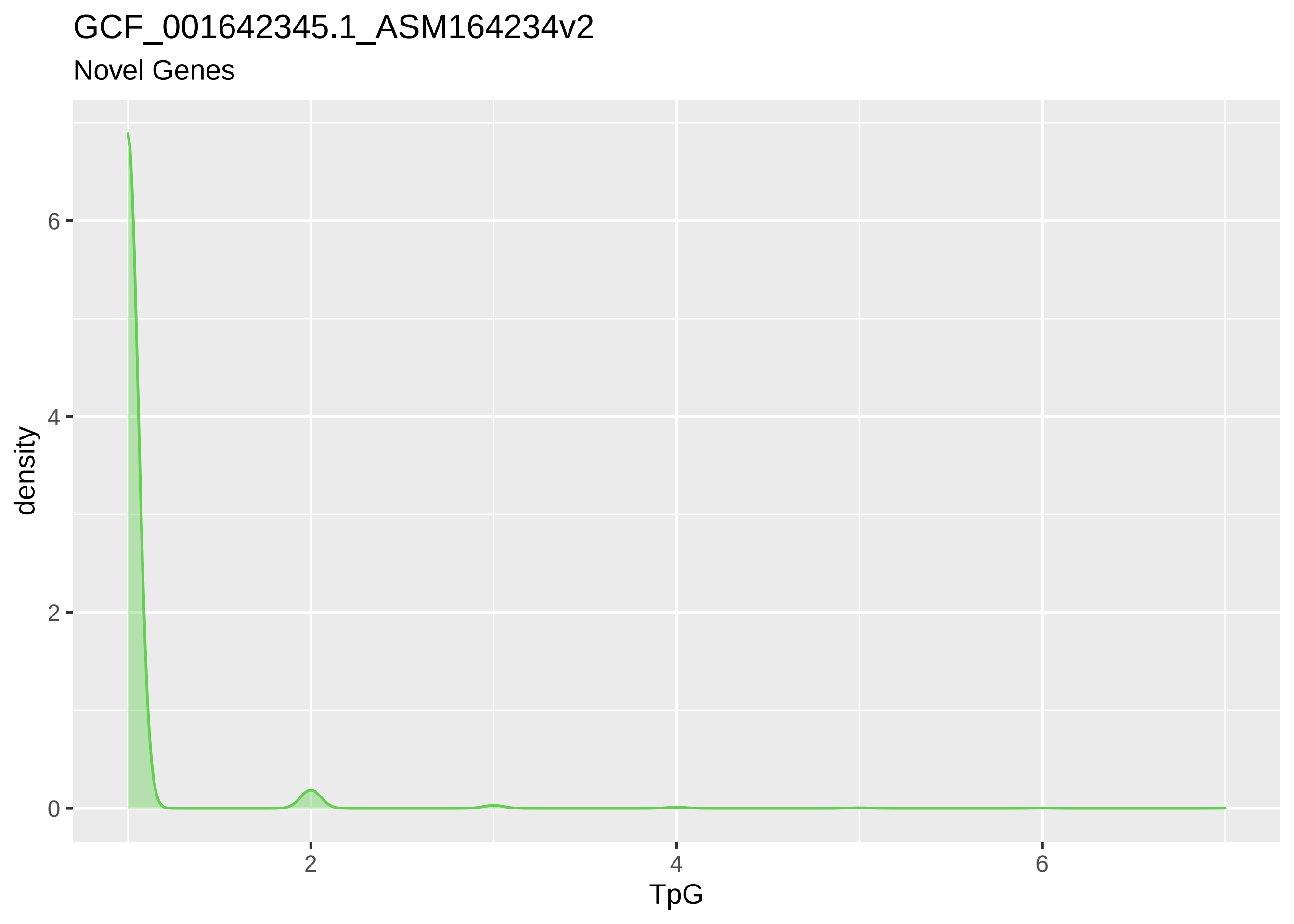
GCF_001642345.1_ASM164234v2
Novel Genes
density
TpG
0
2
4
6
2
4
6

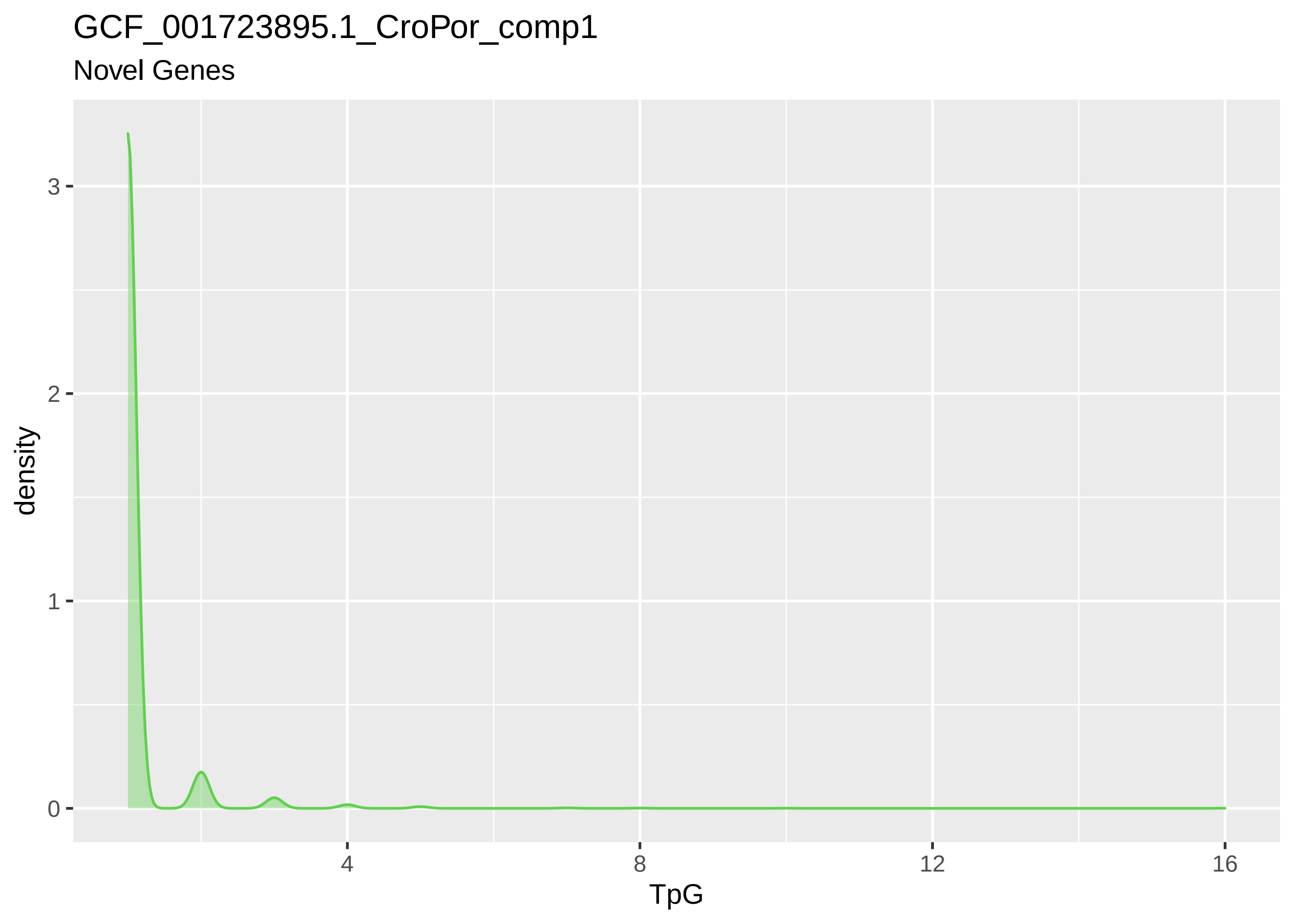
GCF_001723895.1_CroPor_comp1
Novel Genes
density
3
2
1
0
4
8
12
16
TpG

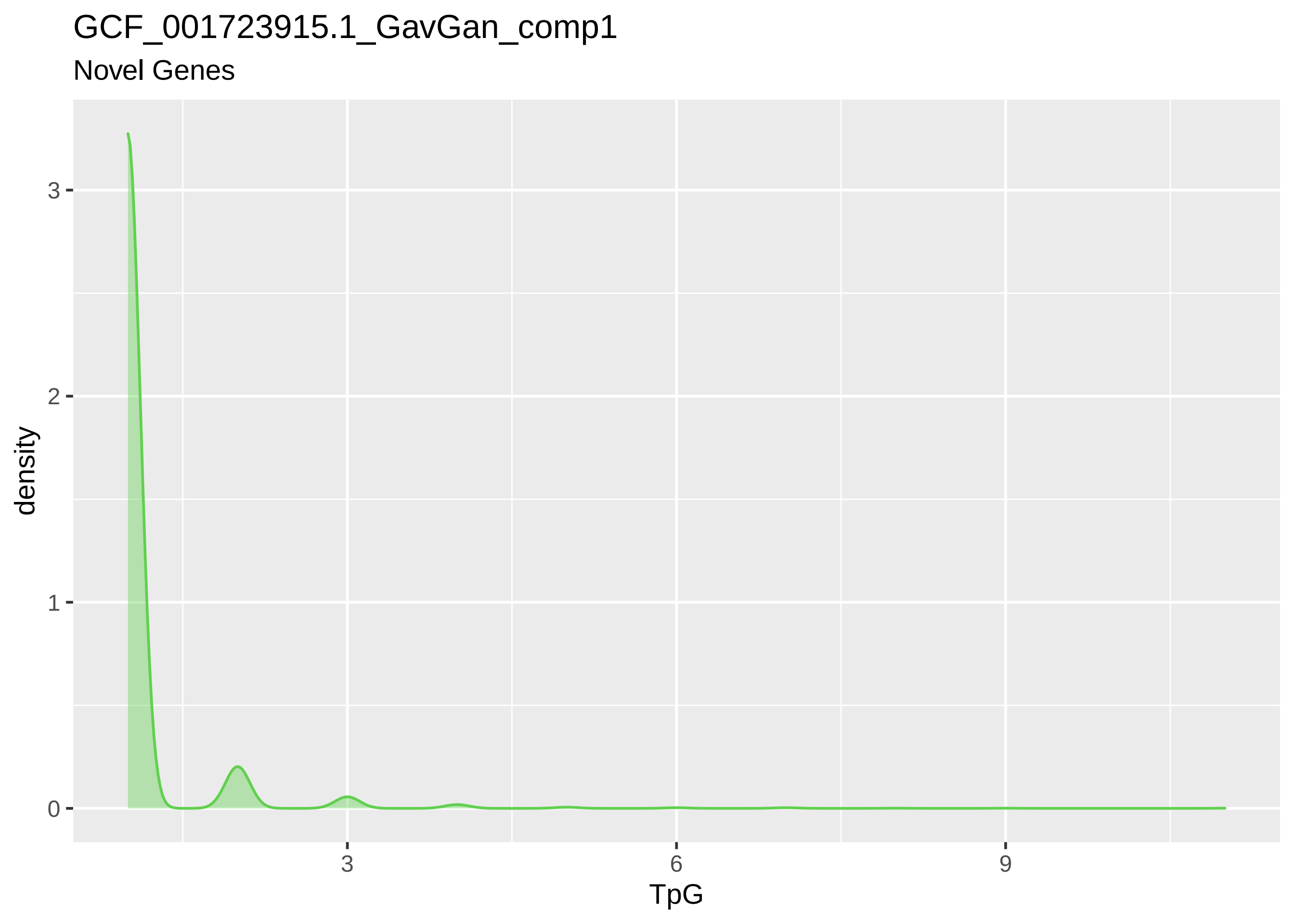

GCF_001723915.1_GavGan_comp1
Novel Genes
density
TpG
0
1
2
3
3
6
9

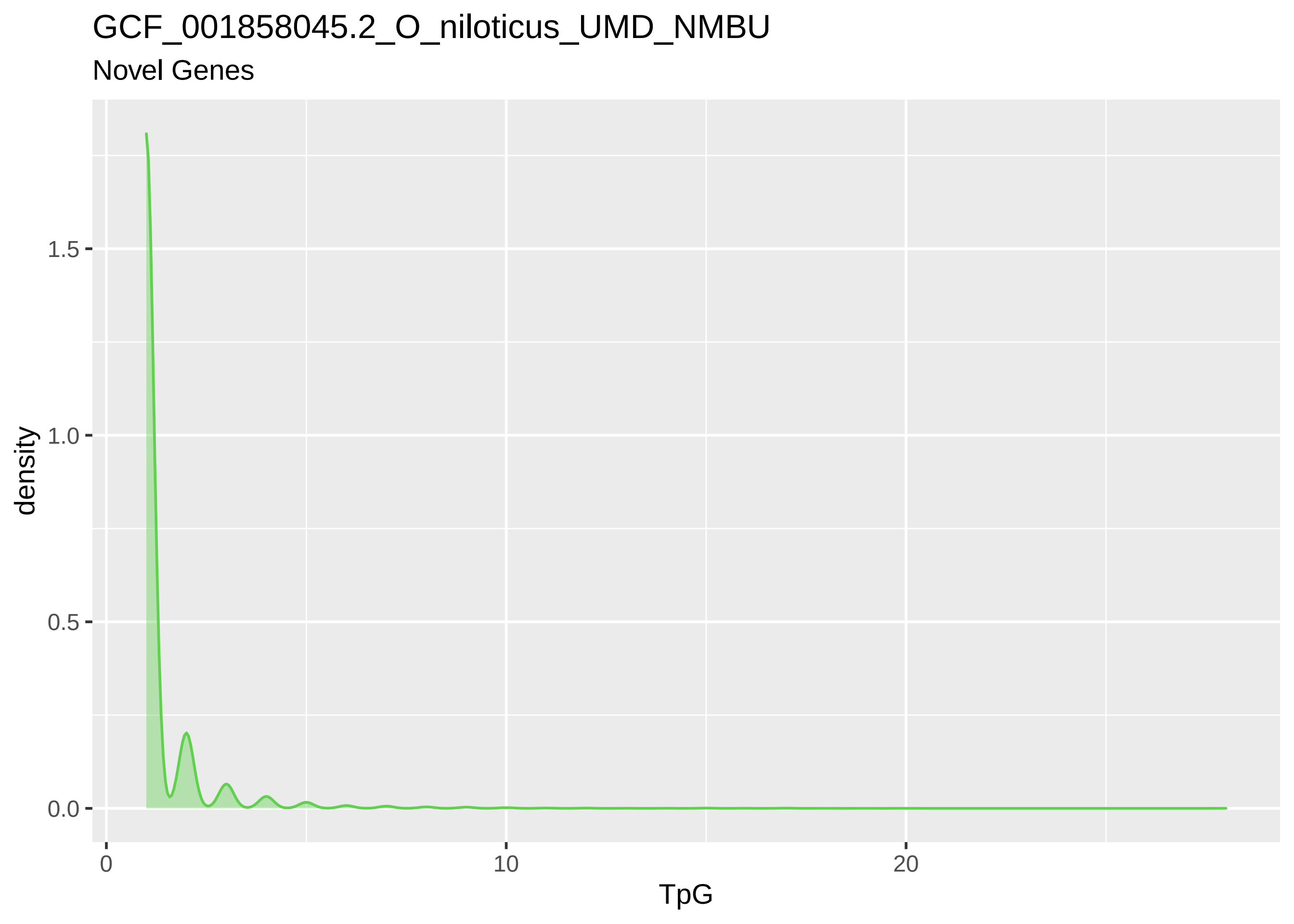

GCF_001858045.2_O_niloticus_UMD_NMBU
Novel Genes
density
1.5
1.0
0.5
0.0
0
10
20
TpG

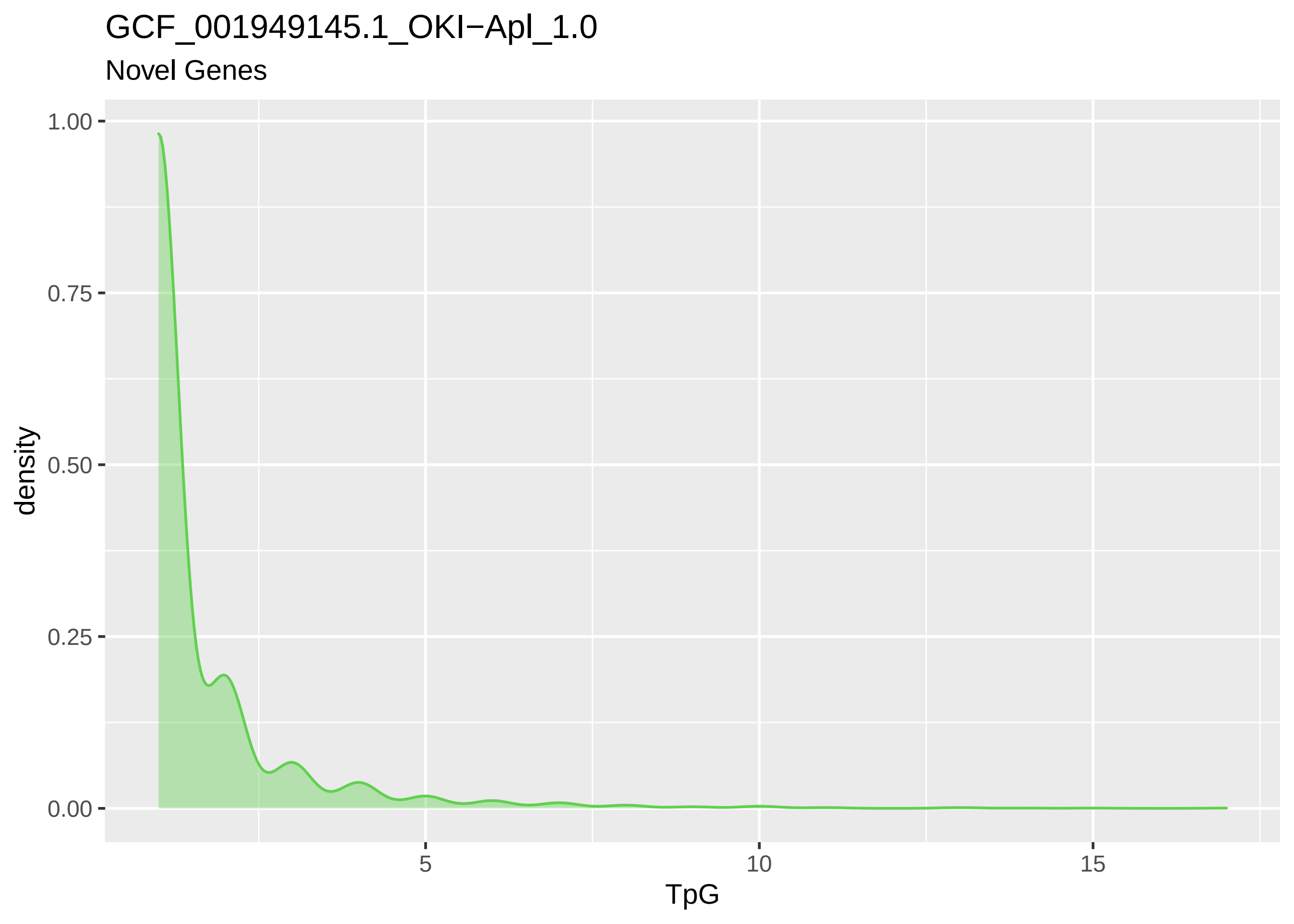
GCF_001949145.1_OKI−Apl_1.0
Novel Genes
density
1.00
0.75
0.50
0.25
0.00
5
10
15
TpG

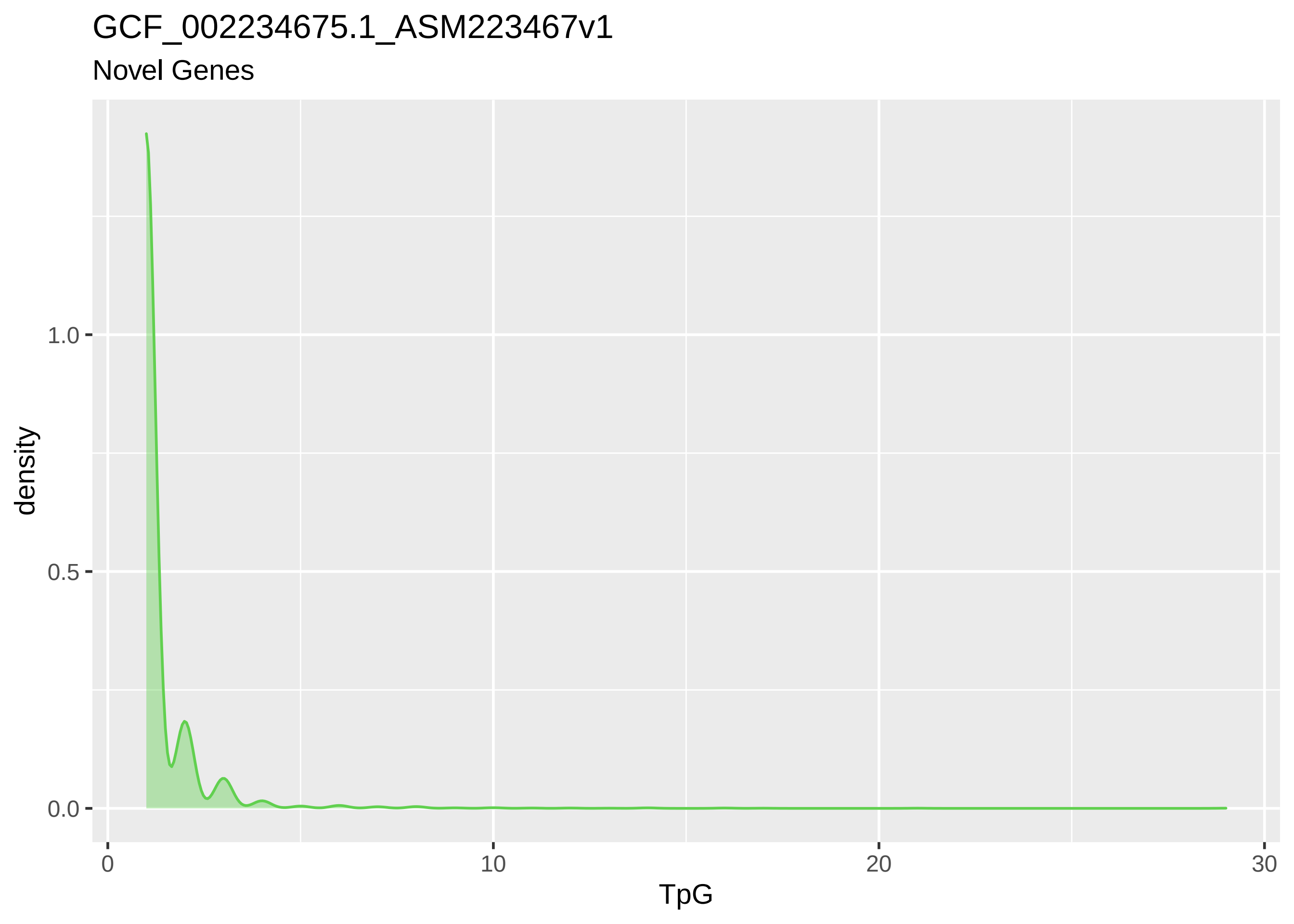
GCF_002234675.1_ASM223467v1
Novel Genes
density
1.0
0.5
0.0
0
10
20
30
TpG

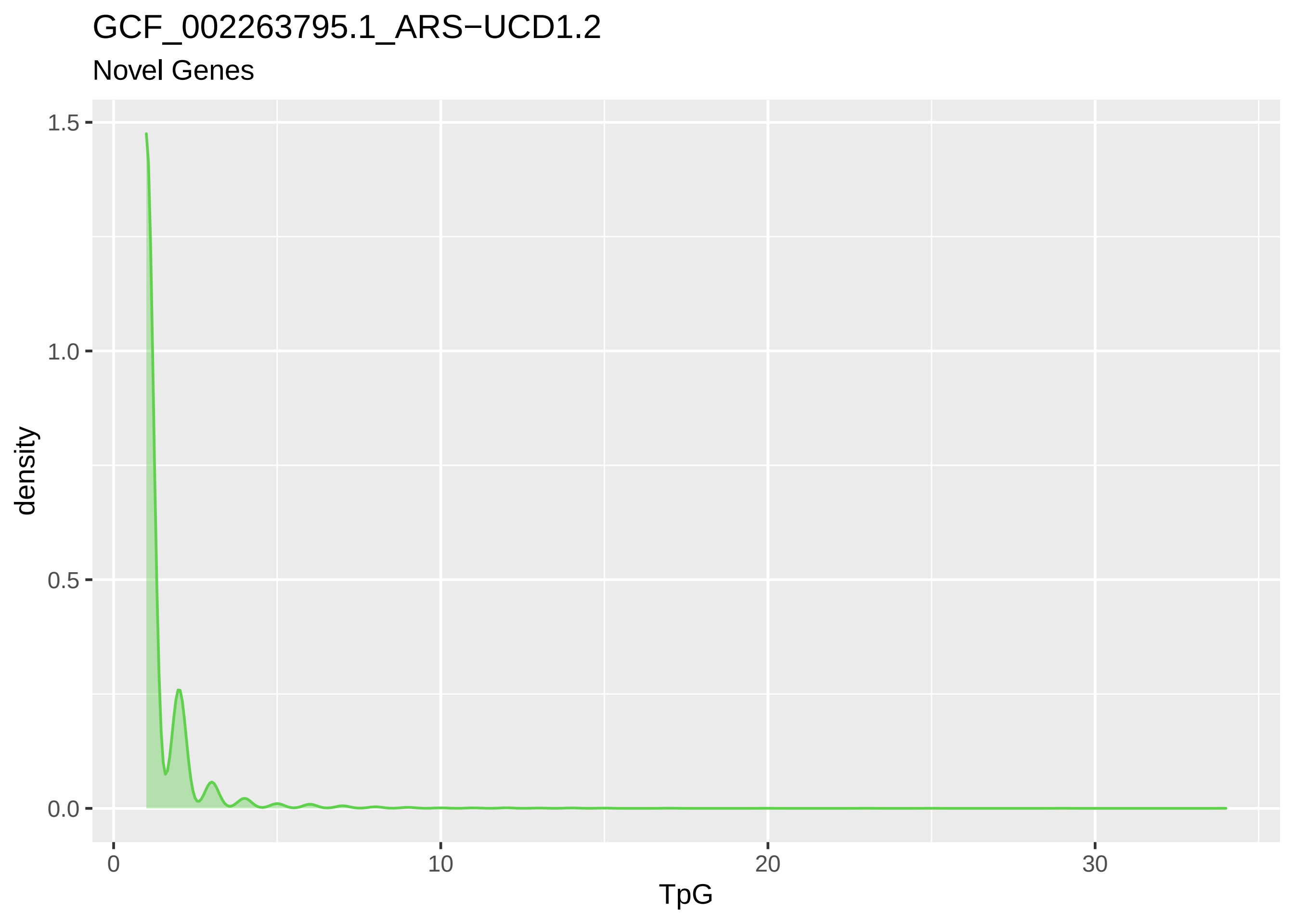
GCF_002263795.1_ARS−UCD1.2
Novel Genes
density
1.5
1.0
0.5
0.0
0
10
20
30
TpG

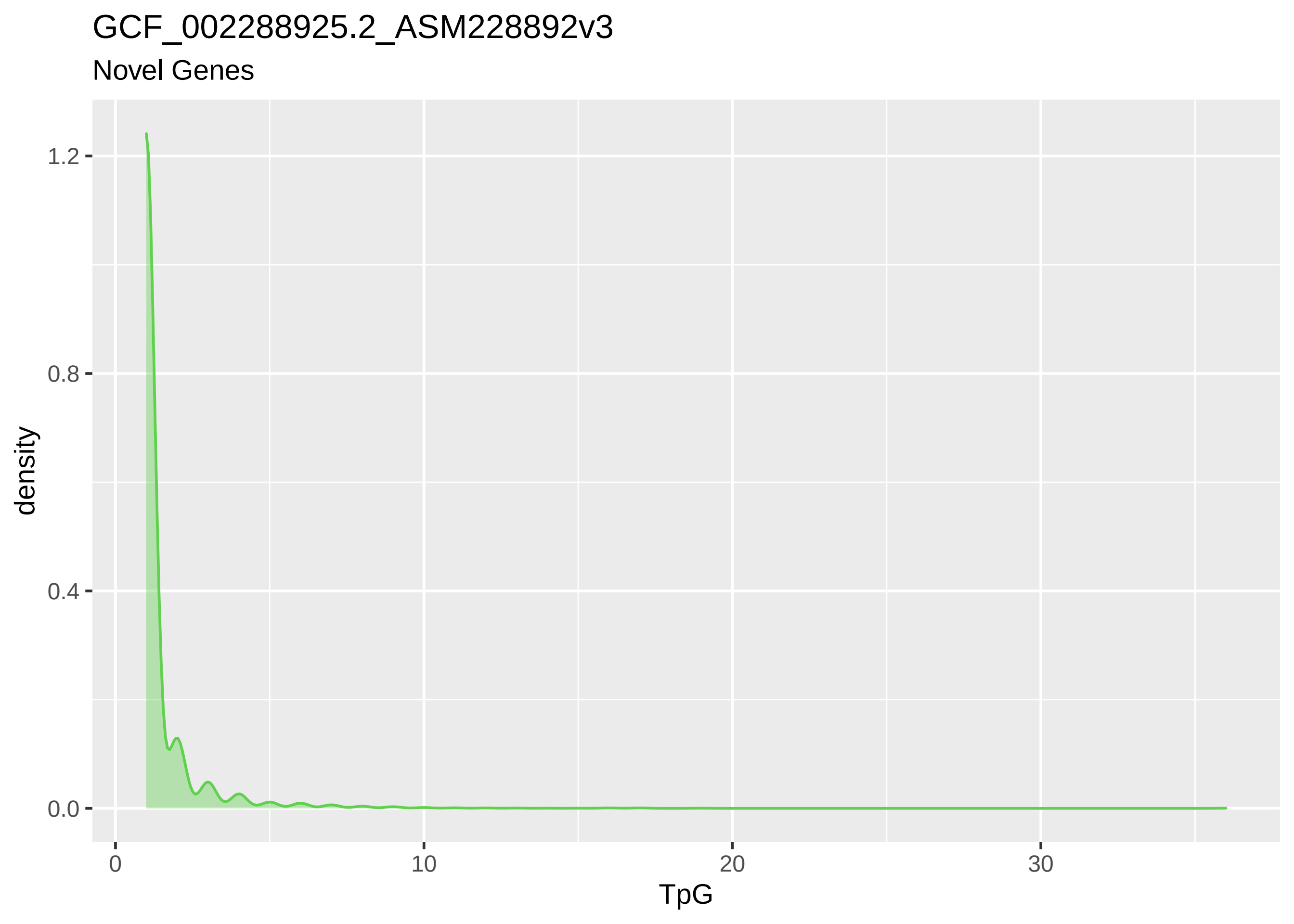
GCF_002288925.2_ASM228892v3
Novel Genes
density
1.2
0.8
0.4
0.0
0
10
20
30
TpG

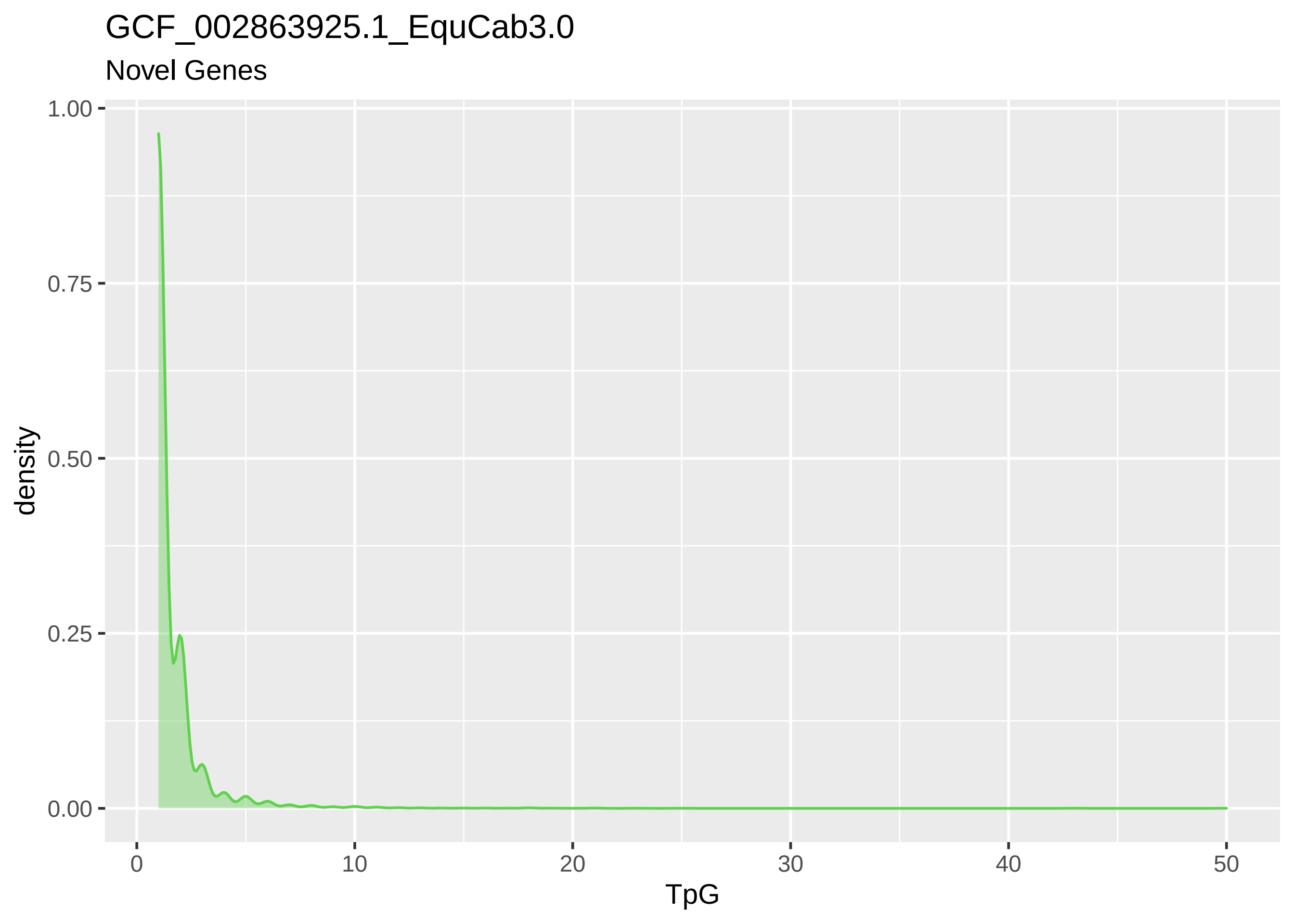
GCF_002863925.1_EquCab3.0
Novel Genes
density
1.00
0.75
0.50
0.25
0.00
0
10
20
30
40
50
TpG

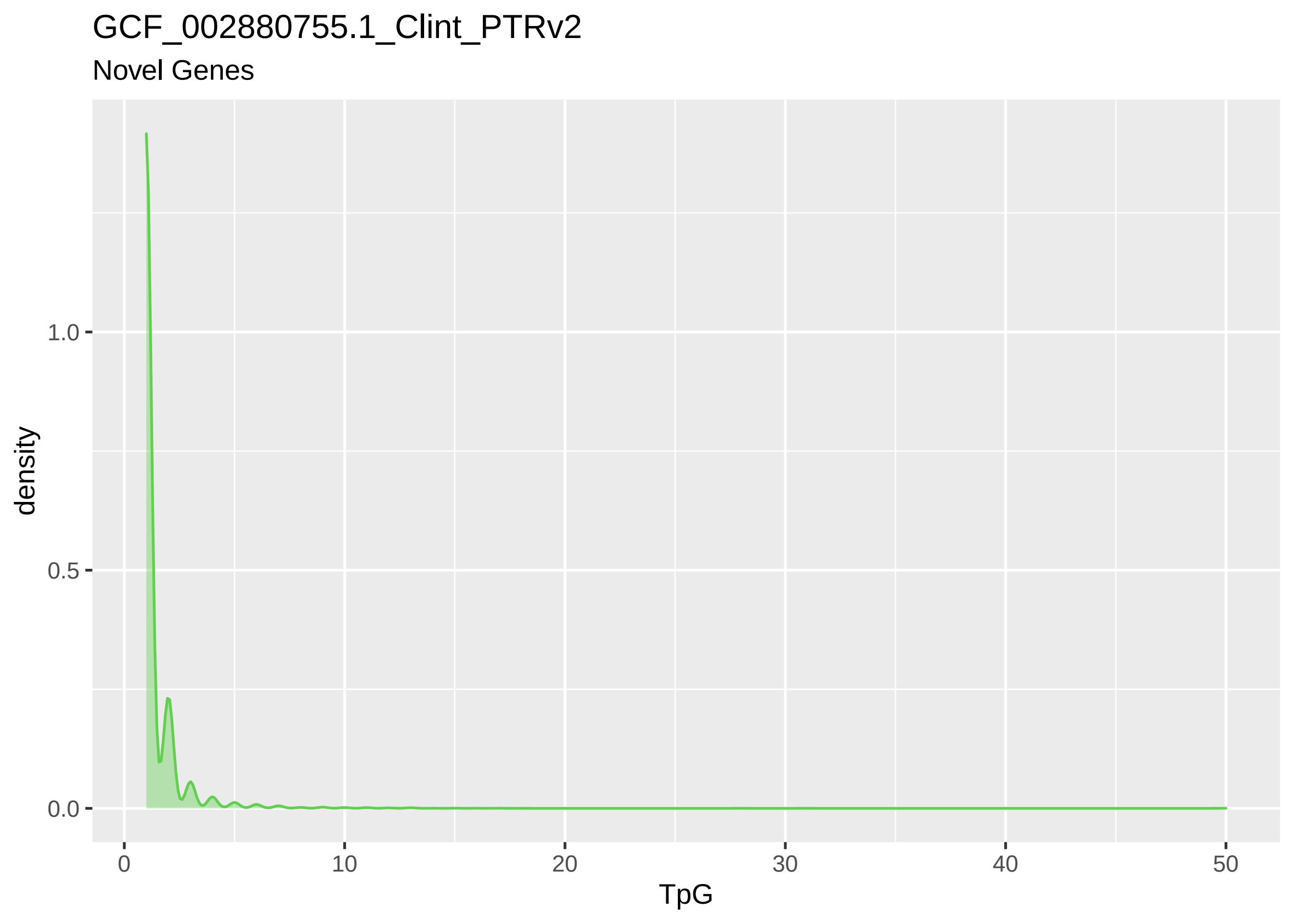
GCF_002880755.1_Clint_PTRv2
Novel Genes
density
1.0
0.5
0.0
0
10
20
30
40
50
TpG

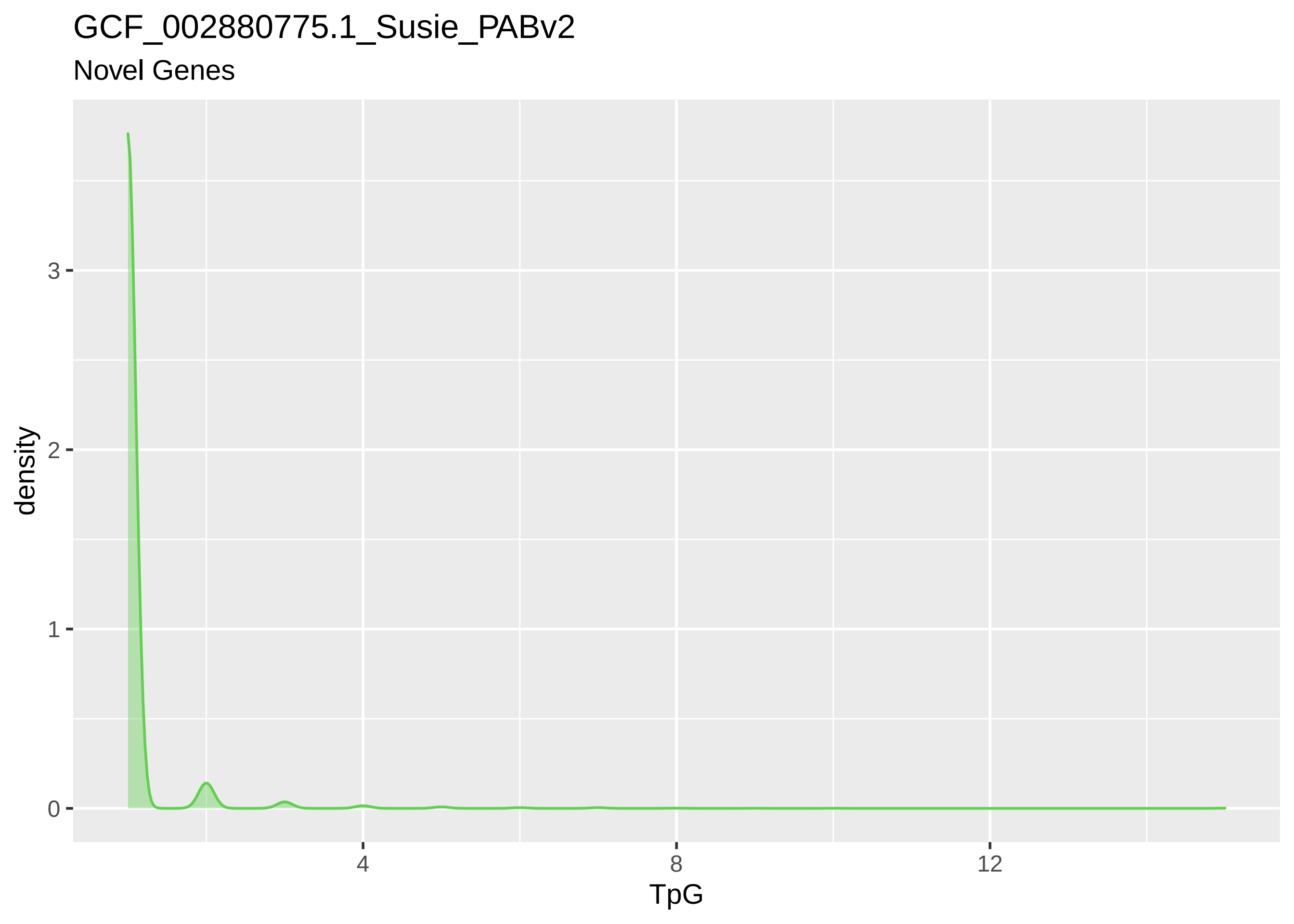

GCF_002880775.1_Susie_PABv2
Novel Genes
density
3
2
1
0
4
8
12
TpG

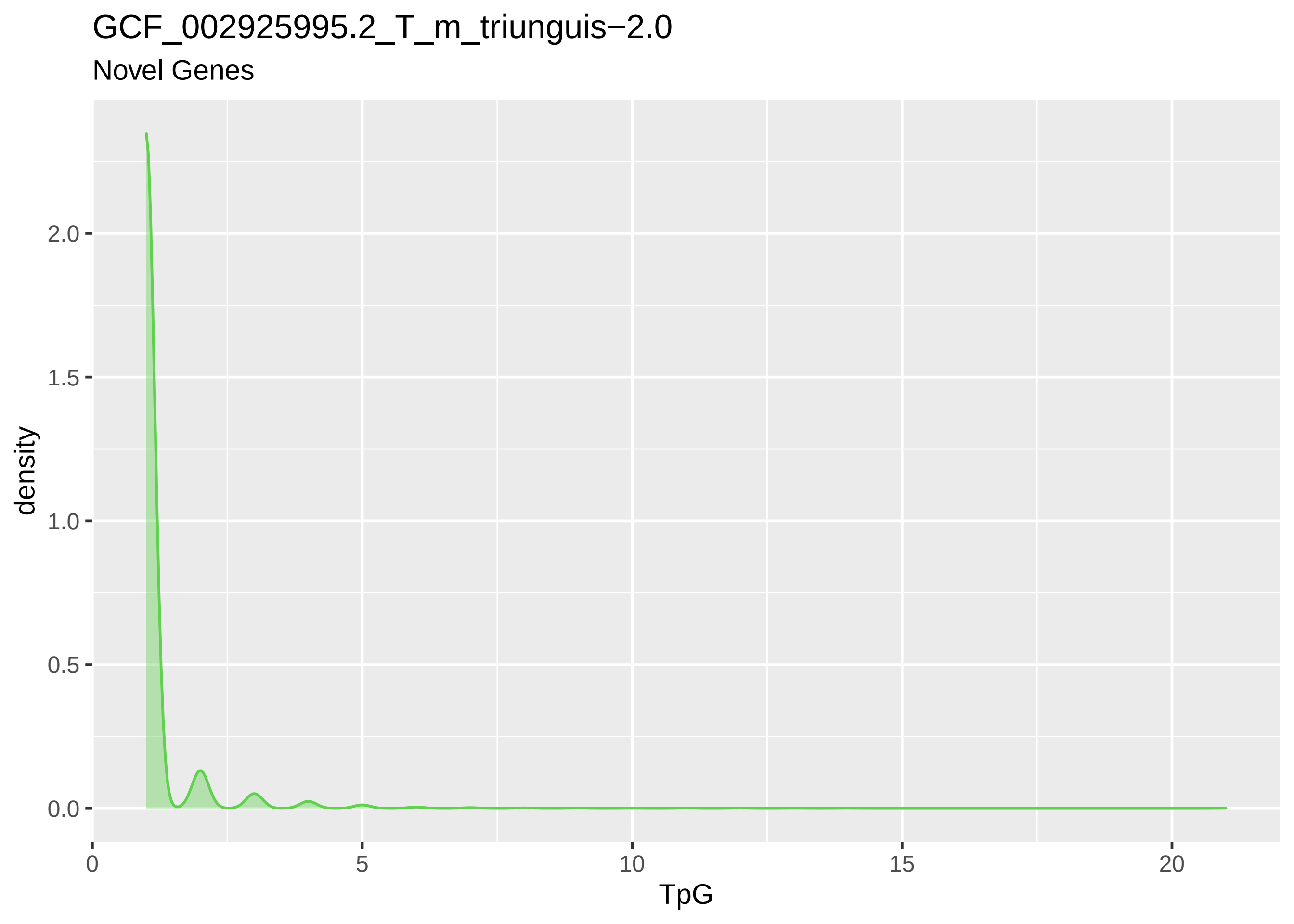
GCF_002925995.2_T_m_triunguis−2.0
Novel Genes
density
2.0
1.5
1.0
0.5
0.0
0
5
10
15
20
TpG

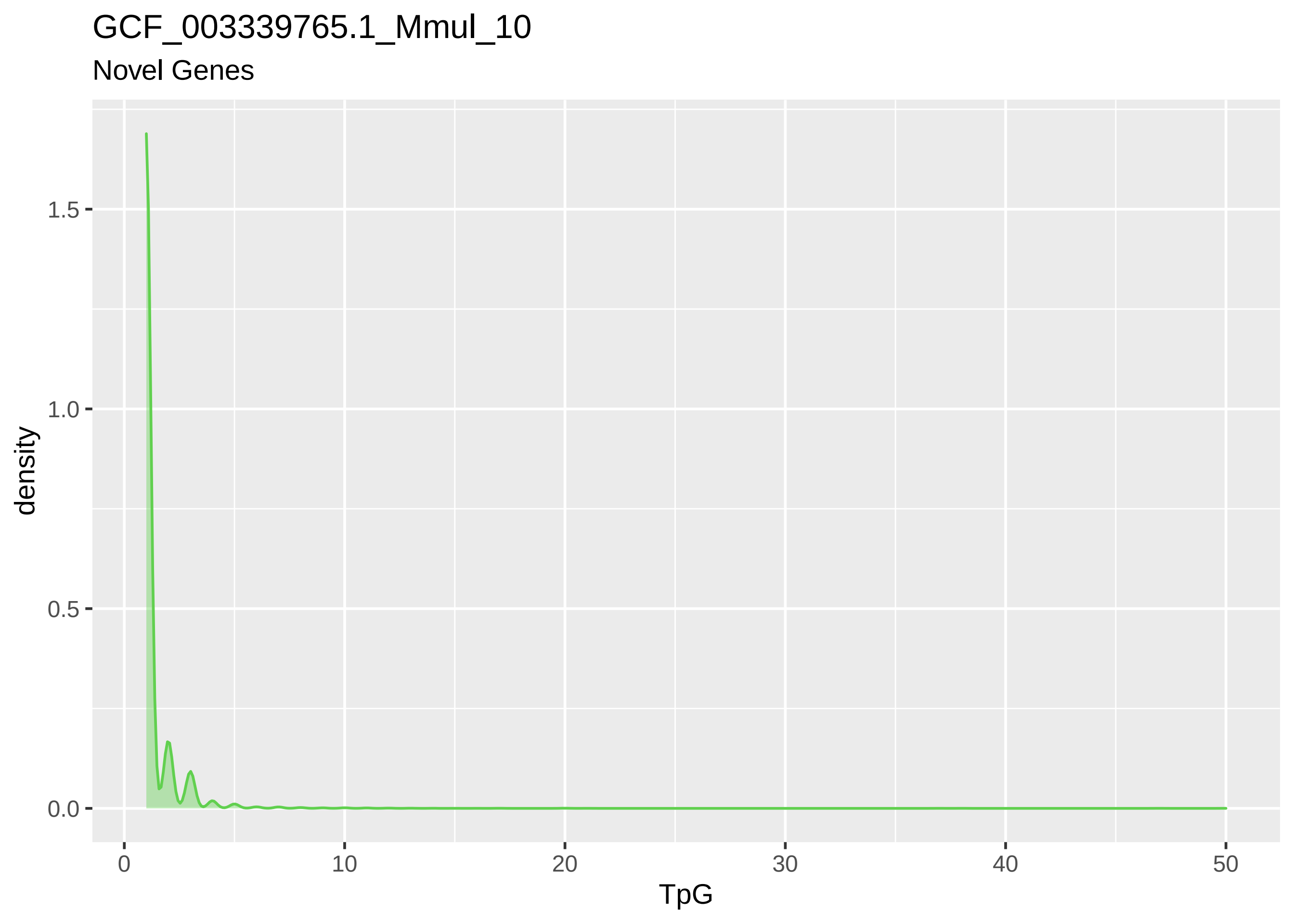

GCF_003339765.1_Mmul_10
Novel Genes
density
1.5
1.0
0.5
0.0
0
10
20
30
40
50
TpG

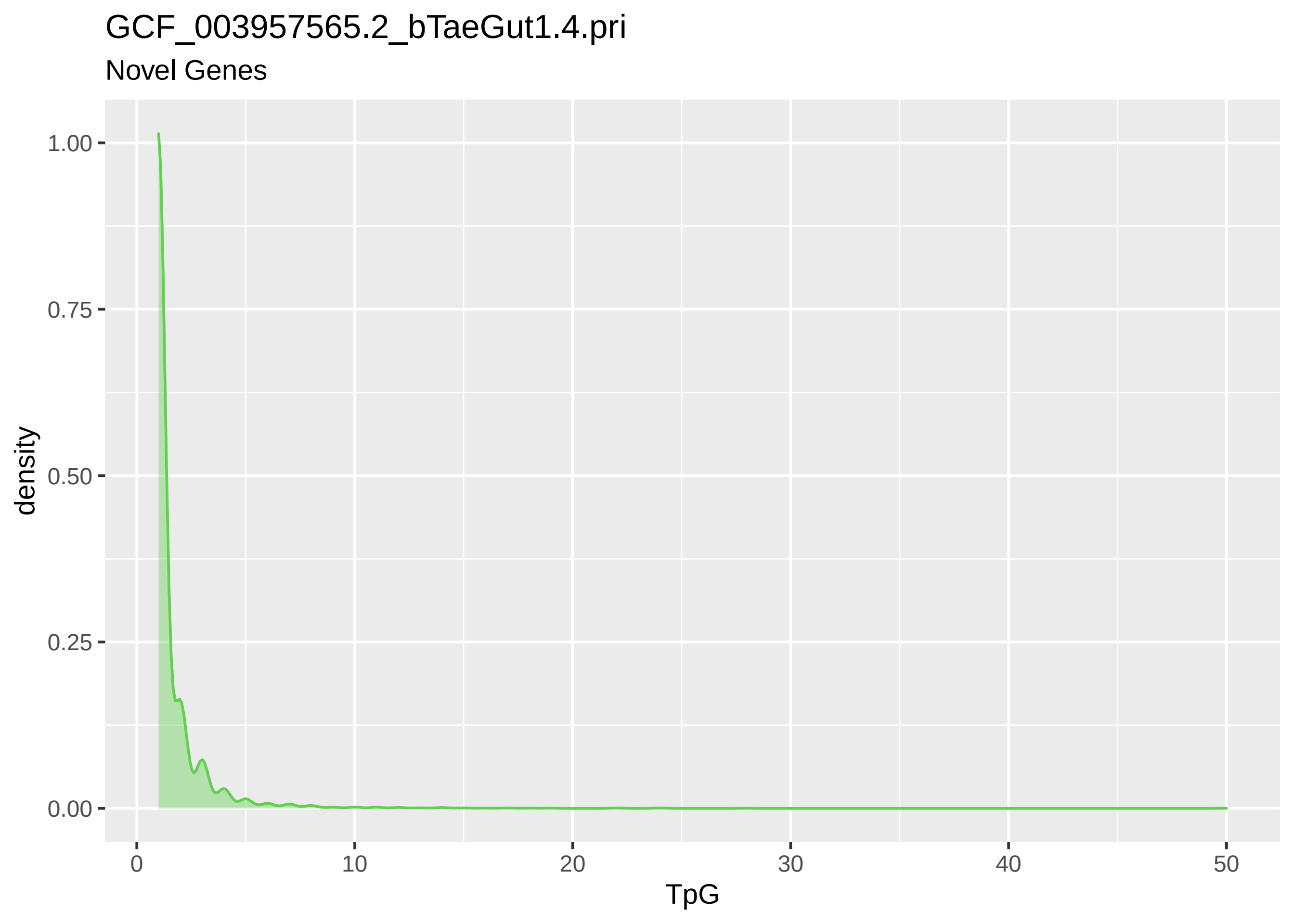
GCF_003957565.2_bTaeGut1.4.pri
Novel Genes
density
1.00
0.75
0.50
0.25
0.00
0
10
20
30
40
50
TpG

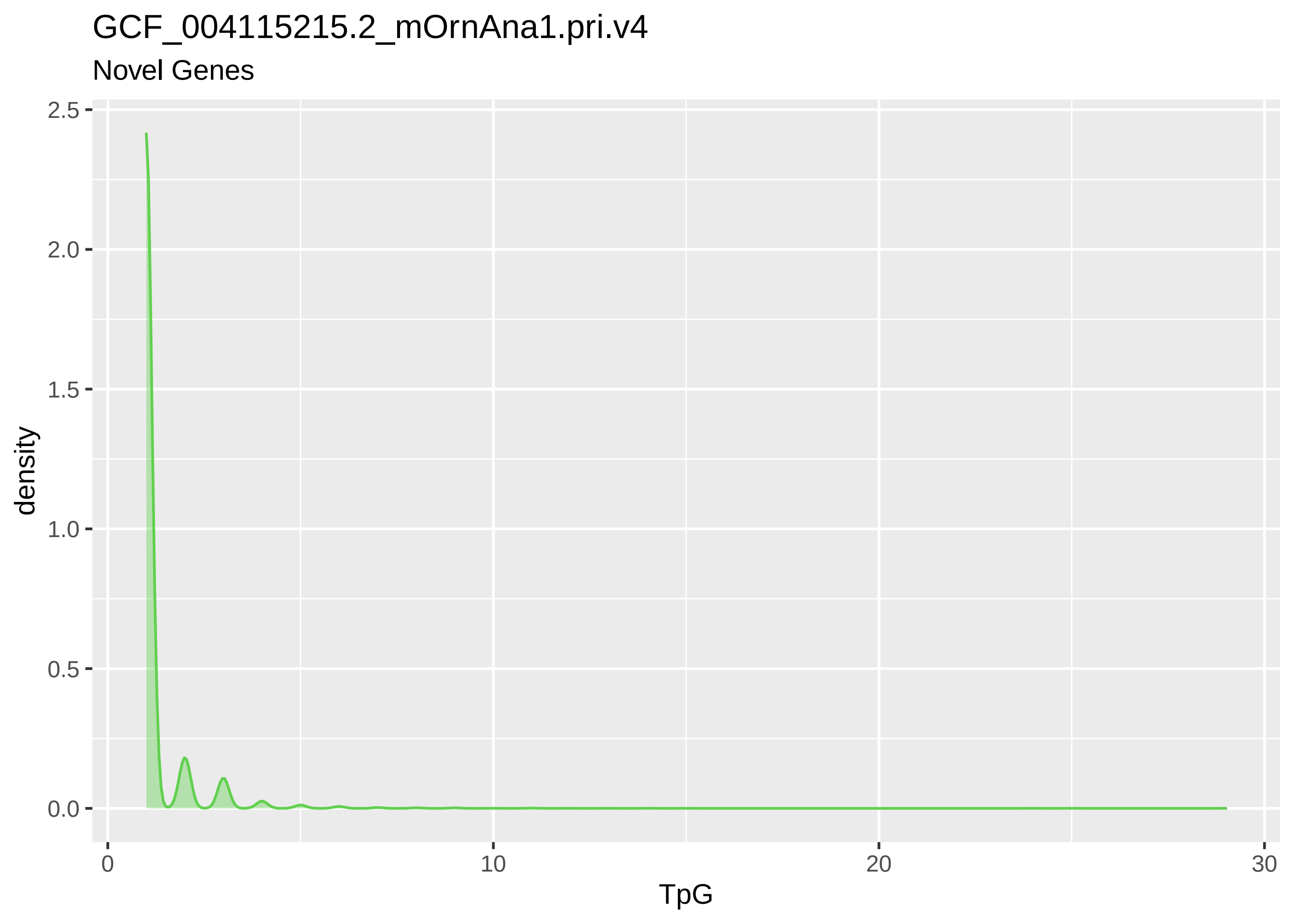
GCF_004115215.2_mOrnAna1.pri.v4
Novel Genes
2.5
2.0
1.5
1.0
0.5
0.0
density
0
10
20
30
TpG

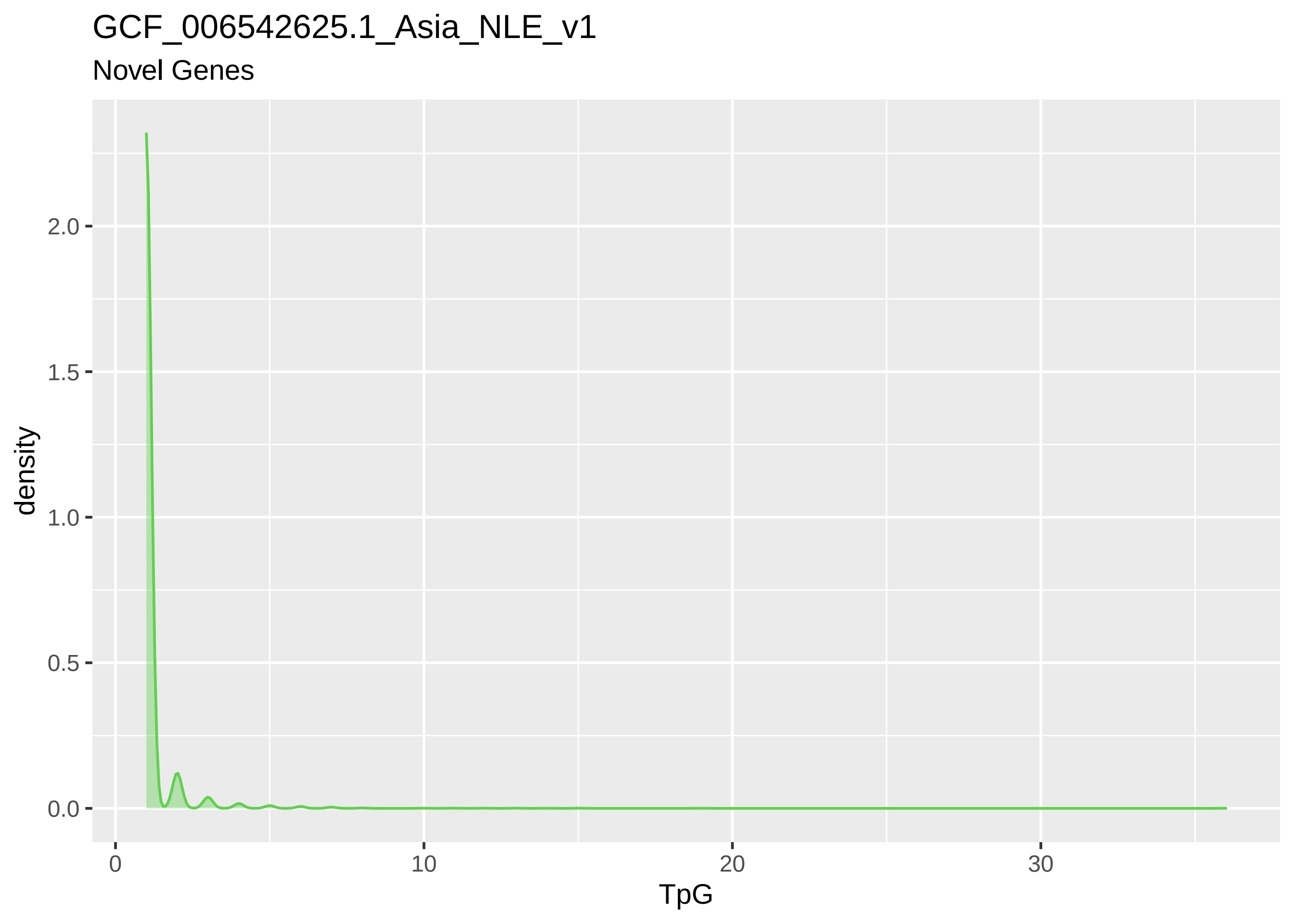
GCF_006542625.1_Asia_NLE_v1
Novel Genes
density
2.0
1.5
1.0
0.5
0.0
0
10
20
30
TpG

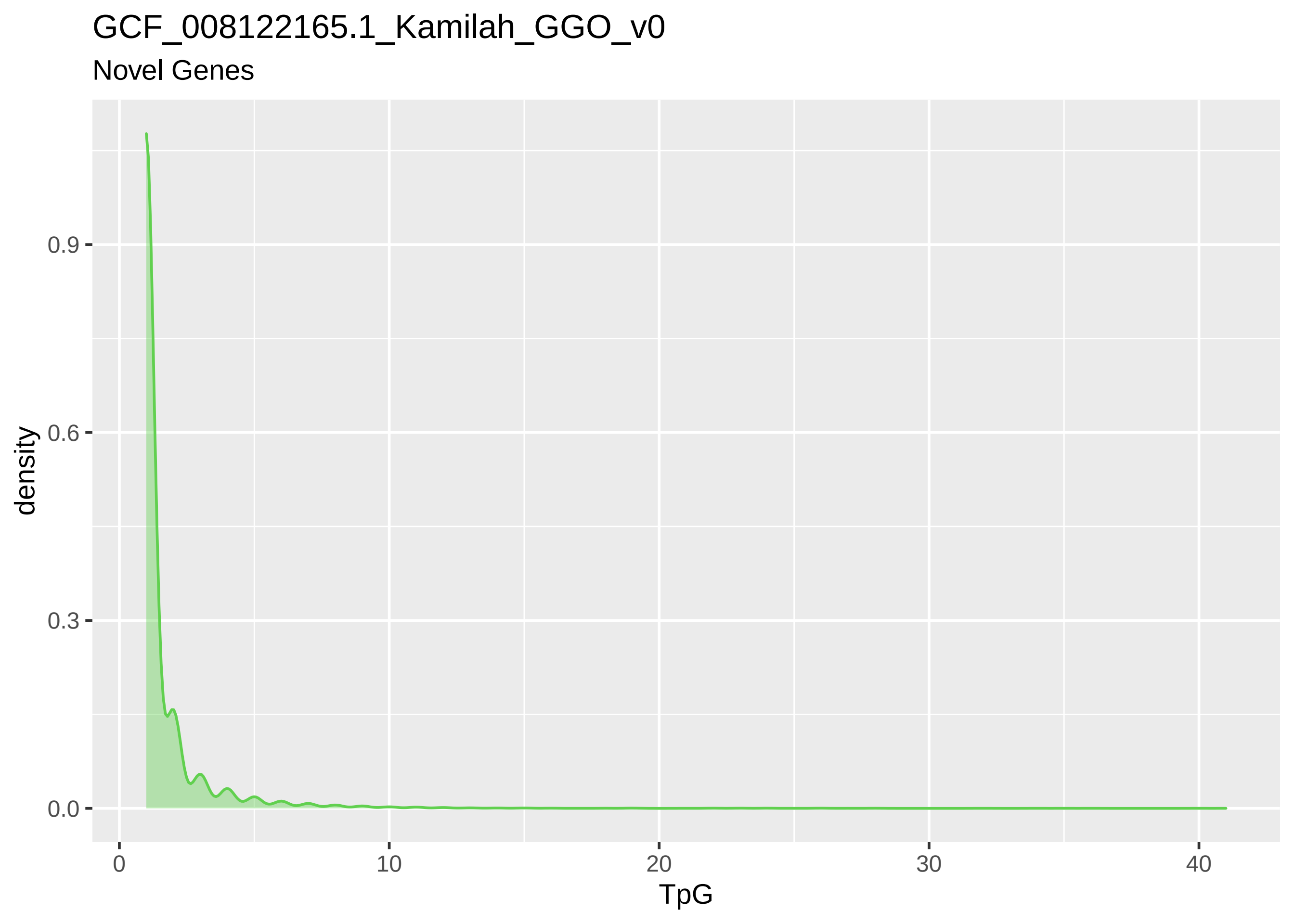
GCF_008122165.1_Kamilah_GGO_v0
Novel Genes
density
0.9
0.6
0.3
0.0
0
10
20
30
40
TpG

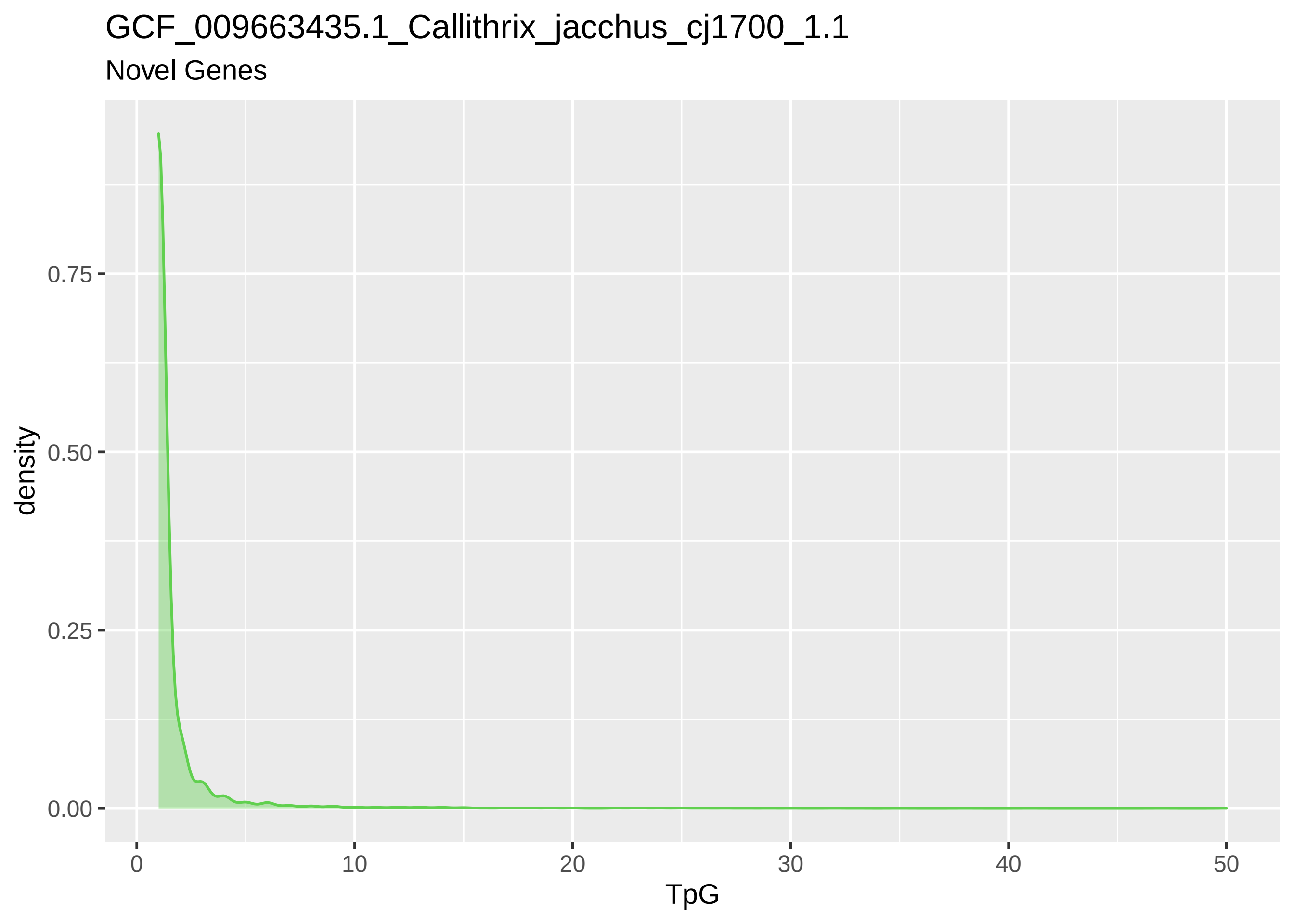
GCF_009663435.1_Callithrix_jacchus_cj1700_1.1
Novel Genes
density
0.00
0.25
0.50
0.75
0
10
20
30
40
50
TpG

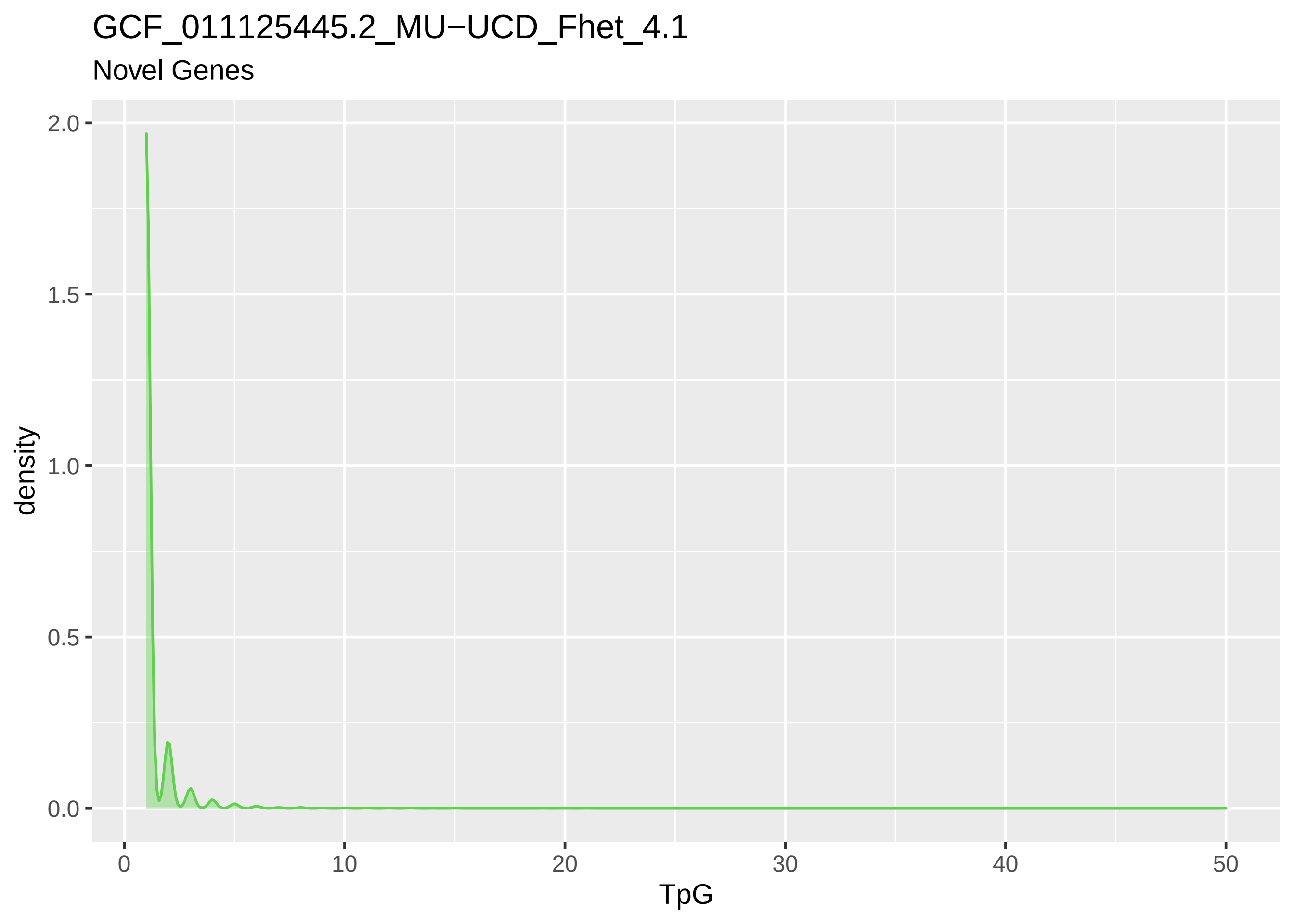
GCF_011125445.2_MU−UCD_Fhet_4.1
Novel Genes
density
2.0
1.5
1.0
0.5
0.0
0
10
20
30
40
50
TpG

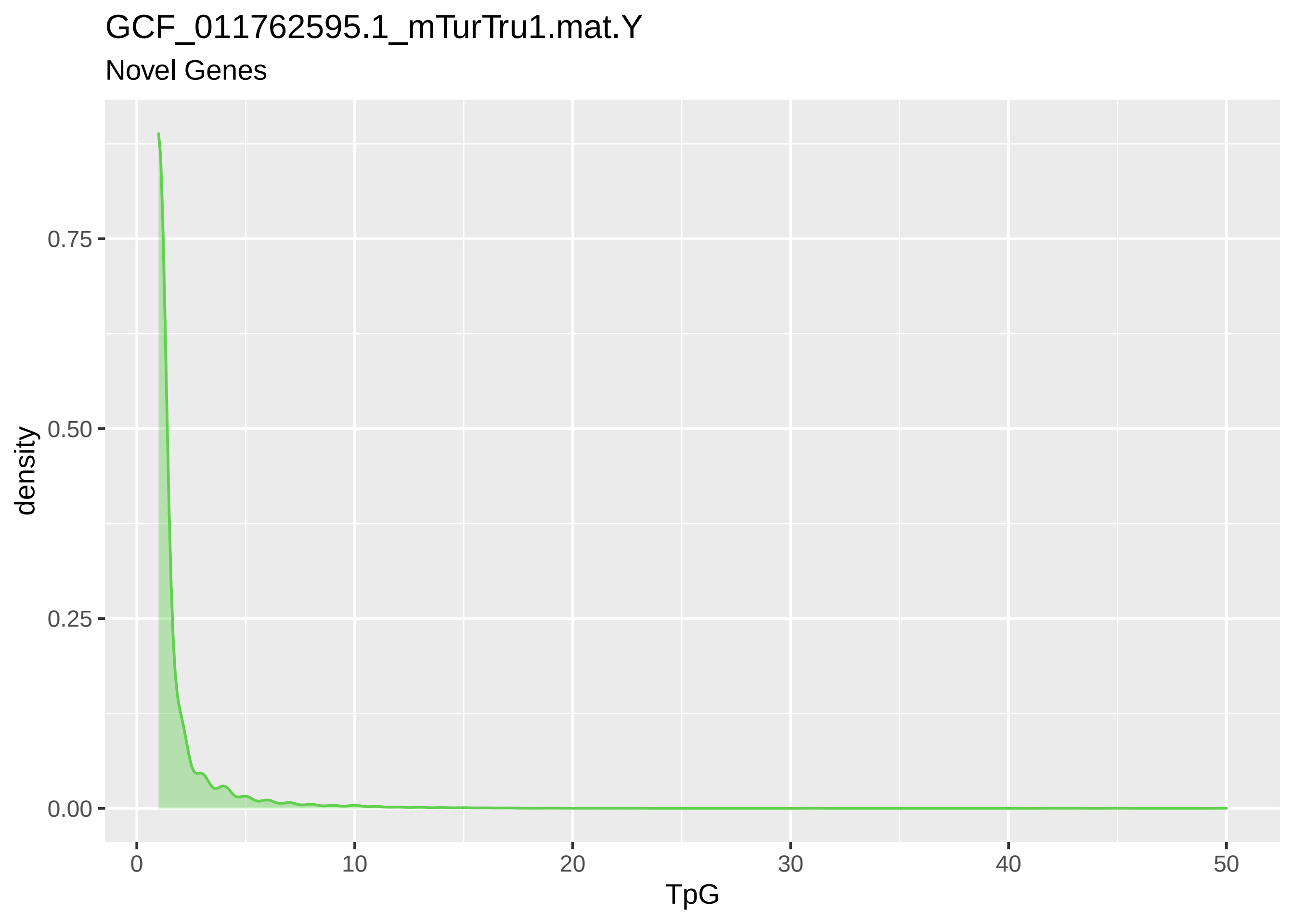
GCF_011762595.1_mTurTru1.mat.Y
Novel Genes
density
0.75
0.50
0.25
0.00
0
10
20
30
40
50
TpG

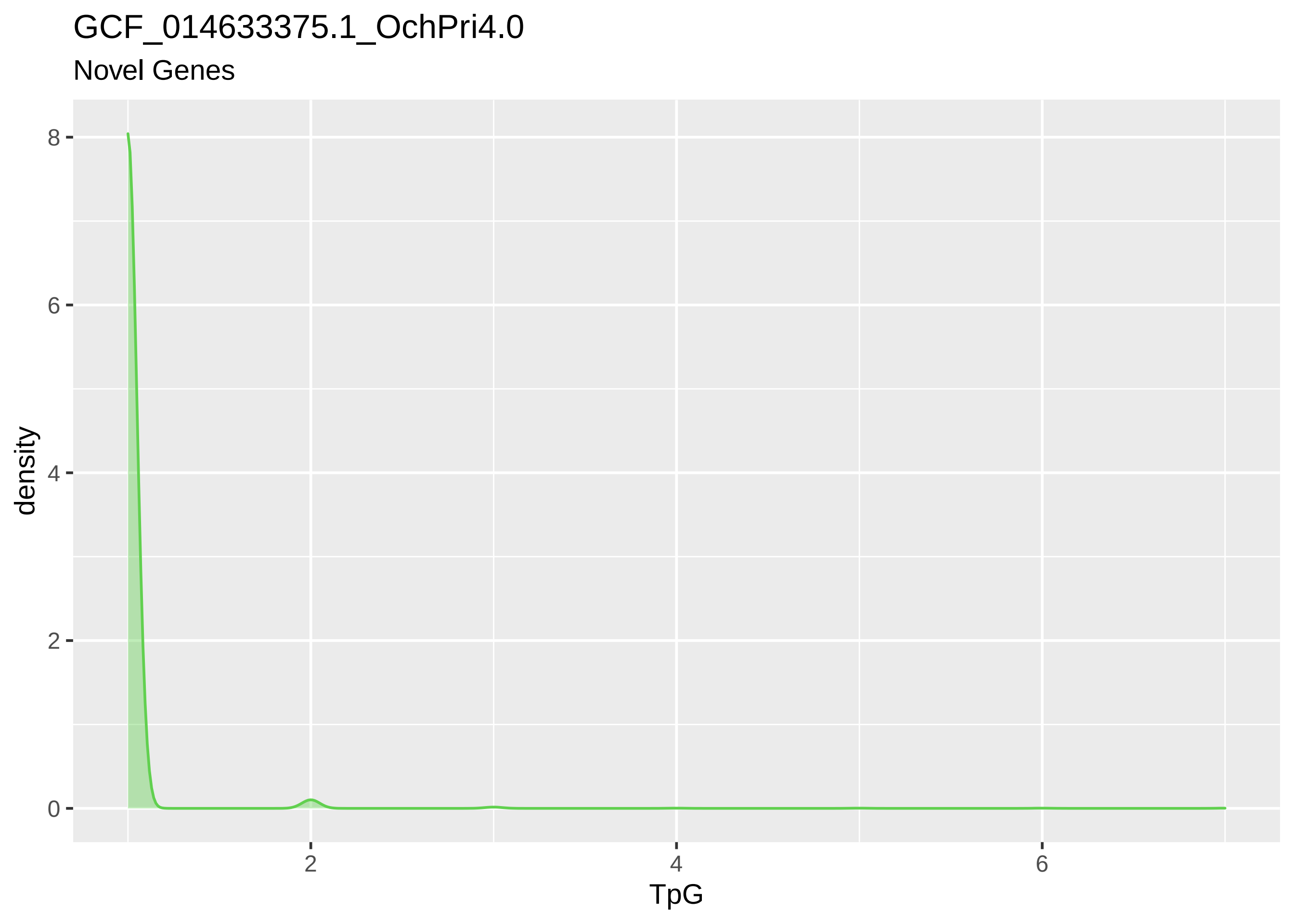

GCF_014633375.1_OchPri4.0
Novel Genes
density
TpG
0
2
4
6
8
2
4
6

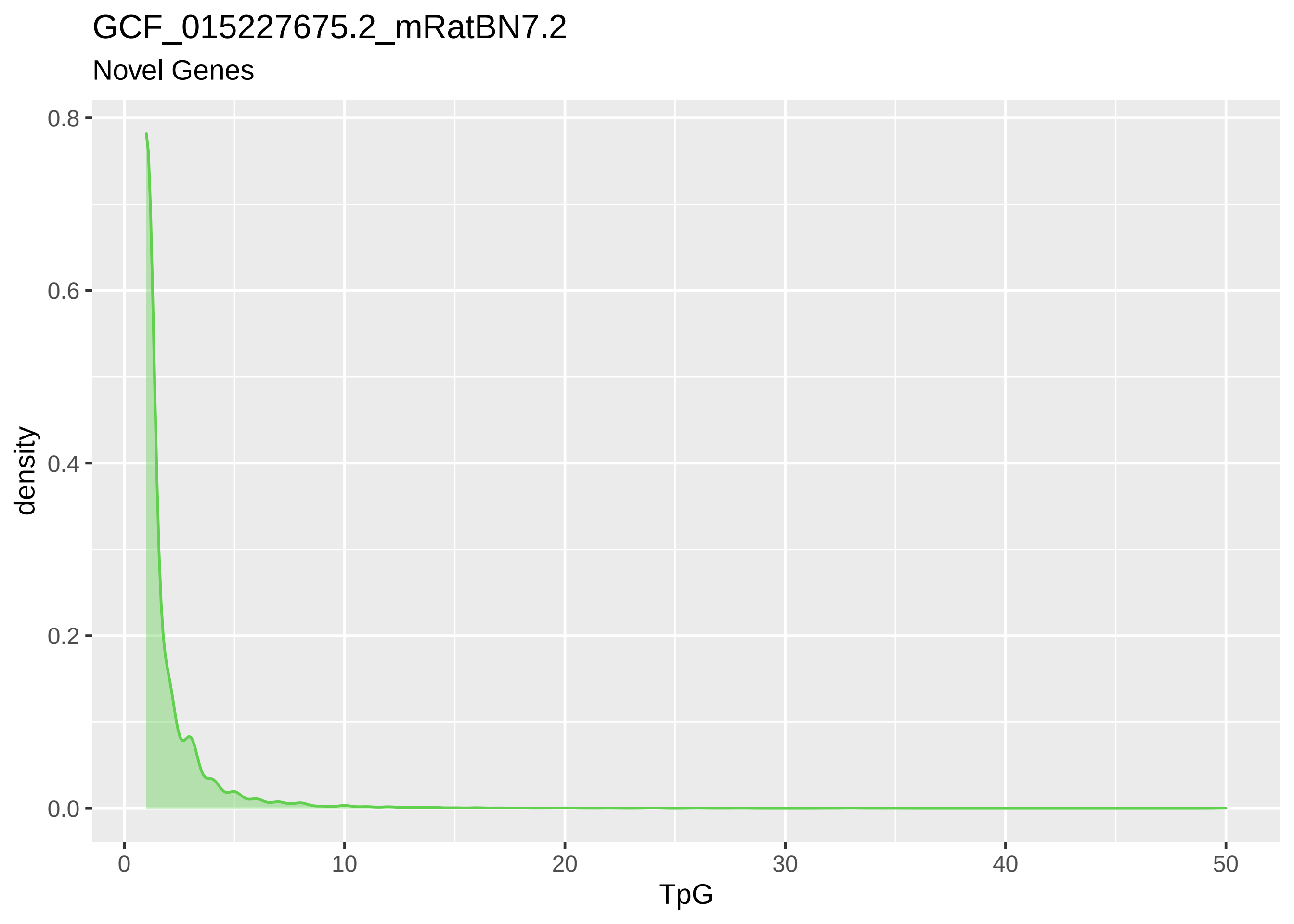
GCF_015227675.2_mRatBN7.2
Novel Genes
density
TpG
0.0
0.2
0.4
0.6
0.8
0
10
20
30
40
50

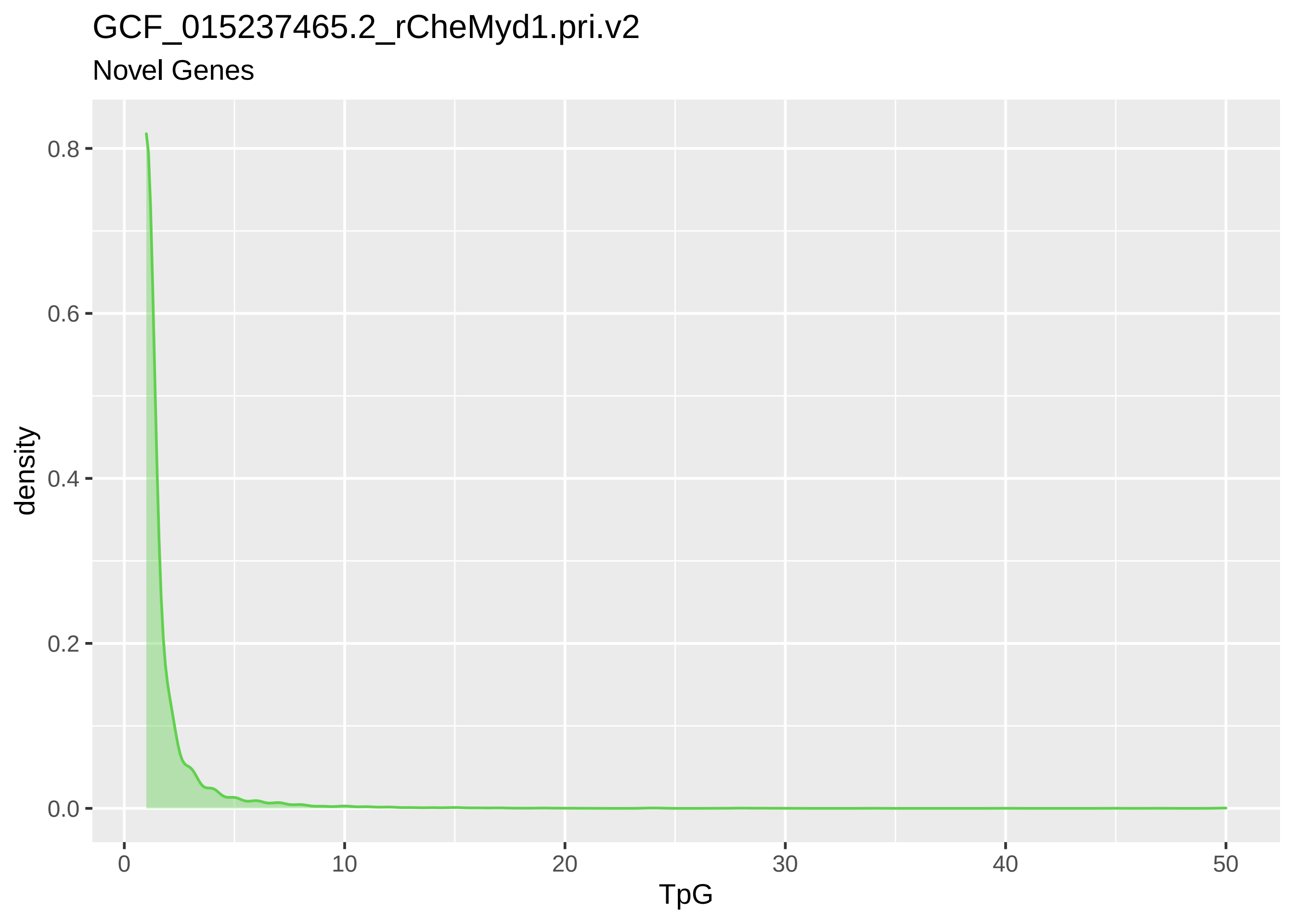
GCF_015237465.2_rCheMyd1.pri.v2
Novel Genes
density
0.0
0.2
0.4
0.6
0.8
0
10
20
30
40
50
TpG

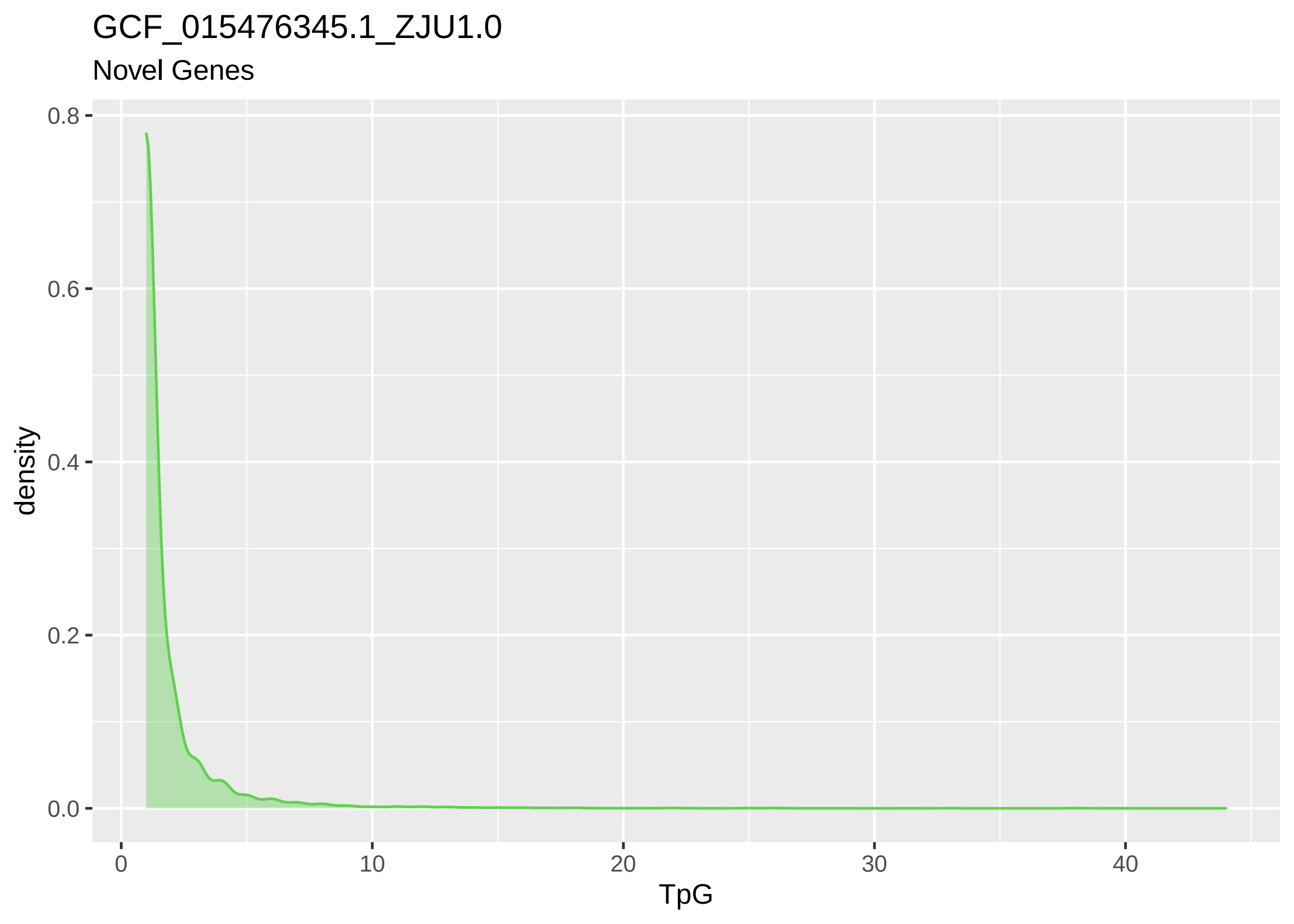
GCF_015476345.1_ZJU1.0
Novel Genes
0.8
0.6
0.4
0.2
0.0
density
0
10
20
30
40
TpG

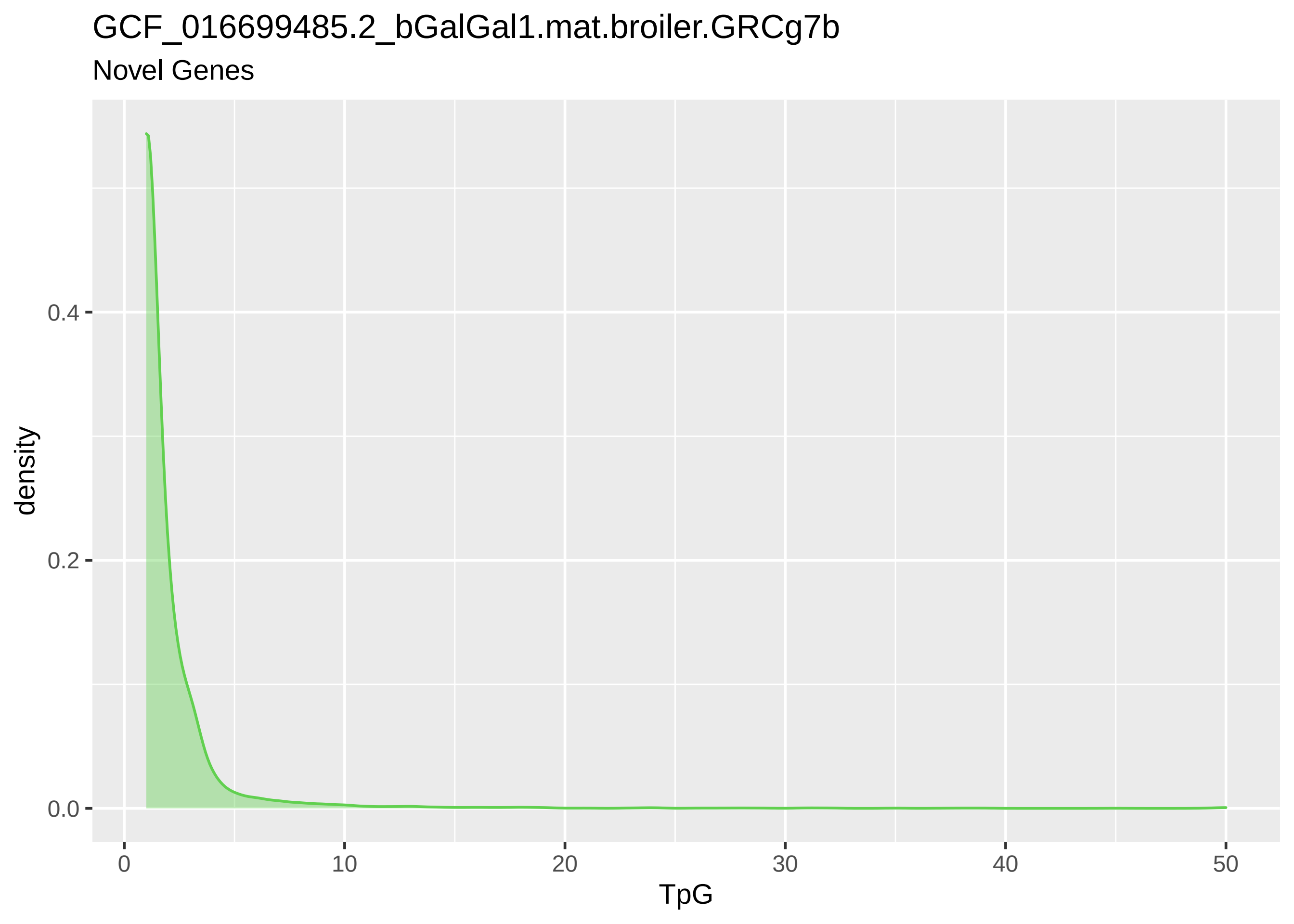
GCF_016699485.2_bGalGal1.mat.broiler.GRCg7b
Novel Genes
density
0.0
0.2
0.4
0
10
20
30
40
50
TpG

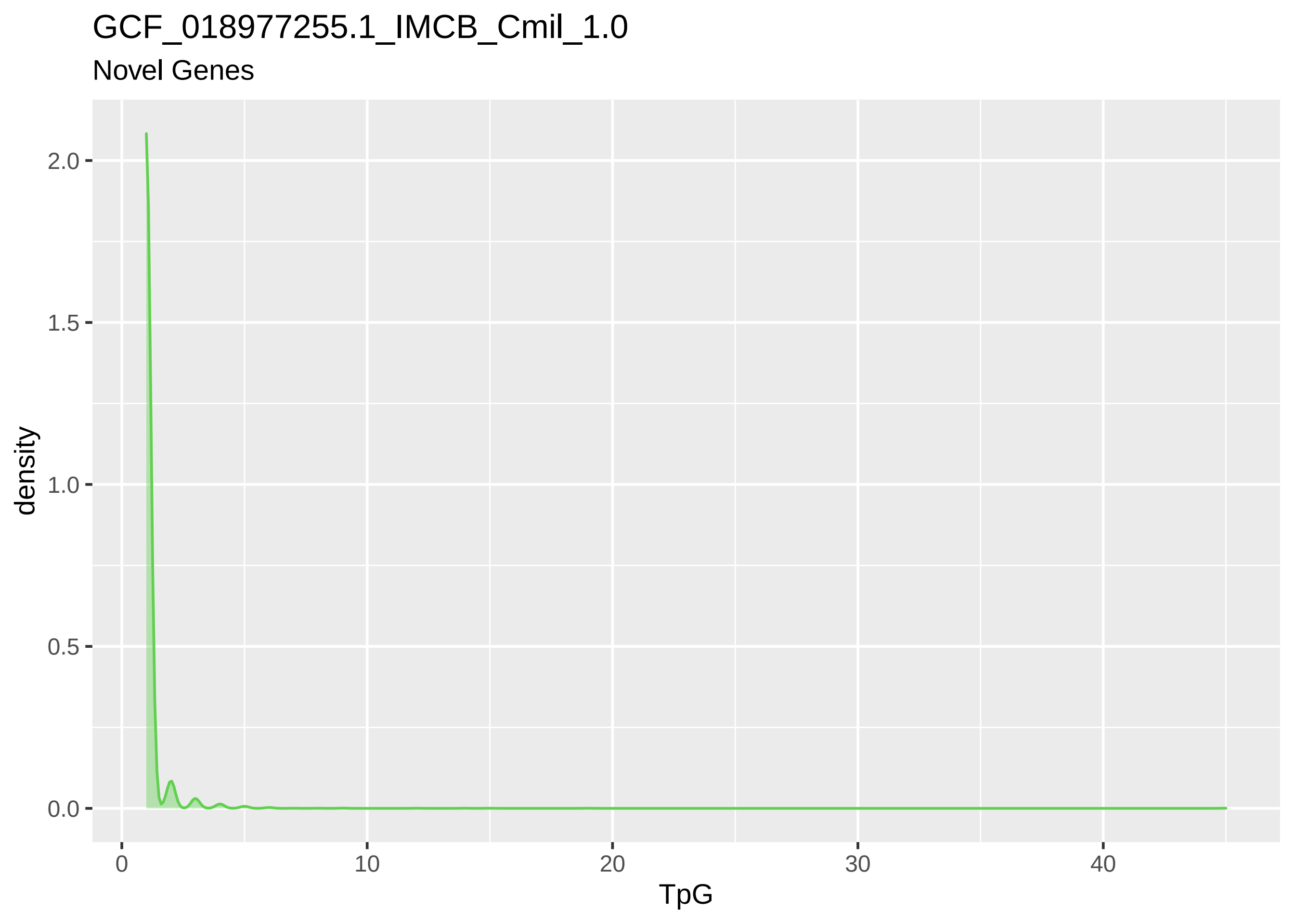

GCF_018977255.1_IMCB_Cmil_1.0
Novel Genes
density
2.0
1.5
1.0
0.5
0.0
0
10
20
30
40
TpG

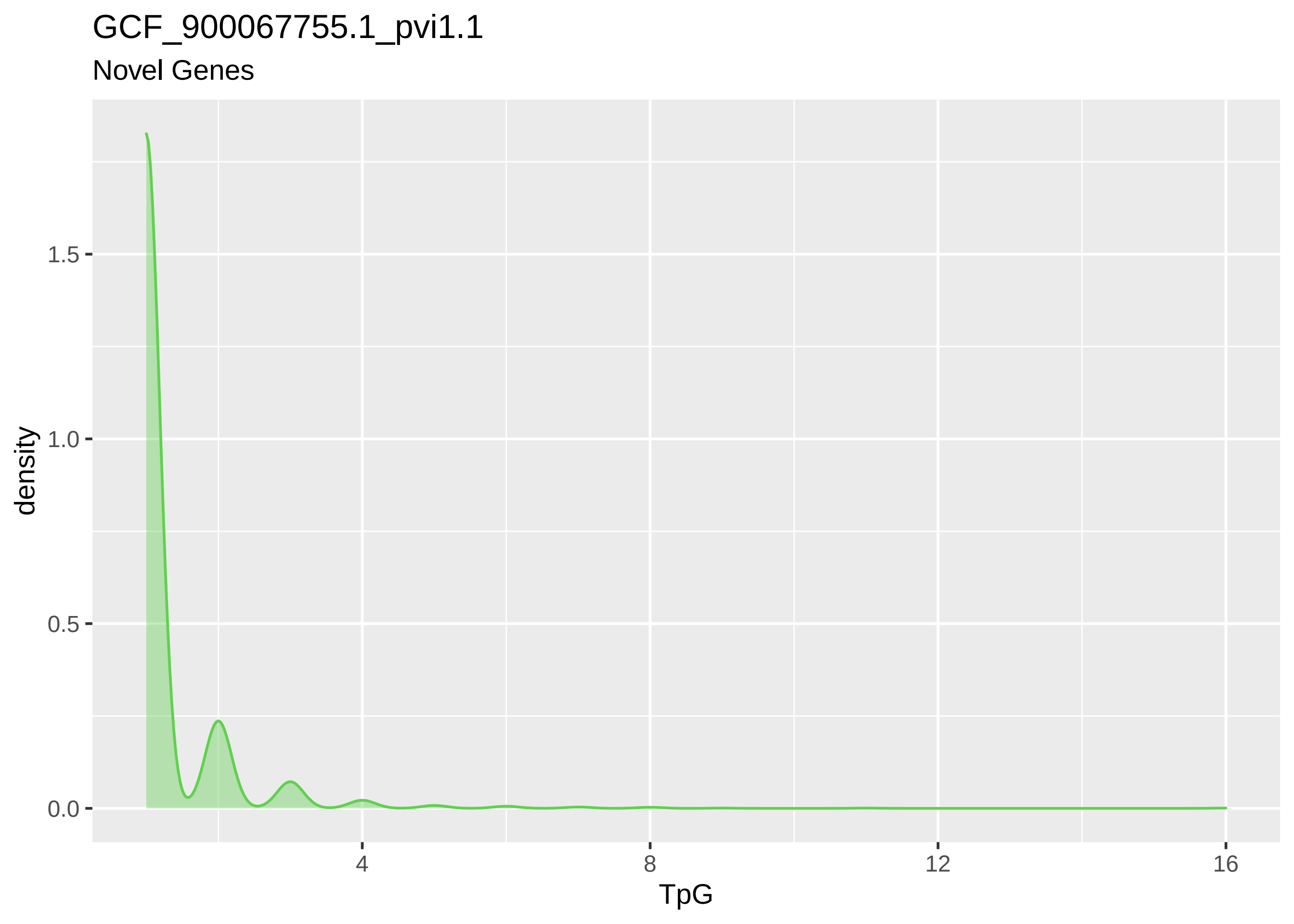
GCF_900067755.1_pvi1.1
Novel Genes
density
1.5
1.0
0.5
0.0
4
8
12
16
TpG

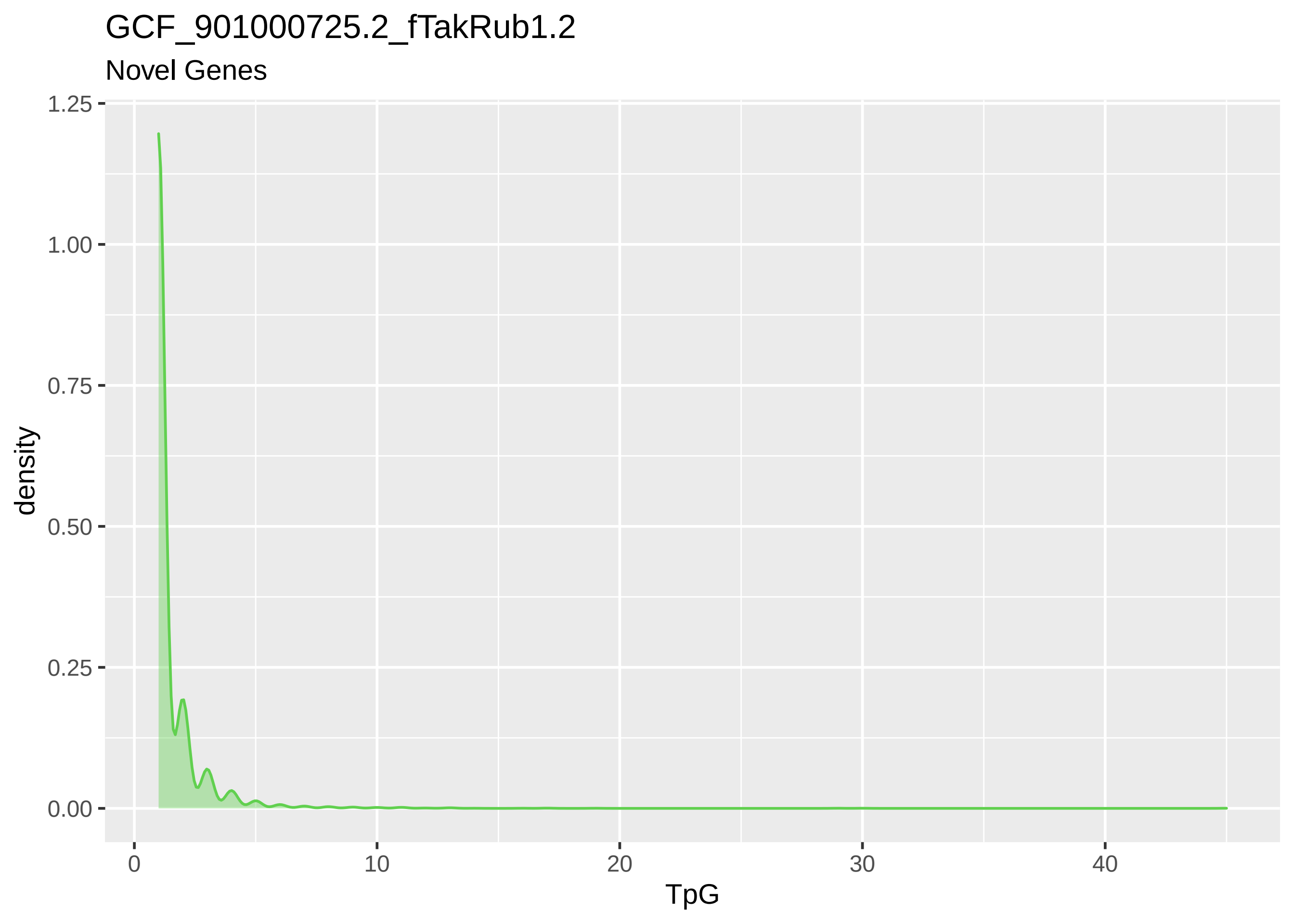
GCF_901000725.2_fTakRub1.2
Novel Genes
density
1.25
1.00
0.75
0.50
0.25
0.00
0
10
20
30
40
TpG

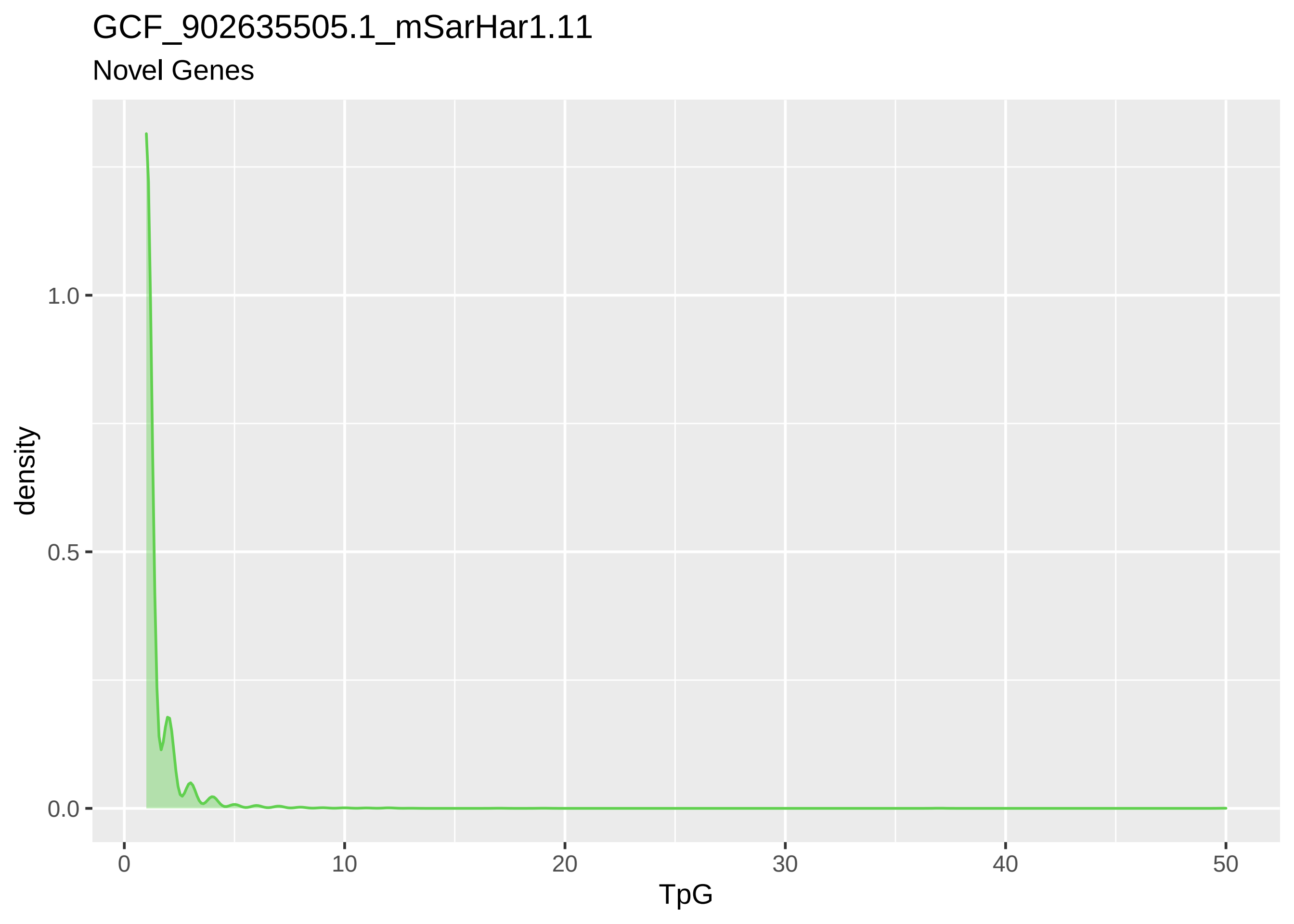

GCF_902635505.1_mSarHar1.11
Novel Genes
density
1.0
0.5
0.0
0
10
20
30
40
50
TpG

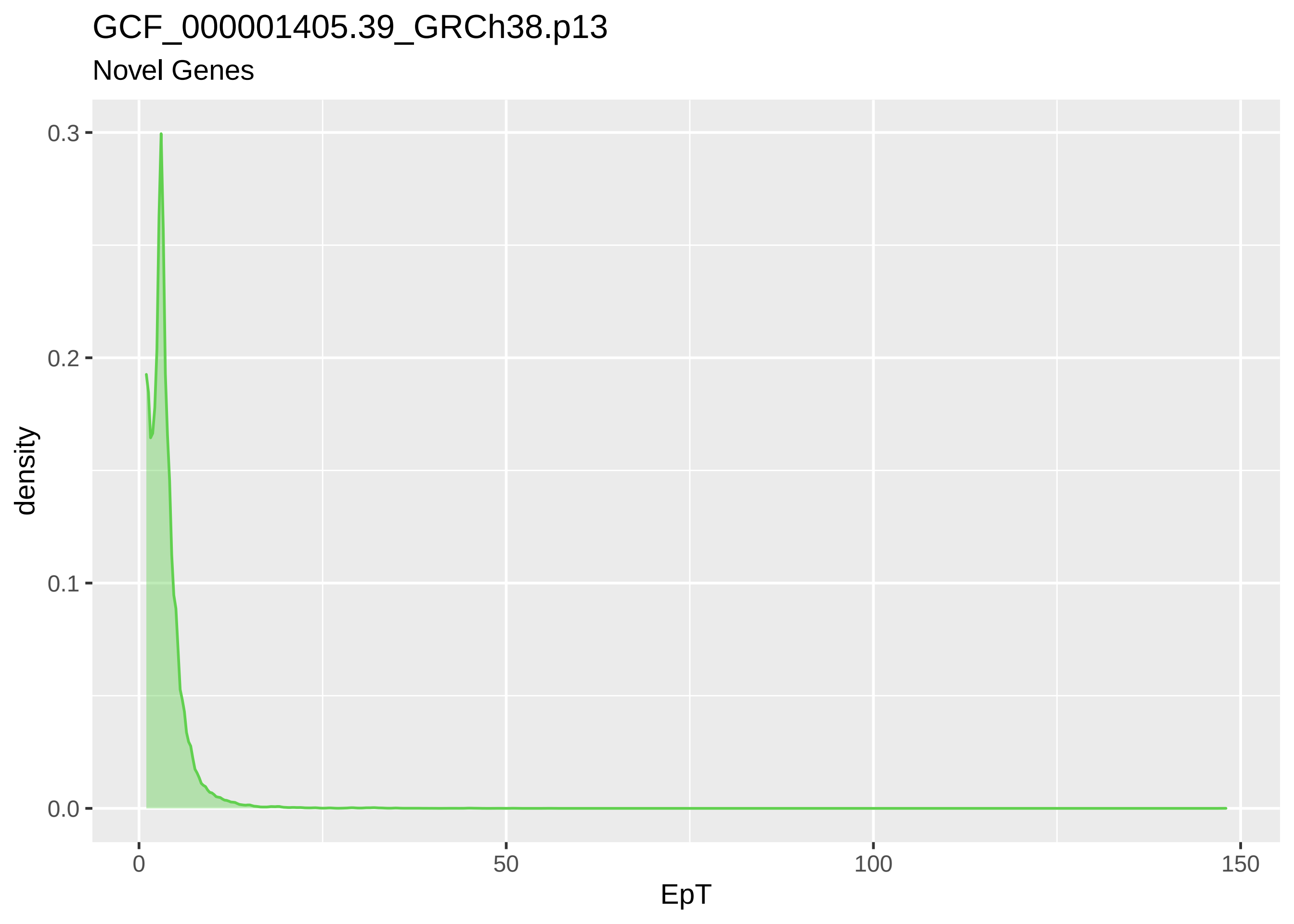
GCF_000001405.39_GRCh38.p13
Novel Genes
density
0.3
0.2
0.1
0.0
0
50
100
150
EpT

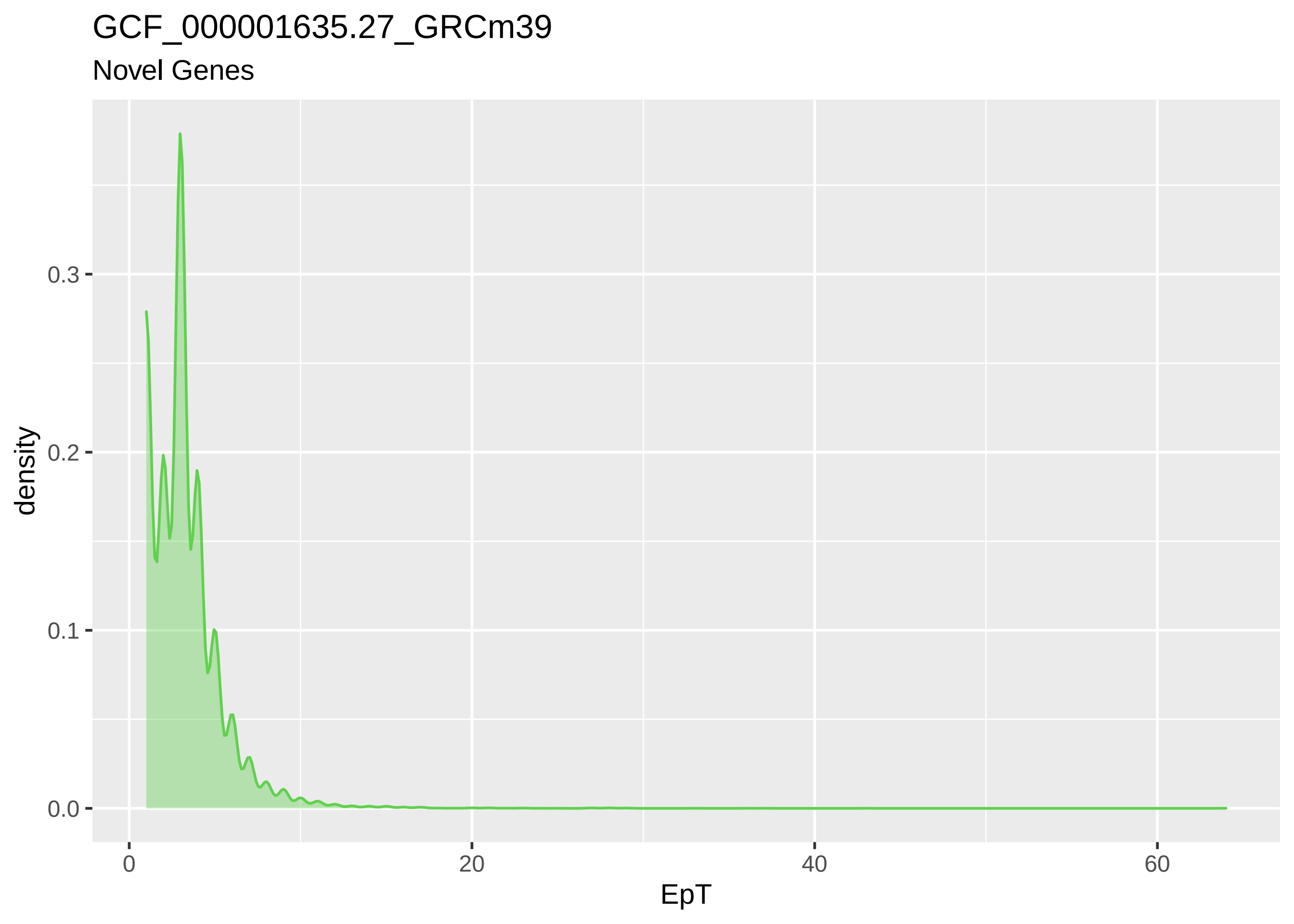
GCF_000001635.27_GRCm39
Novel Genes
density
0.0
0.1
0.2
0.3
0
20
40
60
EpT

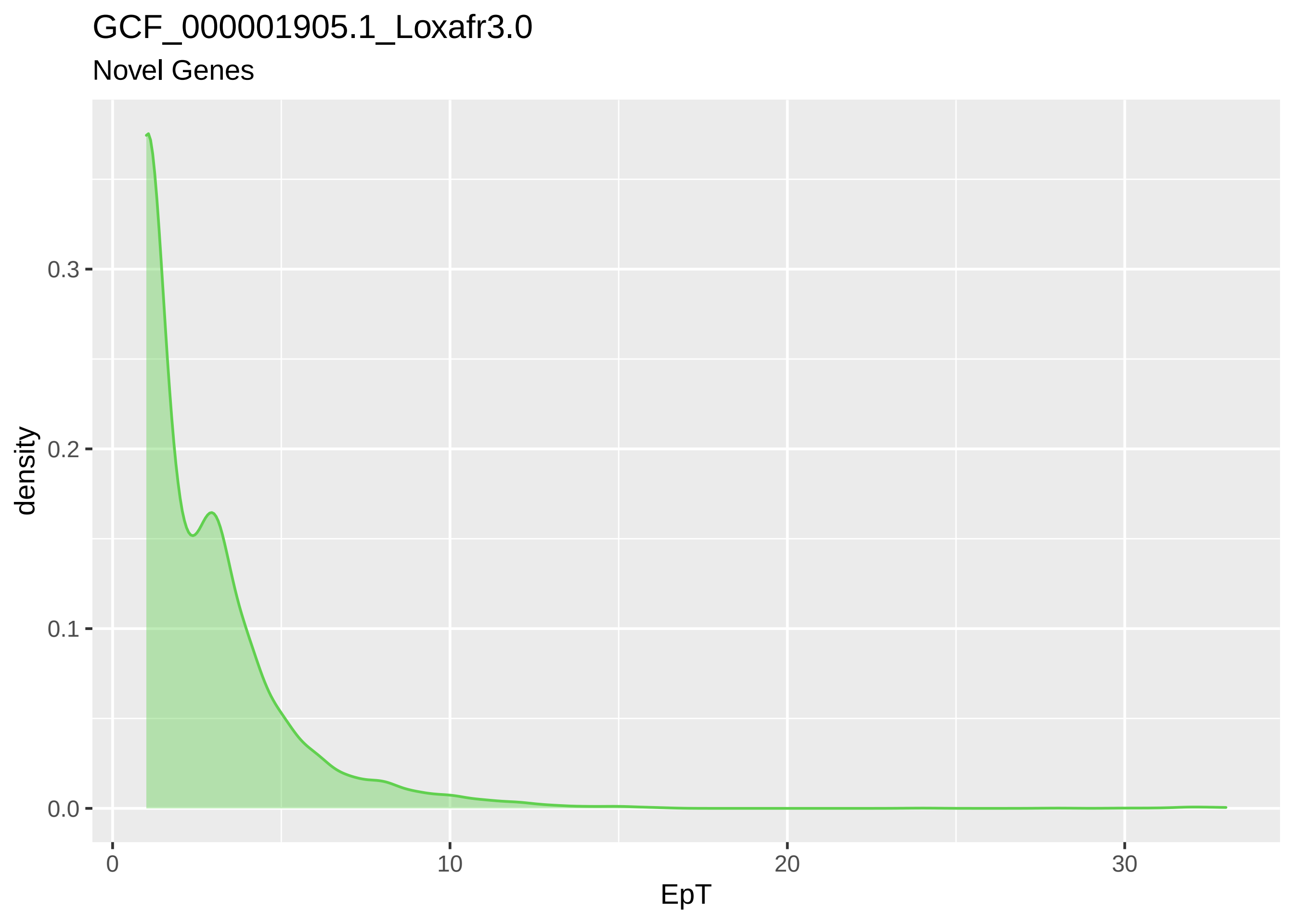
GCF_000001905.1_Loxafr3.0
Novel Genes
density
0.0
0.1
0.2
0.3
0
10
20
30
EpT

GCF_000002035.6_GRCz11

Novel Genes

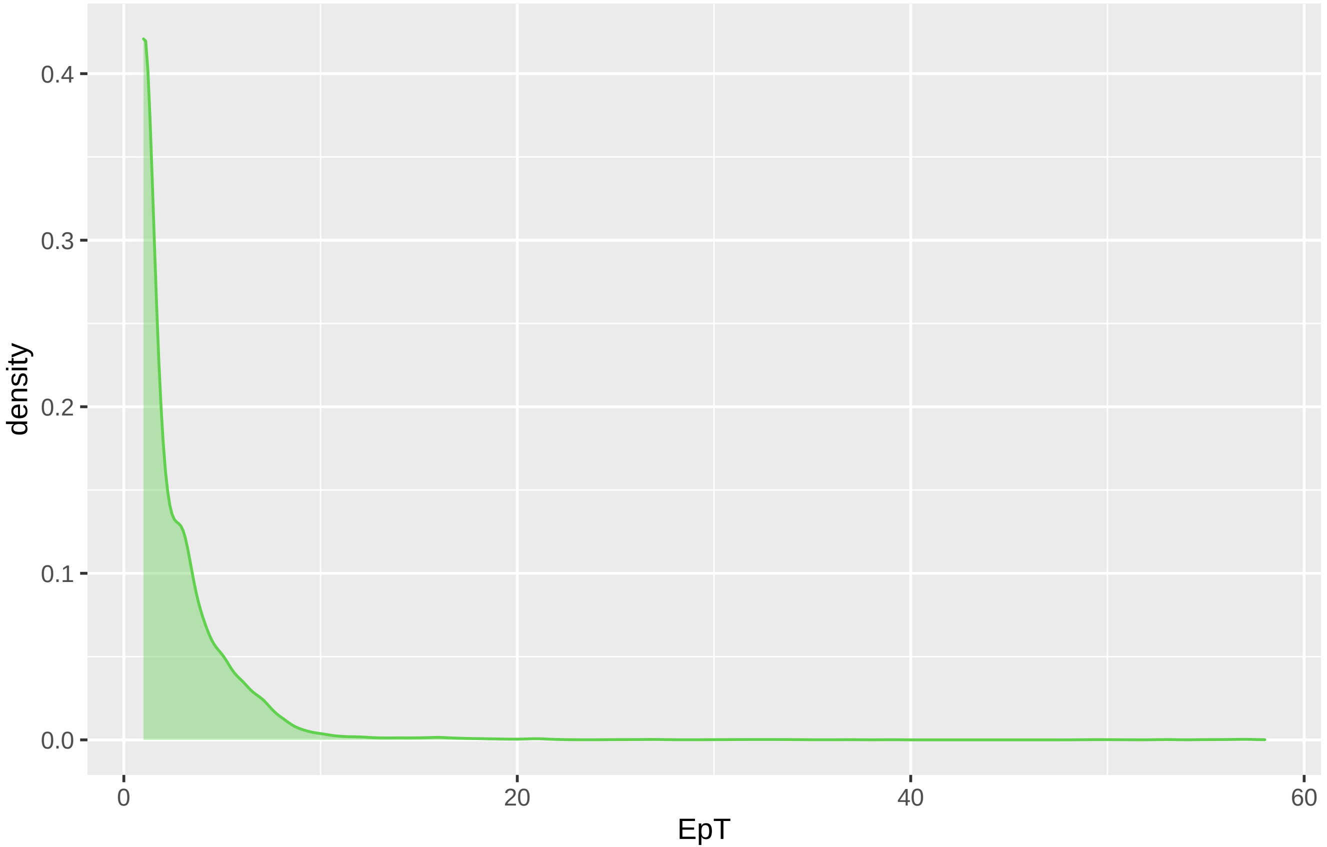

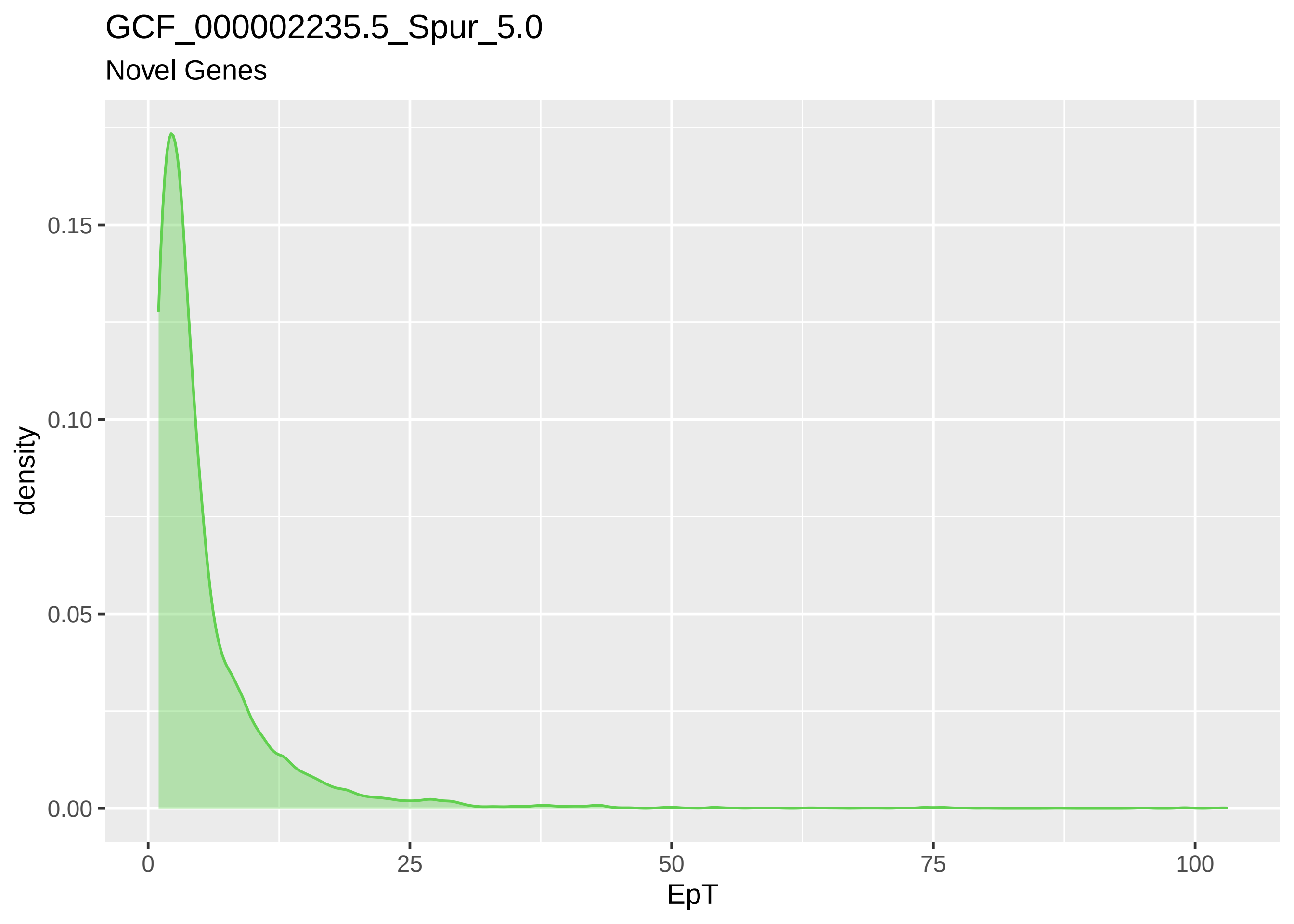
GCF_000002235.5_Spur_5.0
Novel Genes
density
0.15
0.10
0.05
0.00
0
25
50
75
100
EpT

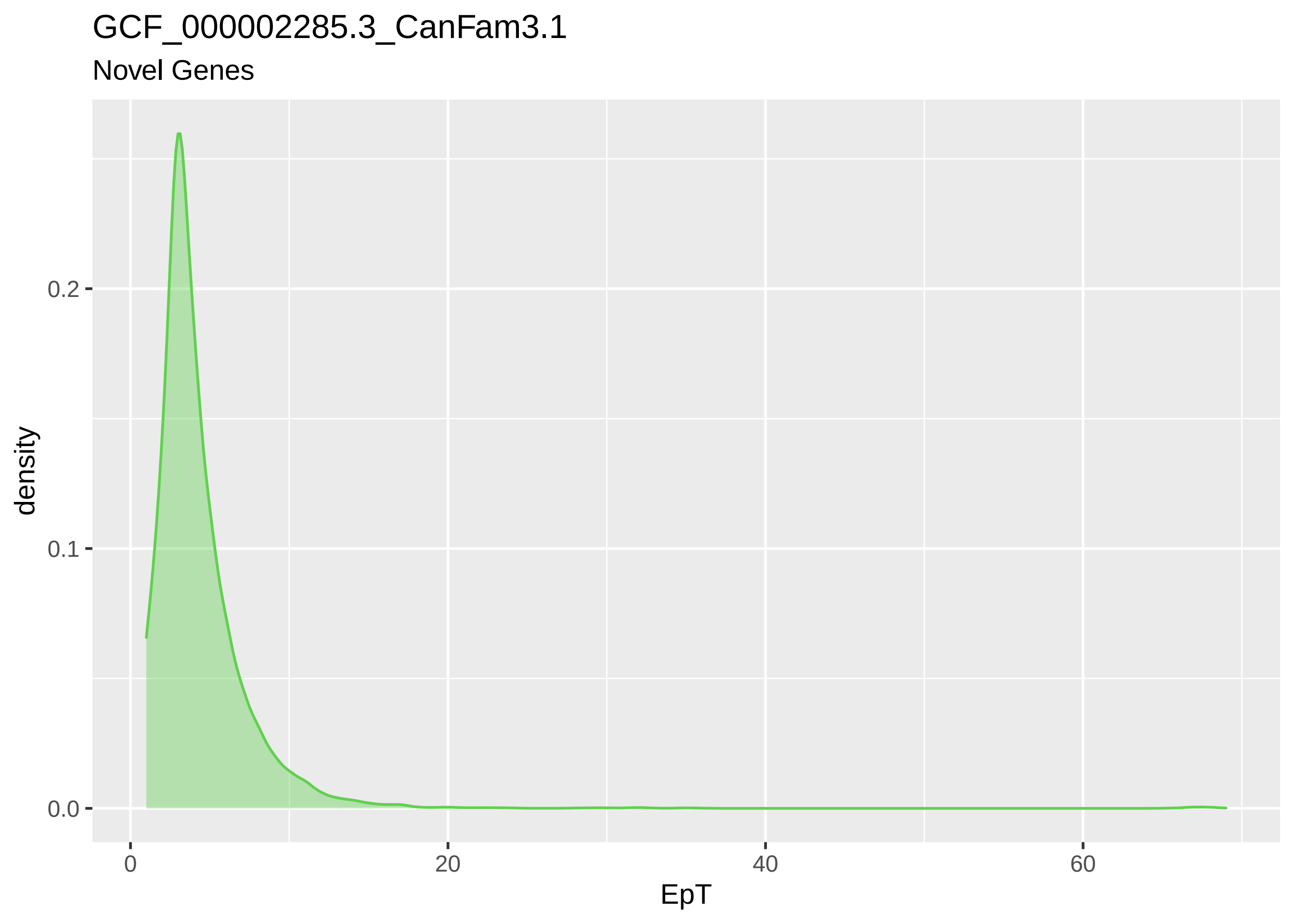
GCF_000002285.3_CanFam3.1
Novel Genes
density
0.2
0.1
0.0
0
20
40
60
EpT

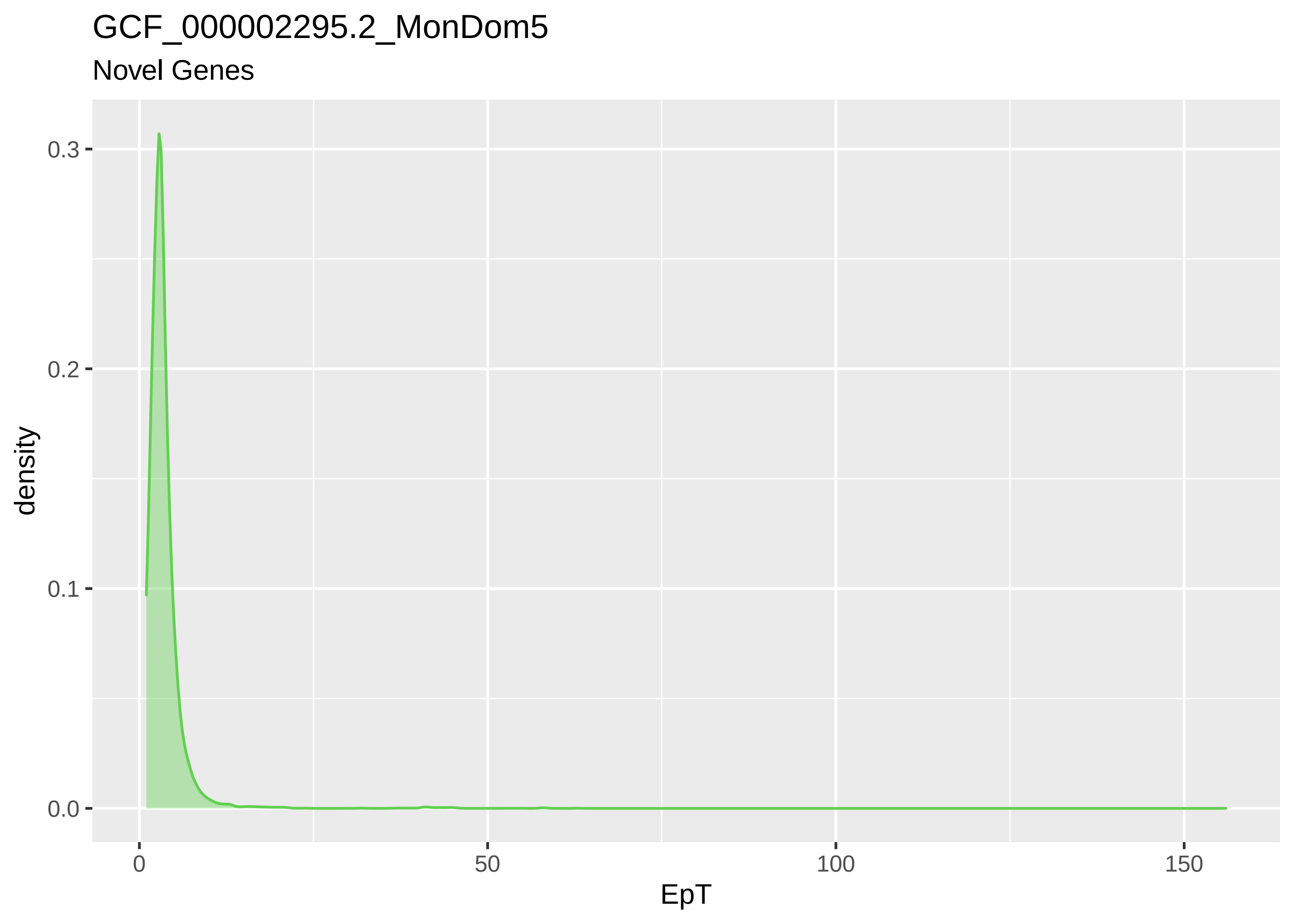

GCF_000002295.2_MonDom5
Novel Genes
density
0.0
0.1
0.2
0.3
0
50
100
150
EpT

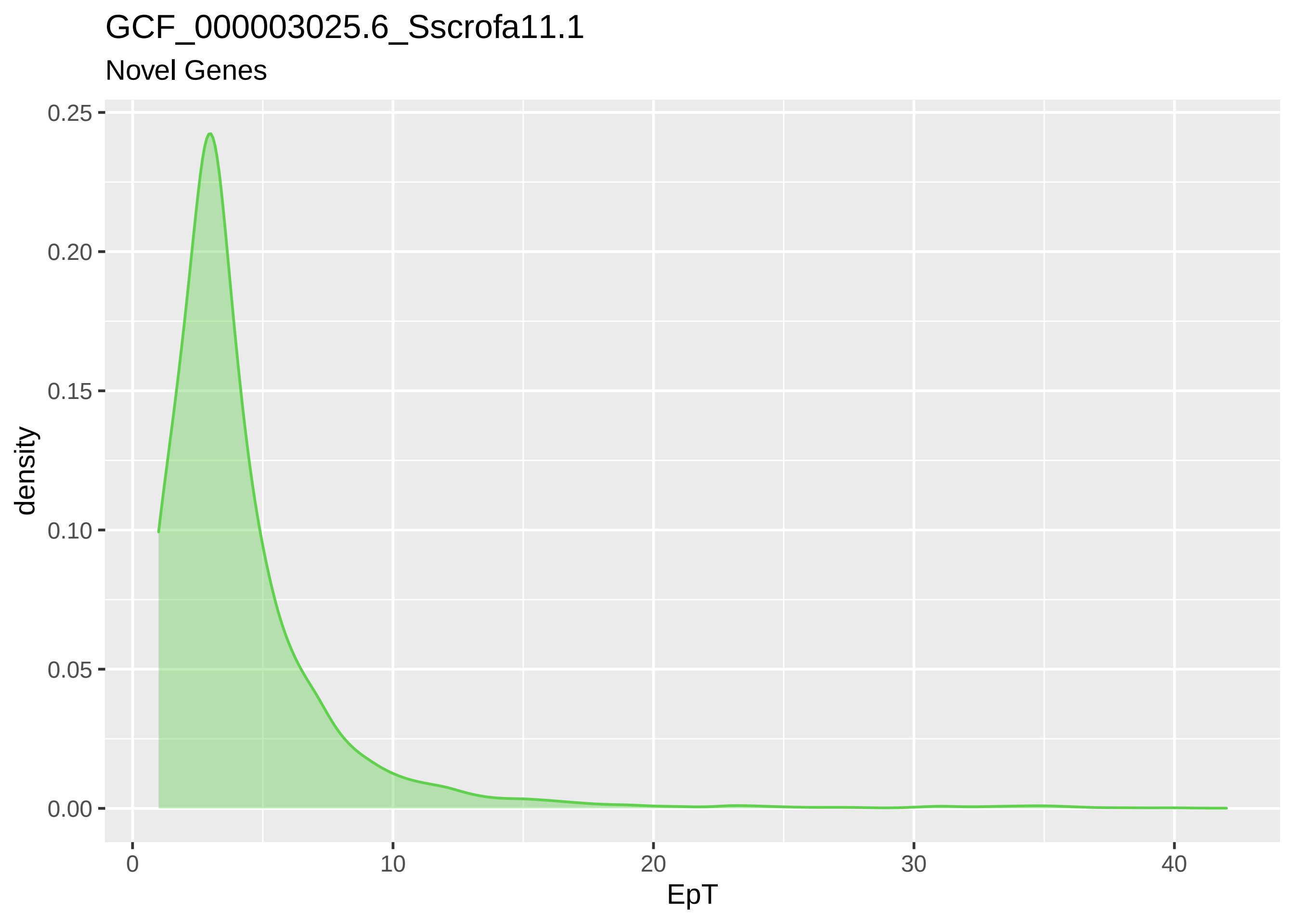

GCF_000003025.6_Sscrofa11.1
Novel Genes
density
0.25
0.20
0.15
0.10
0.05
0.00
0
10
20
30
40
EpT

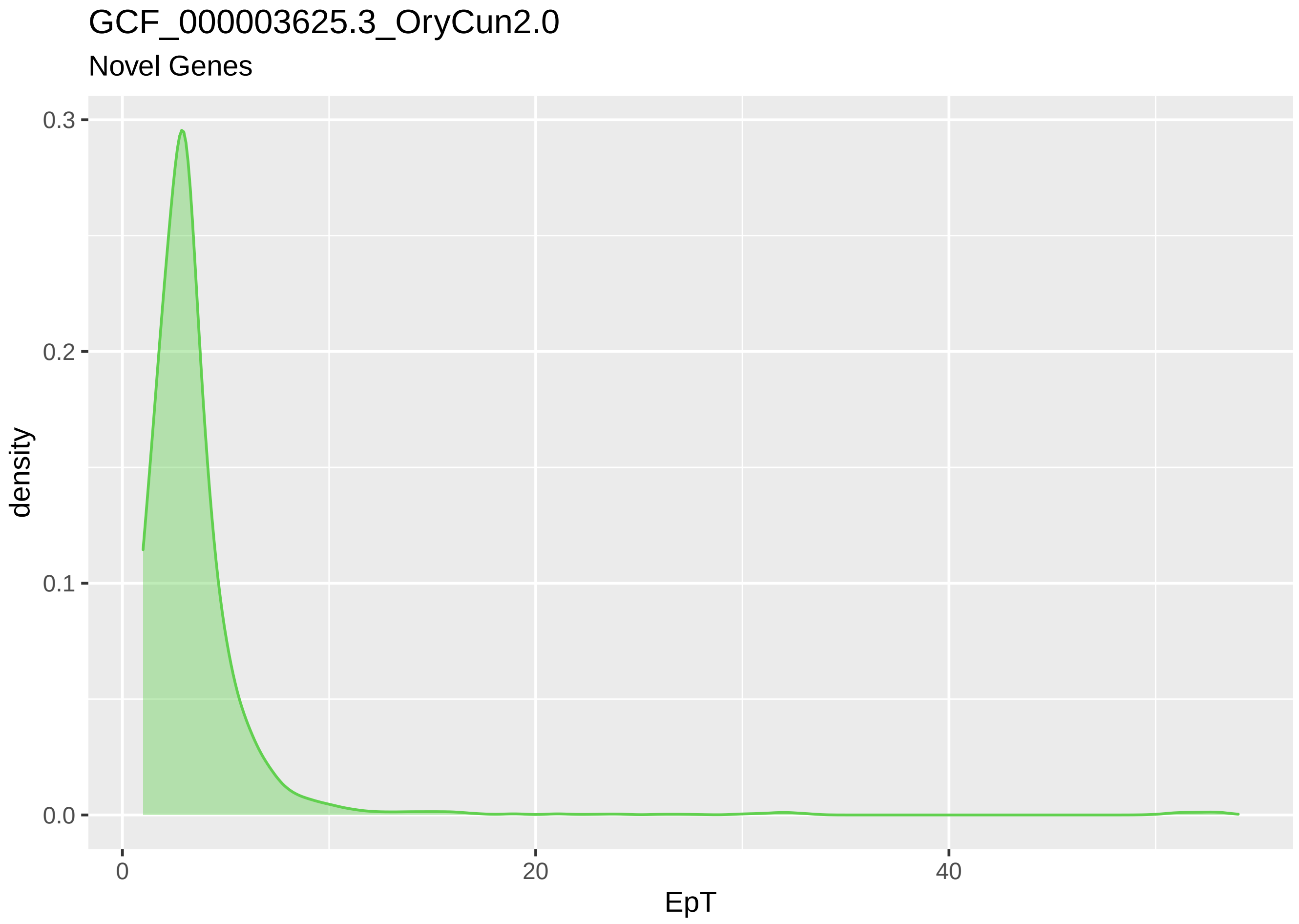

GCF_000003625.3_OryCun2.0
Novel Genes
0.3
0.2
0.1
0.0
density
0
20
40
EpT

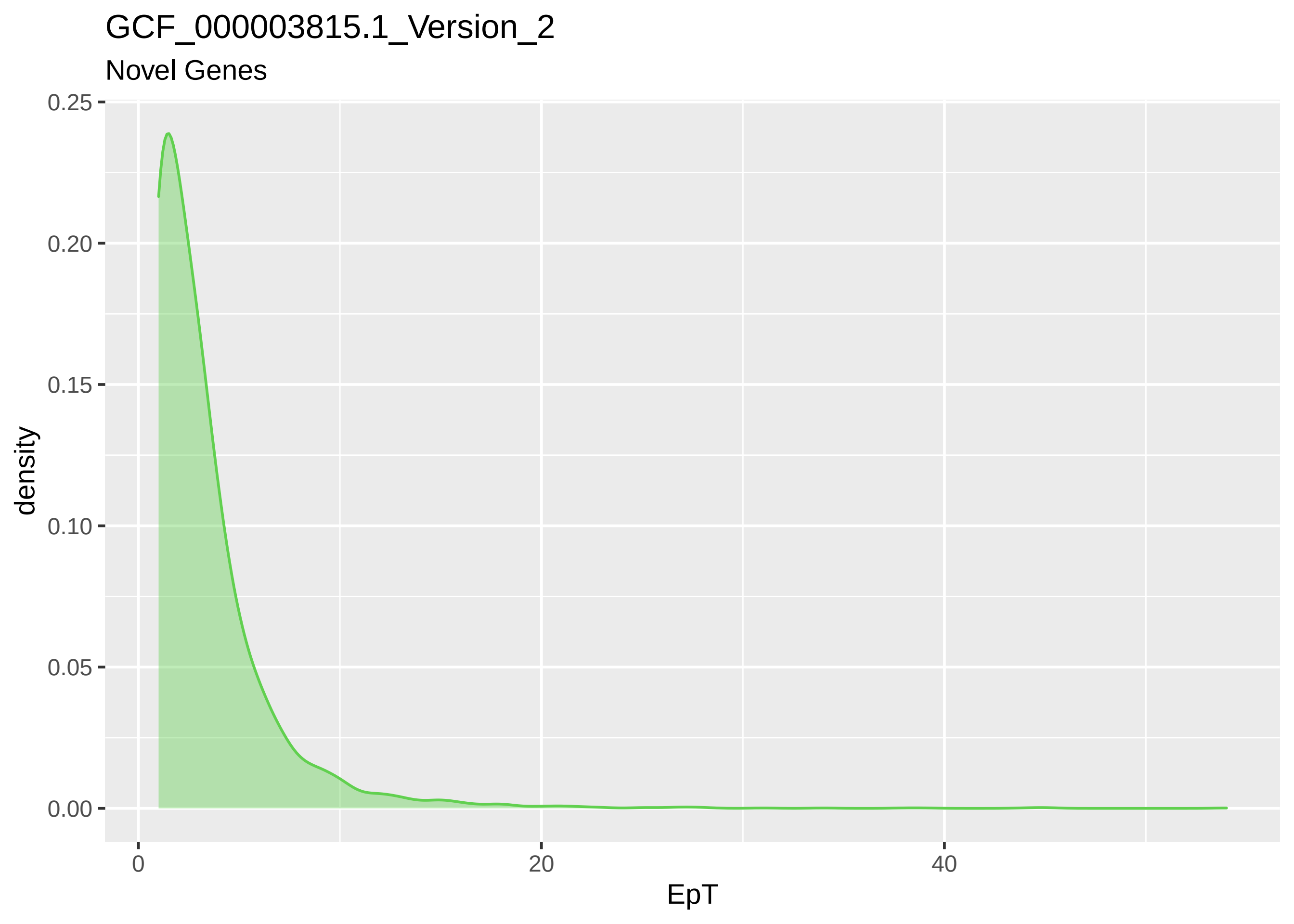
GCF_000003815.1_Version_2
Novel Genes
0.25
0.20
0.15
0.10
0.05
0.00
density
0
20
40
EpT

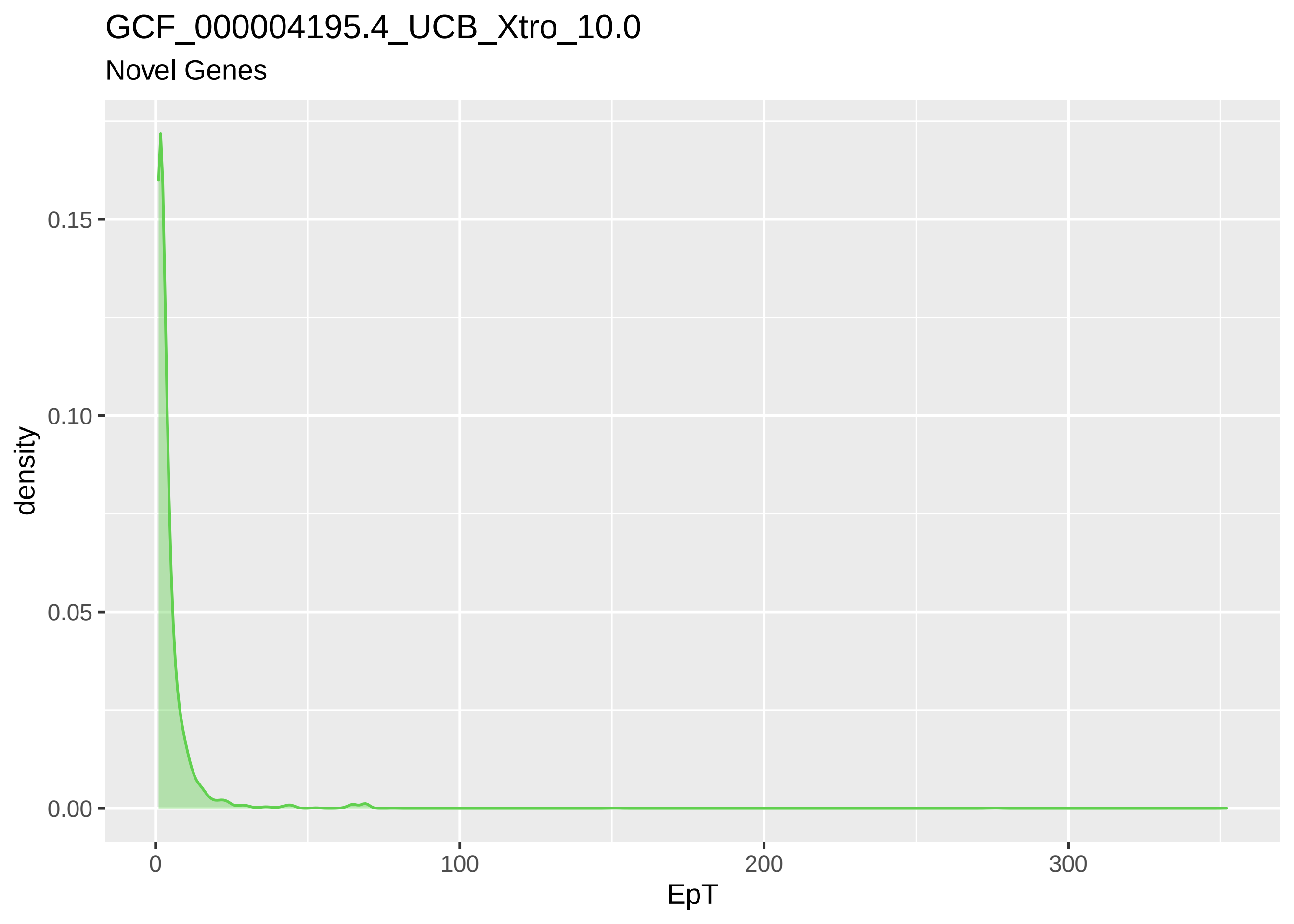
GCF_000004195.4_UCB_Xtro_10.0
Novel Genes
density
0.15
0.10
0.05
0.00
0
100
200
300
EpT

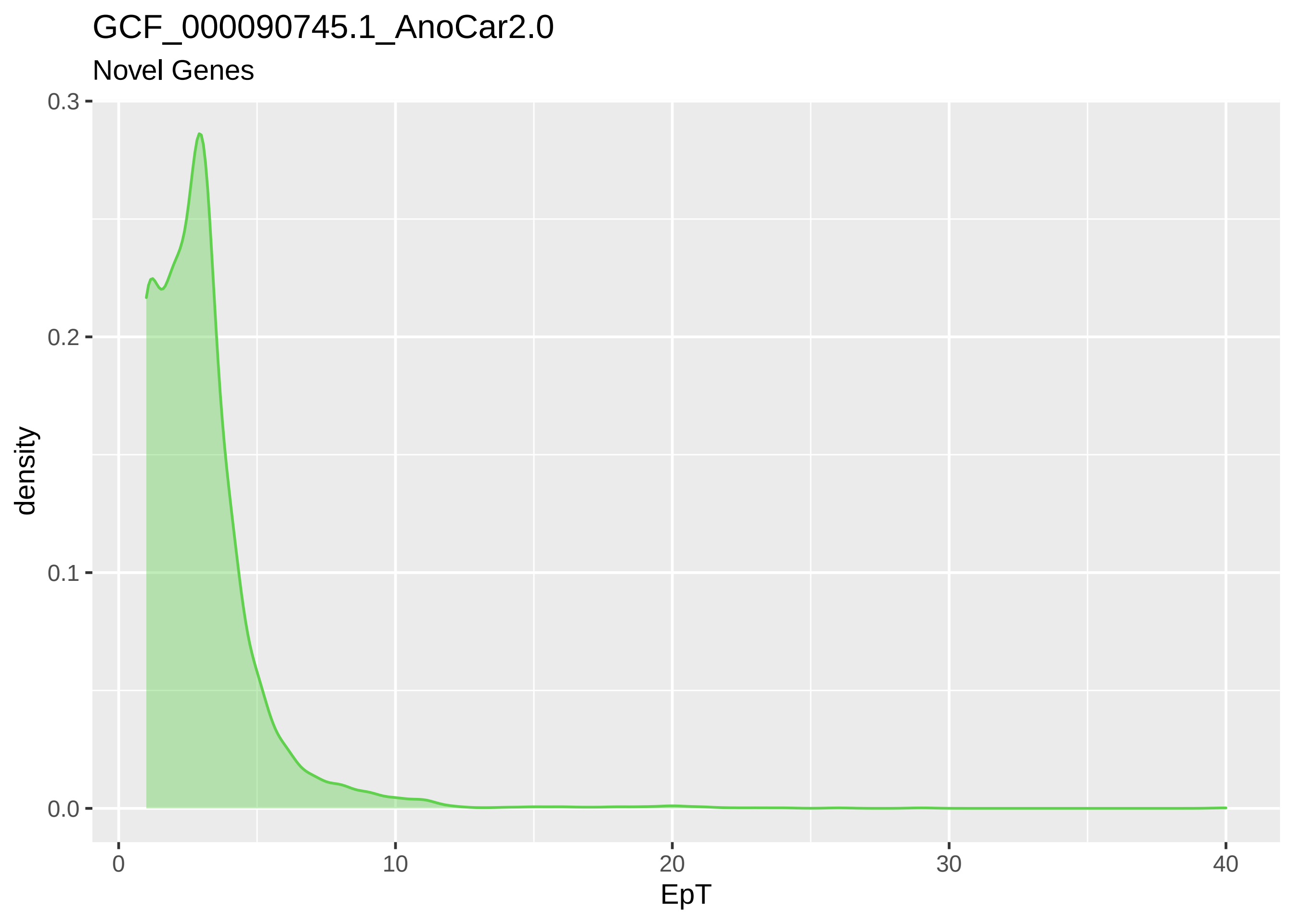
GCF_000090745.1_AnoCar2.0
Novel Genes
0.3
0.2
0.1
0.0
density
0
10
20
30
40
EpT

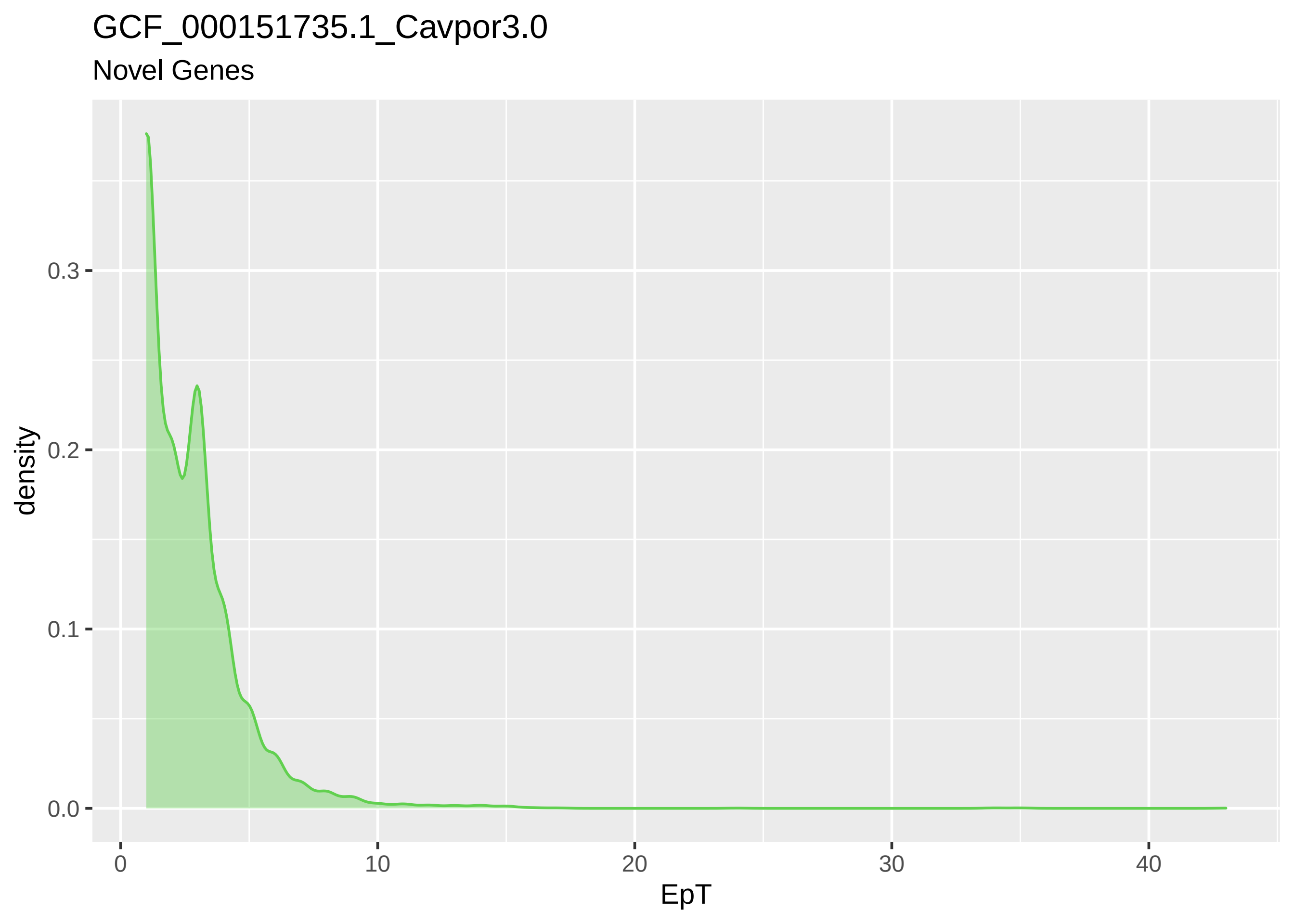

GCF_000151735.1_Cavpor3.0
Novel Genes
density
0.0
0.1
0.2
0.3
0
10
20
30
40
EpT

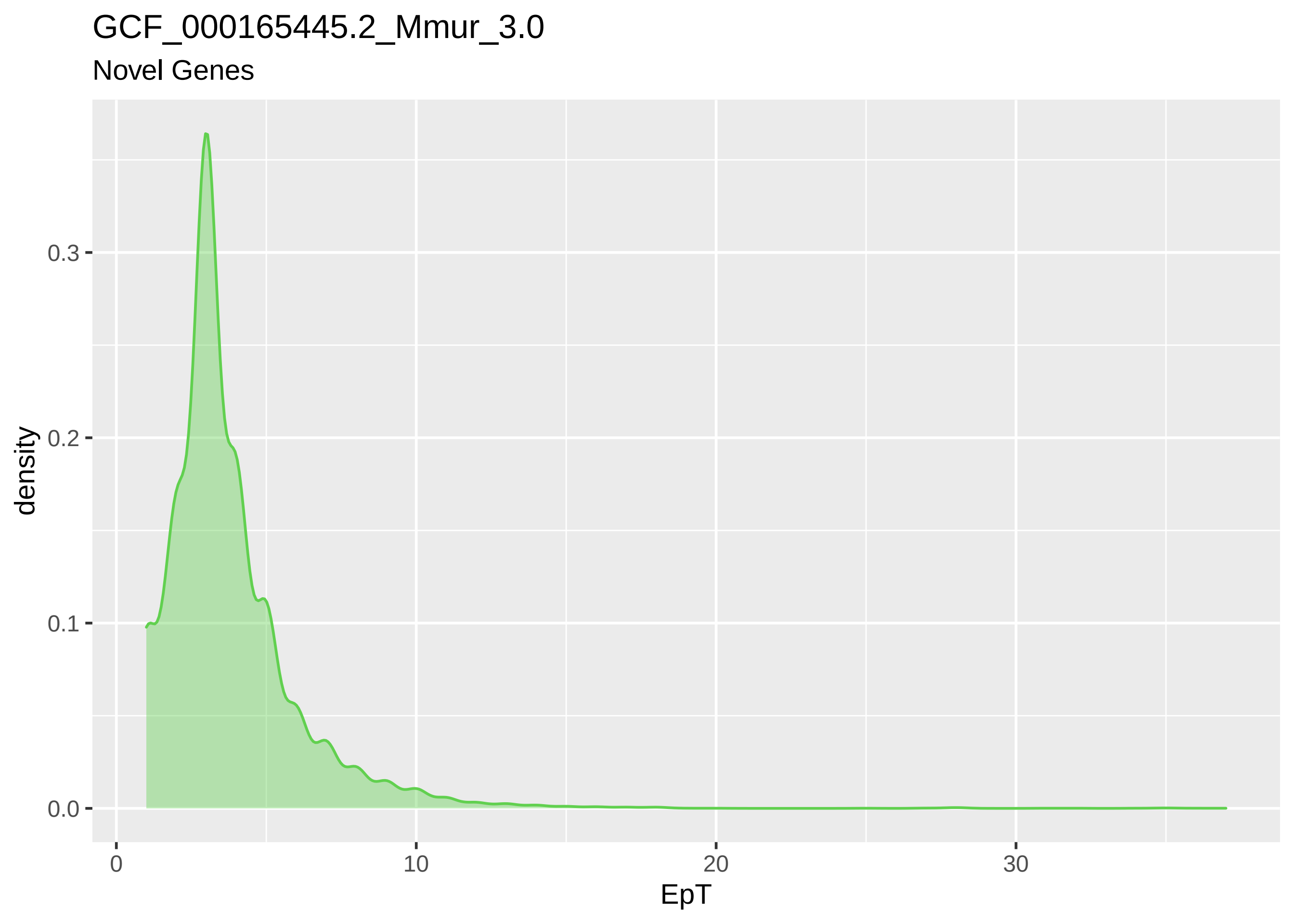
GCF_000165445.2_Mmur_3.0
Novel Genes
density
0.0
0.1
0.2
0.3
0
10
20
30
EpT

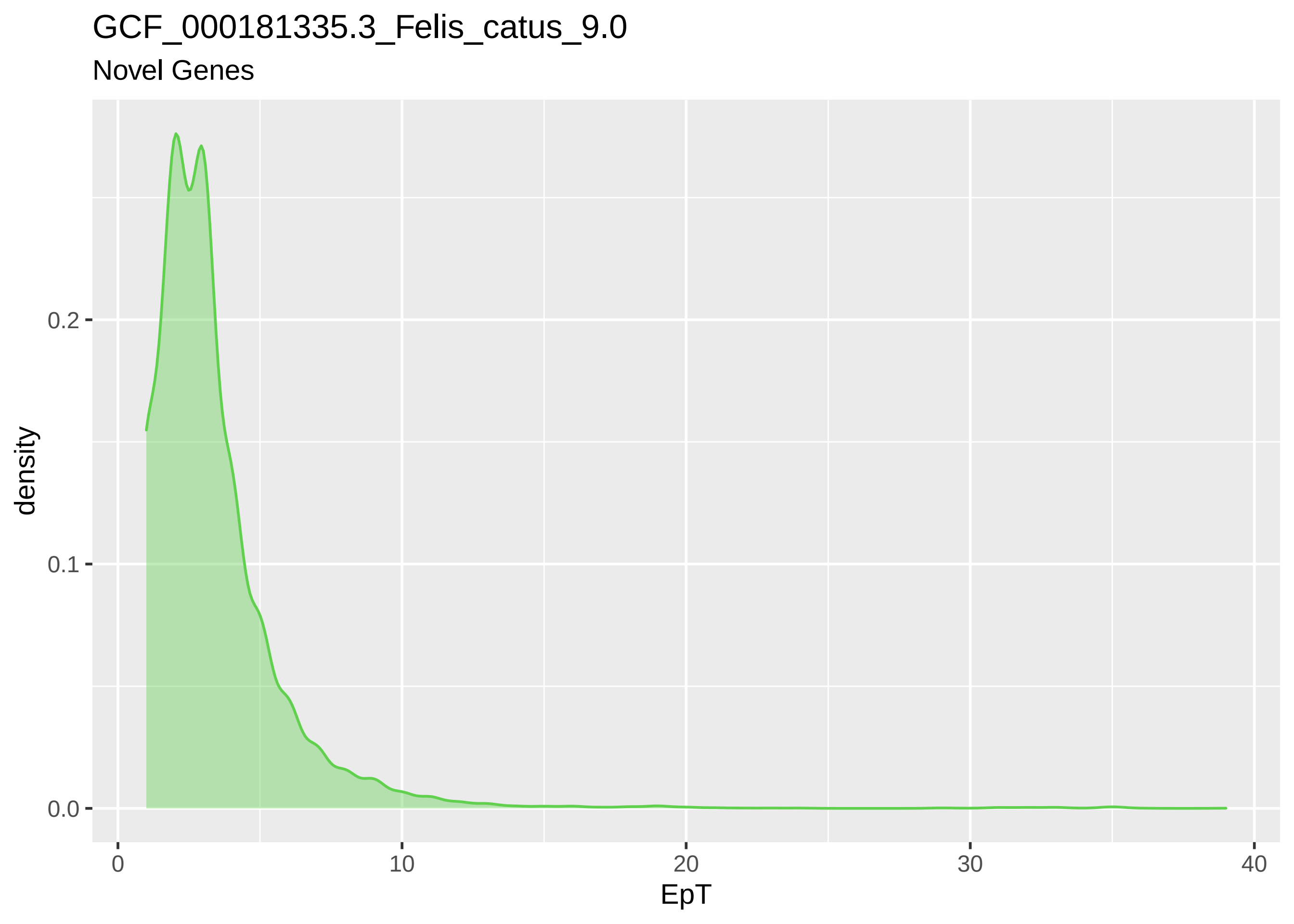
GCF_000181335.3_Felis_catus_9.0
Novel Genes
density
0.0
0.1
0.2
0
10
20
30
40
EpT

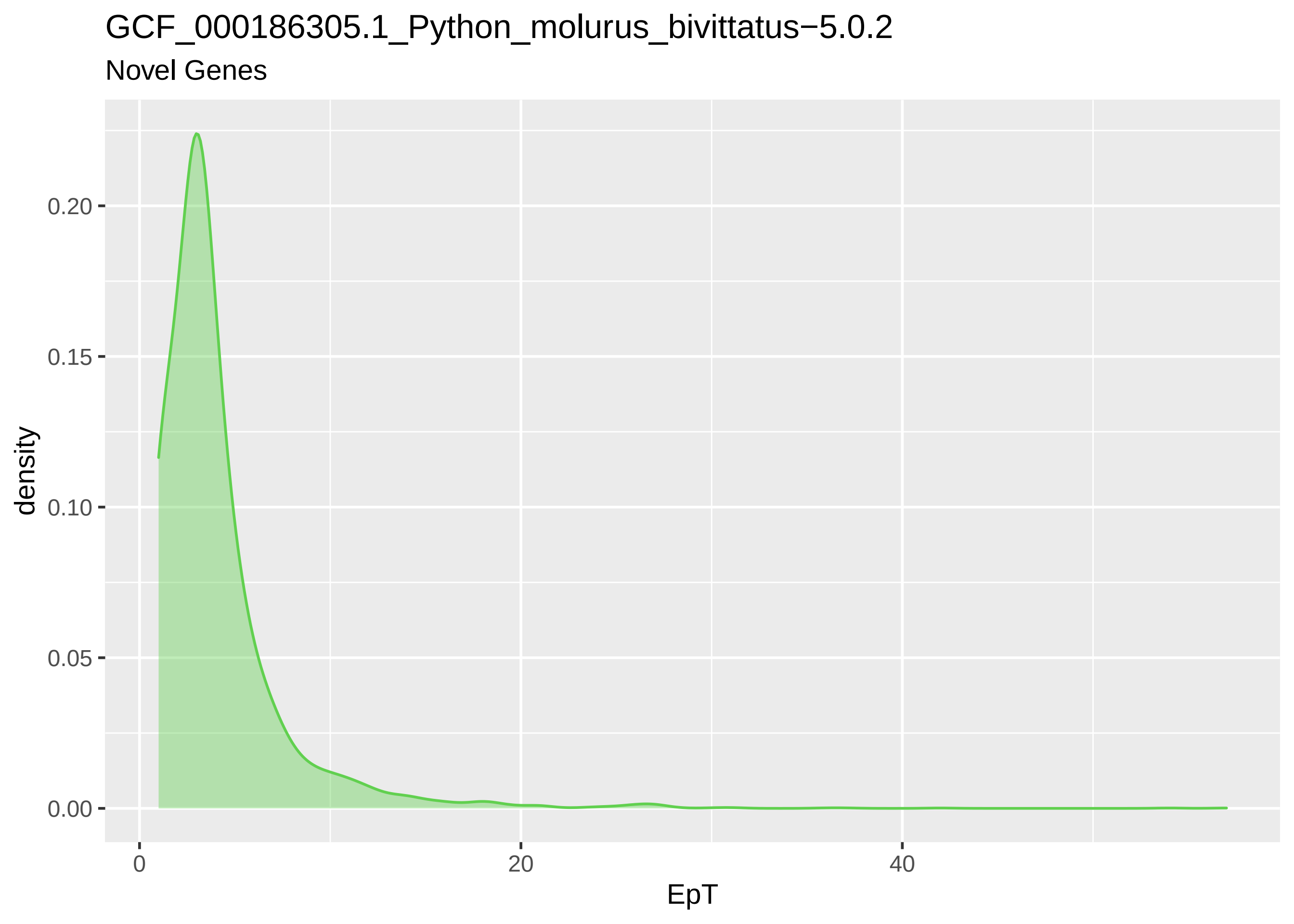
GCF_000186305.1_Python_molurus_bivittatus−5.0.2
Novel Genes
density
0.00
0.05
0.10
0.15
0.20
0
20
40
EpT

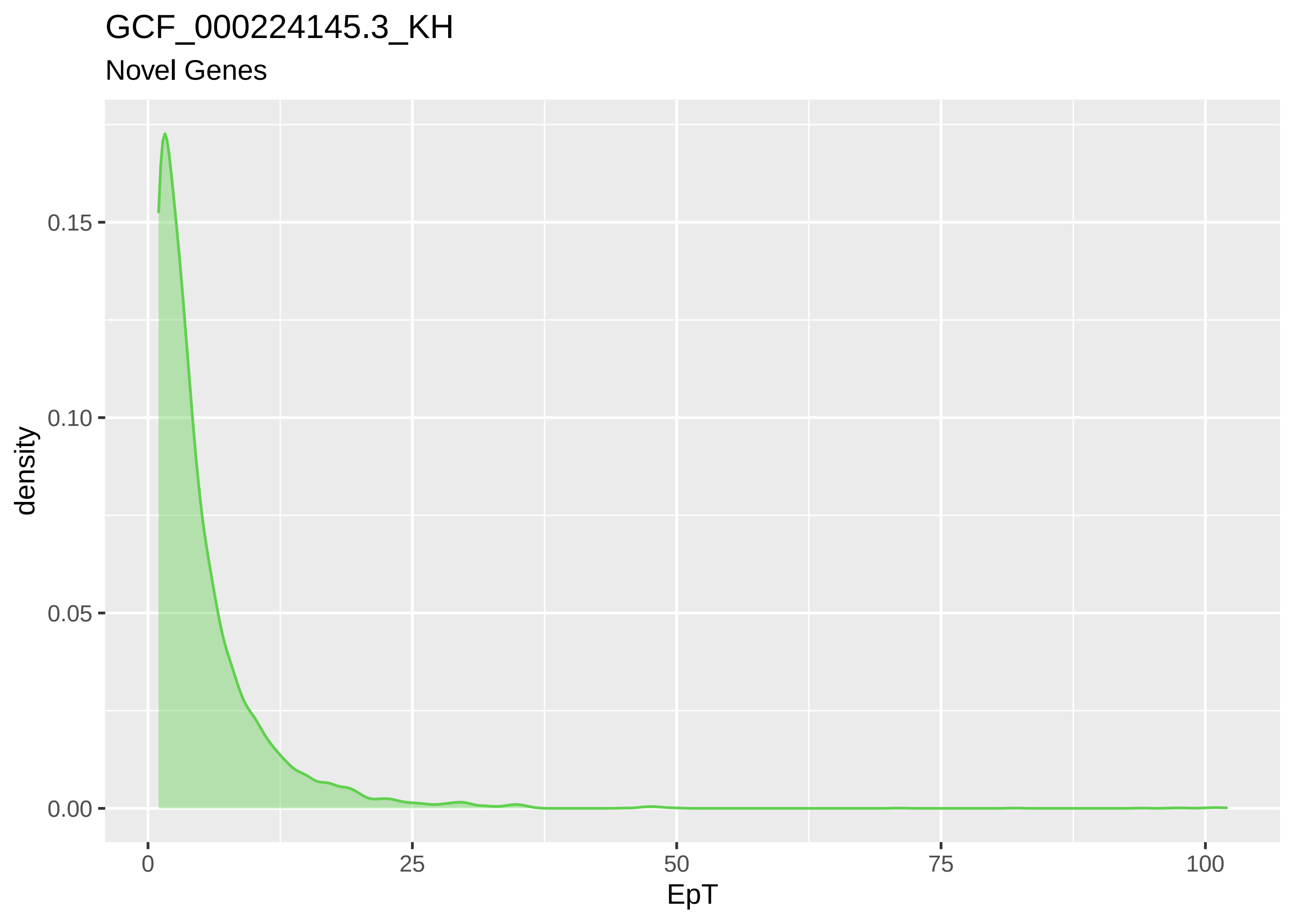
GCF_000224145.3_KH
Novel Genes
density
0.15
0.10
0.05
0.00
0
25
50
75
100
EpT

GCF_000225785.1_LatCha1

Novel Genes

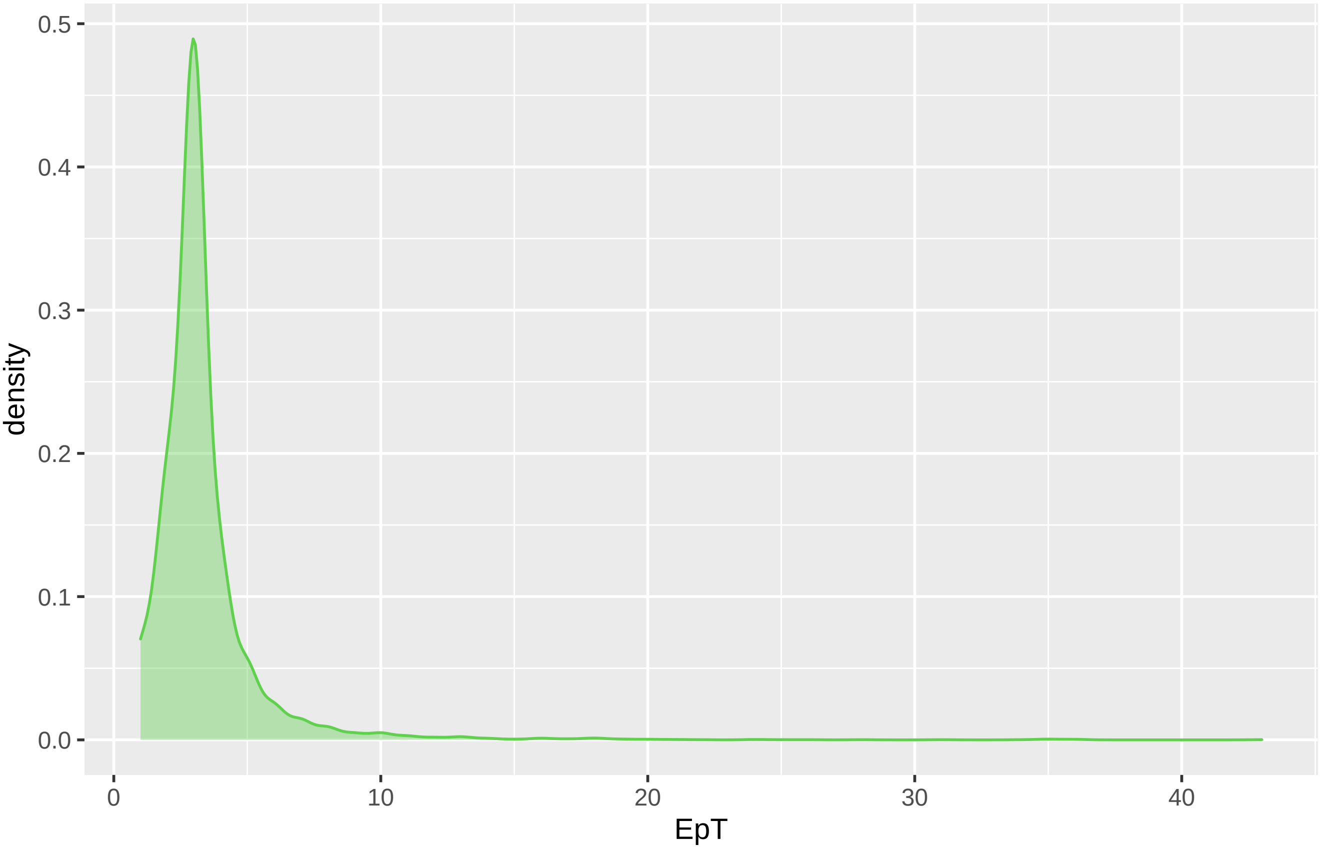

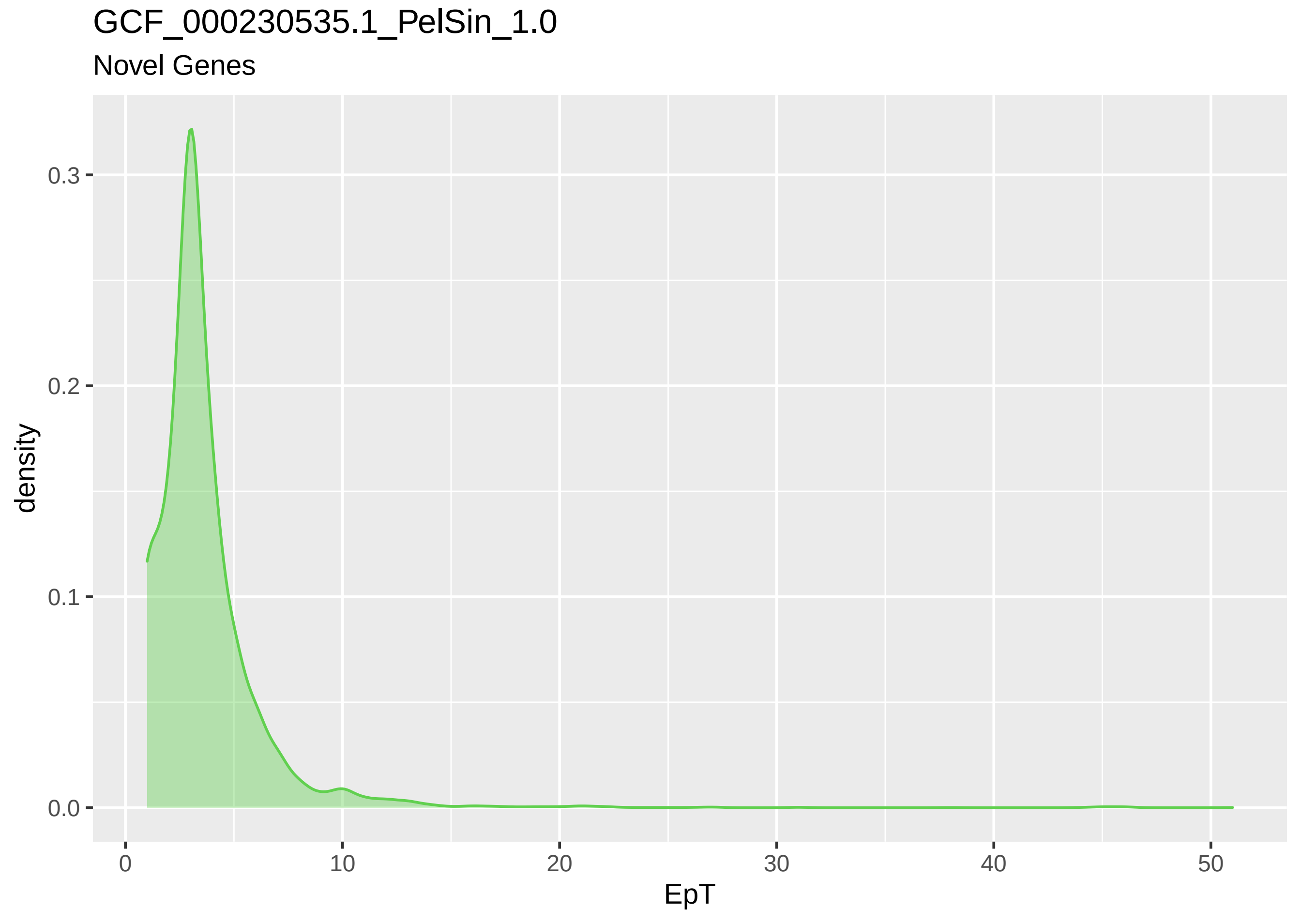
GCF_000230535.1_PelSin_1.0
Novel Genes
density
0.0
0.1
0.2
0.3
0
10
20
30
40
50
EpT

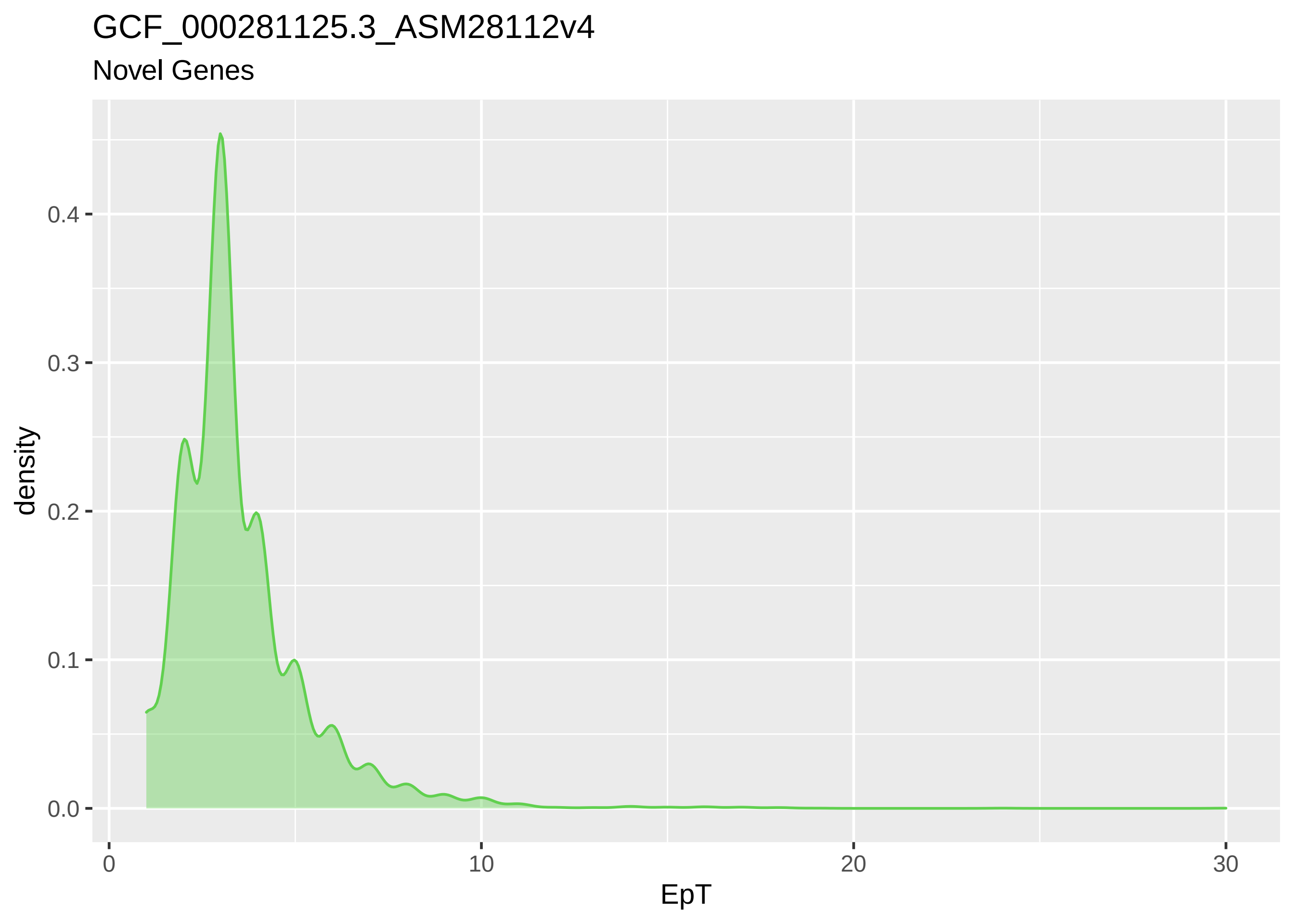
GCF_000281125.3_ASM28112v4
Novel Genes
density
0.0
0.1
0.2
0.3
0.4
0
10
20
30
EpT

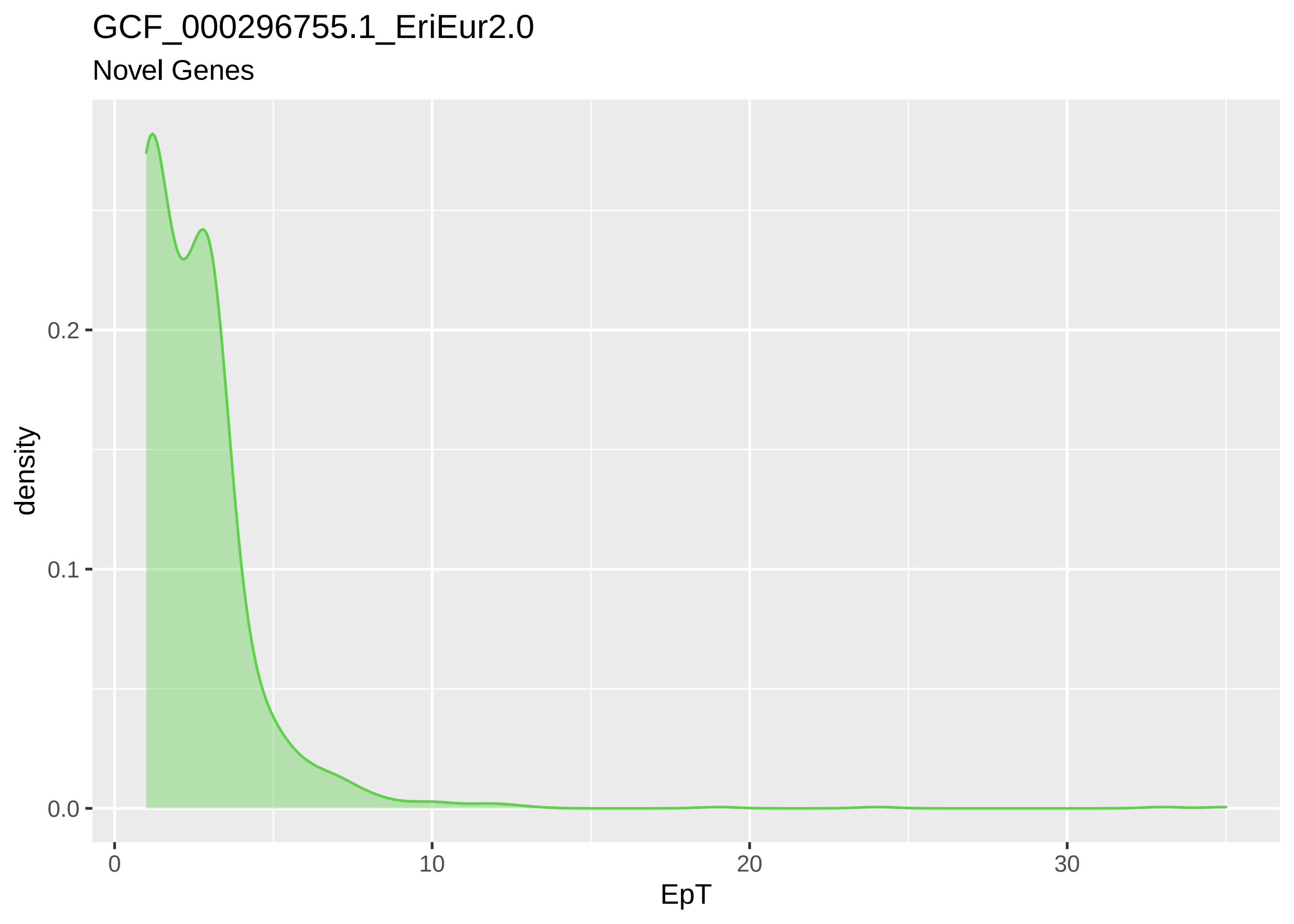
GCF_000296755.1_EriEur2.0
Novel Genes
density
0.2
0.1
0.0
0
10
20
30
EpT

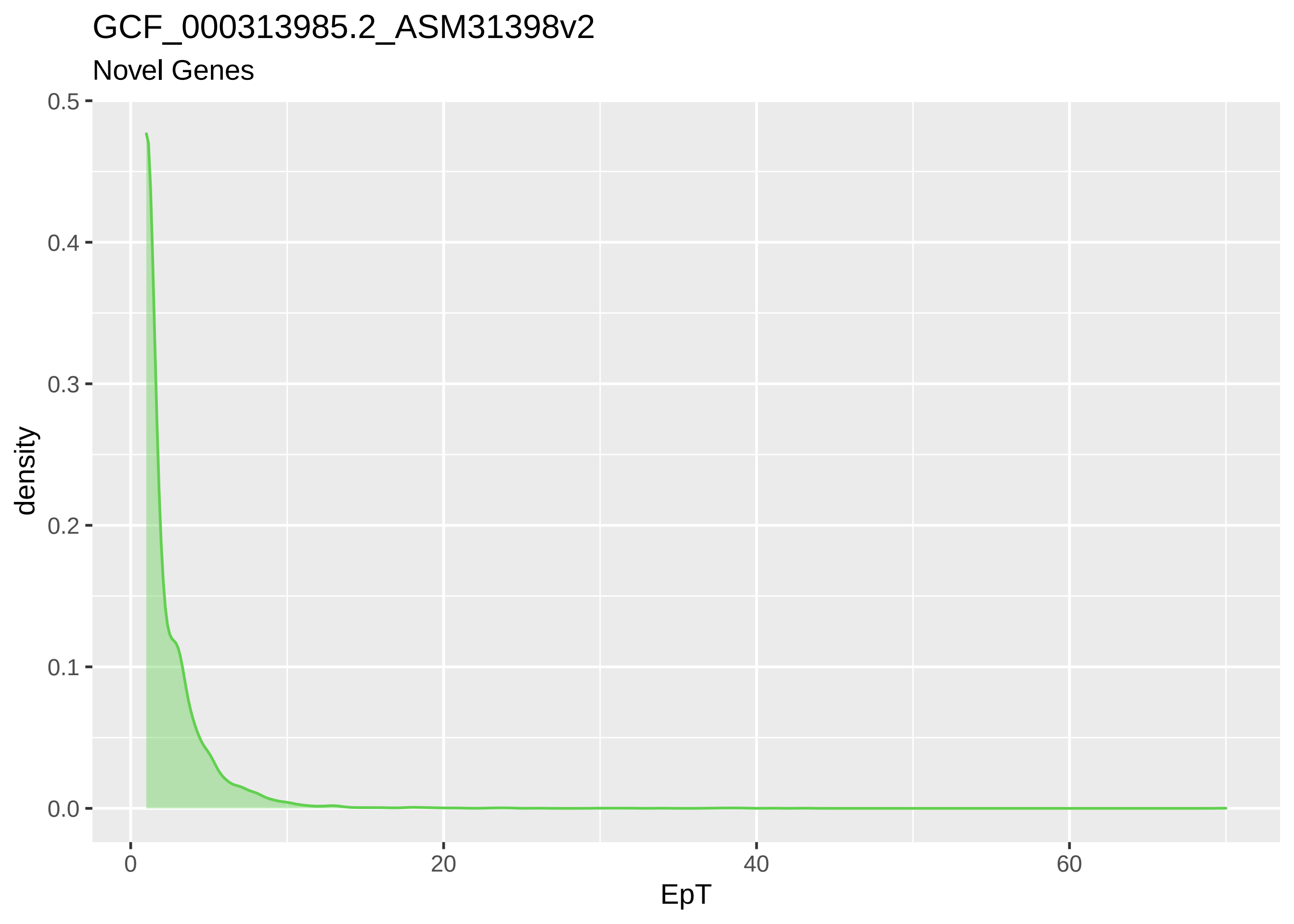
GCF_000313985.2_ASM31398v2
Novel Genes
density
0.0
0.1
0.2
0.3
0.4
0.5
0
20
40
60
EpT

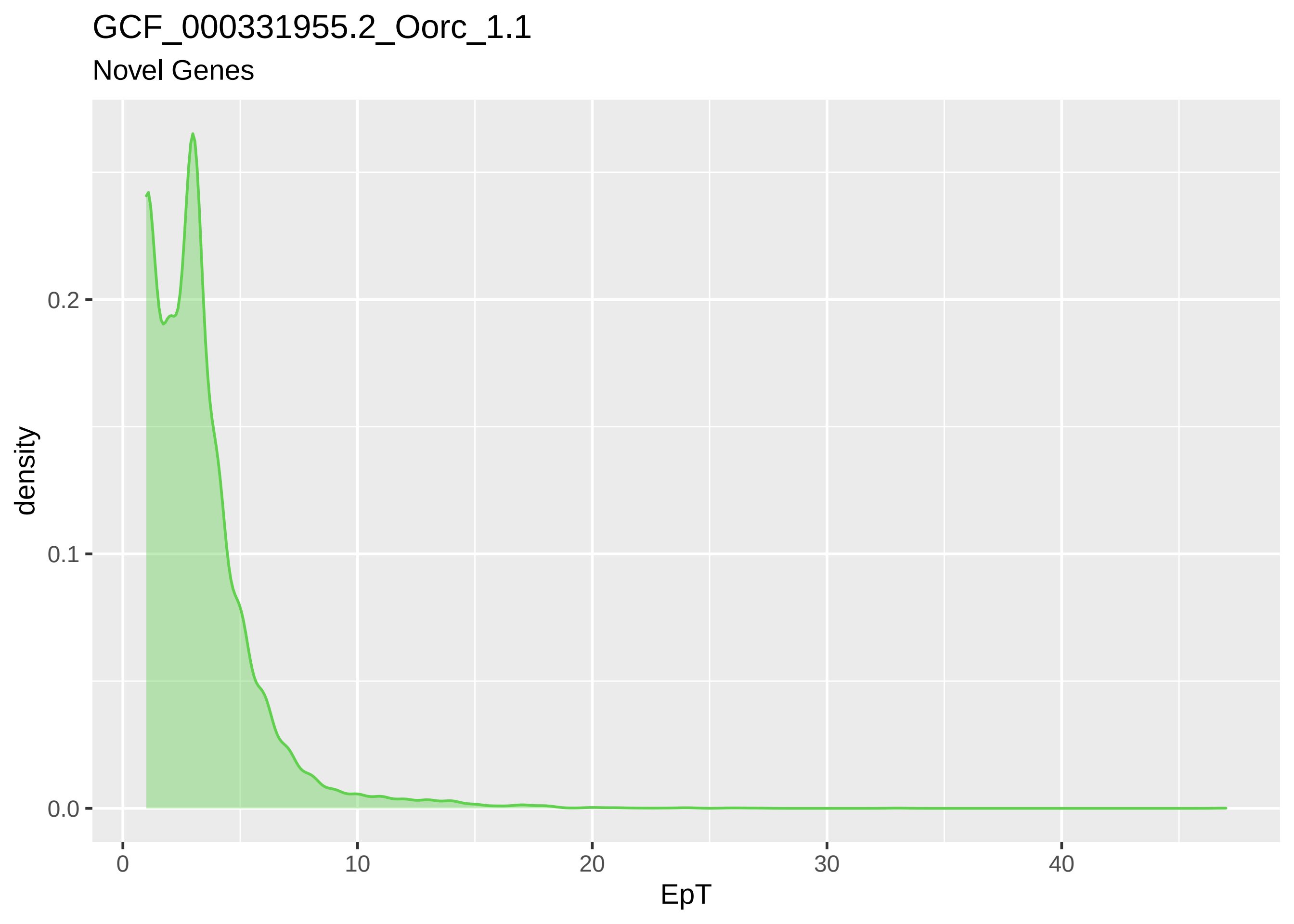
GCF_000331955.2_Oorc_1.1
Novel Genes
density
0.2
0.1
0.0
0
10
20
30
40
EpT

GCF_000334495.1_TupChi_1.0

Novel Genes

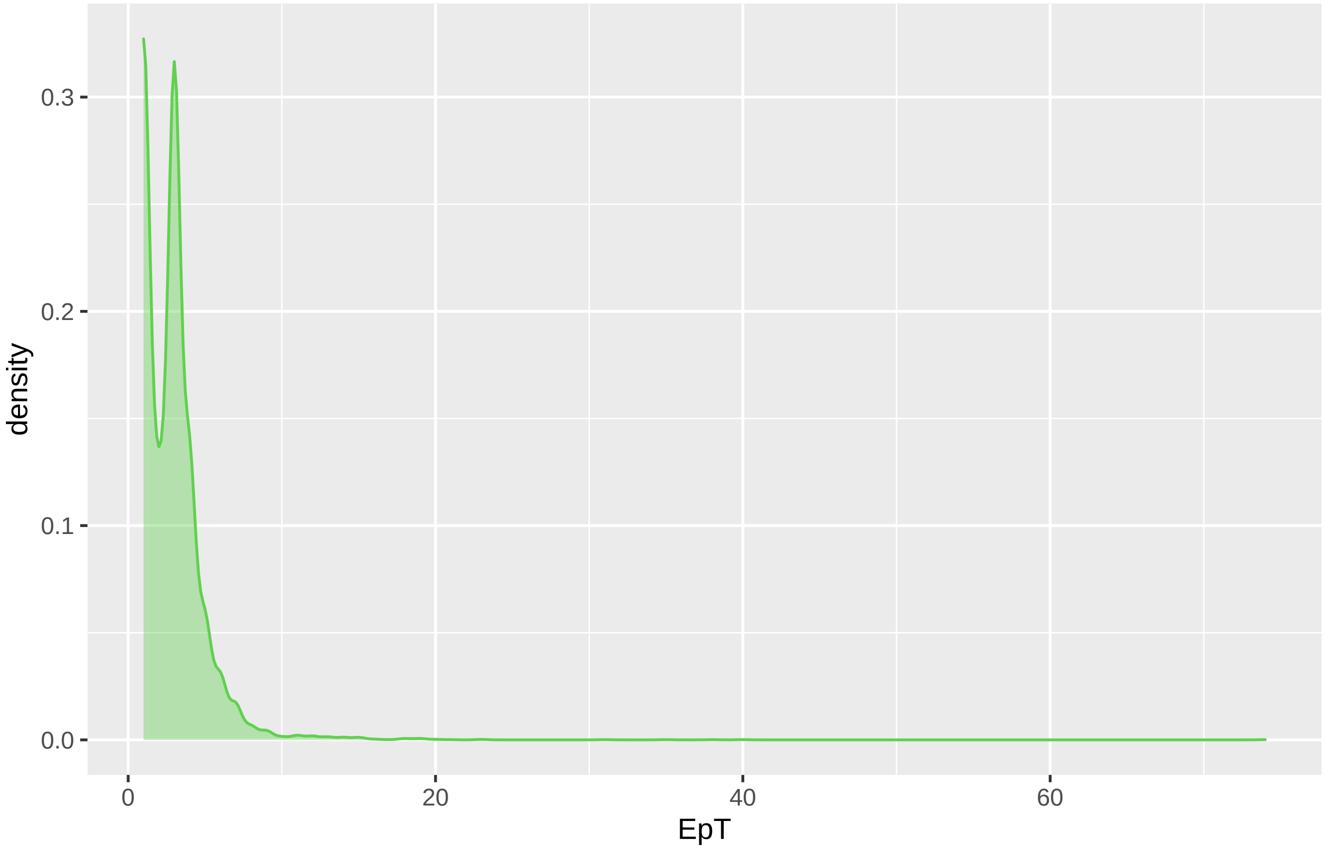

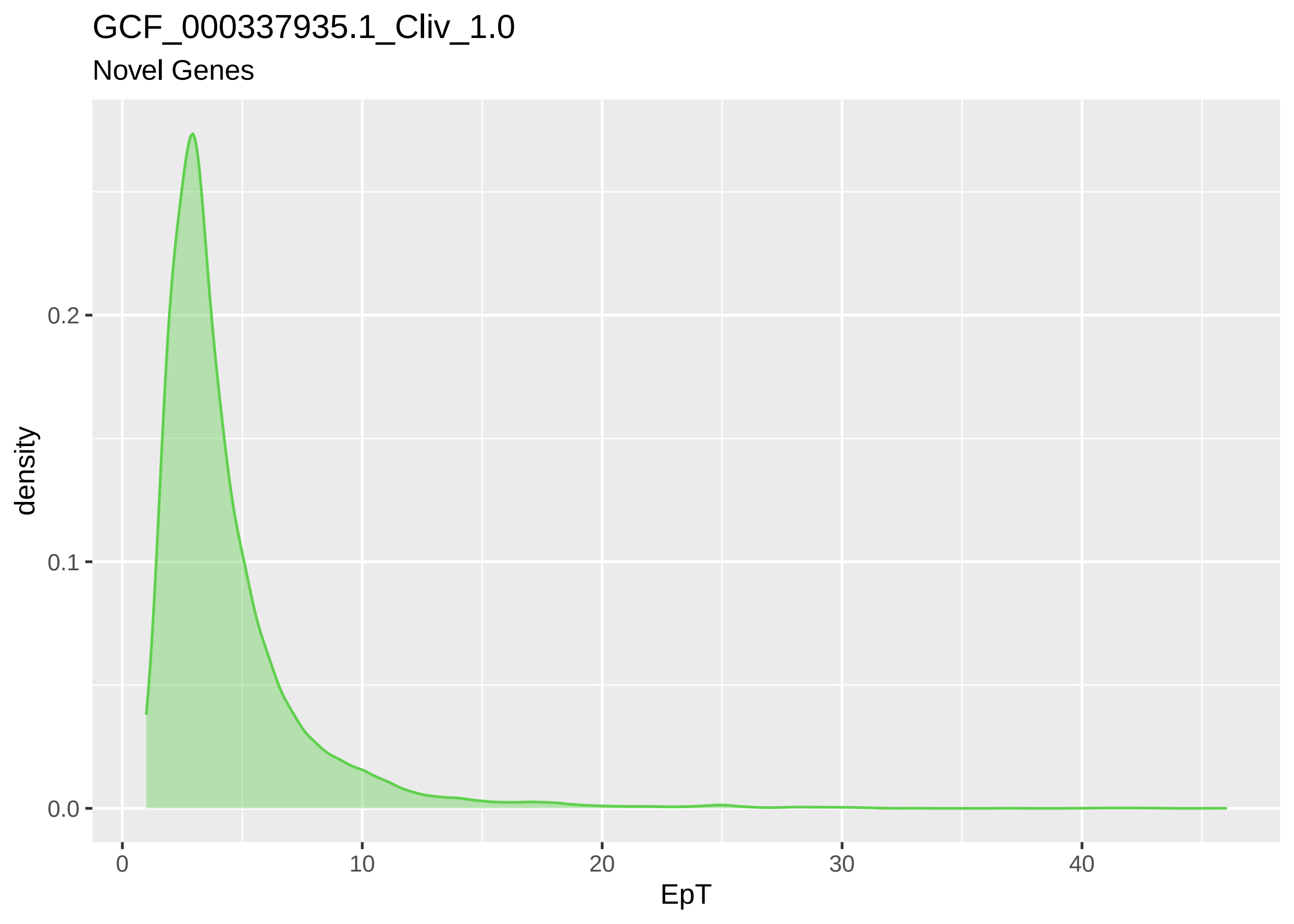
GCF_000337935.1_Cliv_1.0
Novel Genes
density
0.0
0.1
0.2
0
10
20
30
40
EpT

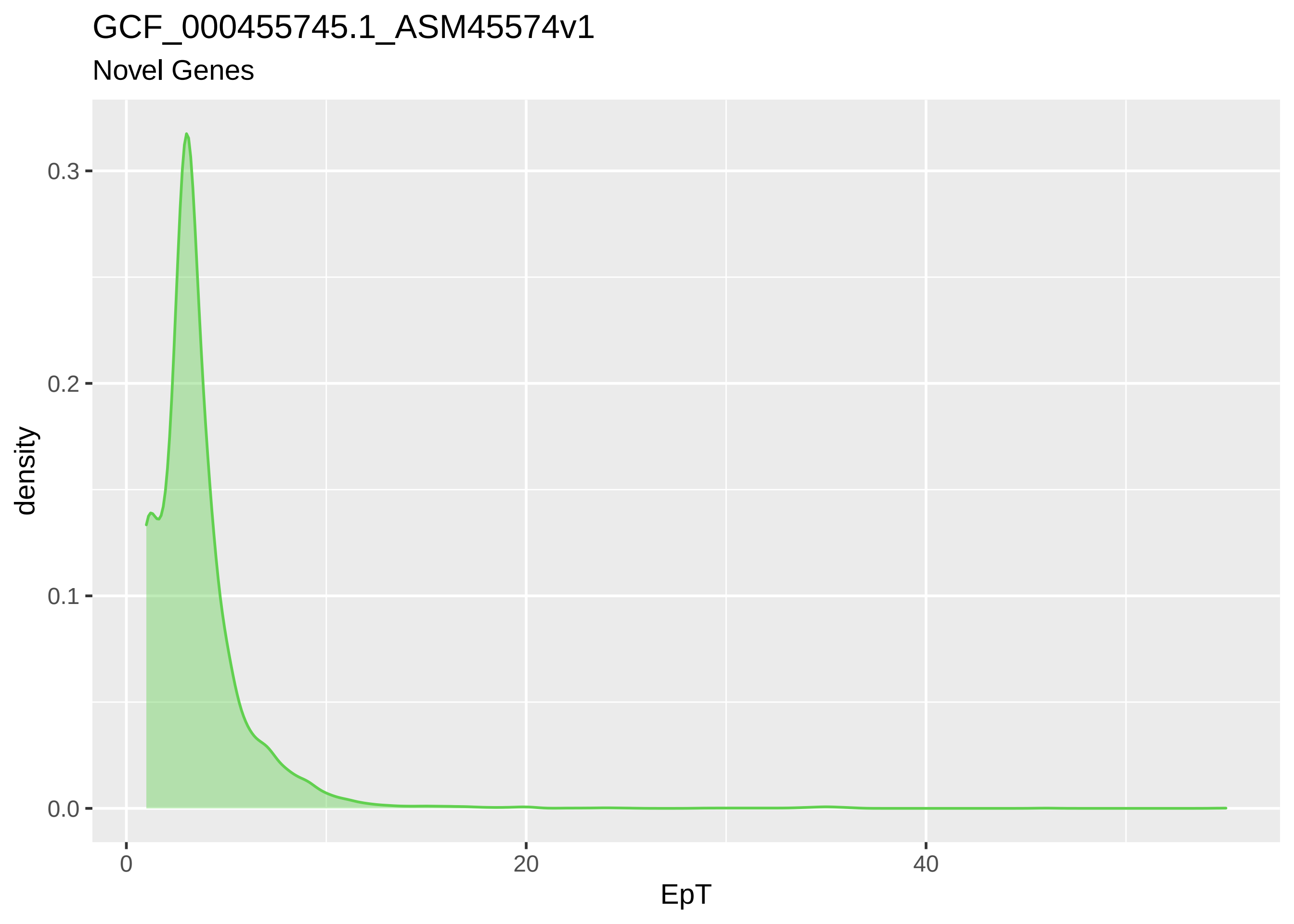
GCF_000455745.1_ASM45574v1
Novel Genes
density
0.3
0.2
0.1
0.0
0
20
40
EpT

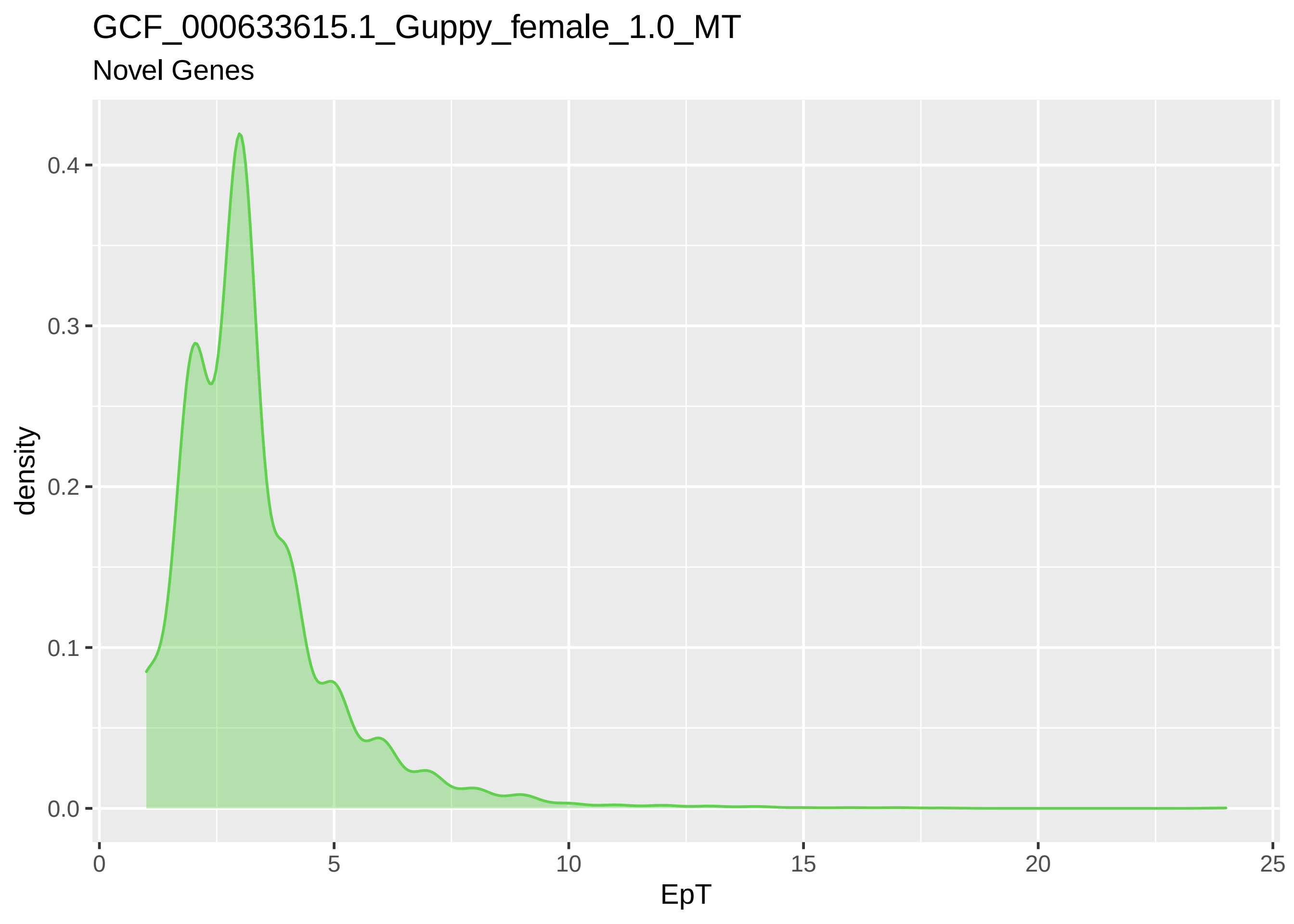
GCF_000633615.1_Guppy_female_1.0_MT
Novel Genes
density
0.0
0.1
0.2
0.3
0.4
0
5
10
15
20
25
EpT

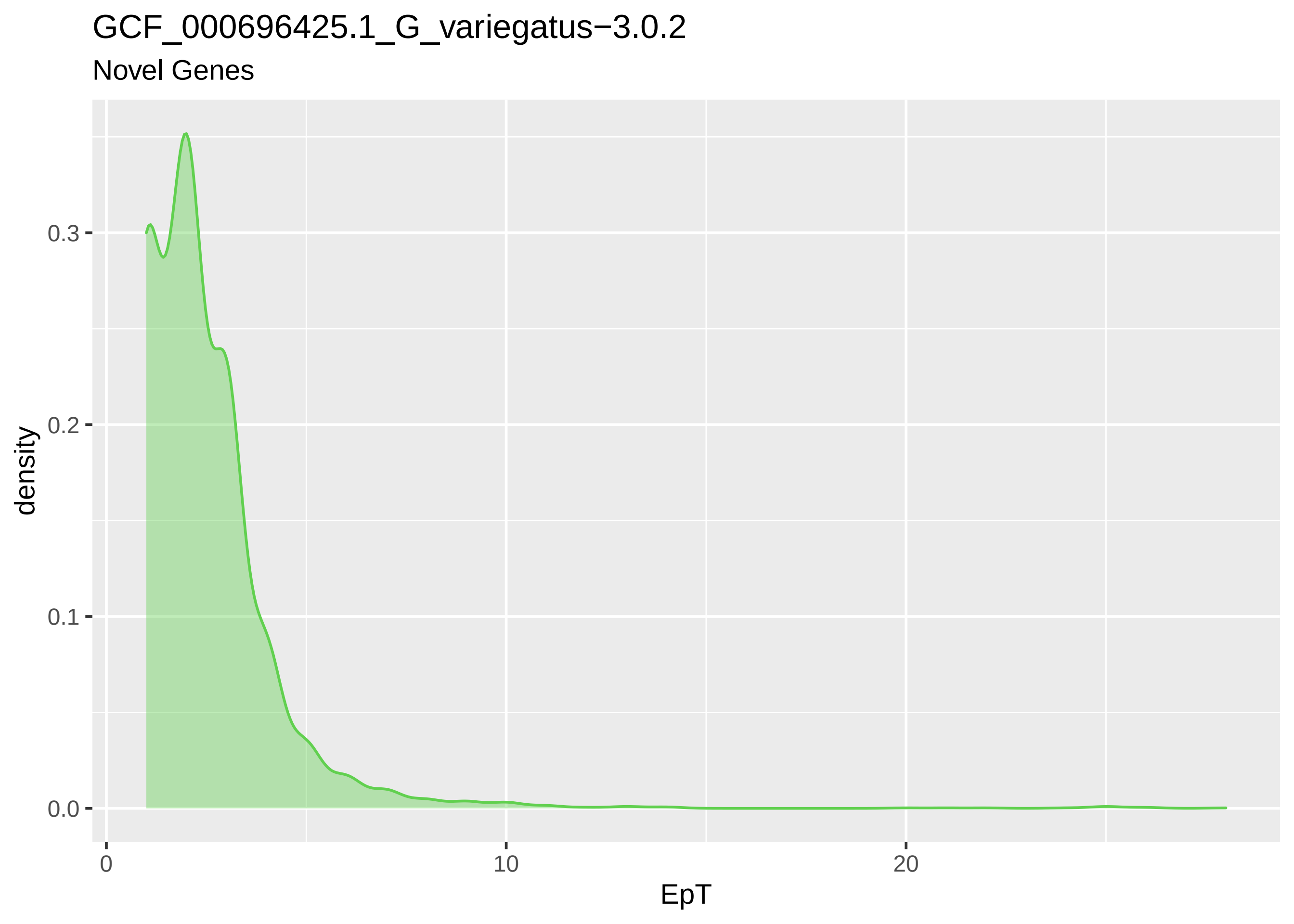
GCF_000696425.1_G_variegatus−3.0.2
Novel Genes
density
0.3
0.2
0.1
0.0
0
10
20
EpT

GCF_000705375.1_ASM70537v2

Novel Genes

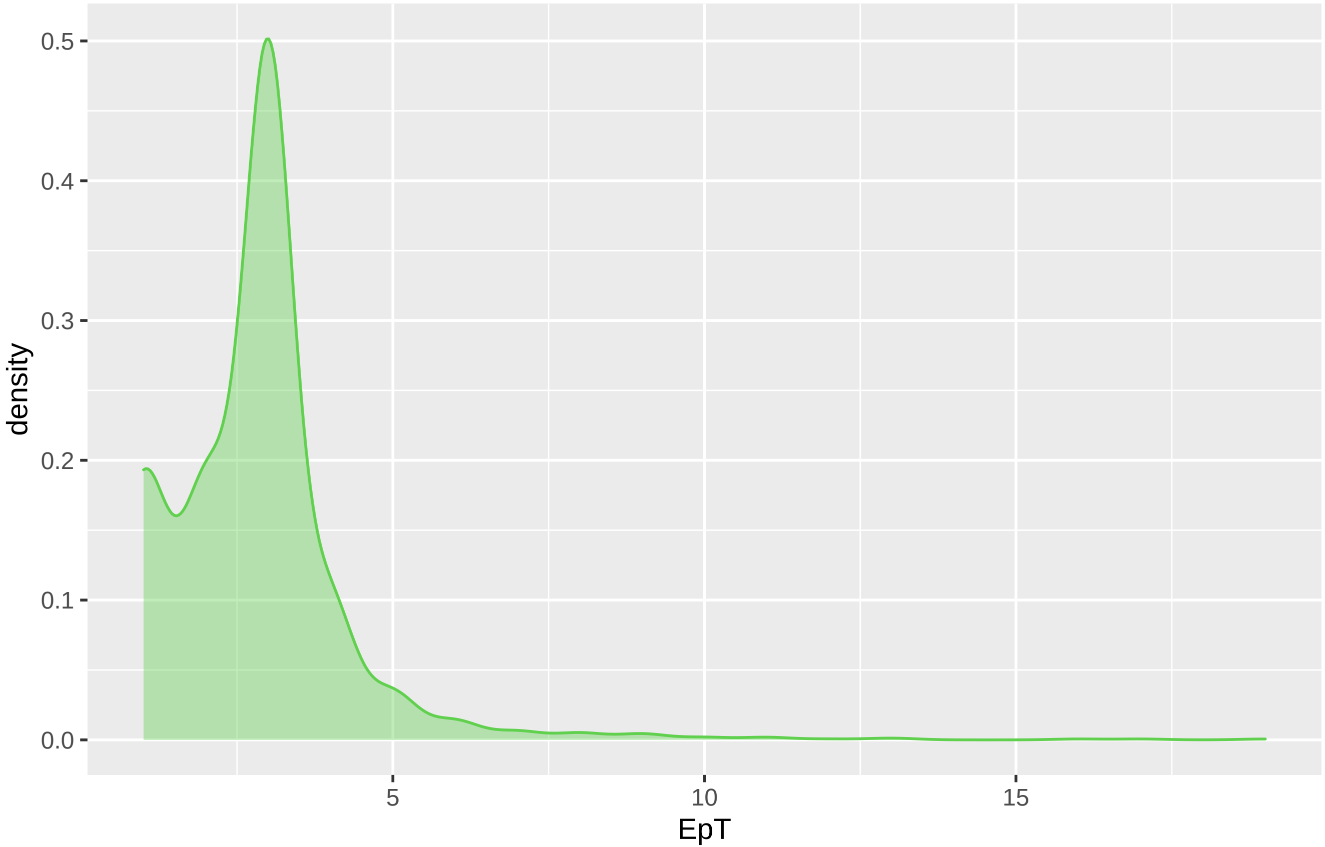

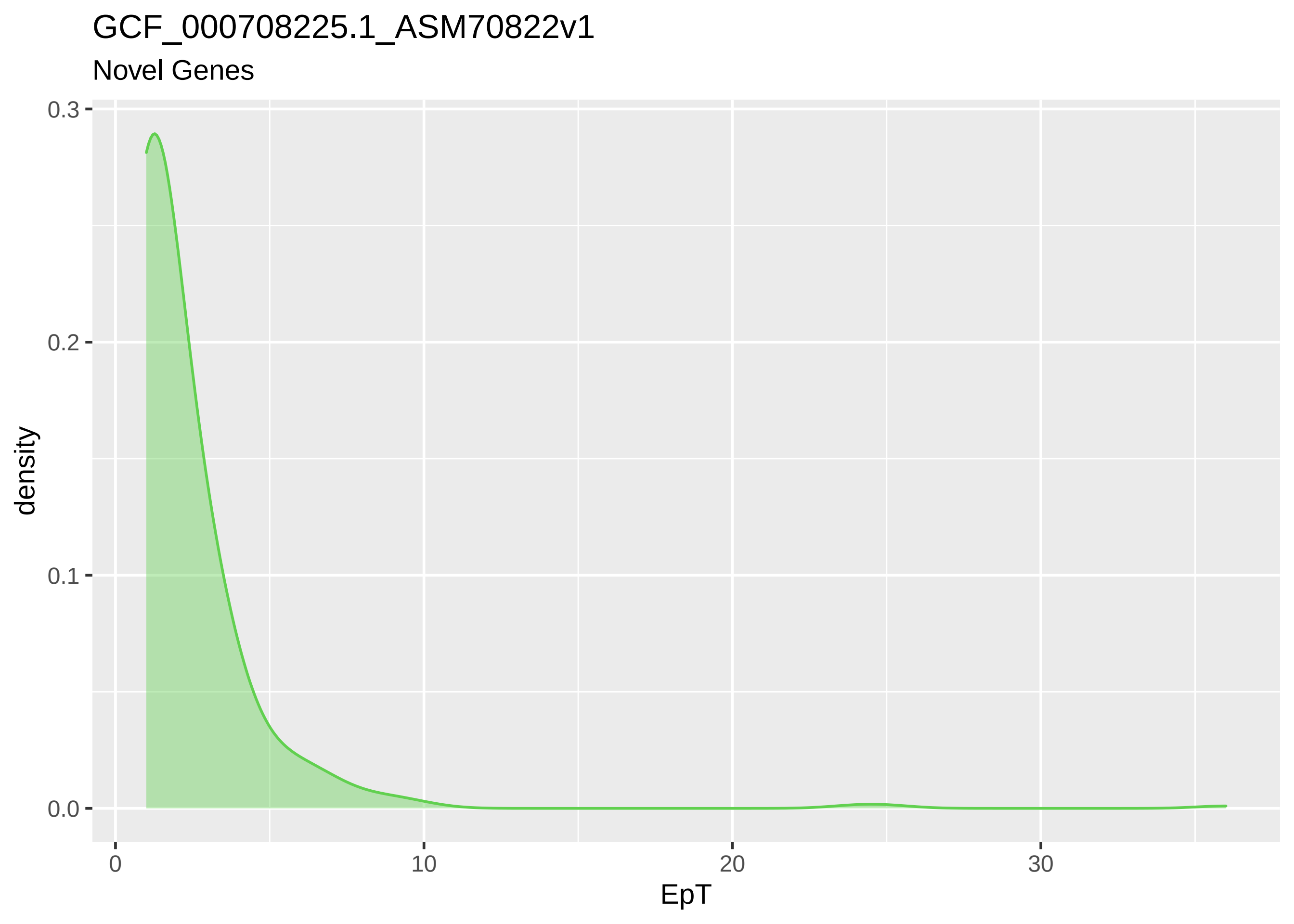

GCF_000708225.1_ASM70822v1
Novel Genes
0.3
0.2
0.1
0.0
density
0
10
20
30
EpT

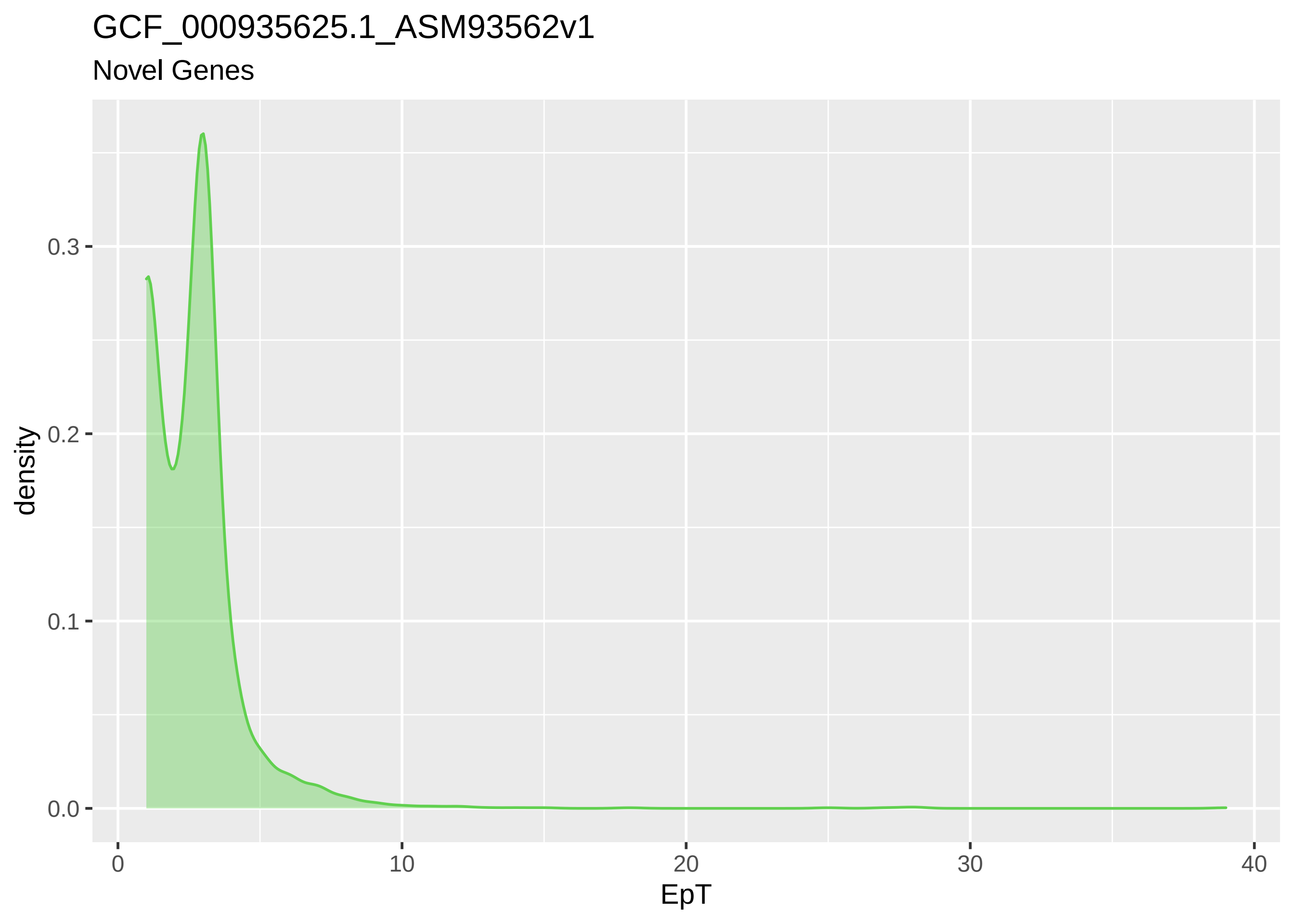

GCF_000935625.1_ASM93562v1
Novel Genes
density
0.0
0.1
0.2
0.3
0
10
20
30
40
EpT

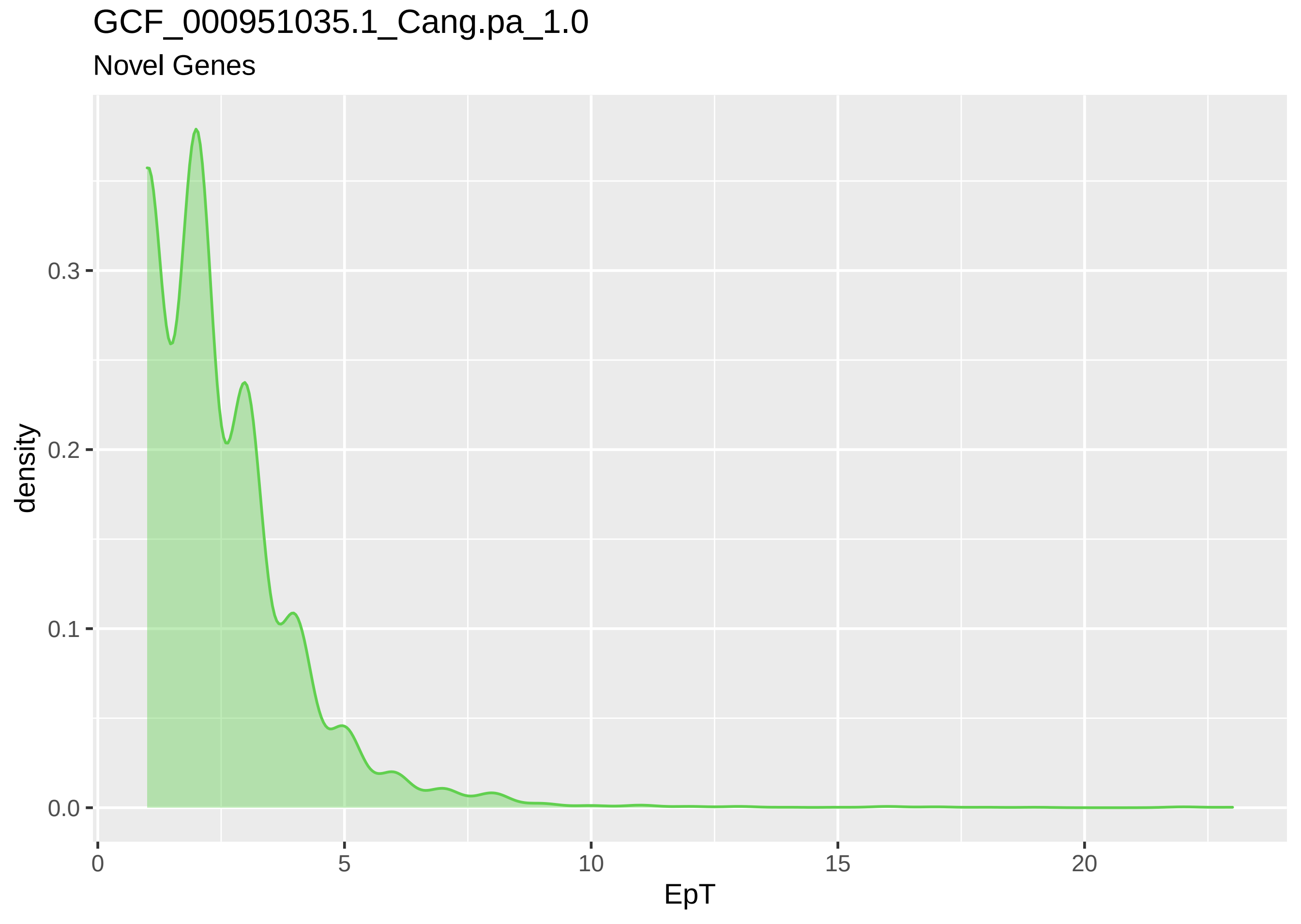
GCF_000951035.1_Cang.pa_1.0
Novel Genes
density
0.0
0.1
0.2
0.3
0
5
10
15
20
EpT

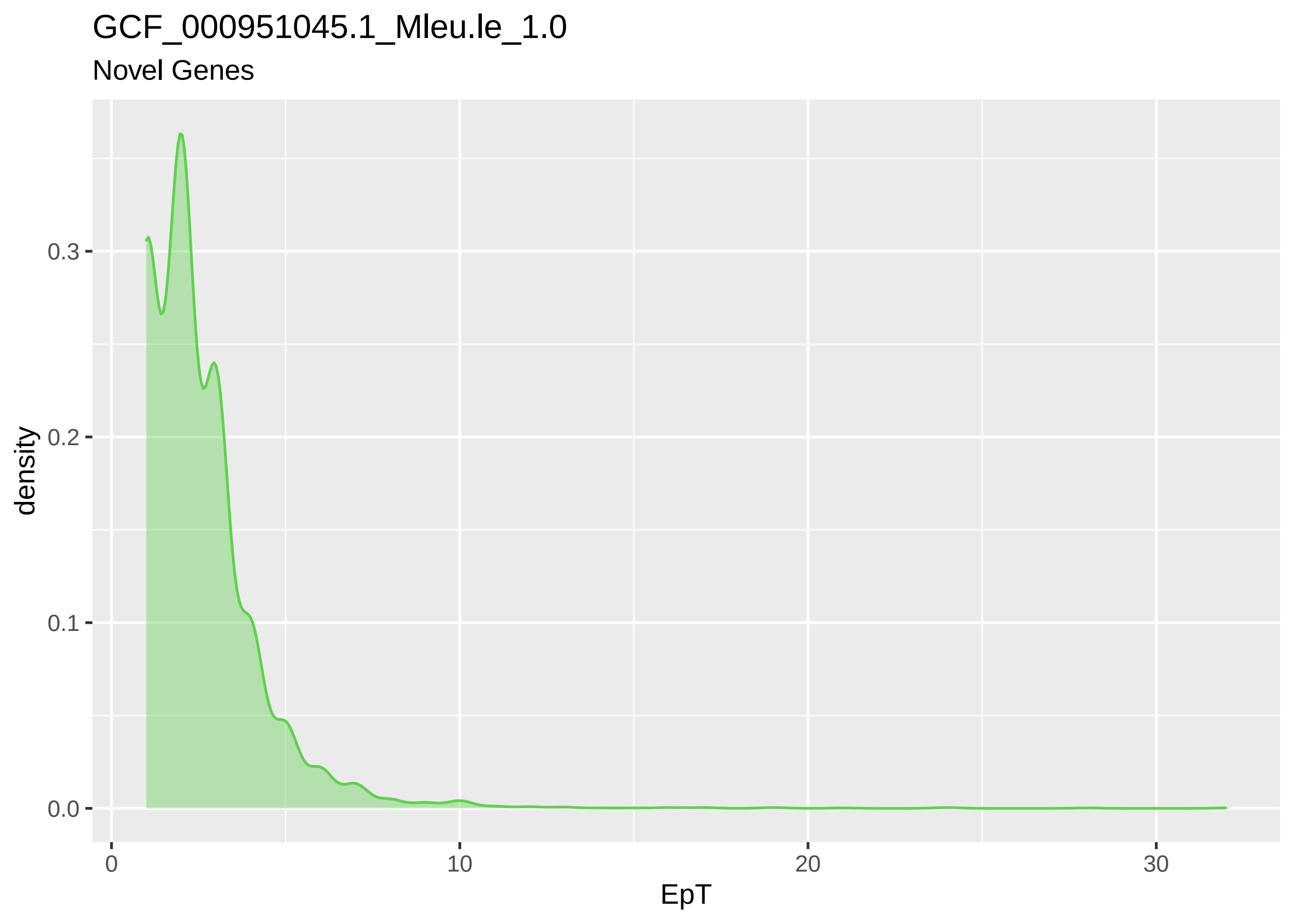
GCF_000951045.1_Mleu.le_1.0
Novel Genes
density
0.0
0.1
0.2
0.3
0
10
20
30
EpT

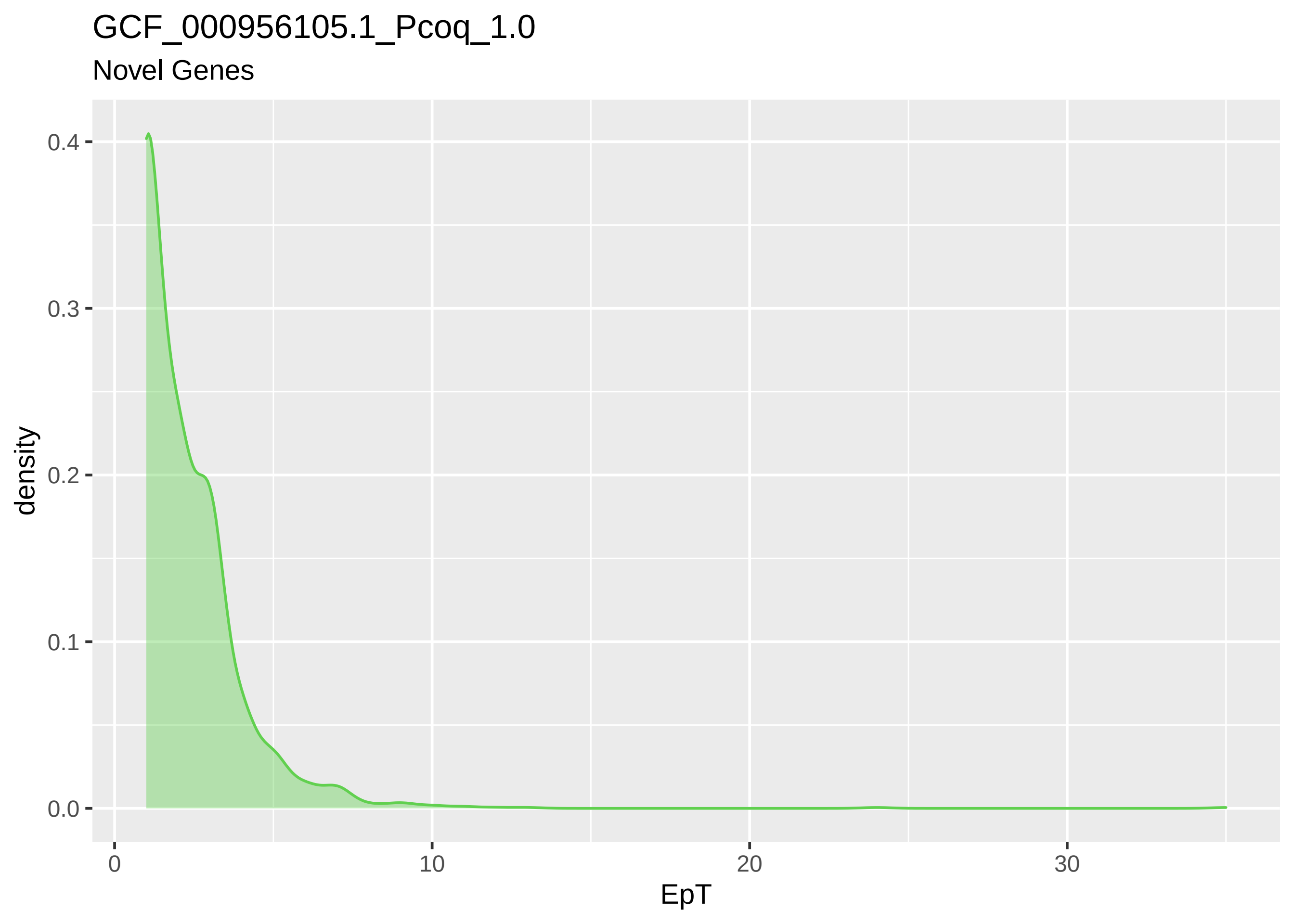
GCF_000956105.1_Pcoq_1.0
Novel Genes
density
EpT
0.0
0.1
0.2
0.3
0.4
0
10
20
30

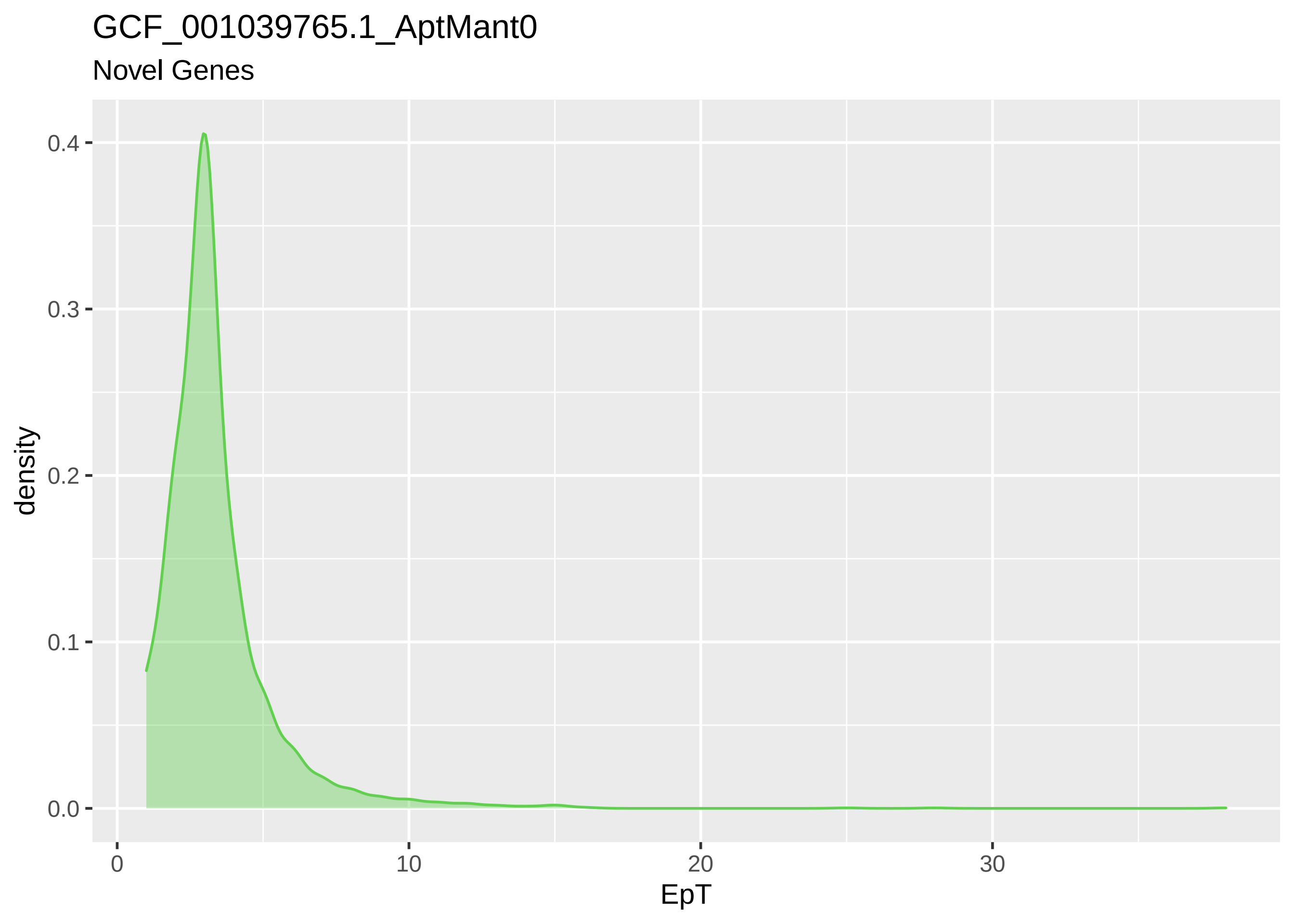
GCF_001039765.1_AptMant0
Novel Genes
density
0.4
0.3
0.2
0.1
0.0
0
10
20
30
EpT

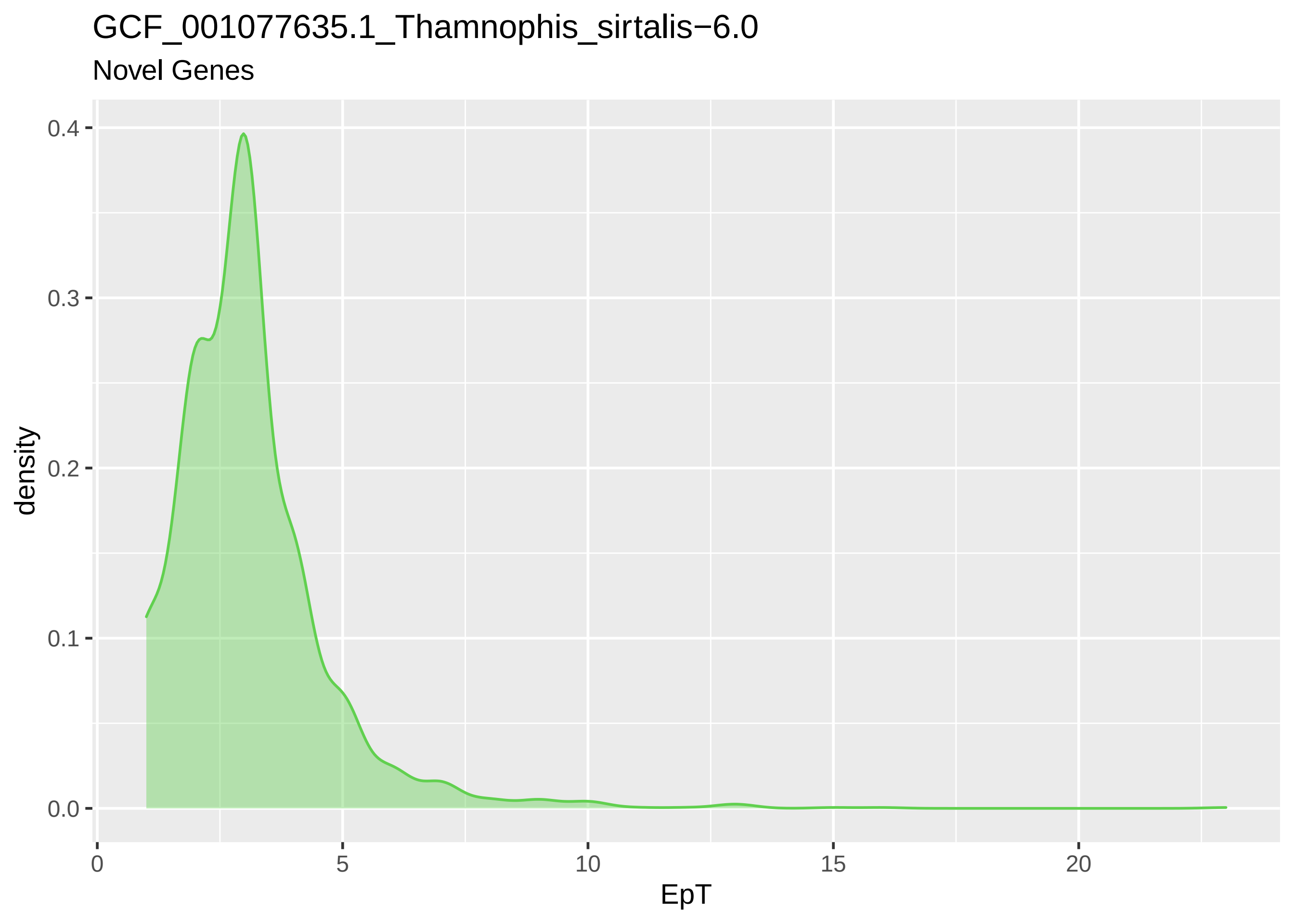
GCF_001077635.1_Thamnophis_sirtalis−6.0
Novel Genes
density
0.4
0.3
0.2
0.1
0.0
0
5
10
15
20
EpT

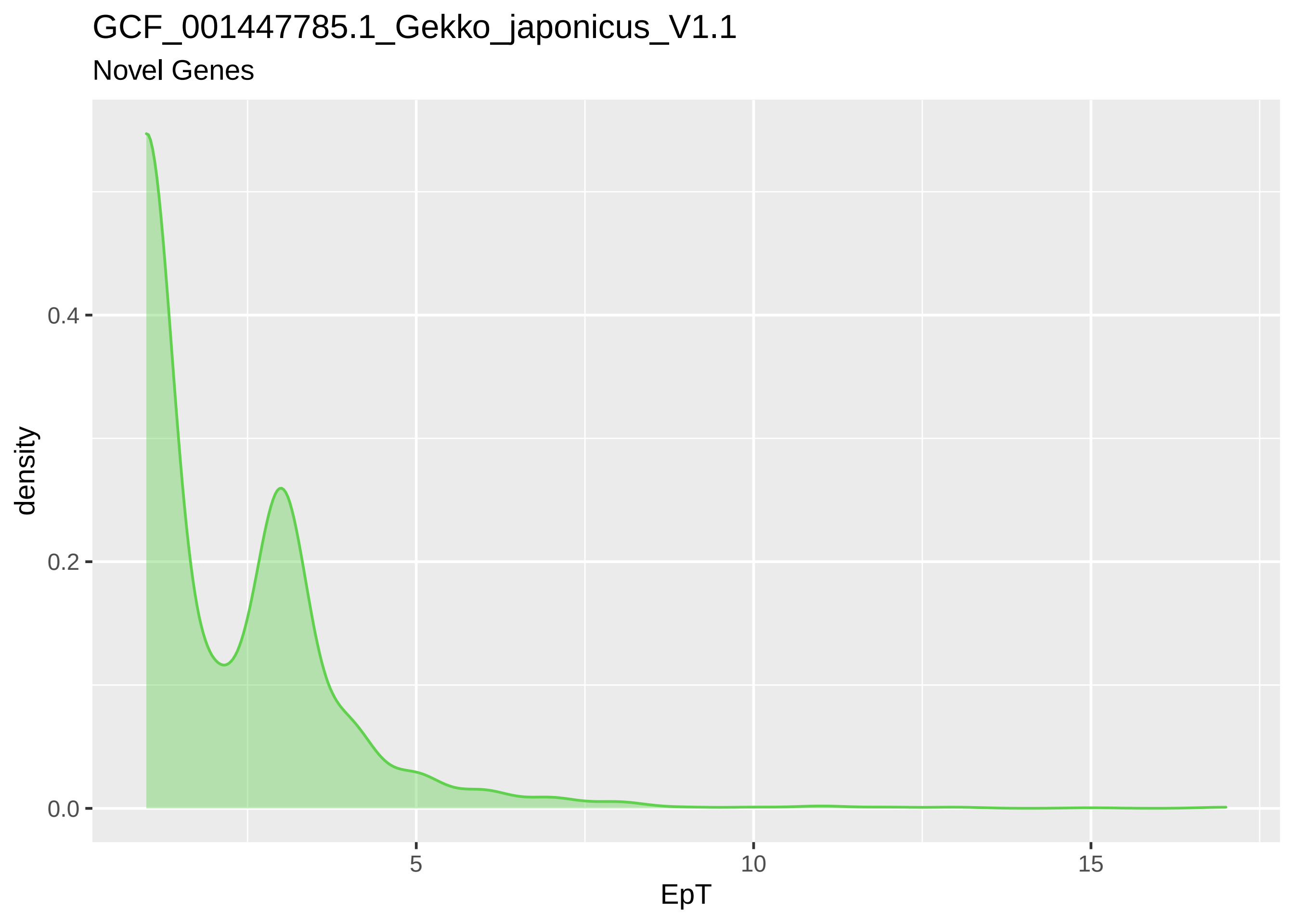
GCF_001447785.1_Gekko_japonicus_V1.1
Novel Genes
density
0.4
0.2
0.0
5
10
15
EpT

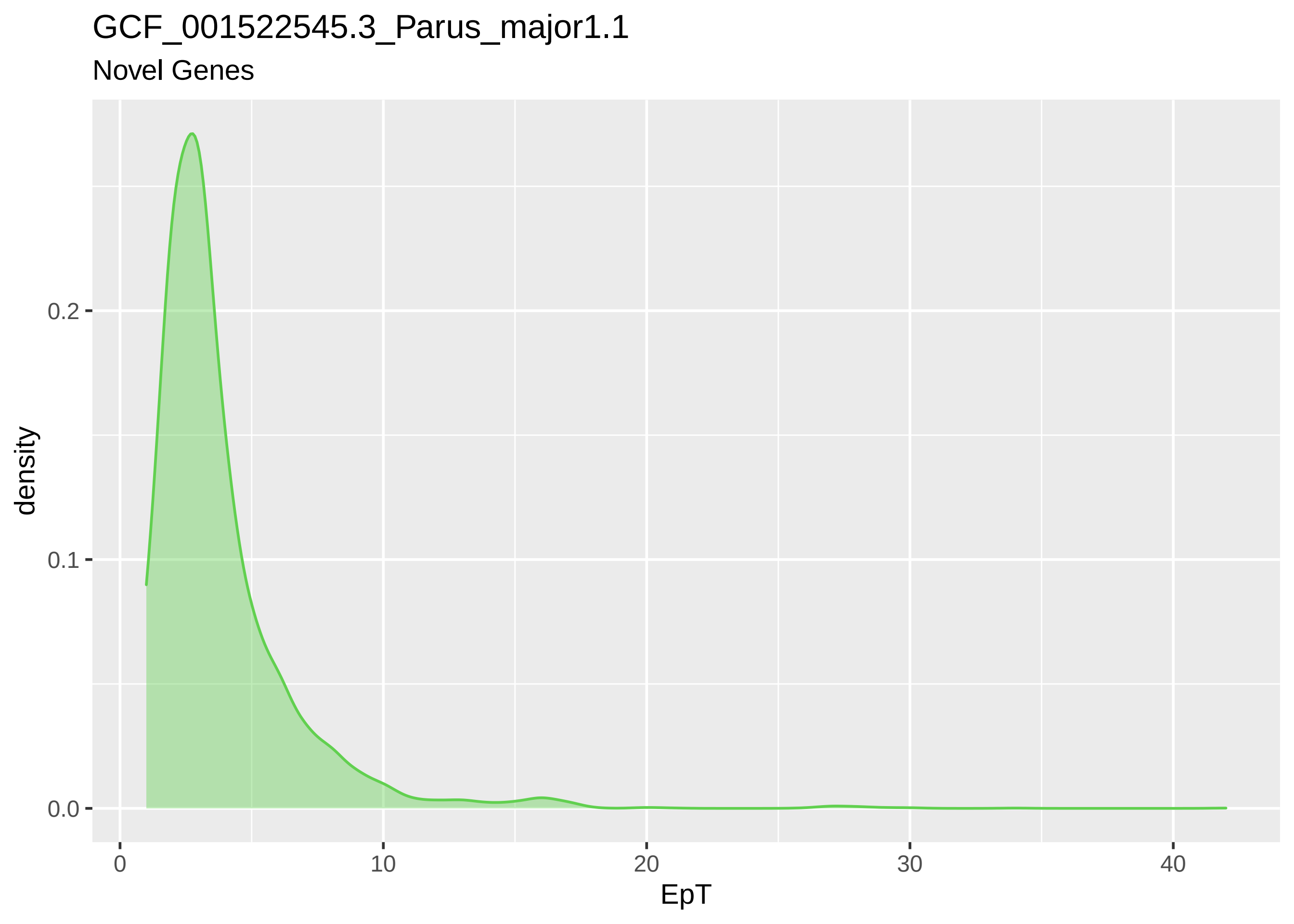
GCF_001522545.3_Parus_major1.1
Novel Genes
density
0.2
0.1
0.0
0
10
20
30
40
EpT

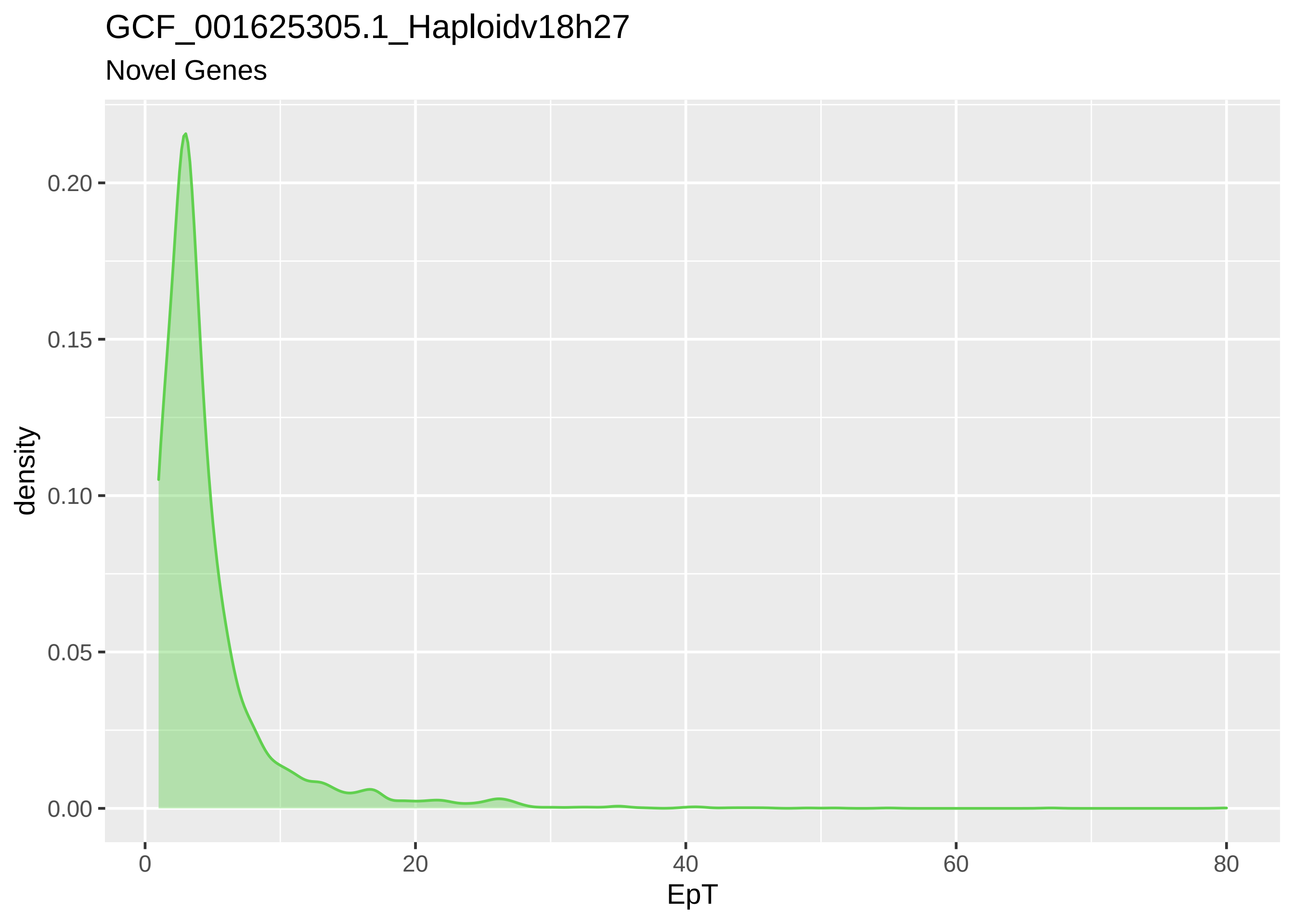

GCF_001625305.1_Haploidv18h27
Novel Genes
density
0.20
0.15
0.10
0.05
0.00
0
20
40
60
80
EpT

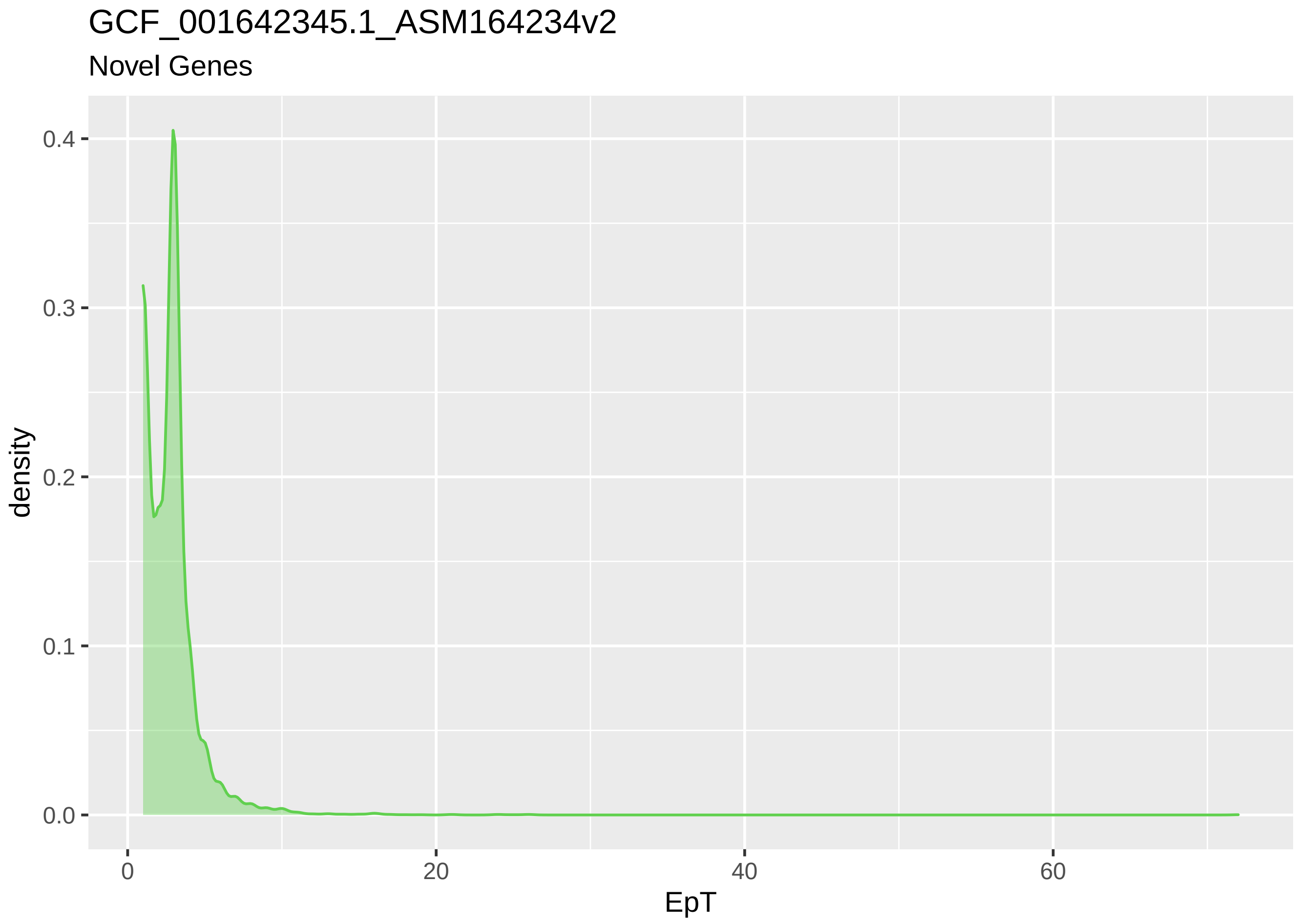
GCF_001642345.1_ASM164234v2
Novel Genes
density
0.4
0.3
0.2
0.1
0.0
0
20
40
60
EpT

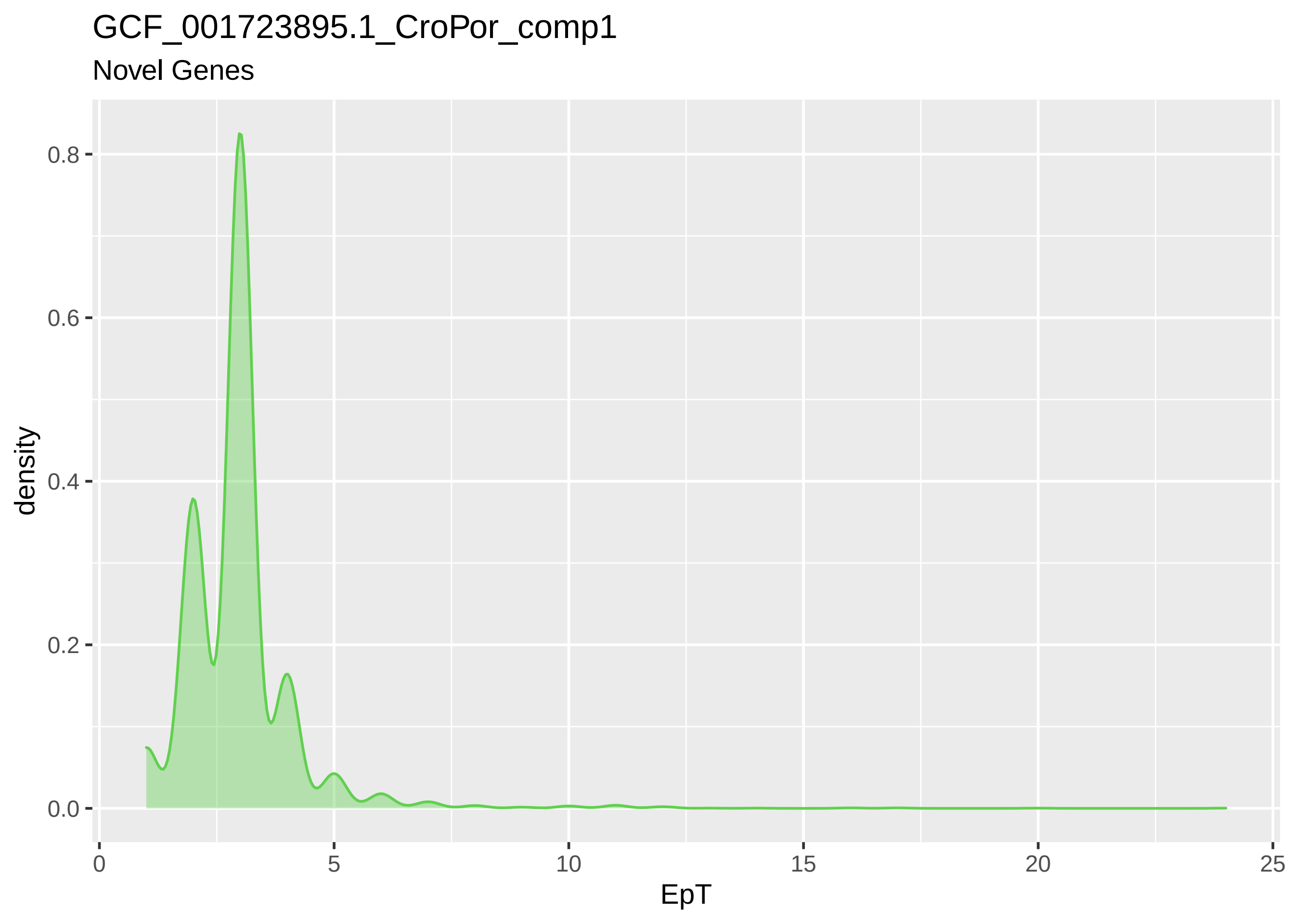
GCF_001723895.1_CroPor_comp1
Novel Genes
density
0.0
0.2
0.4
0.6
0.8
0
5
10
15
20
25
EpT

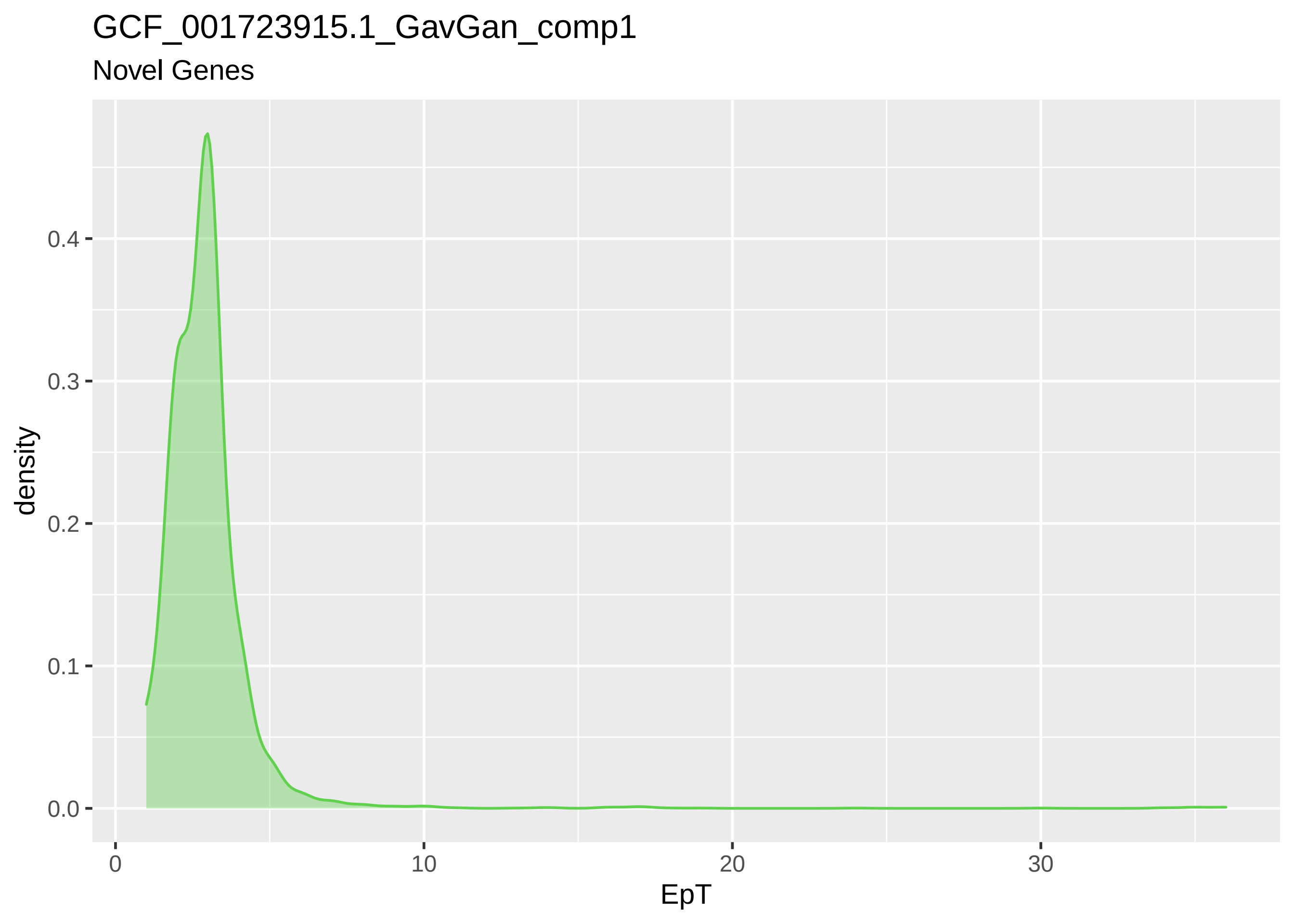
GCF_001723915.1_GavGan_comp1
Novel Genes
density
0.0
0.1
0.2
0.3
0.4
0
10
20
30
EpT

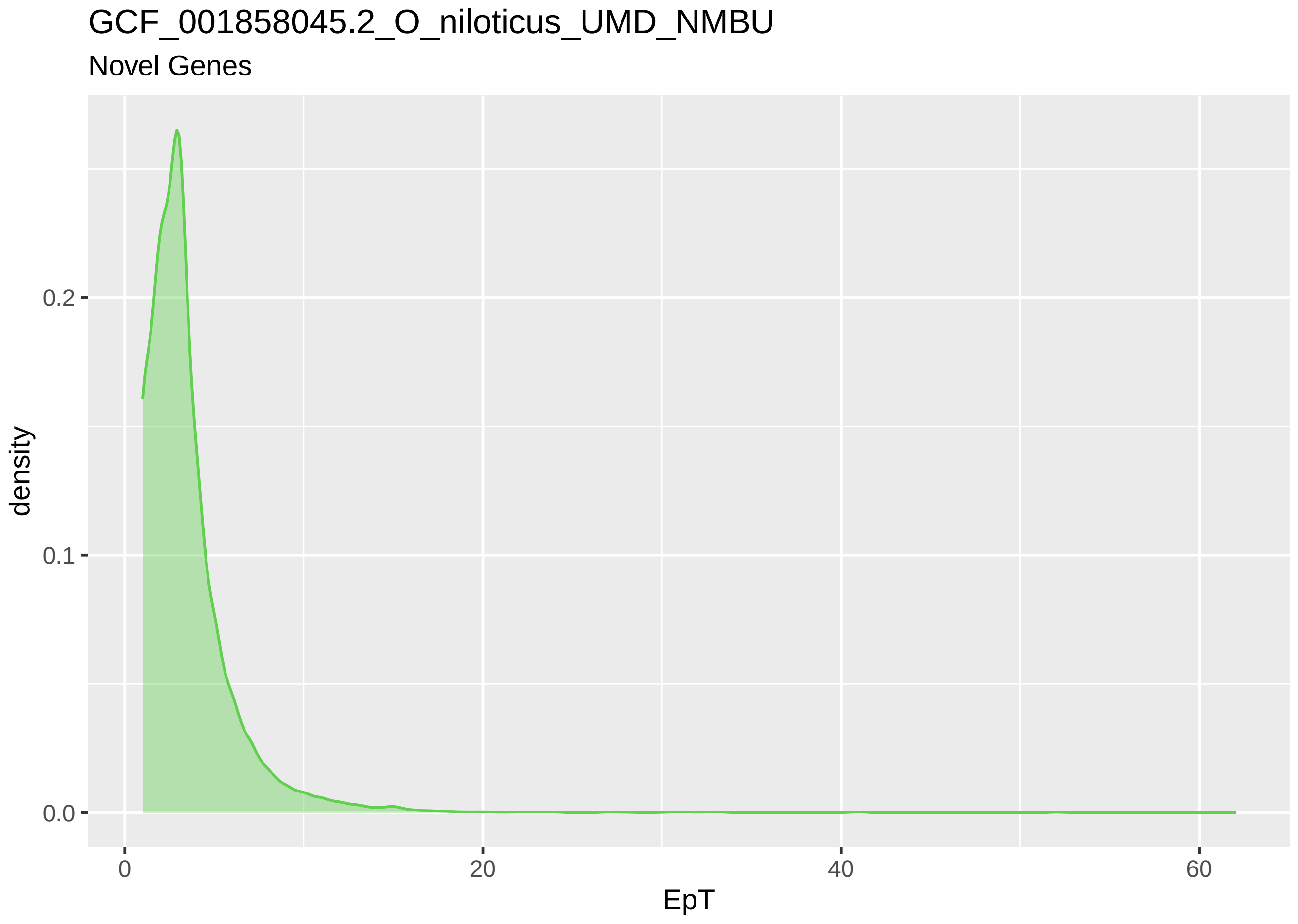
GCF_001858045.2_O_niloticus_UMD_NMBU
Novel Genes
density
0.2
0.1
0.0
0
20
40
60
EpT

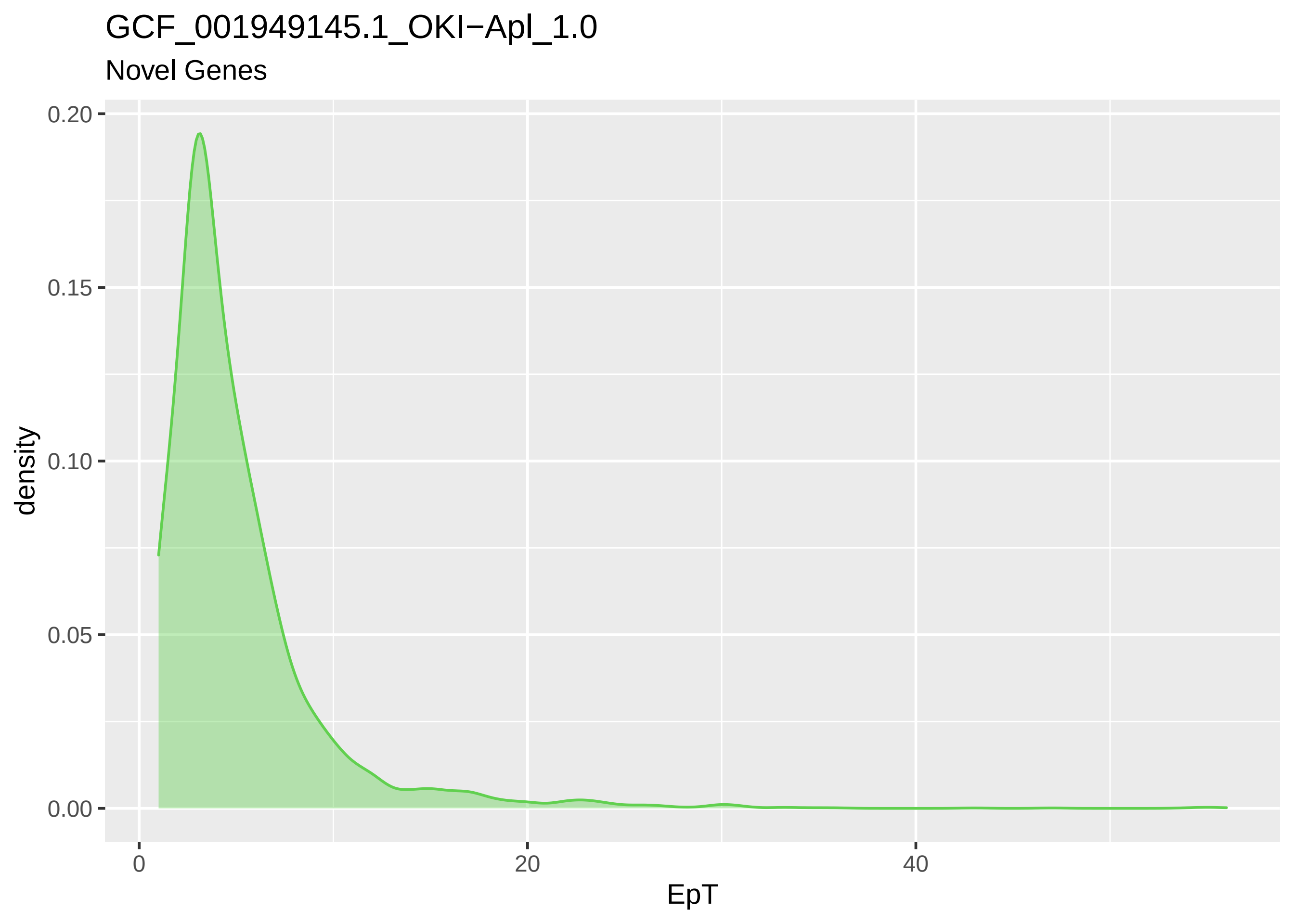
GCF_001949145.1_OKI−Apl_1.0
Novel Genes
0.20
0.15
0.10
0.05
0.00
density
0
20
40
EpT

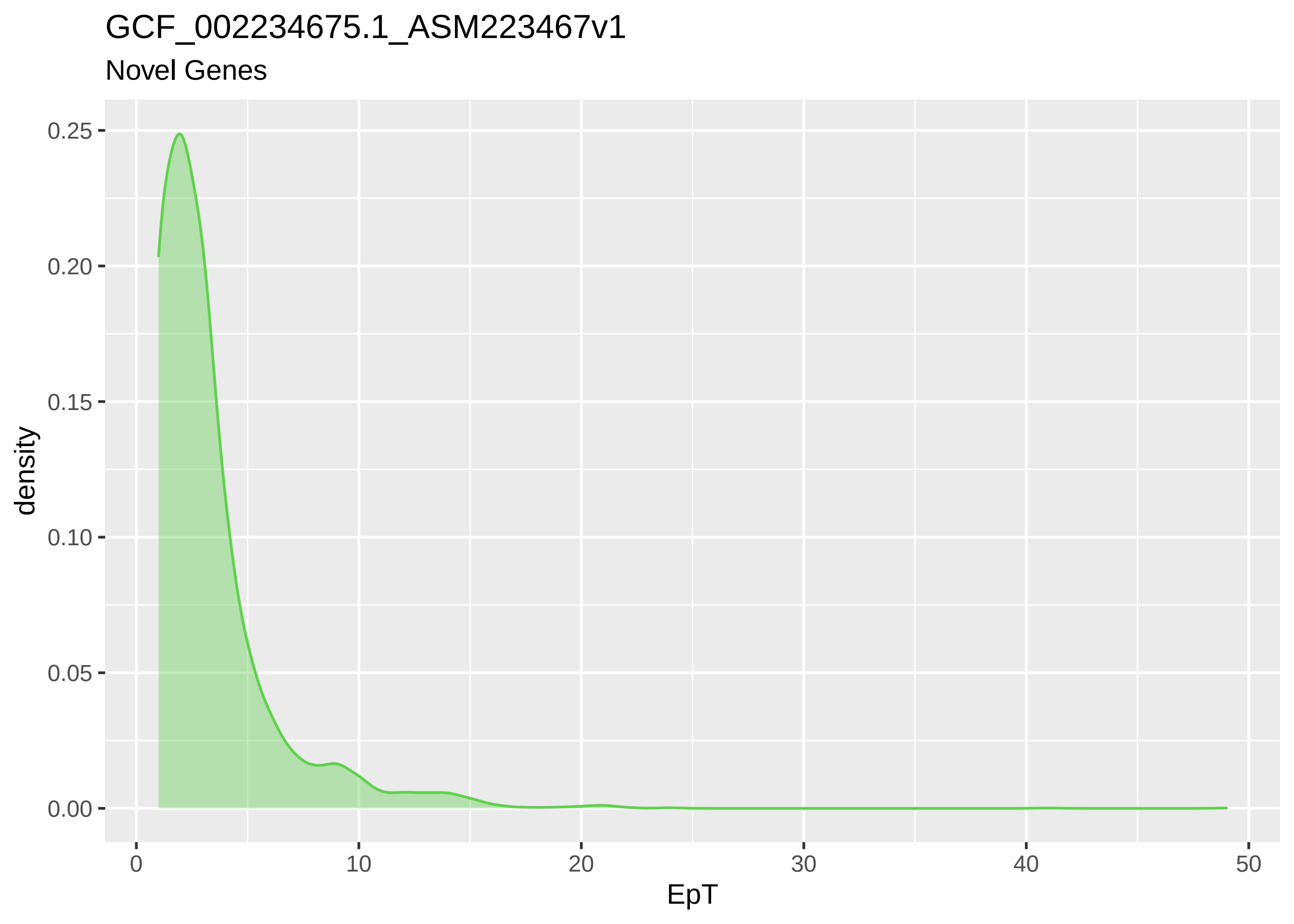

GCF_002234675.1_ASM223467v1
Novel Genes
density
0.25
0.20
0.15
0.10
0.05
0.00
0
10
20
30
40
50
EpT

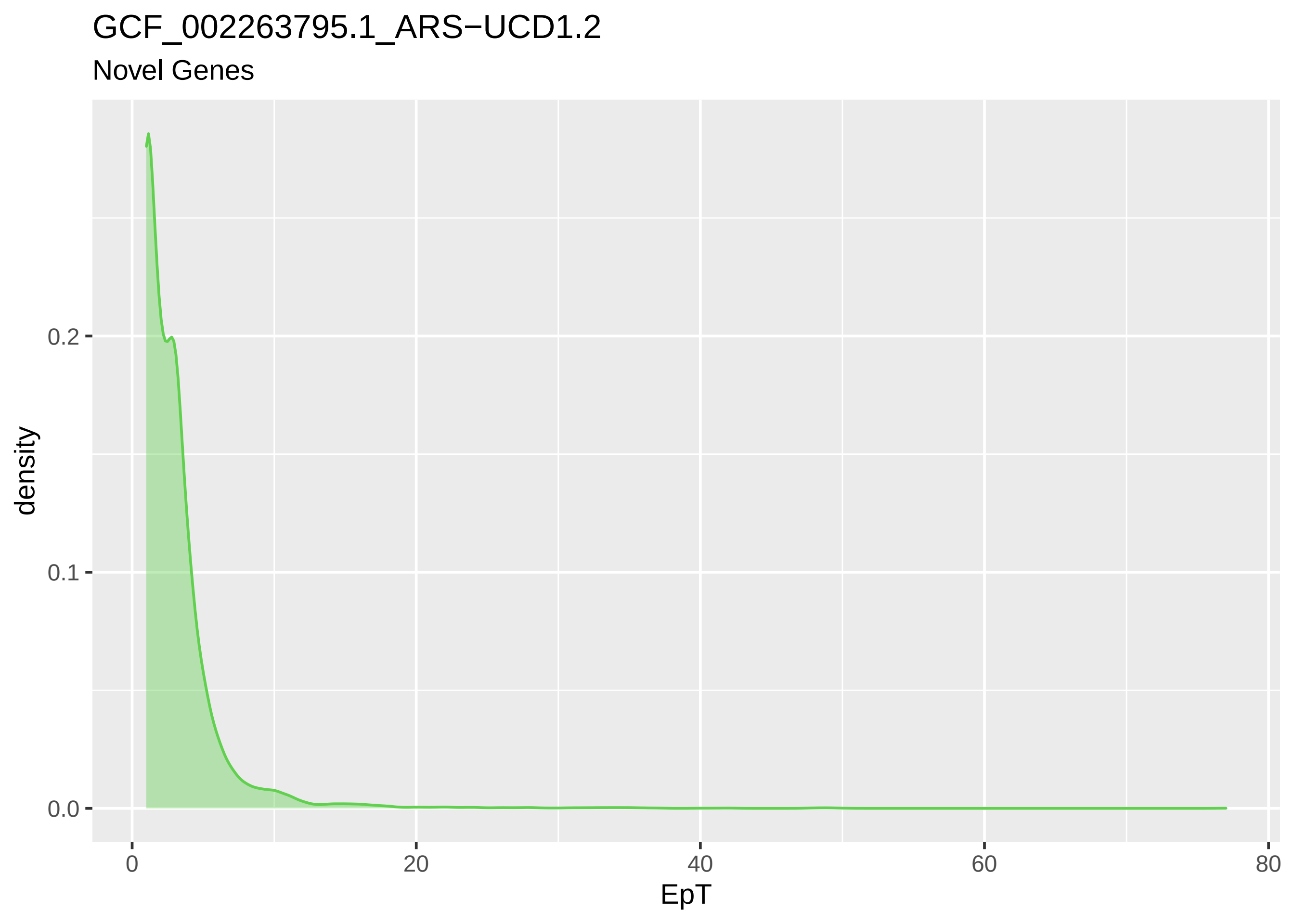
GCF_002263795.1_ARS−UCD1.2
Novel Genes
density
0.0
0.1
0.2
0
20
40
60
80
EpT

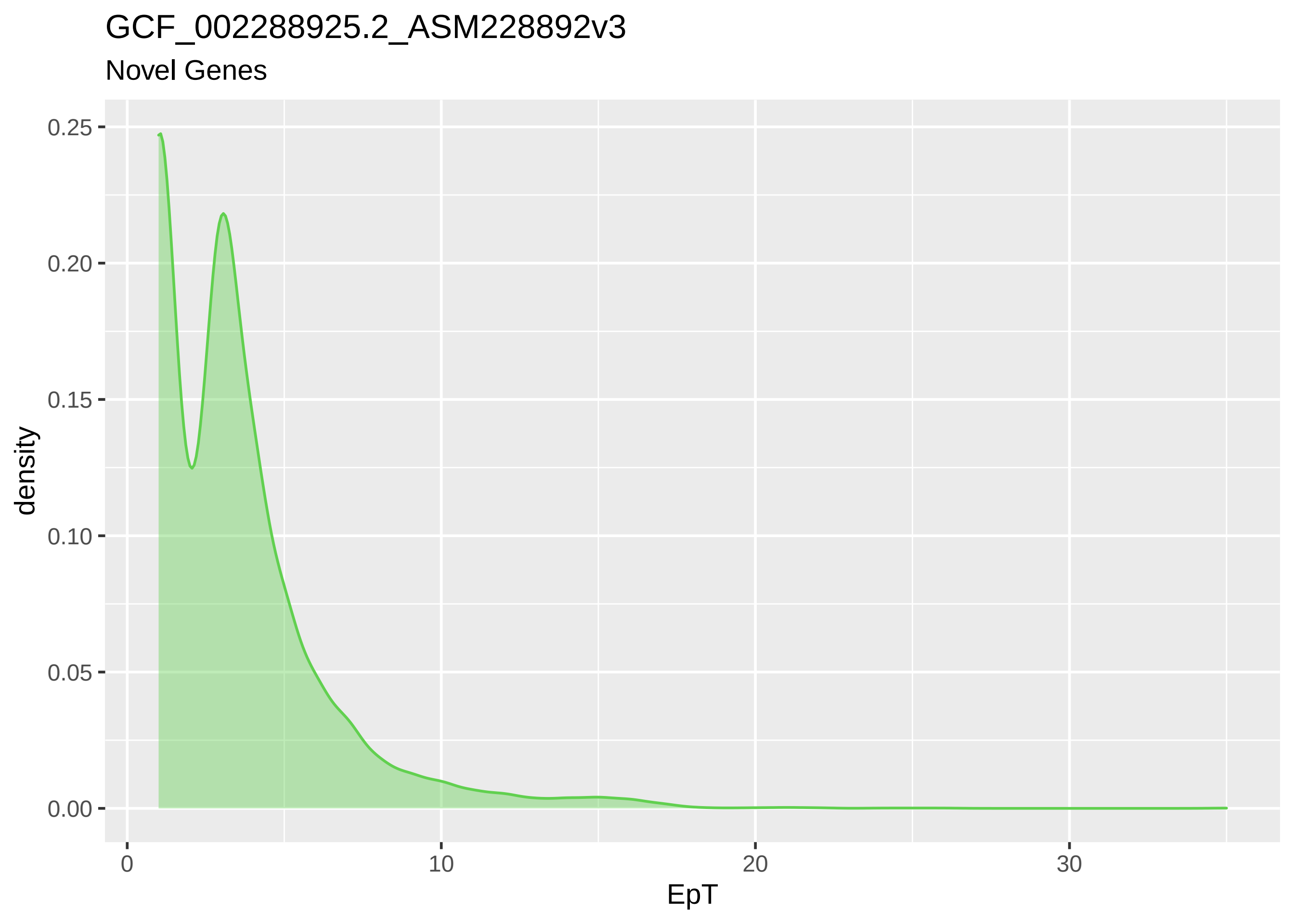
GCF_002288925.2_ASM228892v3
Novel Genes
density
0.25
0.20
0.15
0.10
0.05
0.00
0
10
20
30
EpT

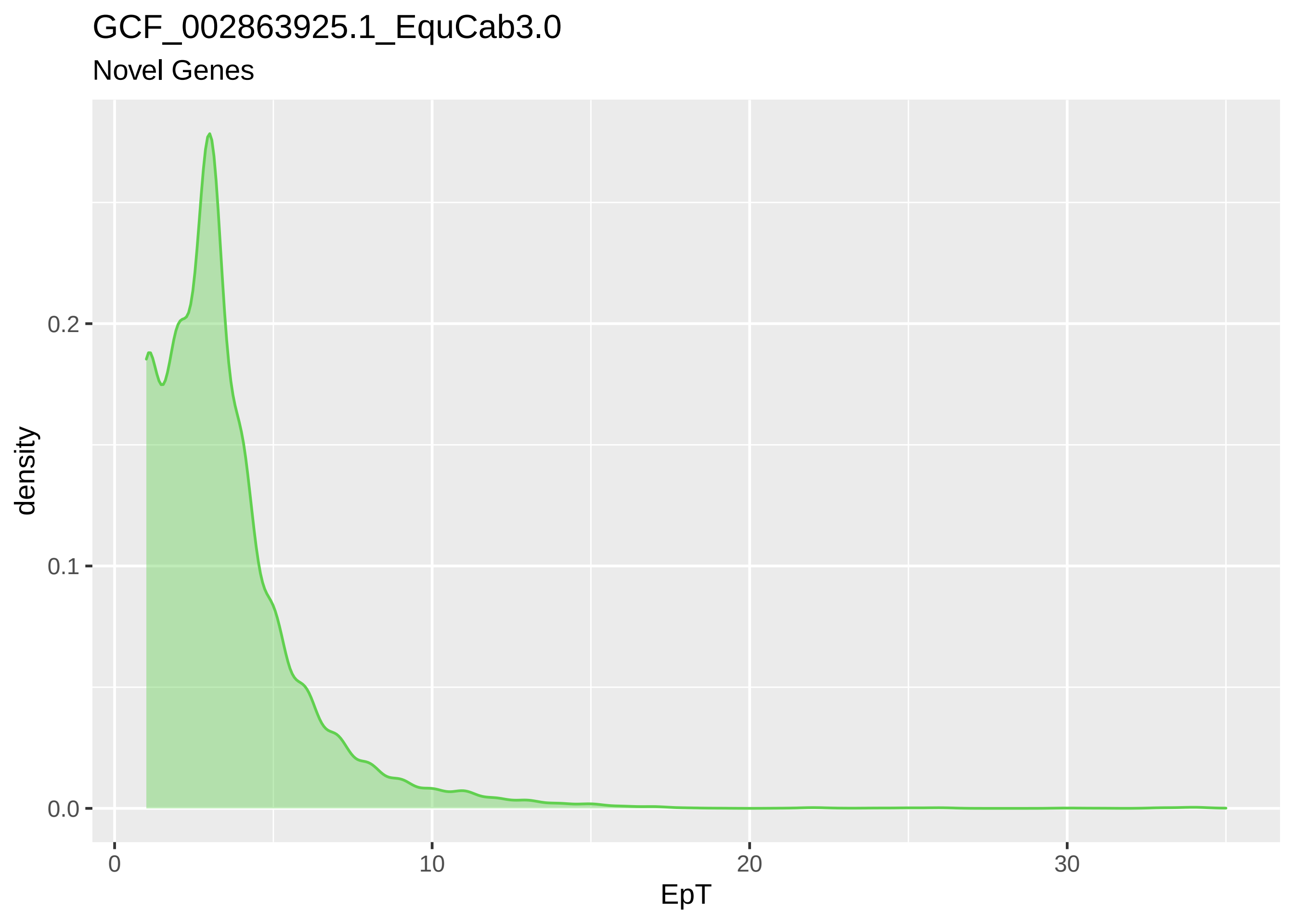
GCF_002863925.1_EquCab3.0
Novel Genes
density
0.2
0.1
0.0
0
10
20
30
EpT

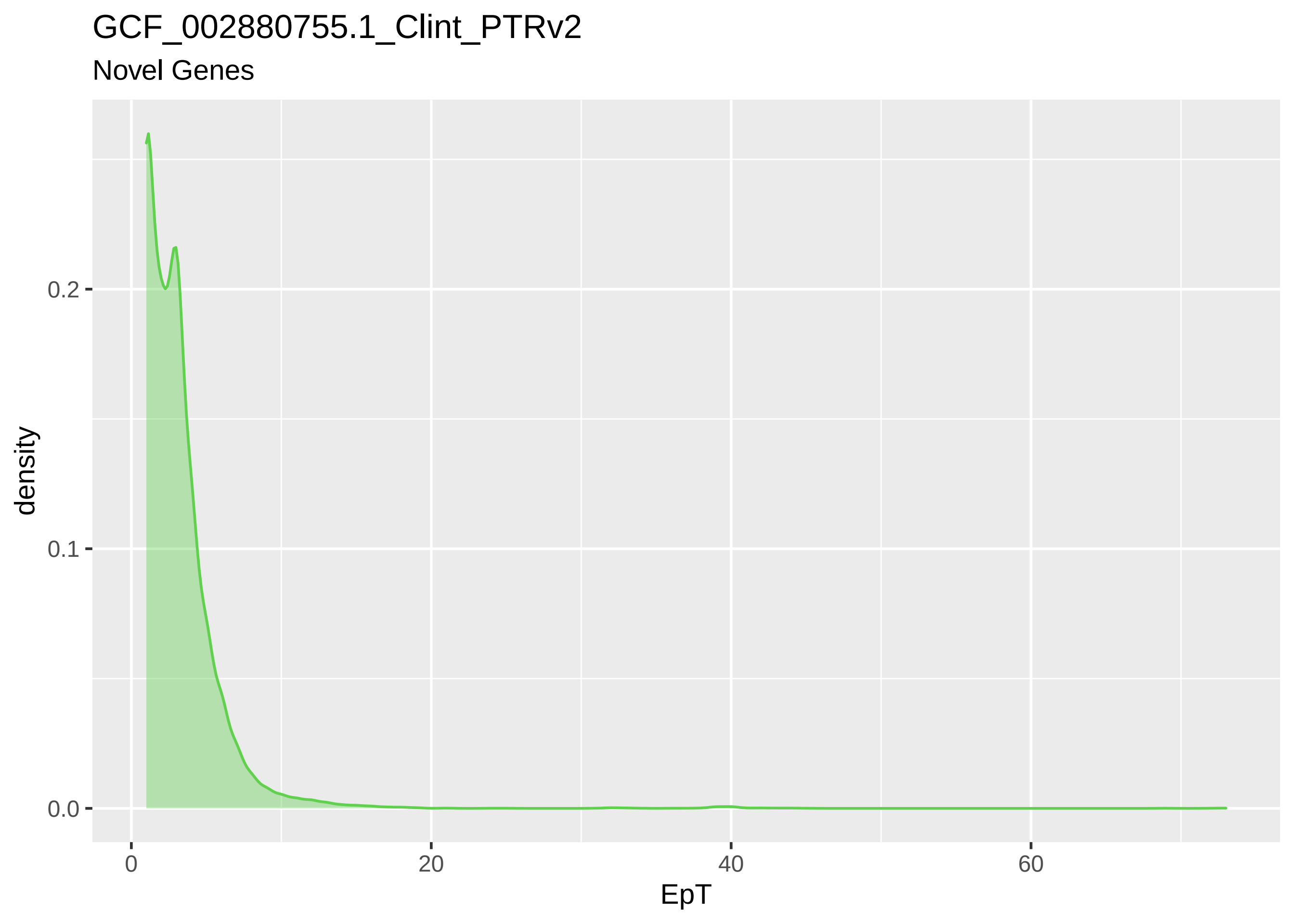
GCF_002880755.1_Clint_PTRv2
Novel Genes
density
0.0
0.1
0.2
0
20
40
60
EpT

GCF_002880775.1_Susie_PABv2

Novel Genes

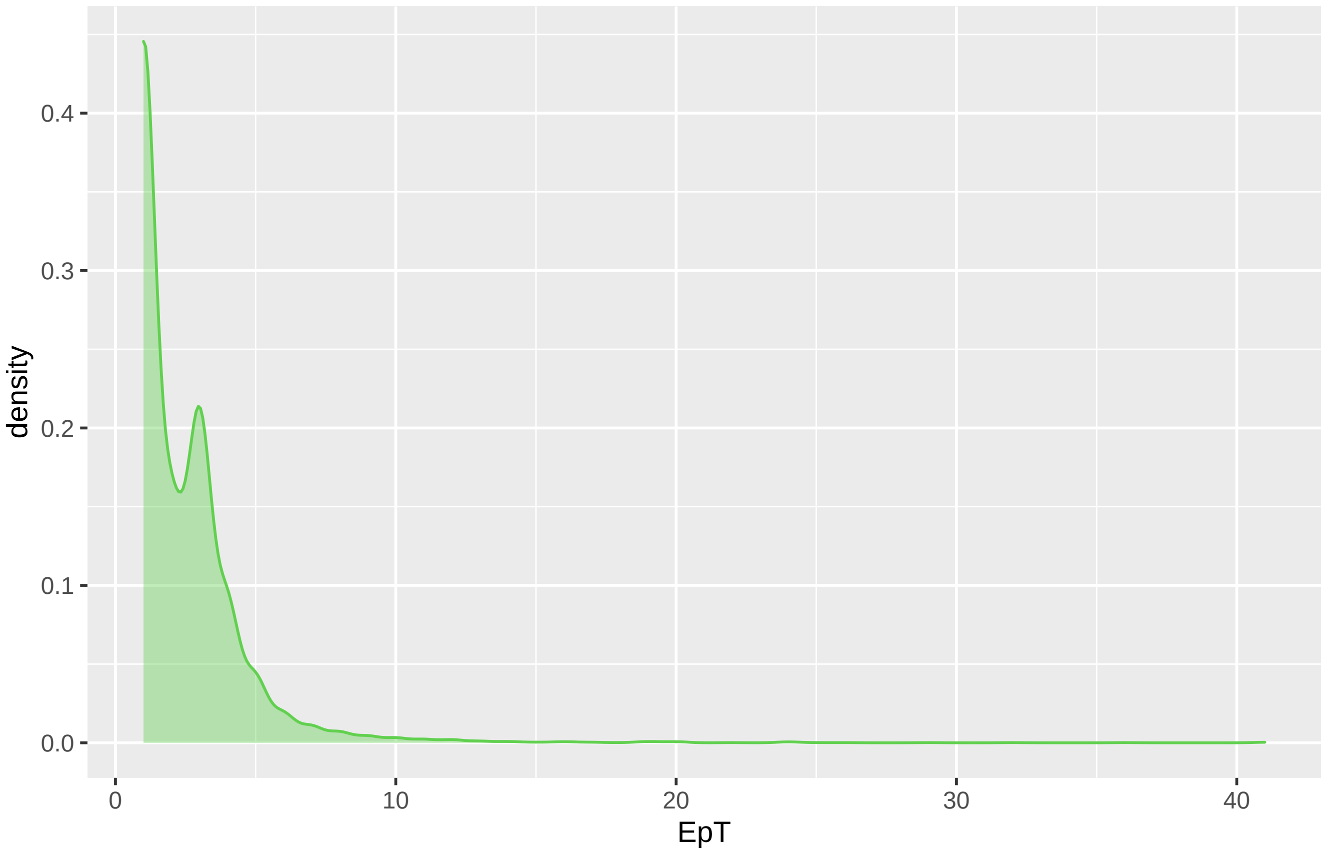

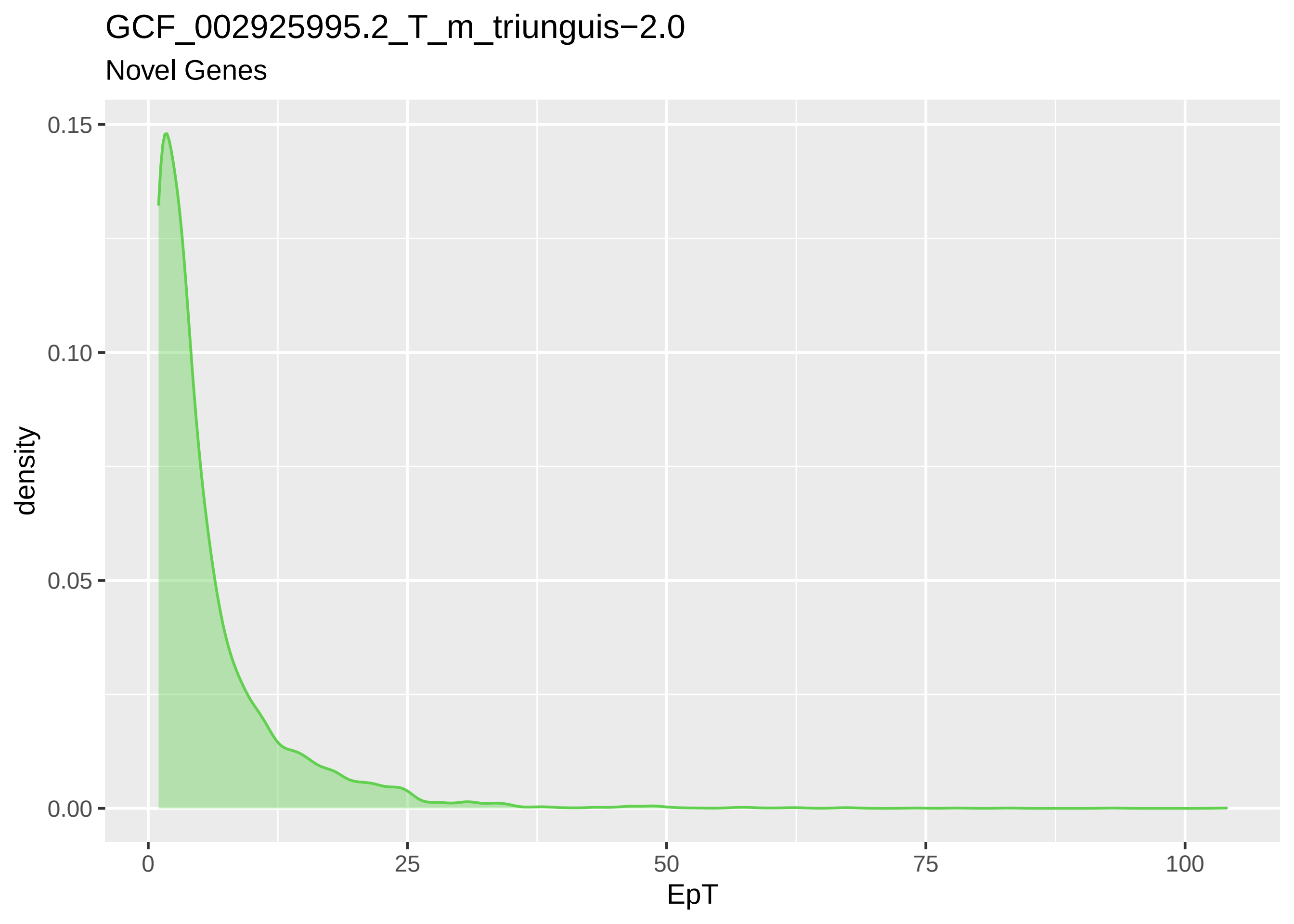
GCF_002925995.2_T_m_triunguis−2.0
Novel Genes
density
0.15
0.10
0.05
0.00
0
25
50
75
100
EpT

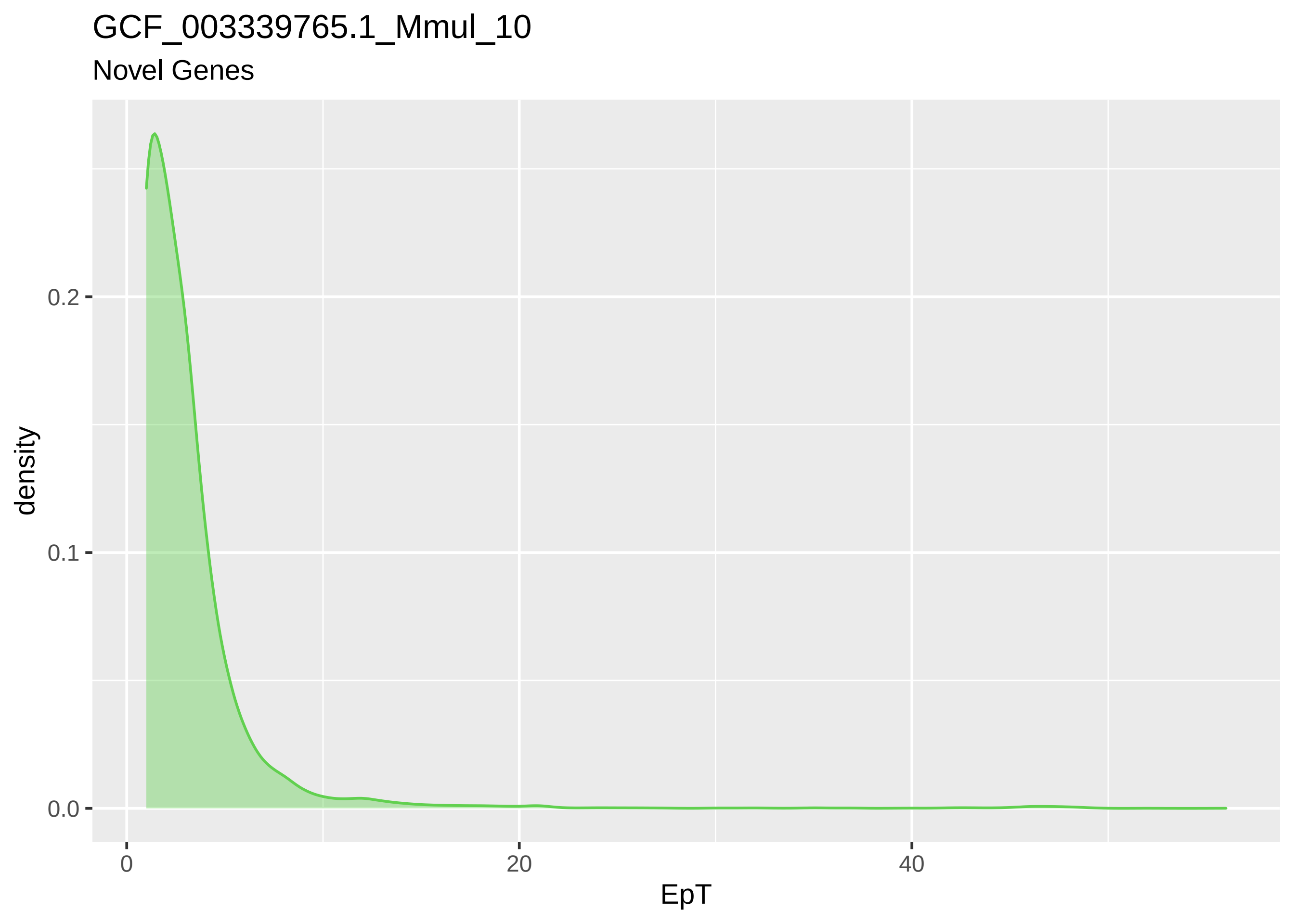
GCF_003339765.1_Mmul_10
Novel Genes
density
0.2
0.1
0.0
0
20
40
EpT

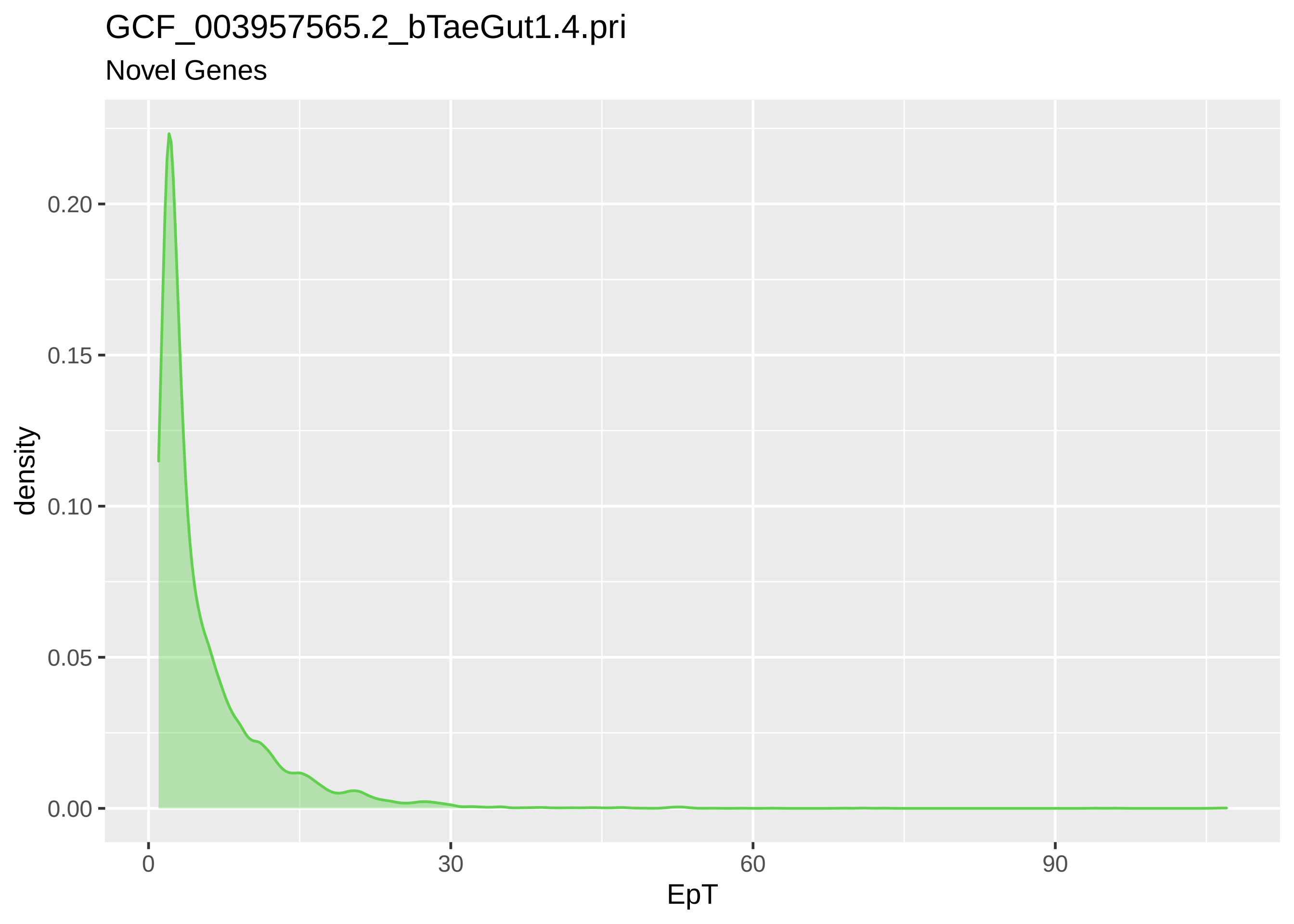

GCF_003957565.2_bTaeGut1.4.pri
Novel Genes
density
0.20
0.15
0.10
0.05
0.00
0
30
60
90
EpT

GCF_004115215.2_mOrnAna1.pri.v4

Novel Genes

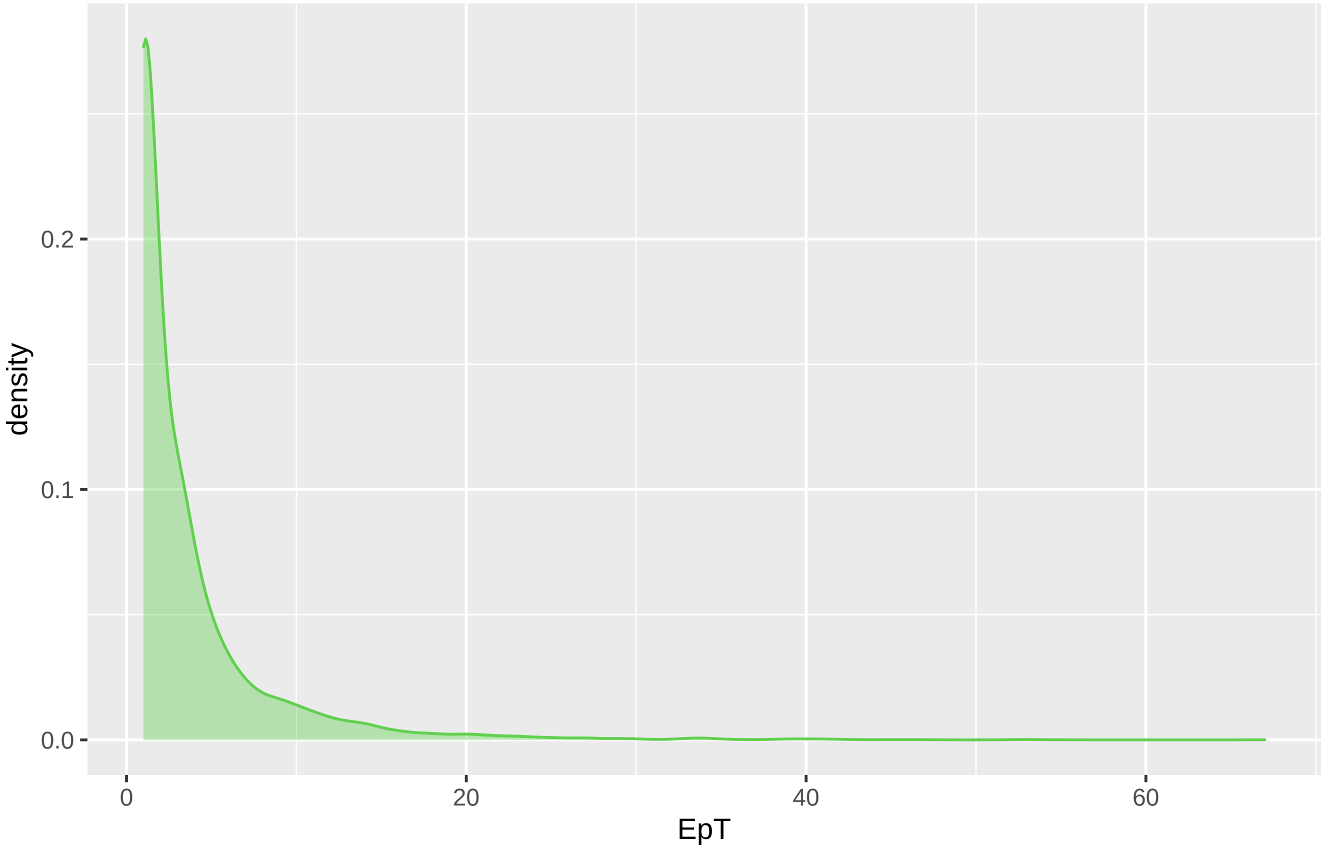

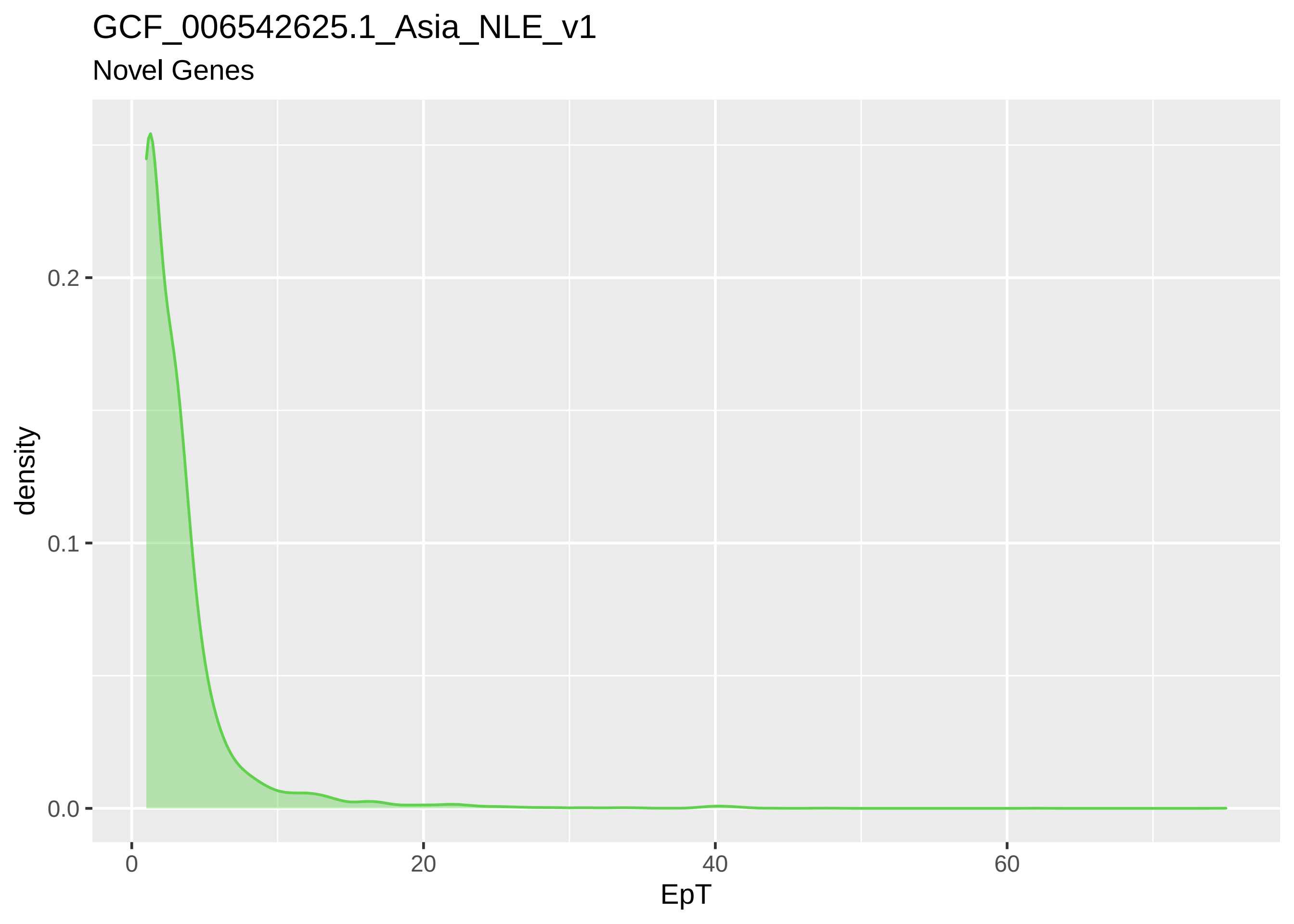

GCF_006542625.1_Asia_NLE_v1
Novel Genes
density
0.2
0.1
0.0
0
20
40
60
EpT

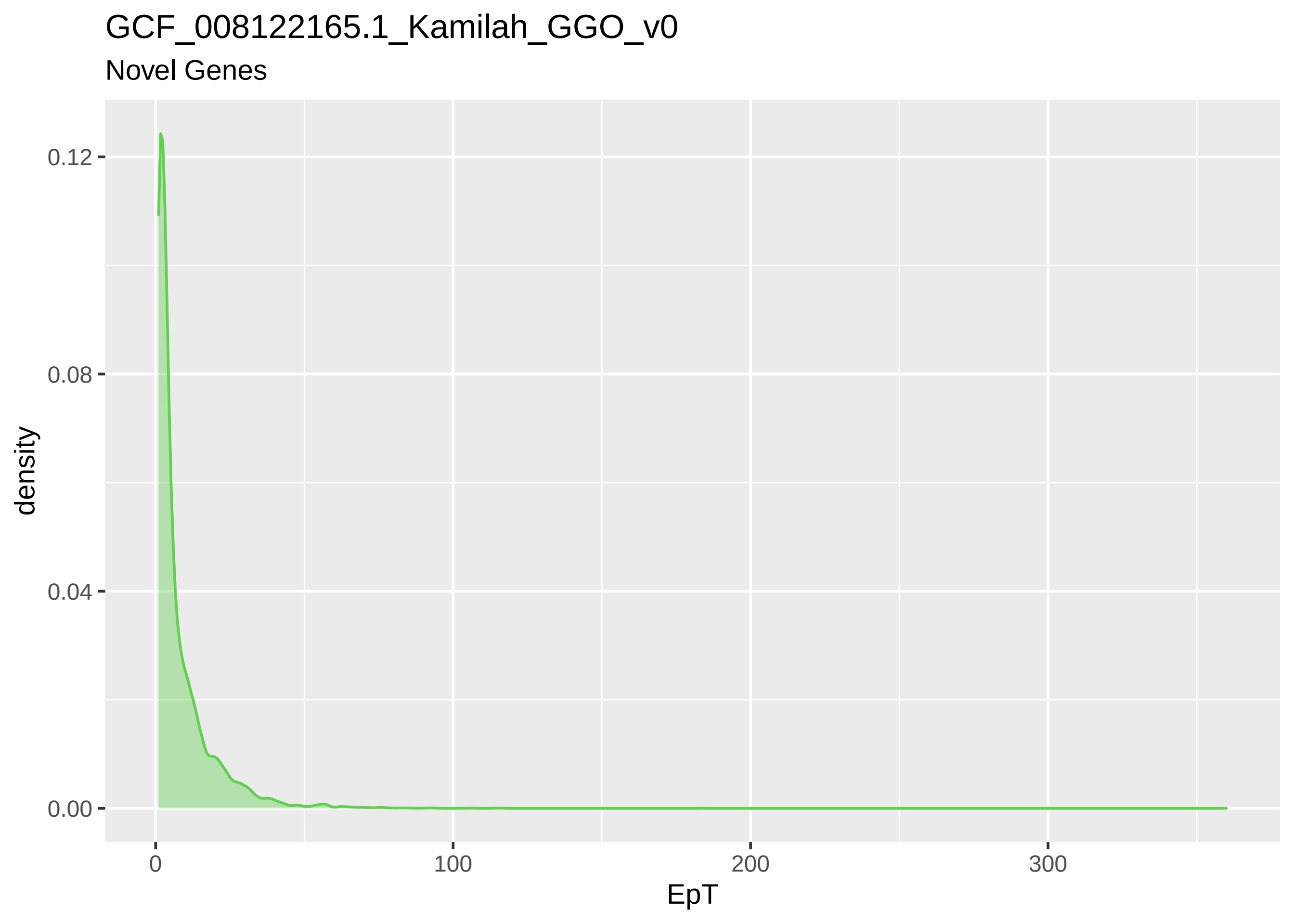
GCF_008122165.1_Kamilah_GGO_v0
Novel Genes
density
0.12
0.08
0.04
0.00
0
100
200
300
EpT

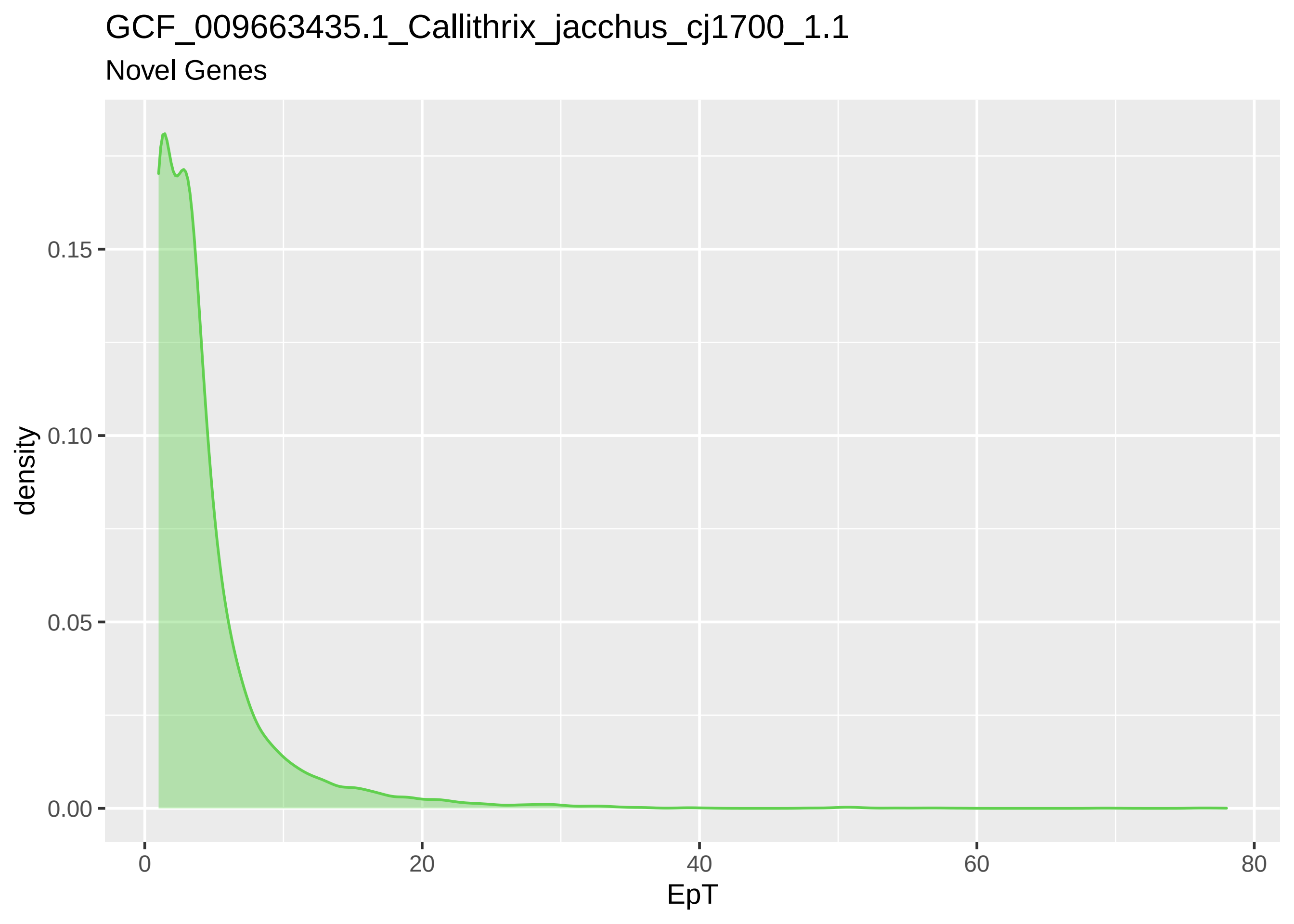

GCF_009663435.1_Callithrix_jacchus_cj1700_1.1
Novel Genes
density
0.15
0.10
0.05
0.00
0
20
40
60
80
EpT

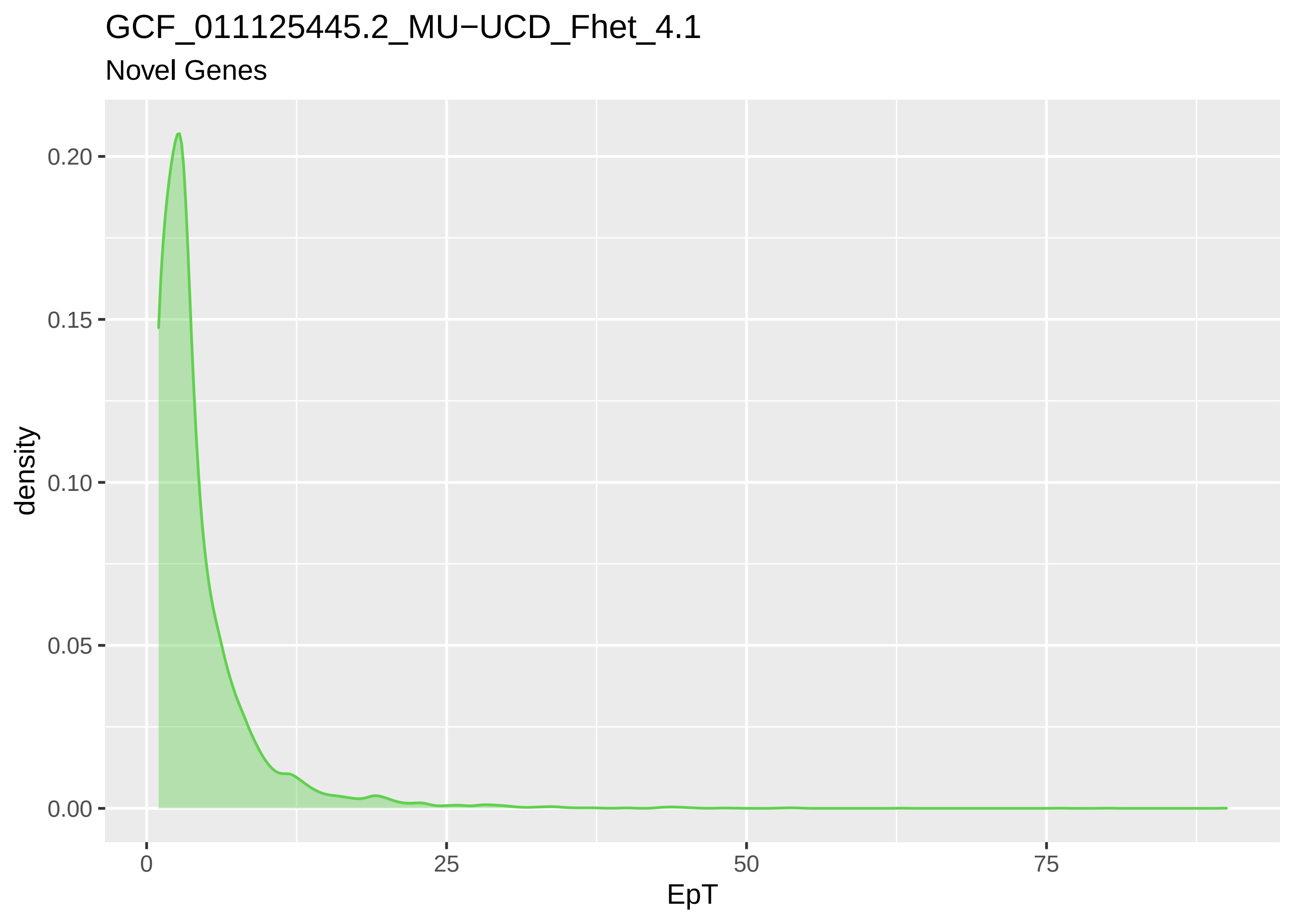
GCF_011125445.2_MU−UCD_Fhet_4.1
Novel Genes
density
0.20
0.15
0.10
0.05
0.00
0
25
50
75
EpT

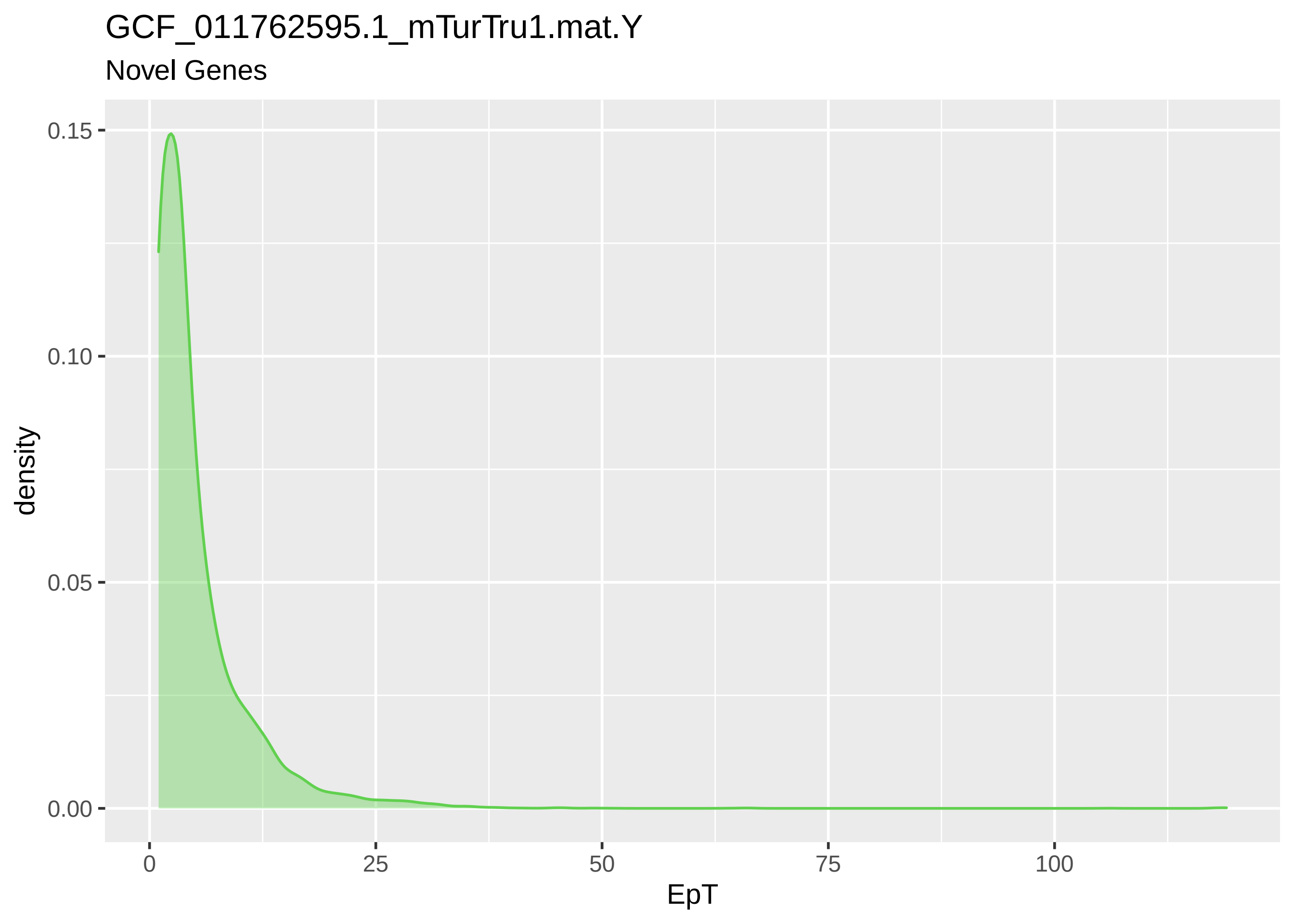
GCF_011762595.1_mTurTru1.mat.Y
Novel Genes
density
0.15
0.10
0.05
0.00
0
25
50
75
100
EpT

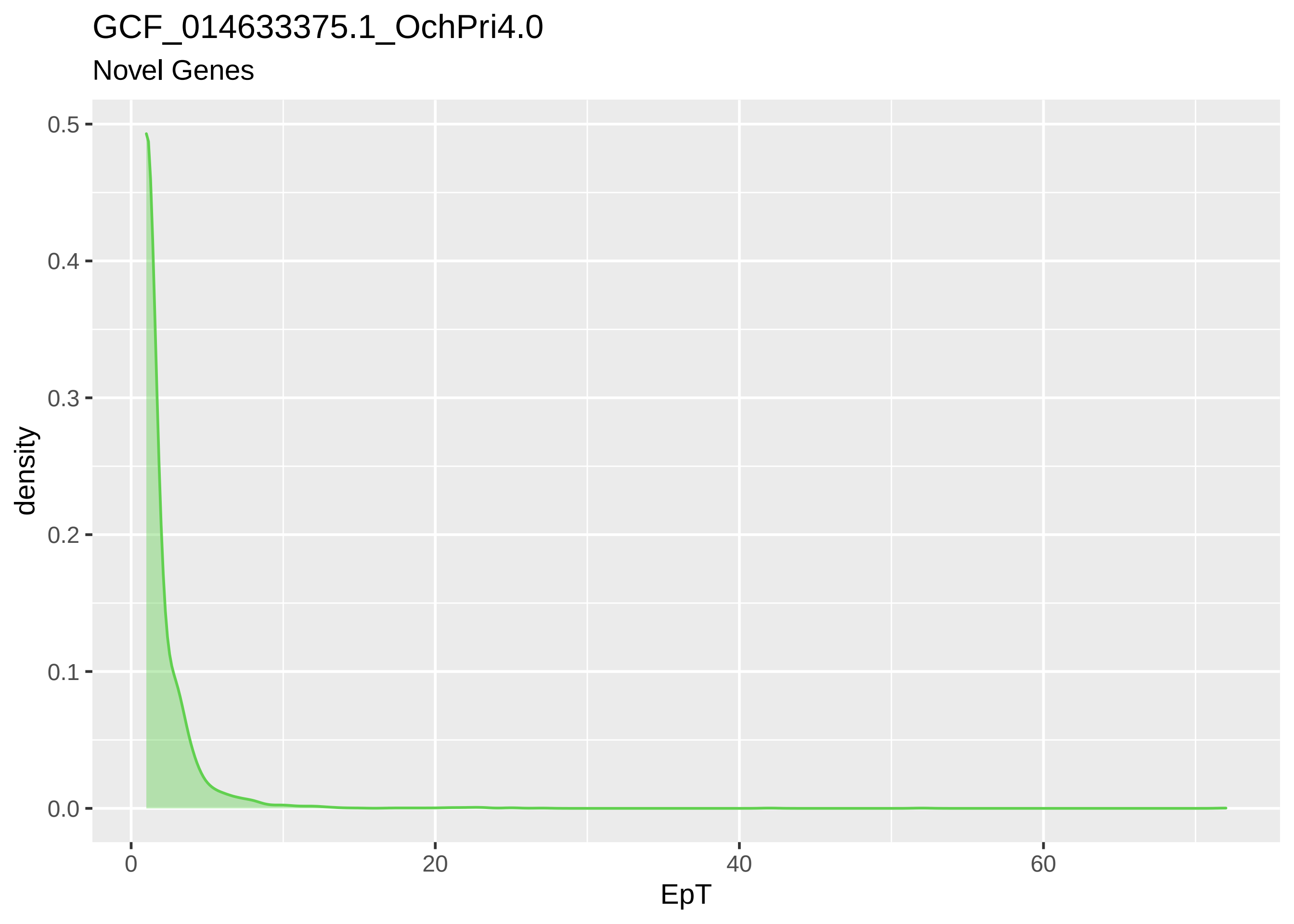
GCF_014633375.1_OchPri4.0
Novel Genes
density
0.0
0.1
0.2
0.3
0.4
0.5
0
20
40
60
EpT

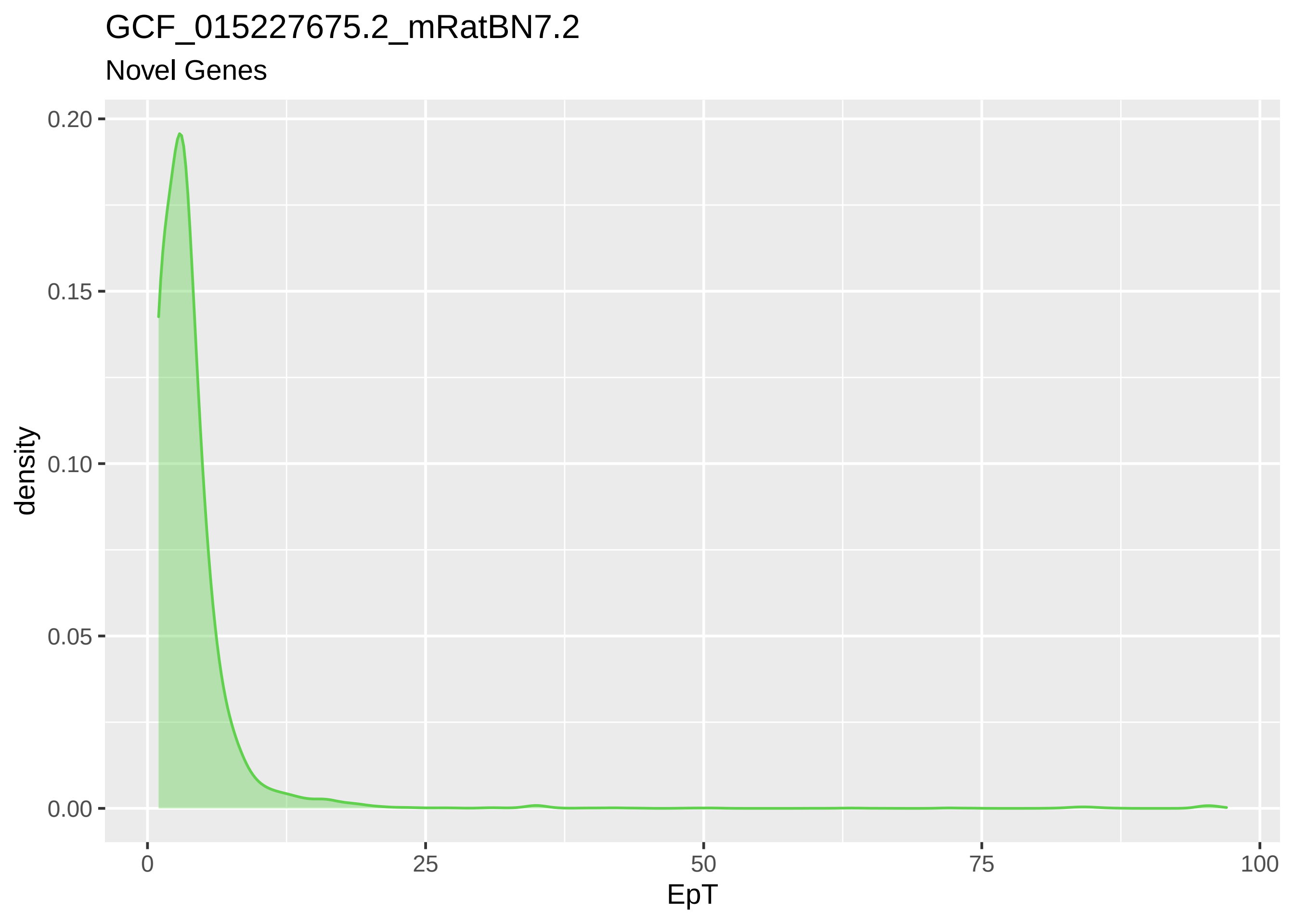

GCF_015227675.2_mRatBN7.2
Novel Genes
density
0.20
0.15
0.10
0.05
0.00
0
25
50
75
100
EpT

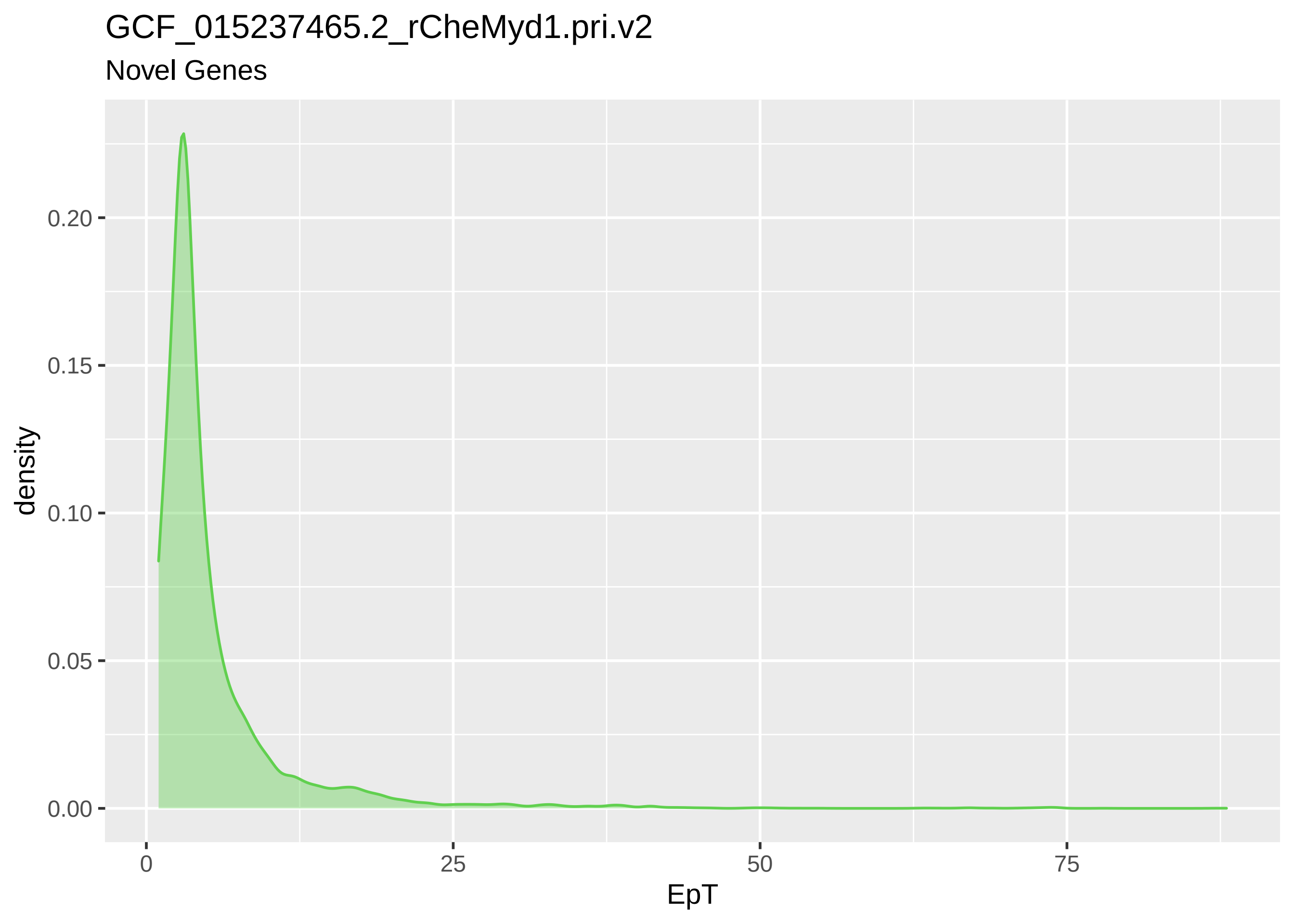
GCF_015237465.2_rCheMyd1.pri.v2
Novel Genes
density
0.20
0.15
0.10
0.05
0.00
0
25
50
75
EpT

GCF_015476345.1_ZJU1.0

Novel Genes

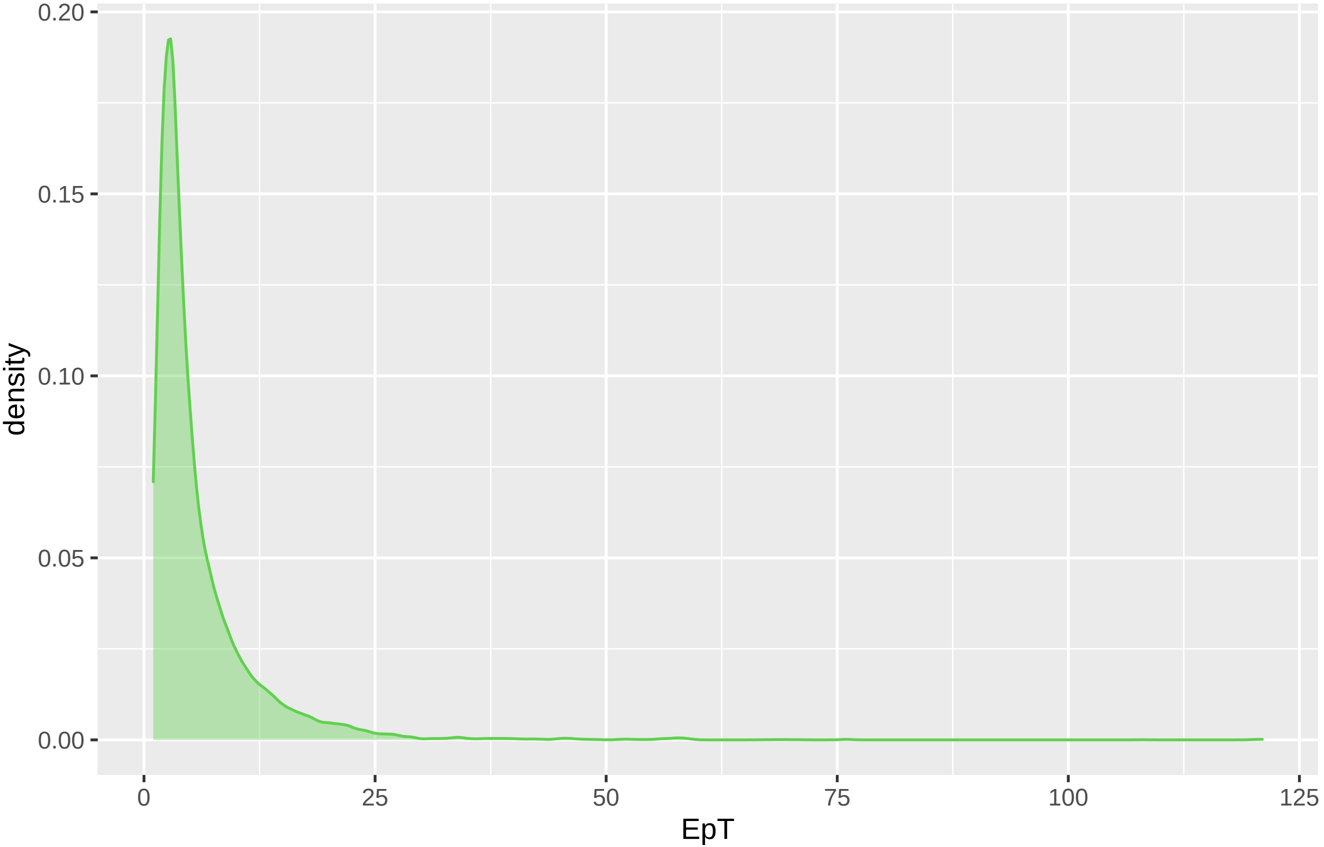

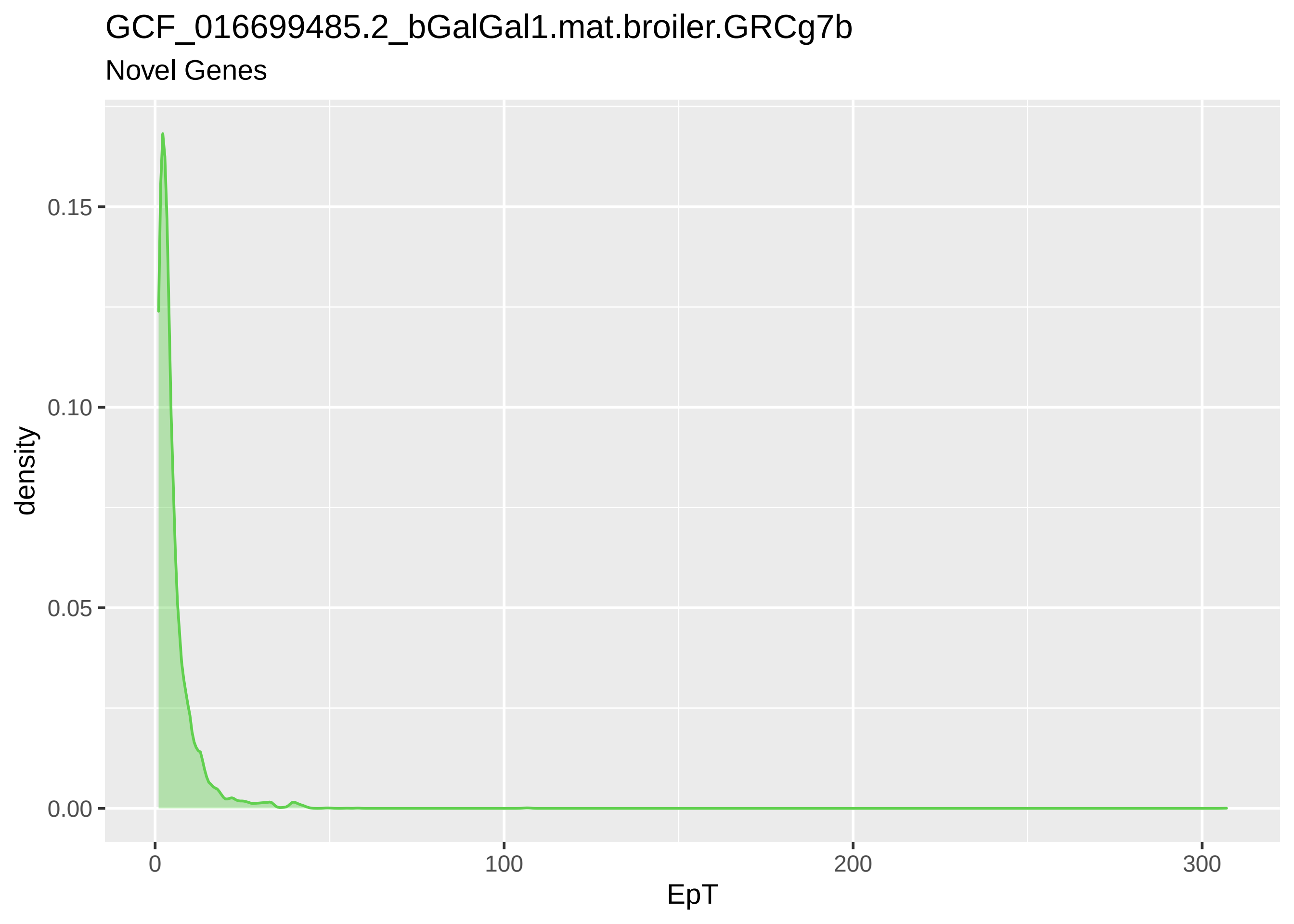
GCF_016699485.2_bGalGal1.mat.broiler.GRCg7b
Novel Genes
density
0.15
0.10
0.05
0.00
0
100
200
300
EpT

GCF_018977255.1_IMCB_Cmil_1.0

Novel Genes

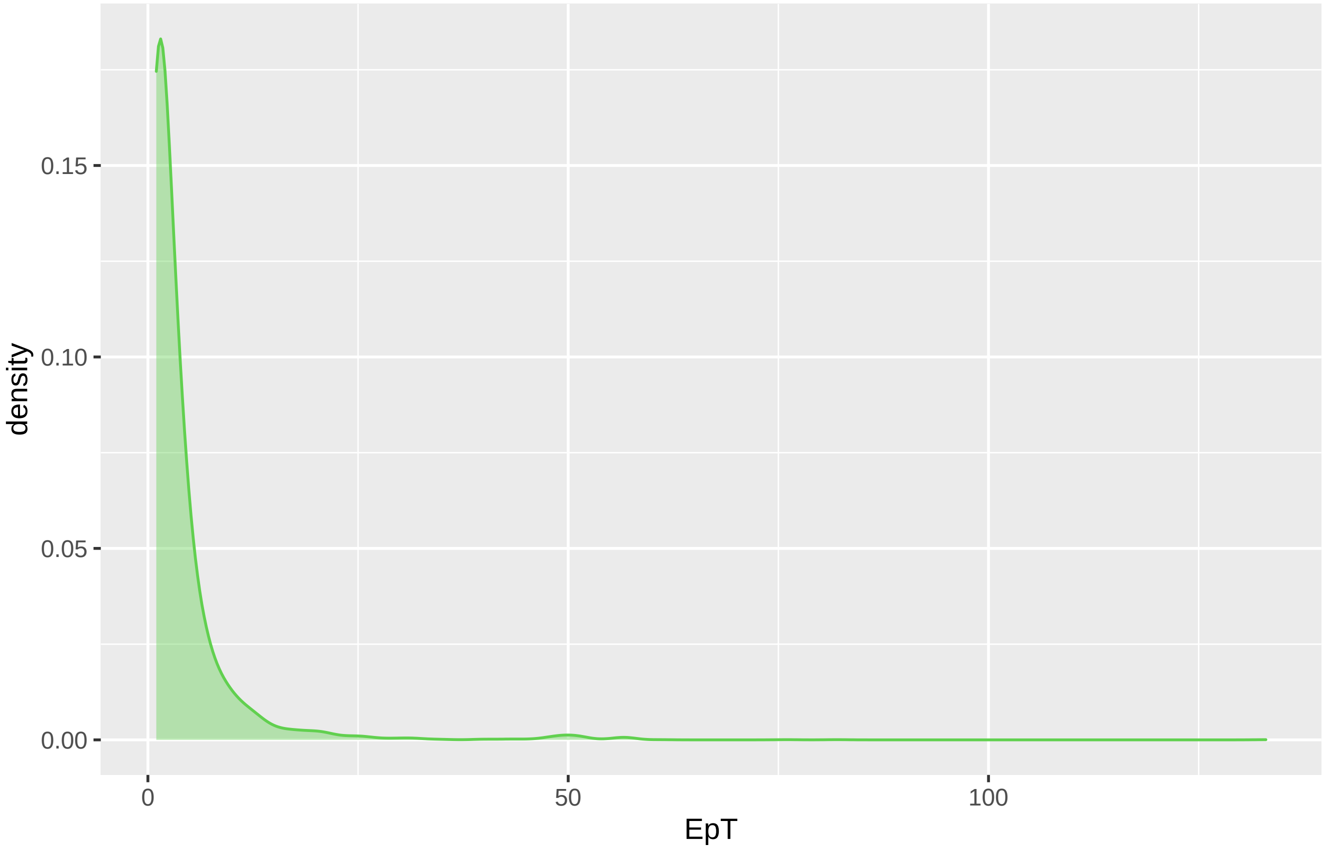

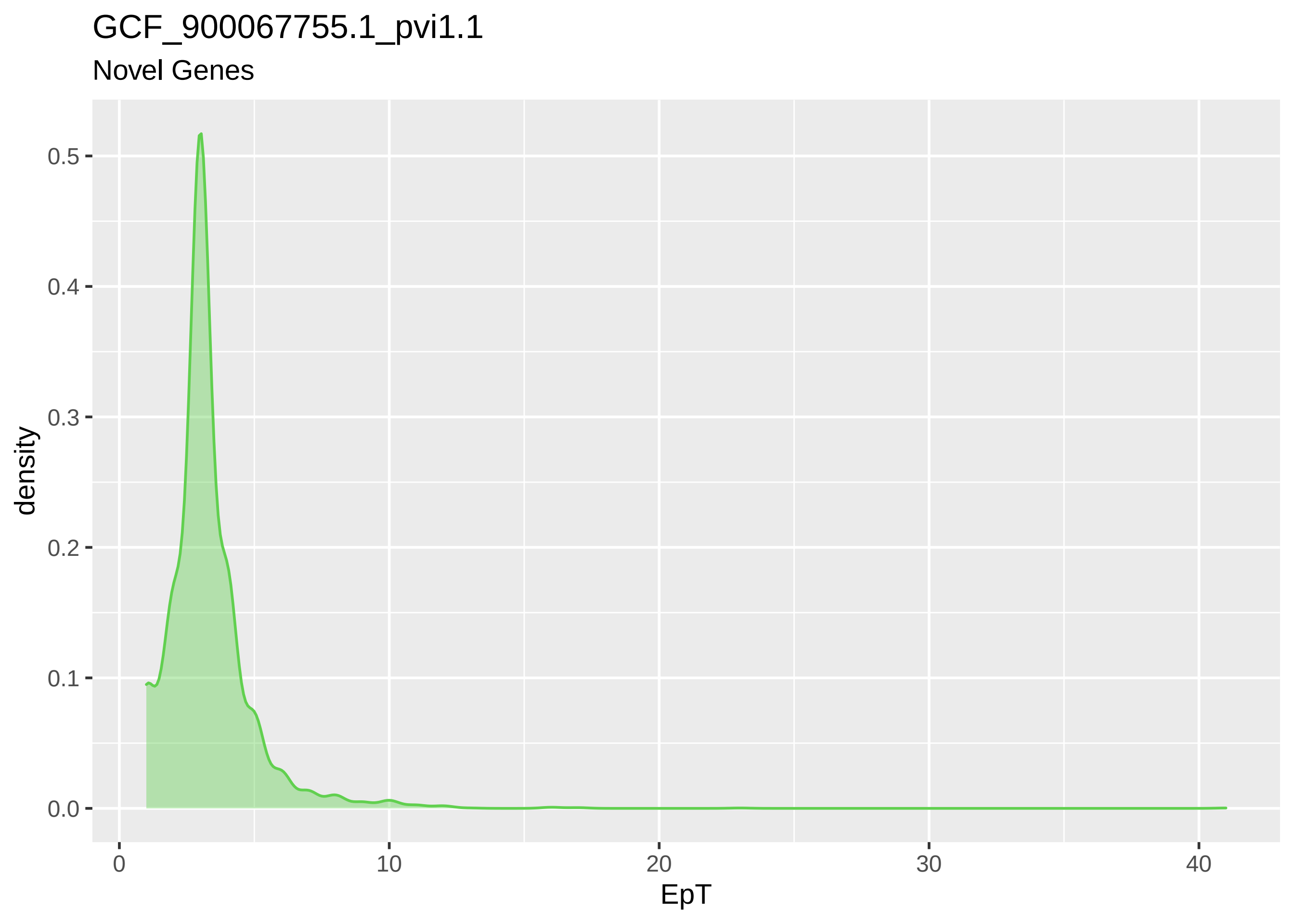
GCF_900067755.1_pvi1.1
Novel Genes
density
0.5
0.4
0.3
0.2
0.1
0.0
0
10
20
30
40
EpT

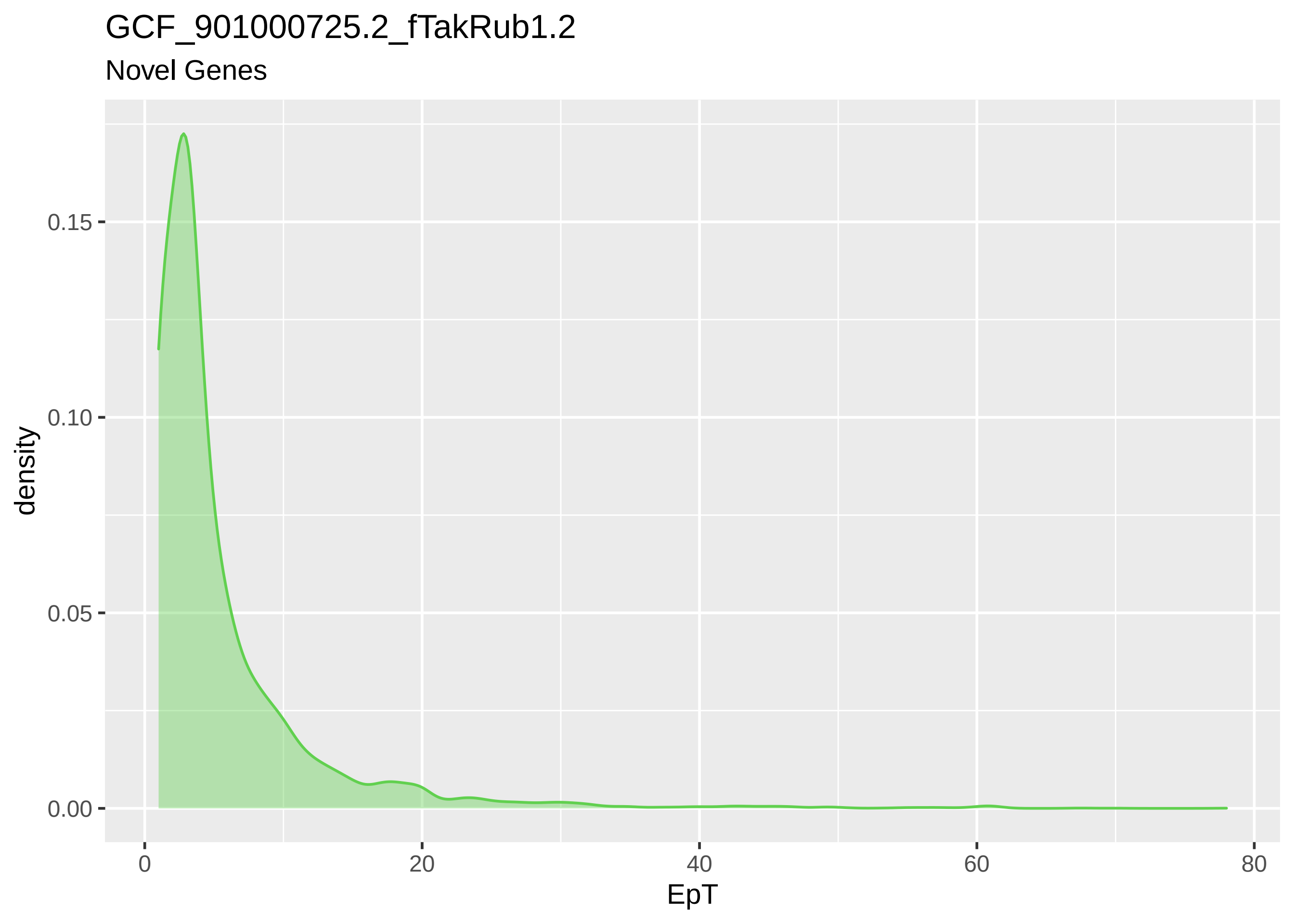
GCF_901000725.2_fTakRub1.2
Novel Genes
density
0.15
0.10
0.05
0.00
0
20
40
60
80
EpT

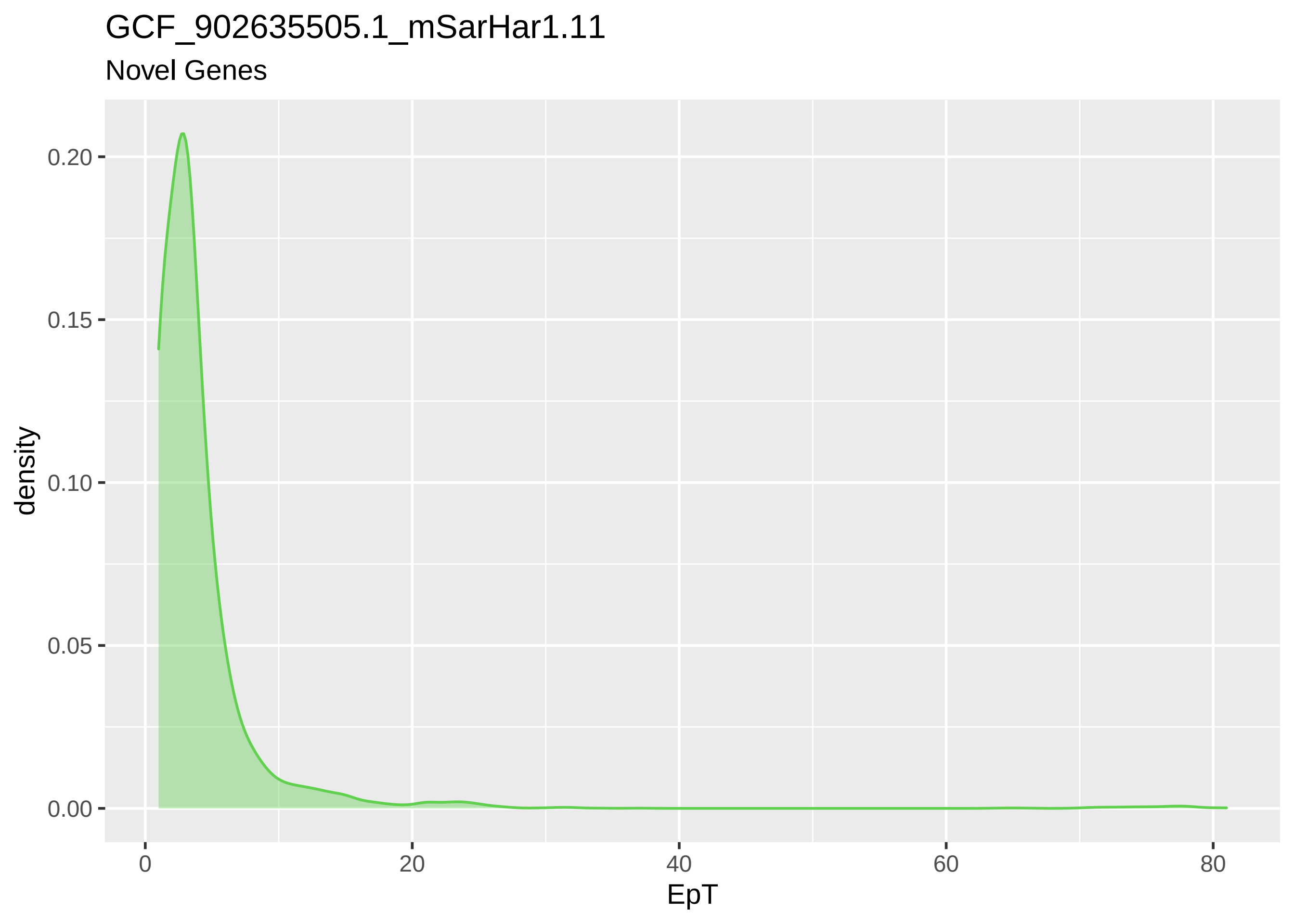
GCF_902635505.1_mSarHar1.11
Novel Genes
density
0.20
0.15
0.10
0.05
0.00
0
20
40
60
80
EpT

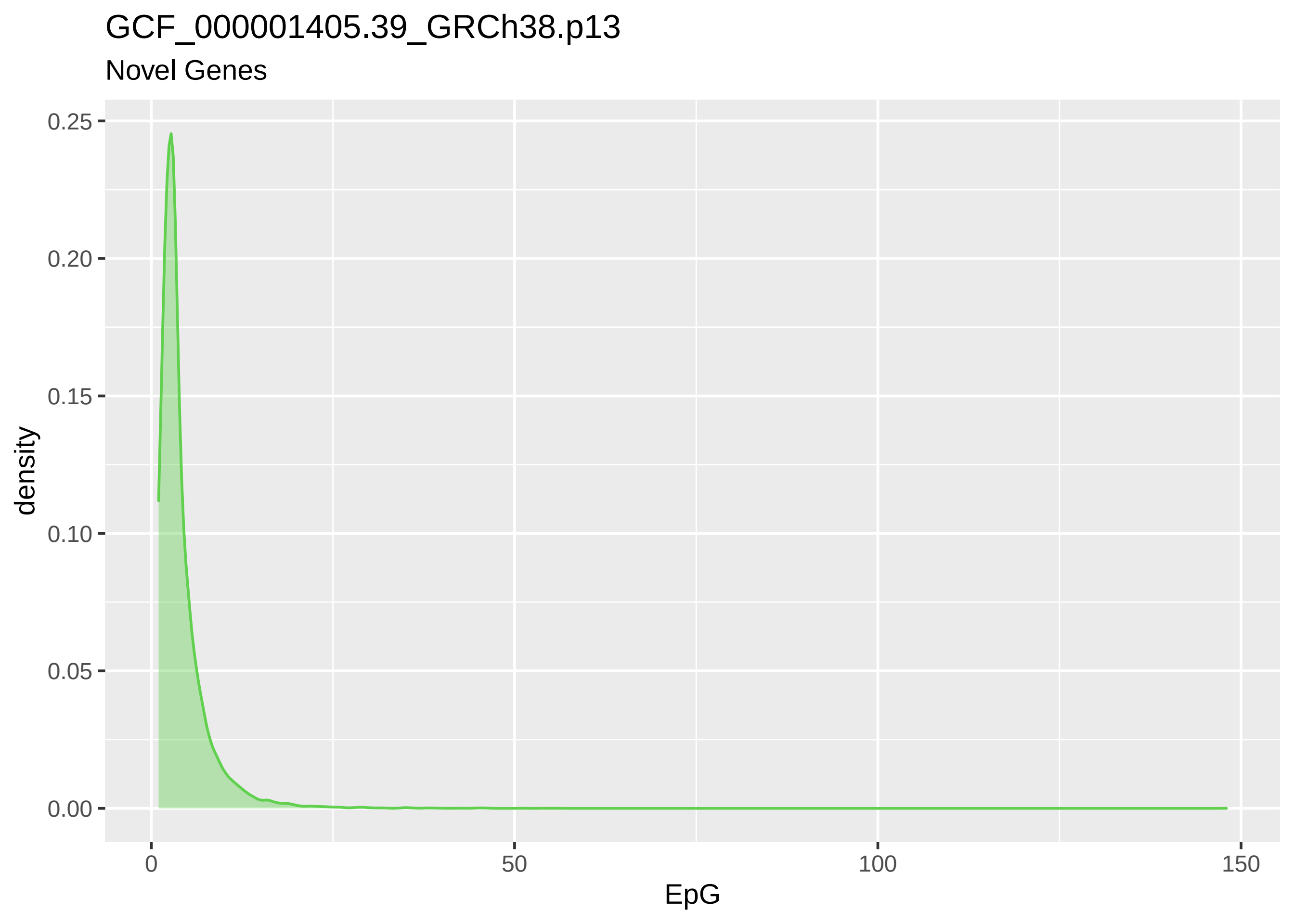
GCF_000001405.39_GRCh38.p13
Novel Genes
density
0.25
0.20
0.15
0.10
0.05
0.00
0
50
100
150
EpG

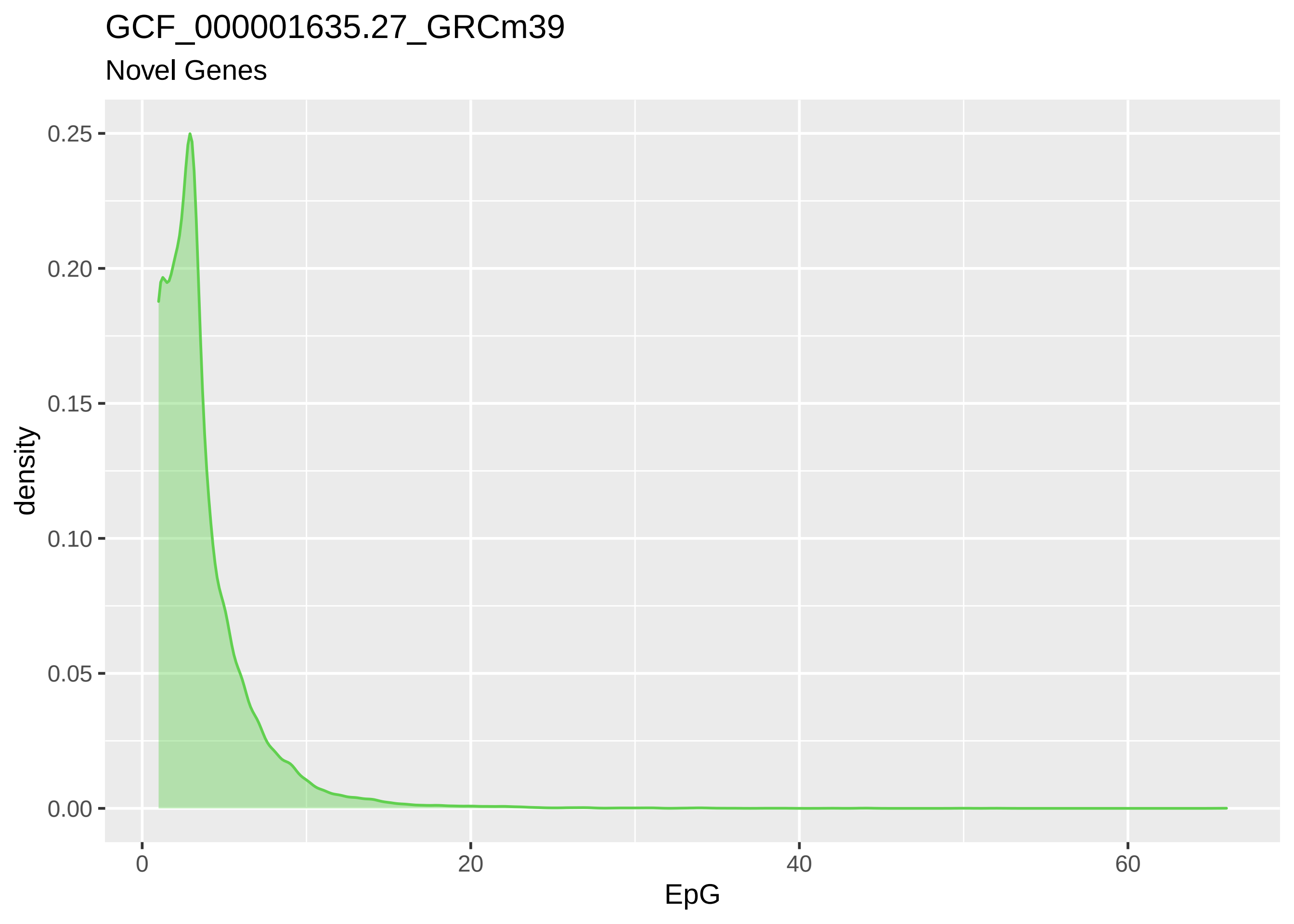
GCF_000001635.27_GRCm39
Novel Genes
density
0.25
0.20
0.15
0.10
0.05
0.00
0
20
40
60
EpG

GCF_000001905.1_Loxafr3.0

Novel Genes

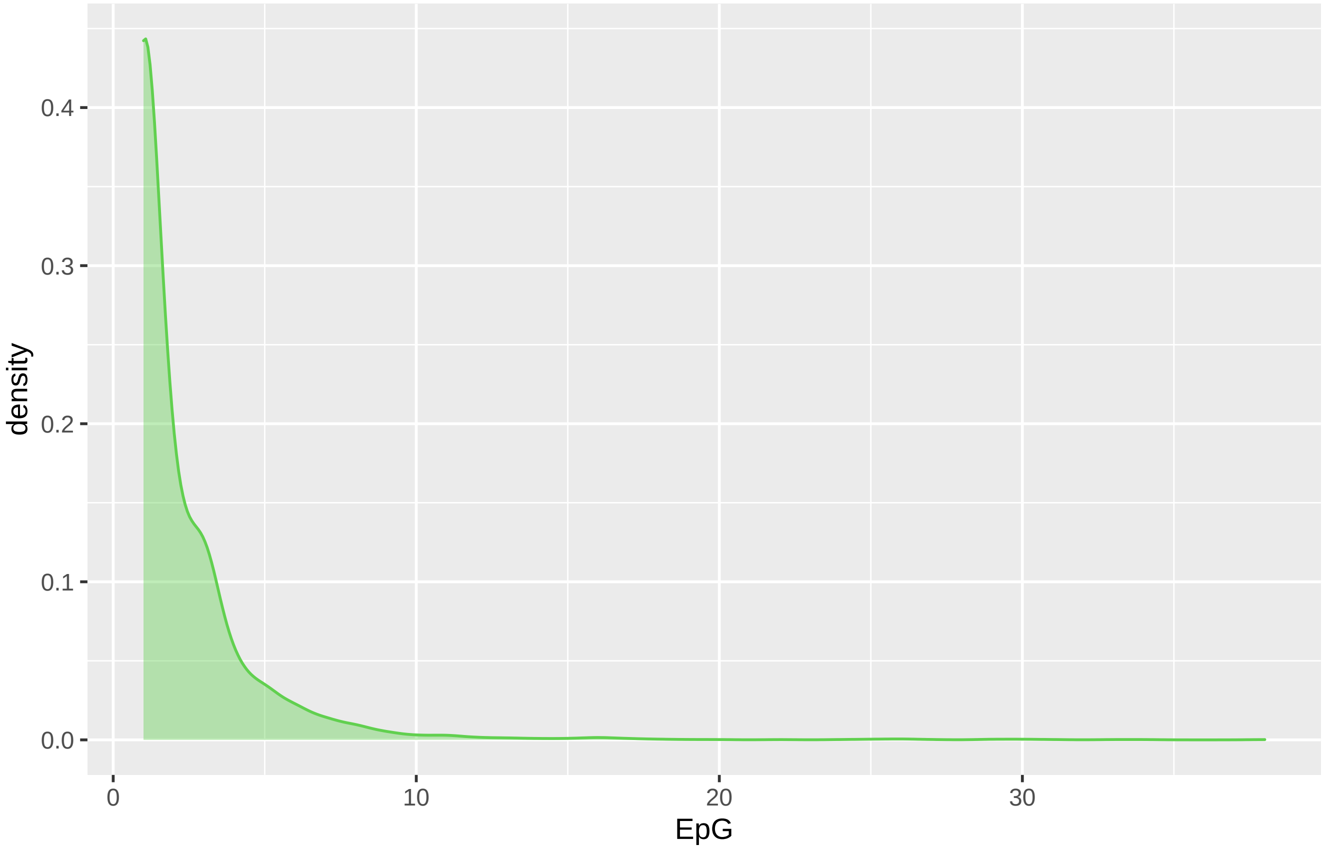

GCF_000002035.6_GRCz11

Novel Genes

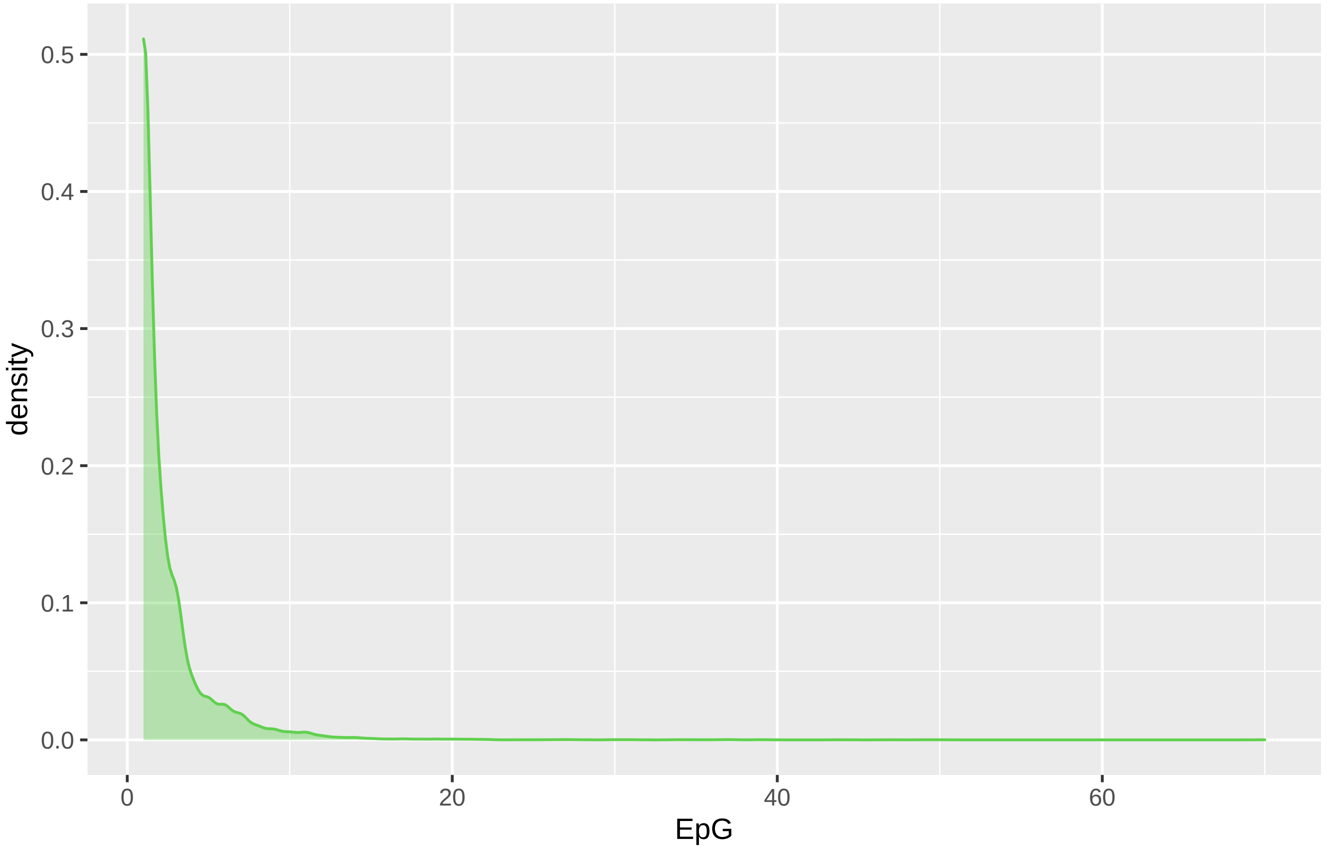

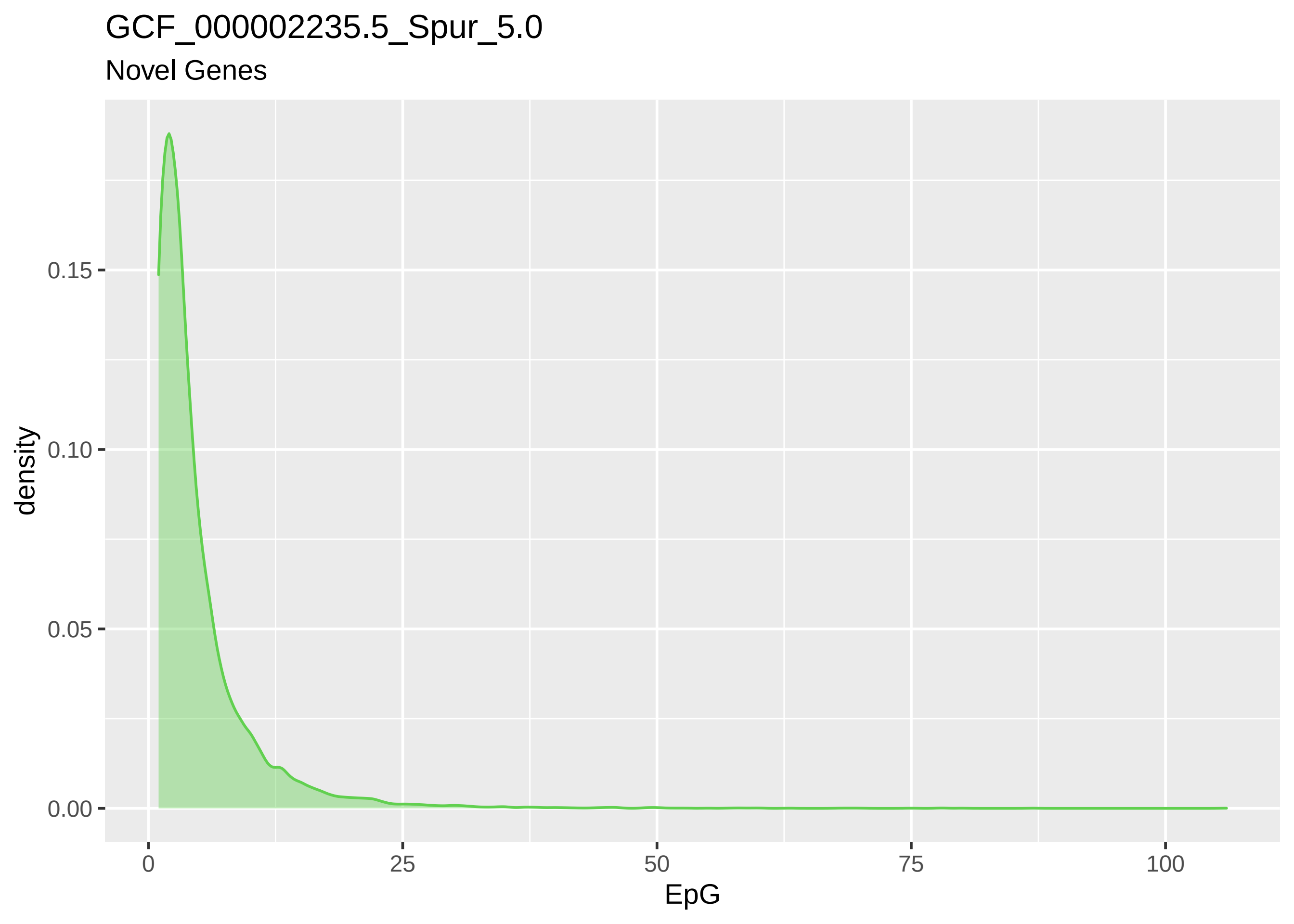

GCF_000002235.5_Spur_5.0
Novel Genes
density
0.15
0.10
0.05
0.00
0
25
50
75
100
EpG

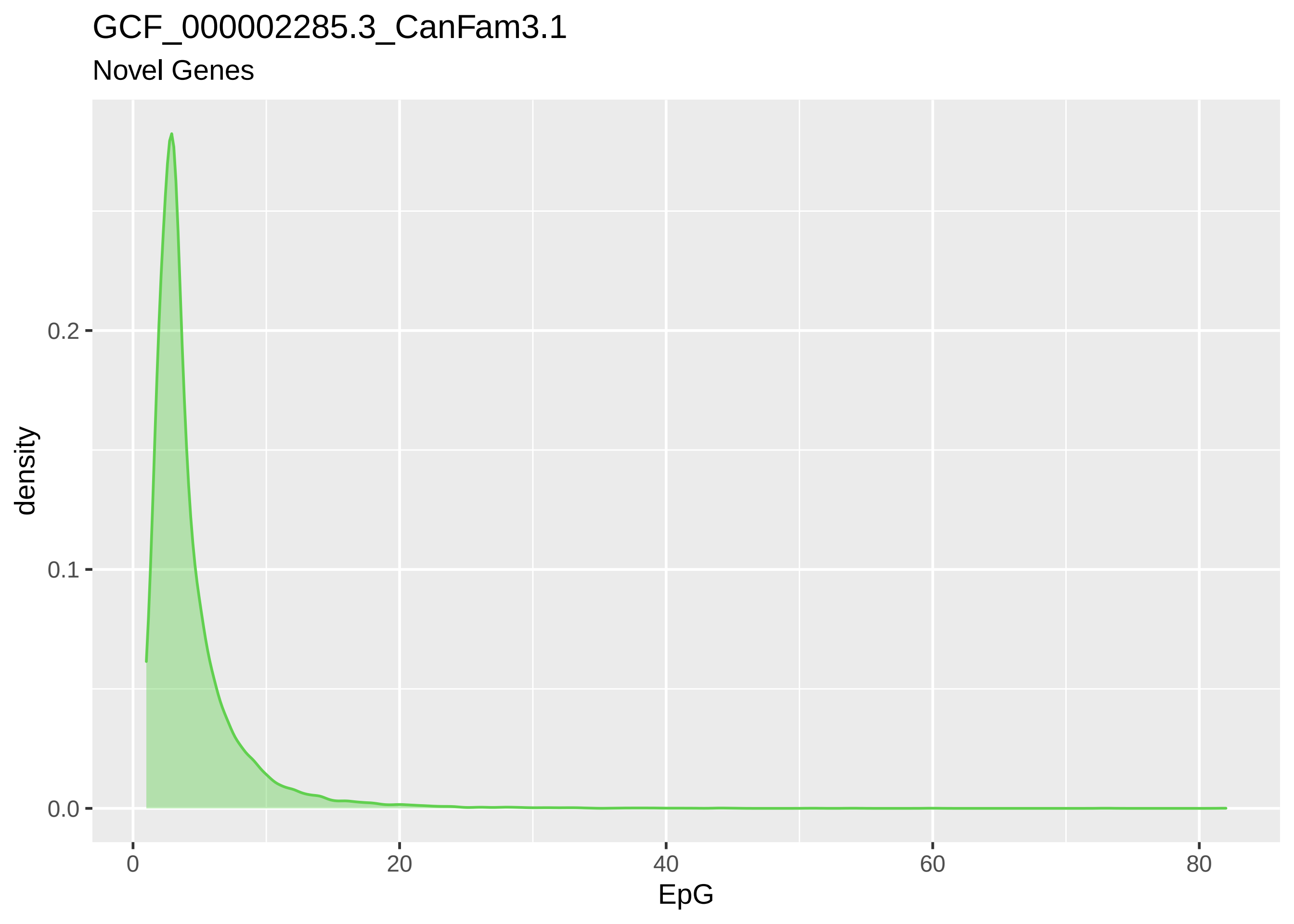
GCF_000002285.3_CanFam3.1
Novel Genes
density
0.2
0.1
0.0
0
20
40
60
80
EpG

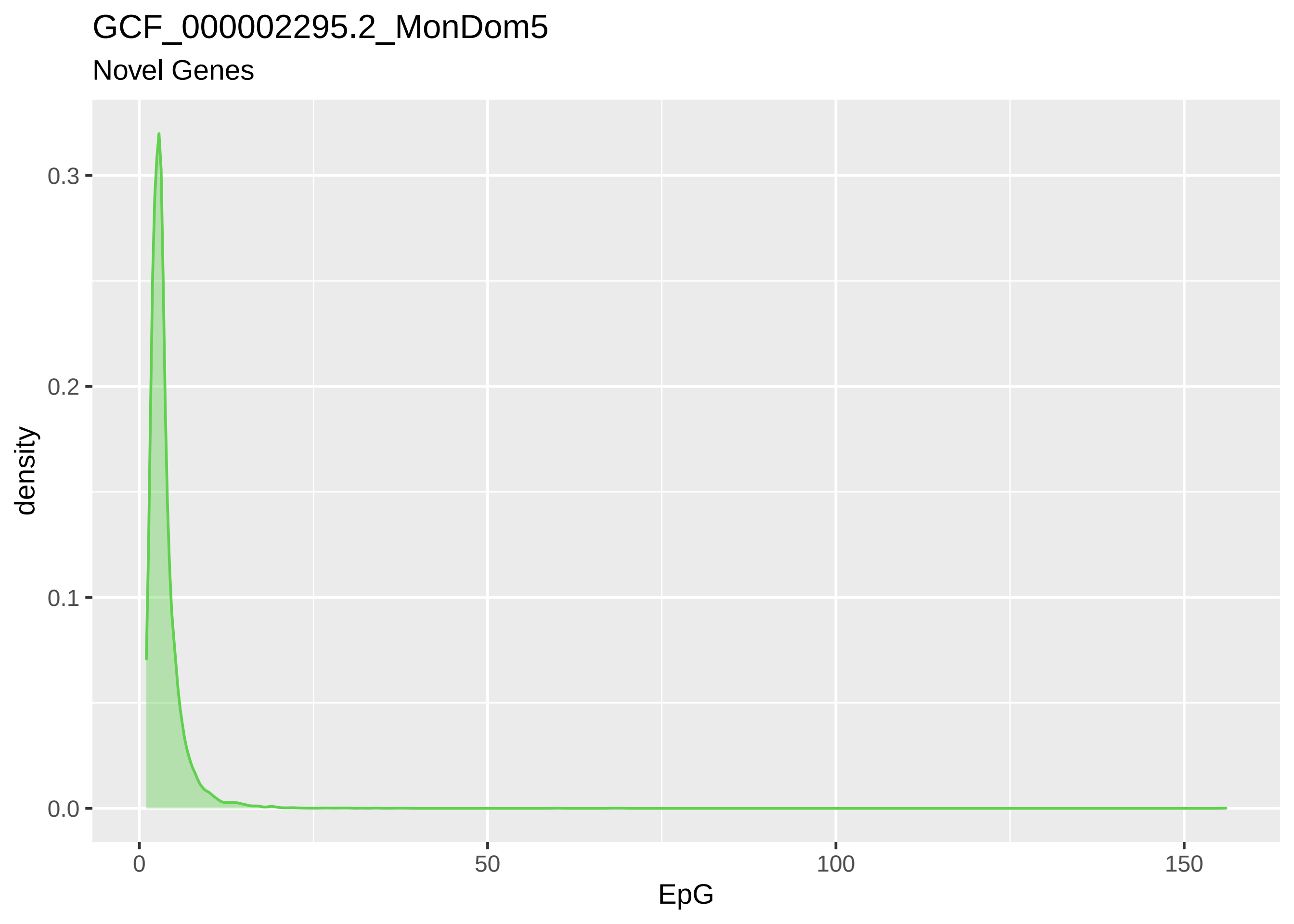
GCF_000002295.2_MonDom5
Novel Genes
density
0.3
0.2
0.1
0.0
0
50
100
150
EpG

GCF_000003025.6_Sscrofa11.1

Novel Genes

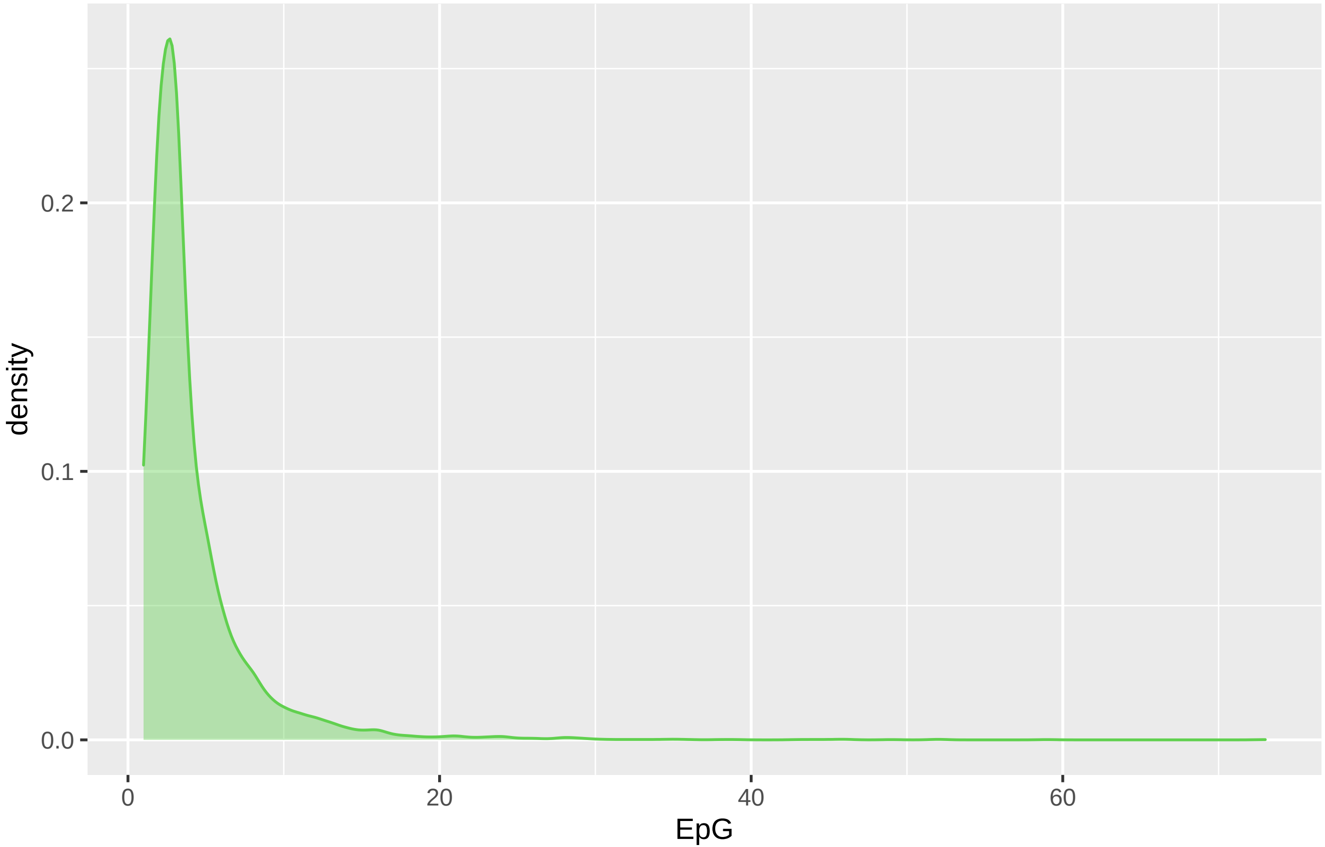

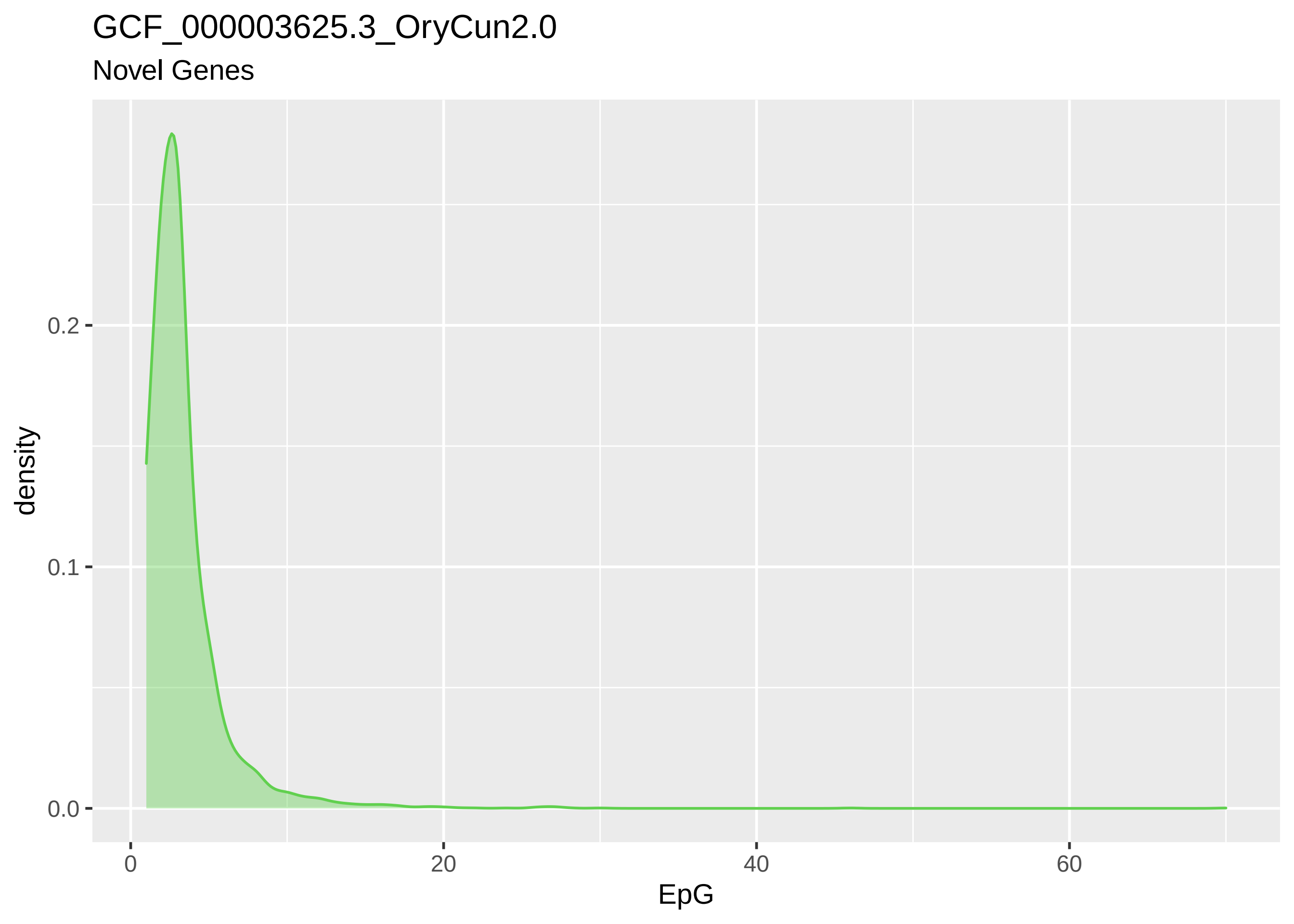
GCF_000003625.3_OryCun2.0
Novel Genes
density
0.0
0.1
0.2
0
20
40
60
EpG

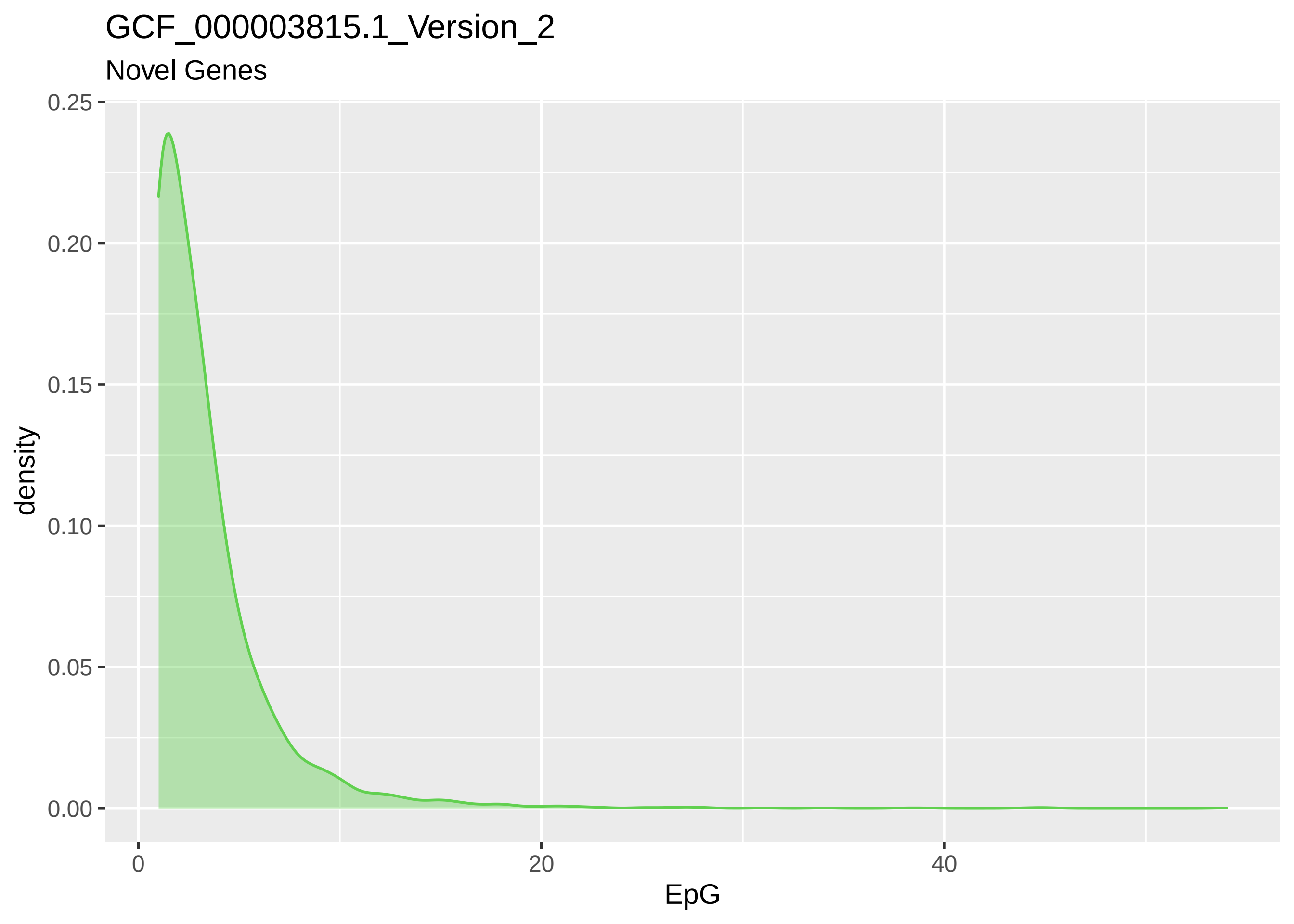
GCF_000003815.1_Version_2
Novel Genes
density
EpG
0.25
0.20
0.15
0.10
0.05
0.00
0
20
40

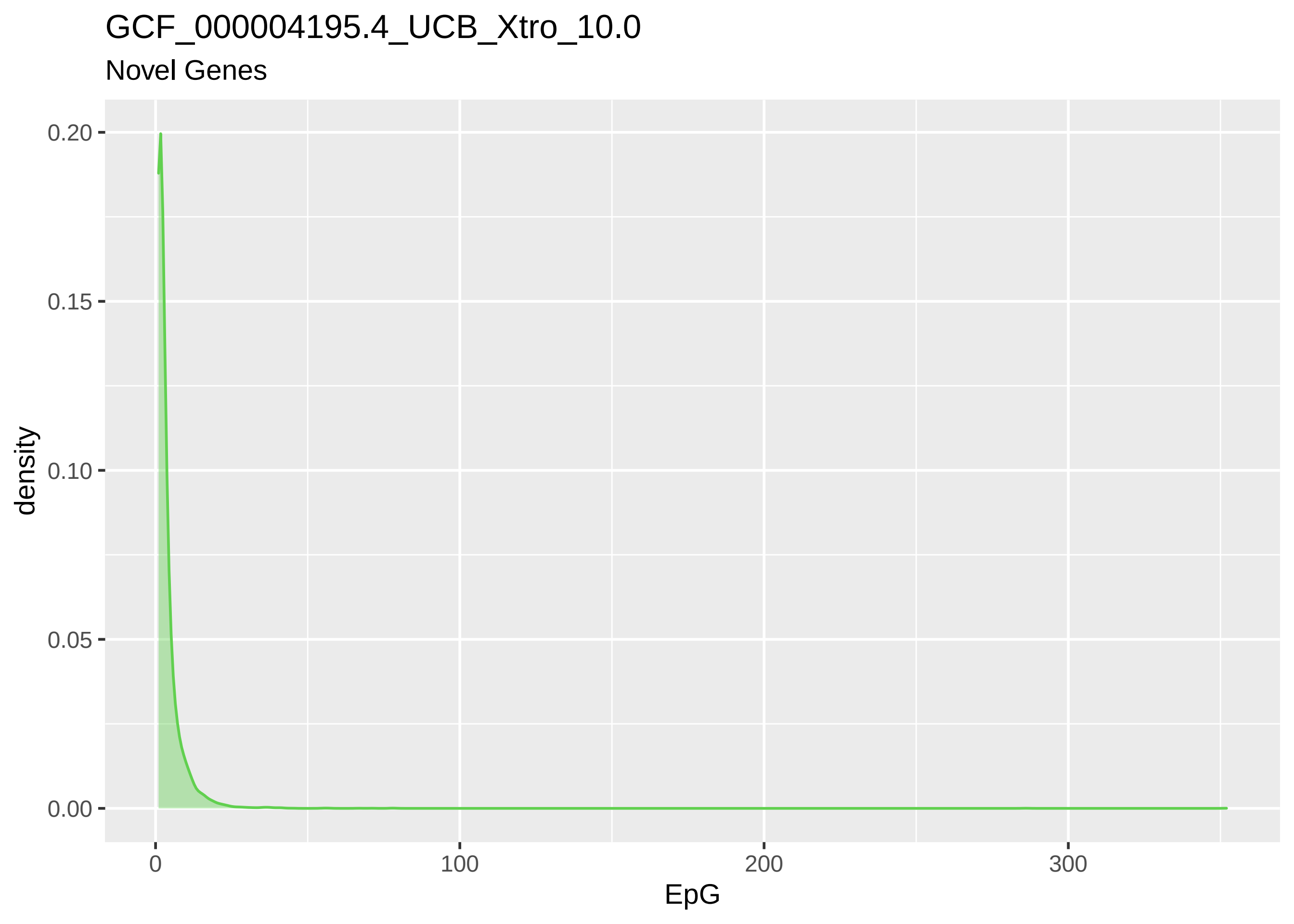
GCF_000004195.4_UCB_Xtro_10.0
Novel Genes
density
0.20
0.15
0.10
0.05
0.00
0
100
200
300
EpG

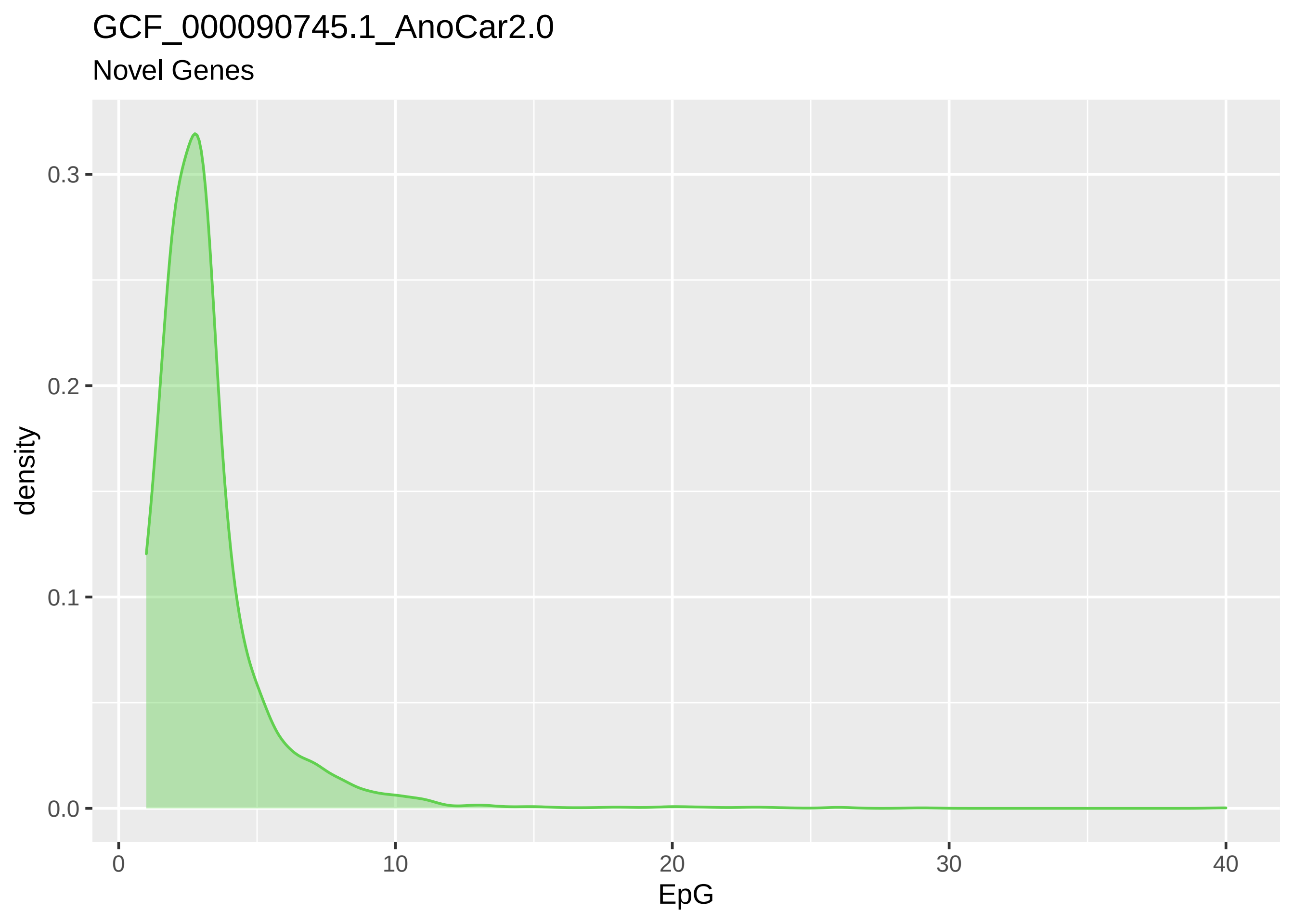
GCF_000090745.1_AnoCar2.0
Novel Genes
density
0.0
0.1
0.2
0.3
0
10
20
30
40
EpG

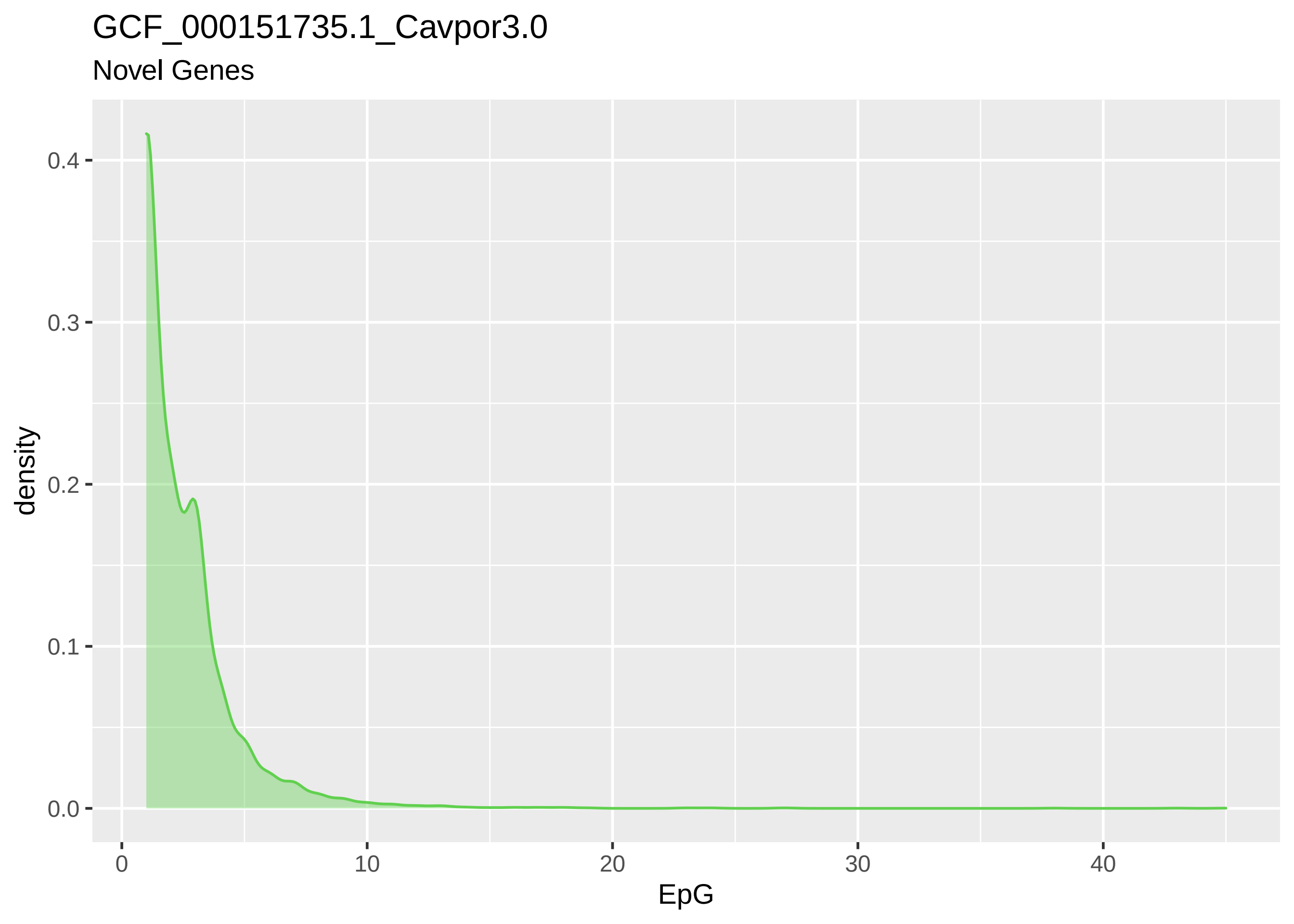
GCF_000151735.1_Cavpor3.0
Novel Genes
density
0.4
0.3
0.2
0.1
0.0
0
10
20
30
40
EpG

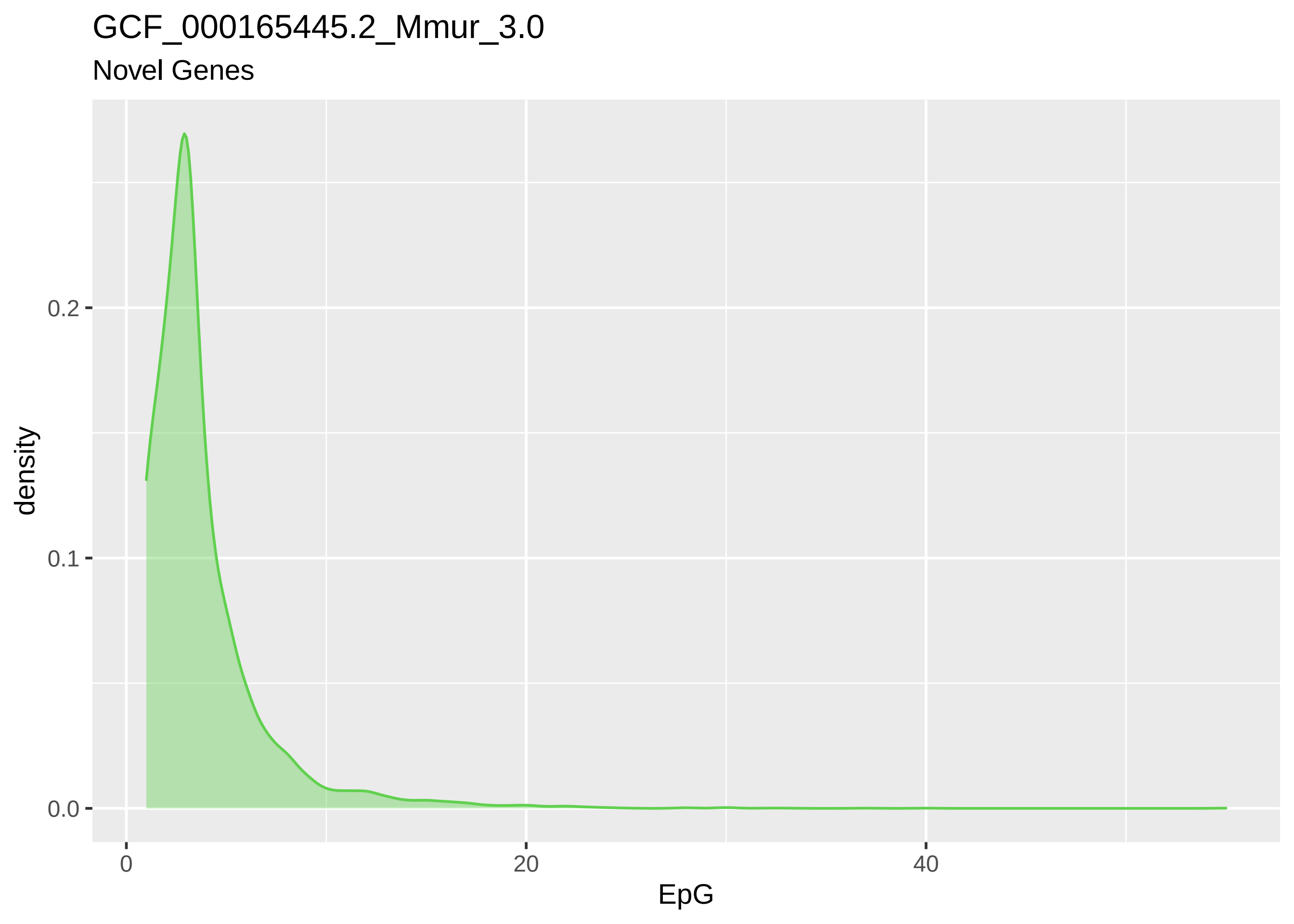
GCF_000165445.2_Mmur_3.0
Novel Genes
density
0.0
0.1
0.2
0
20
40
EpG

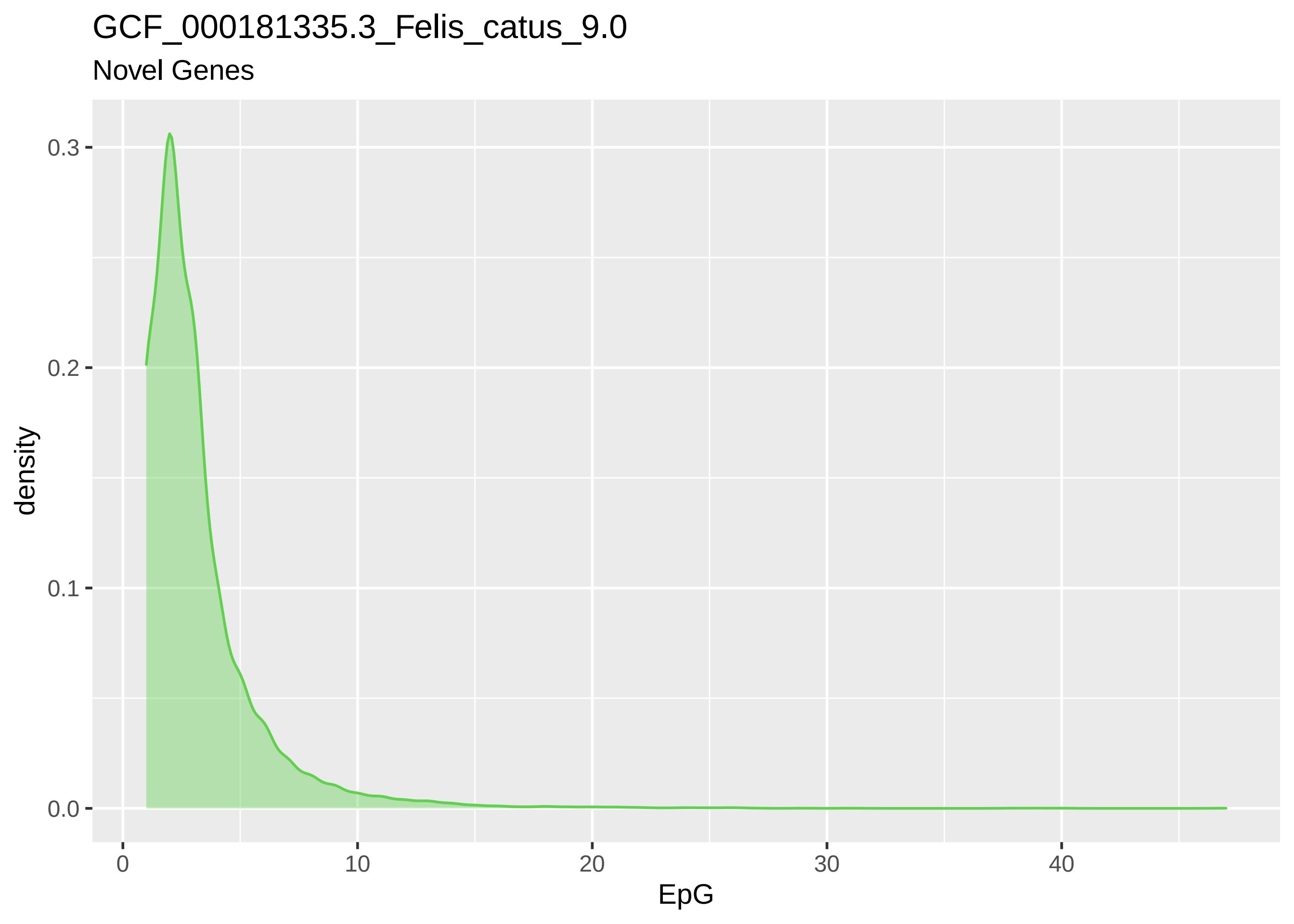

GCF_000181335.3_Felis_catus_9.0
Novel Genes
density
0.3
0.2
0.1
0.0
0
10
20
30
40
EpG

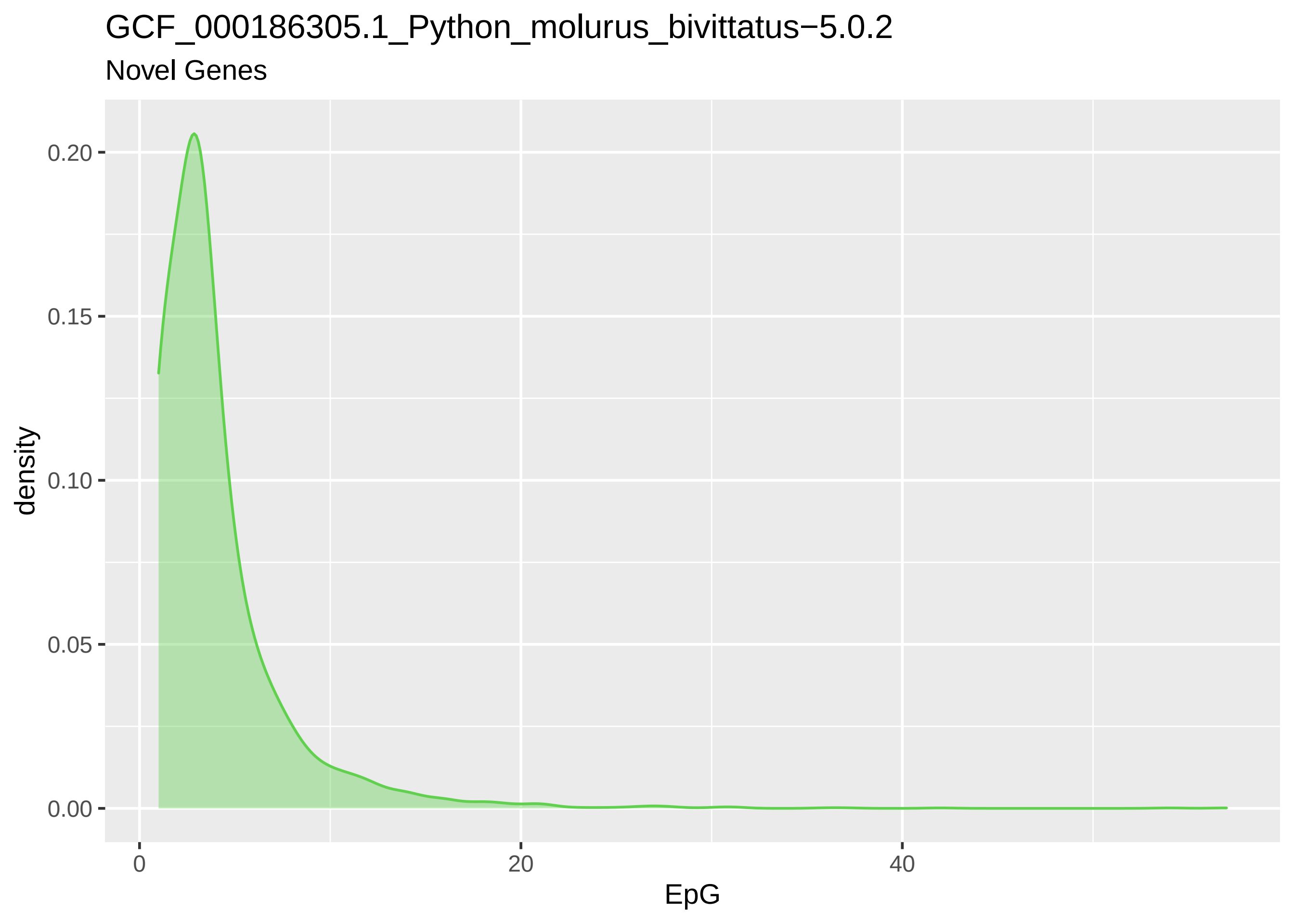
GCF_000186305.1_Python_molurus_bivittatus−5.0.2
Novel Genes
density
0.20
0.15
0.10
0.05
0.00
0
20
40
EpG

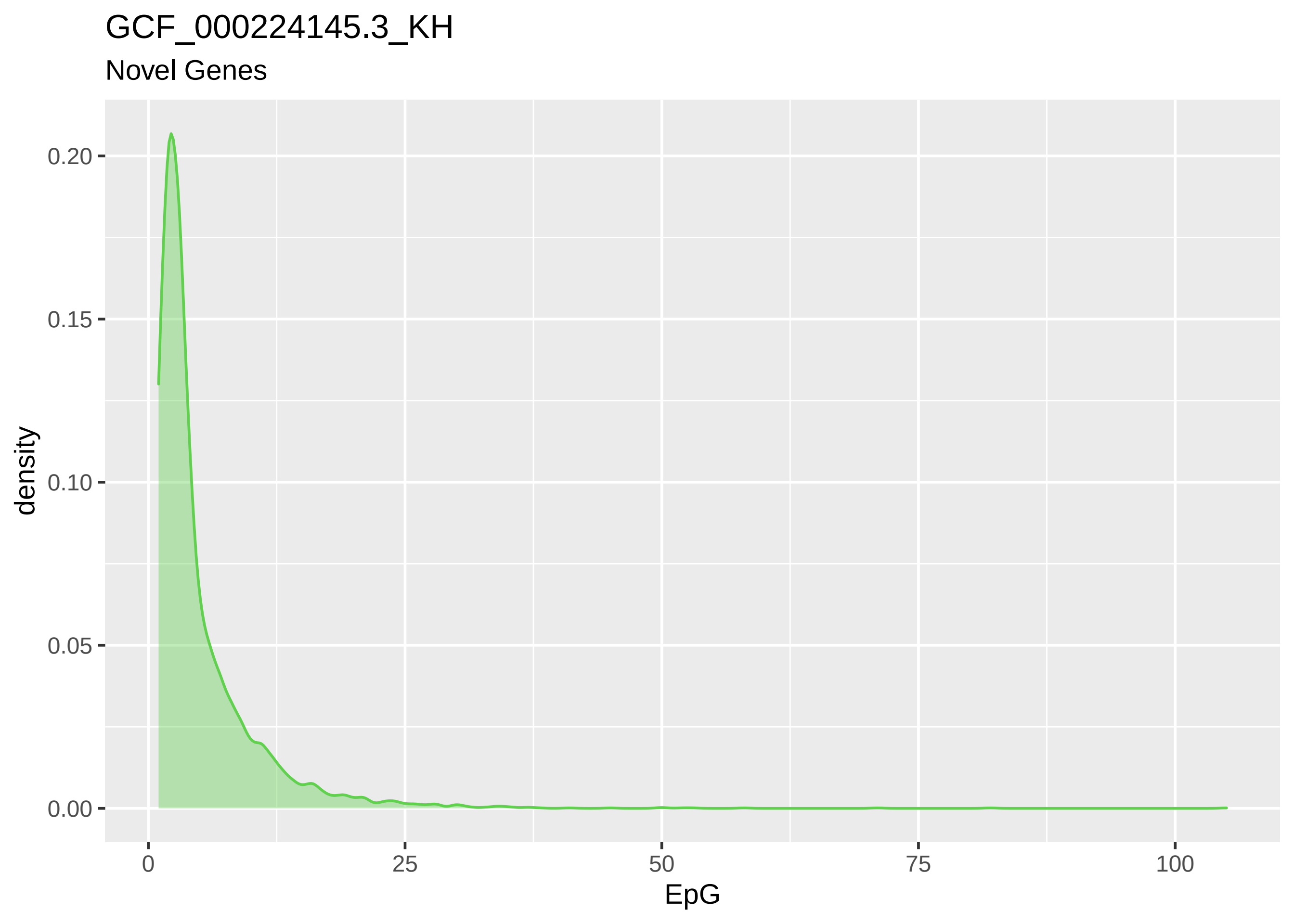
GCF_000224145.3_KH
Novel Genes
density
0.20
0.15
0.10
0.05
0.00
0
25
50
75
100
EpG

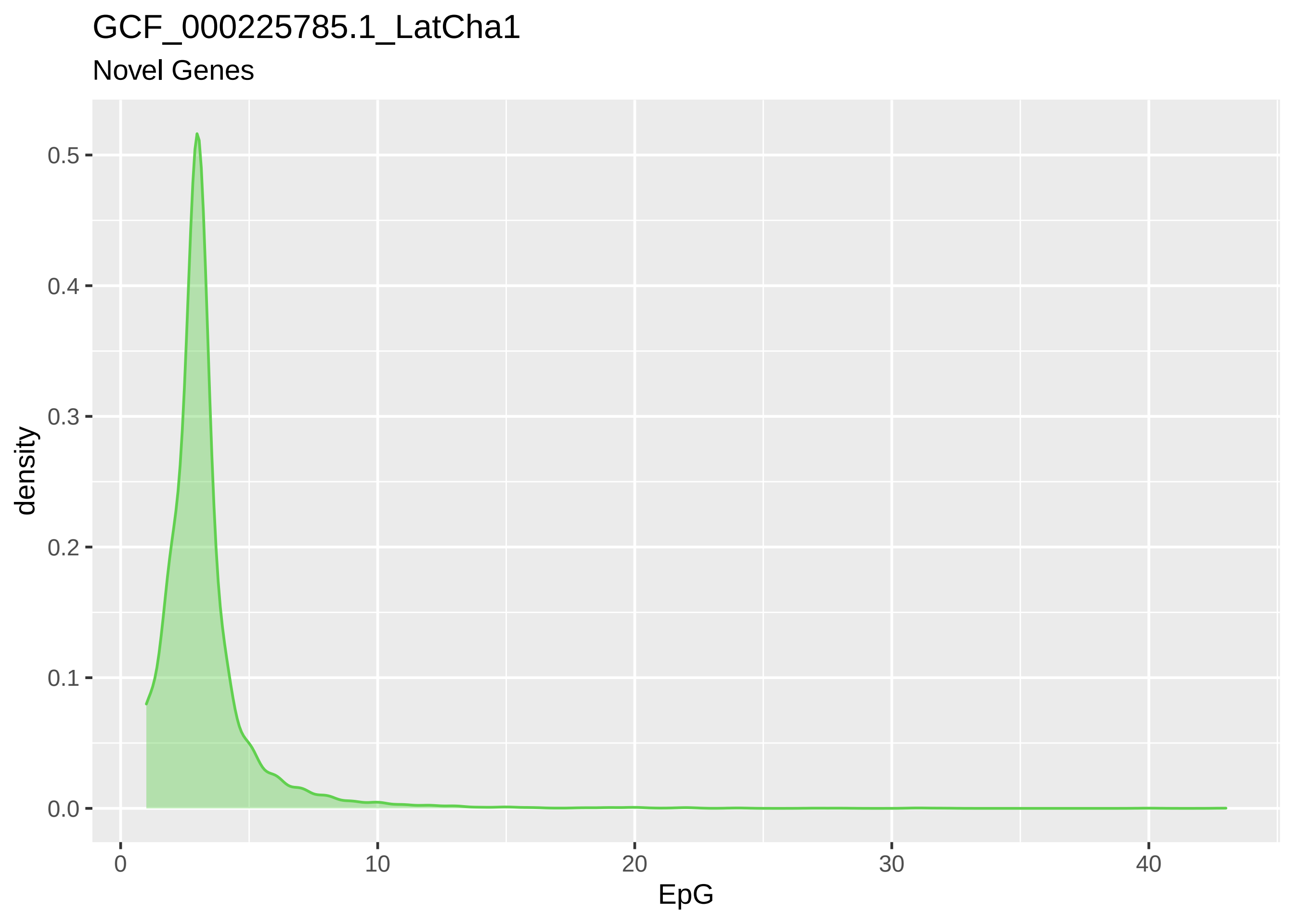
GCF_000225785.1_LatCha1
Novel Genes
density
EpG
0.0
0.1
0.2
0.3
0.4
0.5
0
10
20
30
40

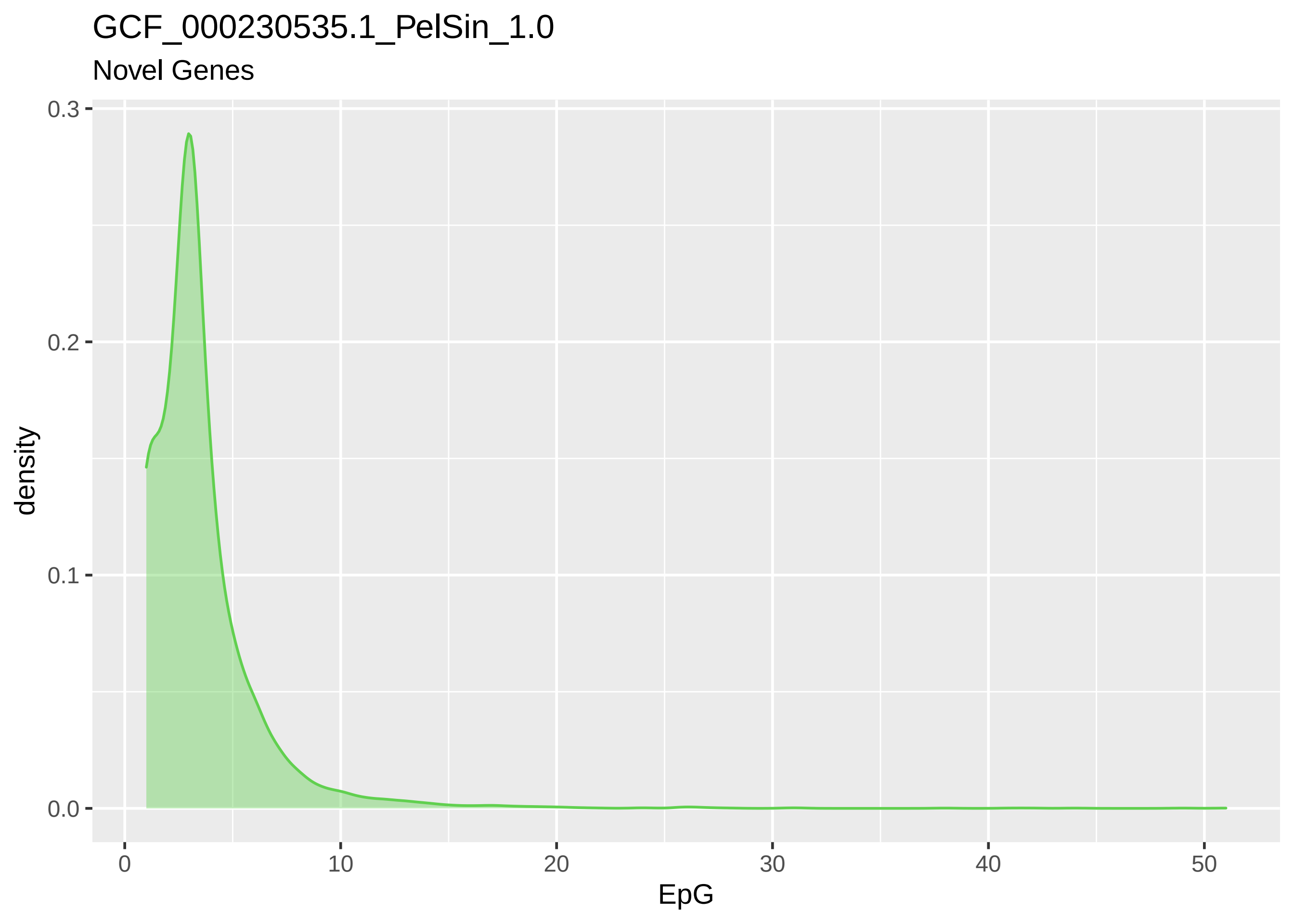

GCF_000230535.1_PelSin_1.0
Novel Genes
density
0.3
0.2
0.1
0.0
0
10
20
30
40
50
EpG

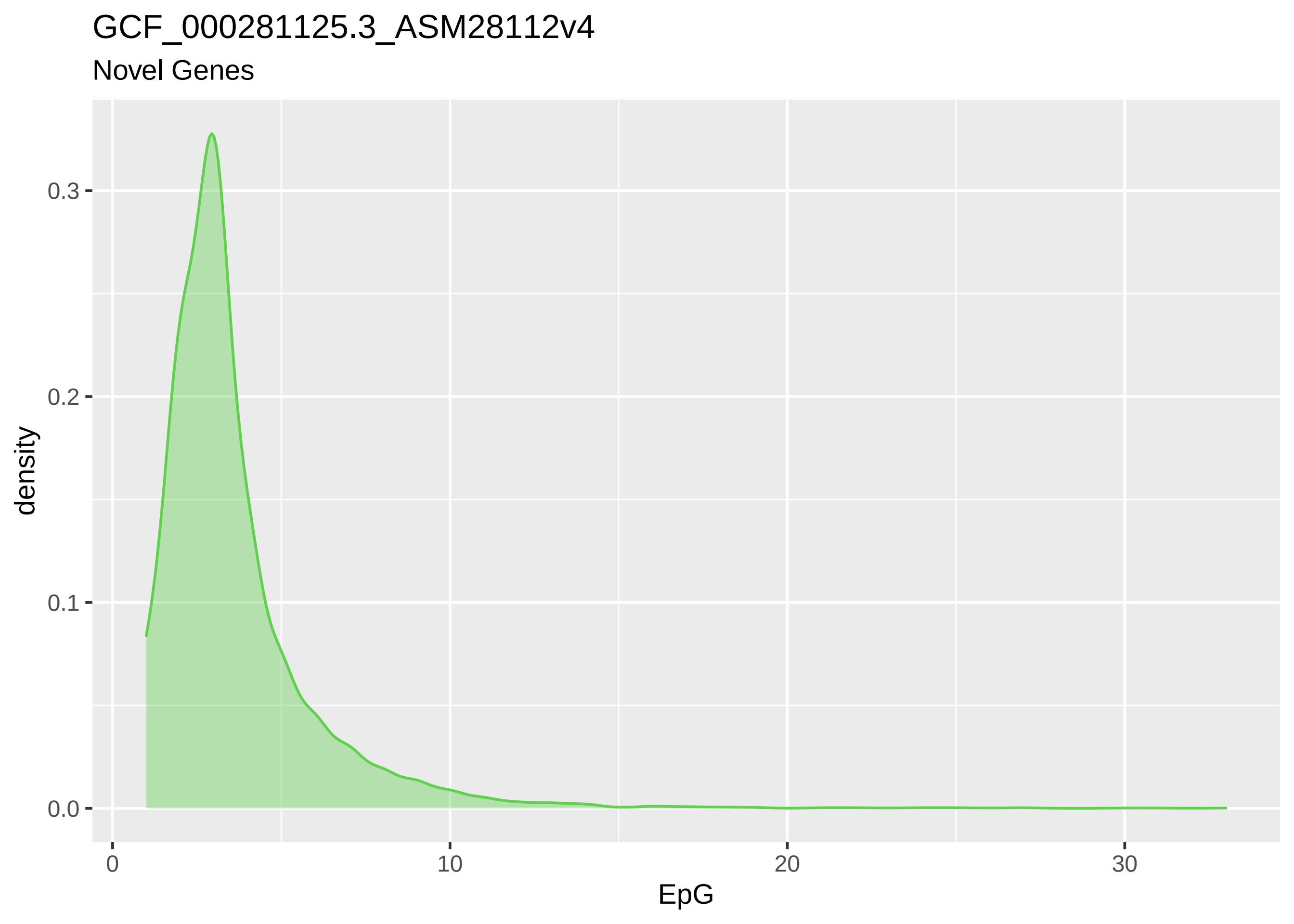
GCF_000281125.3_ASM28112v4
Novel Genes
density
0.0
0.1
0.2
0.3
0
10
20
30
EpG

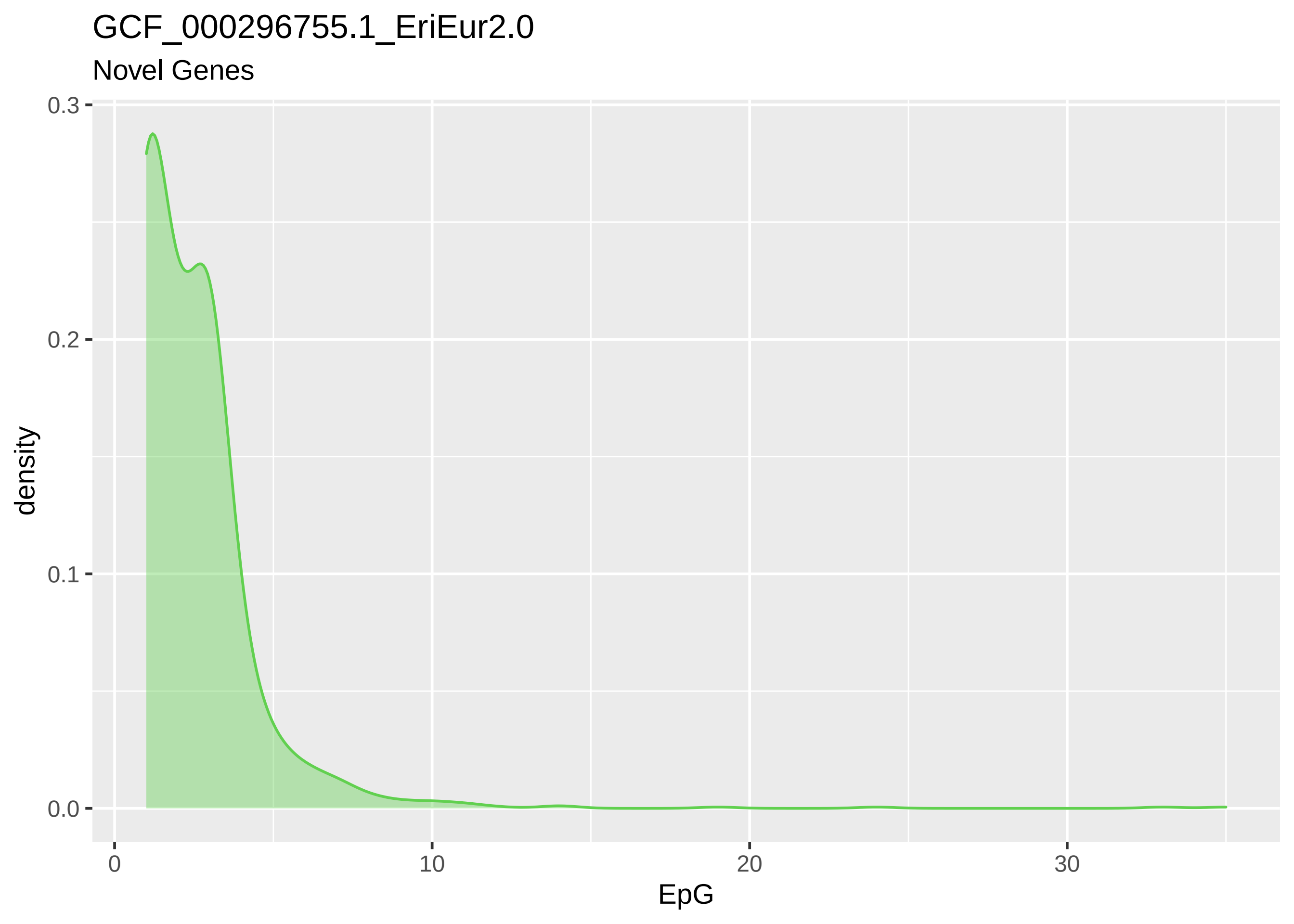

GCF_000296755.1_EriEur2.0
Novel Genes
density
EpG
0.0
0.1
0.2
0.3
0
10
20
30

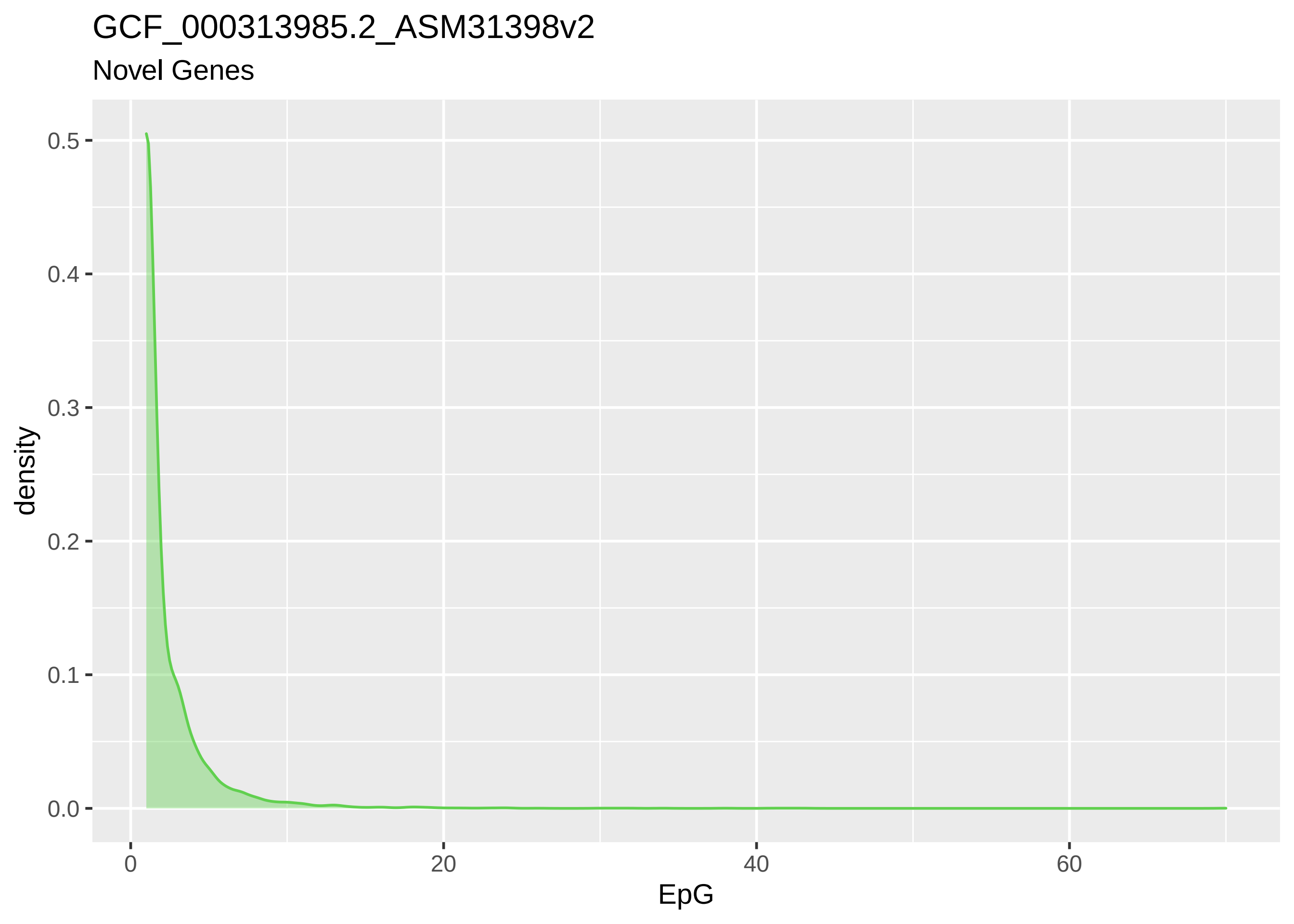
GCF_000313985.2_ASM31398v2
Novel Genes
density
0.5
0.4
0.3
0.2
0.1
0.0
0
20
40
60
EpG

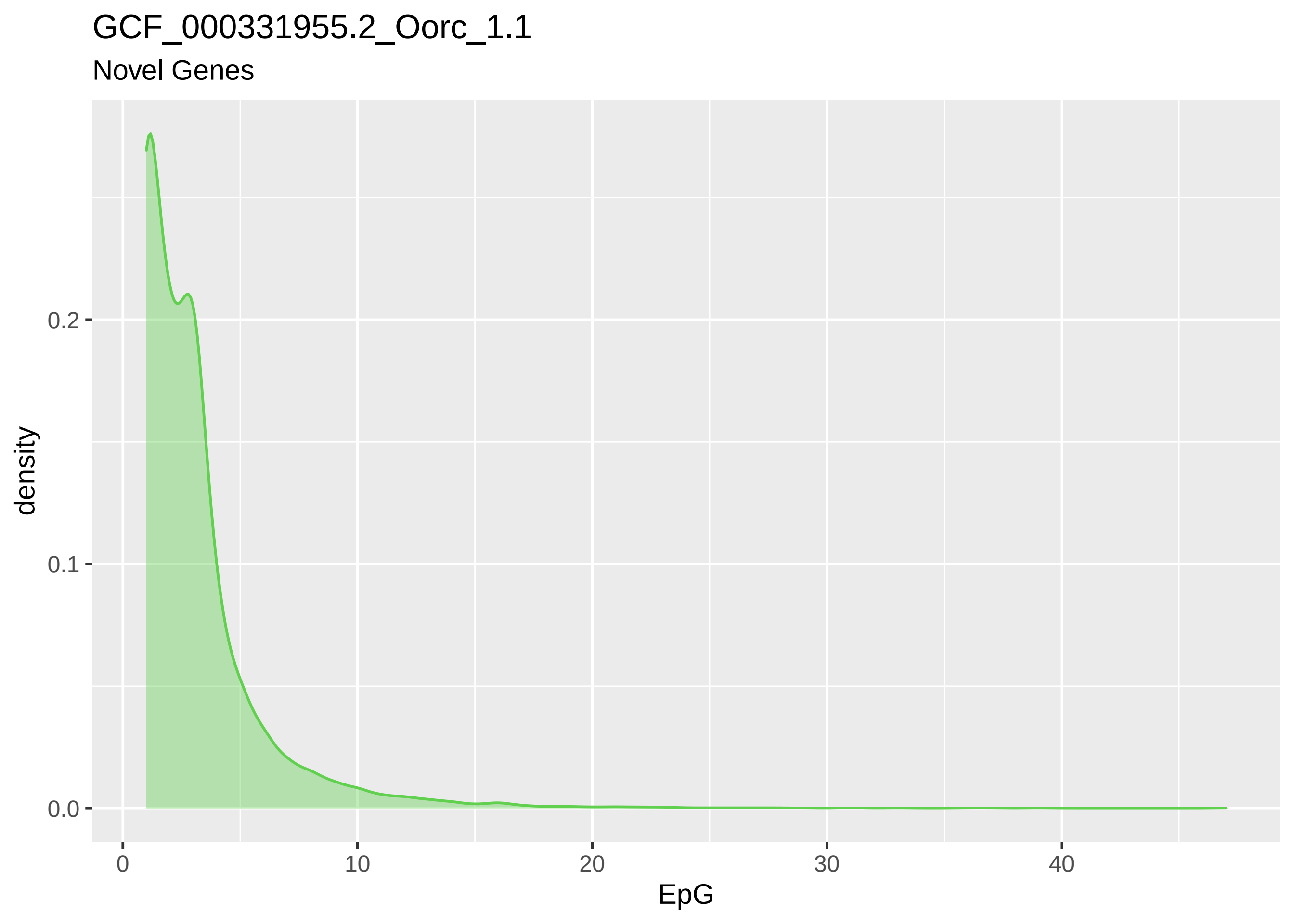
GCF_000331955.2_Oorc_1.1
Novel Genes
density
0.2
0.1
0.0
0
10
20
30
40
EpG

GCF_000334495.1_TupChi_1.0

Novel Genes

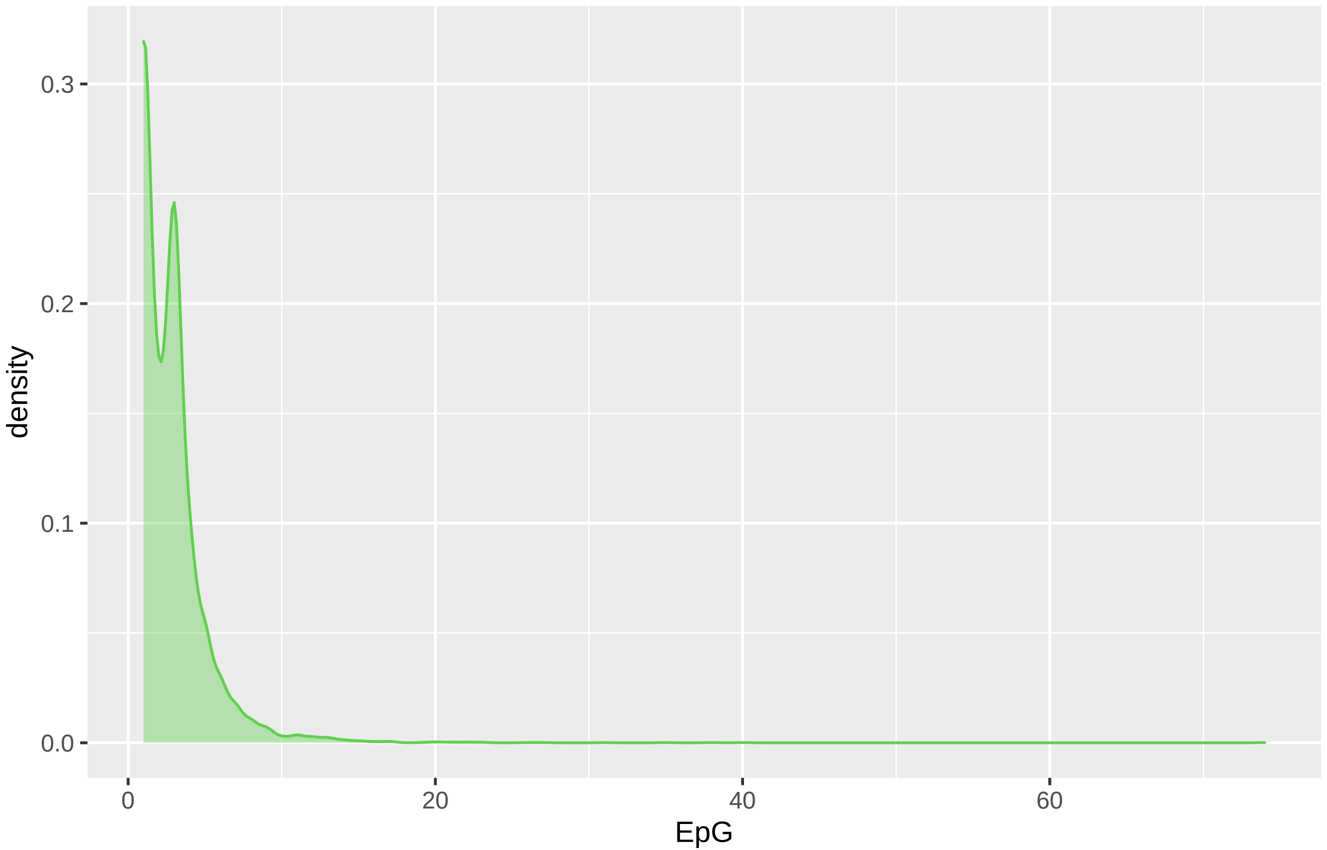

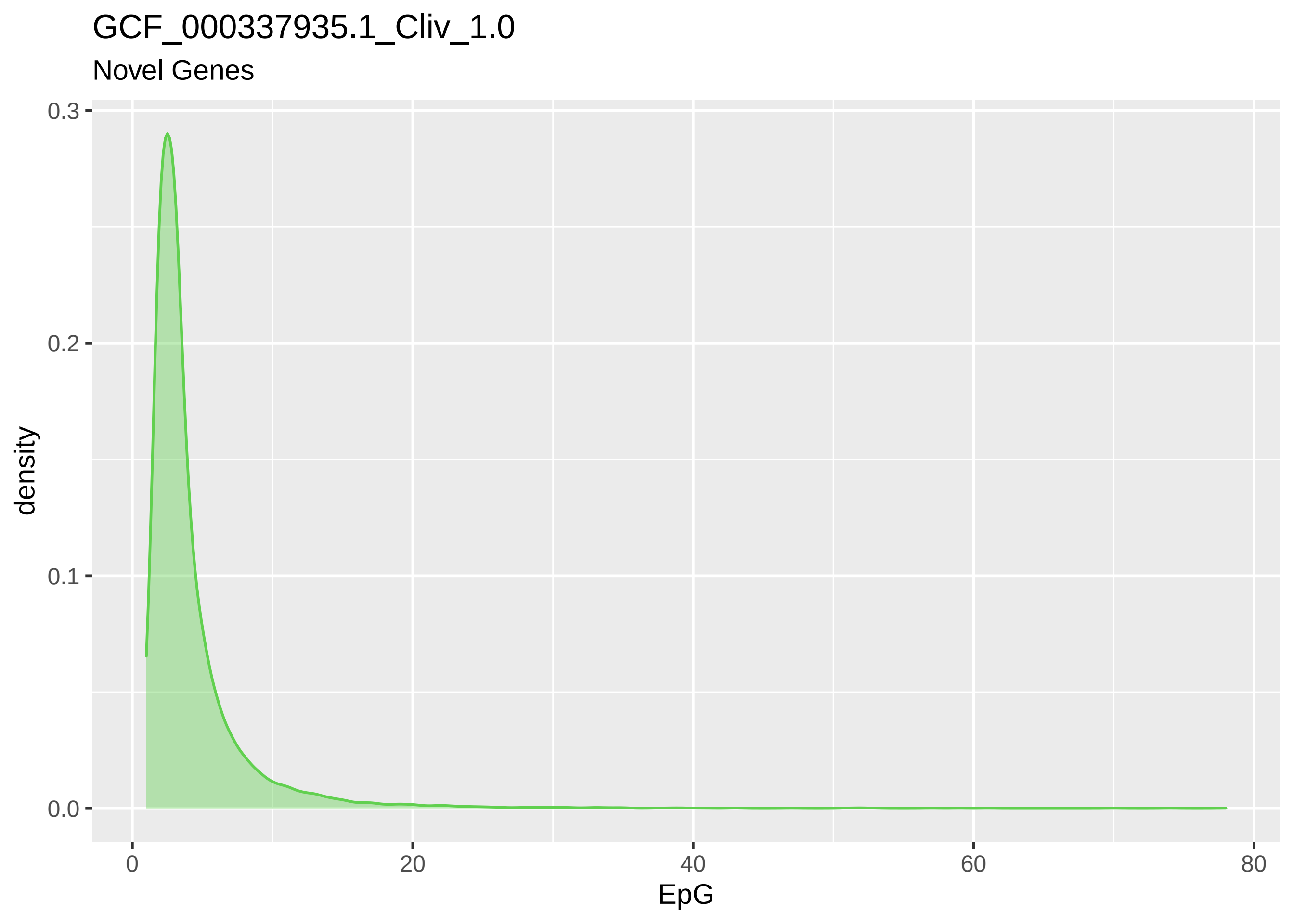

GCF_000337935.1_Cliv_1.0
Novel Genes
density
0.3
0.2
0.1
0.0
0
20
40
60
80
EpG

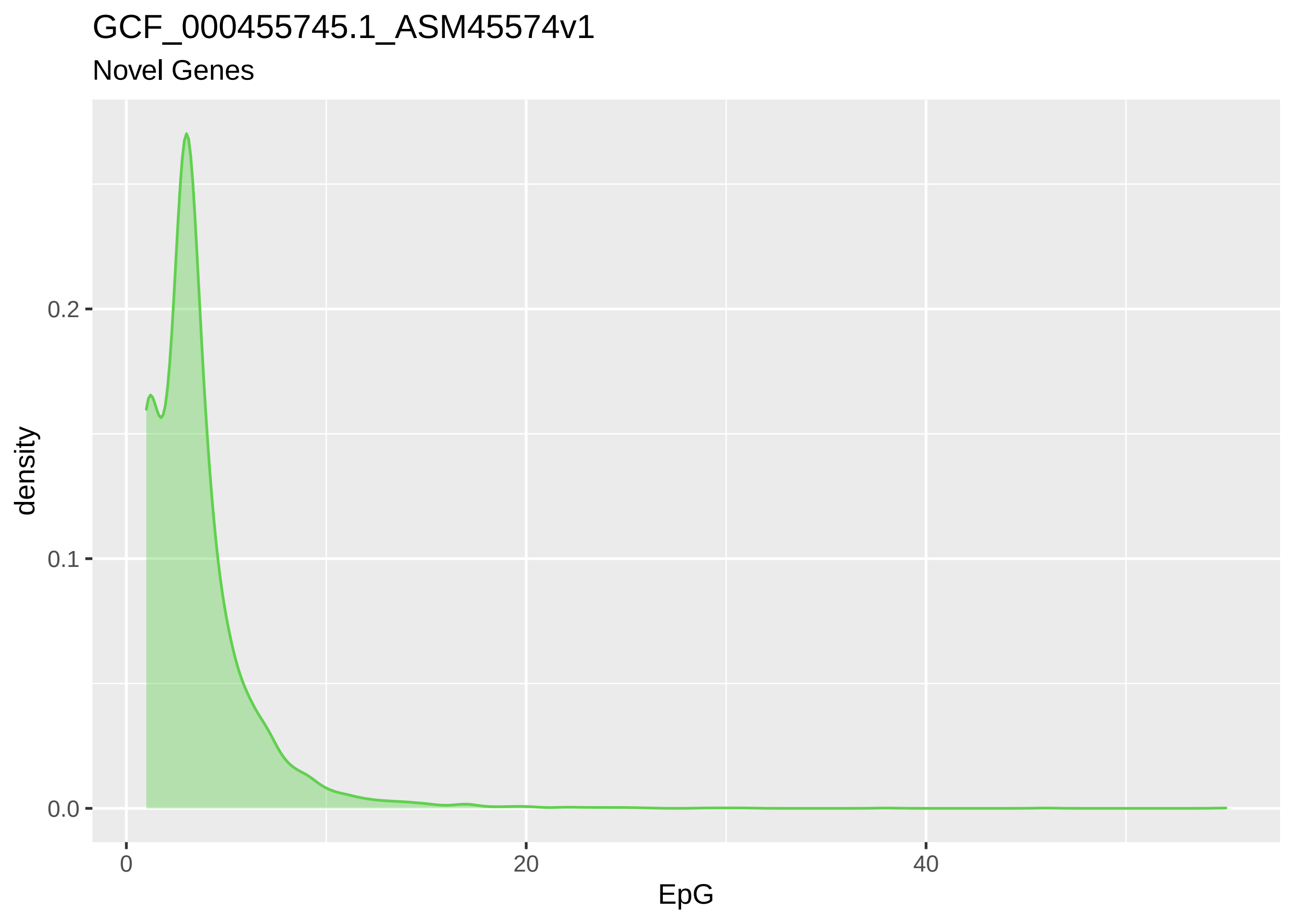

GCF_000455745.1_ASM45574v1
Novel Genes
density
0.2
0.1
0.0
0
20
40
EpG

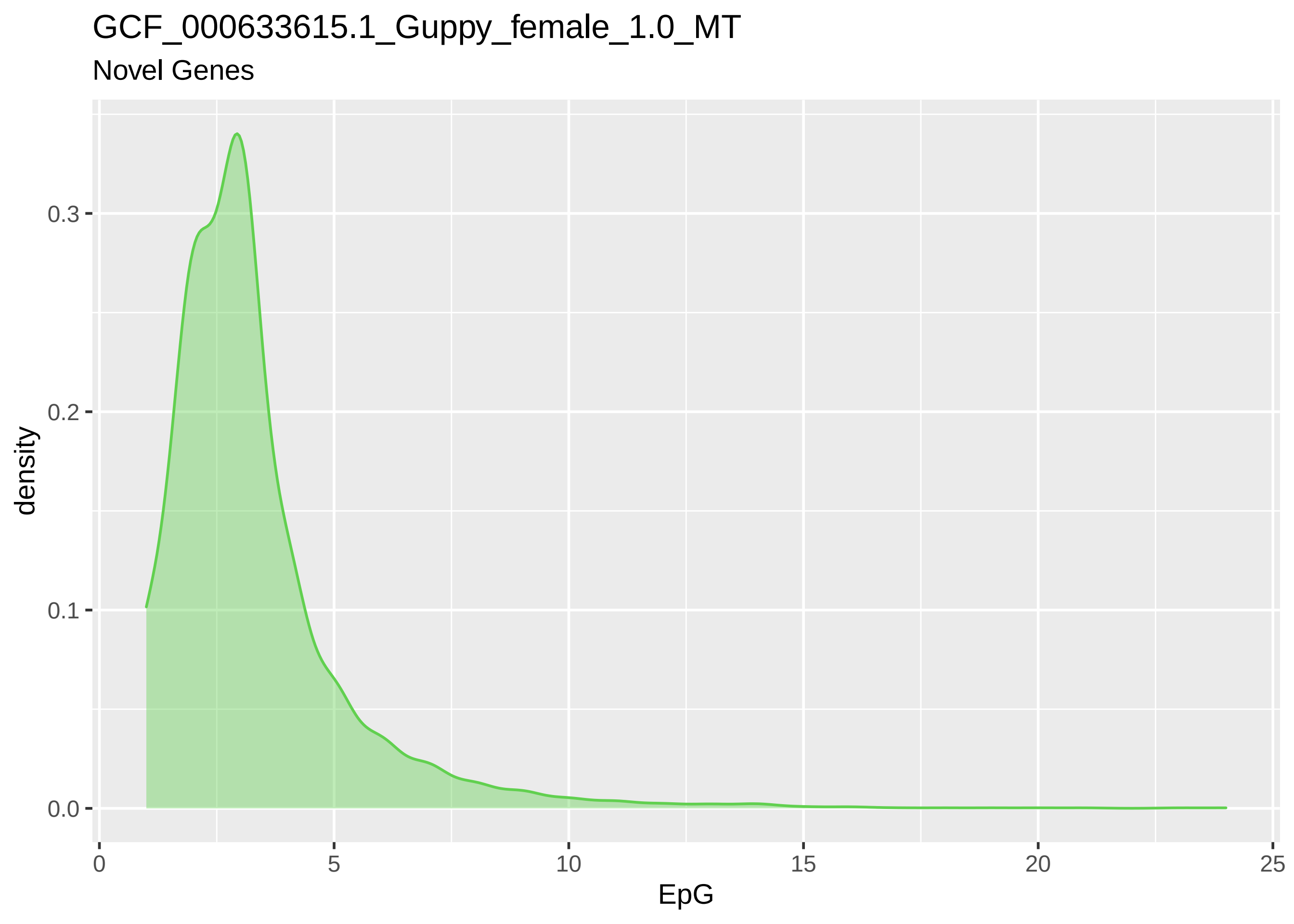
GCF_000633615.1_Guppy_female_1.0_MT
Novel Genes
density
0.0
0.1
0.2
0.3
0
5
10
15
20
25
EpG

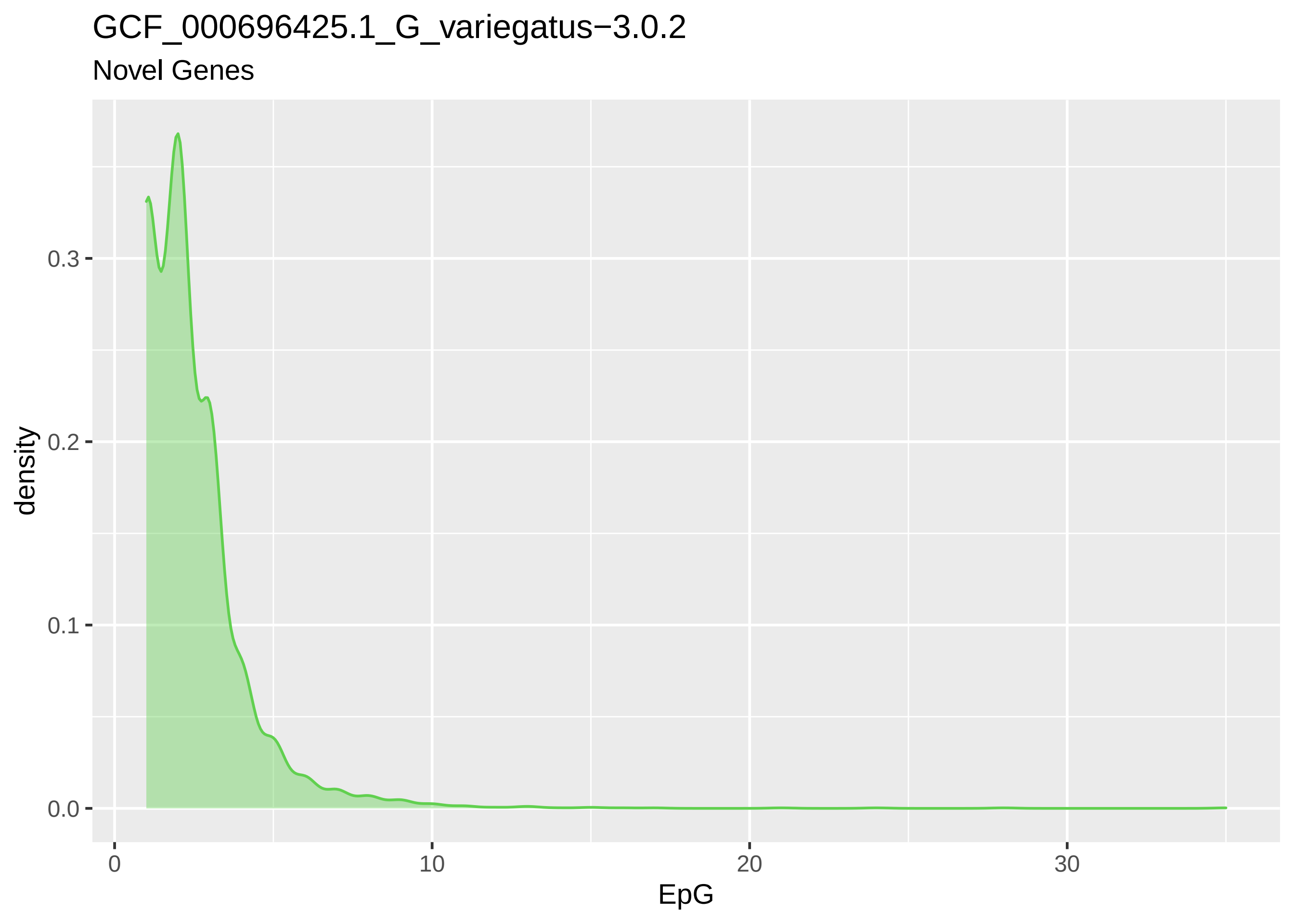

GCF_000696425.1_G_variegatus−3.0.2
Novel Genes
density
0.0
0.1
0.2
0.3
0
10
20
30
EpG

GCF_000705375.1_ASM70537v2

Novel Genes

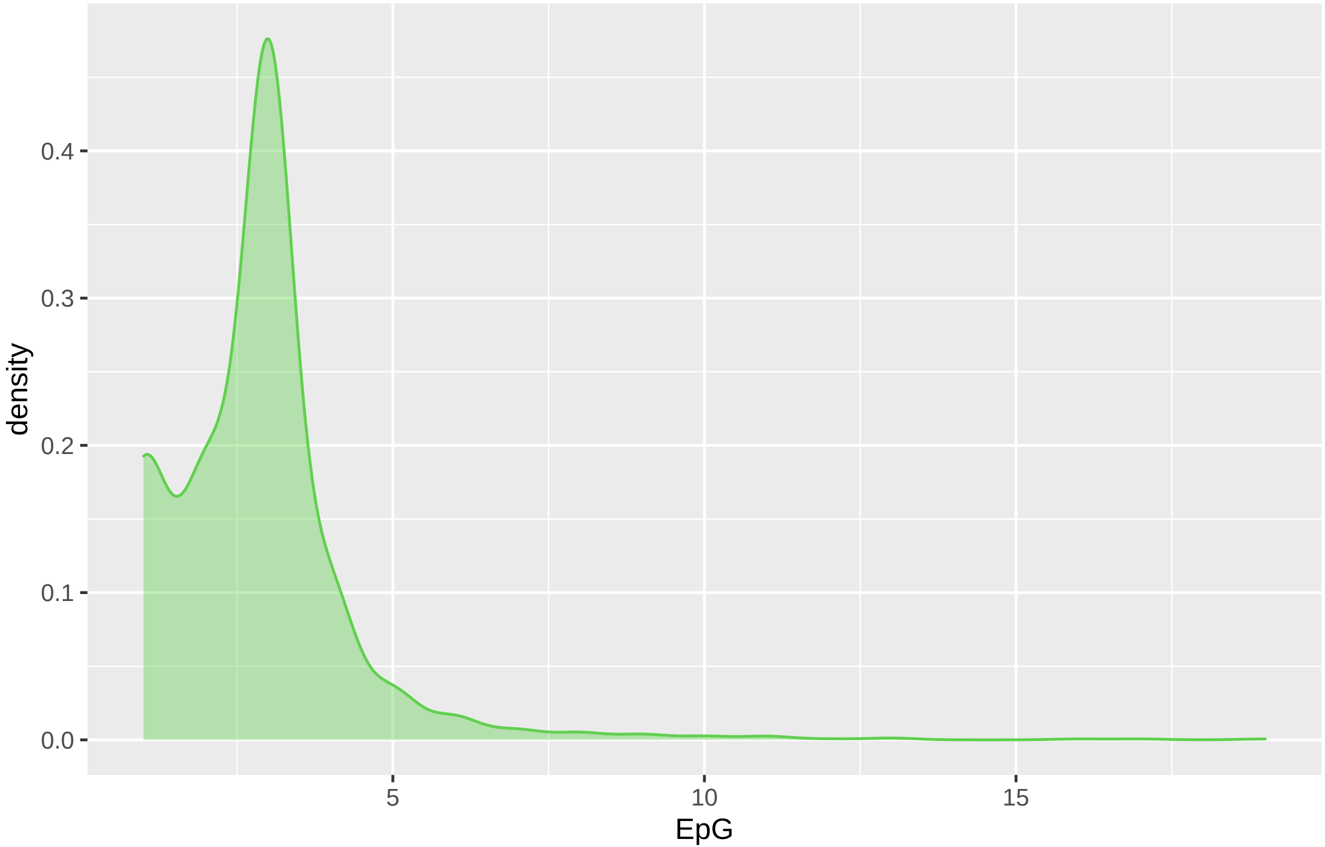

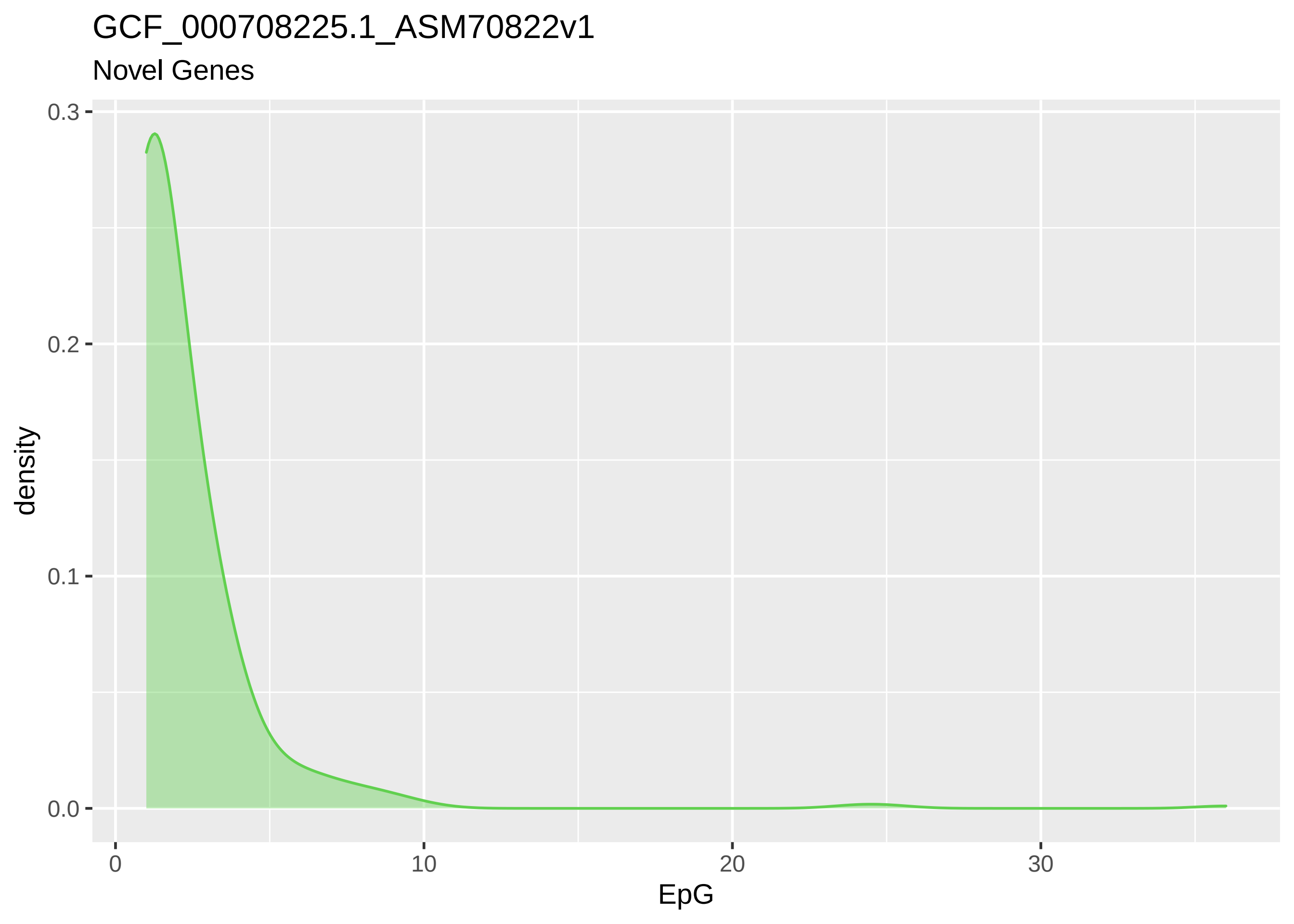
GCF_000708225.1_ASM70822v1
Novel Genes
0.3
0.2
0.1
0.0
density
0
10
20
30
EpG

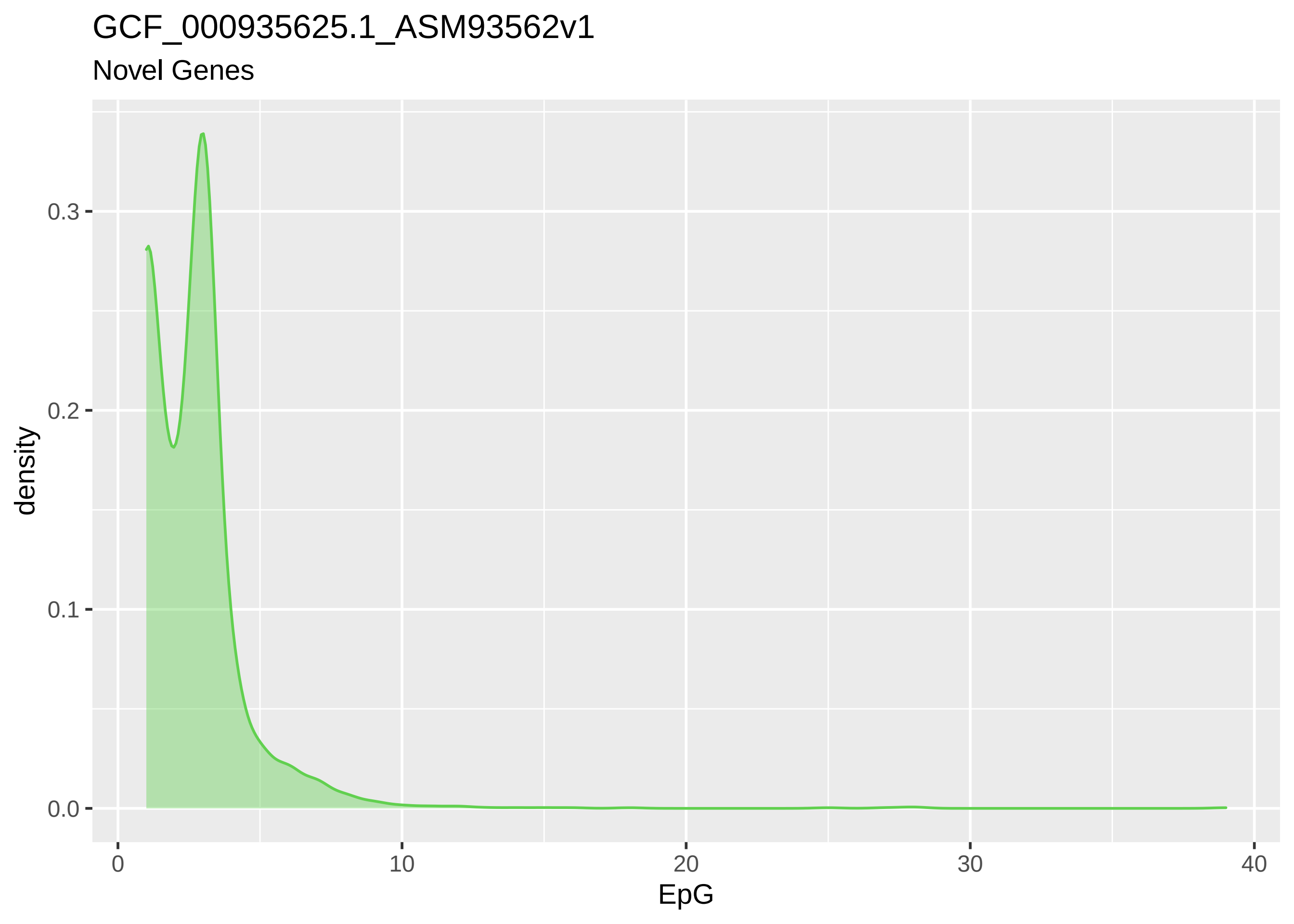
GCF_000935625.1_ASM93562v1
Novel Genes
density
0.3
0.2
0.1
0.0
0
10
20
30
40
EpG

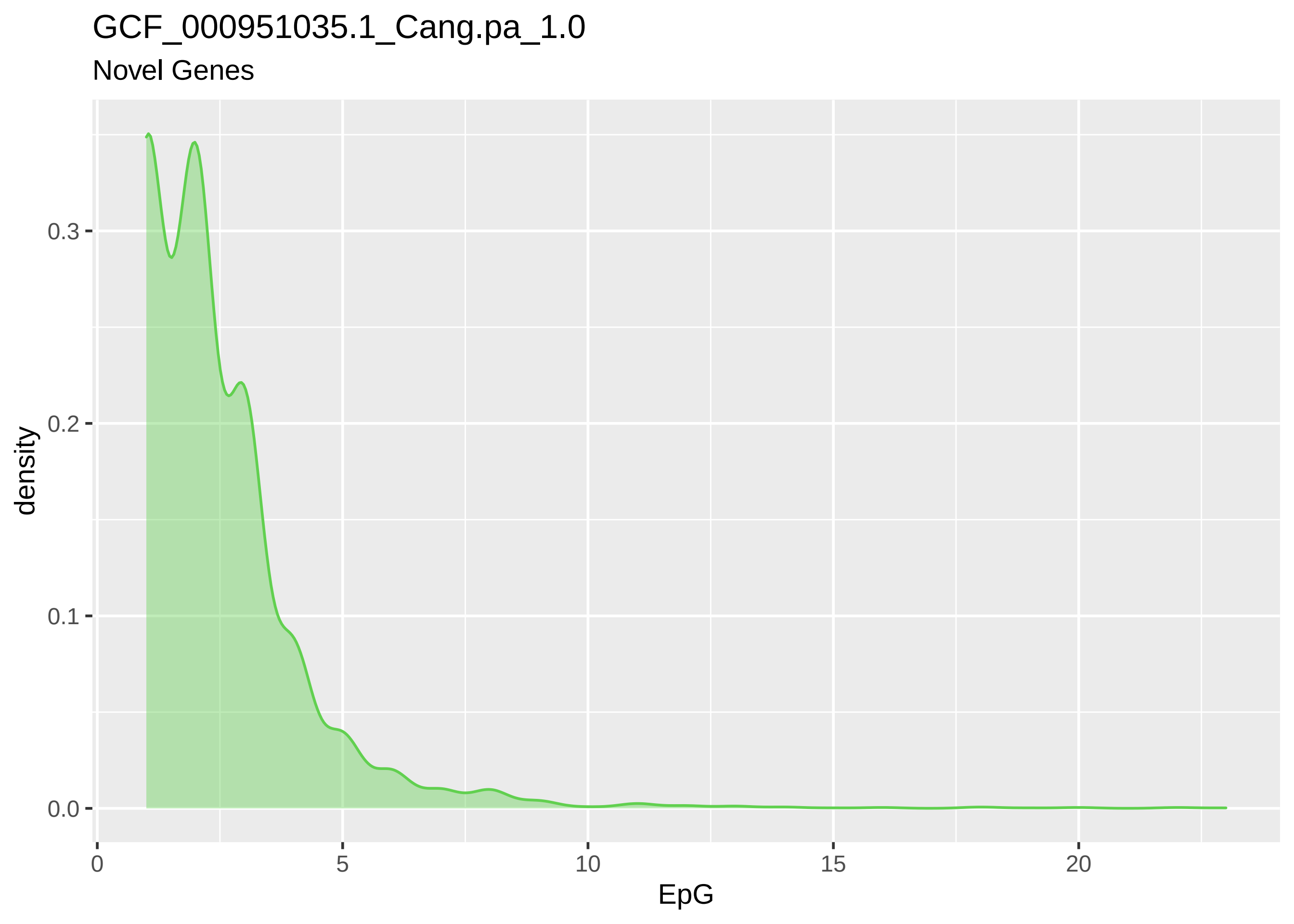
GCF_000951035.1_Cang.pa_1.0
Novel Genes
density
0.0
0.1
0.2
0.3
0
5
10
15
20
EpG

GCF_000951045.1_Mleu.le_1.0

Novel Genes

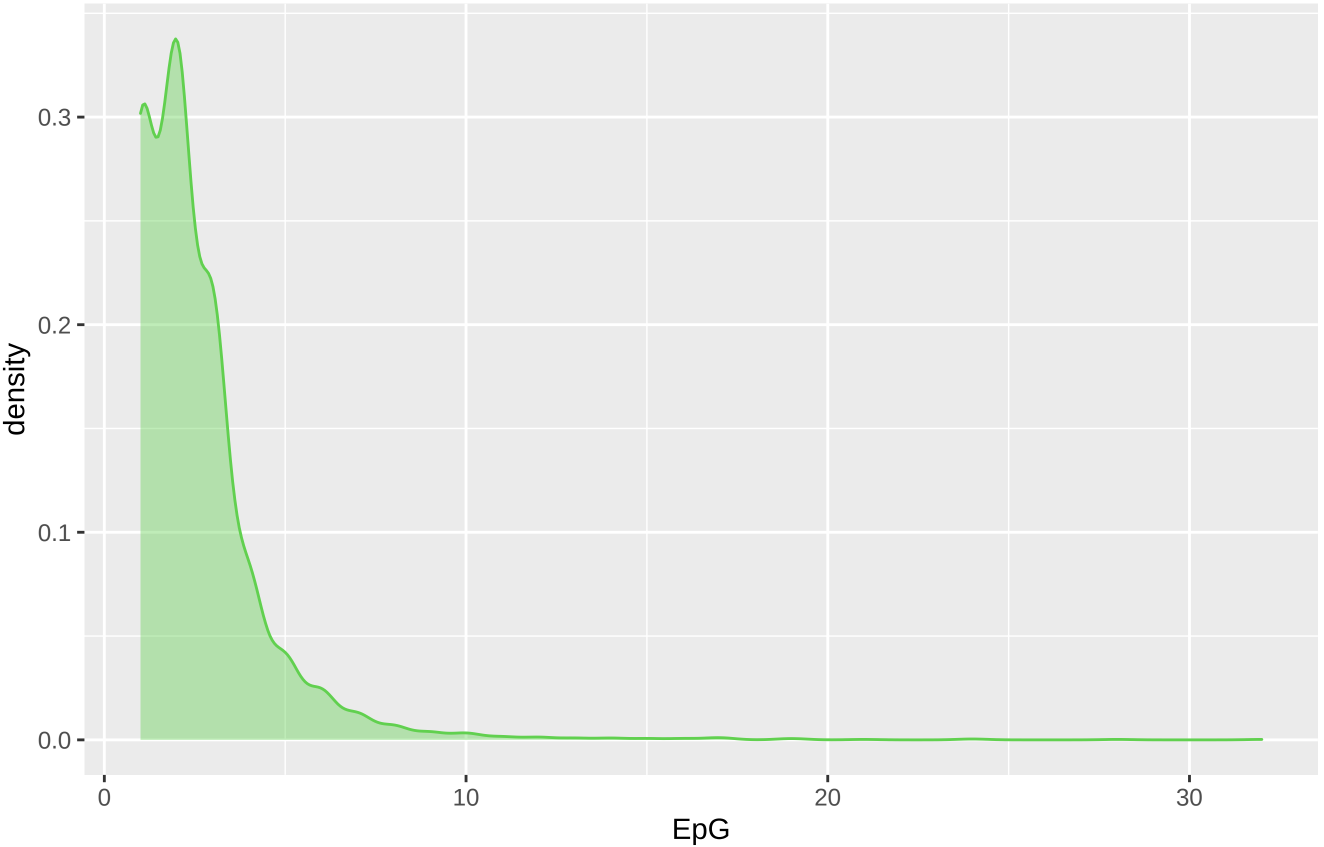

GCF_000956105.1_Pcoq_1.0

Novel Genes

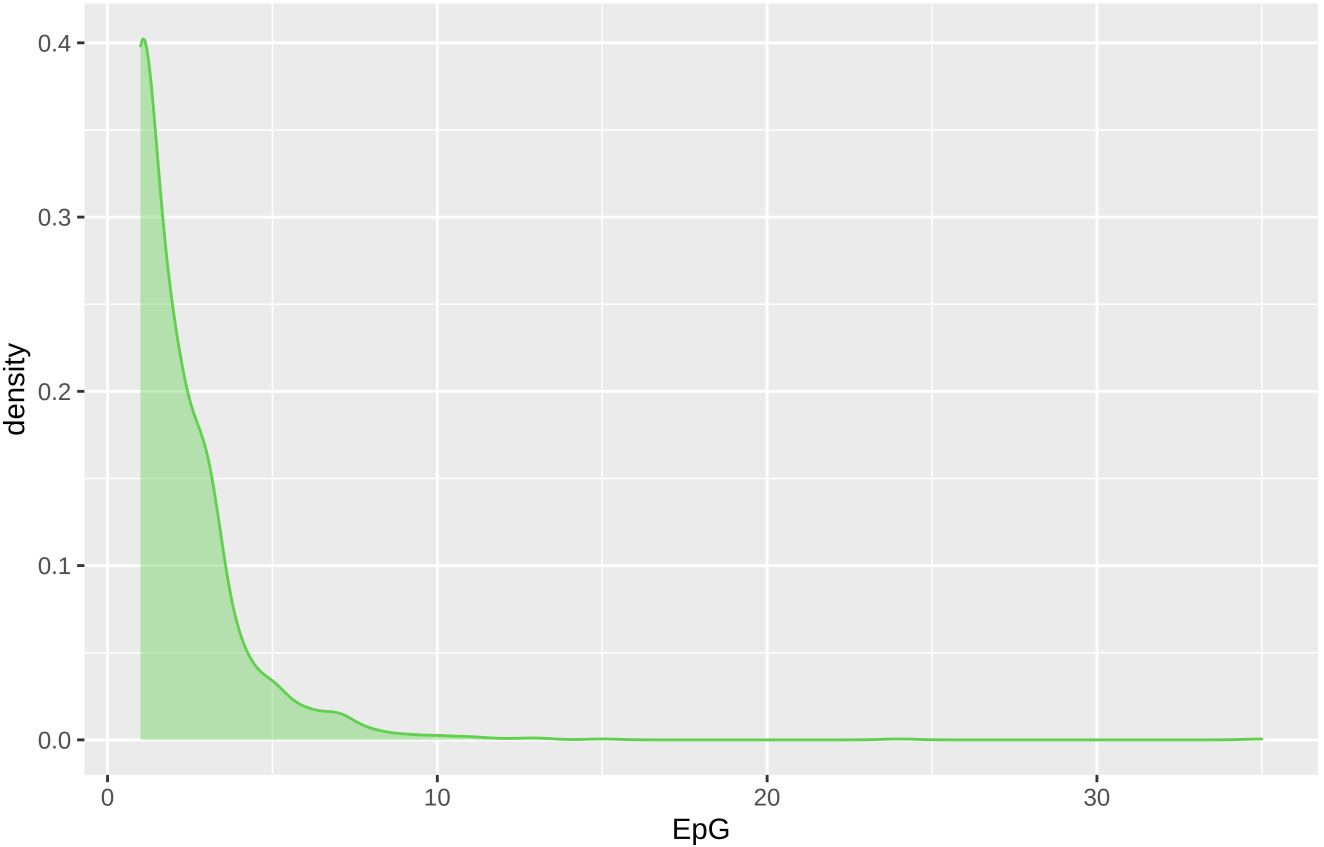

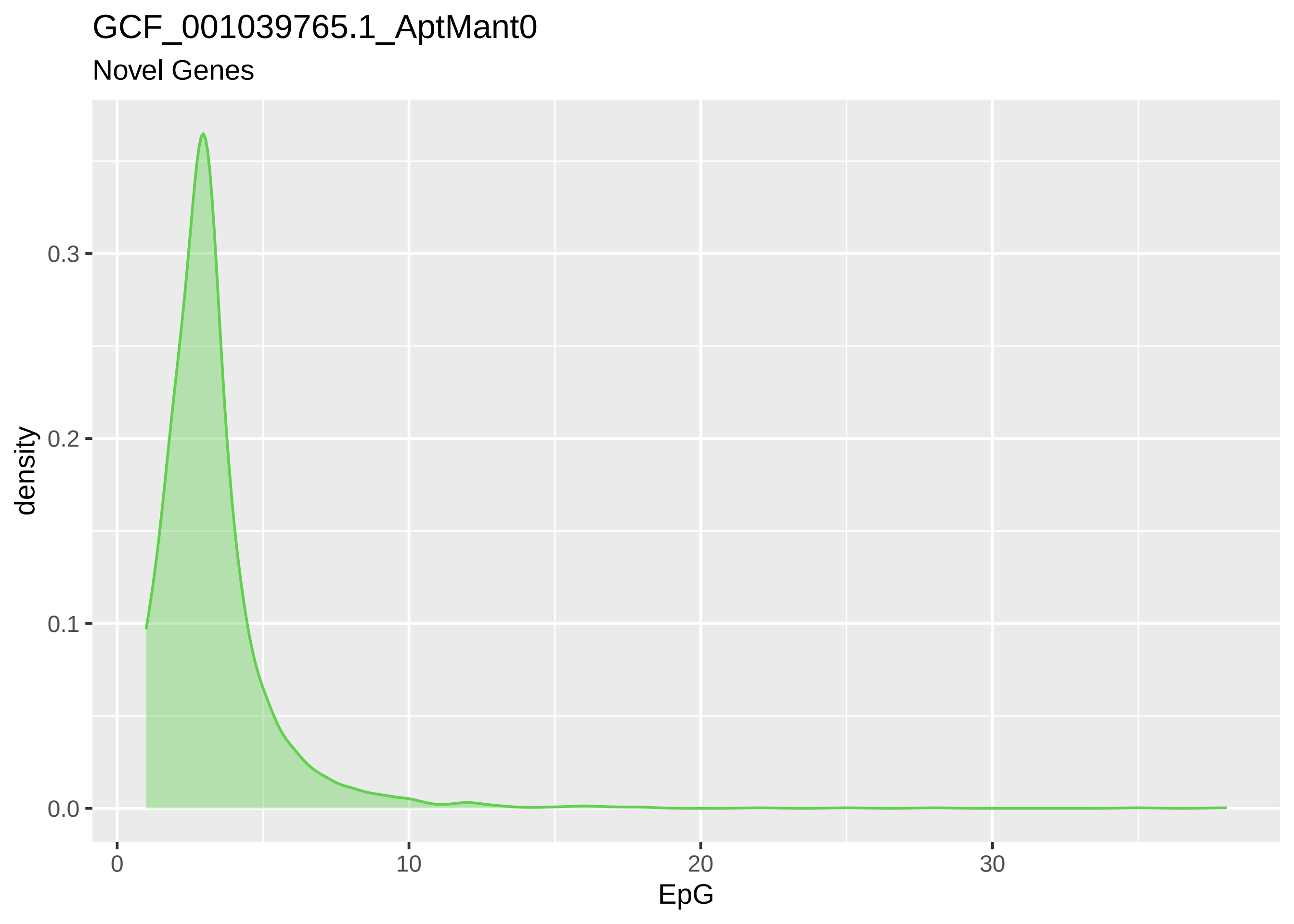
GCF_001039765.1_AptMant0
Novel Genes
density
0.0
0.1
0.2
0.3
0
10
20
30
EpG

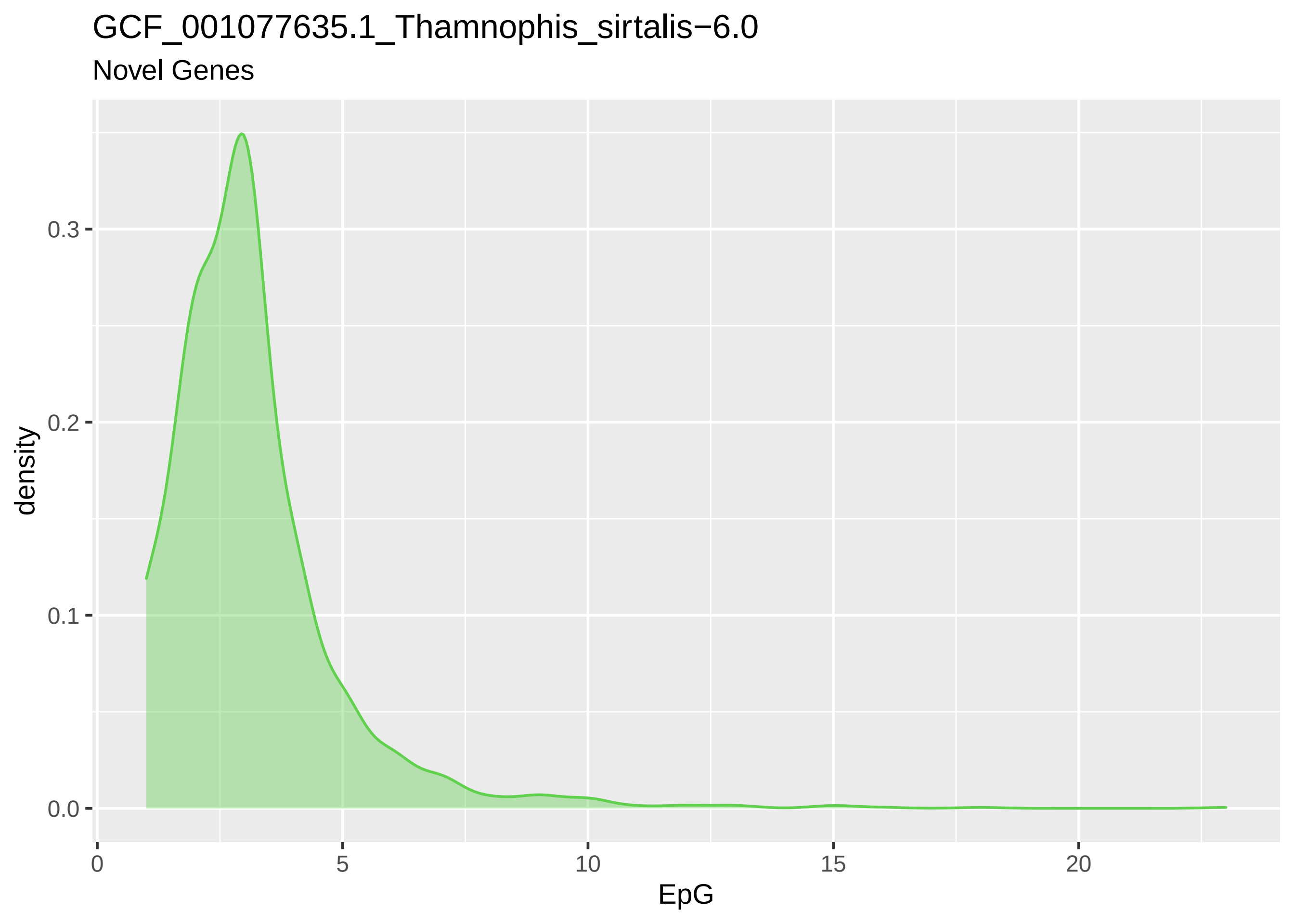
GCF_001077635.1_Thamnophis_sirtalis−6.0
Novel Genes
density
0.0
0.1
0.2
0.3
0
5
10
15
20
EpG

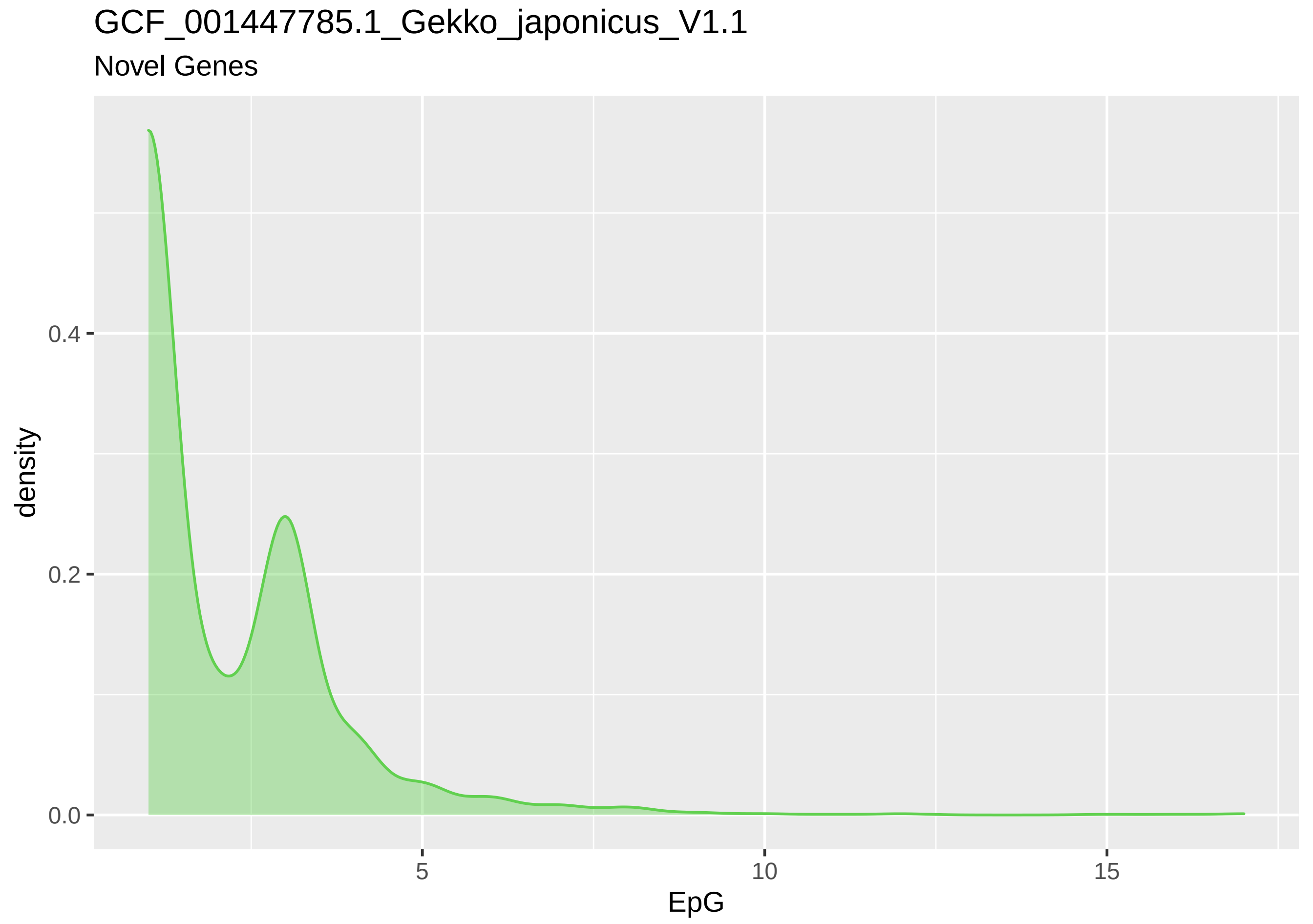
GCF_001447785.1_Gekko_japonicus_V1.1
Novel Genes
density
0.4
0.2
0.0
5
10
15
EpG

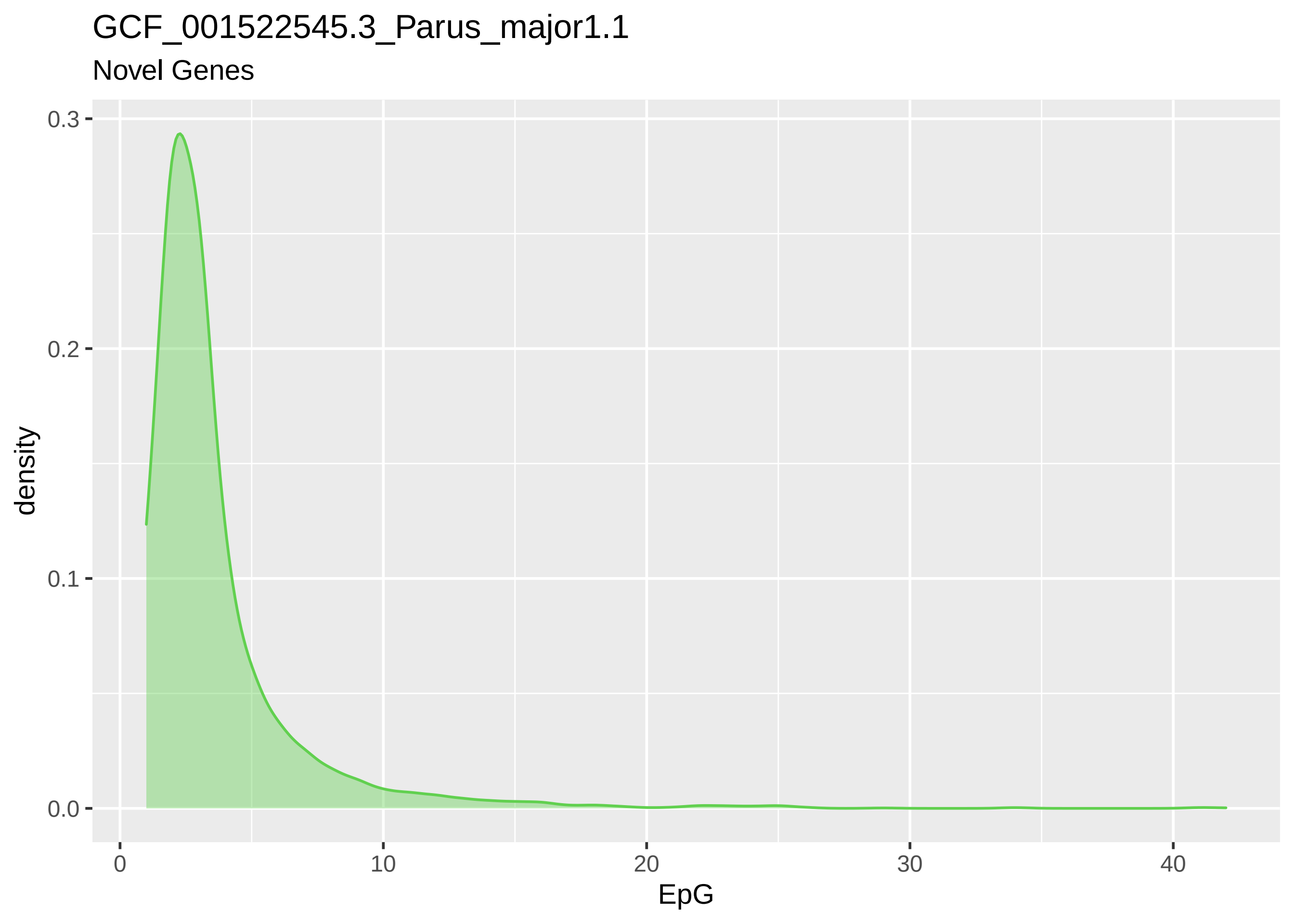
GCF_001522545.3_Parus_major1.1
Novel Genes
density
EpG
0.0
0.1
0.2
0.3
0
10
20
30
40

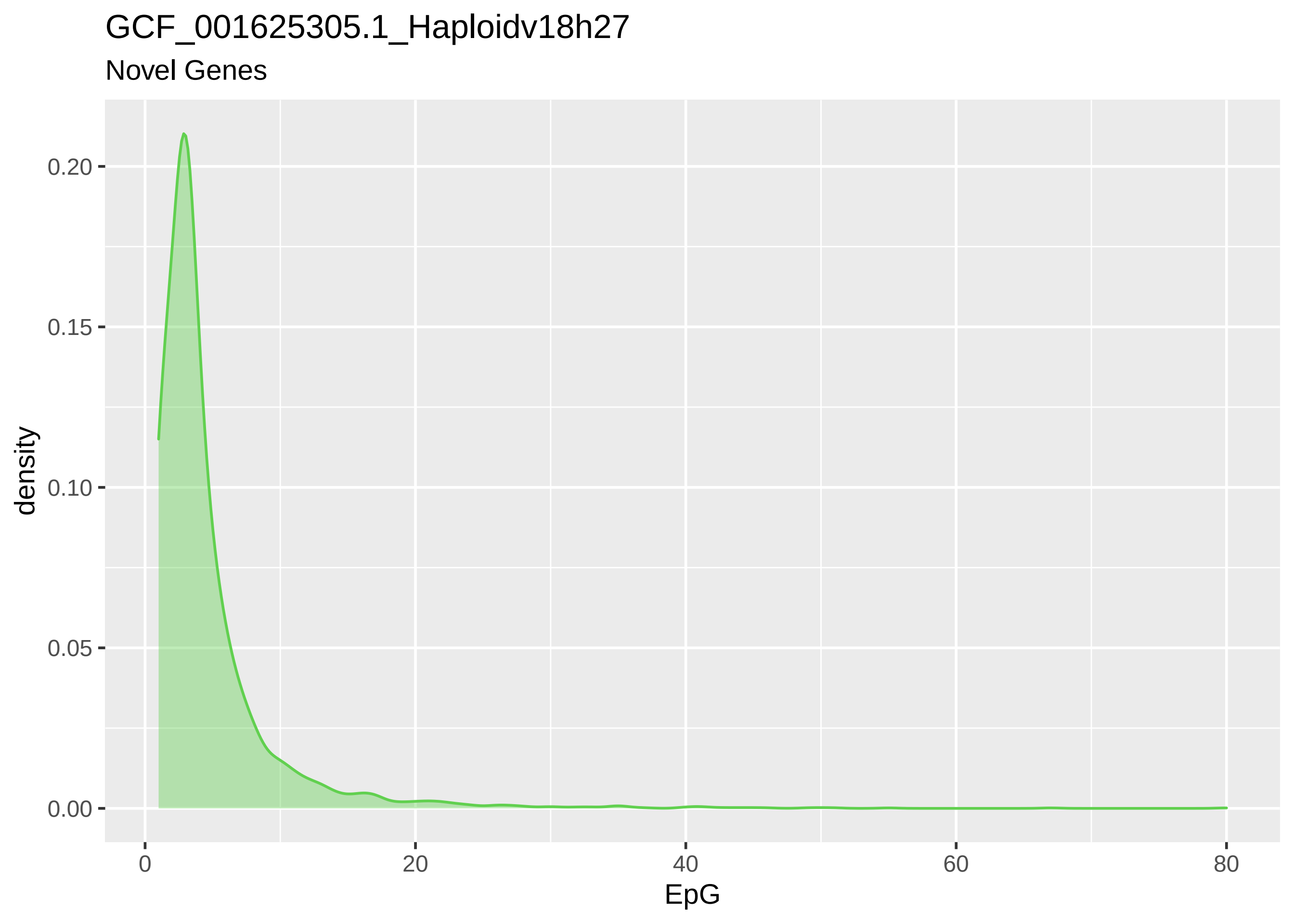
GCF_001625305.1_Haploidv18h27
Novel Genes
density
0.20
0.15
0.10
0.05
0.00
0
20
40
60
80
EpG

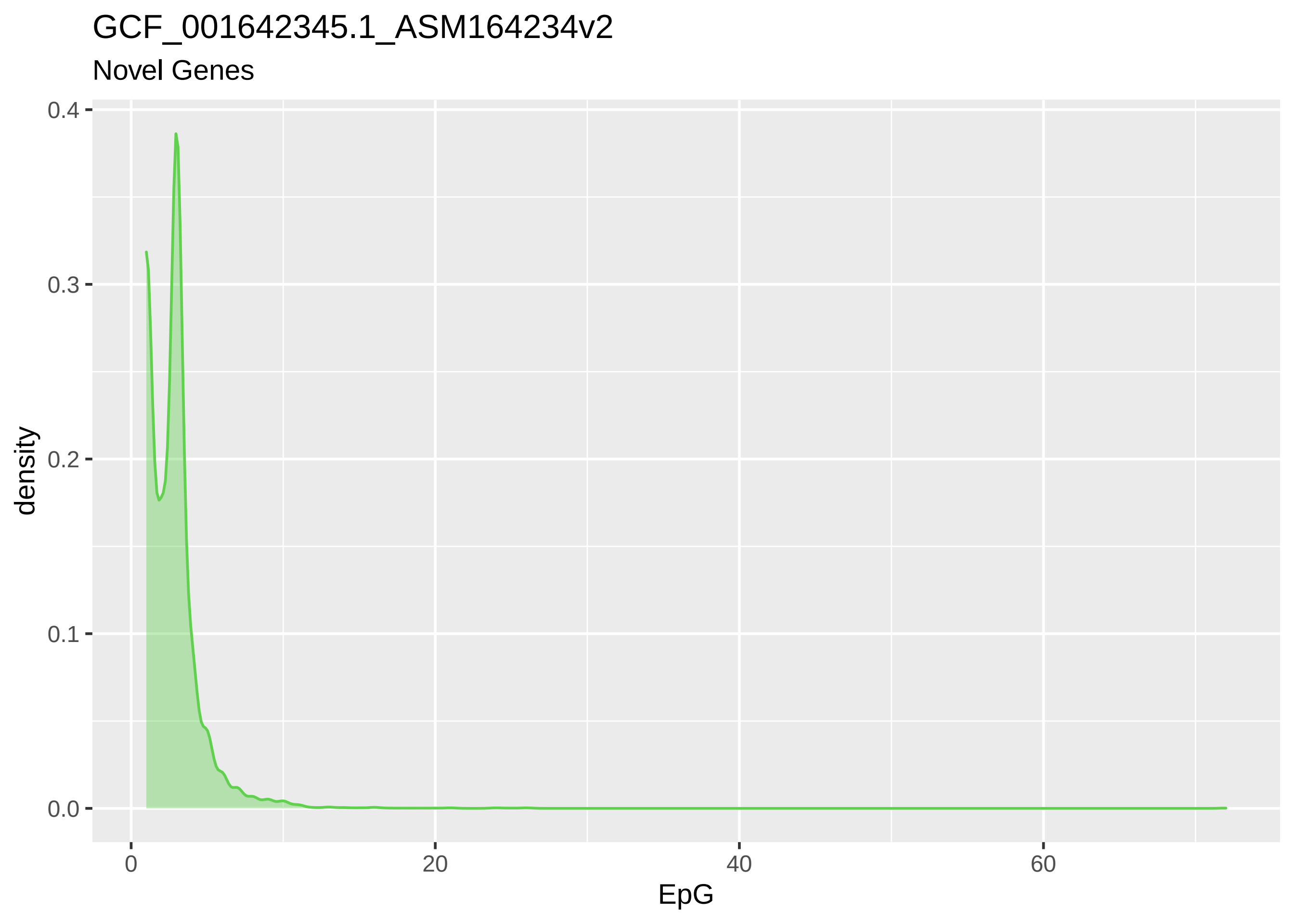
GCF_001642345.1_ASM164234v2
Novel Genes
density
EpG
0.0
0.1
0.2
0.3
0.4
0
20
40
60

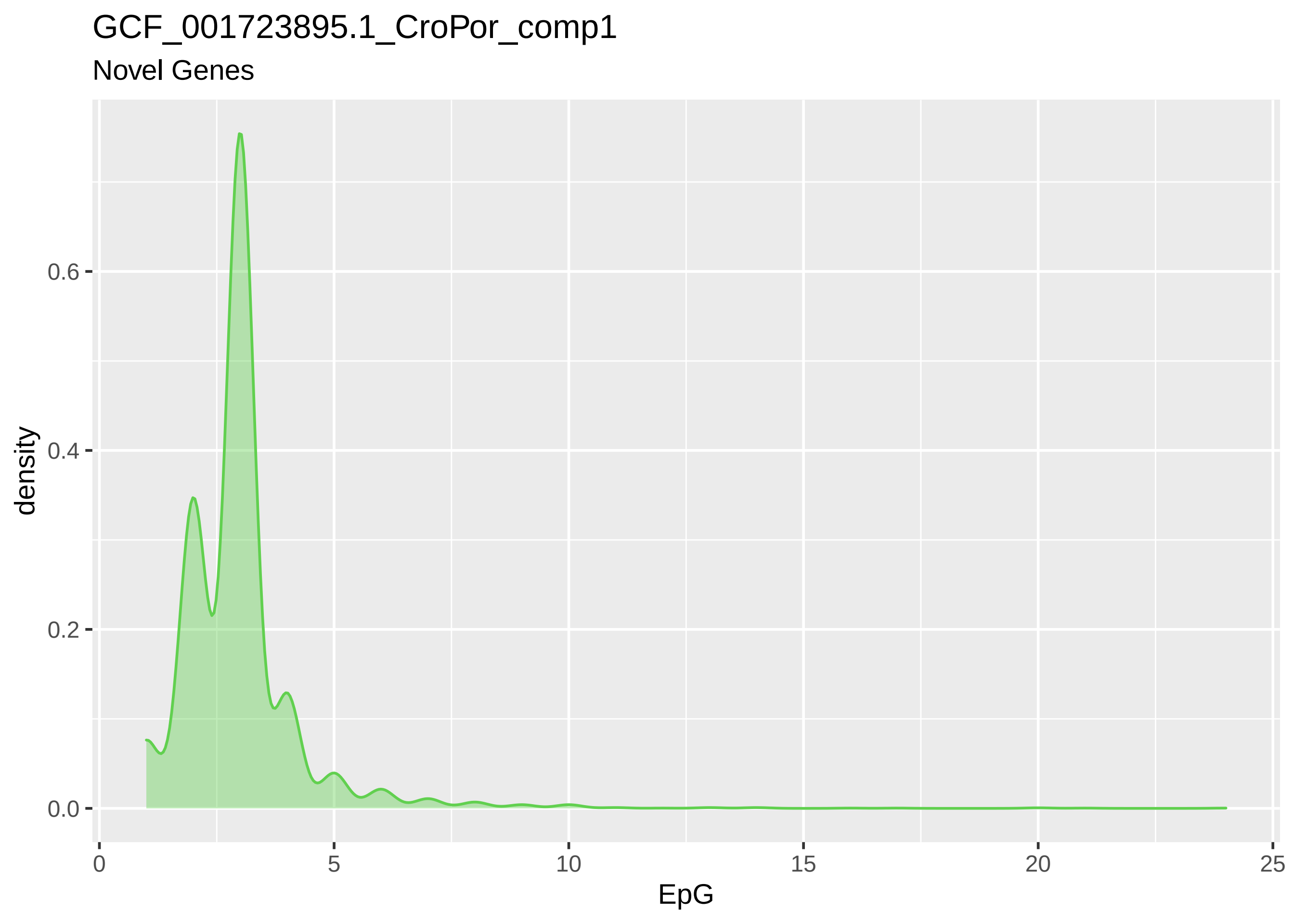
GCF_001723895.1_CroPor_comp1
Novel Genes
density
0.0
0.2
0.4
0.6
0
5
10
15
20
25
EpG

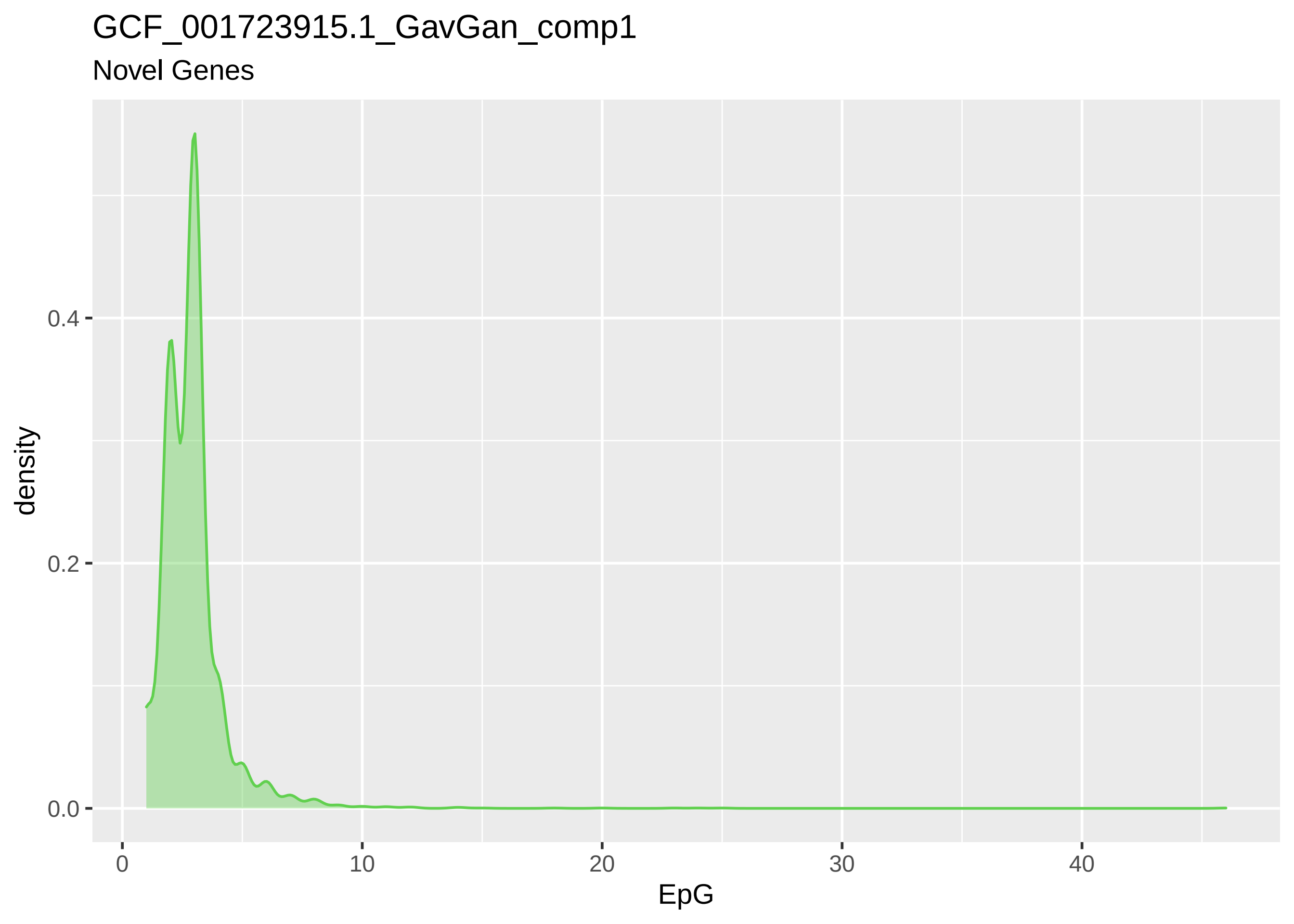
GCF_001723915.1_GavGan_comp1
Novel Genes
density
0.4
0.2
0.0
0
10
20
30
40
EpG

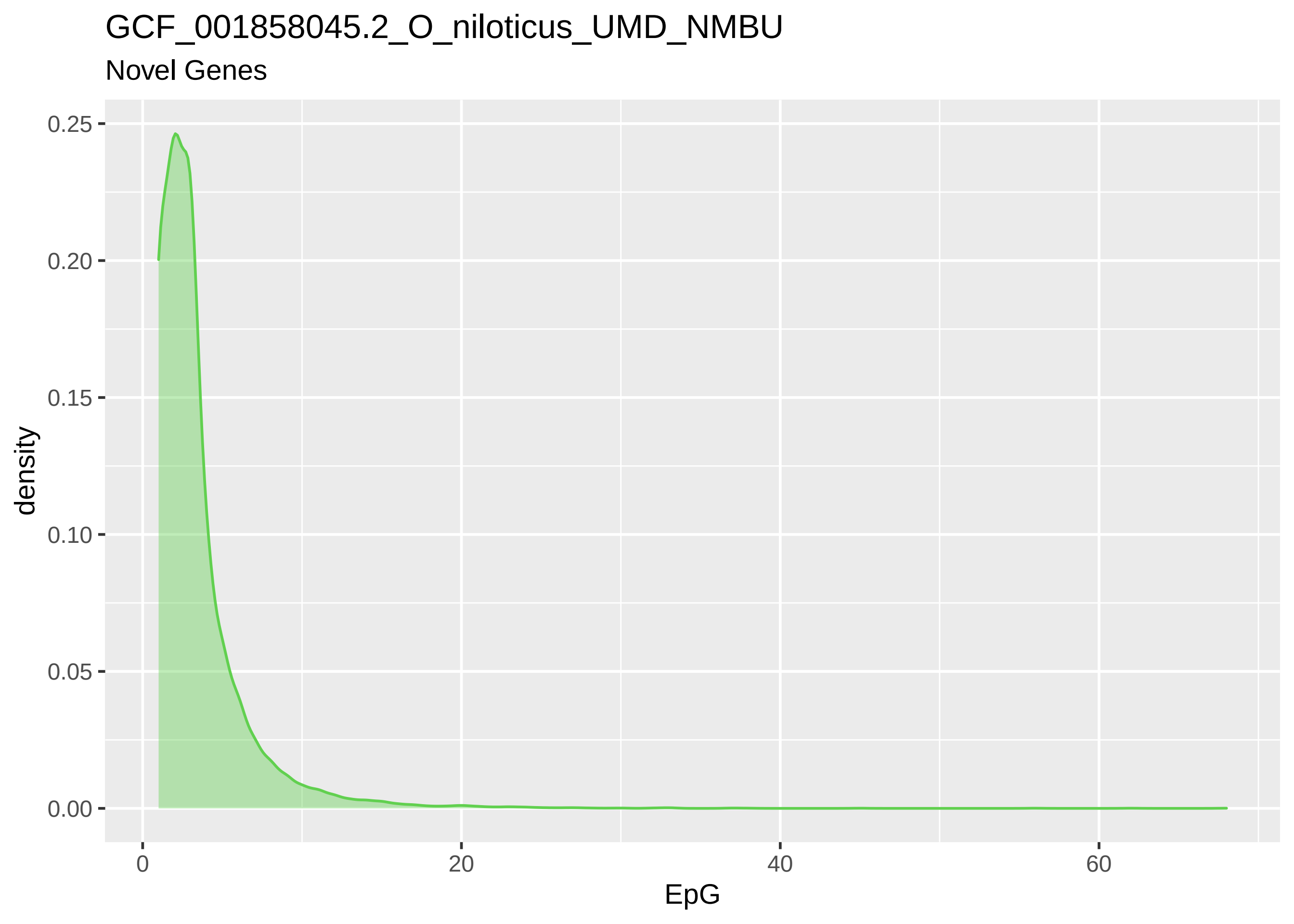

GCF_001858045.2_O_niloticus_UMD_NMBU
Novel Genes
density
0.25
0.20
0.15
0.10
0.05
0.00
0
20
40
60
EpG

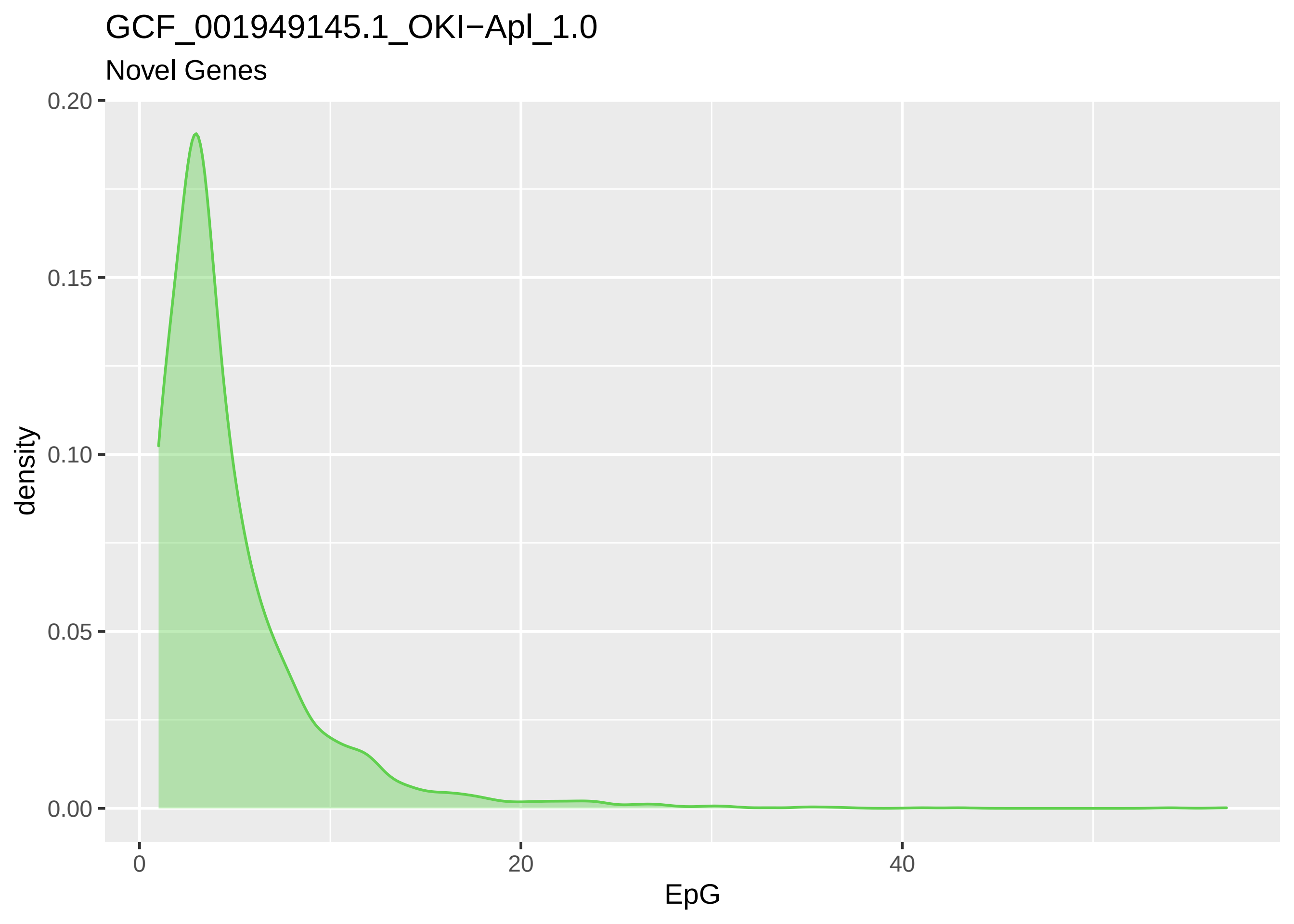
GCF_001949145.1_OKI−Apl_1.0
Novel Genes
density
0.20
0.15
0.10
0.05
0.00
0
20
40
EpG

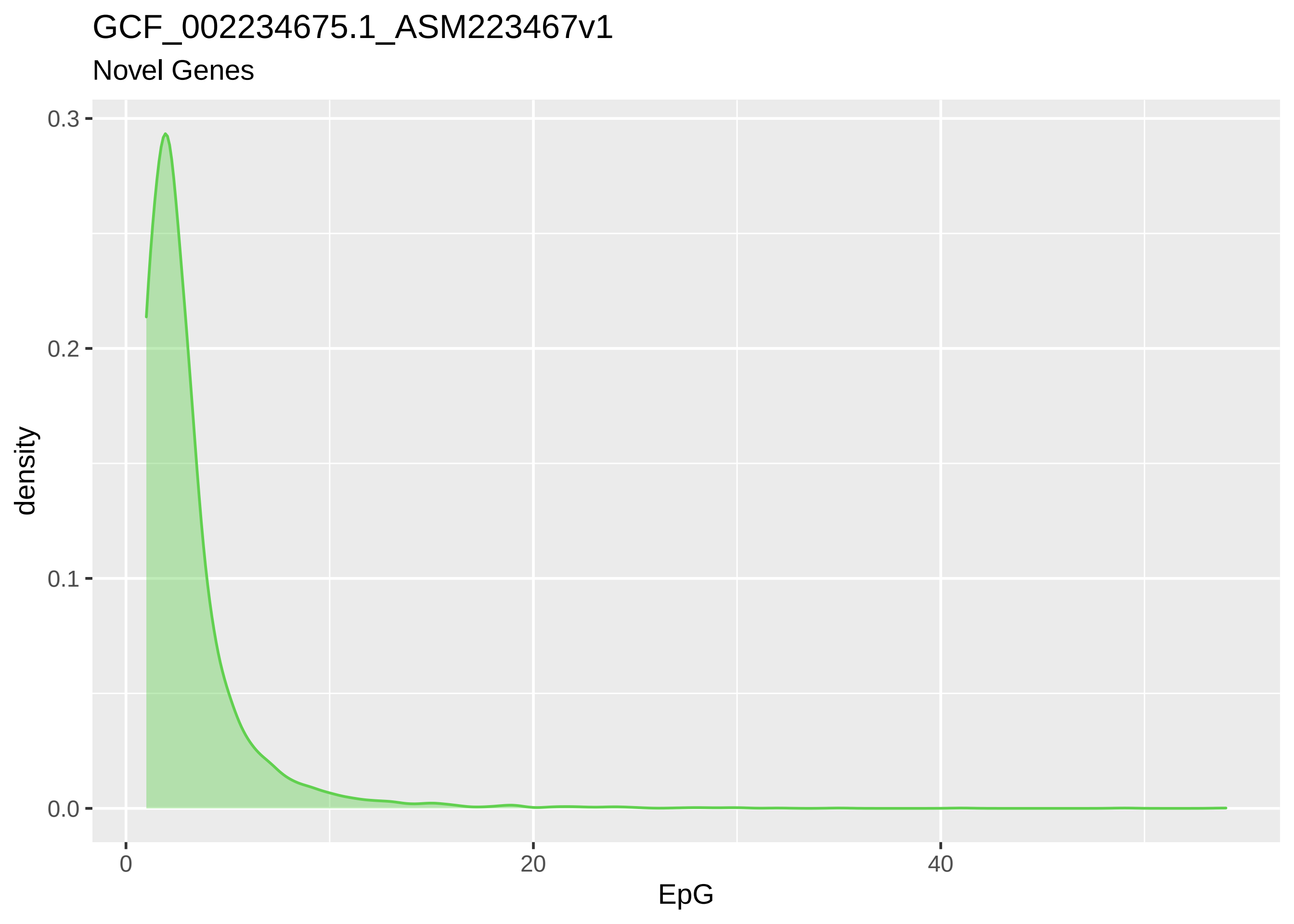
GCF_002234675.1_ASM223467v1
Novel Genes
density
0.3
0.2
0.1
0.0
0
20
40
EpG

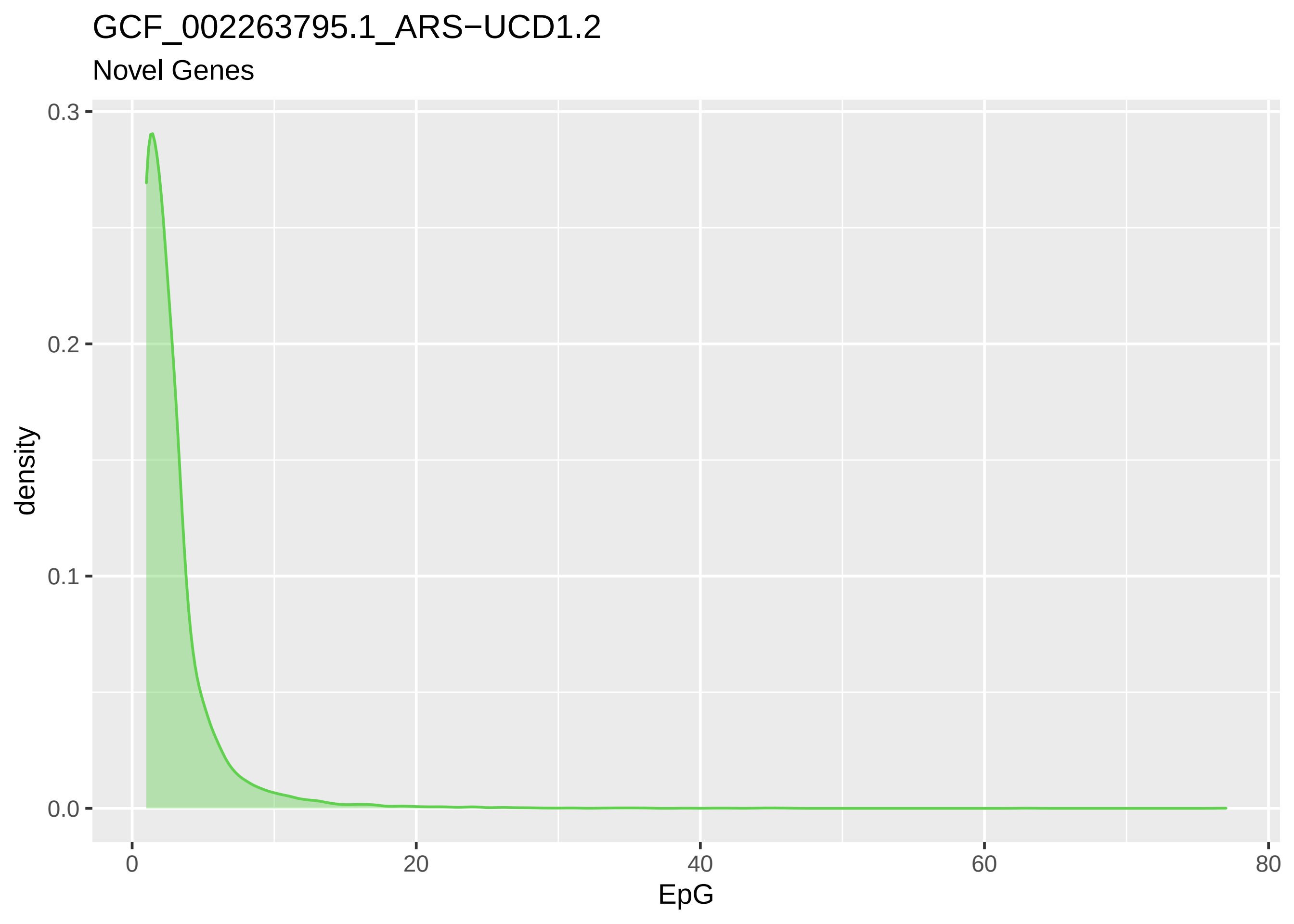

GCF_002263795.1_ARS−UCD1.2
Novel Genes
0.3
0.2
0.1
0.0
density
0
20
40
60
80
EpG

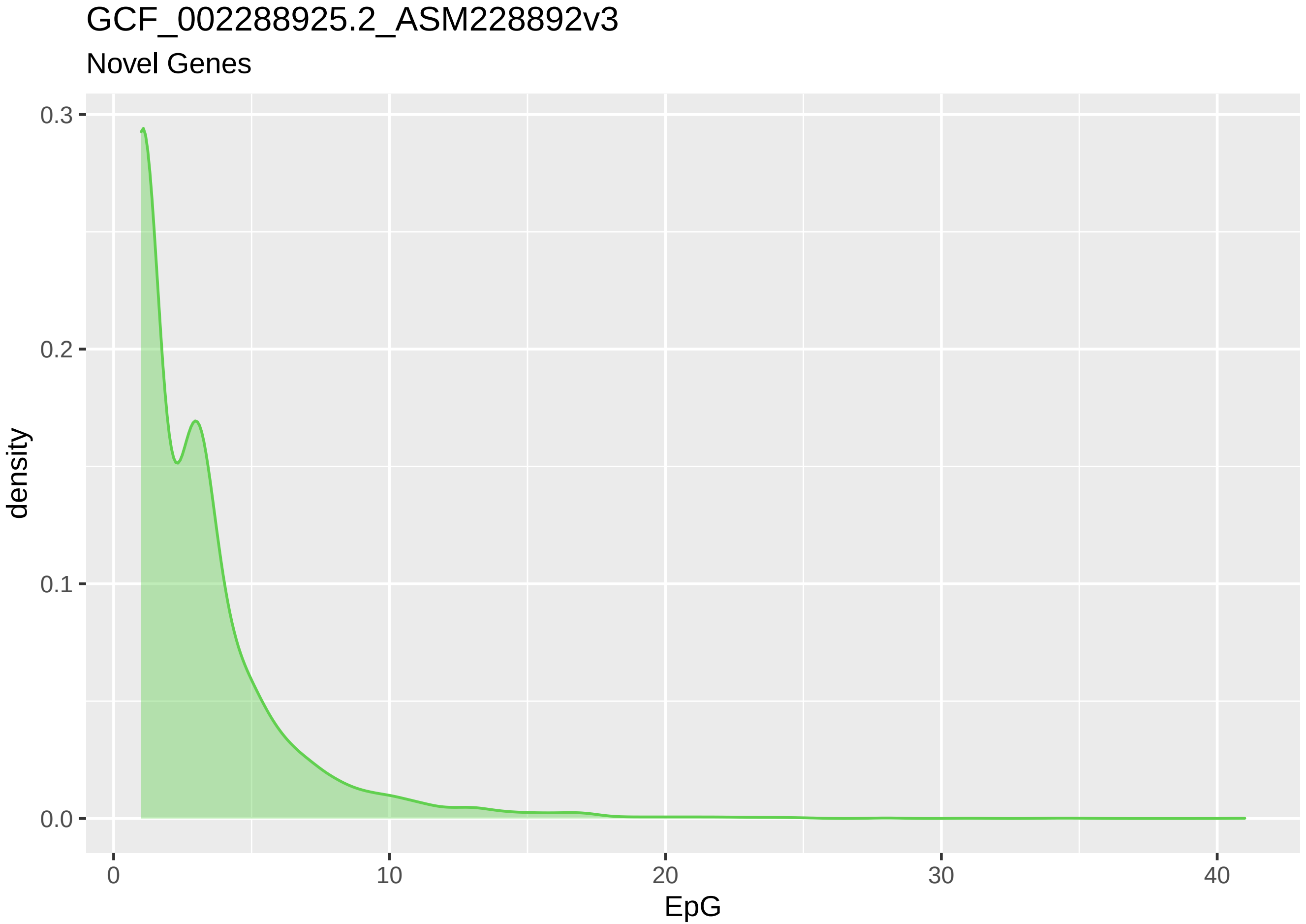

GCF_002288925.2_ASM228892v3
Novel Genes
density
0.3
0.2
0.1
0.0
0
10
20
30
40
EpG

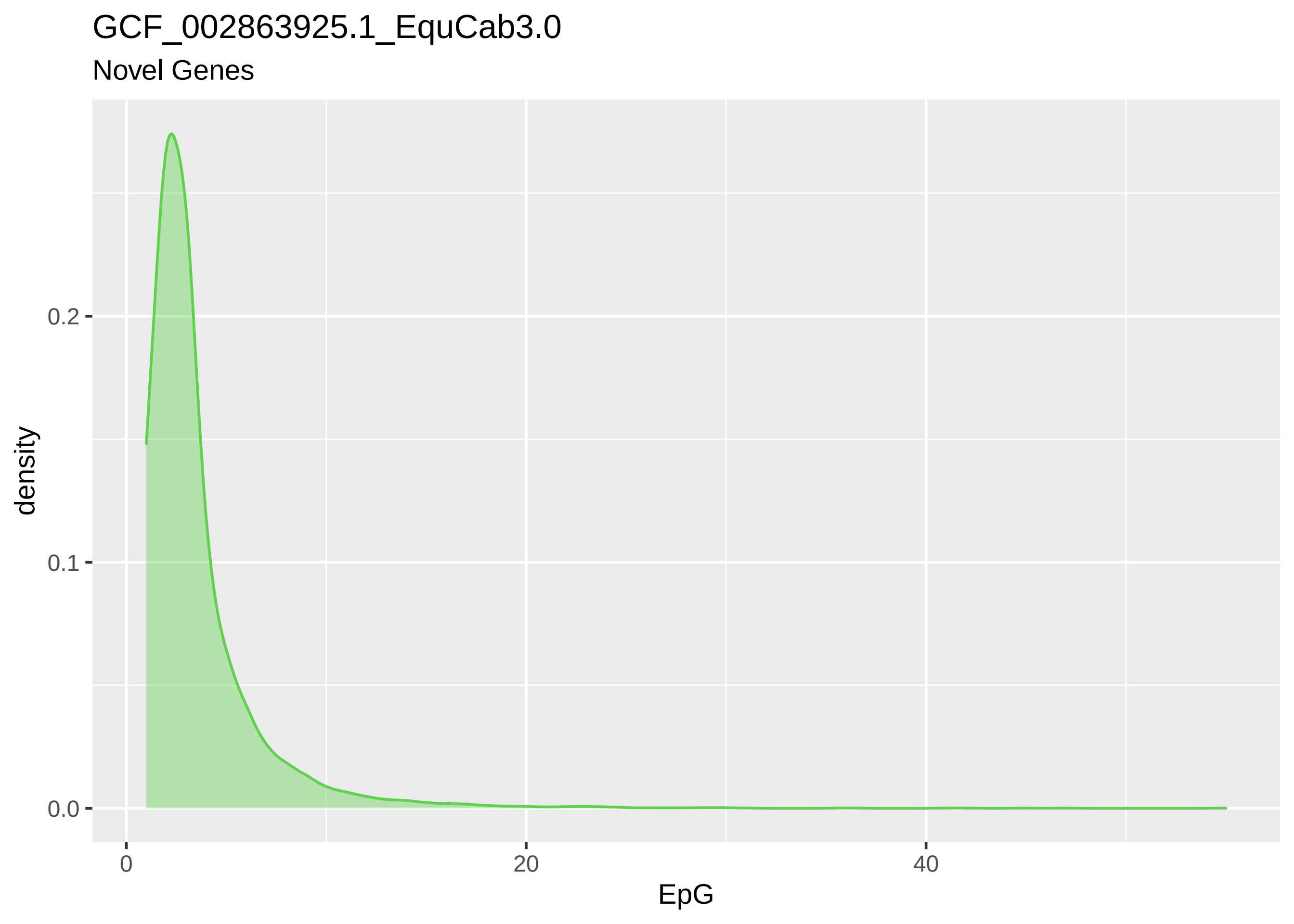
GCF_002863925.1_EquCab3.0
Novel Genes
density
0.0
0.1
0.2
0
20
40
EpG

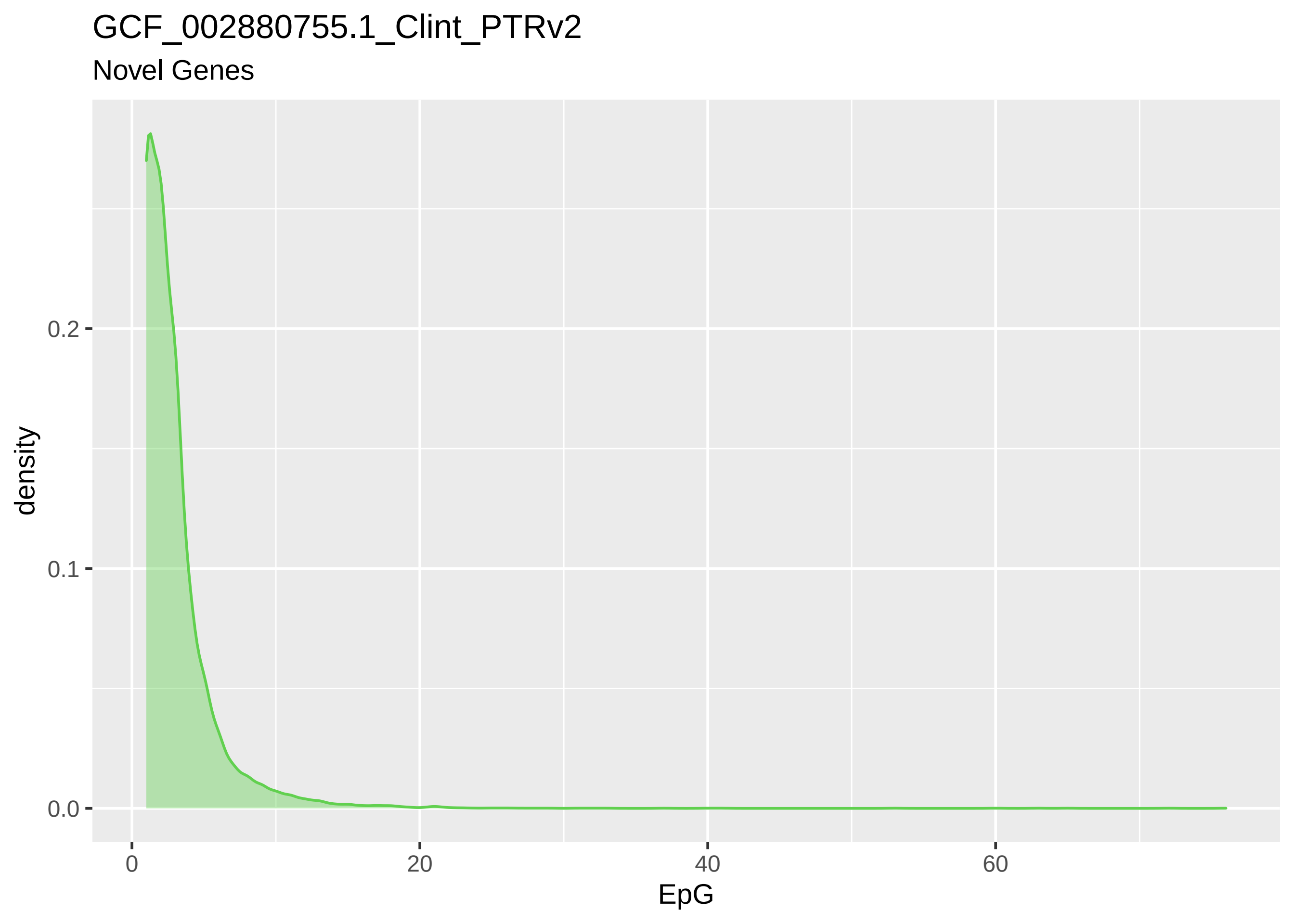
GCF_002880755.1_Clint_PTRv2
Novel Genes
density
0.0
0.1
0.2
0
20
40
60
EpG

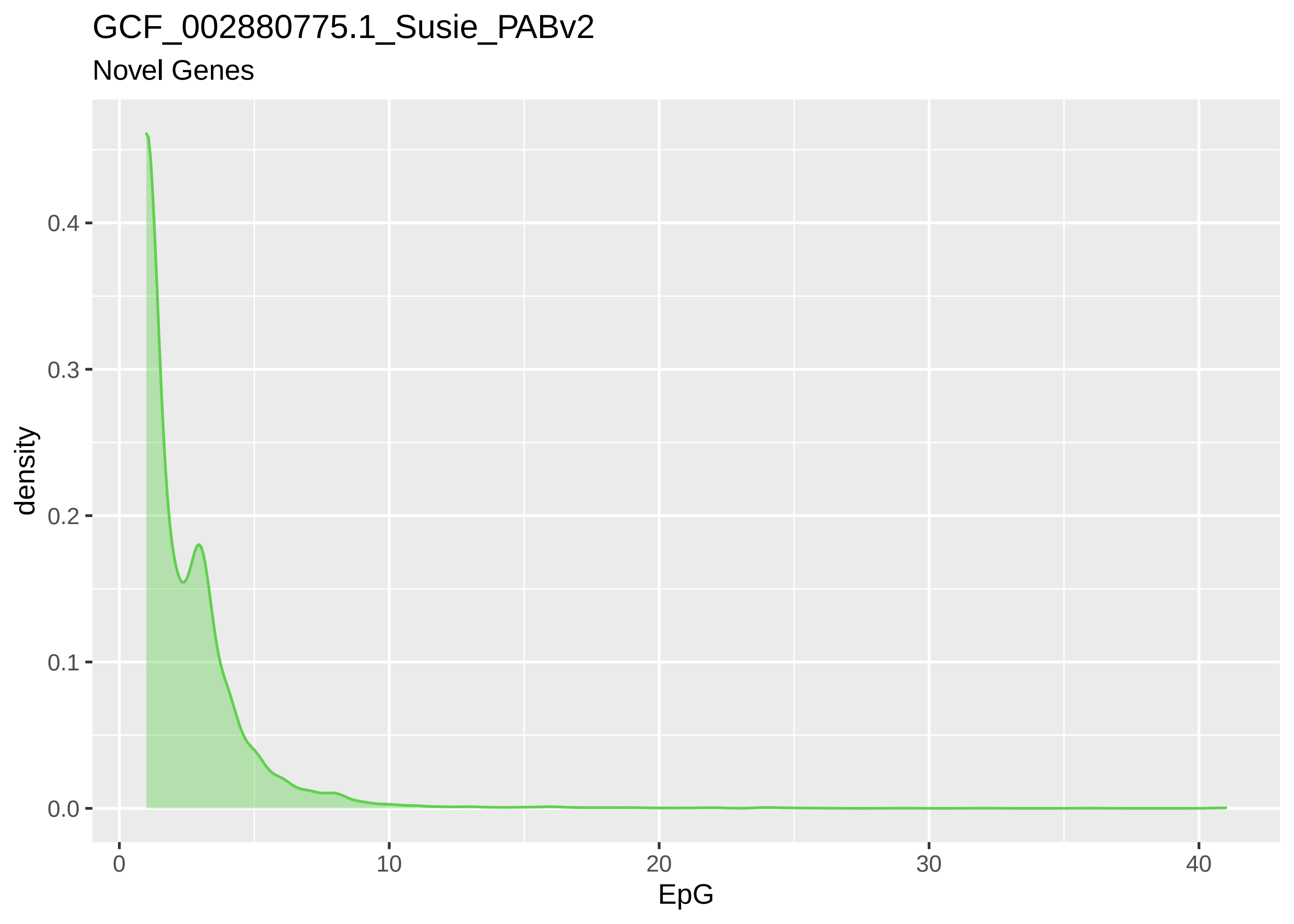
GCF_002880775.1_Susie_PABv2
Novel Genes
density
0.0
0.1
0.2
0.3
0.4
0
10
20
30
40
EpG

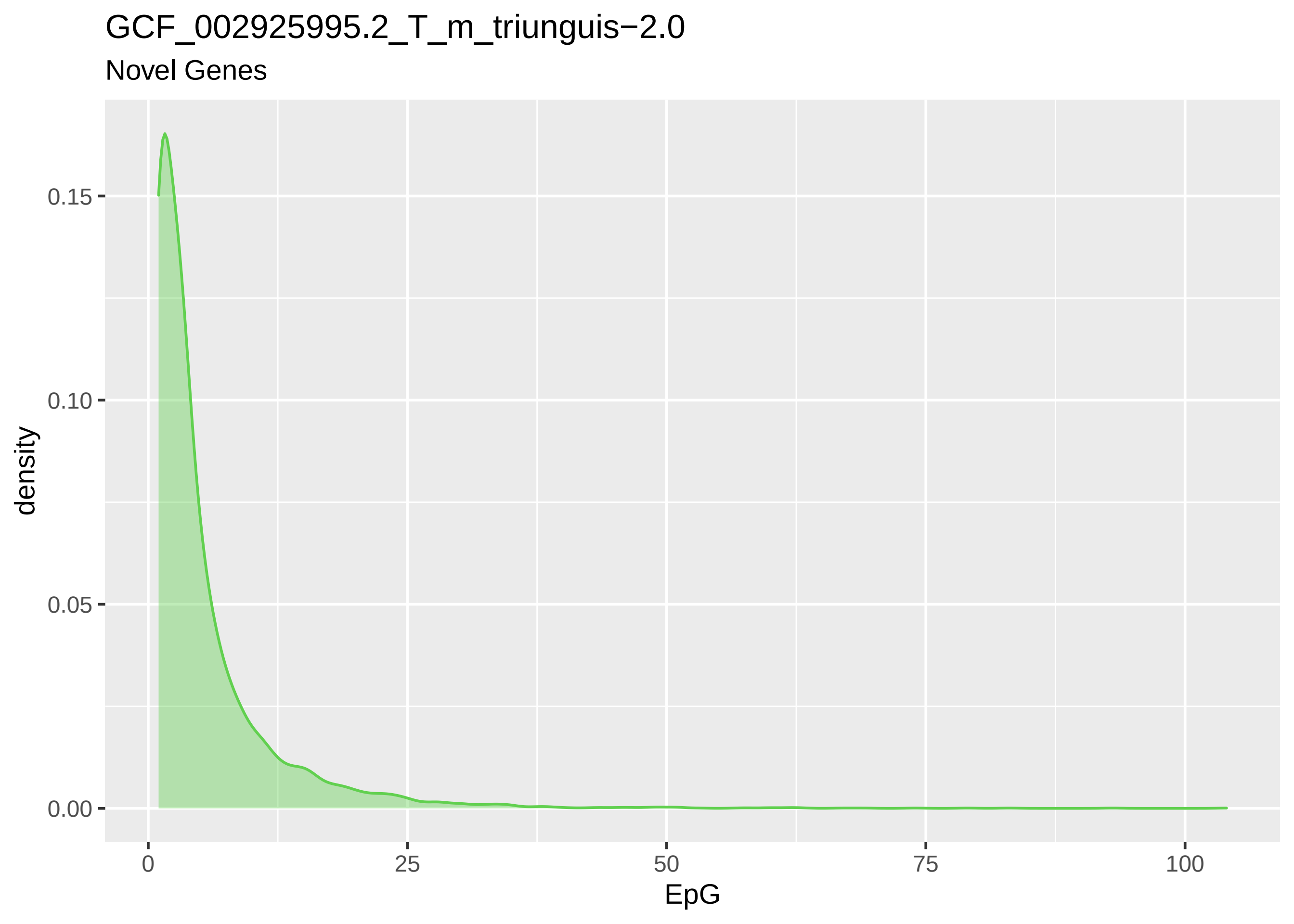
GCF_002925995.2_T_m_triunguis−2.0
Novel Genes
0.15
0.10
0.05
0.00
density
0
25
50
75
100
EpG

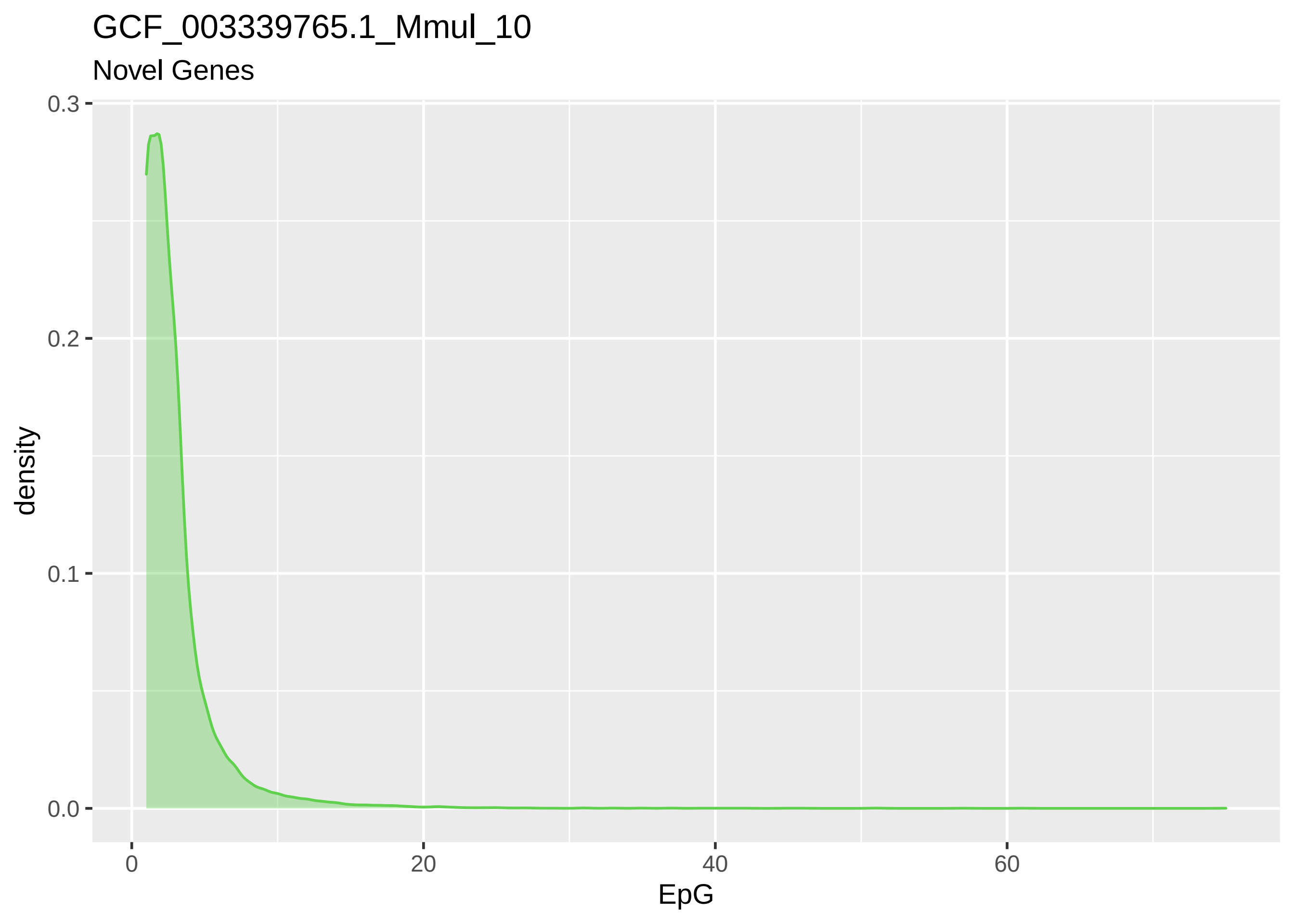
GCF_003339765.1_Mmul_10
Novel Genes
0.3
0.2
0.1
0.0
density
0
20
40
60
EpG

GCF_003957565.2_bTaeGut1.4.pri

Novel Genes

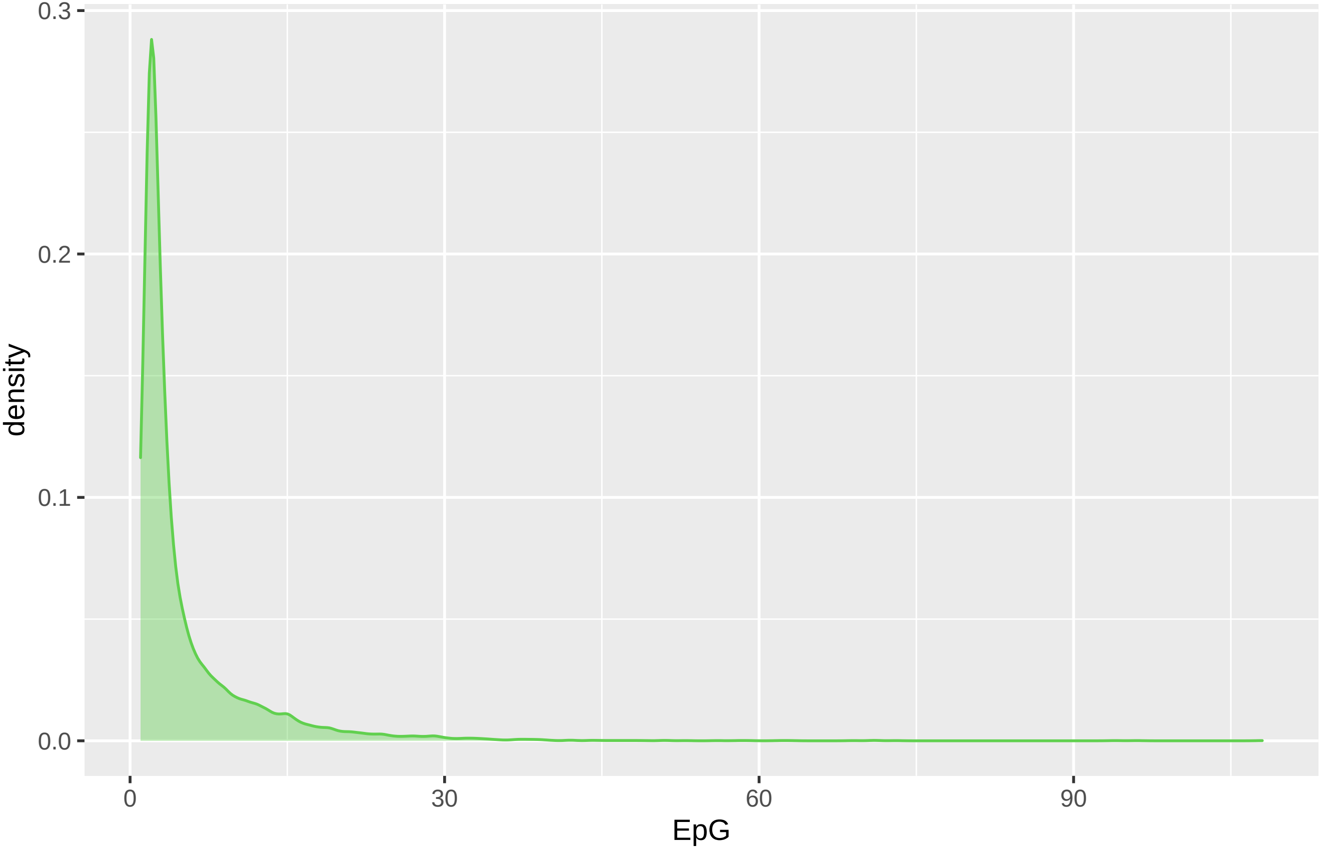

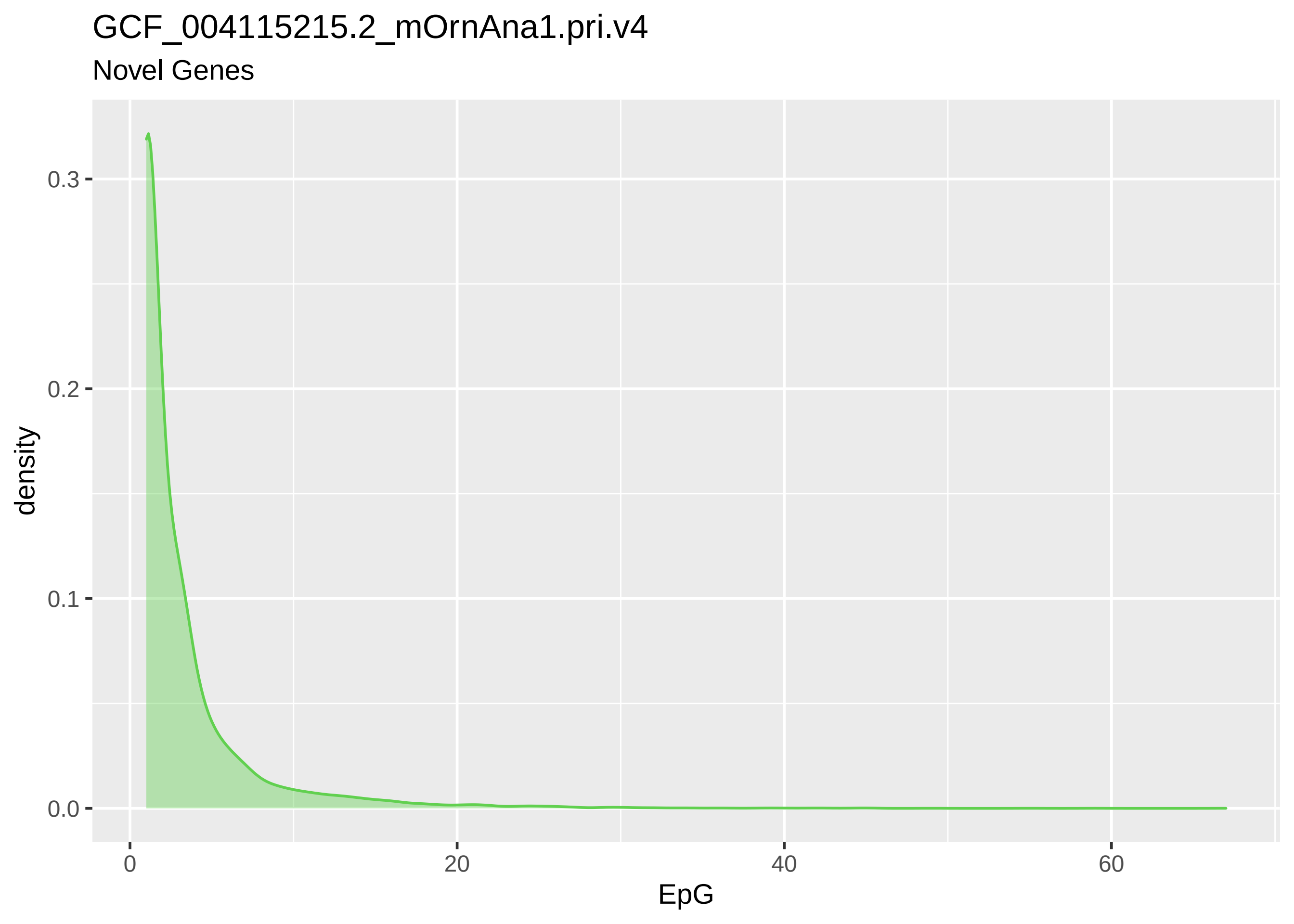

GCF_004115215.2_mOrnAna1.pri.v4
Novel Genes
density
0.3
0.2
0.1
0.0
0
20
40
60
EpG

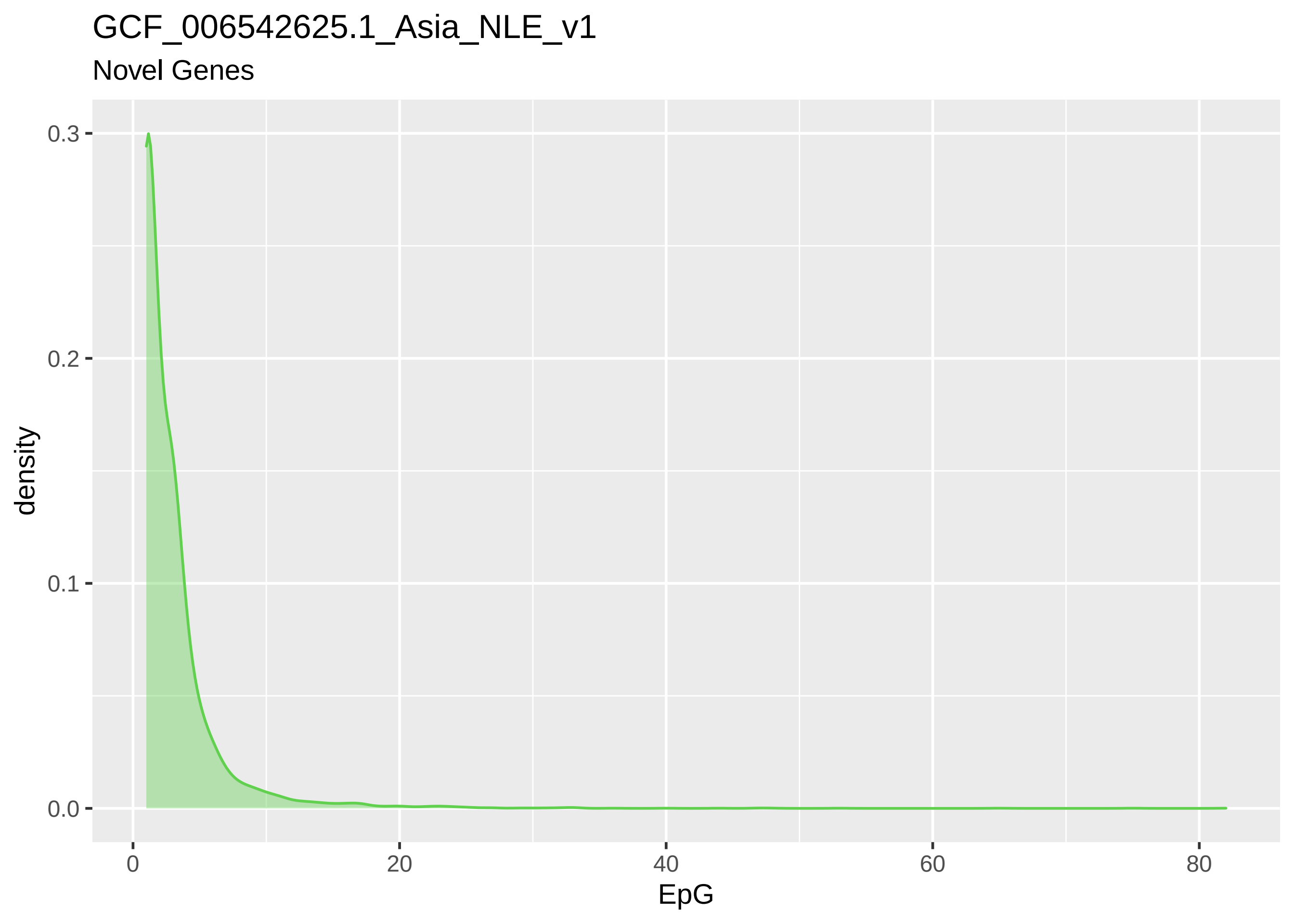
GCF_006542625.1_Asia_NLE_v1
Novel Genes
0.3
0.2
0.1
0.0
density
0
20
40
60
80
EpG

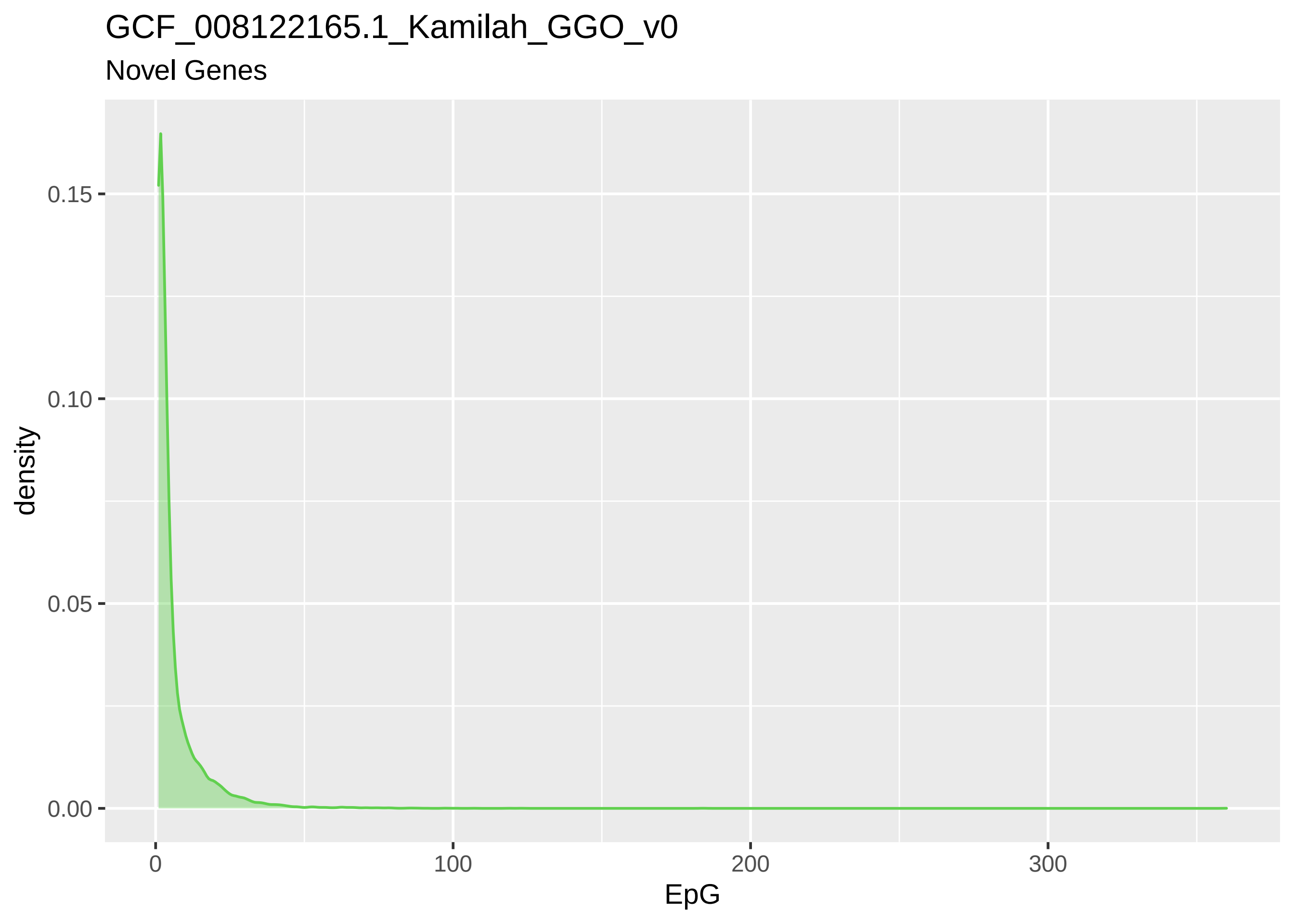
GCF_008122165.1_Kamilah_GGO_v0
Novel Genes
density
0.15
0.10
0.05
0.00
0
100
200
300
EpG

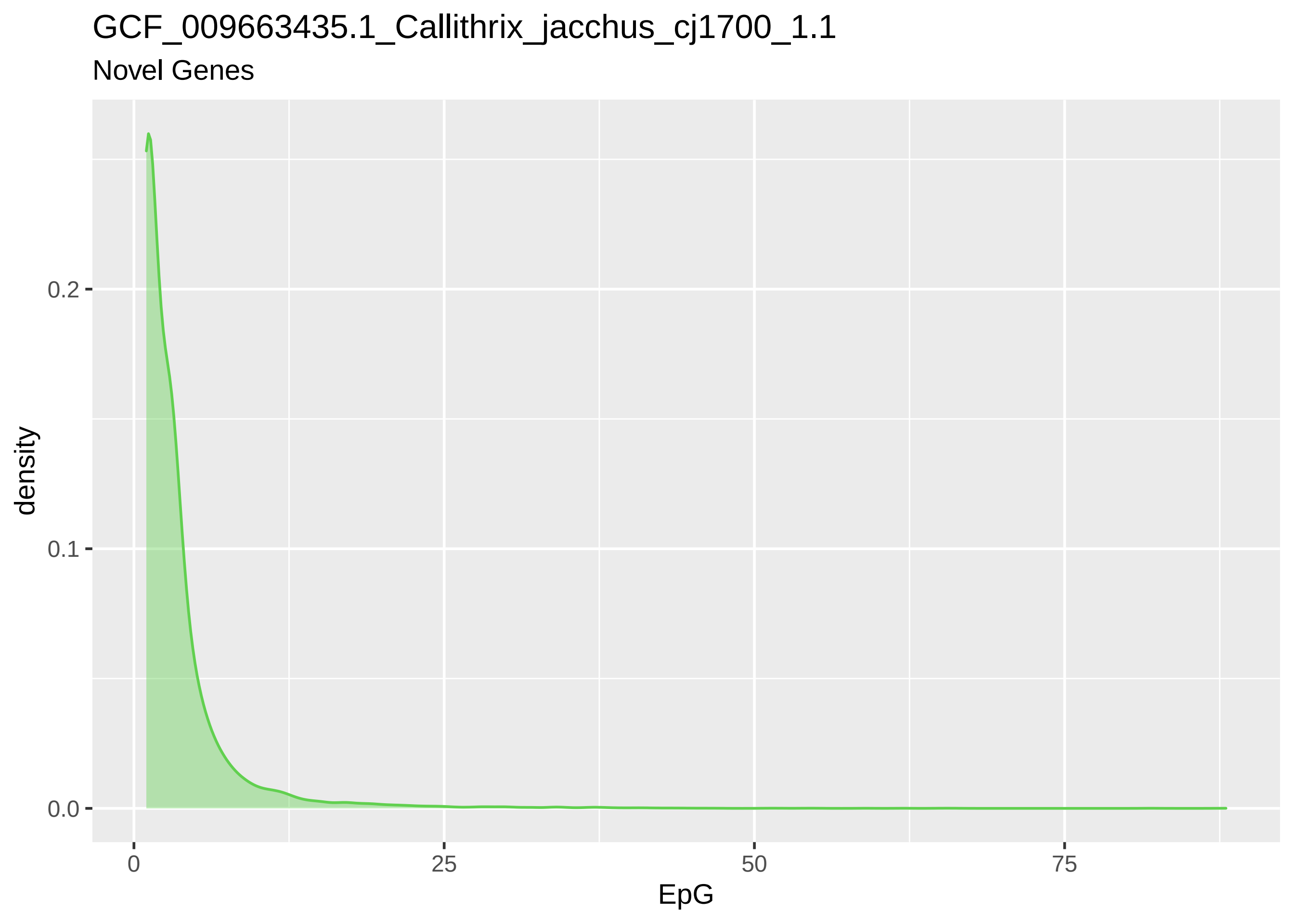
GCF_009663435.1_Callithrix_jacchus_cj1700_1.1
Novel Genes
density
0.2
0.1
0.0
0
25
50
75
EpG

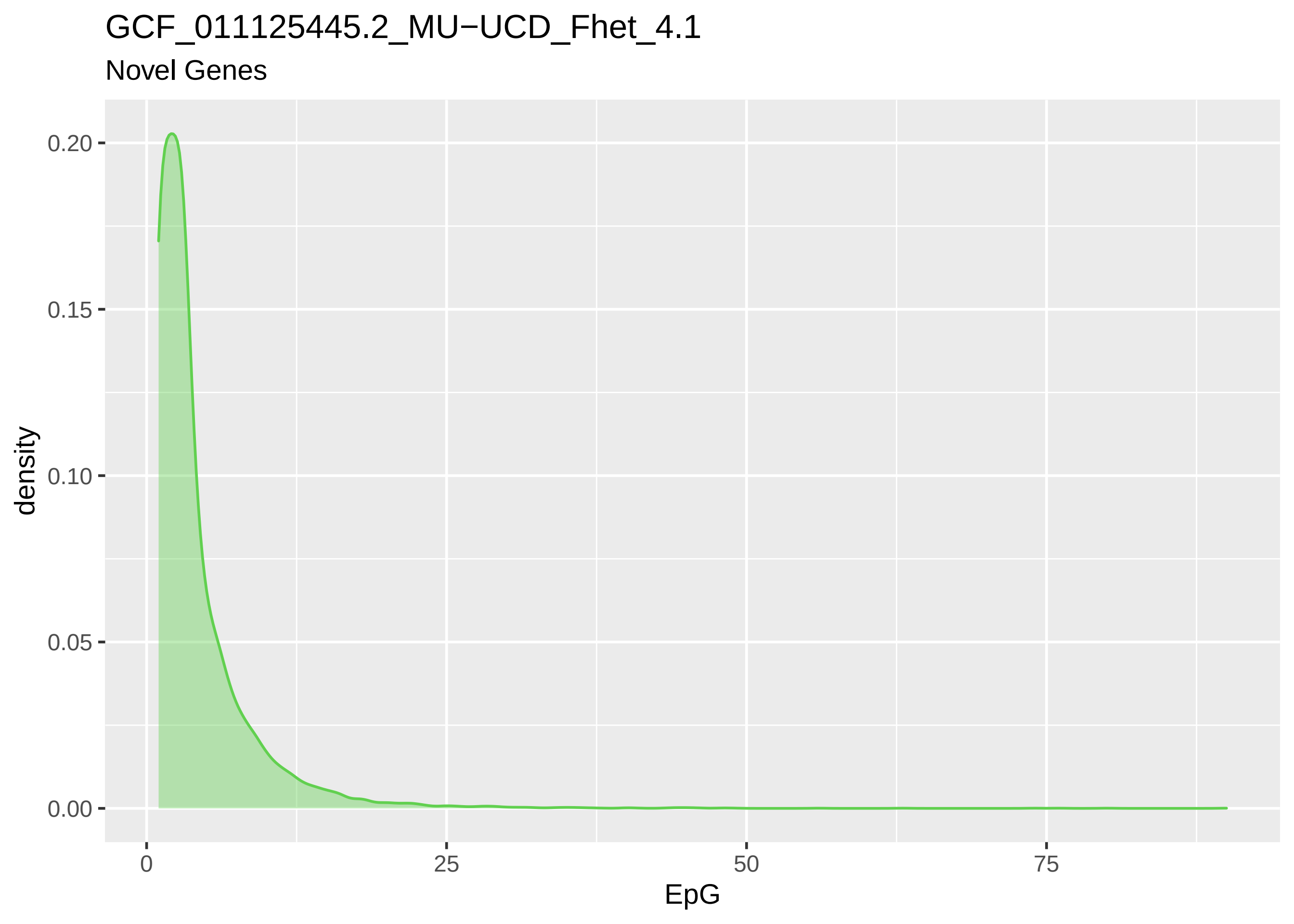
GCF_011125445.2_MU−UCD_Fhet_4.1
Novel Genes
density
0.20
0.15
0.10
0.05
0.00
0
25
50
75
EpG

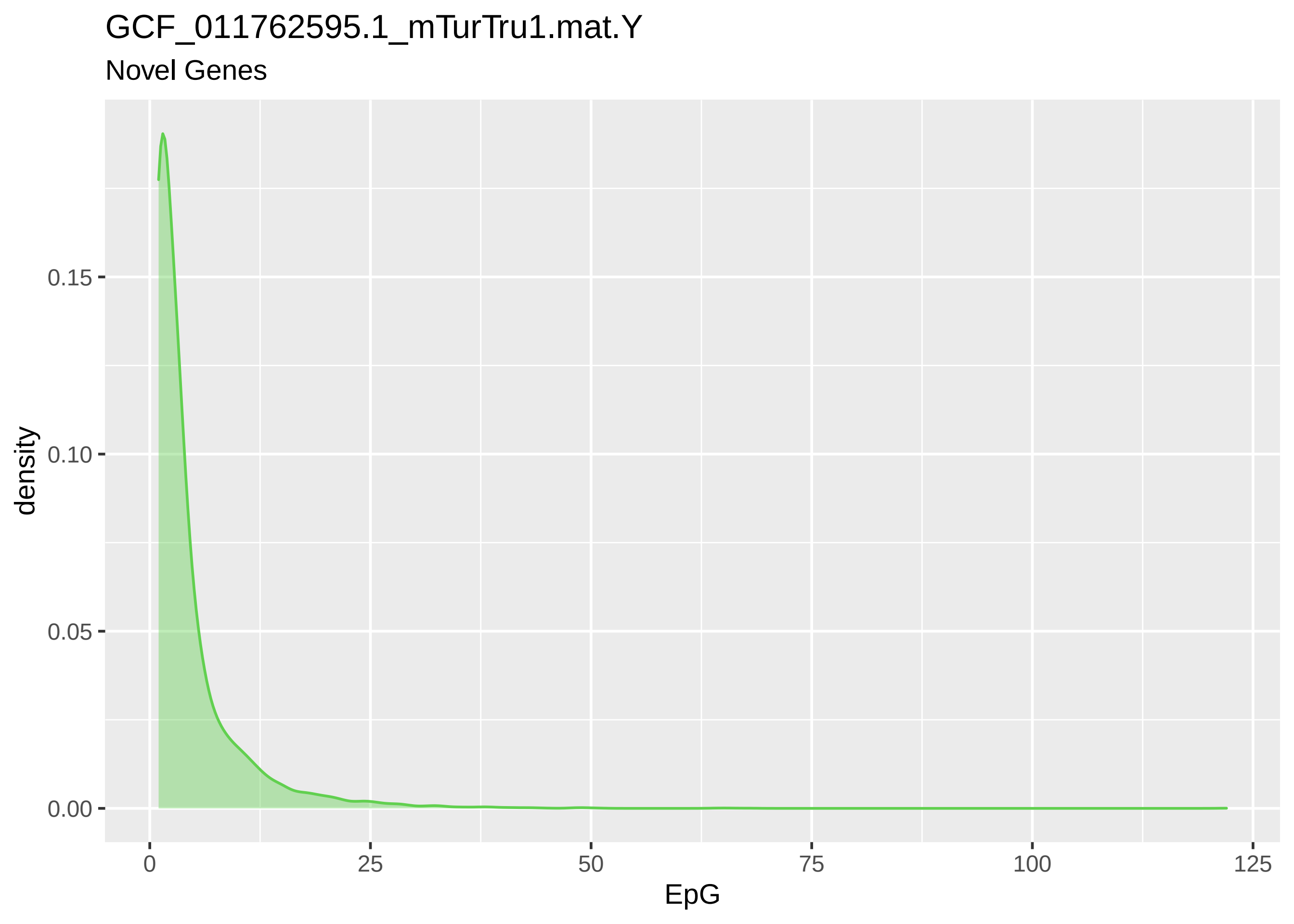
GCF_011762595.1_mTurTru1.mat.Y
Novel Genes
density
0.15
0.10
0.05
0.00
0
25
50
75
100
125
EpG

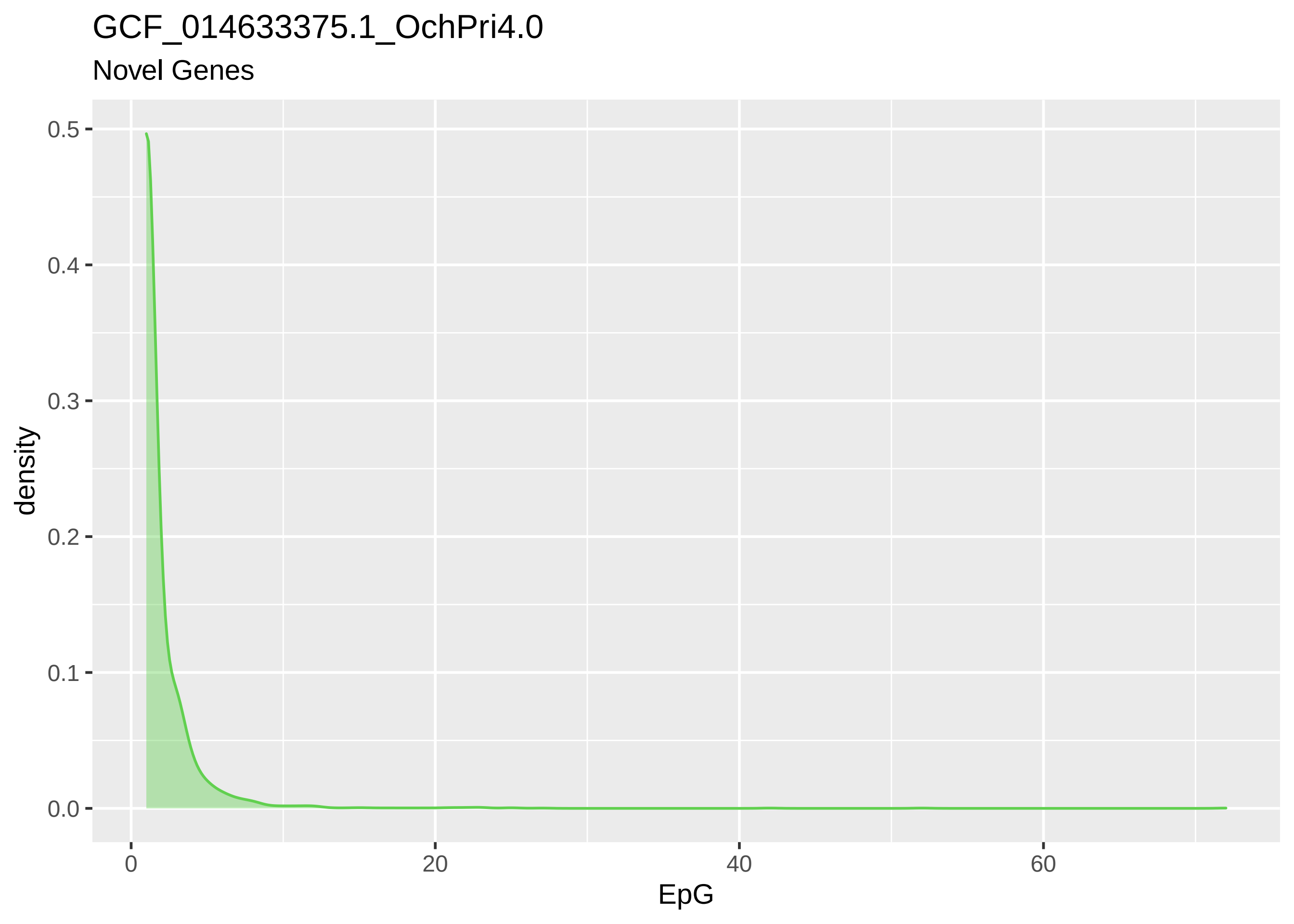

GCF_014633375.1_OchPri4.0
Novel Genes
density
0.5
0.4
0.3
0.2
0.1
0.0
0
20
40
60
EpG

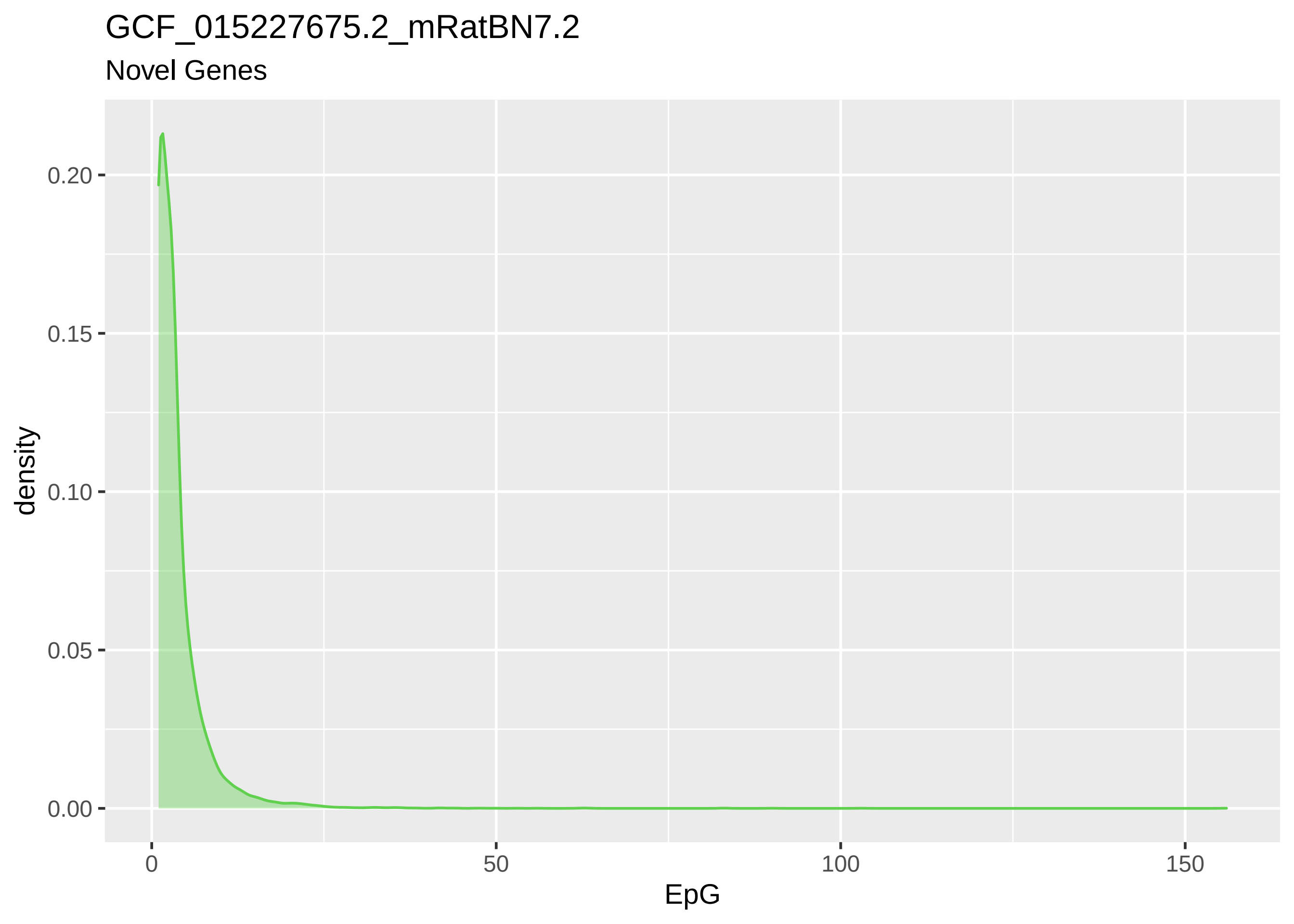

GCF_015227675.2_mRatBN7.2
Novel Genes
density
0.20
0.15
0.10
0.05
0.00
0
50
100
150
EpG

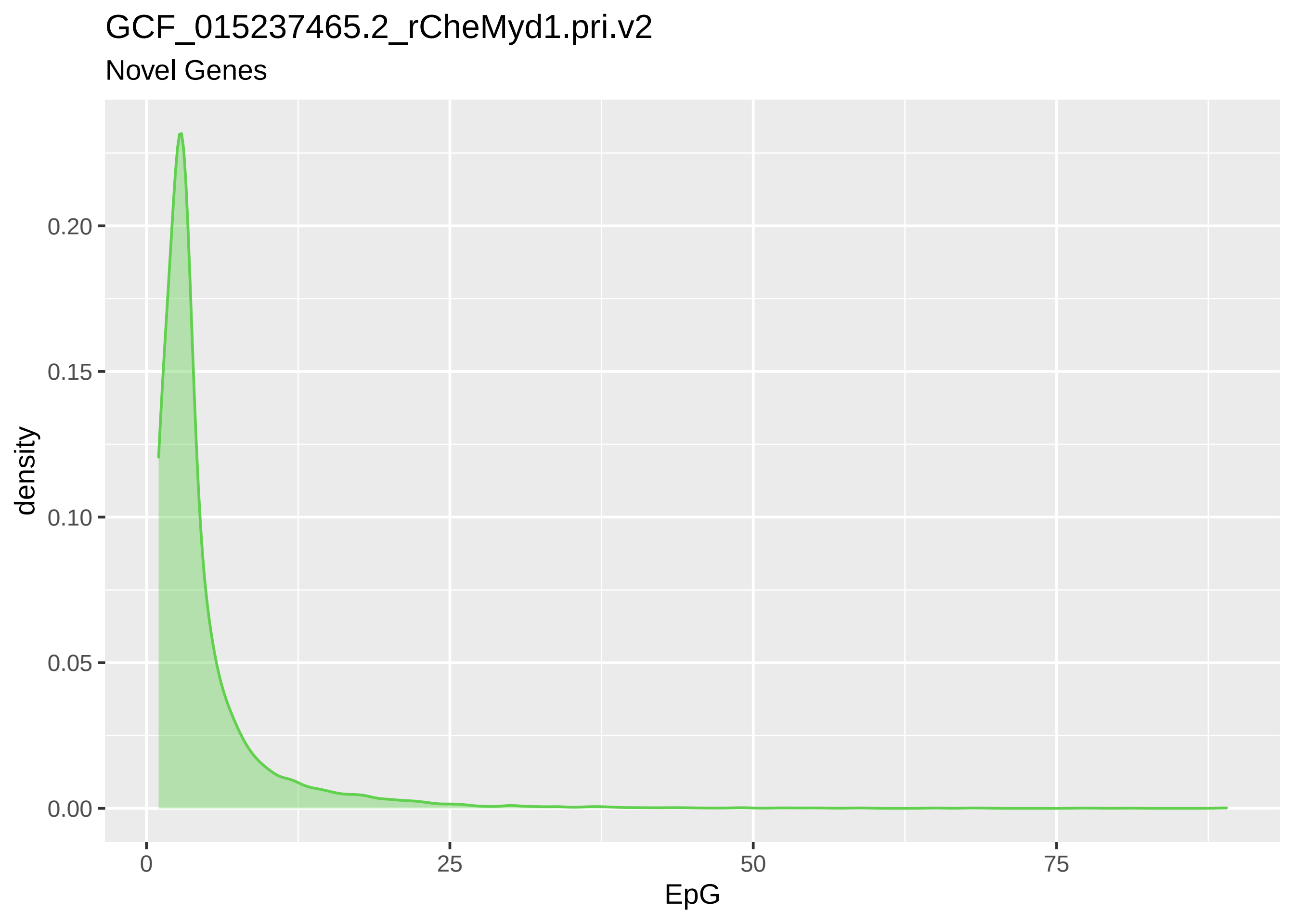
GCF_015237465.2_rCheMyd1.pri.v2
Novel Genes
density
0.20
0.15
0.10
0.05
0.00
0
25
50
75
EpG

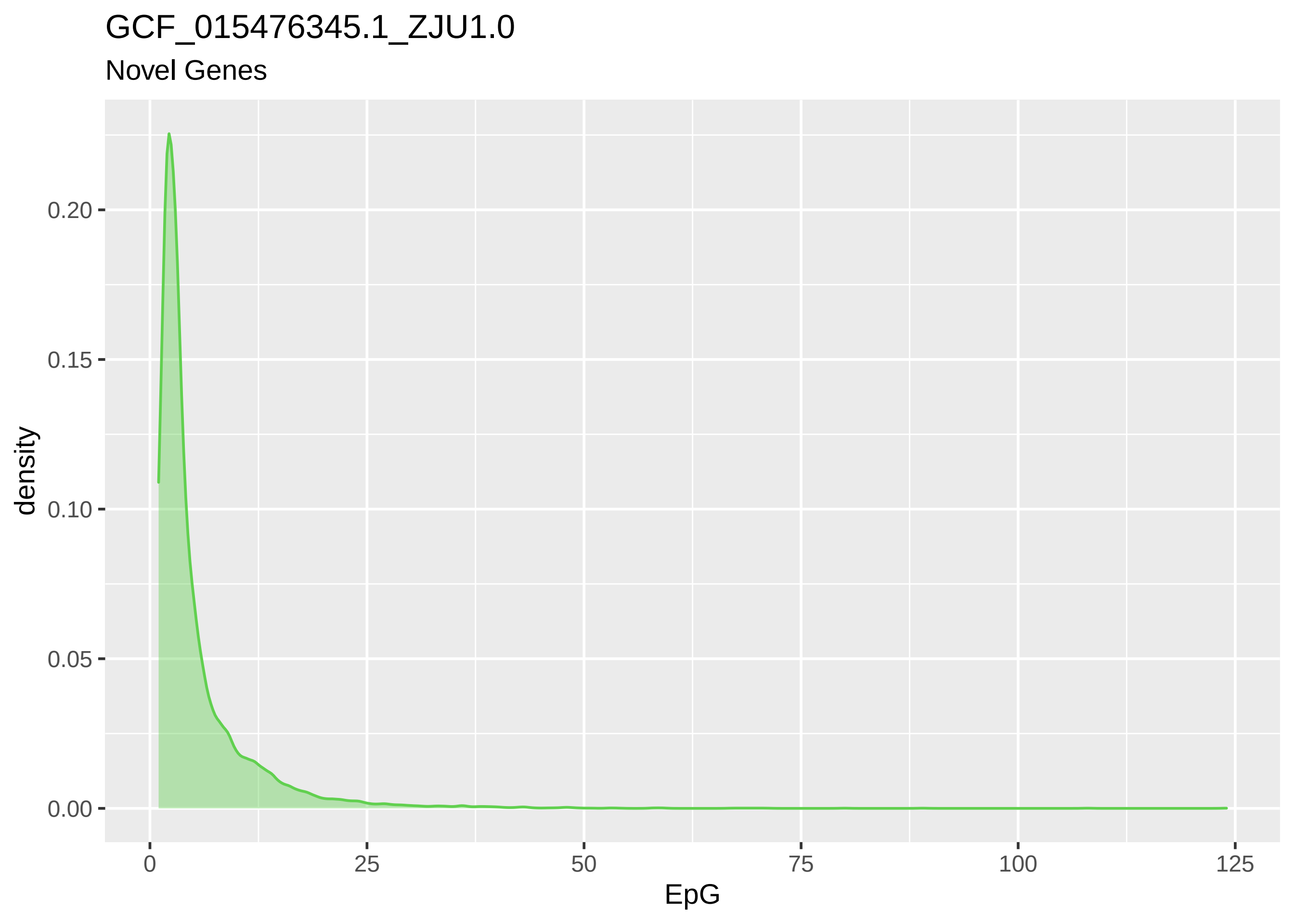
GCF_015476345.1_ZJU1.0
Novel Genes
density
0.00
0.05
0.10
0.15
0.20
0
25
50
75
100
125
EpG

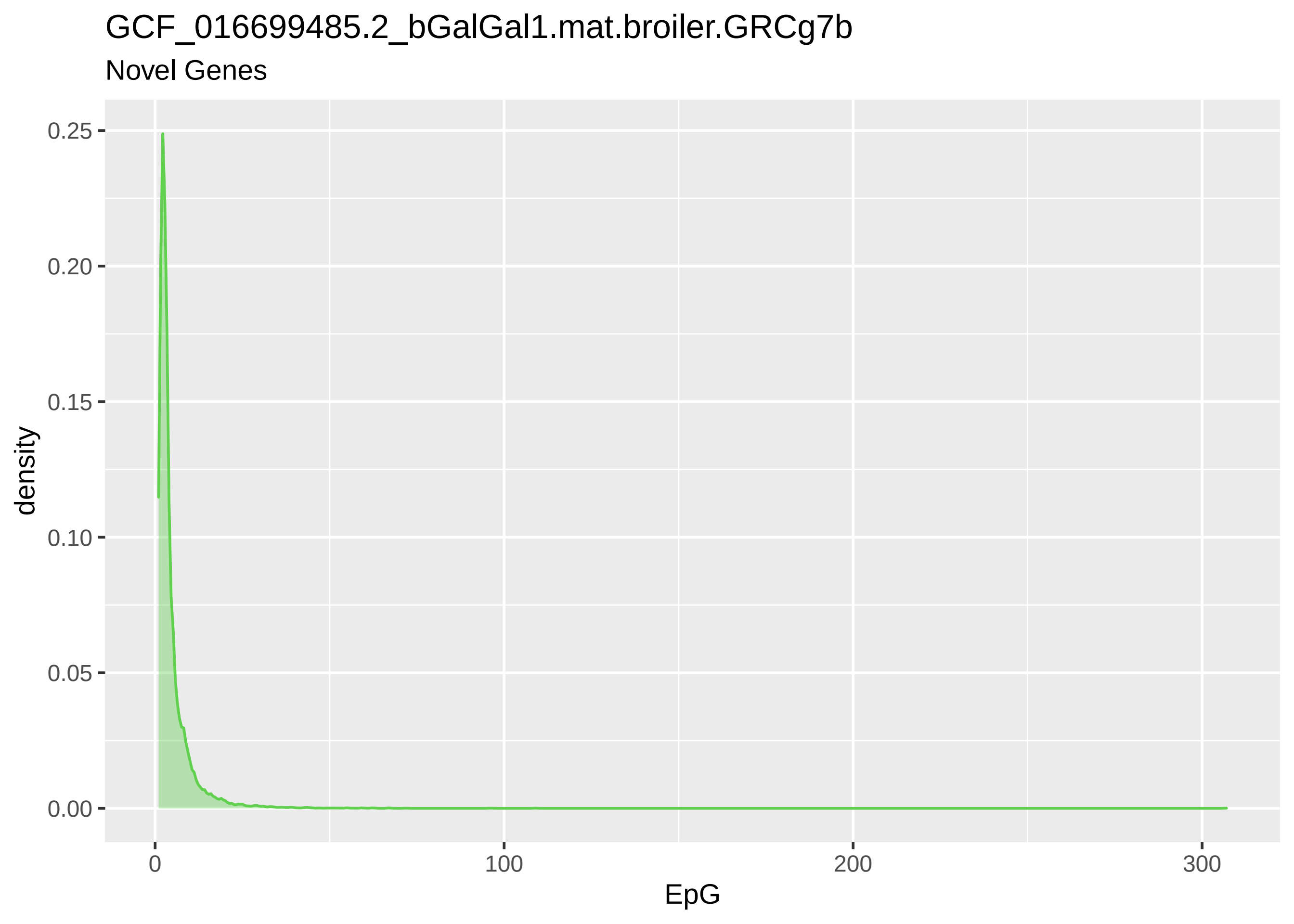

GCF_016699485.2_bGalGal1.mat.broiler.GRCg7b
Novel Genes
density
0.25
0.20
0.15
0.10
0.05
0.00
0
100
200
300
EpG

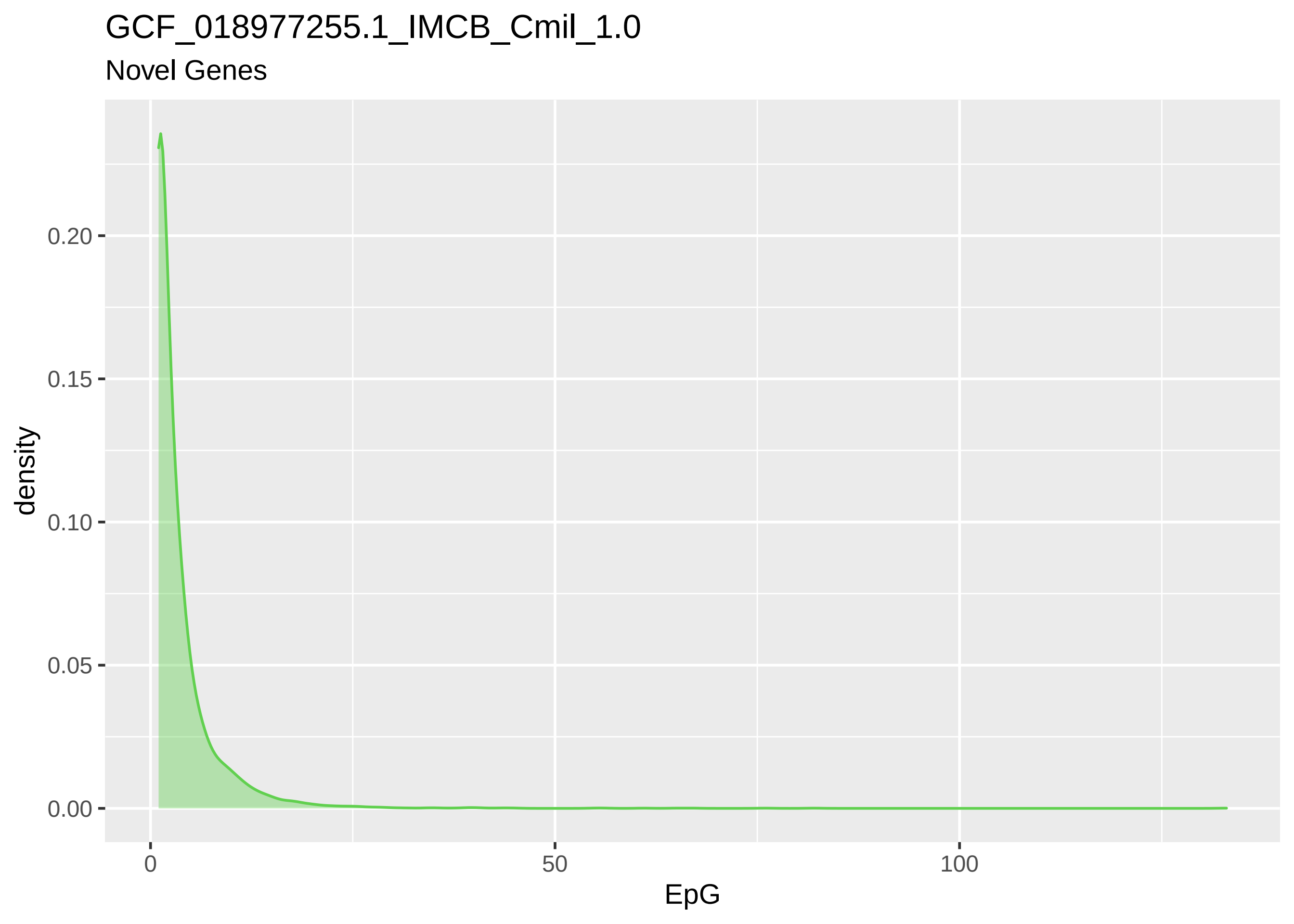
GCF_018977255.1_IMCB_Cmil_1.0
Novel Genes
density
0.20
0.15
0.10
0.05
0.00
0
50
100
EpG

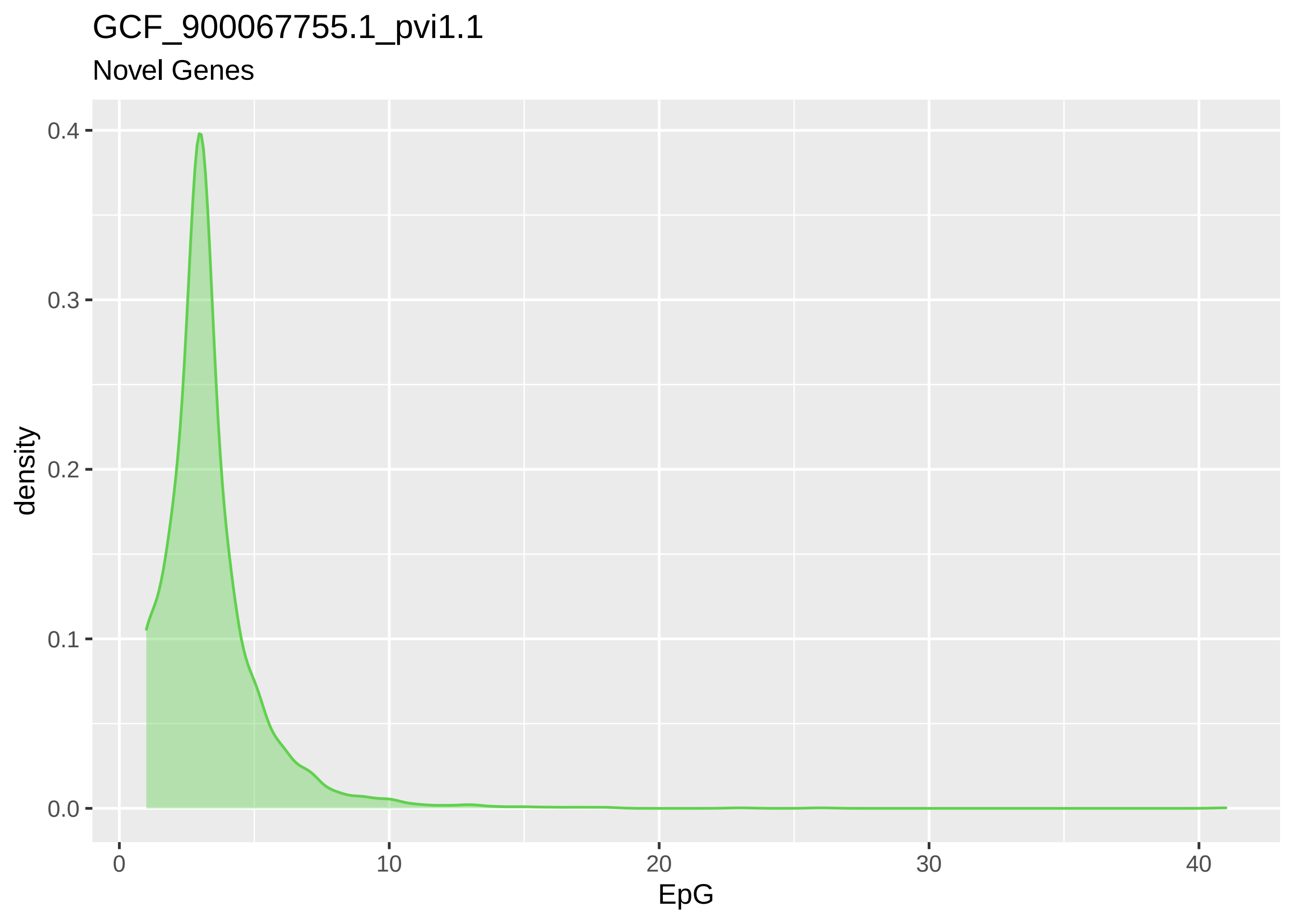
GCF_900067755.1_pvi1.1
Novel Genes
density
0.0
0.1
0.2
0.3
0.4
0
10
20
30
40
EpG

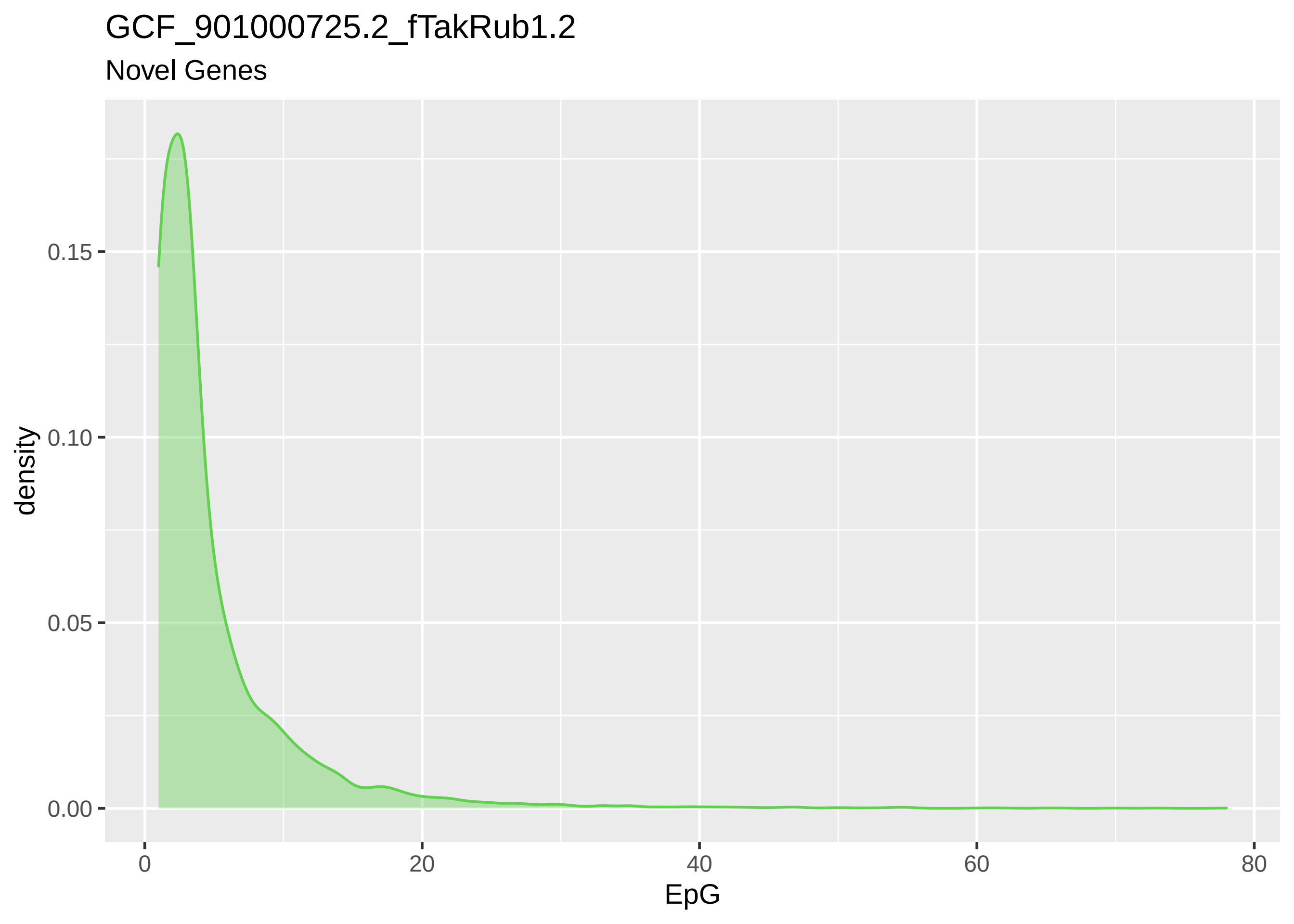
GCF_901000725.2_fTakRub1.2
Novel Genes
density
0.00
0.05
0.10
0.15
0
20
40
60
80
EpG

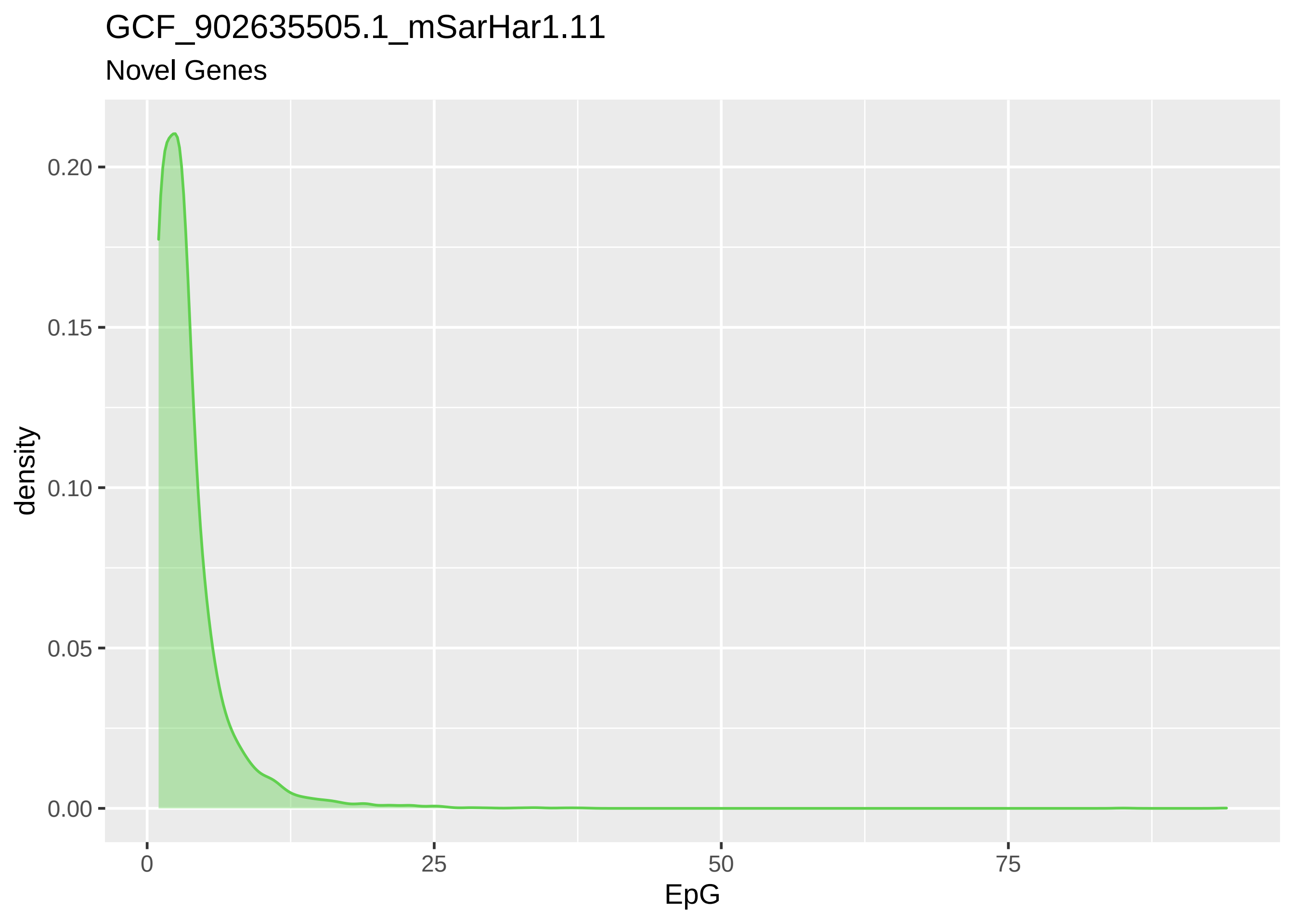
GCF_902635505.1_mSarHar1.11
Novel Genes
density
0.20
0.15
0.10
0.05
0.00
0
25
50
75
EpG

dana−all−r1.04.novel.transcriptome_counts_transcript_level.csv
EpT

Novel Genes

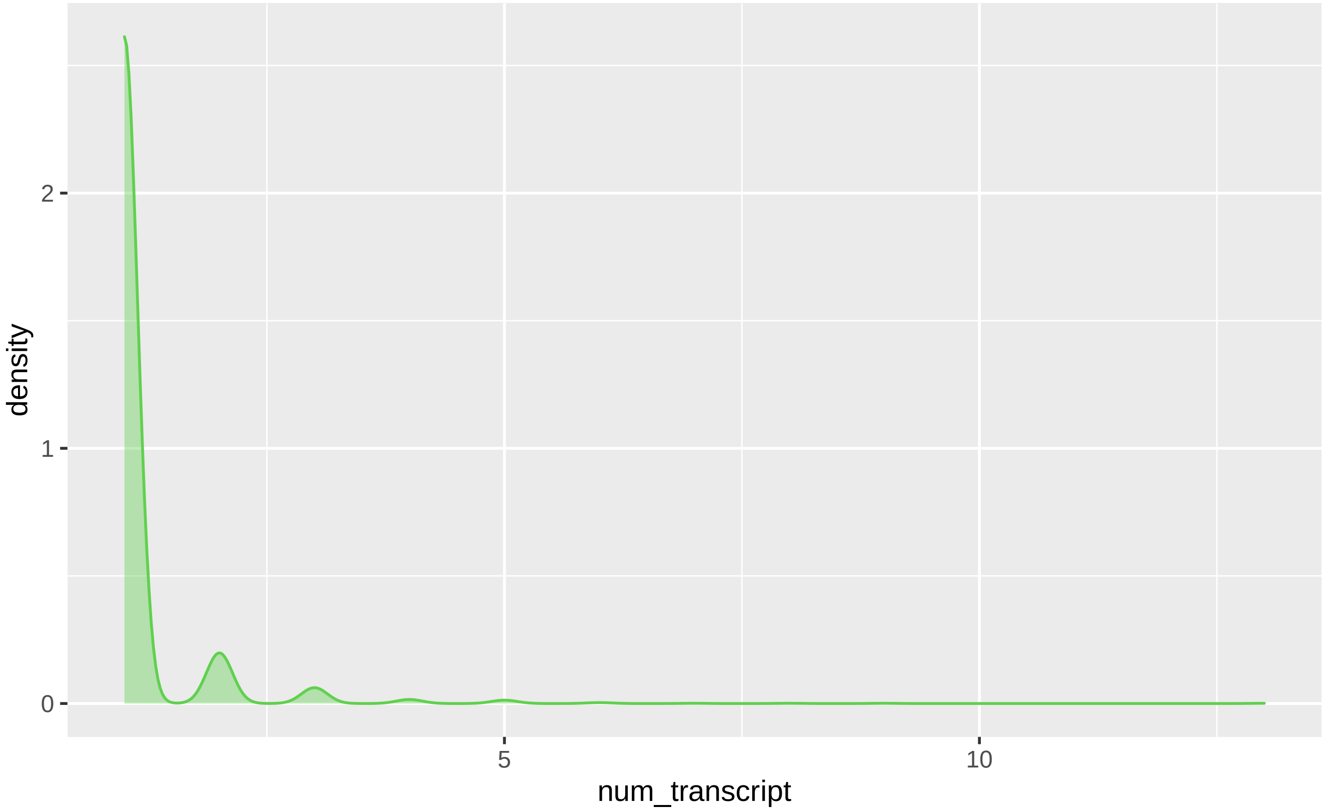

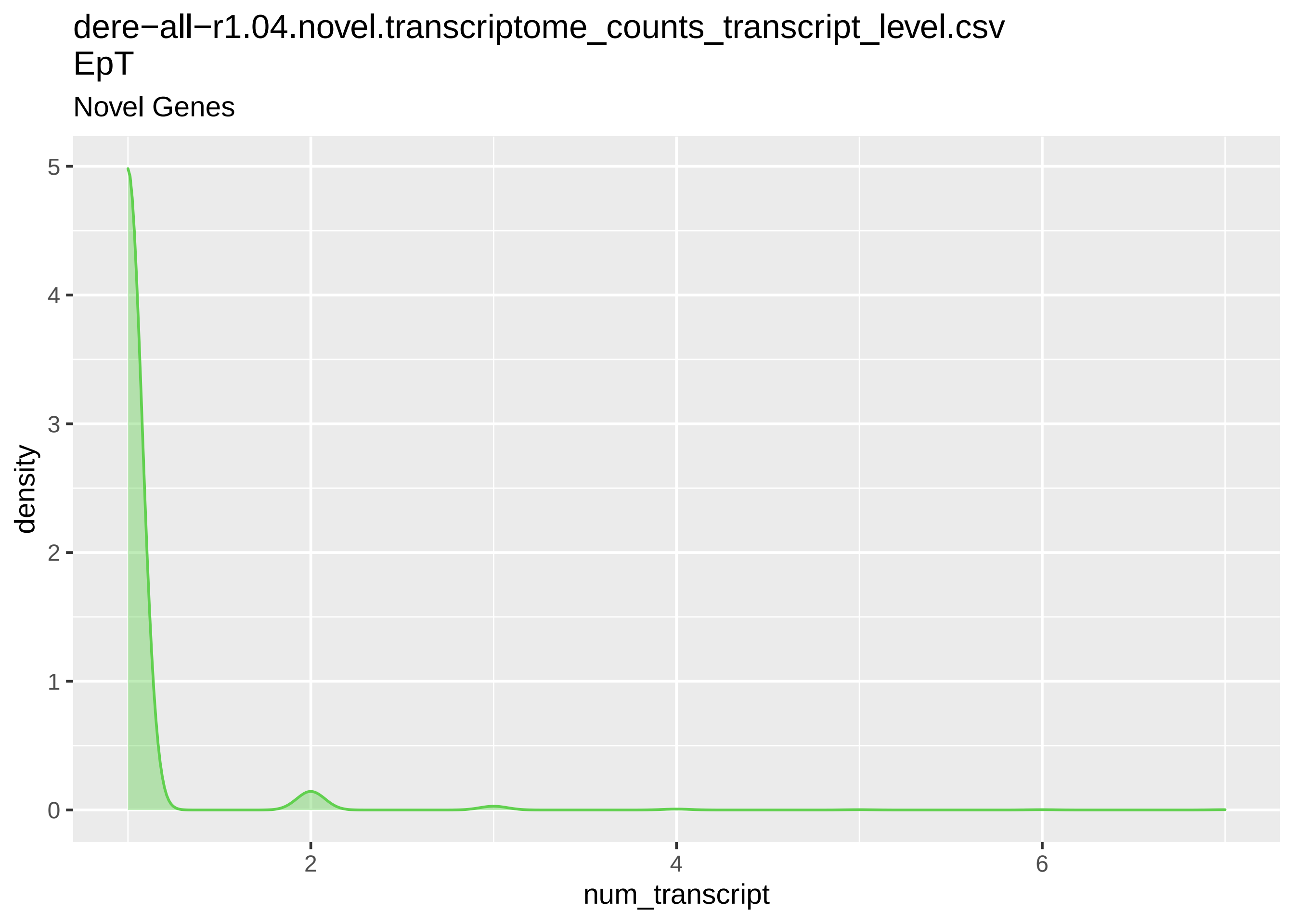
dere−all−r1.04.novel.transcriptome_counts_transcript_level.csv
EpT
Novel Genes
density
num_transcript
0
1
2
3
4
5
2
4
6

dgri−all−r1.3.novel.transcriptome_counts_transcript_level.csv
EpT

Novel Genes

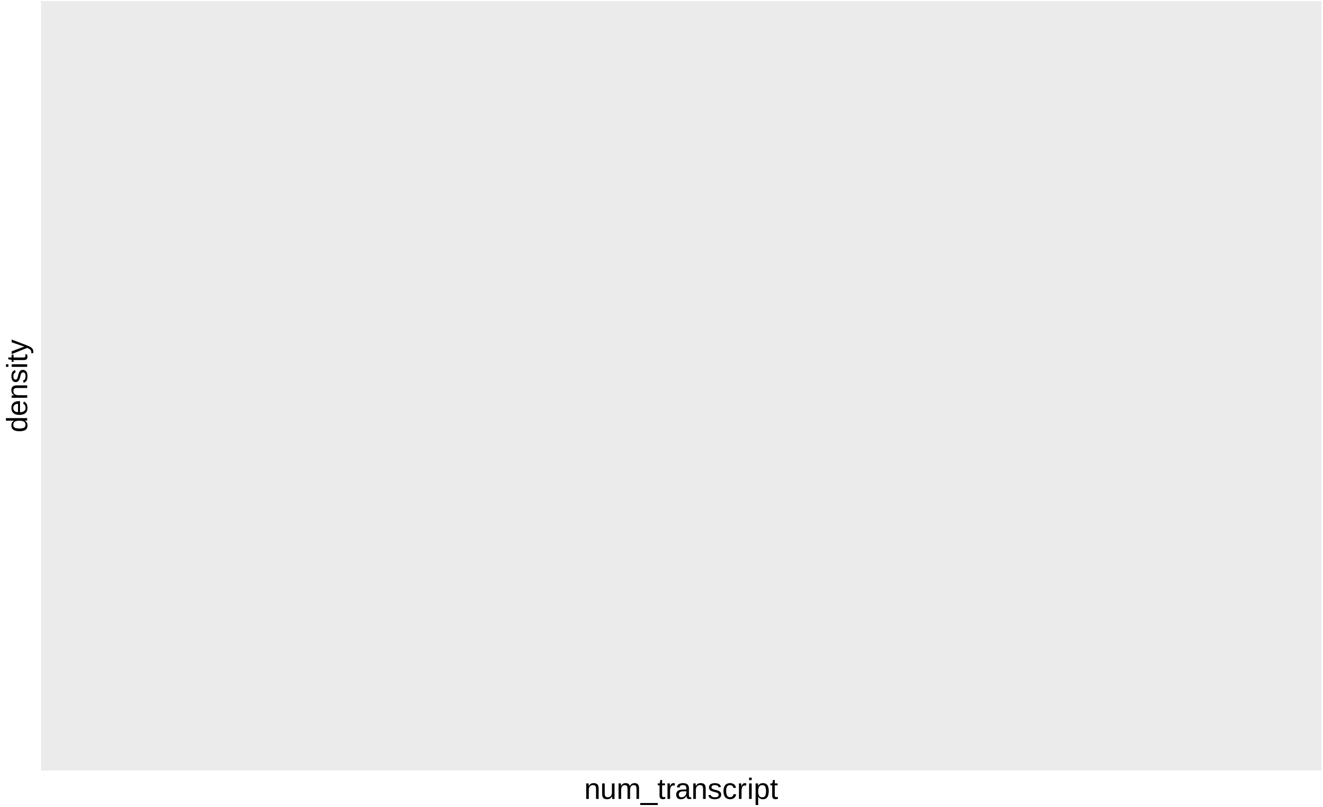

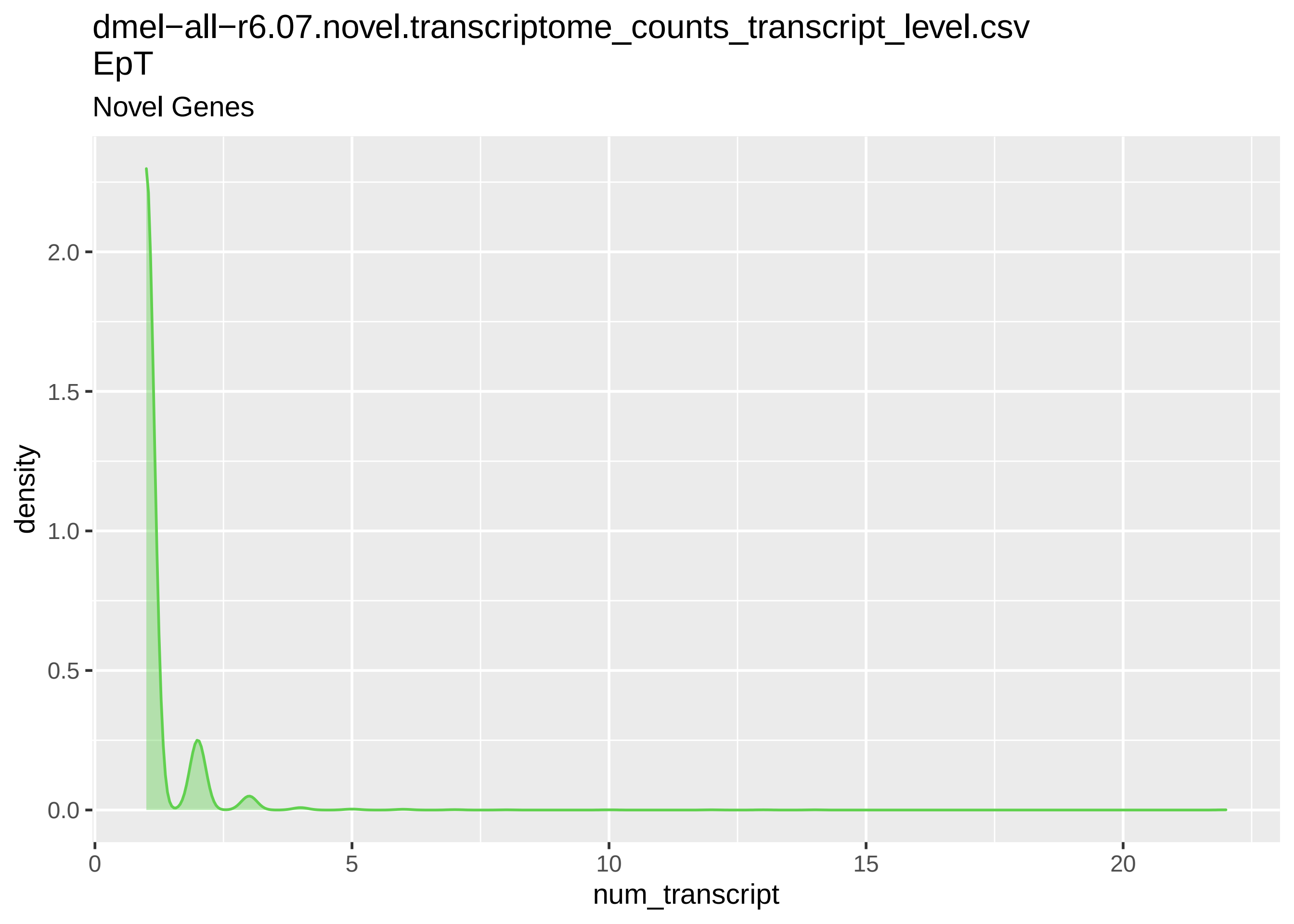
dmel−all−r6.07.novel.transcriptome_counts_transcript_level.csv
EpT
Novel Genes
density
2.0
1.5
1.0
0.5
0.0
0
5
10
15
20
num_transcript

dmoj−all−r1.04.novel.transcriptome_counts_transcript_level.csv
EpT

Novel Genes

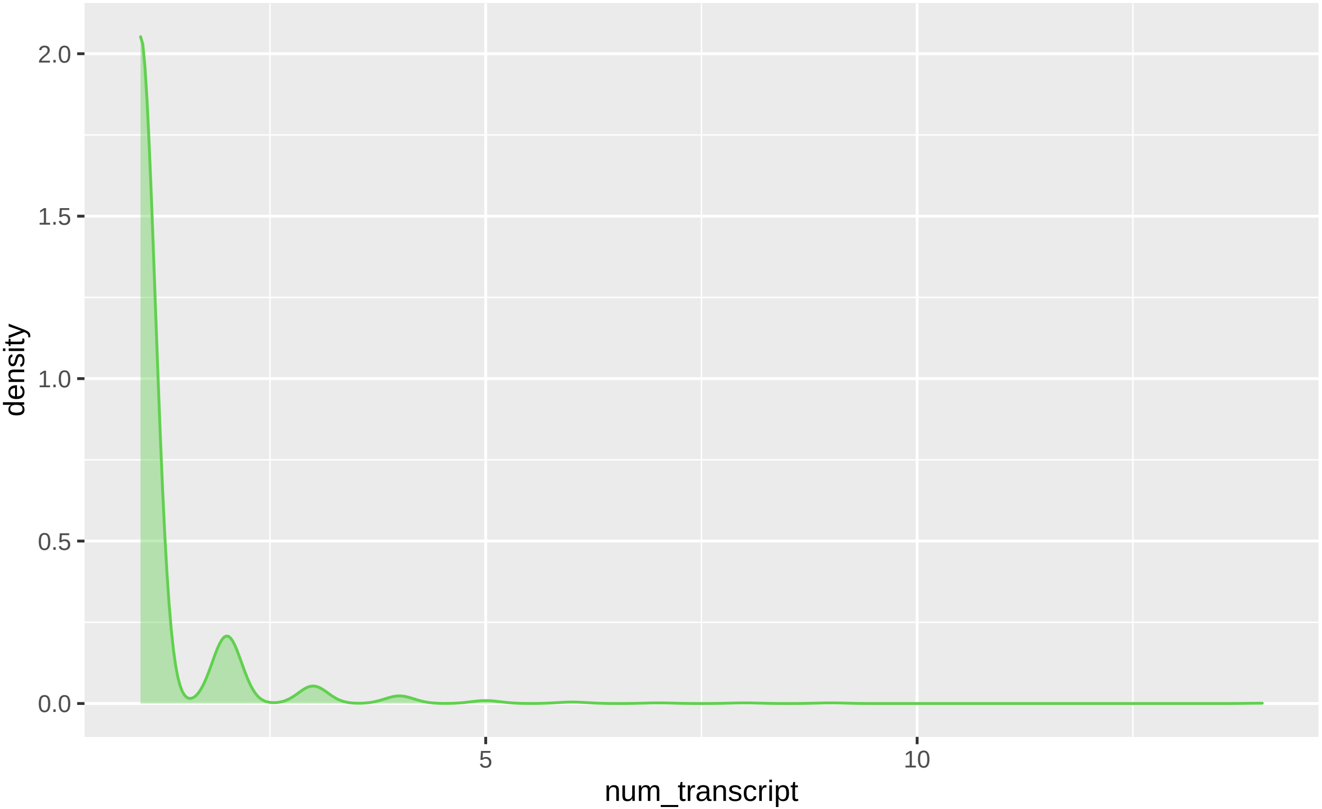

dper−all−r1.3.novel.transcriptome_counts_transcript_level.csv
EpT

Novel Genes

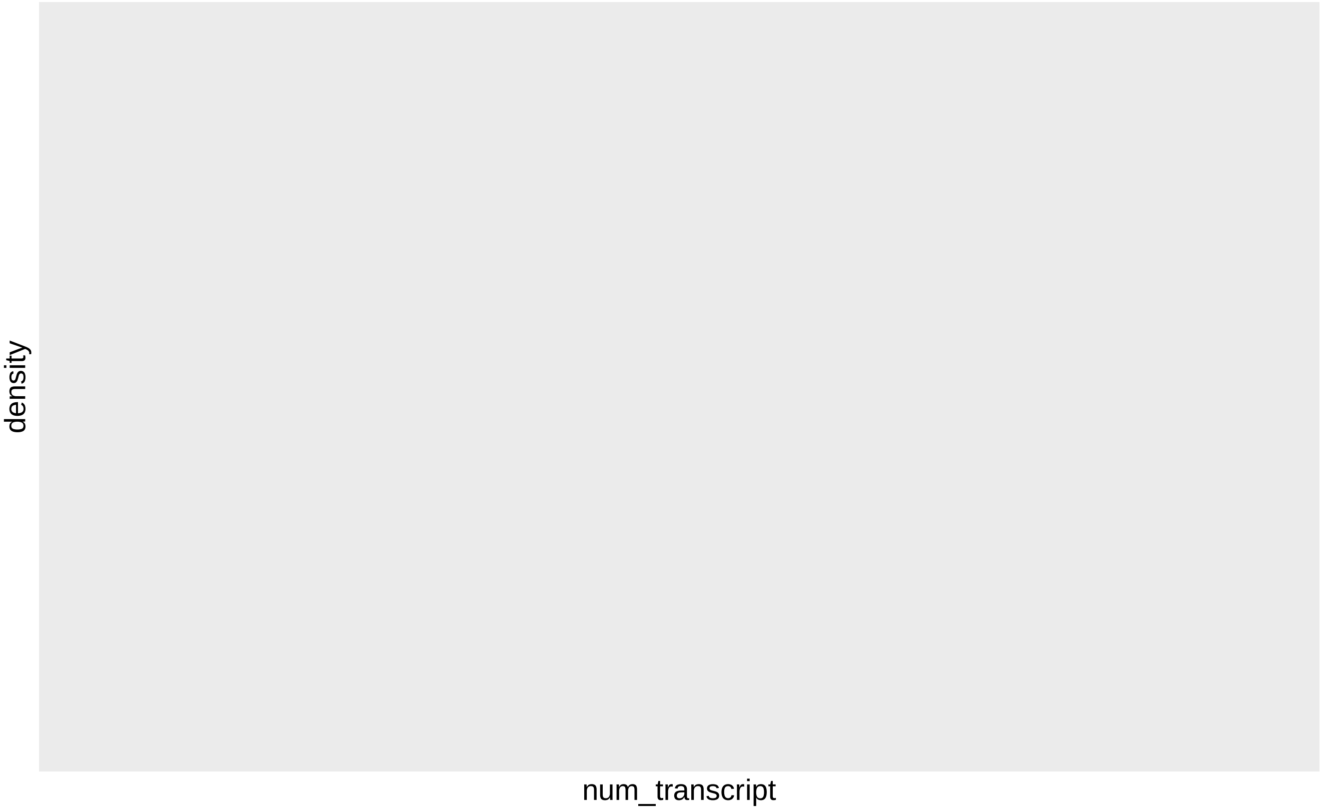

dsec−all−r1.3.novel.transcriptome_counts_transcript_level.csv
EpT

Novel Genes

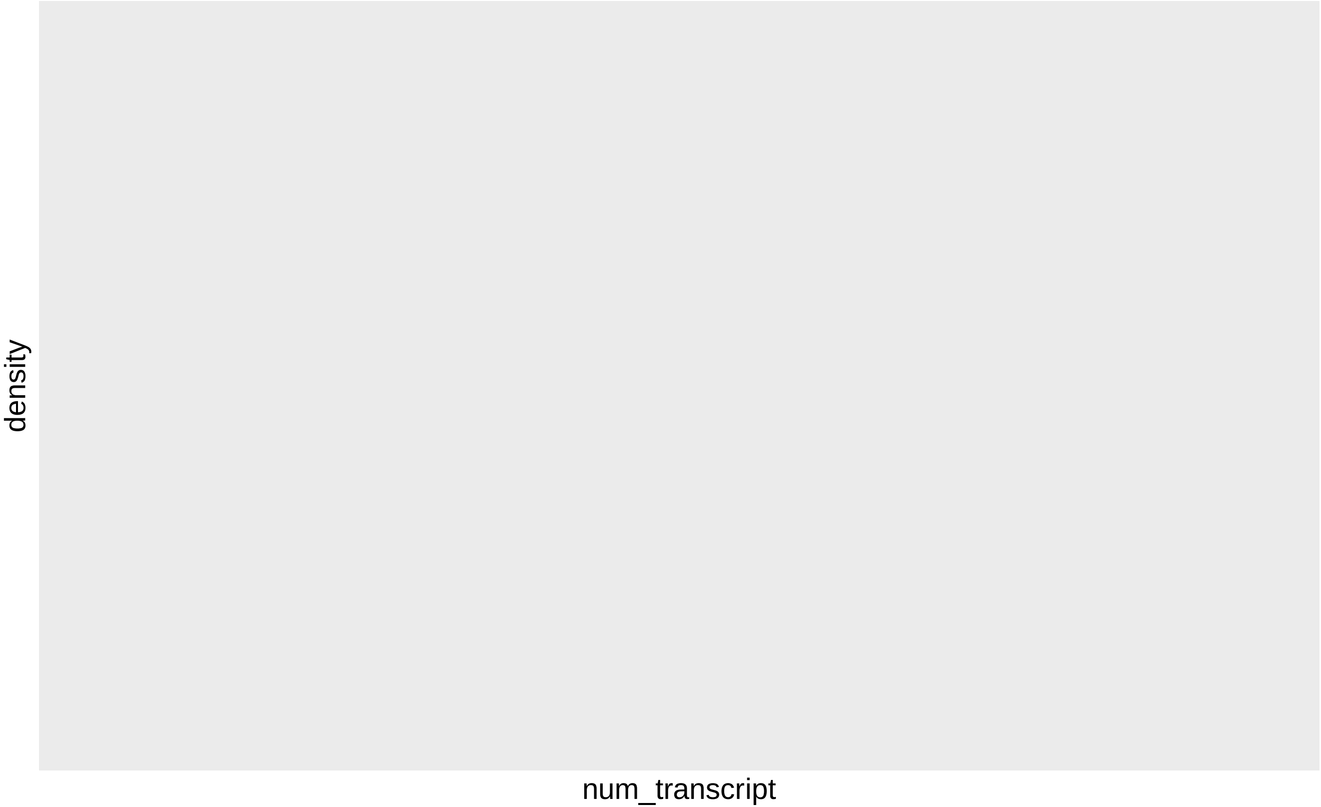

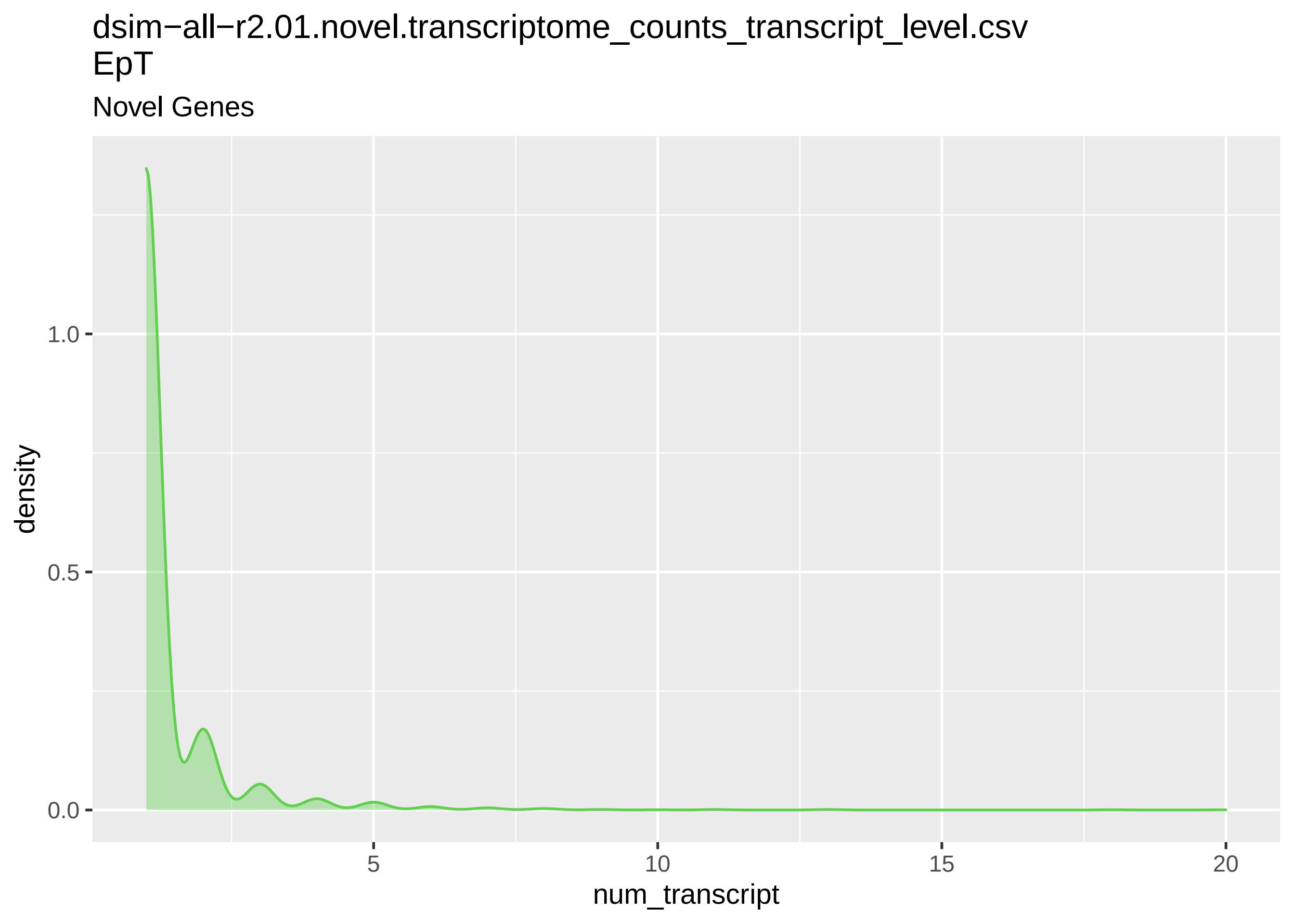
dsim−all−r2.01.novel.transcriptome_counts_transcript_level.csv
EpT
Novel Genes
density
1.0
0.5
0.0
5
10
15
20
num_transcript

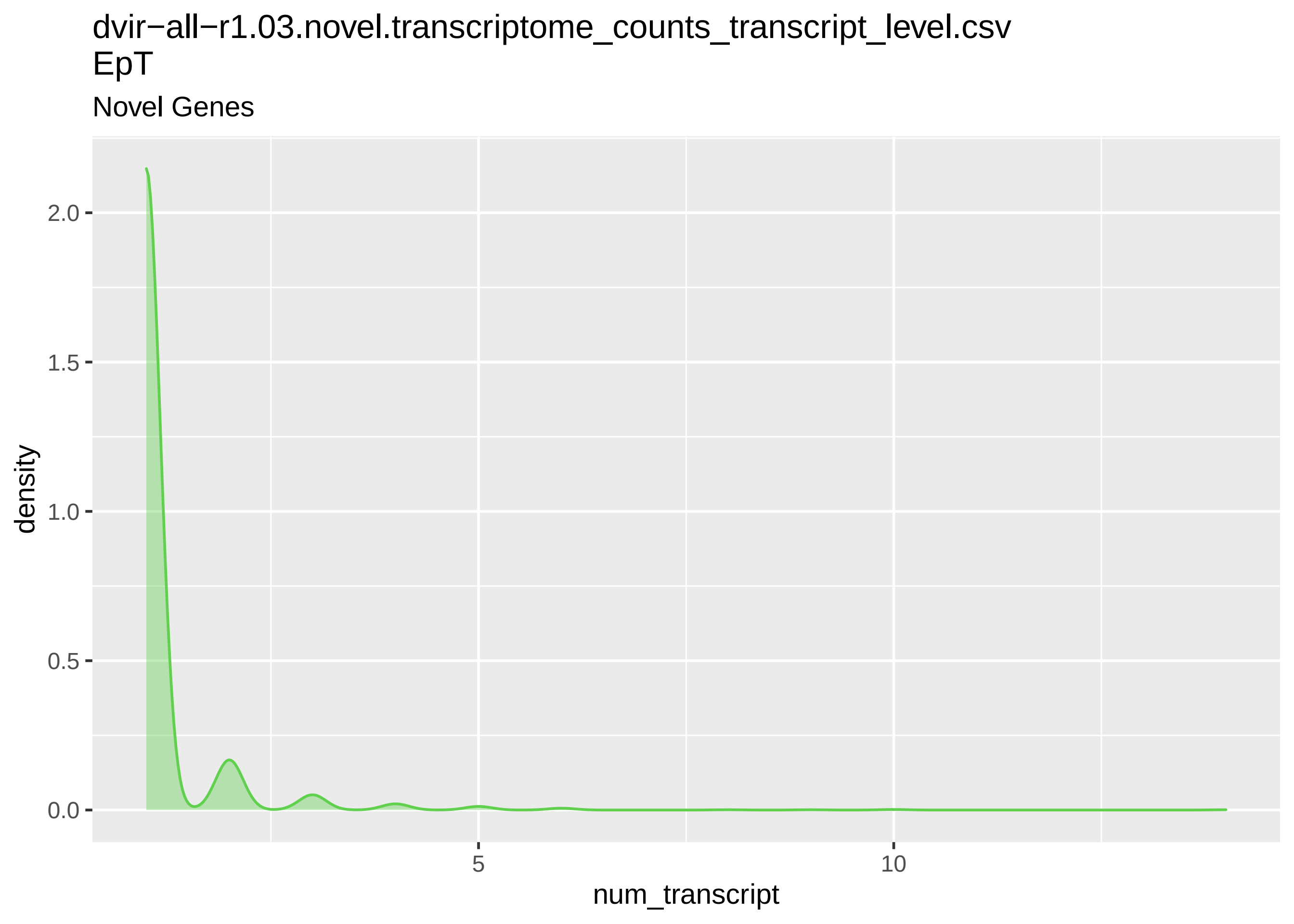
dvir−all−r1.03.novel.transcriptome_counts_transcript_level.csv
EpT
Novel Genes
density
2.0
1.5
1.0
0.5
0.0
5
10
num_transcript

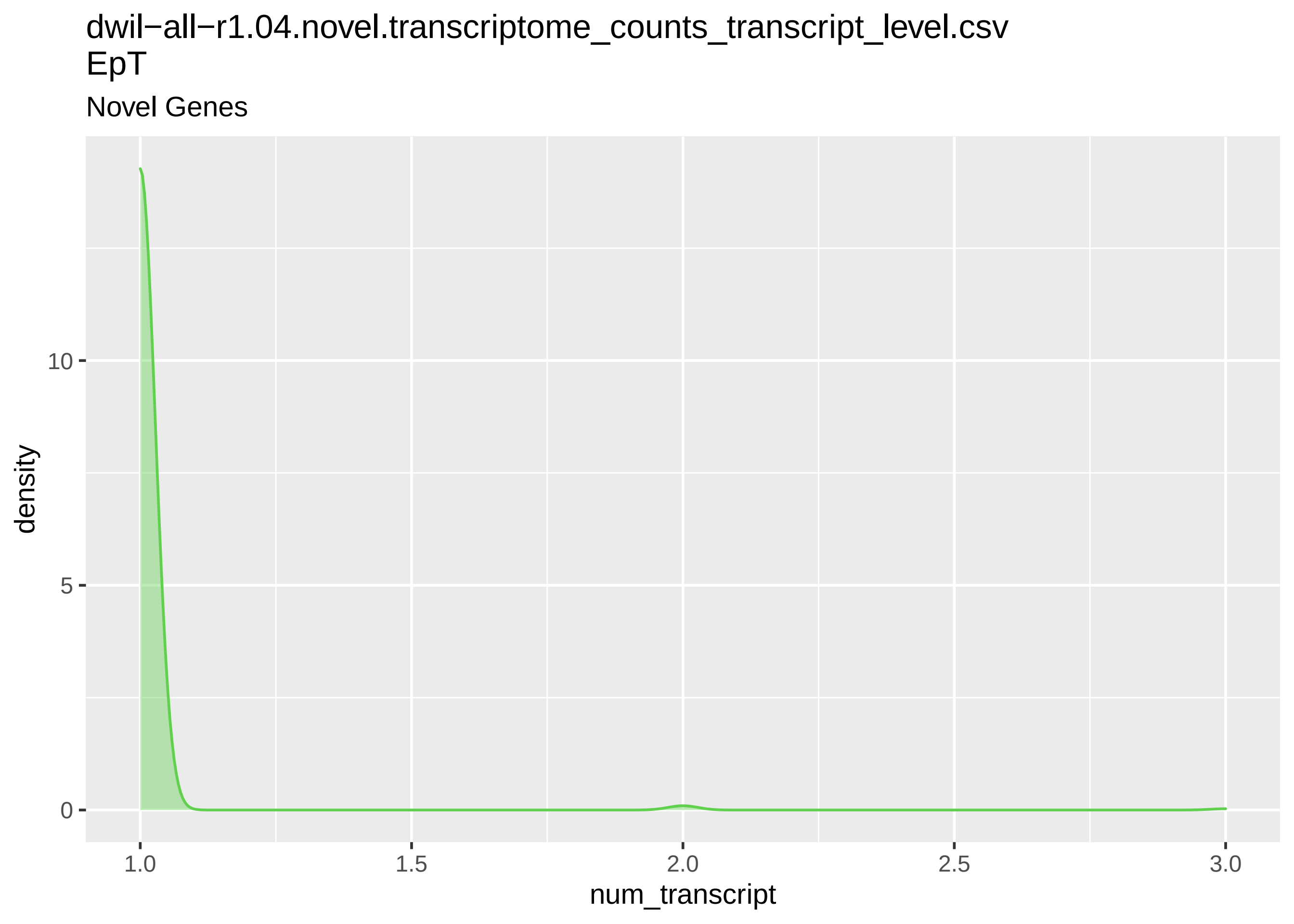
dwil−all−r1.04.novel.transcriptome_counts_transcript_level.csv
EpT
Novel Genes
density
10
5
0
1.0
1.5
2.0
2.5
3.0
num_transcript

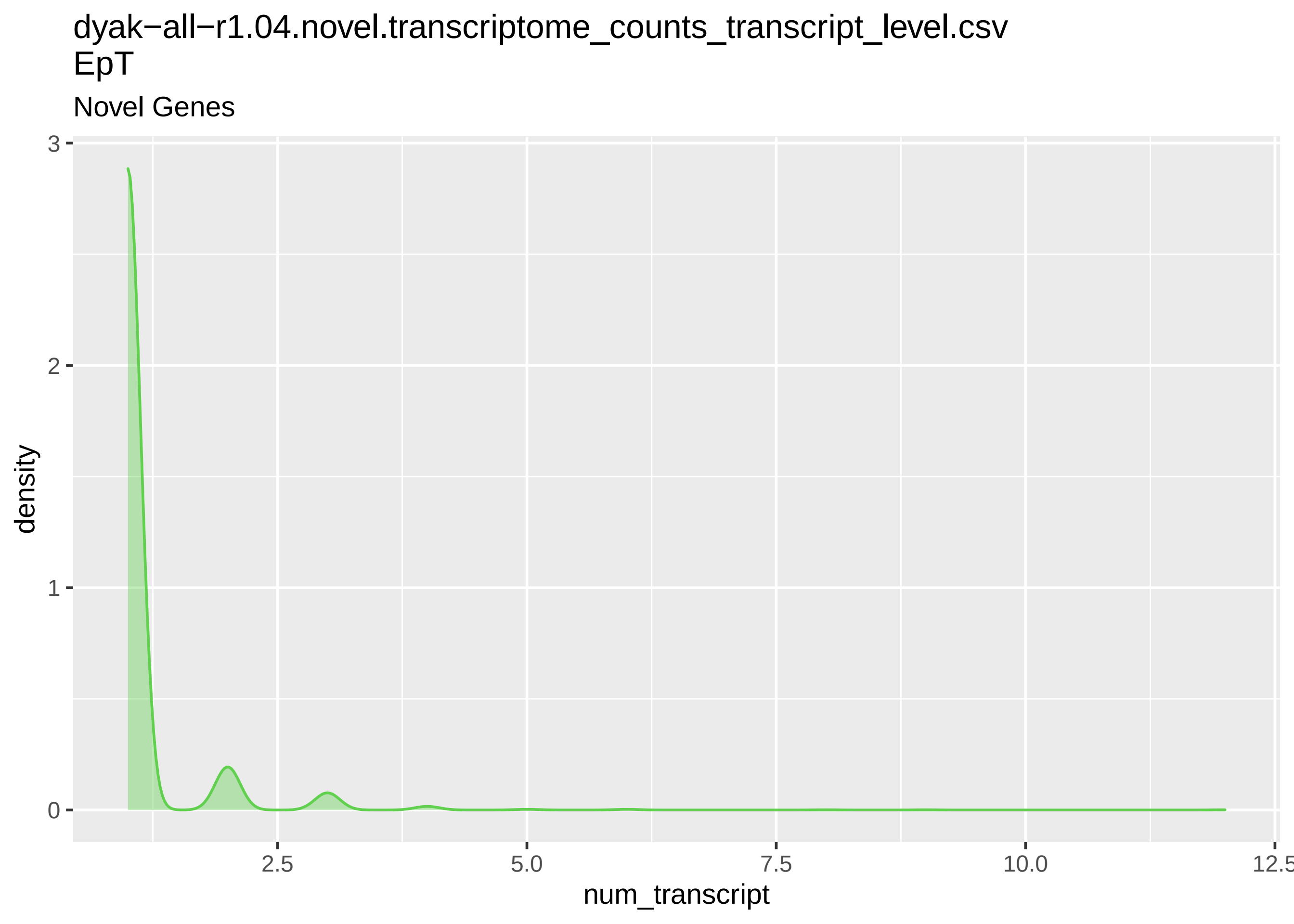
dyak−all−r1.04.novel.transcriptome_counts_transcript_level.csv
EpT
Novel Genes
density
3
2
1
0
2.5
5.0
7.5
10.0
12.5
num_transcript

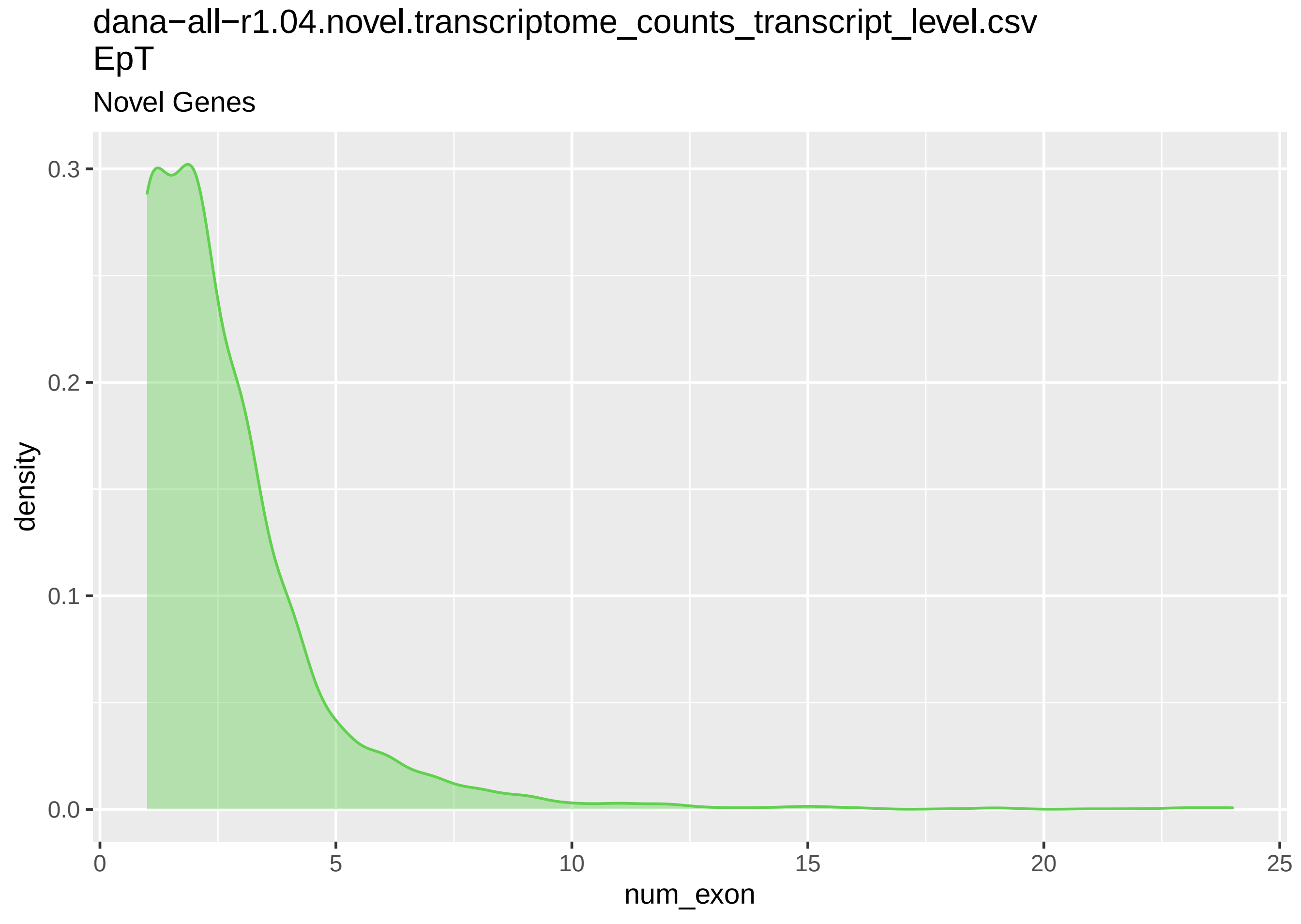
dana−all−r1.04.novel.transcriptome_counts_transcript_level.csv
EpT
Novel Genes
density
0.3
0.2
0.1
0.0
0
5
10
15
20
25
num_exon

dere−all−r1.04.novel.transcriptome_counts_transcript_level.csv
EpT

Novel Genes

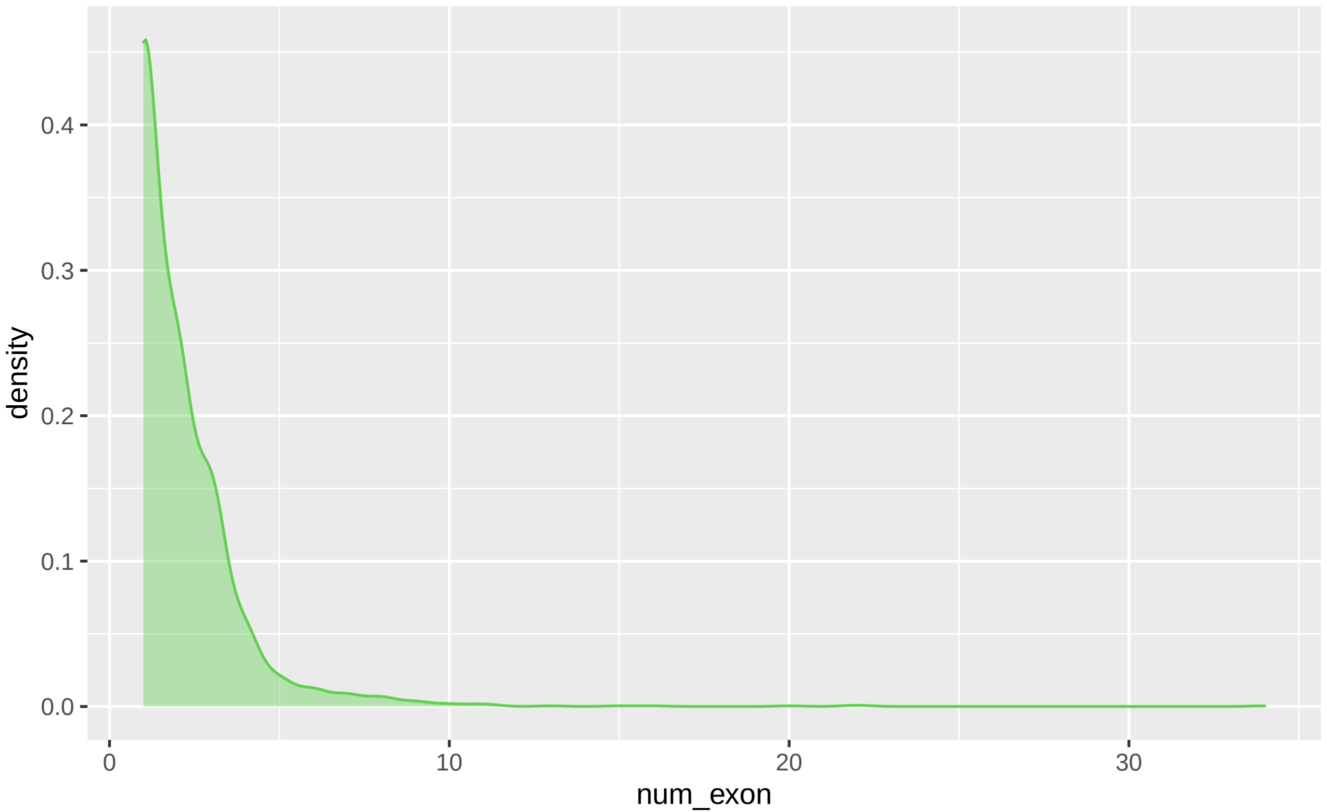

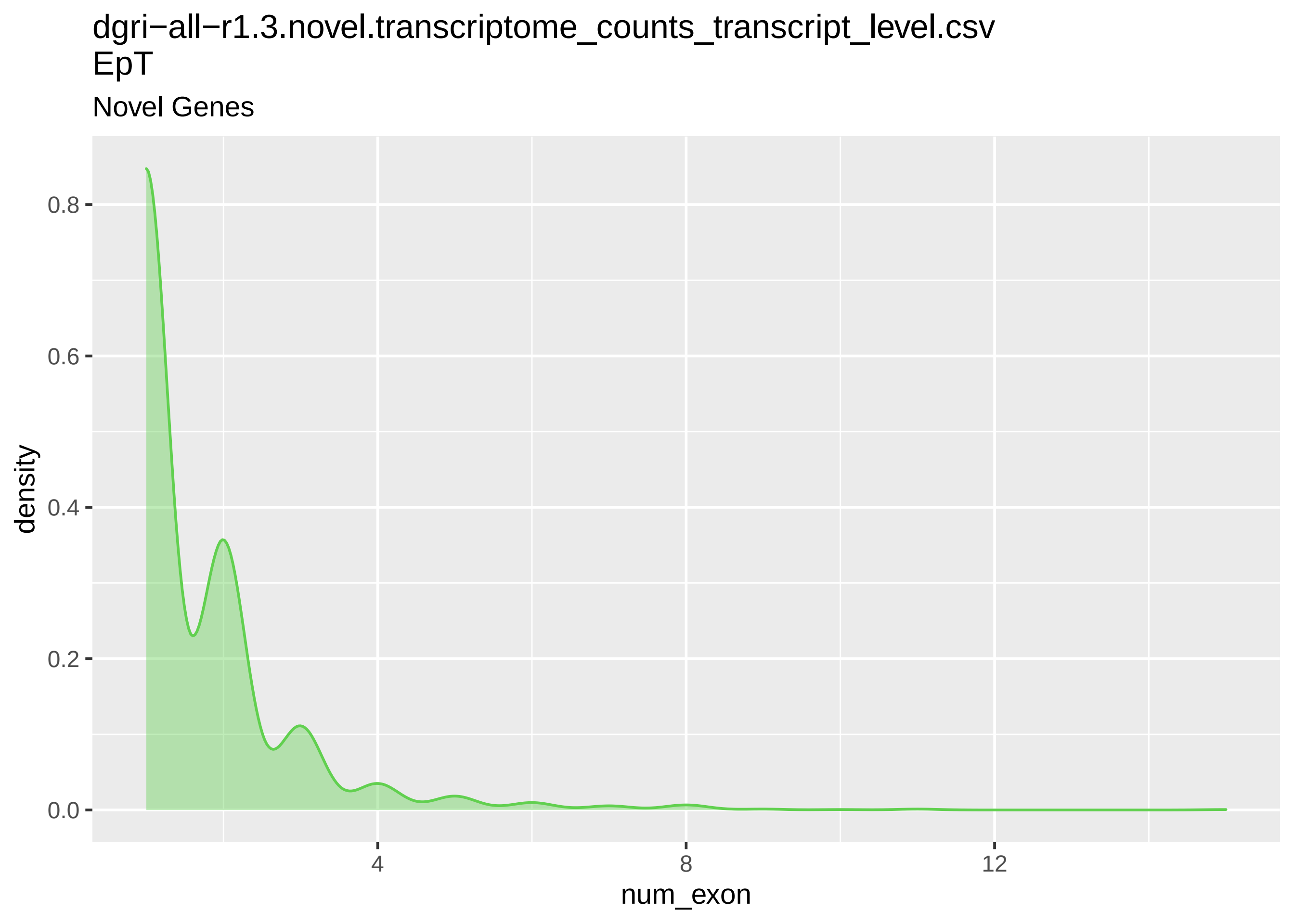
dgri−all−r1.3.novel.transcriptome_counts_transcript_level.csv
EpT
Novel Genes
density
0.0
0.2
0.4
0.6
0.8
4
8
12
num_exon

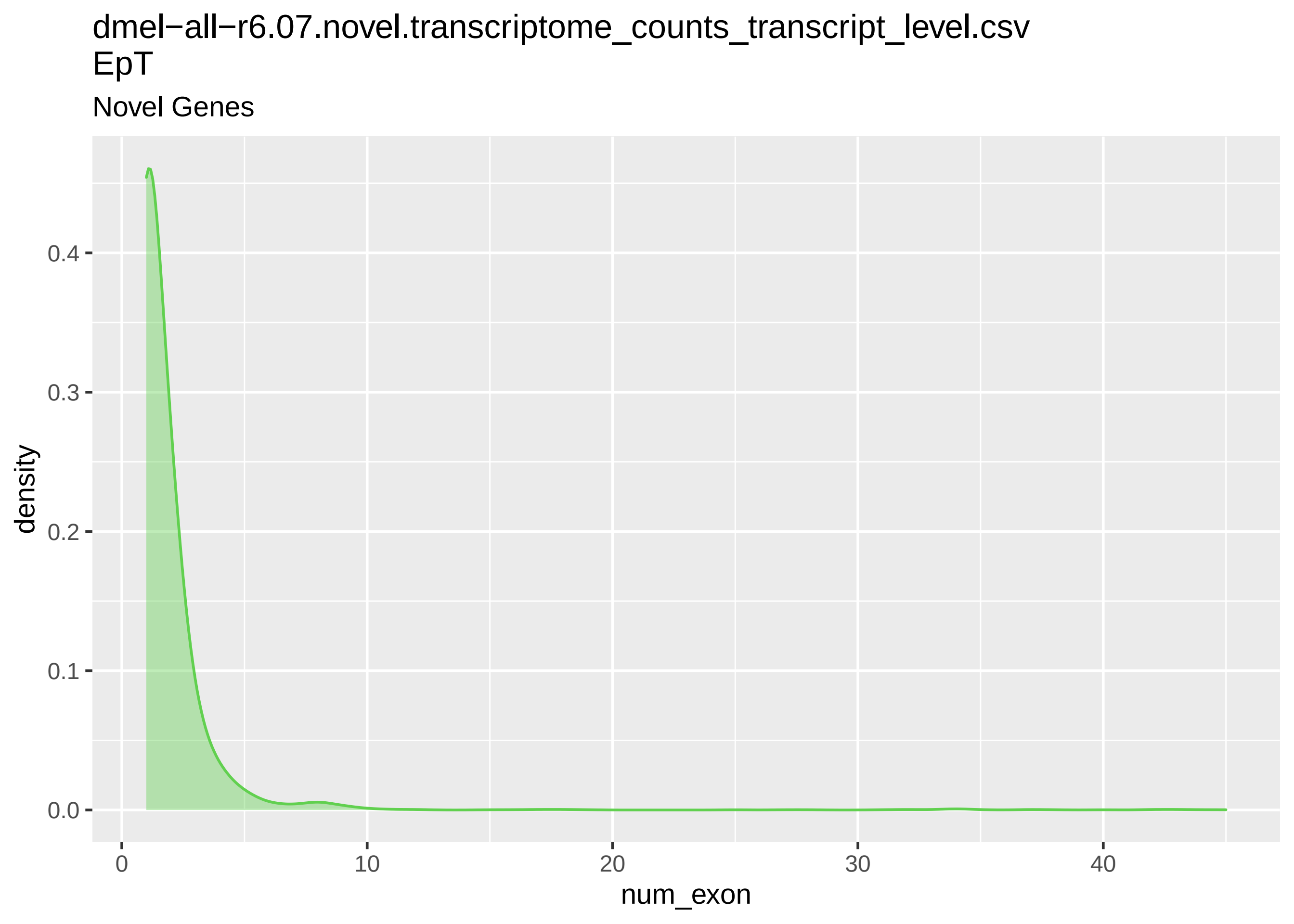
dmel−all−r6.07.novel.transcriptome_counts_transcript_level.csv
EpT
Novel Genes
density
0.4
0.3
0.2
0.1
0.0
0
10
20
30
40
num_exon

dmoj−all−r1.04.novel.transcriptome_counts_transcript_level.csv
EpT

Novel Genes

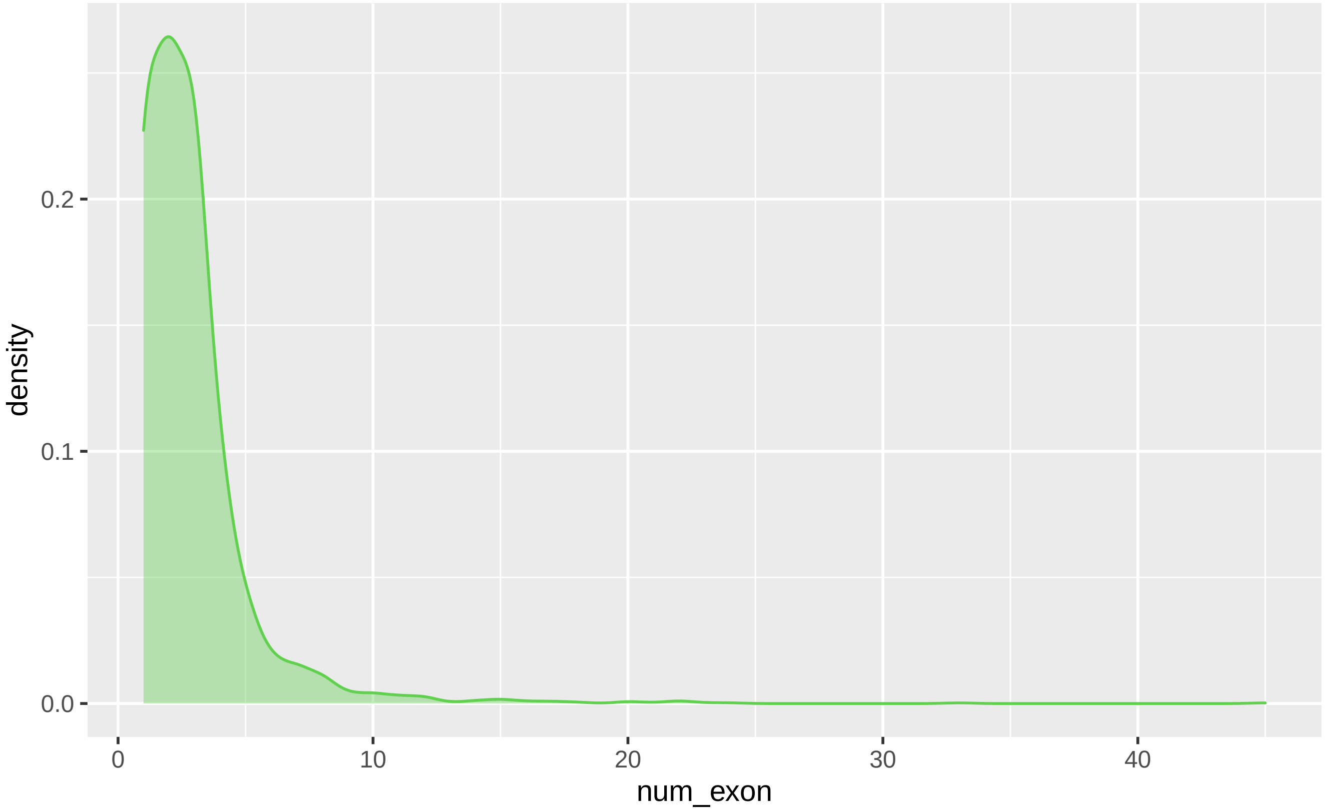

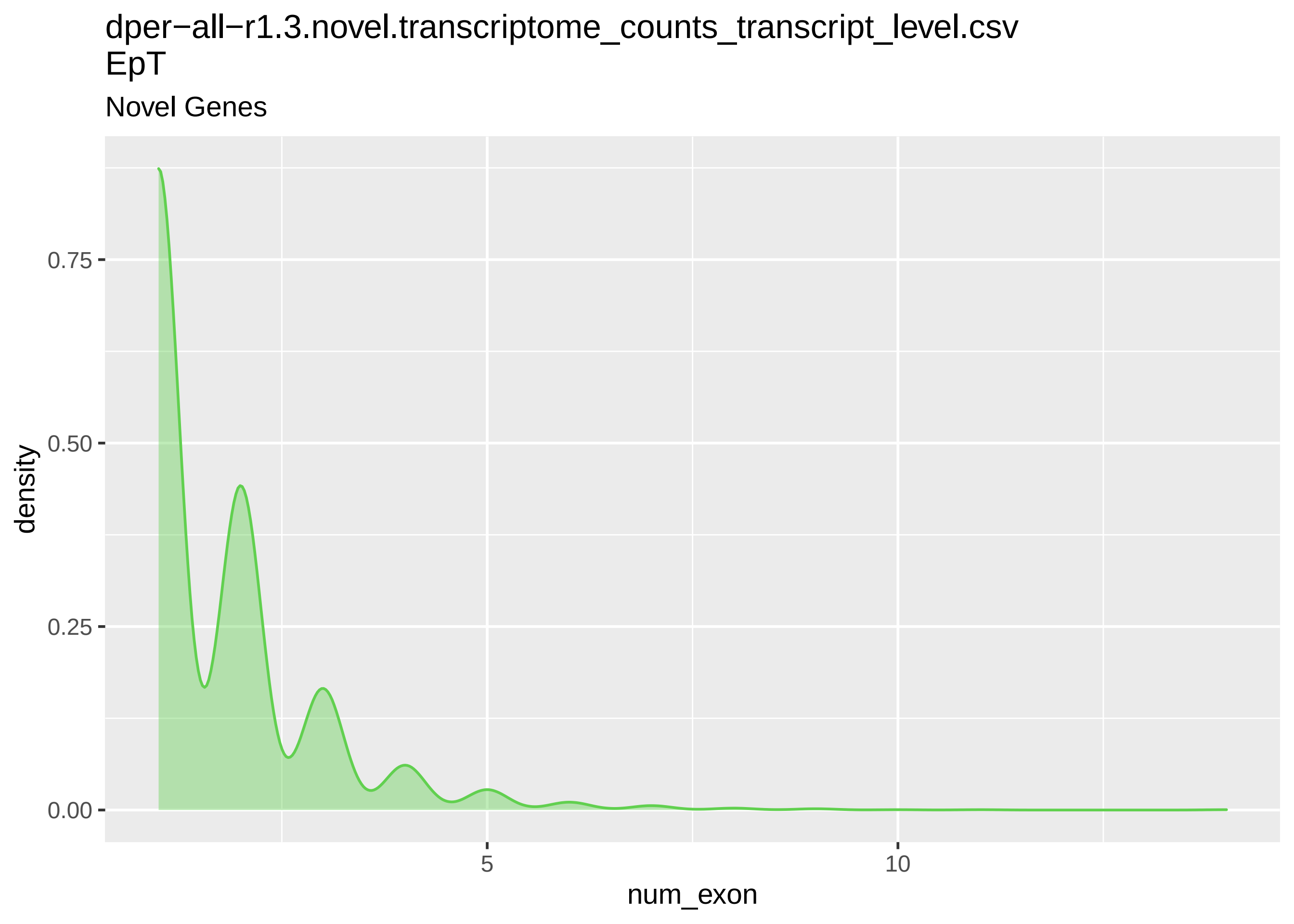
dper−all−r1.3.novel.transcriptome_counts_transcript_level.csv
EpT
Novel Genes
density
0.75
0.50
0.25
0.00
5
10
num_exon

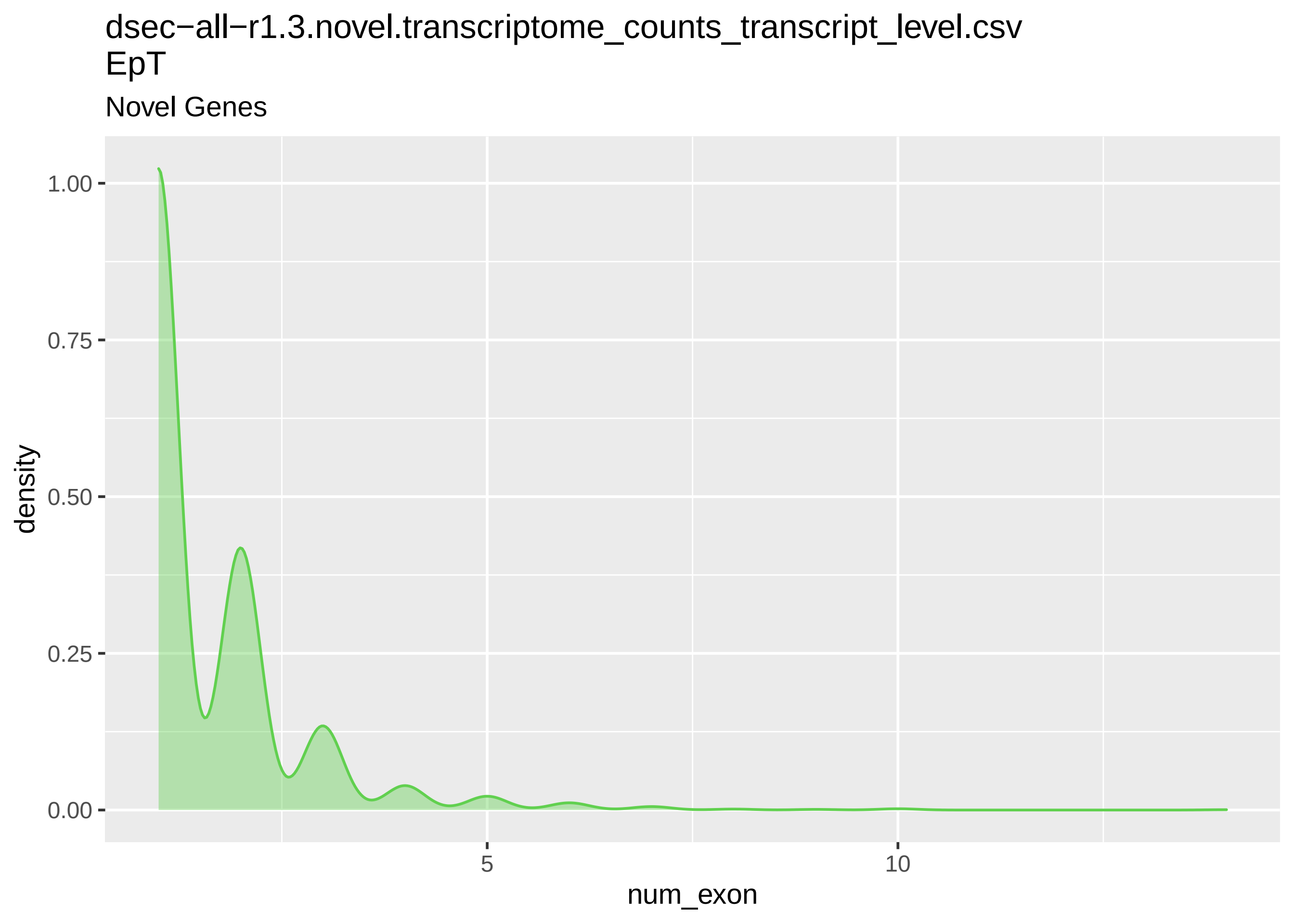
dsec−all−r1.3.novel.transcriptome_counts_transcript_level.csv
EpT
Novel Genes
density
1.00
0.75
0.50
0.25
0.00
5
10
num_exon

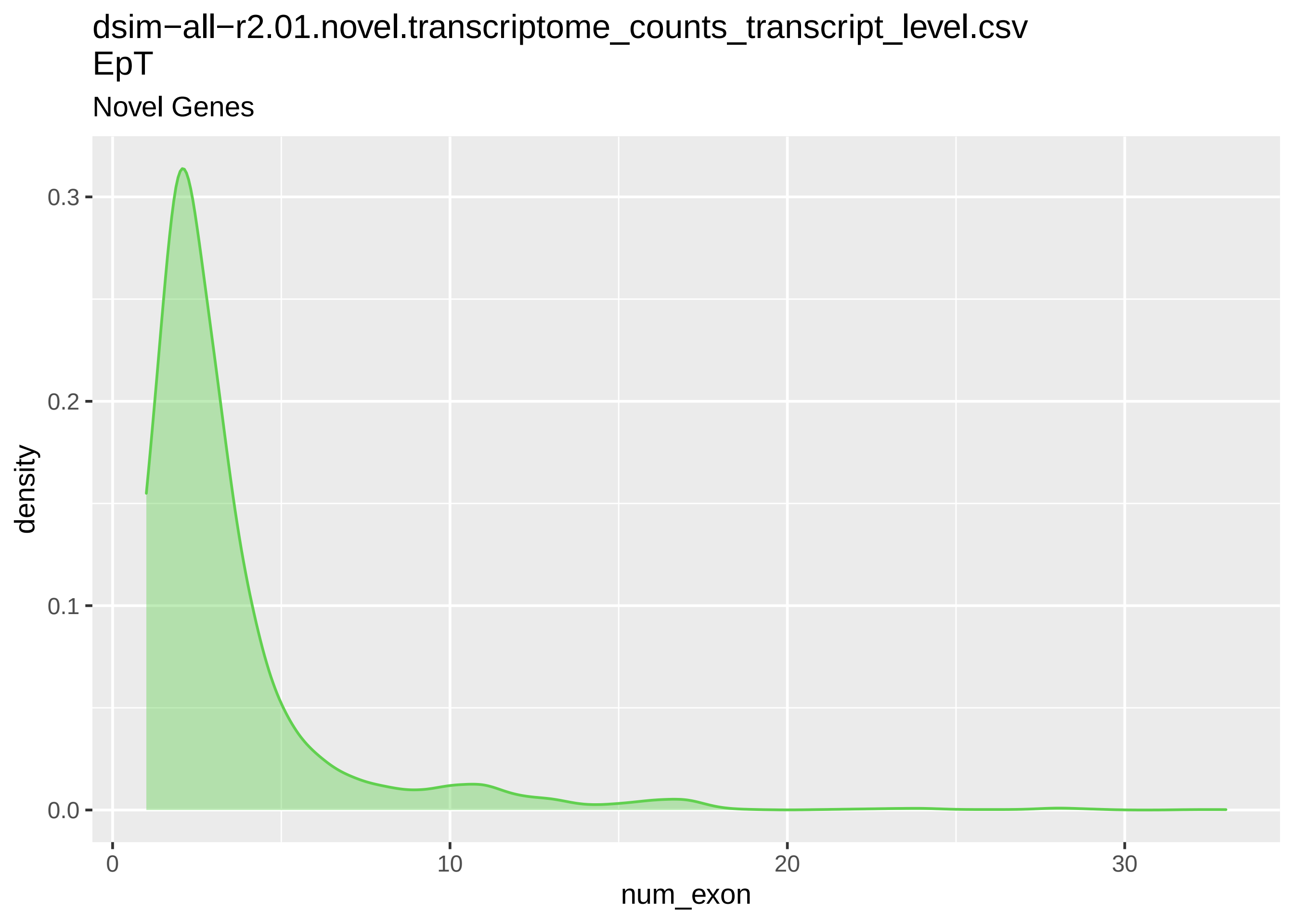
dsim−all−r2.01.novel.transcriptome_counts_transcript_level.csv
EpT
Novel Genes
0.3
0.2
0.1
0.0
density
0
10
20
30
num_exon

dvir−all−r1.03.novel.transcriptome_counts_transcript_level.csv
EpT

Novel Genes

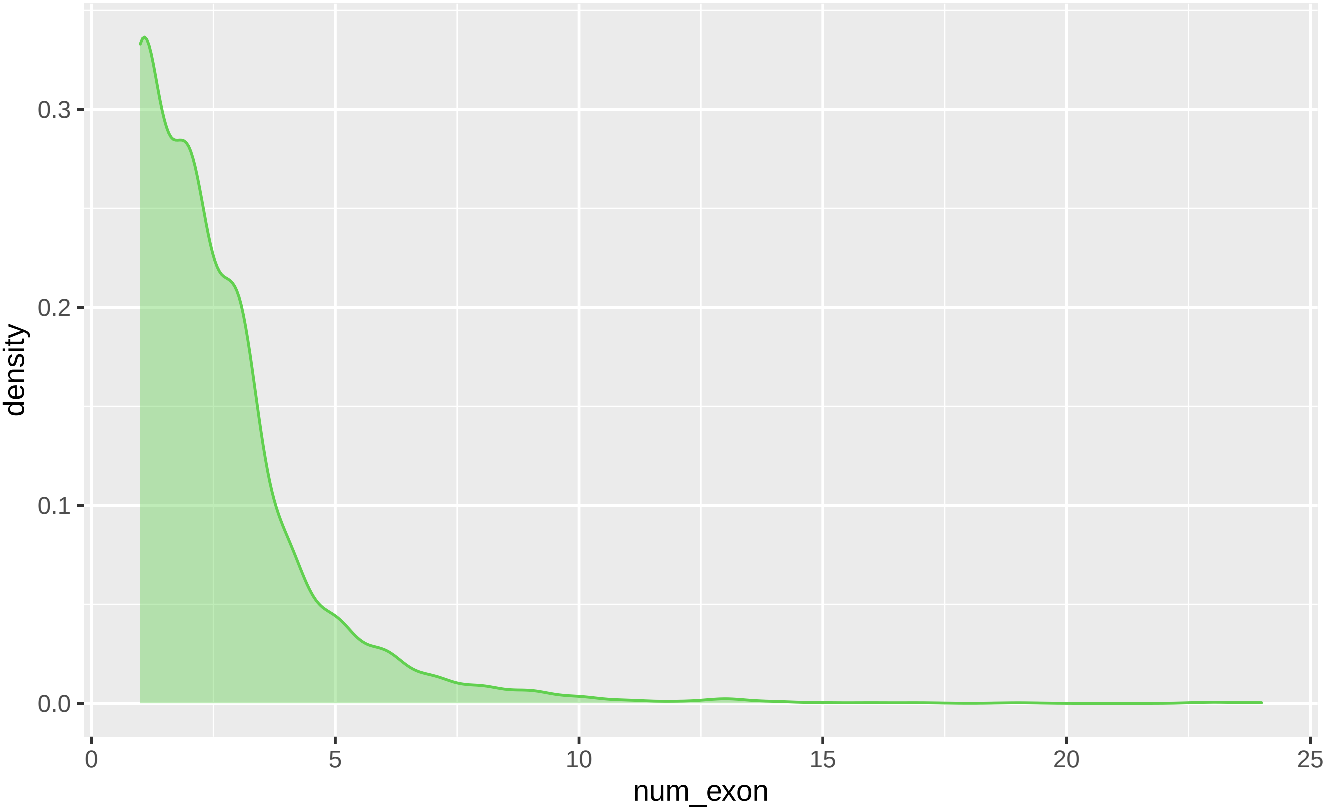

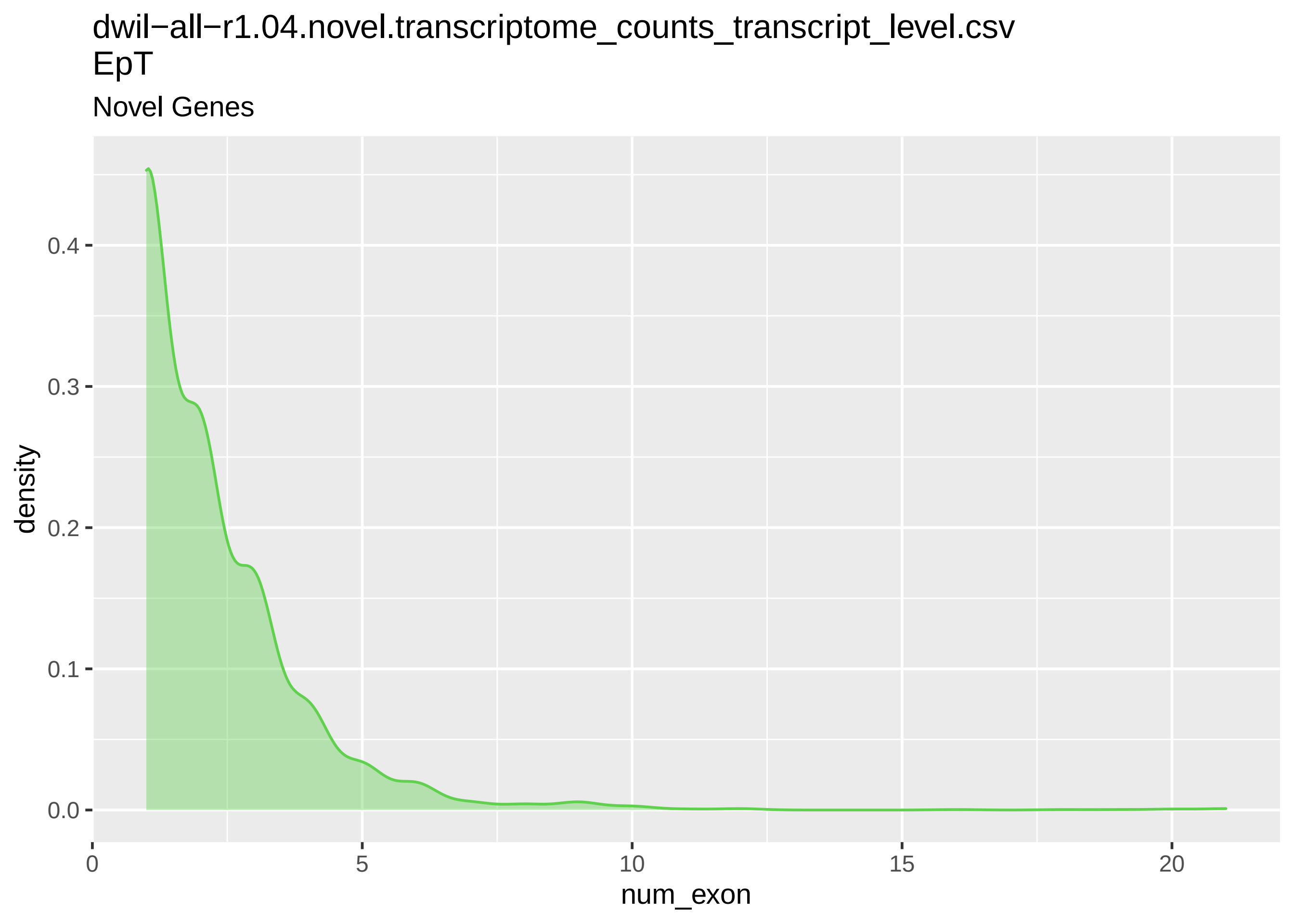
dwil−all−r1.04.novel.transcriptome_counts_transcript_level.csv
EpT
Novel Genes
density
0.4
0.3
0.2
0.1
0.0
0
5
10
15
20
num_exon

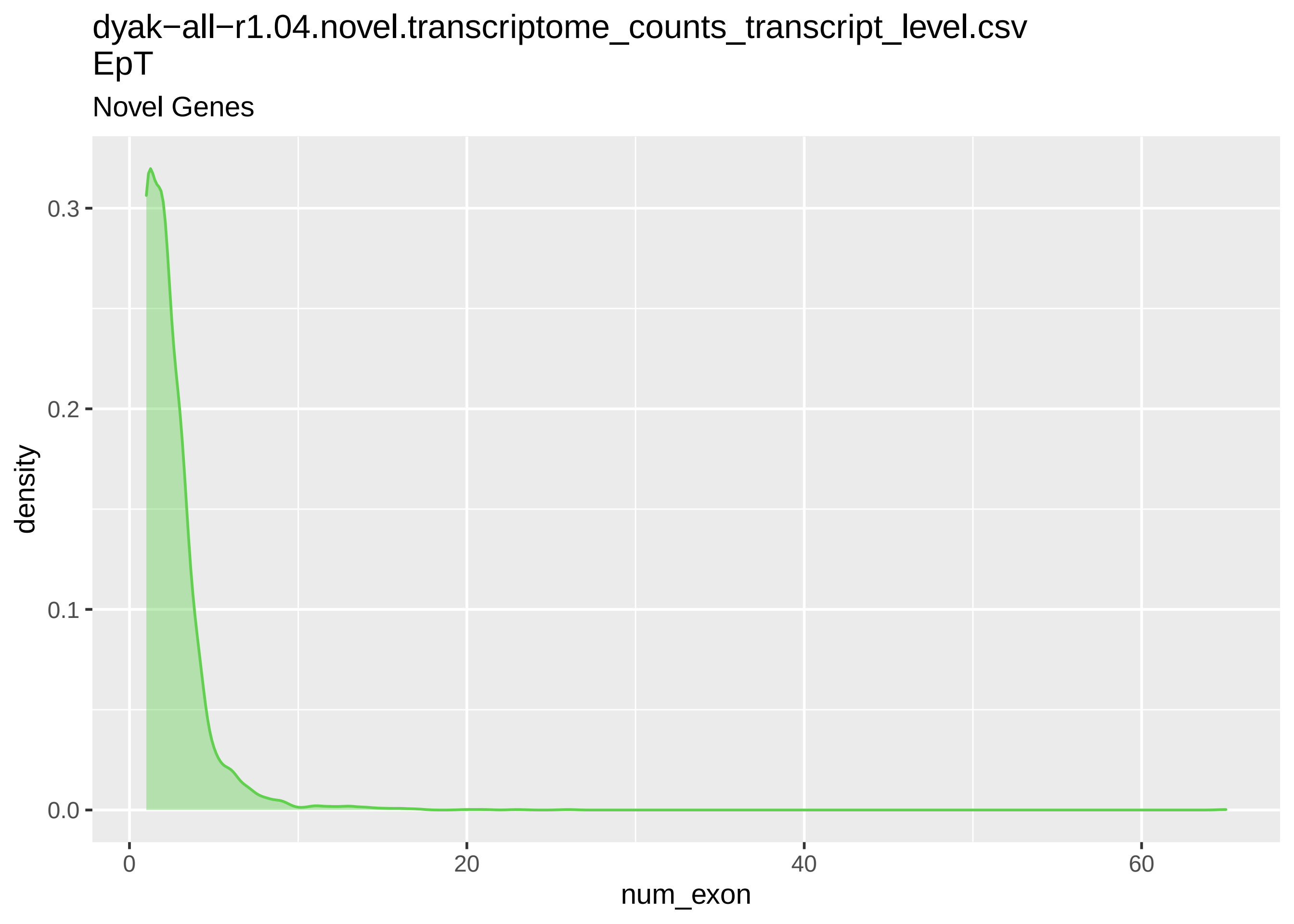

dyak−all−r1.04.novel.transcriptome_counts_transcript_level.csv
EpT
Novel Genes
density
0.3
0.2
0.1
0.0
0
20
40
60
num_exon

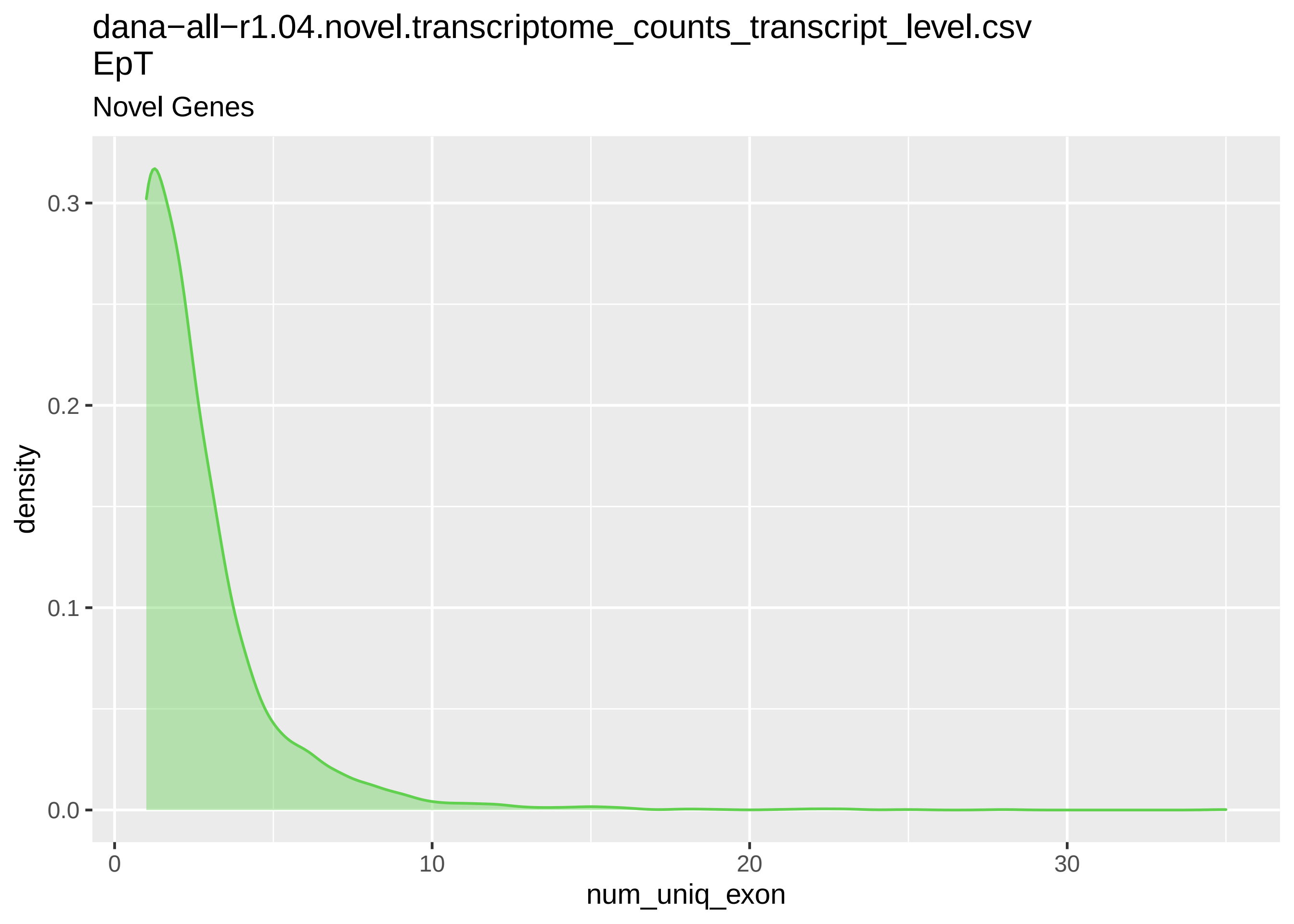
dana−all−r1.04.novel.transcriptome_counts_transcript_level.csv
EpT
Novel Genes
density
0.3
0.2
0.1
0.0
0
10
20
30
num_uniq_exon

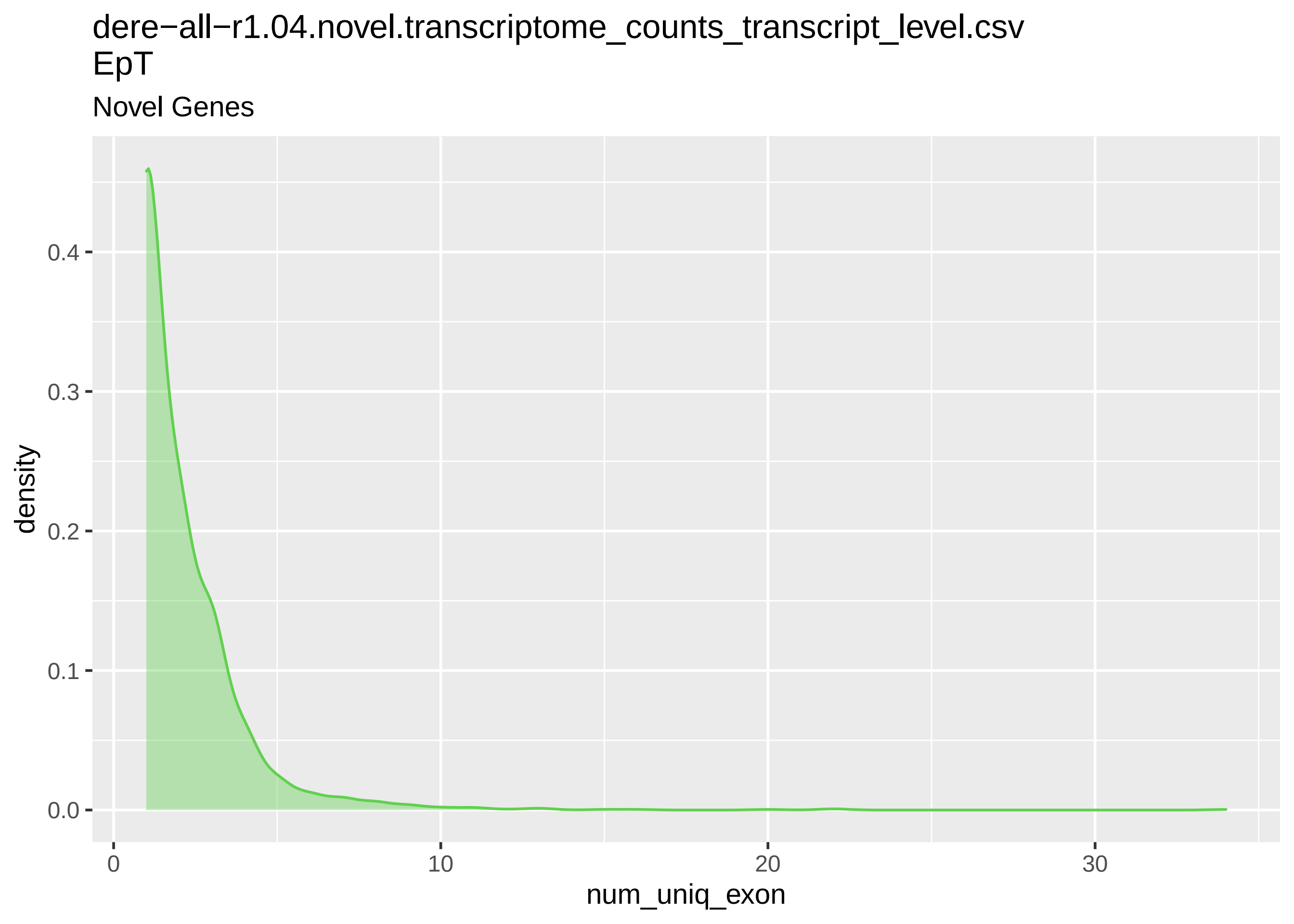
dere−all−r1.04.novel.transcriptome_counts_transcript_level.csv
EpT
Novel Genes
density
0.4
0.3
0.2
0.1
0.0
0
10
20
30
num_uniq_exon

dgri−all−r1.3.novel.transcriptome_counts_transcript_level.csv
EpT

Novel Genes

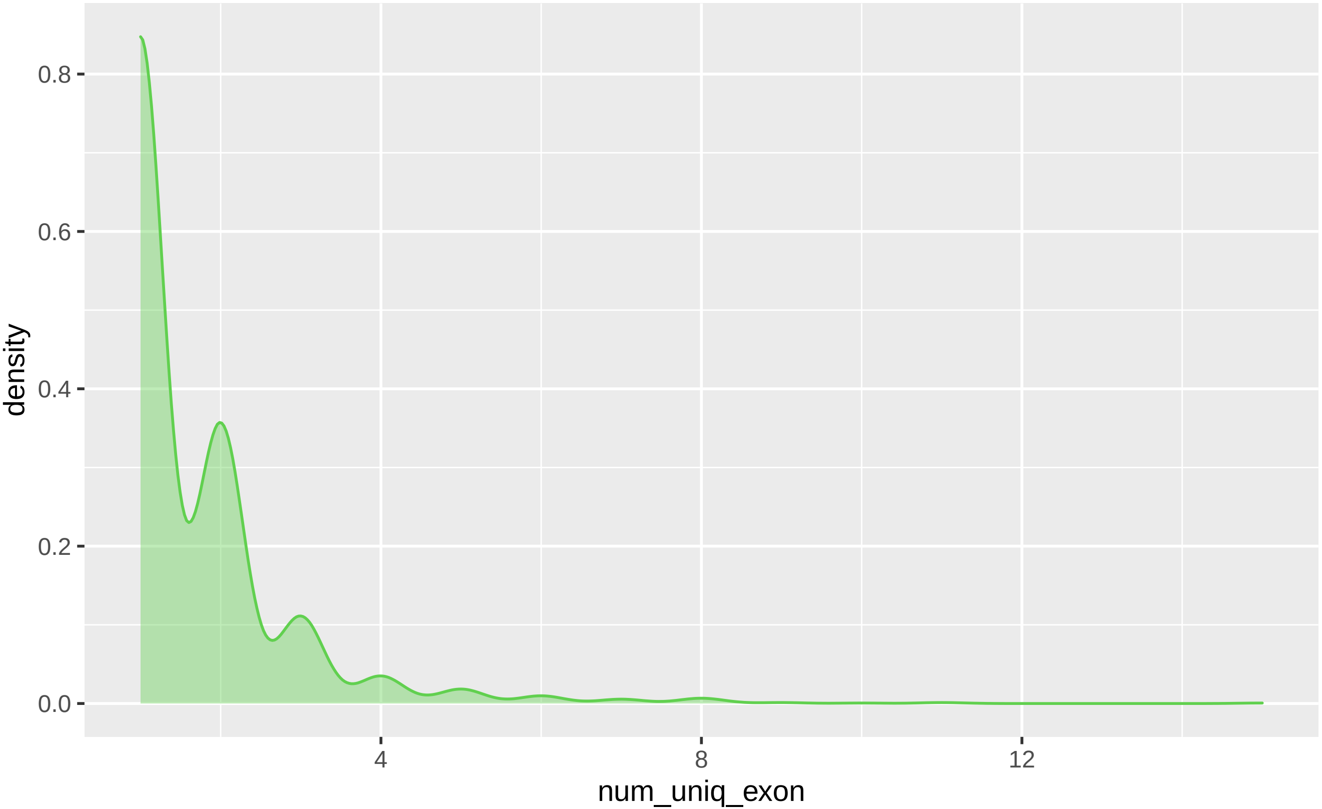

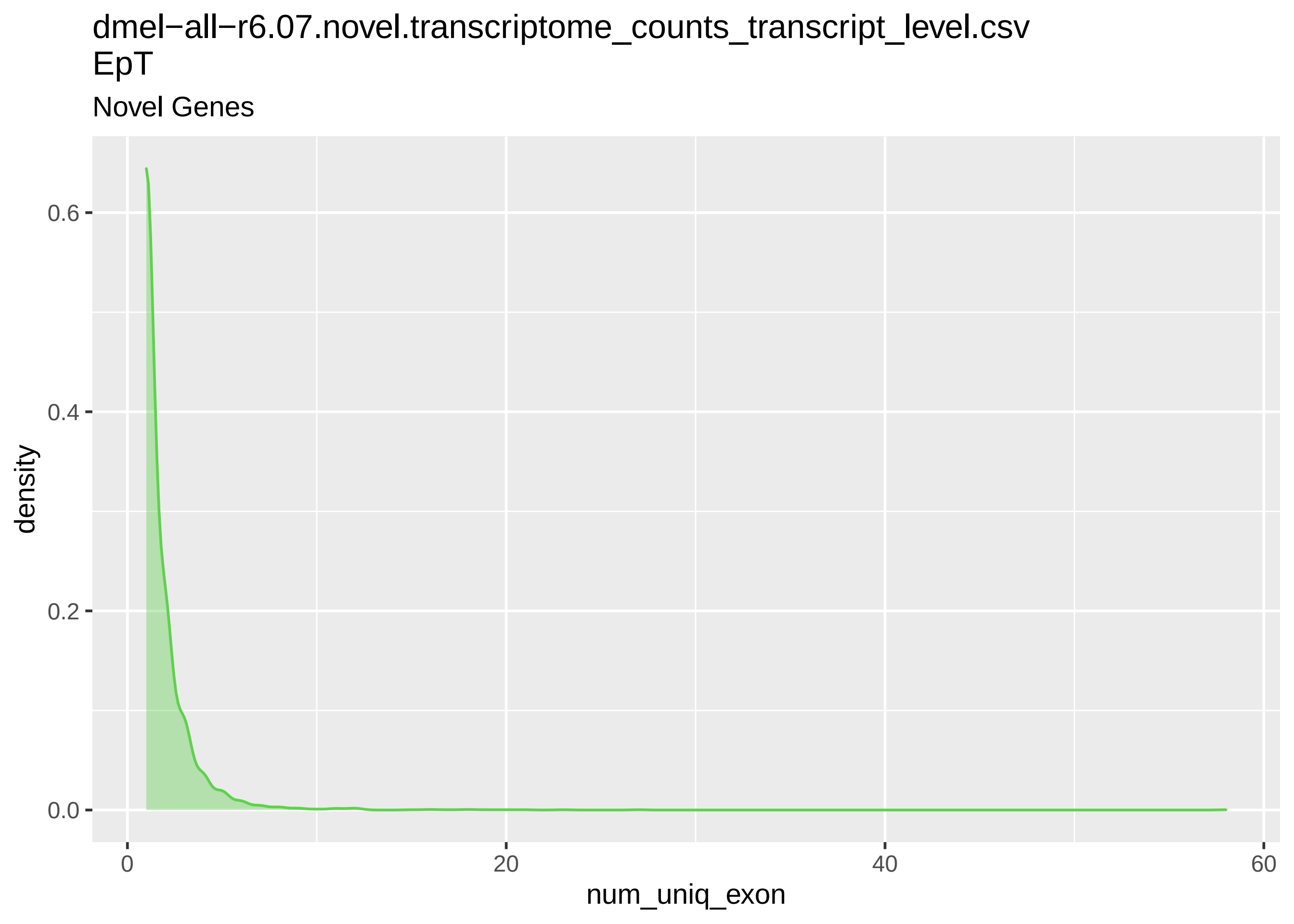
dmel−all−r6.07.novel.transcriptome_counts_transcript_level.csv
EpT
Novel Genes
density
0.6
0.4
0.2
0.0
0
20
40
60
num_uniq_exon

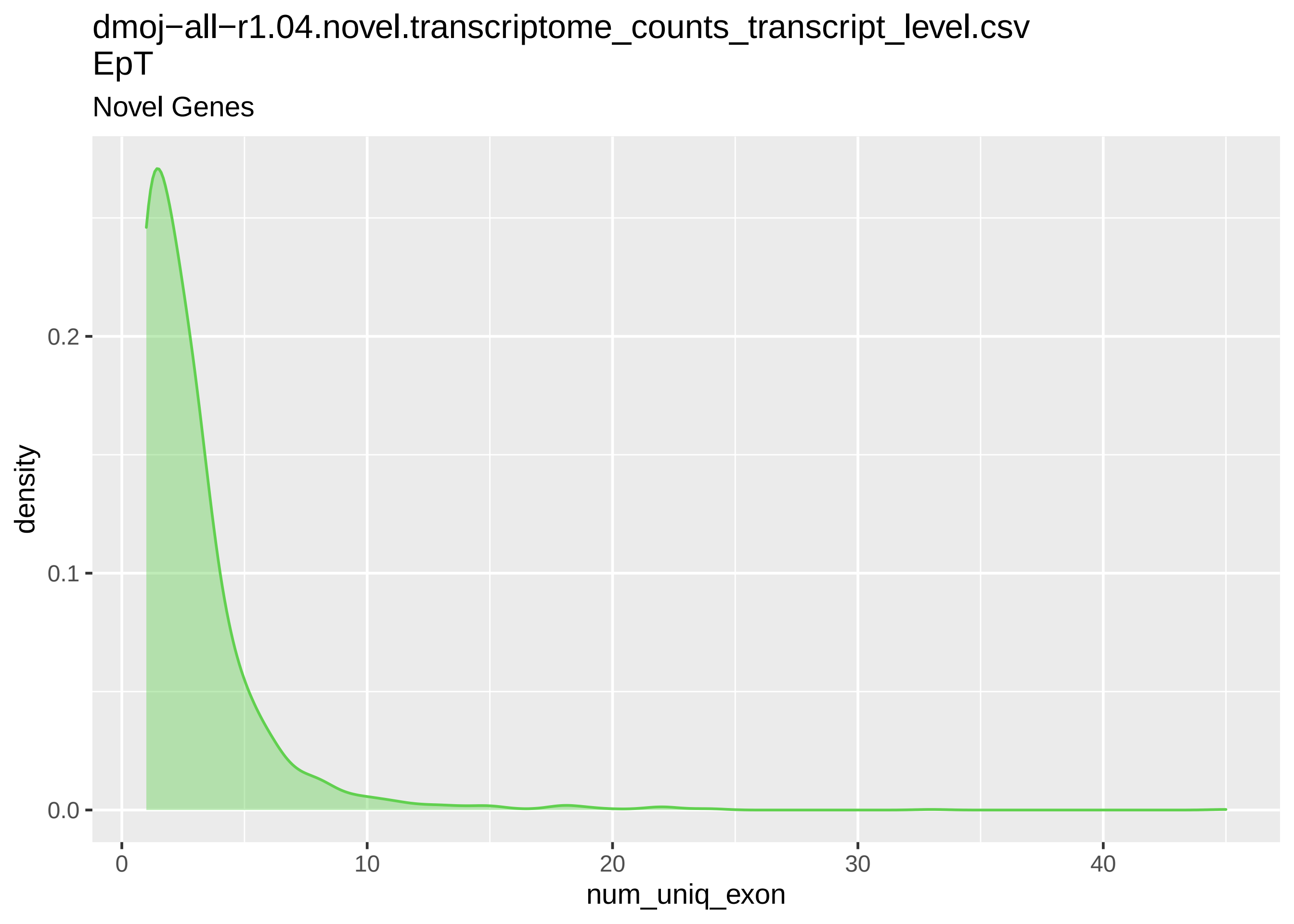
dmoj−all−r1.04.novel.transcriptome_counts_transcript_level.csv
EpT
Novel Genes
density
0.2
0.1
0.0
0
10
20
30
40
num_uniq_exon

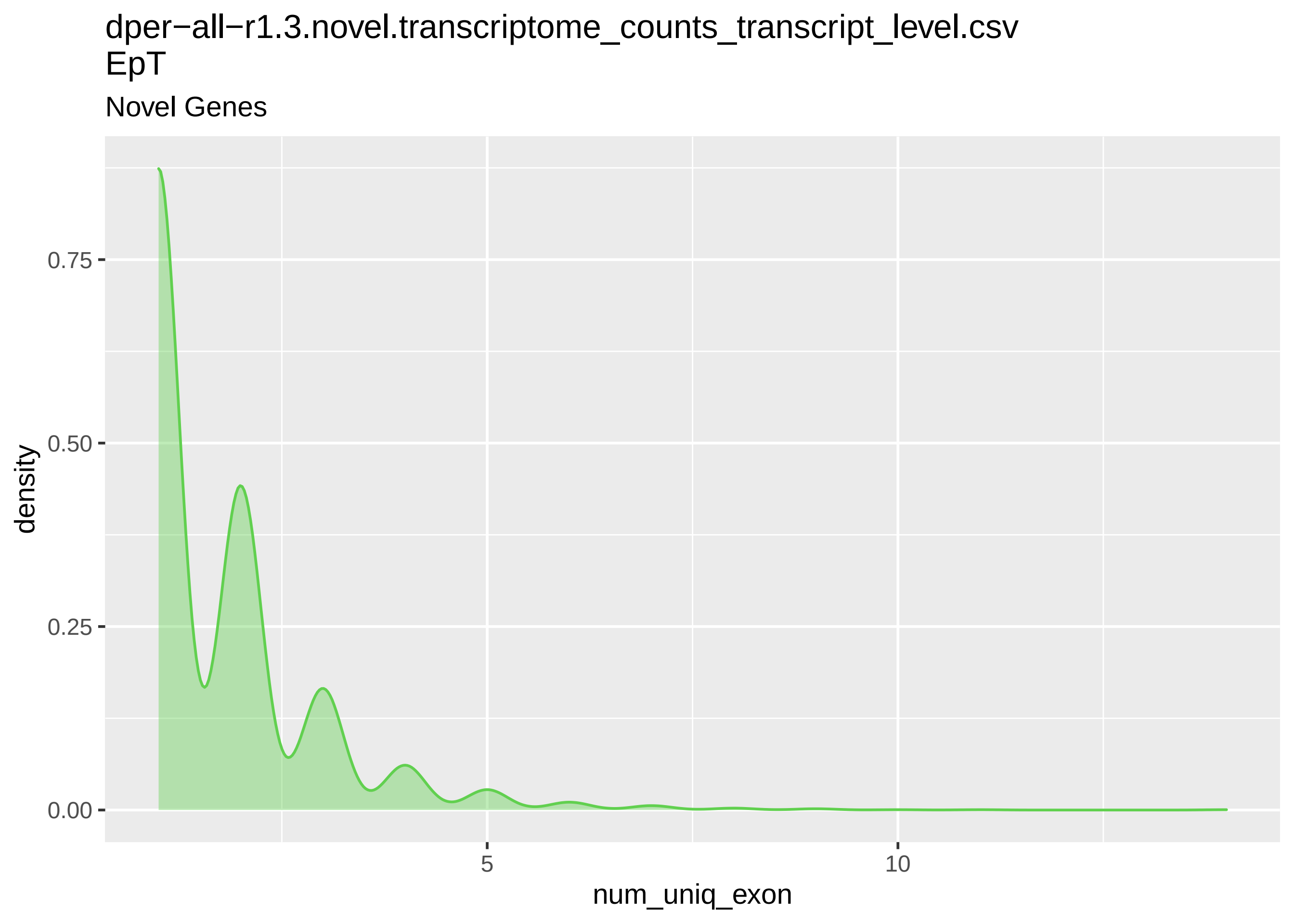
dper−all−r1.3.novel.transcriptome_counts_transcript_level.csv
EpT
Novel Genes
density
0.75
0.50
0.25
0.00
5
10
num_uniq_exon

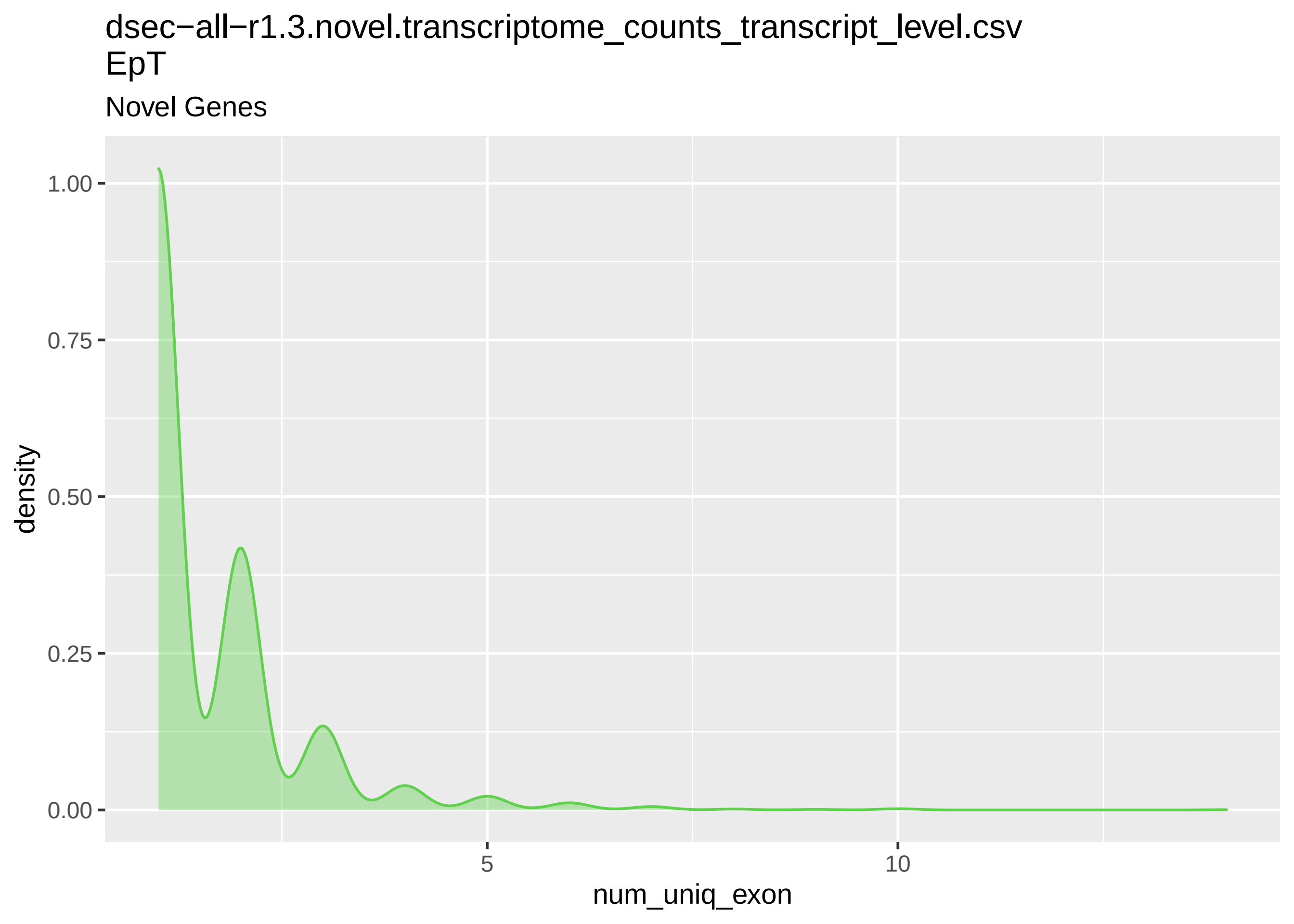

dsec−all−r1.3.novel.transcriptome_counts_transcript_level.csv
EpT
Novel Genes
density
1.00
0.75
0.50
0.25
0.00
5
10
num_uniq_exon

dsim−all−r2.01.novel.transcriptome_counts_transcript_level.csv
EpT

Novel Genes

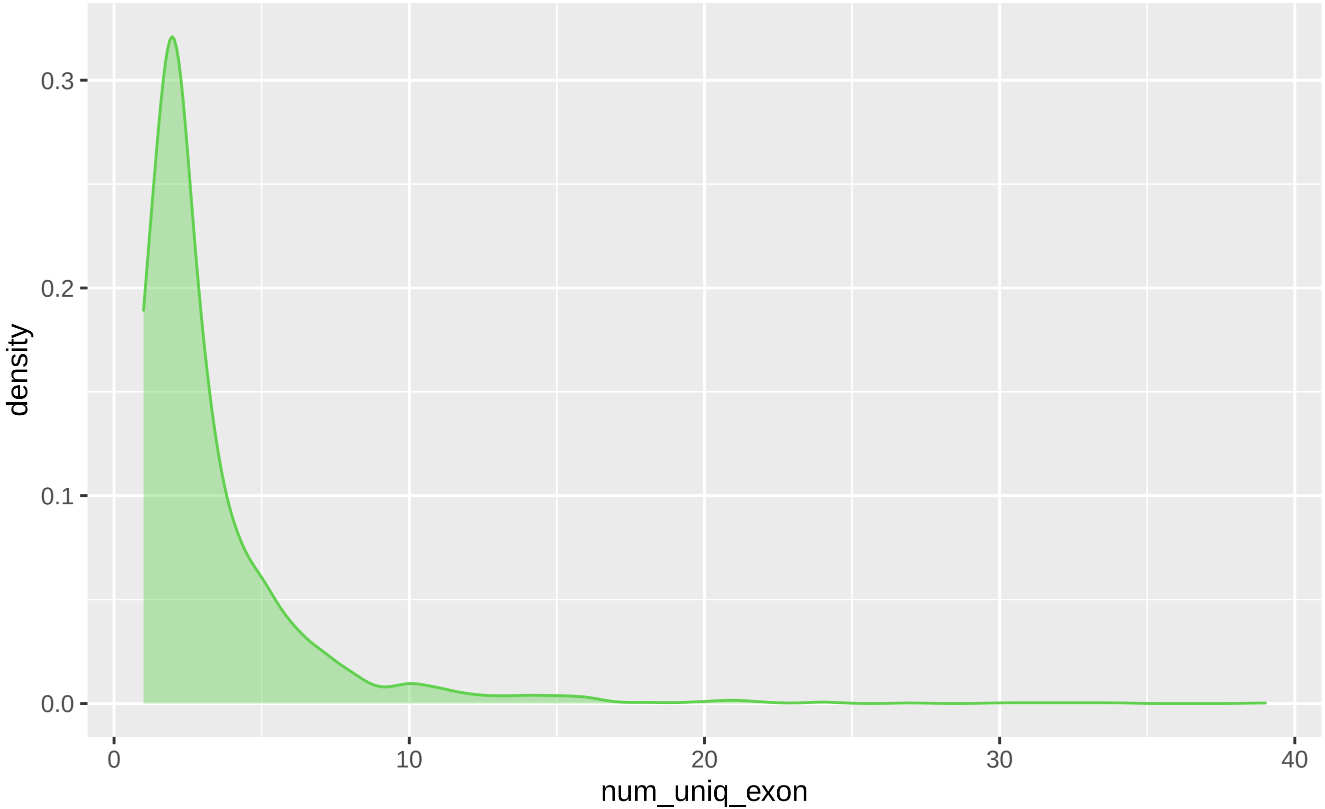

dvir−all−r1.03.novel.transcriptome_counts_transcript_level.csv
EpT

Novel Genes

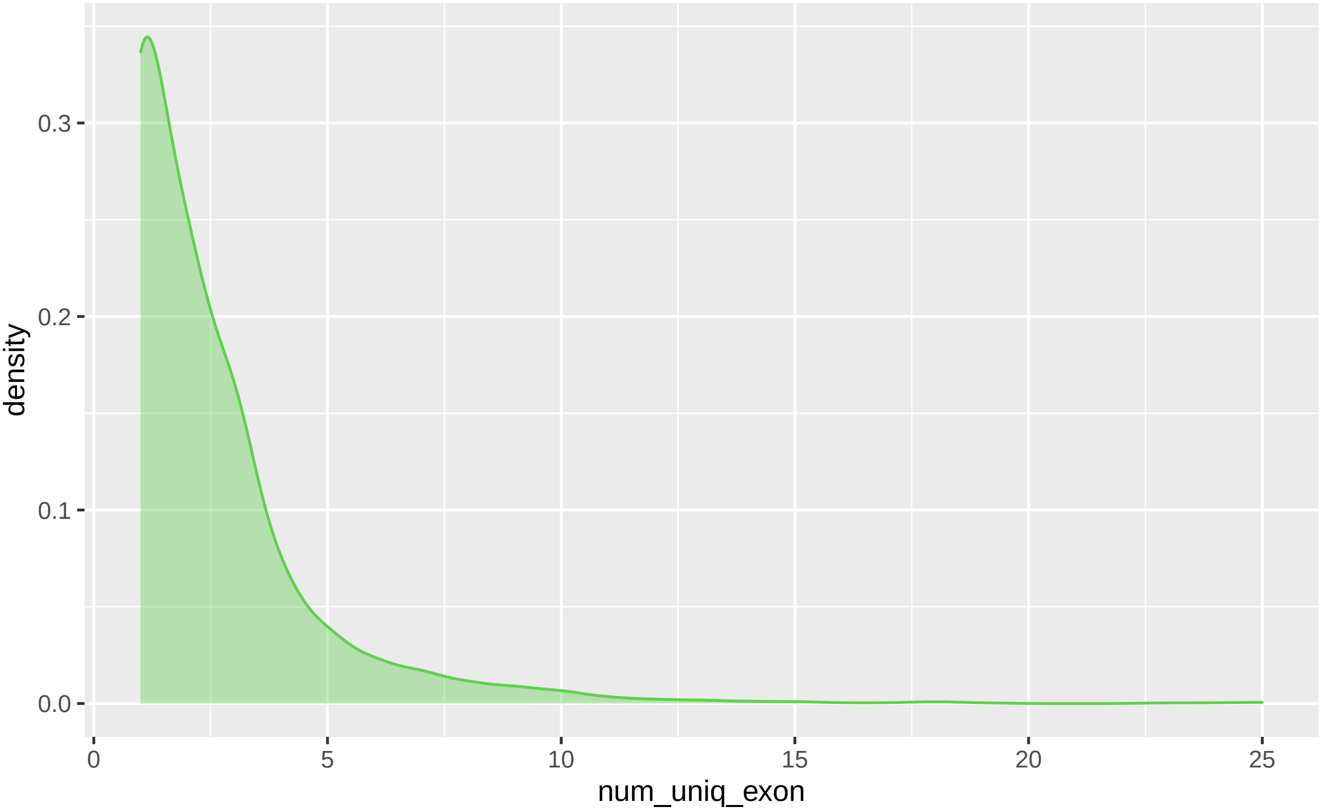

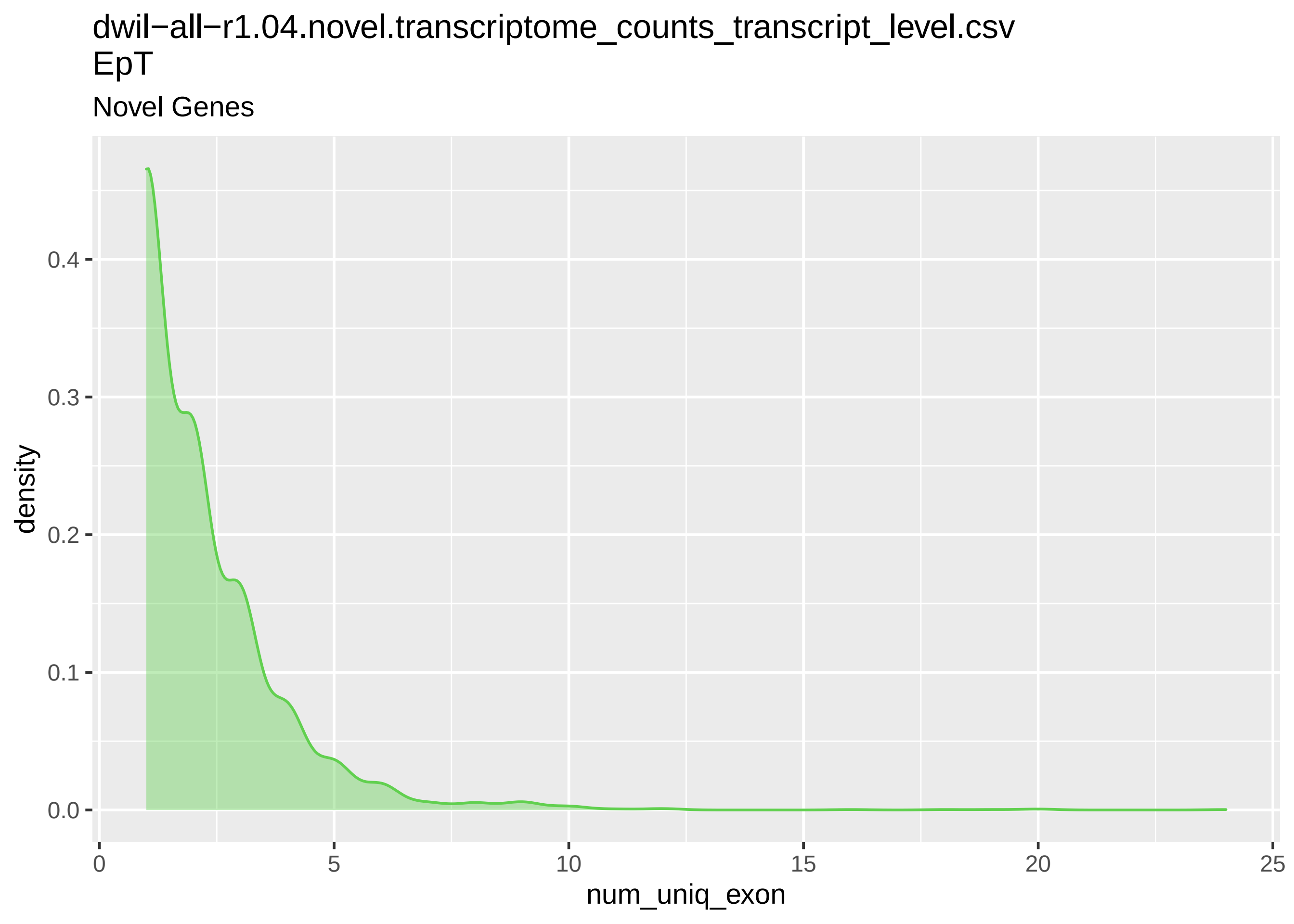
dwil−all−r1.04.novel.transcriptome_counts_transcript_level.csv
EpT
Novel Genes
density
num_uniq_exon
0.0
0.1
0.2
0.3
0.4
0
5
10
15
20
25

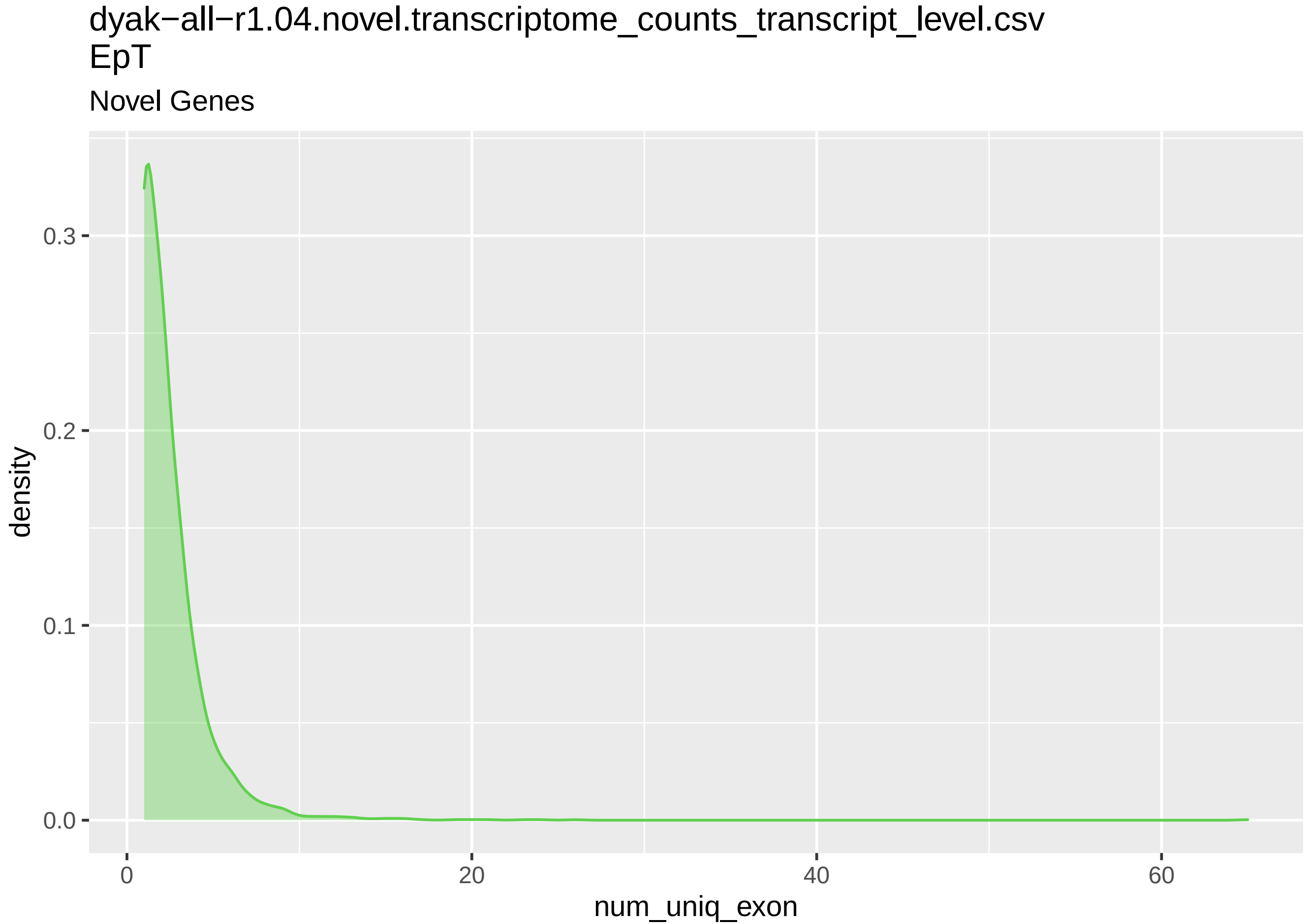
dyak−all−r1.04.novel.transcriptome_counts_transcript_level.csv
EpT
Novel Genes
density
0.3
0.2
0.1
0.0
0
20
40
60
num_uniq_exon

GCF_000001735.4_TAIR10.1
EpT

Novel Genes

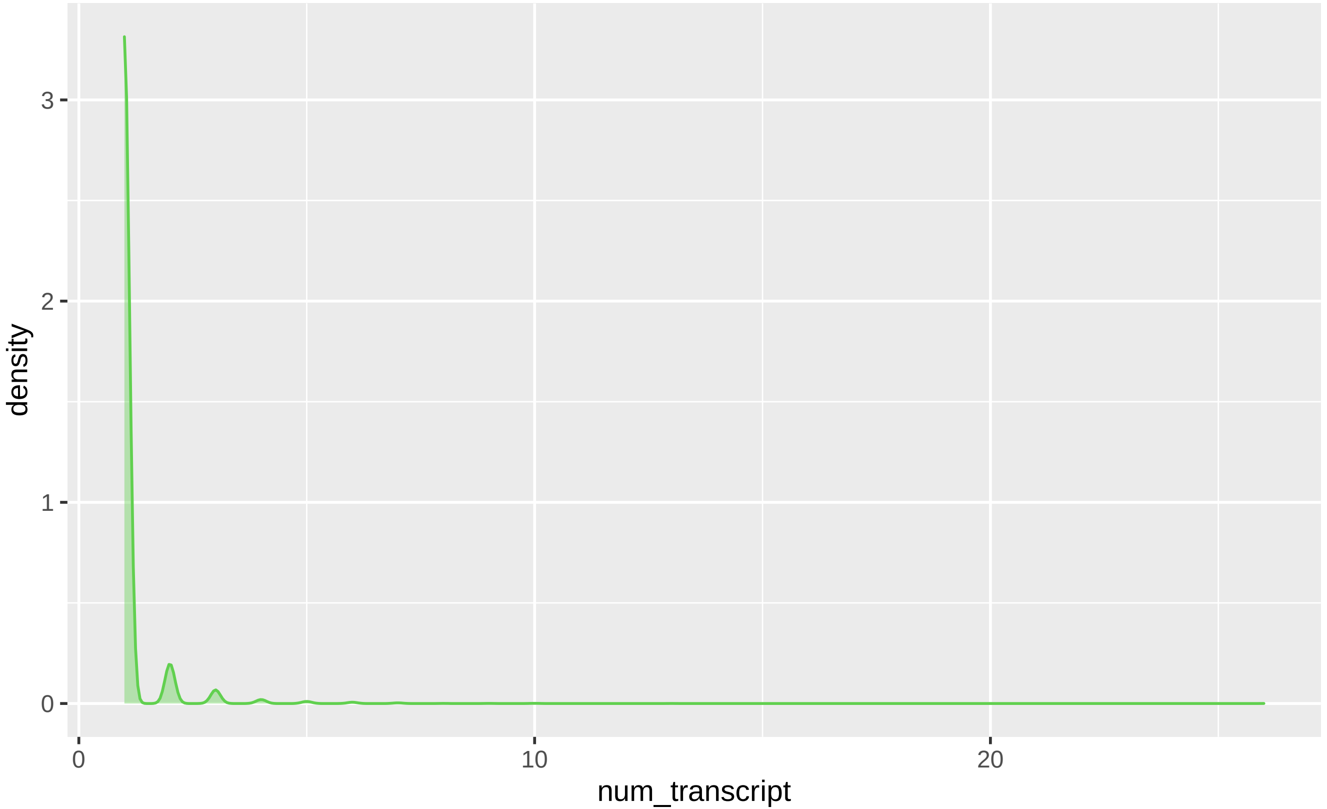

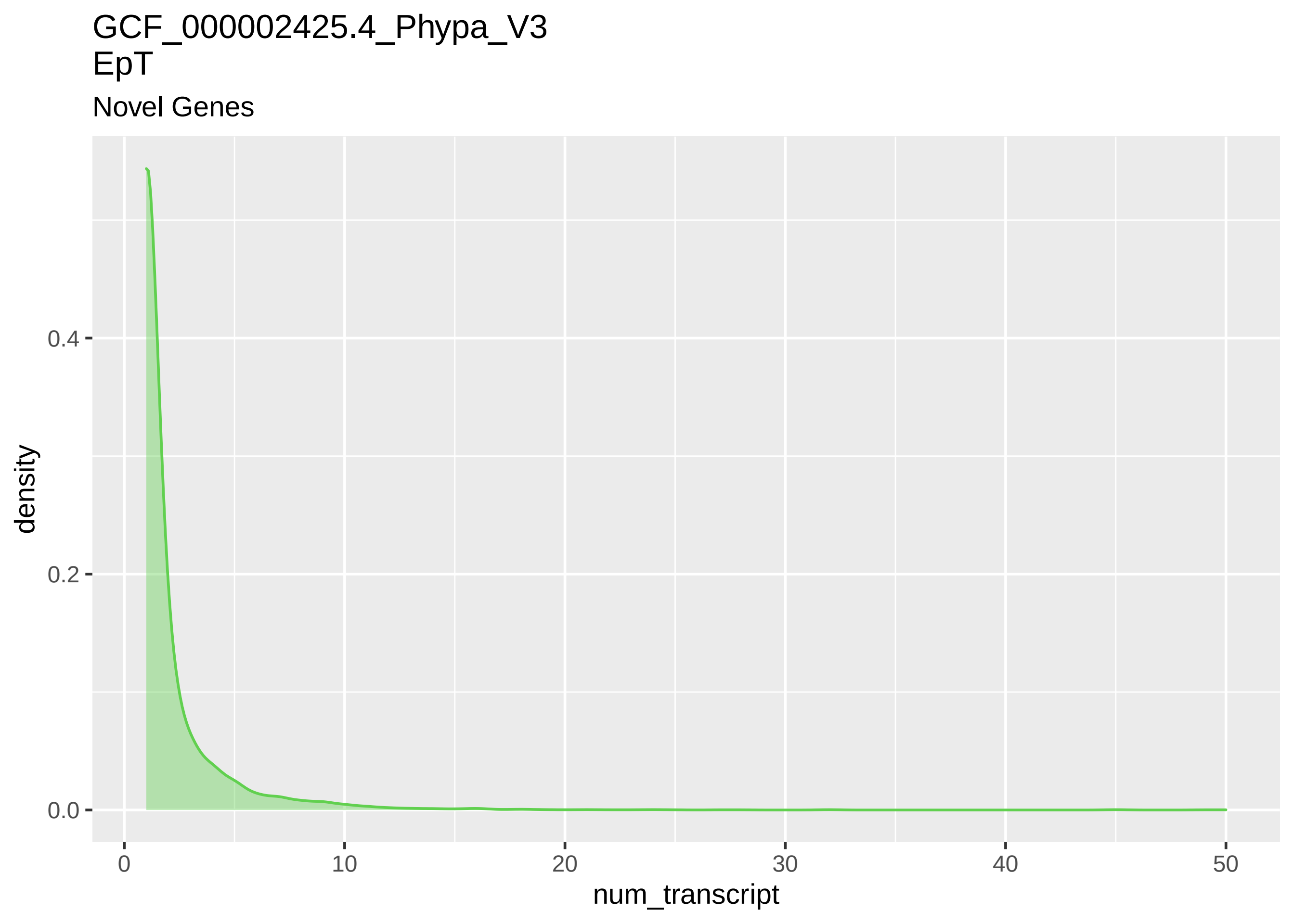
GCF_000002425.4_Phypa_V3
EpT
Novel Genes
density
0.0
0.2
0.4
0
10
20
30
40
50
num_transcript

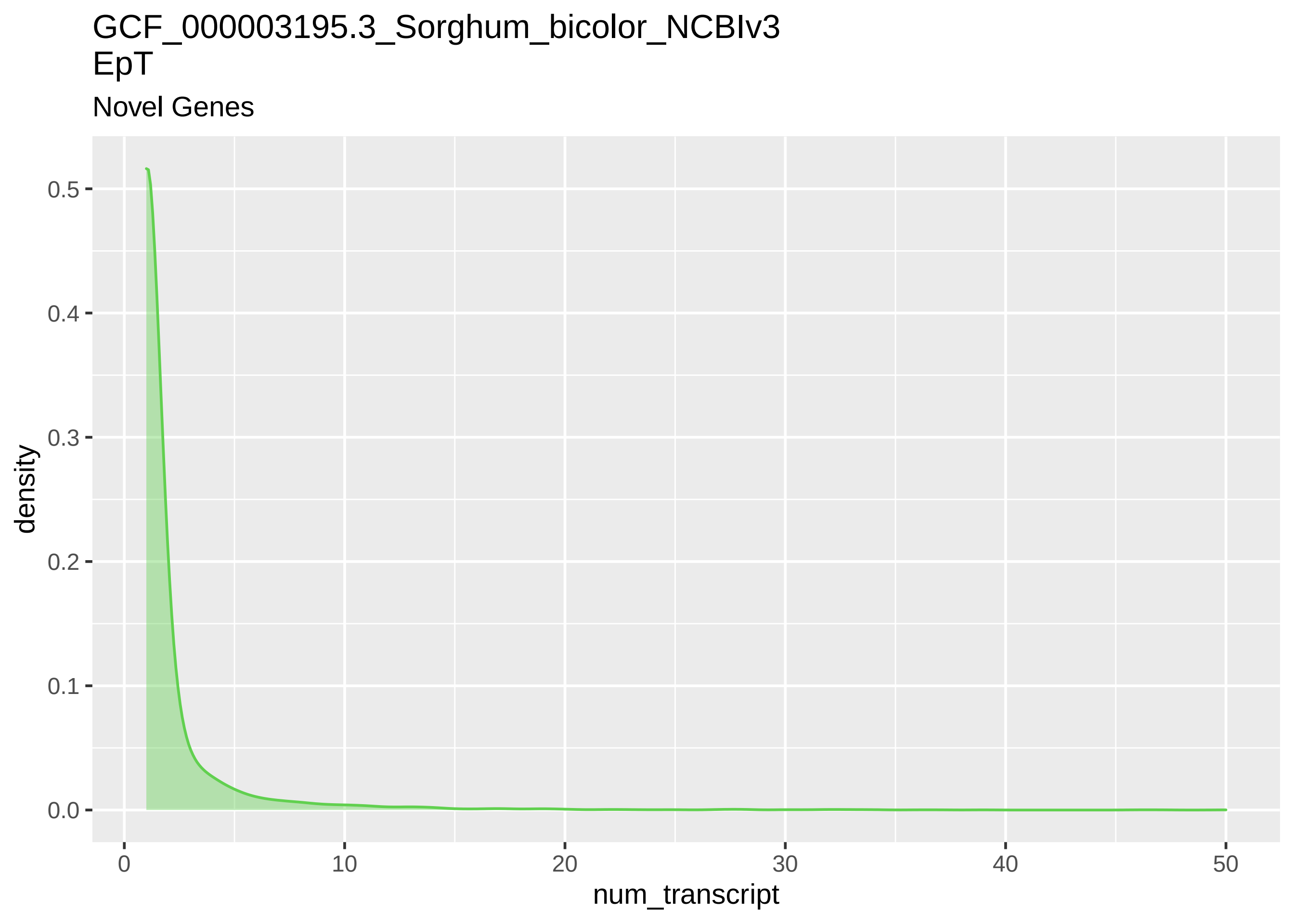
GCF_000003195.3_Sorghum_bicolor_NCBIv3
EpT
Novel Genes
density
0.5
0.4
0.3
0.2
0.1
0.0
0
10
20
30
40
50
num_transcript

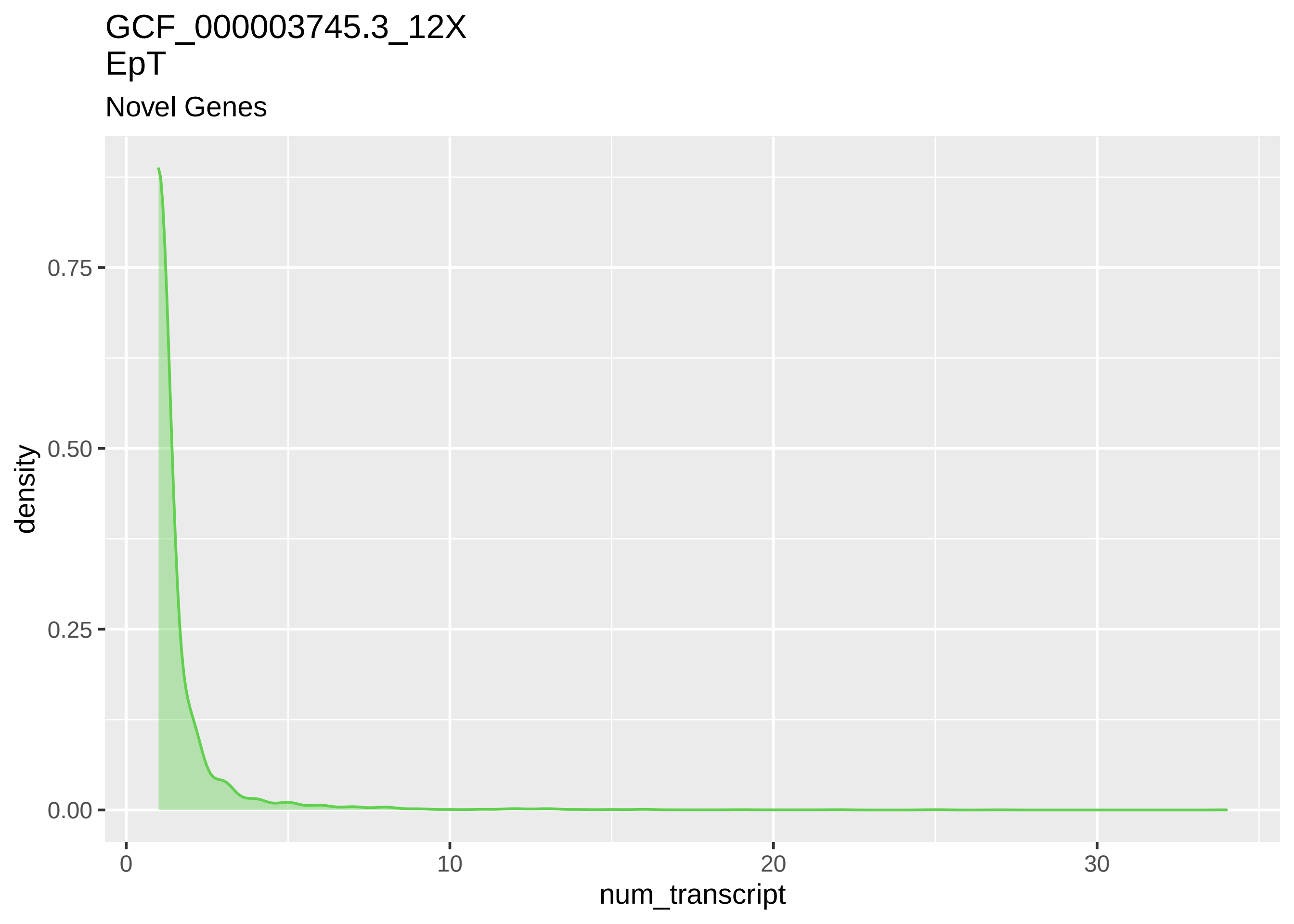
GCF_000003745.3_12X
EpT
Novel Genes
density
0.75
0.50
0.25
0.00
0
10
20
30
num_transcript

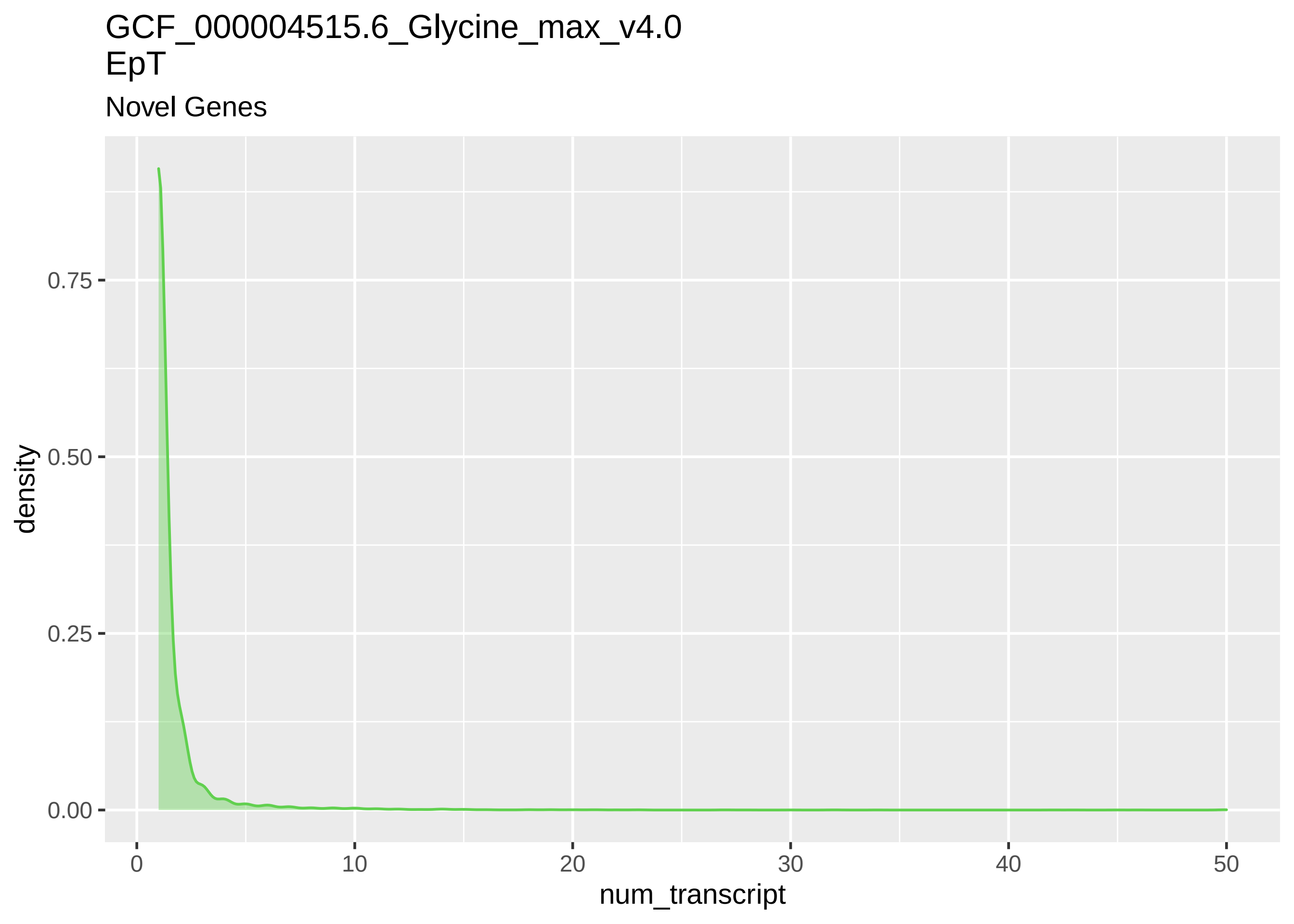
GCF_000004515.6_Glycine_max_v4.0
EpT
Novel Genes
density
0.00
0.25
0.50
0.75
0
10
20
30
40
50
num_transcript

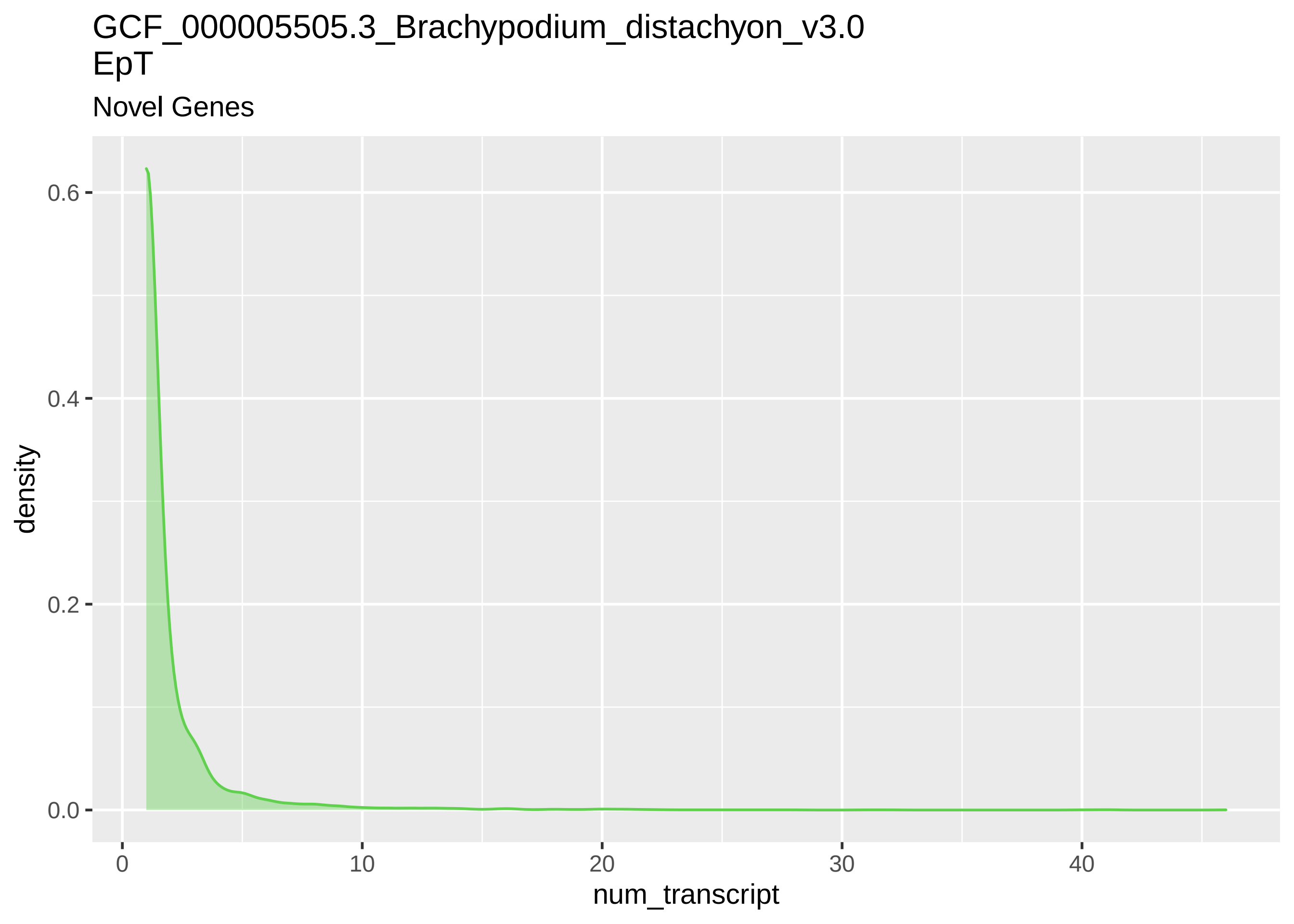
GCF_000005505.3_Brachypodium_distachyon_v3.0
EpT
Novel Genes
density
0.6
0.4
0.2
0.0
0
10
20
30
40
num_transcript

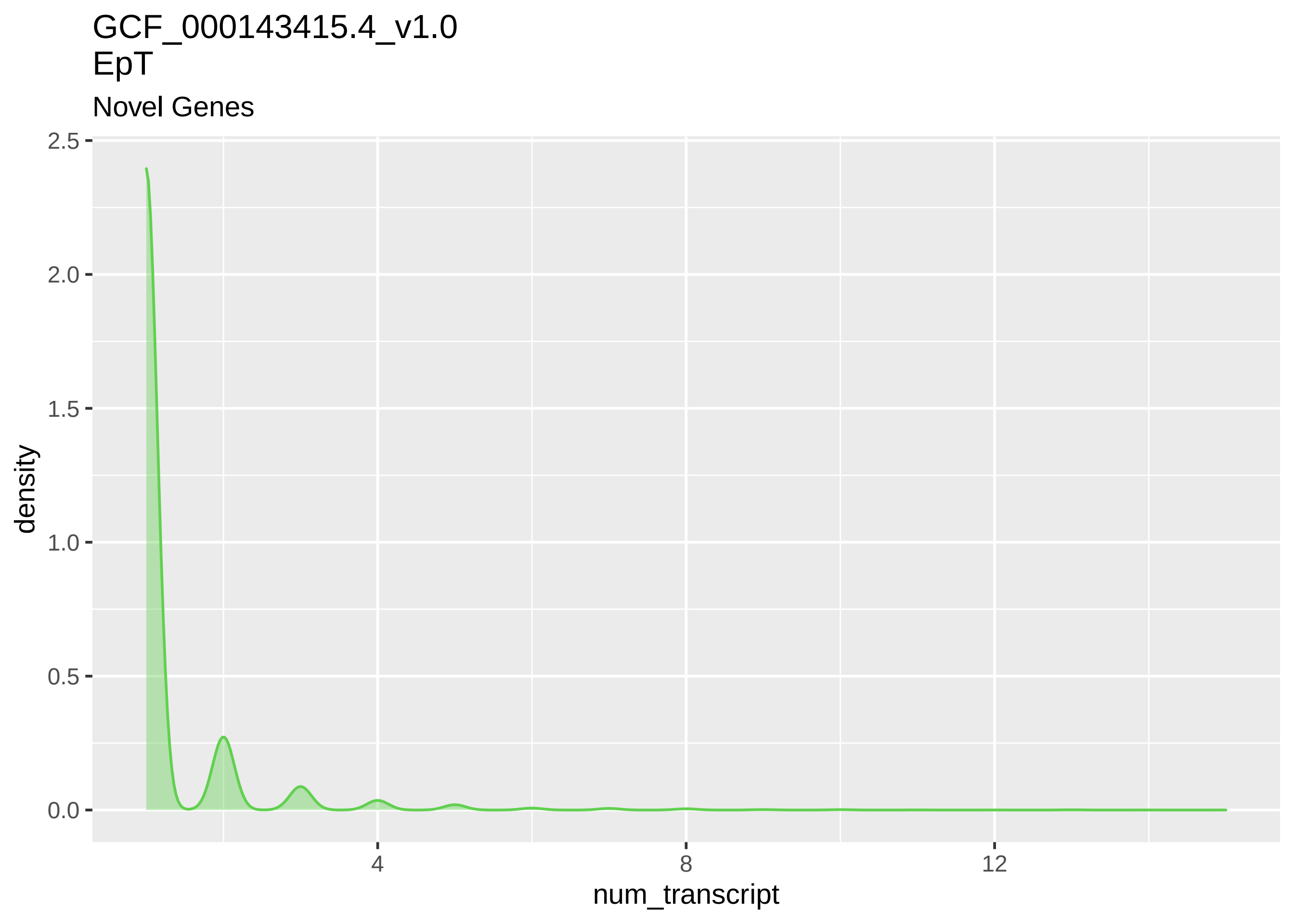
GCF_000143415.4_v1.0
EpT
Novel Genes
density
num_transcript
0.0
0.5
1.0
1.5
2.0
2.5
4
8
12

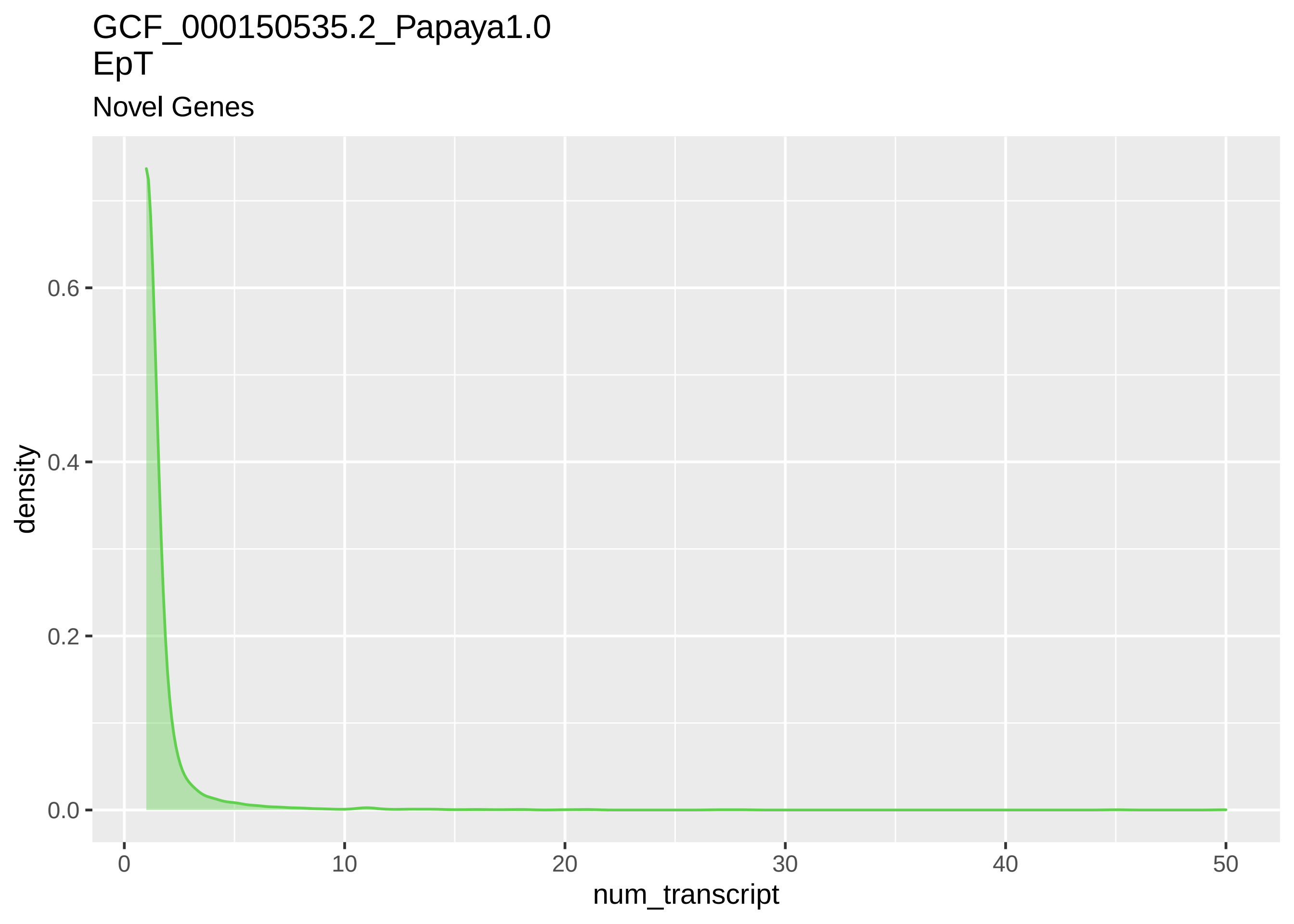

GCF_000150535.2_Papaya1.0
EpT
Novel Genes
density
0.0
0.2
0.4
0.6
0
10
20
30
40
50
num_transcript

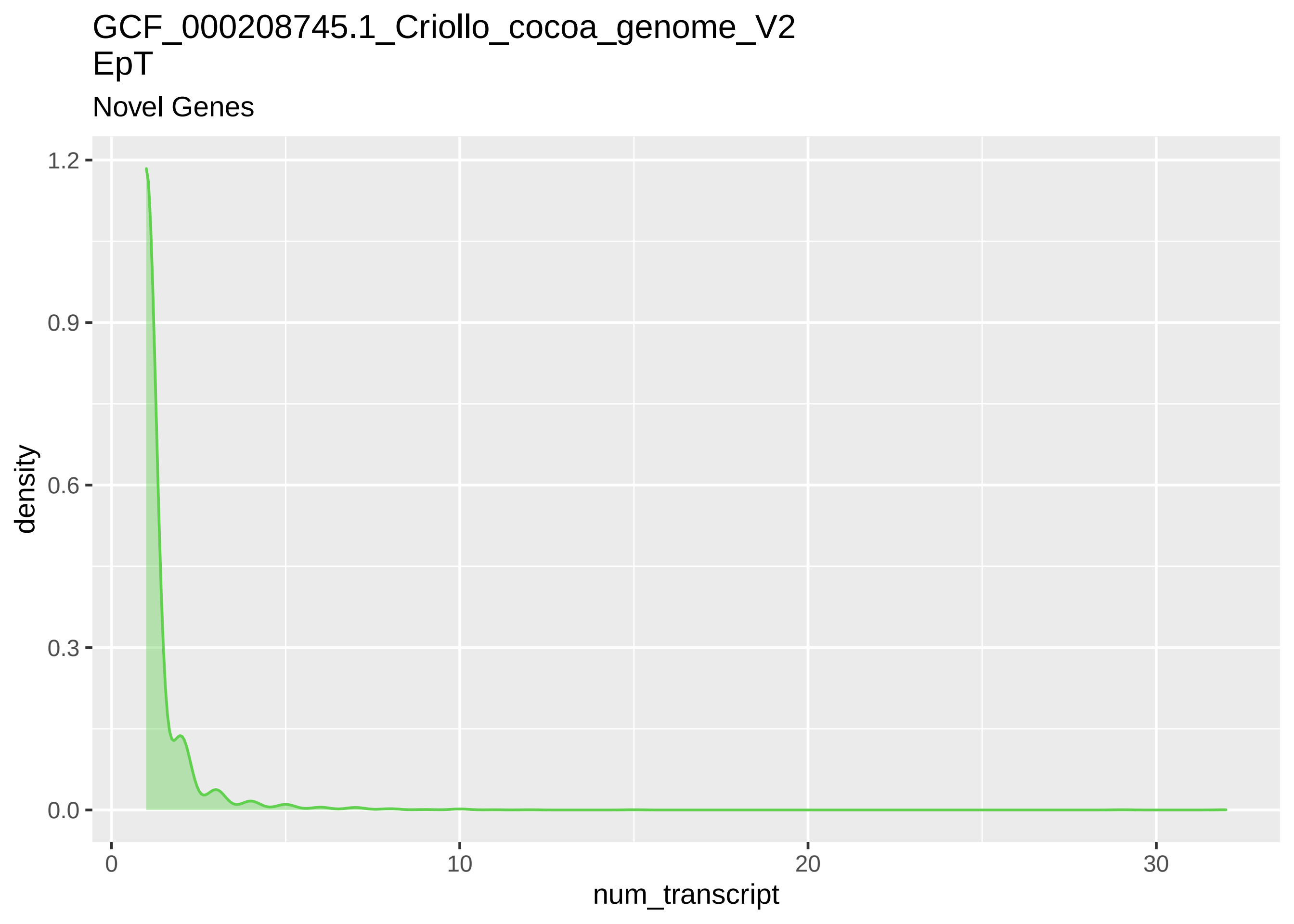

GCF_000208745.1_Criollo_cocoa_genome_V2
EpT
Novel Genes
1.2
0.9
0.6
0.3
0.0
density
0
10
20
30
num_transcript

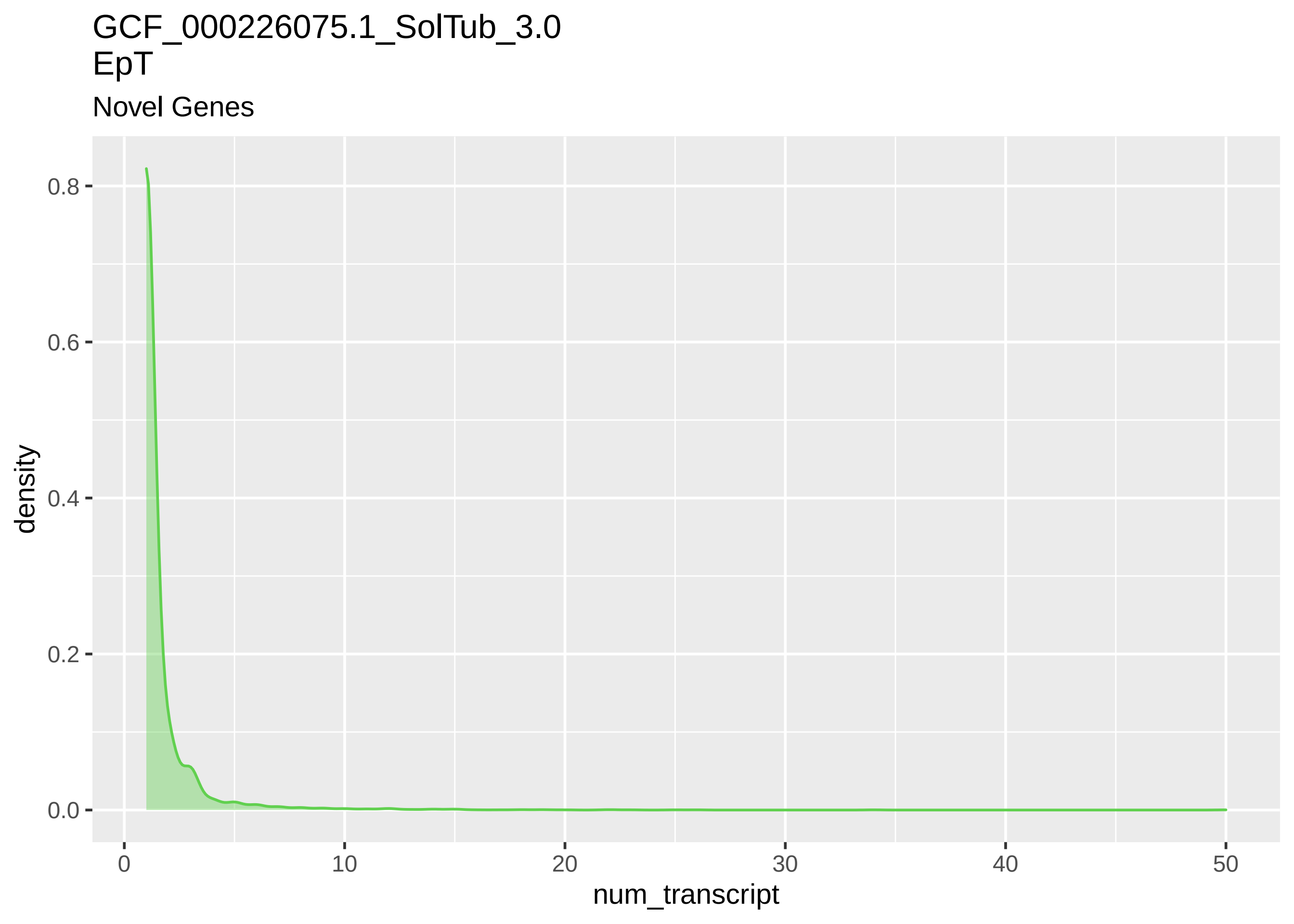
GCF_000226075.1_SolTub_3.0
EpT
Novel Genes
density
0.0
0.2
0.4
0.6
0.8
0
10
20
30
40
50
num_transcript

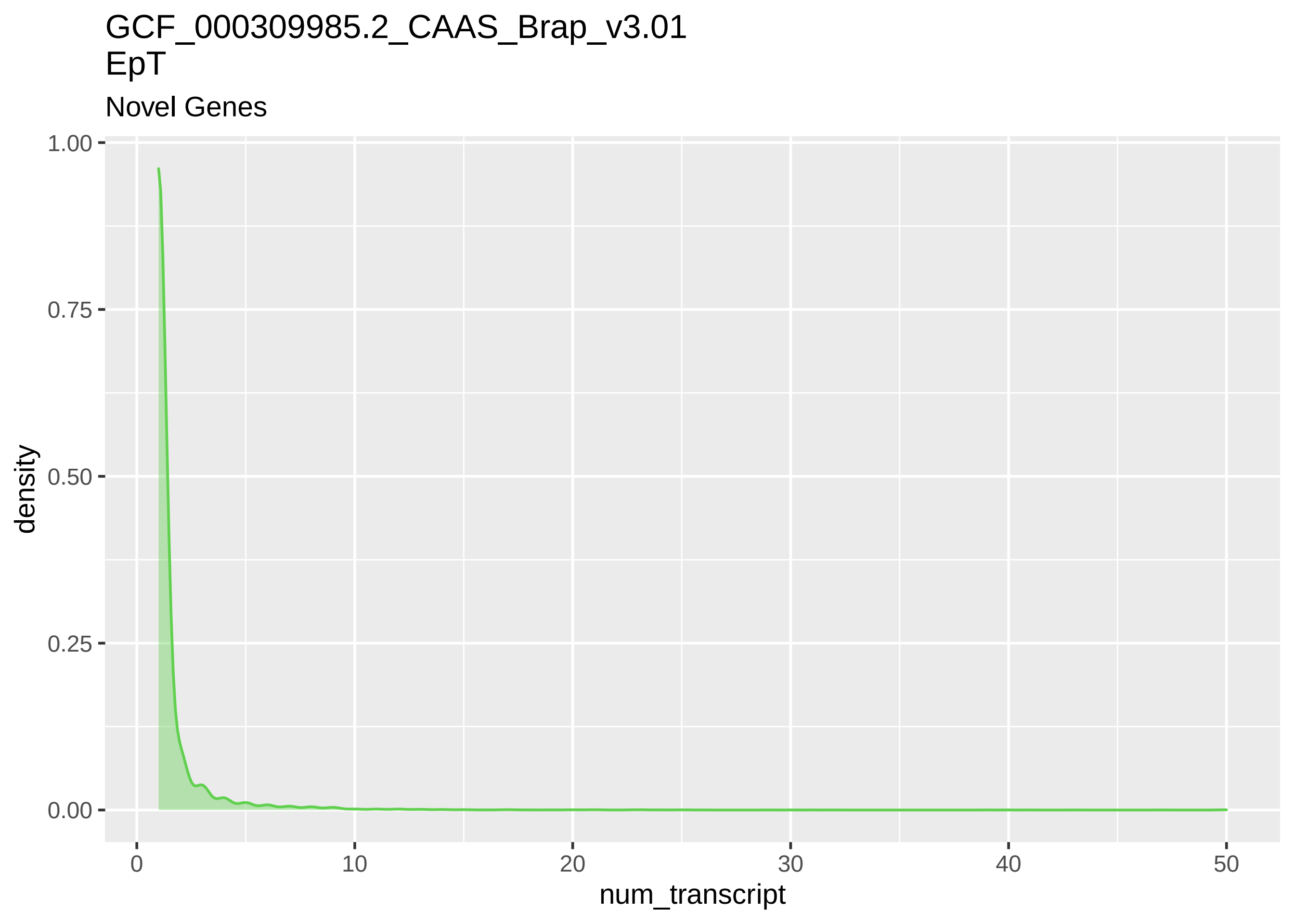
GCF_000309985.2_CAAS_Brap_v3.01
EpT
Novel Genes
density
num_transcript
1.00
0.75
0.50
0.25
0.00
0
10
20
30
40
50

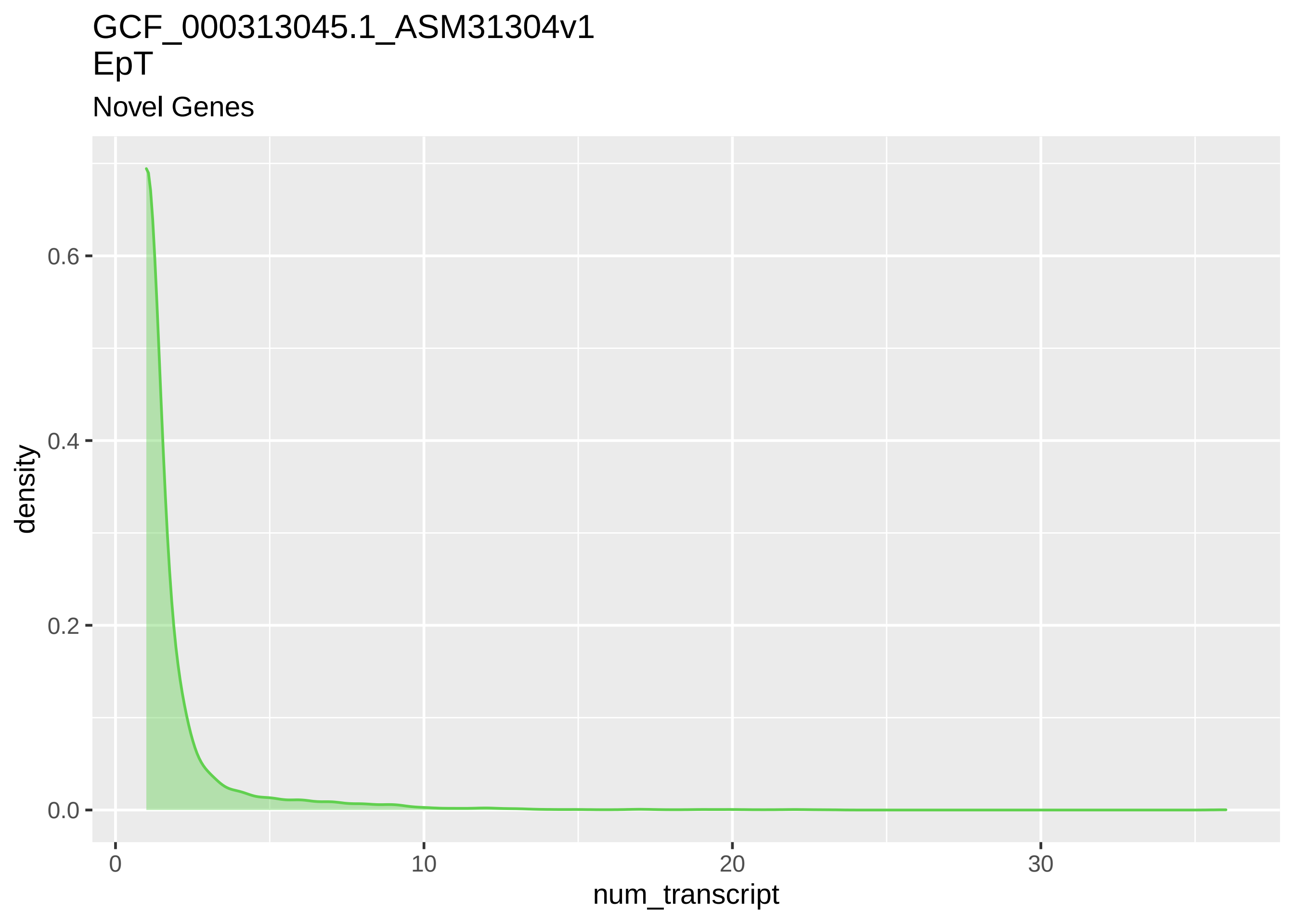
GCF_000313045.1_ASM31304v1
EpT
Novel Genes
density
0.6
0.4
0.2
0.0
0
10
20
30
num_transcript

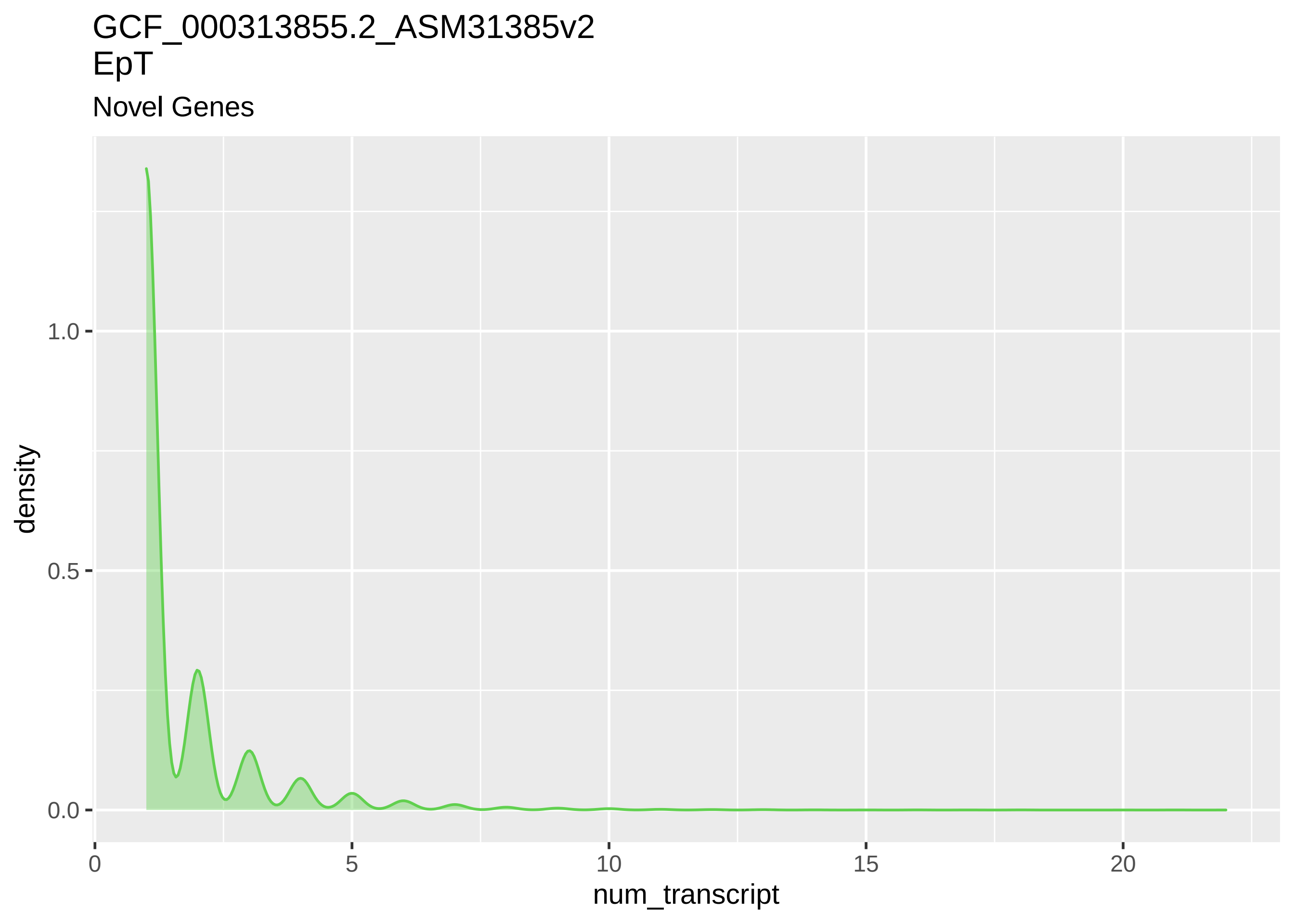
GCF_000313855.2_ASM31385v2
EpT
Novel Genes
density
1.0
0.5
0.0
0
5
10
15
20
num_transcript

GCF_000315295.1_Pbr_v1.0
EpT

Novel Genes

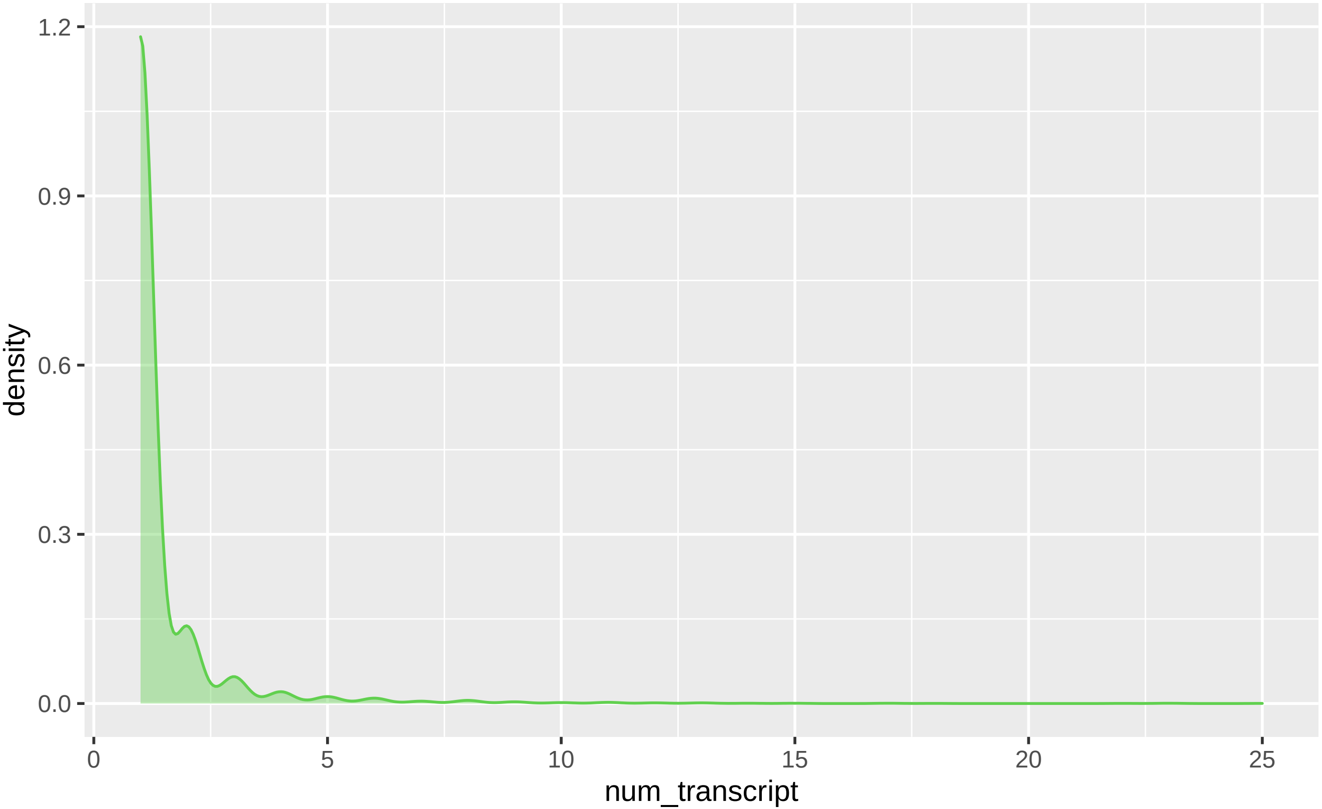

GCF_000317415.1_Csi_valencia_1.0
EpT
Novel Genes

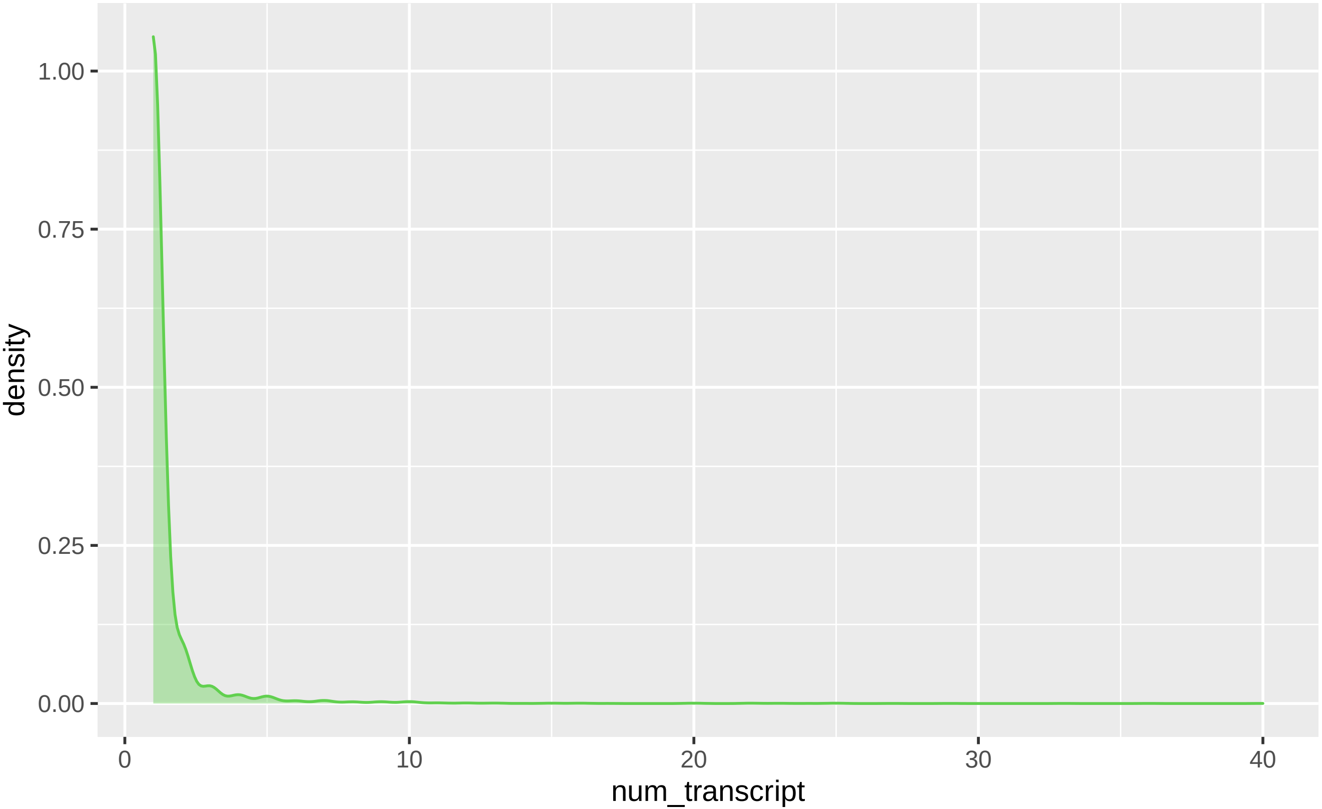

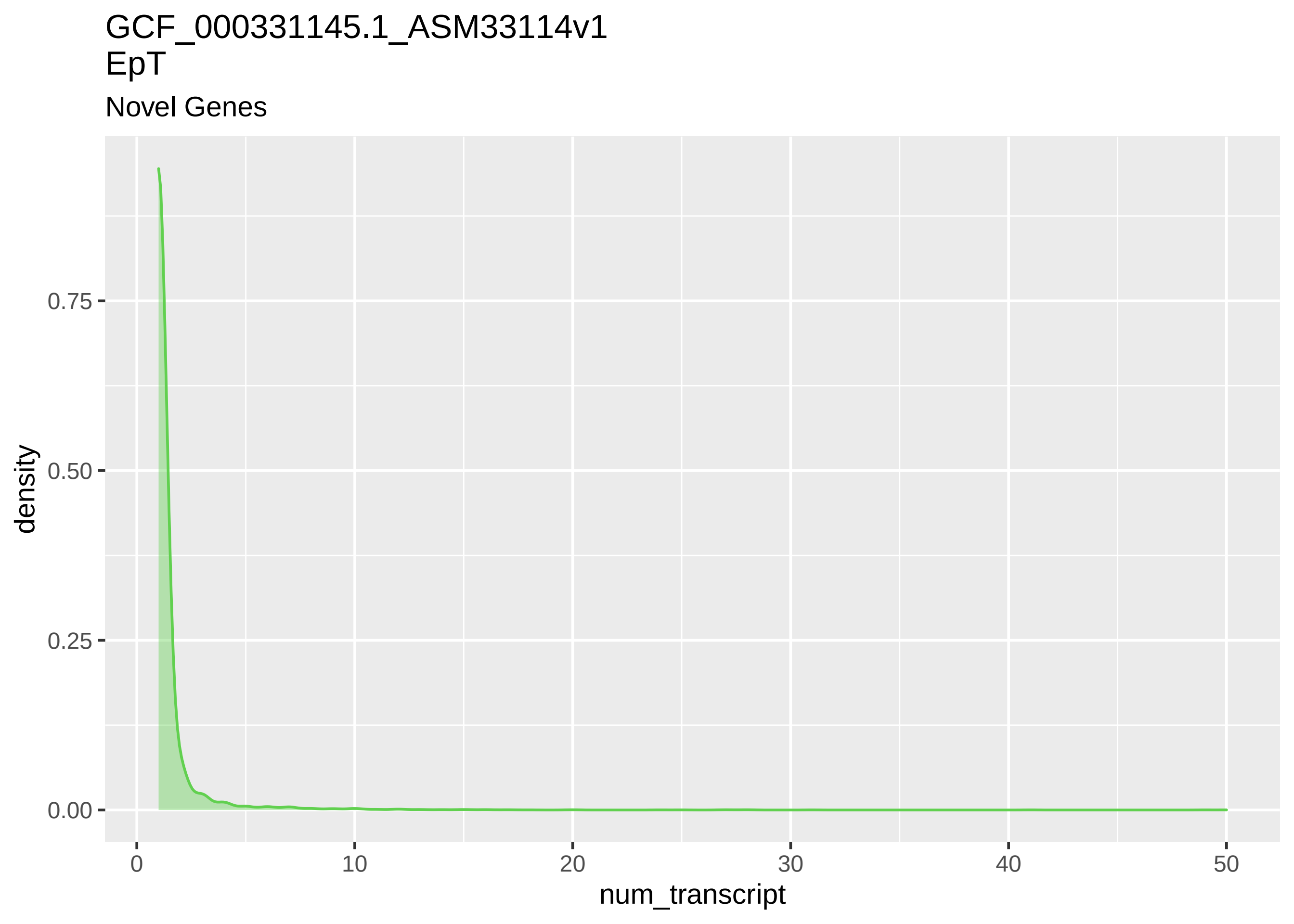
GCF_000331145.1_ASM33114v1
EpT
Novel Genes
density
0.75
0.50
0.25
0.00
0
10
20
30
40
50
num_transcript

GCF_000346465.2_Prunus_persica_NCBIv2
EpT

Novel Genes

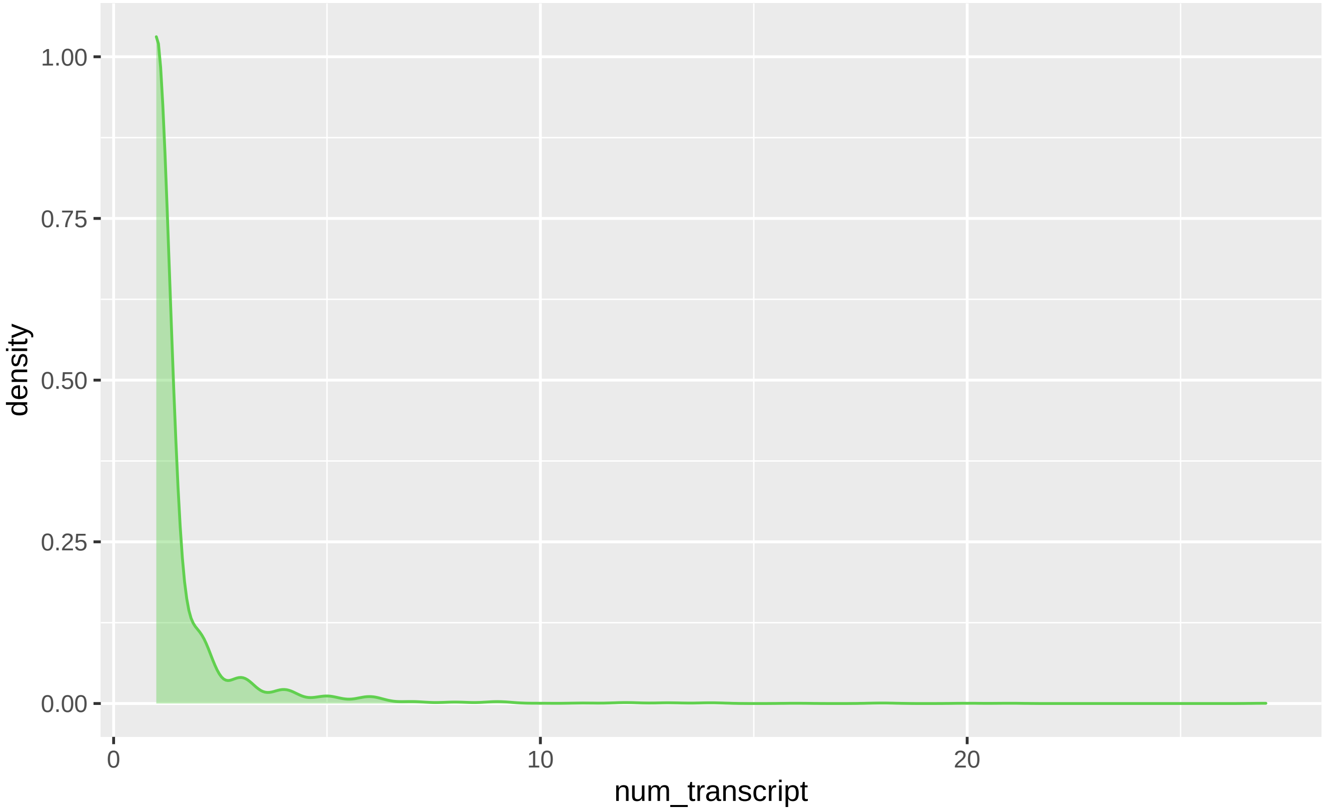

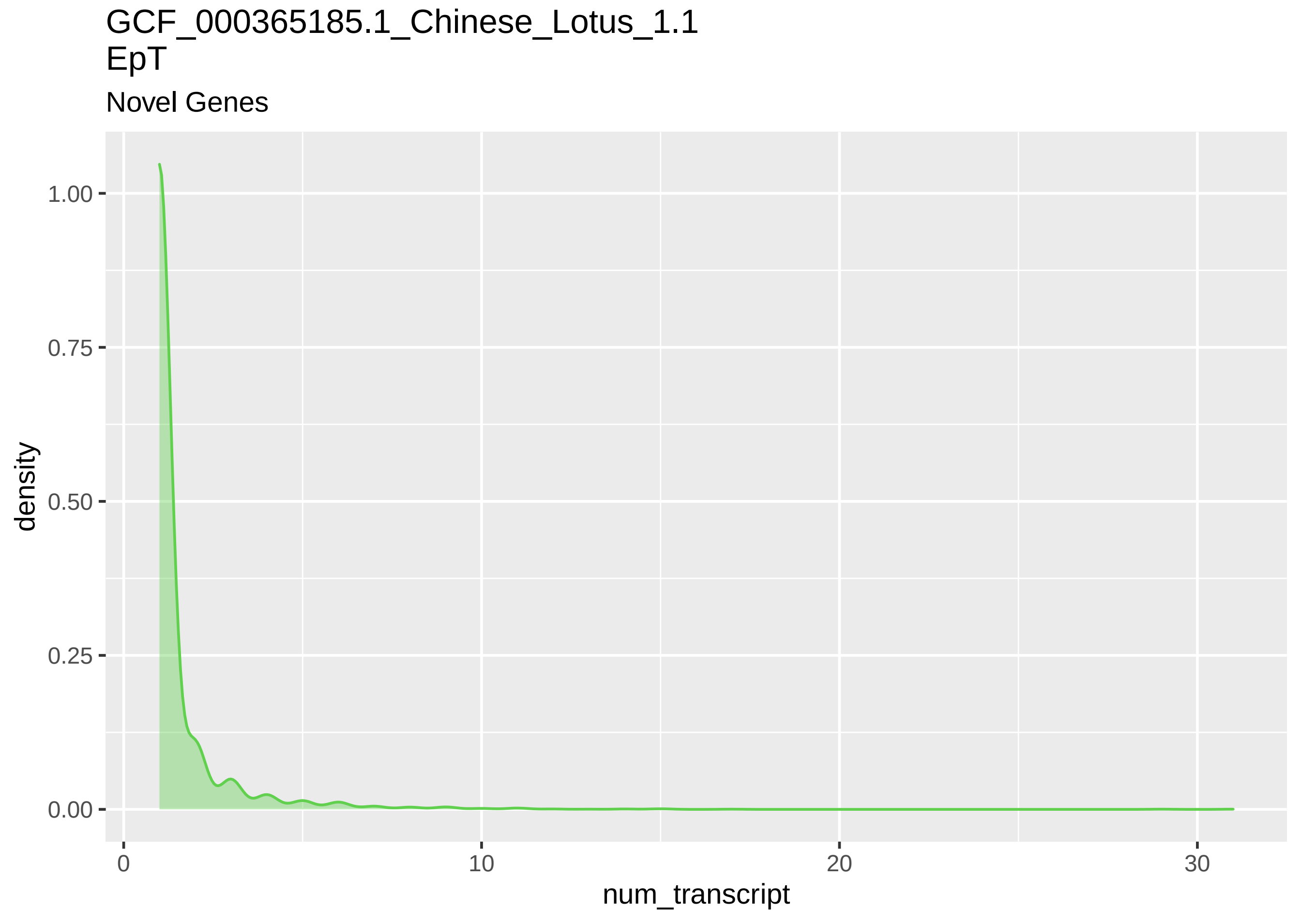
GCF_000365185.1_Chinese_Lotus_1.1
EpT
Novel Genes
density
1.00
0.75
0.50
0.25
0.00
0
10
20
30
num_transcript

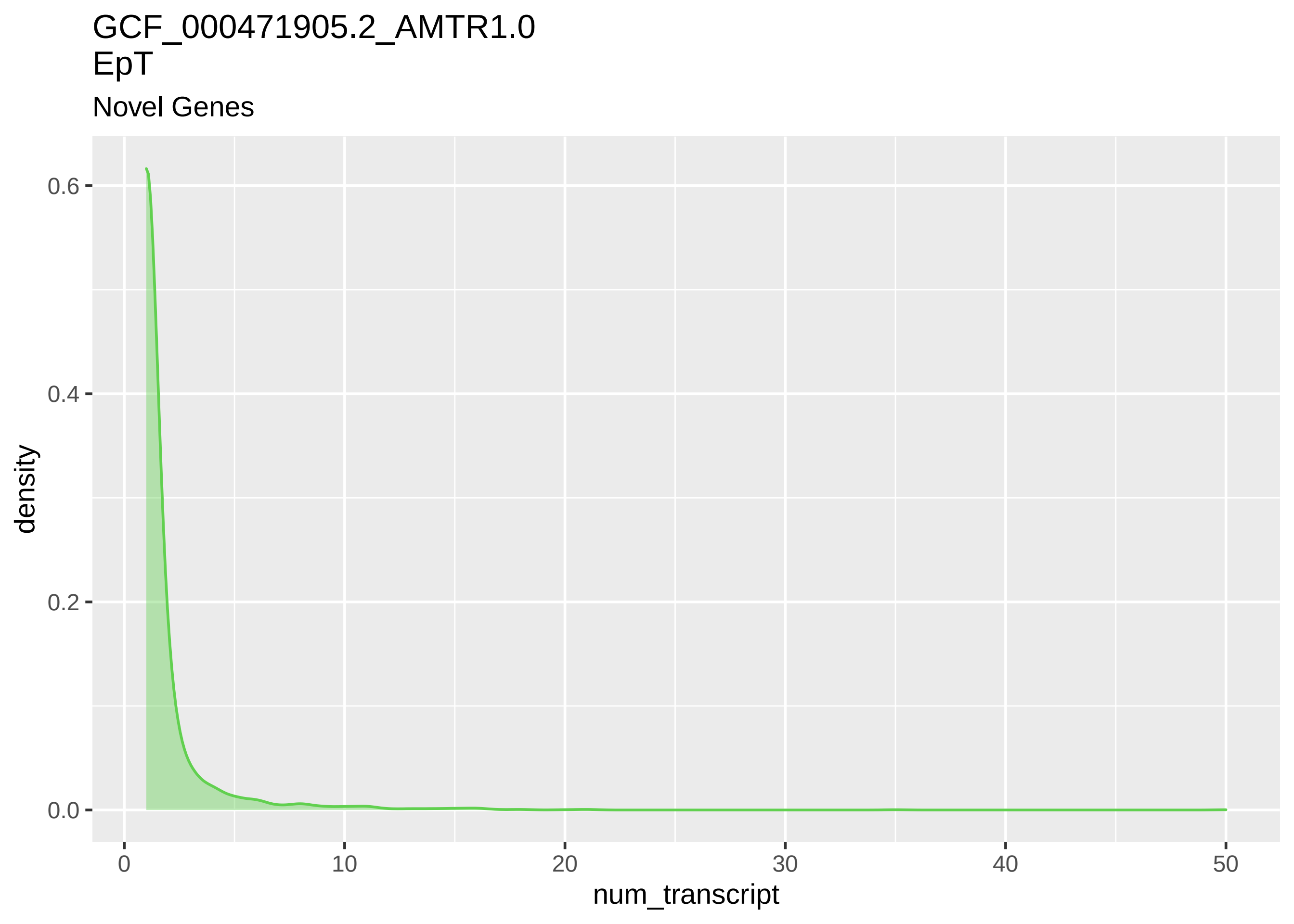
GCF_000471905.2_AMTR1.0
EpT
Novel Genes
density
0.0
0.2
0.4
0.6
0
10
20
30
40
50
num_transcript

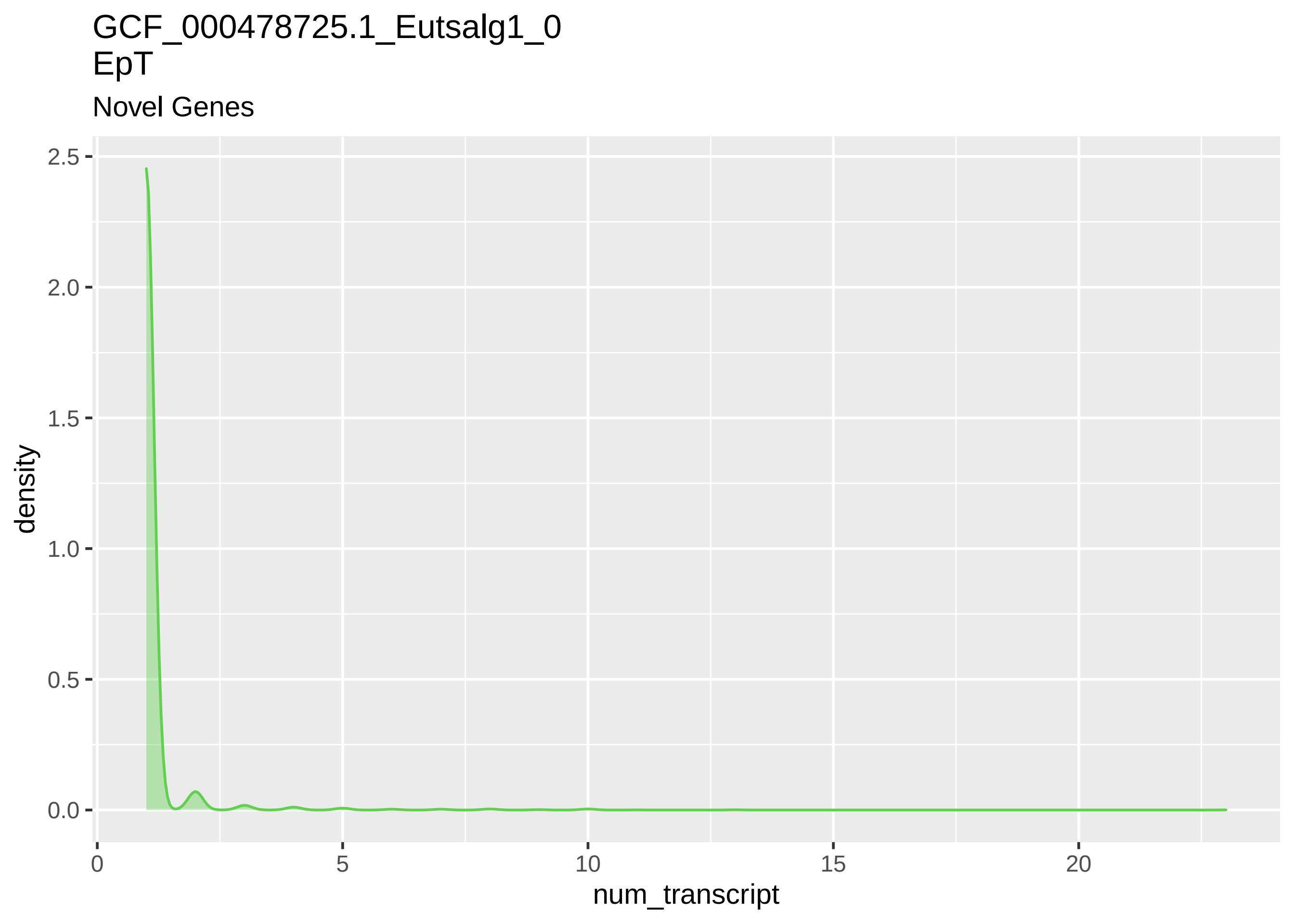
GCF_000478725.1_Eutsalg1_0
EpT
Novel Genes
density
2.5
2.0
1.5
1.0
0.5
0.0
0
5
10
15
20
num_transcript

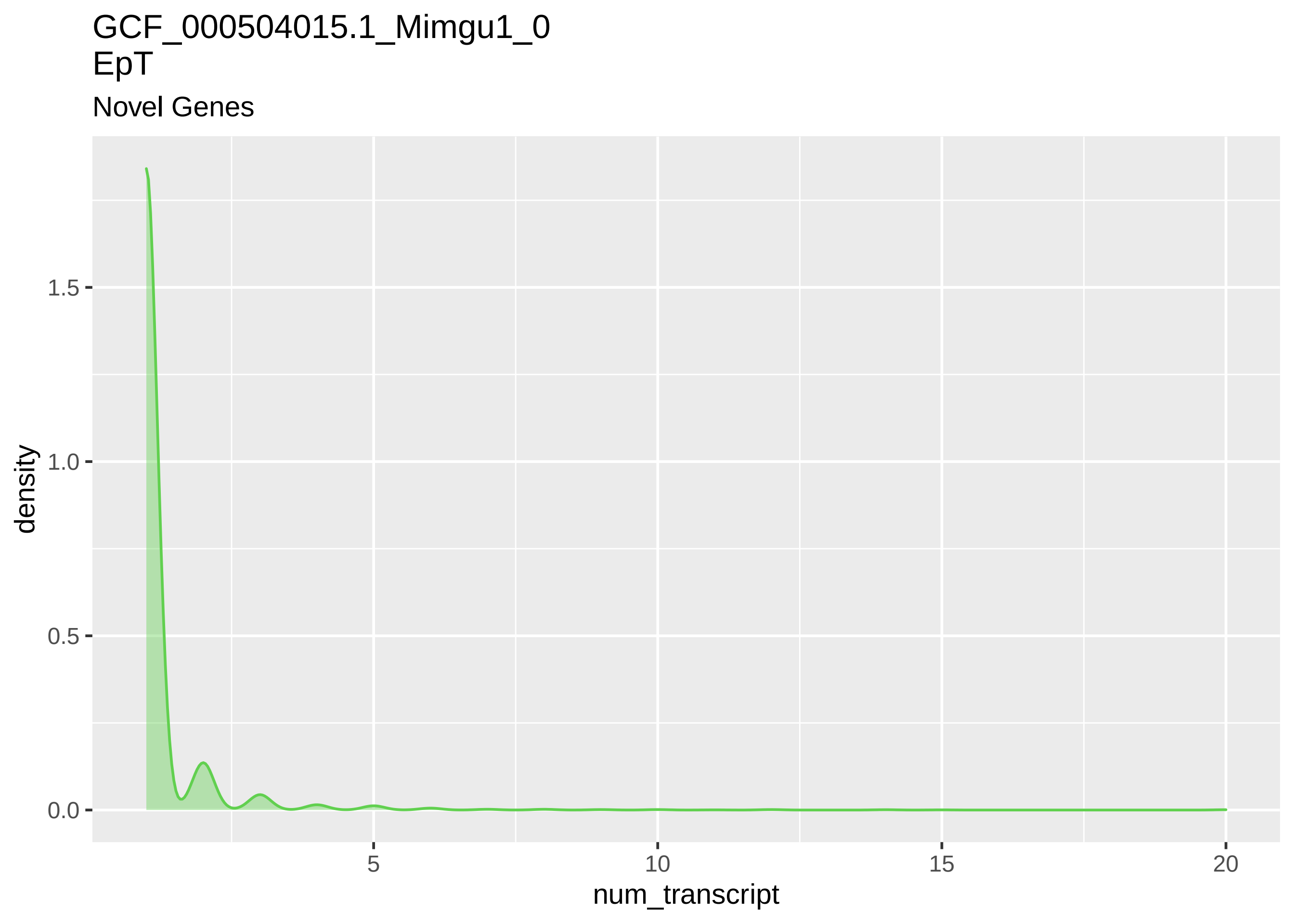
GCF_000504015.1_Mimgu1_0
EpT
Novel Genes
density
1.5
1.0
0.5
0.0
5
10
15
20
num_transcript

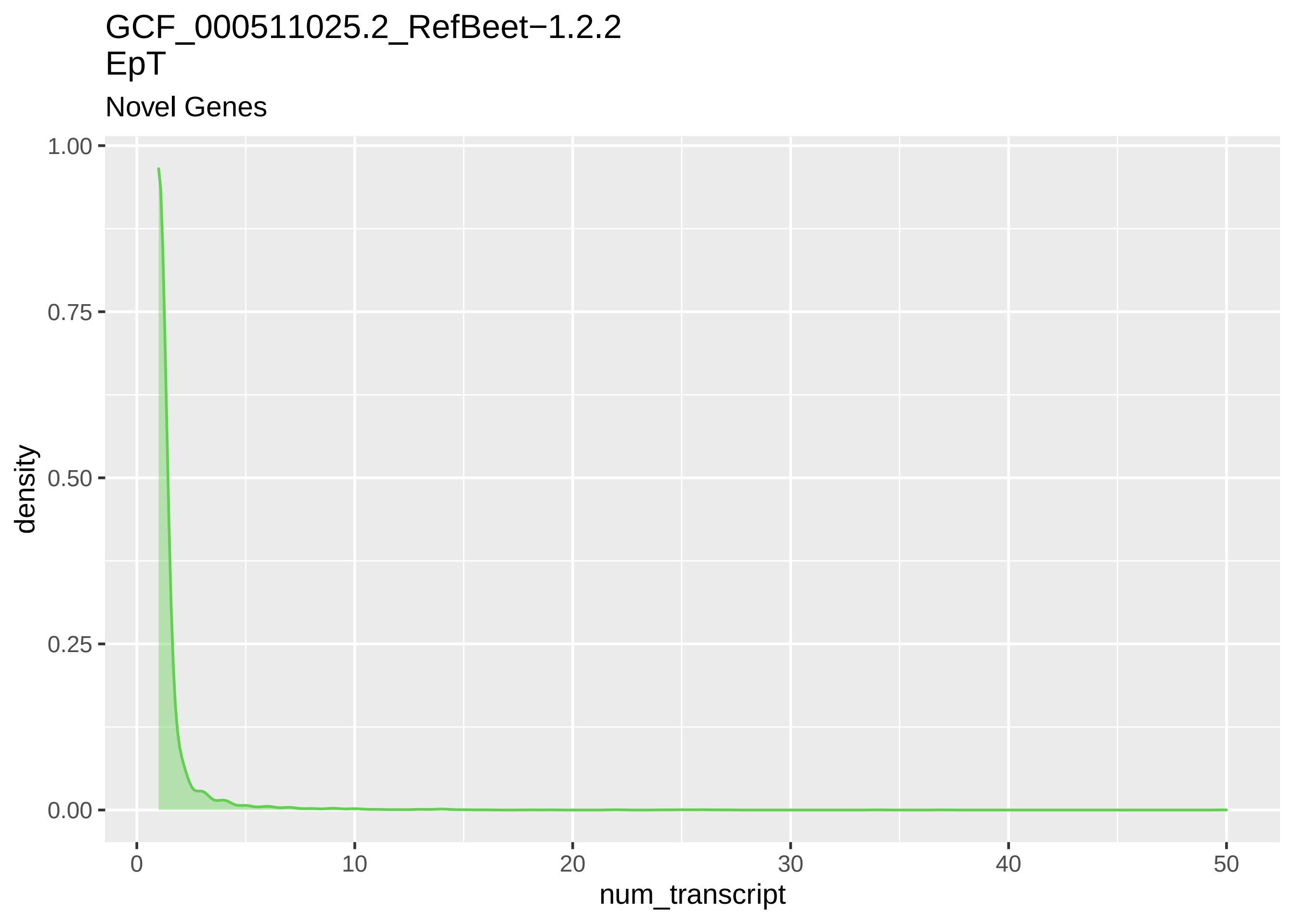
GCF_000511025.2_RefBeet−1.2.2
EpT
Novel Genes
density
num_transcript
1.00
0.75
0.50
0.25
0.00
0
10
20
30
40
50

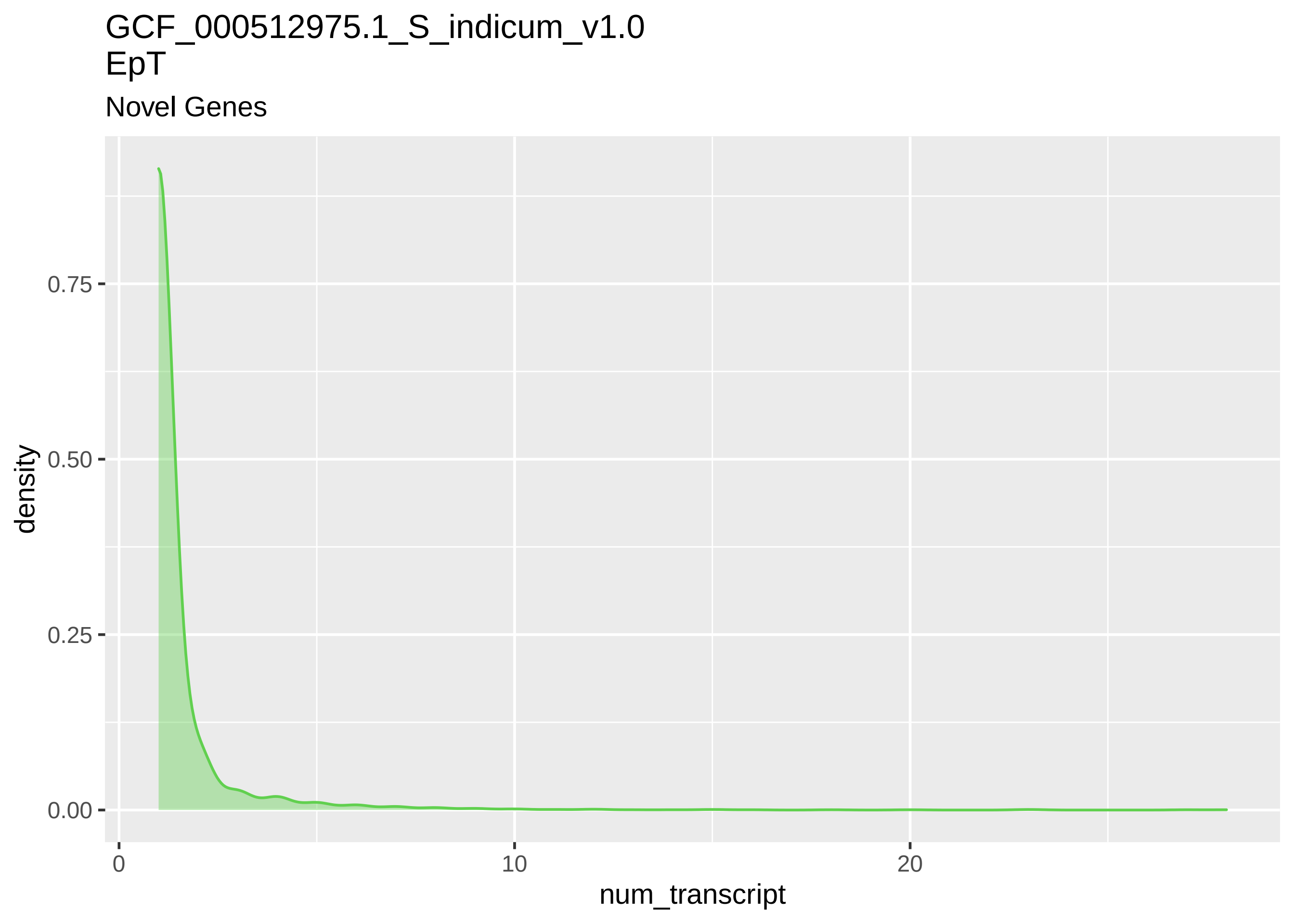
GCF_000512975.1_S_indicum_v1.0
EpT
Novel Genes
density
0.75
0.50
0.25
0.00
0
10
20
num_transcript

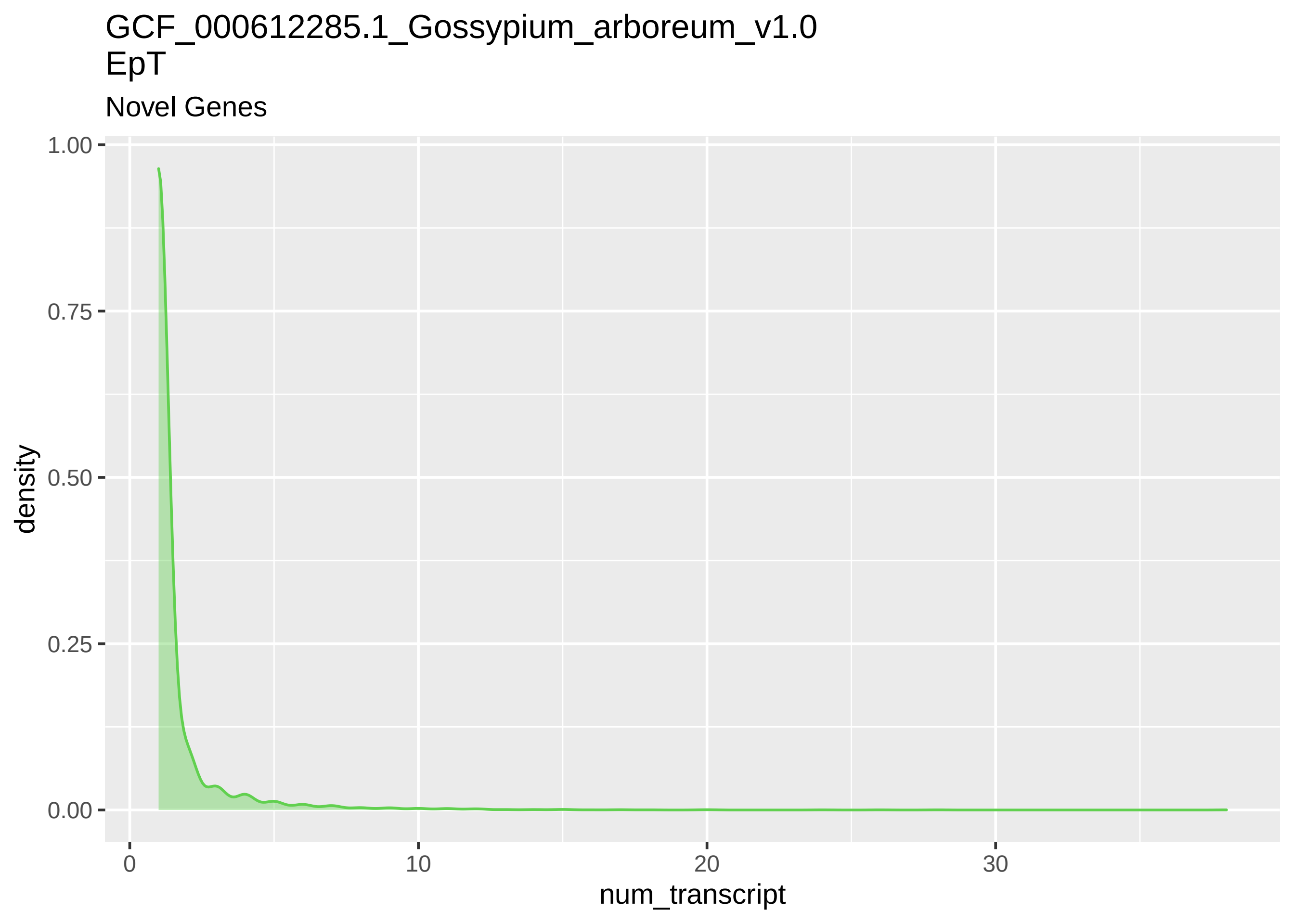
GCF_000612285.1_Gossypium_arboreum_v1.0
EpT
Novel Genes
density
1.00
0.75
0.50
0.25
0.00
0
10
20
30
num_transcript

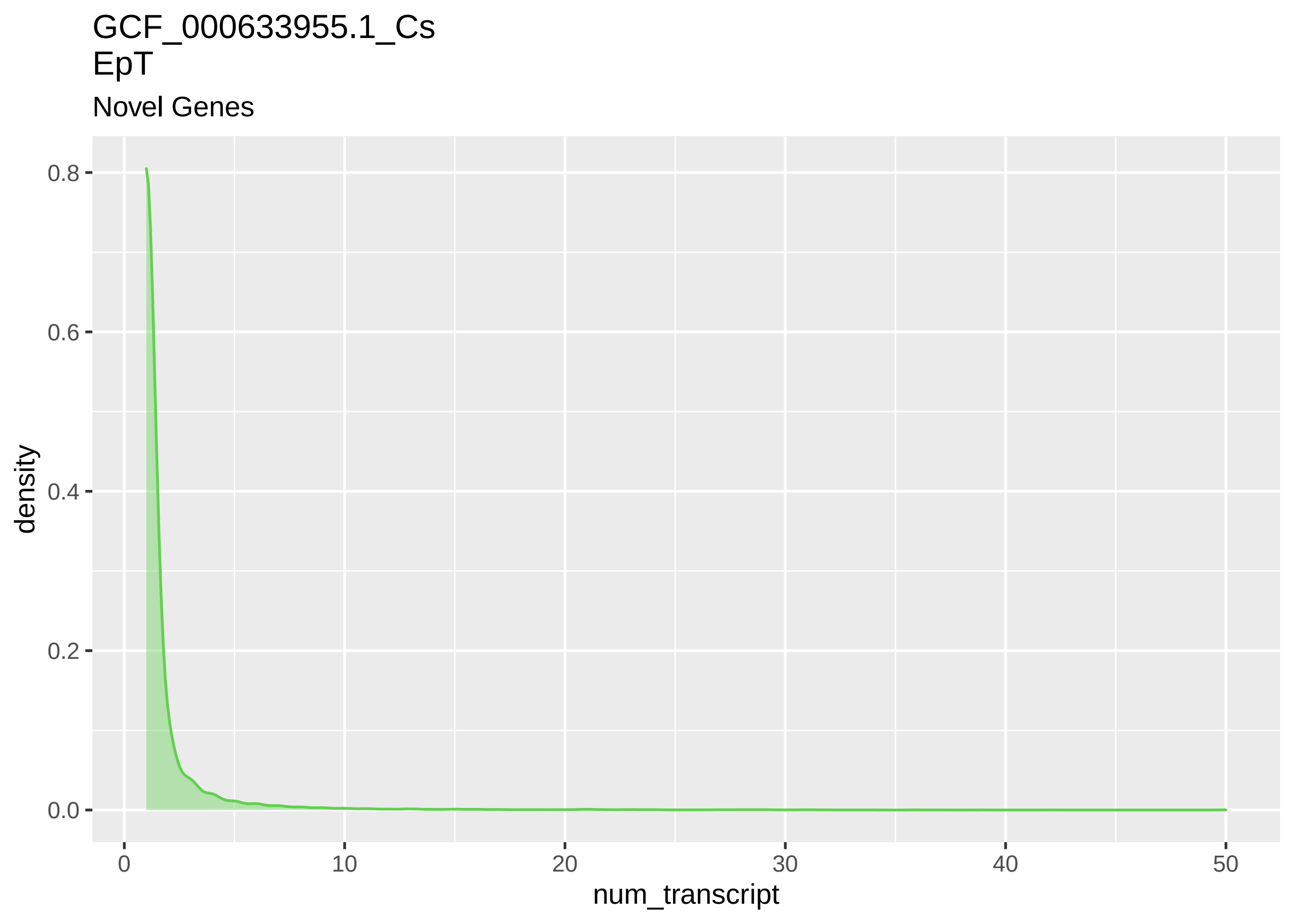
GCF_000633955.1_Cs
EpT
Novel Genes
density
0.8
0.6
0.4
0.2
0.0
0
10
20
30
40
50
num_transcript

GCF_000710875.1_Pepper_Zunla_1_Ref_v1.0
EpT

Novel Genes

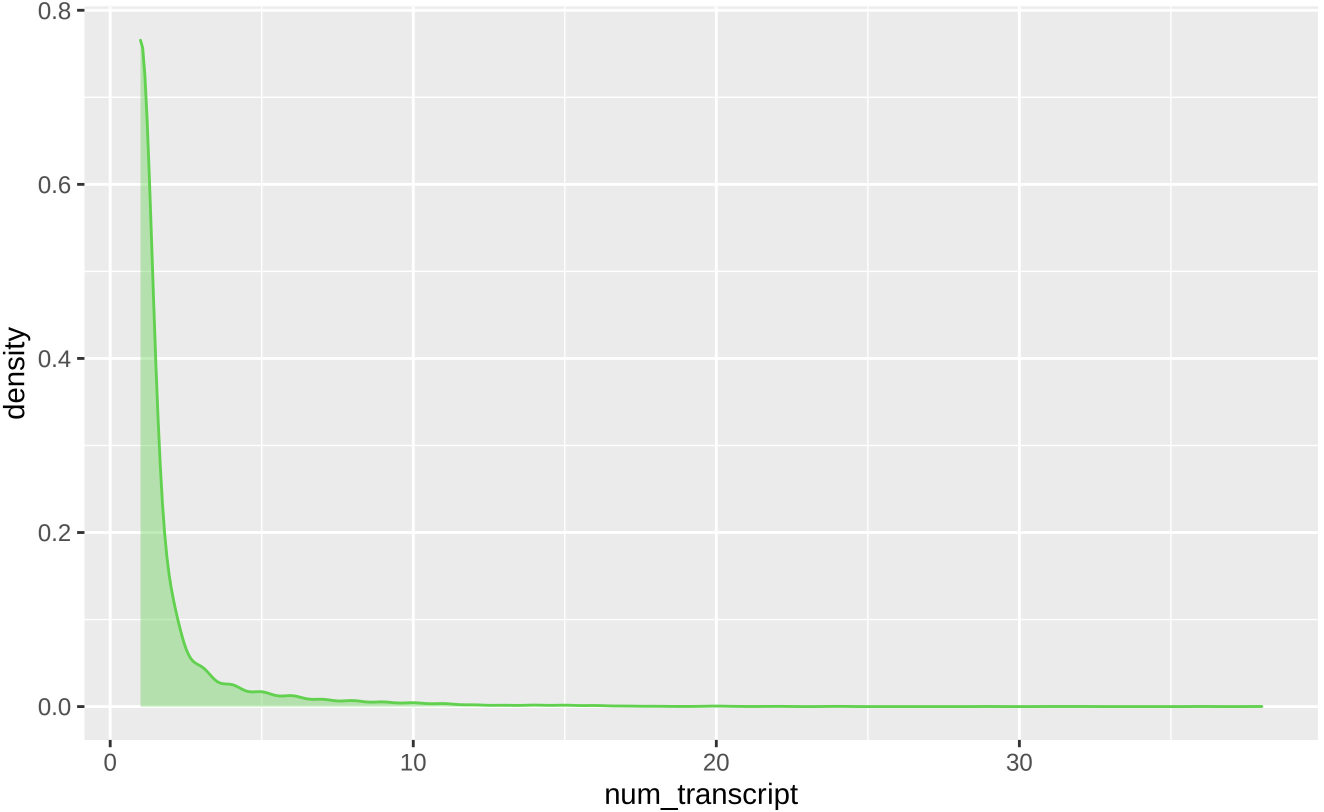

GCF_000715135.1_Ntab−TN90
EpT

Novel Genes

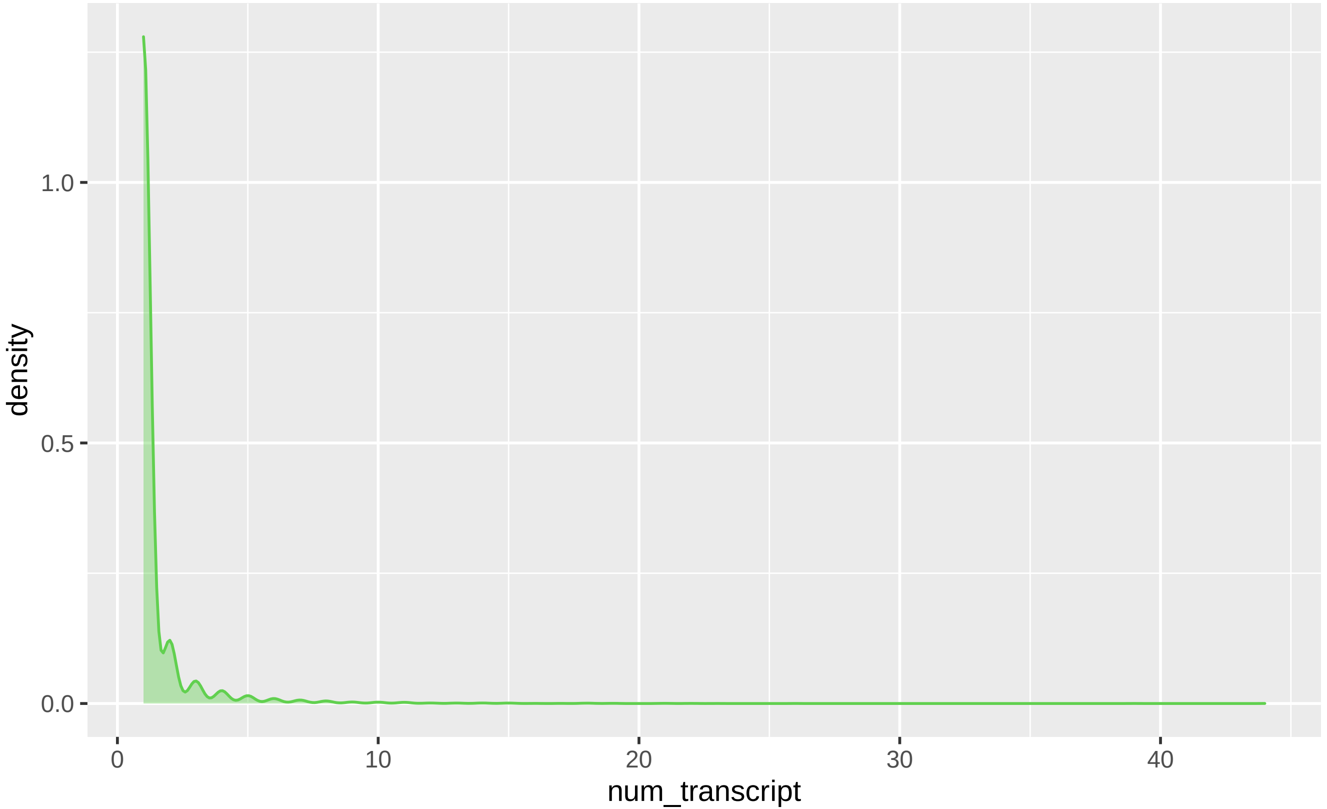

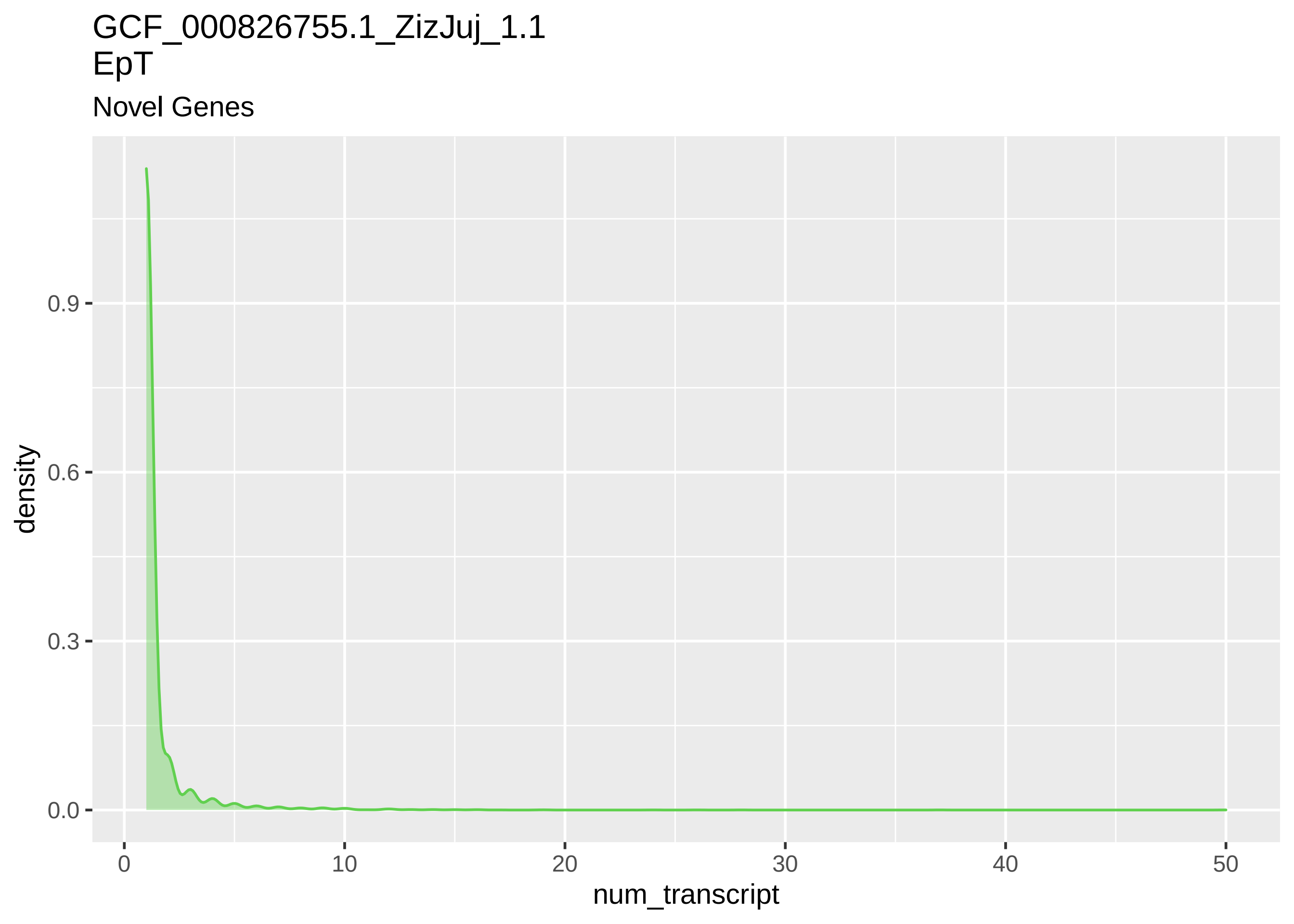
GCF_000826755.1_ZizJuj_1.1
EpT
Novel Genes
density
0.0
0.3
0.6
0.9
0
10
20
30
40
50
num_transcript

GCF_001190045.1_Vigan1.1
EpT

Novel Genes

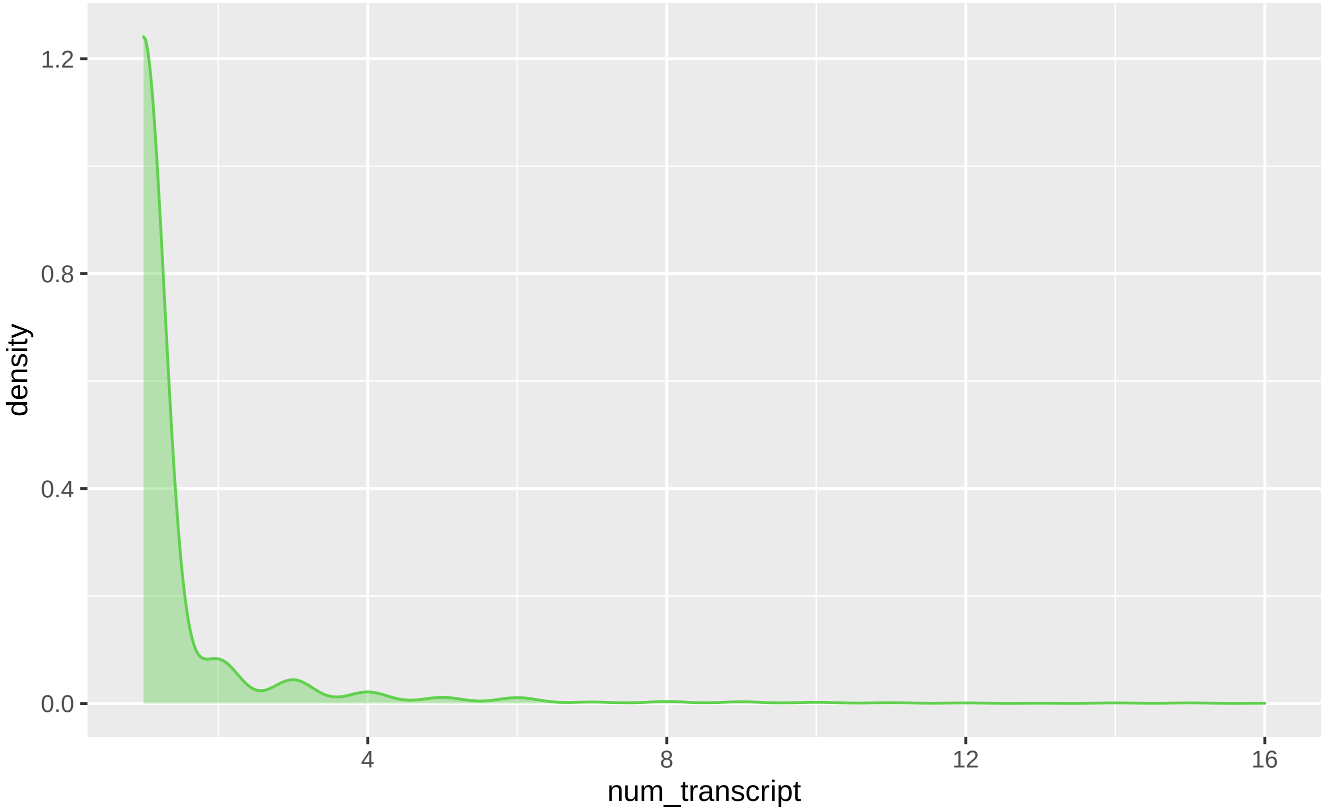

GCF_001433935.1_IRGSP−1.0
EpT

Novel Genes

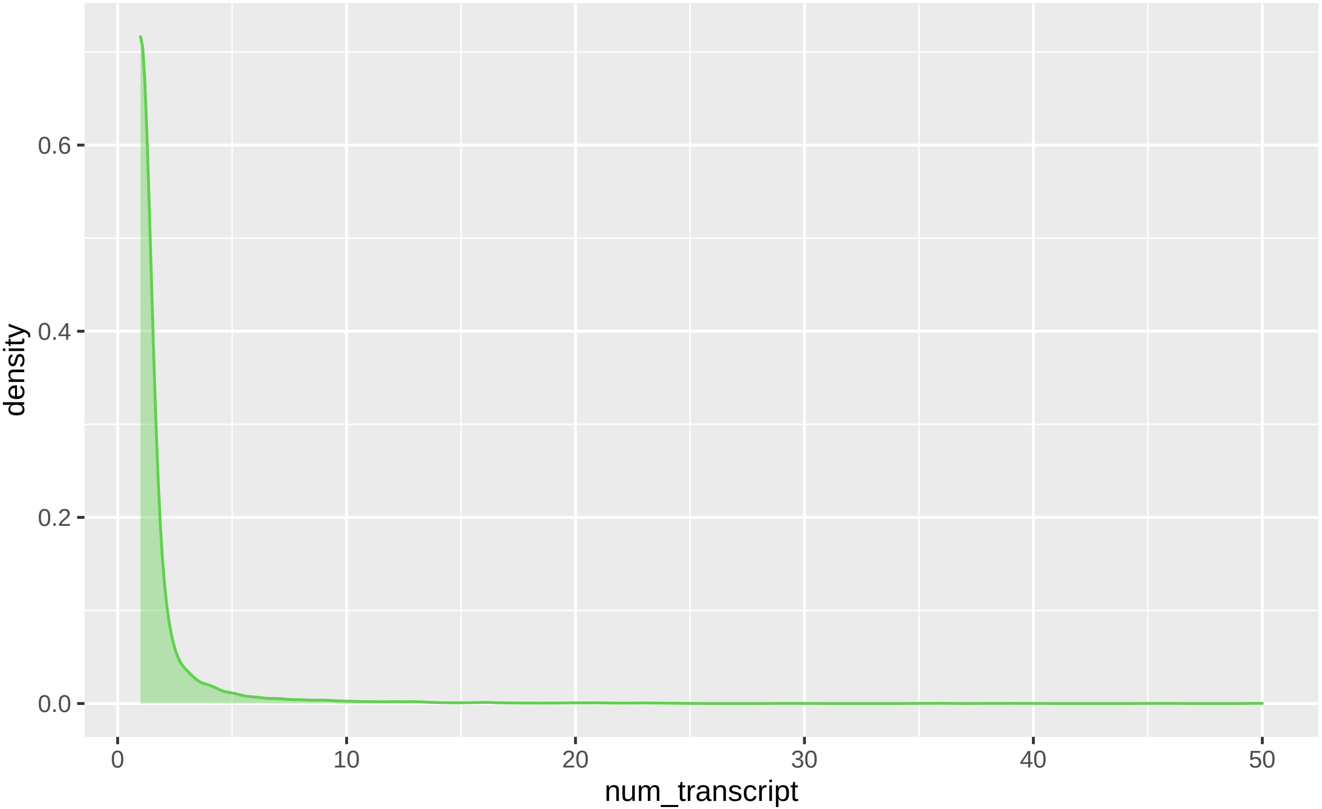

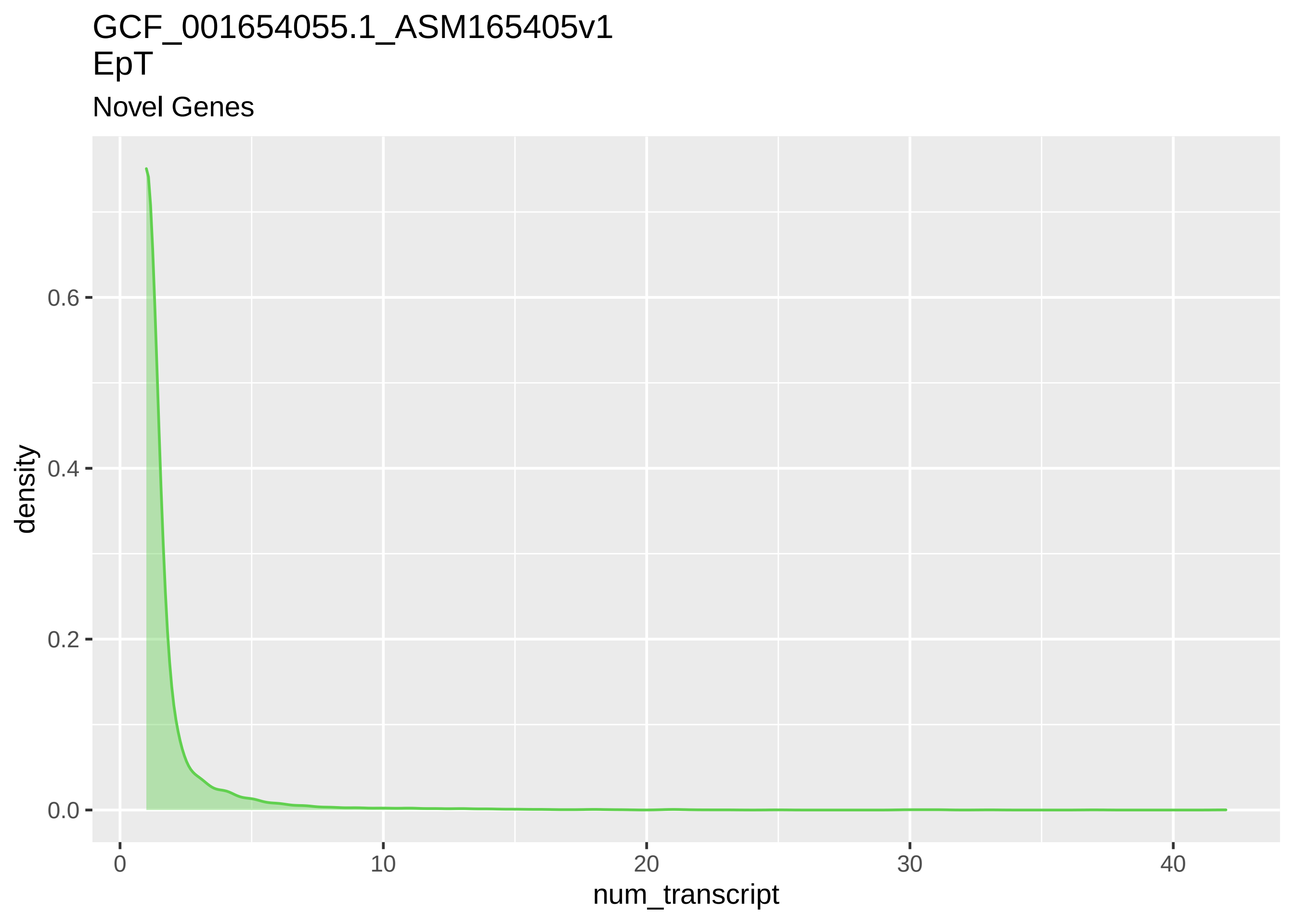
GCF_001654055.1_ASM165405v1
EpT
Novel Genes
density
0.0
0.2
0.4
0.6
0
10
20
30
40
num_transcript

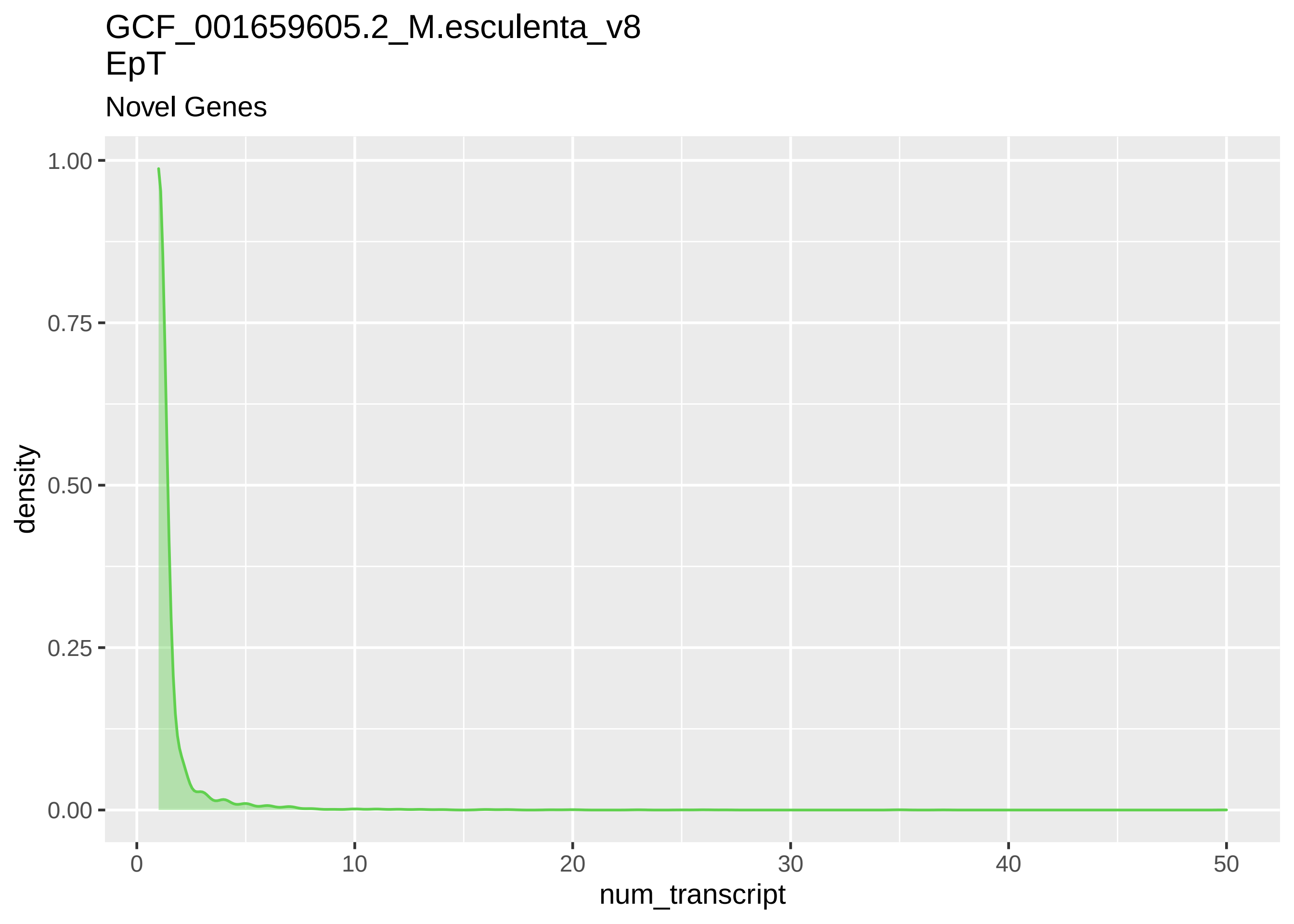
GCF_001659605.2_M.esculenta_v8
EpT
Novel Genes
1.00
0.75
0.50
0.25
0.00
density
0
10
20
30
40
50
num_transcript

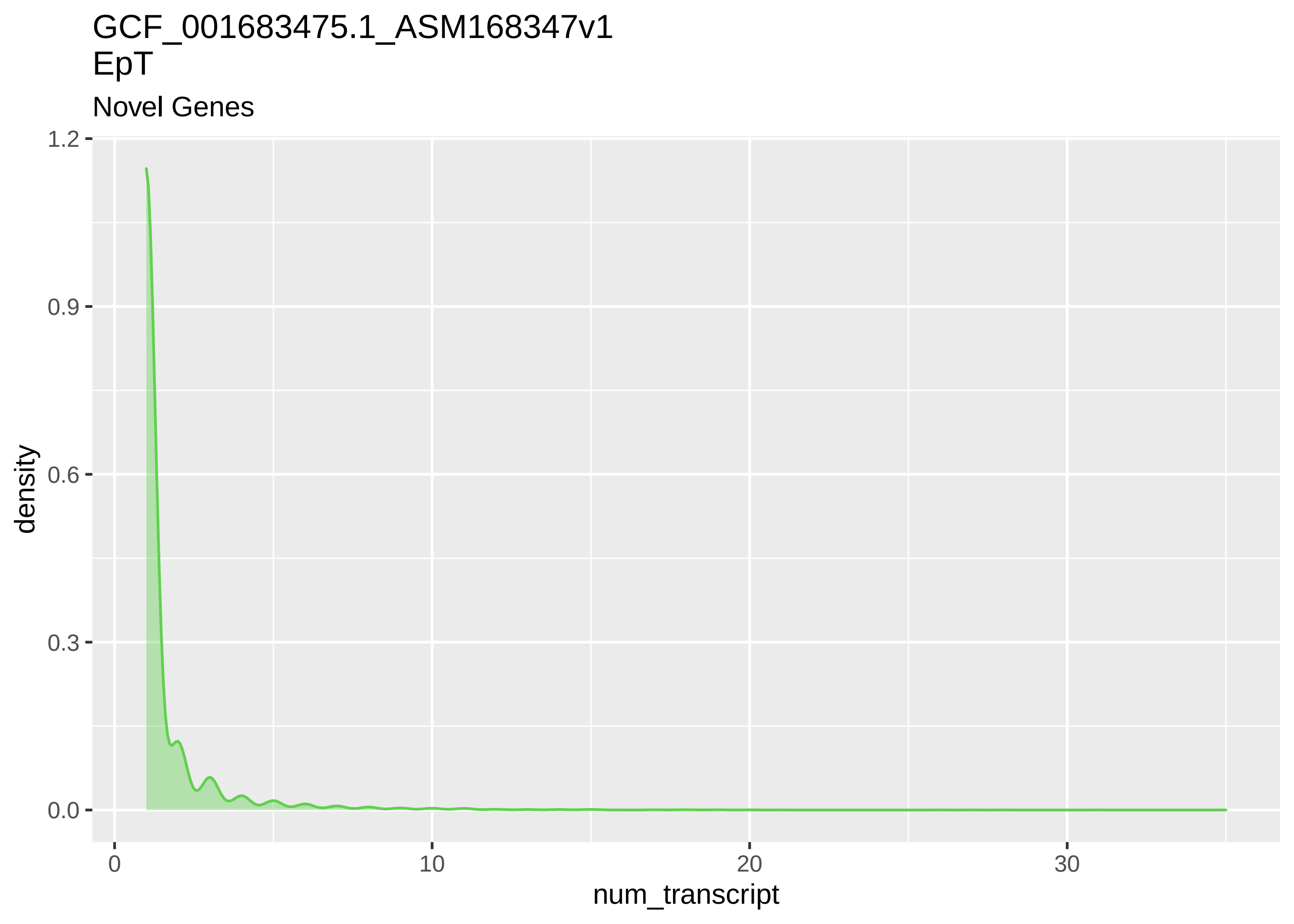
GCF_001683475.1_ASM168347v1
EpT
Novel Genes
density
1.2
0.9
0.6
0.3
0.0
0
10
20
30
num_transcript

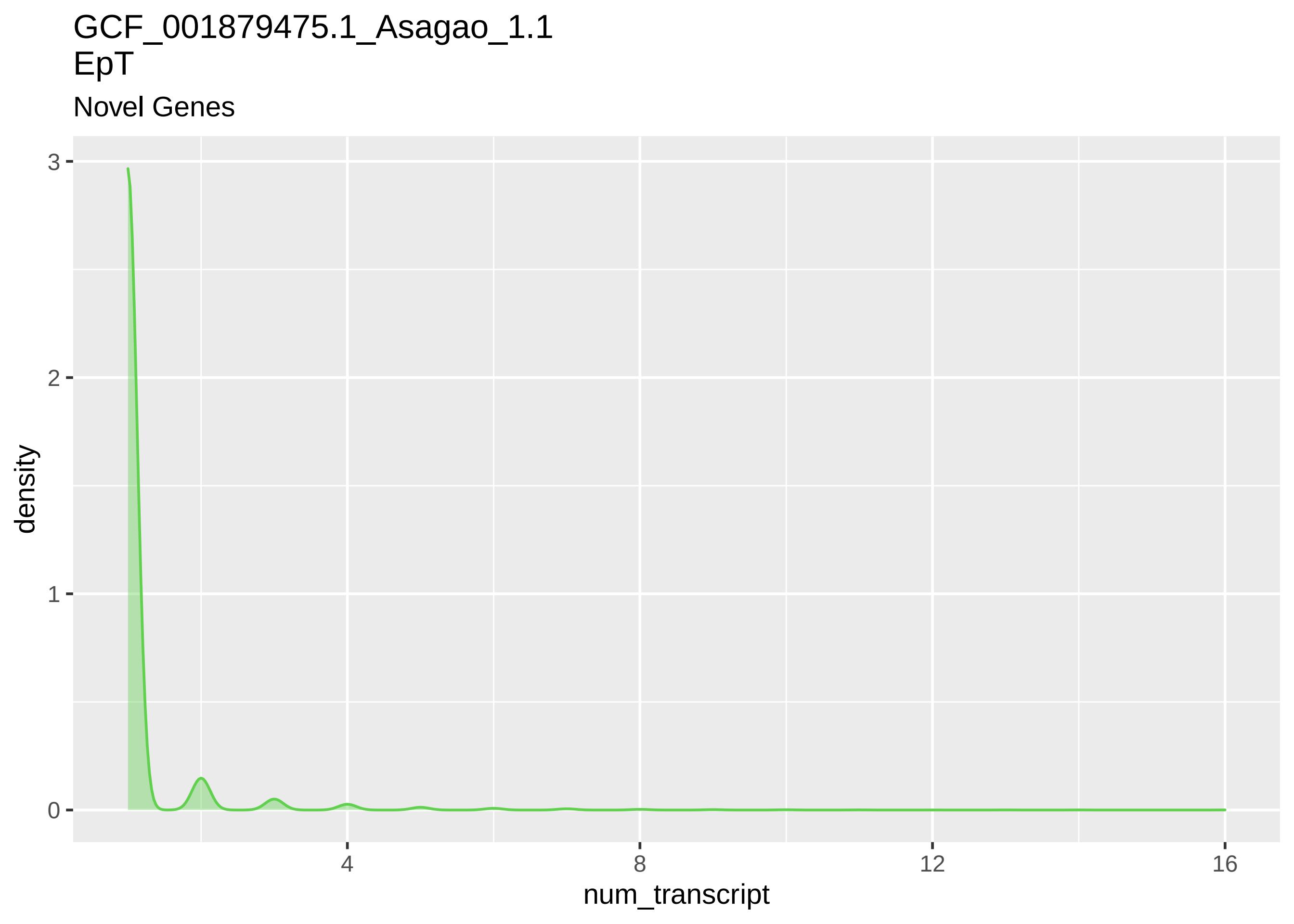
GCF_001879475.1_Asagao_1.1
EpT
Novel Genes
density
num_transcript
0
1
2
3
4
8
12
16

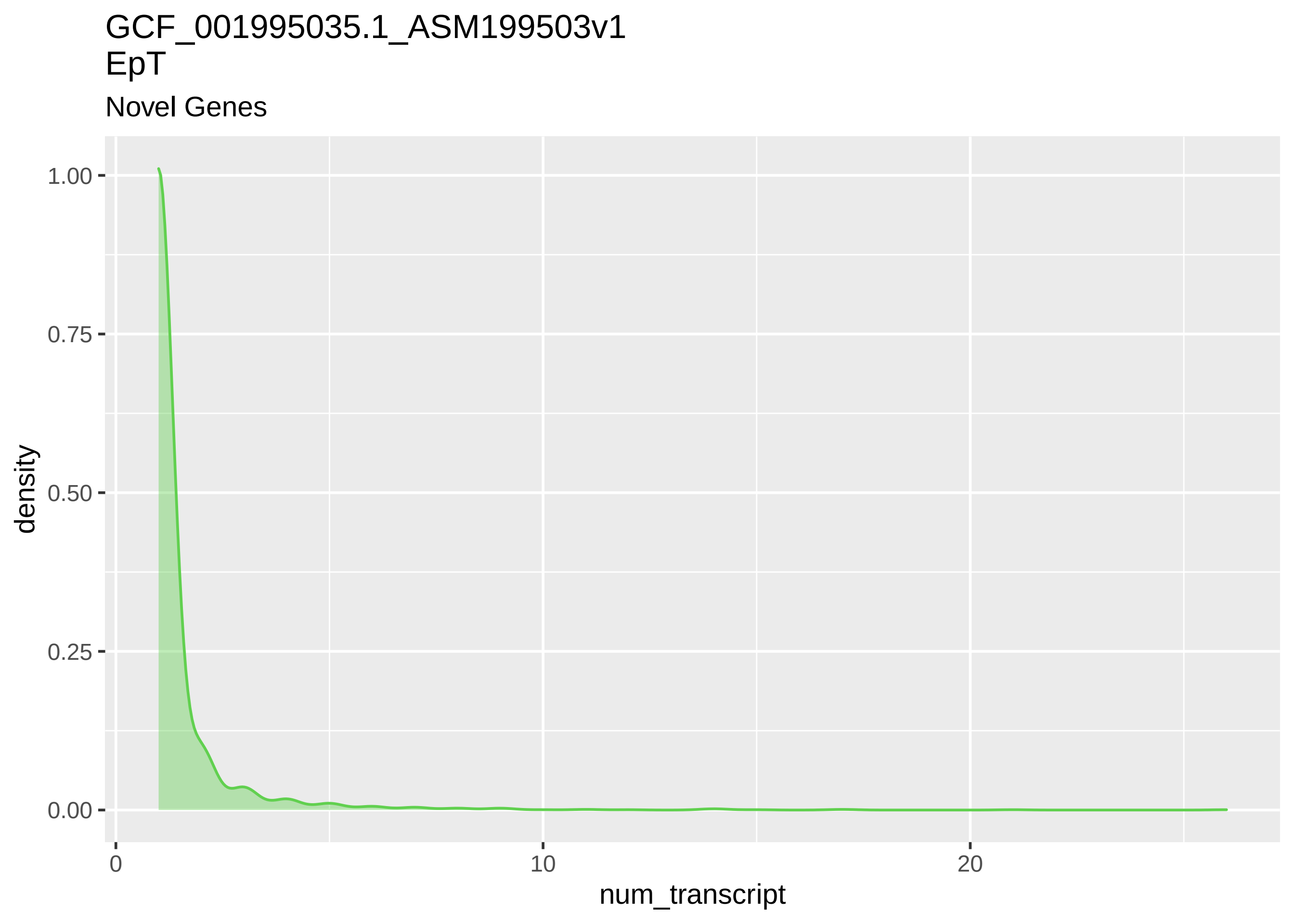

GCF_001995035.1_ASM199503v1
EpT
Novel Genes
density
1.00
0.75
0.50
0.25
0.00
0
10
20
num_transcript

GCF_002114115.1_ASM211411v1
EpT

Novel Genes

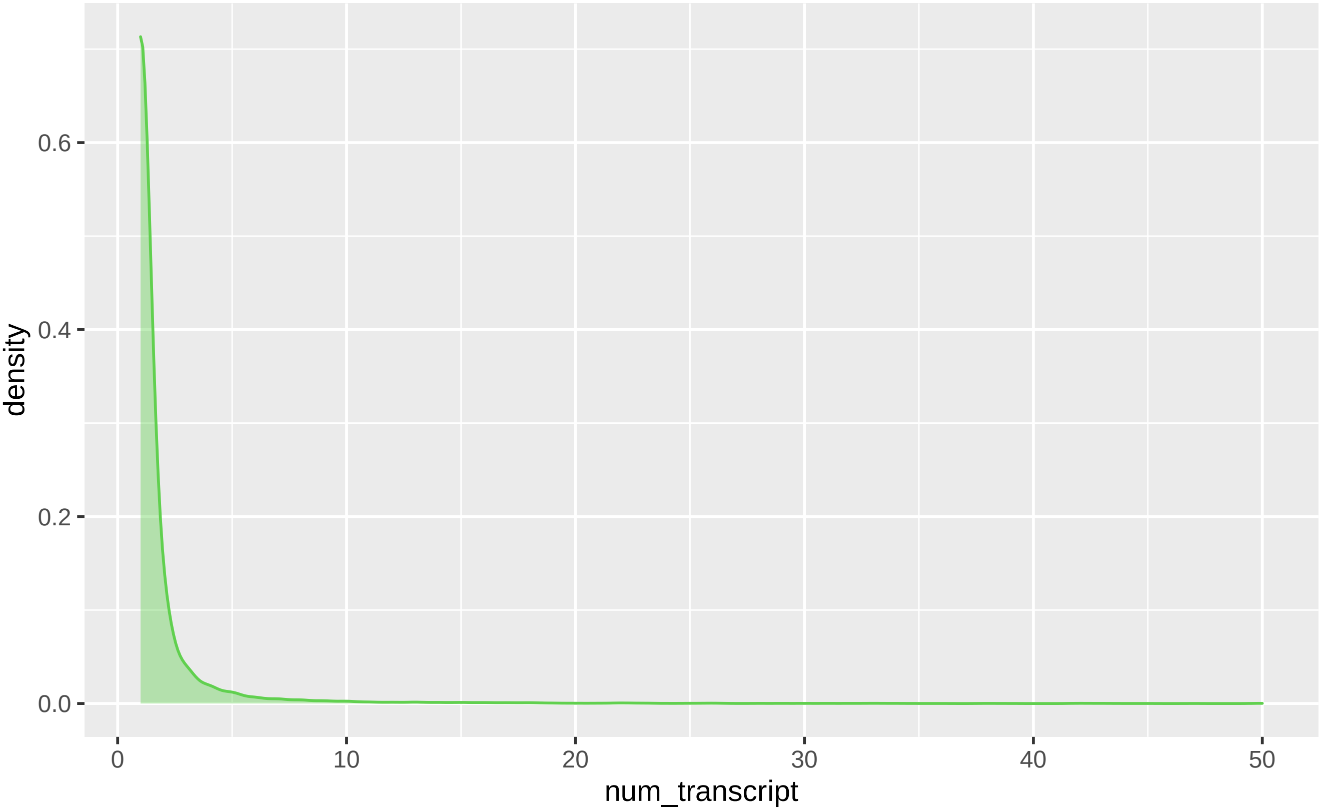

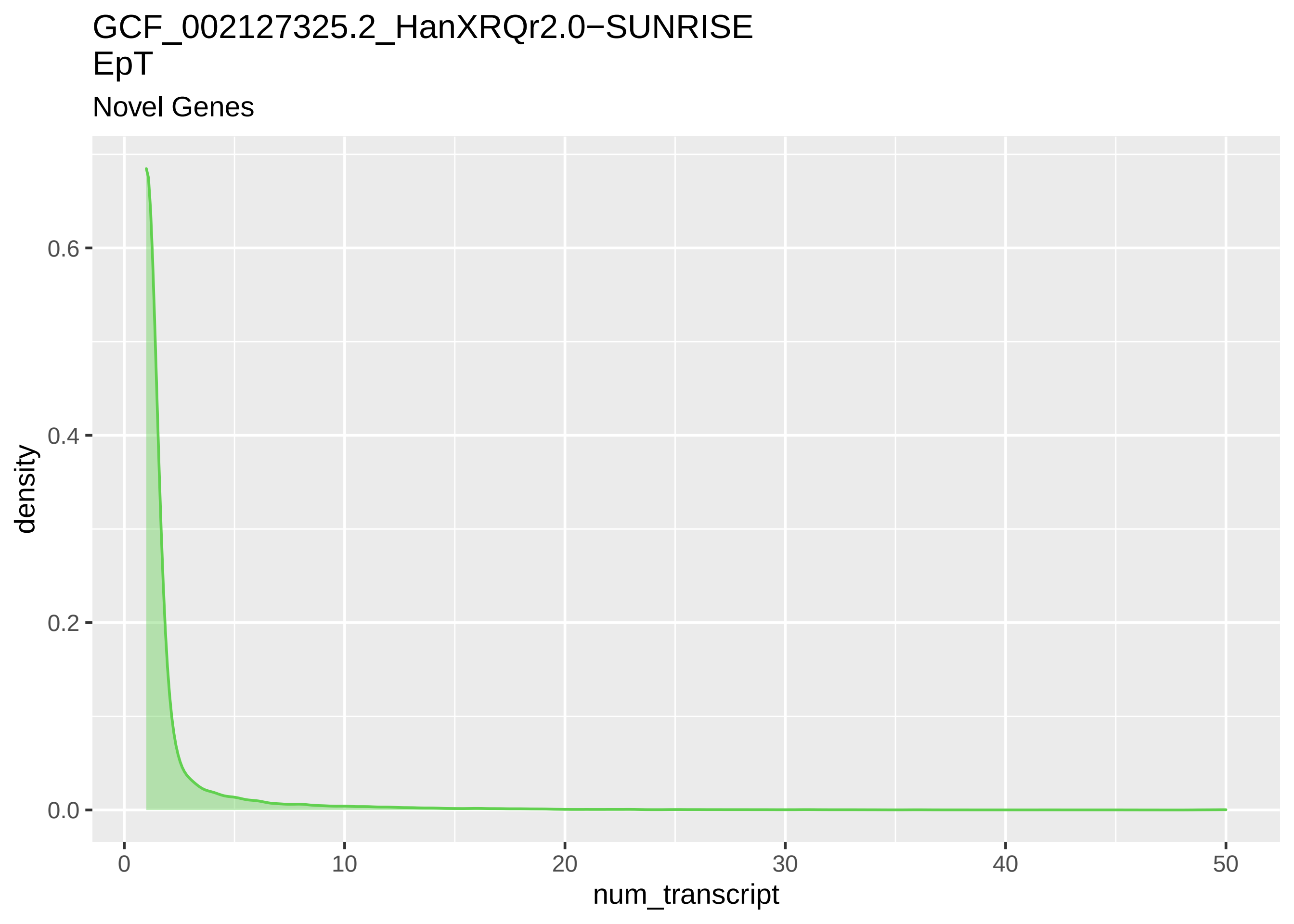
GCF_002127325.2_HanXRQr2.0−SUNRISE
EpT
Novel Genes
density
0.6
0.4
0.2
0.0
0
10
20
30
40
50
num_transcript

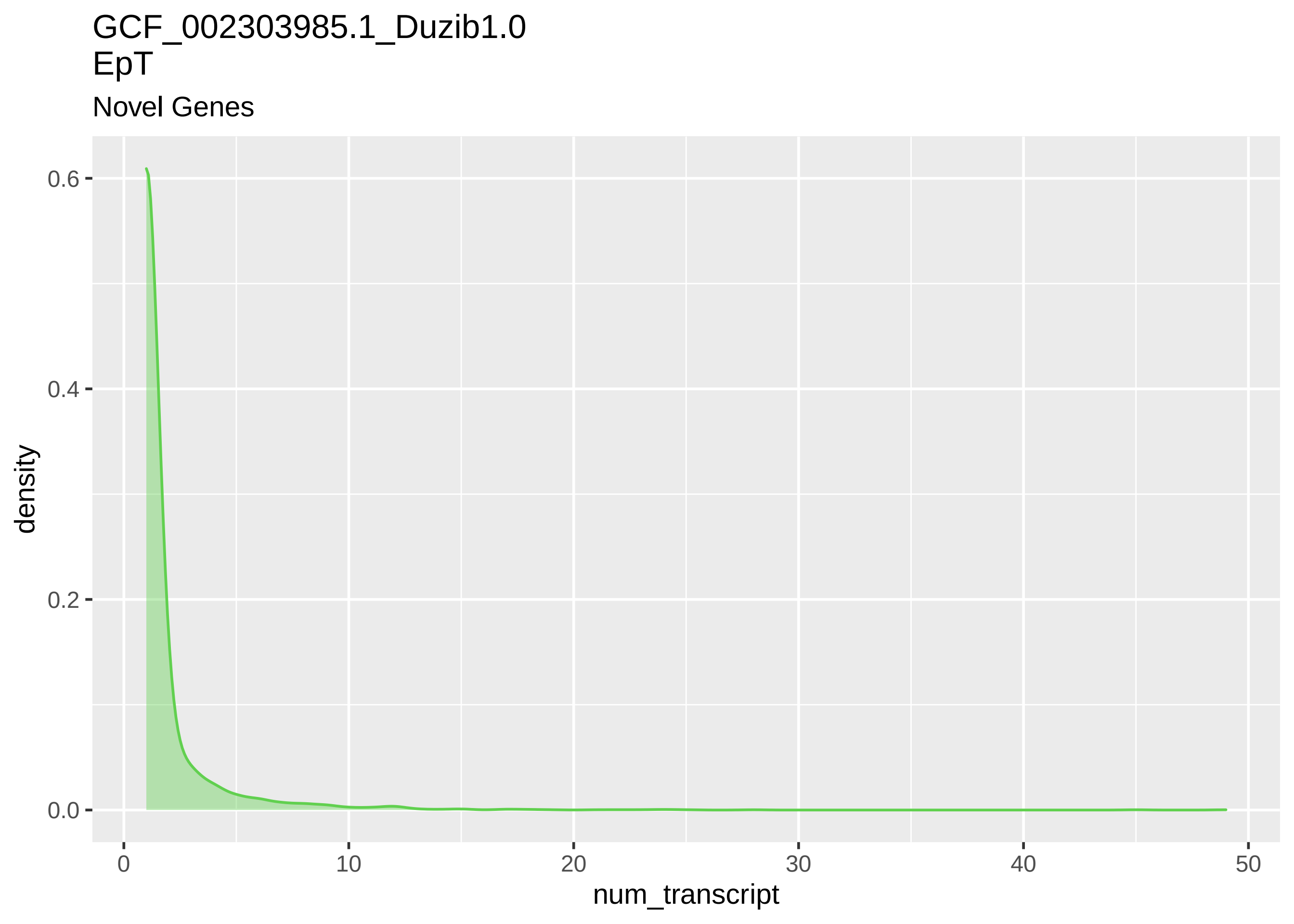
GCF_002303985.1_Duzib1.0
EpT
Novel Genes
density
0.0
0.2
0.4
0.6
0
10
20
30
40
50
num_transcript

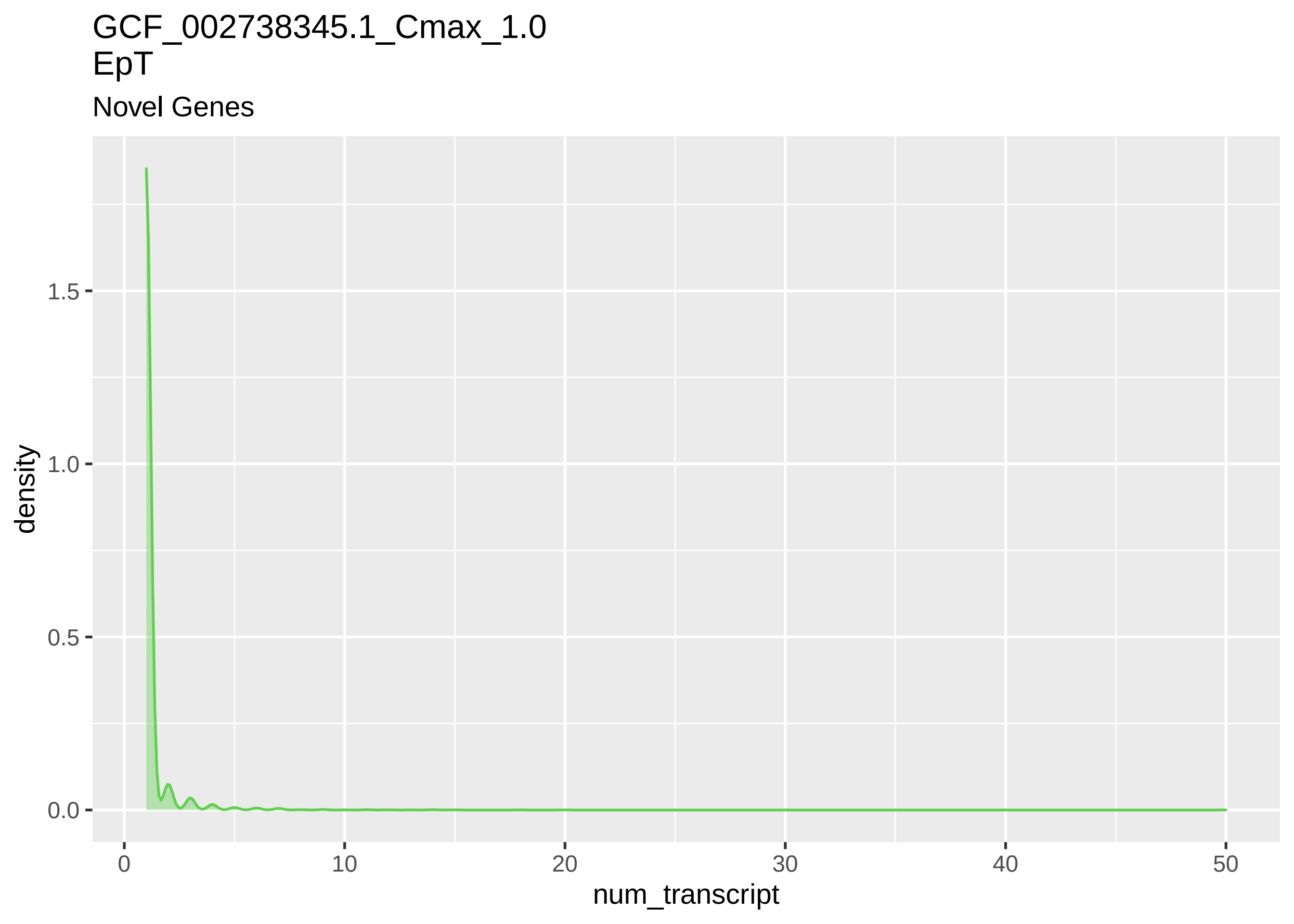
GCF_002738345.1_Cmax_1.0
EpT
Novel Genes
density
num_transcript
0.0
0.5
1.0
1.5
0
10
20
30
40
50

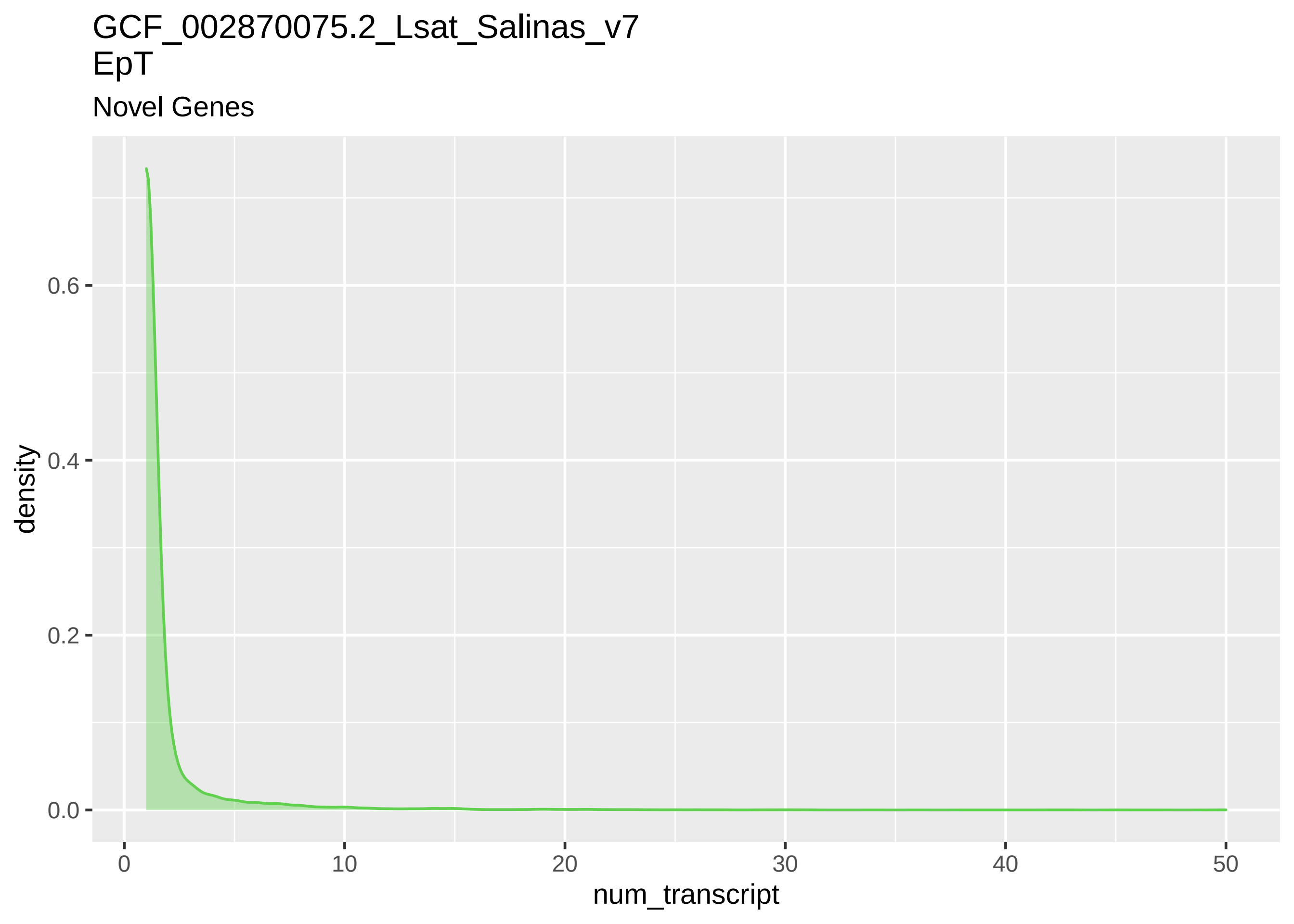
GCF_002870075.2_Lsat_Salinas_v7
EpT
Novel Genes
density
0.0
0.2
0.4
0.6
0
10
20
30
40
50
num_transcript

GCF_002906115.1_CorkOak1.0
EpT

Novel Genes

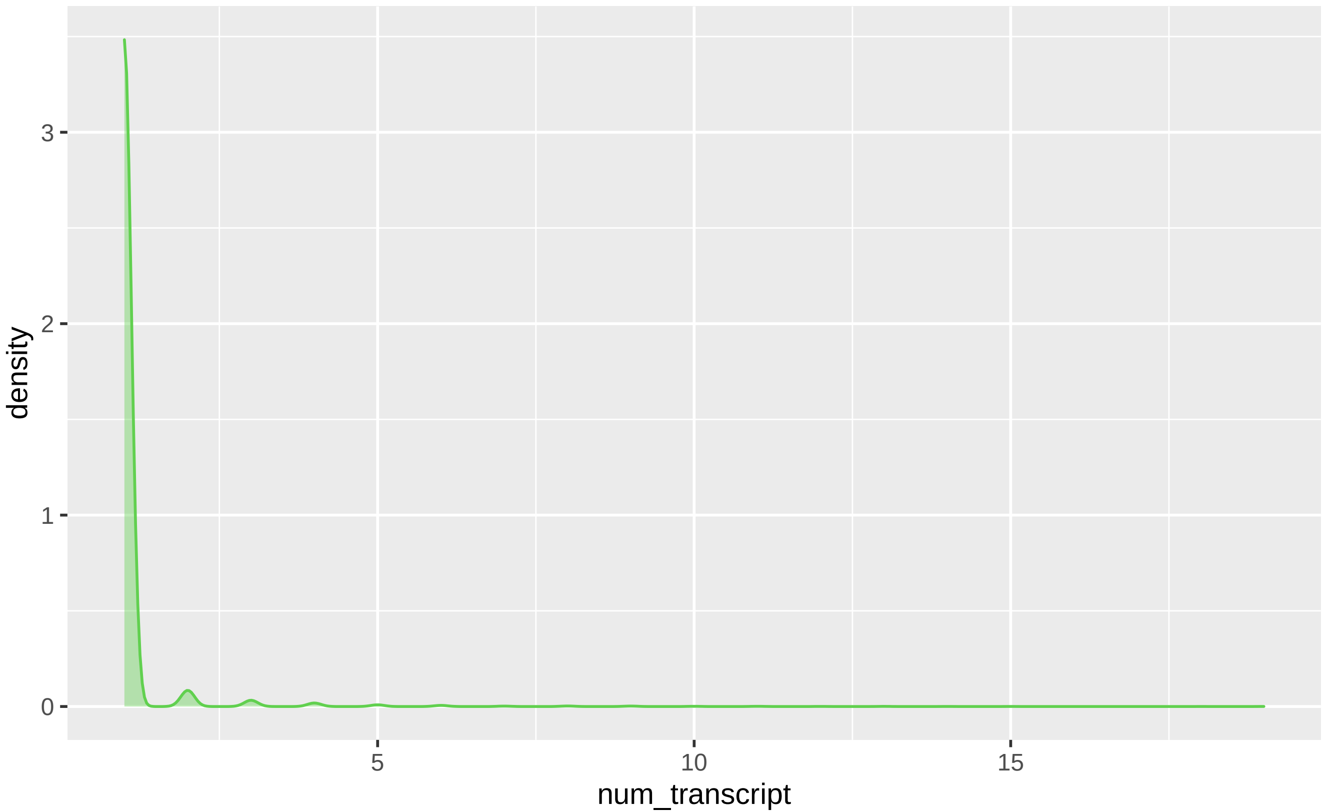

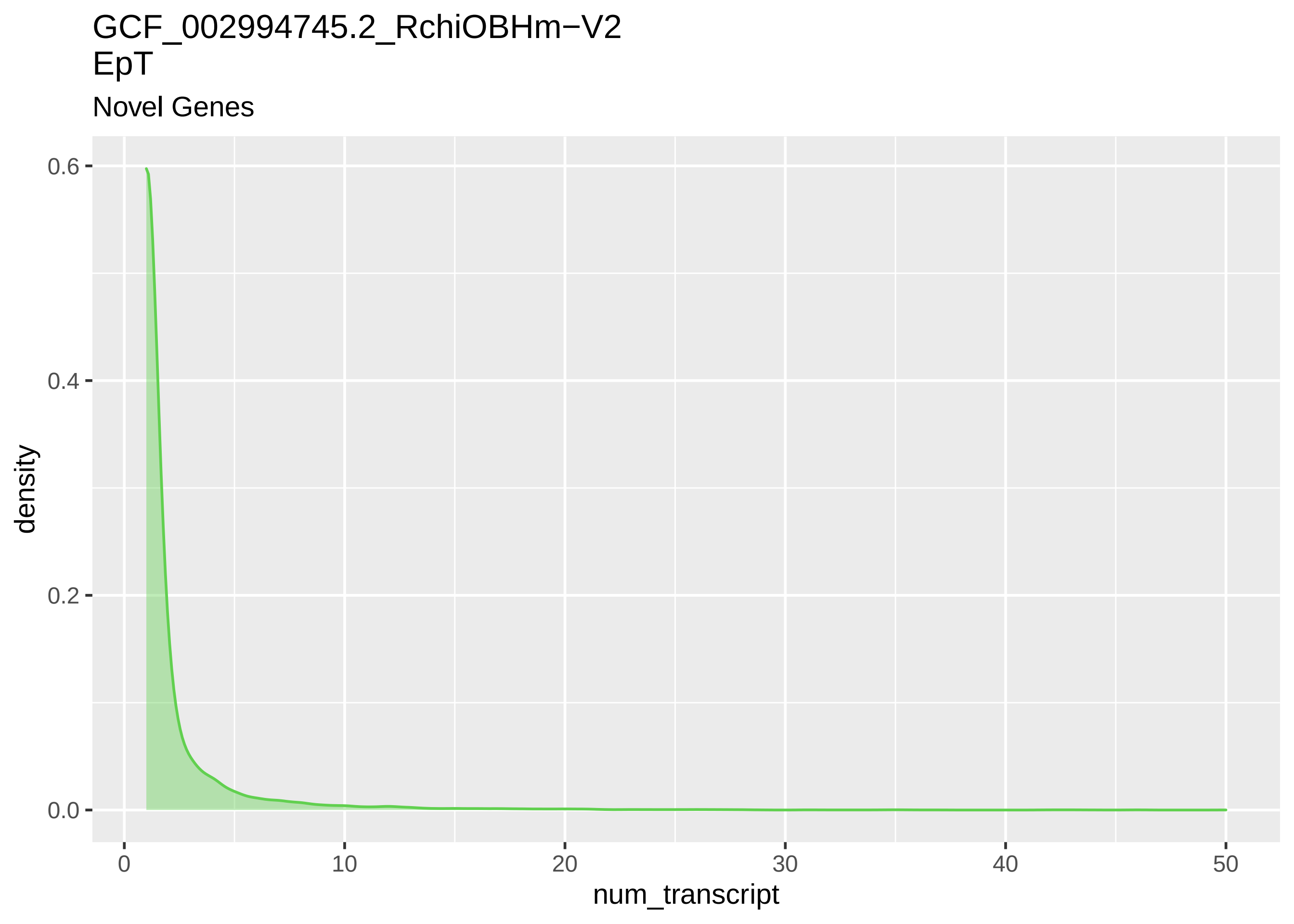
GCF_002994745.2_RchiOBHm−V2
EpT
Novel Genes
density
0.0
0.2
0.4
0.6
0
10
20
30
40
50
num_transcript

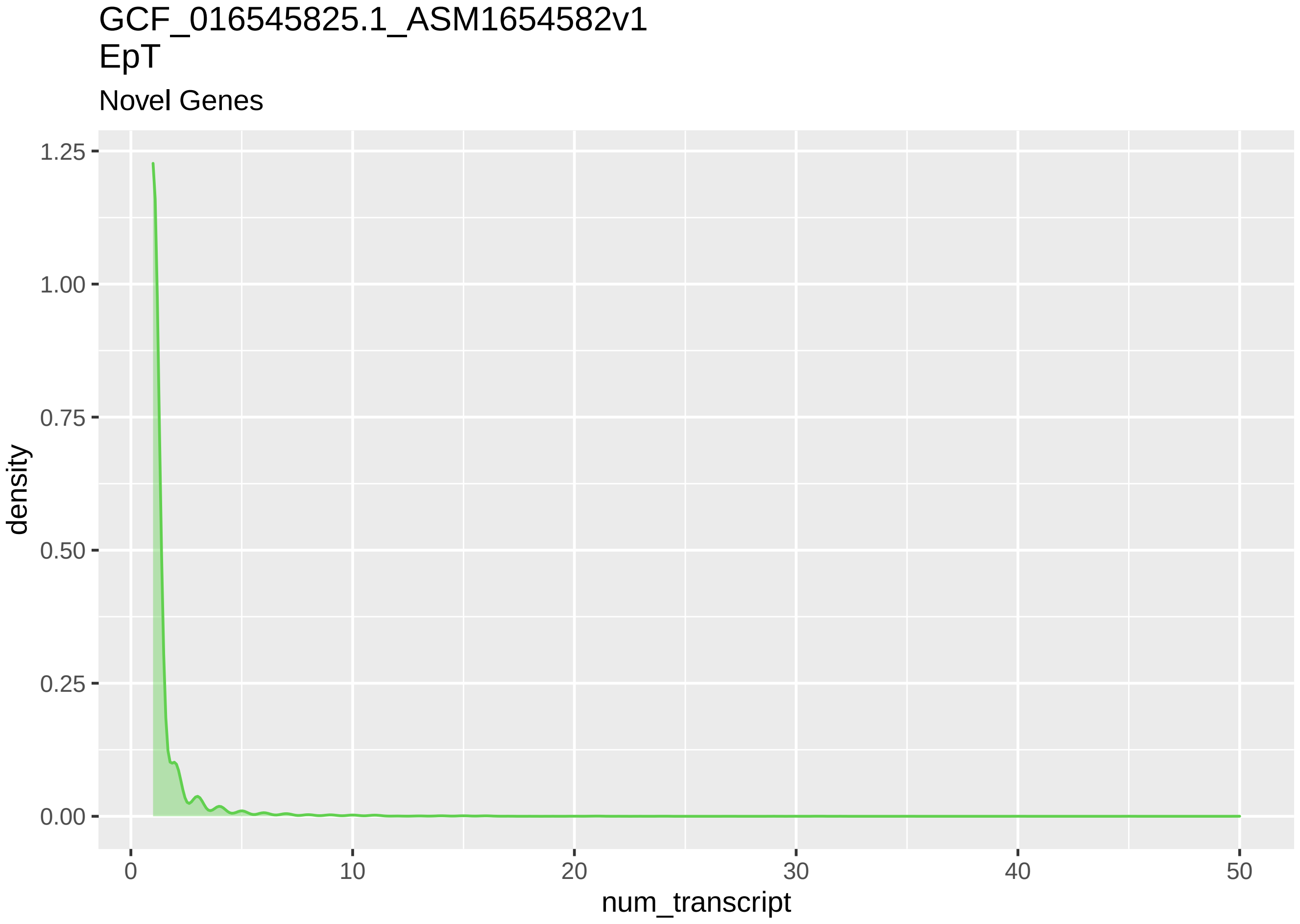
GCF_016545825.1_ASM1654582v1
EpT
Novel Genes
density
1.25
1.00
0.75
0.50
0.25
0.00
0
10
20
30
40
50
num_transcript

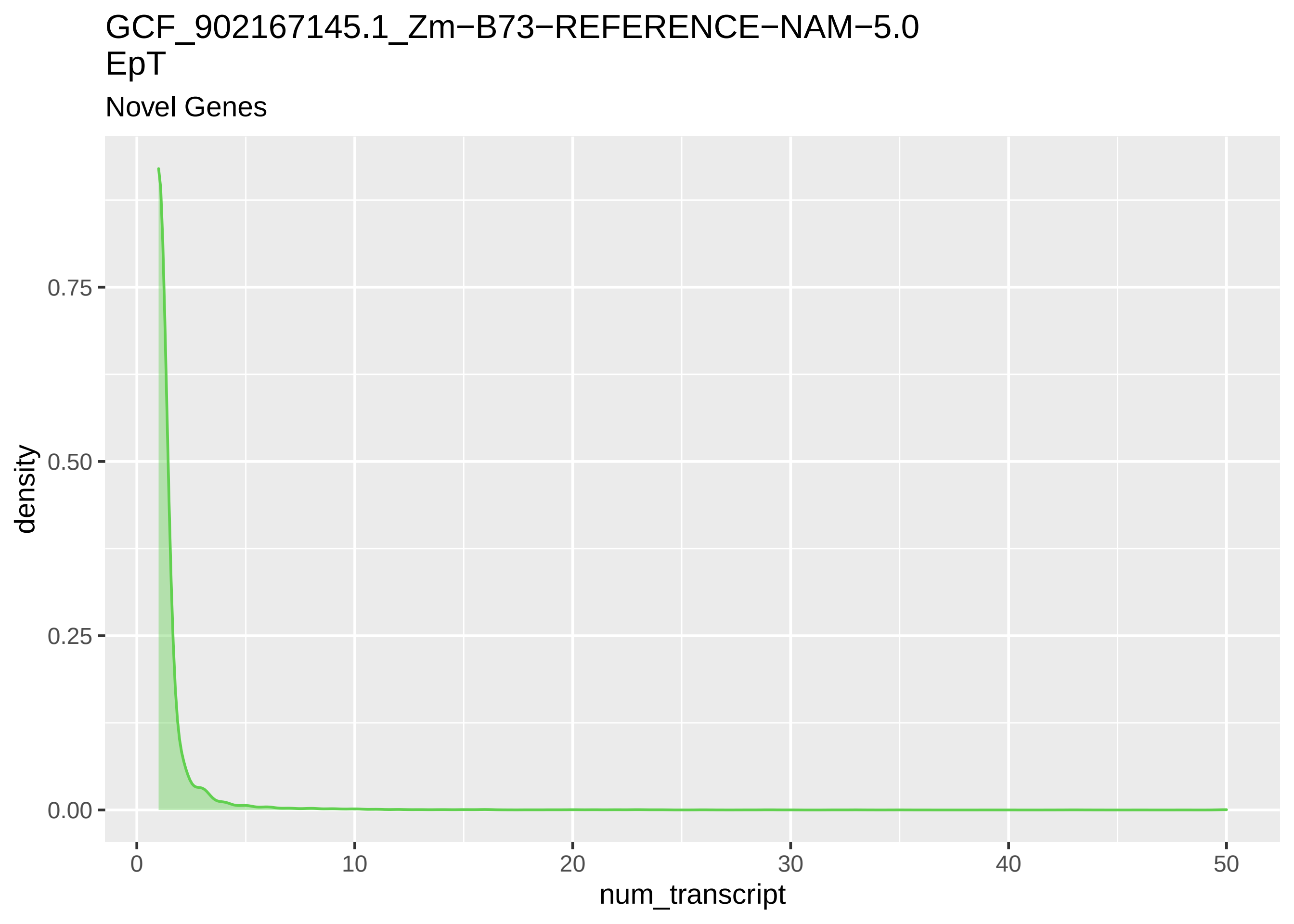
GCF_902167145.1_Zm−B73−REFERENCE−NAM−5.0
EpT
Novel Genes
density
0.75
0.50
0.25
0.00
0
10
20
30
40
50
num_transcript

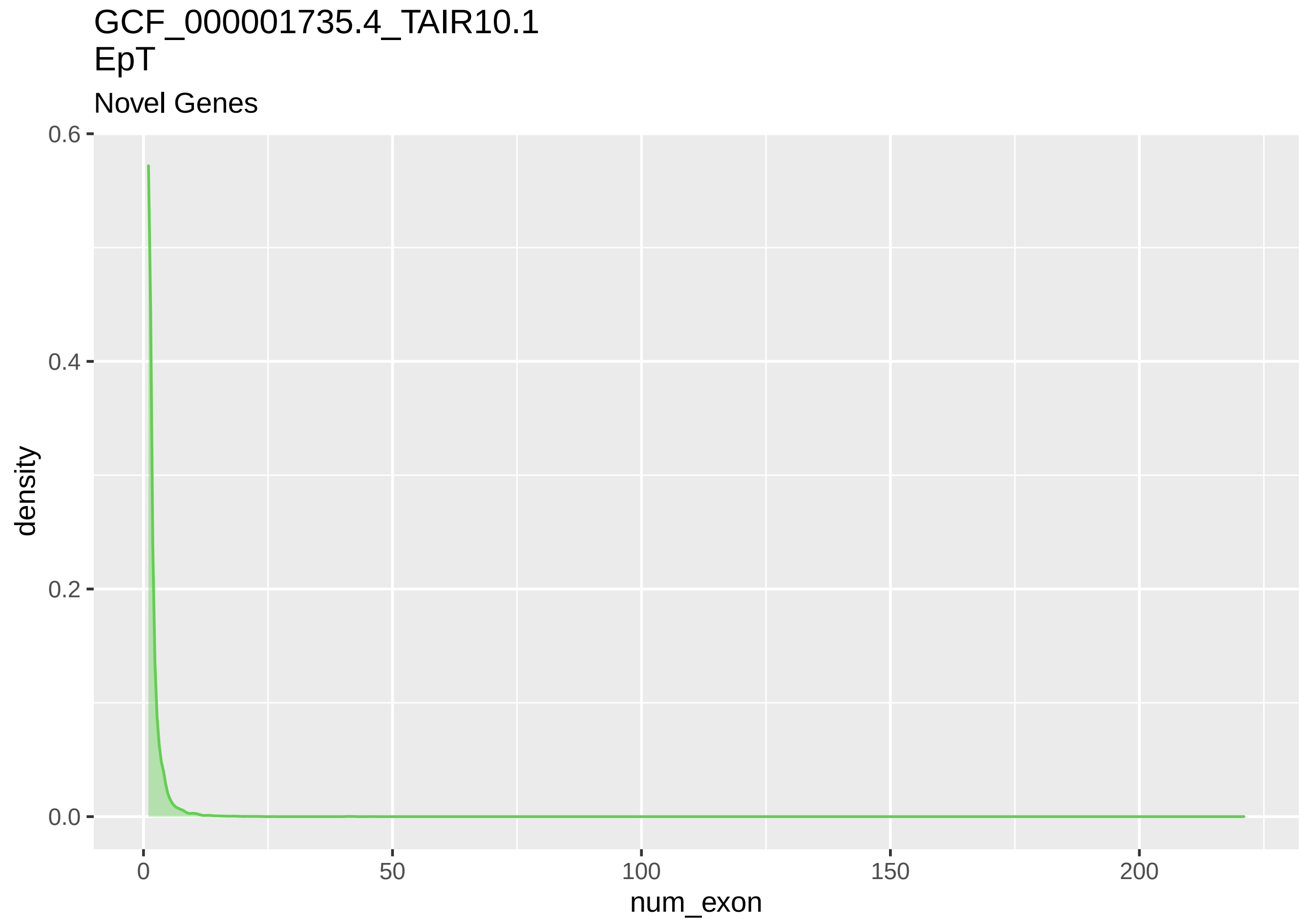
GCF_000001735.4_TAIR10.1
EpT
Novel Genes
density
num_exon
0.6
0.4
0.2
0.0
0
50
100
150
200

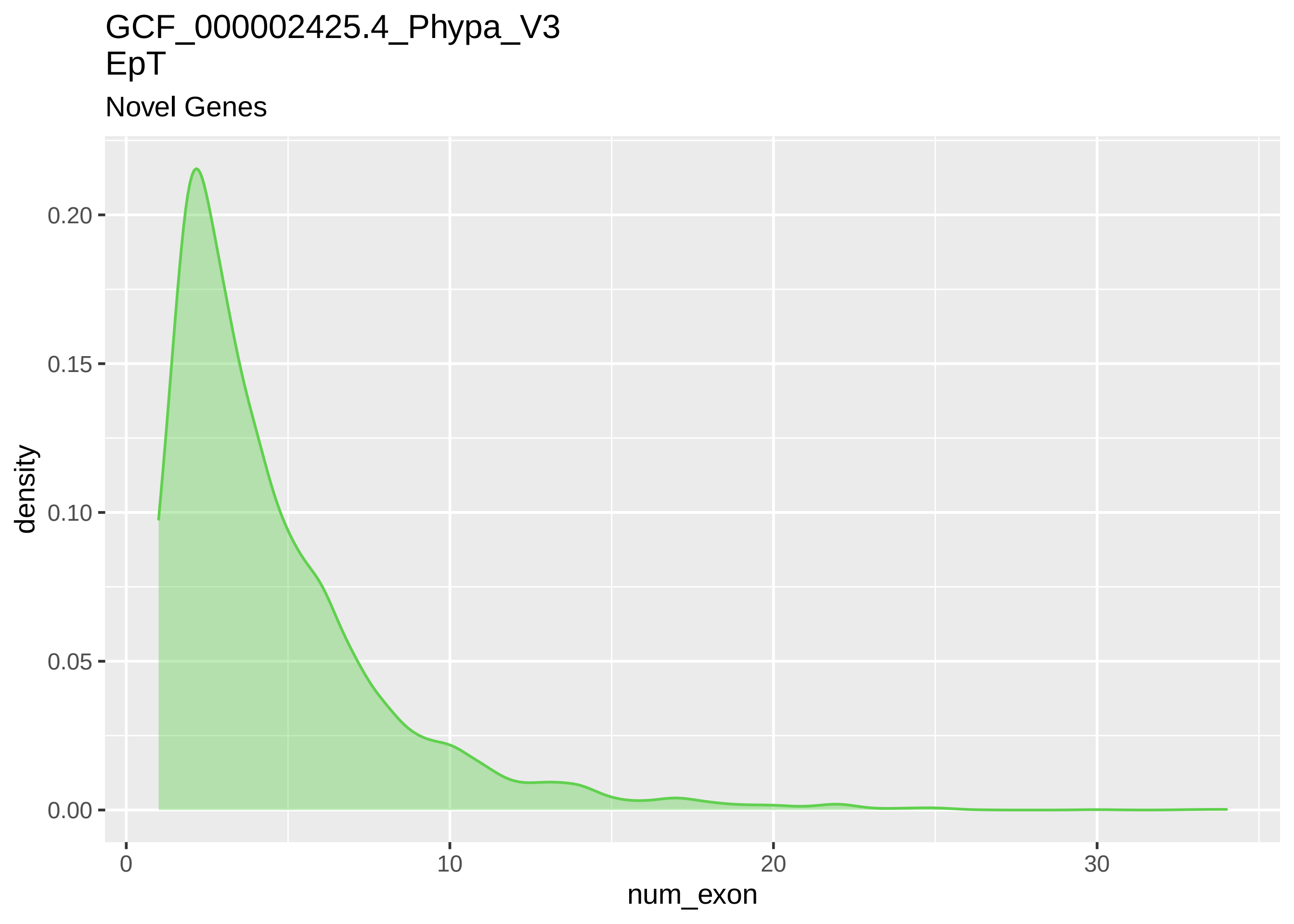
GCF_000002425.4_Phypa_V3
EpT
Novel Genes
density
0.20
0.15
0.10
0.05
0.00
0
10
20
30
num_exon

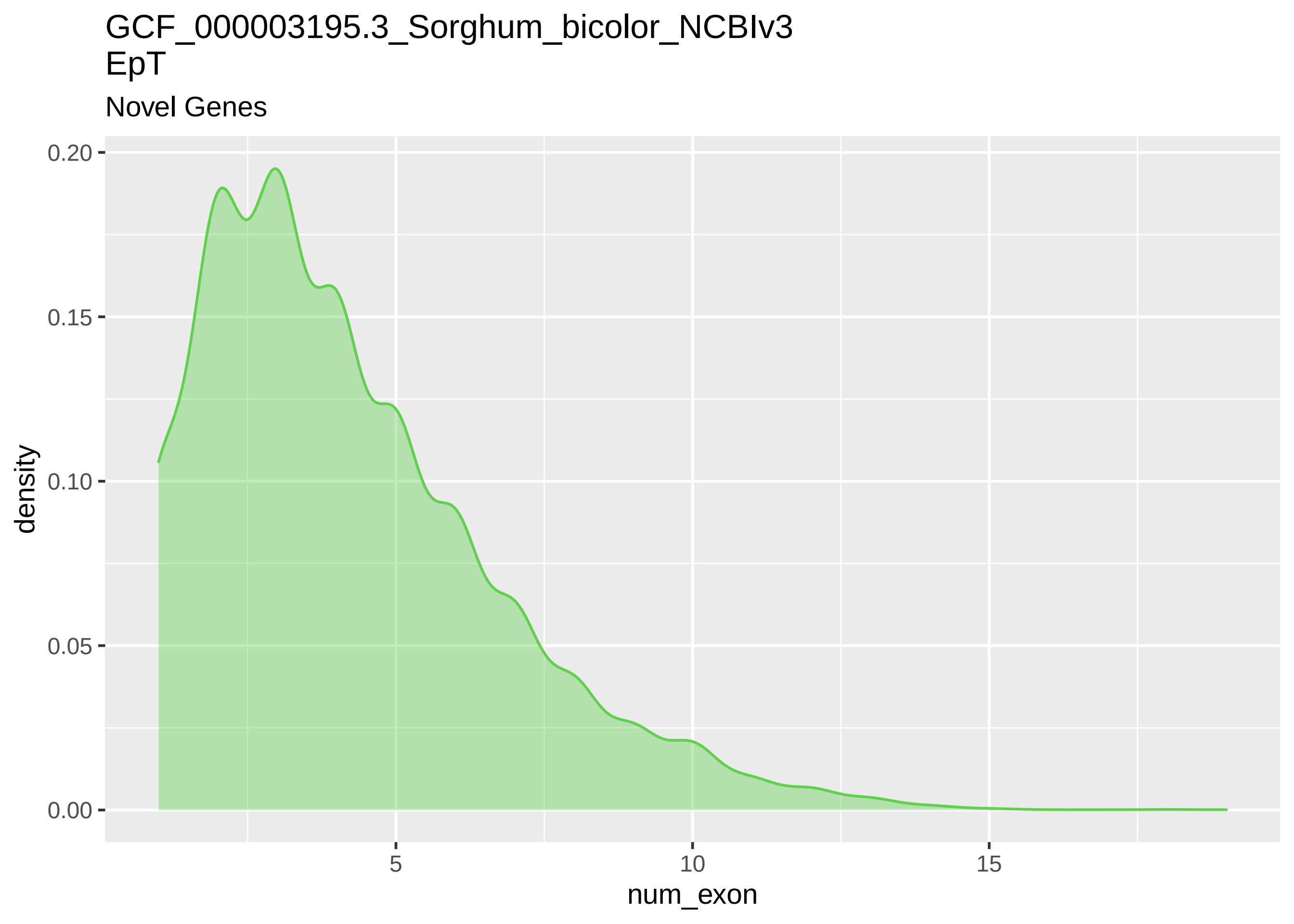

GCF_000003195.3_Sorghum_bicolor_NCBIv3
EpT
Novel Genes
0.20
0.15
0.10
0.05
0.00
density
5
10
15
num_exon

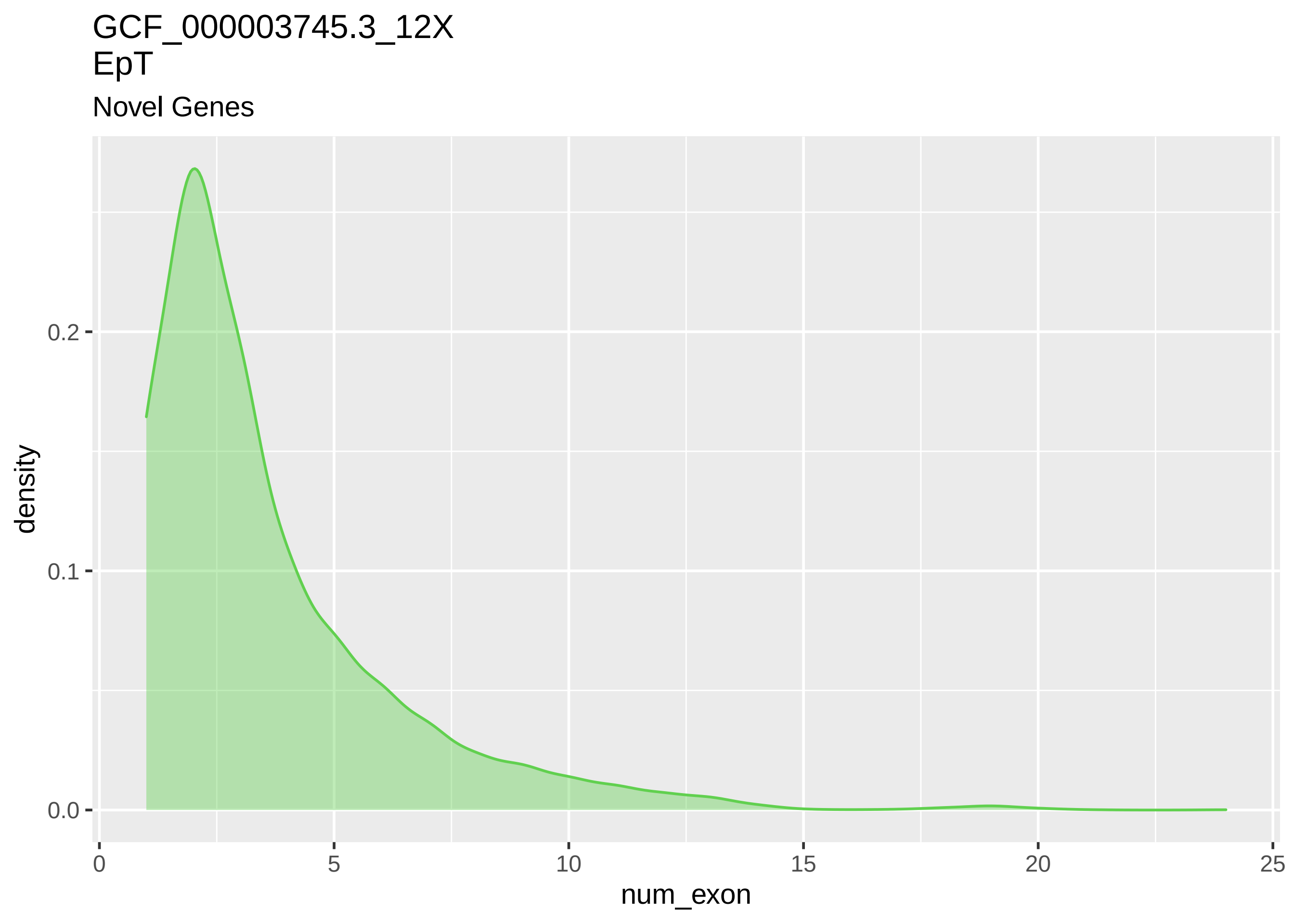
GCF_000003745.3_12X
EpT
Novel Genes
density
0.0
0.1
0.2
0
5
10
15
20
25
num_exon

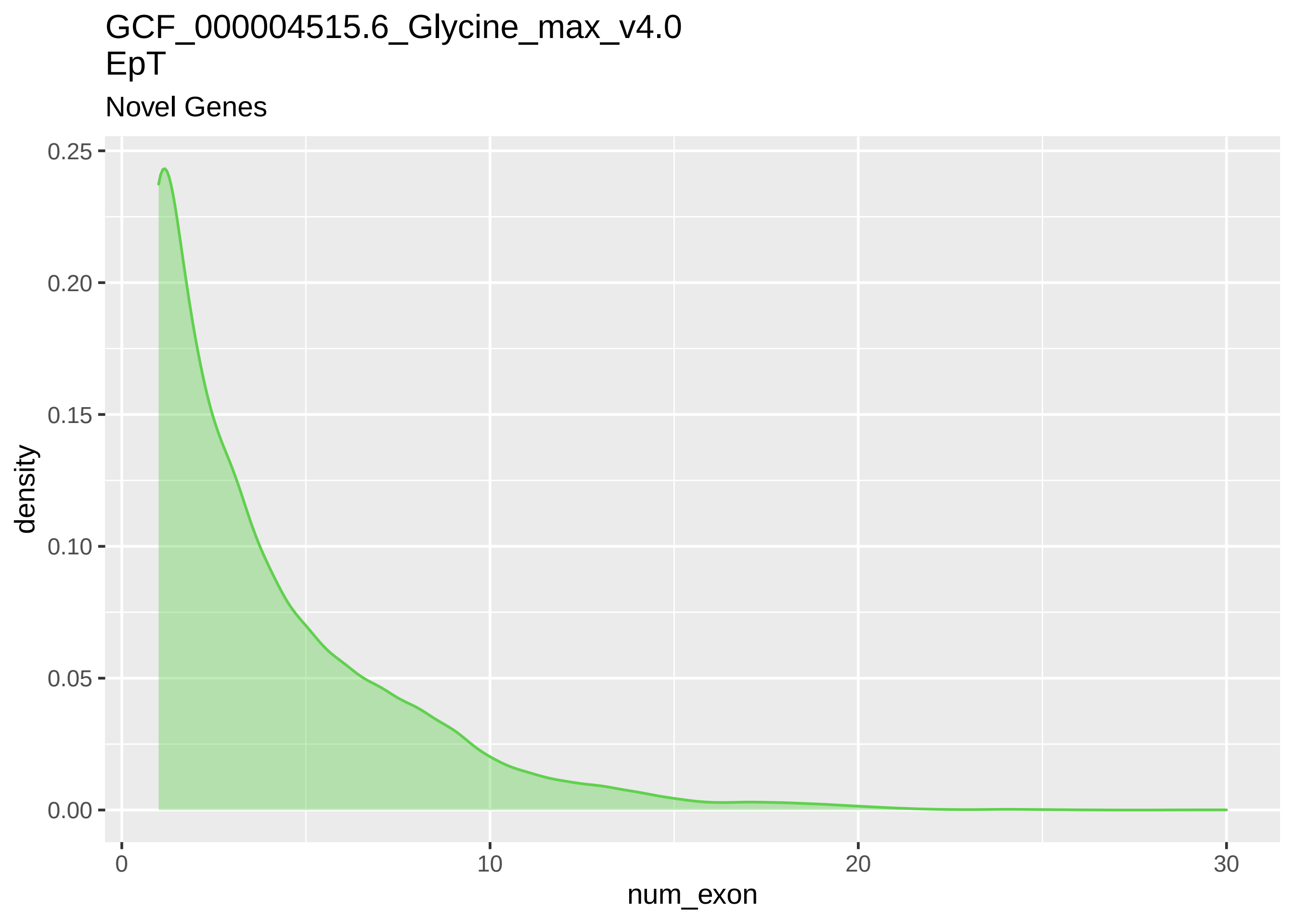
GCF_000004515.6_Glycine_max_v4.0
EpT
Novel Genes
0.25
0.20
0.15
0.10
0.05
0.00
density
0
10
20
30
num_exon

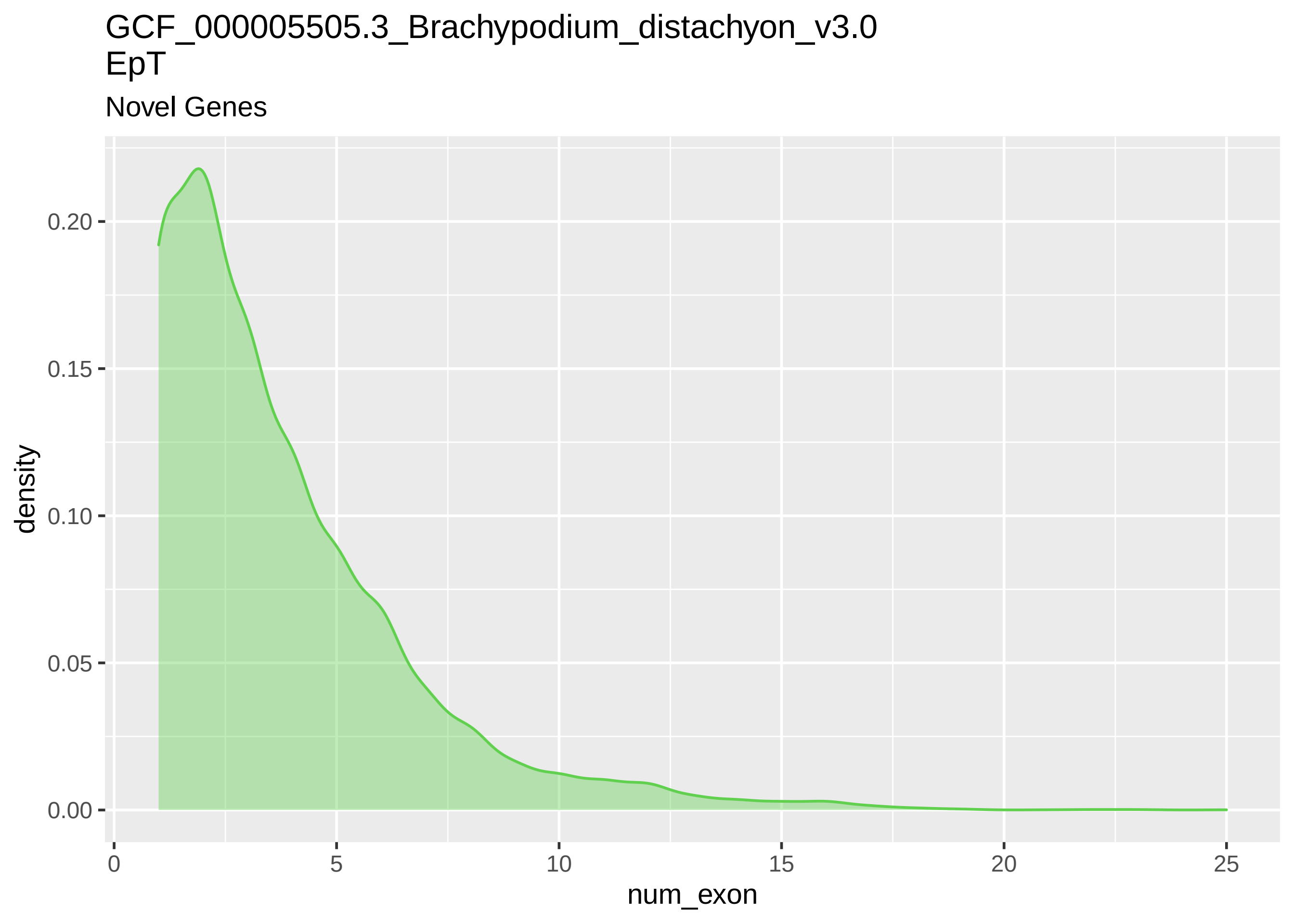
GCF_000005505.3_Brachypodium_distachyon_v3.0
EpT
Novel Genes
density
0.00
0.05
0.10
0.15
0.20
0
5
10
15
20
25
num_exon

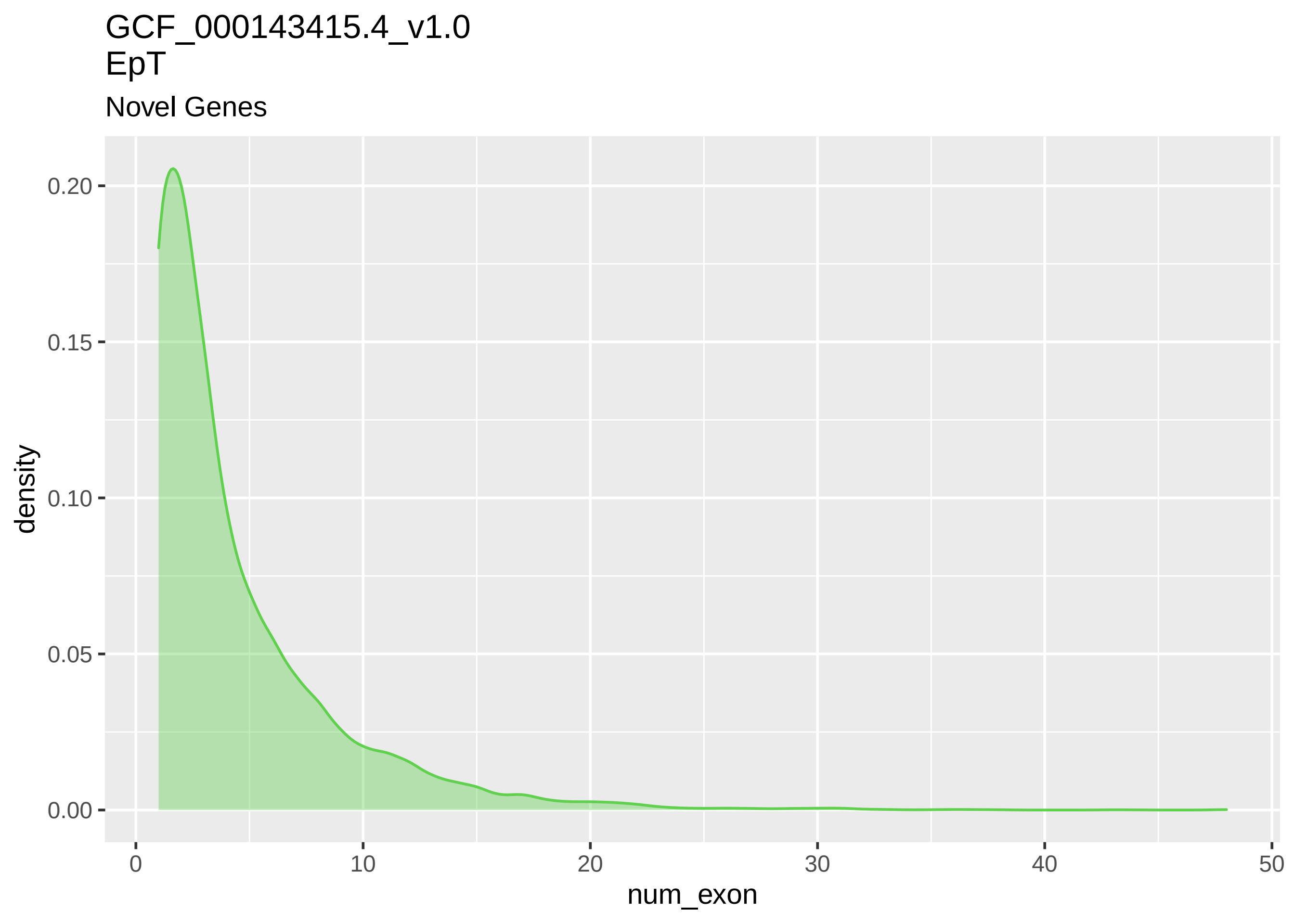
GCF_000143415.4_v1.0
EpT
Novel Genes
density
0.20
0.15
0.10
0.05
0.00
0
10
20
30
40
50
num_exon

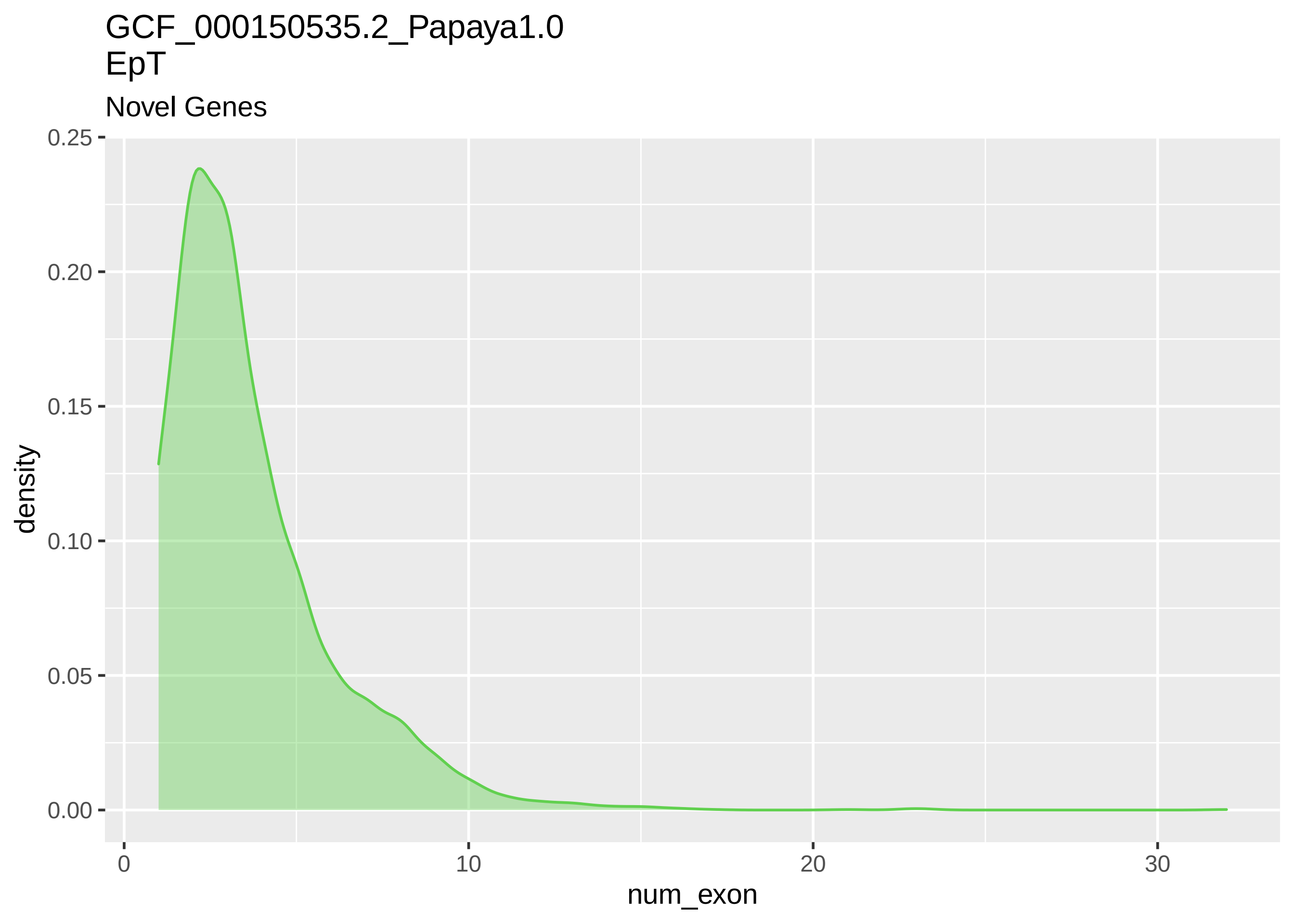
GCF_000150535.2_Papaya1.0
EpT
Novel Genes
density
0.25
0.20
0.15
0.10
0.05
0.00
0
10
20
30
num_exon

GCF_000208745.1_Criollo_cocoa_genome_V2
EpT

Novel Genes

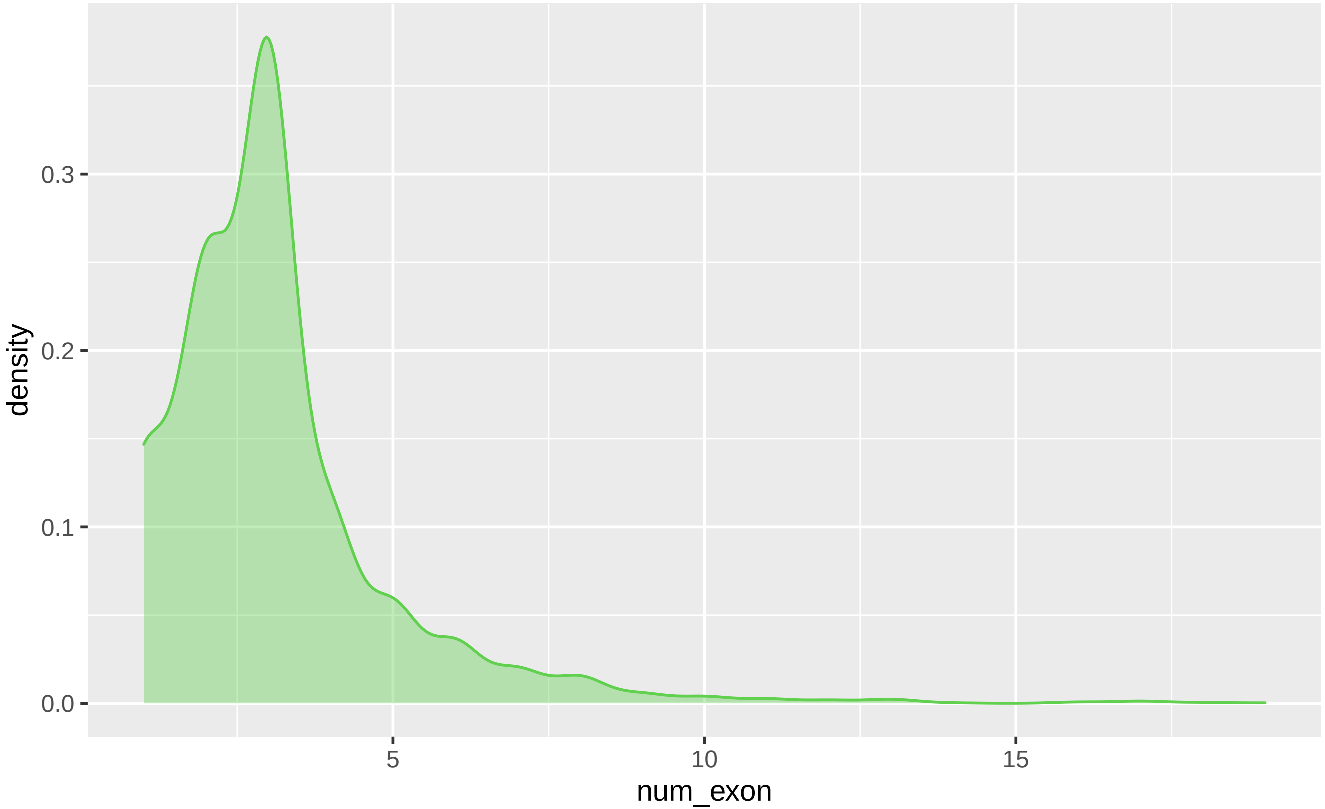

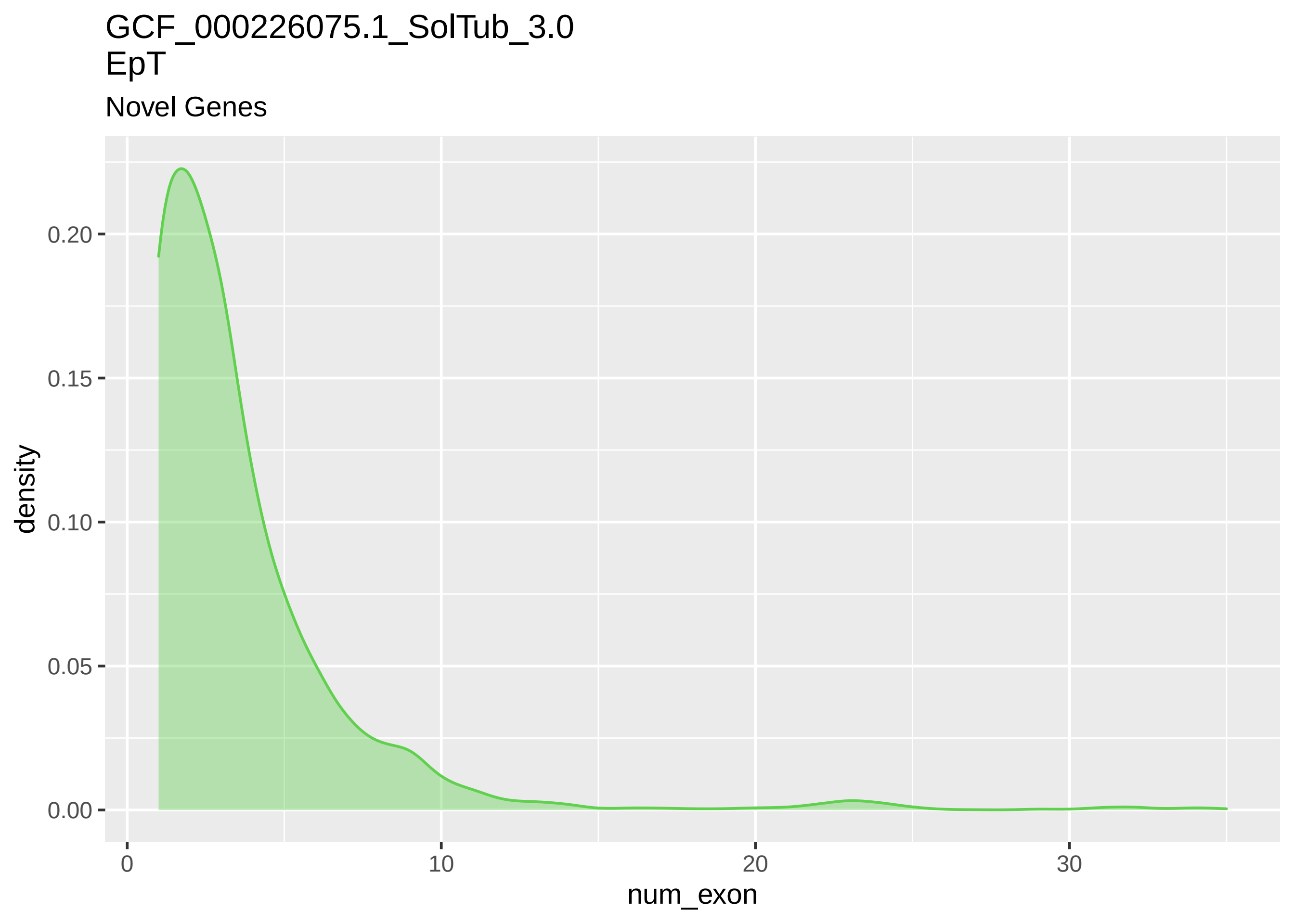
GCF_000226075.1_SolTub_3.0
EpT
Novel Genes
density
0.20
0.15
0.10
0.05
0.00
0
10
20
30
num_exon

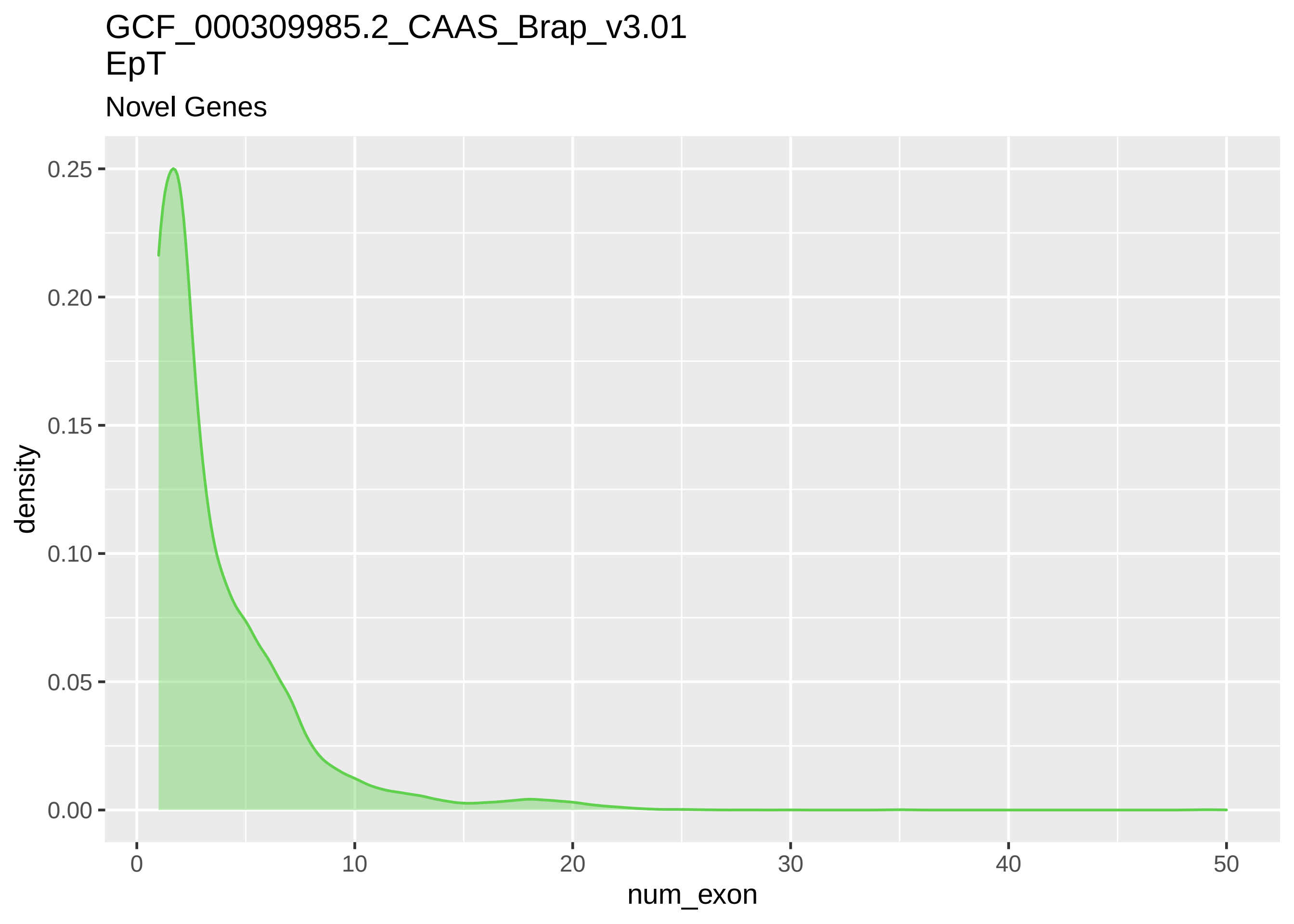
GCF_000309985.2_CAAS_Brap_v3.01
EpT
Novel Genes
density
0.25
0.20
0.15
0.10
0.05
0.00
0
10
20
30
40
50
num_exon

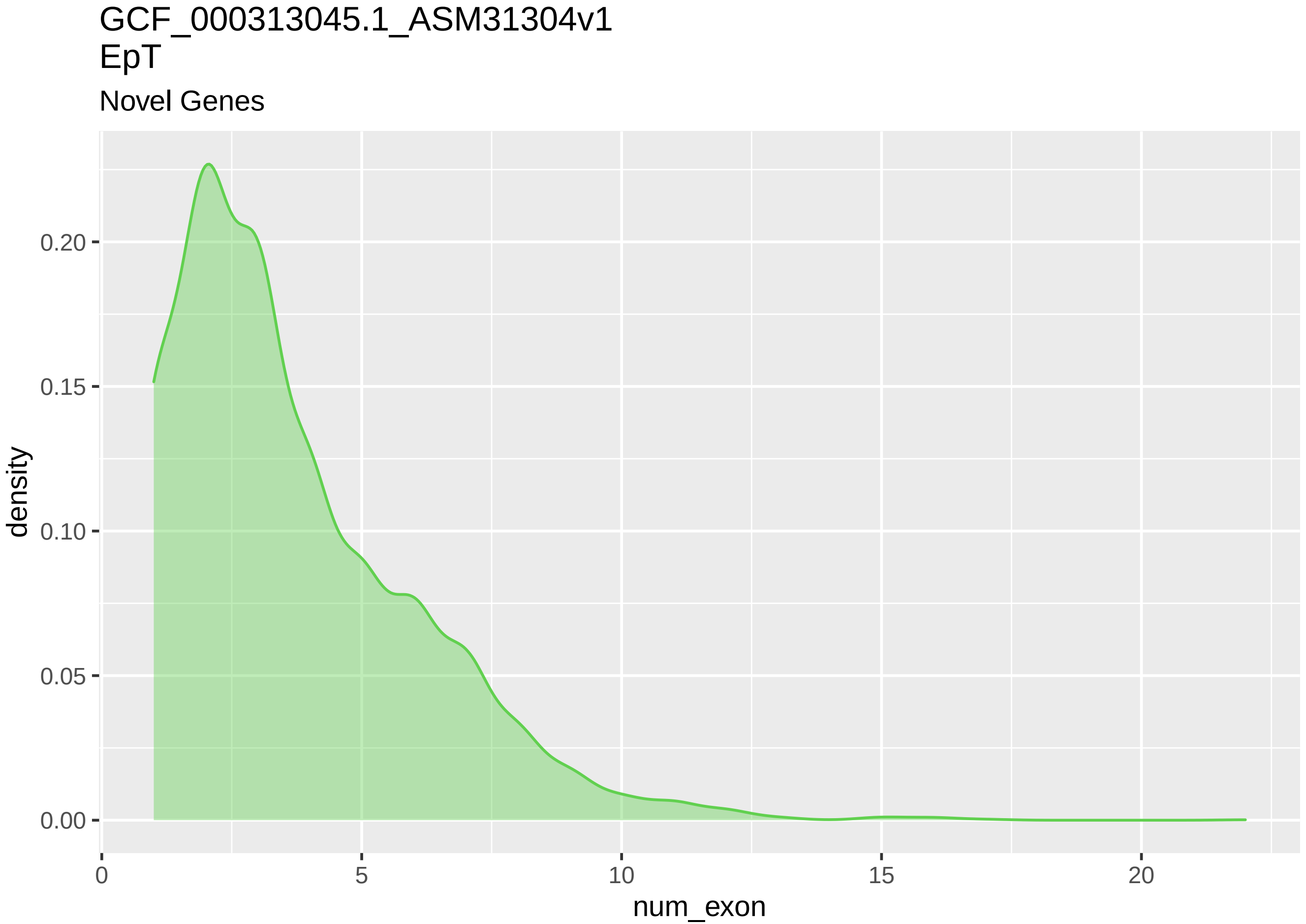
GCF_000313045.1_ASM31304v1
EpT
Novel Genes
density
0.00
0.05
0.10
0.15
0.20
0
5
10
15
20
num_exon

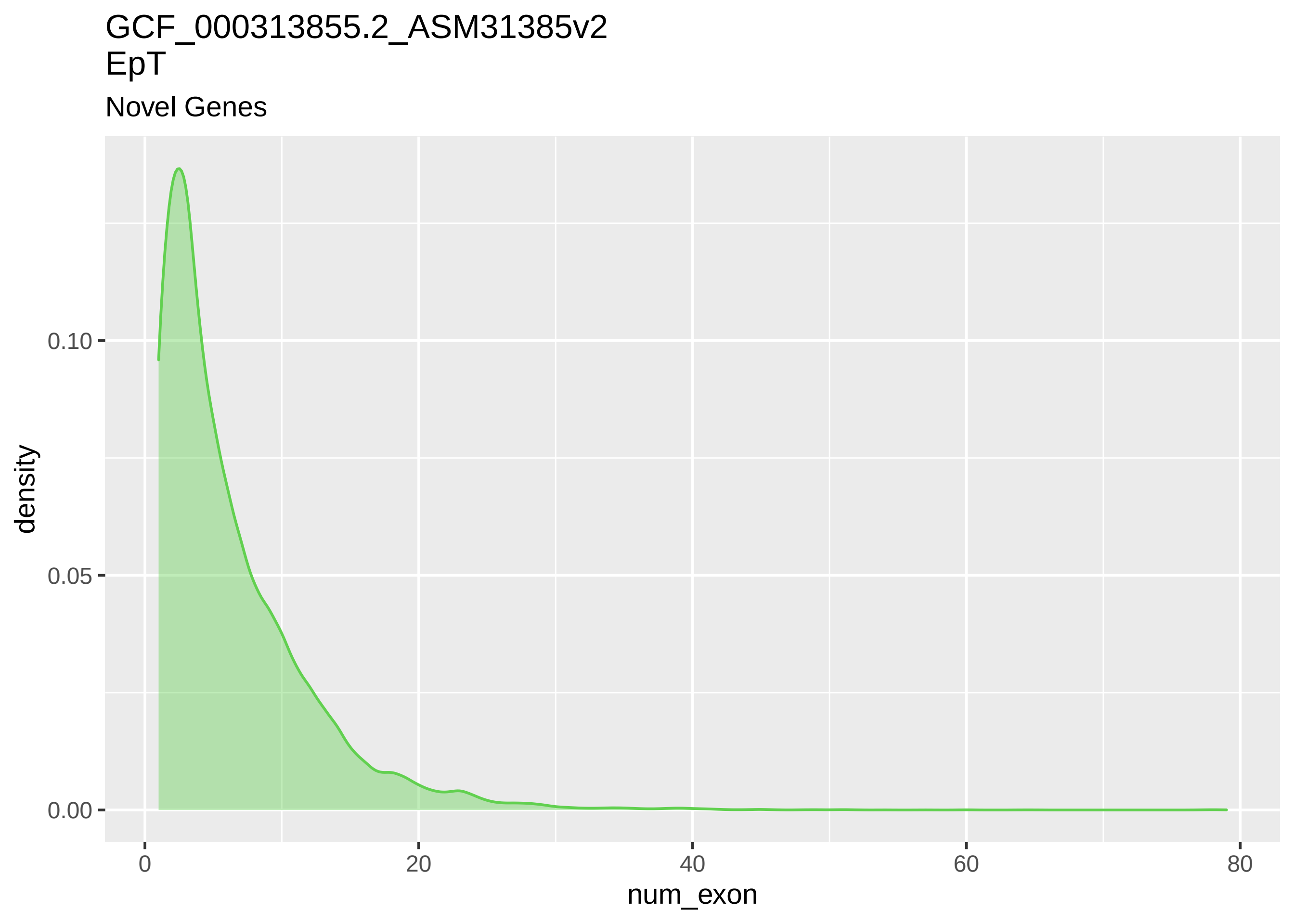
GCF_000313855.2_ASM31385v2
EpT
Novel Genes
density
0.10
0.05
0.00
0
20
40
60
80
num_exon

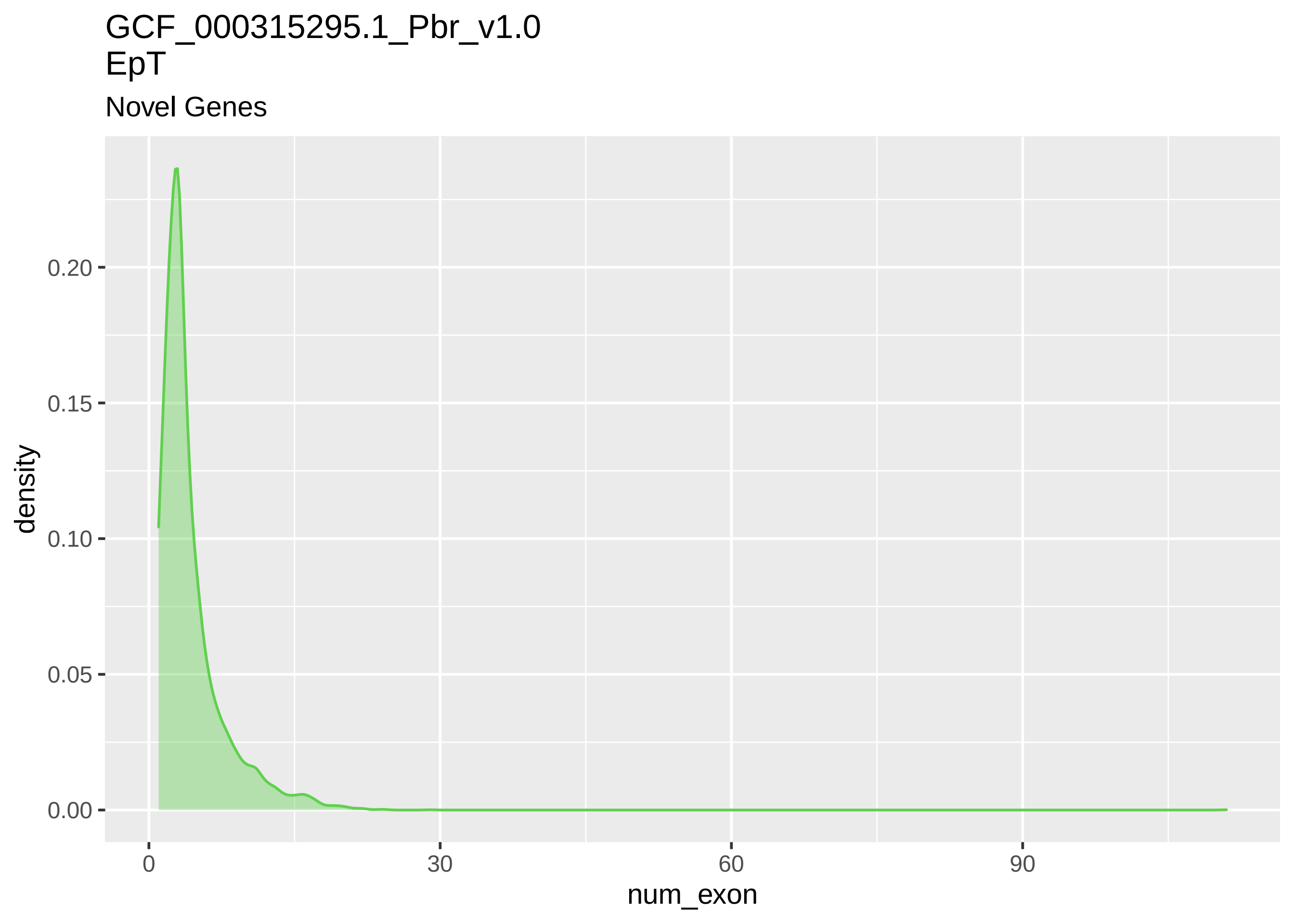

GCF_000315295.1_Pbr_v1.0
EpT
Novel Genes
density
0.00
0.05
0.10
0.15
0.20
0
30
60
90
num_exon

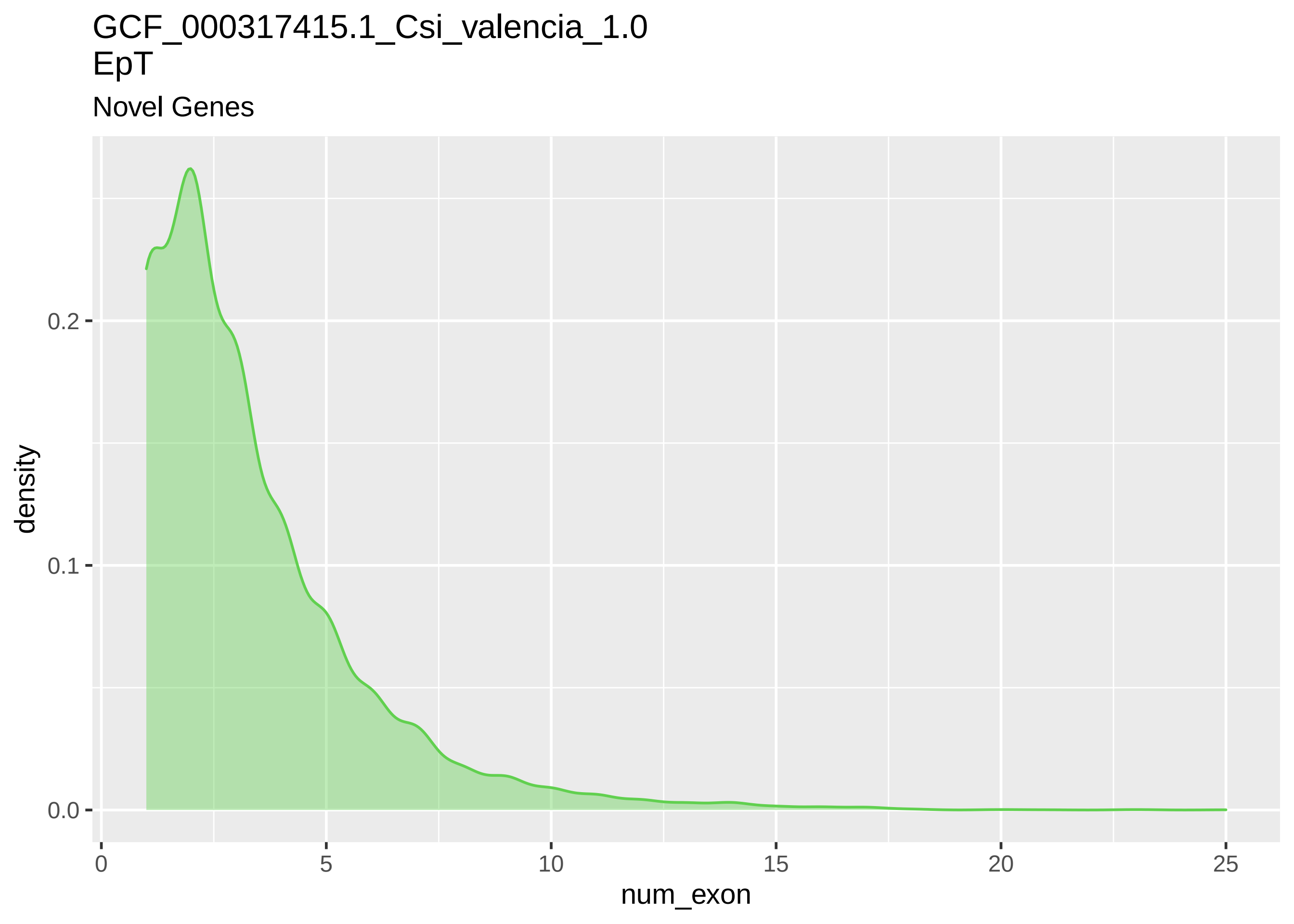
GCF_000317415.1_Csi_valencia_1.0
EpT
Novel Genes
density
0.0
0.1
0.2
0
5
10
15
20
25
num_exon

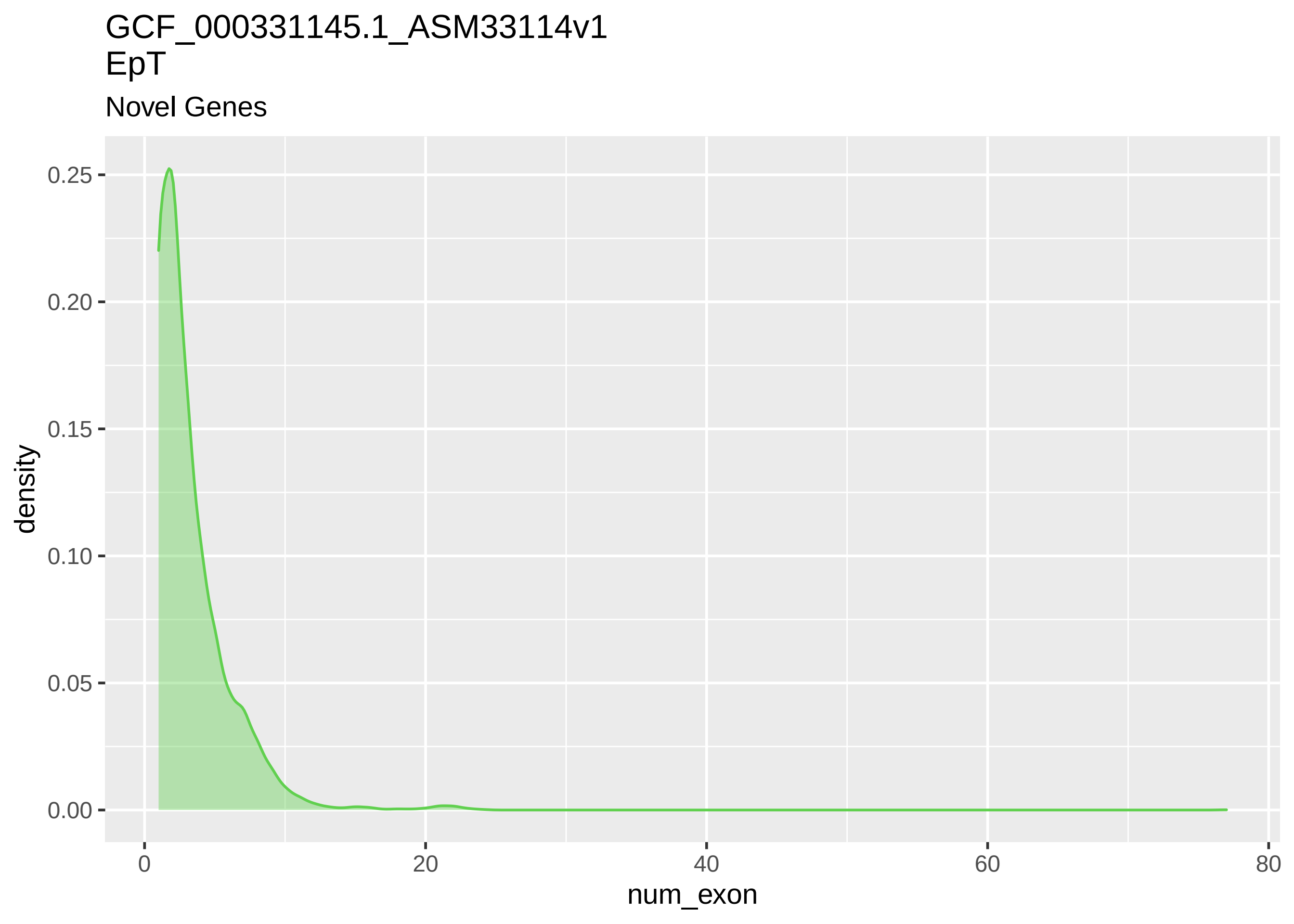
GCF_000331145.1_ASM33114v1
EpT
Novel Genes
density
0.25
0.20
0.15
0.10
0.05
0.00
0
20
40
60
80
num_exon

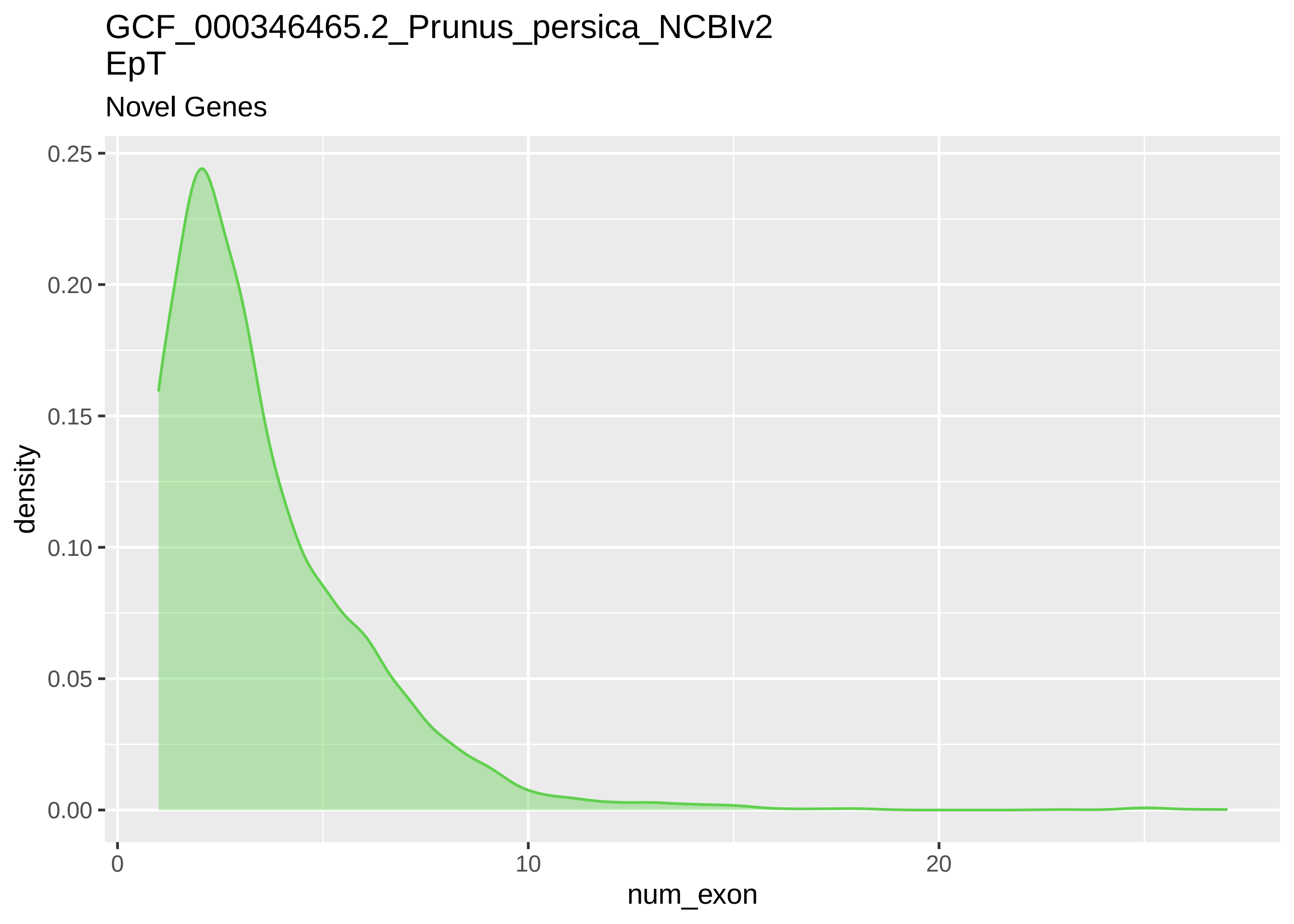
GCF_000346465.2_Prunus_persica_NCBIv2
EpT
Novel Genes
density
0.25
0.20
0.15
0.10
0.05
0.00
0
10
20
num_exon

GCF_000365185.1_Chinese_Lotus_1.1
EpT

Novel Genes

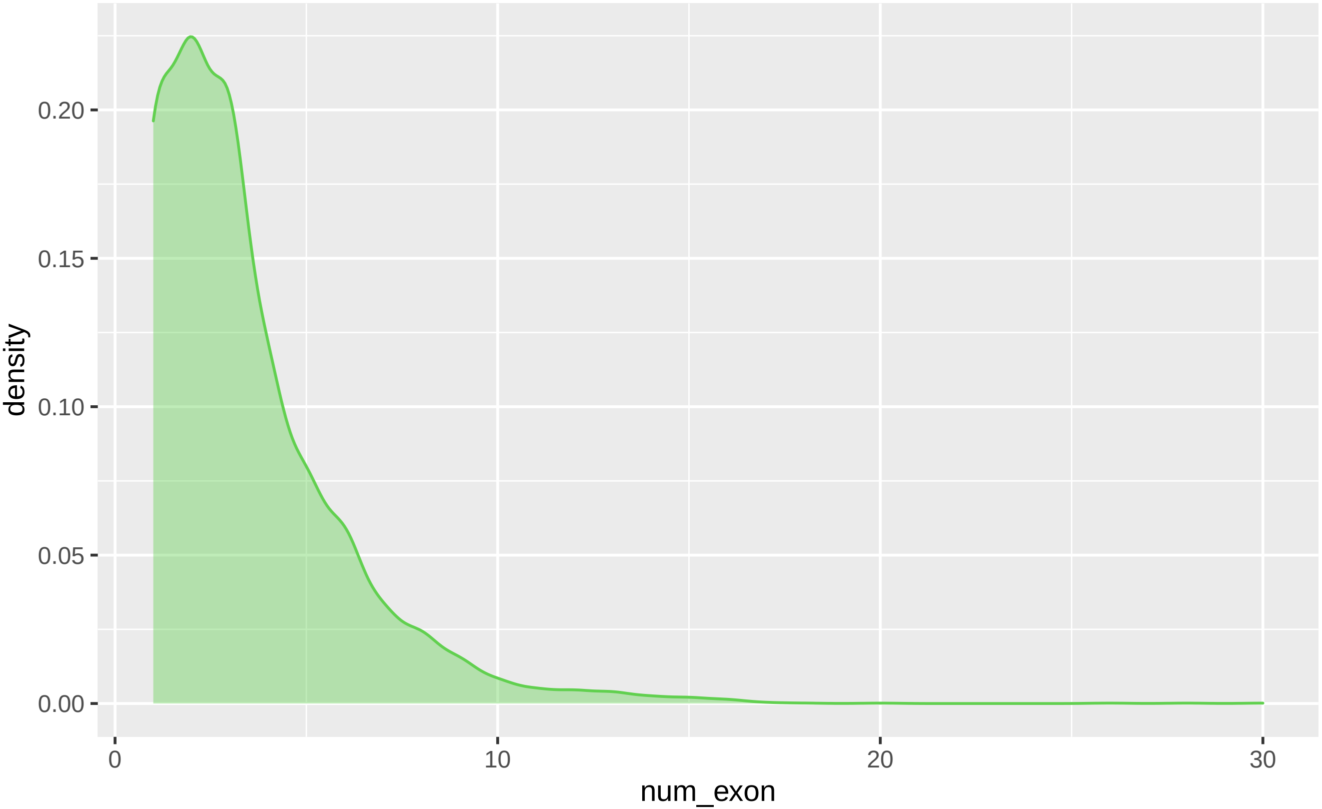

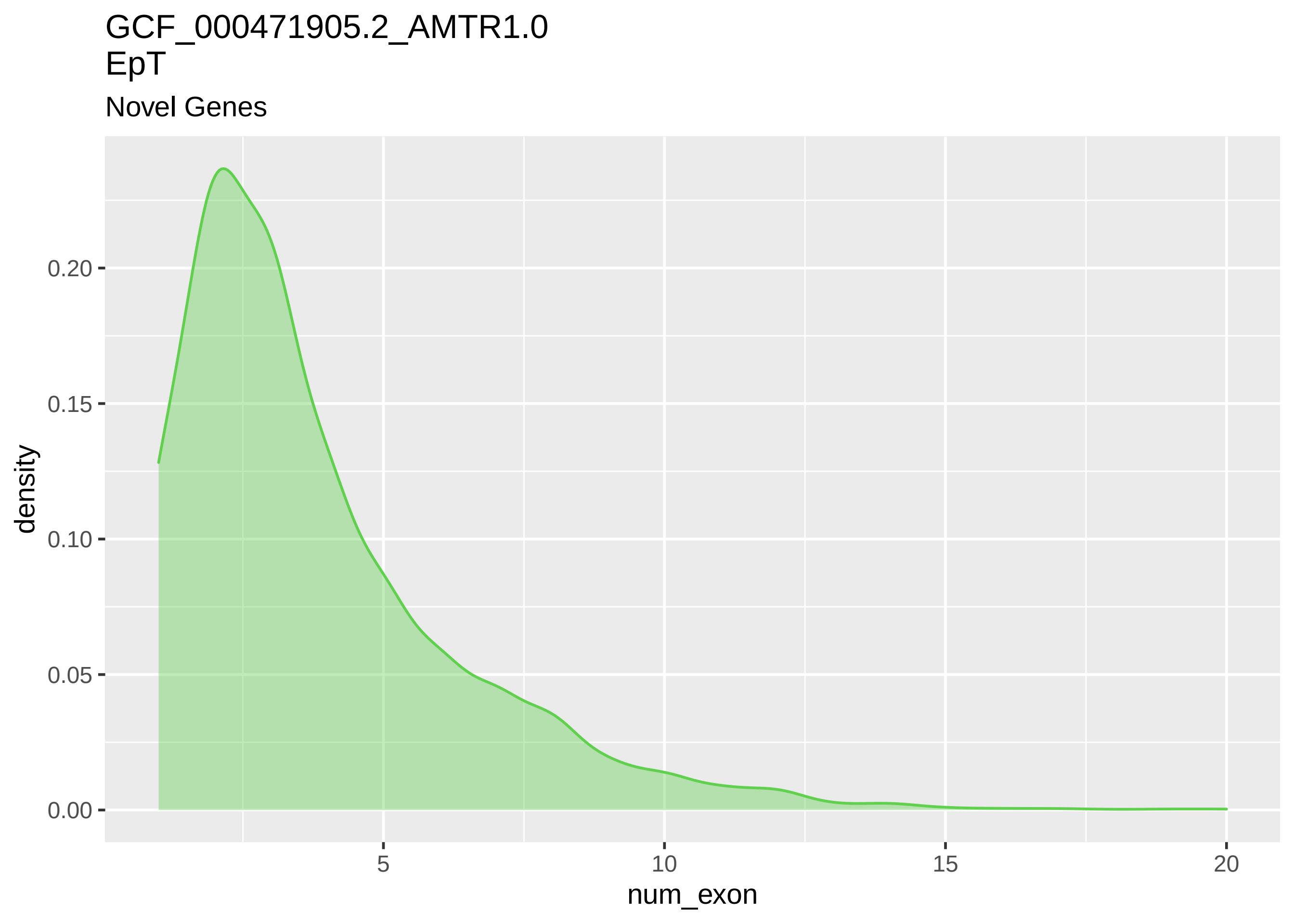
GCF_000471905.2_AMTR1.0
EpT
Novel Genes
density
0.00
0.05
0.10
0.15
0.20
5
10
15
20
num_exon

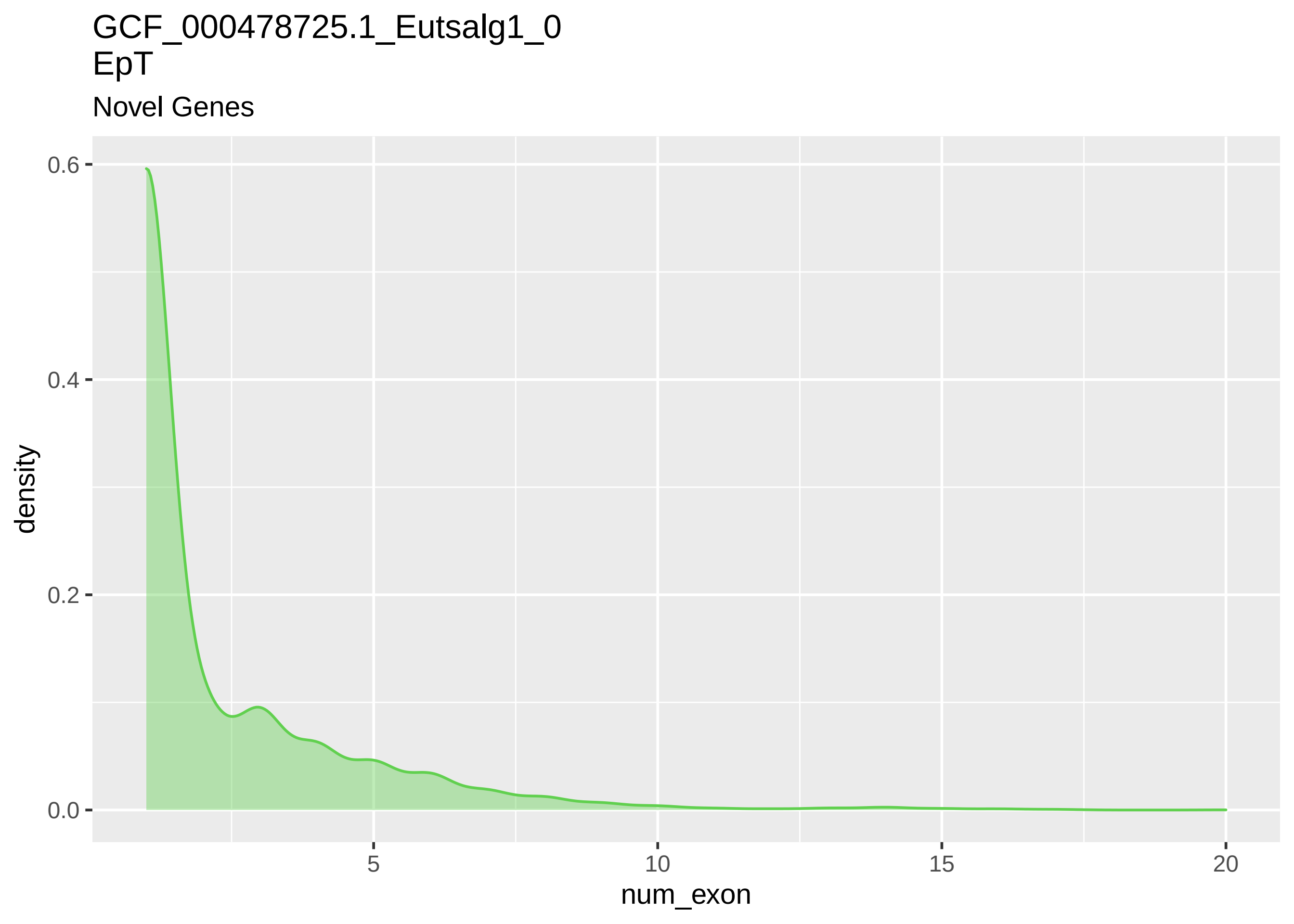

GCF_000478725.1_Eutsalg1_0
EpT
Novel Genes
0.6
0.4
0.2
0.0
density
5
10
15
20
num_exon

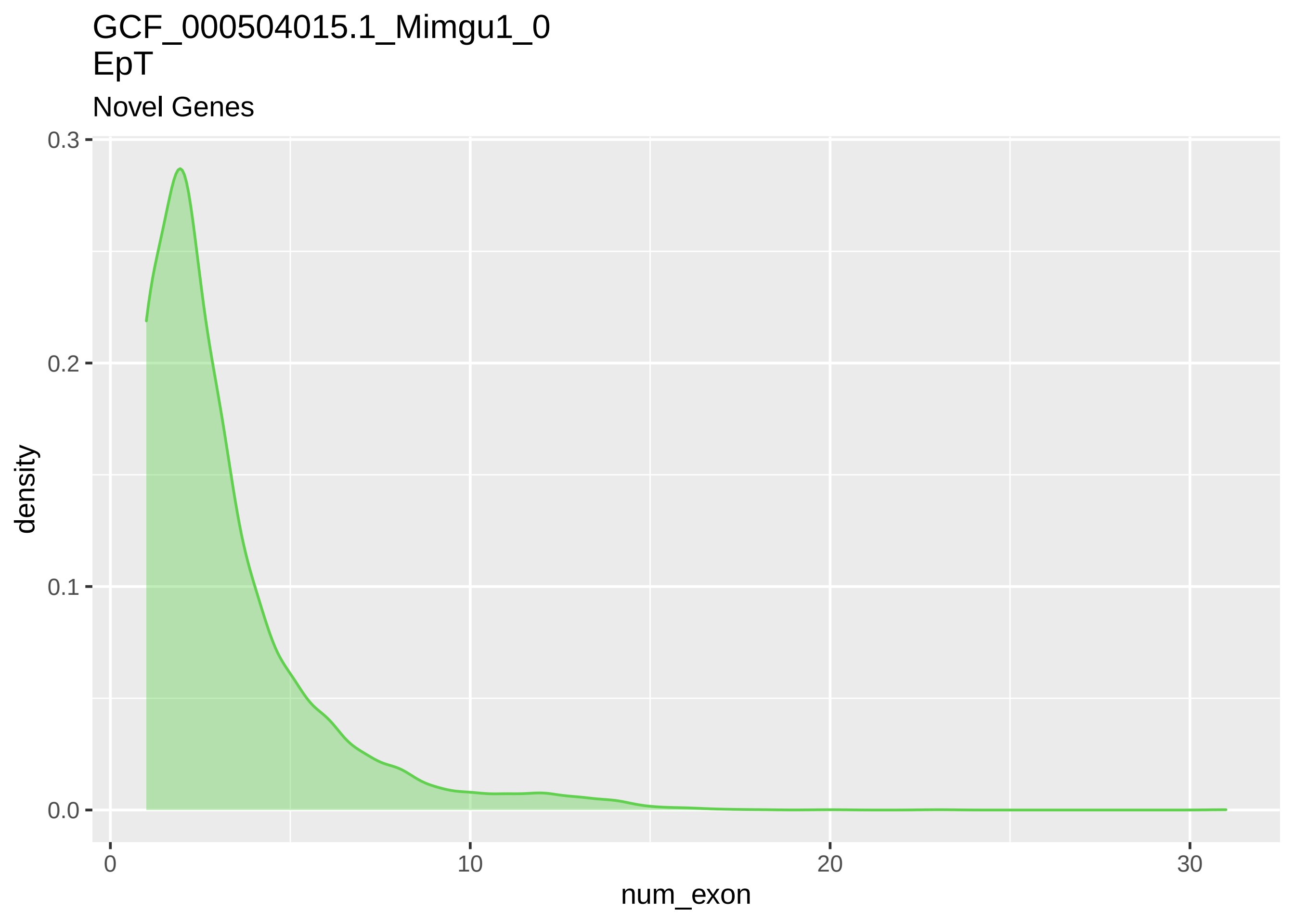
GCF_000504015.1_Mimgu1_0
EpT
Novel Genes
density
num_exon
0.0
0.1
0.2
0.3
0
10
20
30

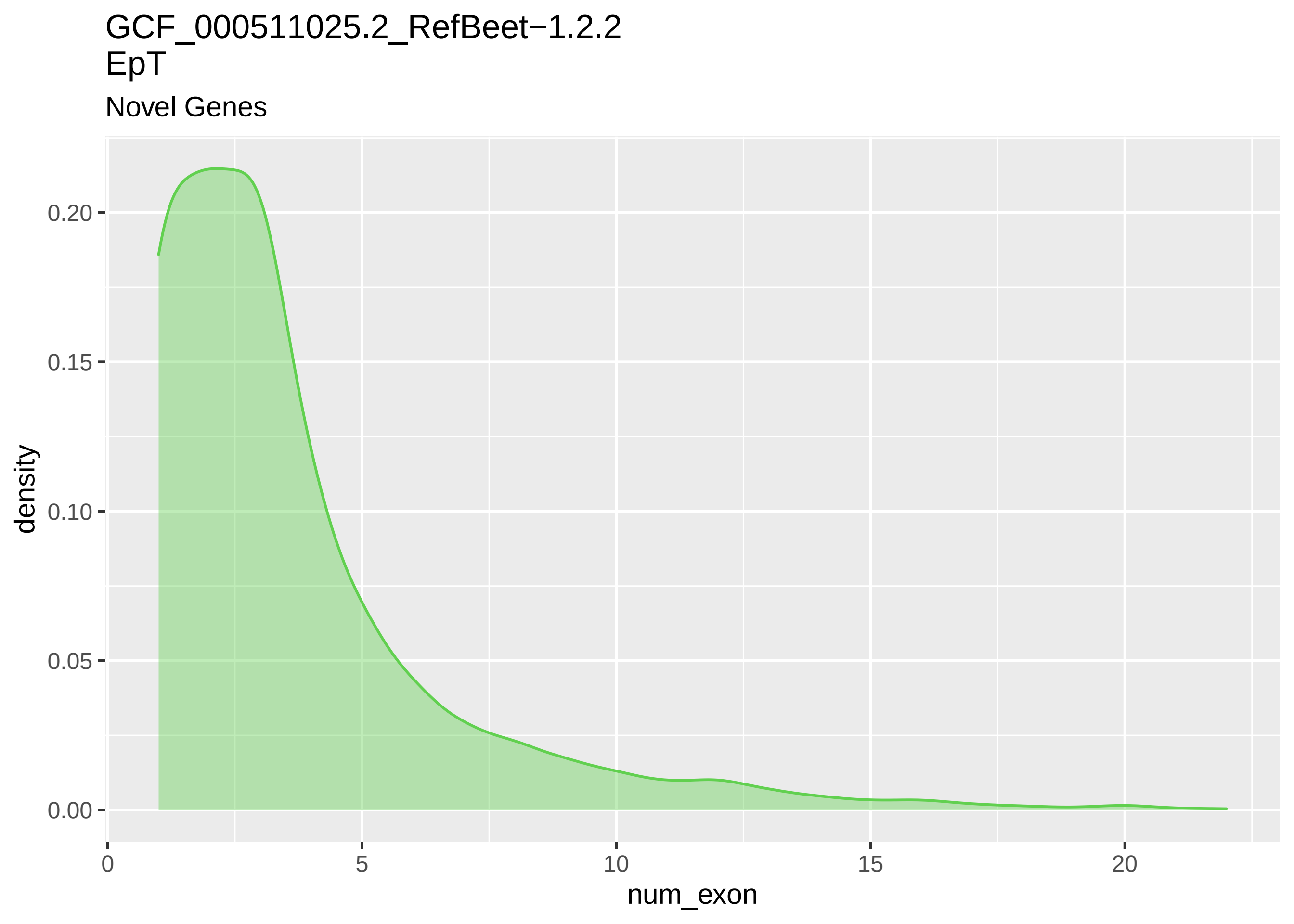
GCF_000511025.2_RefBeet−1.2.2
EpT
Novel Genes
density
0.00
0.05
0.10
0.15
0.20
0
5
10
15
20
num_exon

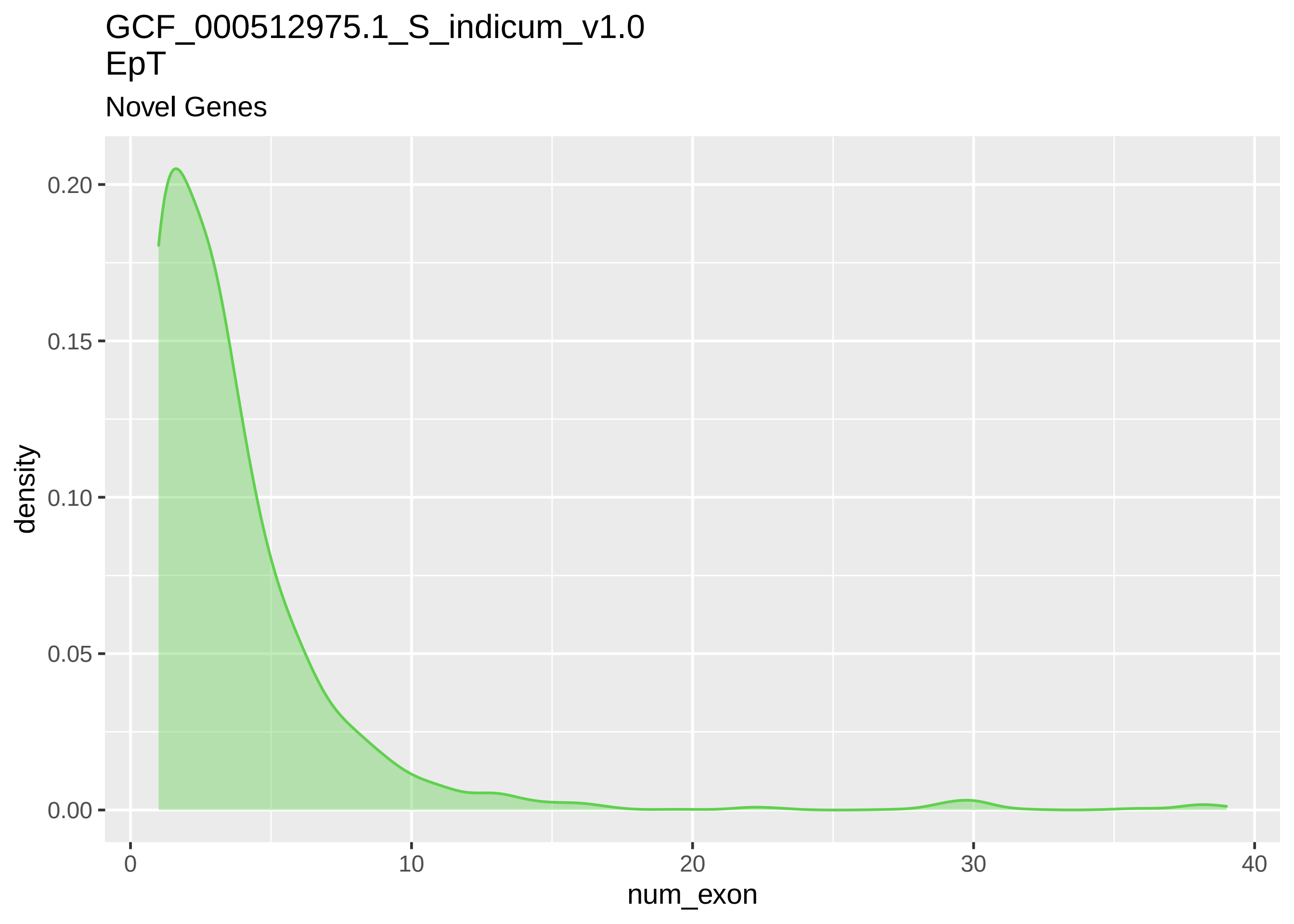
GCF_000512975.1_S_indicum_v1.0
EpT
Novel Genes
density
0.20
0.15
0.10
0.05
0.00
0
10
20
30
40
num_exon

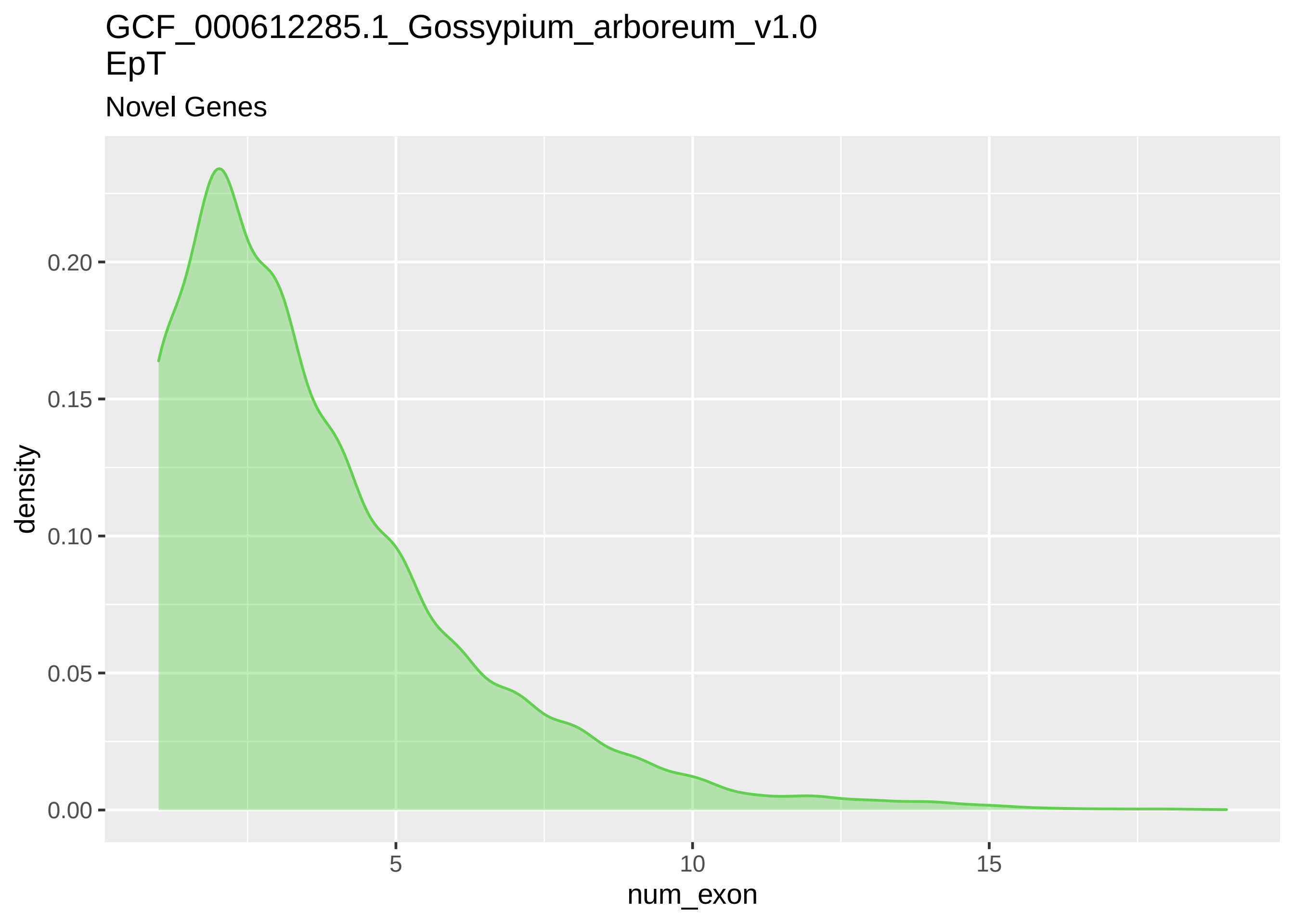

GCF_000612285.1_Gossypium_arboreum_v1.0
EpT
Novel Genes
density
0.00
0.05
0.10
0.15
0.20
5
10
15
num_exon

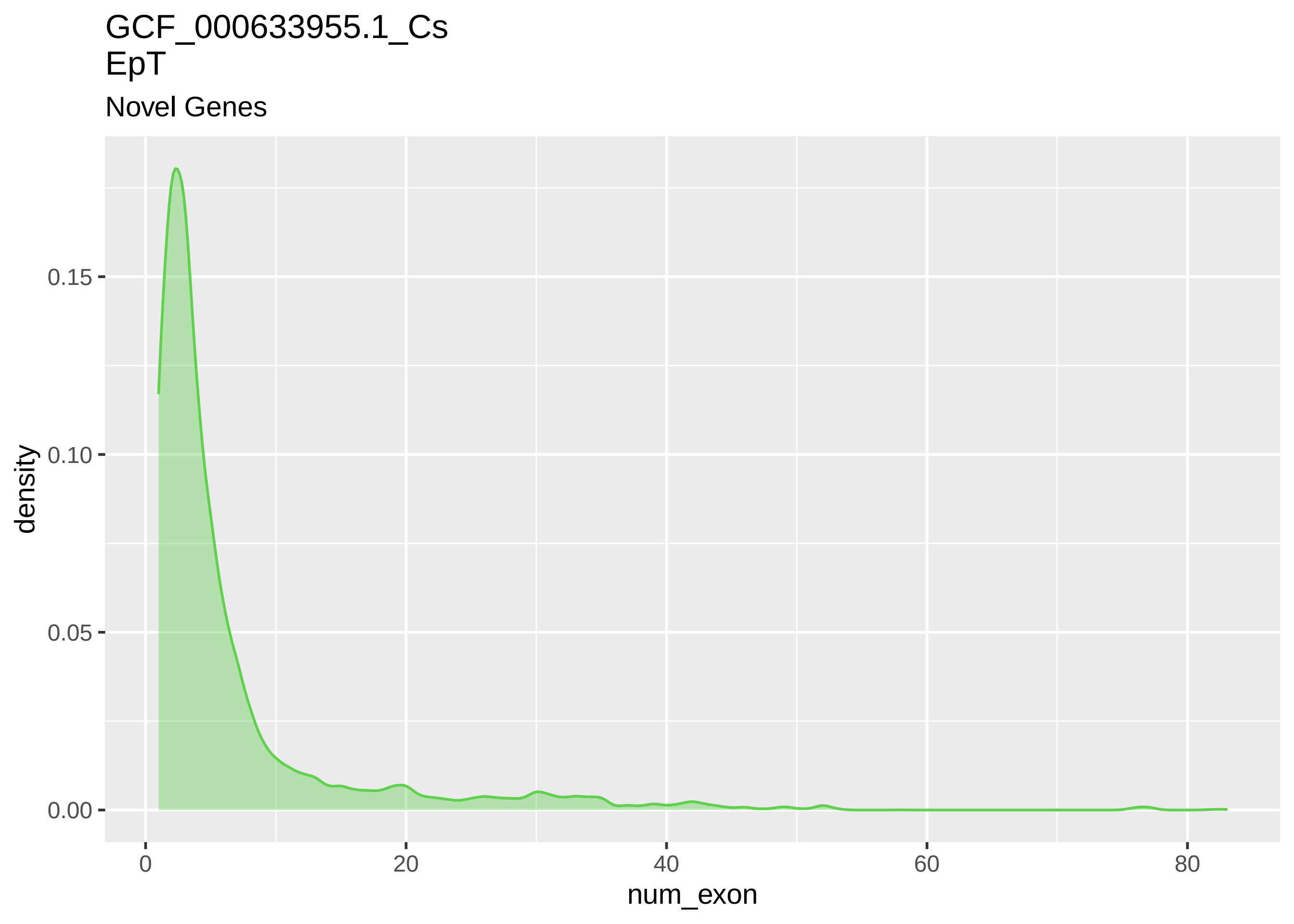
GCF_000633955.1_Cs
EpT
Novel Genes
density
0.15
0.10
0.05
0.00
0
20
40
60
80
num_exon

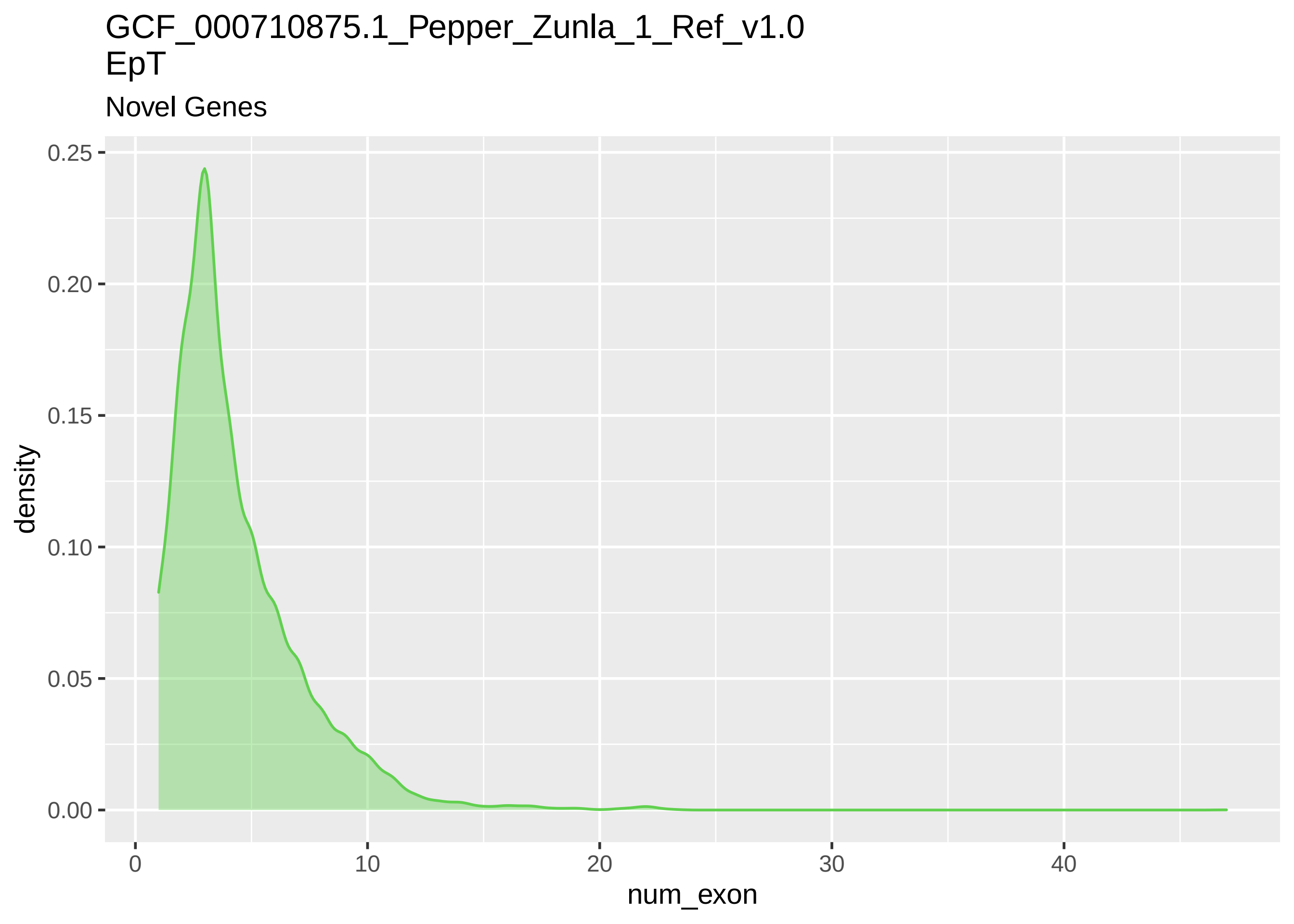

GCF_000710875.1_Pepper_Zunla_1_Ref_v1.0
EpT
Novel Genes
density
0.25
0.20
0.15
0.10
0.05
0.00
0
10
20
30
40
num_exon

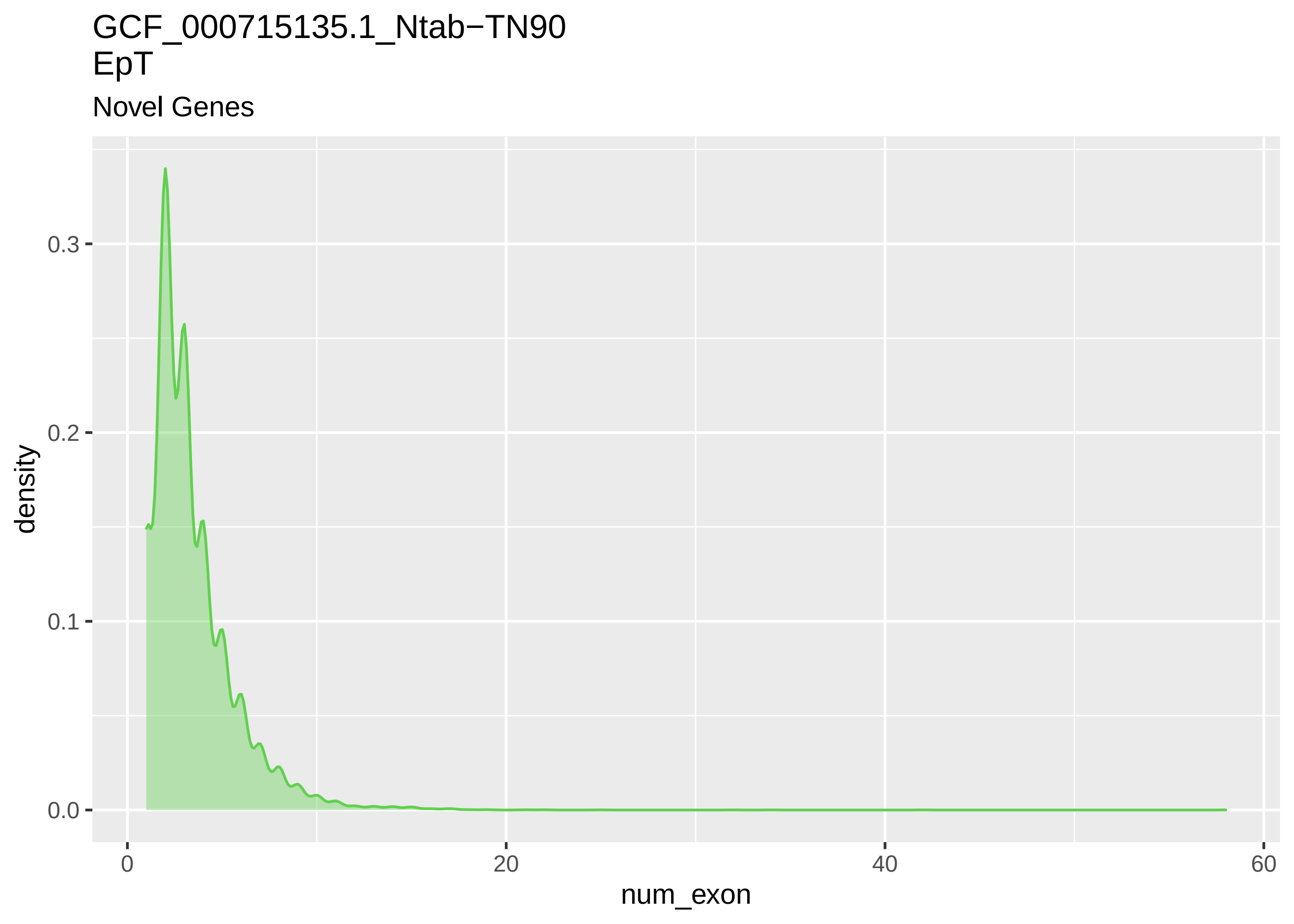
GCF_000715135.1_Ntab−TN90
EpT
Novel Genes
density
0.3
0.2
0.1
0.0
0
20
40
60
num_exon

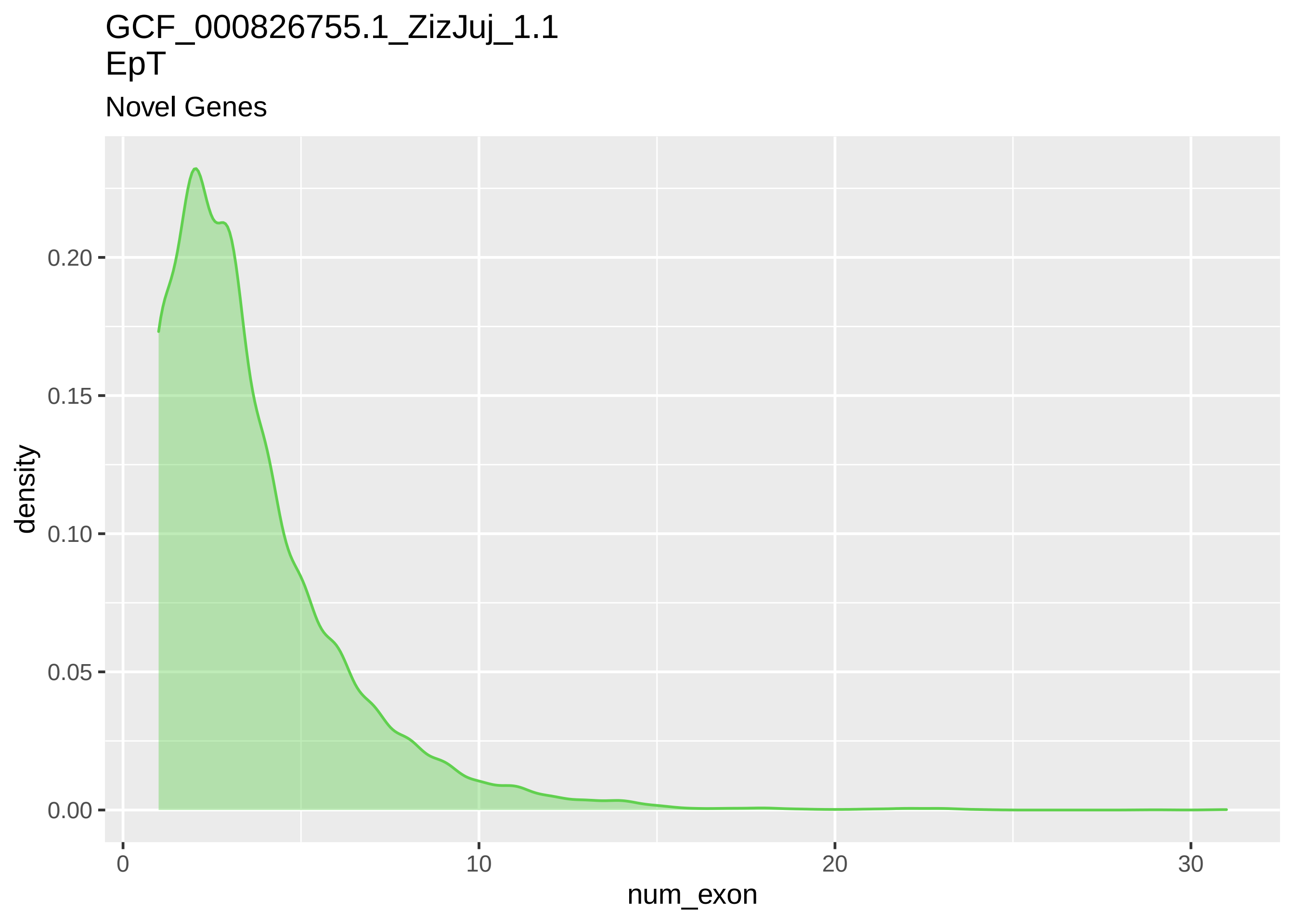

GCF_000826755.1_ZizJuj_1.1
EpT
Novel Genes
0.20
0.15
0.10
0.05
0.00
density
0
10
20
30
num_exon

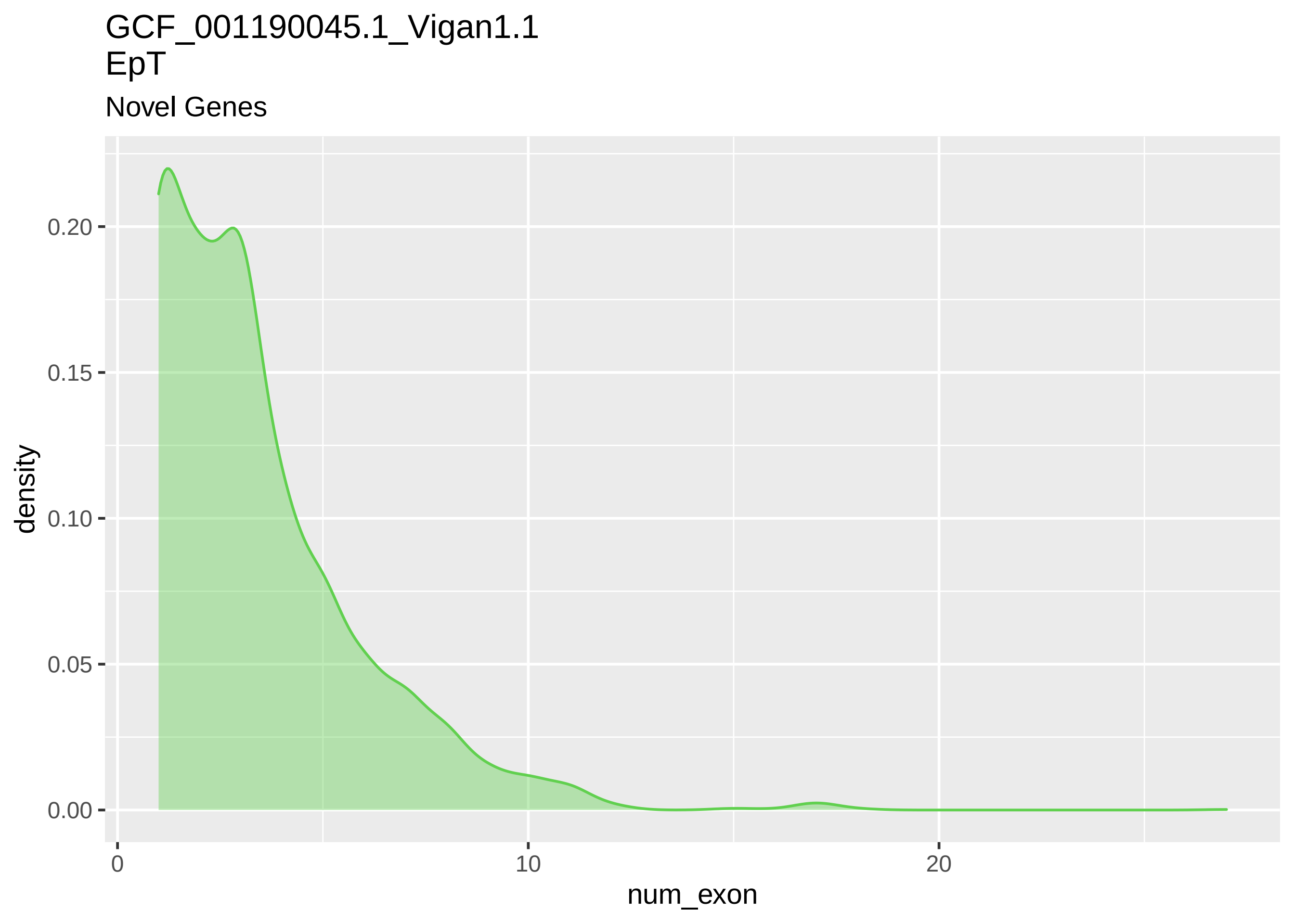
GCF_001190045.1_Vigan1.1
EpT
Novel Genes
density
0.00
0.05
0.10
0.15
0.20
0
10
20
num_exon

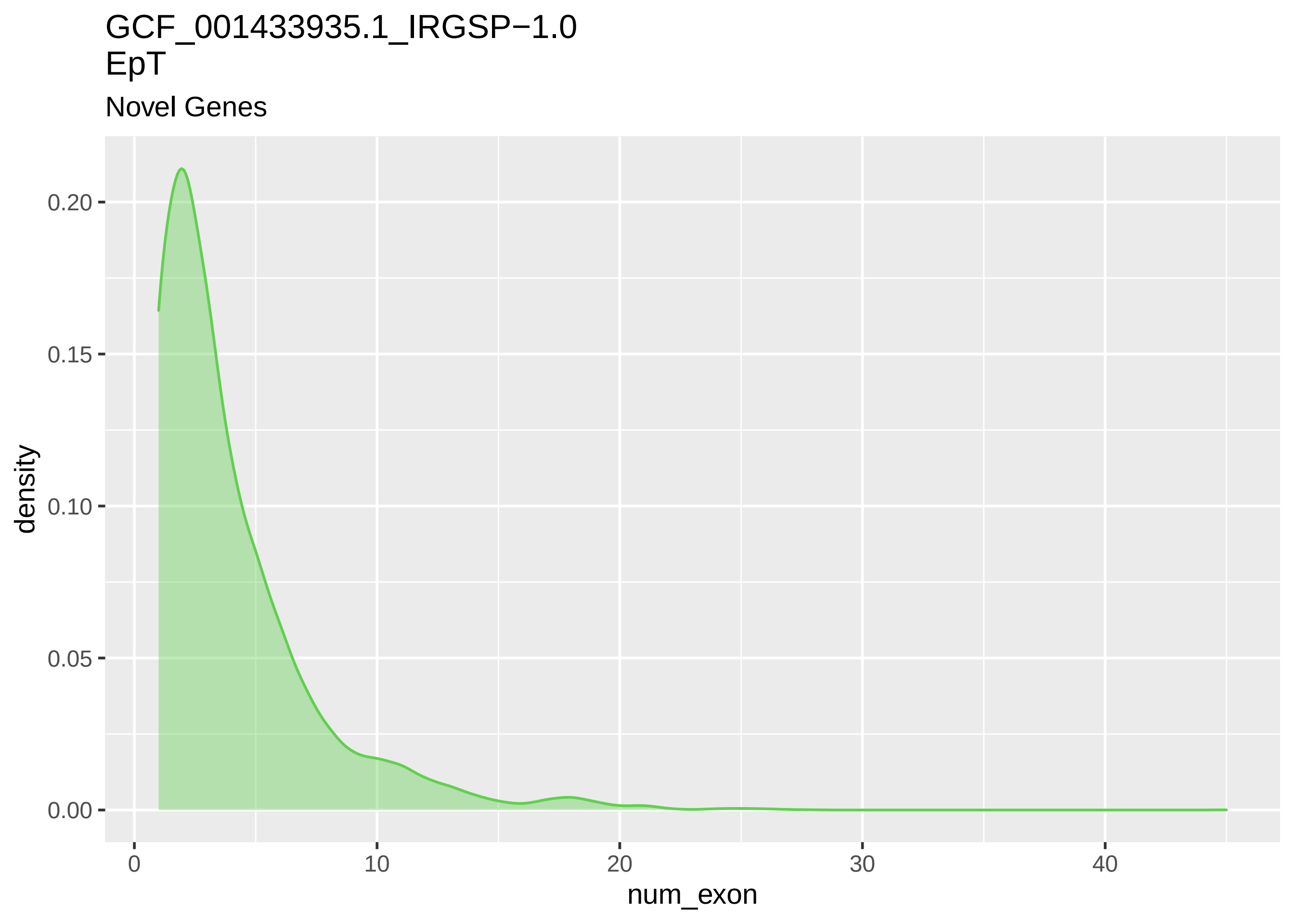
GCF_001433935.1_IRGSP−1.0
EpT
Novel Genes
density
0.20
0.15
0.10
0.05
0.00
0
10
20
30
40
num_exon

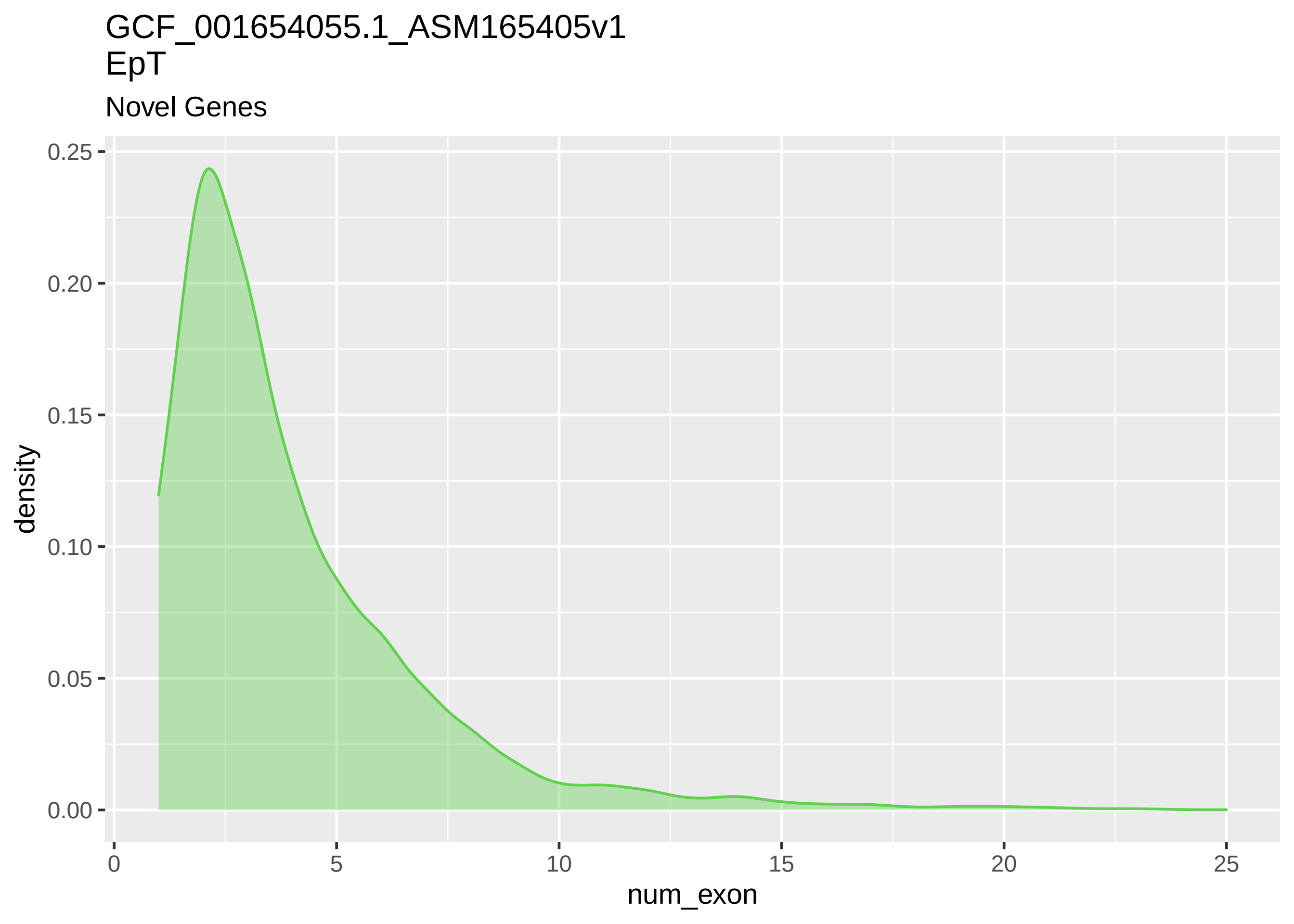
GCF_001654055.1_ASM165405v1
EpT
Novel Genes
density
0.00
0.05
0.10
0.15
0.20
0.25
0
5
10
15
20
25
num_exon

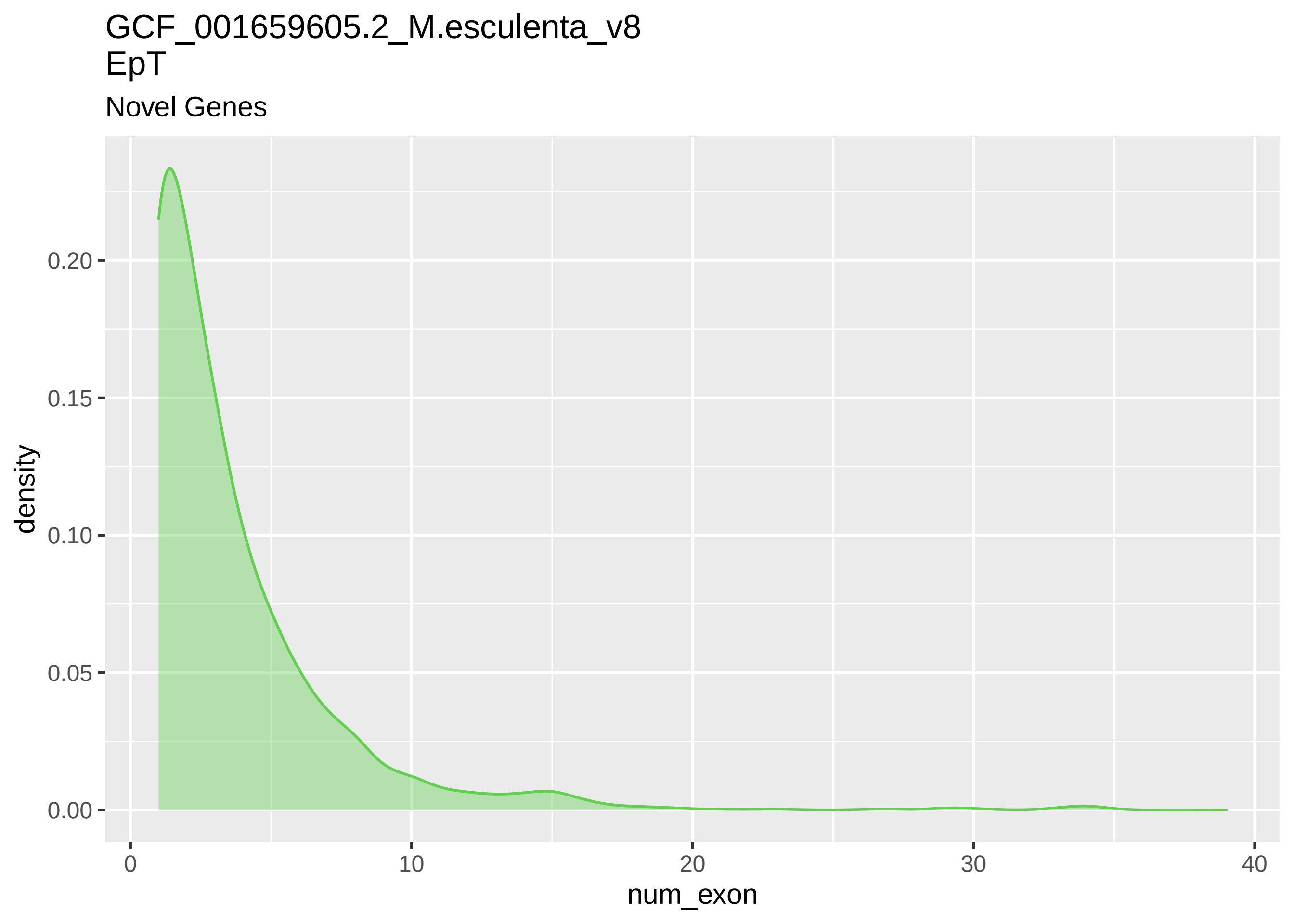
GCF_001659605.2_M.esculenta_v8
EpT
Novel Genes
density
0.00
0.05
0.10
0.15
0.20
0
10
20
30
40
num_exon

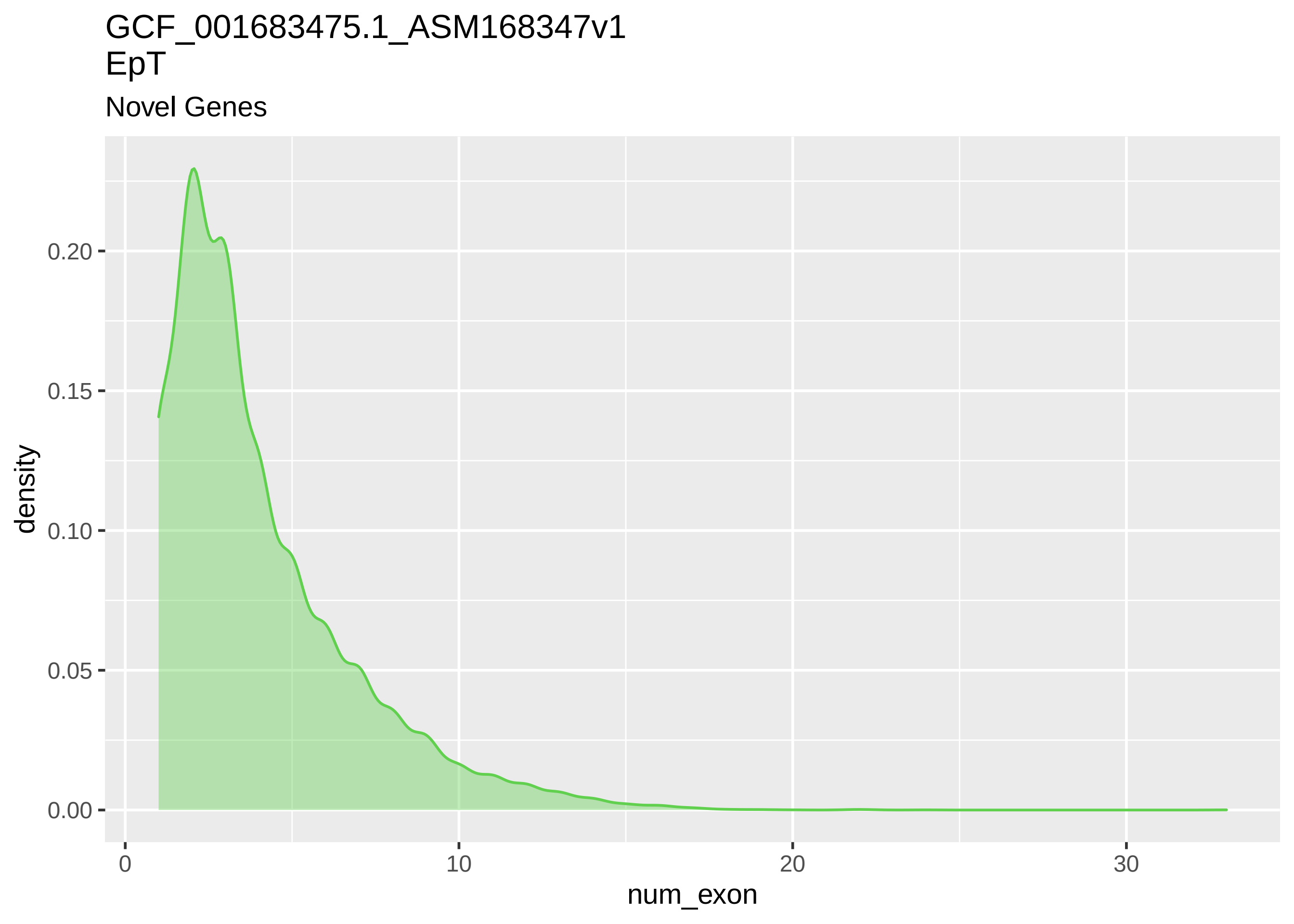
GCF_001683475.1_ASM168347v1
EpT
Novel Genes
density
0.00
0.05
0.10
0.15
0.20
0
10
20
30
num_exon

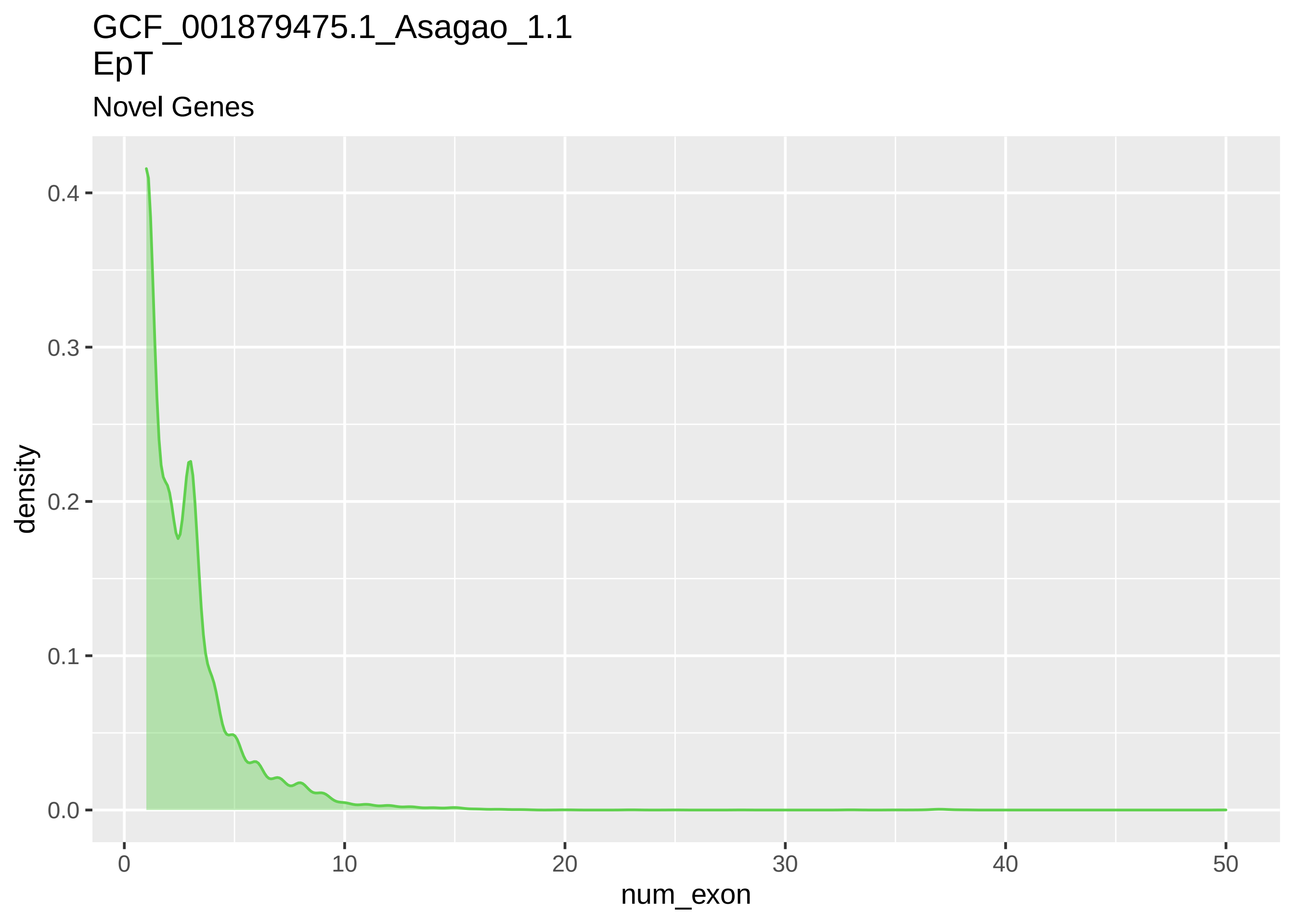
GCF_001879475.1_Asagao_1.1
EpT
Novel Genes
density
0.4
0.3
0.2
0.1
0.0
0
10
20
30
40
50
num_exon

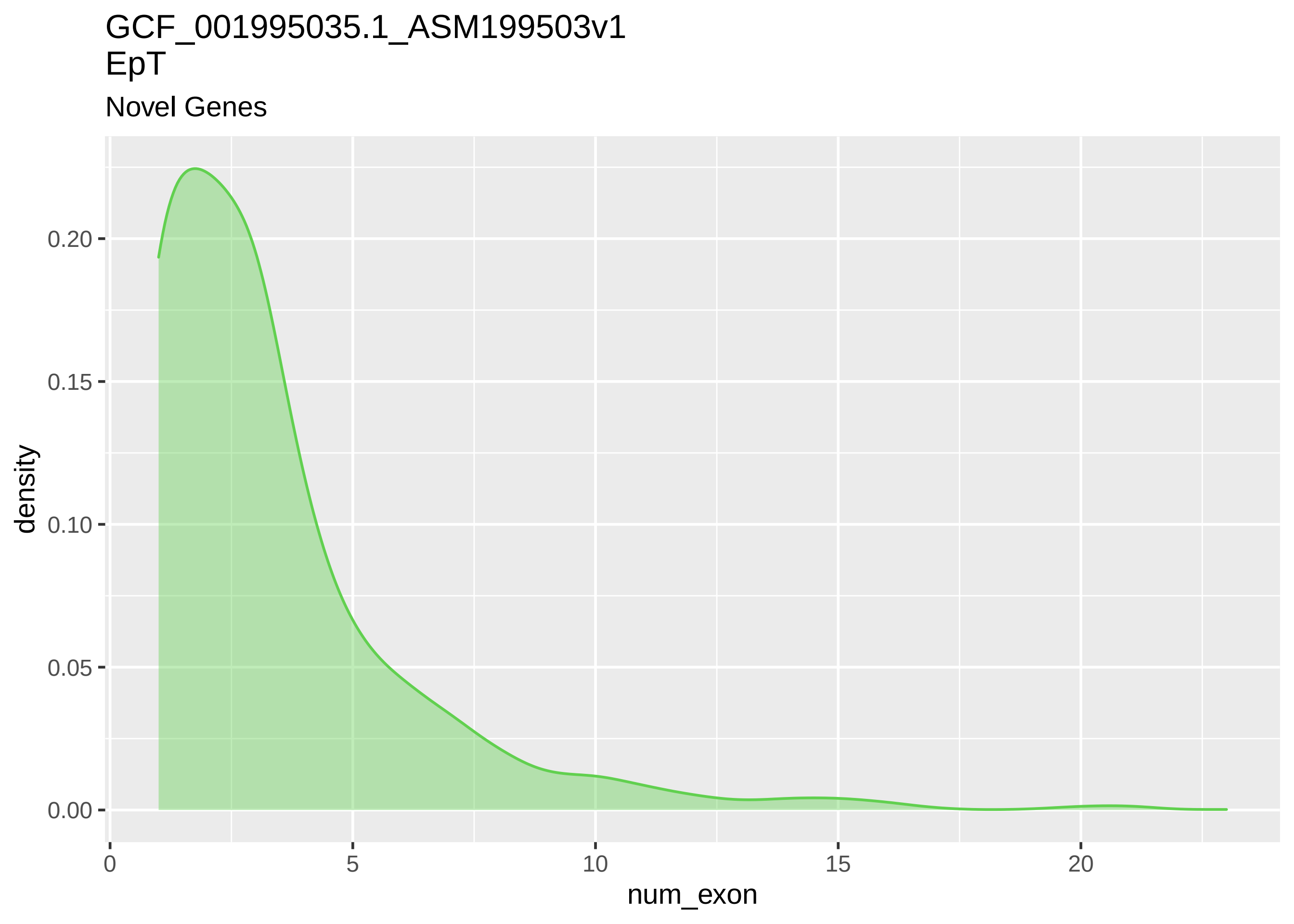
GCF_001995035.1_ASM199503v1
EpT
Novel Genes
density
0.00
0.05
0.10
0.15
0.20
0
5
10
15
20
num_exon

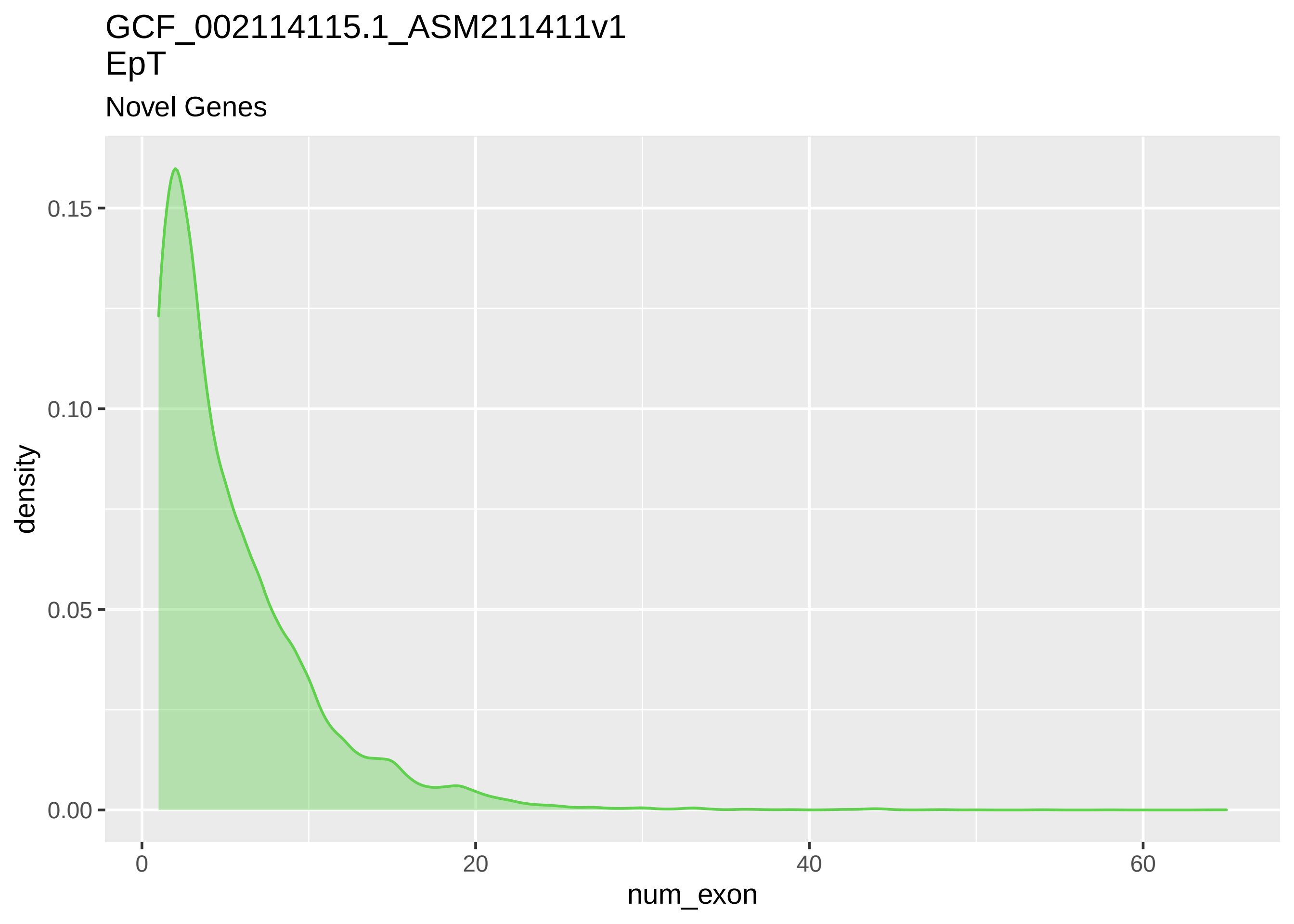
GCF_002114115.1_ASM211411v1
EpT
Novel Genes
density
0.15
0.10
0.05
0.00
0
20
40
60
num_exon

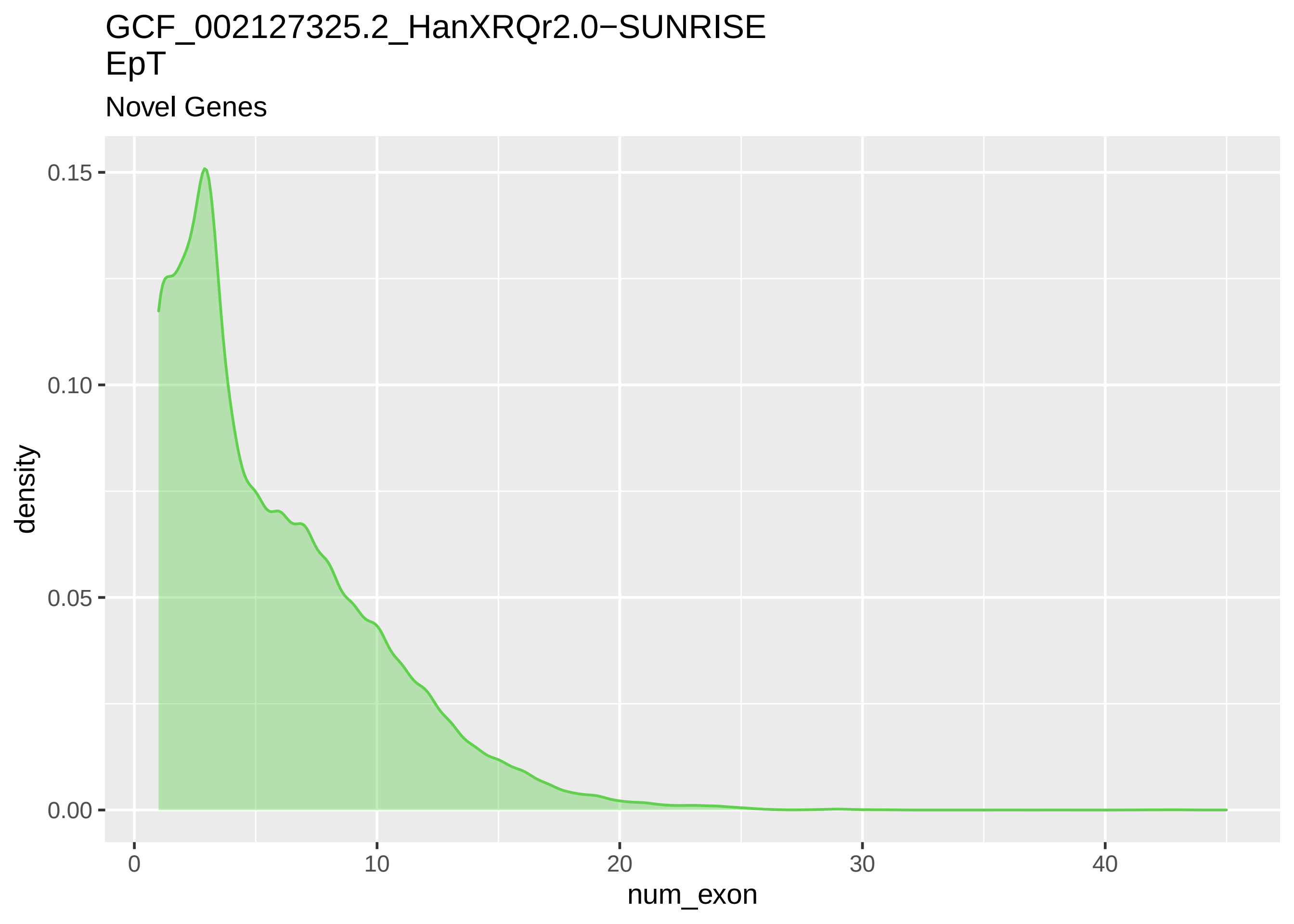
GCF_002127325.2_HanXRQr2.0−SUNRISE
EpT
Novel Genes
0.15
0.10
0.05
0.00
density
0
10
20
30
40
num_exon

GCF_002303985.1_Duzib1.0
EpT

Novel Genes

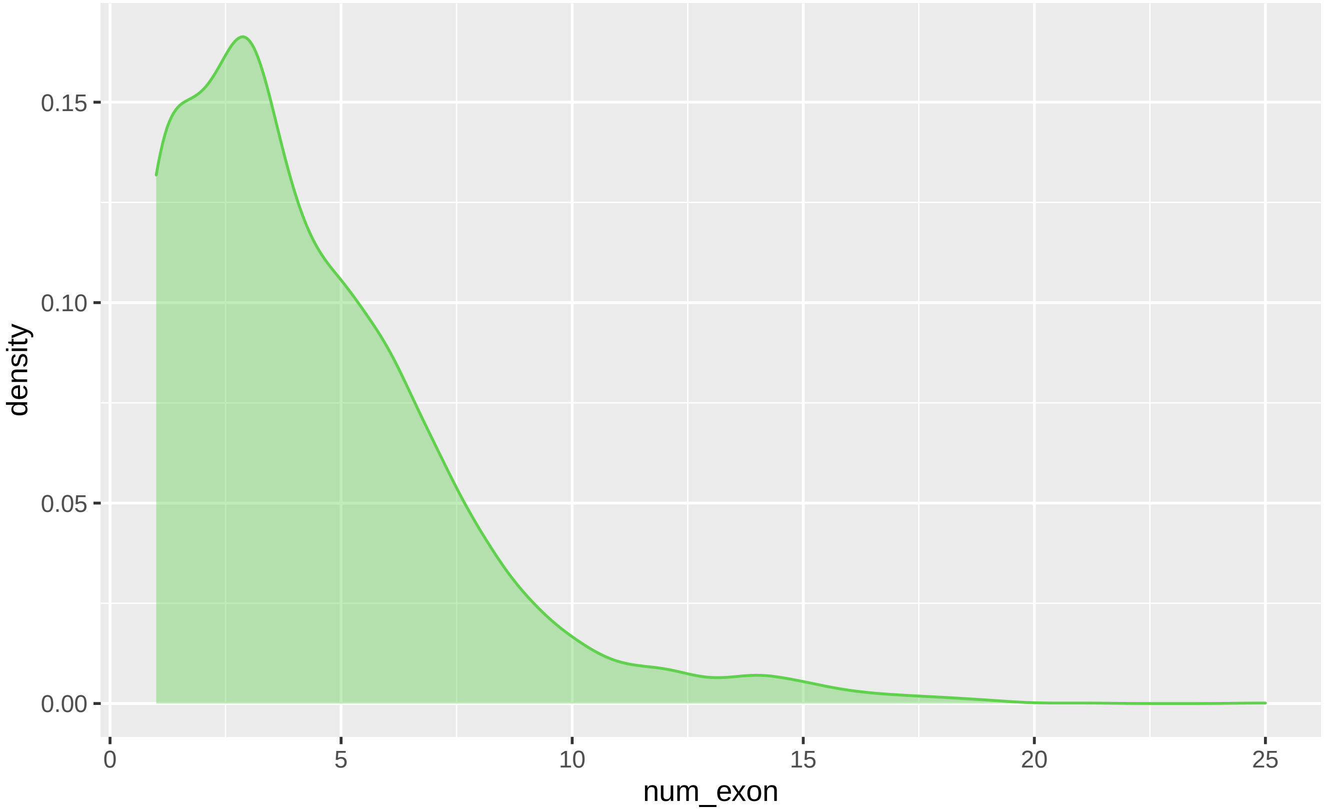

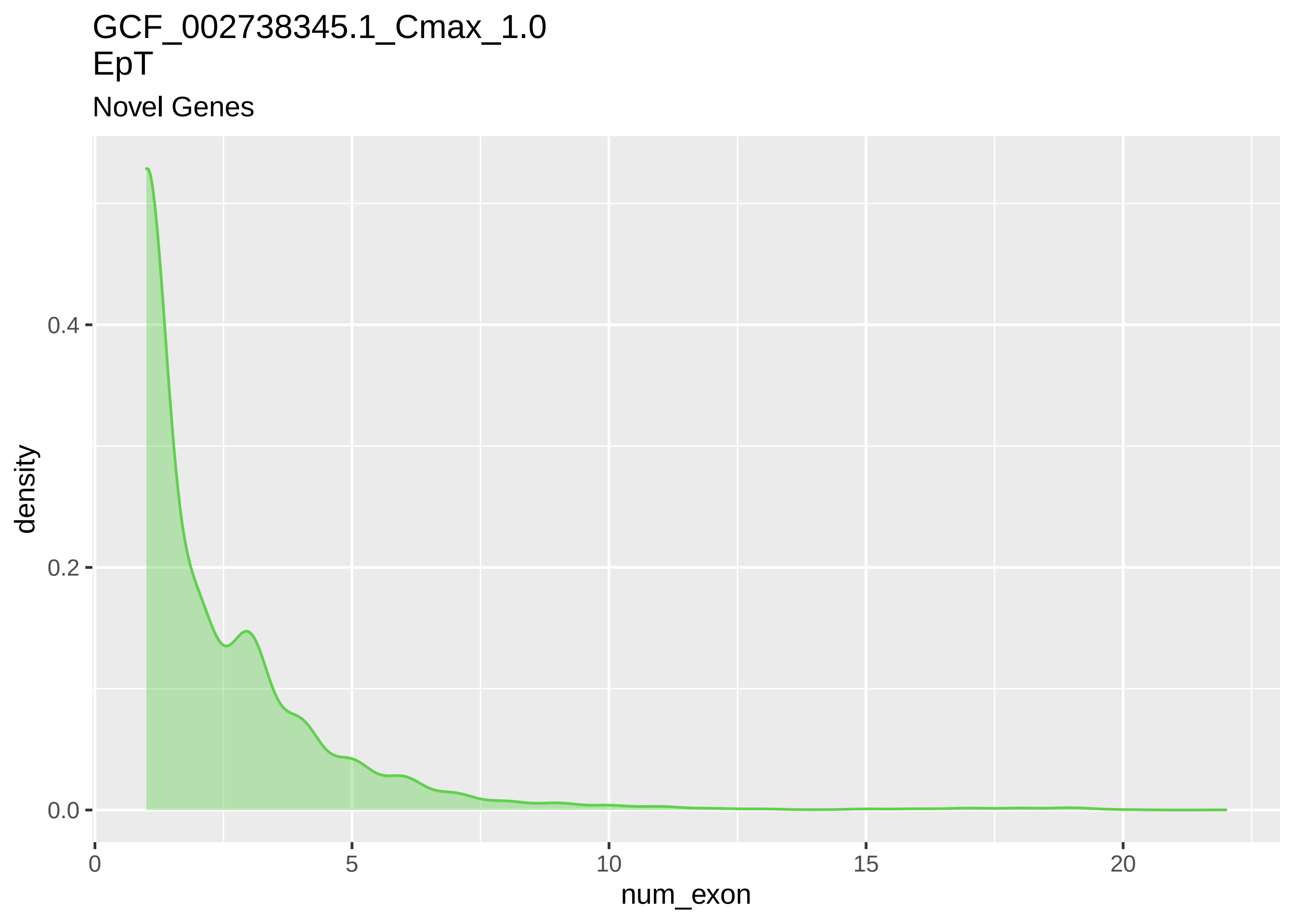

GCF_002738345.1_Cmax_1.0
EpT
Novel Genes
density
0.0
0.2
0.4
0
5
10
15
20
num_exon

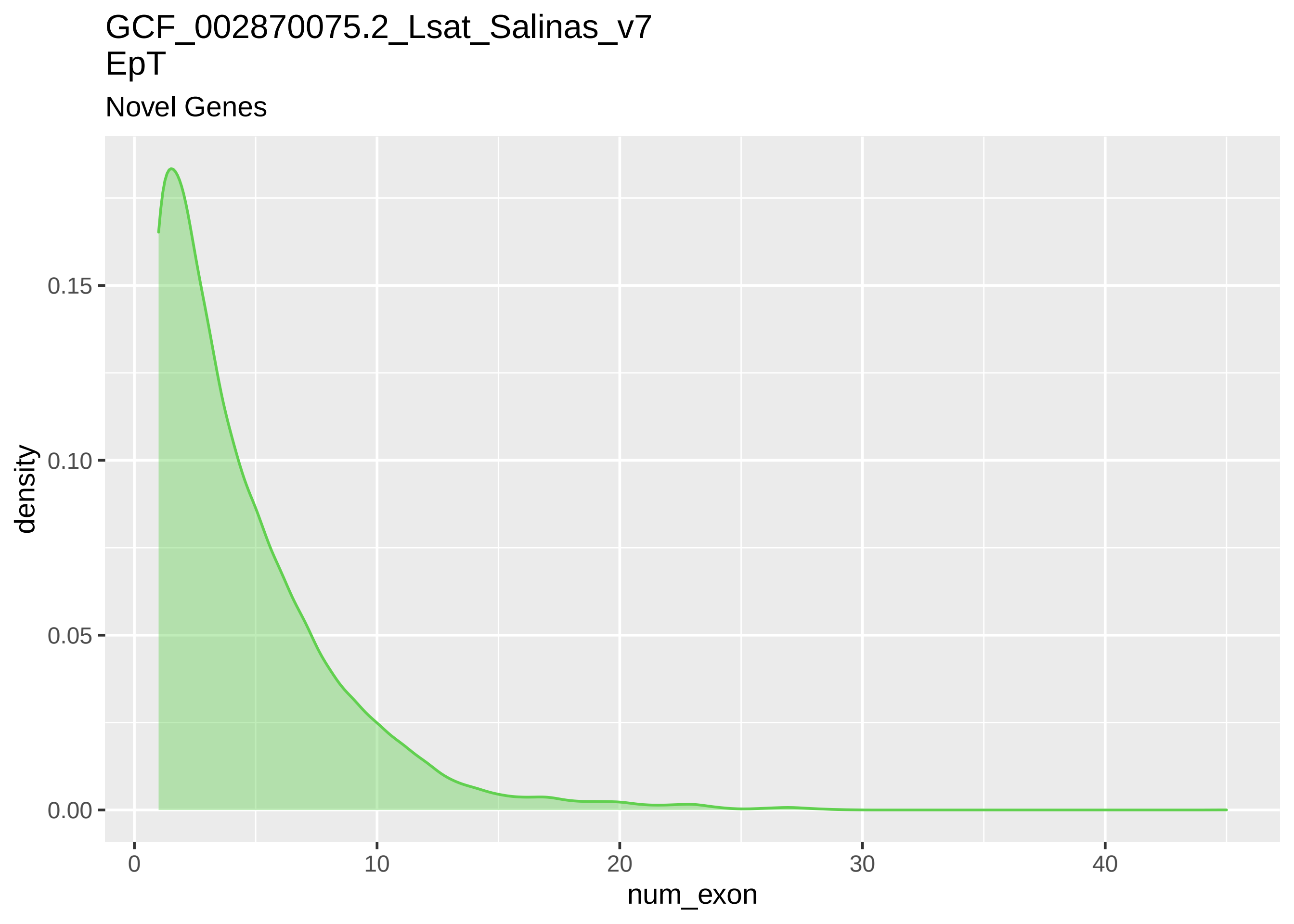
GCF_002870075.2_Lsat_Salinas_v7
EpT
Novel Genes
density
0.15
0.10
0.05
0.00
0
10
20
30
40
num_exon

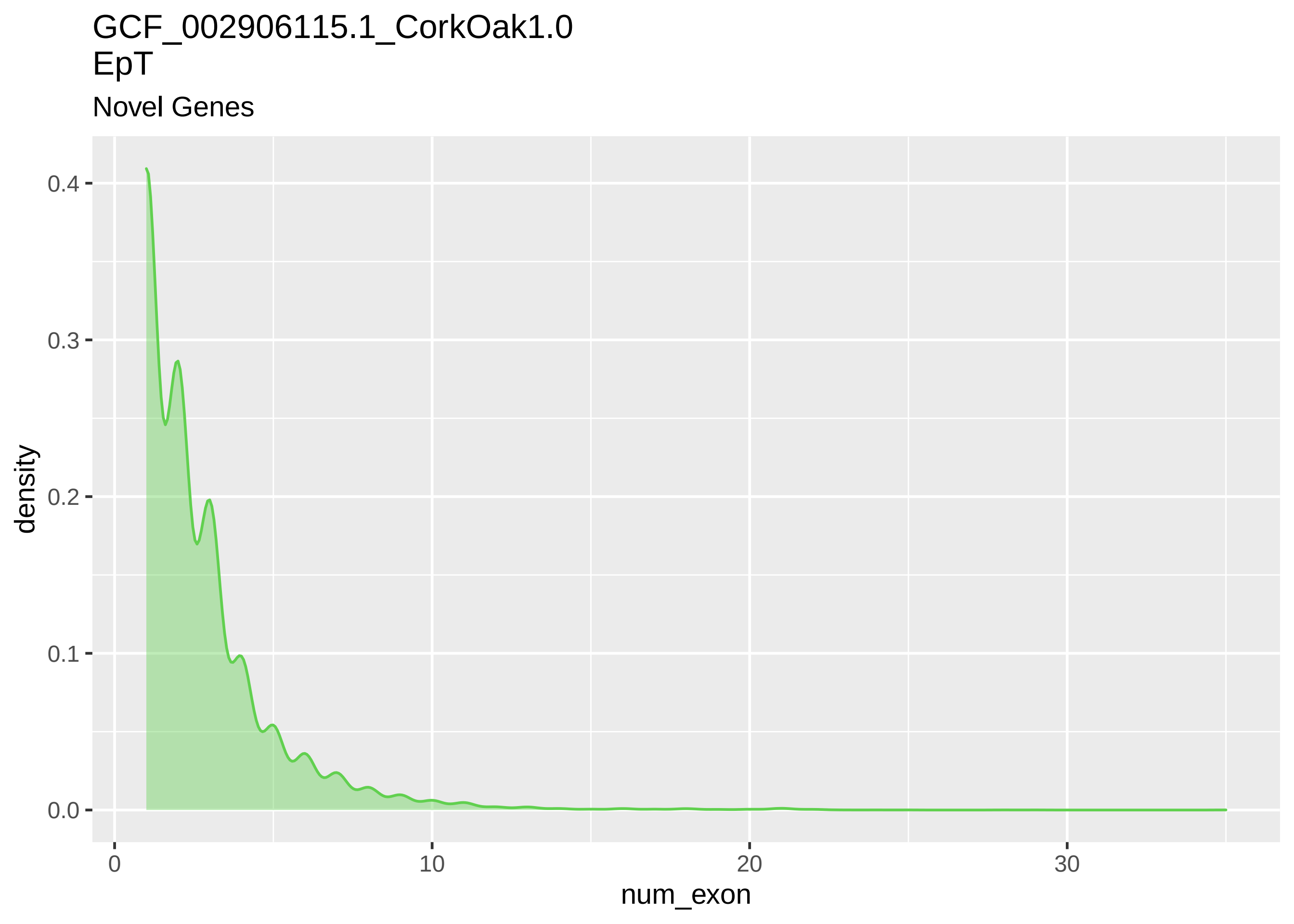
GCF_002906115.1_CorkOak1.0
EpT
Novel Genes
density
0.4
0.3
0.2
0.1
0.0
0
10
20
30
num_exon

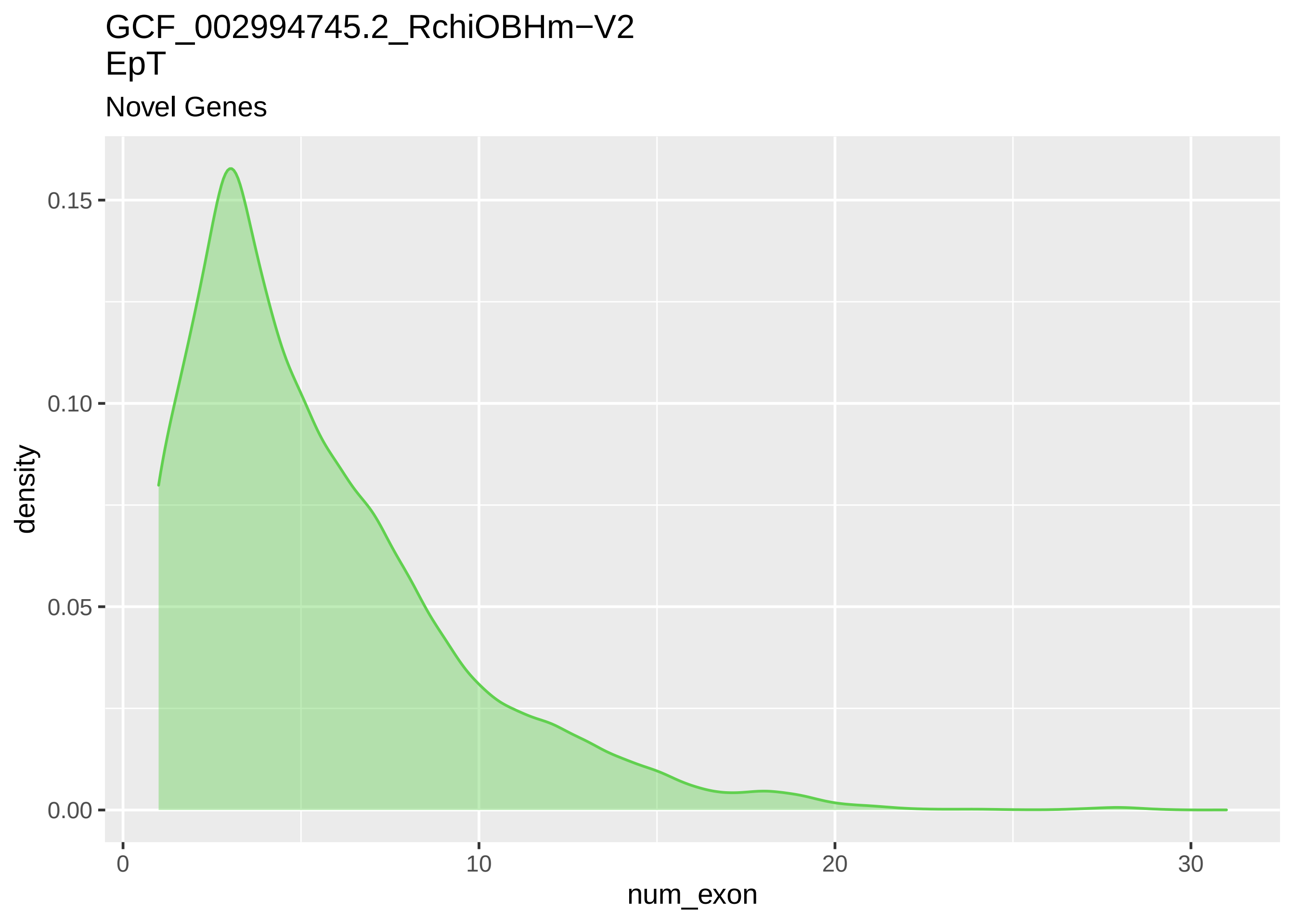

GCF_002994745.2_RchiOBHm−V2
EpT
Novel Genes
density
0.15
0.10
0.05
0.00
0
10
20
30
num_exon

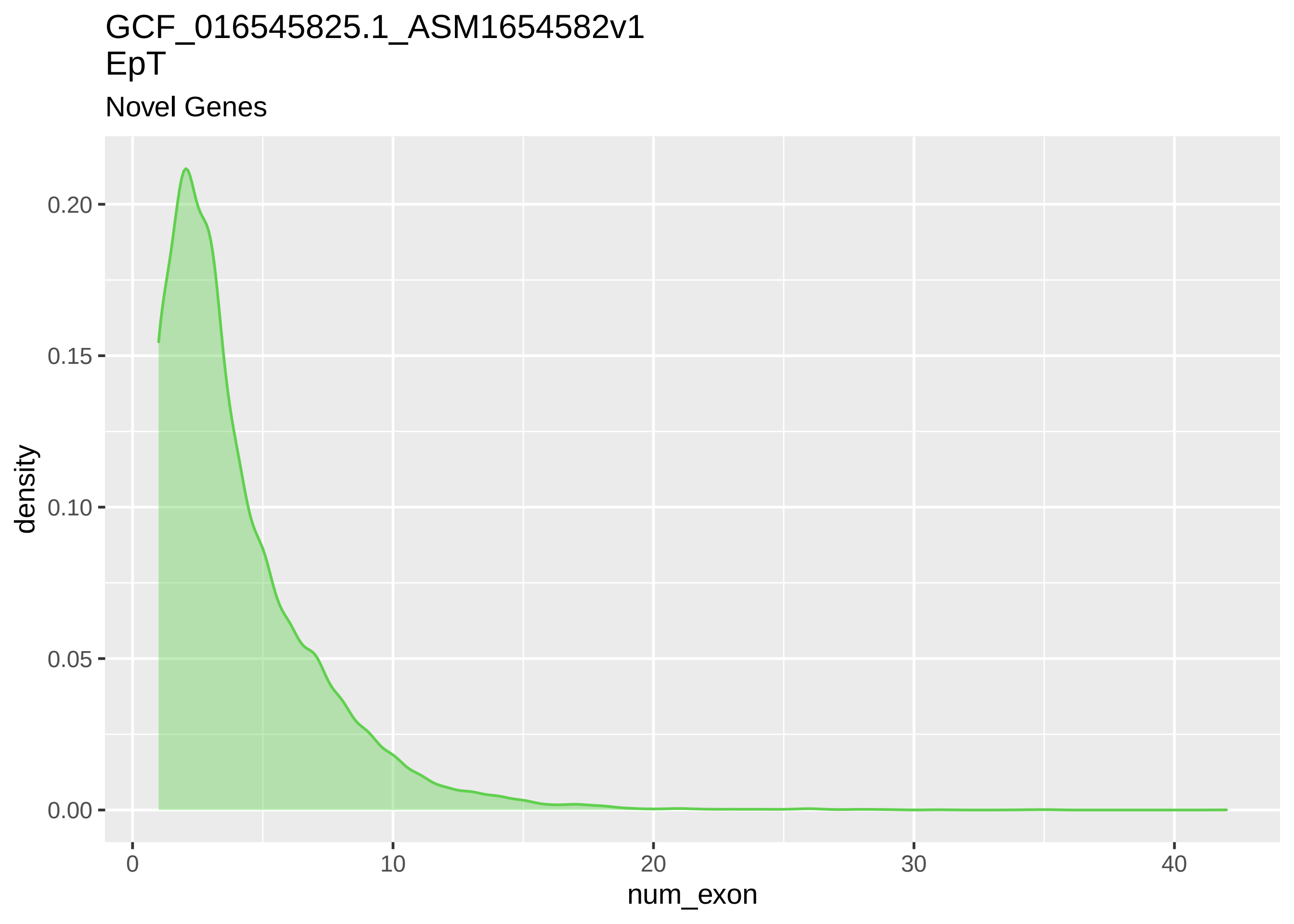
GCF_016545825.1_ASM1654582v1
EpT
Novel Genes
density
0.20
0.15
0.10
0.05
0.00
0
10
20
30
40
num_exon

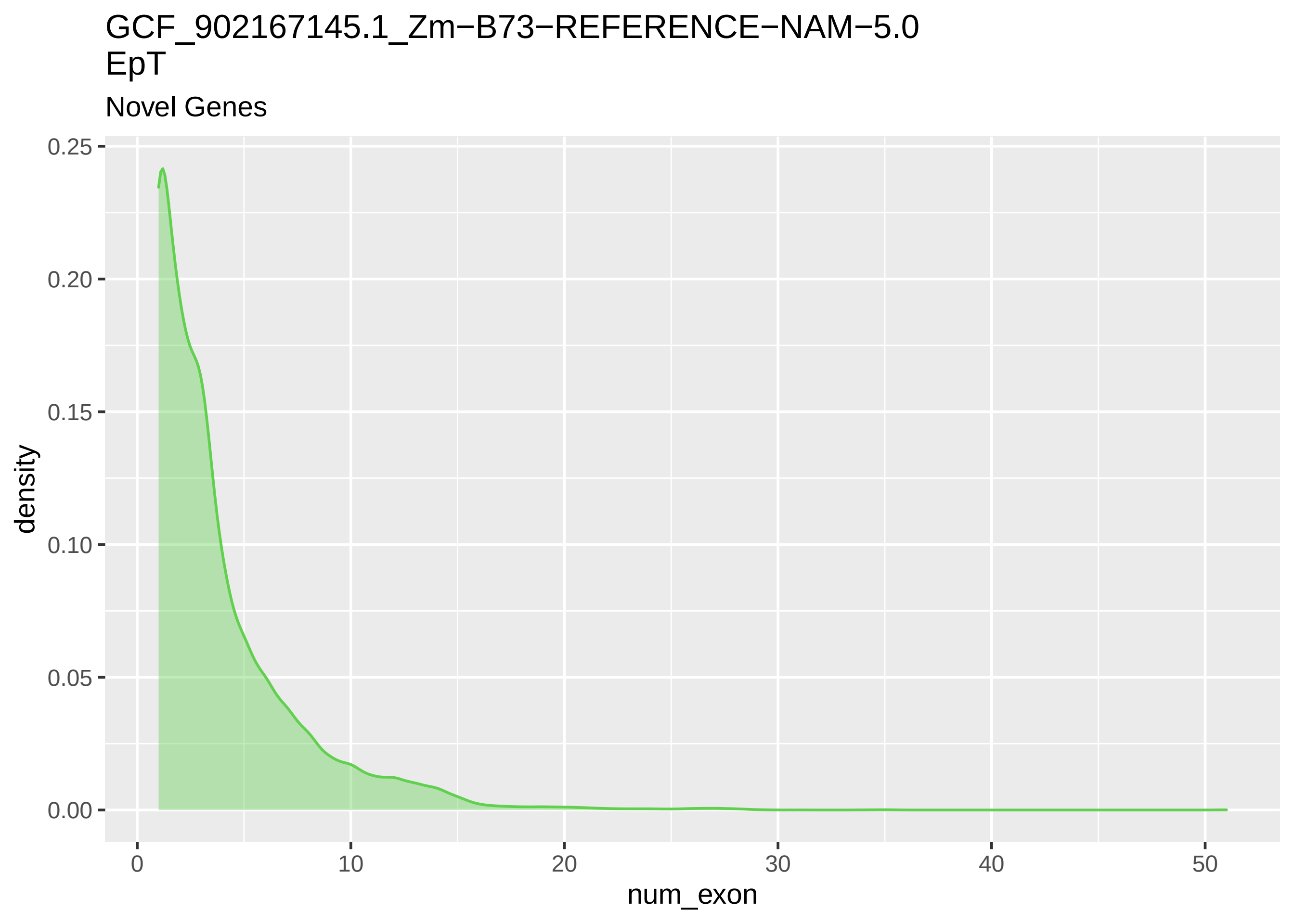
GCF_902167145.1_Zm−B73−REFERENCE−NAM−5.0
EpT
Novel Genes
density
0.25
0.20
0.15
0.10
0.05
0.00
0
10
20
30
40
50
num_exon

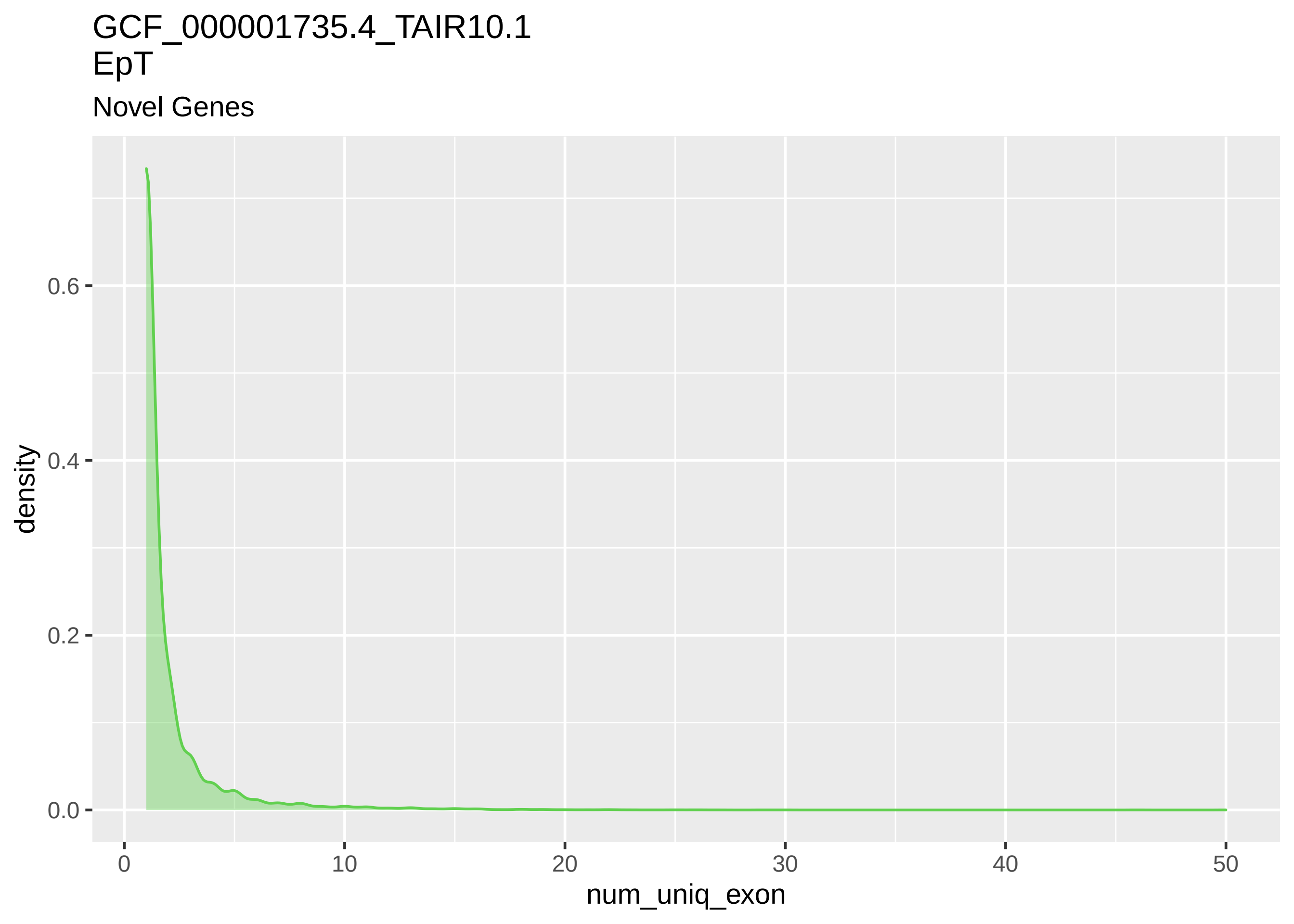

GCF_000001735.4_TAIR10.1
EpT
Novel Genes
density
0.0
0.2
0.4
0.6
0
10
20
30
40
50
num_uniq_exon

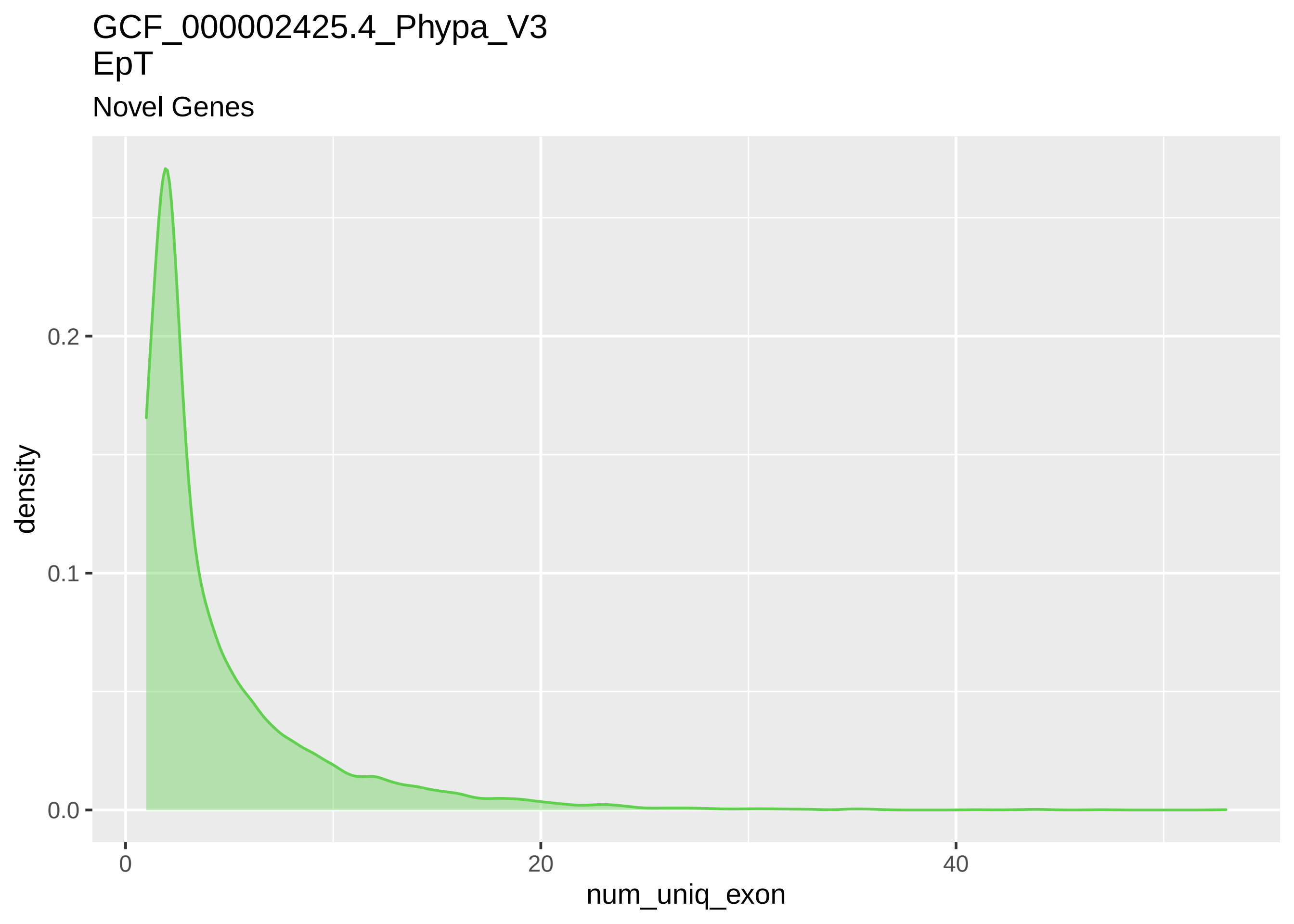
GCF_000002425.4_Phypa_V3
EpT
Novel Genes
density
0.2
0.1
0.0
0
20
40
num_uniq_exon

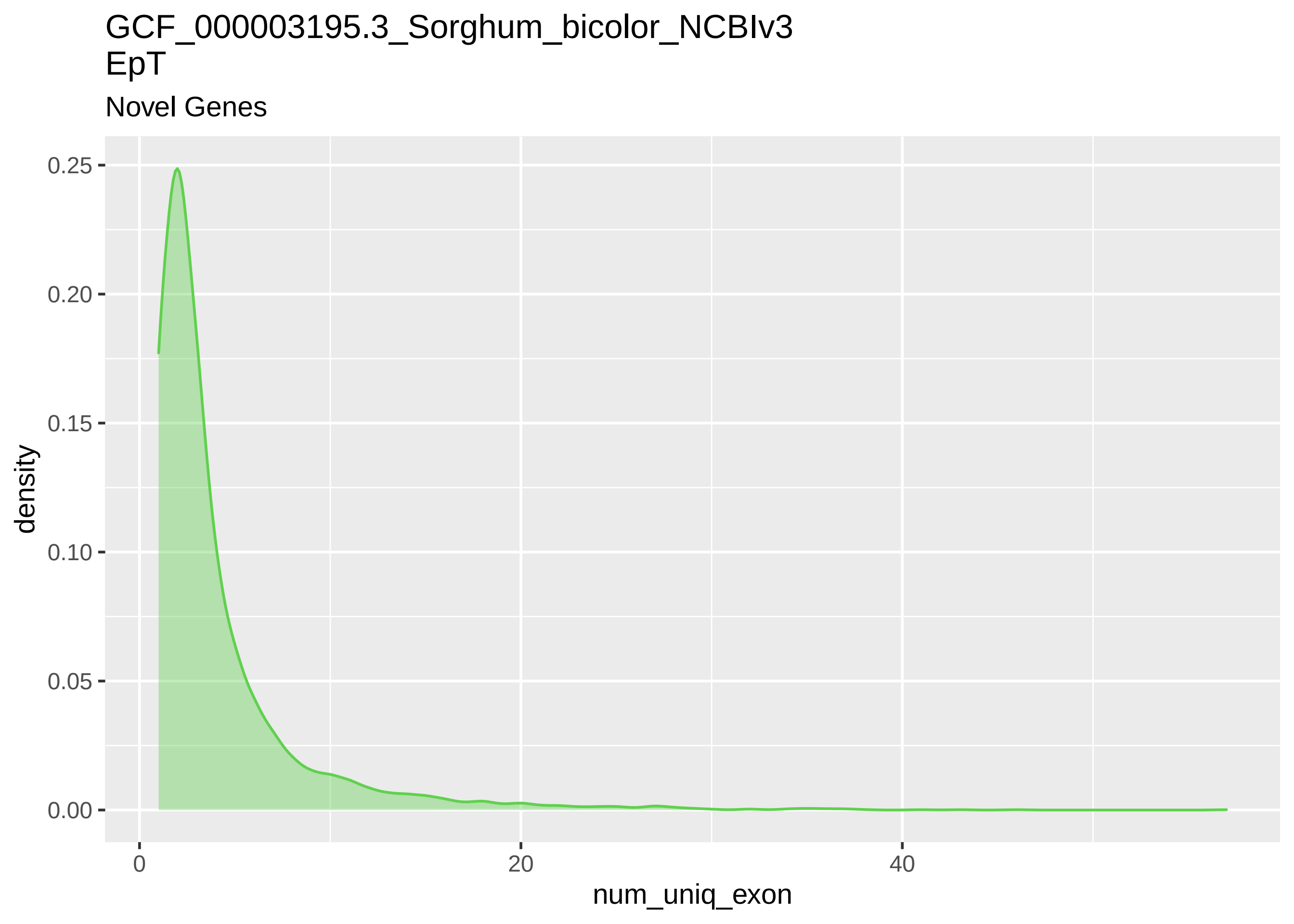

GCF_000003195.3_Sorghum_bicolor_NCBIv3
EpT
Novel Genes
density
0.25
0.20
0.15
0.10
0.05
0.00
0
20
40
num_uniq_exon

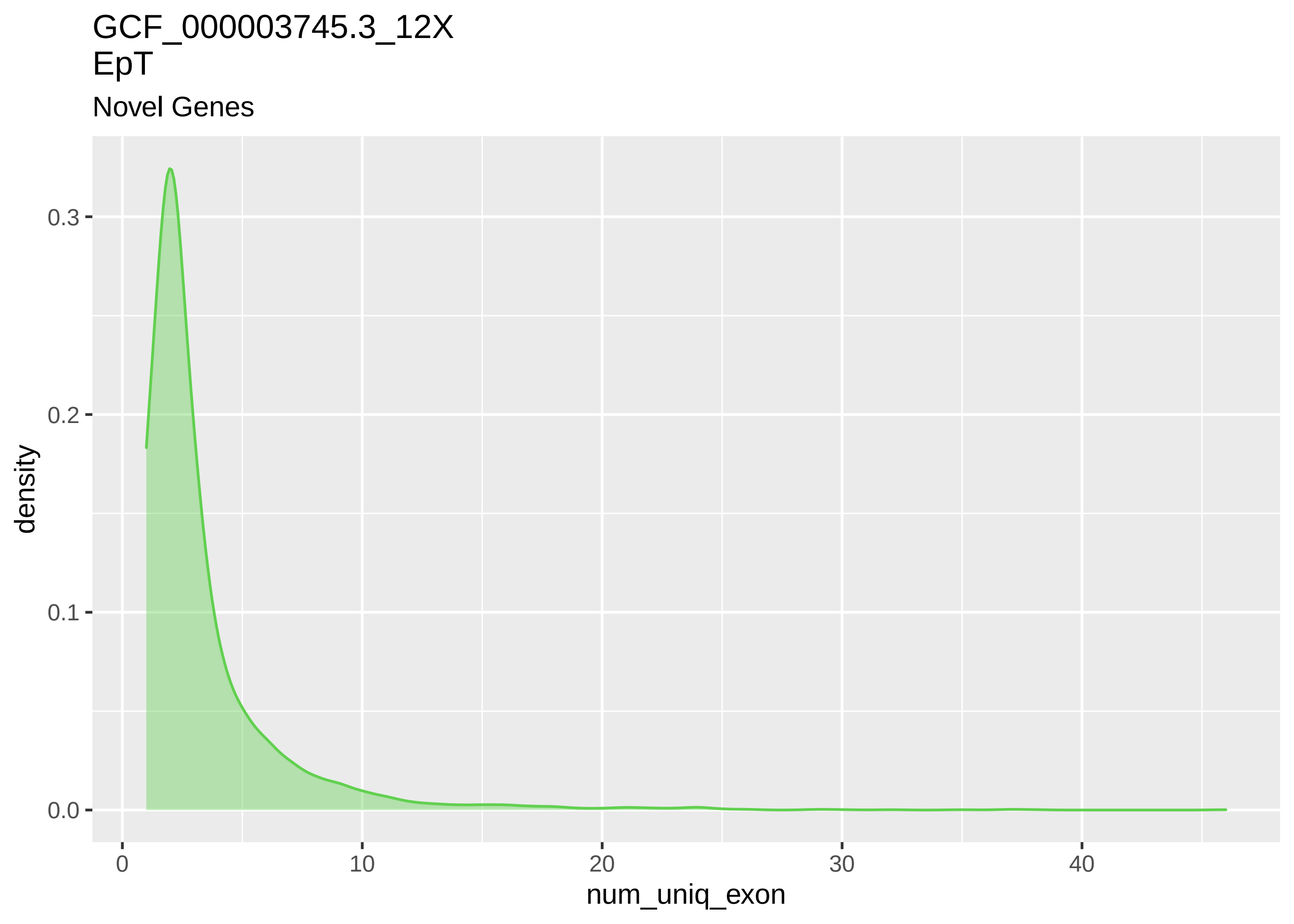

GCF_000003745.3_12X
EpT
Novel Genes
density
0.0
0.1
0.2
0.3
0
10
20
30
40
num_uniq_exon

GCF_000004515.6_Glycine_max_v4.0
EpT

Novel Genes

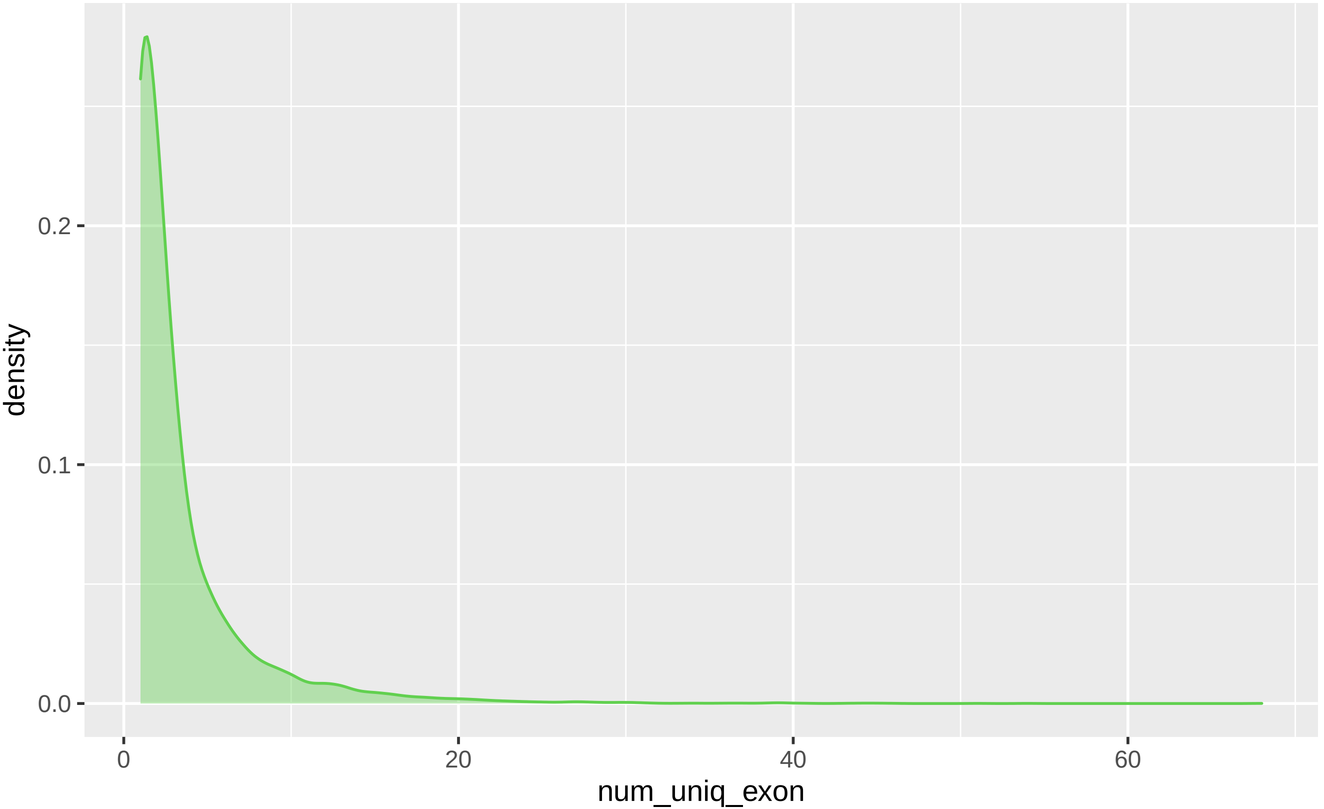

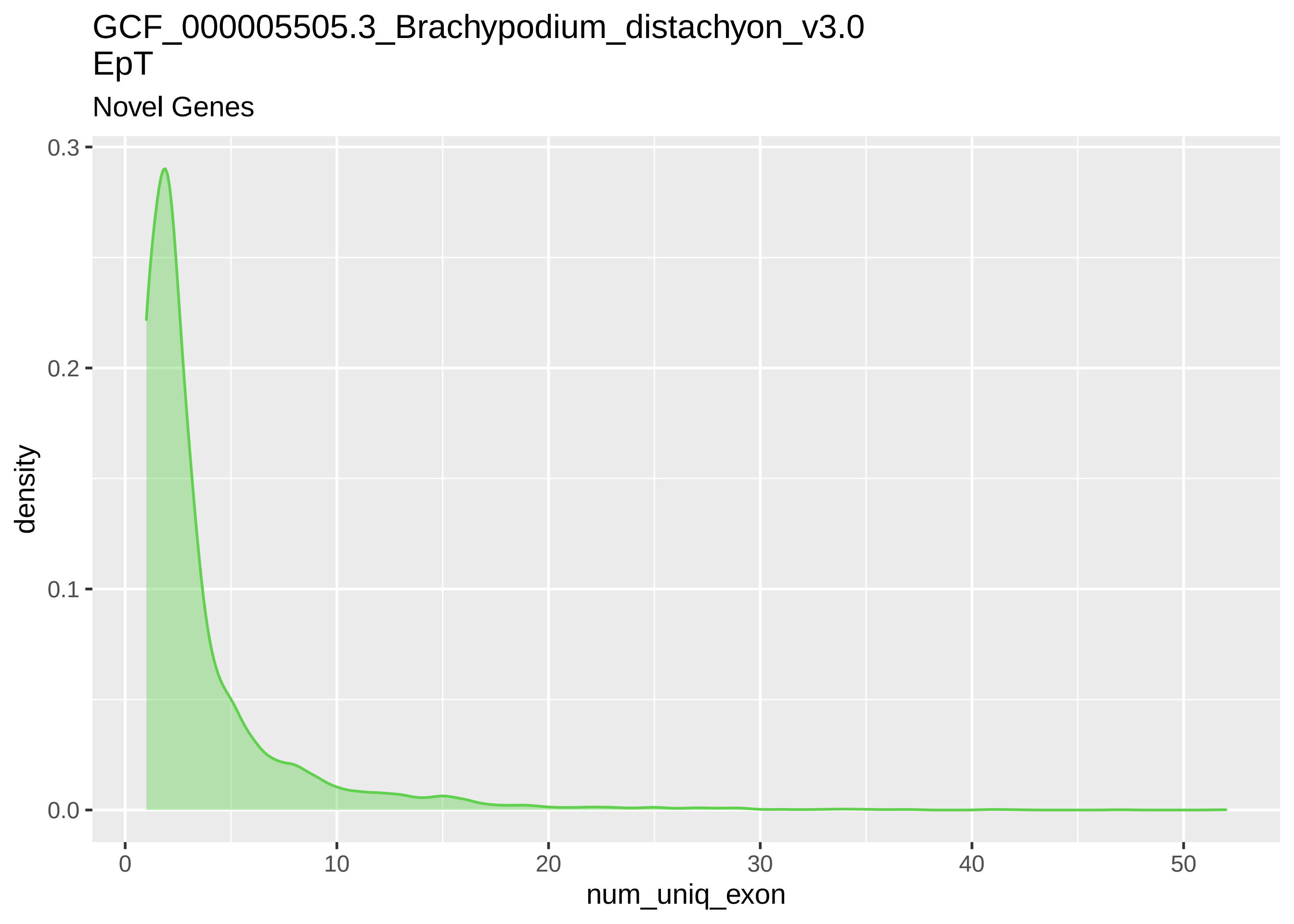
GCF_000005505.3_Brachypodium_distachyon_v3.0
EpT
Novel Genes
density
0.3
0.2
0.1
0.0
0
10
20
30
40
50
num_uniq_exon

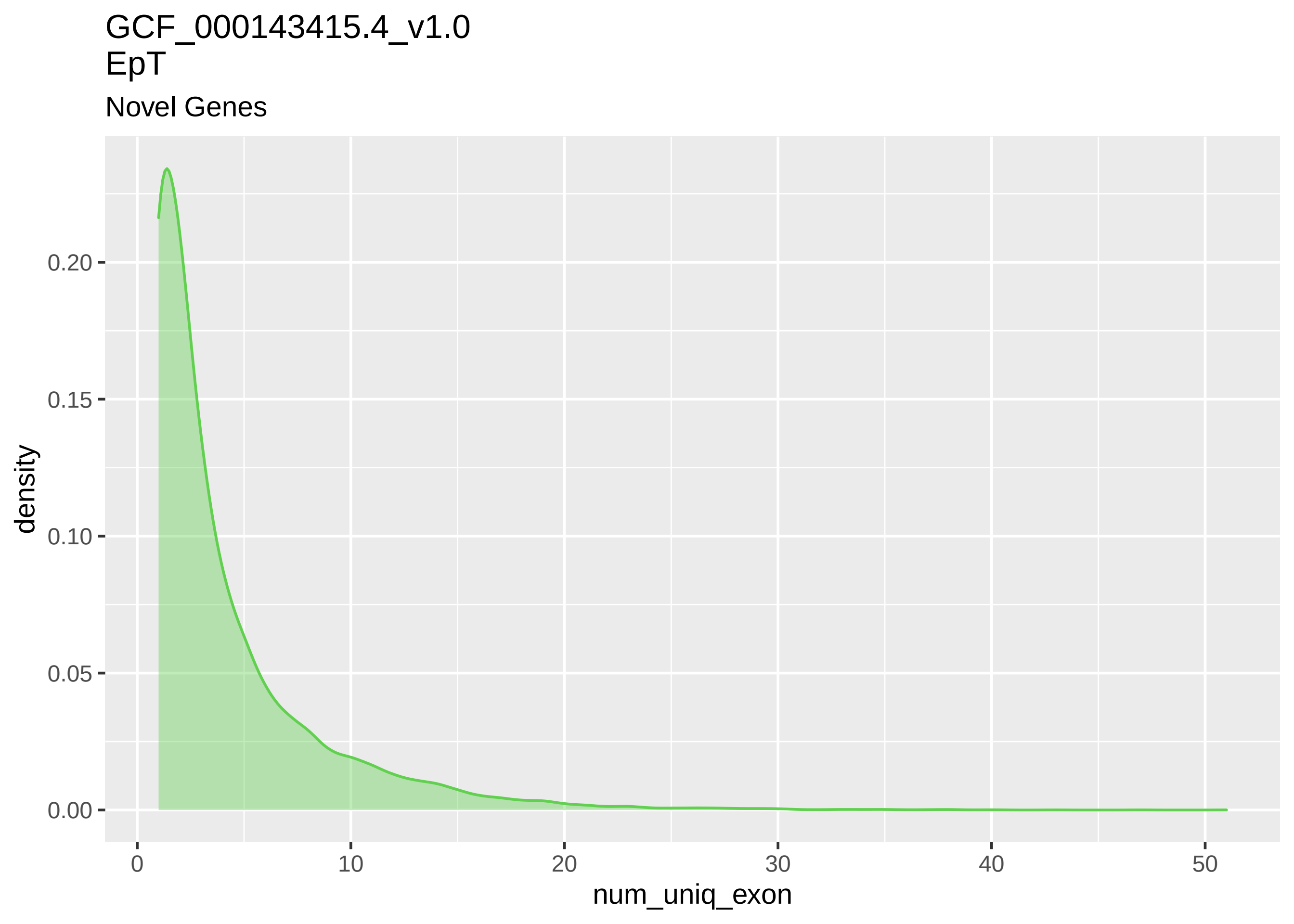
GCF_000143415.4_v1.0
EpT
Novel Genes
density
0.00
0.05
0.10
0.15
0.20
0
10
20
30
40
50
num_uniq_exon

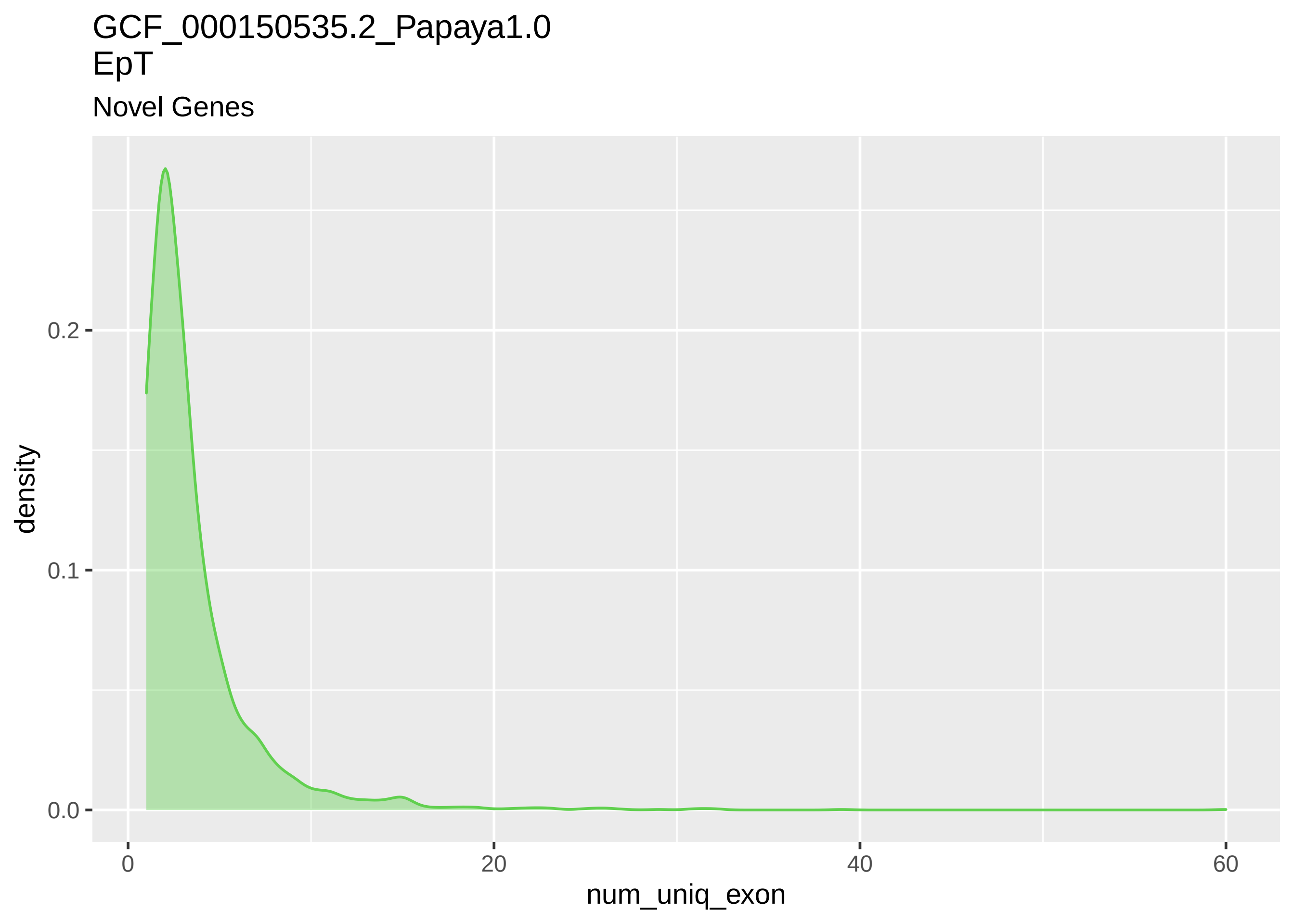
GCF_000150535.2_Papaya1.0
EpT
Novel Genes
density
0.2
0.1
0.0
0
20
40
60
num_uniq_exon

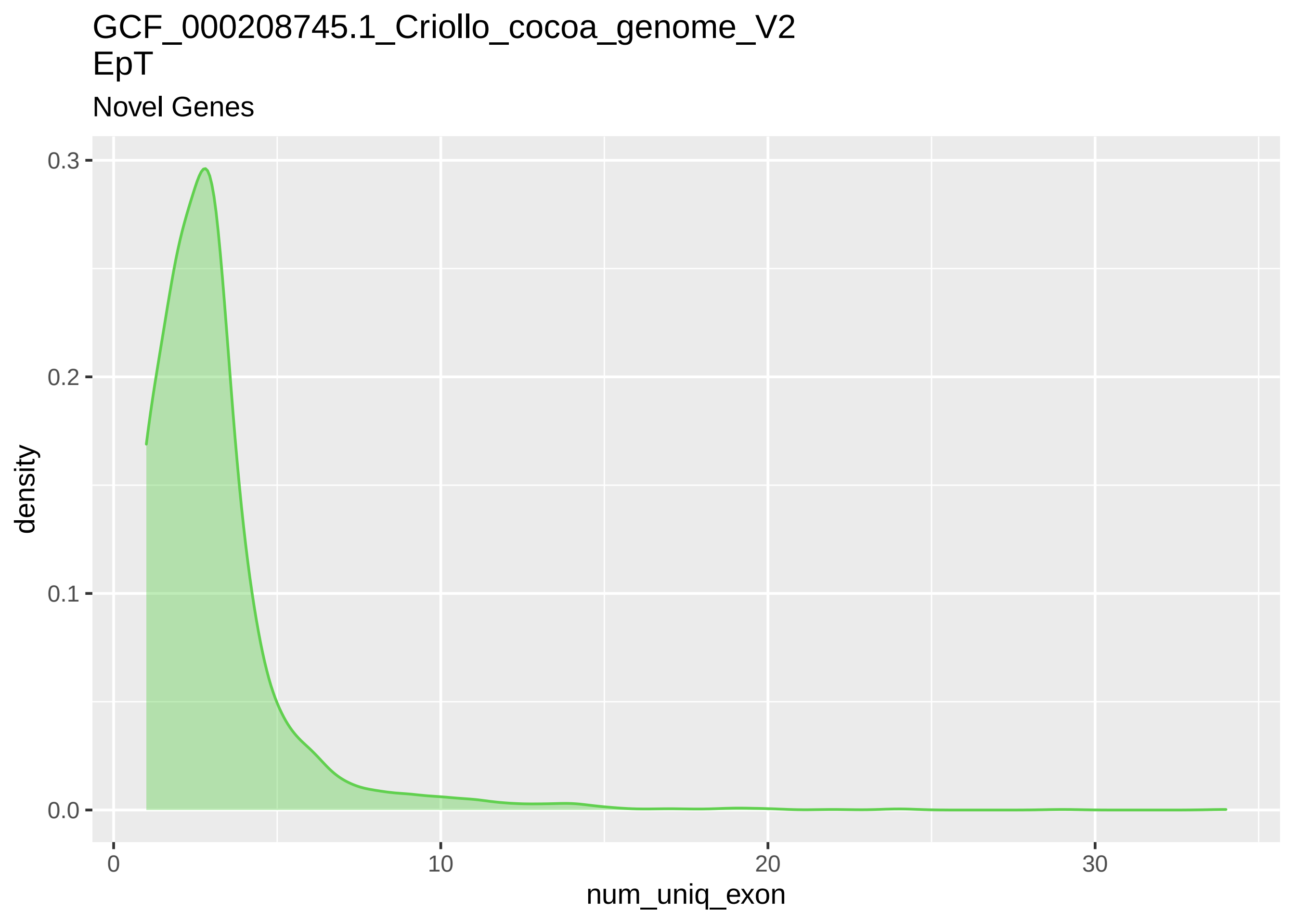

GCF_000208745.1_Criollo_cocoa_genome_V2
EpT
Novel Genes
0.3
0.2
0.1
0.0
density
0
10
20
30
num_uniq_exon

GCF_000226075.1_SolTub_3.0
EpT

Novel Genes

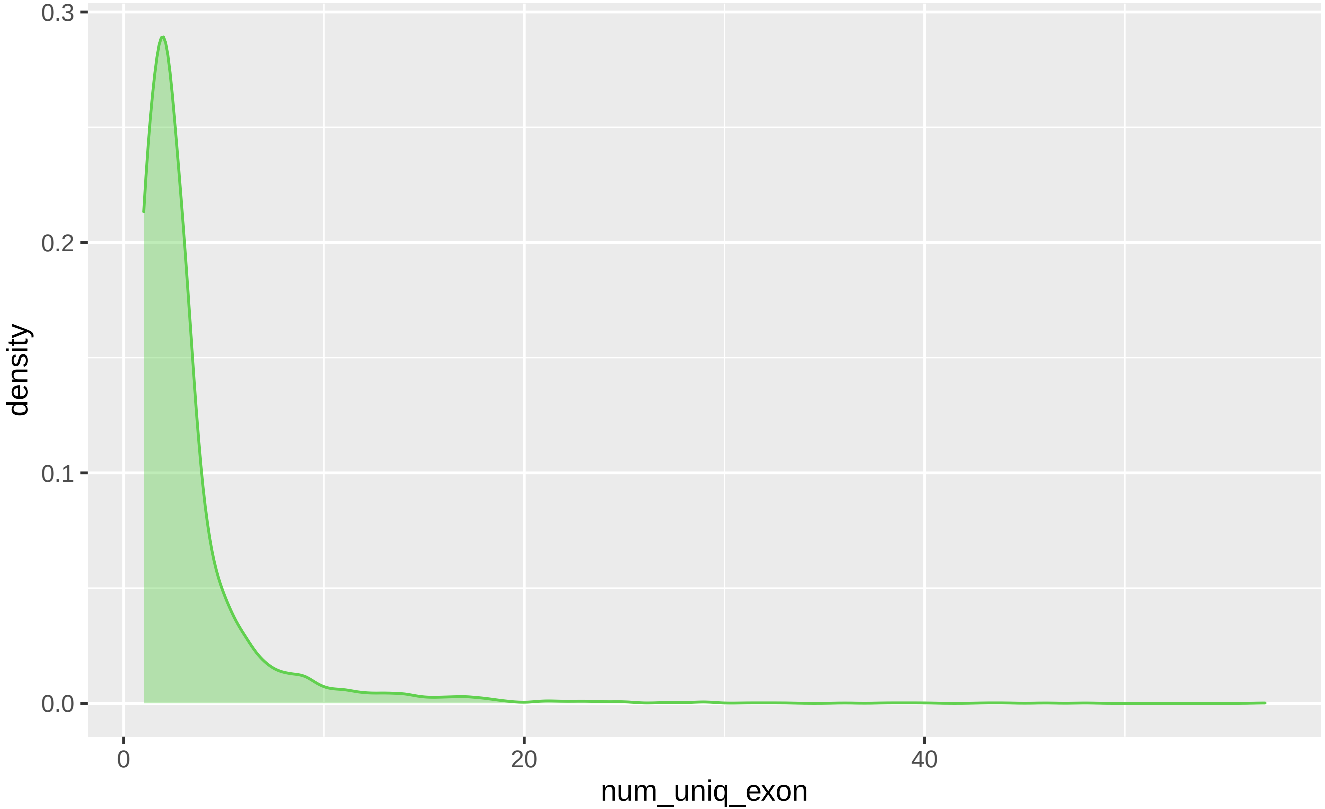

GCF_000309985.2_CAAS_Brap_v3.01
EpT

Novel Genes

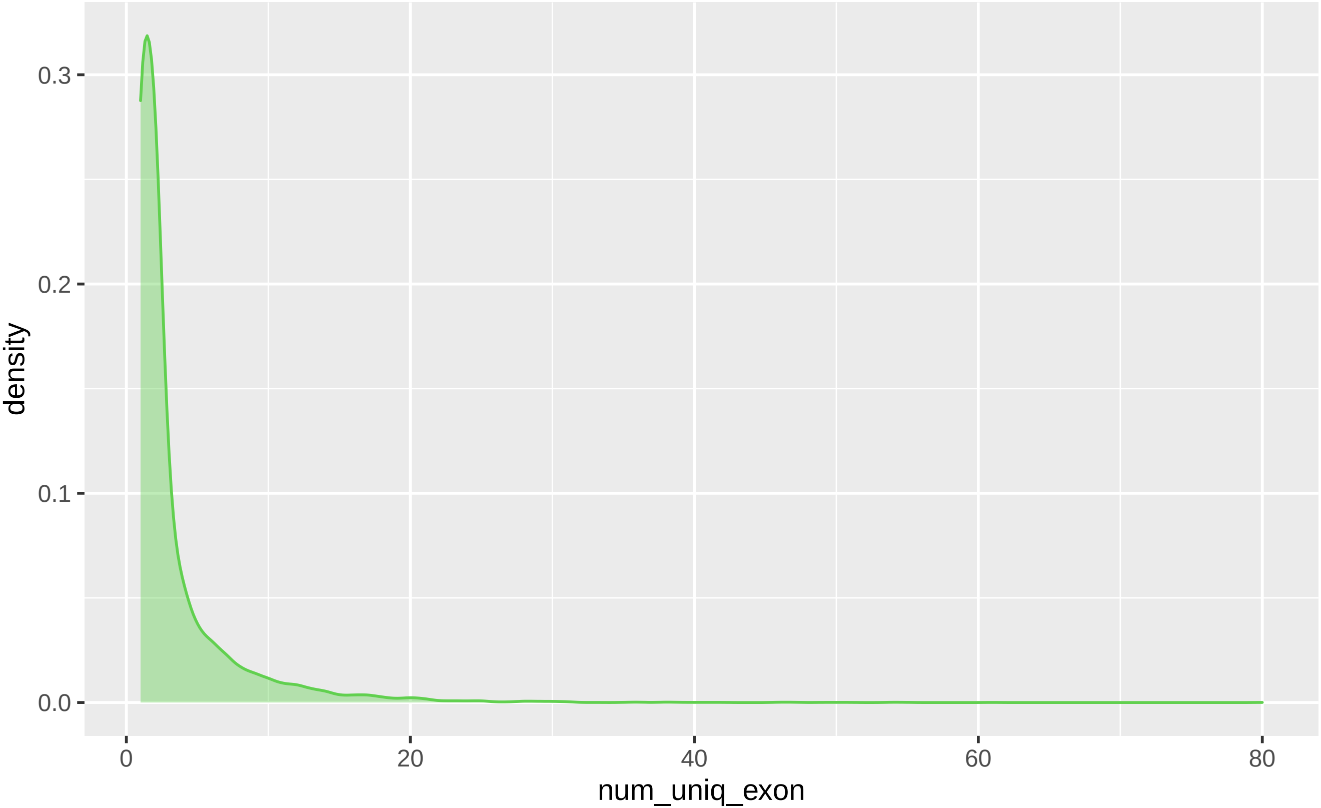

GCF_000313045.1_ASM31304v1
EpT

Novel Genes

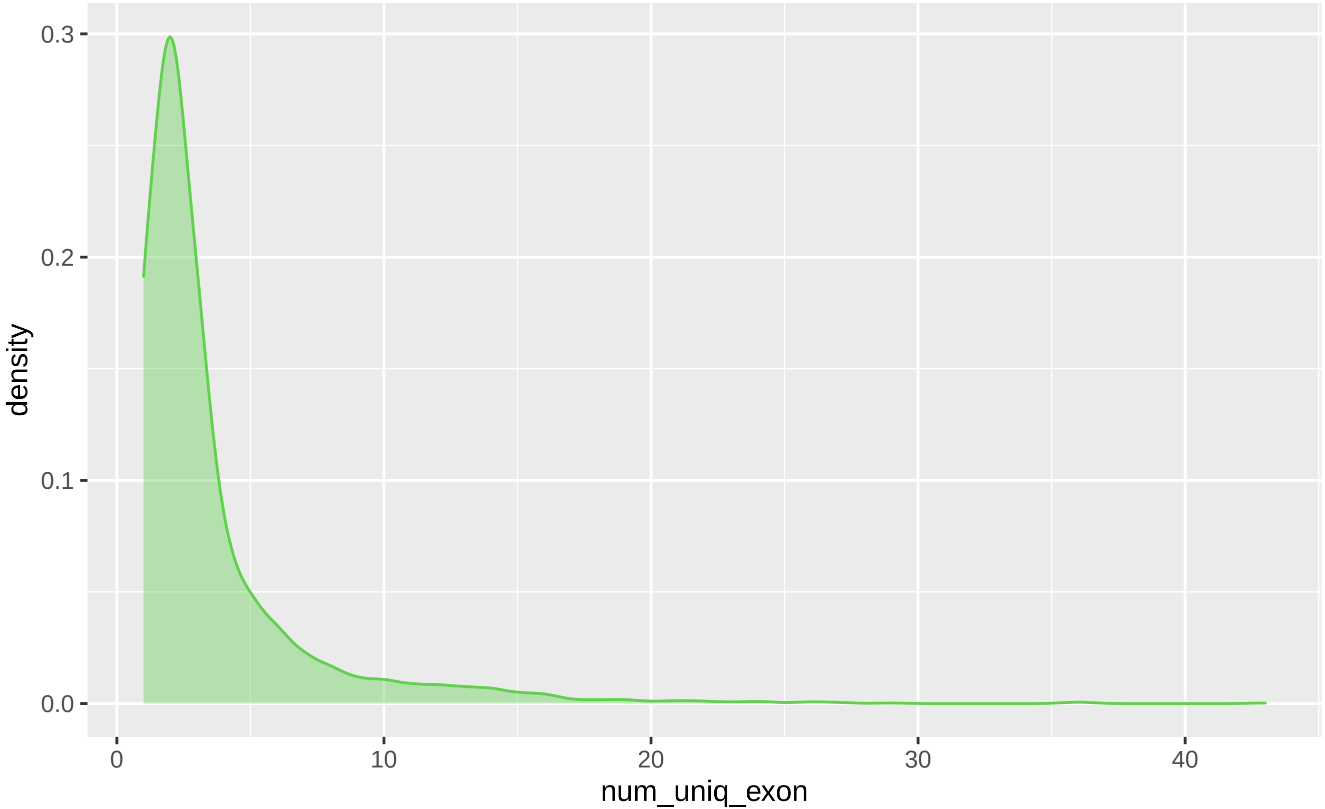

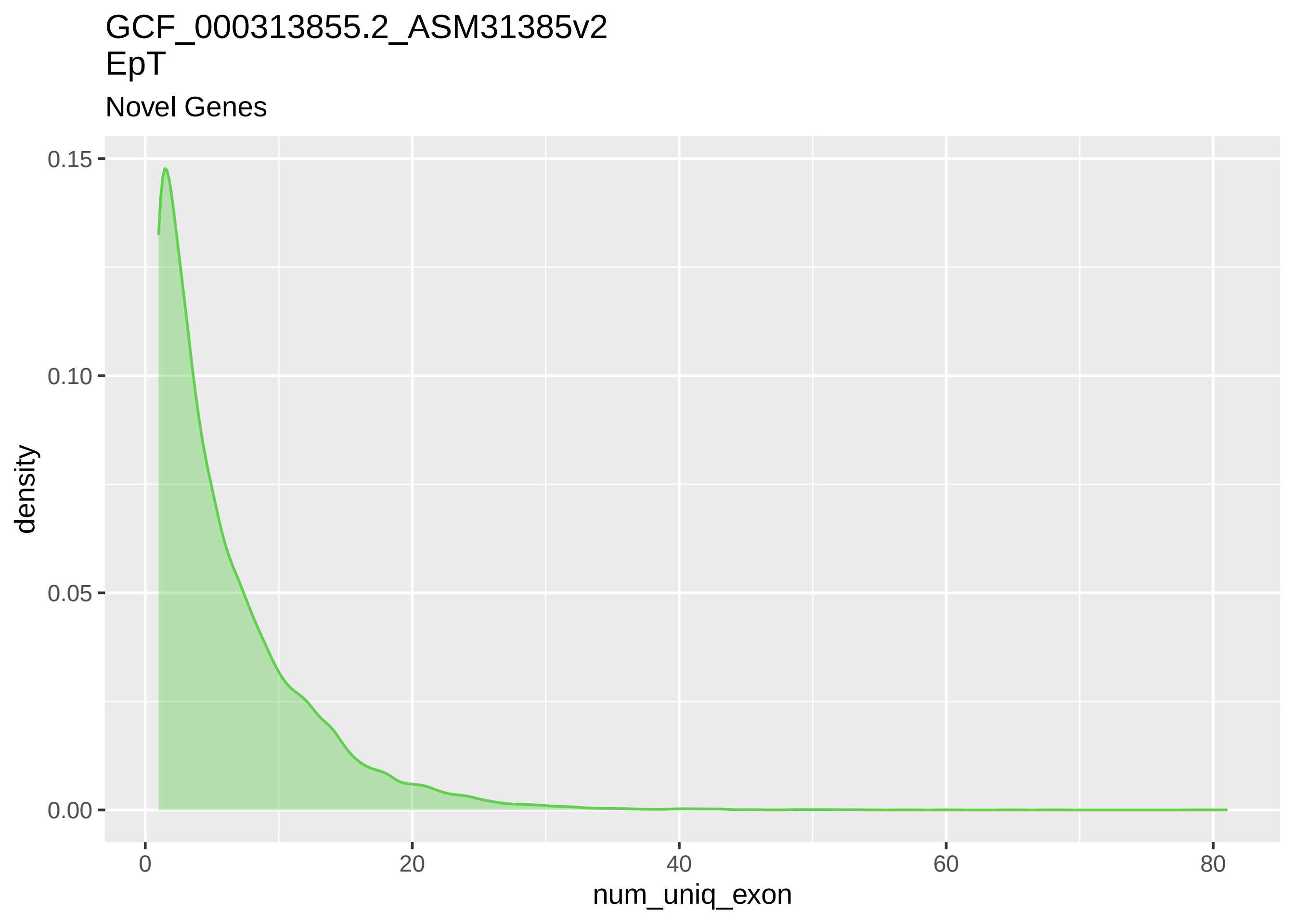
GCF_000313855.2_ASM31385v2
EpT
Novel Genes
density
0.15
0.10
0.05
0.00
0
20
40
60
80
num_uniq_exon

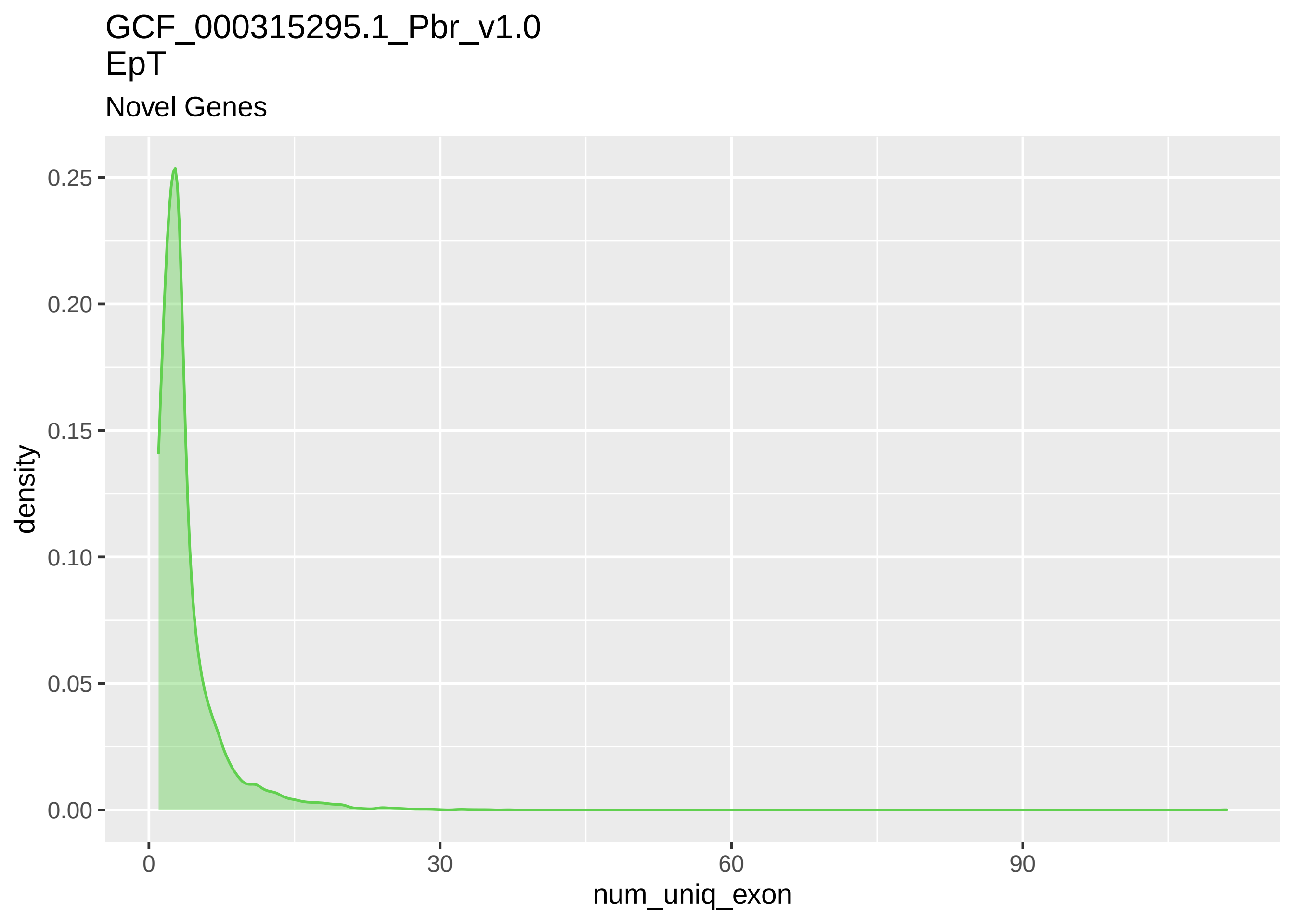

GCF_000315295.1_Pbr_v1.0
EpT
Novel Genes
density
0.25
0.20
0.15
0.10
0.05
0.00
0
30
60
90
num_uniq_exon

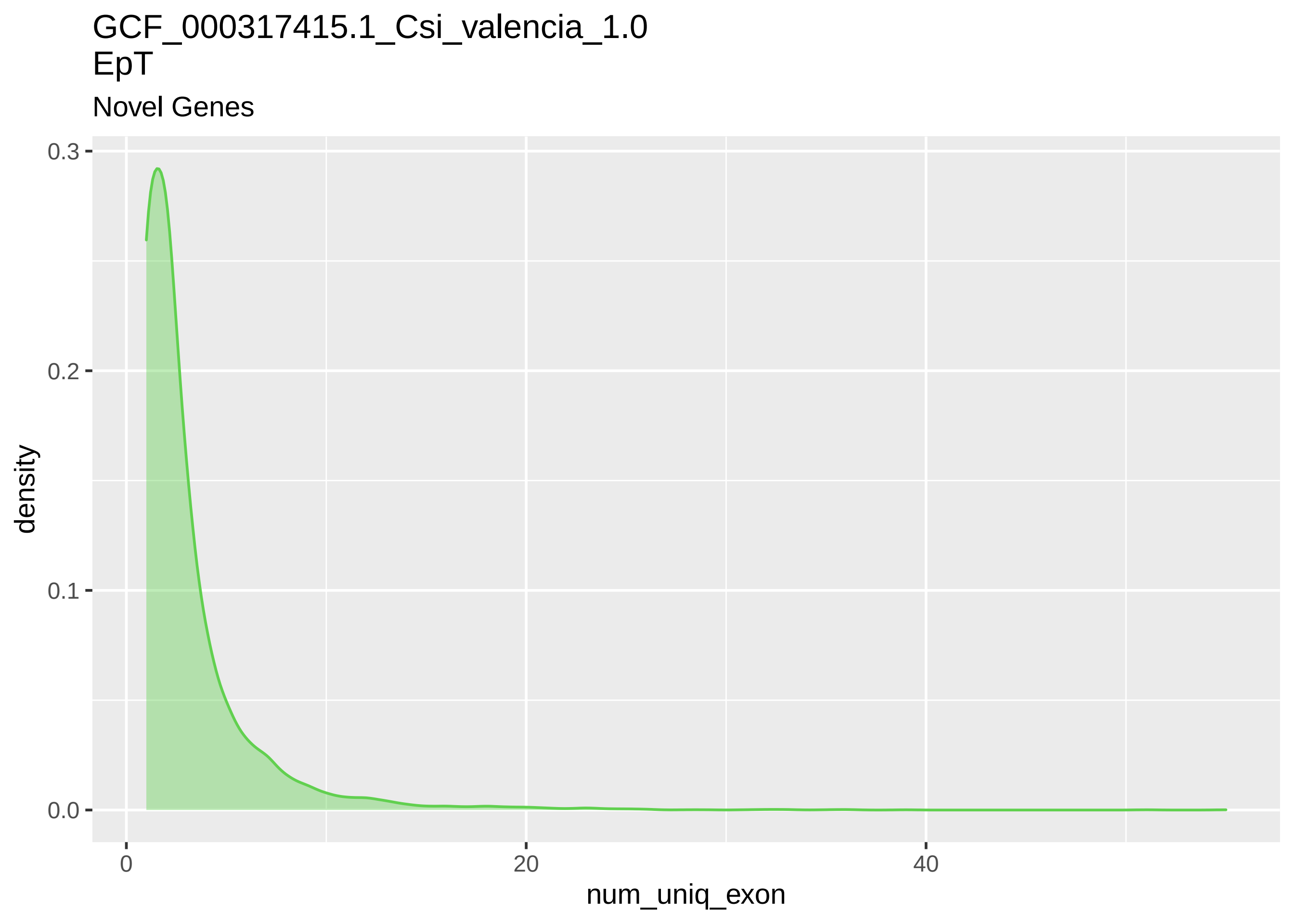
GCF_000317415.1_Csi_valencia_1.0
EpT
Novel Genes
density
0.3
0.2
0.1
0.0
0
20
40
num_uniq_exon

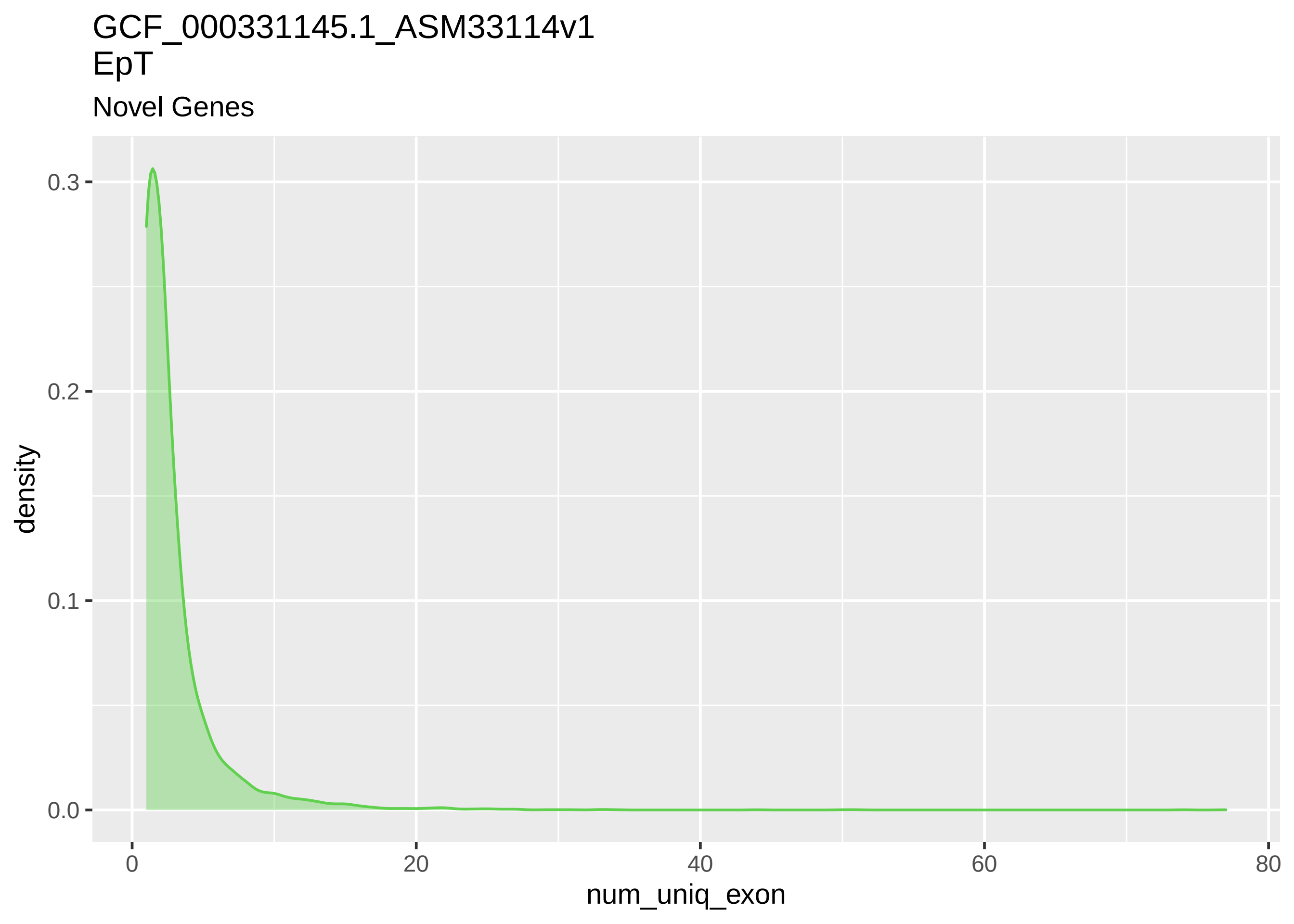
GCF_000331145.1_ASM33114v1
EpT
Novel Genes
density
0.3
0.2
0.1
0.0
0
20
40
60
80
num_uniq_exon

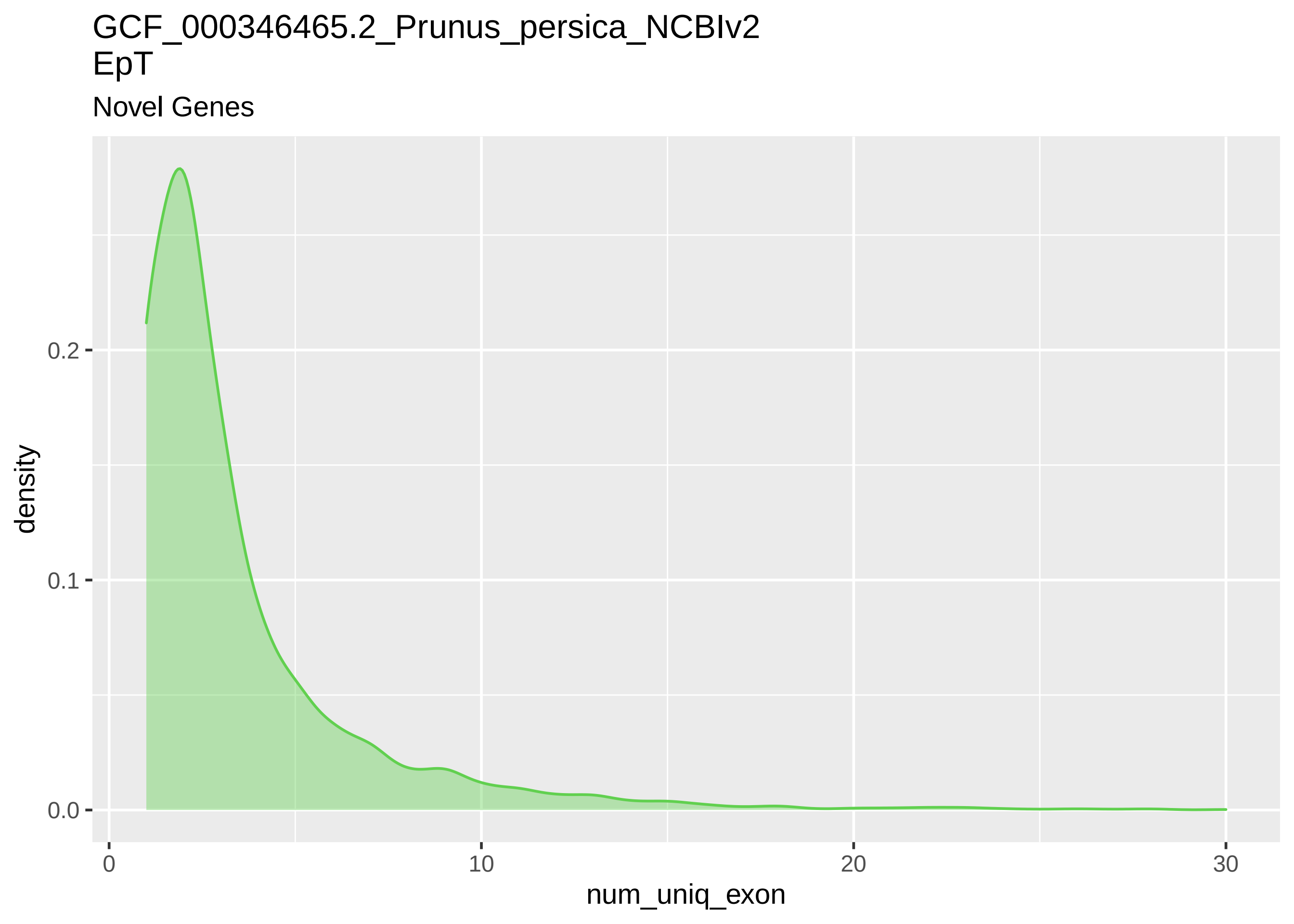

GCF_000346465.2_Prunus_persica_NCBIv2
EpT
Novel Genes
density
0.2
0.1
0.0
0
10
20
30
num_uniq_exon

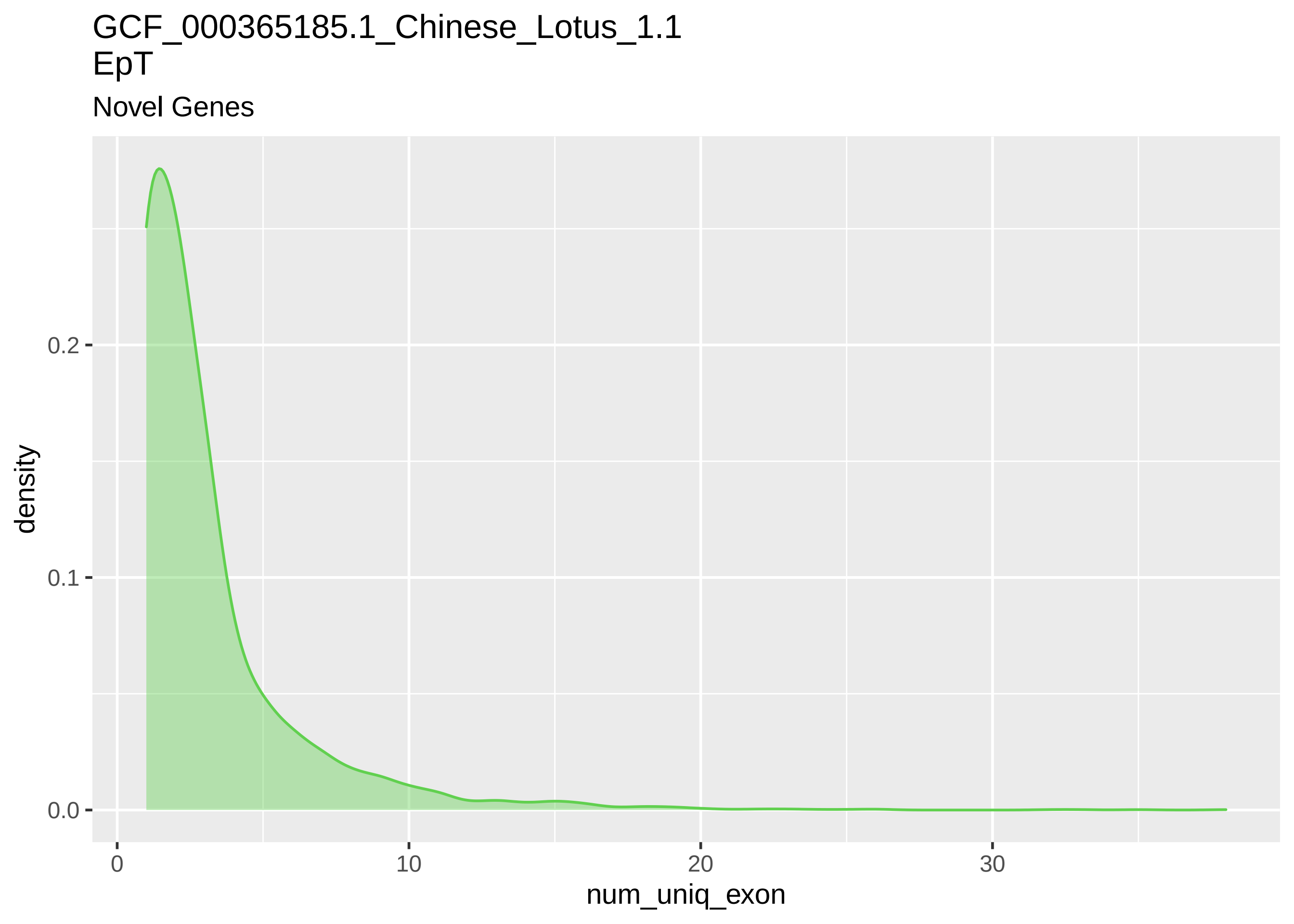

GCF_000365185.1_Chinese_Lotus_1.1
EpT
Novel Genes
density
0.0
0.1
0.2
0
10
20
30
num_uniq_exon

GCF_000471905.2_AMTR1.0
EpT

Novel Genes

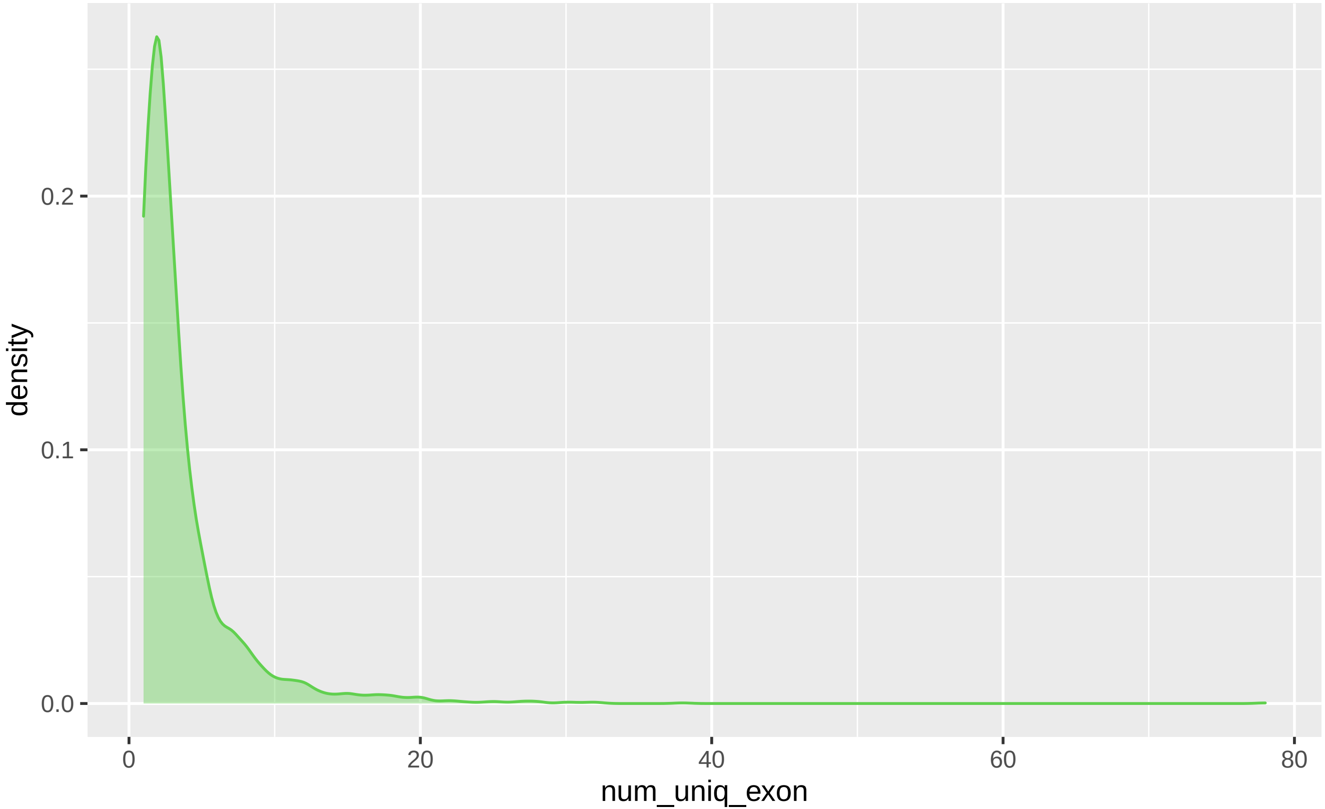

GCF_000478725.1_Eutsalg1_0
EpT

Novel Genes

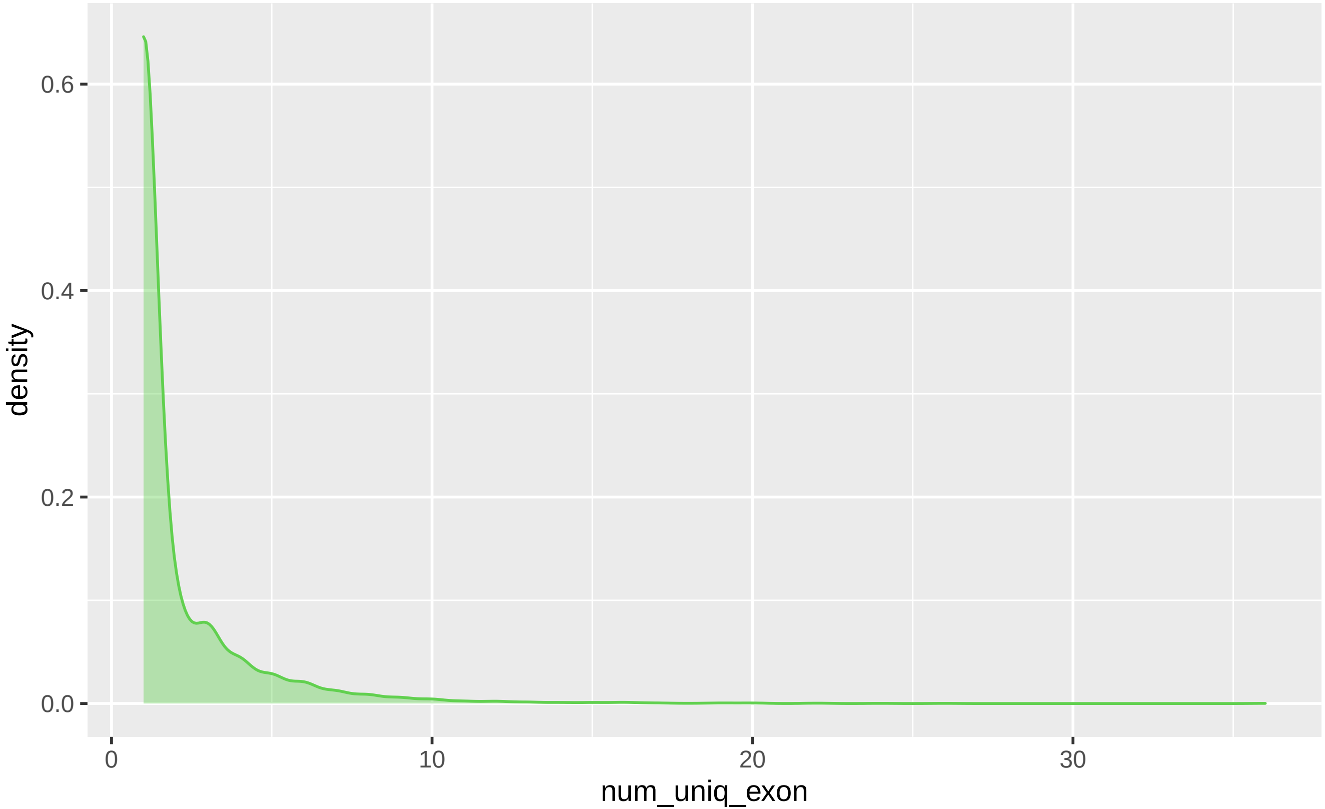

GCF_000504015.1_Mimgu1_0
EpT

Novel Genes

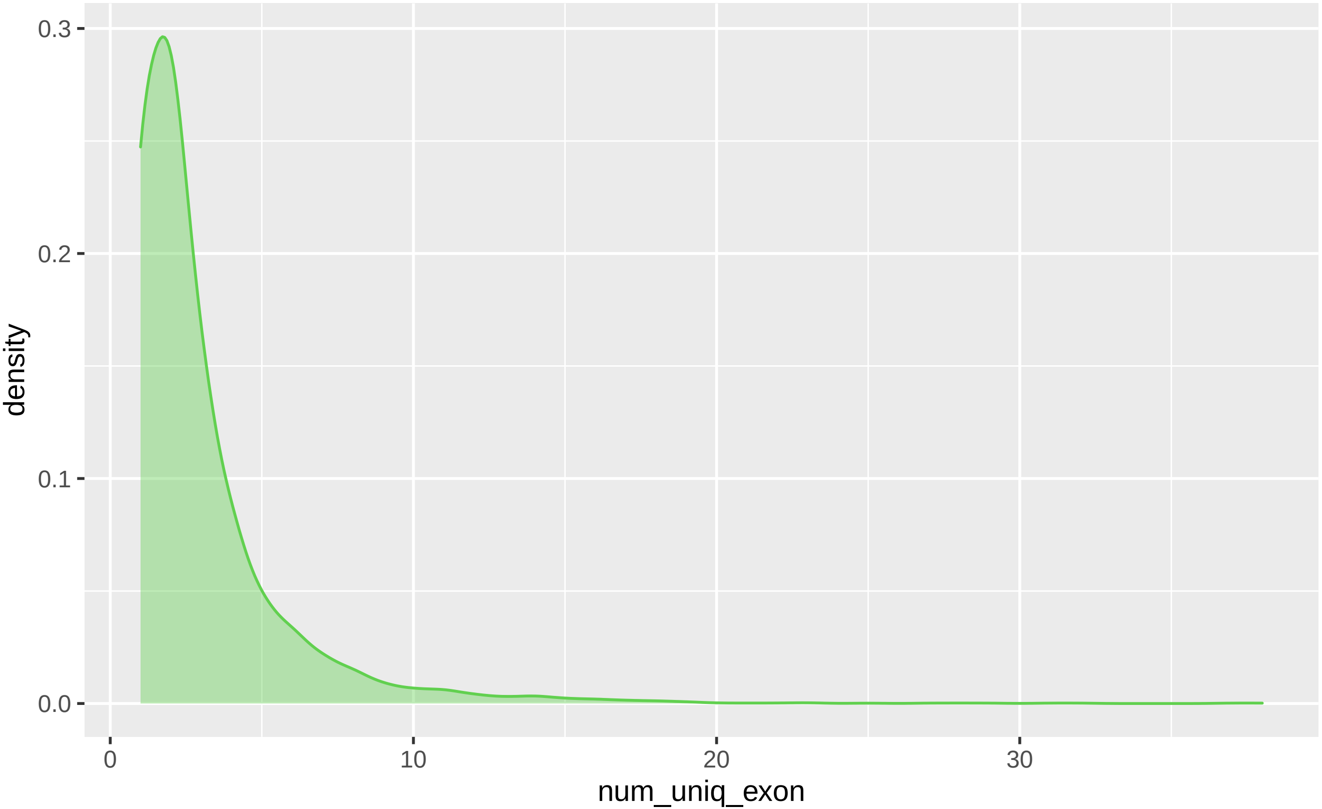

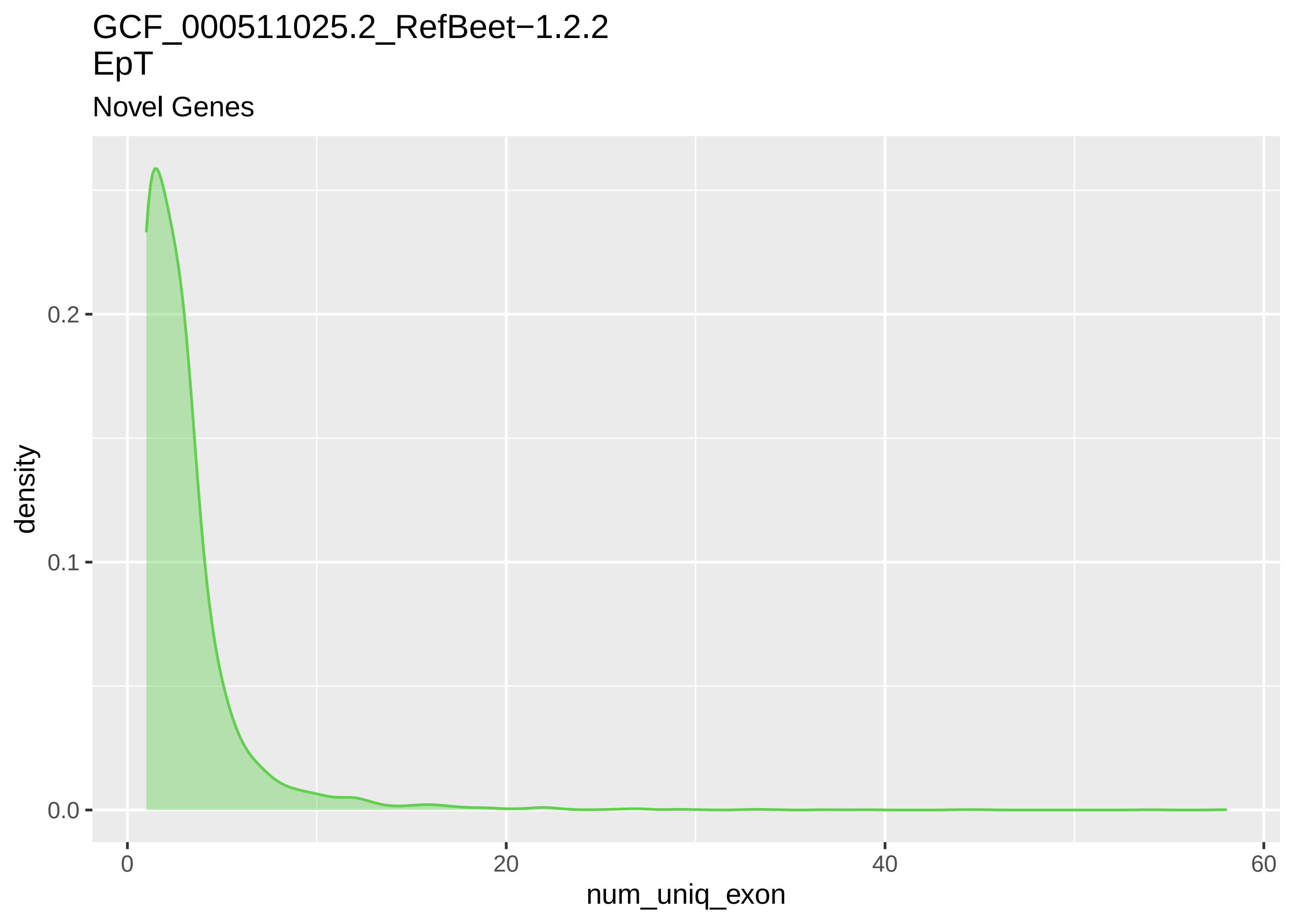
GCF_000511025.2_RefBeet−1.2.2
EpT
Novel Genes
density
0.2
0.1
0.0
0
20
40
60
num_uniq_exon

GCF_000512975.1_S_indicum_v1.0
EpT

Novel Genes

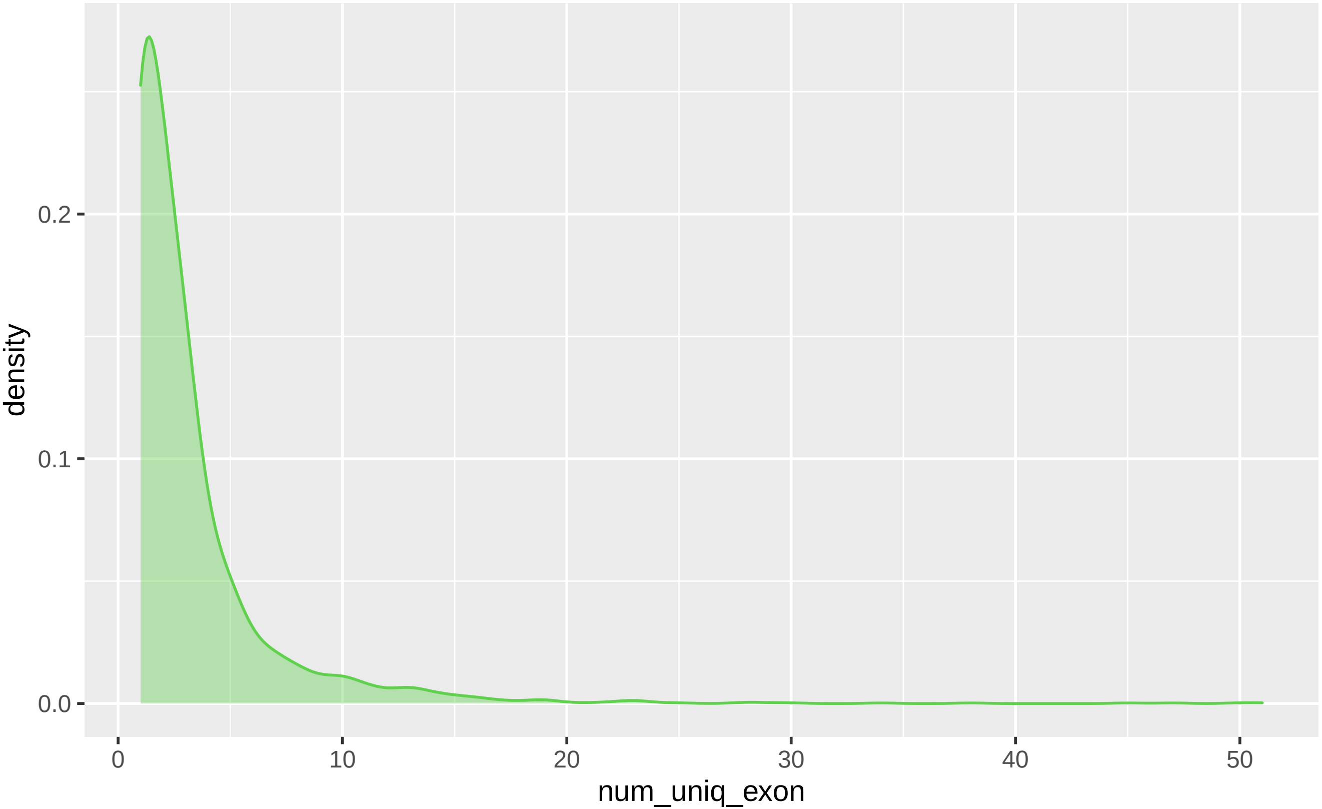

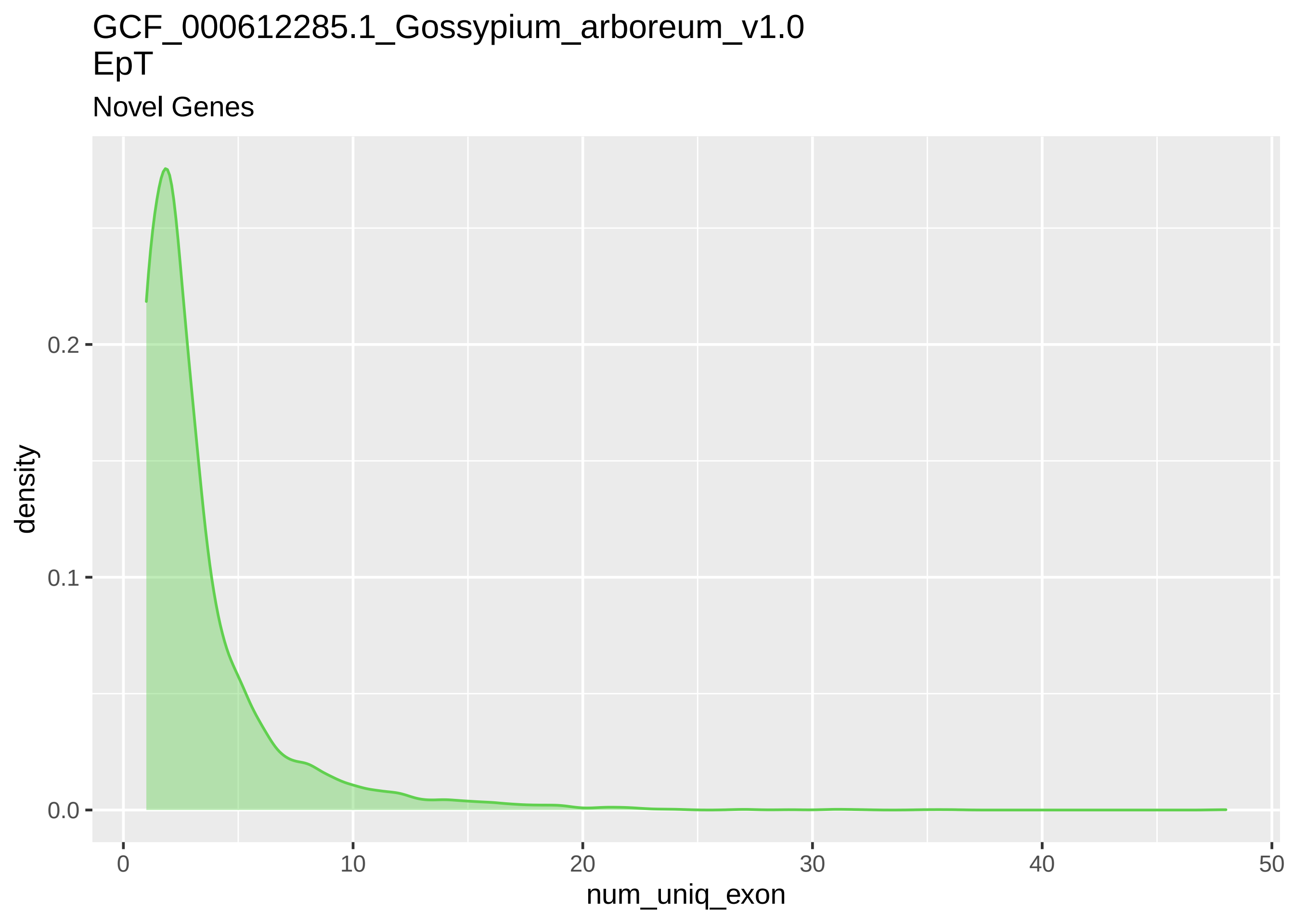
GCF_000612285.1_Gossypium_arboreum_v1.0
EpT
Novel Genes
density
0.0
0.1
0.2
0
10
20
30
40
50
num_uniq_exon

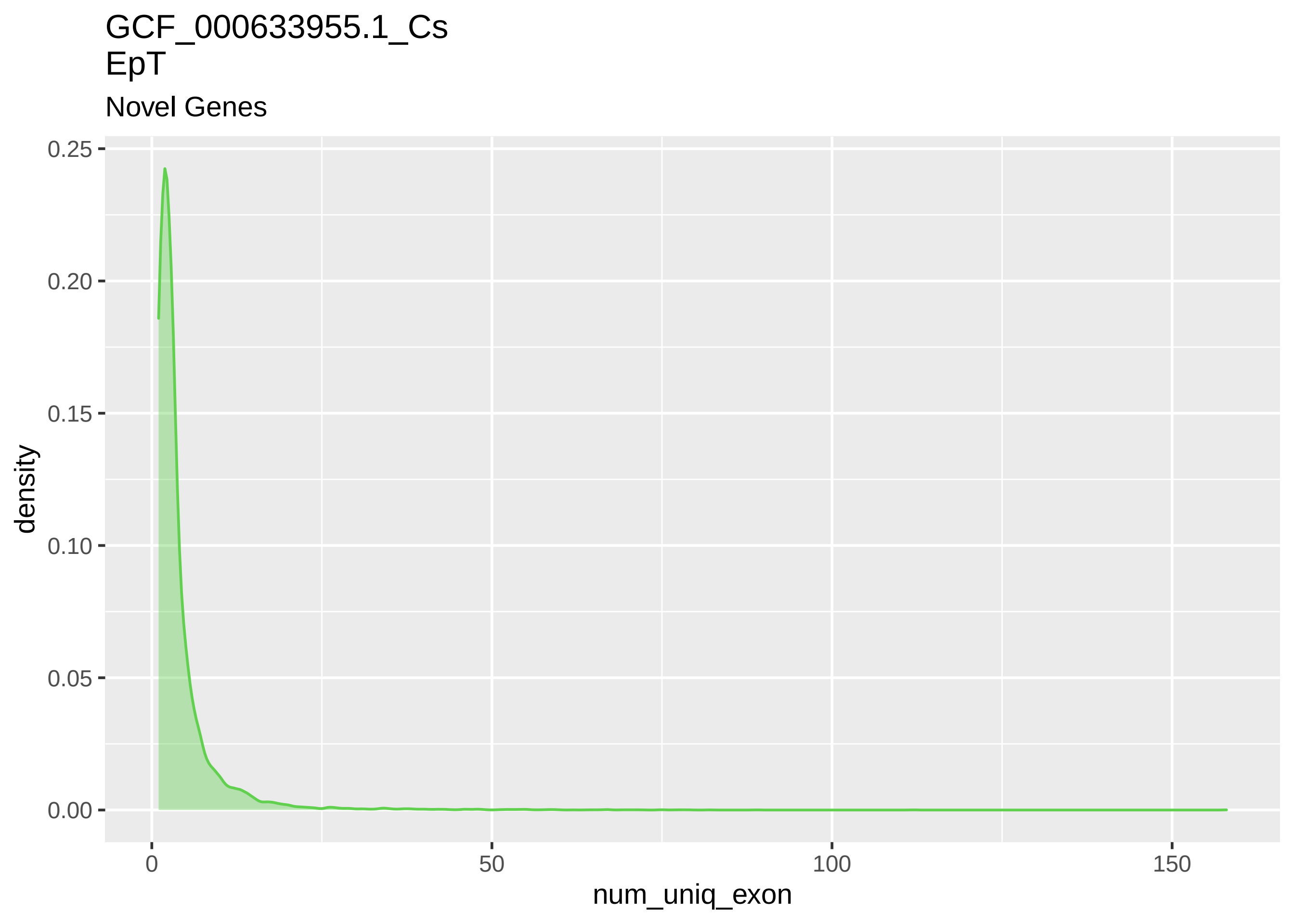
GCF_000633955.1_Cs
EpT
Novel Genes
density
0.25
0.20
0.15
0.10
0.05
0.00
0
50
100
150
num_uniq_exon

GCF_000710875.1_Pepper_Zunla_1_Ref_v1.0
EpT

Novel Genes

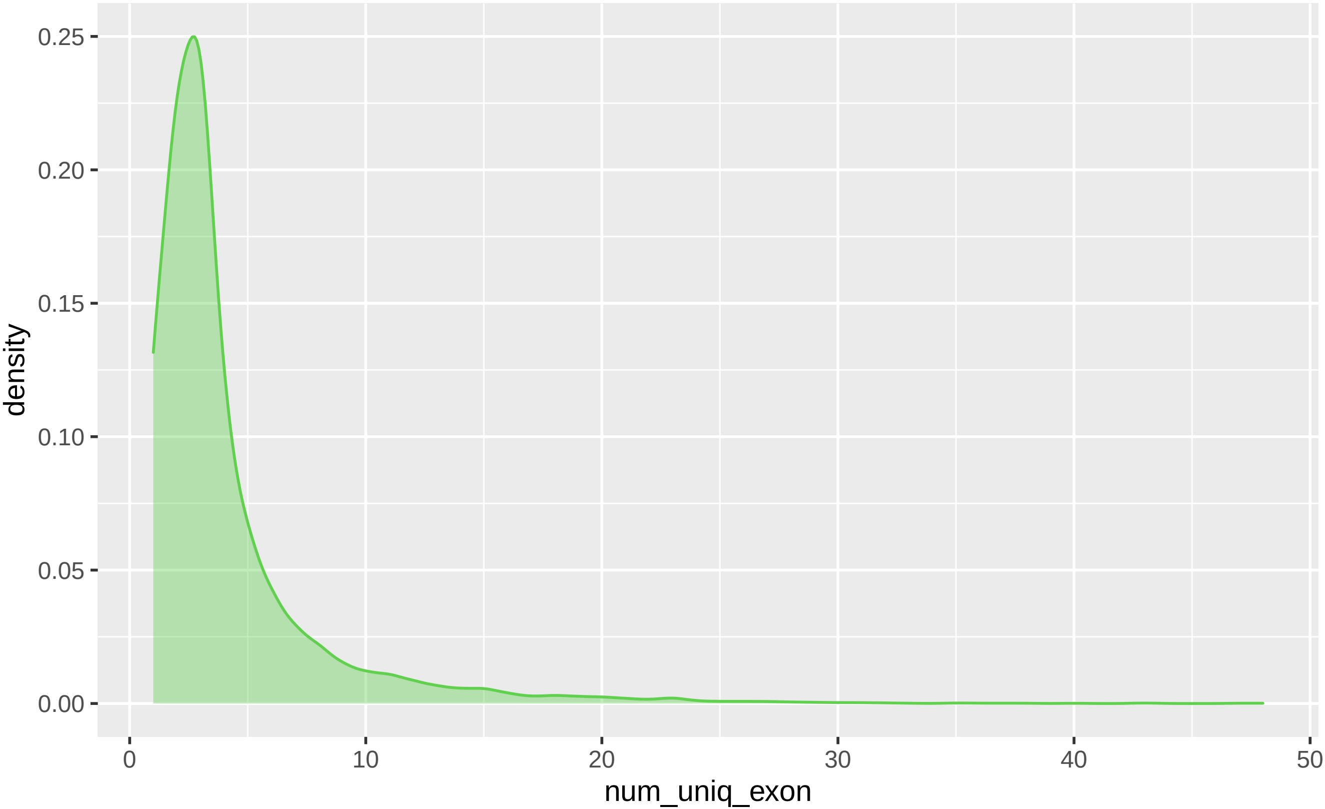

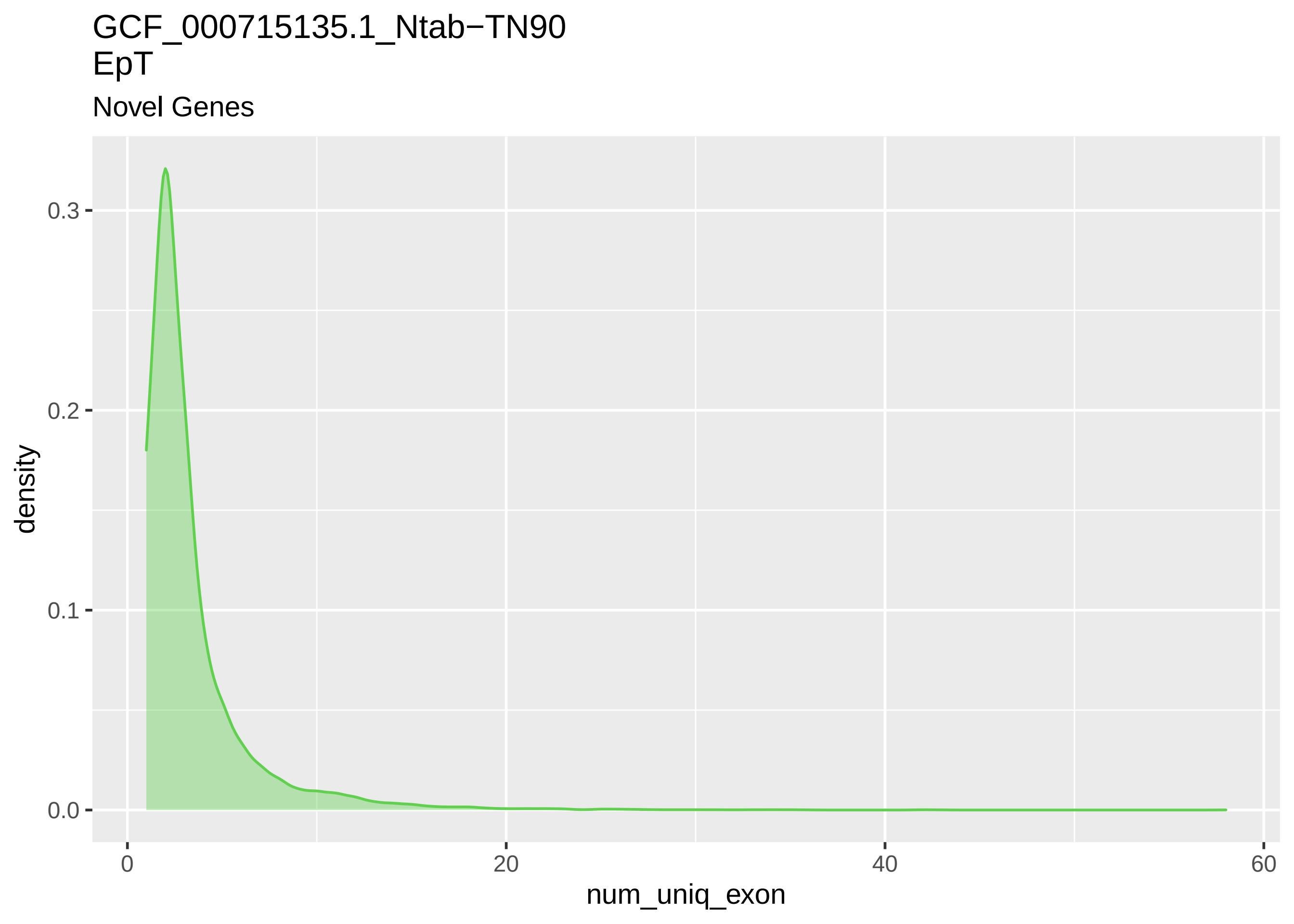
GCF_000715135.1_Ntab−TN90
EpT
Novel Genes
0.3
0.2
0.1
0.0
density
0
20
40
60
num_uniq_exon

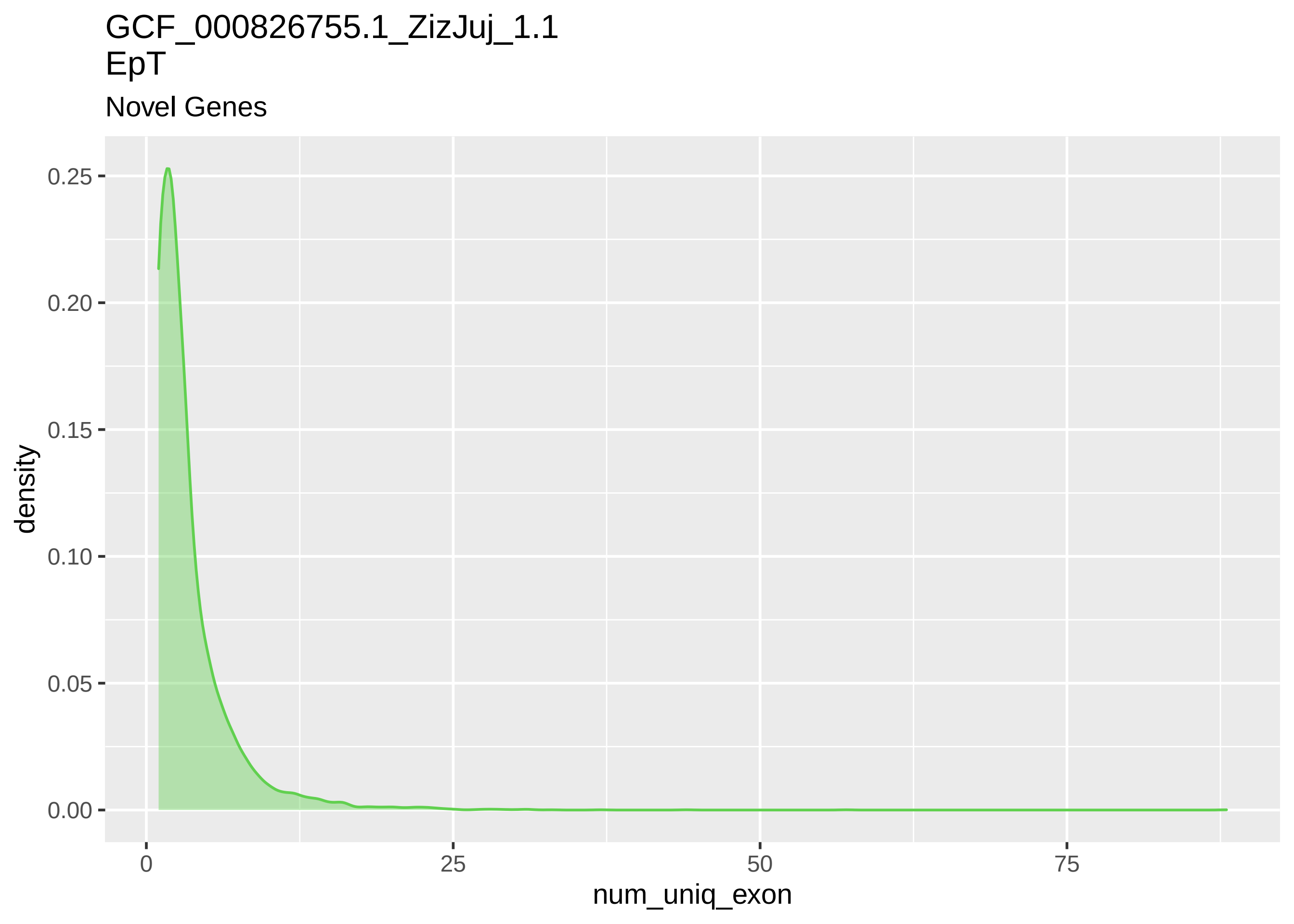

GCF_000826755.1_ZizJuj_1.1
EpT
Novel Genes
density
0.25
0.20
0.15
0.10
0.05
0.00
0
25
50
75
num_uniq_exon

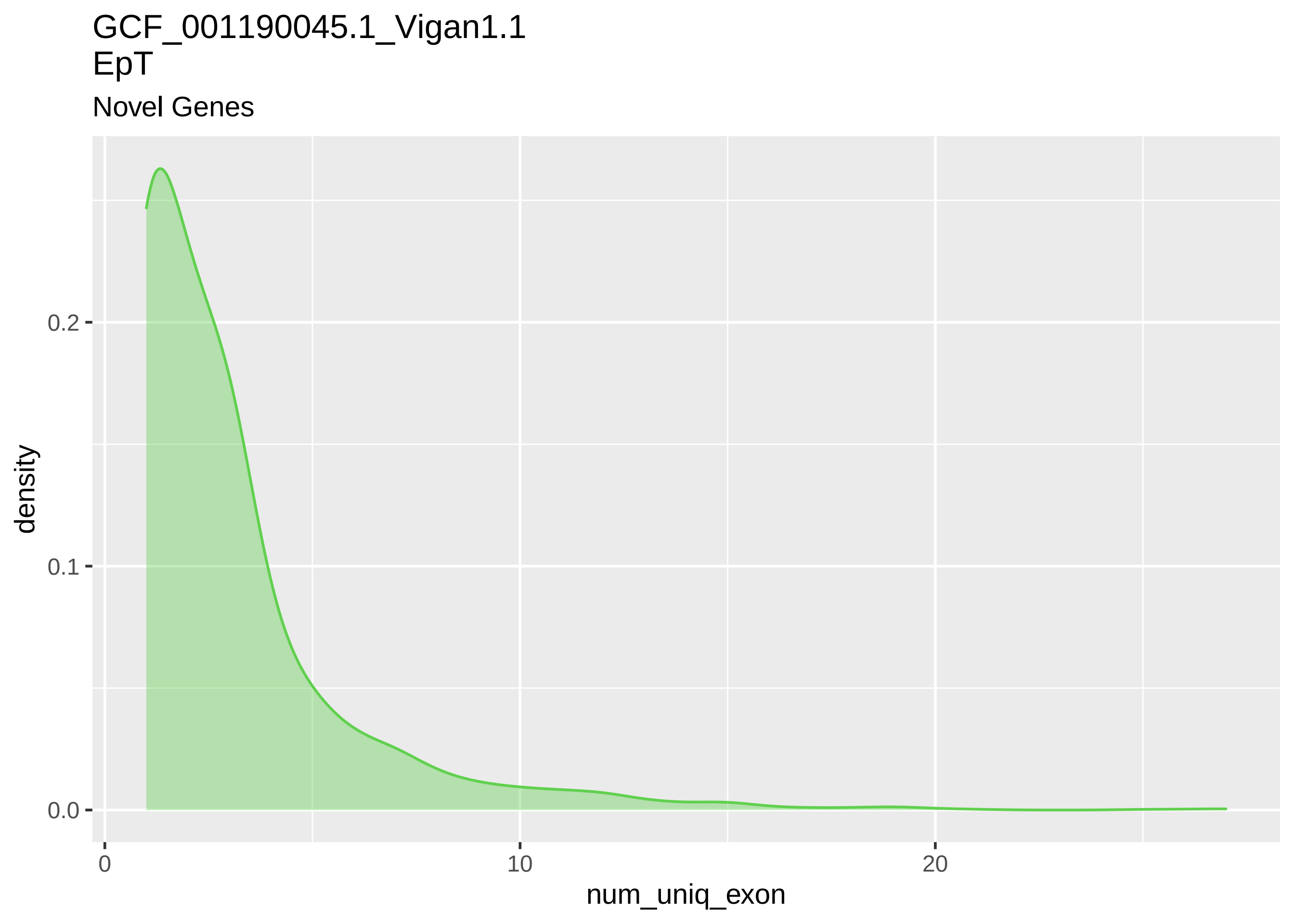

GCF_001190045.1_Vigan1.1
EpT
Novel Genes
density
0.0
0.1
0.2
0
10
20
num_uniq_exon

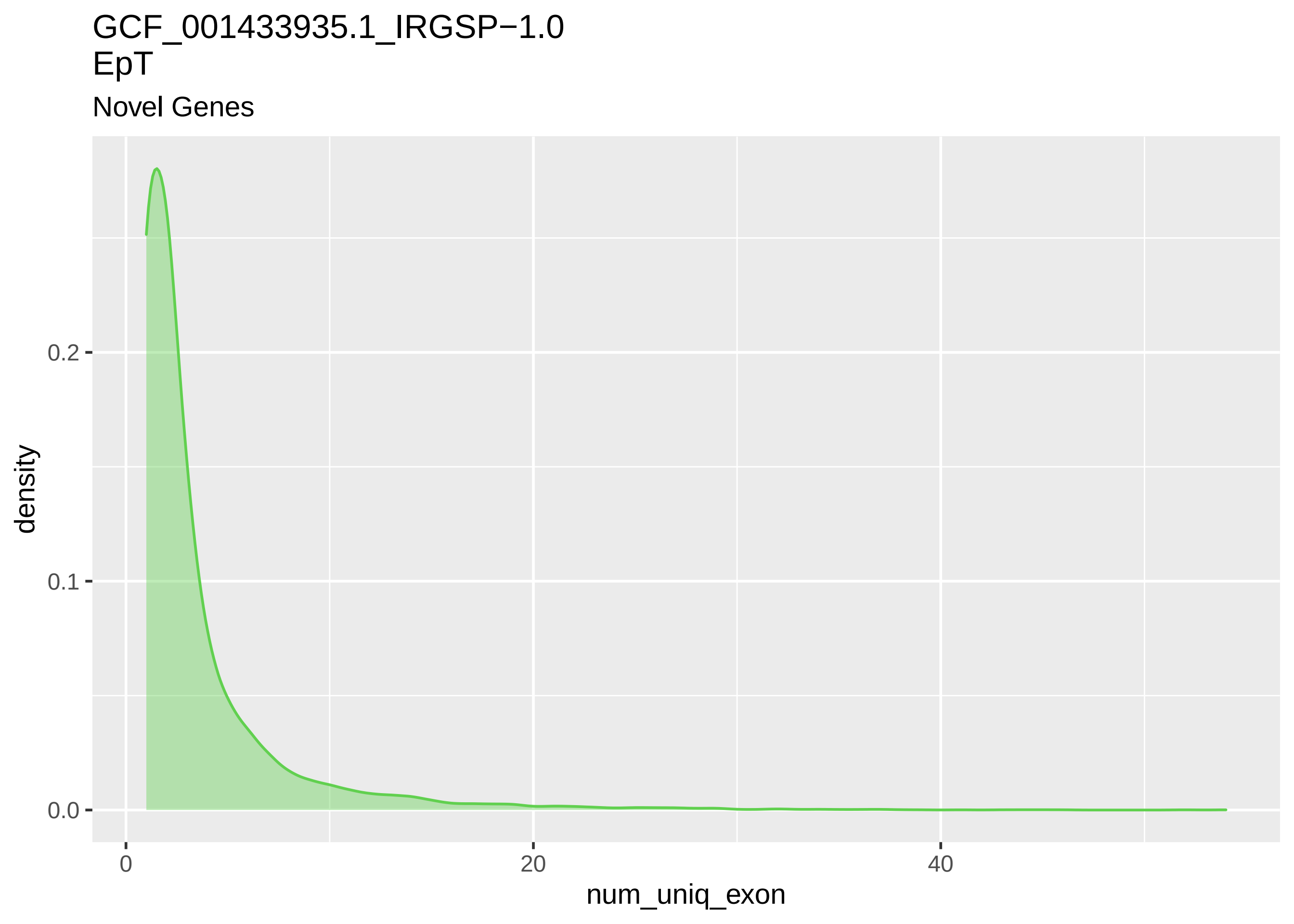
GCF_001433935.1_IRGSP−1.0
EpT
Novel Genes
density
0.0
0.1
0.2
0
20
40
num_uniq_exon

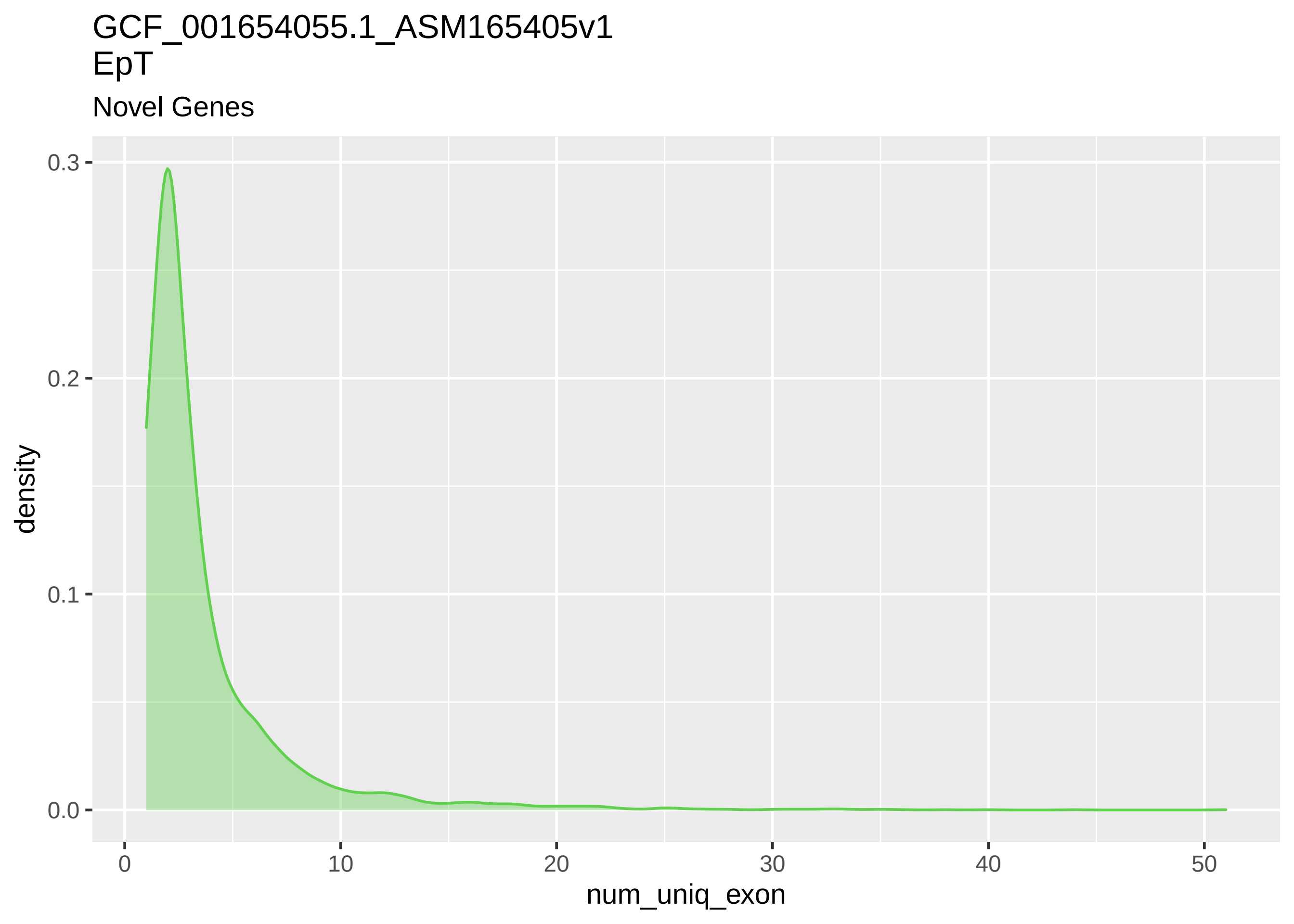
GCF_001654055.1_ASM165405v1
EpT
Novel Genes
density
0.3
0.2
0.1
0.0
0
10
20
30
40
50
num_uniq_exon

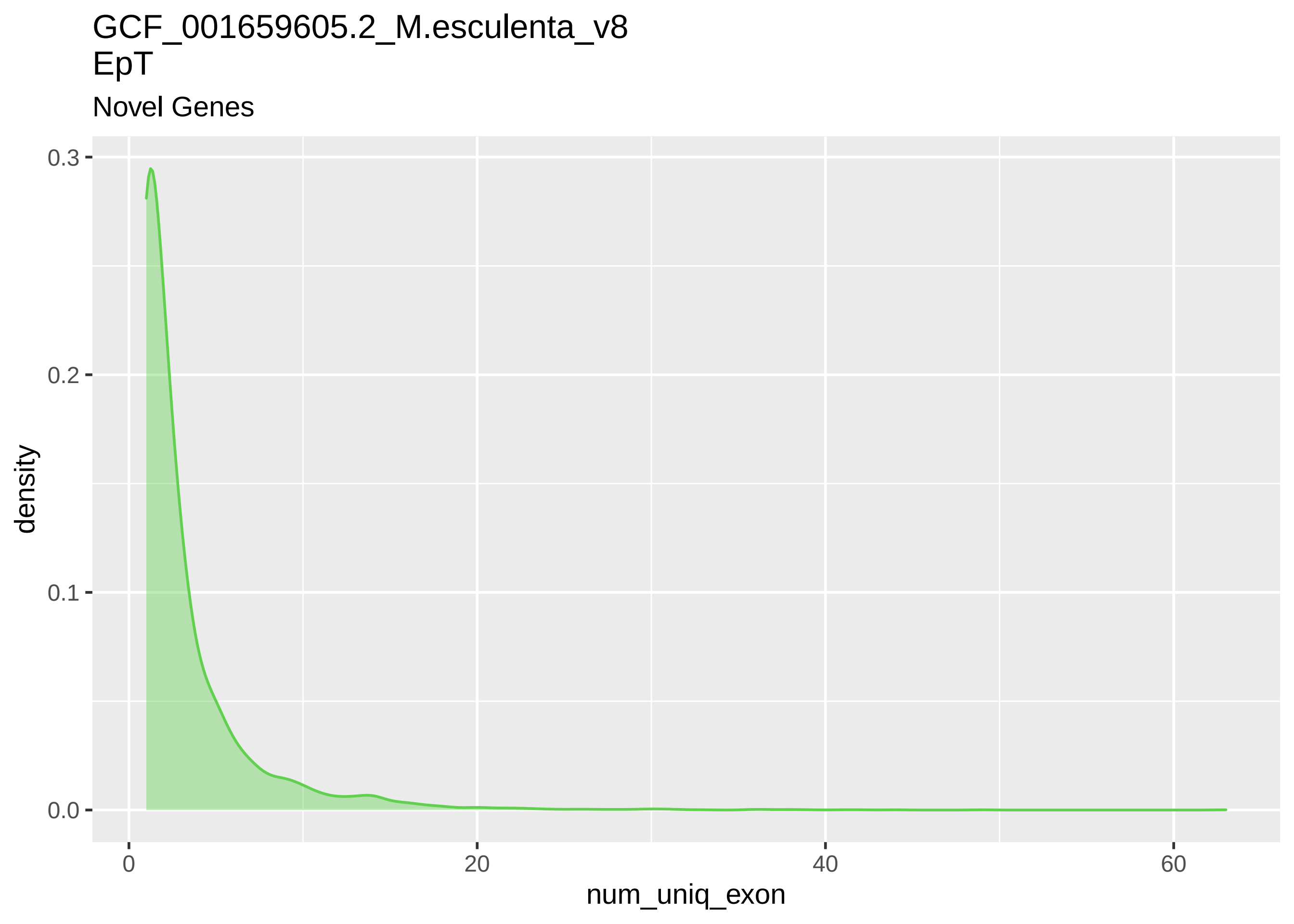
GCF_001659605.2_M.esculenta_v8
EpT
Novel Genes
density
0.3
0.2
0.1
0.0
0
20
40
60
num_uniq_exon

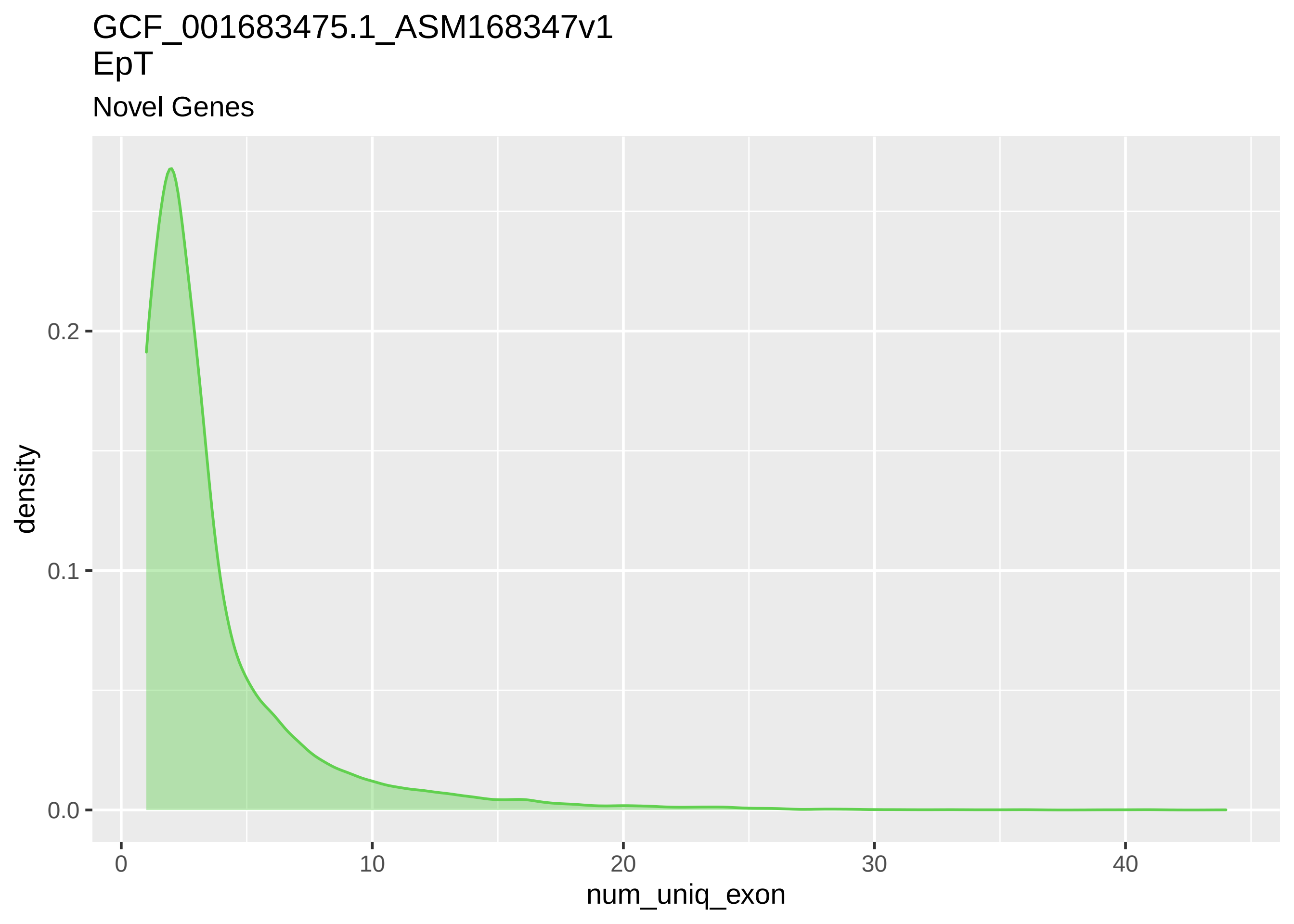

GCF_001683475.1_ASM168347v1
EpT
Novel Genes
density
0.0
0.1
0.2
0
10
20
30
40
num_uniq_exon

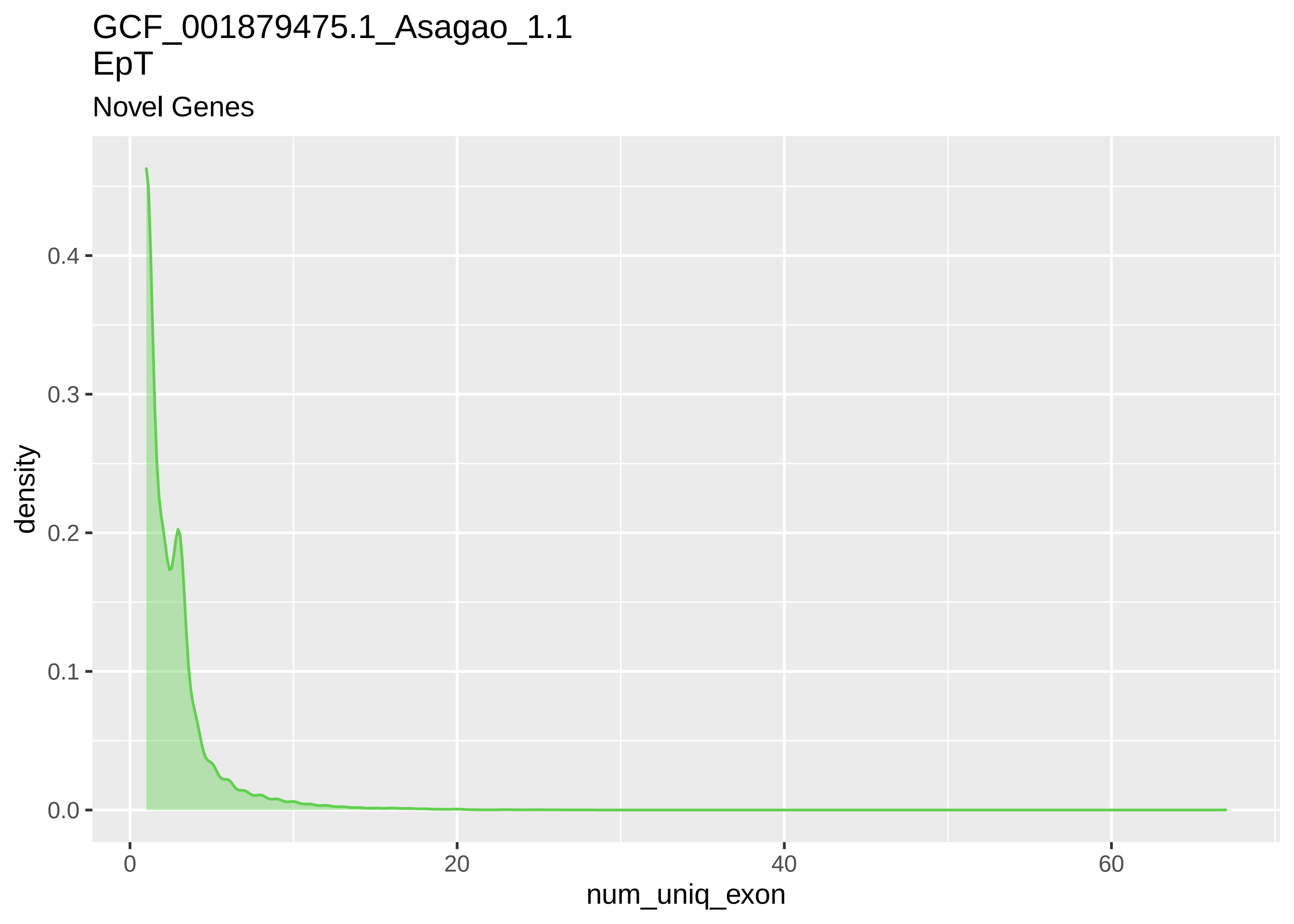
GCF_001879475.1_Asagao_1.1
EpT
Novel Genes
density
0.0
0.1
0.2
0.3
0.4
0
20
40
60
num_uniq_exon

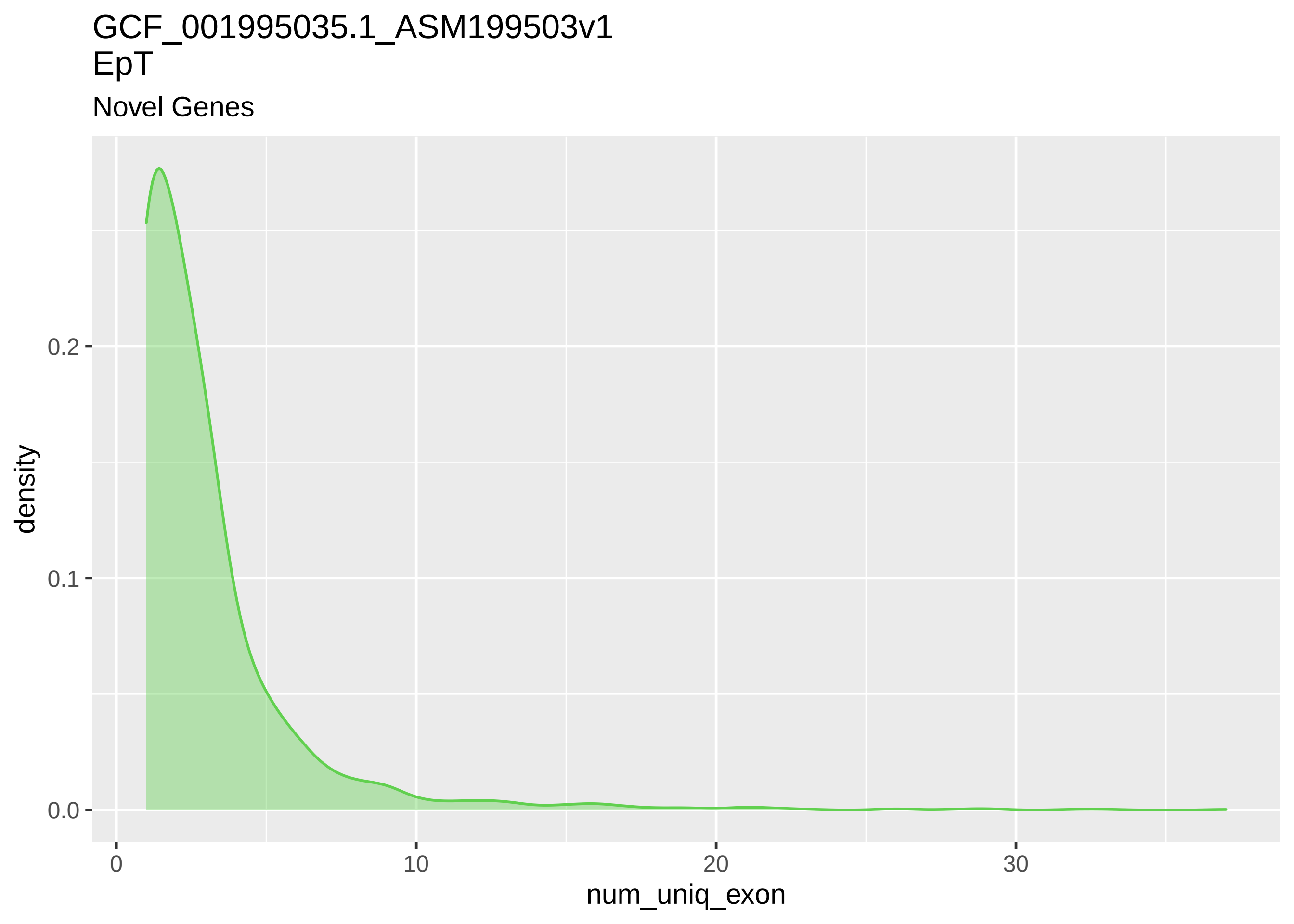

GCF_001995035.1_ASM199503v1
EpT
Novel Genes
density
0.2
0.1
0.0
0
10
20
30
num_uniq_exon

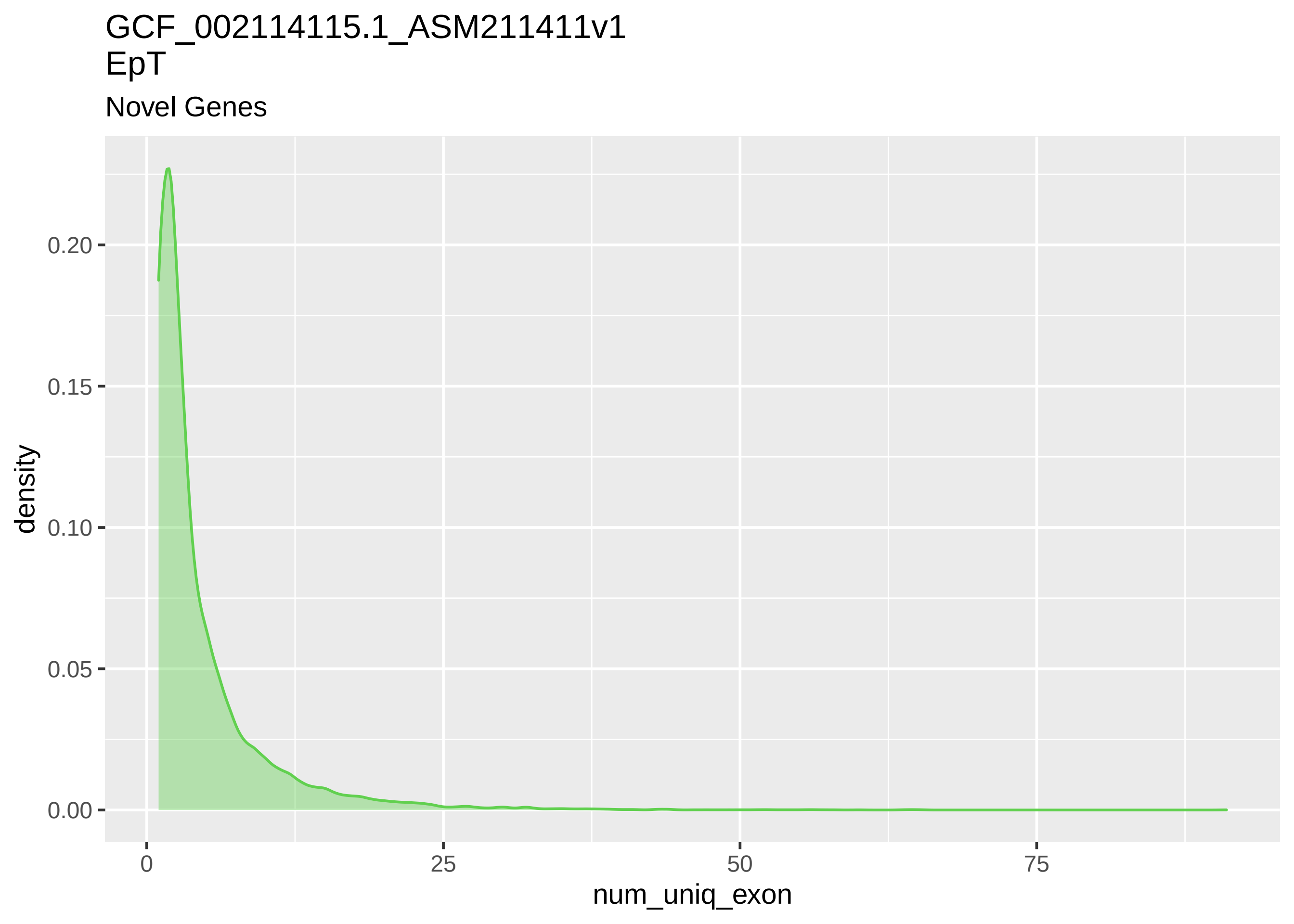
GCF_002114115.1_ASM211411v1
EpT
Novel Genes
density
0.00
0.05
0.10
0.15
0.20
0
25
50
75
num_uniq_exon

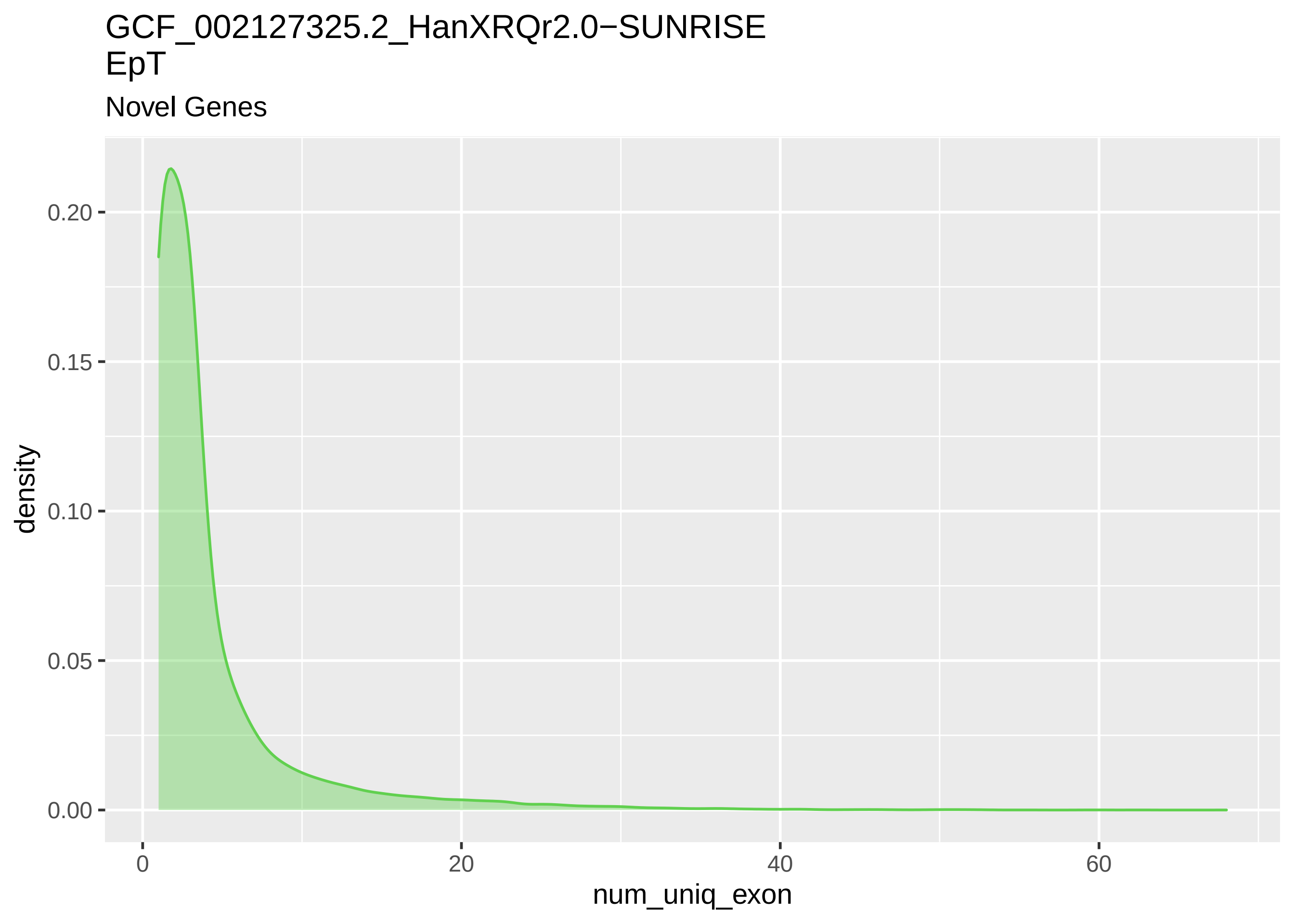
GCF_002127325.2_HanXRQr2.0−SUNRISE
EpT
Novel Genes
0.20
0.15
0.10
0.05
0.00
density
0
20
40
60
num_uniq_exon

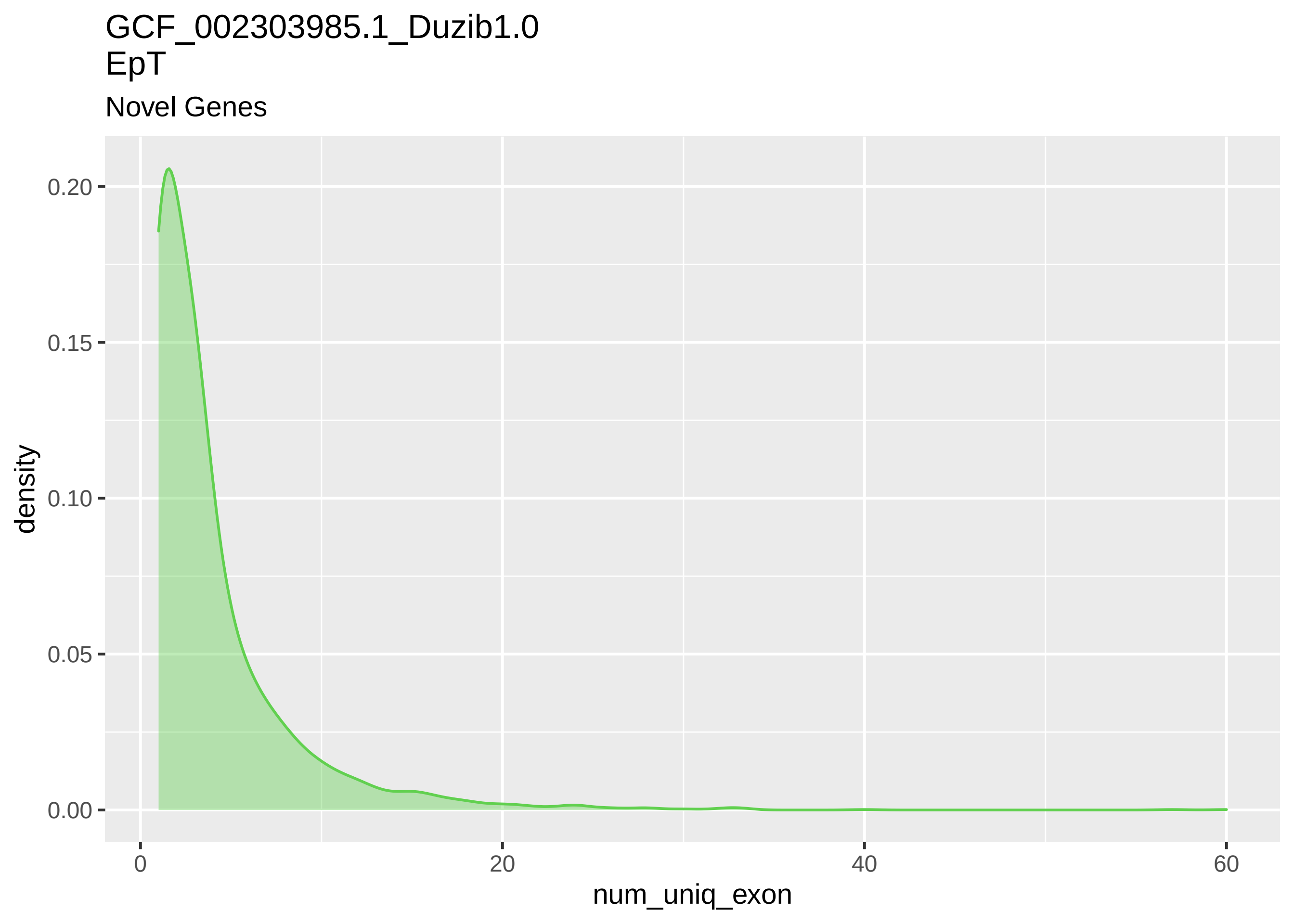
GCF_002303985.1_Duzib1.0
EpT
Novel Genes
density
0.20
0.15
0.10
0.05
0.00
0
20
40
60
num_uniq_exon

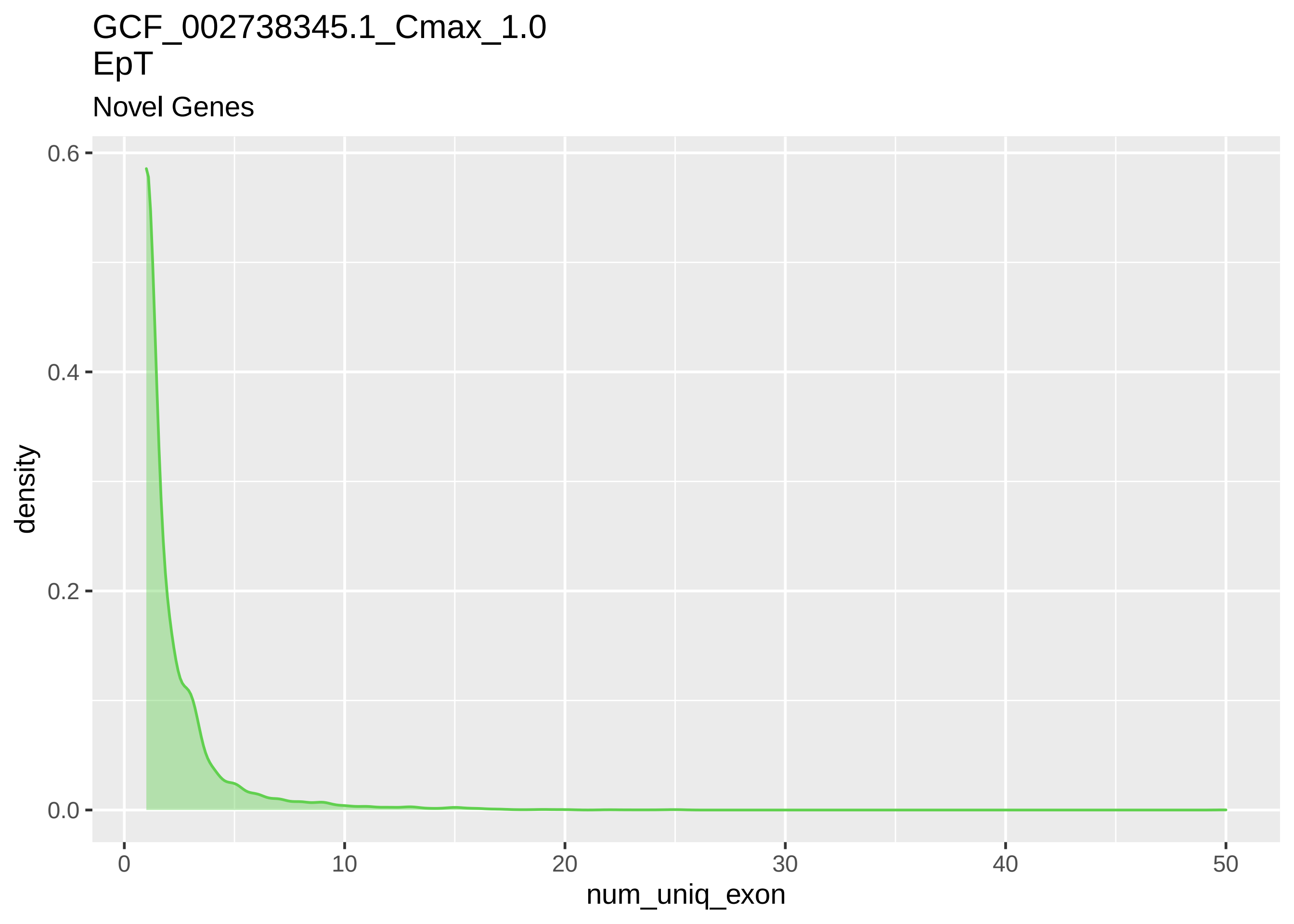

GCF_002738345.1_Cmax_1.0
EpT
Novel Genes
density
0.6
0.4
0.2
0.0
0
10
20
30
40
50
num_uniq_exon

GCF_002870075.2_Lsat_Salinas_v7
EpT

Novel Genes

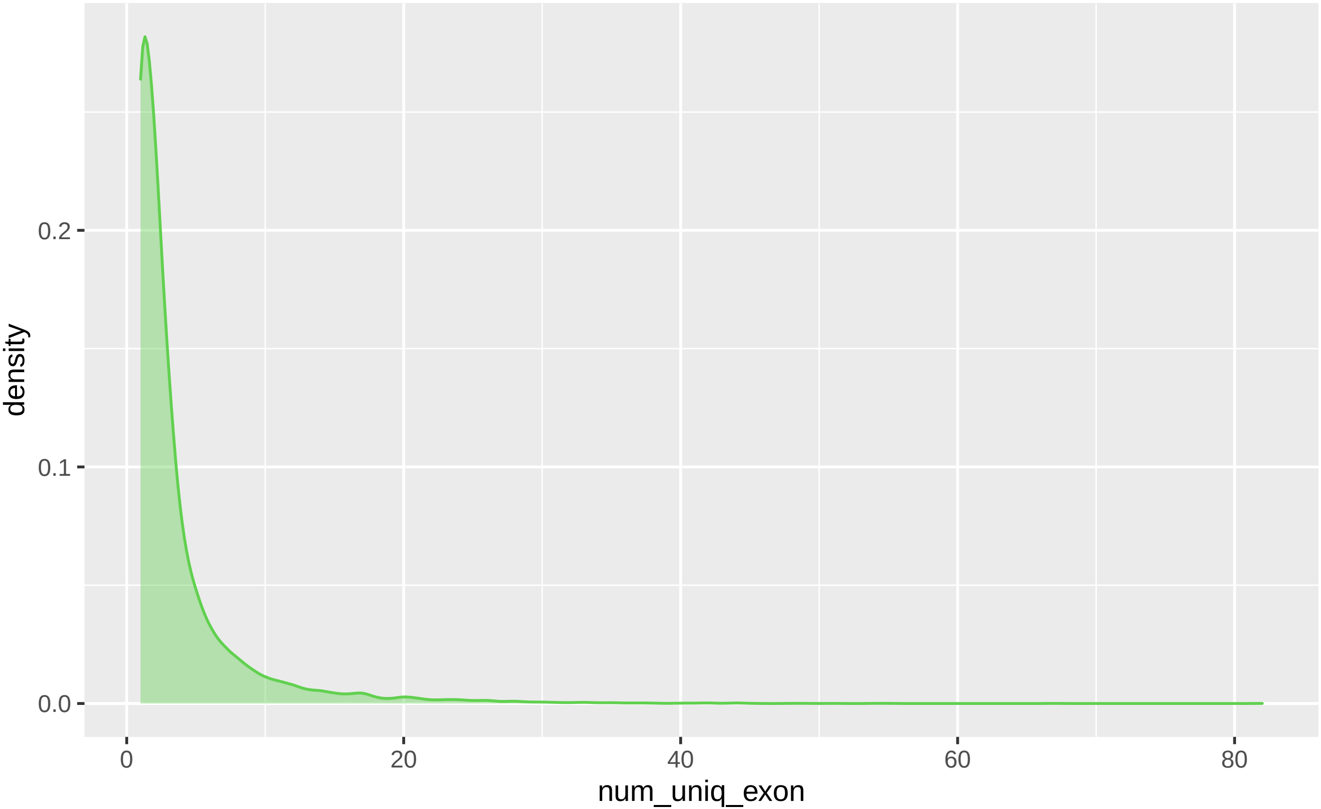

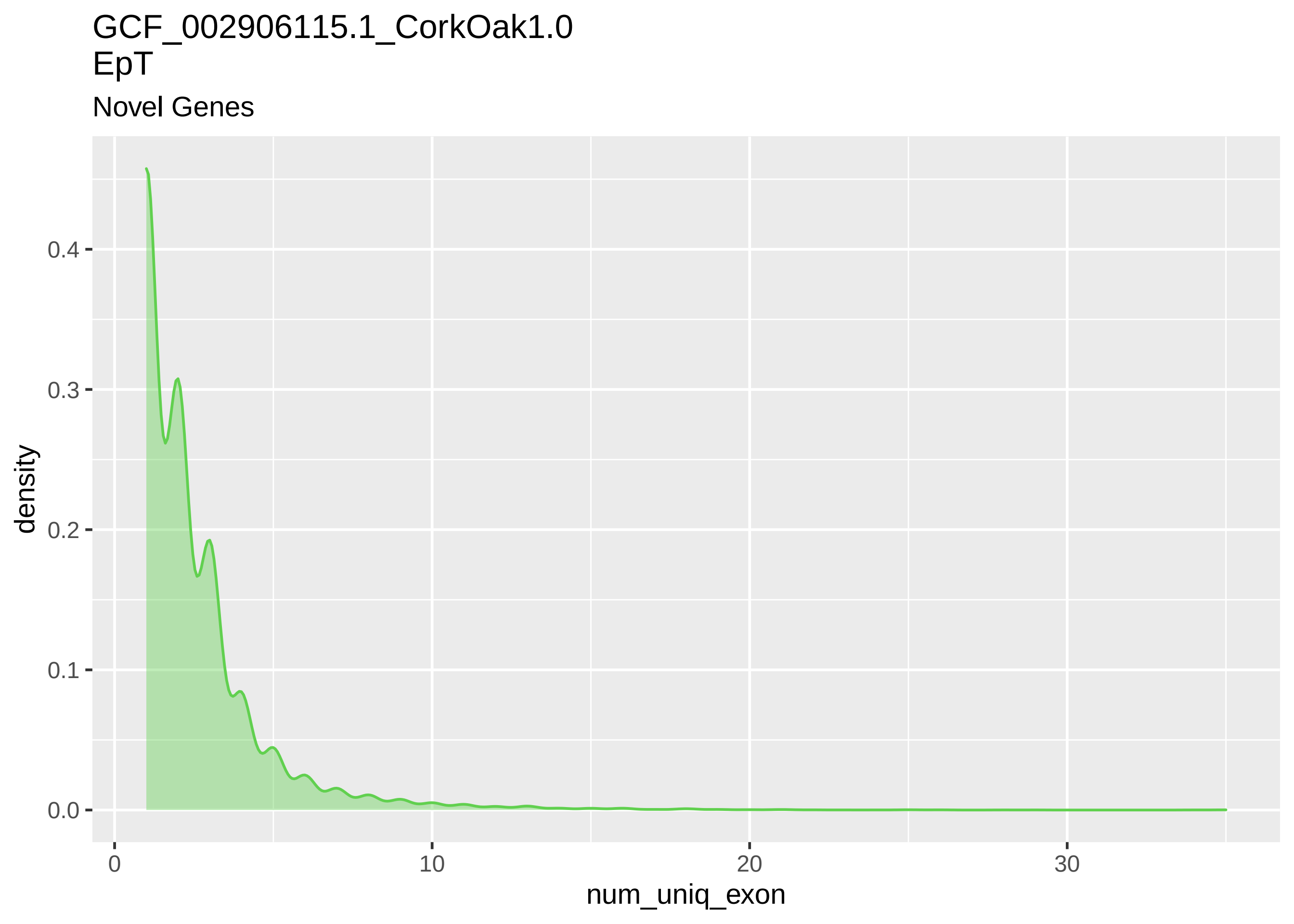
GCF_002906115.1_CorkOak1.0
EpT
Novel Genes
density
0.0
0.1
0.2
0.3
0.4
0
10
20
30
num_uniq_exon

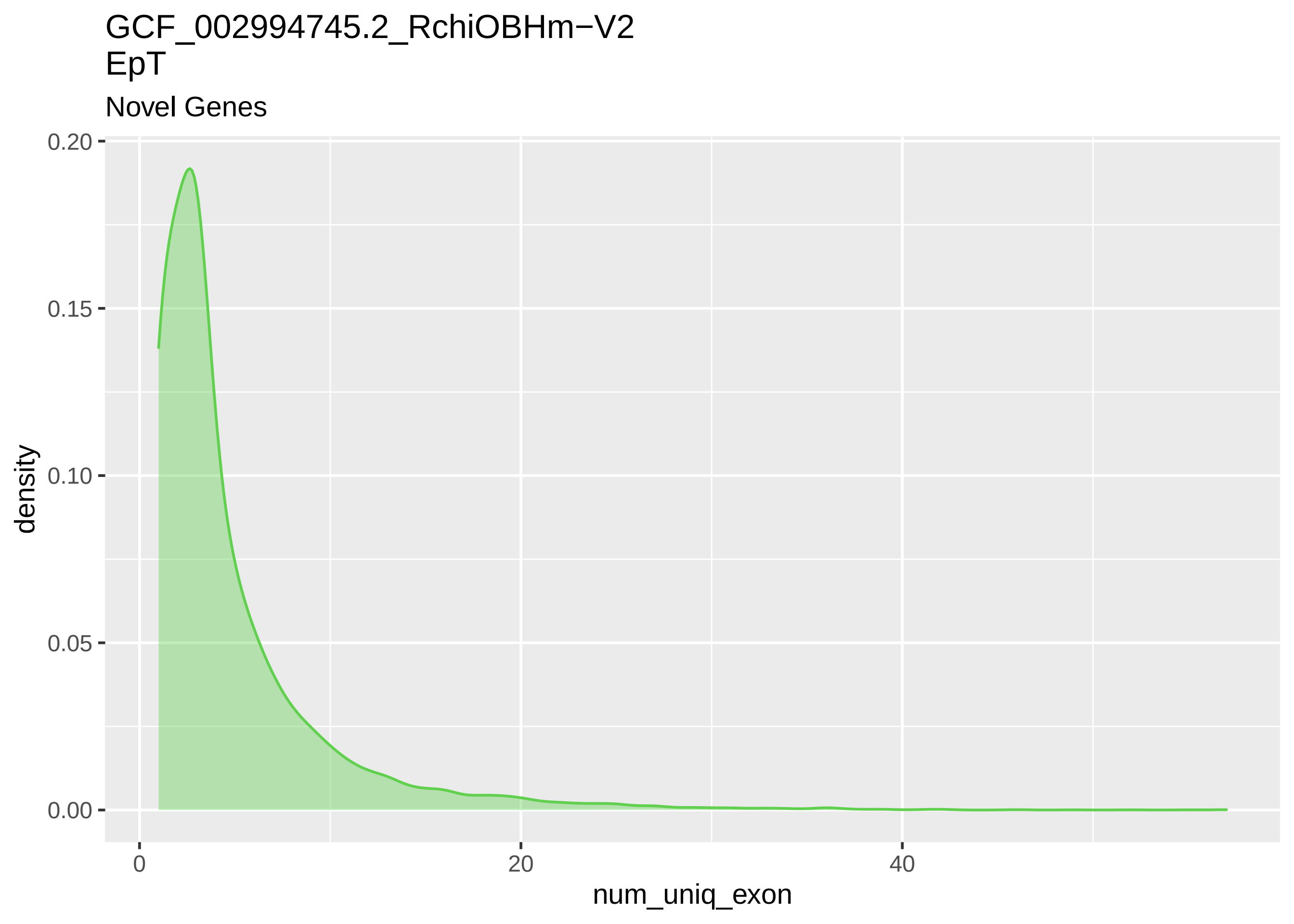
GCF_002994745.2_RchiOBHm−V2
EpT
Novel Genes
density
0.20
0.15
0.10
0.05
0.00
0
20
40
num_uniq_exon

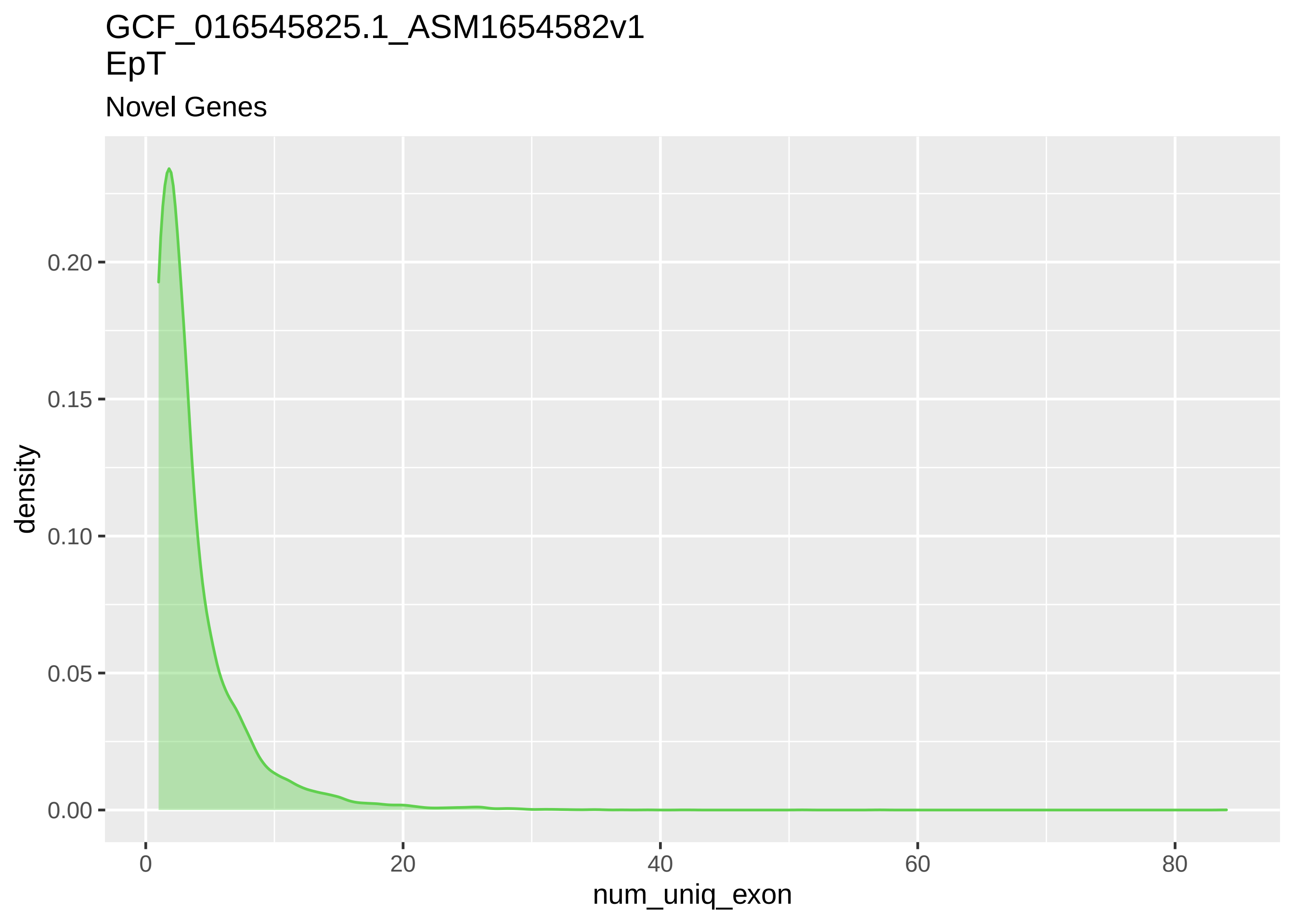
GCF_016545825.1_ASM1654582v1
EpT
Novel Genes
density
num_uniq_exon
0.00
0.05
0.10
0.15
0.20
0
20
40
60
80

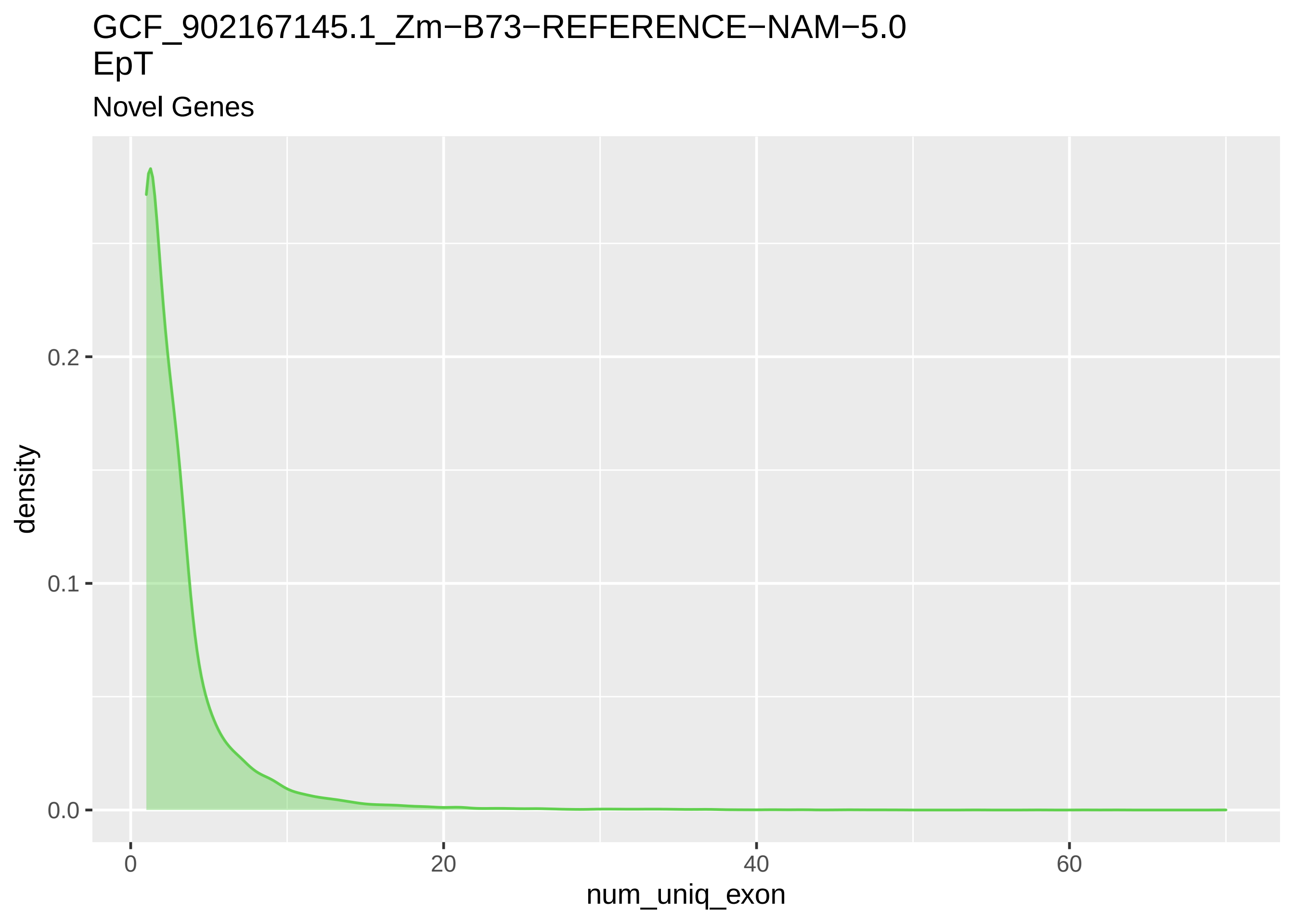
GCF_902167145.1_Zm−B73−REFERENCE−NAM−5.0
EpT
Novel Genes
density
0.0
0.1
0.2
0
20
40
60
num_uniq_exon

GCA_000003515.2_ASM351v2
EpT

Novel Genes

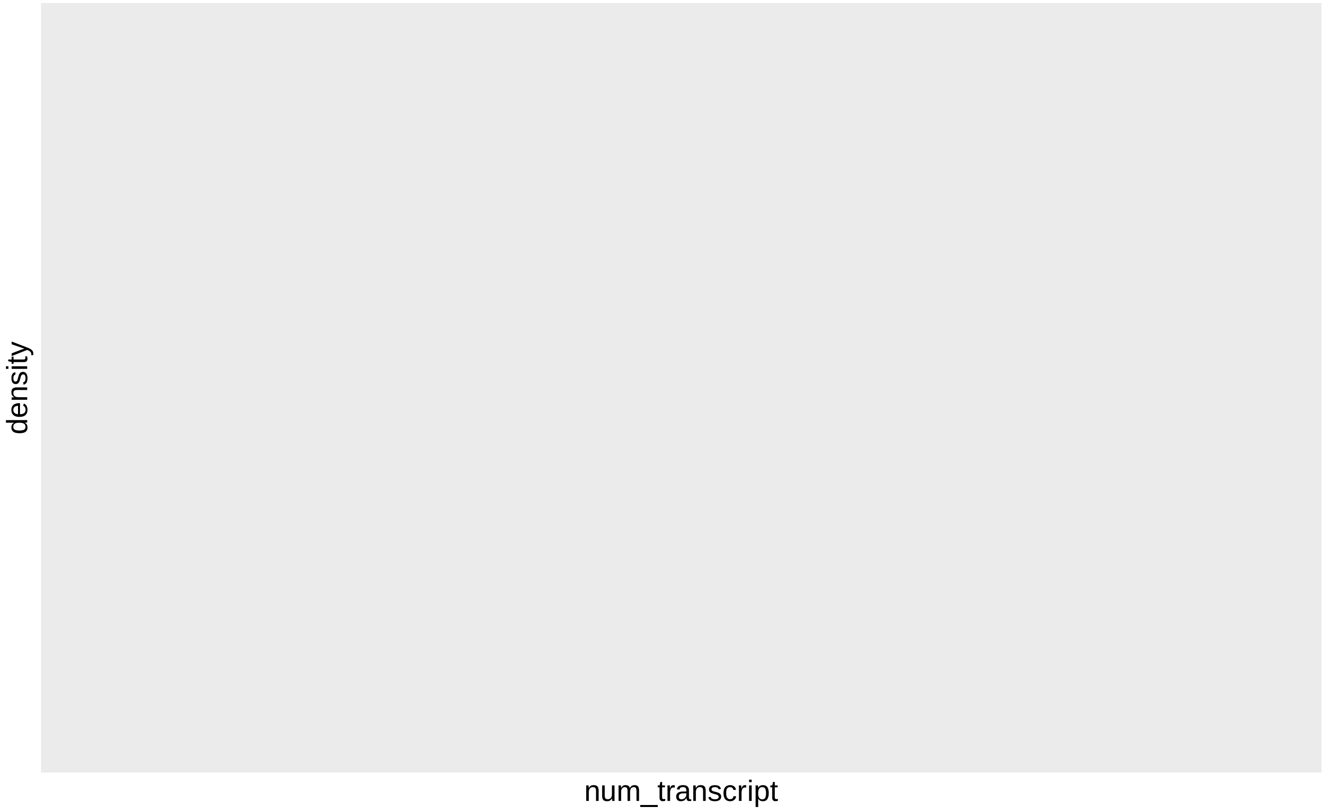

GCA_000365165.2_Clad_carr_CBS_160_54_V1
EpT

Novel Genes

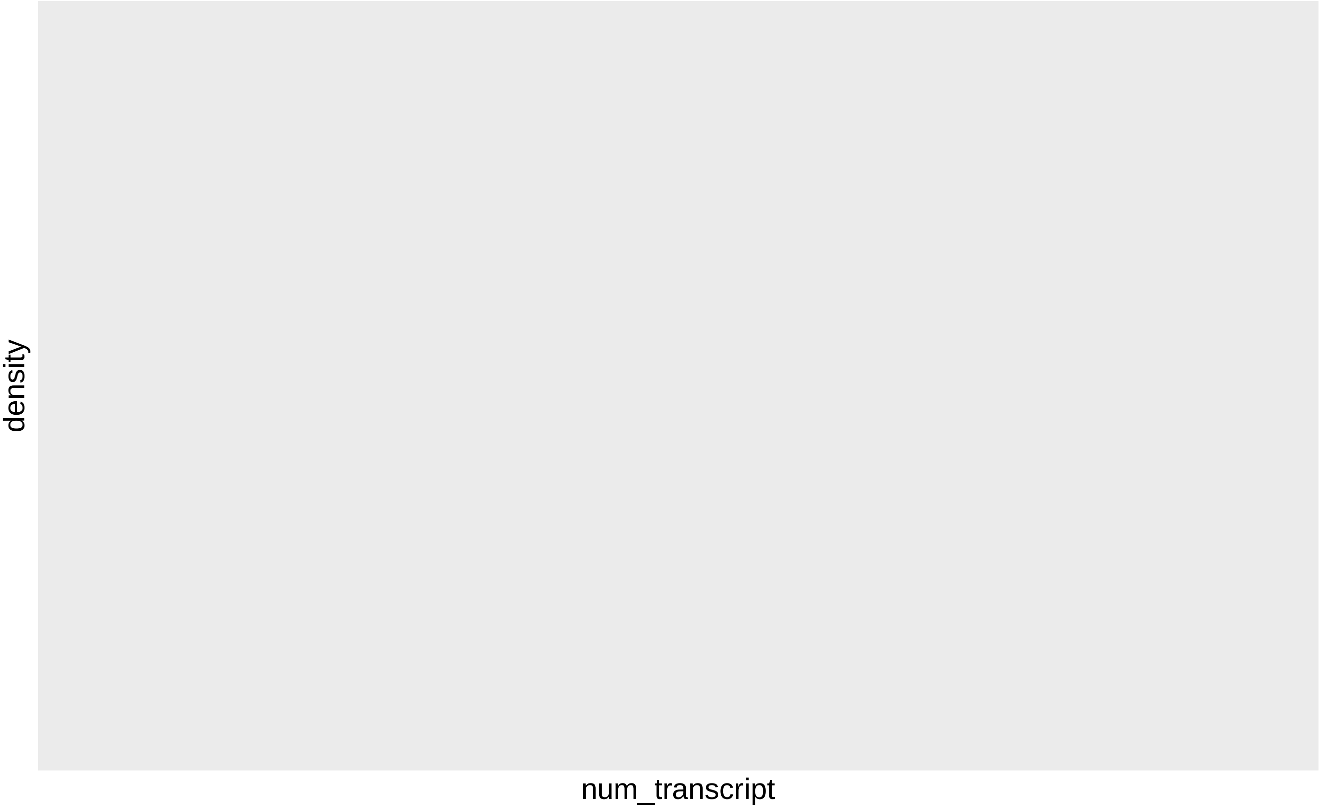

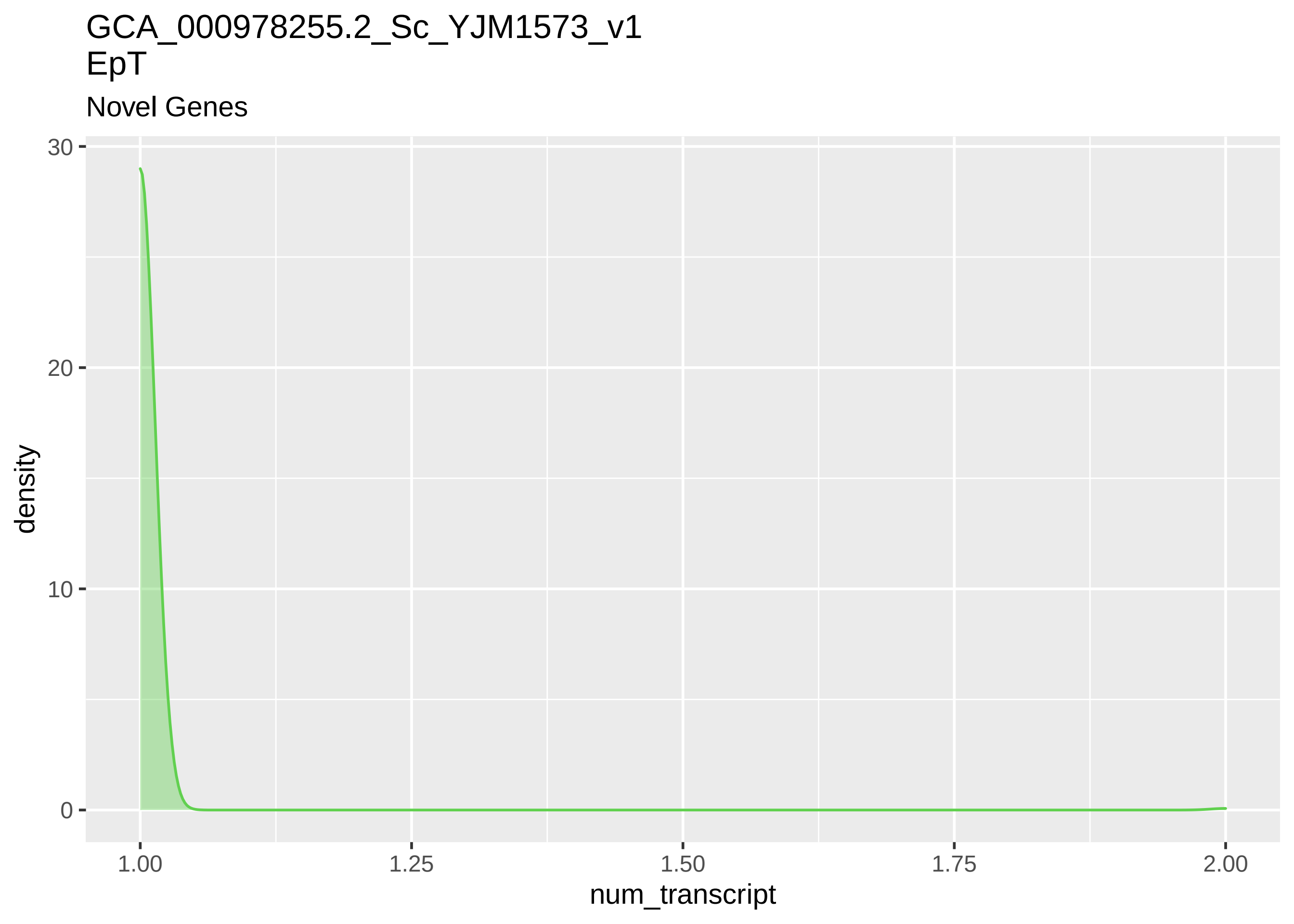
GCA_000978255.2_Sc_YJM1573_v1
EpT
Novel Genes
30
20
10
0
density
1.00
1.25
1.50
1.75
2.00
num_transcript

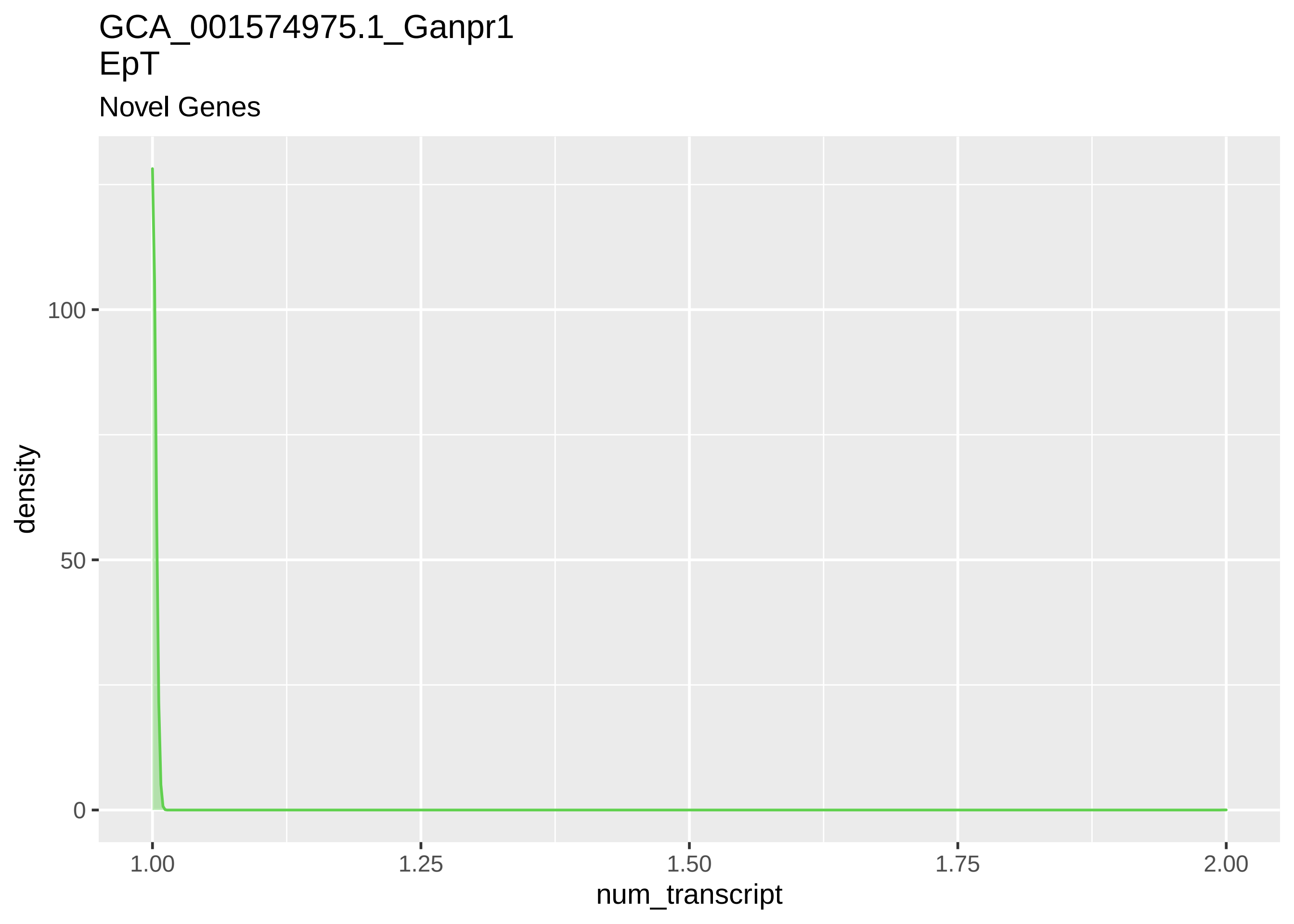

GCA_001574975.1_Ganpr1
EpT
Novel Genes
density
100
50
0
1.00
1.25
1.50
1.75
2.00
num_transcript

GCA_001636715.1_AAP_1.0
EpT

Novel Genes

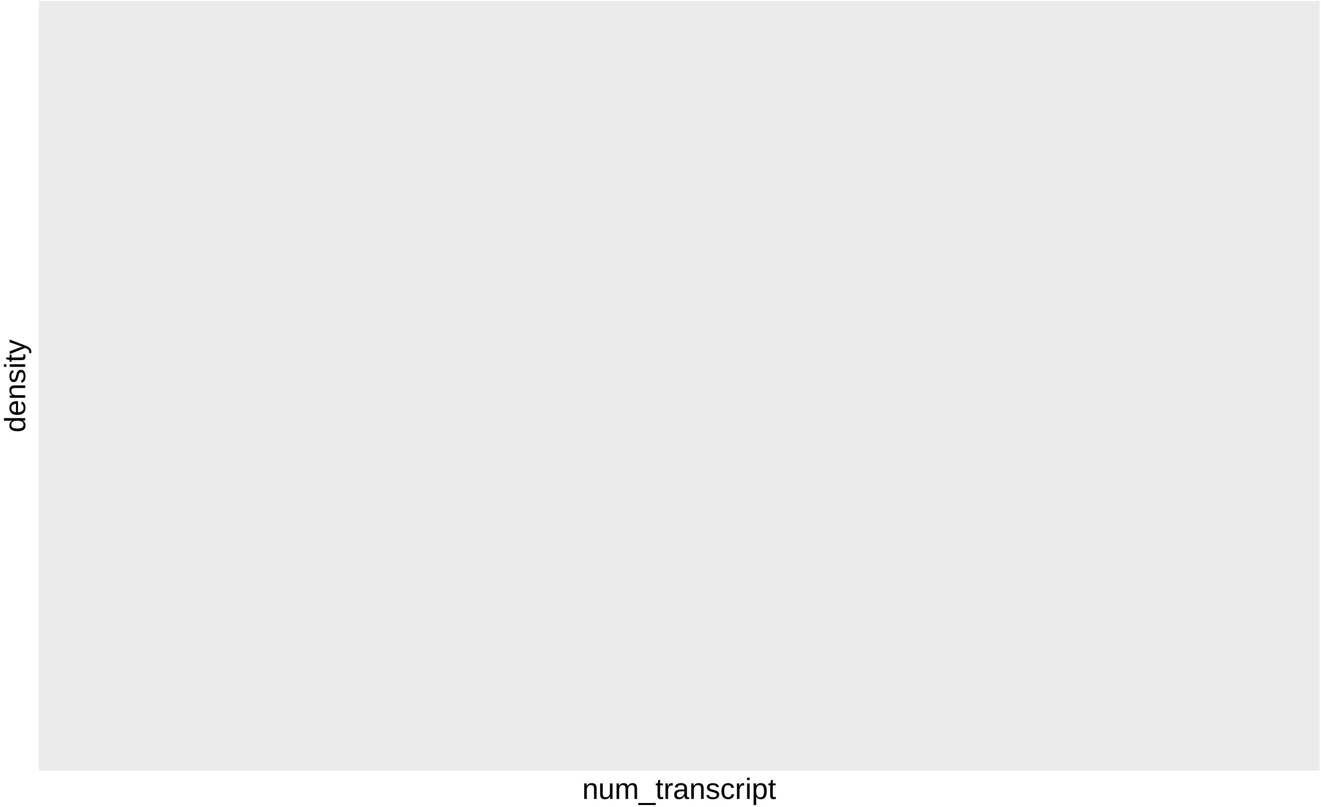

GCA_001747045.1_ASM174704v1
EpT

Novel Genes

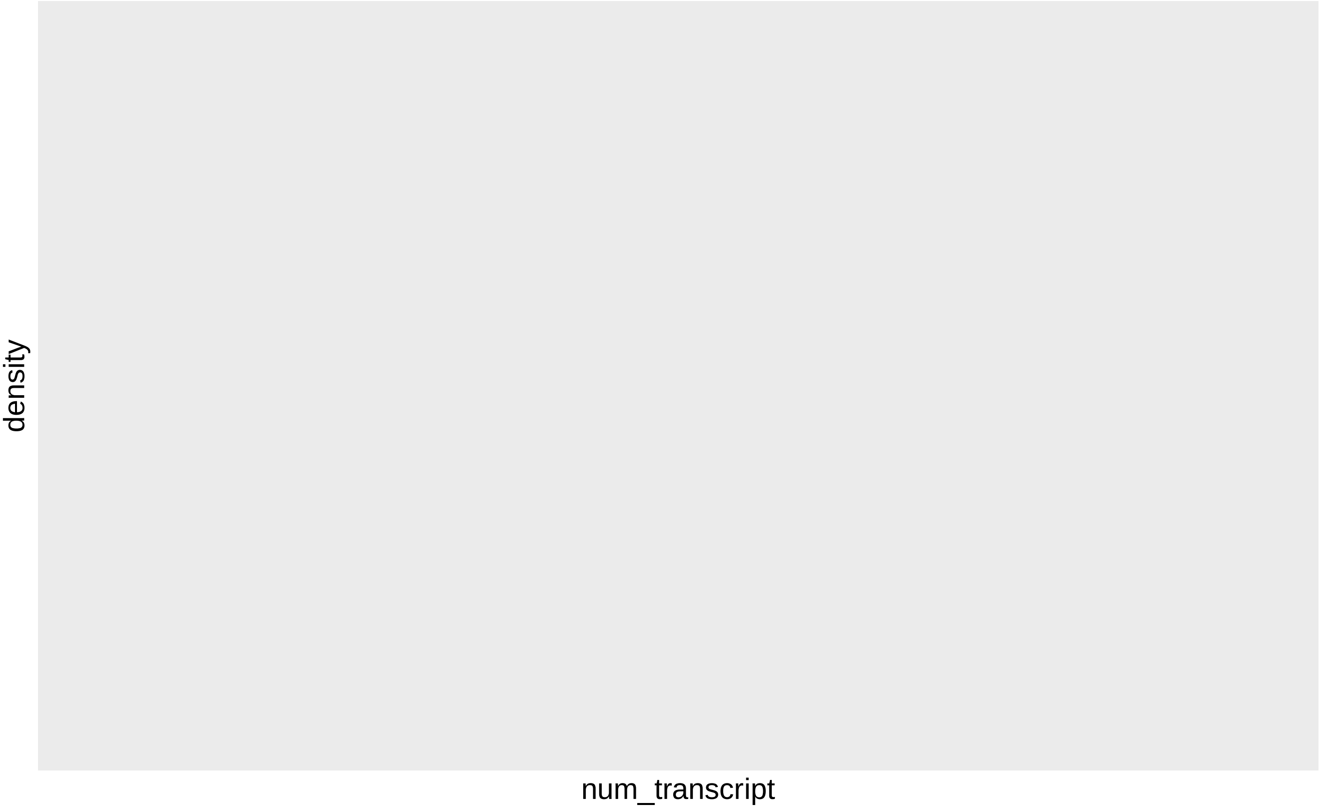

GCA_001883825.1_Emmo_past_UAMH9510_V1
EpT

Novel Genes

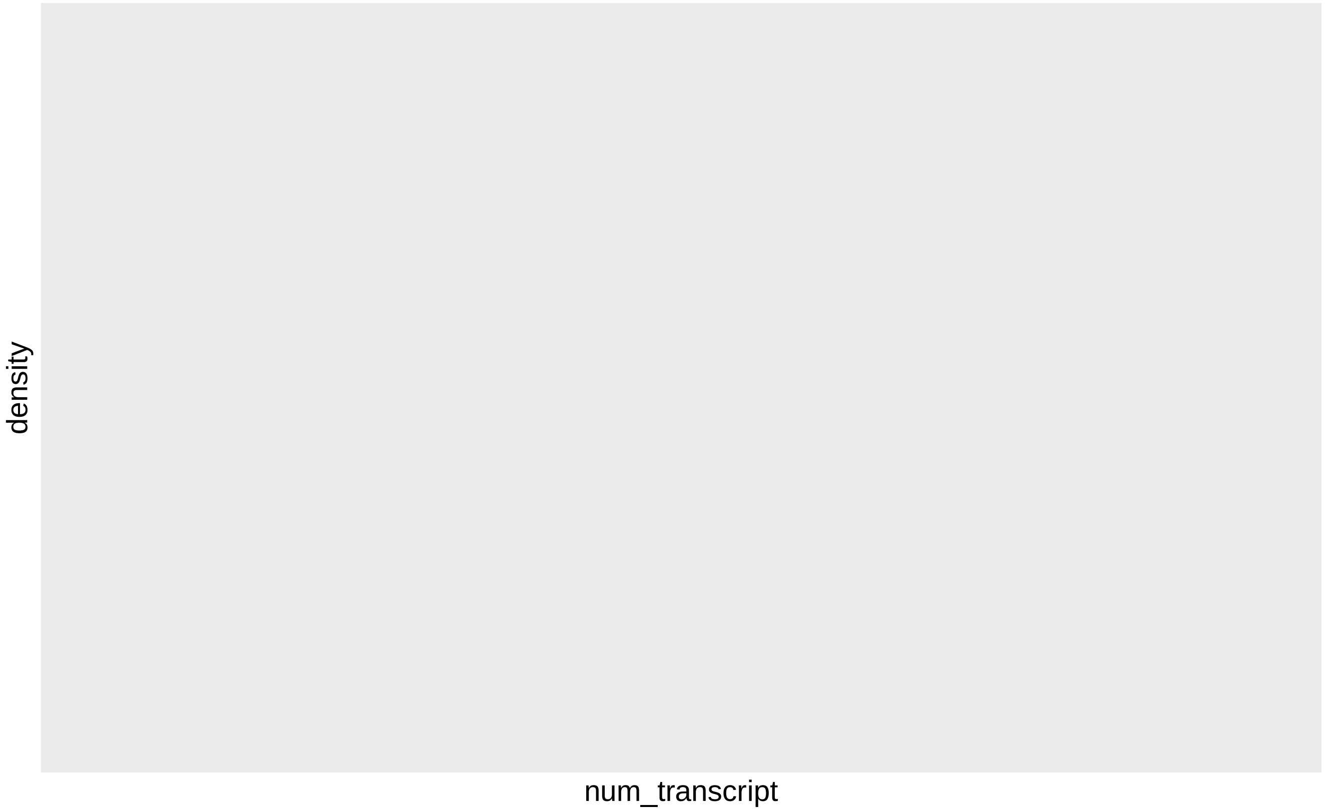

GCA_001929475.1_NeoIirr1.0
EpT

Novel Genes

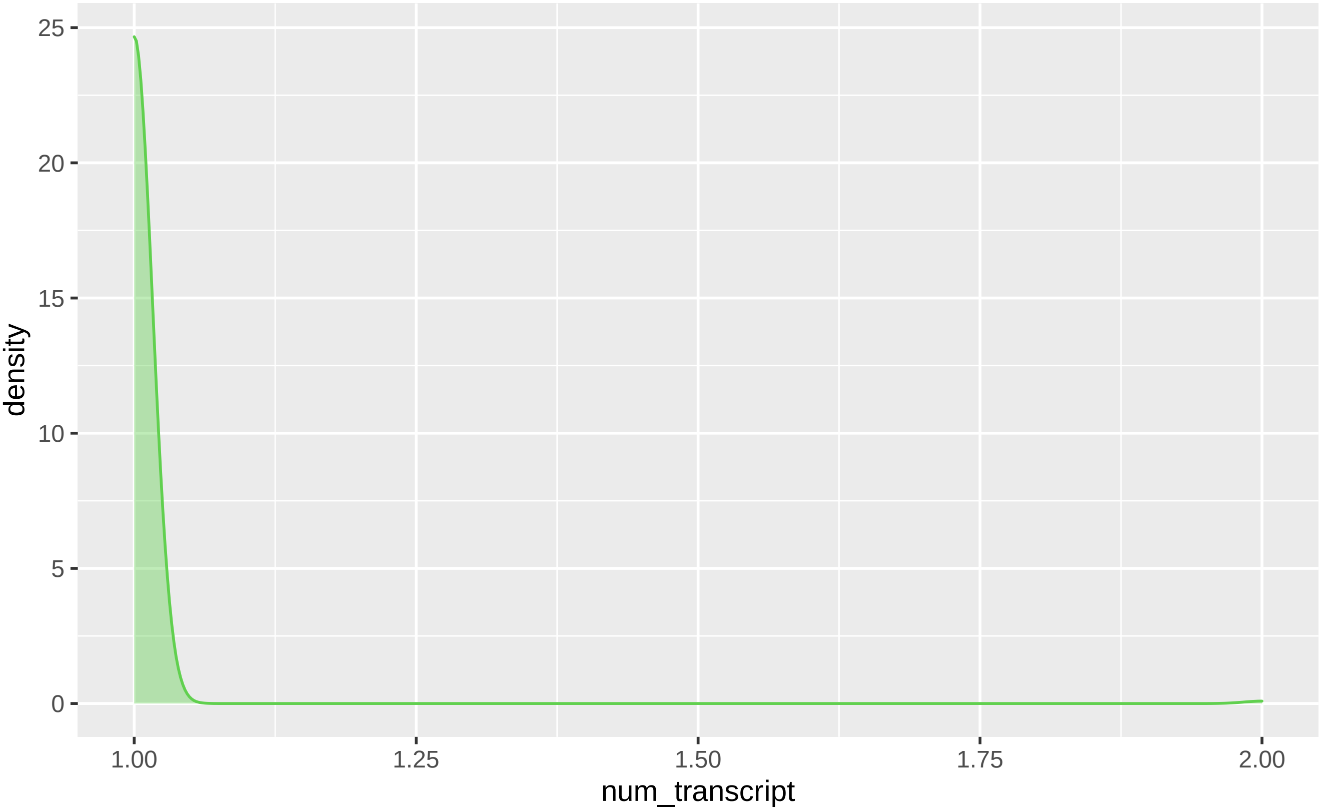

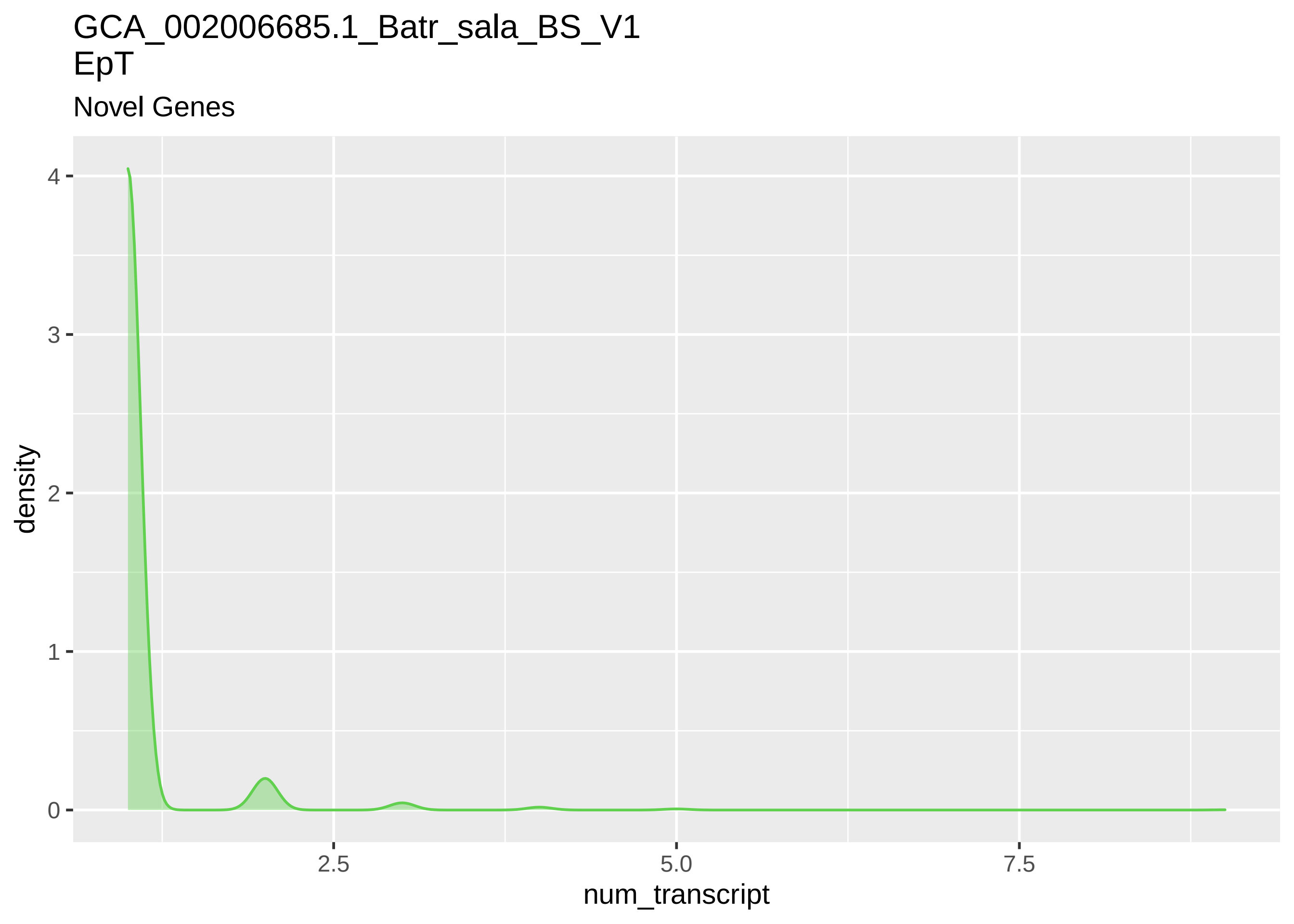

GCA_002006685.1_Batr_sala_BS_V1
EpT
Novel Genes
density
num_transcript
0
1
2
3
4
2.5
5.0
7.5

GCA_002104895.1_Anaeromyces_sp._S4_v1.0
EpT

Novel Genes

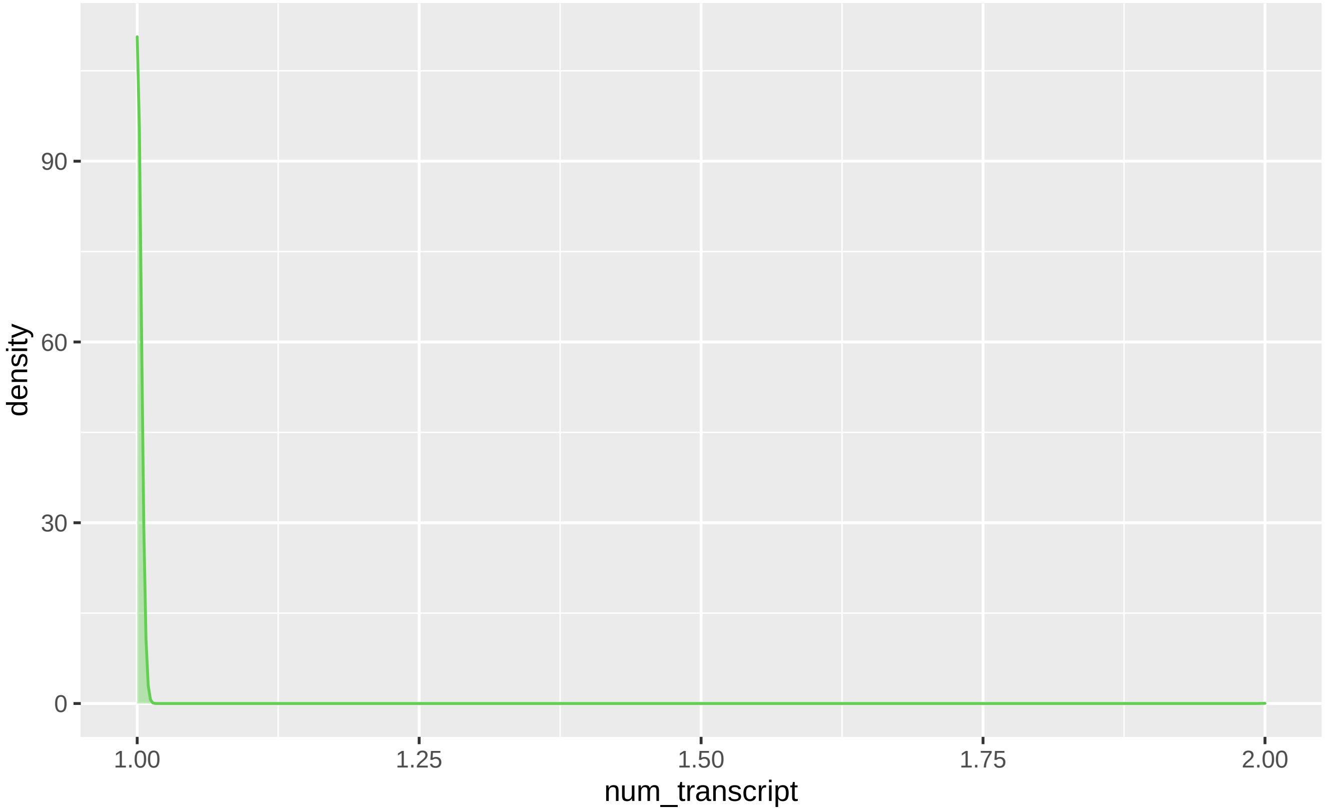

GCA_002104945.1_Piromyces_sp._finnis_v3.0
EpT

Novel Genes

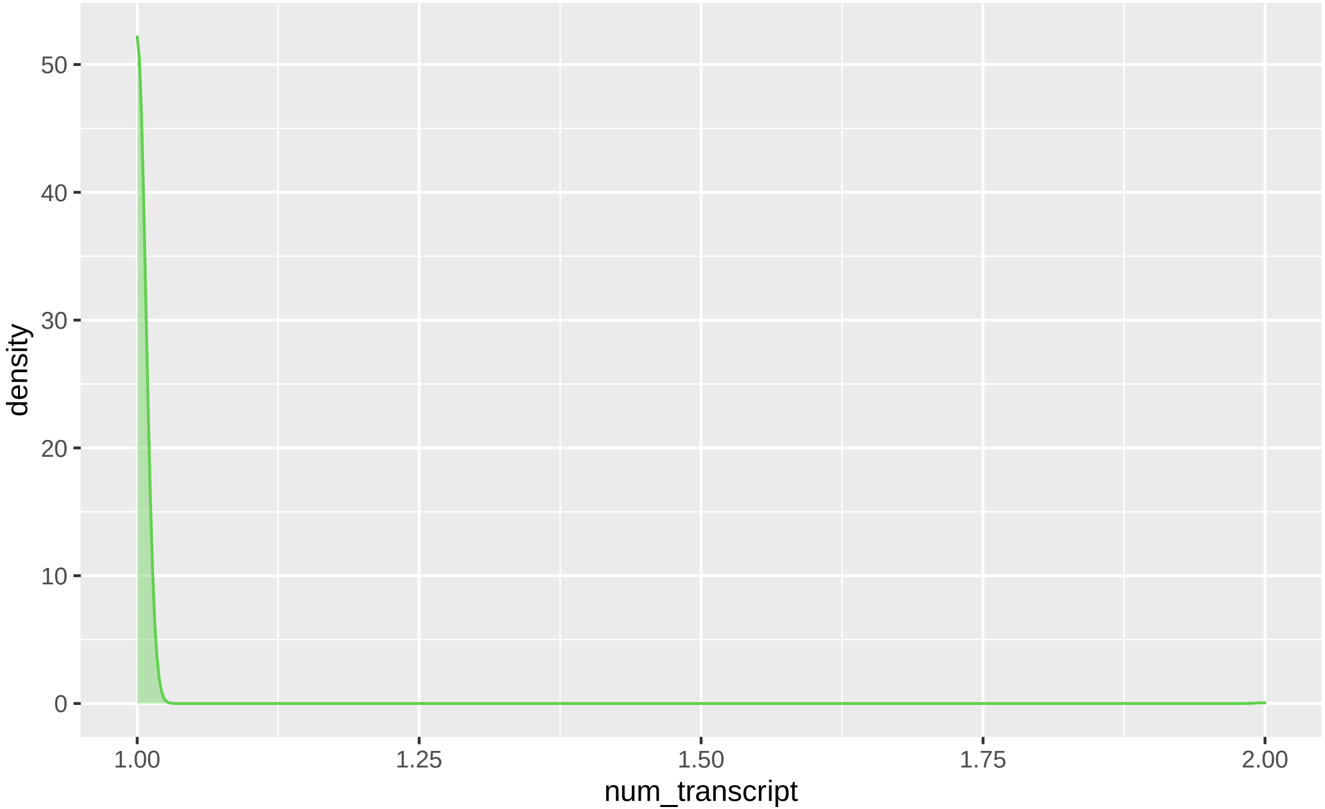

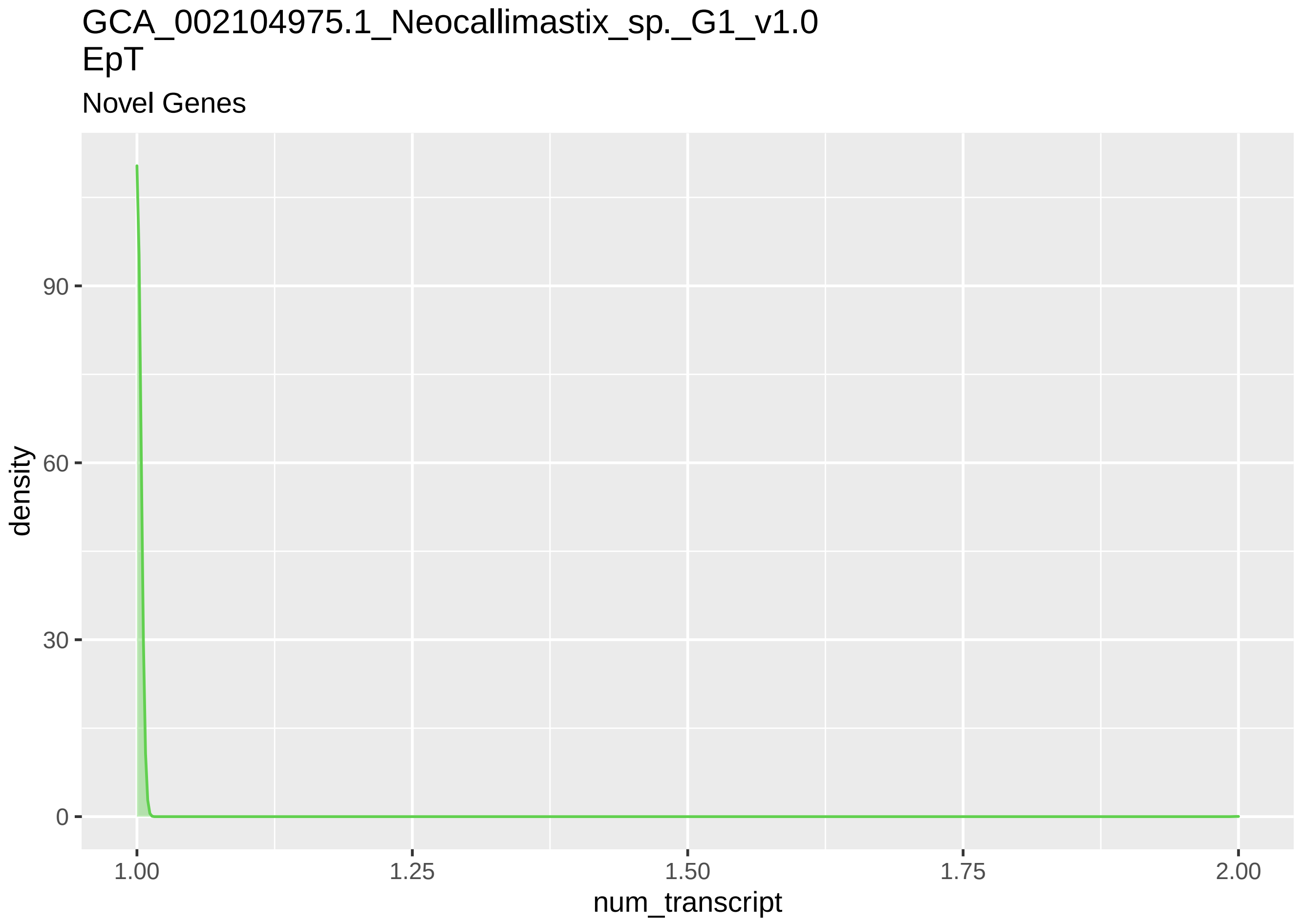

GCA_002104975.1_Neocallimastix_sp._G1_v1.0
EpT
Novel Genes
density
0
30
60
90
1.00
1.25
1.50
1.75
2.00
num_transcript

GCA_002104985.1_Rhihy1
EpT

Novel Genes

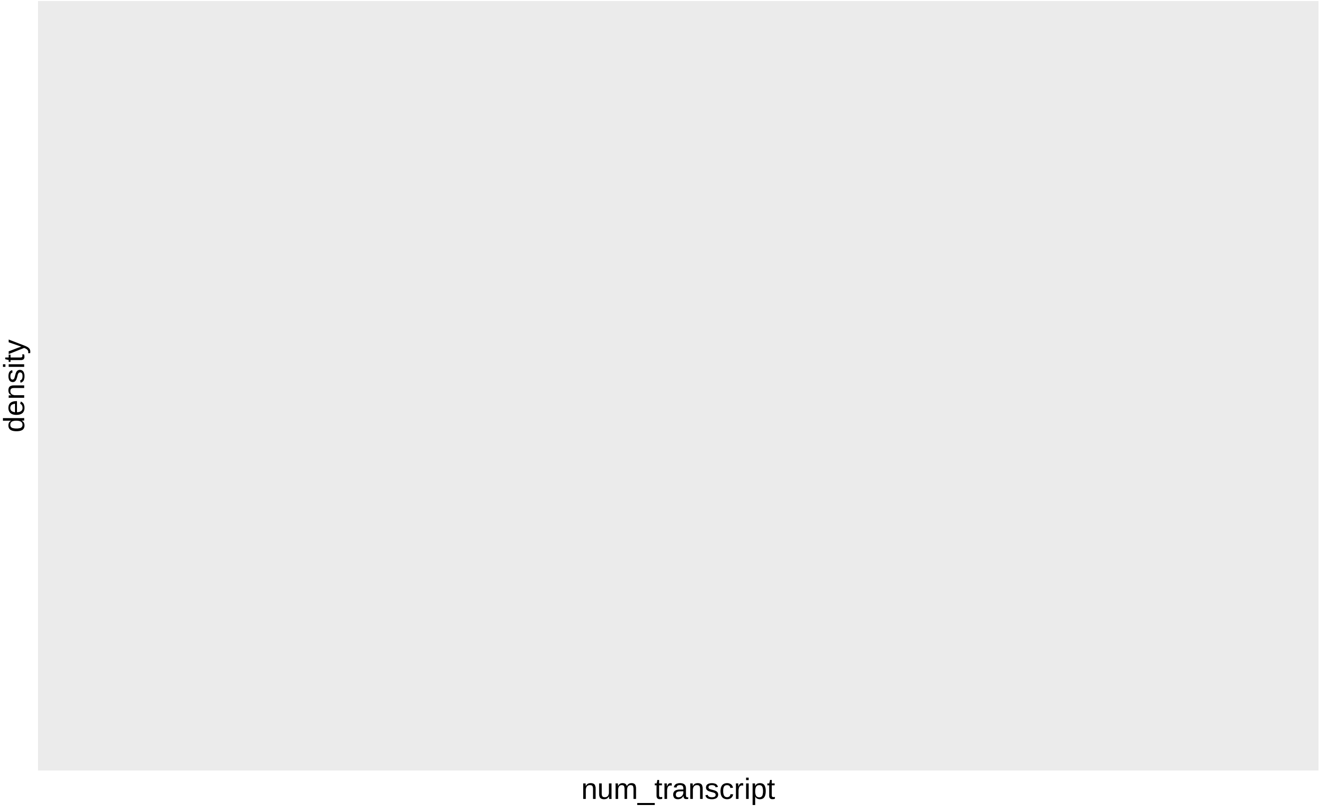

GCA_002918395.1_ASM291839v1
EpT

Novel Genes

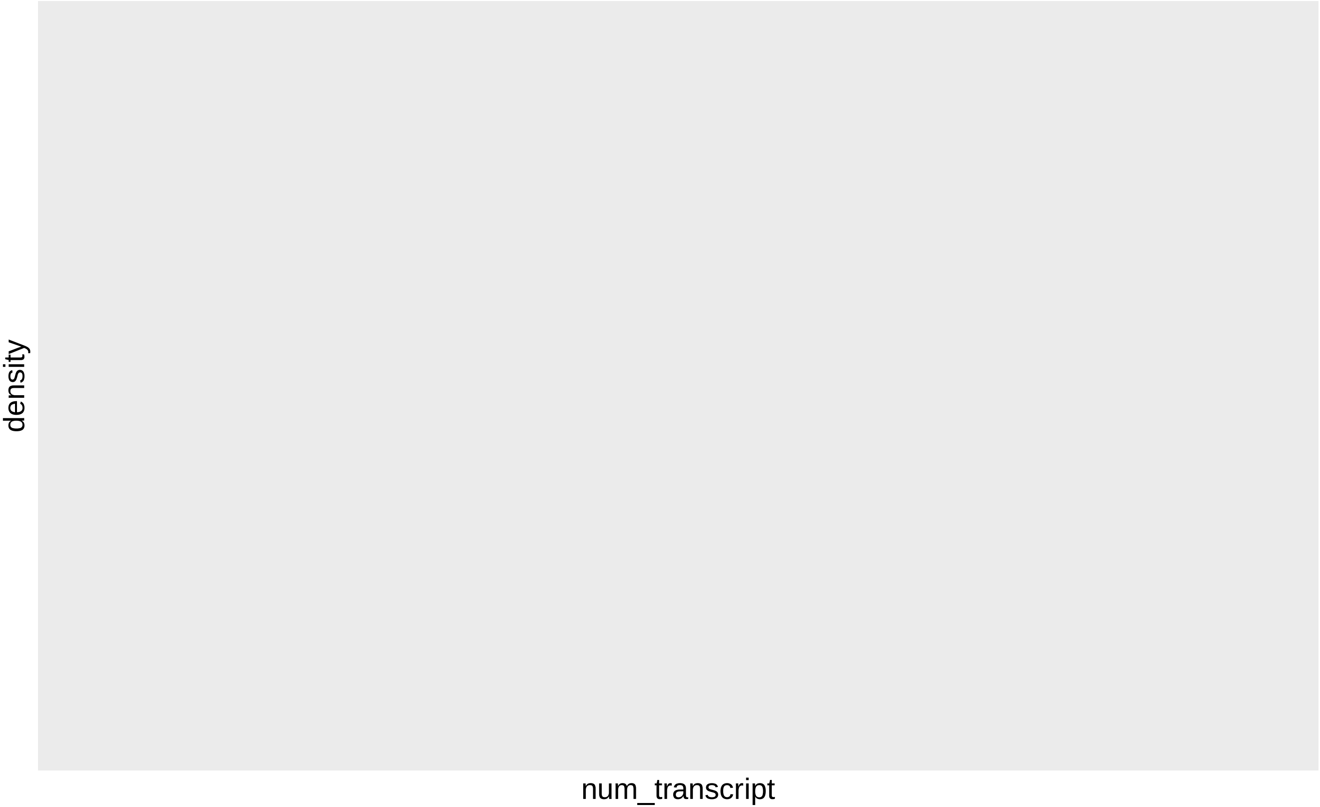

GCA_002938375.1_Psicy2
EpT

Novel Genes

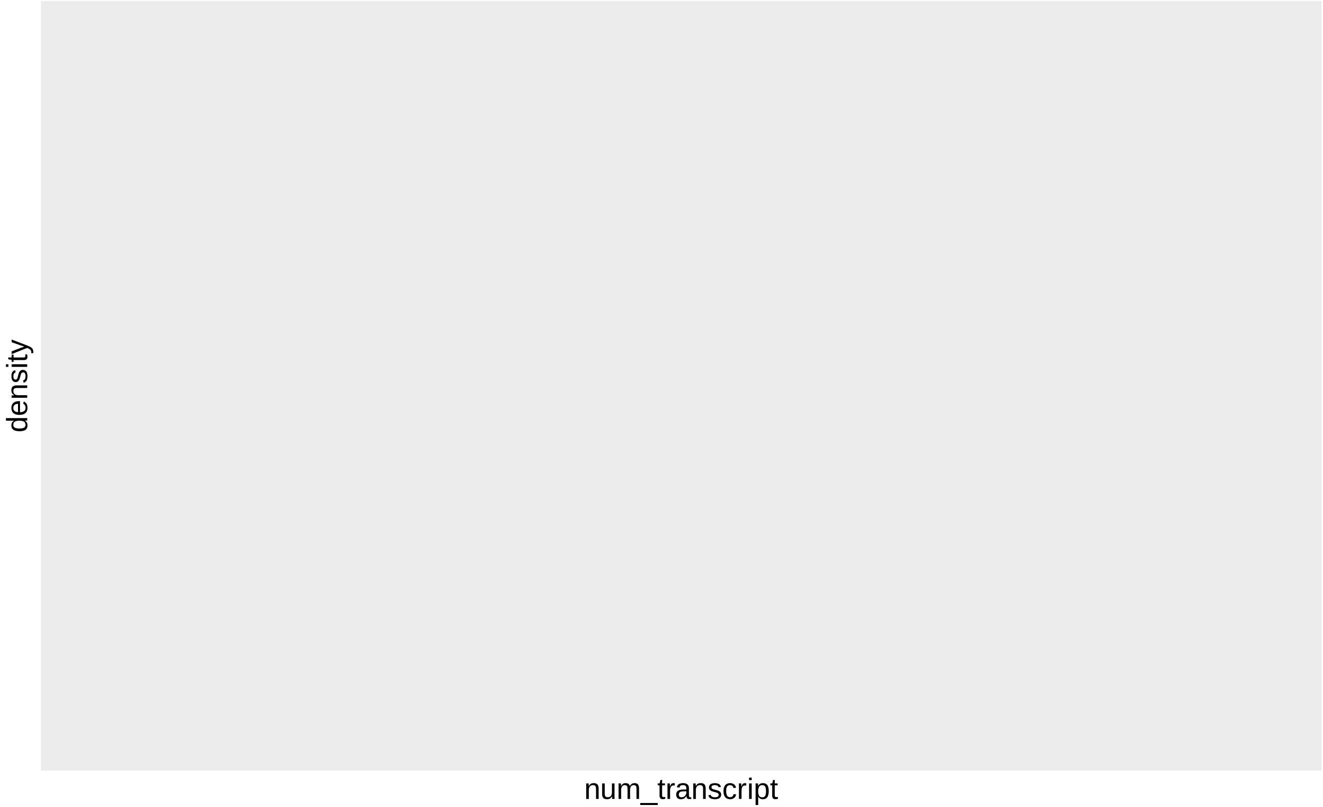

GCA_900106115.1_CBS_141442_assembly
EpT

Novel Genes

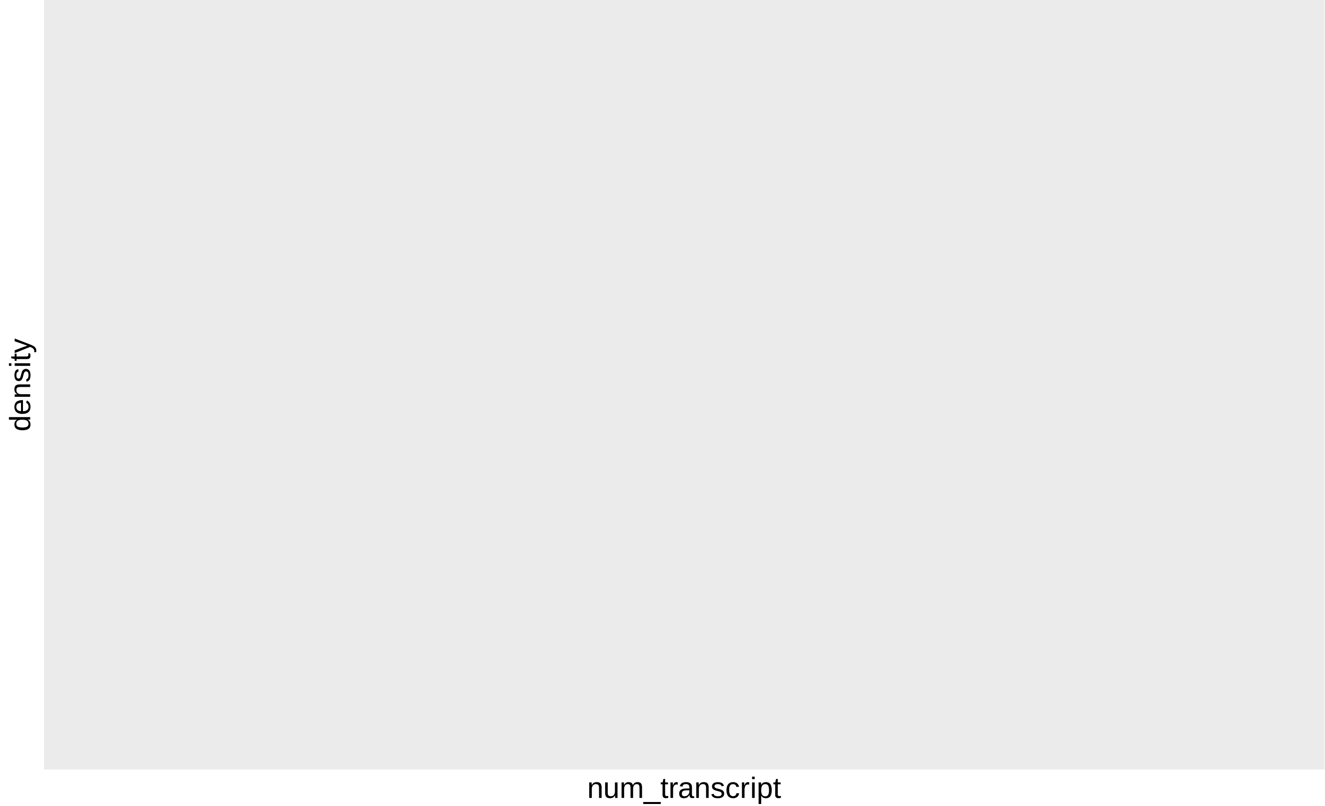

GCF_000001985.1_JCVI−PMFA1−2.0
EpT

Novel Genes

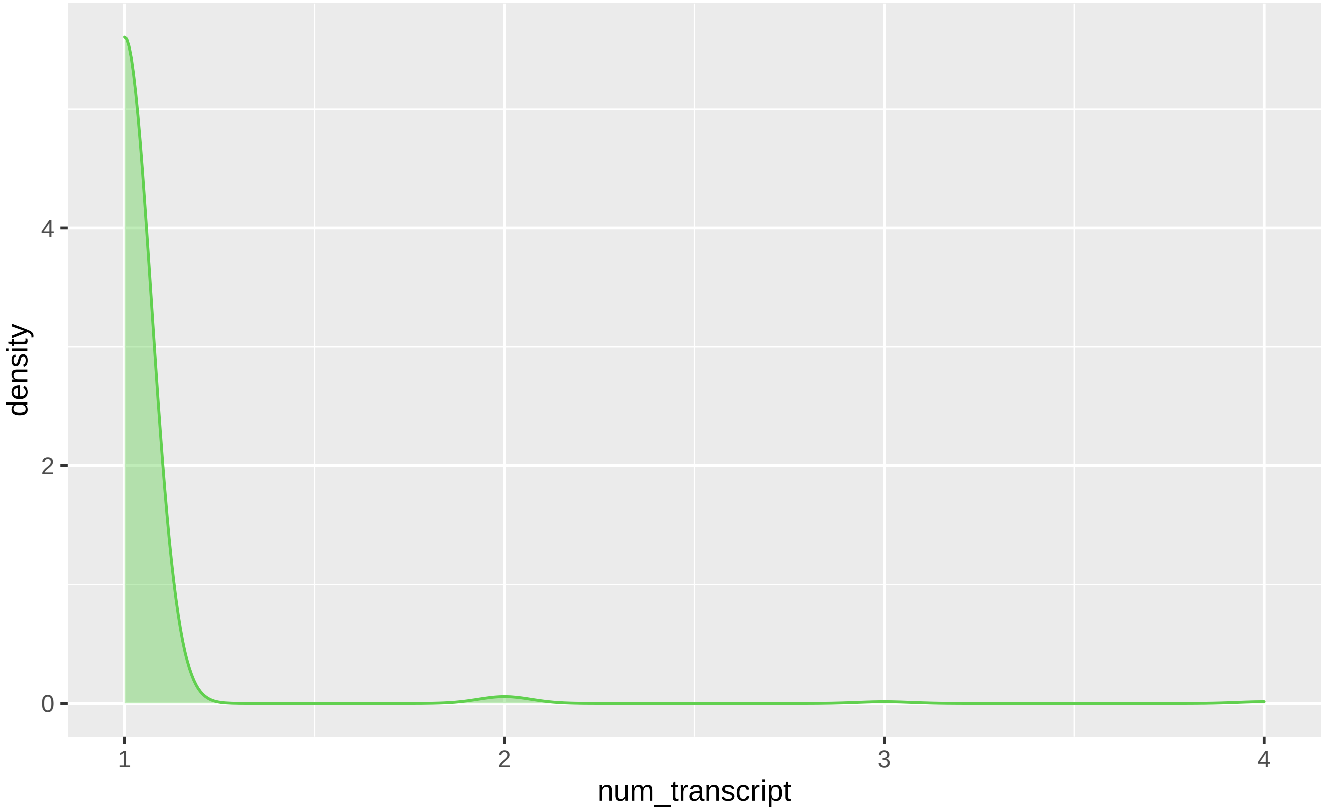

GCF_000002545.3_ASM254v2
EpT

Novel Genes

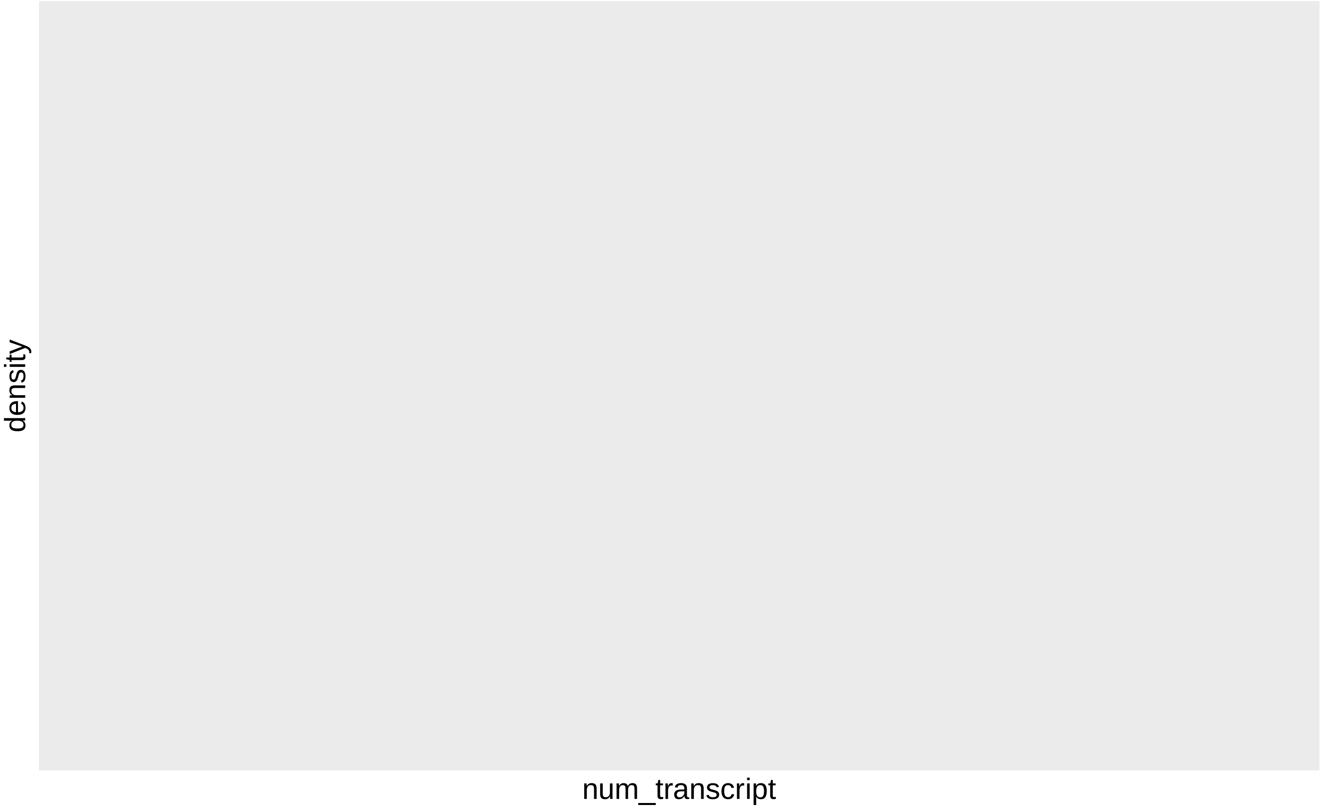

GCF_000026945.1_ASM2694v1
EpT

Novel Genes

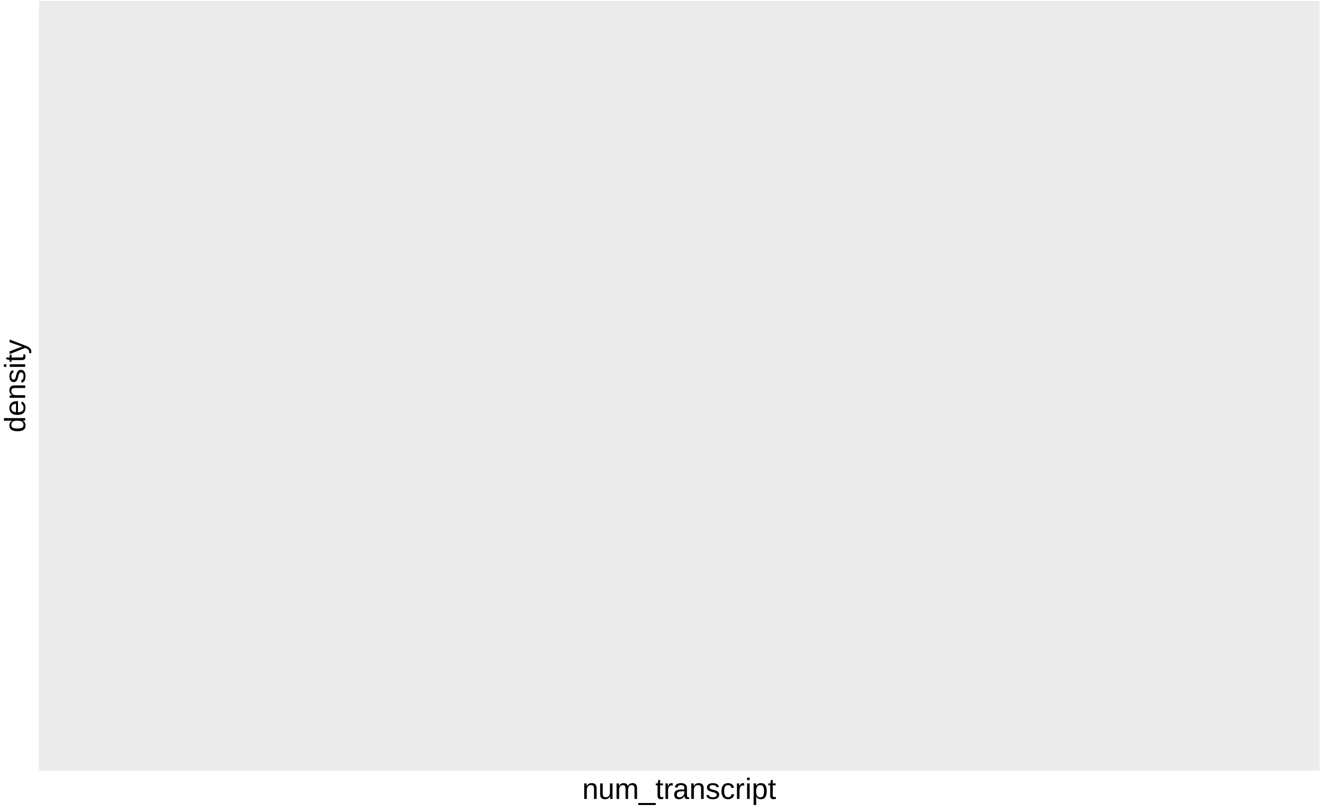

GCF_000091045.1_ASM9104v1
EpT

Novel Genes

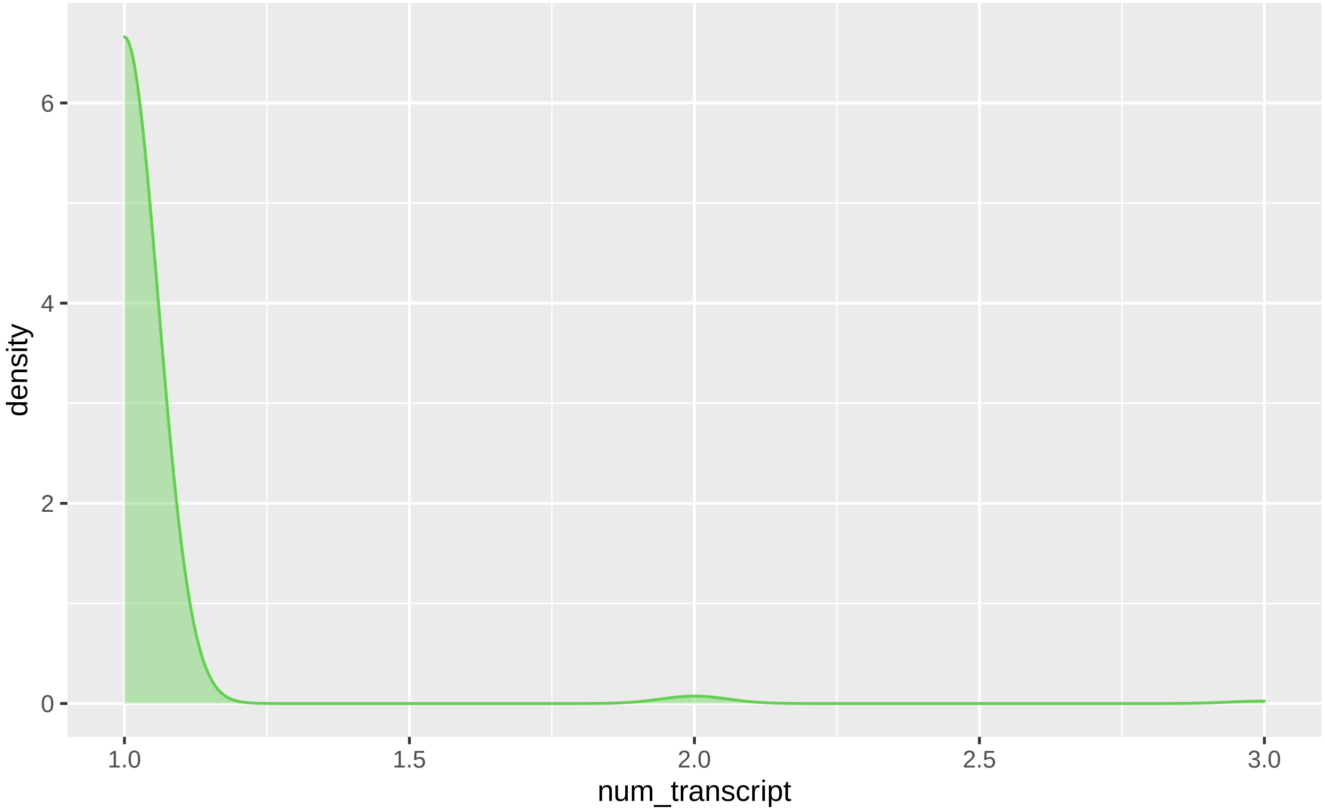

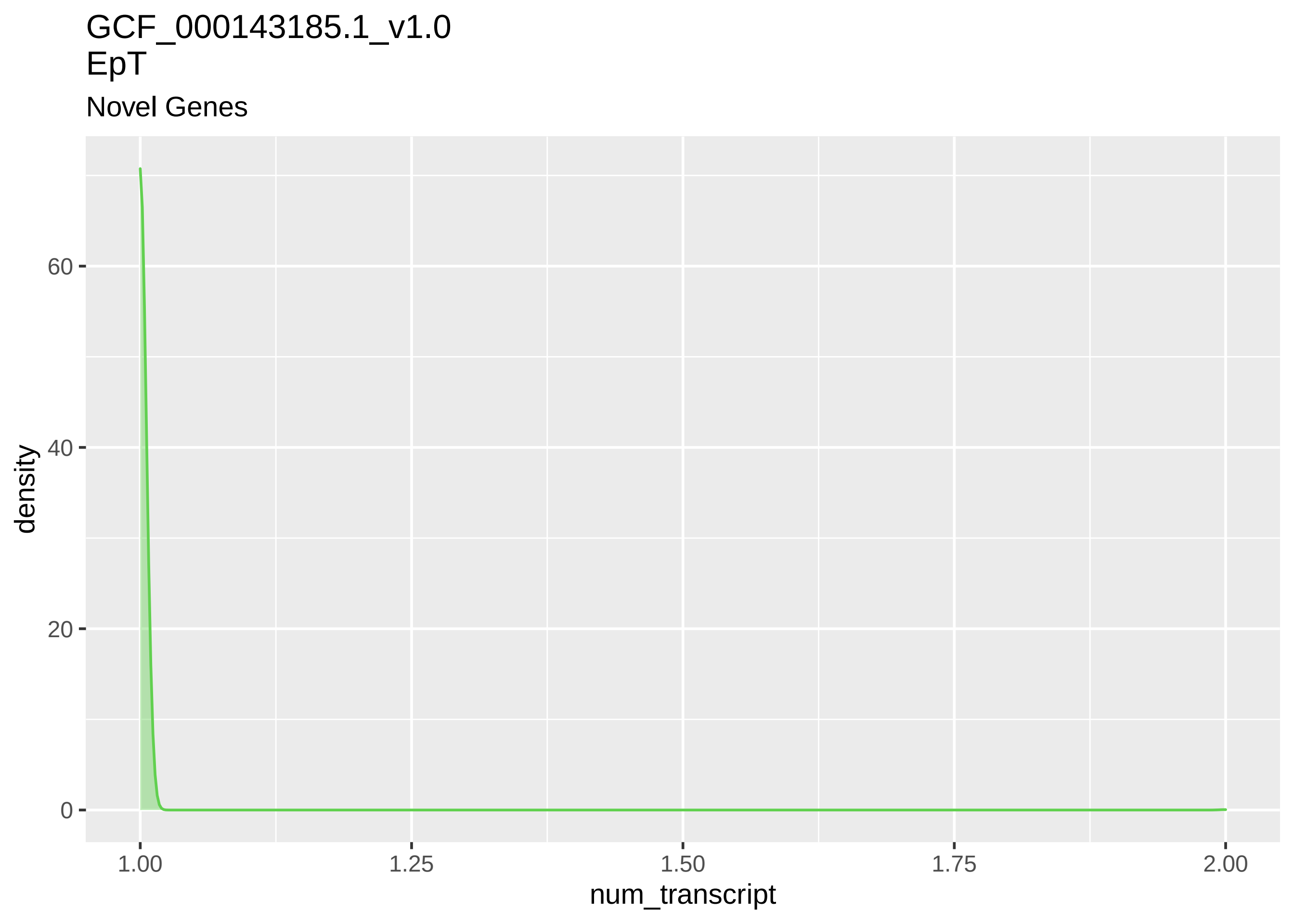
GCF_000143185.1_v1.0
EpT
Novel Genes
density
60
40
20
0
1.00
1.25
1.50
1.75
2.00
num_transcript

GCF_000149035.1_C_graminicola_M1_001_V1
EpT

Novel Genes

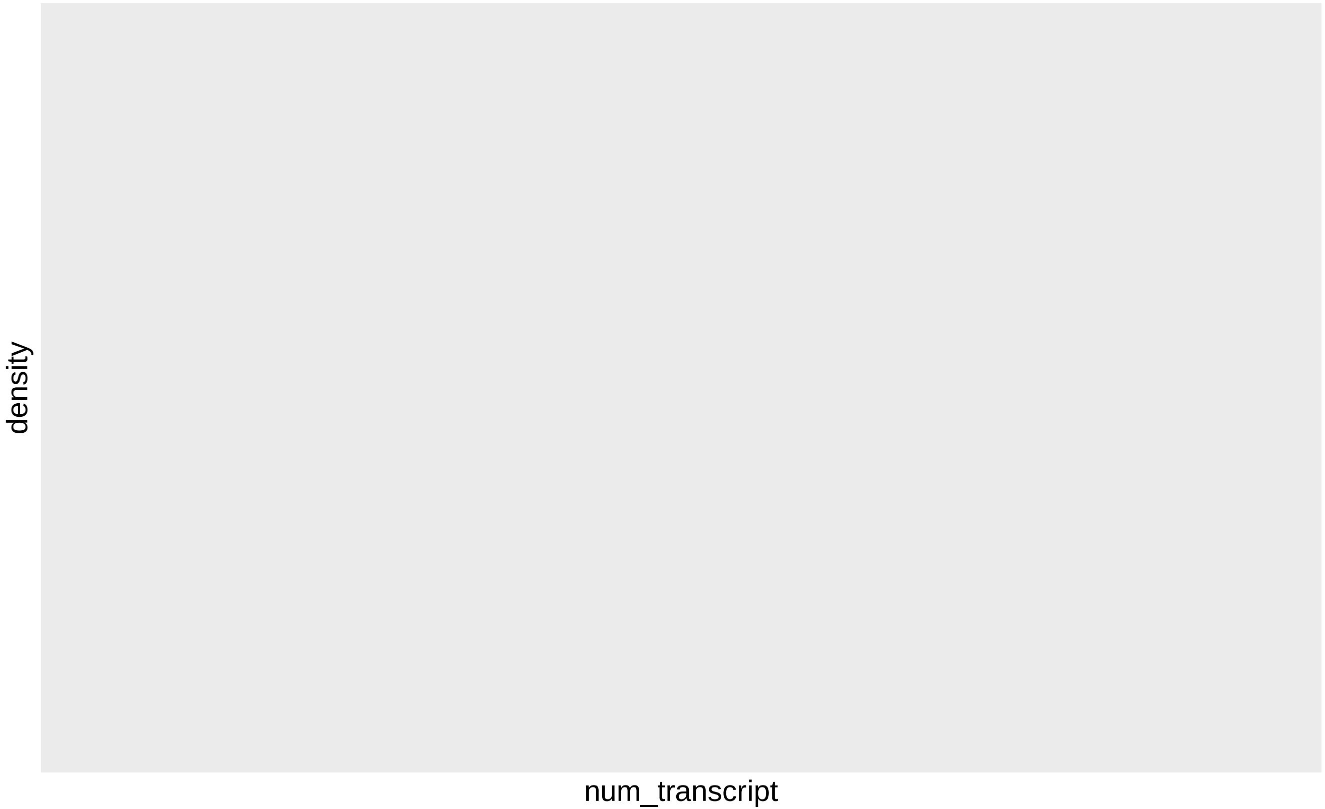

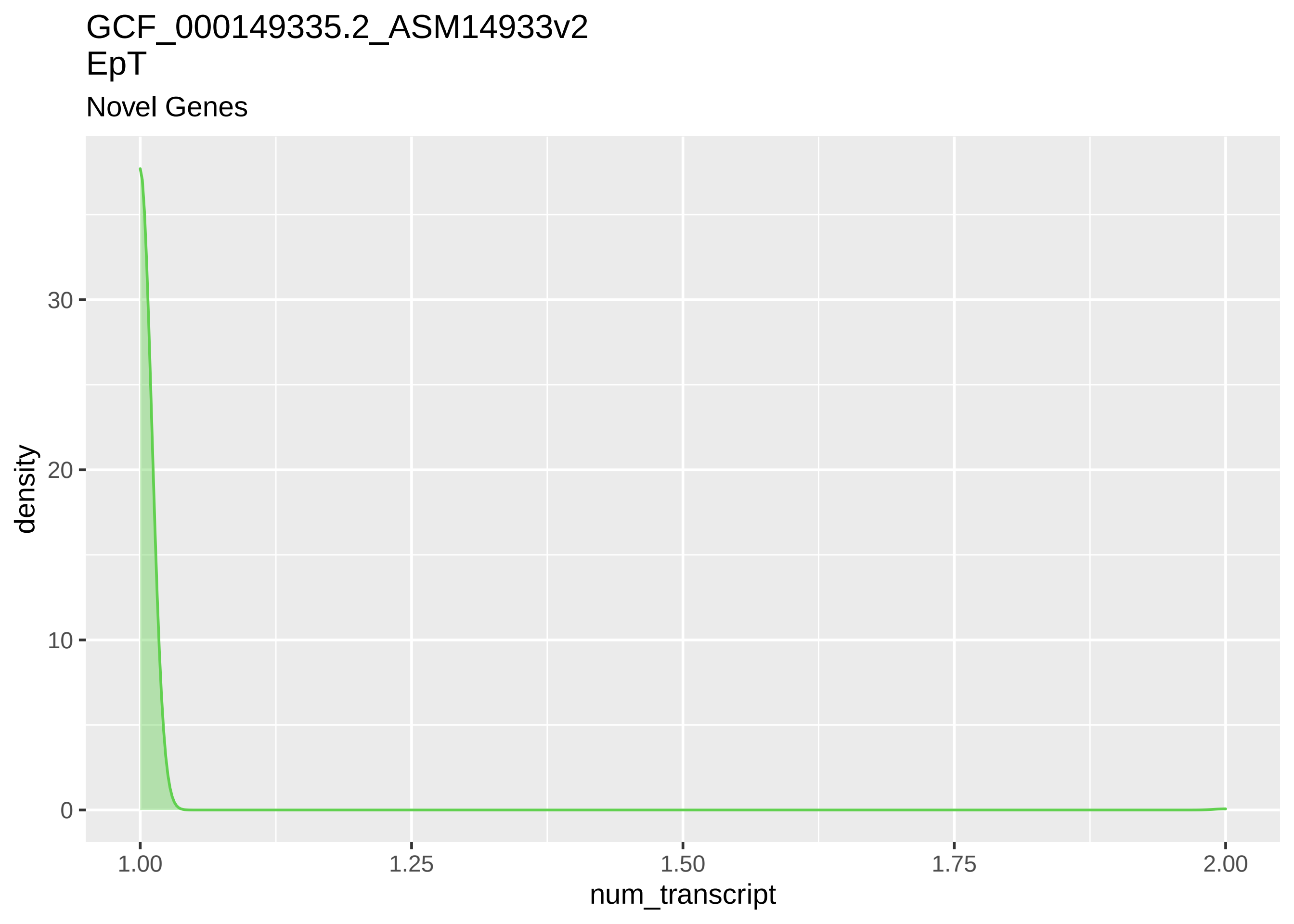
GCF_000149335.2_ASM14933v2
EpT
Novel Genes
density
30
20
10
0
1.00
1.25
1.50
1.75
2.00
num_transcript

GCF_000149555.1_ASM14955v1
EpT

Novel Genes

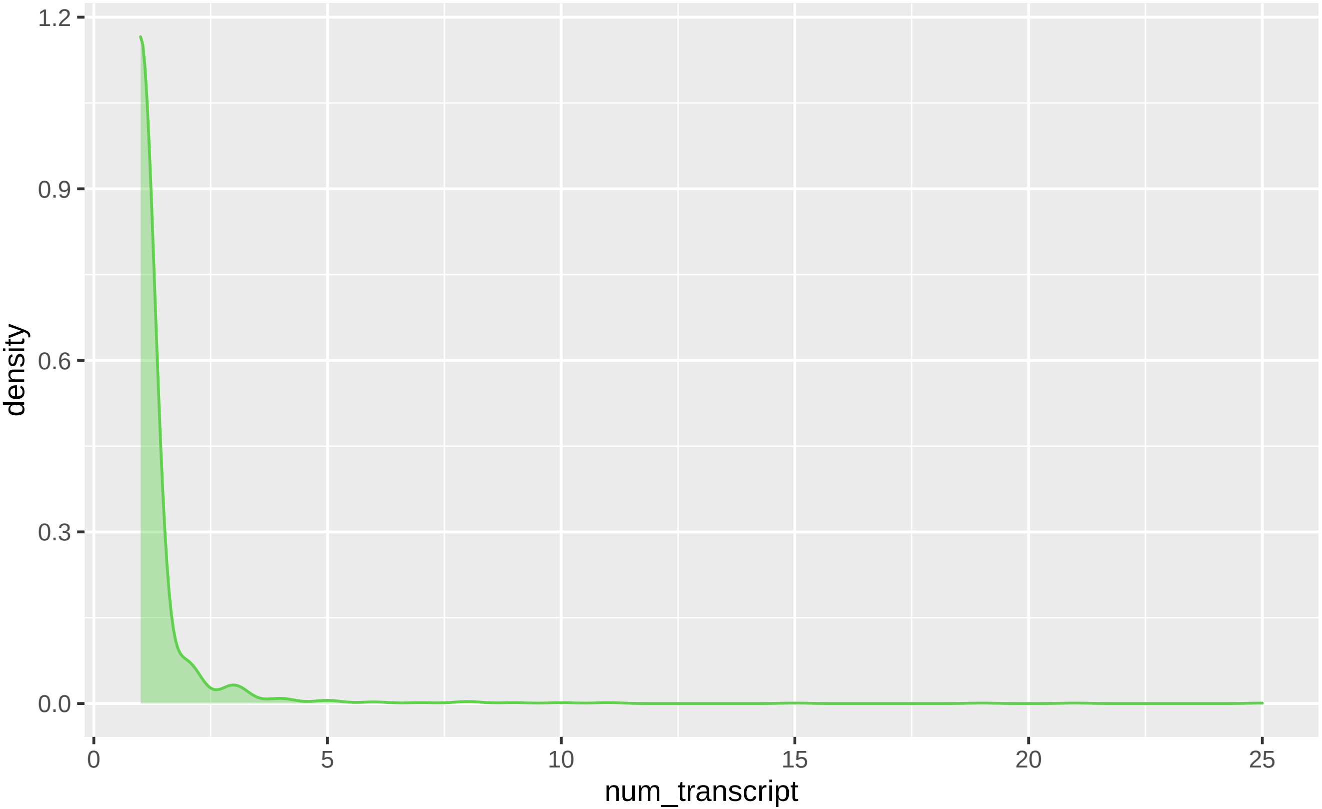

GCF_000150505.1_SO6
EpT

Novel Genes

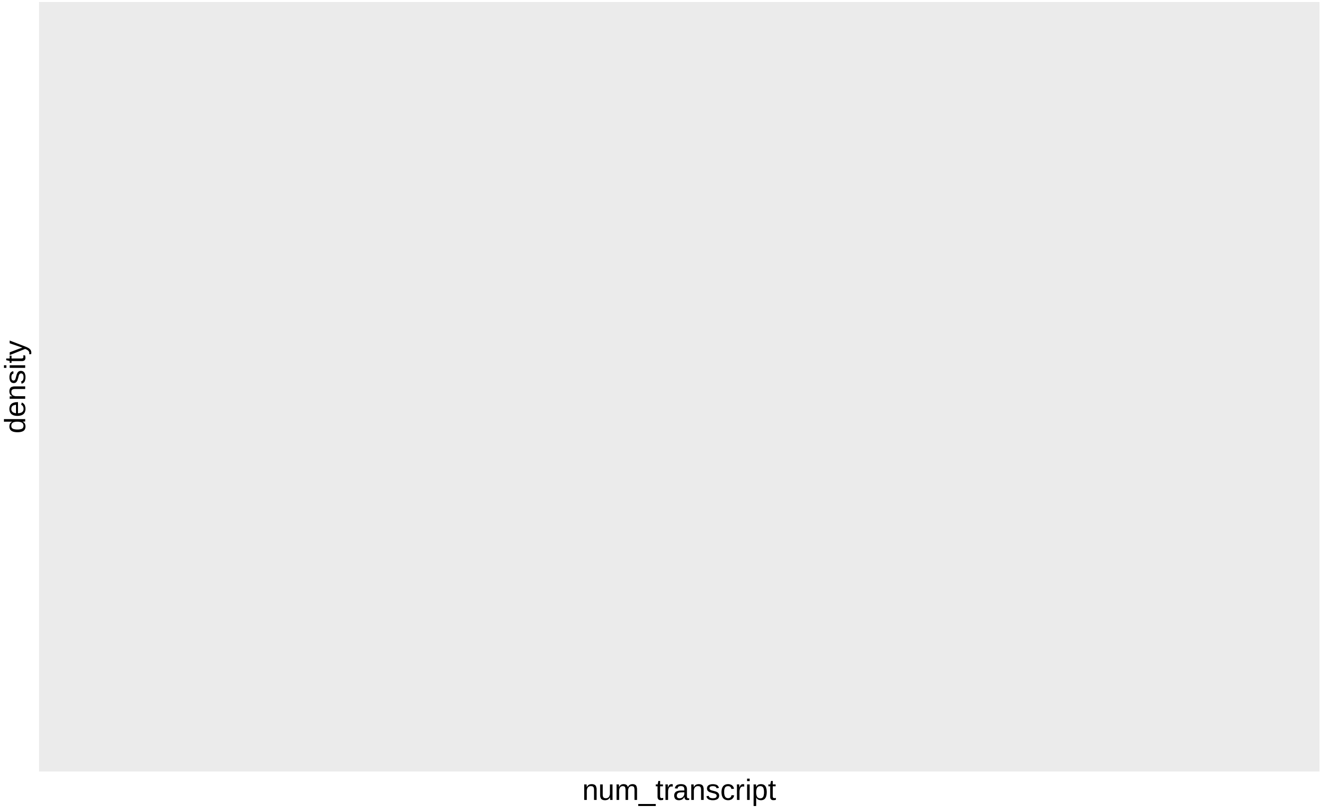

GCF_000150705.2_Paracocci_br_Pb01_V2
EpT

Novel Genes

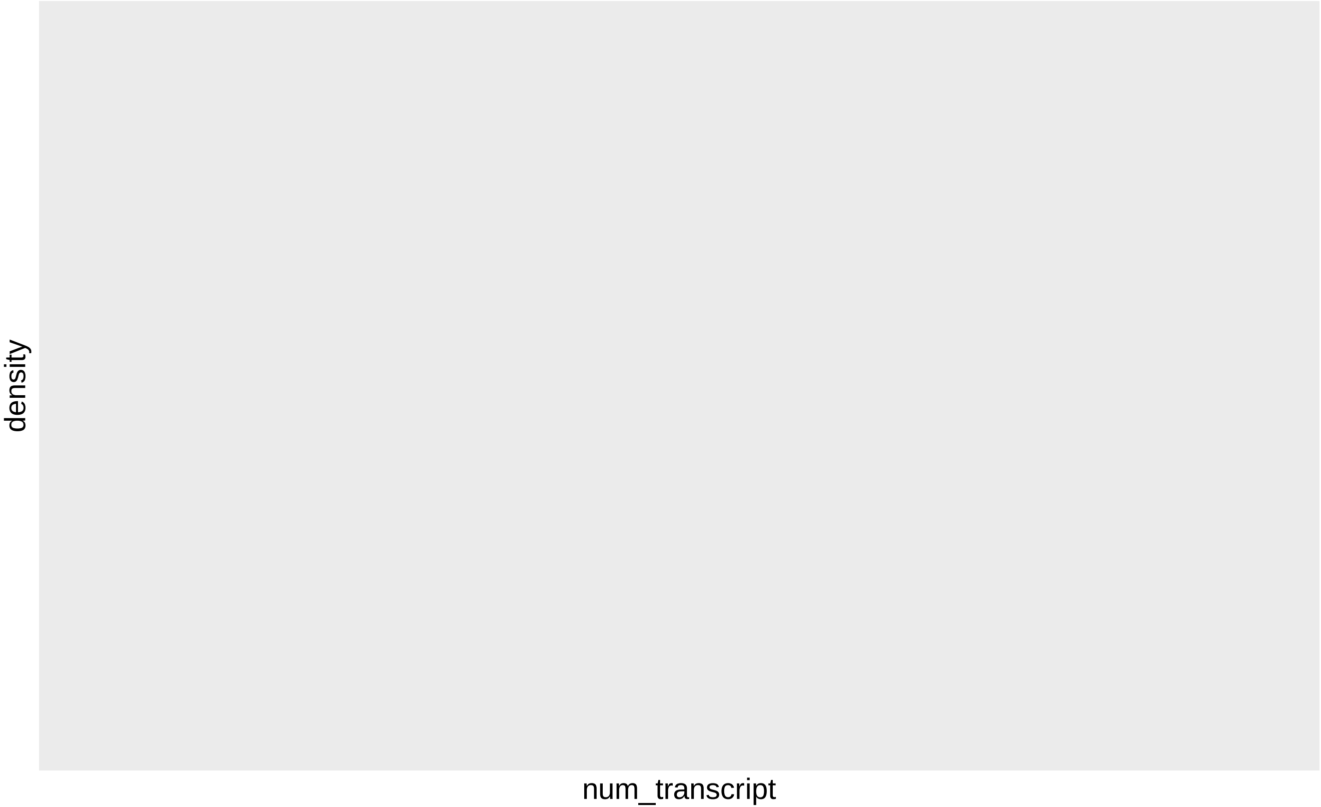

GCF_000171015.1_TRIAT_v2.0
EpT

Novel Genes

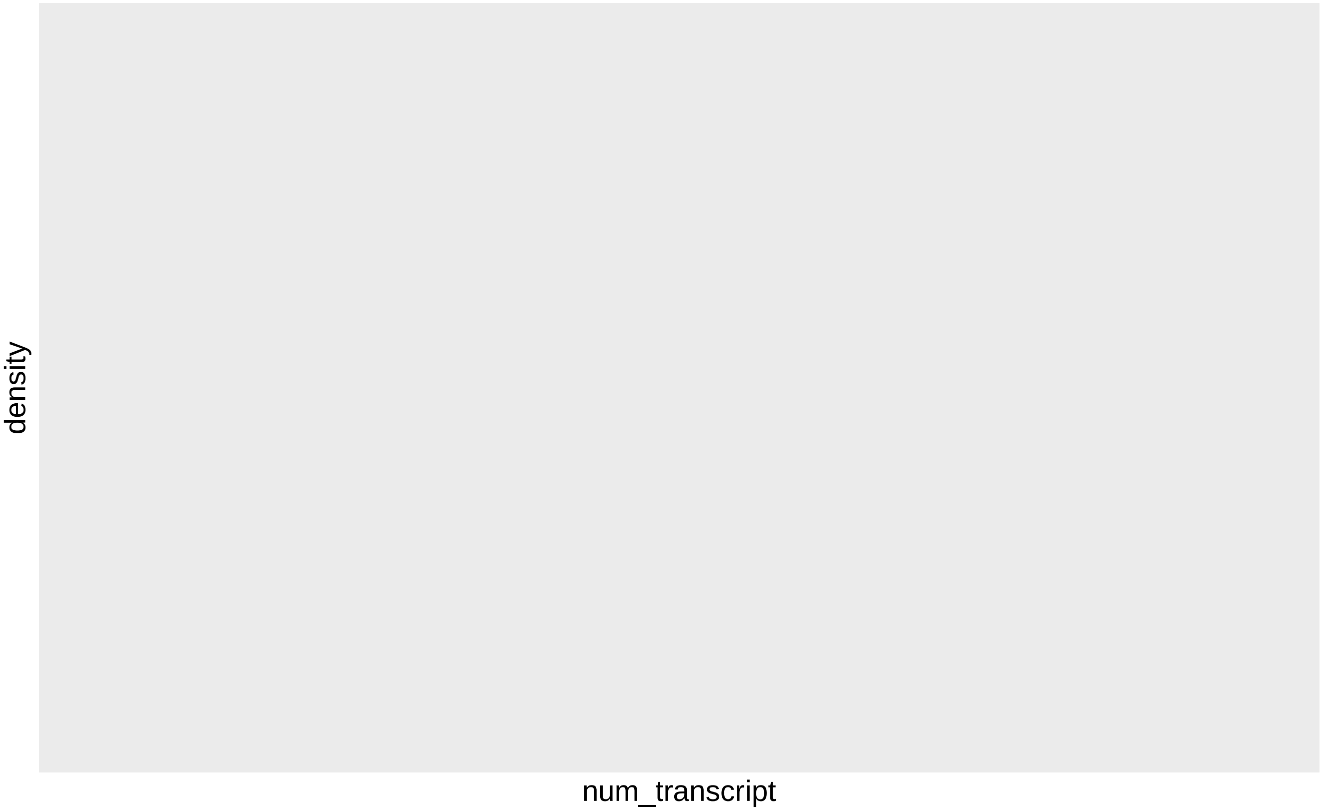

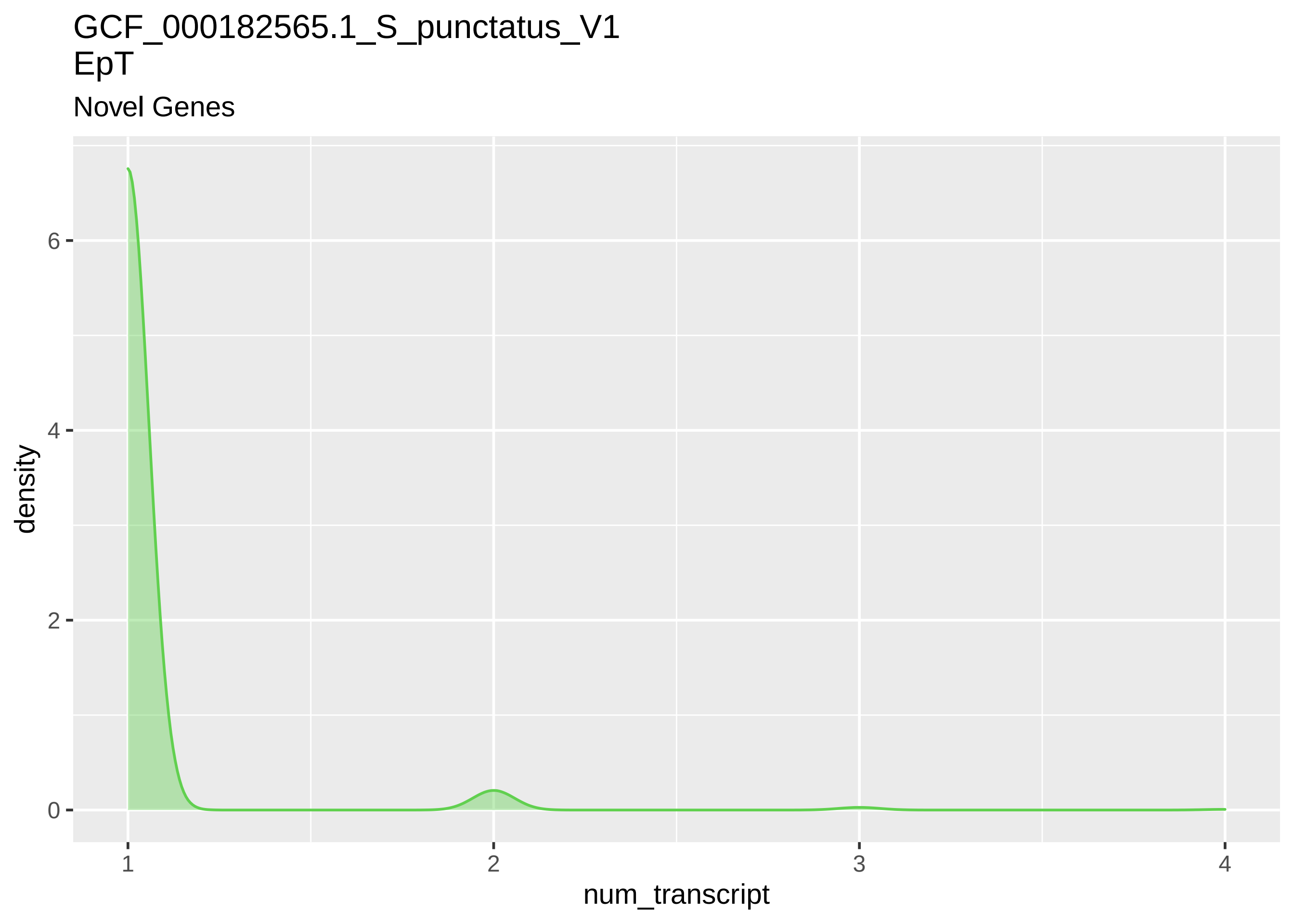

GCF_000182565.1_S_punctatus_V1
EpT
Novel Genes
density
num_transcript
0
2
4
6
1
2
3
4

GCF_000182805.2_ASM18280v2
EpT

Novel Genes

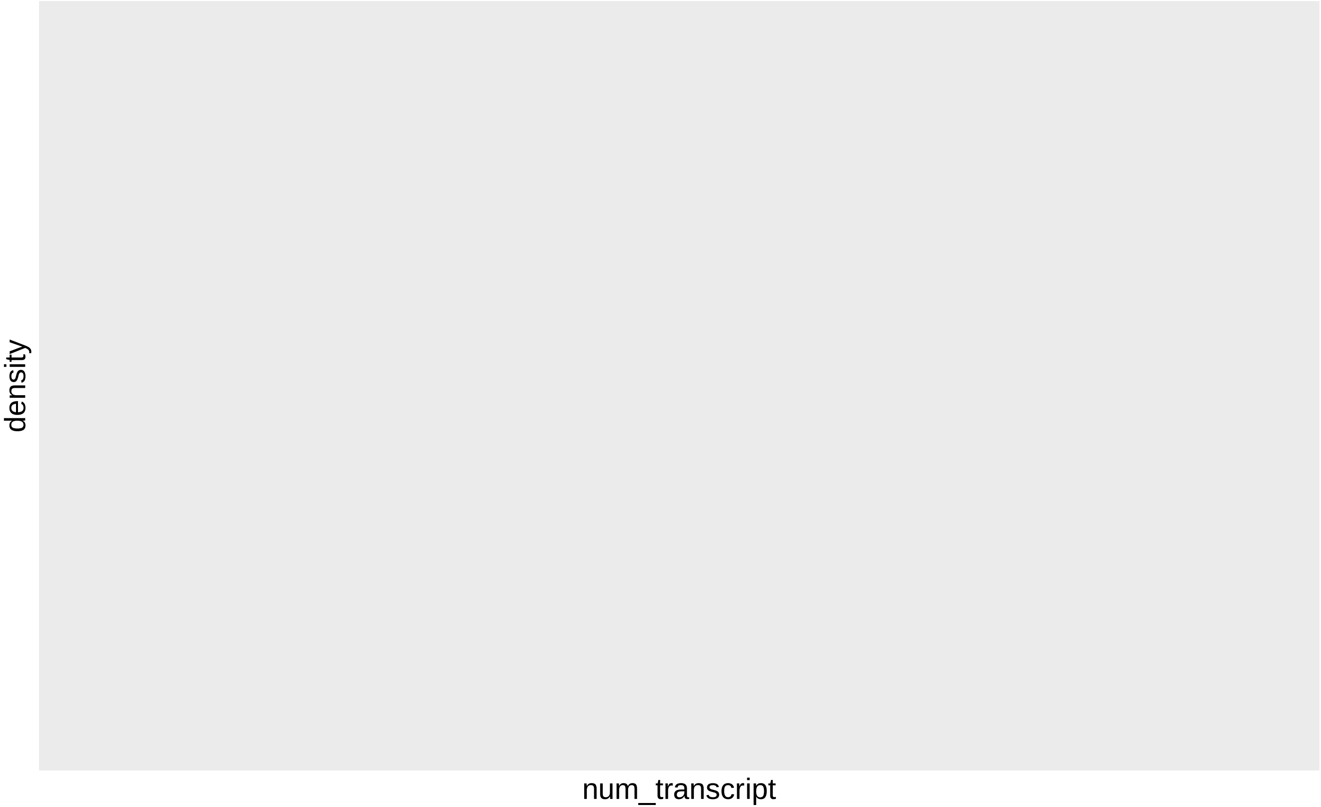

GCF_000182895.1_CC3
EpT

Novel Genes

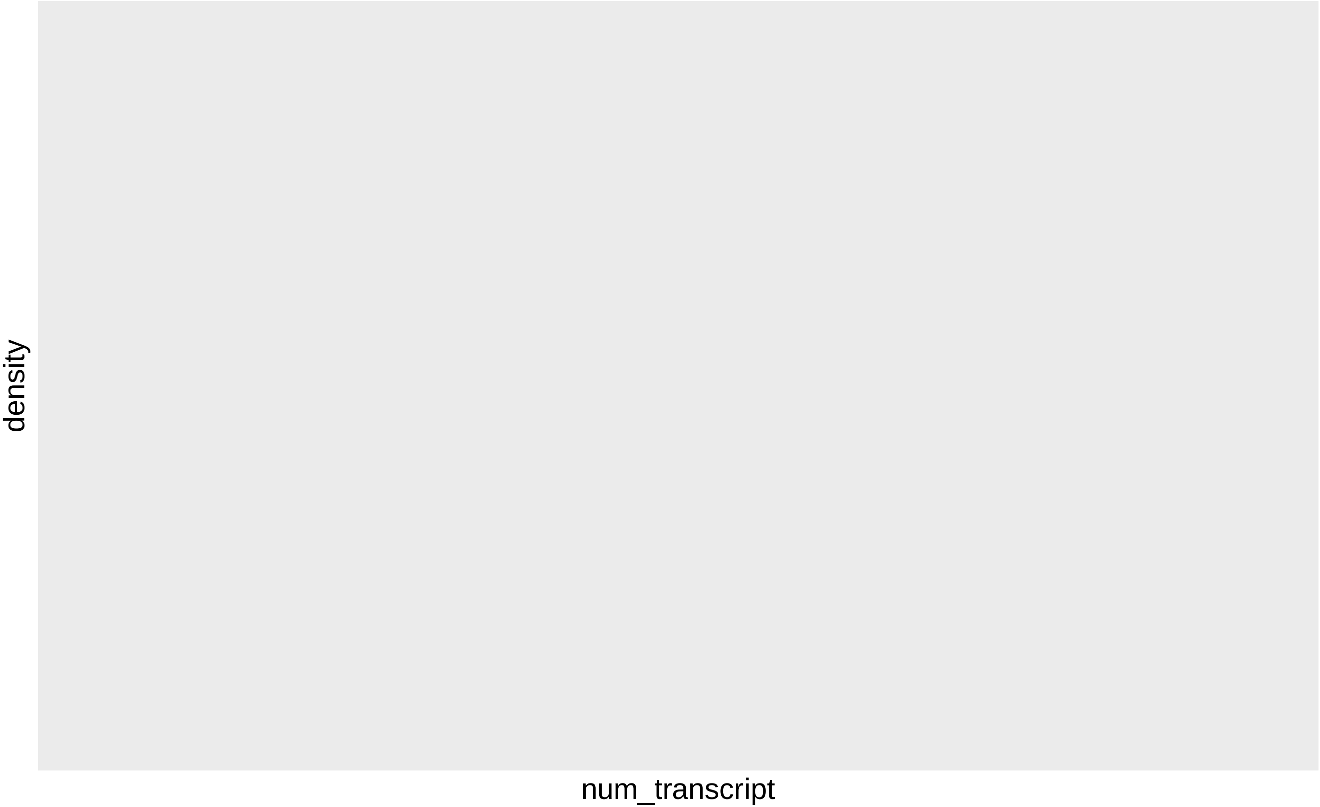

GCF_000203795.1_v1.0
EpT

Novel Genes

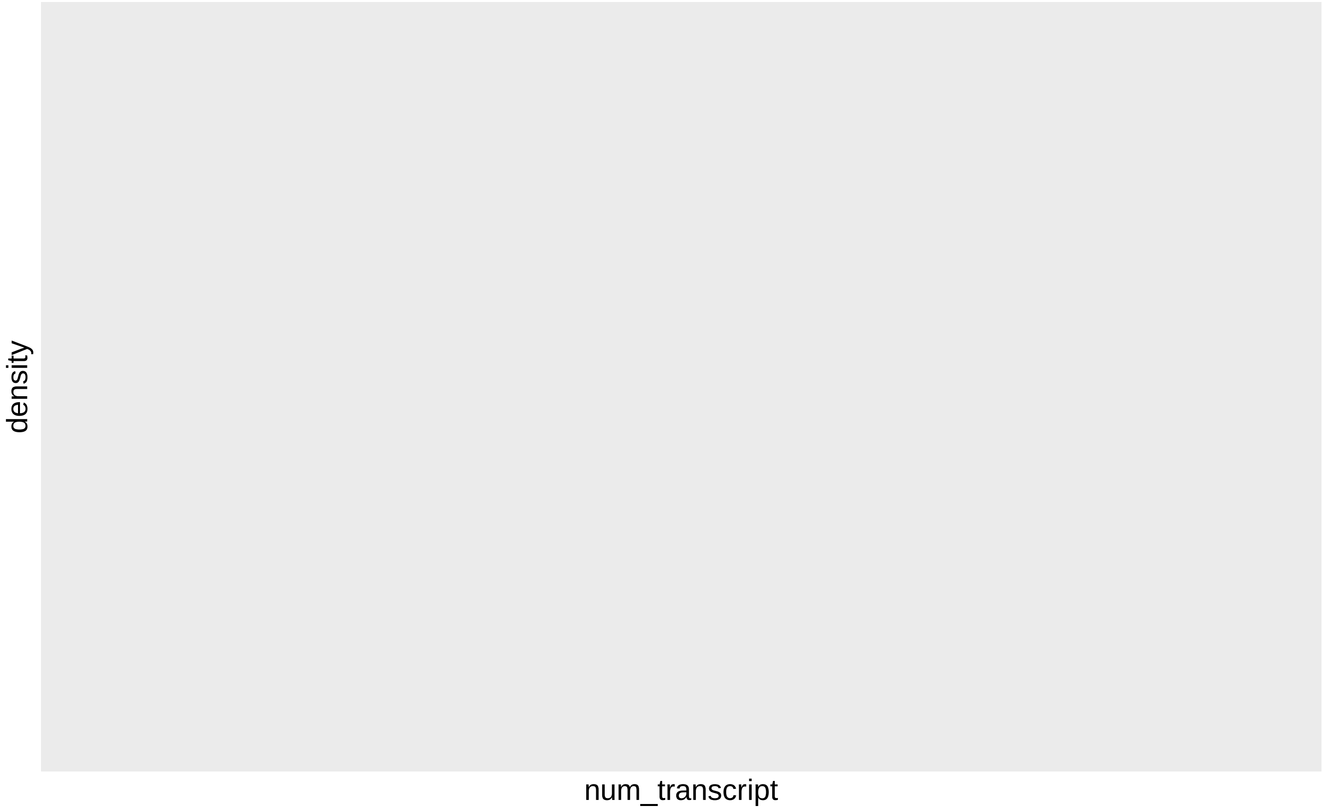

GCF_000204055.1_v1.0
EpT

Novel Genes

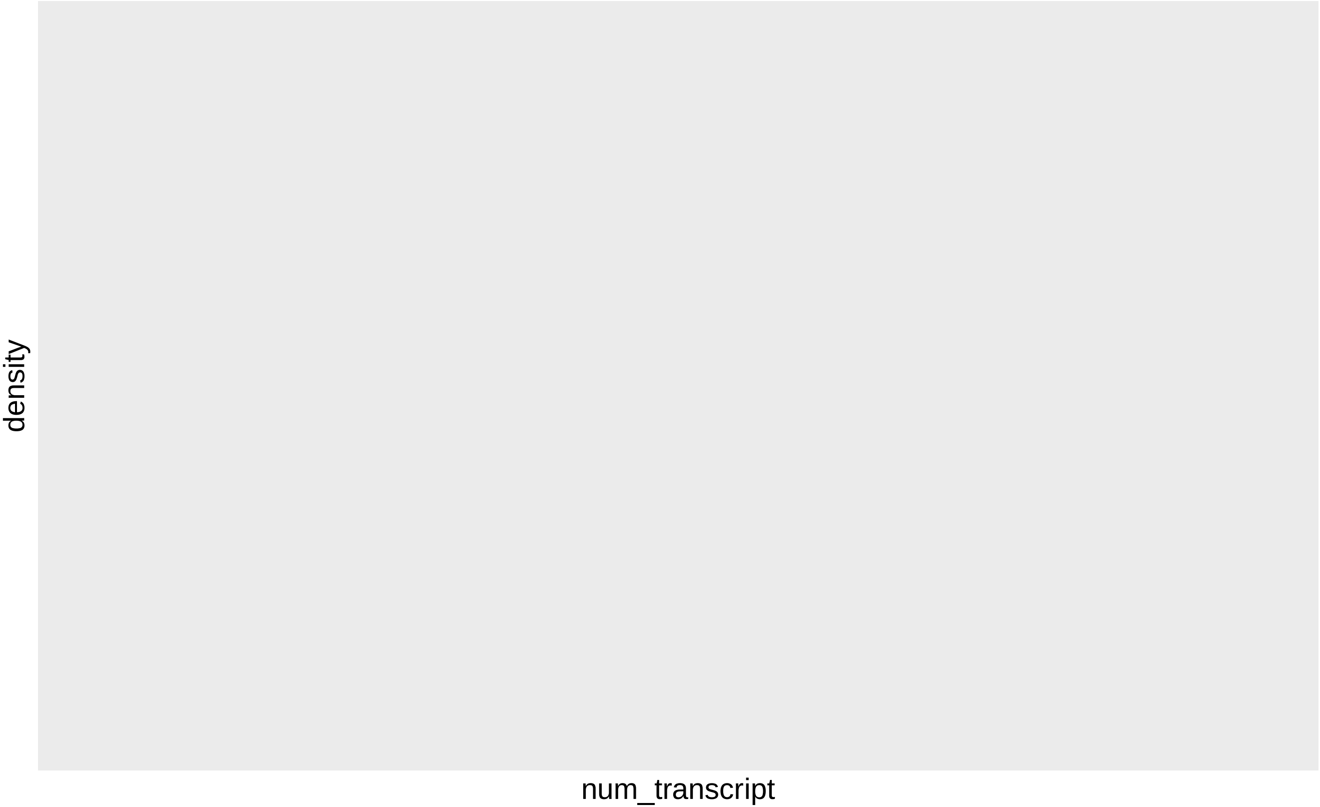

GCF_000221225.1_CTHT_3.0
EpT

Novel Genes

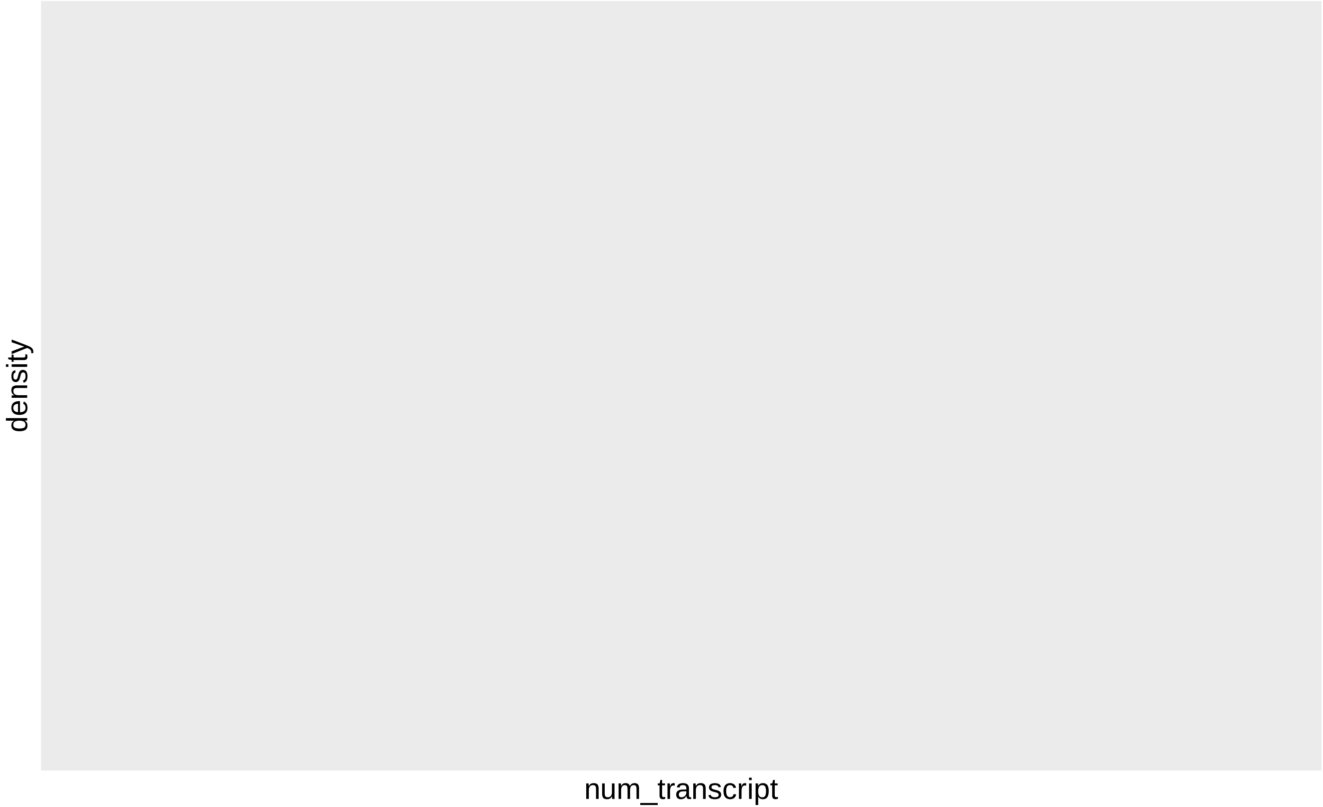

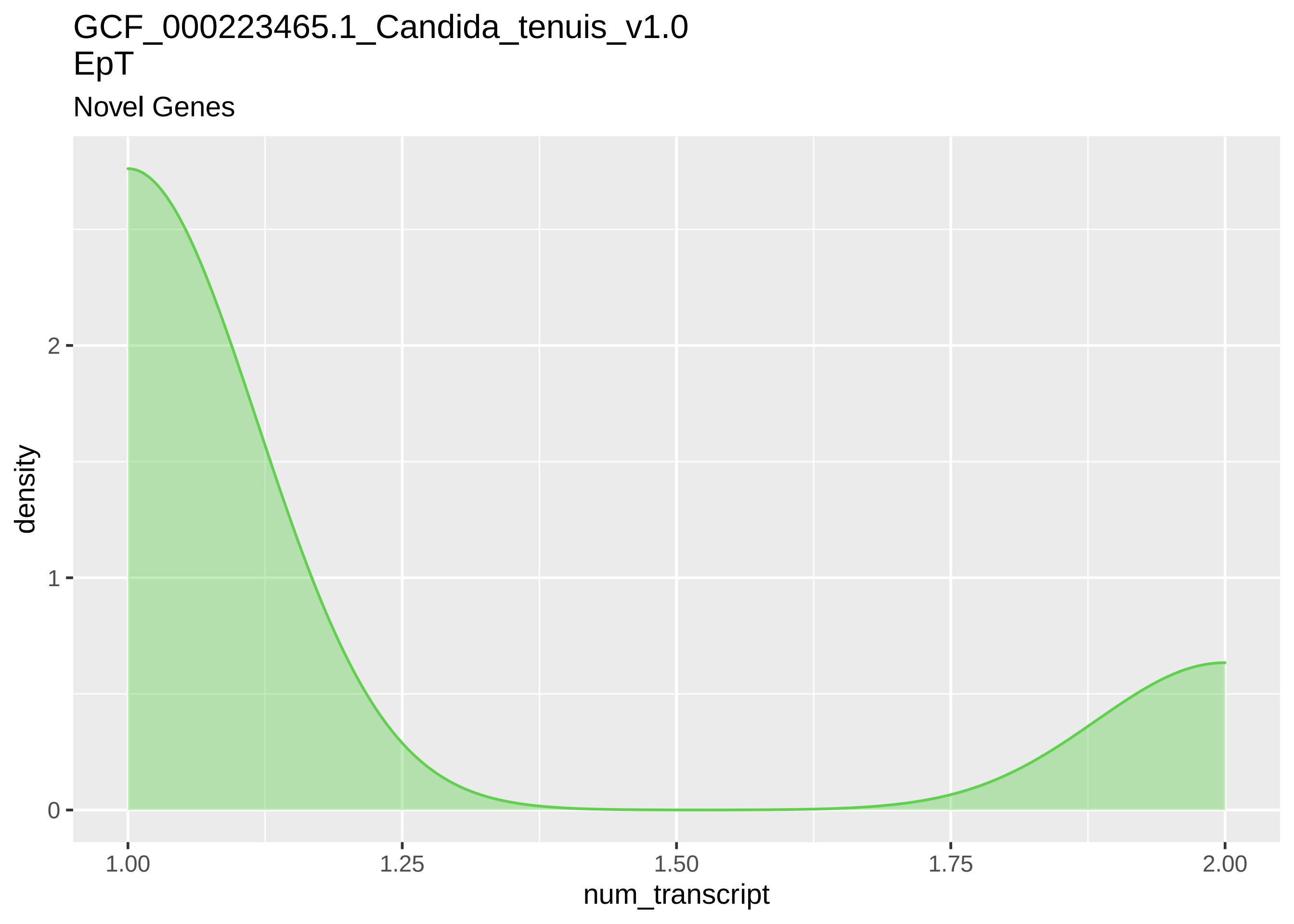
GCF_000223465.1_Candida_tenuis_v1.0
EpT
Novel Genes
density
2
1
0
1.00
1.25
1.50
1.75
2.00
num_transcript

GCF_000230375.1_ASM23037v1
EpT

Novel Genes

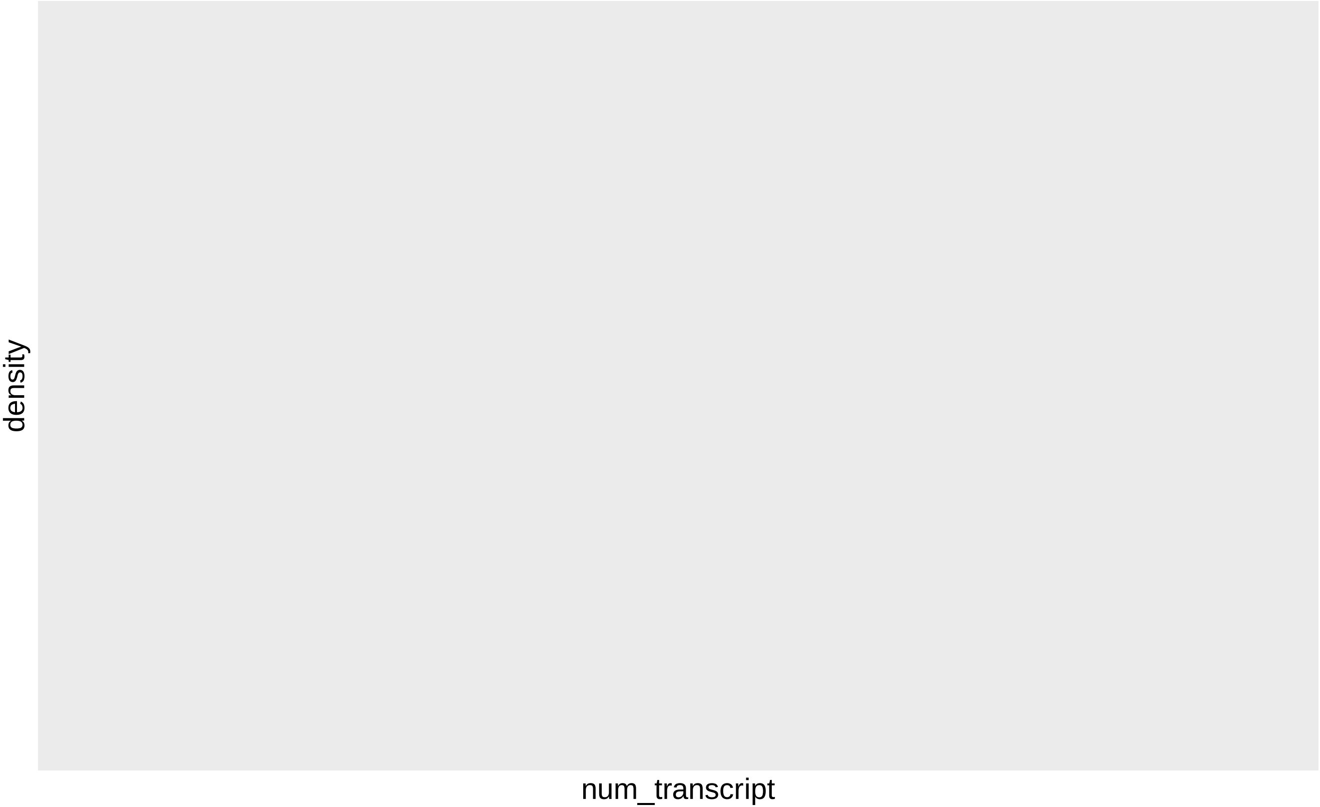

GCF_000264905.1_Stehi1
EpT

Novel Genes

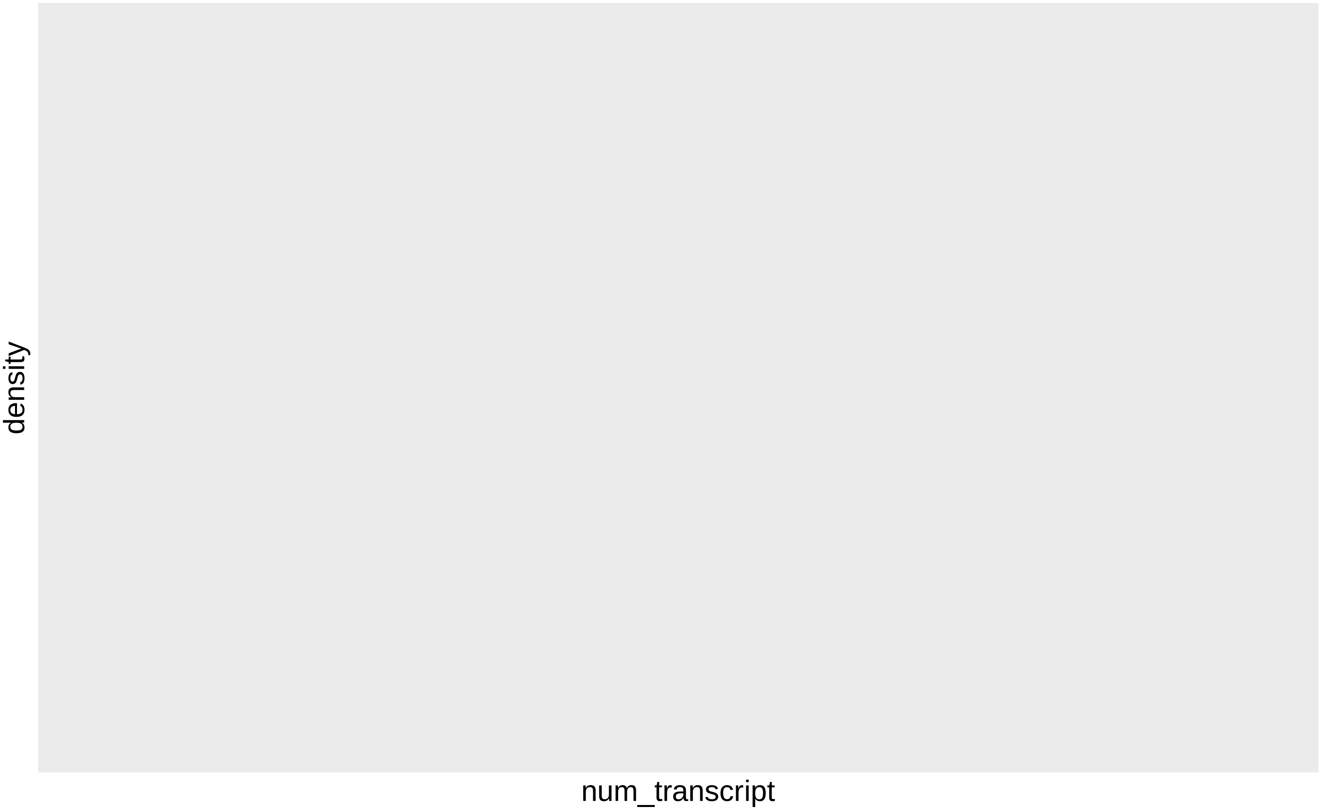

GCF_000264995.1_Punctularia_strigosozonata_v1.0
EpT

Novel Genes

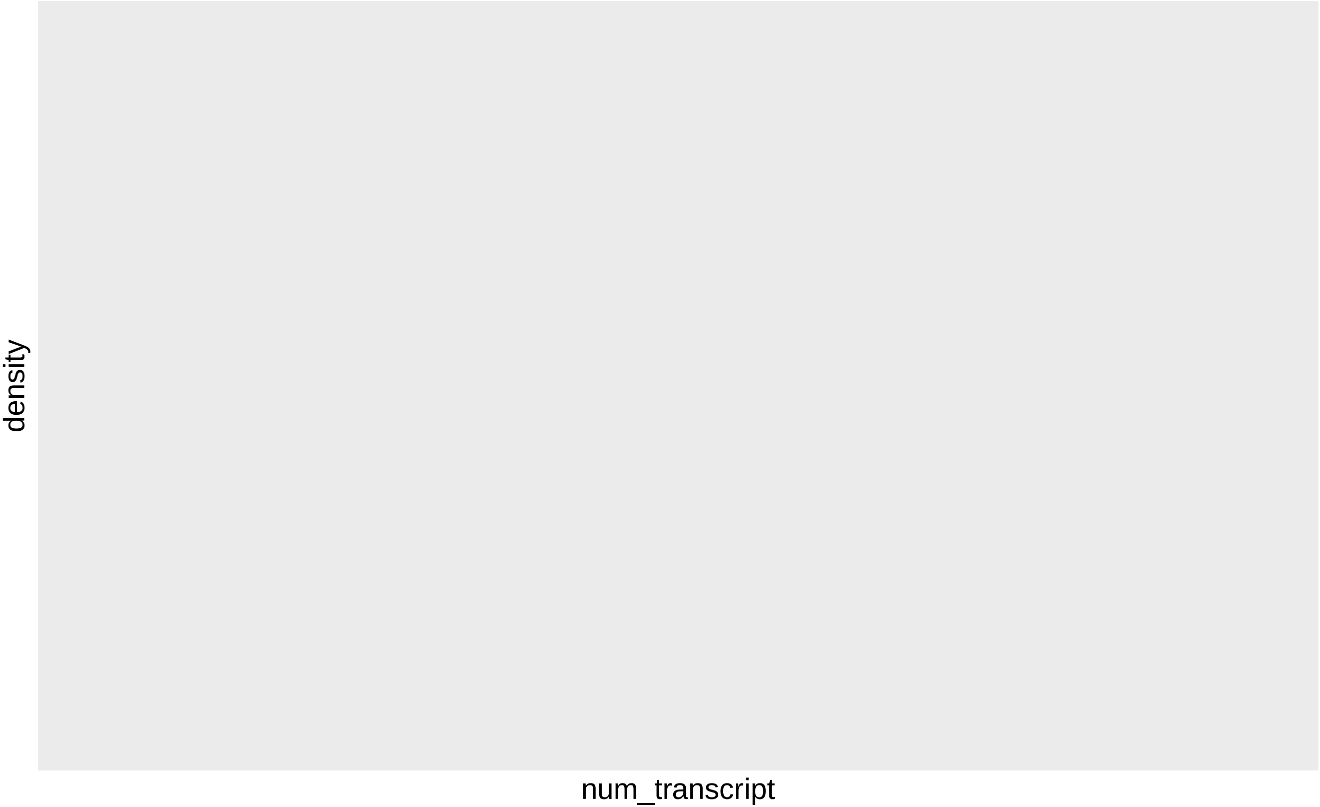

GCF_000271605.1_Fomme1
EpT

Novel Genes

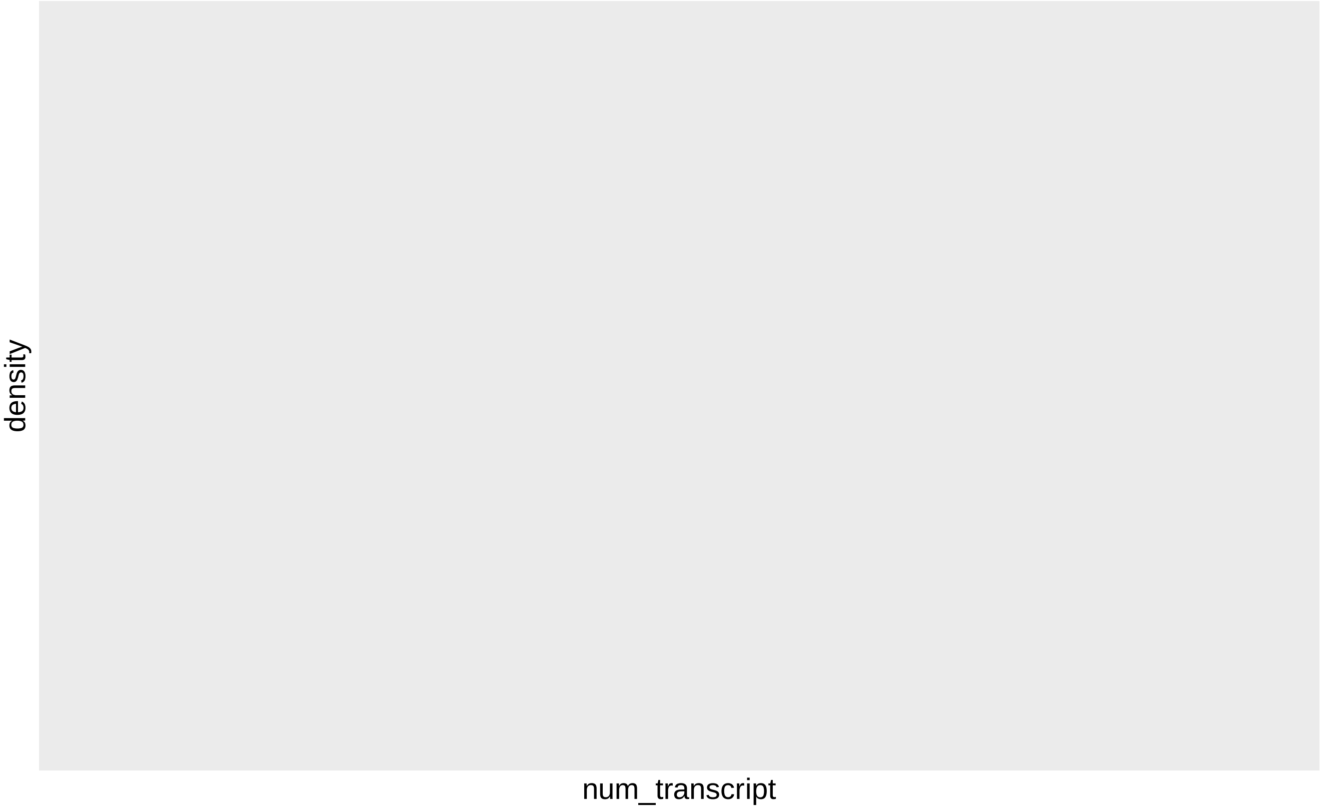

GCF_000271625.1_Conpu1
EpT

Novel Genes

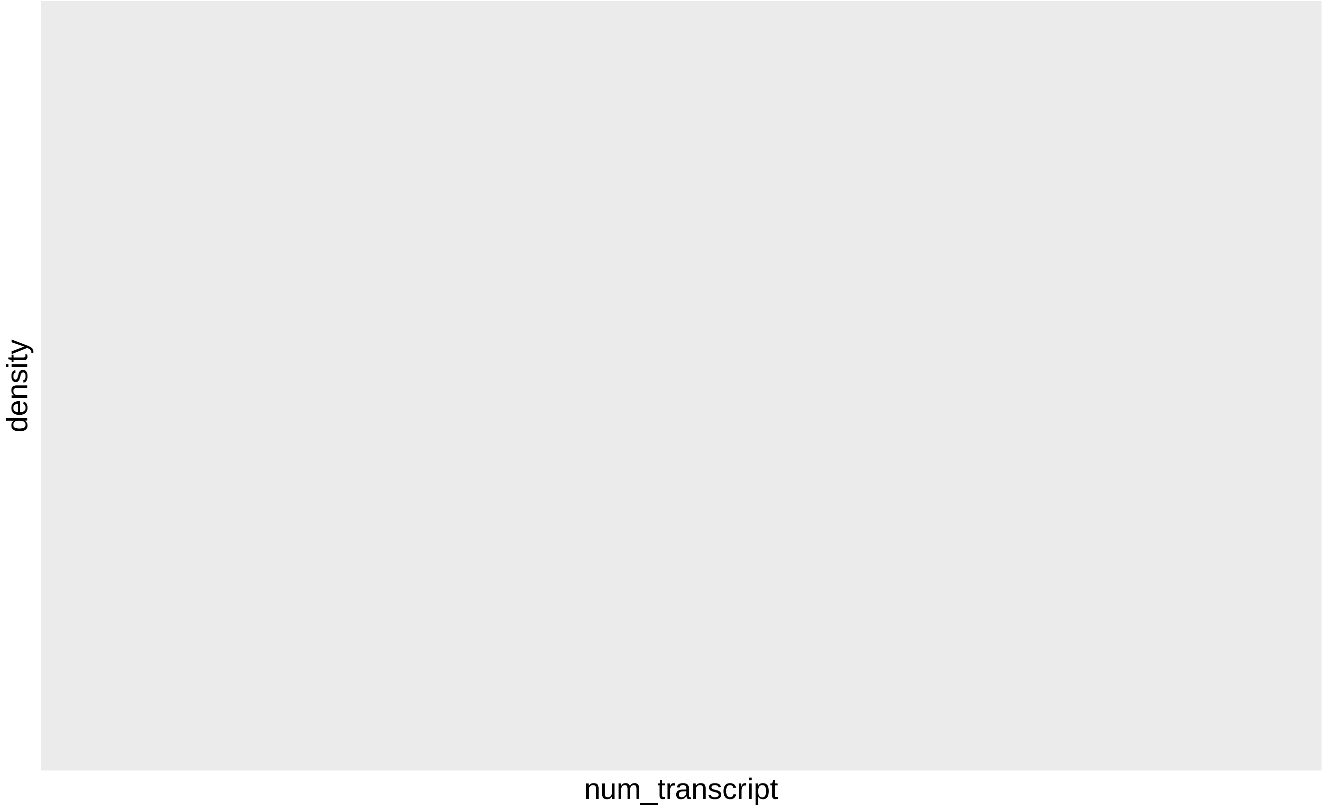

GCF_000271645.1_Treme1
EpT

Novel Genes

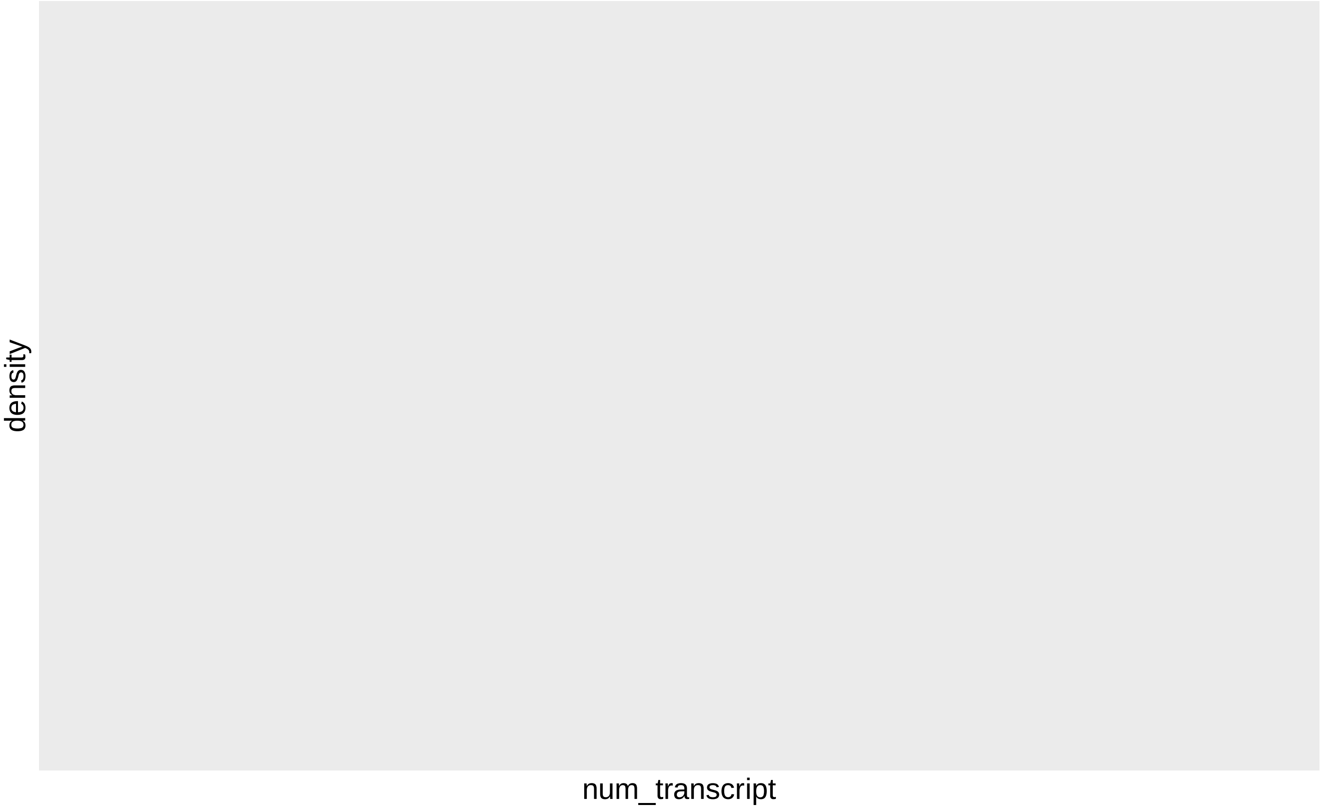

GCF_000275845.1_Dichomitus_squalens_v1.0
EpT

Novel Genes

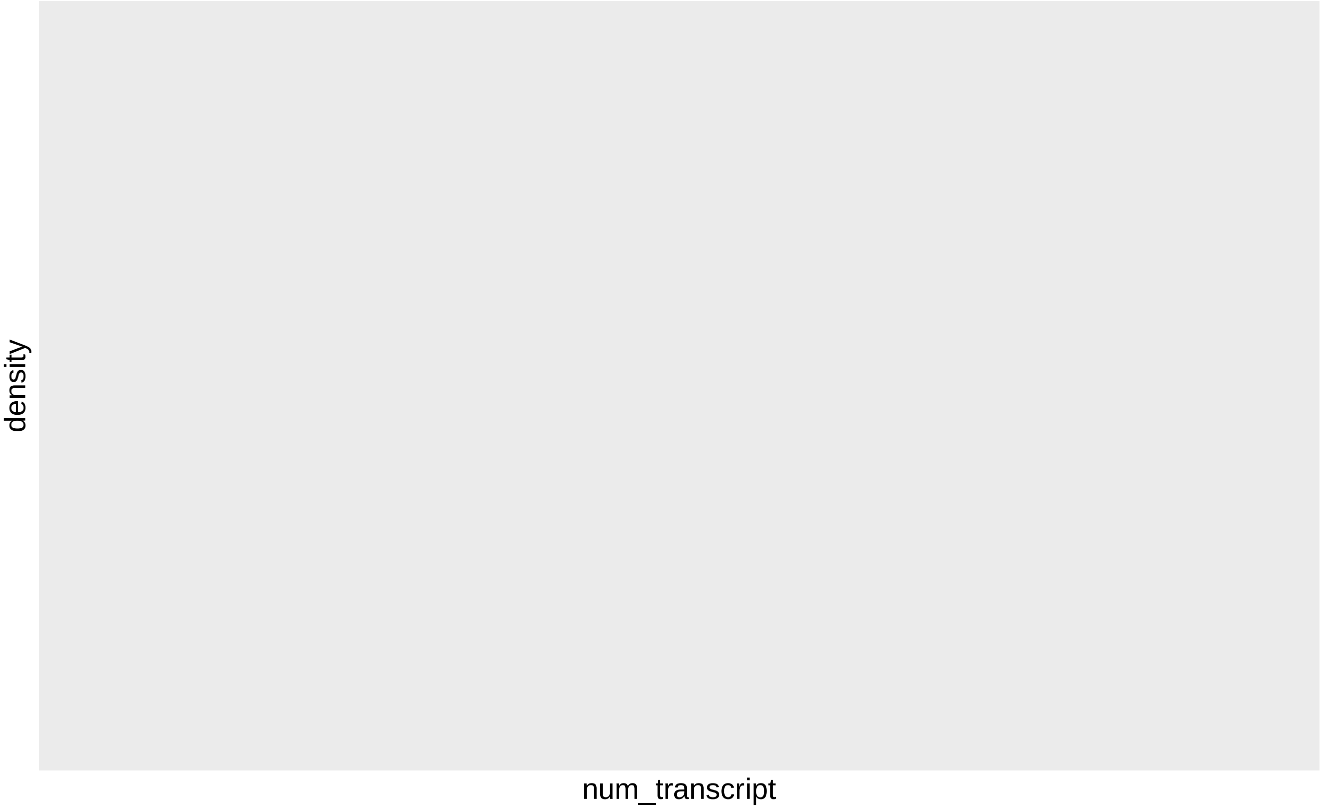

GCF_000281105.1_Coni_apol_CBS100218_V1
EpT

Novel Genes

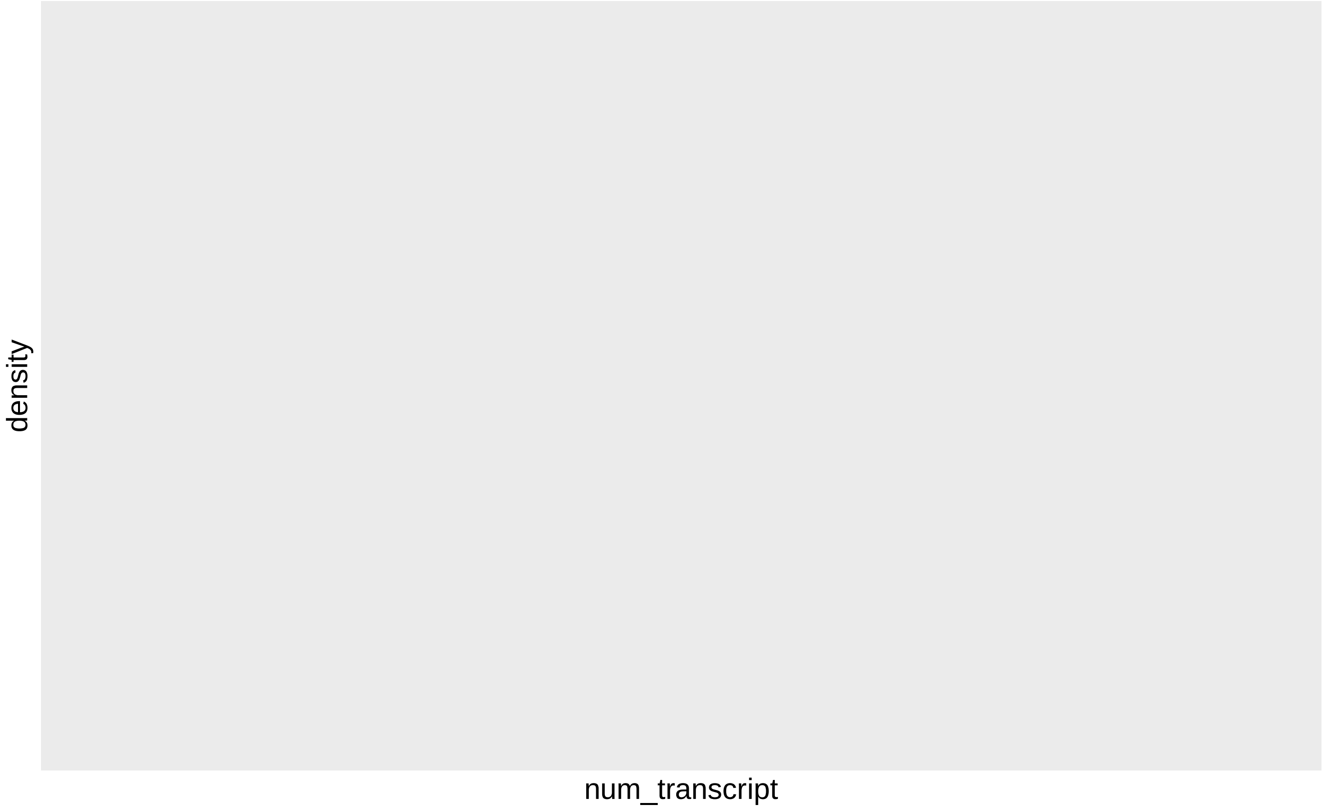

GCF_000300595.1_Phanerochaete_carnosa_HHB−10118−Sp_v1.0
EpT

Novel Genes

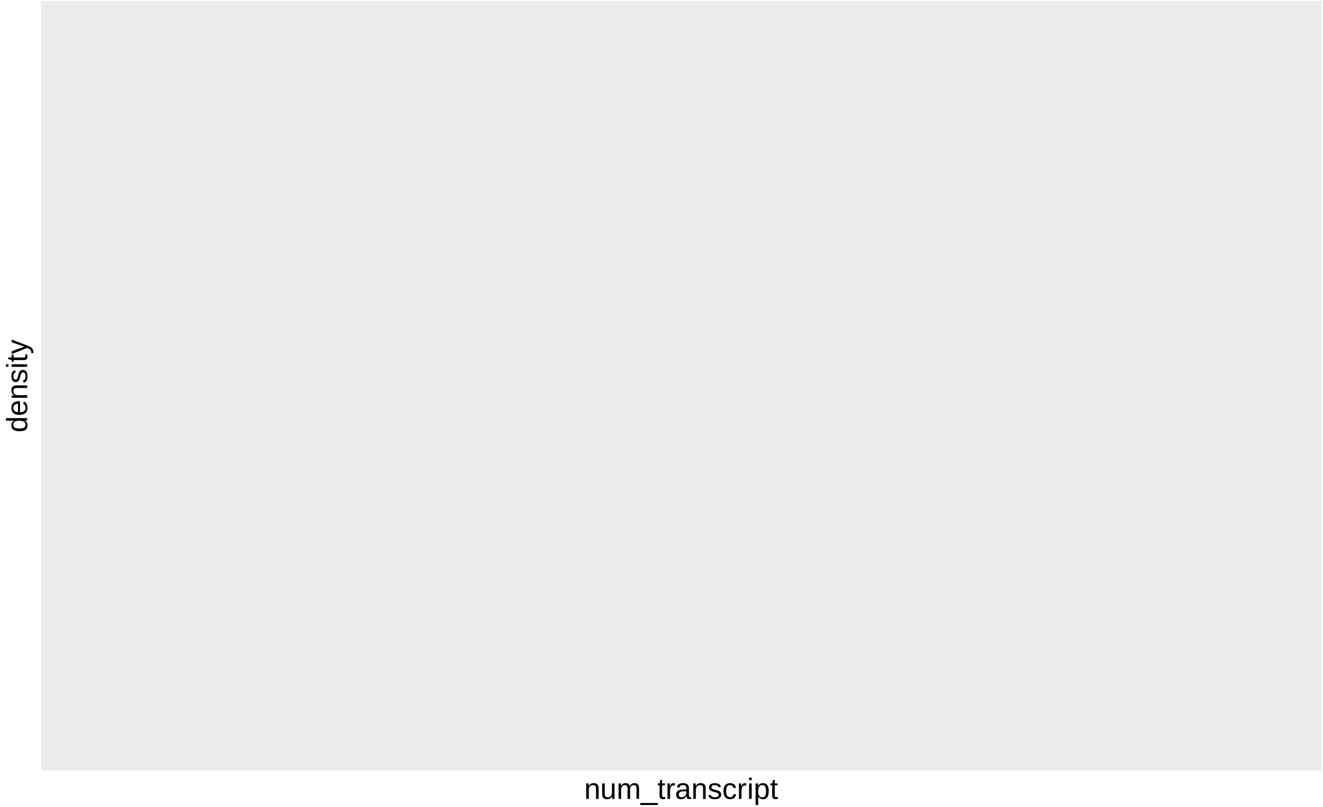

GCF_000313525.1_ASM31352v1
EpT

Novel Genes

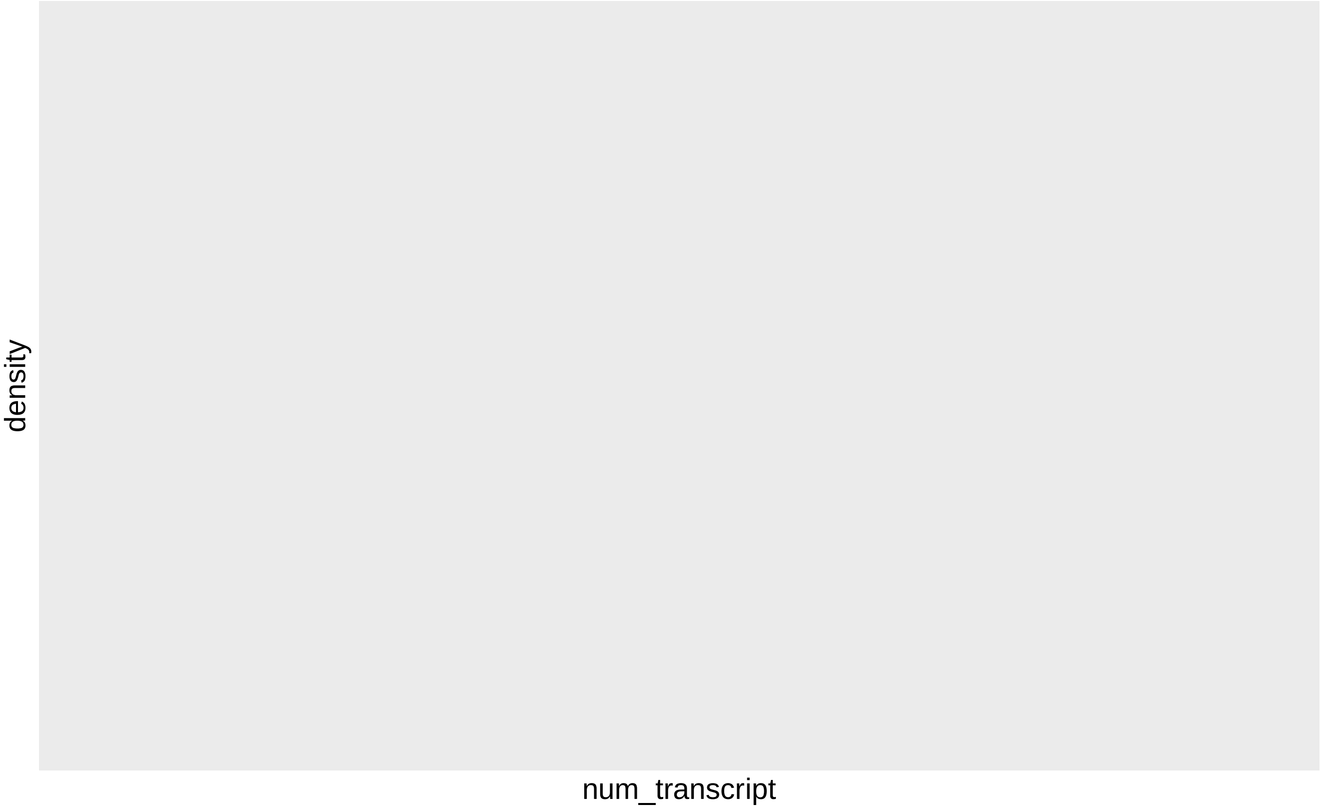

GCF_000320585.1_Heterobasidion_irregulare_v2.0
EpT

Novel Genes

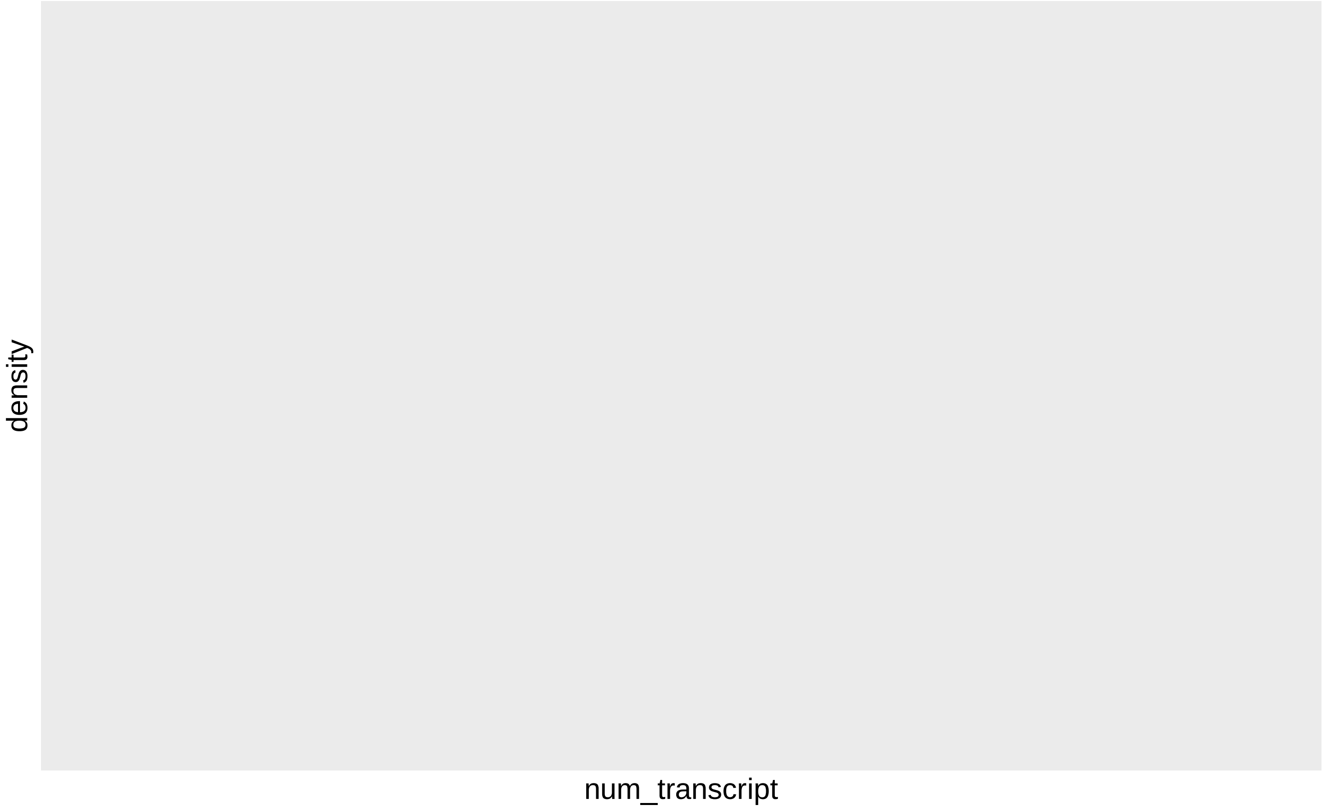

GCF_000328475.2_Umaydis521_2.0
EpT

Novel Genes

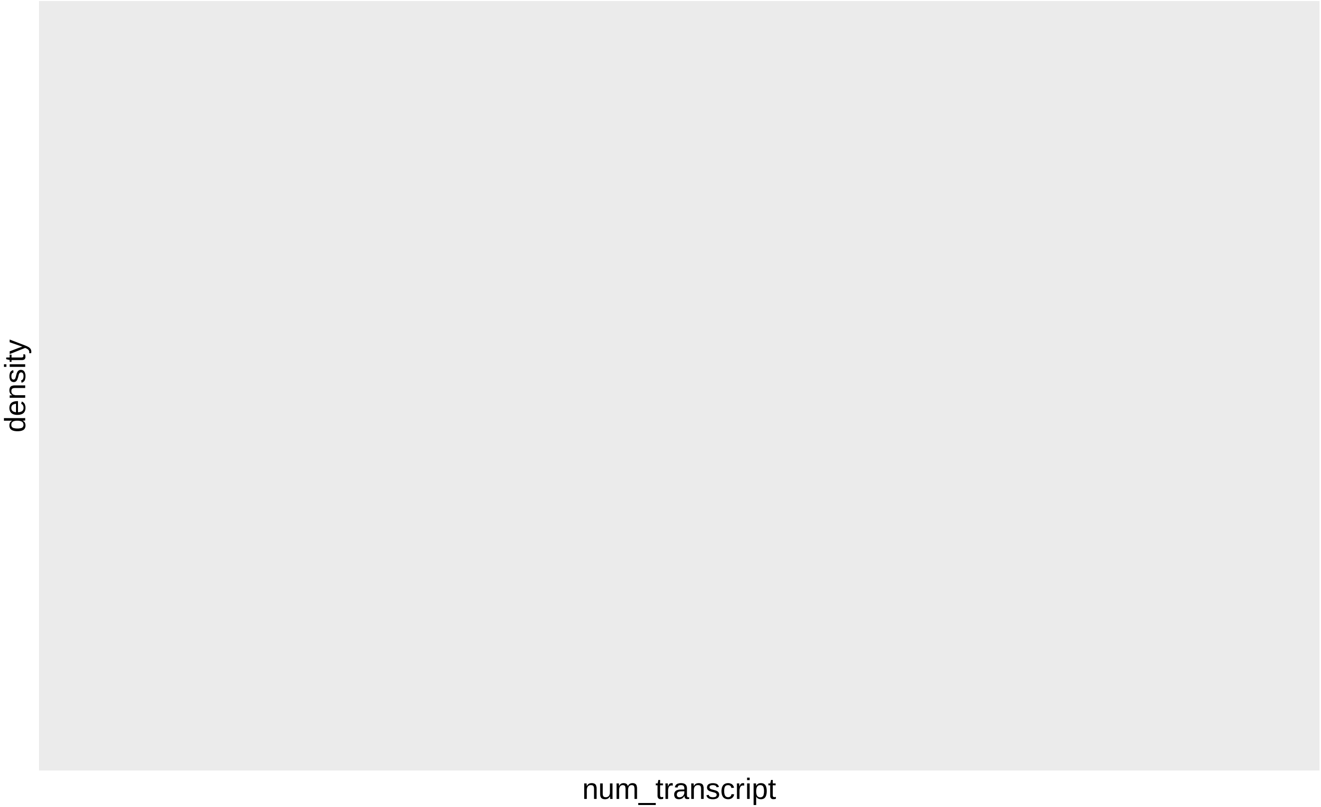

GCF_000344685.1_Glotr1_1
EpT

Novel Genes

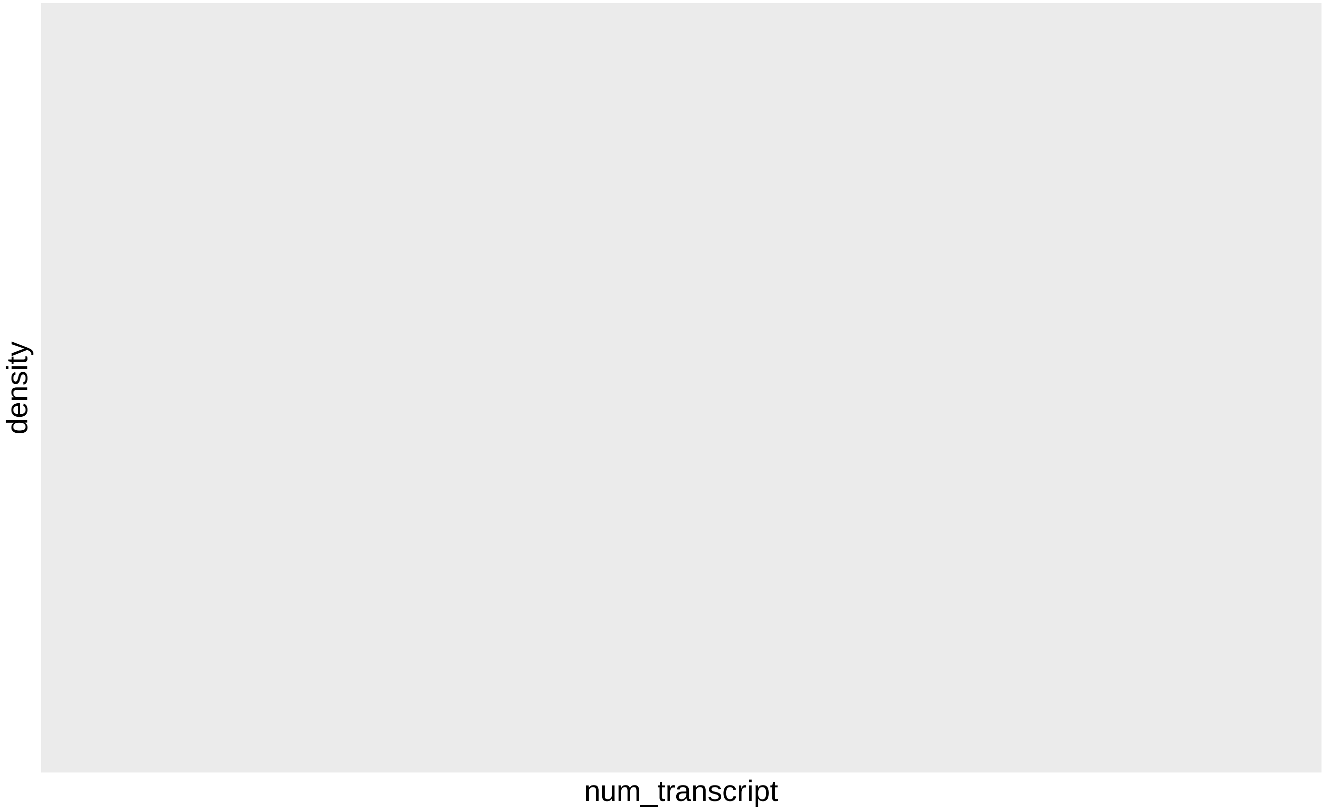

GCF_000354255.1_CocheC4_1
EpT

Novel Genes

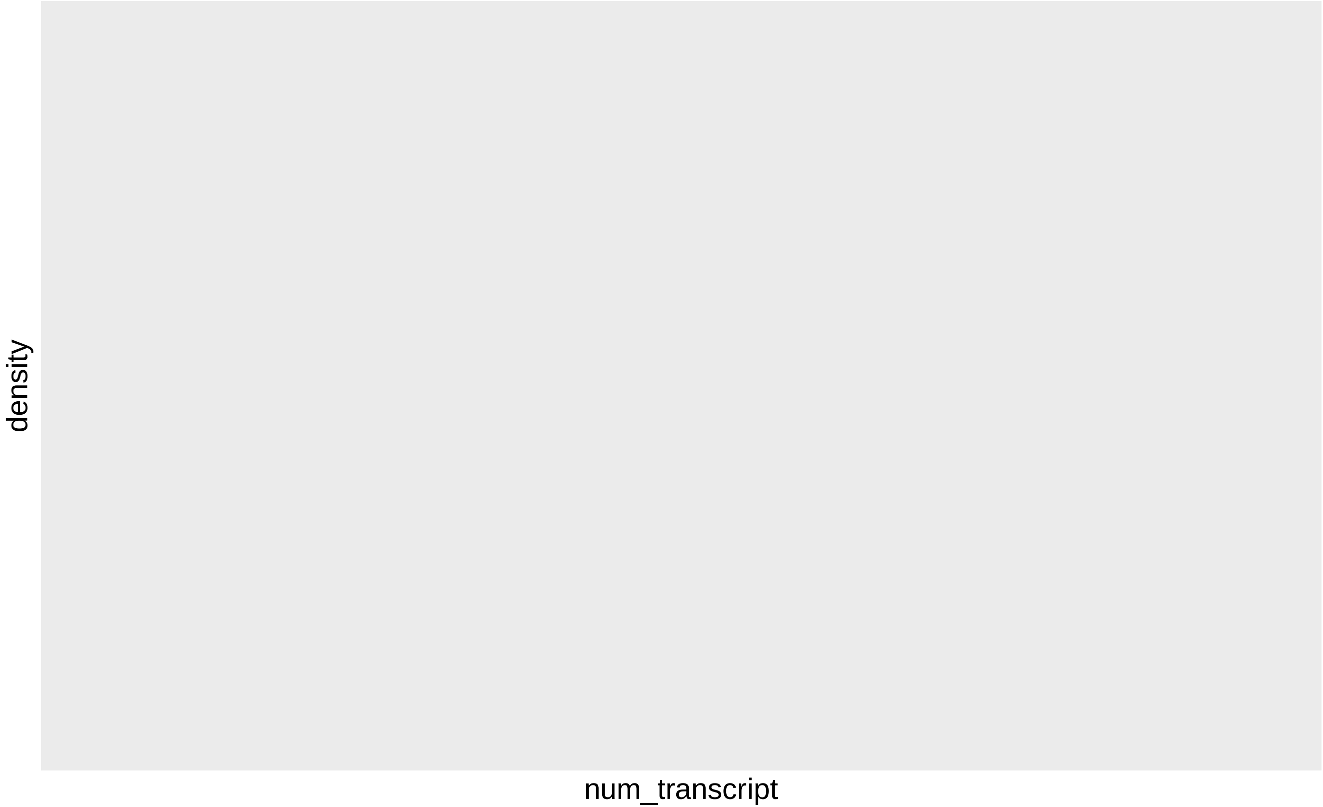

GCF_000400465.1_Wallemia_ichthyophaga_version_1.0
EpT

Novel Genes

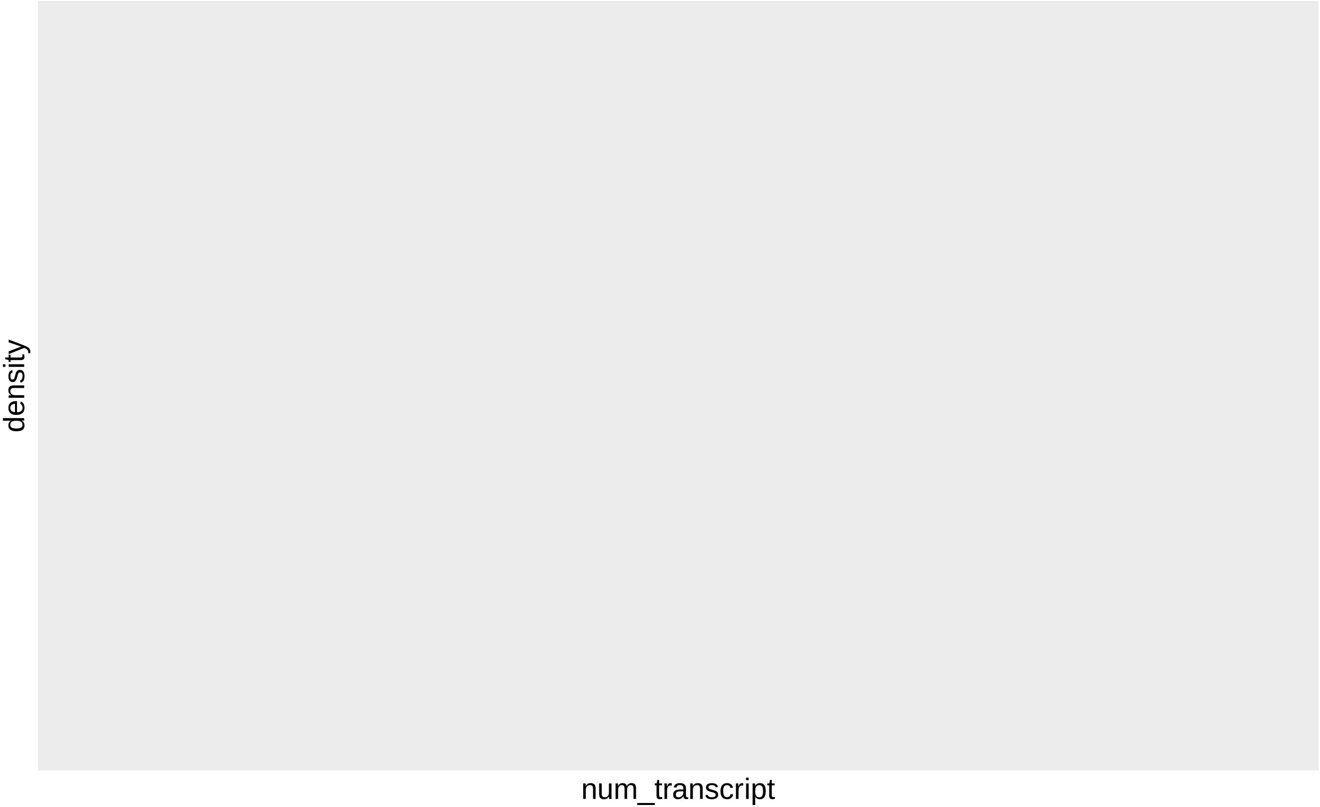

GCF_000409485.1_GLAREA
EpT

Novel Genes

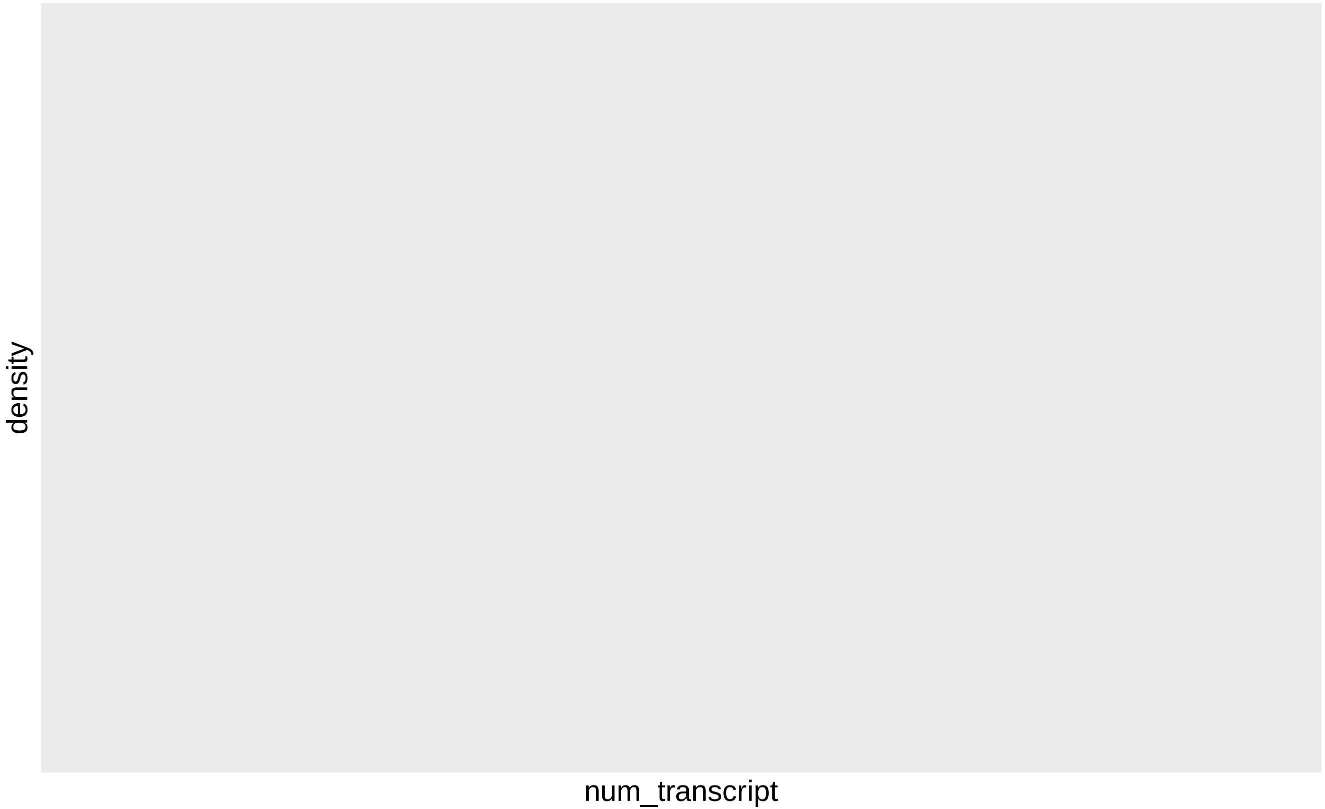

GCF_000497045.1_PSEUBRA1
EpT

Novel Genes

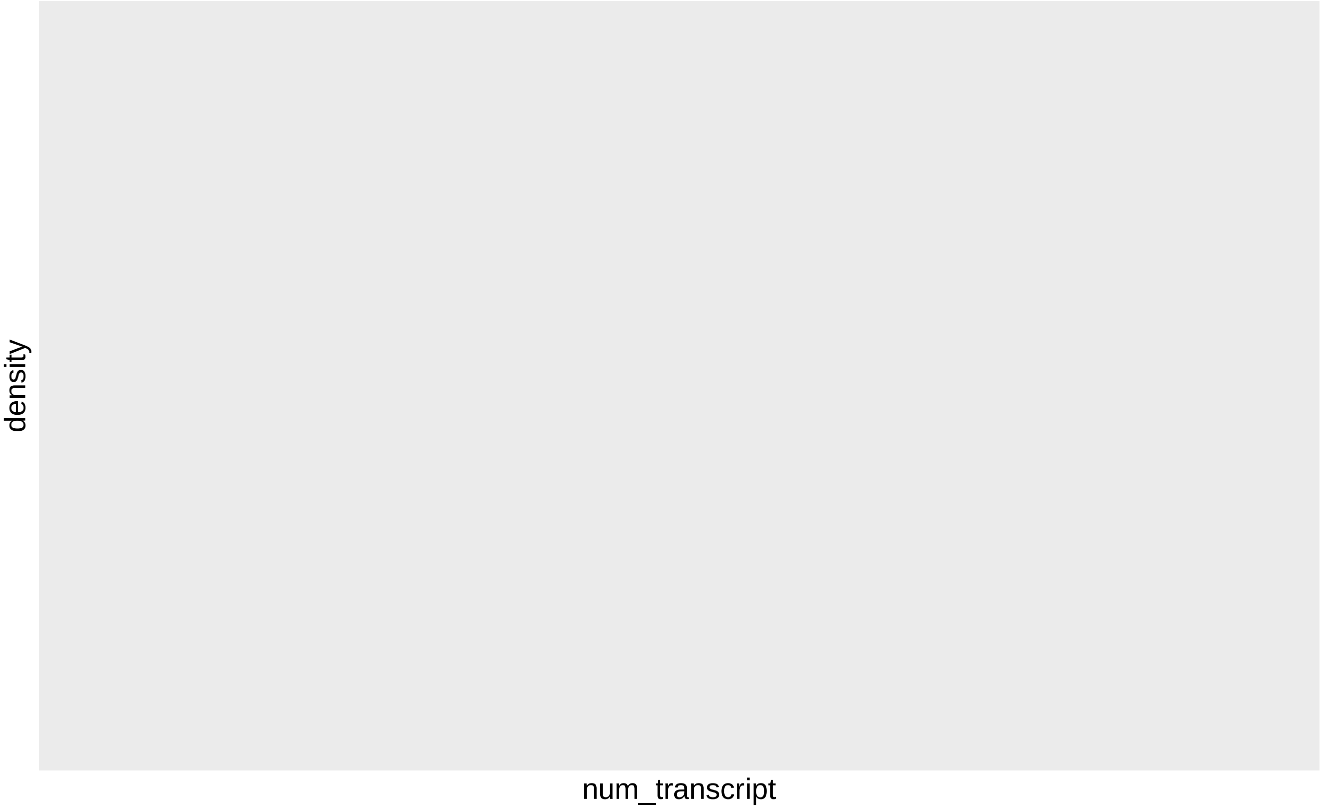

GCF_000512605.1_Cryp_pinu_CBS10737_V1
EpT

Novel Genes

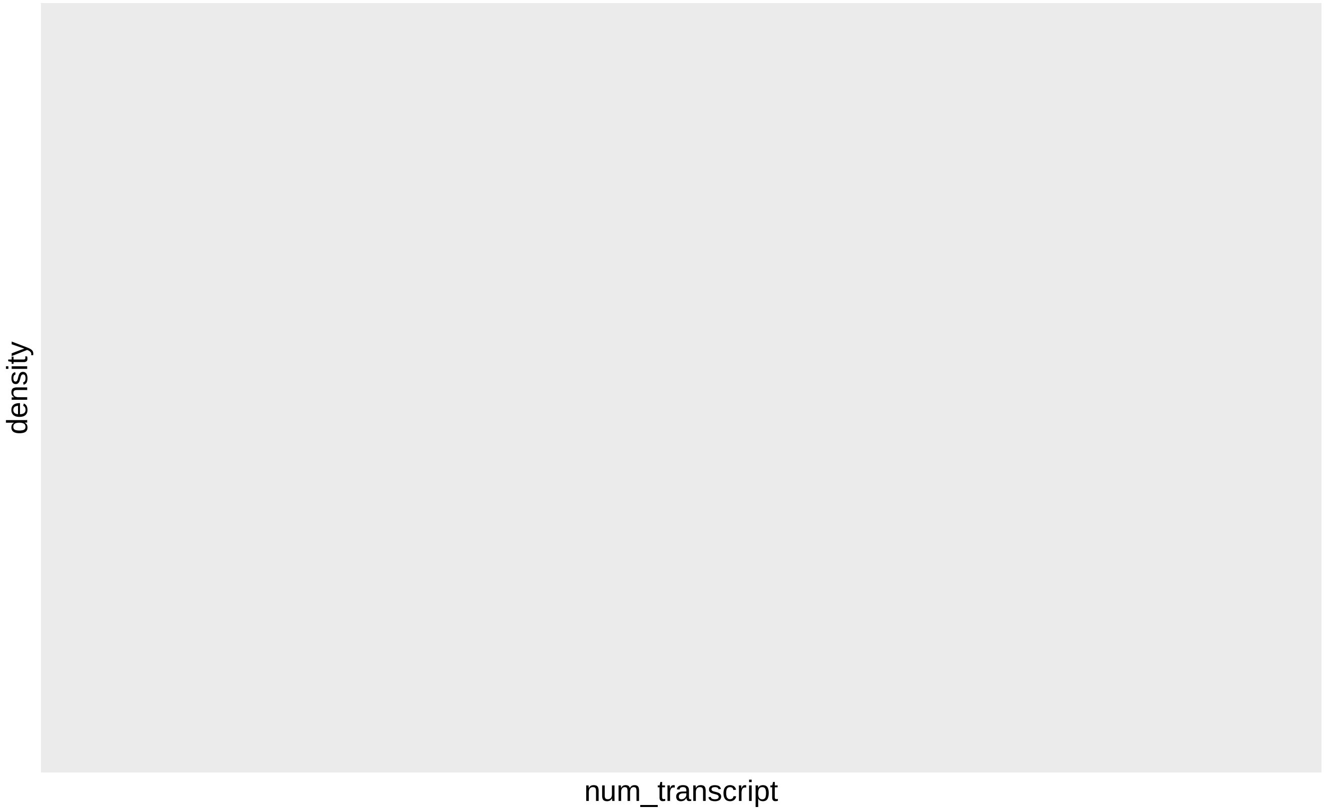

GCF_000516985.1_PFICI
EpT

Novel Genes

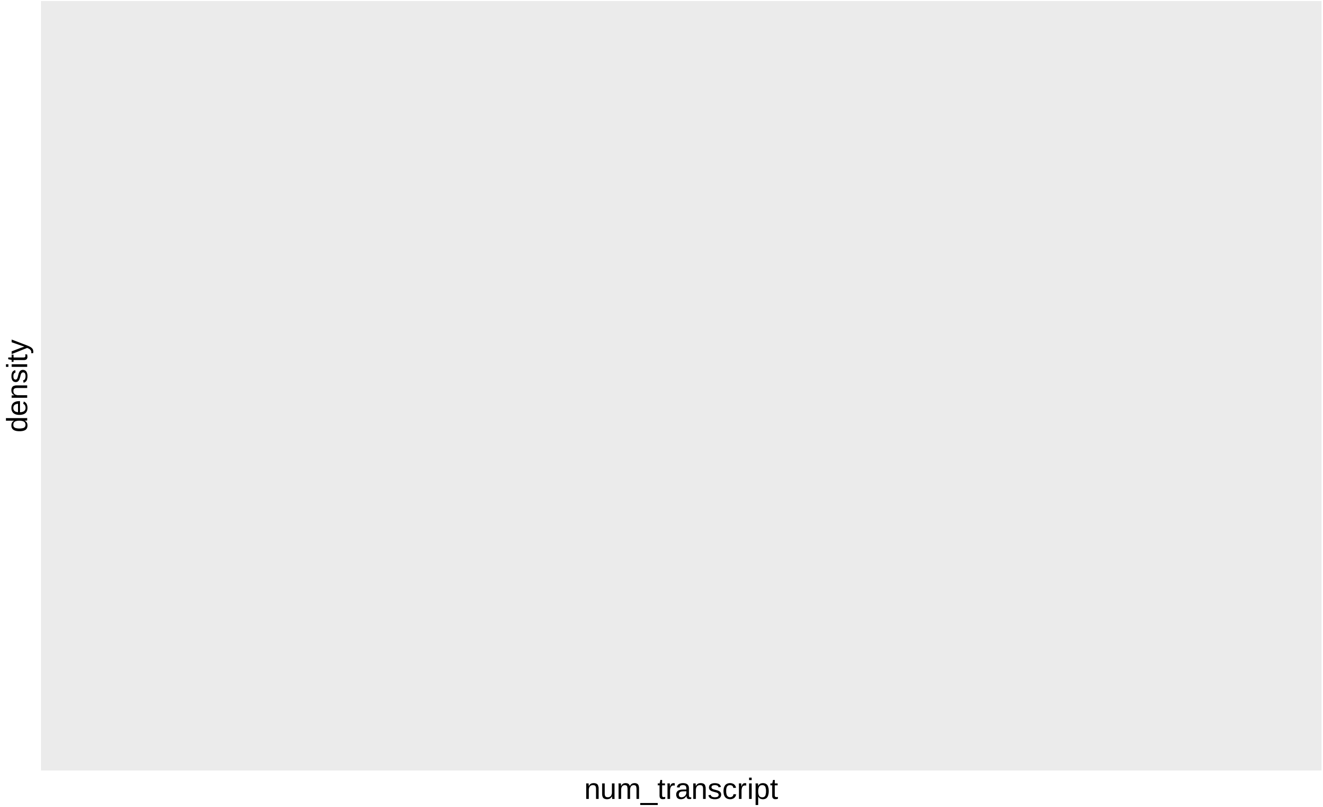

GCF_000576695.1_AUH_PRJEB4427_v1
EpT

Novel Genes

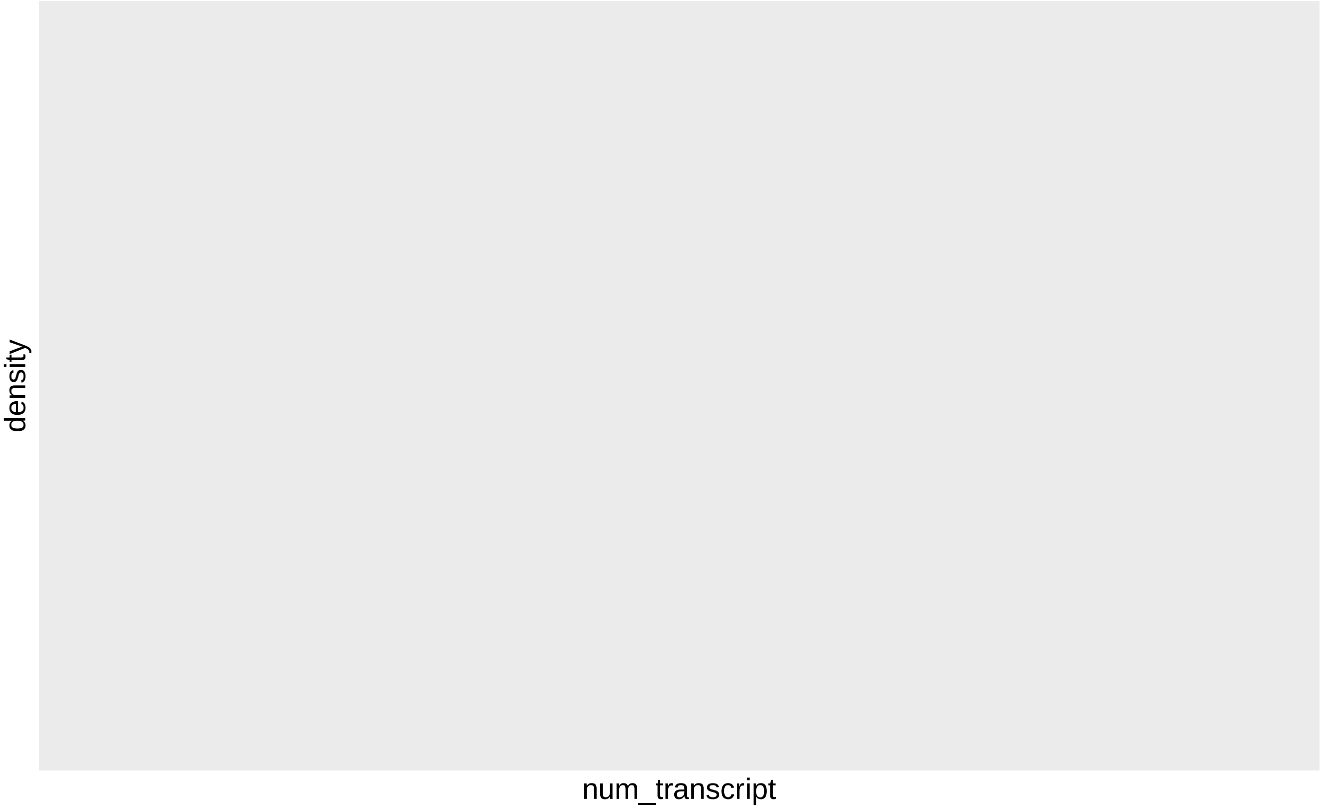

GCF_000709125.1_Exop_aqua_CBS_119918_V1
EpT

Novel Genes

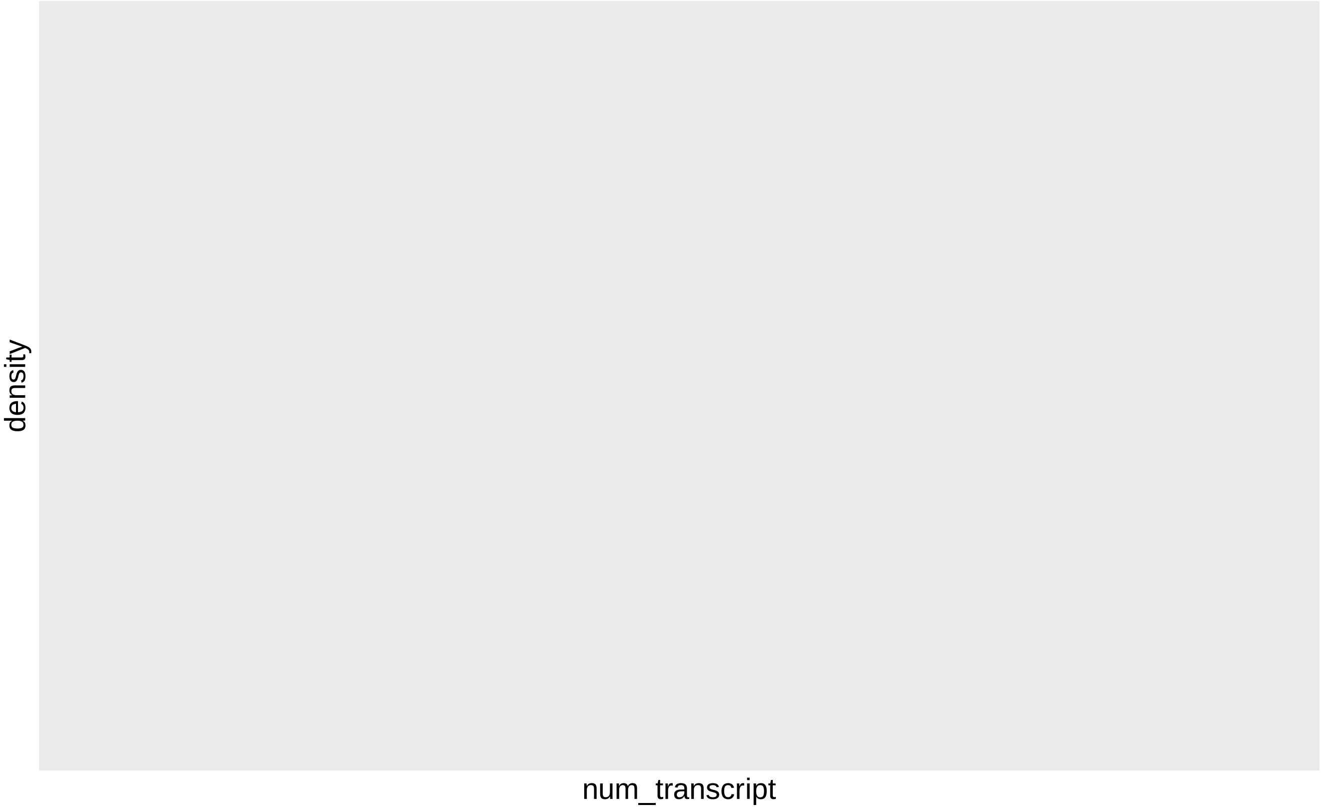

GCF_000835455.1_Fons_pedr_CBS_271_37_V1
EpT

Novel Genes

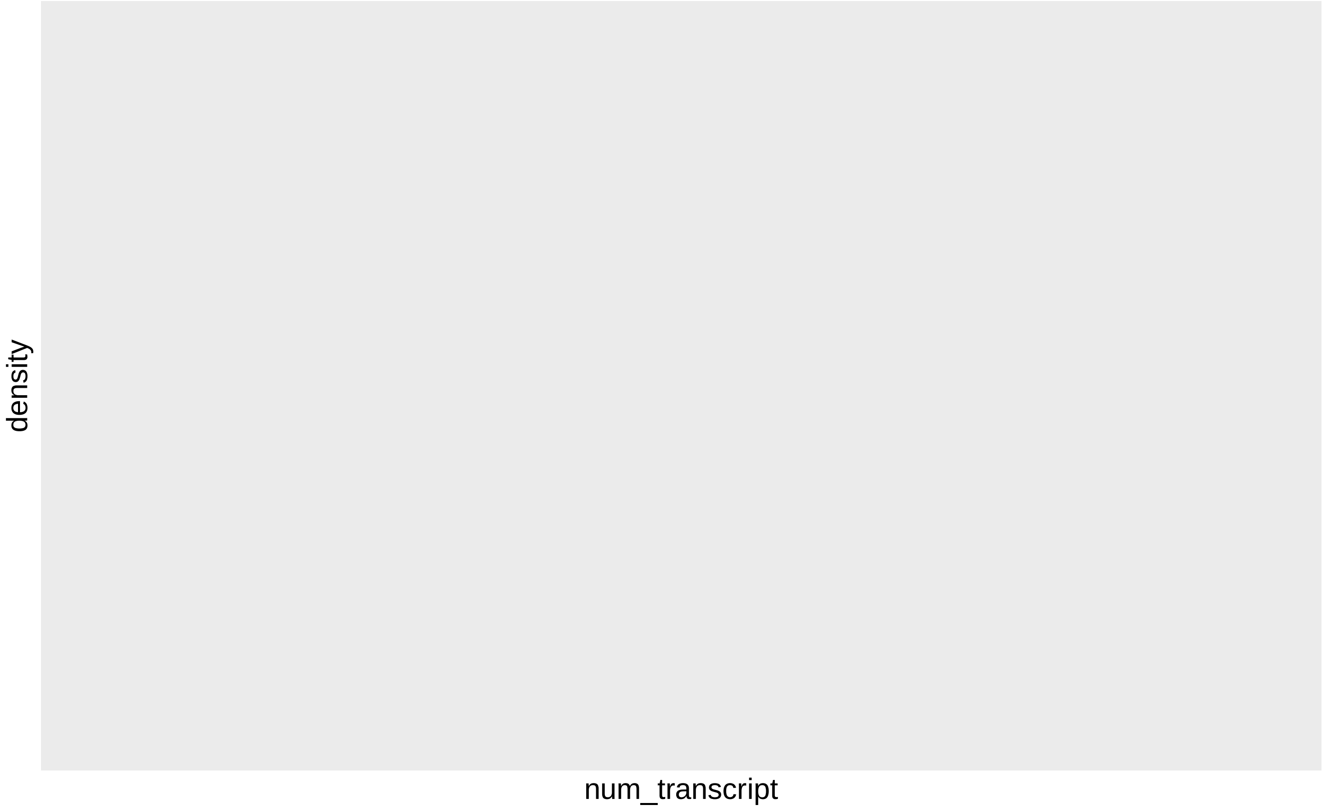

GCF_000835555.1_Rhin_mack_CBS_650_93_V1
EpT

Novel Genes

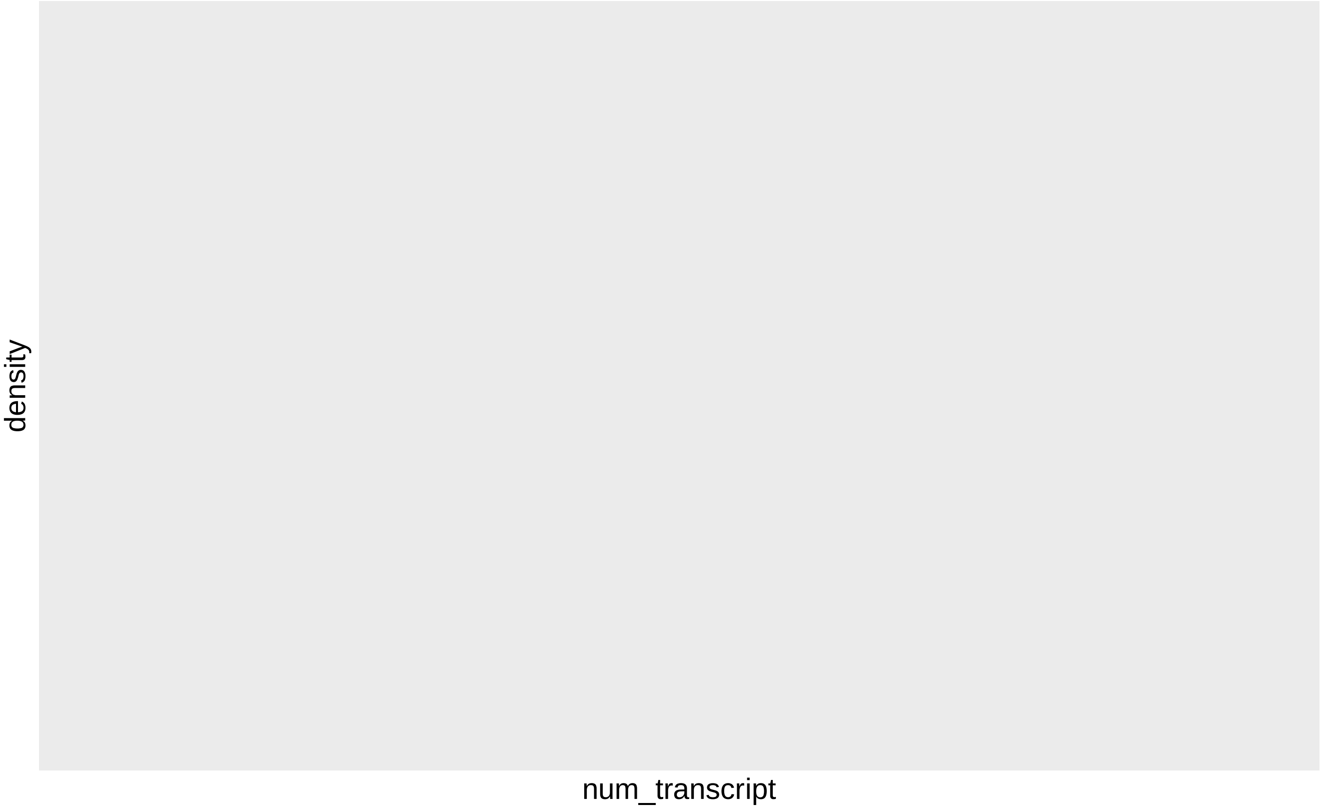

GCF_000836295.1_O_gall_CBS43764
EpT

Novel Genes

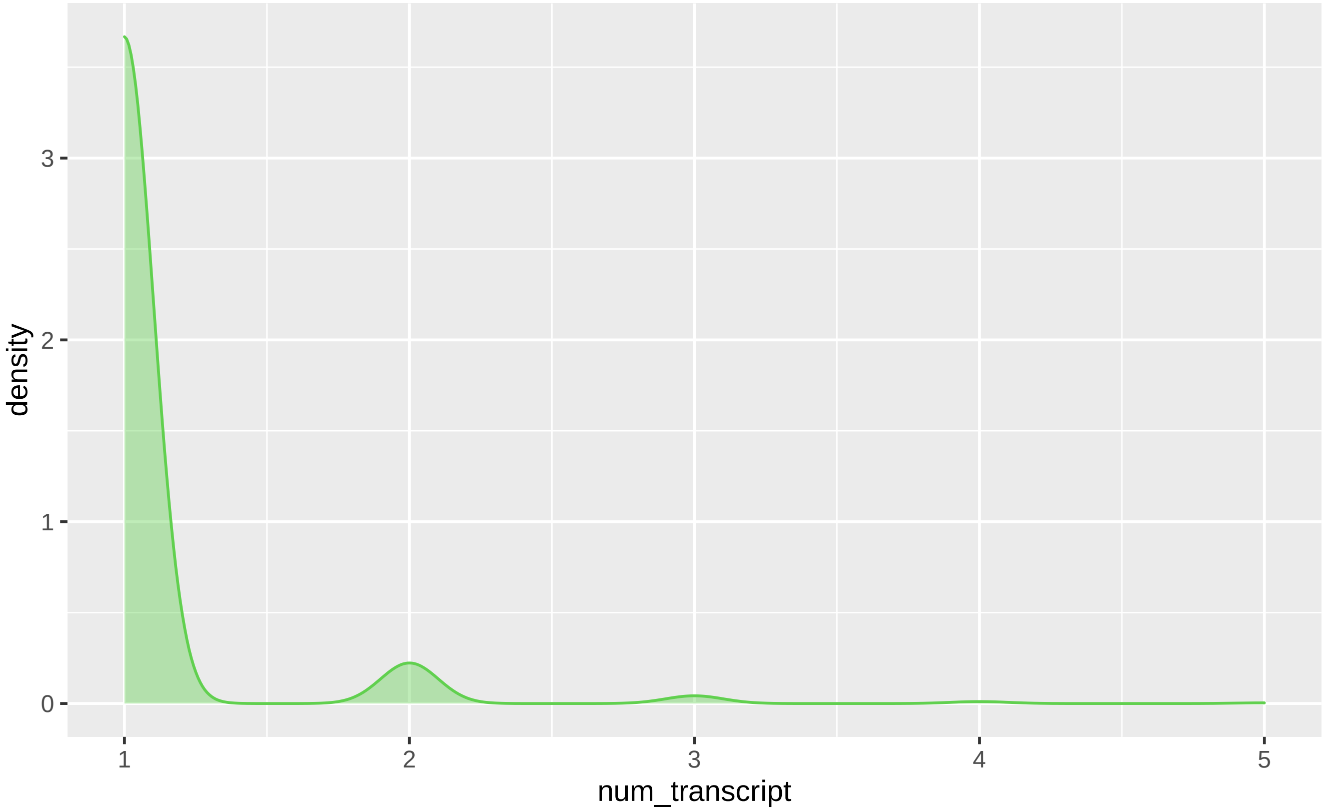

GCF_000938715.1_LALA0
EpT

Novel Genes

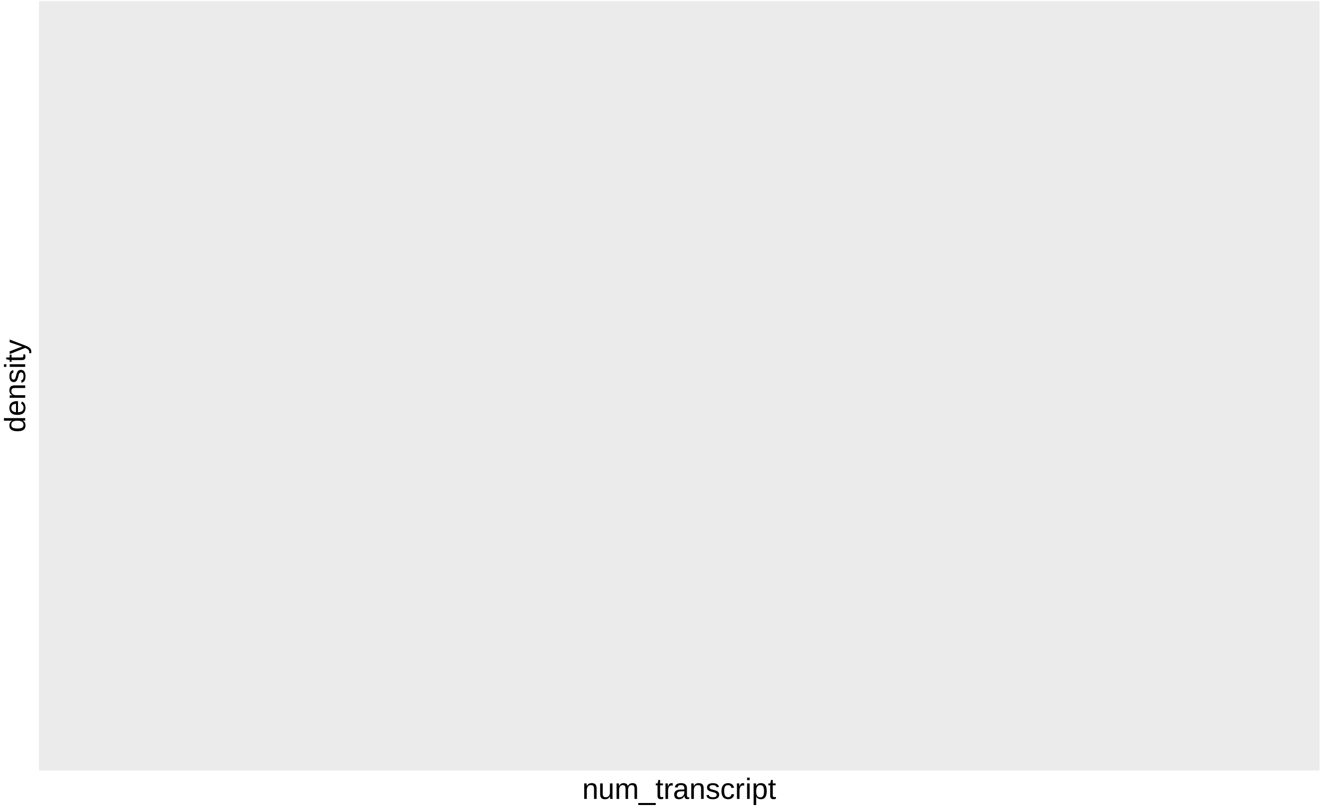

GCF_001027345.1_Triol1
EpT

Novel Genes

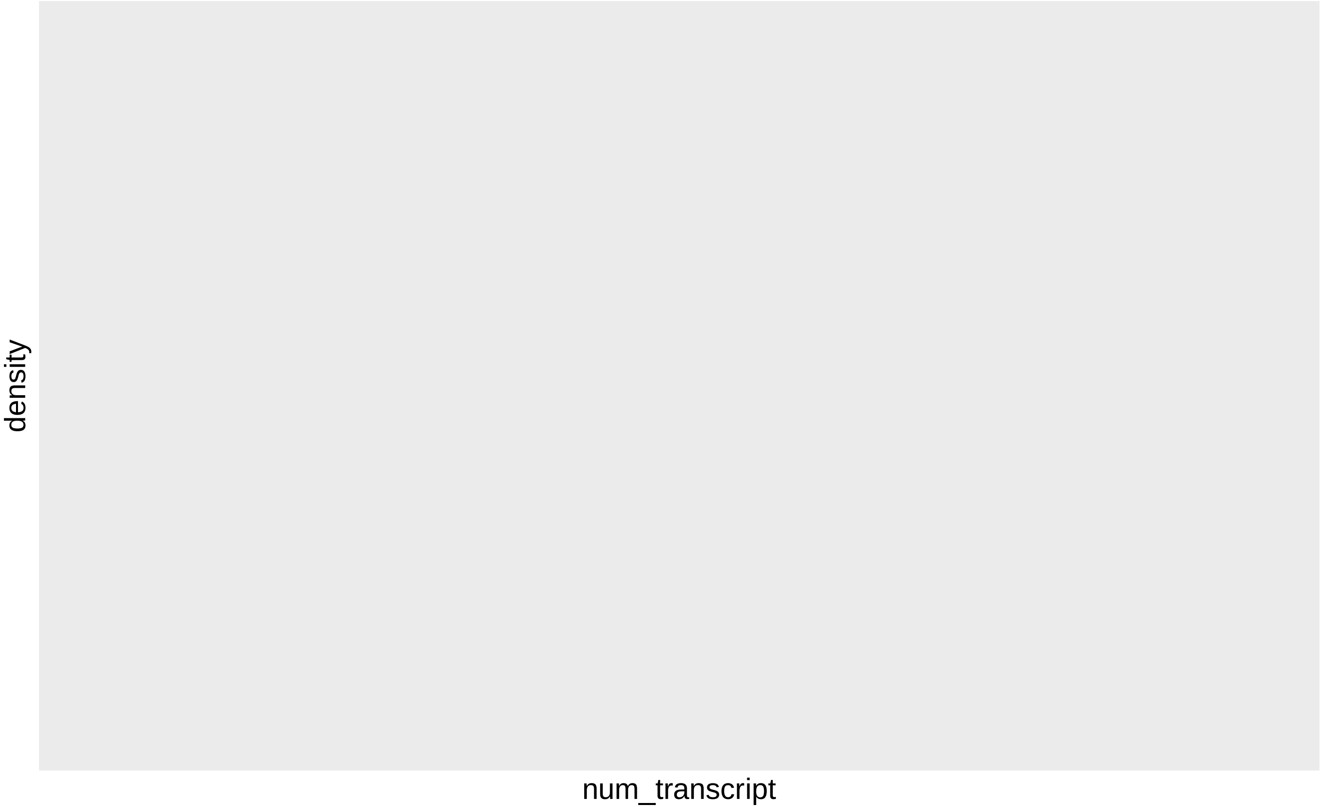

GCF_001278385.1_MalaPachy
EpT

Novel Genes

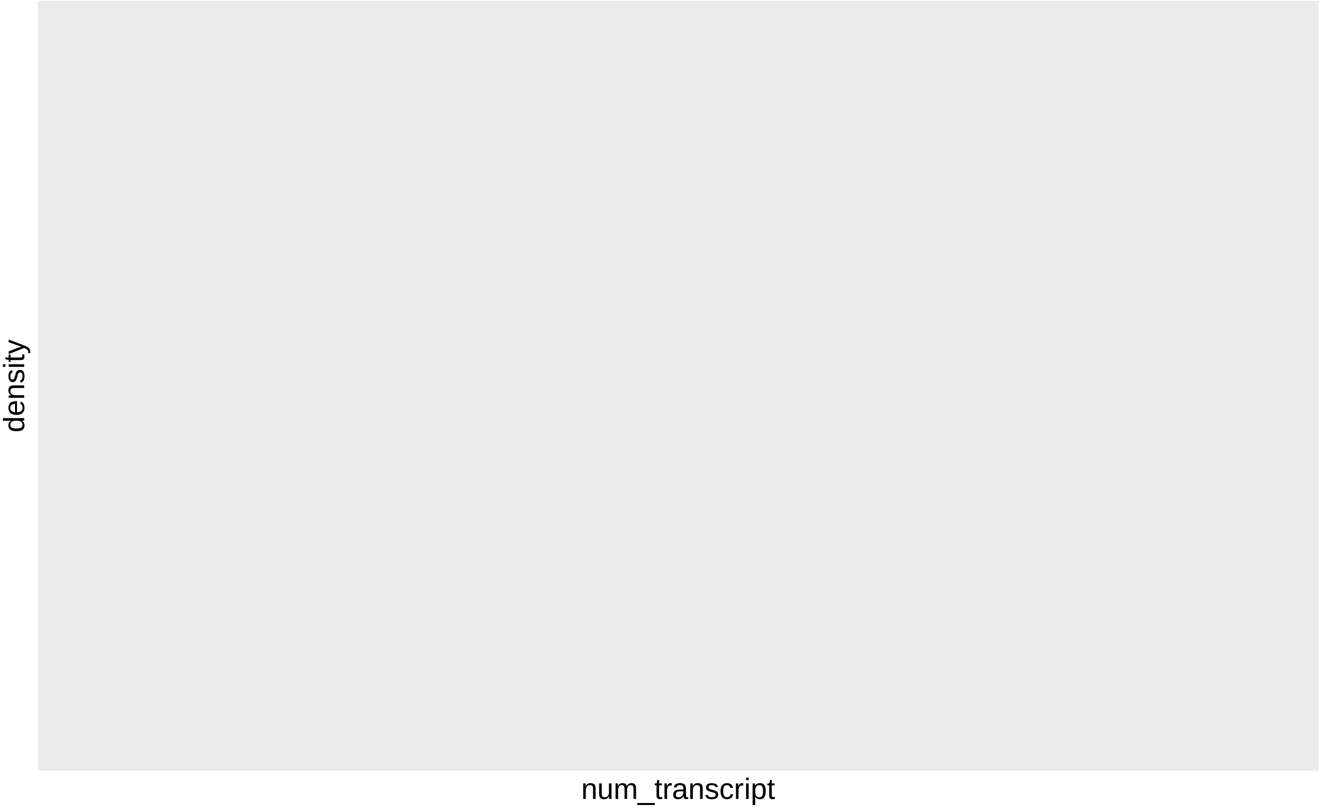

GCF_001329695.1_Rhoba1_1
EpT

Novel Genes

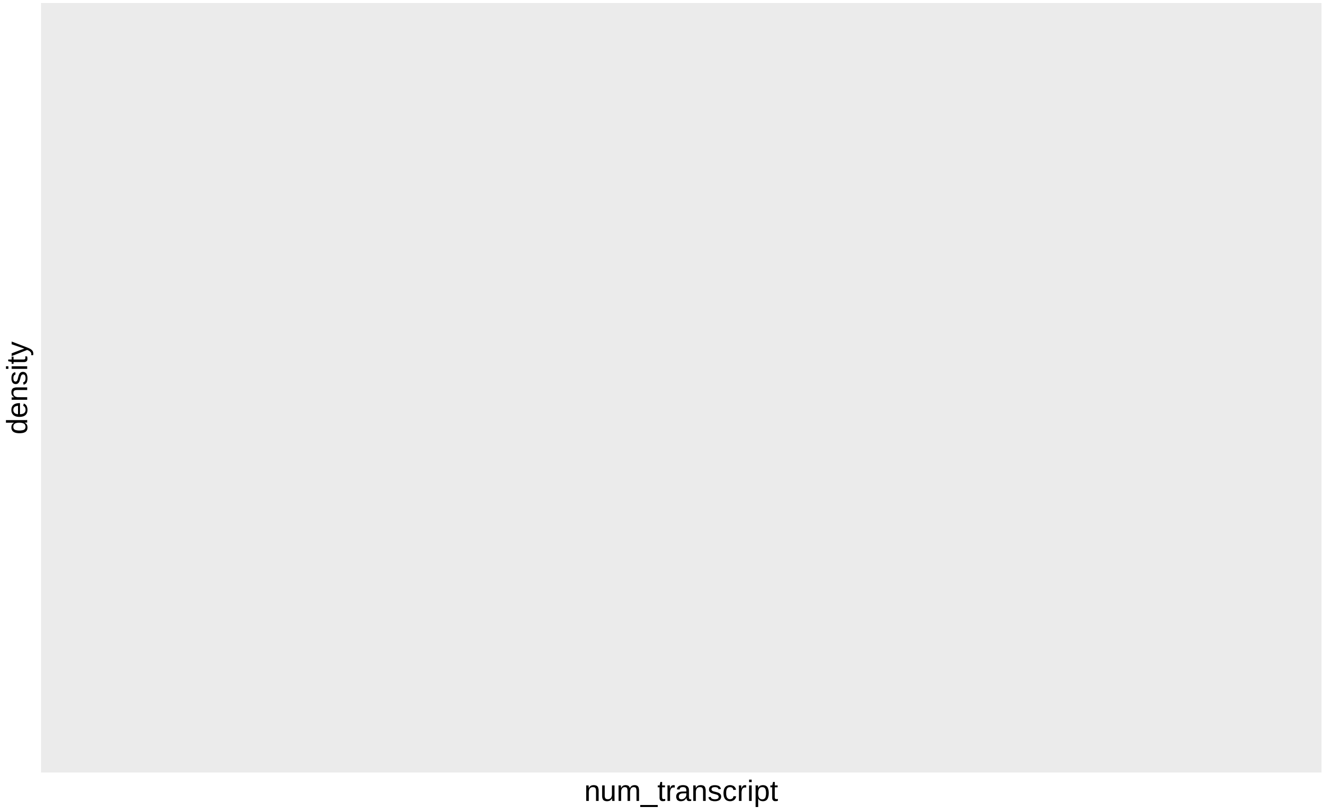

GCF_001477535.1_Pneu_jiro_RU7_V2
EpT

Novel Genes

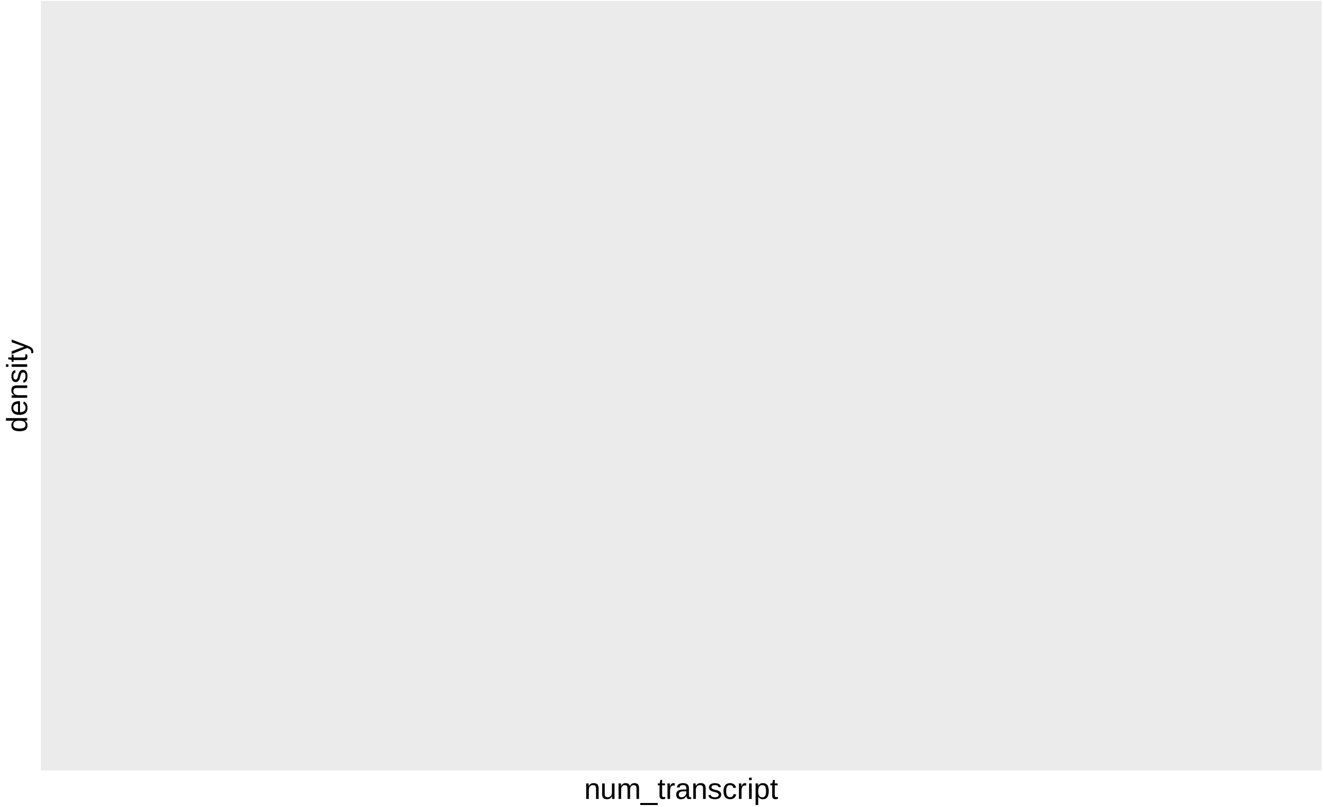

GCF_001619985.1_Xylona_heveae_TC161_v1.0
EpT

Novel Genes

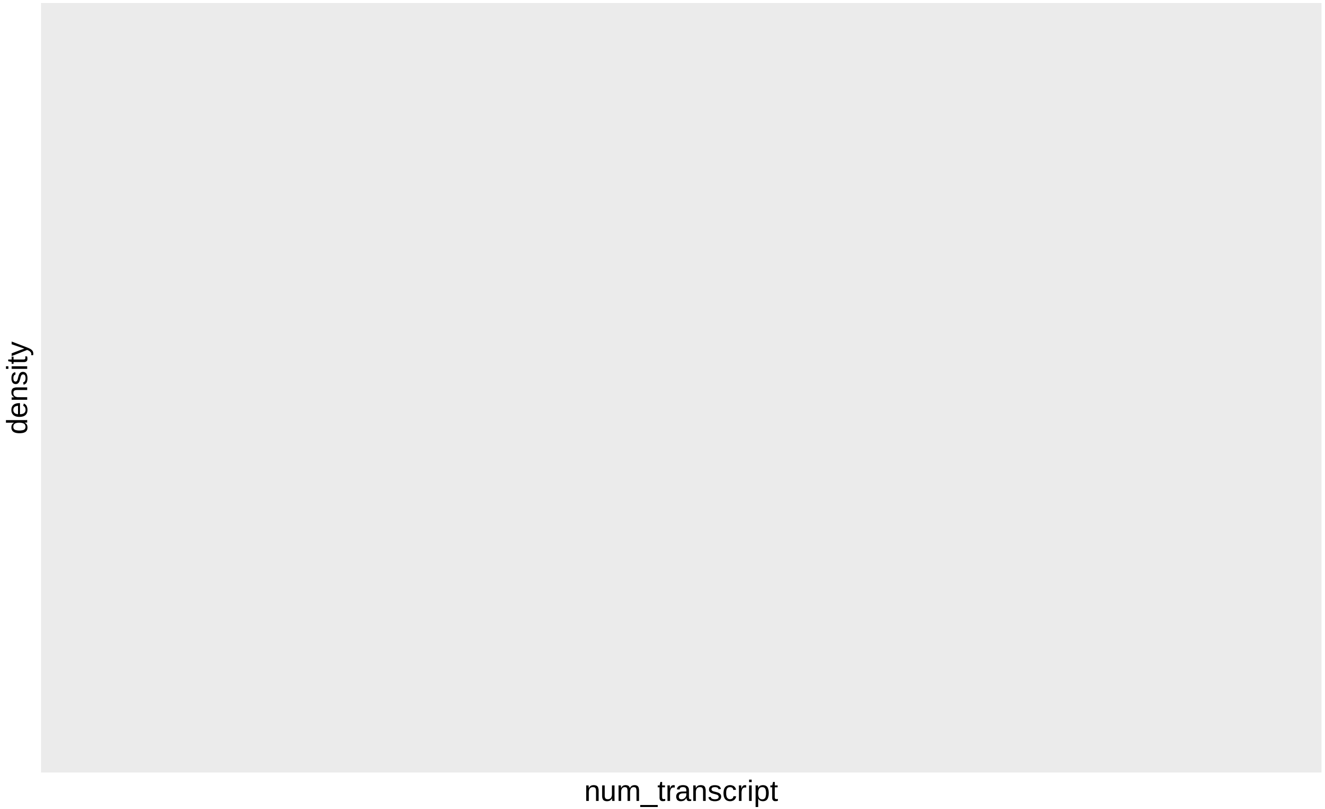

GCF_001636725.1_ISF_1.0
EpT

Novel Genes

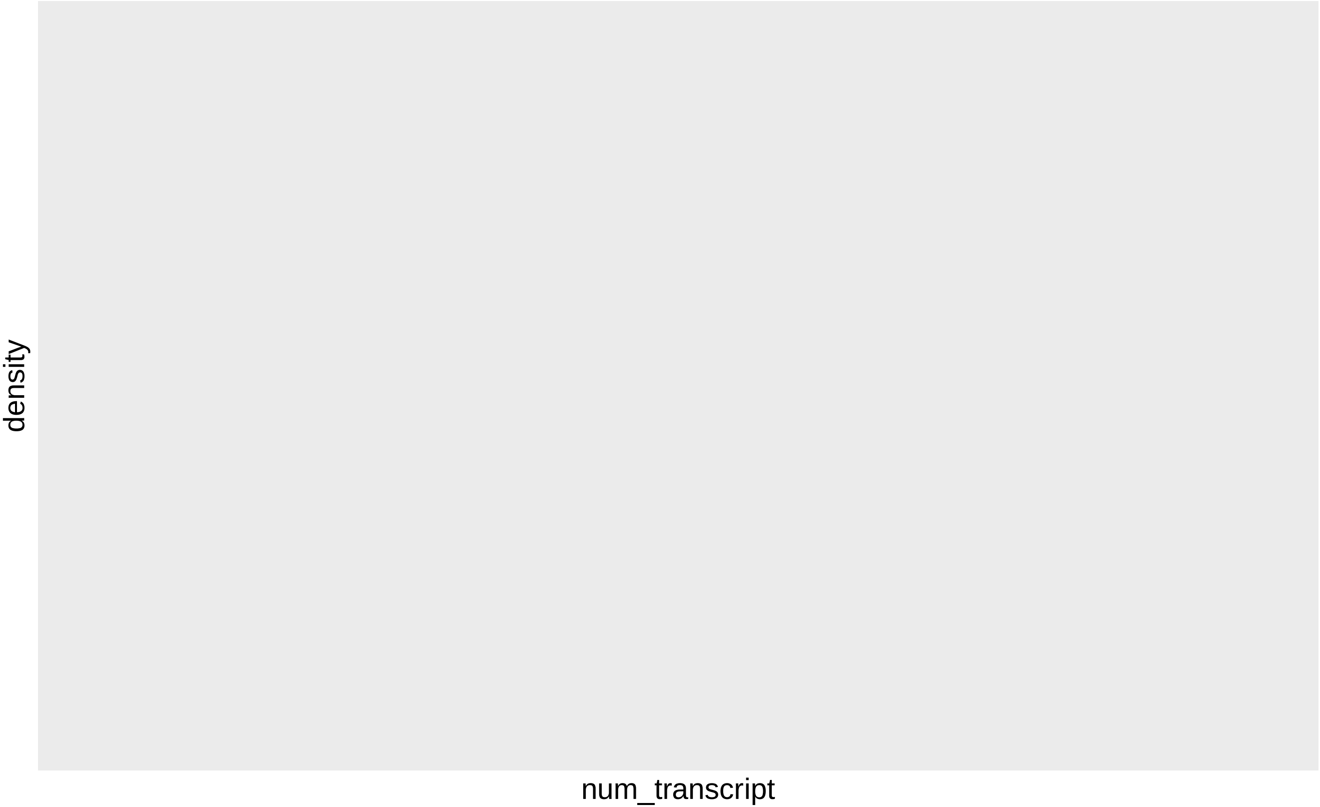

GCF_001638985.1_Phybl2
EpT

Novel Genes

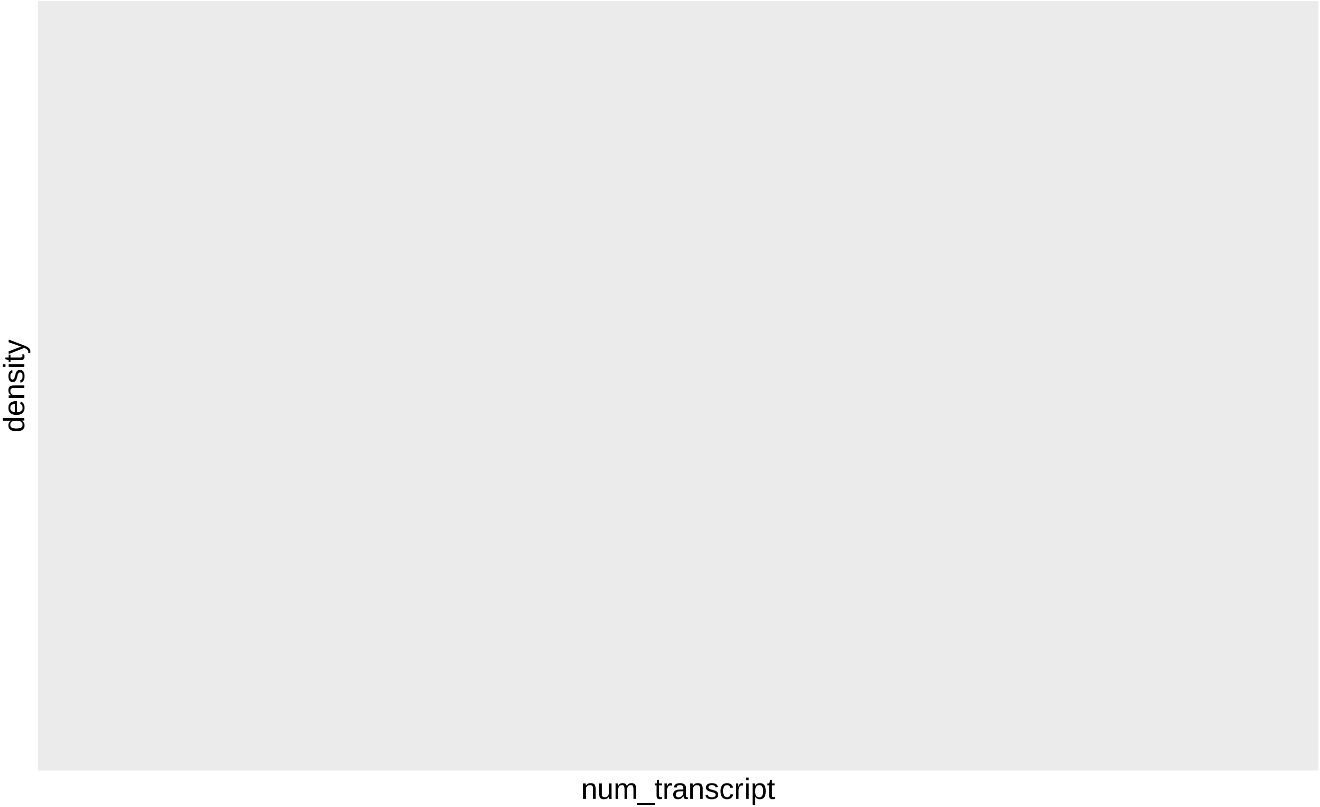

GCF_001661235.1_Picme2
EpT

Novel Genes

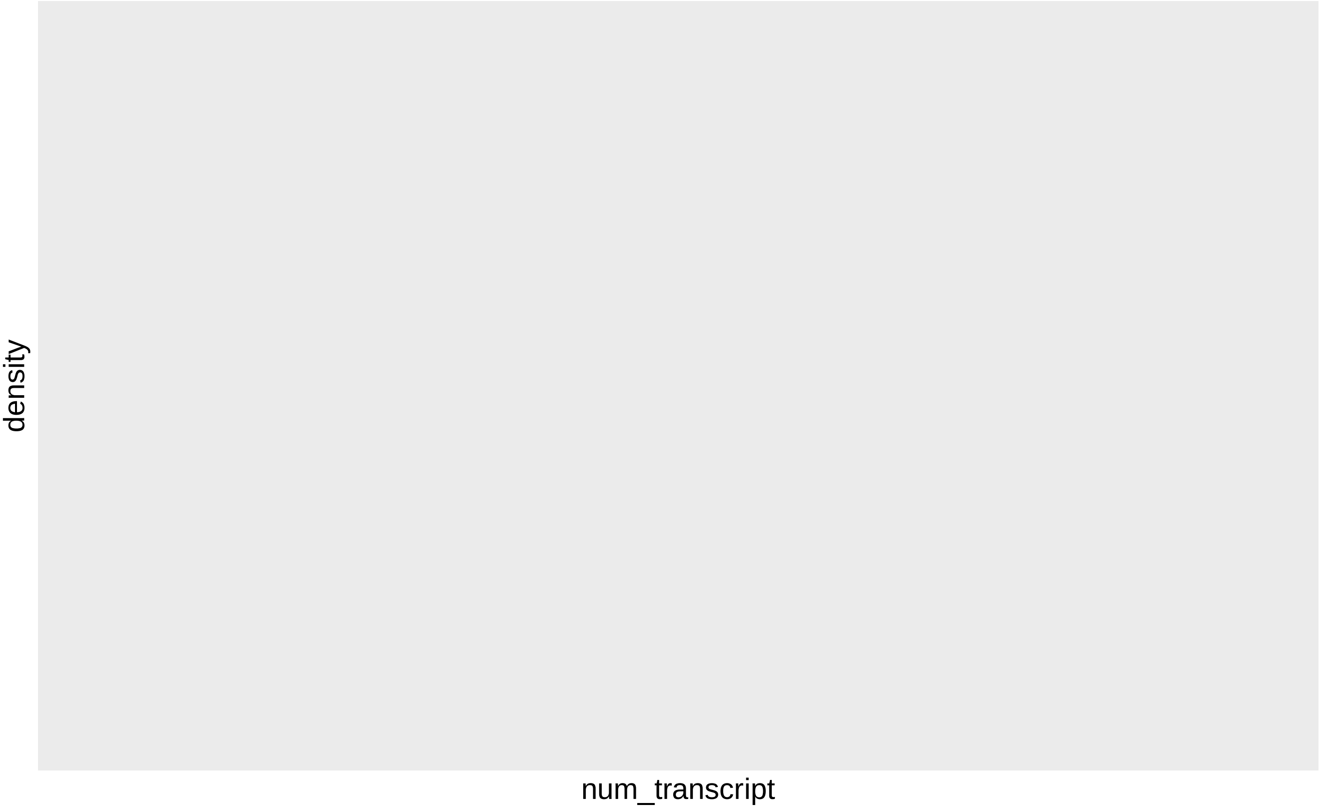

GCF_001661335.1_Babin1
EpT

Novel Genes

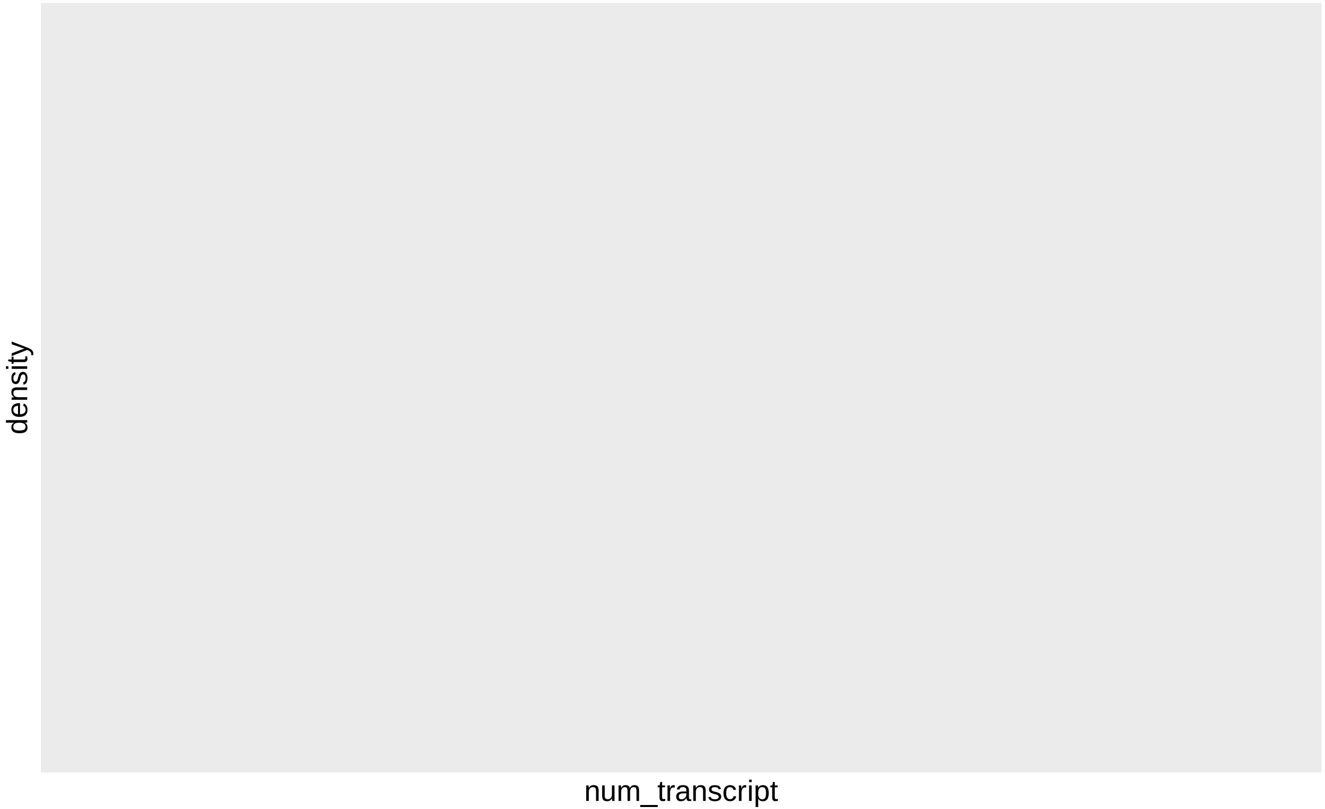

GCF_001661345.1_Ascru1
EpT

Novel Genes

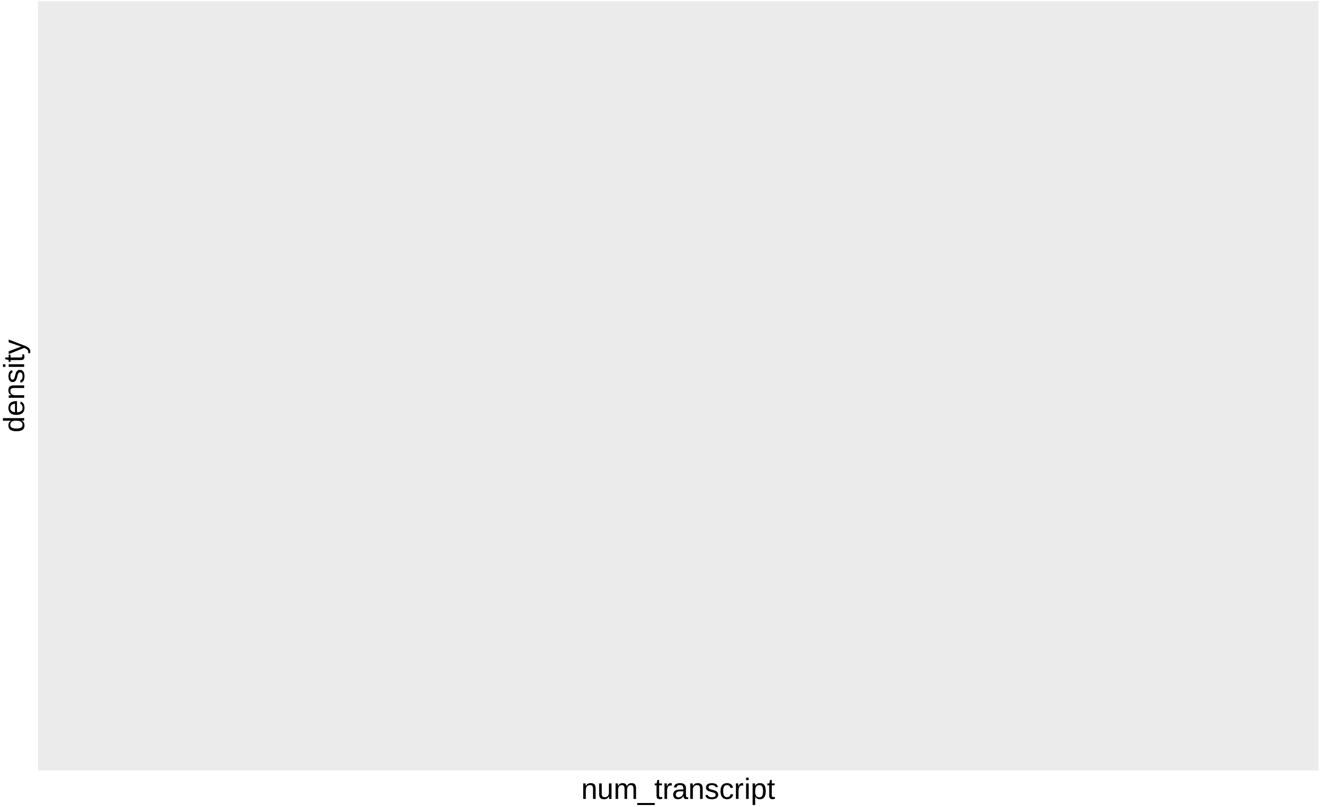

GCF_001661405.1_Cybja1
EpT

Novel Genes

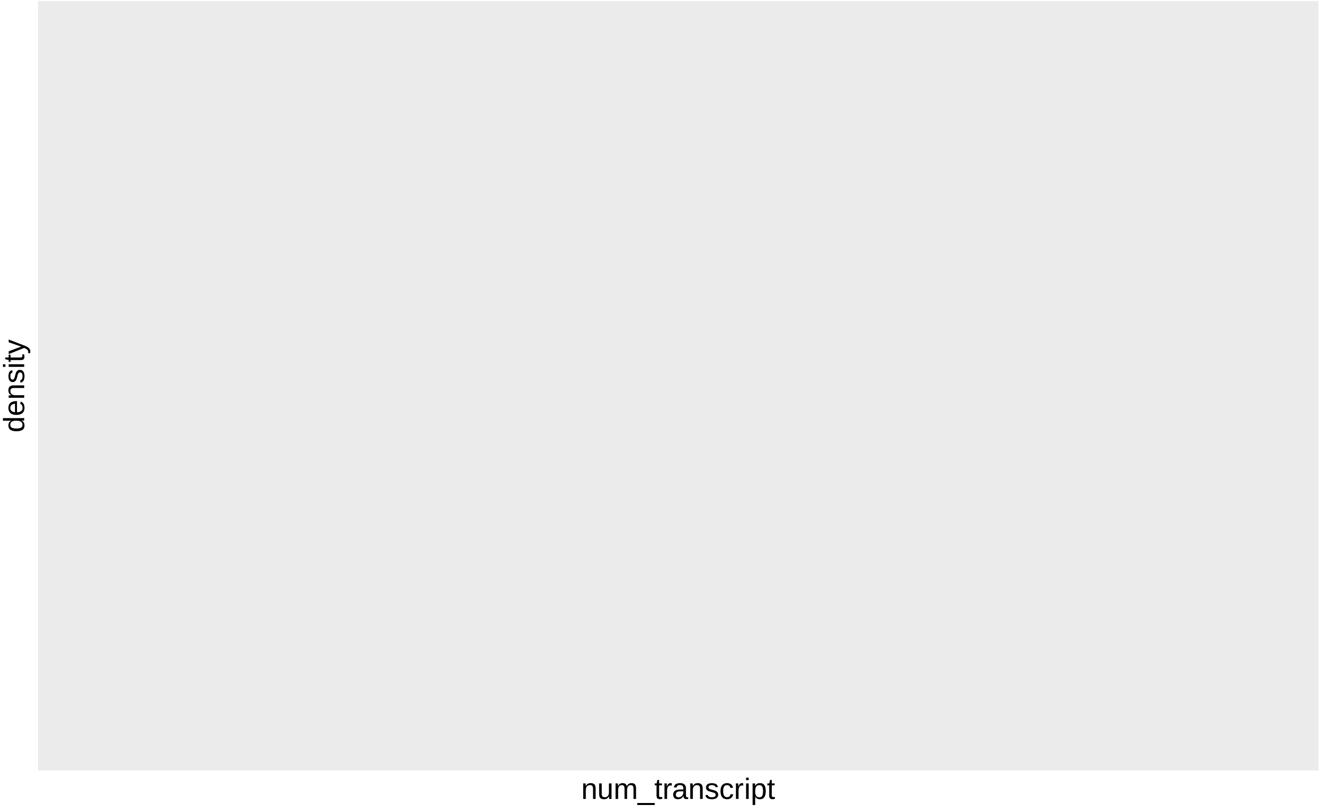

GCF_001664035.1_Metbi1
EpT

Novel Genes

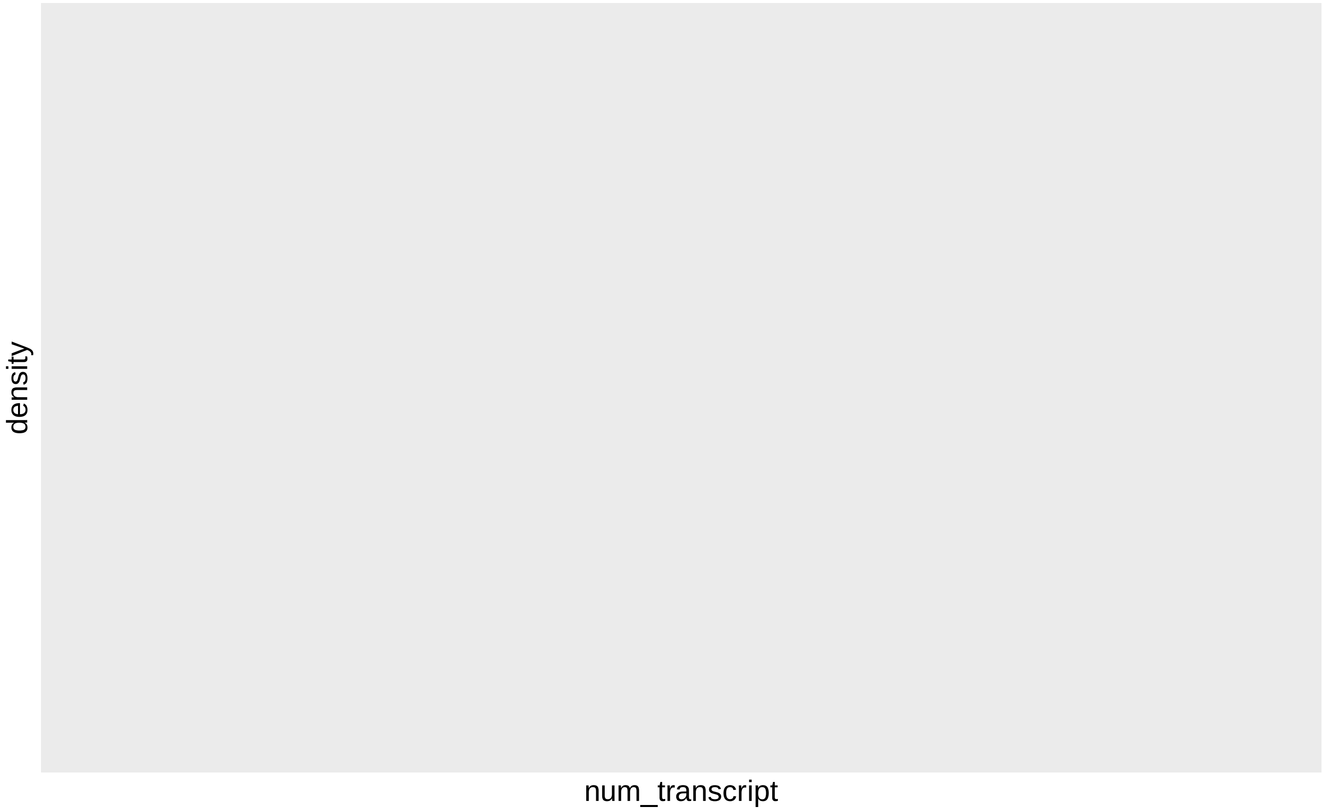

GCF_001883845.1_ASM188384v1
EpT

Novel Genes

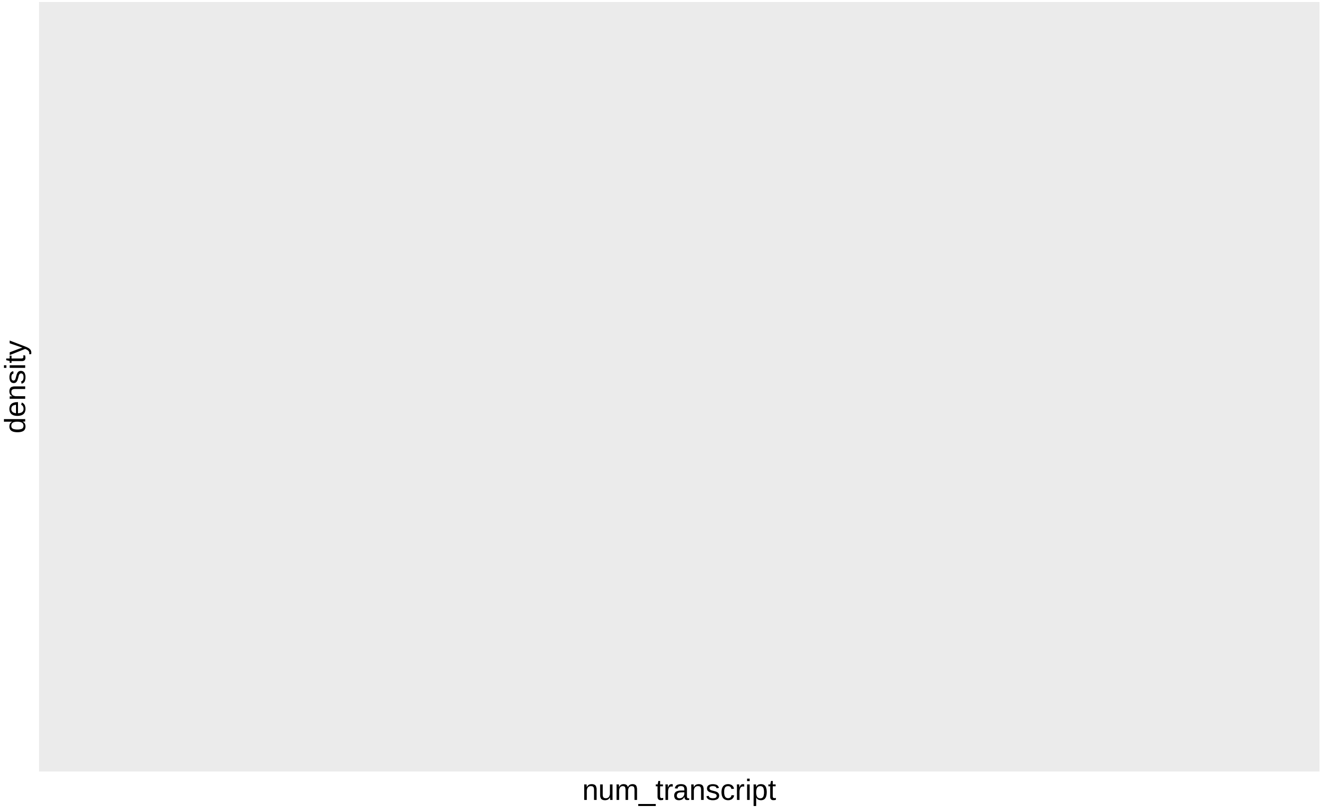

GCF_001890105.1_Aspzo1
EpT

Novel Genes

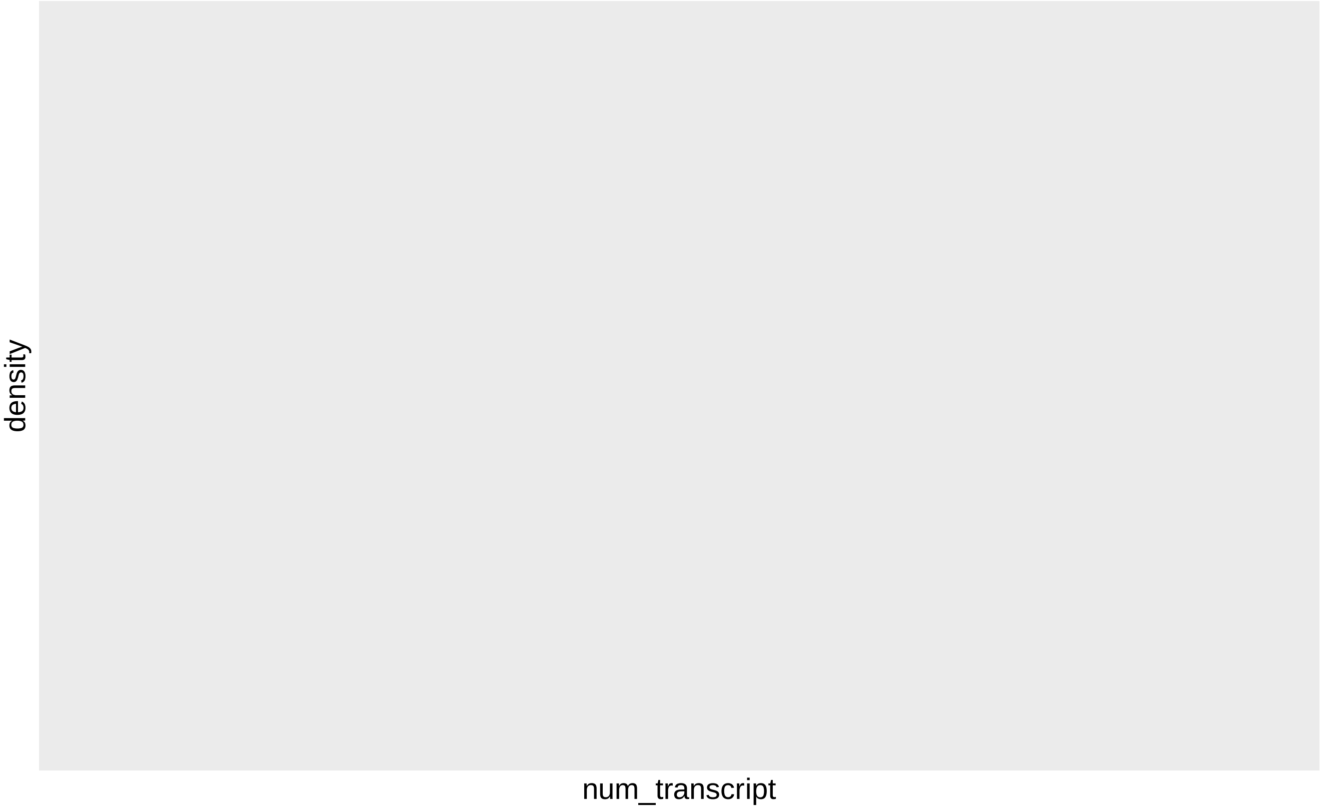

GCF_002102565.1_Kocim1
EpT

Novel Genes

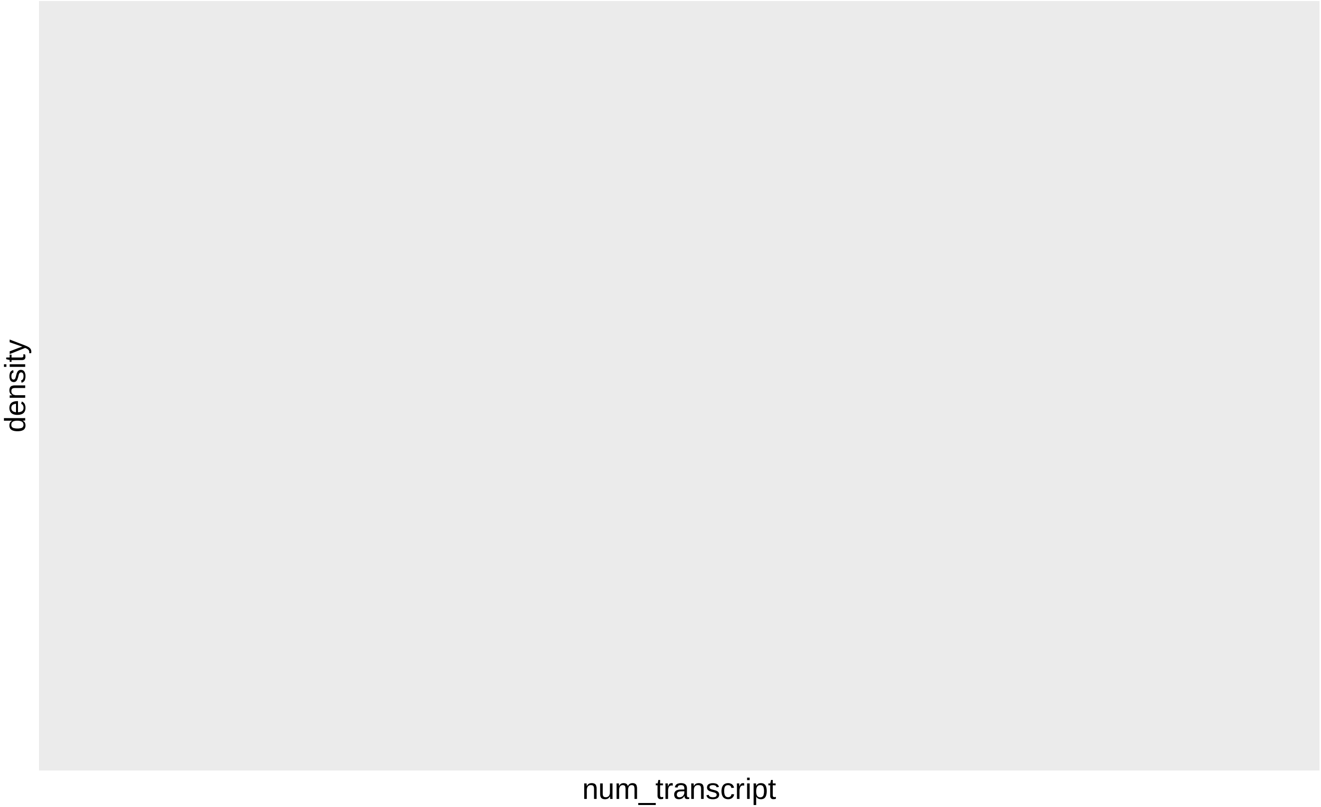

GCF_002105155.1_Lobtra1
EpT

Novel Genes

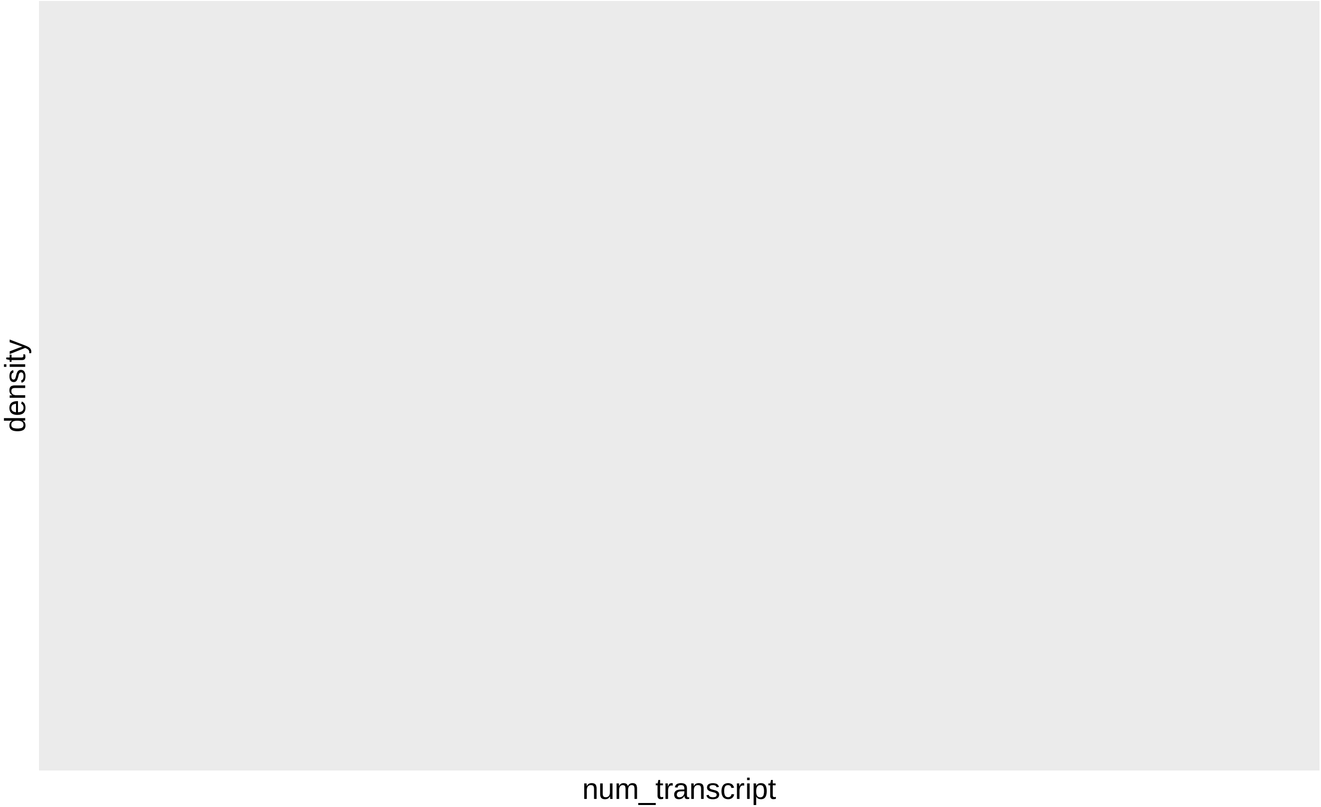

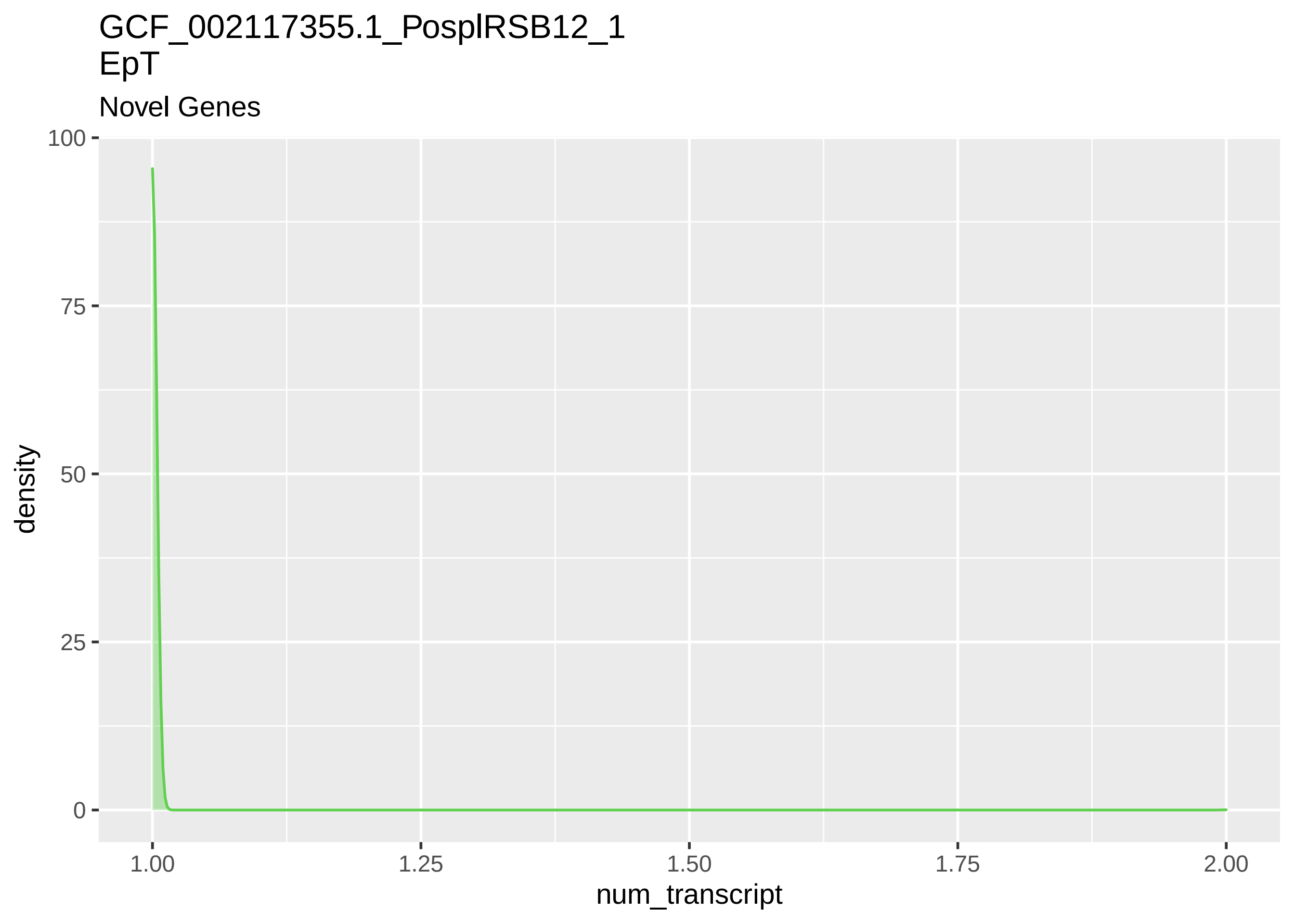
GCF_002117355.1_PosplRSB12_1
EpT
Novel Genes
100
75
50
25
0
density
1.00
1.25
1.50
1.75
2.00
num_transcript

GCF_002847465.1_Aspnov1
EpT

Novel Genes

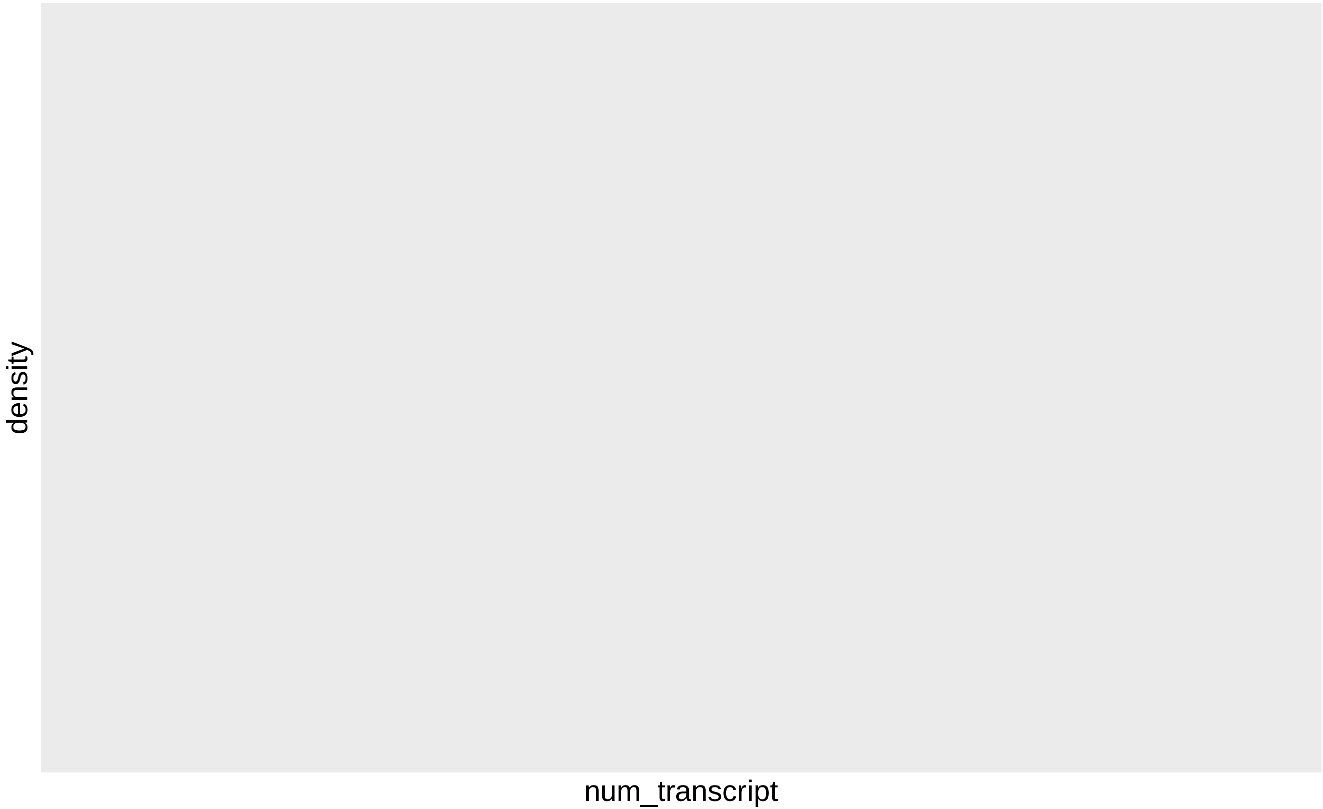

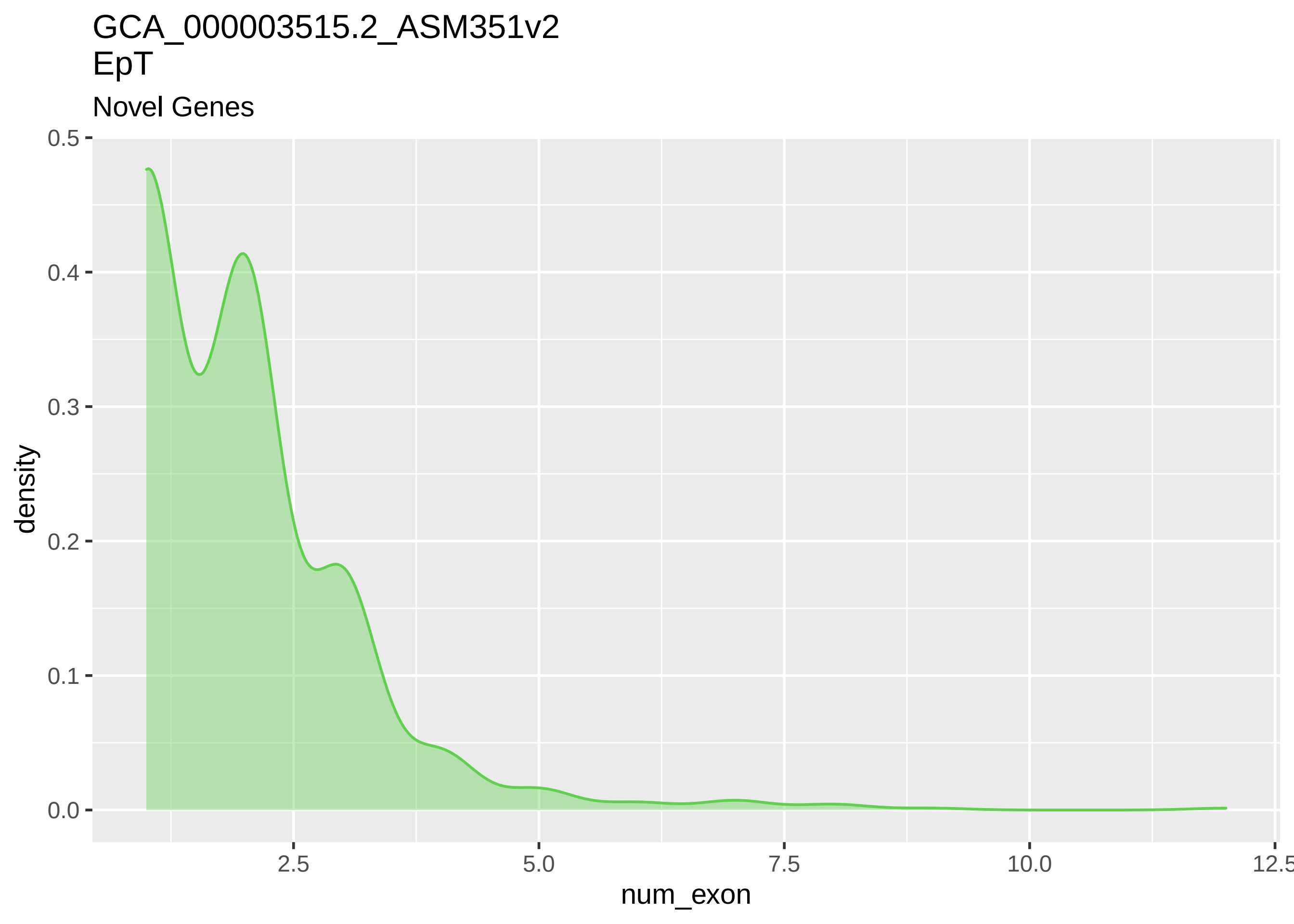
GCA_000003515.2_ASM351v2
EpT
Novel Genes
density
0.0
0.1
0.2
0.3
0.4
0.5
2.5
5.0
7.5
10.0
12.5
num_exon

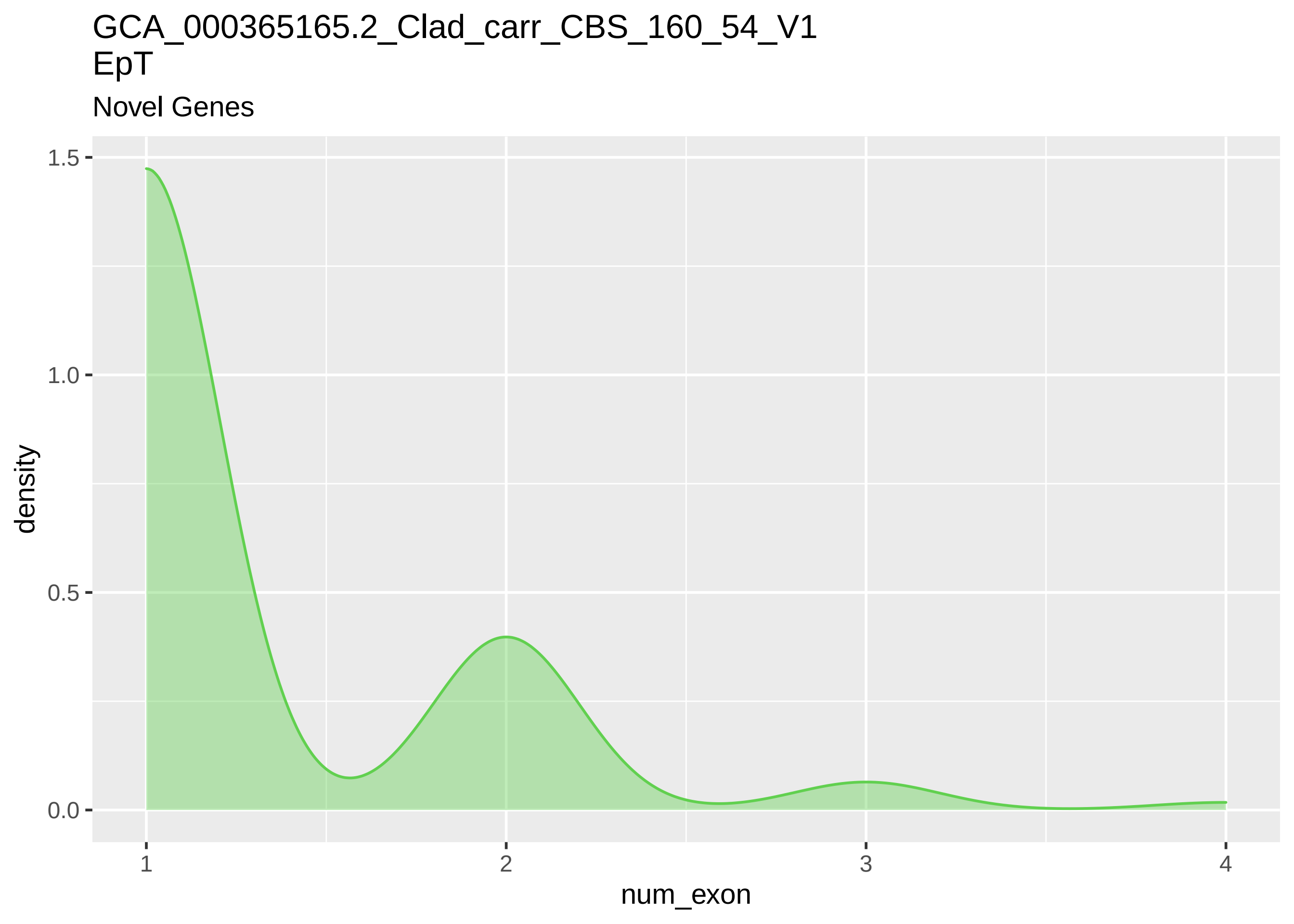

GCA_000365165.2_Clad_carr_CBS_160_54_V1
EpT
Novel Genes
density
num_exon
1.5
1.0
0.5
0.0
1
2
3
4

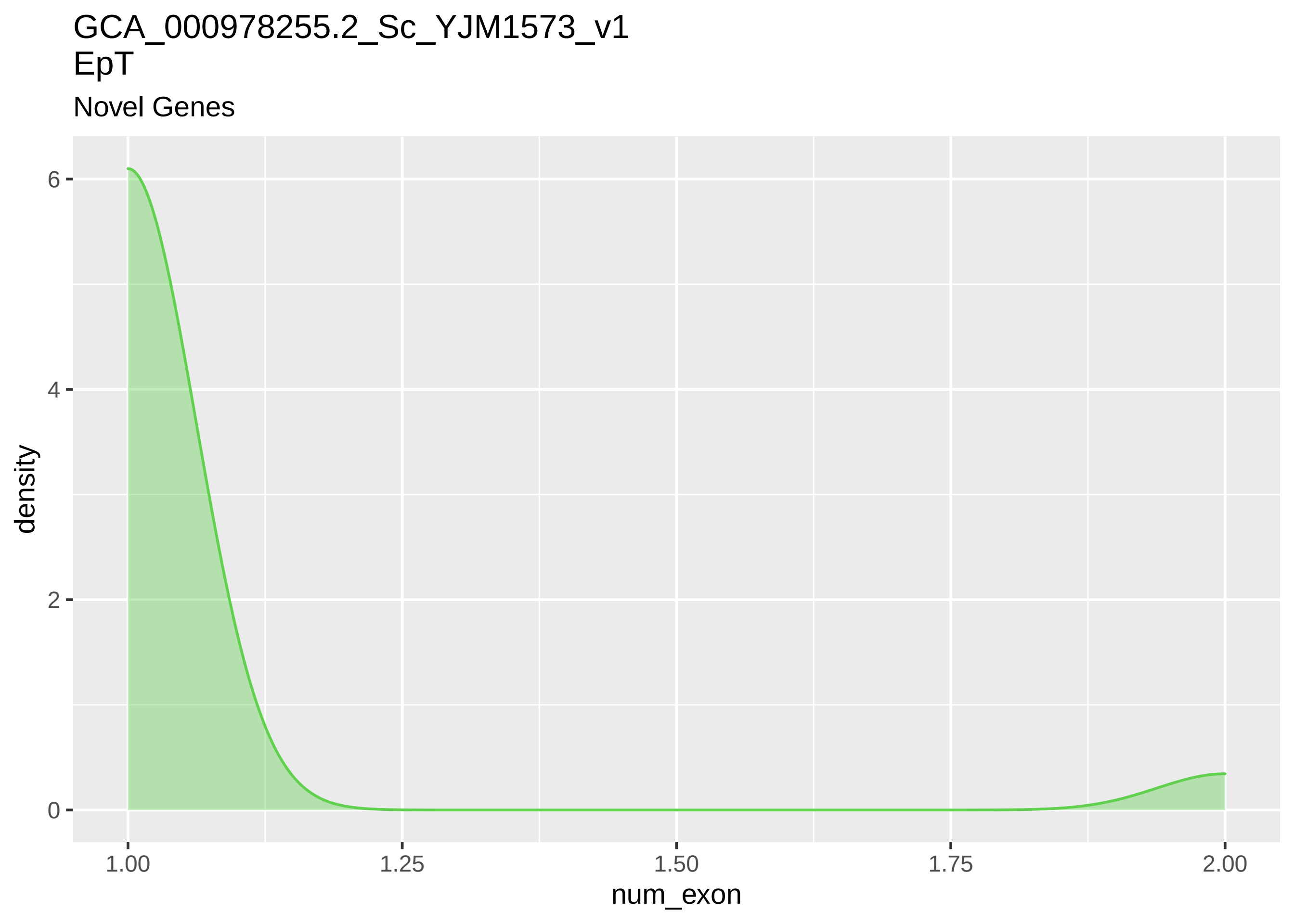
GCA_000978255.2_Sc_YJM1573_v1
EpT
Novel Genes
density
num_exon
0
2
4
6
1.00
1.25
1.50
1.75
2.00

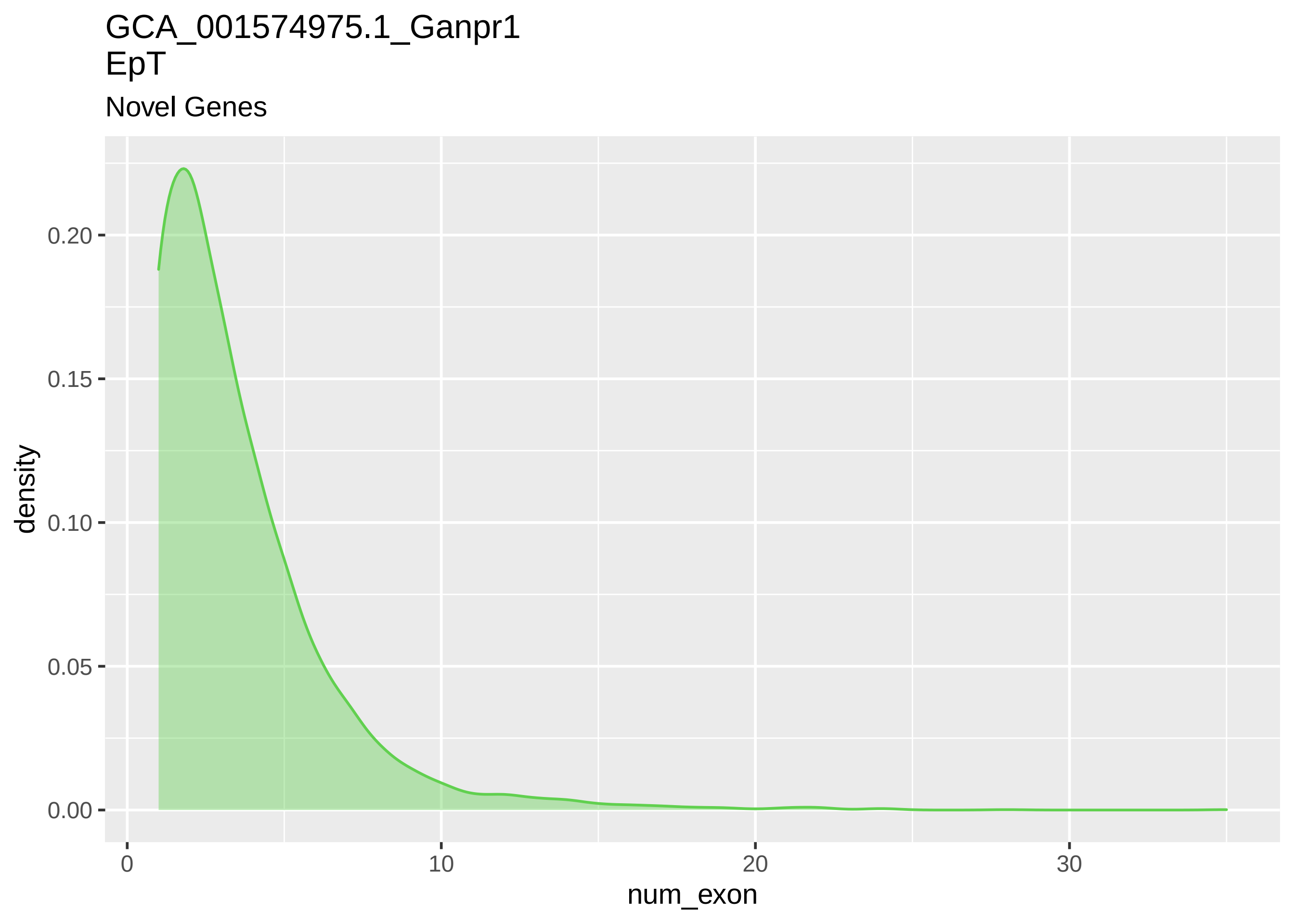

GCA_001574975.1_Ganpr1
EpT
Novel Genes
density
0.20
0.15
0.10
0.05
0.00
0
10
20
30
num_exon

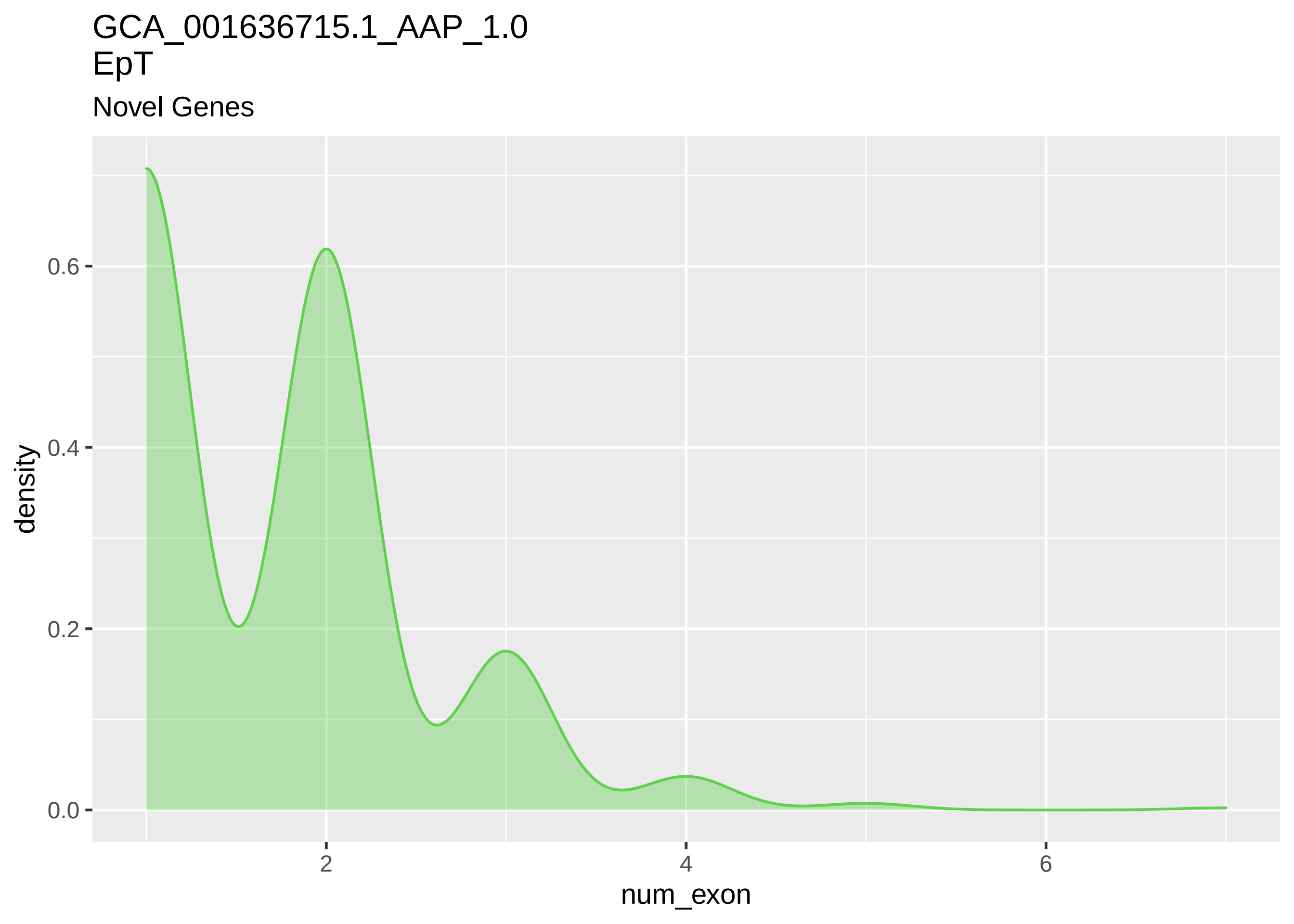
GCA_001636715.1_AAP_1.0
EpT
Novel Genes
density
0.0
0.2
0.4
0.6
2
4
6
num_exon

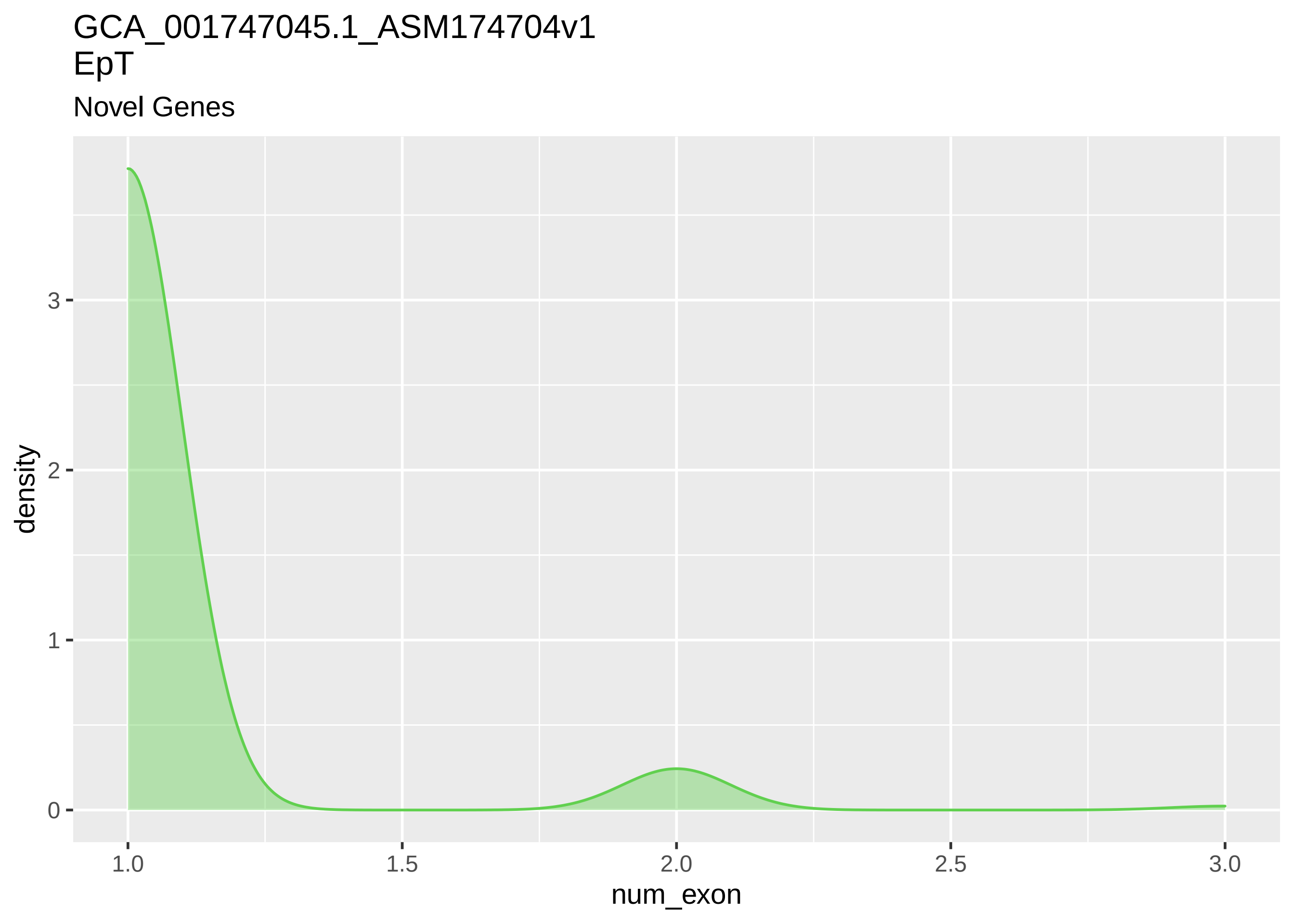
GCA_001747045.1_ASM174704v1
EpT
Novel Genes
density
num_exon
0
1
2
3
1.0
1.5
2.0
2.5
3.0

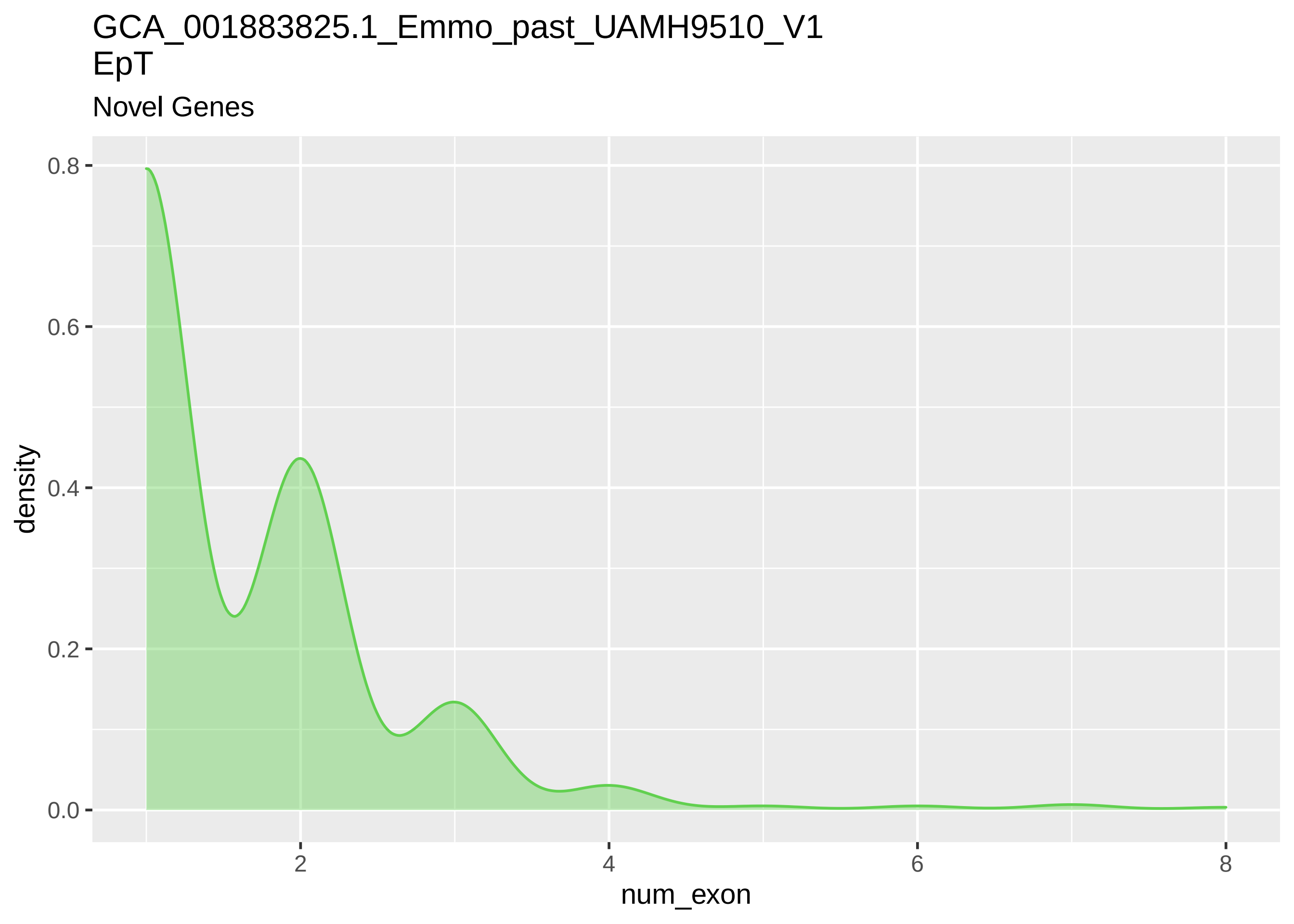
GCA_001883825.1_Emmo_past_UAMH9510_V1
EpT
Novel Genes
density
0.0
0.2
0.4
0.6
0.8
2
4
6
8
num_exon

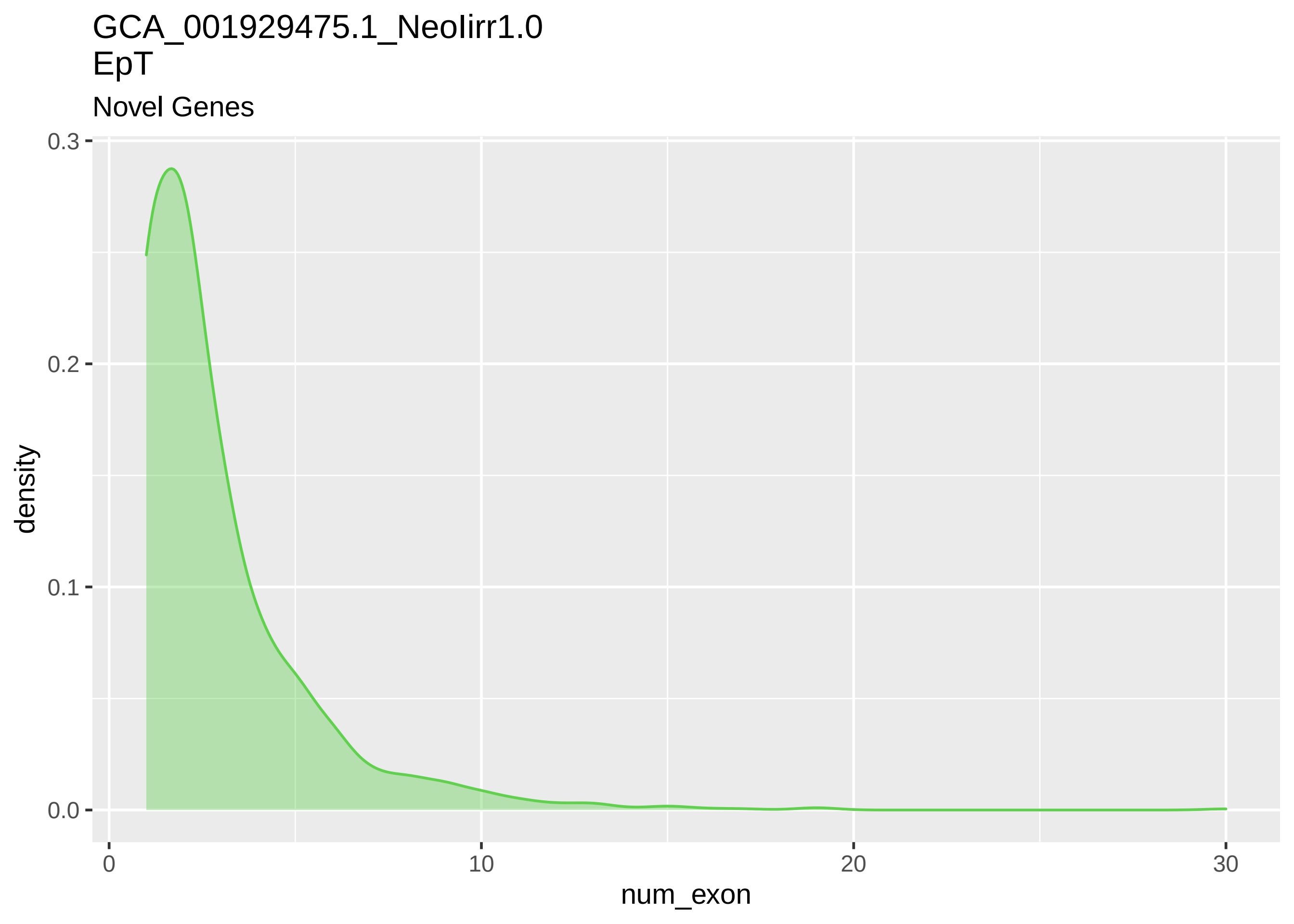
GCA_001929475.1_NeoIirr1.0
EpT
Novel Genes
0.3
0.2
0.1
0.0
density
0
10
20
30
num_exon

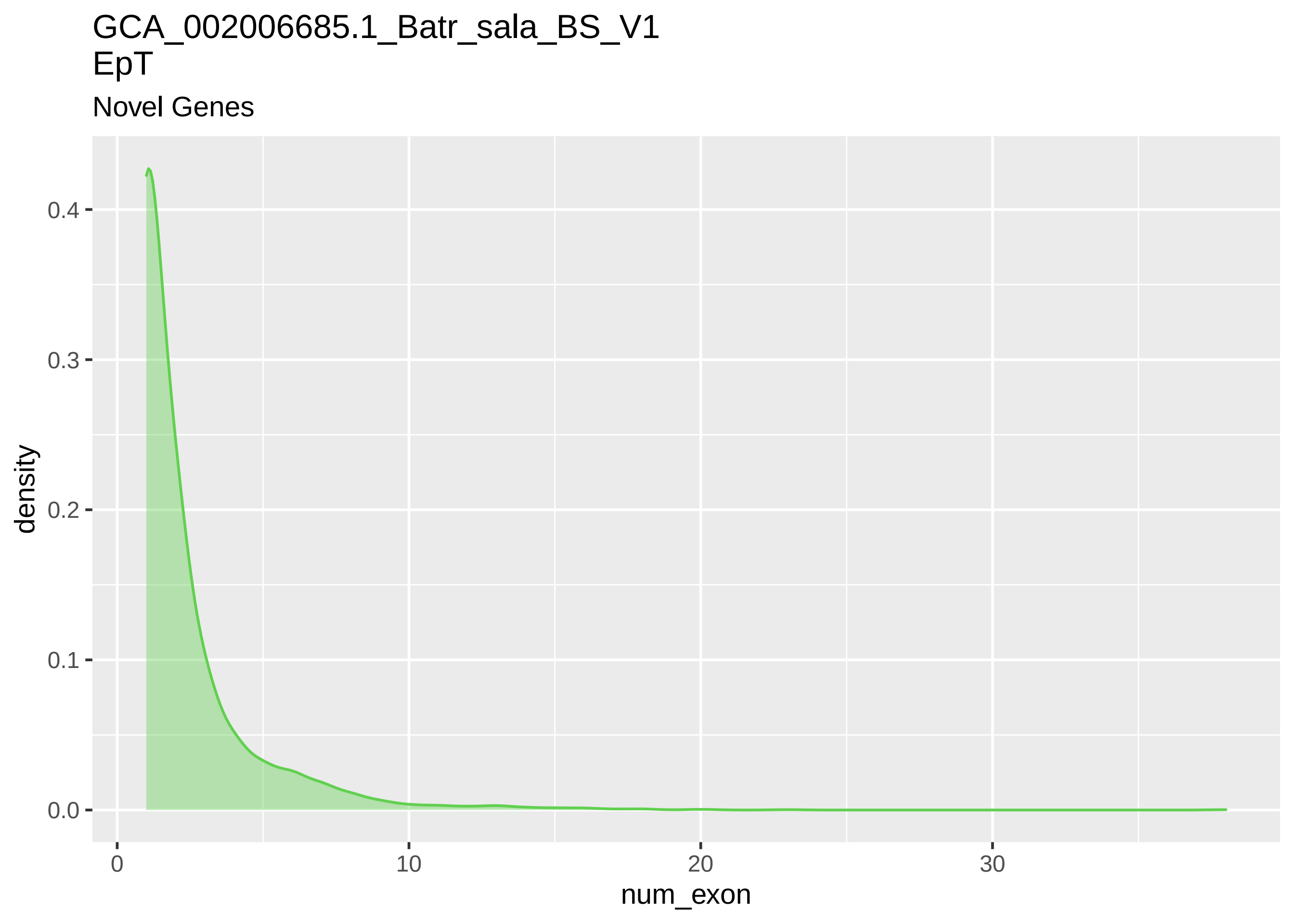
GCA_002006685.1_Batr_sala_BS_V1
EpT
Novel Genes
density
0.0
0.1
0.2
0.3
0.4
0
10
20
30
num_exon

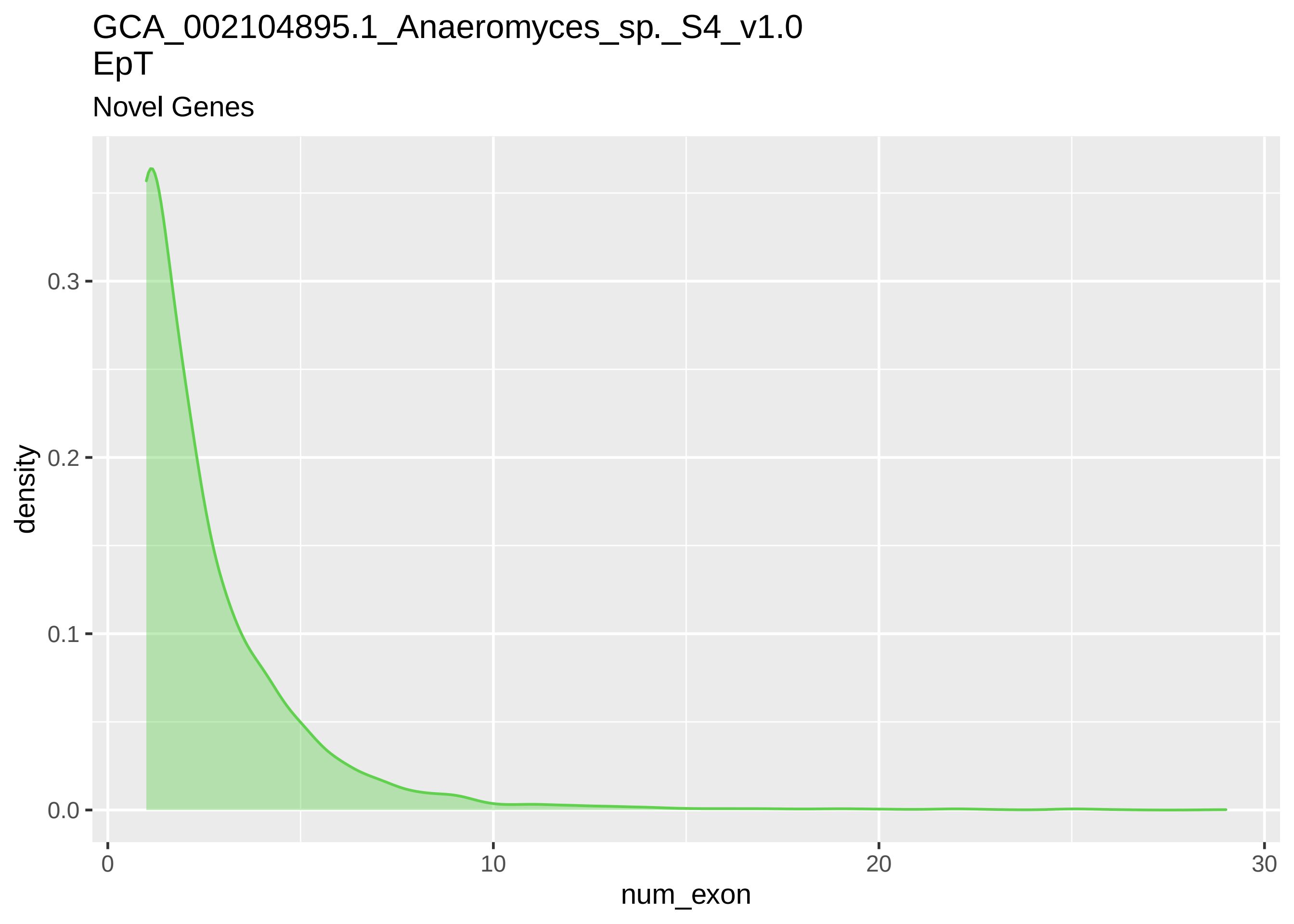
GCA_002104895.1_Anaeromyces_sp._S4_v1.0
EpT
Novel Genes
density
0.0
0.1
0.2
0.3
0
10
20
30
num_exon

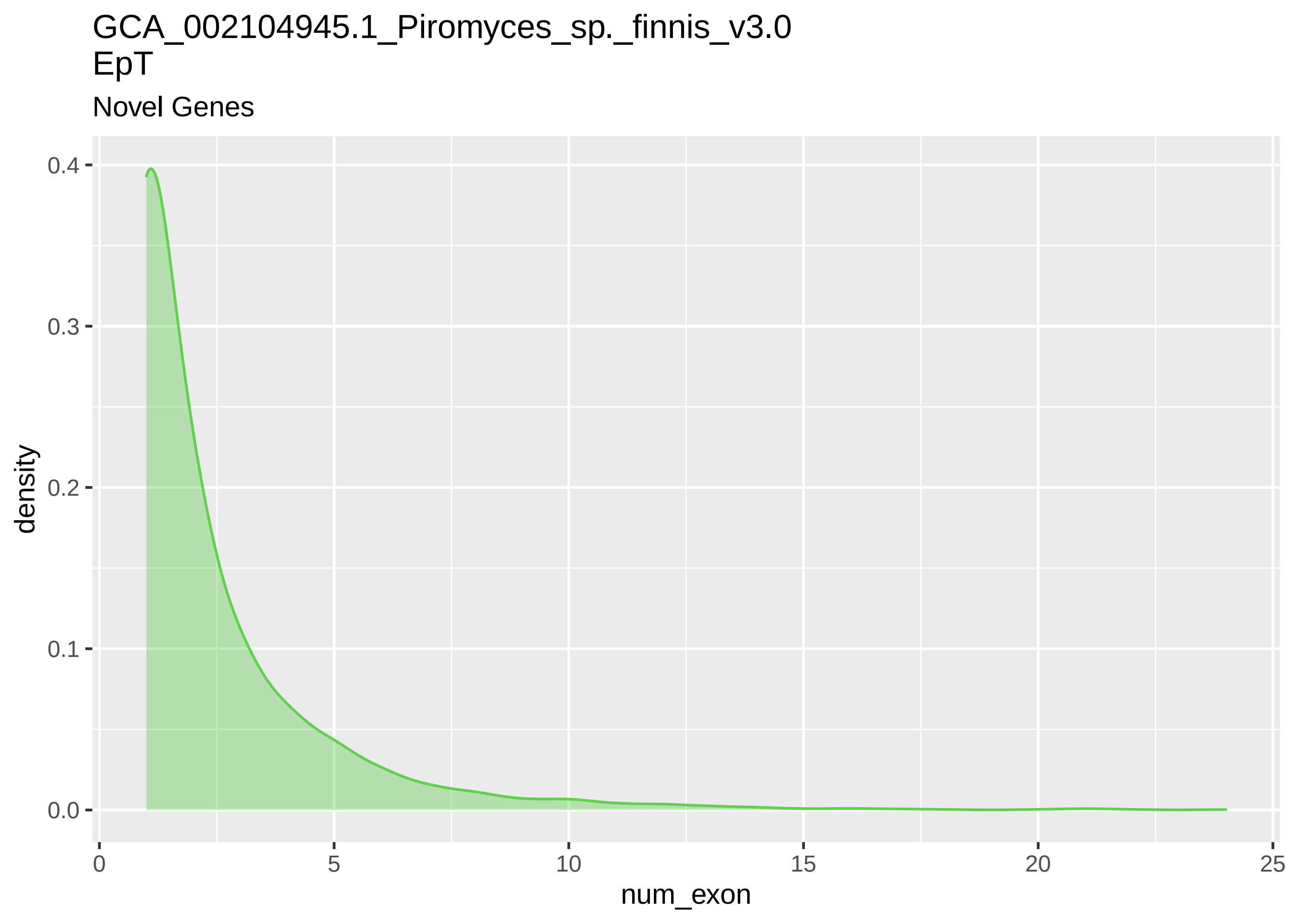
GCA_002104945.1_Piromyces_sp._finnis_v3.0
EpT
Novel Genes
density
0.4
0.3
0.2
0.1
0.0
0
5
10
15
20
25
num_exon

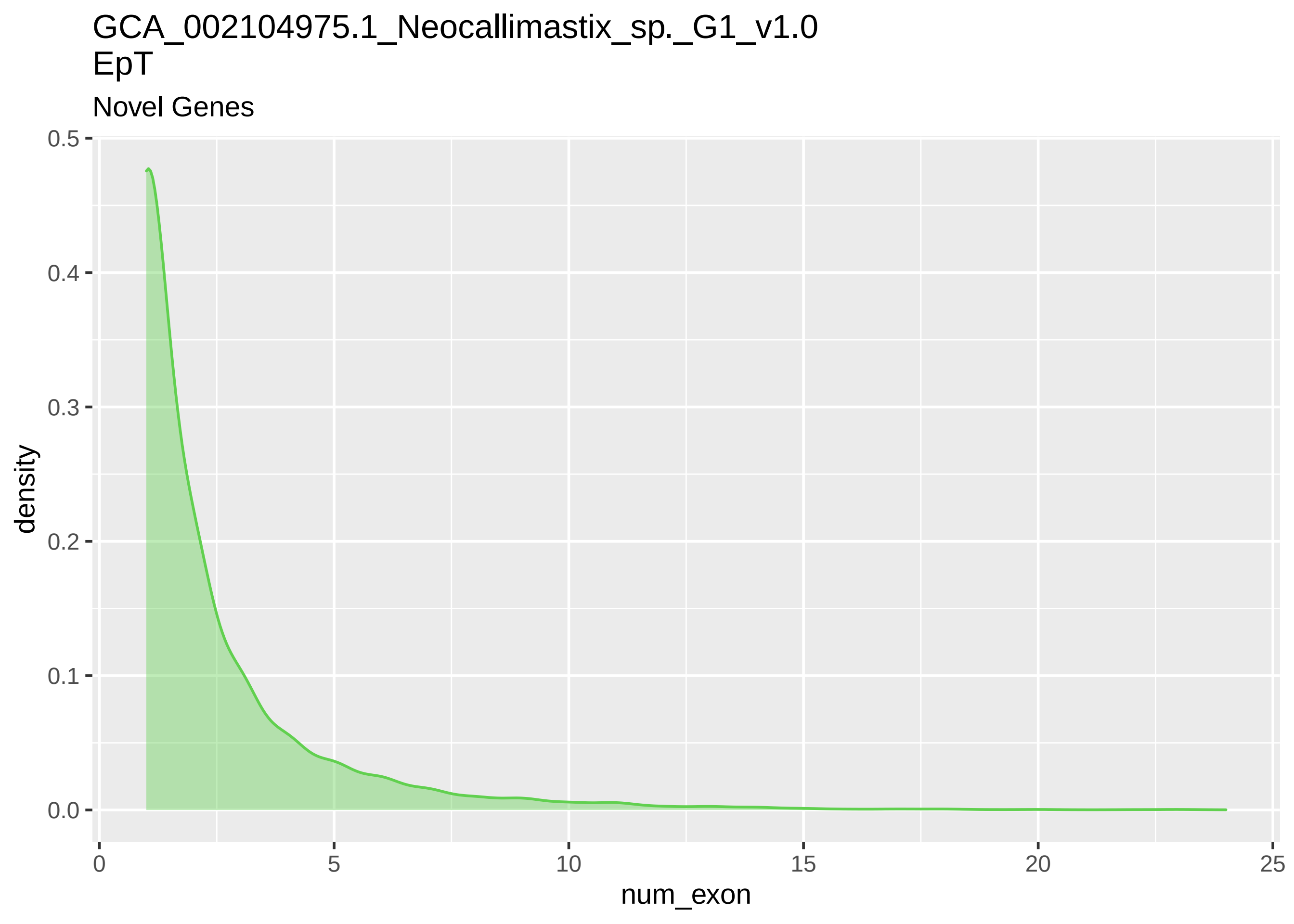
GCA_002104975.1_Neocallimastix_sp._G1_v1.0
EpT
Novel Genes
density
num_exon
0.0
0.1
0.2
0.3
0.4
0.5
0
5
10
15
20
25

GCA_002104985.1_Rhihy1
EpT

Novel Genes

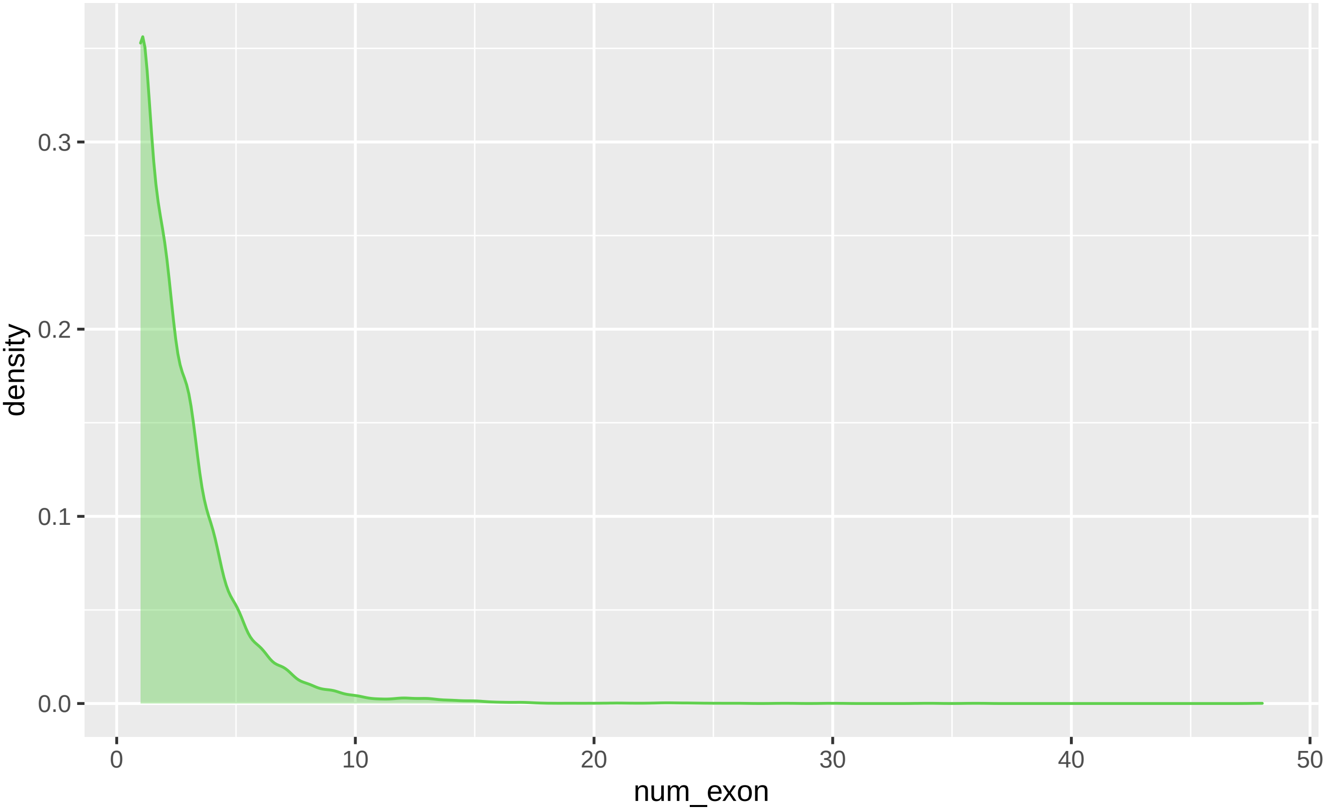

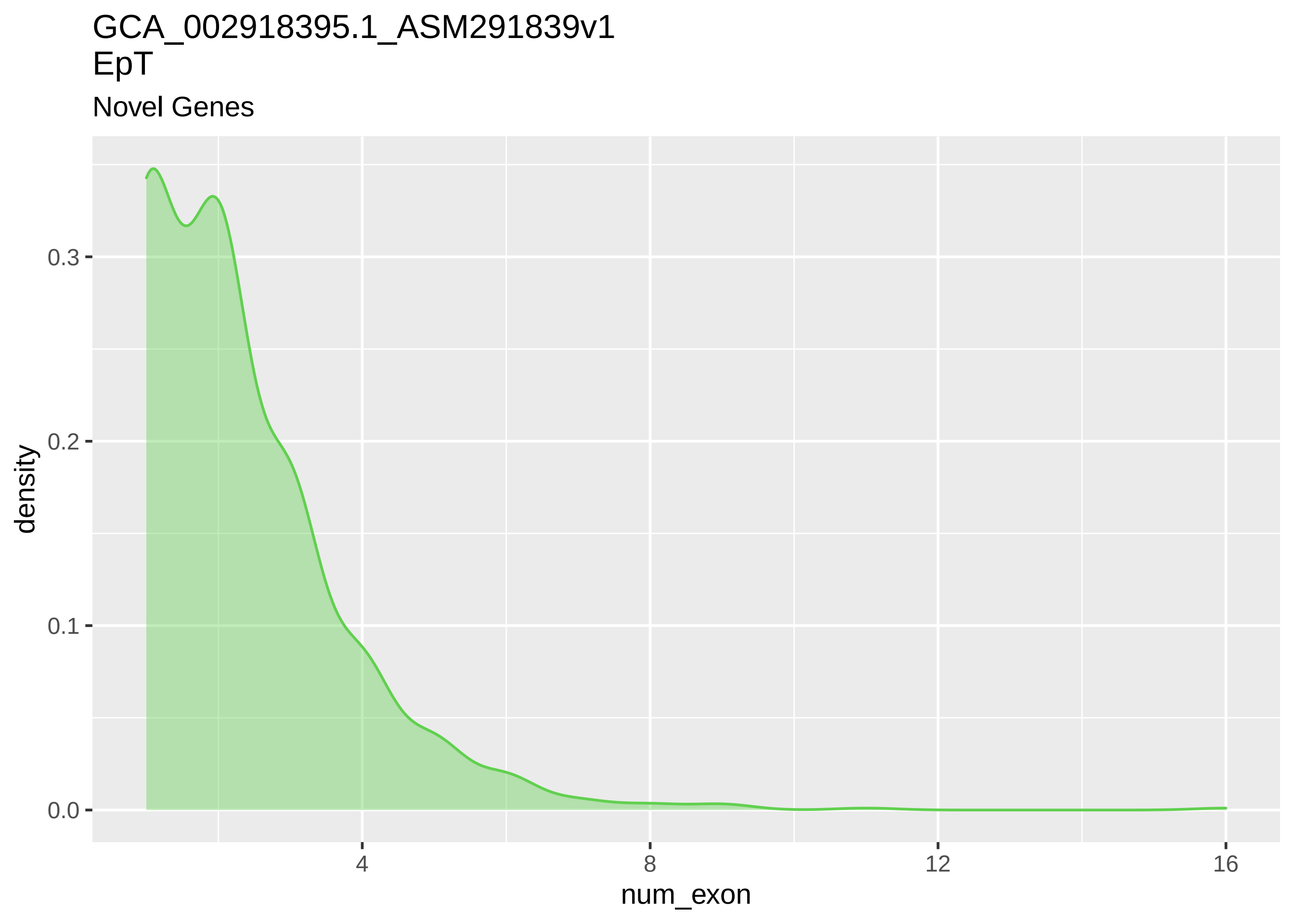
GCA_002918395.1_ASM291839v1
EpT
Novel Genes
density
0.0
0.1
0.2
0.3
4
8
12
16
num_exon

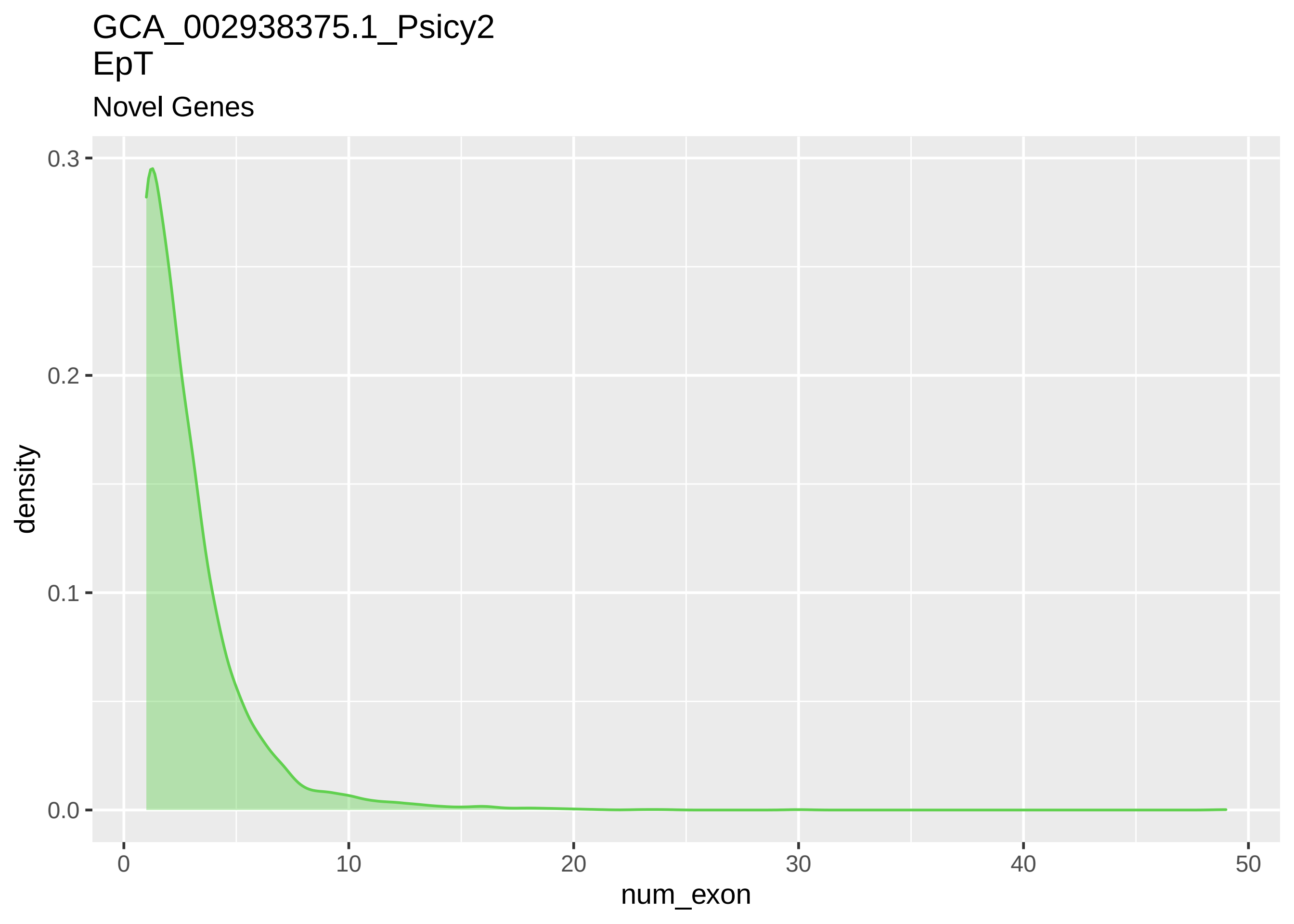
GCA_002938375.1_Psicy2
EpT
Novel Genes
density
0.0
0.1
0.2
0.3
0
10
20
30
40
50
num_exon

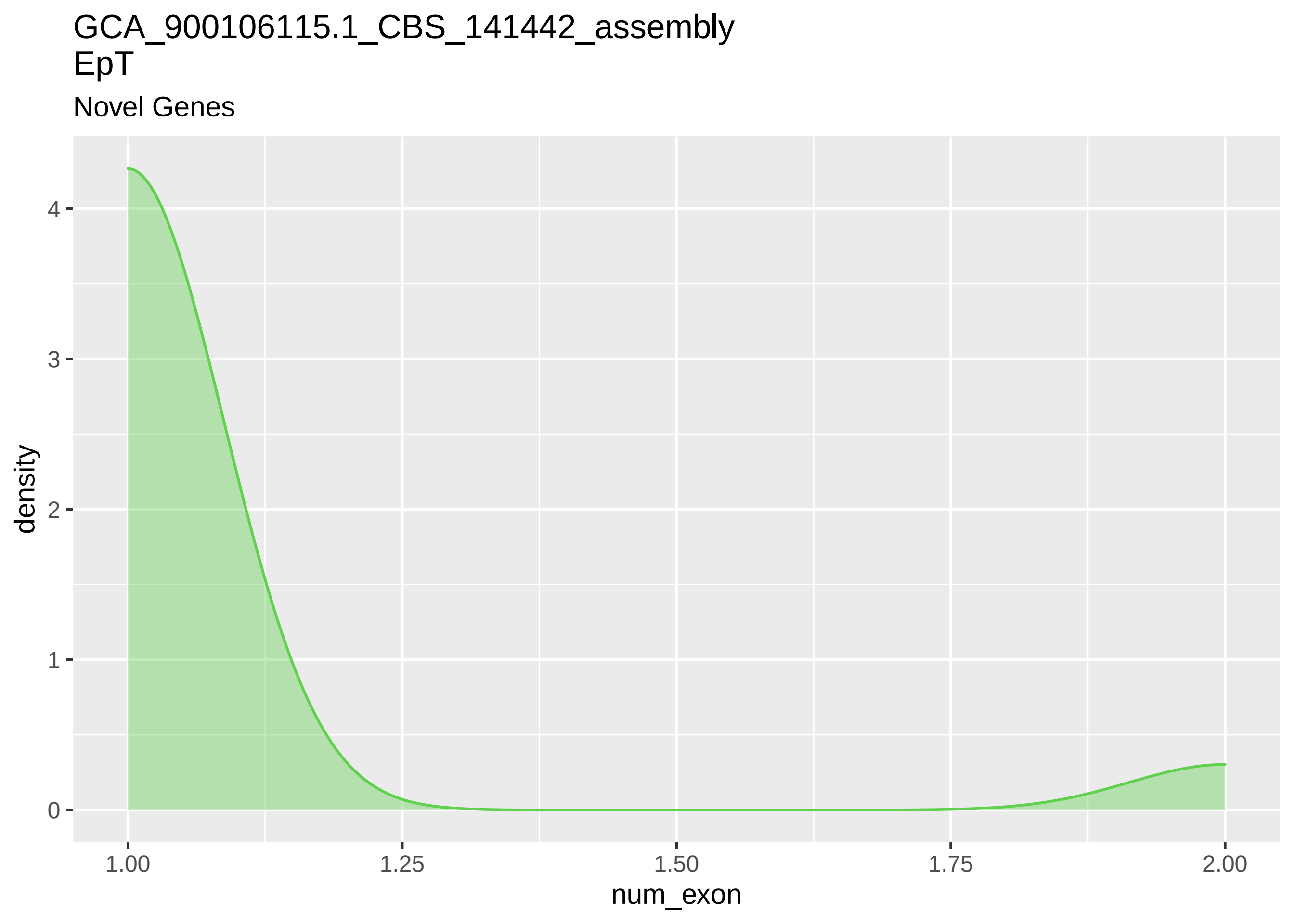
GCA_900106115.1_CBS_141442_assembly
EpT
Novel Genes
density
num_exon
0
1
2
3
4
1.00
1.25
1.50
1.75
2.00

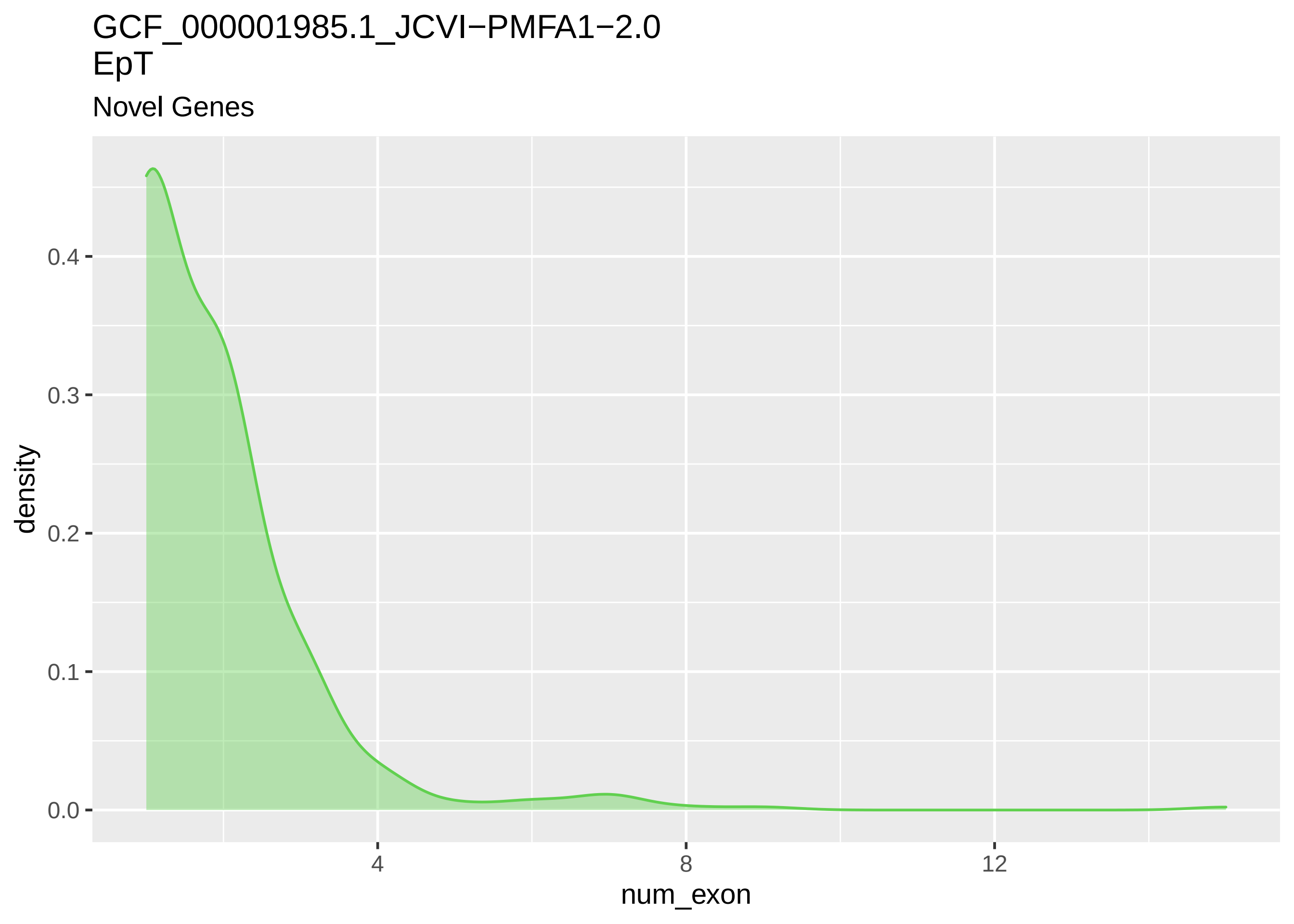

GCF_000001985.1_JCVI−PMFA1−2.0
EpT
Novel Genes
density
0.0
0.1
0.2
0.3
0.4
4
8
12
num_exon

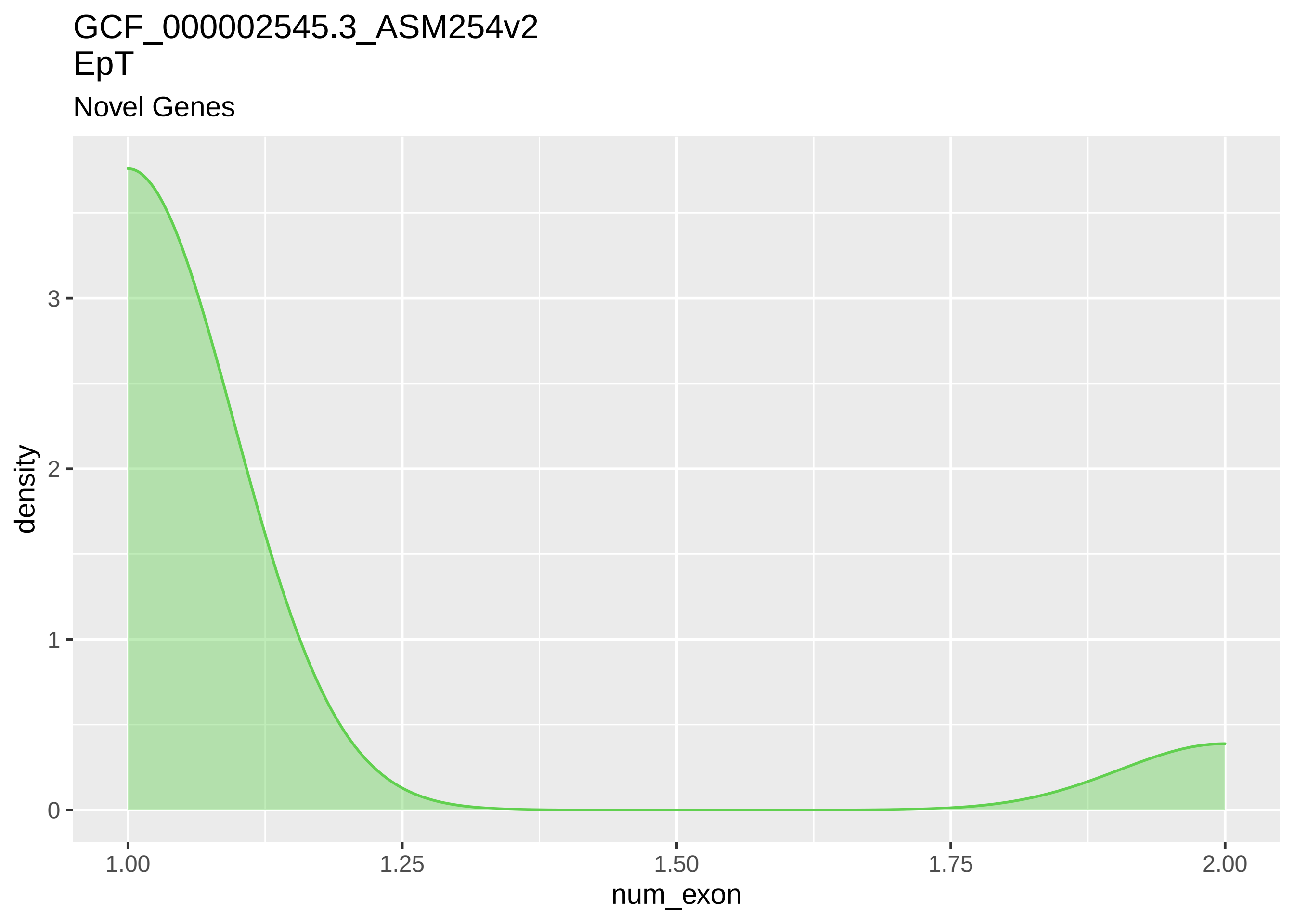

GCF_000002545.3_ASM254v2
EpT
Novel Genes
density
num_exon
0
1
2
3
1.00
1.25
1.50
1.75
2.00

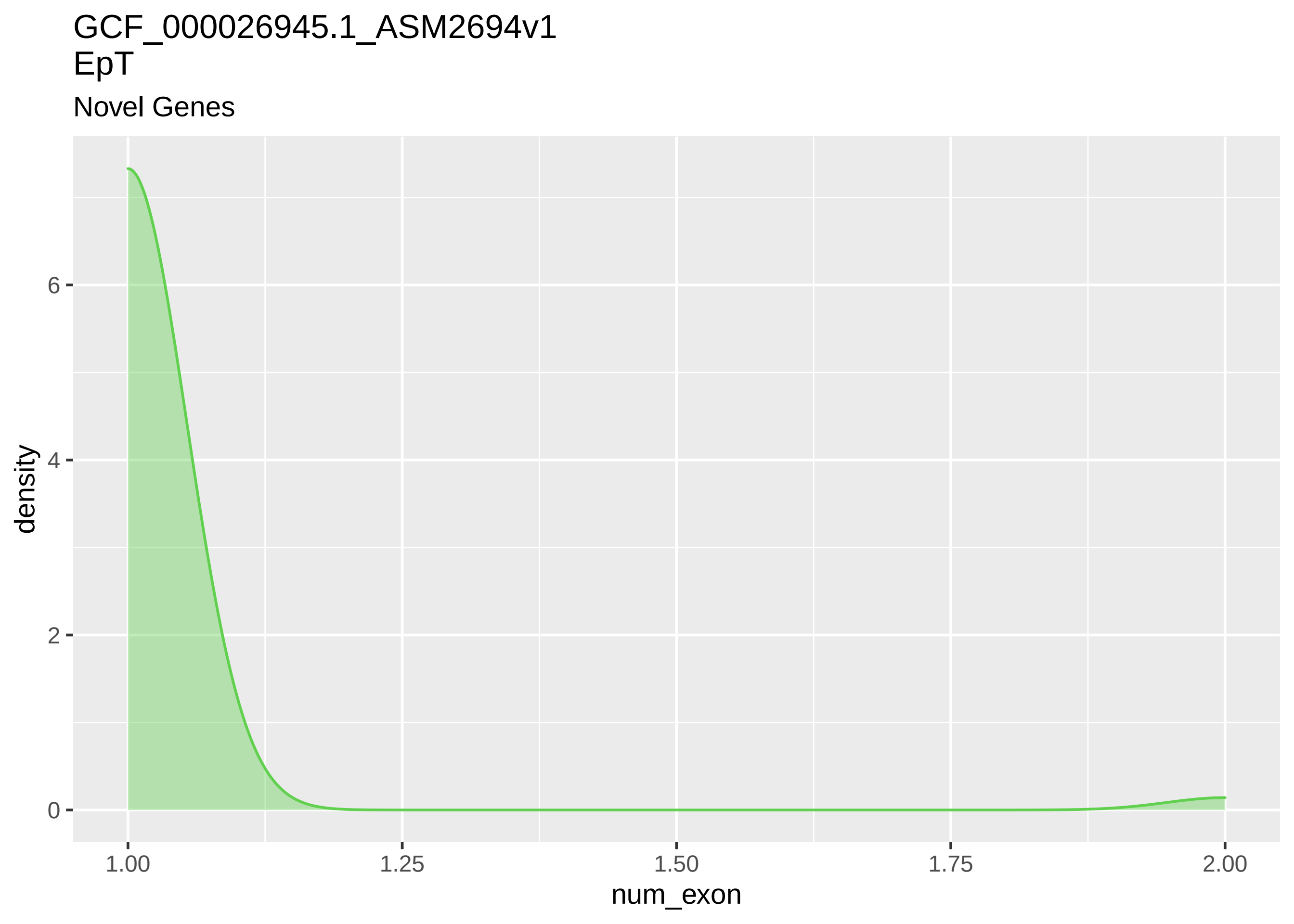
GCF_000026945.1_ASM2694v1
EpT
Novel Genes
density
num_exon
0
2
4
6
1.00
1.25
1.50
1.75
2.00

GCF_000091045.1_ASM9104v1
EpT

Novel Genes

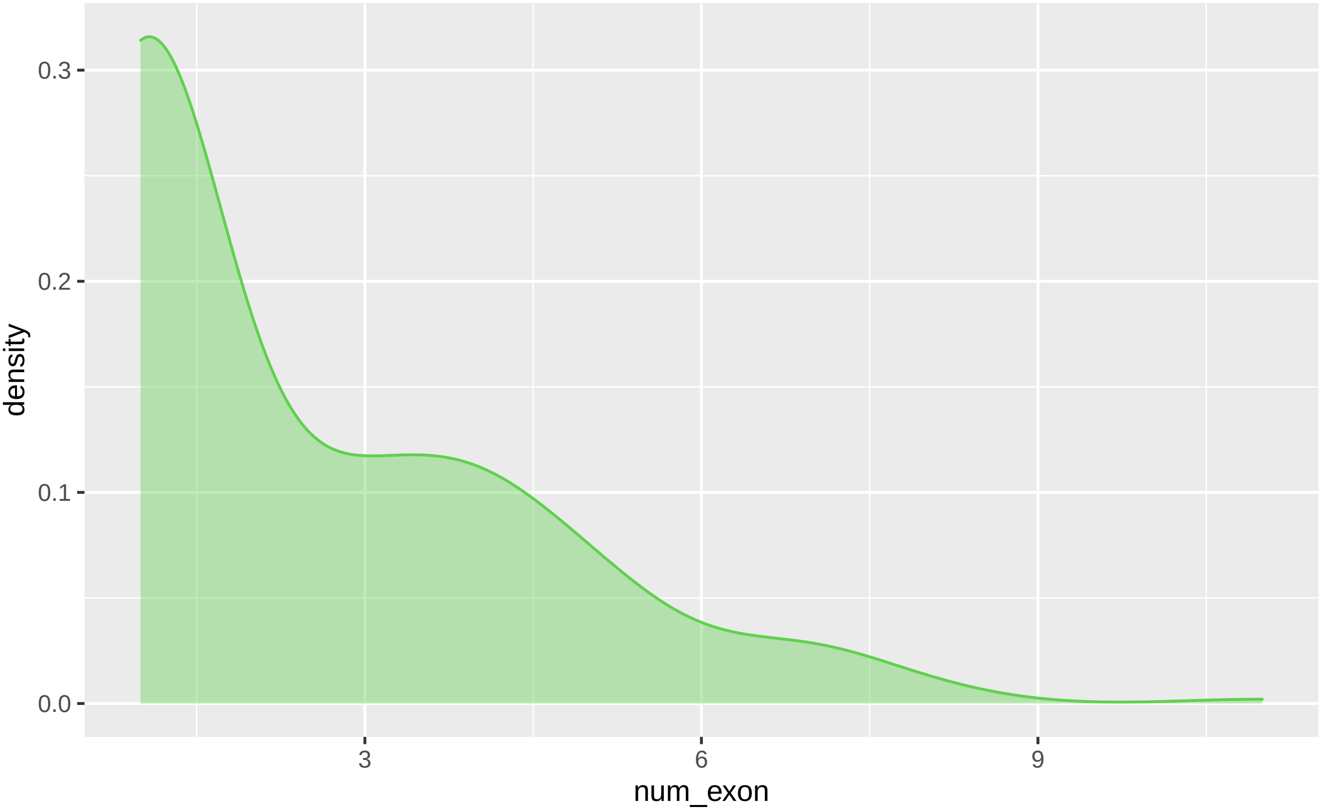

GCF_000143185.1_v1.0
EpT

Novel Genes

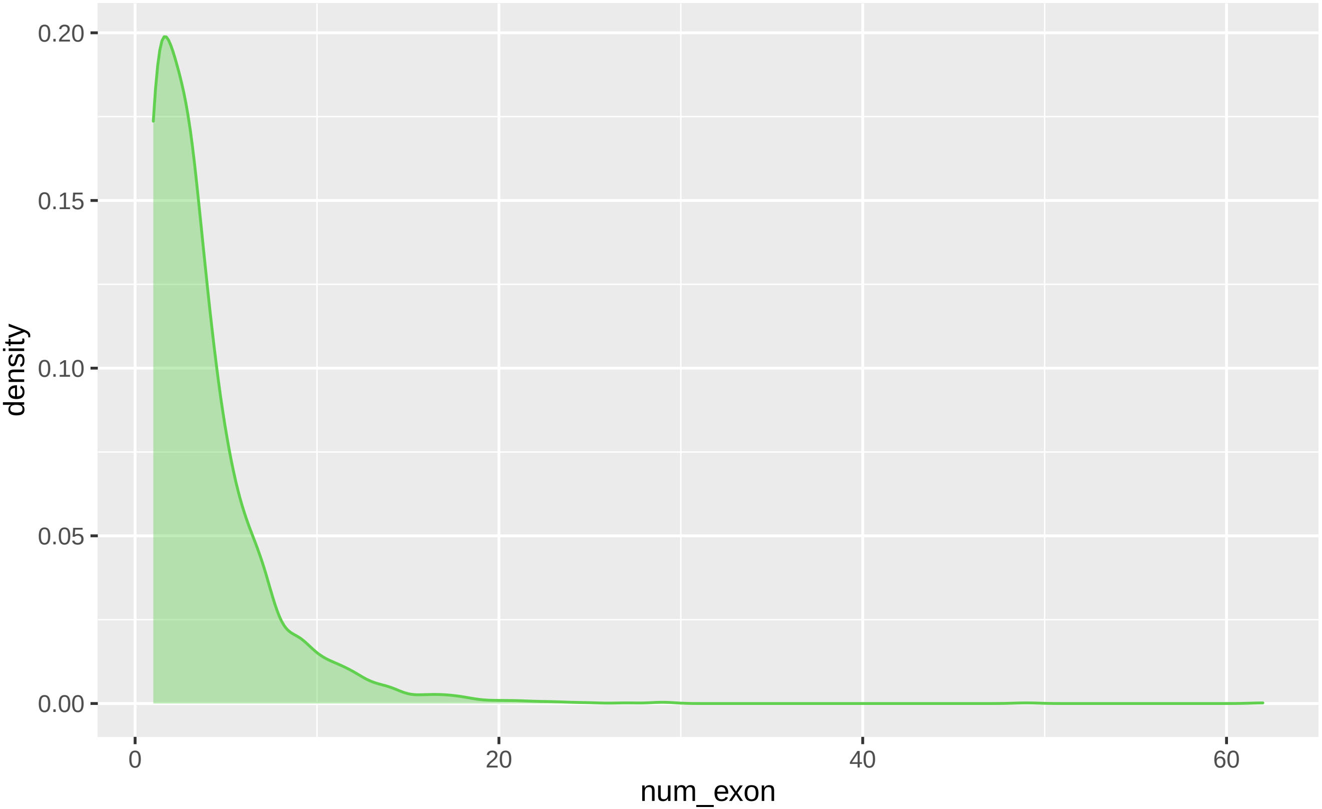

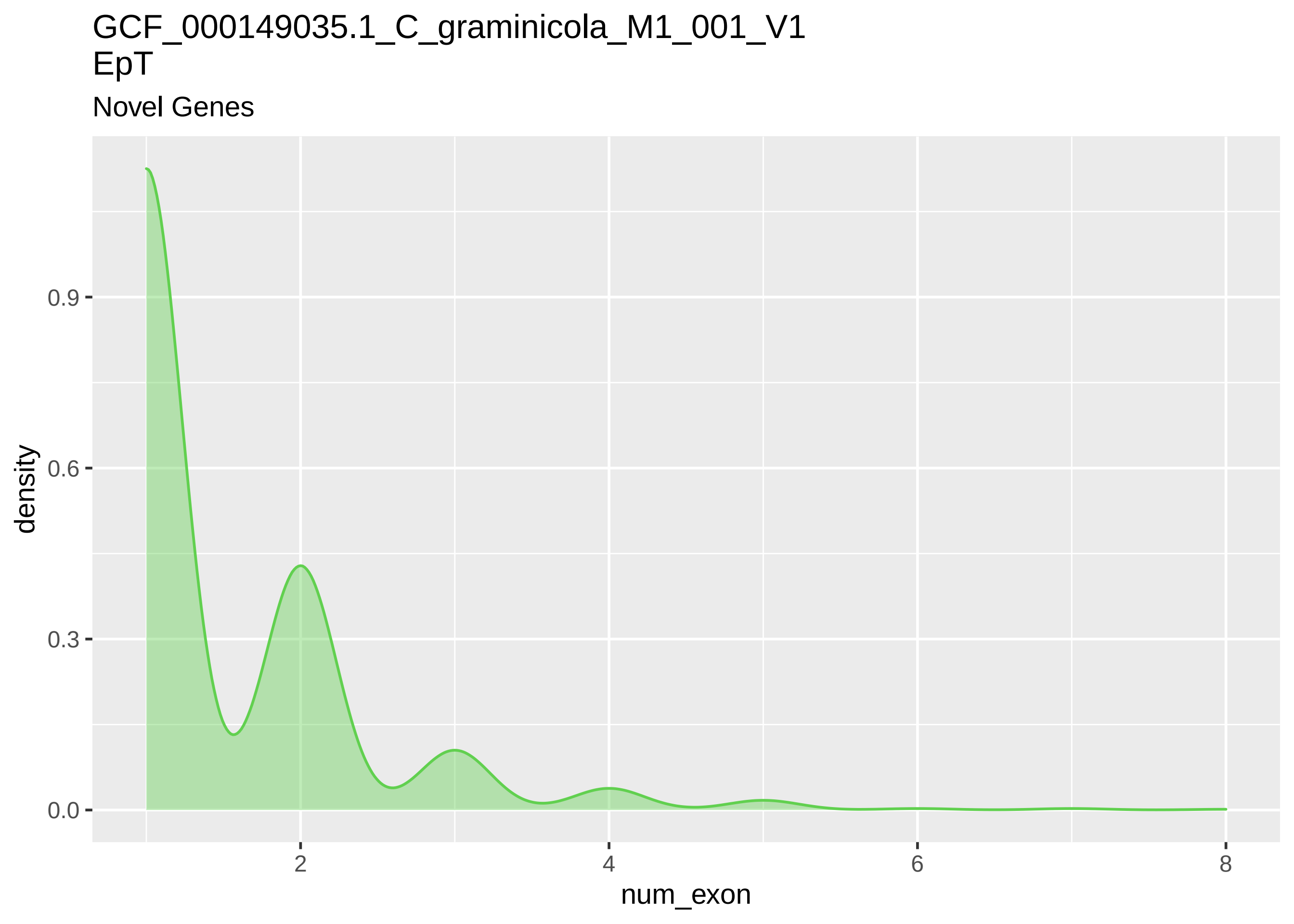
GCF_000149035.1_C_graminicola_M1_001_V1
EpT
Novel Genes
density
0.9
0.6
0.3
0.0
2
4
6
8
num_exon

GCF_000149335.2_ASM14933v2
EpT

Novel Genes

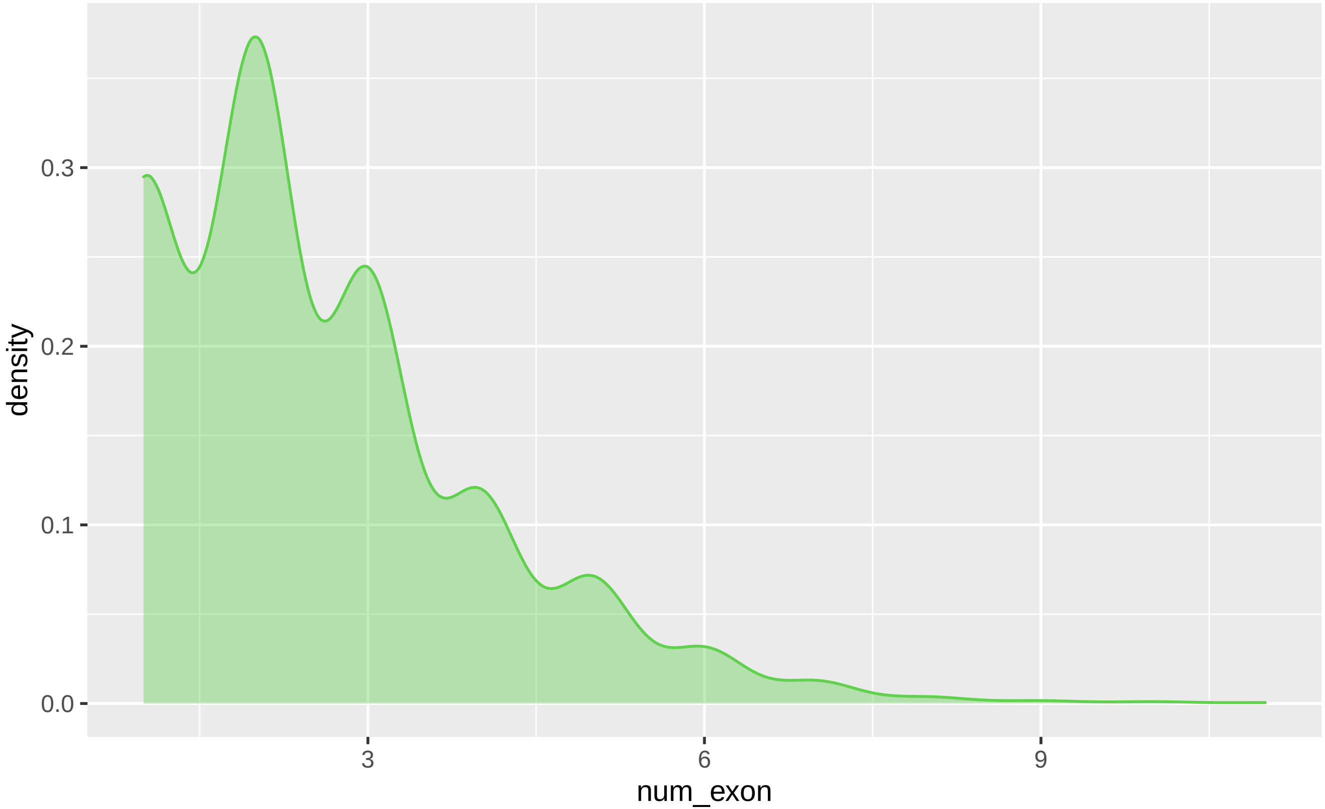

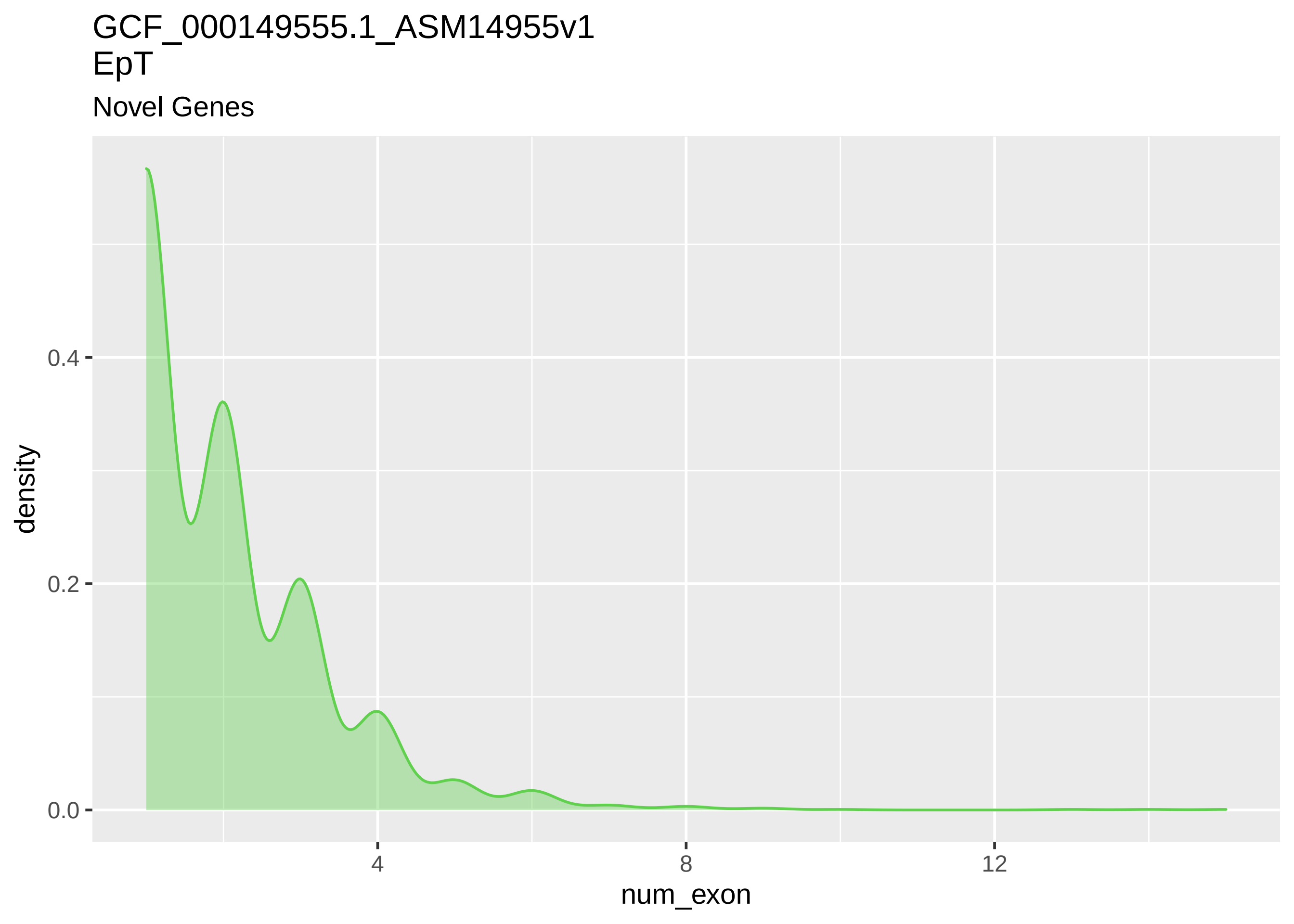
GCF_000149555.1_ASM14955v1
EpT
Novel Genes
density
0.0
0.2
0.4
4
8
12
num_exon

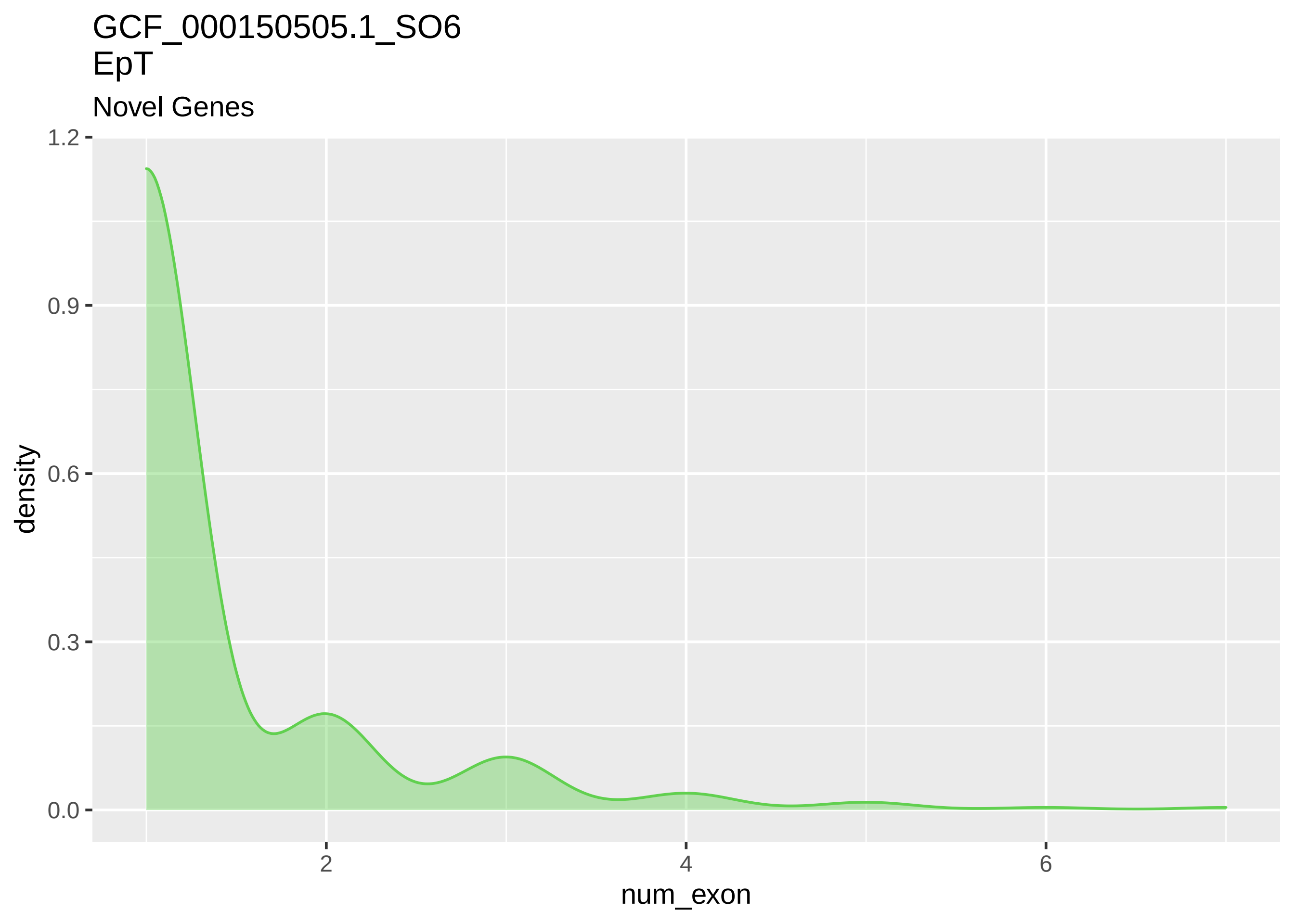
GCF_000150505.1_SO6
EpT
Novel Genes
1.2
0.9
0.6
0.3
0.0
density
2
4
6
num_exon

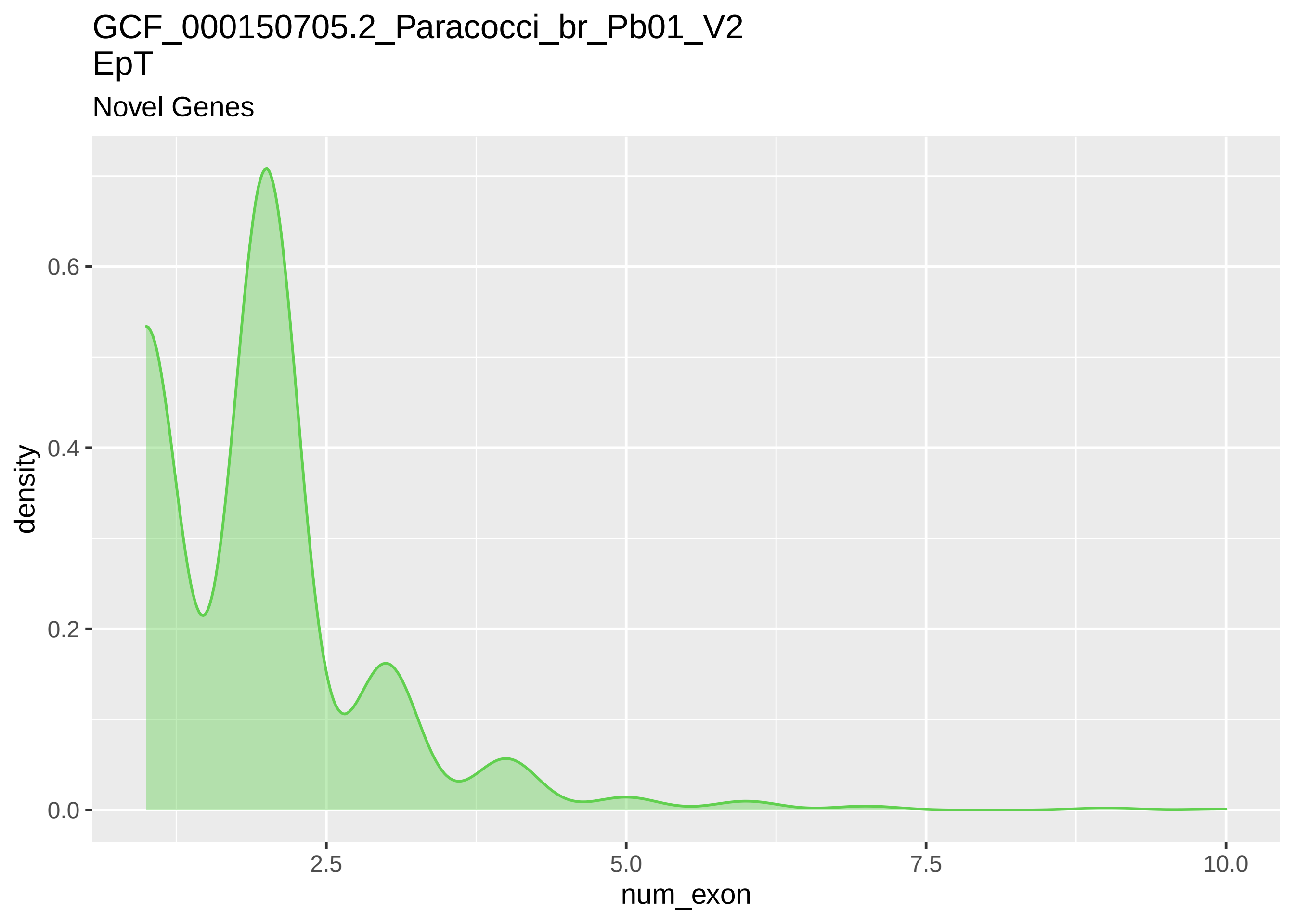
GCF_000150705.2_Paracocci_br_Pb01_V2
EpT
Novel Genes
density
0.0
0.2
0.4
0.6
2.5
5.0
7.5
10.0
num_exon

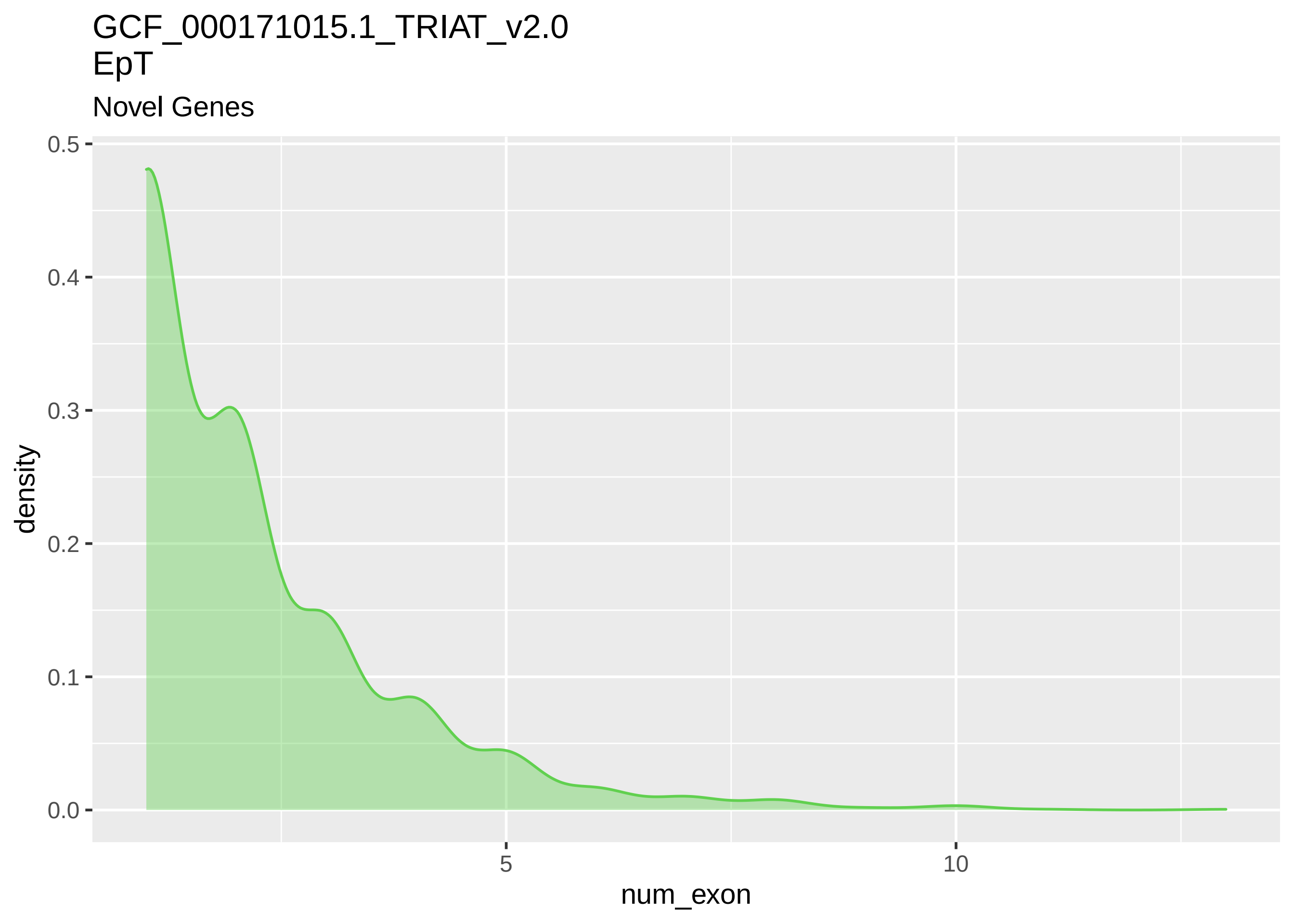

GCF_000171015.1_TRIAT_v2.0
EpT
Novel Genes
0.5
0.4
0.3
0.2
0.1
0.0
density
5
10
num_exon

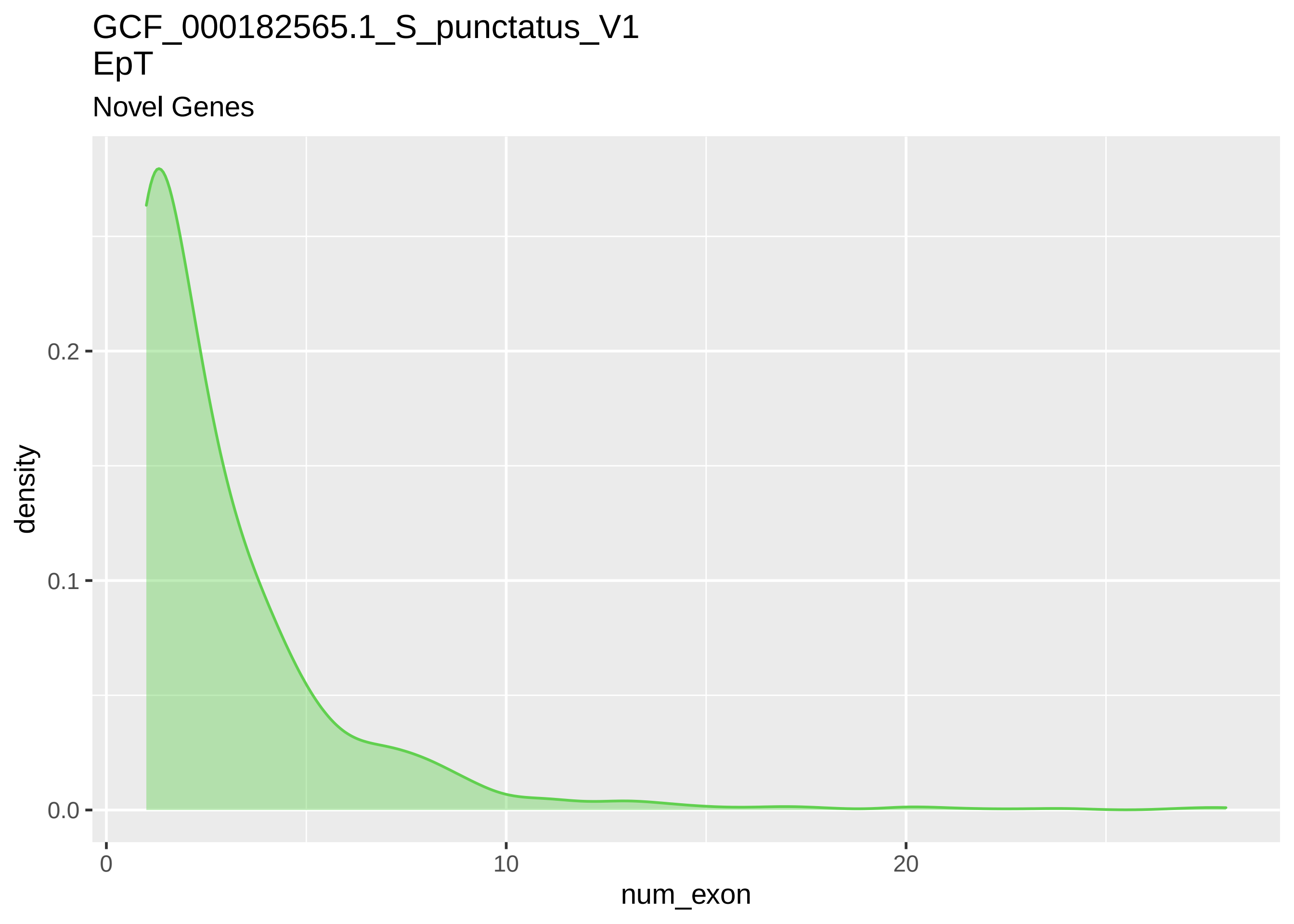

GCF_000182565.1_S_punctatus_V1
EpT
Novel Genes
density
0.2
0.1
0.0
0
10
20
num_exon

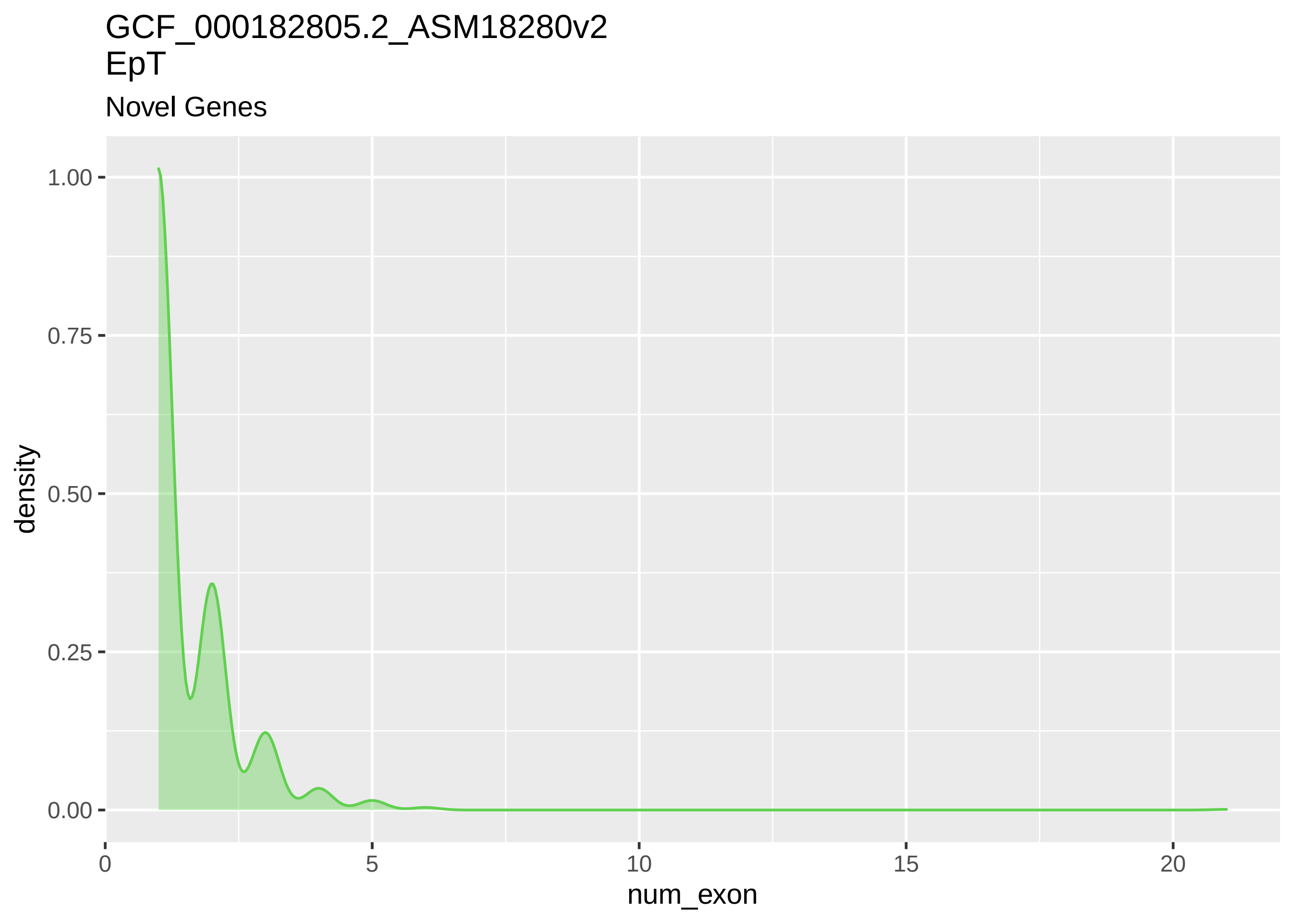
GCF_000182805.2_ASM18280v2
EpT
Novel Genes
density
1.00
0.75
0.50
0.25
0.00
0
5
10
15
20
num_exon

GCF_000182895.1_CC3
EpT

Novel Genes

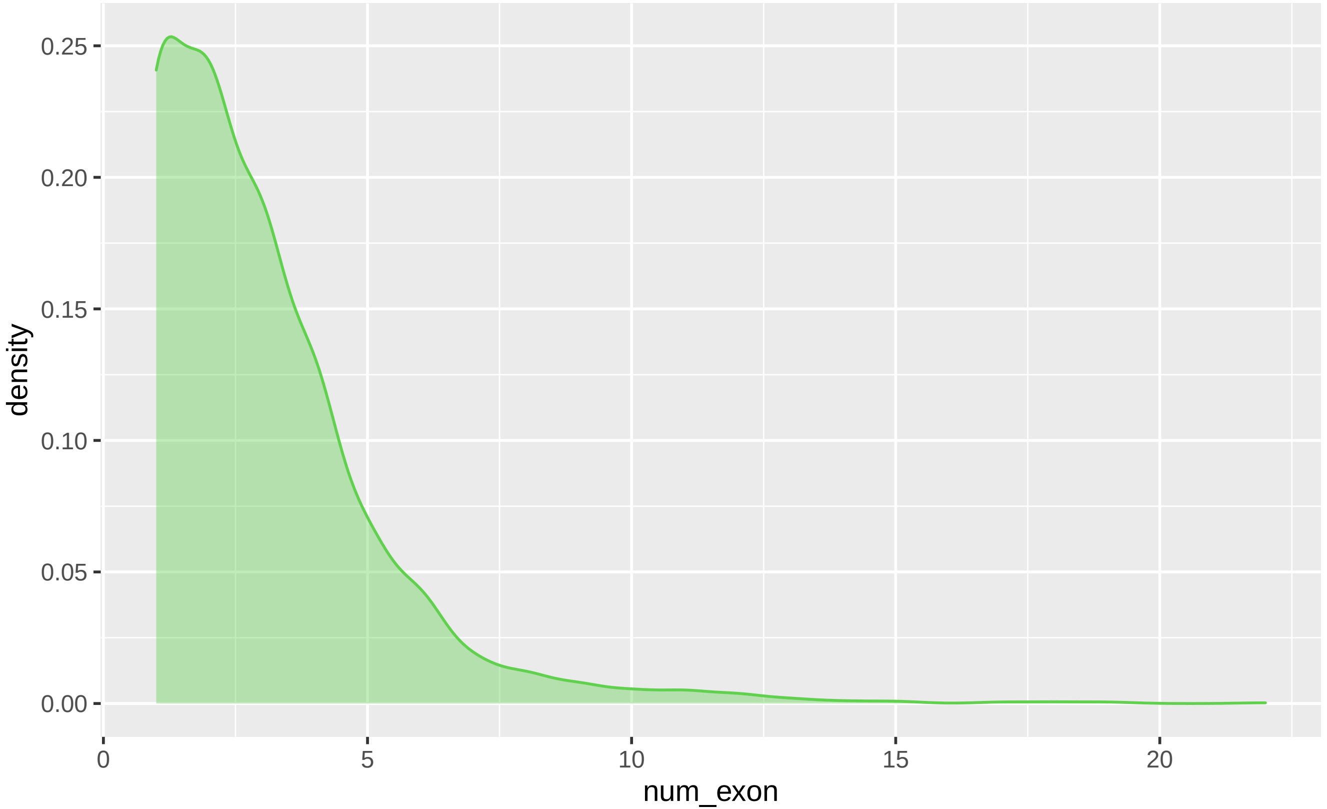

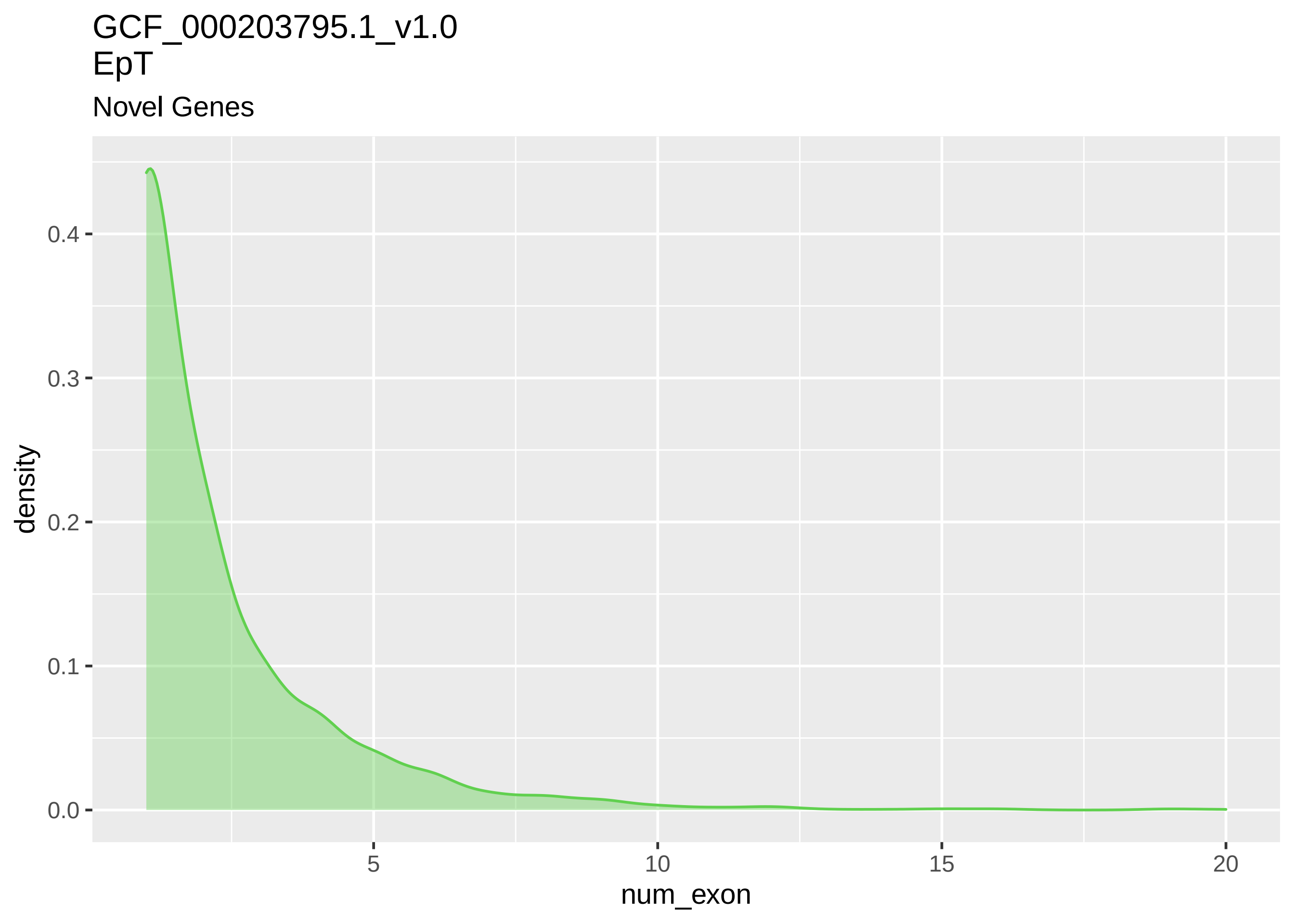
GCF_000203795.1_v1.0
EpT
Novel Genes
density
0.0
0.1
0.2
0.3
0.4
5
10
15
20
num_exon

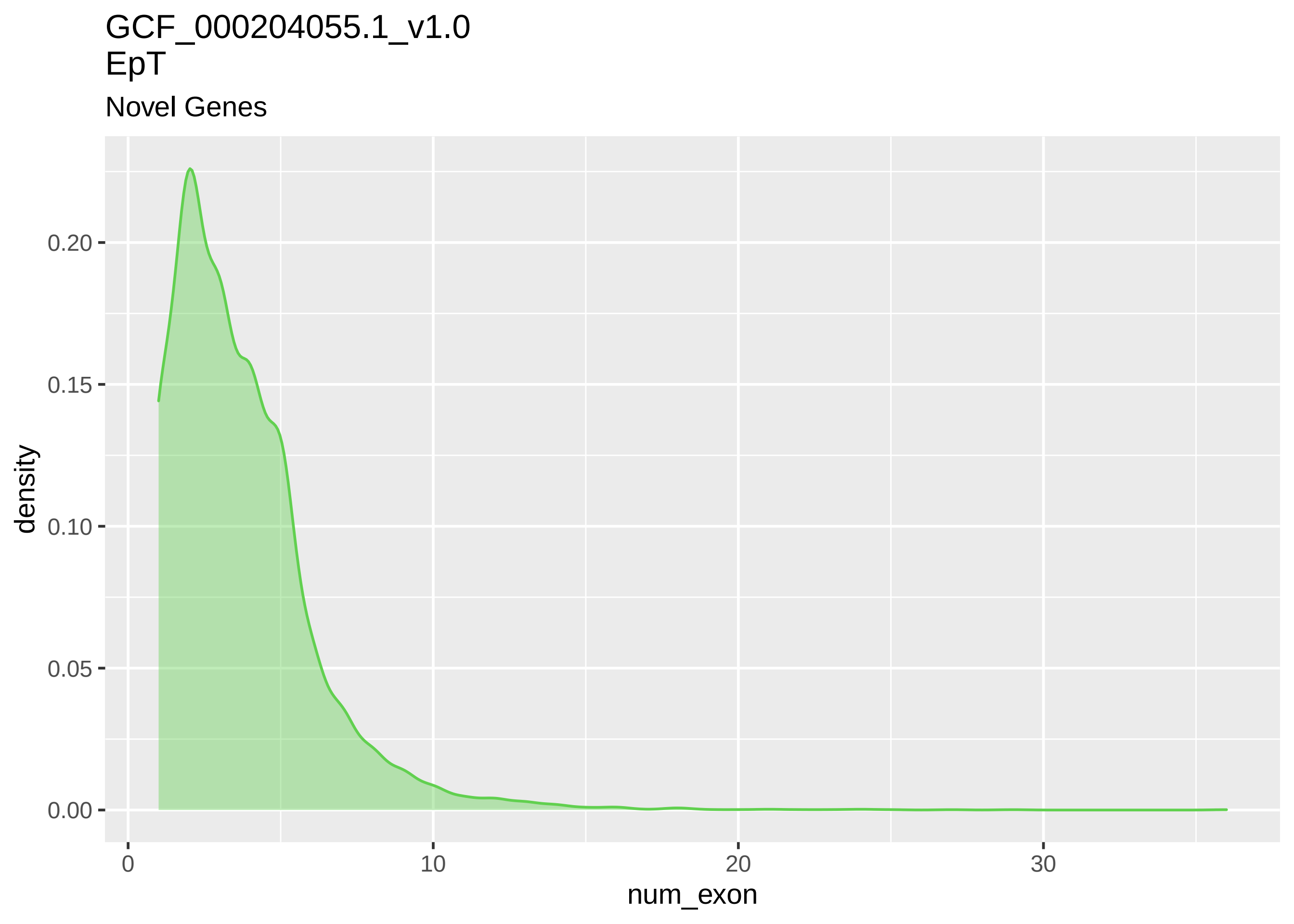
GCF_000204055.1_v1.0
EpT
Novel Genes
density
0.00
0.05
0.10
0.15
0.20
0
10
20
30
num_exon

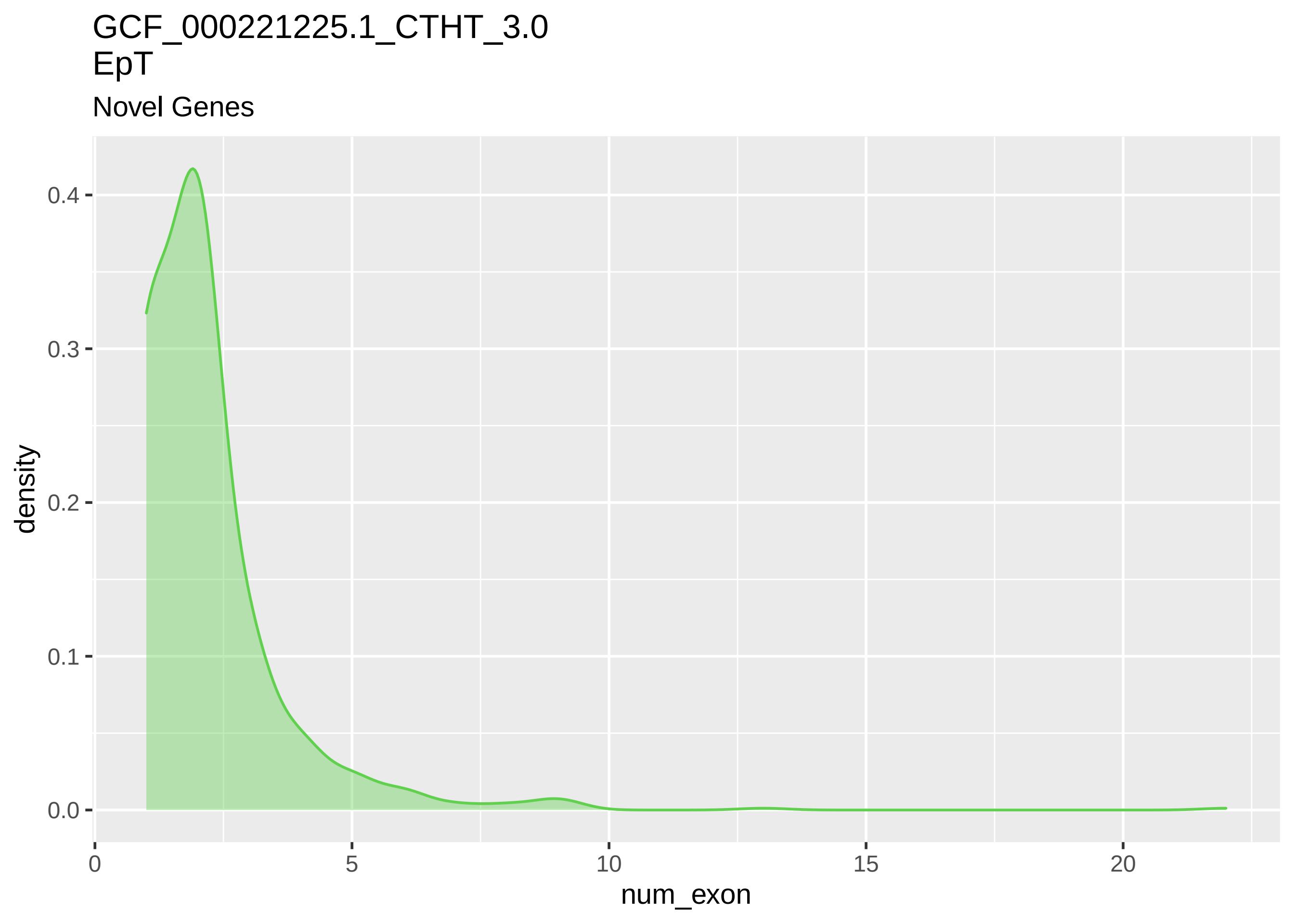
GCF_000221225.1_CTHT_3.0
EpT
Novel Genes
density
num_exon
0.0
0.1
0.2
0.3
0.4
0
5
10
15
20

GCF_000223465.1_Candida_tenuis_v1.0
EpT

Novel Genes

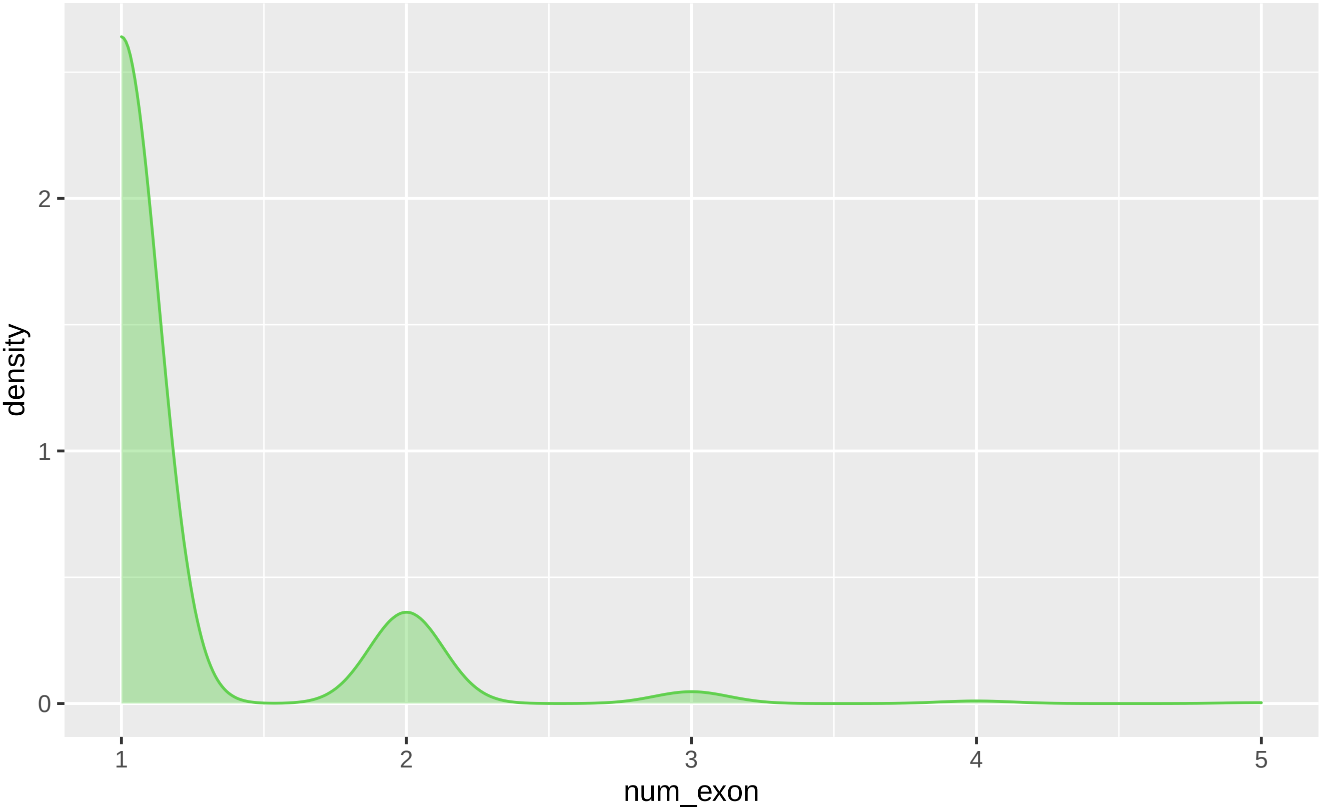

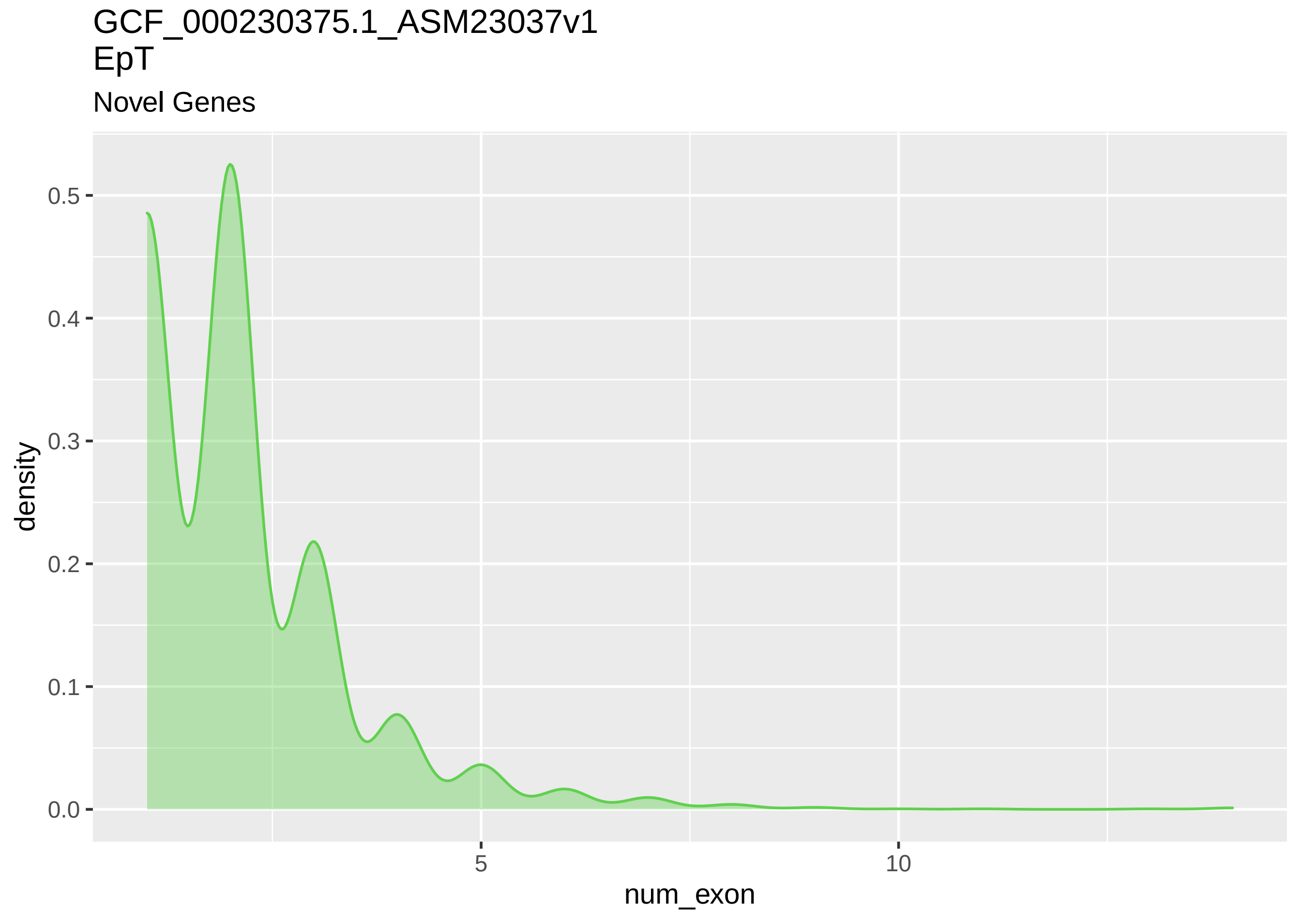
GCF_000230375.1_ASM23037v1
EpT
Novel Genes
density
0.0
0.1
0.2
0.3
0.4
0.5
5
10
num_exon

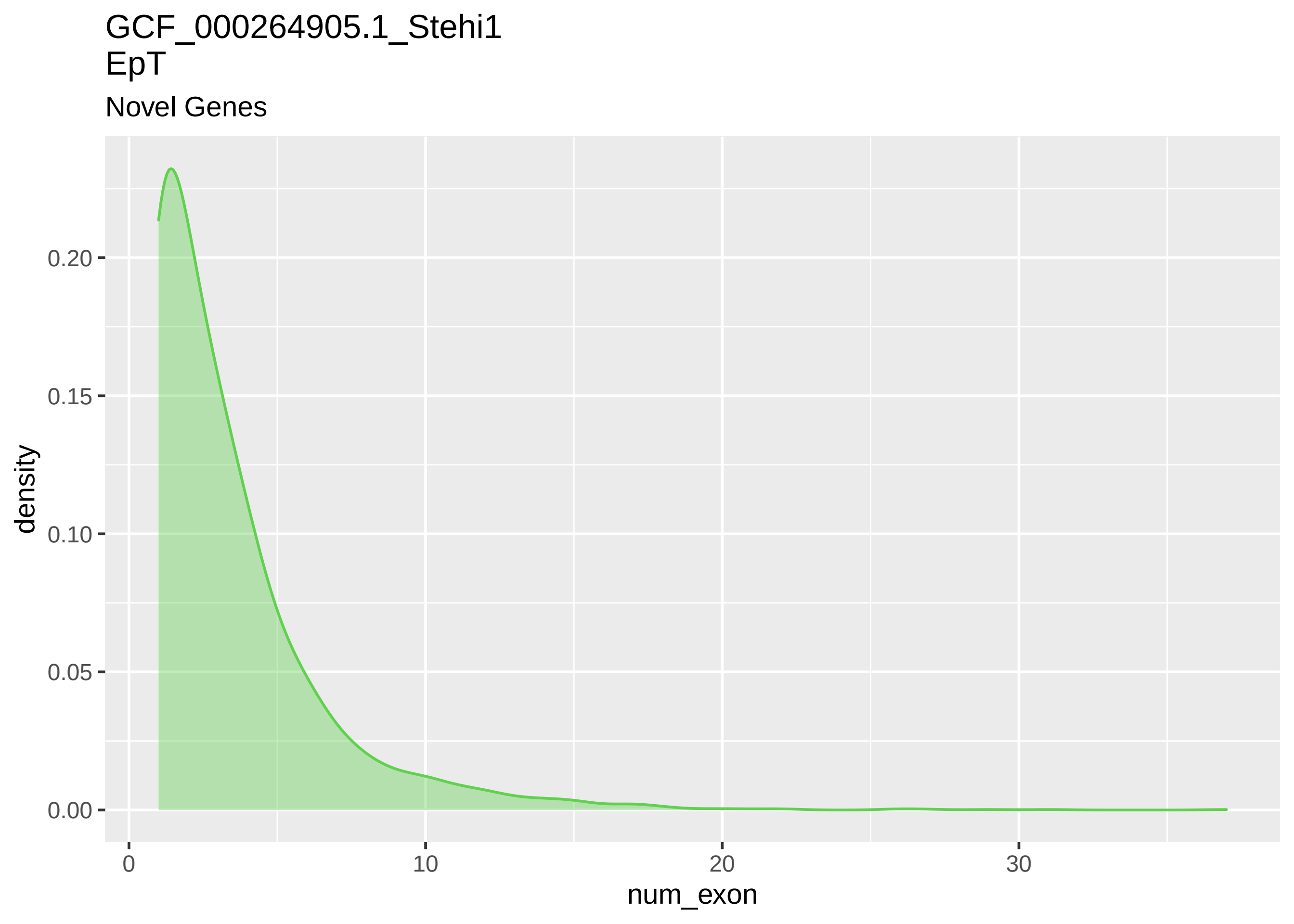

GCF_000264905.1_Stehi1
EpT
Novel Genes
density
0.00
0.05
0.10
0.15
0.20
0
10
20
30
num_exon

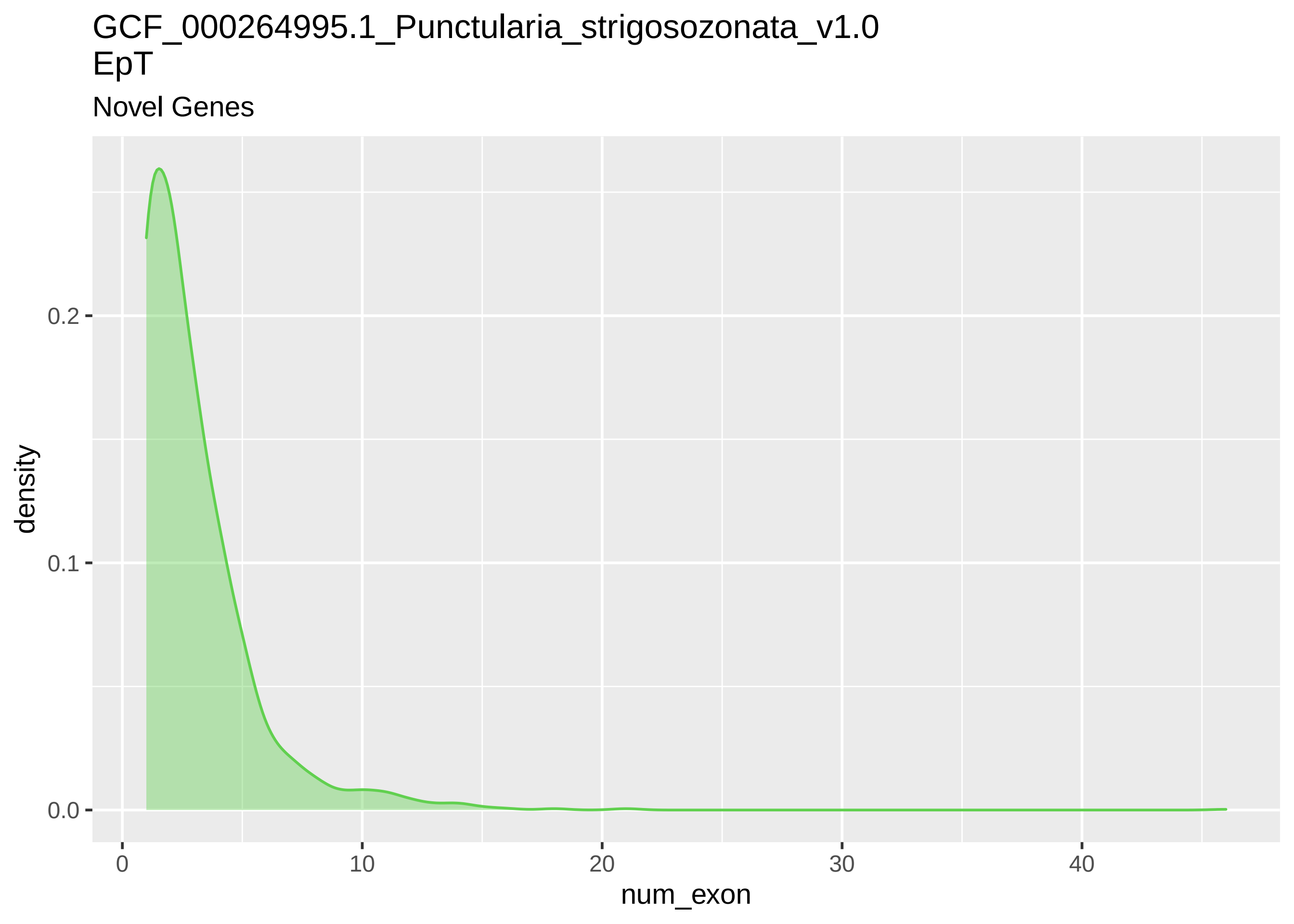
GCF_000264995.1_Punctularia_strigosozonata_v1.0
EpT
Novel Genes
density
0.2
0.1
0.0
0
10
20
30
40
num_exon

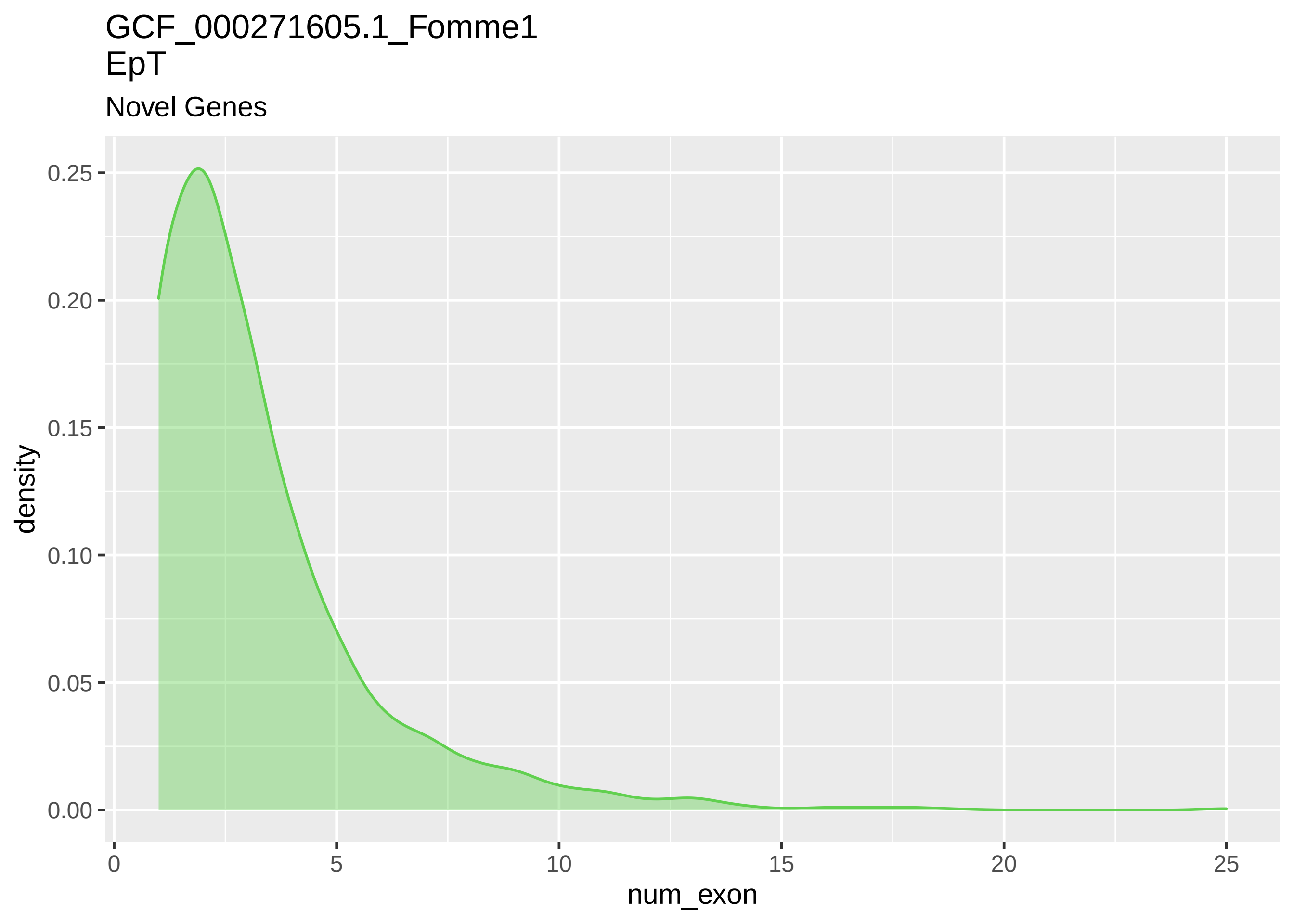

GCF_000271605.1_Fomme1
EpT
Novel Genes
density
num_exon
0.00
0.05
0.10
0.15
0.20
0.25
0
5
10
15
20
25

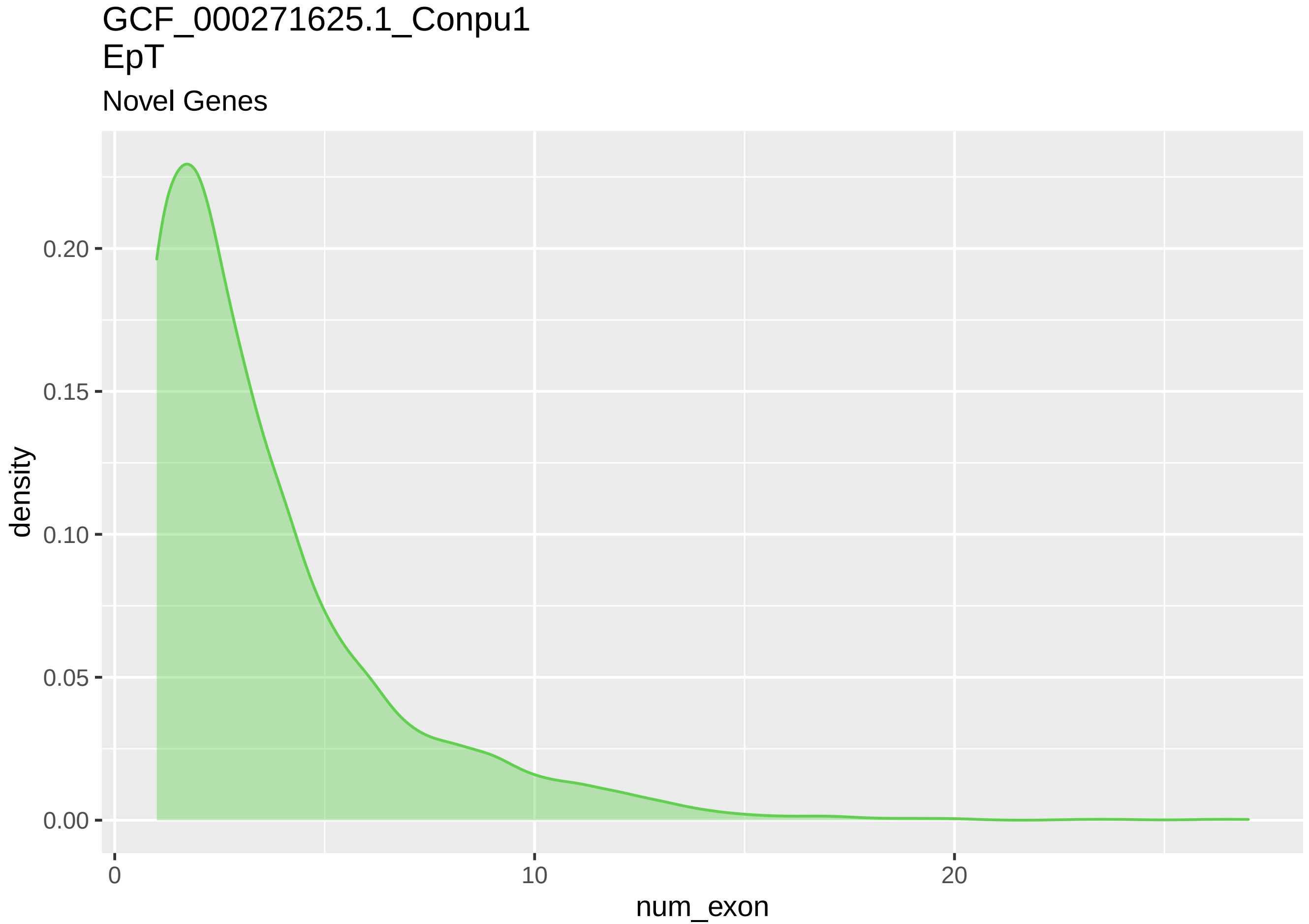
GCF_000271625.1_Conpu1
EpT
Novel Genes
density
0.20
0.15
0.10
0.05
0.00
0
10
20
num_exon

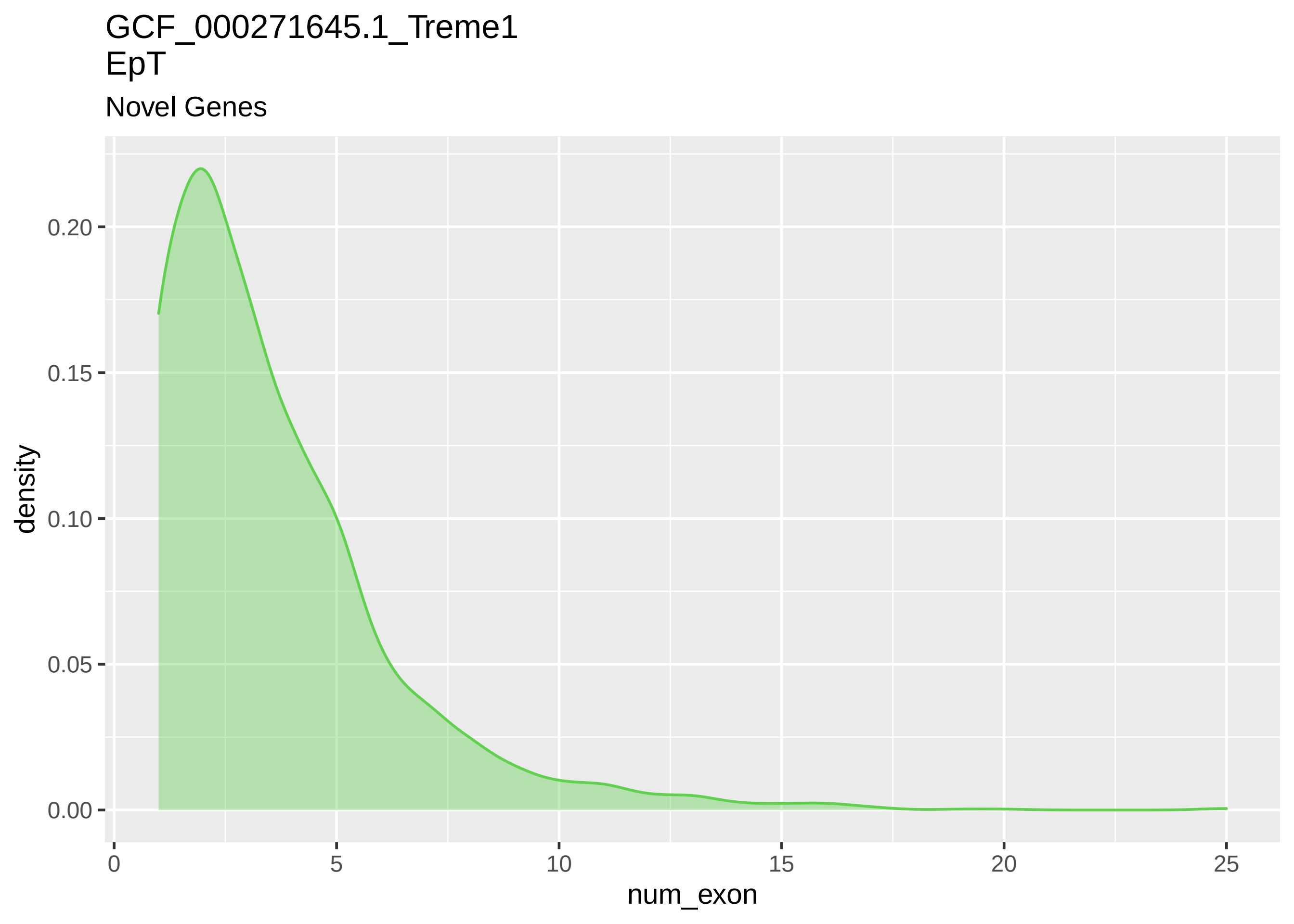
GCF_000271645.1_Treme1
EpT
Novel Genes
density
0.00
0.05
0.10
0.15
0.20
0
5
10
15
20
25
num_exon

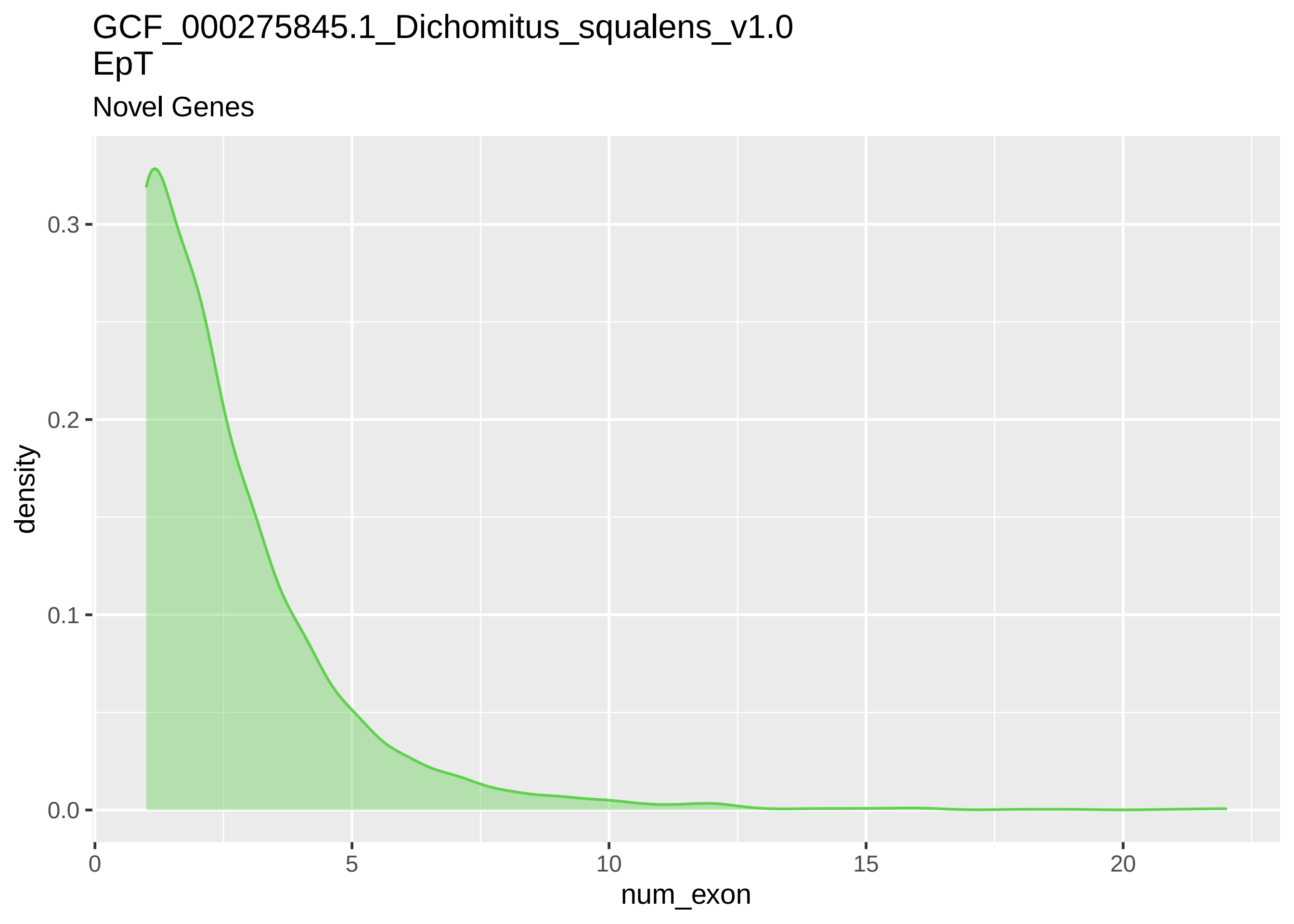
GCF_000275845.1_Dichomitus_squalens_v1.0
EpT
Novel Genes
density
0.0
0.1
0.2
0.3
0
5
10
15
20
num_exon

GCF_000281105.1_Coni_apol_CBS100218_V1
EpT

Novel Genes

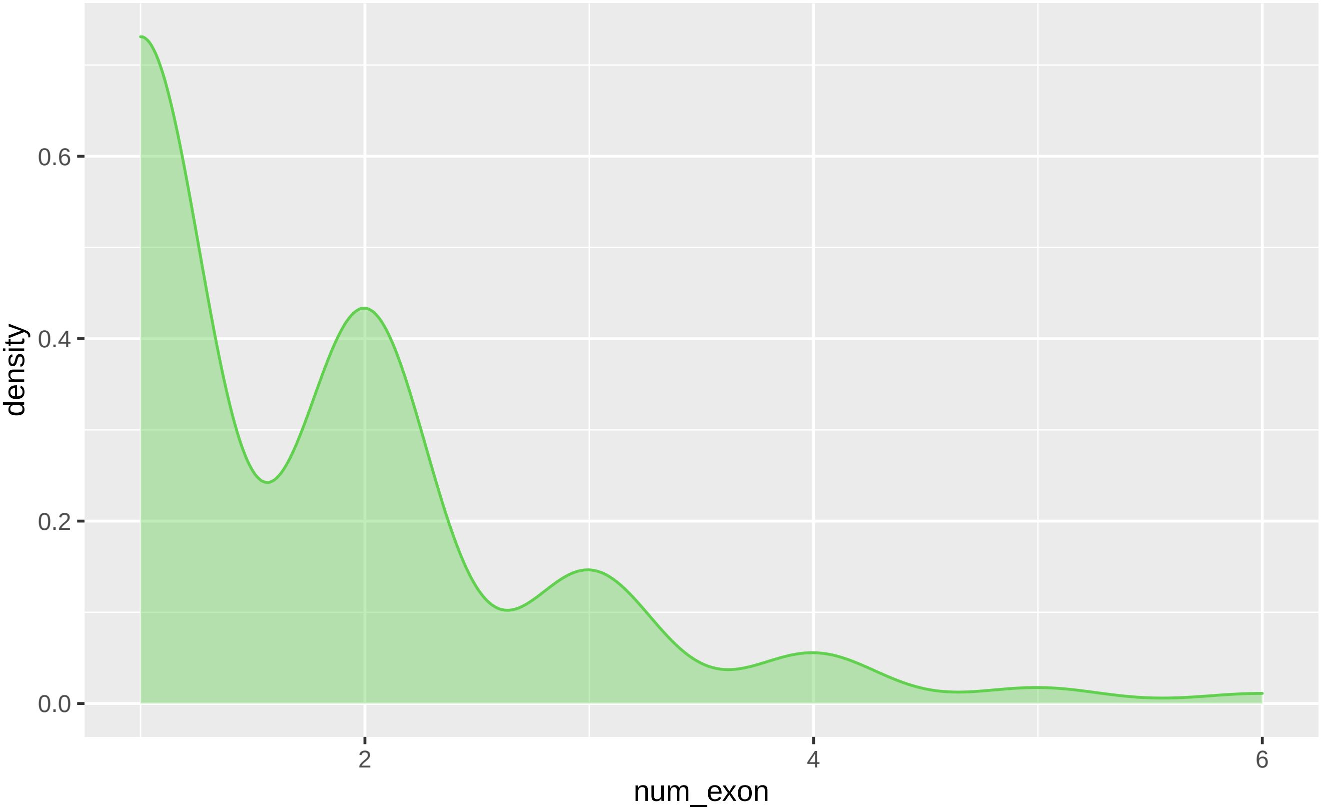

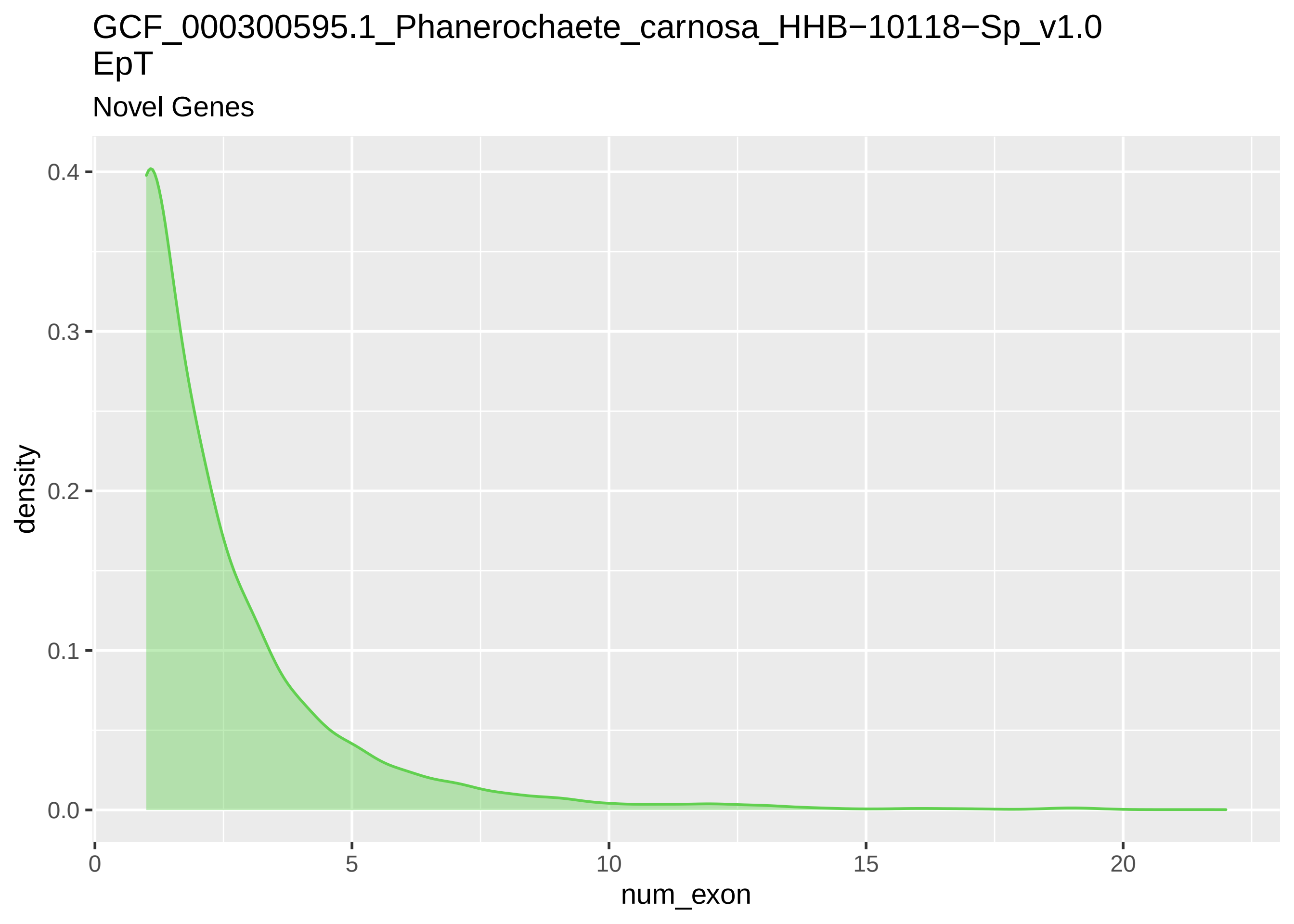
GCF_000300595.1_Phanerochaete_carnosa_HHB−10118−Sp_v1.0
EpT
Novel Genes
density
0.0
0.1
0.2
0.3
0.4
0
5
10
15
20
num_exon

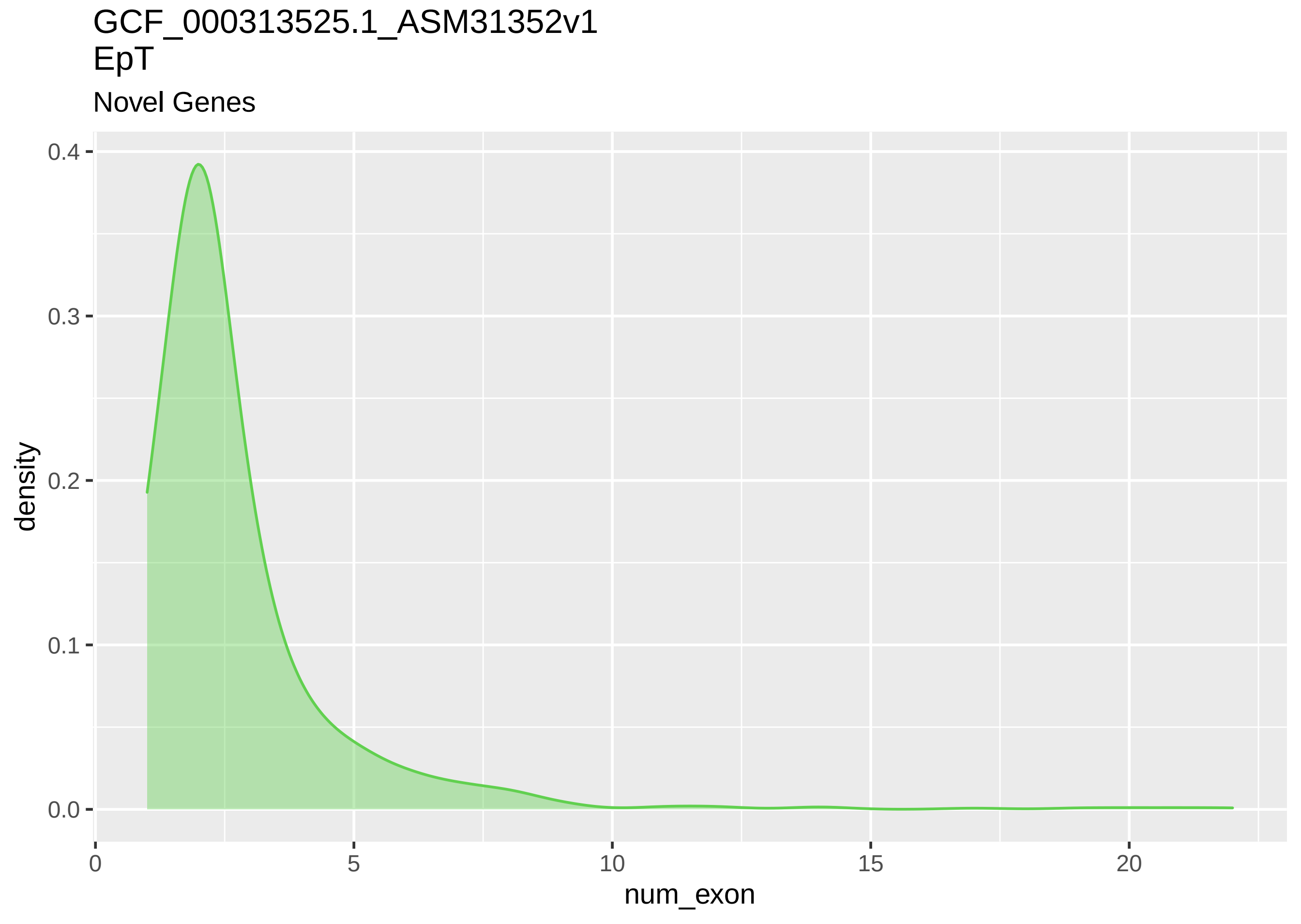
GCF_000313525.1_ASM31352v1
EpT
Novel Genes
density
0.0
0.1
0.2
0.3
0.4
0
5
10
15
20
num_exon

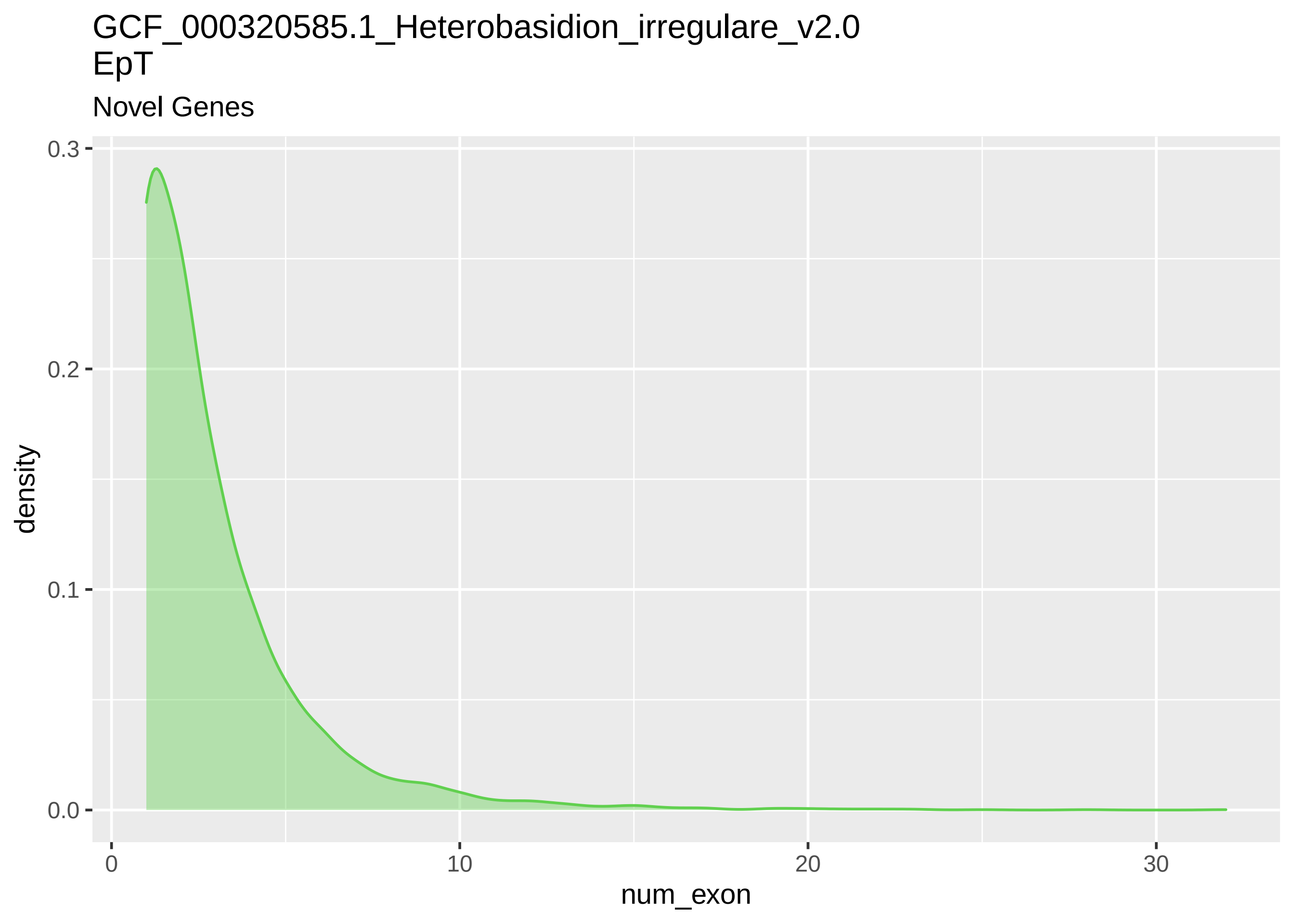
GCF_000320585.1_Heterobasidion_irregulare_v2.0
EpT
Novel Genes
density
0.3
0.2
0.1
0.0
0
10
20
30
num_exon

GCF_000328475.2_Umaydis521_2.0
EpT

Novel Genes

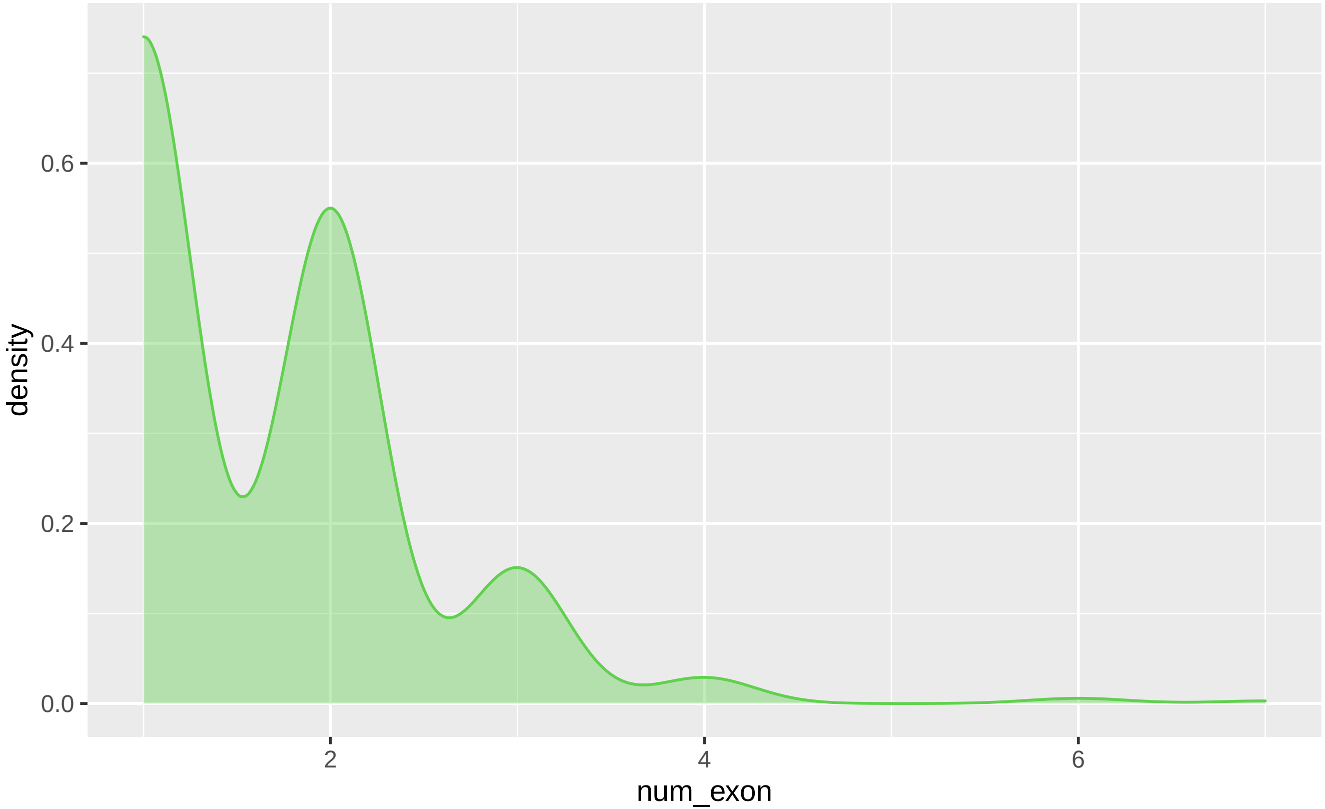

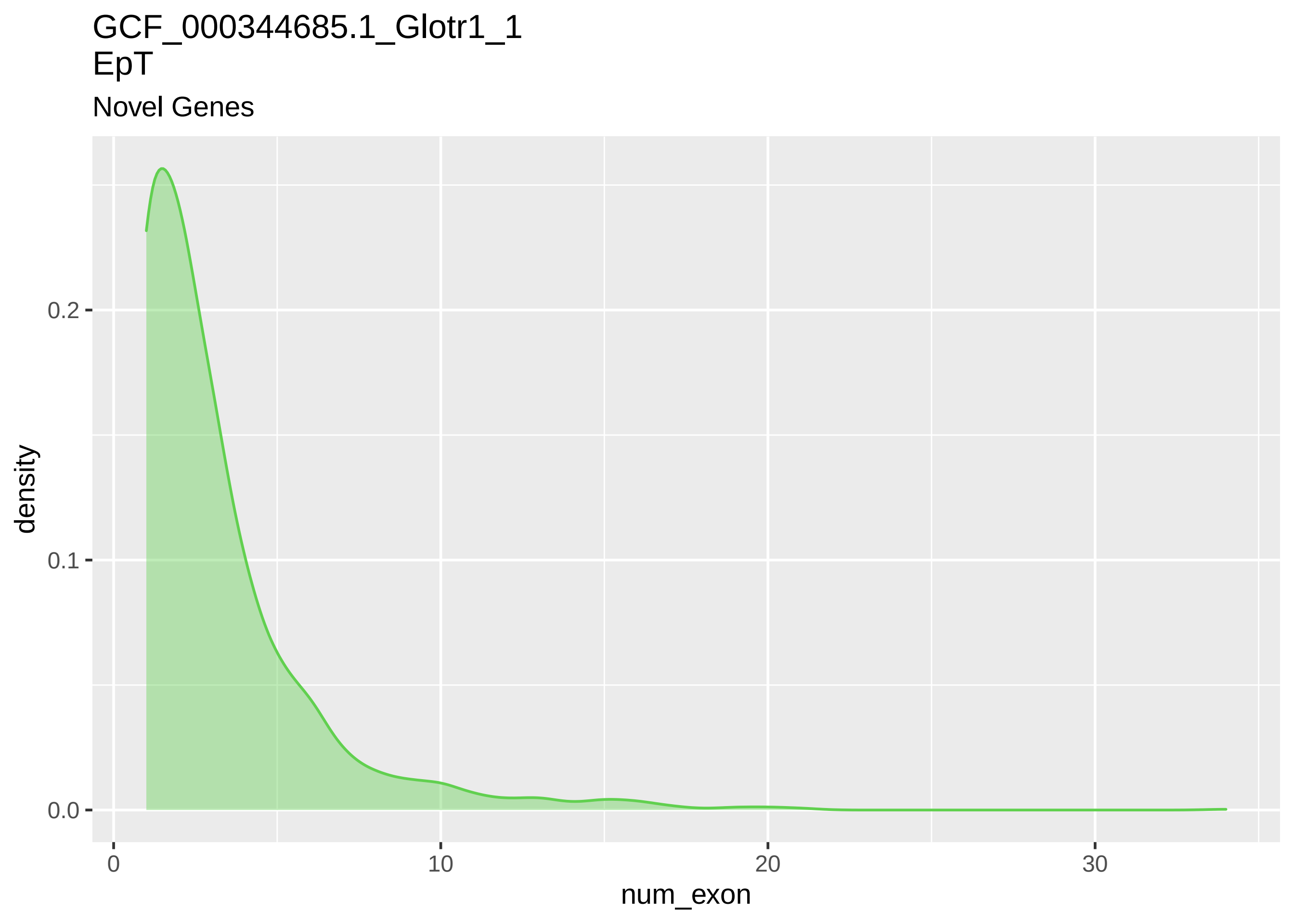
GCF_000344685.1_Glotr1_1
EpT
Novel Genes
density
0.2
0.1
0.0
0
10
20
30
num_exon

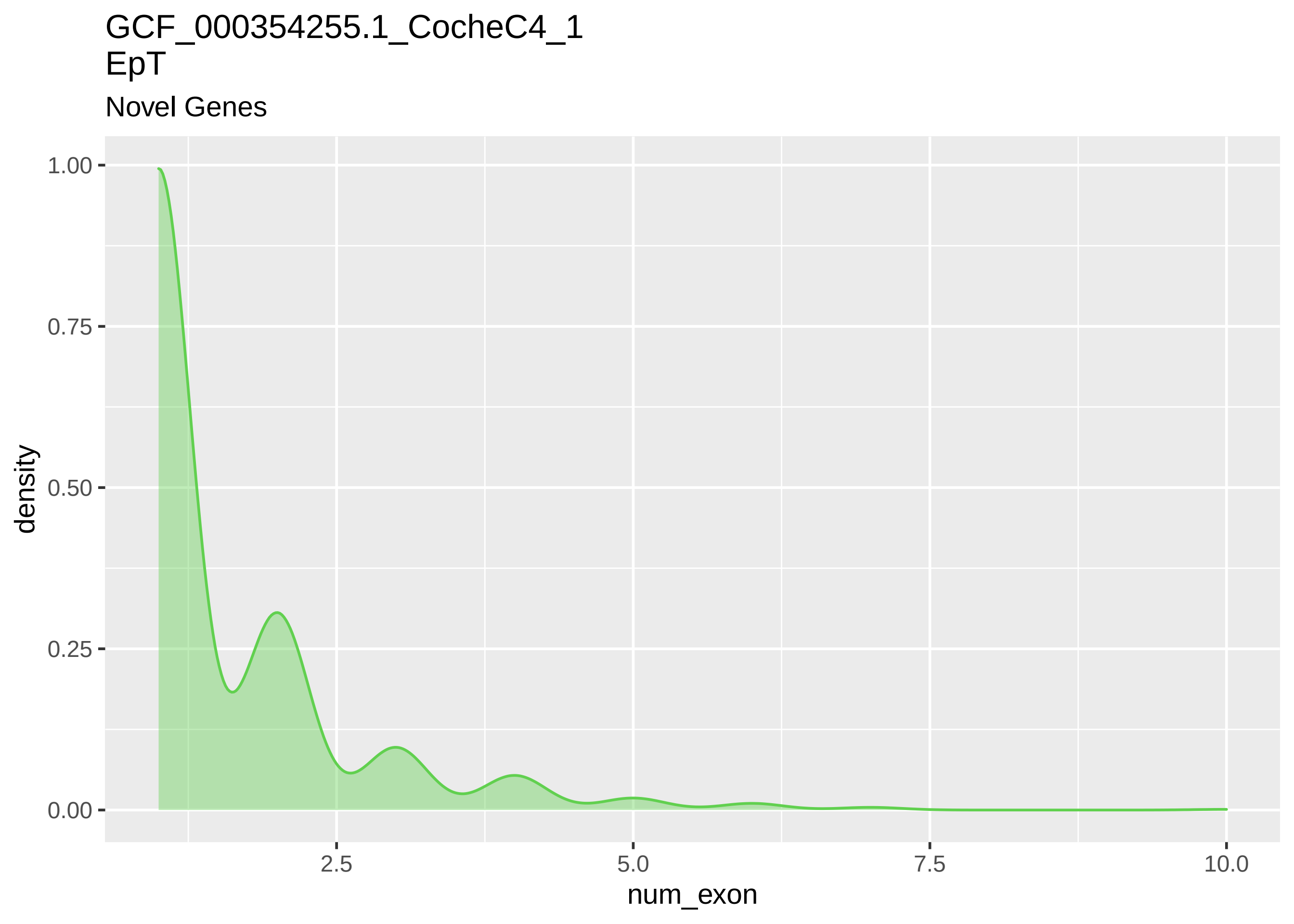

GCF_000354255.1_CocheC4_1
EpT
Novel Genes
density
num_exon
1.00
0.75
0.50
0.25
0.00
2.5
5.0
7.5
10.0

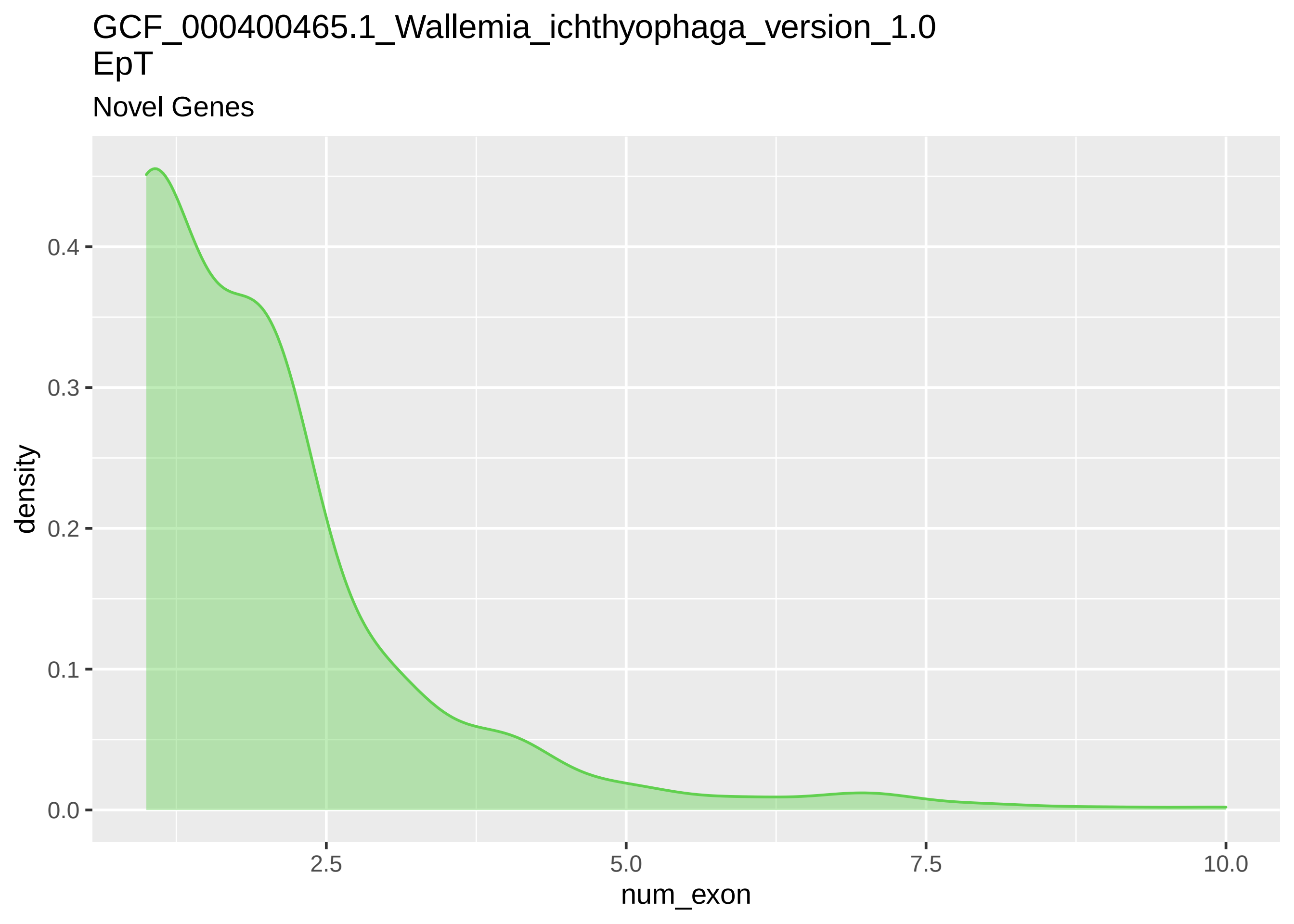
GCF_000400465.1_Wallemia_ichthyophaga_version_1.0
EpT
Novel Genes
density
0.0
0.1
0.2
0.3
0.4
2.5
5.0
7.5
10.0
num_exon

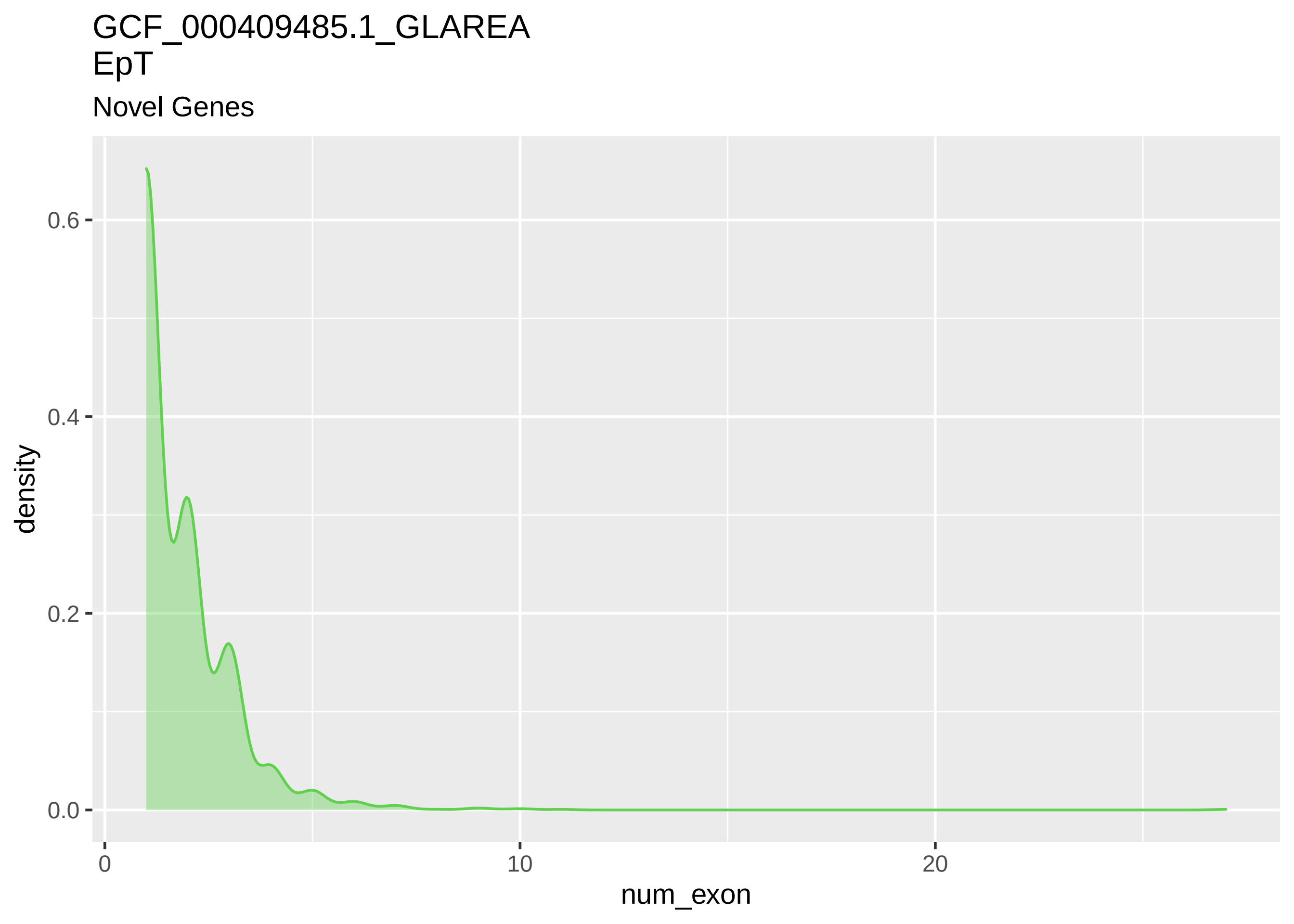
GCF_000409485.1_GLAREA
EpT
Novel Genes
density
0.0
0.2
0.4
0.6
0
10
20
num_exon

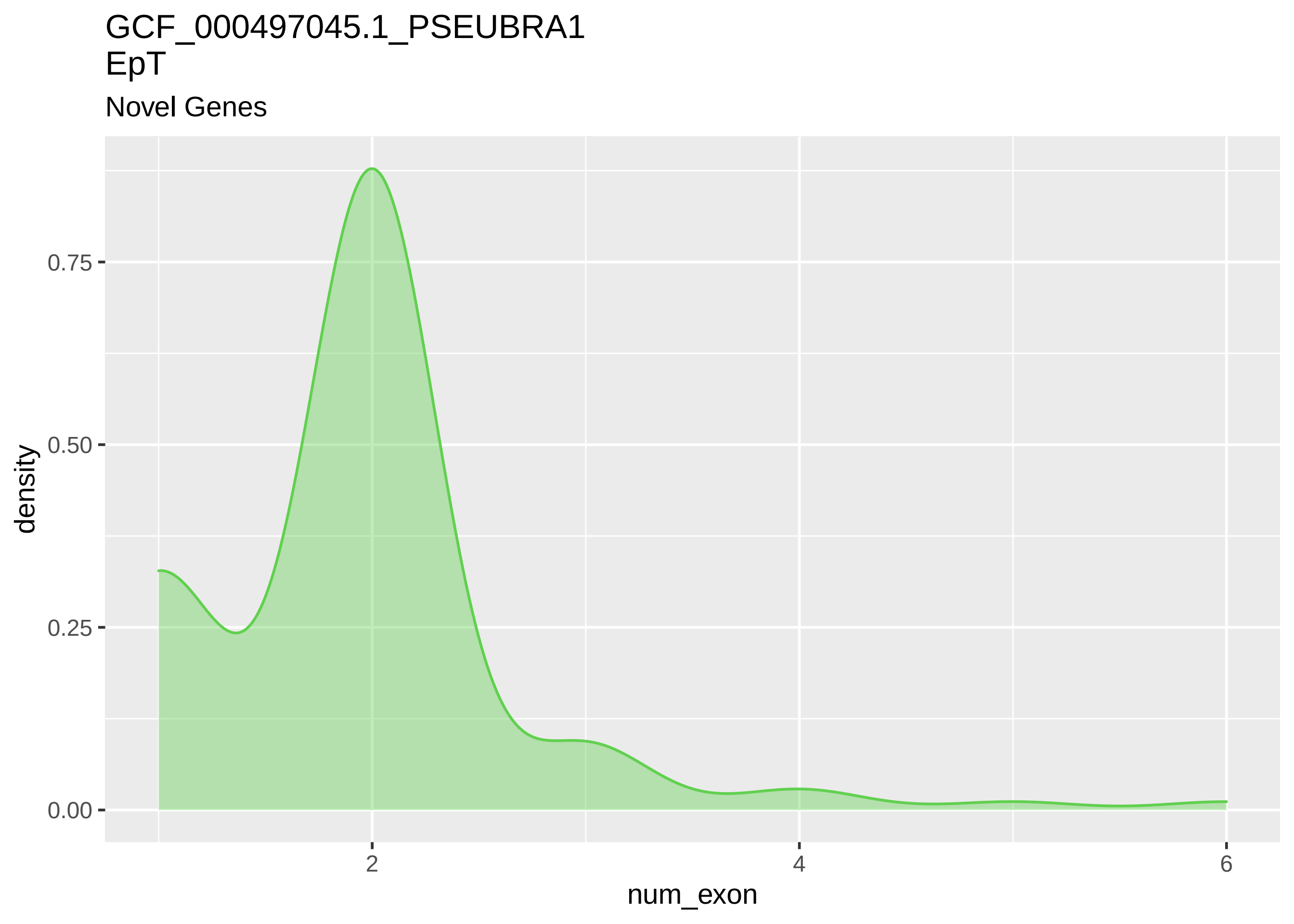

GCF_000497045.1_PSEUBRA1
EpT
Novel Genes
density
0.75
0.50
0.25
0.00
2
4
6
num_exon

GCF_000512605.1_Cryp_pinu_CBS10737_V1
EpT

Novel Genes

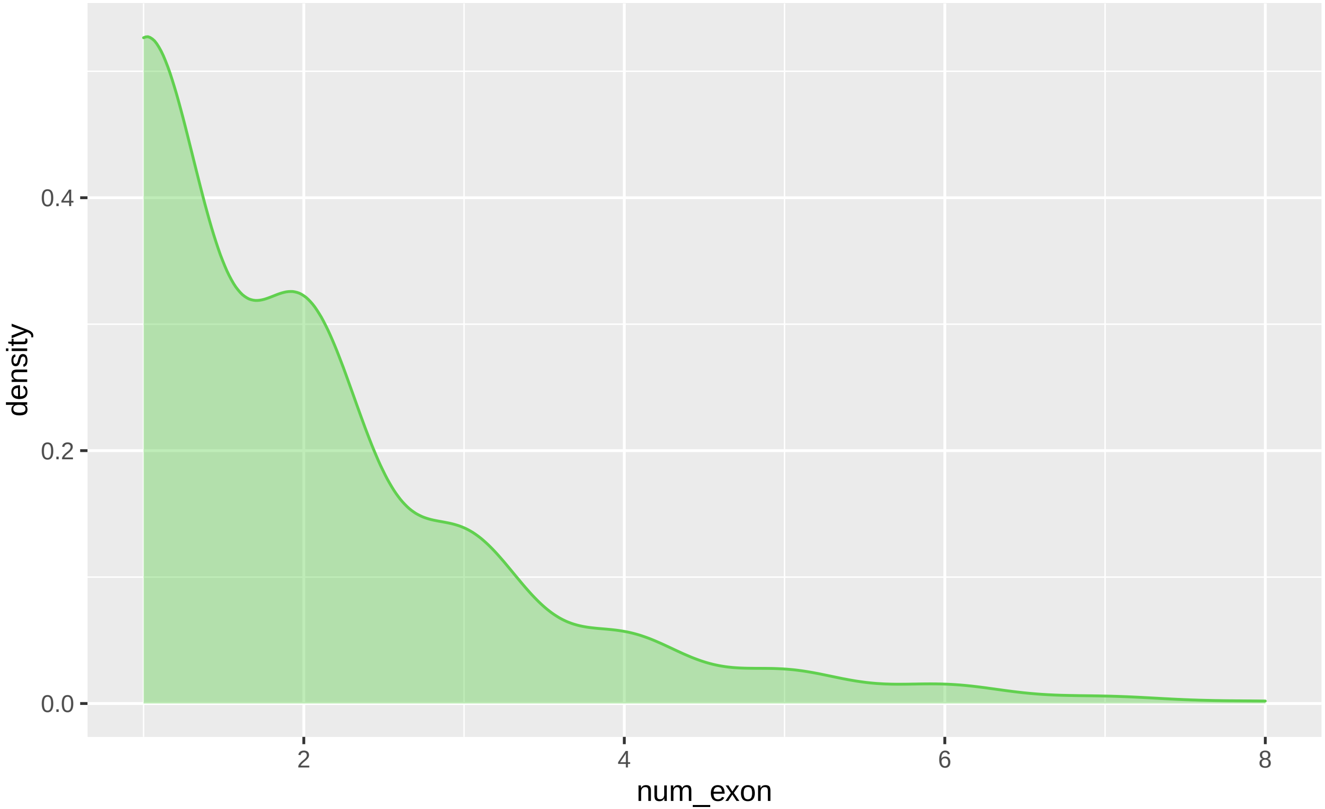

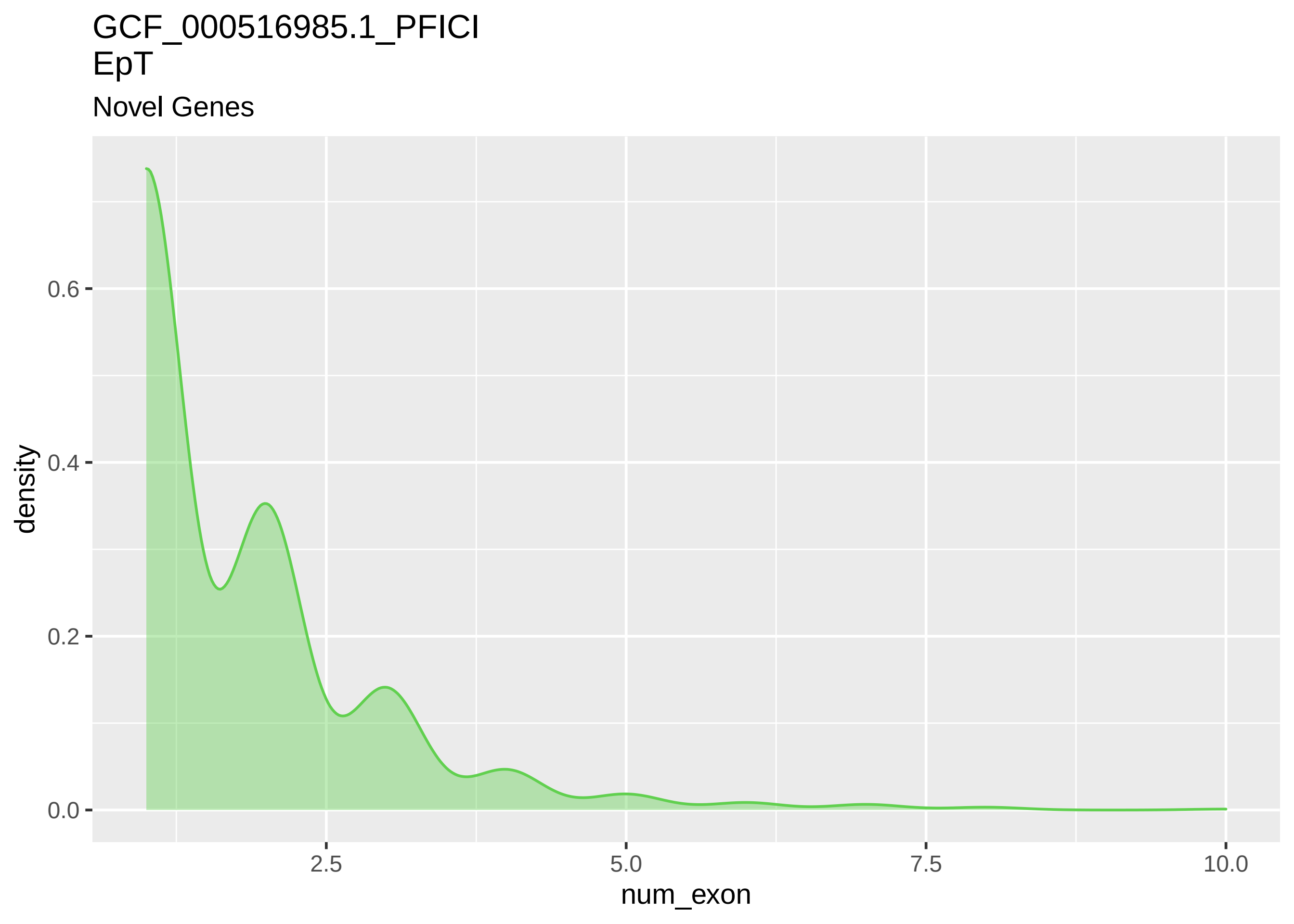
GCF_000516985.1_PFICI
EpT
Novel Genes
density
0.0
0.2
0.4
0.6
2.5
5.0
7.5
10.0
num_exon

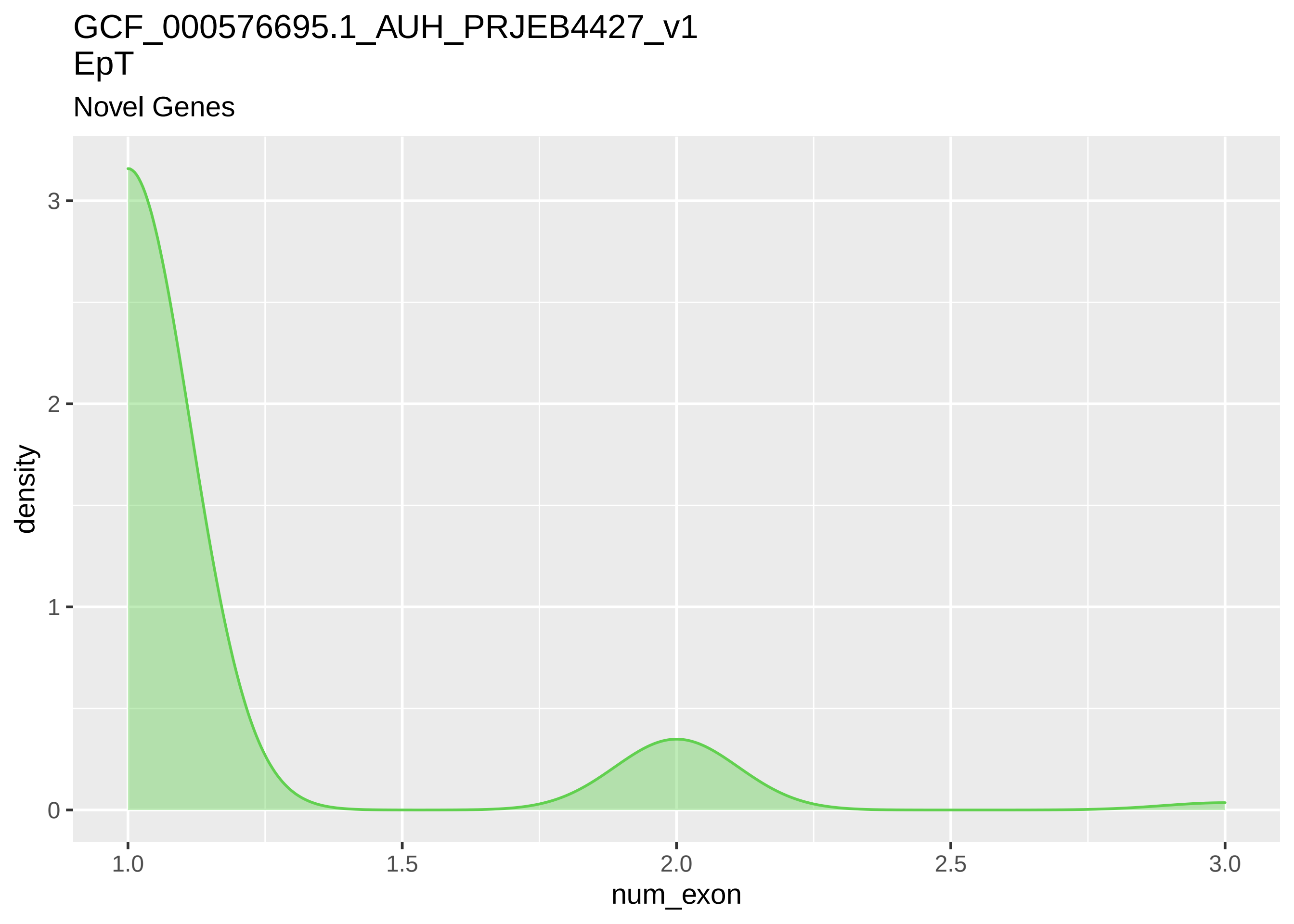
GCF_000576695.1_AUH_PRJEB4427_v1
EpT
Novel Genes
density
3
2
1
0
1.0
1.5
2.0
2.5
3.0
num_exon

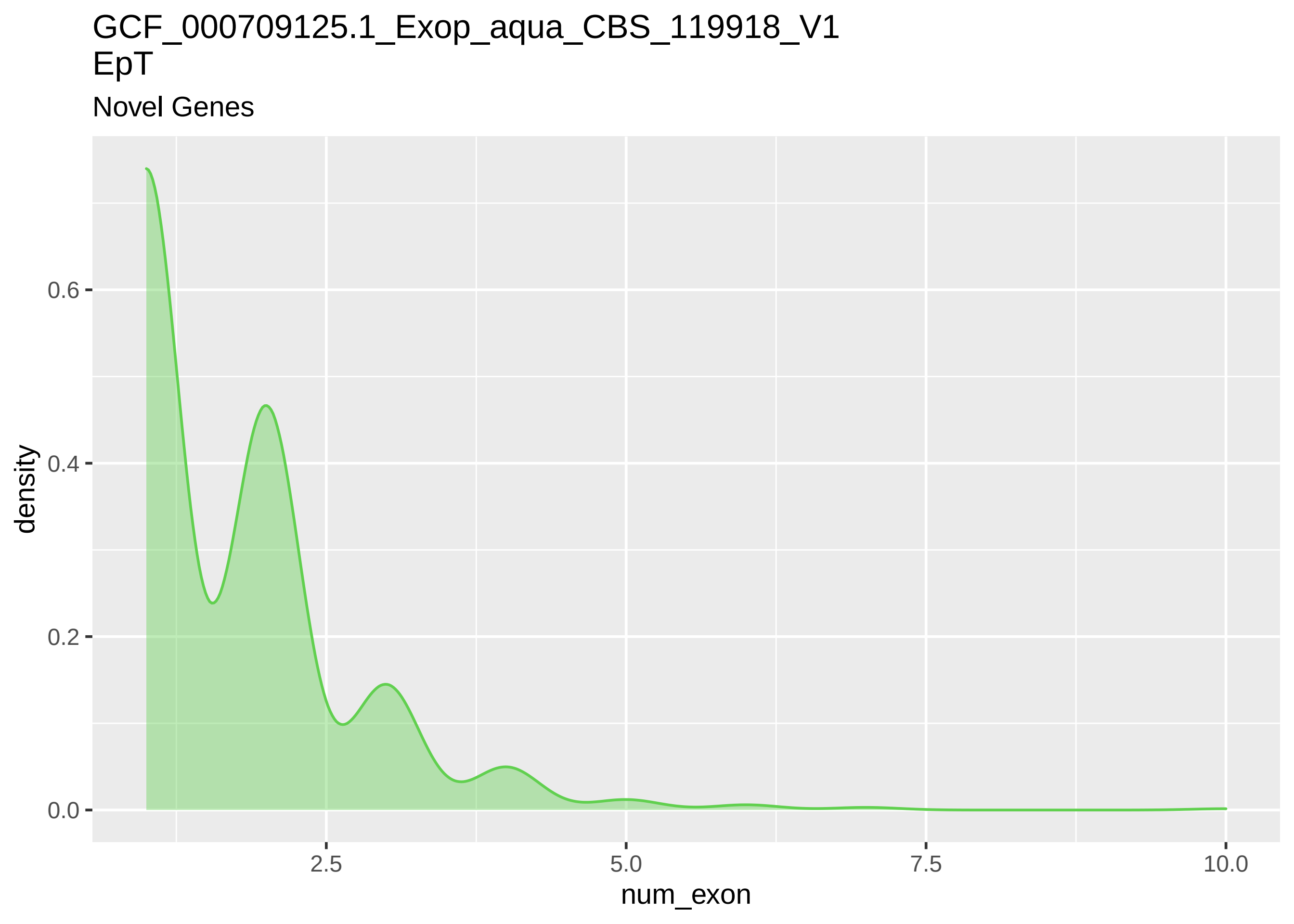
GCF_000709125.1_Exop_aqua_CBS_119918_V1
EpT
Novel Genes
density
0.0
0.2
0.4
0.6
2.5
5.0
7.5
10.0
num_exon

GCF_000835455.1_Fons_pedr_CBS_271_37_V1
EpT

Novel Genes

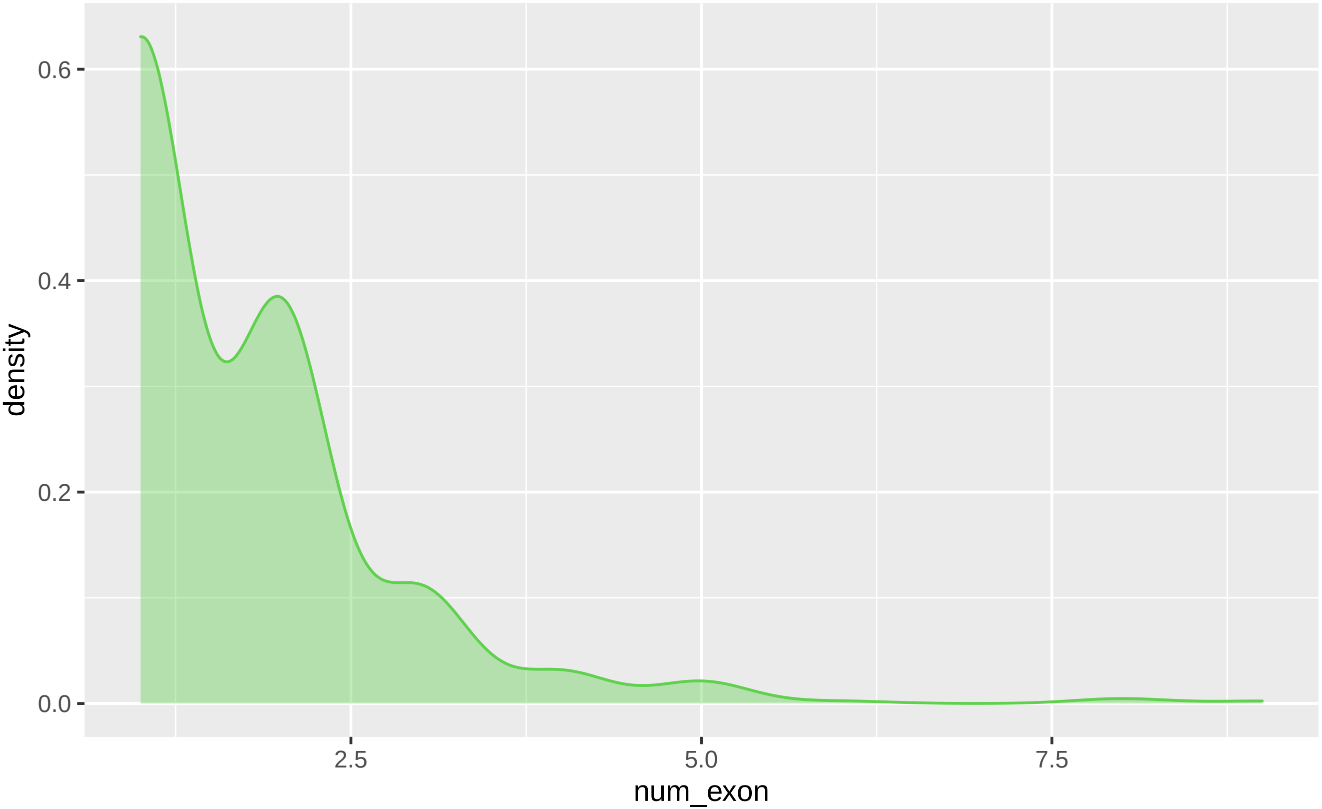

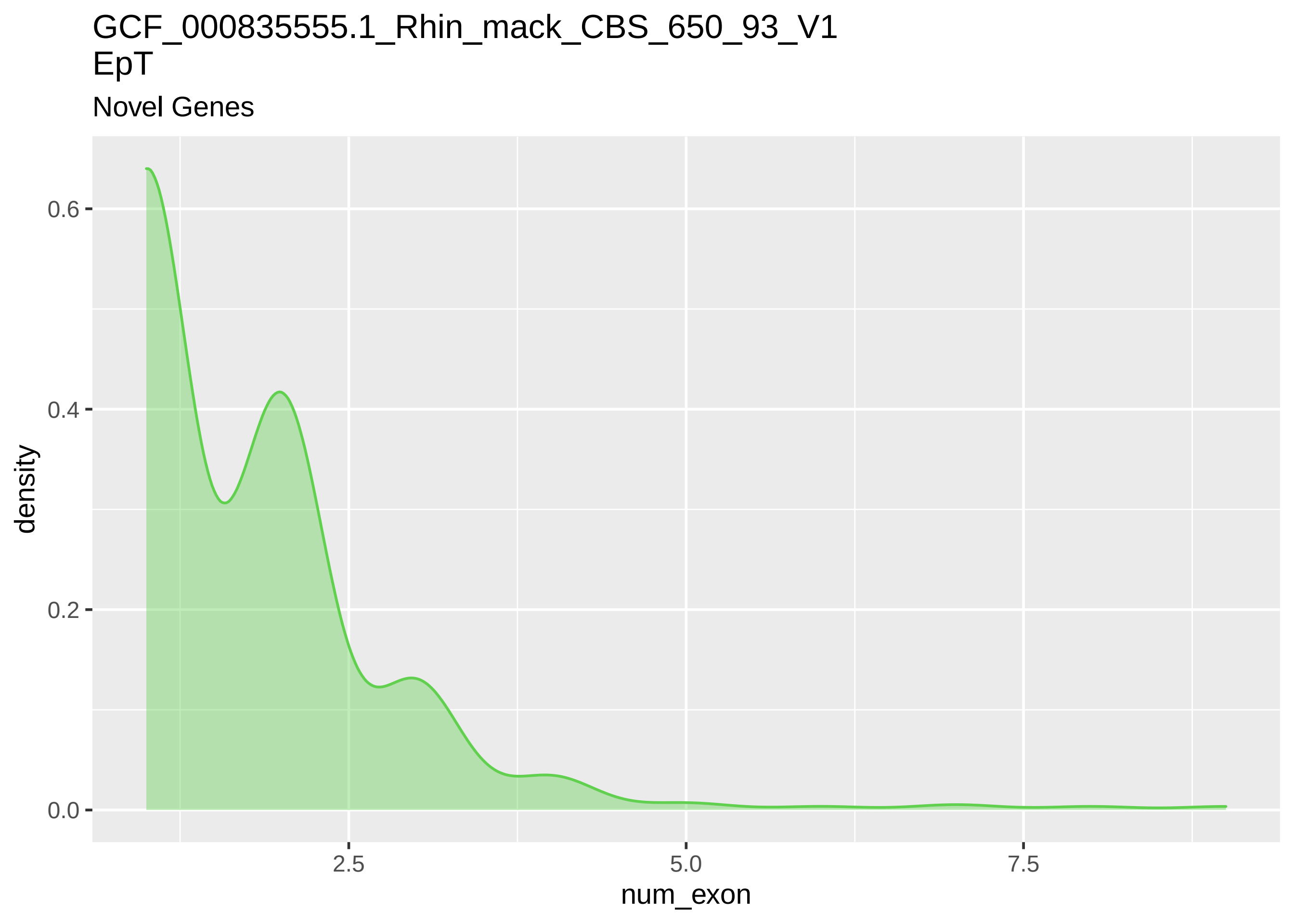
GCF_000835555.1_Rhin_mack_CBS_650_93_V1
EpT
Novel Genes
density
0.0
0.2
0.4
0.6
2.5
5.0
7.5
num_exon

GCF_000836295.1_O_gall_CBS43764
EpT

Novel Genes

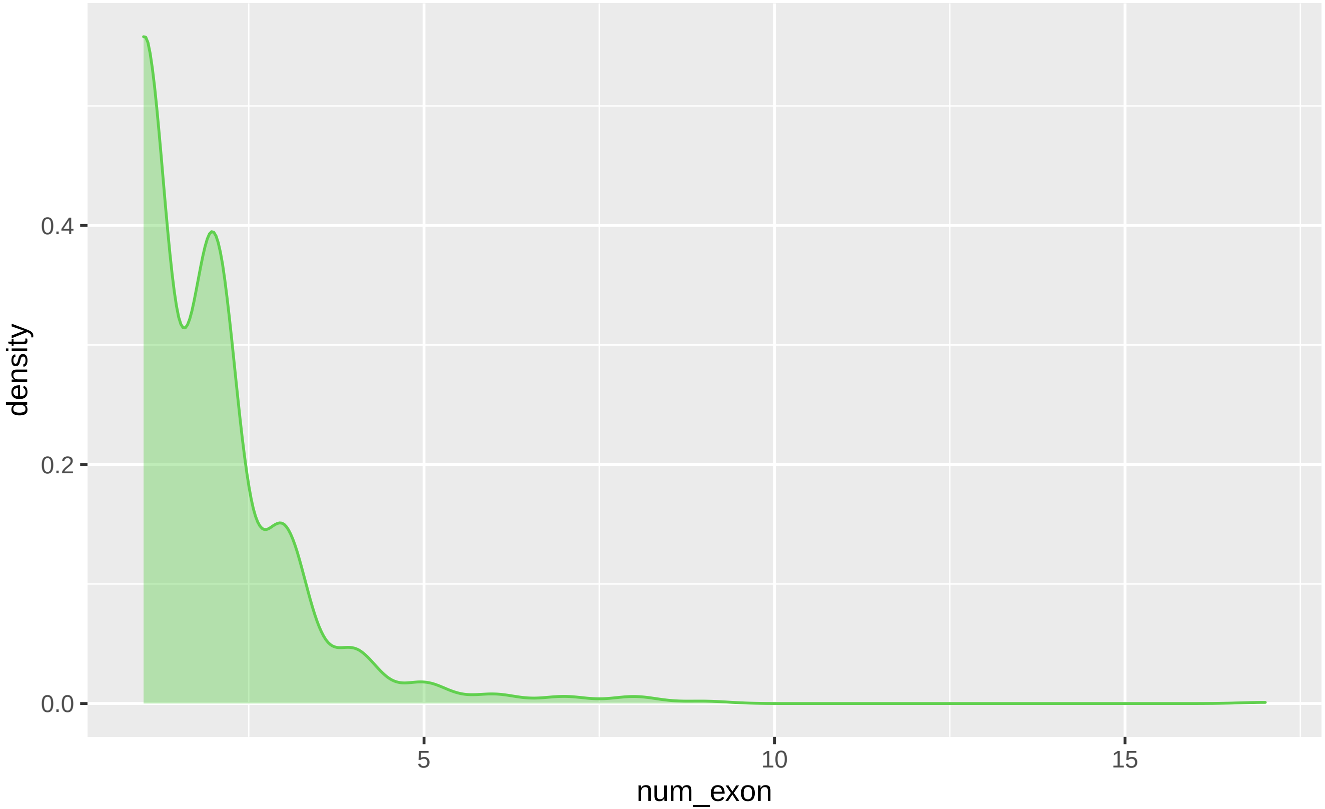

GCF_000938715.1_LALA0
EpT

Novel Genes

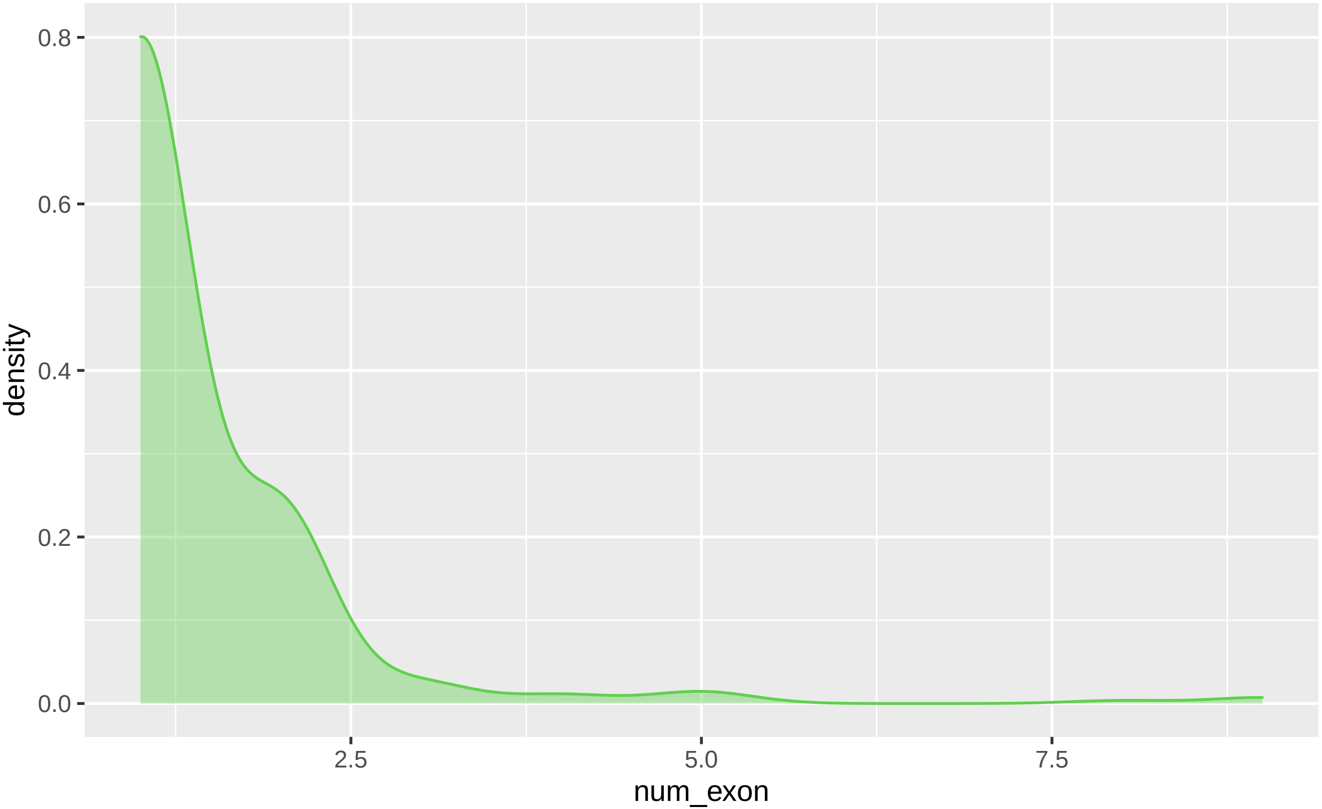

GCF_001027345.1_Triol1
EpT

Novel Genes

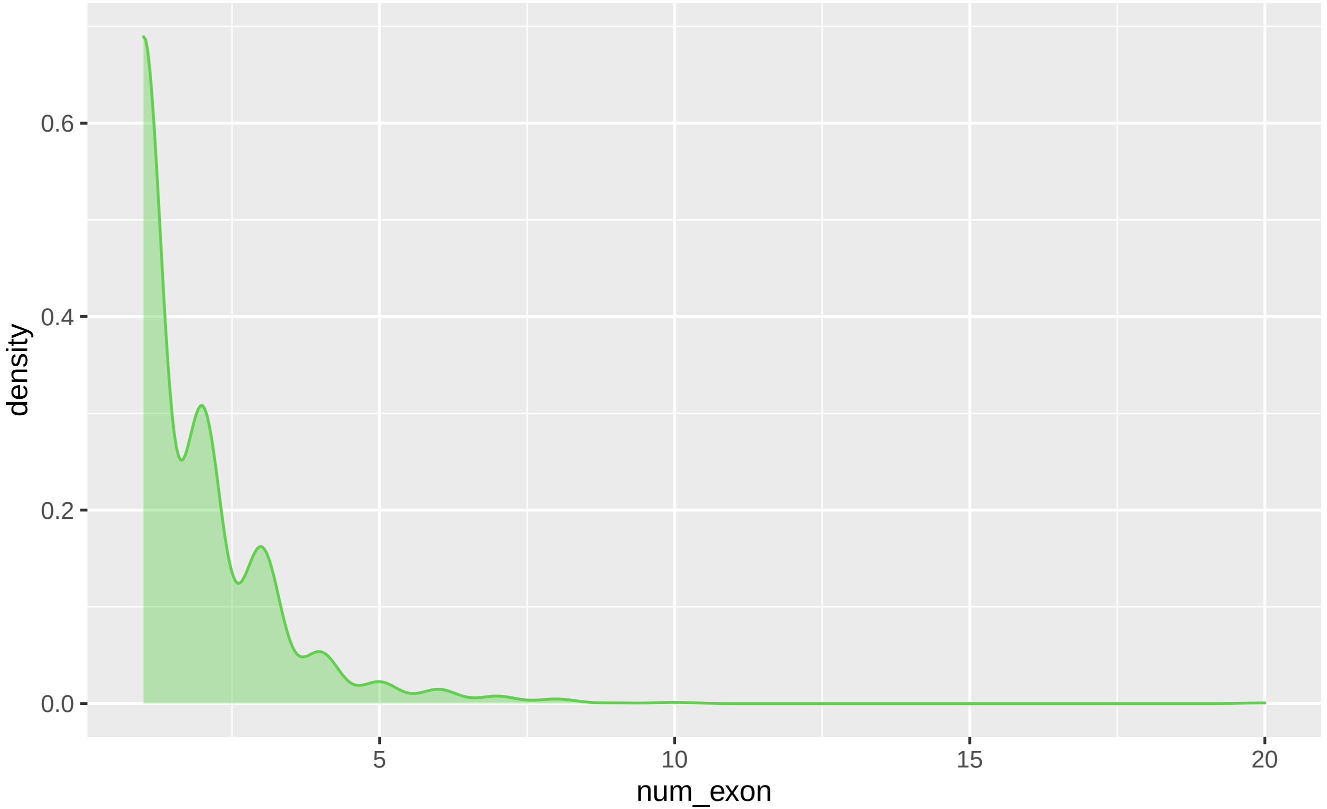

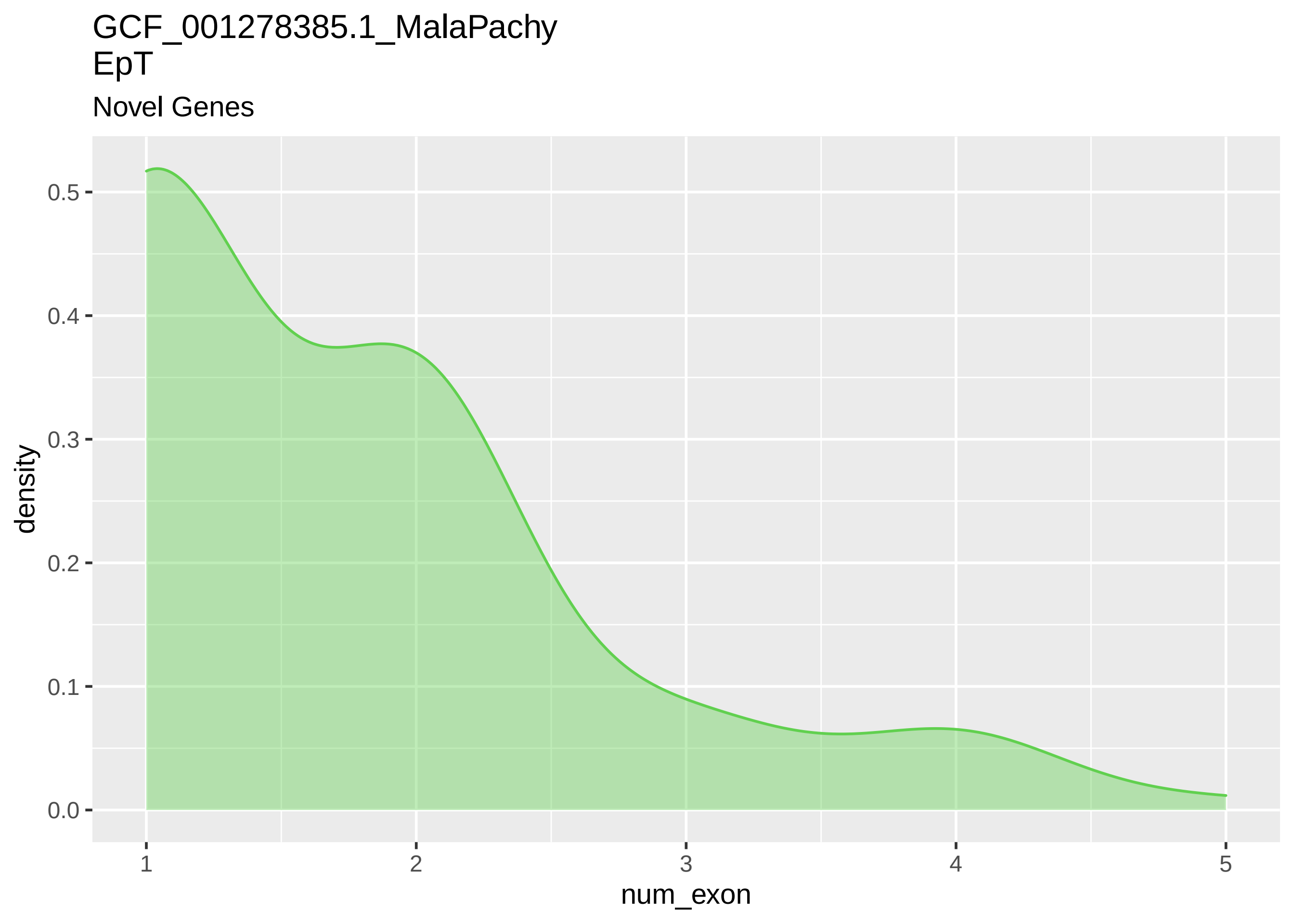
GCF_001278385.1_MalaPachy
EpT
Novel Genes
density
0.0
0.1
0.2
0.3
0.4
0.5
1
2
3
4
5
num_exon

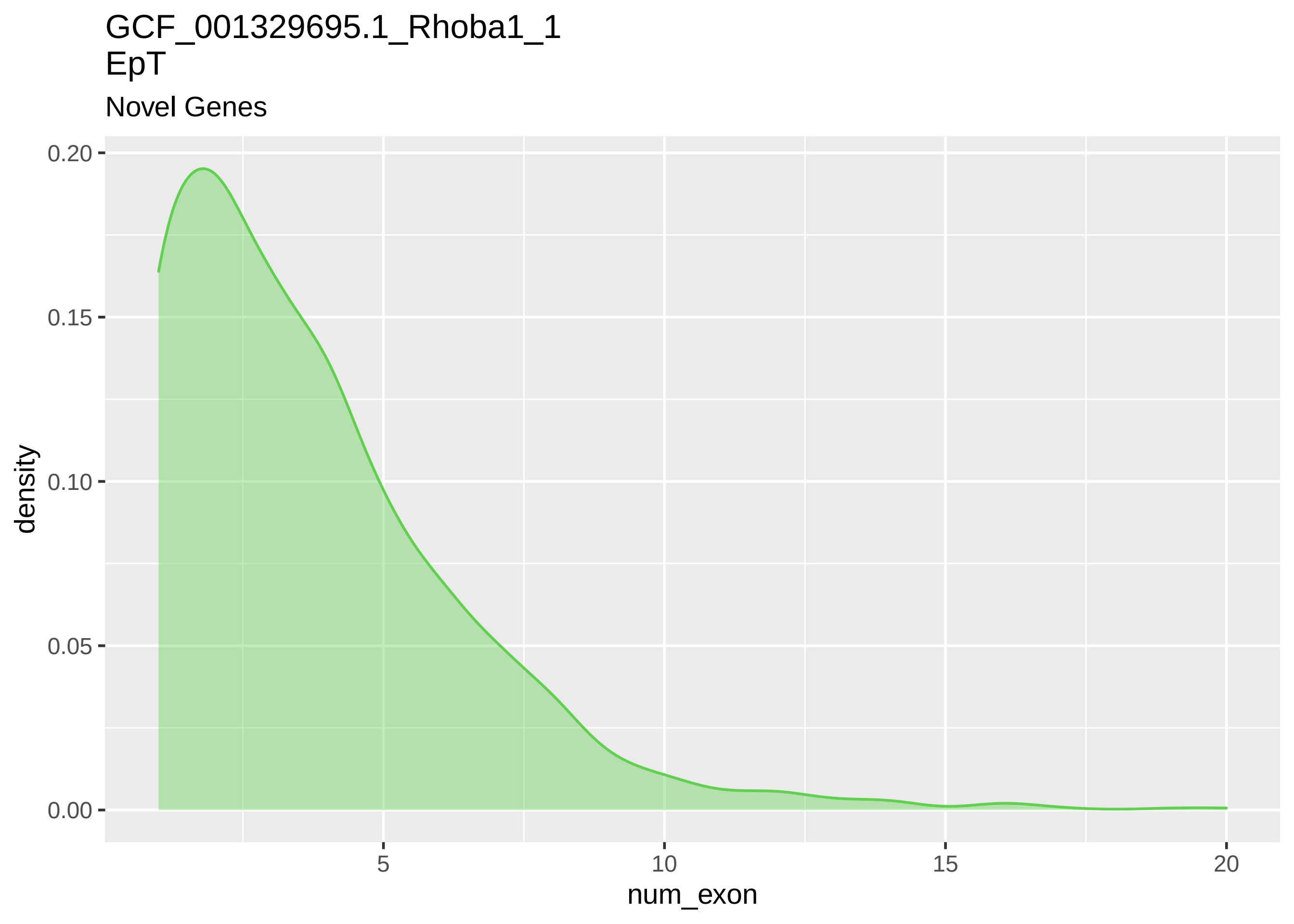
GCF_001329695.1_Rhoba1_1
EpT
Novel Genes
density
0.20
0.15
0.10
0.05
0.00
5
10
15
20
num_exon

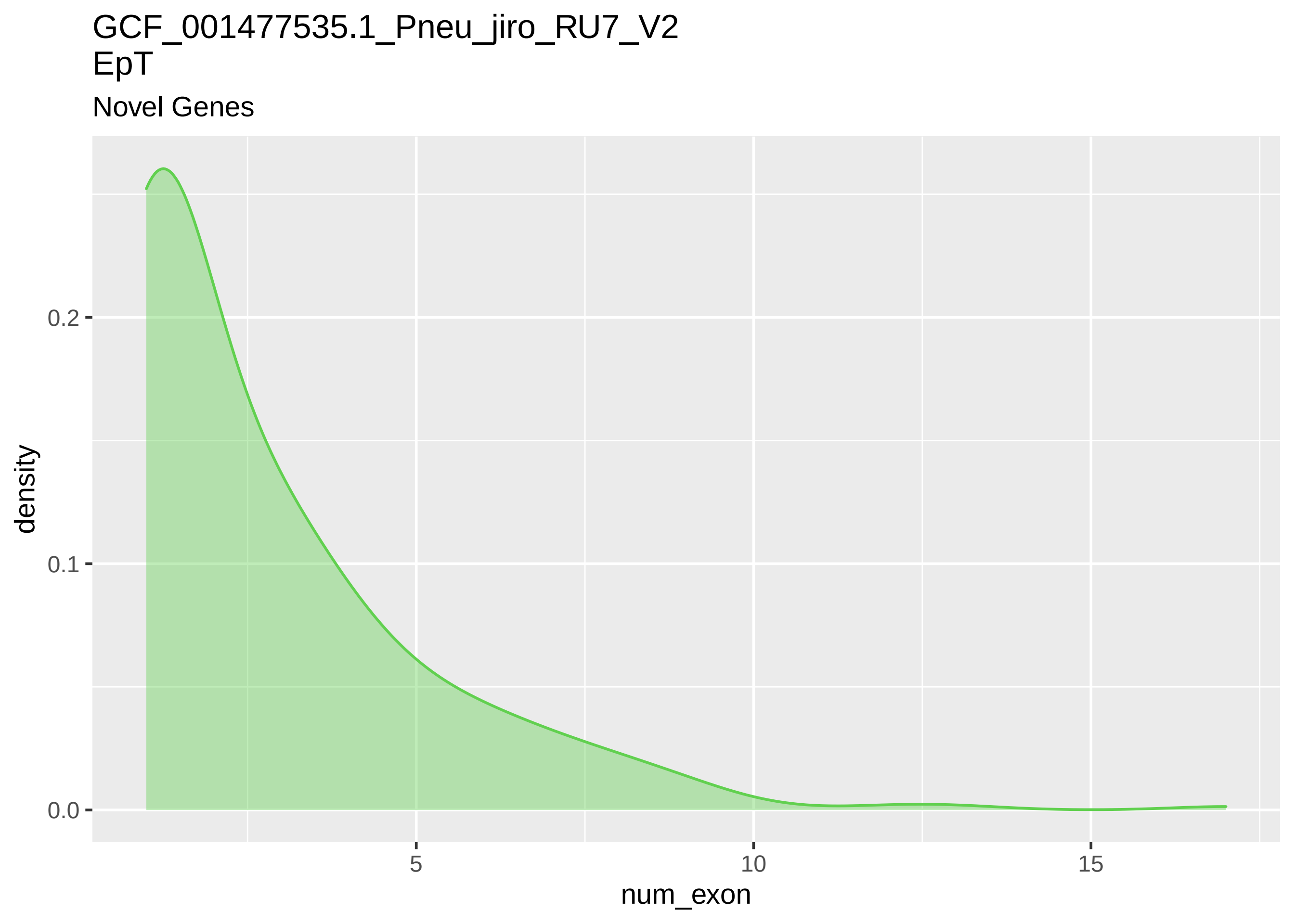

GCF_001477535.1_Pneu_jiro_RU7_V2
EpT
Novel Genes
density
0.0
0.1
0.2
5
10
15
num_exon

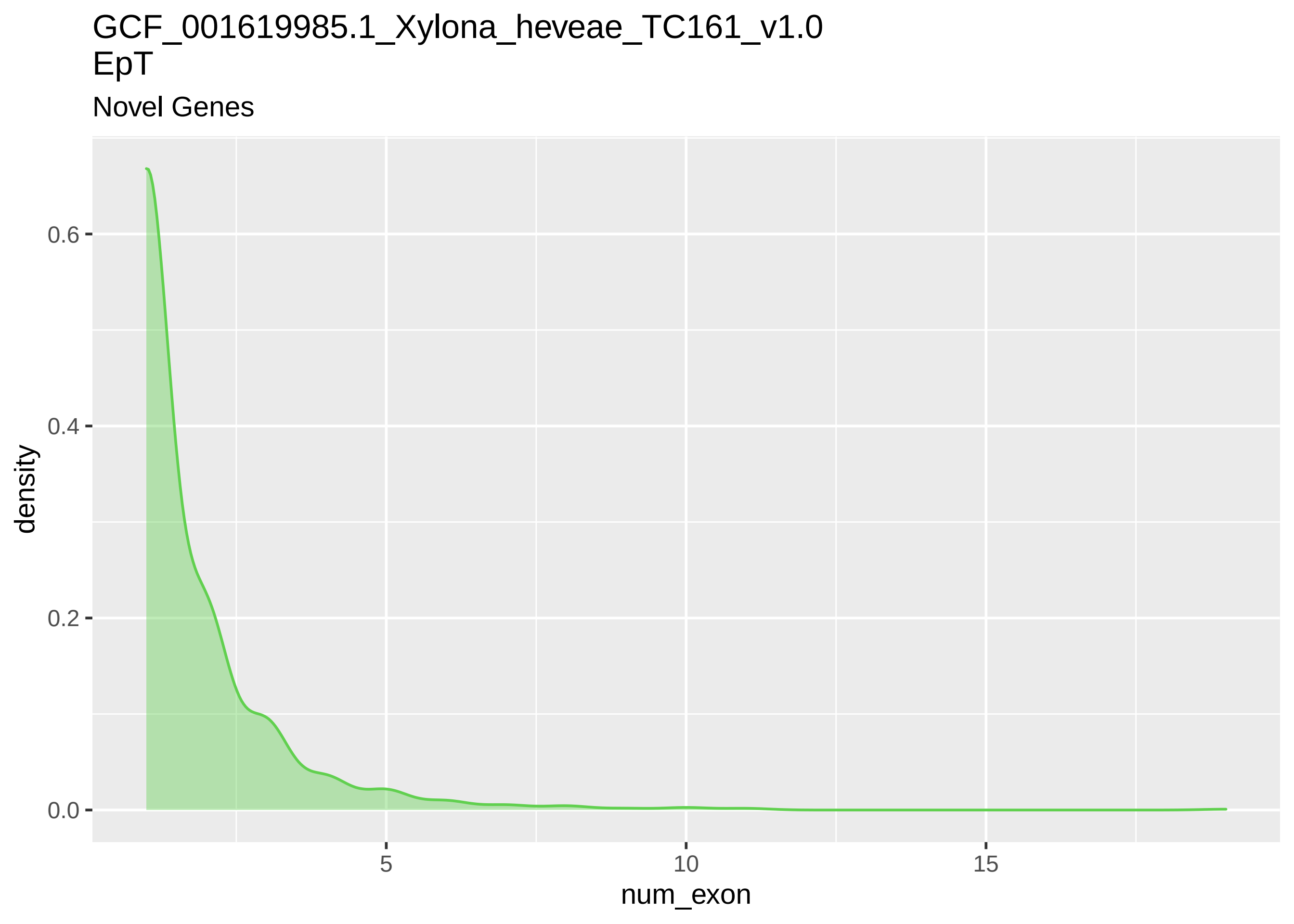
GCF_001619985.1_Xylona_heveae_TC161_v1.0
EpT
Novel Genes
density
0.6
0.4
0.2
0.0
5
10
15
num_exon

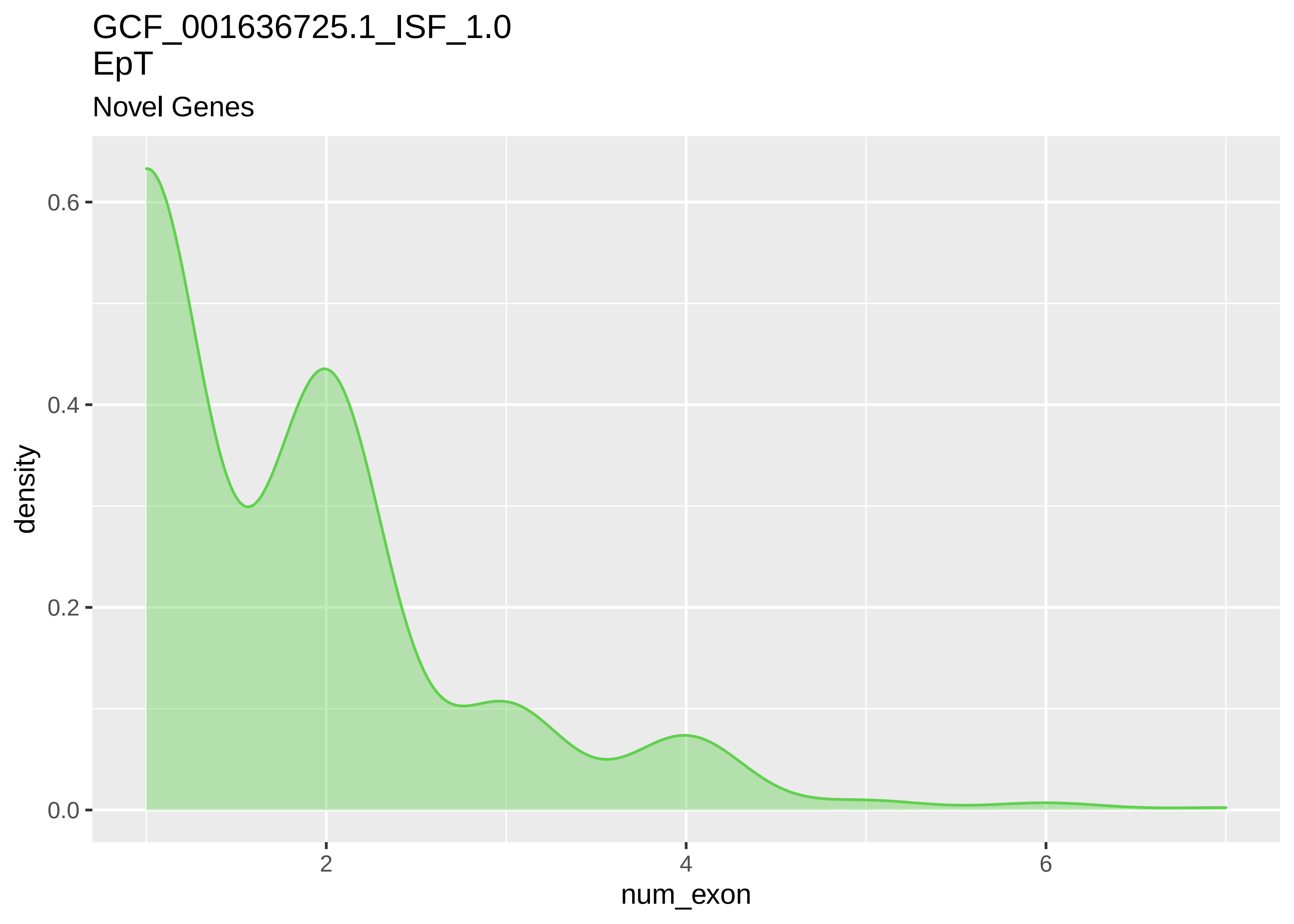
GCF_001636725.1_ISF_1.0
EpT
Novel Genes
density
0.0
0.2
0.4
0.6
2
4
6
num_exon

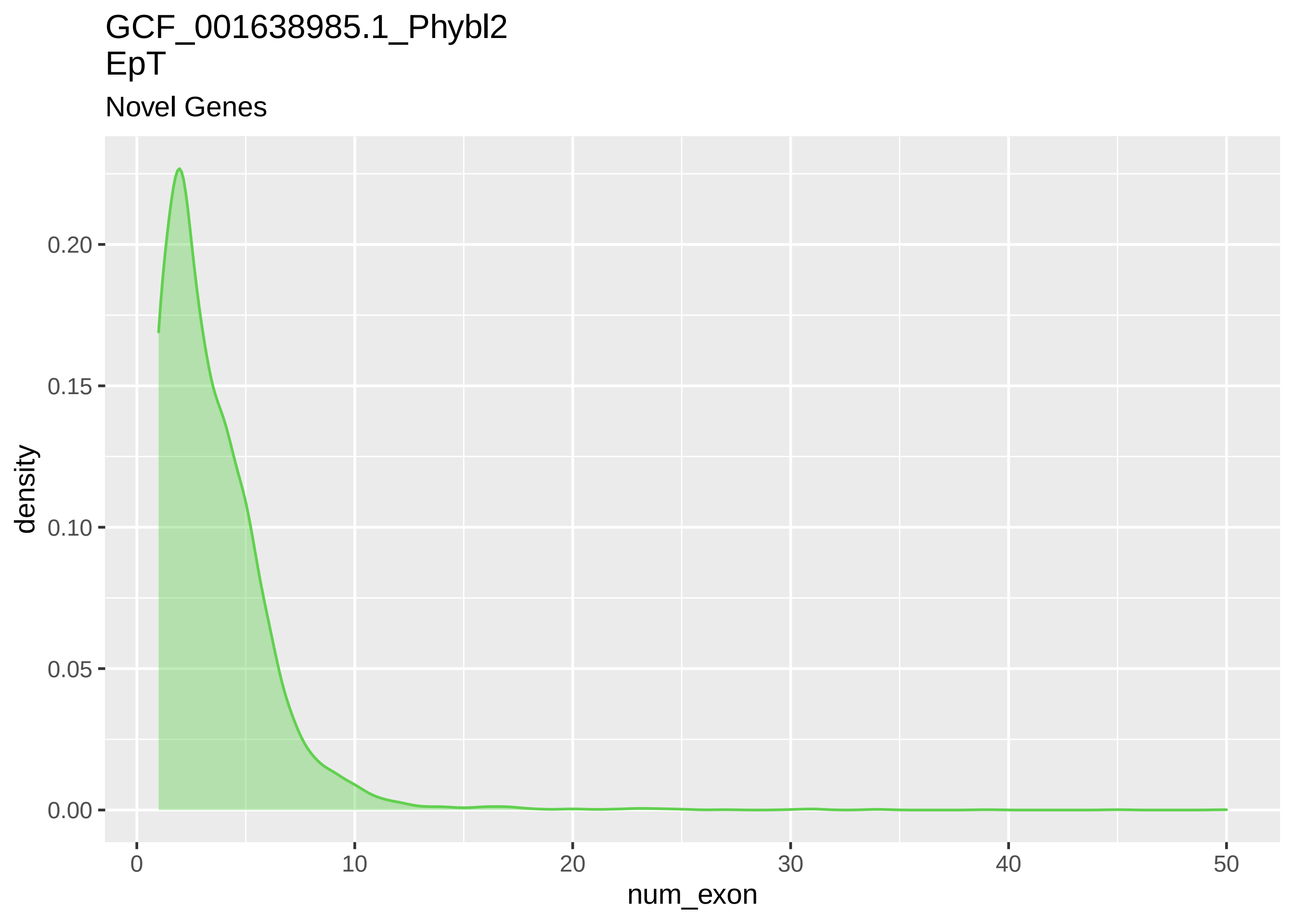
GCF_001638985.1_Phybl2
EpT
Novel Genes
density
0.00
0.05
0.10
0.15
0.20
0
10
20
30
40
50
num_exon

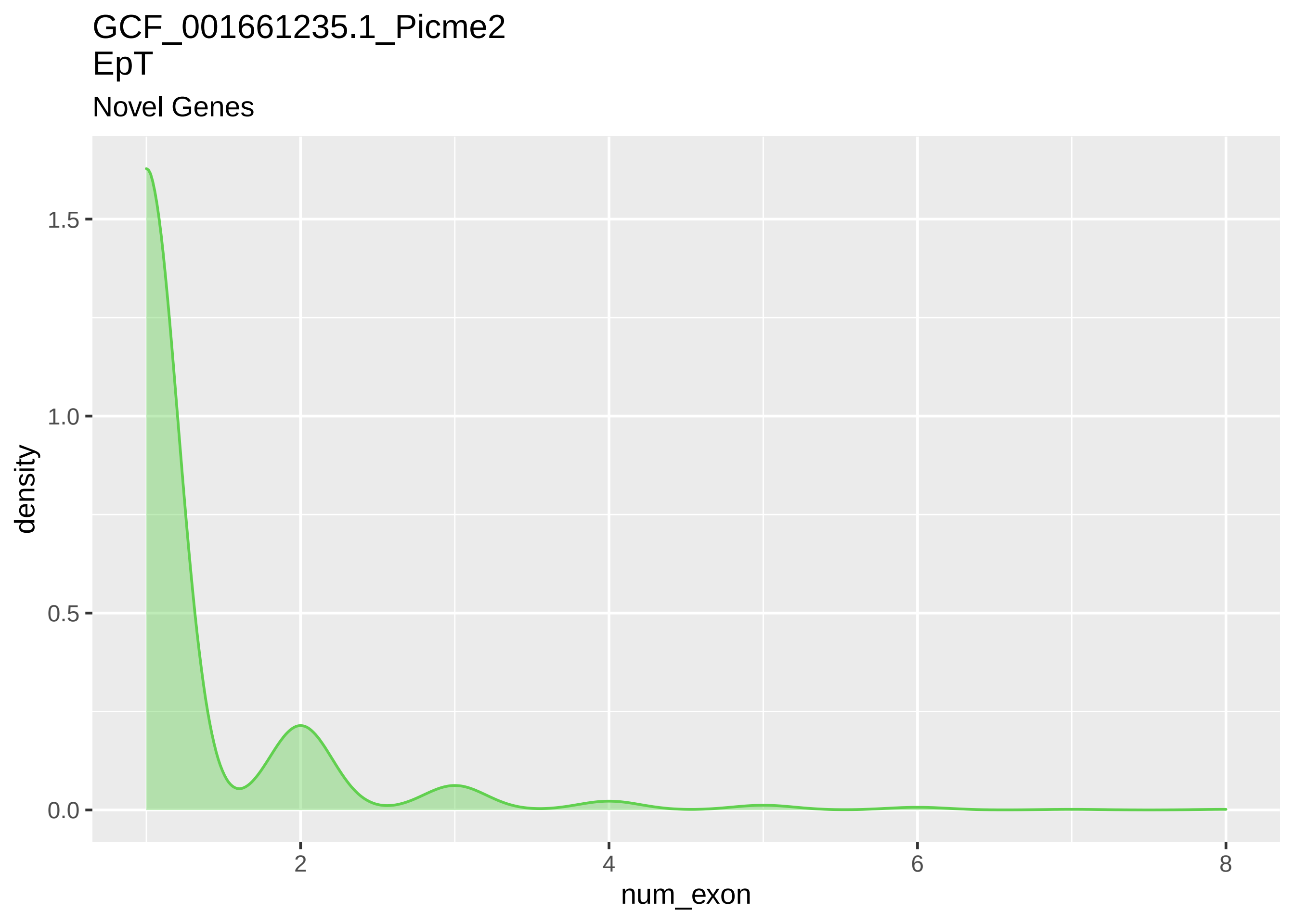
GCF_001661235.1_Picme2
EpT
Novel Genes
density
1.5
1.0
0.5
0.0
2
4
6
8
num_exon

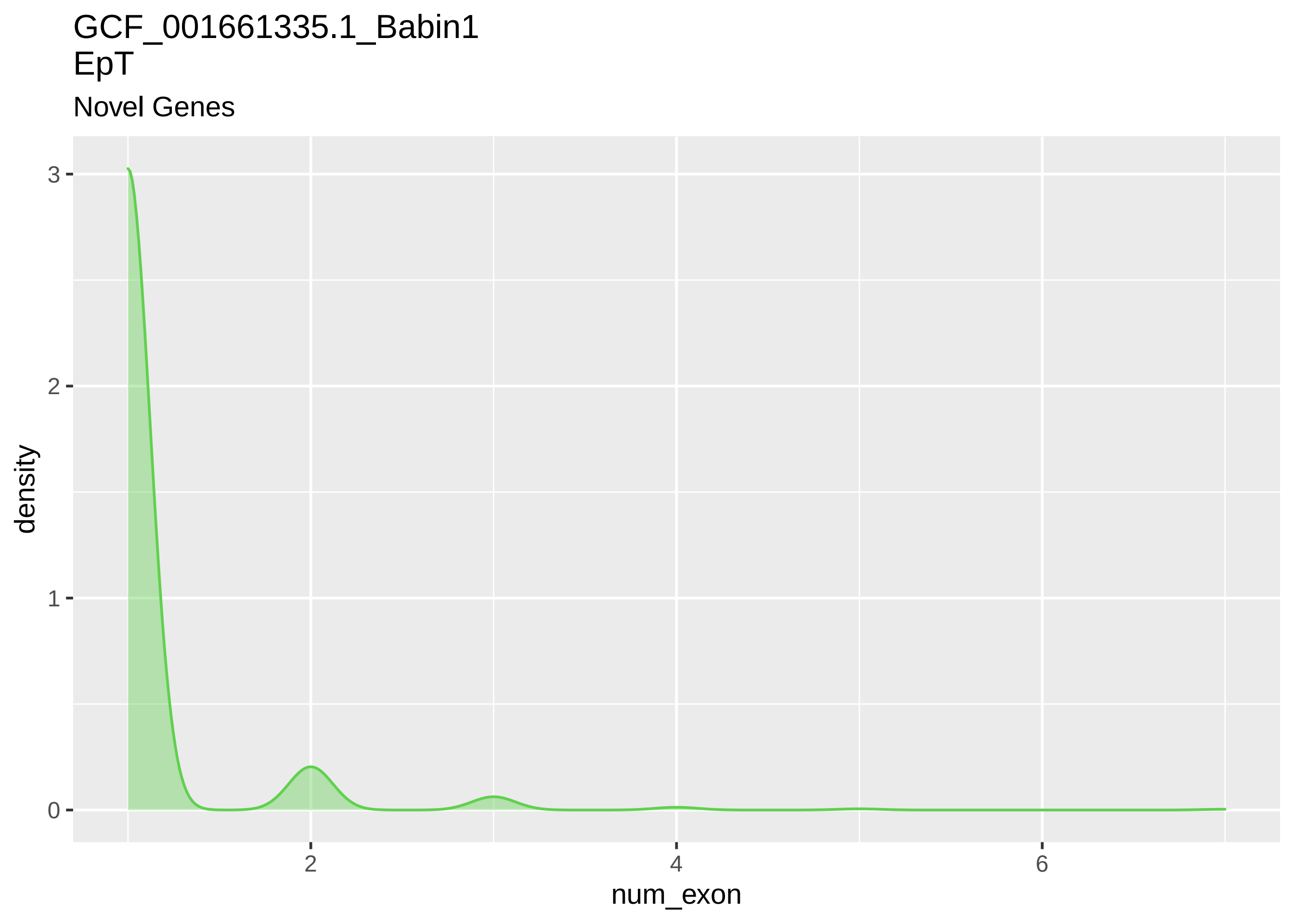
GCF_001661335.1_Babin1
EpT
Novel Genes
density
num_exon
0
1
2
3
2
4
6

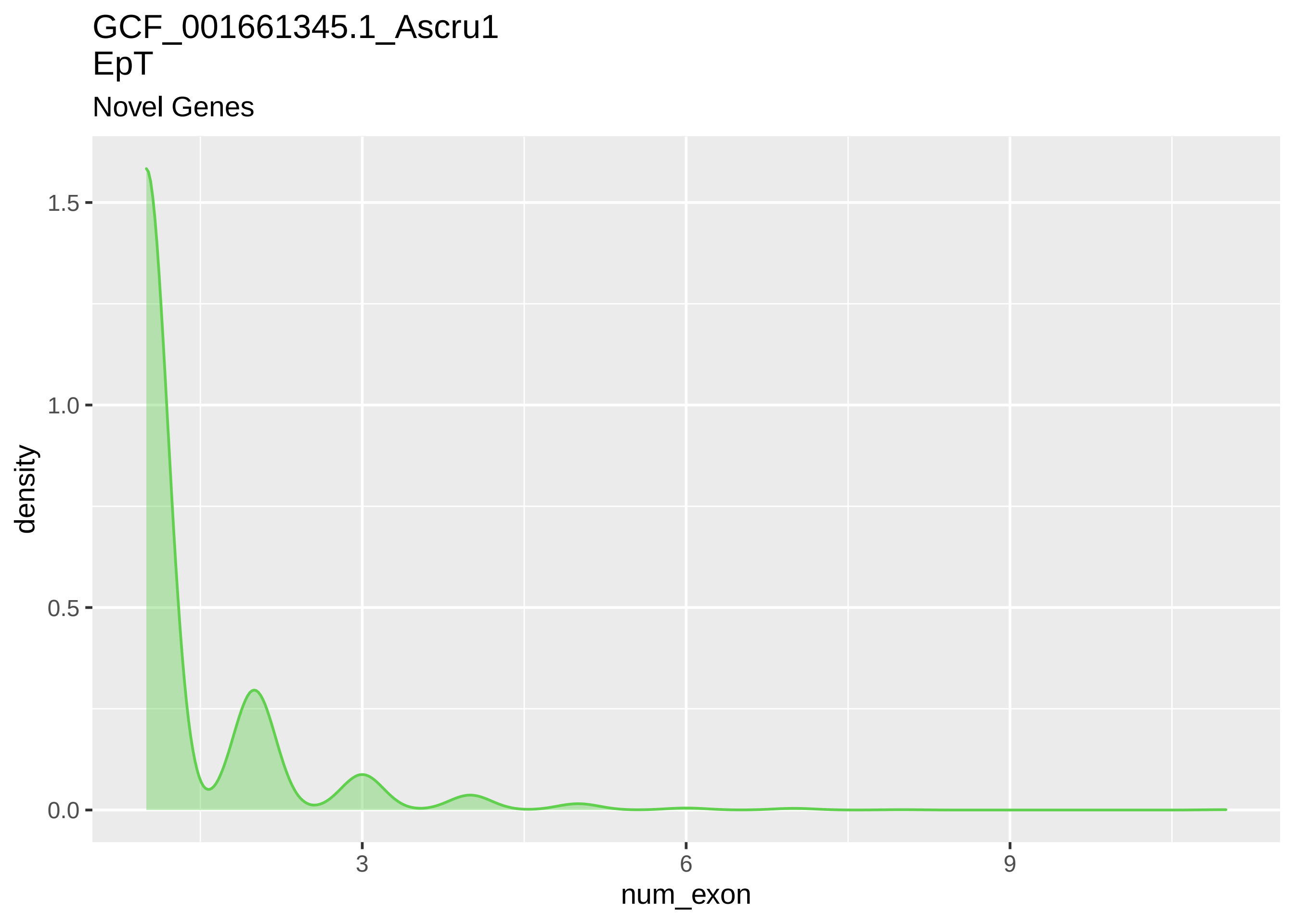
GCF_001661345.1_Ascru1
EpT
Novel Genes
density
1.5
1.0
0.5
0.0
3
6
9
num_exon

GCF_001661405.1_Cybja1
EpT

Novel Genes

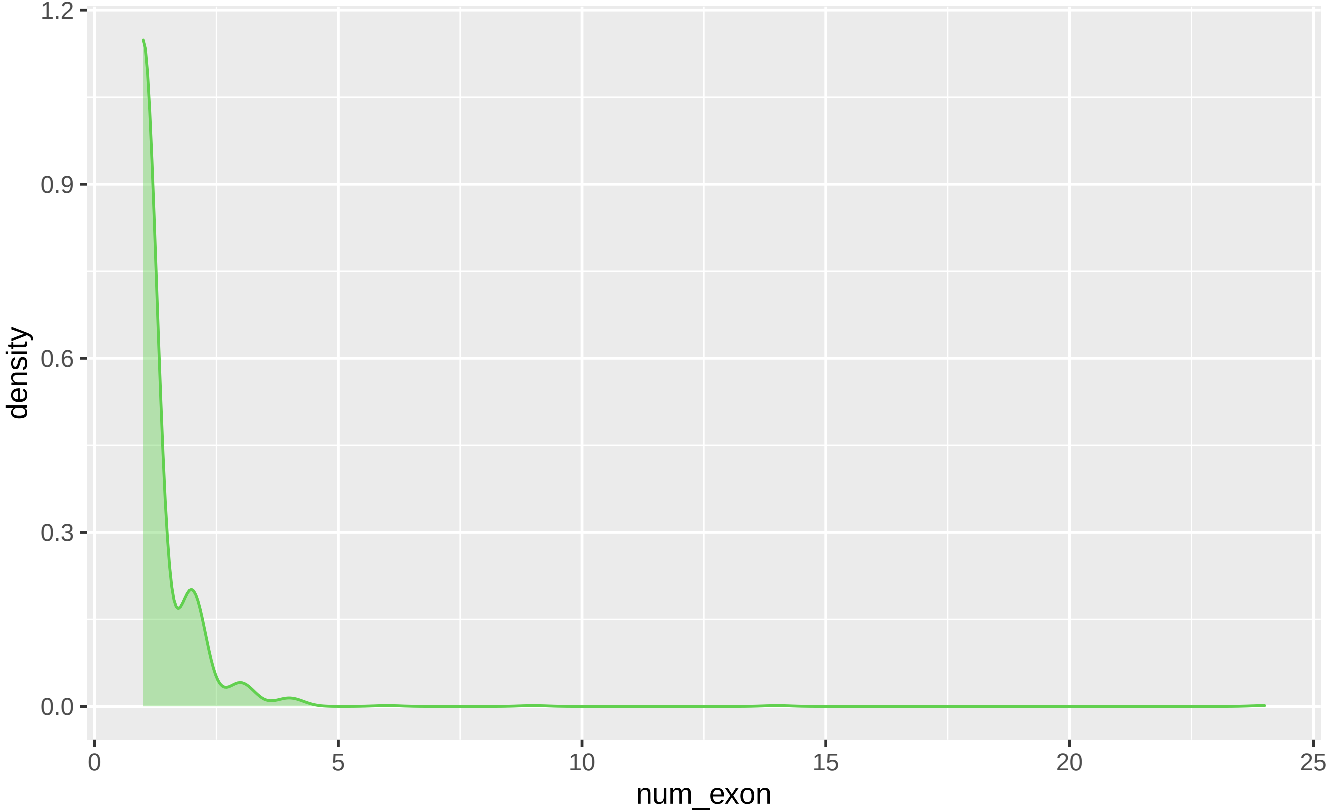

GCF_001664035.1_Metbi1
EpT

Novel Genes

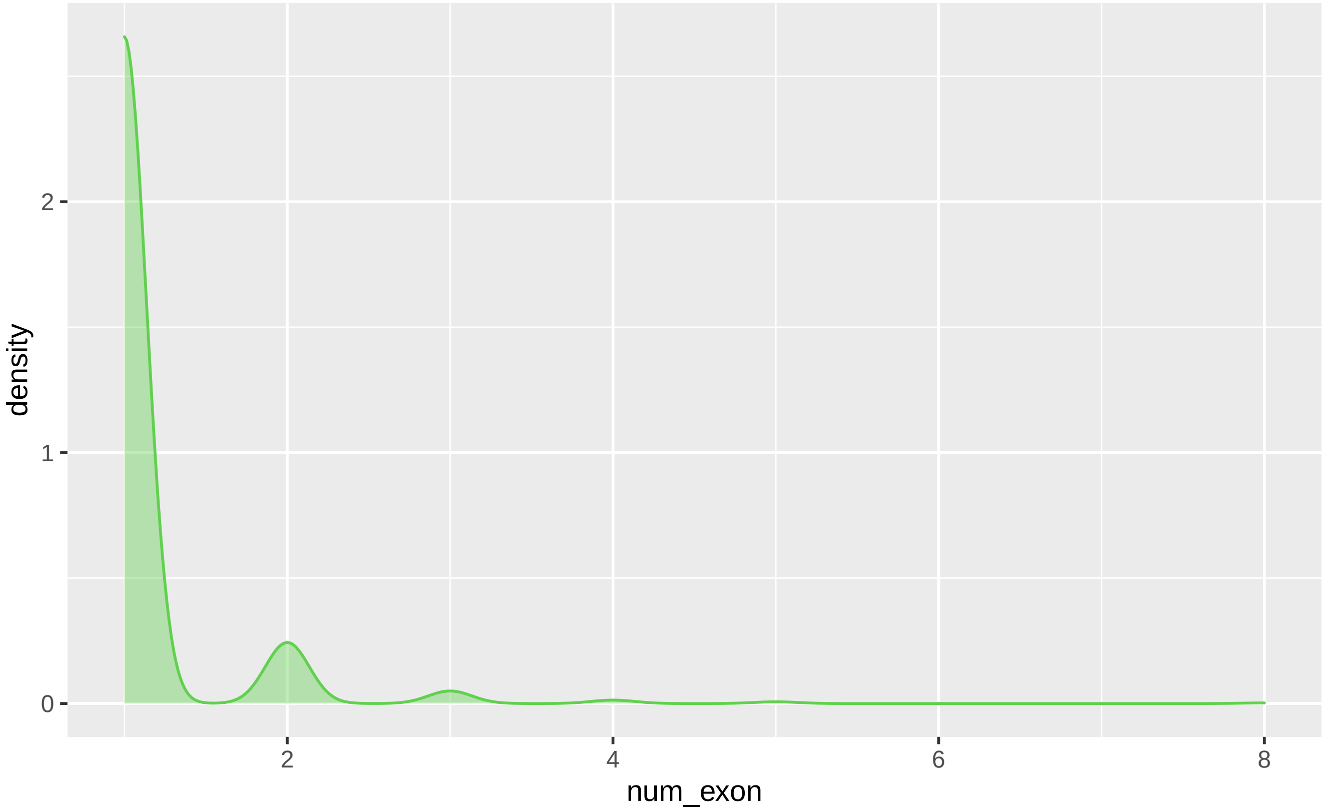

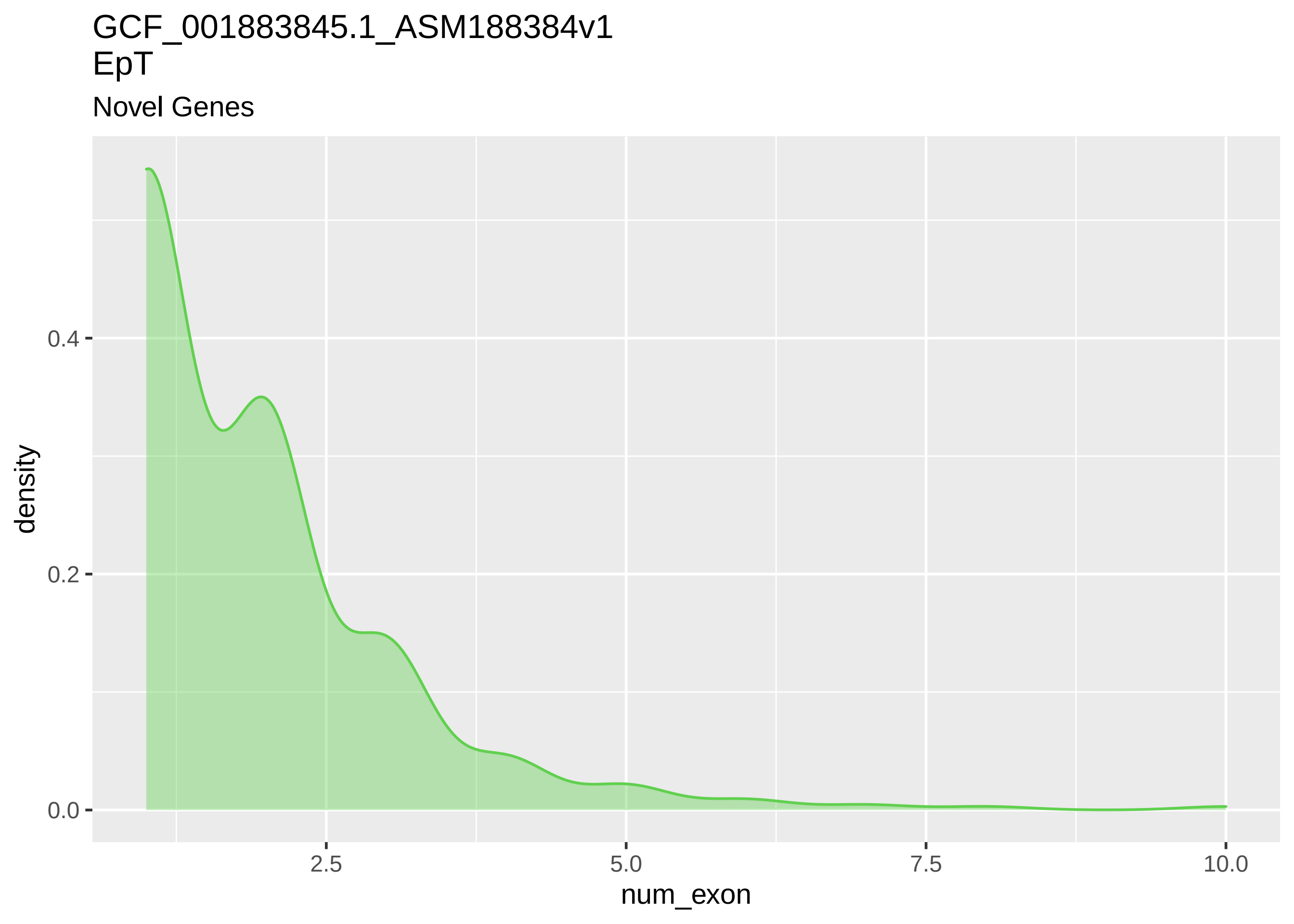
GCF_001883845.1_ASM188384v1
EpT
Novel Genes
density
0.0
0.2
0.4
2.5
5.0
7.5
10.0
num_exon

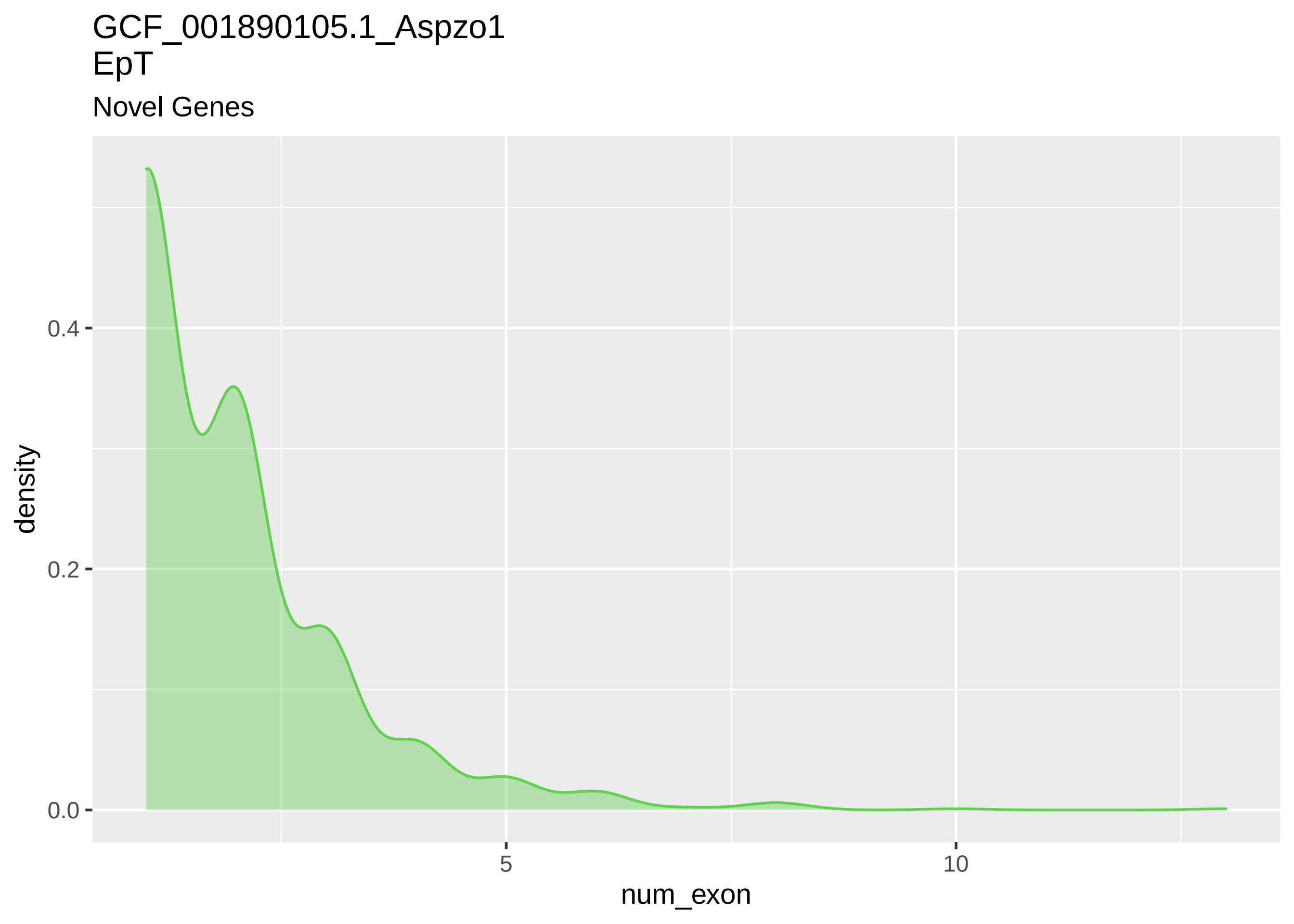
GCF_001890105.1_Aspzo1
EpT
Novel Genes
density
0.4
0.2
0.0
5
10
num_exon

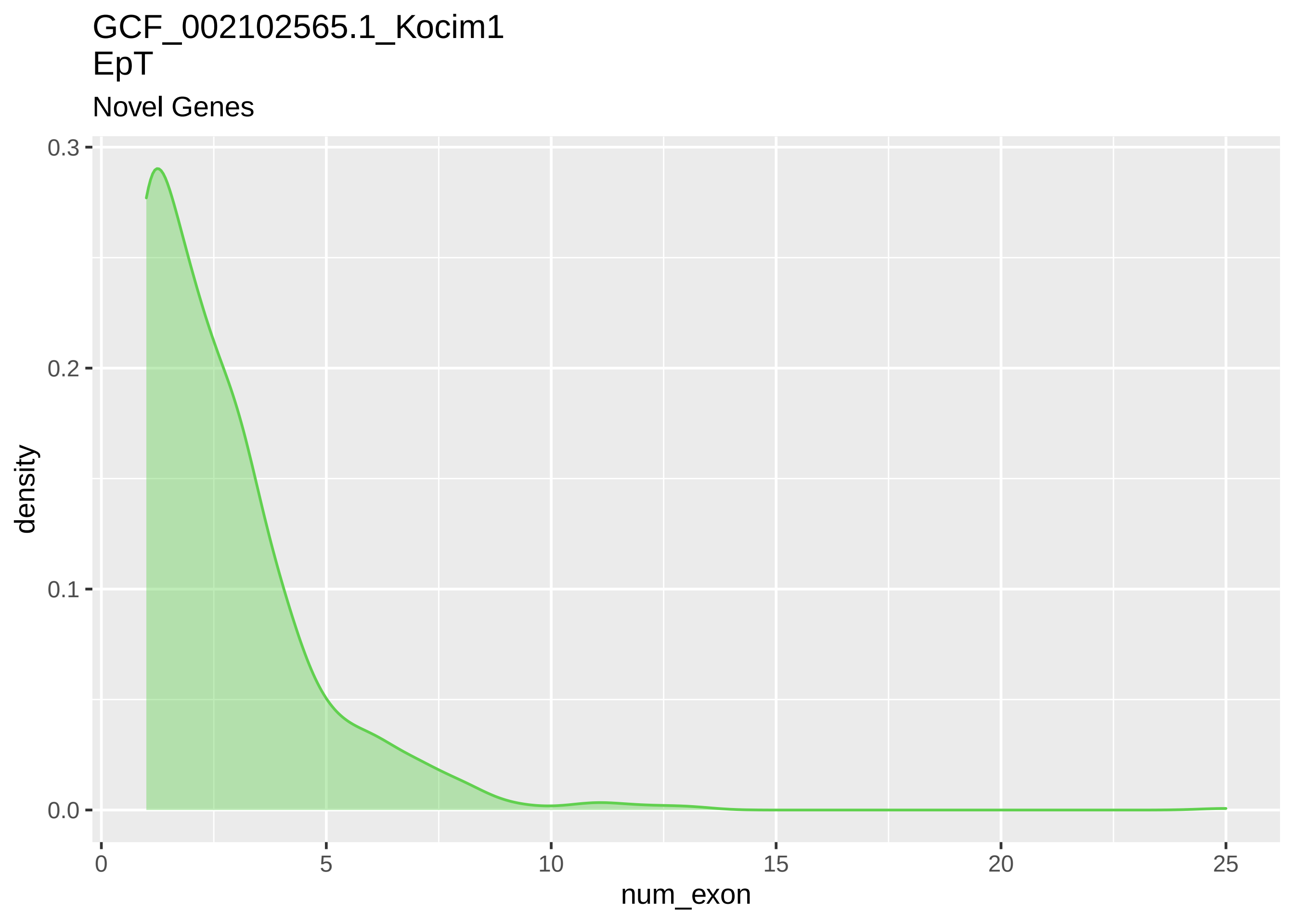
GCF_002102565.1_Kocim1
EpT
Novel Genes
0.3
0.2
0.1
0.0
density
0
5
10
15
20
25
num_exon

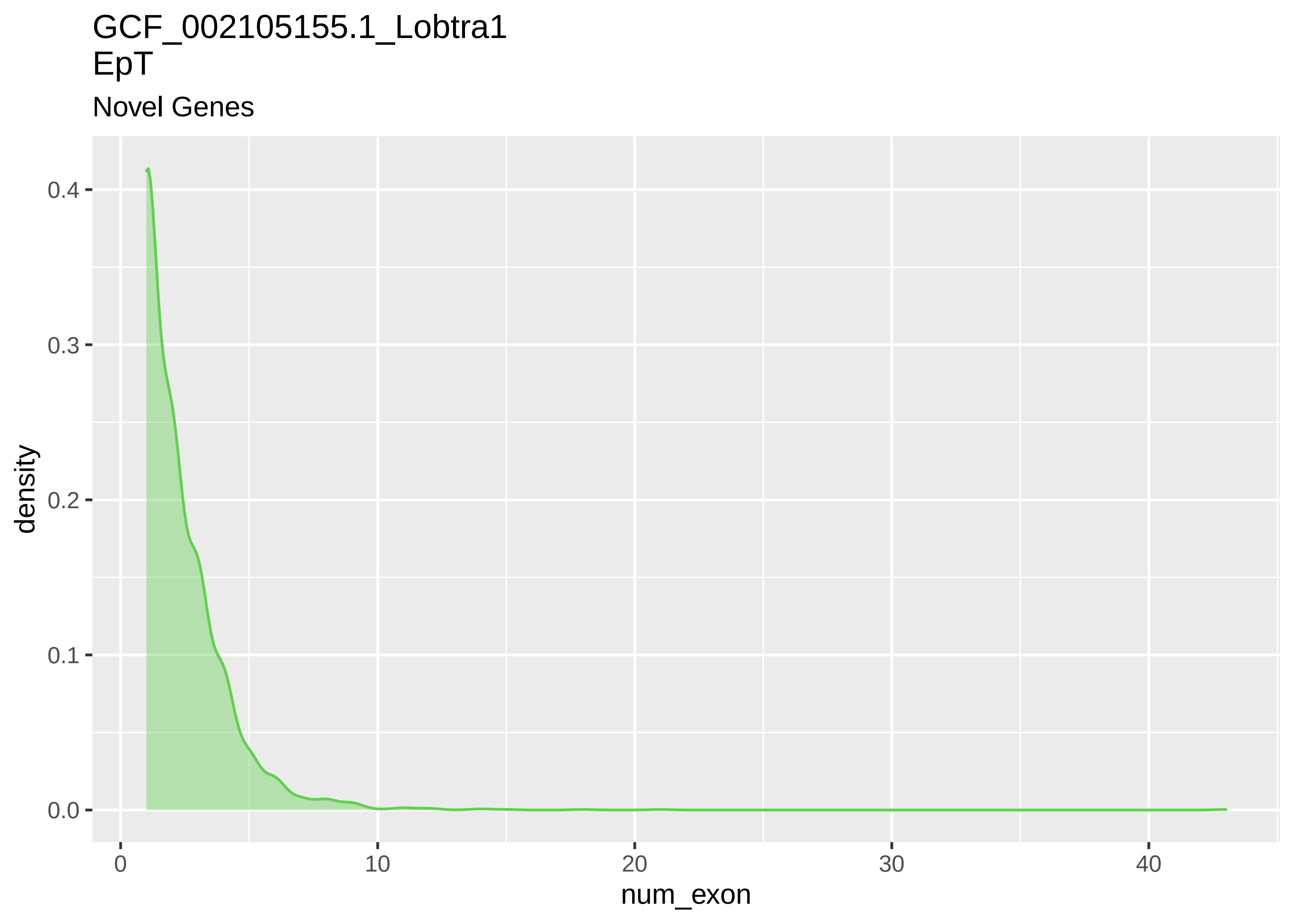
GCF_002105155.1_Lobtra1
EpT
Novel Genes
density
num_exon
0.0
0.1
0.2
0.3
0.4
0
10
20
30
40

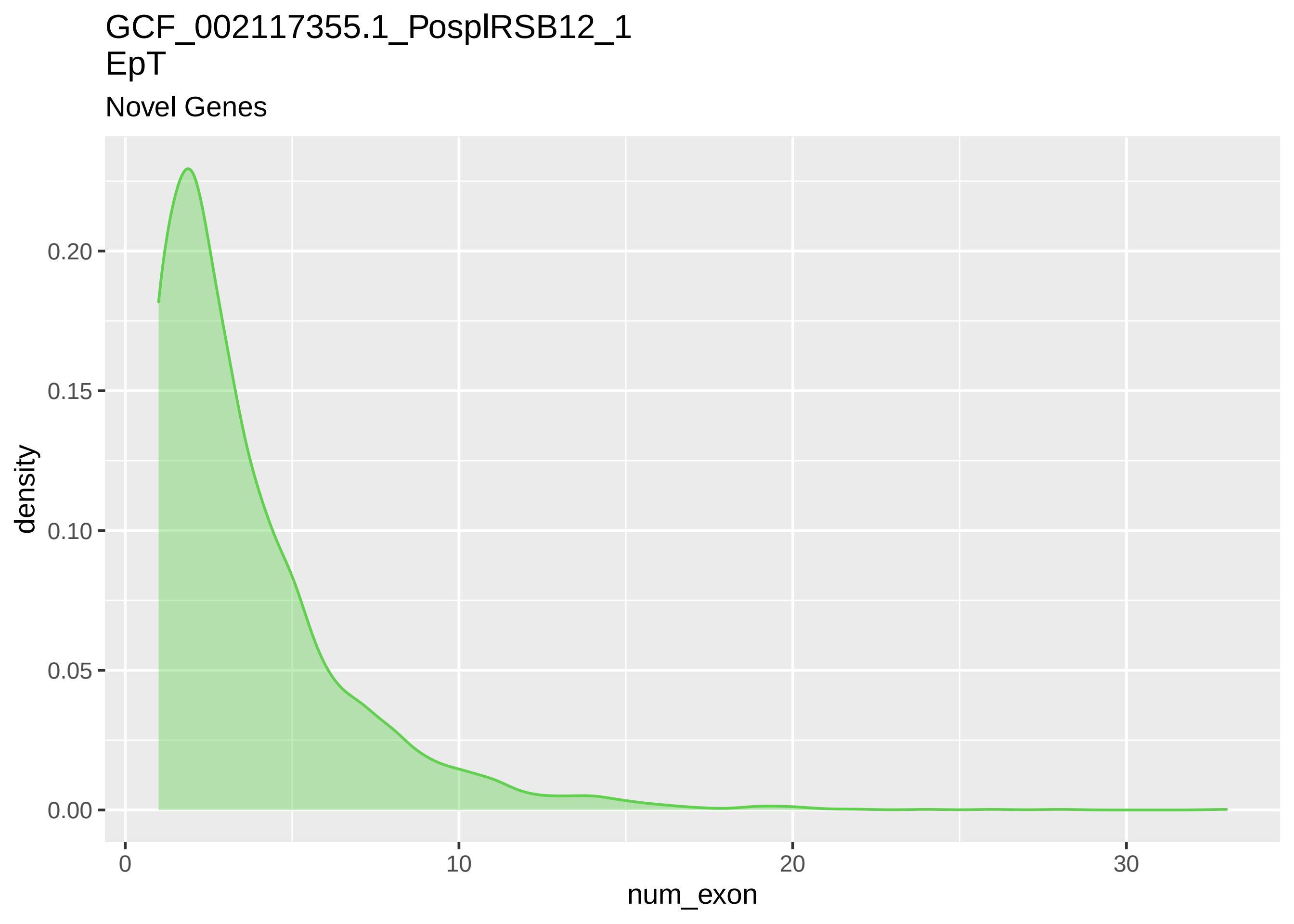
GCF_002117355.1_PosplRSB12_1
EpT
Novel Genes
density
0.20
0.15
0.10
0.05
0.00
0
10
20
30
num_exon

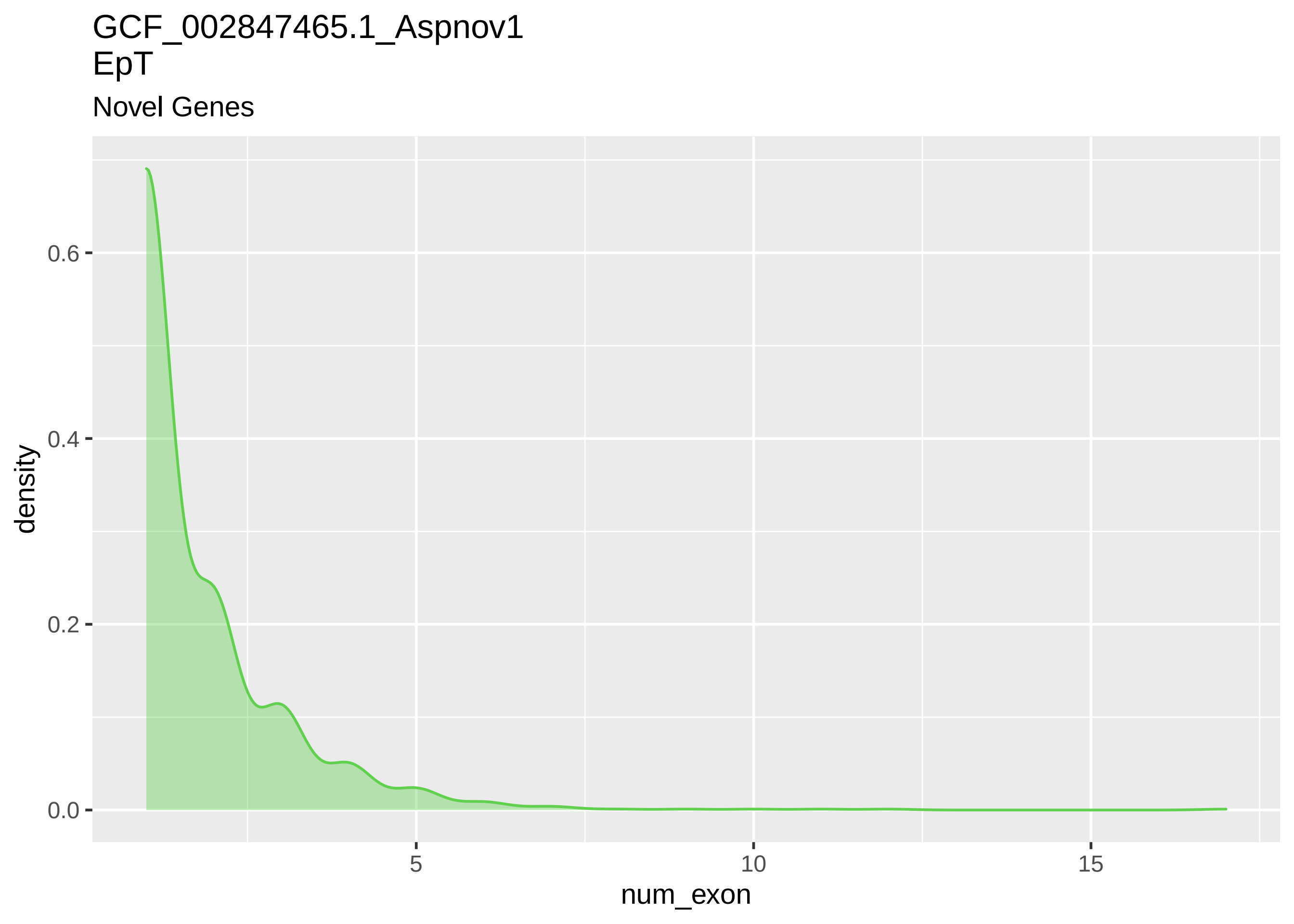
GCF_002847465.1_Aspnov1
EpT
Novel Genes
density
0.0
0.2
0.4
0.6
5
10
15
num_exon

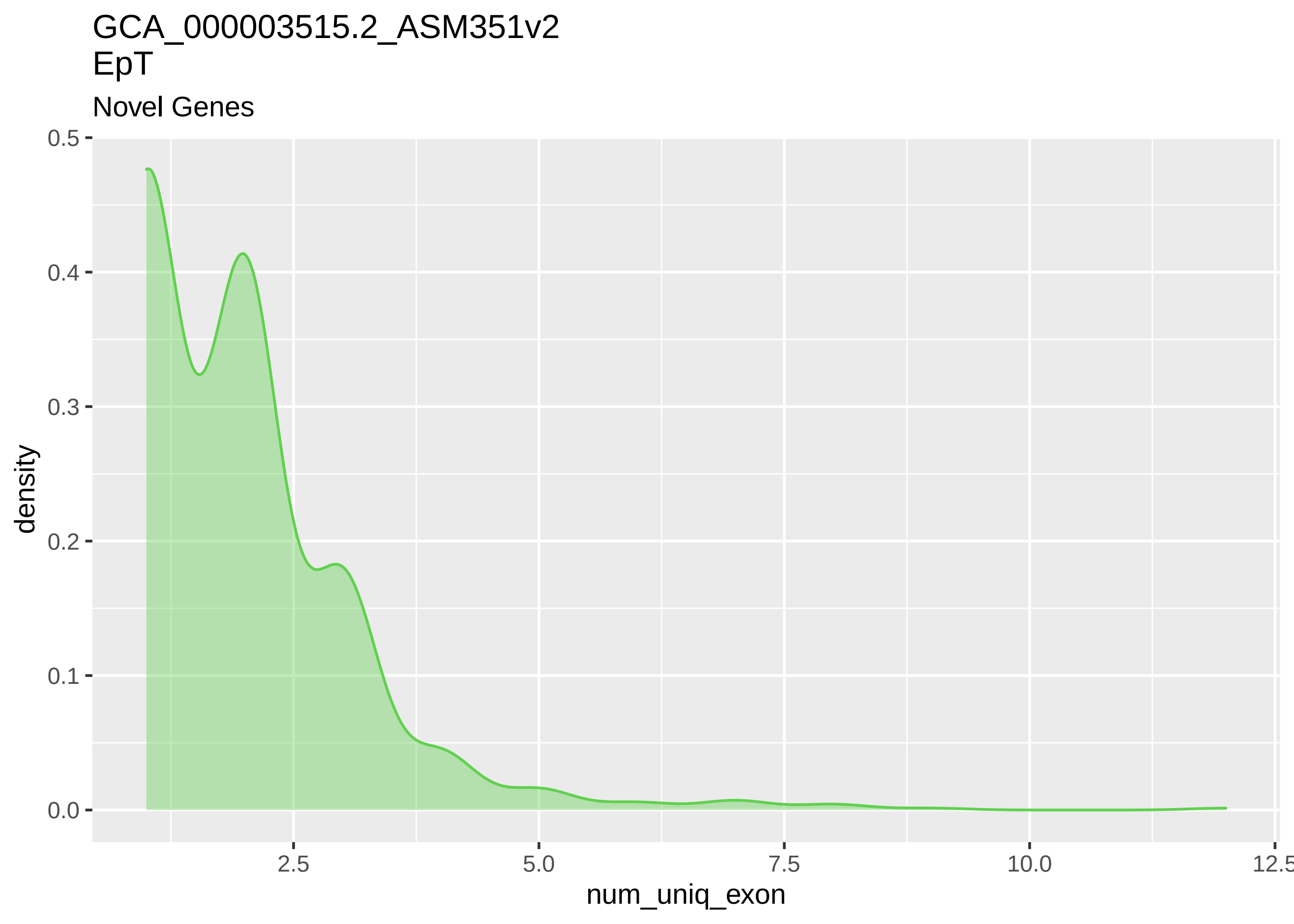
GCA_000003515.2_ASM351v2
EpT
Novel Genes
density
num_uniq_exon
0.0
0.1
0.2
0.3
0.4
0.5
2.5
5.0
7.5
10.0
12.5

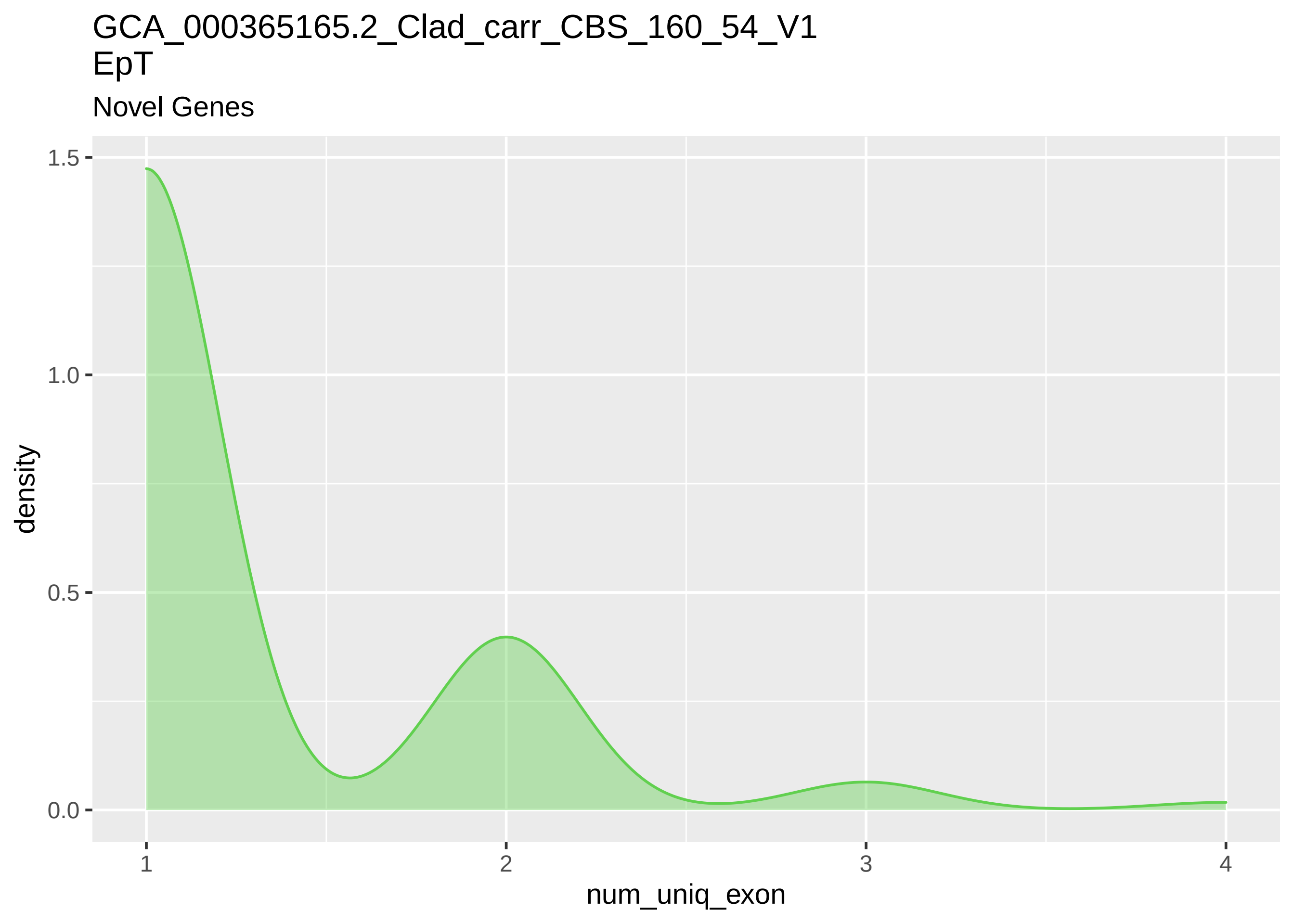
GCA_000365165.2_Clad_carr_CBS_160_54_V1
EpT
Novel Genes
density
num_uniq_exon
0.0
0.5
1.0
1.5
1
2
3
4

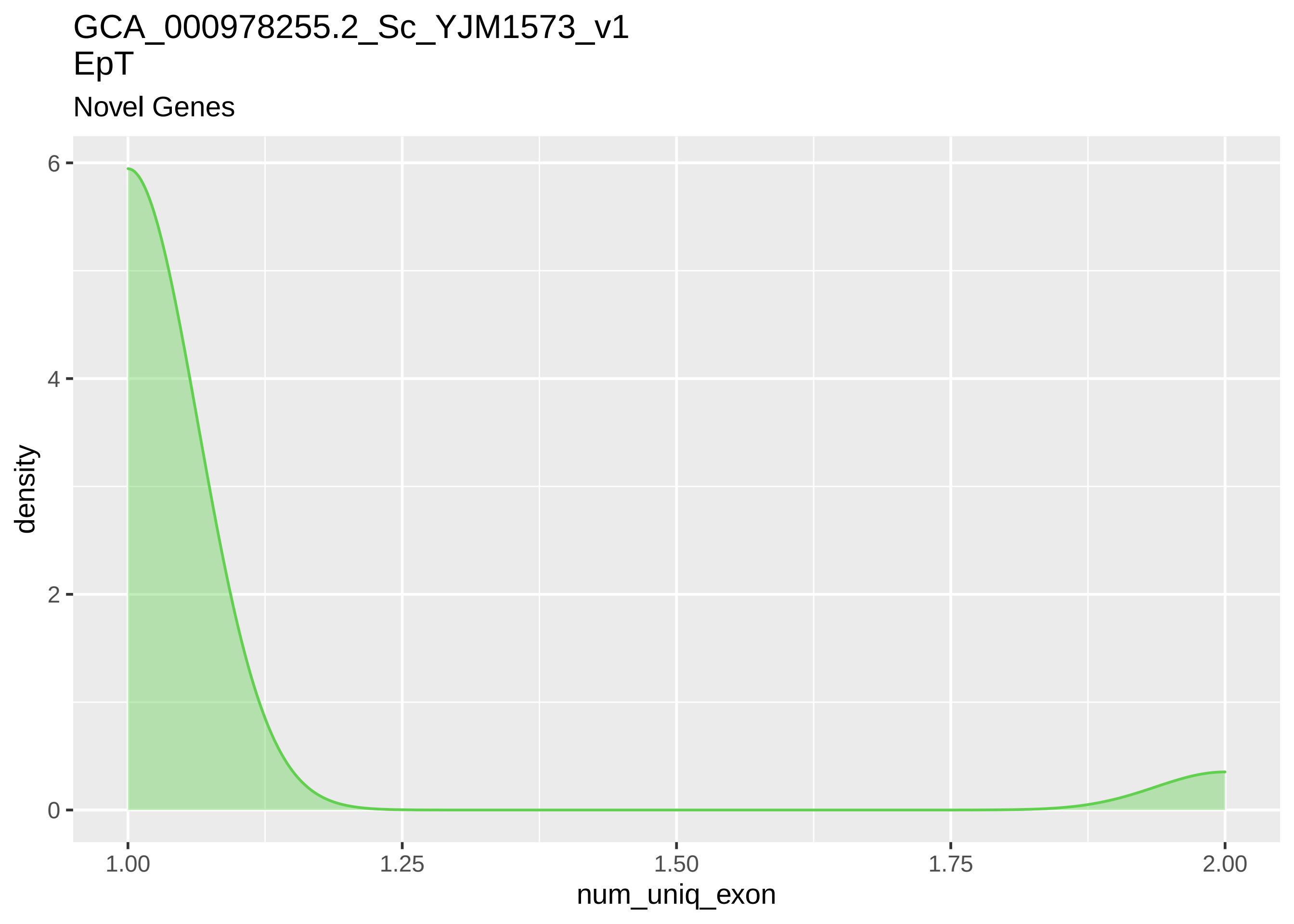
GCA_000978255.2_Sc_YJM1573_v1
EpT
Novel Genes
density
num_uniq_exon
0
2
4
6
1.00
1.25
1.50
1.75
2.00

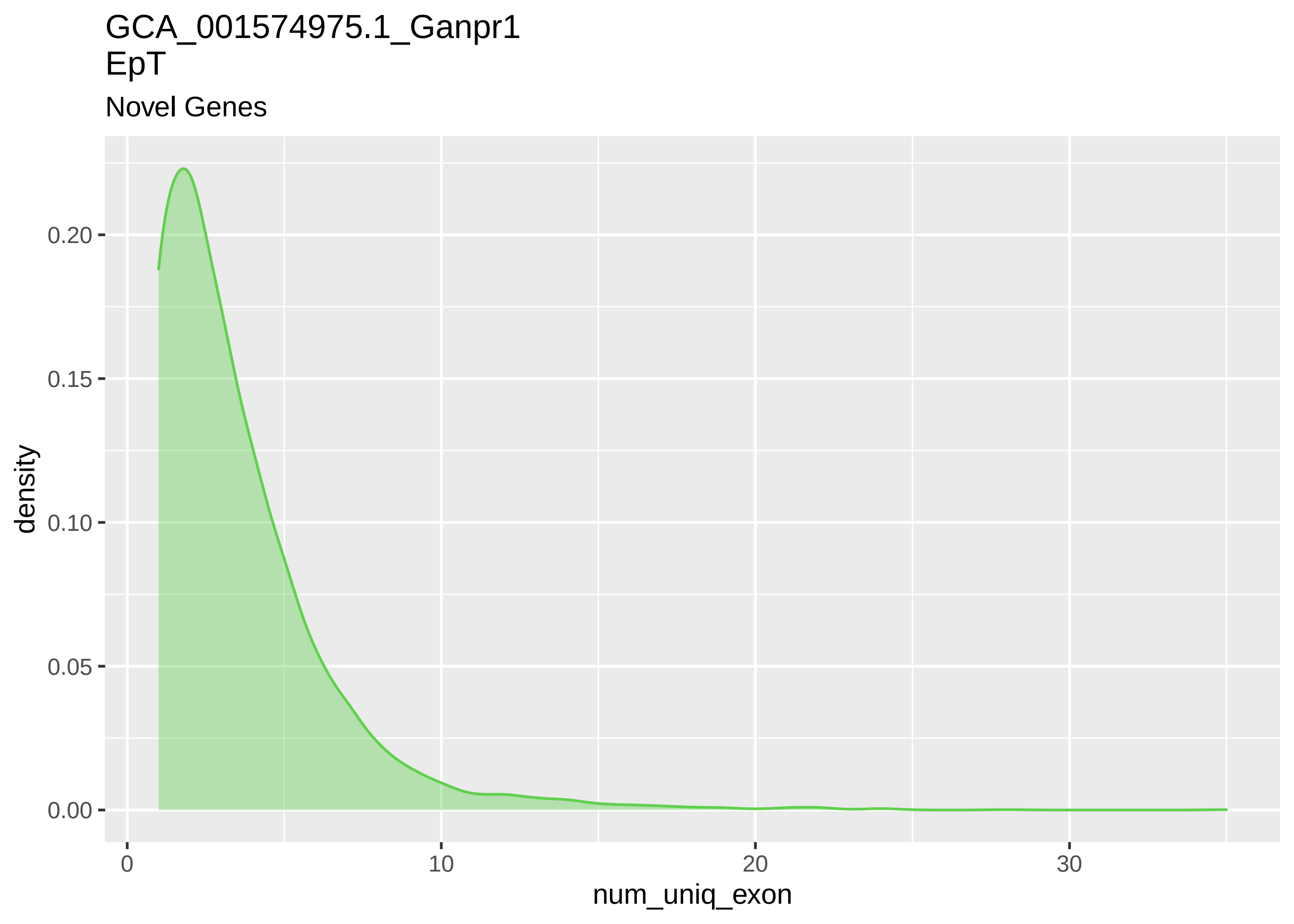
GCA_001574975.1_Ganpr1
EpT
Novel Genes
density
0.20
0.15
0.10
0.05
0.00
0
10
20
30
num_uniq_exon

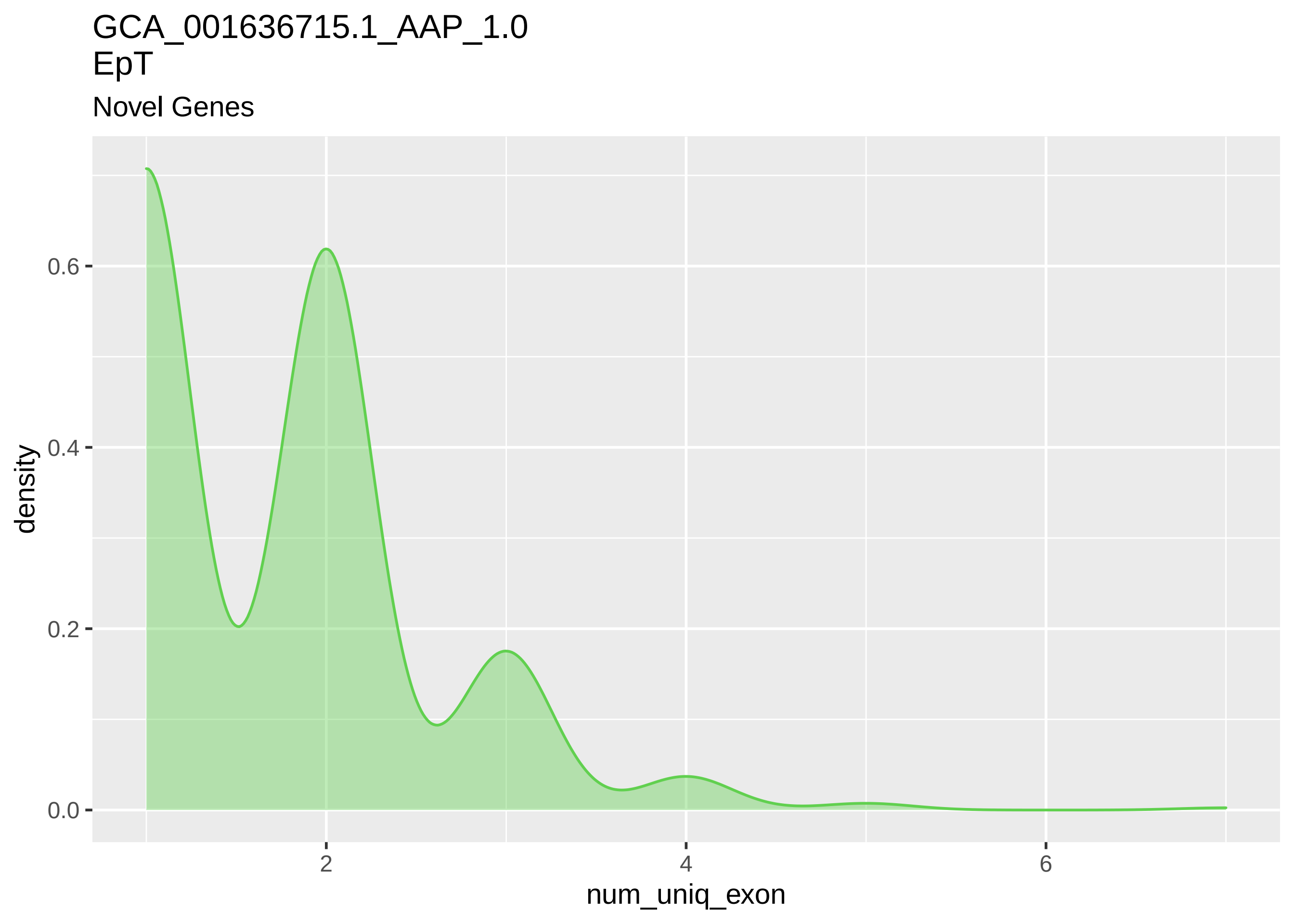
GCA_001636715.1_AAP_1.0
EpT
Novel Genes
density
0.0
0.2
0.4
0.6
2
4
6
num_uniq_exon

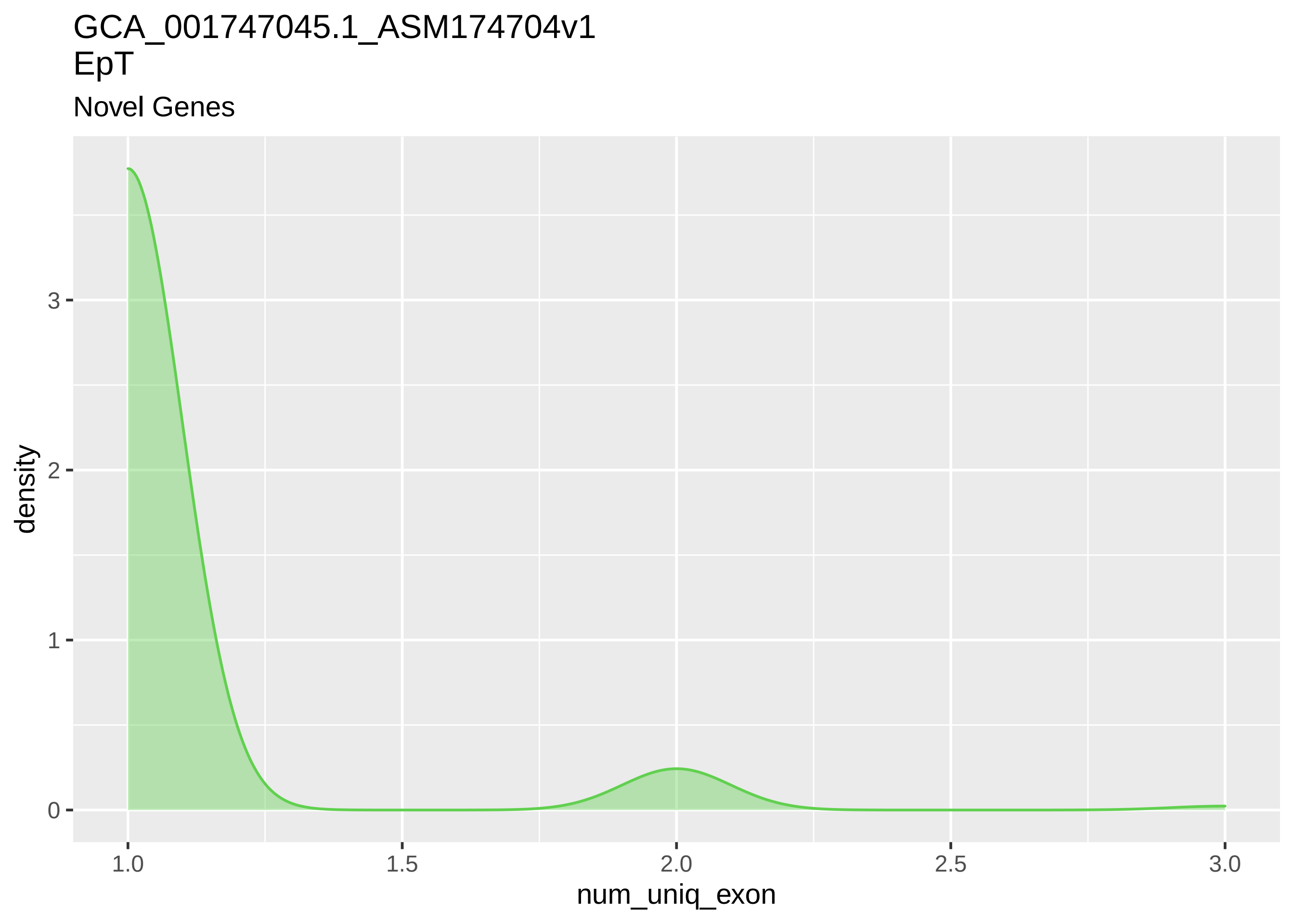
GCA_001747045.1_ASM174704v1
EpT
Novel Genes
density
0
1
2
3
1.0
1.5
2.0
2.5
3.0
num_uniq_exon

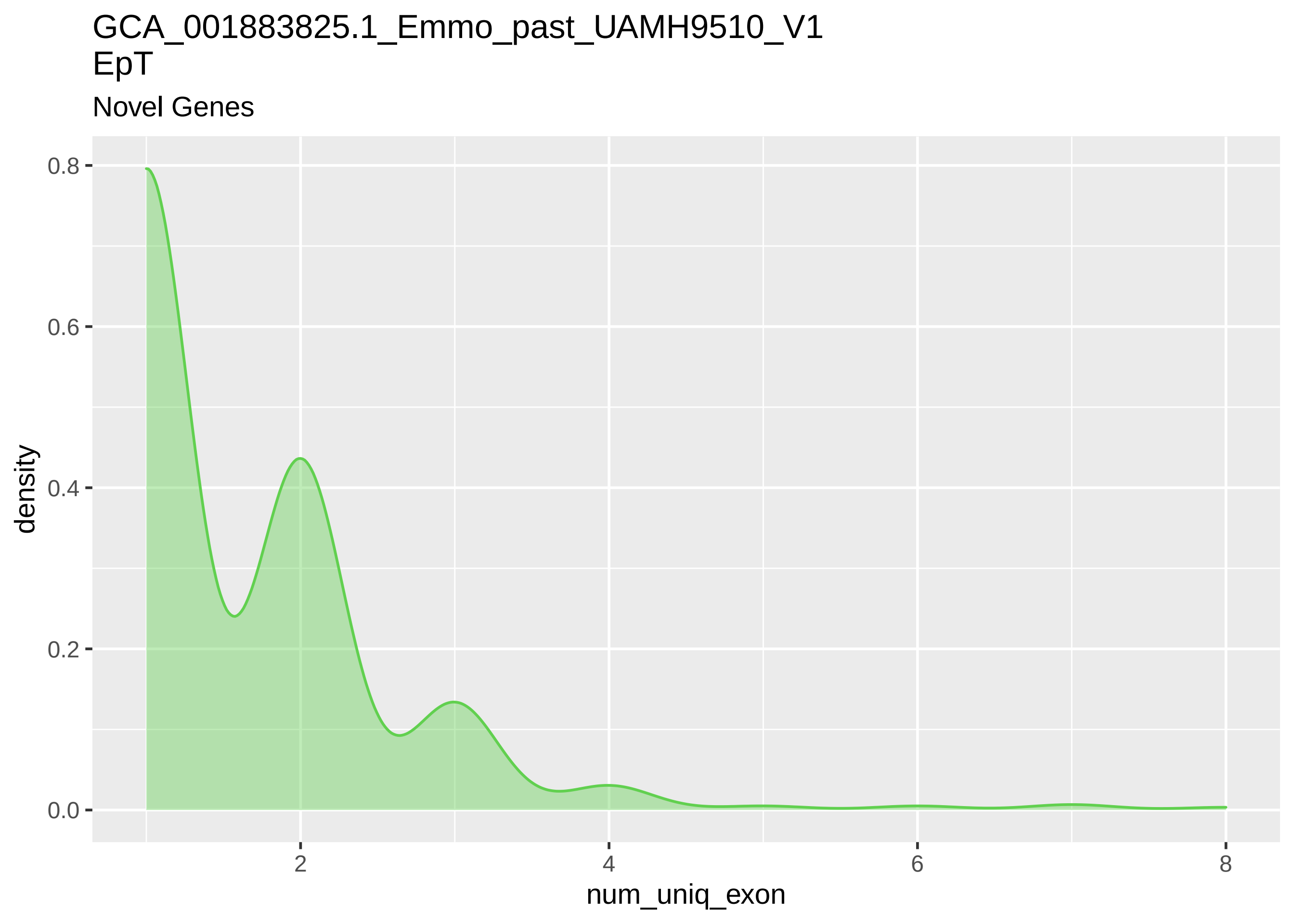

GCA_001883825.1_Emmo_past_UAMH9510_V1
EpT
Novel Genes
density
0.8
0.6
0.4
0.2
0.0
2
4
6
8
num_uniq_exon

GCA_001929475.1_NeoIirr1.0
EpT

Novel Genes

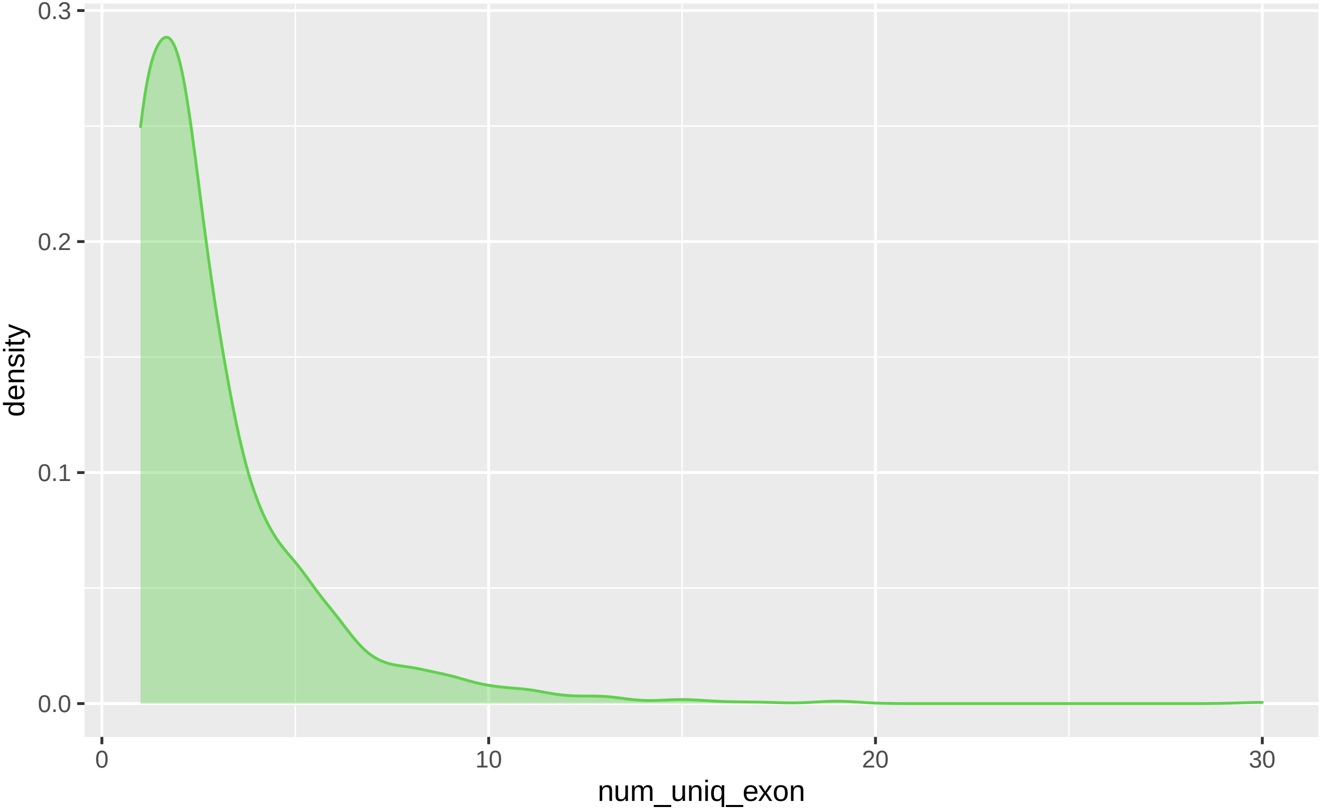

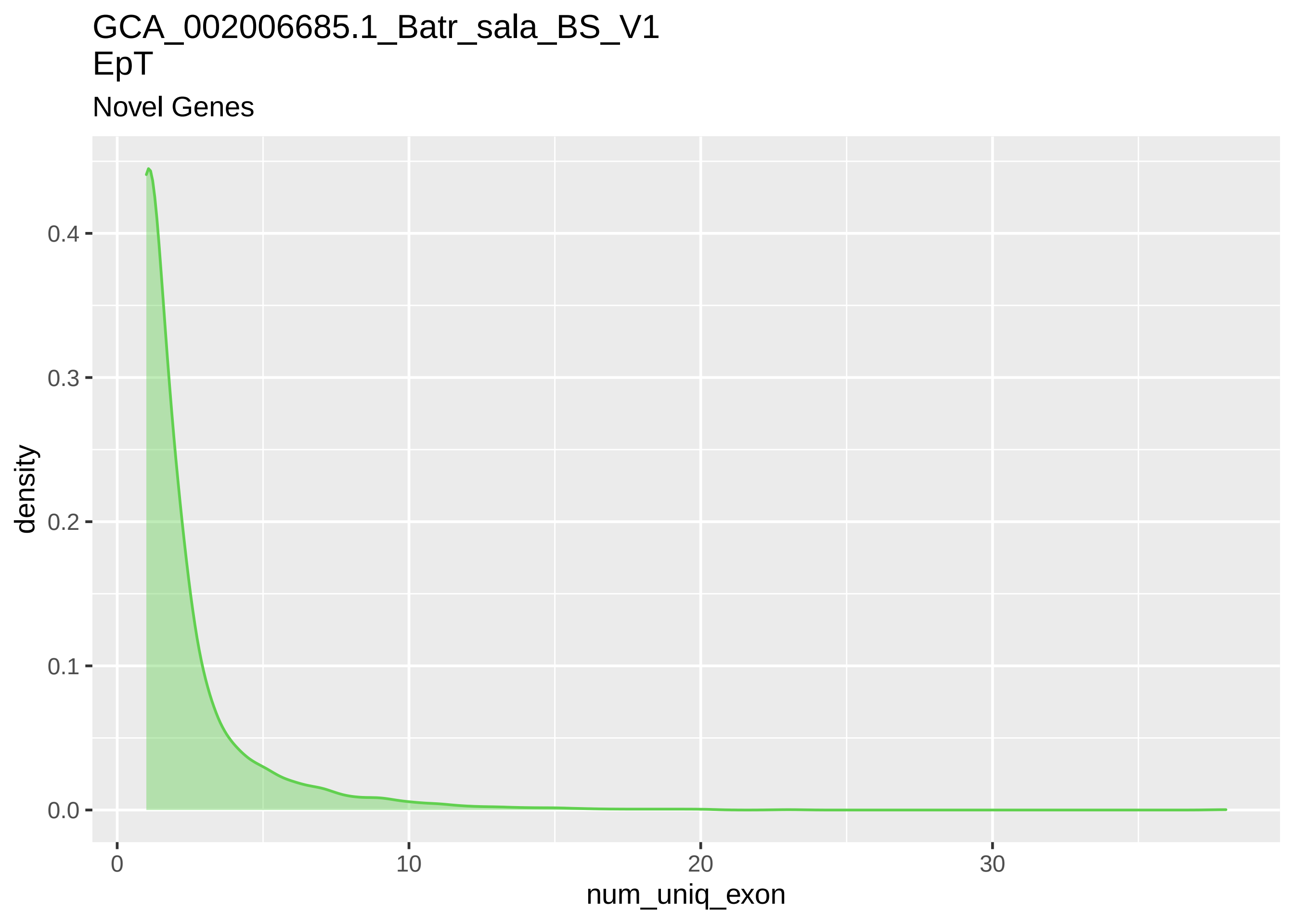
GCA_002006685.1_Batr_sala_BS_V1
EpT
Novel Genes
density
0.0
0.1
0.2
0.3
0.4
0
10
20
30
num_uniq_exon

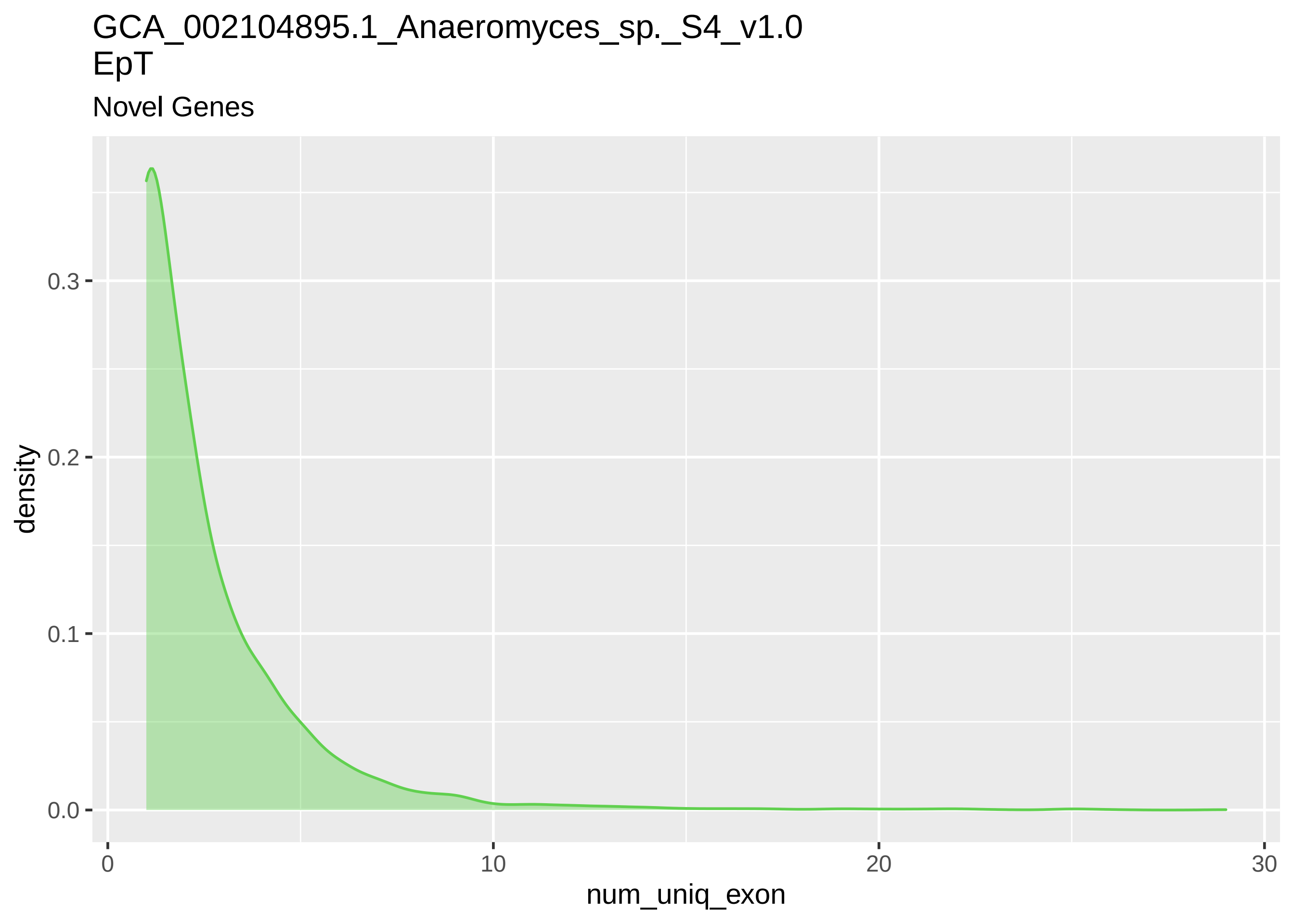

GCA_002104895.1_Anaeromyces_sp._S4_v1.0
EpT
Novel Genes
density
0.0
0.1
0.2
0.3
0
10
20
30
num_uniq_exon

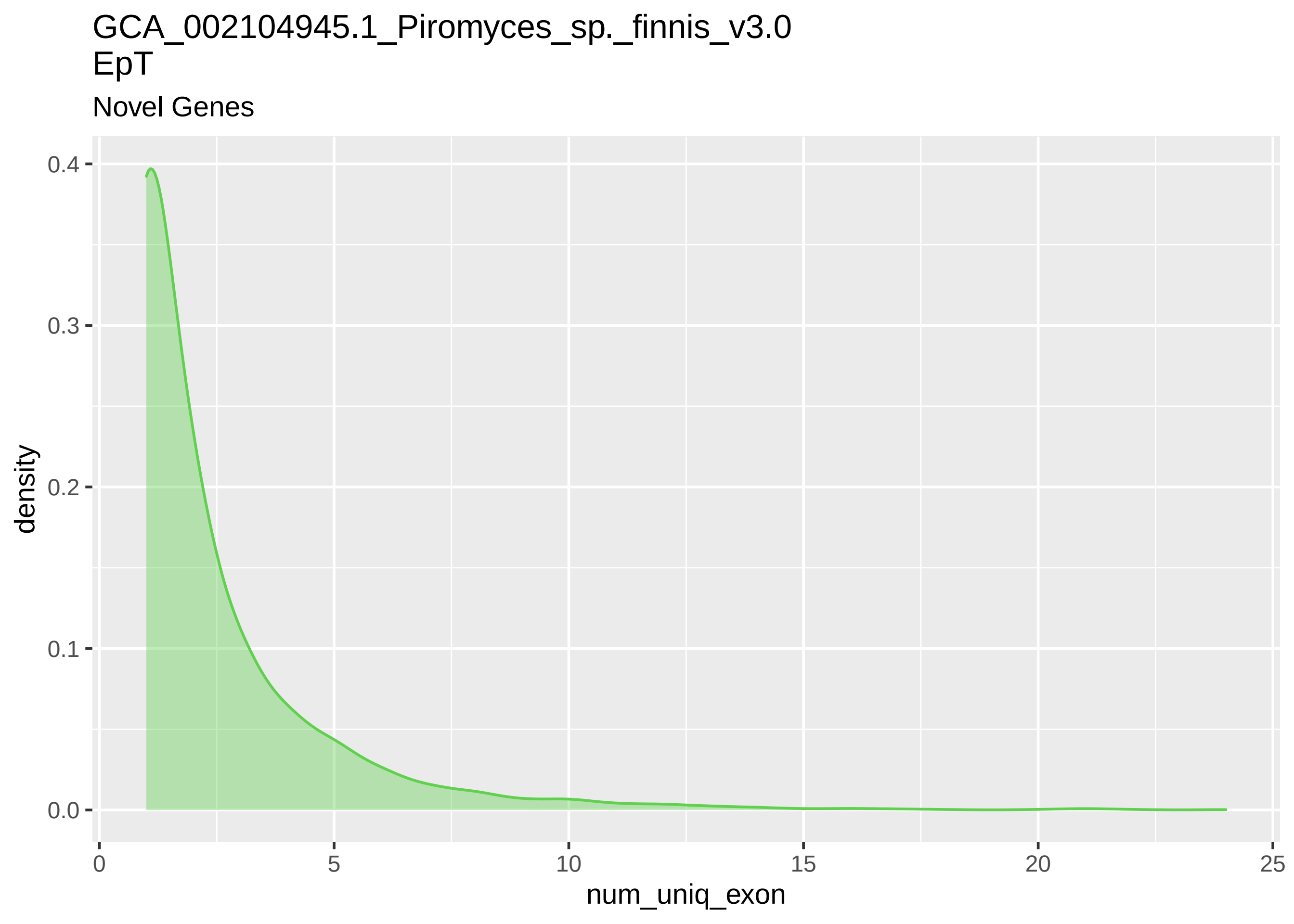
GCA_002104945.1_Piromyces_sp._finnis_v3.0
EpT
Novel Genes
density
0.4
0.3
0.2
0.1
0.0
0
5
10
15
20
25
num_uniq_exon

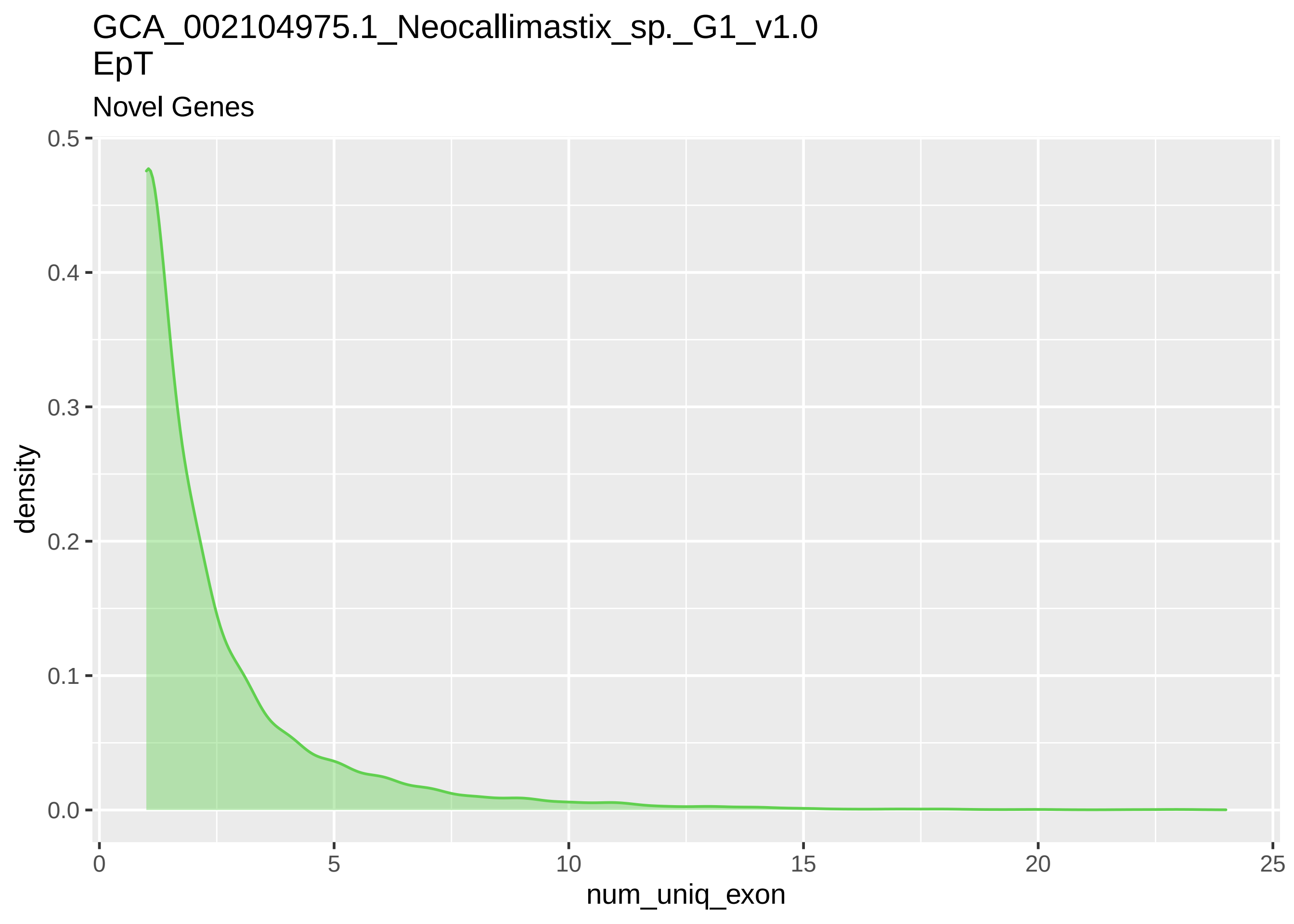
GCA_002104975.1_Neocallimastix_sp._G1_v1.0
EpT
Novel Genes
density
num_uniq_exon
0.0
0.1
0.2
0.3
0.4
0.5
0
5
10
15
20
25

GCA_002104985.1_Rhihy1
EpT

Novel Genes

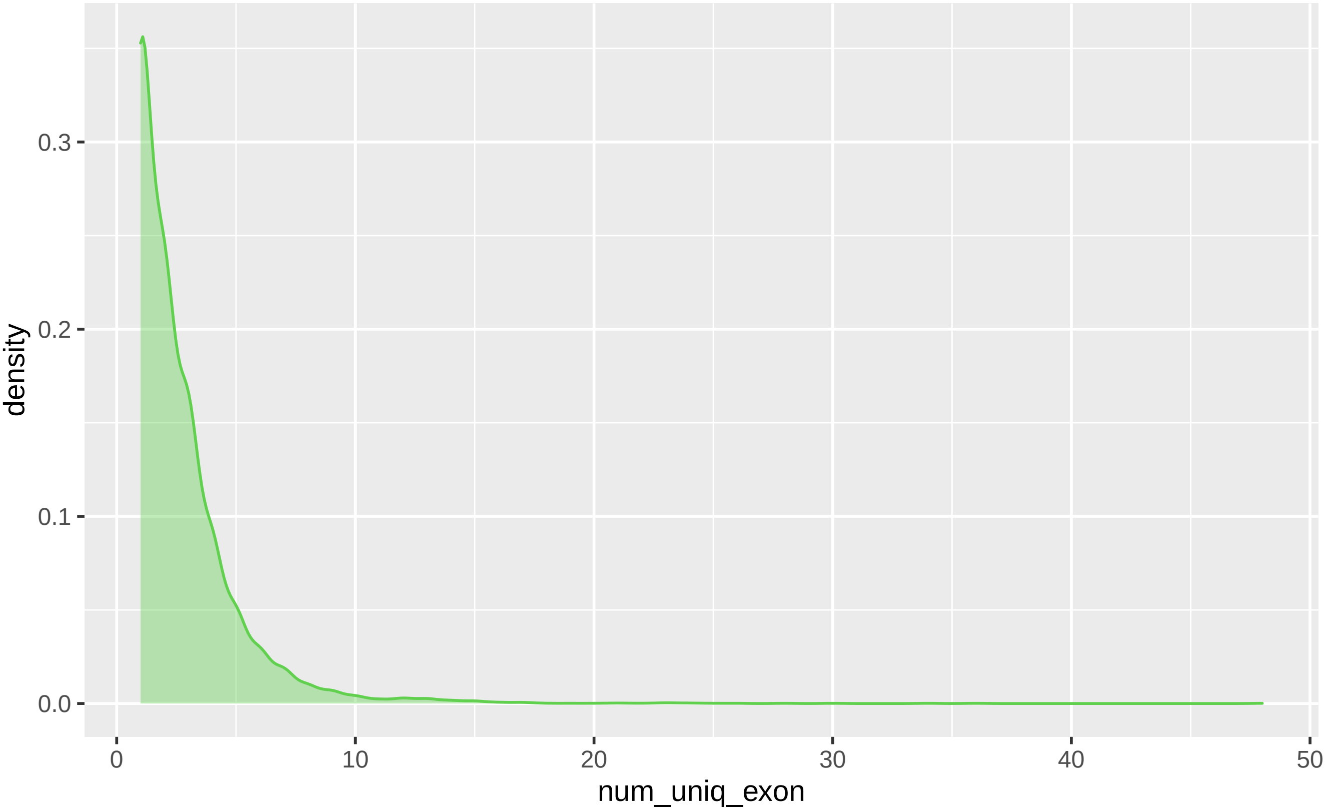

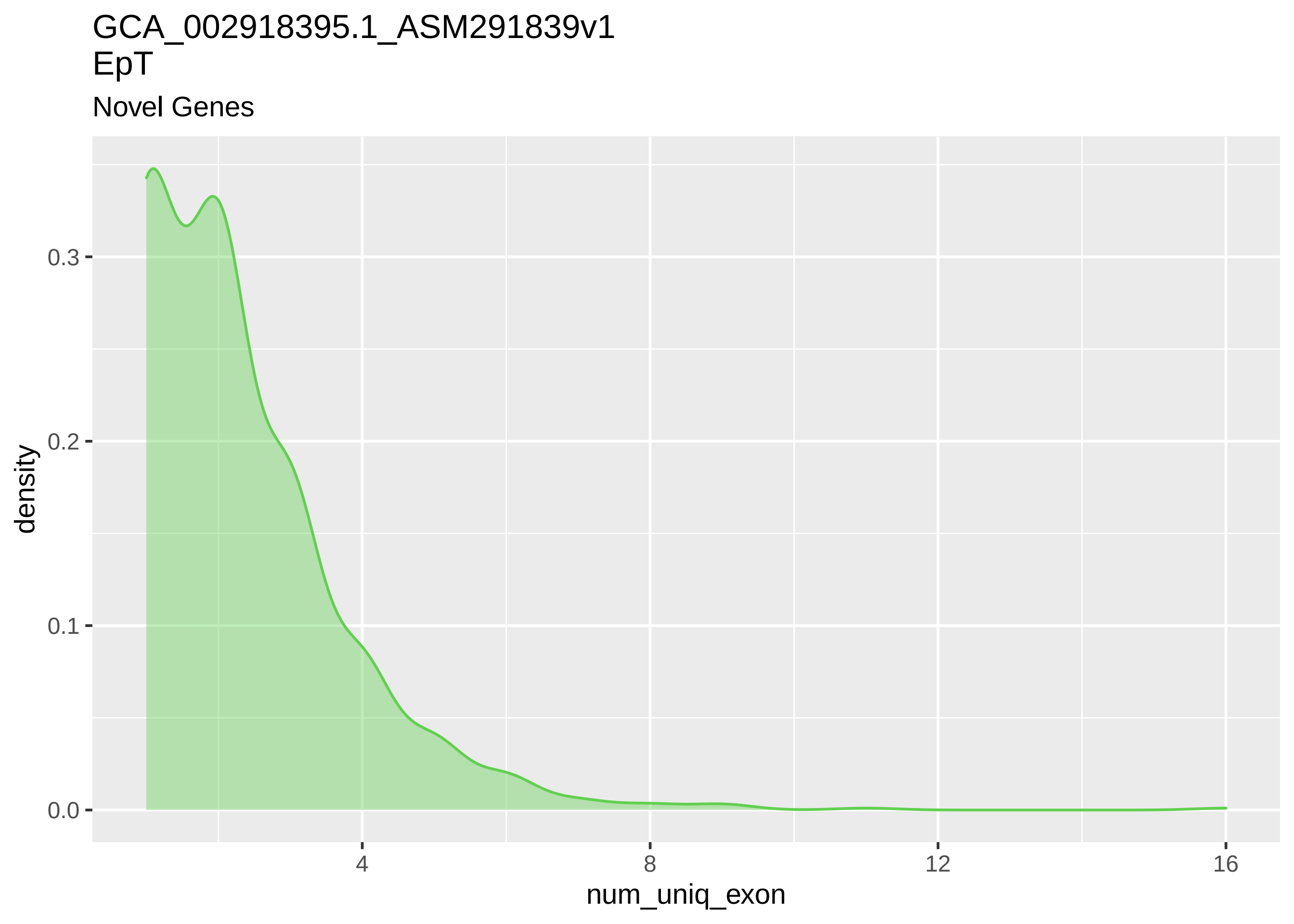
GCA_002918395.1_ASM291839v1
EpT
Novel Genes
density
0.3
0.2
0.1
0.0
4
8
12
16
num_uniq_exon

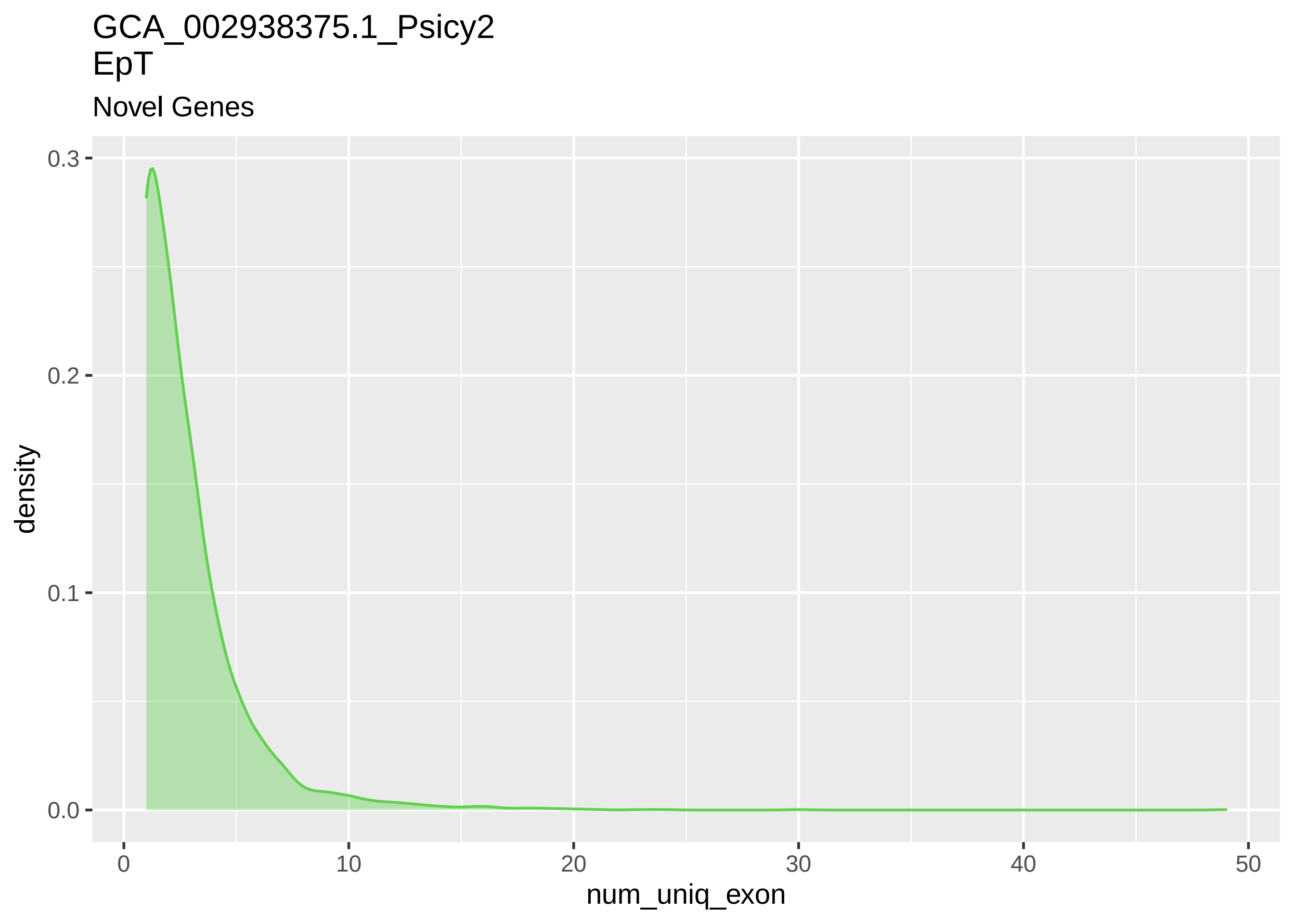
GCA_002938375.1_Psicy2
EpT
Novel Genes
density
num_uniq_exon
0.0
0.1
0.2
0.3
0
10
20
30
40
50

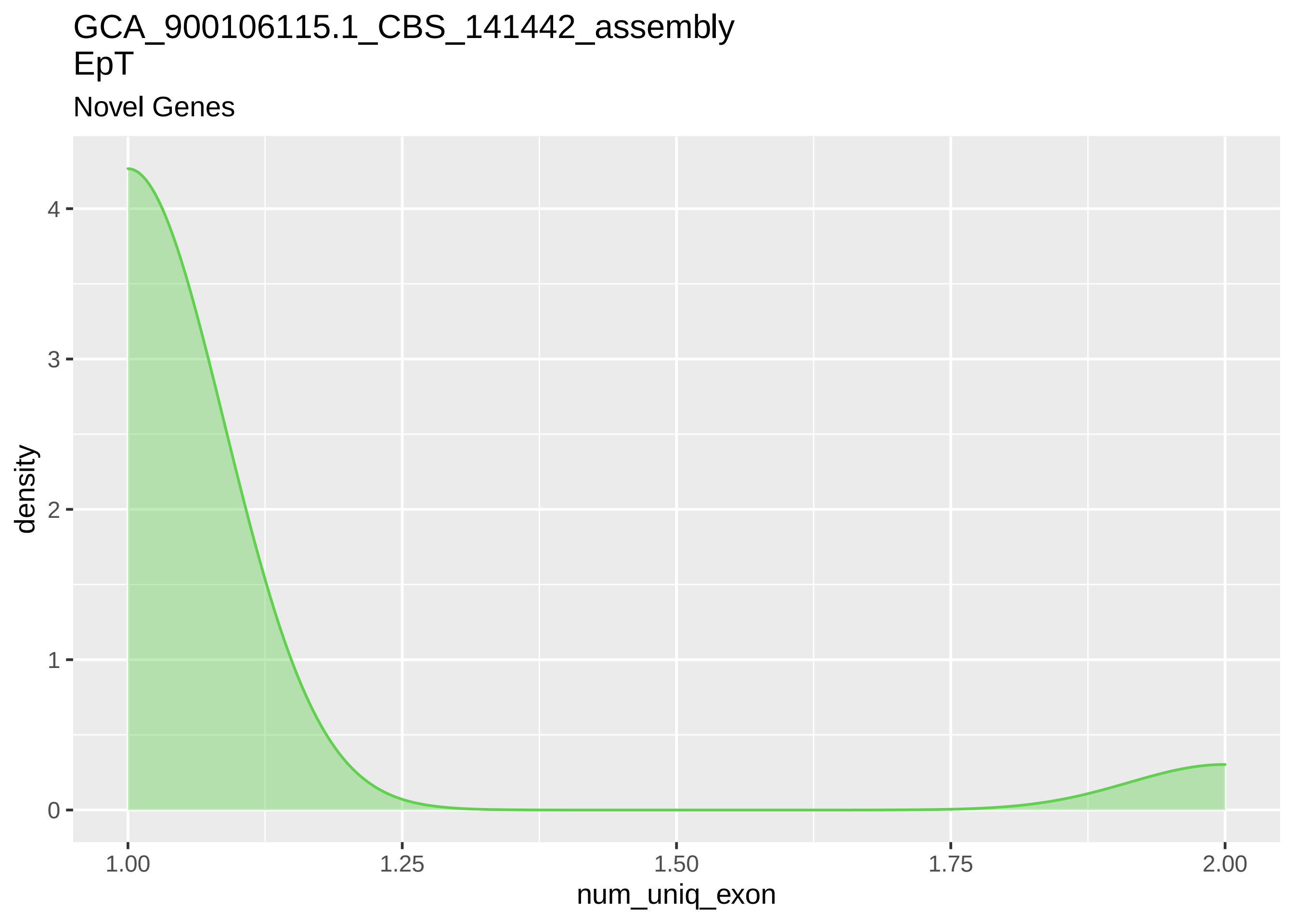
GCA_900106115.1_CBS_141442_assembly
EpT
Novel Genes
density
num_uniq_exon
0
1
2
3
4
1.00
1.25
1.50
1.75
2.00

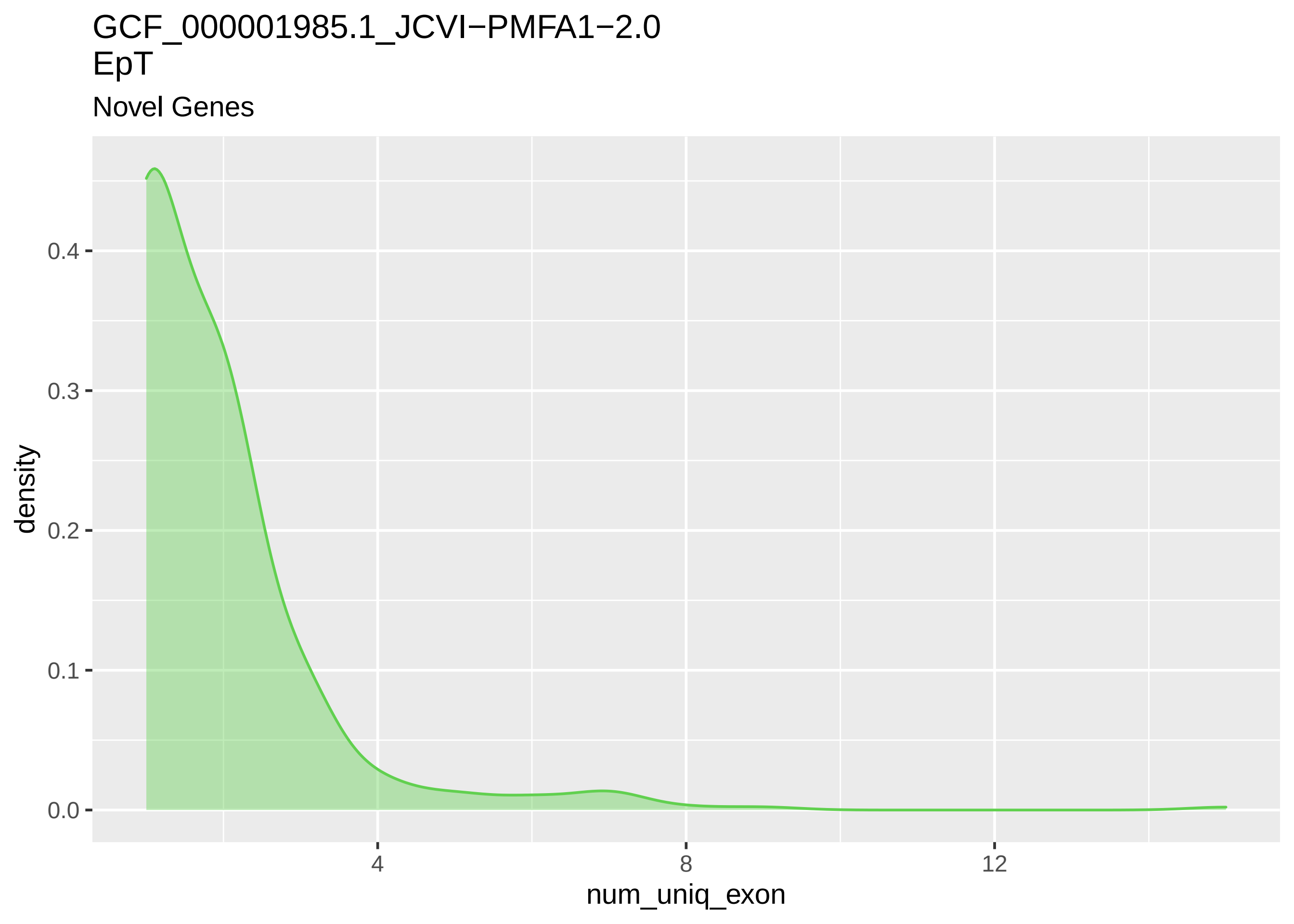
GCF_000001985.1_JCVI−PMFA1−2.0
EpT
Novel Genes
density
0.0
0.1
0.2
0.3
0.4
4
8
12
num_uniq_exon

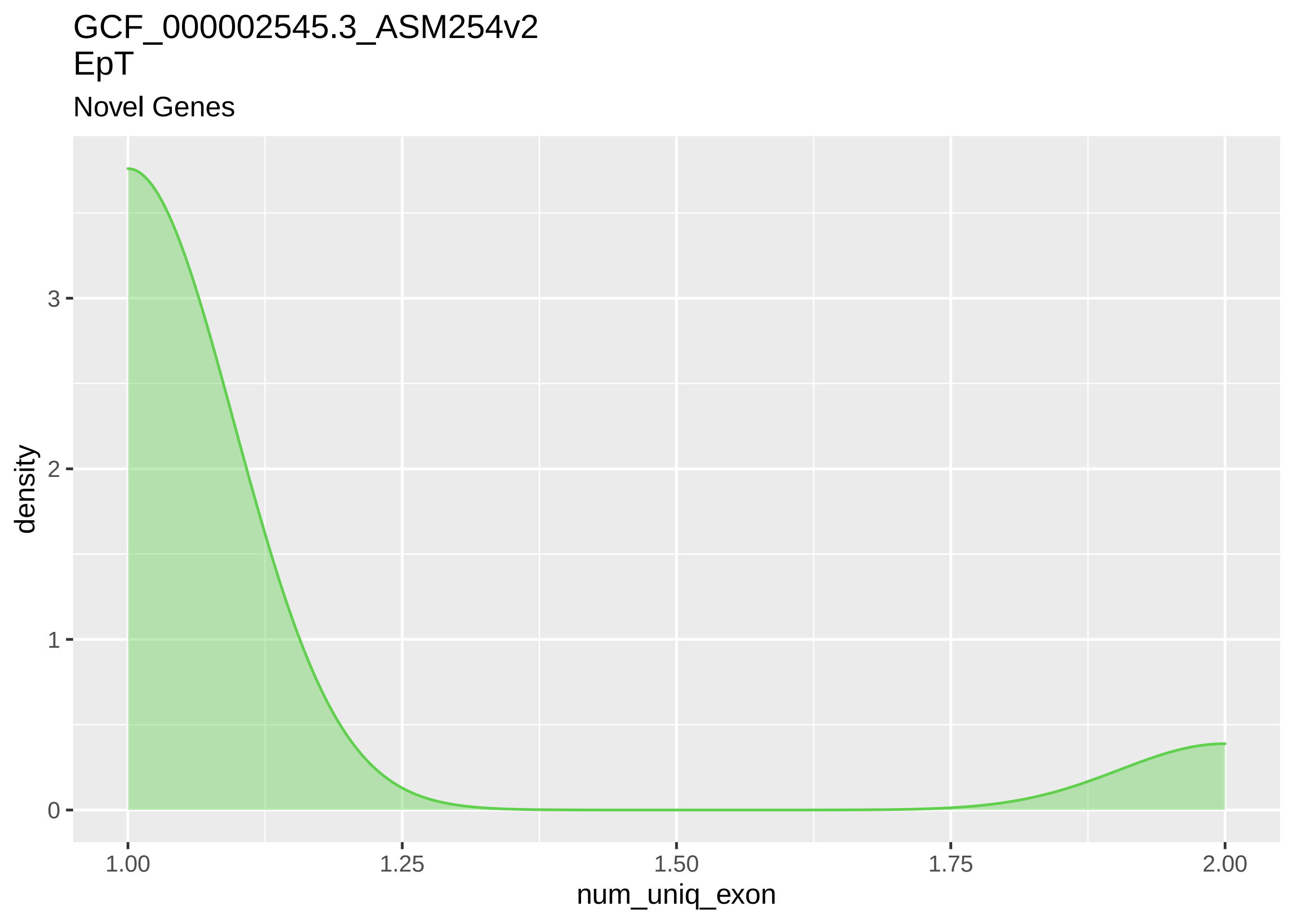
GCF_000002545.3_ASM254v2
EpT
Novel Genes
density
3
2
1
0
1.00
1.25
1.50
1.75
2.00
num_uniq_exon

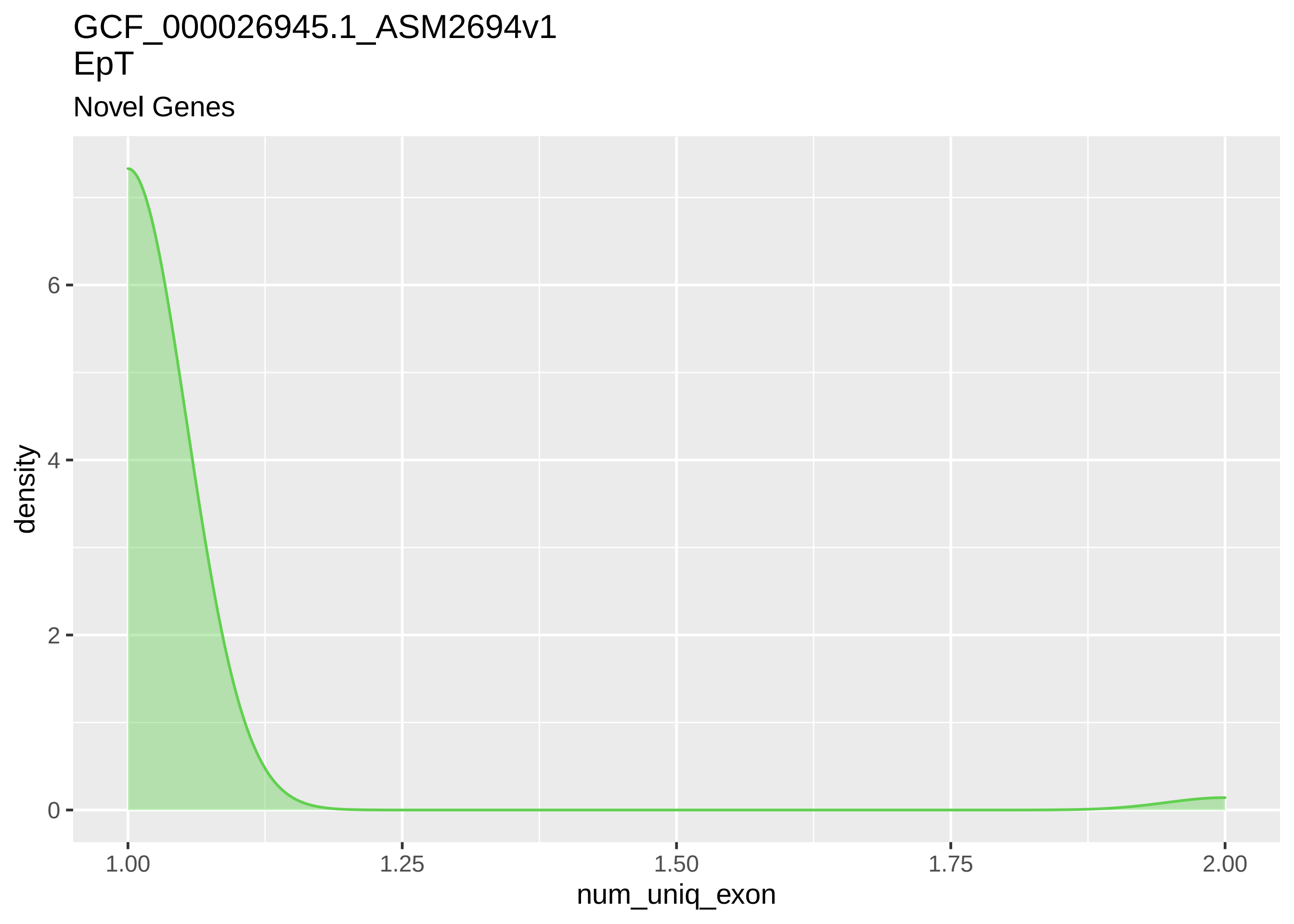
GCF_000026945.1_ASM2694v1
EpT
Novel Genes
density
num_uniq_exon
0
2
4
6
1.00
1.25
1.50
1.75
2.00

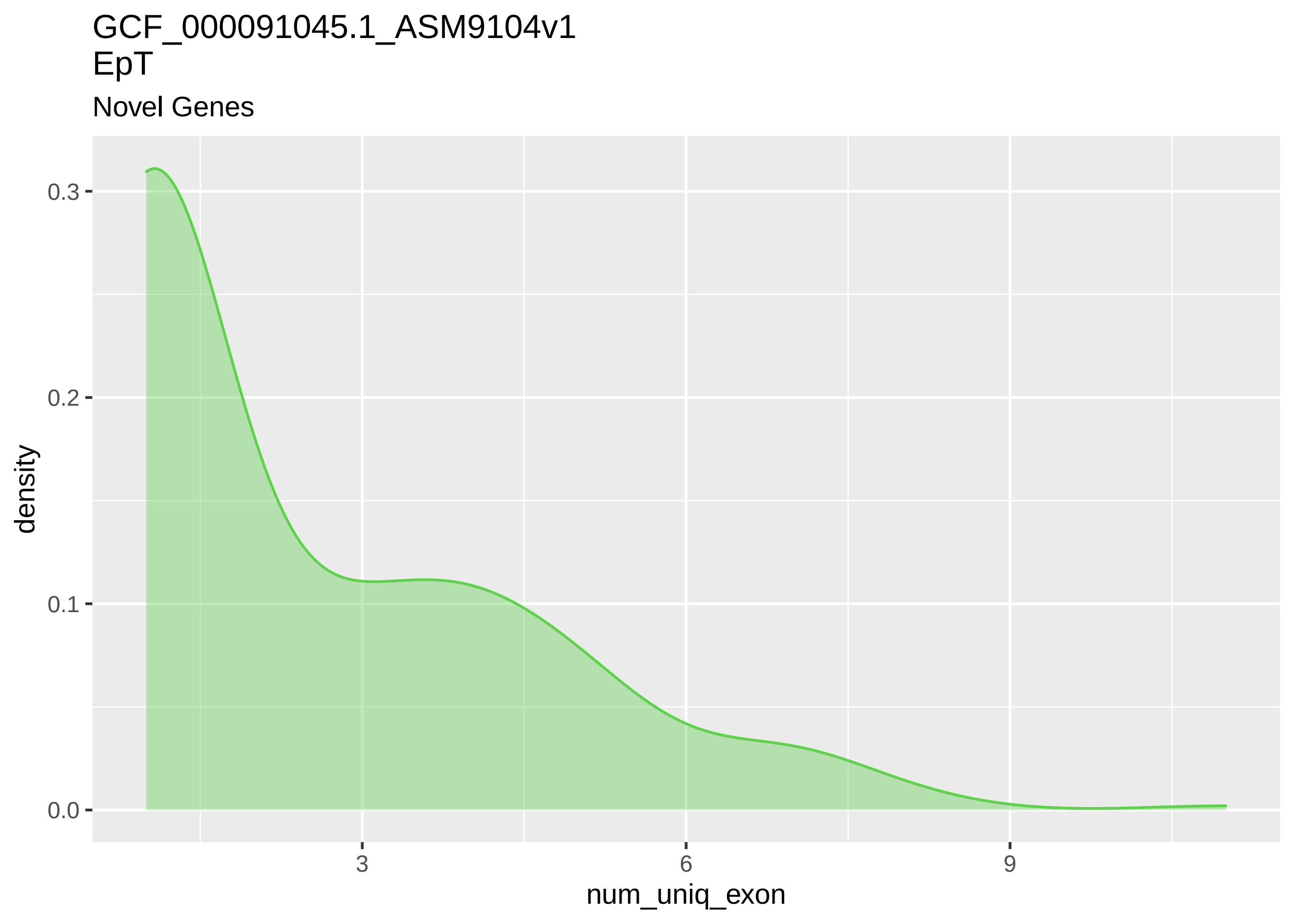
GCF_000091045.1_ASM9104v1
EpT
Novel Genes
density
0.0
0.1
0.2
0.3
3
6
9
num_uniq_exon

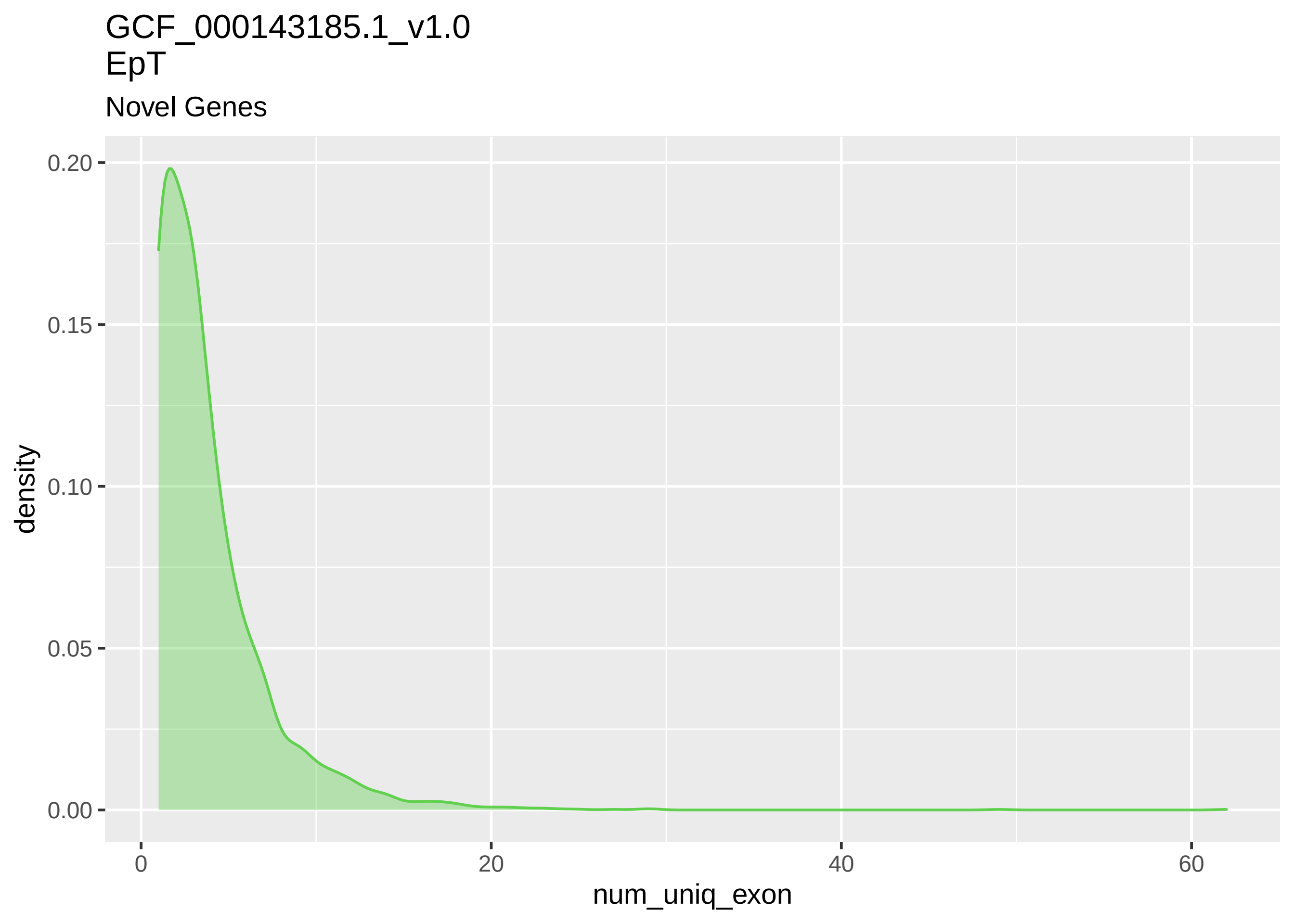
GCF_000143185.1_v1.0
EpT
Novel Genes
density
0.20
0.15
0.10
0.05
0.00
0
20
40
60
num_uniq_exon

GCF_000149035.1_C_graminicola_M1_001_V1
EpT

Novel Genes

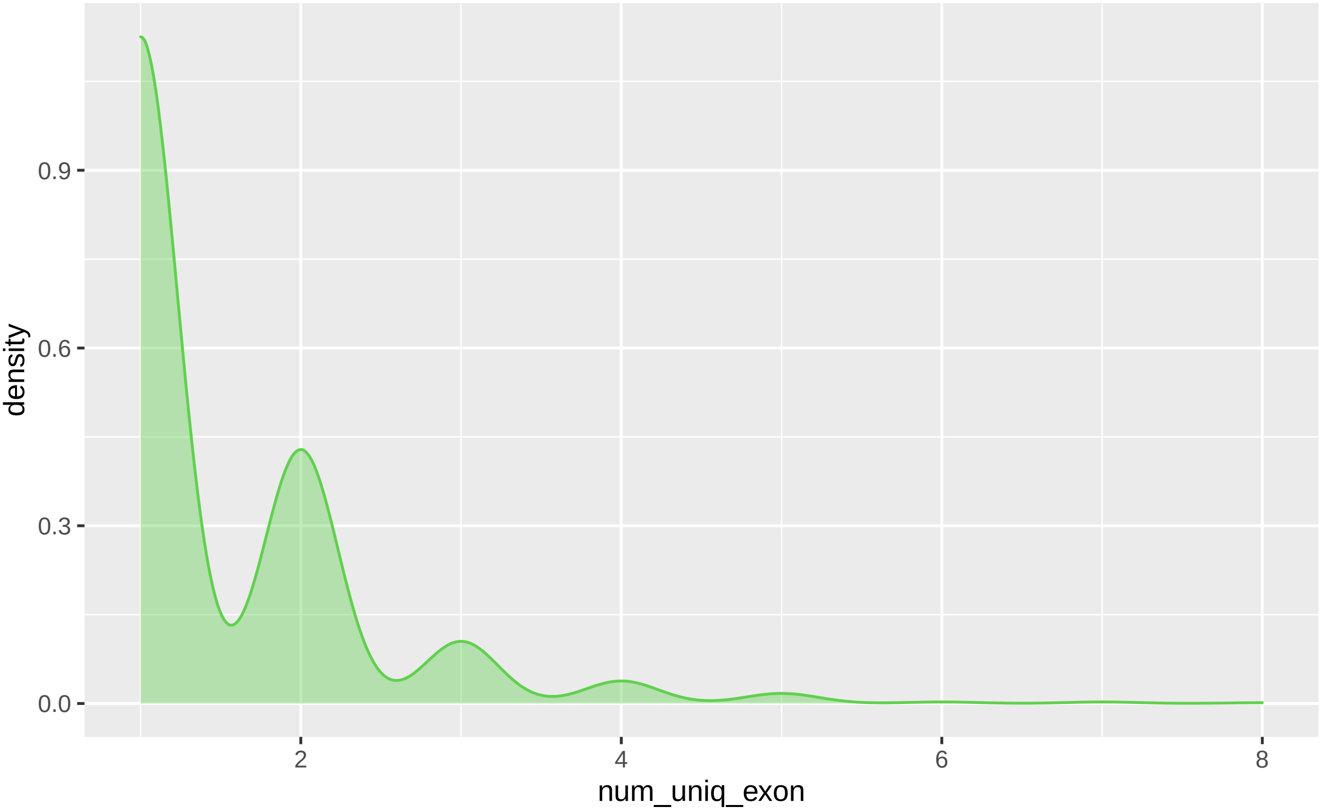

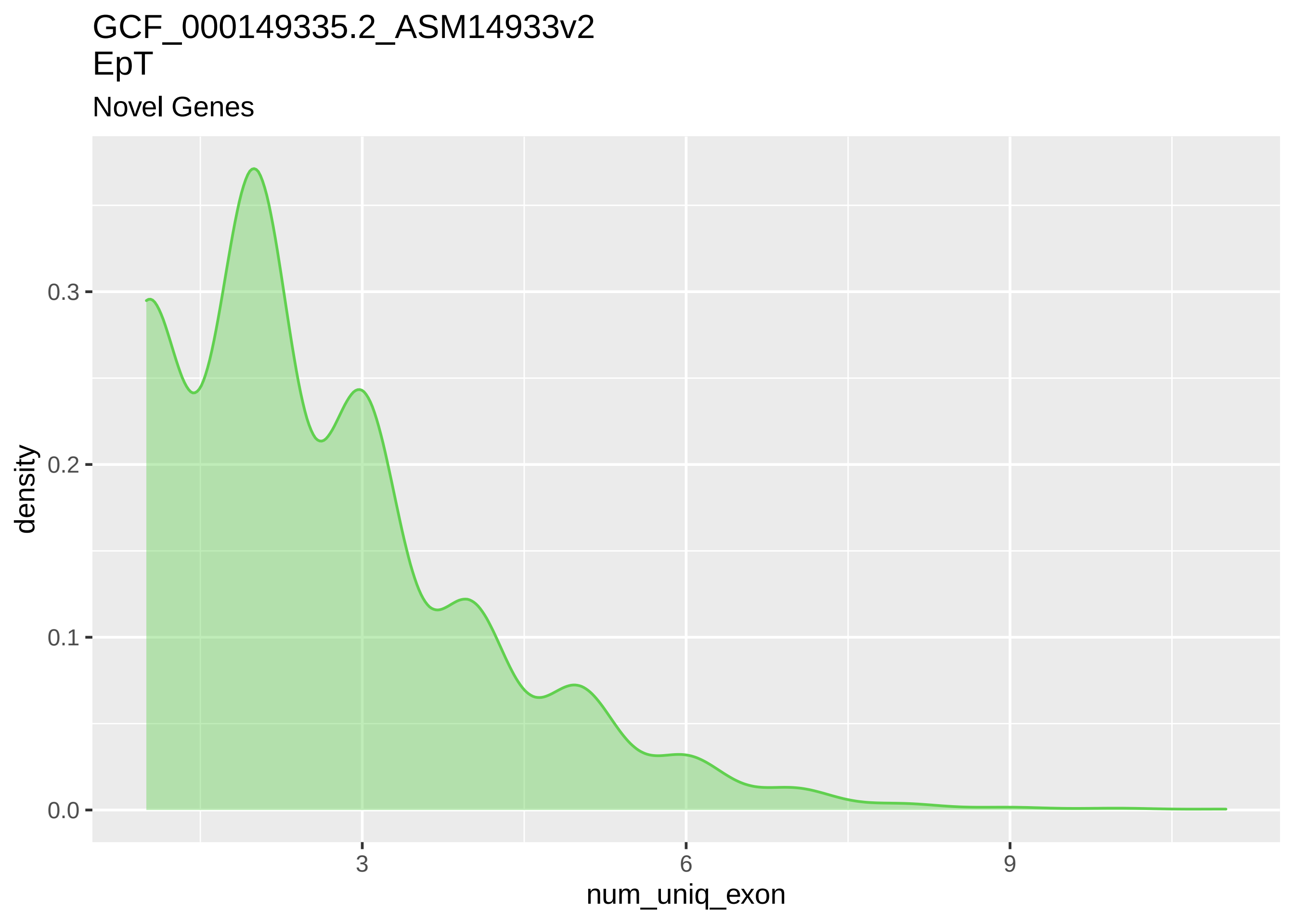

GCF_000149335.2_ASM14933v2
EpT
Novel Genes
density
0.3
0.2
0.1
0.0
3
6
9
num_uniq_exon

GCF_000149555.1_ASM14955v1
EpT

Novel Genes

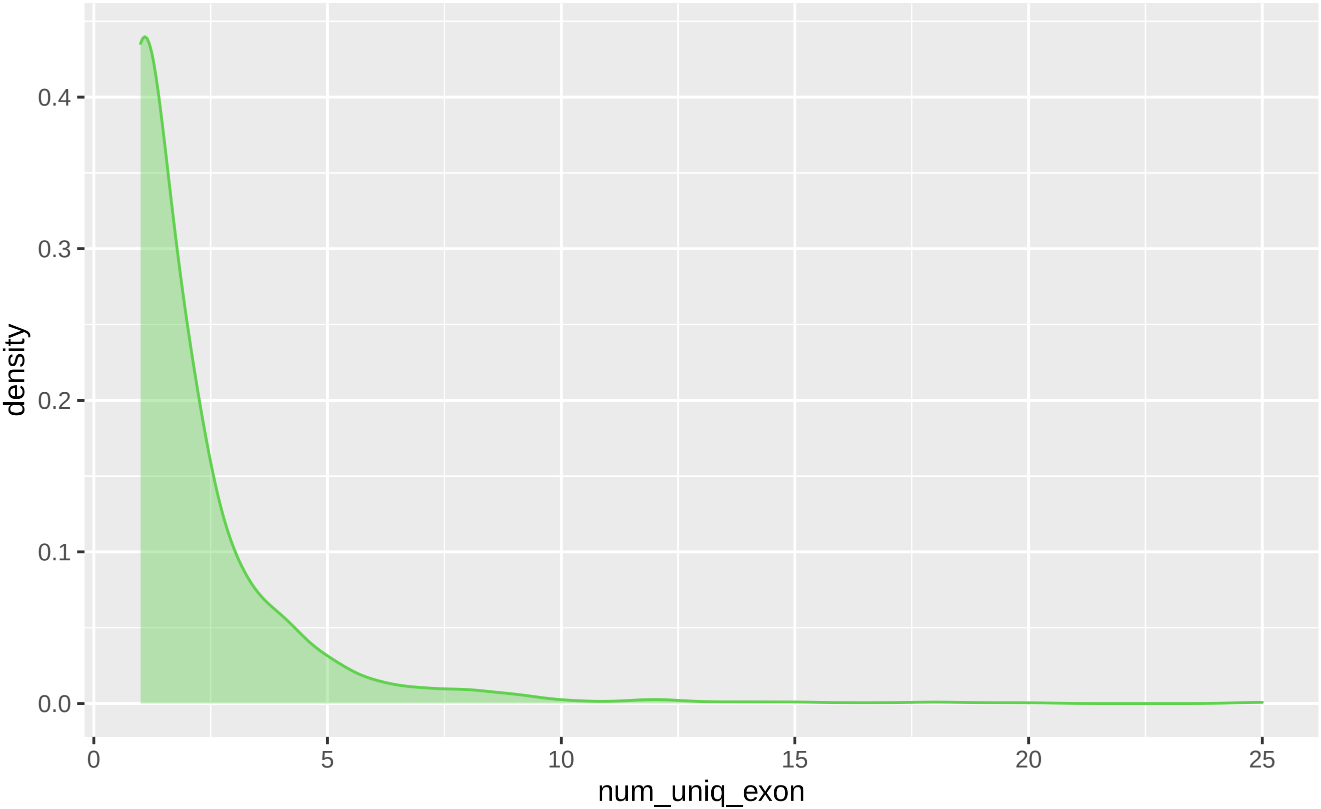

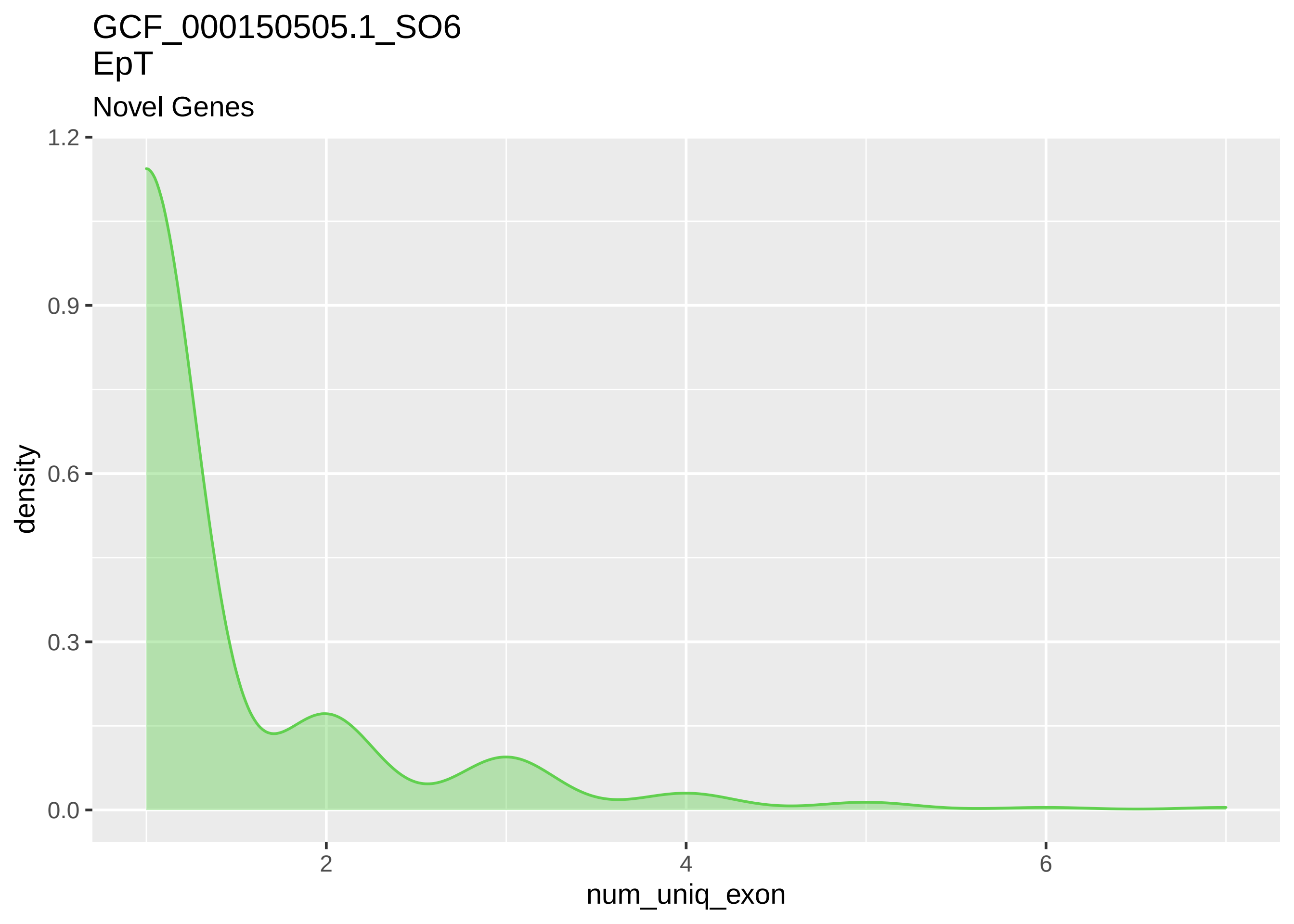
GCF_000150505.1_SO6
EpT
Novel Genes
1.2
0.9
0.6
0.3
0.0
density
2
4
6
num_uniq_exon

GCF_000150705.2_Paracocci_br_Pb01_V2
EpT

Novel Genes

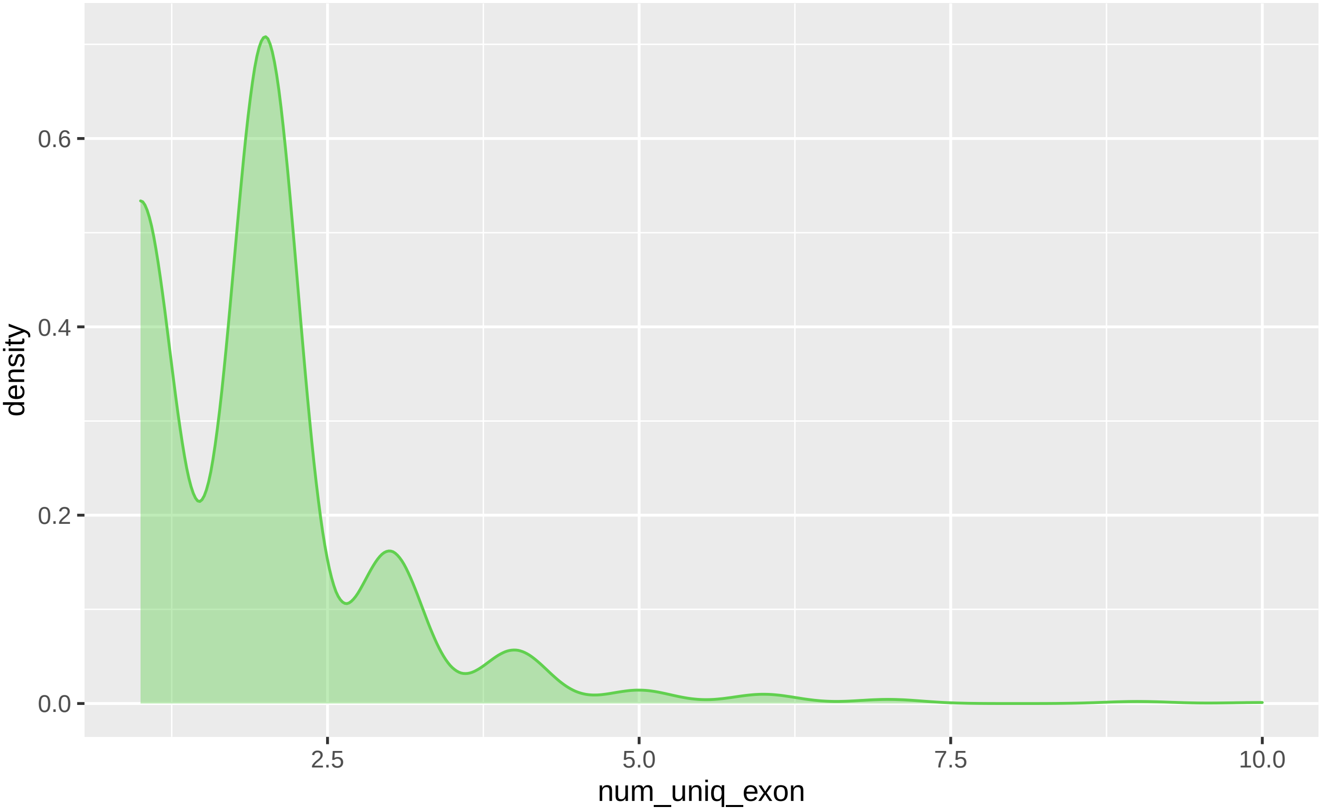

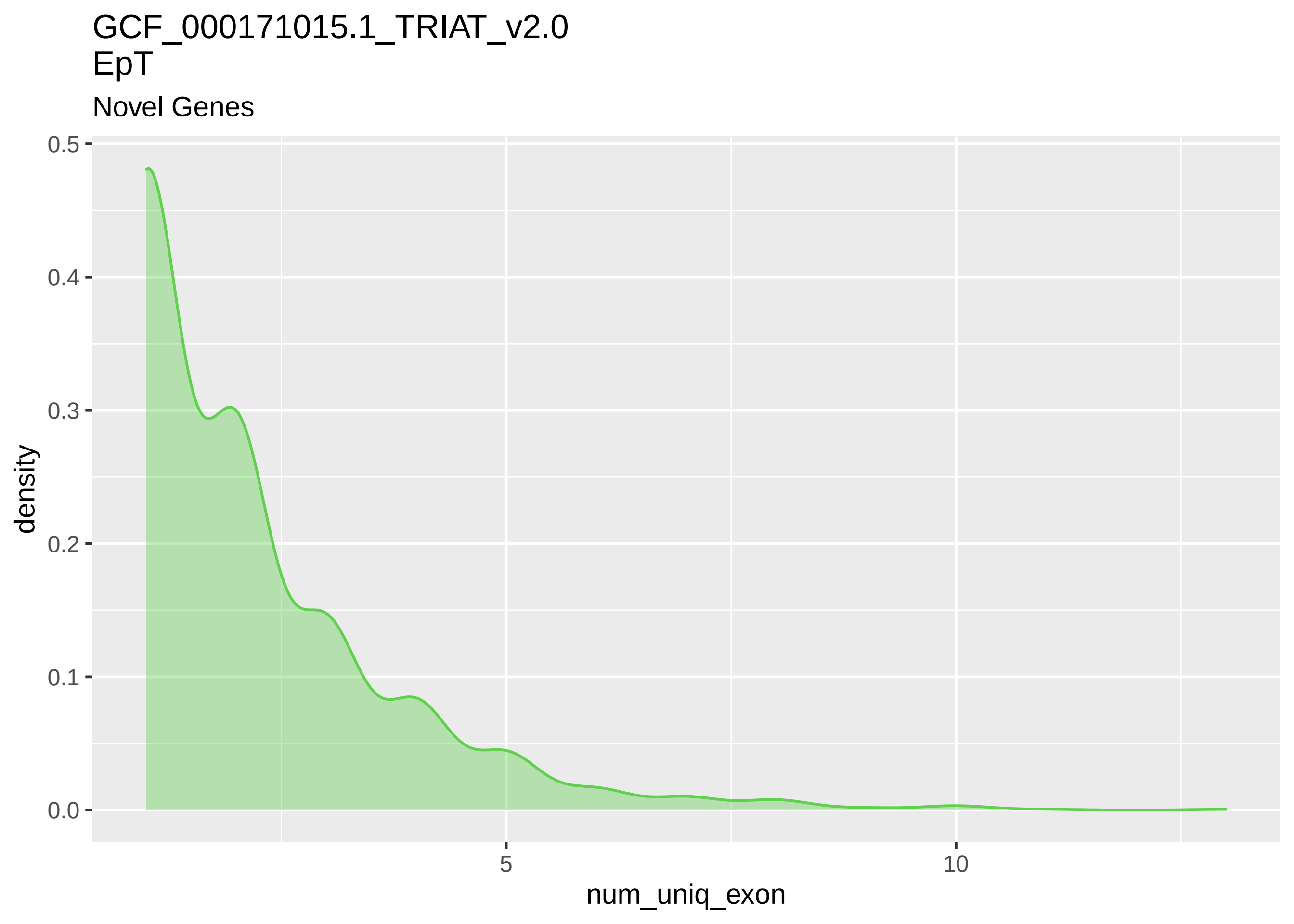

GCF_000171015.1_TRIAT_v2.0
EpT
Novel Genes
density
num_uniq_exon
0.5
0.4
0.3
0.2
0.1
0.0
5
10

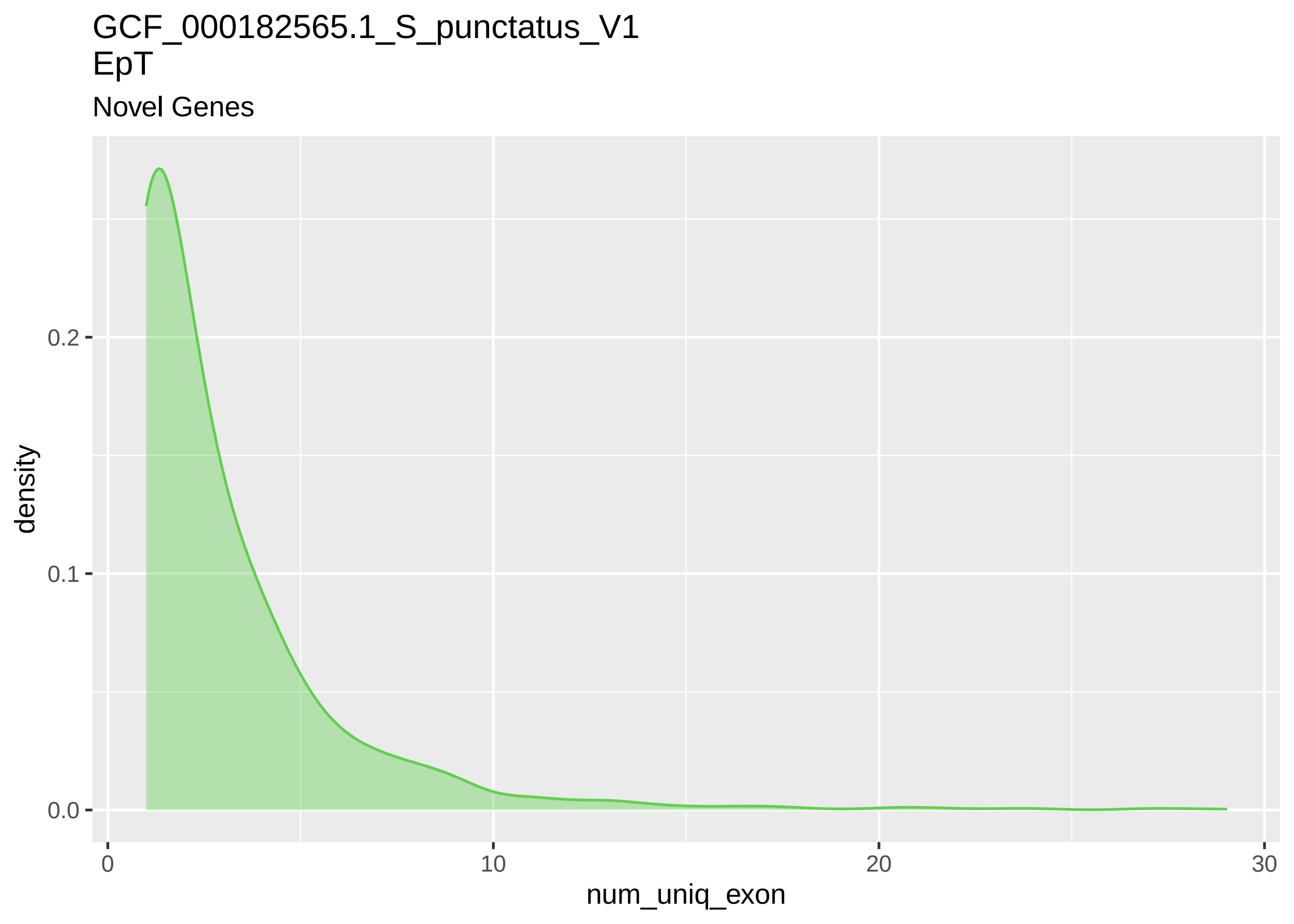
GCF_000182565.1_S_punctatus_V1
EpT
Novel Genes
density
0.0
0.1
0.2
0
10
20
30
num_uniq_exon

GCF_000182805.2_ASM18280v2
EpT

Novel Genes

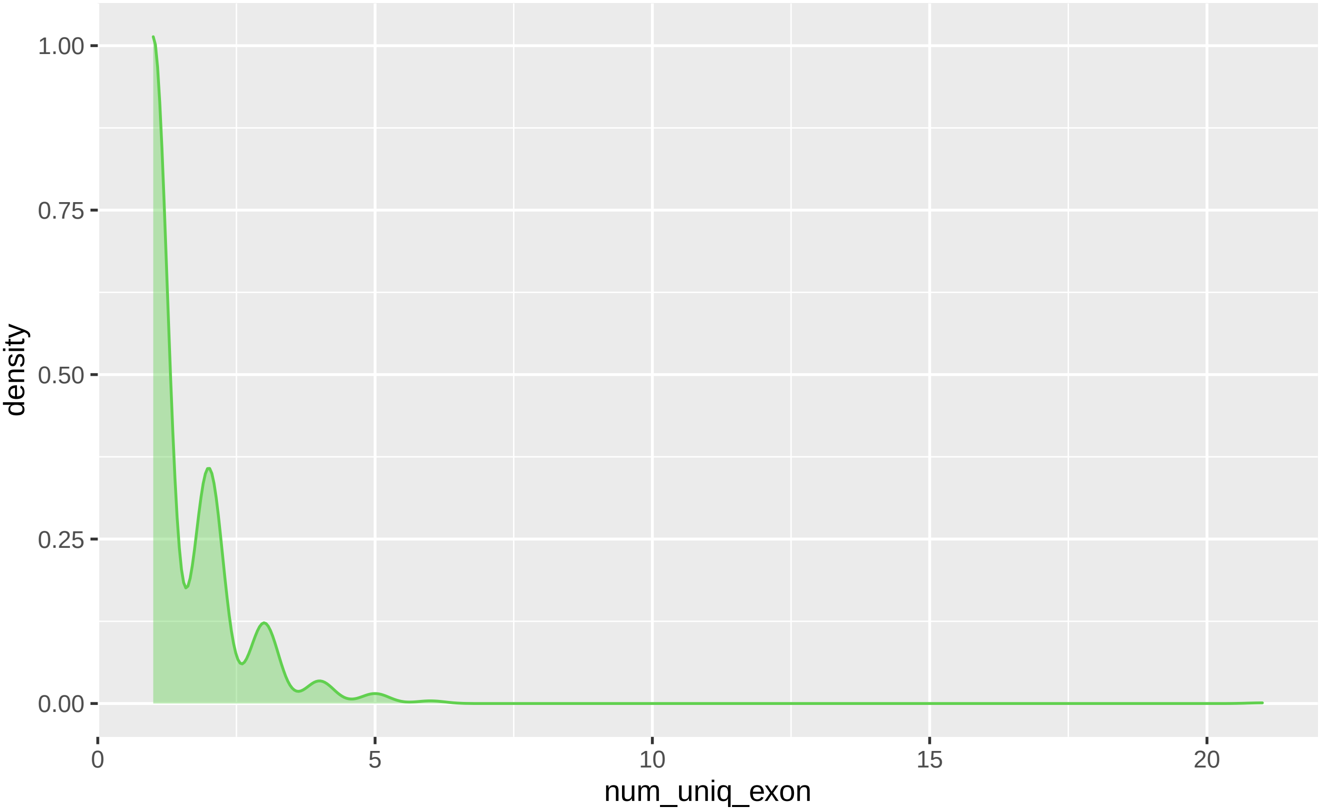

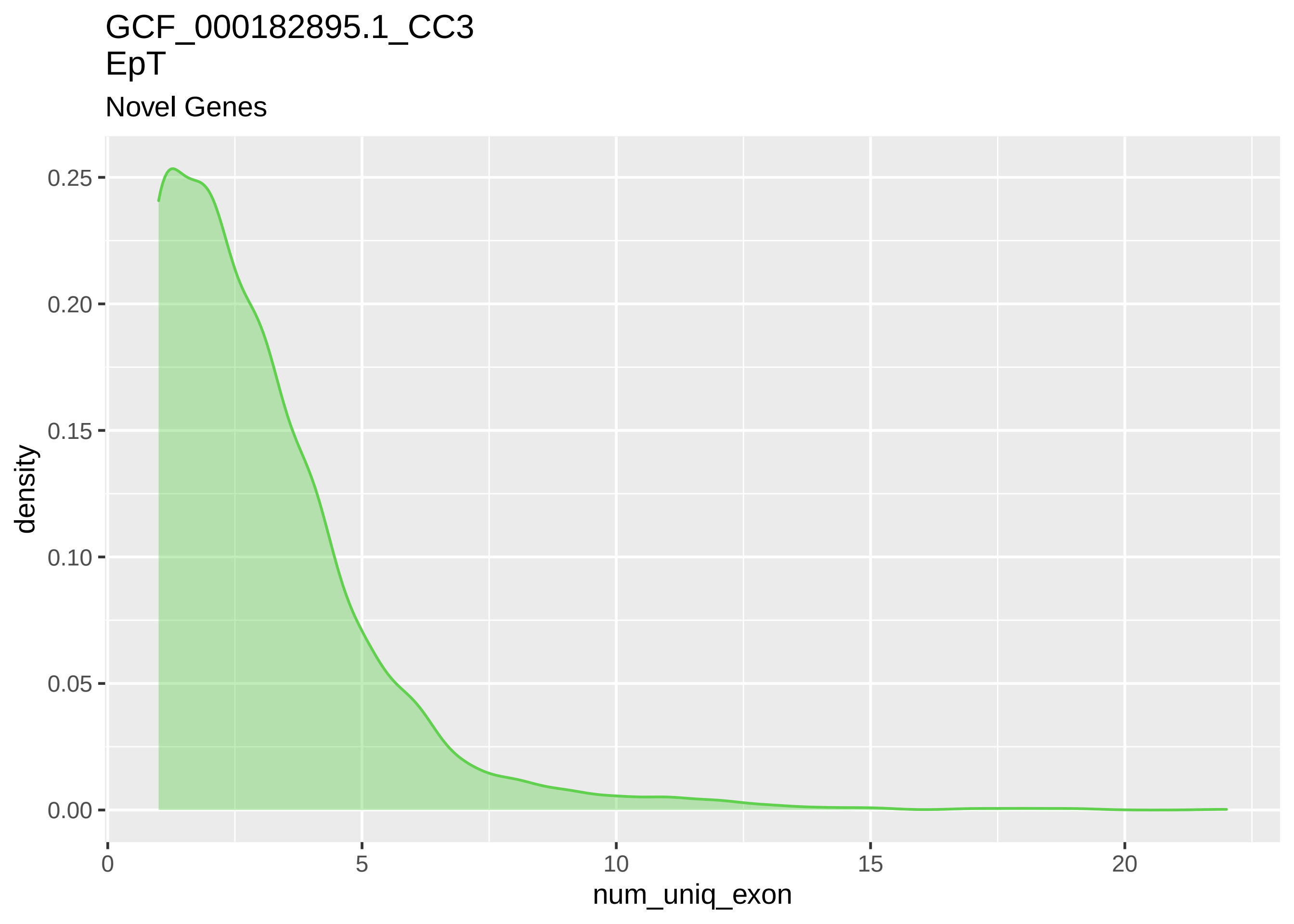
GCF_000182895.1_CC3
EpT
Novel Genes
density
0.25
0.20
0.15
0.10
0.05
0.00
0
5
10
15
20
num_uniq_exon

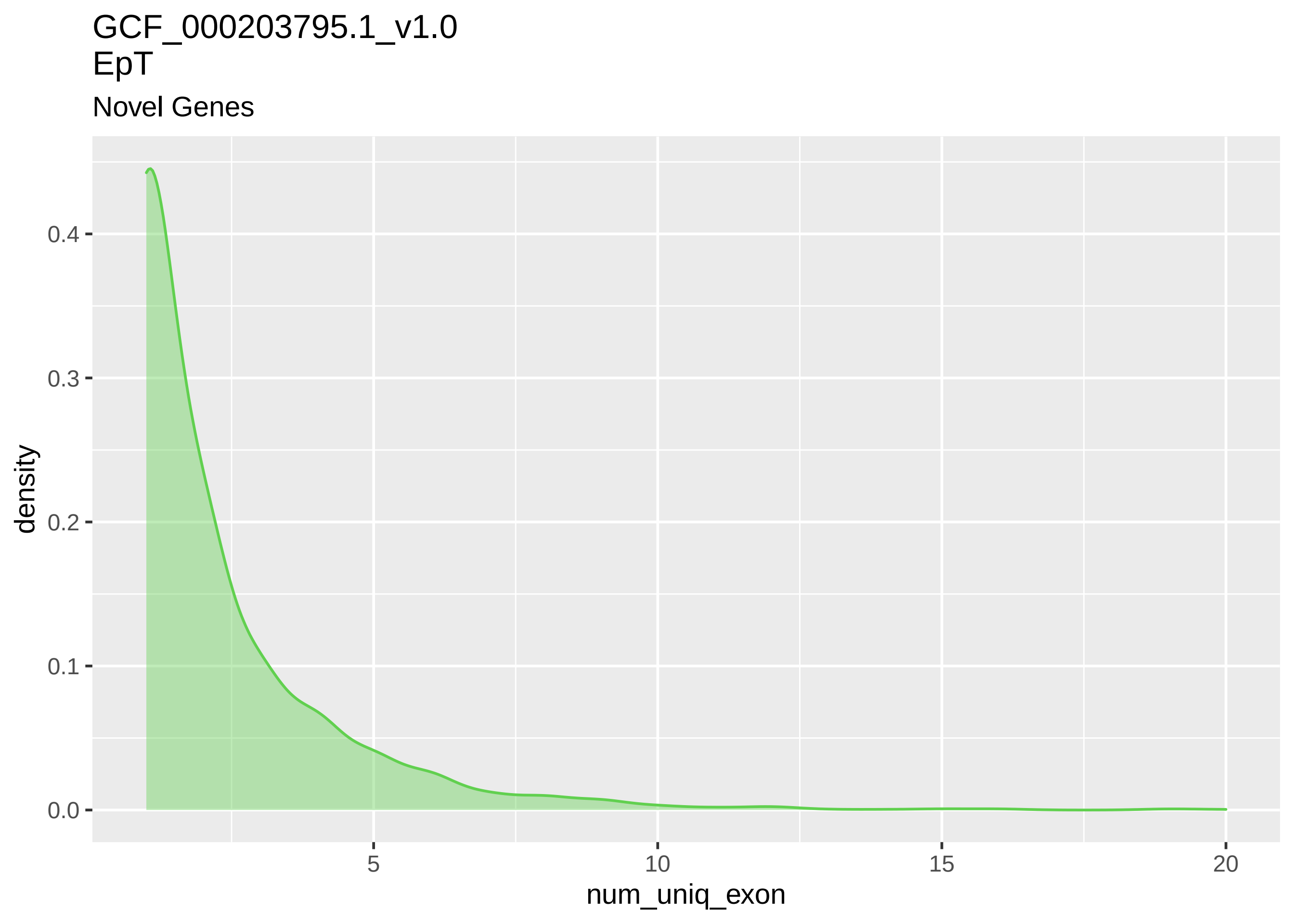

GCF_000203795.1_v1.0
EpT
Novel Genes
density
0.0
0.1
0.2
0.3
0.4
5
10
15
20
num_uniq_exon

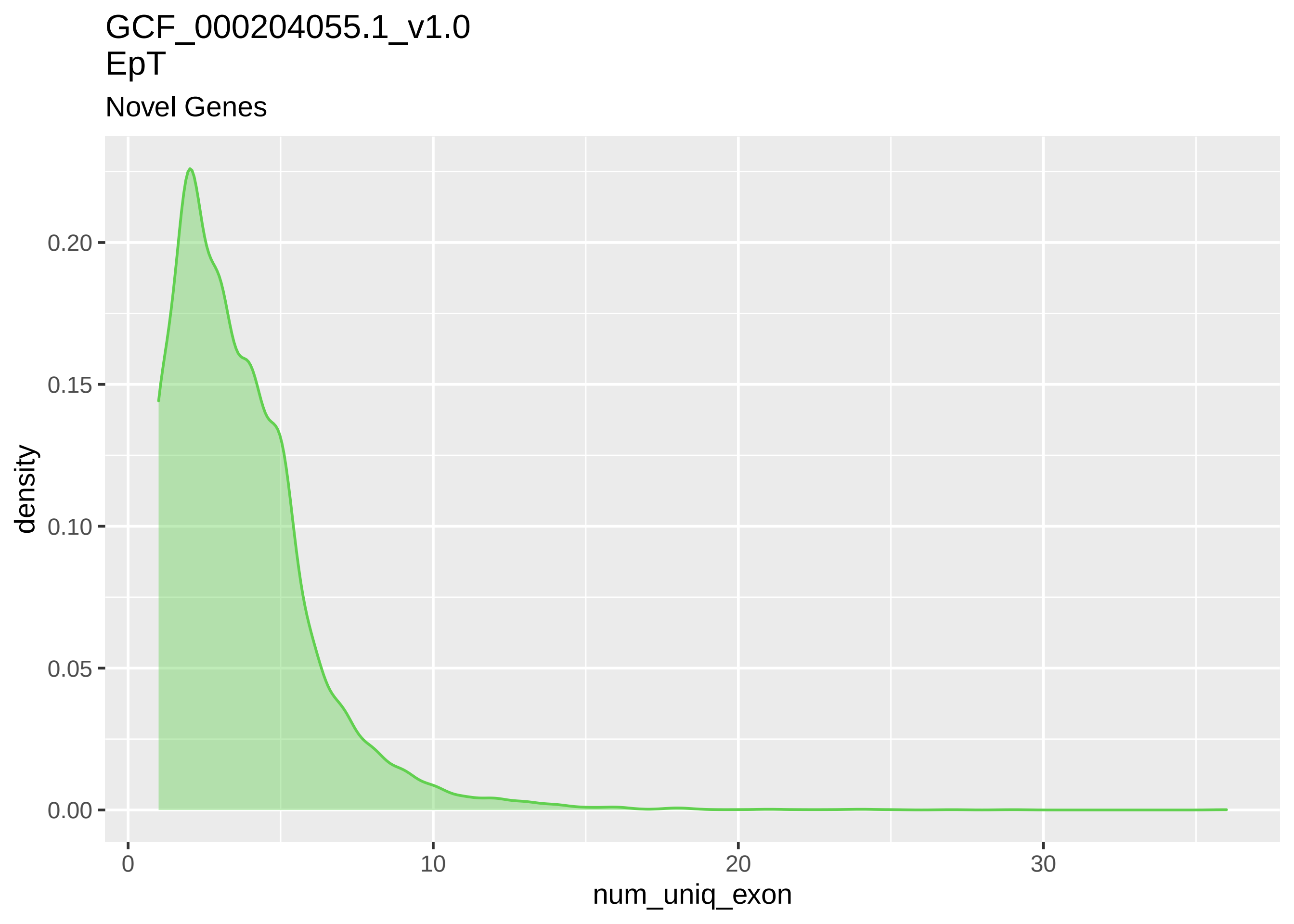
GCF_000204055.1_v1.0
EpT
Novel Genes
density
0.20
0.15
0.10
0.05
0.00
0
10
20
30
num_uniq_exon

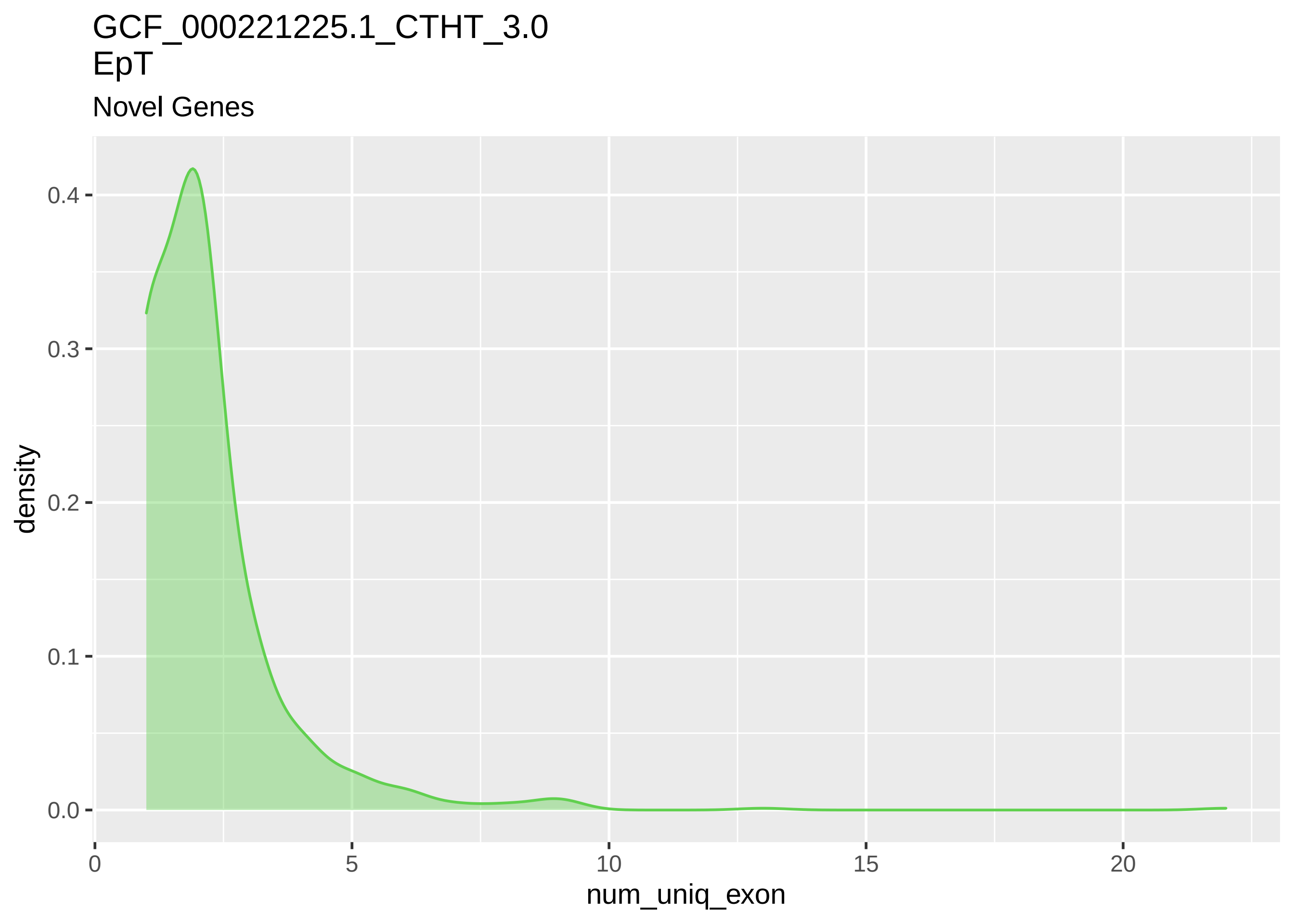
GCF_000221225.1_CTHT_3.0
EpT
Novel Genes
density
0.0
0.1
0.2
0.3
0.4
0
5
10
15
20
num_uniq_exon

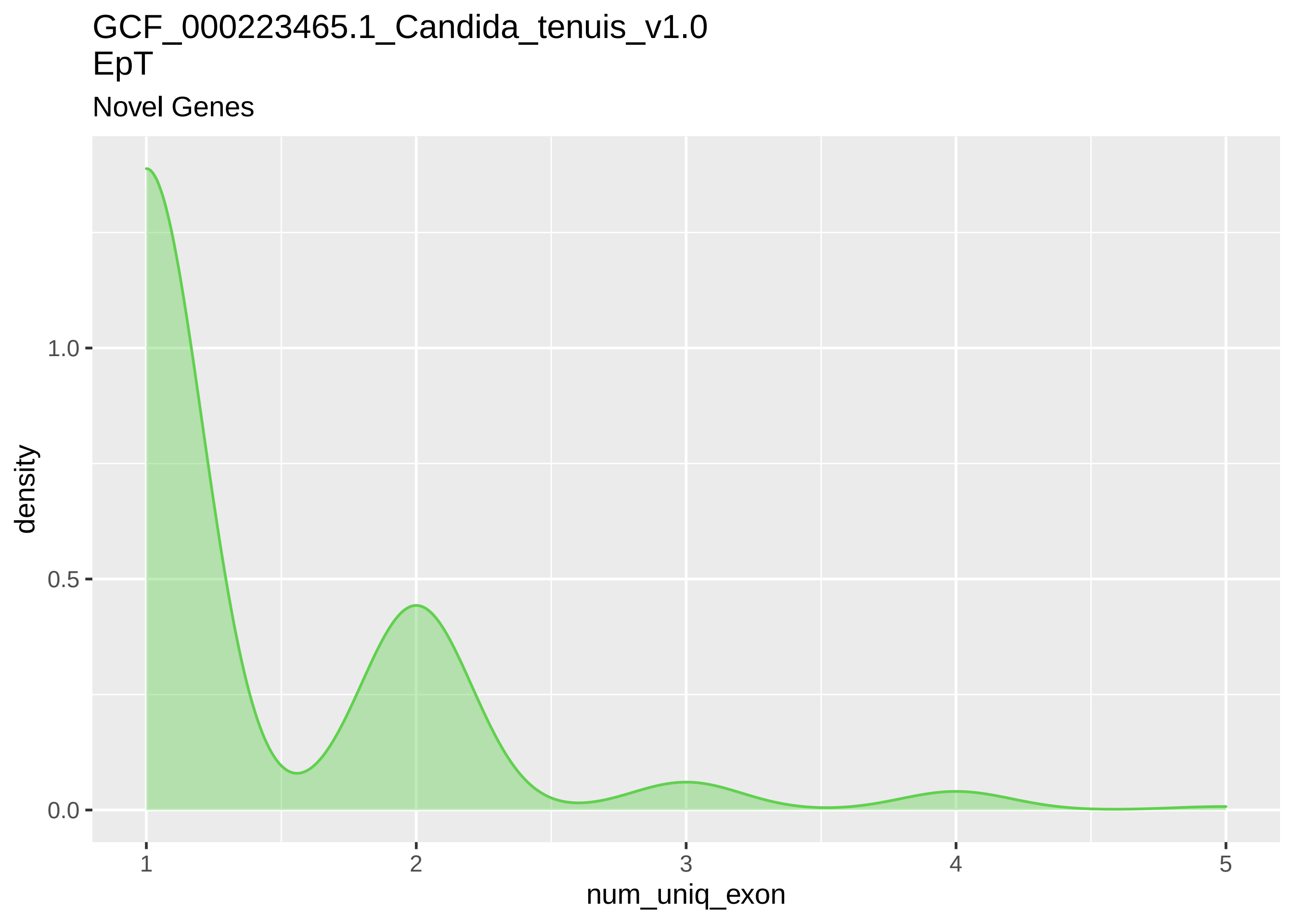

GCF_000223465.1_Candida_tenuis_v1.0
EpT
Novel Genes
density
1.0
0.5
0.0
1
2
3
4
5
num_uniq_exon

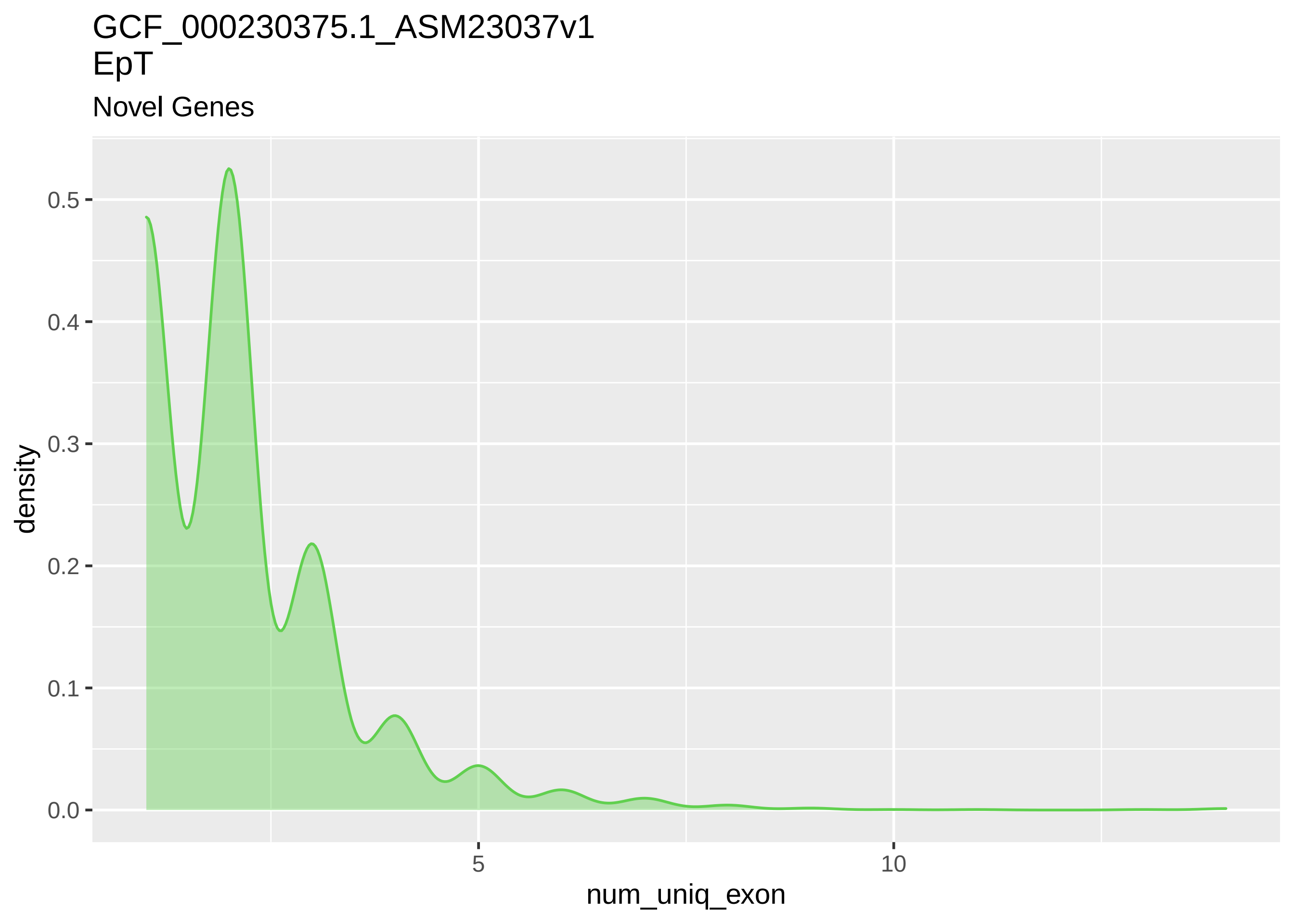
GCF_000230375.1_ASM23037v1
EpT
Novel Genes
density
0.5
0.4
0.3
0.2
0.1
0.0
5
10
num_uniq_exon

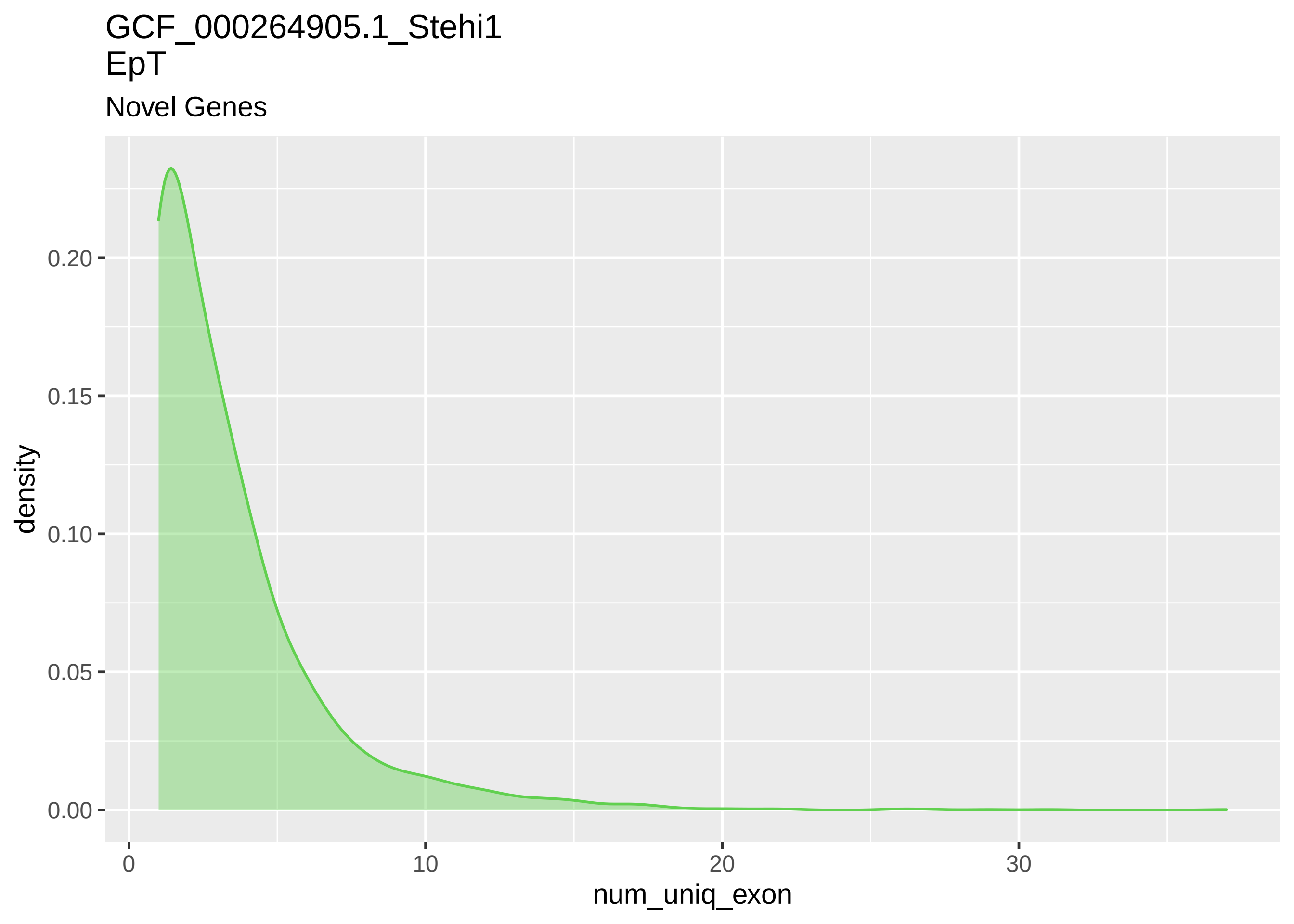
GCF_000264905.1_Stehi1
EpT
Novel Genes
density
0.00
0.05
0.10
0.15
0.20
0
10
20
30
num_uniq_exon

GCF_000264995.1_Punctularia_strigosozonata_v1.0
EpT

Novel Genes

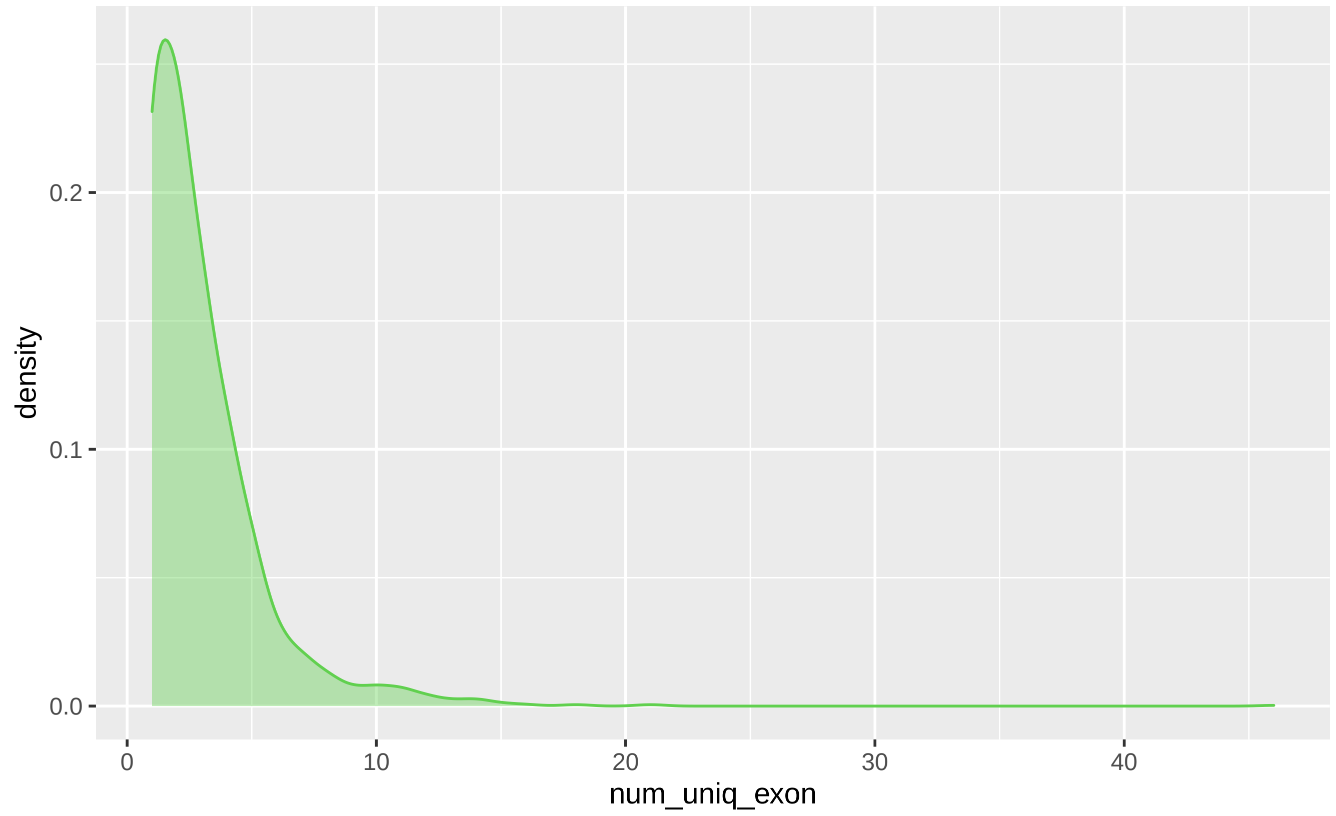

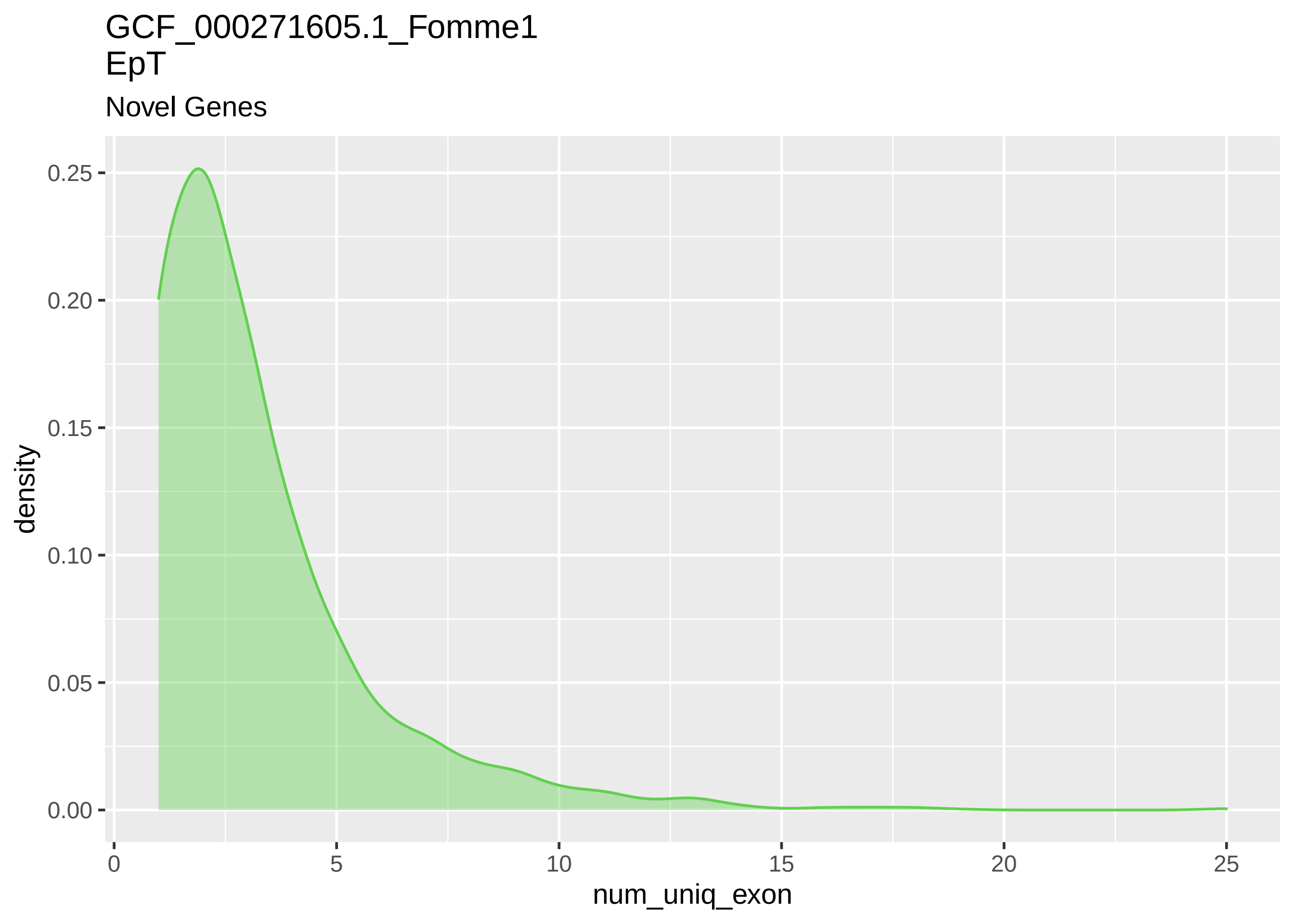
GCF_000271605.1_Fomme1
EpT
Novel Genes
density
0.25
0.20
0.15
0.10
0.05
0.00
0
5
10
15
20
25
num_uniq_exon

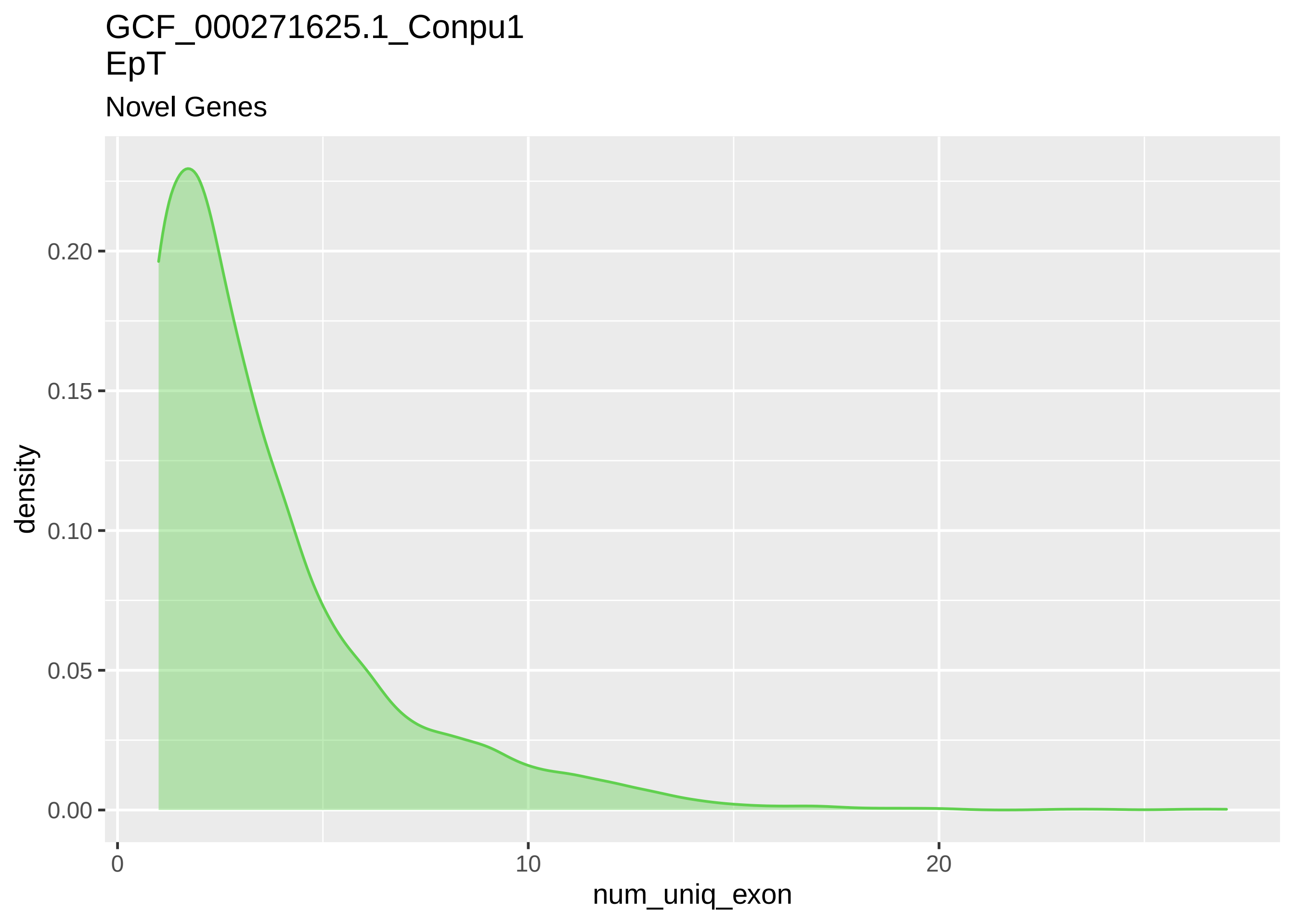
GCF_000271625.1_Conpu1
EpT
Novel Genes
density
0.20
0.15
0.10
0.05
0.00
0
10
20
num_uniq_exon

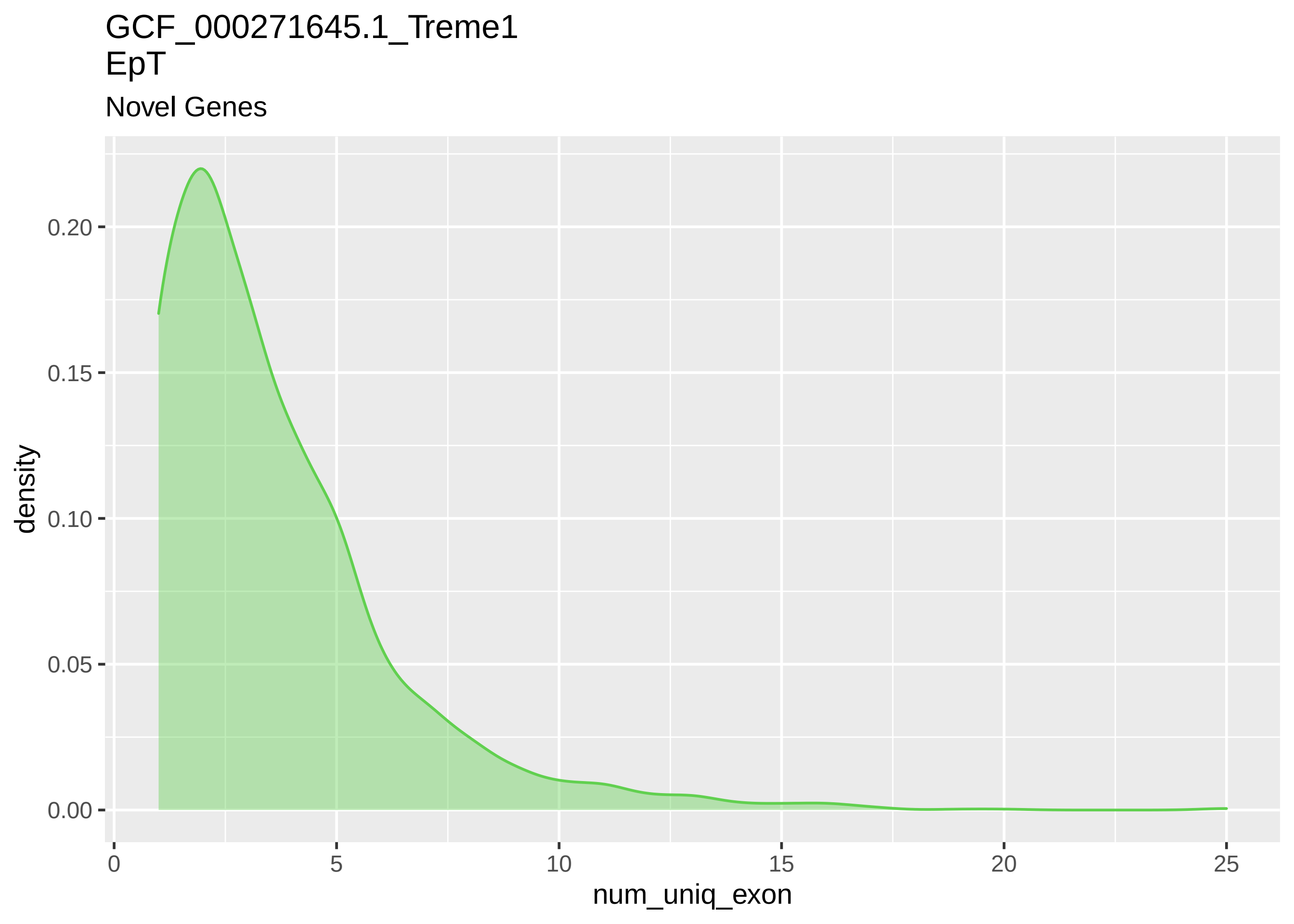
GCF_000271645.1_Treme1
EpT
Novel Genes
density
0.20
0.15
0.10
0.05
0.00
0
5
10
15
20
25
num_uniq_exon

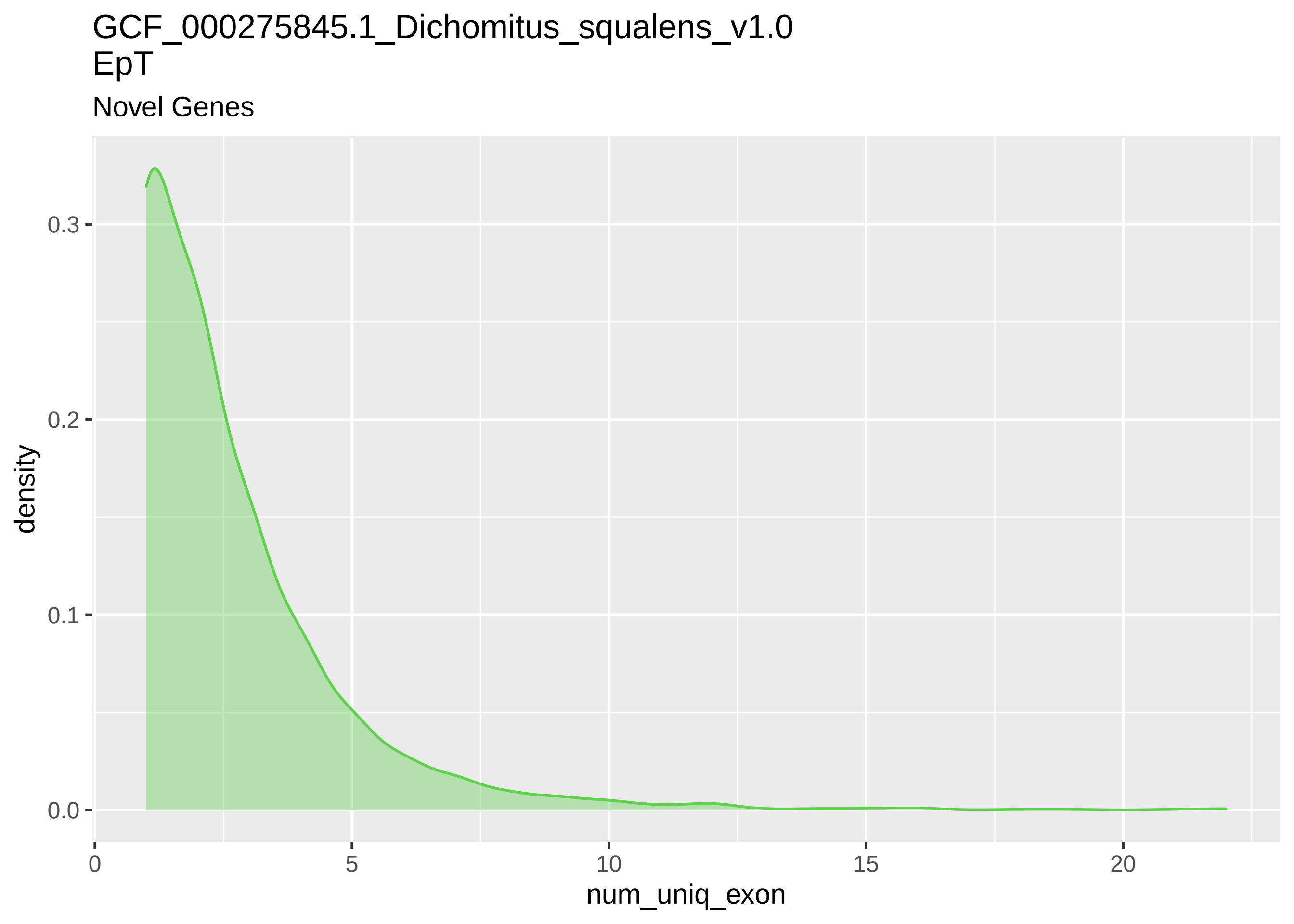
GCF_000275845.1_Dichomitus_squalens_v1.0
EpT
Novel Genes
density
0.0
0.1
0.2
0.3
0
5
10
15
20
num_uniq_exon

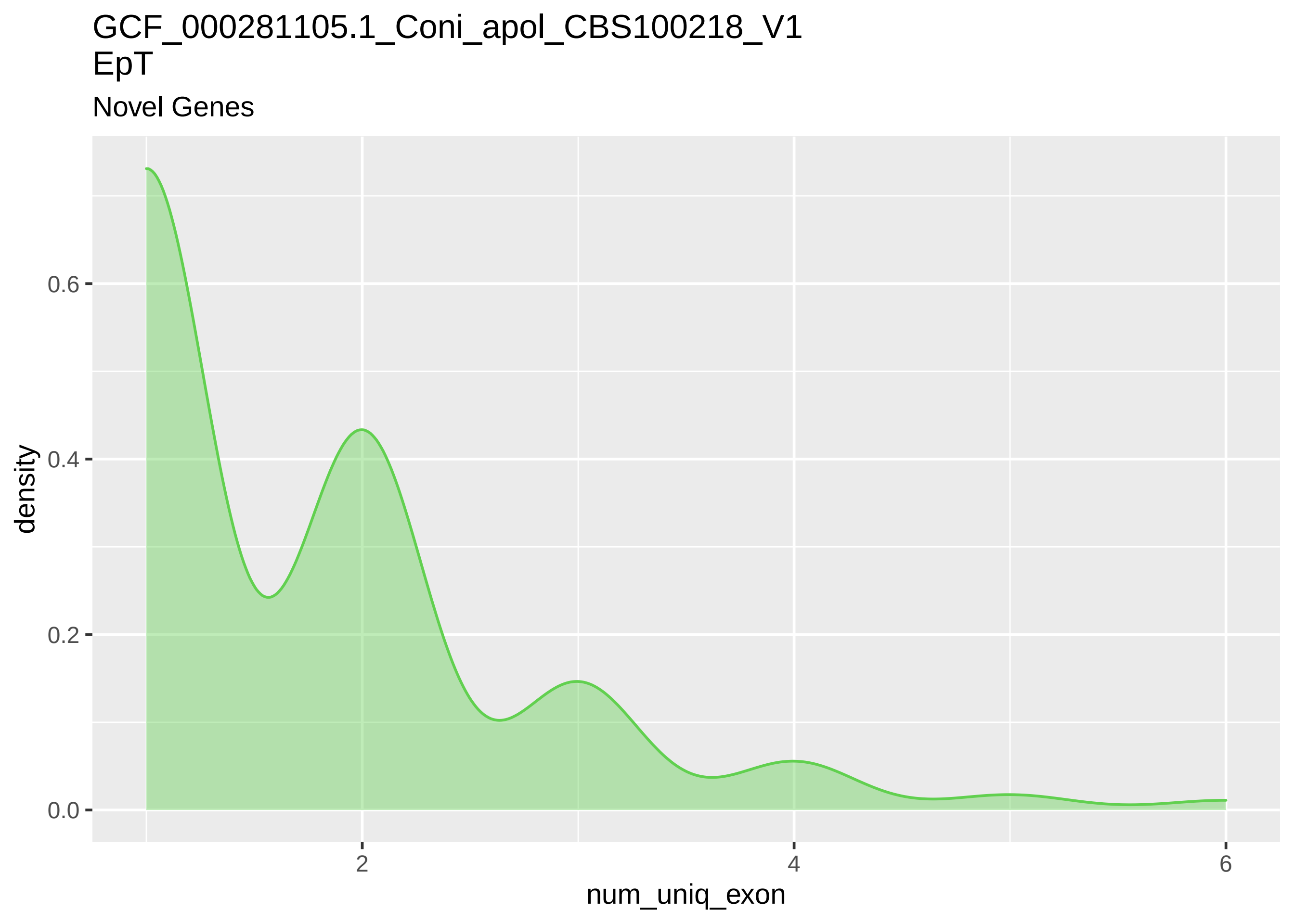
GCF_000281105.1_Coni_apol_CBS100218_V1
EpT
Novel Genes
density
0.6
0.4
0.2
0.0
2
4
6
num_uniq_exon

GCF_000300595.1_Phanerochaete_carnosa_HHB−10118−Sp_v1.0
EpT

Novel Genes

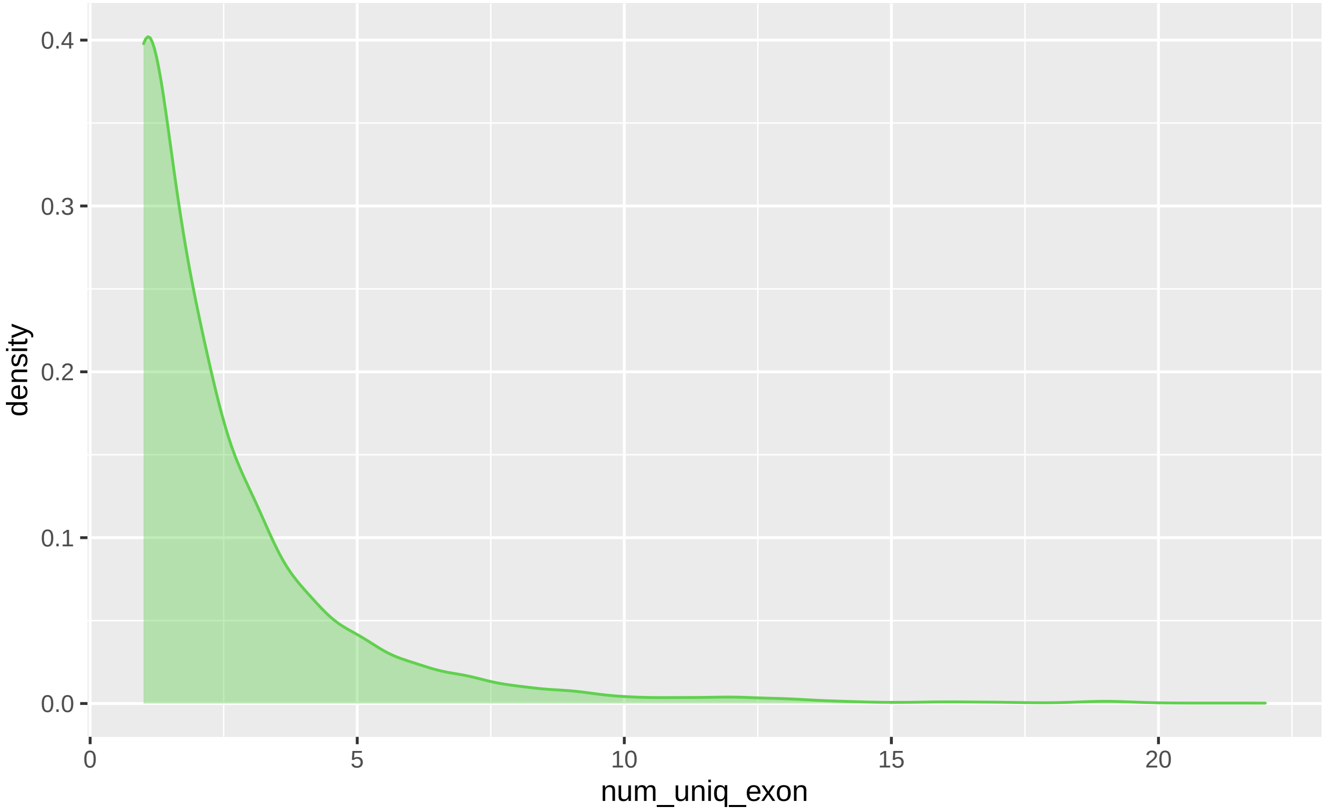

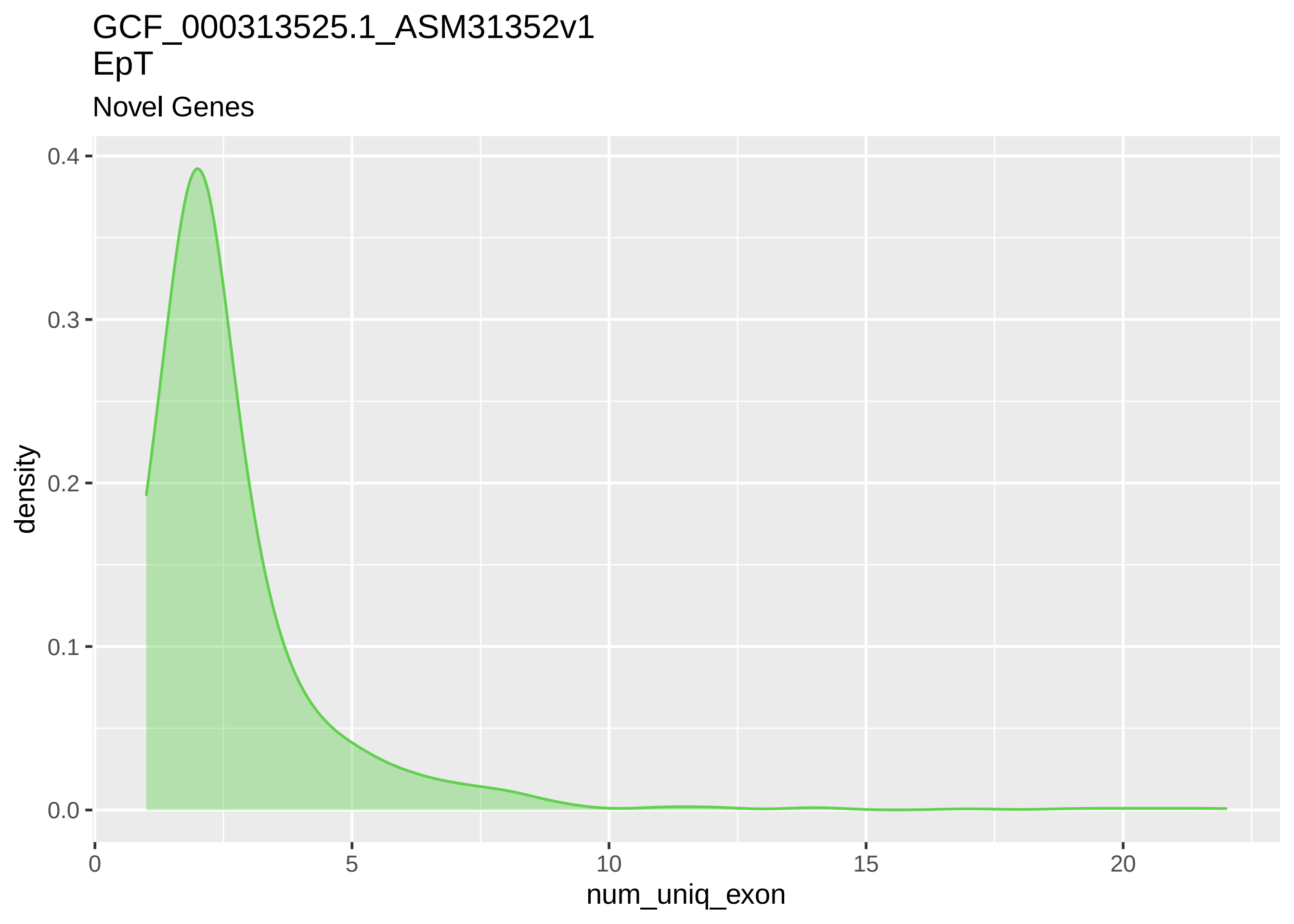
GCF_000313525.1_ASM31352v1
EpT
Novel Genes
density
0.0
0.1
0.2
0.3
0.4
0
5
10
15
20
num_uniq_exon

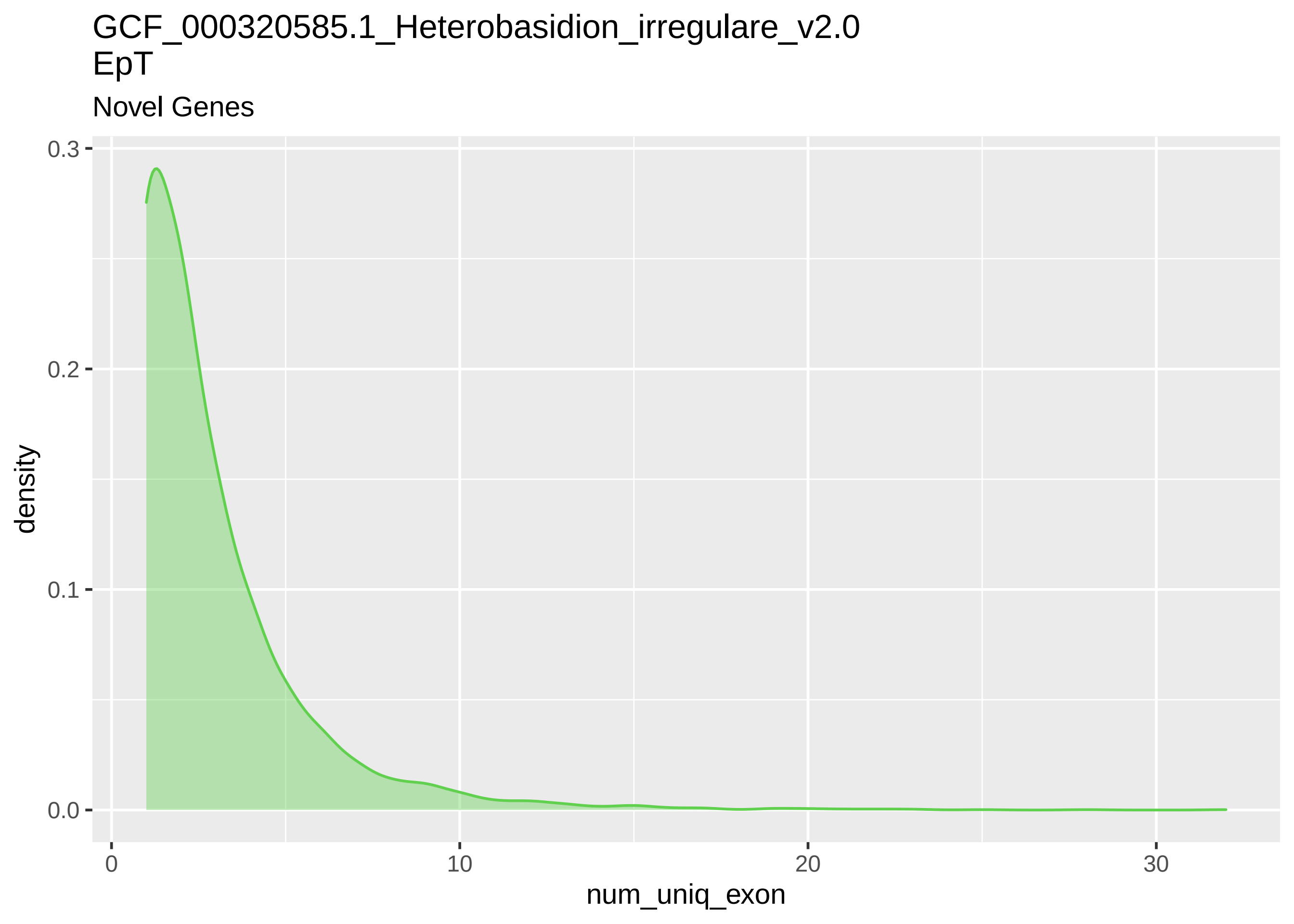
GCF_000320585.1_Heterobasidion_irregulare_v2.0
EpT
Novel Genes
density
0.3
0.2
0.1
0.0
0
10
20
30
num_uniq_exon

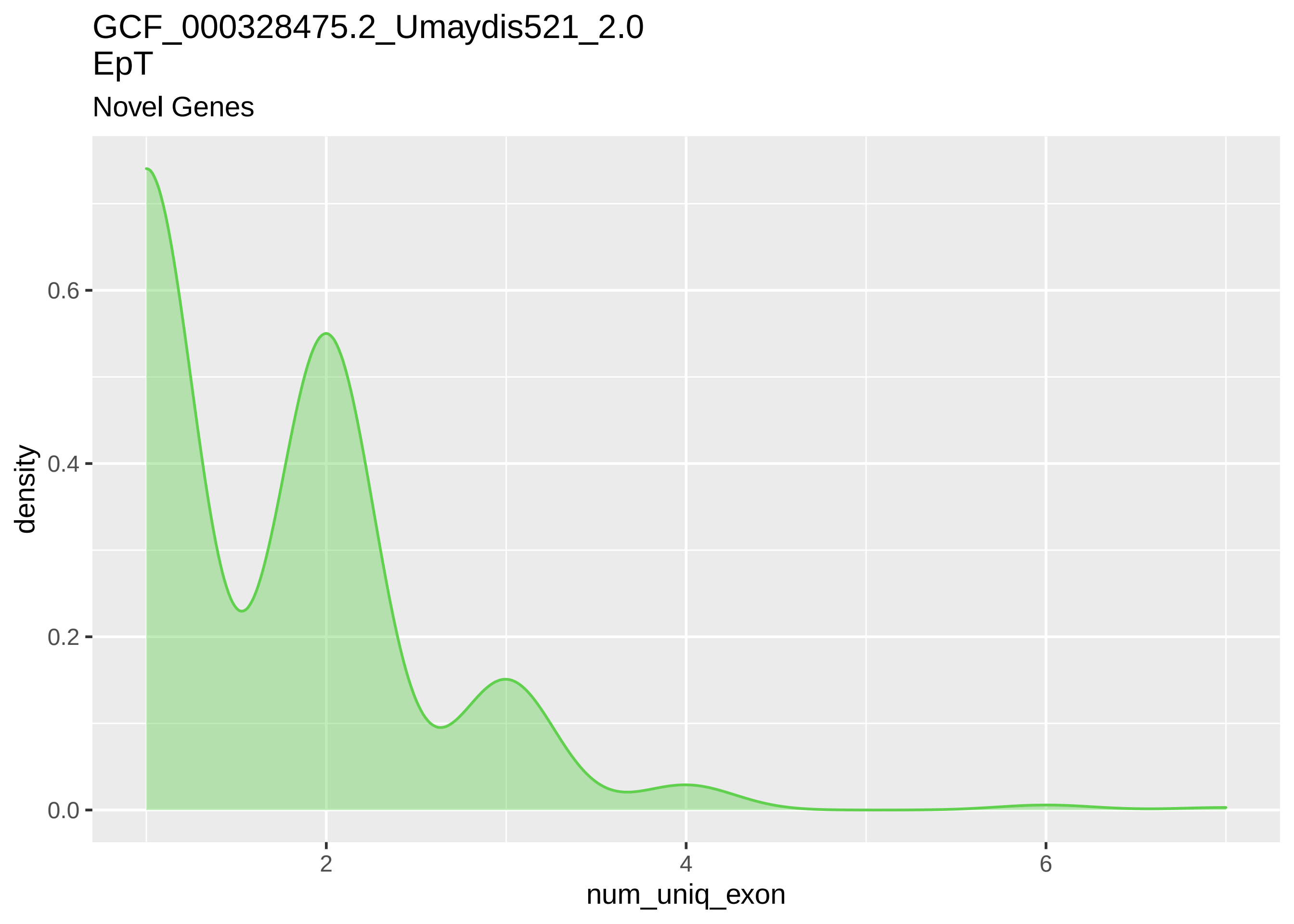
GCF_000328475.2_Umaydis521_2.0
EpT
Novel Genes
density
0.0
0.2
0.4
0.6
2
4
6
num_uniq_exon

GCF_000344685.1_Glotr1_1
EpT

Novel Genes

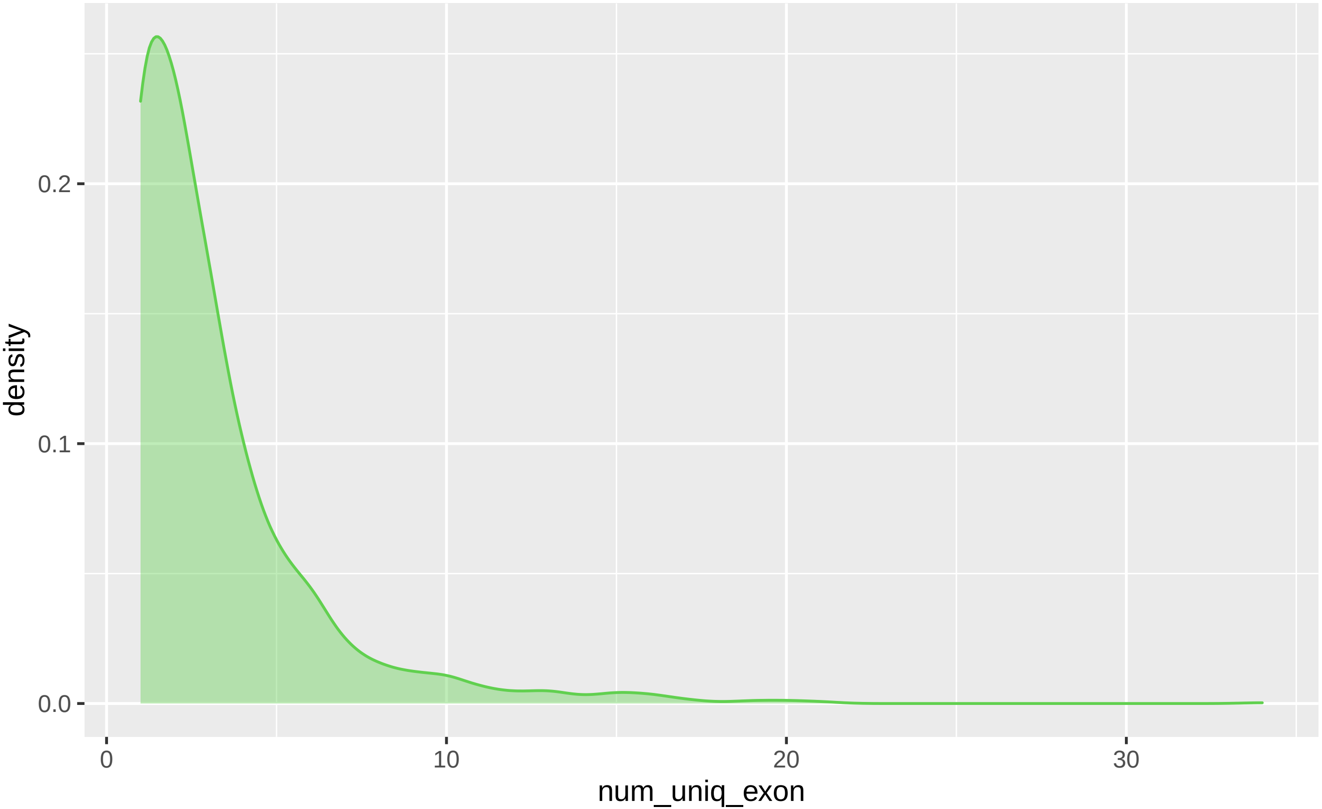

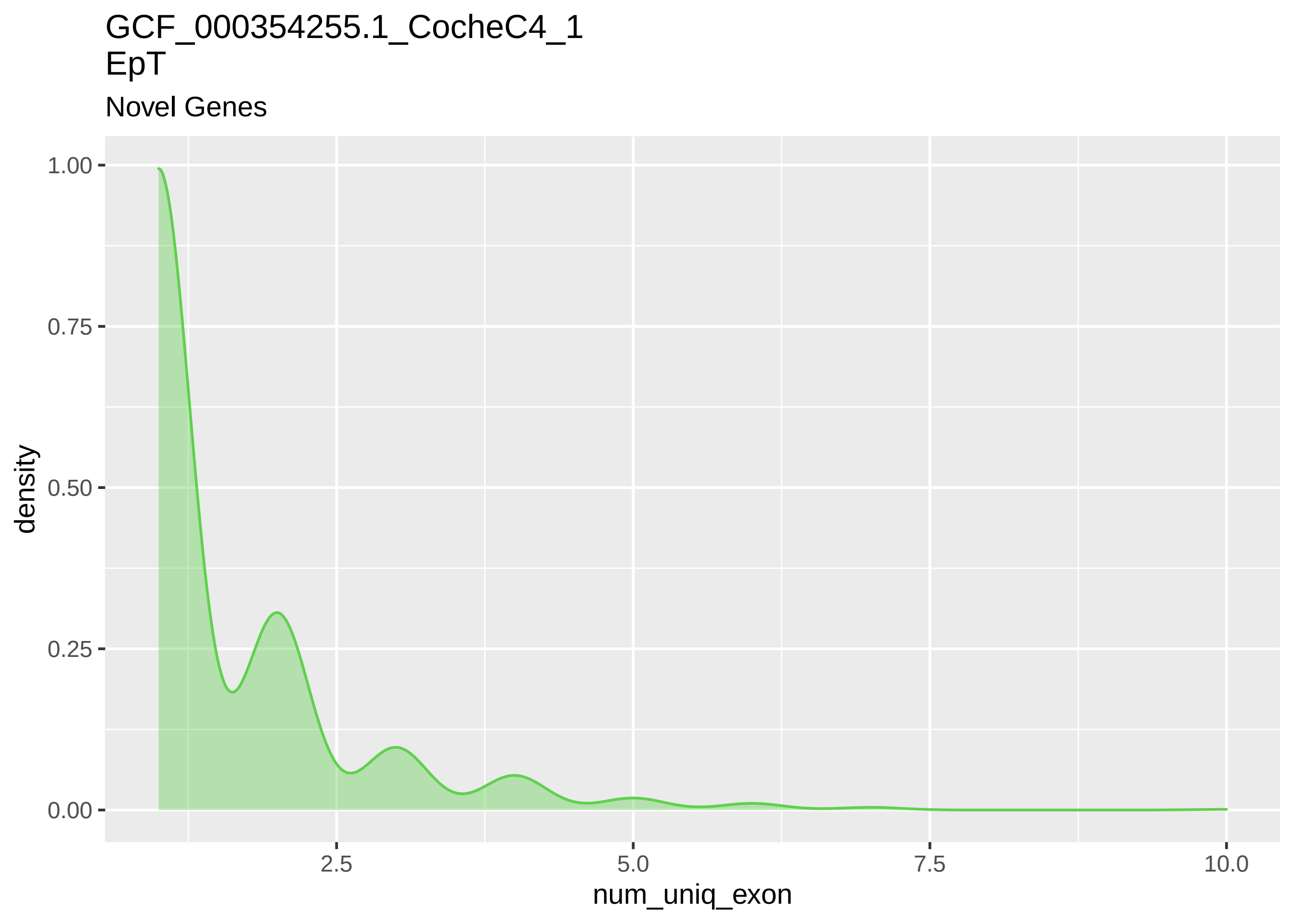
GCF_000354255.1_CocheC4_1
EpT
Novel Genes
density
1.00
0.75
0.50
0.25
0.00
2.5
5.0
7.5
10.0
num_uniq_exon

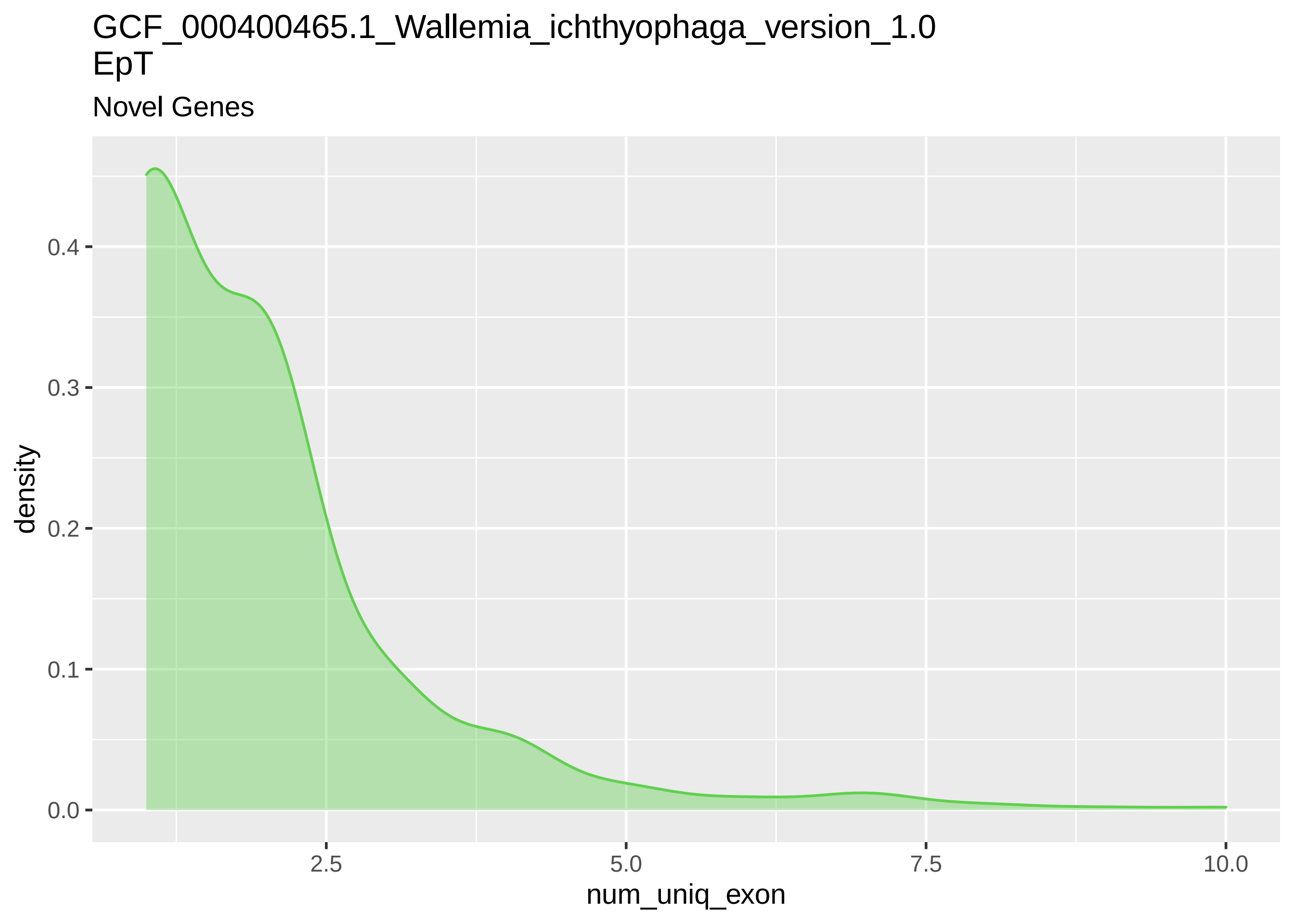
GCF_000400465.1_Wallemia_ichthyophaga_version_1.0
EpT
Novel Genes
density
0.0
0.1
0.2
0.3
0.4
2.5
5.0
7.5
10.0
num_uniq_exon

GCF_000409485.1_GLAREA
EpT

Novel Genes

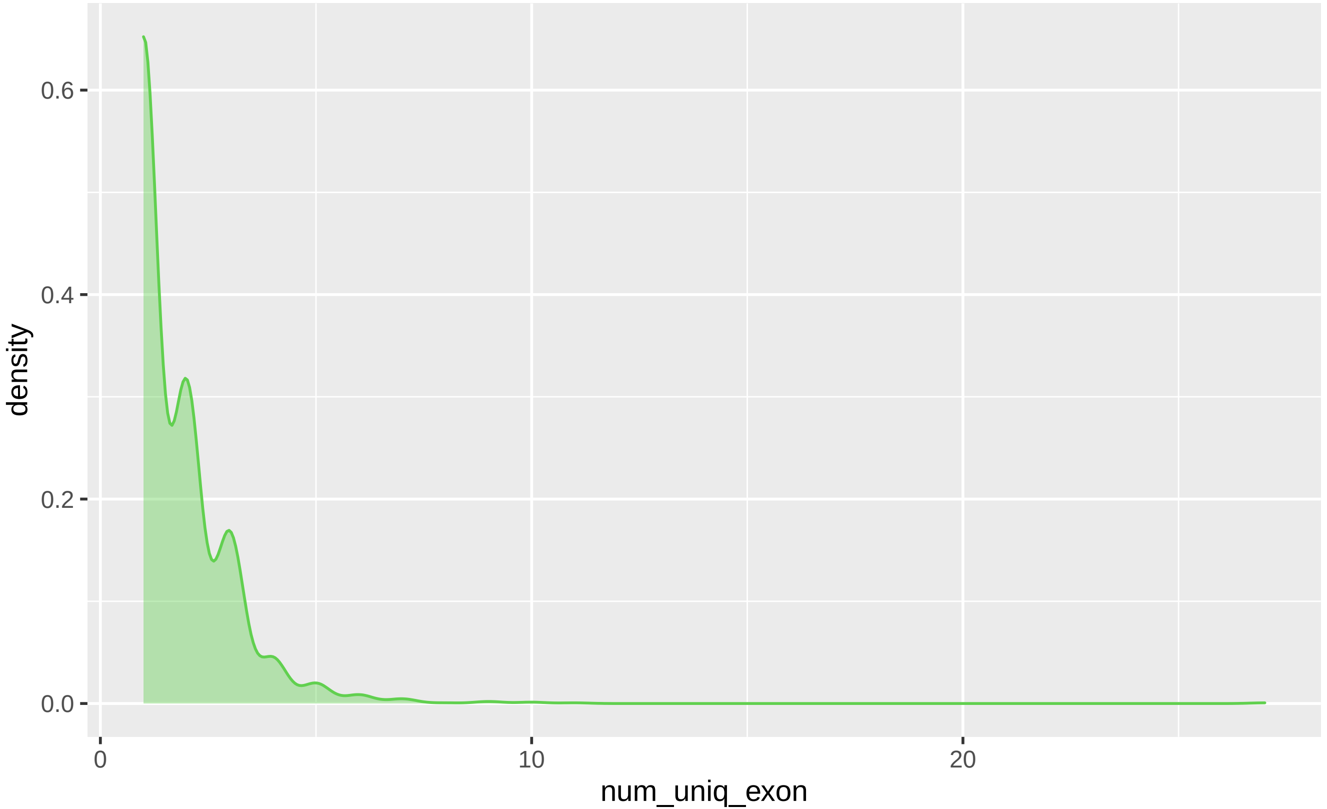

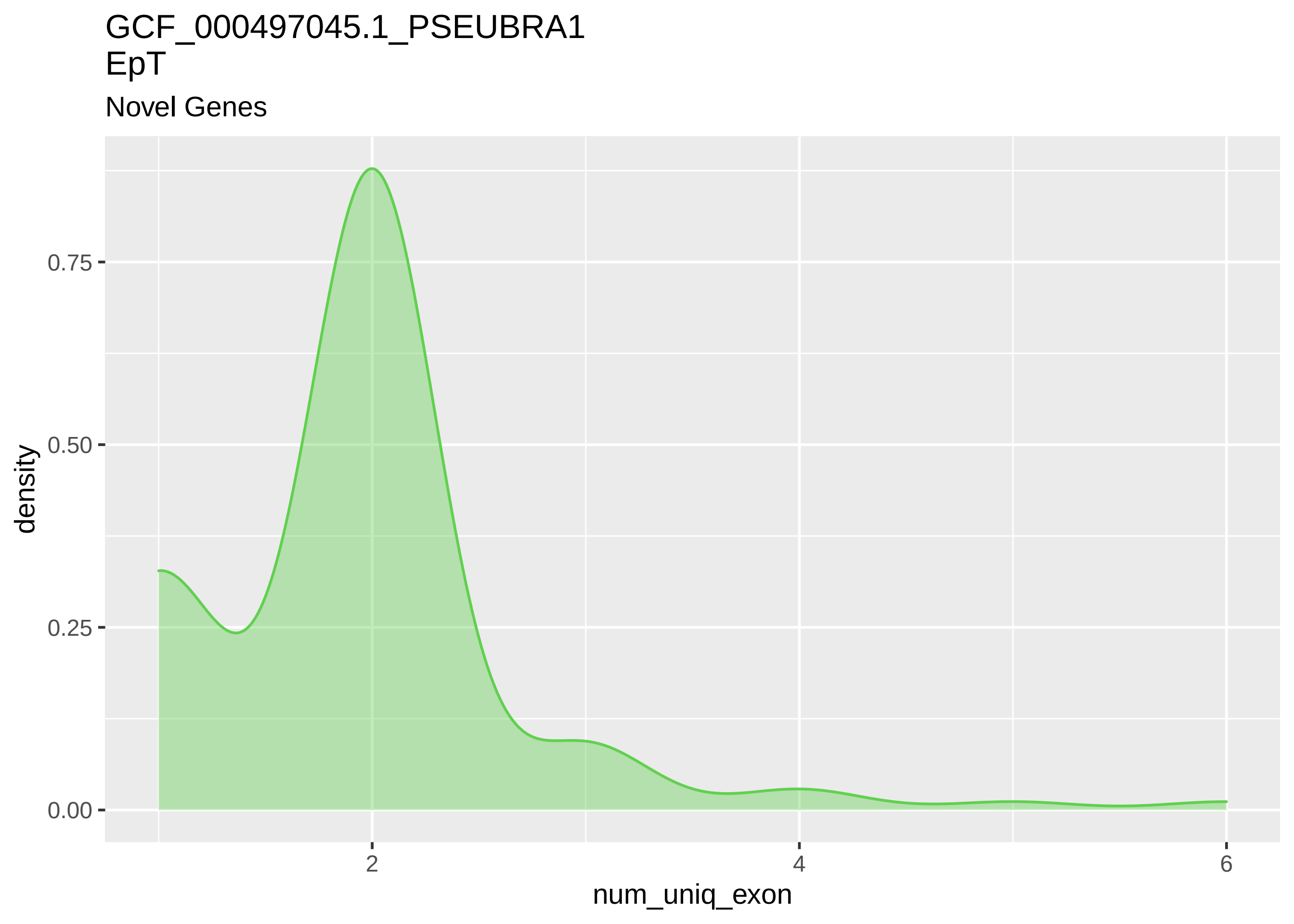

GCF_000497045.1_PSEUBRA1
EpT
Novel Genes
density
0.75
0.50
0.25
0.00
2
4
6
num_uniq_exon

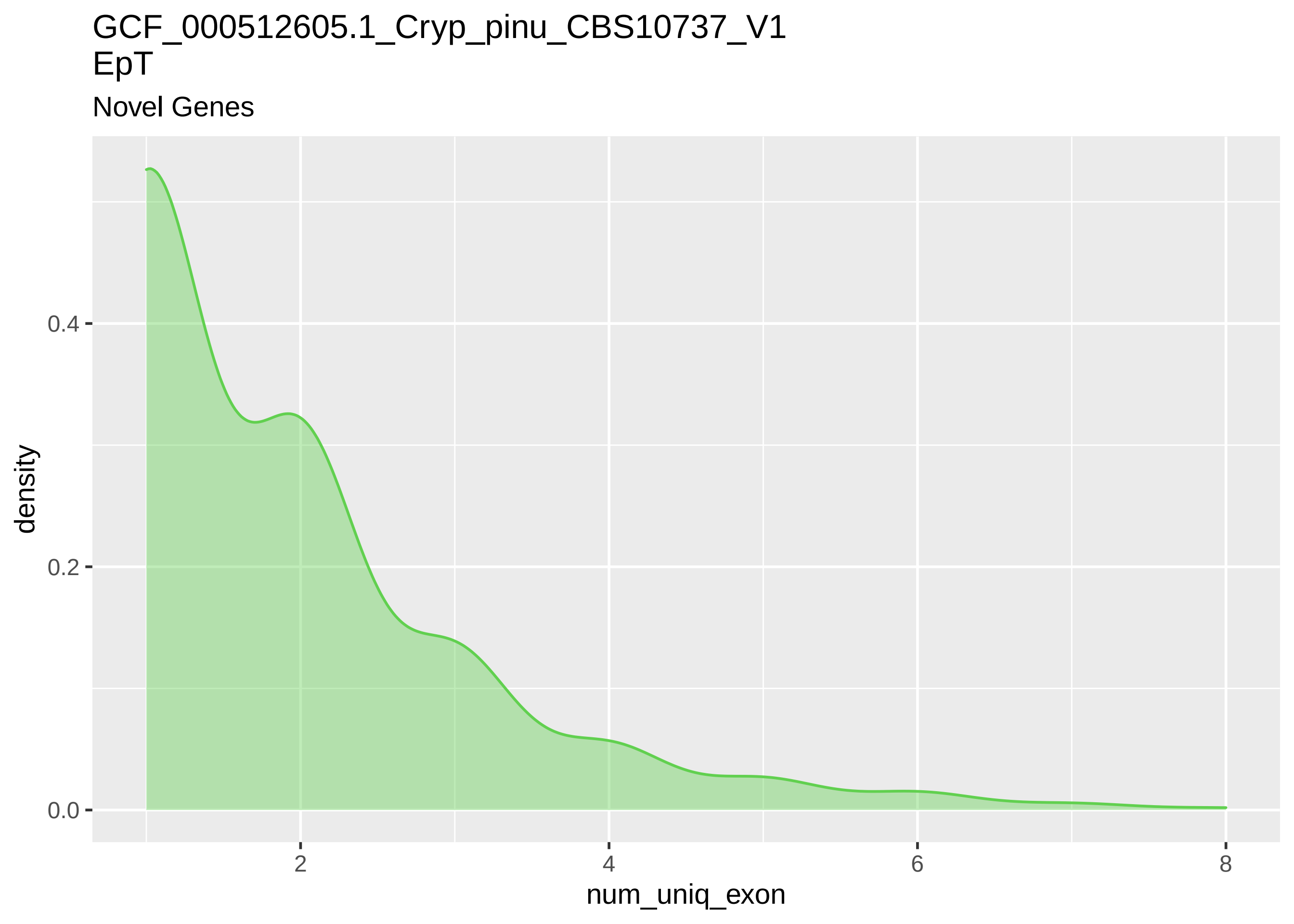
GCF_000512605.1_Cryp_pinu_CBS10737_V1
EpT
Novel Genes
density
0.0
0.2
0.4
2
4
6
8
num_uniq_exon

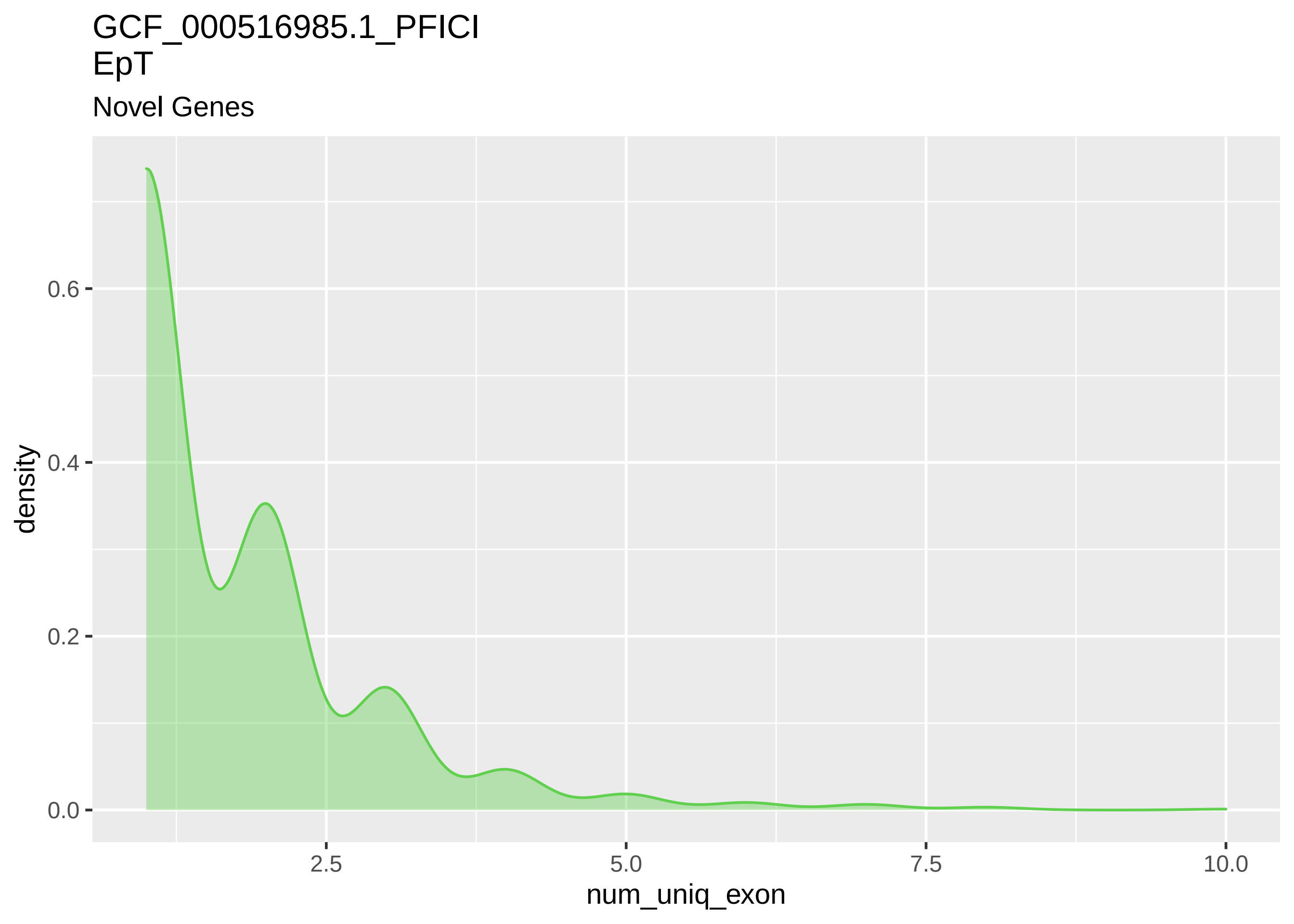

GCF_000516985.1_PFICI
EpT
Novel Genes
density
0.0
0.2
0.4
0.6
2.5
5.0
7.5
10.0
num_uniq_exon

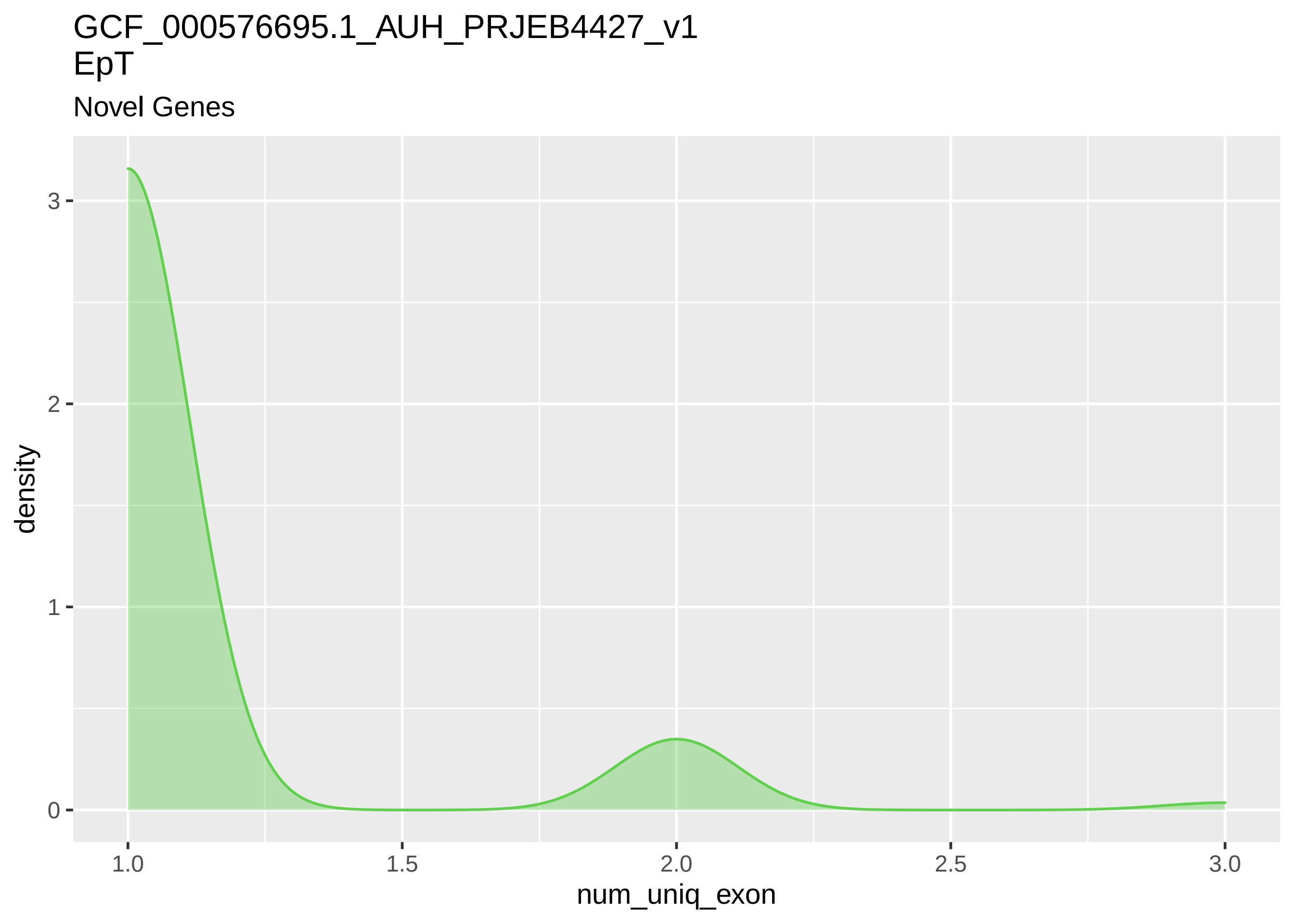
GCF_000576695.1_AUH_PRJEB4427_v1
EpT
Novel Genes
density
num_uniq_exon
0
1
2
3
1.0
1.5
2.0
2.5
3.0

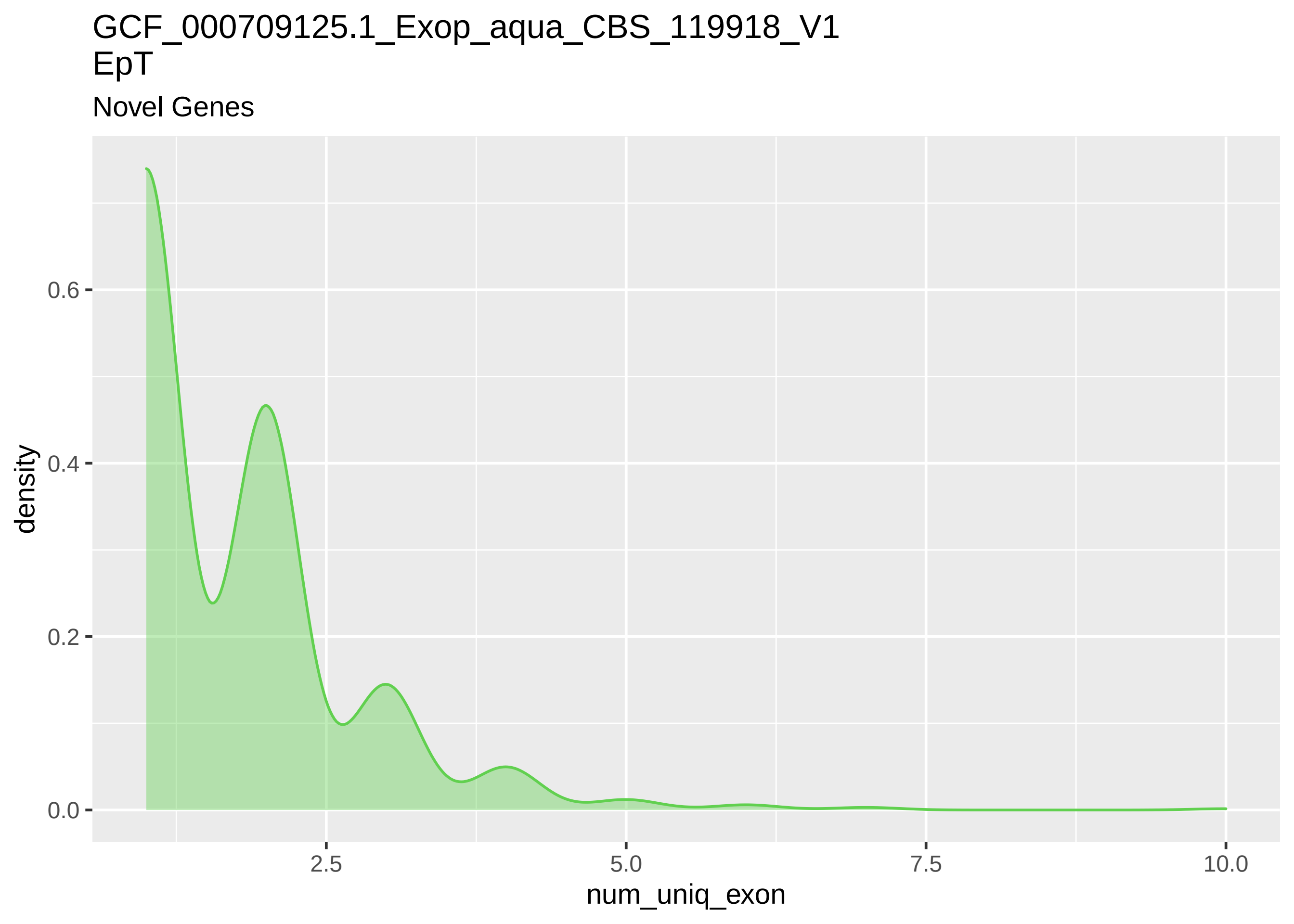
GCF_000709125.1_Exop_aqua_CBS_119918_V1
EpT
Novel Genes
density
0.0
0.2
0.4
0.6
2.5
5.0
7.5
10.0
num_uniq_exon

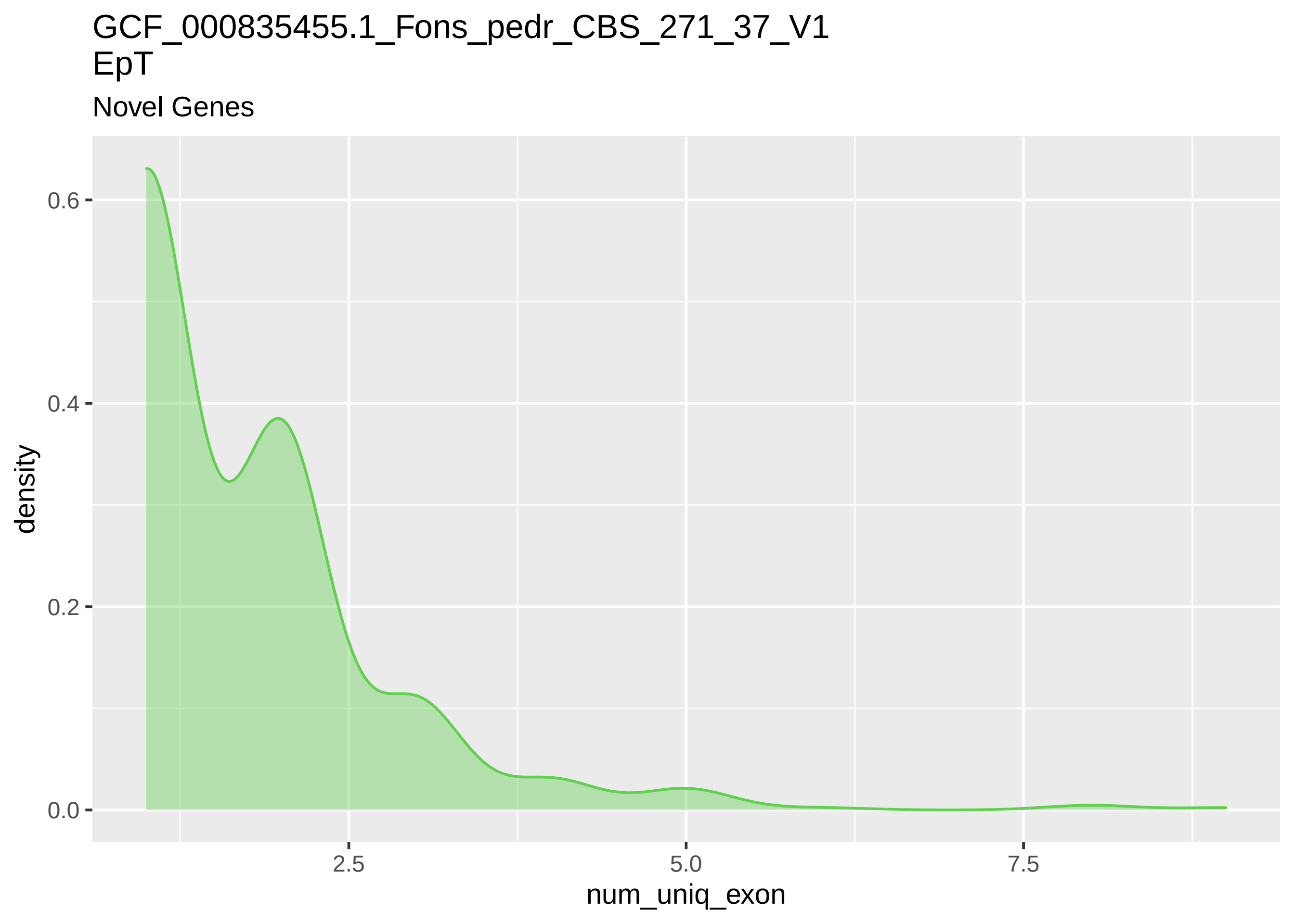

GCF_000835455.1_Fons_pedr_CBS_271_37_V1
EpT
Novel Genes
density
0.6
0.4
0.2
0.0
2.5
5.0
7.5
num_uniq_exon

GCF_000835555.1_Rhin_mack_CBS_650_93_V1
EpT

Novel Genes

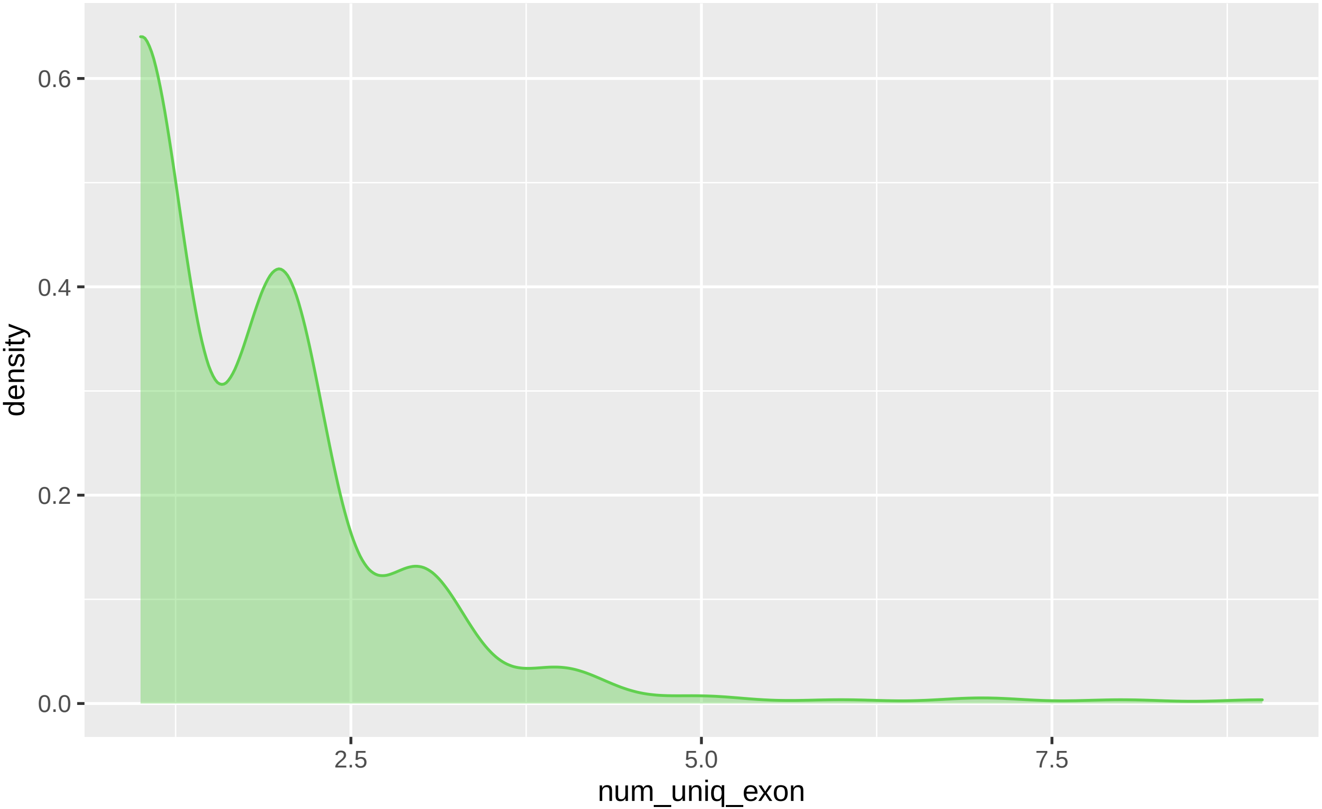

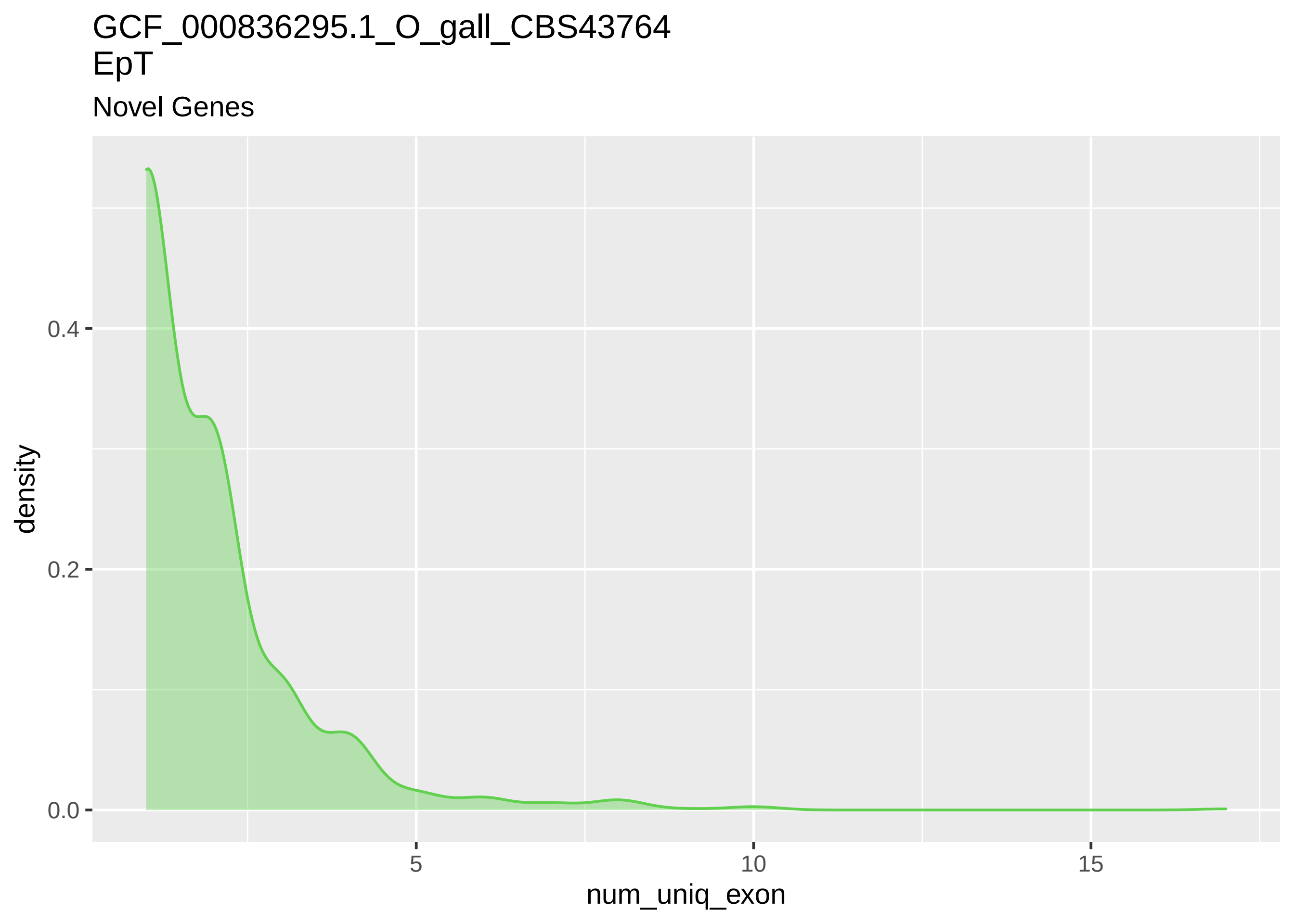

GCF_000836295.1_O_gall_CBS43764
EpT
Novel Genes
density
0.4
0.2
0.0
5
10
15
num_uniq_exon

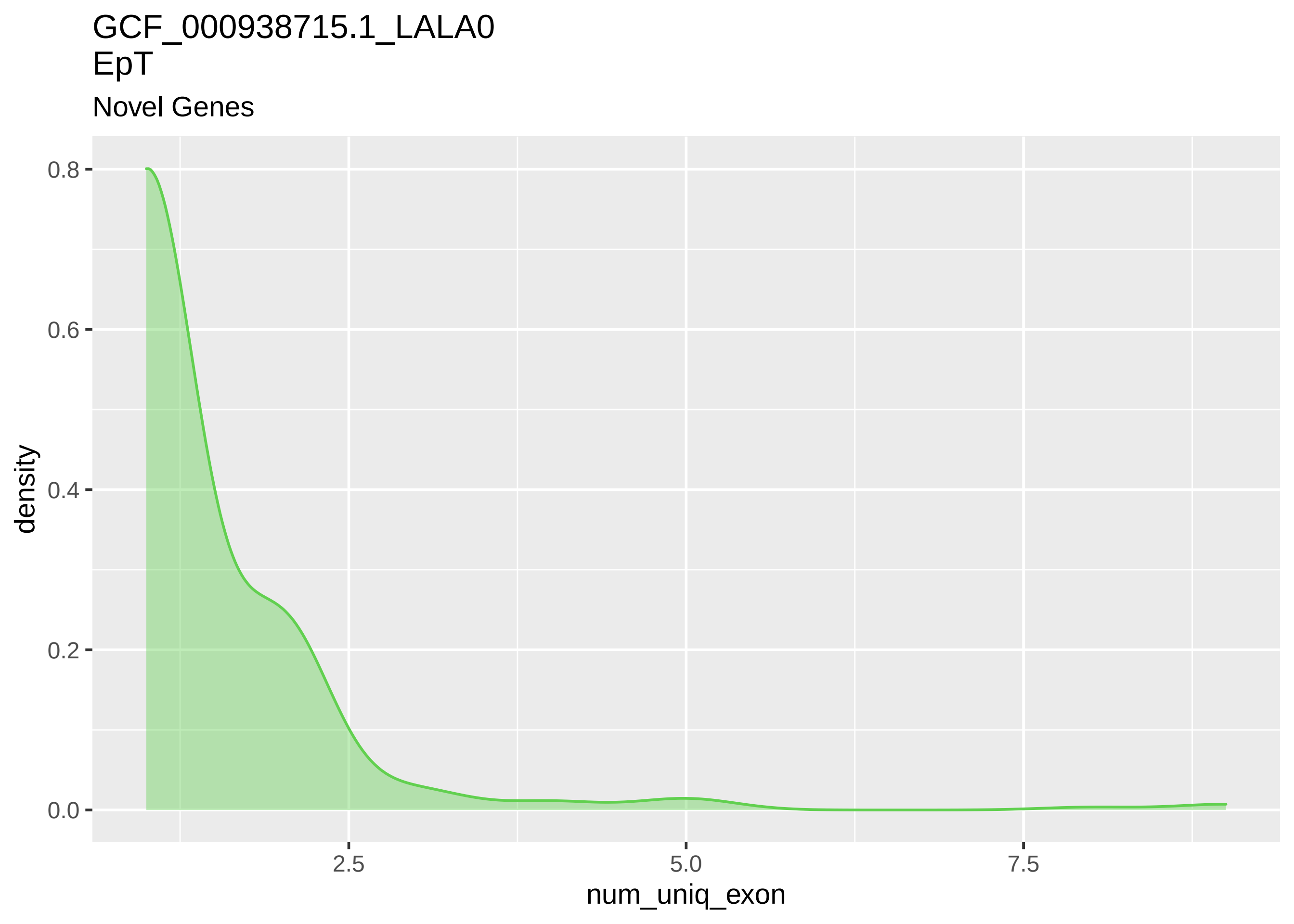
GCF_000938715.1_LALA0
EpT
Novel Genes
density
0.0
0.2
0.4
0.6
0.8
2.5
5.0
7.5
num_uniq_exon

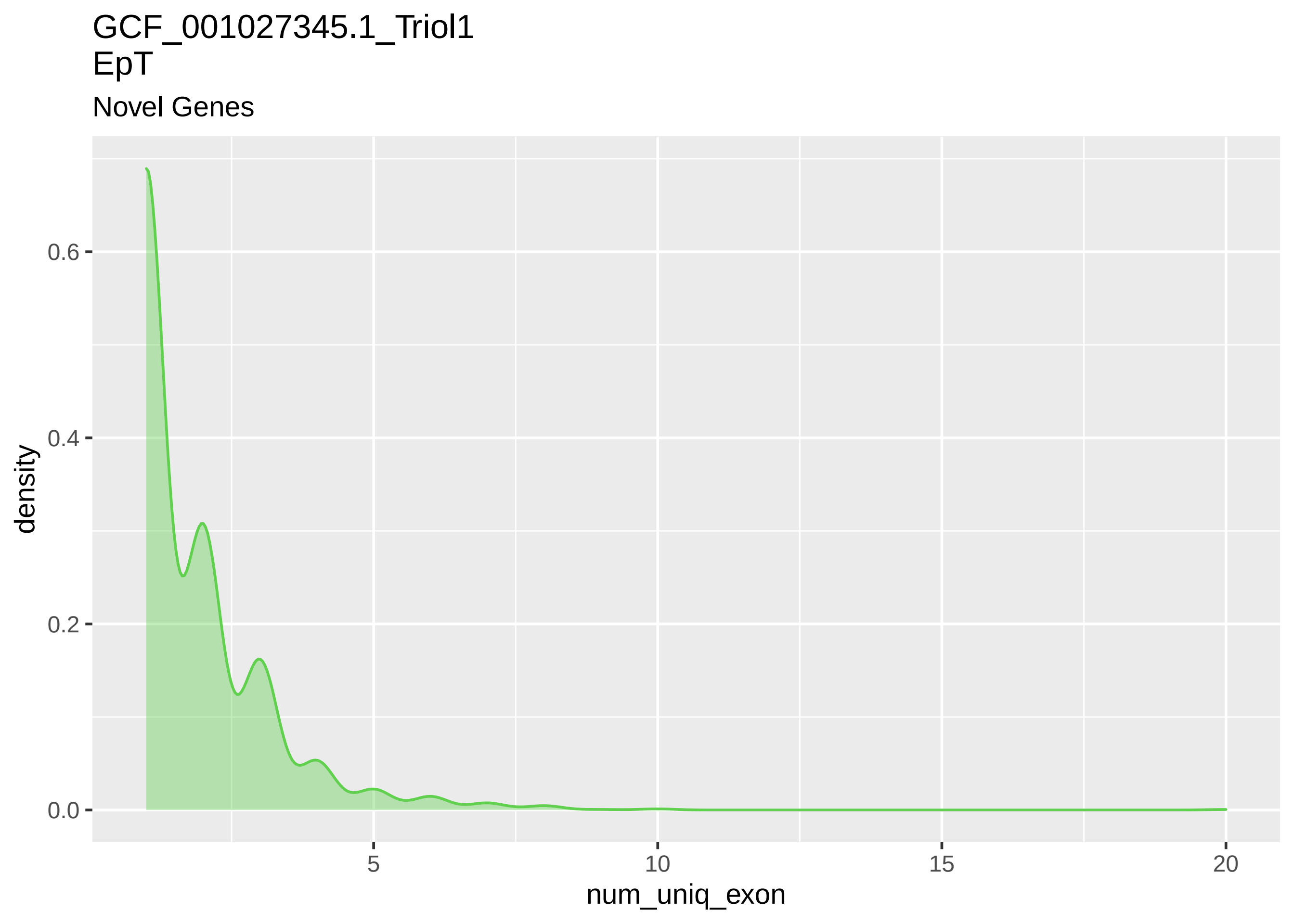
GCF_001027345.1_Triol1
EpT
Novel Genes
density
0.6
0.4
0.2
0.0
5
10
15
20
num_uniq_exon

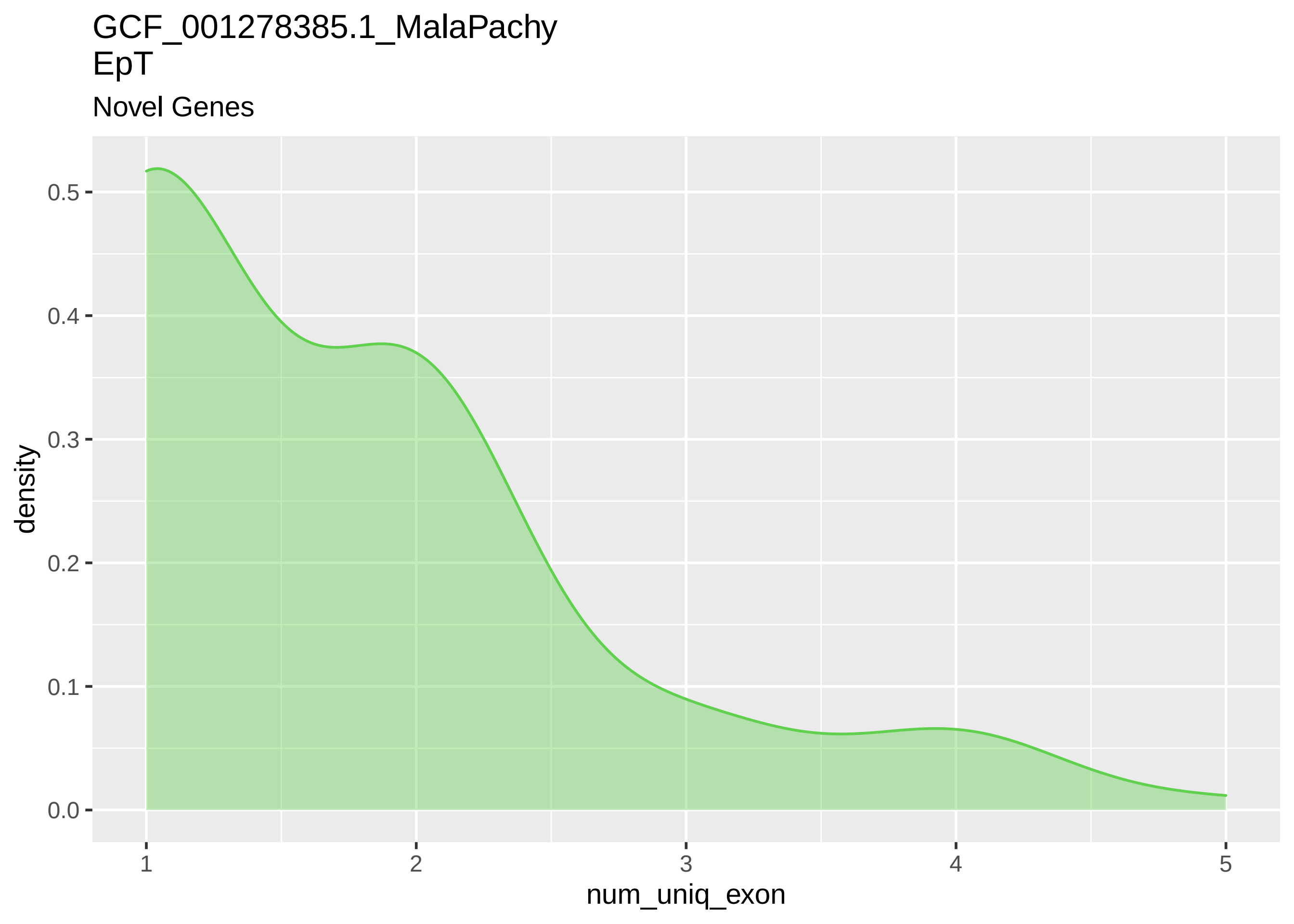

GCF_001278385.1_MalaPachy
EpT
Novel Genes
density
0.5
0.4
0.3
0.2
0.1
0.0
1
2
3
4
5
num_uniq_exon

GCF_001329695.1_Rhoba1_1
EpT

Novel Genes

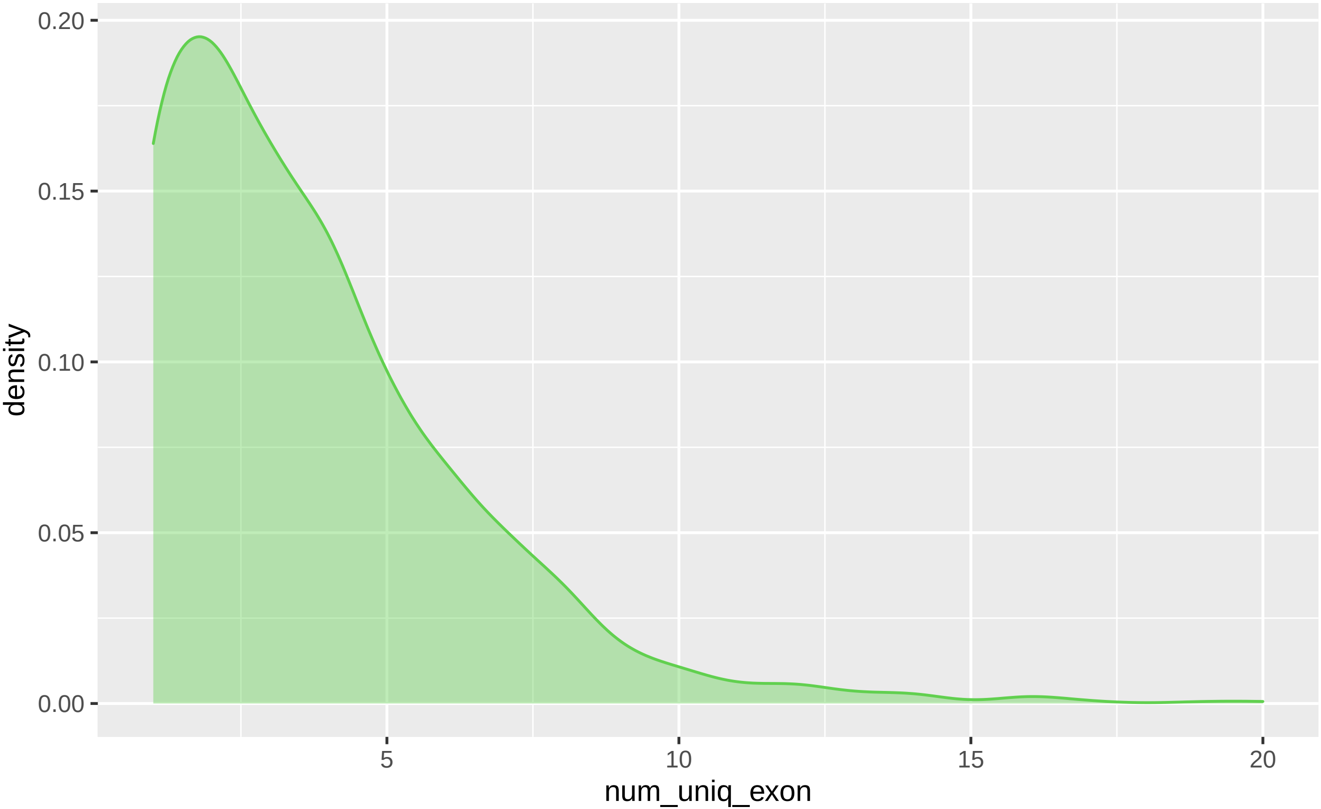

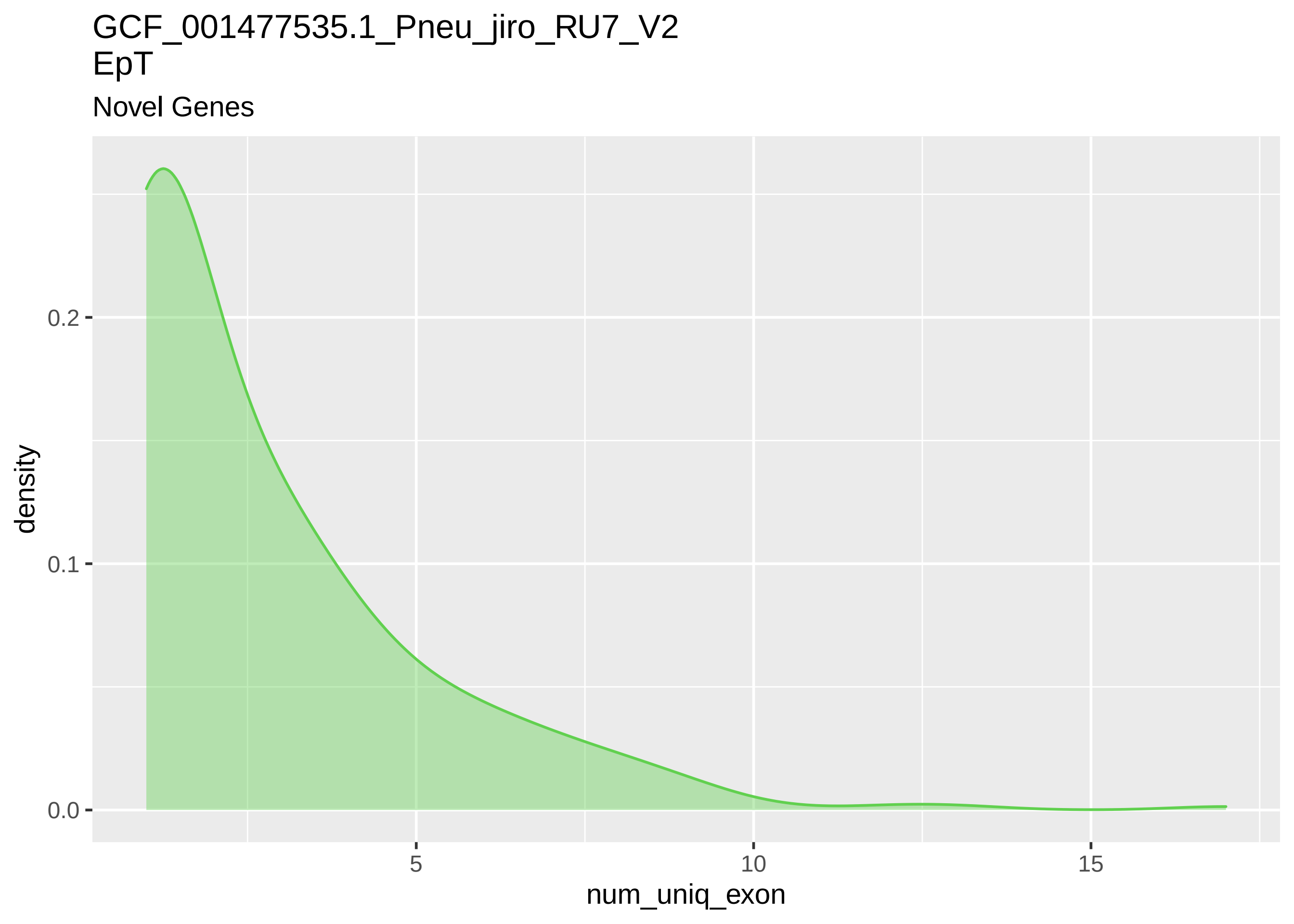
GCF_001477535.1_Pneu_jiro_RU7_V2
EpT
Novel Genes
density
0.0
0.1
0.2
5
10
15
num_uniq_exon

GCF_001619985.1_Xylona_heveae_TC161_v1.0
EpT

Novel Genes

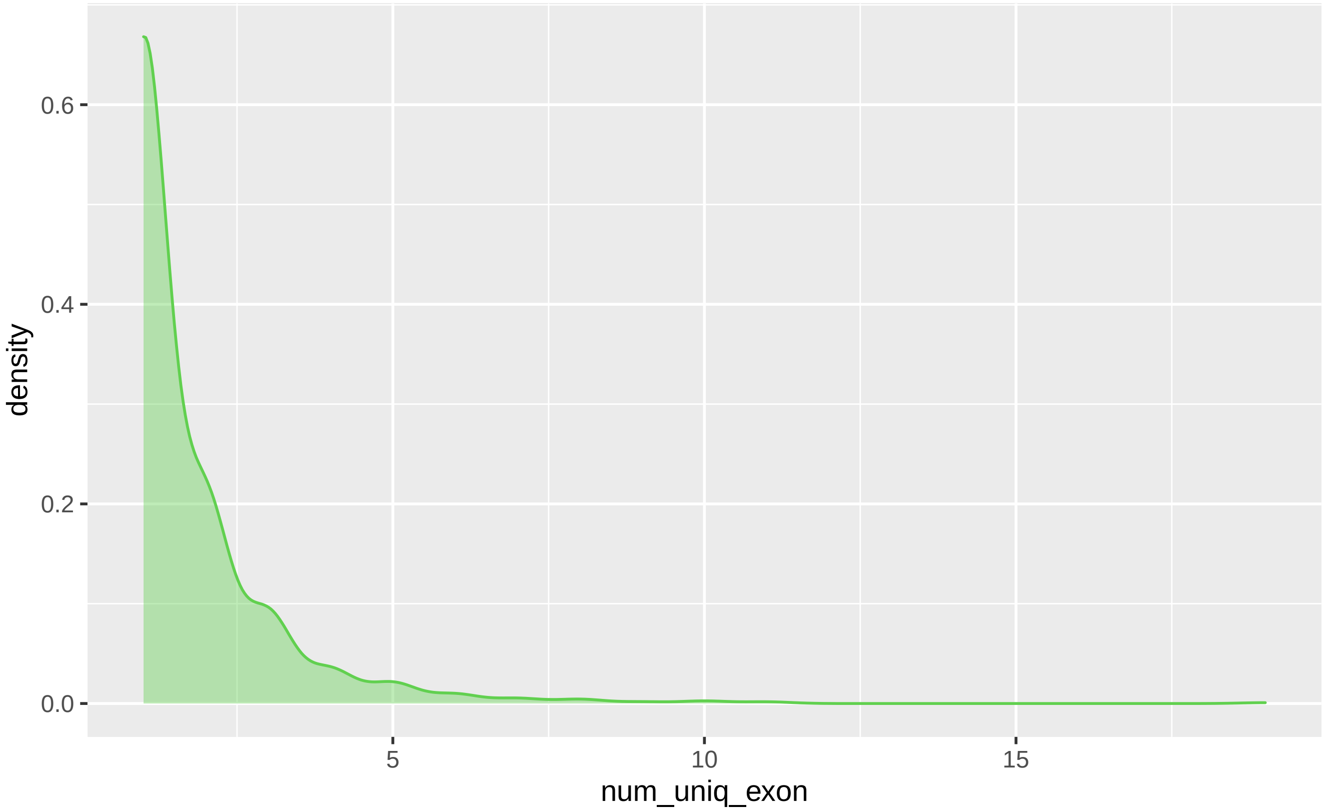

GCF_001636725.1_ISF_1.0
EpT

Novel Genes

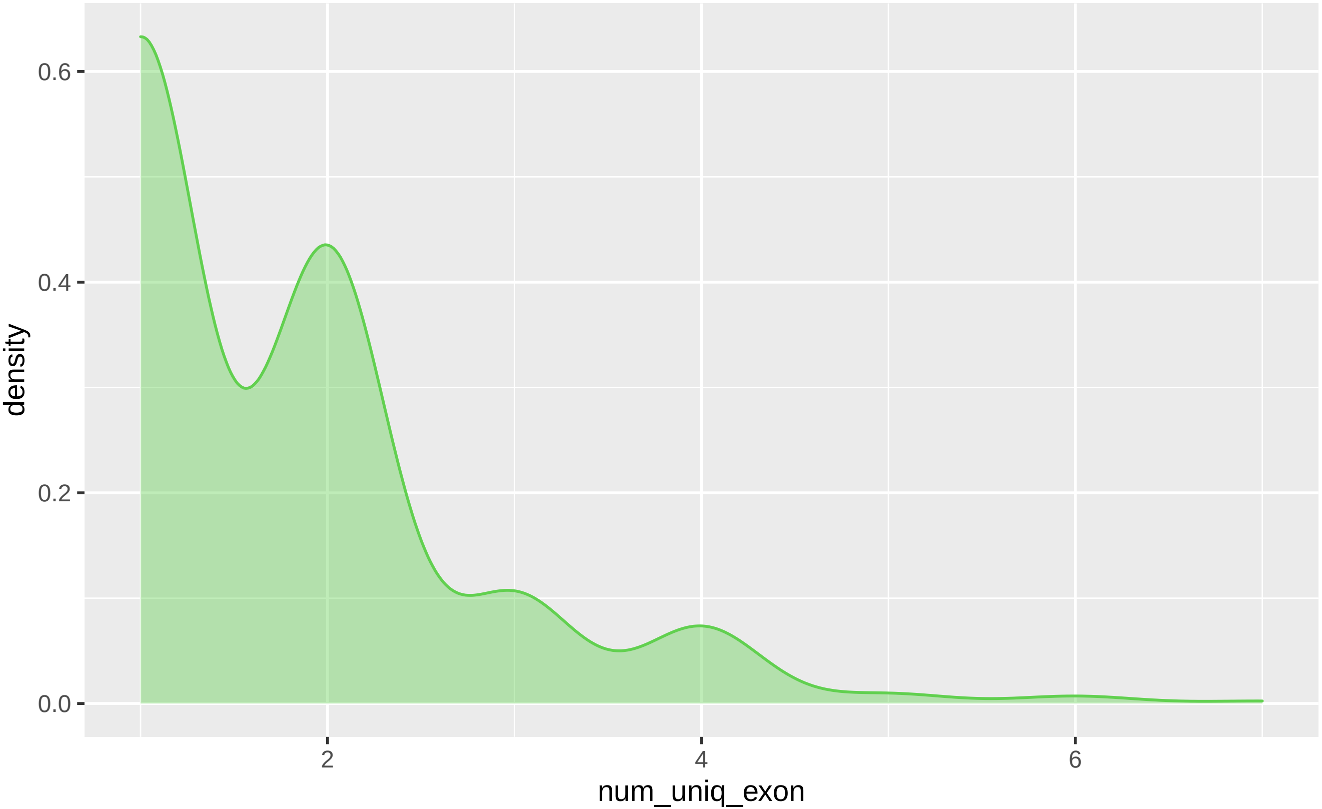

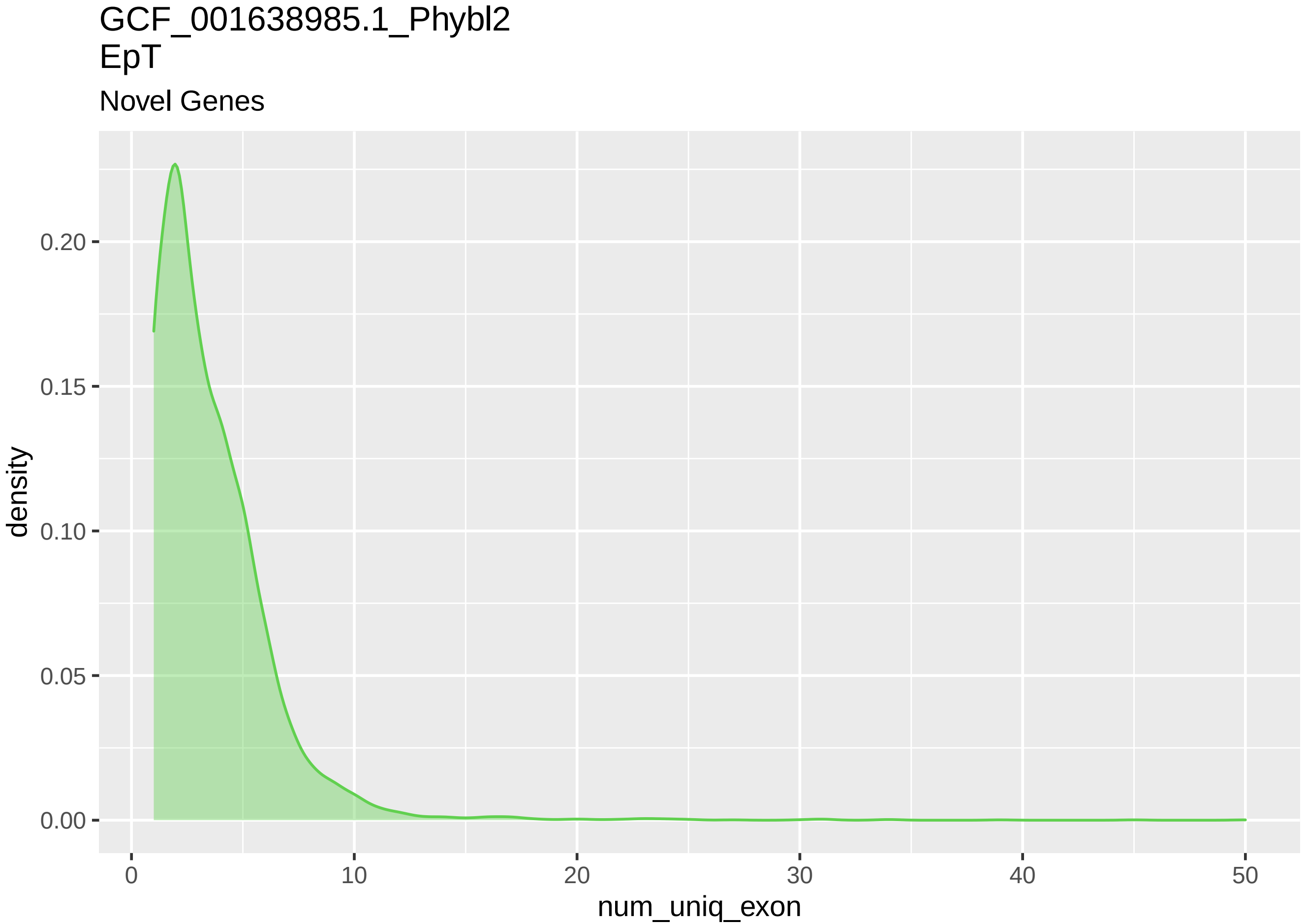
GCF_001638985.1_Phybl2
EpT
Novel Genes
density
0.00
0.05
0.10
0.15
0.20
0
10
20
30
40
50
num_uniq_exon

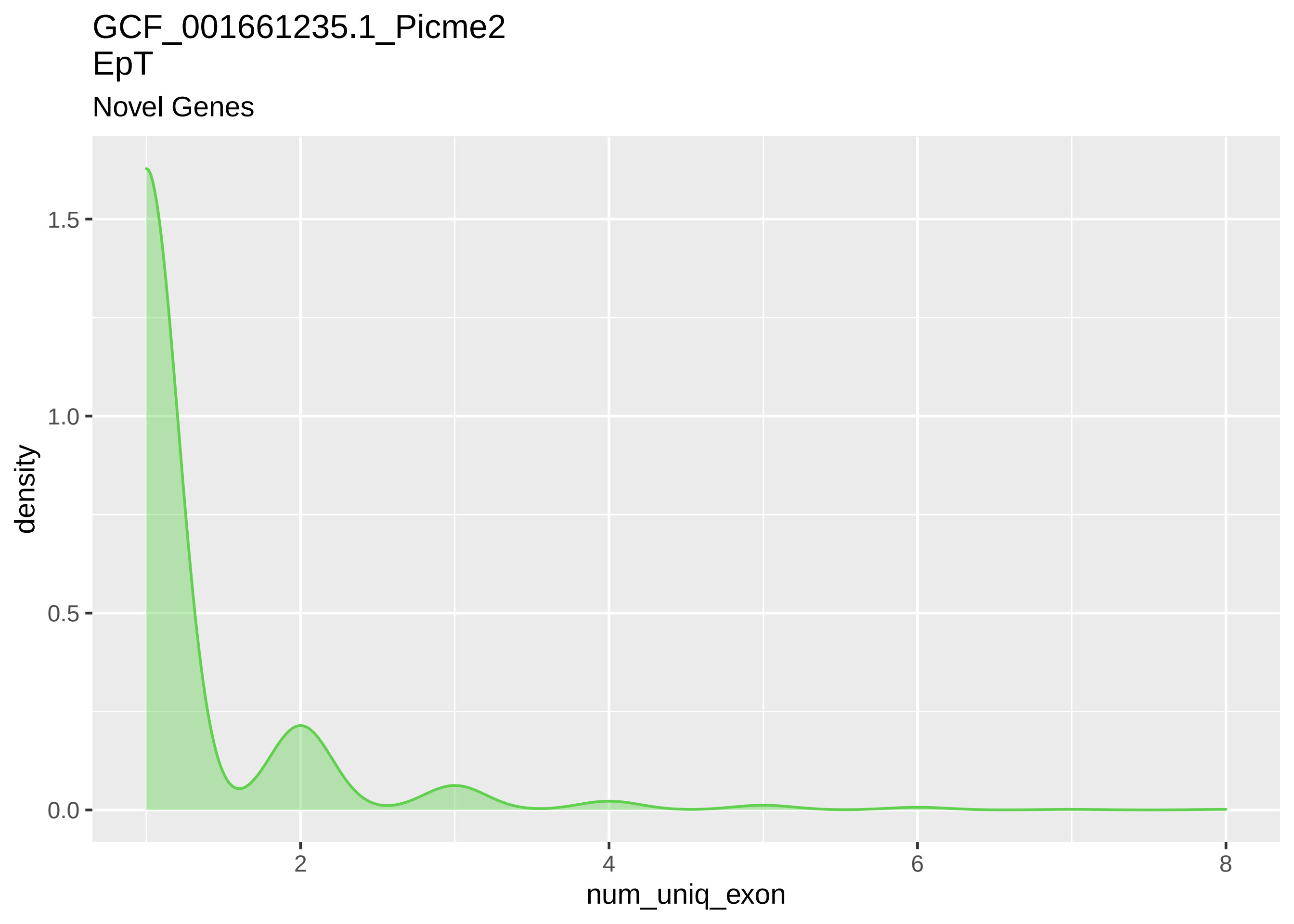

GCF_001661235.1_Picme2
EpT
Novel Genes
density
1.5
1.0
0.5
0.0
2
4
6
8
num_uniq_exon

GCF_001661335.1_Babin1
EpT

Novel Genes

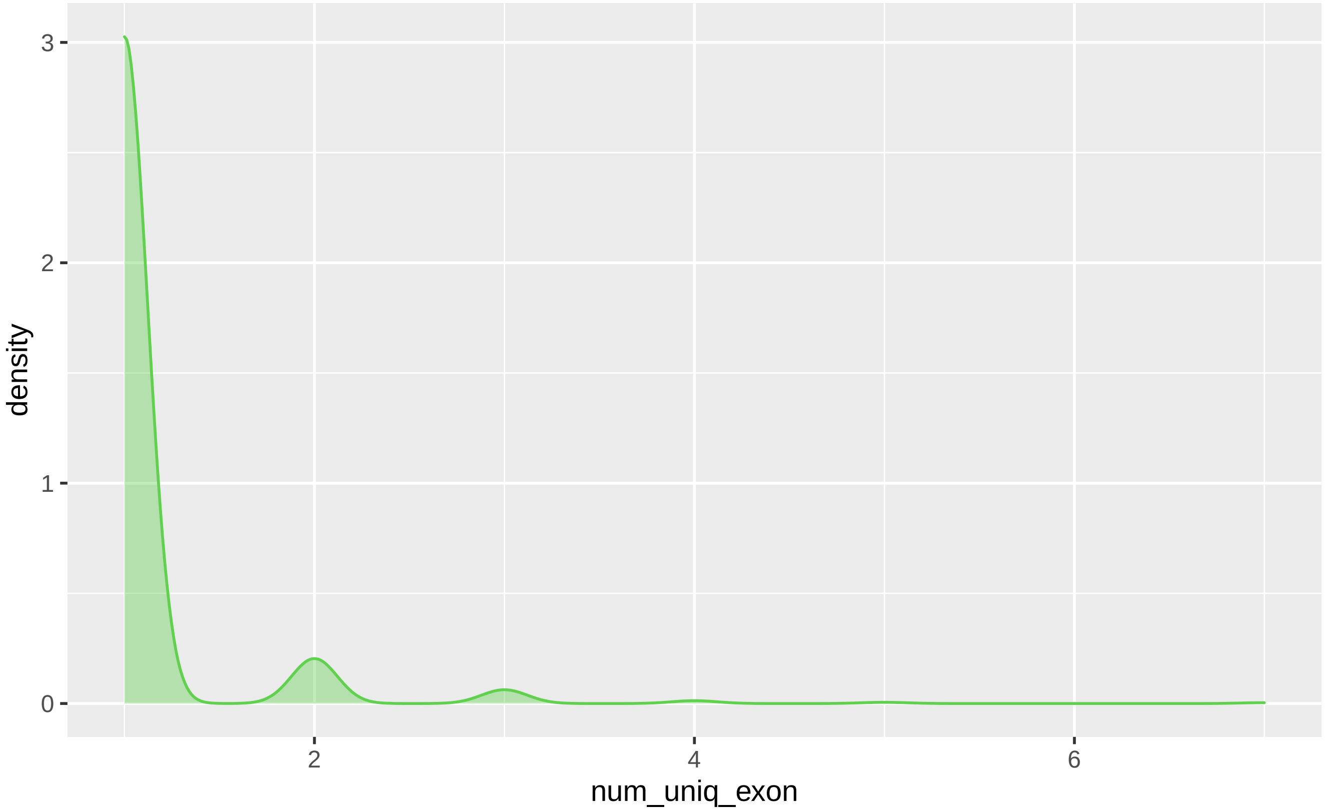

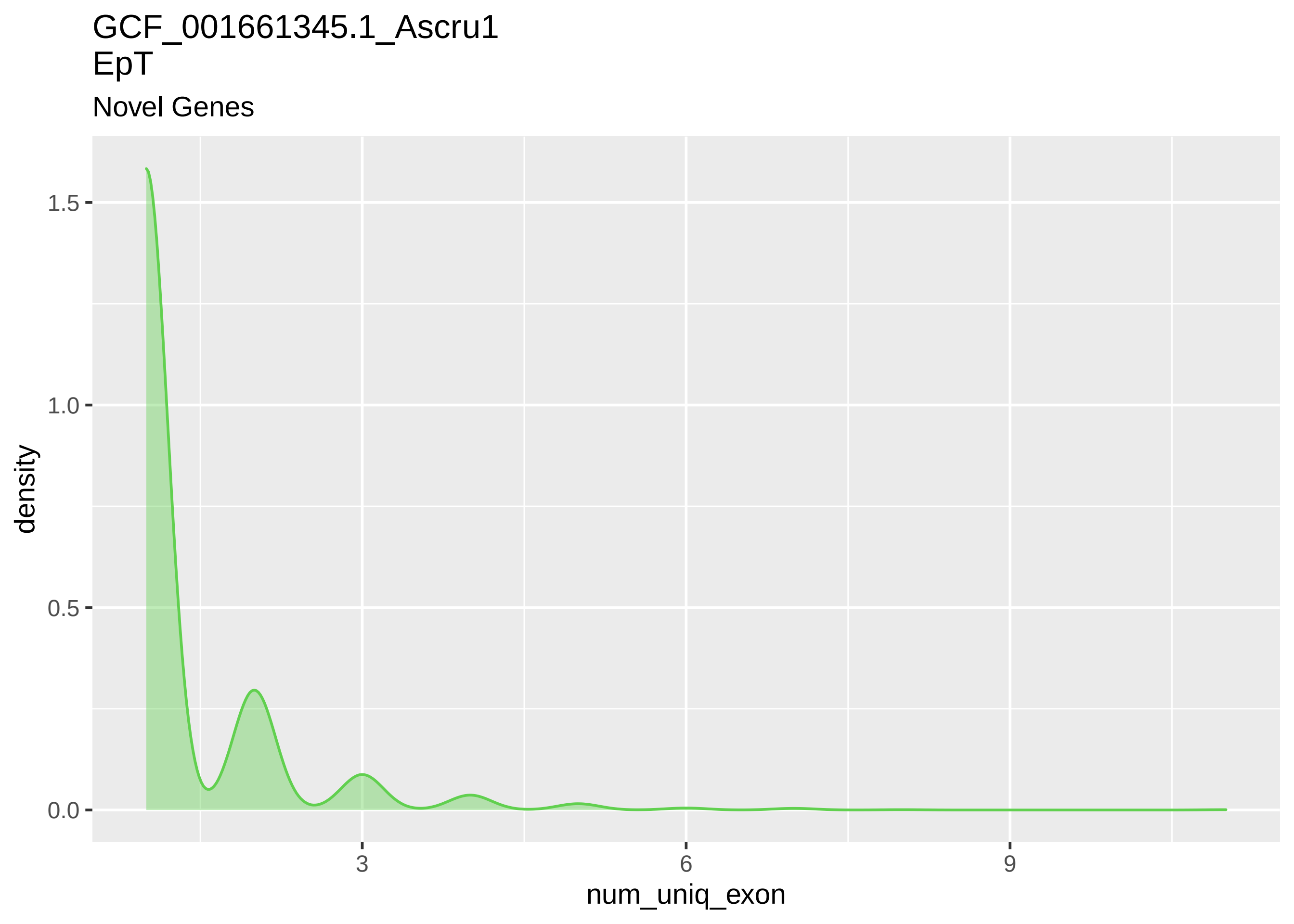

GCF_001661345.1_Ascru1
EpT
Novel Genes
density
1.5
1.0
0.5
0.0
3
6
9
num_uniq_exon

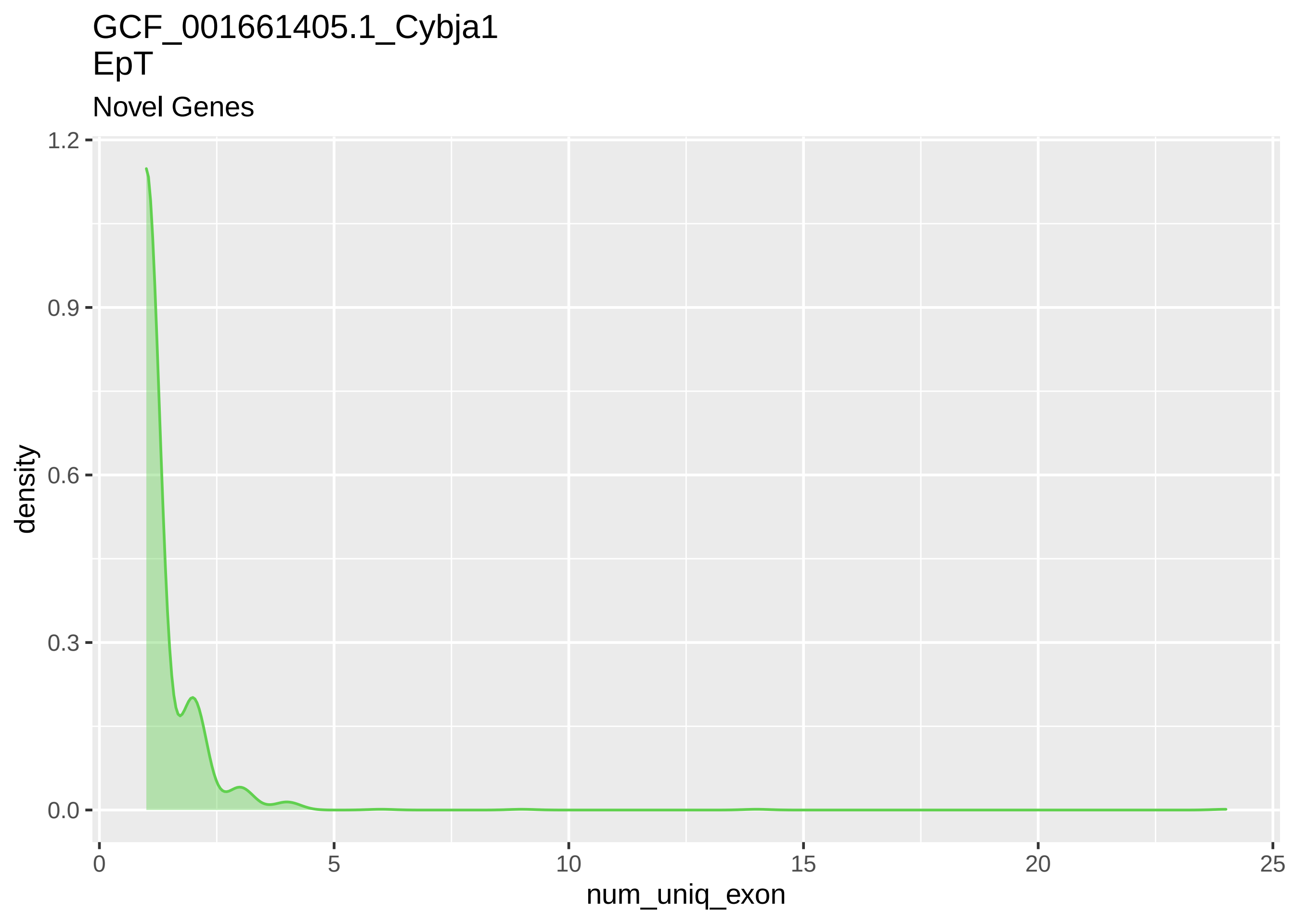

GCF_001661405.1_Cybja1
EpT
Novel Genes
density
1.2
0.9
0.6
0.3
0.0
0
5
10
15
20
25
num_uniq_exon

GCF_001664035.1_Metbi1
EpT

Novel Genes

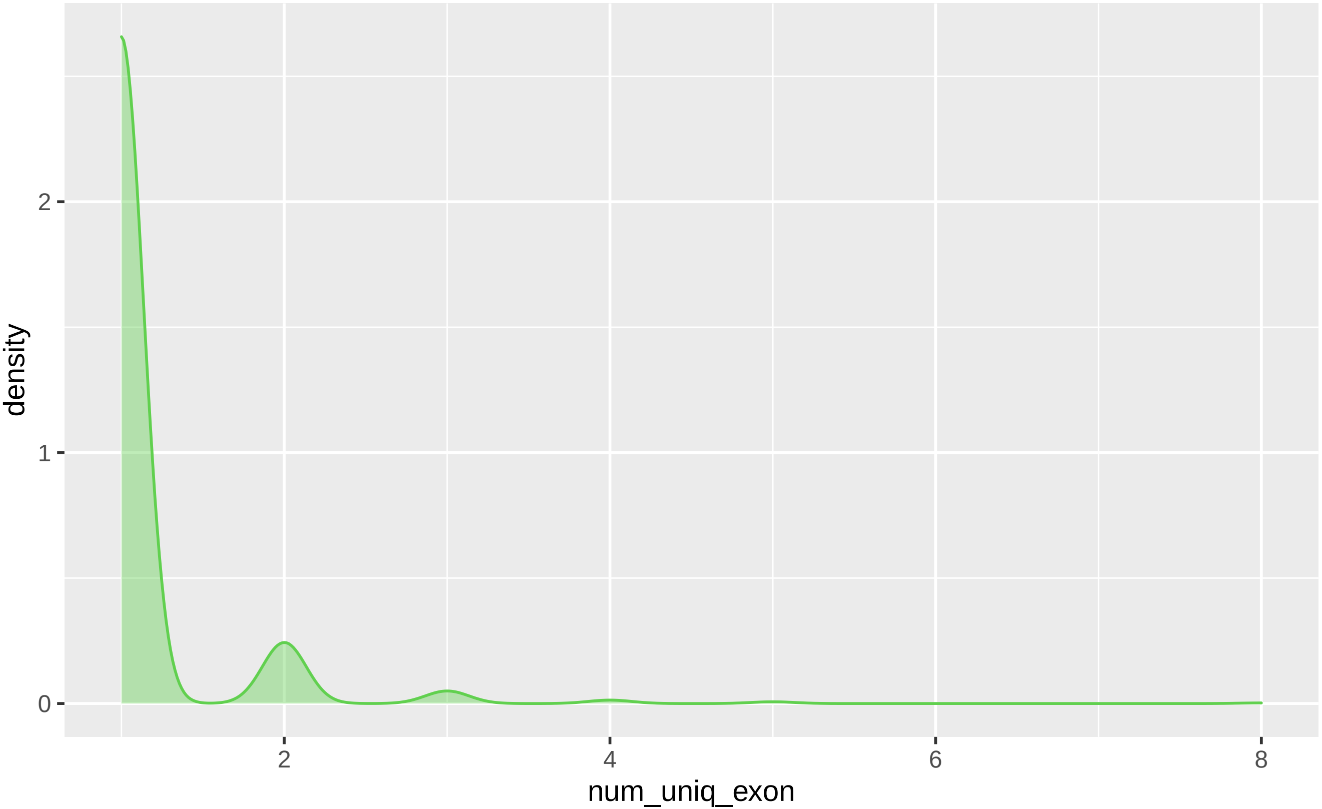

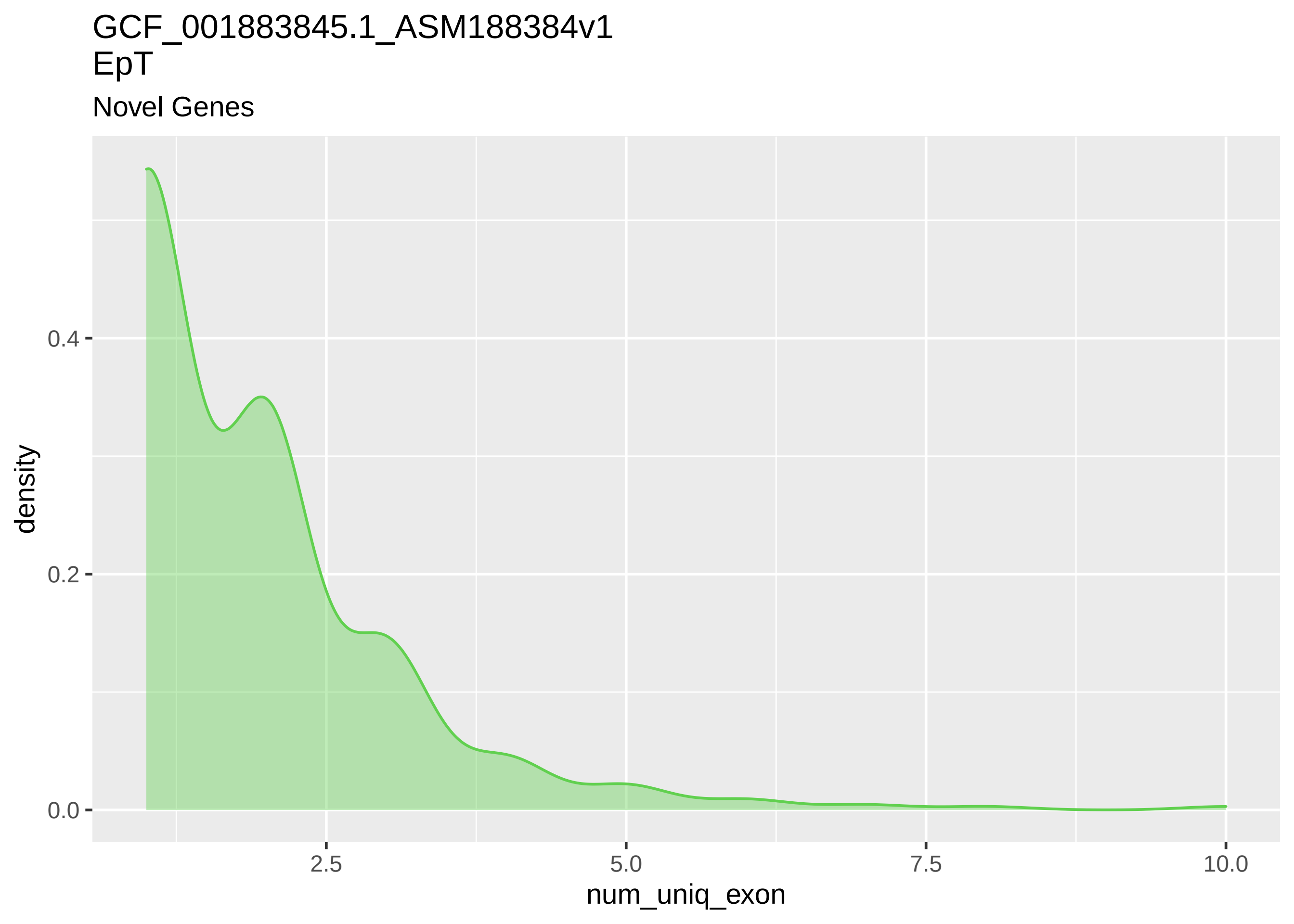
GCF_001883845.1_ASM188384v1
EpT
Novel Genes
density
0.0
0.2
0.4
2.5
5.0
7.5
10.0
num_uniq_exon

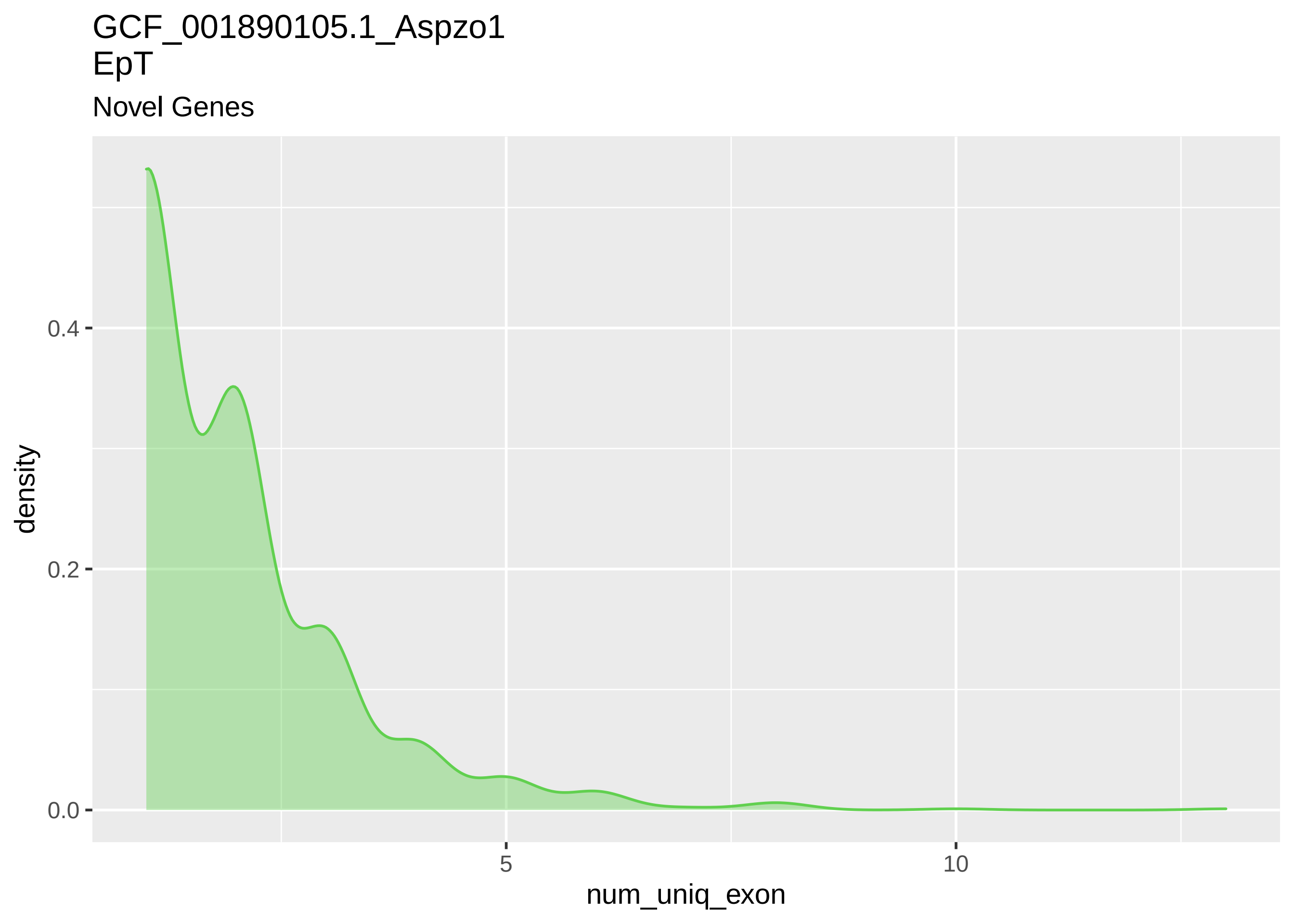
GCF_001890105.1_Aspzo1
EpT
Novel Genes
density
0.4
0.2
0.0
5
10
num_uniq_exon

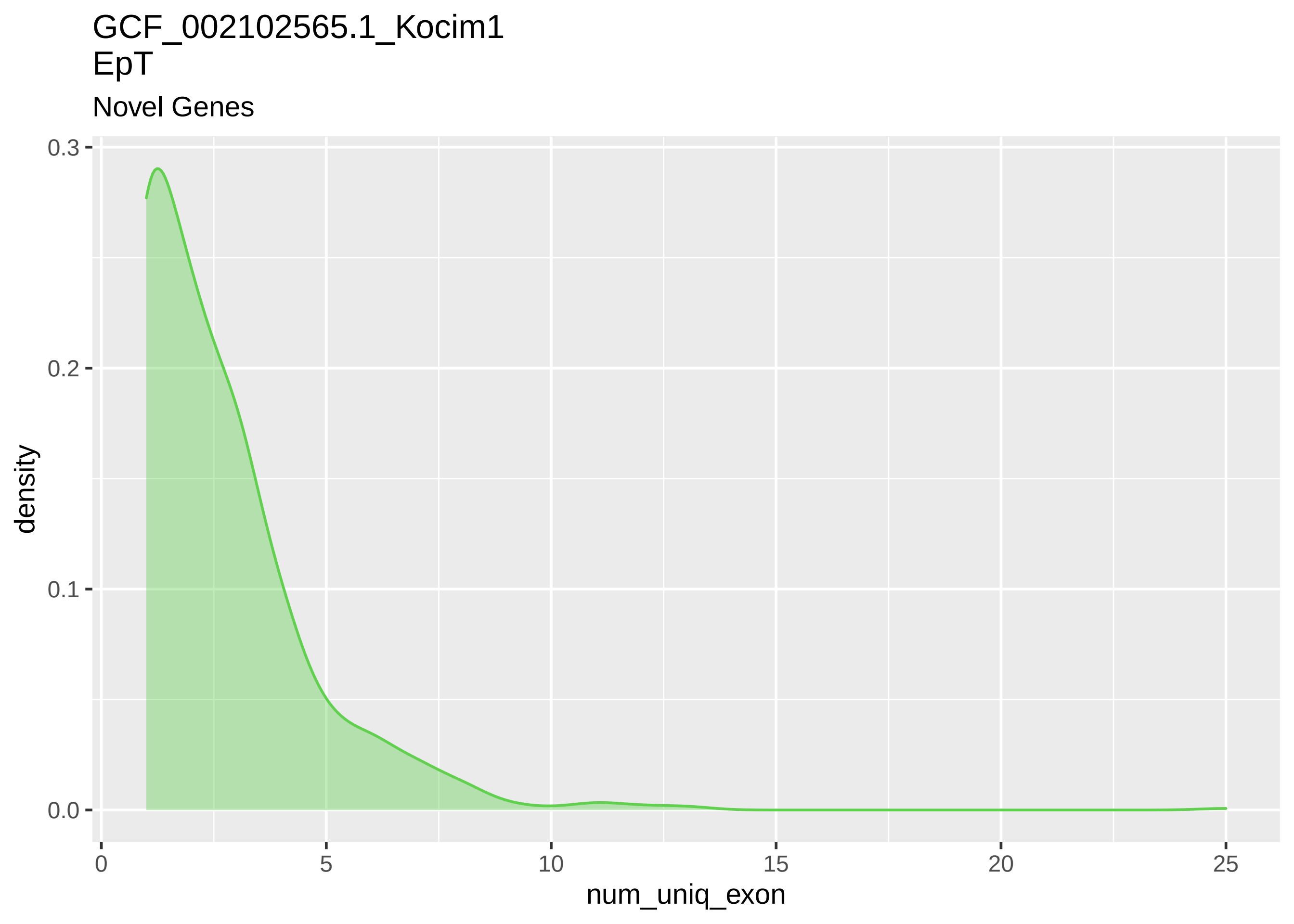
GCF_002102565.1_Kocim1
EpT
Novel Genes
density
0.3
0.2
0.1
0.0
0
5
10
15
20
25
num_uniq_exon

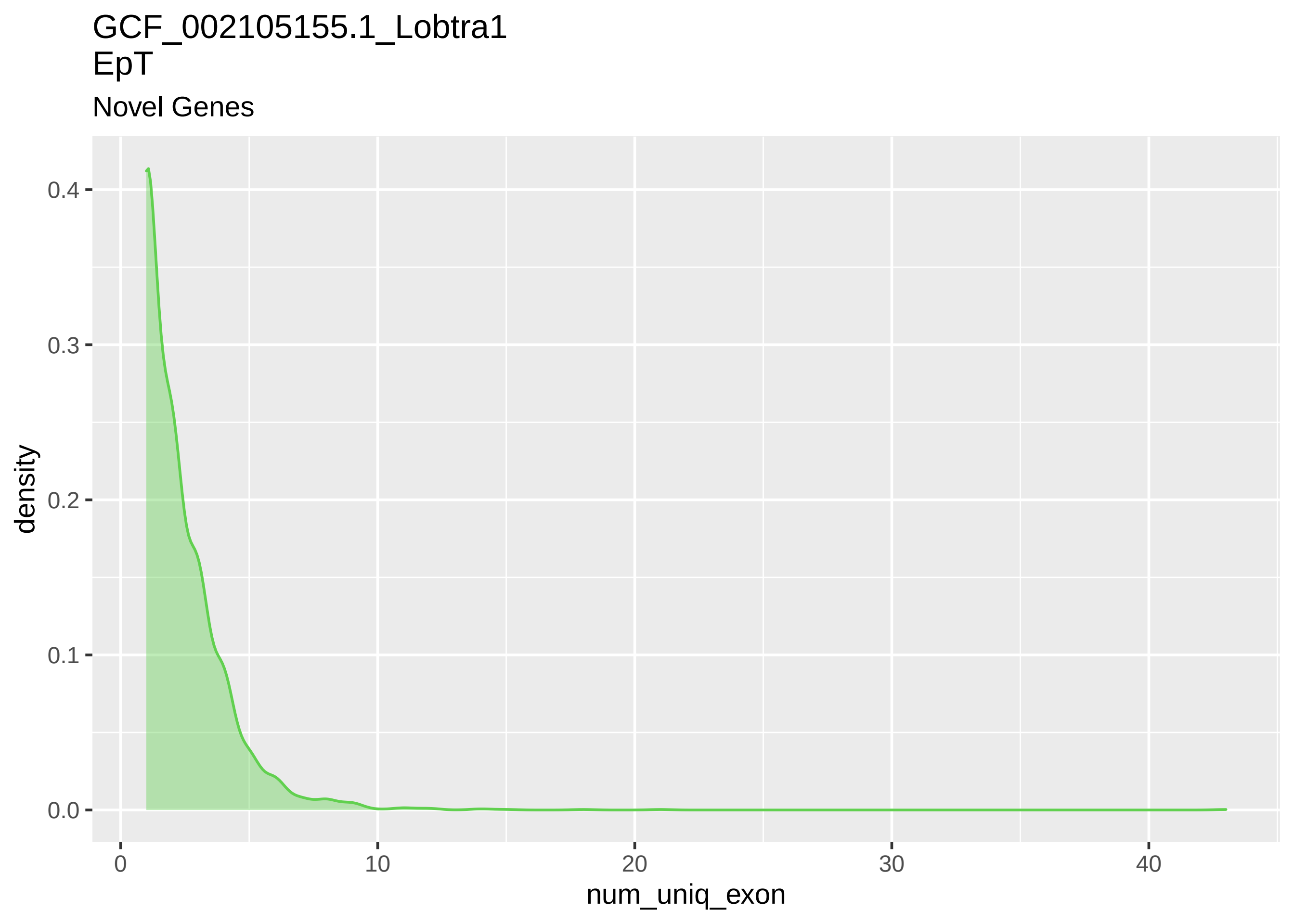

GCF_002105155.1_Lobtra1
EpT
Novel Genes
density
0.0
0.1
0.2
0.3
0.4
0
10
20
30
40
num_uniq_exon

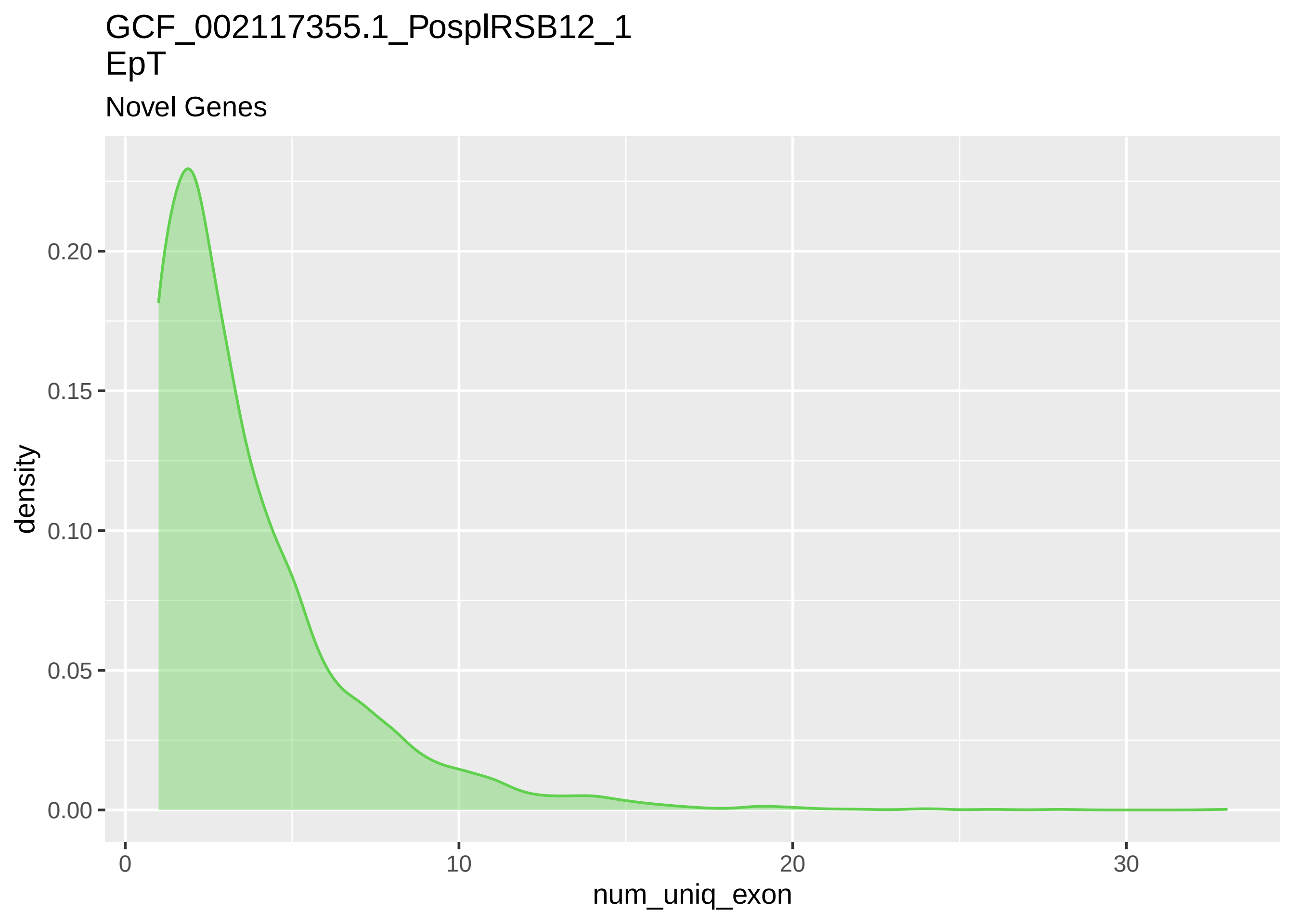
GCF_002117355.1_PosplRSB12_1
EpT
Novel Genes
density
0.00
0.05
0.10
0.15
0.20
0
10
20
30
num_uniq_exon

GCF_002847465.1_Aspnov1
EpT

Novel Genes

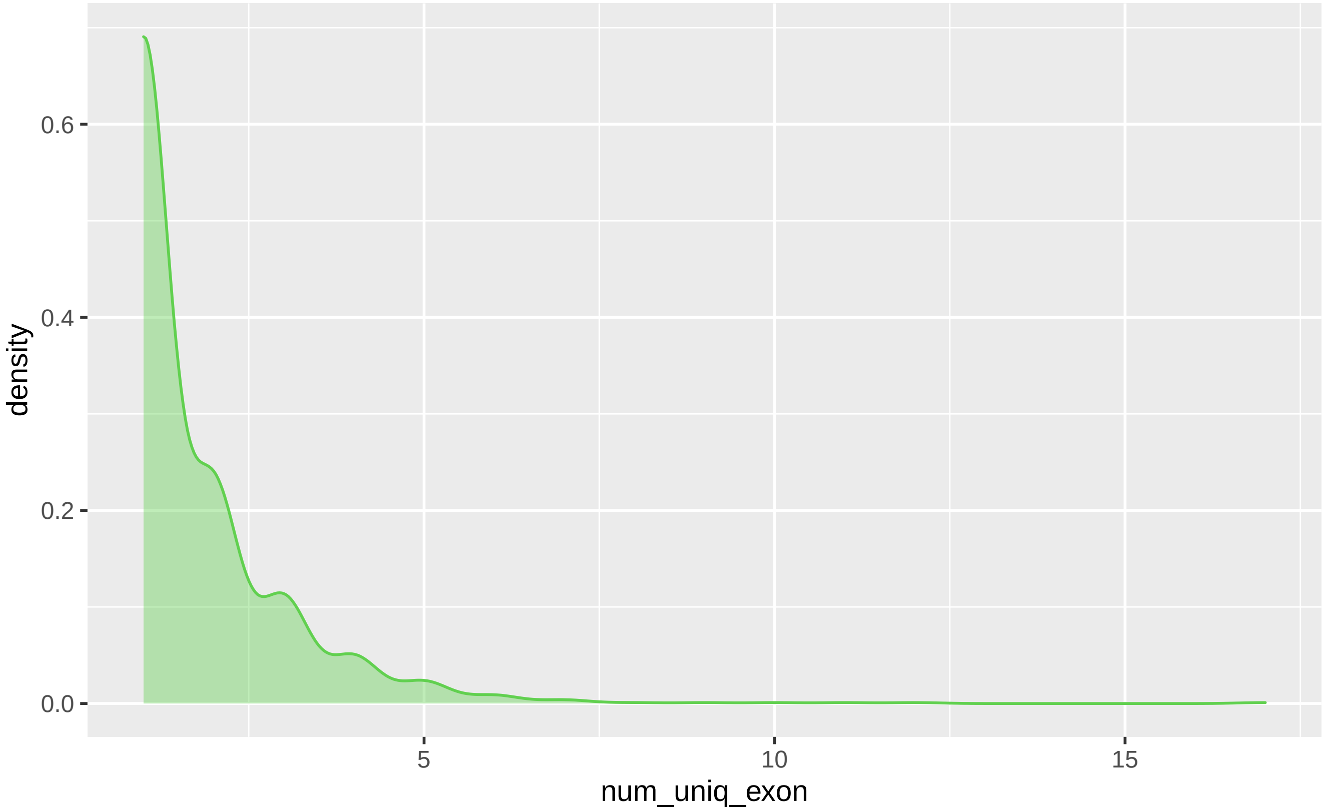

Supplement: Supplementary file 1 — Additional file 1. [file 12864_2023_9326_MOESM1_ESM.zip › Supp. Mat. Novel Gene Density Plots_ESM.pdf]
