# Supplementary material for: Estimating transcriptome complexities across eukaryotes
Source: BMC Genomics. 2023 May 11;24:254. doi: 10.1186/s12864-023-09326-0 (PMC10173493; doi:10.1186/s12864-023-09326-0)
Supplement: Supplementary file 1 — Additional file 1. [file 12864_2023_9326_MOESM1_ESM.zip › Supp. Mat. Whole-Transcriptome Vs Ortholog Density Plots_ESM.pdf]

WHOLE-TRANSCRIPTOME VS ORTHOLOG DENSITY PLOTS

Figures below are density plots between whole-genome (red) and orthologs (light blue) for complexity metrics (TpG, EpT, and EpG) with densities on the y-axis and genetic element counts on the x-axis. Plots include every organism used in this study.

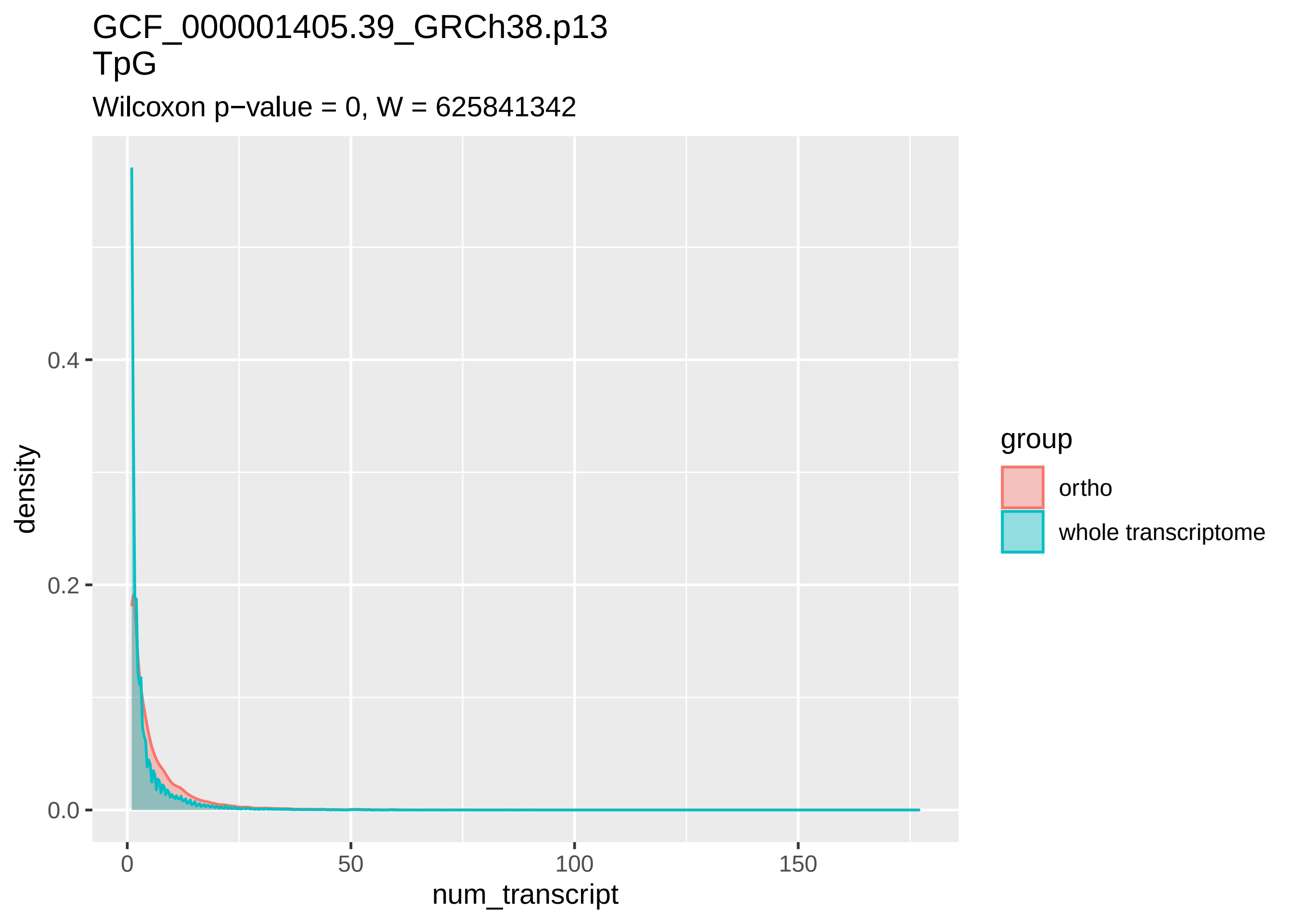

GCF_000001405.39_GRCh38.p13
TpG
Wilcoxon p−value = 0, W = 625841342
density
0.0
0.2
0.4
0
50
100
150
num_transcript
group
ortho
whole transcriptome

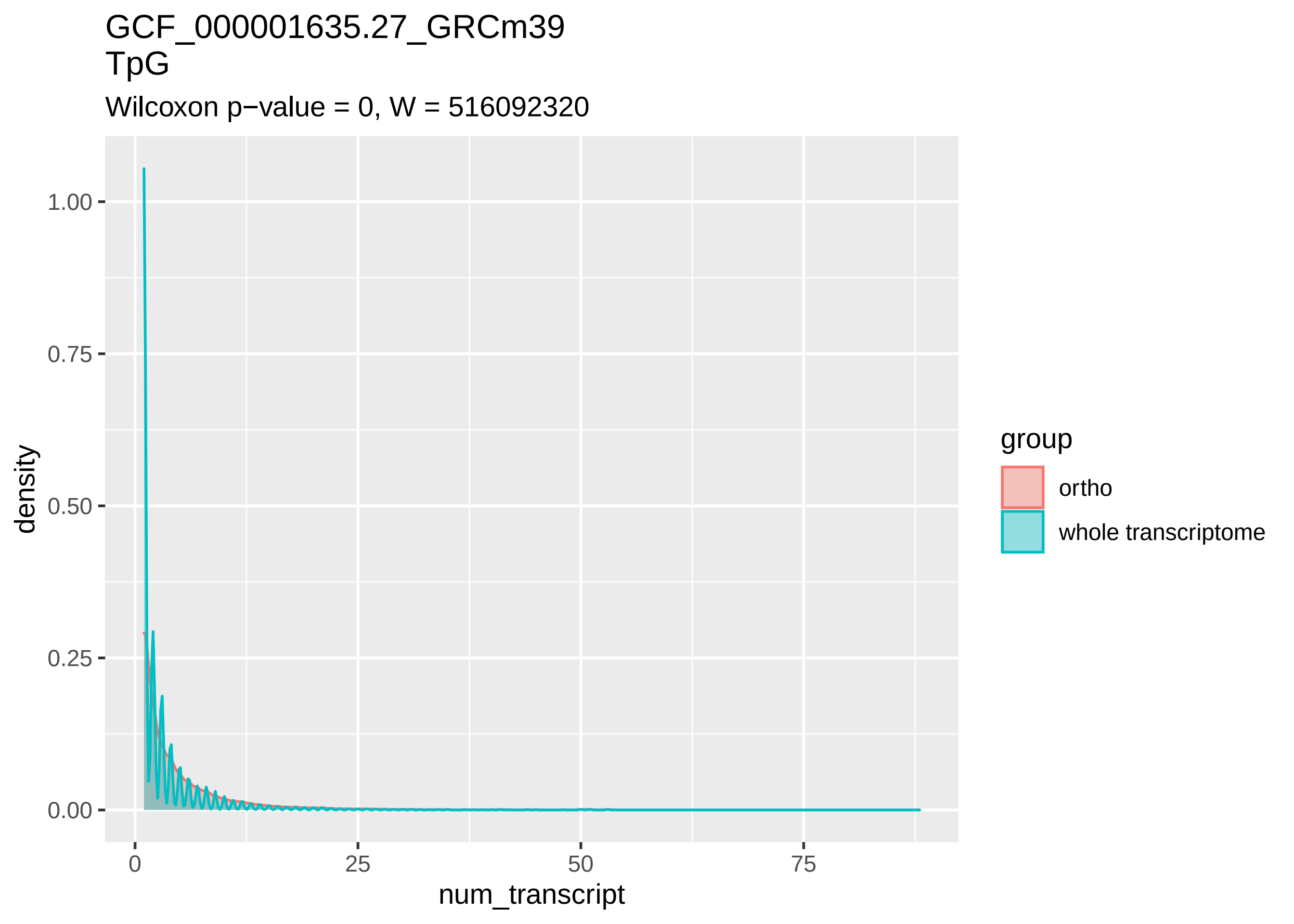

GCF_000001635.27_GRCm39
TpG
Wilcoxon p−value = 0, W = 516092320
density
1.00
0.75
0.50
0.25
0.00
0
25
50
75
num_transcript
group
ortho
whole transcriptome

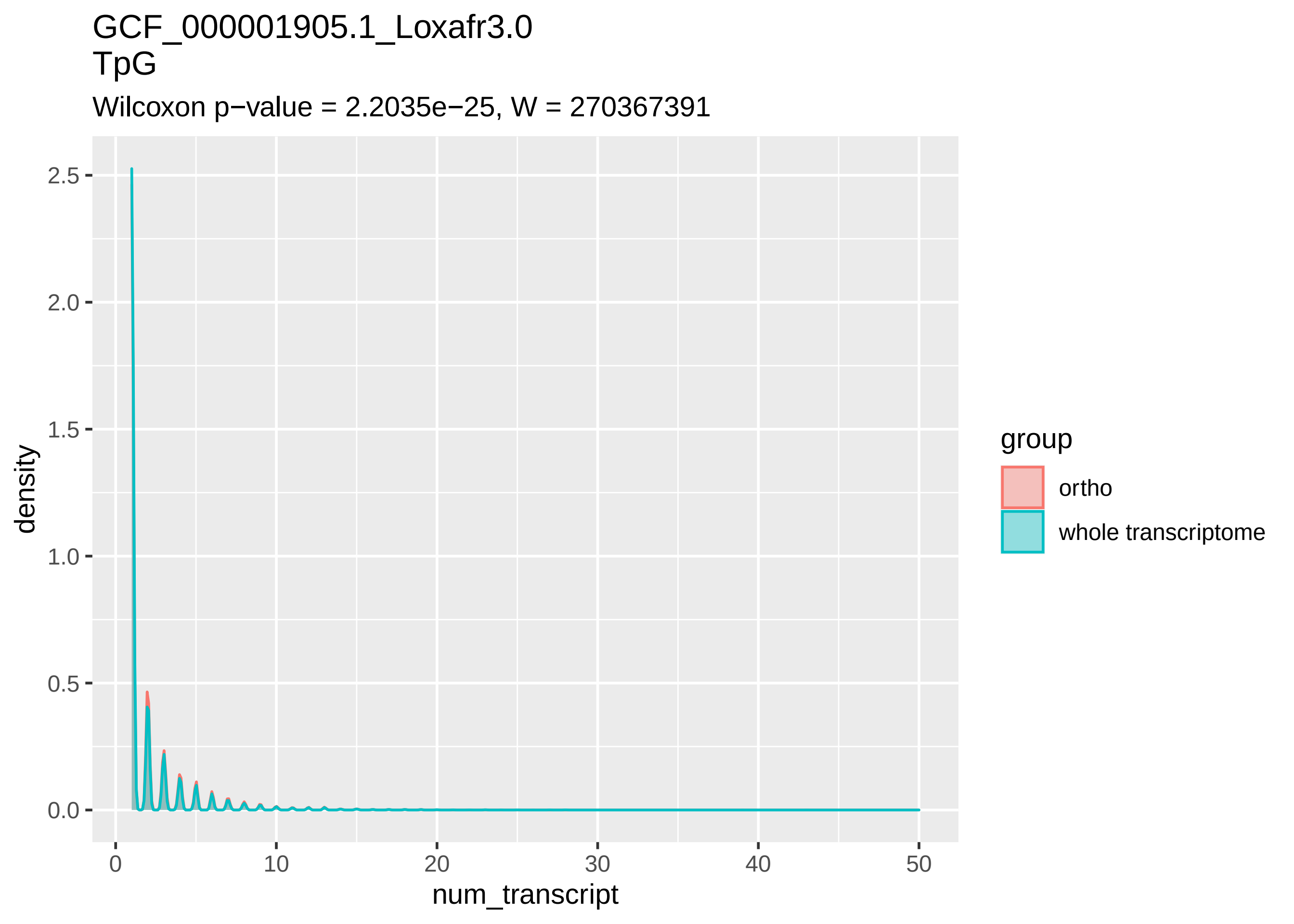
GCF_000001905.1_Loxafr3.0
TpG
Wilcoxon p−value = 2.2035e−25, W = 270367391
density
2.5
2.0
1.5
1.0
0.5
0.0
0
10
20
30
40
50
num_transcript
group
ortho
whole transcriptome

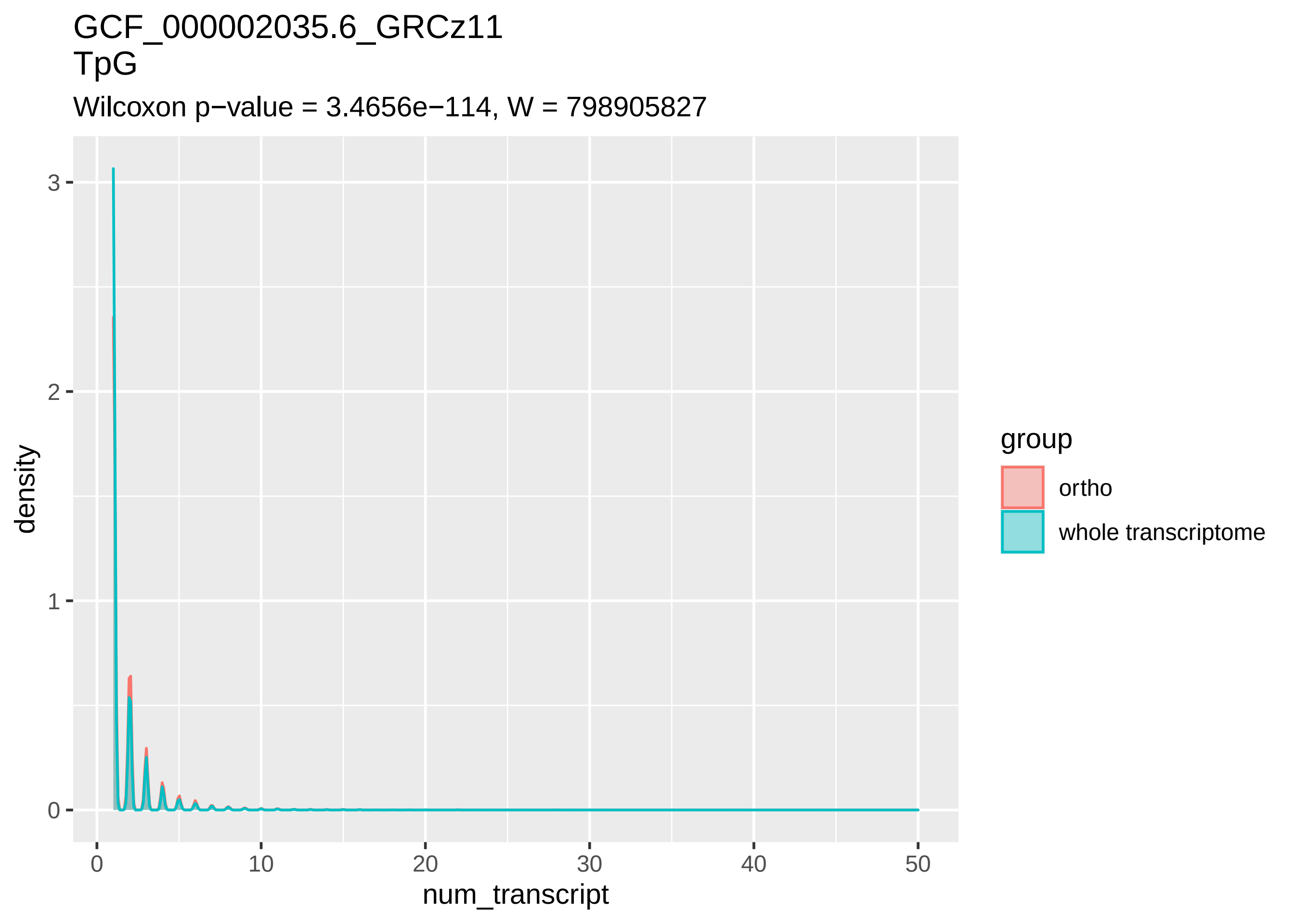

GCF_000002035.6_GRCz11
TpG
Wilcoxon p−value = 3.4656e−114, W = 798905827
density
num_transcript
0
1
2
3
0
10
20
30
40
50
group
ortho
whole transcriptome

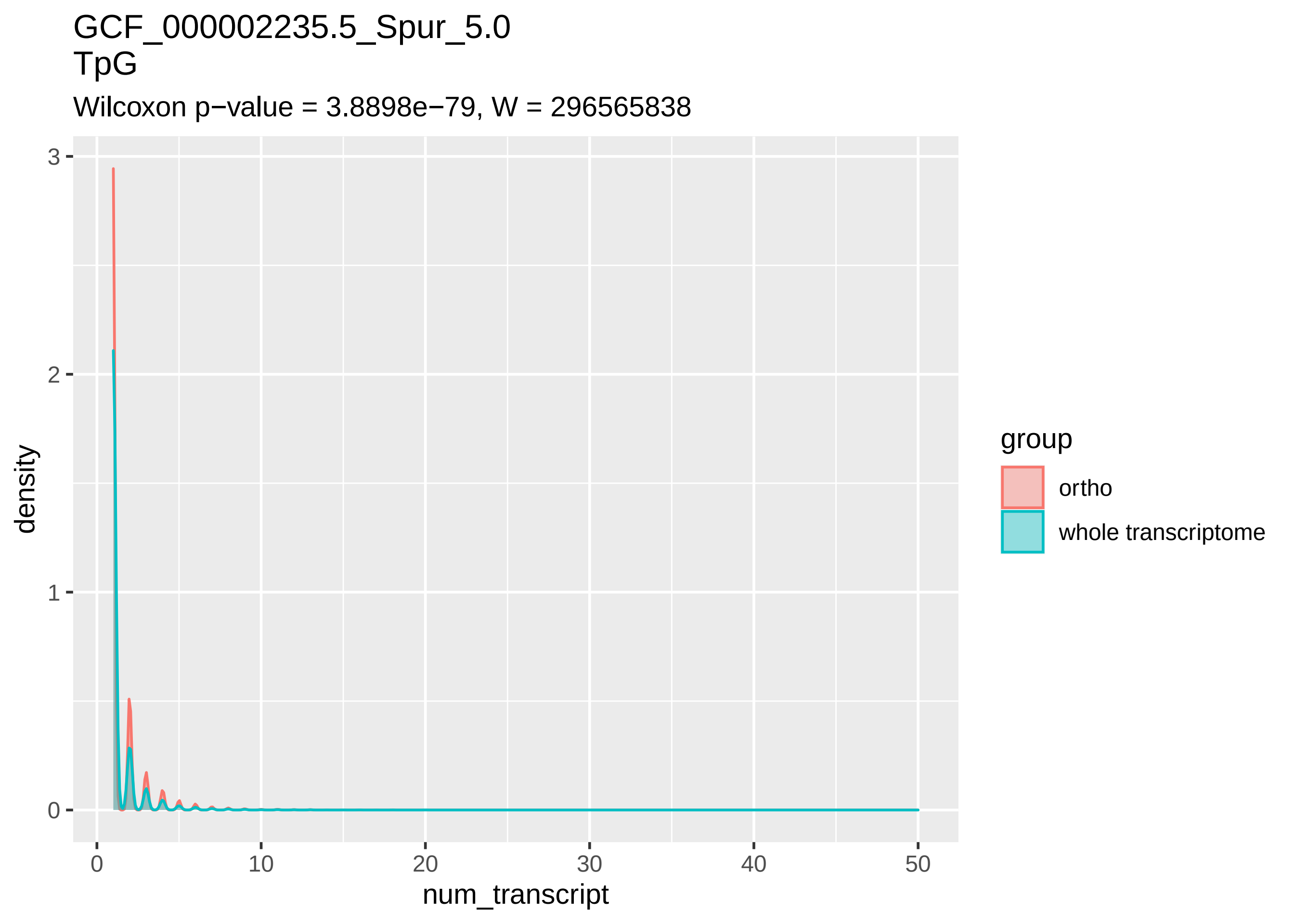
GCF_000002235.5_Spur_5.0
TpG
Wilcoxon p−value = 3.8898e−79, W = 296565838
density
num_transcript
0
1
2
3
0
10
20
30
40
50
group
ortho
whole transcriptome

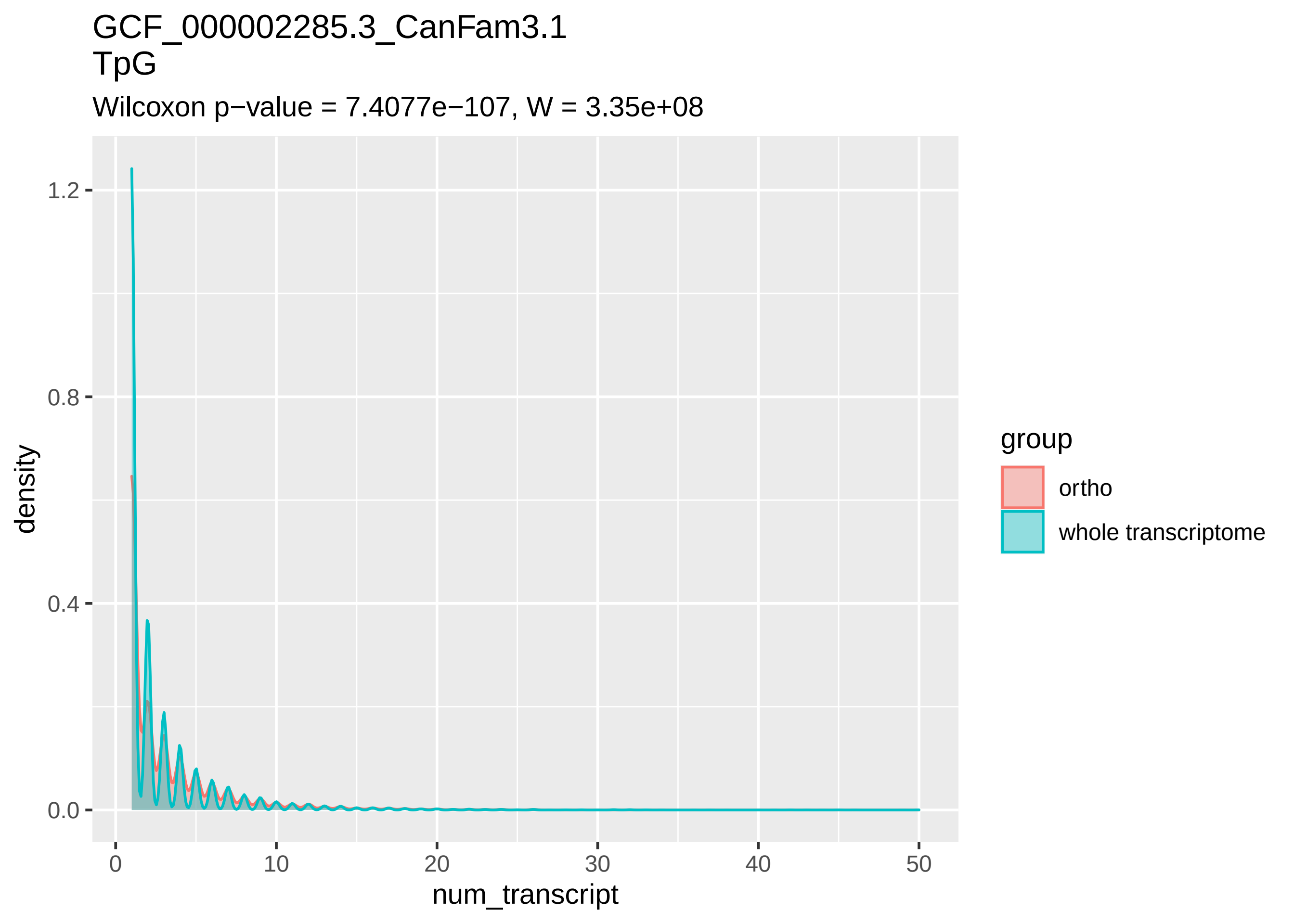
GCF_000002285.3_CanFam3.1
TpG
Wilcoxon p−value = 7.4077e−107, W = 3.35e+08
density
1.2
0.8
0.4
0.0
0
10
20
30
40
50
num_transcript
group
ortho
whole transcriptome

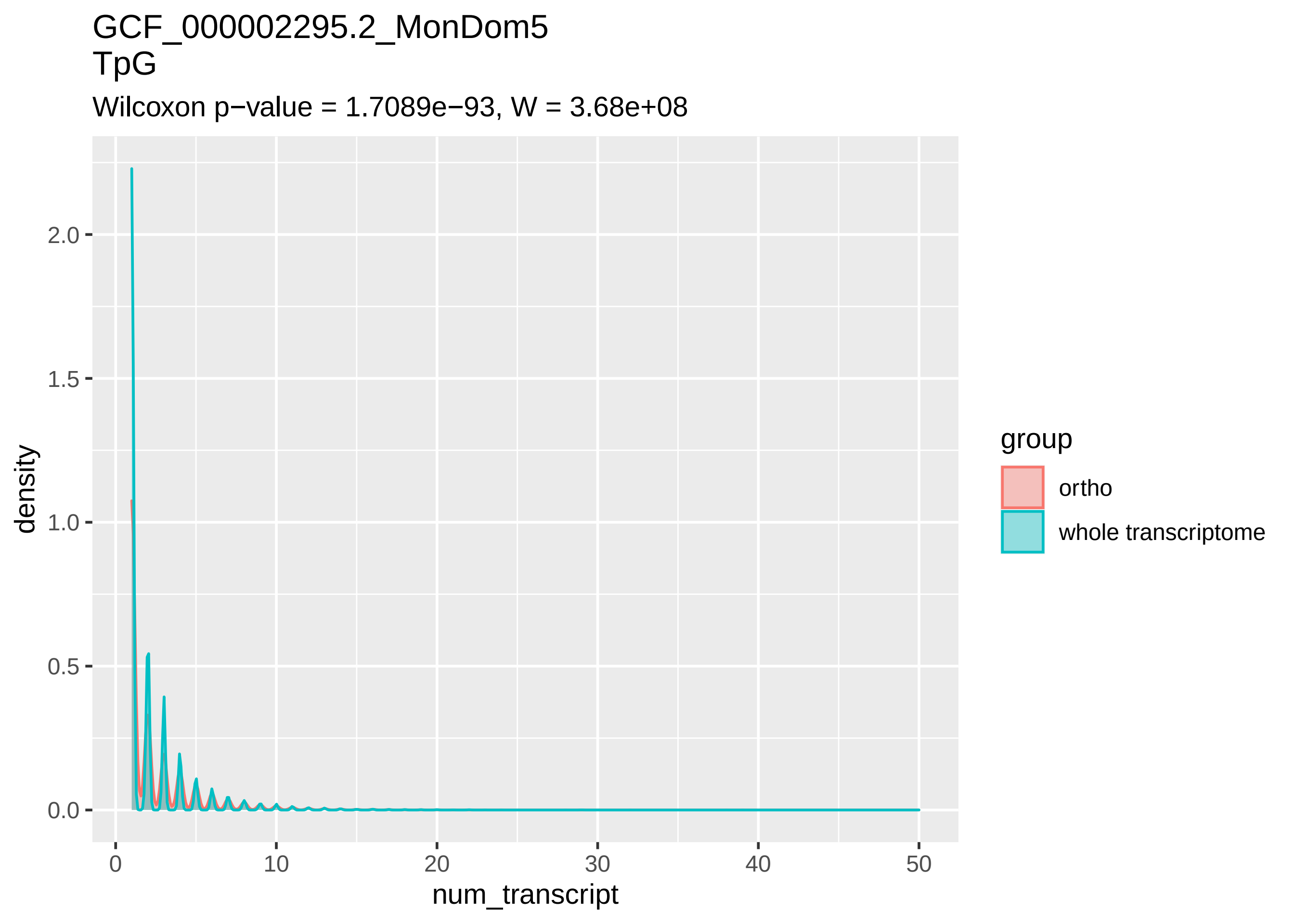

GCF_000002295.2_MonDom5
TpG
Wilcoxon p−value = 1.7089e−93, W = 3.68e+08
density
2.0
1.5
1.0
0.5
0.0
0
10
20
30
40
50
num_transcript
group
ortho
whole transcriptome

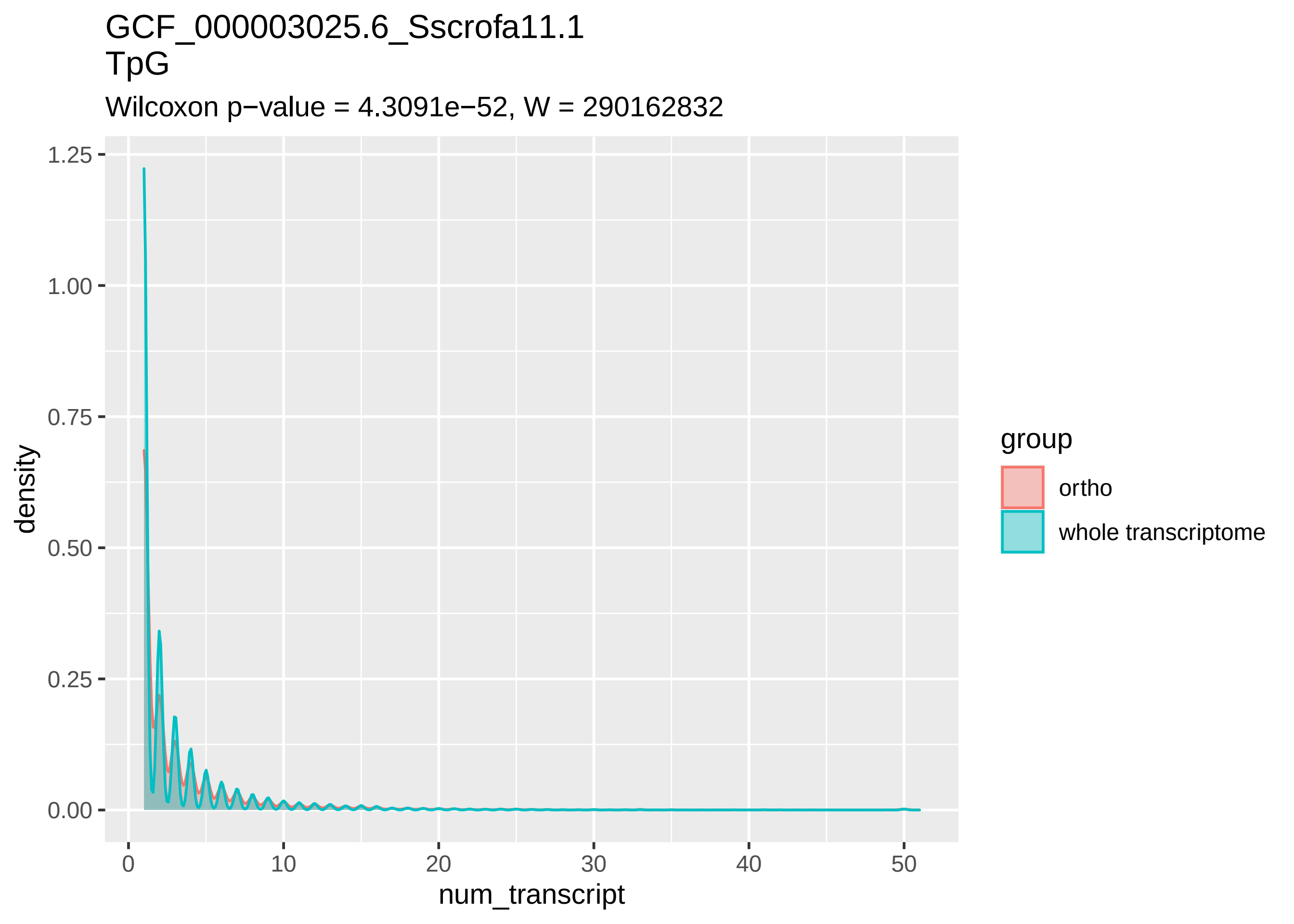

GCF_000003025.6_Sscrofa11.1
TpG
Wilcoxon p−value = 4.3091e−52, W = 290162832
density
1.25
1.00
0.75
0.50
0.25
0.00
0
10
20
30
40
50
num_transcript
group
ortho
whole transcriptome

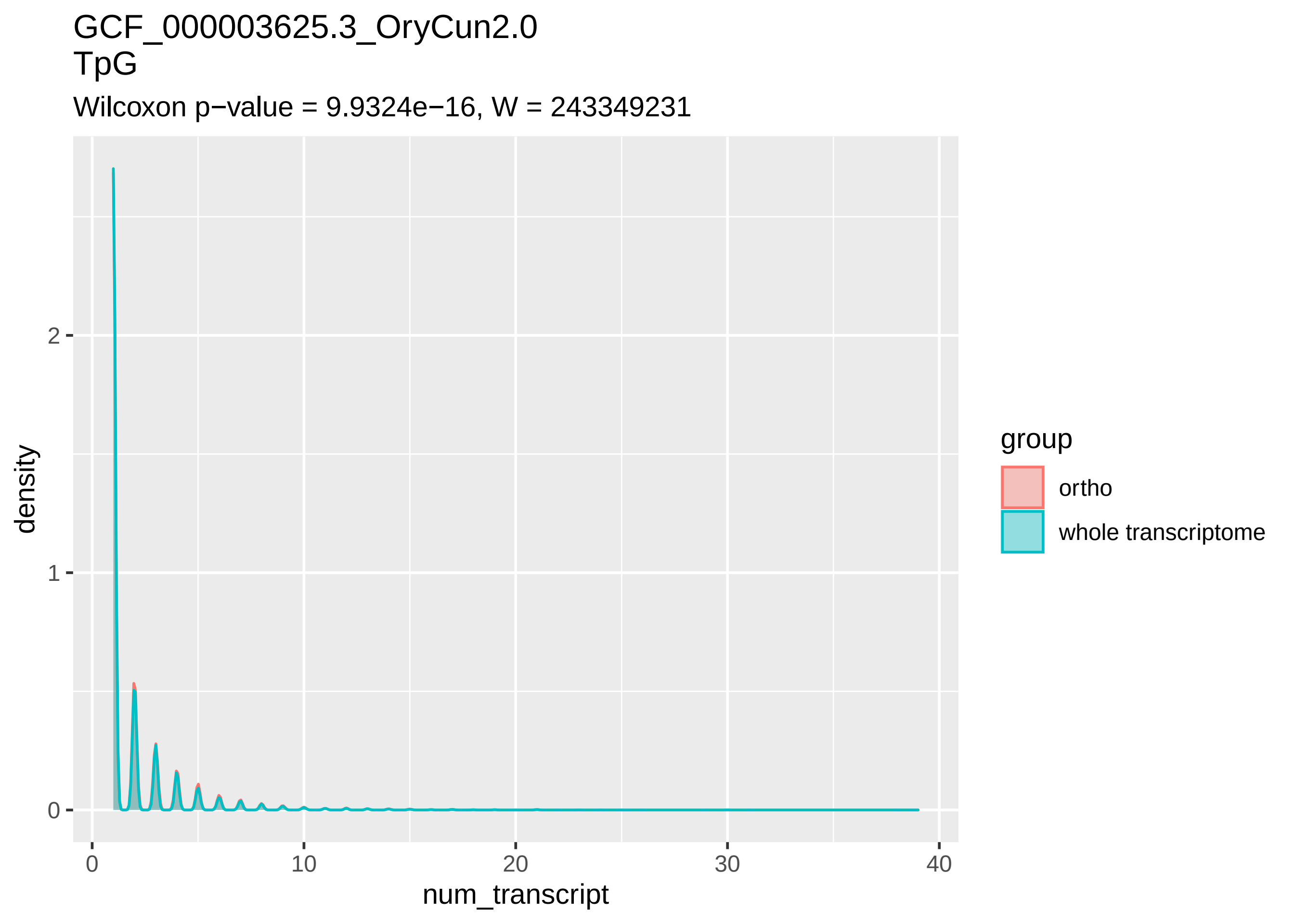

GCF_000003625.3_OryCun2.0
TpG
Wilcoxon p−value = 9.9324e−16, W = 243349231
density
num_transcript
0
1
2
0
10
20
30
40
group
ortho
whole transcriptome

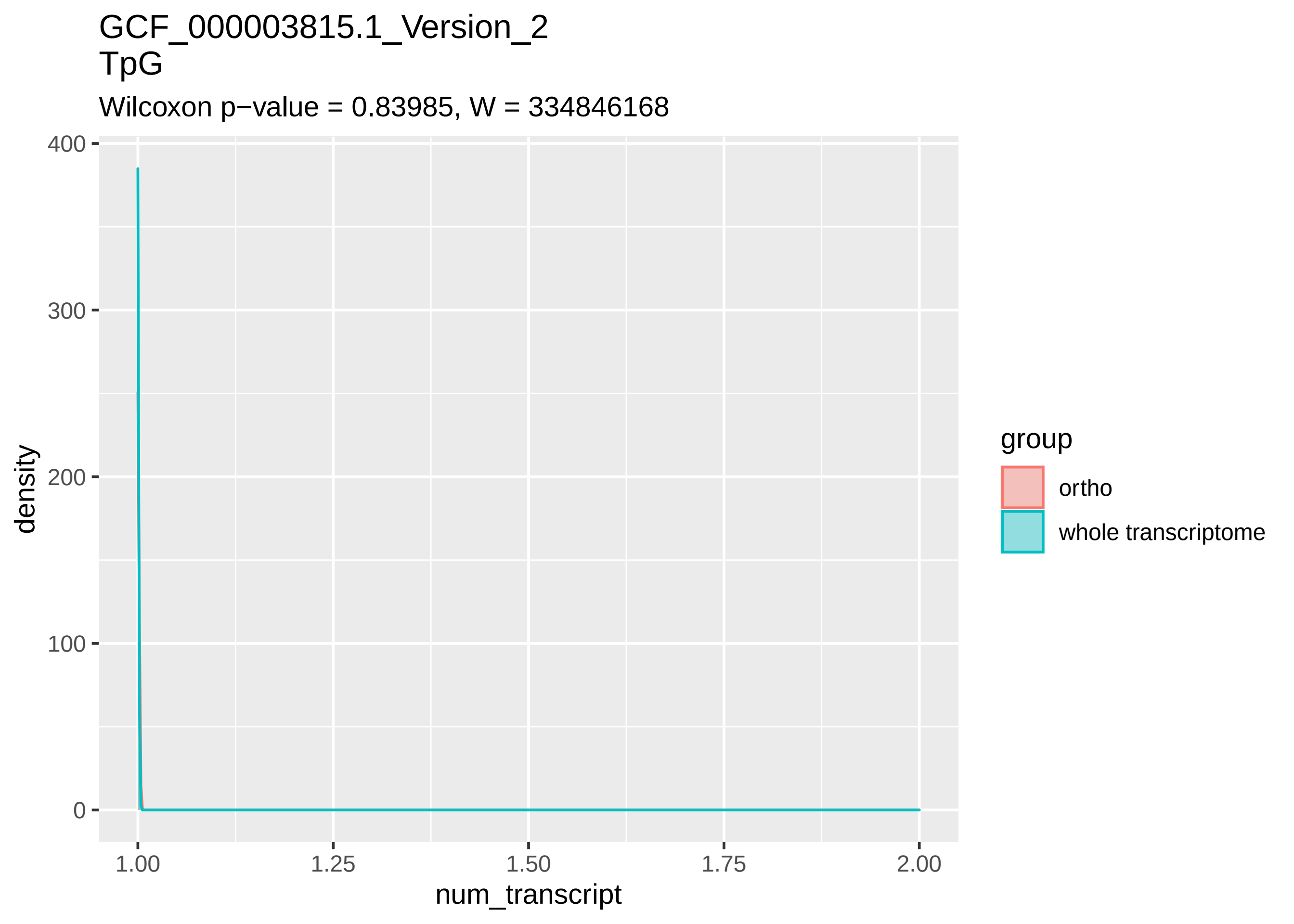
GCF_000003815.1_Version_2
TpG
Wilcoxon p−value = 0.83985, W = 334846168
400
300
200
100
0
density
1.00
1.25
1.50
1.75
2.00
num_transcript
group
ortho
whole transcriptome

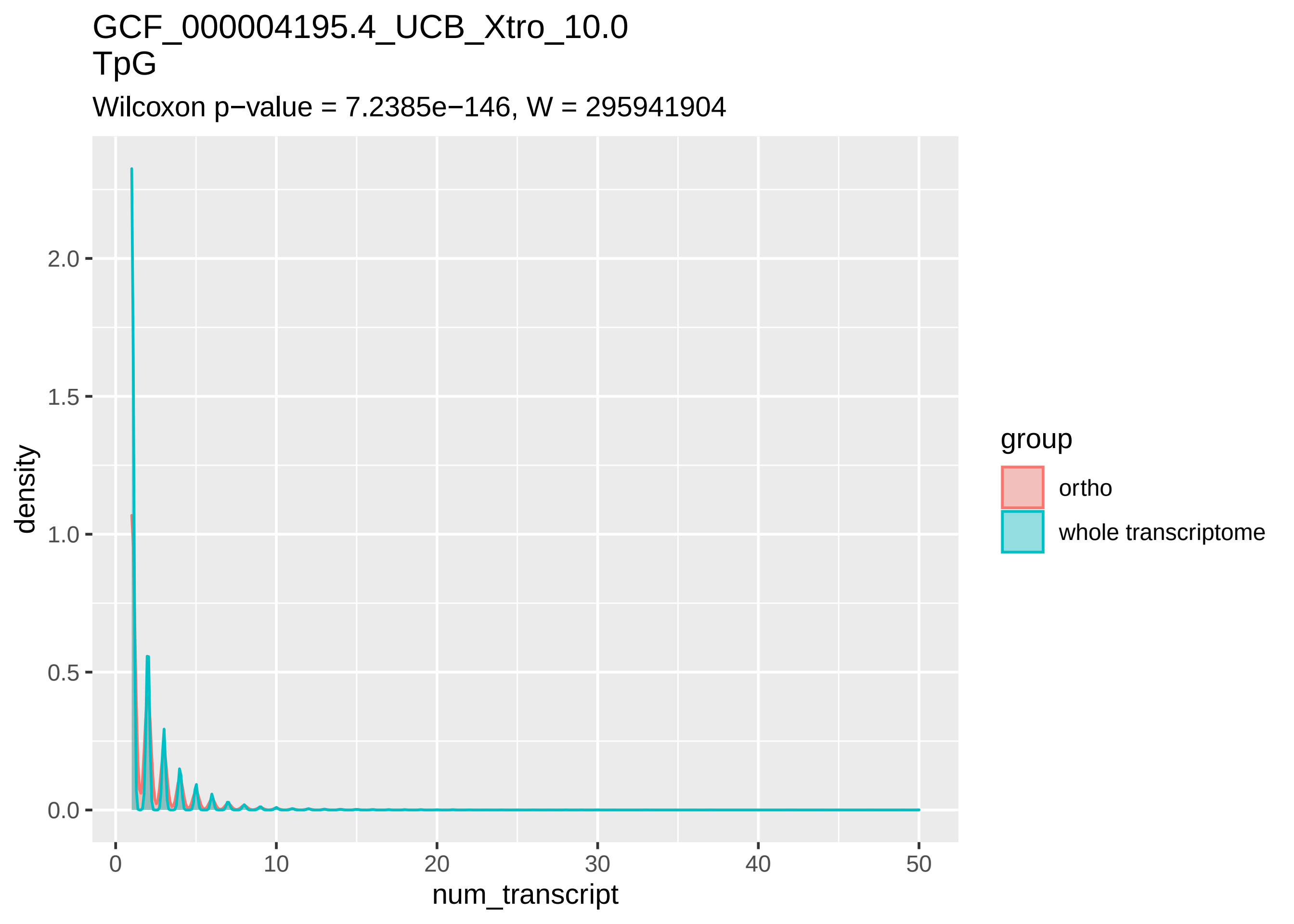

GCF_000004195.4_UCB_Xtro_10.0
TpG
Wilcoxon p−value = 7.2385e−146, W = 295941904
density
2.0
1.5
1.0
0.5
0.0
0
10
20
30
40
50
num_transcript
group
ortho
whole transcriptome

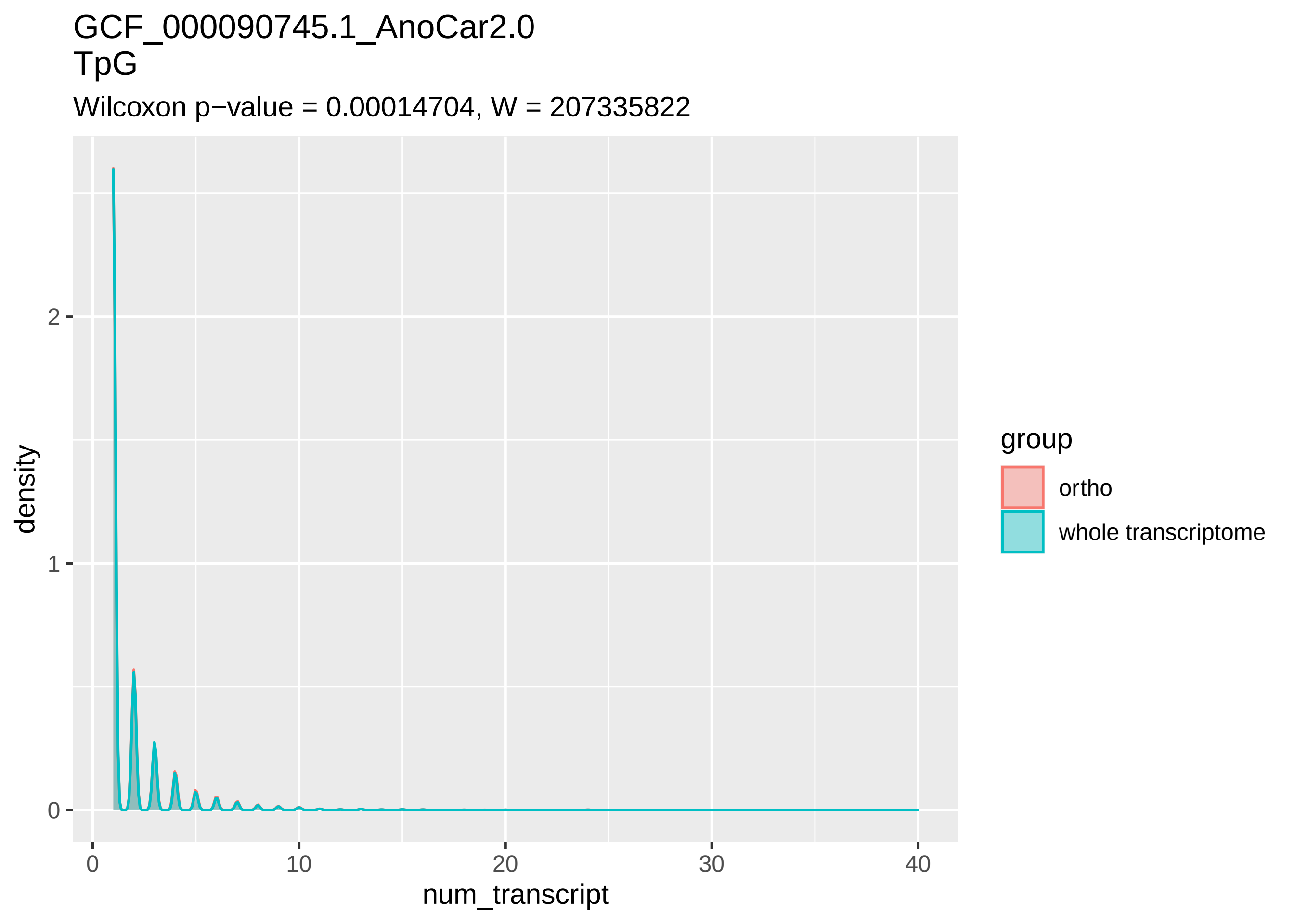
GCF_000090745.1_AnoCar2.0
TpG
Wilcoxon p−value = 0.00014704, W = 207335822
density
num_transcript
0
1
2
0
10
20
30
40
group
ortho
whole transcriptome

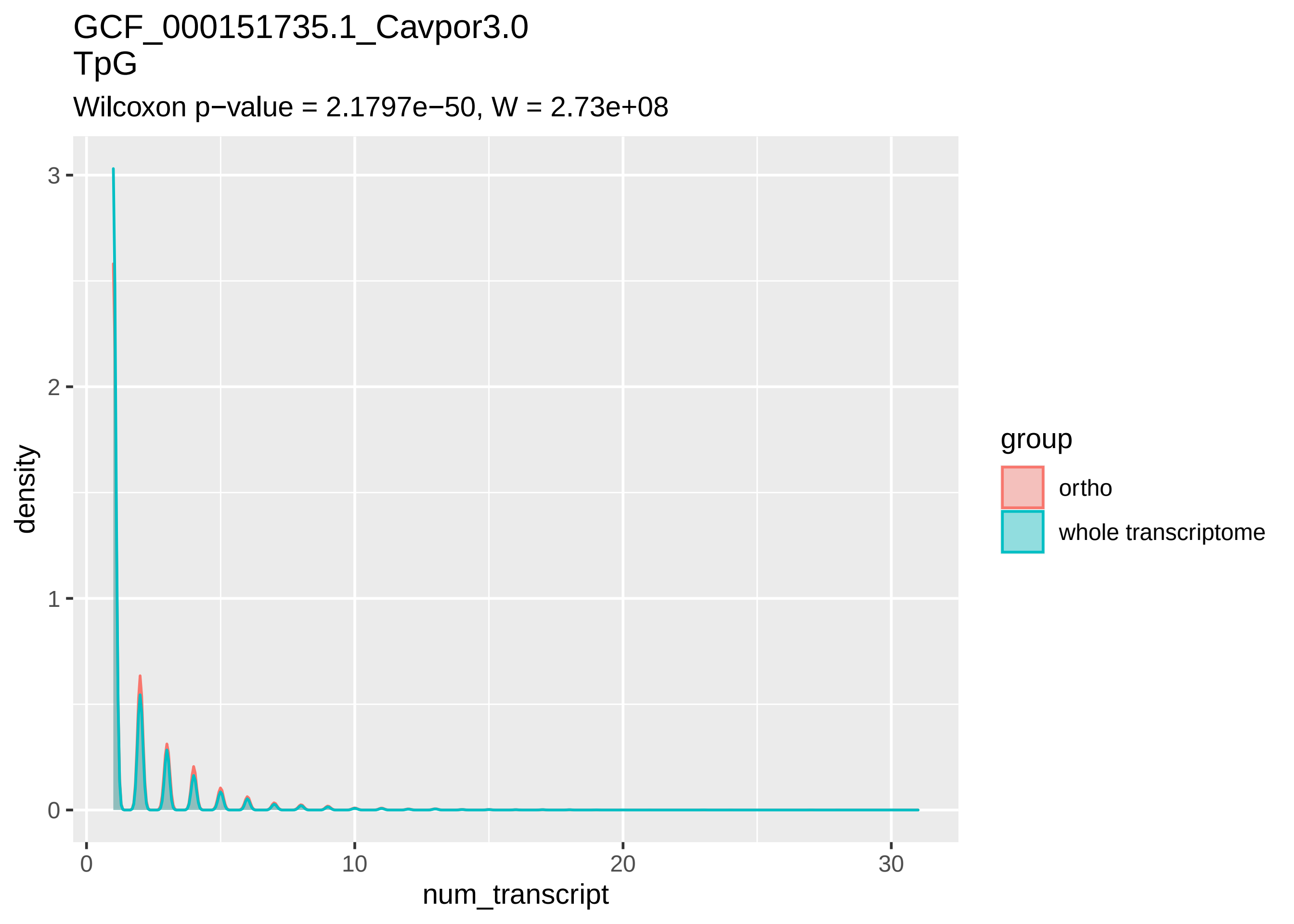
GCF_000151735.1_Cavpor3.0
TpG
Wilcoxon p−value = 2.1797e−50, W = 2.73e+08
density
3
2
1
0
0
10
20
30
num_transcript
group
ortho
whole transcriptome

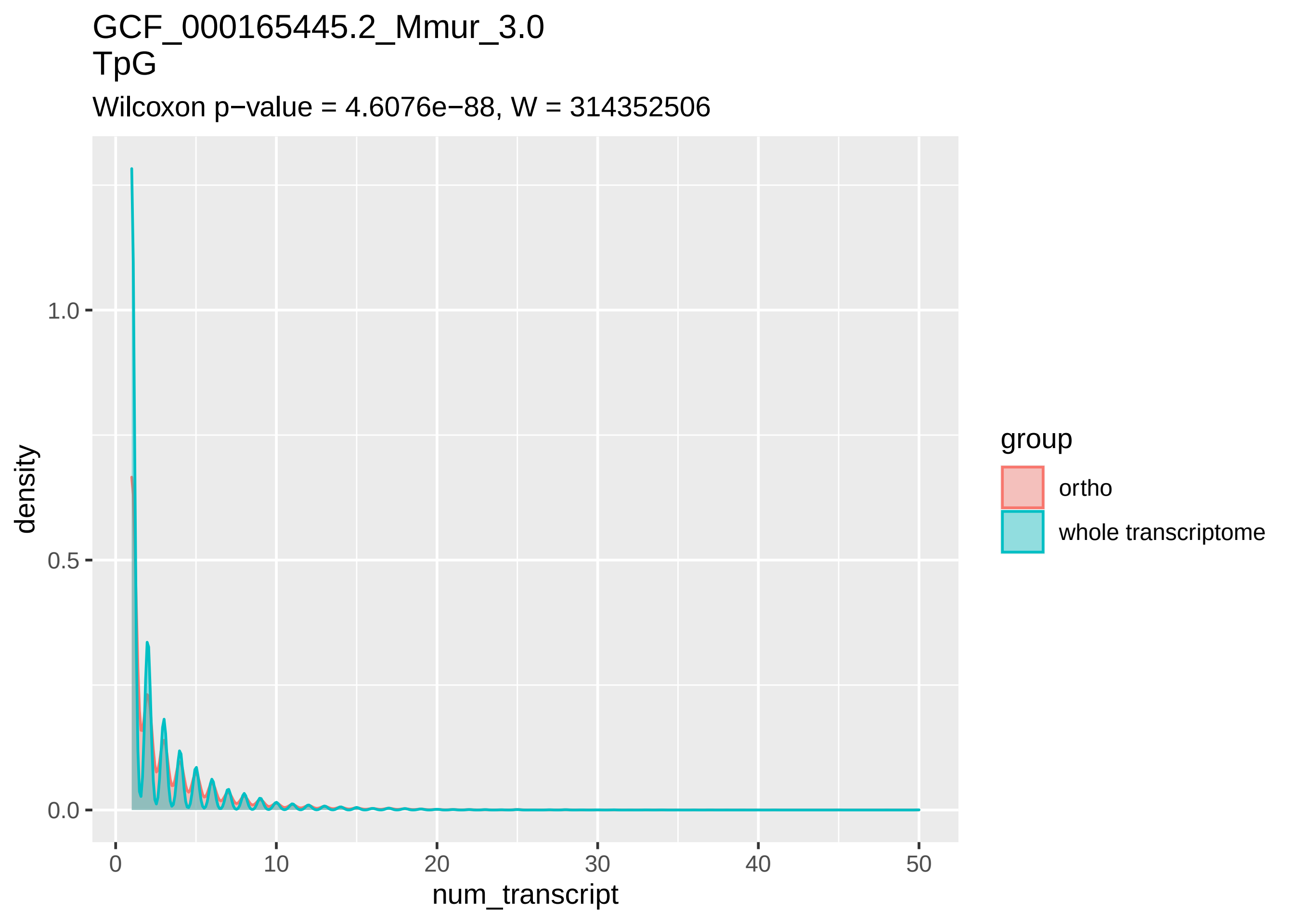
GCF_000165445.2_Mmur_3.0
TpG
Wilcoxon p−value = 4.6076e−88, W = 314352506
density
1.0
0.5
0.0
0
10
20
30
40
50
num_transcript
group
ortho
whole transcriptome

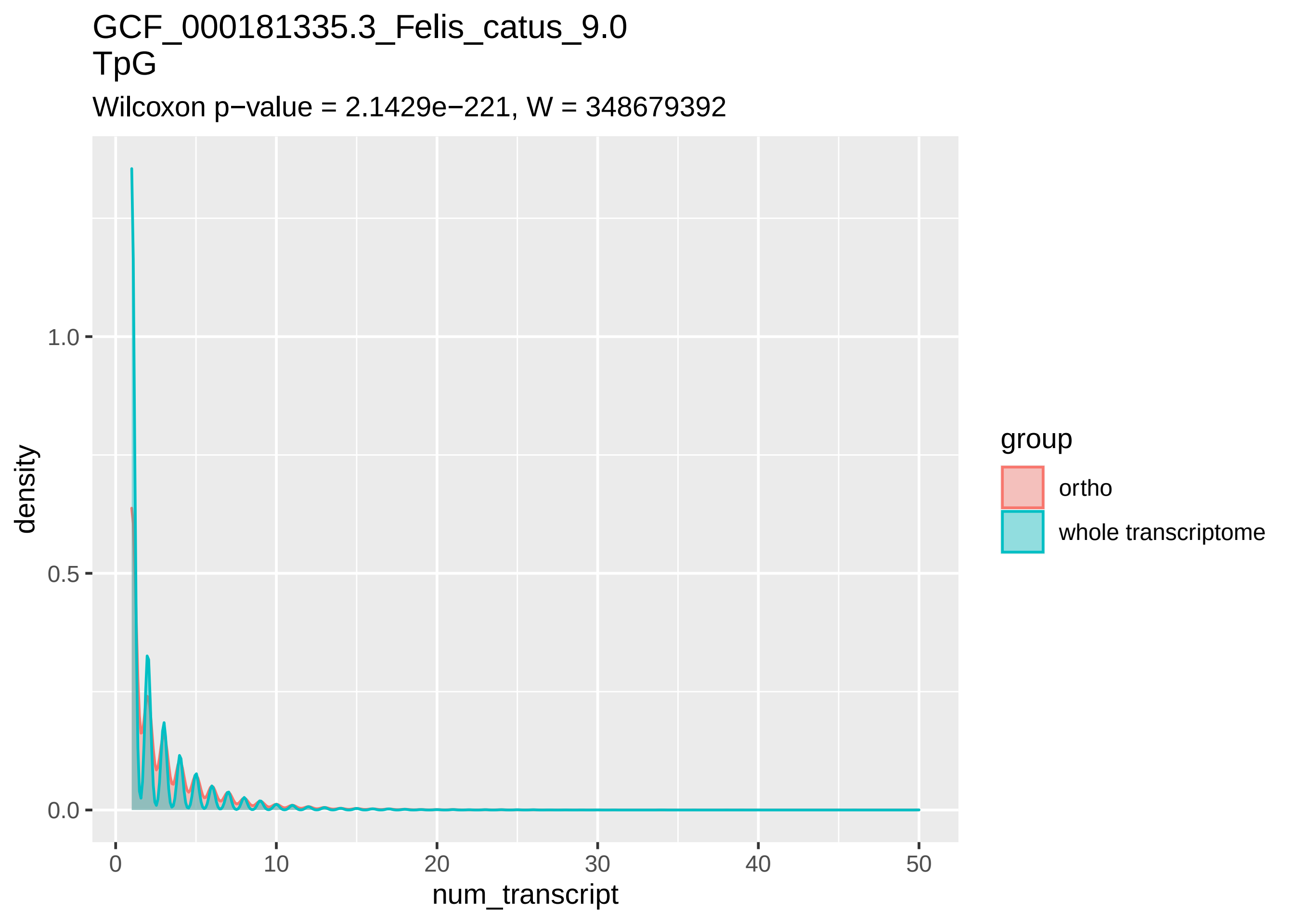
GCF_000181335.3_Felis_catus_9.0
TpG
Wilcoxon p−value = 2.1429e−221, W = 348679392
density
1.0
0.5
0.0
0
10
20
30
40
50
num_transcript
group
ortho
whole transcriptome

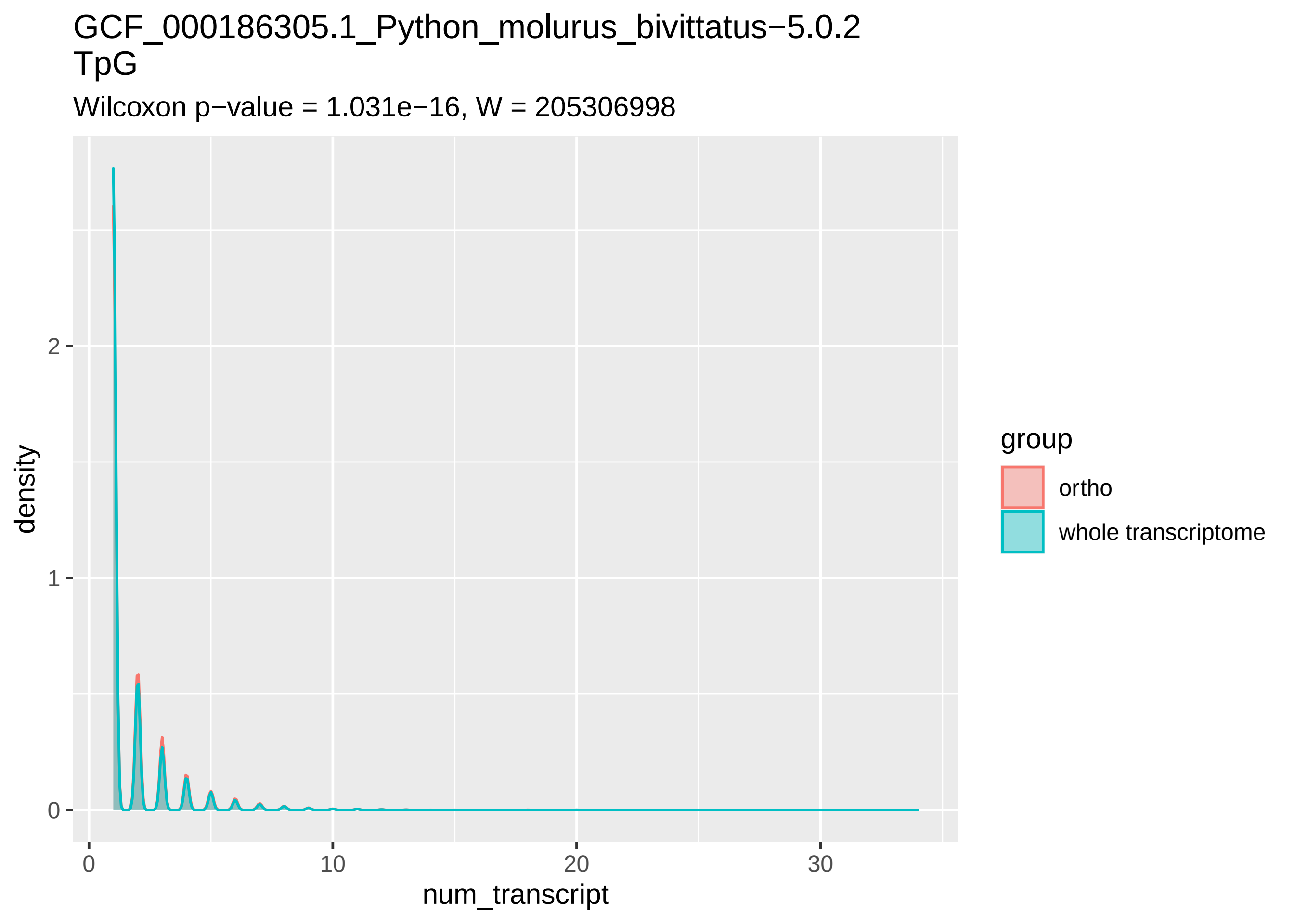
GCF_000186305.1_Python_molurus_bivittatus−5.0.2
TpG
Wilcoxon p−value = 1.031e−16, W = 205306998
density
2
1
0
0
10
20
30
num_transcript
group
ortho
whole transcriptome

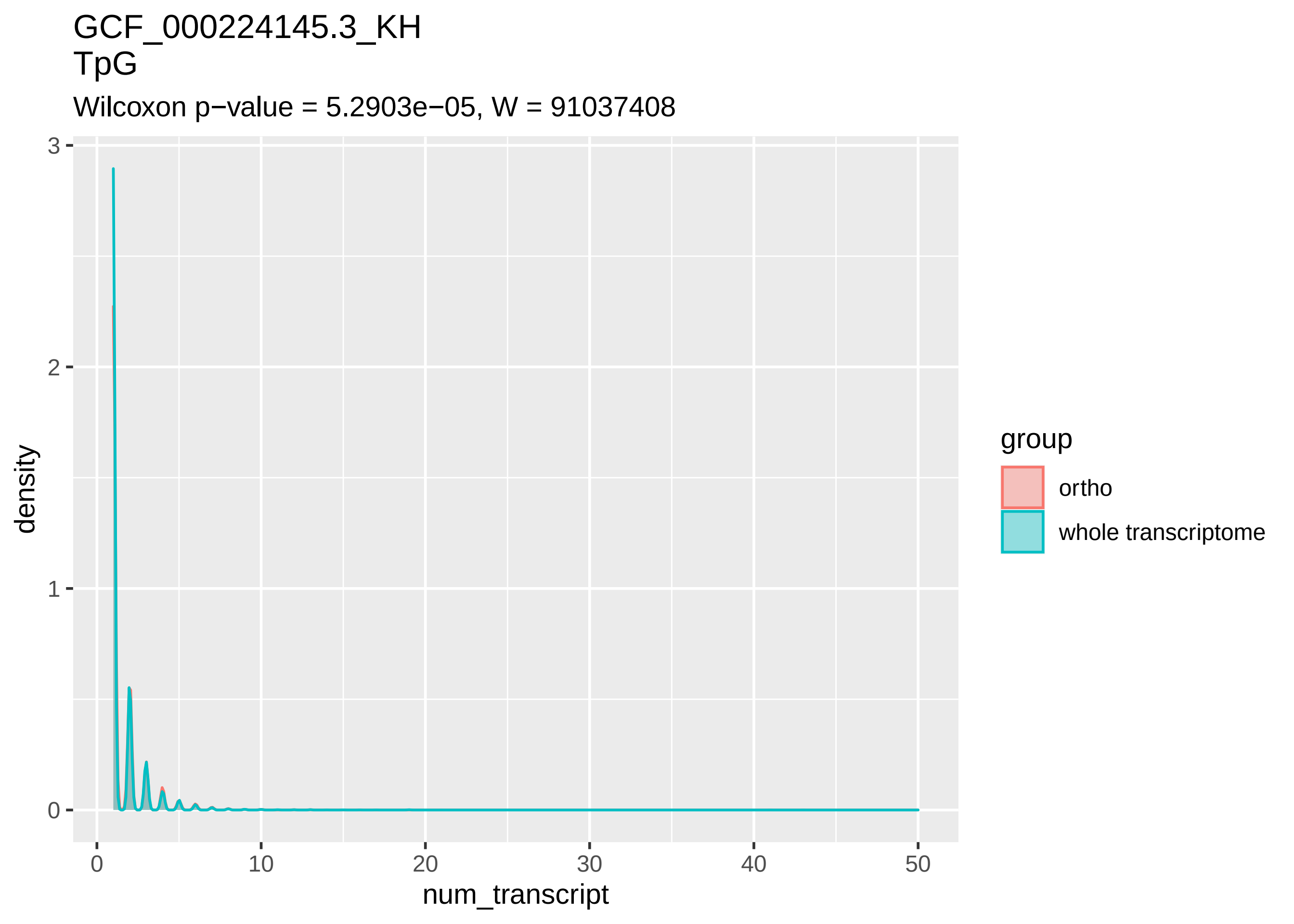

GCF_000224145.3_KH
TpG
Wilcoxon p−value = 5.2903e−05, W = 91037408
density
num_transcript
0
1
2
3
0
10
20
30
40
50
group
ortho
whole transcriptome

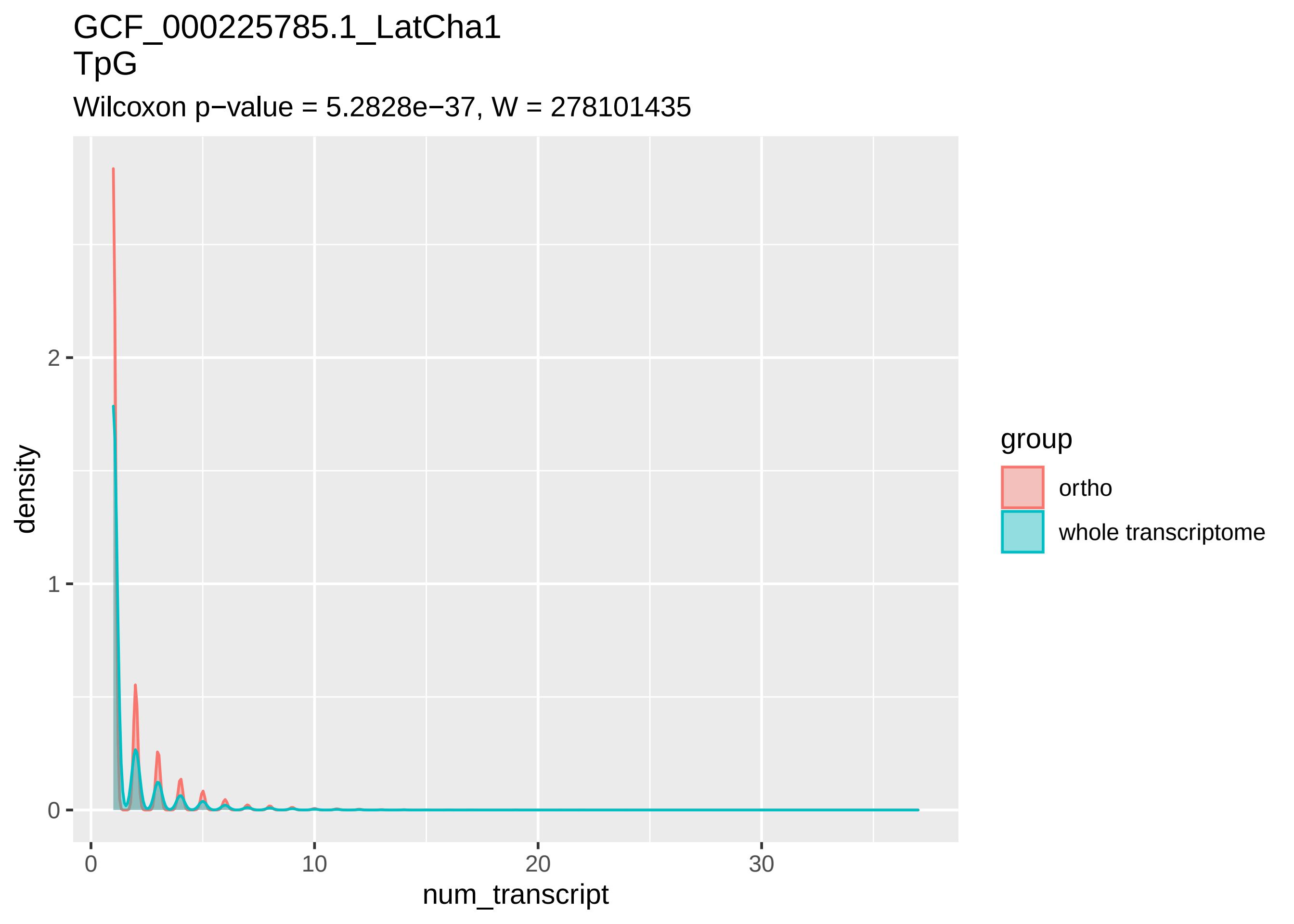
GCF_000225785.1_LatCha1
TpG
Wilcoxon p−value = 5.2828e−37, W = 278101435
density
num_transcript
0
1
2
0
10
20
30
group
ortho
whole transcriptome

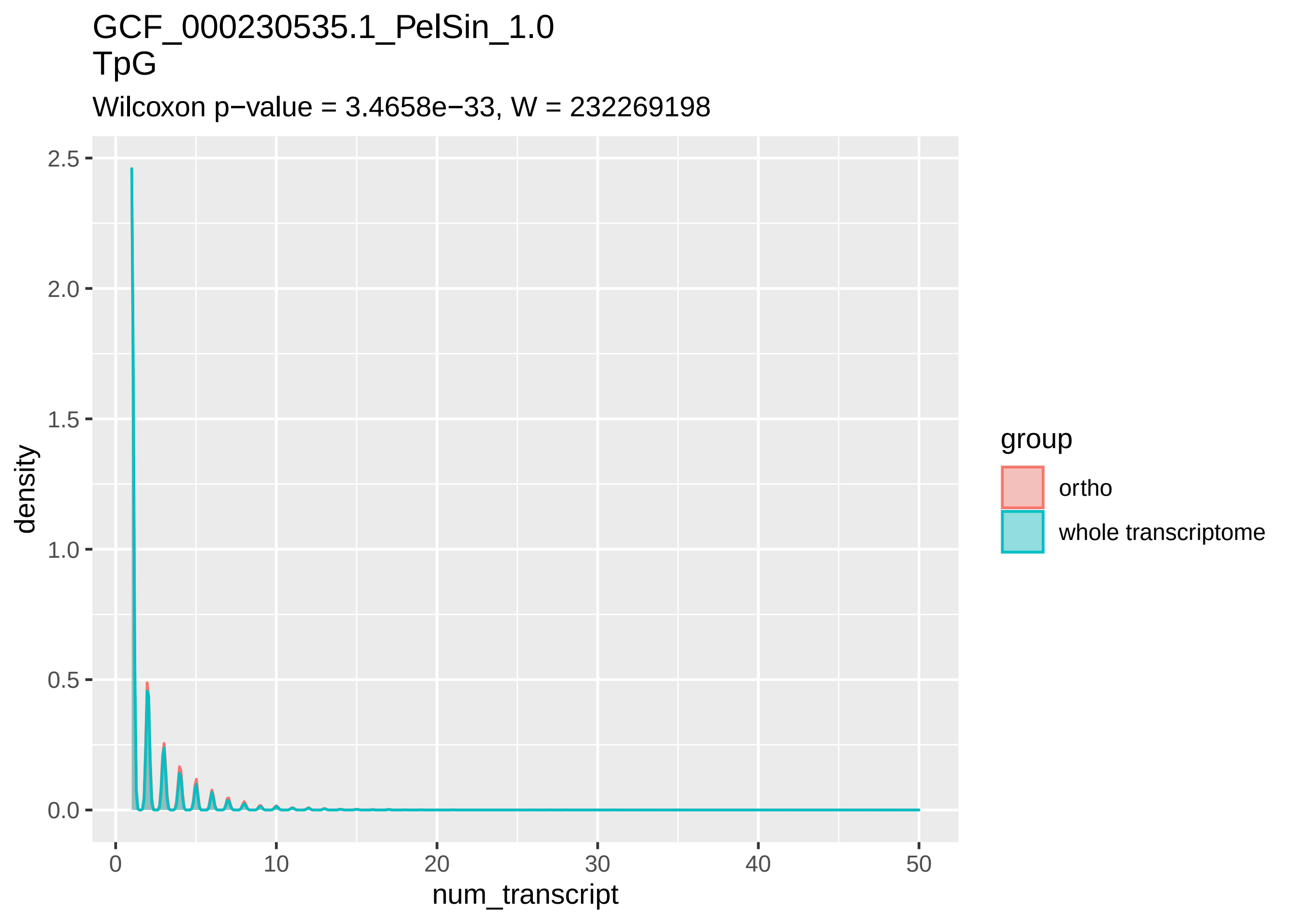

GCF_000230535.1_PelSin_1.0
TpG
Wilcoxon p−value = 3.4658e−33, W = 232269198
density
2.5
2.0
1.5
1.0
0.5
0.0
0
10
20
30
40
50
num_transcript
group
ortho
whole transcriptome

GCF_000281125.3_ASM28112v4
TpG

Wilcoxon p−value = 3.6583e−46, W = 2.46e+08

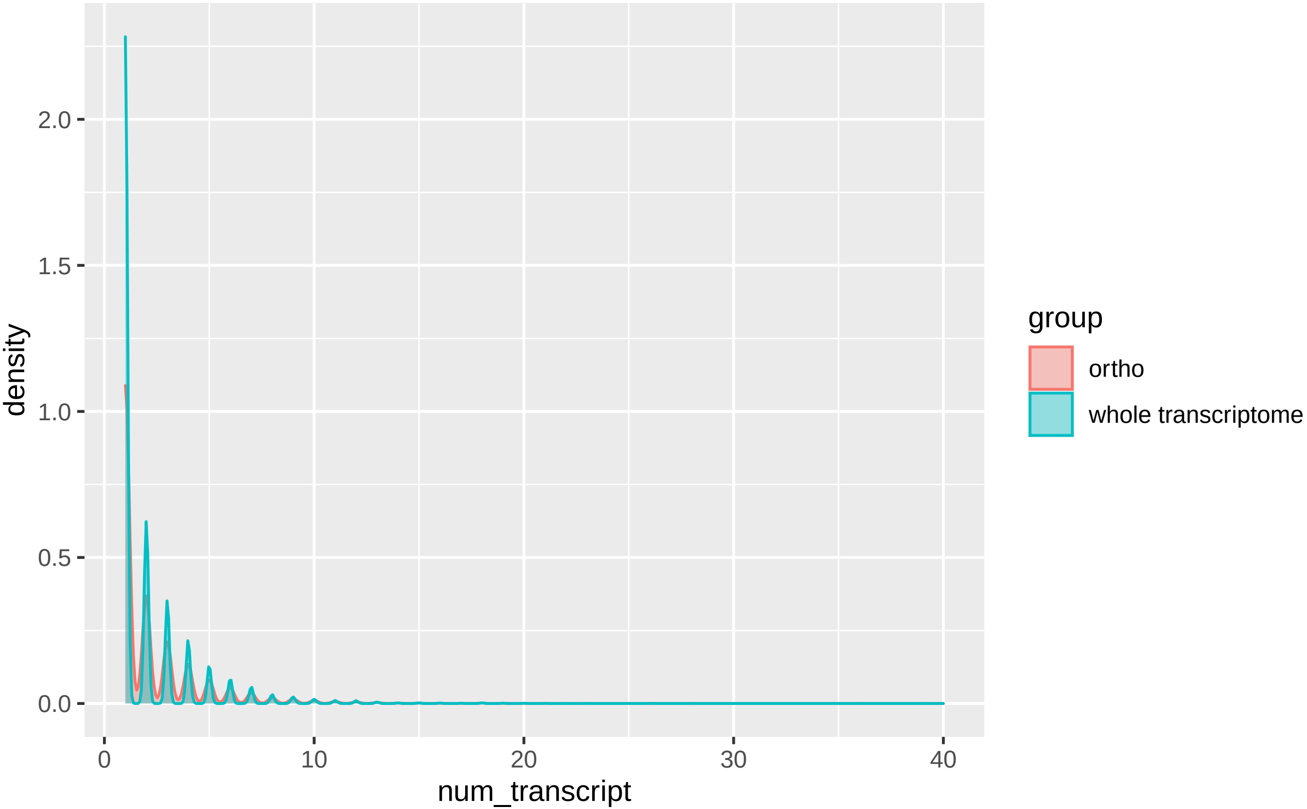

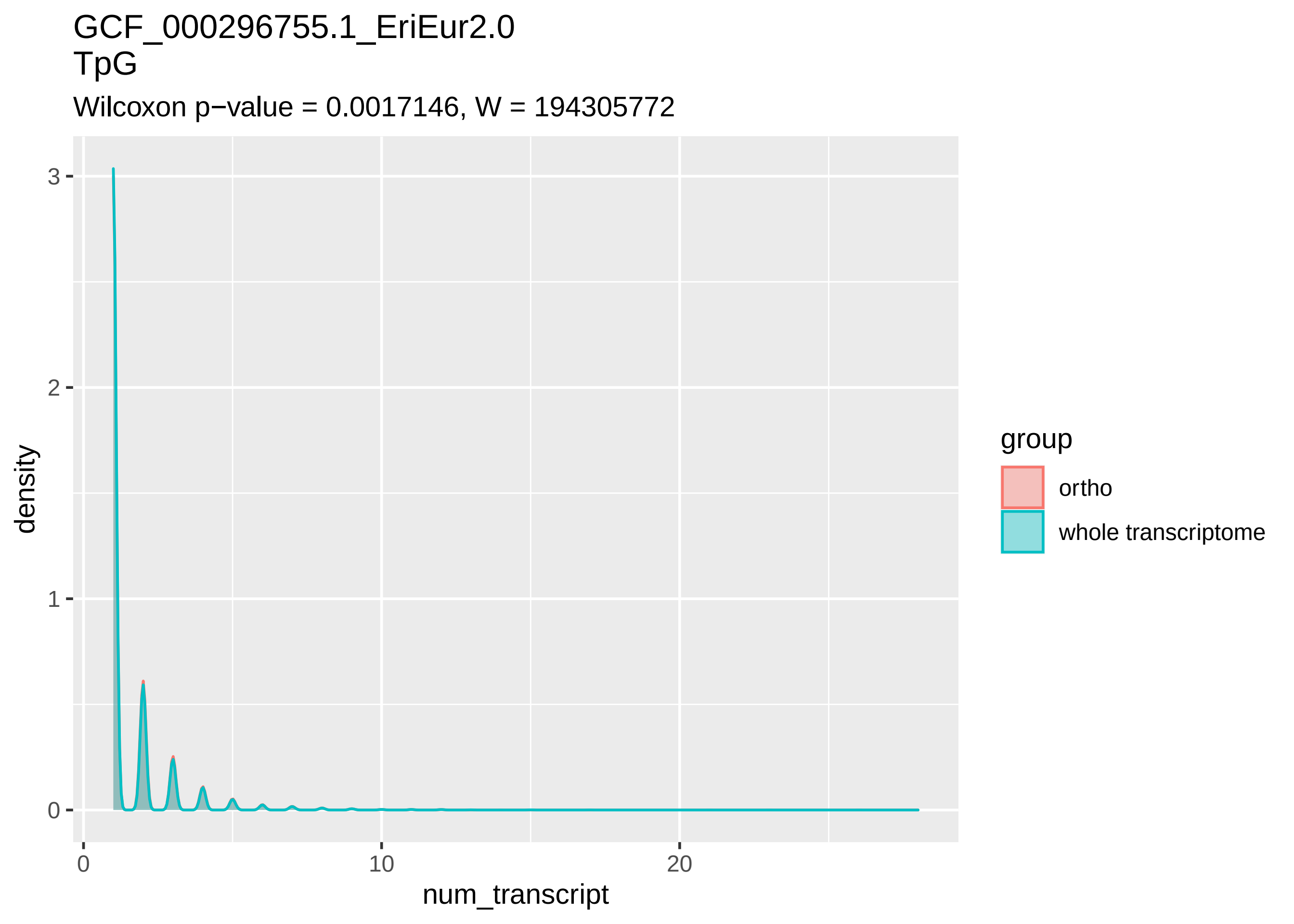

GCF_000296755.1_EriEur2.0
TpG
Wilcoxon p−value = 0.0017146, W = 194305772
density
3
2
1
0
0
10
20
num_transcript
group
ortho
whole transcriptome

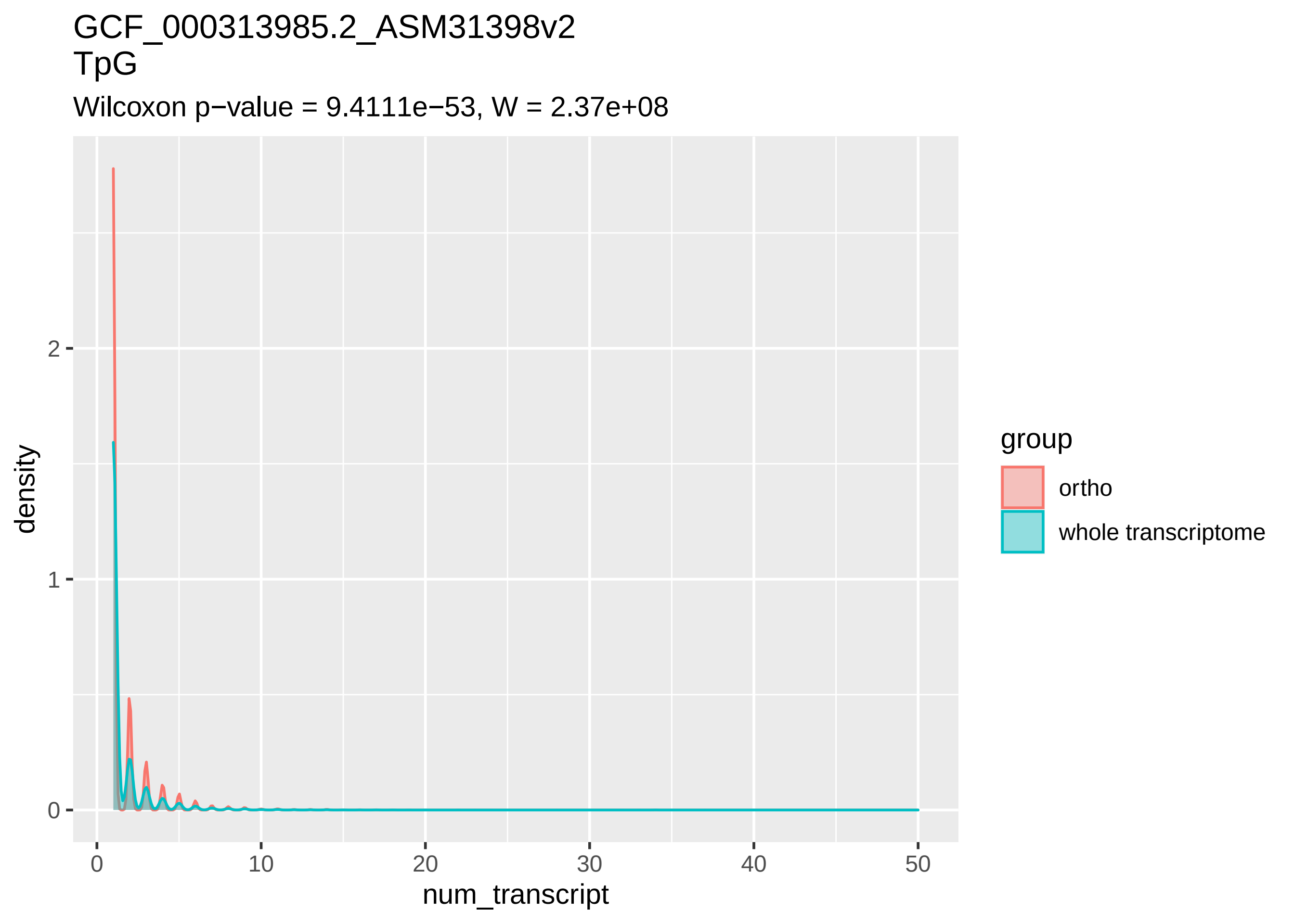

GCF_000313985.2_ASM31398v2
TpG
Wilcoxon p−value = 9.4111e−53, W = 2.37e+08
density
num_transcript
0
1
2
0
10
20
30
40
50
group
ortho
whole transcriptome

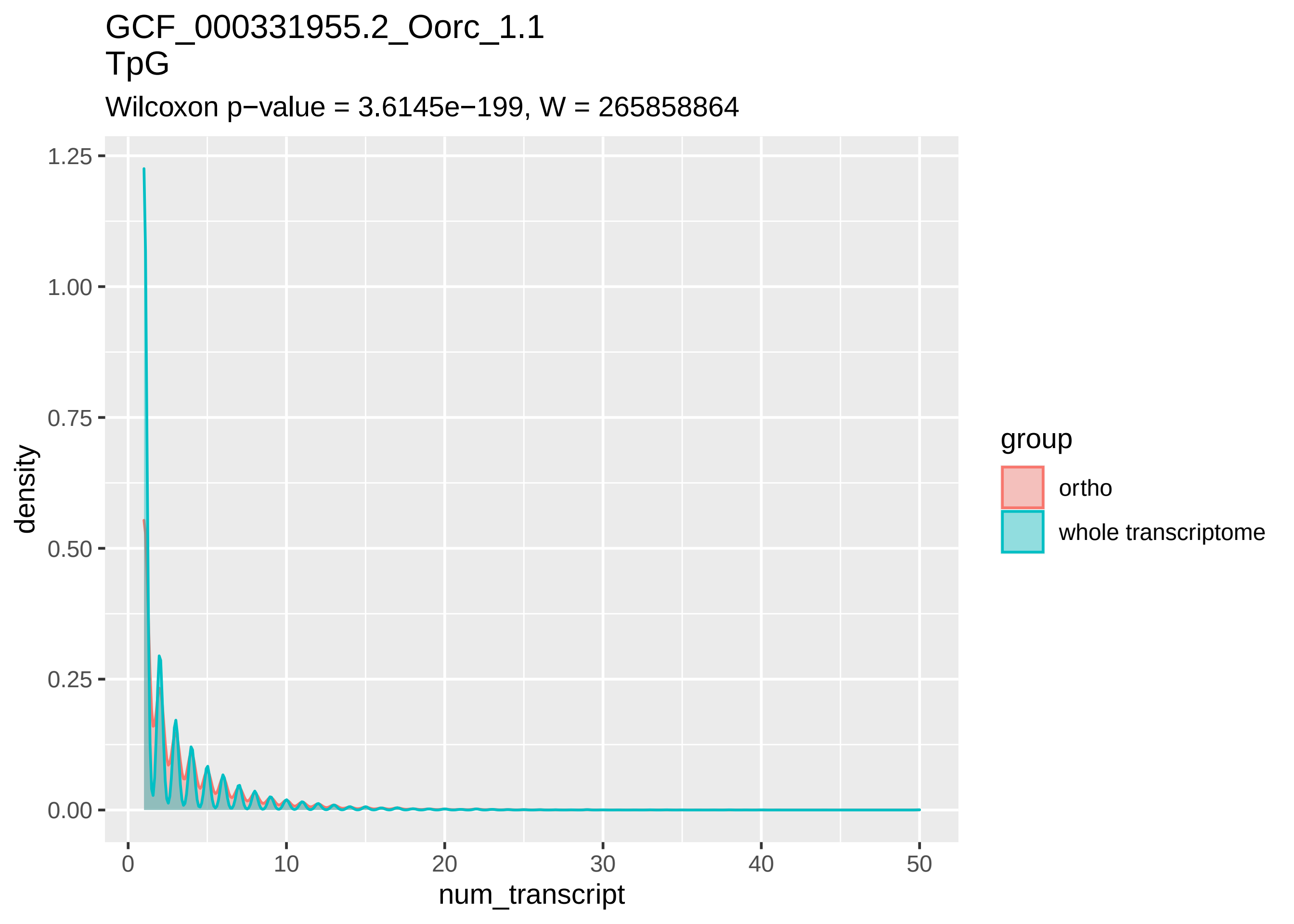
GCF_000331955.2_Oorc_1.1
TpG
Wilcoxon p−value = 3.6145e−199, W = 265858864
density
num_transcript
1.25
1.00
0.75
0.50
0.25
0.00
0
10
20
30
40
50
group
ortho
whole transcriptome

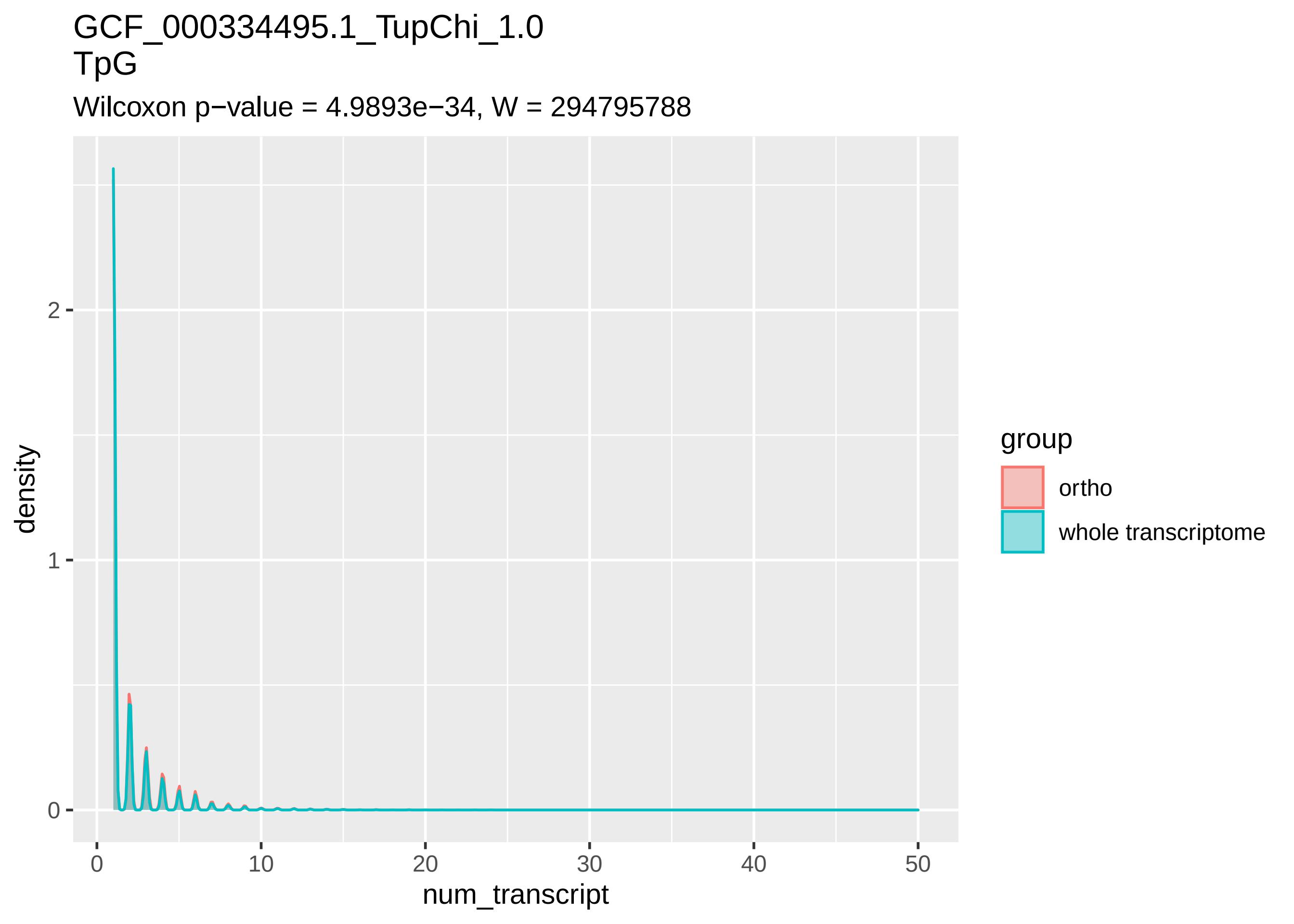
GCF_000334495.1_TupChi_1.0
TpG
Wilcoxon p−value = 4.9893e−34, W = 294795788
density
num_transcript
0
1
2
0
10
20
30
40
50
group
ortho
whole transcriptome

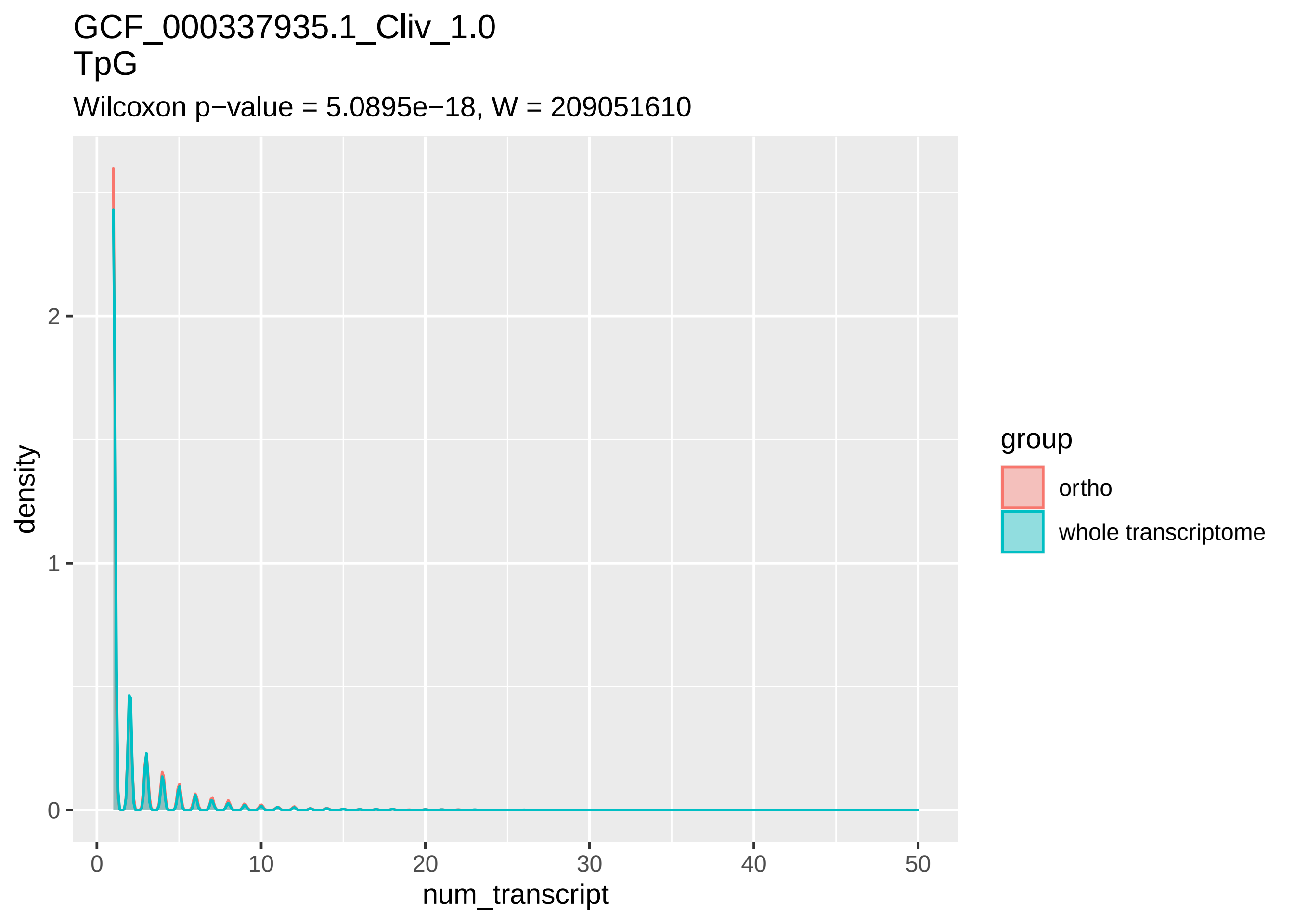
GCF_000337935.1_Cliv_1.0
TpG
Wilcoxon p−value = 5.0895e−18, W = 209051610
density
num_transcript
0
1
2
0
10
20
30
40
50
group
ortho
whole transcriptome

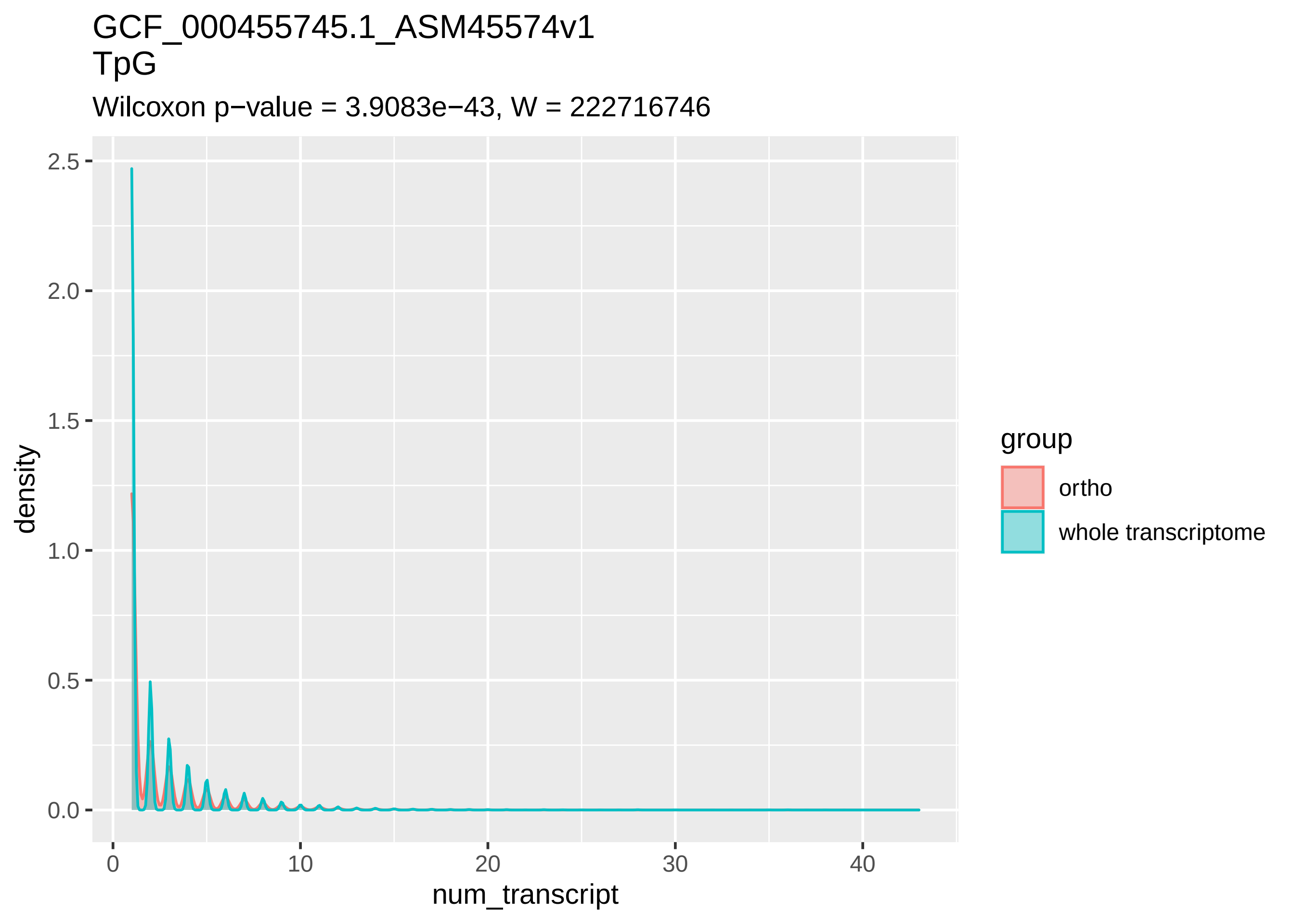
GCF_000455745.1_ASM45574v1
TpG
Wilcoxon p−value = 3.9083e−43, W = 222716746
density
2.5
2.0
1.5
1.0
0.5
0.0
0
10
20
30
40
num_transcript
group
ortho
whole transcriptome

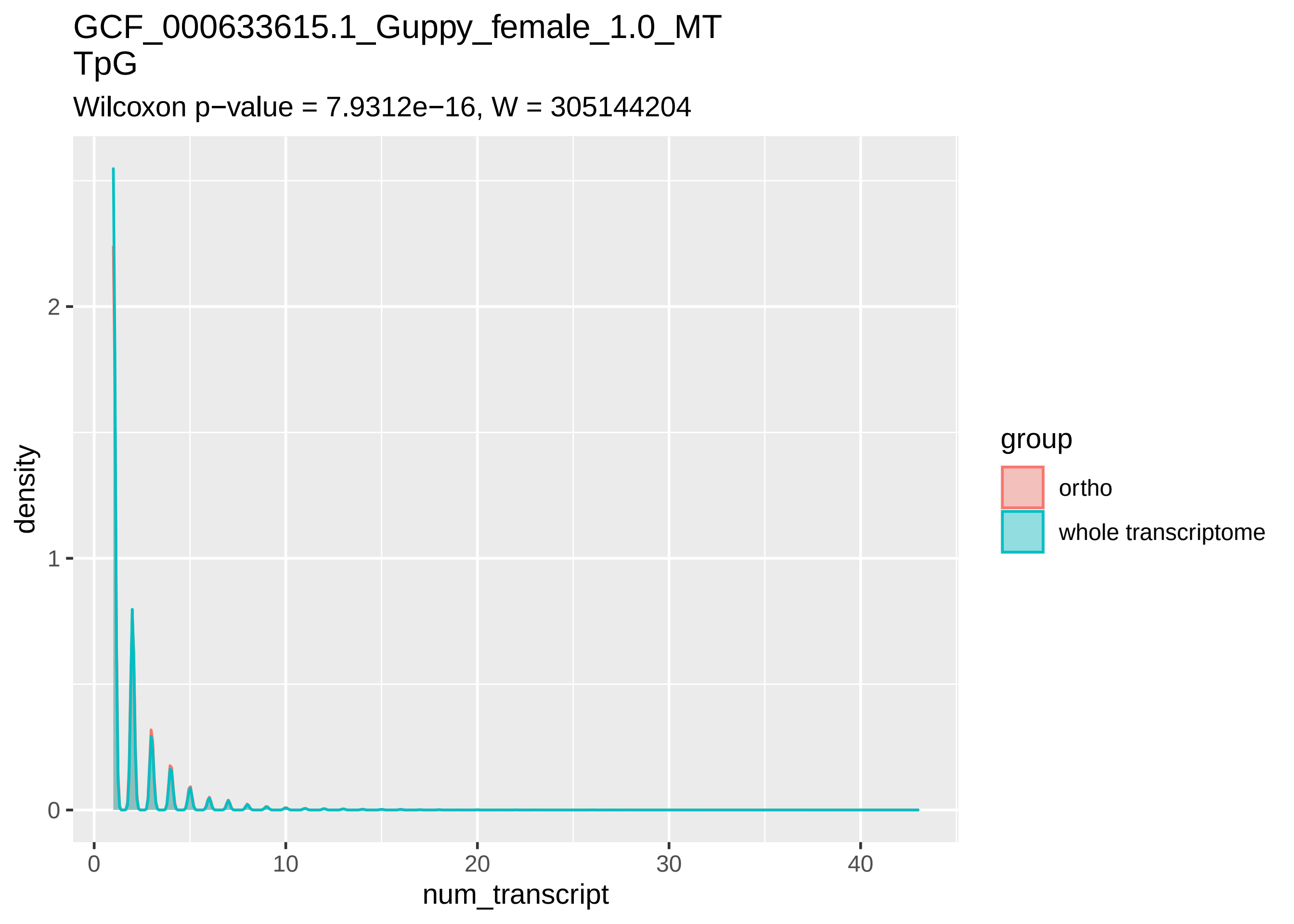
GCF_000633615.1_Guppy_female_1.0_MT
TpG
Wilcoxon p−value = 7.9312e−16, W = 305144204
density
num_transcript
0
1
2
0
10
20
30
40
group
ortho
whole transcriptome

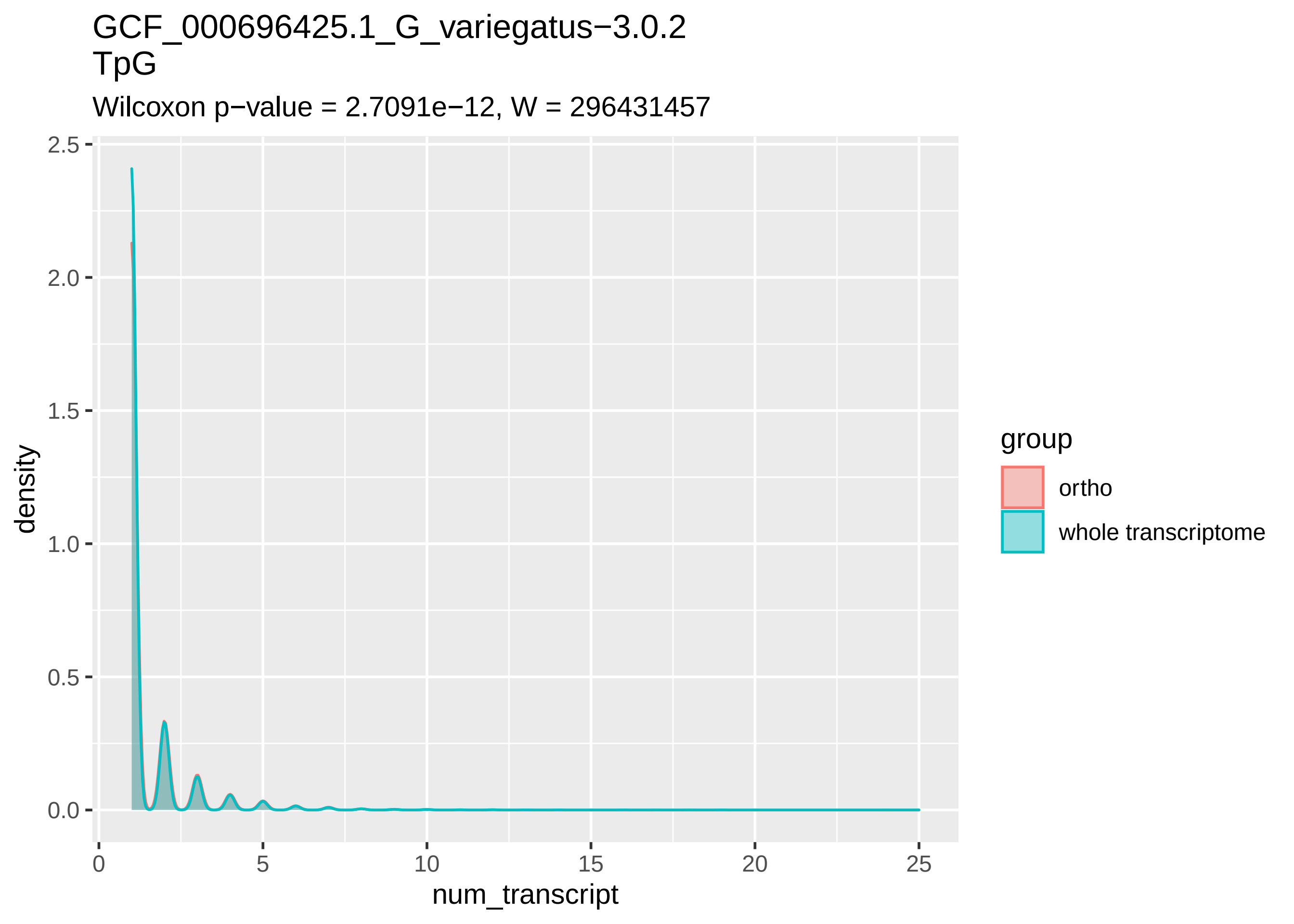
GCF_000696425.1_G_variegatus−3.0.2
TpG
Wilcoxon p−value = 2.7091e−12, W = 296431457
density
2.5
2.0
1.5
1.0
0.5
0.0
0
5
10
15
20
25
num_transcript
group
ortho
whole transcriptome

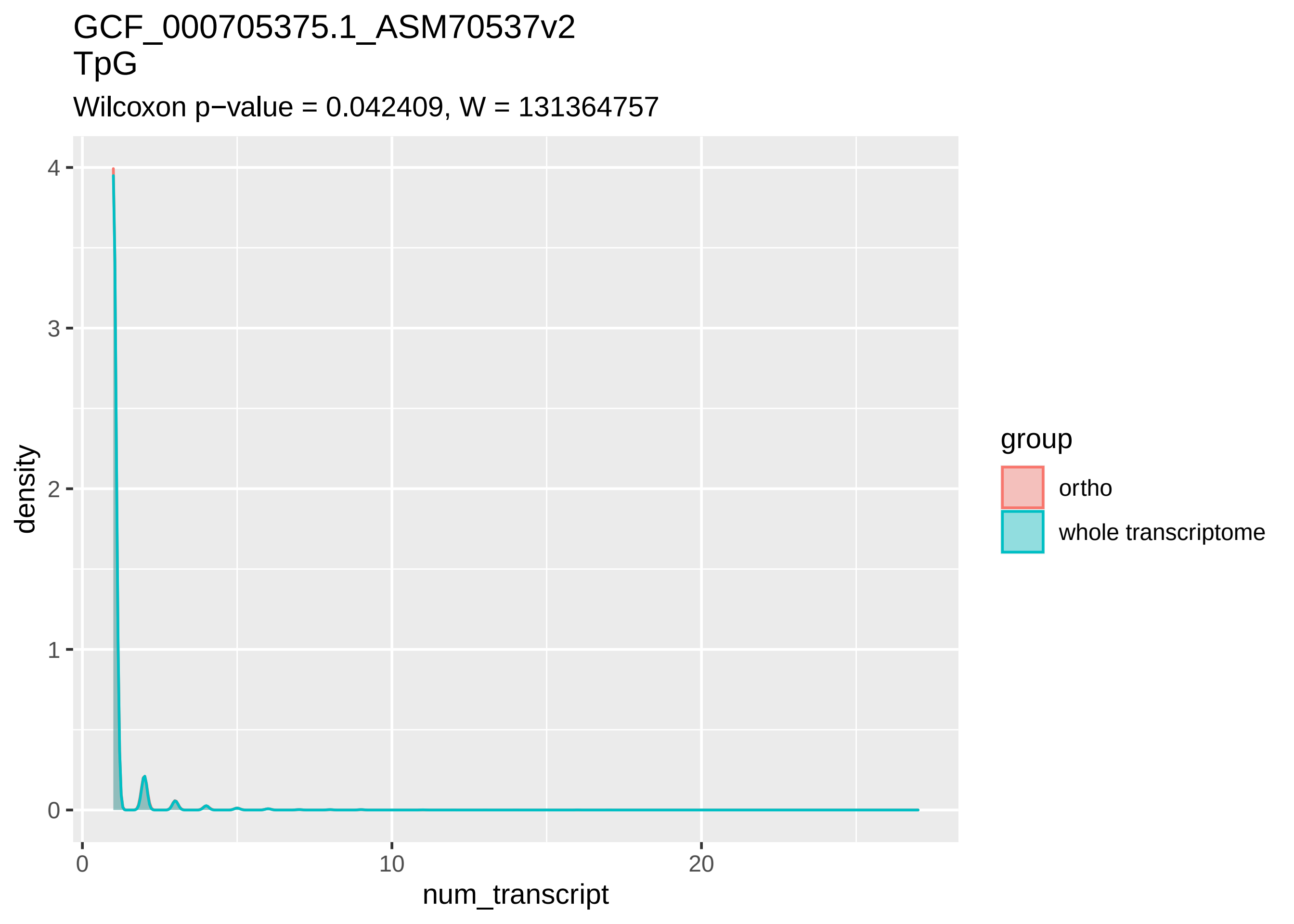
GCF_000705375.1_ASM70537v2
TpG
Wilcoxon p−value = 0.042409, W = 131364757
density
4
3
2
1
0
0
10
20
num_transcript
group
ortho
whole transcriptome

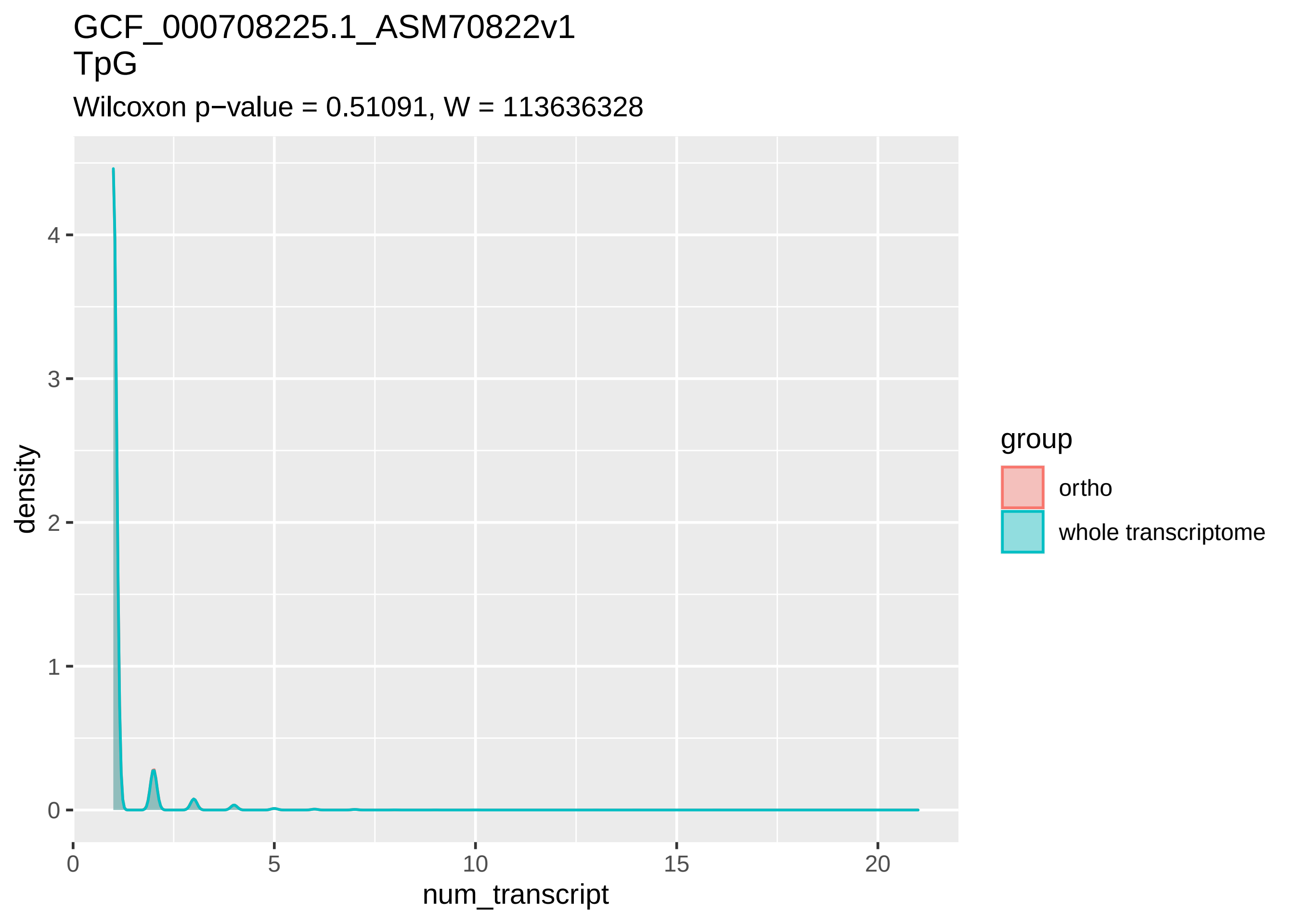
GCF_000708225.1_ASM70822v1
TpG
Wilcoxon p−value = 0.51091, W = 113636328
density
num_transcript
0
1
2
3
4
0
5
10
15
20
group
ortho
whole transcriptome

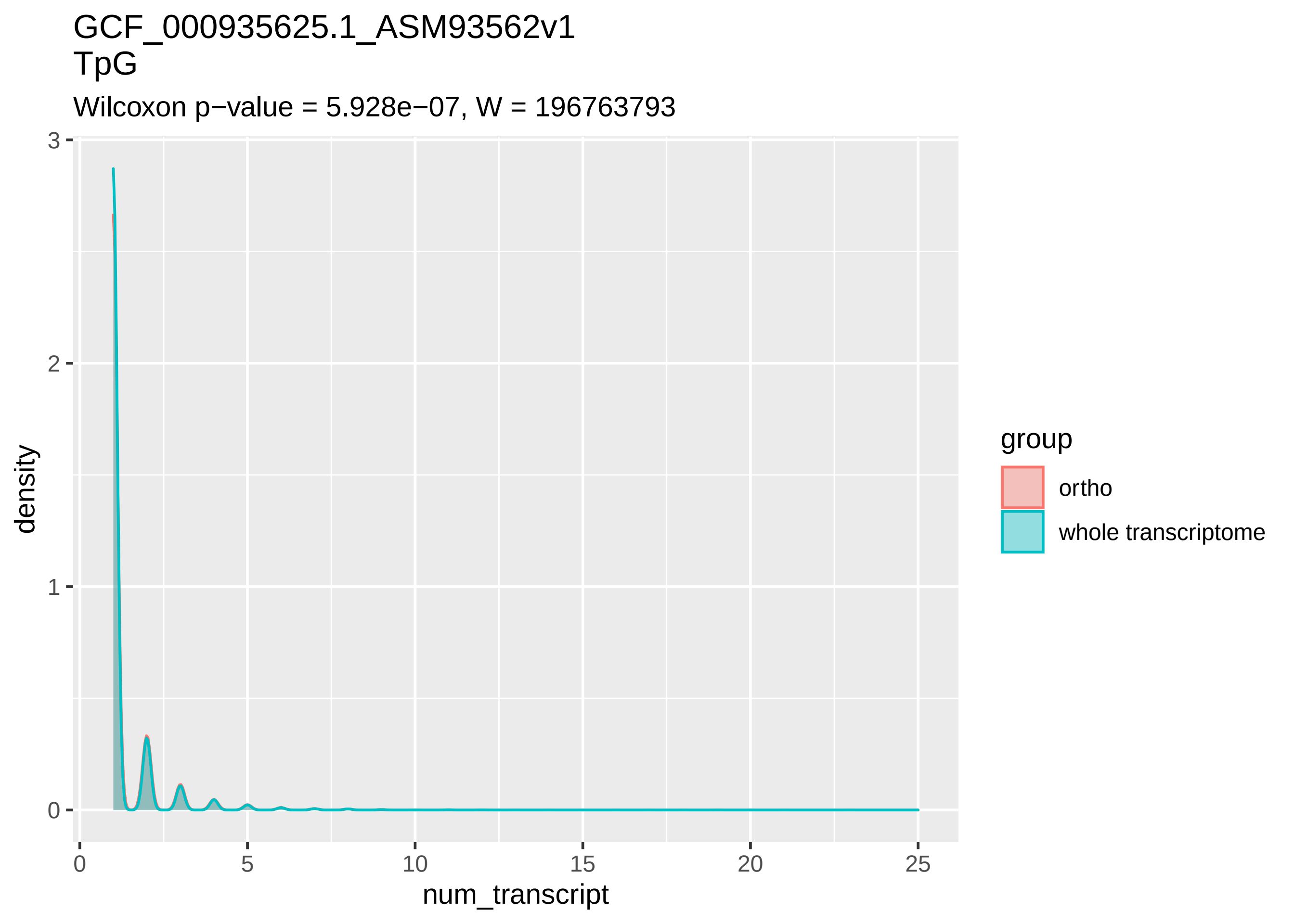
GCF_000935625.1_ASM93562v1
TpG
Wilcoxon p−value = 5.928e−07, W = 196763793
density
num_transcript
0
1
2
3
0
5
10
15
20
25
group
ortho
whole transcriptome

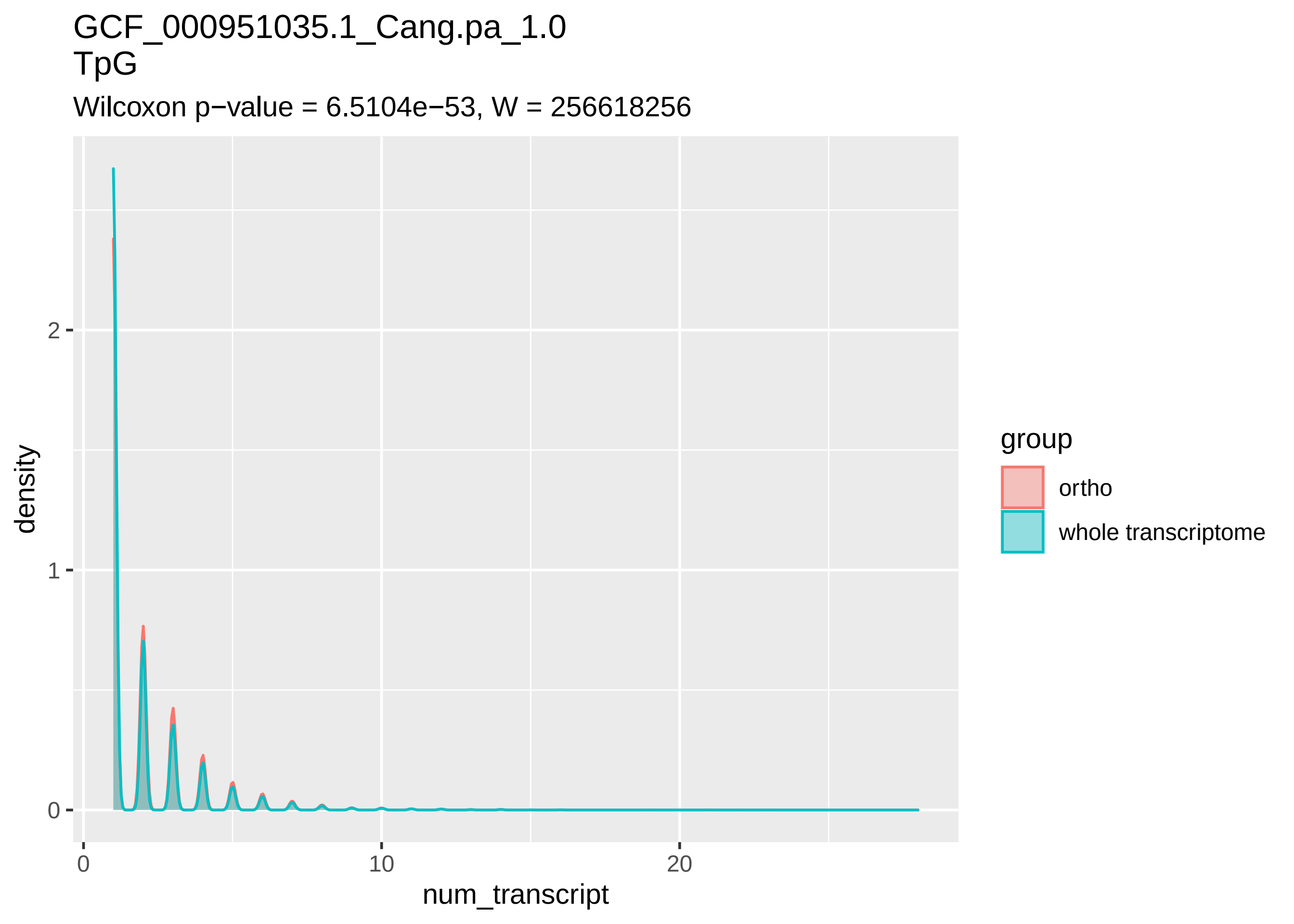

GCF_000951035.1_Cang.pa_1.0
TpG
Wilcoxon p−value = 6.5104e−53, W = 256618256
density
num_transcript
0
1
2
0
10
20
group
ortho
whole transcriptome

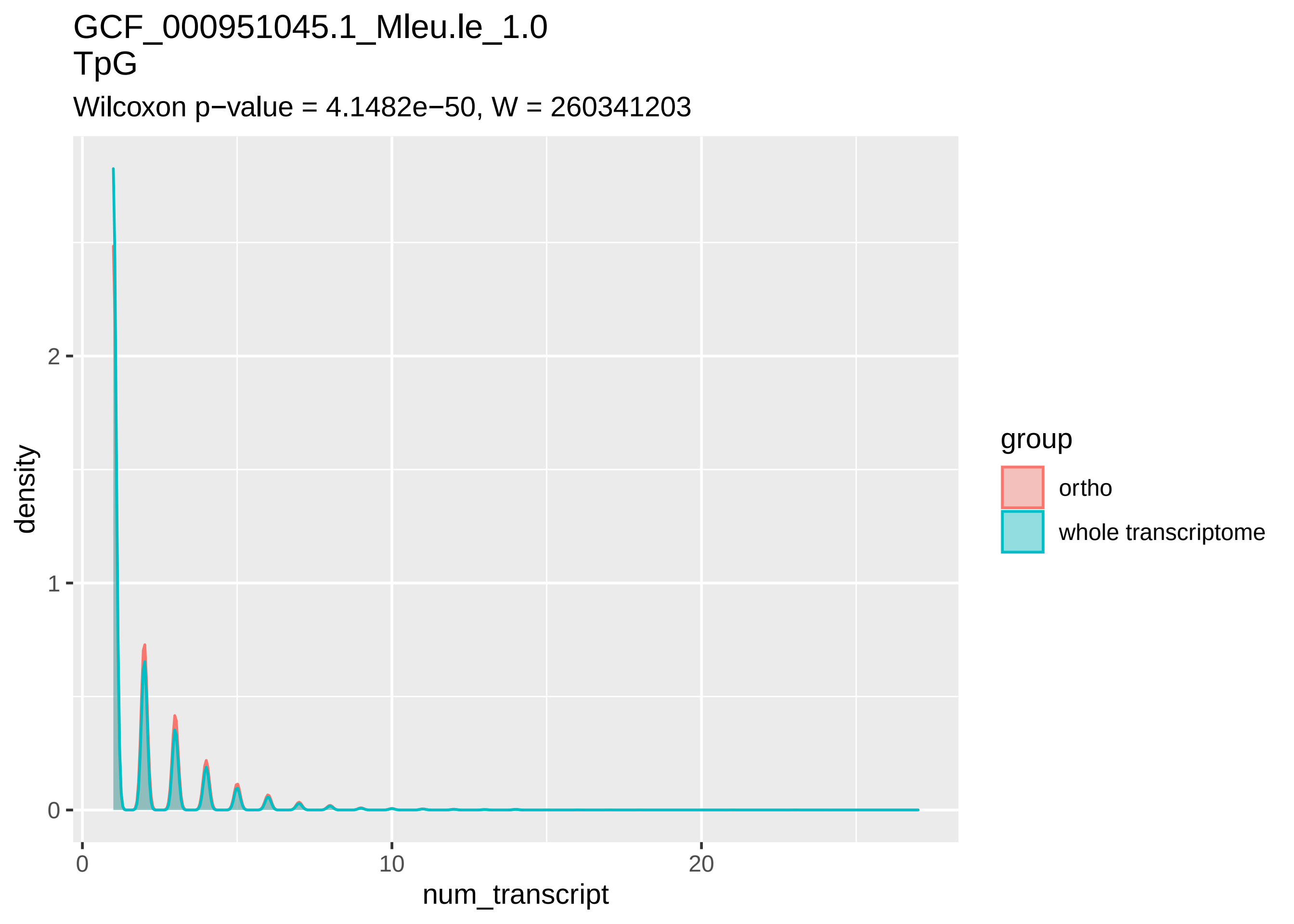
GCF_000951045.1_Mleu.le_1.0
TpG
Wilcoxon p−value = 4.1482e−50, W = 260341203
density
num_transcript
0
1
2
0
10
20
group
ortho
whole transcriptome

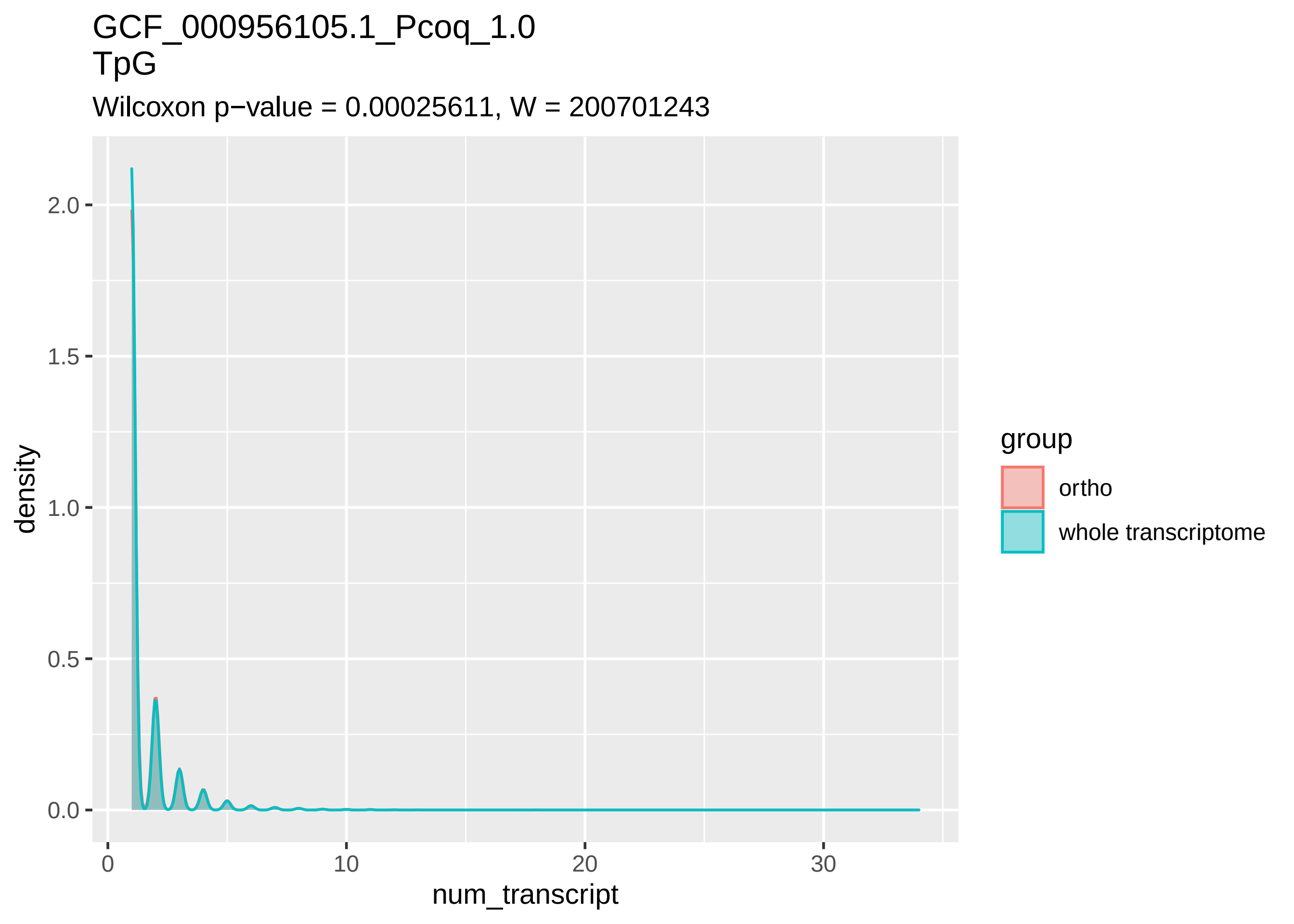
GCF_000956105.1_Pcoq_1.0
TpG
Wilcoxon p−value = 0.00025611, W = 200701243
density
2.0
1.5
1.0
0.5
0.0
0
10
20
30
num_transcript
group
ortho
whole transcriptome

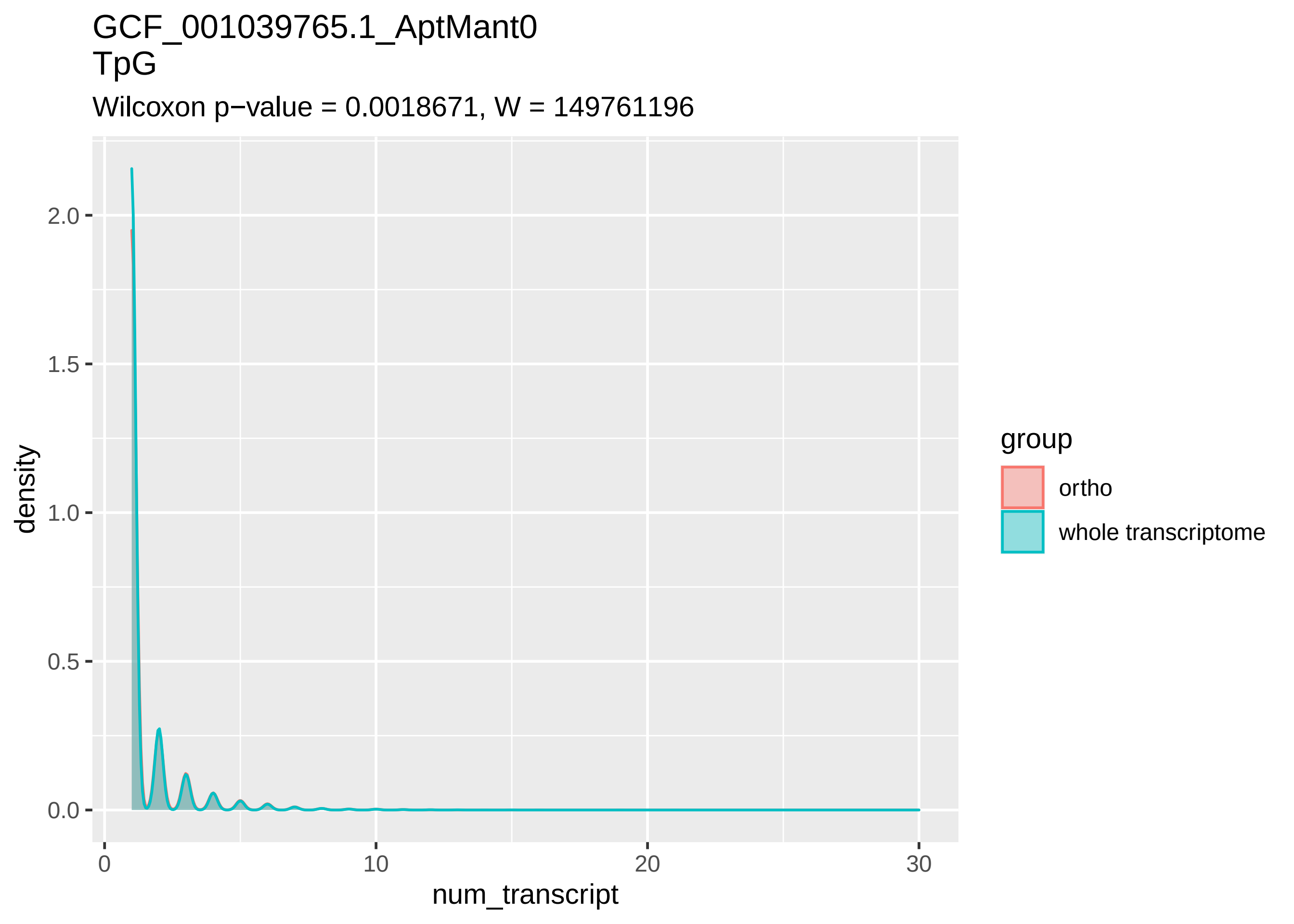
GCF_001039765.1_AptMant0
TpG
Wilcoxon p−value = 0.0018671, W = 149761196
density
2.0
1.5
1.0
0.5
0.0
0
10
20
30
num_transcript
group
ortho
whole transcriptome

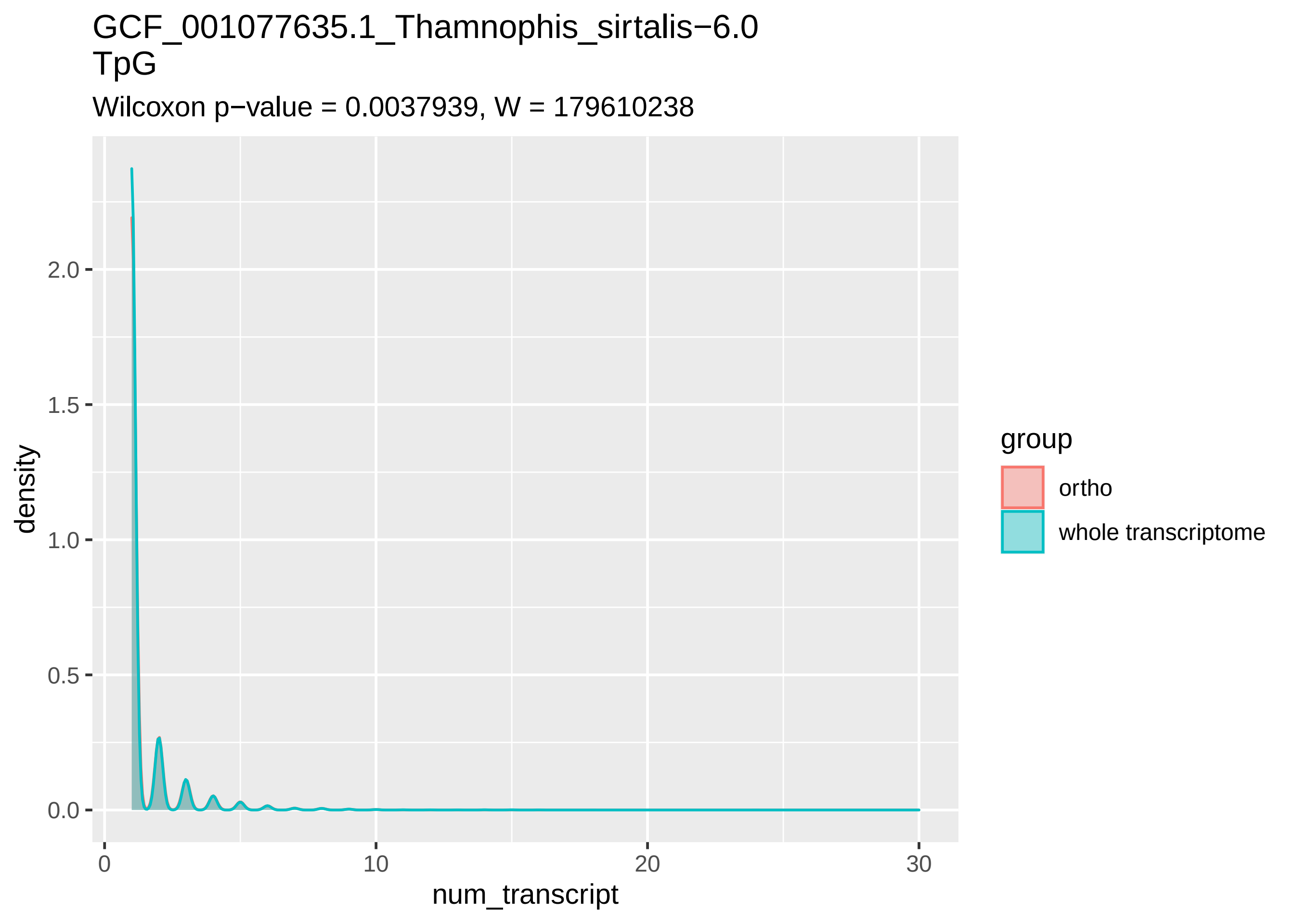

GCF_001077635.1_Thamnophis_sirtalis−6.0
TpG
Wilcoxon p−value = 0.0037939, W = 179610238
density
2.0
1.5
1.0
0.5
0.0
0
10
20
30
num_transcript
group
ortho
whole transcriptome

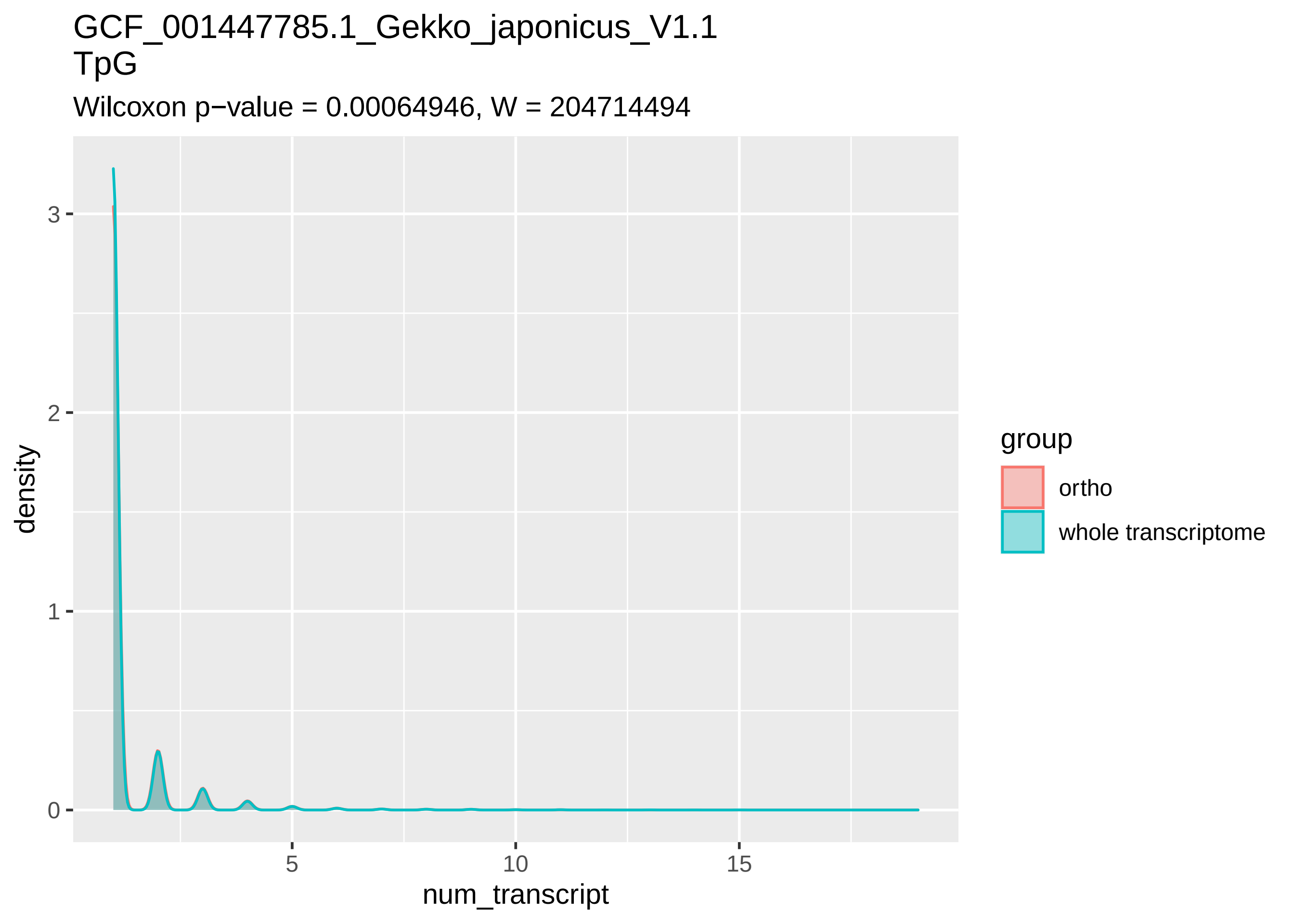
GCF_001447785.1_Gekko_japonicus_V1.1
TpG
Wilcoxon p−value = 0.00064946, W = 204714494
density
num_transcript
0
1
2
3
5
10
15
group
ortho
whole transcriptome

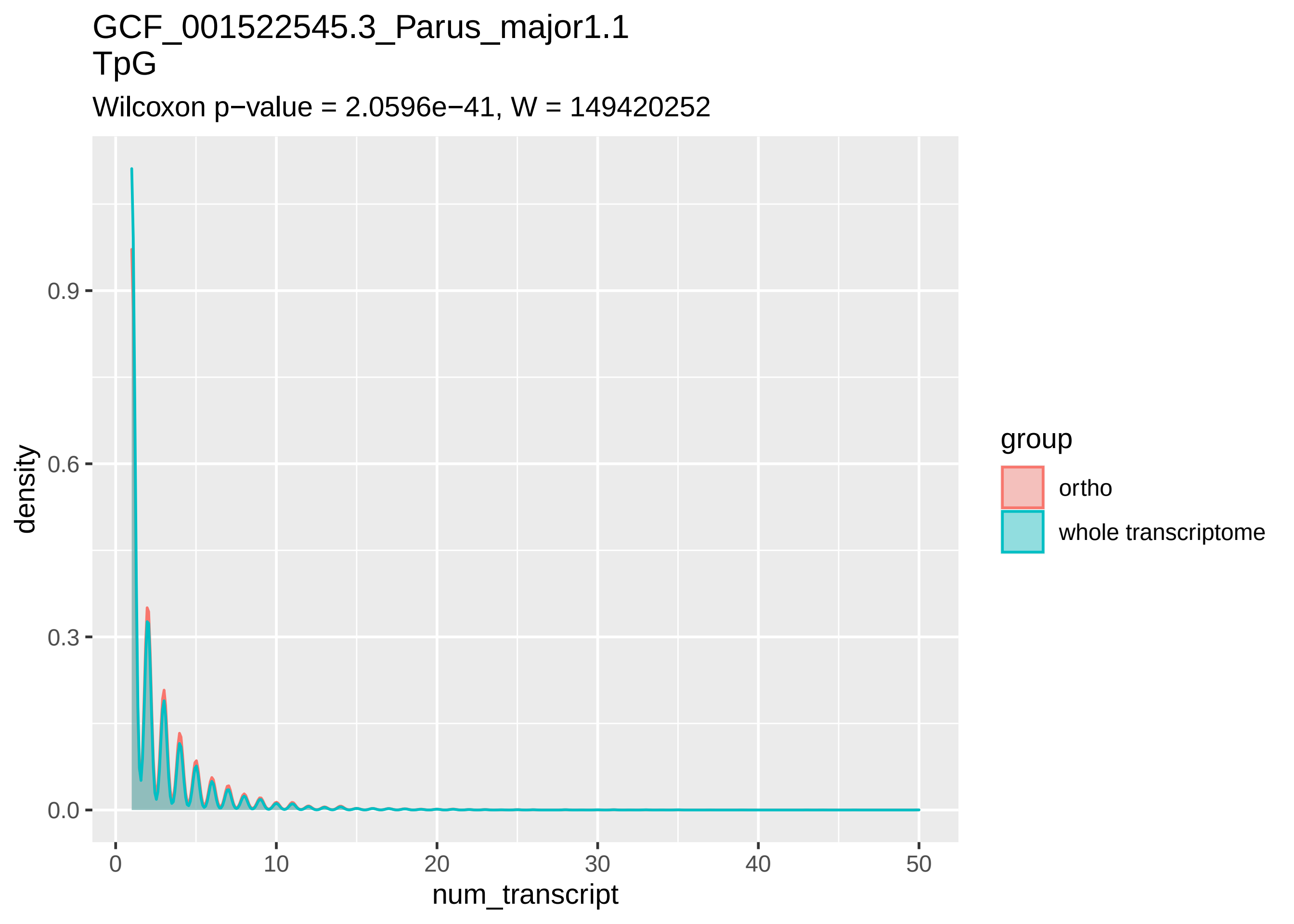

GCF_001522545.3_Parus_major1.1
TpG
Wilcoxon p−value = 2.0596e−41, W = 149420252
density
0.9
0.6
0.3
0.0
0
10
20
30
40
50
num_transcript
group
ortho
whole transcriptome

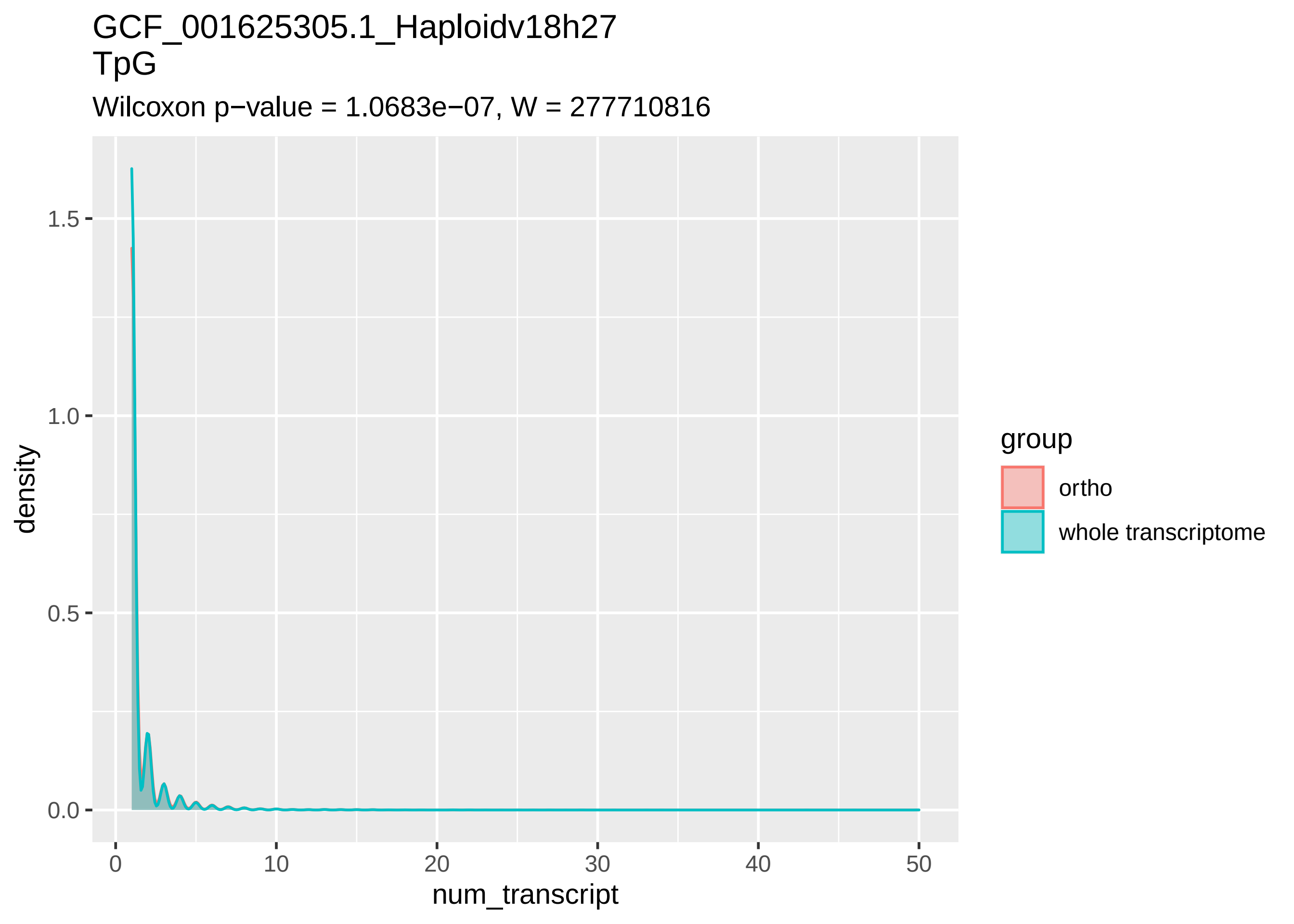
GCF_001625305.1_Haploidv18h27
TpG
Wilcoxon p−value = 1.0683e−07, W = 277710816
density
1.5
1.0
0.5
0.0
0
10
20
30
40
50
num_transcript
group
ortho
whole transcriptome

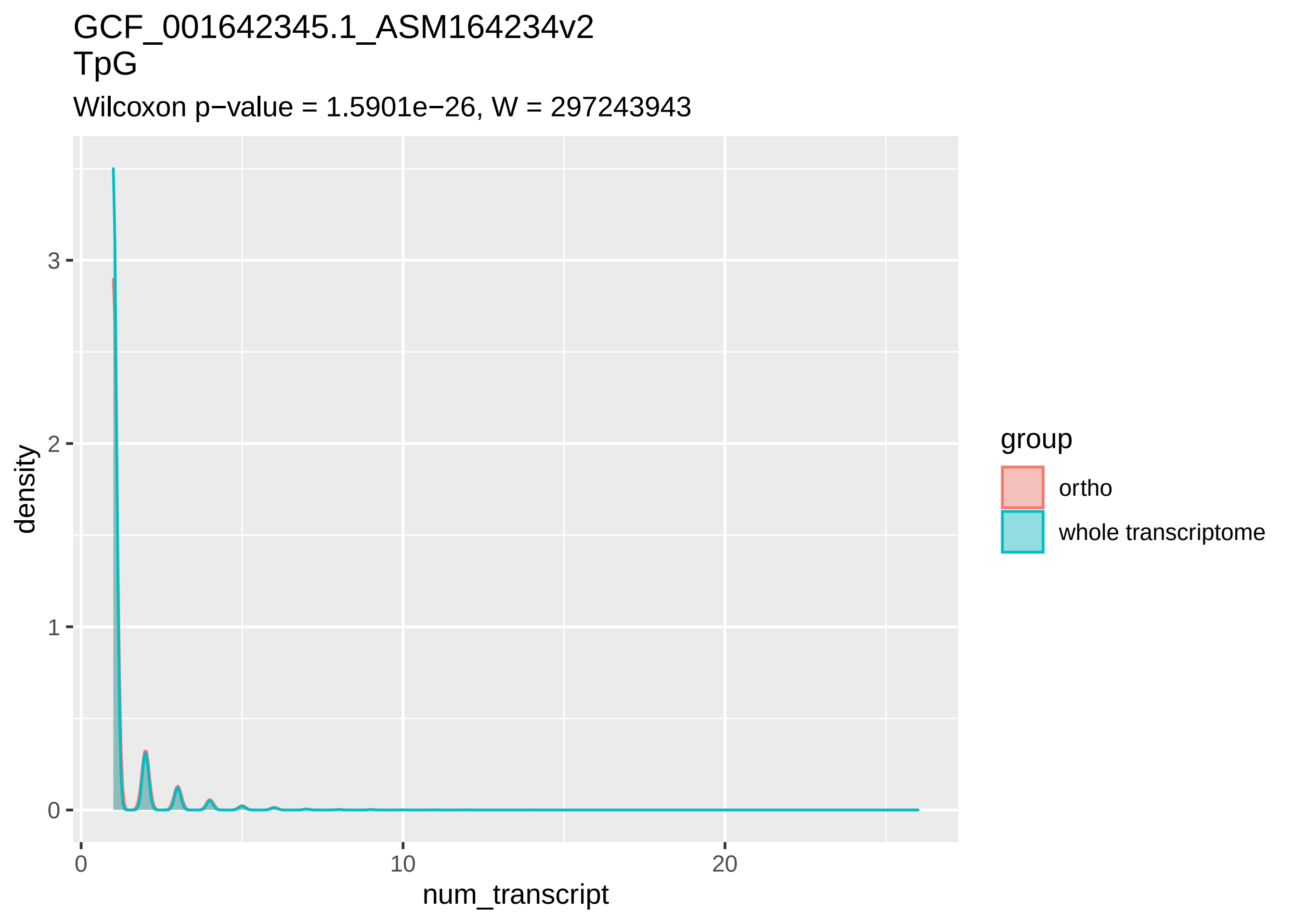
GCF_001642345.1_ASM164234v2
TpG
Wilcoxon p−value = 1.5901e−26, W = 297243943
density
num_transcript
0
1
2
3
0
10
20
group
ortho
whole transcriptome

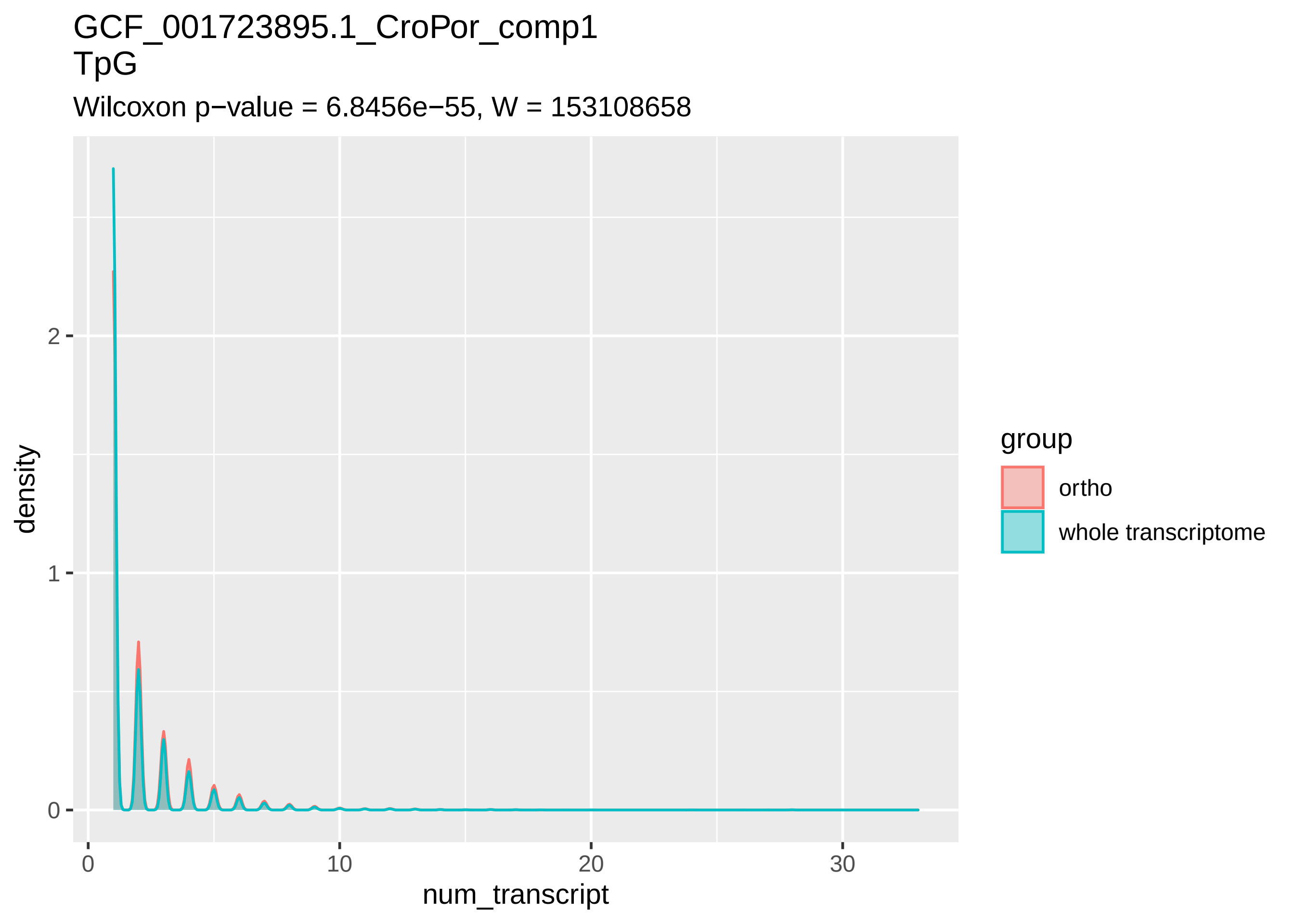
GCF_001723895.1_CroPor_comp1
TpG
Wilcoxon p−value = 6.8456e−55, W = 153108658
density
num_transcript
0
1
2
0
10
20
30
group
ortho
whole transcriptome

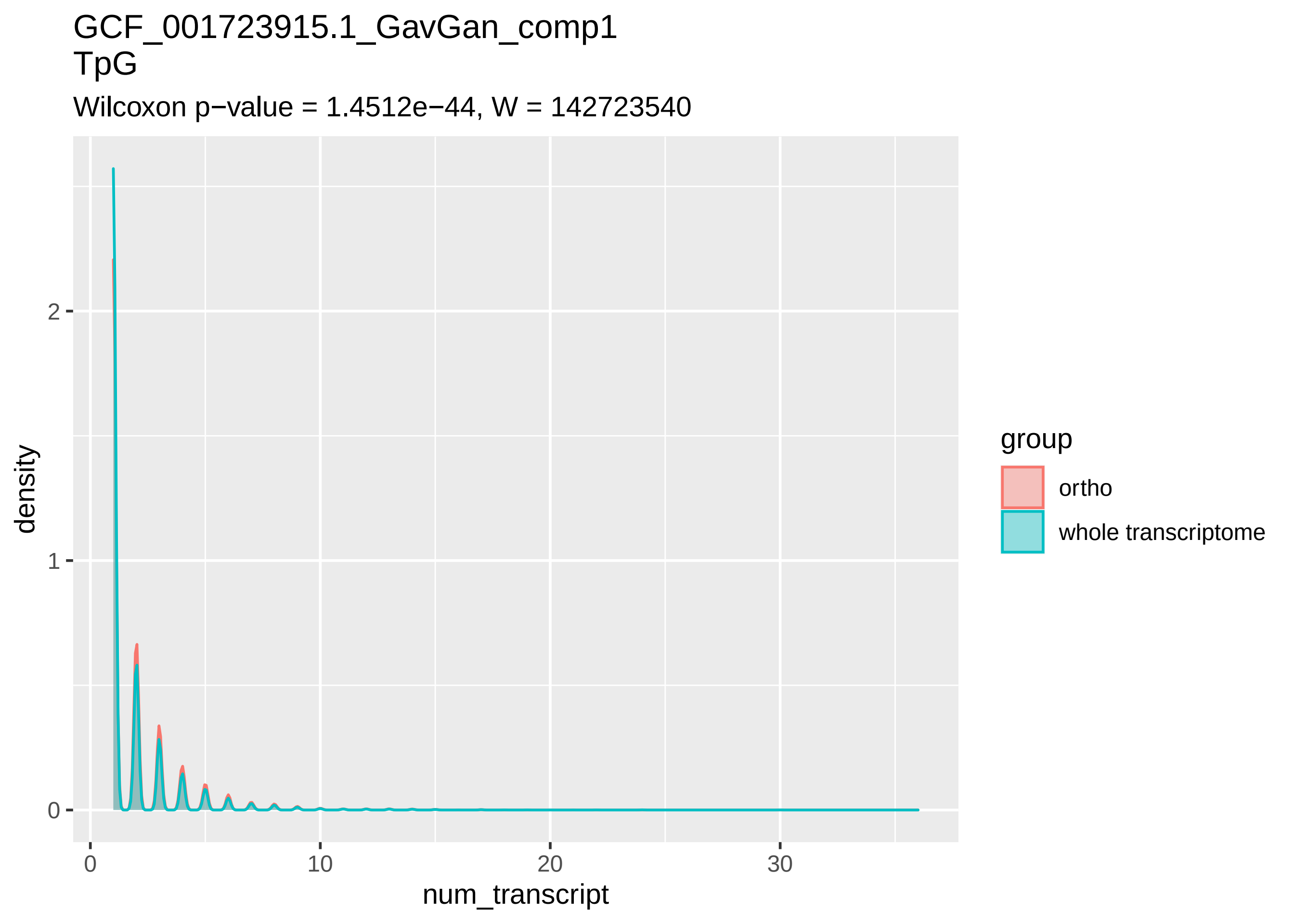
GCF_001723915.1_GavGan_comp1
TpG
Wilcoxon p−value = 1.4512e−44, W = 142723540
density
2
1
0
0
10
20
30
num_transcript
group
ortho
whole transcriptome

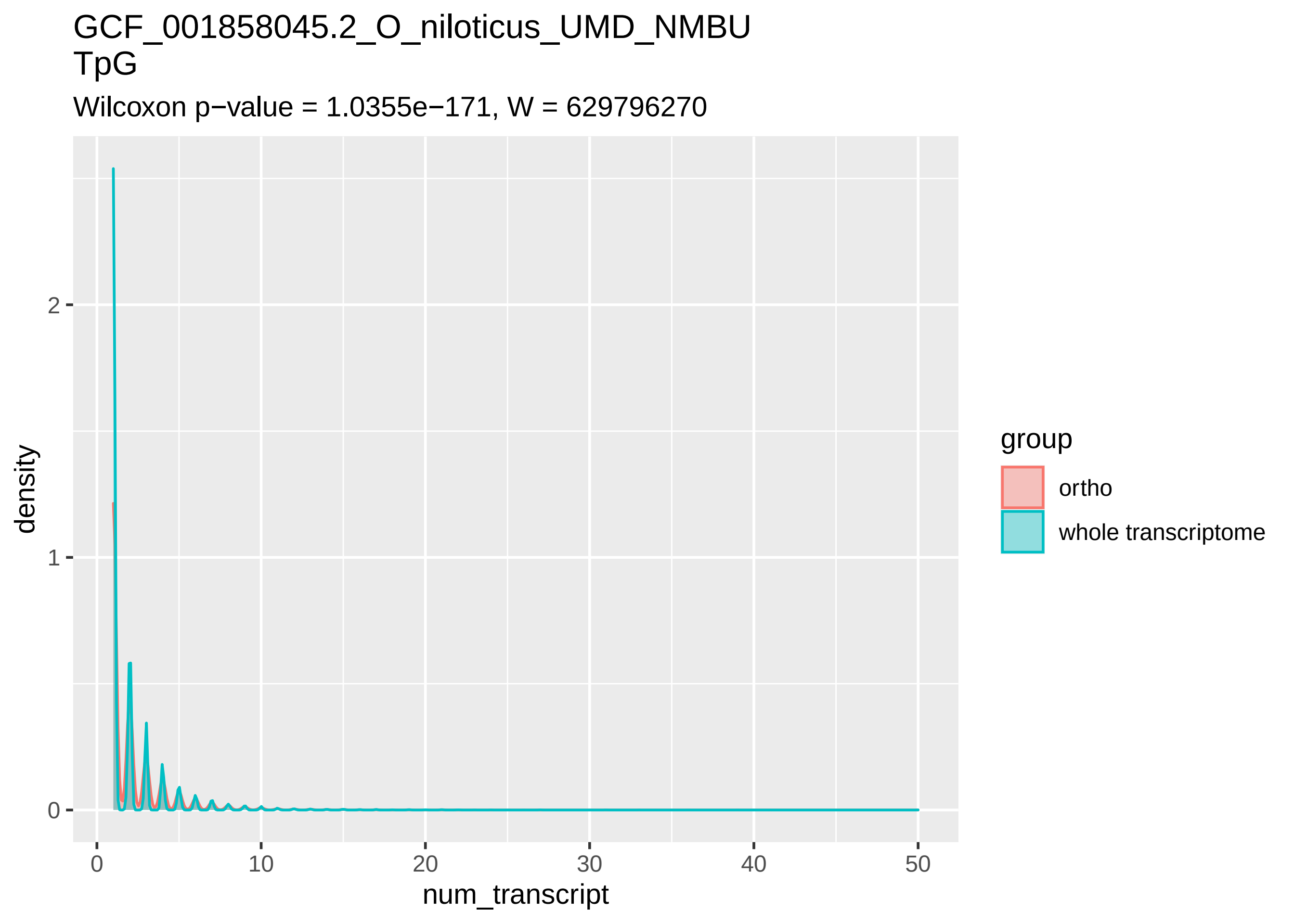
GCF_001858045.2_O_niloticus_UMD_NMBU
TpG
Wilcoxon p−value = 1.0355e−171, W = 629796270
density
2
1
0
0
10
20
30
40
50
num_transcript
group
ortho
whole transcriptome

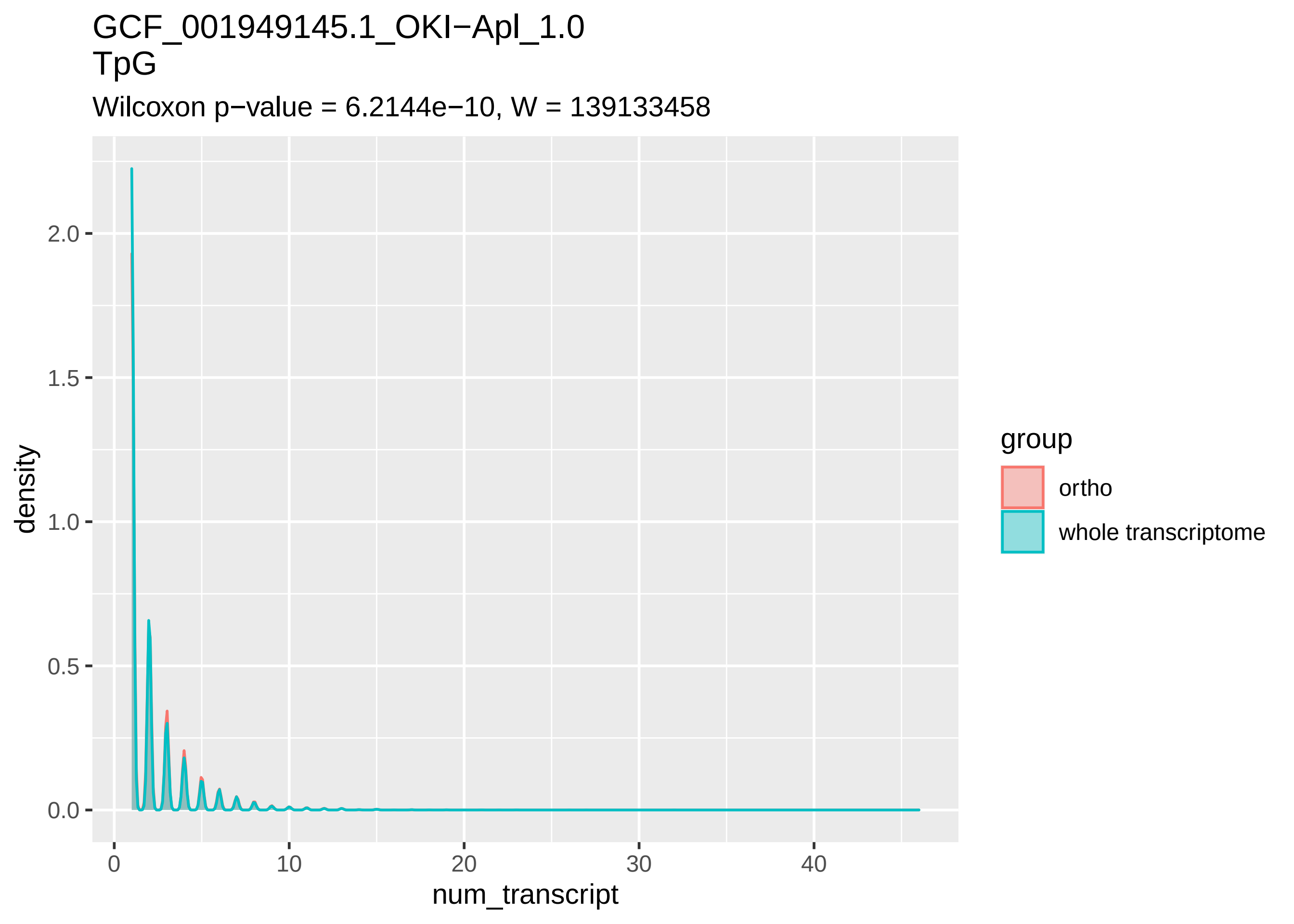
GCF_001949145.1_OKI−Apl_1.0
TpG
Wilcoxon p−value = 6.2144e−10, W = 139133458
density
2.0
1.5
1.0
0.5
0.0
0
10
20
30
40
num_transcript
group
ortho
whole transcriptome

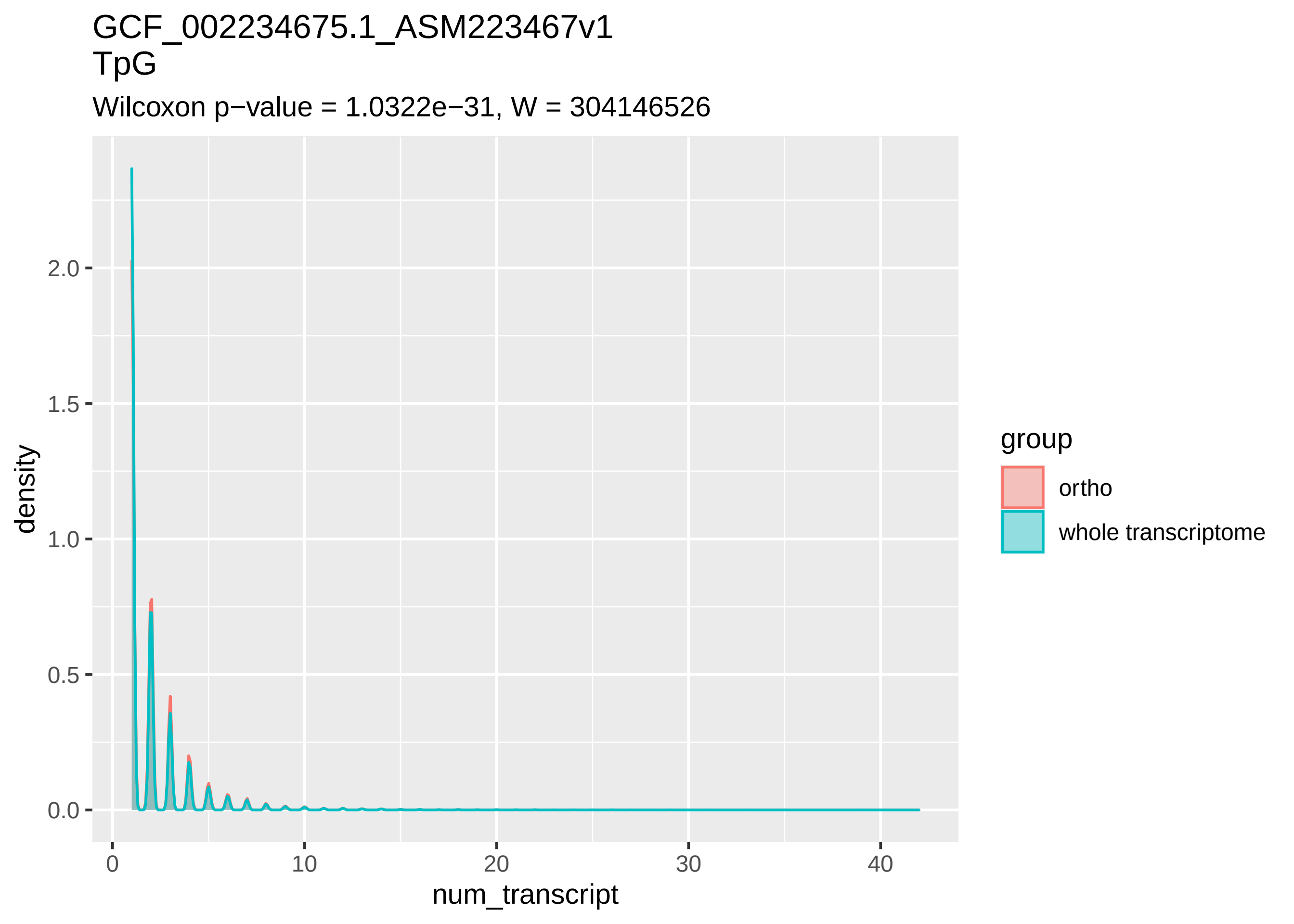
GCF_002234675.1_ASM223467v1
TpG
Wilcoxon p−value = 1.0322e−31, W = 304146526
density
2.0
1.5
1.0
0.5
0.0
0
10
20
30
40
num_transcript
group
ortho
whole transcriptome

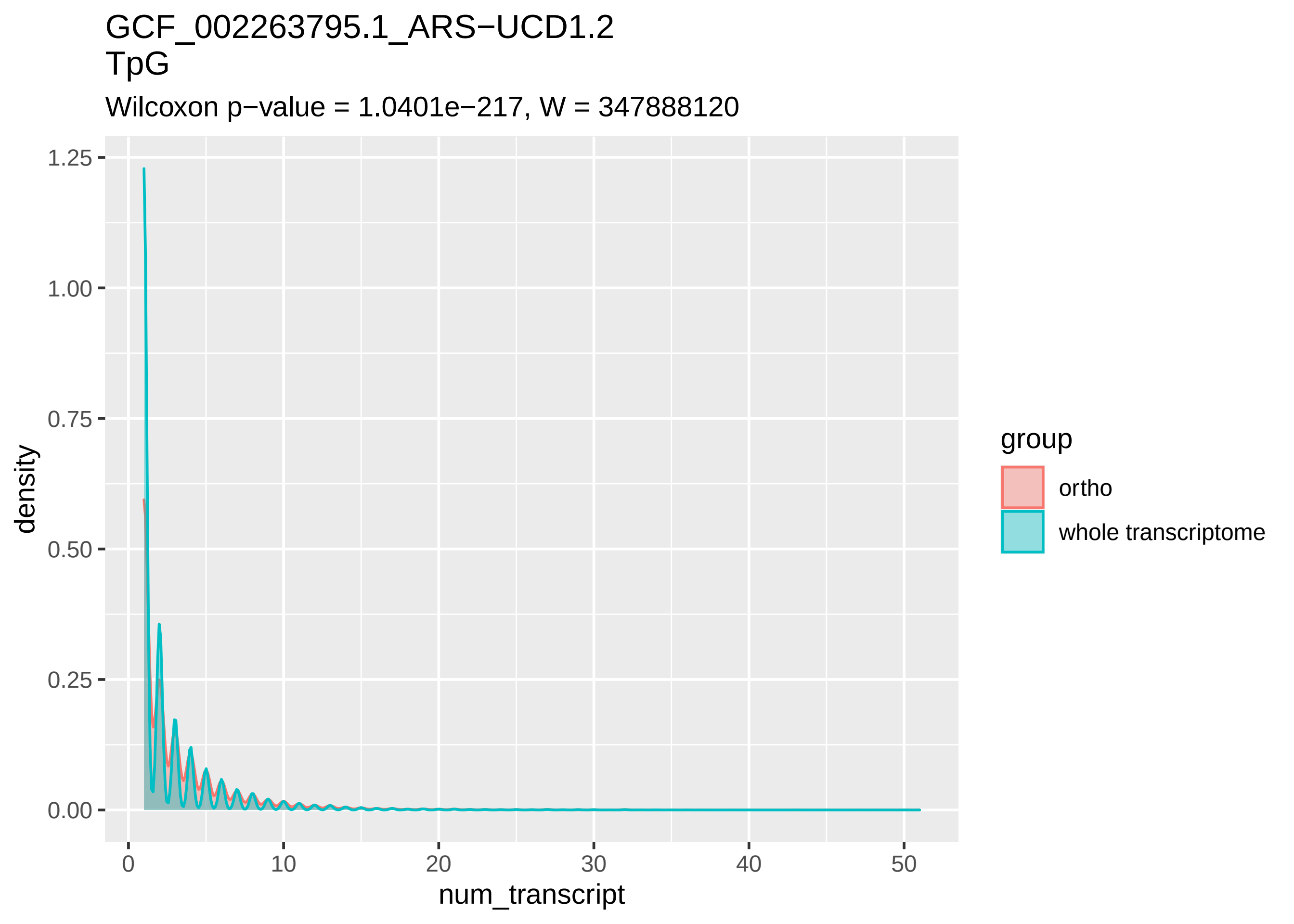
GCF_002263795.1_ARS−UCD1.2
TpG
Wilcoxon p−value = 1.0401e−217, W = 347888120
density
1.25
1.00
0.75
0.50
0.25
0.00
0
10
20
30
40
50
num_transcript
group
ortho
whole transcriptome

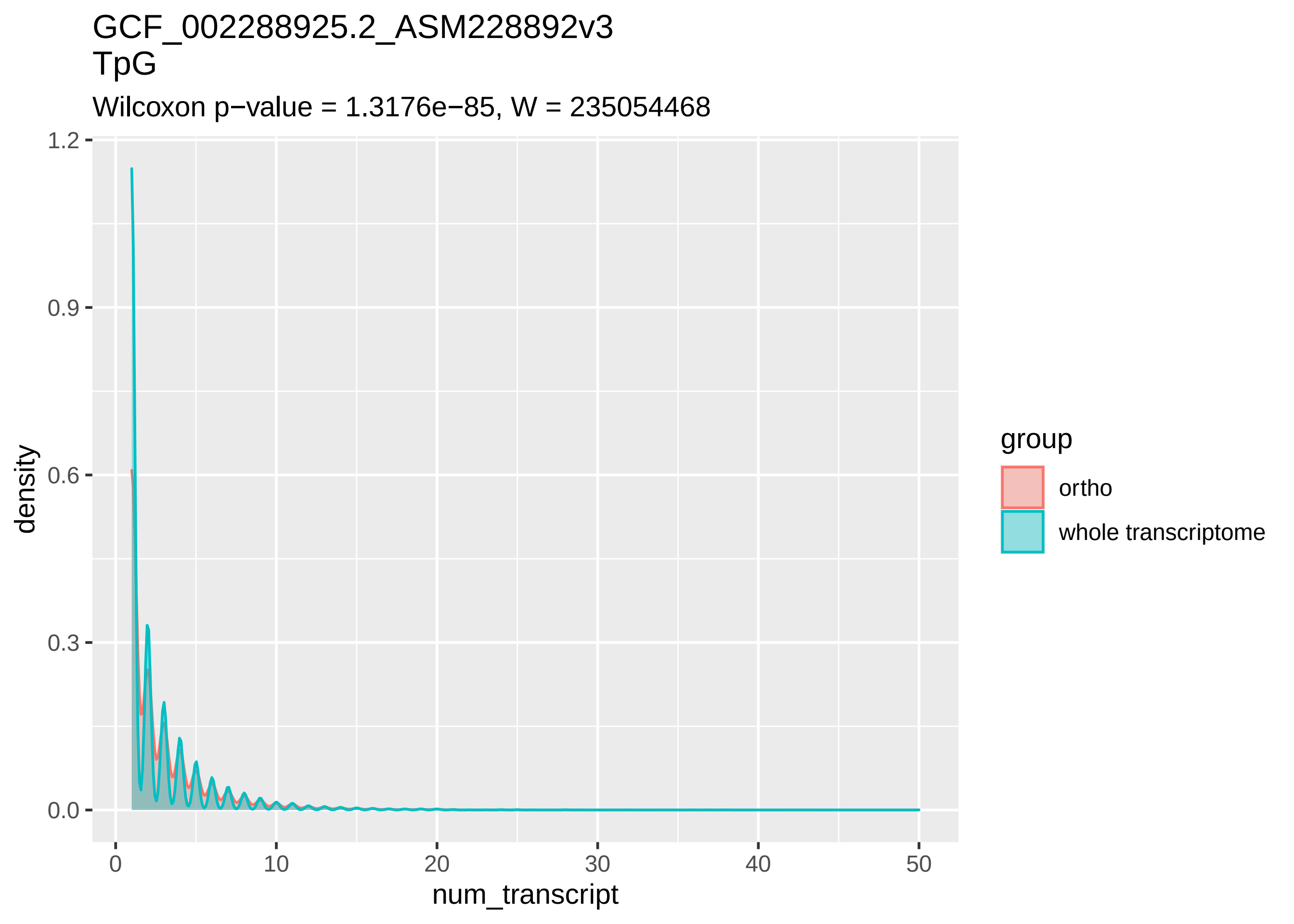

GCF_002288925.2_ASM228892v3
TpG
Wilcoxon p−value = 1.3176e−85, W = 235054468
1.2
0.9
0.6
0.3
0.0
density
0
10
20
30
40
50
num_transcript
group
ortho
whole transcriptome

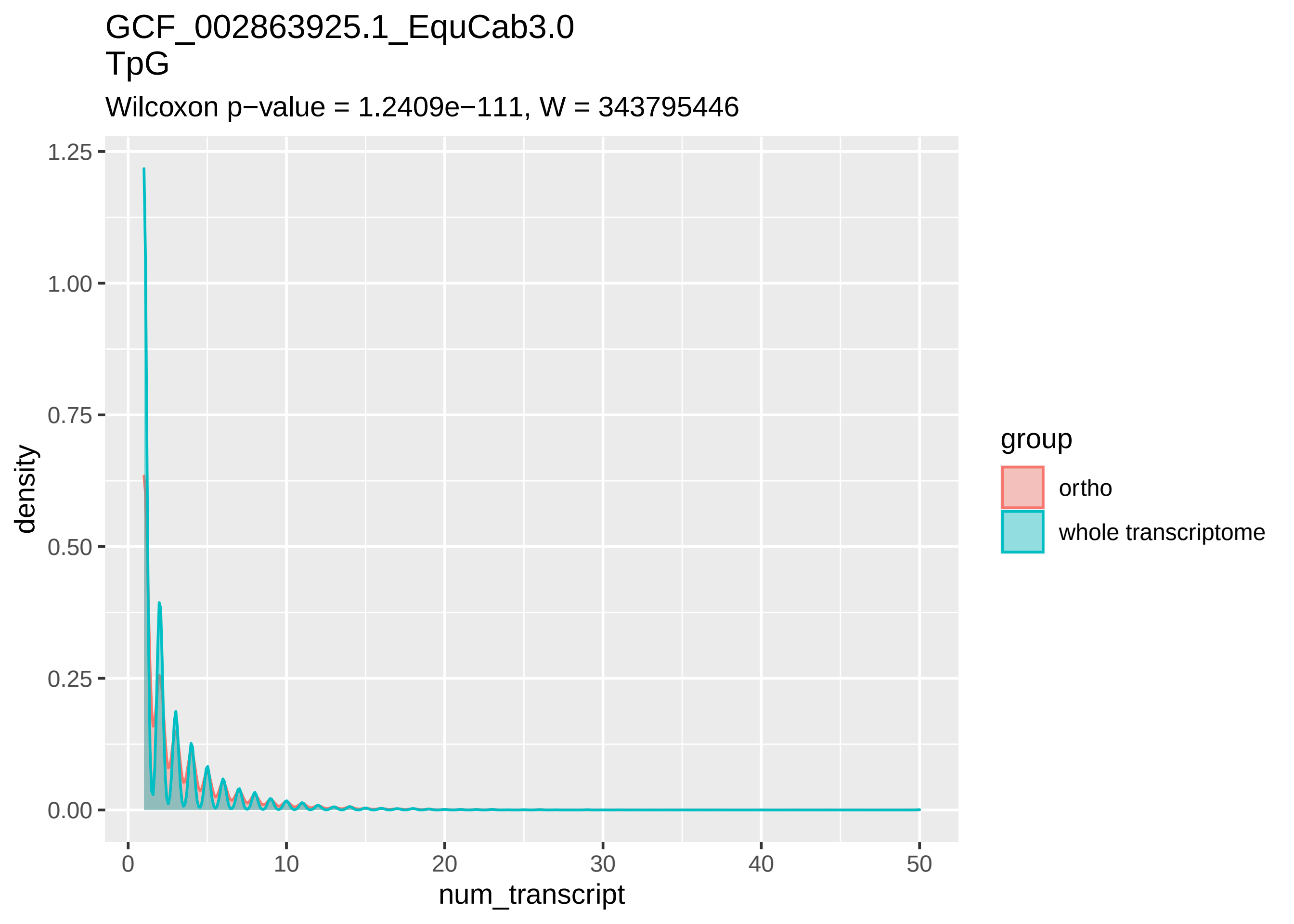
GCF_002863925.1_EquCab3.0
TpG
Wilcoxon p−value = 1.2409e−111, W = 343795446
density
1.25
1.00
0.75
0.50
0.25
0.00
0
10
20
30
40
50
num_transcript
group
ortho
whole transcriptome

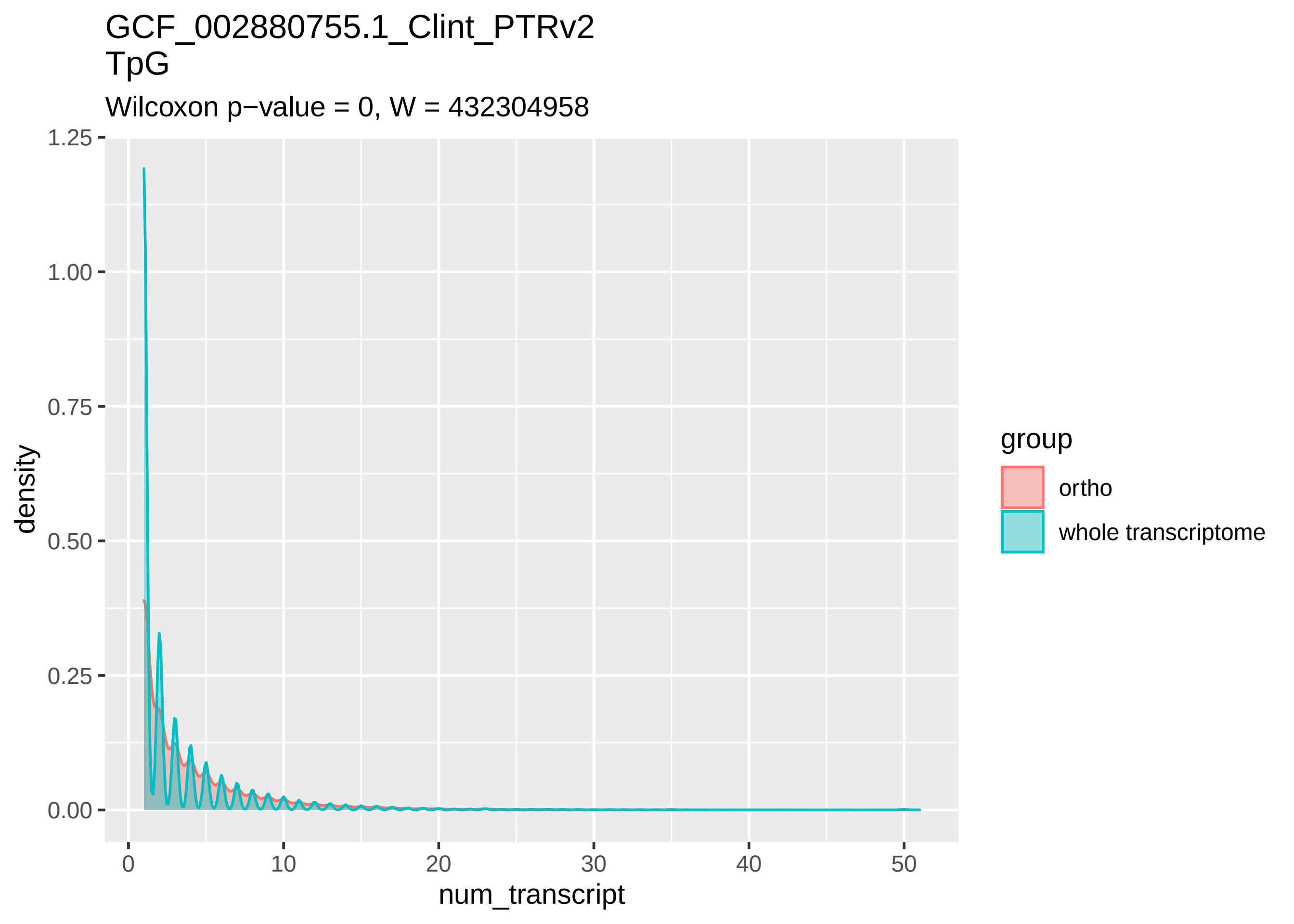

GCF_002880755.1_Clint_PTRv2
TpG
Wilcoxon p−value = 0, W = 432304958
density
1.25
1.00
0.75
0.50
0.25
0.00
0
10
20
30
40
50
num_transcript
group
ortho
whole transcriptome

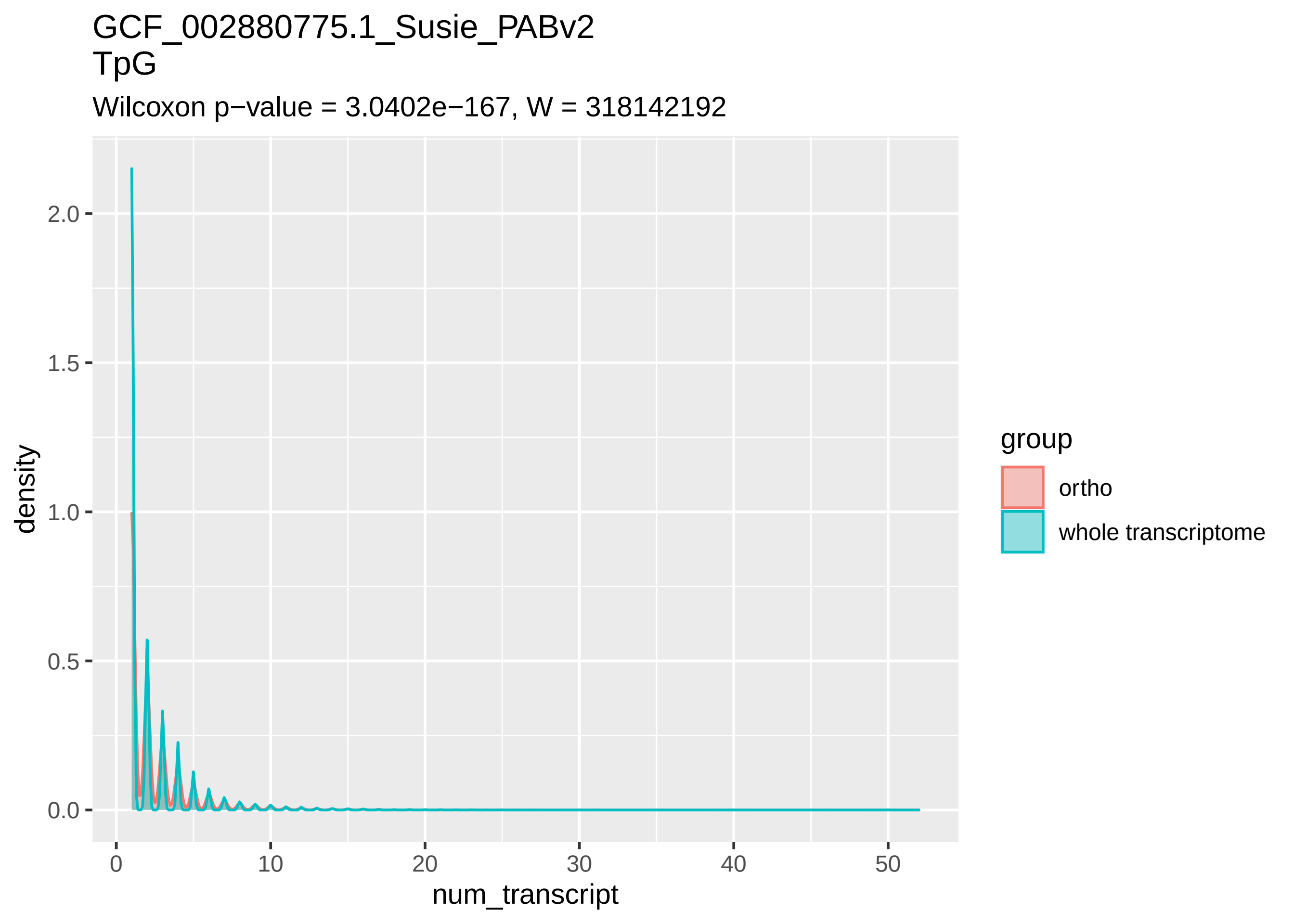
GCF_002880775.1_Susie_PABv2
TpG
Wilcoxon p−value = 3.0402e−167, W = 318142192
density
2.0
1.5
1.0
0.5
0.0
0
10
20
30
40
50
num_transcript
group
ortho
whole transcriptome

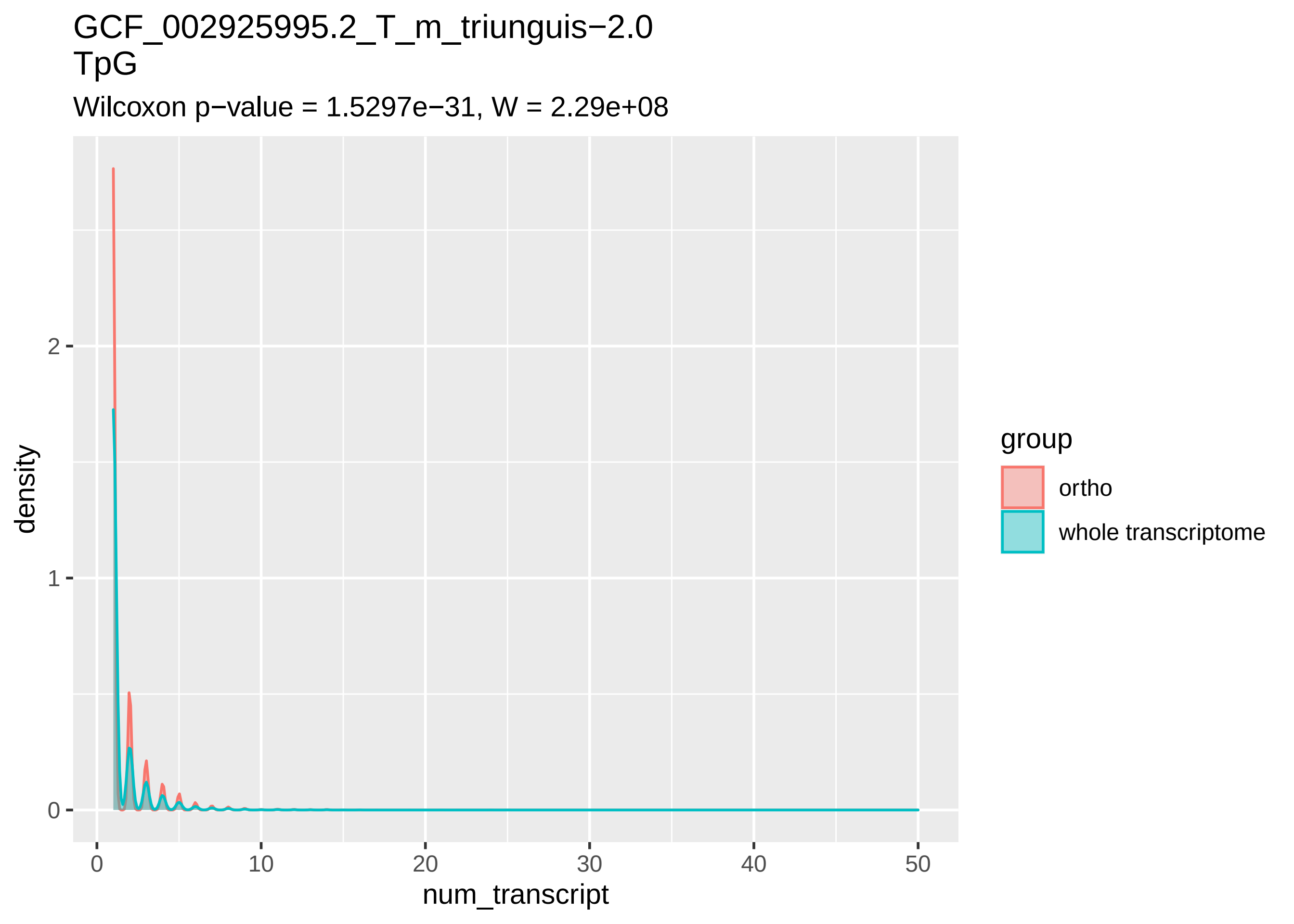
GCF_002925995.2_T_m_triunguis−2.0
TpG
Wilcoxon p−value = 1.5297e−31, W = 2.29e+08
density
num_transcript
0
1
2
0
10
20
30
40
50
group
ortho
whole transcriptome

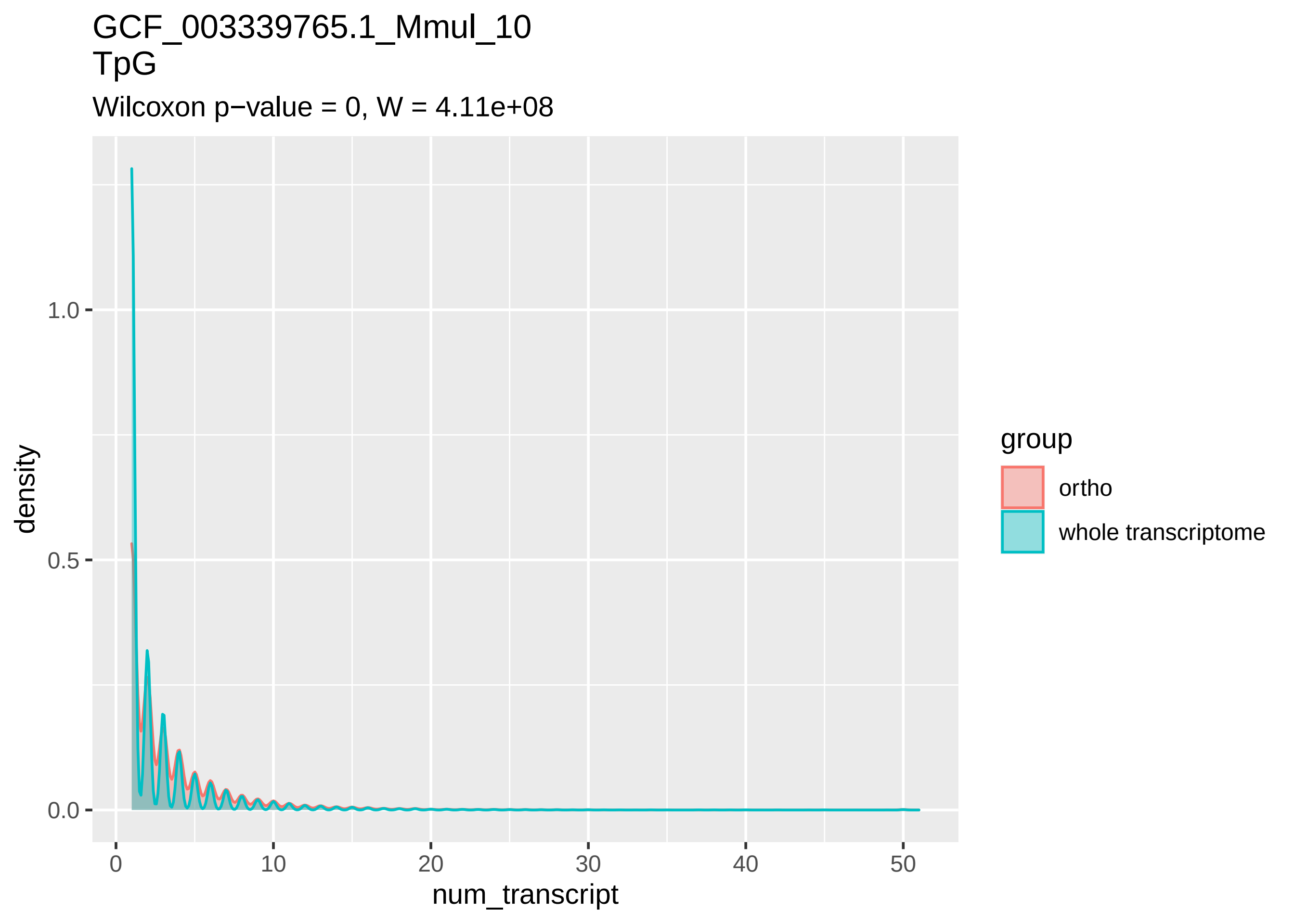

GCF_003339765.1_Mmul_10
TpG
Wilcoxon p−value = 0, W = 4.11e+08
density
1.0
0.5
0.0
0
10
20
30
40
50
num_transcript
group
ortho
whole transcriptome

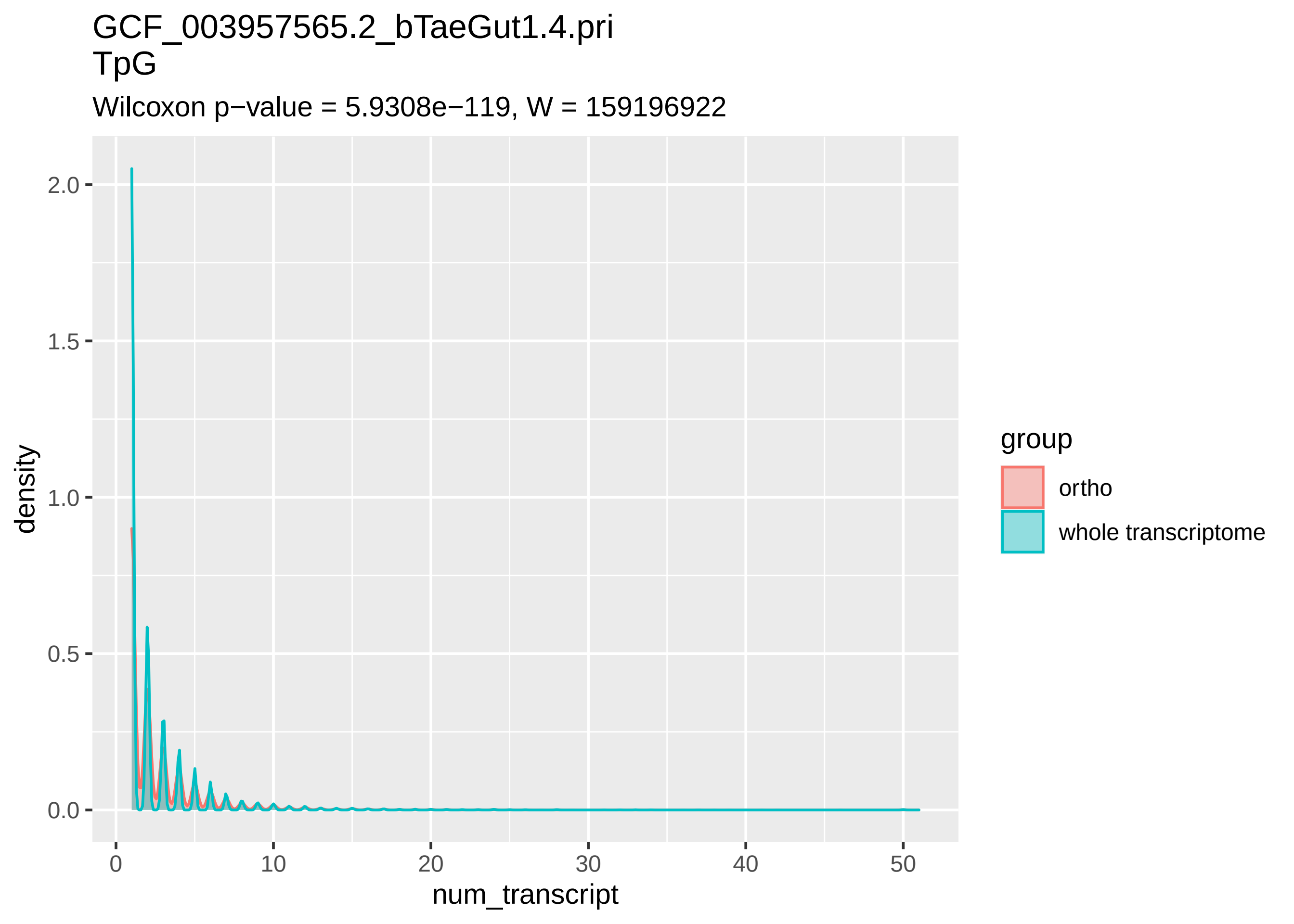

GCF_003957565.2_bTaeGut1.4.pri
TpG
Wilcoxon p−value = 5.9308e−119, W = 159196922
density
2.0
1.5
1.0
0.5
0.0
0
10
20
30
40
50
num_transcript
group
ortho
whole transcriptome

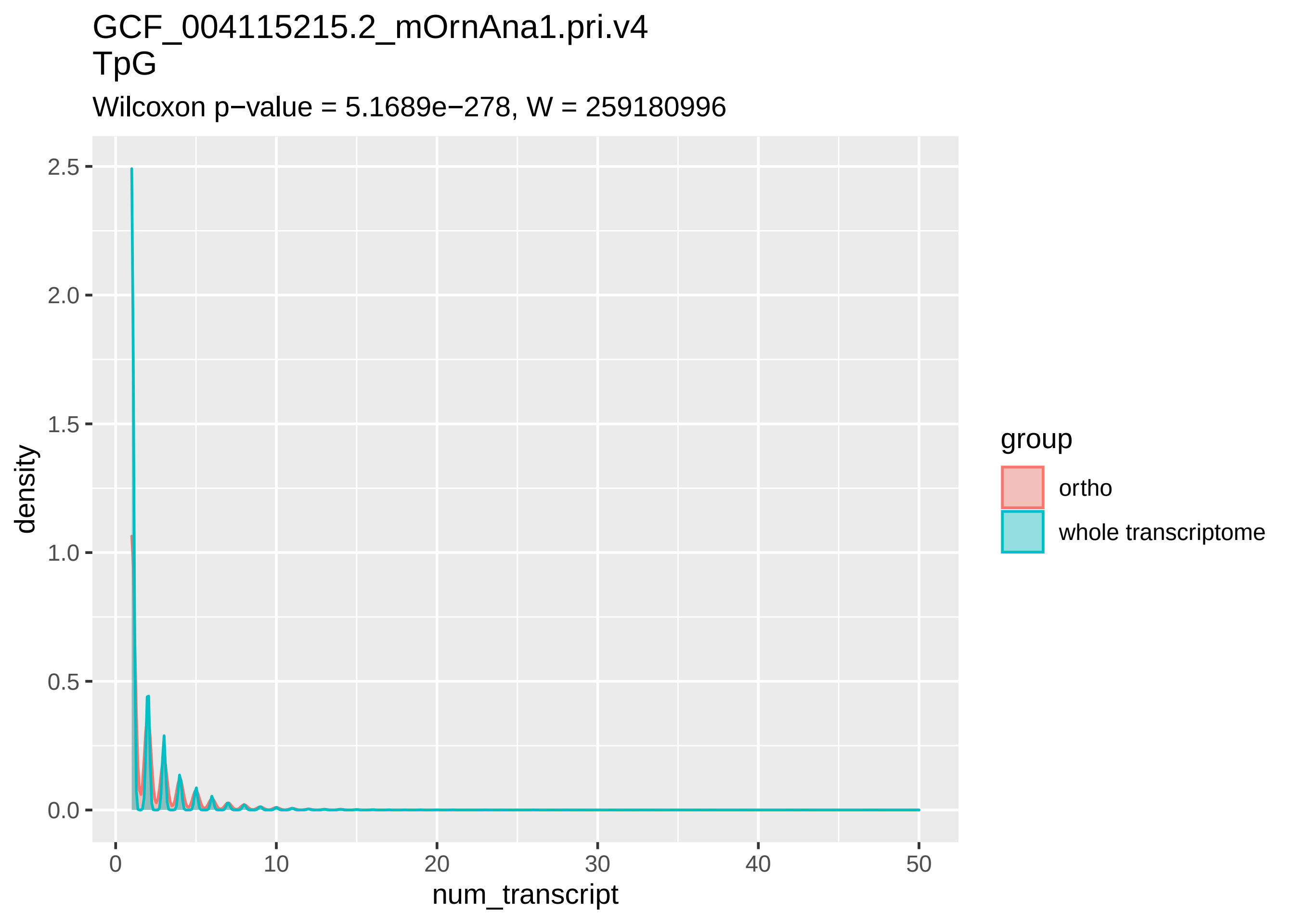
GCF_004115215.2_mOrnAna1.pri.v4
TpG
Wilcoxon p−value = 5.1689e−278, W = 259180996
density
2.5
2.0
1.5
1.0
0.5
0.0
0
10
20
30
40
50
num_transcript
group
ortho
whole transcriptome

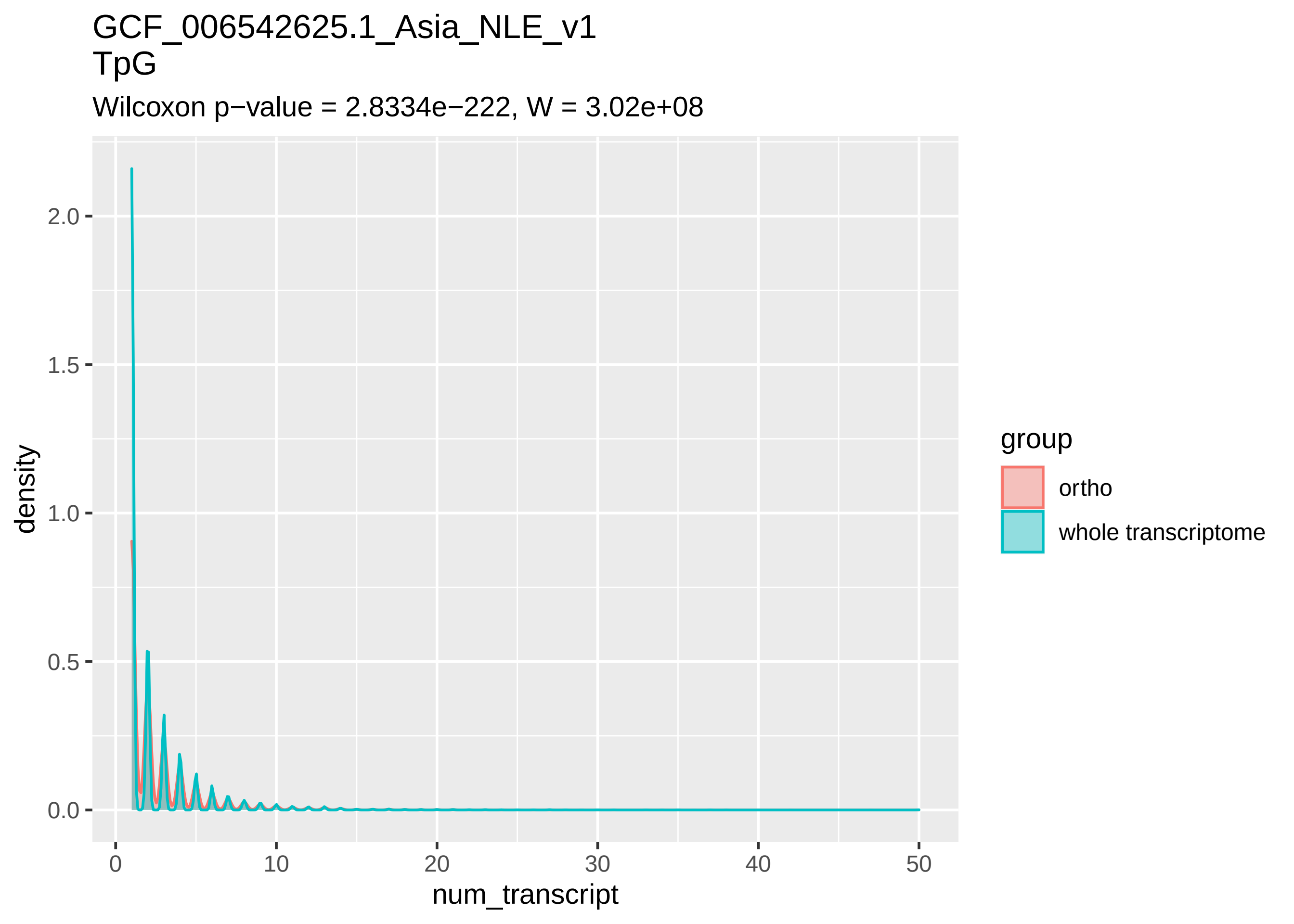
GCF_006542625.1_Asia_NLE_v1
TpG
Wilcoxon p−value = 2.8334e−222, W = 3.02e+08
density
num_transcript
0.0
0.5
1.0
1.5
2.0
0
10
20
30
40
50
group
ortho
whole transcriptome

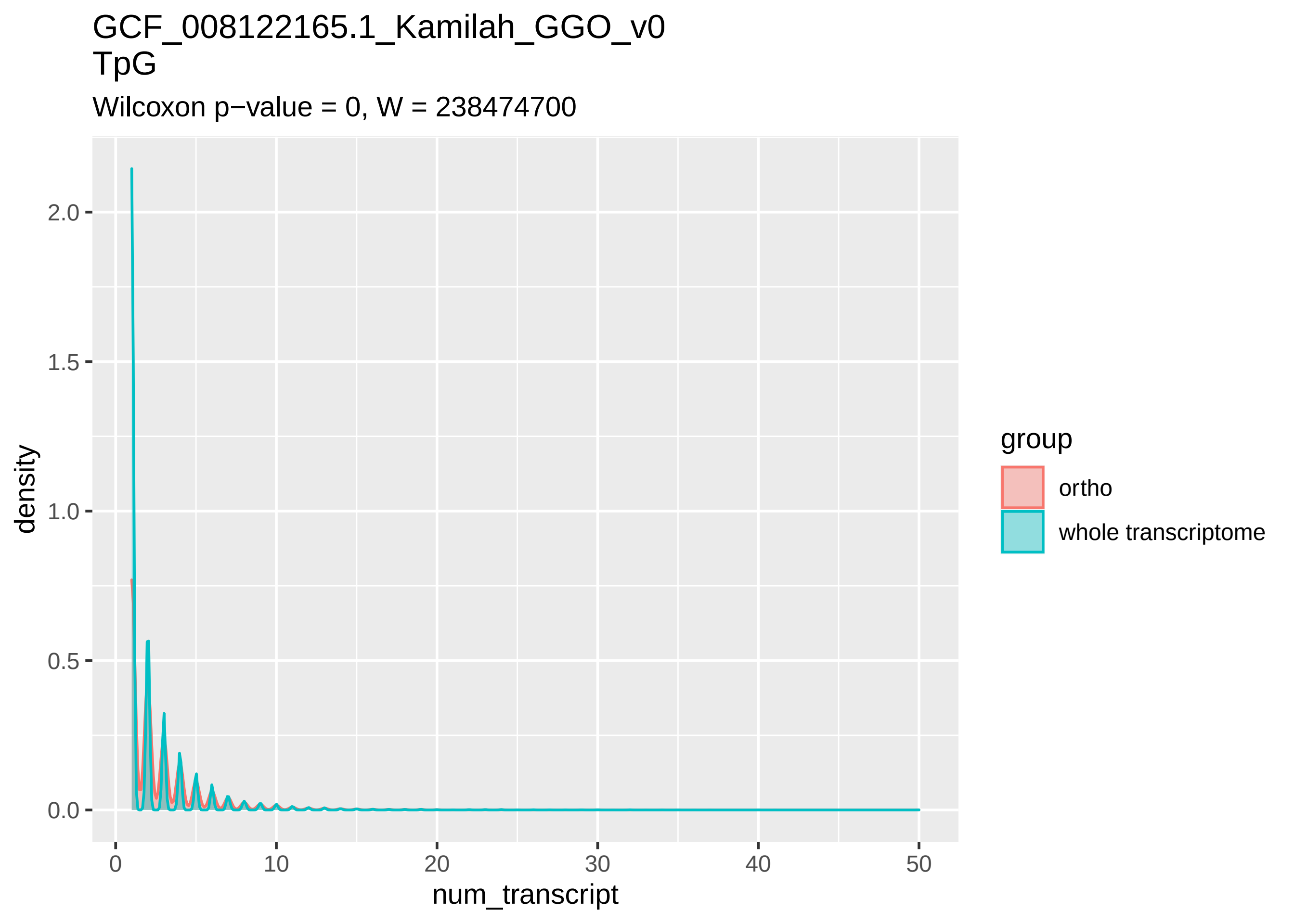

GCF_008122165.1_Kamilah_GGO_v0
TpG
Wilcoxon p−value = 0, W = 238474700
density
2.0
1.5
1.0
0.5
0.0
0
10
20
30
40
50
num_transcript
group
ortho
whole transcriptome

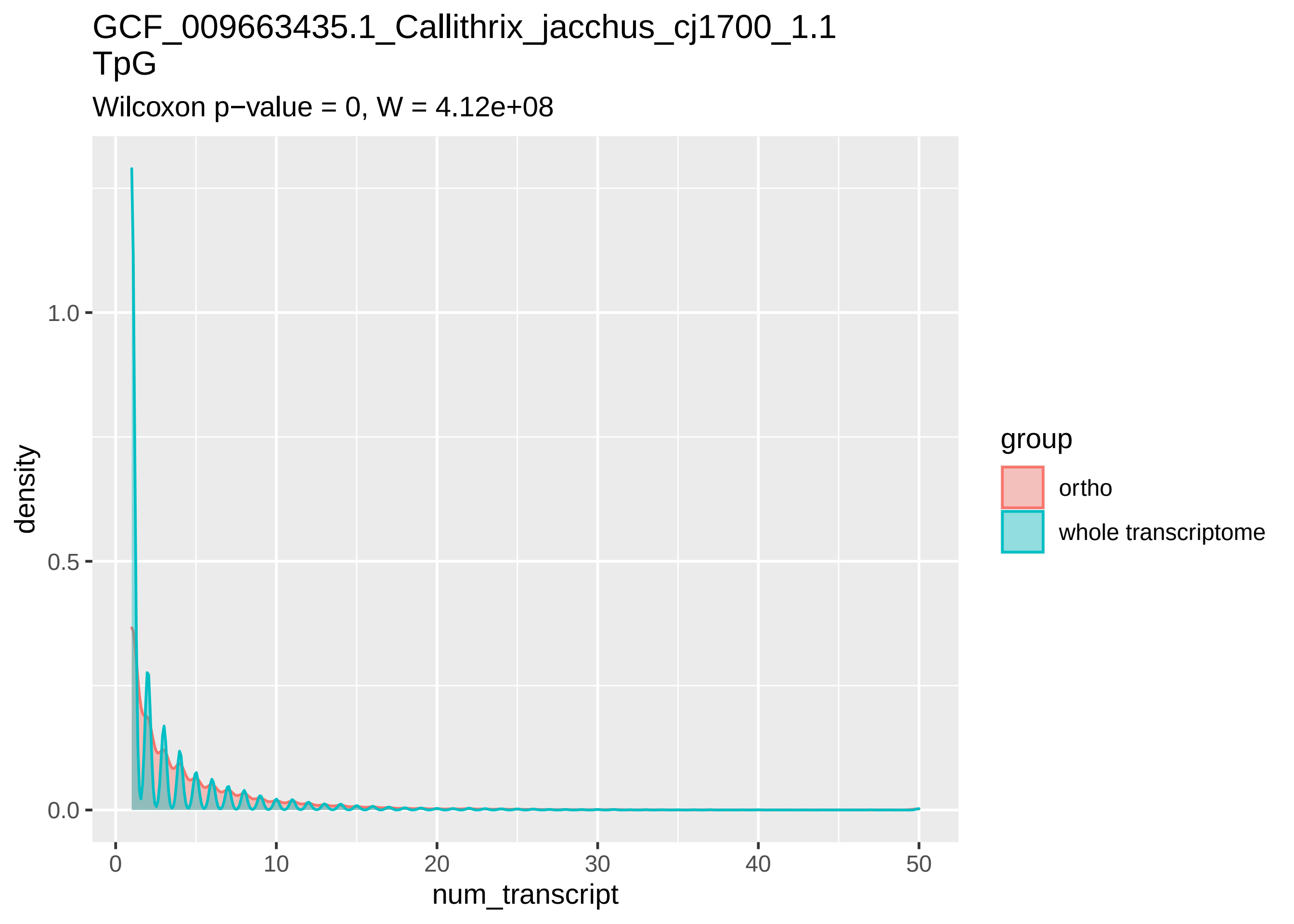

GCF_009663435.1_Callithrix_jacchus_cj1700_1.1
TpG
Wilcoxon p−value = 0, W = 4.12e+08
density
1.0
0.5
0.0
0
10
20
30
40
50
num_transcript
group
ortho
whole transcriptome

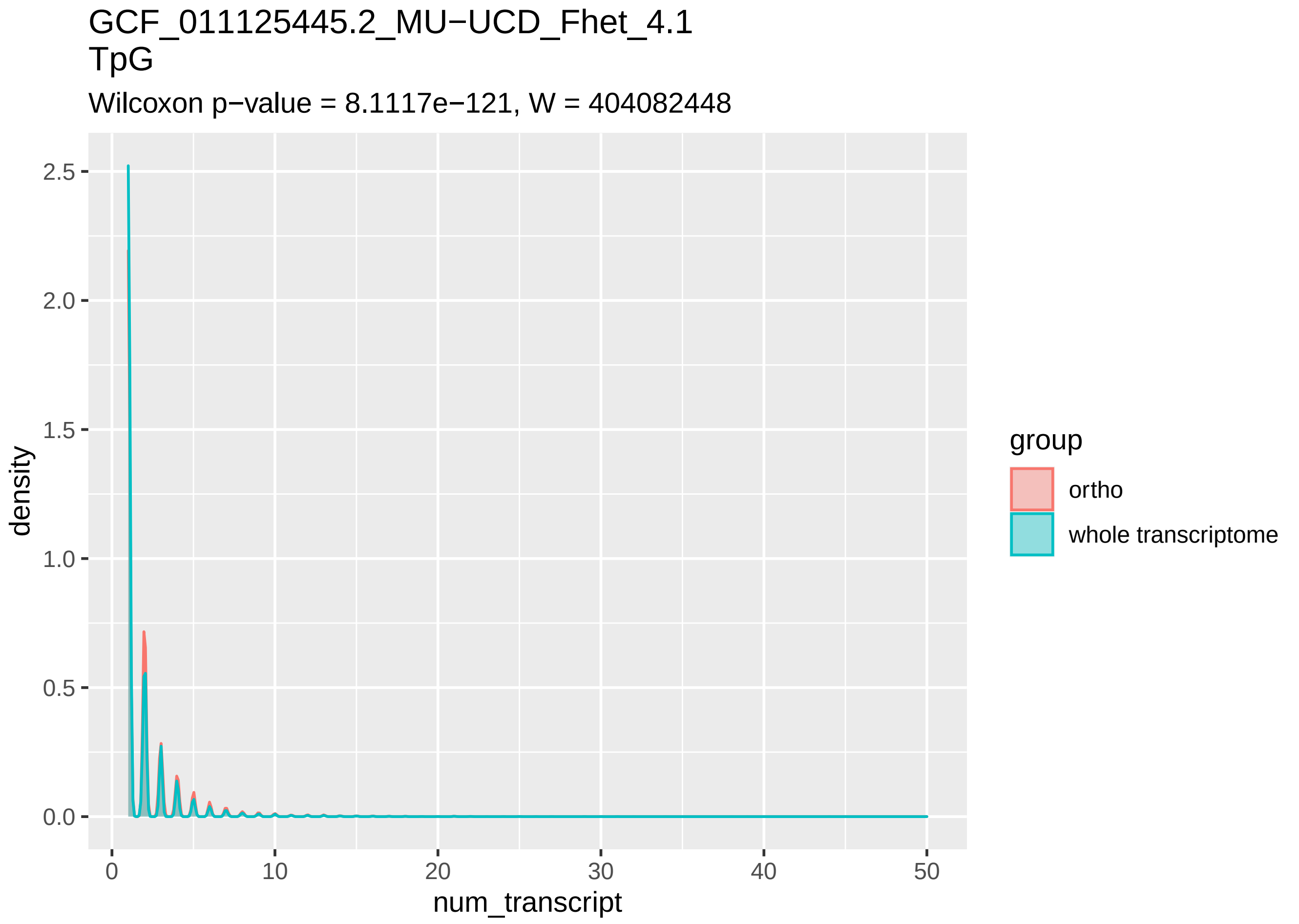
GCF_011125445.2_MU−UCD_Fhet_4.1
TpG
Wilcoxon p−value = 8.1117e−121, W = 404082448
density
num_transcript
2.5
2.0
1.5
1.0
0.5
0.0
0
10
20
30
40
50
group
ortho
whole transcriptome

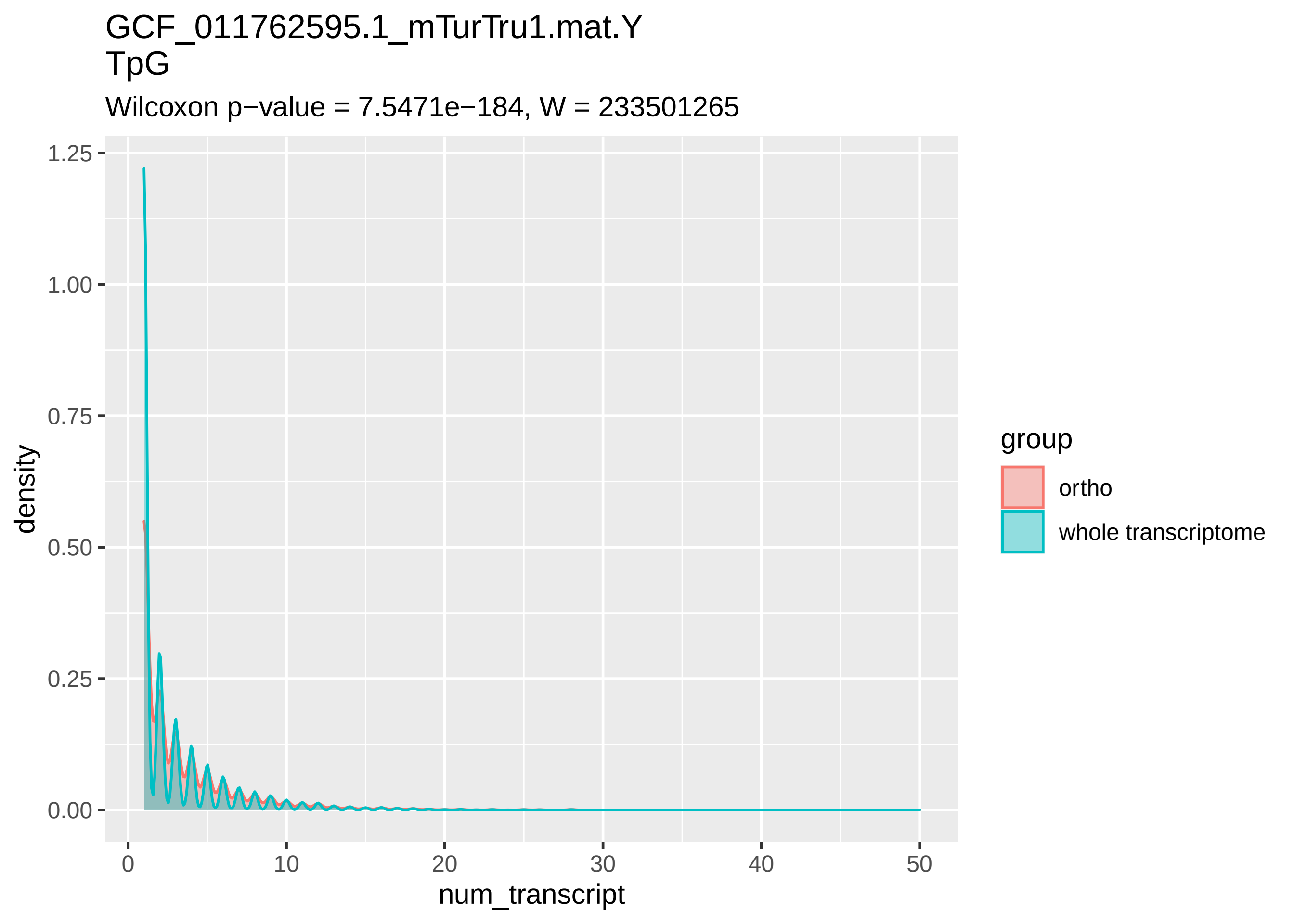

GCF_011762595.1_mTurTru1.mat.Y
TpG
Wilcoxon p−value = 7.5471e−184, W = 233501265
density
1.25
1.00
0.75
0.50
0.25
0.00
0
10
20
30
40
50
num_transcript
group
ortho
whole transcriptome

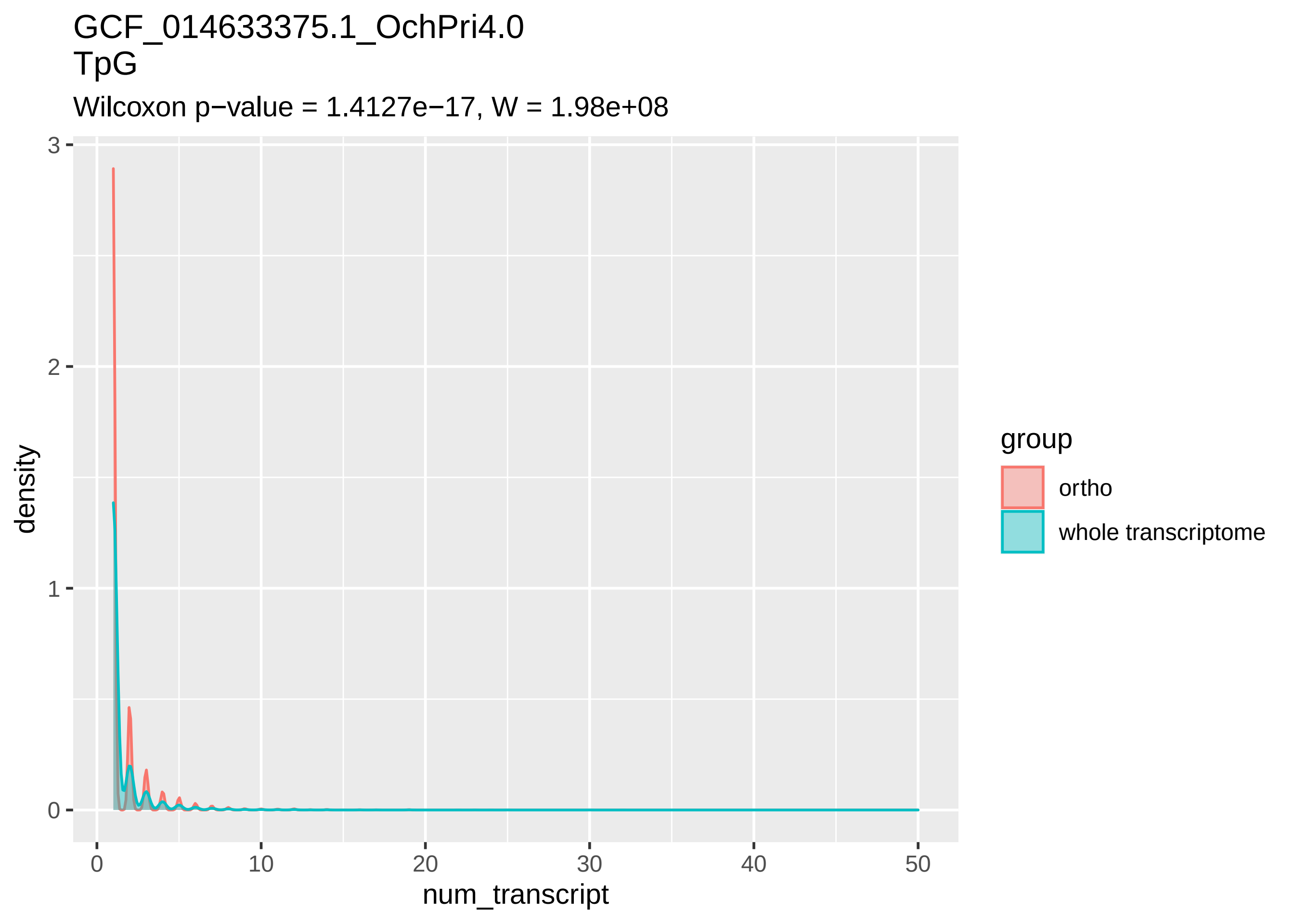
GCF_014633375.1_OchPri4.0
TpG
Wilcoxon p−value = 1.4127e−17, W = 1.98e+08
density
3
2
1
0
0
10
20
30
40
50
num_transcript
group
ortho
whole transcriptome

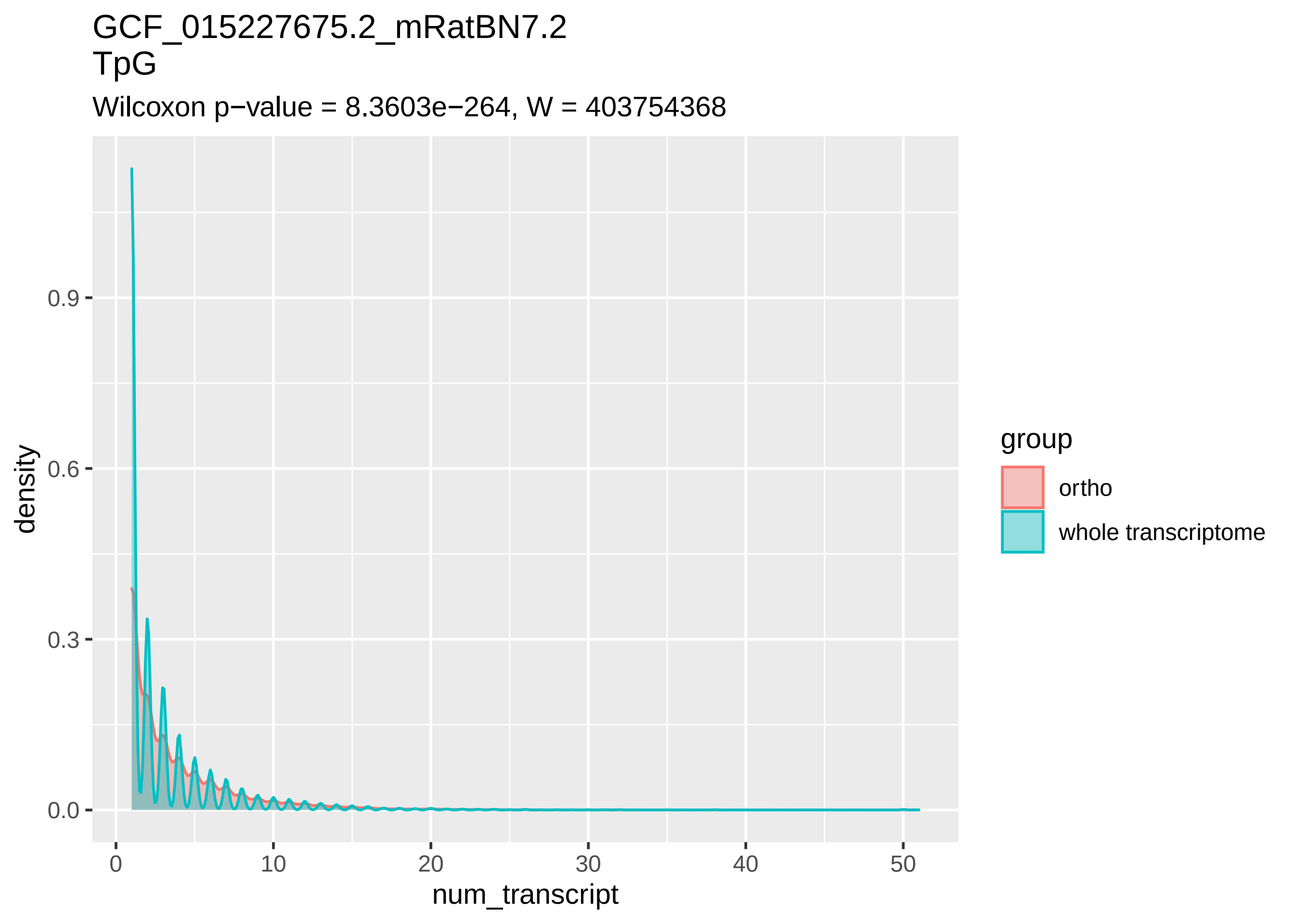

GCF_015227675.2_mRatBN7.2
TpG
Wilcoxon p−value = 8.3603e−264, W = 403754368
density
0.0
0.3
0.6
0.9
0
10
20
30
40
50
num_transcript
group
ortho
whole transcriptome

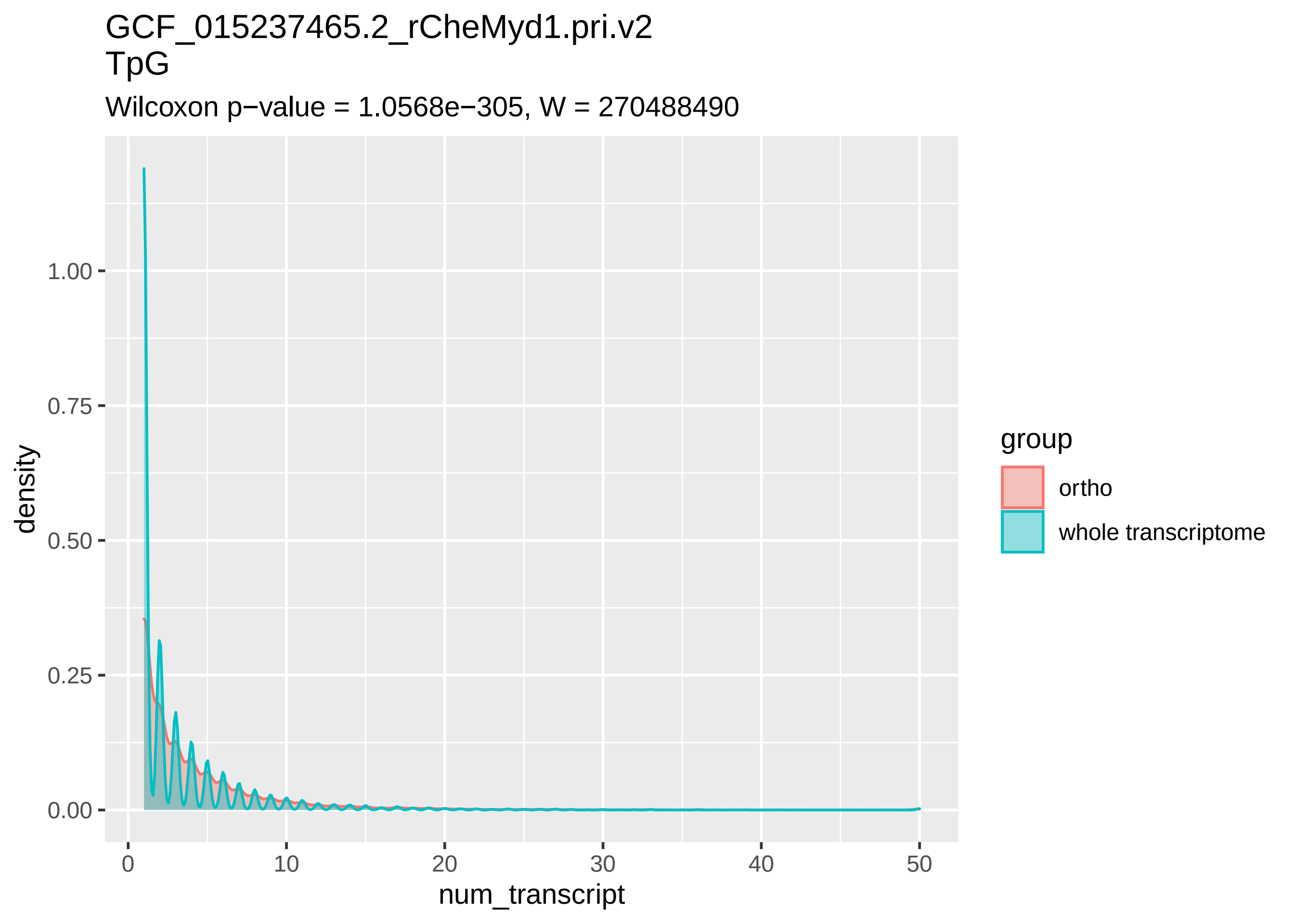

GCF_015237465.2_rCheMyd1.pri.v2
TpG
Wilcoxon p−value = 1.0568e−305, W = 270488490
density
1.00
0.75
0.50
0.25
0.00
0
10
20
30
40
50
num_transcript
group
ortho
whole transcriptome

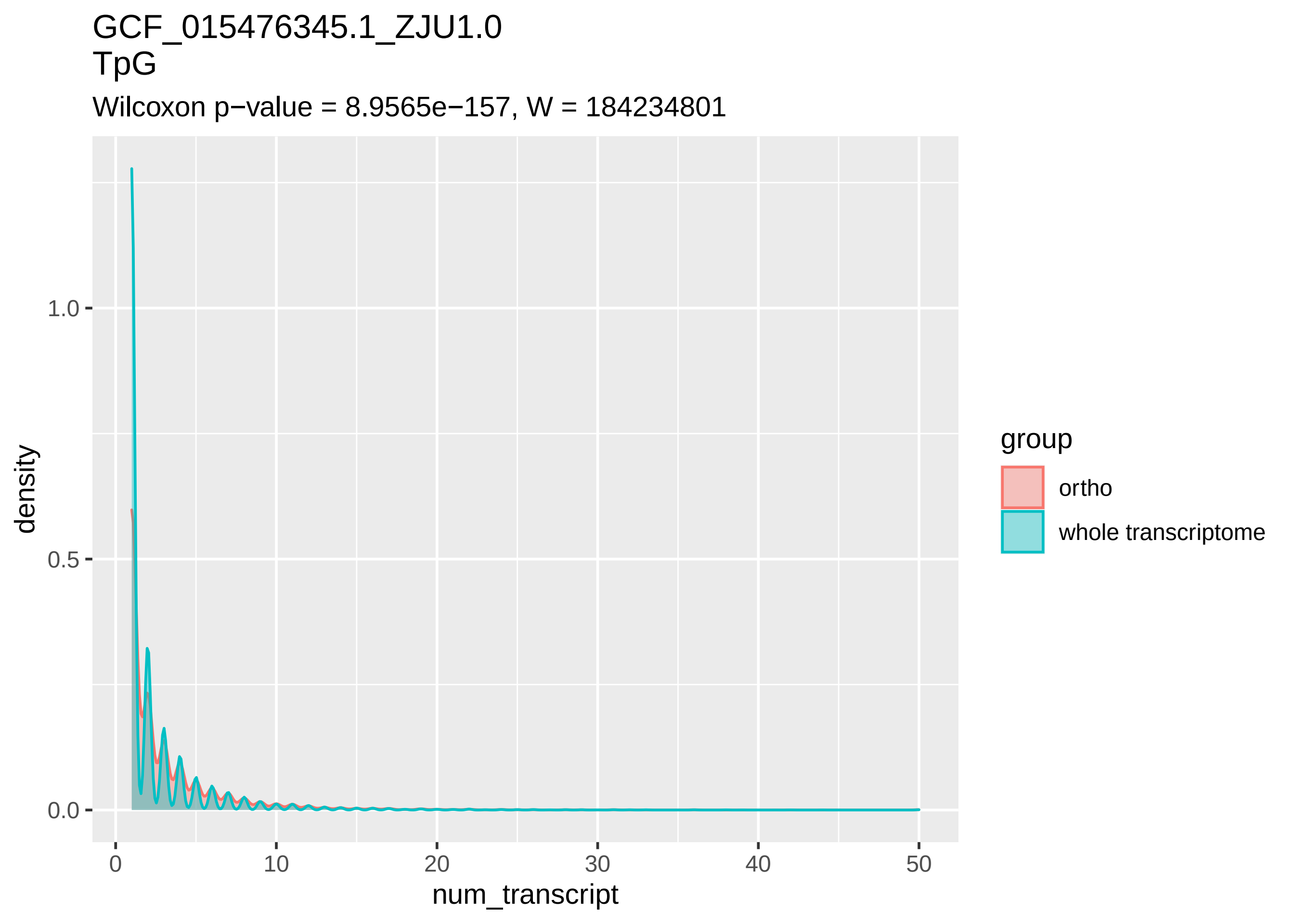
GCF_015476345.1_ZJU1.0
TpG
Wilcoxon p−value = 8.9565e−157, W = 184234801
density
1.0
0.5
0.0
0
10
20
30
40
50
num_transcript
group
ortho
whole transcriptome

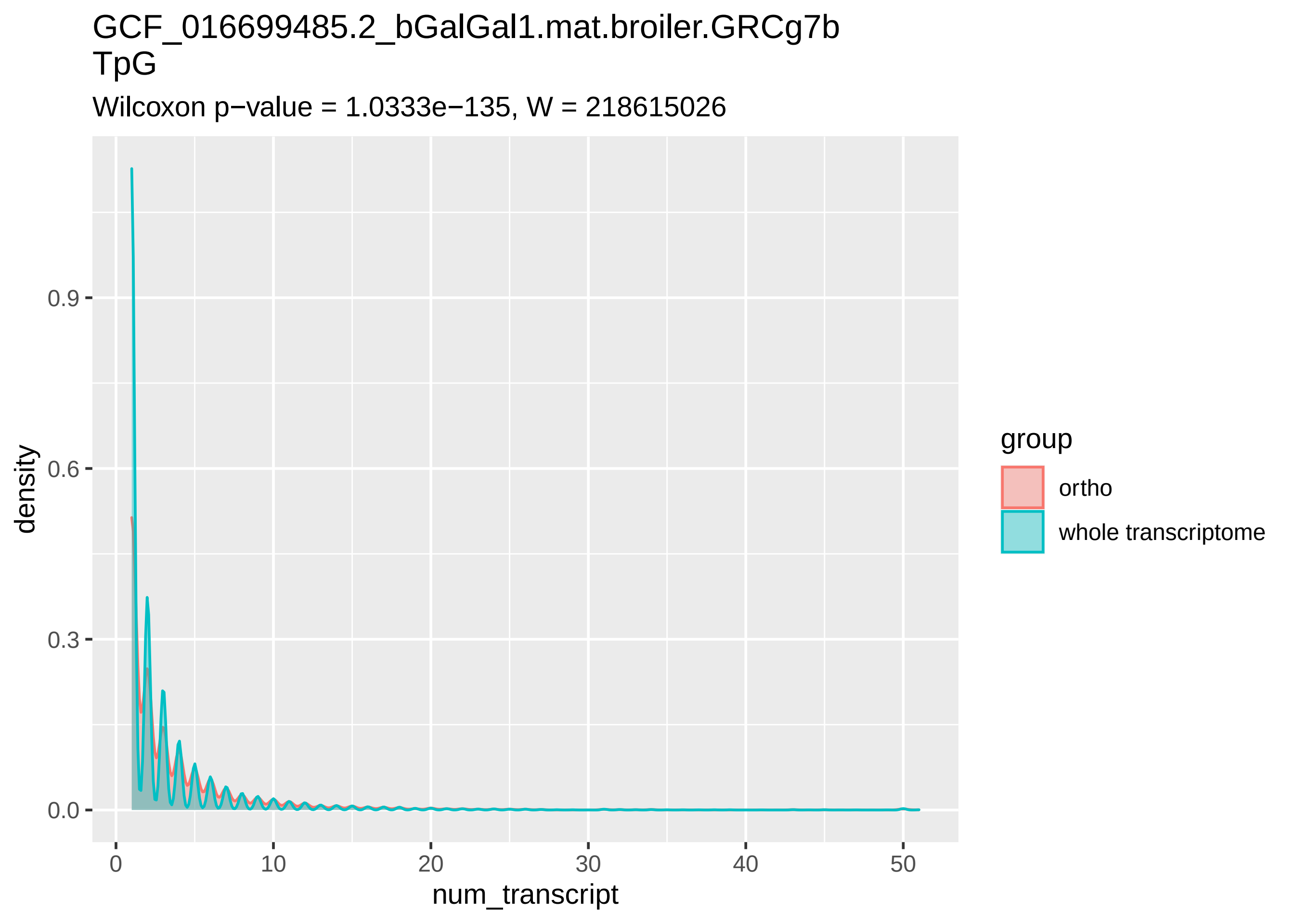

GCF_016699485.2_bGalGal1.mat.broiler.GRCg7b
TpG
Wilcoxon p−value = 1.0333e−135, W = 218615026
density
0.0
0.3
0.6
0.9
0
10
20
30
40
50
num_transcript
group
ortho
whole transcriptome

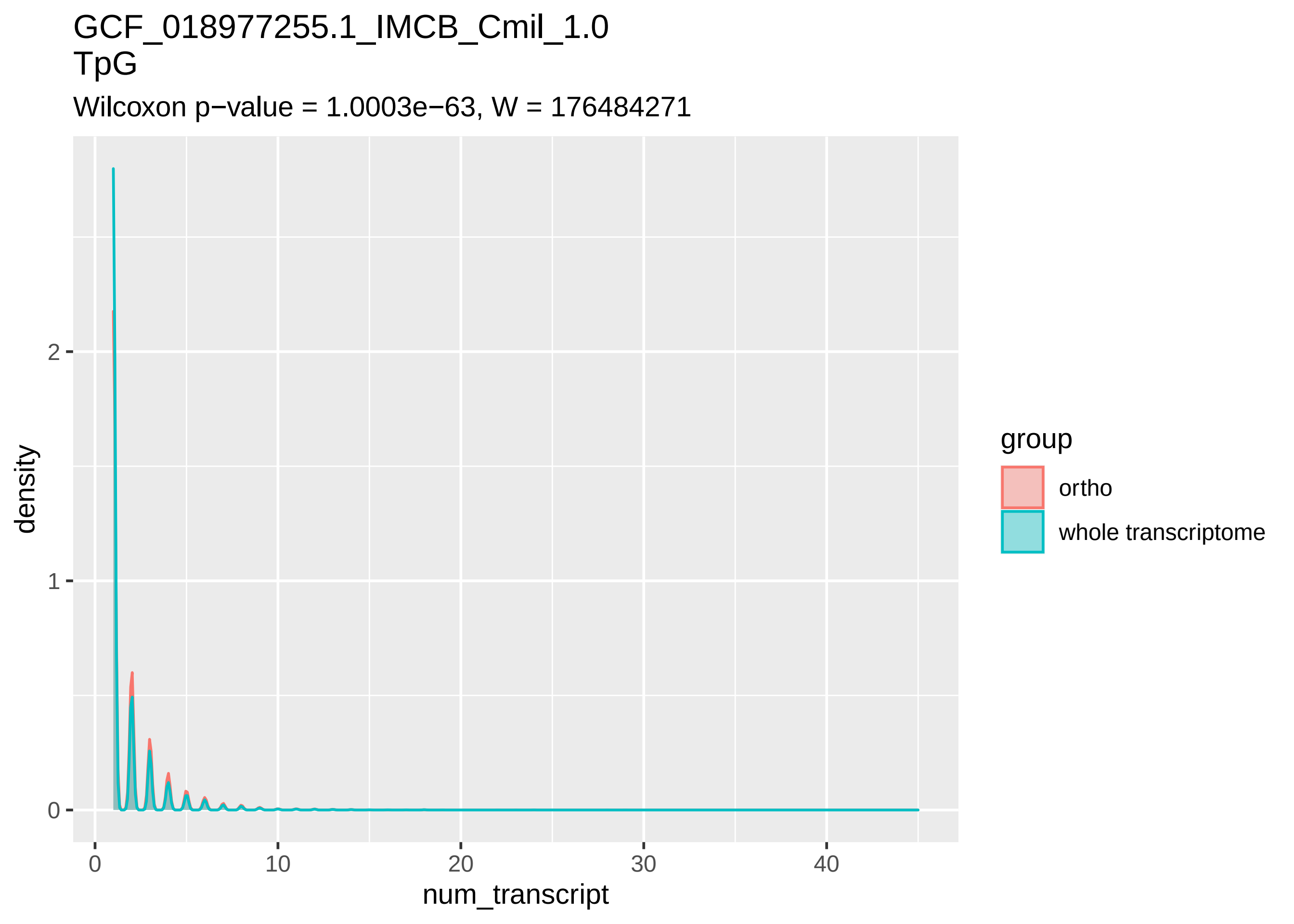

GCF_018977255.1_IMCB_Cmil_1.0
TpG
Wilcoxon p−value = 1.0003e−63, W = 176484271
density
0
1
2
0
10
20
30
40
num_transcript
group
ortho
whole transcriptome

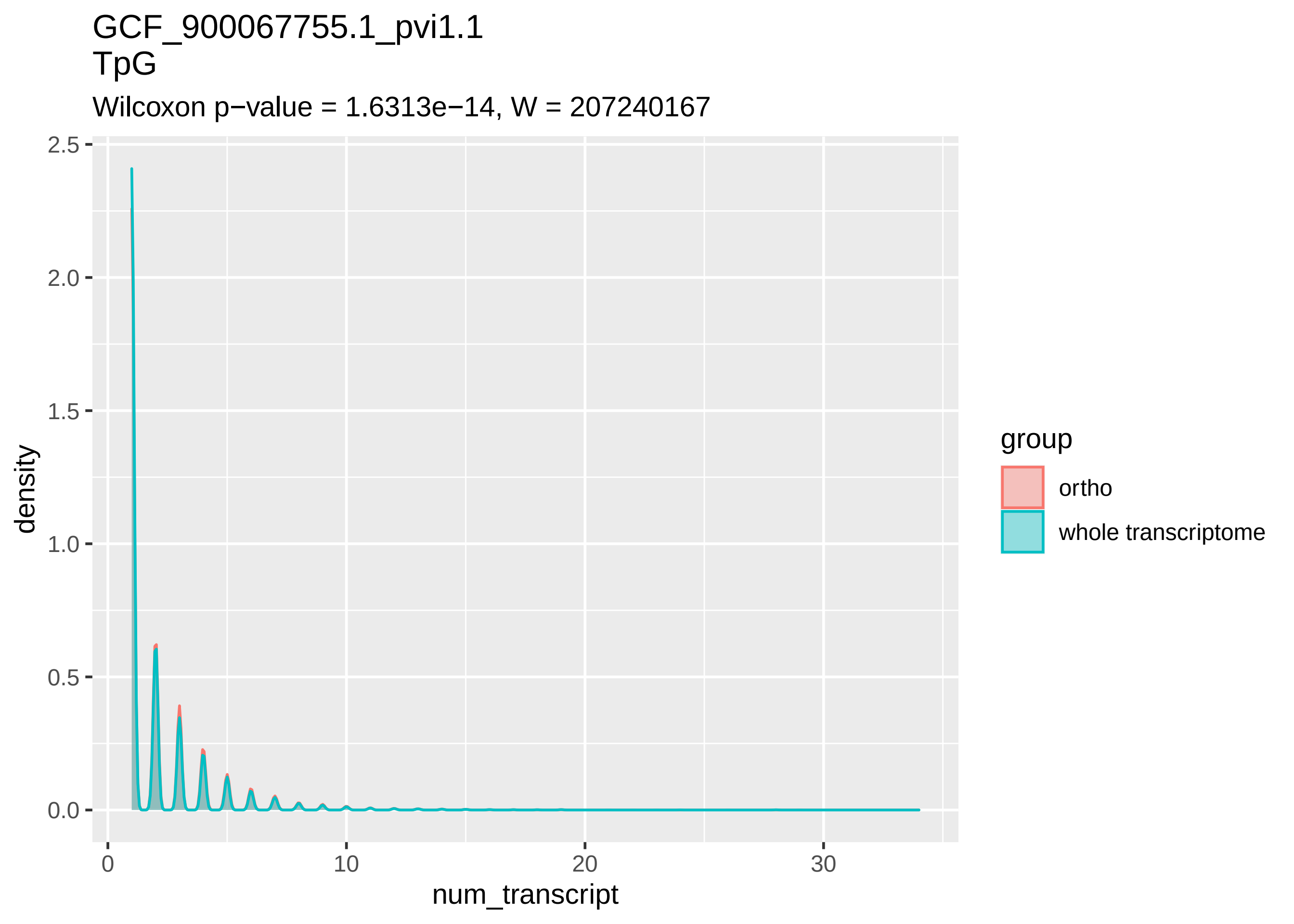
GCF_900067755.1_pvi1.1
TpG
Wilcoxon p−value = 1.6313e−14, W = 207240167
density
2.5
2.0
1.5
1.0
0.5
0.0
0
10
20
30
num_transcript
group
ortho
whole transcriptome

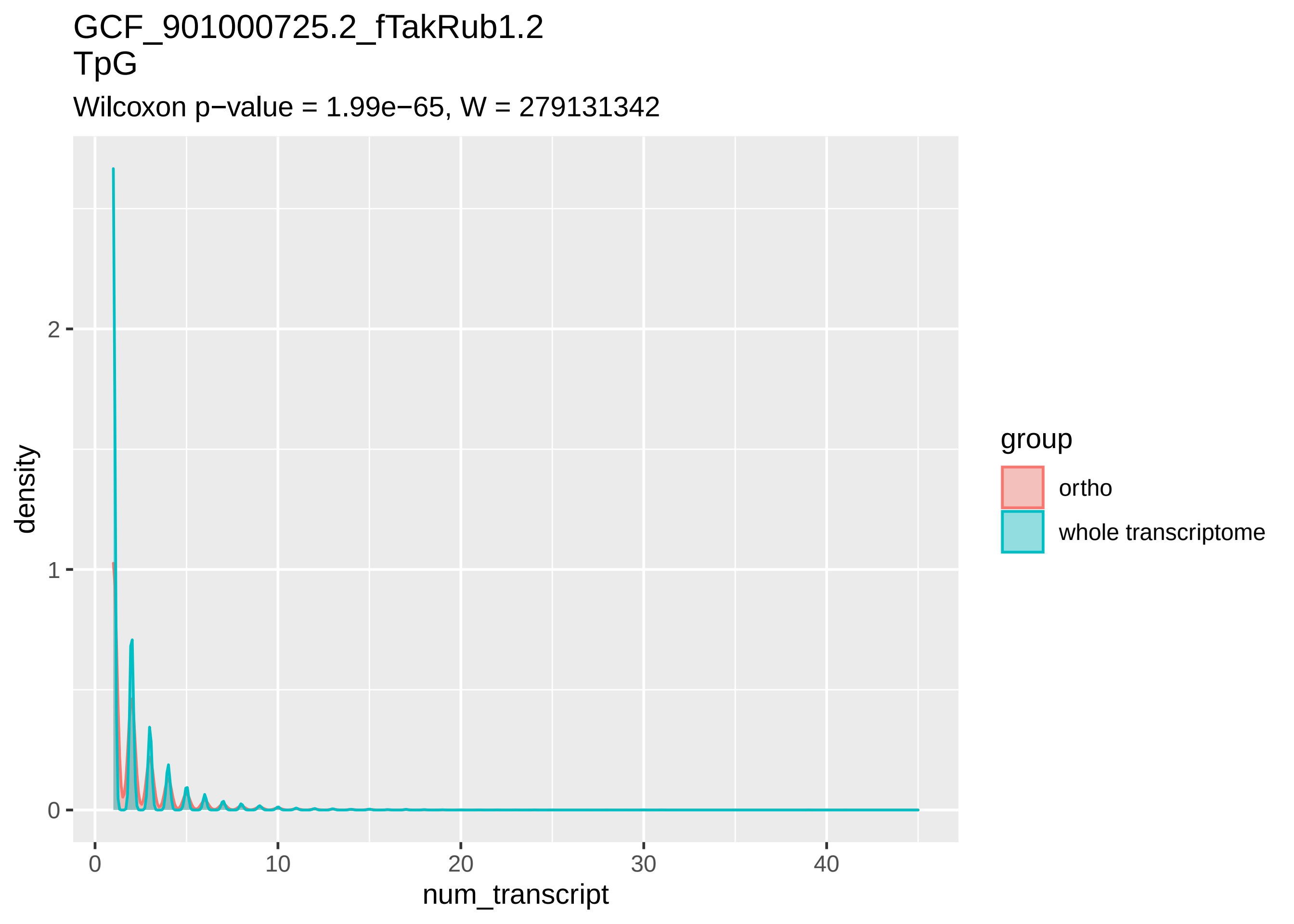
GCF_901000725.2_fTakRub1.2
TpG
Wilcoxon p−value = 1.99e−65, W = 279131342
density
num_transcript
0
1
2
0
10
20
30
40
group
ortho
whole transcriptome

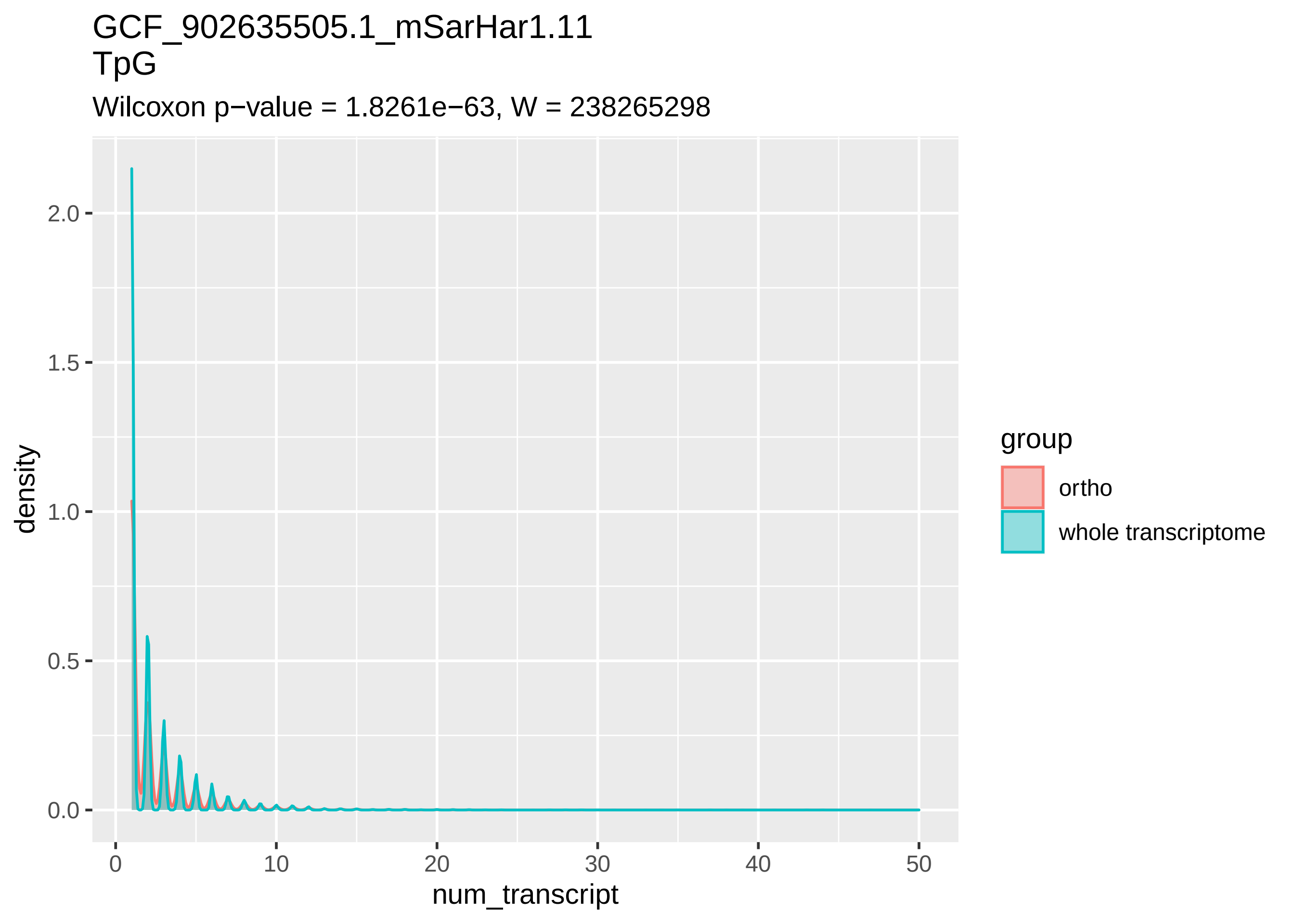

GCF_902635505.1_mSarHar1.11
TpG
Wilcoxon p−value = 1.8261e−63, W = 238265298
density
2.0
1.5
1.0
0.5
0.0
0
10
20
30
40
50
num_transcript
group
ortho
whole transcriptome

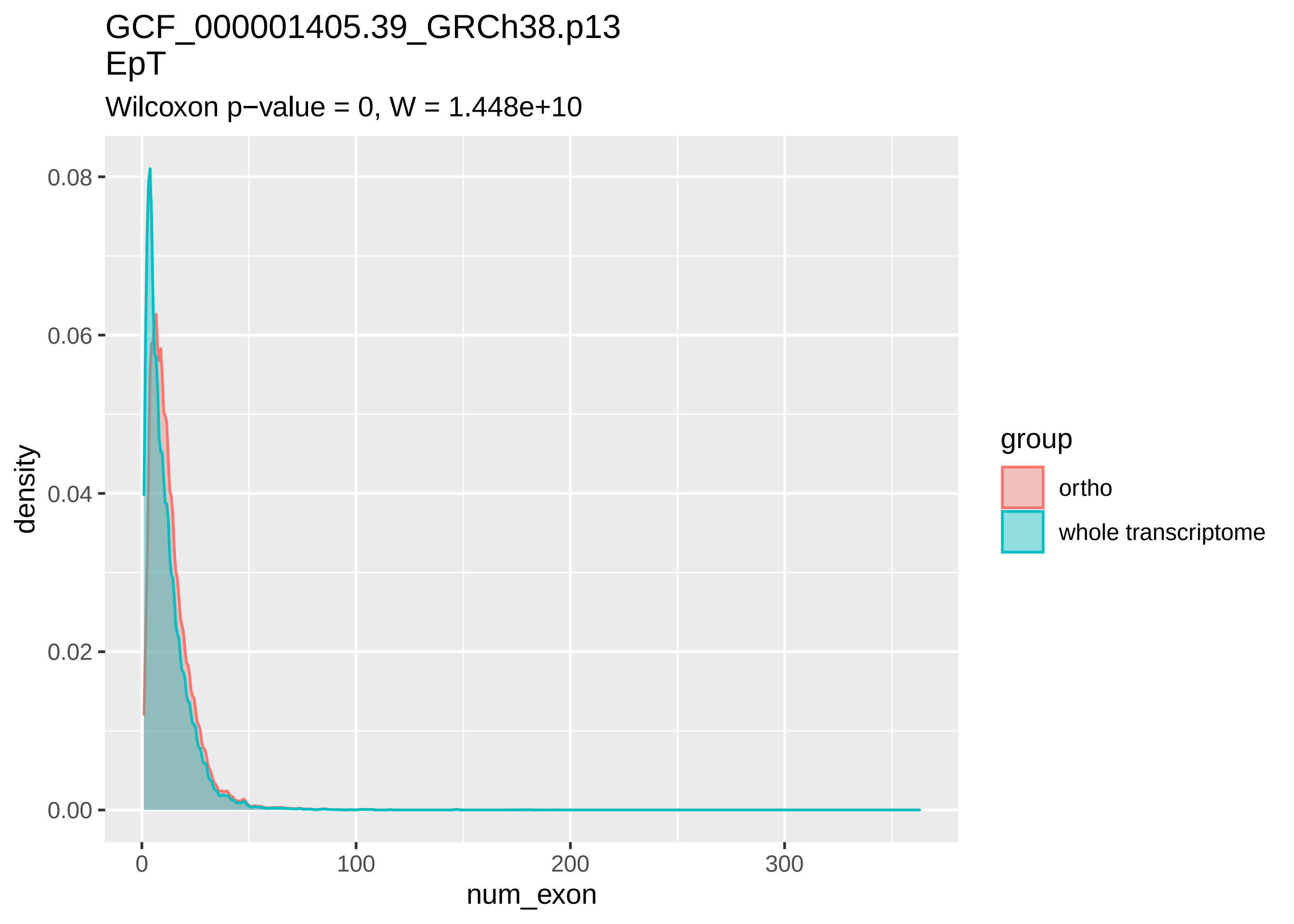
GCF_000001405.39_GRCh38.p13
EpT
Wilcoxon p−value = 0, W = 1.448e+10
0.08
0.06
0.04
0.02
0.00
density
0
100
200
300
num_exon
group
ortho
whole transcriptome

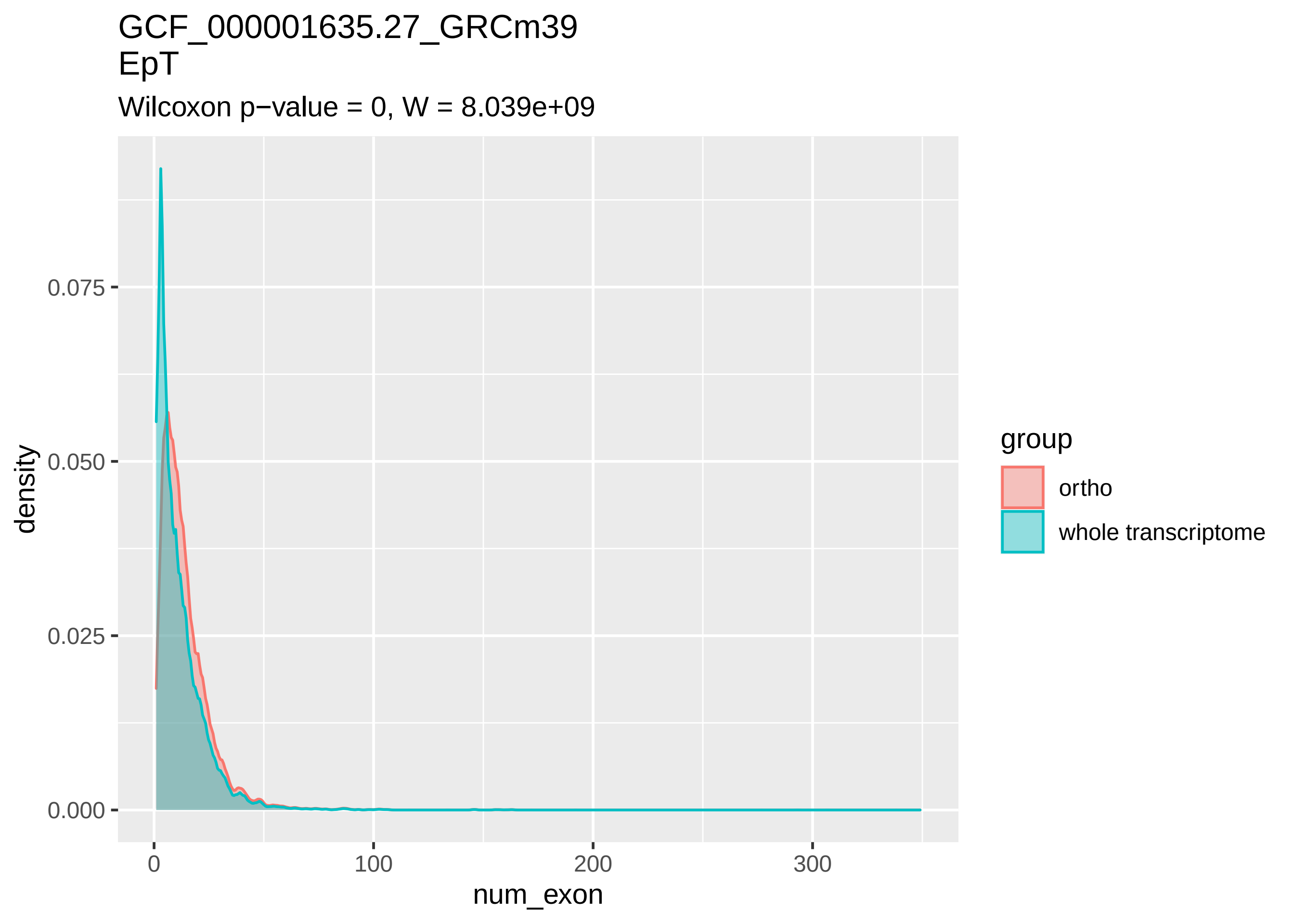

GCF_000001635.27_GRCm39
EpT
Wilcoxon p−value = 0, W = 8.039e+09
density
0.000
0.025
0.050
0.075
0
100
200
300
num_exon
group
ortho
whole transcriptome

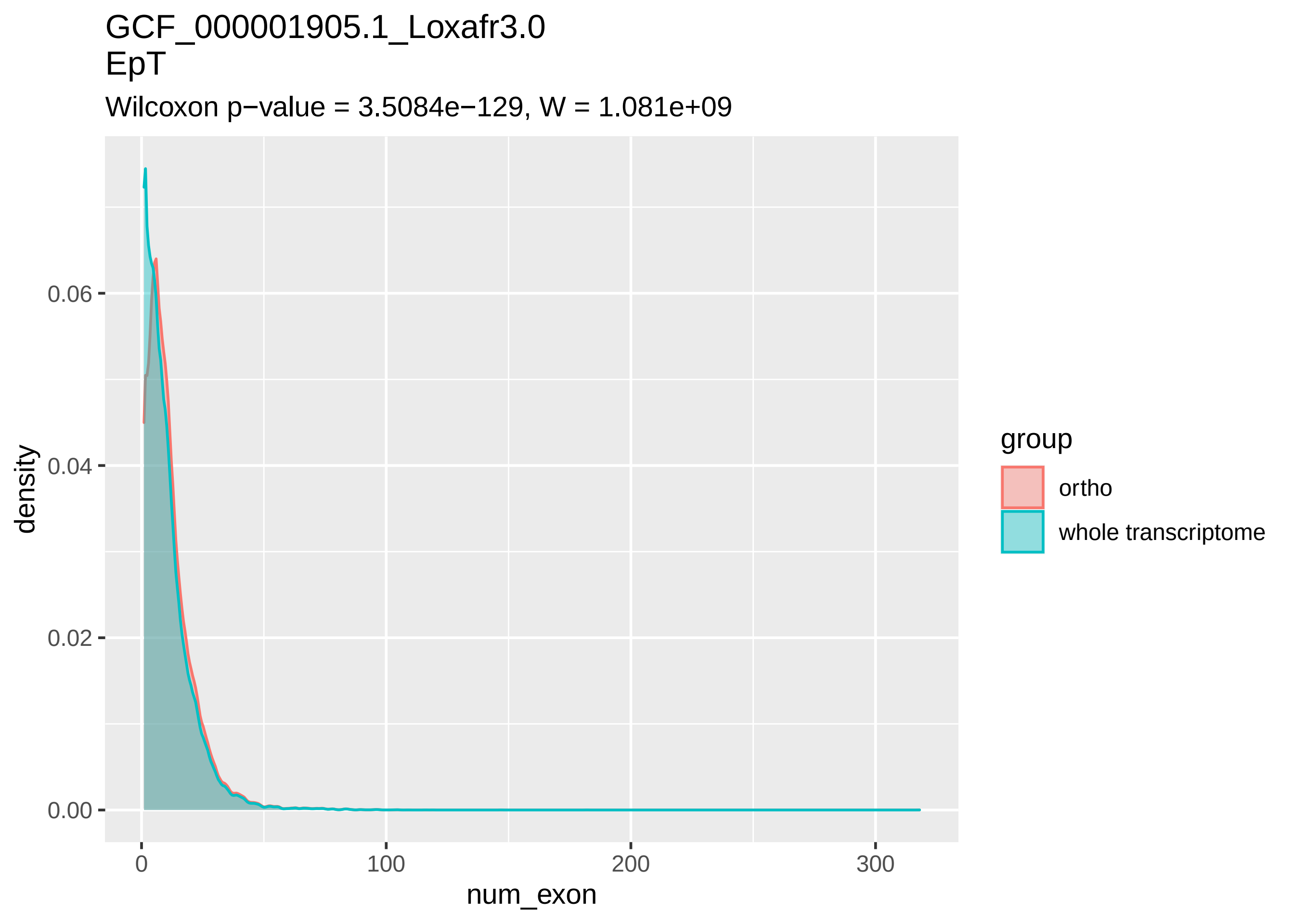
GCF_000001905.1_Loxafr3.0
EpT
Wilcoxon p−value = 3.5084e−129, W = 1.081e+09
density
0.00
0.02
0.04
0.06
0
100
200
300
num_exon
group
ortho
whole transcriptome

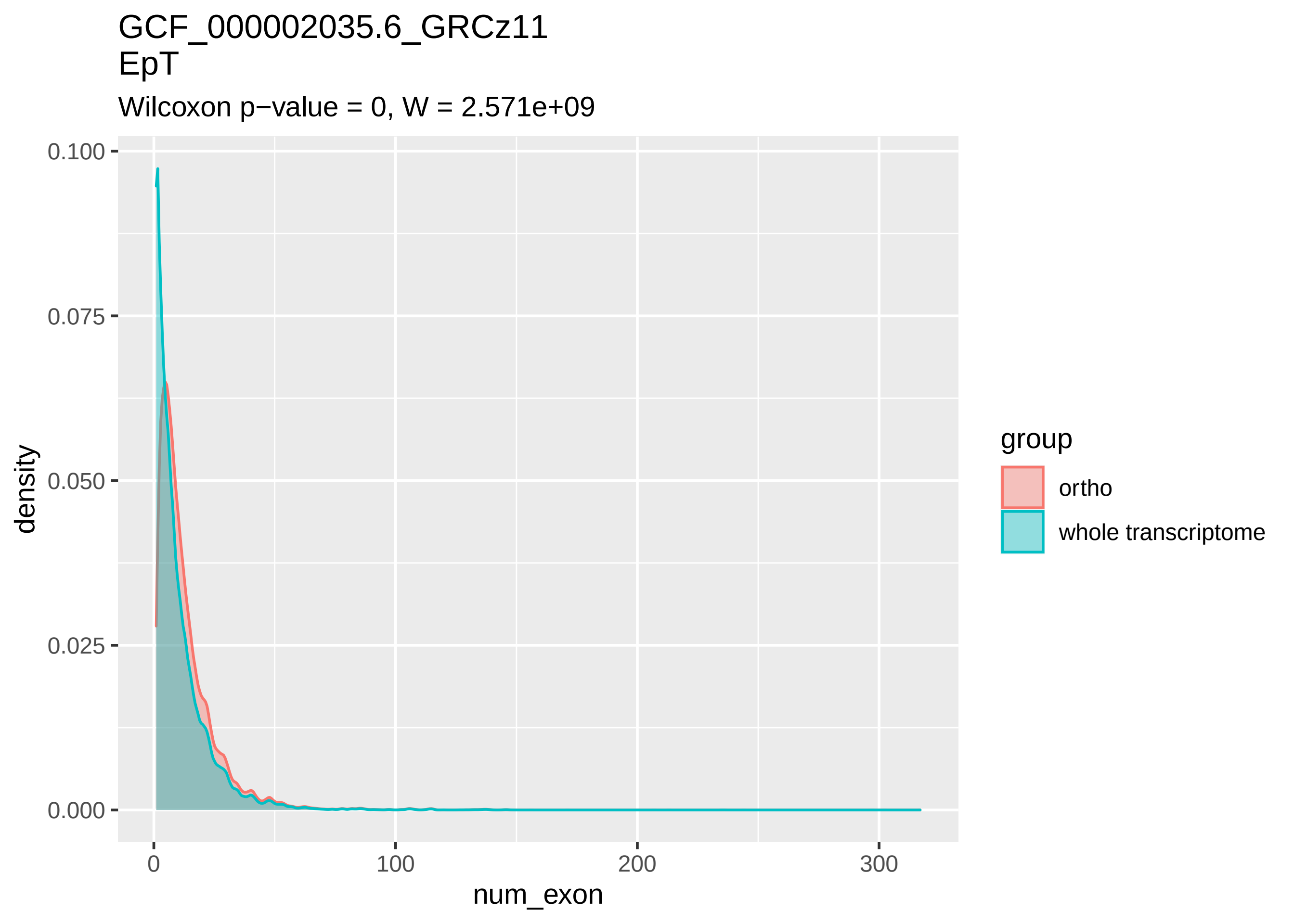
GCF_000002035.6_GRCz11
EpT
Wilcoxon p−value = 0, W = 2.571e+09
density
0.100
0.075
0.050
0.025
0.000
0
100
200
300
num_exon
group
ortho
whole transcriptome

GCF_000002235.5_Spur_5.0
EpT

Wilcoxon p−value = 0, W = 711586317

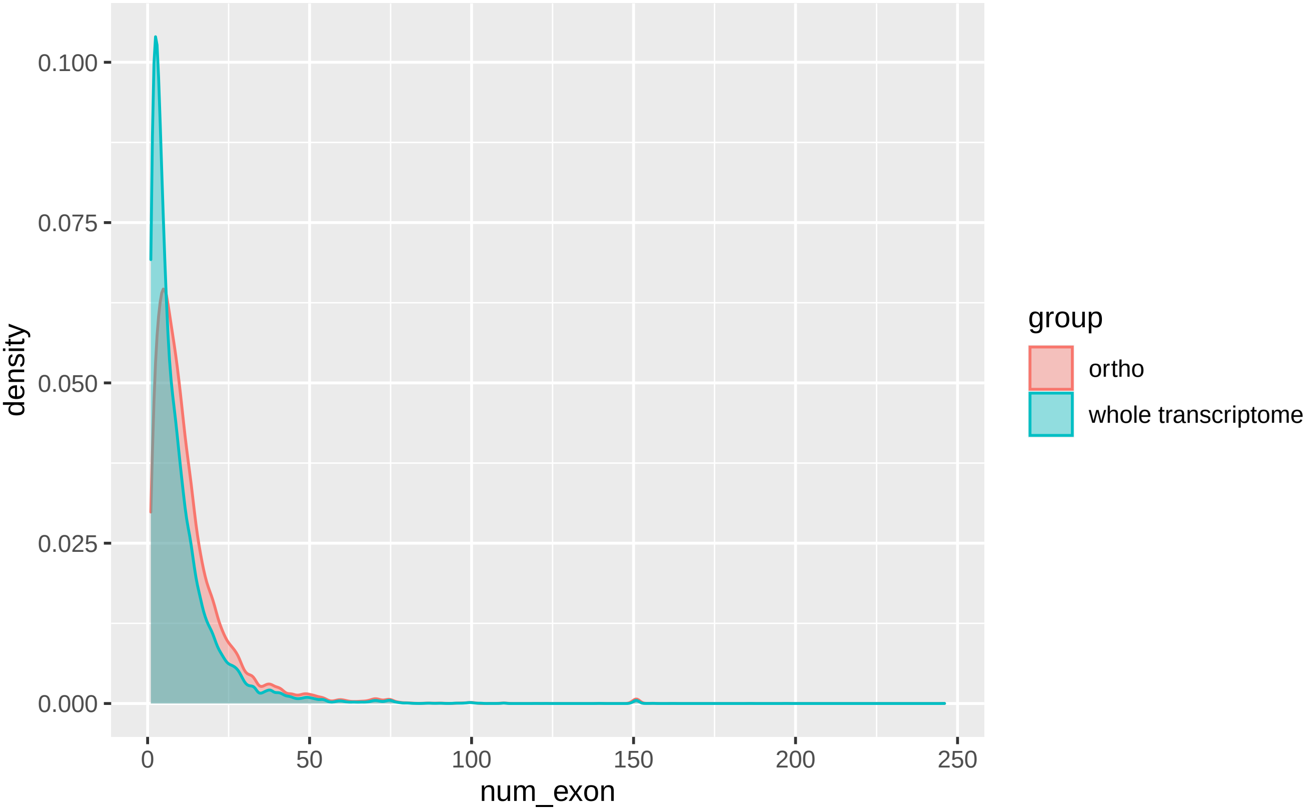

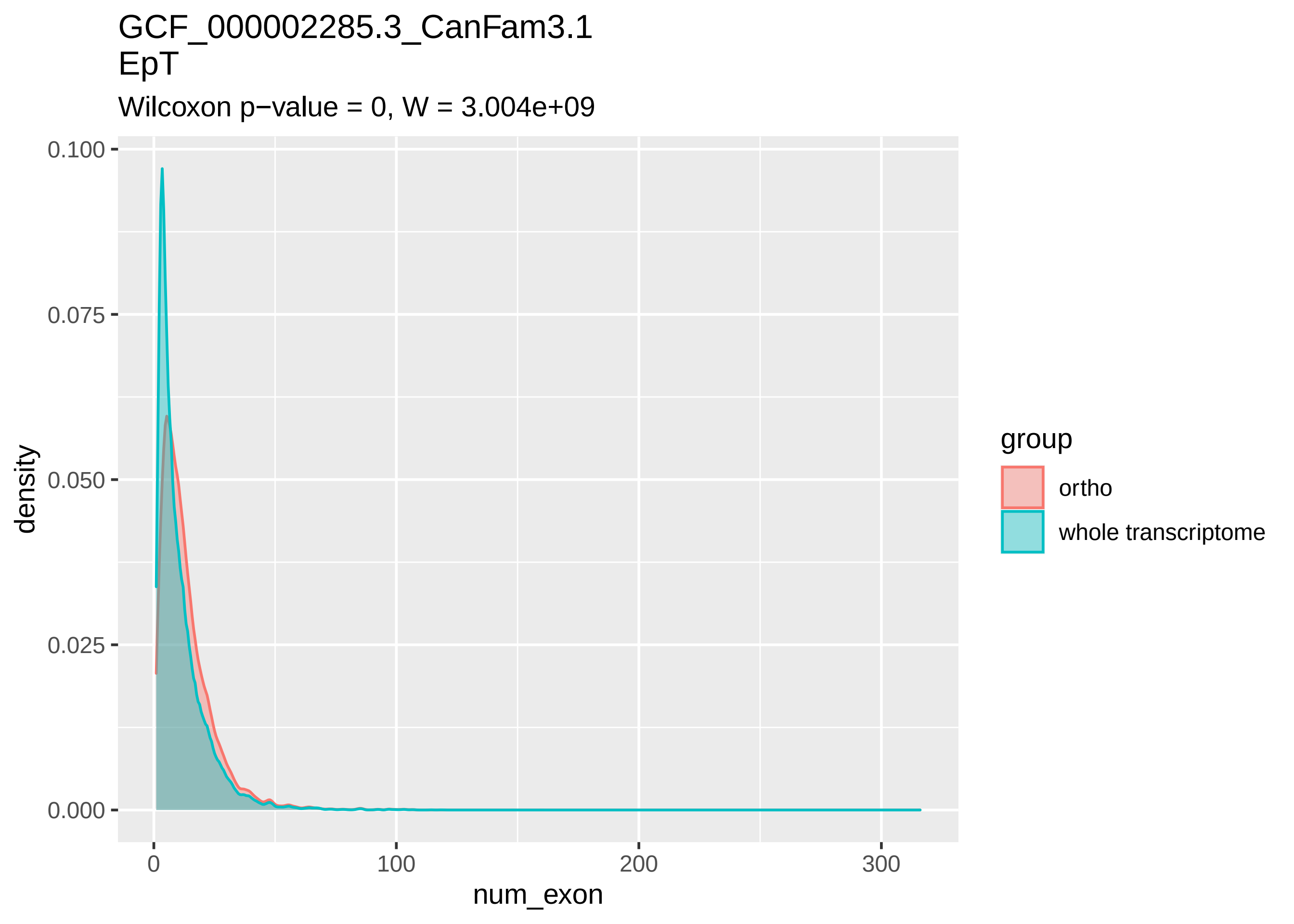
GCF_000002285.3_CanFam3.1
EpT
Wilcoxon p−value = 0, W = 3.004e+09
0.100
0.075
0.050
0.025
0.000
density
0
100
200
300
num_exon
group
ortho
whole transcriptome

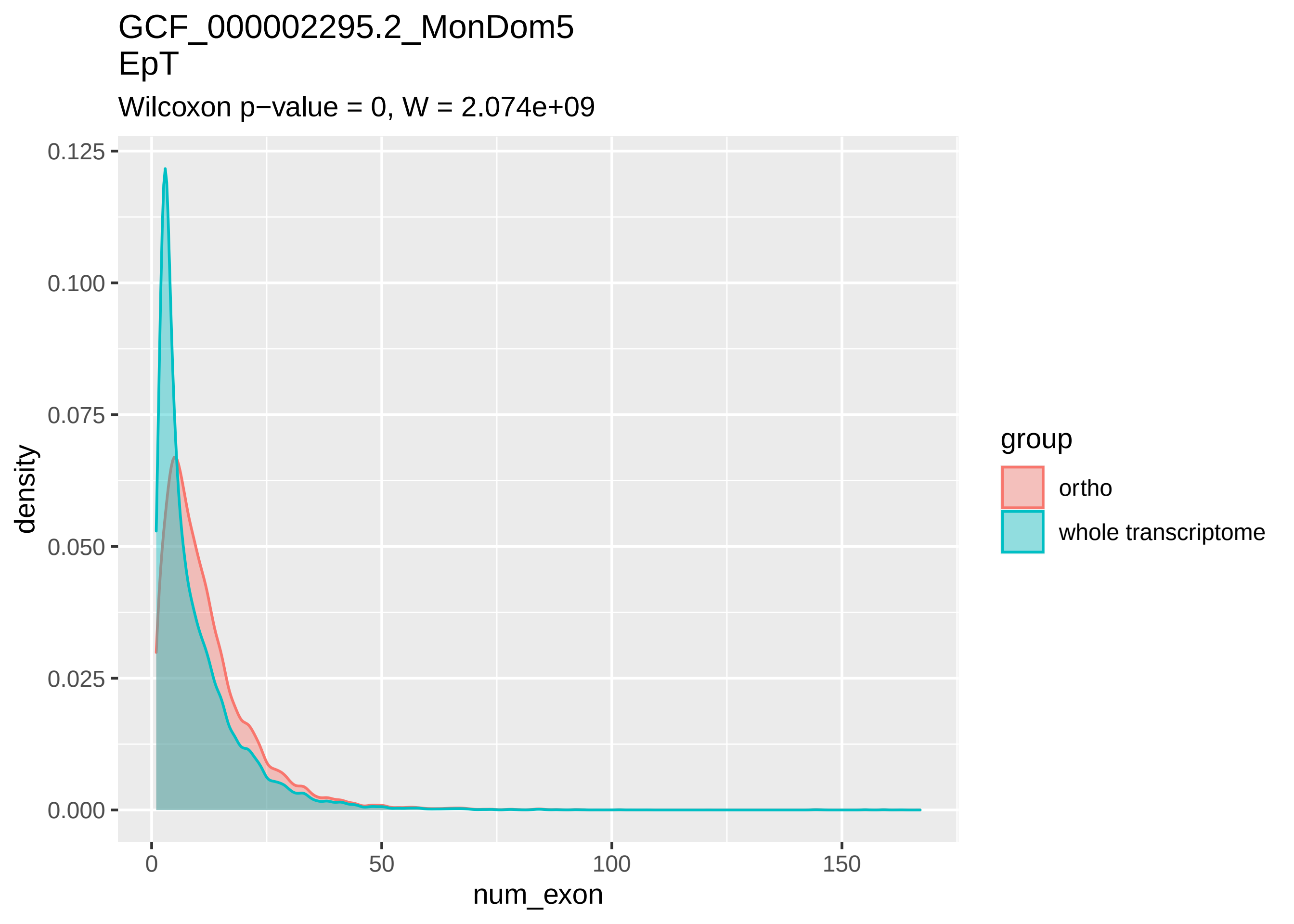
GCF_000002295.2_MonDom5
EpT
Wilcoxon p−value = 0, W = 2.074e+09
0.125
0.100
0.075
0.050
0.025
0.000
density
0
50
100
150
num_exon
group
ortho
whole transcriptome

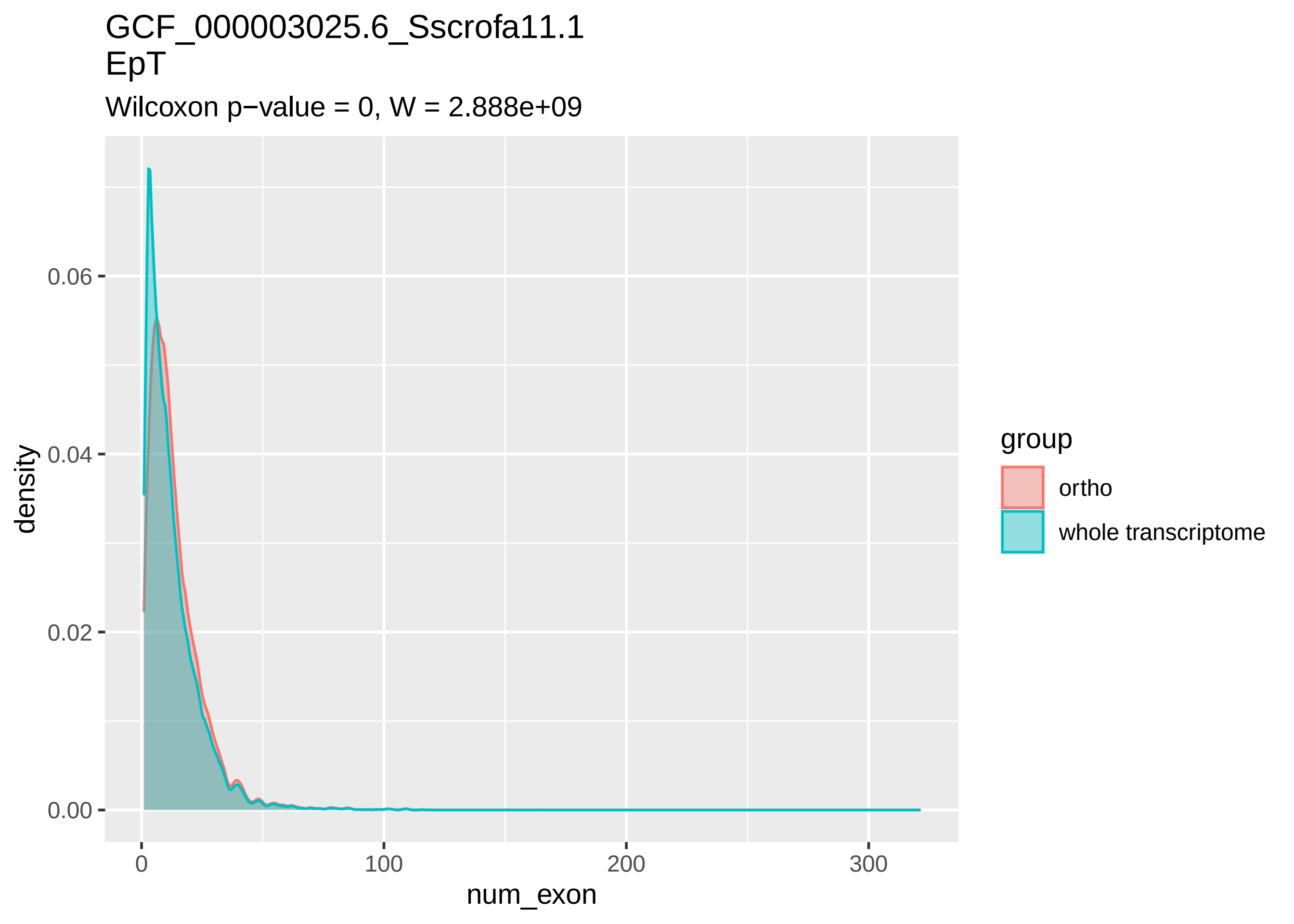

GCF_000003025.6_Sscrofa11.1
EpT
Wilcoxon p−value = 0, W = 2.888e+09
density
0.06
0.04
0.02
0.00
0
100
200
300
num_exon
group
ortho
whole transcriptome

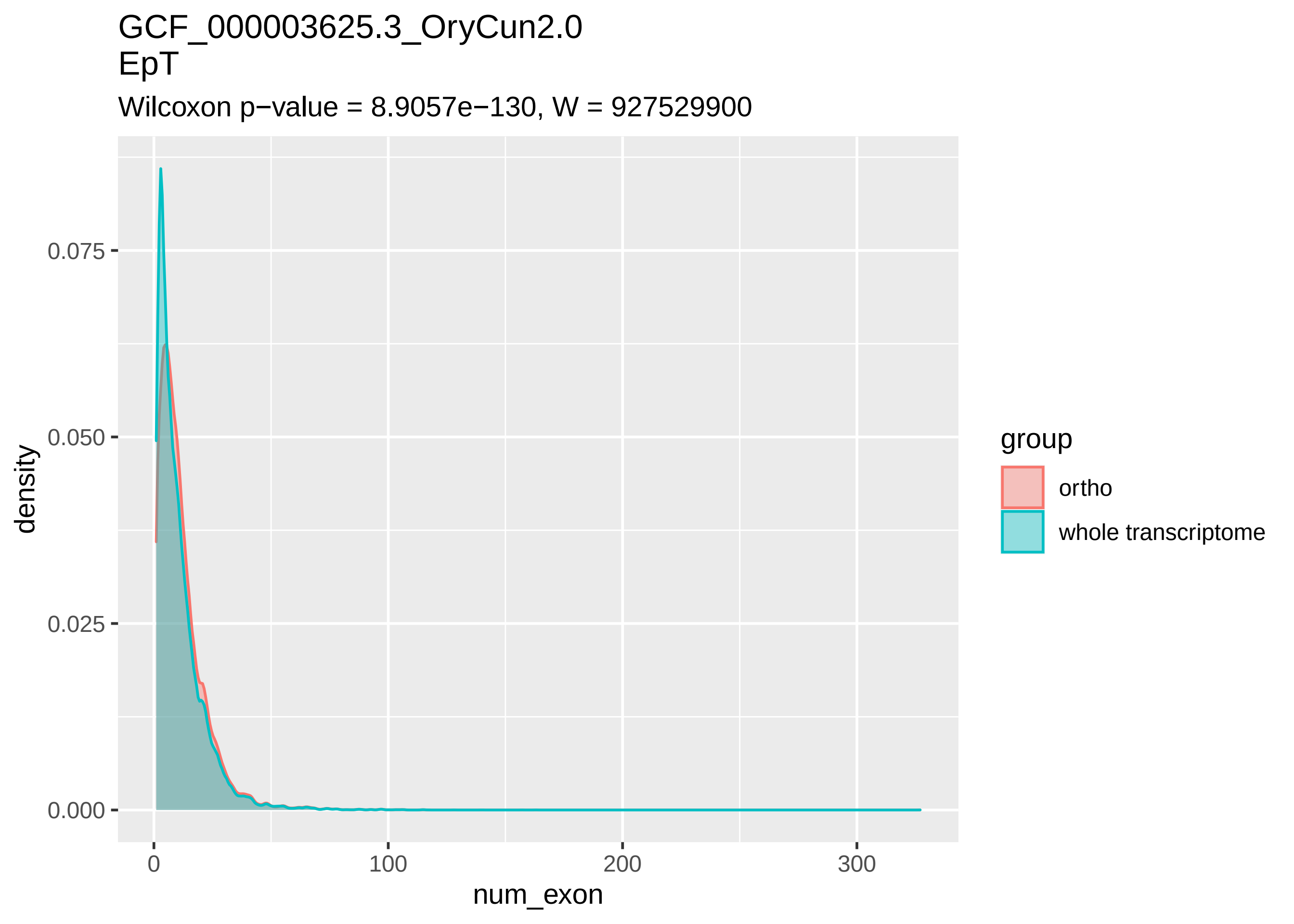

GCF_000003625.3_OryCun2.0
EpT
Wilcoxon p−value = 8.9057e−130, W = 927529900
density
0.075
0.050
0.025
0.000
0
100
200
300
num_exon
group
ortho
whole transcriptome

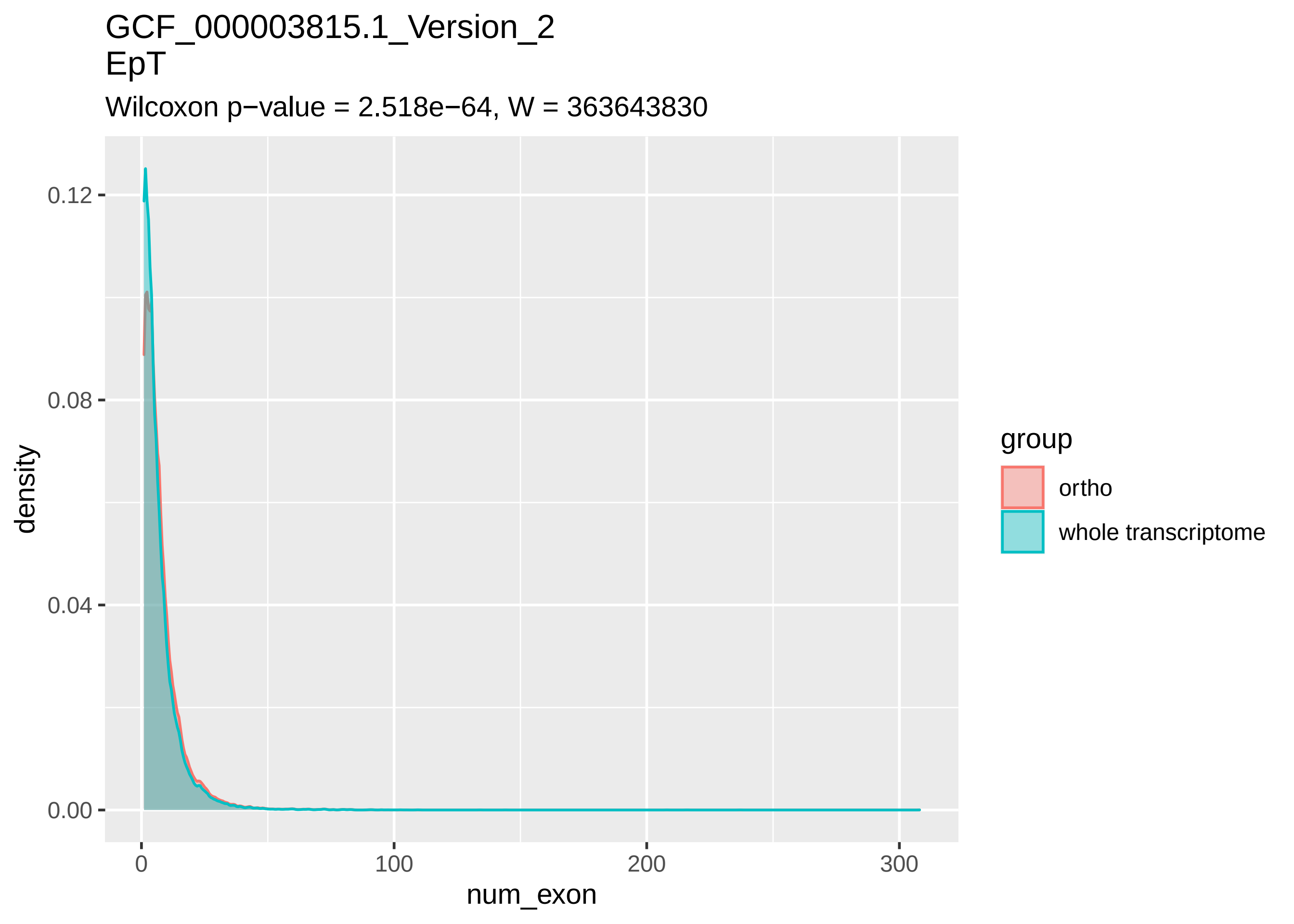
GCF_000003815.1_Version_2
EpT
Wilcoxon p−value = 2.518e−64, W = 363643830
density
0.12
0.08
0.04
0.00
0
100
200
300
num_exon
group
ortho
whole transcriptome

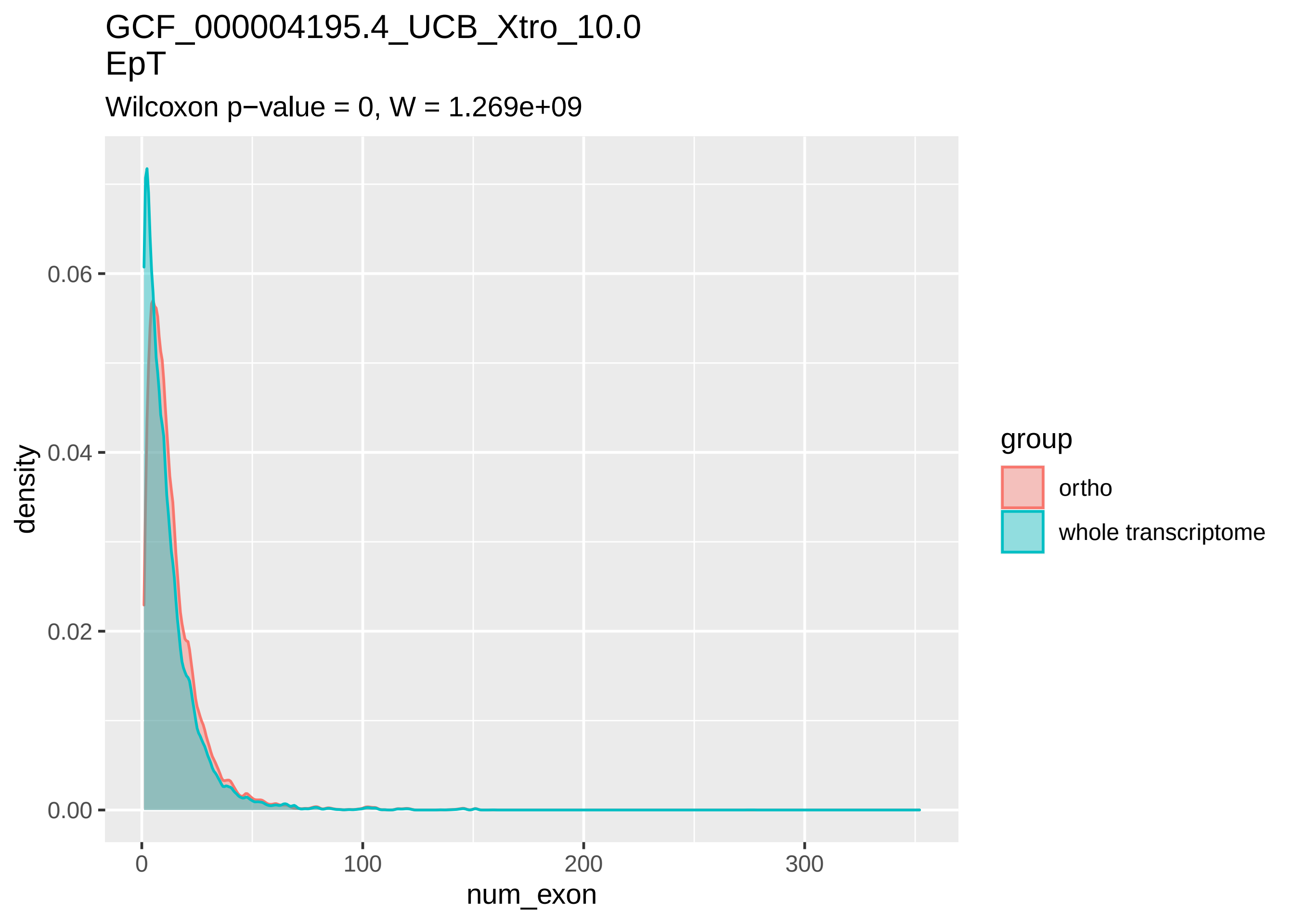
GCF_000004195.4_UCB_Xtro_10.0
EpT
Wilcoxon p−value = 0, W = 1.269e+09
density
0.06
0.04
0.02
0.00
0
100
200
300
num_exon
group
ortho
whole transcriptome

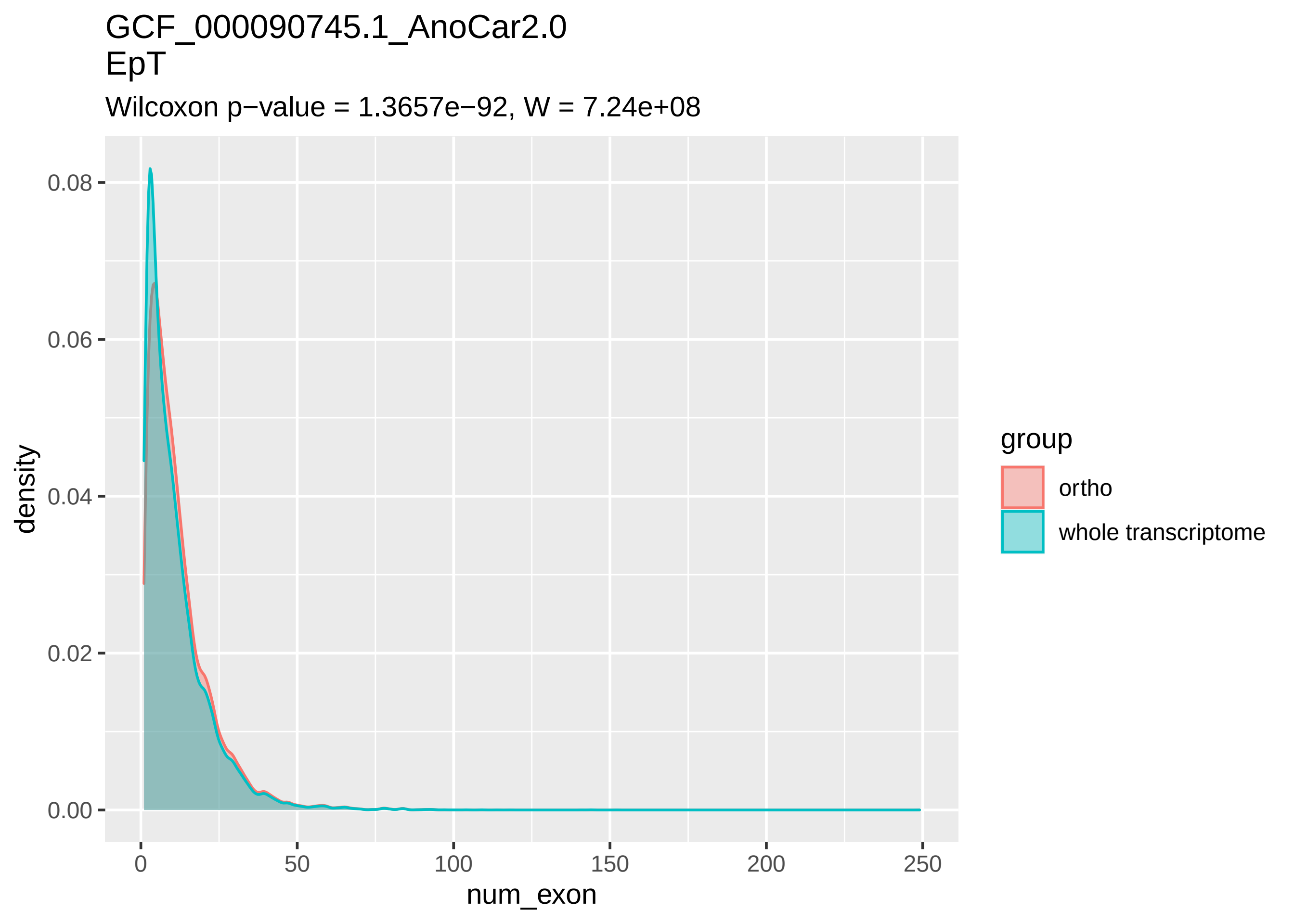
GCF_000090745.1_AnoCar2.0
EpT
Wilcoxon p−value = 1.3657e−92, W = 7.24e+08
density
0.08
0.06
0.04
0.02
0.00
0
50
100
150
200
250
num_exon
group
ortho
whole transcriptome

GCF_000151735.1_Cavpor3.0
EpT

Wilcoxon p−value = 3.917e−271, W = 9.66e+08

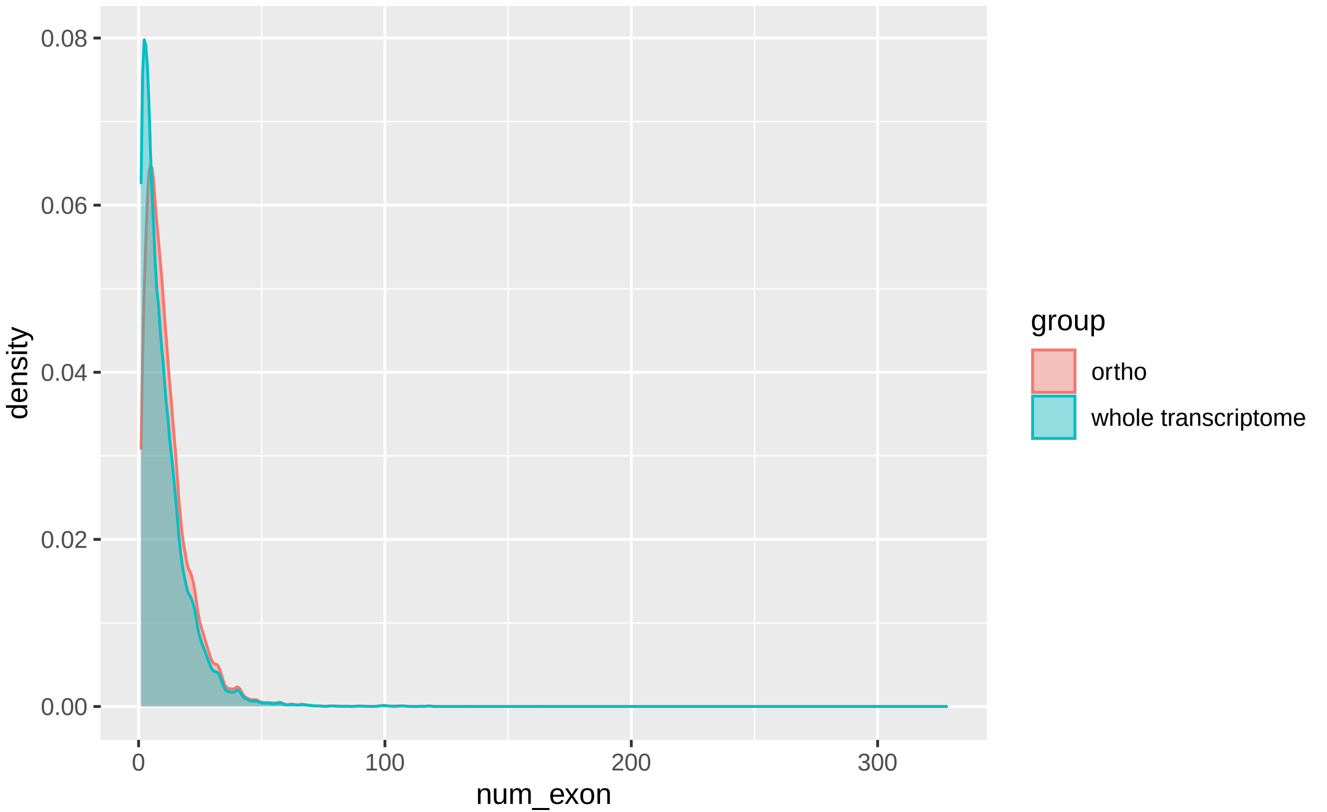

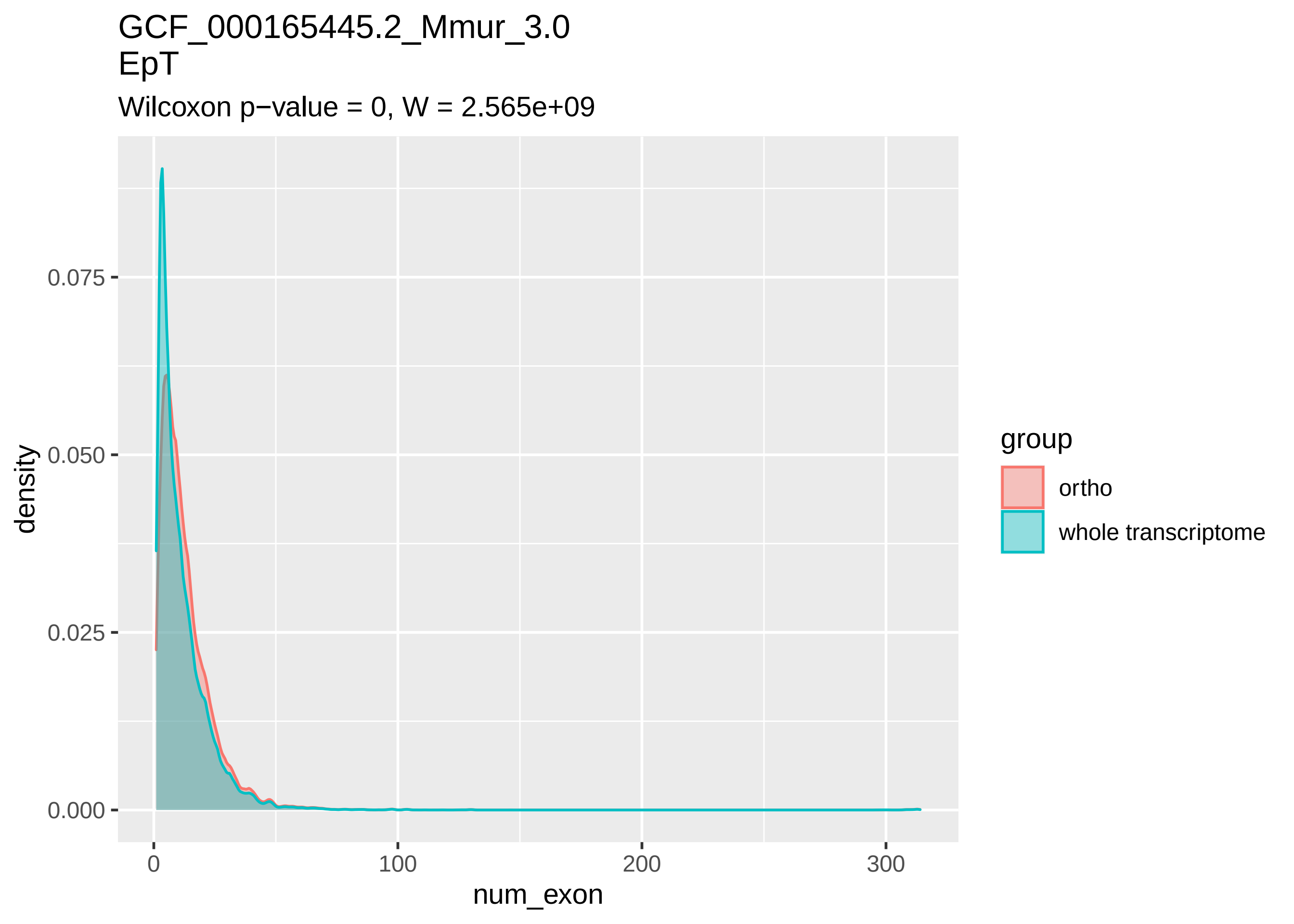

GCF_000165445.2_Mmur_3.0
EpT
Wilcoxon p−value = 0, W = 2.565e+09
density
0.000
0.025
0.050
0.075
0
100
200
300
num_exon
group
ortho
whole transcriptome

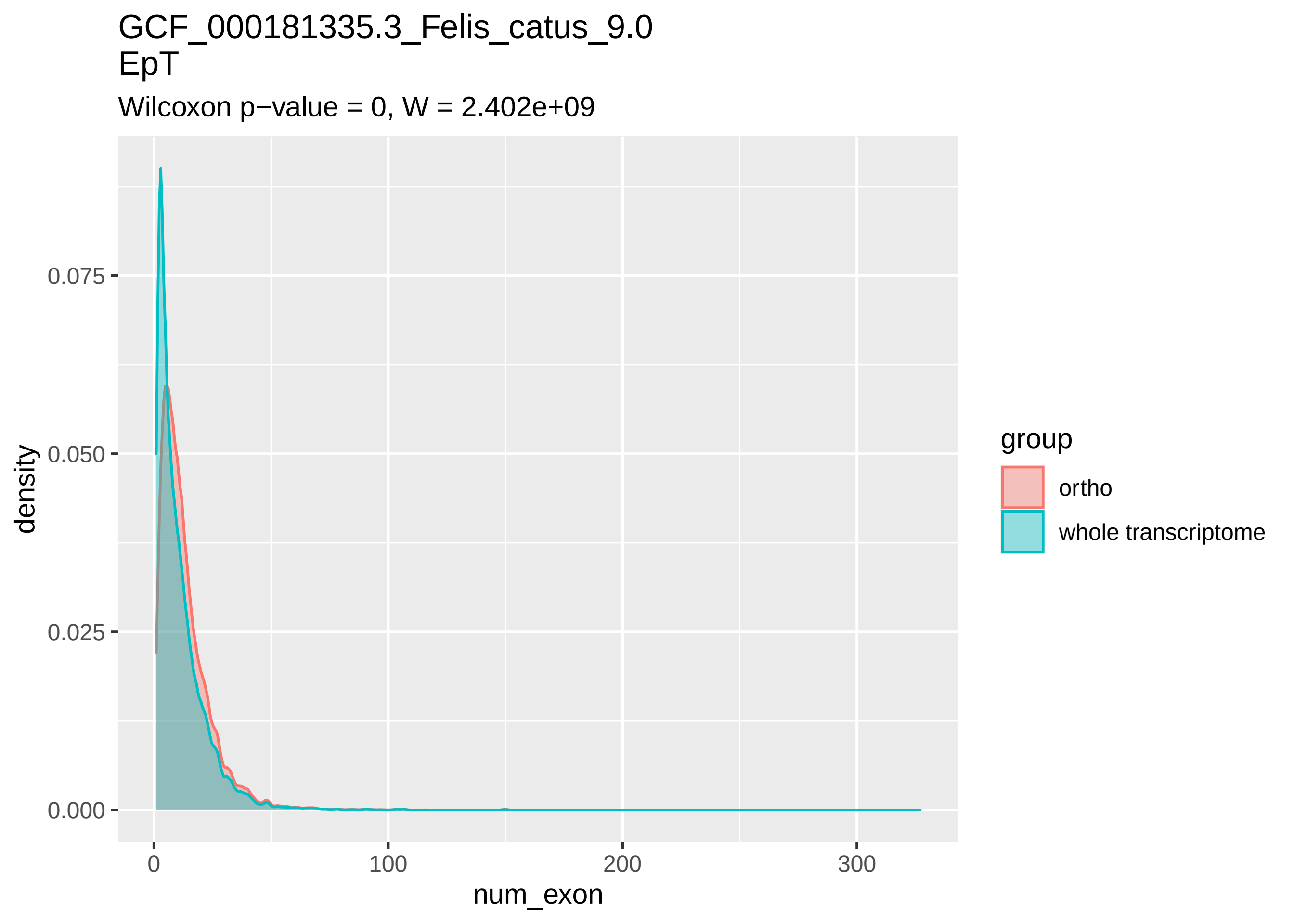
GCF_000181335.3_Felis_catus_9.0
EpT
Wilcoxon p−value = 0, W = 2.402e+09
density
0.075
0.050
0.025
0.000
0
100
200
300
num_exon
group
ortho
whole transcriptome

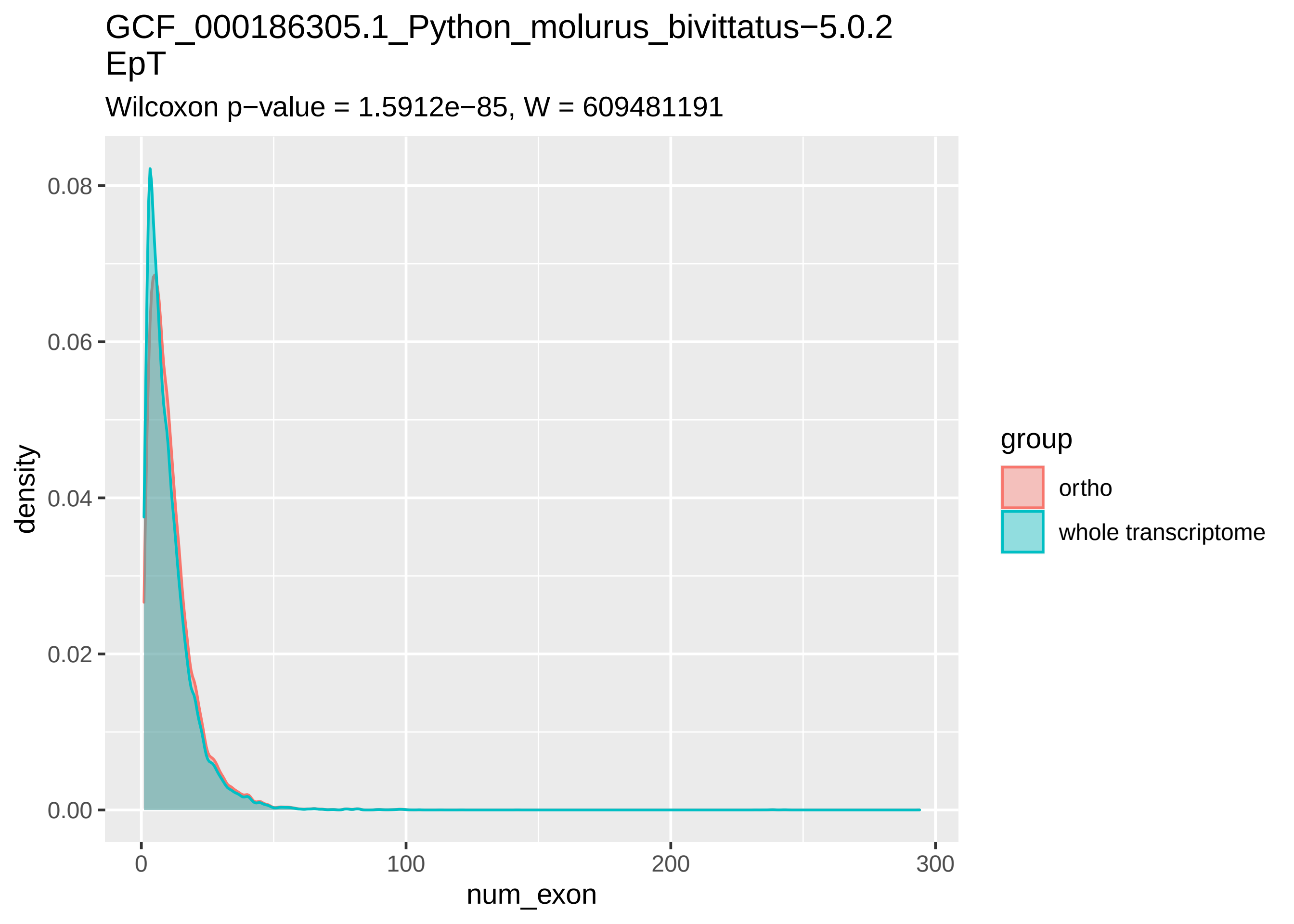

GCF_000186305.1_Python_molurus_bivittatus−5.0.2
EpT
Wilcoxon p−value = 1.5912e−85, W = 609481191
density
0.08
0.06
0.04
0.02
0.00
0
100
200
300
num_exon
group
ortho
whole transcriptome

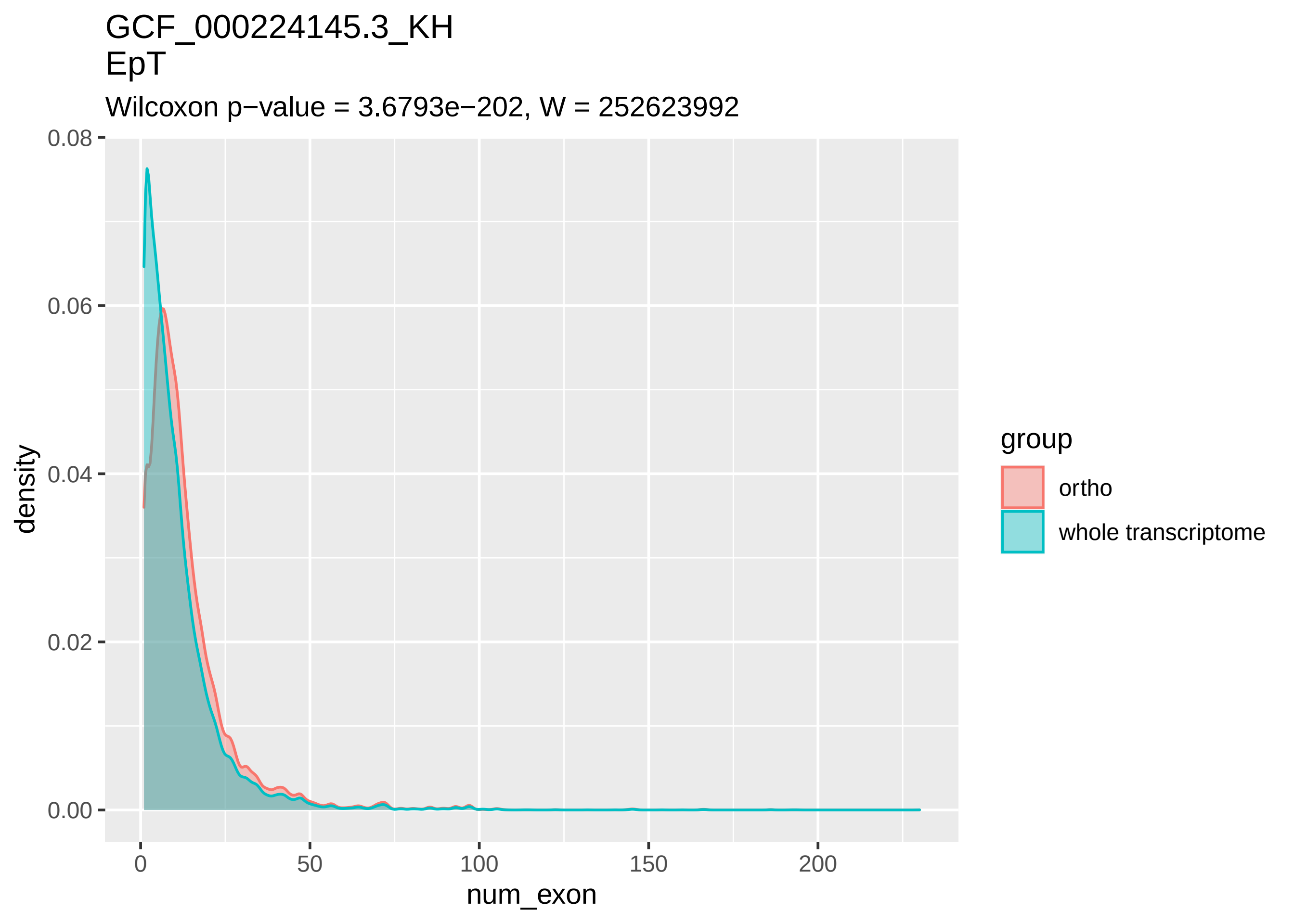
GCF_000224145.3_KH
EpT
Wilcoxon p−value = 3.6793e−202, W = 252623992
0.08
0.06
0.04
0.02
0.00
density
0
50
100
150
200
num_exon
group
ortho
whole transcriptome

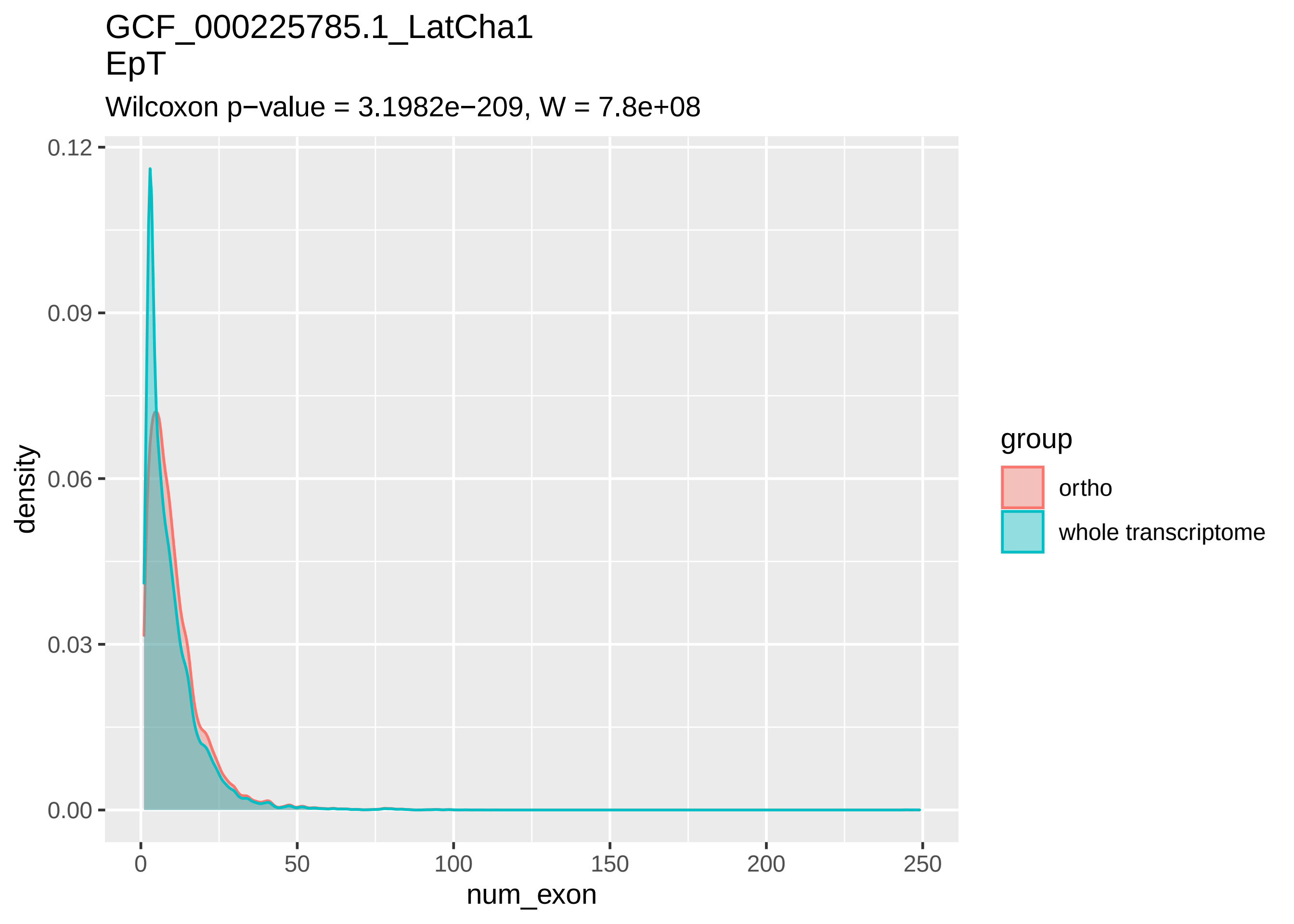
GCF_000225785.1_LatCha1
EpT
Wilcoxon p−value = 3.1982e−209, W = 7.8e+08
0.12
0.09
0.06
0.03
0.00
density
0
50
100
150
200
250
num_exon
group
ortho
whole transcriptome

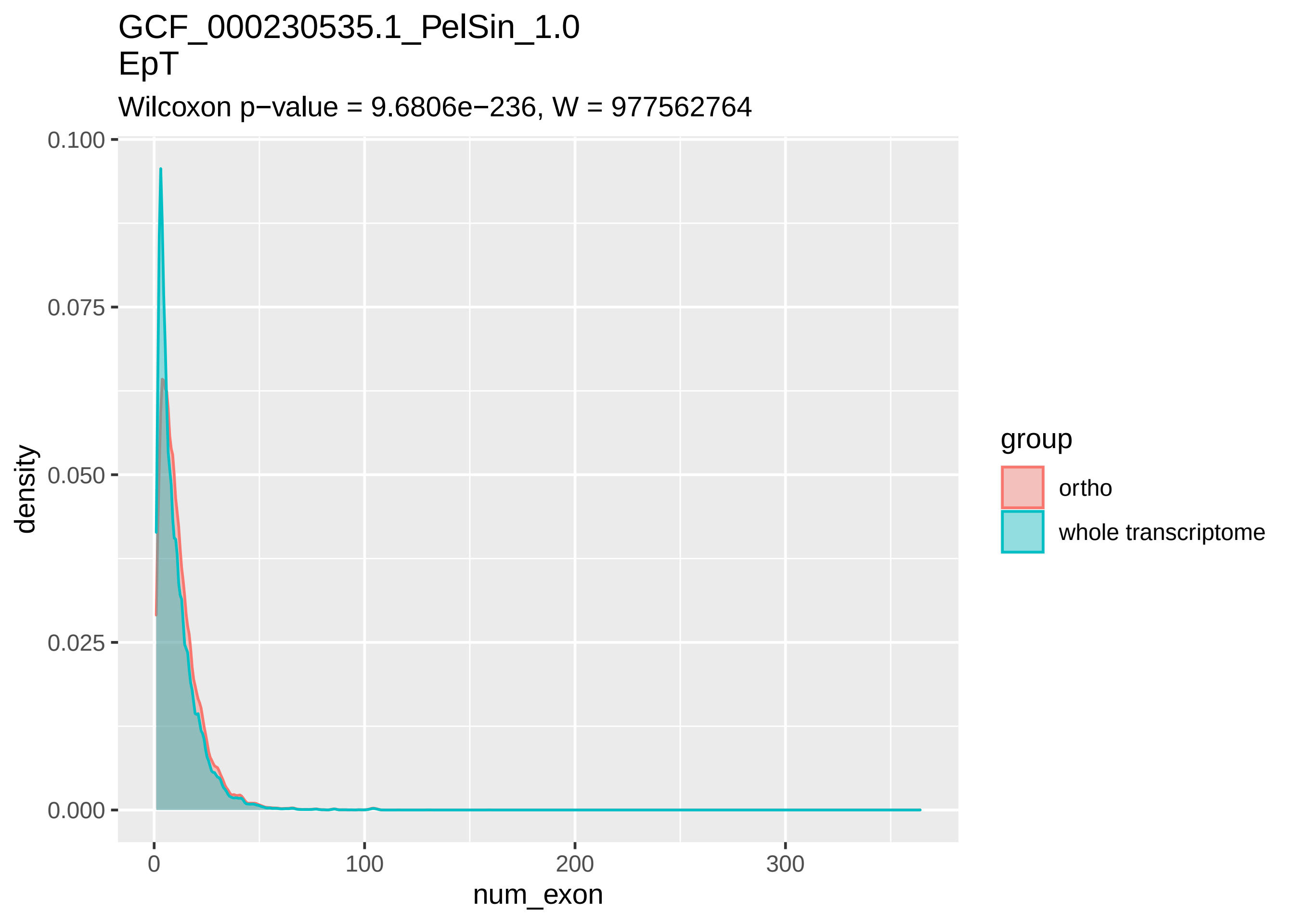
GCF_000230535.1_PelSin_1.0
EpT
Wilcoxon p−value = 9.6806e−236, W = 977562764
density
0.100
0.075
0.050
0.025
0.000
0
100
200
300
num_exon
group
ortho
whole transcriptome

GCF_000281125.3_ASM28112v4
EpT

Wilcoxon p−value = 5.8896e−261, W = 1.245e+09

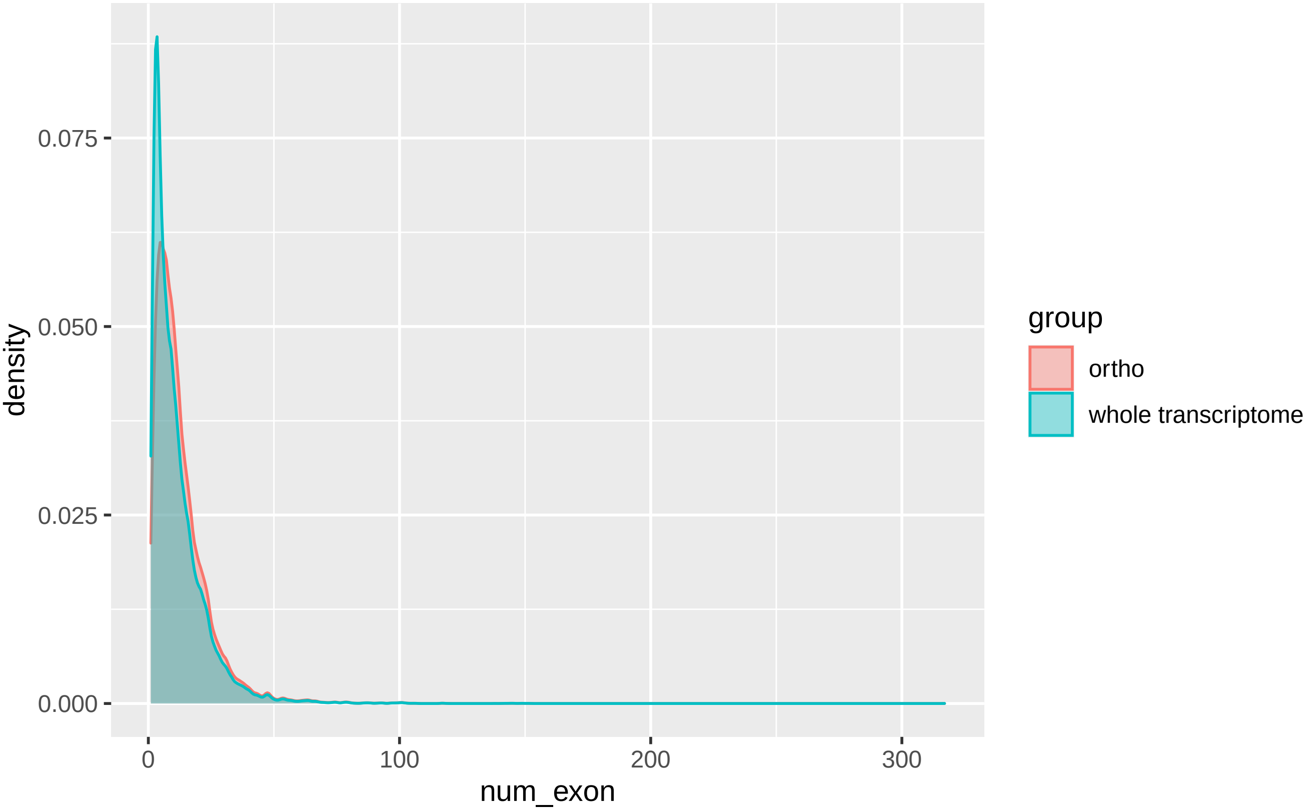

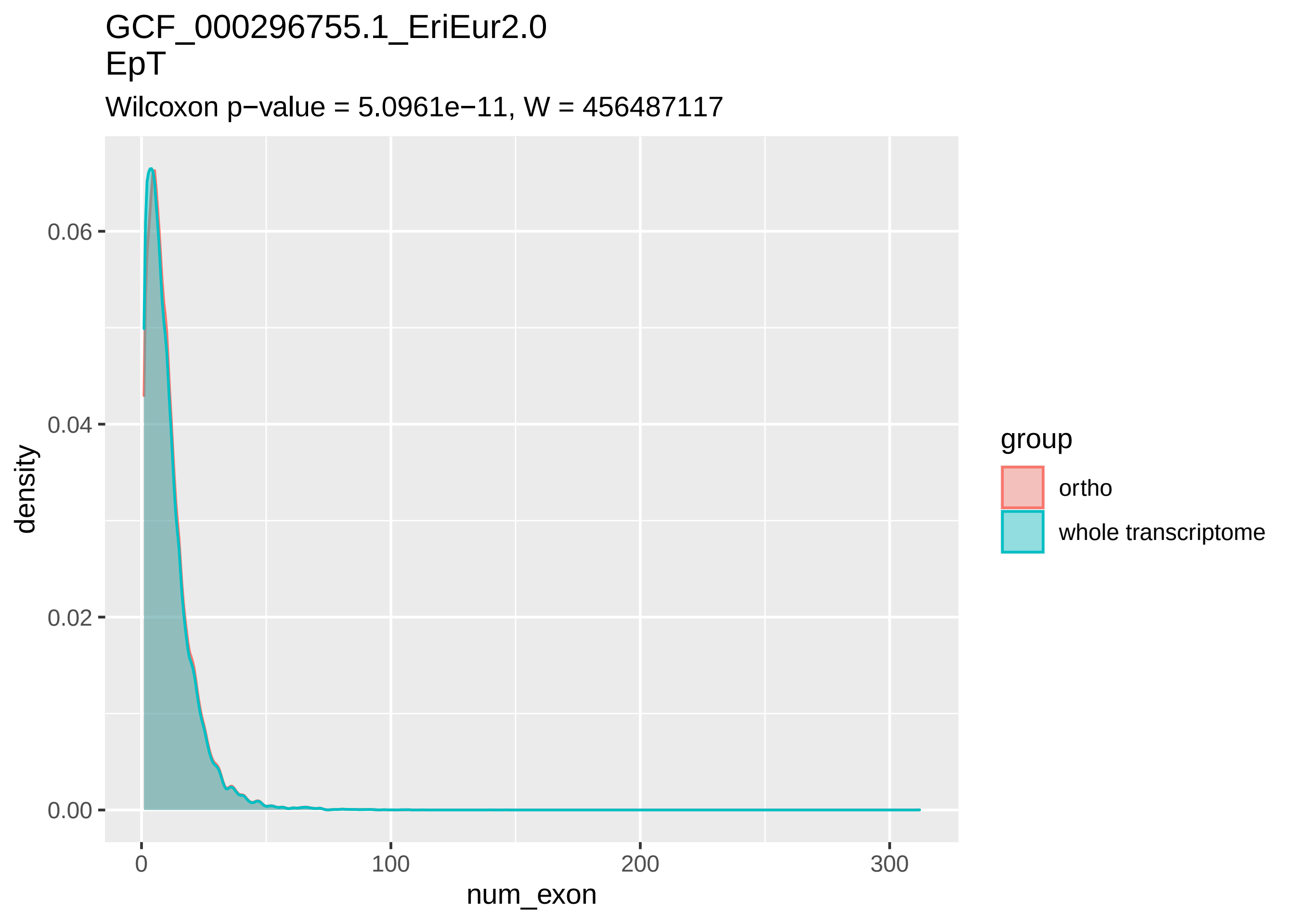

GCF_000296755.1_EriEur2.0
EpT
Wilcoxon p−value = 5.0961e−11, W = 456487117
density
0.06
0.04
0.02
0.00
0
100
200
300
num_exon
group
ortho
whole transcriptome

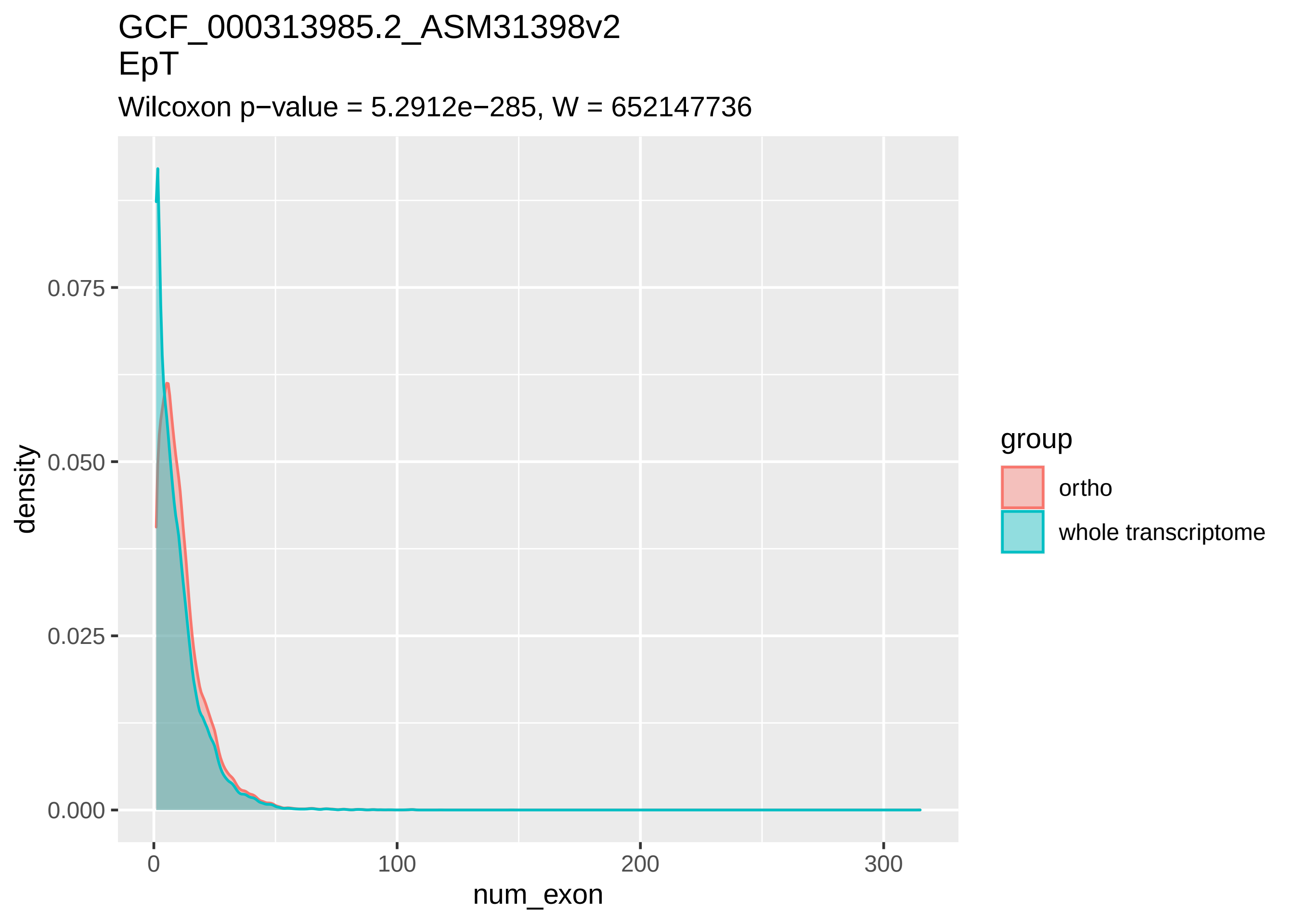

GCF_000313985.2_ASM31398v2
EpT
Wilcoxon p−value = 5.2912e−285, W = 652147736
density
0.000
0.025
0.050
0.075
0
100
200
300
num_exon
group
ortho
whole transcriptome

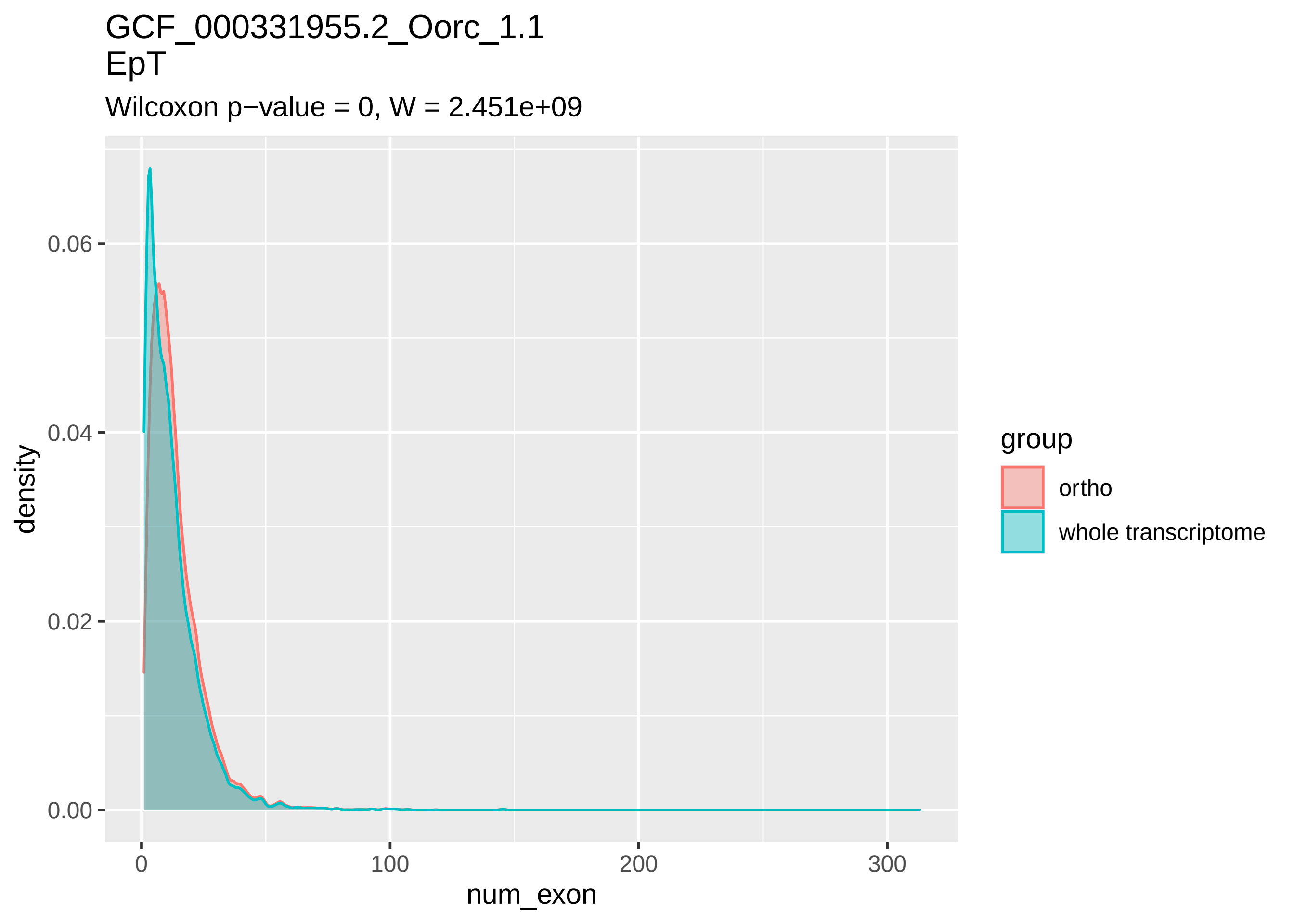
GCF_000331955.2_Oorc_1.1
EpT
Wilcoxon p−value = 0, W = 2.451e+09
density
0.06
0.04
0.02
0.00
0
100
200
300
num_exon
group
ortho
whole transcriptome

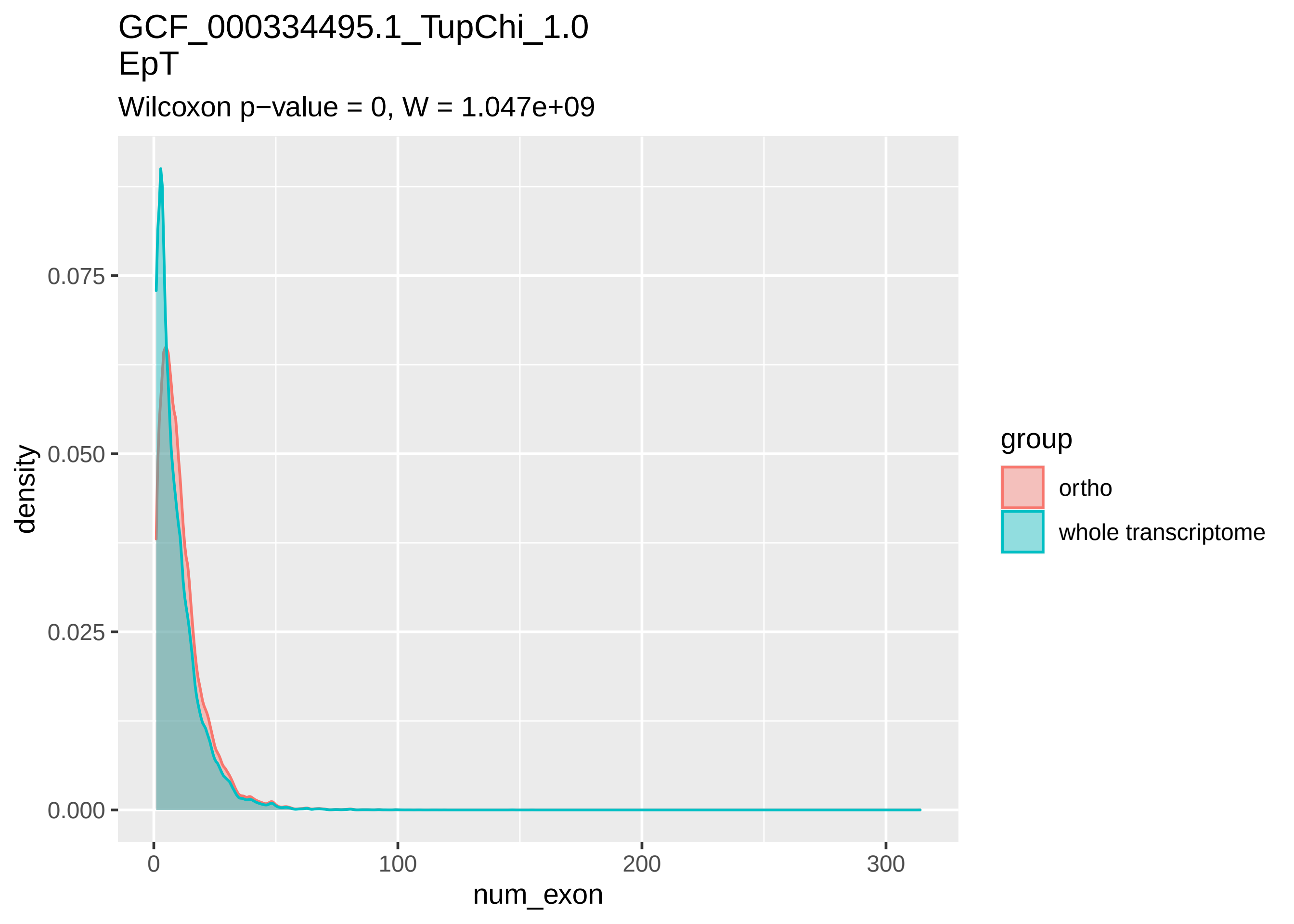

GCF_000334495.1_TupChi_1.0
EpT
Wilcoxon p−value = 0, W = 1.047e+09
density
0.000
0.025
0.050
0.075
0
100
200
300
num_exon
group
ortho
whole transcriptome

GCF_000337935.1_Cliv_1.0
EpT

Wilcoxon p−value = 0, W = 1.087e+09

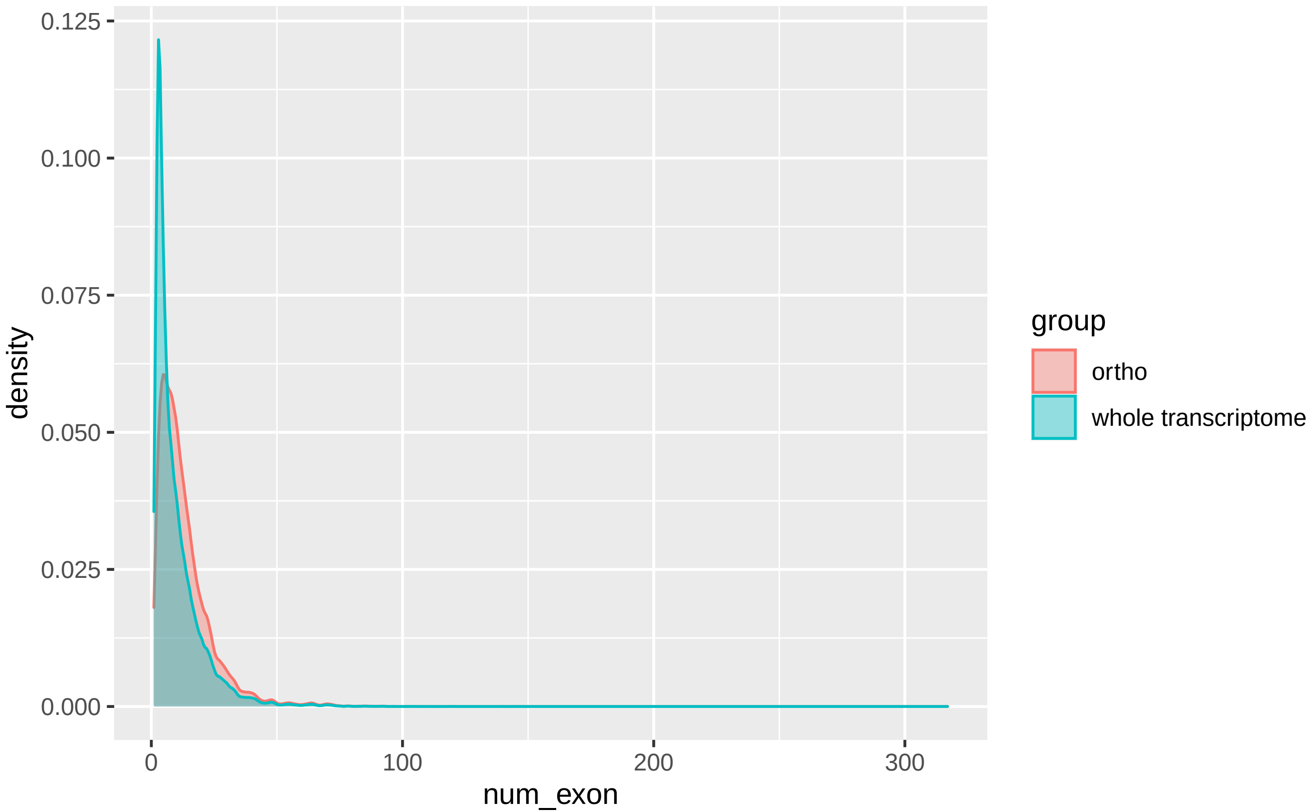

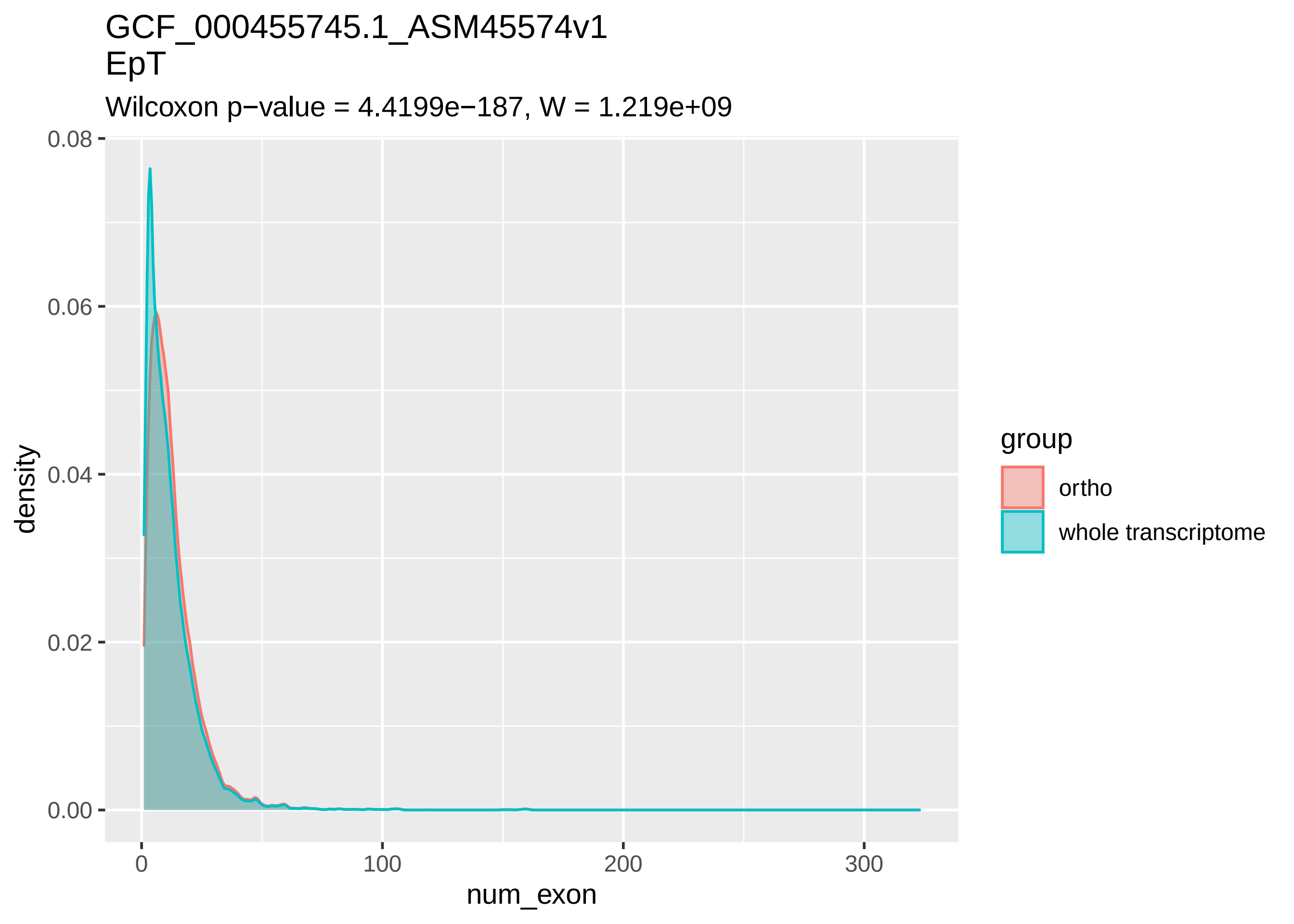

GCF_000455745.1_ASM45574v1
EpT
Wilcoxon p−value = 4.4199e−187, W = 1.219e+09
density
0.00
0.02
0.04
0.06
0.08
0
100
200
300
num_exon
group
ortho
whole transcriptome

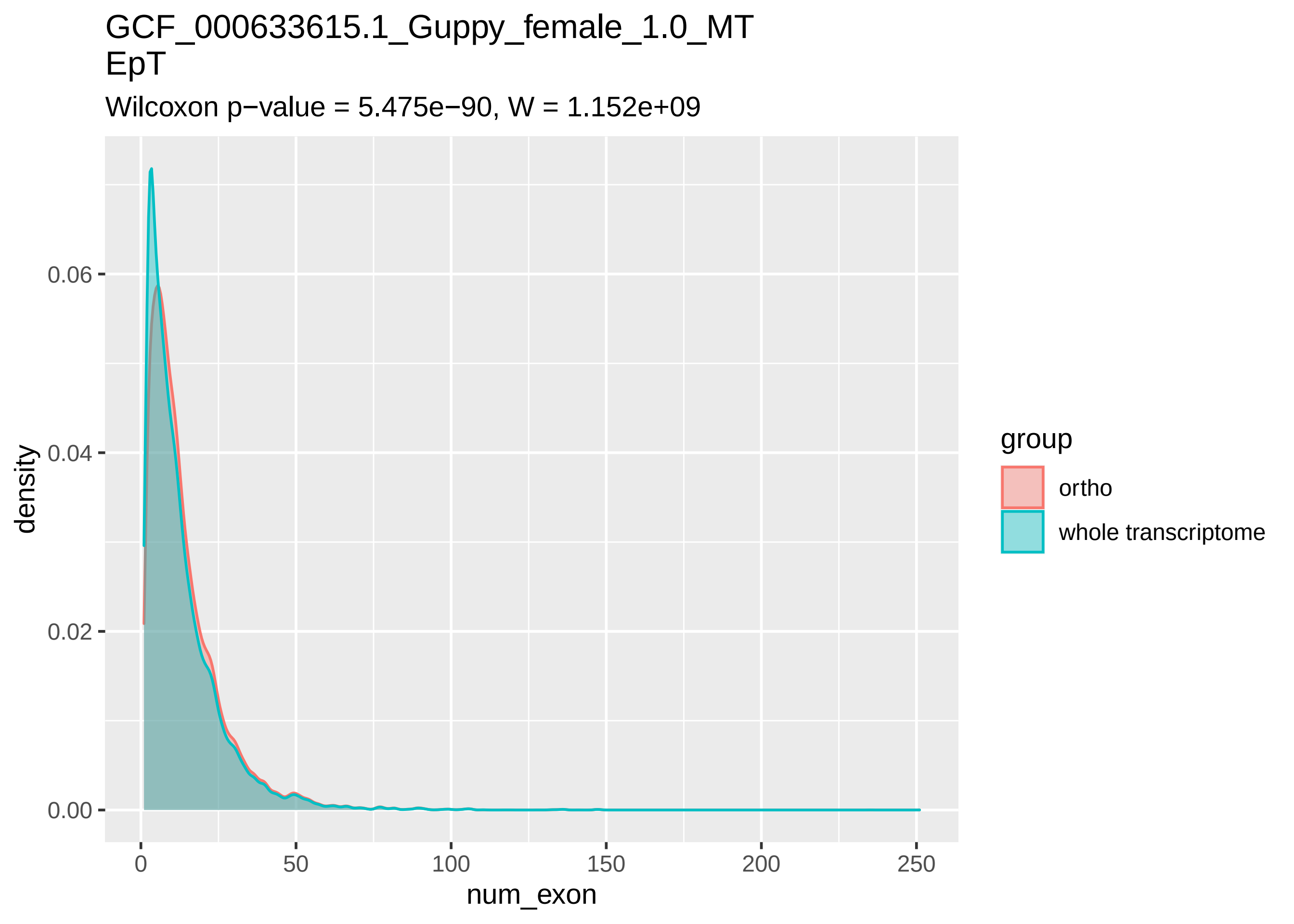

GCF_000633615.1_Guppy_female_1.0_MT
EpT
Wilcoxon p−value = 5.475e−90, W = 1.152e+09
density
0.06
0.04
0.02
0.00
0
50
100
150
200
250
num_exon
group
ortho
whole transcriptome

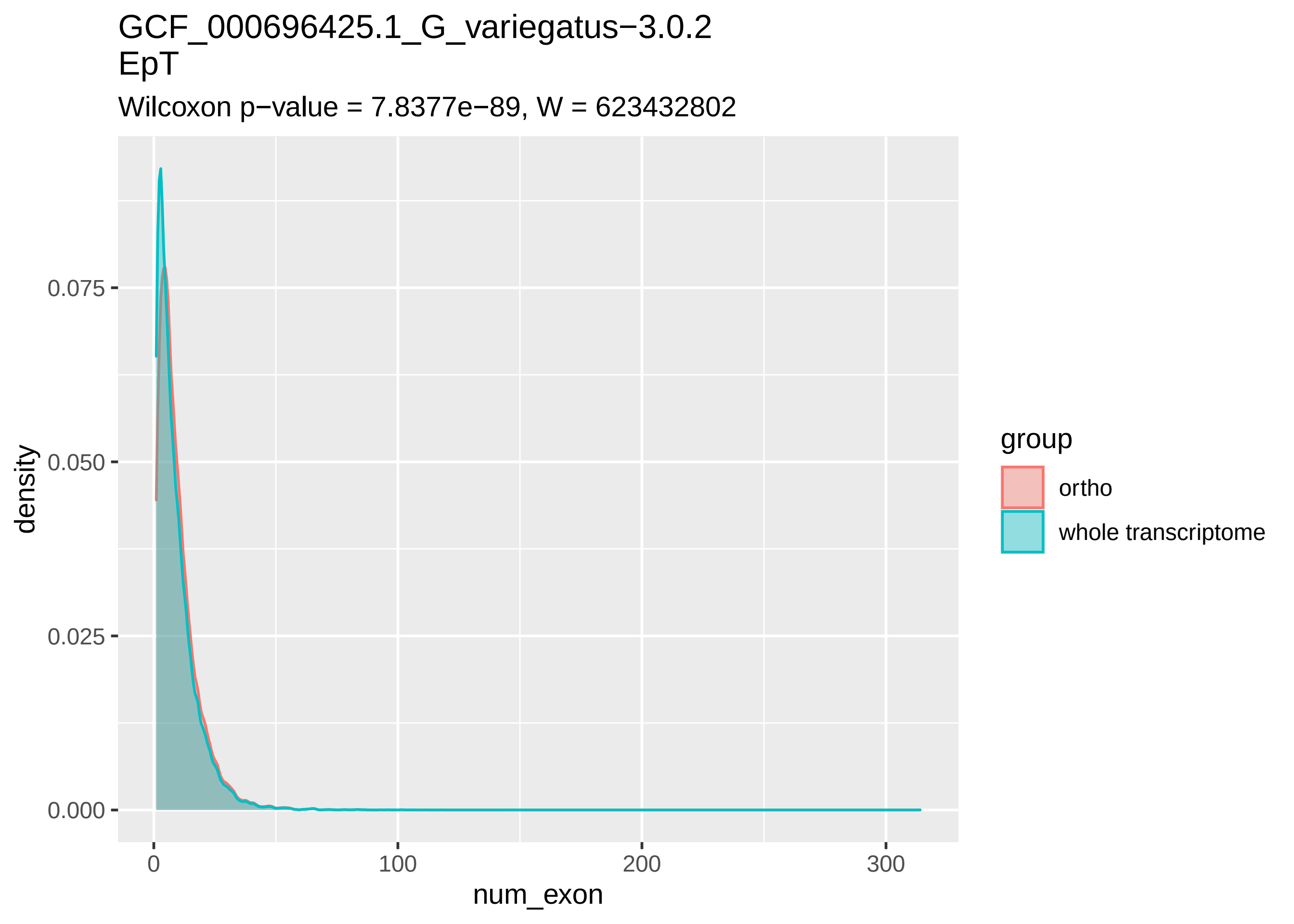
GCF_000696425.1_G_variegatus−3.0.2
EpT
Wilcoxon p−value = 7.8377e−89, W = 623432802
density
0.075
0.050
0.025
0.000
0
100
200
300
num_exon
group
ortho
whole transcriptome

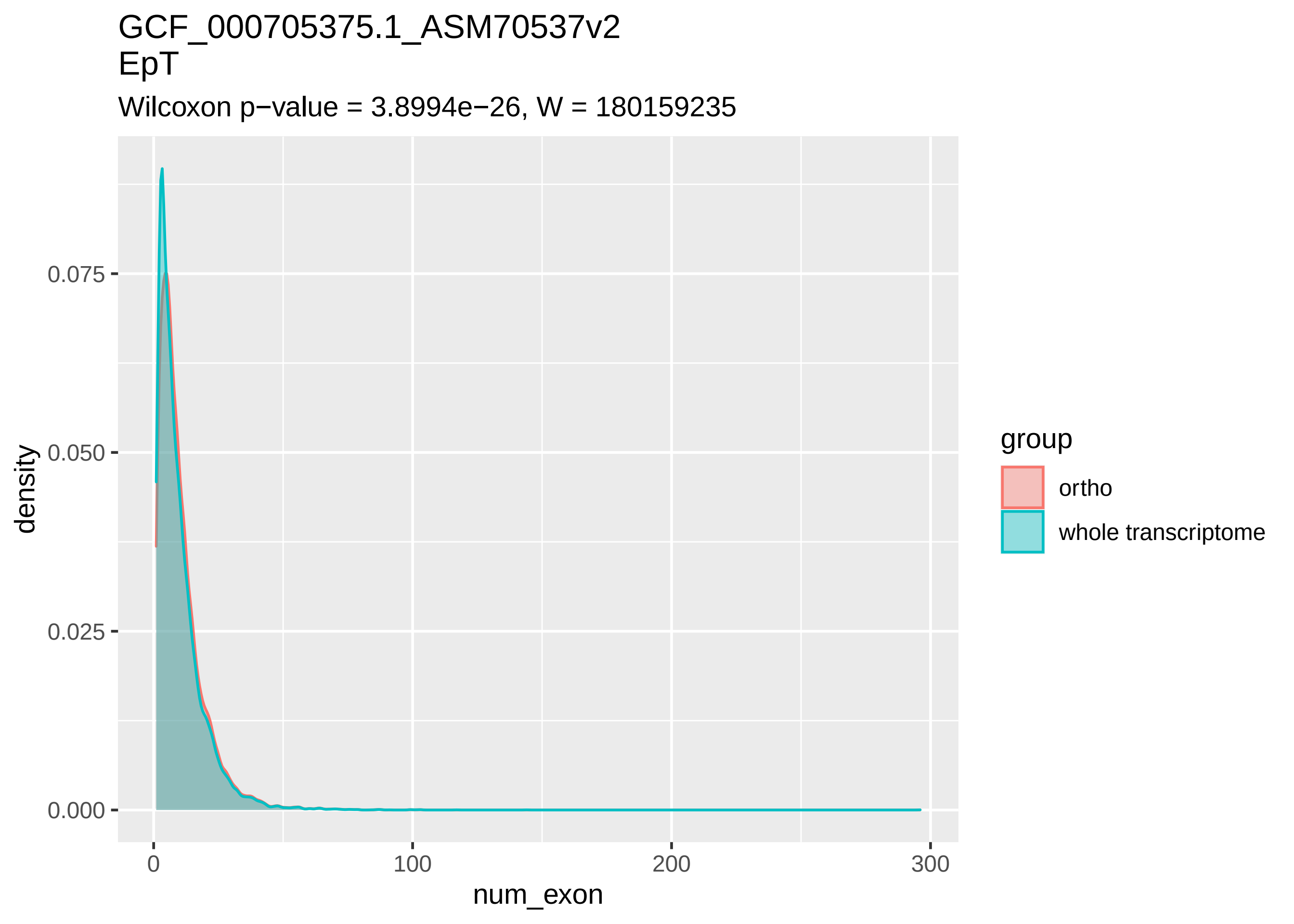

GCF_000705375.1_ASM70537v2
EpT
Wilcoxon p−value = 3.8994e−26, W = 180159235
density
0.075
0.050
0.025
0.000
0
100
200
300
num_exon
group
ortho
whole transcriptome

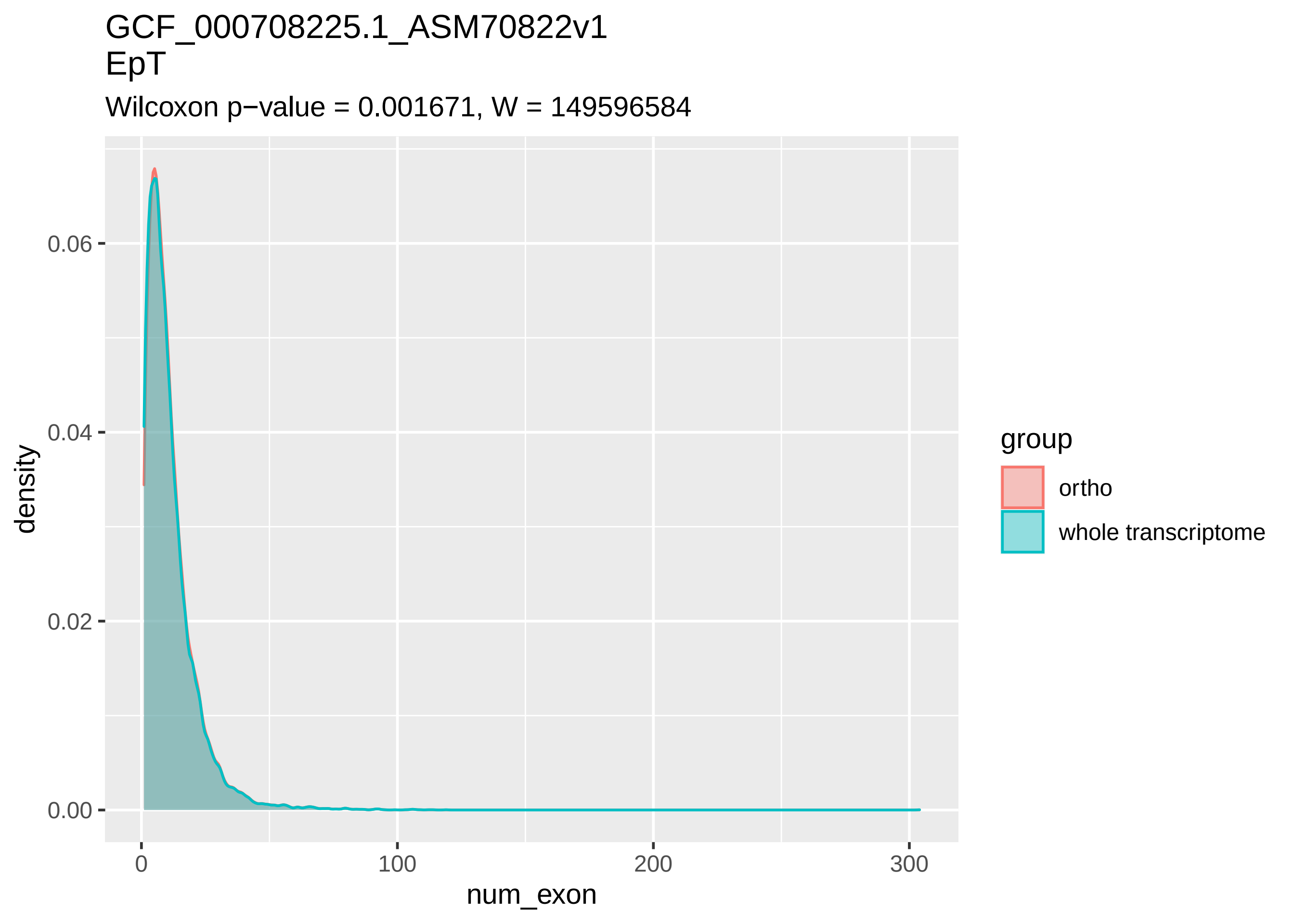

GCF_000708225.1_ASM70822v1
EpT
Wilcoxon p−value = 0.001671, W = 149596584
density
0.00
0.02
0.04
0.06
0
100
200
300
num_exon
group
ortho
whole transcriptome

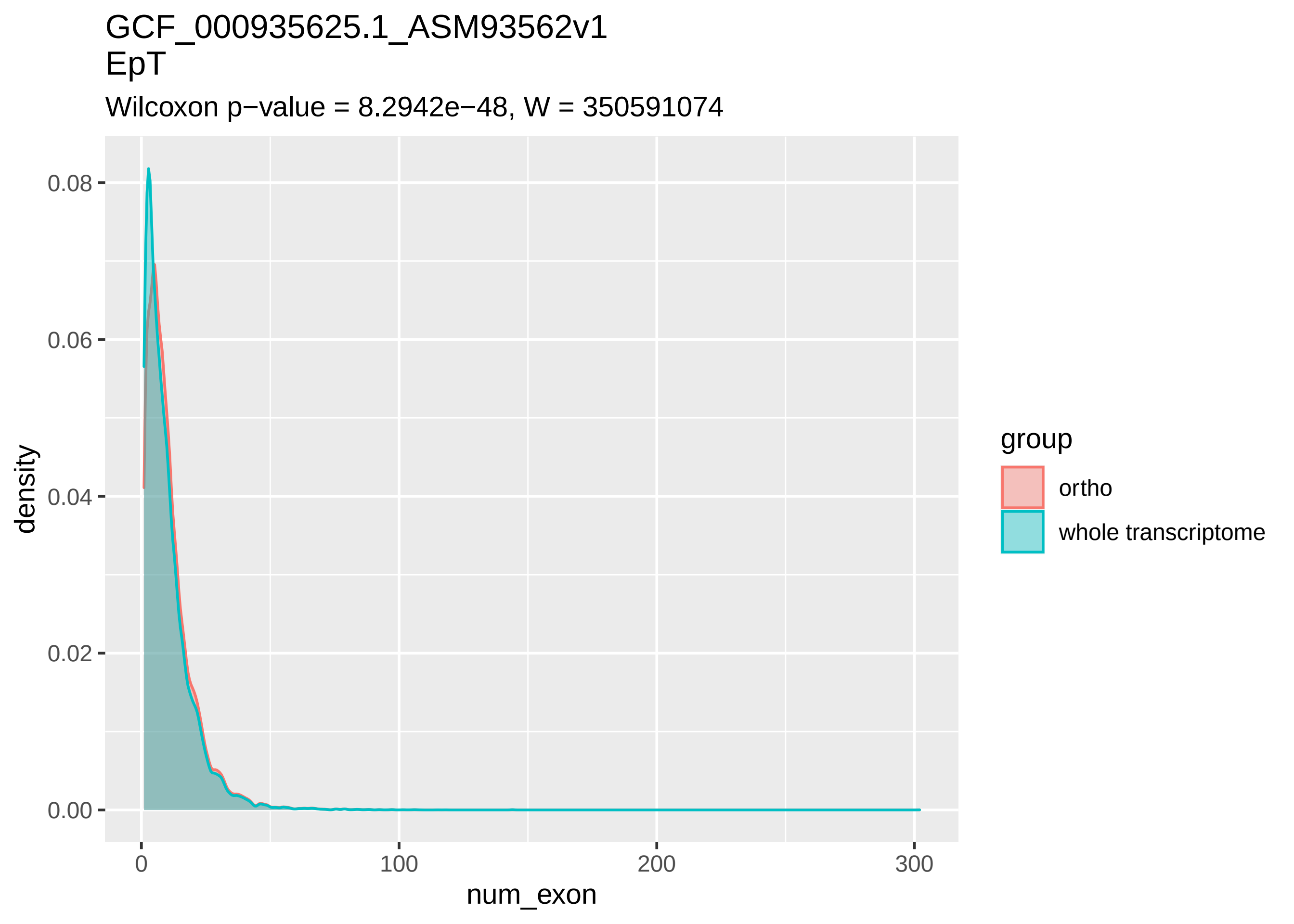
GCF_000935625.1_ASM93562v1
EpT
Wilcoxon p−value = 8.2942e−48, W = 350591074
density
0.08
0.06
0.04
0.02
0.00
0
100
200
300
num_exon
group
ortho
whole transcriptome

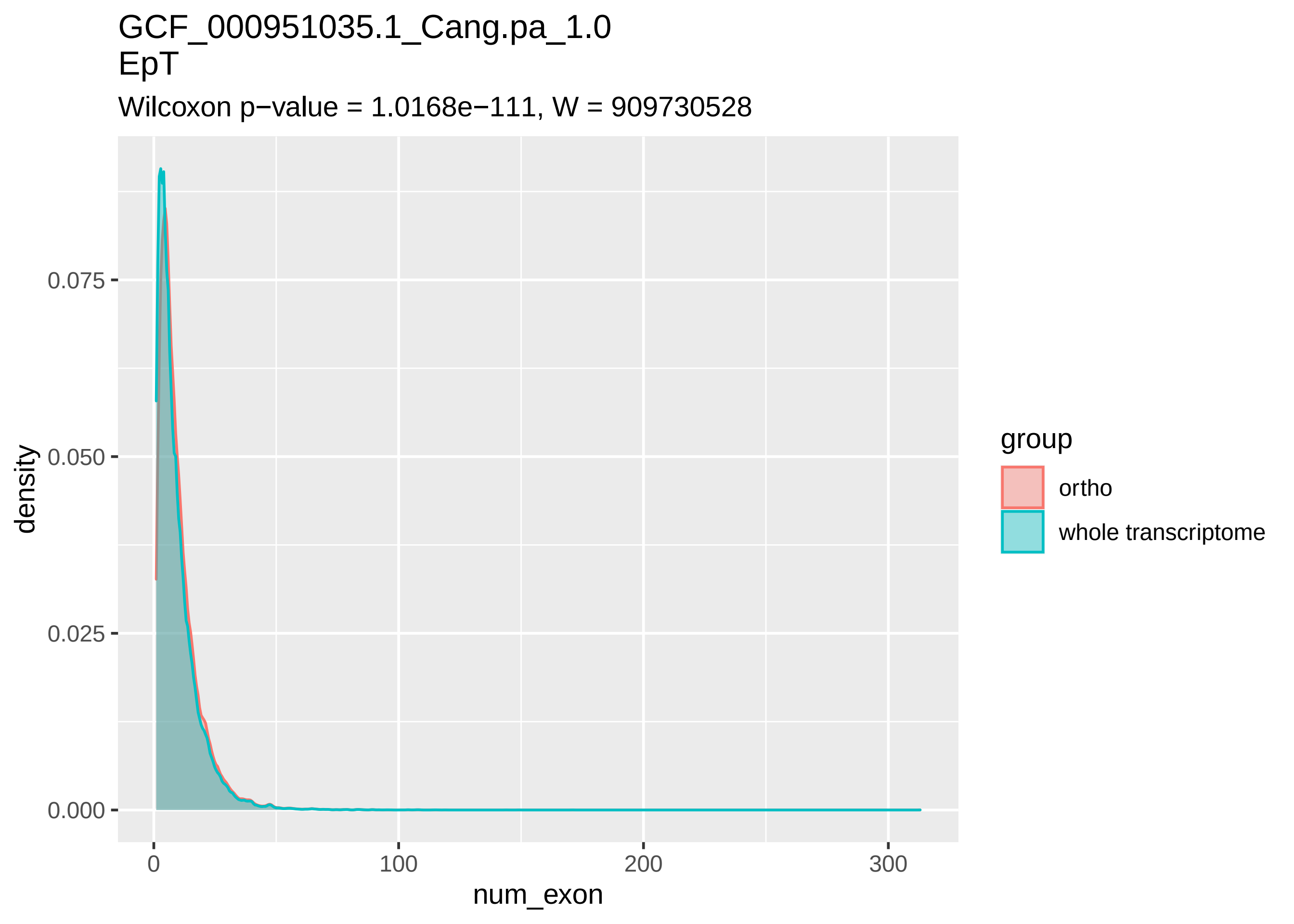
GCF_000951035.1_Cang.pa_1.0
EpT
Wilcoxon p−value = 1.0168e−111, W = 909730528
density
0.000
0.025
0.050
0.075
0
100
200
300
num_exon
group
ortho
whole transcriptome

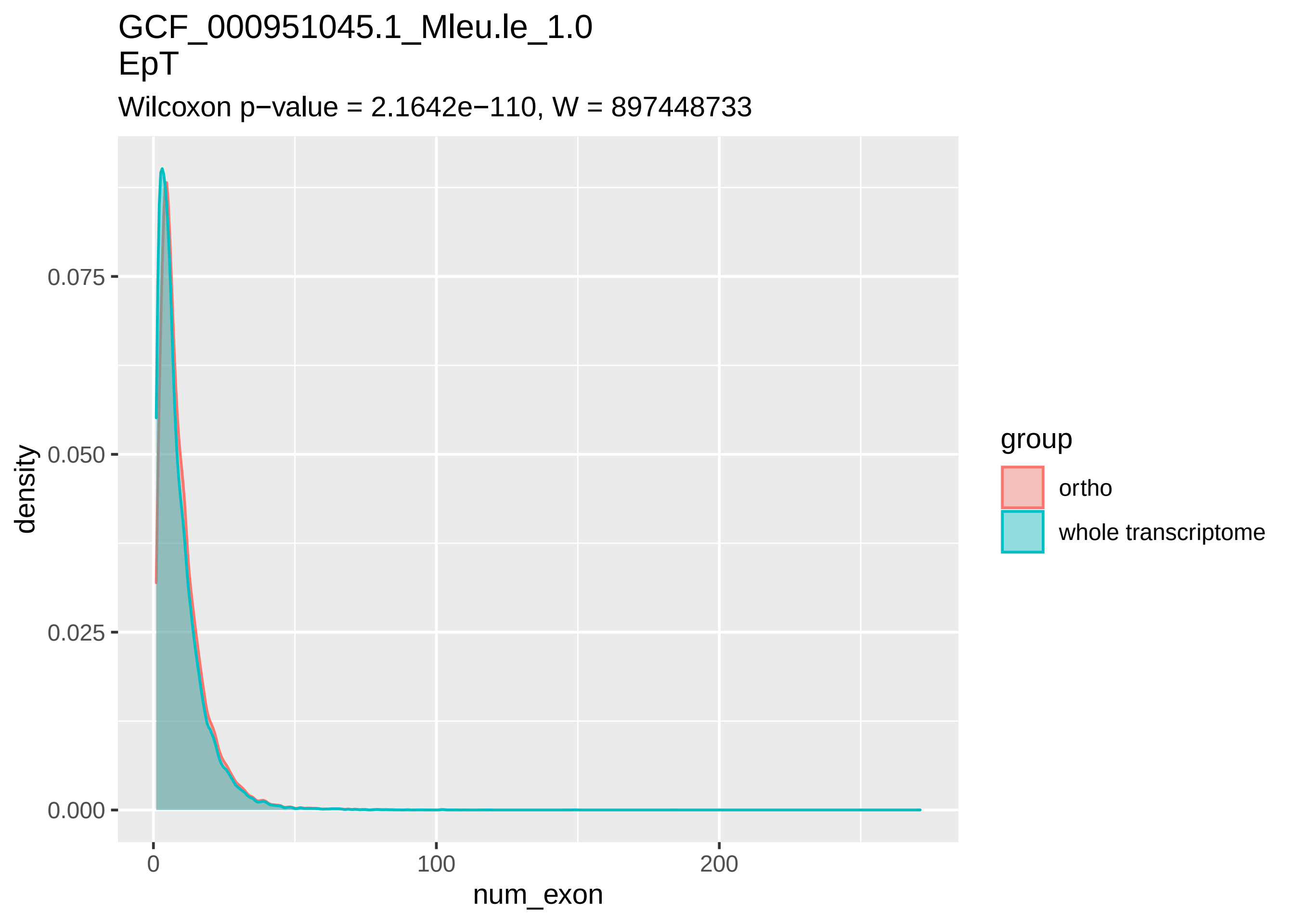
GCF_000951045.1_Mleu.le_1.0
EpT
Wilcoxon p−value = 2.1642e−110, W = 897448733
density
0.000
0.025
0.050
0.075
0
100
200
num_exon
group
ortho
whole transcriptome

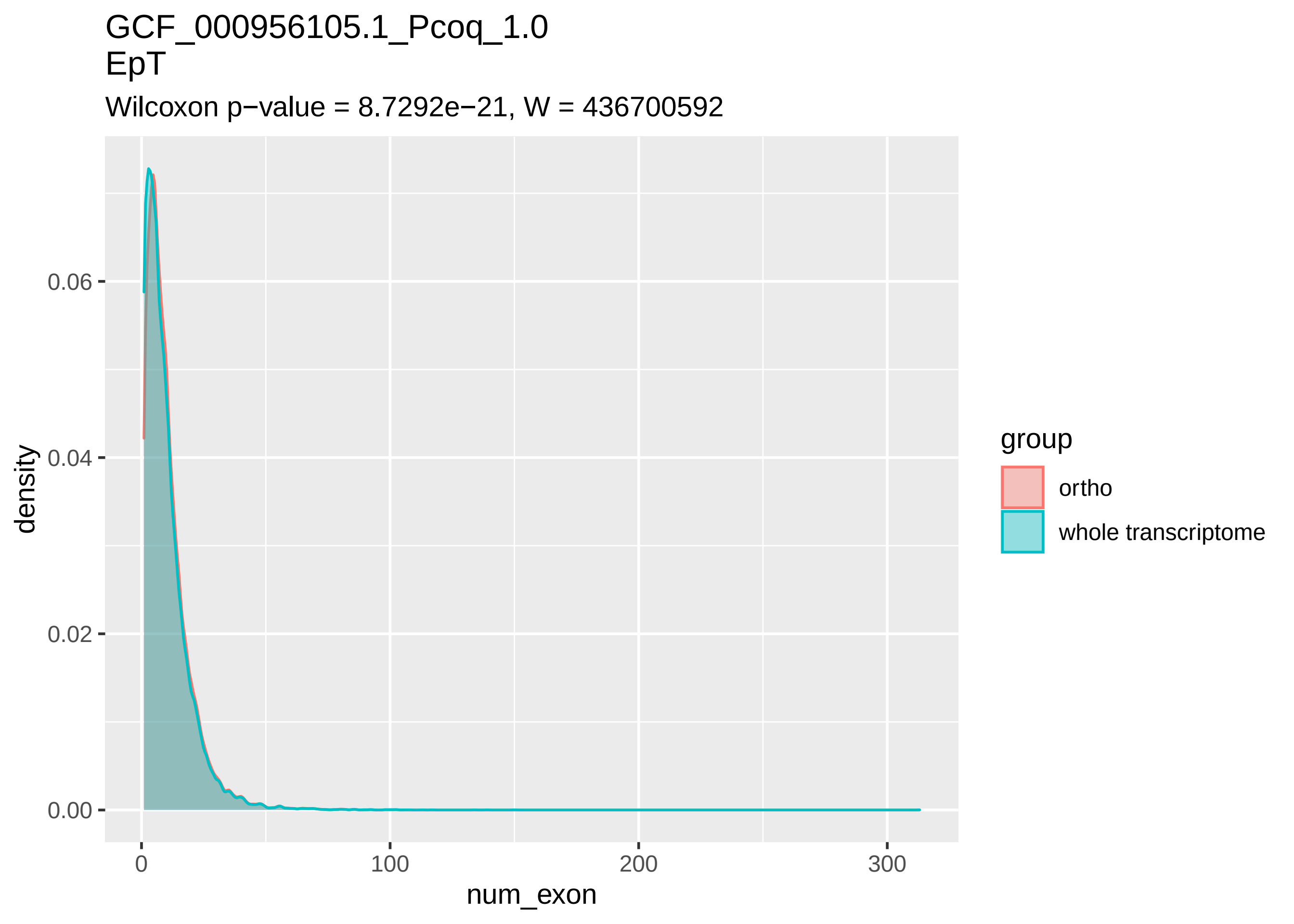
GCF_000956105.1_Pcoq_1.0
EpT
Wilcoxon p−value = 8.7292e−21, W = 436700592
density
0.06
0.04
0.02
0.00
0
100
200
300
num_exon
group
ortho
whole transcriptome

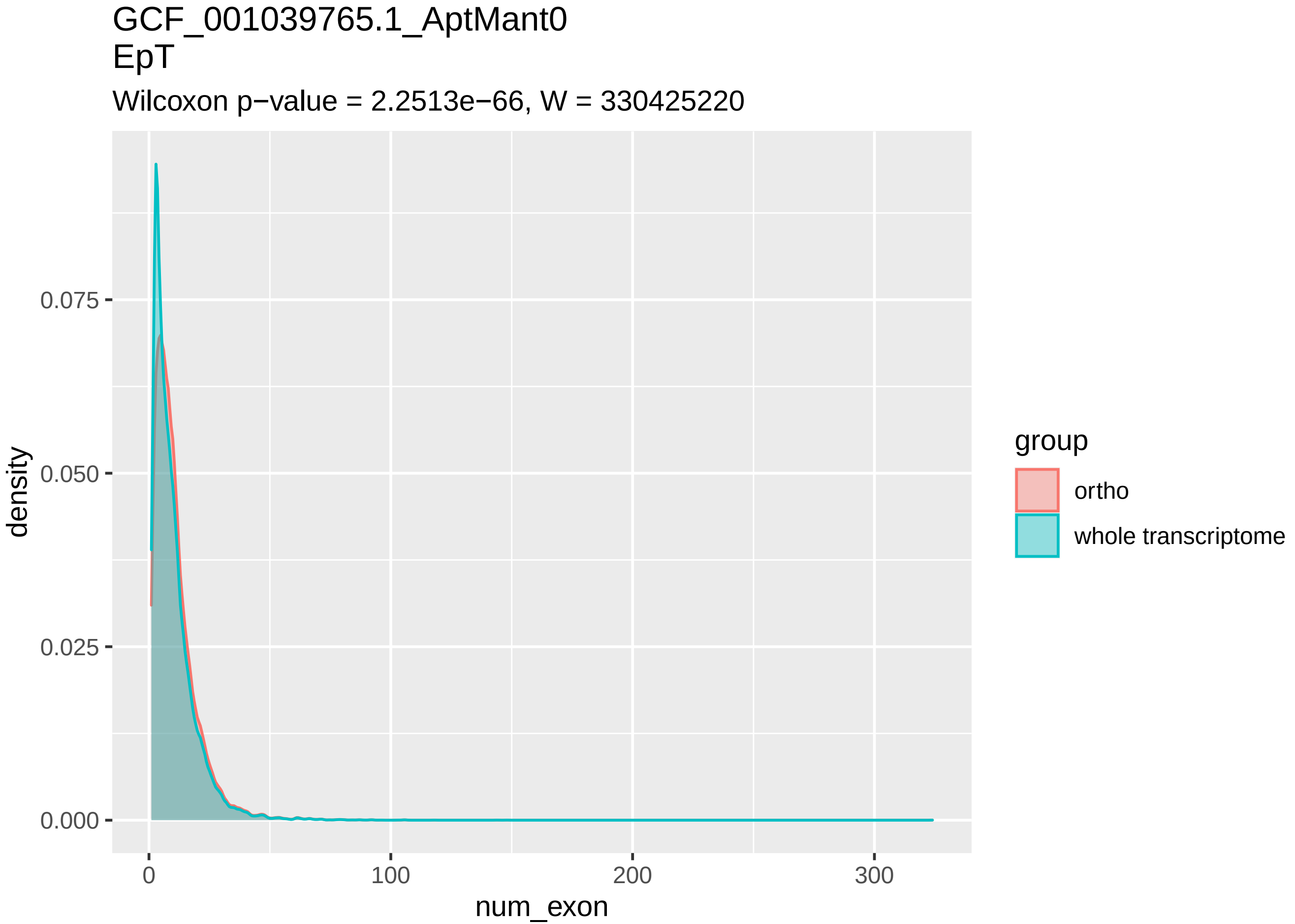
GCF_001039765.1_AptMant0
EpT
Wilcoxon p−value = 2.2513e−66, W = 330425220
density
0.075
0.050
0.025
0.000
0
100
200
300
num_exon
group
ortho
whole transcriptome

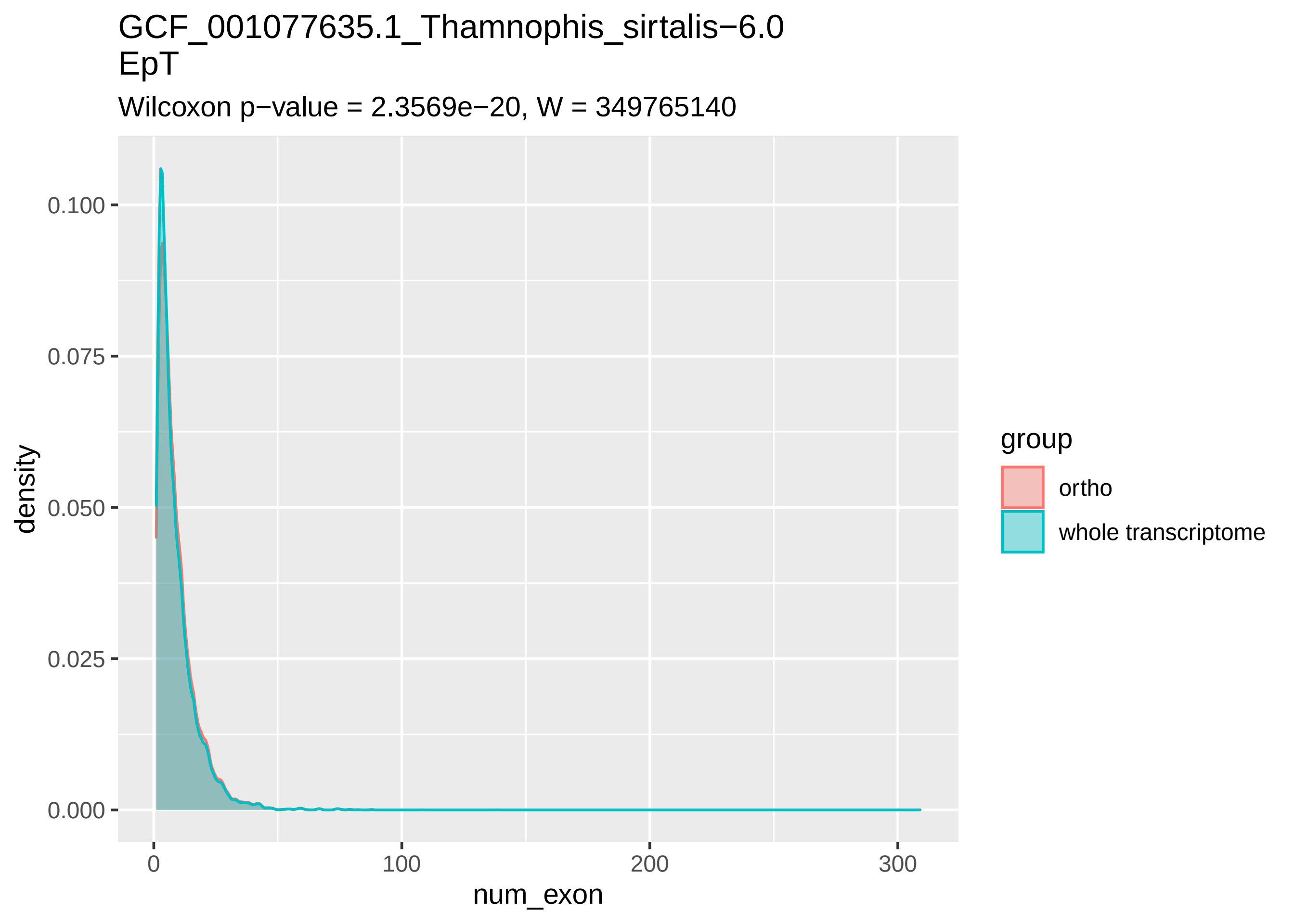
GCF_001077635.1_Thamnophis_sirtalis−6.0
EpT
Wilcoxon p−value = 2.3569e−20, W = 349765140
density
0.100
0.075
0.050
0.025
0.000
0
100
200
300
num_exon
group
ortho
whole transcriptome

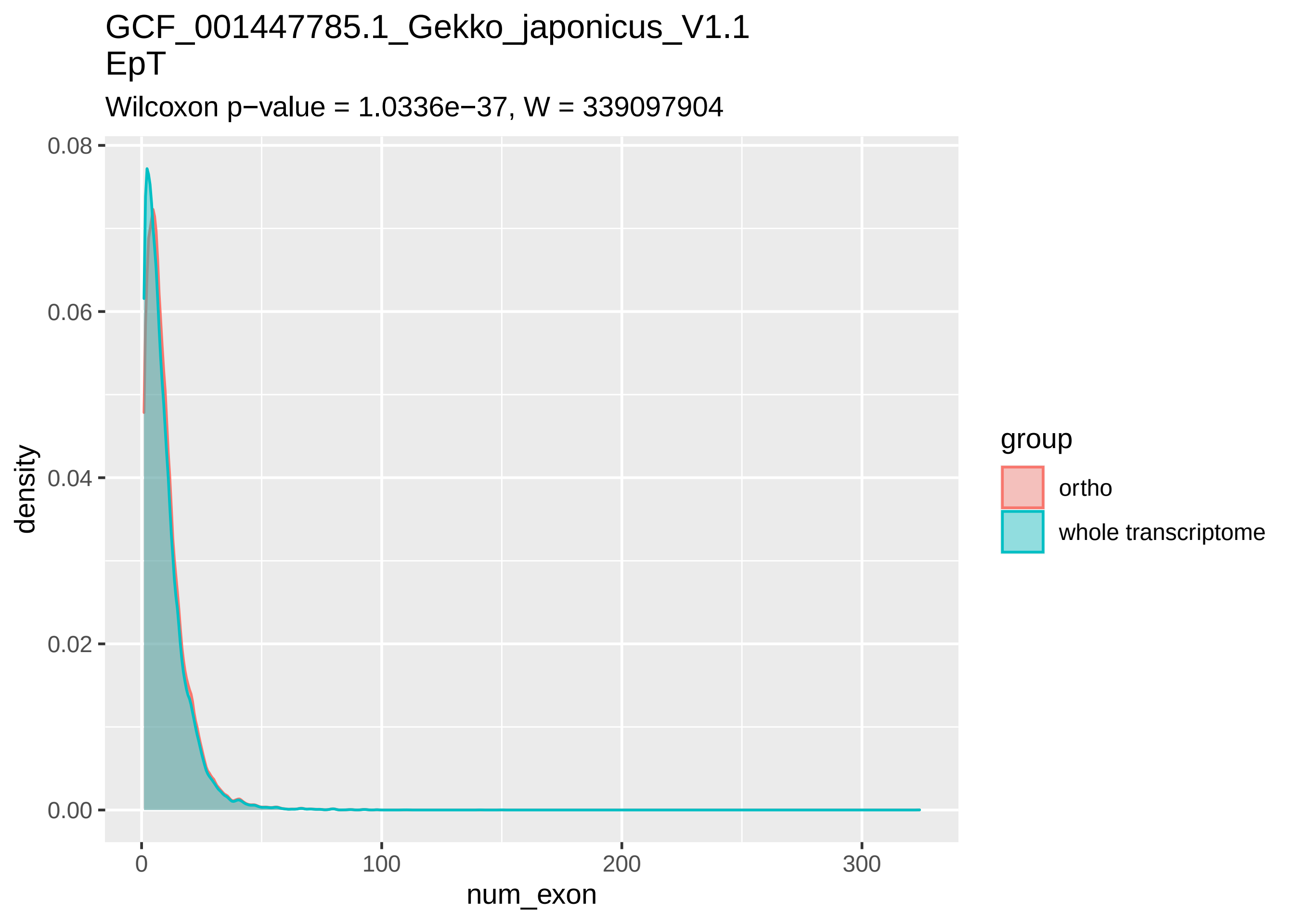
GCF_001447785.1_Gekko_japonicus_V1.1
EpT
Wilcoxon p−value = 1.0336e−37, W = 339097904
density
0.08
0.06
0.04
0.02
0.00
0
100
200
300
num_exon
group
ortho
whole transcriptome

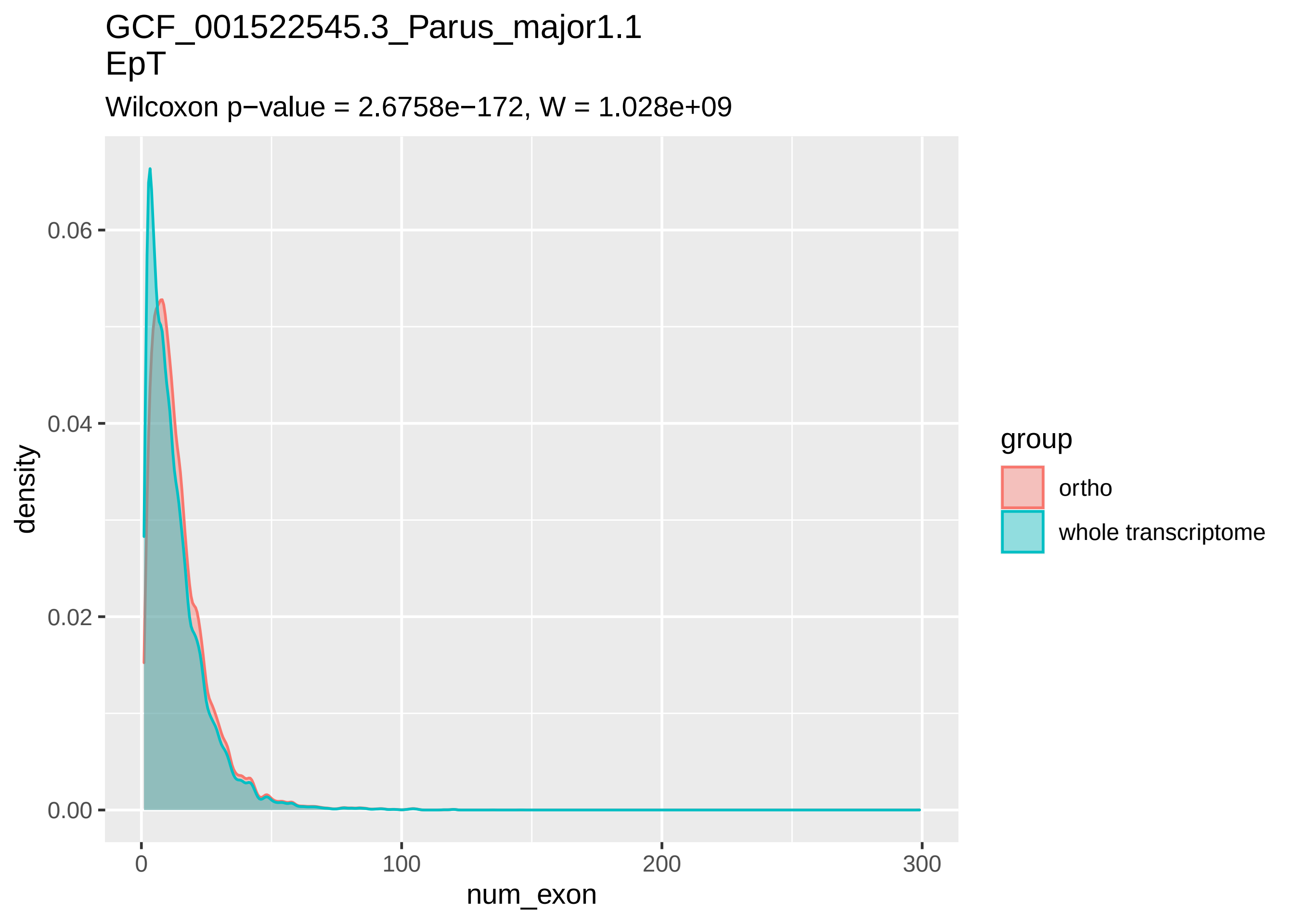
GCF_001522545.3_Parus_major1.1
EpT
Wilcoxon p−value = 2.6758e−172, W = 1.028e+09
density
0.00
0.02
0.04
0.06
0
100
200
300
num_exon
group
ortho
whole transcriptome

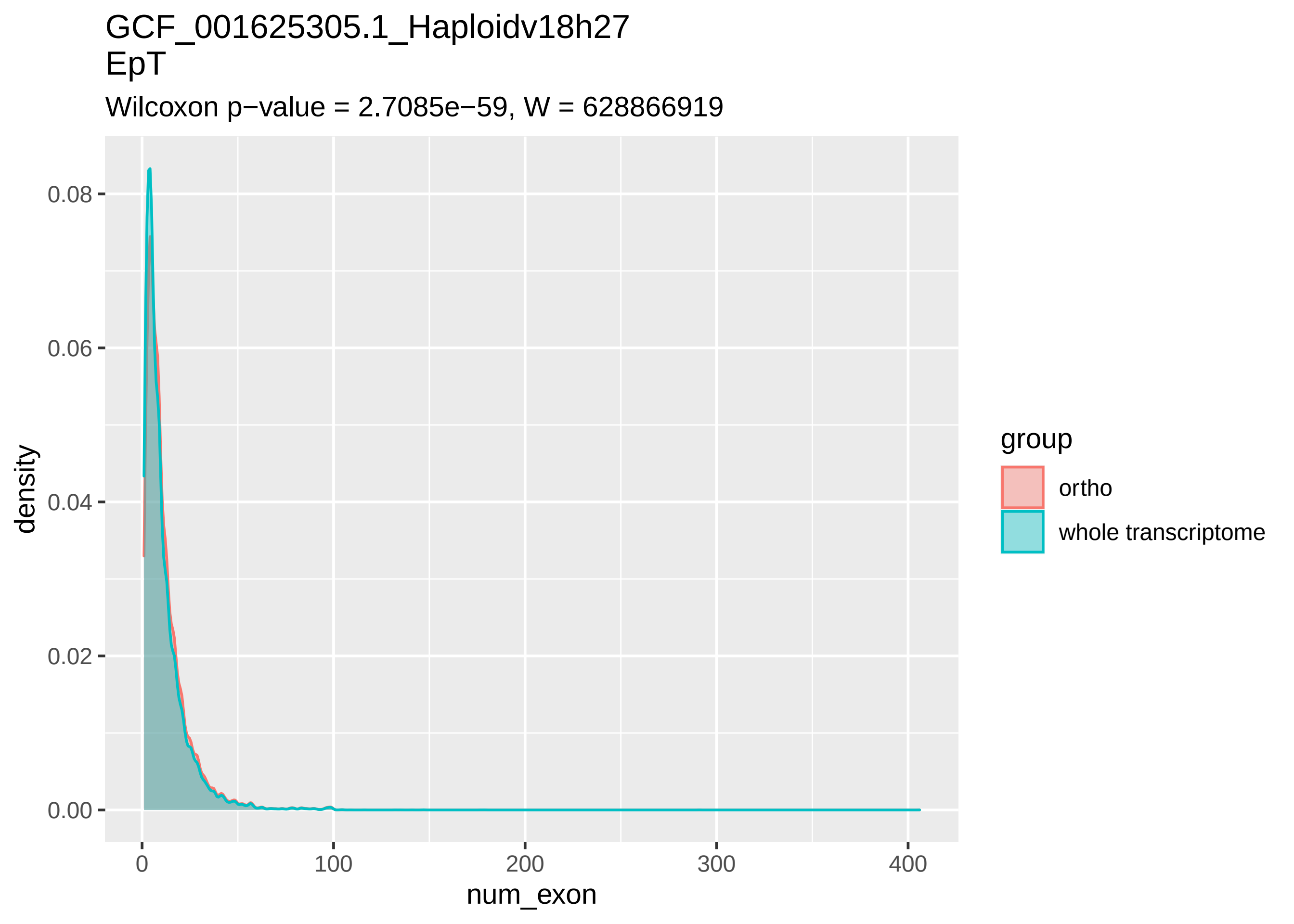

GCF_001625305.1_Haploidv18h27
EpT
Wilcoxon p−value = 2.7085e−59, W = 628866919
density
0.00
0.02
0.04
0.06
0.08
0
100
200
300
400
num_exon
group
ortho
whole transcriptome

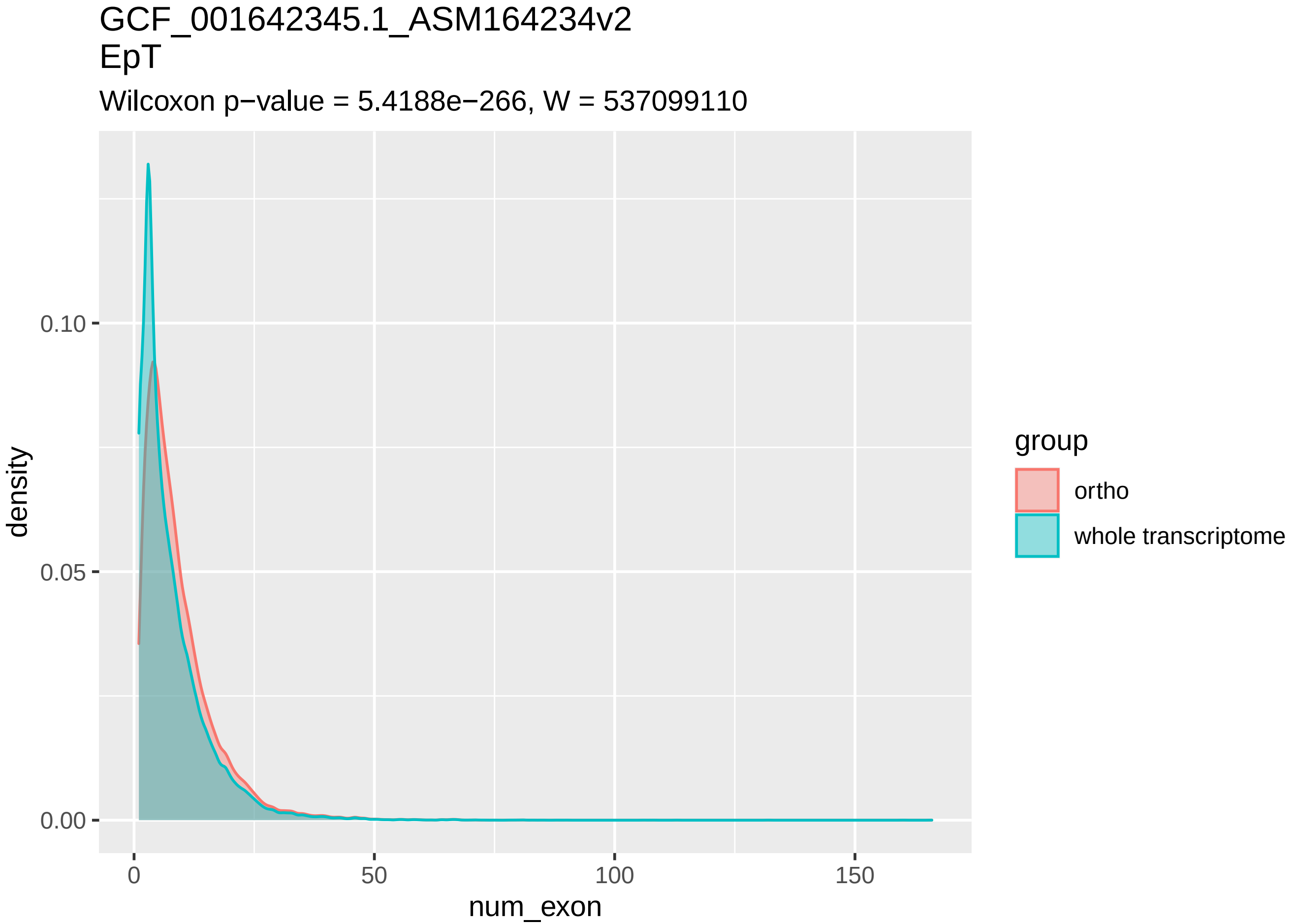
GCF_001642345.1_ASM164234v2
EpT
Wilcoxon p−value = 5.4188e−266, W = 537099110
density
0.10
0.05
0.00
0
50
100
150
num_exon
group
ortho
whole transcriptome

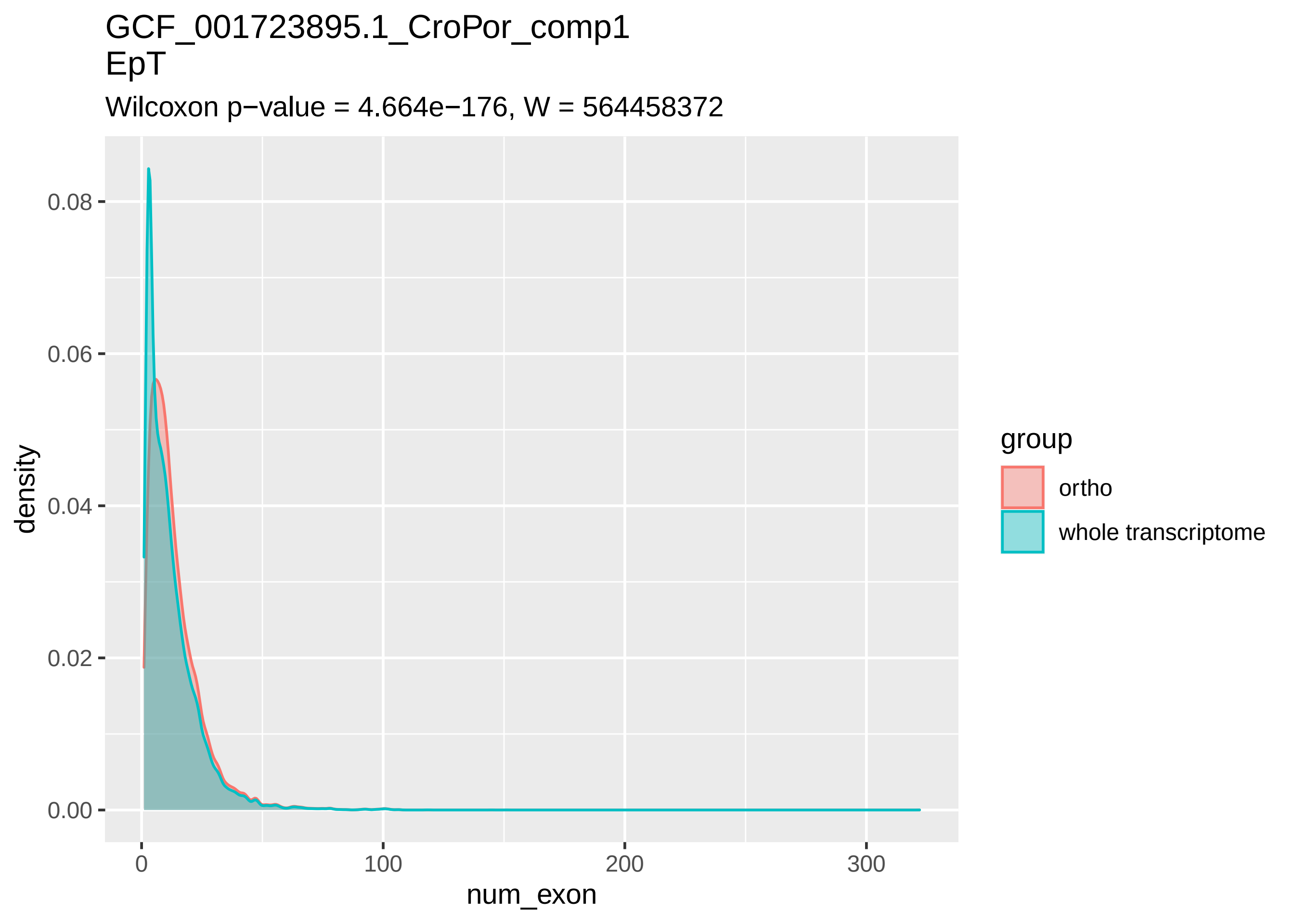
GCF_001723895.1_CroPor_comp1
EpT
Wilcoxon p−value = 4.664e−176, W = 564458372
density
0.08
0.06
0.04
0.02
0.00
0
100
200
300
num_exon
group
ortho
whole transcriptome

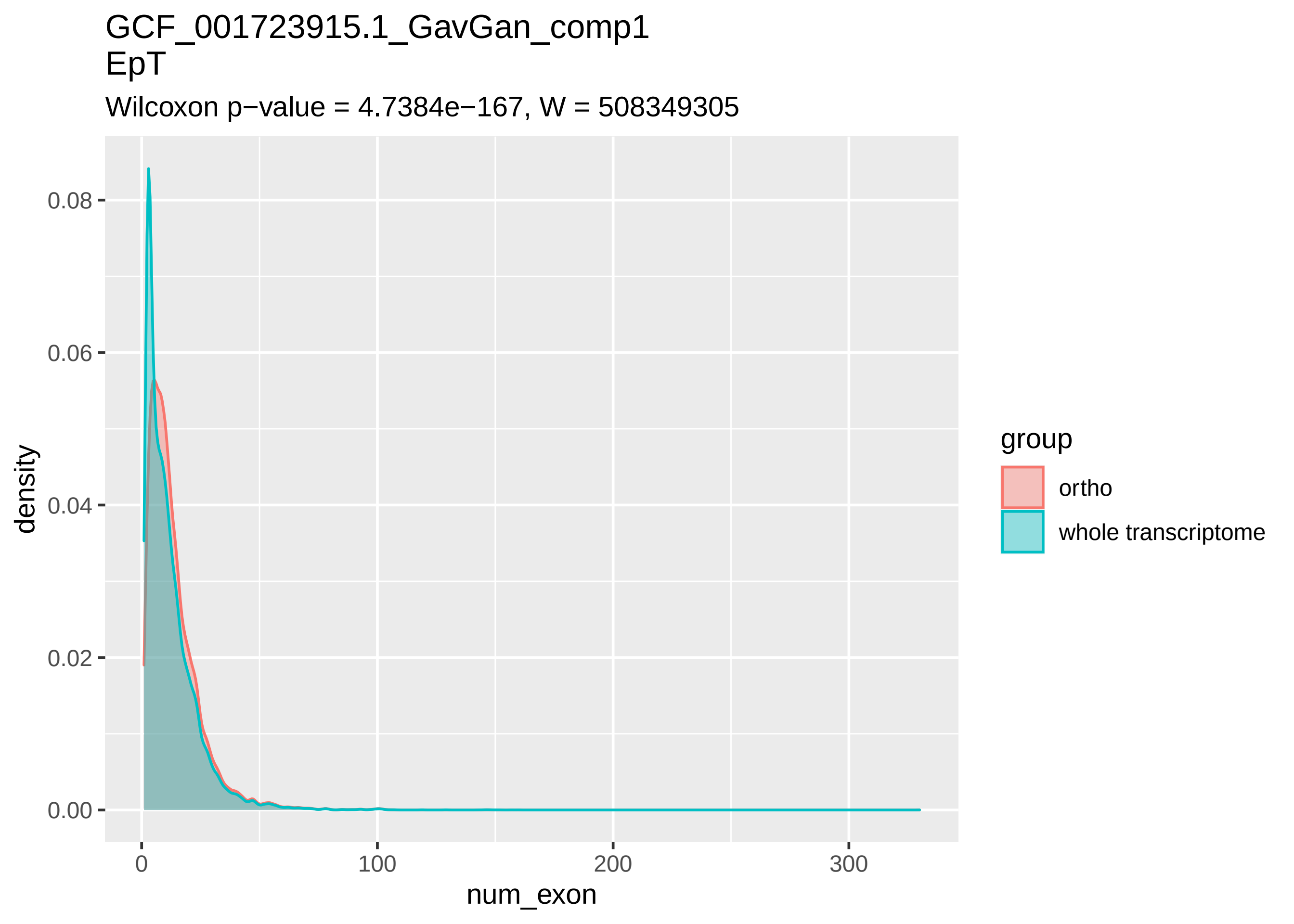

GCF_001723915.1_GavGan_comp1
EpT
Wilcoxon p−value = 4.7384e−167, W = 508349305
density
0.00
0.02
0.04
0.06
0.08
0
100
200
300
num_exon
group
ortho
whole transcriptome

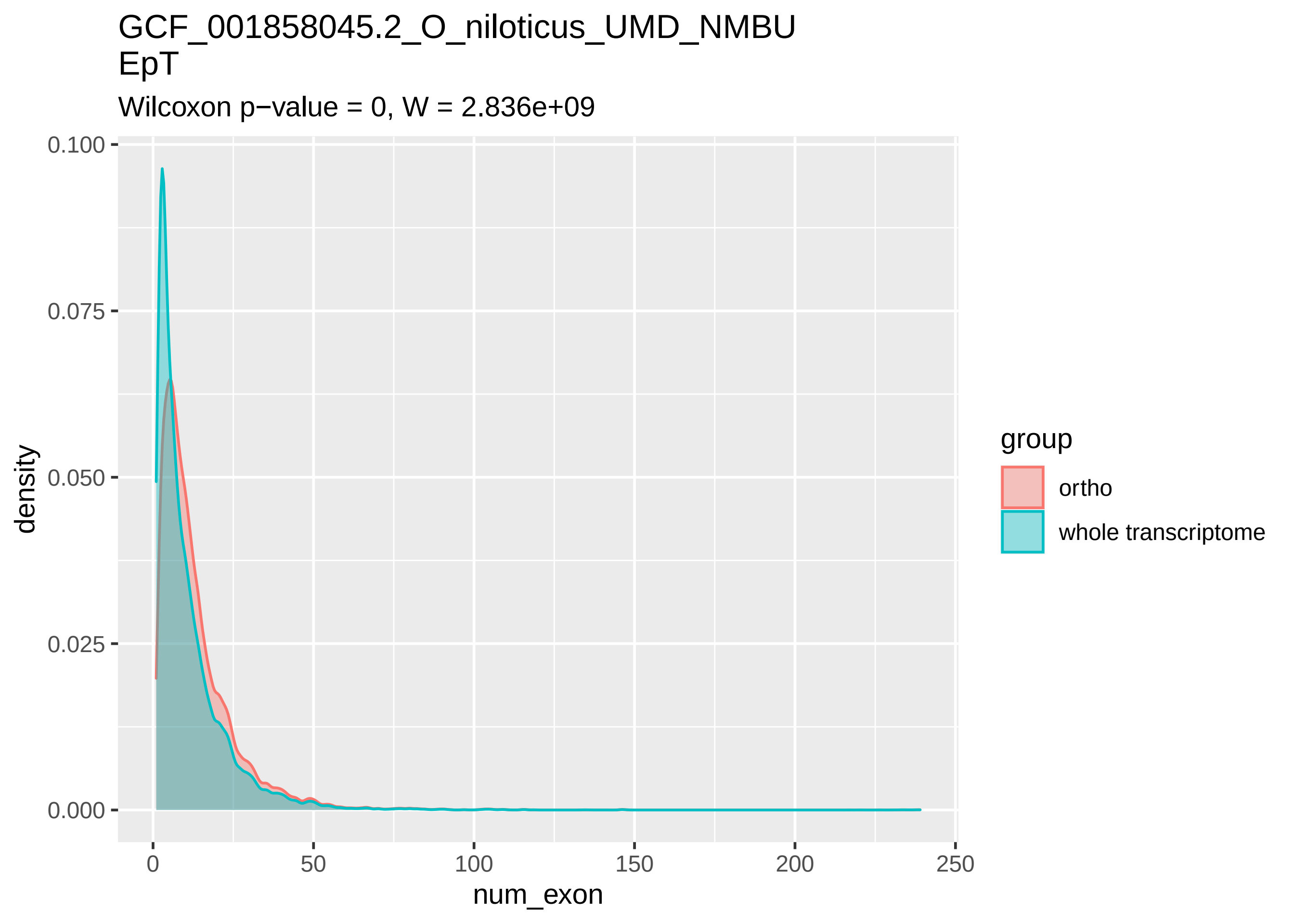
GCF_001858045.2_O_niloticus_UMD_NMBU
EpT
Wilcoxon p−value = 0, W = 2.836e+09
density
0.100
0.075
0.050
0.025
0.000
0
50
100
150
200
250
num_exon
group
ortho
whole transcriptome

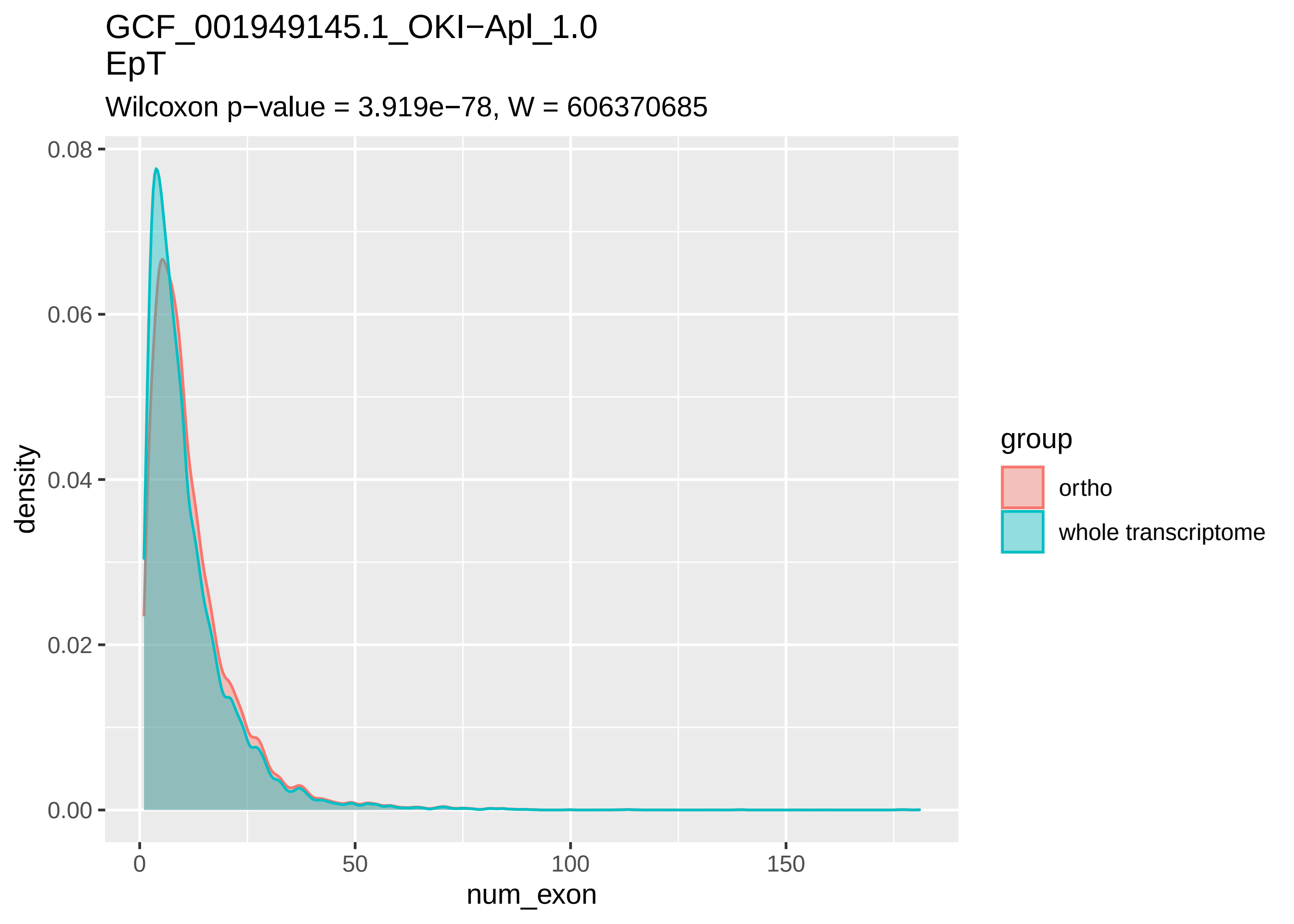
GCF_001949145.1_OKI−Apl_1.0
EpT
Wilcoxon p−value = 3.919e−78, W = 606370685
density
0.00
0.02
0.04
0.06
0.08
0
50
100
150
num_exon
group
ortho
whole transcriptome

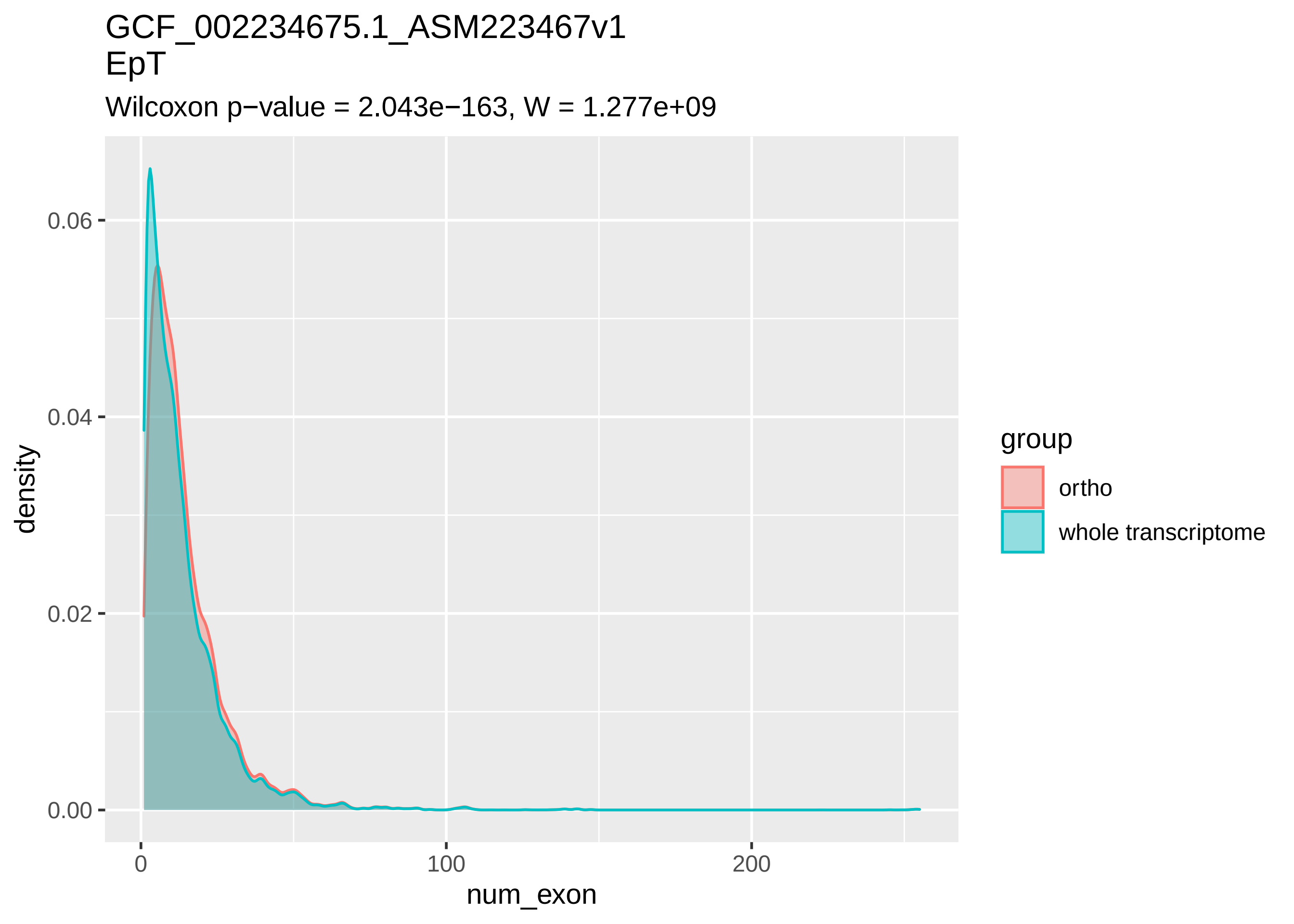
GCF_002234675.1_ASM223467v1
EpT
Wilcoxon p−value = 2.043e−163, W = 1.277e+09
density
0.06
0.04
0.02
0.00
0
100
200
num_exon
group
ortho
whole transcriptome

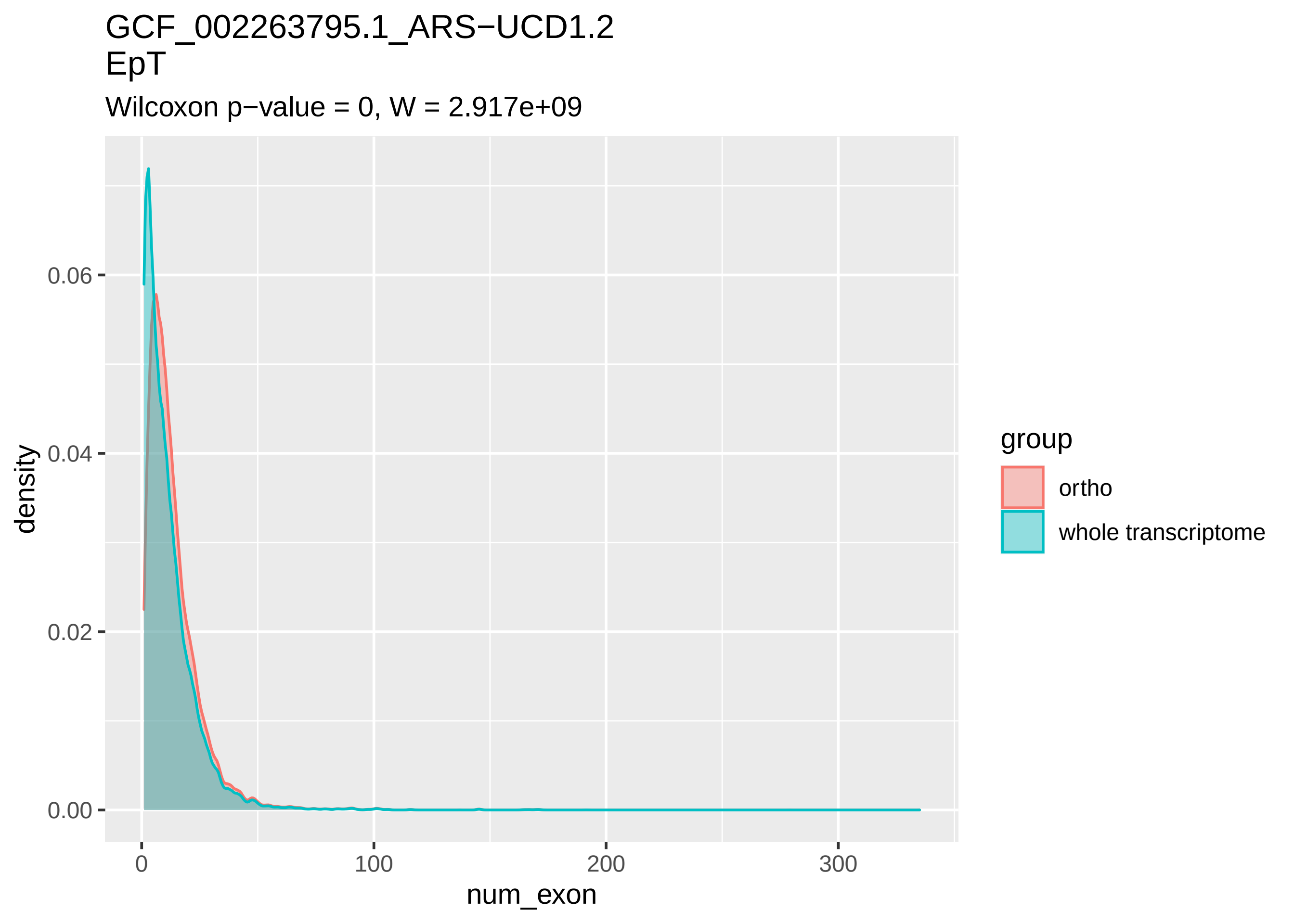

GCF_002263795.1_ARS−UCD1.2
EpT
Wilcoxon p−value = 0, W = 2.917e+09
density
0.00
0.02
0.04
0.06
0
100
200
300
num_exon
group
ortho
whole transcriptome

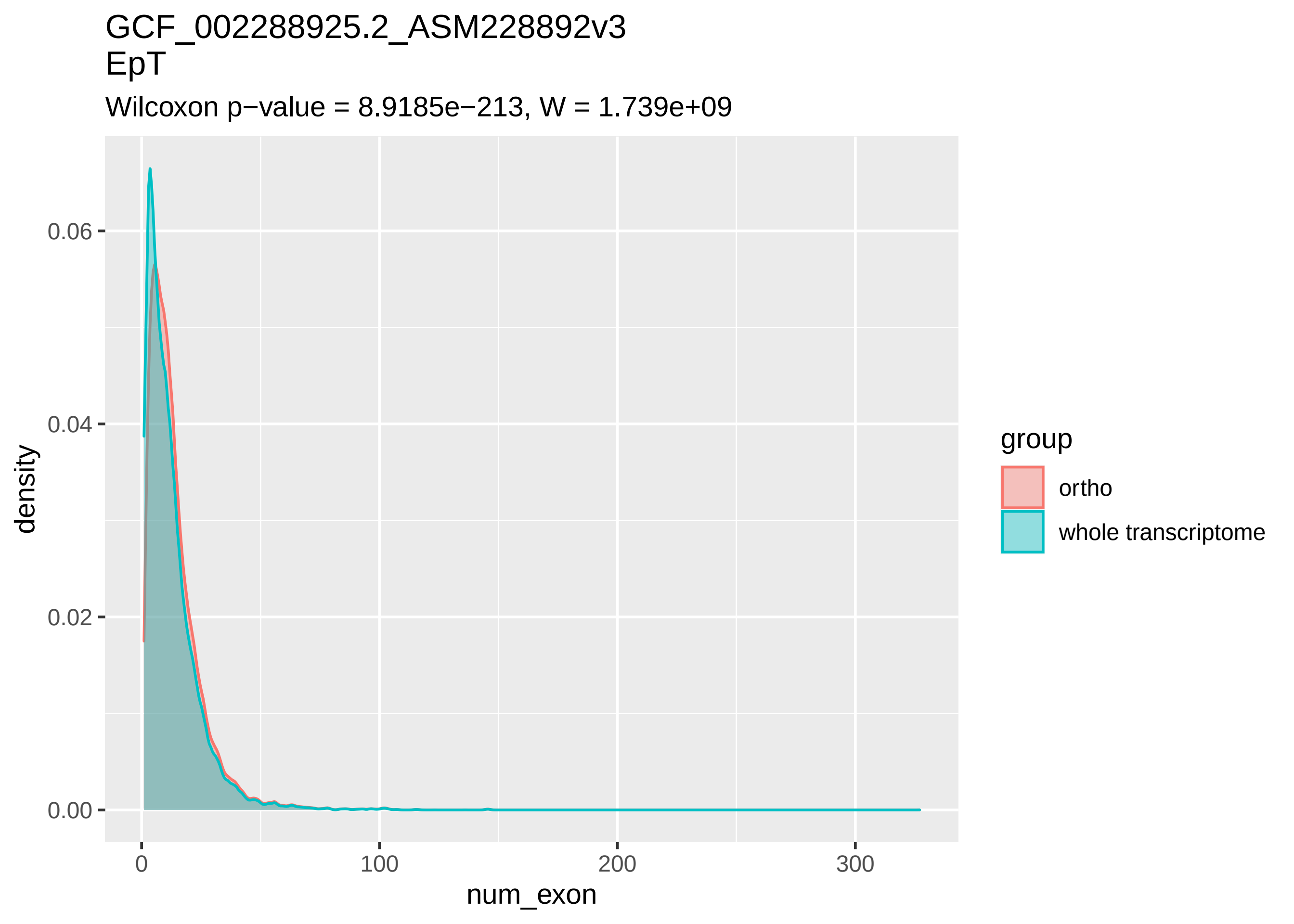
GCF_002288925.2_ASM228892v3
EpT
Wilcoxon p−value = 8.9185e−213, W = 1.739e+09
density
0.06
0.04
0.02
0.00
0
100
200
300
num_exon
group
ortho
whole transcriptome

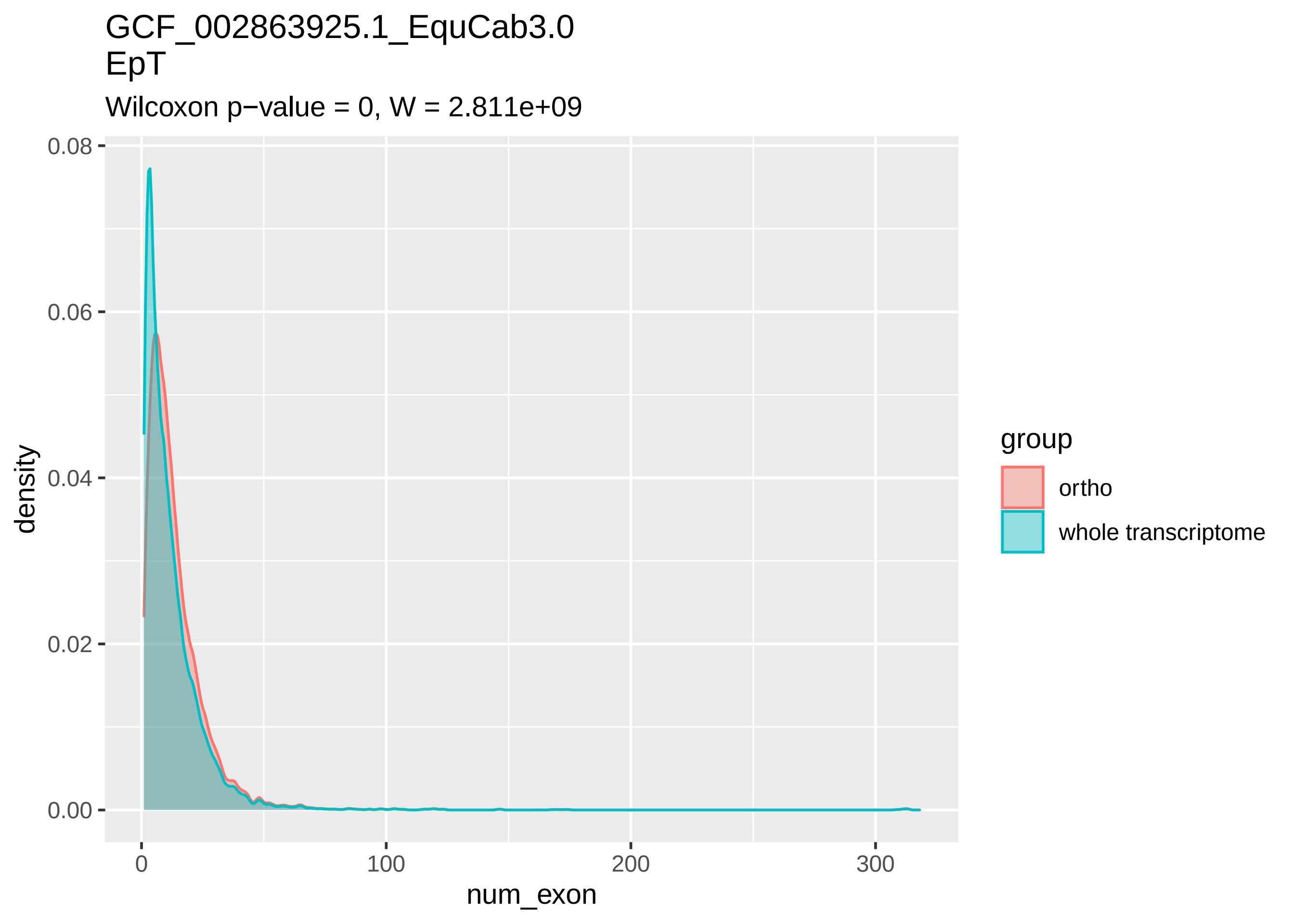

GCF_002863925.1_EquCab3.0
EpT
Wilcoxon p−value = 0, W = 2.811e+09
0.08
0.06
0.04
0.02
0.00
density
0
100
200
300
num_exon
group
ortho
whole transcriptome

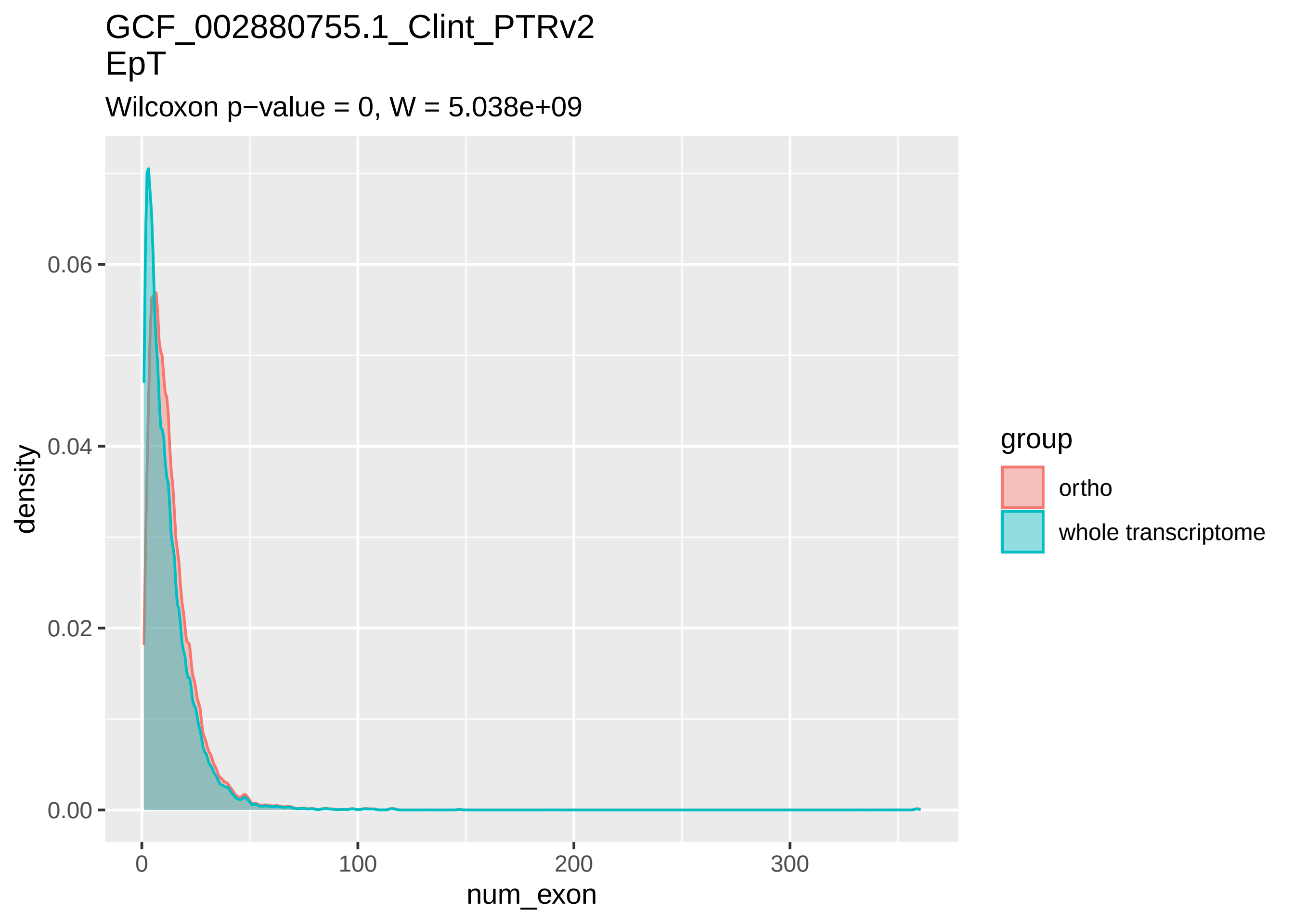
GCF_002880755.1_Clint_PTRv2
EpT
Wilcoxon p−value = 0, W = 5.038e+09
density
0.06
0.04
0.02
0.00
0
100
200
300
num_exon
group
ortho
whole transcriptome

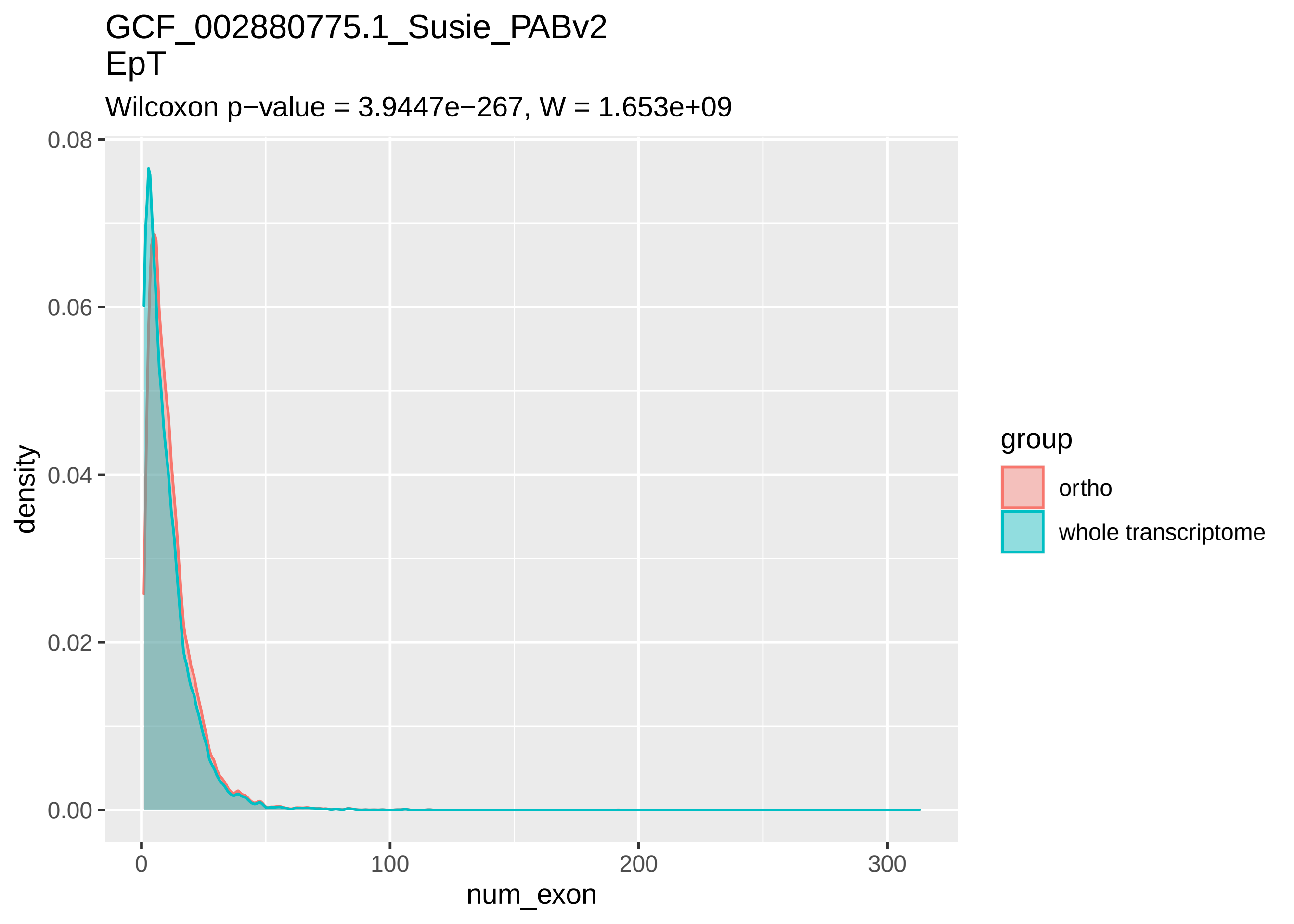
GCF_002880775.1_Susie_PABv2
EpT
Wilcoxon p−value = 3.9447e−267, W = 1.653e+09
0.08
0.06
0.04
0.02
0.00
density
0
100
200
300
num_exon
group
ortho
whole transcriptome

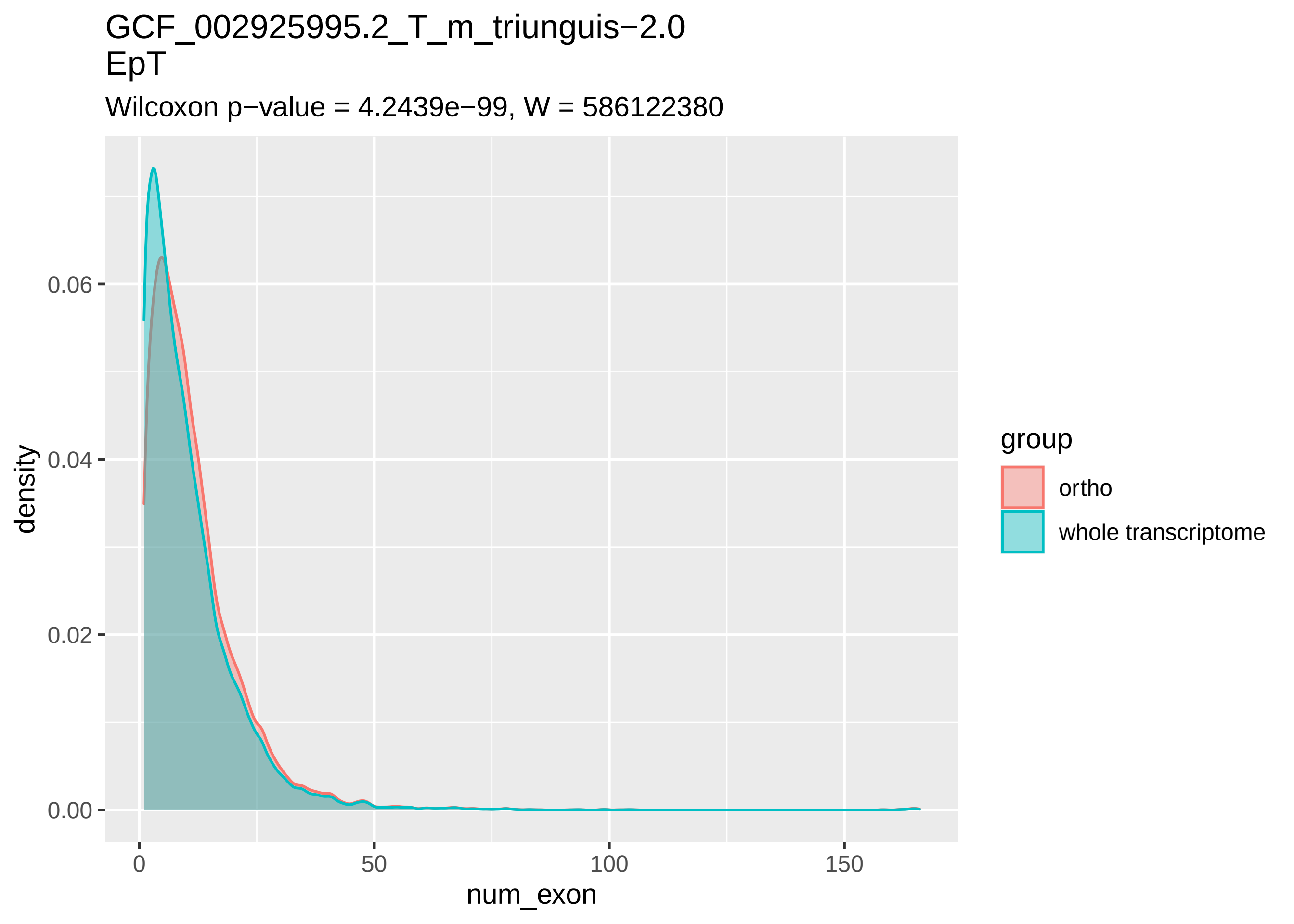
GCF_002925995.2_T_m_triunguis−2.0
EpT
Wilcoxon p−value = 4.2439e−99, W = 586122380
density
0.00
0.02
0.04
0.06
0
50
100
150
num_exon
group
ortho
whole transcriptome

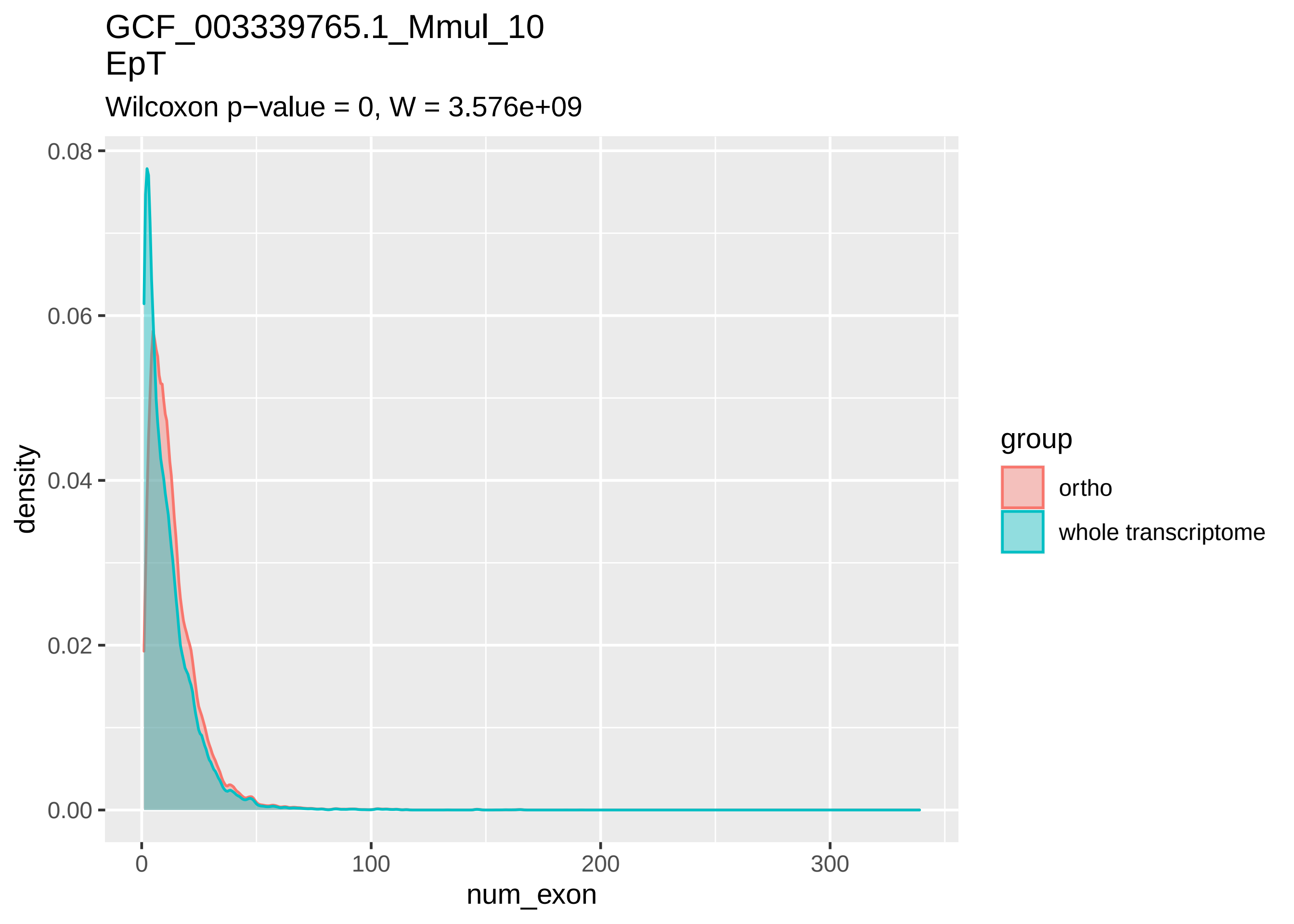
GCF_003339765.1_Mmul_10
EpT
Wilcoxon p−value = 0, W = 3.576e+09
0.08
0.06
0.04
0.02
0.00
density
0
100
200
300
num_exon
group
ortho
whole transcriptome

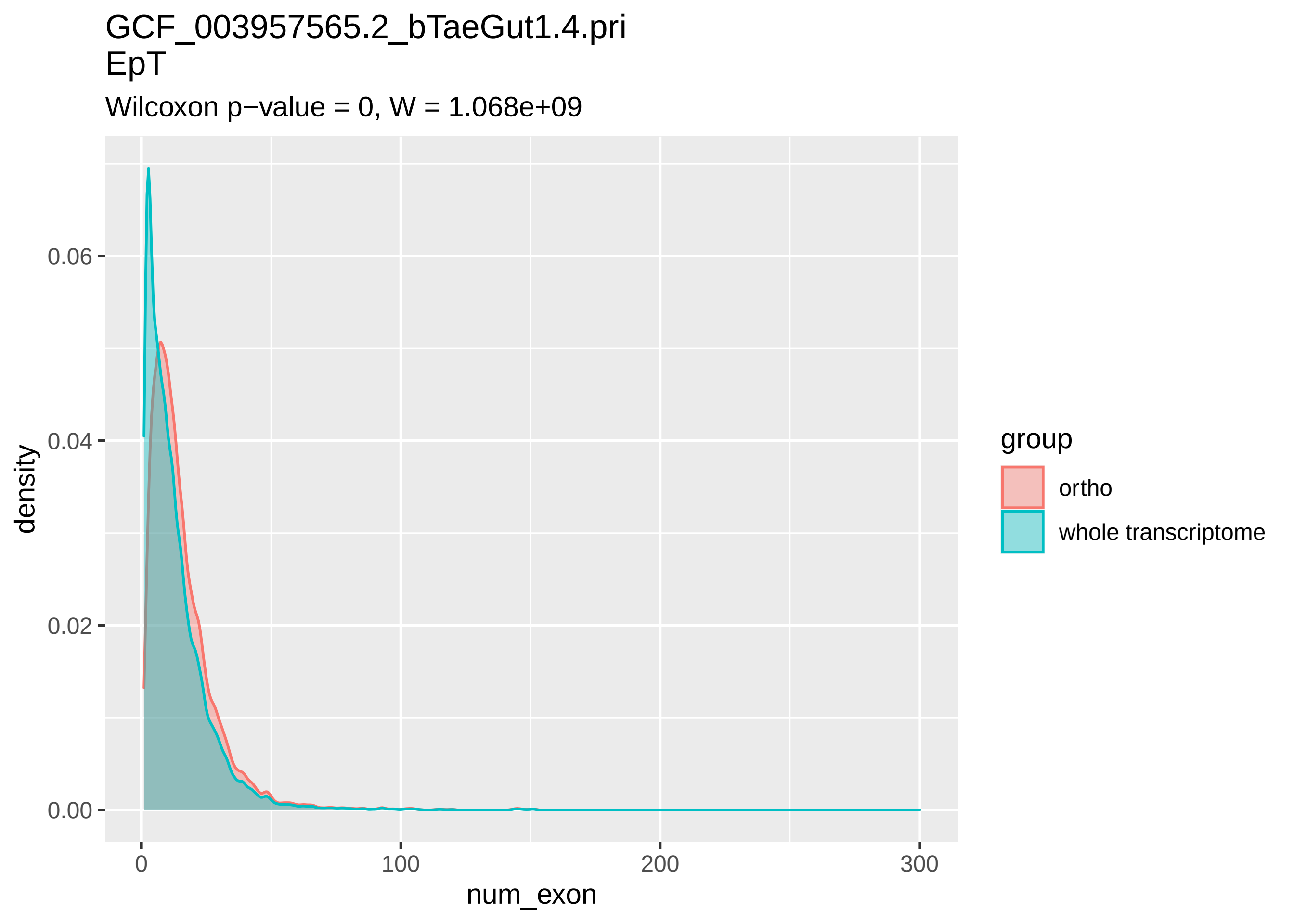
GCF_003957565.2_bTaeGut1.4.pri
EpT
Wilcoxon p−value = 0, W = 1.068e+09
density
0.06
0.04
0.02
0.00
0
100
200
300
num_exon
group
ortho
whole transcriptome

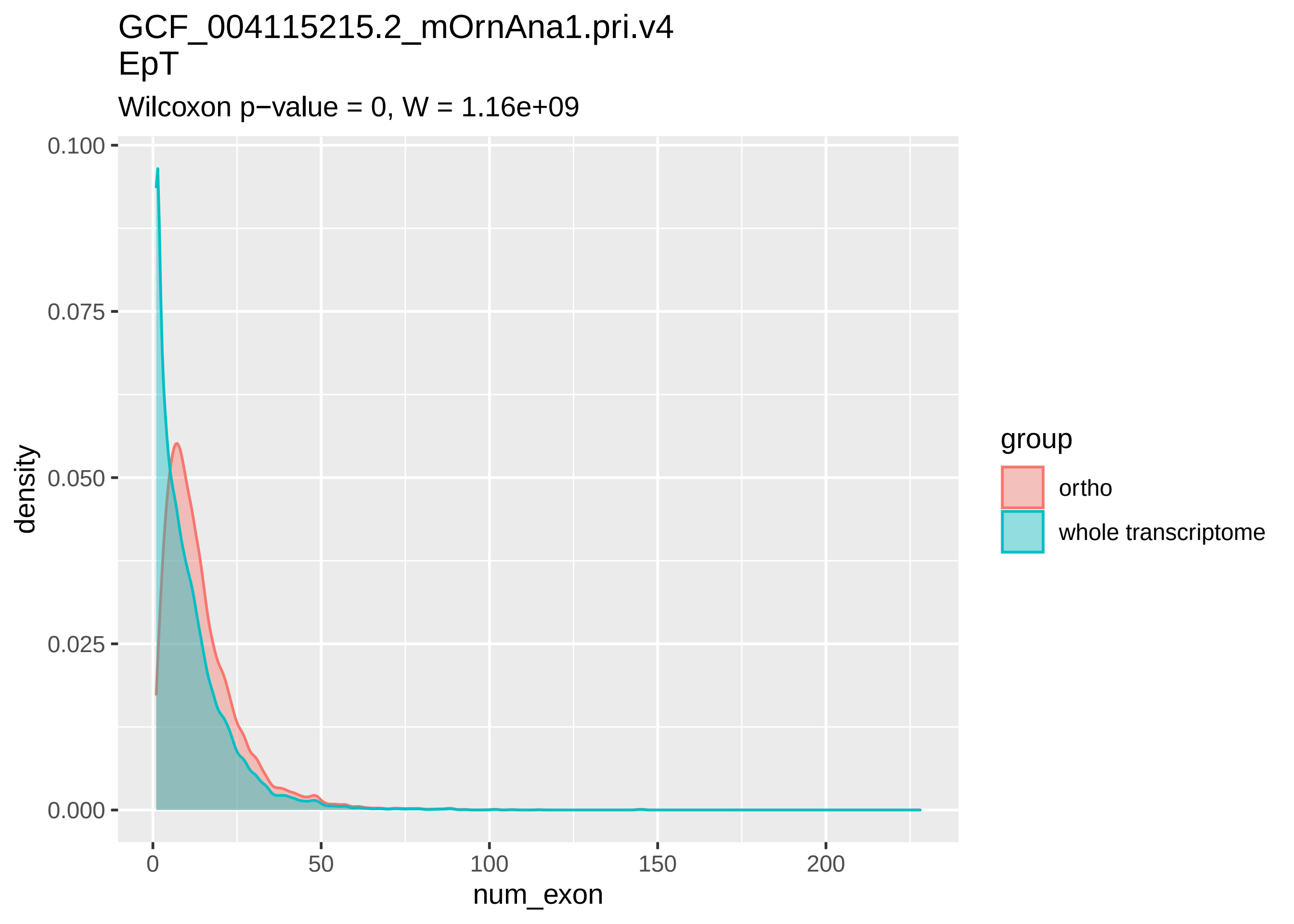

GCF_004115215.2_mOrnAna1.pri.v4
EpT
Wilcoxon p−value = 0, W = 1.16e+09
density
0.100
0.075
0.050
0.025
0.000
0
50
100
150
200
num_exon
group
ortho
whole transcriptome

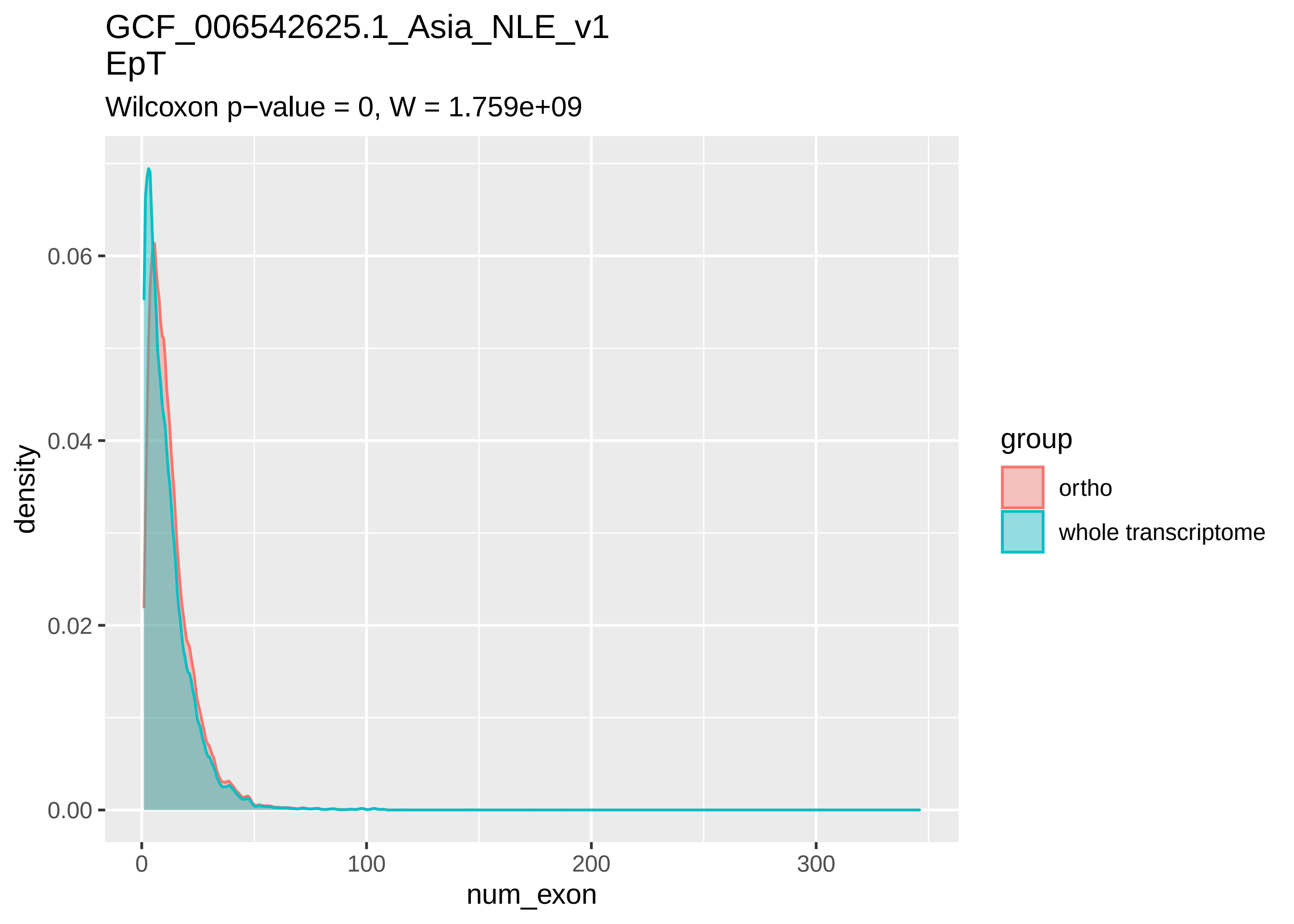

GCF_006542625.1_Asia_NLE_v1
EpT
Wilcoxon p−value = 0, W = 1.759e+09
density
0.06
0.04
0.02
0.00
0
100
200
300
num_exon
group
ortho
whole transcriptome

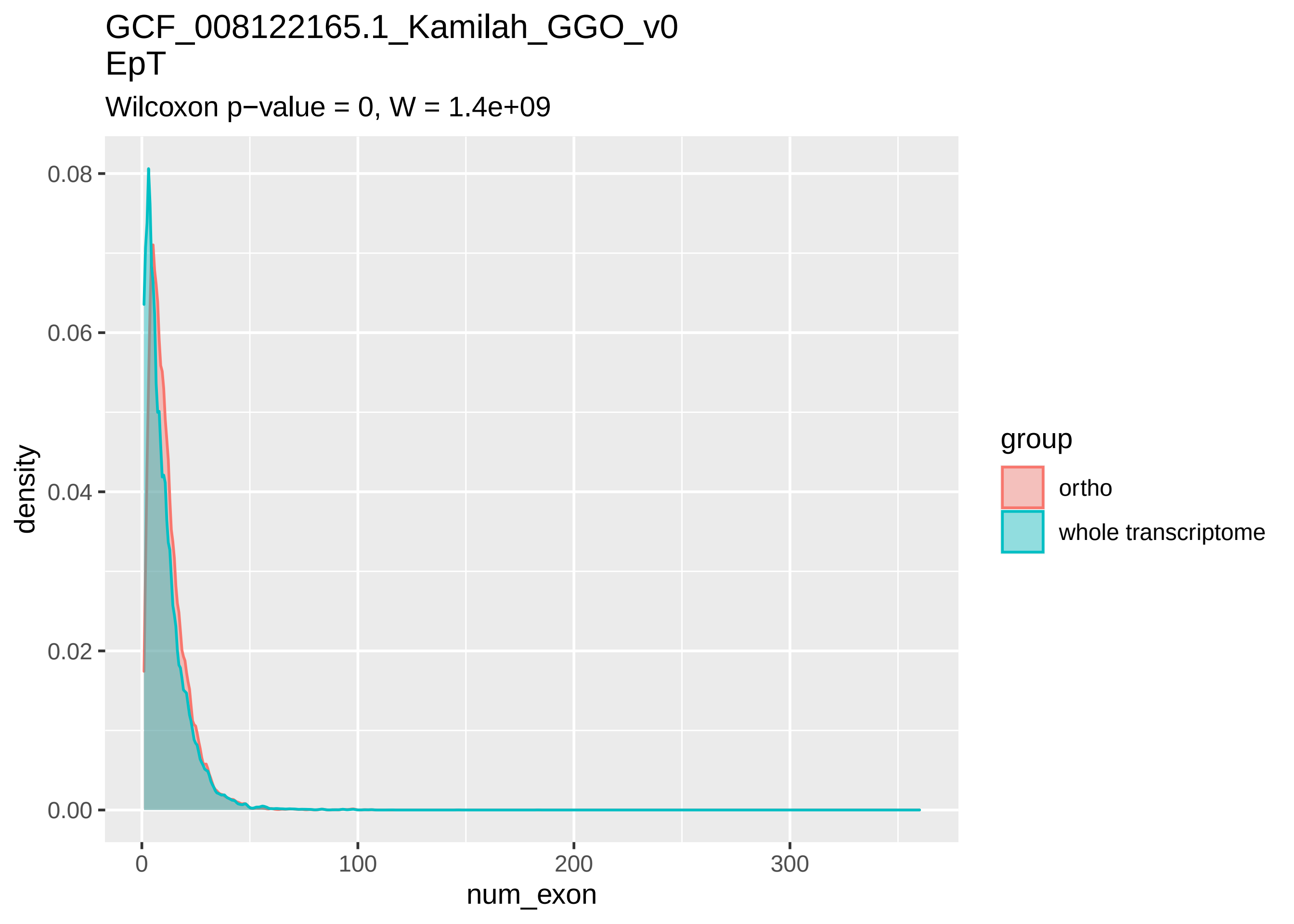
GCF_008122165.1_Kamilah_GGO_v0
EpT
Wilcoxon p−value = 0, W = 1.4e+09
density
0.08
0.06
0.04
0.02
0.00
0
100
200
300
num_exon
group
ortho
whole transcriptome

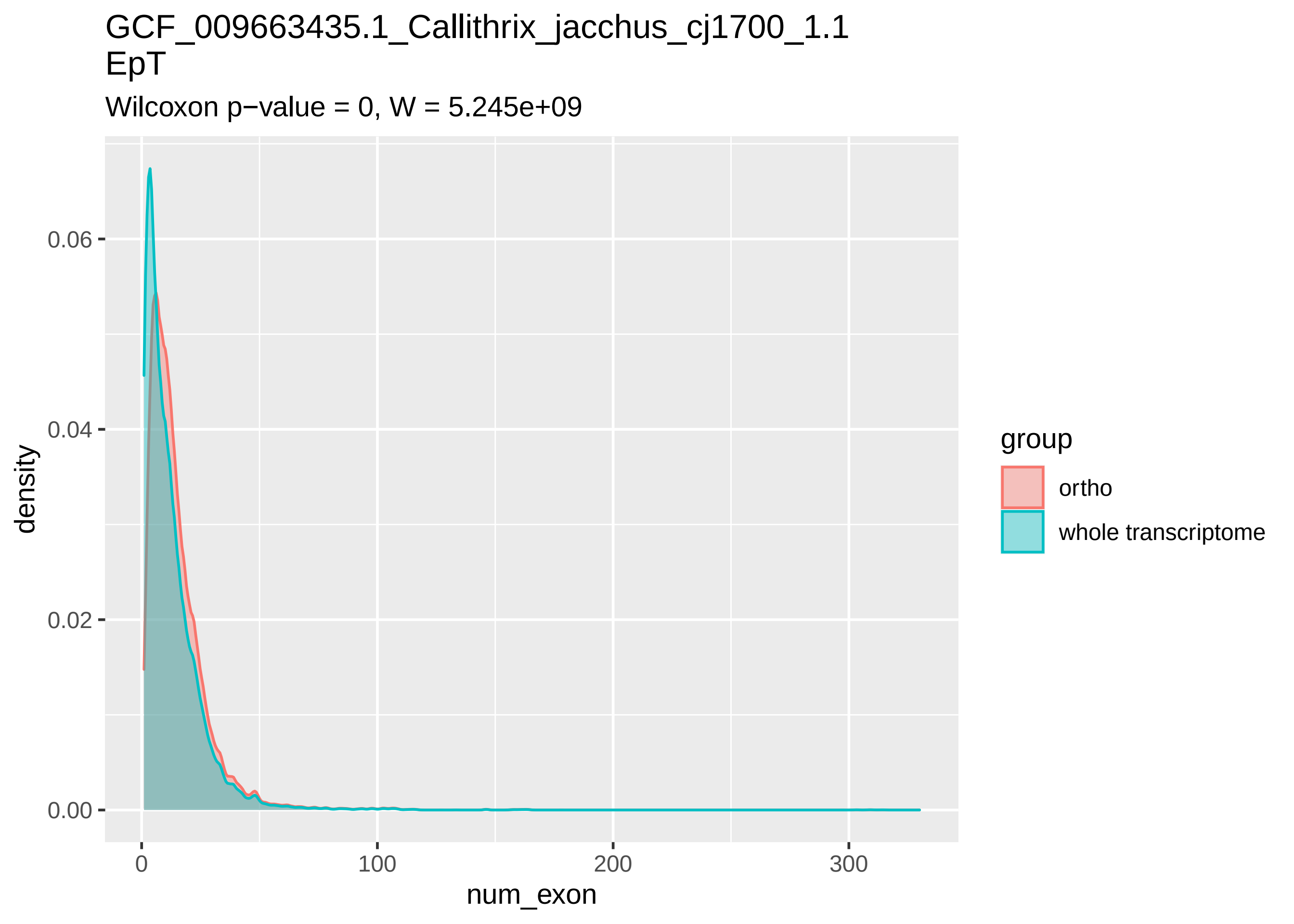
GCF_009663435.1_Callithrix_jacchus_cj1700_1.1
EpT
Wilcoxon p−value = 0, W = 5.245e+09
density
0.06
0.04
0.02
0.00
0
100
200
300
num_exon
group
ortho
whole transcriptome

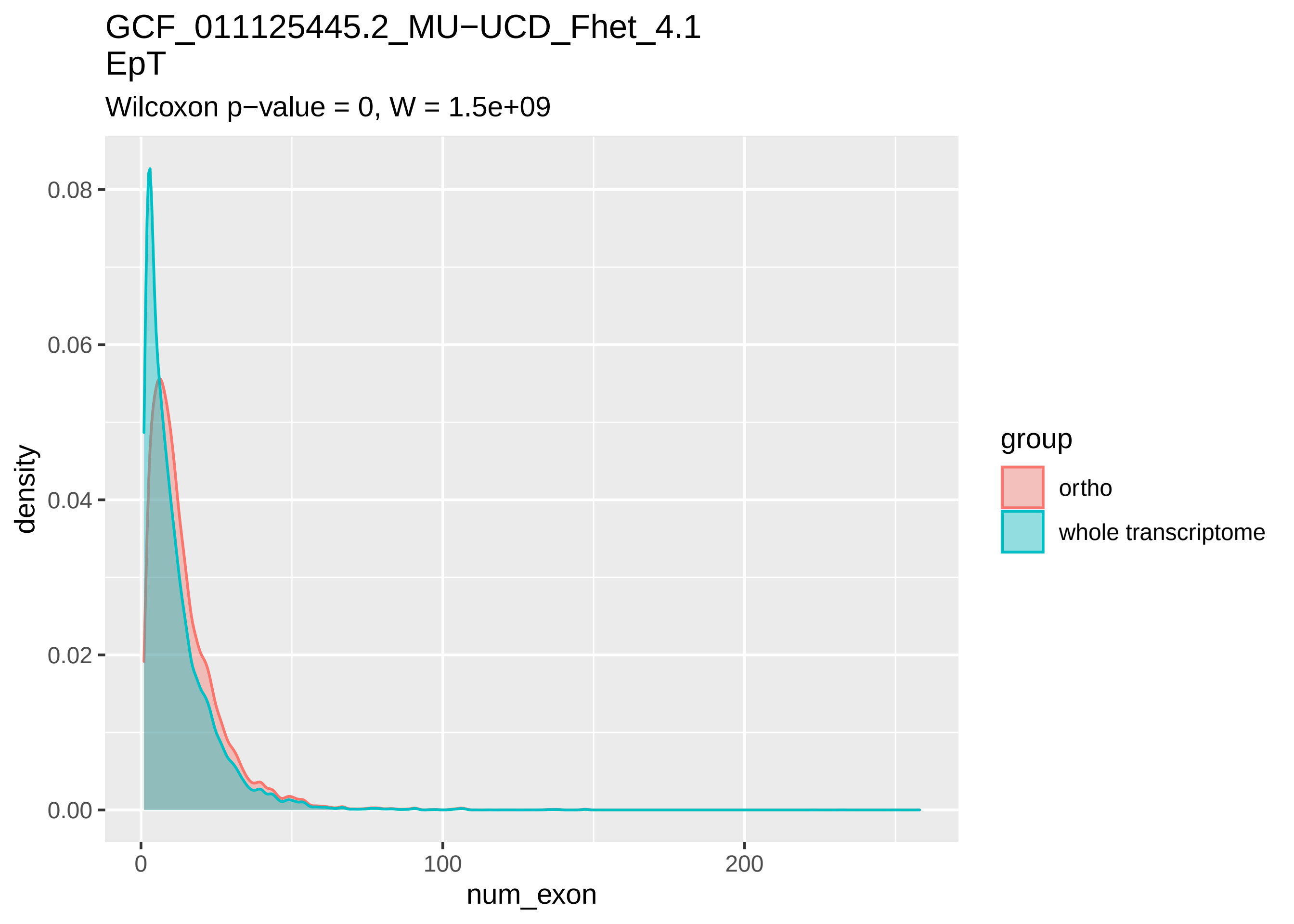
GCF_011125445.2_MU−UCD_Fhet_4.1
EpT
Wilcoxon p−value = 0, W = 1.5e+09
density
0.08
0.06
0.04
0.02
0.00
0
100
200
num_exon
group
ortho
whole transcriptome

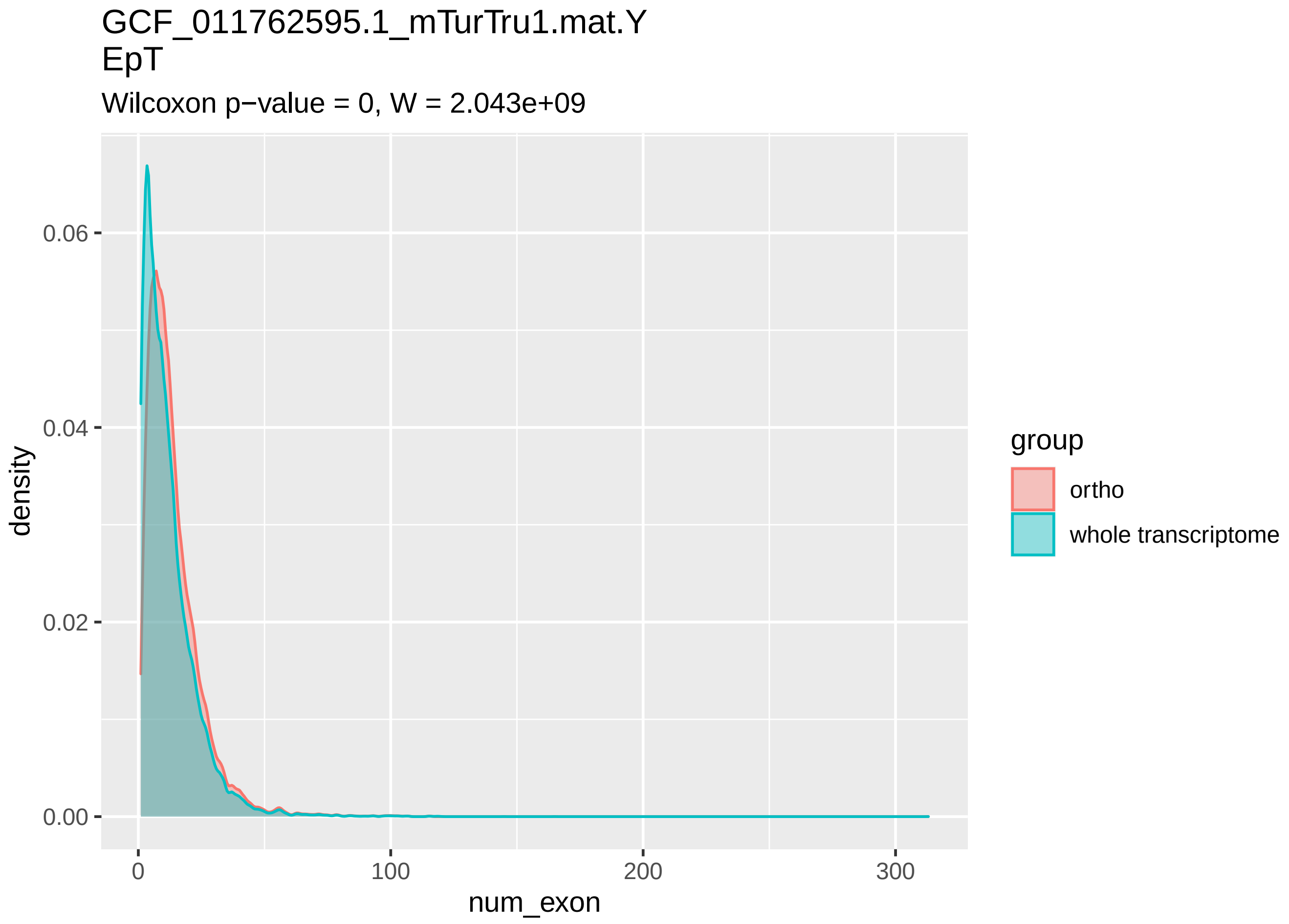
GCF_011762595.1_mTurTru1.mat.Y
EpT
Wilcoxon p−value = 0, W = 2.043e+09
density
0.06
0.04
0.02
0.00
0
100
200
300
num_exon
group
ortho
whole transcriptome

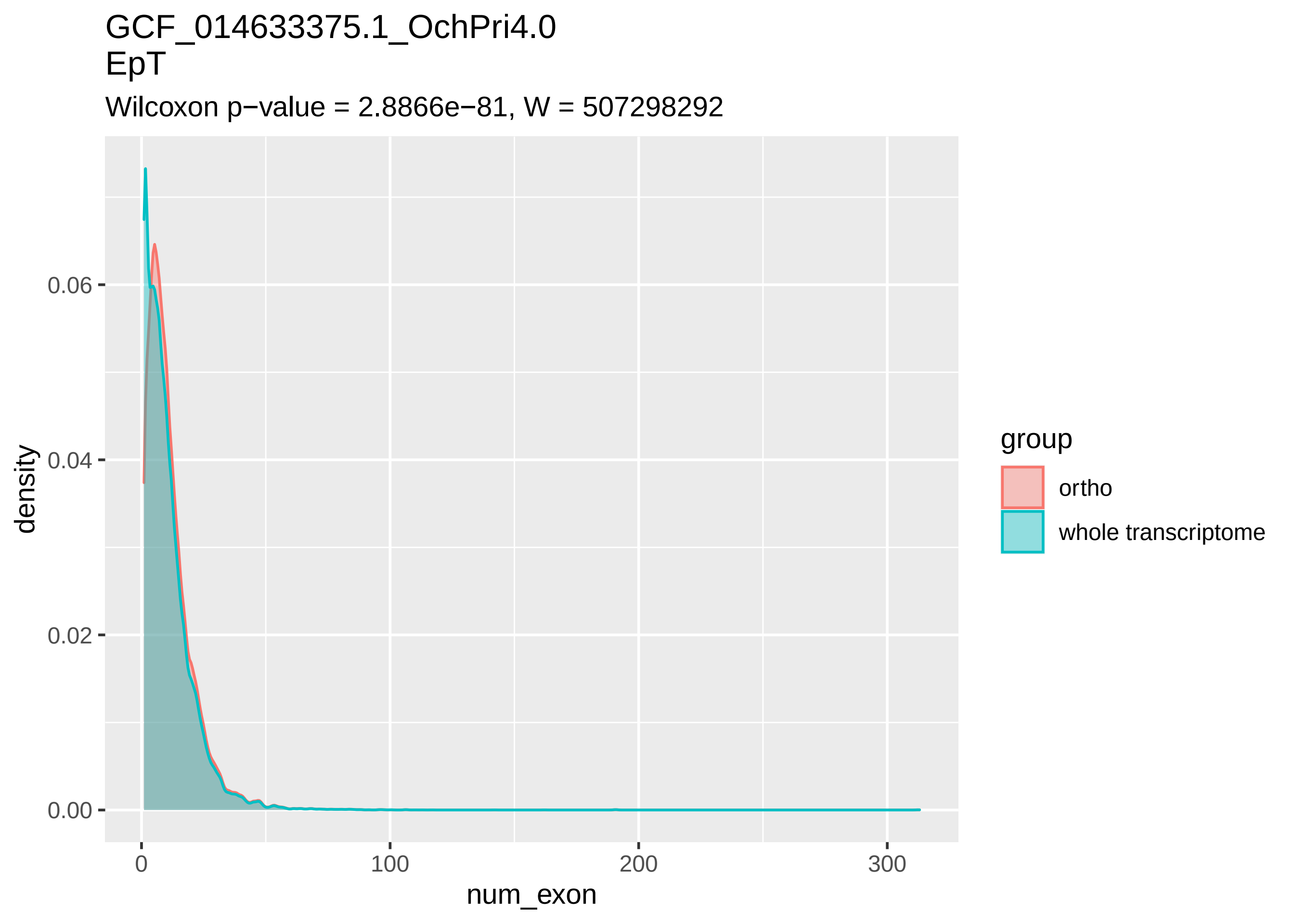

GCF_014633375.1_OchPri4.0
EpT
Wilcoxon p−value = 2.8866e−81, W = 507298292
density
0.06
0.04
0.02
0.00
0
100
200
300
num_exon
group
ortho
whole transcriptome

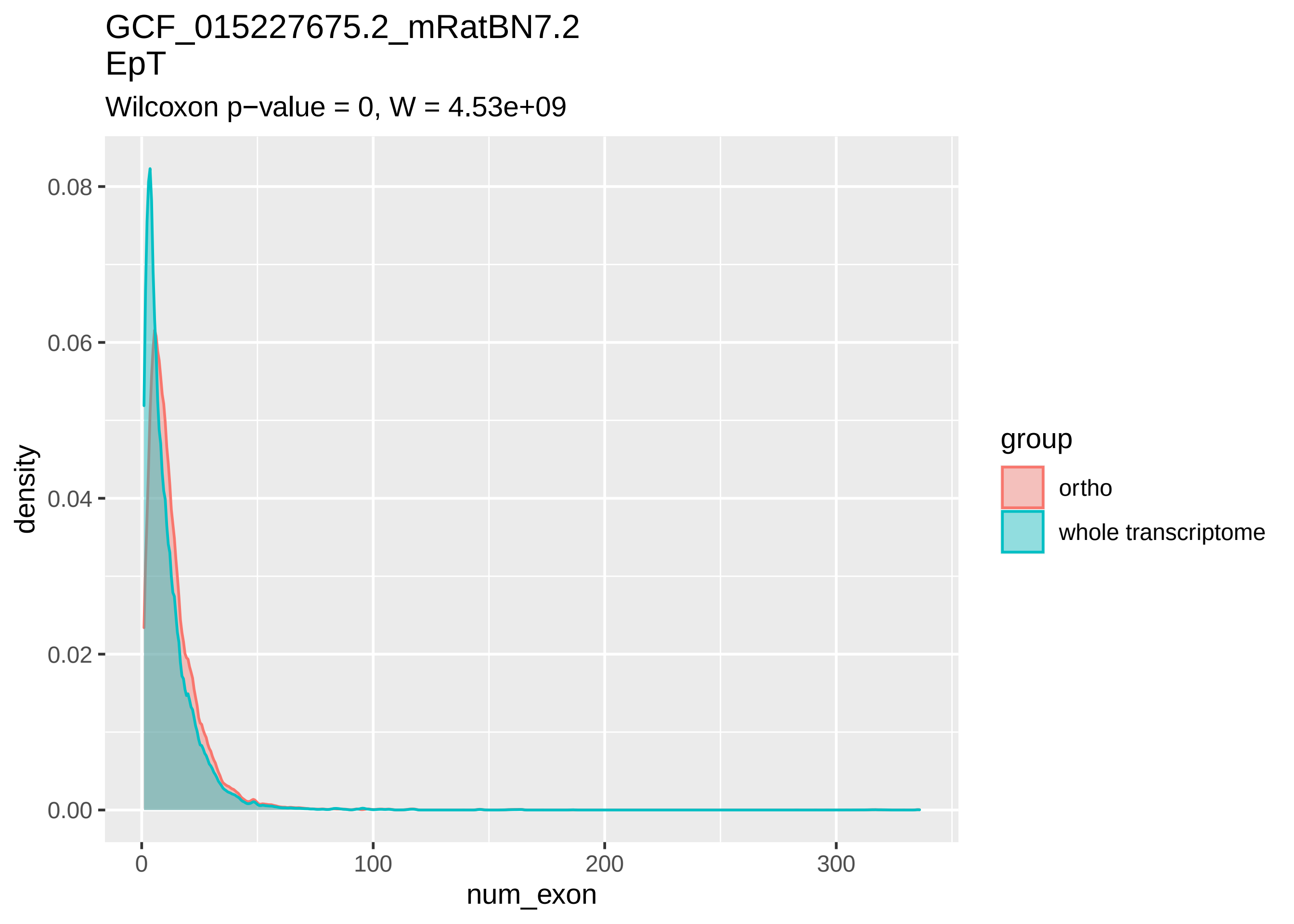
GCF_015227675.2_mRatBN7.2
EpT
Wilcoxon p−value = 0, W = 4.53e+09
density
0.08
0.06
0.04
0.02
0.00
0
100
200
300
num_exon
group
ortho
whole transcriptome

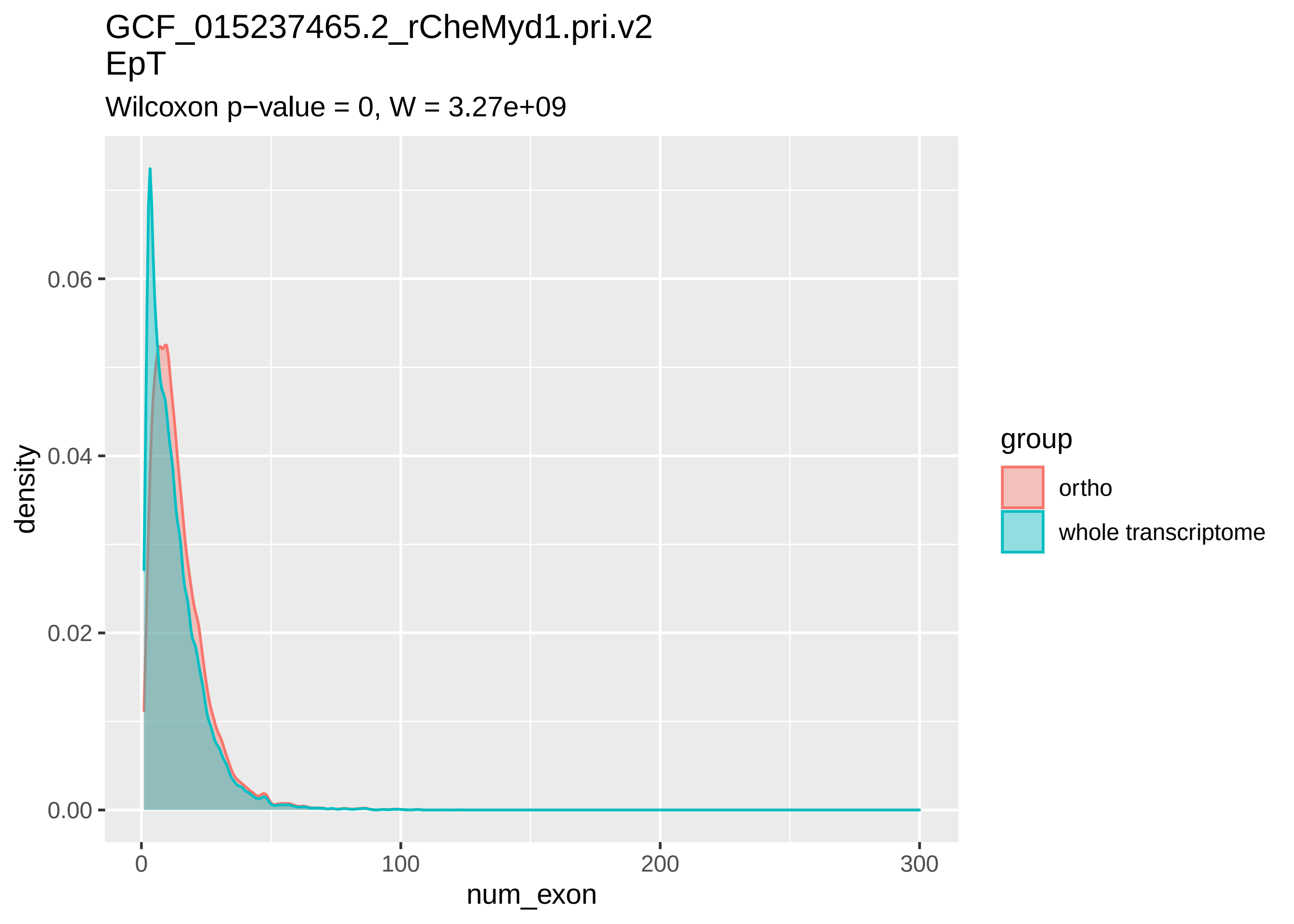
GCF_015237465.2_rCheMyd1.pri.v2
EpT
Wilcoxon p−value = 0, W = 3.27e+09
density
0.06
0.04
0.02
0.00
0
100
200
300
num_exon
group
ortho
whole transcriptome

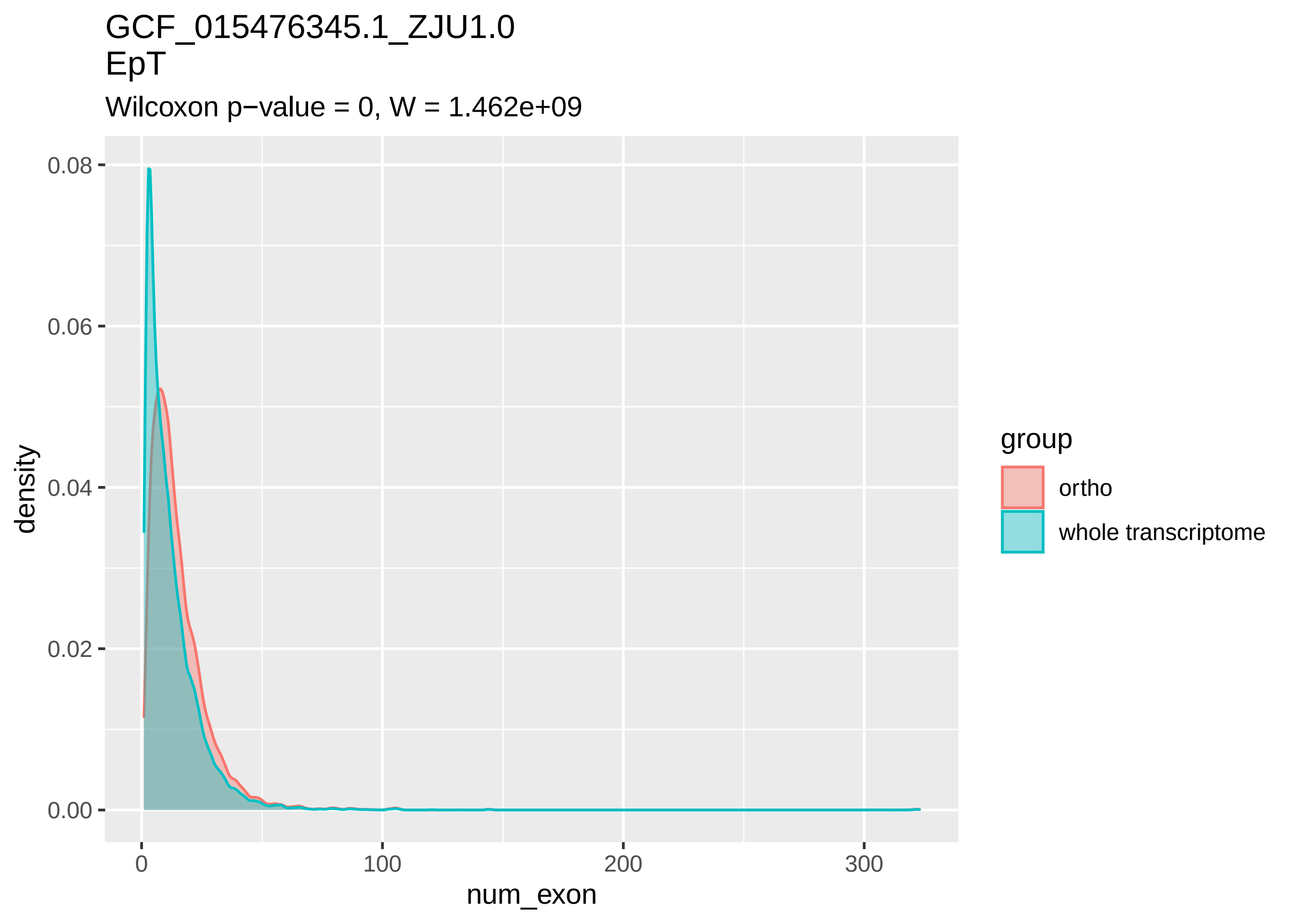

GCF_015476345.1_ZJU1.0
EpT
Wilcoxon p−value = 0, W = 1.462e+09
density
0.08
0.06
0.04
0.02
0.00
0
100
200
300
num_exon
group
ortho
whole transcriptome

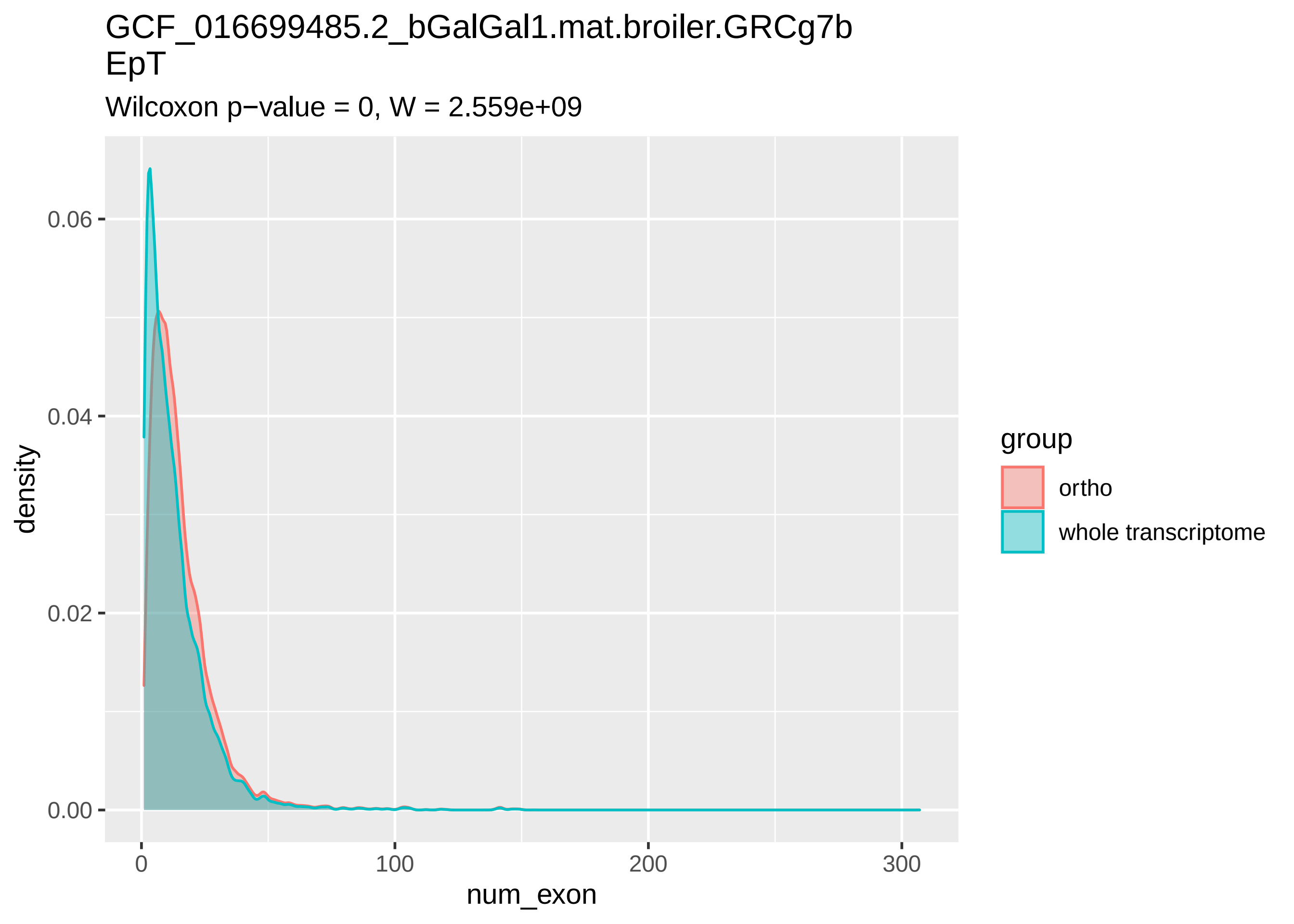

GCF_016699485.2_bGalGal1.mat.broiler.GRCg7b
EpT
Wilcoxon p−value = 0, W = 2.559e+09
0.06
0.04
0.02
0.00
density
0
100
200
300
num_exon
group
ortho
whole transcriptome

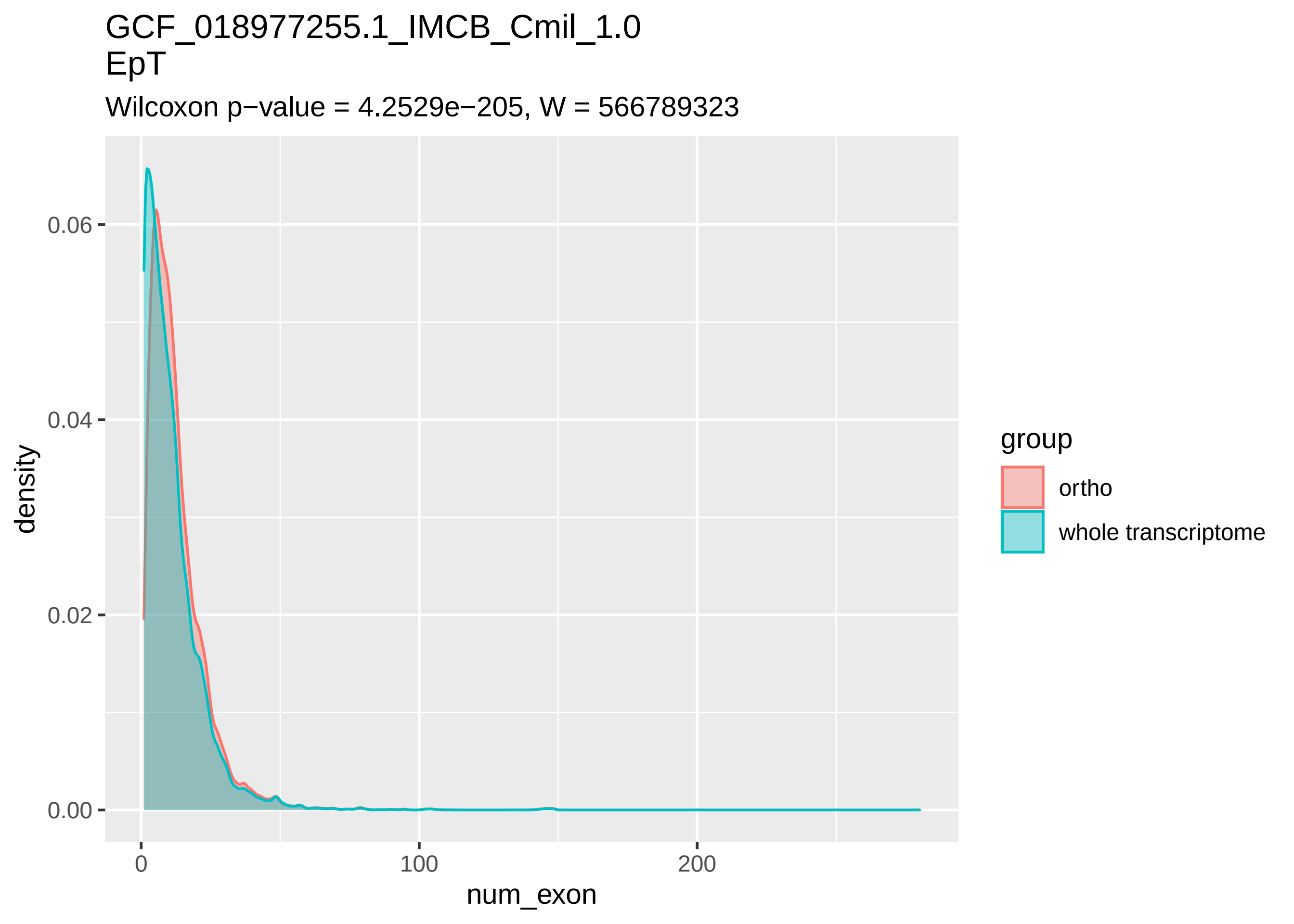
GCF_018977255.1_IMCB_Cmil_1.0
EpT
Wilcoxon p−value = 4.2529e−205, W = 566789323
0.06
0.04
0.02
0.00
density
0
100
200
num_exon
group
ortho
whole transcriptome

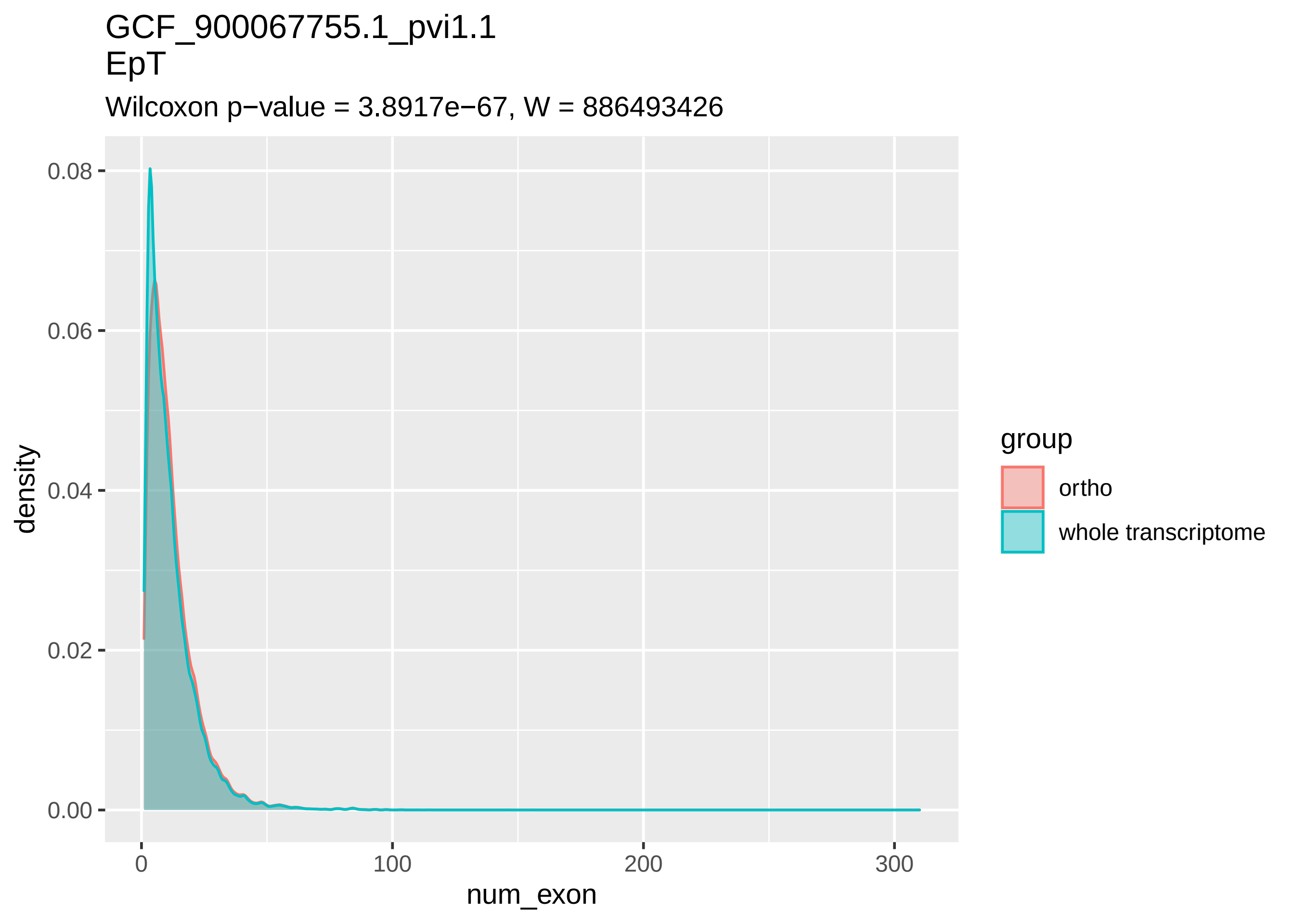

GCF_900067755.1_pvi1.1
EpT
Wilcoxon p−value = 3.8917e−67, W = 886493426
density
0.08
0.06
0.04
0.02
0.00
0
100
200
300
num_exon
group
ortho
whole transcriptome

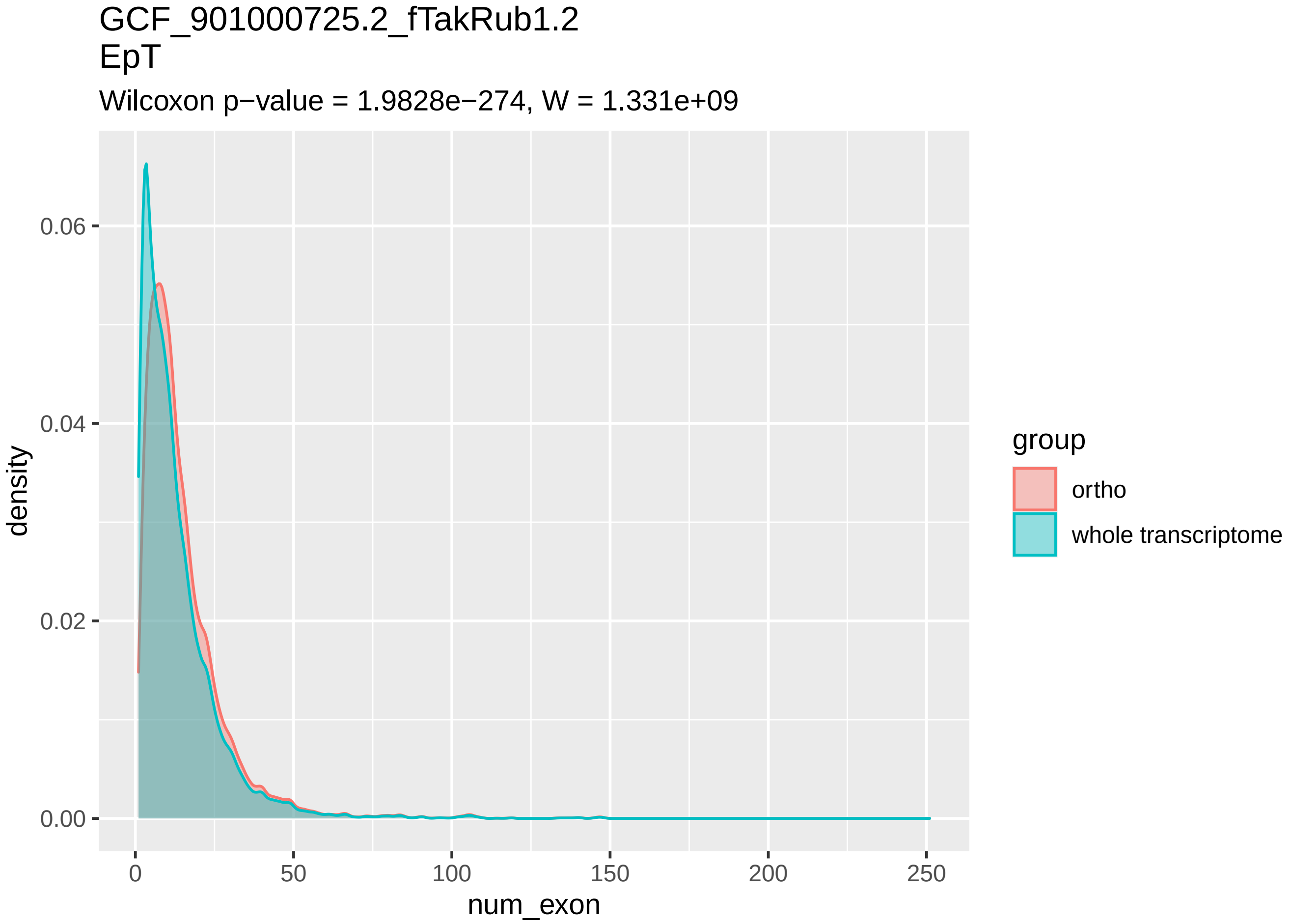
GCF_901000725.2_fTakRub1.2
EpT
Wilcoxon p−value = 1.9828e−274, W = 1.331e+09
density
0.06
0.04
0.02
0.00
0
50
100
150
200
250
num_exon
group
ortho
whole transcriptome

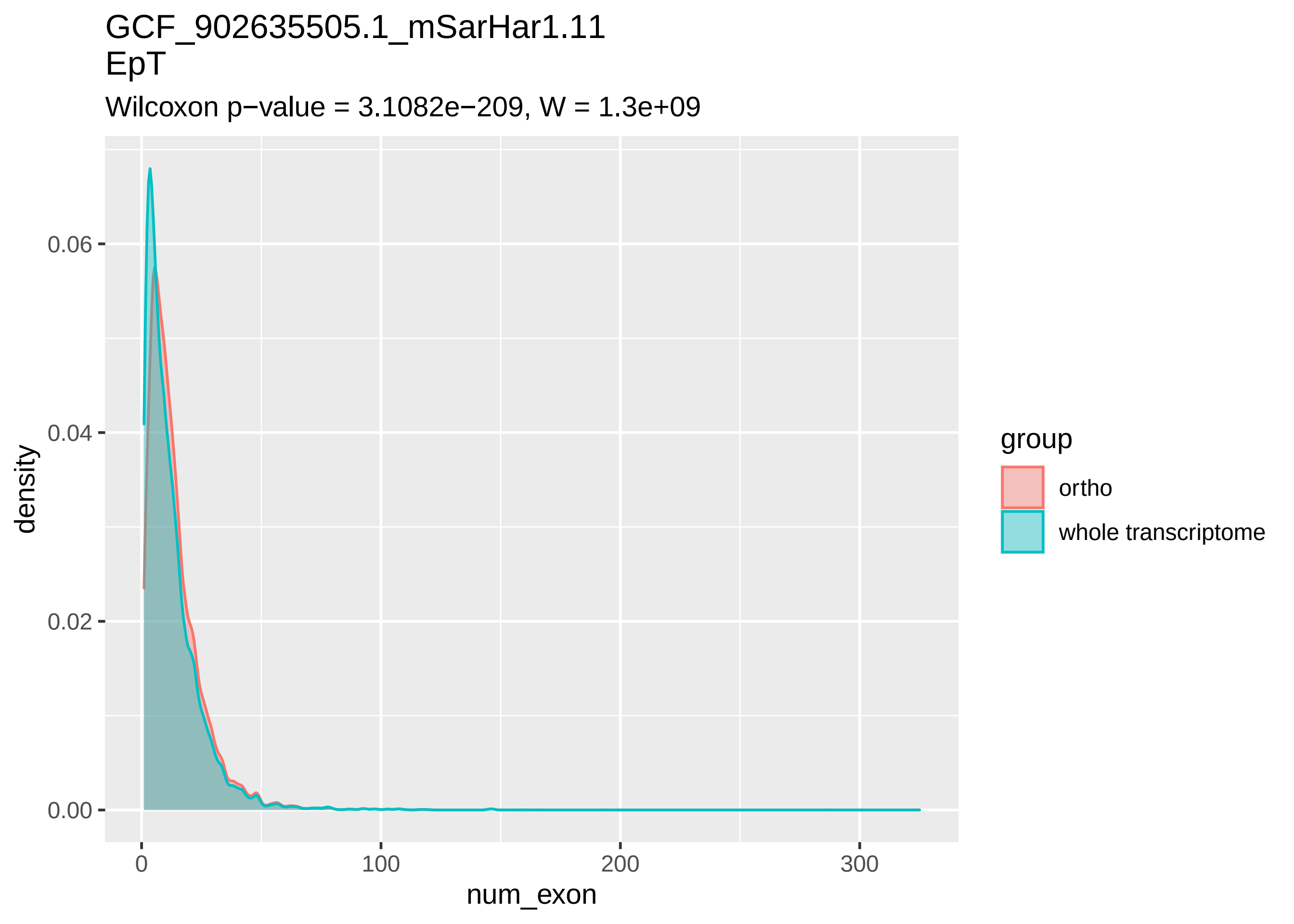
GCF_902635505.1_mSarHar1.11
EpT
Wilcoxon p−value = 3.1082e−209, W = 1.3e+09
density
0.00
0.02
0.04
0.06
0
100
200
300
num_exon
group
ortho
whole transcriptome

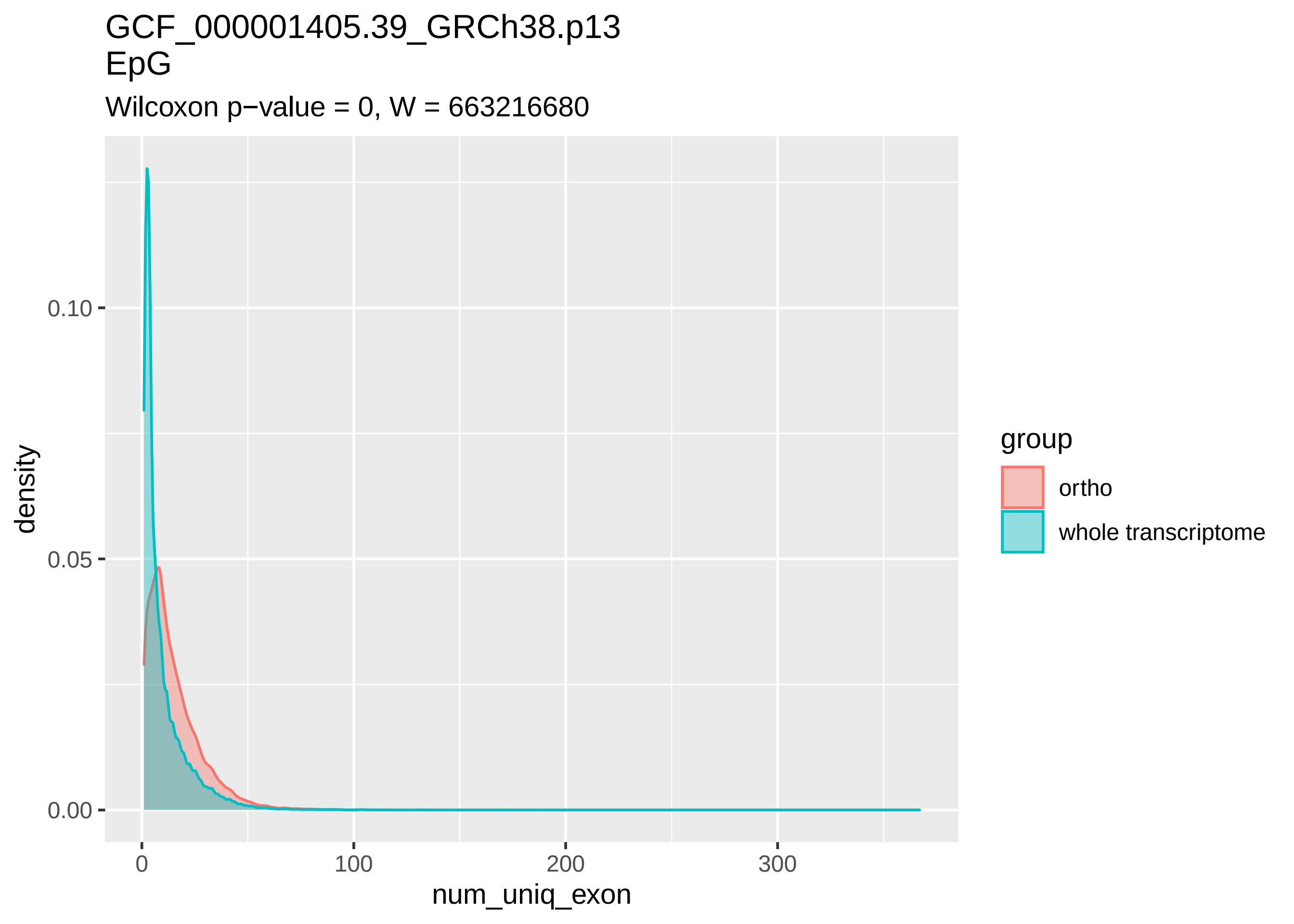

GCF_000001405.39_GRCh38.p13
EpG
Wilcoxon p−value = 0, W = 663216680
0.10
0.05
0.00
density
0
100
200
300
num_uniq_exon
group
ortho
whole transcriptome

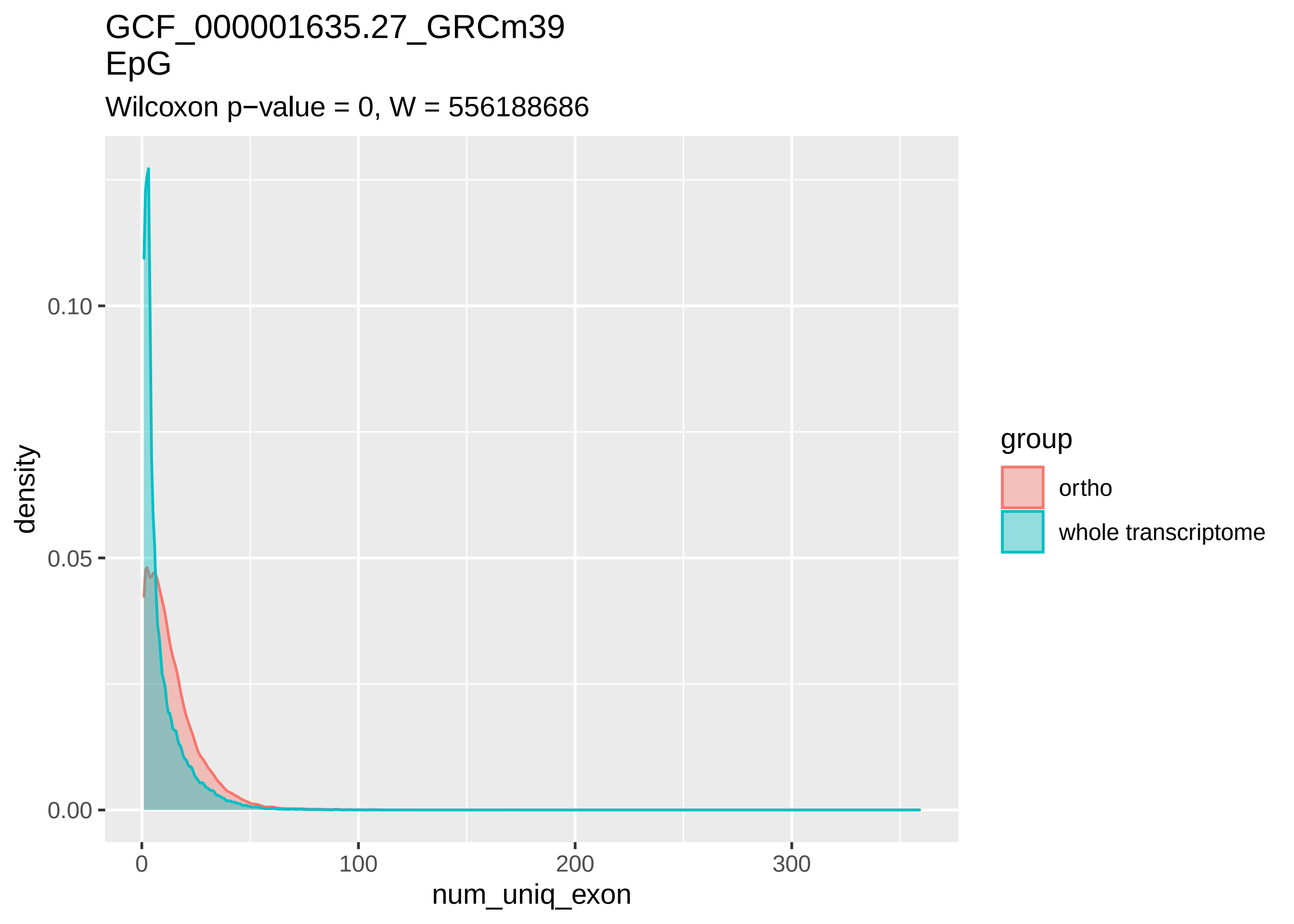
GCF_000001635.27_GRCm39
EpG
Wilcoxon p−value = 0, W = 556188686
density
0.10
0.05
0.00
0
100
200
300
num_uniq_exon
group
ortho
whole transcriptome

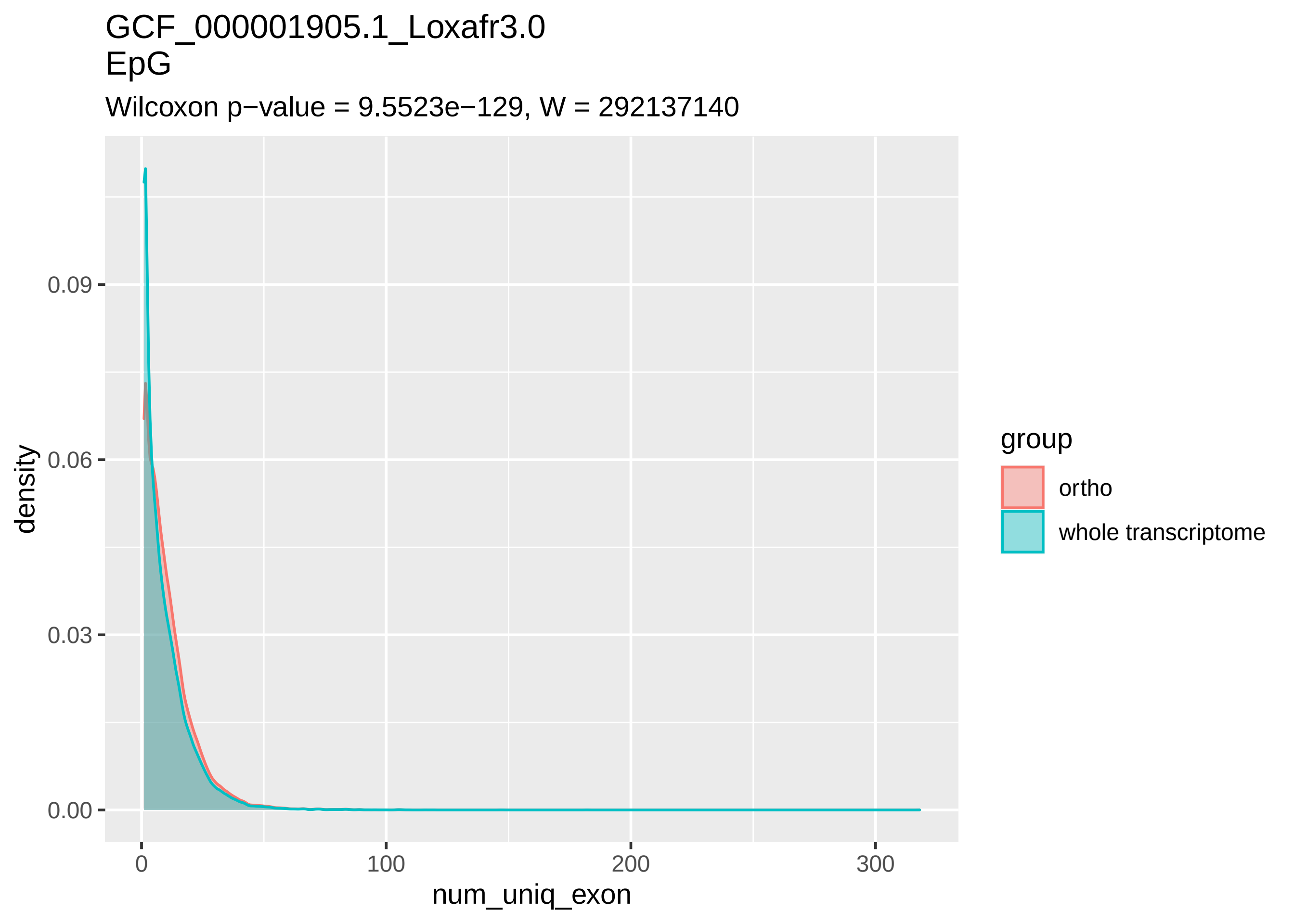

GCF_000001905.1_Loxafr3.0
EpG
Wilcoxon p−value = 9.5523e−129, W = 292137140
0.09
0.06
0.03
0.00
density
0
100
200
300
num_uniq_exon
group
ortho
whole transcriptome

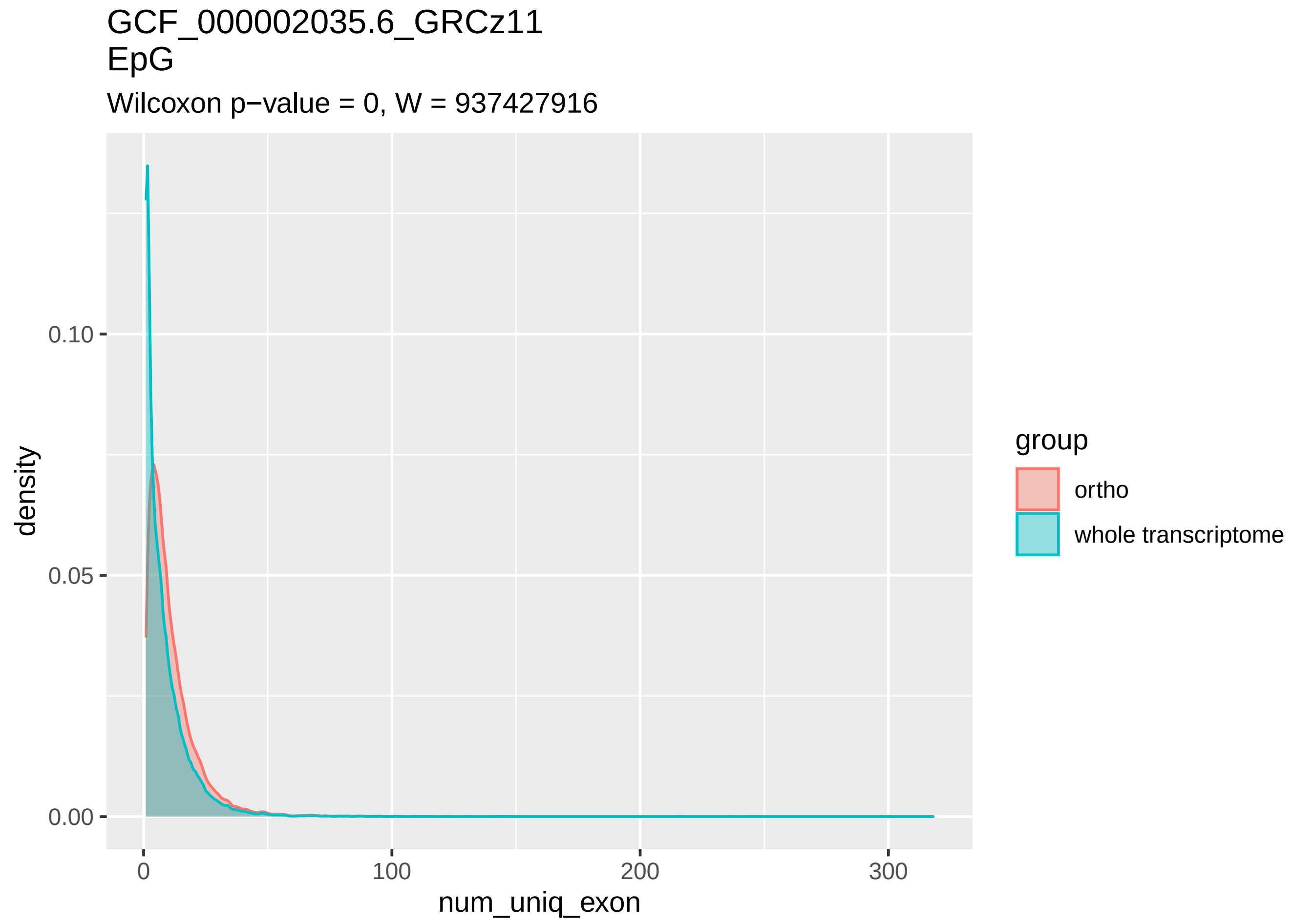
GCF_000002035.6_GRCz11
EpG
Wilcoxon p−value = 0, W = 937427916
density
0.10
0.05
0.00
0
100
200
300
num_uniq_exon
group
ortho
whole transcriptome

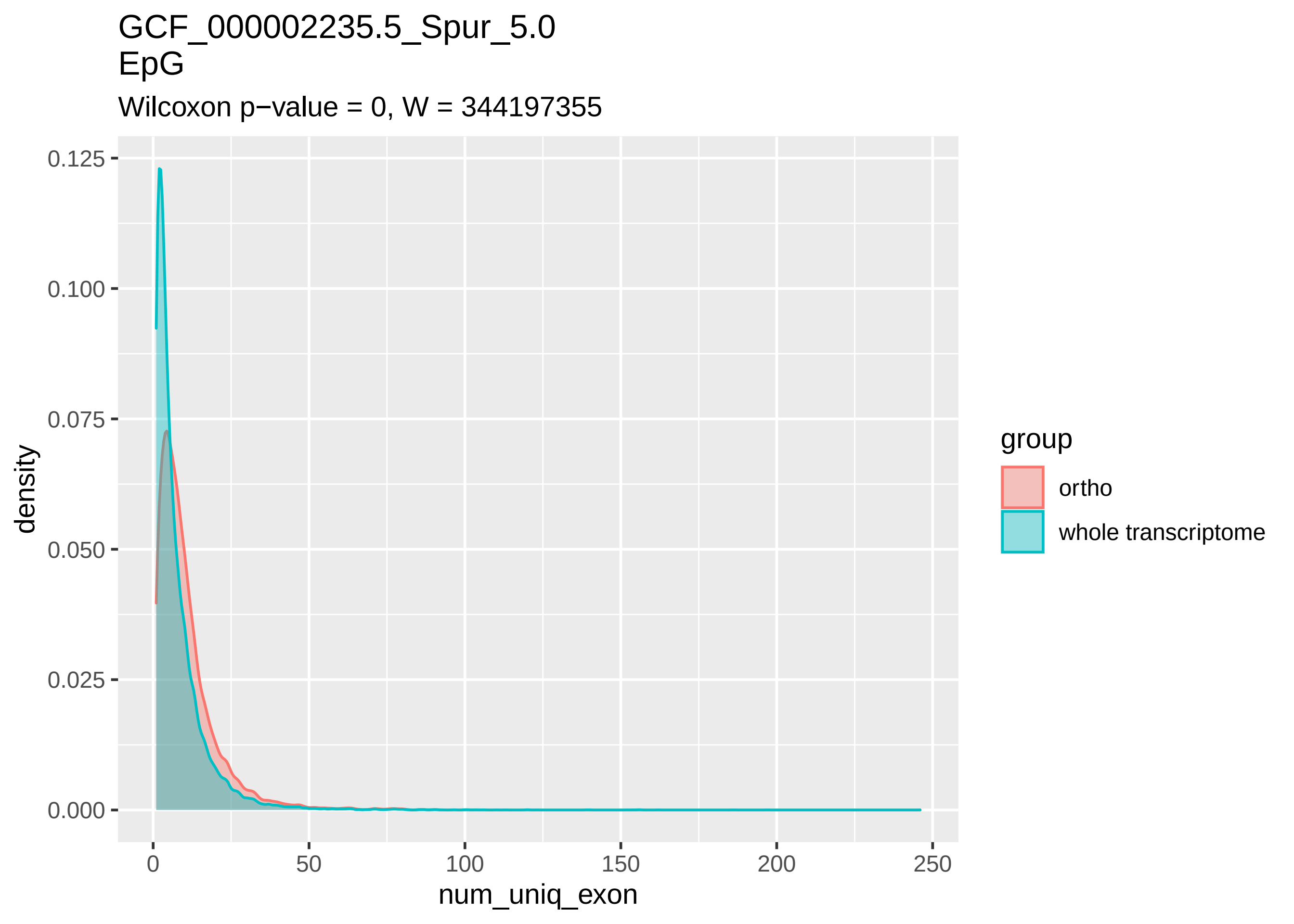
GCF_000002235.5_Spur_5.0
EpG
Wilcoxon p−value = 0, W = 344197355
0.125
0.100
0.075
0.050
0.025
0.000
density
0
50
100
150
200
250
num_uniq_exon
group
ortho
whole transcriptome

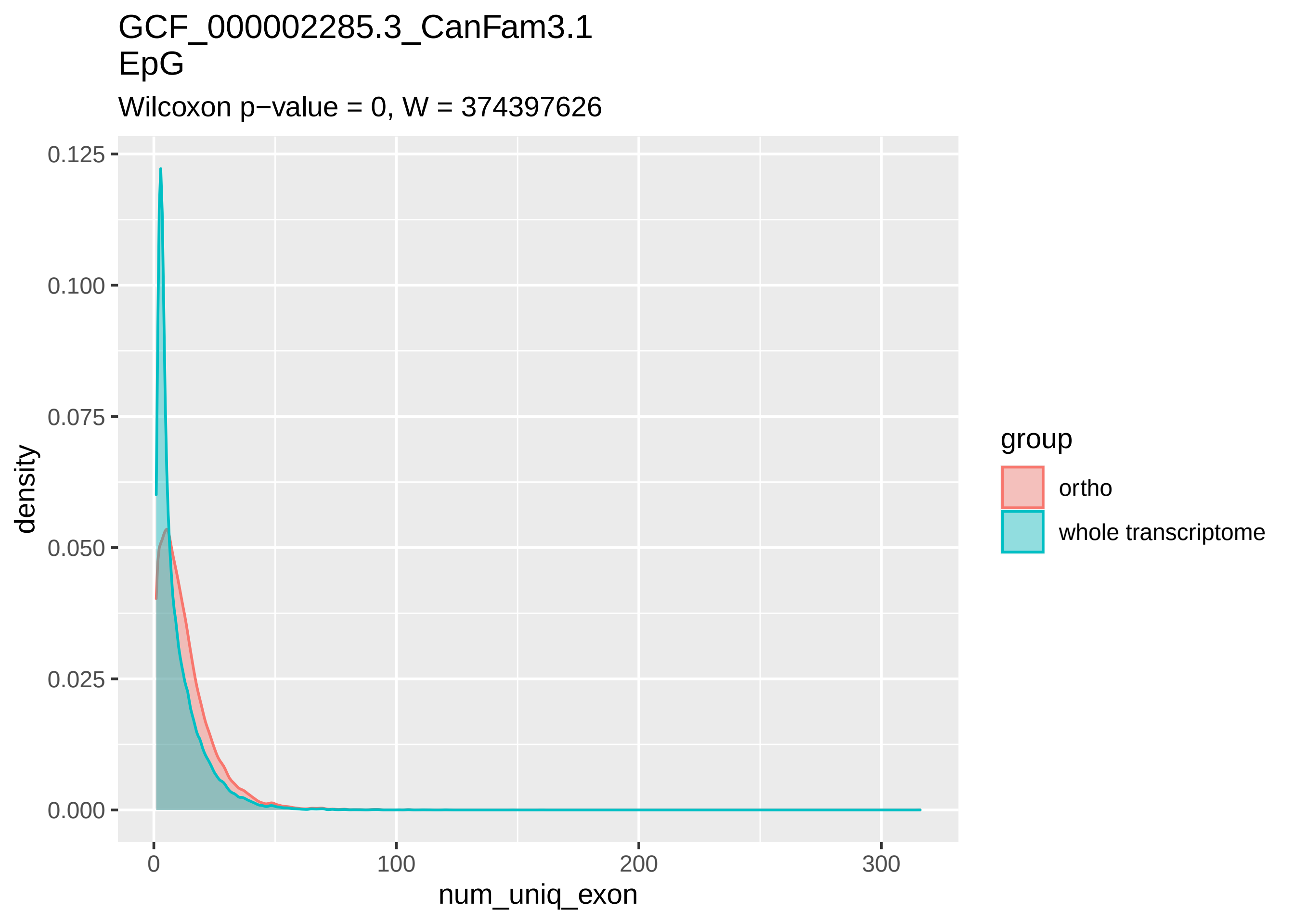
GCF_000002285.3_CanFam3.1
EpG
Wilcoxon p−value = 0, W = 374397626
density
0.125
0.100
0.075
0.050
0.025
0.000
0
100
200
300
num_uniq_exon
group
ortho
whole transcriptome

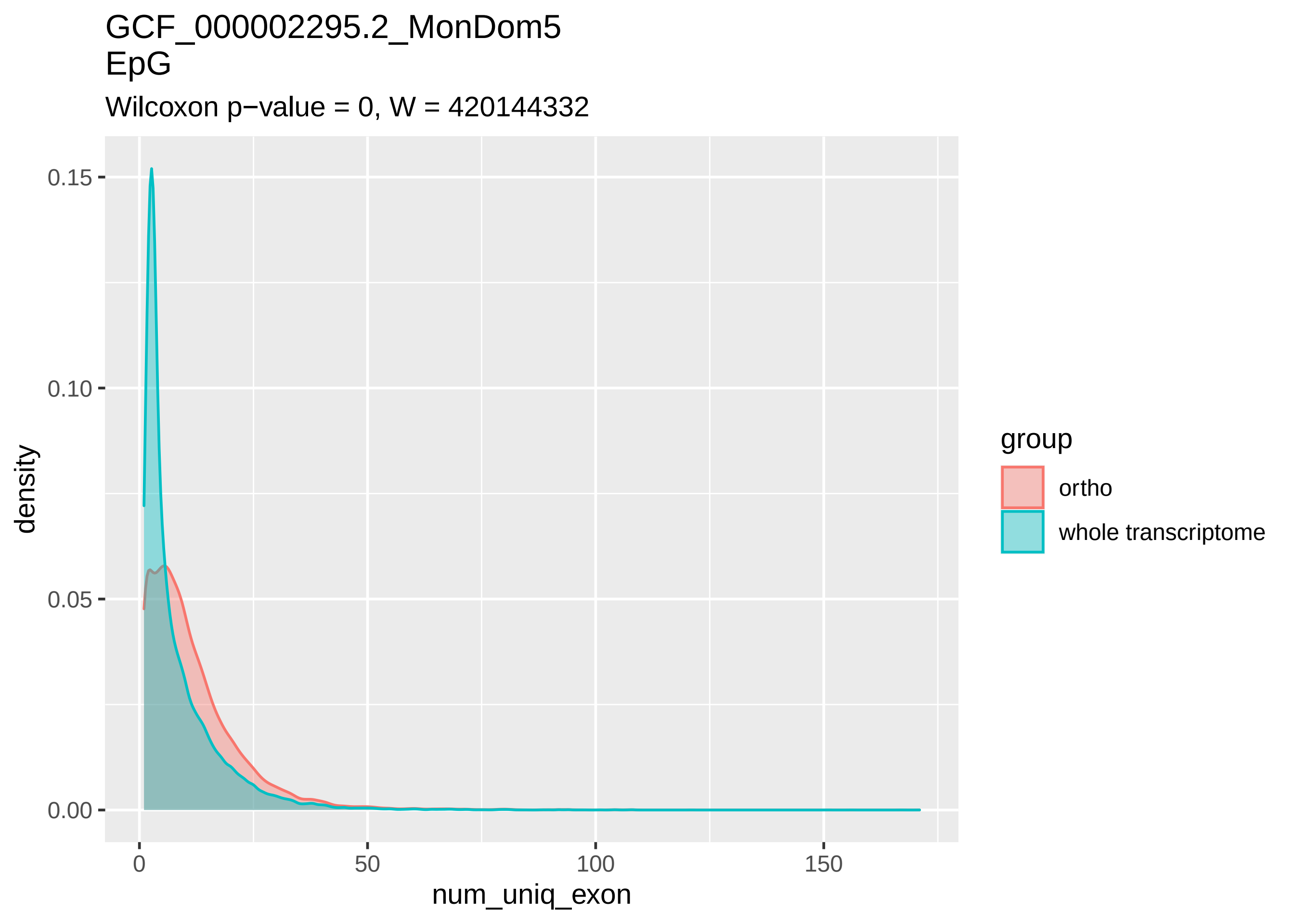
GCF_000002295.2_MonDom5
EpG
Wilcoxon p−value = 0, W = 420144332
0.15
0.10
0.05
0.00
density
0
50
100
150
num_uniq_exon
group
ortho
whole transcriptome

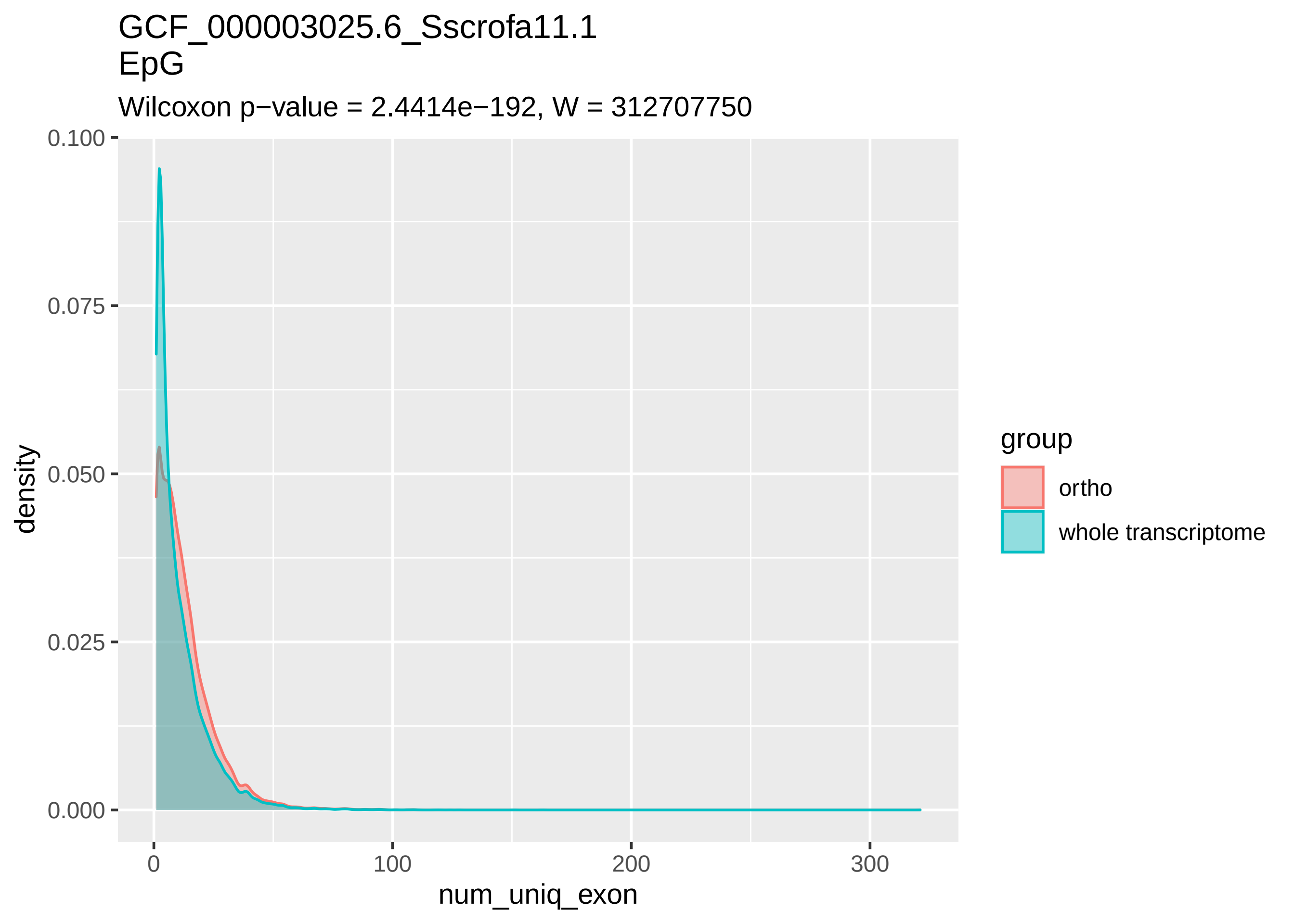

GCF_000003025.6_Sscrofa11.1
EpG
Wilcoxon p−value = 2.4414e−192, W = 312707750
density
0.100
0.075
0.050
0.025
0.000
0
100
200
300
num_uniq_exon
group
ortho
whole transcriptome

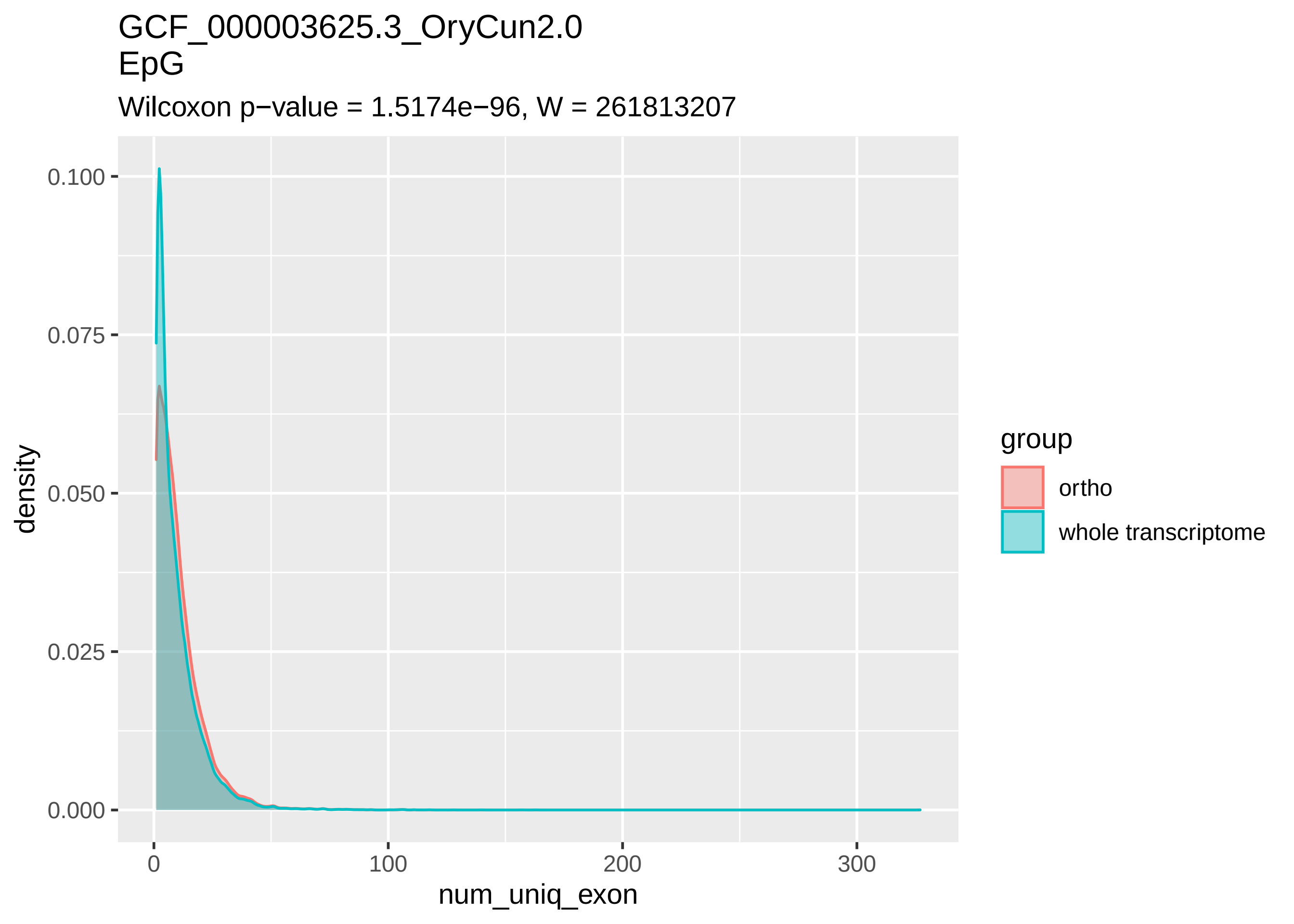
GCF_000003625.3_OryCun2.0
EpG
Wilcoxon p−value = 1.5174e−96, W = 261813207
0.100
0.075
0.050
0.025
0.000
density
0
100
200
300
num_uniq_exon
group
ortho
whole transcriptome

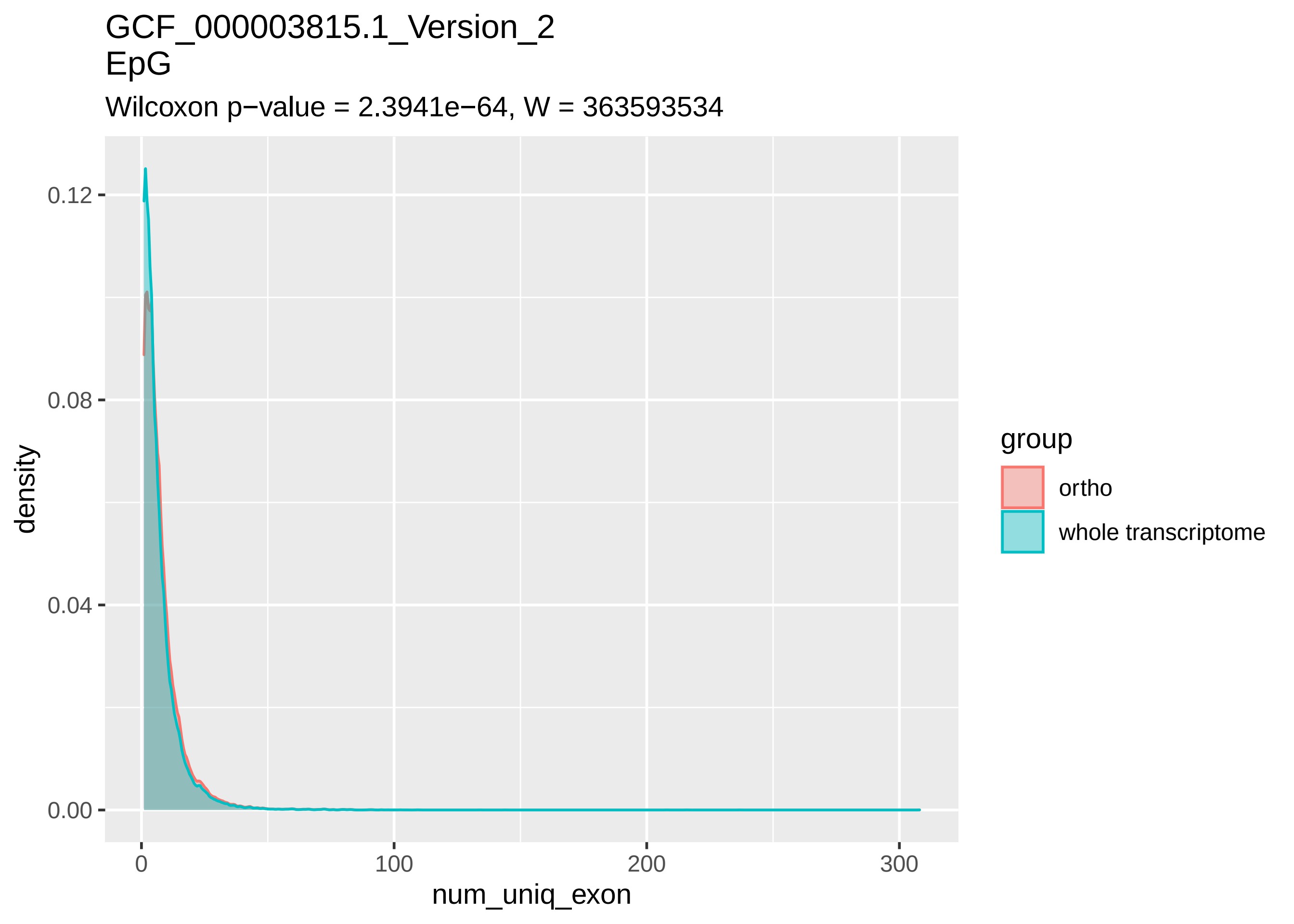
GCF_000003815.1_Version_2
EpG
Wilcoxon p−value = 2.3941e−64, W = 363593534
density
0.12
0.08
0.04
0.00
0
100
200
300
num_uniq_exon
group
ortho
whole transcriptome

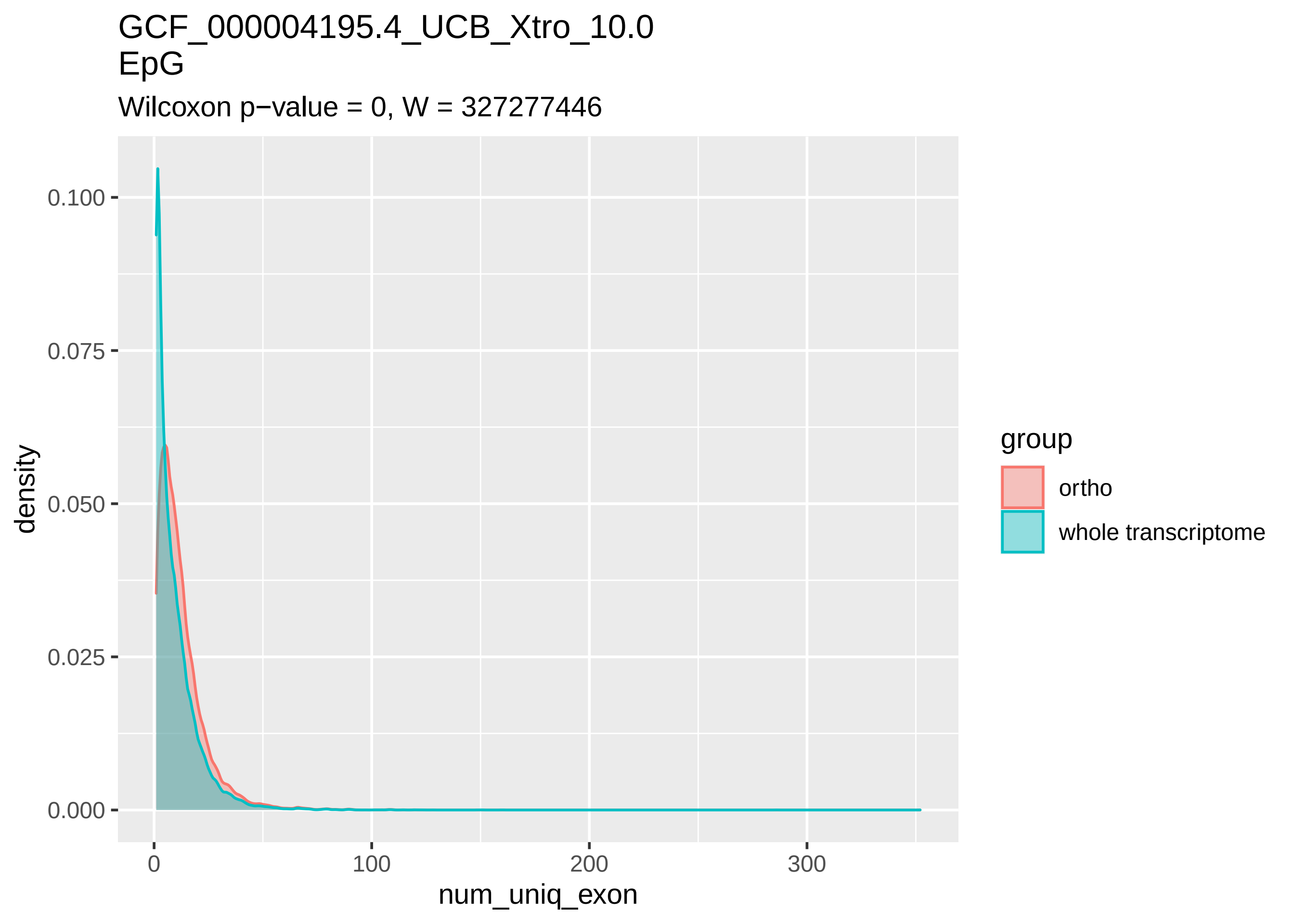
GCF_000004195.4_UCB_Xtro_10.0
EpG
Wilcoxon p−value = 0, W = 327277446
density
0.100
0.075
0.050
0.025
0.000
0
100
200
300
num_uniq_exon
group
ortho
whole transcriptome

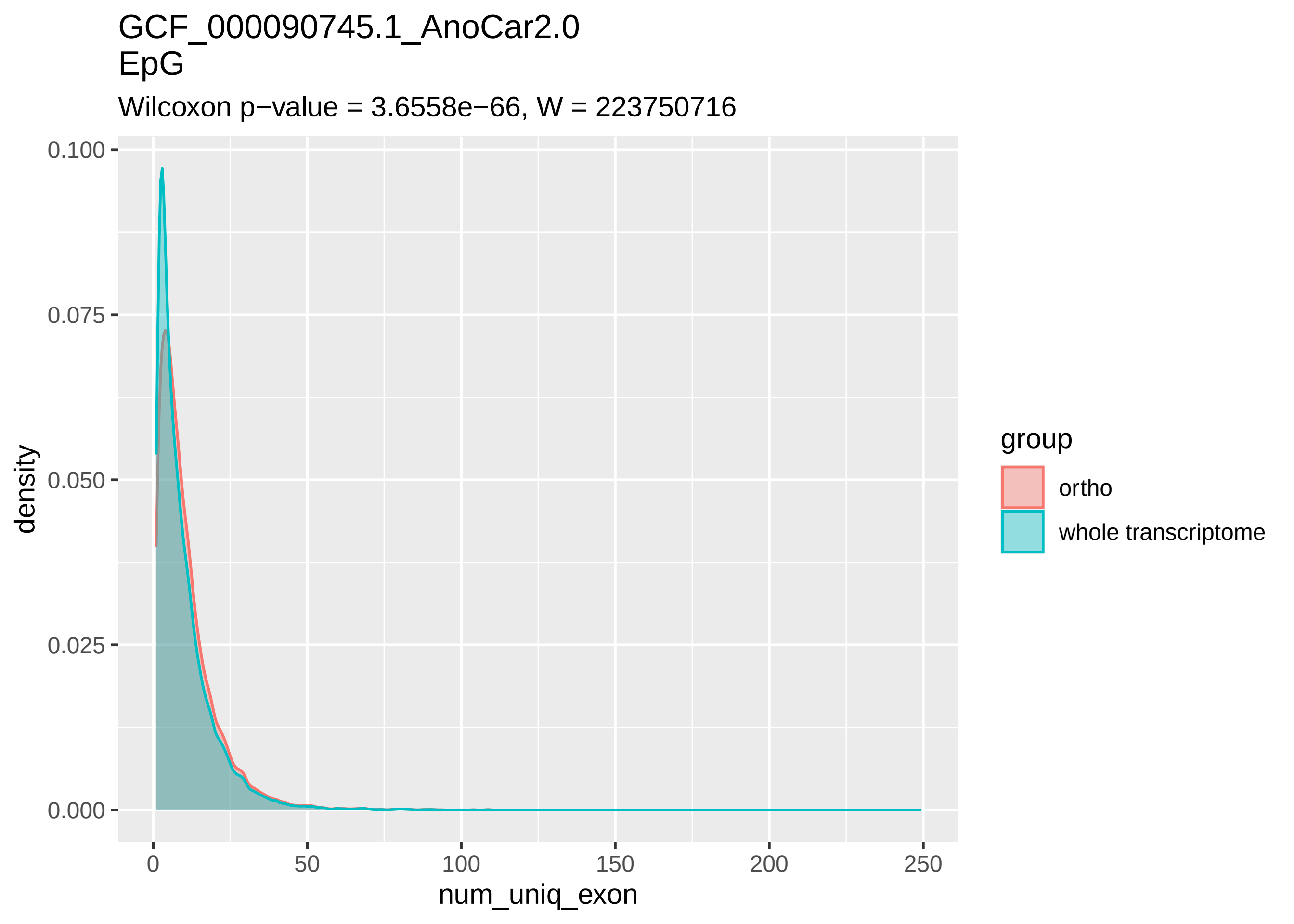

GCF_000090745.1_AnoCar2.0
EpG
Wilcoxon p−value = 3.6558e−66, W = 223750716
0.100
0.075
0.050
0.025
0.000
density
0
50
100
150
200
250
num_uniq_exon
group
ortho
whole transcriptome

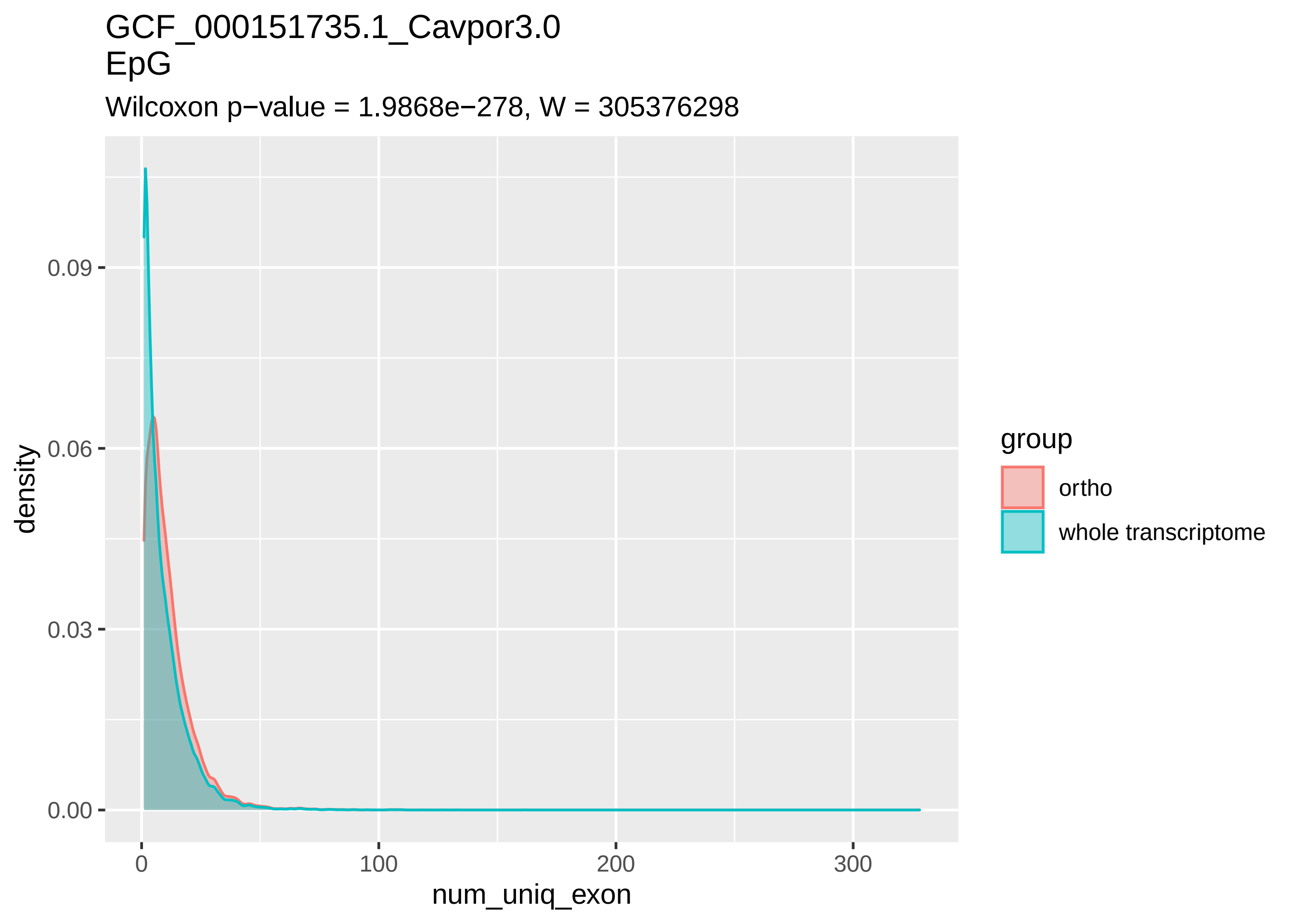
GCF_000151735.1_Cavpor3.0
EpG
Wilcoxon p−value = 1.9868e−278, W = 305376298
density
0.09
0.06
0.03
0.00
0
100
200
300
num_uniq_exon
group
ortho
whole transcriptome

GCF_000165445.2_Mmur_3.0
EpG

Wilcoxon p−value = 0, W = 344130102

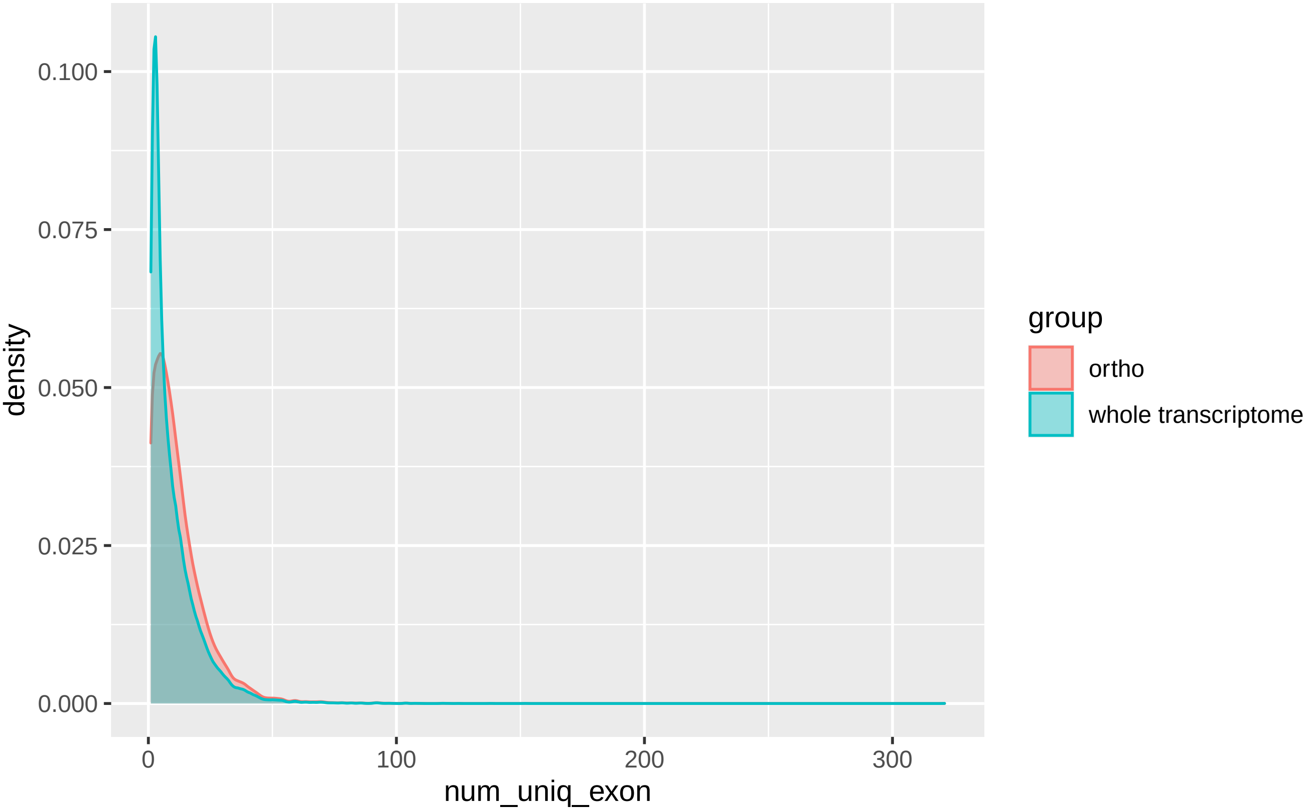

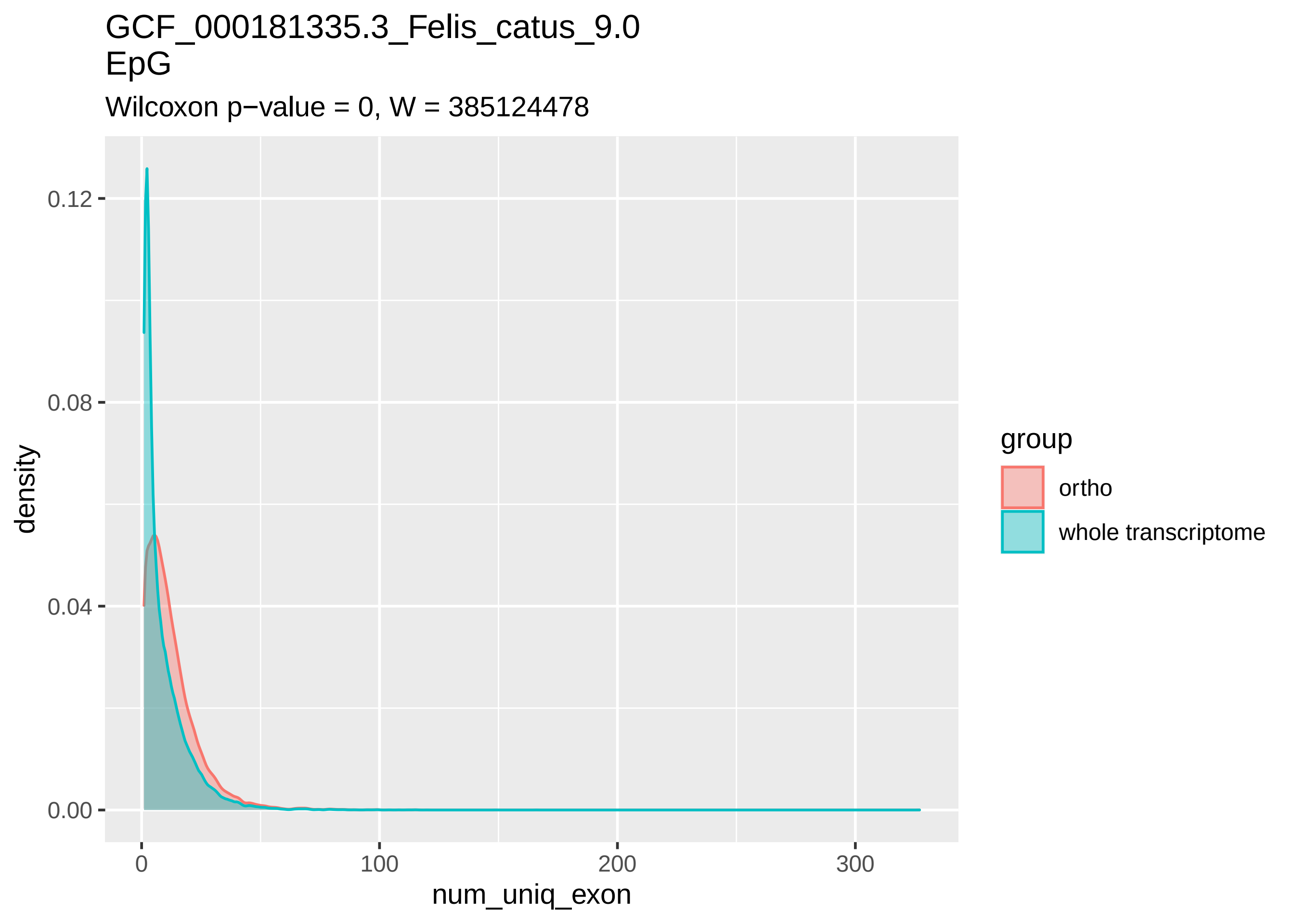
GCF_000181335.3_Felis_catus_9.0
EpG
Wilcoxon p−value = 0, W = 385124478
density
0.12
0.08
0.04
0.00
0
100
200
300
num_uniq_exon
group
ortho
whole transcriptome

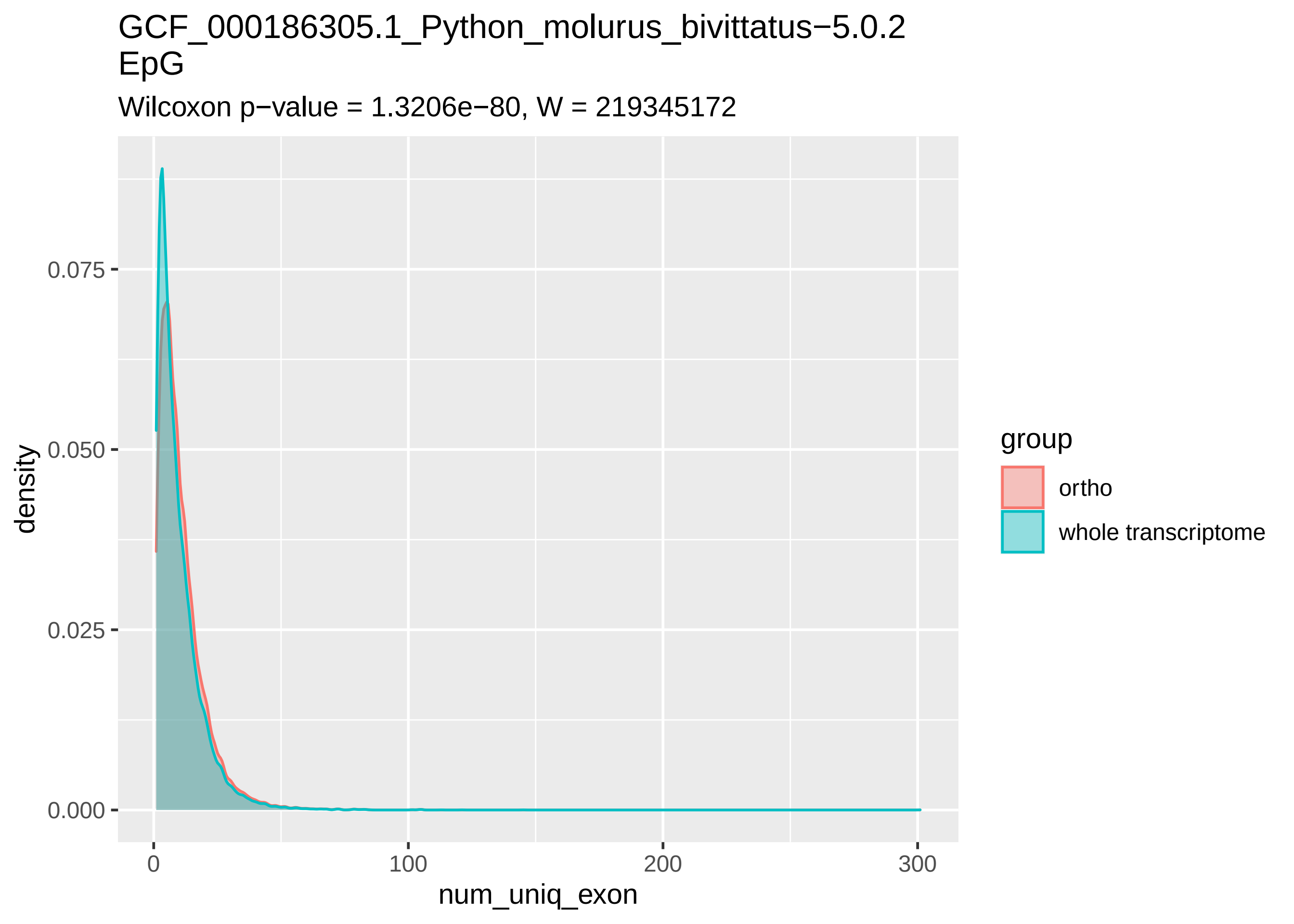
GCF_000186305.1_Python_molurus_bivittatus−5.0.2
EpG
Wilcoxon p−value = 1.3206e−80, W = 219345172
density
0.075
0.050
0.025
0.000
0
100
200
300
num_uniq_exon
group
ortho
whole transcriptome

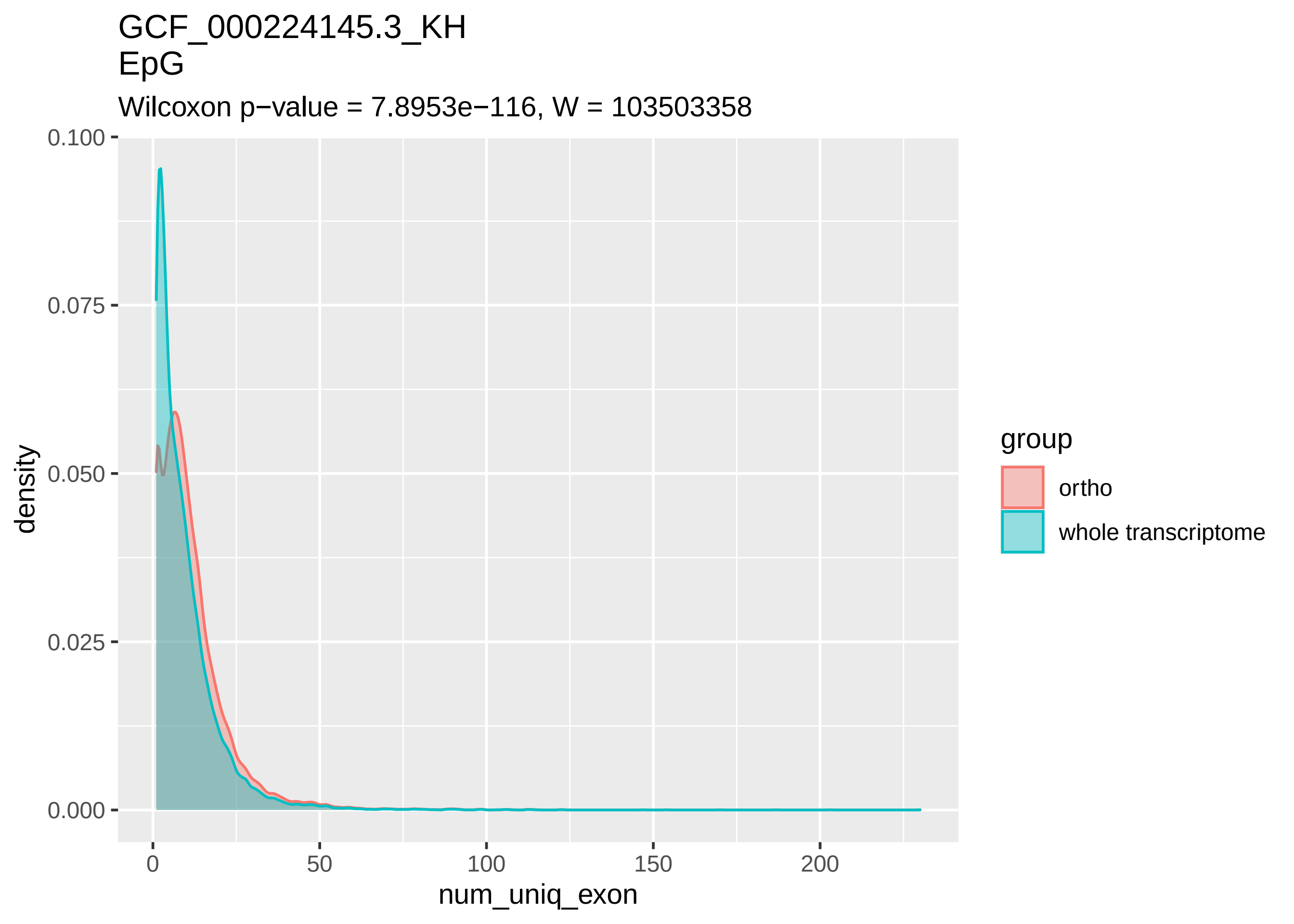
GCF_000224145.3_KH
EpG
Wilcoxon p−value = 7.8953e−116, W = 103503358
density
0.100
0.075
0.050
0.025
0.000
0
50
100
150
200
num_uniq_exon
group
ortho
whole transcriptome

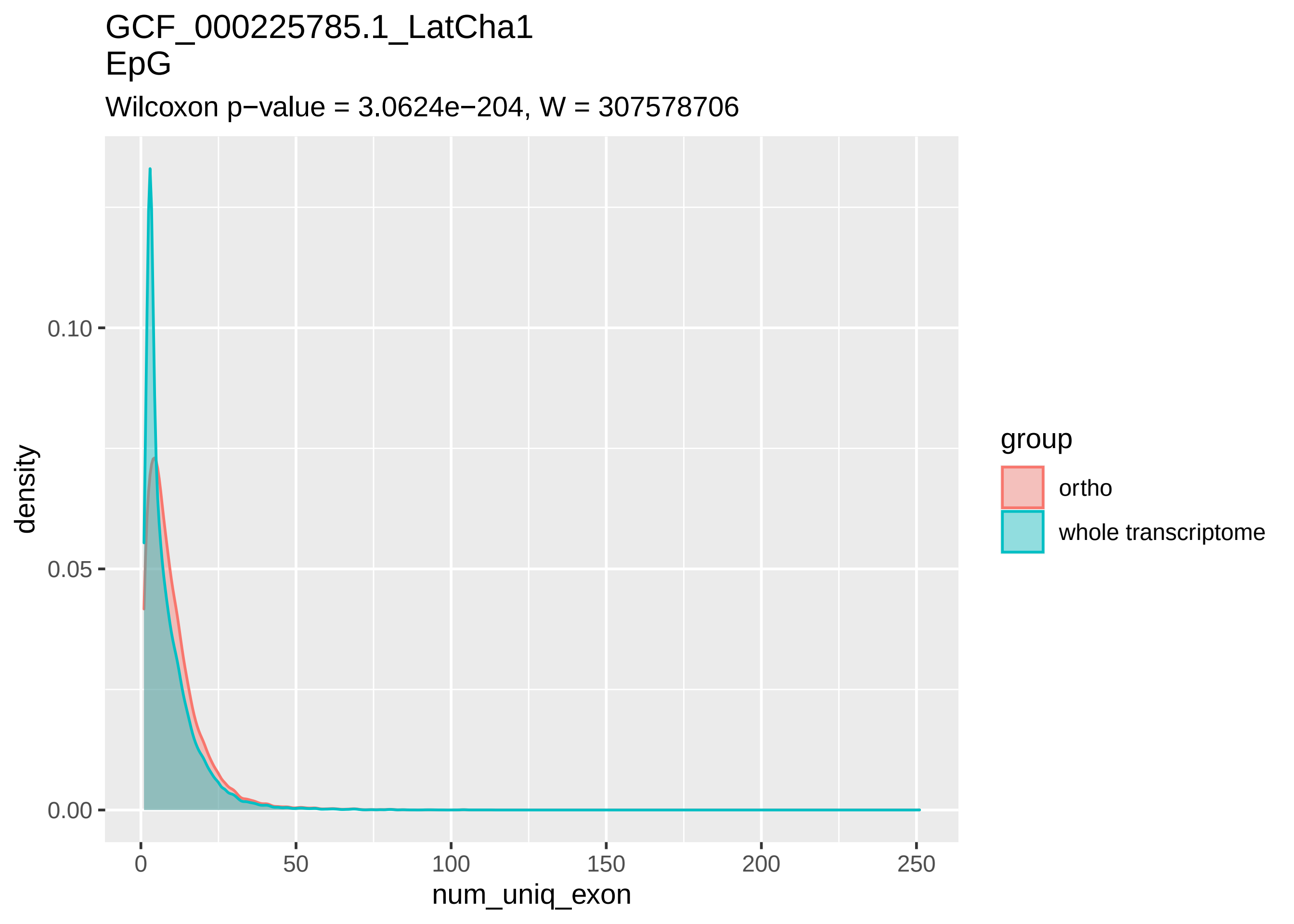

GCF_000225785.1_LatCha1
EpG
Wilcoxon p−value = 3.0624e−204, W = 307578706
density
0.10
0.05
0.00
0
50
100
150
200
250
num_uniq_exon
group
ortho
whole transcriptome

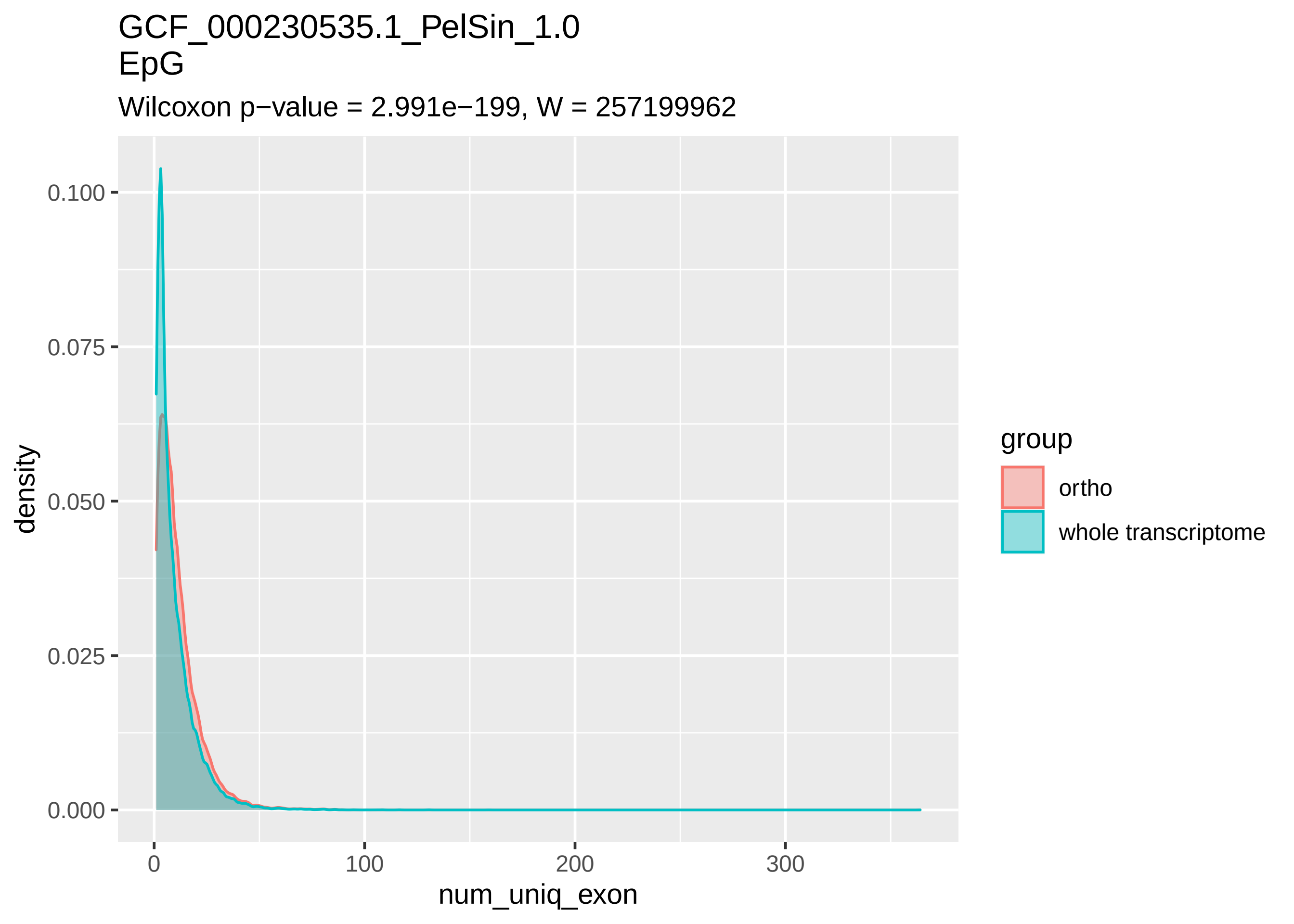
GCF_000230535.1_PelSin_1.0
EpG
Wilcoxon p−value = 2.991e−199, W = 257199962
0.100
0.075
0.050
0.025
0.000
density
0
100
200
300
num_uniq_exon
group
ortho
whole transcriptome

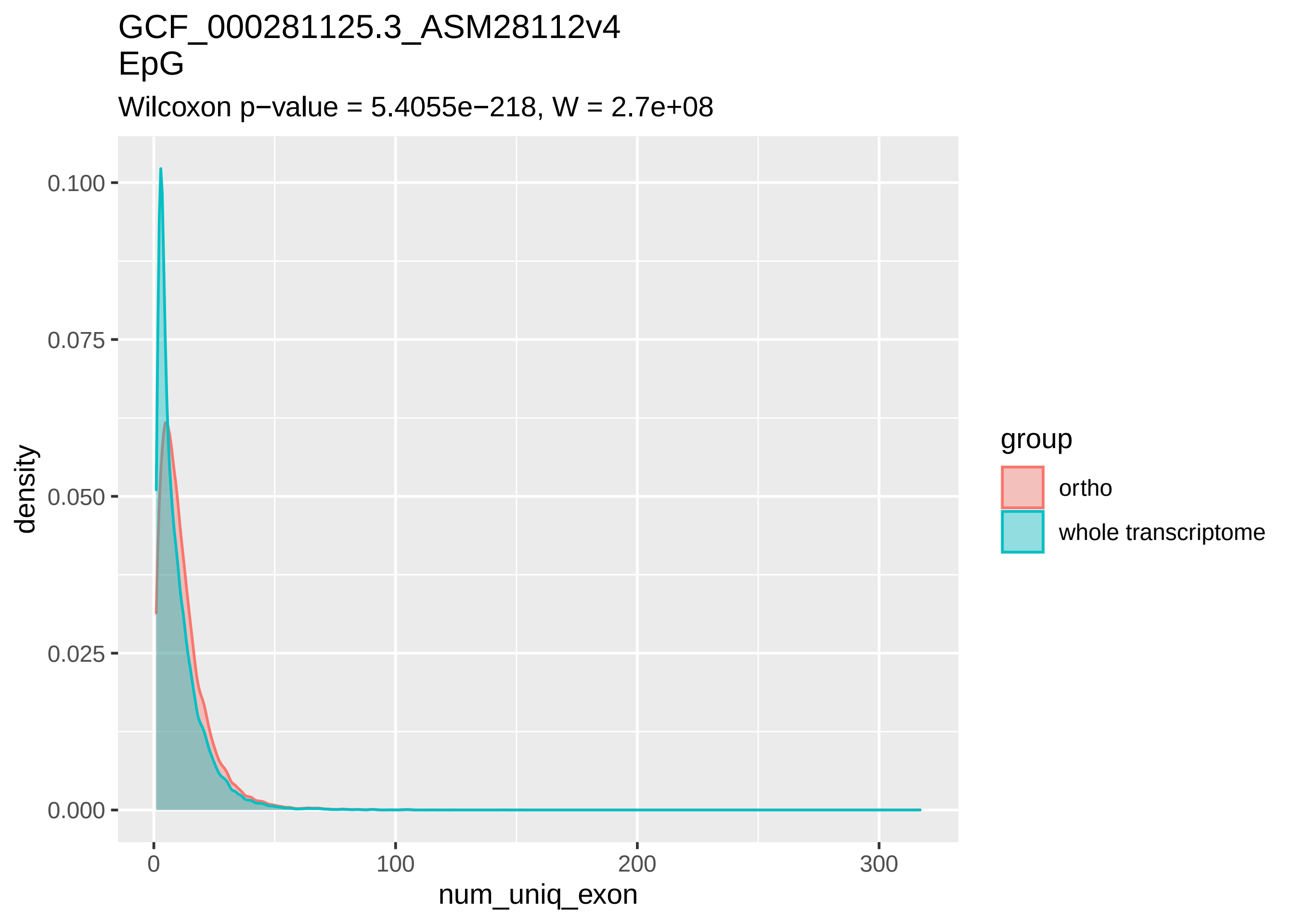
GCF_000281125.3_ASM28112v4
EpG
Wilcoxon p−value = 5.4055e−218, W = 2.7e+08
density
0.100
0.075
0.050
0.025
0.000
0
100
200
300
num_uniq_exon
group
ortho
whole transcriptome

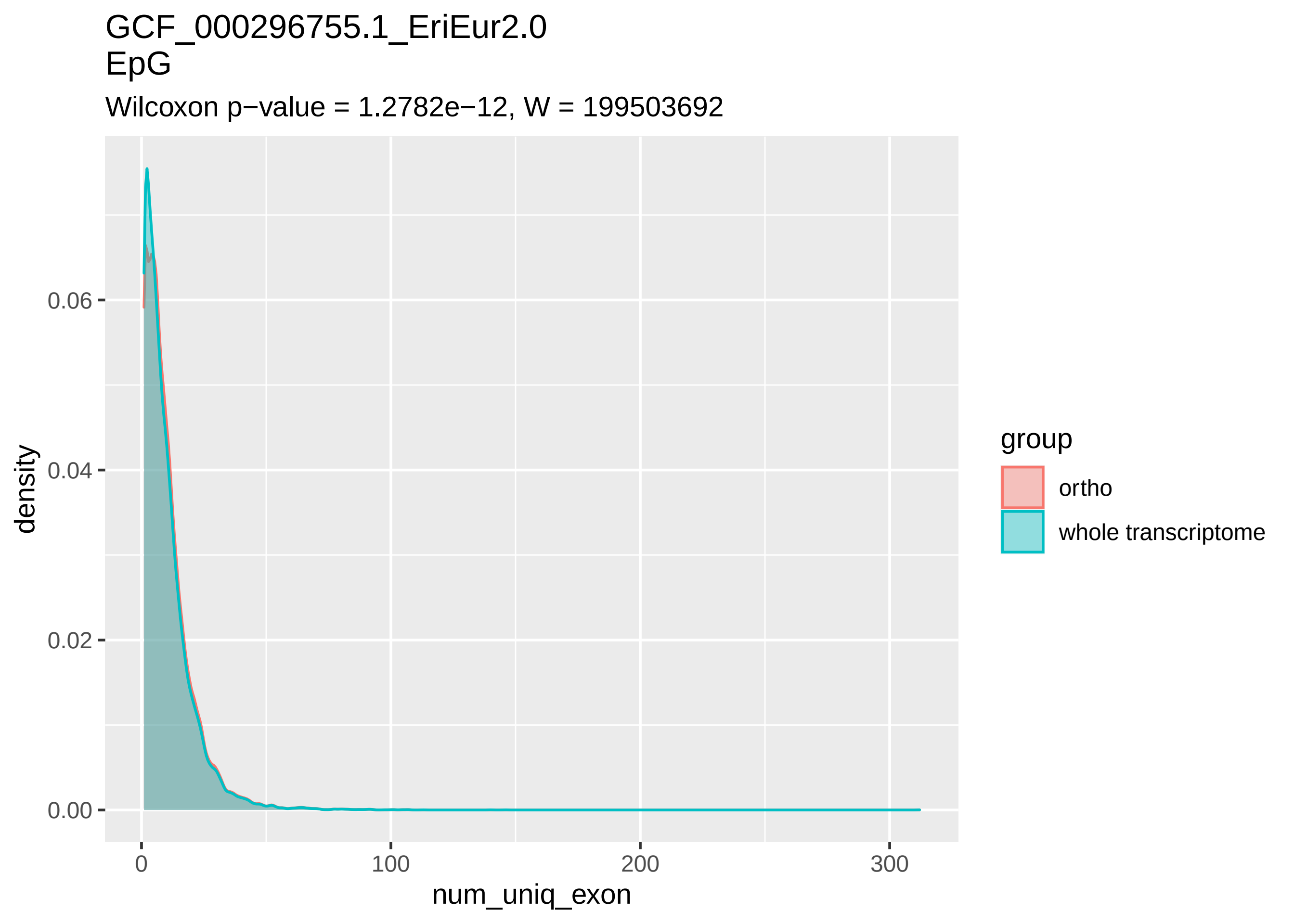
GCF_000296755.1_EriEur2.0
EpG
Wilcoxon p−value = 1.2782e−12, W = 199503692
density
0.00
0.02
0.04
0.06
0
100
200
300
num_uniq_exon
group
ortho
whole transcriptome

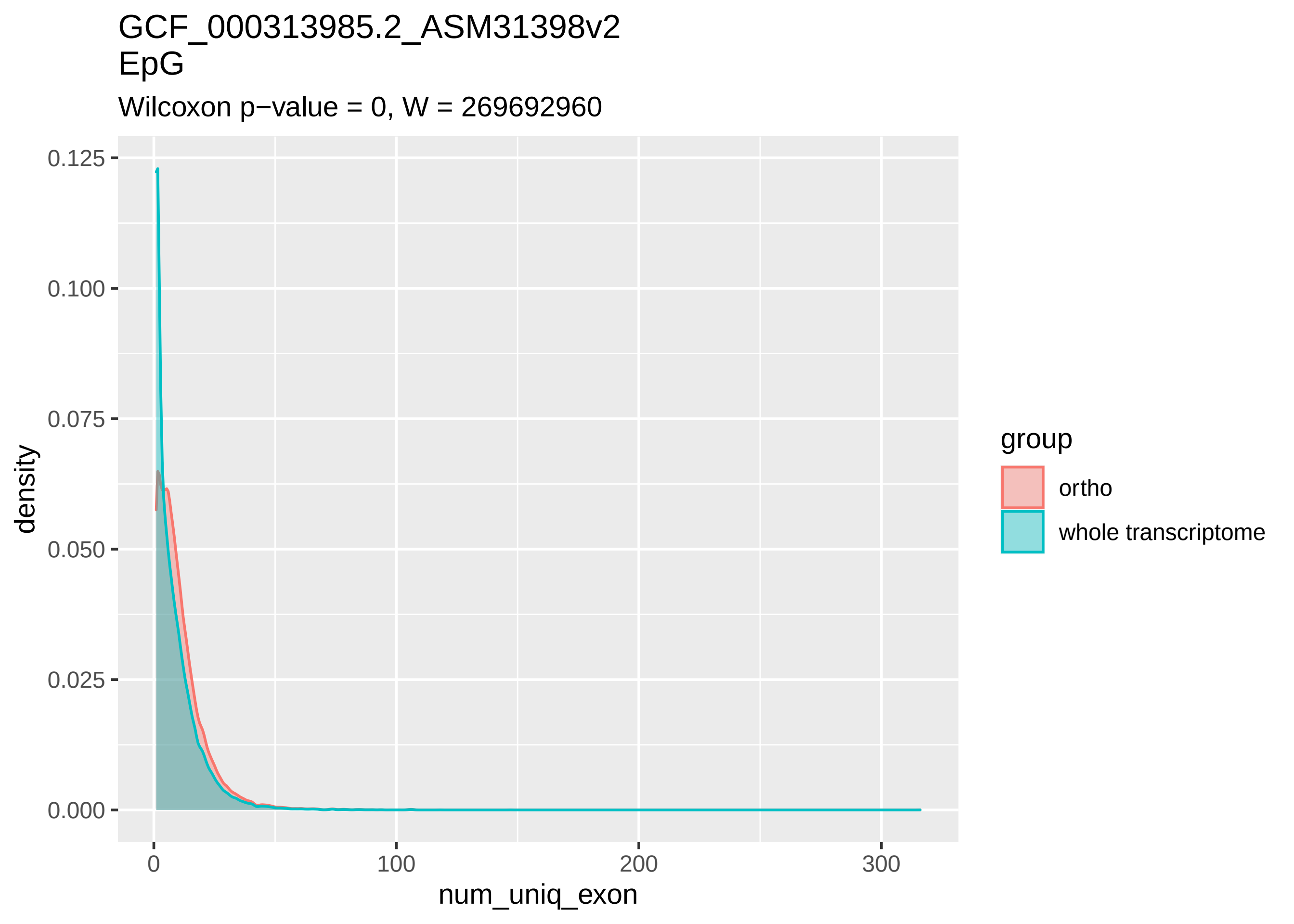
GCF_000313985.2_ASM31398v2
EpG
Wilcoxon p−value = 0, W = 269692960
density
0.125
0.100
0.075
0.050
0.025
0.000
0
100
200
300
num_uniq_exon
group
ortho
whole transcriptome

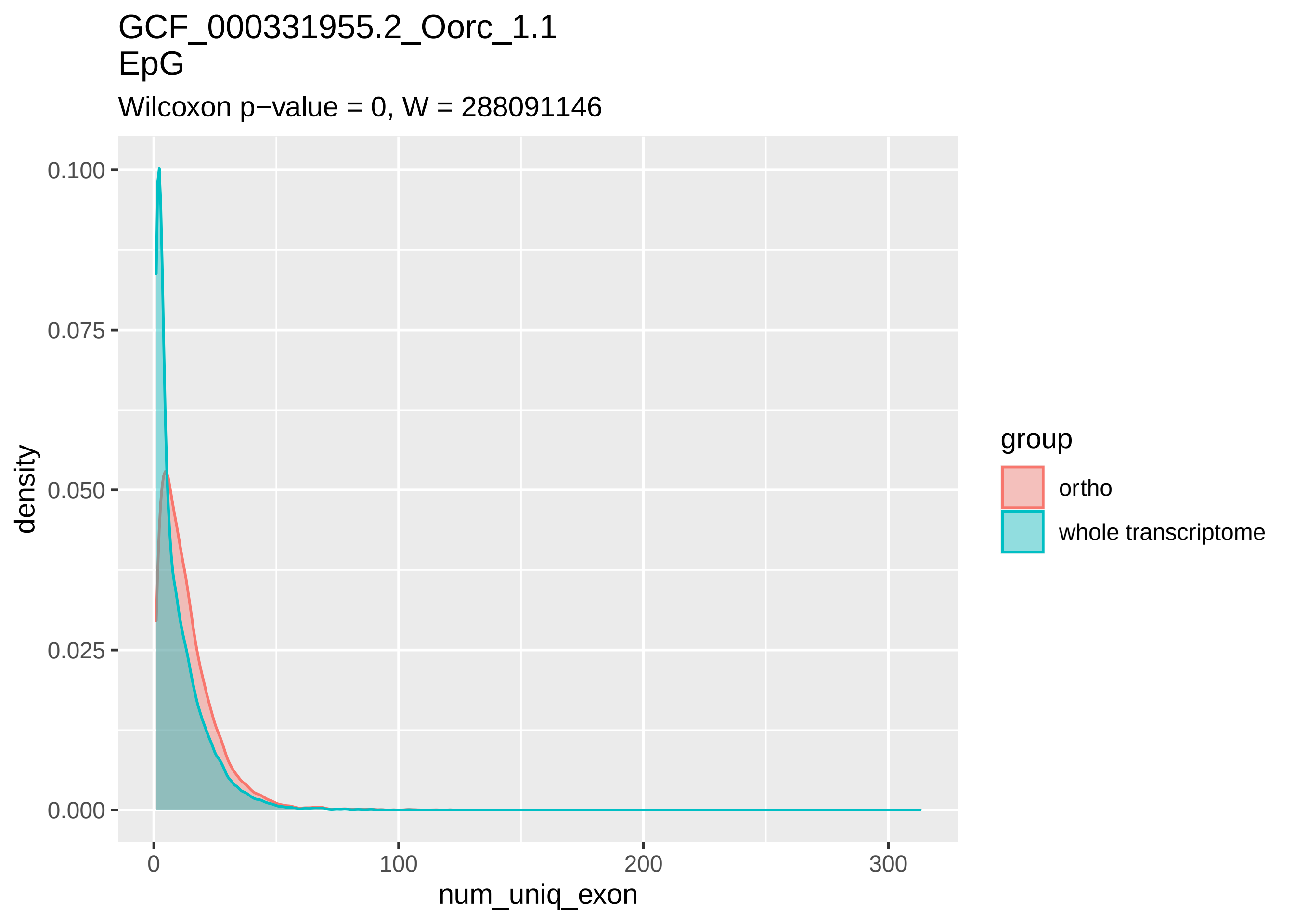
GCF_000331955.2_Oorc_1.1
EpG
Wilcoxon p−value = 0, W = 288091146
density
0.100
0.075
0.050
0.025
0.000
0
100
200
300
num_uniq_exon
group
ortho
whole transcriptome

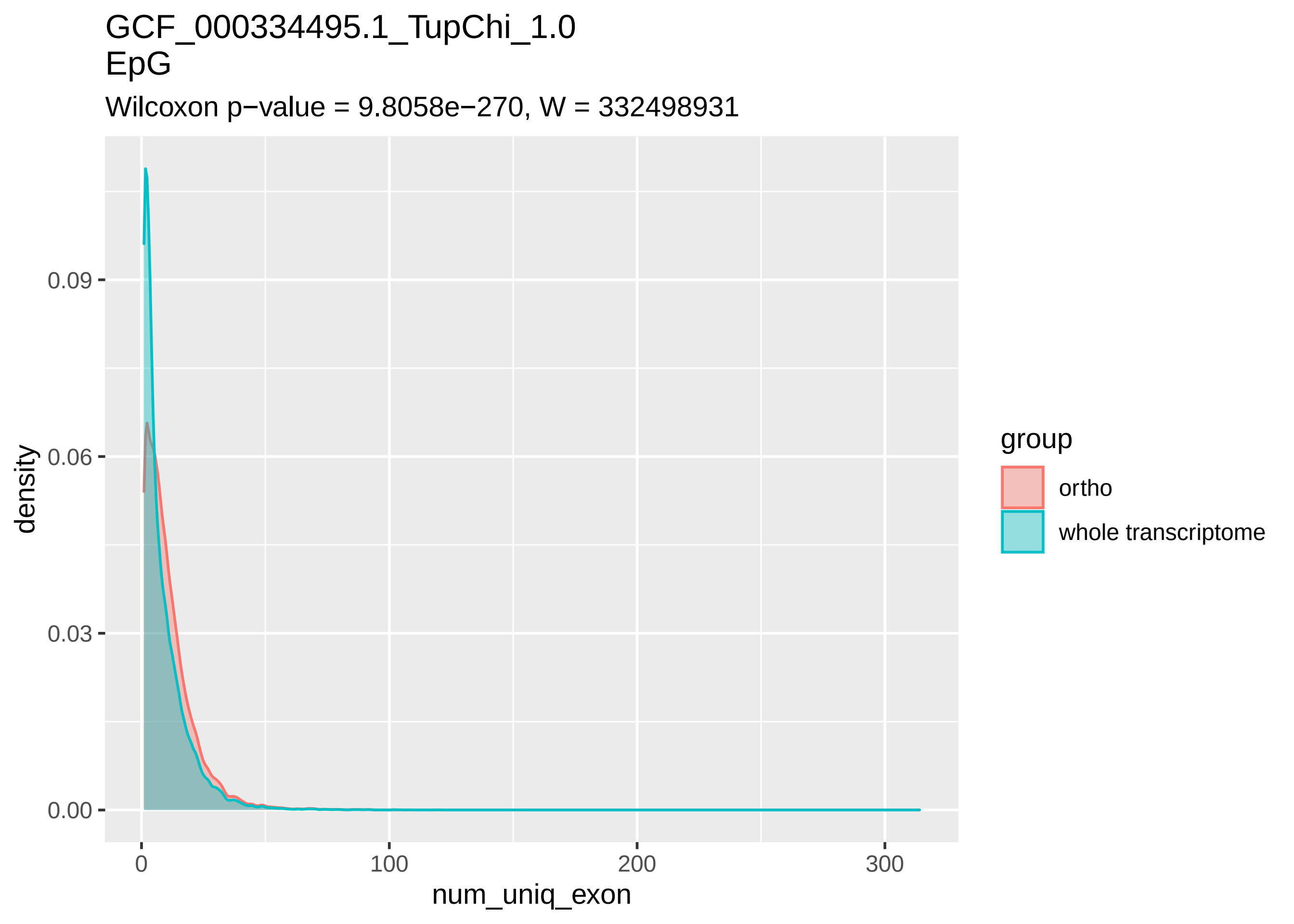

GCF_000334495.1_TupChi_1.0
EpG
Wilcoxon p−value = 9.8058e−270, W = 332498931
density
0.00
0.03
0.06
0.09
0
100
200
300
num_uniq_exon
group
ortho
whole transcriptome

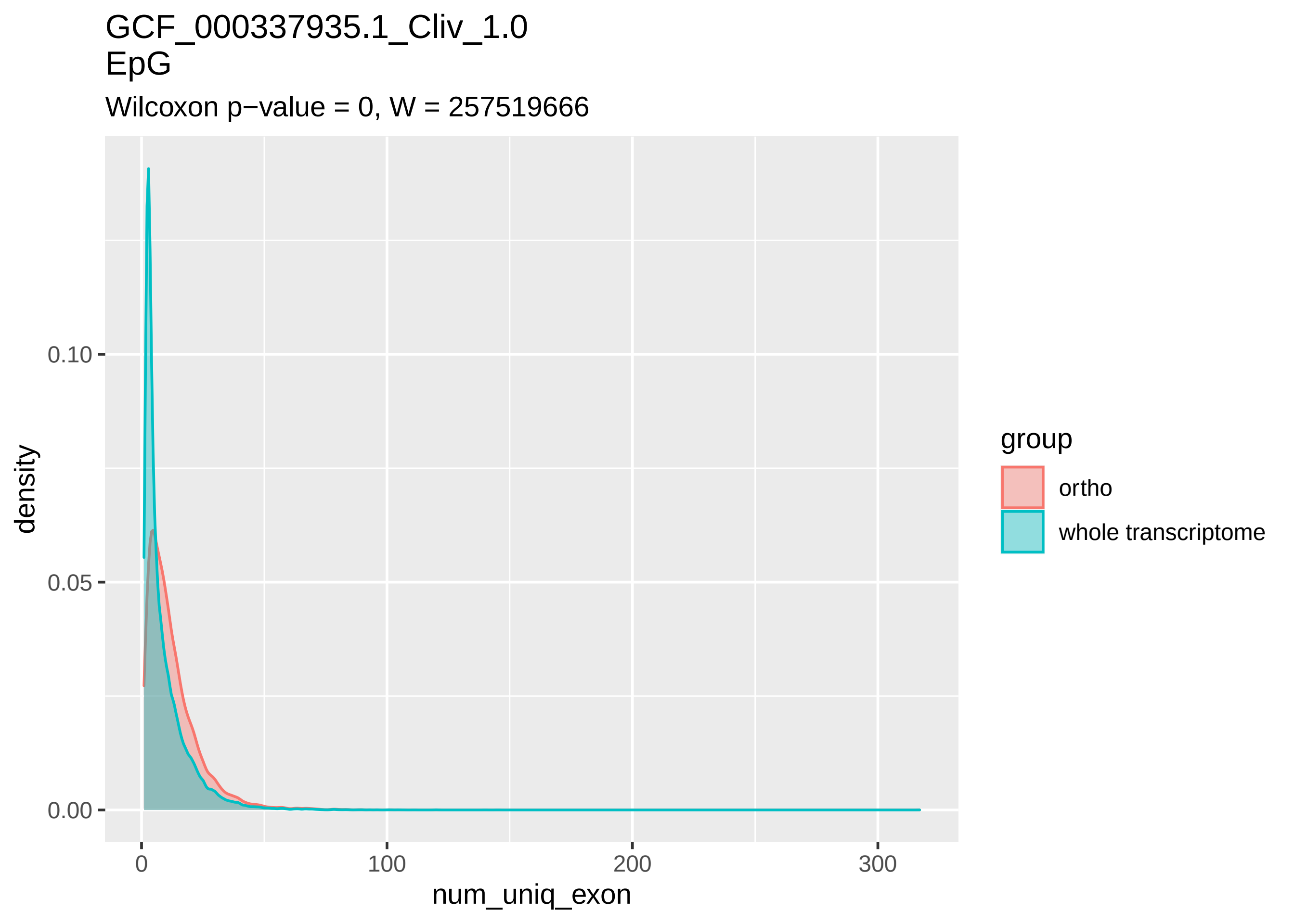

GCF_000337935.1_Cliv_1.0
EpG
Wilcoxon p−value = 0, W = 257519666
density
0.00
0.05
0.10
0
100
200
300
num_uniq_exon
group
ortho
whole transcriptome

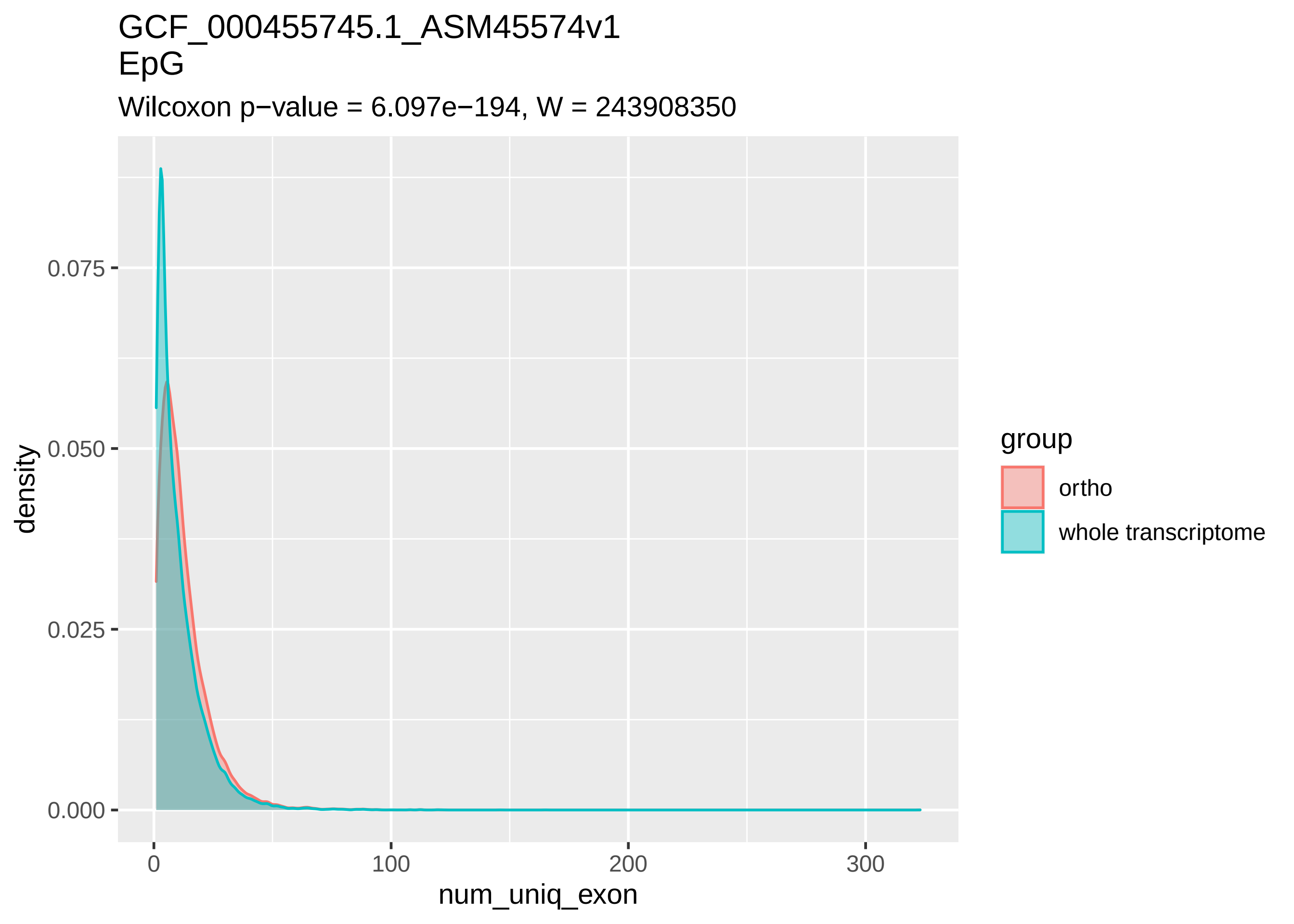

GCF_000455745.1_ASM45574v1
EpG
Wilcoxon p−value = 6.097e−194, W = 243908350
density
0.075
0.050
0.025
0.000
0
100
200
300
num_uniq_exon
group
ortho
whole transcriptome

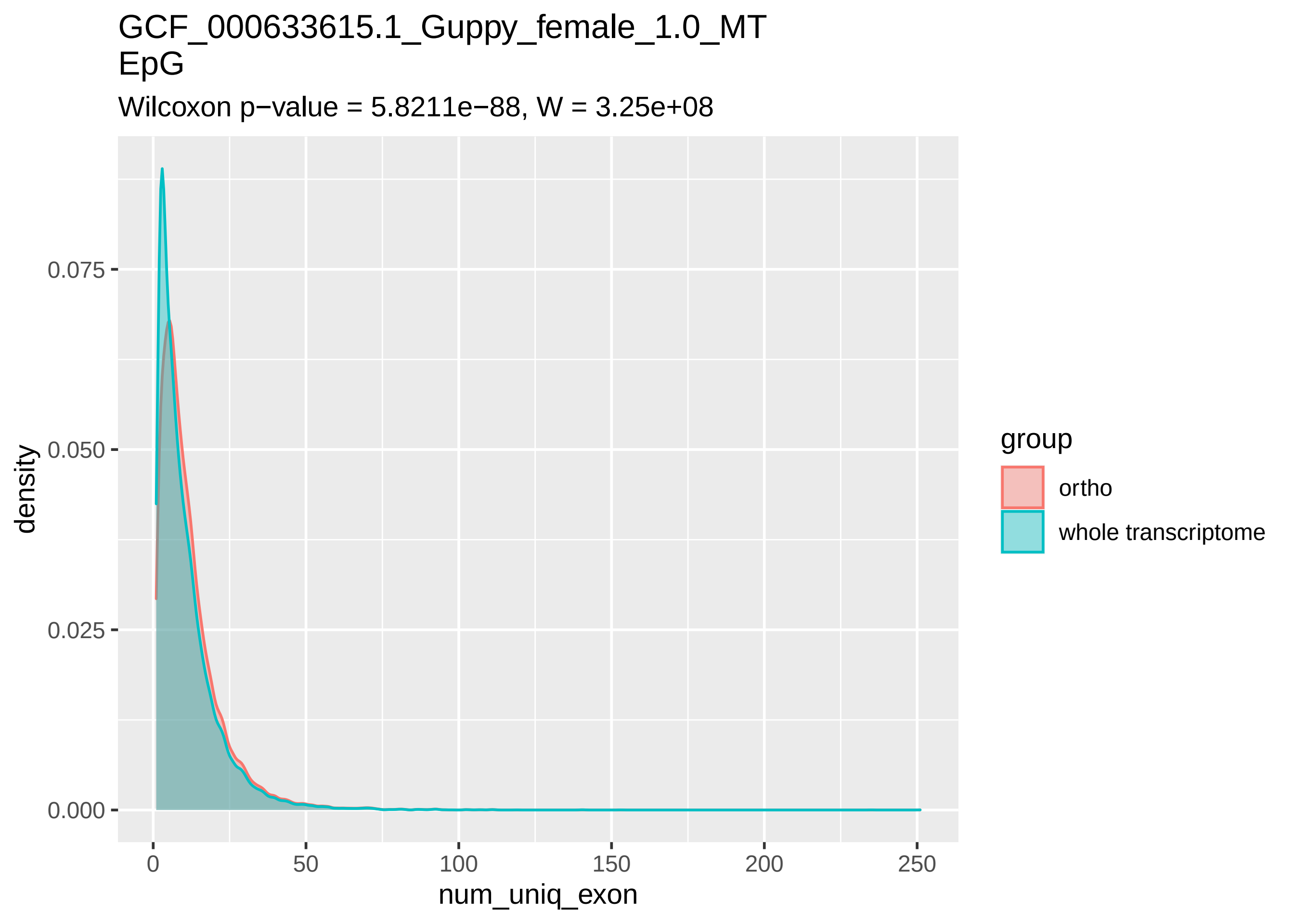

GCF_000633615.1_Guppy_female_1.0_MT
EpG
Wilcoxon p−value = 5.8211e−88, W = 3.25e+08
density
0.000
0.025
0.050
0.075
0
50
100
150
200
250
num_uniq_exon
group
ortho
whole transcriptome

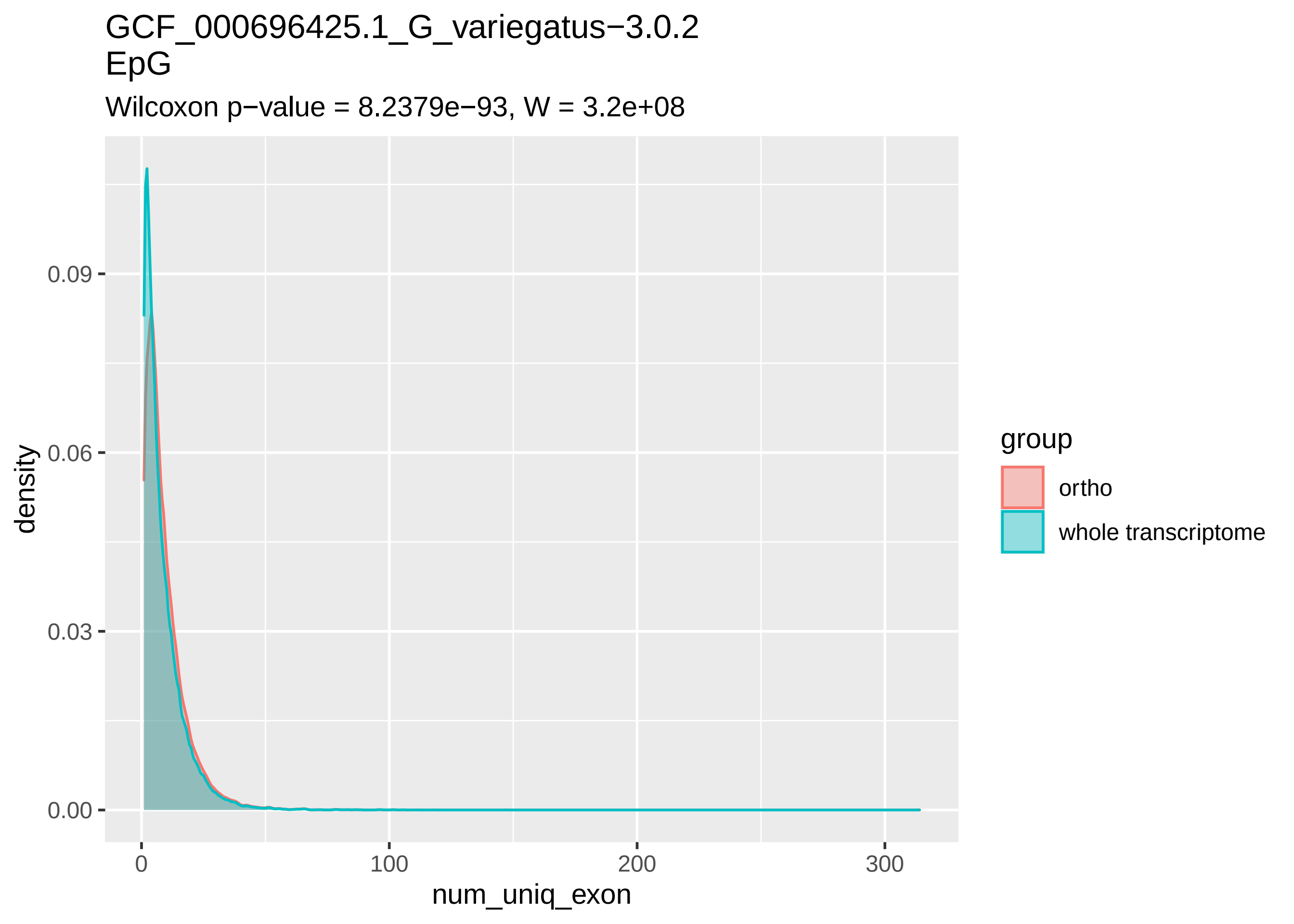
GCF_000696425.1_G_variegatus−3.0.2
EpG
Wilcoxon p−value = 8.2379e−93, W = 3.2e+08
density
0.09
0.06
0.03
0.00
0
100
200
300
num_uniq_exon
group
ortho
whole transcriptome

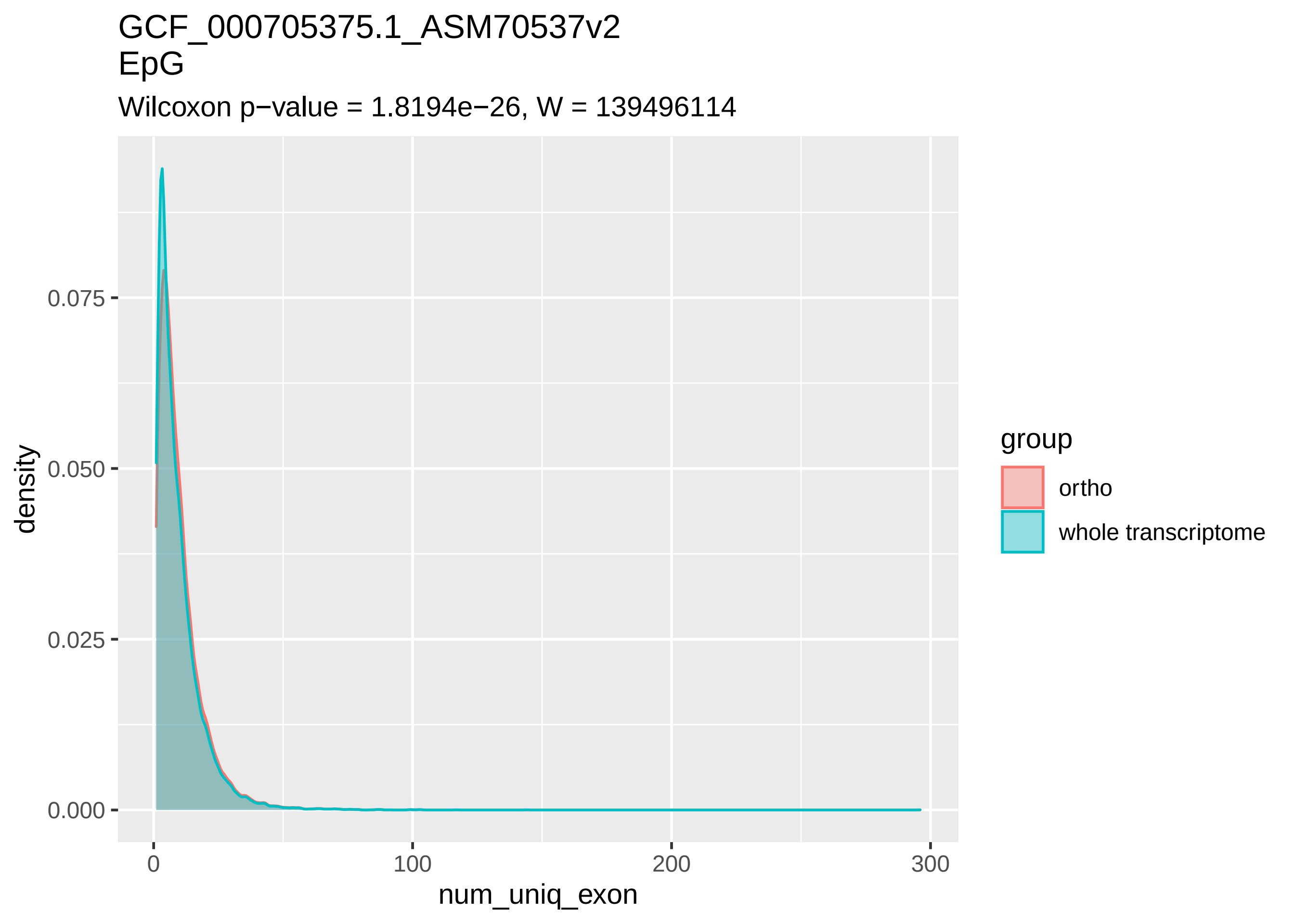

GCF_000705375.1_ASM70537v2
EpG
Wilcoxon p−value = 1.8194e−26, W = 139496114
0.075
0.050
0.025
0.000
density
0
100
200
300
num_uniq_exon
group
ortho
whole transcriptome

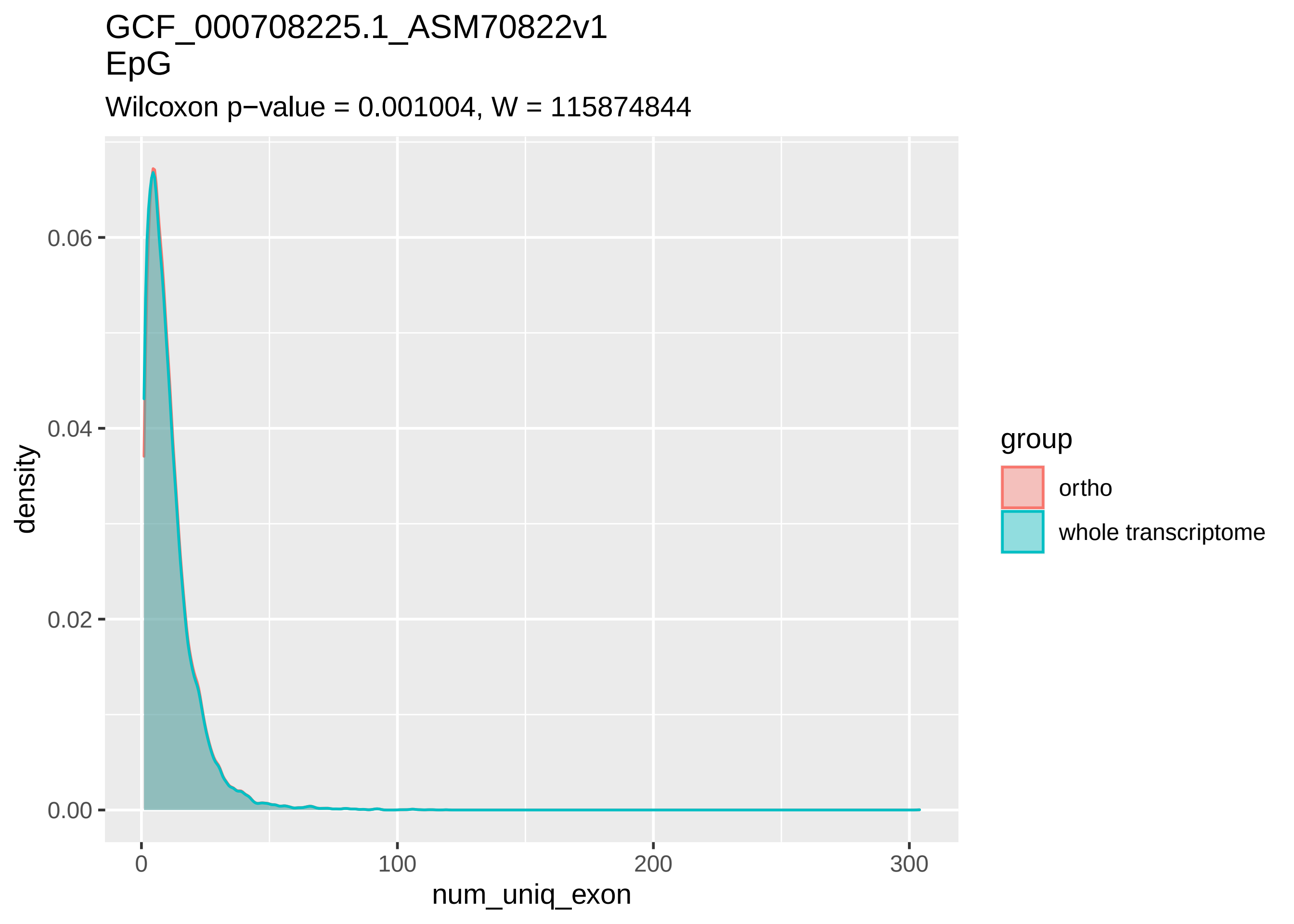
GCF_000708225.1_ASM70822v1
EpG
Wilcoxon p−value = 0.001004, W = 115874844
density
0.00
0.02
0.04
0.06
0
100
200
300
num_uniq_exon
group
ortho
whole transcriptome

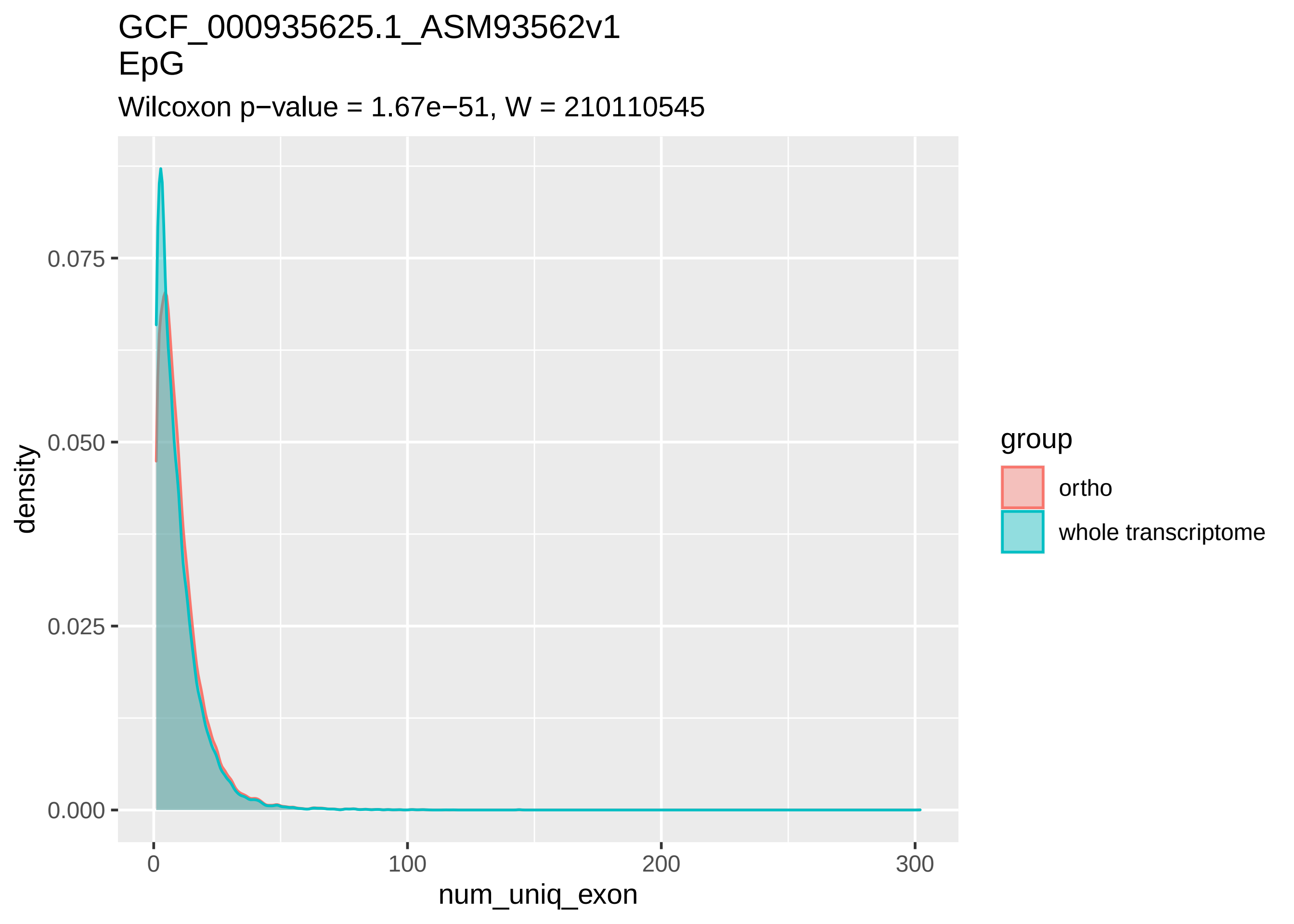
GCF_000935625.1_ASM93562v1
EpG
Wilcoxon p−value = 1.67e−51, W = 210110545
density
0.075
0.050
0.025
0.000
0
100
200
300
num_uniq_exon
group
ortho
whole transcriptome

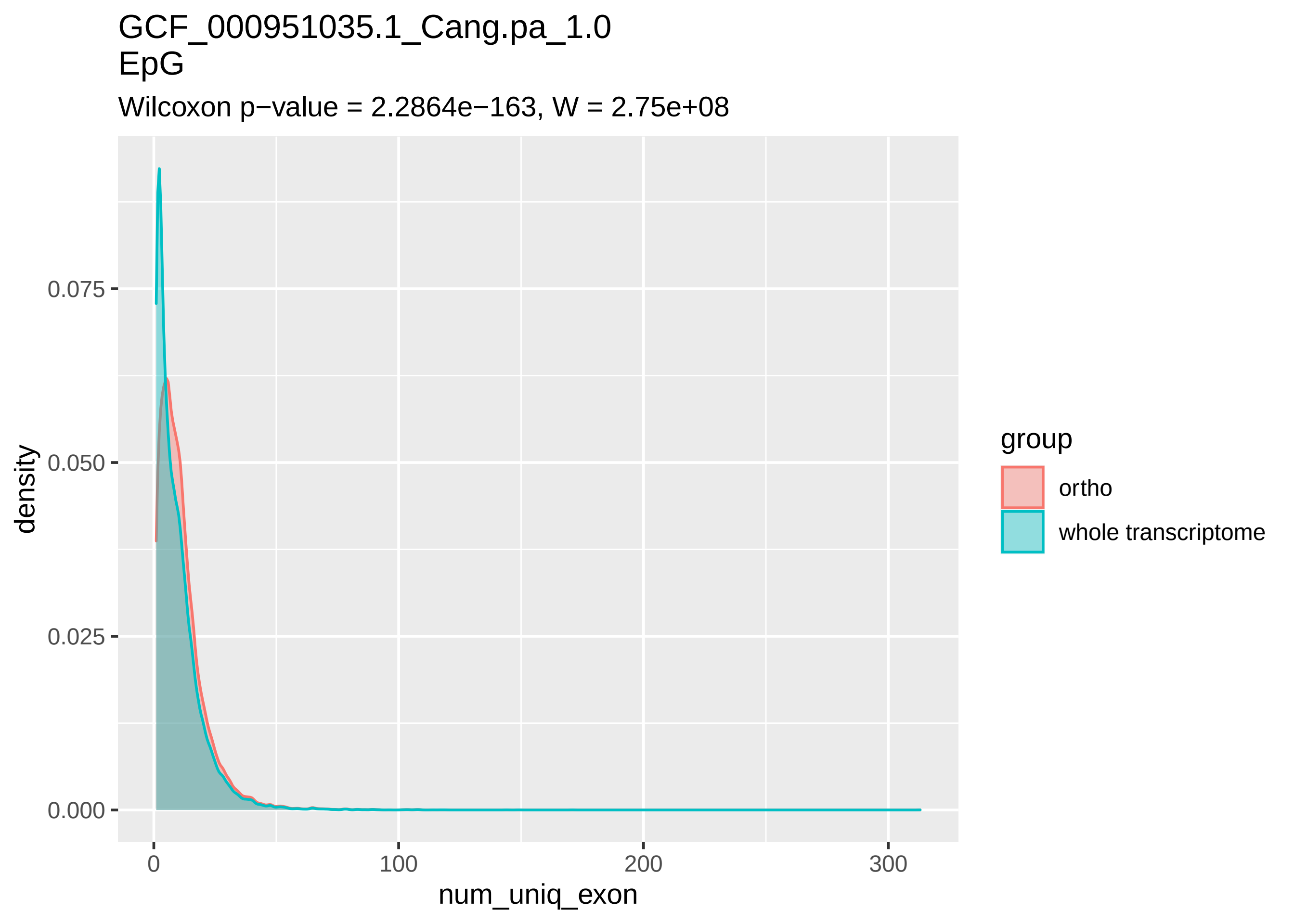
GCF_000951035.1_Cang.pa_1.0
EpG
Wilcoxon p−value = 2.2864e−163, W = 2.75e+08
density
0.075
0.050
0.025
0.000
0
100
200
300
num_uniq_exon
group
ortho
whole transcriptome

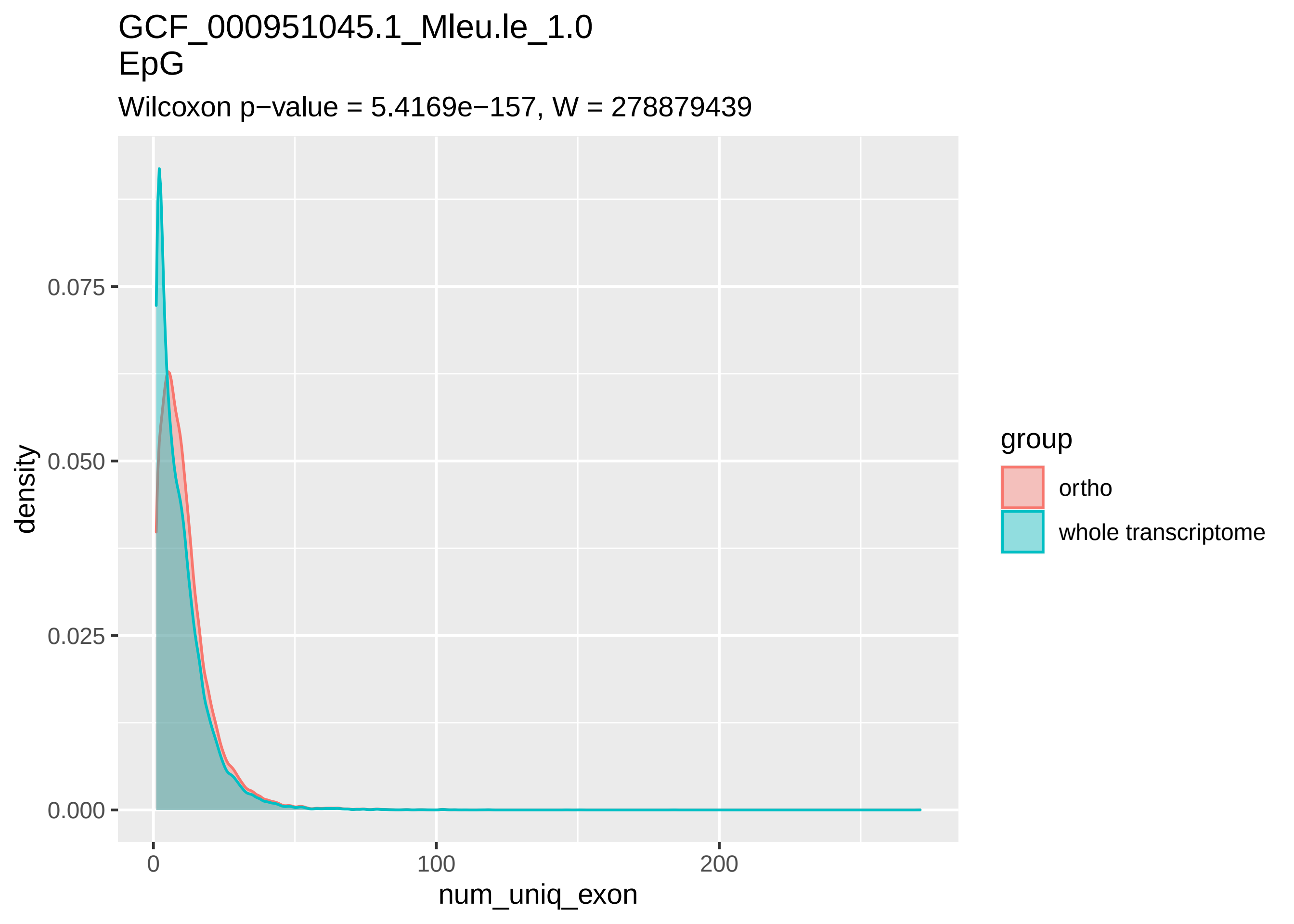
GCF_000951045.1_Mleu.le_1.0
EpG
Wilcoxon p−value = 5.4169e−157, W = 278879439
density
0.000
0.025
0.050
0.075
0
100
200
num_uniq_exon
group
ortho
whole transcriptome

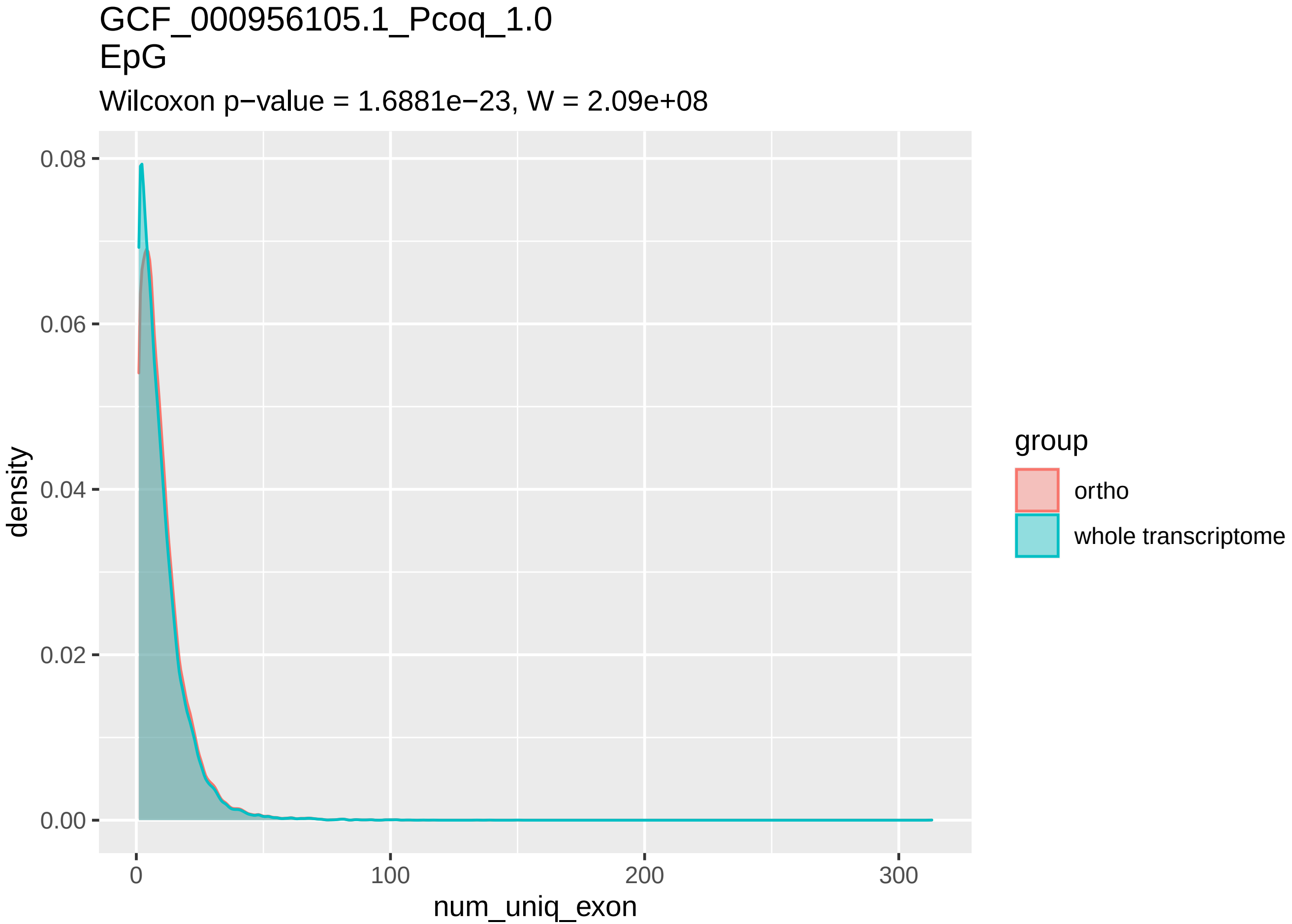
GCF_000956105.1_Pcoq_1.0
EpG
Wilcoxon p−value = 1.6881e−23, W = 2.09e+08
0.08
0.06
0.04
0.02
0.00
density
0
100
200
300
num_uniq_exon
group
ortho
whole transcriptome

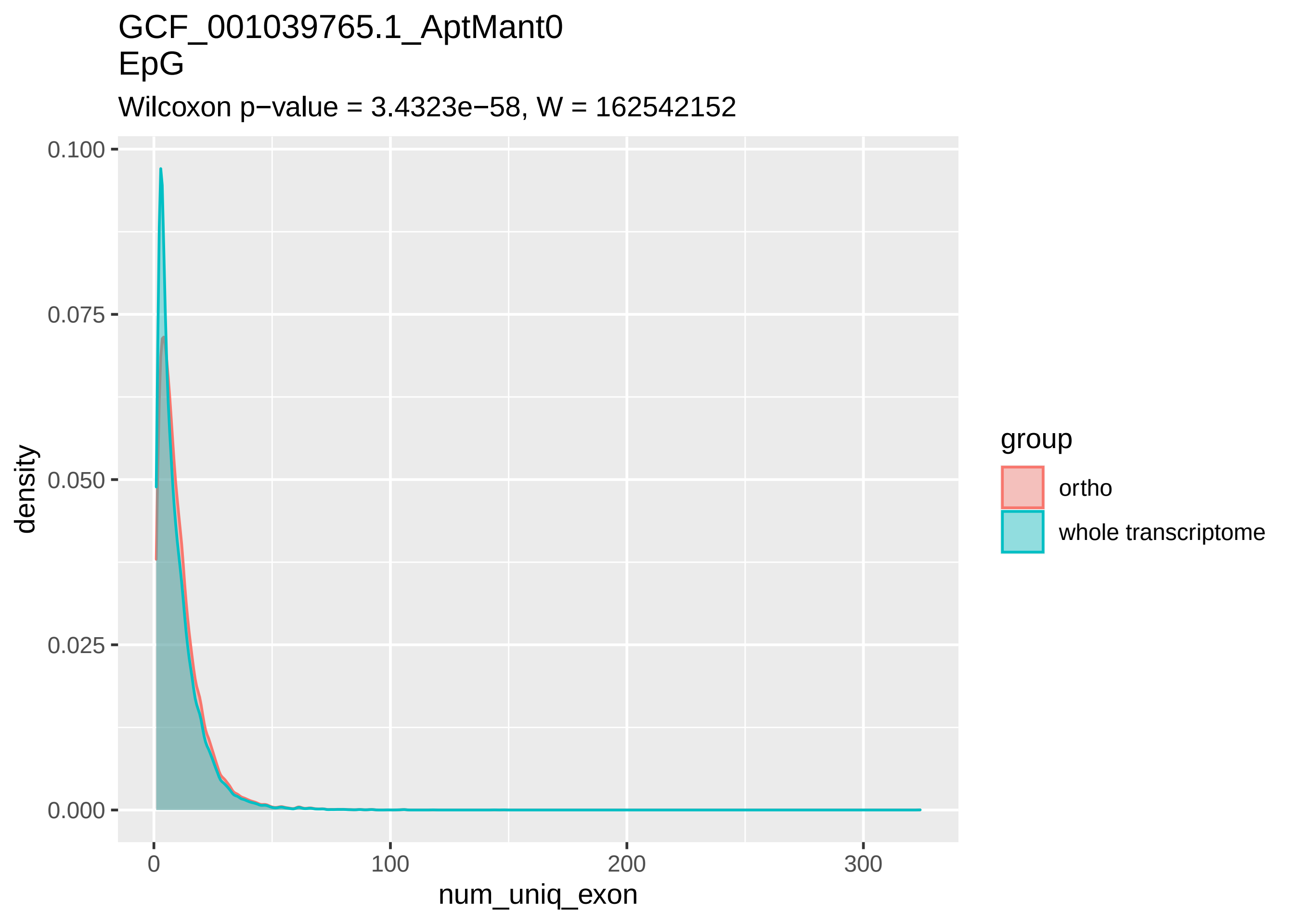

GCF_001039765.1_AptMant0
EpG
Wilcoxon p−value = 3.4323e−58, W = 162542152
density
0.100
0.075
0.050
0.025
0.000
0
100
200
300
num_uniq_exon
group
ortho
whole transcriptome

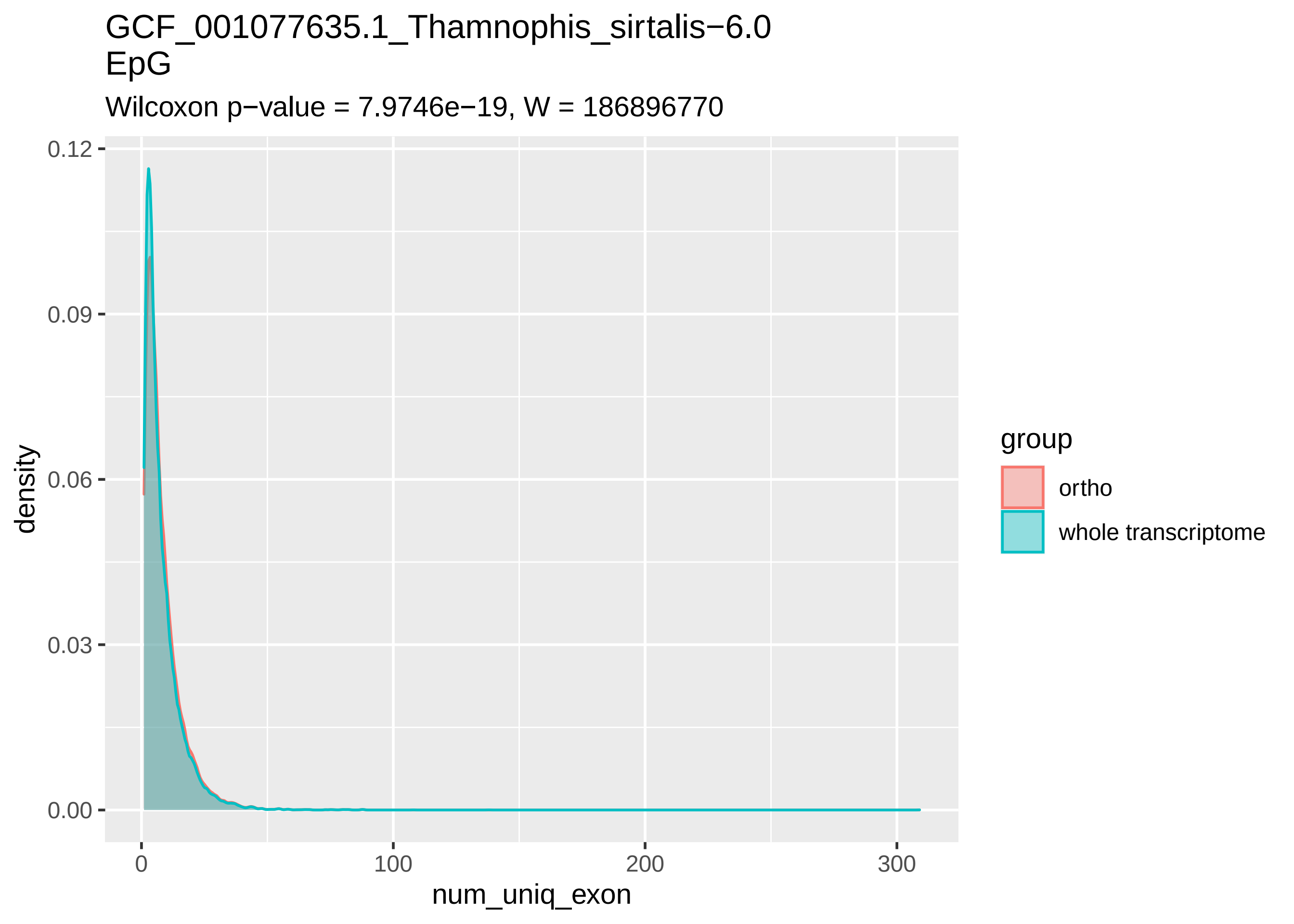
GCF_001077635.1_Thamnophis_sirtalis−6.0
EpG
Wilcoxon p−value = 7.9746e−19, W = 186896770
0.12
0.09
0.06
0.03
0.00
density
0
100
200
300
num_uniq_exon
group
ortho
whole transcriptome

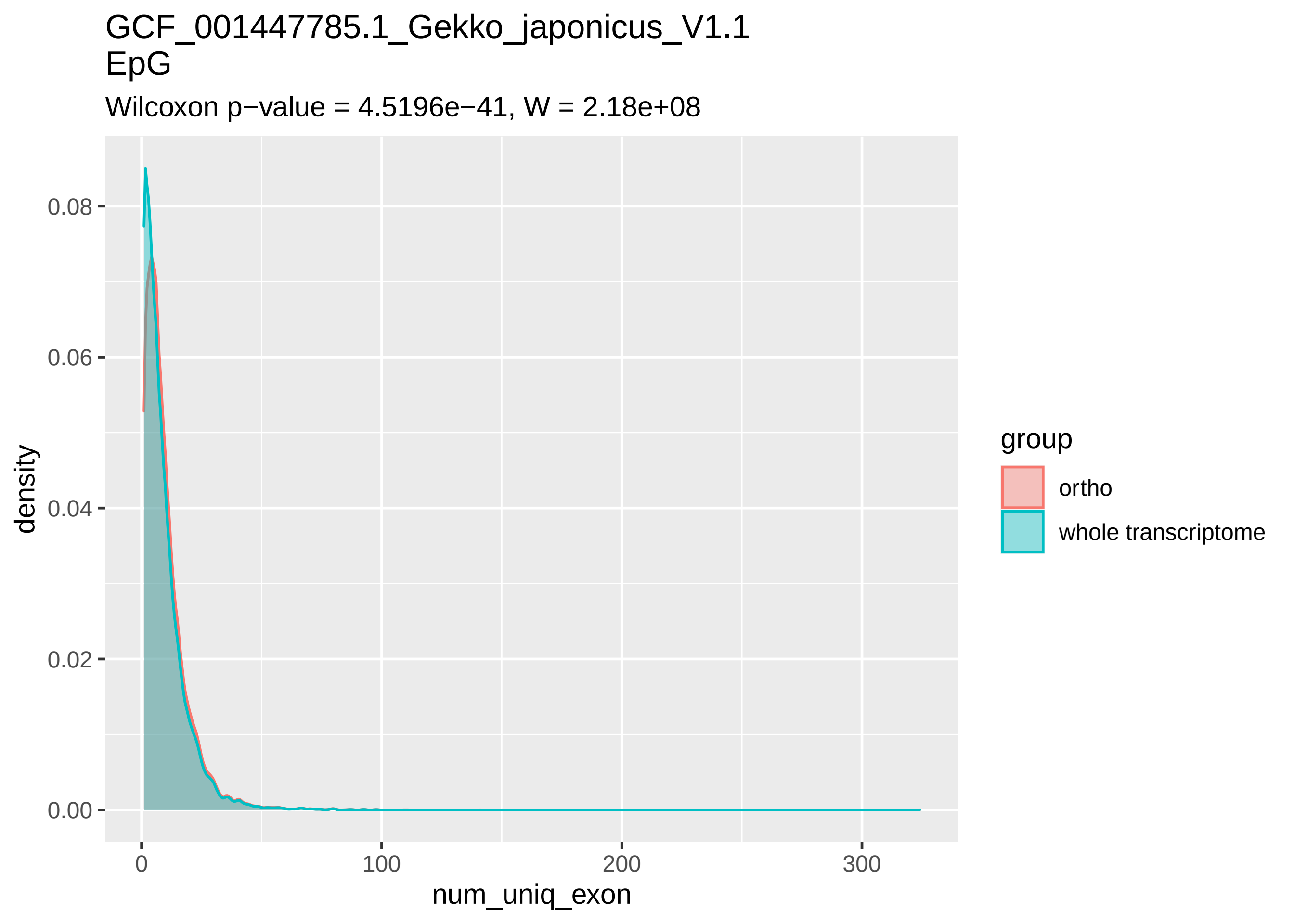

GCF_001447785.1_Gekko_japonicus_V1.1
EpG
Wilcoxon p−value = 4.5196e−41, W = 2.18e+08
density
0.08
0.06
0.04
0.02
0.00
0
100
200
300
num_uniq_exon
group
ortho
whole transcriptome

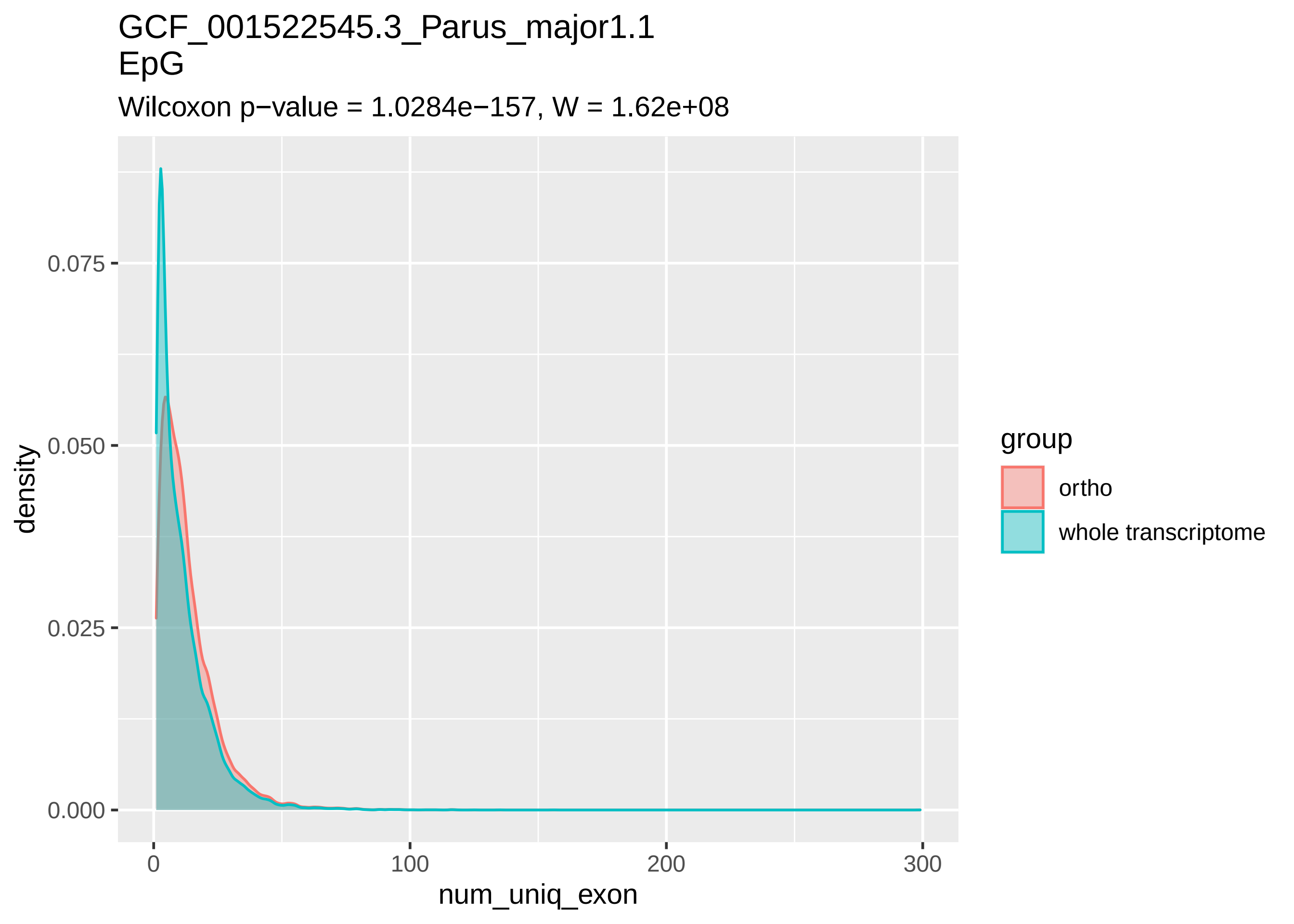
GCF_001522545.3_Parus_major1.1
EpG
Wilcoxon p−value = 1.0284e−157, W = 1.62e+08
density
0.000
0.025
0.050
0.075
0
100
200
300
num_uniq_exon
group
ortho
whole transcriptome

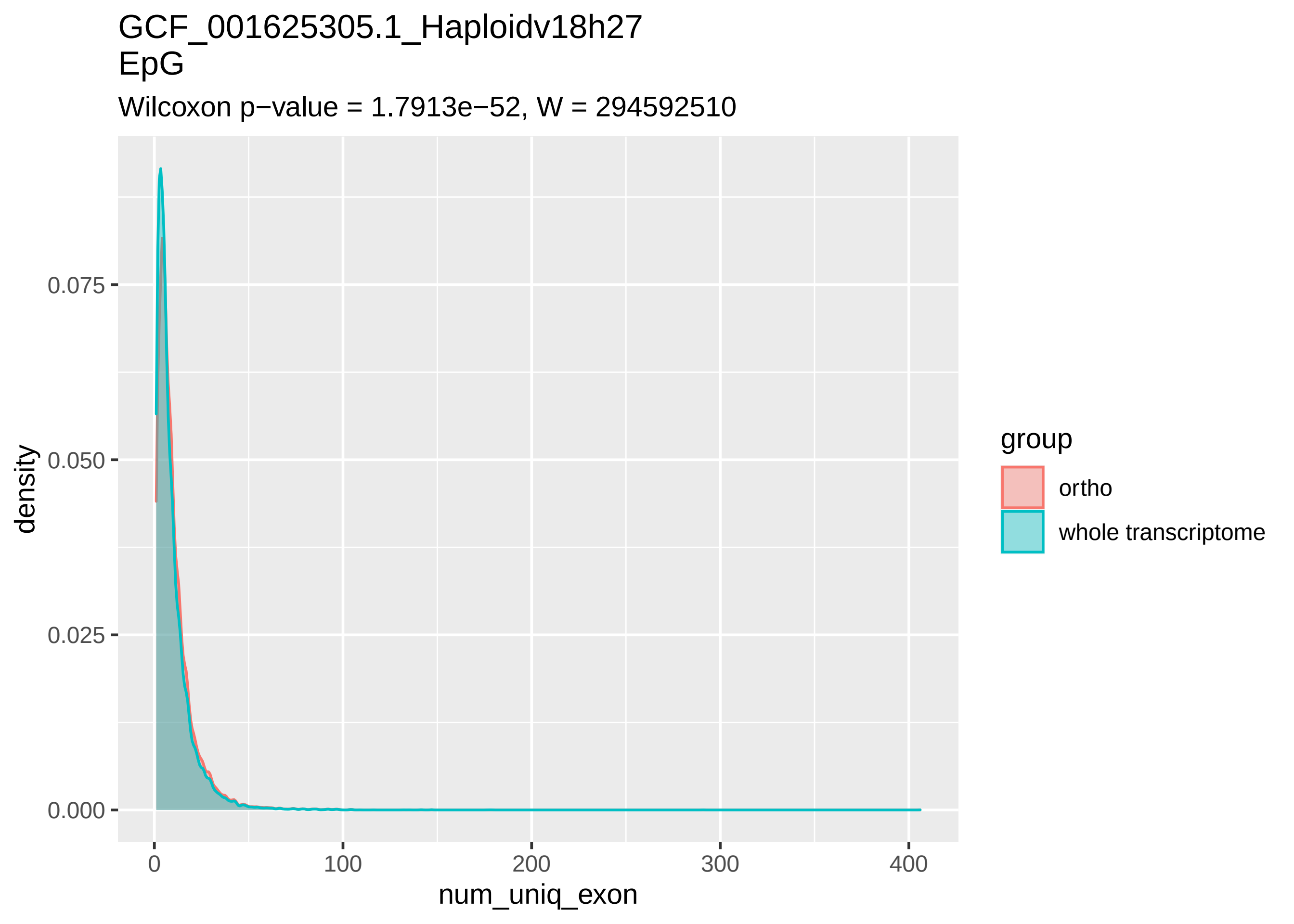

GCF_001625305.1_Haploidv18h27
EpG
Wilcoxon p−value = 1.7913e−52, W = 294592510
density
0.000
0.025
0.050
0.075
0
100
200
300
400
num_uniq_exon
group
ortho
whole transcriptome

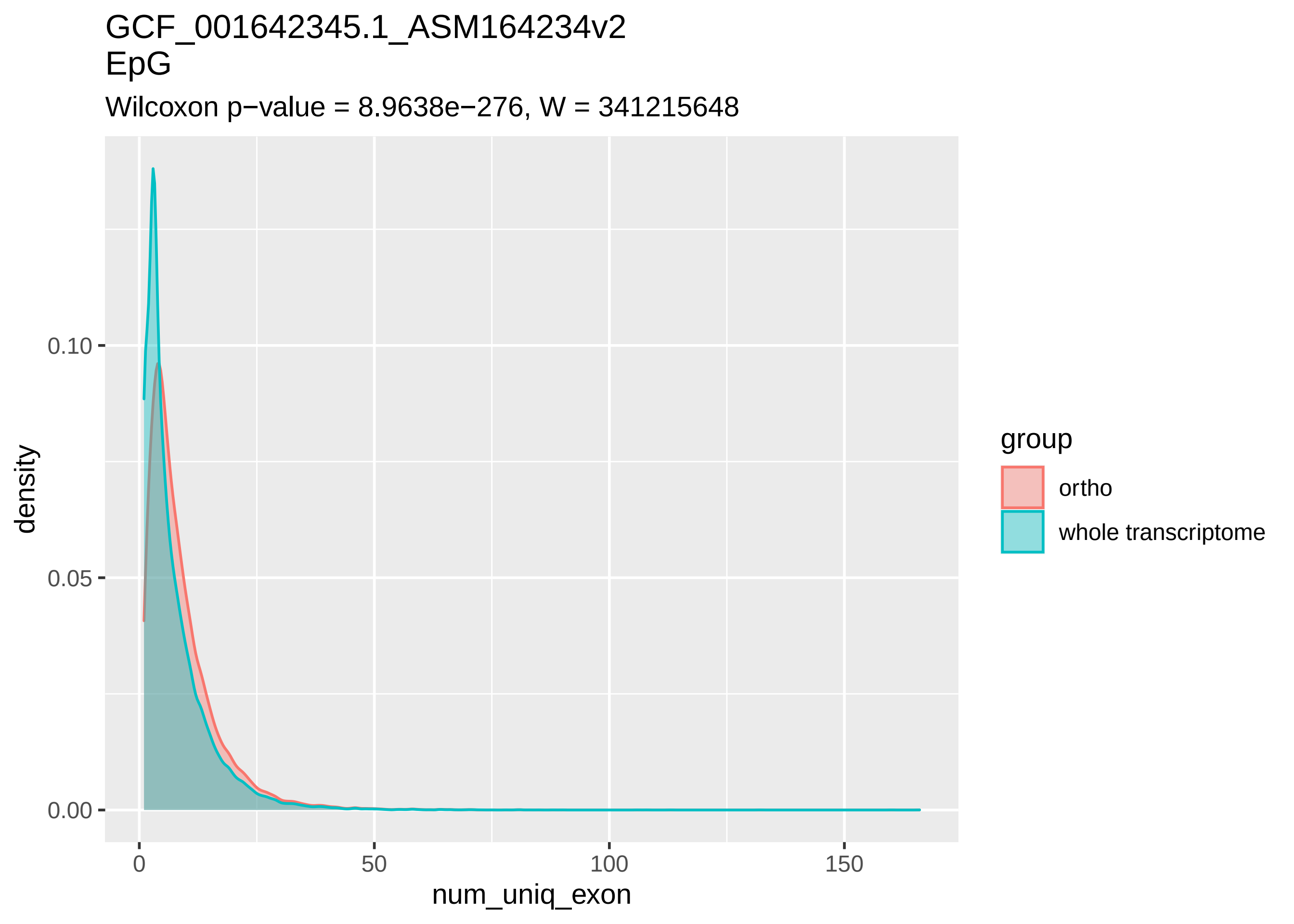

GCF_001642345.1_ASM164234v2
EpG
Wilcoxon p−value = 8.9638e−276, W = 341215648
density
0.10
0.05
0.00
0
50
100
150
num_uniq_exon
group
ortho
whole transcriptome

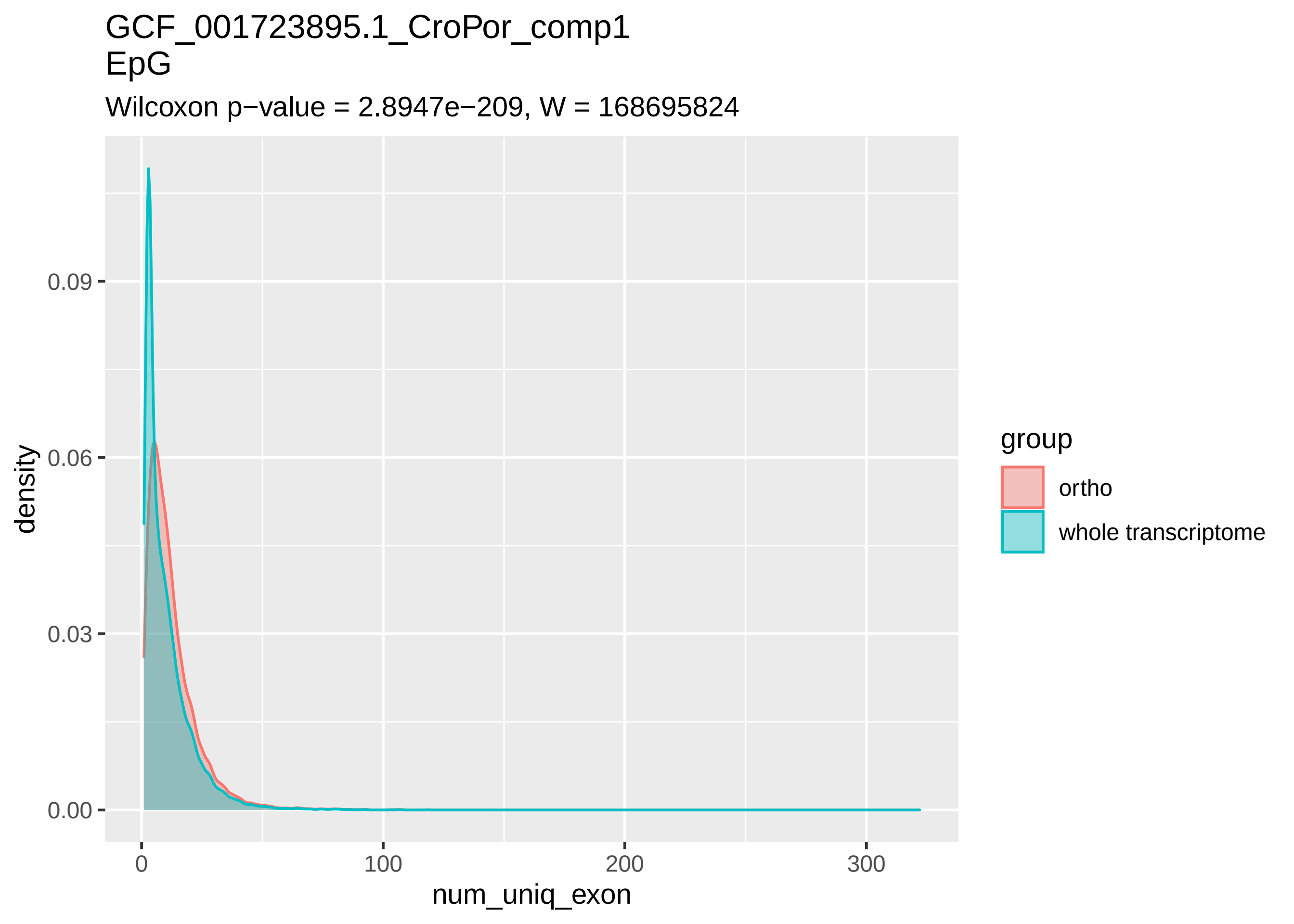

GCF_001723895.1_CroPor_comp1
EpG
Wilcoxon p−value = 2.8947e−209, W = 168695824
density
0.09
0.06
0.03
0.00
0
100
200
300
num_uniq_exon
group
ortho
whole transcriptome

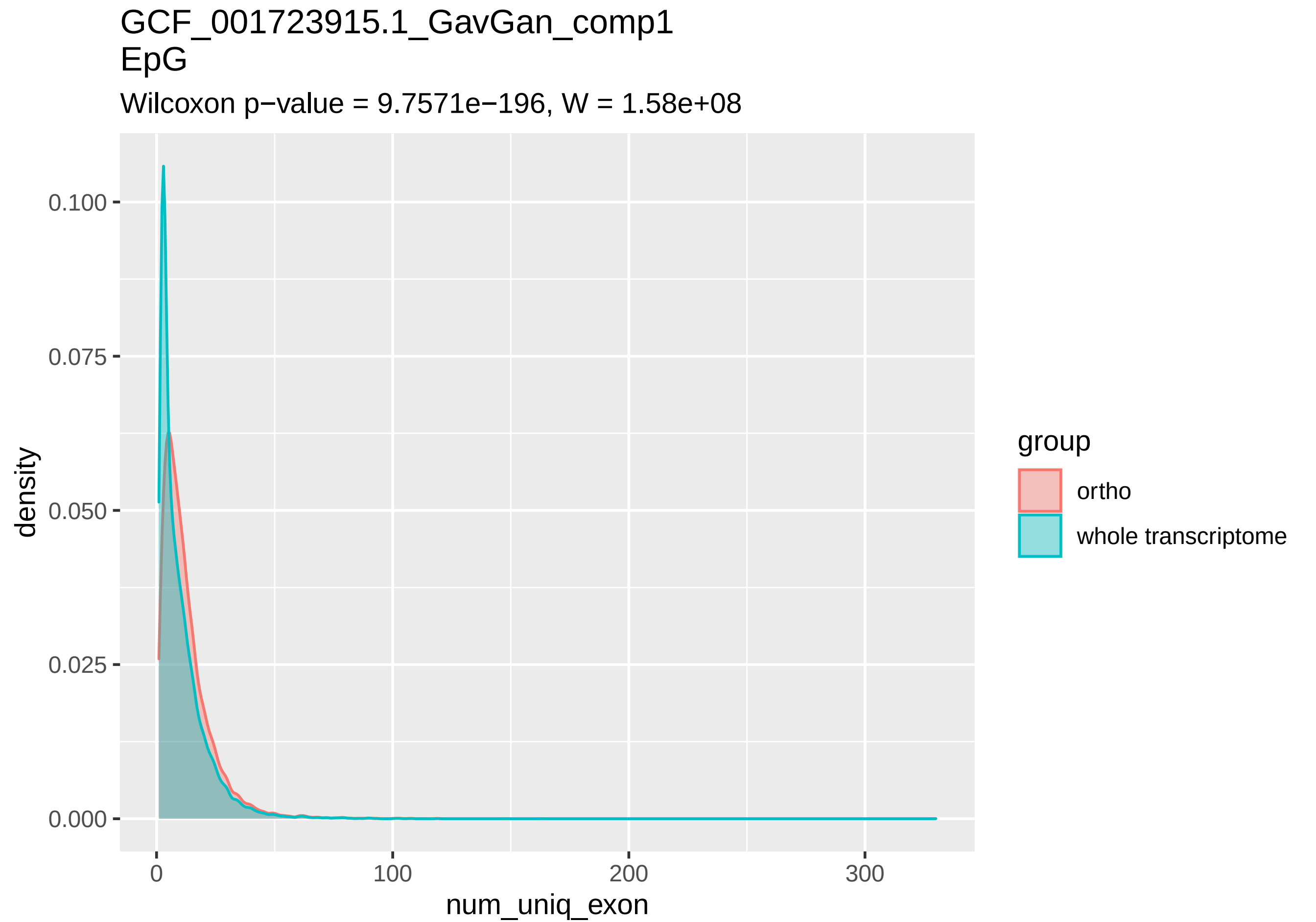
GCF_001723915.1_GavGan_comp1
EpG
Wilcoxon p−value = 9.7571e−196, W = 1.58e+08
density
0.100
0.075
0.050
0.025
0.000
0
100
200
300
num_uniq_exon
group
ortho
whole transcriptome

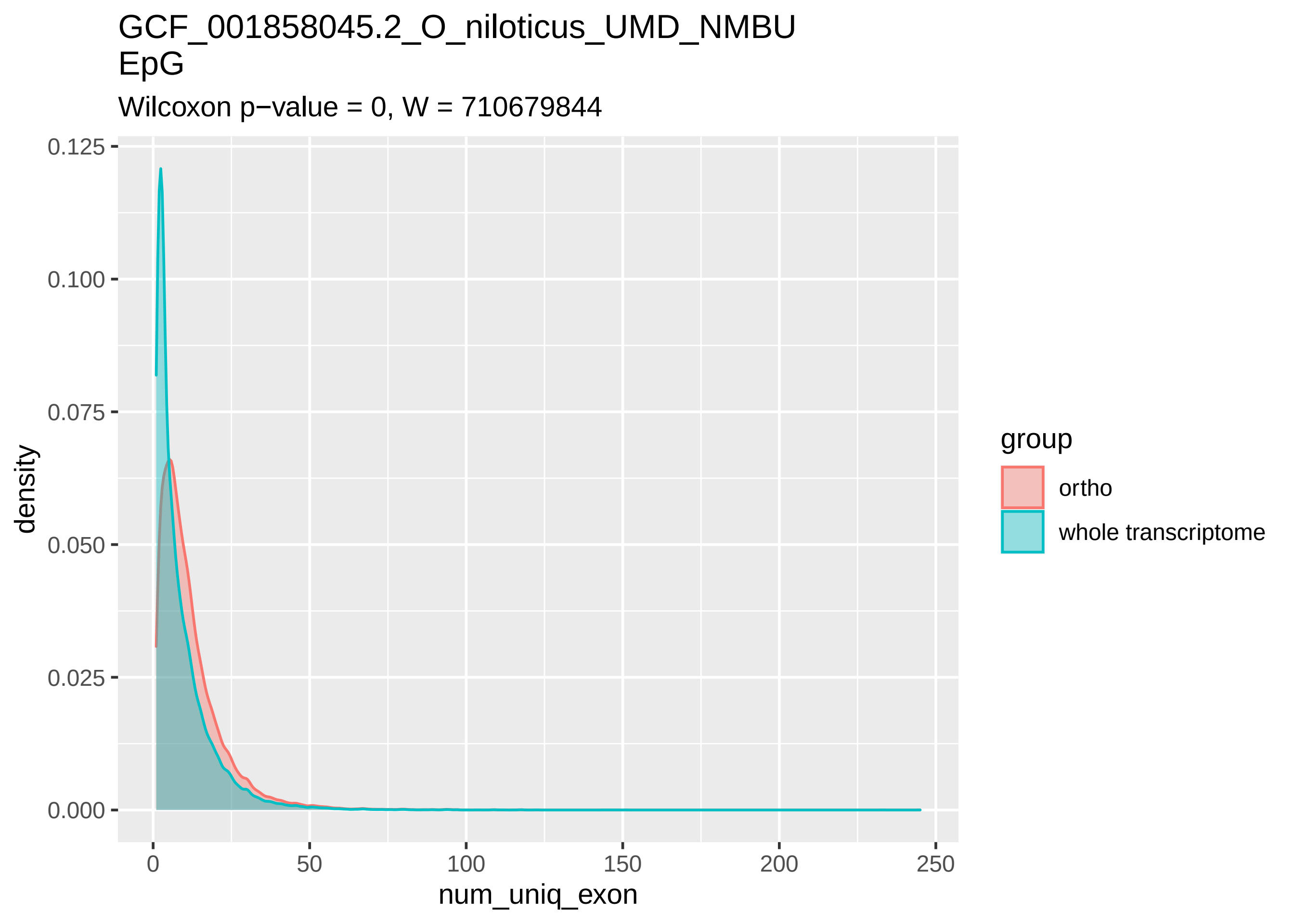

GCF_001858045.2_O_niloticus_UMD_NMBU
EpG
Wilcoxon p−value = 0, W = 710679844
0.125
0.100
0.075
0.050
0.025
0.000
density
0
50
100
150
200
250
num_uniq_exon
group
ortho
whole transcriptome

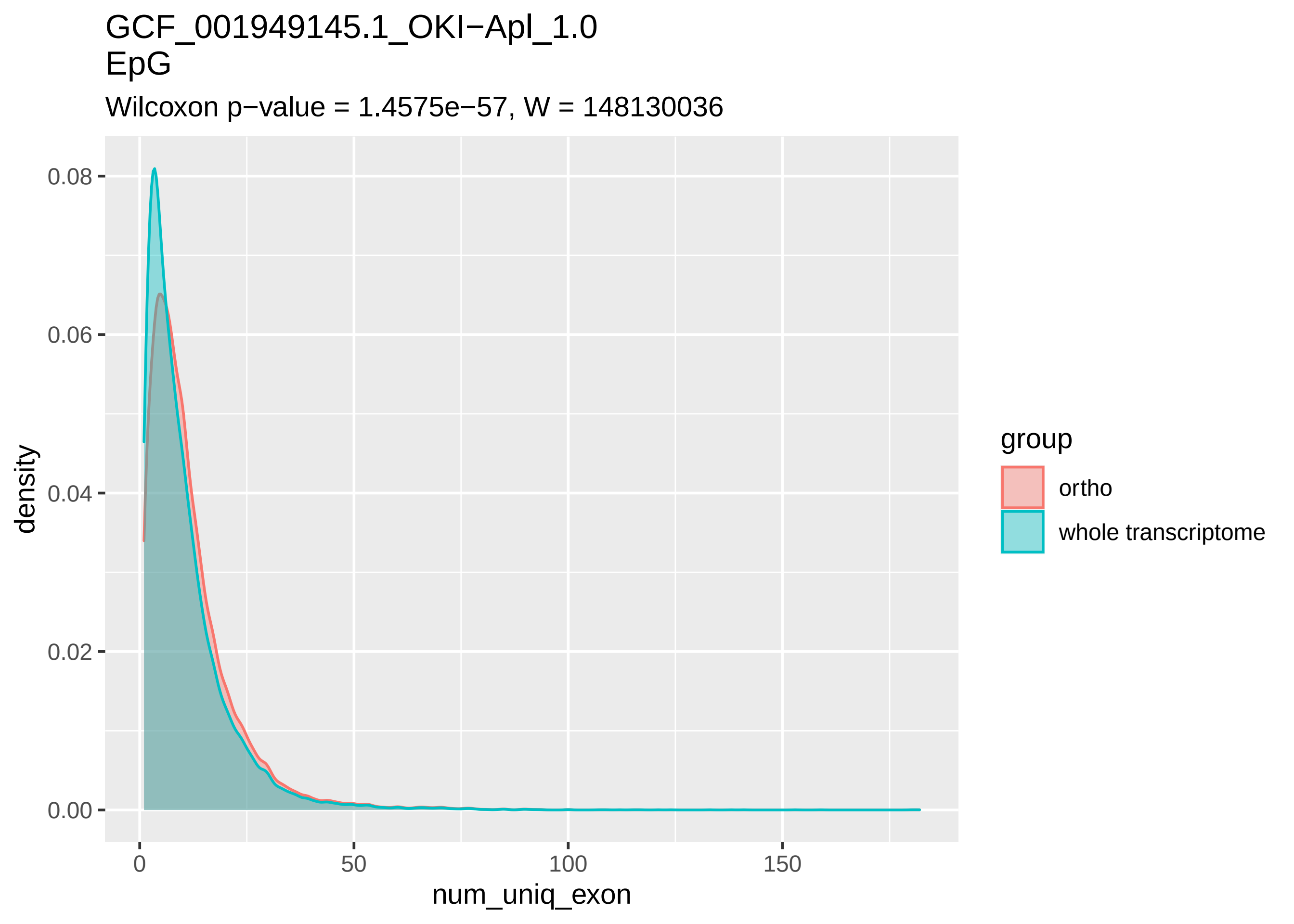
GCF_001949145.1_OKI−Apl_1.0
EpG
Wilcoxon p−value = 1.4575e−57, W = 148130036
density
0.08
0.06
0.04
0.02
0.00
0
50
100
150
num_uniq_exon
group
ortho
whole transcriptome

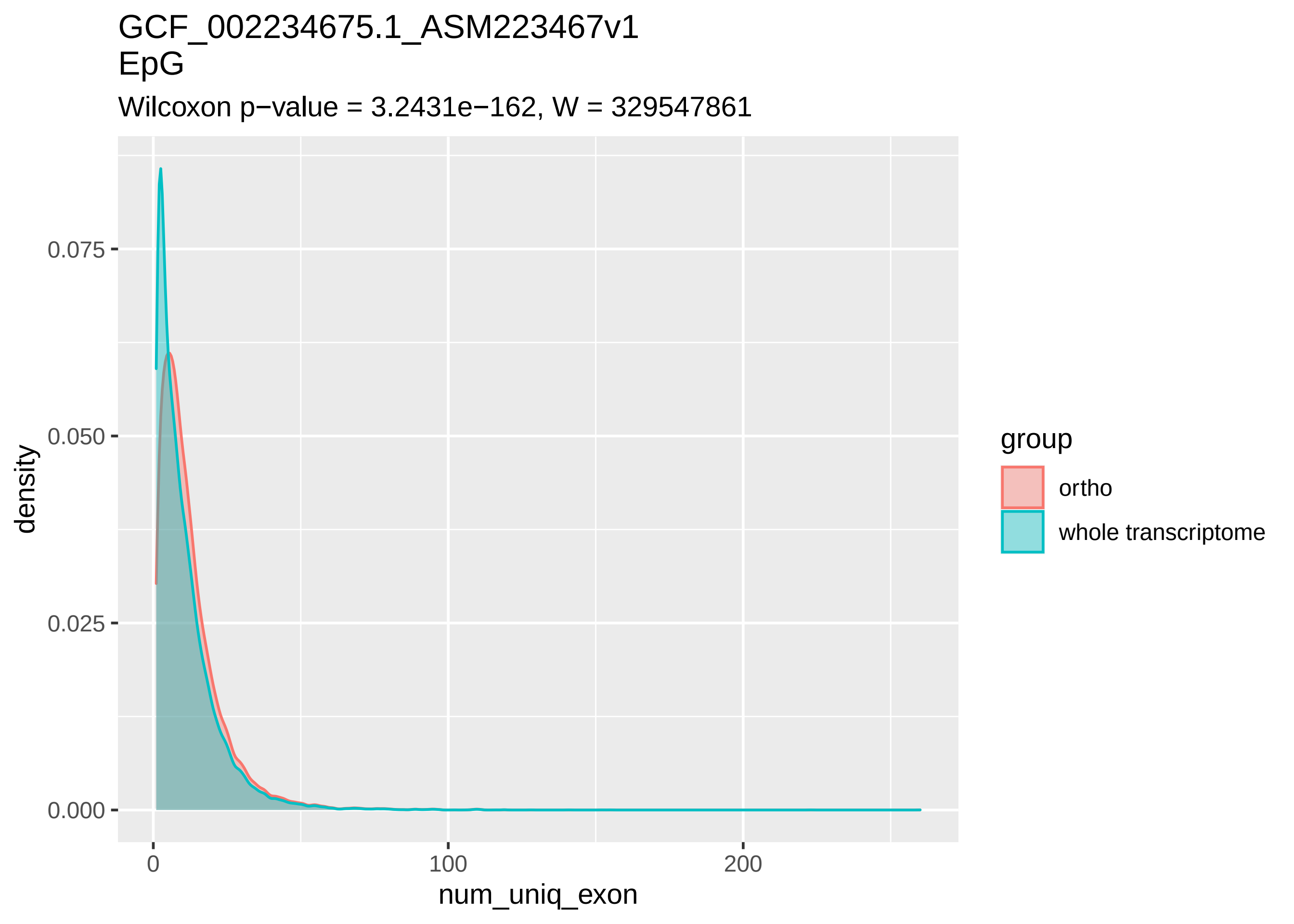
GCF_002234675.1_ASM223467v1
EpG
Wilcoxon p−value = 3.2431e−162, W = 329547861
0.075
0.050
0.025
0.000
density
0
100
200
num_uniq_exon
group
ortho
whole transcriptome

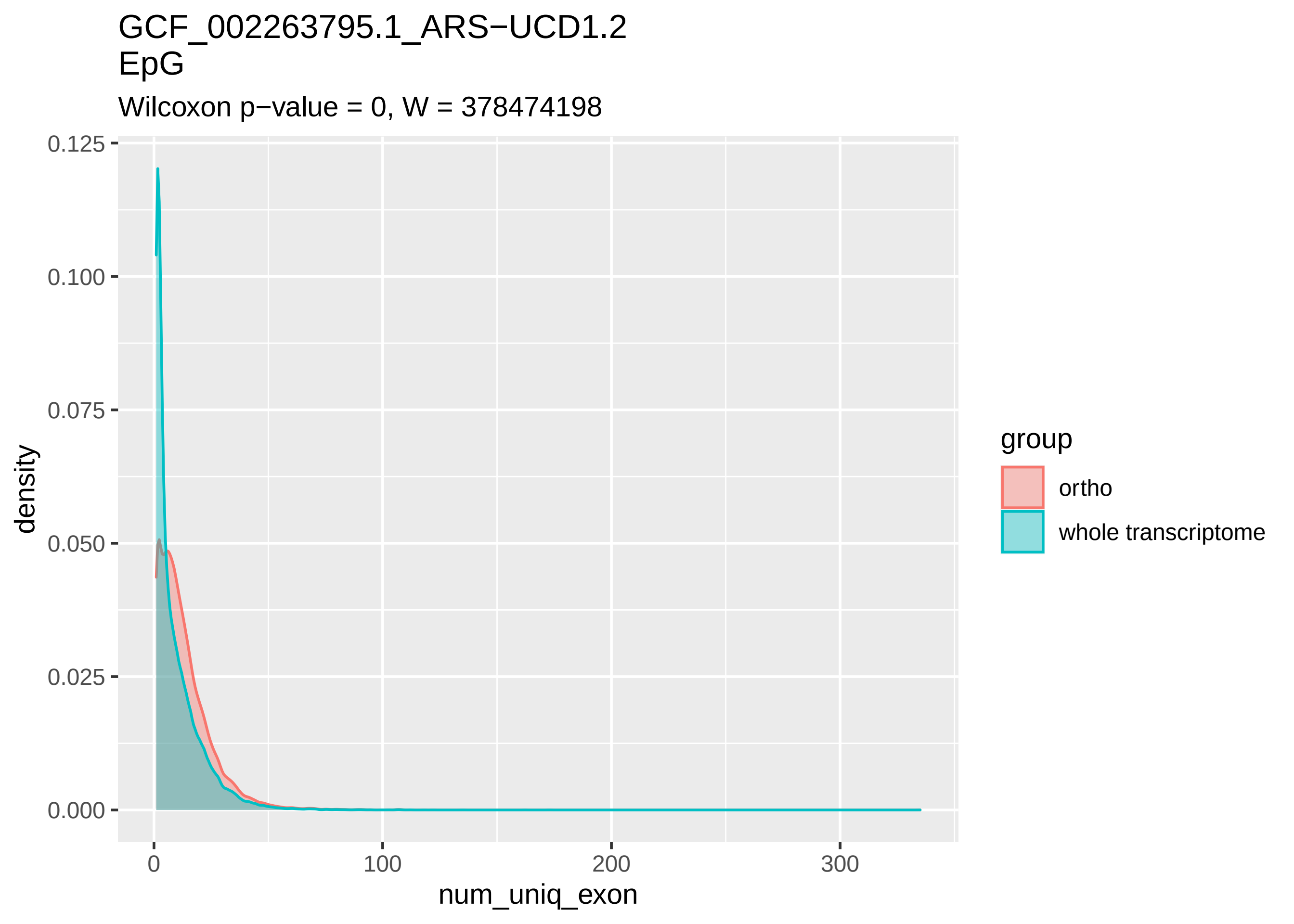
GCF_002263795.1_ARS−UCD1.2
EpG
Wilcoxon p−value = 0, W = 378474198
density
0.125
0.100
0.075
0.050
0.025
0.000
0
100
200
300
num_uniq_exon
group
ortho
whole transcriptome

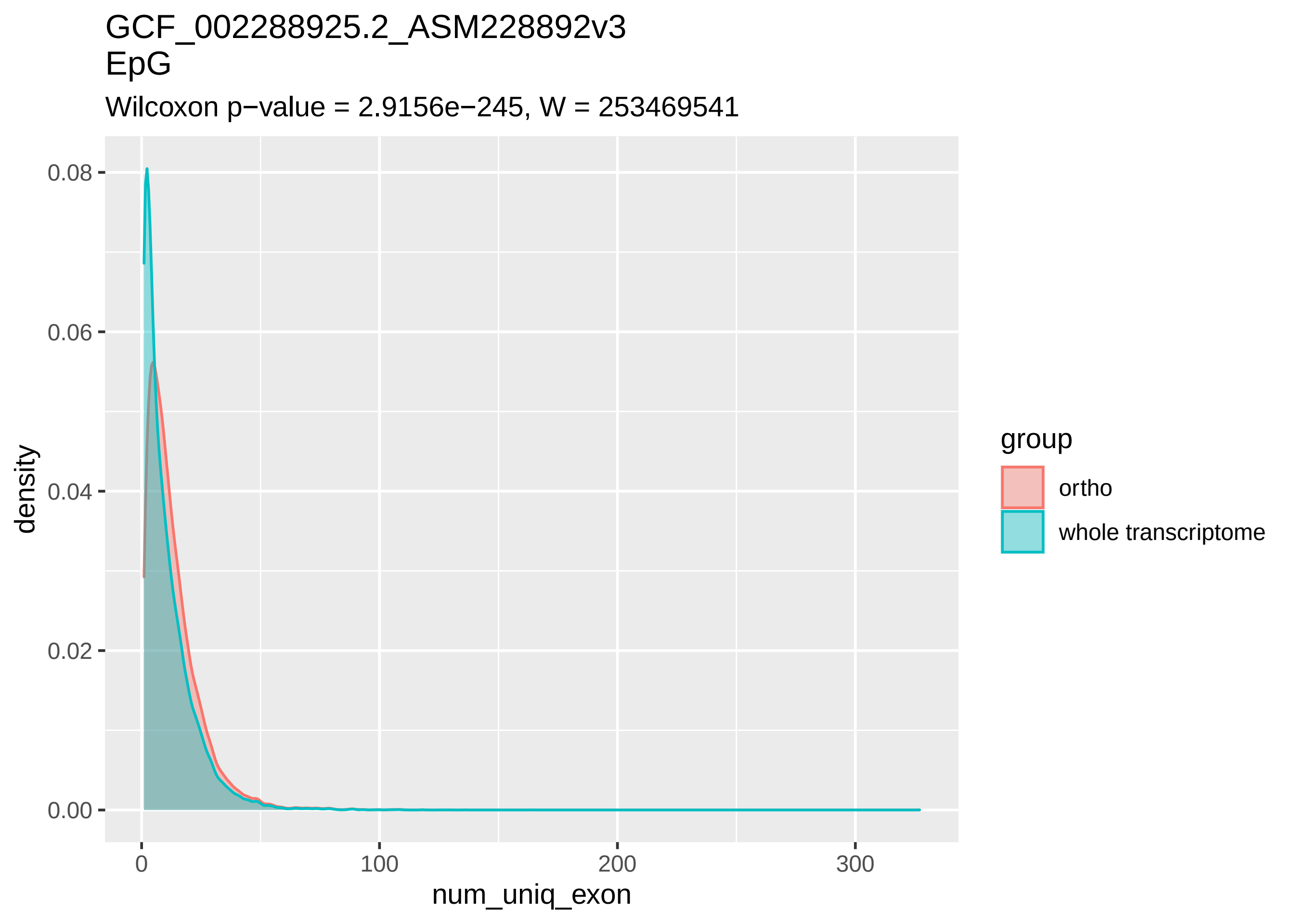

GCF_002288925.2_ASM228892v3
EpG
Wilcoxon p−value = 2.9156e−245, W = 253469541
density
0.08
0.06
0.04
0.02
0.00
0
100
200
300
num_uniq_exon
group
ortho
whole transcriptome

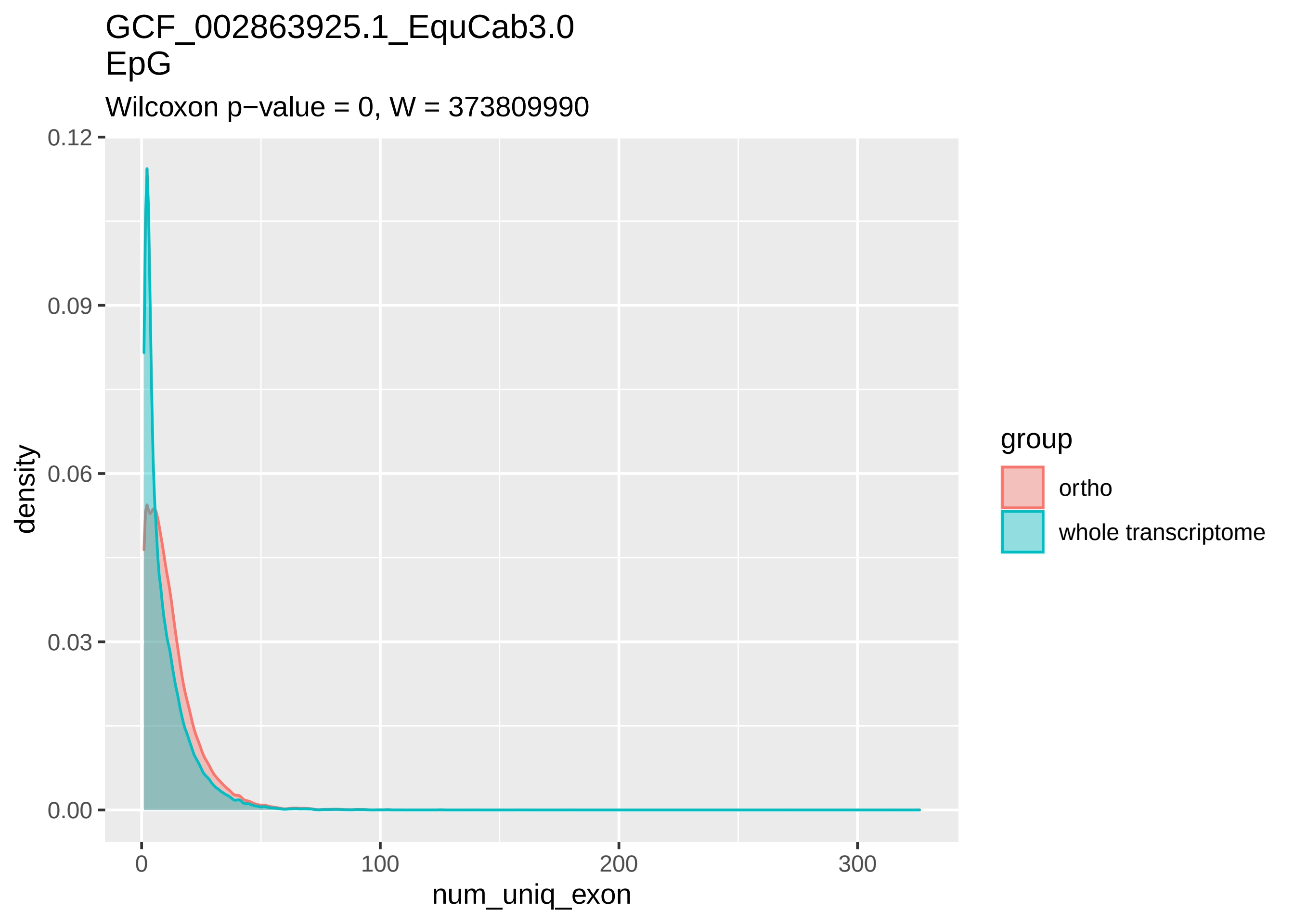
GCF_002863925.1_EquCab3.0
EpG
Wilcoxon p−value = 0, W = 373809990
0.12
0.09
0.06
0.03
0.00
density
0
100
200
300
num_uniq_exon
group
ortho
whole transcriptome

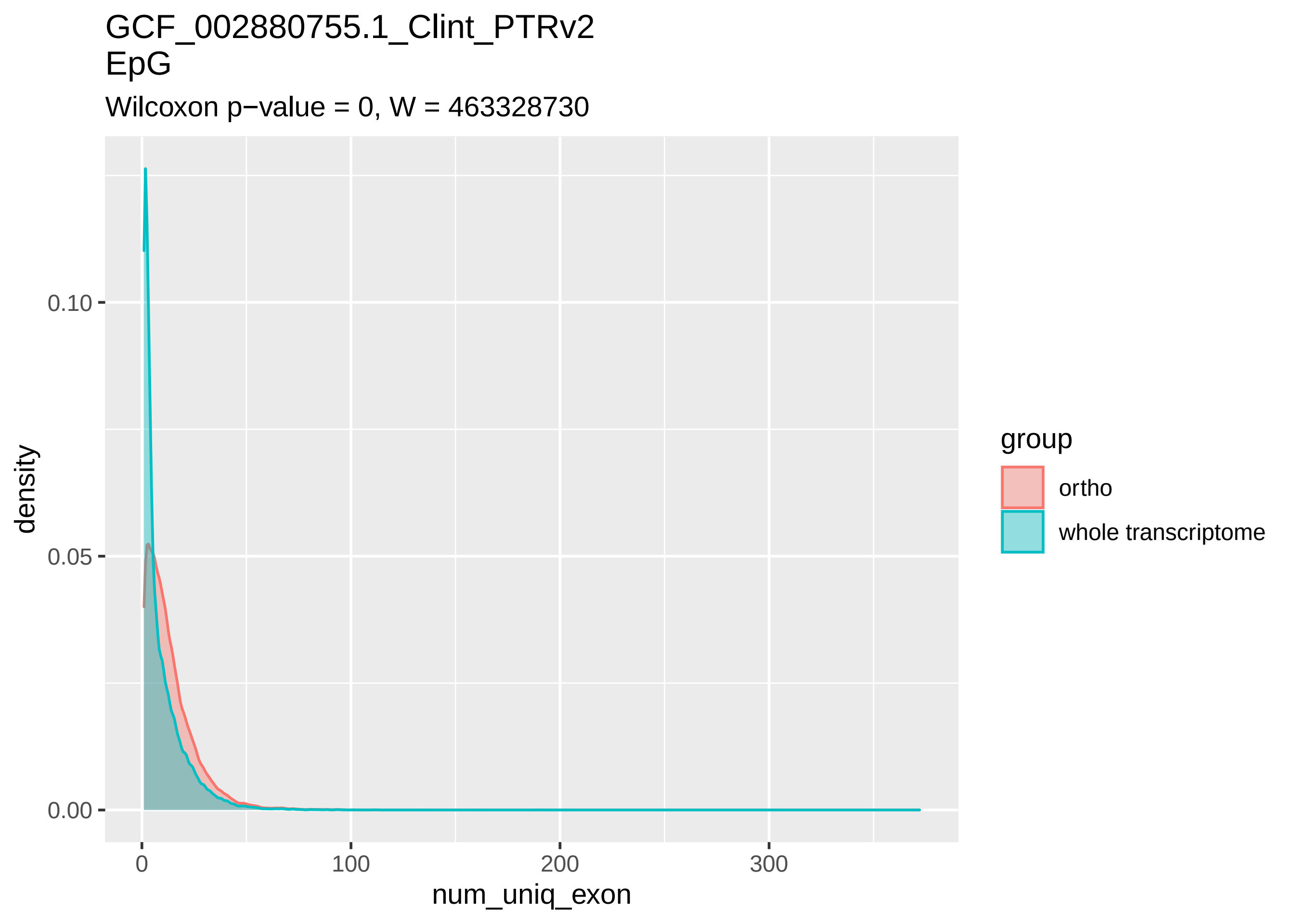
GCF_002880755.1_Clint_PTRv2
EpG
Wilcoxon p−value = 0, W = 463328730
density
0.10
0.05
0.00
0
100
200
300
num_uniq_exon
group
ortho
whole transcriptome

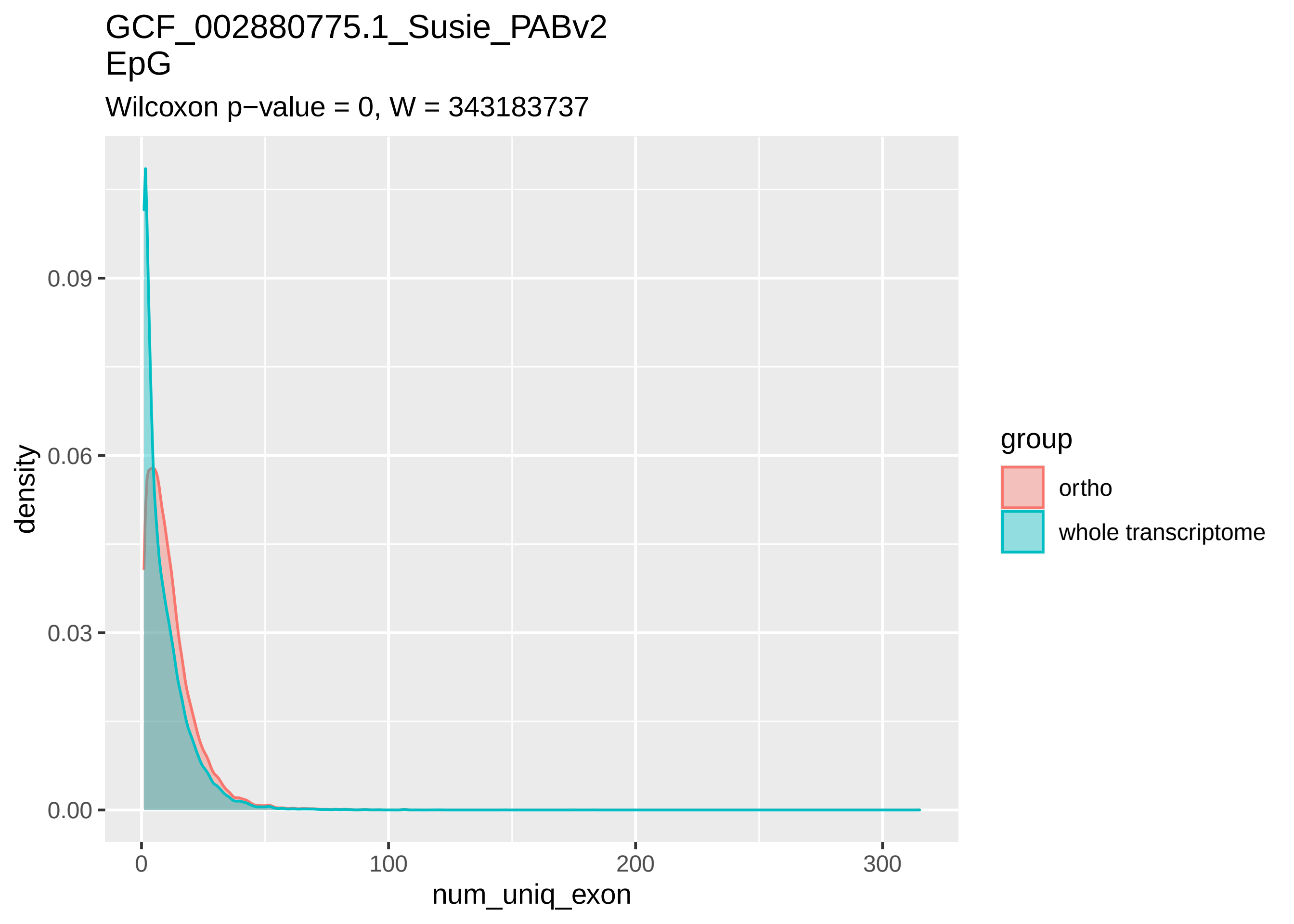
GCF_002880775.1_Susie_PABv2
EpG
Wilcoxon p−value = 0, W = 343183737
density
0.09
0.06
0.03
0.00
0
100
200
300
num_uniq_exon
group
ortho
whole transcriptome

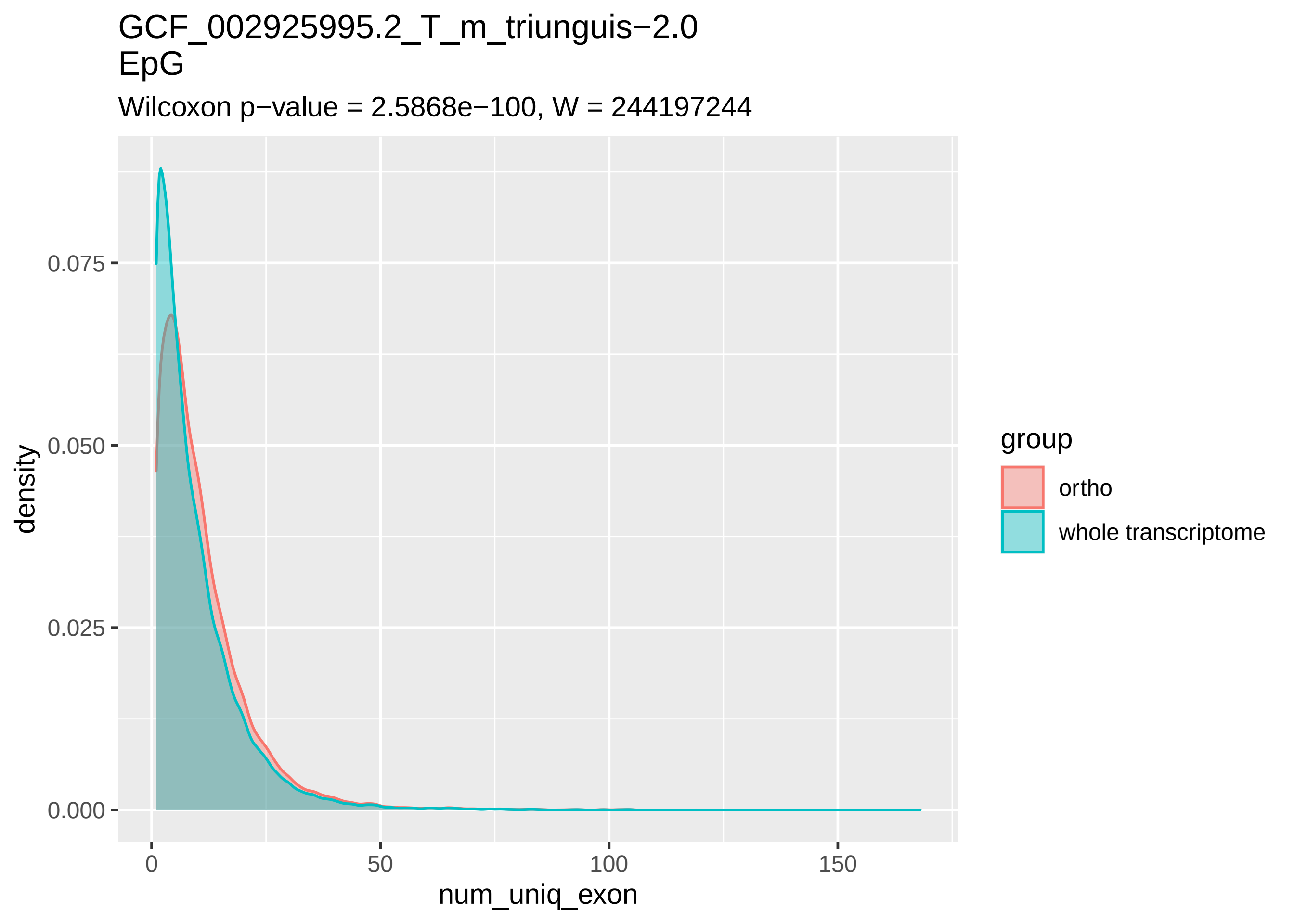
GCF_002925995.2_T_m_triunguis−2.0
EpG
Wilcoxon p−value = 2.5868e−100, W = 244197244
density
0.075
0.050
0.025
0.000
0
50
100
150
num_uniq_exon
group
ortho
whole transcriptome

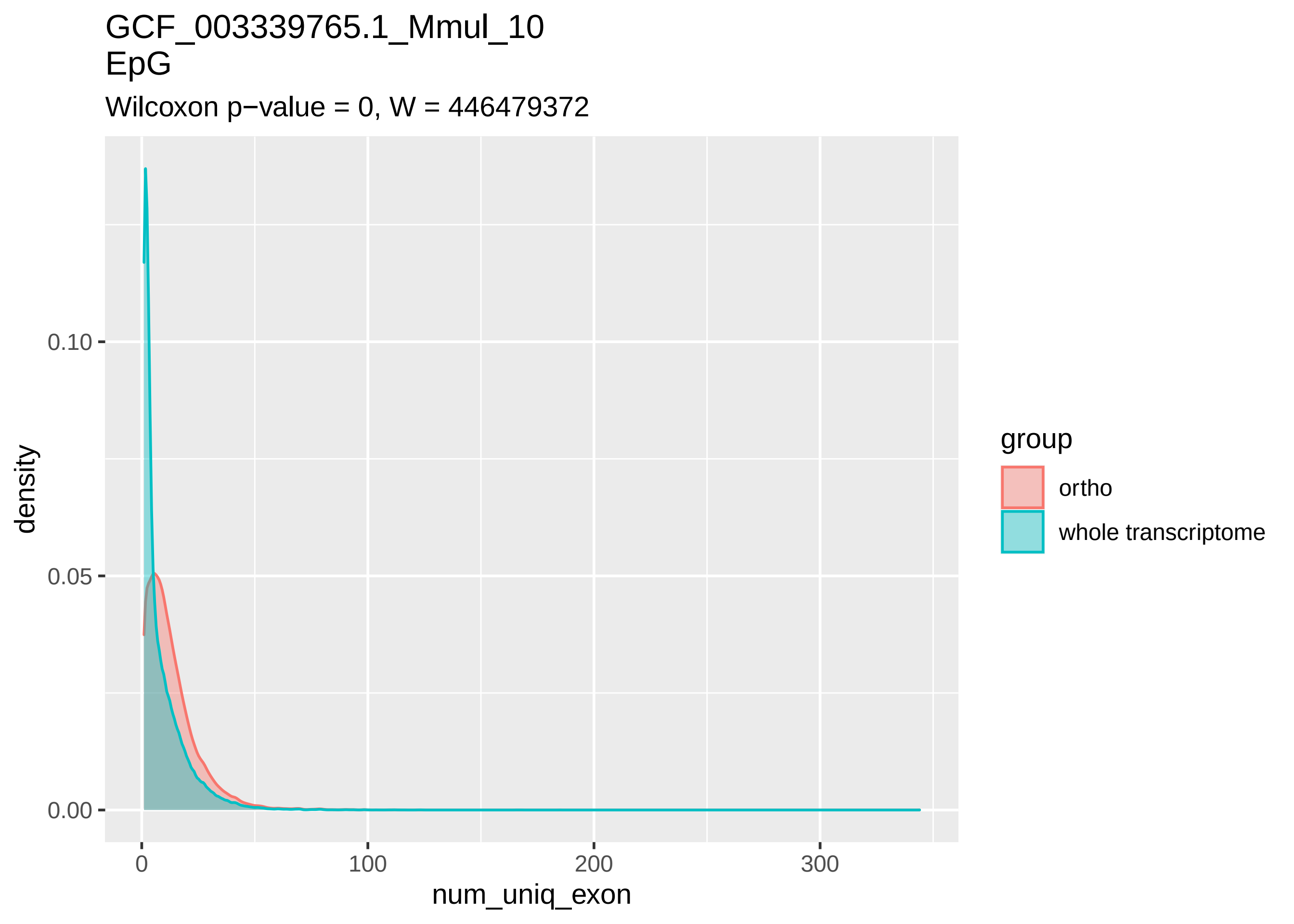

GCF_003339765.1_Mmul_10
EpG
Wilcoxon p−value = 0, W = 446479372
density
0.10
0.05
0.00
0
100
200
300
num_uniq_exon
group
ortho
whole transcriptome

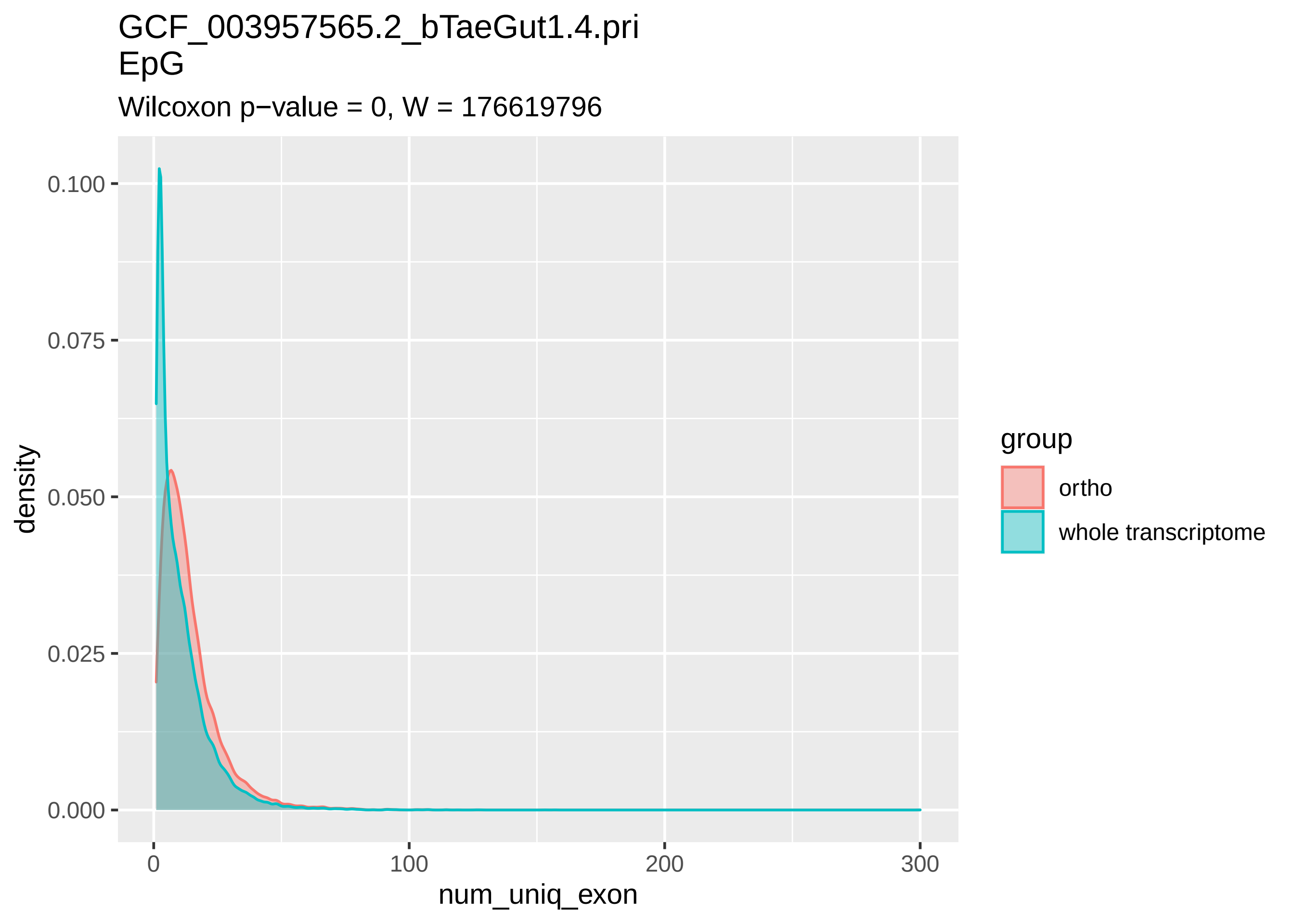

GCF_003957565.2_bTaeGut1.4.pri
EpG
Wilcoxon p−value = 0, W = 176619796
0.100
0.075
0.050
0.025
0.000
density
0
100
200
300
num_uniq_exon
group
ortho
whole transcriptome

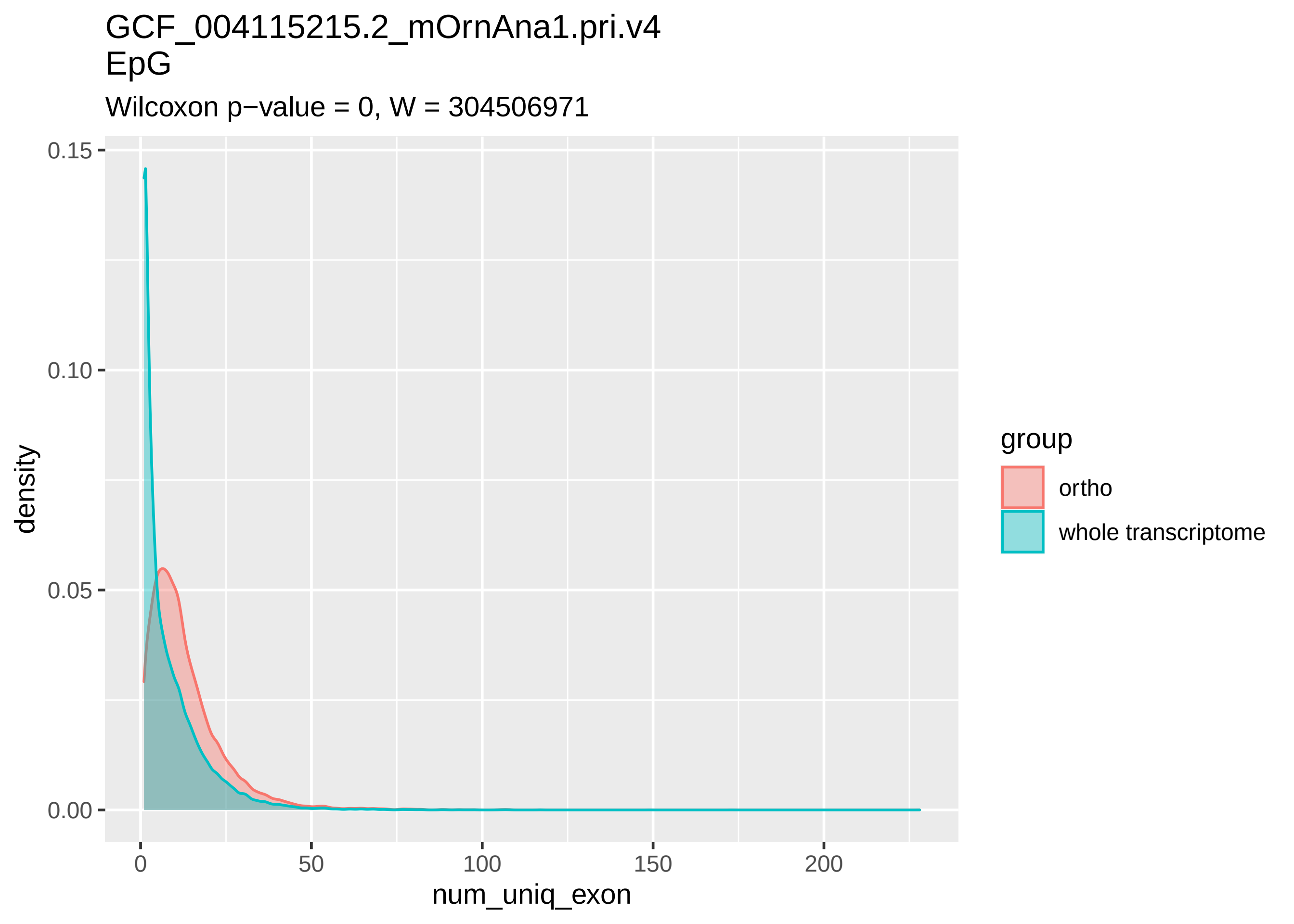

GCF_004115215.2_mOrnAna1.pri.v4
EpG
Wilcoxon p−value = 0, W = 304506971
0.15
0.10
0.05
0.00
density
0
50
100
150
200
num_uniq_exon
group
ortho
whole transcriptome

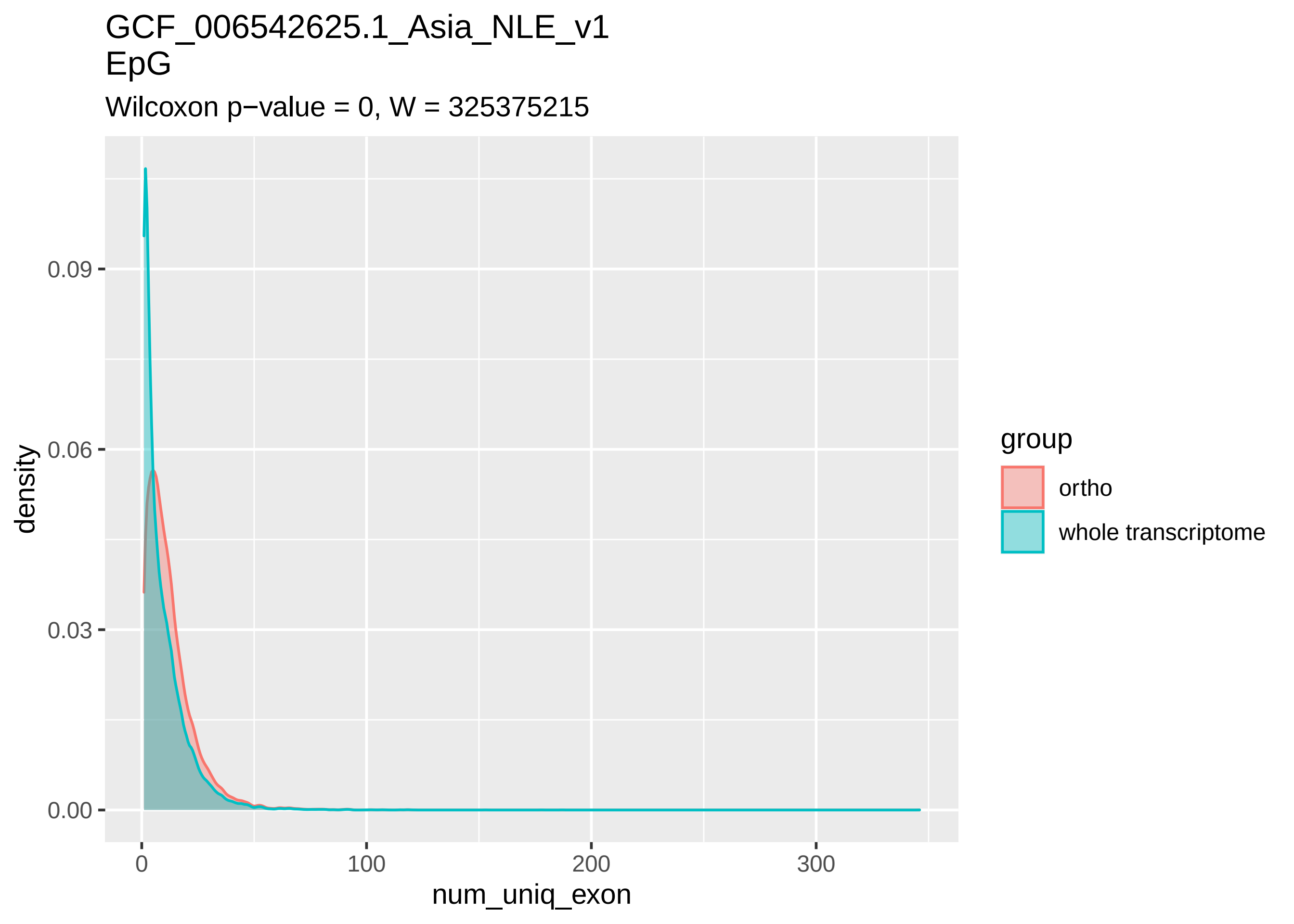
GCF_006542625.1_Asia_NLE_v1
EpG
Wilcoxon p−value = 0, W = 325375215
density
0.09
0.06
0.03
0.00
0
100
200
300
num_uniq_exon
group
ortho
whole transcriptome

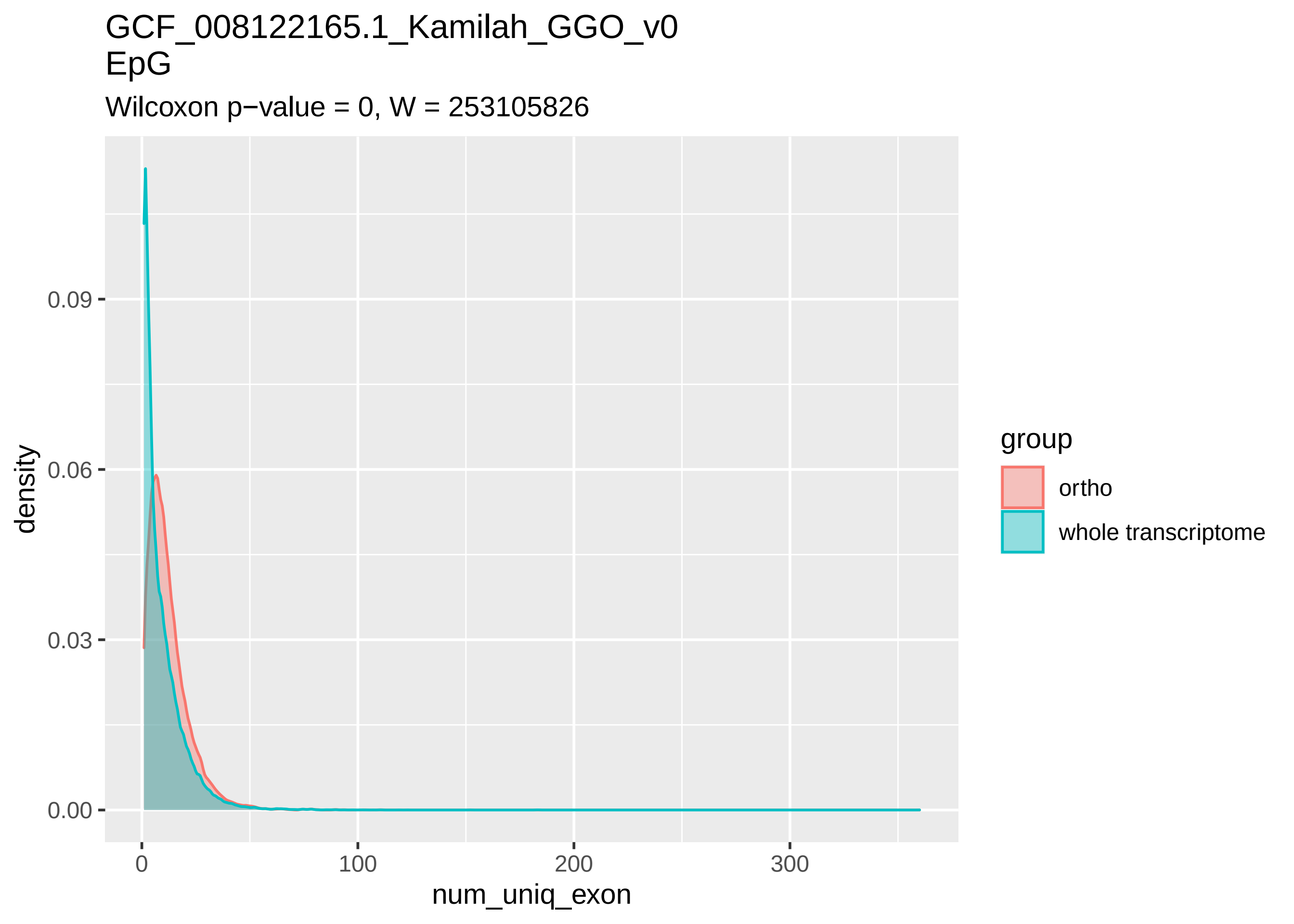
GCF_008122165.1_Kamilah_GGO_v0
EpG
Wilcoxon p−value = 0, W = 253105826
density
0.09
0.06
0.03
0.00
0
100
200
300
num_uniq_exon
group
ortho
whole transcriptome

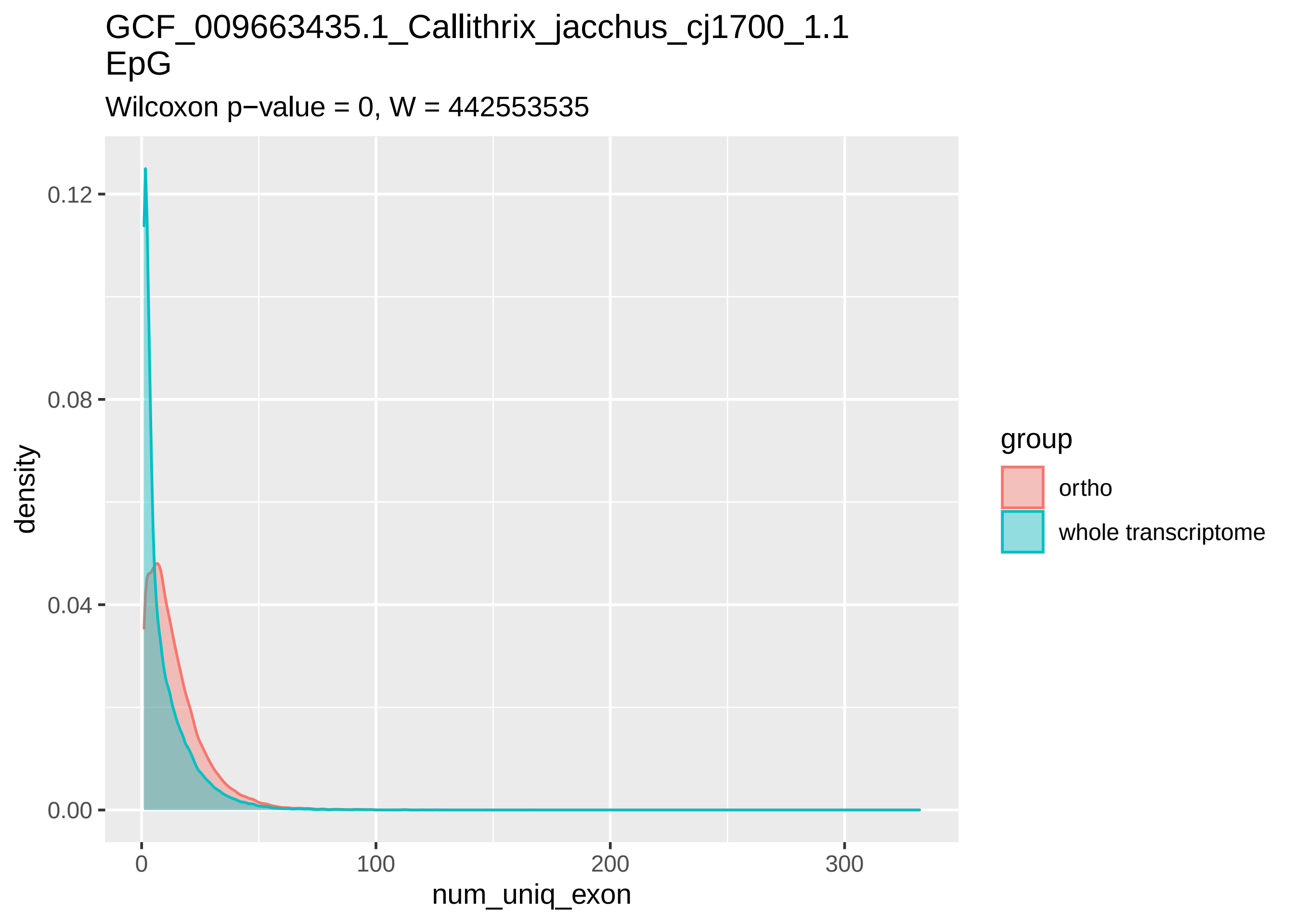

GCF_009663435.1_Callithrix_jacchus_cj1700_1.1
EpG
Wilcoxon p−value = 0, W = 442553535
density
0.12
0.08
0.04
0.00
0
100
200
300
num_uniq_exon
group
ortho
whole transcriptome

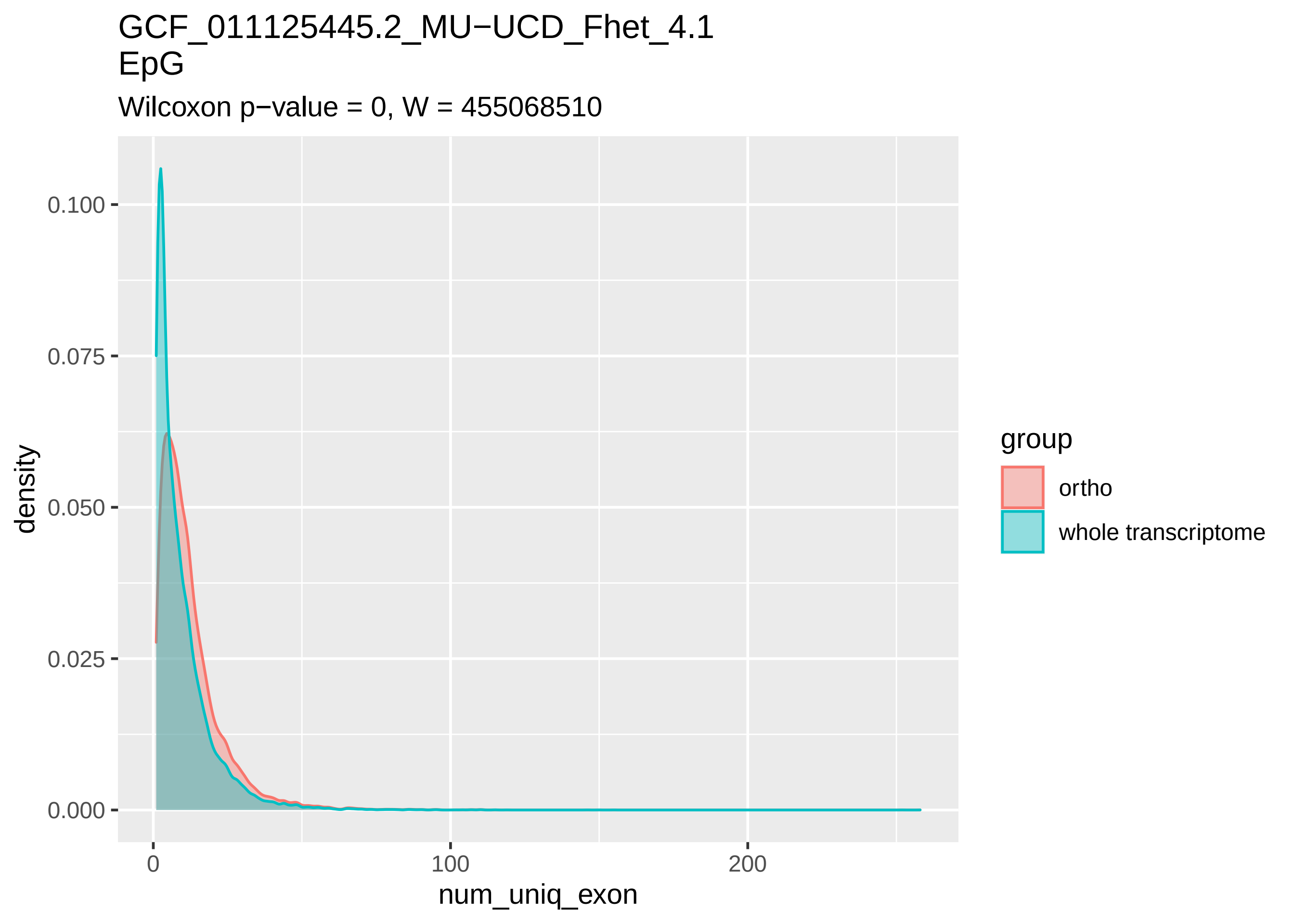

GCF_011125445.2_MU−UCD_Fhet_4.1
EpG
Wilcoxon p−value = 0, W = 455068510
0.100
0.075
0.050
0.025
0.000
density
0
100
200
num_uniq_exon
group
ortho
whole transcriptome

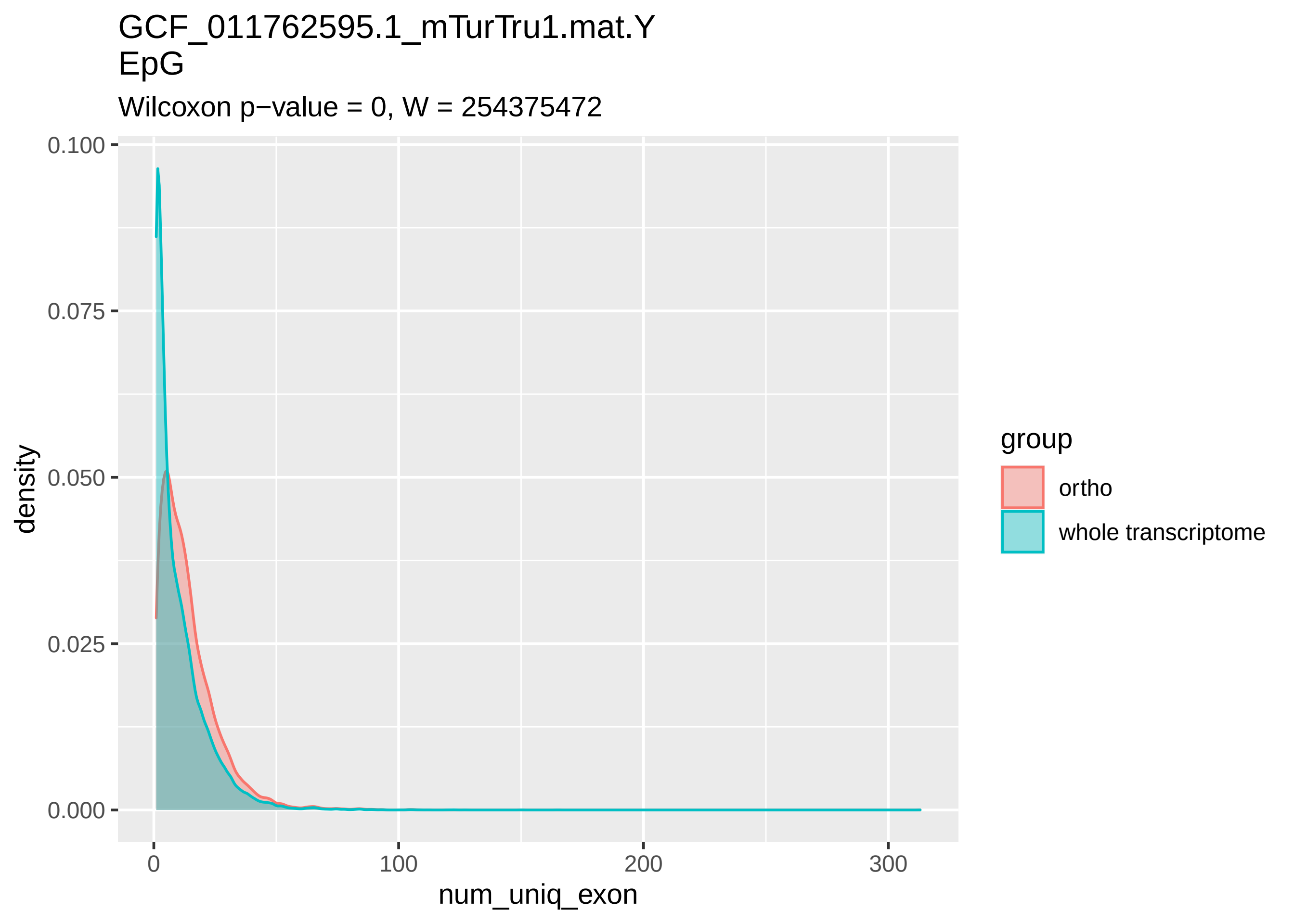

GCF_011762595.1_mTurTru1.mat.Y
EpG
Wilcoxon p−value = 0, W = 254375472
density
0.100
0.075
0.050
0.025
0.000
0
100
200
300
num_uniq_exon
group
ortho
whole transcriptome

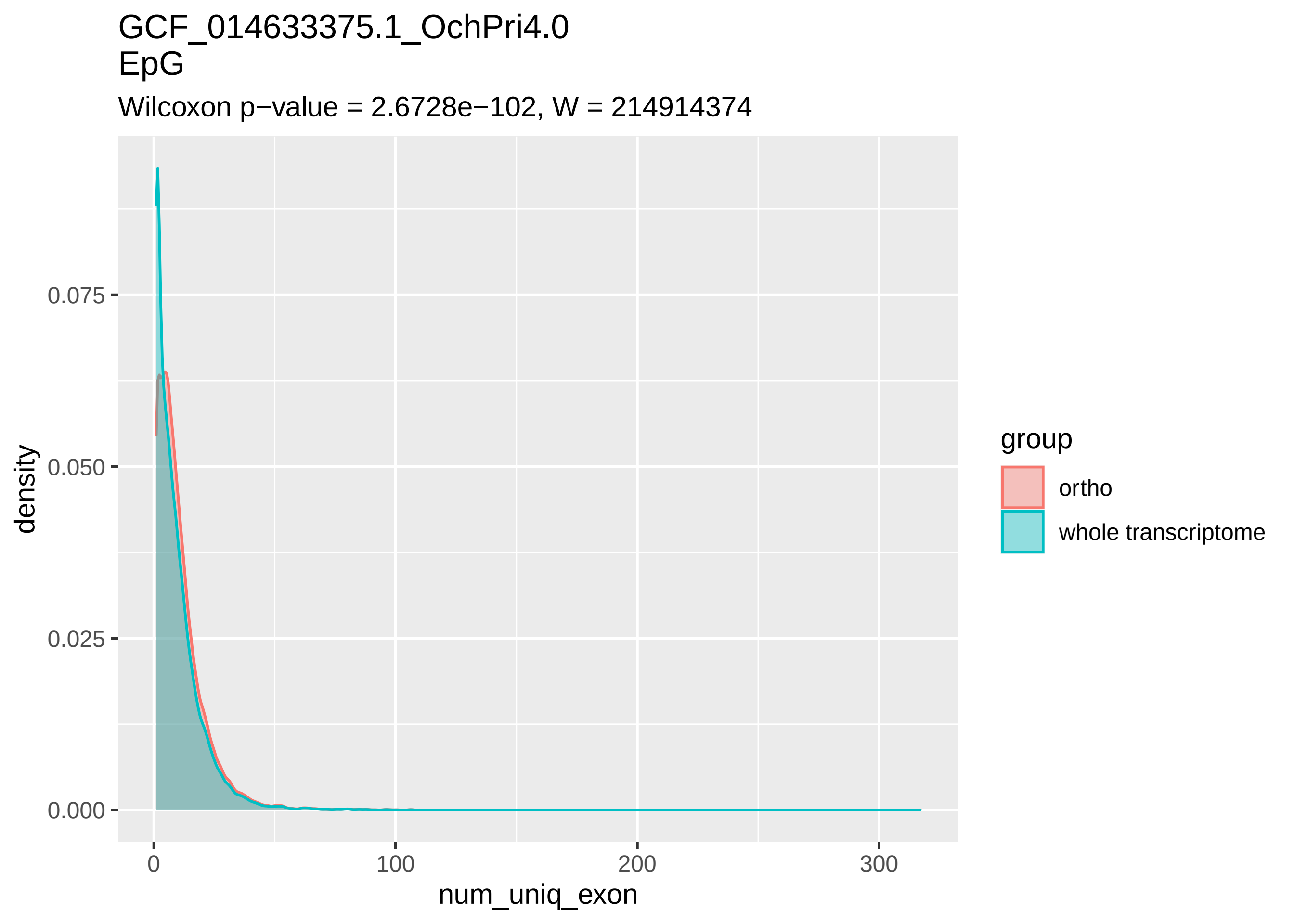
GCF_014633375.1_OchPri4.0
EpG
Wilcoxon p−value = 2.6728e−102, W = 214914374
density
0.000
0.025
0.050
0.075
0
100
200
300
num_uniq_exon
group
ortho
whole transcriptome

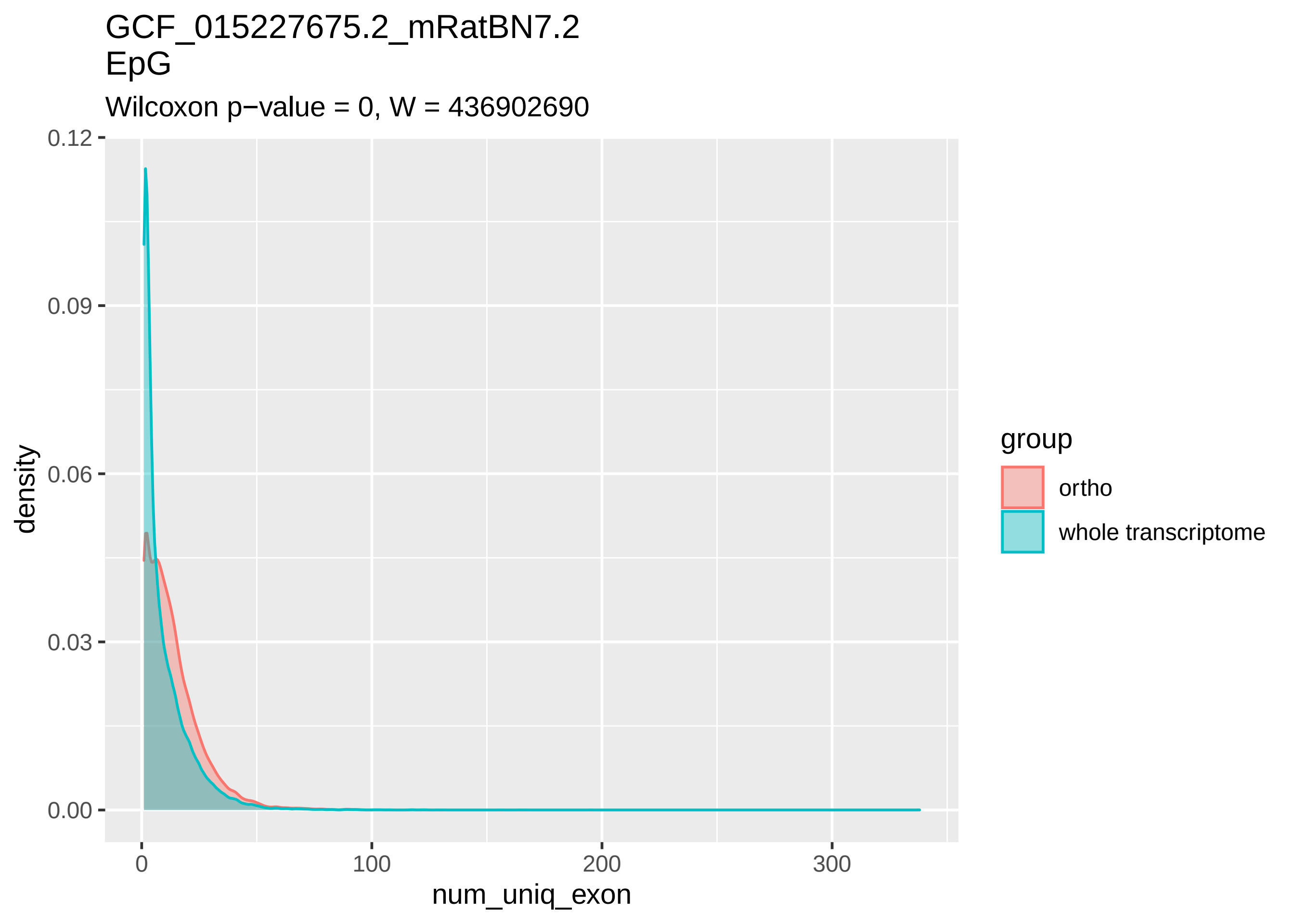
GCF_015227675.2_mRatBN7.2
EpG
Wilcoxon p−value = 0, W = 436902690
0.12
0.09
0.06
0.03
0.00
density
0
100
200
300
num_uniq_exon
group
ortho
whole transcriptome

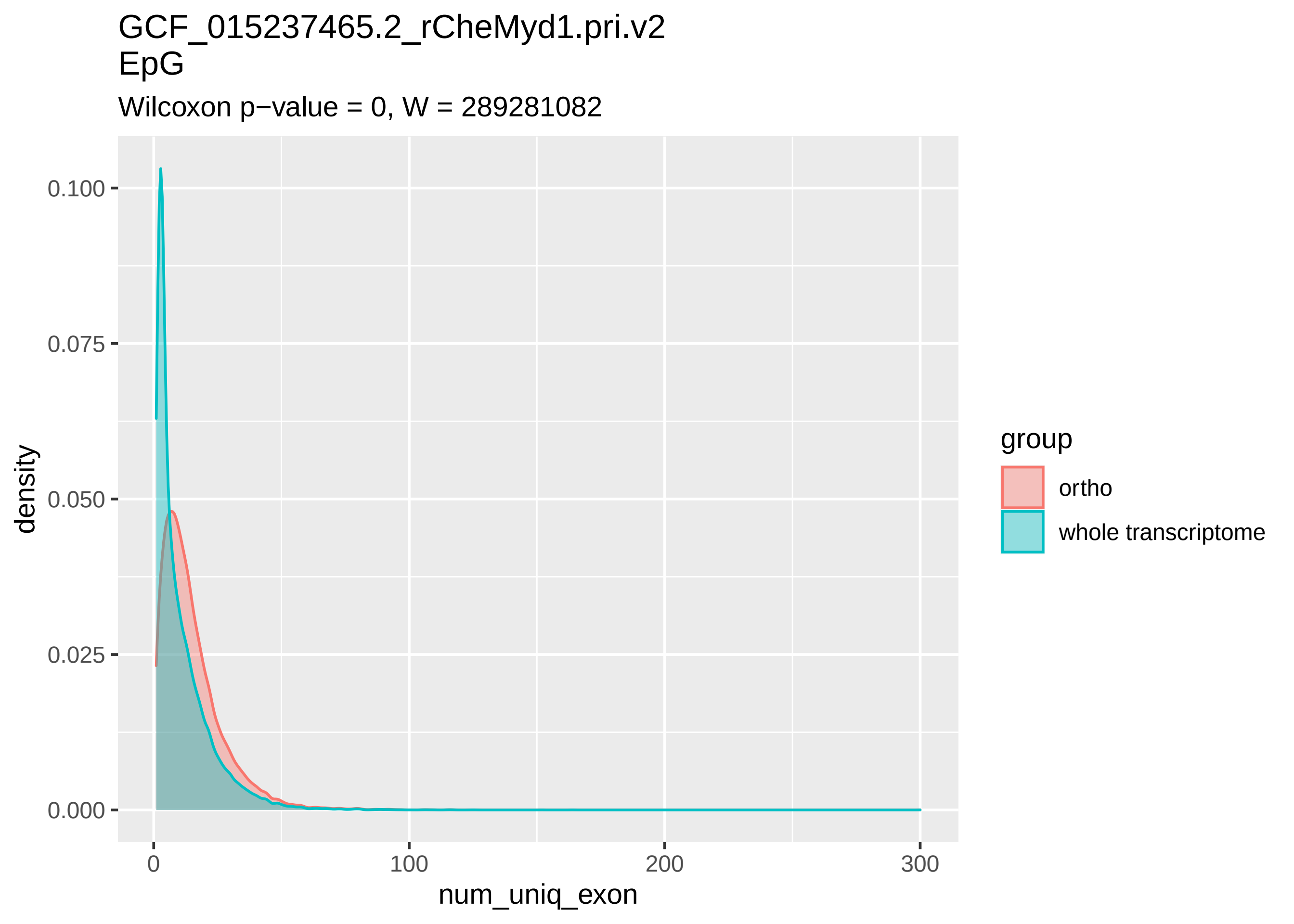
GCF_015237465.2_rCheMyd1.pri.v2
EpG
Wilcoxon p−value = 0, W = 289281082
density
0.100
0.075
0.050
0.025
0.000
0
100
200
300
num_uniq_exon
group
ortho
whole transcriptome

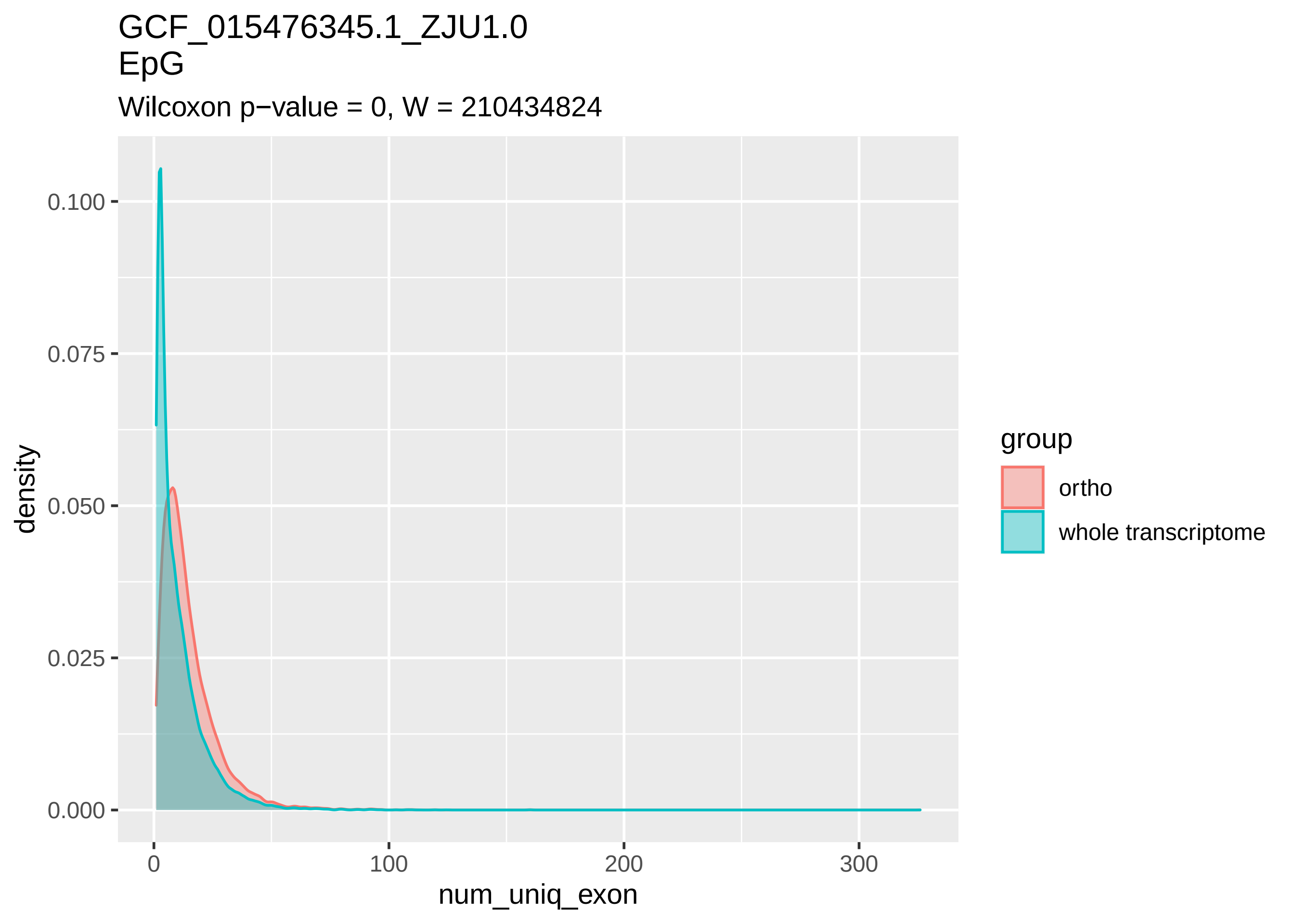
GCF_015476345.1_ZJU1.0
EpG
Wilcoxon p−value = 0, W = 210434824
density
0.100
0.075
0.050
0.025
0.000
0
100
200
300
num_uniq_exon
group
ortho
whole transcriptome

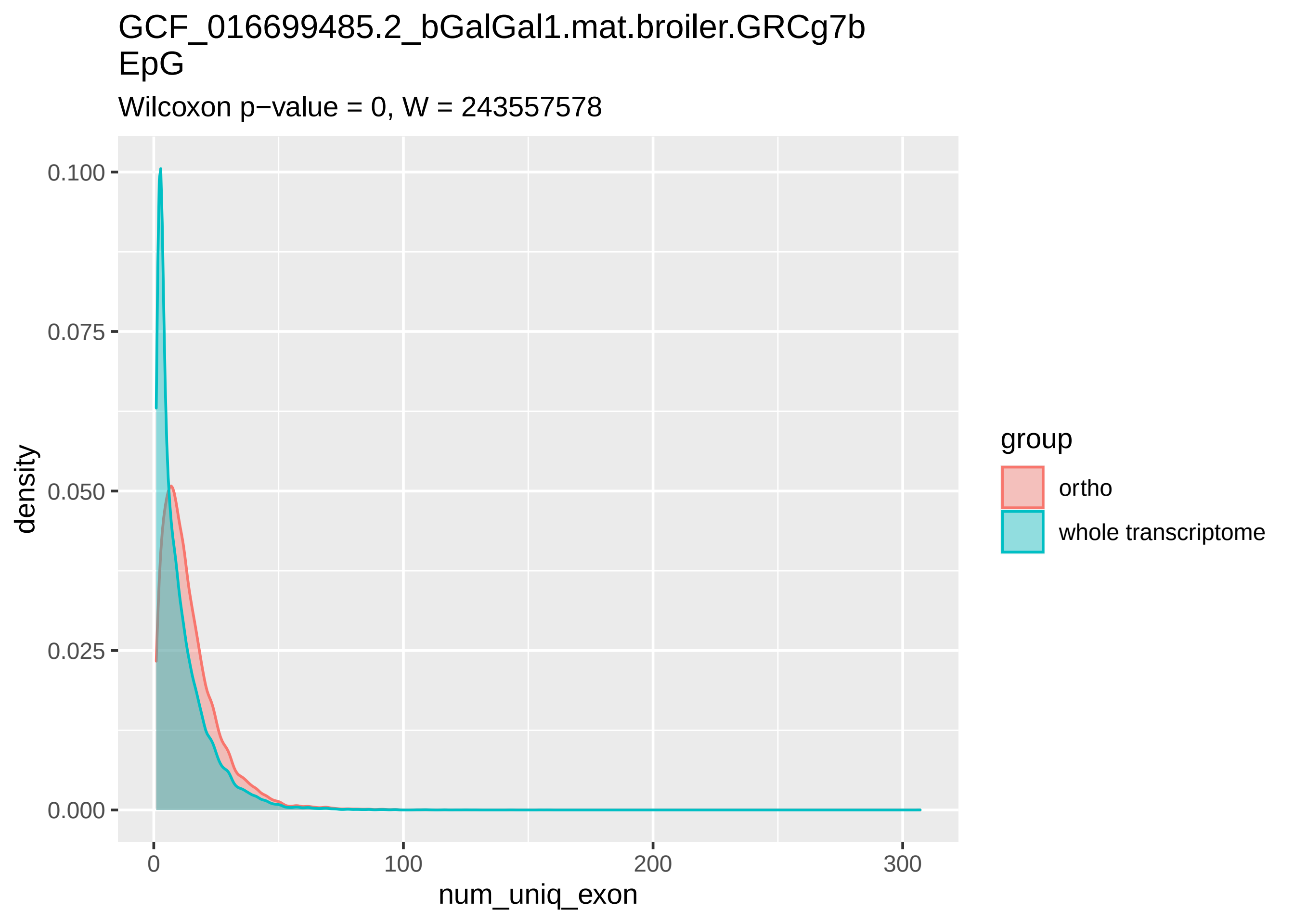
GCF_016699485.2_bGalGal1.mat.broiler.GRCg7b
EpG
Wilcoxon p−value = 0, W = 243557578
0.100
0.075
0.050
0.025
0.000
density
0
100
200
300
num_uniq_exon
group
ortho
whole transcriptome

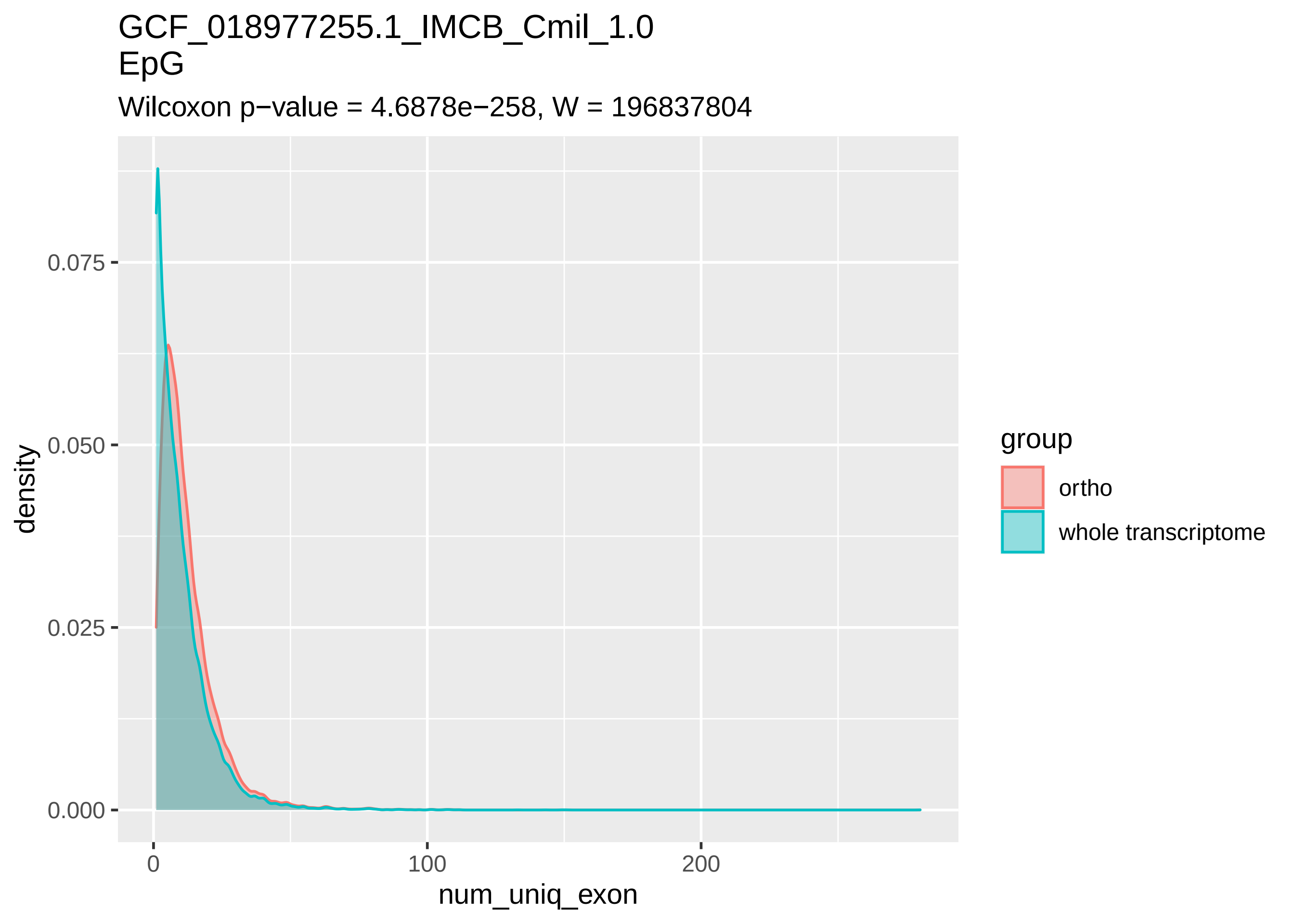
GCF_018977255.1_IMCB_Cmil_1.0
EpG
Wilcoxon p−value = 4.6878e−258, W = 196837804
density
0.075
0.050
0.025
0.000
0
100
200
num_uniq_exon
group
ortho
whole transcriptome

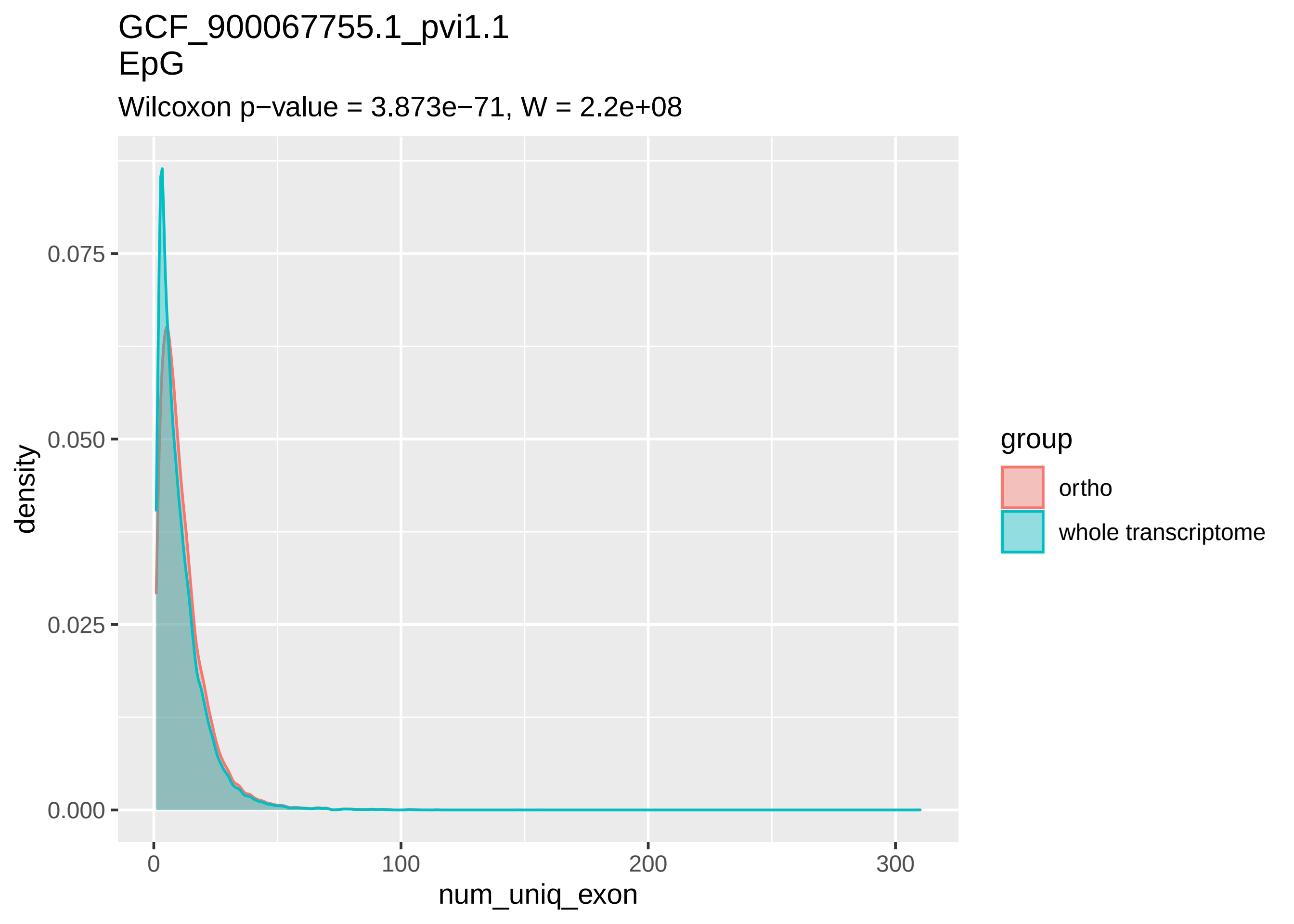
GCF_900067755.1_pvi1.1
EpG
Wilcoxon p−value = 3.873e−71, W = 2.2e+08
density
0.000
0.025
0.050
0.075
0
100
200
300
num_uniq_exon
group
ortho
whole transcriptome

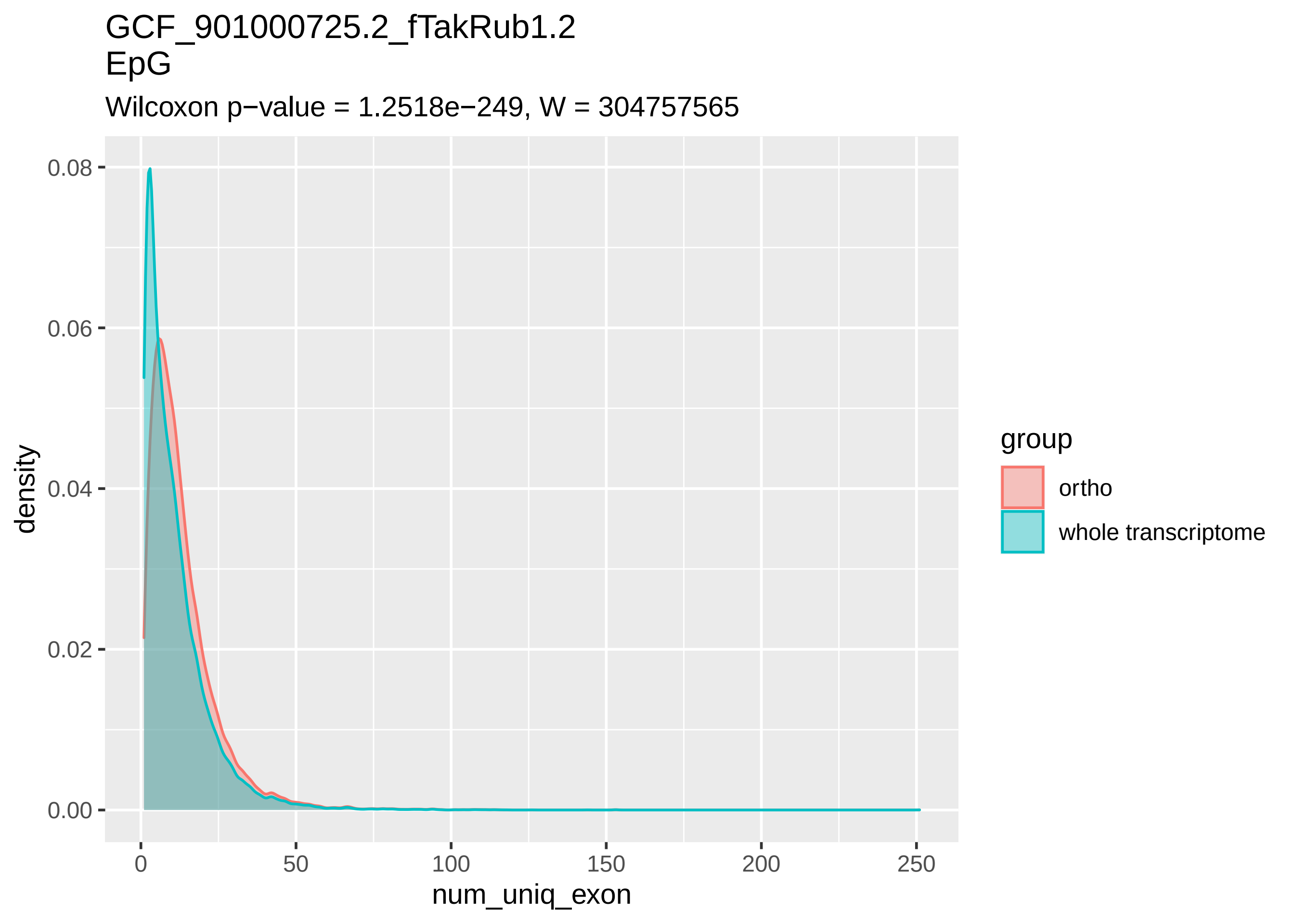
GCF_901000725.2_fTakRub1.2
EpG
Wilcoxon p−value = 1.2518e−249, W = 304757565
density
0.08
0.06
0.04
0.02
0.00
0
50
100
150
200
250
num_uniq_exon
group
ortho
whole transcriptome

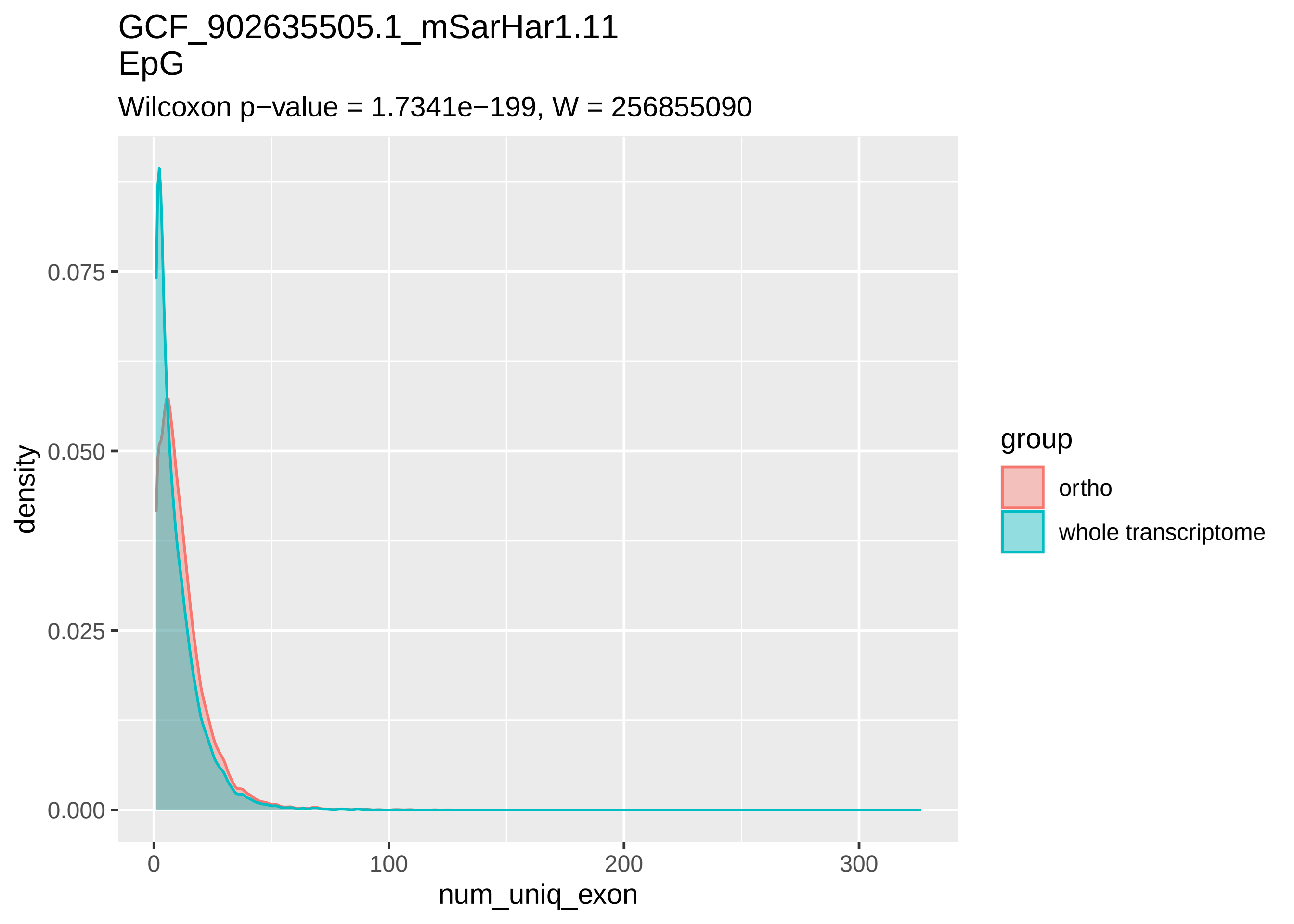
GCF_902635505.1_mSarHar1.11
EpG
Wilcoxon p−value = 1.7341e−199, W = 256855090
density
0.000
0.025
0.050
0.075
0
100
200
300
num_uniq_exon
group
ortho
whole transcriptome

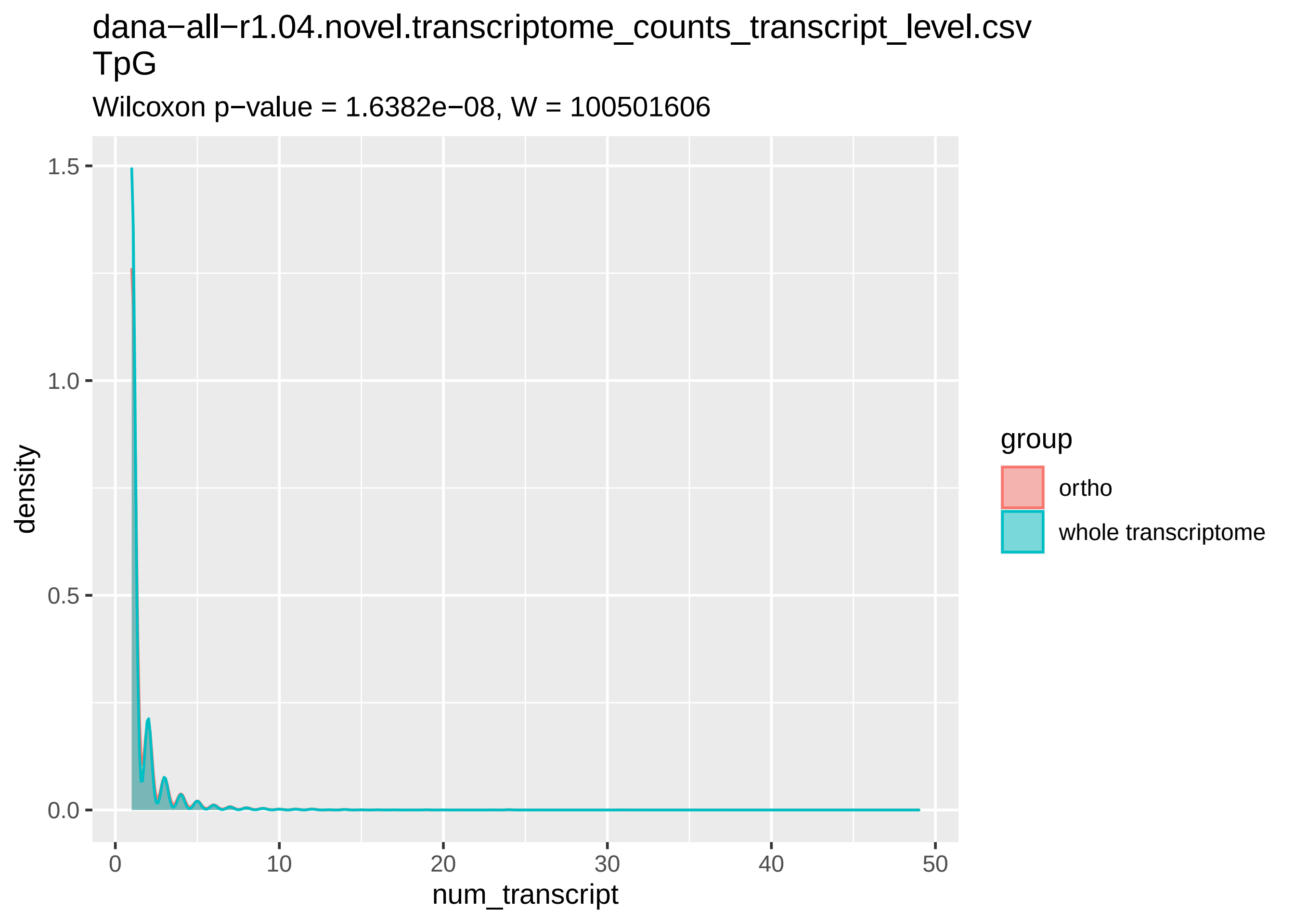
dana−all−r1.04.novel.transcriptome_counts_transcript_level.csv
TpG
Wilcoxon p−value = 1.6382e−08, W = 100501606
density
1.5
1.0
0.5
0.0
0
10
20
30
40
50
num_transcript
group
ortho
whole transcriptome

dere−all−r1.04.novel.transcriptome_counts_transcript_level.csv
TpG

Wilcoxon p−value = 7.7457e−07, W = 94359921

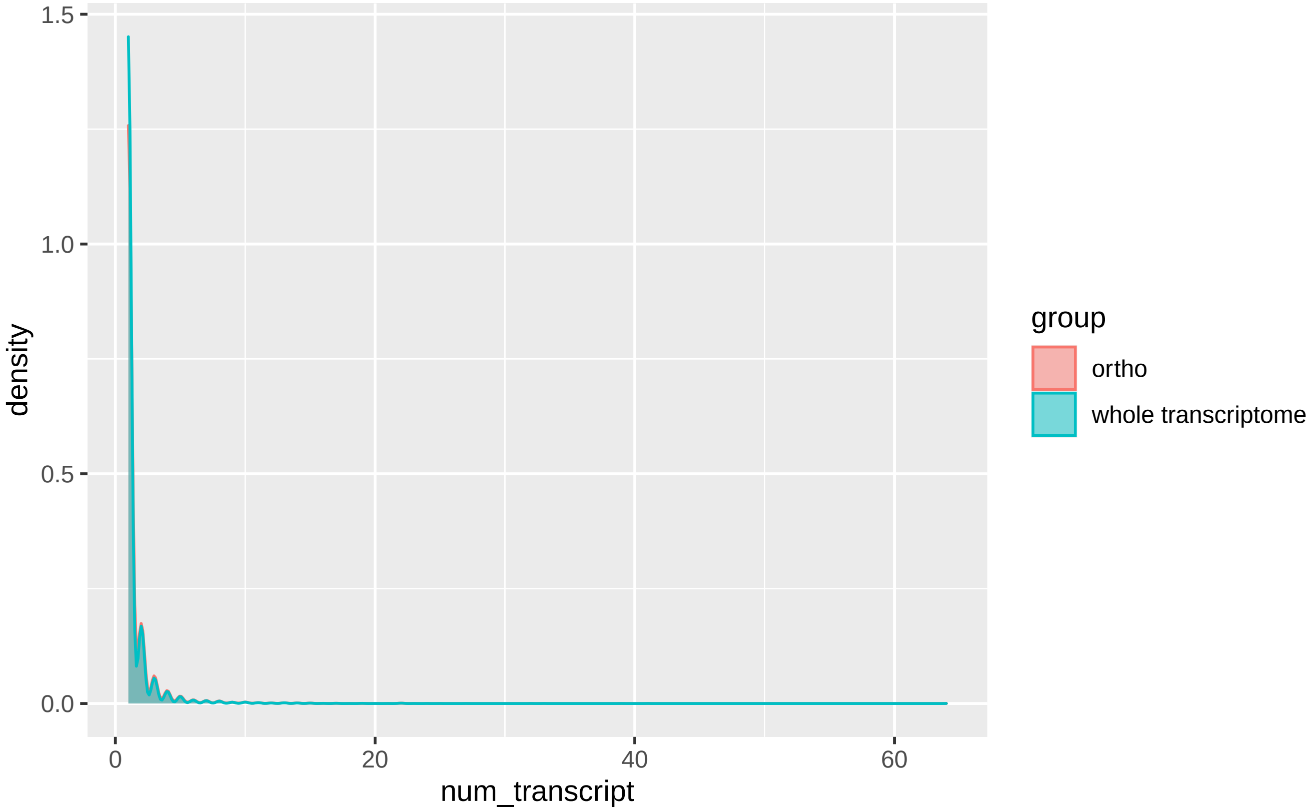

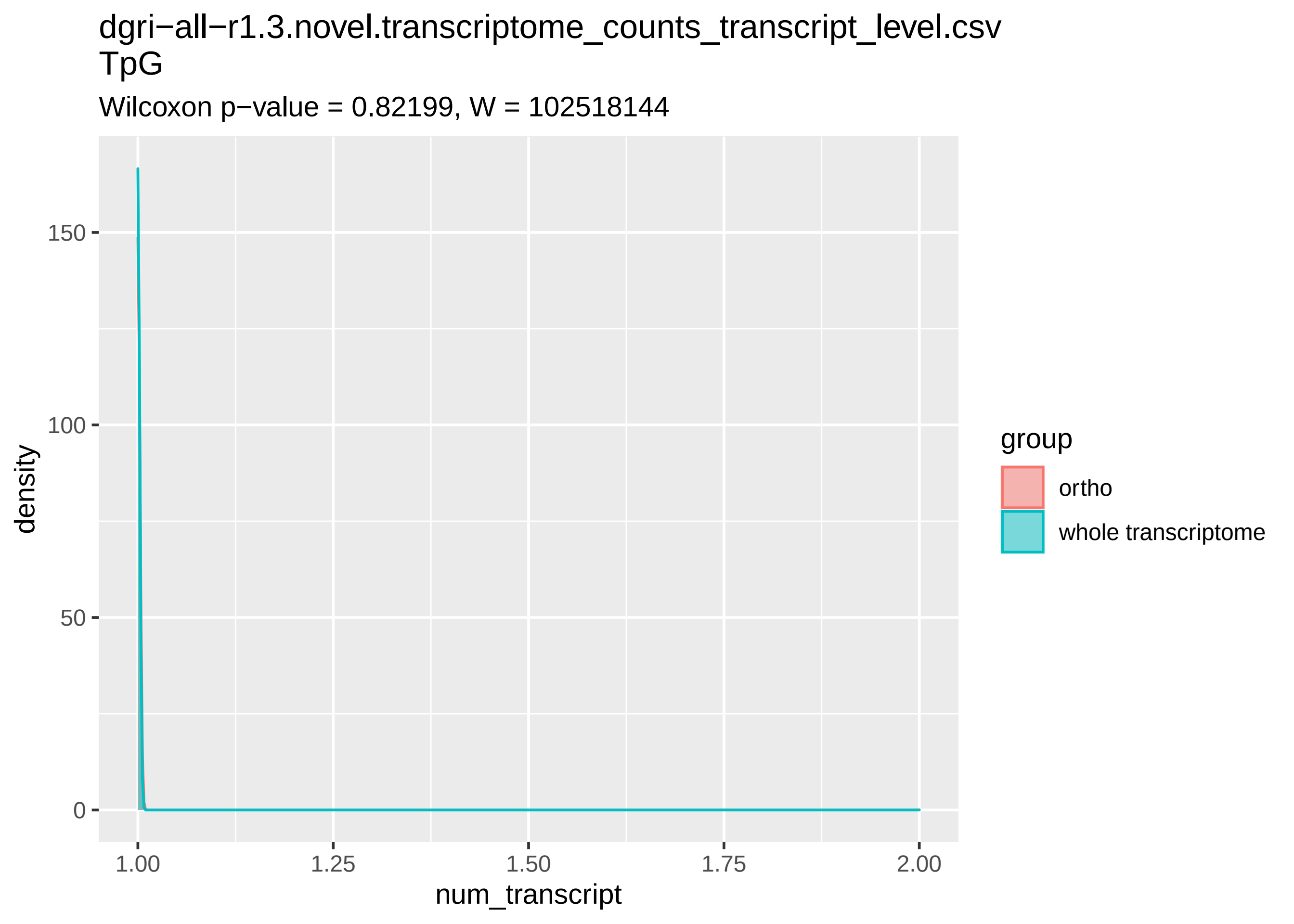
dgri−all−r1.3.novel.transcriptome_counts_transcript_level.csv
TpG
Wilcoxon p−value = 0.82199, W = 102518144
density
150
100
50
0
1.00
1.25
1.50
1.75
2.00
num_transcript
group
ortho
whole transcriptome

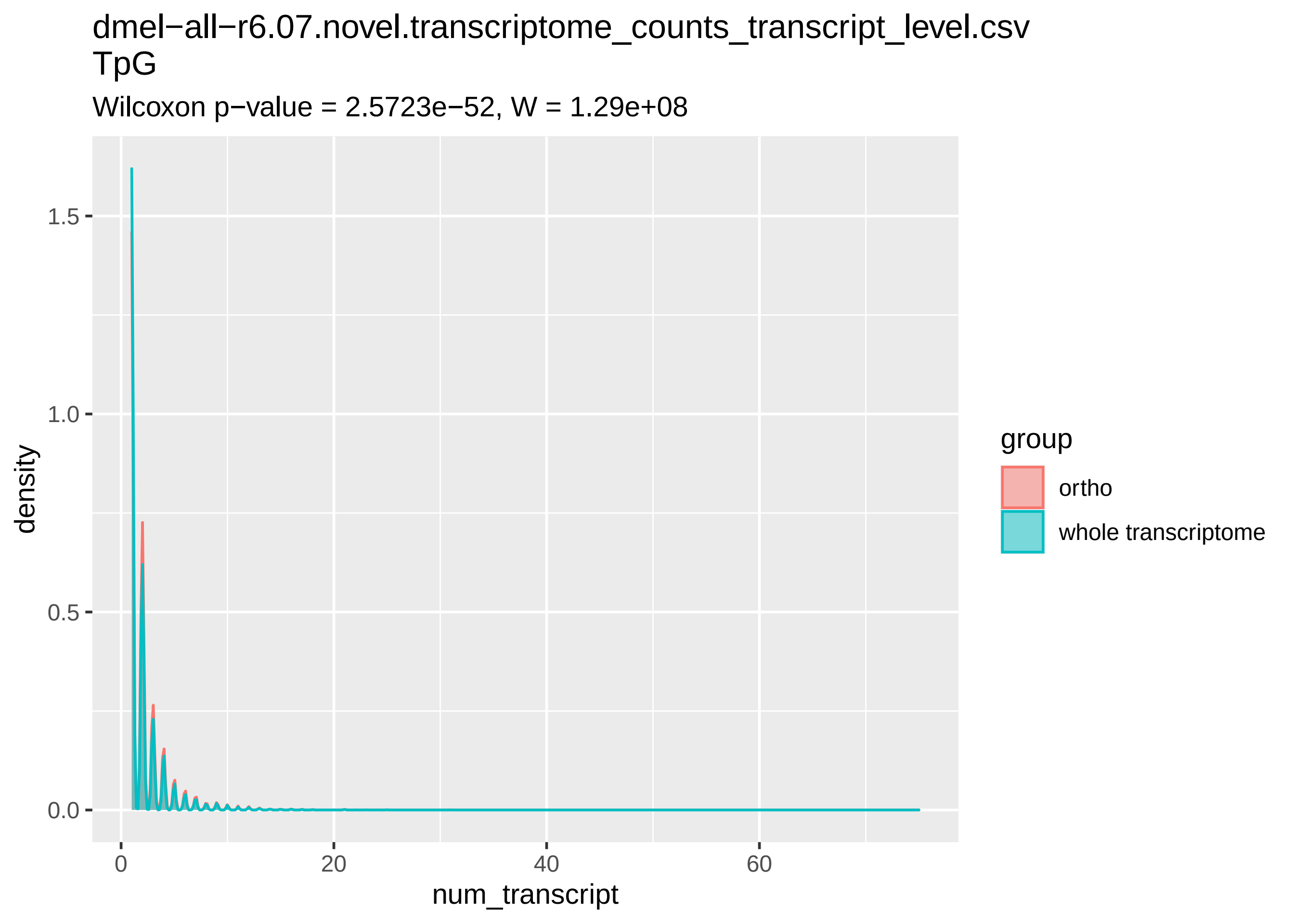
dmel−all−r6.07.novel.transcriptome_counts_transcript_level.csv
TpG
Wilcoxon p−value = 2.5723e−52, W = 1.29e+08
density
1.5
1.0
0.5
0.0
0
20
40
60
num_transcript
group
ortho
whole transcriptome

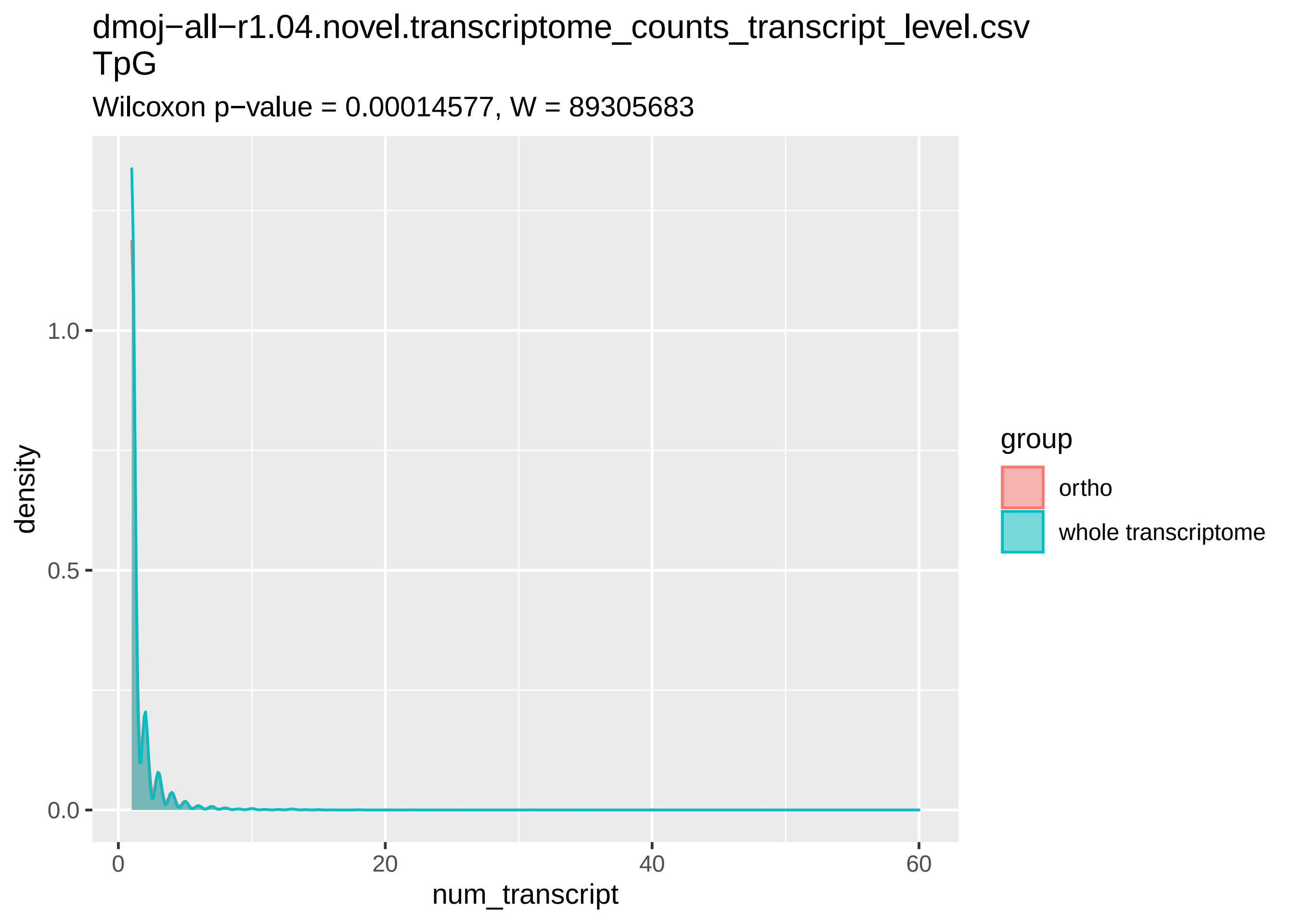
dmoj−all−r1.04.novel.transcriptome_counts_transcript_level.csv
TpG
Wilcoxon p−value = 0.00014577, W = 89305683
density
1.0
0.5
0.0
0
20
40
60
num_transcript
group
ortho
whole transcriptome

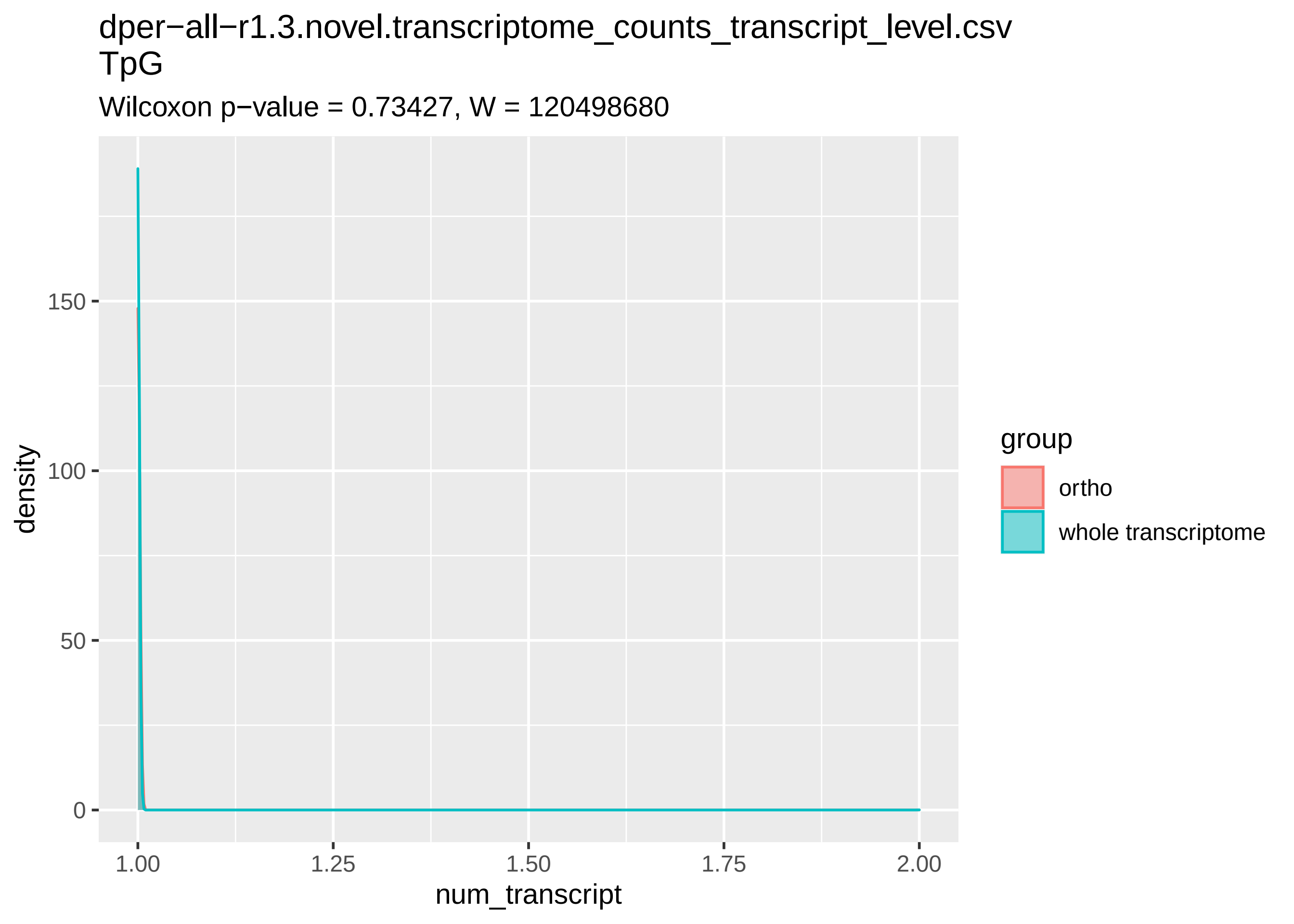

dper−all−r1.3.novel.transcriptome_counts_transcript_level.csv
TpG
Wilcoxon p−value = 0.73427, W = 120498680
density
150
100
50
0
1.00
1.25
1.50
1.75
2.00
num_transcript
group
ortho
whole transcriptome

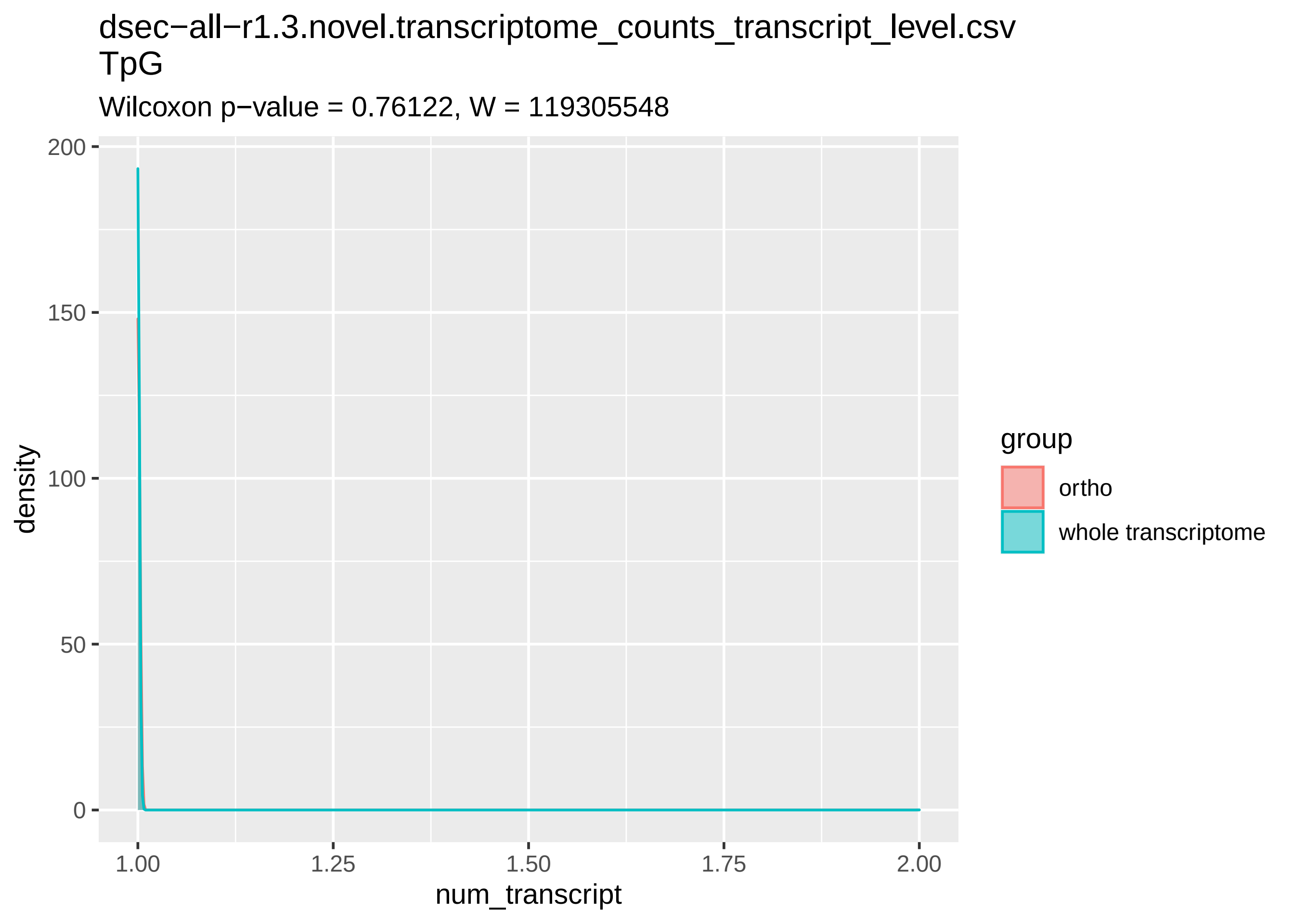
dsec−all−r1.3.novel.transcriptome_counts_transcript_level.csv
TpG
Wilcoxon p−value = 0.76122, W = 119305548
200
150
100
50
0
density
1.00
1.25
1.50
1.75
2.00
num_transcript
group
ortho
whole transcriptome

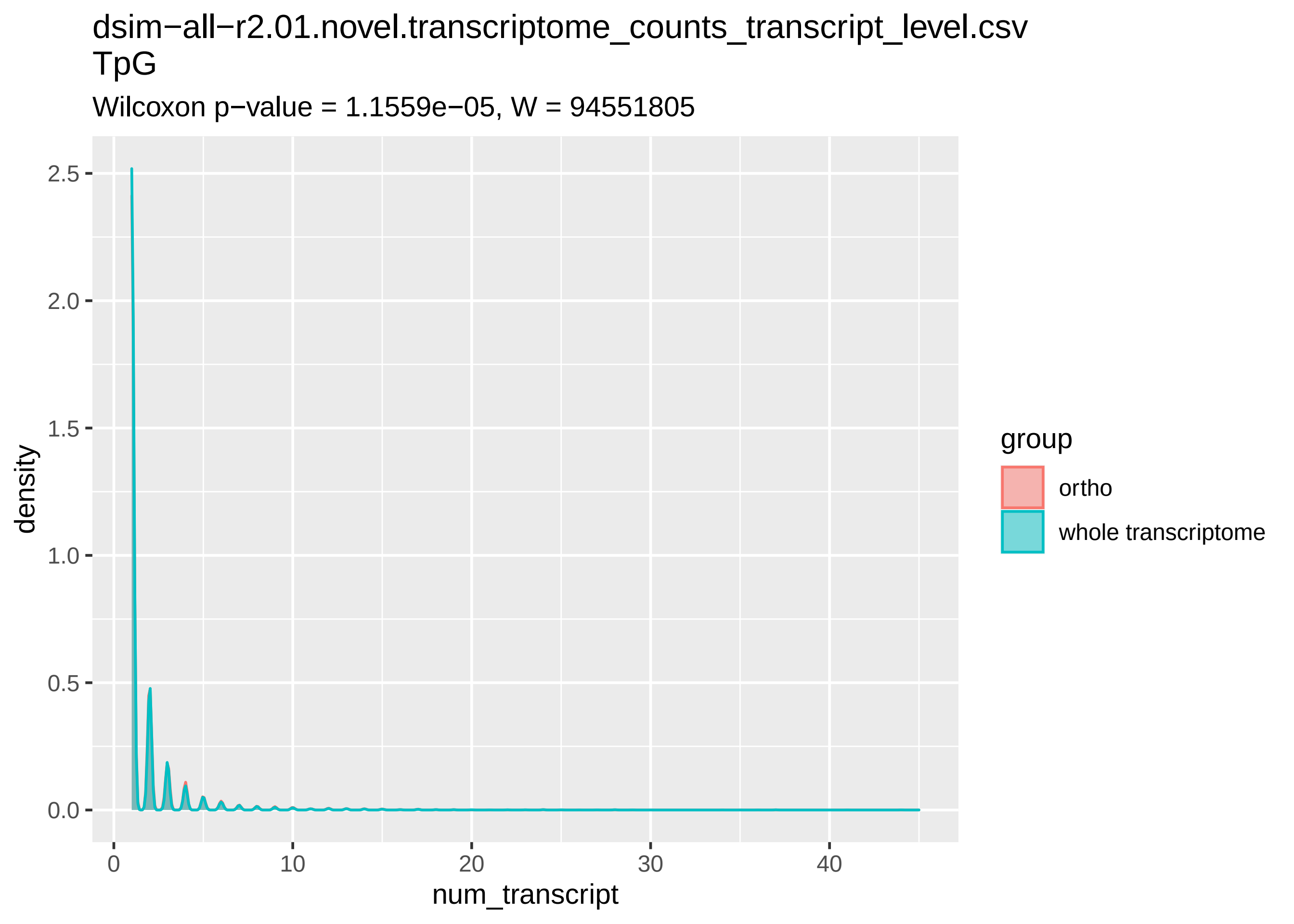
dsim−all−r2.01.novel.transcriptome_counts_transcript_level.csv
TpG
Wilcoxon p−value = 1.1559e−05, W = 94551805
density
num_transcript
group
ortho
whole transcriptome
0.0
0.5
1.0
1.5
2.0
2.5
0
10
20
30
40

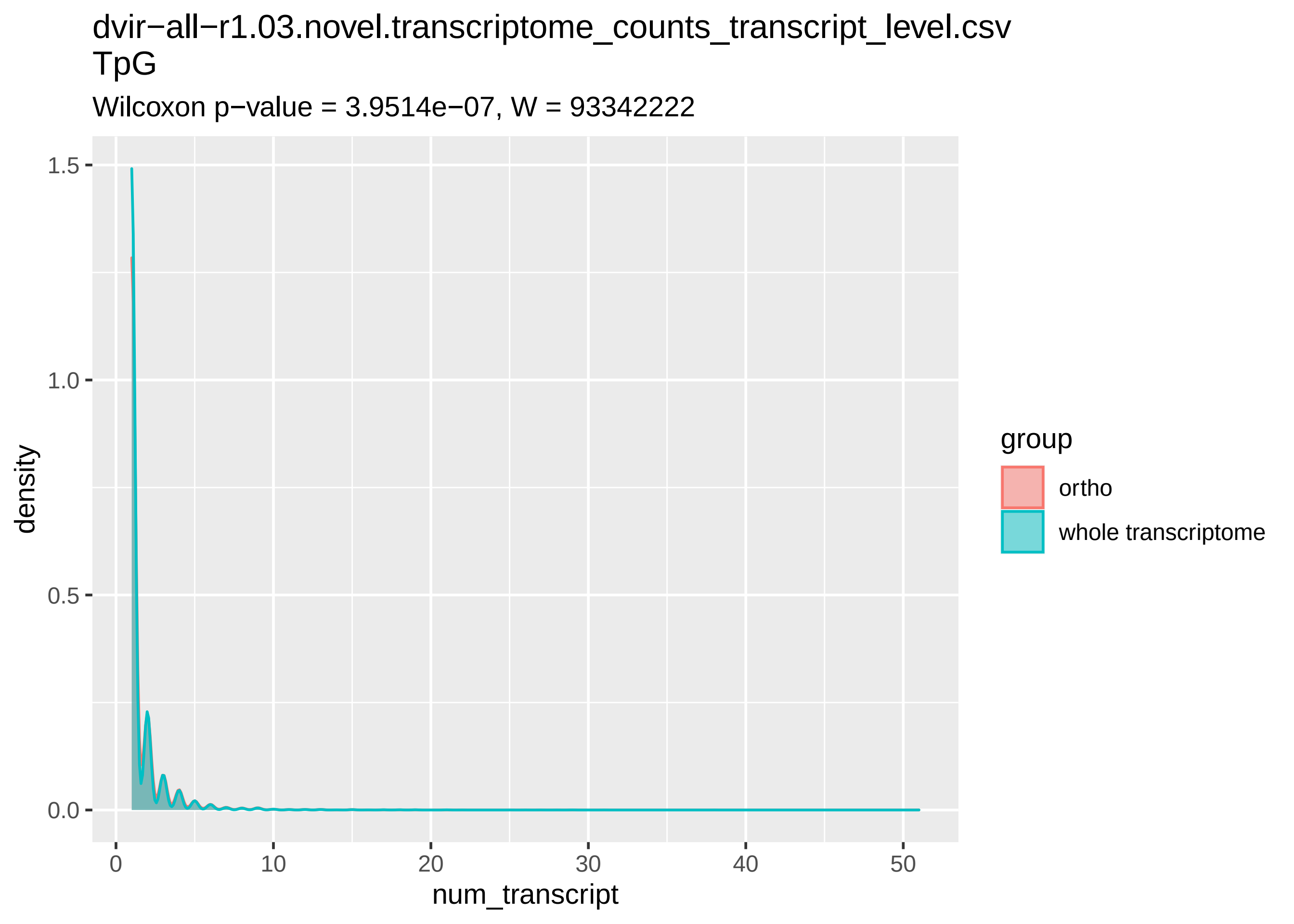
dvir−all−r1.03.novel.transcriptome_counts_transcript_level.csv
TpG
Wilcoxon p−value = 3.9514e−07, W = 93342222
density
1.5
1.0
0.5
0.0
0
10
20
30
40
50
num_transcript
group
ortho
whole transcriptome

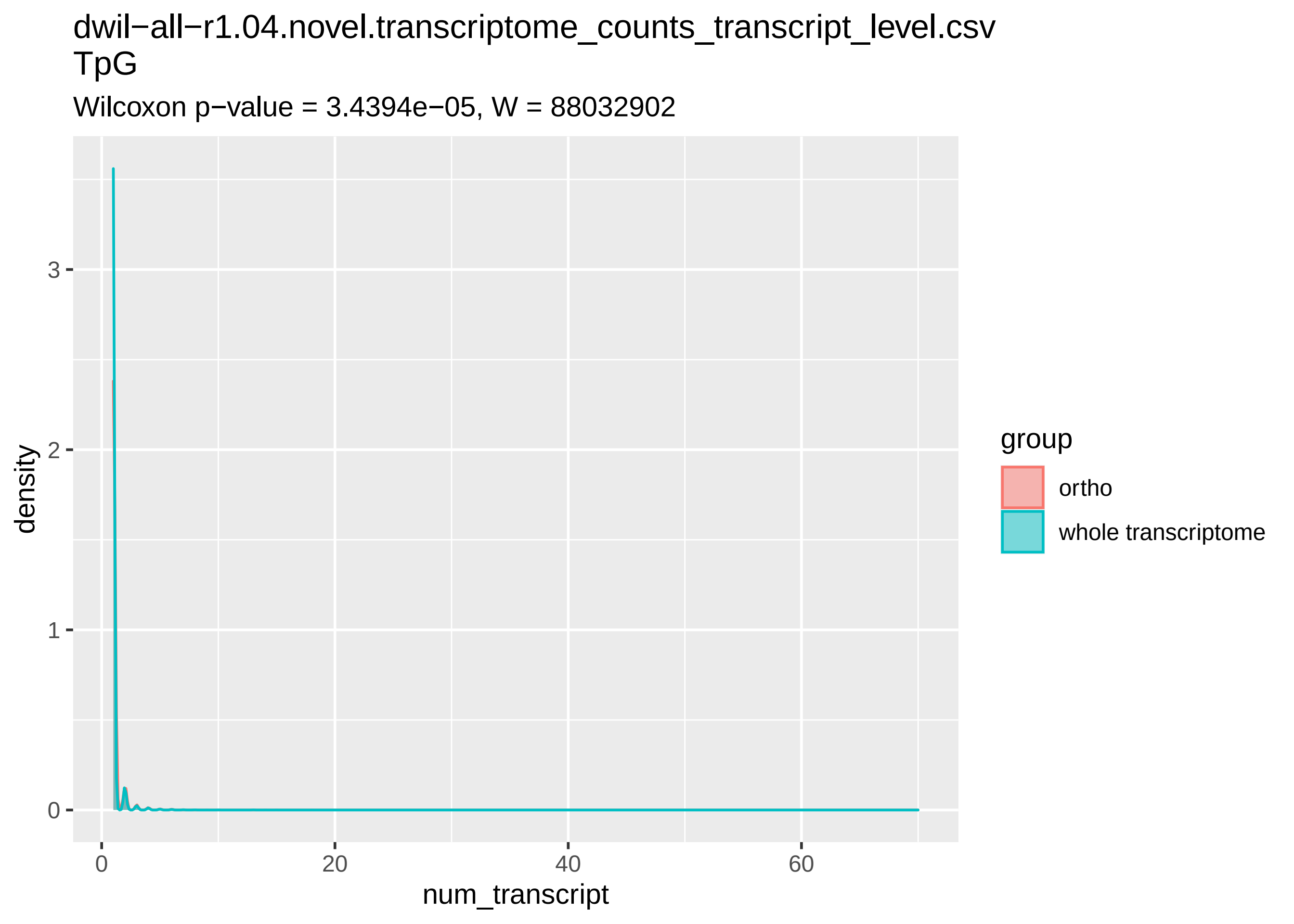
dwil−all−r1.04.novel.transcriptome_counts_transcript_level.csv
TpG
Wilcoxon p−value = 3.4394e−05, W = 88032902
density
3
2
1
0
0
20
40
60
num_transcript
group
ortho
whole transcriptome

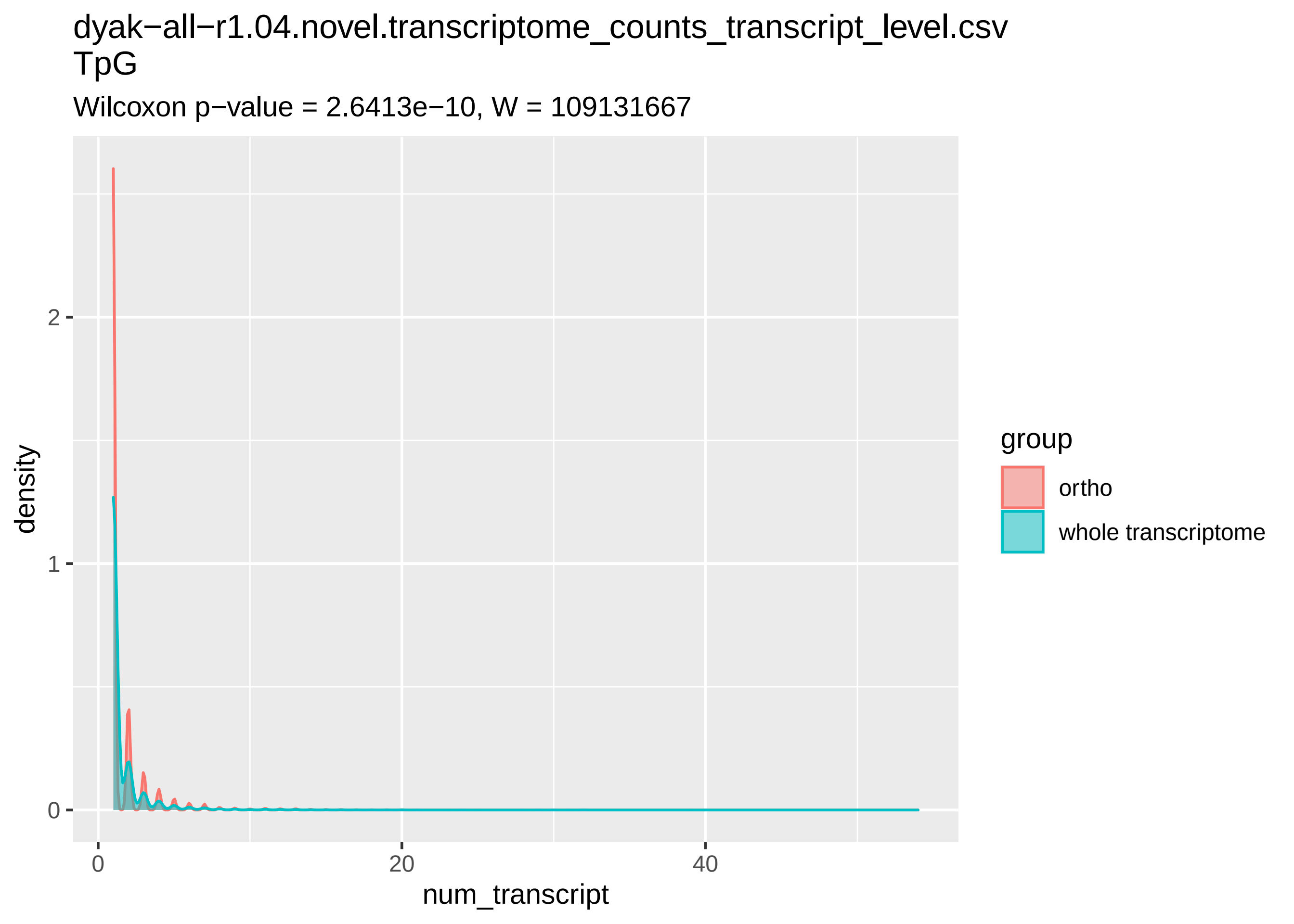
dyak−all−r1.04.novel.transcriptome_counts_transcript_level.csv
TpG
Wilcoxon p−value = 2.6413e−10, W = 109131667
density
2
1
0
0
20
40
num_transcript
group
ortho
whole transcriptome

dana−all−r1.04.novel.transcriptome_counts_transcript_level.csv
EpT

Wilcoxon p−value = 3.6461e−55, W = 242074453

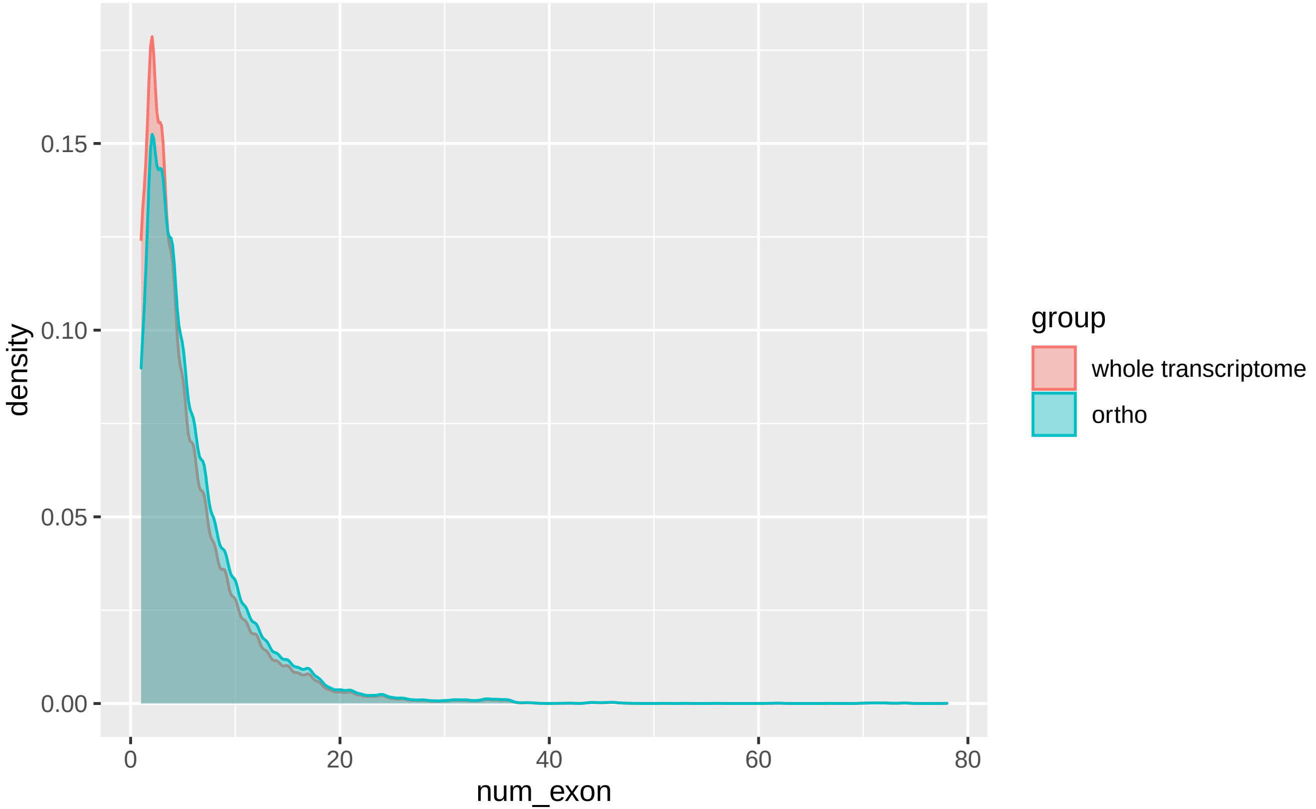

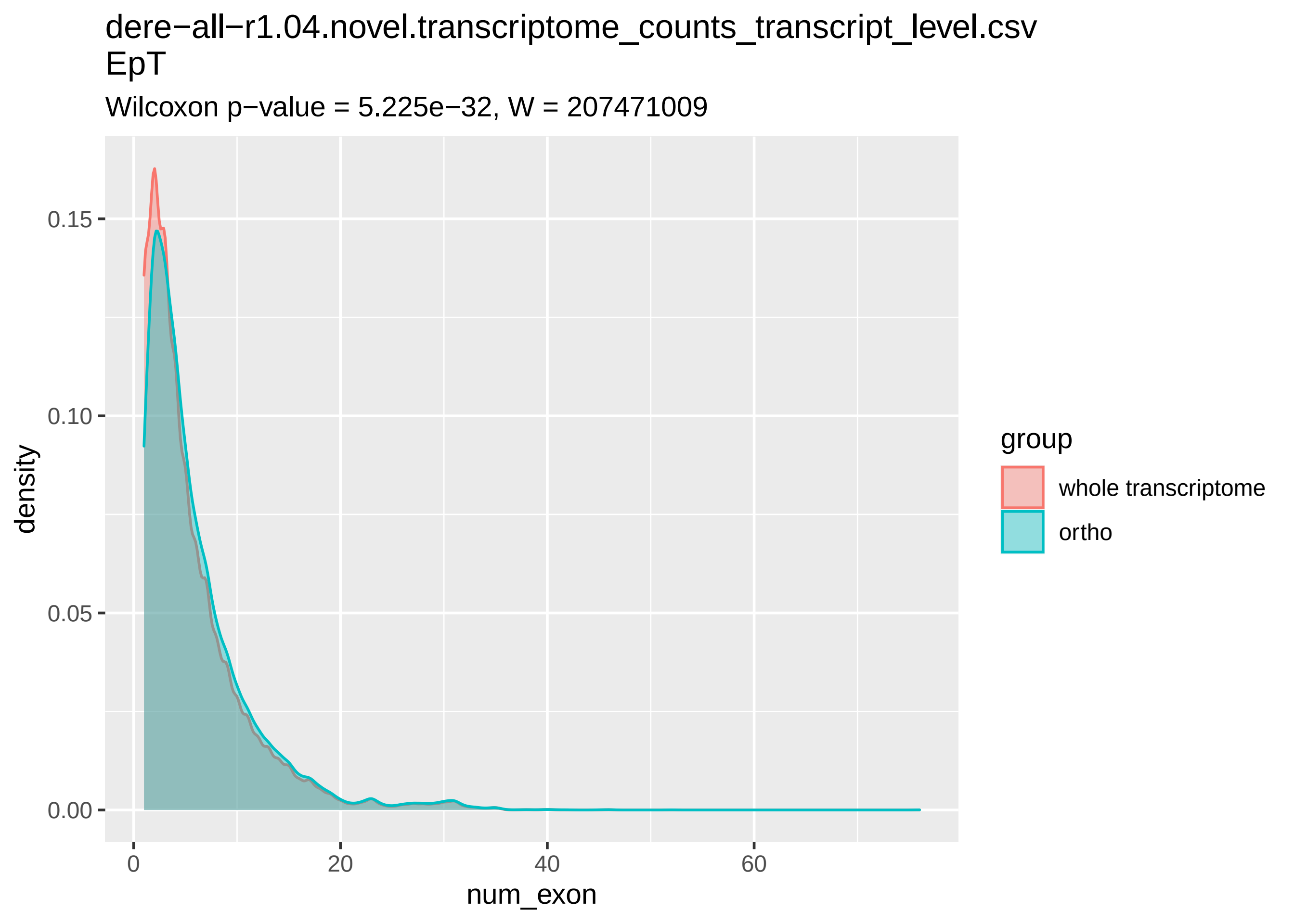
dere−all−r1.04.novel.transcriptome_counts_transcript_level.csv
EpT
Wilcoxon p−value = 5.225e−32, W = 207471009
density
0.15
0.10
0.05
0.00
0
20
40
60
num_exon
group
whole transcriptome
ortho

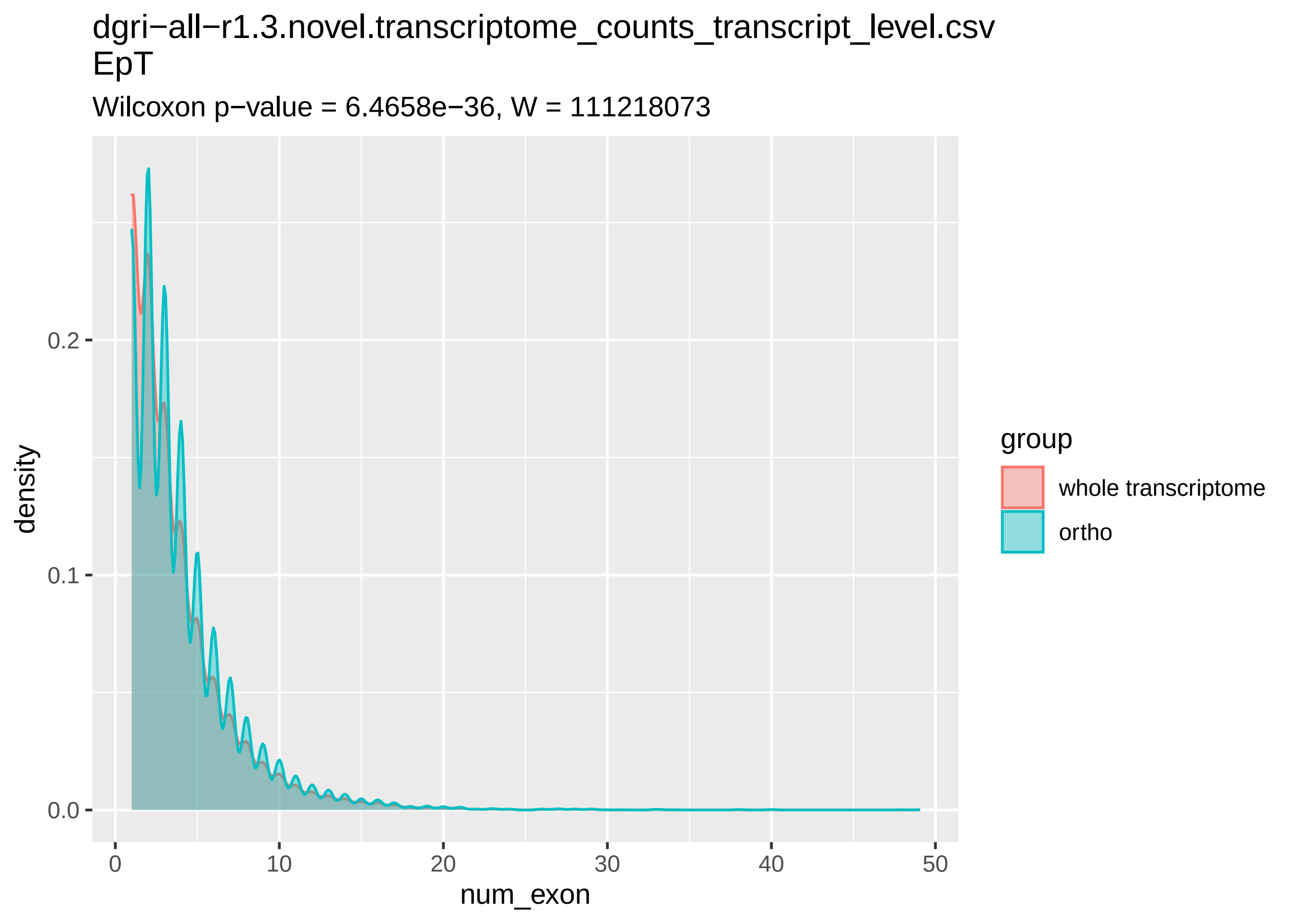
dgri−all−r1.3.novel.transcriptome_counts_transcript_level.csv
EpT
Wilcoxon p−value = 6.4658e−36, W = 111218073
density
0.0
0.1
0.2
0
10
20
30
40
50
num_exon
group
whole transcriptome
ortho

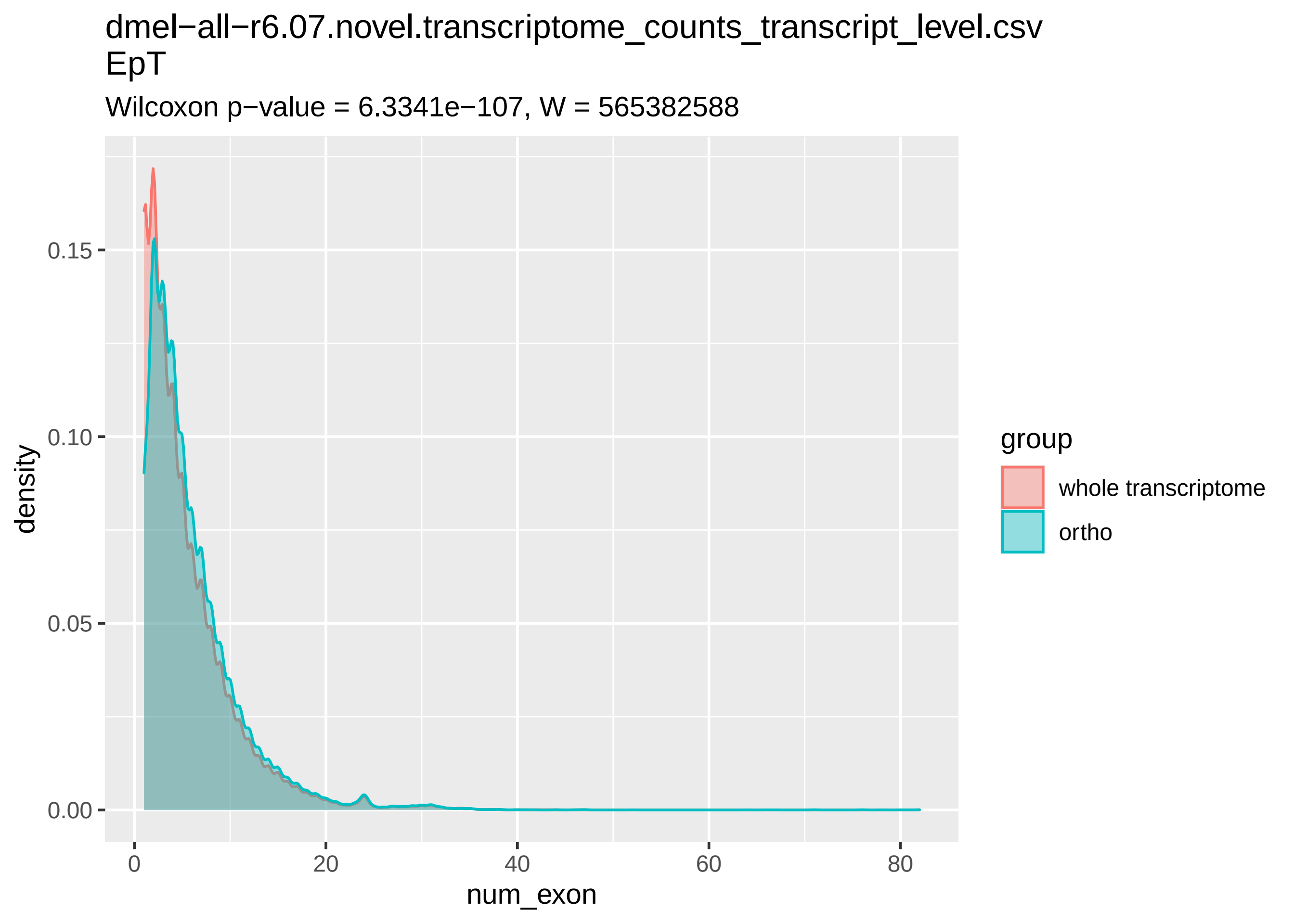
dmel−all−r6.07.novel.transcriptome_counts_transcript_level.csv
EpT
Wilcoxon p−value = 6.3341e−107, W = 565382588
density
0.00
0.05
0.10
0.15
0
20
40
60
80
num_exon
group
whole transcriptome
ortho

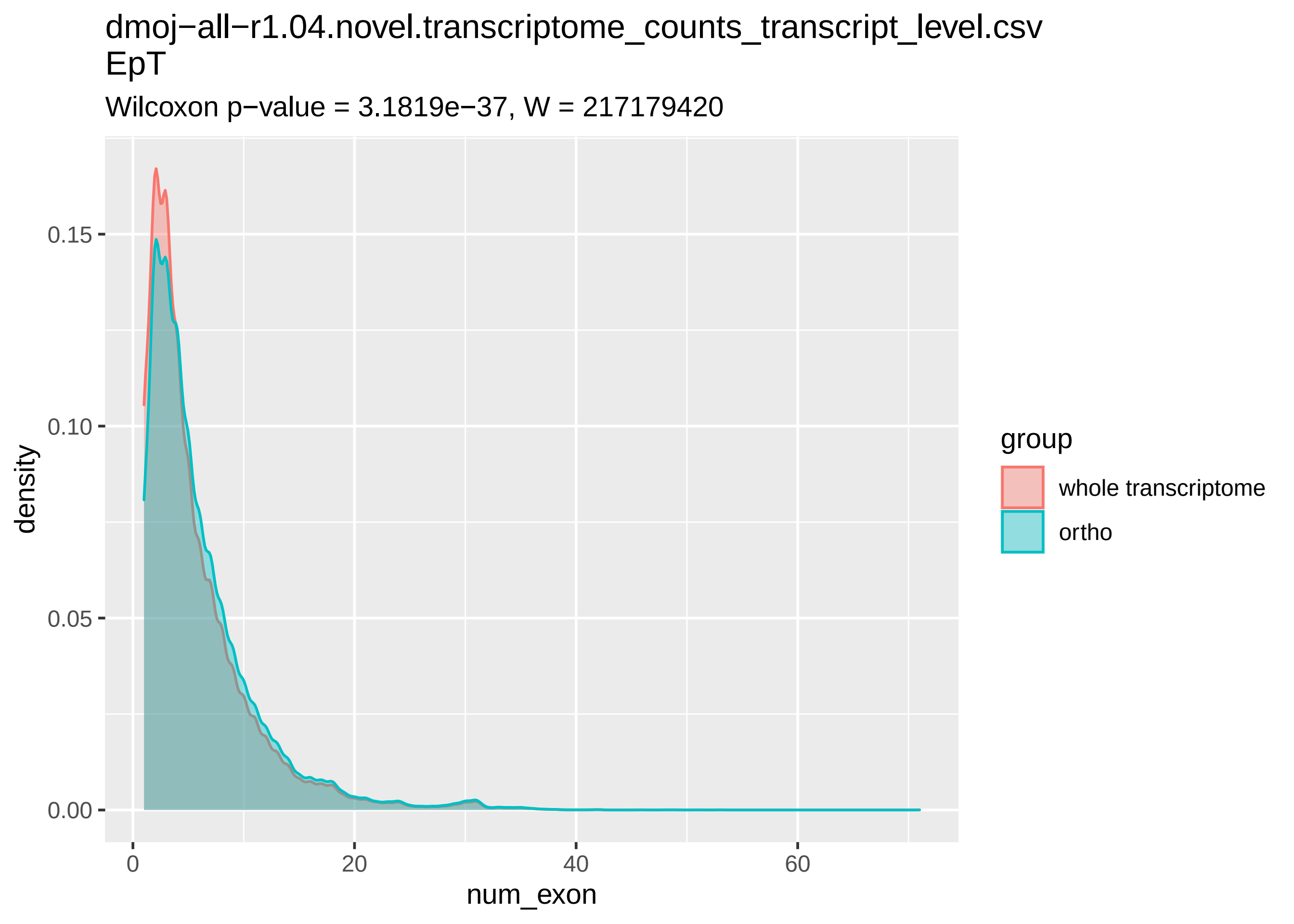

dmoj−all−r1.04.novel.transcriptome_counts_transcript_level.csv
EpT
Wilcoxon p−value = 3.1819e−37, W = 217179420
density
0.15
0.10
0.05
0.00
0
20
40
60
num_exon
group
whole transcriptome
ortho

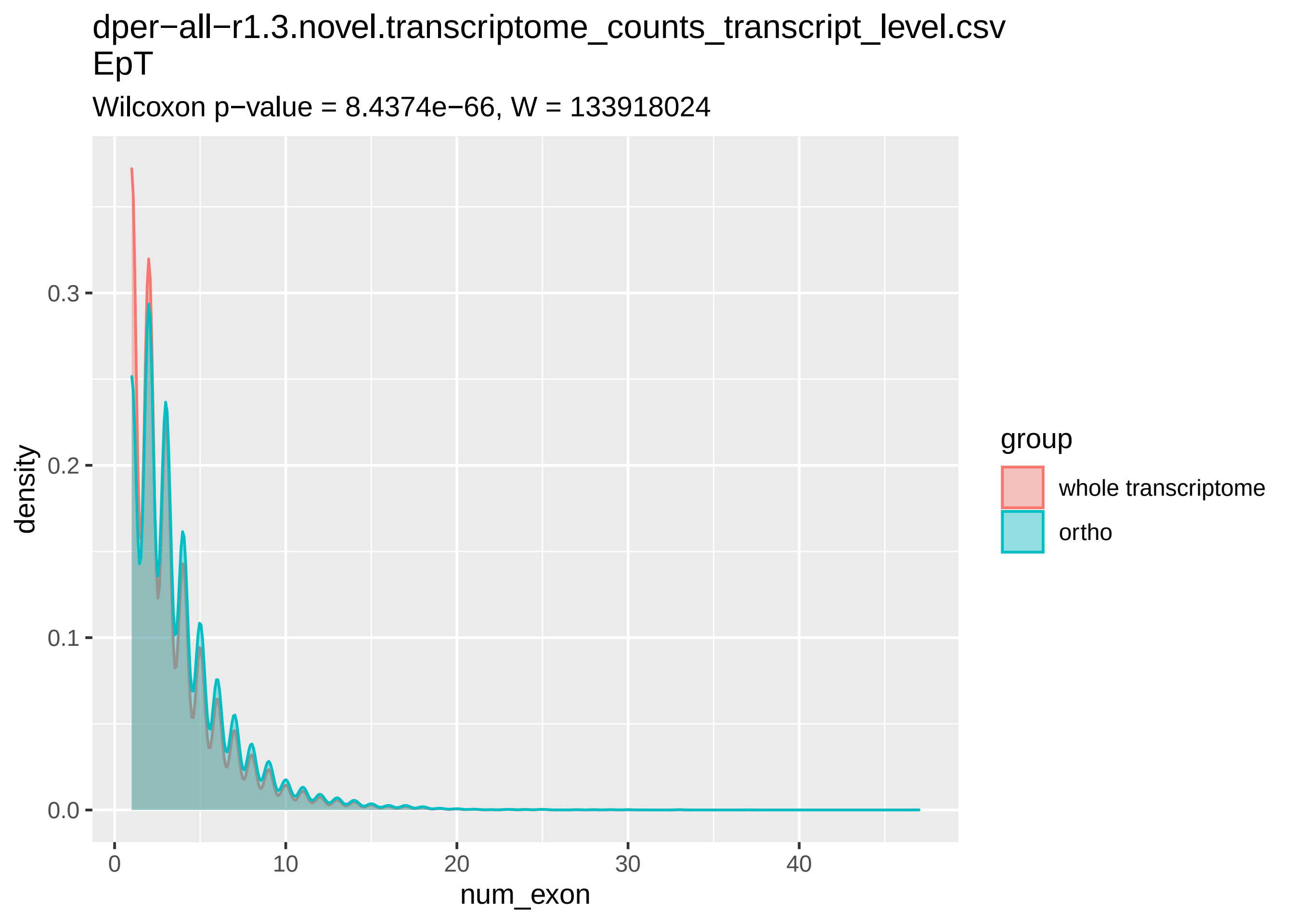
dper−all−r1.3.novel.transcriptome_counts_transcript_level.csv
EpT
Wilcoxon p−value = 8.4374e−66, W = 133918024
density
0.0
0.1
0.2
0.3
0
10
20
30
40
num_exon
group
whole transcriptome
ortho

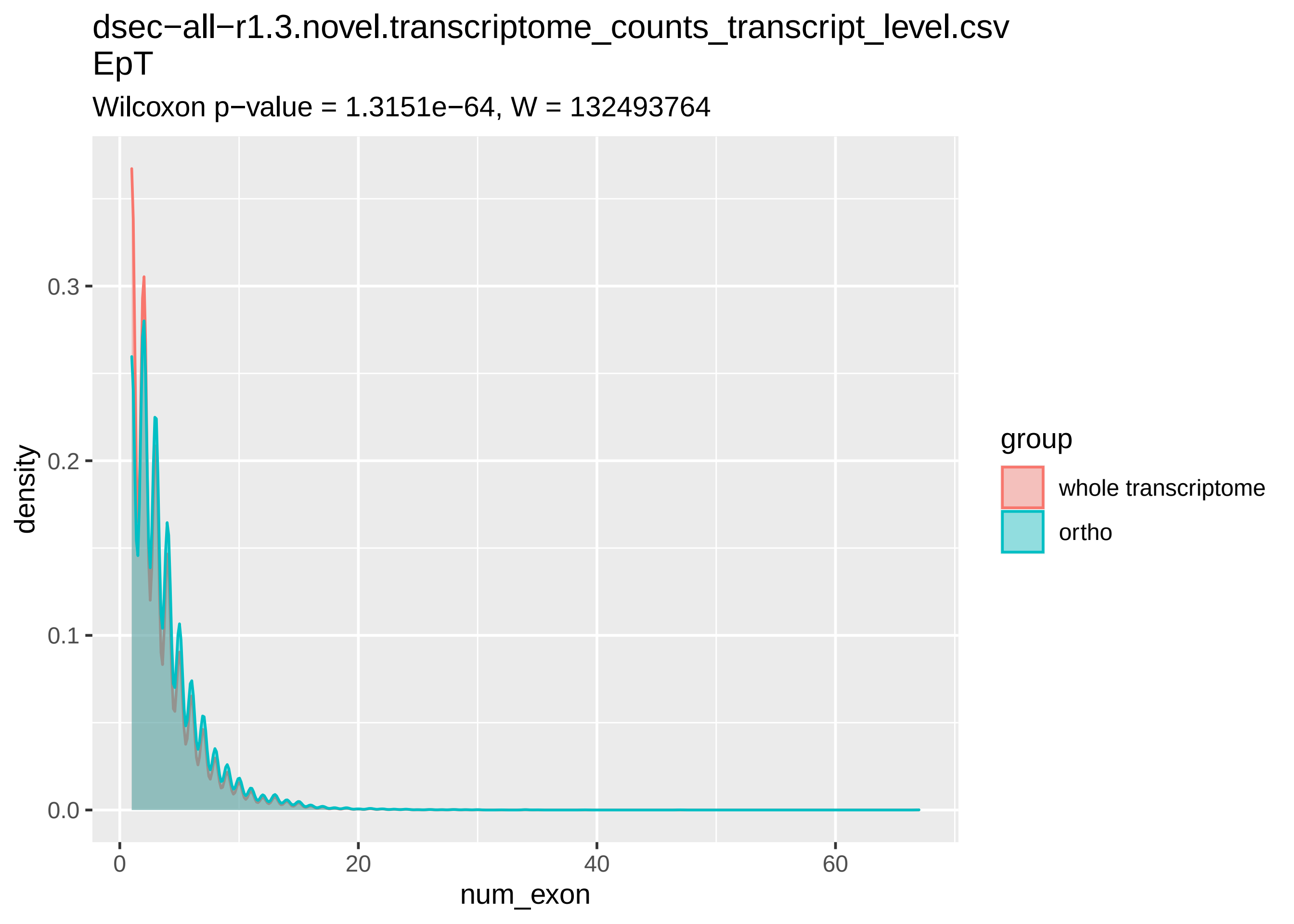
dsec−all−r1.3.novel.transcriptome_counts_transcript_level.csv
EpT
Wilcoxon p−value = 1.3151e−64, W = 132493764
density
0.0
0.1
0.2
0.3
0
20
40
60
num_exon
group
whole transcriptome
ortho

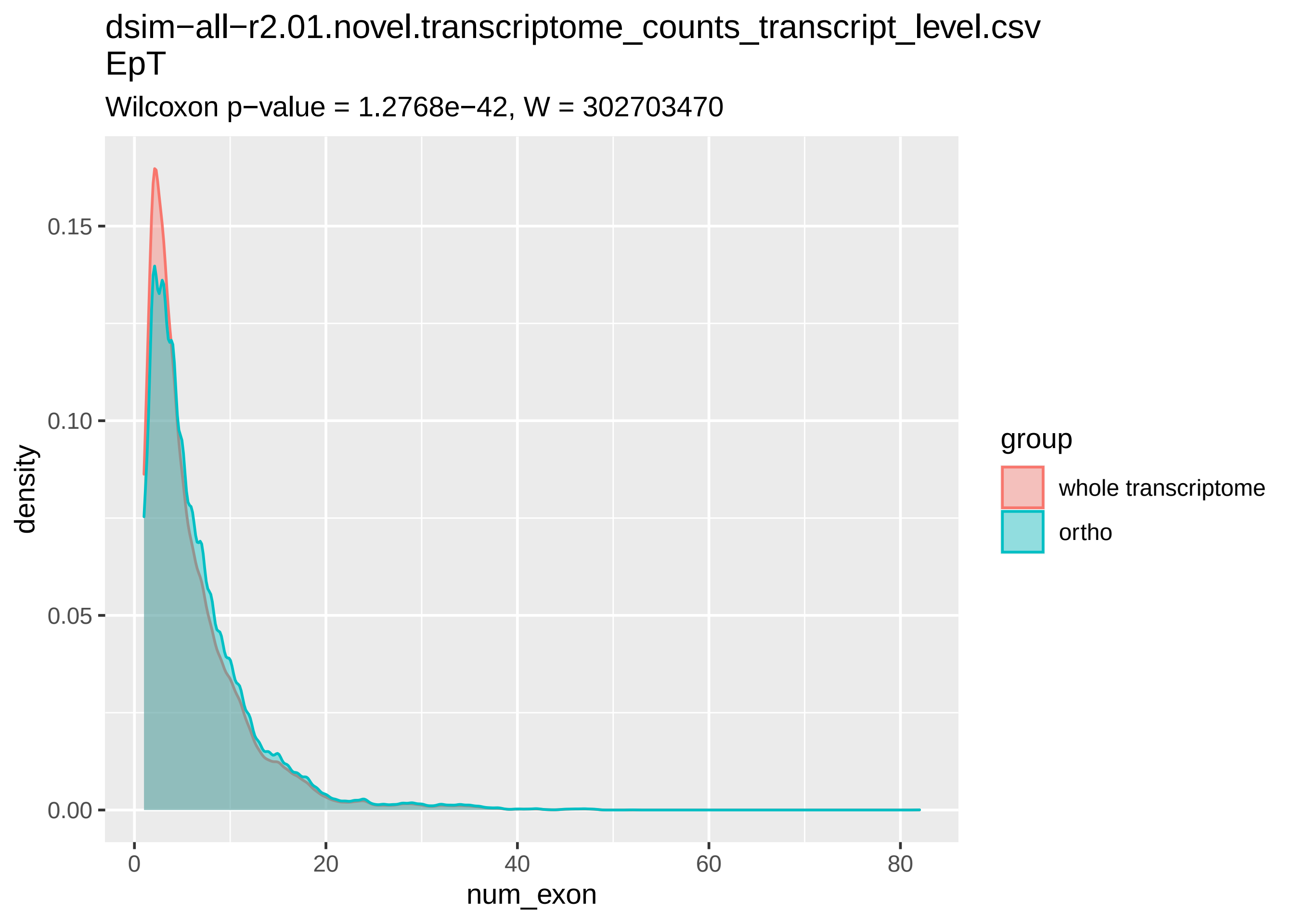
dsim−all−r2.01.novel.transcriptome_counts_transcript_level.csv
EpT
Wilcoxon p−value = 1.2768e−42, W = 302703470
density
0.15
0.10
0.05
0.00
0
20
40
60
80
num_exon
group
whole transcriptome
ortho

dvir−all−r1.03.novel.transcriptome_counts_transcript_level.csv
EpT

Wilcoxon p−value = 8.2806e−49, W = 230739586

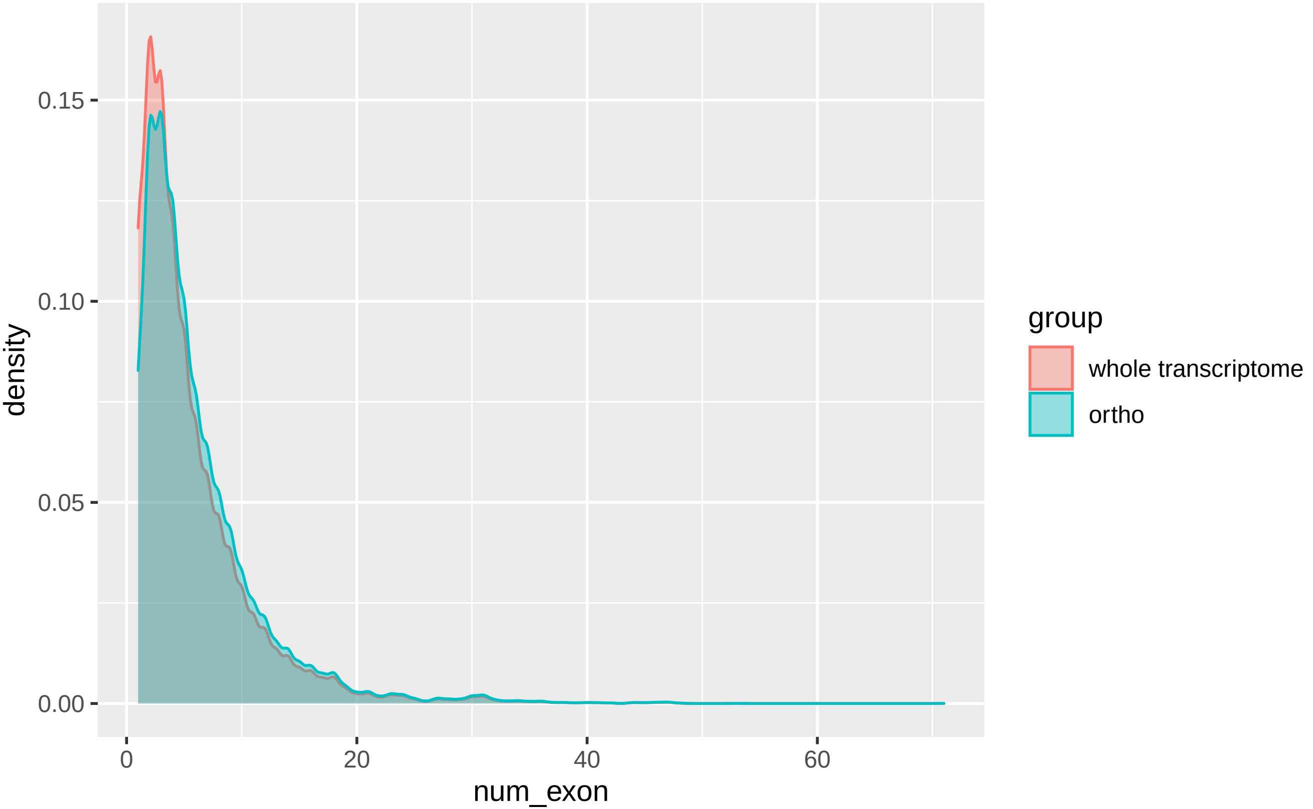

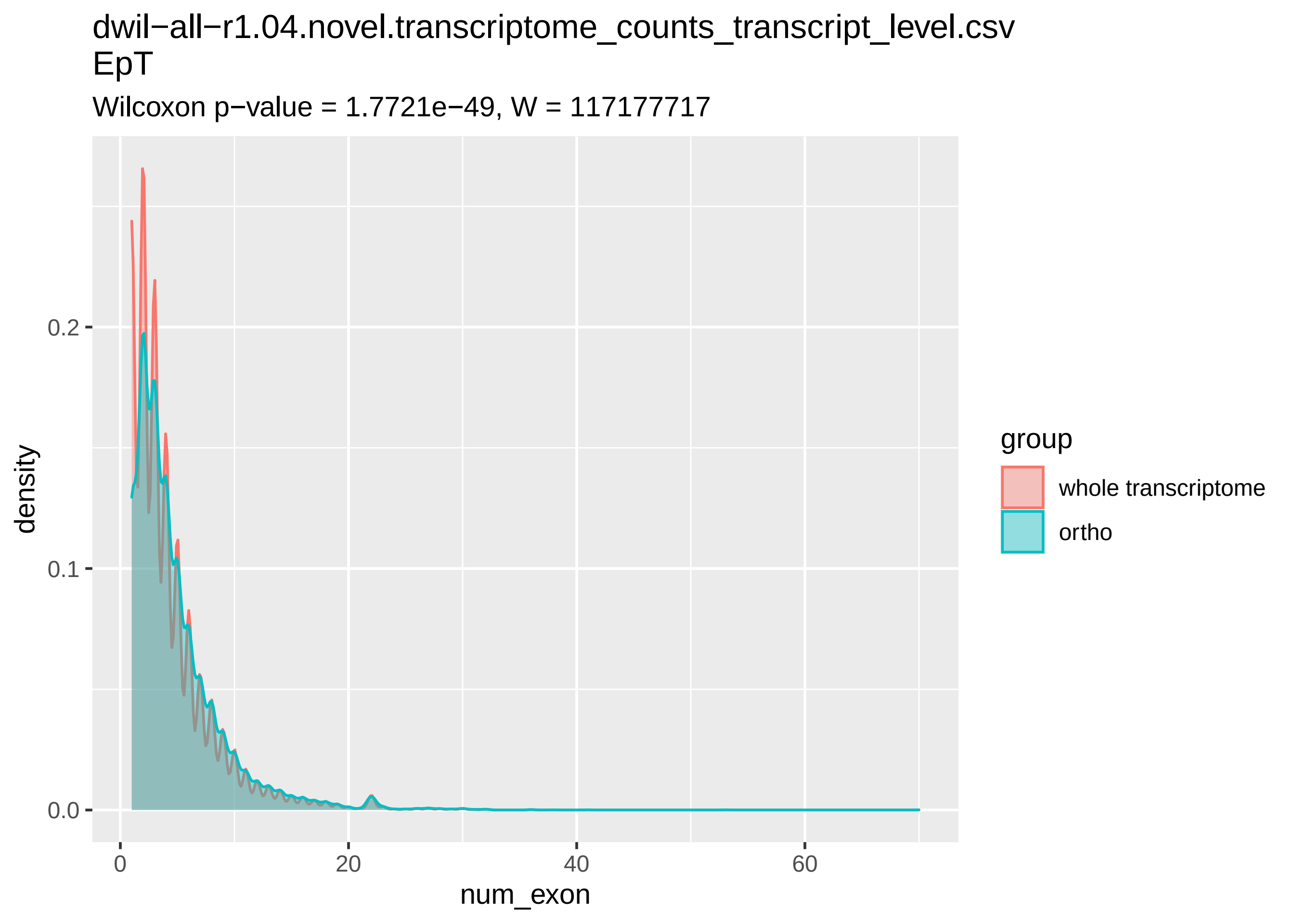
dwil−all−r1.04.novel.transcriptome_counts_transcript_level.csv
EpT
Wilcoxon p−value = 1.7721e−49, W = 117177717
density
0.2
0.1
0.0
0
20
40
60
num_exon
group
whole transcriptome
ortho

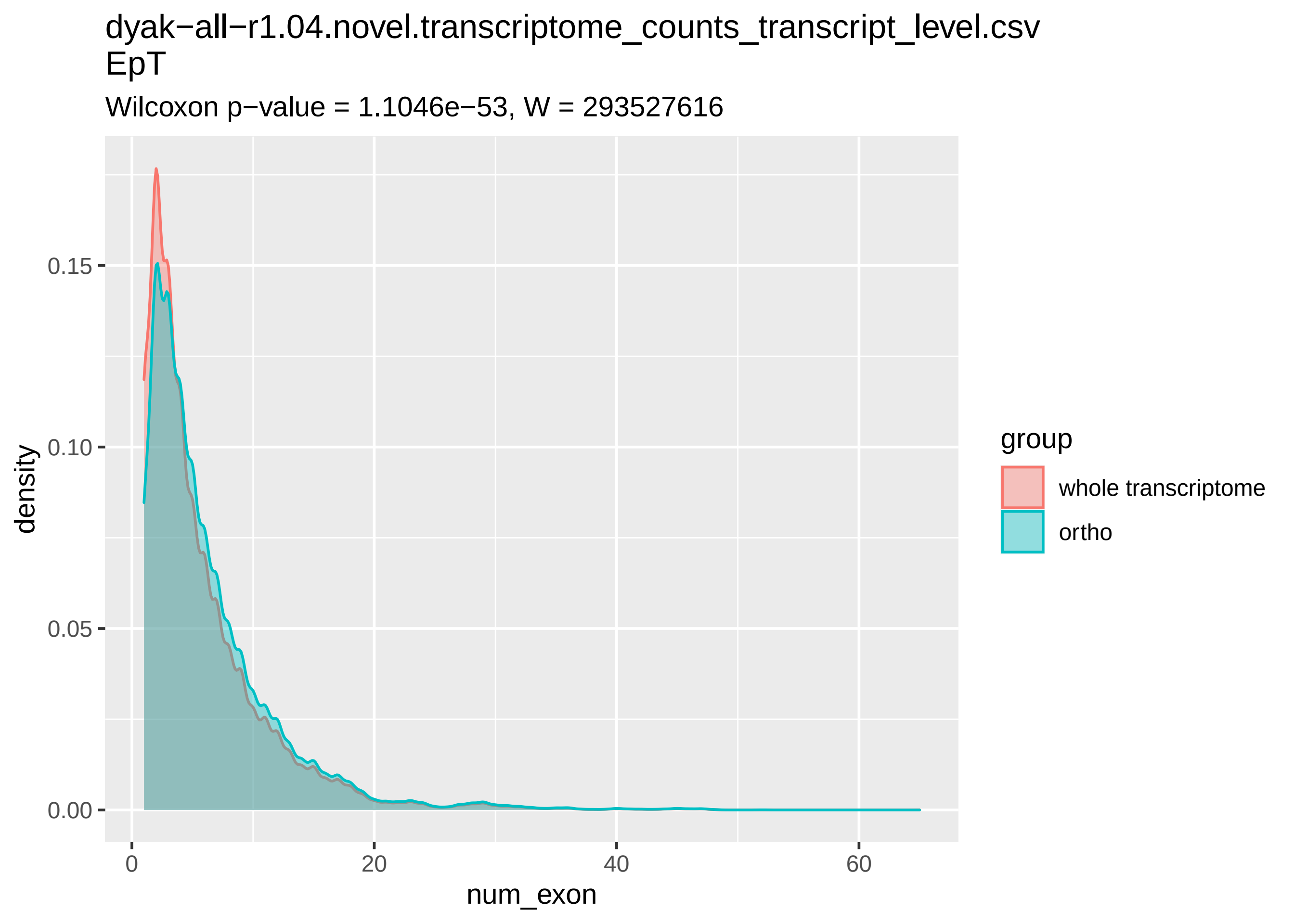
dyak−all−r1.04.novel.transcriptome_counts_transcript_level.csv
EpT
Wilcoxon p−value = 1.1046e−53, W = 293527616
density
0.15
0.10
0.05
0.00
0
20
40
60
num_exon
group
whole transcriptome
ortho

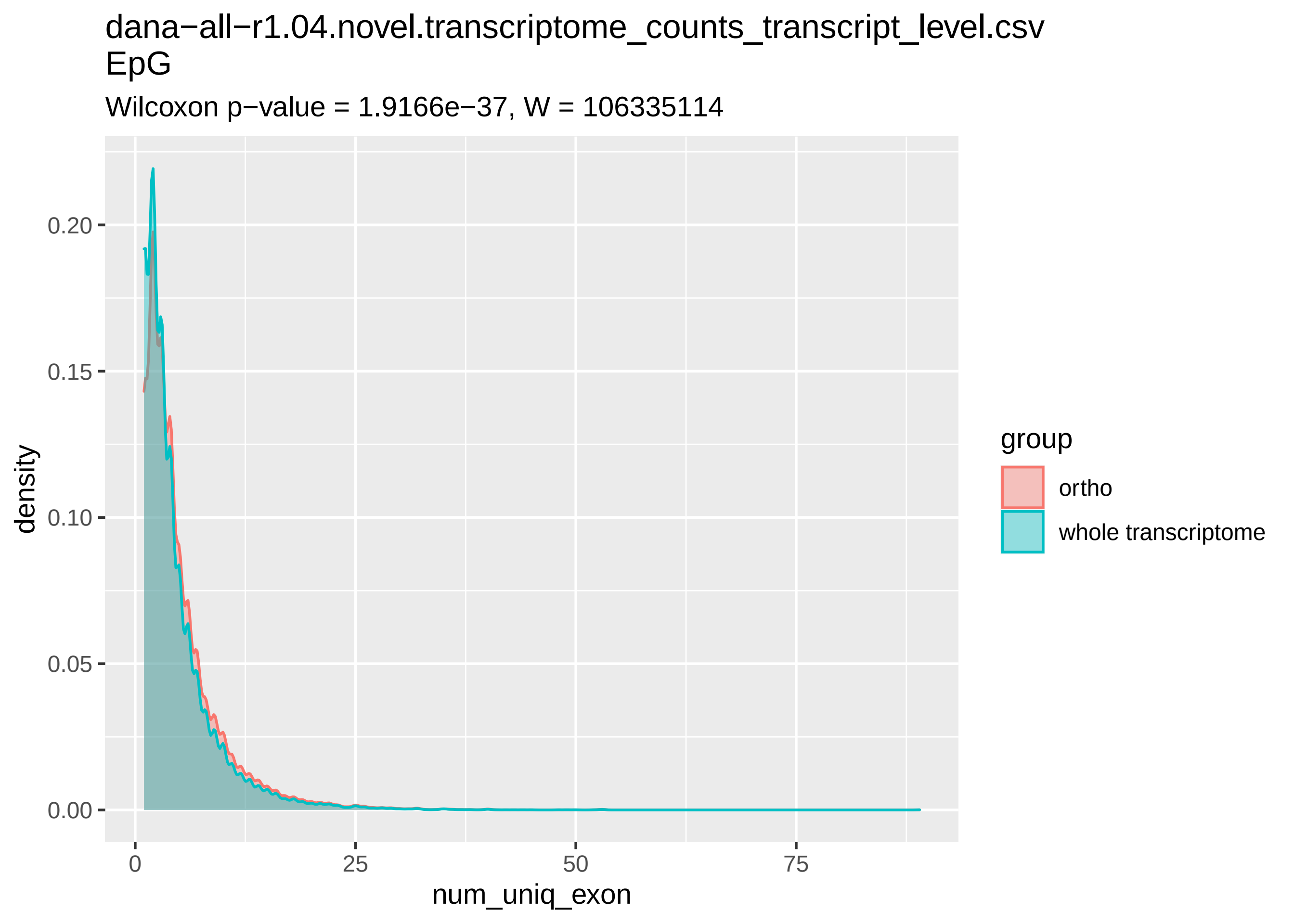
dana−all−r1.04.novel.transcriptome_counts_transcript_level.csv
EpG
Wilcoxon p−value = 1.9166e−37, W = 106335114
density
0.00
0.05
0.10
0.15
0.20
0
25
50
75
num_uniq_exon
group
ortho
whole transcriptome

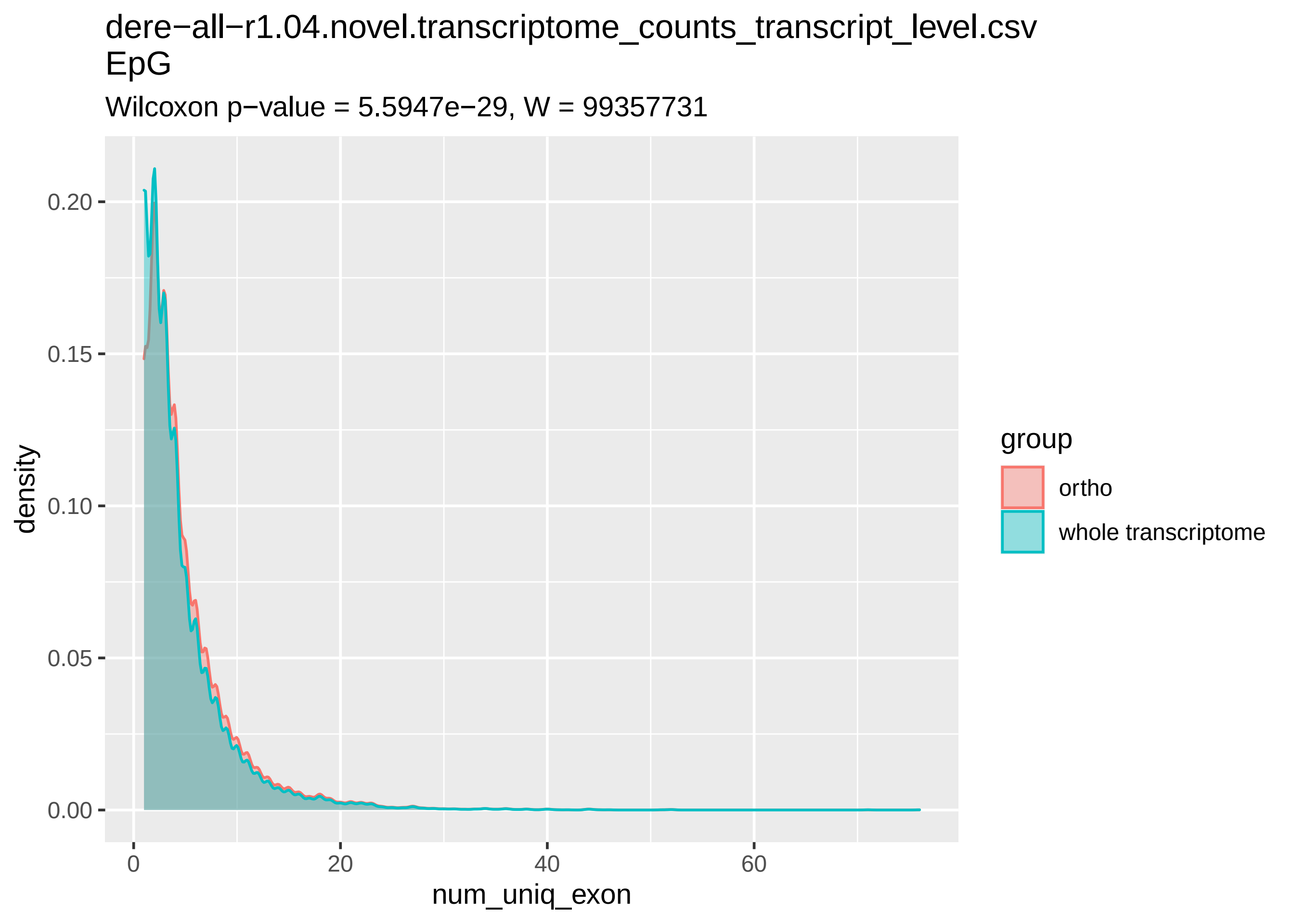

dere−all−r1.04.novel.transcriptome_counts_transcript_level.csv
EpG
Wilcoxon p−value = 5.5947e−29, W = 99357731
0.20
0.15
0.10
0.05
0.00
density
0
20
40
60
num_uniq_exon
group
ortho
whole transcriptome

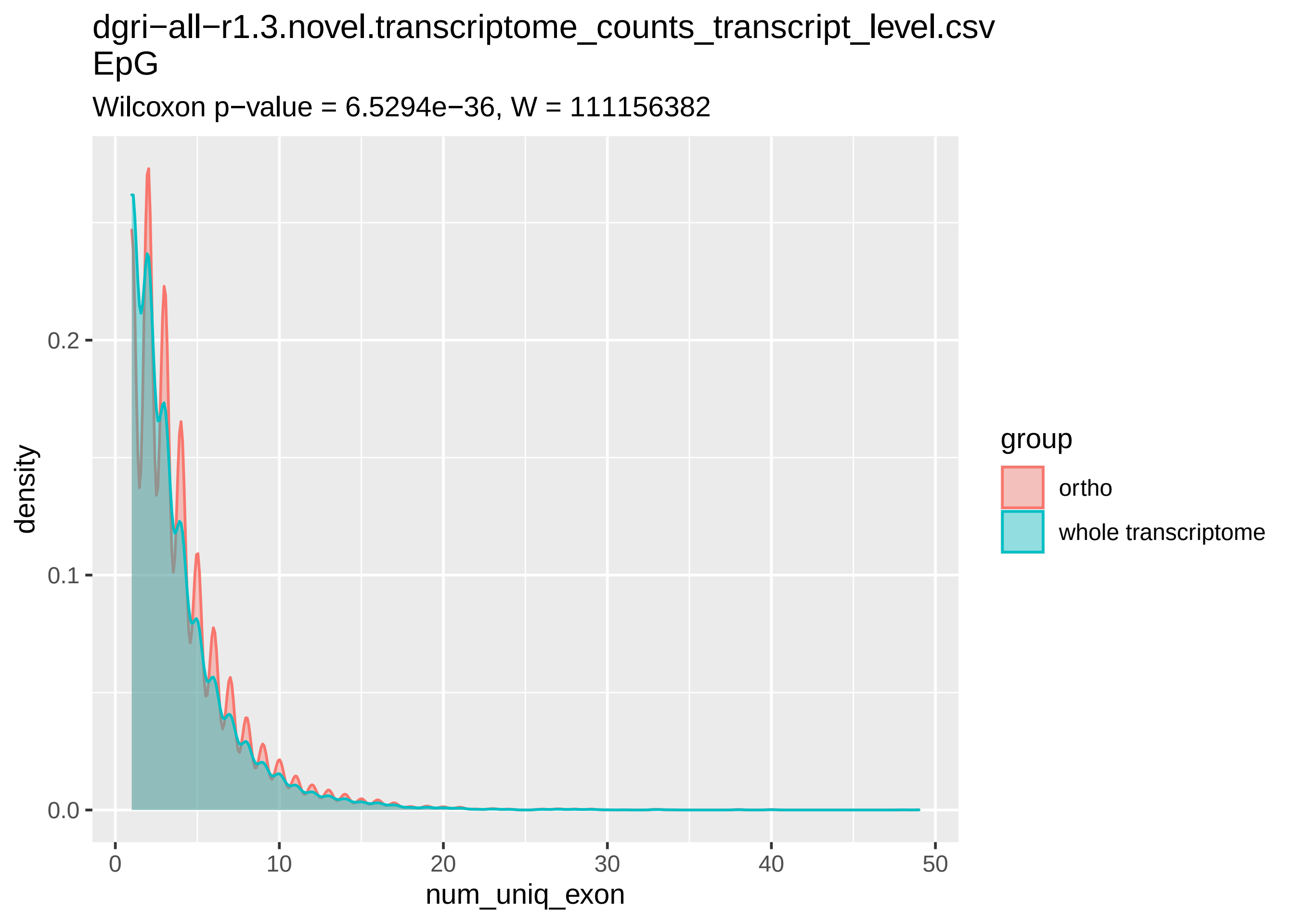
dgri−all−r1.3.novel.transcriptome_counts_transcript_level.csv
EpG
Wilcoxon p−value = 6.5294e−36, W = 111156382
density
0.0
0.1
0.2
0
10
20
30
40
50
num_uniq_exon
group
ortho
whole transcriptome

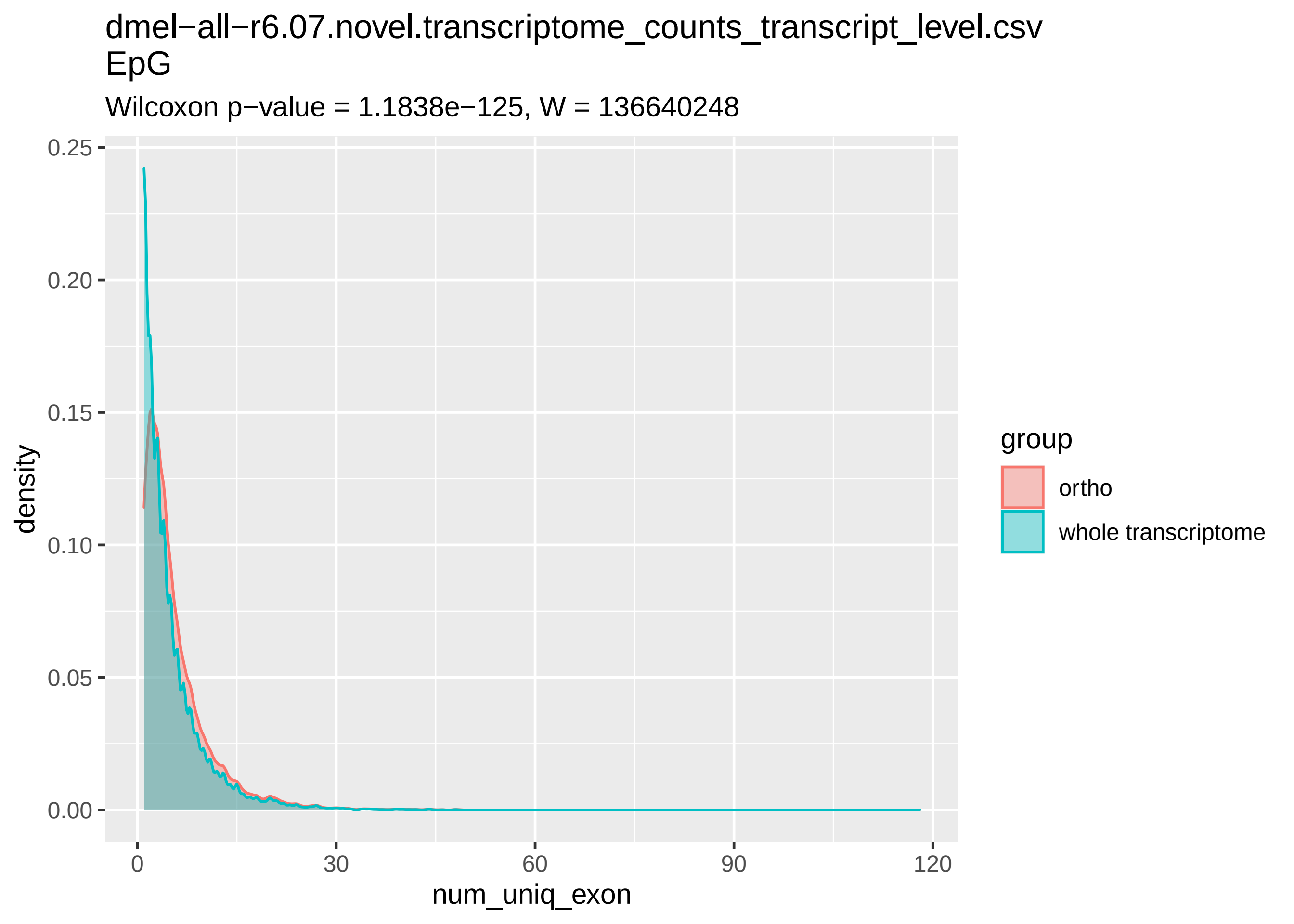
dmel−all−r6.07.novel.transcriptome_counts_transcript_level.csv
EpG
Wilcoxon p−value = 1.1838e−125, W = 136640248
density
0.25
0.20
0.15
0.10
0.05
0.00
0
30
60
90
120
num_uniq_exon
group
ortho
whole transcriptome

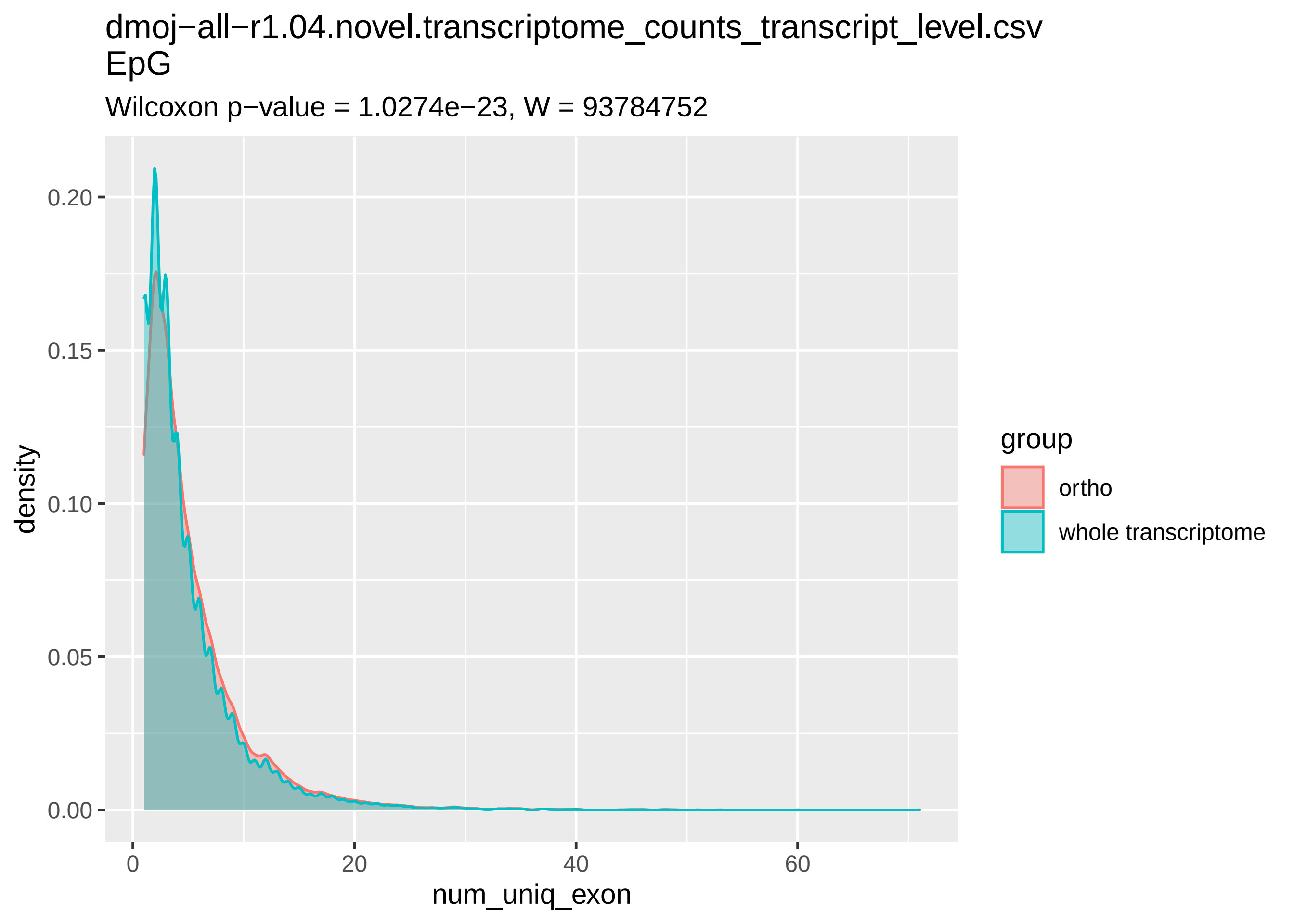
dmoj−all−r1.04.novel.transcriptome_counts_transcript_level.csv
EpG
Wilcoxon p−value = 1.0274e−23, W = 93784752
density
0.00
0.05
0.10
0.15
0.20
0
20
40
60
num_uniq_exon
group
ortho
whole transcriptome

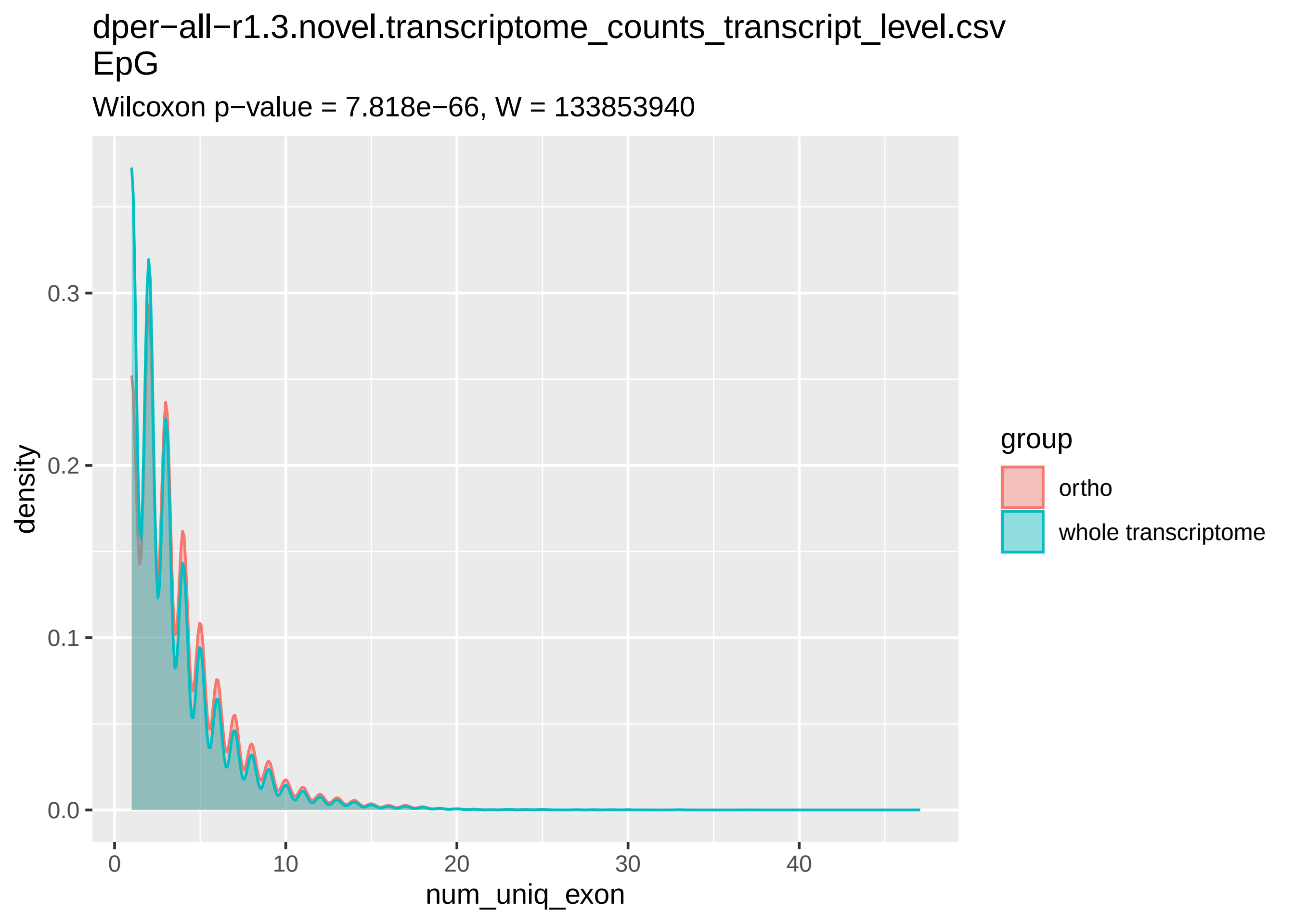

dper−all−r1.3.novel.transcriptome_counts_transcript_level.csv
EpG
Wilcoxon p−value = 7.818e−66, W = 133853940
density
0.0
0.1
0.2
0.3
0
10
20
30
40
num_uniq_exon
group
ortho
whole transcriptome

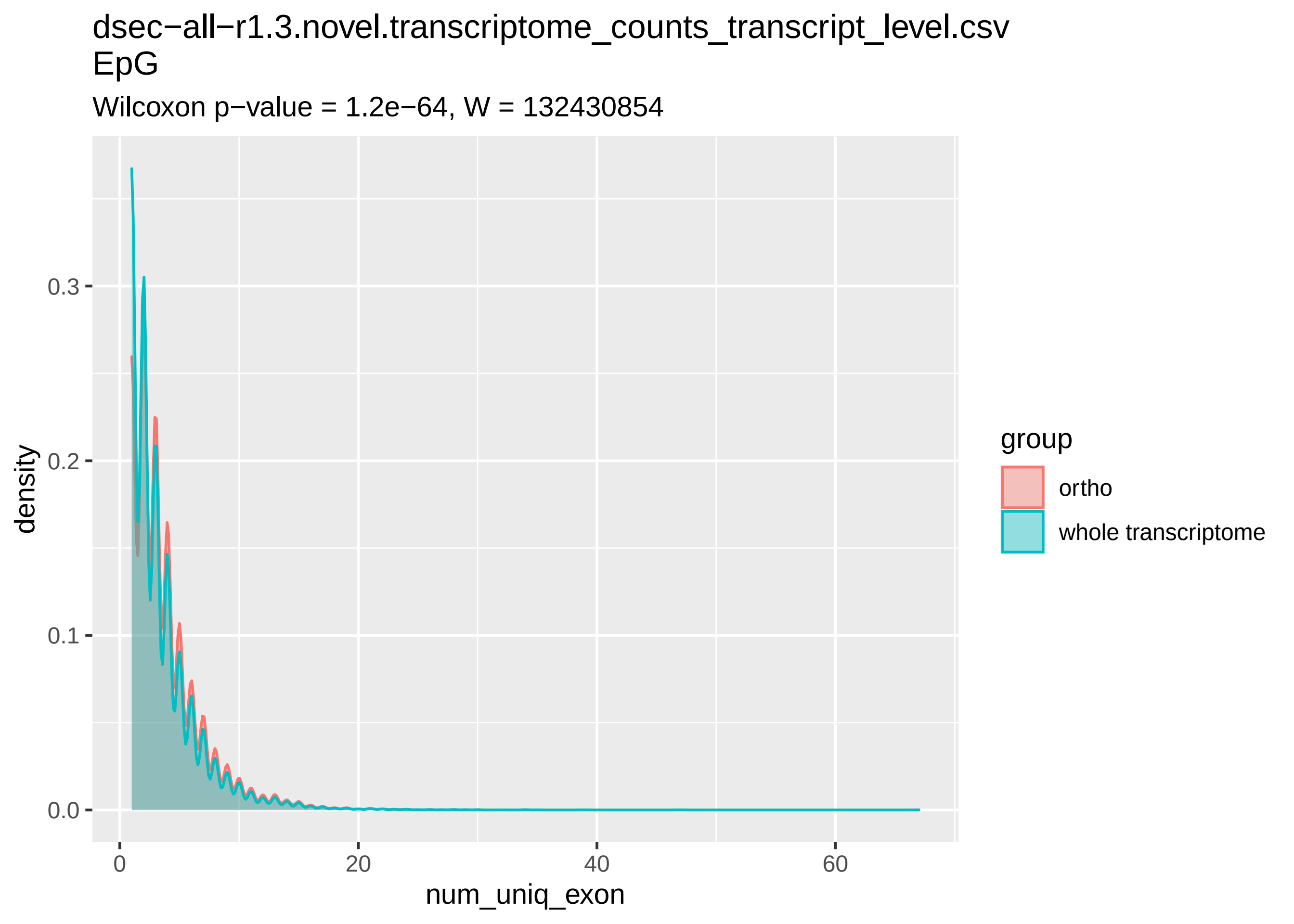
dsec−all−r1.3.novel.transcriptome_counts_transcript_level.csv
EpG
Wilcoxon p−value = 1.2e−64, W = 132430854
density
0.0
0.1
0.2
0.3
0
20
40
60
num_uniq_exon
group
ortho
whole transcriptome

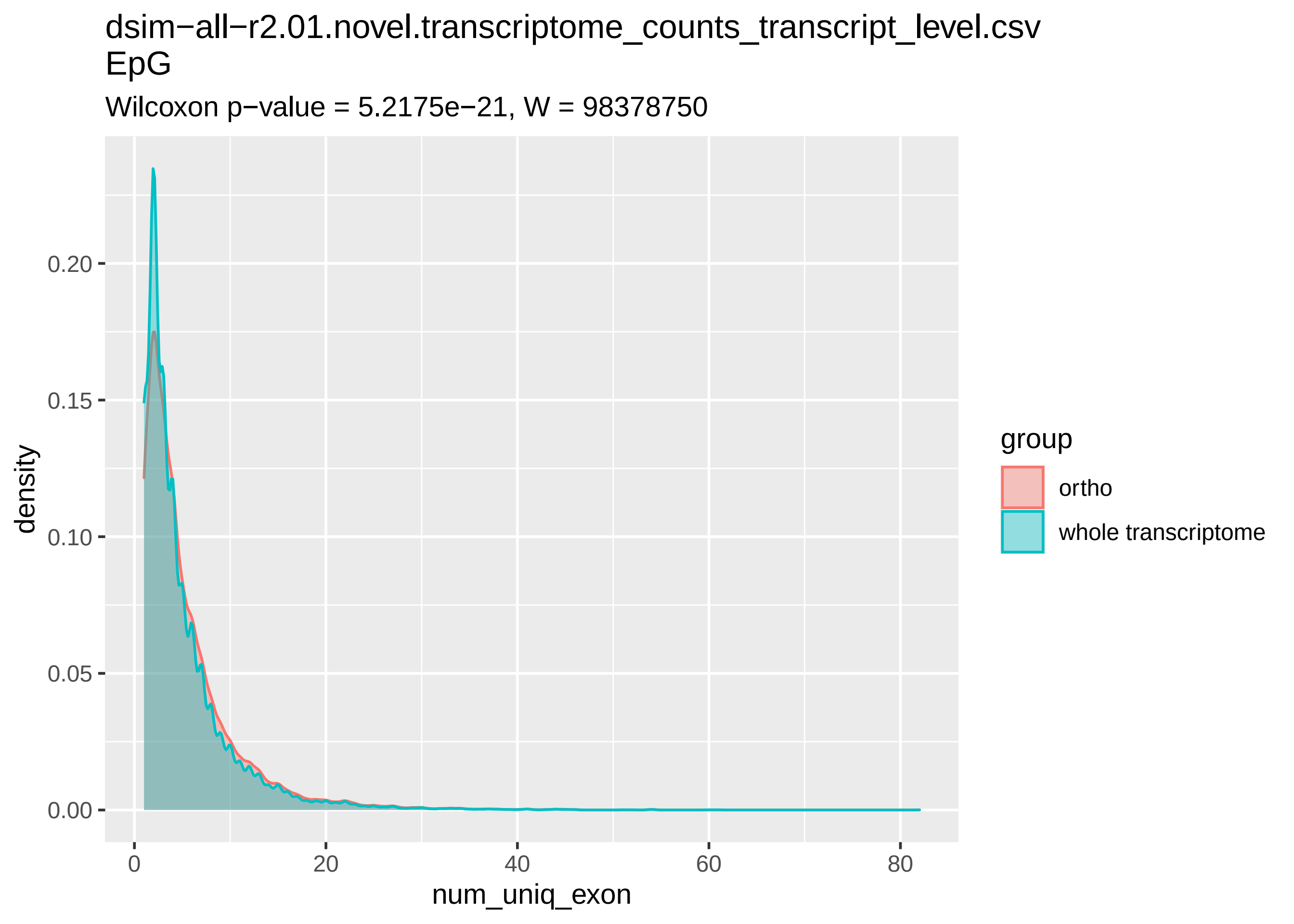
dsim−all−r2.01.novel.transcriptome_counts_transcript_level.csv
EpG
Wilcoxon p−value = 5.2175e−21, W = 98378750
density
0.20
0.15
0.10
0.05
0.00
0
20
40
60
80
num_uniq_exon
group
ortho
whole transcriptome

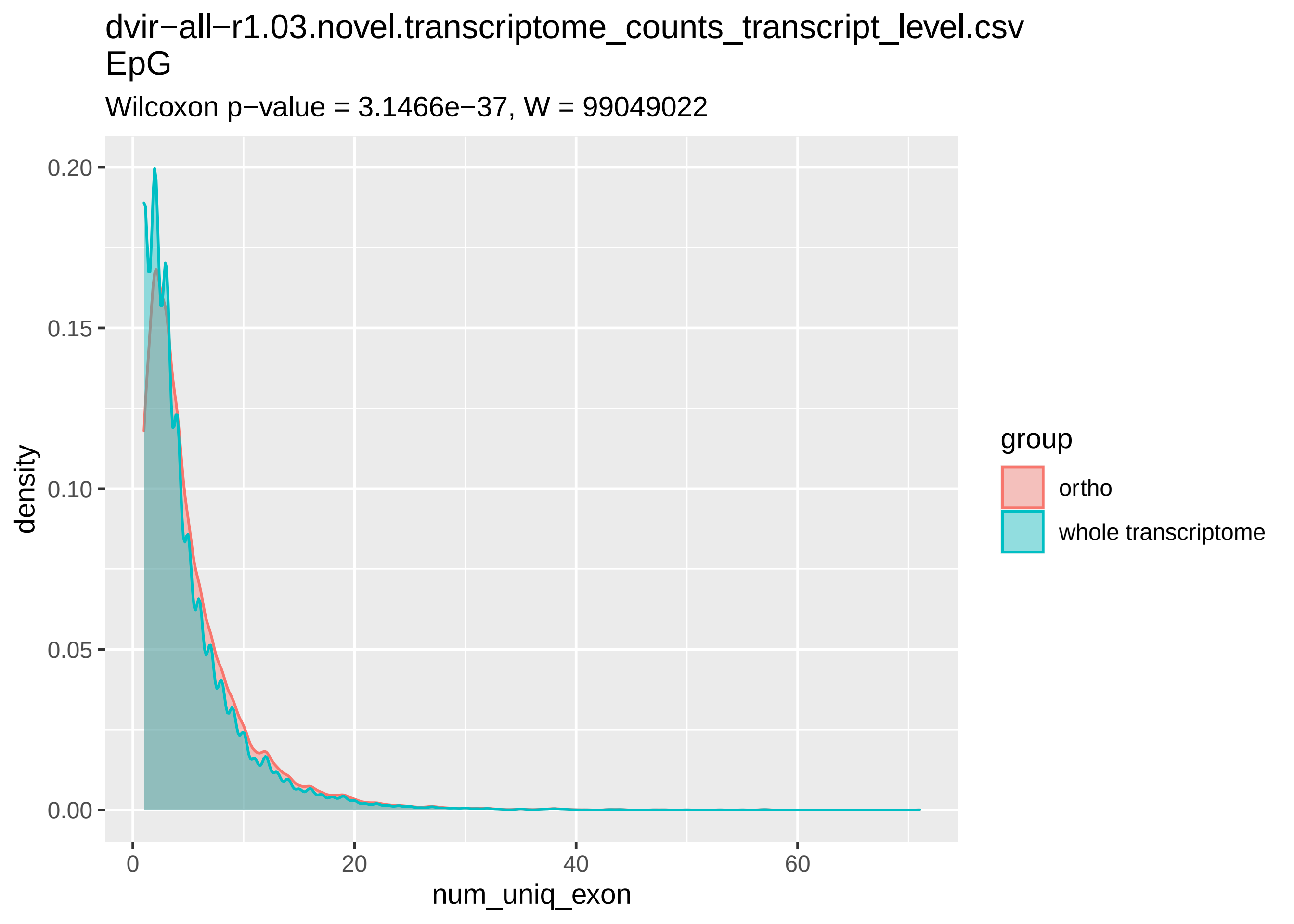

dvir−all−r1.03.novel.transcriptome_counts_transcript_level.csv
EpG
Wilcoxon p−value = 3.1466e−37, W = 99049022
density
0.20
0.15
0.10
0.05
0.00
0
20
40
60
num_uniq_exon
group
ortho
whole transcriptome

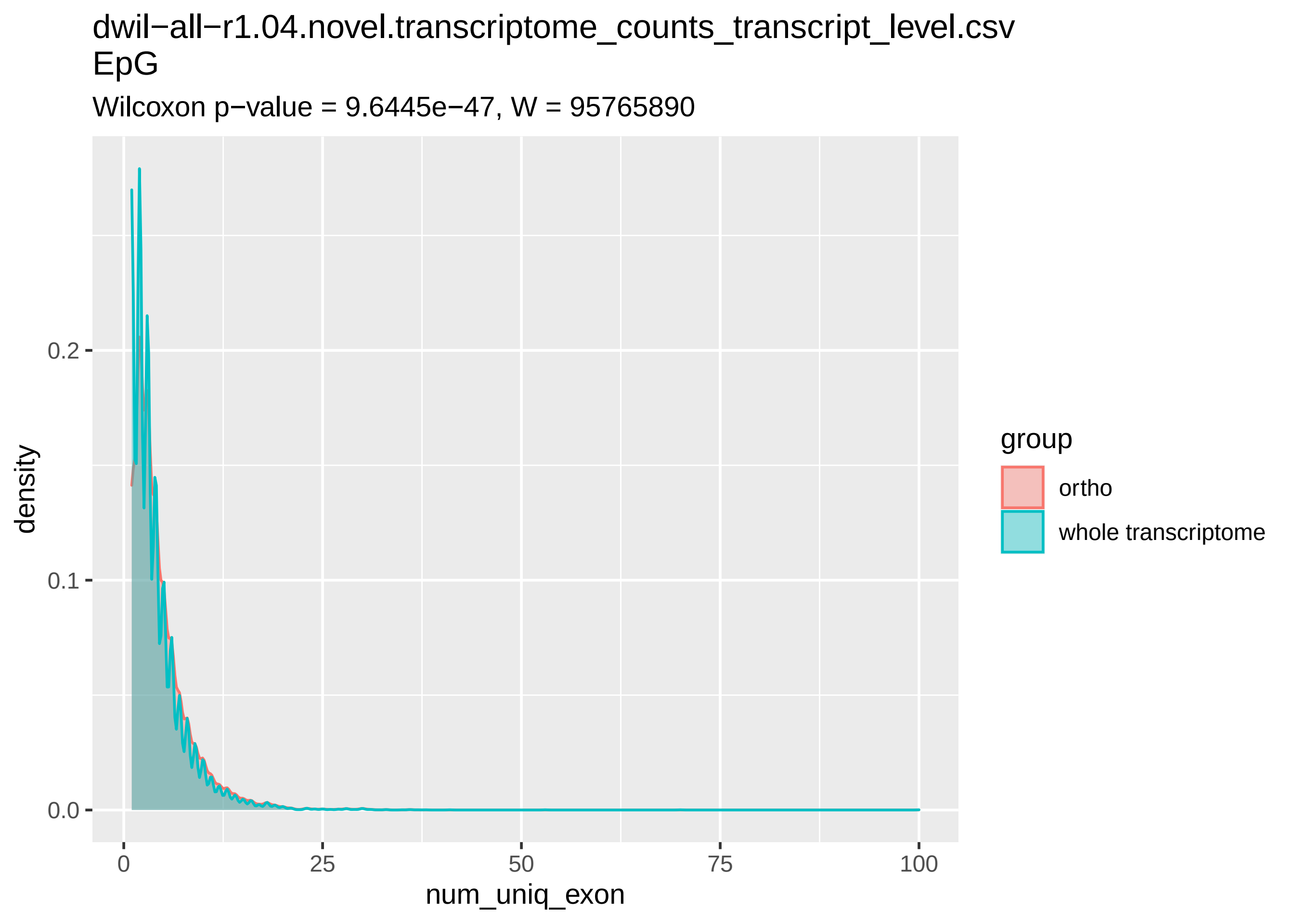

dwil−all−r1.04.novel.transcriptome_counts_transcript_level.csv
EpG
Wilcoxon p−value = 9.6445e−47, W = 95765890
density
0.0
0.1
0.2
0
25
50
75
100
num_uniq_exon
group
ortho
whole transcriptome

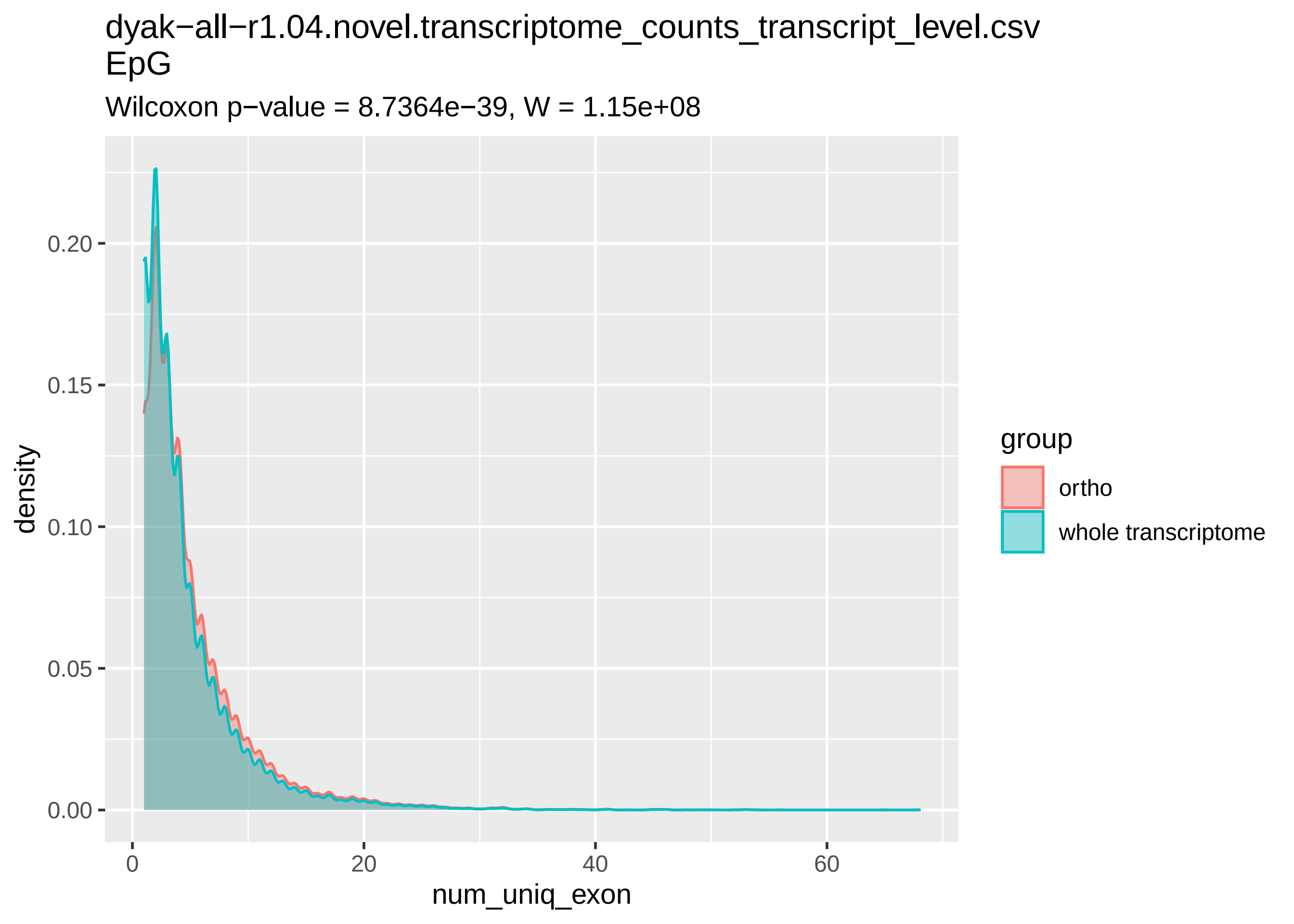
dyak−all−r1.04.novel.transcriptome_counts_transcript_level.csv
EpG
Wilcoxon p−value = 8.7364e−39, W = 1.15e+08
density
0.20
0.15
0.10
0.05
0.00
0
20
40
60
num_uniq_exon
group
ortho
whole transcriptome

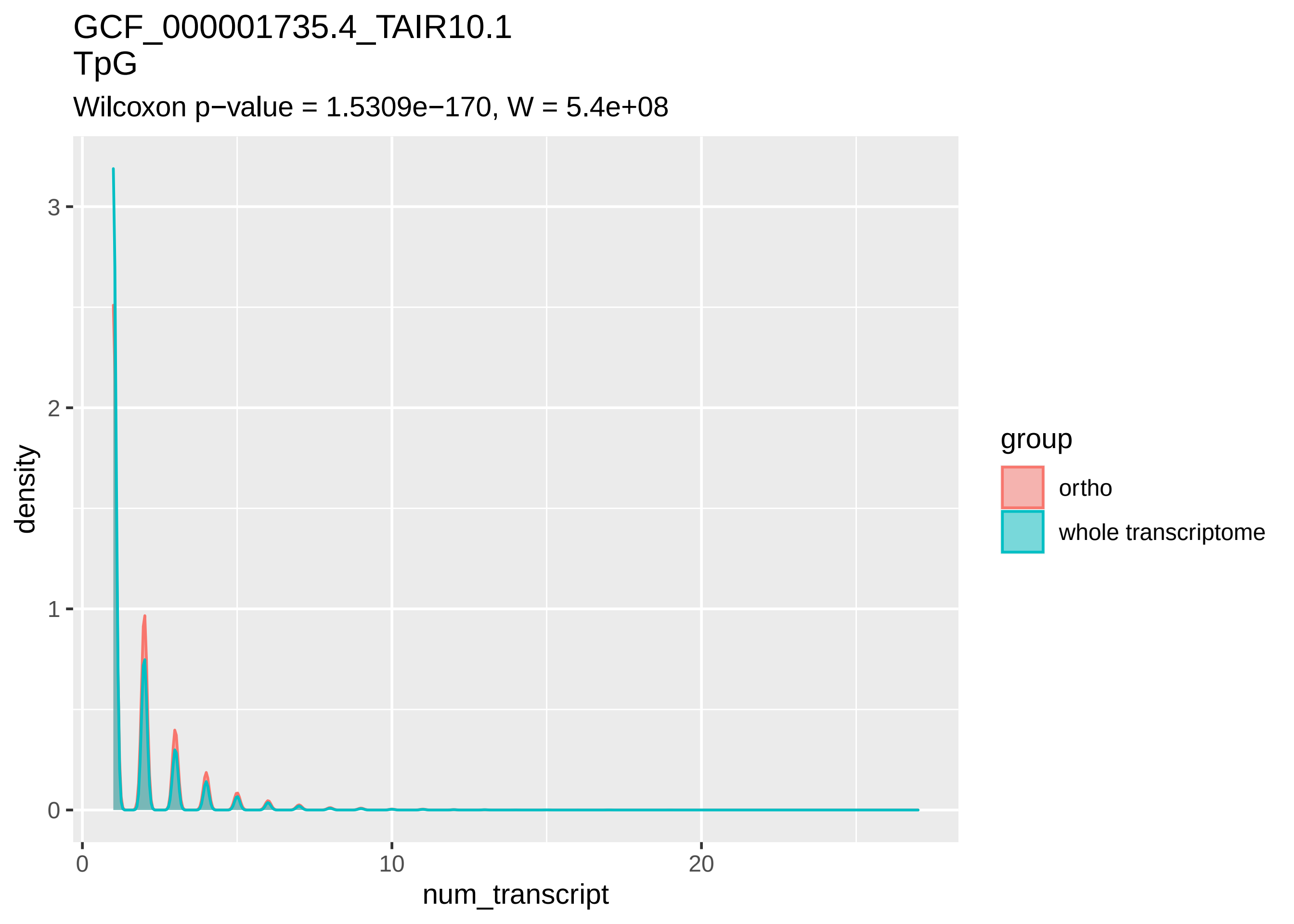
GCF_000001735.4_TAIR10.1
TpG
Wilcoxon p−value = 1.5309e−170, W = 5.4e+08
density
num_transcript
0
1
2
3
0
10
20
group
ortho
whole transcriptome

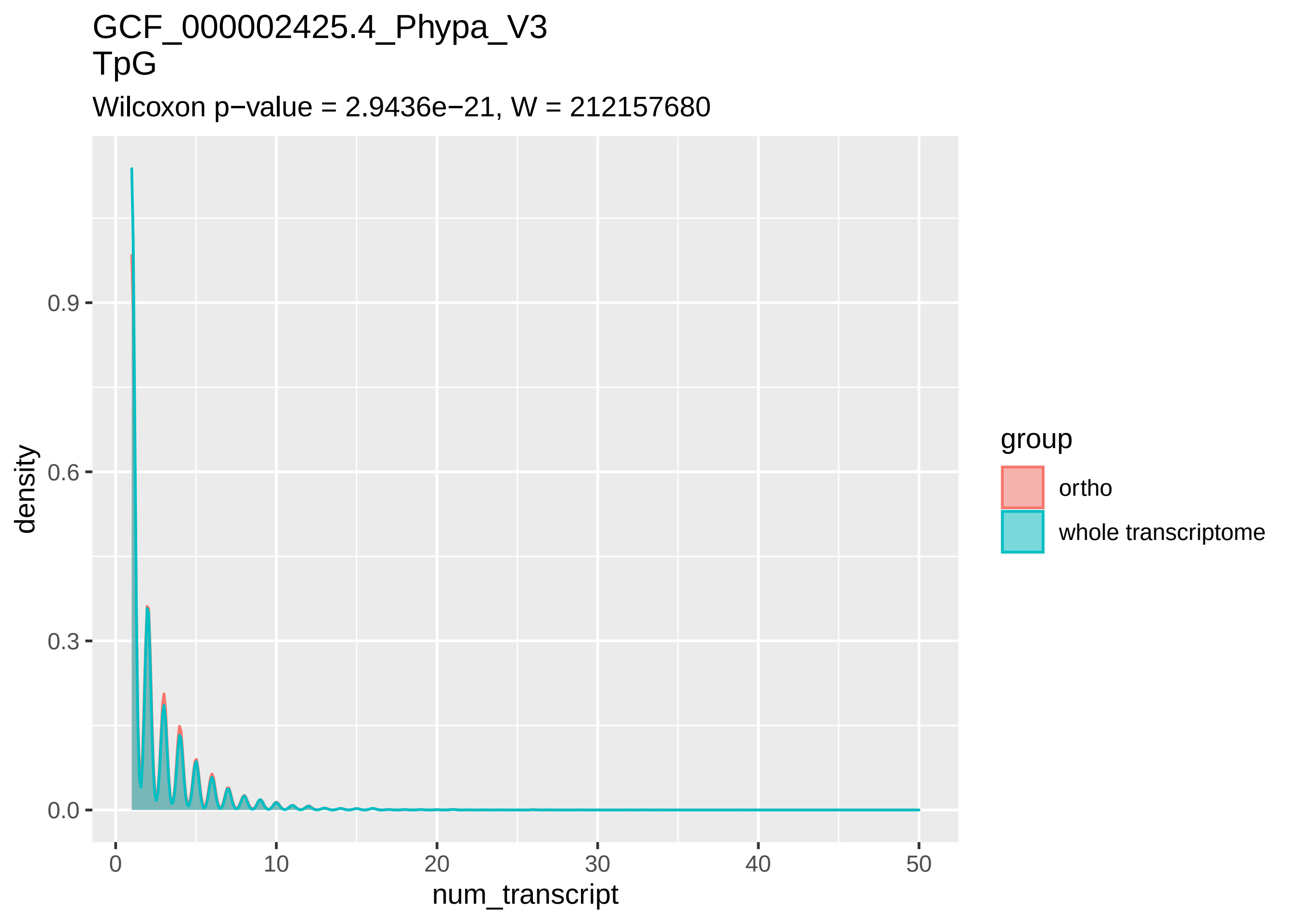
GCF_000002425.4_Phypa_V3
TpG
Wilcoxon p−value = 2.9436e−21, W = 212157680
density
0.0
0.3
0.6
0.9
0
10
20
30
40
50
num_transcript
group
ortho
whole transcriptome

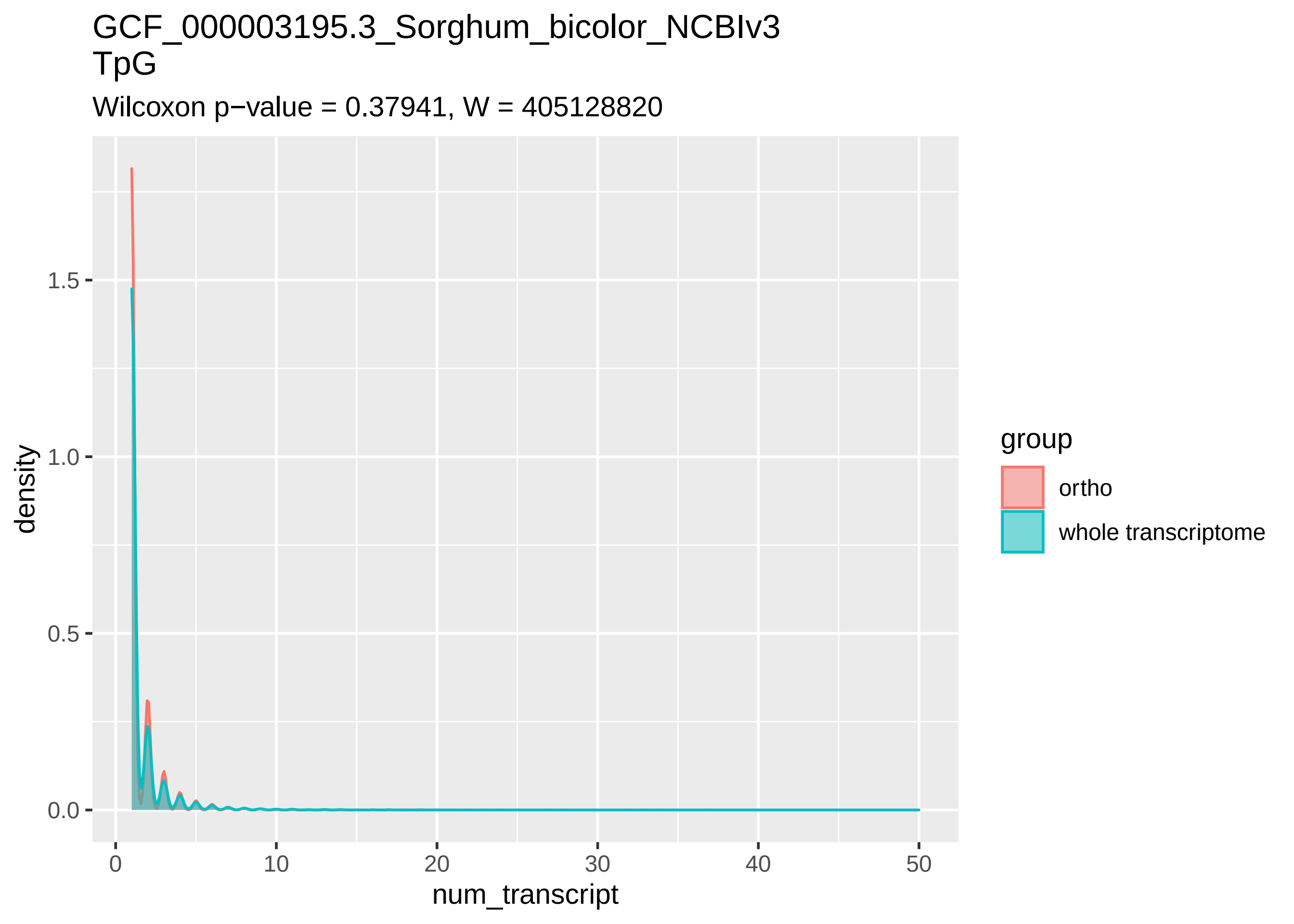
GCF_000003195.3_Sorghum_bicolor_NCBIv3
TpG
Wilcoxon p−value = 0.37941, W = 405128820
density
1.5
1.0
0.5
0.0
0
10
20
30
40
50
num_transcript
group
ortho
whole transcriptome

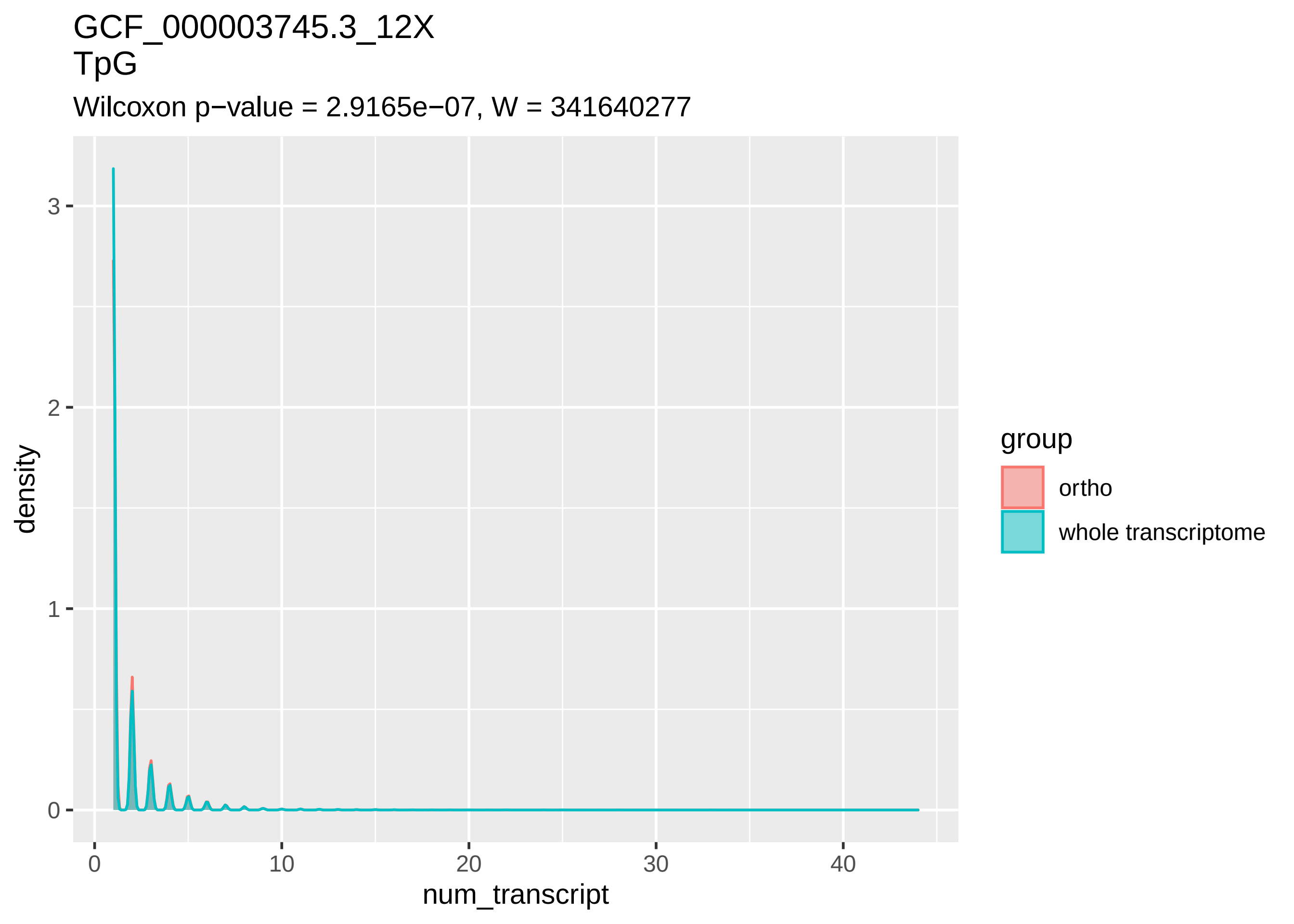
GCF_000003745.3_12X
TpG
Wilcoxon p−value = 2.9165e−07, W = 341640277
density
num_transcript
0
1
2
3
0
10
20
30
40
group
ortho
whole transcriptome

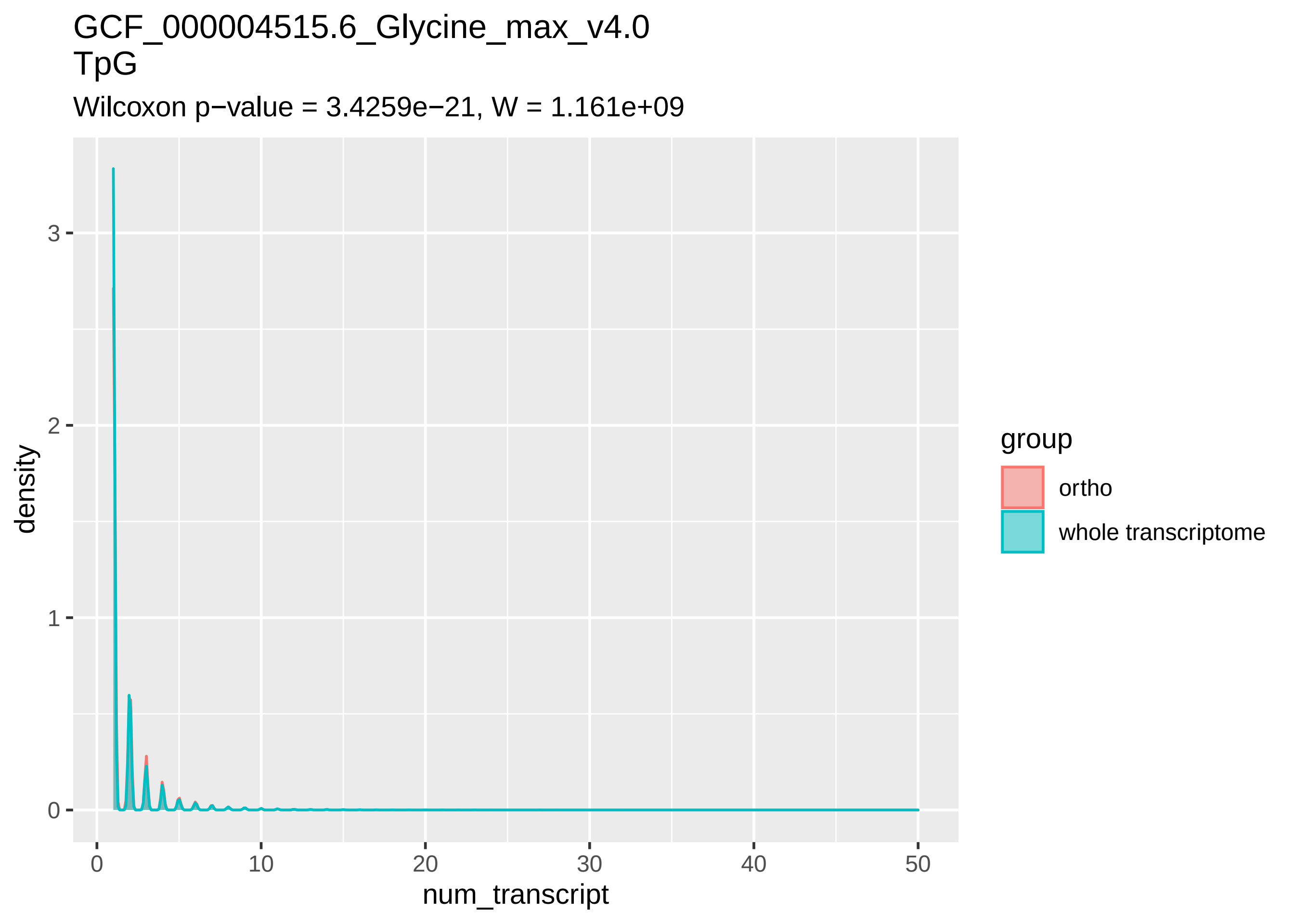
GCF_000004515.6_Glycine_max_v4.0
TpG
Wilcoxon p−value = 3.4259e−21, W = 1.161e+09
density
3
2
1
0
0
10
20
30
40
50
num_transcript
group
ortho
whole transcriptome

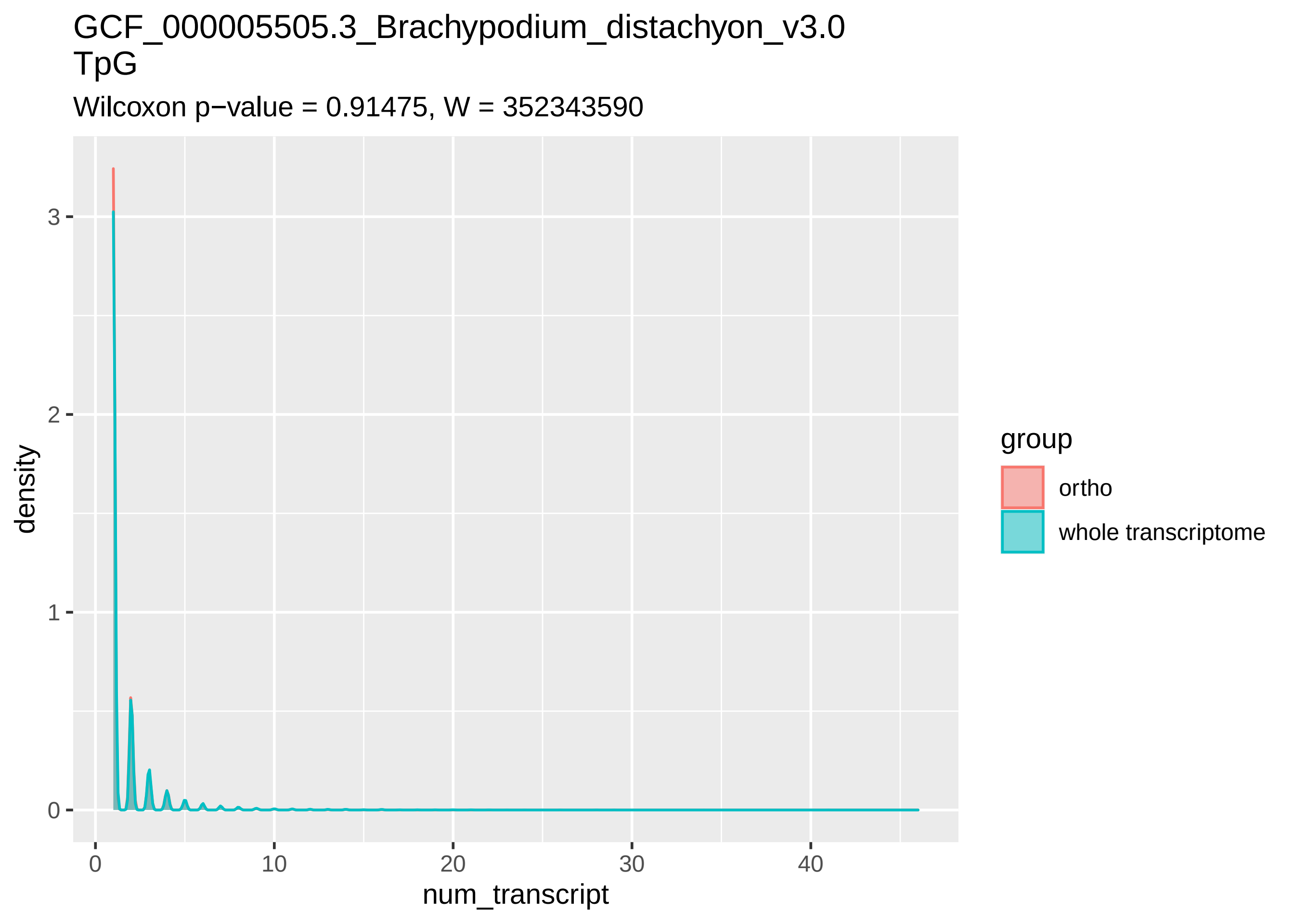
GCF_000005505.3_Brachypodium_distachyon_v3.0
TpG
Wilcoxon p−value = 0.91475, W = 352343590
density
num_transcript
0
1
2
3
0
10
20
30
40
group
ortho
whole transcriptome

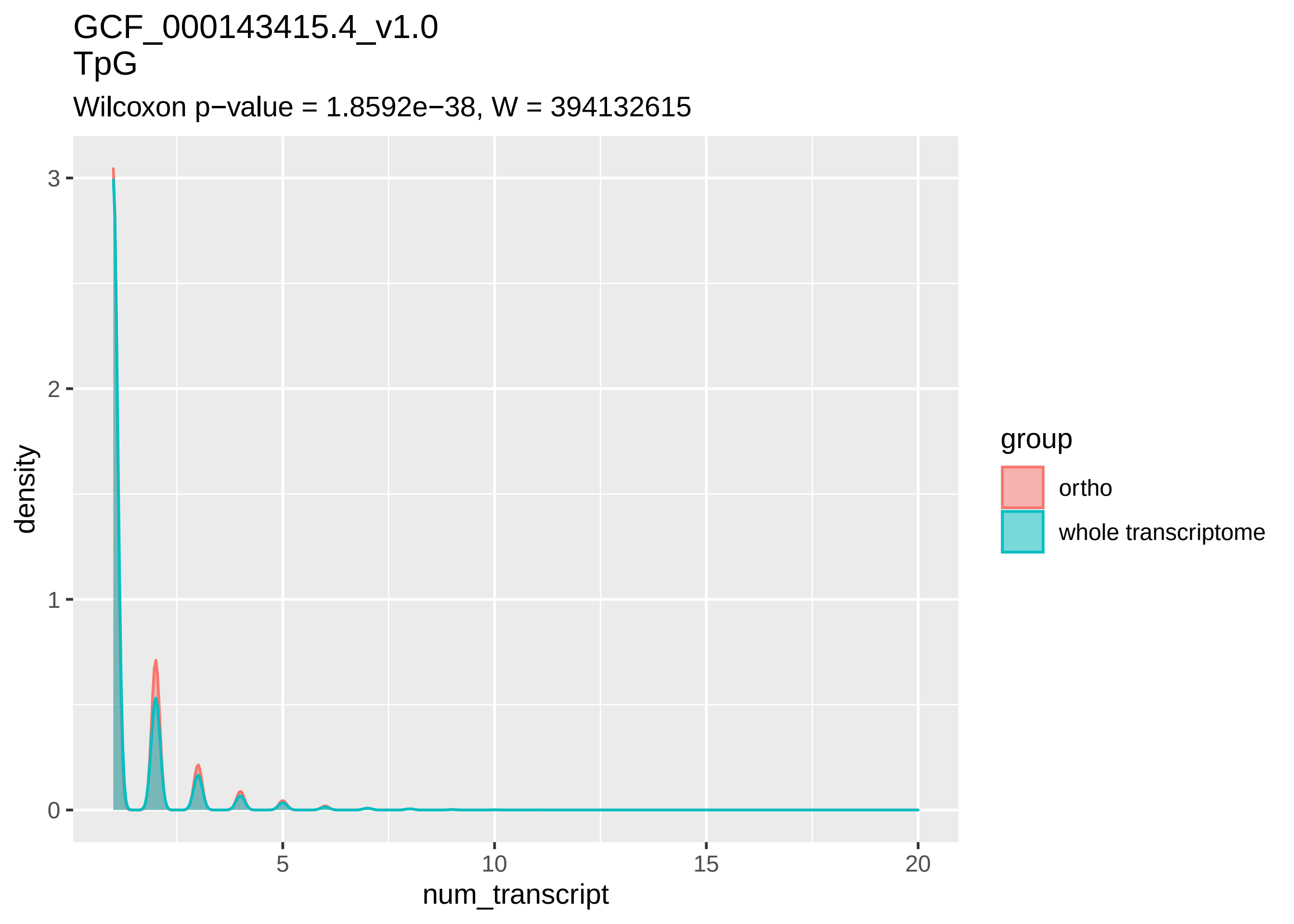

GCF_000143415.4_v1.0
TpG
Wilcoxon p−value = 1.8592e−38, W = 394132615
density
num_transcript
0
1
2
3
5
10
15
20
group
ortho
whole transcriptome

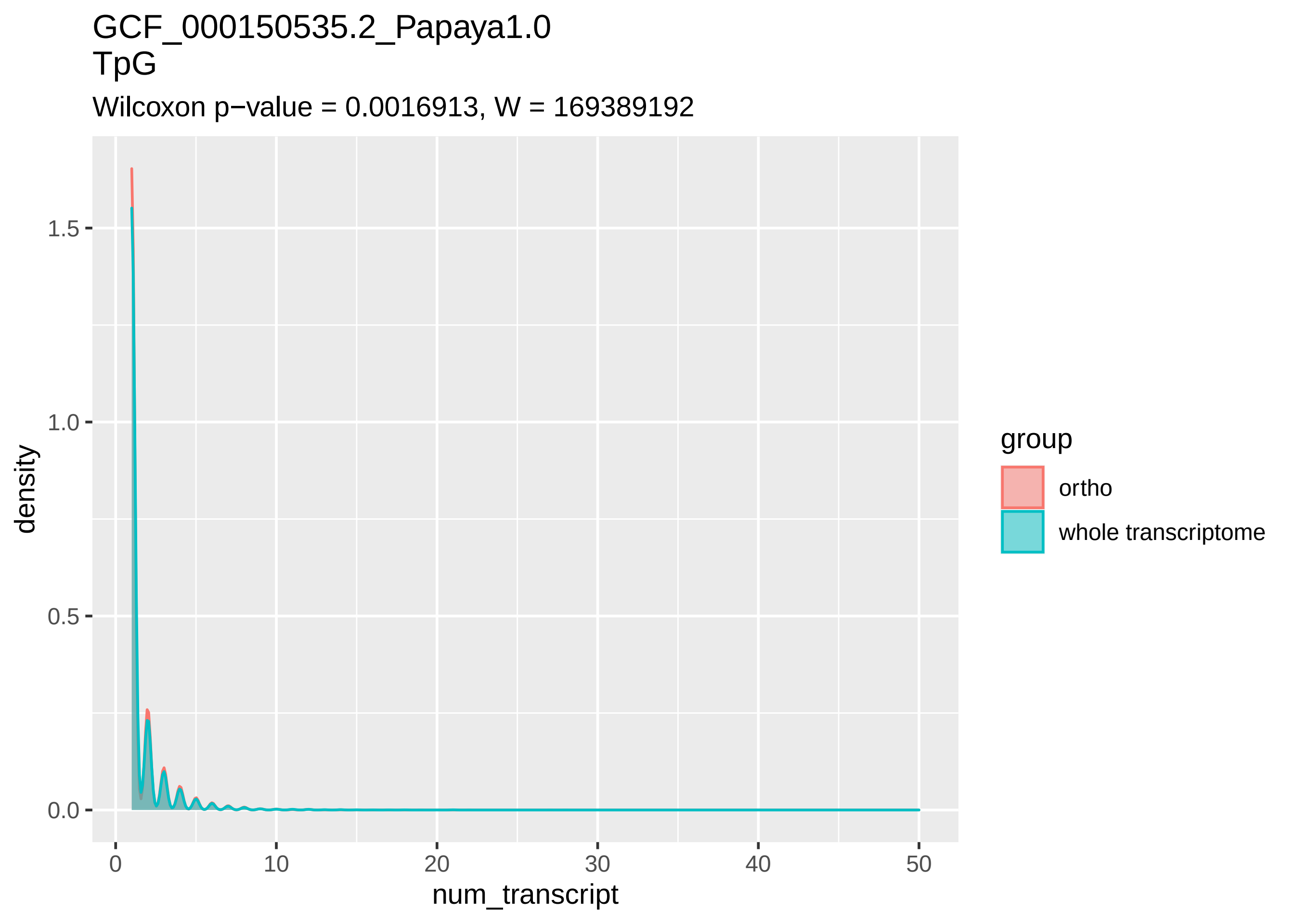
GCF_000150535.2_Papaya1.0
TpG
Wilcoxon p−value = 0.0016913, W = 169389192
density
1.5
1.0
0.5
0.0
0
10
20
30
40
50
num_transcript
group
ortho
whole transcriptome

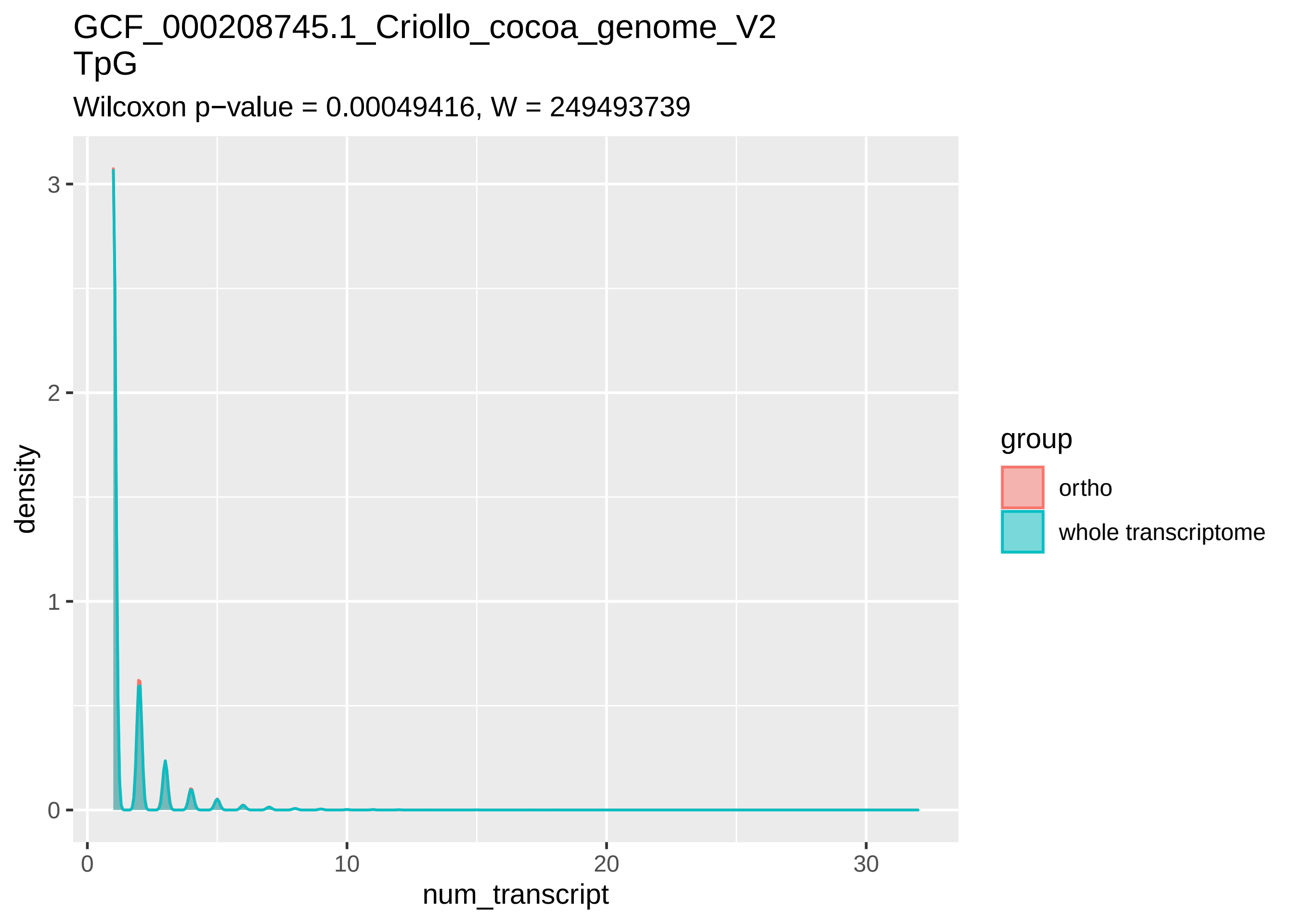

GCF_000208745.1_Criollo_cocoa_genome_V2
TpG
Wilcoxon p−value = 0.00049416, W = 249493739
density
3
2
1
0
0
10
20
30
num_transcript
group
ortho
whole transcriptome

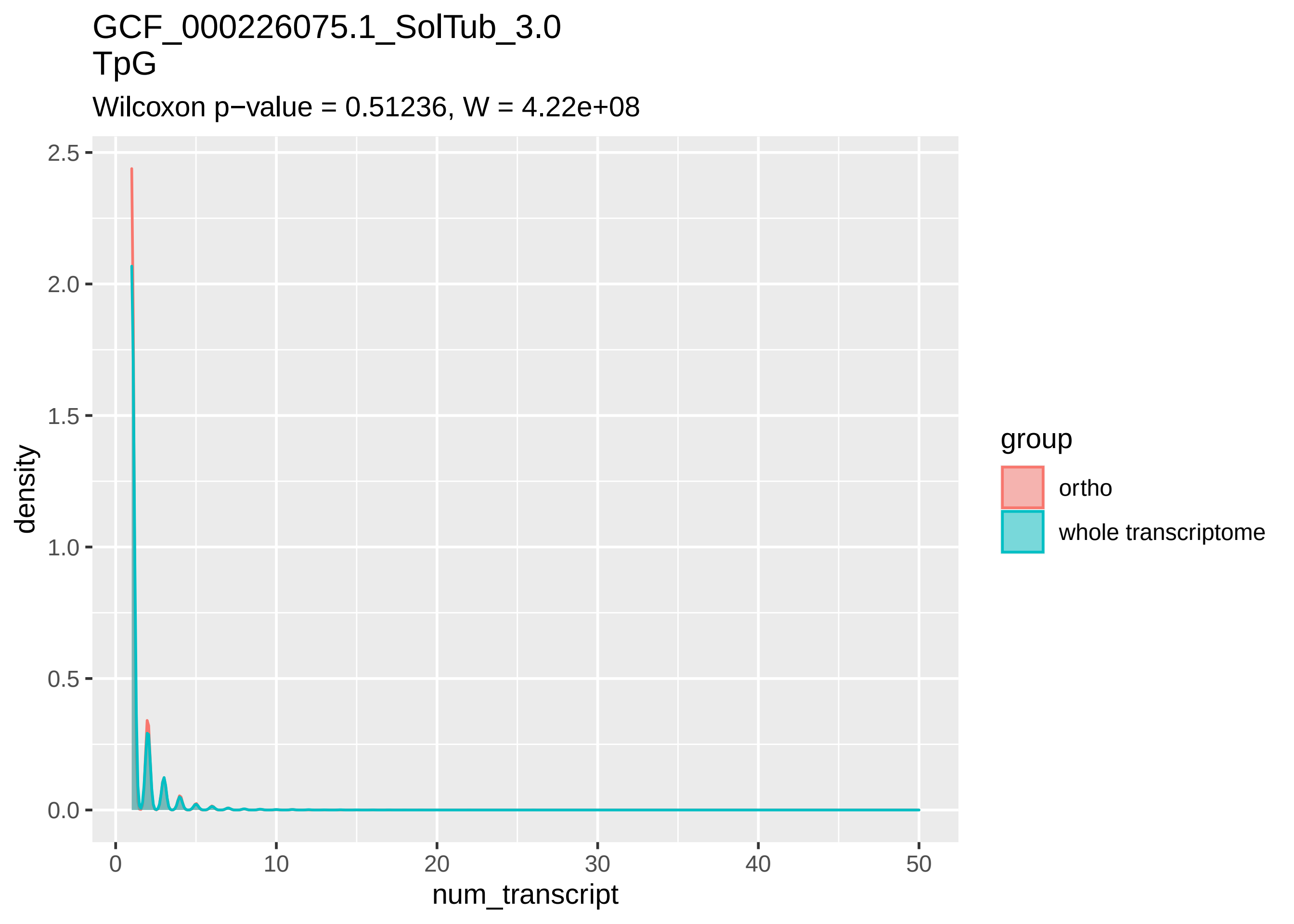
GCF_000226075.1_SolTub_3.0
TpG
Wilcoxon p−value = 0.51236, W = 4.22e+08
density
2.5
2.0
1.5
1.0
0.5
0.0
0
10
20
30
40
50
num_transcript
group
ortho
whole transcriptome

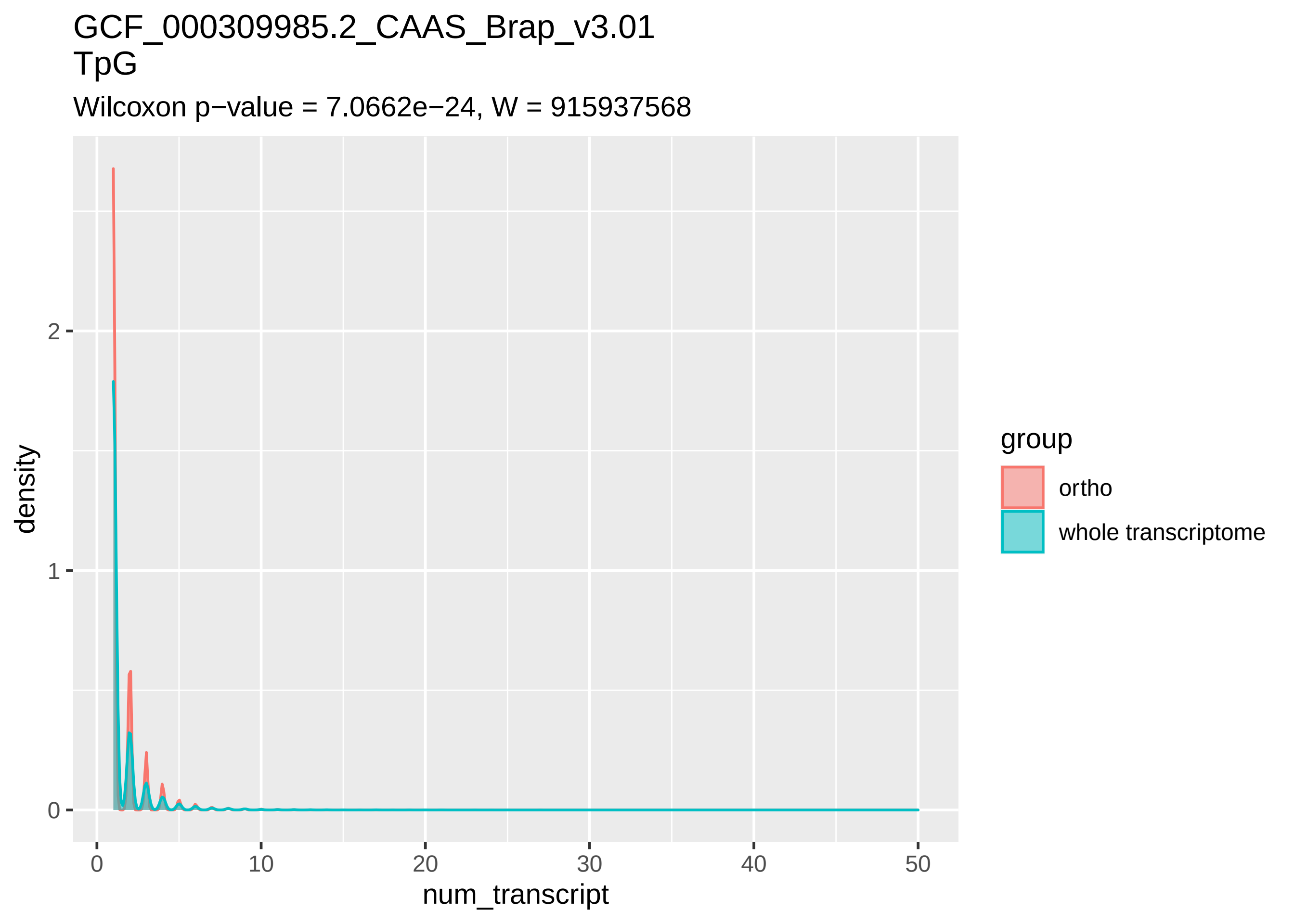
GCF_000309985.2_CAAS_Brap_v3.01
TpG
Wilcoxon p−value = 7.0662e−24, W = 915937568
density
0
1
2
0
10
20
30
40
50
num_transcript
group
ortho
whole transcriptome

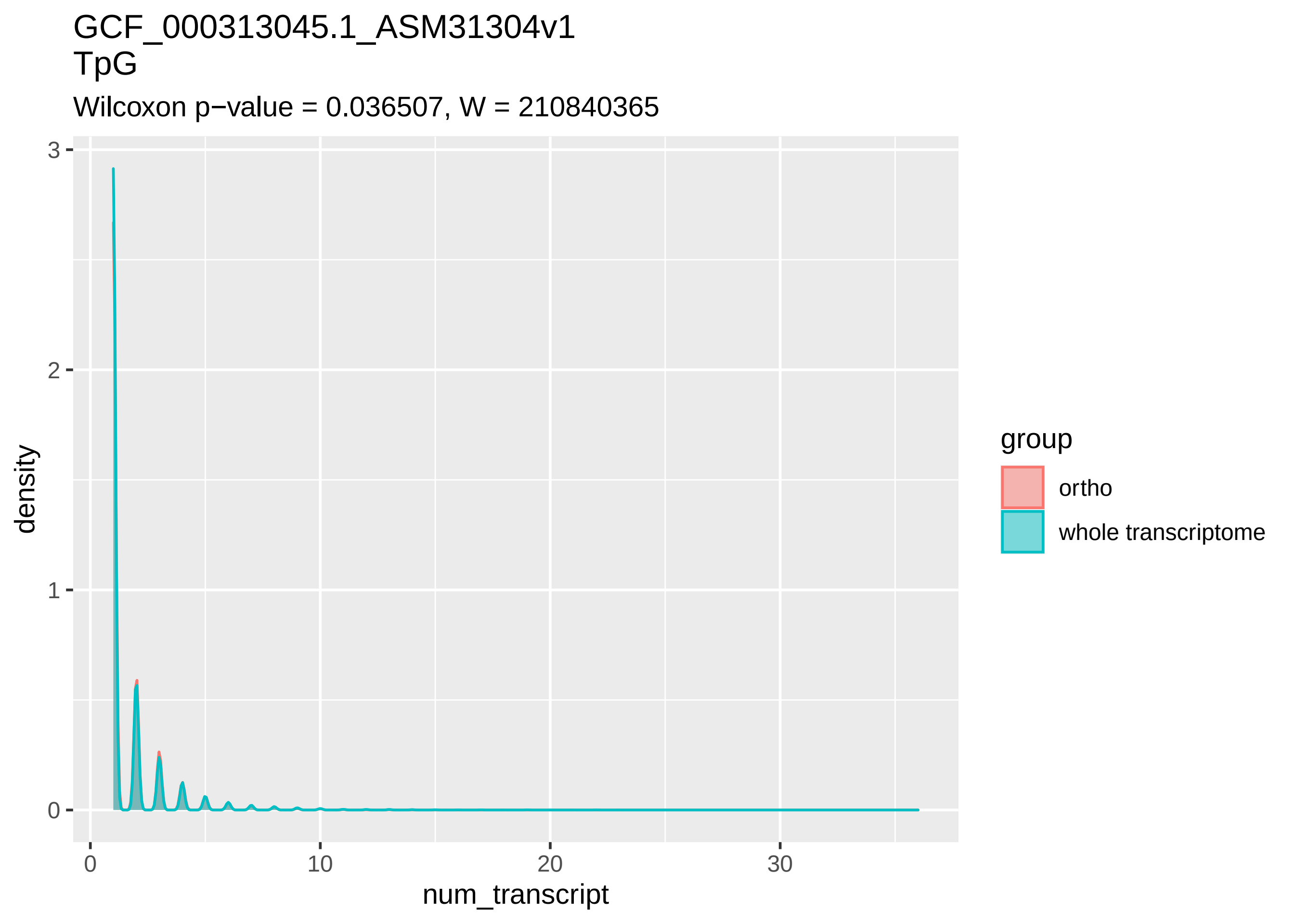
GCF_000313045.1_ASM31304v1
TpG
Wilcoxon p−value = 0.036507, W = 210840365
density
num_transcript
0
1
2
3
0
10
20
30
group
ortho
whole transcriptome

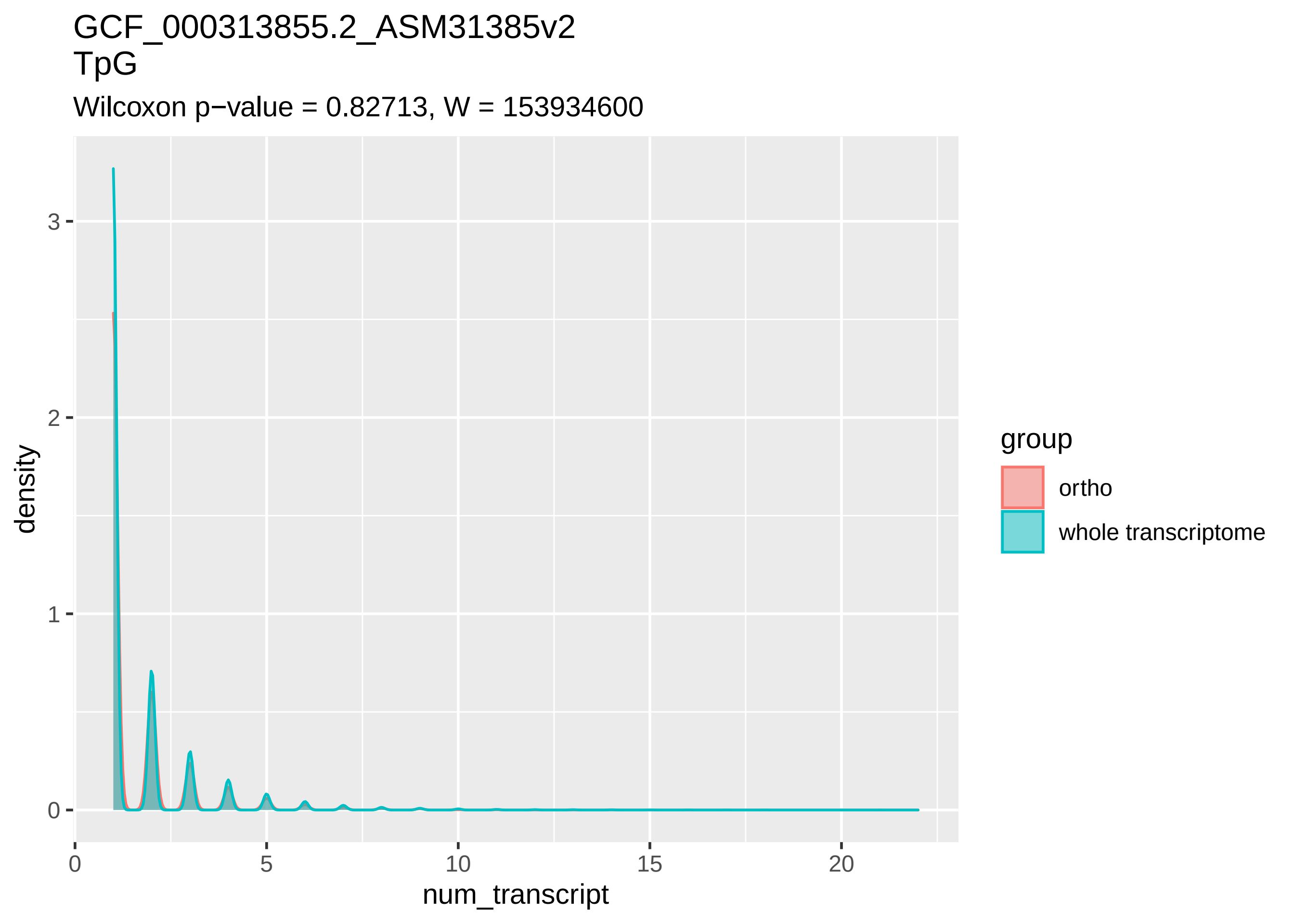

GCF_000313855.2_ASM31385v2
TpG
Wilcoxon p−value = 0.82713, W = 153934600
density
3
2
1
0
0
5
10
15
20
num_transcript
group
ortho
whole transcriptome

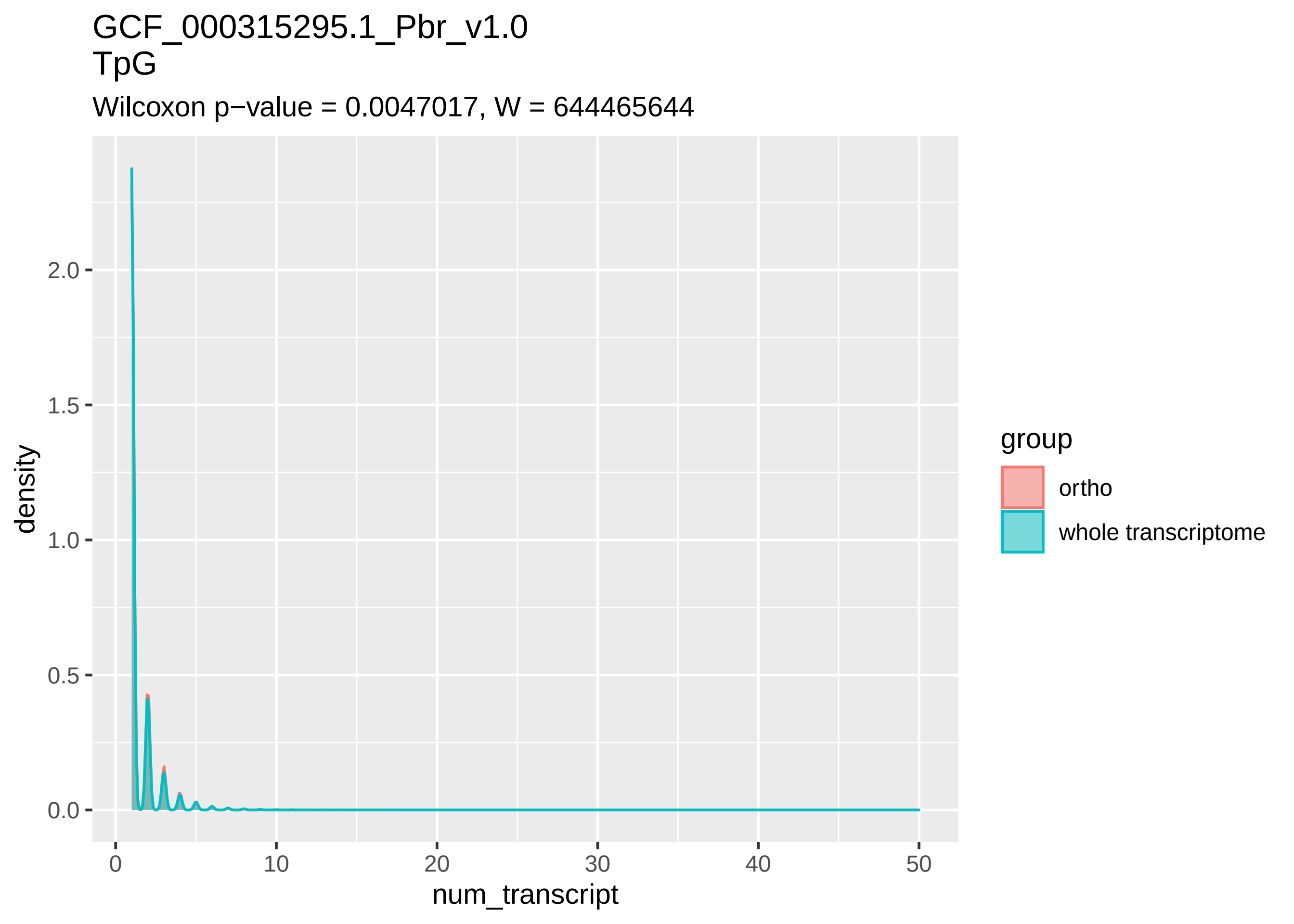
GCF_000315295.1_Pbr_v1.0
TpG
Wilcoxon p−value = 0.0047017, W = 644465644
density
num_transcript
0.0
0.5
1.0
1.5
2.0
0
10
20
30
40
50
group
ortho
whole transcriptome

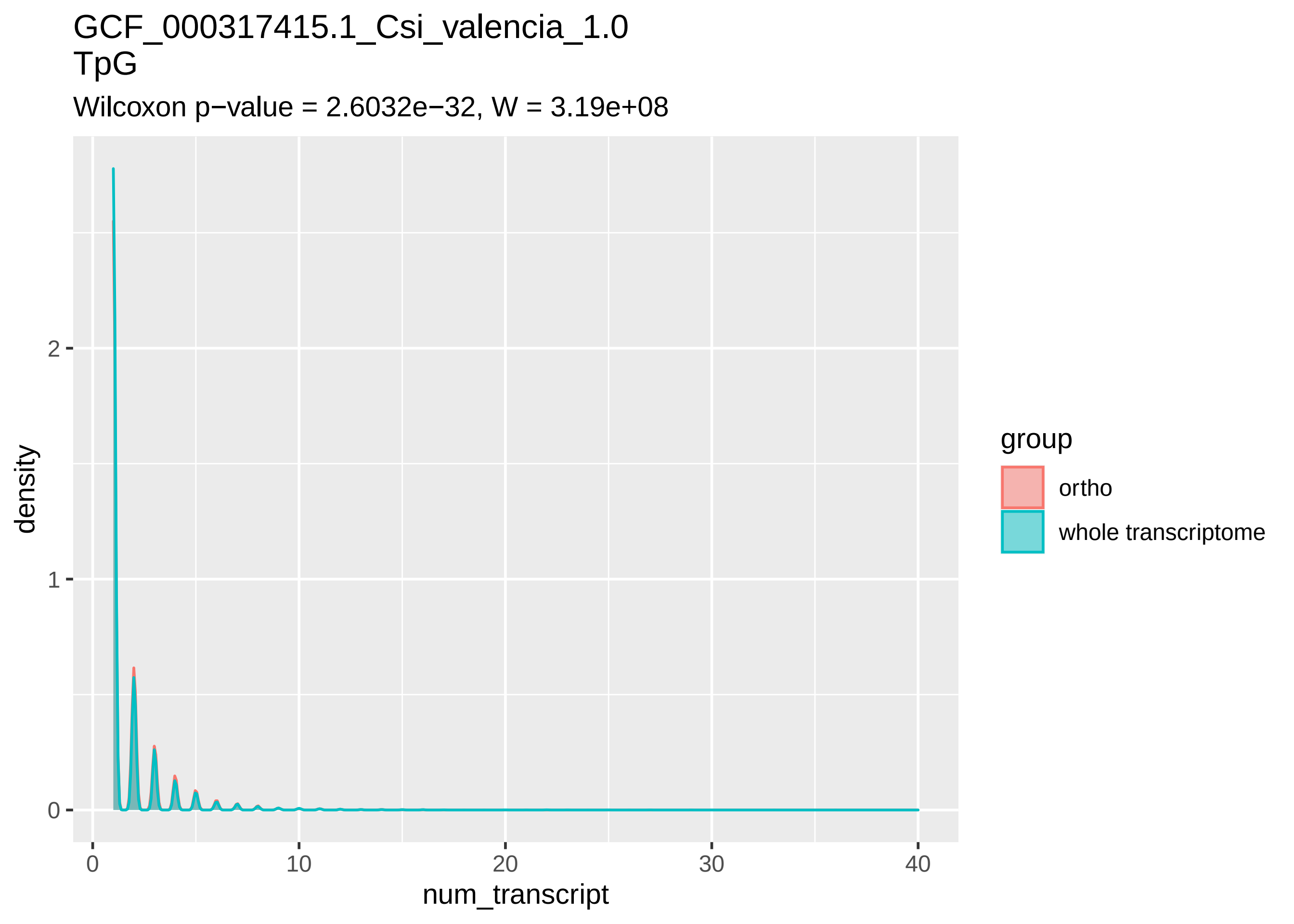
GCF_000317415.1_Csi_valencia_1.0
TpG
Wilcoxon p−value = 2.6032e−32, W = 3.19e+08
density
num_transcript
0
1
2
0
10
20
30
40
group
ortho
whole transcriptome

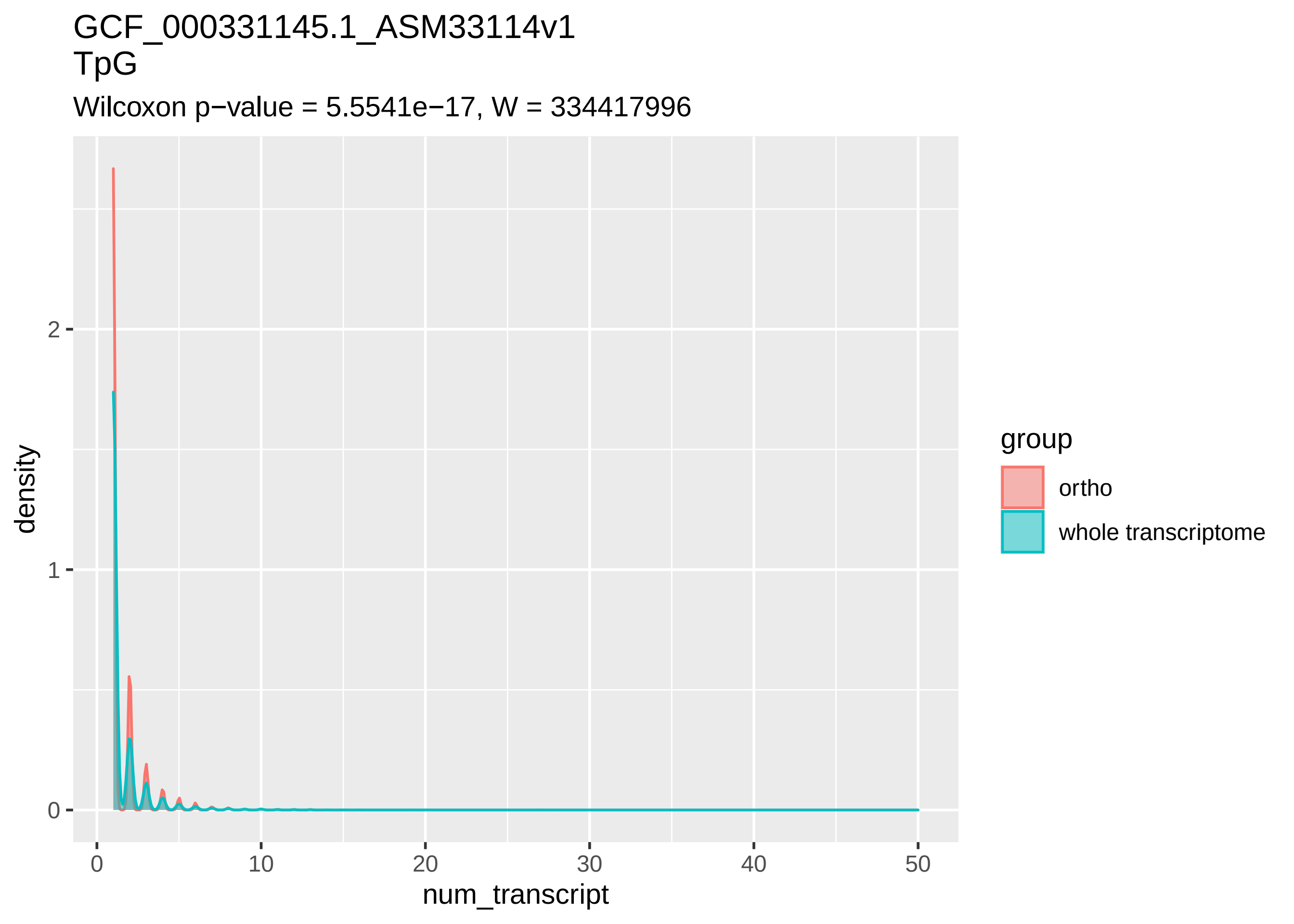
GCF_000331145.1_ASM33114v1
TpG
Wilcoxon p−value = 5.5541e−17, W = 334417996
density
2
1
0
0
10
20
30
40
50
num_transcript
group
ortho
whole transcriptome

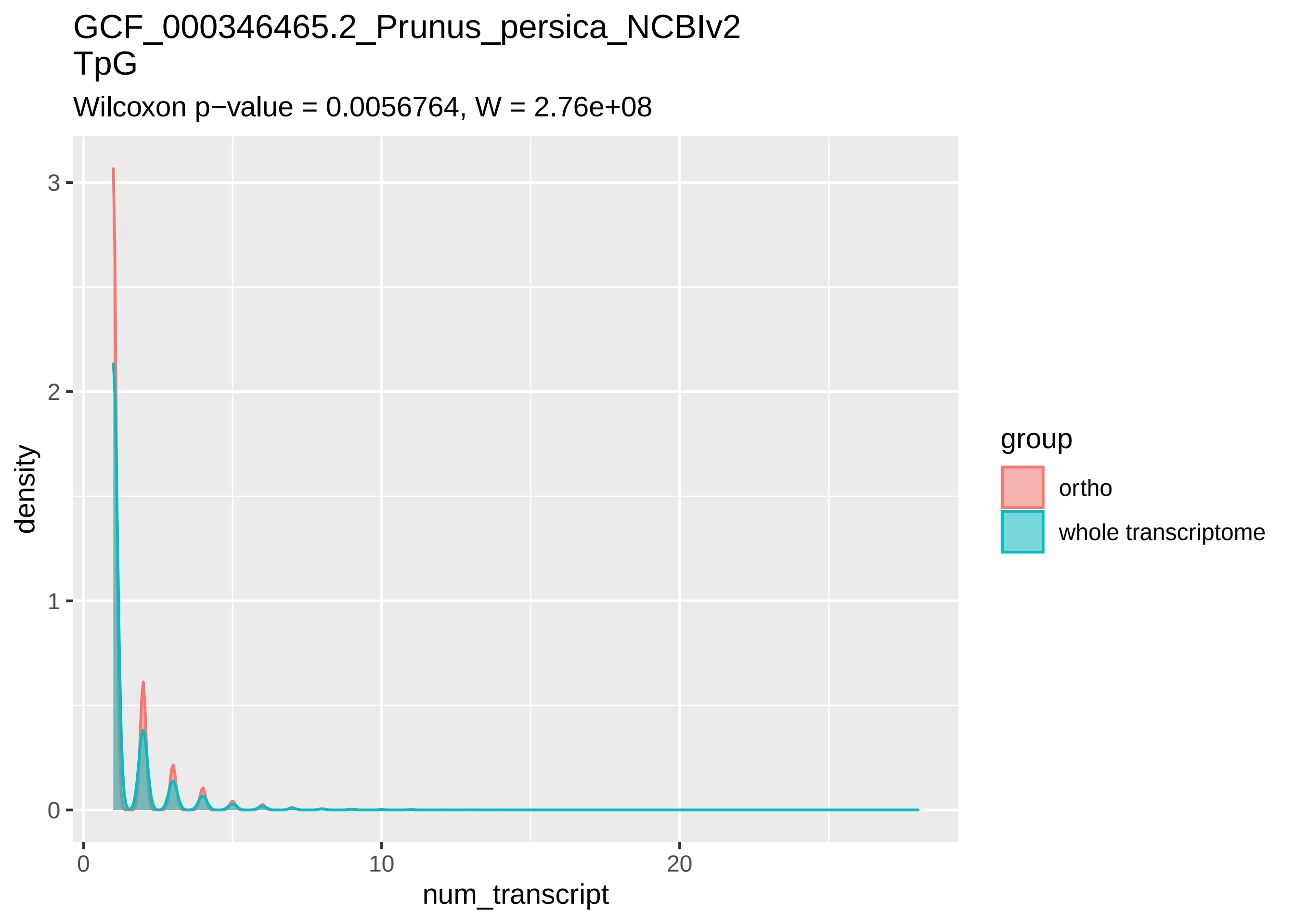

GCF_000346465.2_Prunus_persica_NCBIv2
TpG
Wilcoxon p−value = 0.0056764, W = 2.76e+08
density
num_transcript
0
1
2
3
0
10
20
group
ortho
whole transcriptome

GCF_000365185.1_Chinese_Lotus_1.1
TpG

Wilcoxon p−value = 1.268e−07, W = 307747197

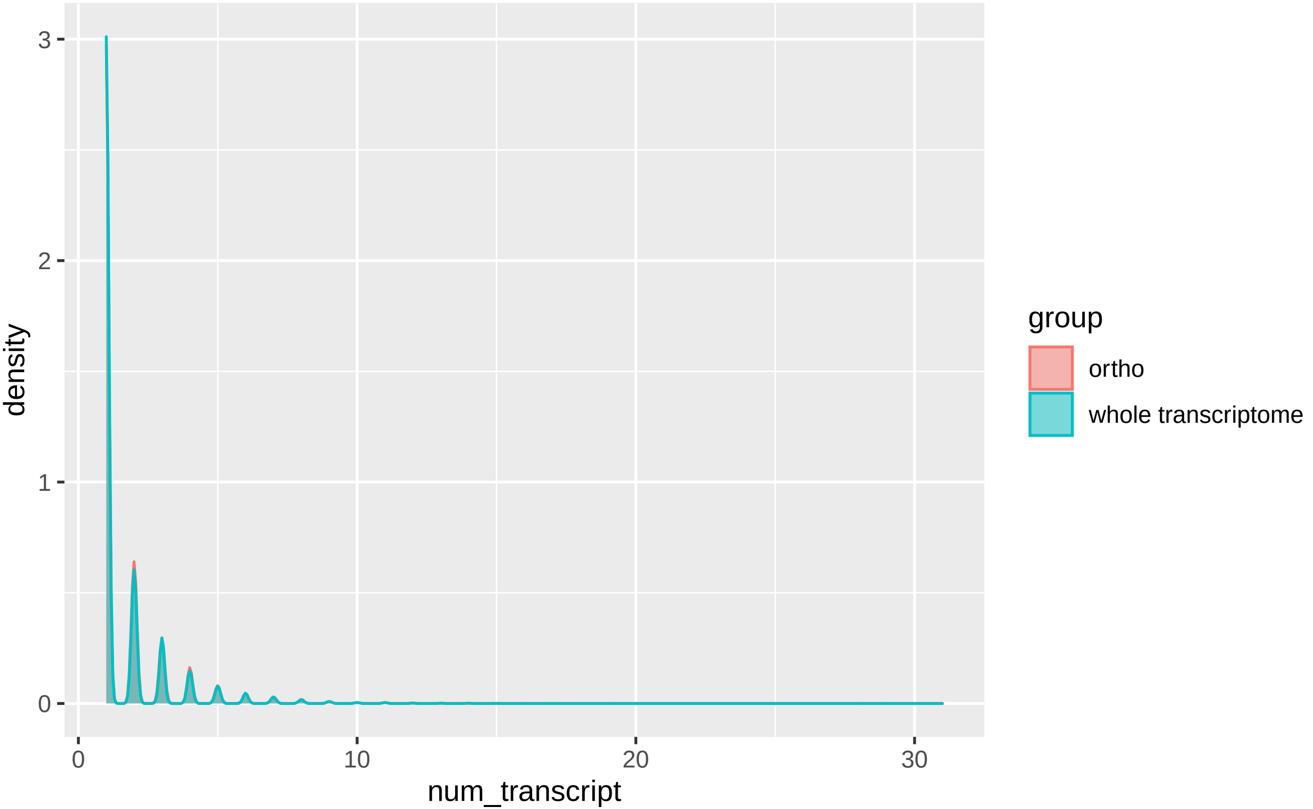

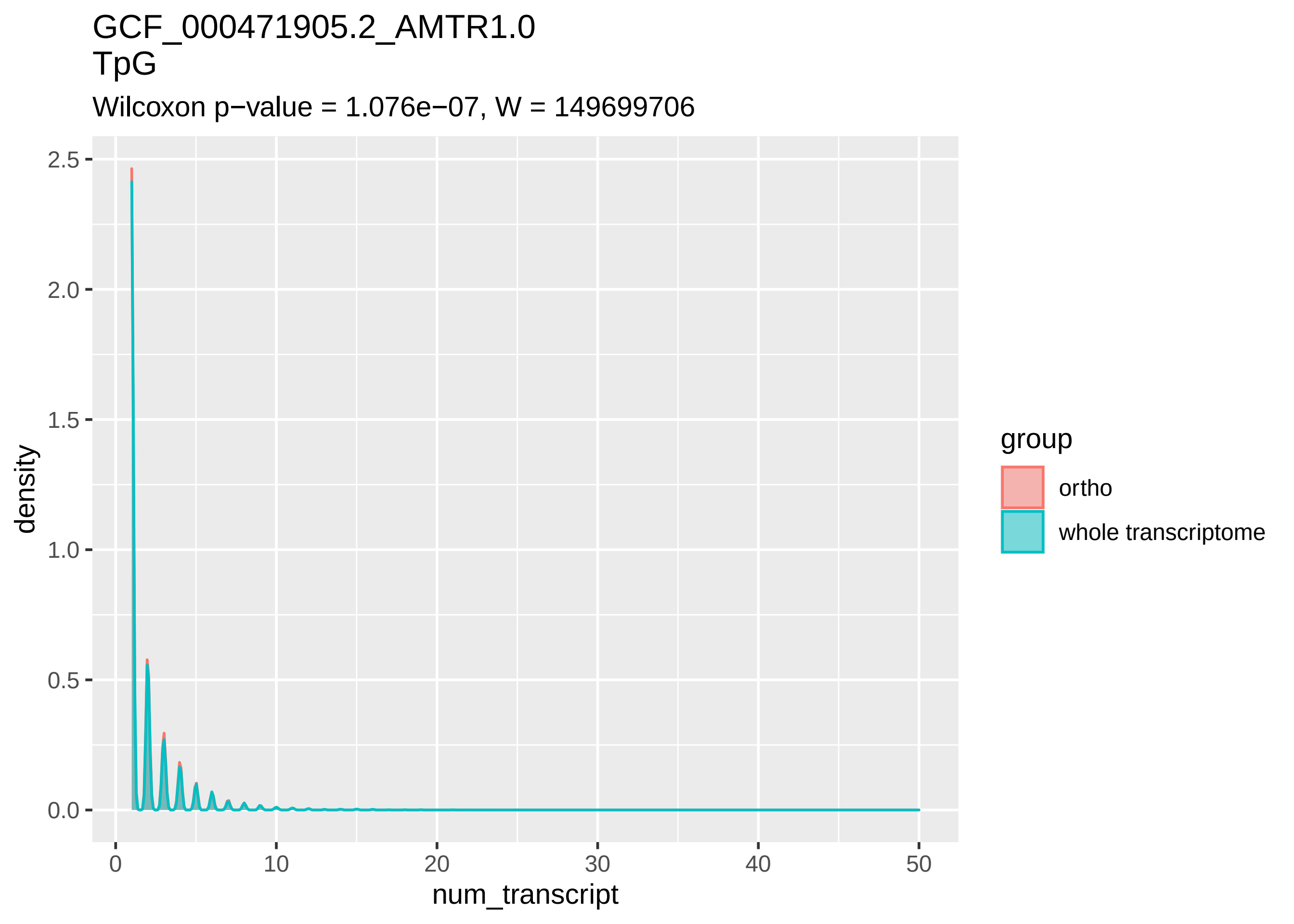
GCF_000471905.2_AMTR1.0
TpG
Wilcoxon p−value = 1.076e−07, W = 149699706
density
2.5
2.0
1.5
1.0
0.5
0.0
0
10
20
30
40
50
num_transcript
group
ortho
whole transcriptome

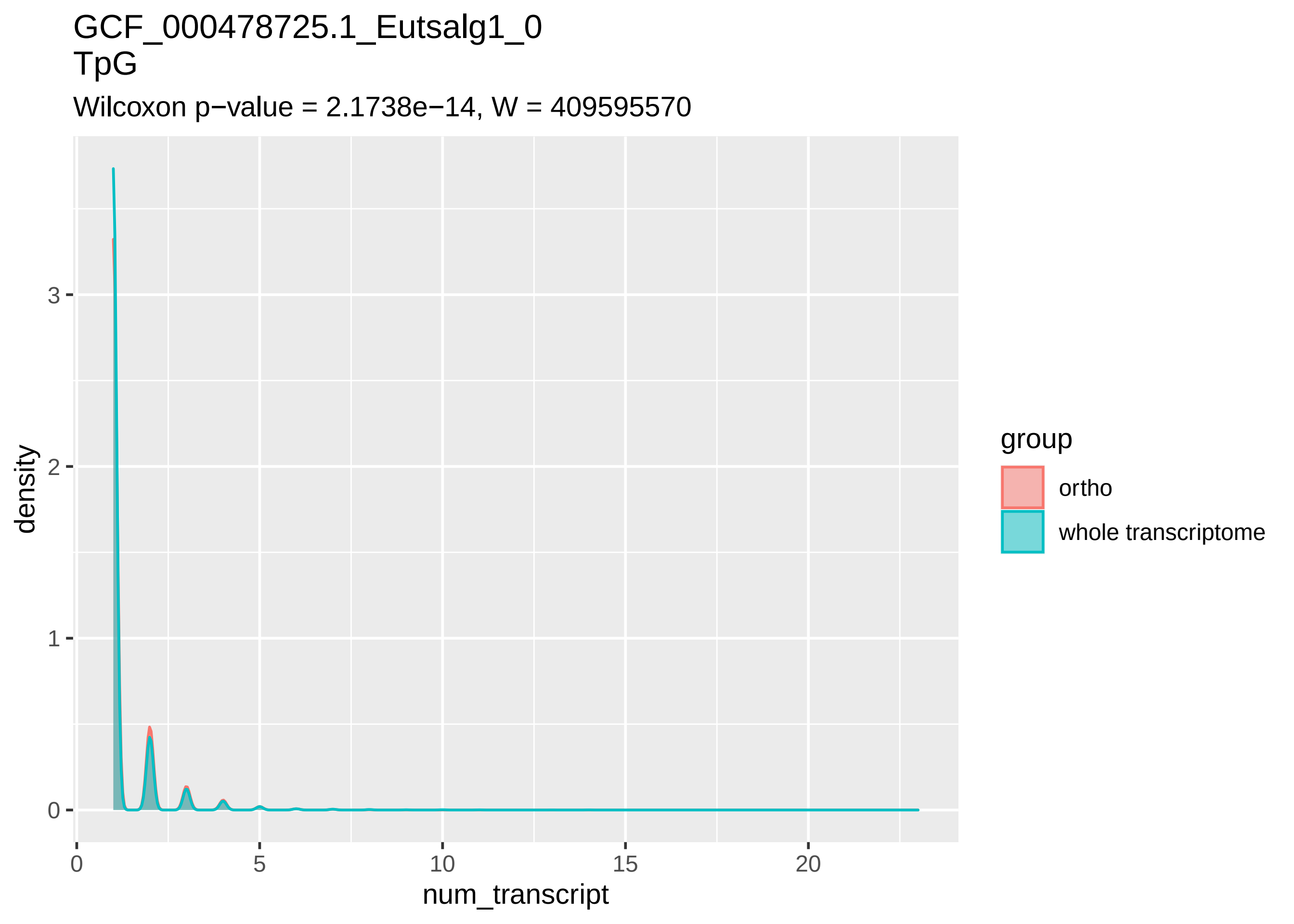
GCF_000478725.1_Eutsalg1_0
TpG
Wilcoxon p−value = 2.1738e−14, W = 409595570
density
num_transcript
0
1
2
3
0
5
10
15
20
group
ortho
whole transcriptome

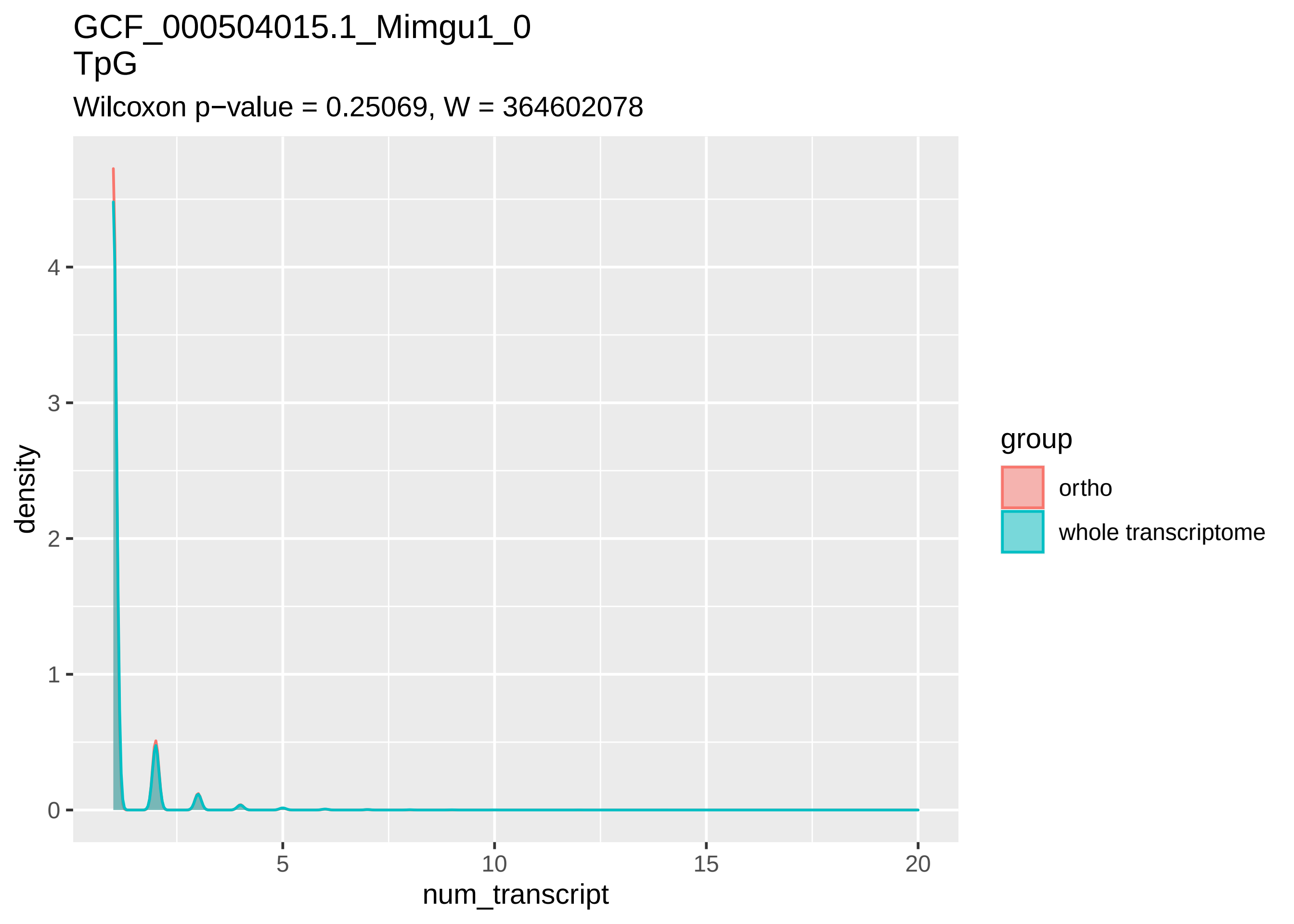
GCF_000504015.1_Mimgu1_0
TpG
Wilcoxon p−value = 0.25069, W = 364602078
density
num_transcript
0
1
2
3
4
5
10
15
20
group
ortho
whole transcriptome

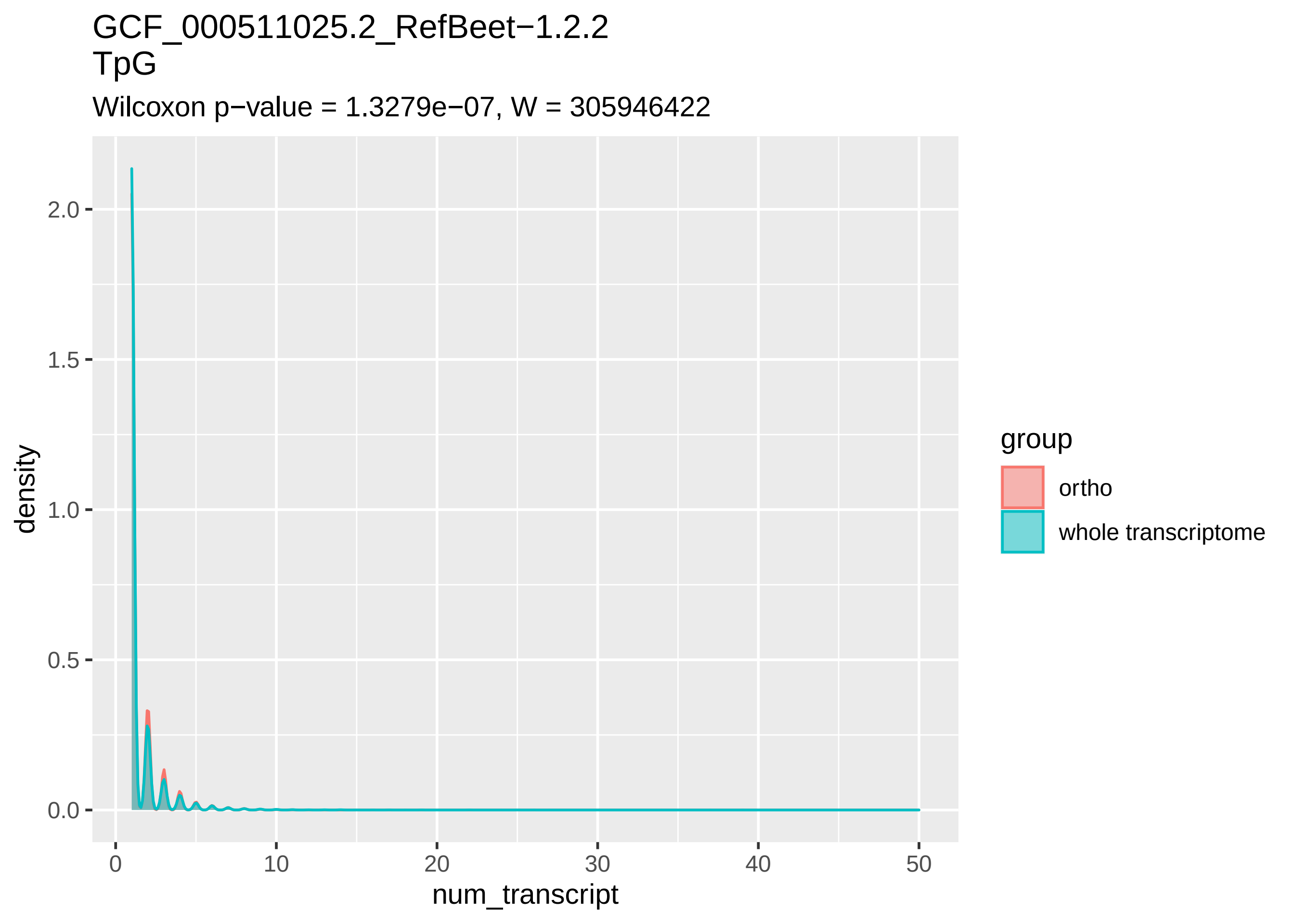
GCF_000511025.2_RefBeet−1.2.2
TpG
Wilcoxon p−value = 1.3279e−07, W = 305946422
density
2.0
1.5
1.0
0.5
0.0
0
10
20
30
40
50
num_transcript
group
ortho
whole transcriptome

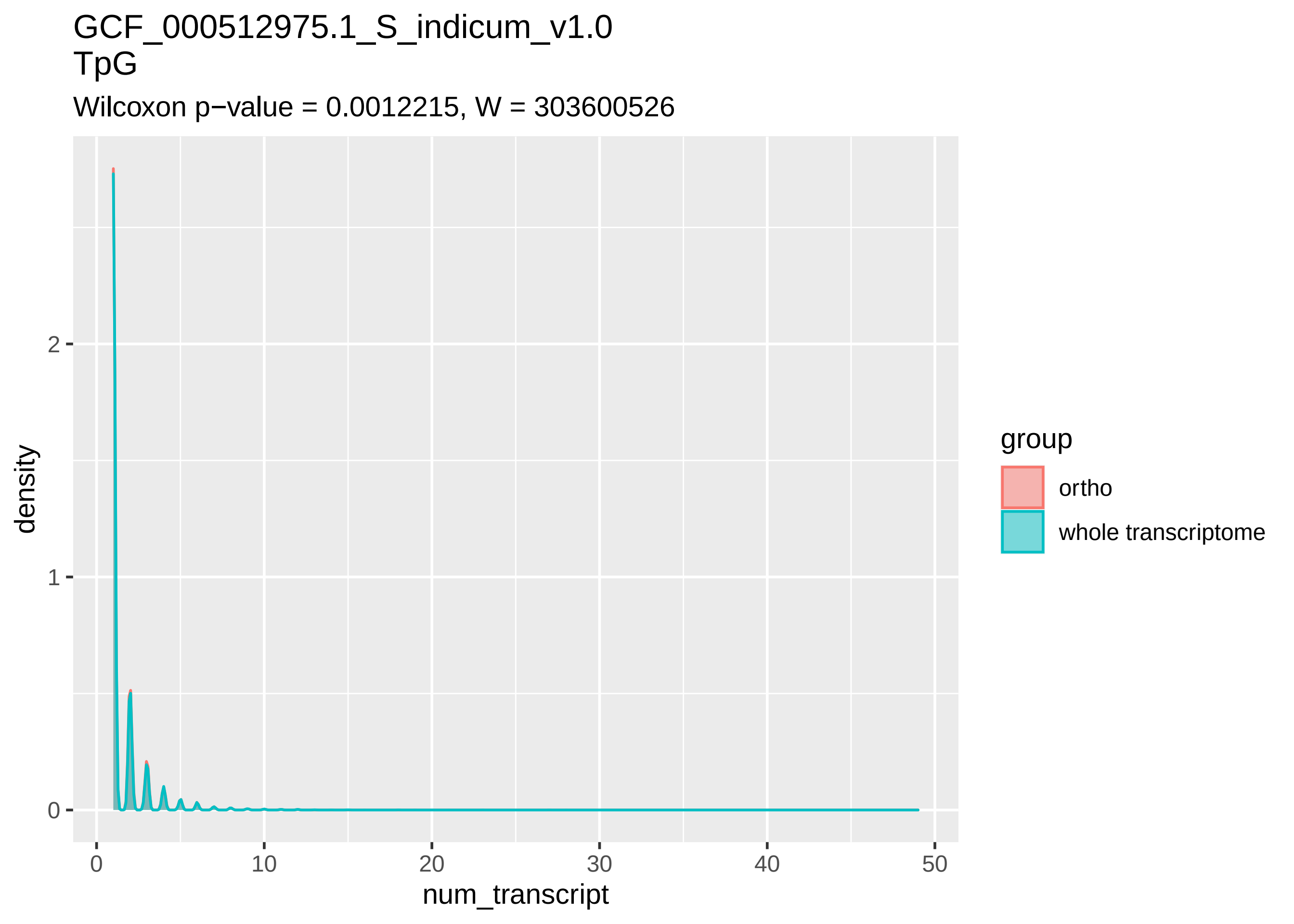
GCF_000512975.1_S_indicum_v1.0
TpG
Wilcoxon p−value = 0.0012215, W = 303600526
density
2
1
0
0
10
20
30
40
50
num_transcript
group
ortho
whole transcriptome

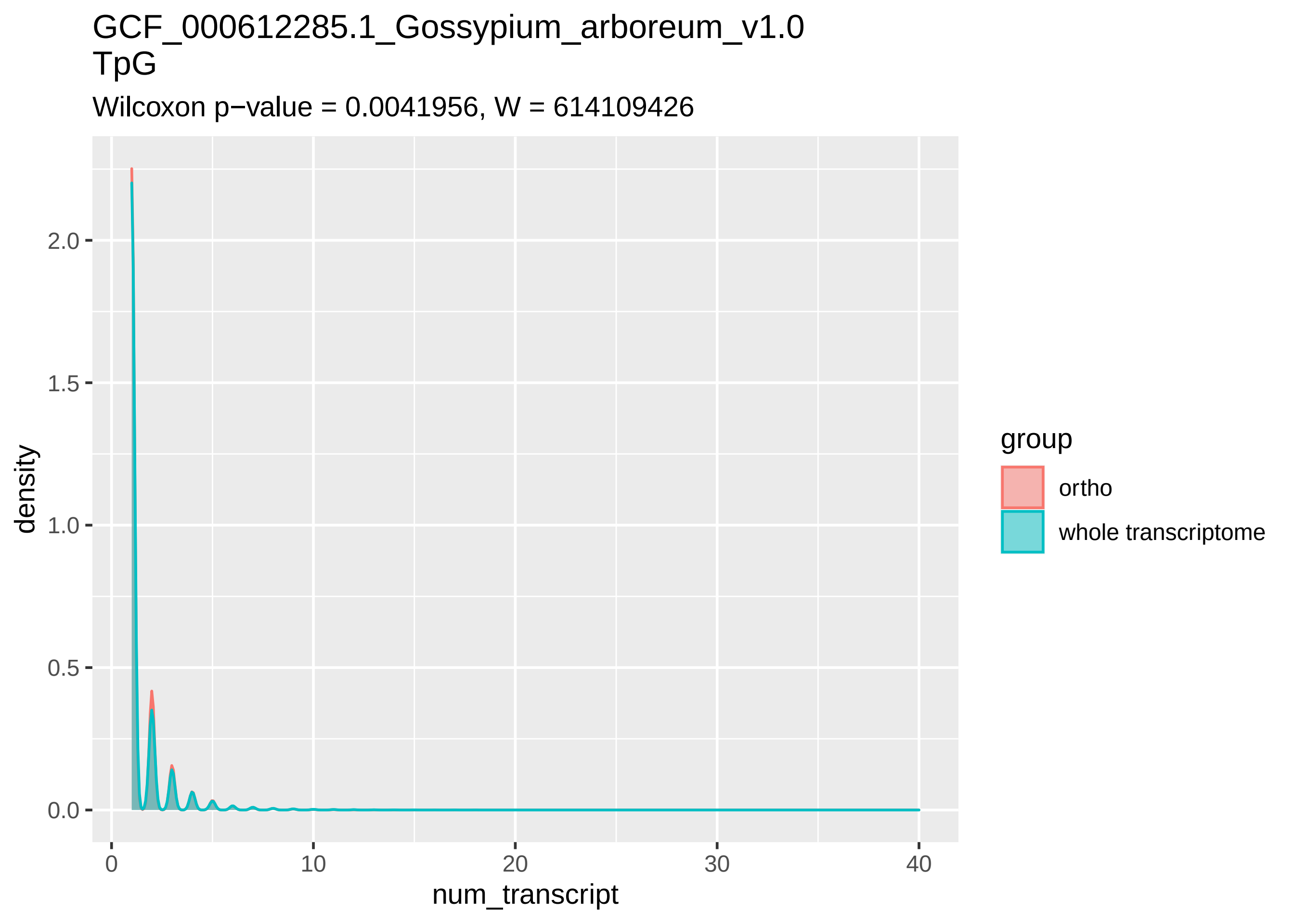
GCF_000612285.1_Gossypium_arboreum_v1.0
TpG
Wilcoxon p−value = 0.0041956, W = 614109426
density
2.0
1.5
1.0
0.5
0.0
0
10
20
30
40
num_transcript
group
ortho
whole transcriptome

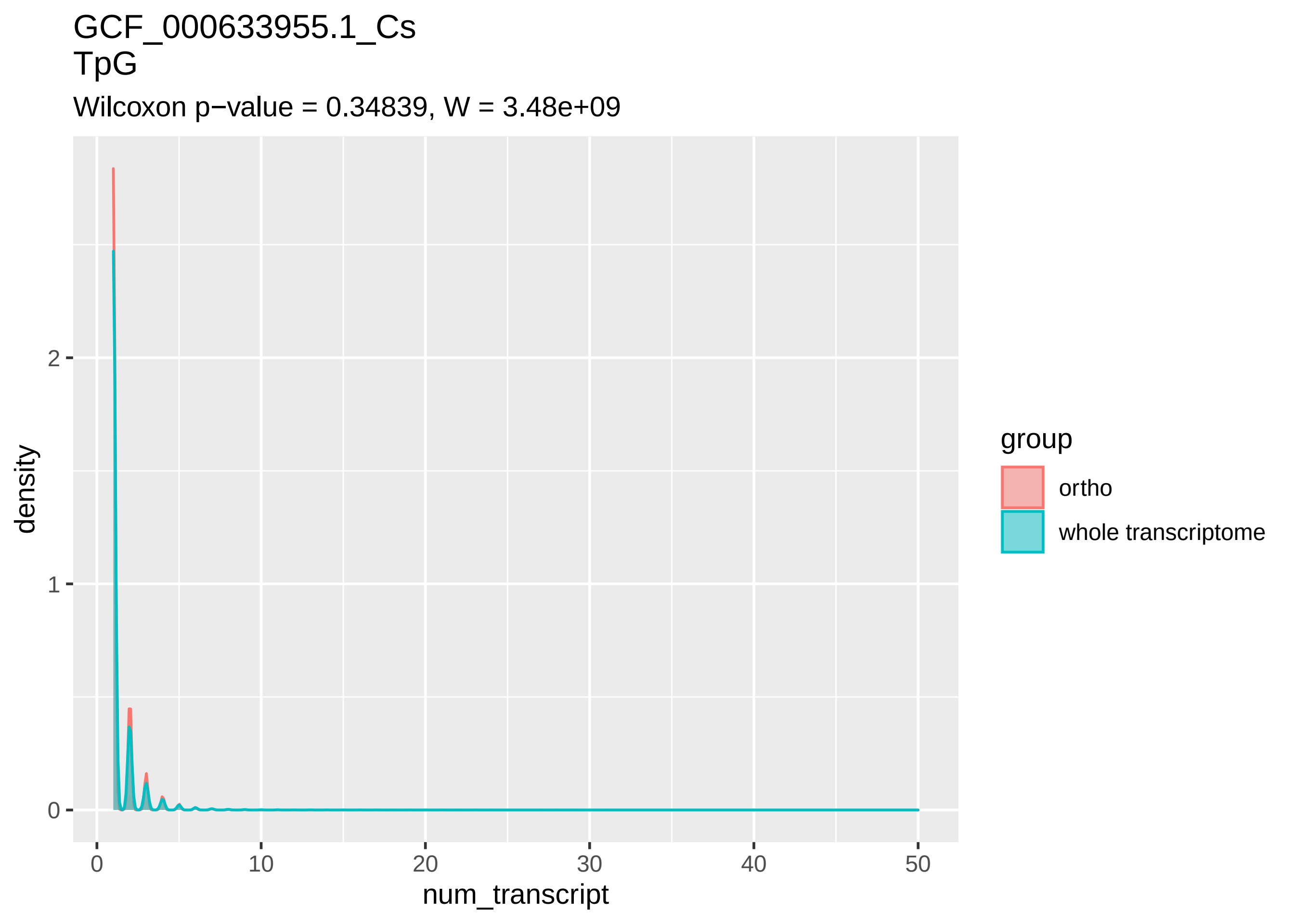
GCF_000633955.1_Cs
TpG
Wilcoxon p−value = 0.34839, W = 3.48e+09
density
2
1
0
0
10
20
30
40
50
num_transcript
group
ortho
whole transcriptome

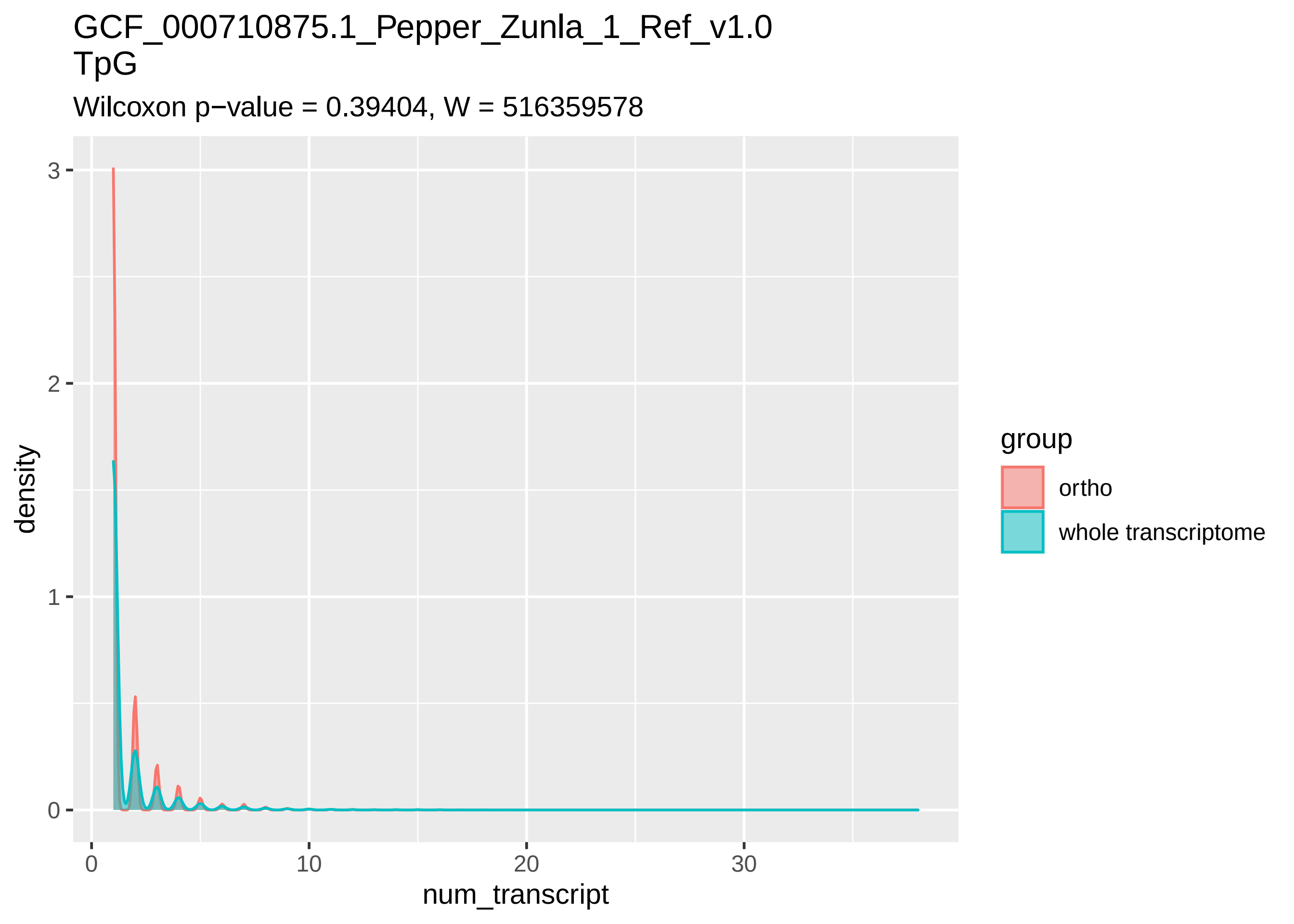

GCF_000710875.1_Pepper_Zunla_1_Ref_v1.0
TpG
Wilcoxon p−value = 0.39404, W = 516359578
density
num_transcript
0
1
2
3
0
10
20
30
group
ortho
whole transcriptome

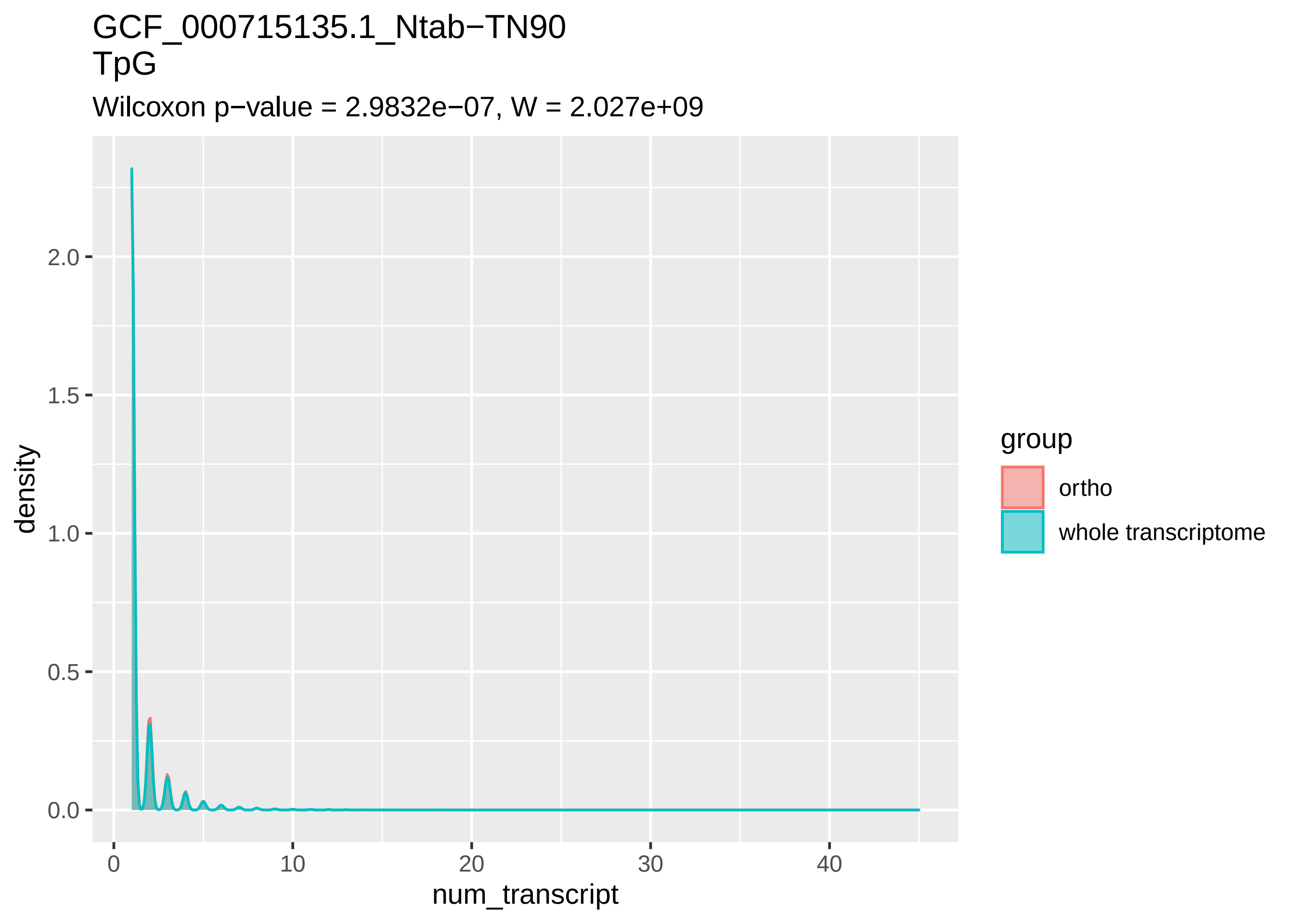

GCF_000715135.1_Ntab−TN90
TpG
Wilcoxon p−value = 2.9832e−07, W = 2.027e+09
density
2.0
1.5
1.0
0.5
0.0
0
10
20
30
40
num_transcript
group
ortho
whole transcriptome

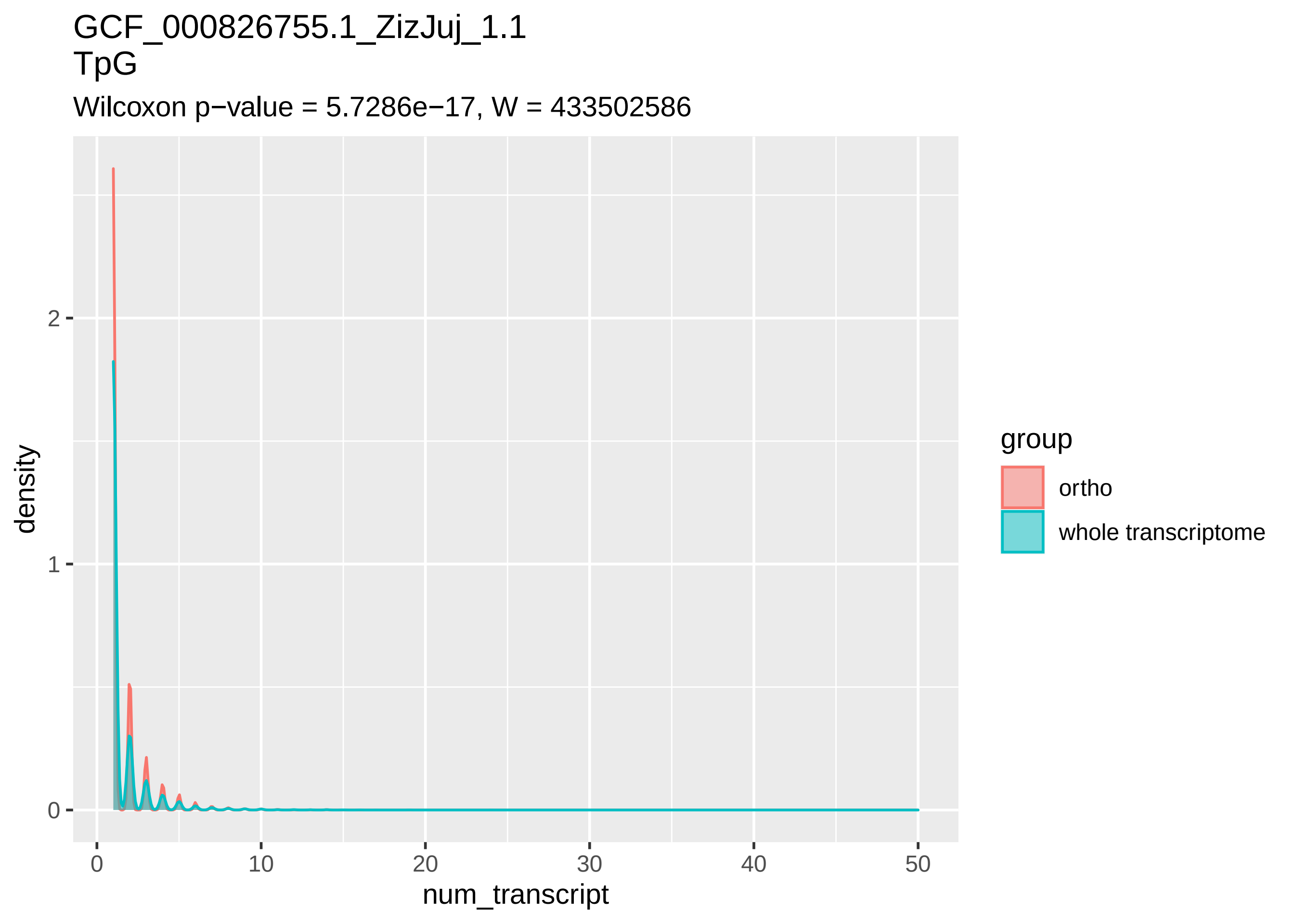
GCF_000826755.1_ZizJuj_1.1
TpG
Wilcoxon p−value = 5.7286e−17, W = 433502586
density
2
1
0
0
10
20
30
40
50
num_transcript
group
ortho
whole transcriptome

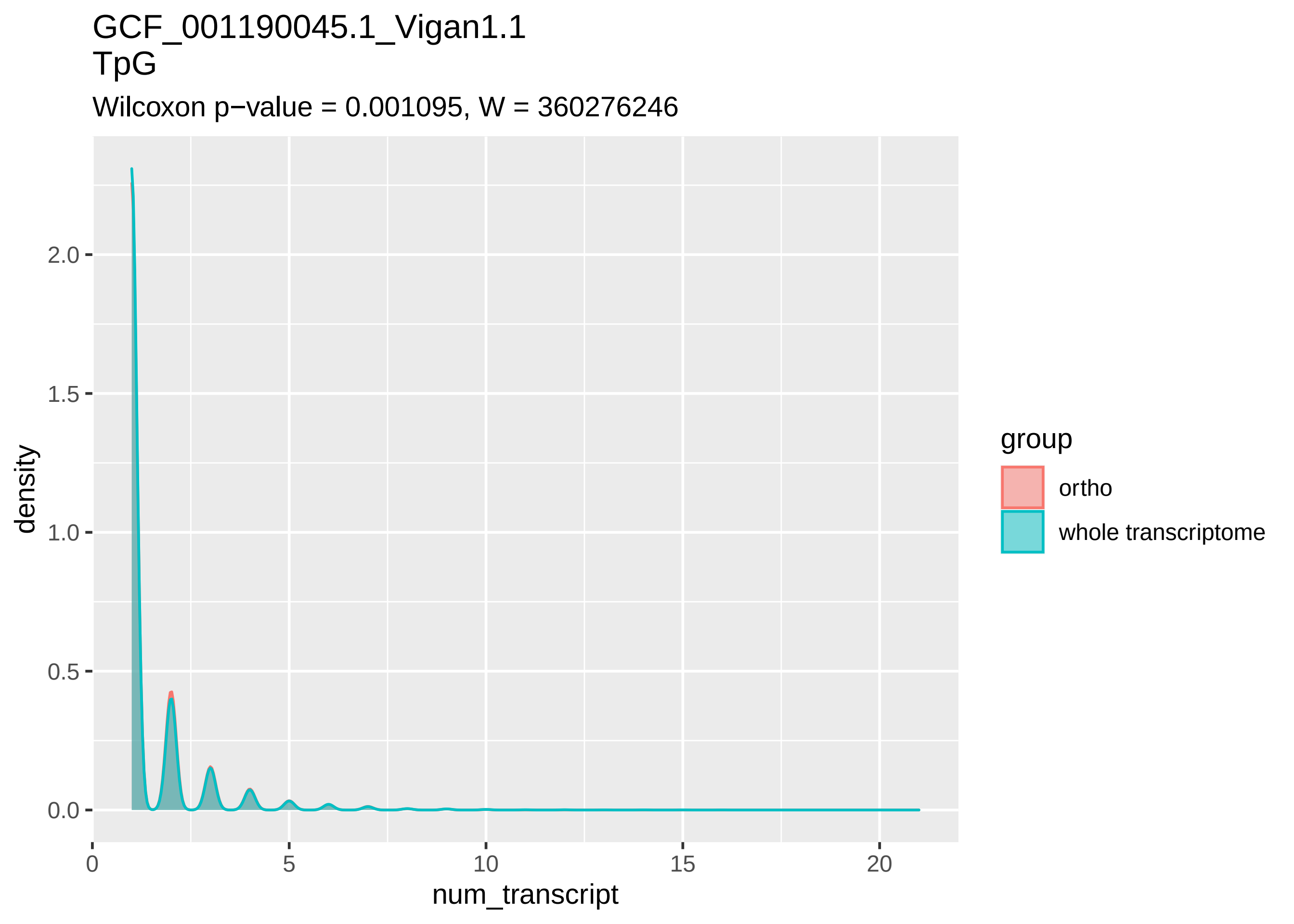

GCF_001190045.1_Vigan1.1
TpG
Wilcoxon p−value = 0.001095, W = 360276246
density
2.0
1.5
1.0
0.5
0.0
0
5
10
15
20
num_transcript
group
ortho
whole transcriptome

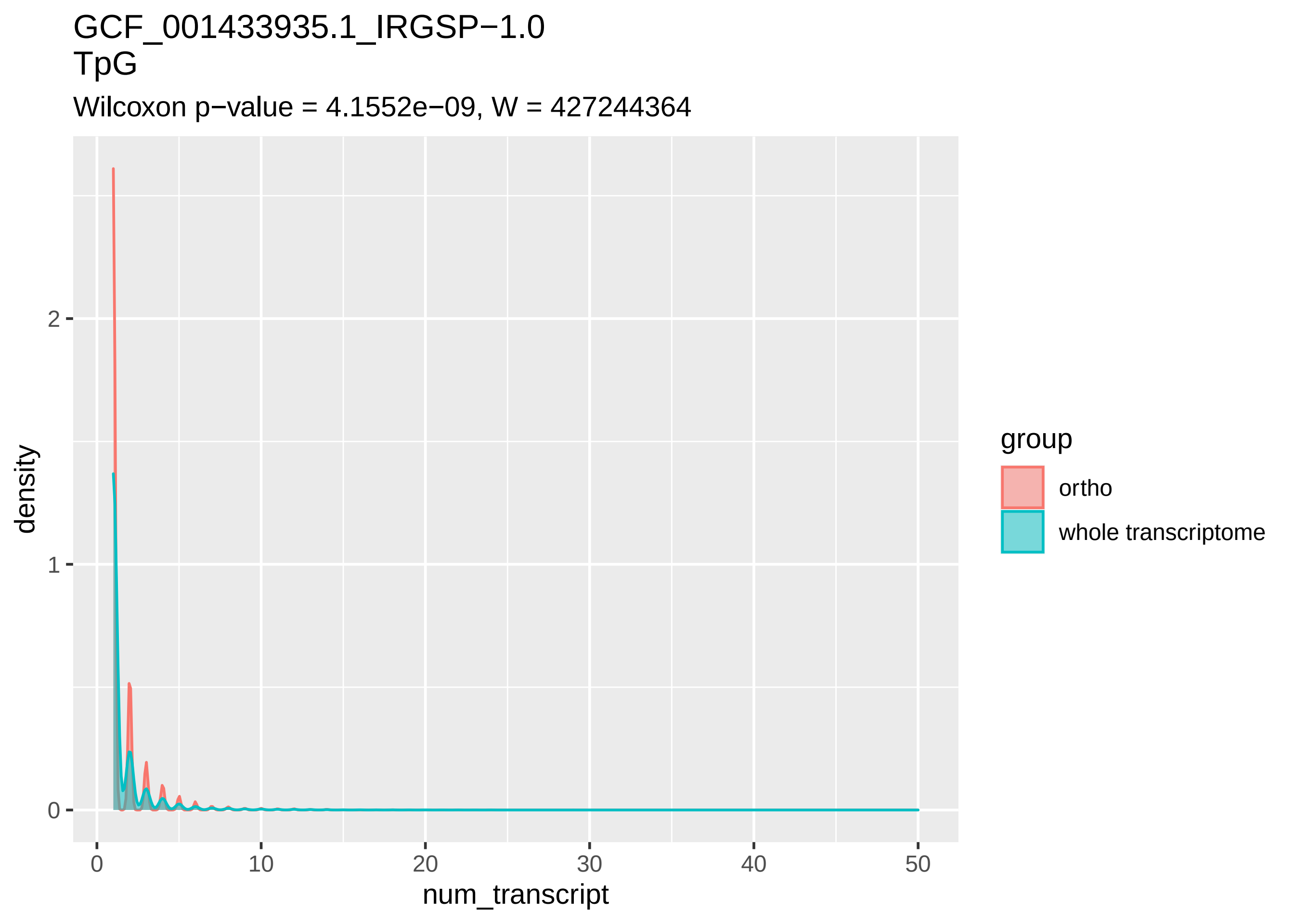
GCF_001433935.1_IRGSP−1.0
TpG
Wilcoxon p−value = 4.1552e−09, W = 427244364
density
2
1
0
0
10
20
30
40
50
num_transcript
group
ortho
whole transcriptome

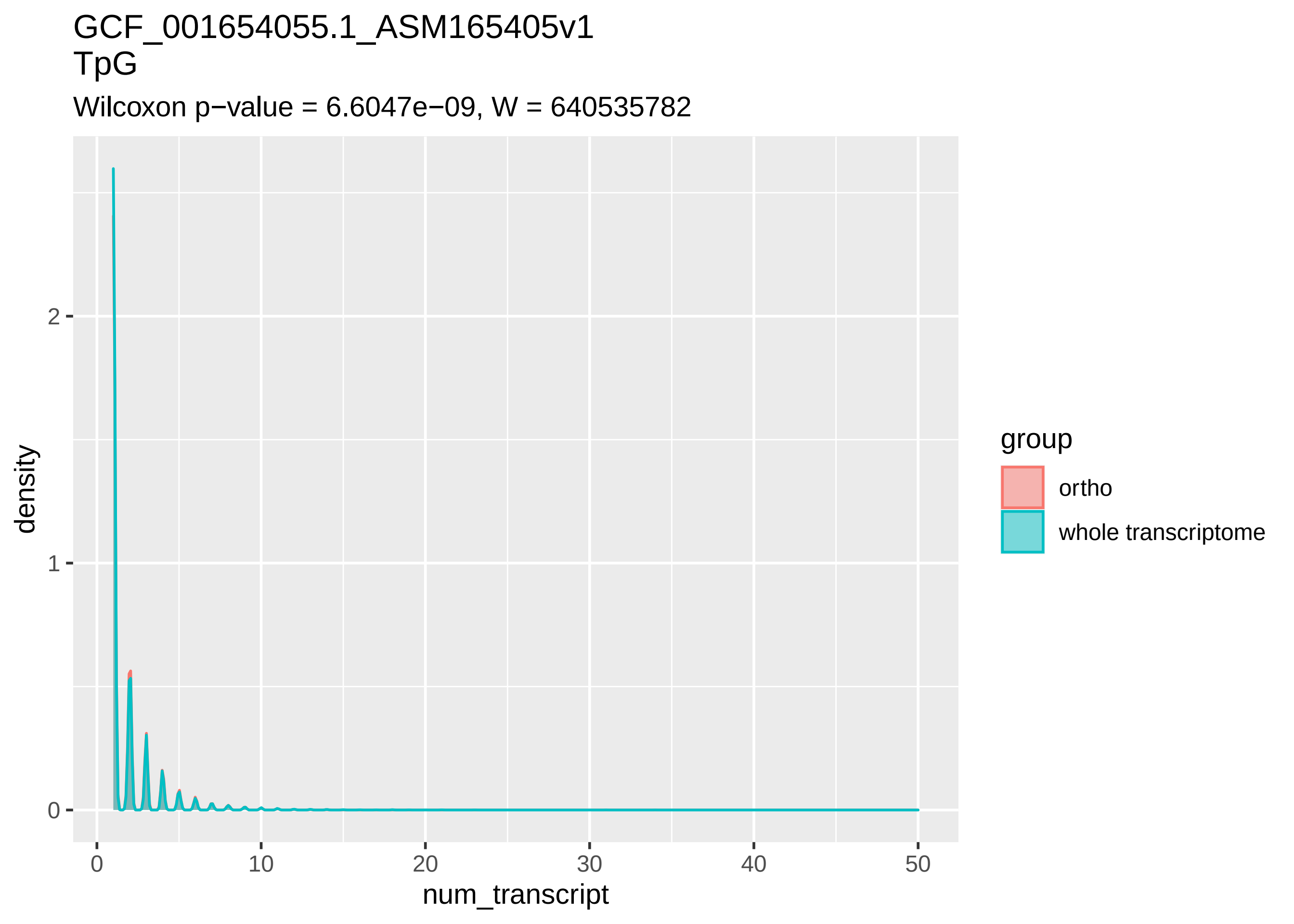
GCF_001654055.1_ASM165405v1
TpG
Wilcoxon p−value = 6.6047e−09, W = 640535782
density
num_transcript
0
1
2
0
10
20
30
40
50
group
ortho
whole transcriptome

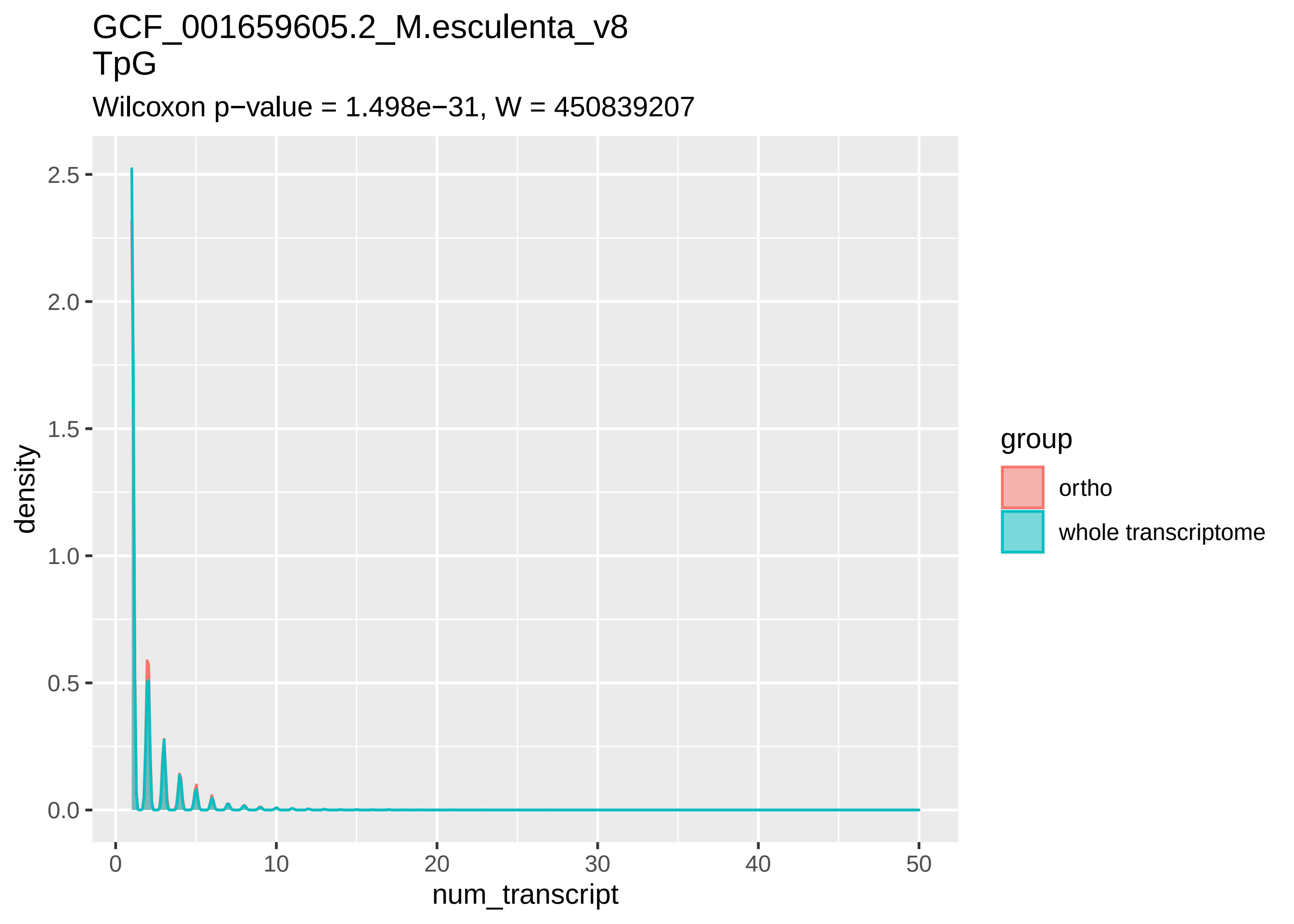

GCF_001659605.2_M.esculenta_v8
TpG
Wilcoxon p−value = 1.498e−31, W = 450839207
density
2.5
2.0
1.5
1.0
0.5
0.0
0
10
20
30
40
50
num_transcript
group
ortho
whole transcriptome

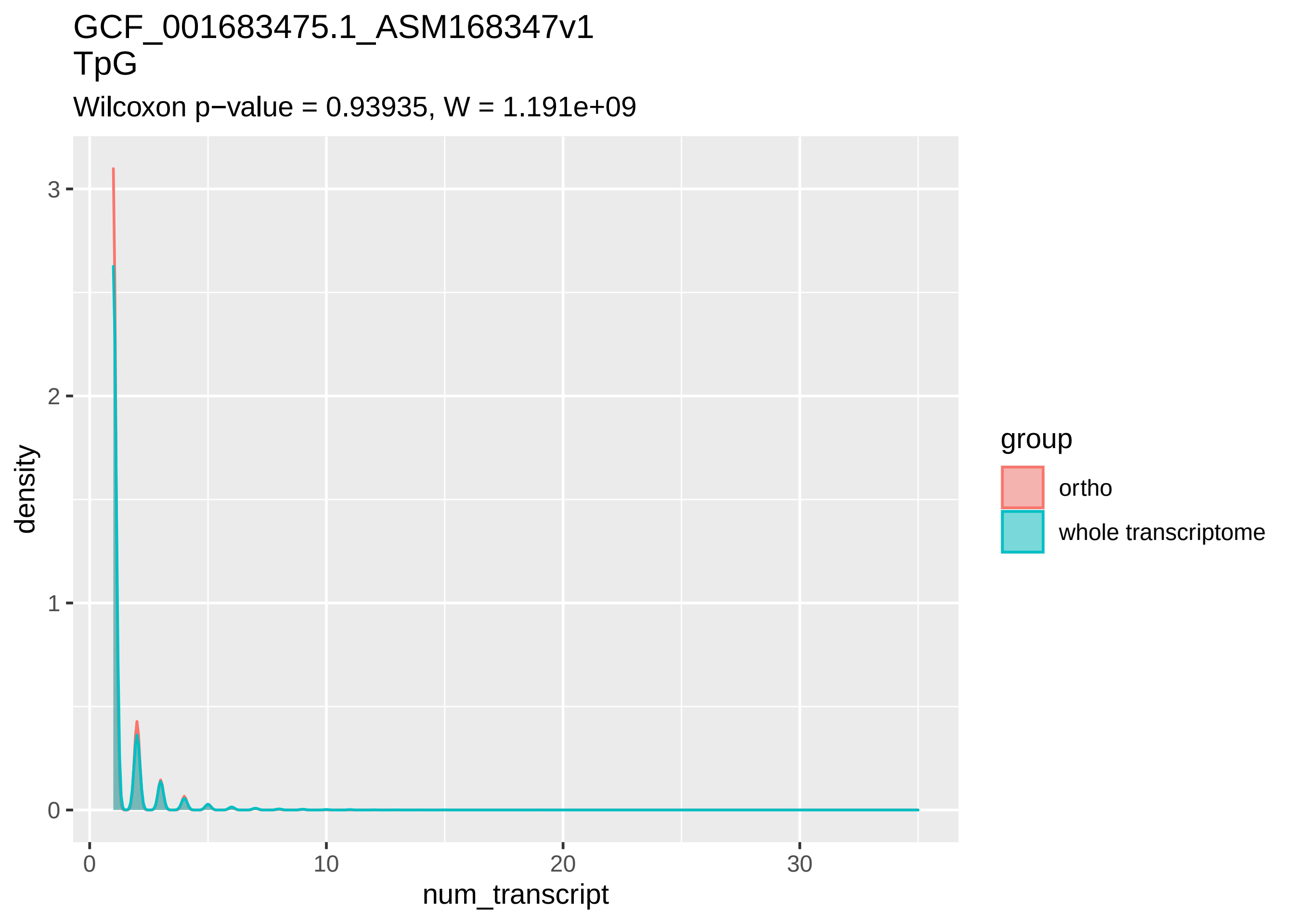

GCF_001683475.1_ASM168347v1
TpG
Wilcoxon p−value = 0.93935, W = 1.191e+09
density
num_transcript
0
1
2
3
0
10
20
30
group
ortho
whole transcriptome

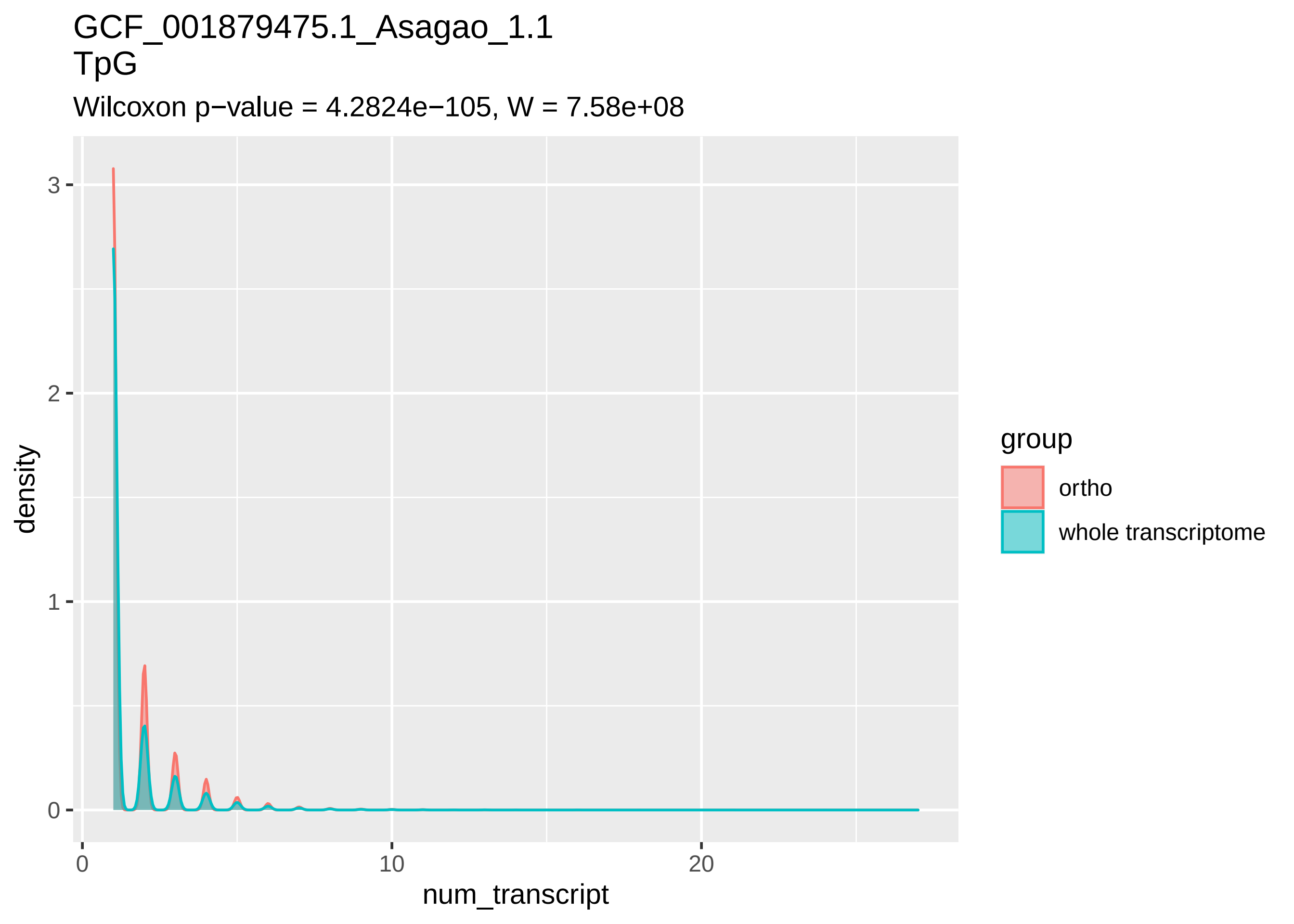
GCF_001879475.1_Asagao_1.1
TpG
Wilcoxon p−value = 4.2824e−105, W = 7.58e+08
density
3
2
1
0
0
10
20
num_transcript
group
ortho
whole transcriptome

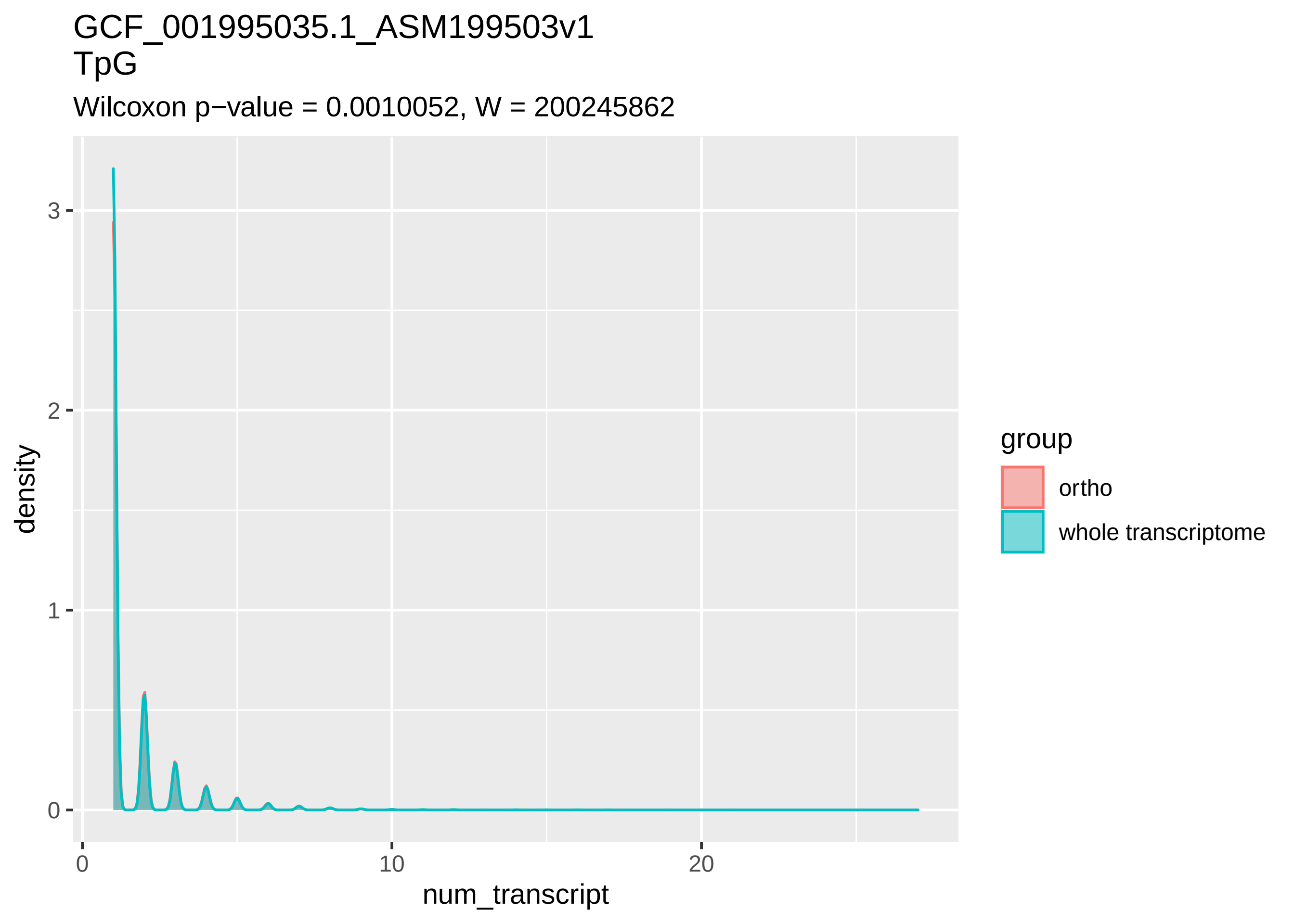

GCF_001995035.1_ASM199503v1
TpG
Wilcoxon p−value = 0.0010052, W = 200245862
density
num_transcript
0
1
2
3
0
10
20
group
ortho
whole transcriptome

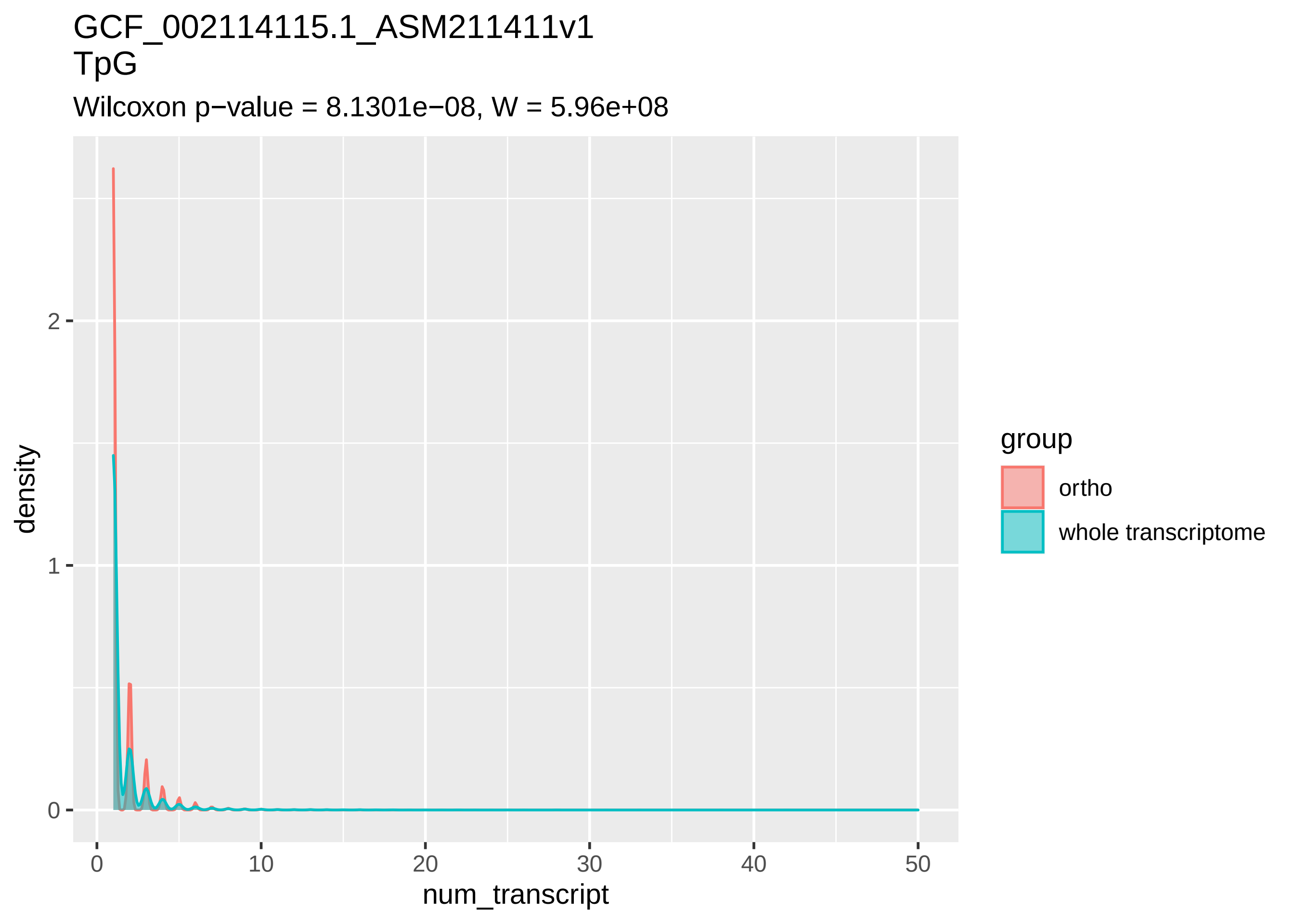

GCF_002114115.1_ASM211411v1
TpG
Wilcoxon p−value = 8.1301e−08, W = 5.96e+08
density
num_transcript
0
1
2
0
10
20
30
40
50
group
ortho
whole transcriptome

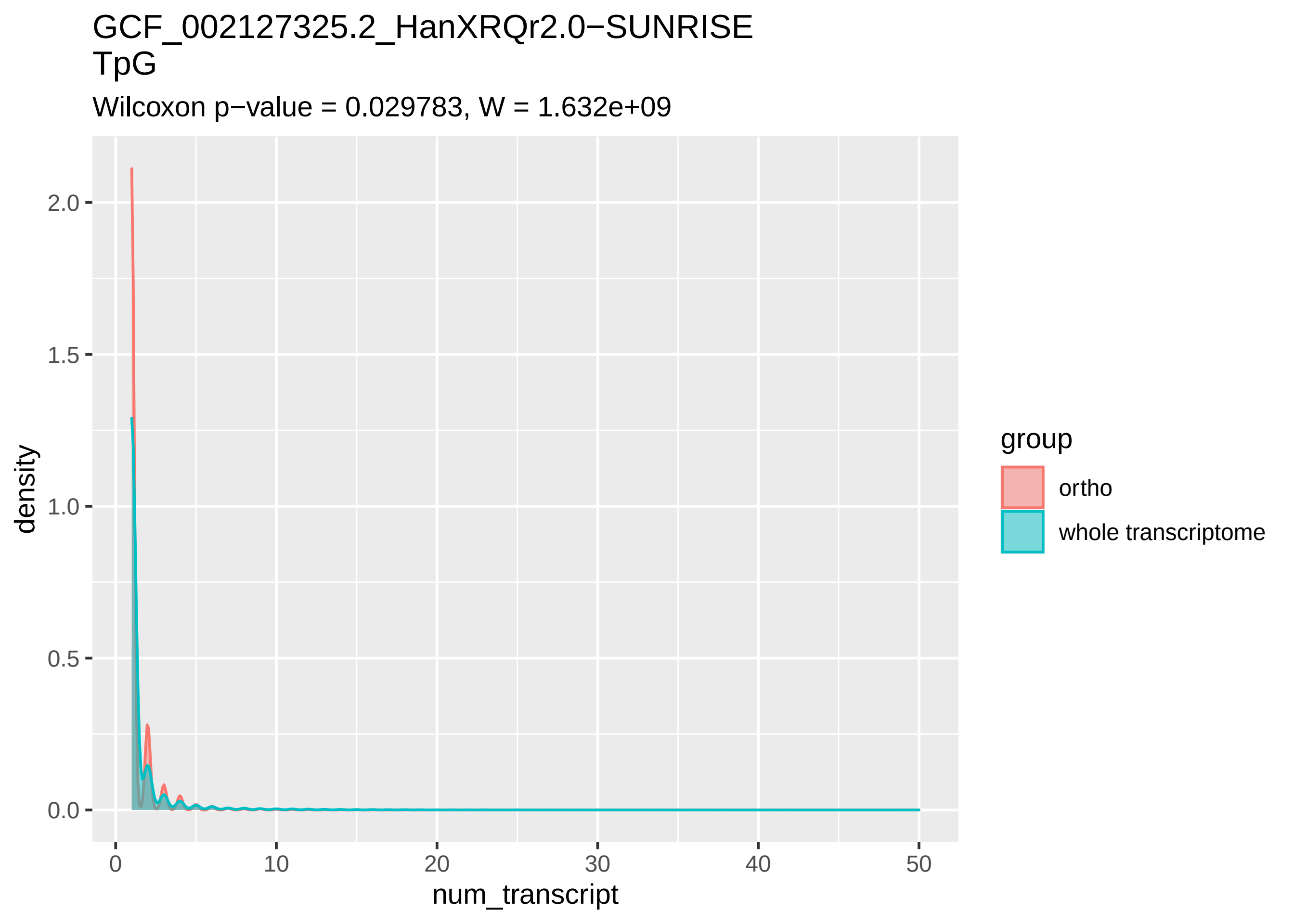
GCF_002127325.2_HanXRQr2.0−SUNRISE
TpG
Wilcoxon p−value = 0.029783, W = 1.632e+09
density
2.0
1.5
1.0
0.5
0.0
0
10
20
30
40
50
num_transcript
group
ortho
whole transcriptome

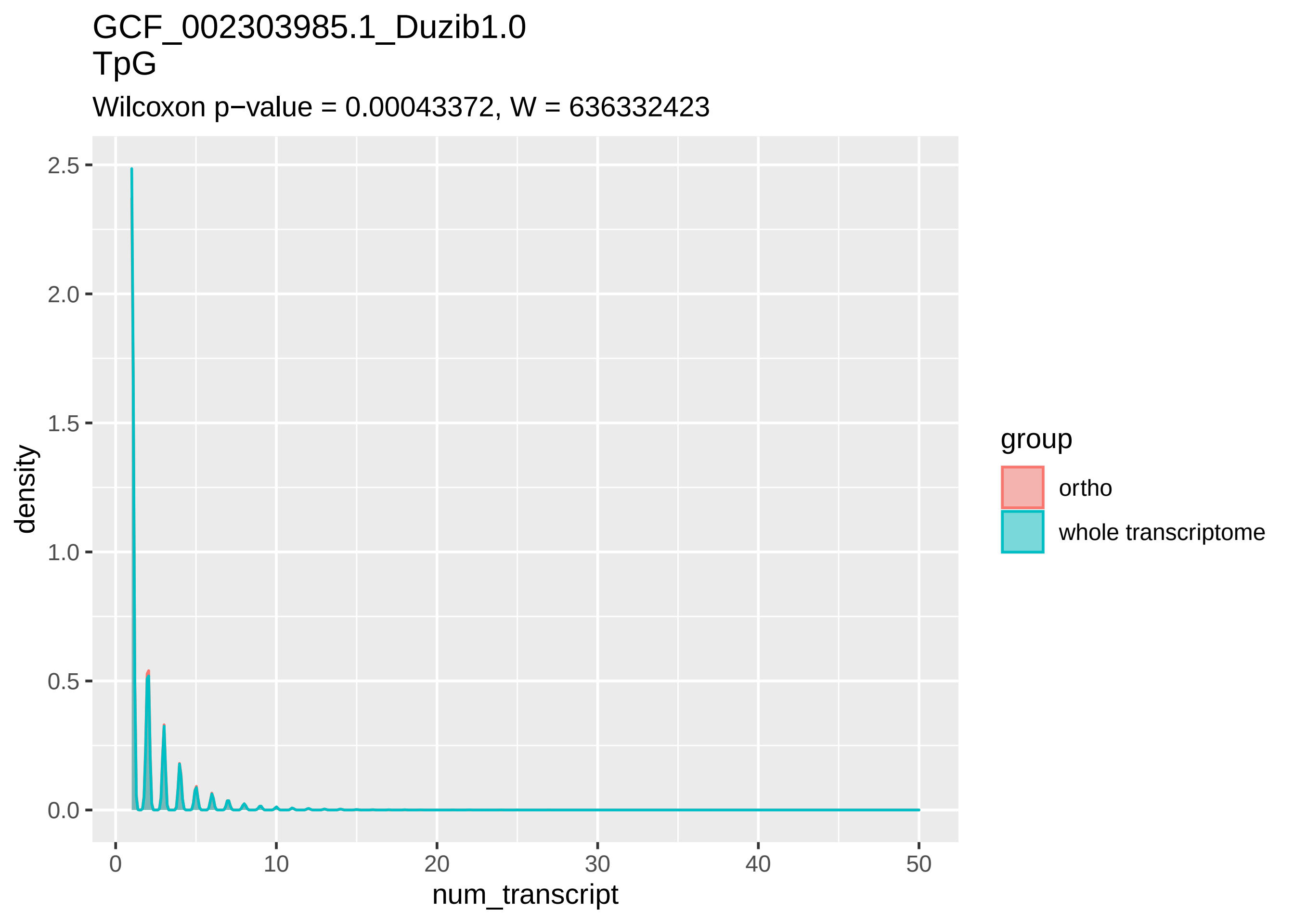
GCF_002303985.1_Duzib1.0
TpG
Wilcoxon p−value = 0.00043372, W = 636332423
density
num_transcript
2.5
2.0
1.5
1.0
0.5
0.0
0
10
20
30
40
50
group
ortho
whole transcriptome

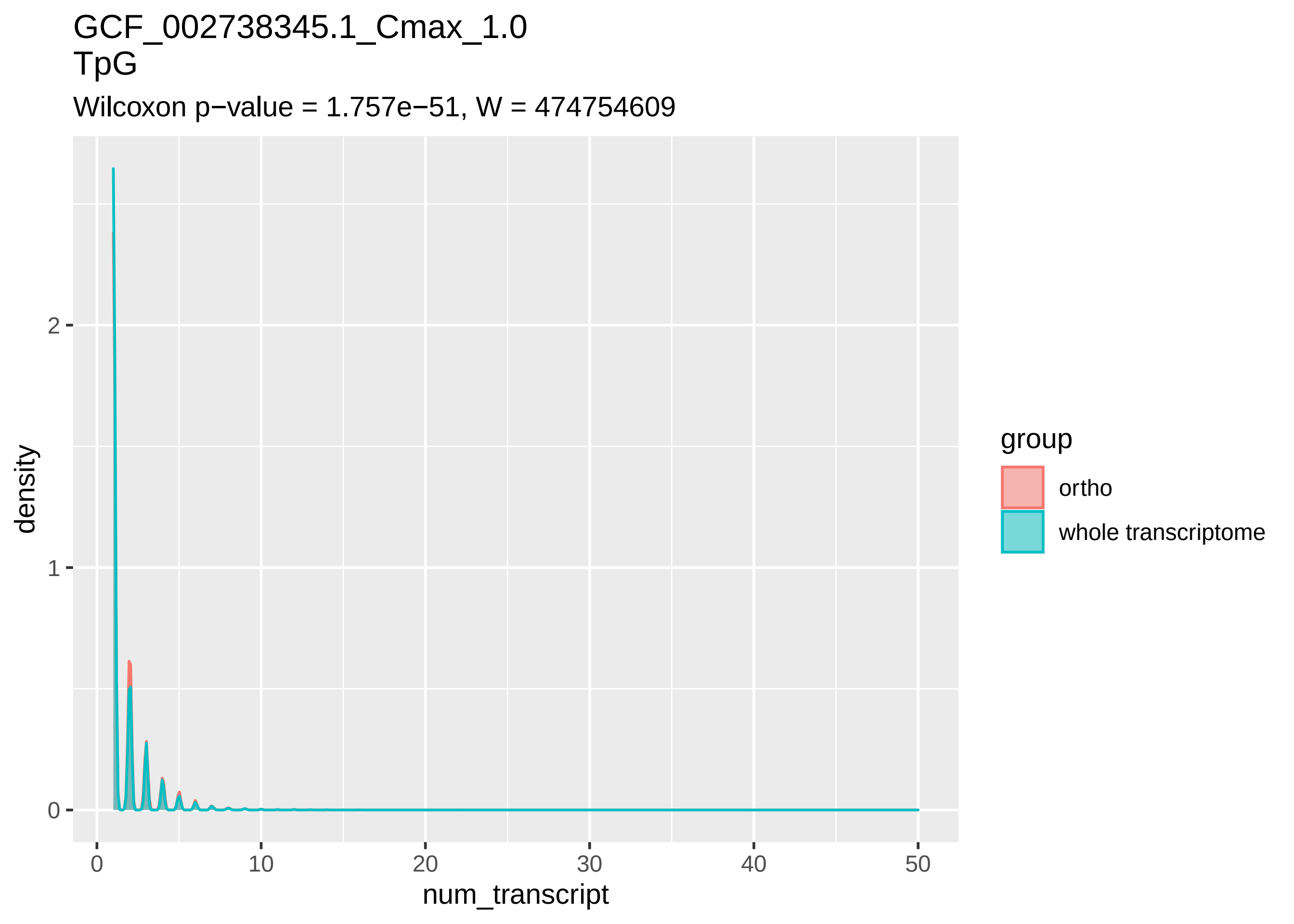
GCF_002738345.1_Cmax_1.0
TpG
Wilcoxon p−value = 1.757e−51, W = 474754609
density
num_transcript
0
1
2
0
10
20
30
40
50
group
ortho
whole transcriptome

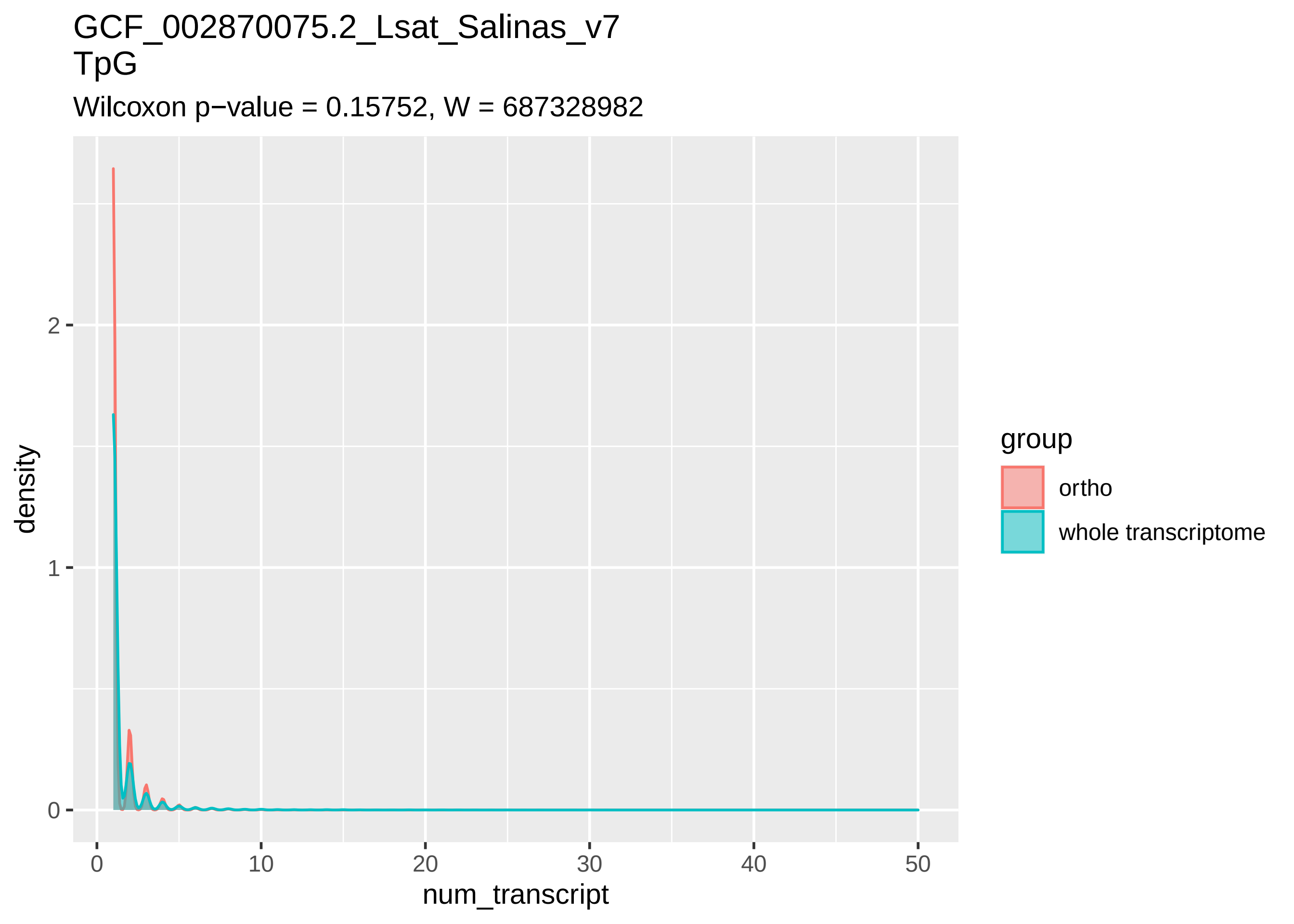
GCF_002870075.2_Lsat_Salinas_v7
TpG
Wilcoxon p−value = 0.15752, W = 687328982
density
2
1
0
0
10
20
30
40
50
num_transcript
group
ortho
whole transcriptome

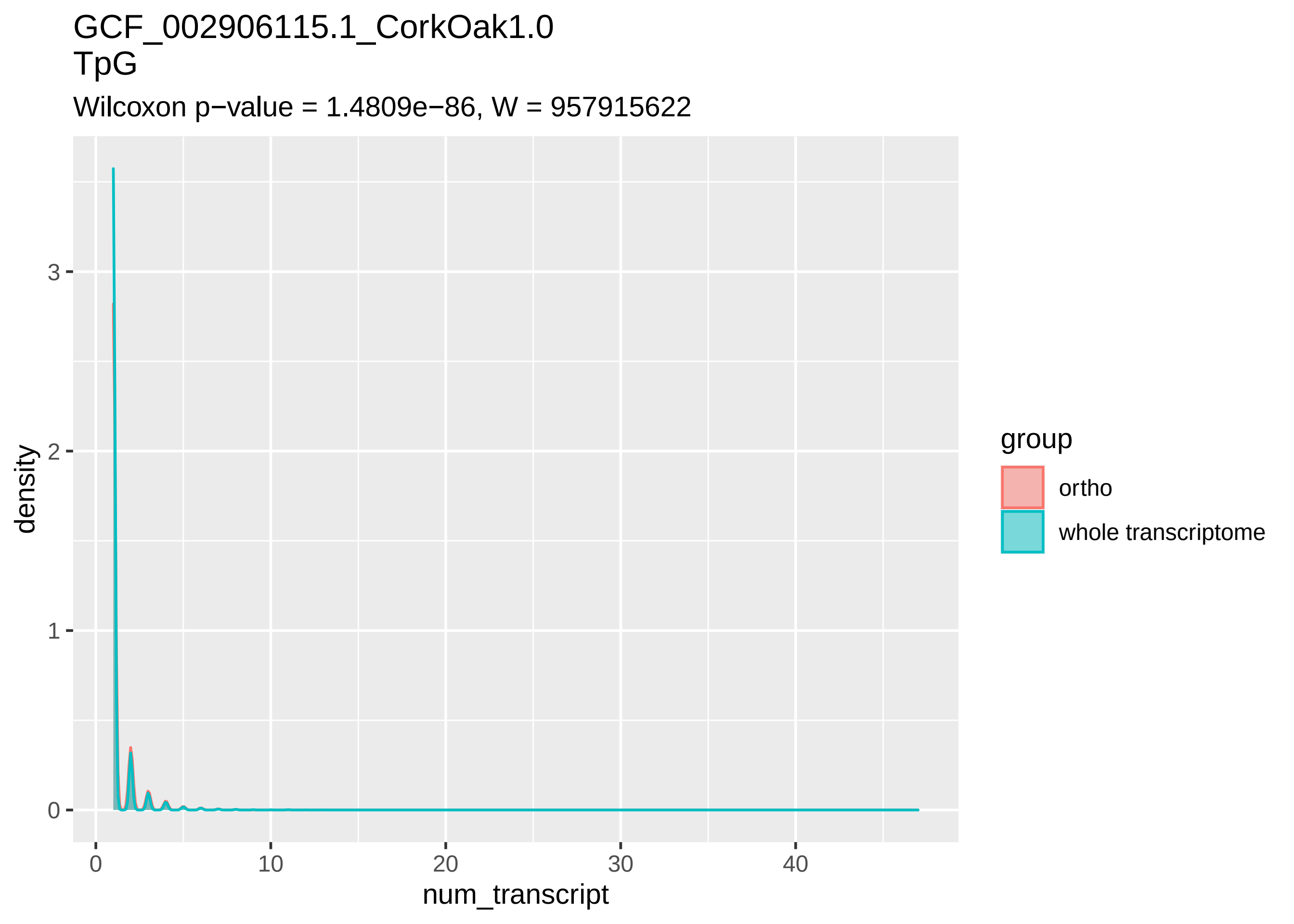

GCF_002906115.1_CorkOak1.0
TpG
Wilcoxon p−value = 1.4809e−86, W = 957915622
density
num_transcript
0
1
2
3
0
10
20
30
40
group
ortho
whole transcriptome

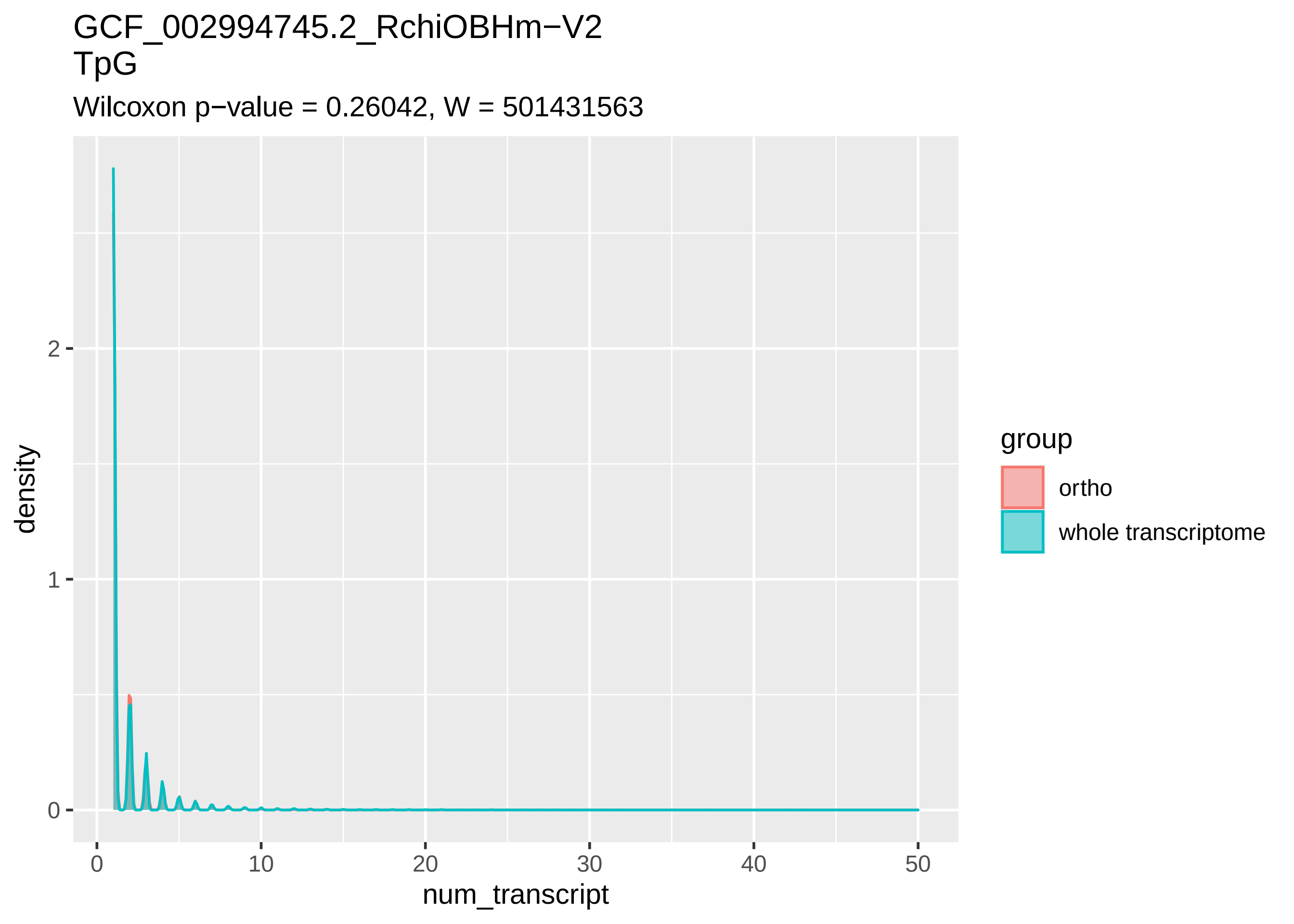
GCF_002994745.2_RchiOBHm−V2
TpG
Wilcoxon p−value = 0.26042, W = 501431563
density
0
1
2
0
10
20
30
40
50
num_transcript
group
ortho
whole transcriptome

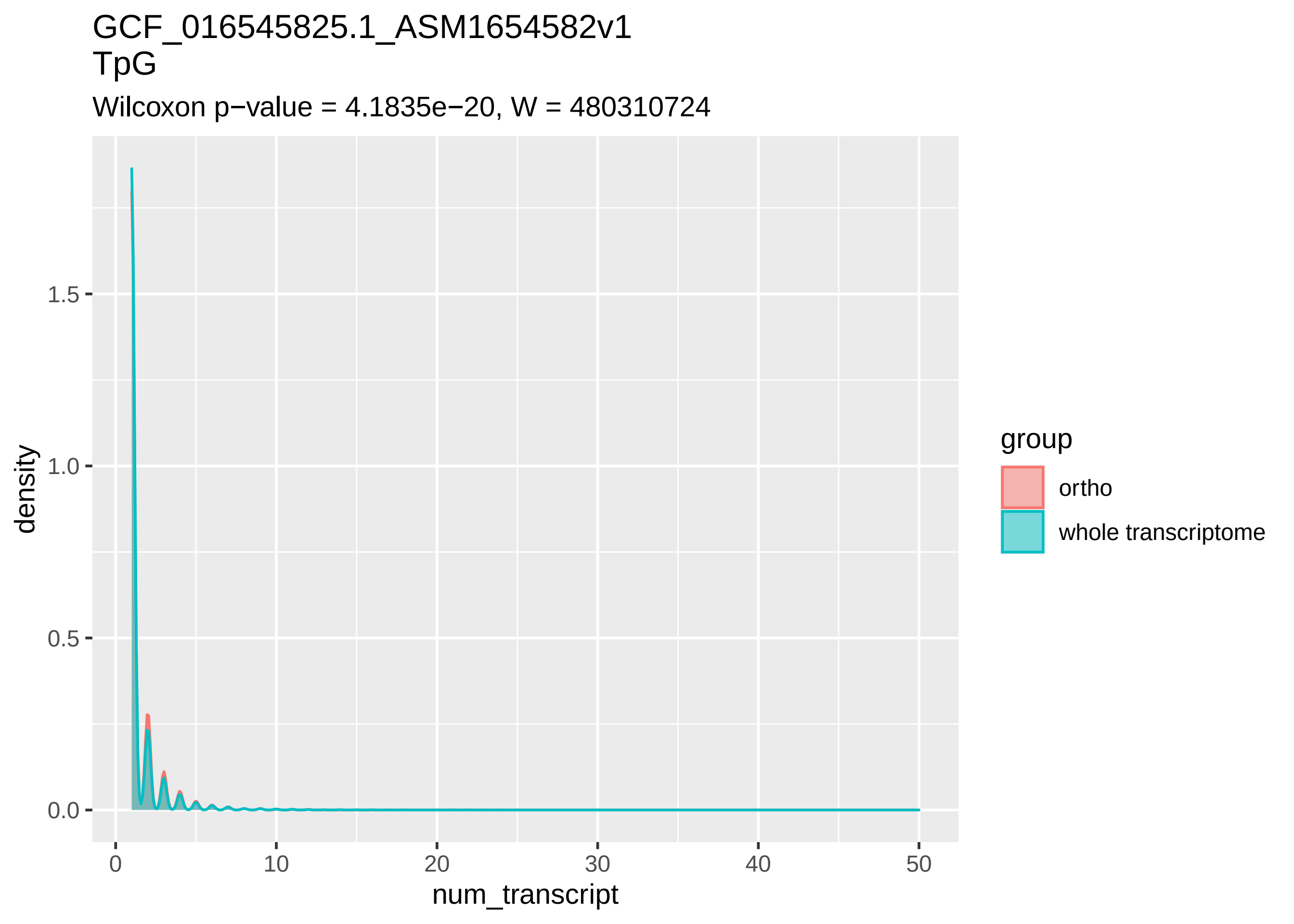

GCF_016545825.1_ASM1654582v1
TpG
Wilcoxon p−value = 4.1835e−20, W = 480310724
density
num_transcript
group
ortho
whole transcriptome
0.0
0.5
1.0
1.5
0
10
20
30
40
50

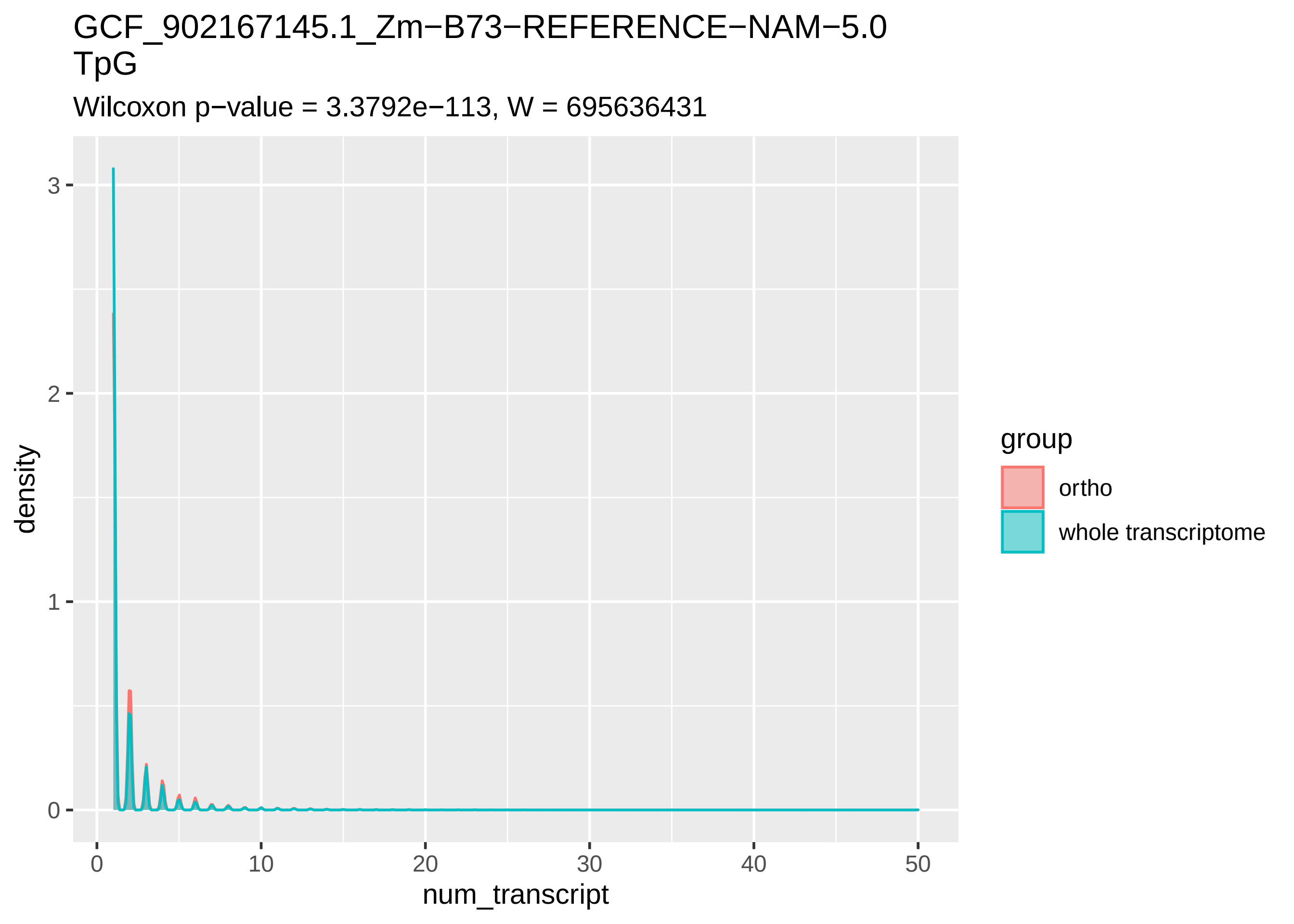
GCF_902167145.1_Zm−B73−REFERENCE−NAM−5.0
TpG
Wilcoxon p−value = 3.3792e−113, W = 695636431
density
num_transcript
0
1
2
3
0
10
20
30
40
50
group
ortho
whole transcriptome

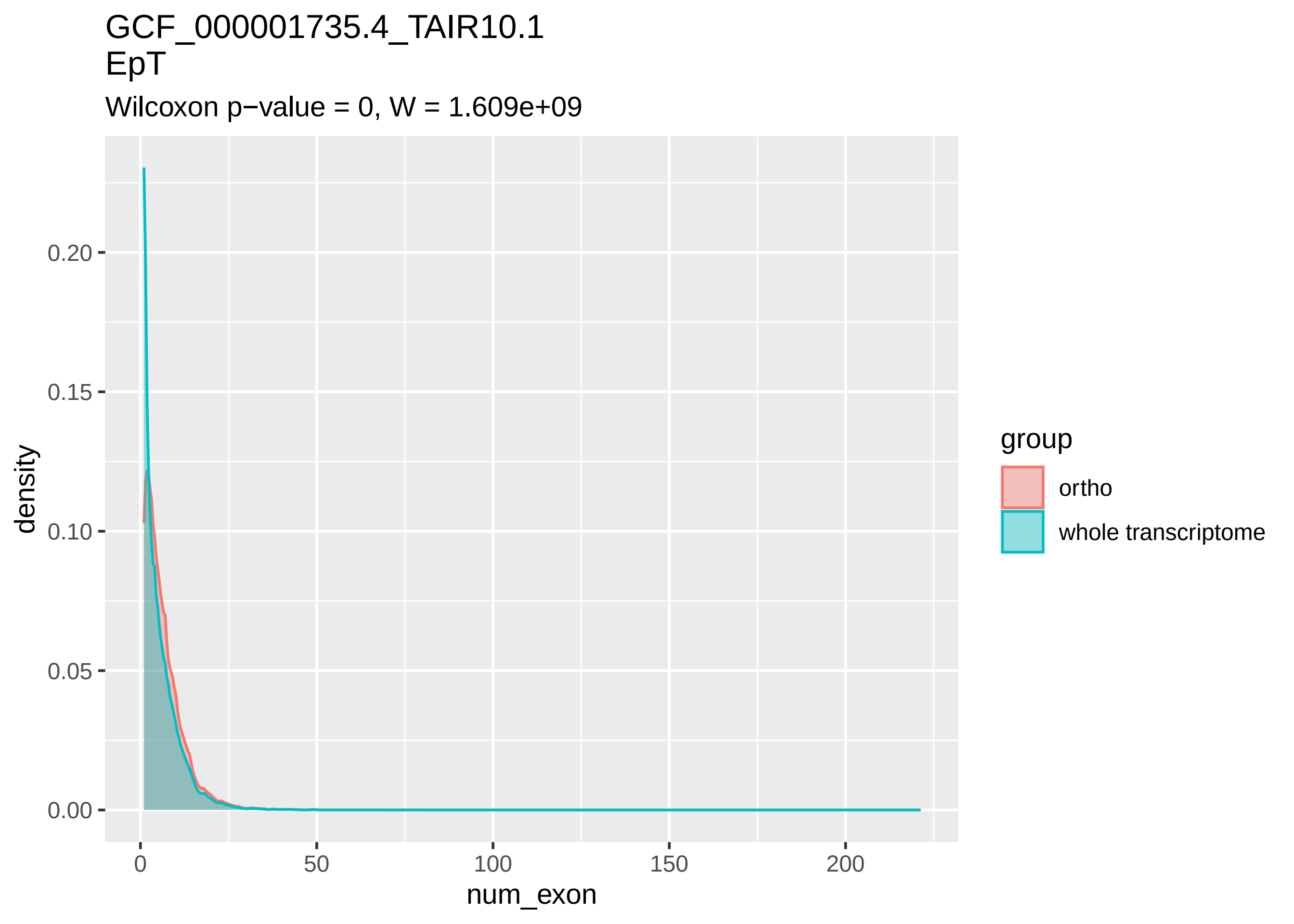
GCF_000001735.4_TAIR10.1
EpT
Wilcoxon p−value = 0, W = 1.609e+09
density
0.20
0.15
0.10
0.05
0.00
0
50
100
150
200
num_exon
group
ortho
whole transcriptome

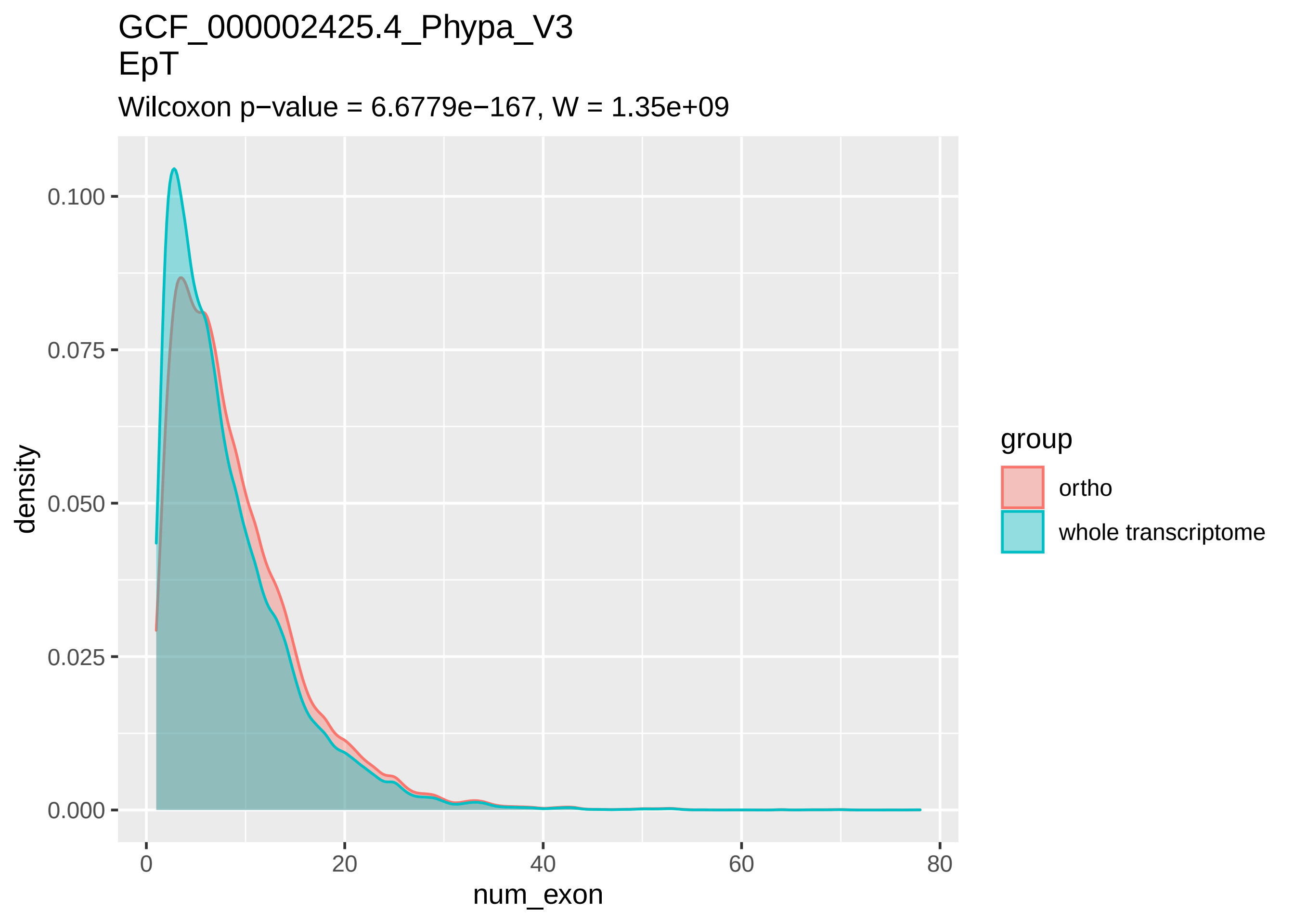

GCF_000002425.4_Phypa_V3
EpT
Wilcoxon p−value = 6.6779e−167, W = 1.35e+09
0.100
0.075
0.050
0.025
0.000
density
0
20
40
60
80
num_exon
group
ortho
whole transcriptome

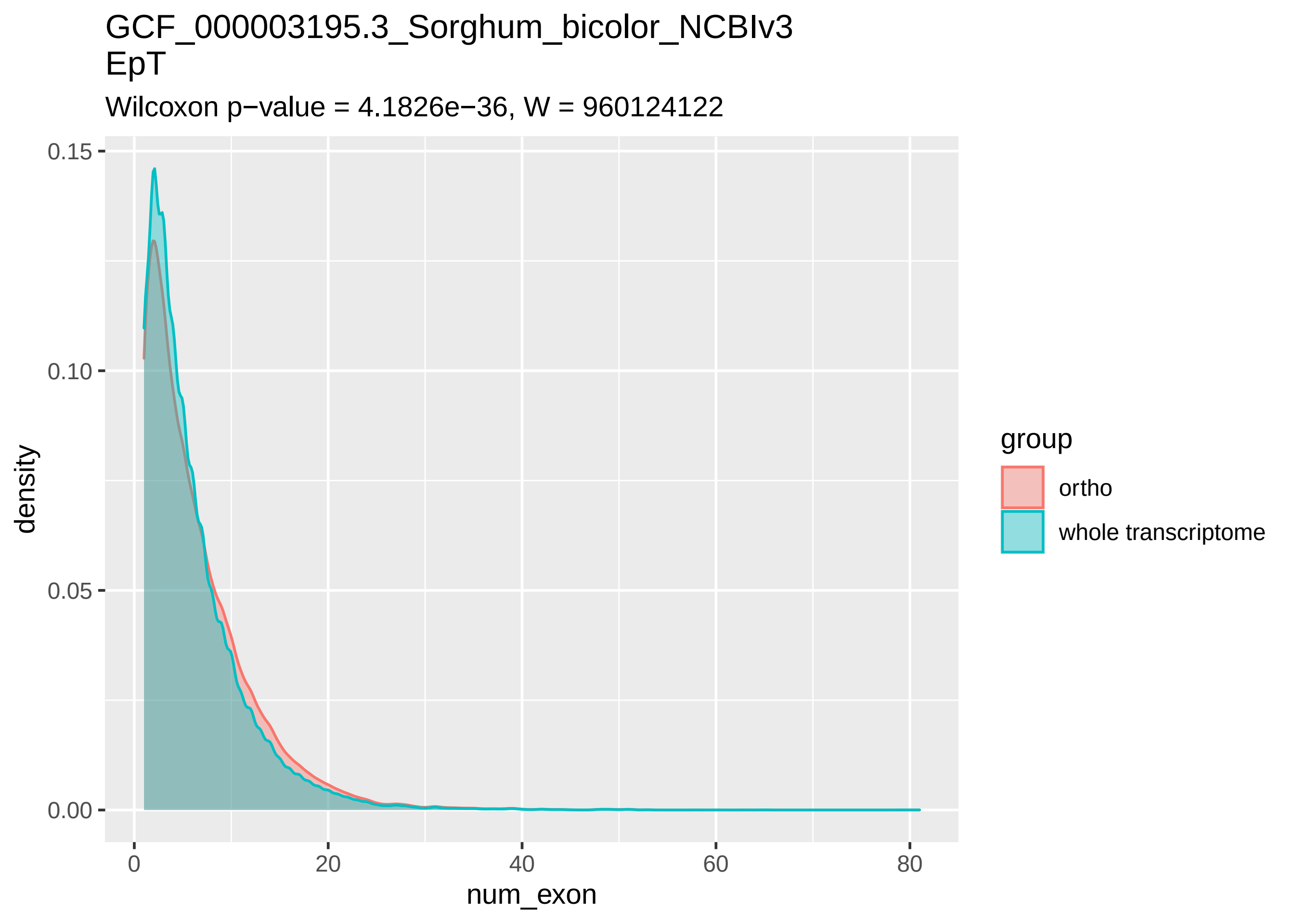
GCF_000003195.3_Sorghum_bicolor_NCBIv3
EpT
Wilcoxon p−value = 4.1826e−36, W = 960124122
0.15
0.10
0.05
0.00
density
0
20
40
60
80
num_exon
group
ortho
whole transcriptome

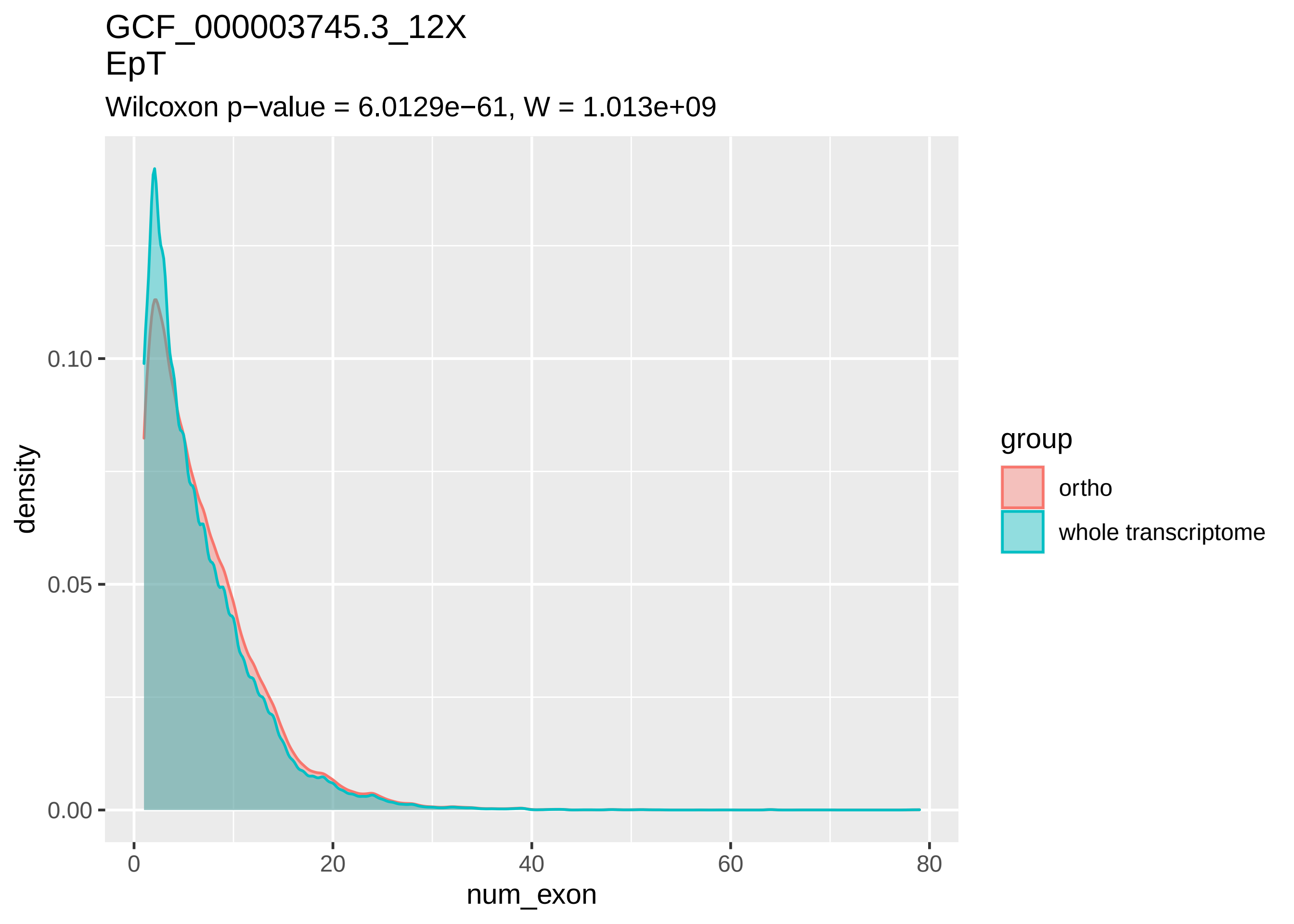
GCF_000003745.3_12X
EpT
Wilcoxon p−value = 6.0129e−61, W = 1.013e+09
density
0.00
0.05
0.10
0
20
40
60
80
num_exon
group
ortho
whole transcriptome

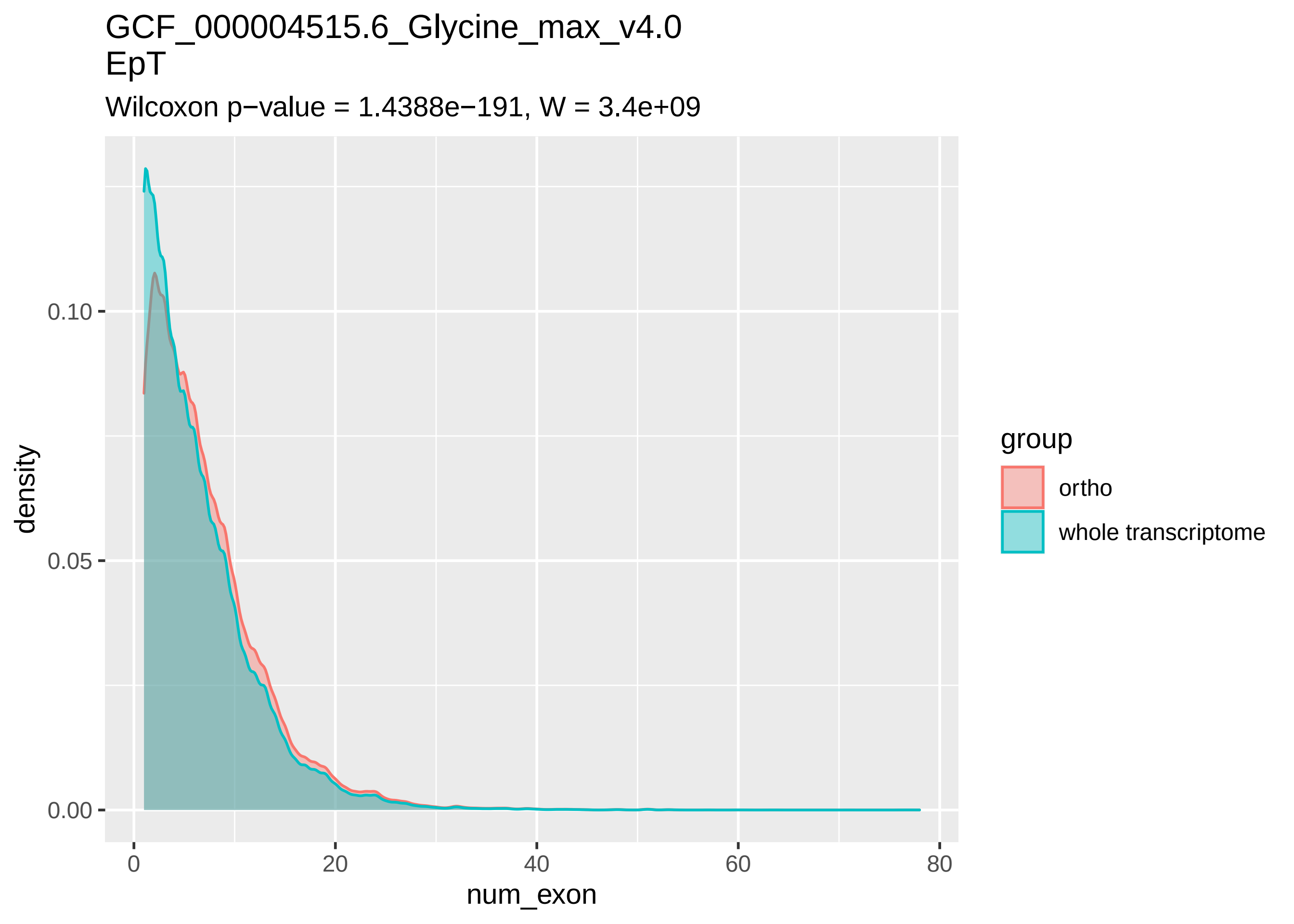

GCF_000004515.6_Glycine_max_v4.0
EpT
Wilcoxon p−value = 1.4388e−191, W = 3.4e+09
density
0.10
0.05
0.00
0
20
40
60
80
num_exon
group
ortho
whole transcriptome

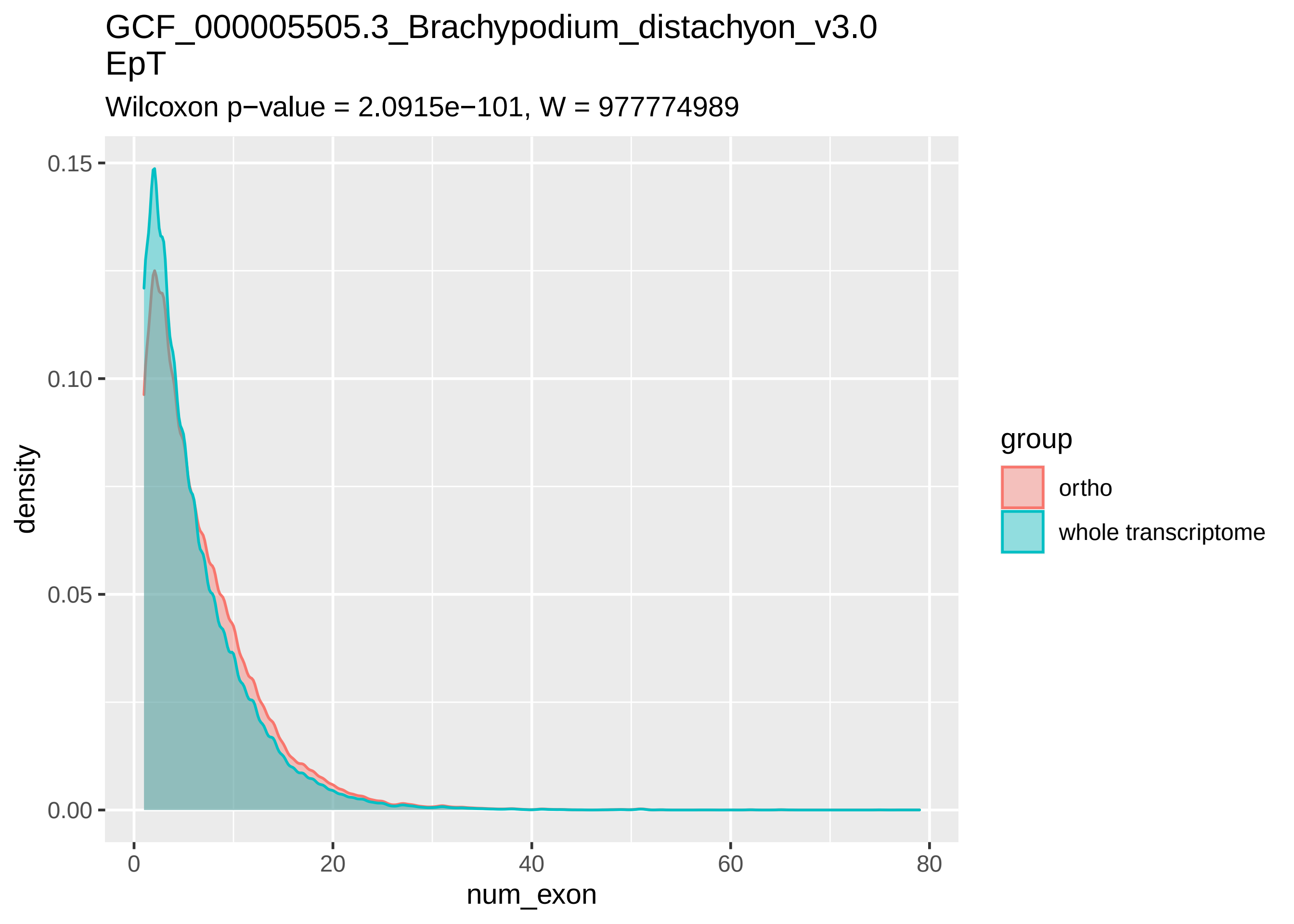

GCF_000005505.3_Brachypodium_distachyon_v3.0
EpT
Wilcoxon p−value = 2.0915e−101, W = 977774989
0.15
0.10
0.05
0.00
density
0
20
40
60
80
num_exon
group
ortho
whole transcriptome

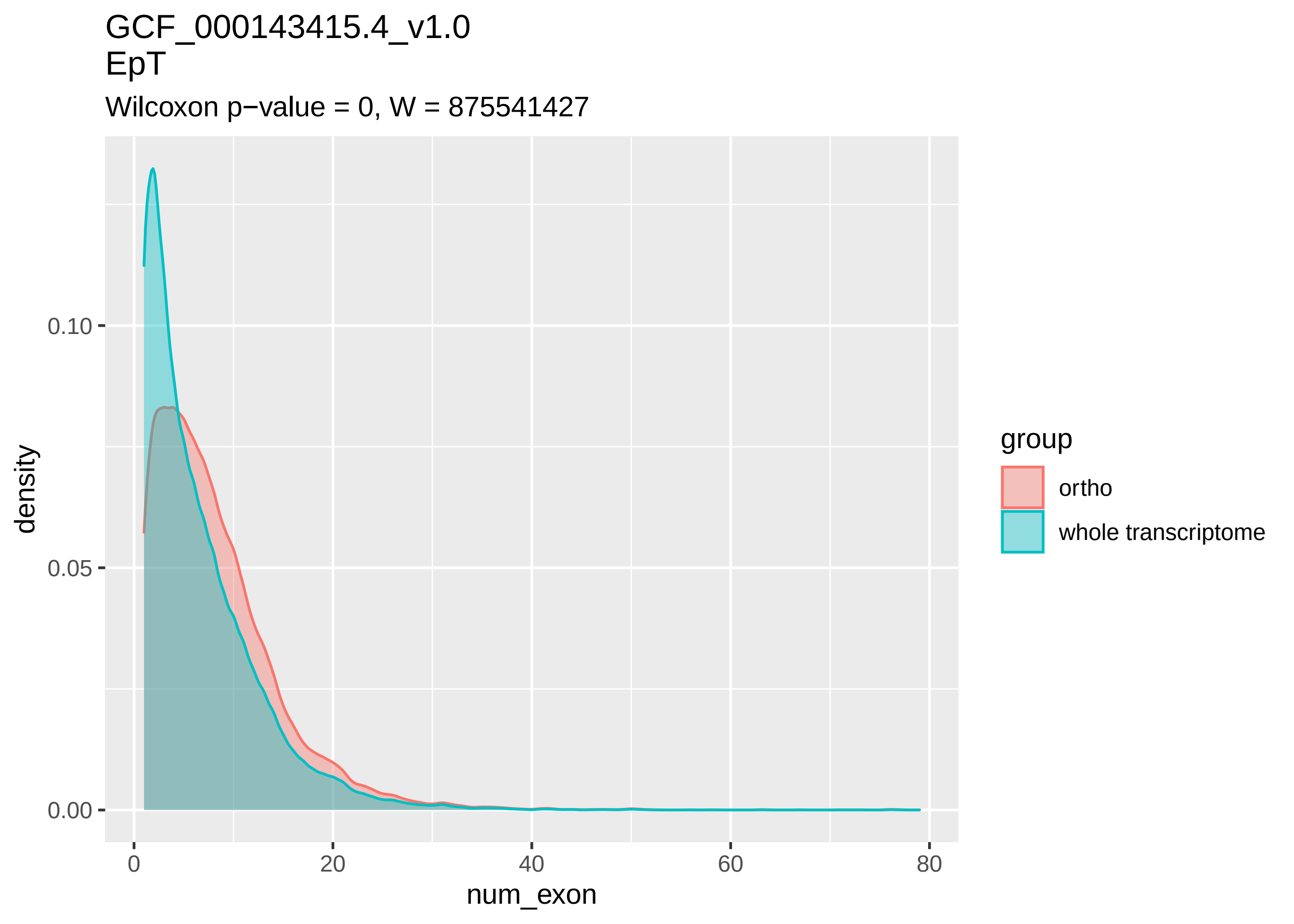

GCF_000143415.4_v1.0
EpT
Wilcoxon p−value = 0, W = 875541427
density
0.10
0.05
0.00
0
20
40
60
80
num_exon
group
ortho
whole transcriptome

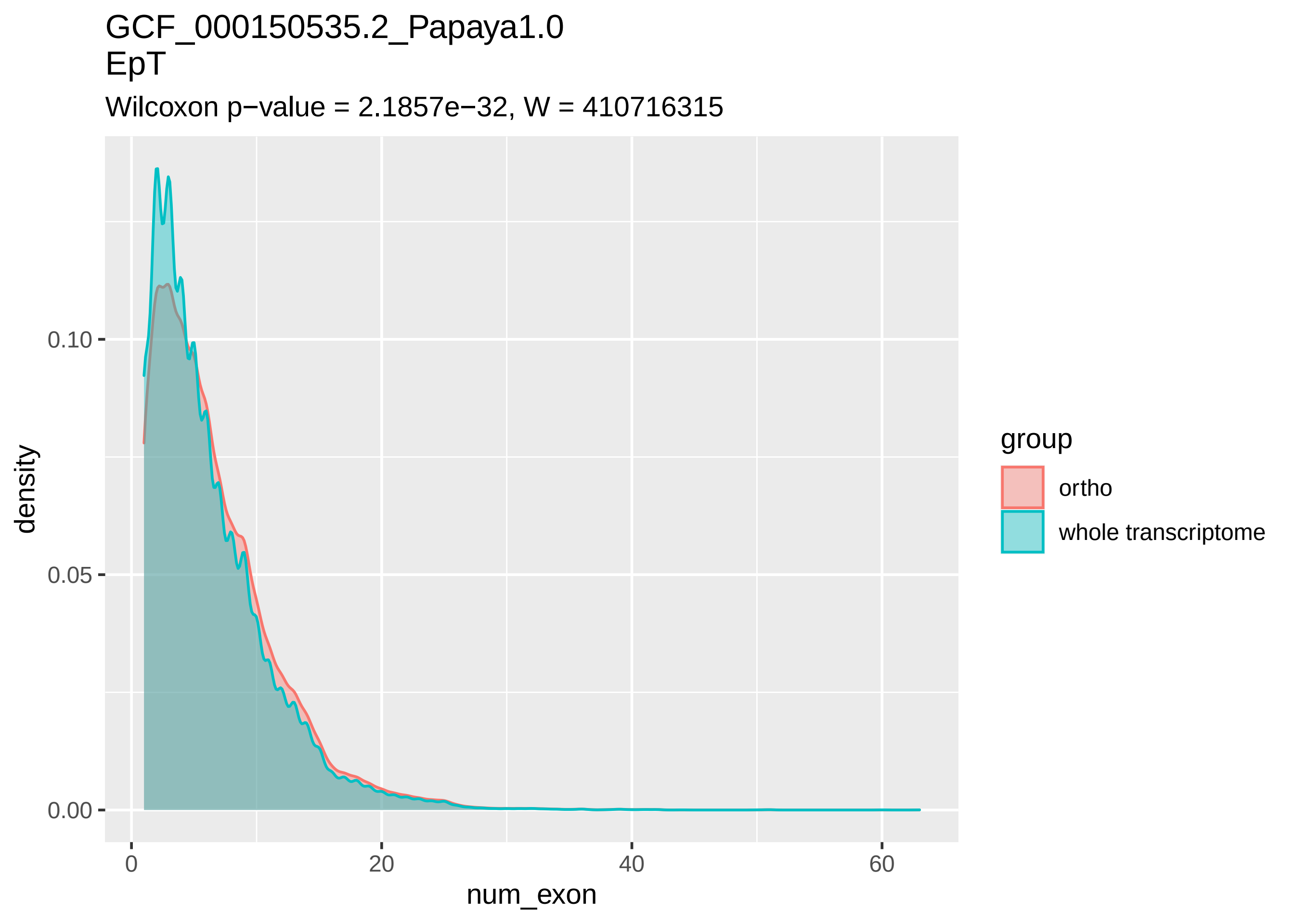
GCF_000150535.2_Papaya1.0
EpT
Wilcoxon p−value = 2.1857e−32, W = 410716315
density
0.10
0.05
0.00
0
20
40
60
num_exon
group
ortho
whole transcriptome

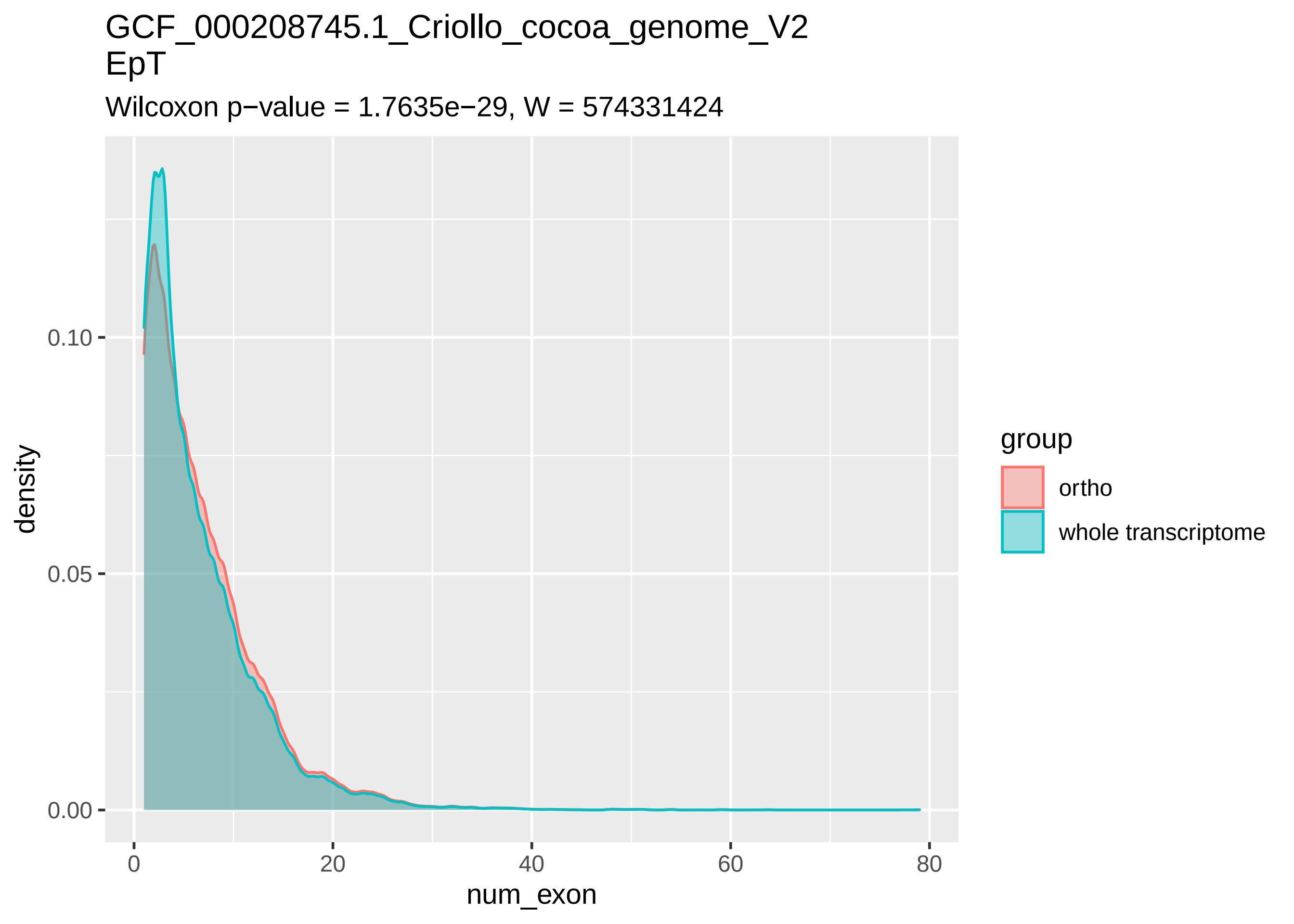
GCF_000208745.1_Criollo_cocoa_genome_V2
EpT
Wilcoxon p−value = 1.7635e−29, W = 574331424
density
0.10
0.05
0.00
0
20
40
60
80
num_exon
group
ortho
whole transcriptome

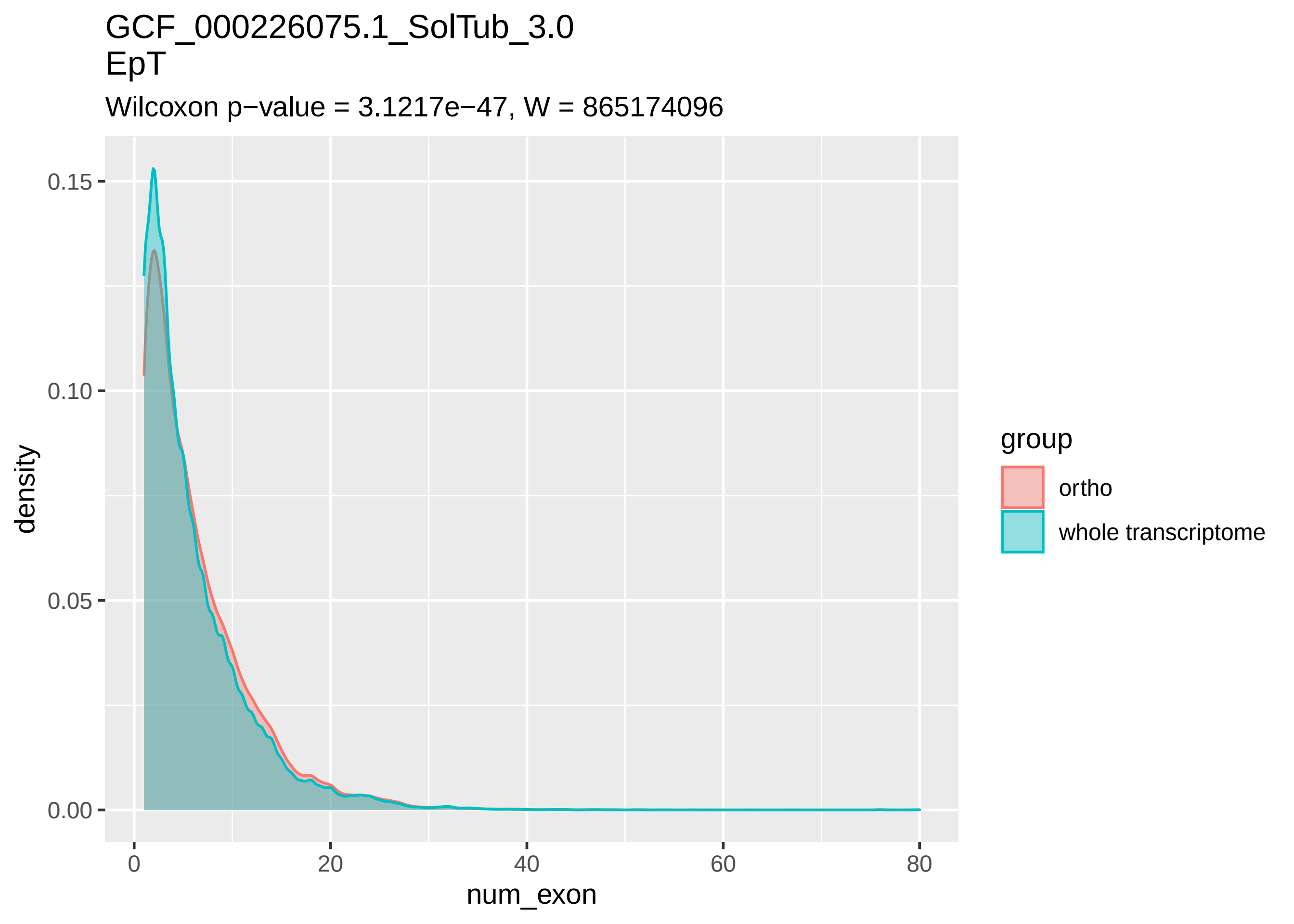
GCF_000226075.1_SolTub_3.0
EpT
Wilcoxon p−value = 3.1217e−47, W = 865174096
0.15
0.10
0.05
0.00
density
0
20
40
60
80
num_exon
group
ortho
whole transcriptome

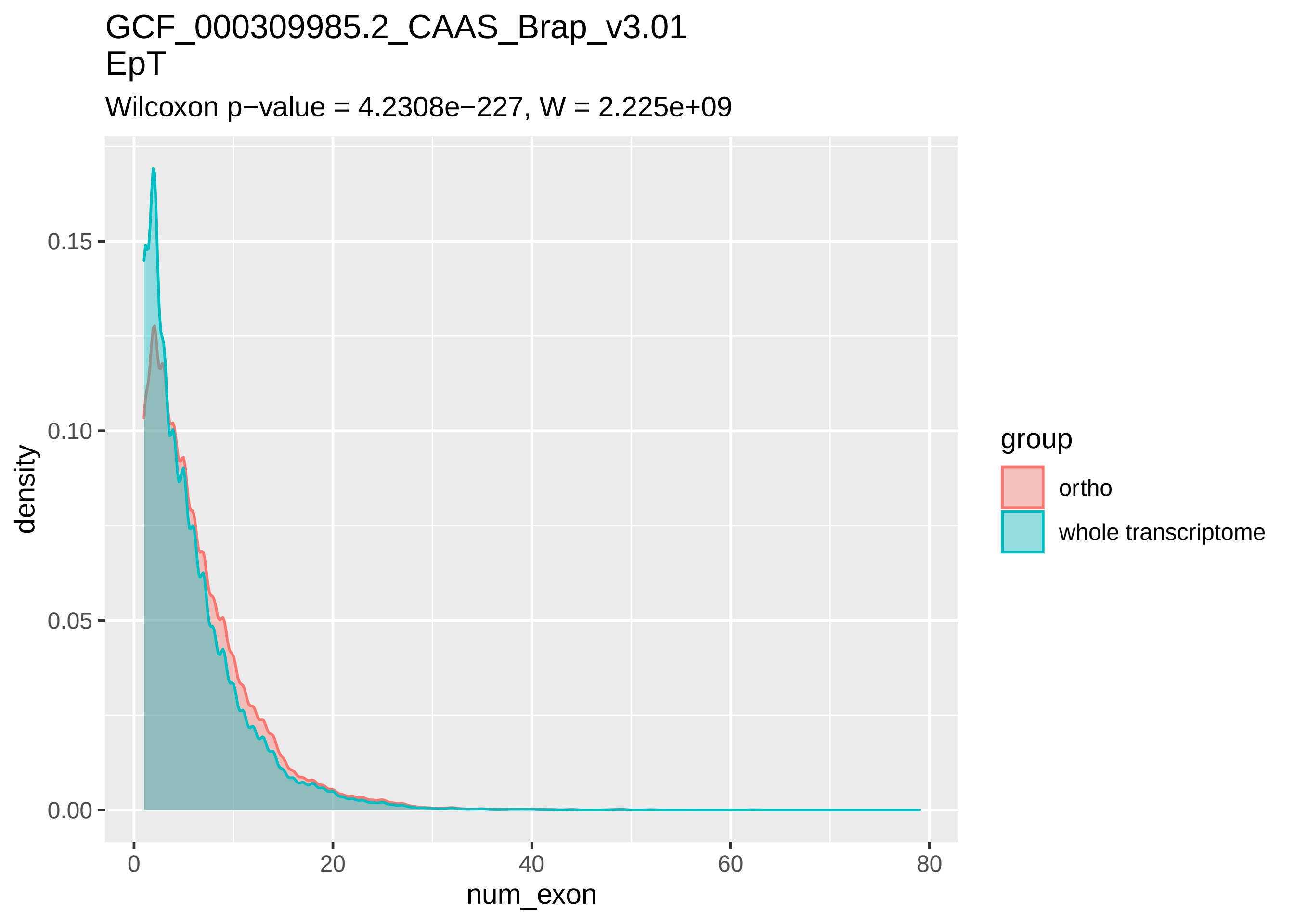
GCF_000309985.2_CAAS_Brap_v3.01
EpT
Wilcoxon p−value = 4.2308e−227, W = 2.225e+09
0.15
0.10
0.05
0.00
density
0
20
40
60
80
num_exon
group
ortho
whole transcriptome

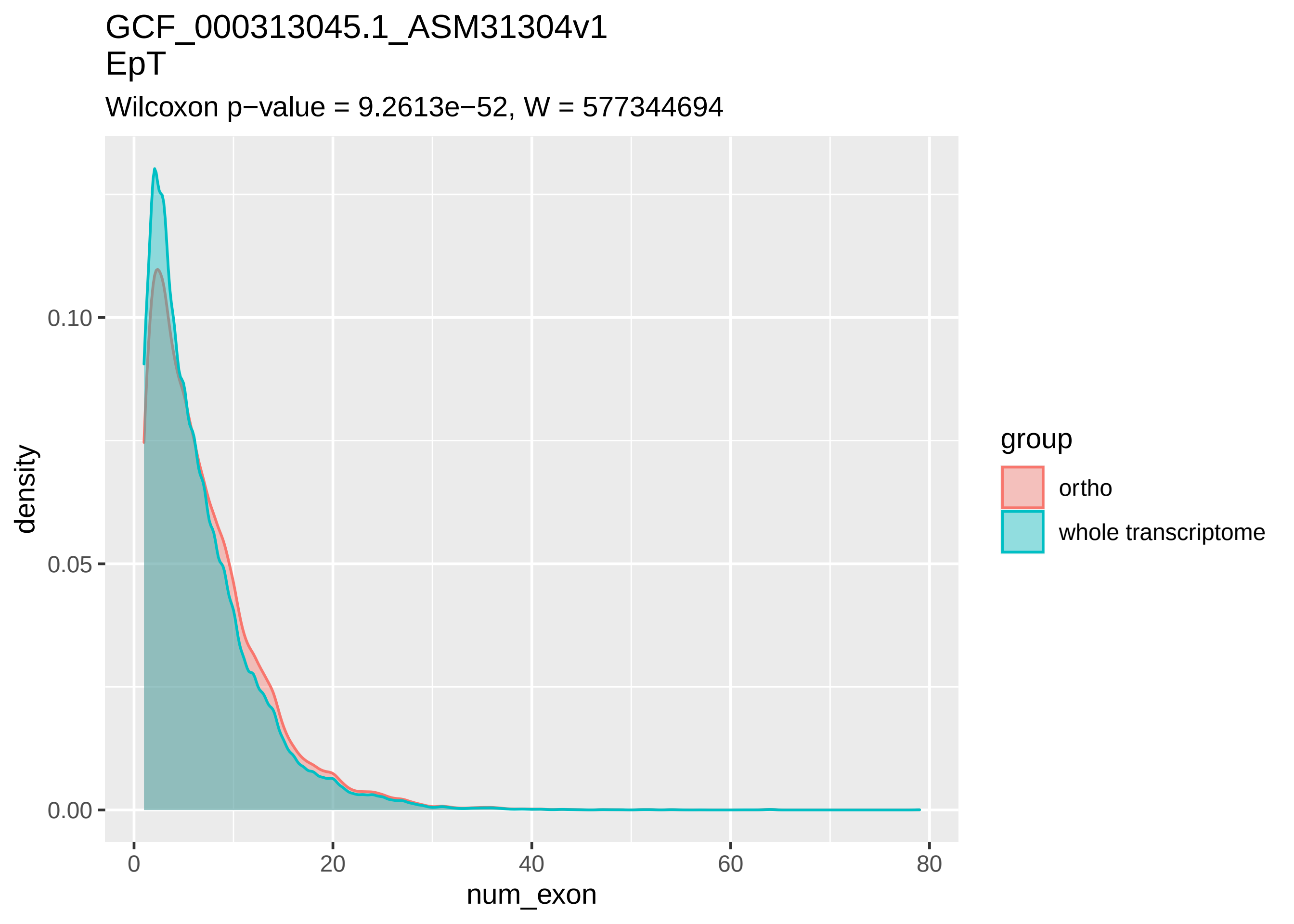
GCF_000313045.1_ASM31304v1
EpT
Wilcoxon p−value = 9.2613e−52, W = 577344694
density
0.10
0.05
0.00
0
20
40
60
80
num_exon
group
ortho
whole transcriptome

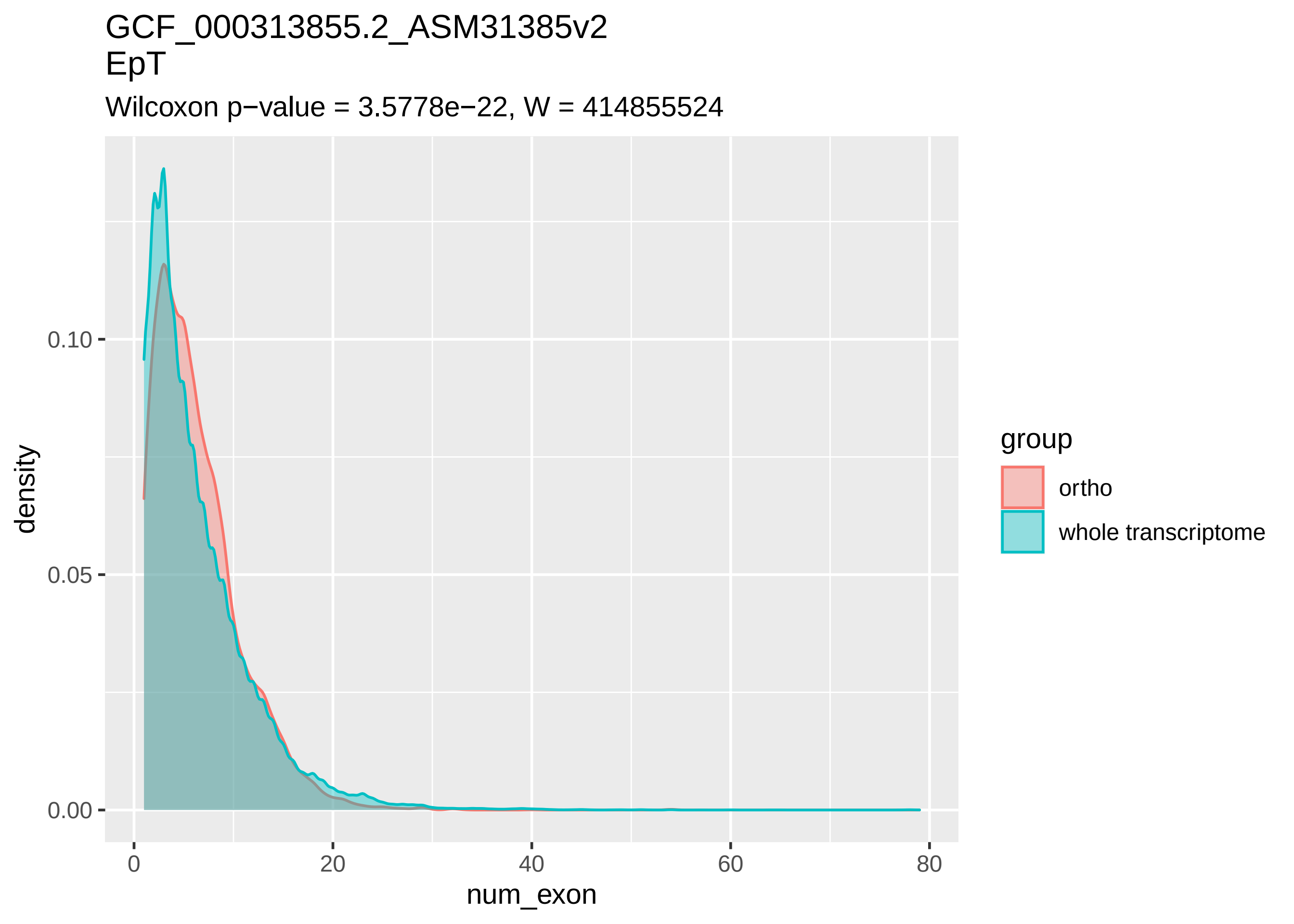
GCF_000313855.2_ASM31385v2
EpT
Wilcoxon p−value = 3.5778e−22, W = 414855524
density
0.10
0.05
0.00
0
20
40
60
80
num_exon
group
ortho
whole transcriptome

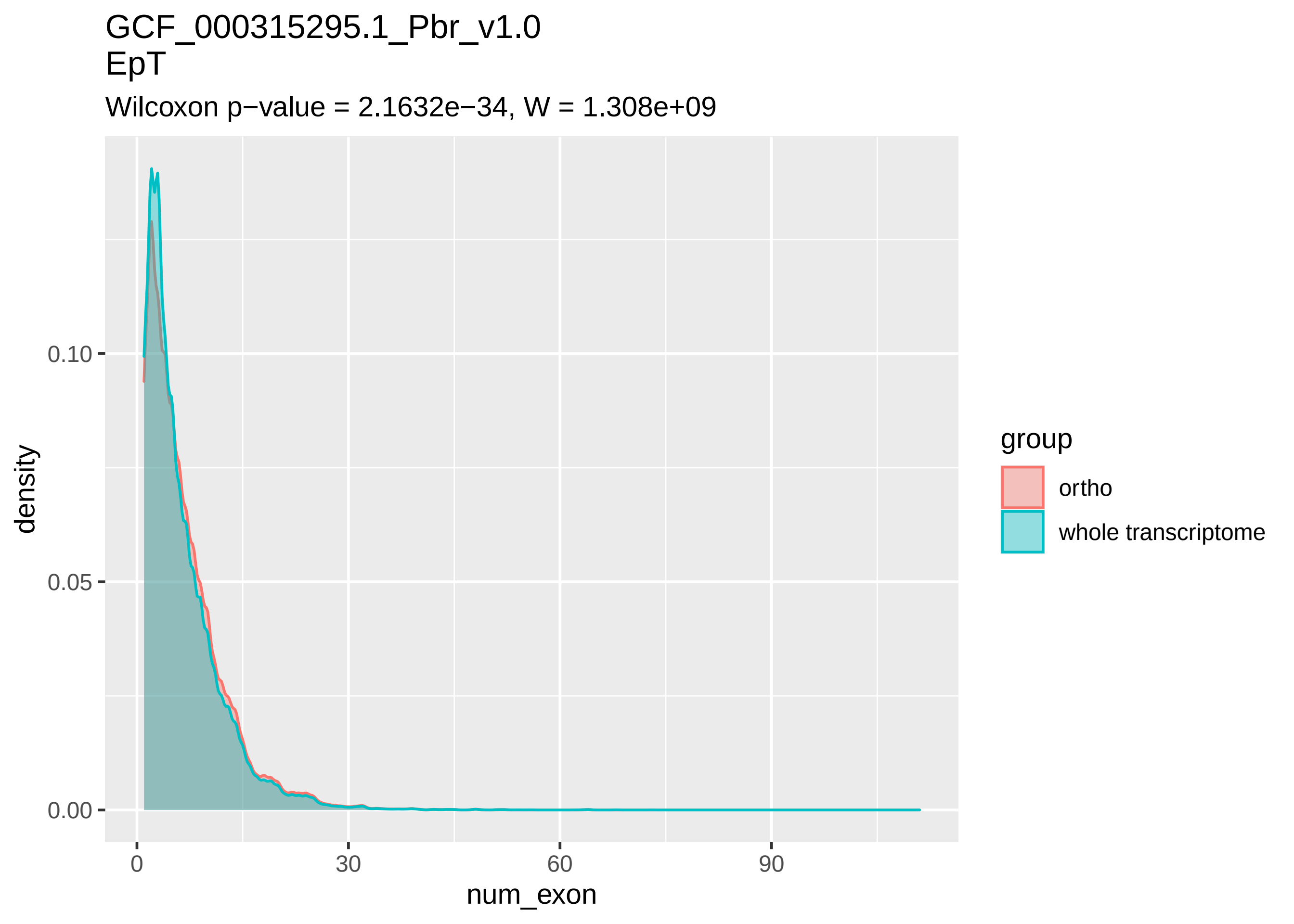

GCF_000315295.1_Pbr_v1.0
EpT
Wilcoxon p−value = 2.1632e−34, W = 1.308e+09
density
0.10
0.05
0.00
0
30
60
90
num_exon
group
ortho
whole transcriptome

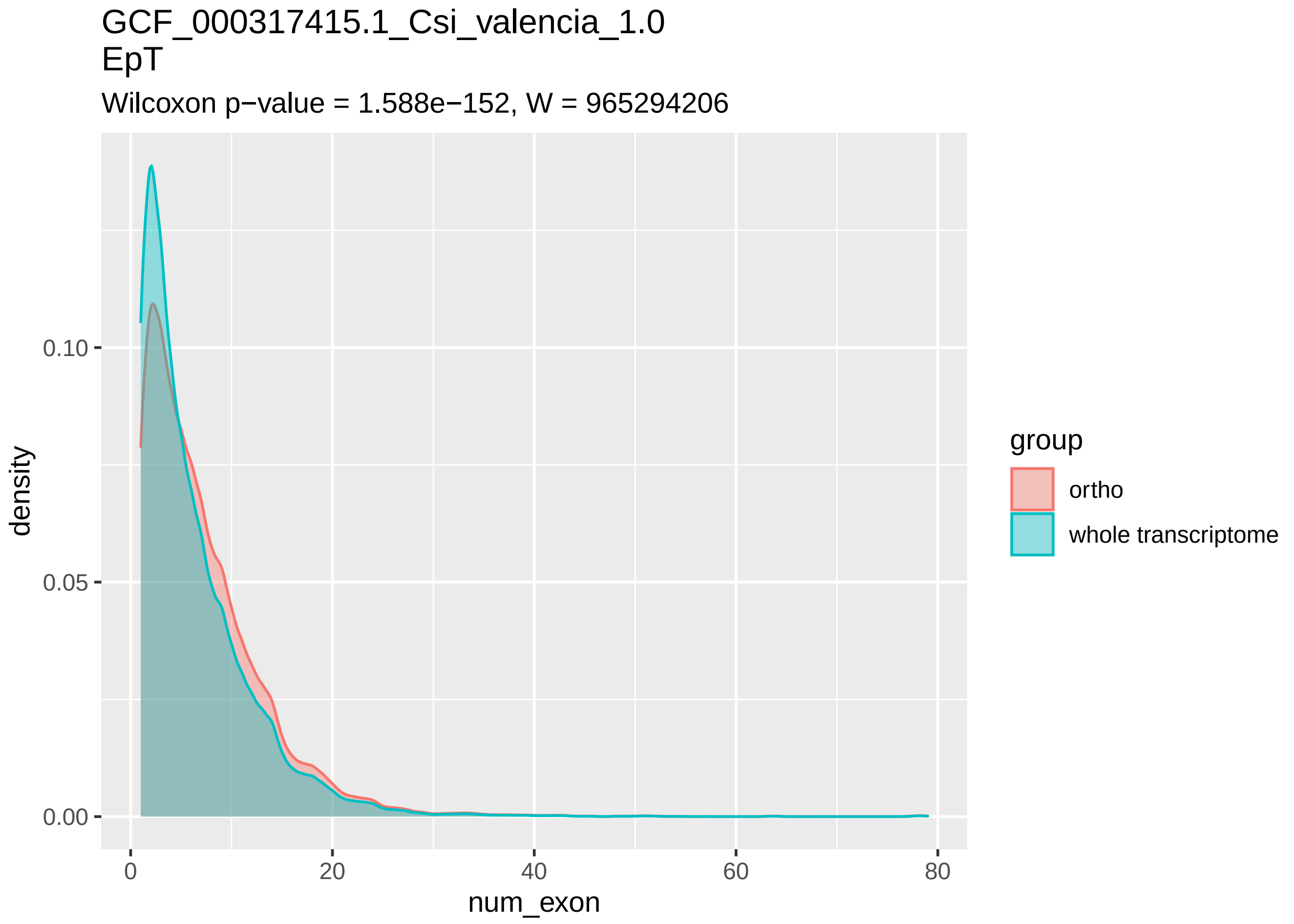
GCF_000317415.1_Csi_valencia_1.0
EpT
Wilcoxon p−value = 1.588e−152, W = 965294206
density
0.10
0.05
0.00
0
20
40
60
80
num_exon
group
ortho
whole transcriptome

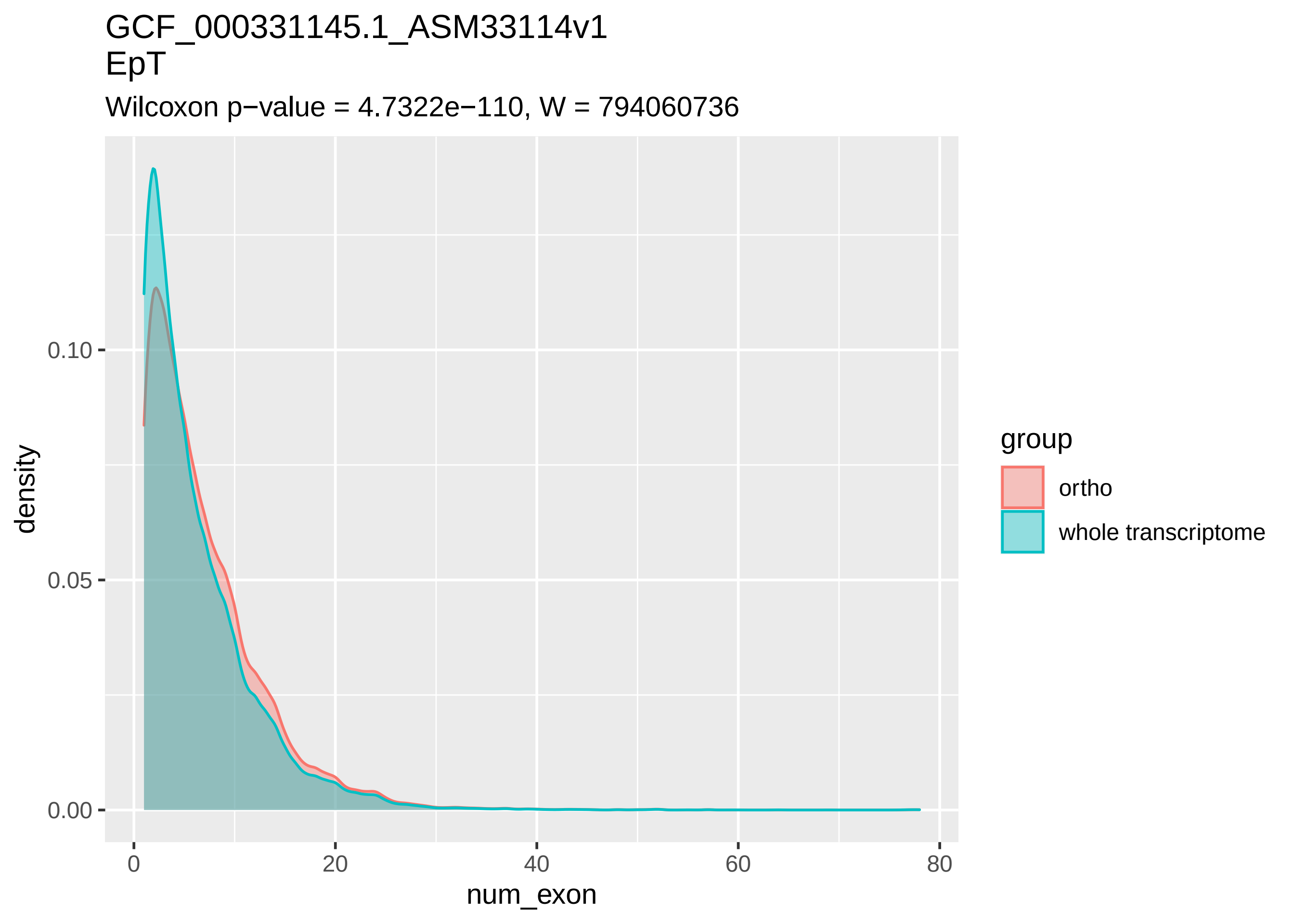
GCF_000331145.1_ASM33114v1
EpT
Wilcoxon p−value = 4.7322e−110, W = 794060736
density
0.10
0.05
0.00
0
20
40
60
80
num_exon
group
ortho
whole transcriptome

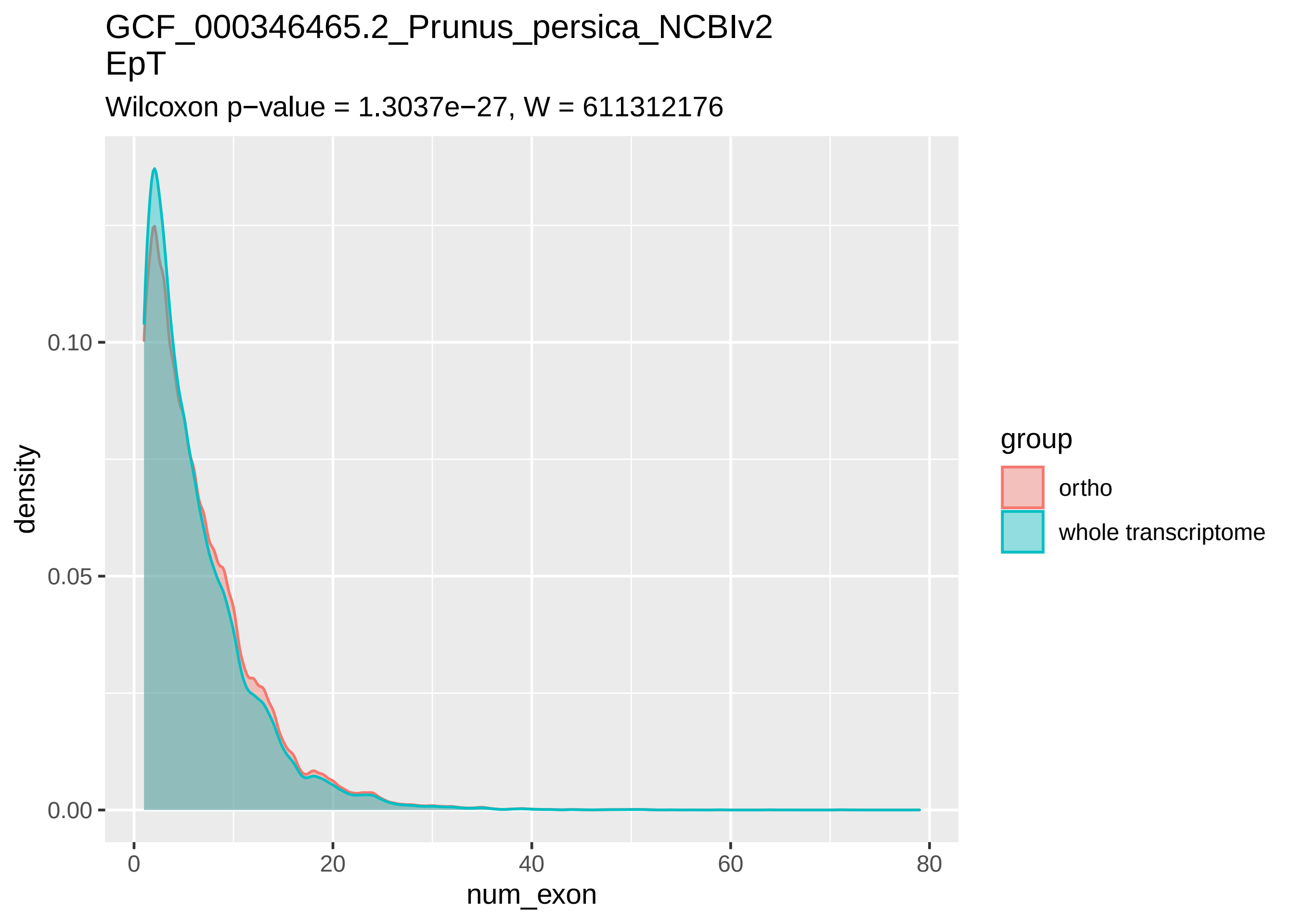

GCF_000346465.2_Prunus_persica_NCBIv2
EpT
Wilcoxon p−value = 1.3037e−27, W = 611312176
density
0.10
0.05
0.00
0
20
40
60
80
num_exon
group
ortho
whole transcriptome

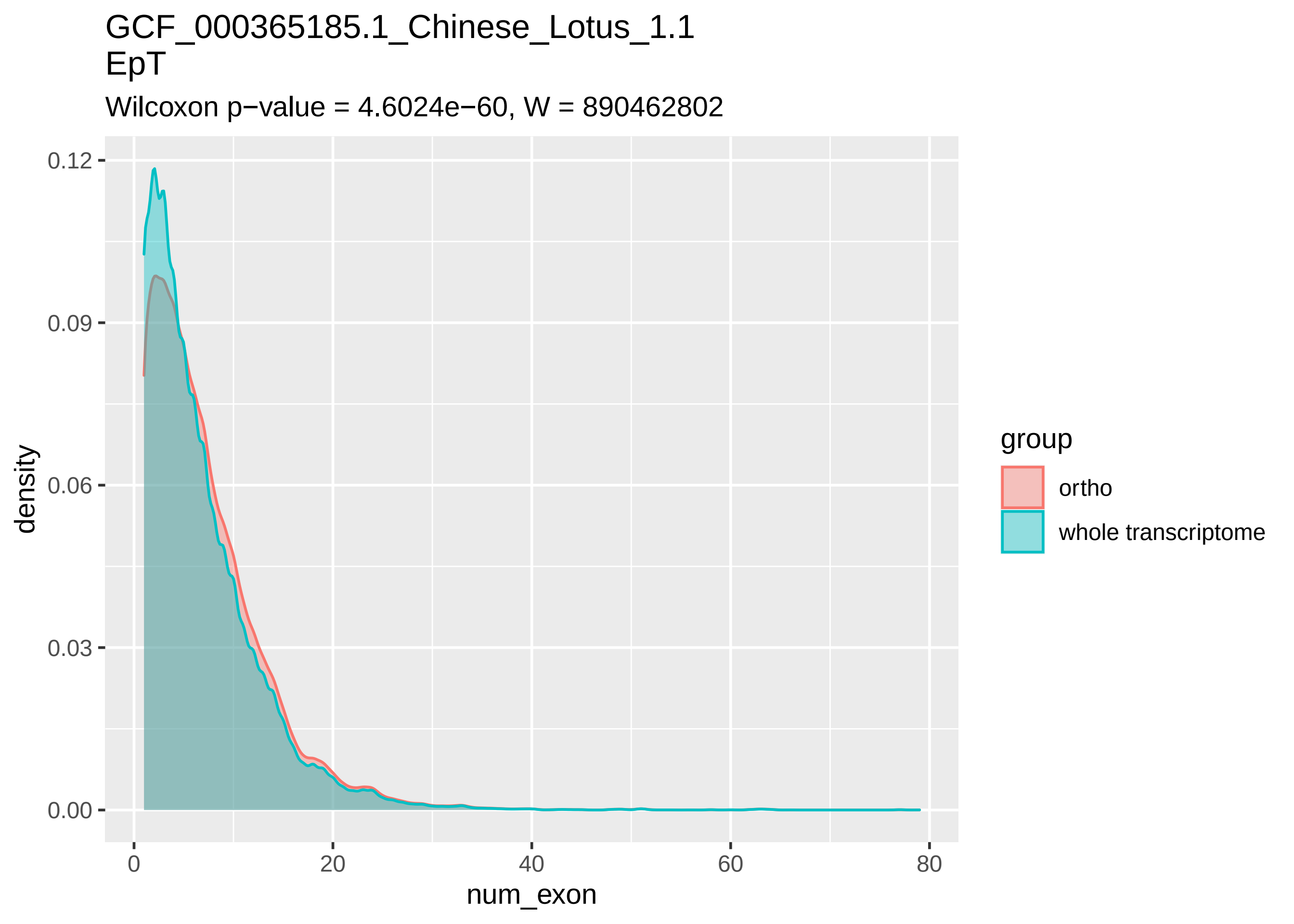
GCF_000365185.1_Chinese_Lotus_1.1
EpT
Wilcoxon p−value = 4.6024e−60, W = 890462802
density
0.12
0.09
0.06
0.03
0.00
0
20
40
60
80
num_exon
group
ortho
whole transcriptome

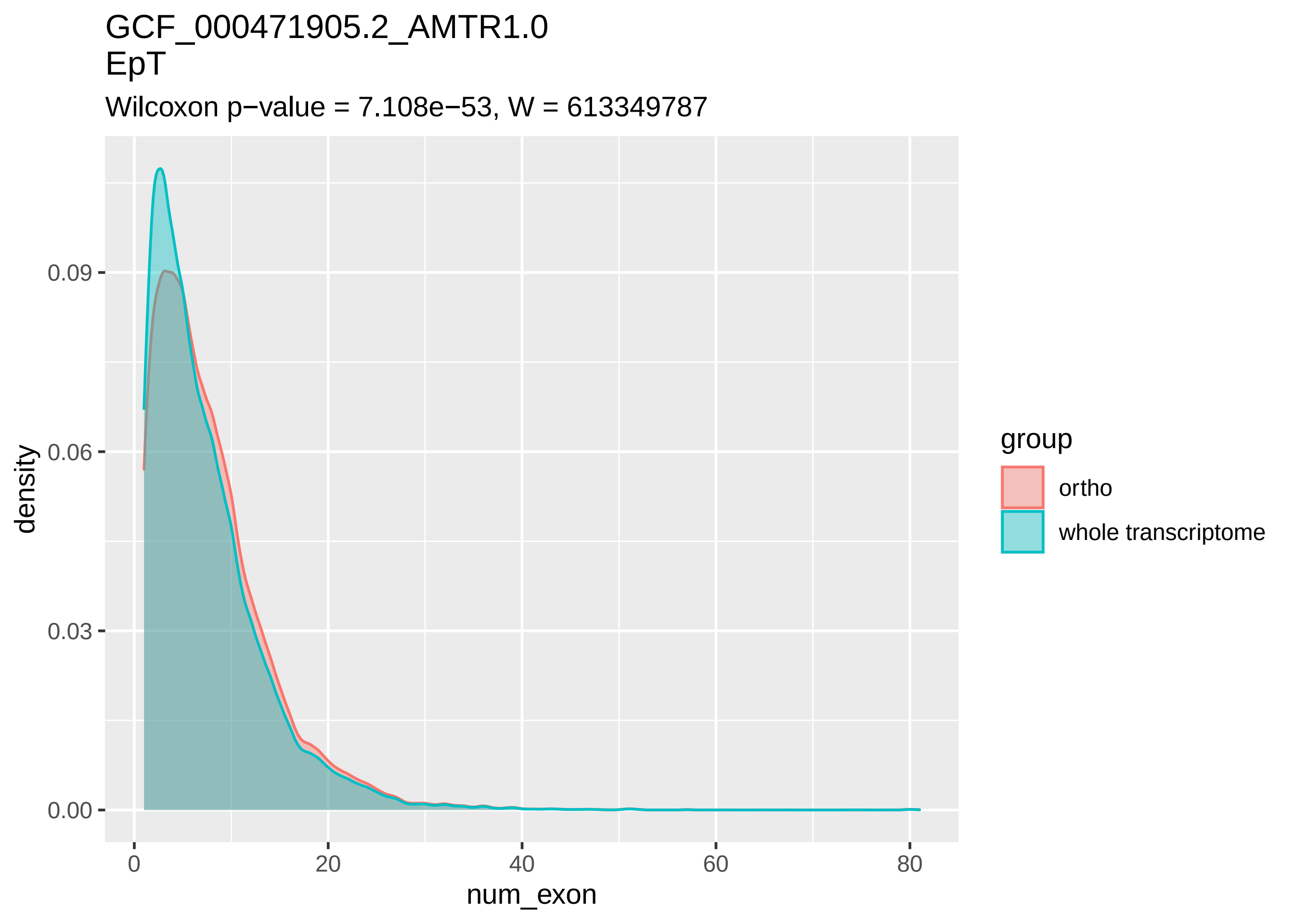

GCF_000471905.2_AMTR1.0
EpT
Wilcoxon p−value = 7.108e−53, W = 613349787
density
0.09
0.06
0.03
0.00
0
20
40
60
80
num_exon
group
ortho
whole transcriptome

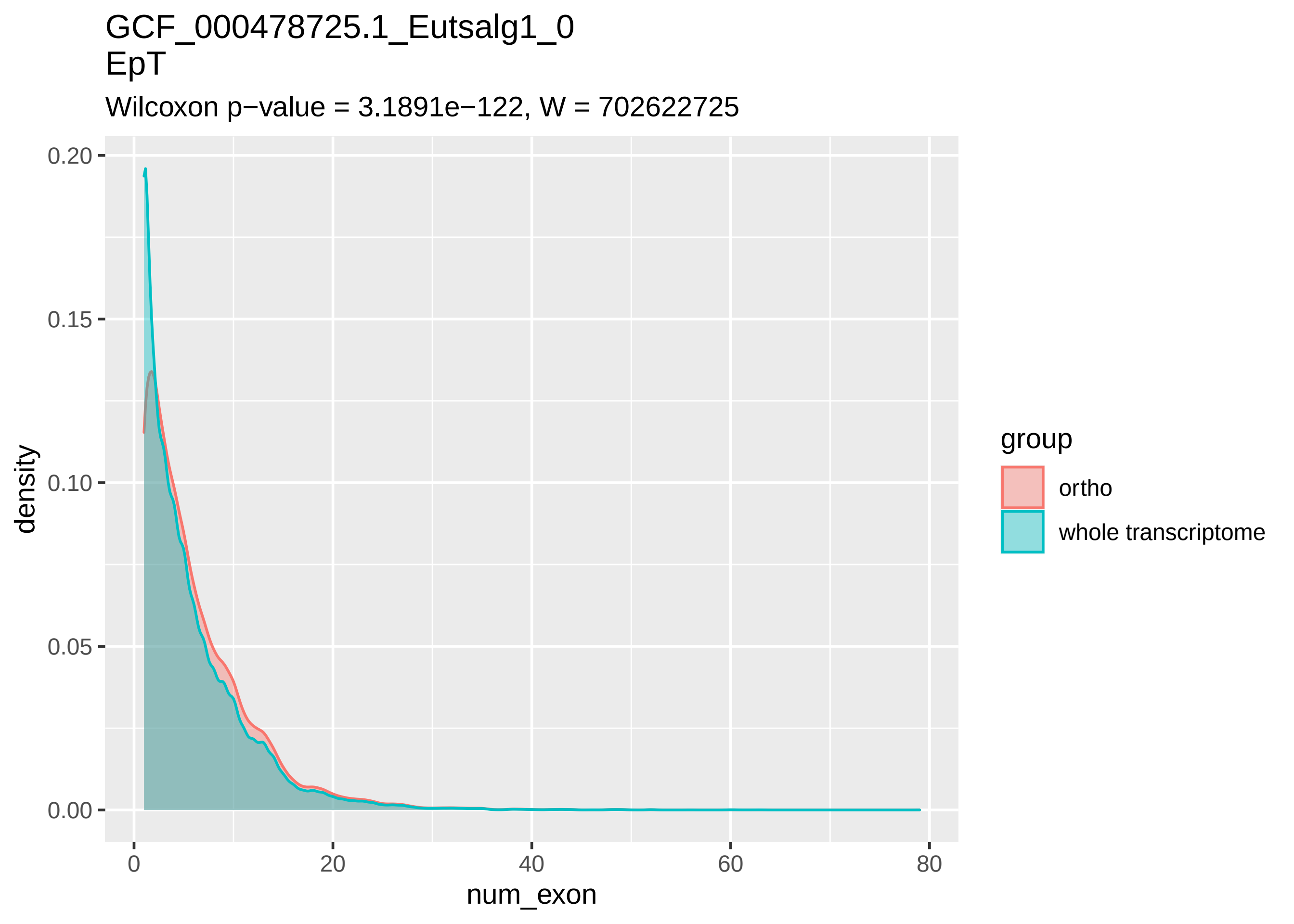

GCF_000478725.1_Eutsalg1_0
EpT
Wilcoxon p−value = 3.1891e−122, W = 702622725
density
0.20
0.15
0.10
0.05
0.00
0
20
40
60
80
num_exon
group
ortho
whole transcriptome

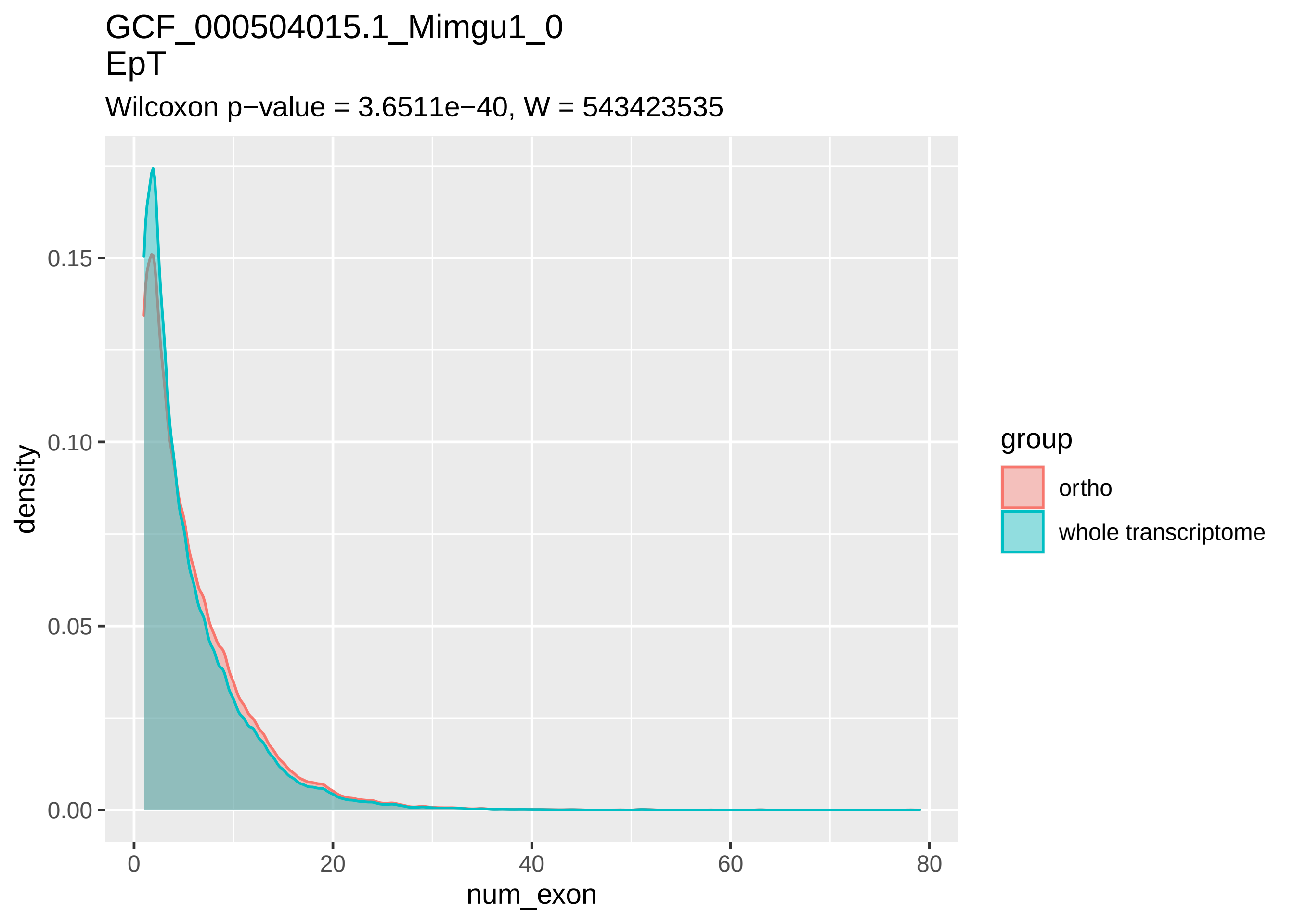

GCF_000504015.1_Mimgu1_0
EpT
Wilcoxon p−value = 3.6511e−40, W = 543423535
0.15
0.10
0.05
0.00
density
0
20
40
60
80
num_exon
group
ortho
whole transcriptome

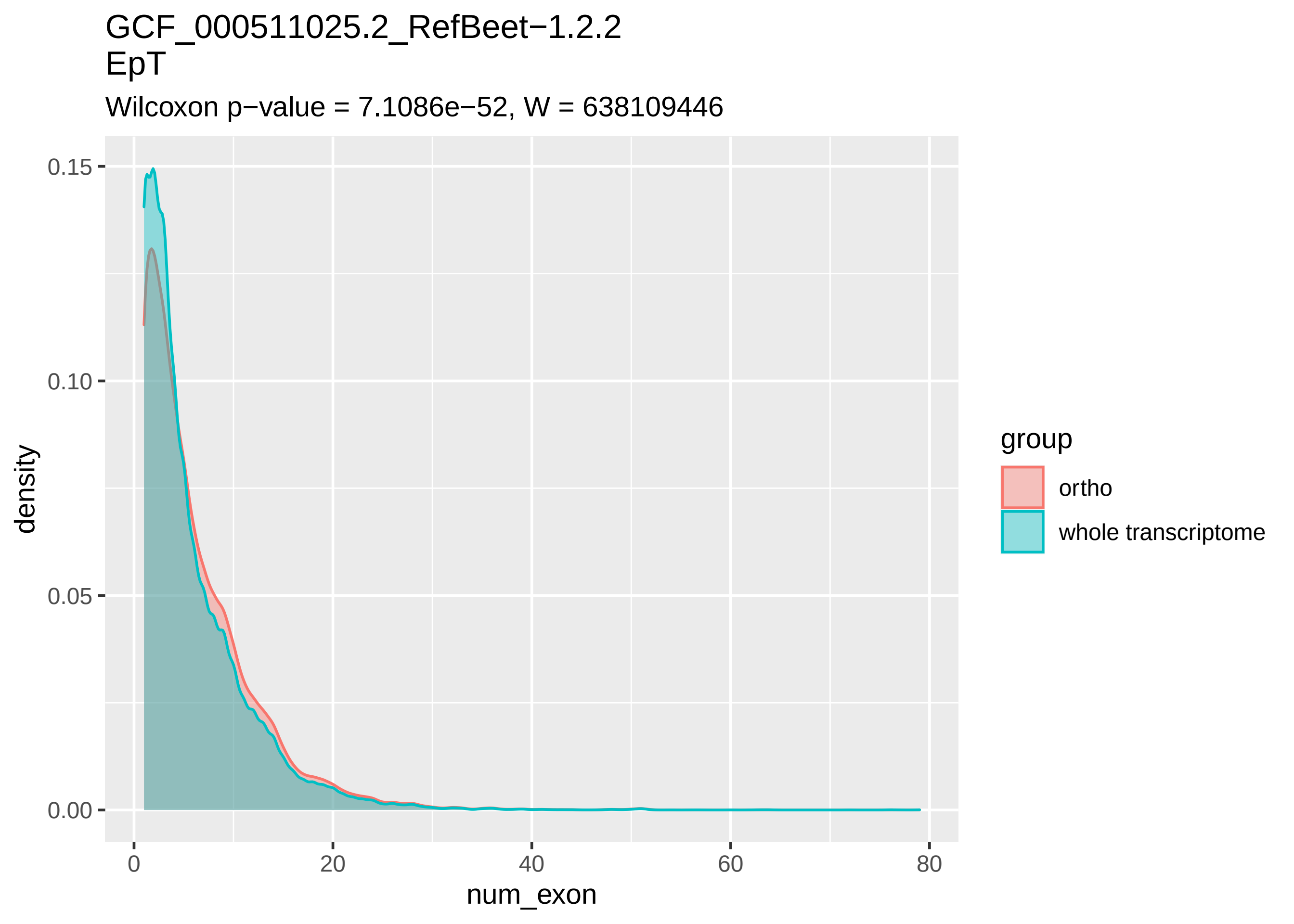
GCF_000511025.2_RefBeet−1.2.2
EpT
Wilcoxon p−value = 7.1086e−52, W = 638109446
0.15
0.10
0.05
0.00
density
0
20
40
60
80
num_exon
group
ortho
whole transcriptome

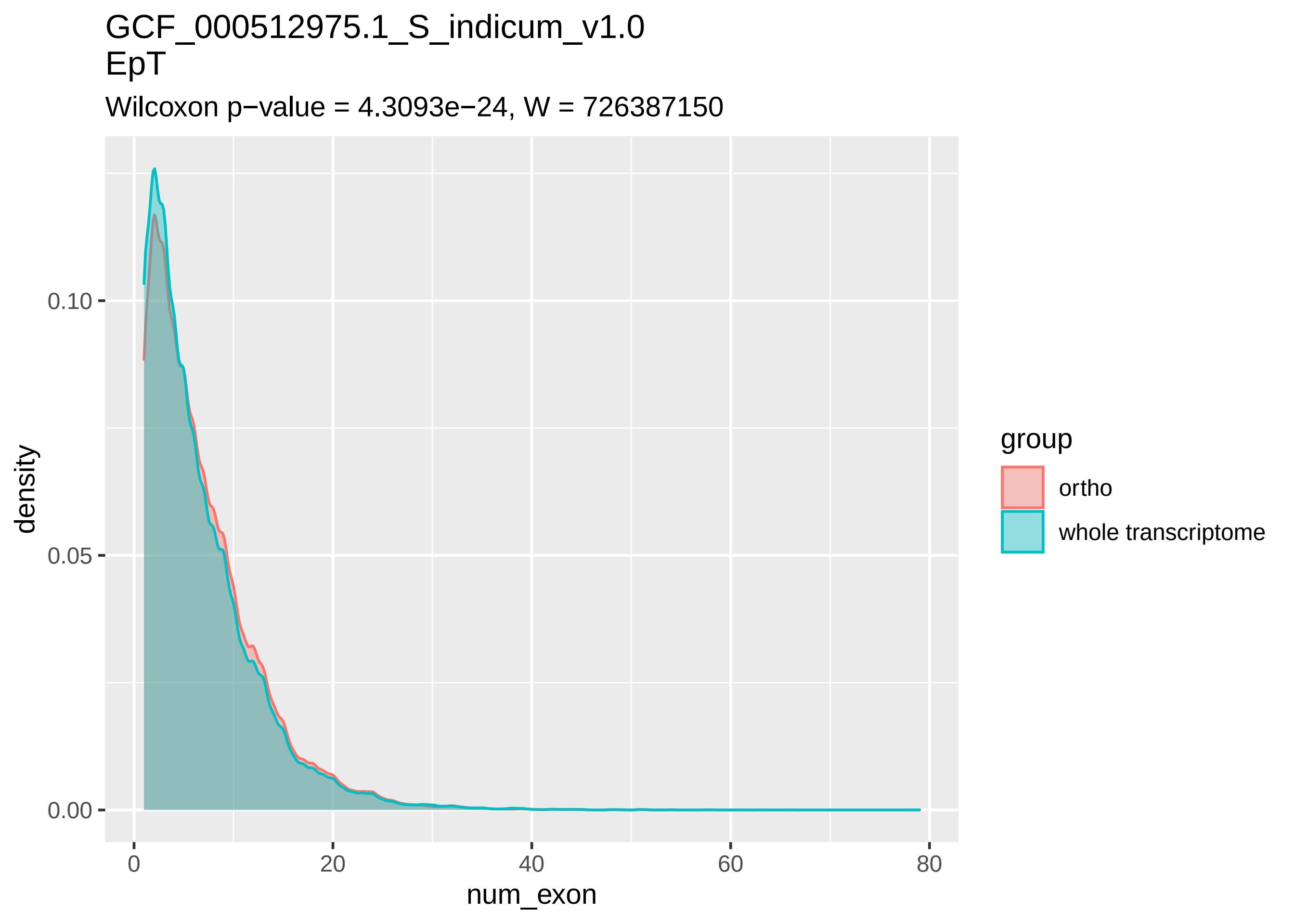
GCF_000512975.1_S_indicum_v1.0
EpT
Wilcoxon p−value = 4.3093e−24, W = 726387150
density
0.10
0.05
0.00
0
20
40
60
80
num_exon
group
ortho
whole transcriptome

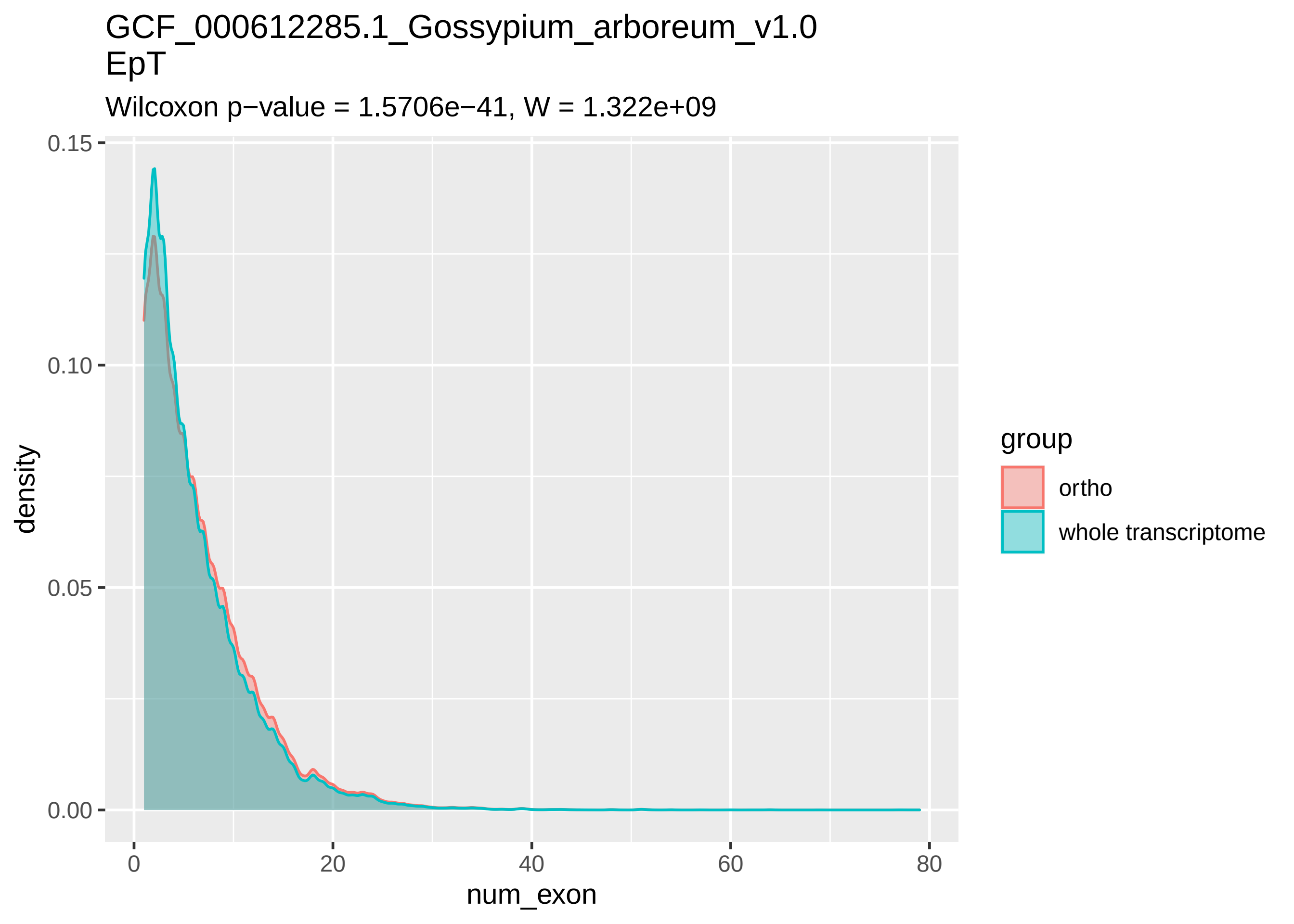

GCF_000612285.1_Gossypium_arboreum_v1.0
EpT
Wilcoxon p−value = 1.5706e−41, W = 1.322e+09
density
0.15
0.10
0.05
0.00
0
20
40
60
80
num_exon
group
ortho
whole transcriptome

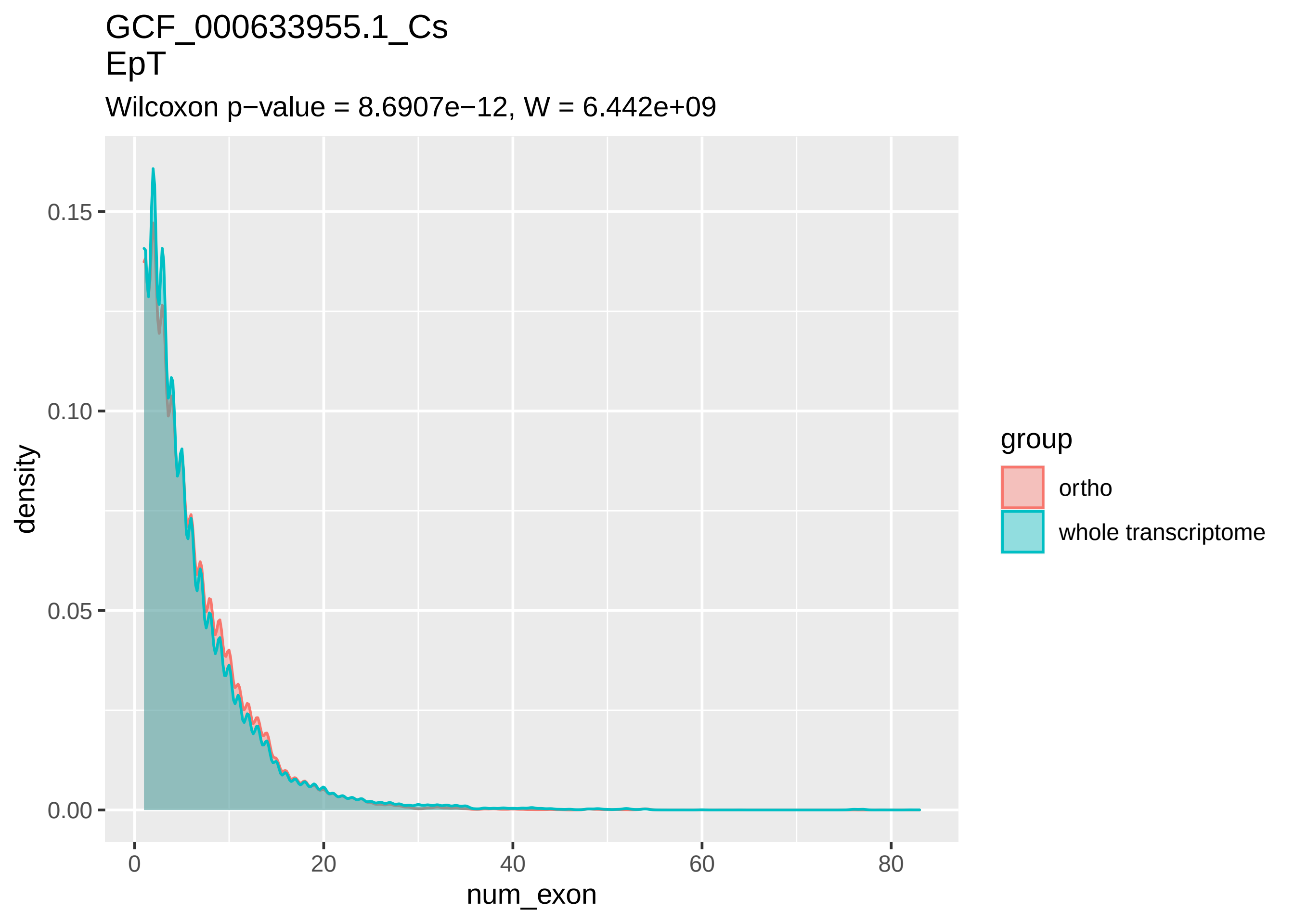
GCF_000633955.1_Cs
EpT
Wilcoxon p−value = 8.6907e−12, W = 6.442e+09
density
0.15
0.10
0.05
0.00
0
20
40
60
80
num_exon
group
ortho
whole transcriptome

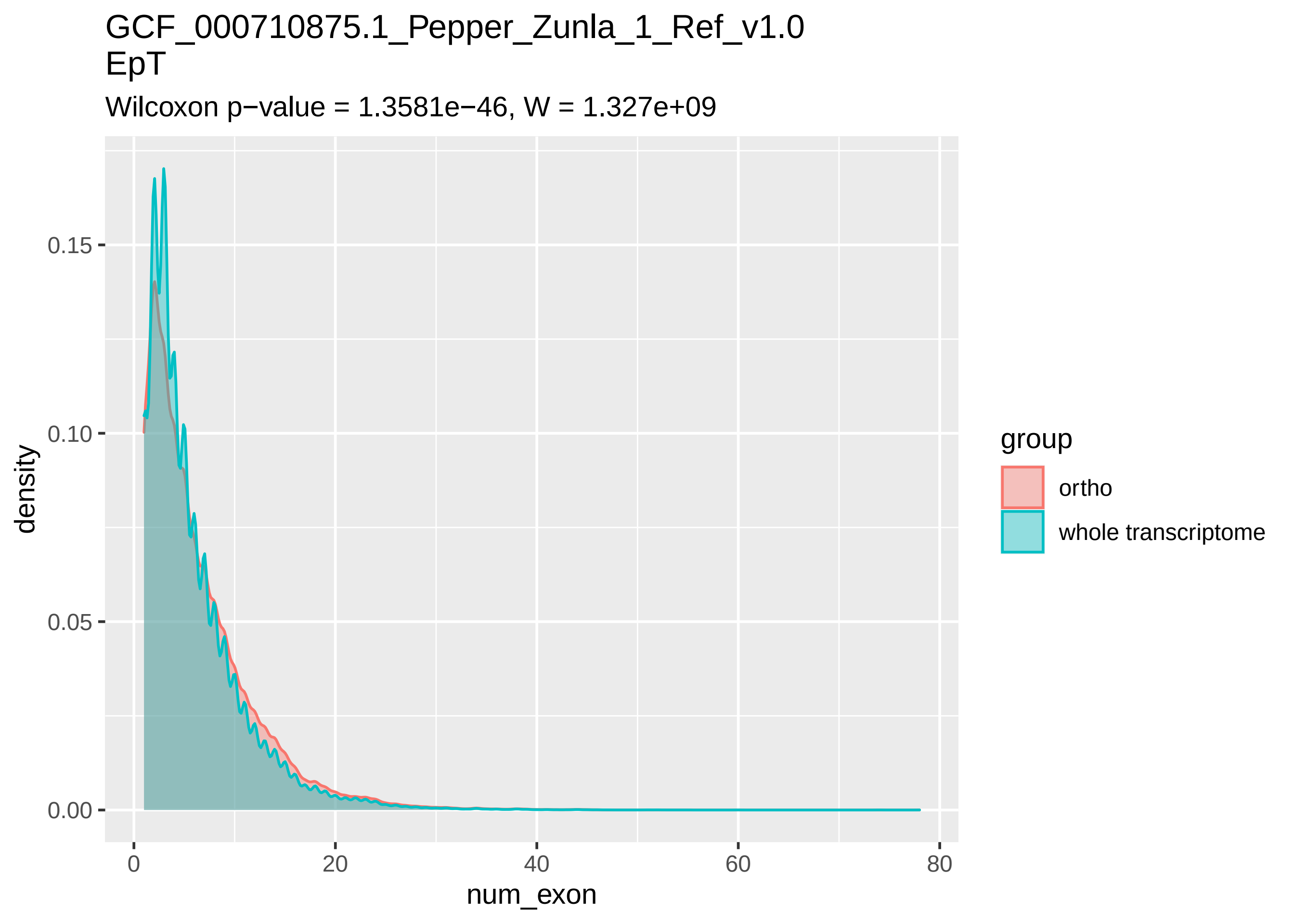
GCF_000710875.1_Pepper_Zunla_1_Ref_v1.0
EpT
Wilcoxon p−value = 1.3581e−46, W = 1.327e+09
density
0.15
0.10
0.05
0.00
0
20
40
60
80
num_exon
group
ortho
whole transcriptome

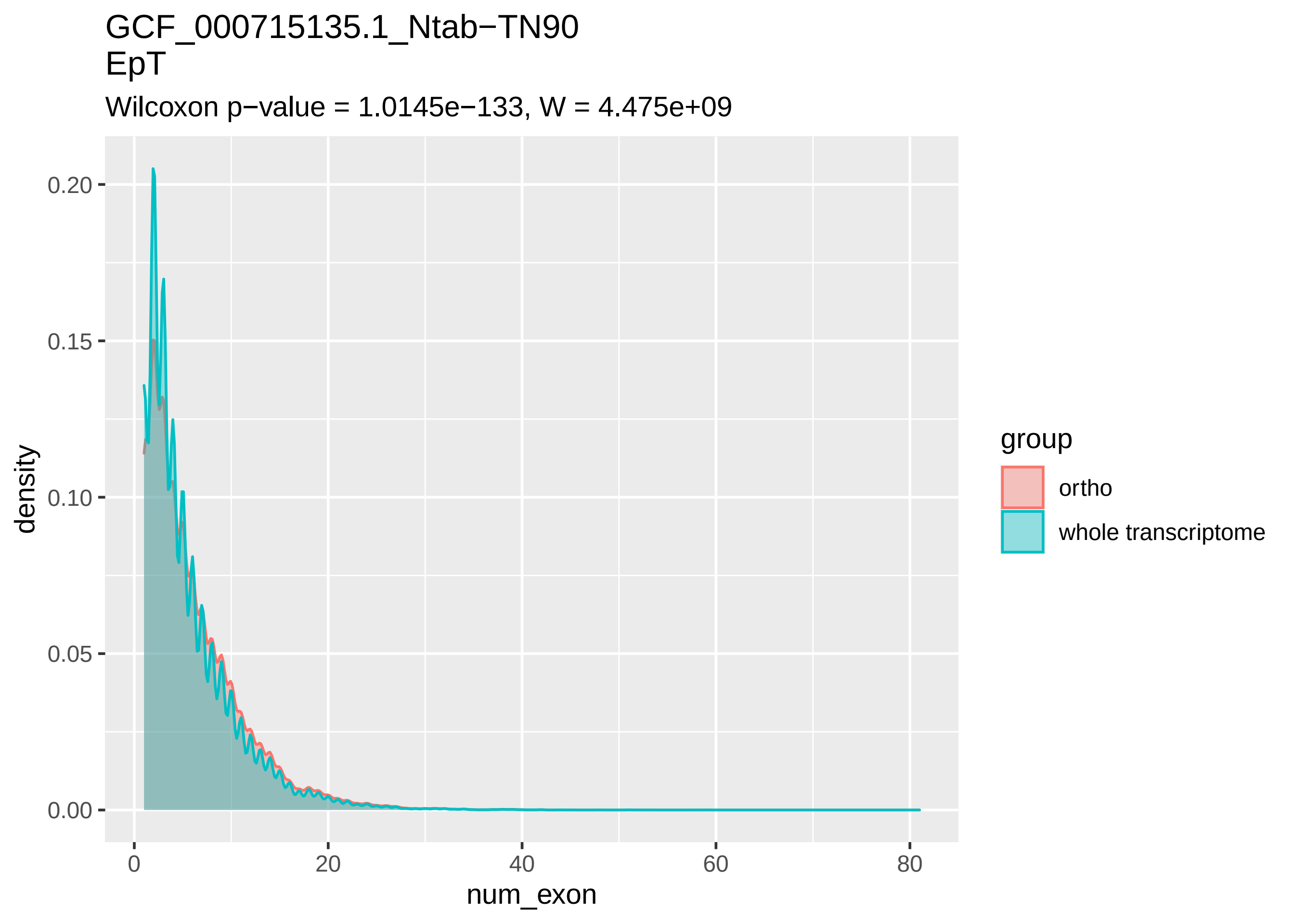
GCF_000715135.1_Ntab−TN90
EpT
Wilcoxon p−value = 1.0145e−133, W = 4.475e+09
0.20
0.15
0.10
0.05
0.00
density
0
20
40
60
80
num_exon
group
ortho
whole transcriptome

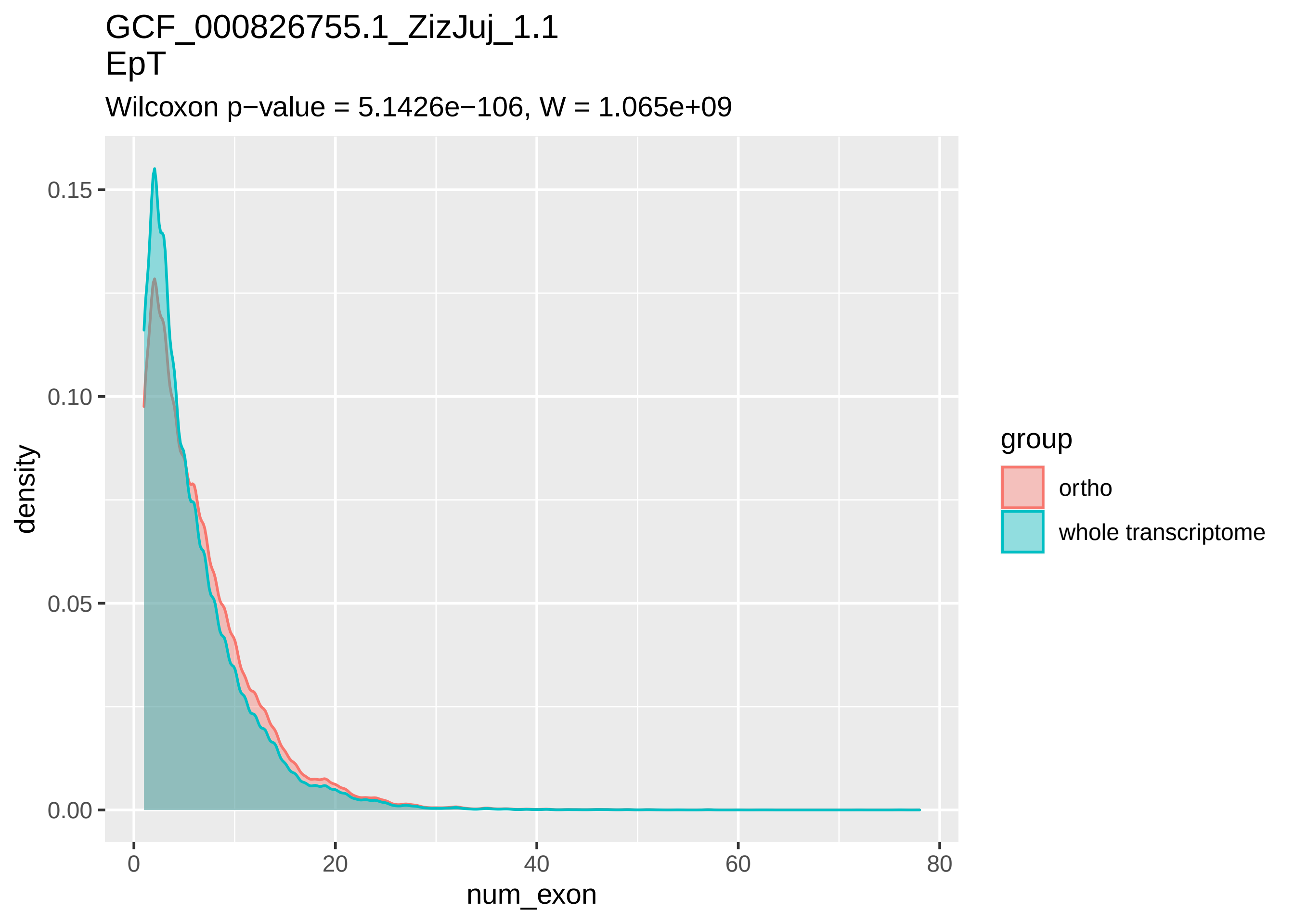

GCF_000826755.1_ZizJuj_1.1
EpT
Wilcoxon p−value = 5.1426e−106, W = 1.065e+09
density
0.00
0.05
0.10
0.15
0
20
40
60
80
num_exon
group
ortho
whole transcriptome

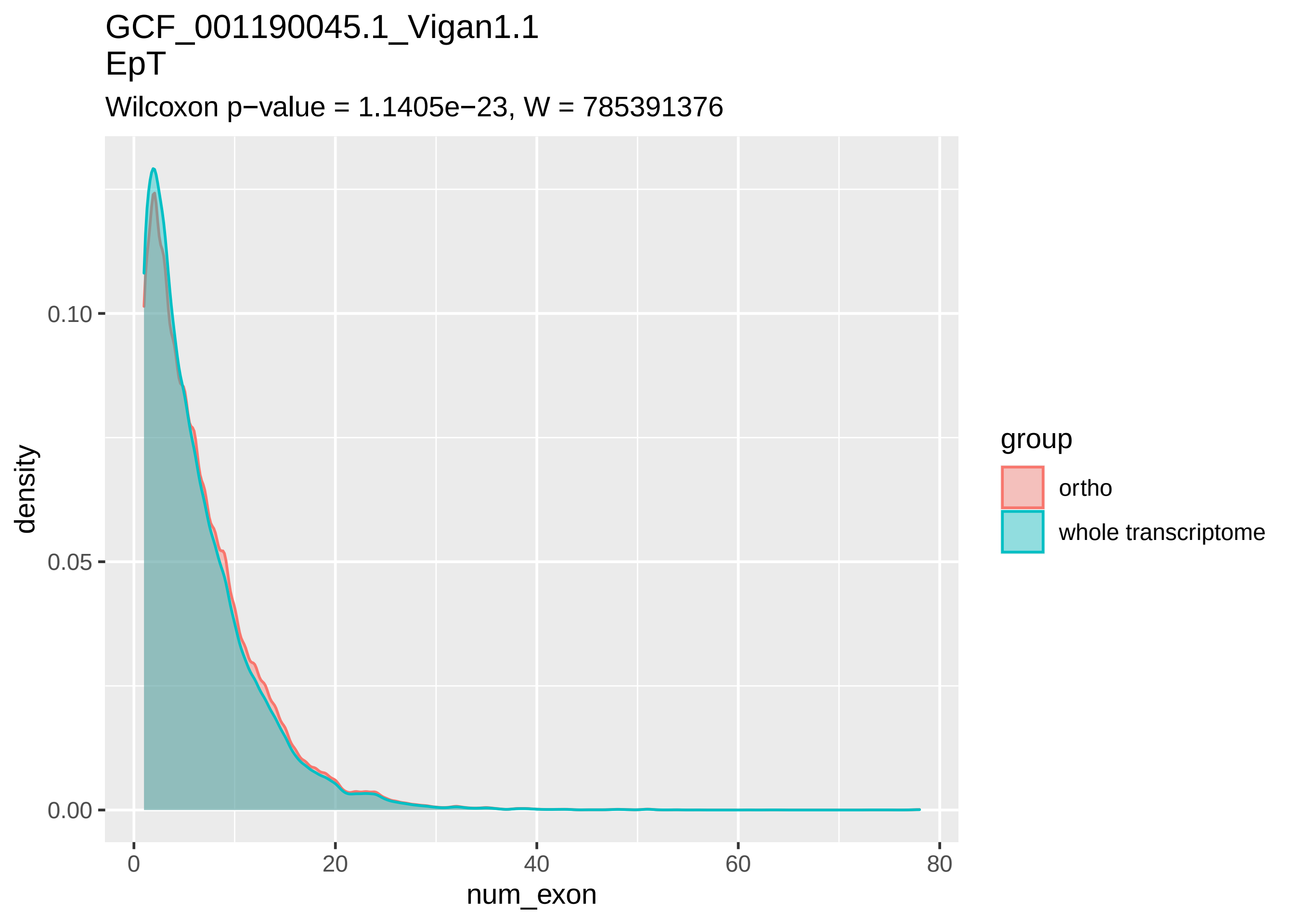
GCF_001190045.1_Vigan1.1
EpT
Wilcoxon p−value = 1.1405e−23, W = 785391376
density
0.10
0.05
0.00
0
20
40
60
80
num_exon
group
ortho
whole transcriptome

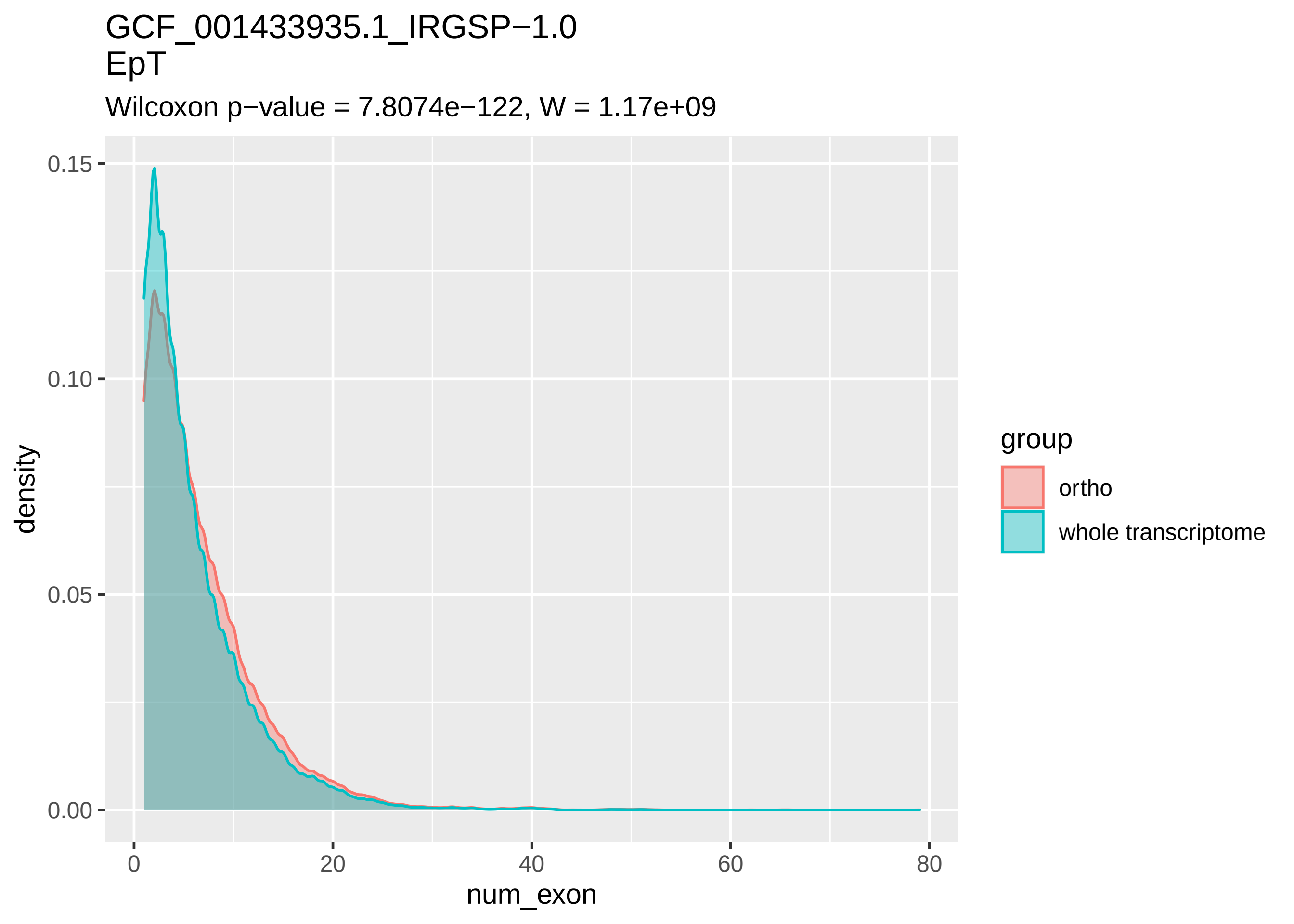

GCF_001433935.1_IRGSP−1.0
EpT
Wilcoxon p−value = 7.8074e−122, W = 1.17e+09
density
0.15
0.10
0.05
0.00
0
20
40
60
80
num_exon
group
ortho
whole transcriptome

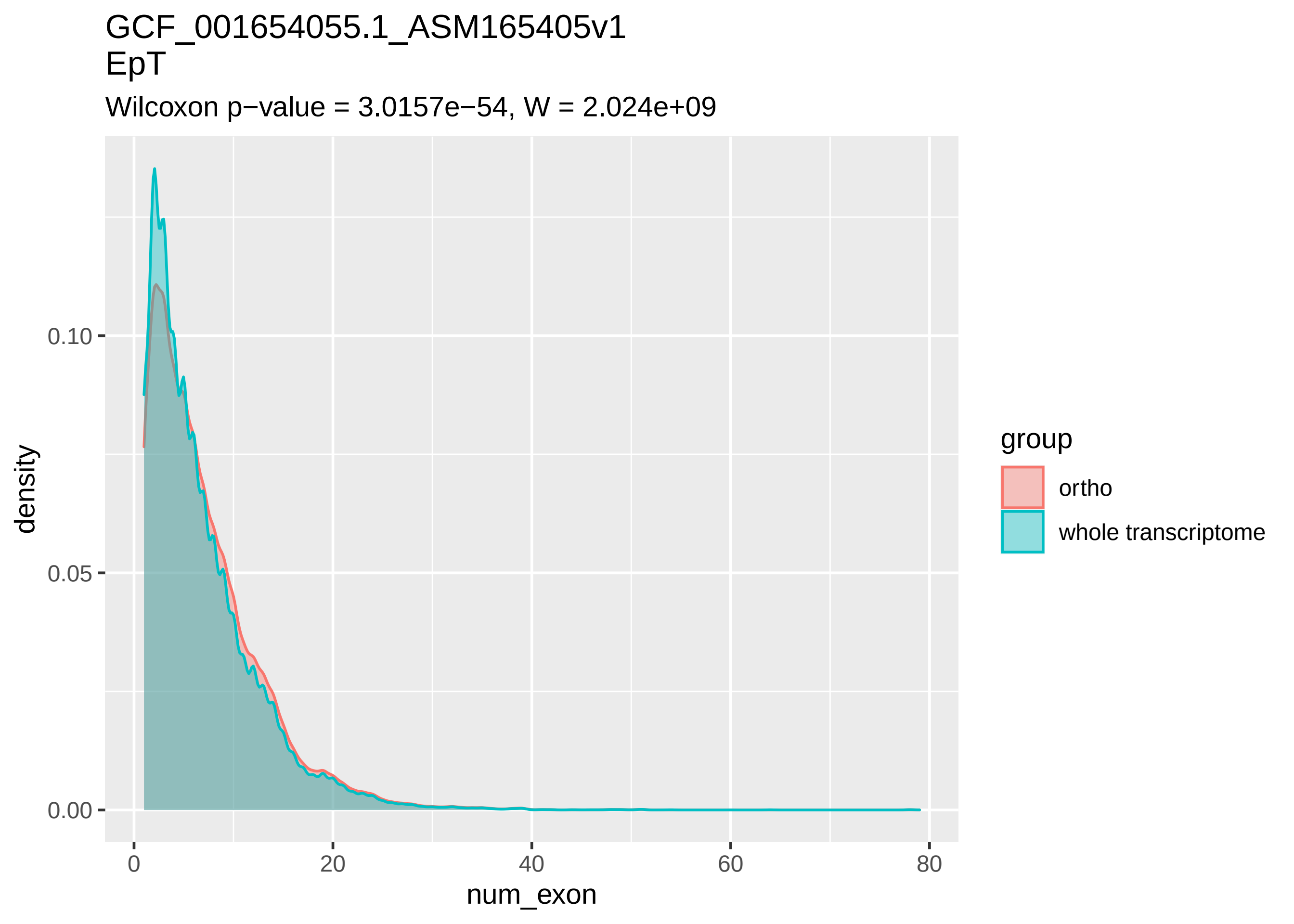
GCF_001654055.1_ASM165405v1
EpT
Wilcoxon p−value = 3.0157e−54, W = 2.024e+09
density
0.10
0.05
0.00
0
20
40
60
80
num_exon
group
ortho
whole transcriptome

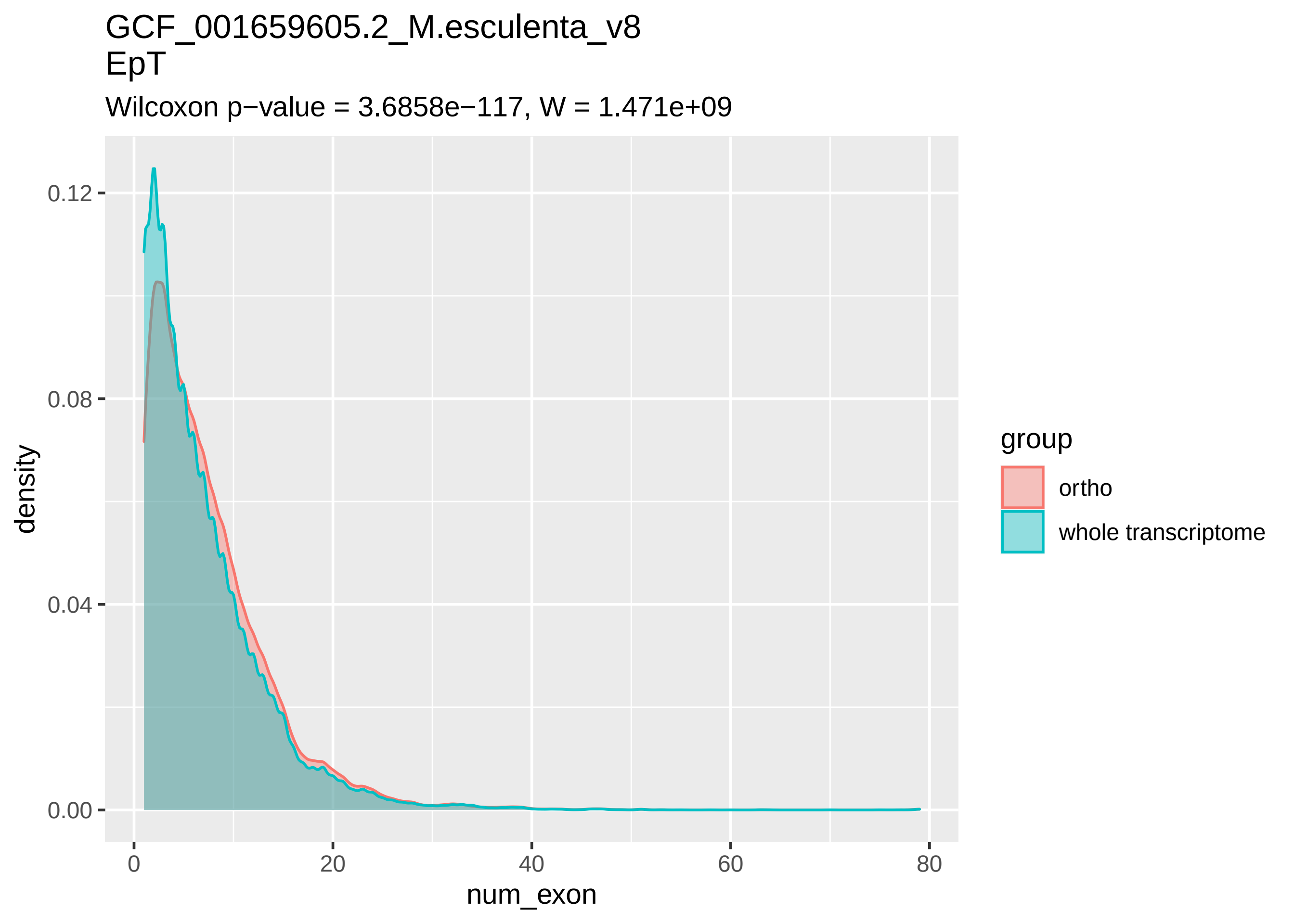

GCF_001659605.2_M.esculenta_v8
EpT
Wilcoxon p−value = 3.6858e−117, W = 1.471e+09
density
0.12
0.08
0.04
0.00
0
20
40
60
80
num_exon
group
ortho
whole transcriptome

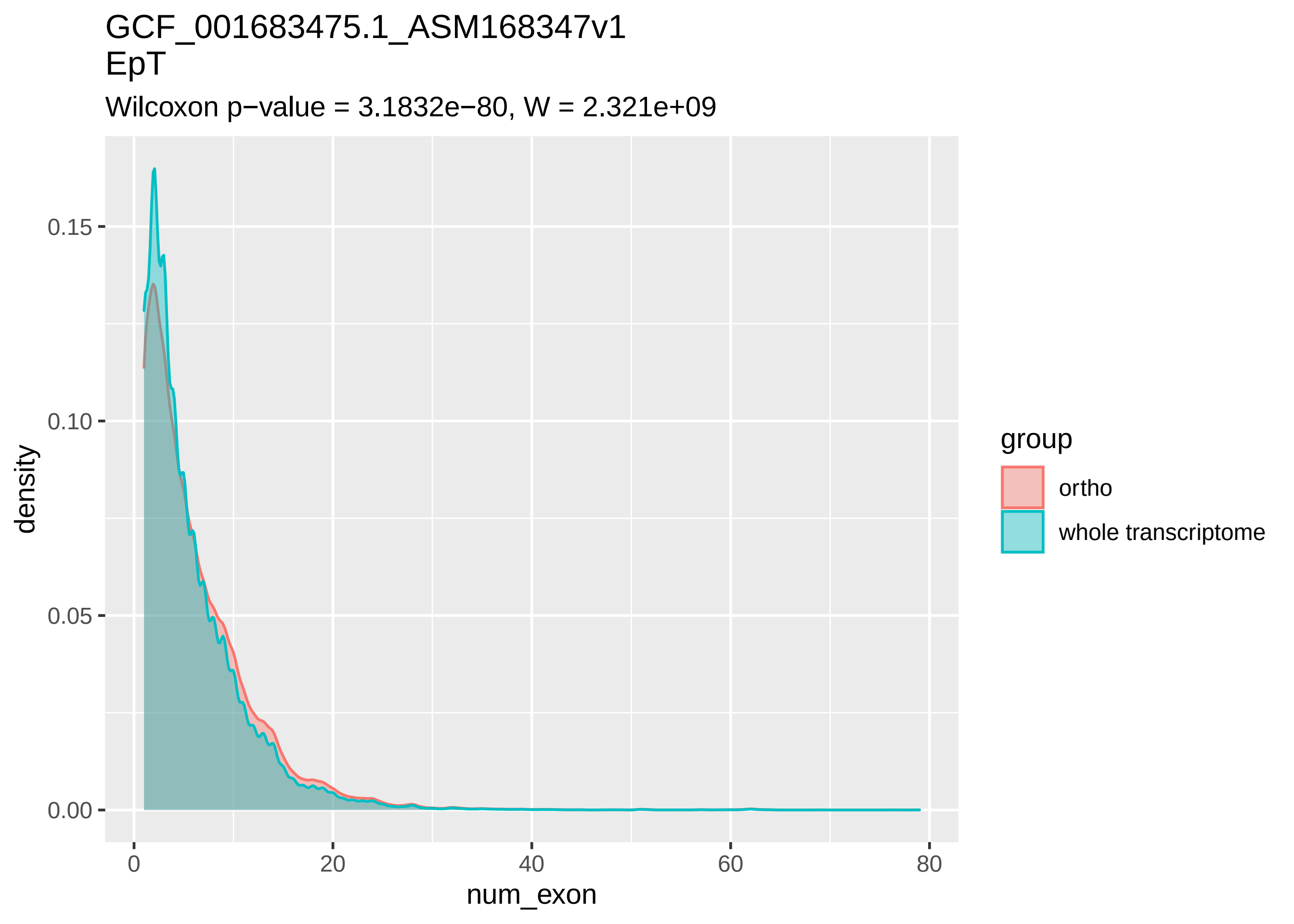
GCF_001683475.1_ASM168347v1
EpT
Wilcoxon p−value = 3.1832e−80, W = 2.321e+09
density
0.15
0.10
0.05
0.00
0
20
40
60
80
num_exon
group
ortho
whole transcriptome

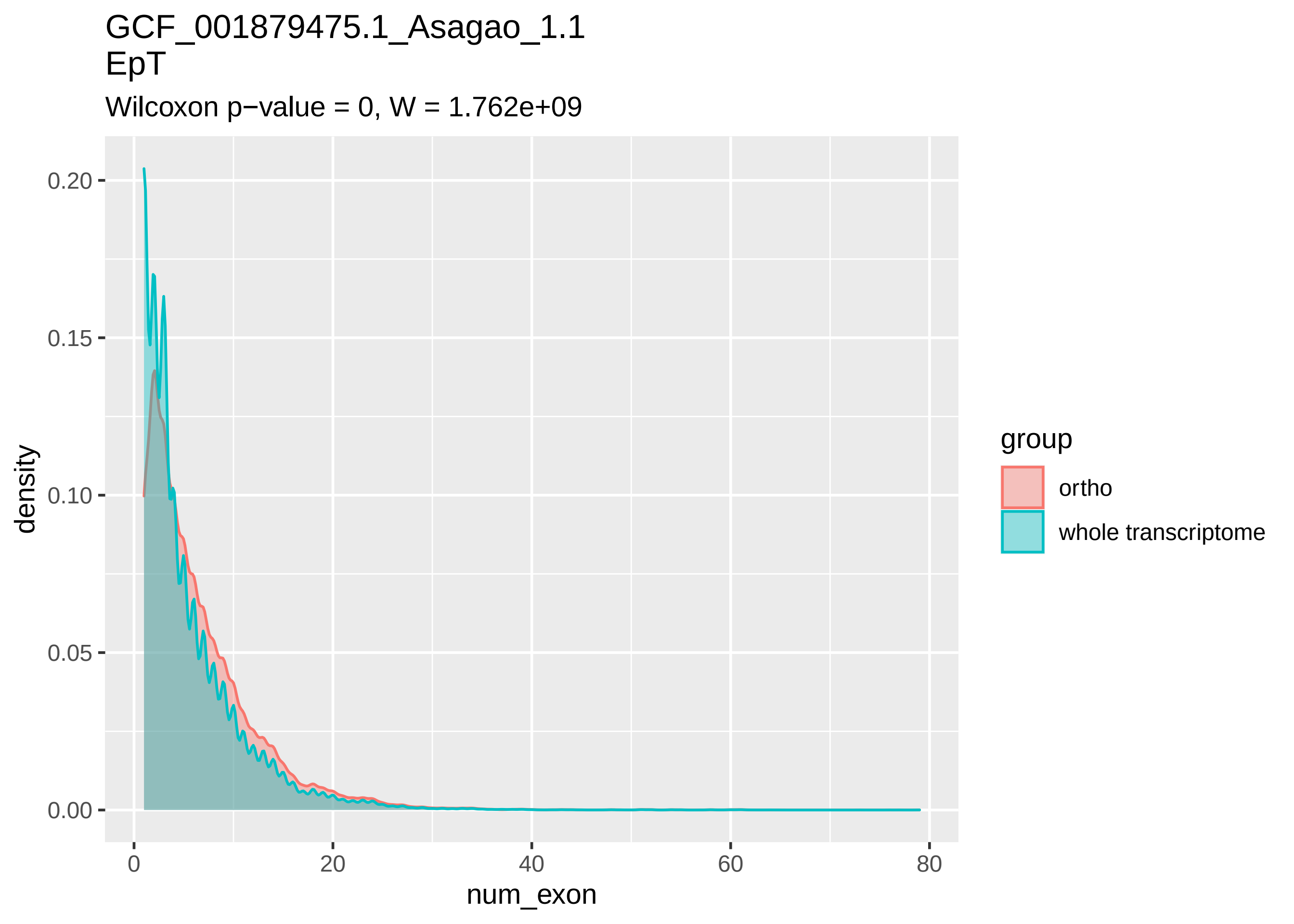

GCF_001879475.1_Asagao_1.1
EpT
Wilcoxon p−value = 0, W = 1.762e+09
density
0.20
0.15
0.10
0.05
0.00
0
20
40
60
80
num_exon
group
ortho
whole transcriptome

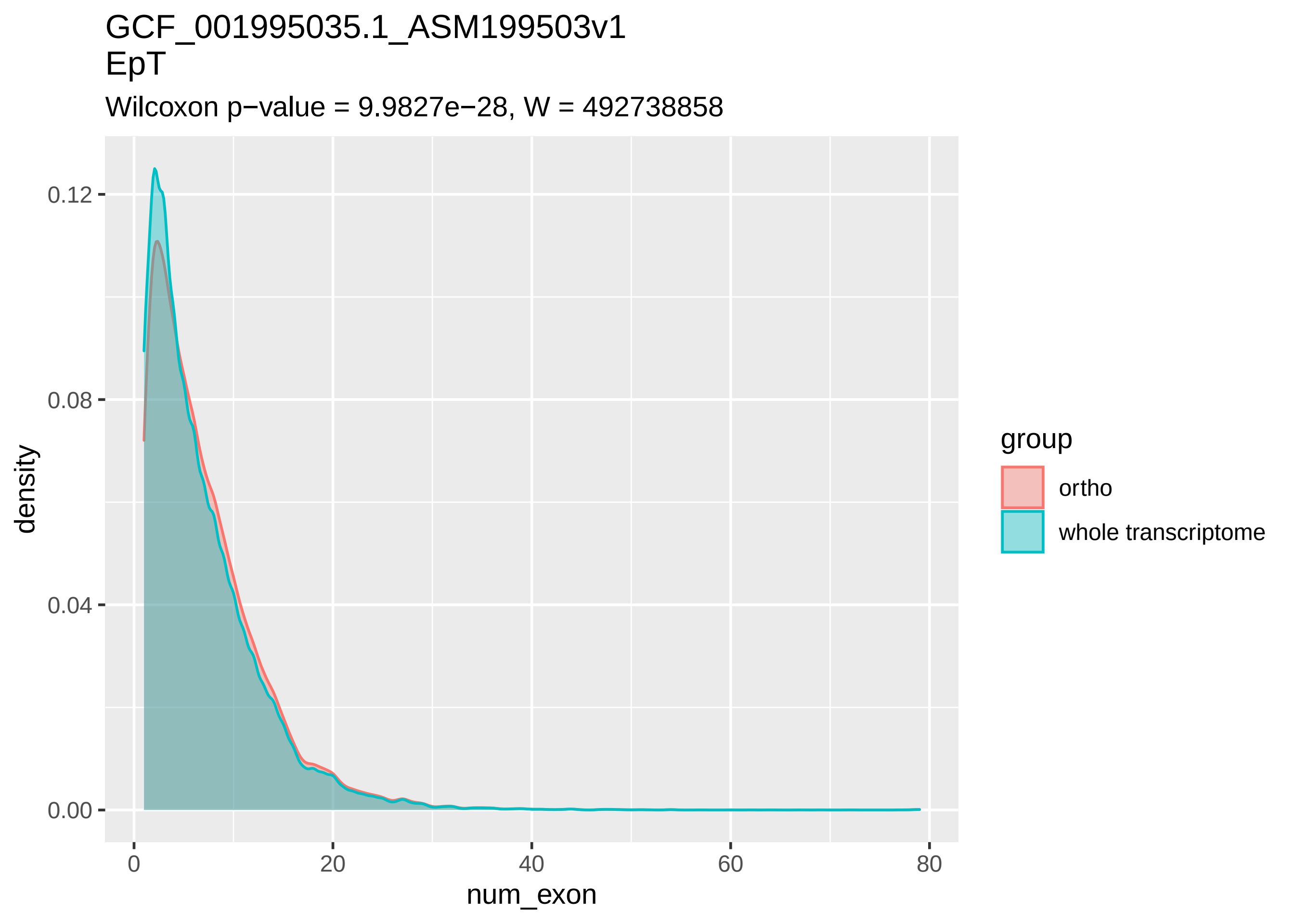
GCF_001995035.1_ASM199503v1
EpT
Wilcoxon p−value = 9.9827e−28, W = 492738858
density
0.12
0.08
0.04
0.00
0
20
40
60
80
num_exon
group
ortho
whole transcriptome

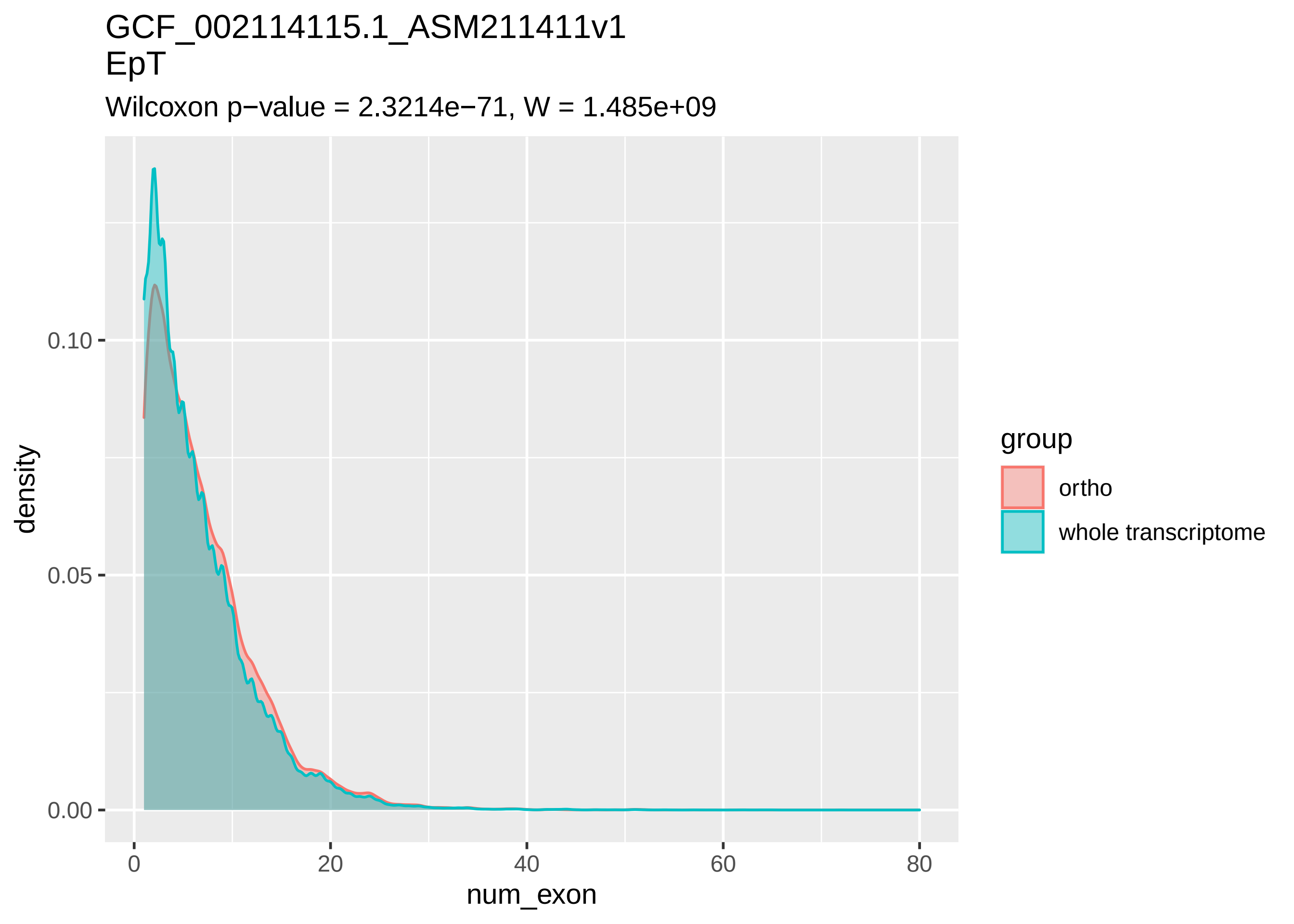
GCF_002114115.1_ASM211411v1
EpT
Wilcoxon p−value = 2.3214e−71, W = 1.485e+09
density
0.10
0.05
0.00
0
20
40
60
80
num_exon
group
ortho
whole transcriptome

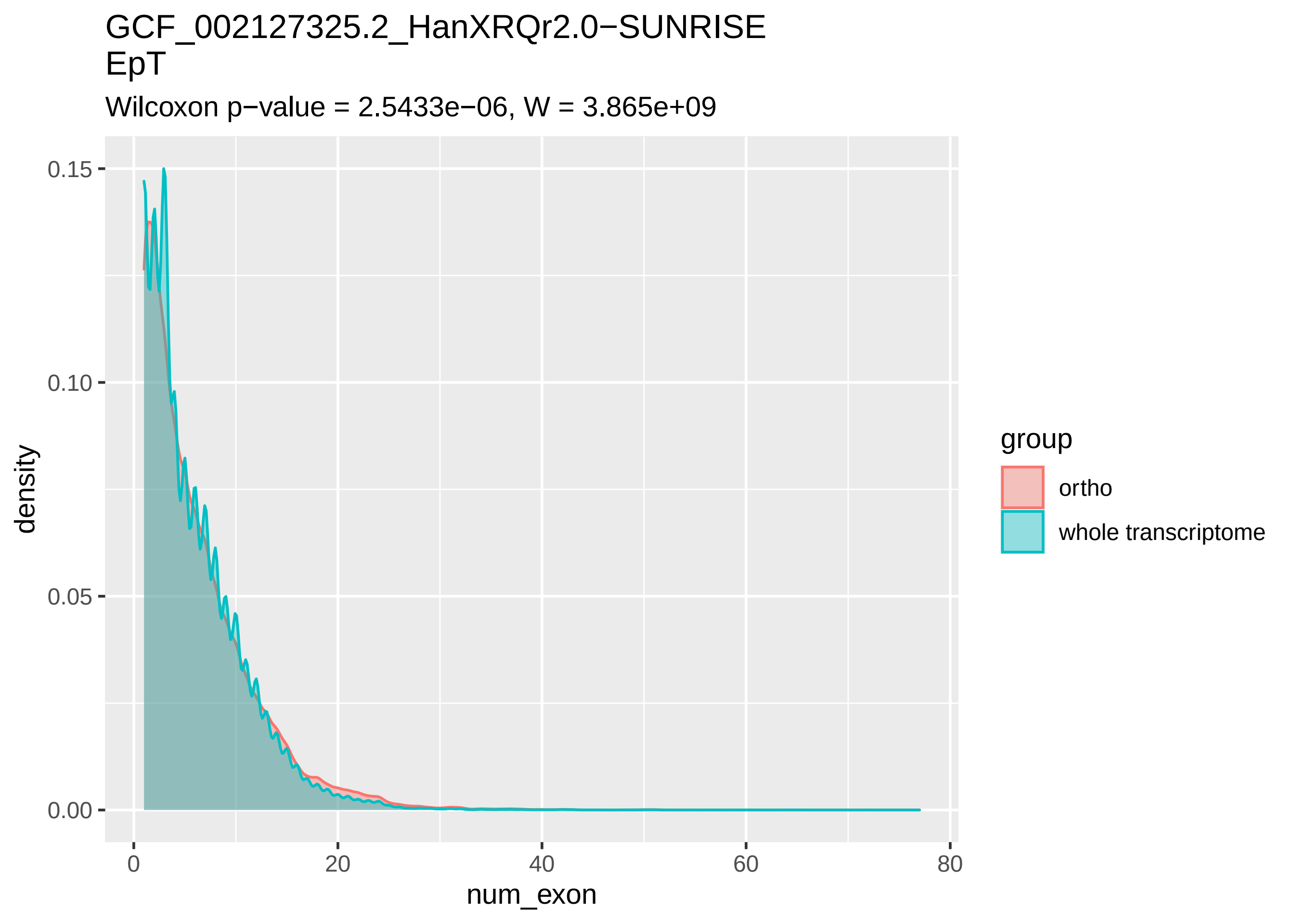
GCF_002127325.2_HanXRQr2.0−SUNRISE
EpT
Wilcoxon p−value = 2.5433e−06, W = 3.865e+09
density
0.15
0.10
0.05
0.00
0
20
40
60
80
num_exon
group
ortho
whole transcriptome

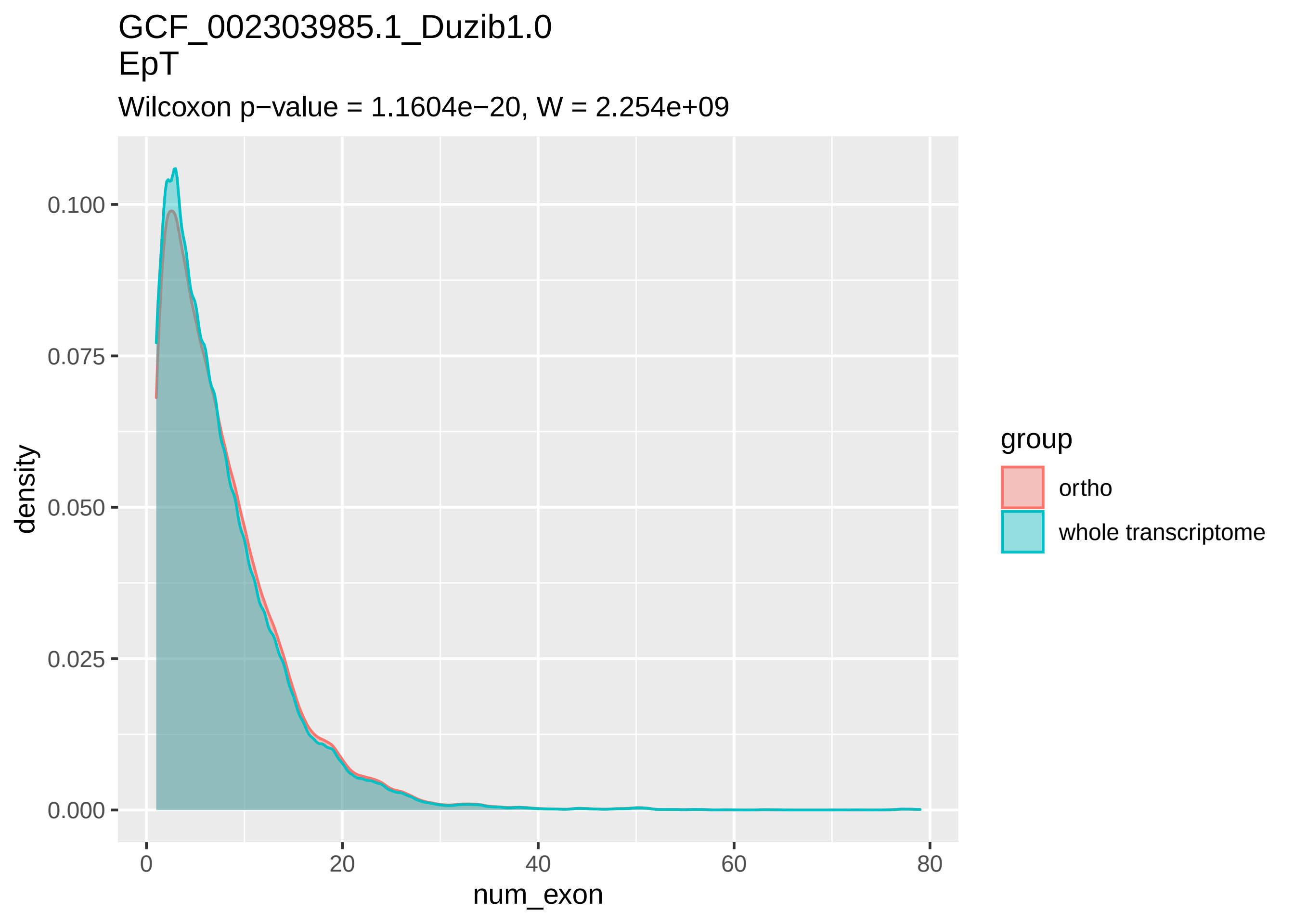

GCF_002303985.1_Duzib1.0
EpT
Wilcoxon p−value = 1.1604e−20, W = 2.254e+09
density
0.100
0.075
0.050
0.025
0.000
0
20
40
60
80
num_exon
group
ortho
whole transcriptome

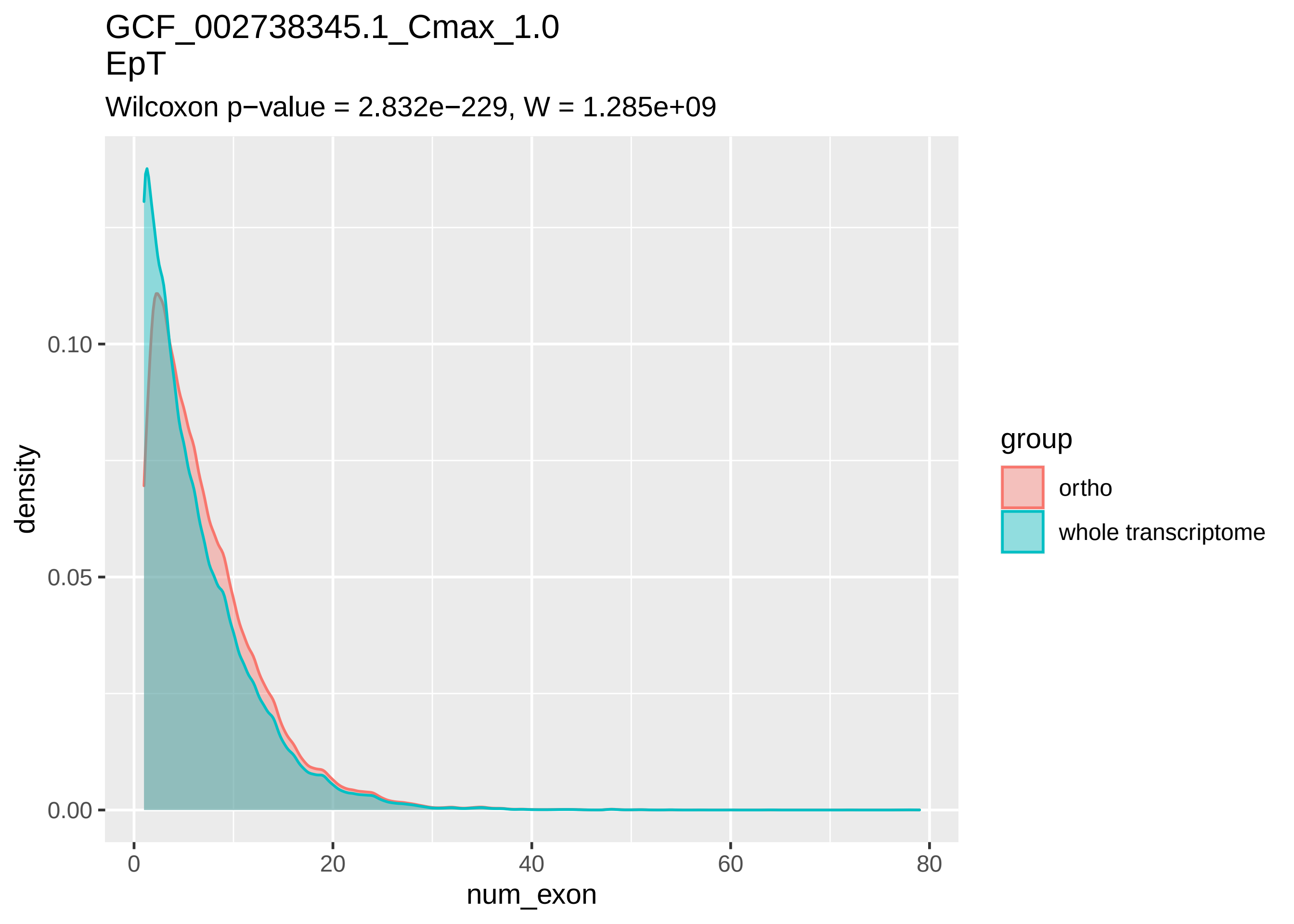
GCF_002738345.1_Cmax_1.0
EpT
Wilcoxon p−value = 2.832e−229, W = 1.285e+09
density
0.10
0.05
0.00
0
20
40
60
80
num_exon
group
ortho
whole transcriptome

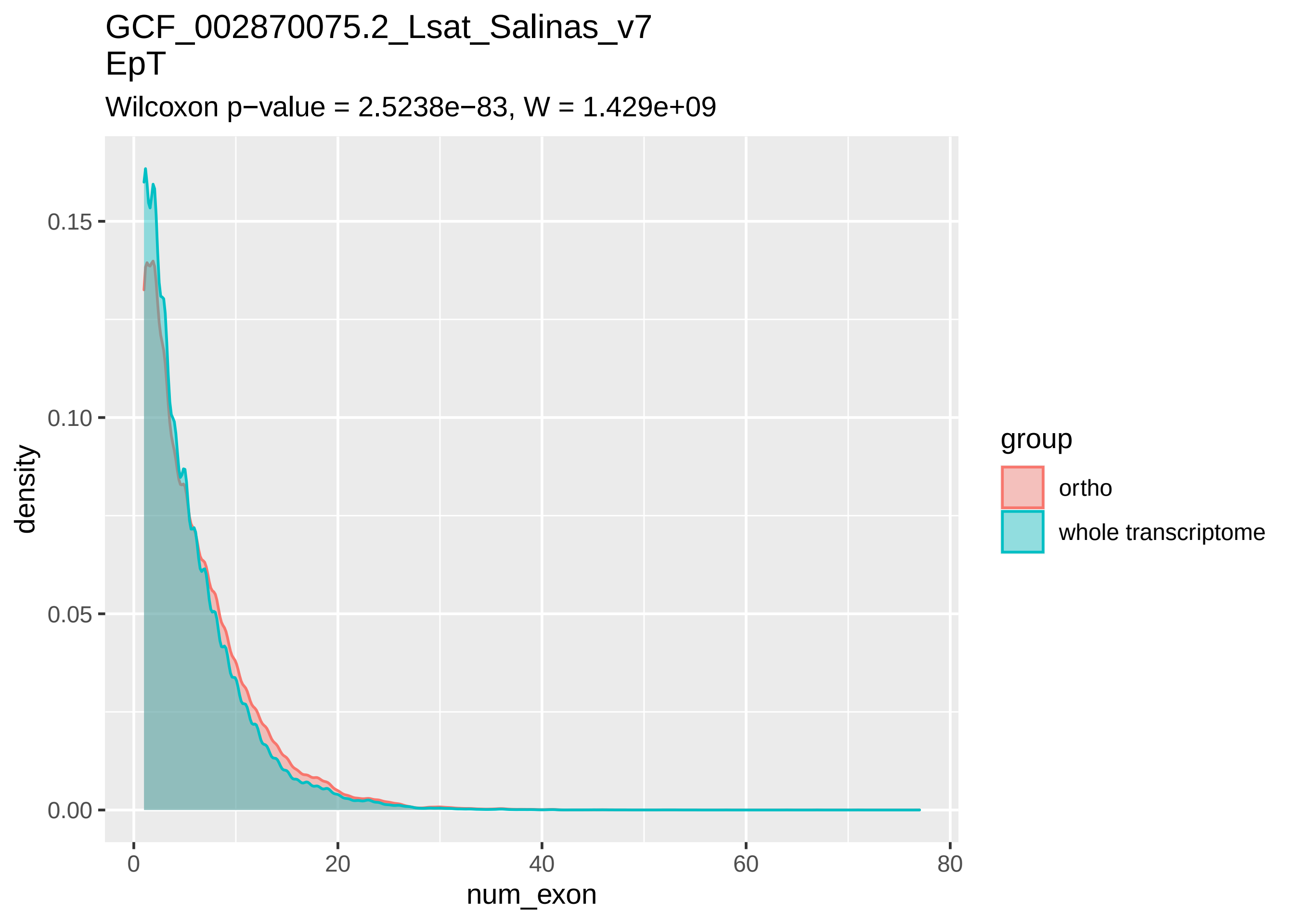

GCF_002870075.2_Lsat_Salinas_v7
EpT
Wilcoxon p−value = 2.5238e−83, W = 1.429e+09
density
0.15
0.10
0.05
0.00
0
20
40
60
80
num_exon
group
ortho
whole transcriptome

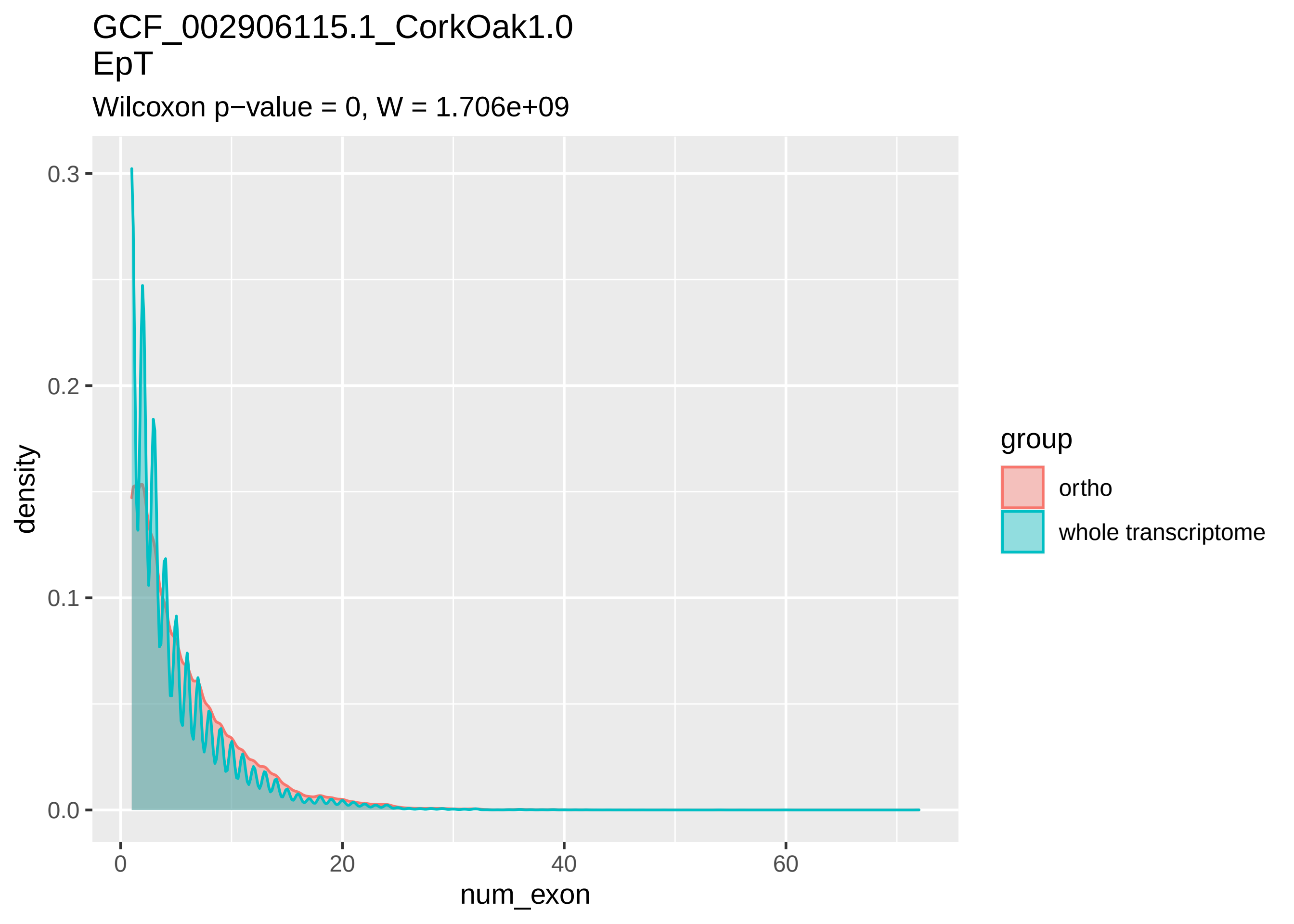
GCF_002906115.1_CorkOak1.0
EpT
Wilcoxon p−value = 0, W = 1.706e+09
0.3
0.2
0.1
0.0
density
0
20
40
60
num_exon
group
ortho
whole transcriptome

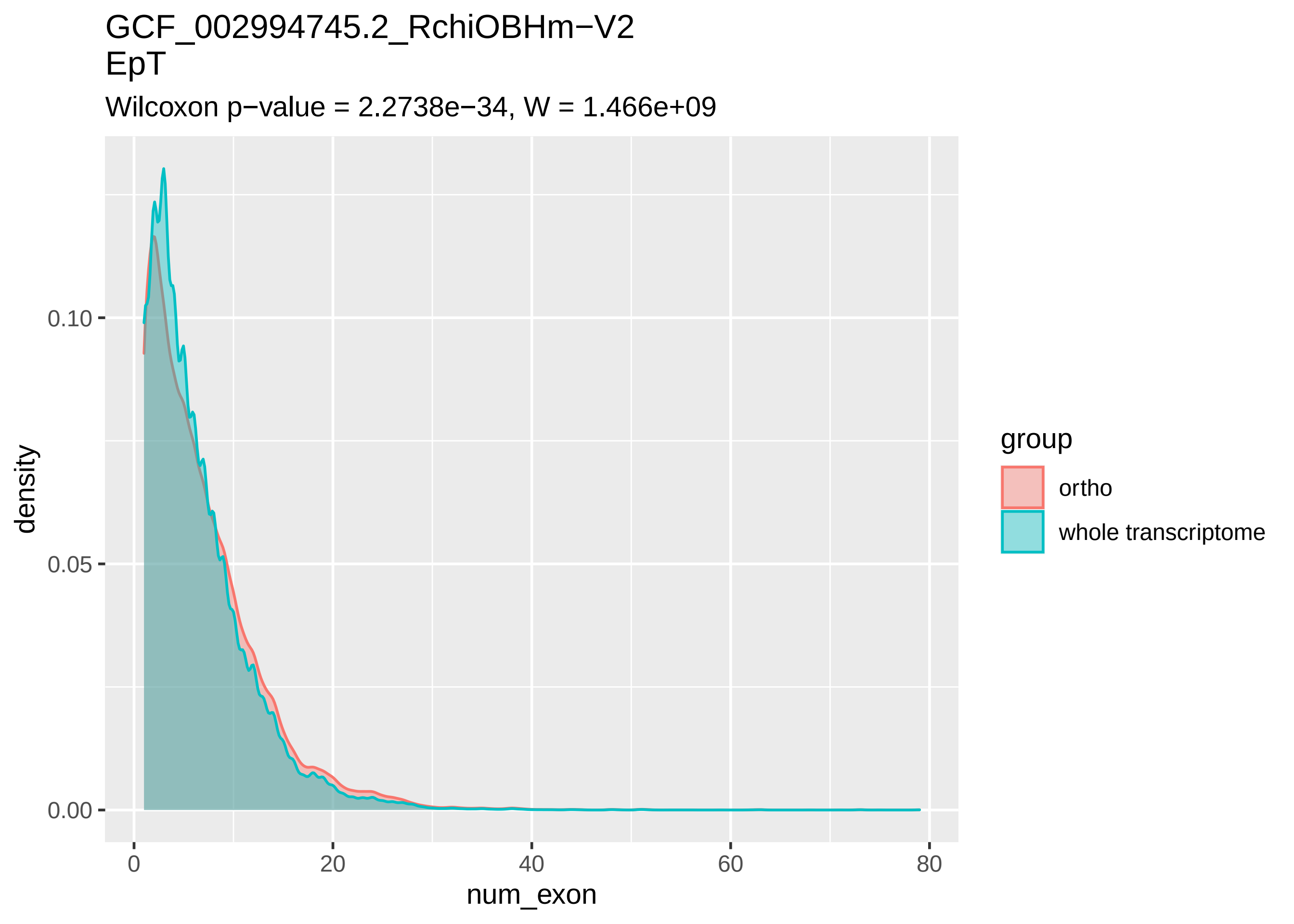

GCF_002994745.2_RchiOBHm−V2
EpT
Wilcoxon p−value = 2.2738e−34, W = 1.466e+09
density
0.10
0.05
0.00
0
20
40
60
80
num_exon
group
ortho
whole transcriptome

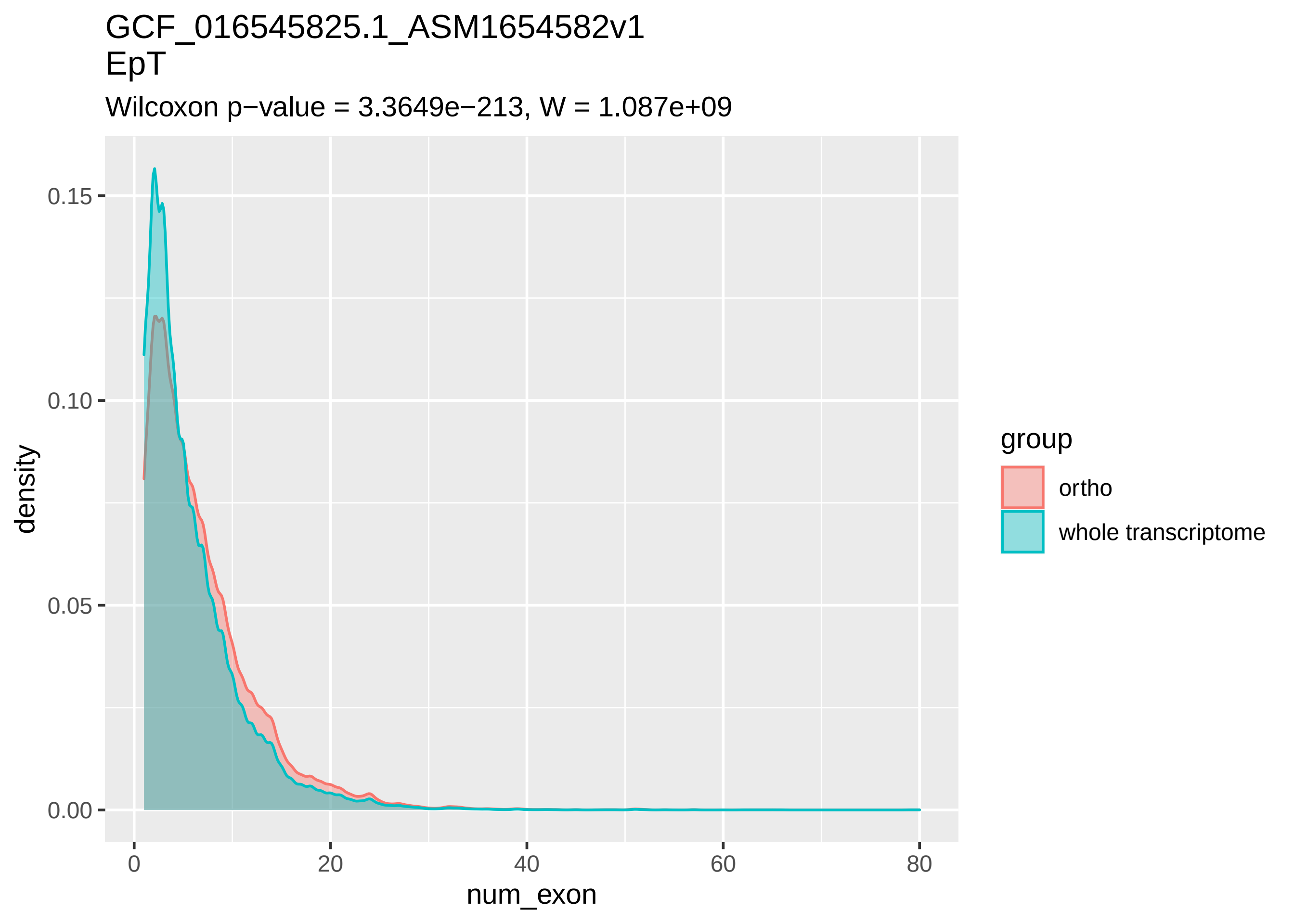

GCF_016545825.1_ASM1654582v1
EpT
Wilcoxon p−value = 3.3649e−213, W = 1.087e+09
density
0.15
0.10
0.05
0.00
0
20
40
60
80
num_exon
group
ortho
whole transcriptome

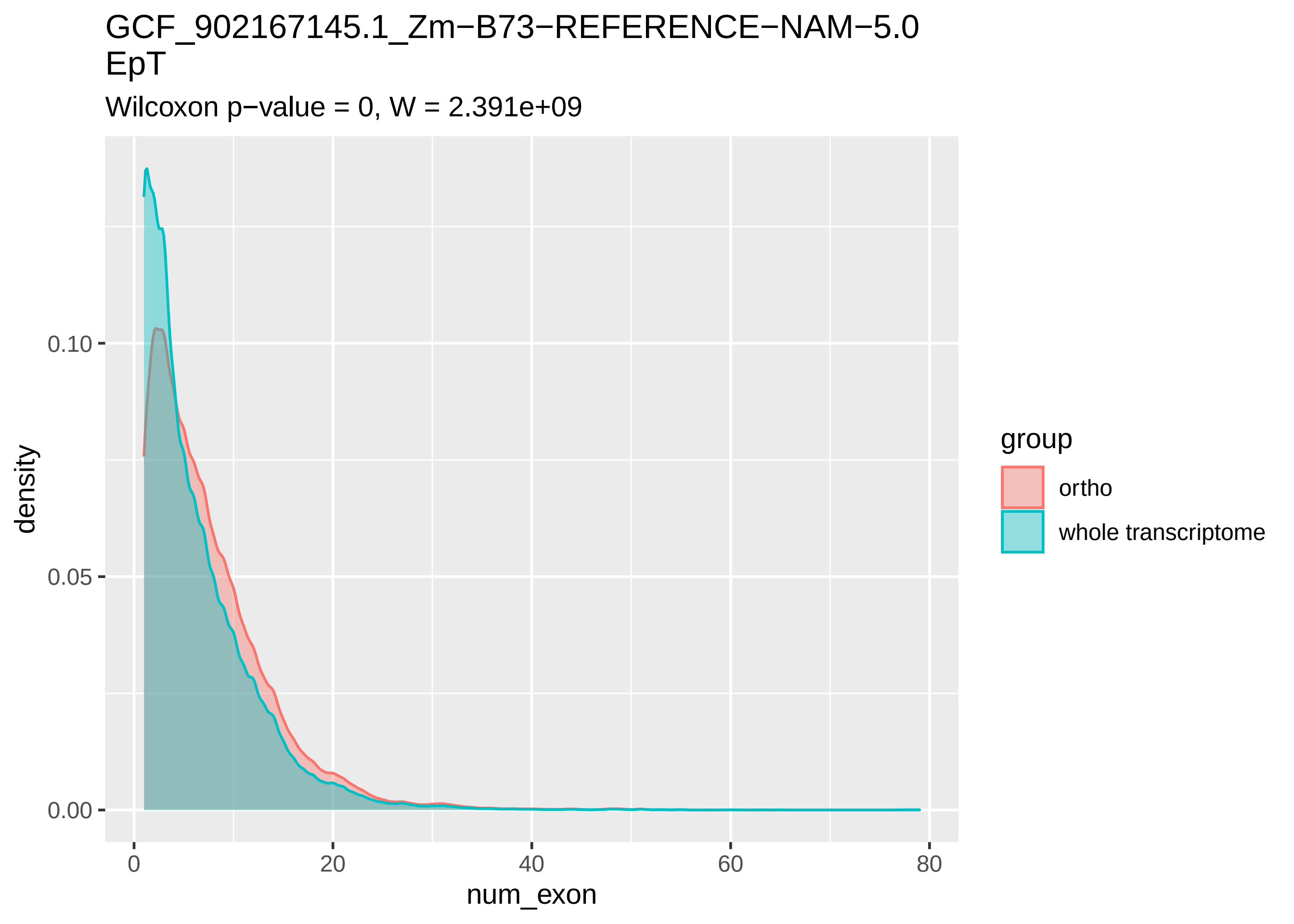

GCF_902167145.1_Zm−B73−REFERENCE−NAM−5.0
EpT
Wilcoxon p−value = 0, W = 2.391e+09
density
0.10
0.05
0.00
0
20
40
60
80
num_exon
group
ortho
whole transcriptome

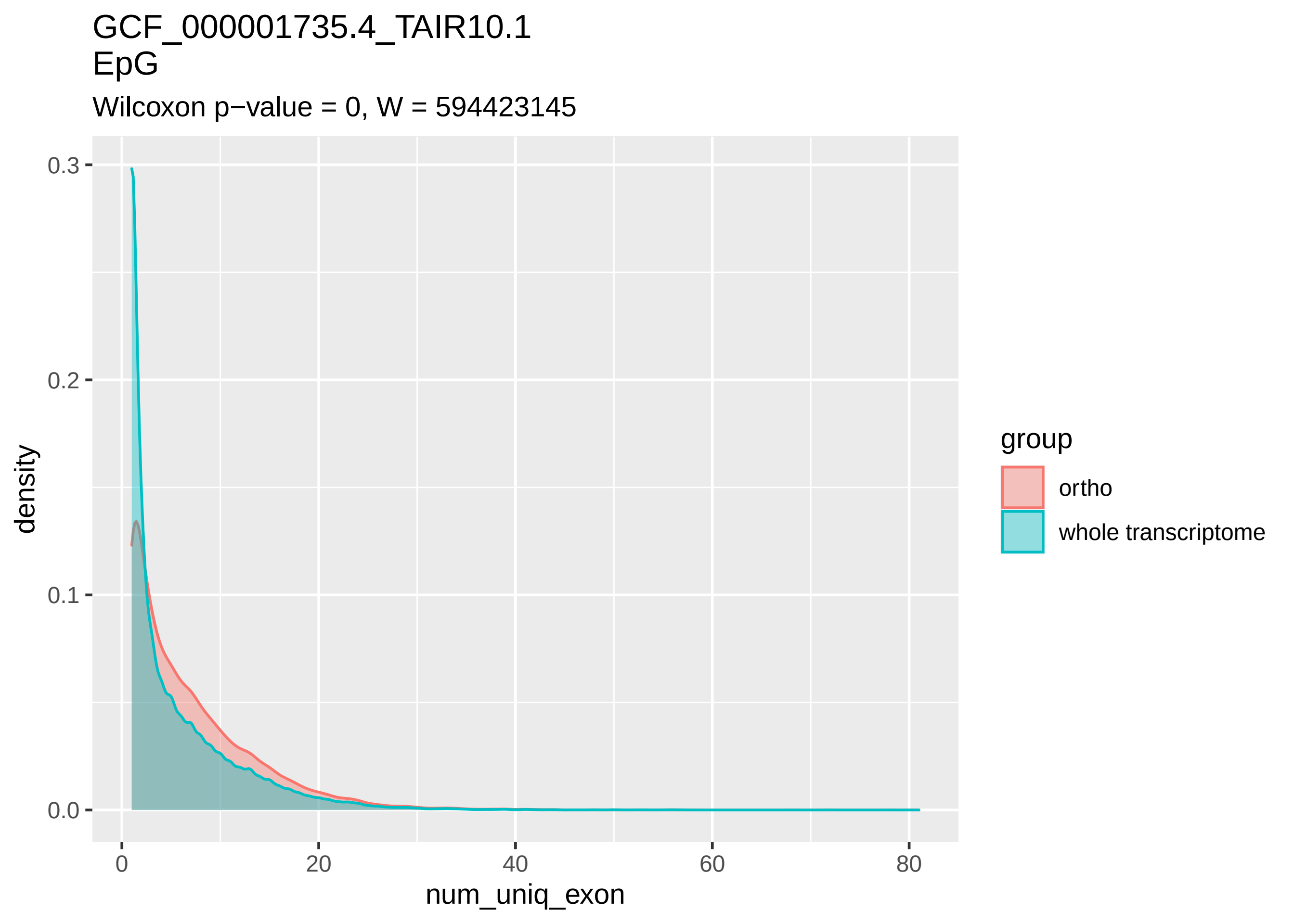
GCF_000001735.4_TAIR10.1
EpG
Wilcoxon p−value = 0, W = 594423145
density
0.3
0.2
0.1
0.0
0
20
40
60
80
num_uniq_exon
group
ortho
whole transcriptome

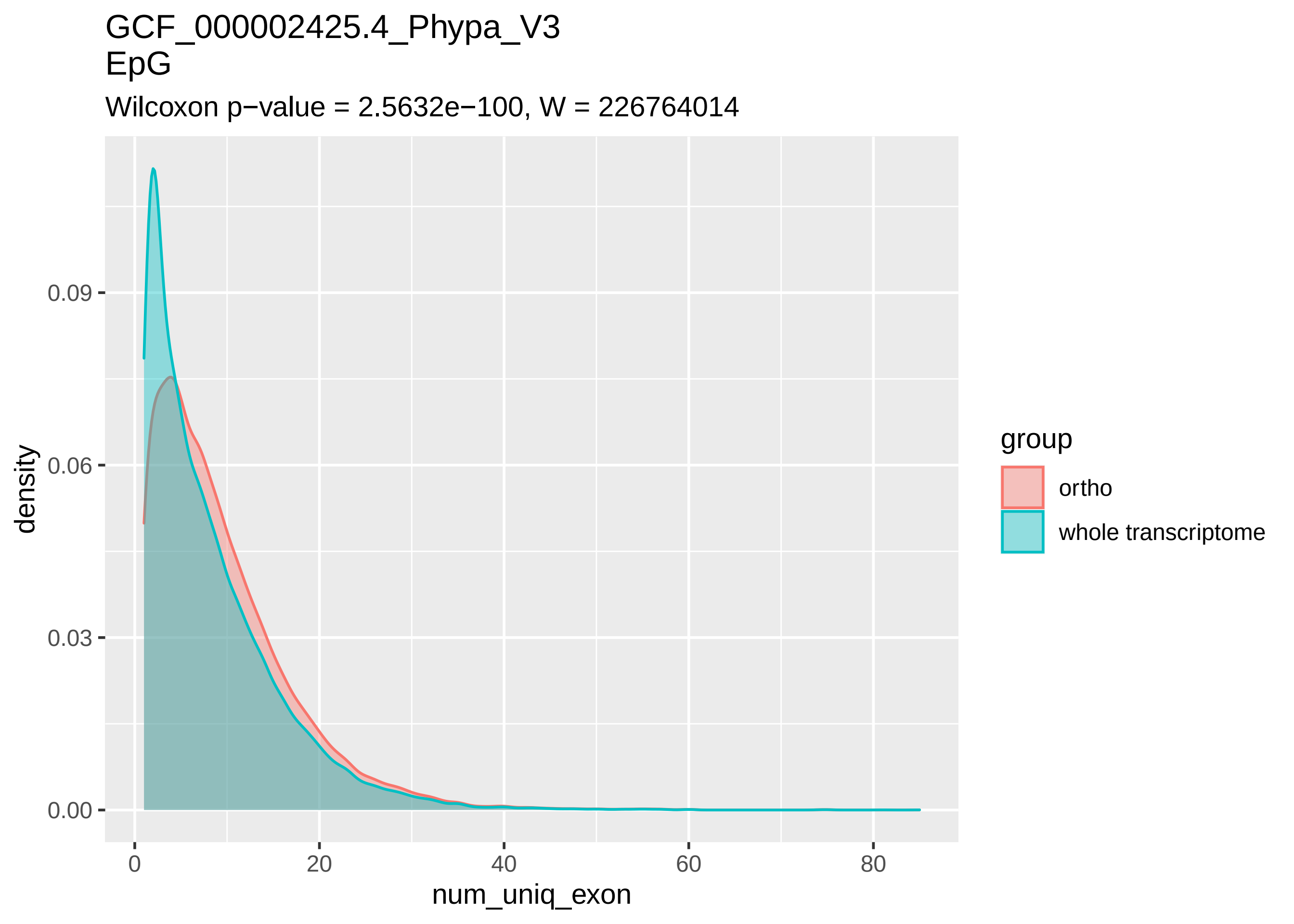

GCF_000002425.4_Phypa_V3
EpG
Wilcoxon p−value = 2.5632e−100, W = 226764014
density
0.09
0.06
0.03
0.00
0
20
40
60
80
num_uniq_exon
group
ortho
whole transcriptome

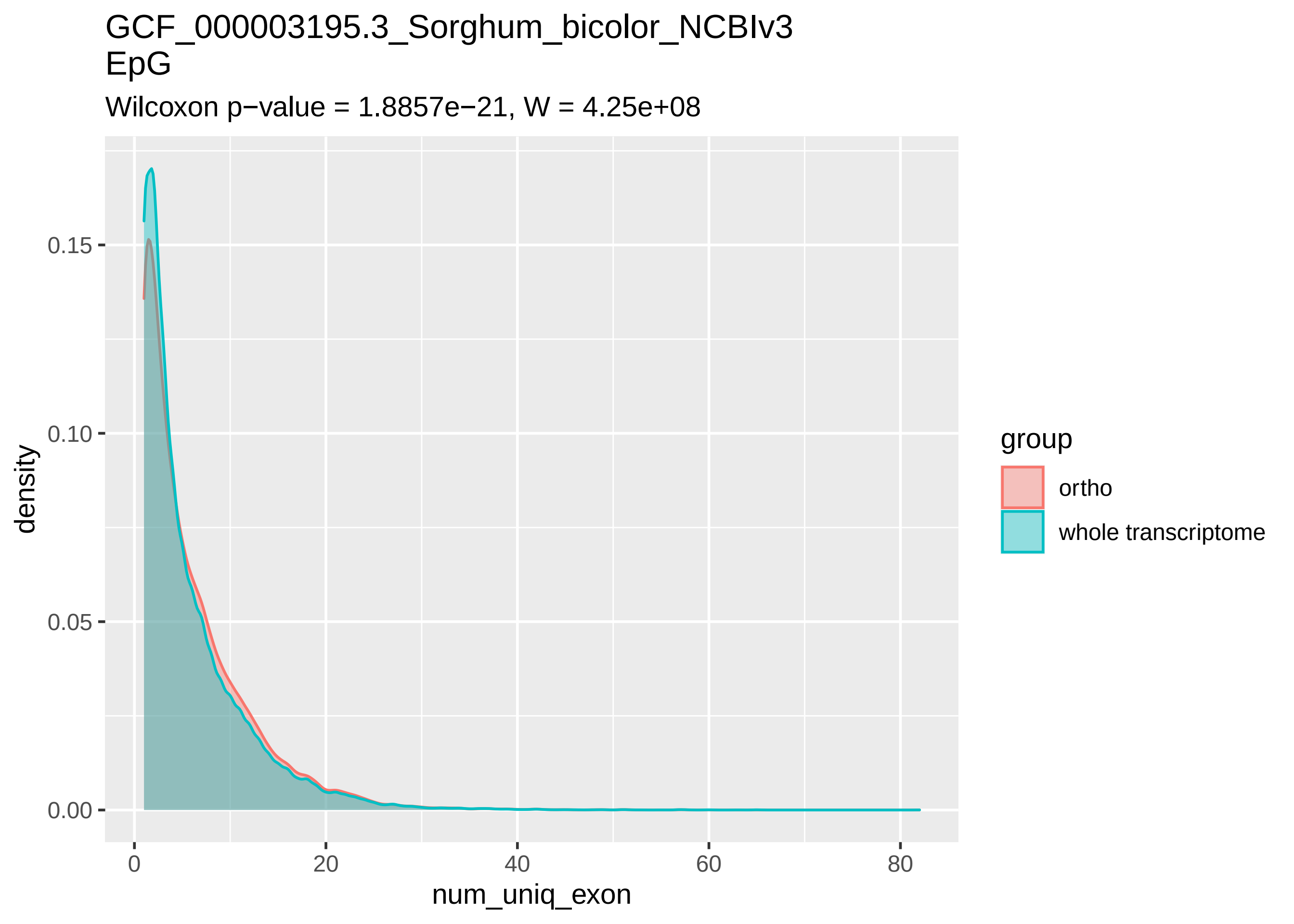
GCF_000003195.3_Sorghum_bicolor_NCBIv3
EpG
Wilcoxon p−value = 1.8857e−21, W = 4.25e+08
density
0.15
0.10
0.05
0.00
0
20
40
60
80
num_uniq_exon
group
ortho
whole transcriptome

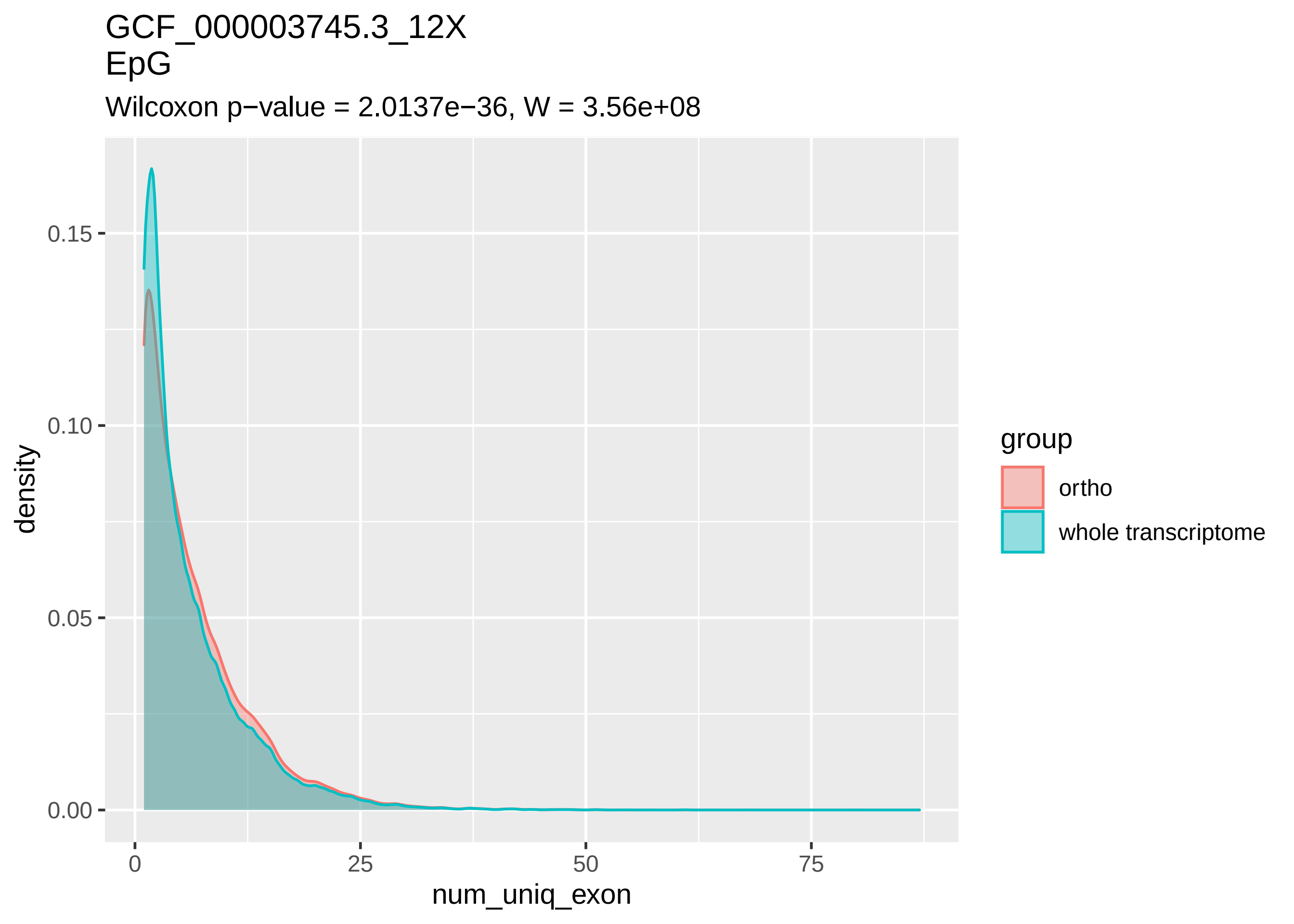
GCF_000003745.3_12X
EpG
Wilcoxon p−value = 2.0137e−36, W = 3.56e+08
density
0.15
0.10
0.05
0.00
0
25
50
75
num_uniq_exon
group
ortho
whole transcriptome

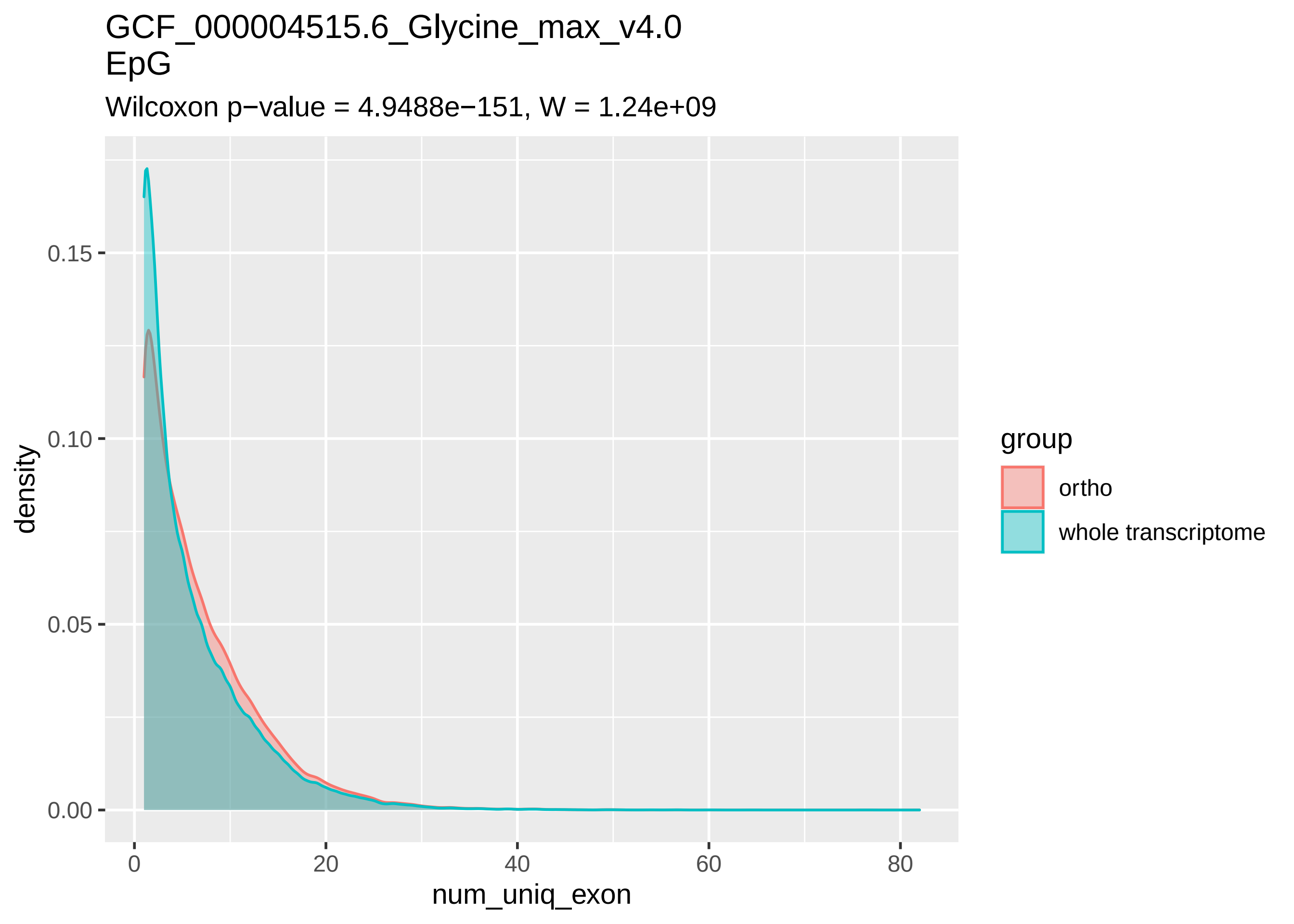
GCF_000004515.6_Glycine_max_v4.0
EpG
Wilcoxon p−value = 4.9488e−151, W = 1.24e+09
density
0.15
0.10
0.05
0.00
0
20
40
60
80
num_uniq_exon
group
ortho
whole transcriptome

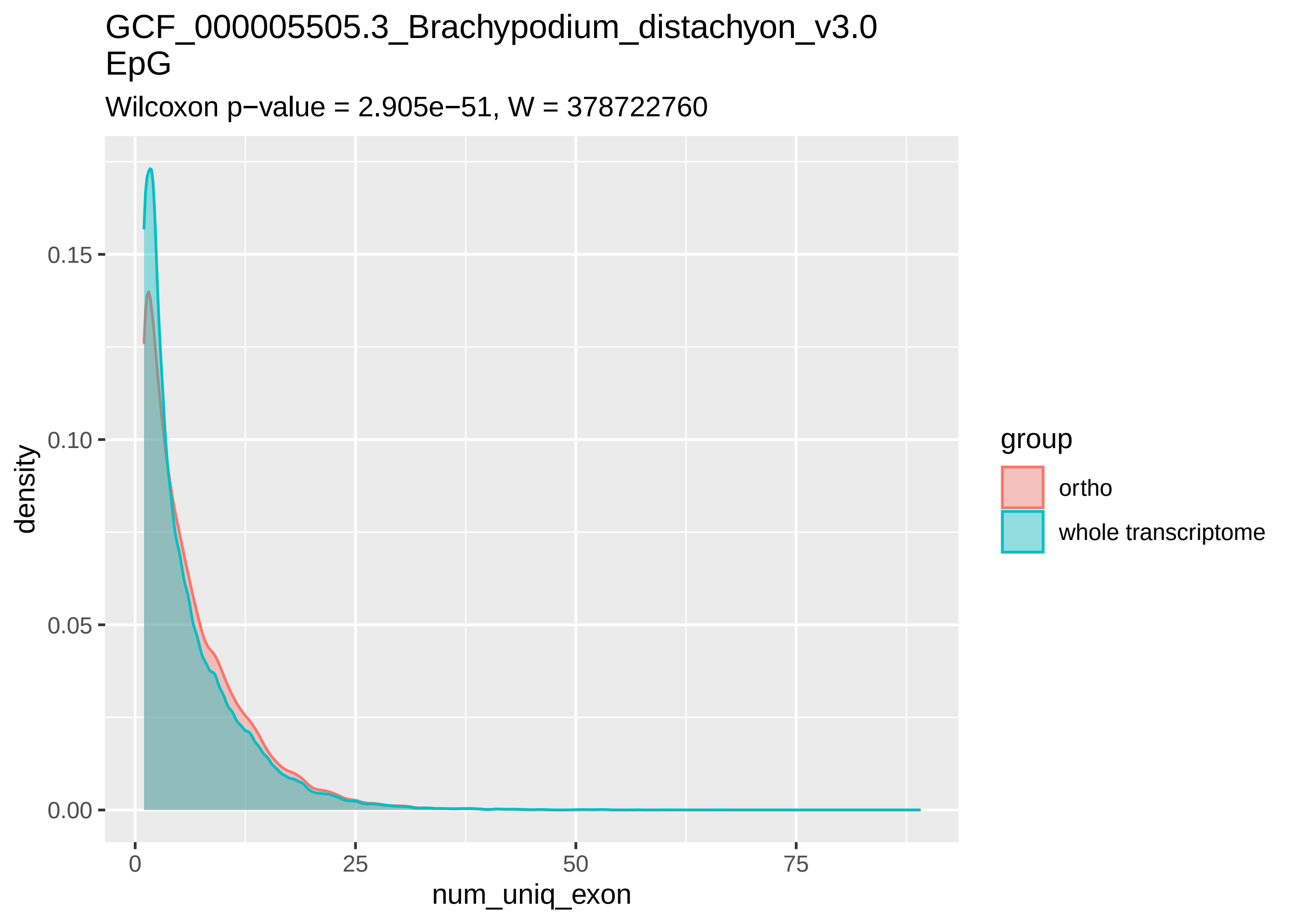

GCF_000005505.3_Brachypodium_distachyon_v3.0
EpG
Wilcoxon p−value = 2.905e−51, W = 378722760
density
0.15
0.10
0.05
0.00
0
25
50
75
num_uniq_exon
group
ortho
whole transcriptome

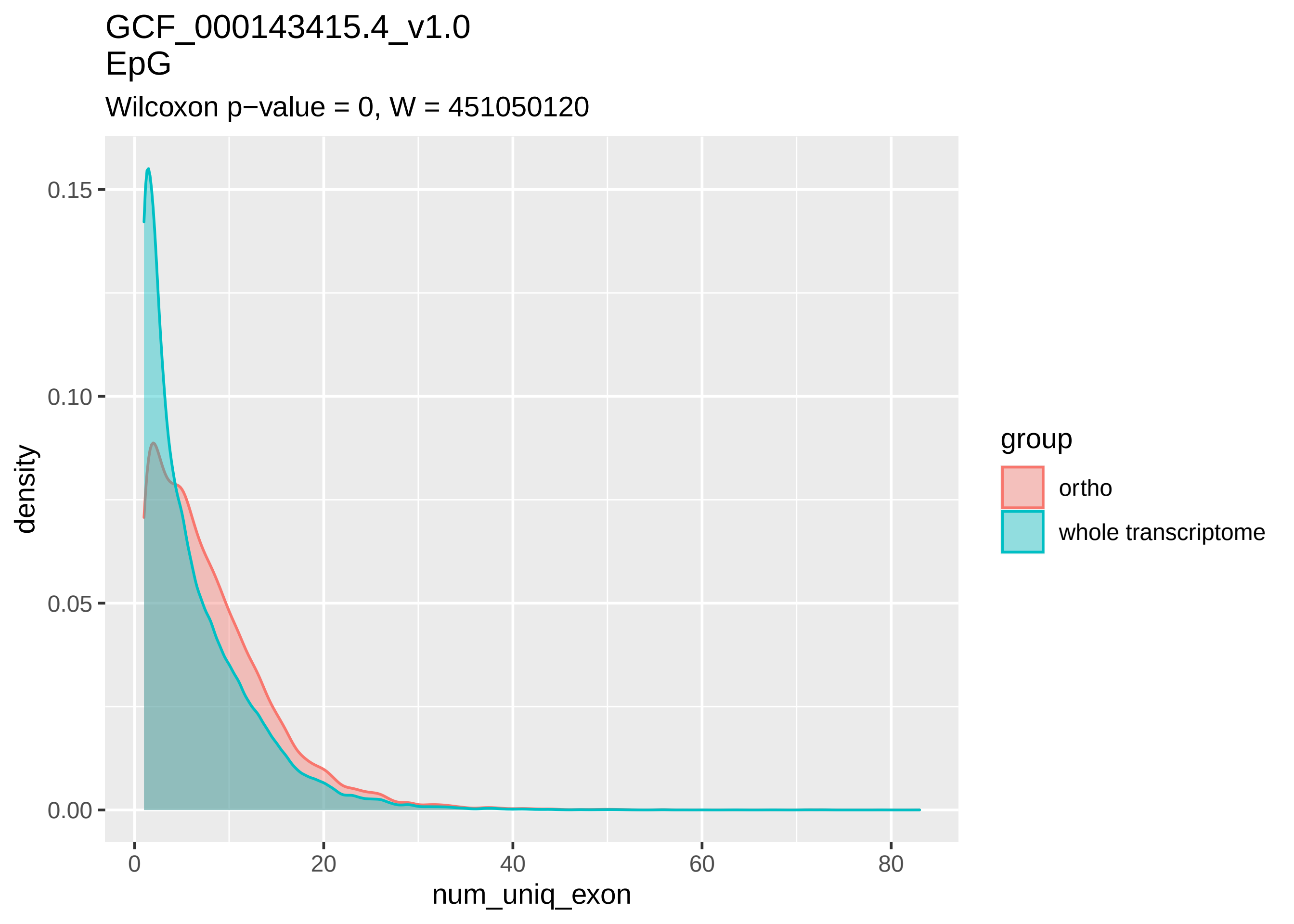

GCF_000143415.4_v1.0
EpG
Wilcoxon p−value = 0, W = 451050120
0.15
0.10
0.05
0.00
density
0
20
40
60
80
num_uniq_exon
group
ortho
whole transcriptome

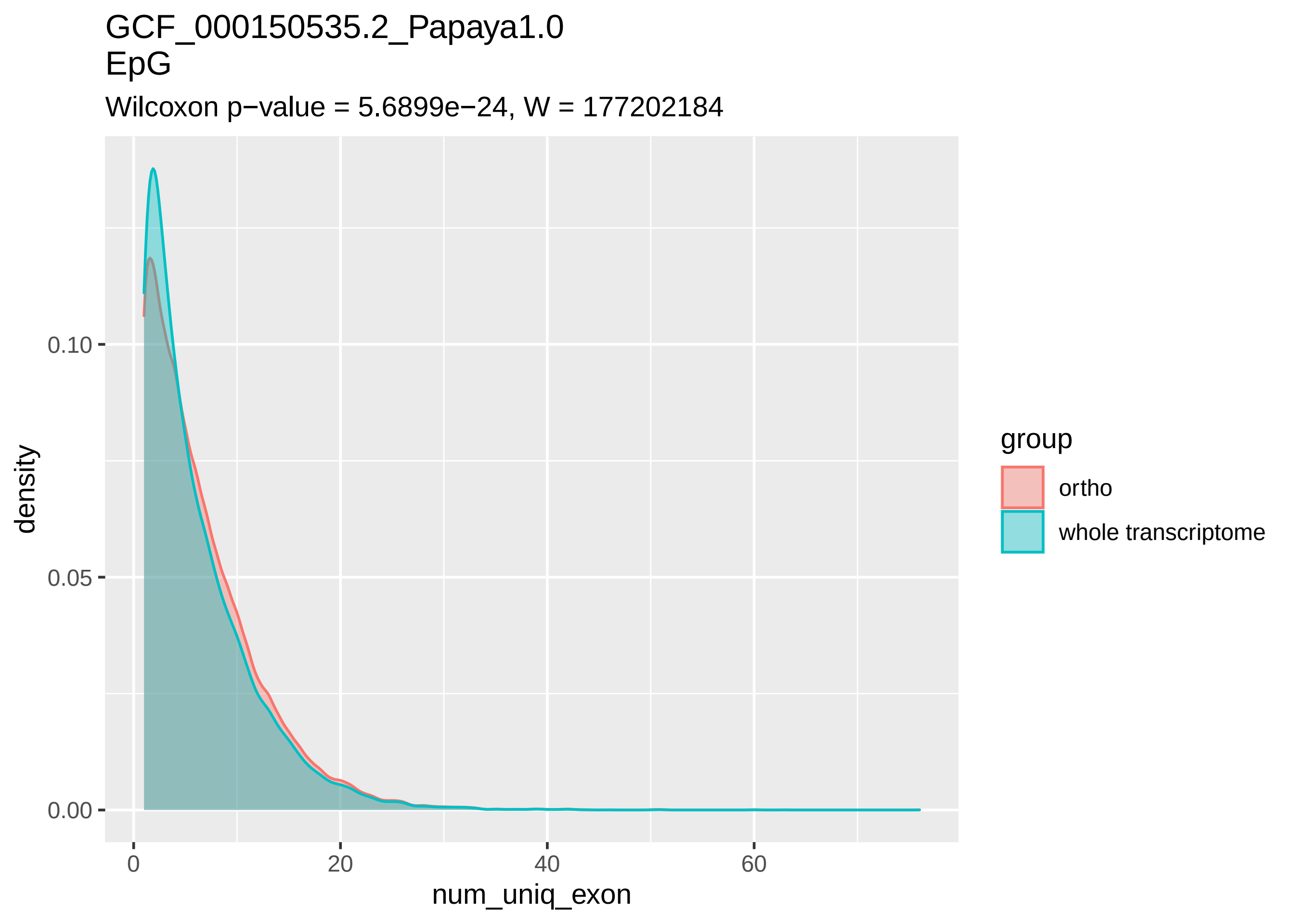
GCF_000150535.2_Papaya1.0
EpG
Wilcoxon p−value = 5.6899e−24, W = 177202184
0.10
0.05
0.00
density
0
20
40
60
num_uniq_exon
group
ortho
whole transcriptome

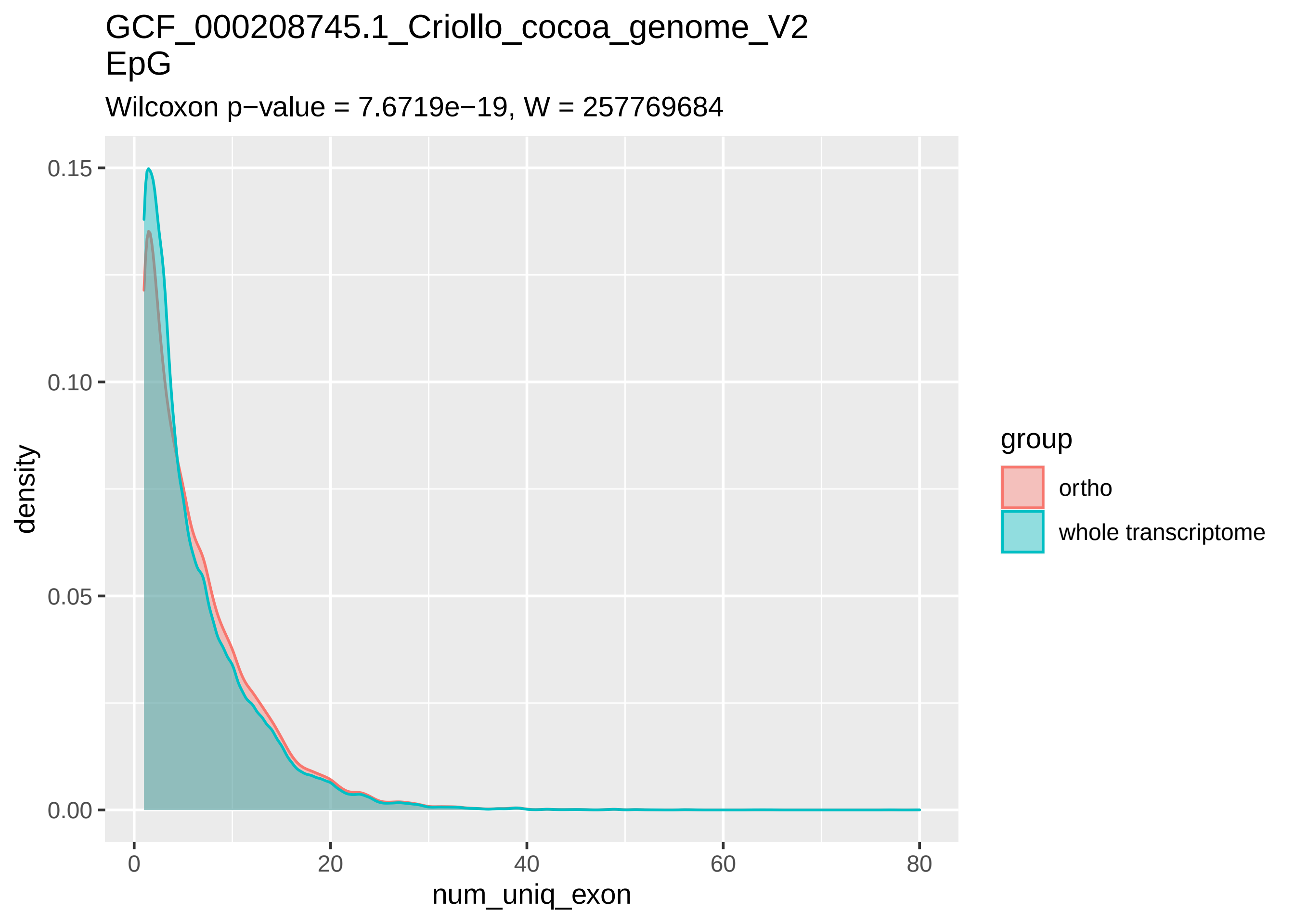
GCF_000208745.1_Criollo_cocoa_genome_V2
EpG
Wilcoxon p−value = 7.6719e−19, W = 257769684
density
0.15
0.10
0.05
0.00
0
20
40
60
80
num_uniq_exon
group
ortho
whole transcriptome

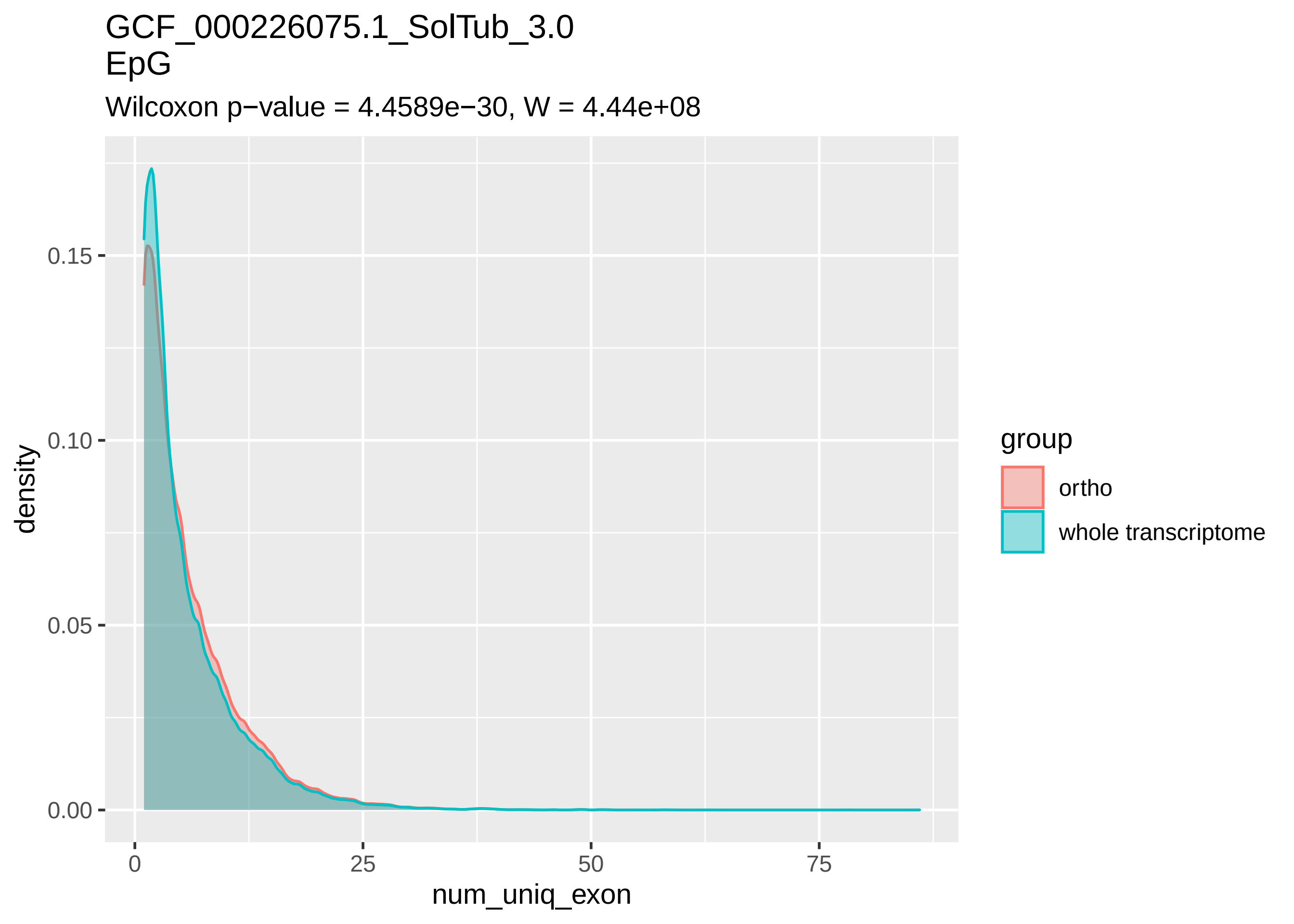

GCF_000226075.1_SolTub_3.0
EpG
Wilcoxon p−value = 4.4589e−30, W = 4.44e+08
density
0.00
0.05
0.10
0.15
0
25
50
75
num_uniq_exon
group
ortho
whole transcriptome

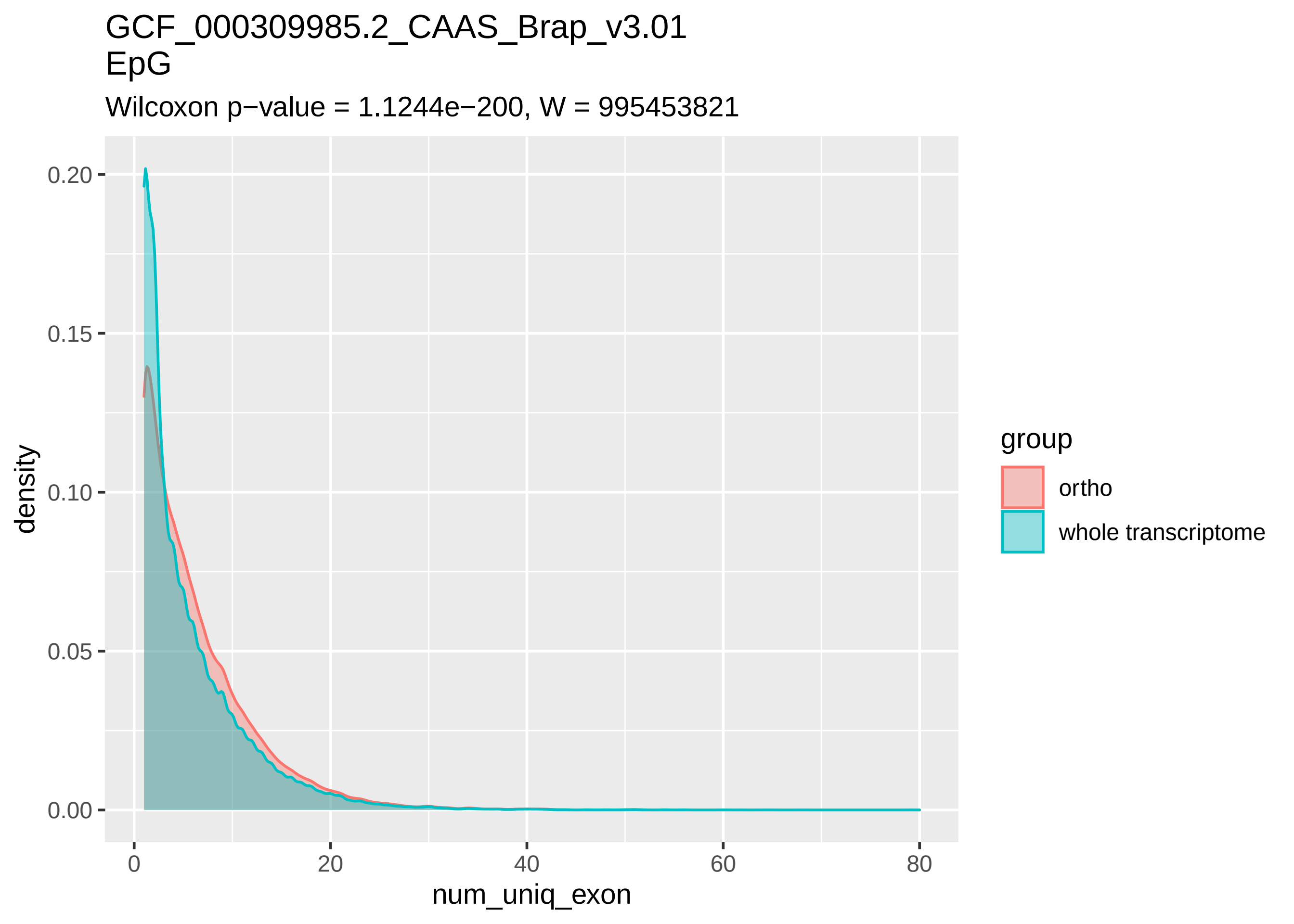
GCF_000309985.2_CAAS_Brap_v3.01
EpG
Wilcoxon p−value = 1.1244e−200, W = 995453821
density
0.20
0.15
0.10
0.05
0.00
0
20
40
60
80
num_uniq_exon
group
ortho
whole transcriptome

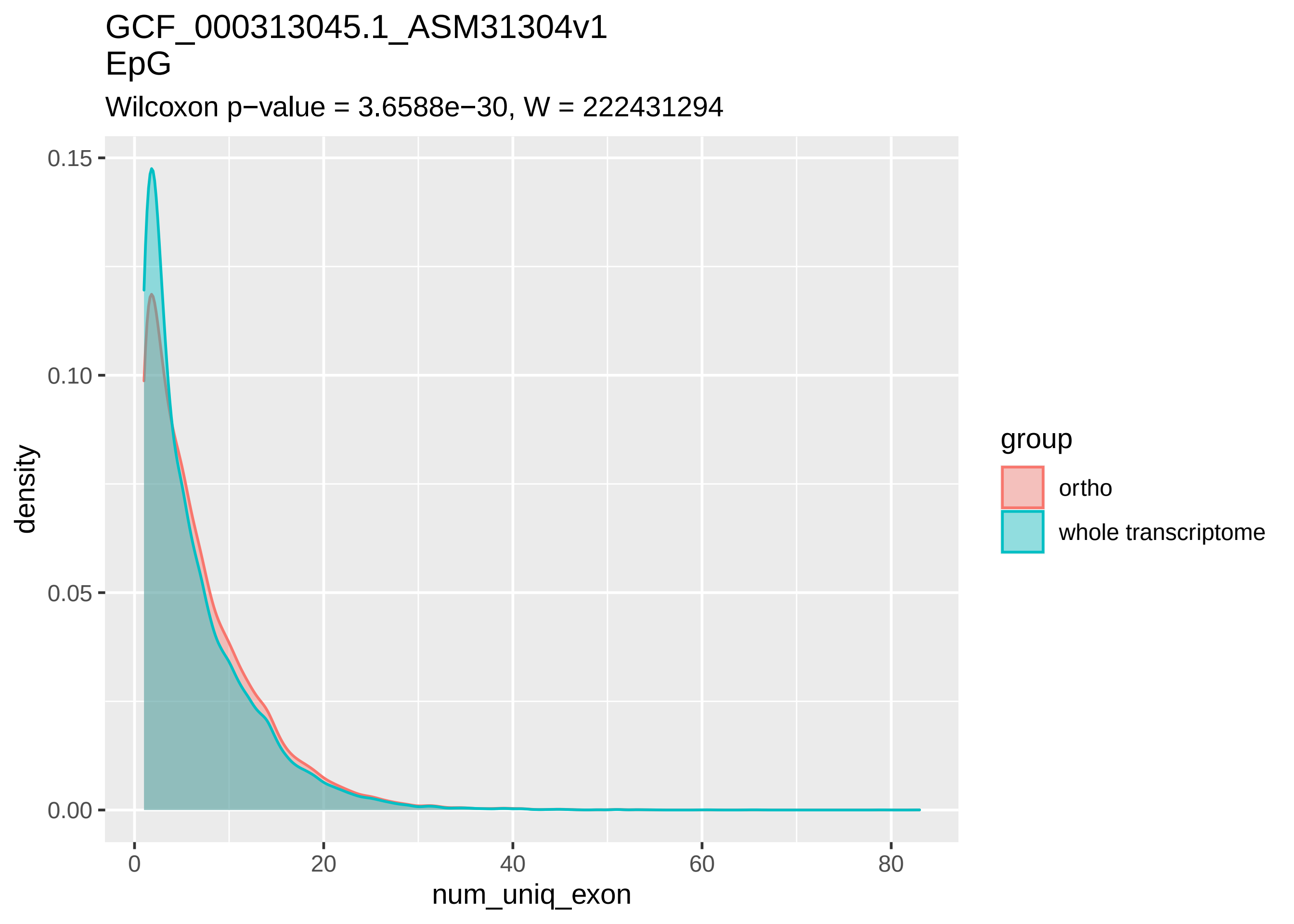

GCF_000313045.1_ASM31304v1
EpG
Wilcoxon p−value = 3.6588e−30, W = 222431294
density
0.15
0.10
0.05
0.00
0
20
40
60
80
num_uniq_exon
group
ortho
whole transcriptome

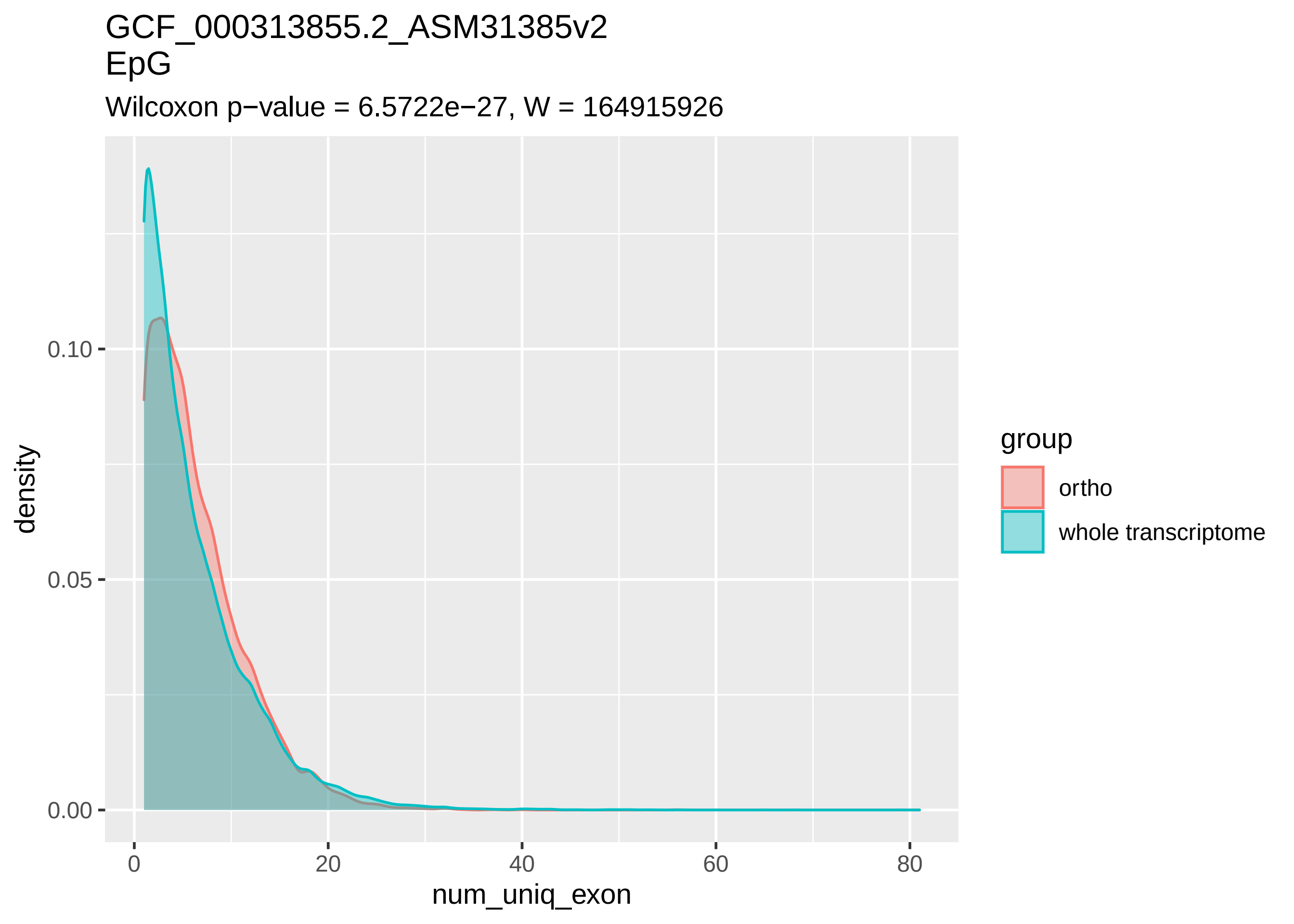

GCF_000313855.2_ASM31385v2
EpG
Wilcoxon p−value = 6.5722e−27, W = 164915926
density
0.00
0.05
0.10
0
20
40
60
80
num_uniq_exon
group
ortho
whole transcriptome

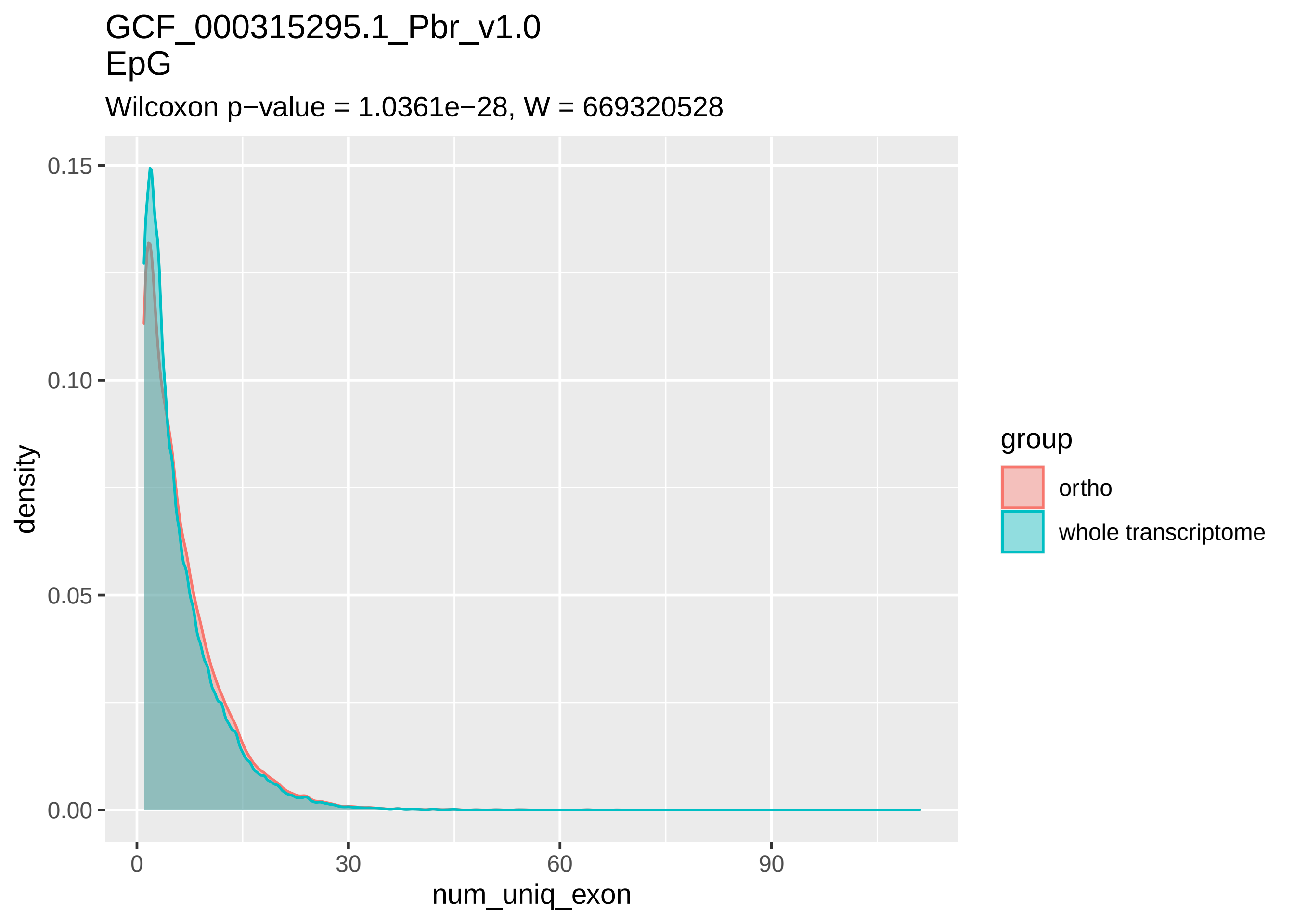
GCF_000315295.1_Pbr_v1.0
EpG
Wilcoxon p−value = 1.0361e−28, W = 669320528
density
0.15
0.10
0.05
0.00
0
30
60
90
num_uniq_exon
group
ortho
whole transcriptome

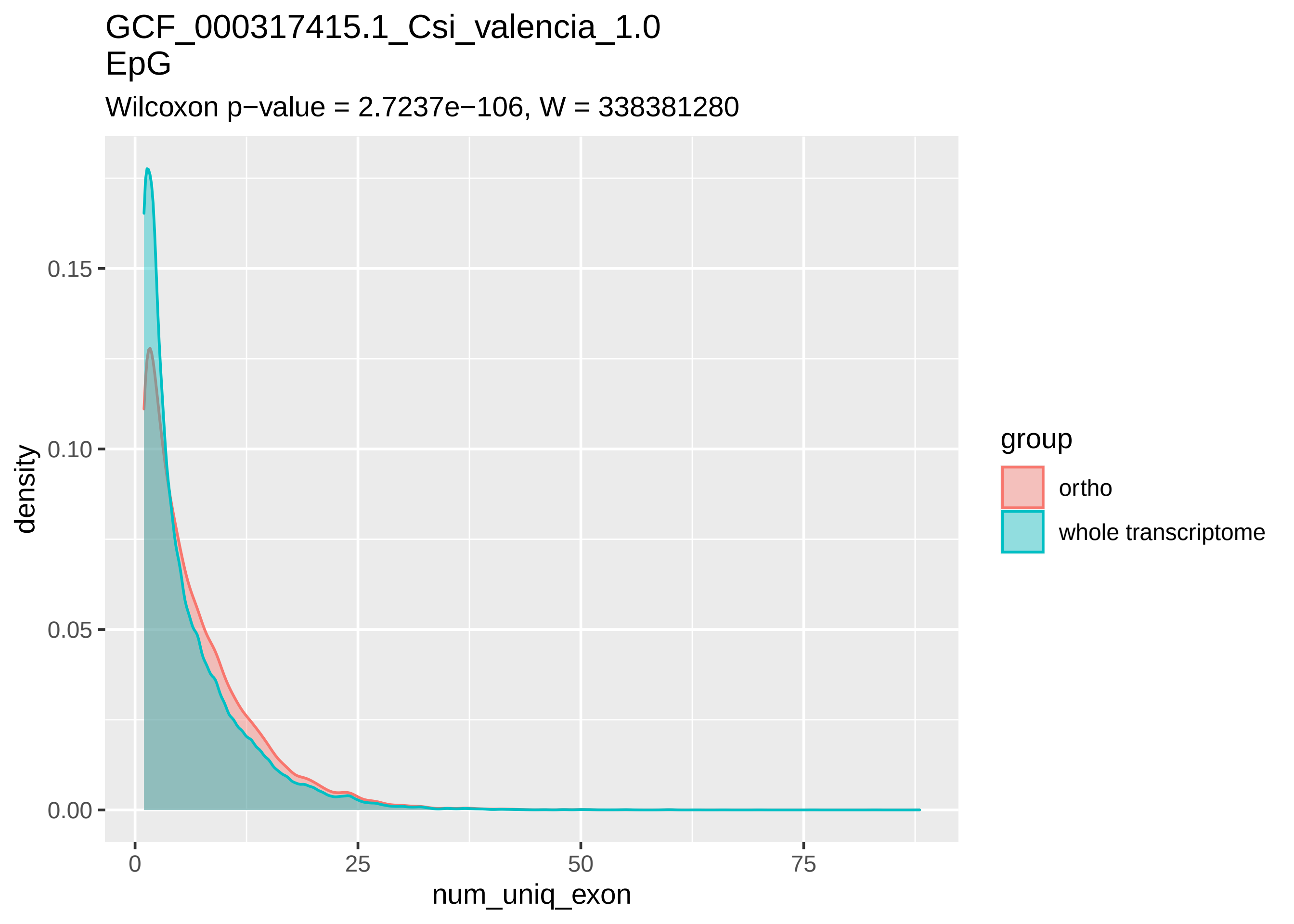

GCF_000317415.1_Csi_valencia_1.0
EpG
Wilcoxon p−value = 2.7237e−106, W = 338381280
density
0.00
0.05
0.10
0.15
0
25
50
75
num_uniq_exon
group
ortho
whole transcriptome

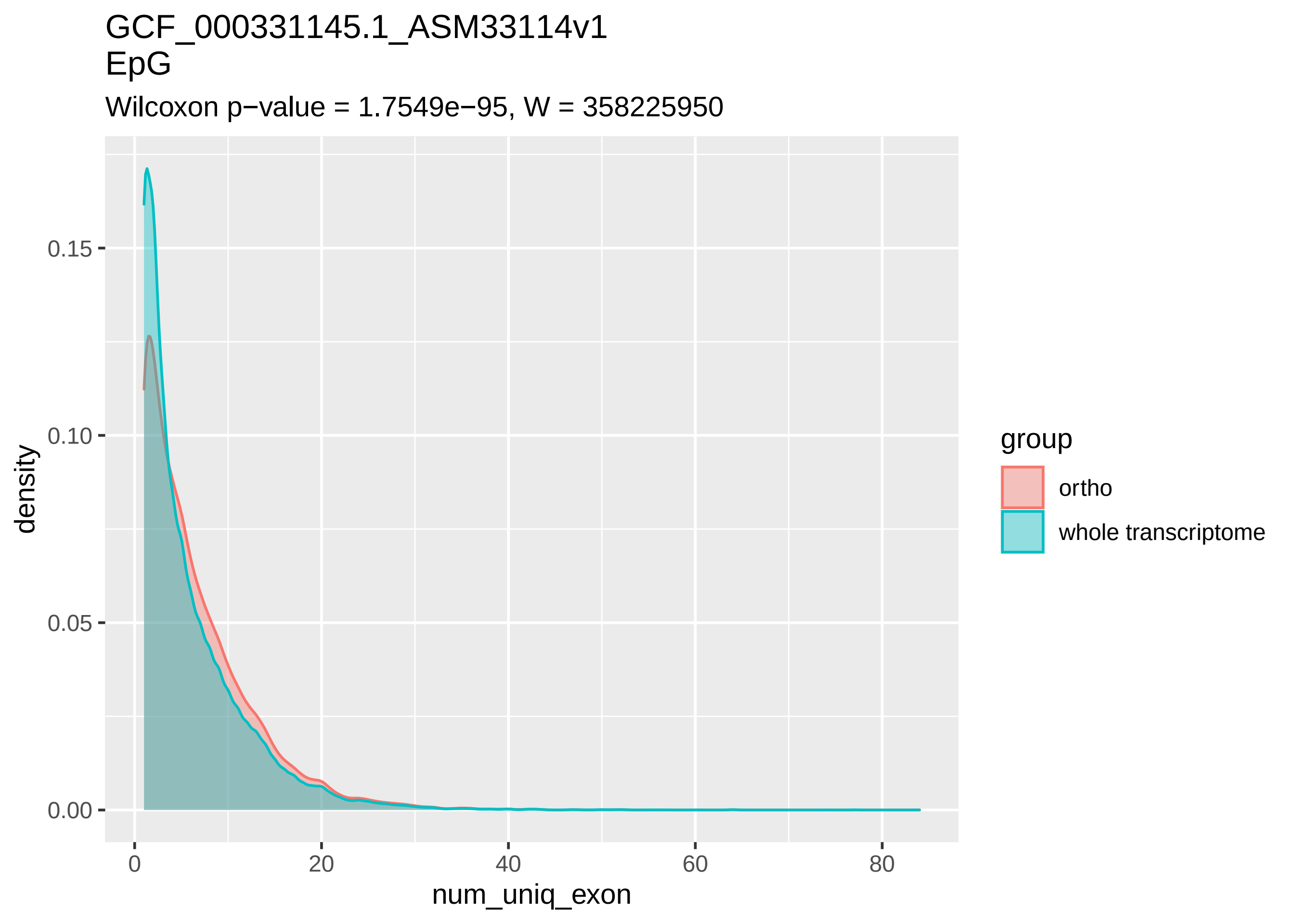

GCF_000331145.1_ASM33114v1
EpG
Wilcoxon p−value = 1.7549e−95, W = 358225950
density
0.15
0.10
0.05
0.00
0
20
40
60
80
num_uniq_exon
group
ortho
whole transcriptome

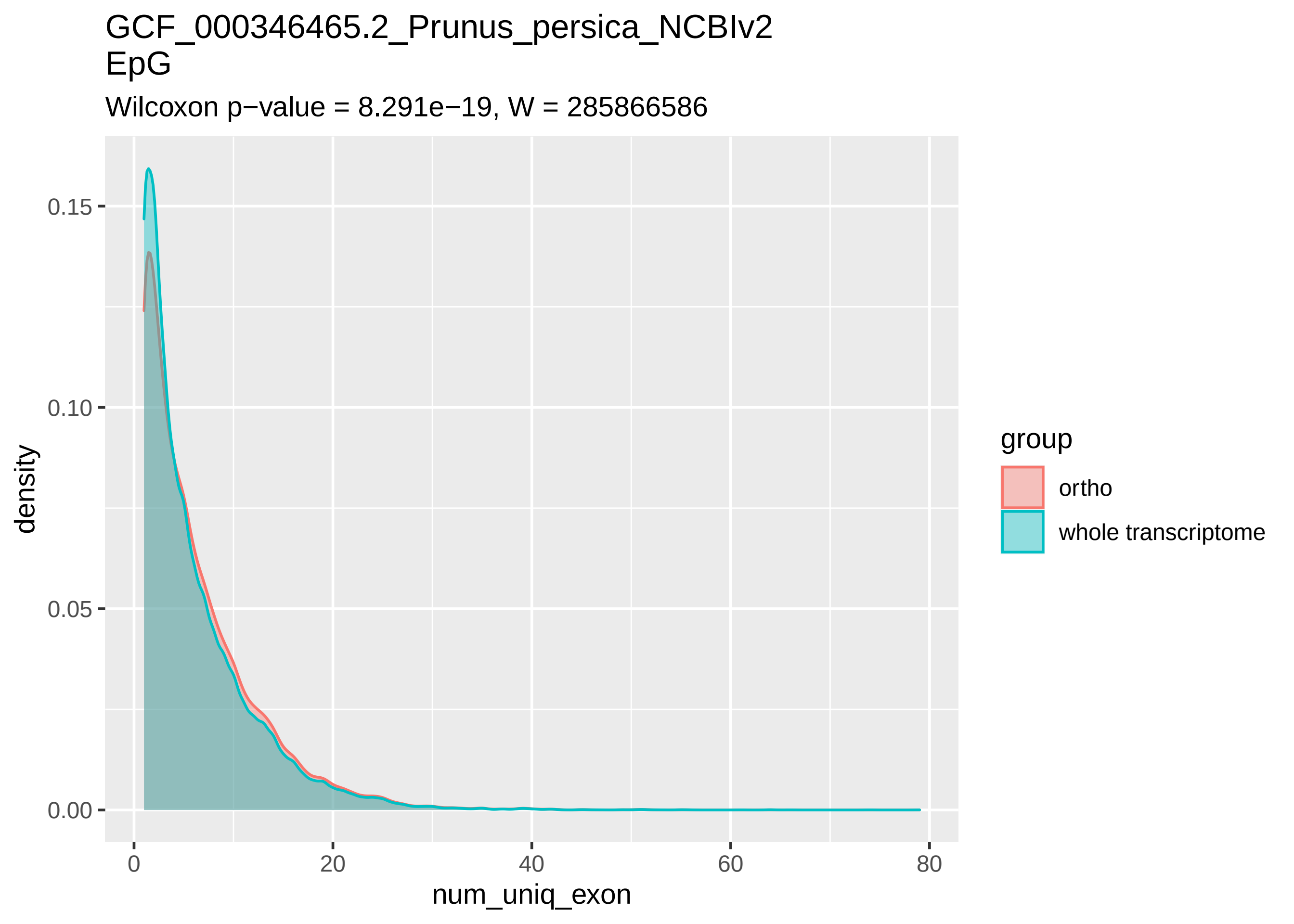
GCF_000346465.2_Prunus_persica_NCBIv2
EpG
Wilcoxon p−value = 8.291e−19, W = 285866586
density
0.15
0.10
0.05
0.00
0
20
40
60
80
num_uniq_exon
group
ortho
whole transcriptome

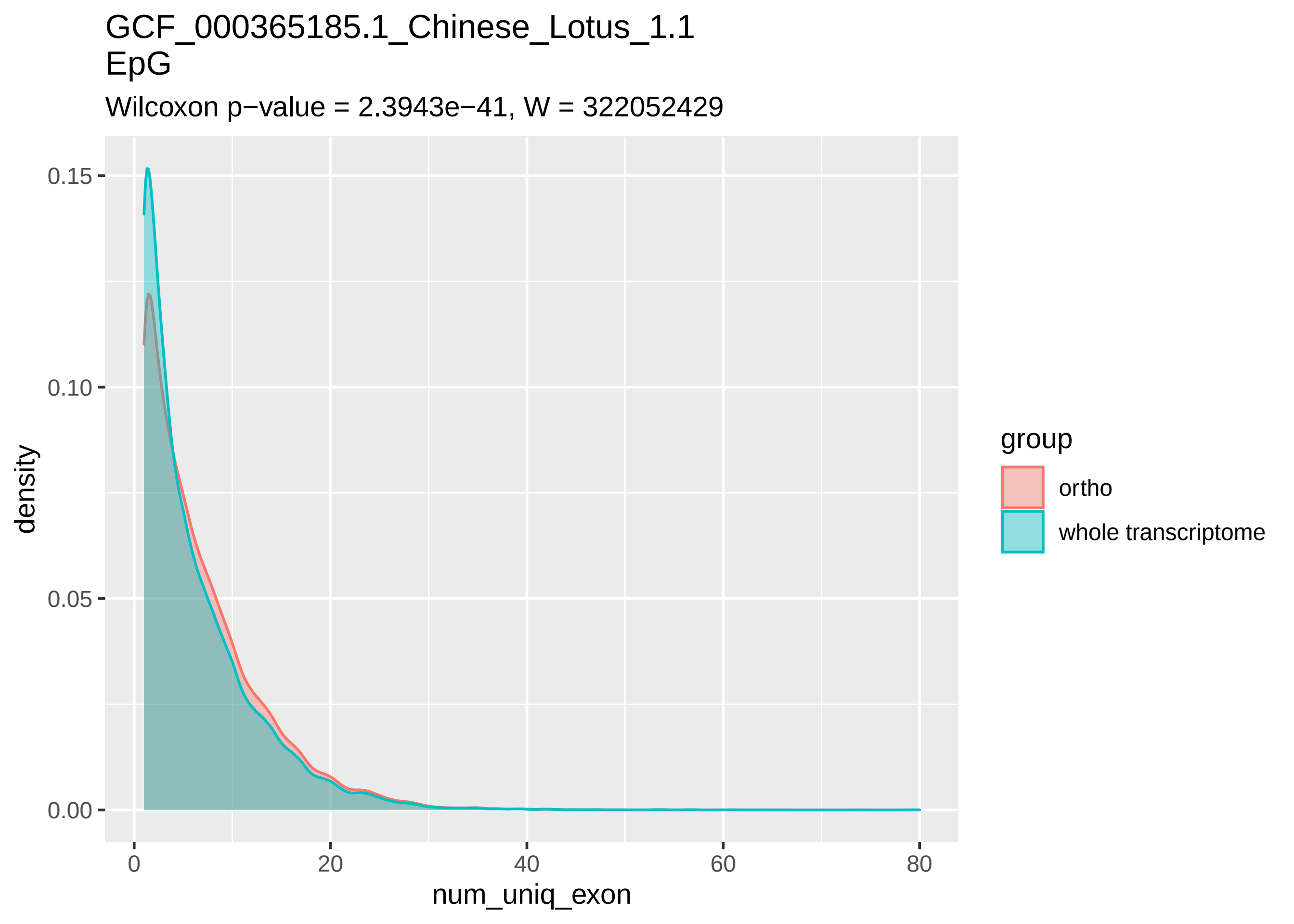
GCF_000365185.1_Chinese_Lotus_1.1
EpG
Wilcoxon p−value = 2.3943e−41, W = 322052429
0.15
0.10
0.05
0.00
density
0
20
40
60
80
num_uniq_exon
group
ortho
whole transcriptome

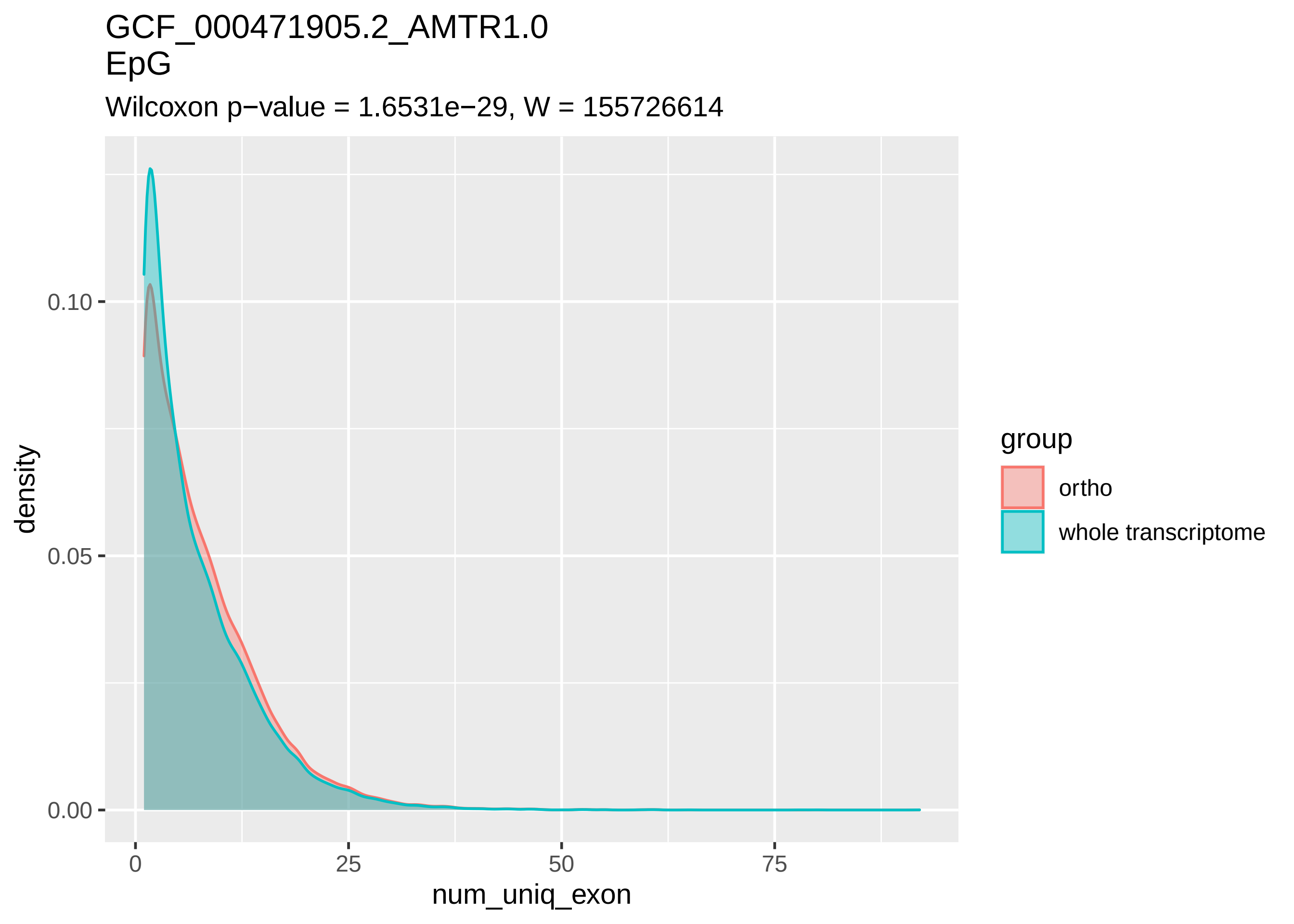

GCF_000471905.2_AMTR1.0
EpG
Wilcoxon p−value = 1.6531e−29, W = 155726614
density
0.10
0.05
0.00
0
25
50
75
num_uniq_exon
group
ortho
whole transcriptome

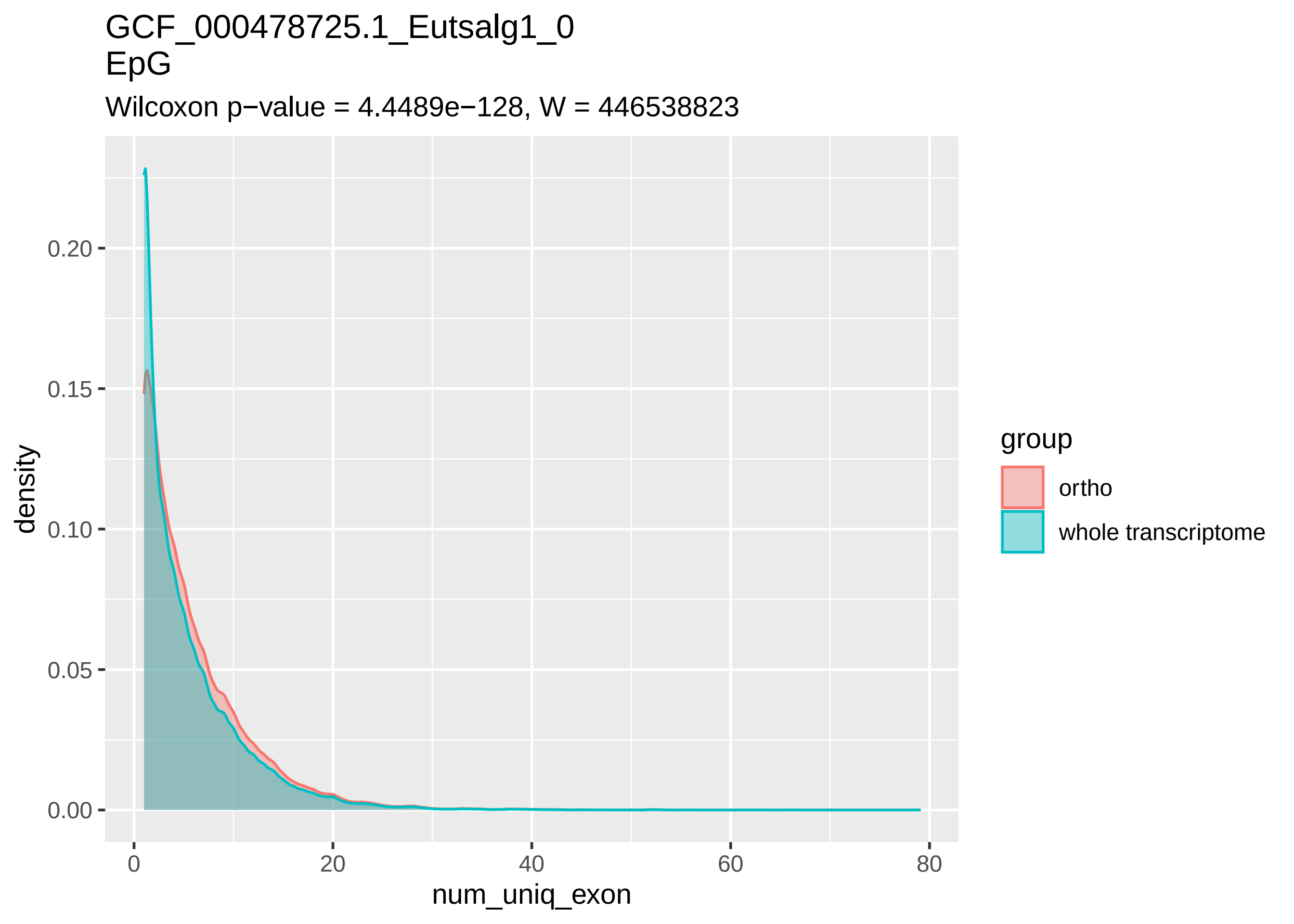

GCF_000478725.1_Eutsalg1_0
EpG
Wilcoxon p−value = 4.4489e−128, W = 446538823
density
0.20
0.15
0.10
0.05
0.00
0
20
40
60
80
num_uniq_exon
group
ortho
whole transcriptome

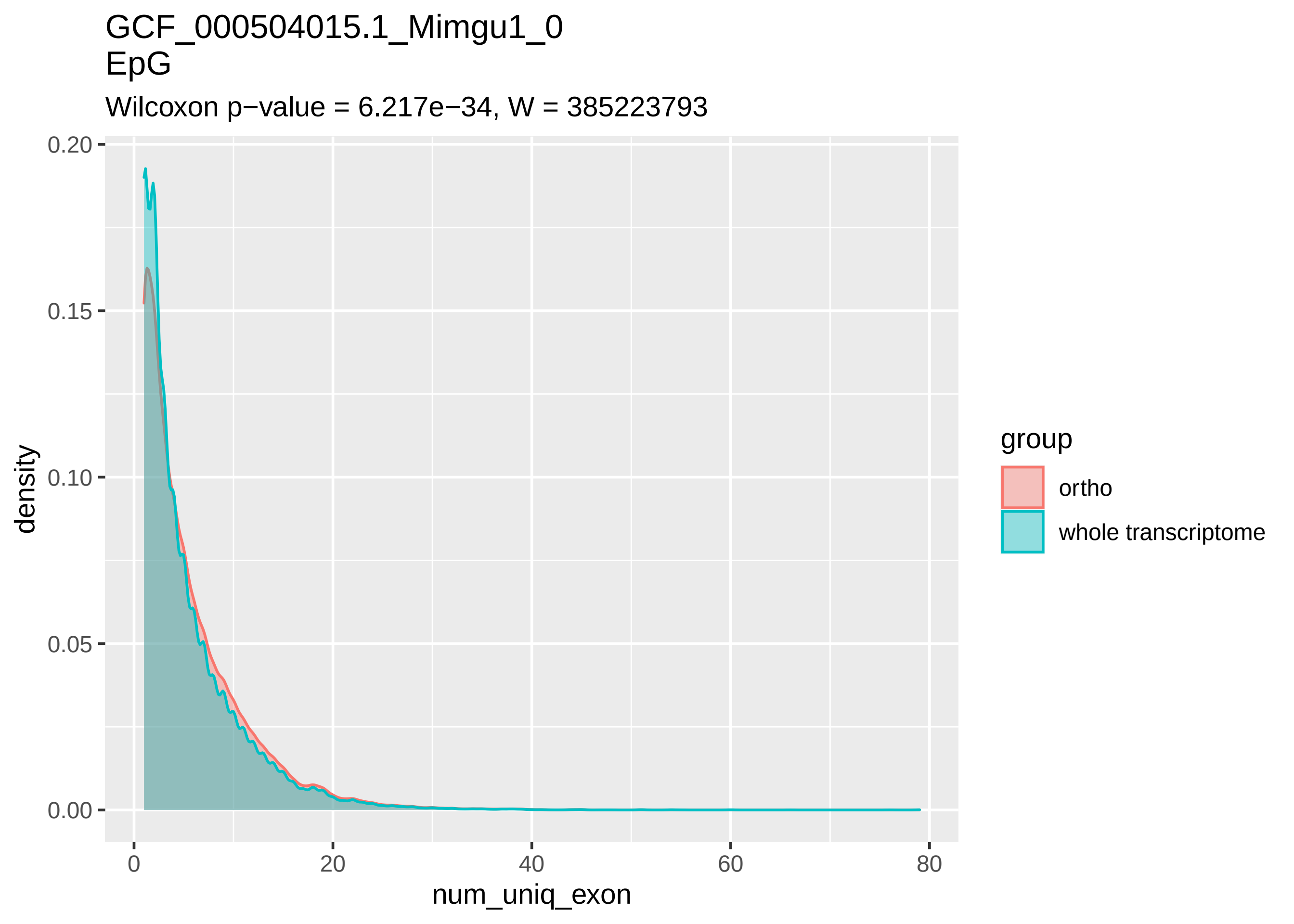

GCF_000504015.1_Mimgu1_0
EpG
Wilcoxon p−value = 6.217e−34, W = 385223793
density
0.20
0.15
0.10
0.05
0.00
0
20
40
60
80
num_uniq_exon
group
ortho
whole transcriptome

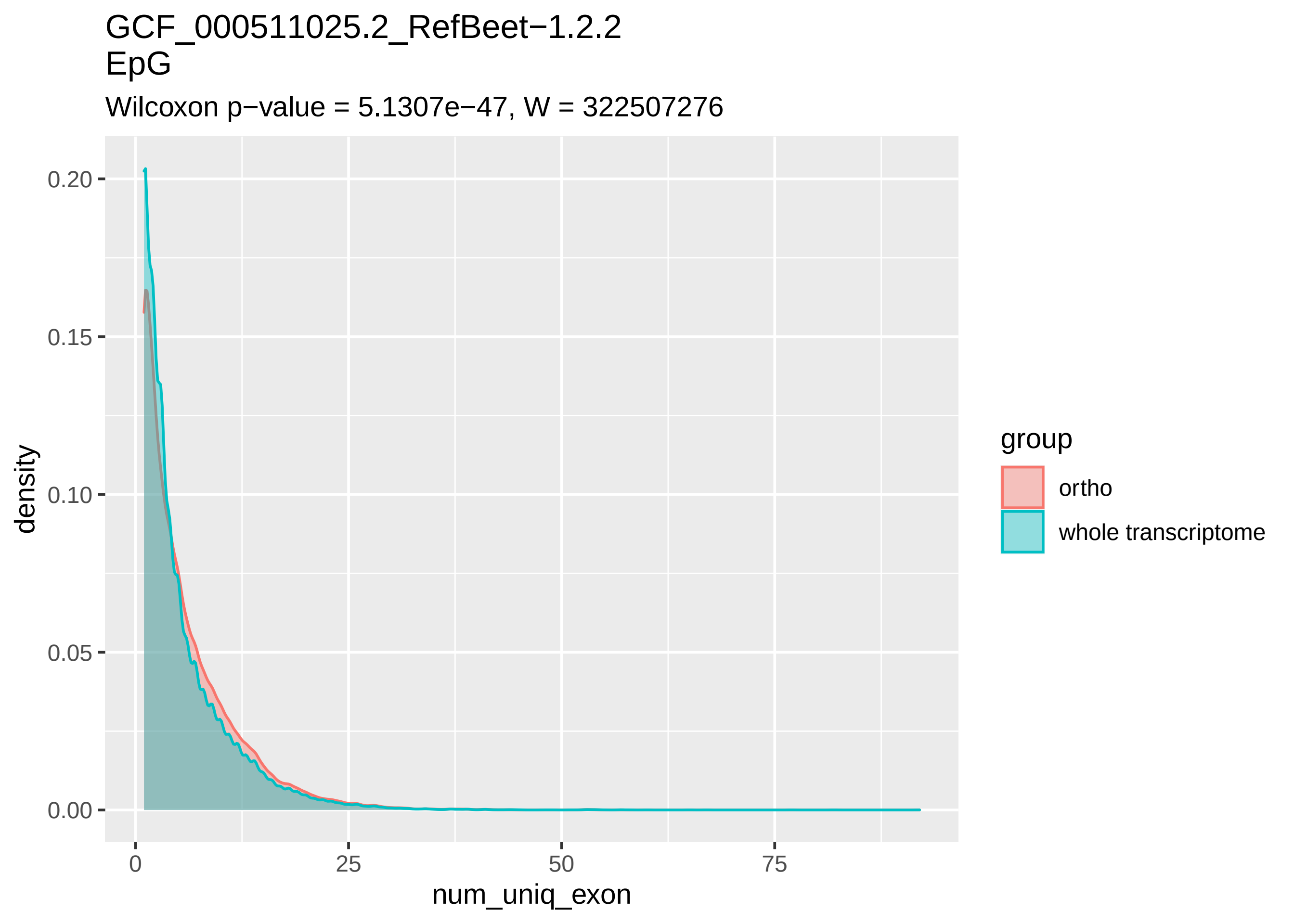
GCF_000511025.2_RefBeet−1.2.2
EpG
Wilcoxon p−value = 5.1307e−47, W = 322507276
density
0.20
0.15
0.10
0.05
0.00
0
25
50
75
num_uniq_exon
group
ortho
whole transcriptome

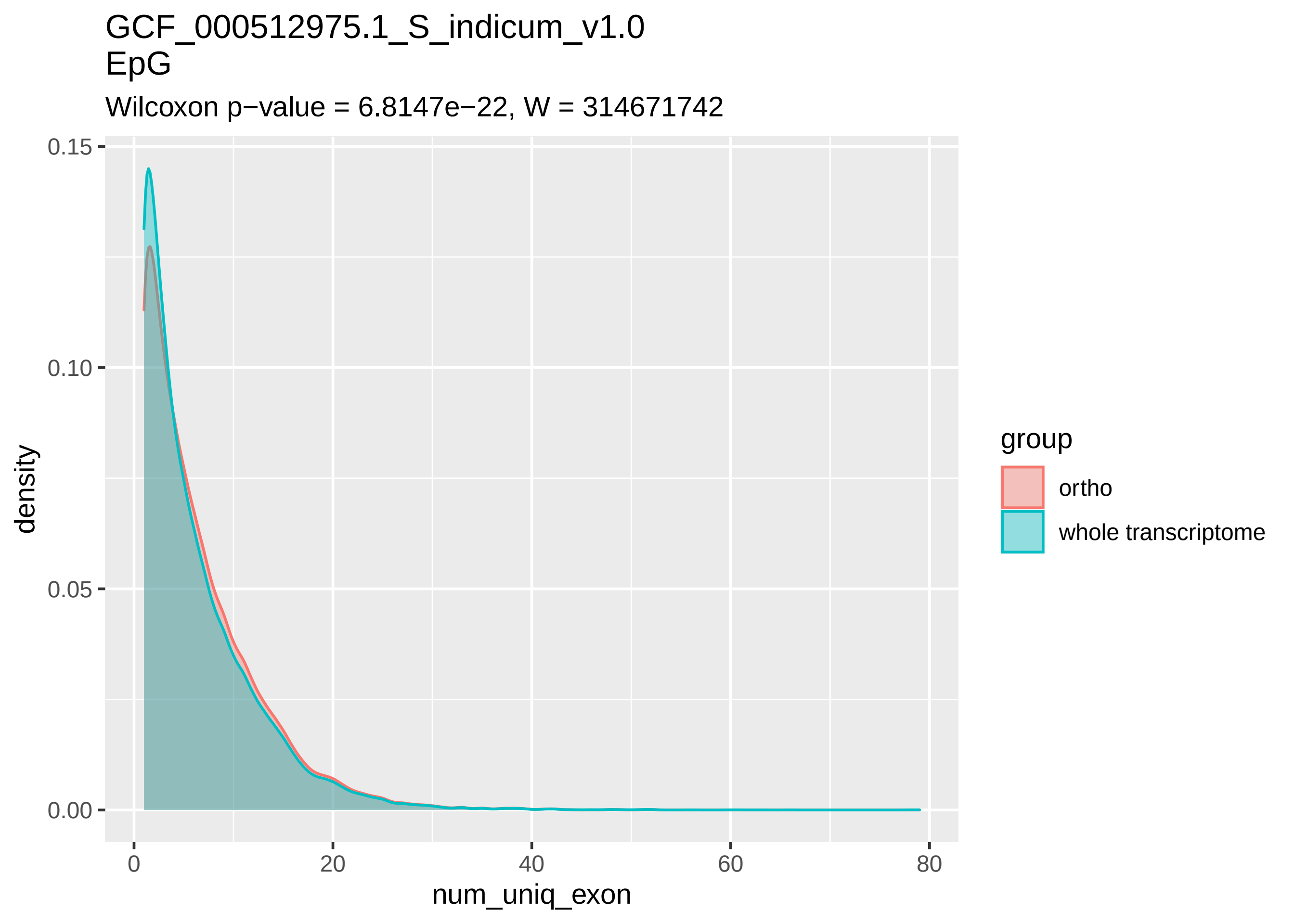
GCF_000512975.1_S_indicum_v1.0
EpG
Wilcoxon p−value = 6.8147e−22, W = 314671742
density
0.15
0.10
0.05
0.00
0
20
40
60
80
num_uniq_exon
group
ortho
whole transcriptome

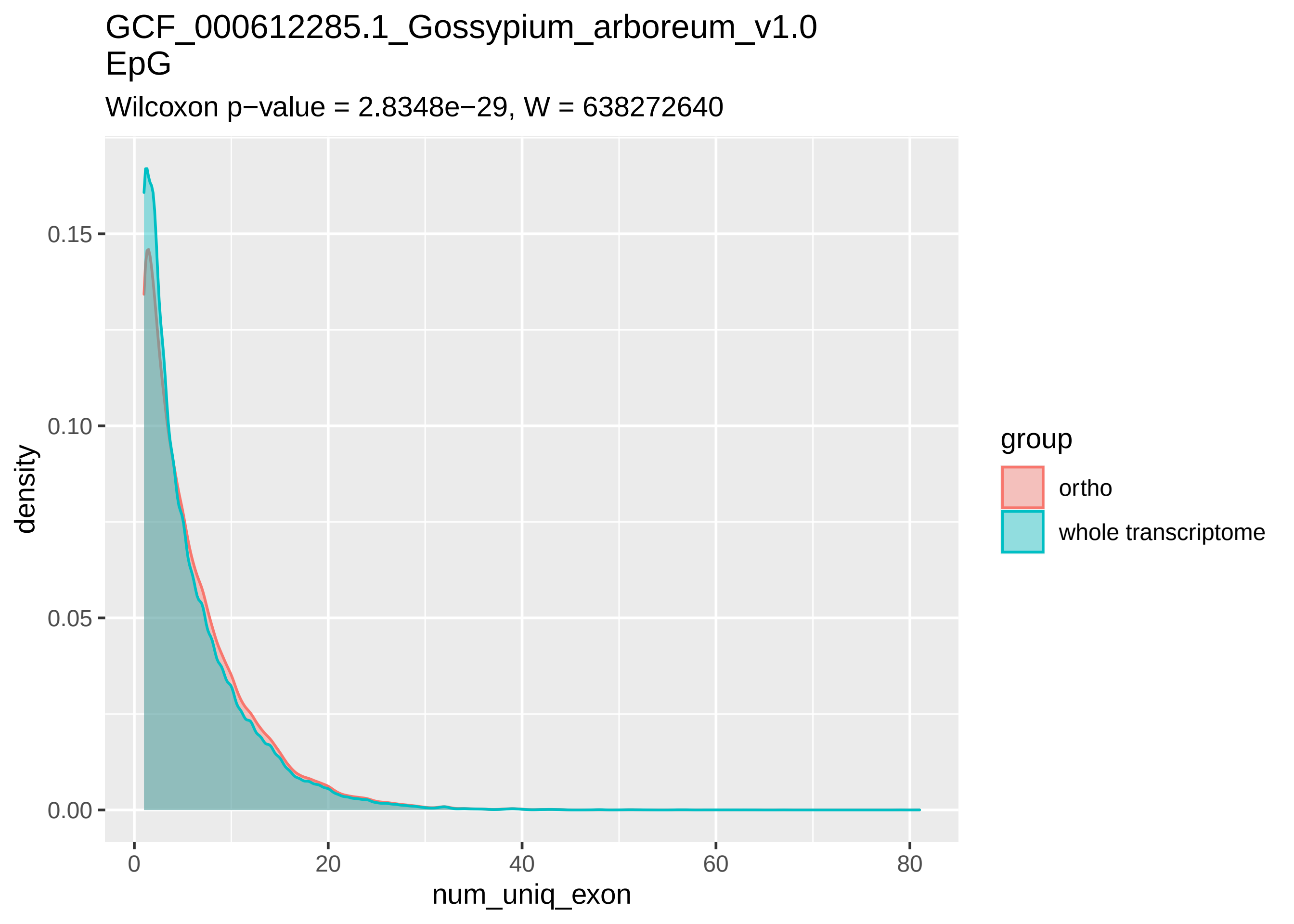
GCF_000612285.1_Gossypium_arboreum_v1.0
EpG
Wilcoxon p−value = 2.8348e−29, W = 638272640
density
0.15
0.10
0.05
0.00
0
20
40
60
80
num_uniq_exon
group
ortho
whole transcriptome

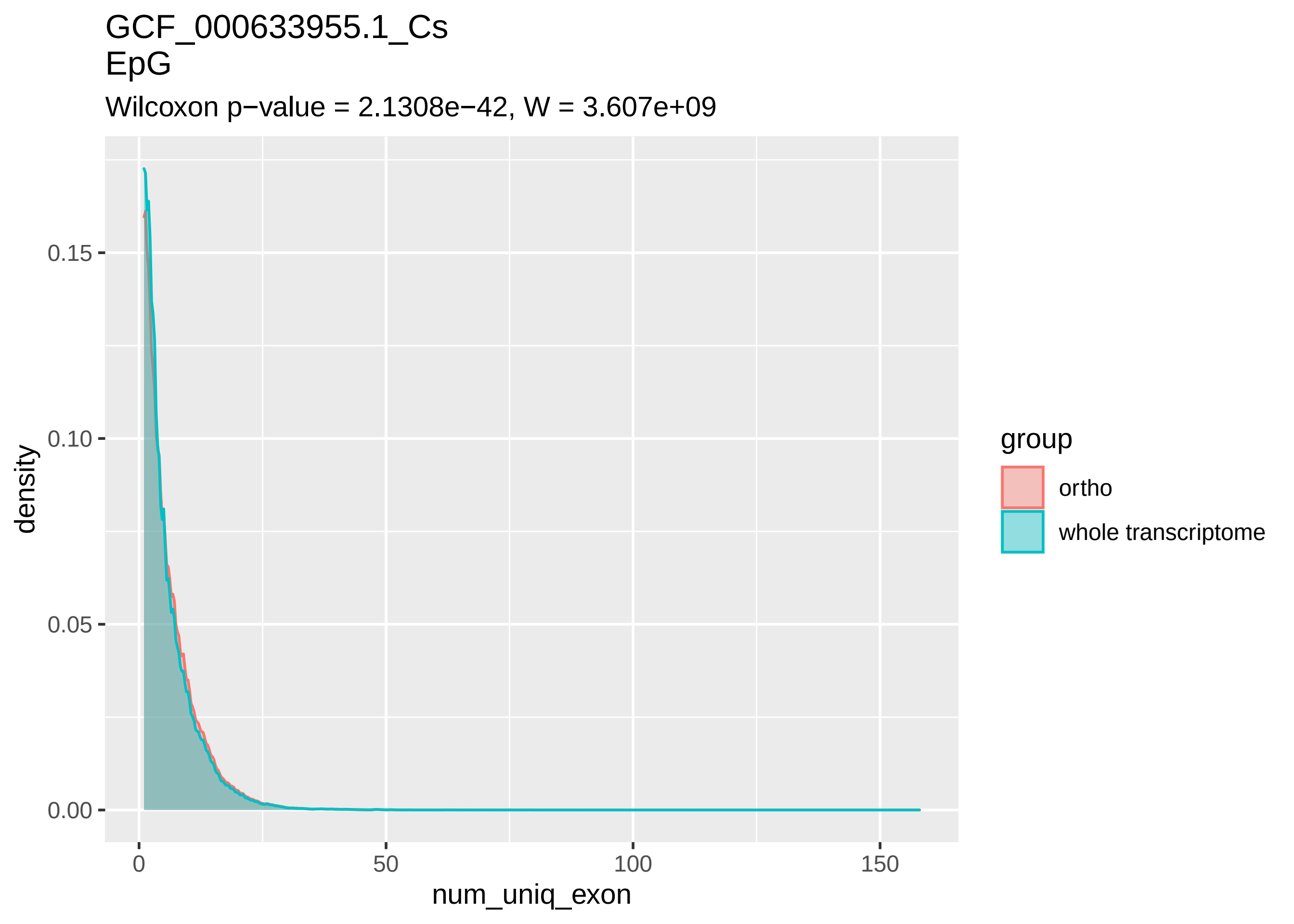

GCF_000633955.1_Cs
EpG
Wilcoxon p−value = 2.1308e−42, W = 3.607e+09
density
0.15
0.10
0.05
0.00
0
50
100
150
num_uniq_exon
group
ortho
whole transcriptome

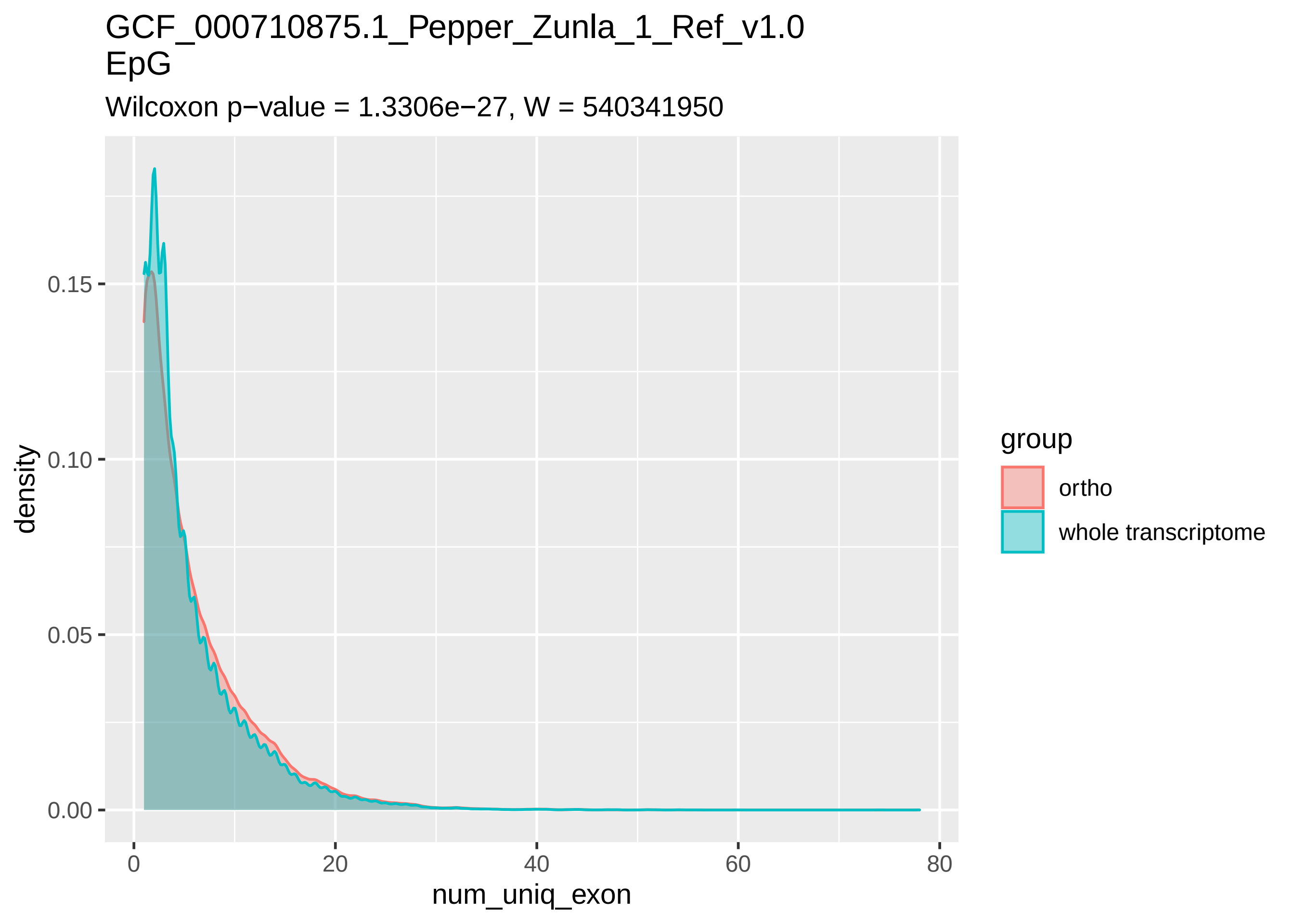

GCF_000710875.1_Pepper_Zunla_1_Ref_v1.0
EpG
Wilcoxon p−value = 1.3306e−27, W = 540341950
density
0.00
0.05
0.10
0.15
0
20
40
60
80
num_uniq_exon
group
ortho
whole transcriptome

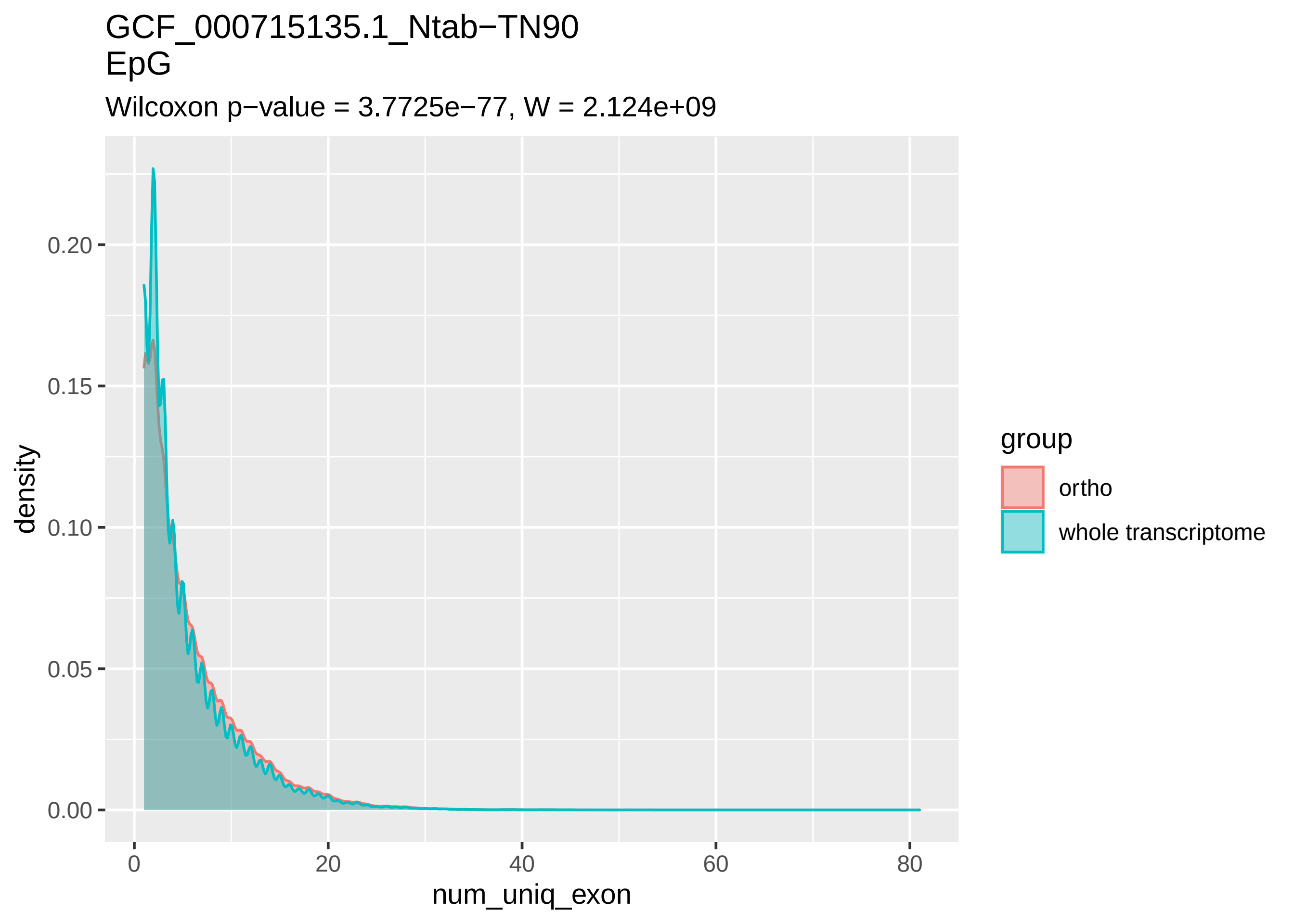
GCF_000715135.1_Ntab−TN90
EpG
Wilcoxon p−value = 3.7725e−77, W = 2.124e+09
density
0.20
0.15
0.10
0.05
0.00
0
20
40
60
80
num_uniq_exon
group
ortho
whole transcriptome

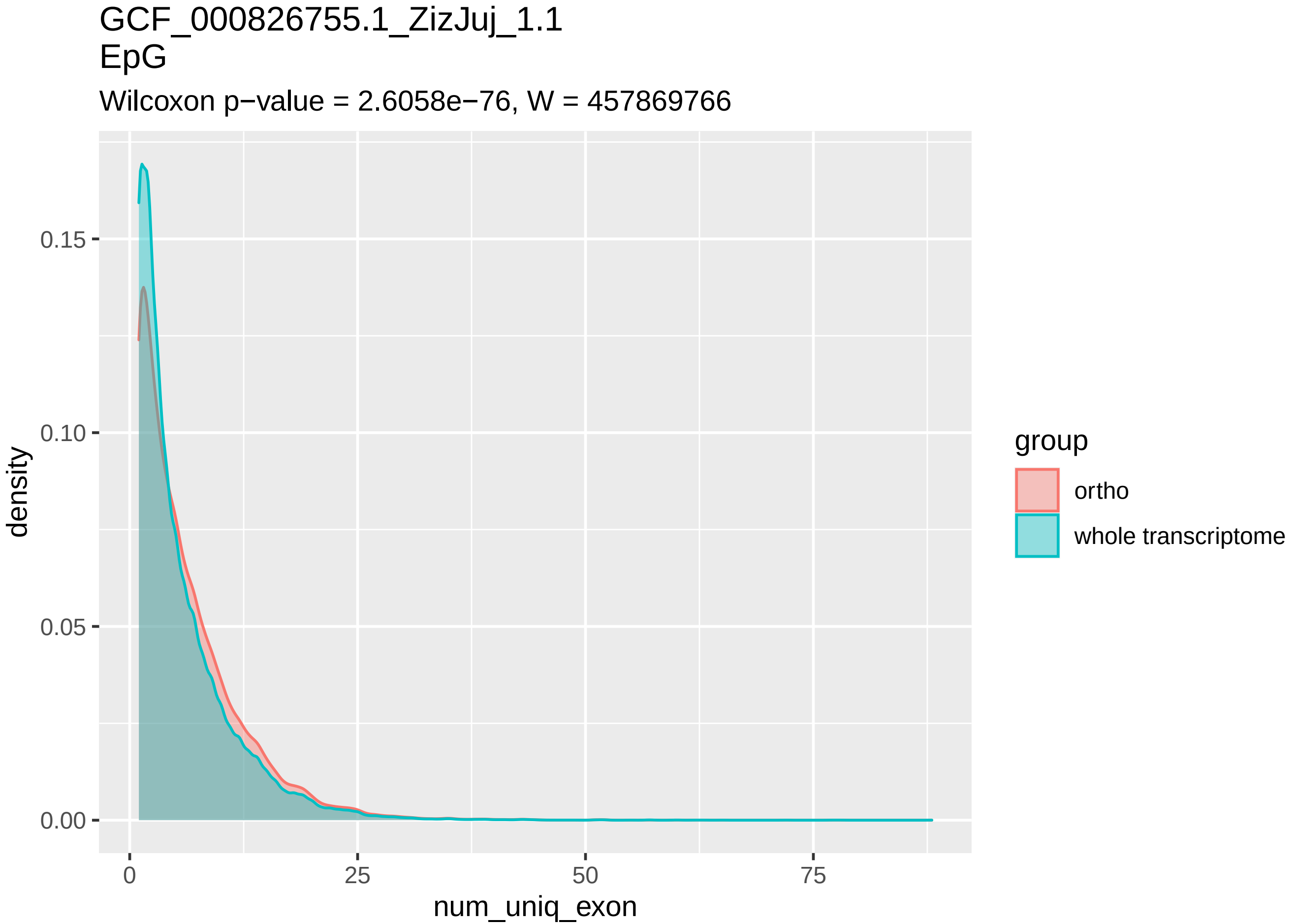
GCF_000826755.1_ZizJuj_1.1
EpG
Wilcoxon p−value = 2.6058e−76, W = 457869766
density
0.15
0.10
0.05
0.00
0
25
50
75
num_uniq_exon
group
ortho
whole transcriptome

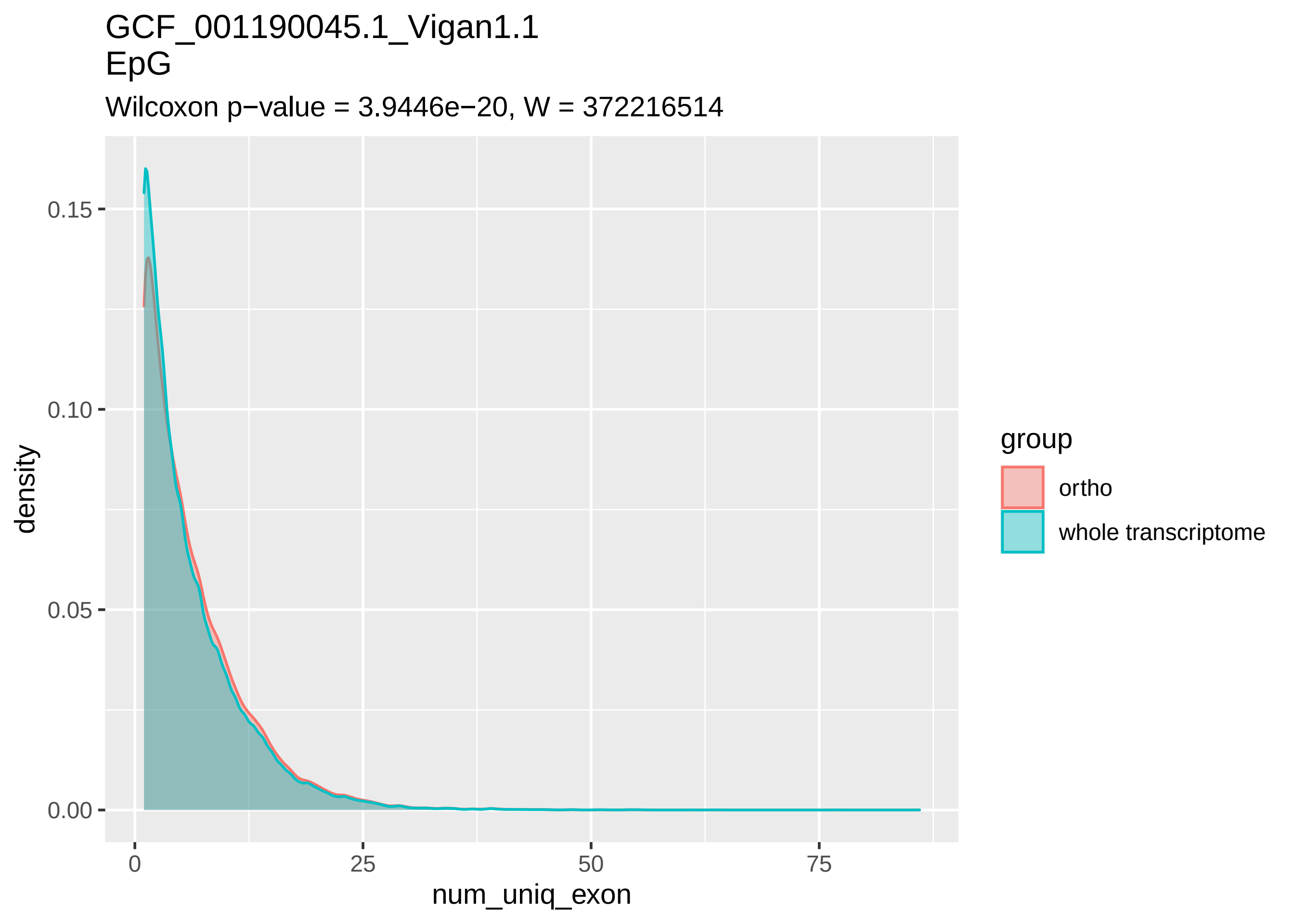
GCF_001190045.1_Vigan1.1
EpG
Wilcoxon p−value = 3.9446e−20, W = 372216514
density
0.15
0.10
0.05
0.00
0
25
50
75
num_uniq_exon
group
ortho
whole transcriptome

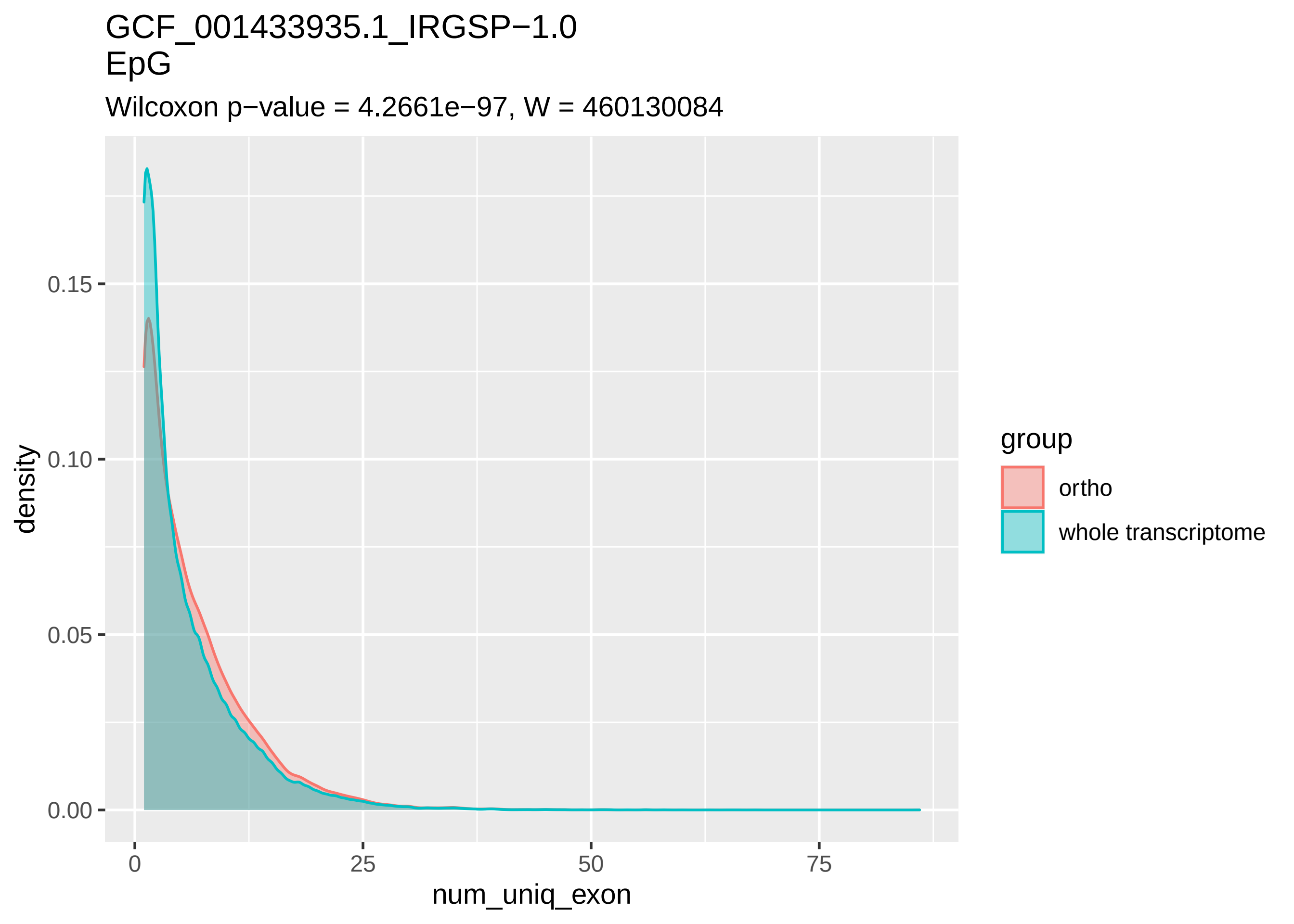

GCF_001433935.1_IRGSP−1.0
EpG
Wilcoxon p−value = 4.2661e−97, W = 460130084
density
0.15
0.10
0.05
0.00
0
25
50
75
num_uniq_exon
group
ortho
whole transcriptome

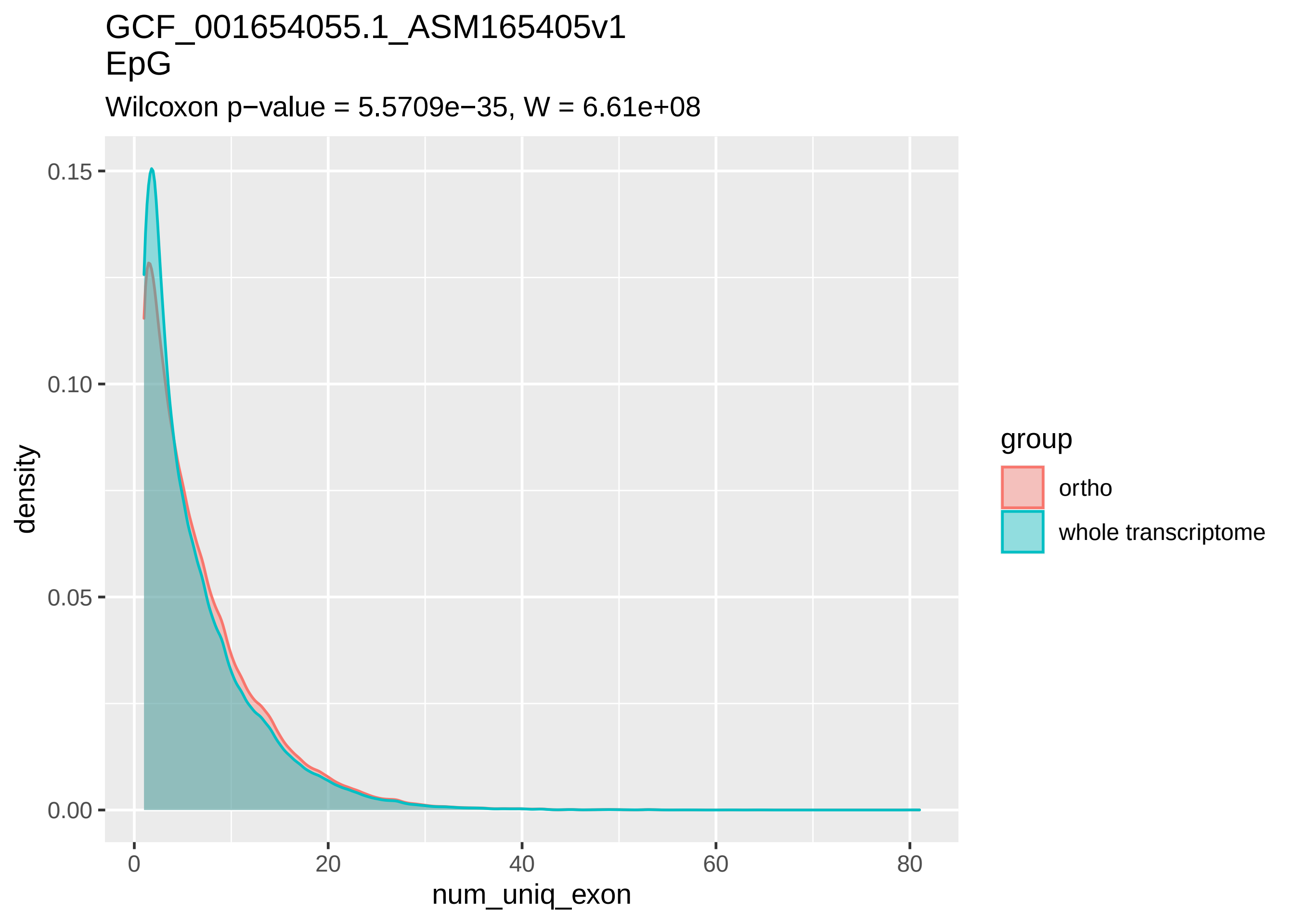
GCF_001654055.1_ASM165405v1
EpG
Wilcoxon p−value = 5.5709e−35, W = 6.61e+08
density
0.15
0.10
0.05
0.00
0
20
40
60
80
num_uniq_exon
group
ortho
whole transcriptome

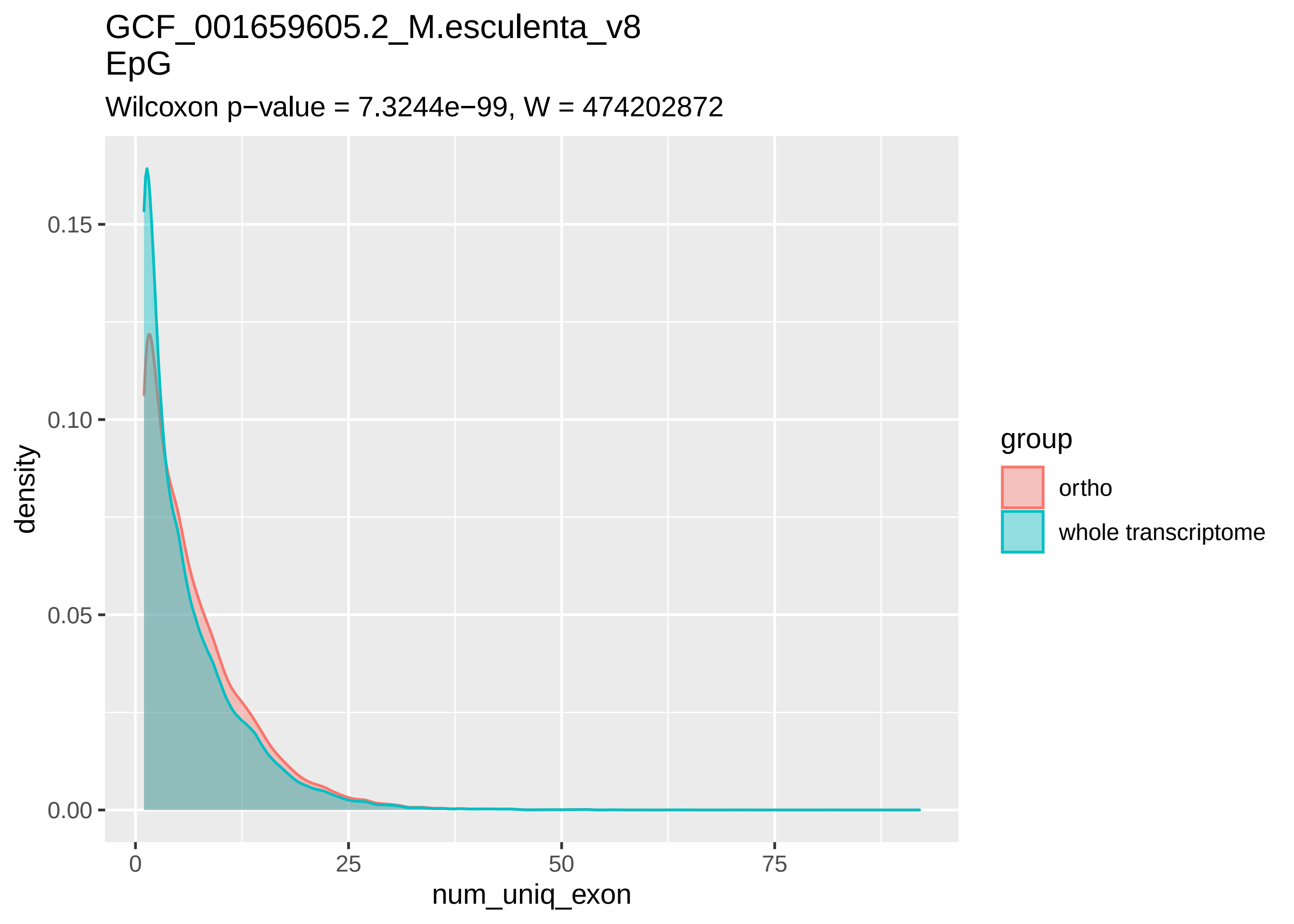

GCF_001659605.2_M.esculenta_v8
EpG
Wilcoxon p−value = 7.3244e−99, W = 474202872
density
0.15
0.10
0.05
0.00
0
25
50
75
num_uniq_exon
group
ortho
whole transcriptome

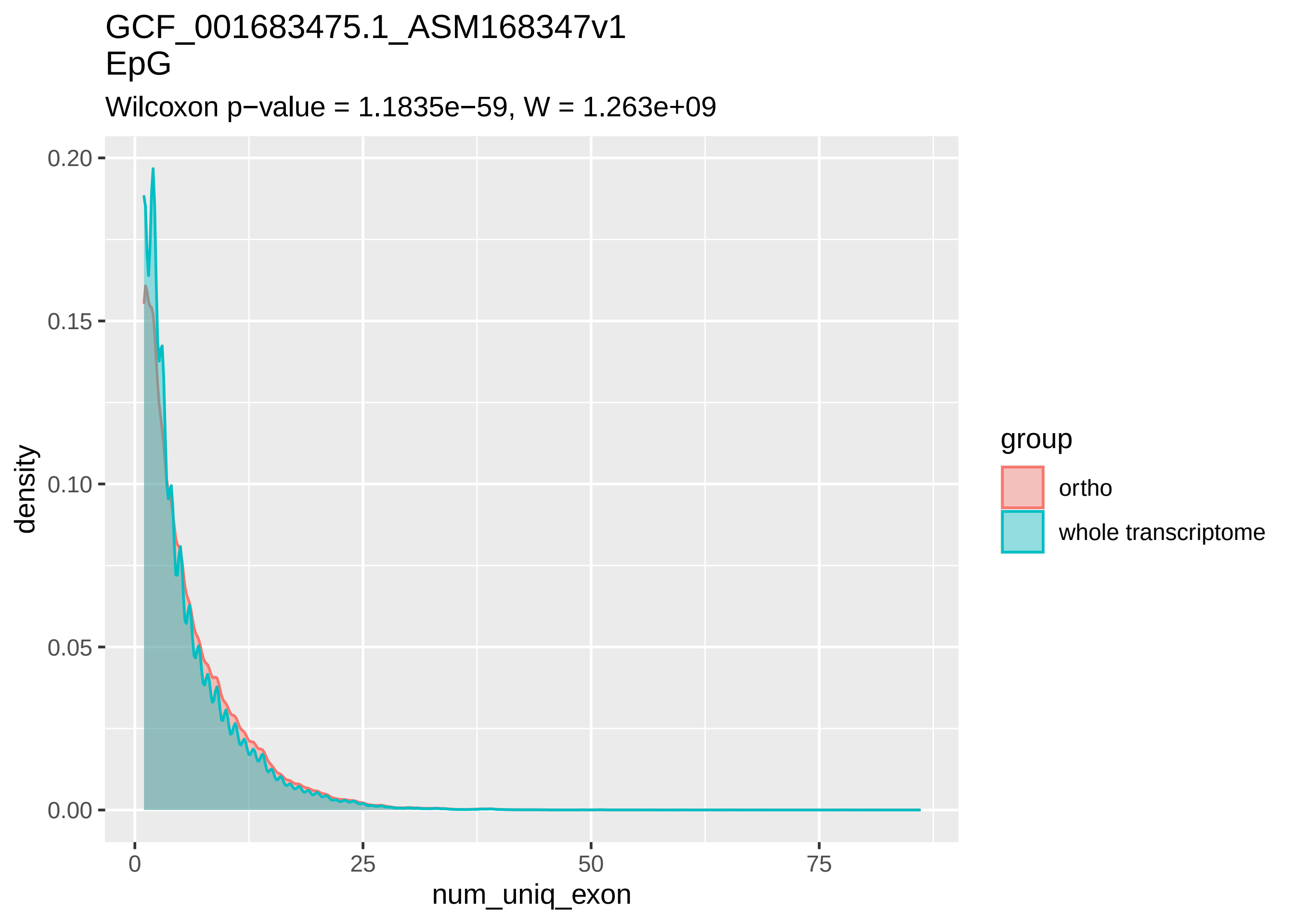
GCF_001683475.1_ASM168347v1
EpG
Wilcoxon p−value = 1.1835e−59, W = 1.263e+09
density
0.20
0.15
0.10
0.05
0.00
0
25
50
75
num_uniq_exon
group
ortho
whole transcriptome

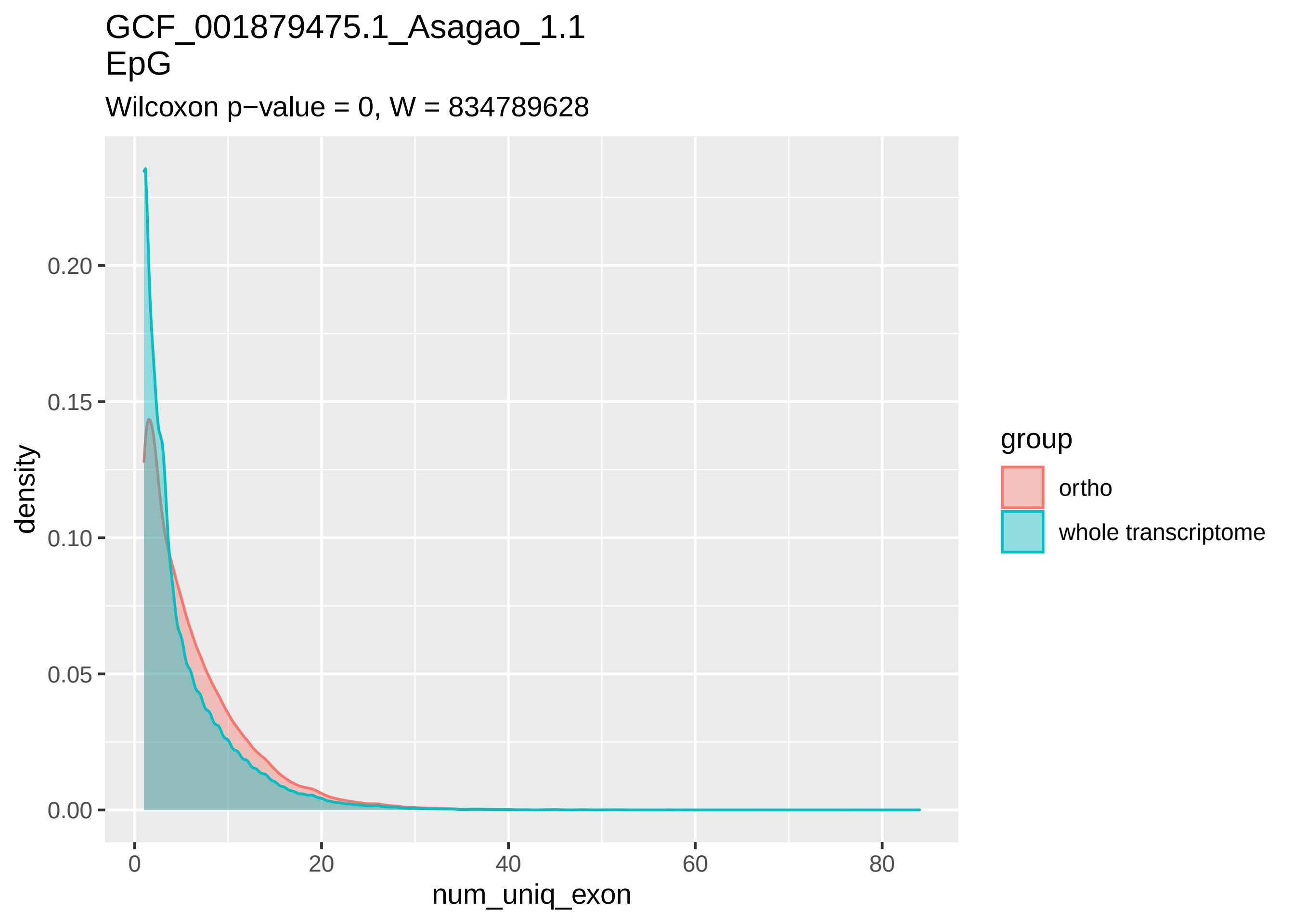
GCF_001879475.1_Asagao_1.1
EpG
Wilcoxon p−value = 0, W = 834789628
density
0.00
0.05
0.10
0.15
0.20
0
20
40
60
80
num_uniq_exon
group
ortho
whole transcriptome

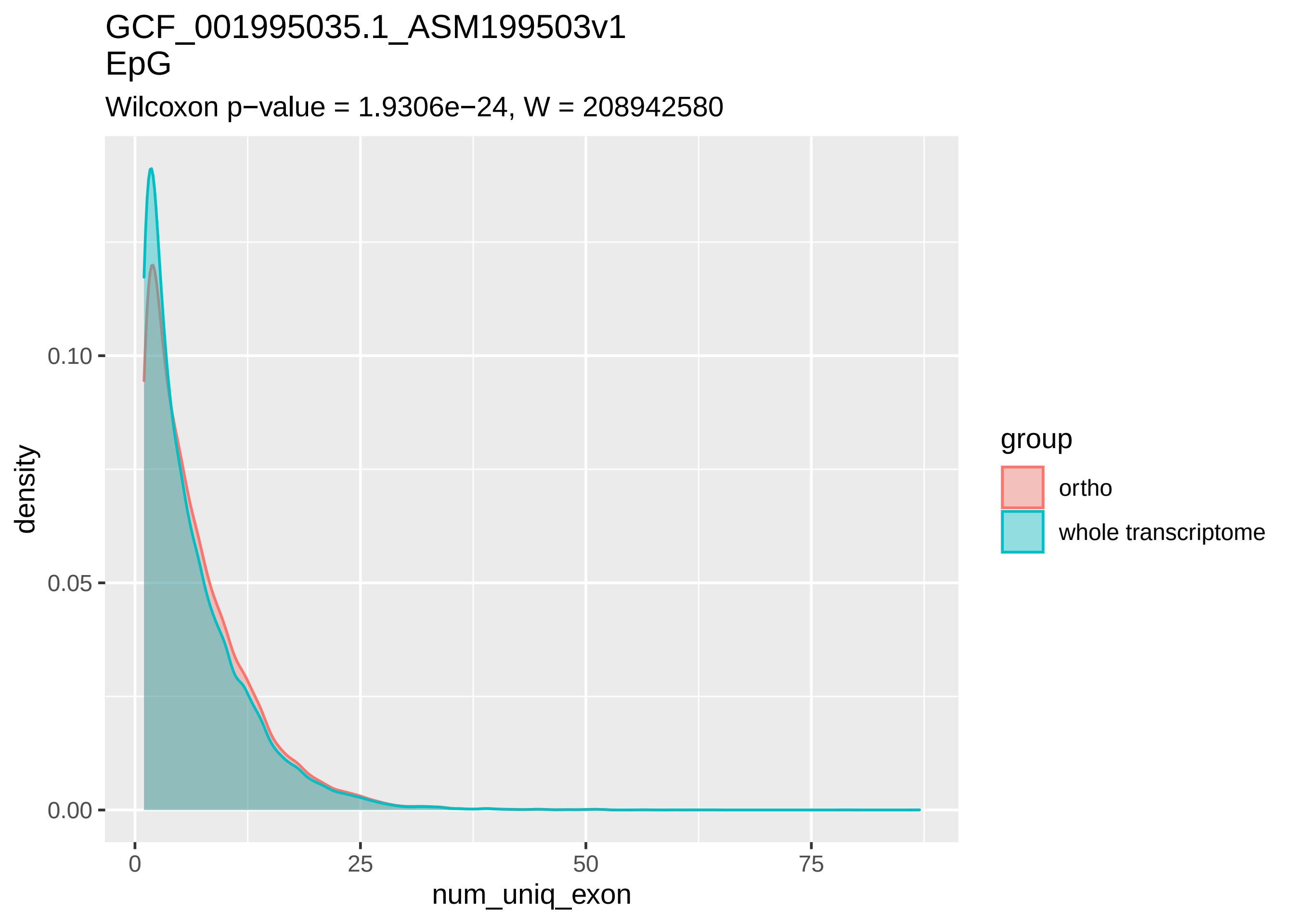

GCF_001995035.1_ASM199503v1
EpG
Wilcoxon p−value = 1.9306e−24, W = 208942580
density
0.10
0.05
0.00
0
25
50
75
num_uniq_exon
group
ortho
whole transcriptome

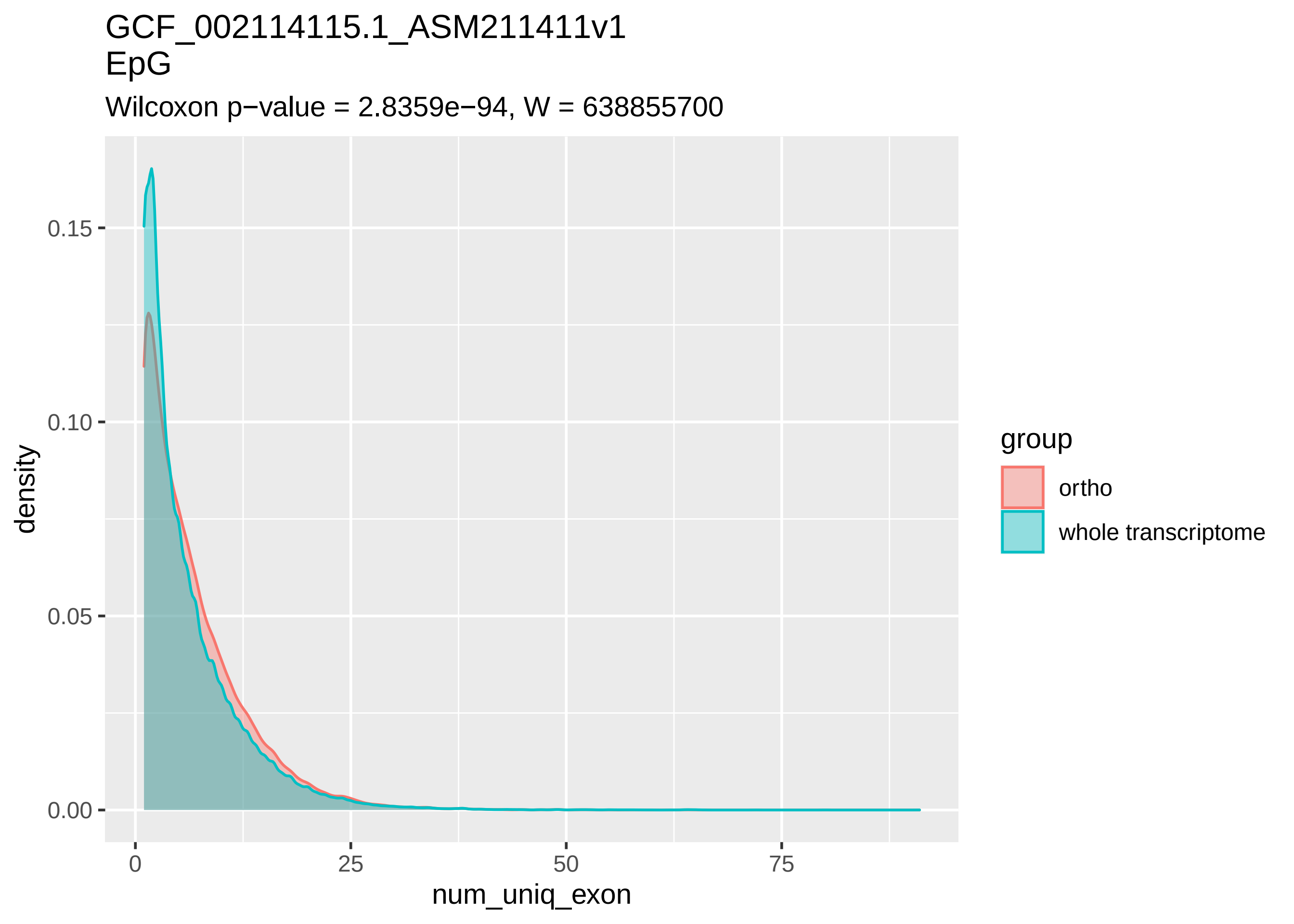
GCF_002114115.1_ASM211411v1
EpG
Wilcoxon p−value = 2.8359e−94, W = 638855700
density
0.15
0.10
0.05
0.00
0
25
50
75
num_uniq_exon
group
ortho
whole transcriptome

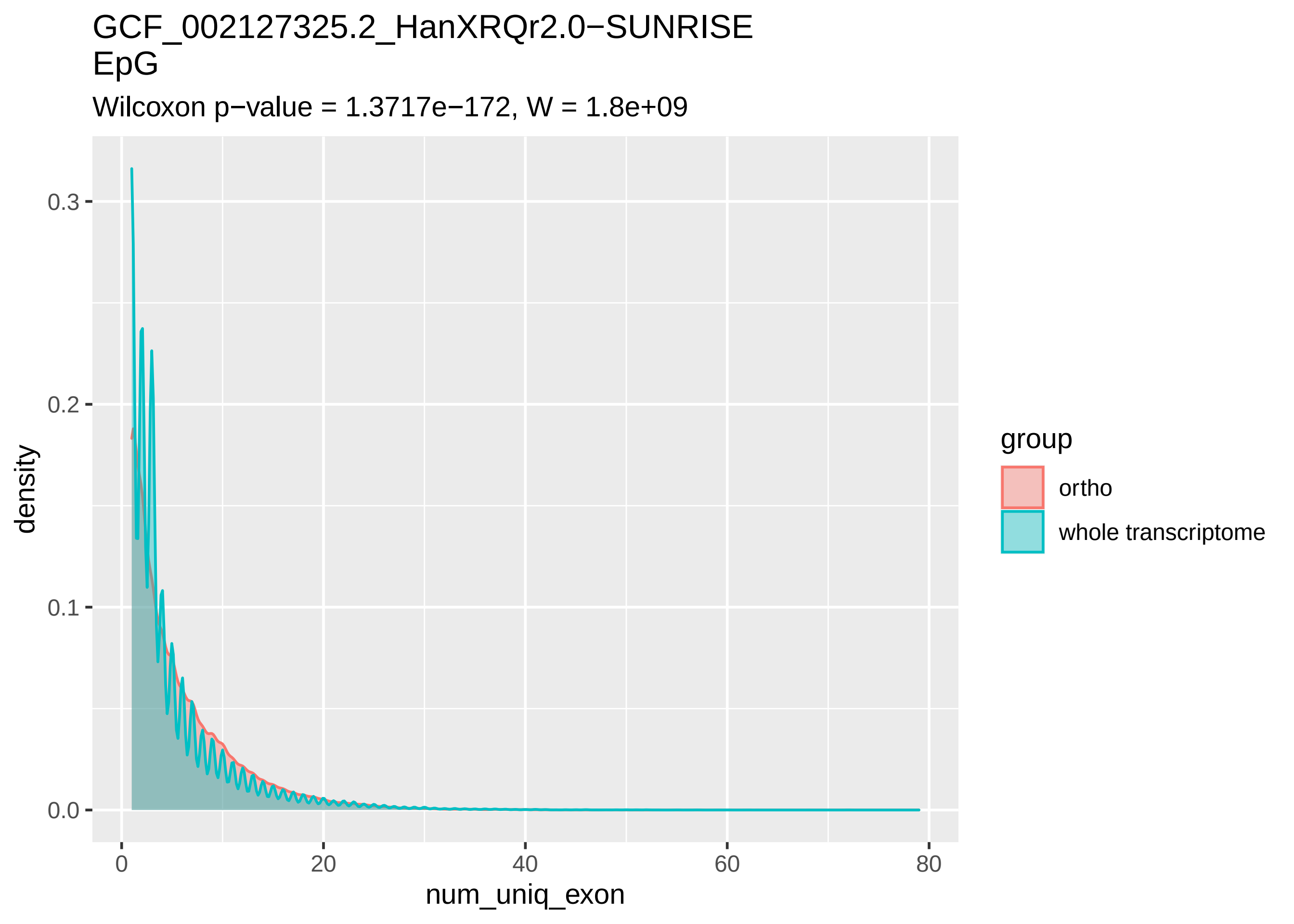

GCF_002127325.2_HanXRQr2.0−SUNRISE
EpG
Wilcoxon p−value = 1.3717e−172, W = 1.8e+09
density
0.0
0.1
0.2
0.3
0
20
40
60
80
num_uniq_exon
group
ortho
whole transcriptome

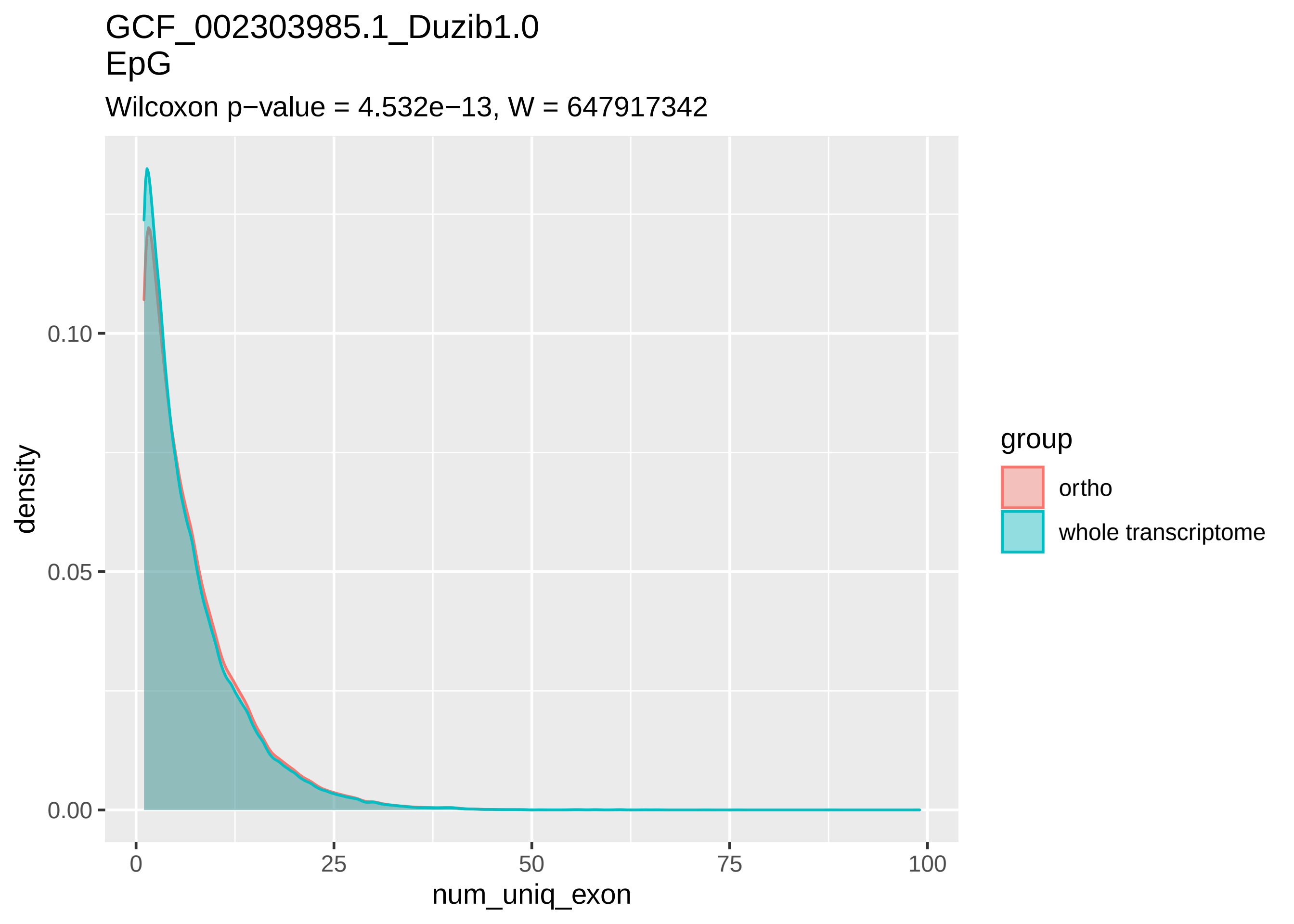
GCF_002303985.1_Duzib1.0
EpG
Wilcoxon p−value = 4.532e−13, W = 647917342
density
0.10
0.05
0.00
0
25
50
75
100
num_uniq_exon
group
ortho
whole transcriptome

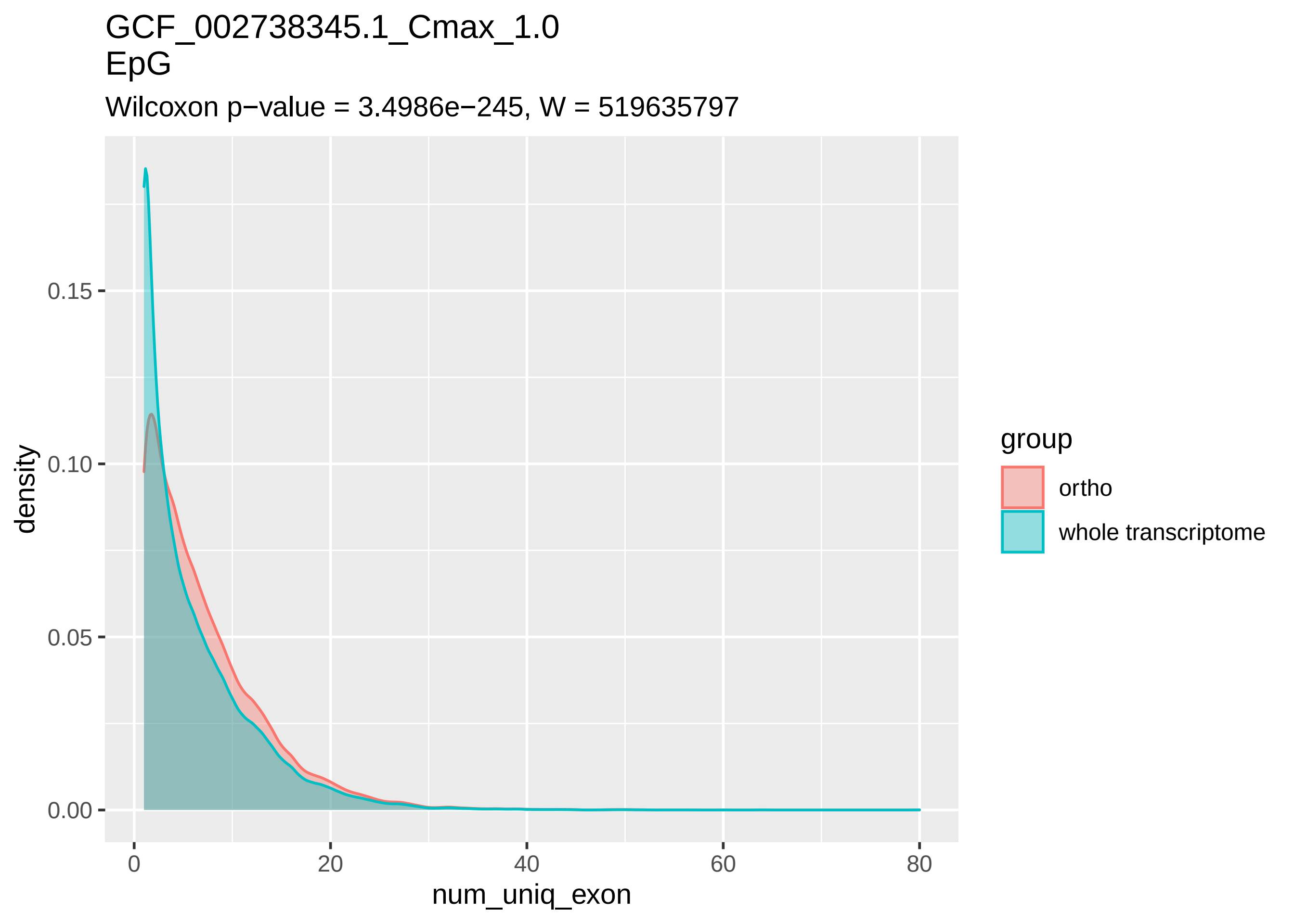
GCF_002738345.1_Cmax_1.0
EpG
Wilcoxon p−value = 3.4986e−245, W = 519635797
density
0.00
0.05
0.10
0.15
0
20
40
60
80
num_uniq_exon
group
ortho
whole transcriptome

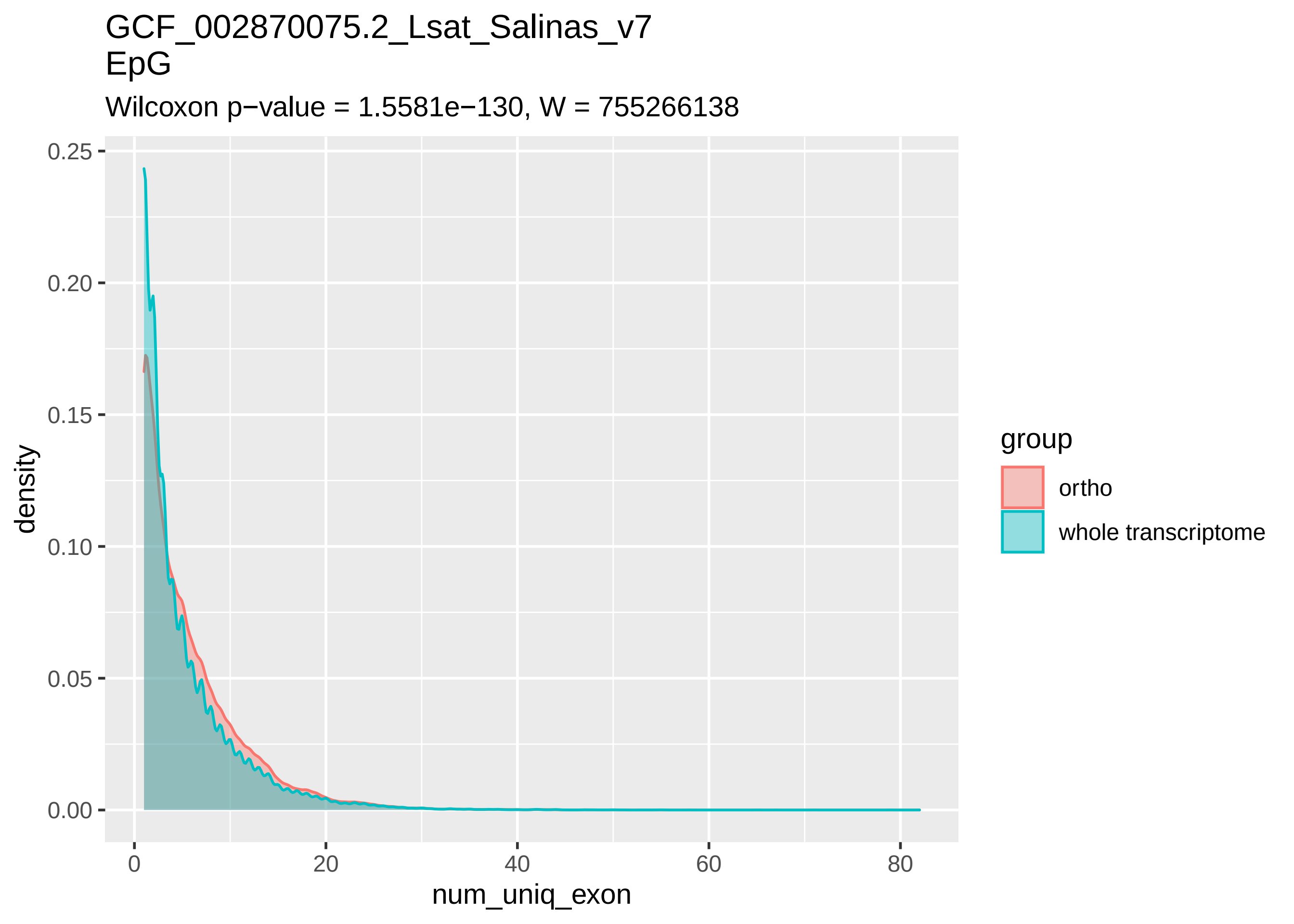

GCF_002870075.2_Lsat_Salinas_v7
EpG
Wilcoxon p−value = 1.5581e−130, W = 755266138
density
0.25
0.20
0.15
0.10
0.05
0.00
0
20
40
60
80
num_uniq_exon
group
ortho
whole transcriptome

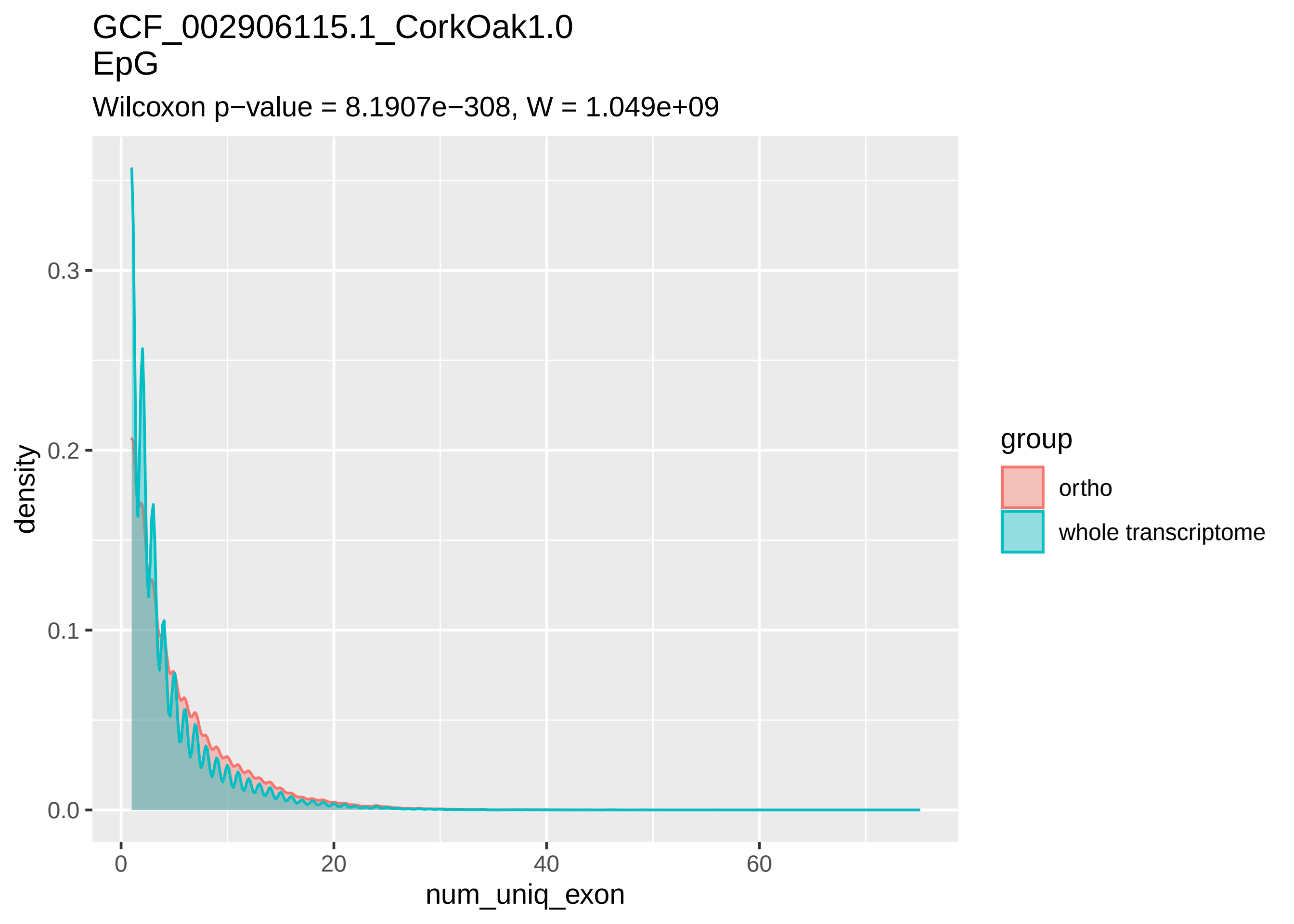
GCF_002906115.1_CorkOak1.0
EpG
Wilcoxon p−value = 8.1907e−308, W = 1.049e+09
density
0.0
0.1
0.2
0.3
0
20
40
60
num_uniq_exon
group
ortho
whole transcriptome

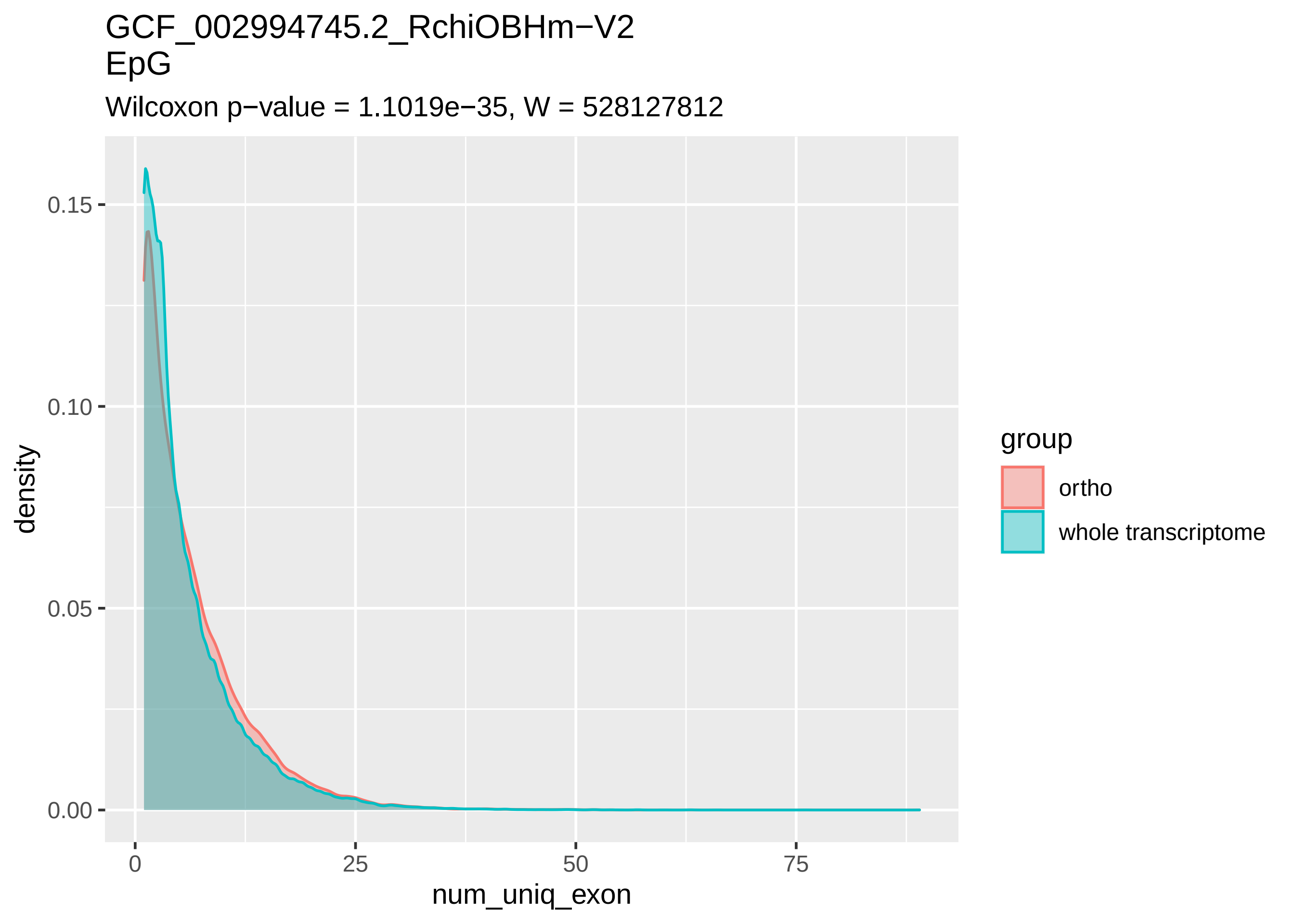
GCF_002994745.2_RchiOBHm−V2
EpG
Wilcoxon p−value = 1.1019e−35, W = 528127812
0.15
0.10
0.05
0.00
density
0
25
50
75
num_uniq_exon
group
ortho
whole transcriptome

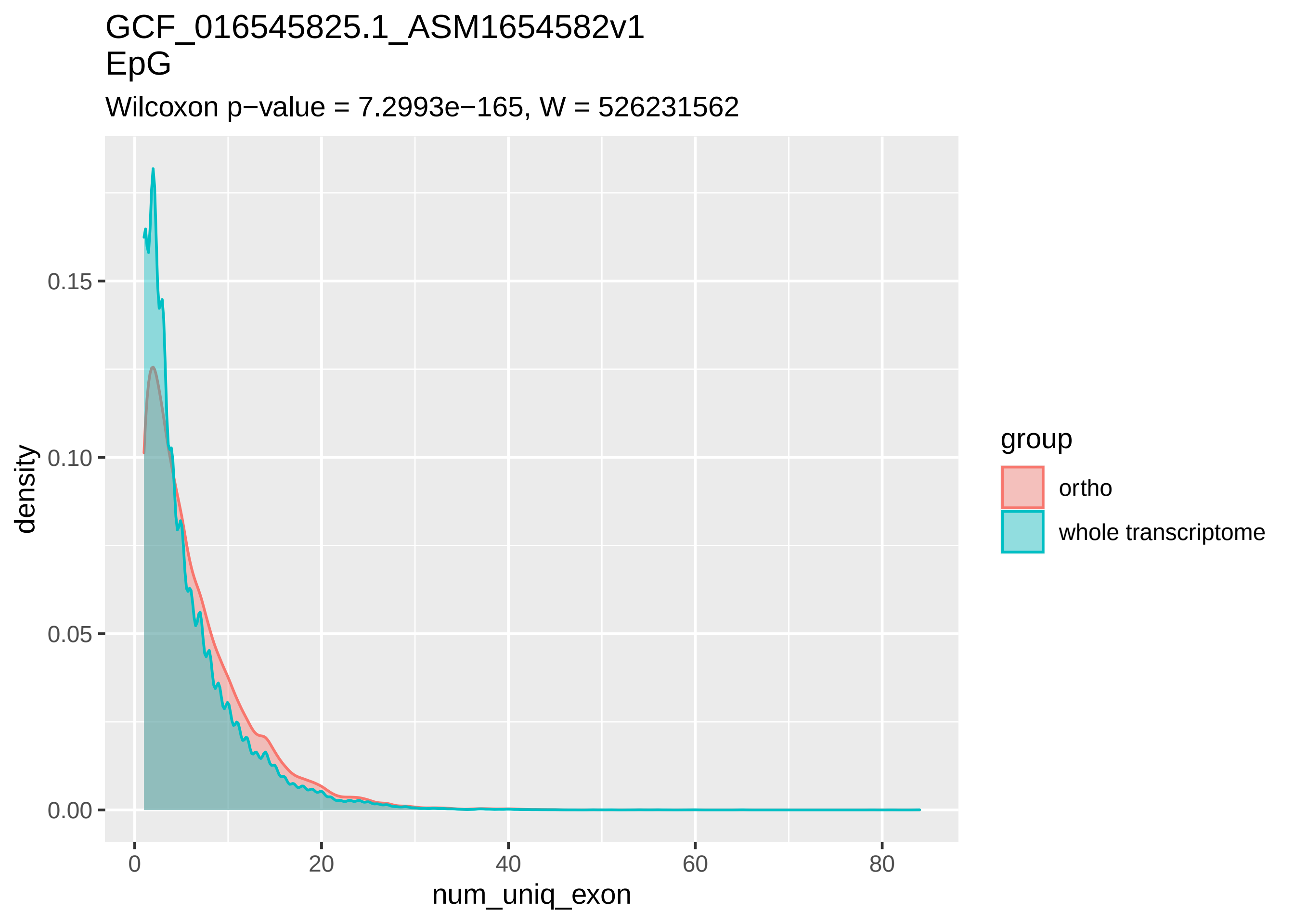
GCF_016545825.1_ASM1654582v1
EpG
Wilcoxon p−value = 7.2993e−165, W = 526231562
density
0.00
0.05
0.10
0.15
0
20
40
60
80
num_uniq_exon
group
ortho
whole transcriptome

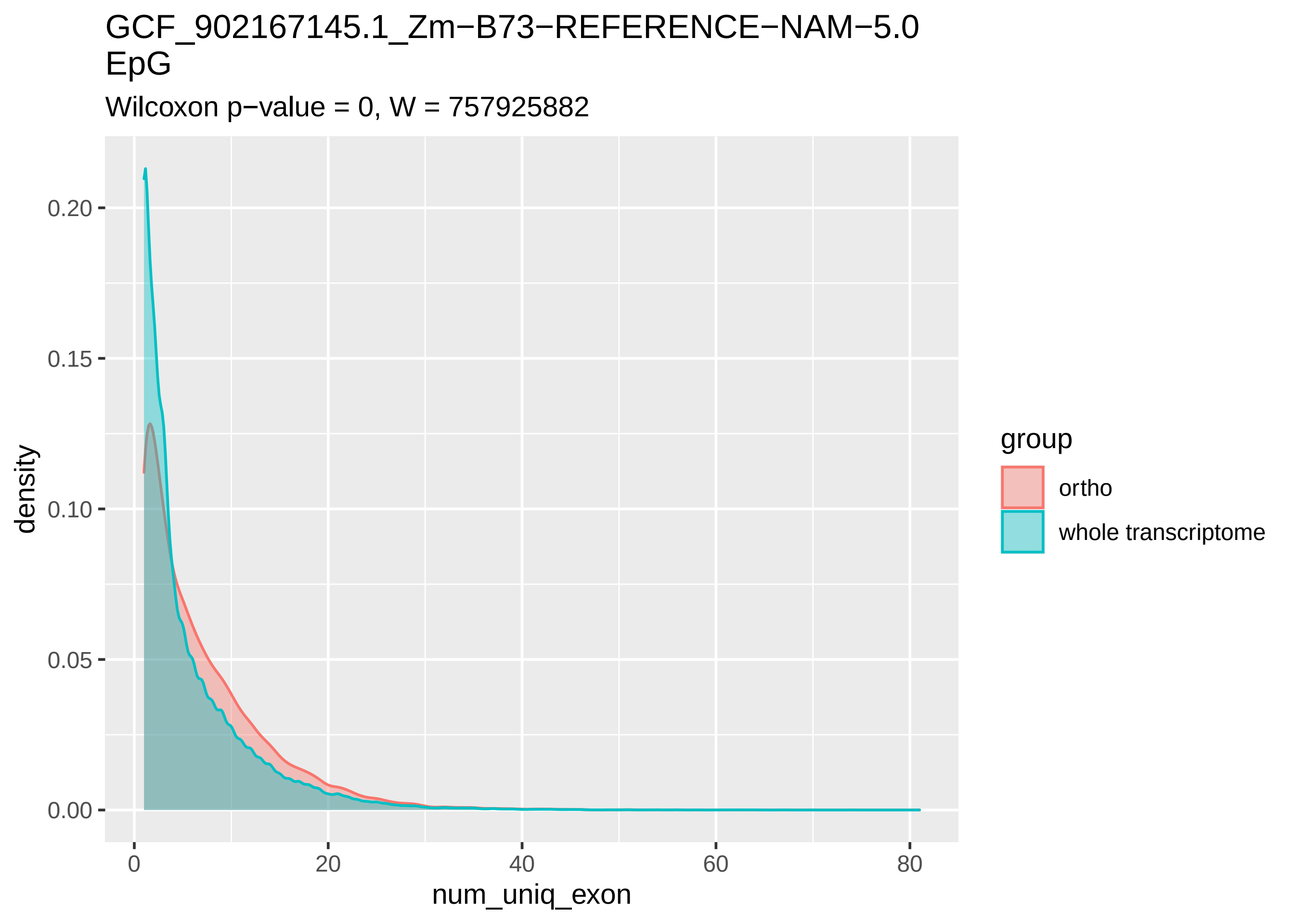
GCF_902167145.1_Zm−B73−REFERENCE−NAM−5.0
EpG
Wilcoxon p−value = 0, W = 757925882
density
0.00
0.05
0.10
0.15
0.20
0
20
40
60
80
num_uniq_exon
group
ortho
whole transcriptome

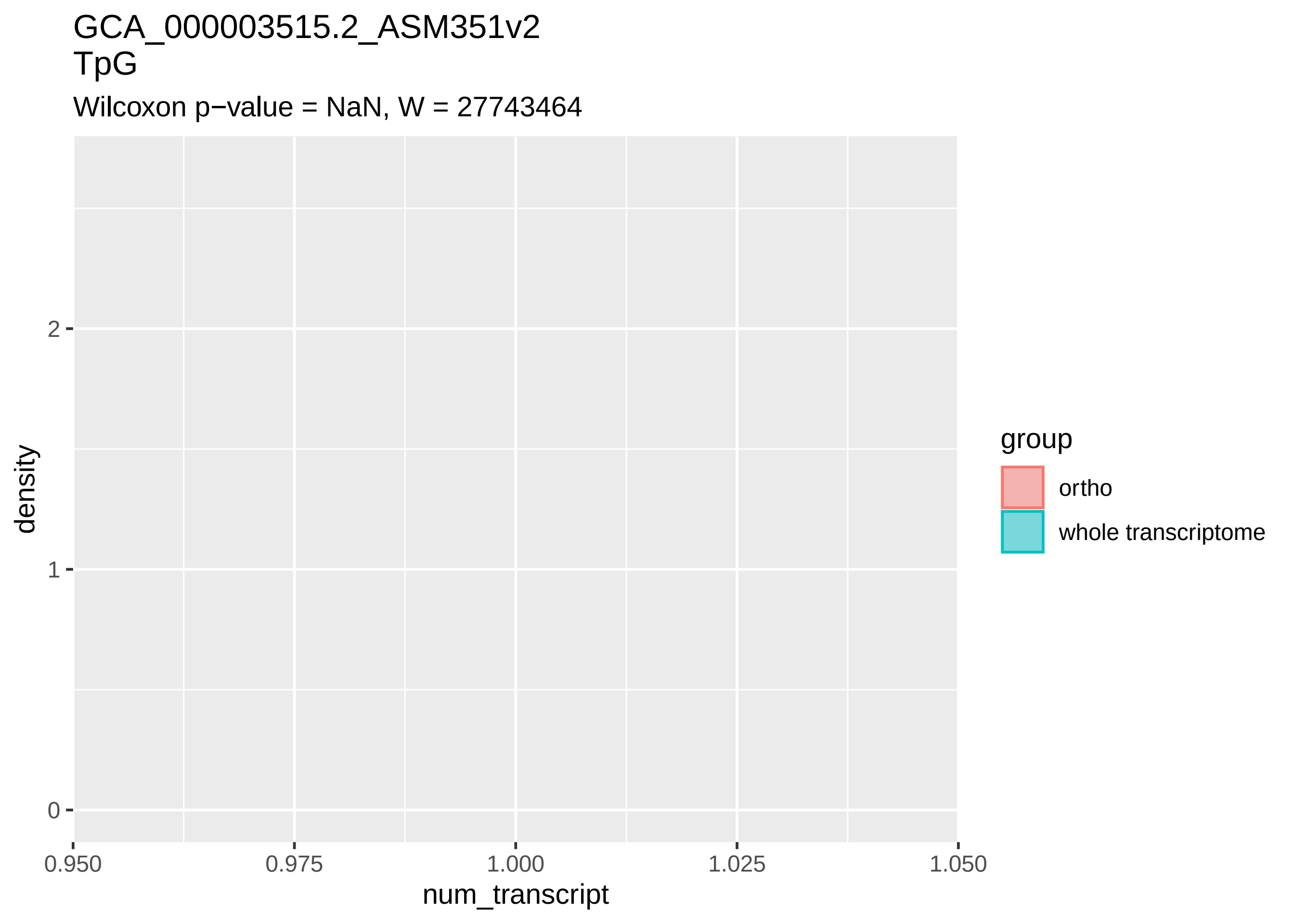
GCA_000003515.2_ASM351v2
TpG
Wilcoxon p−value = NaN, W = 27743464
density
2
1
0
0.950
0.975
1.000
1.025
1.050
num_transcript
group
ortho
whole transcriptome

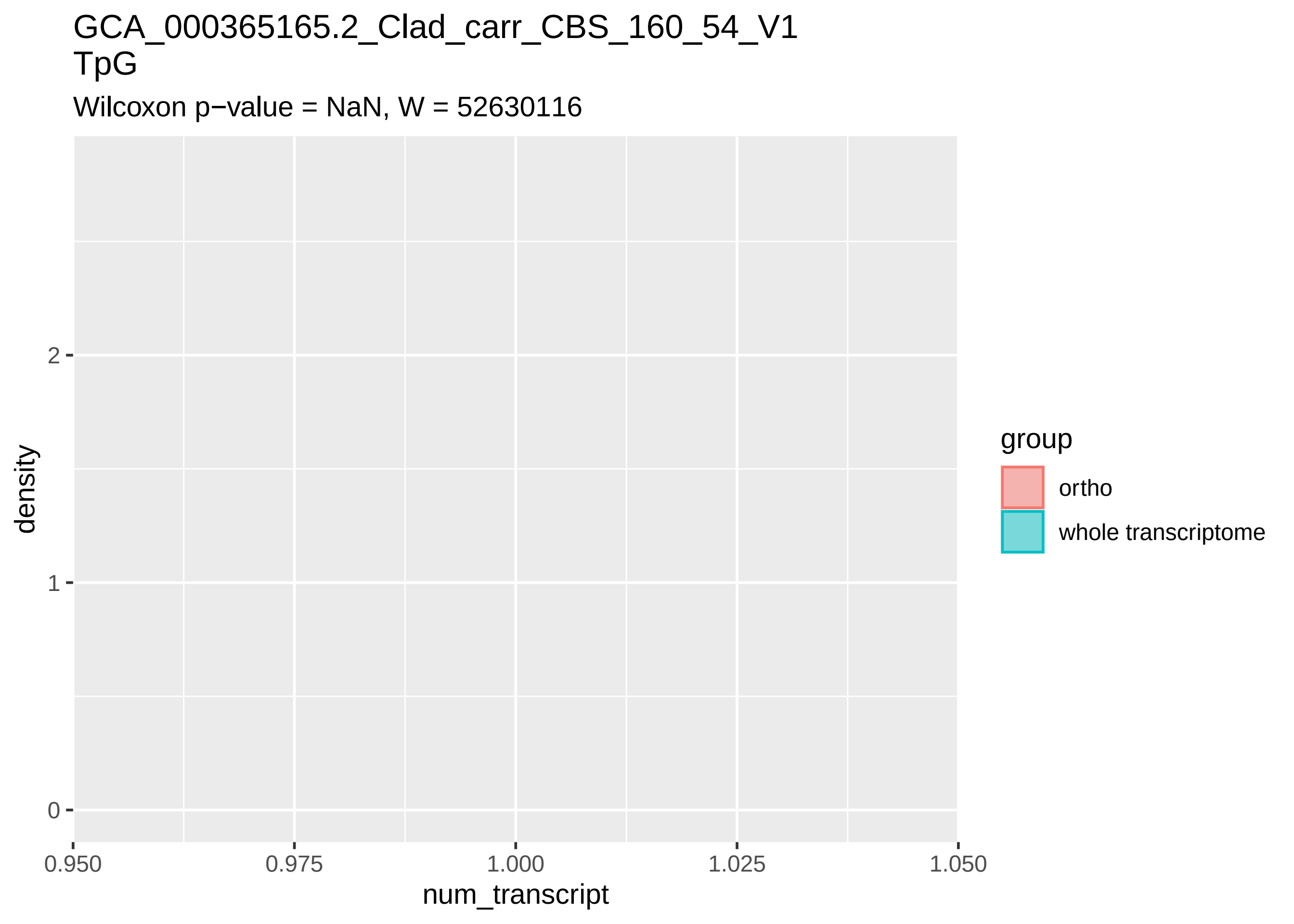

GCA_000365165.2_Clad_carr_CBS_160_54_V1
TpG
Wilcoxon p−value = NaN, W = 52630116
density
2
1
0
0.950
0.975
1.000
1.025
1.050
num_transcript
group
ortho
whole transcriptome

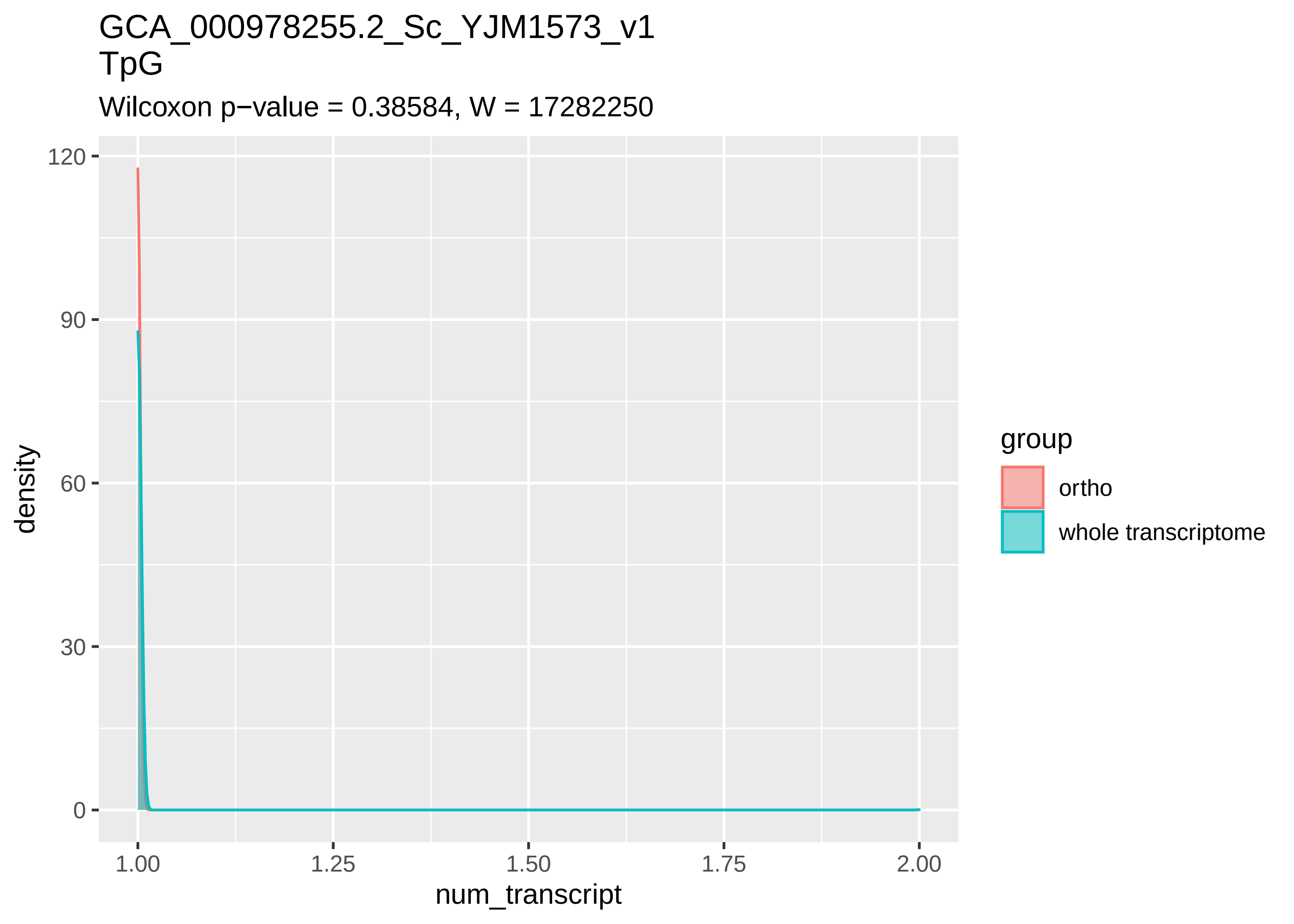
GCA_000978255.2_Sc_YJM1573_v1
TpG
Wilcoxon p−value = 0.38584, W = 17282250
density
120
90
60
30
0
1.00
1.25
1.50
1.75
2.00
num_transcript
group
ortho
whole transcriptome

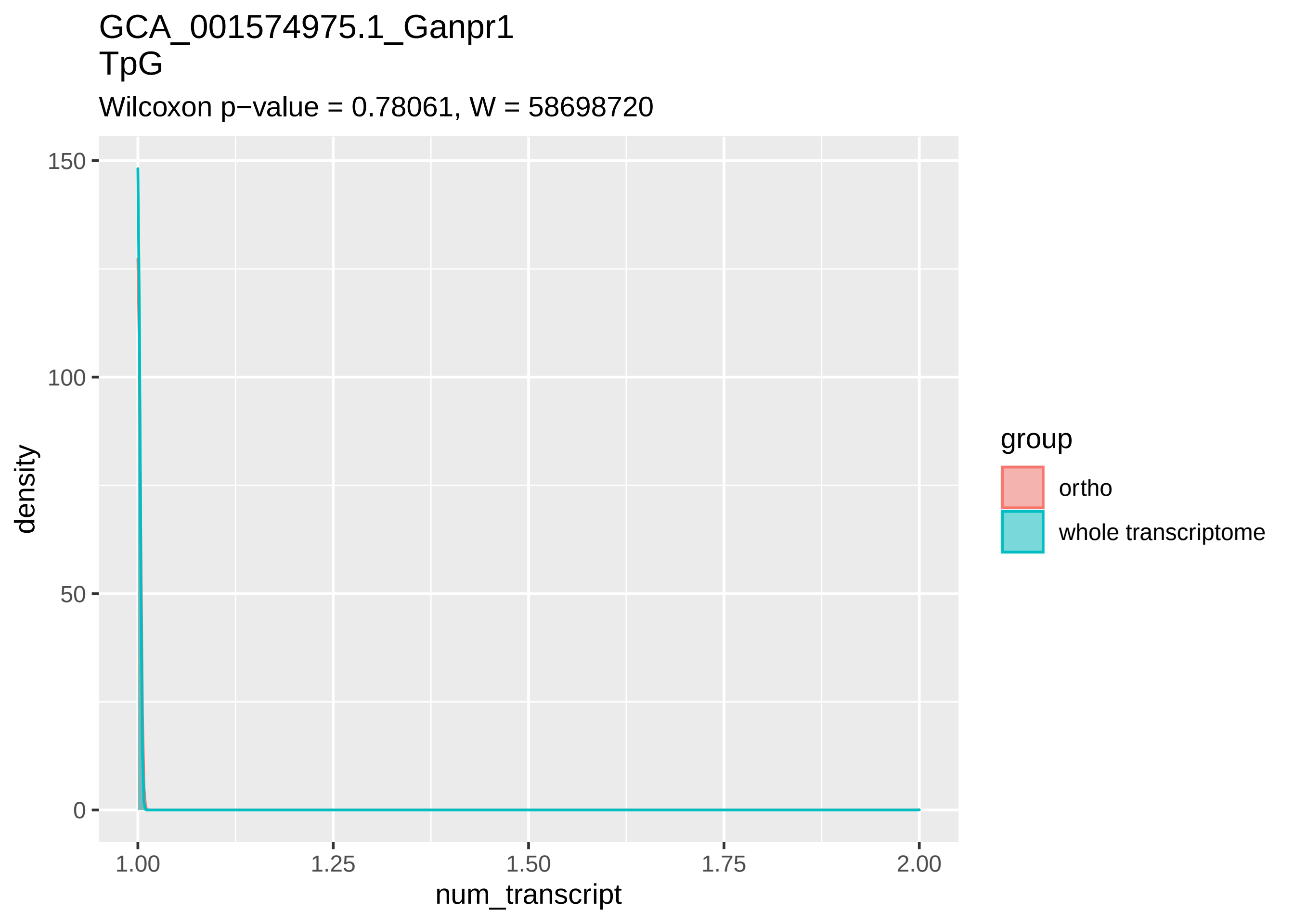
GCA_001574975.1_Ganpr1
TpG
Wilcoxon p−value = 0.78061, W = 58698720
150
100
50
0
density
1.00
1.25
1.50
1.75
2.00
num_transcript
group
ortho
whole transcriptome

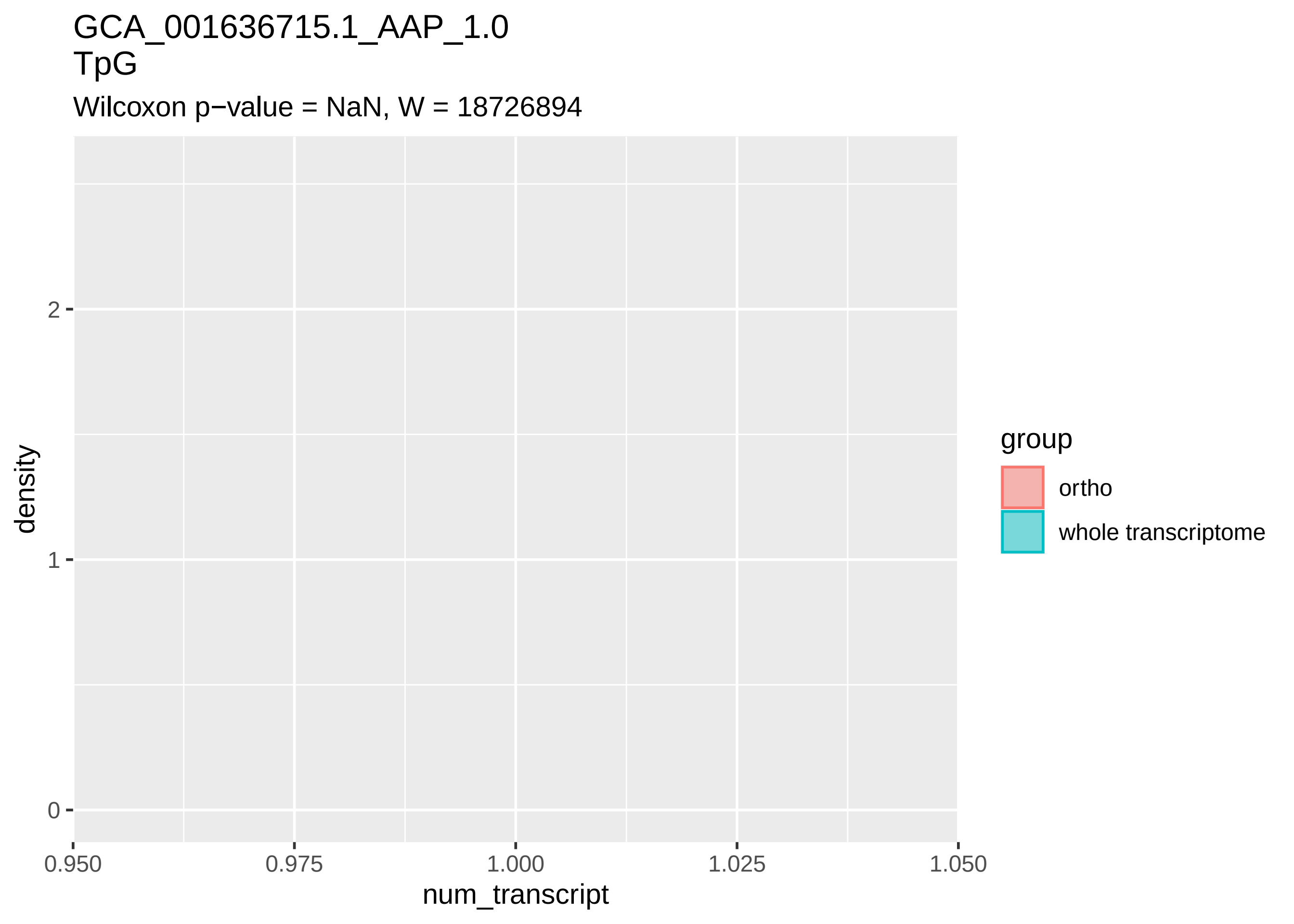

GCA_001636715.1_AAP_1.0
TpG
Wilcoxon p−value = NaN, W = 18726894
density
2
1
0
0.950
0.975
1.000
1.025
1.050
num_transcript
group
ortho
whole transcriptome

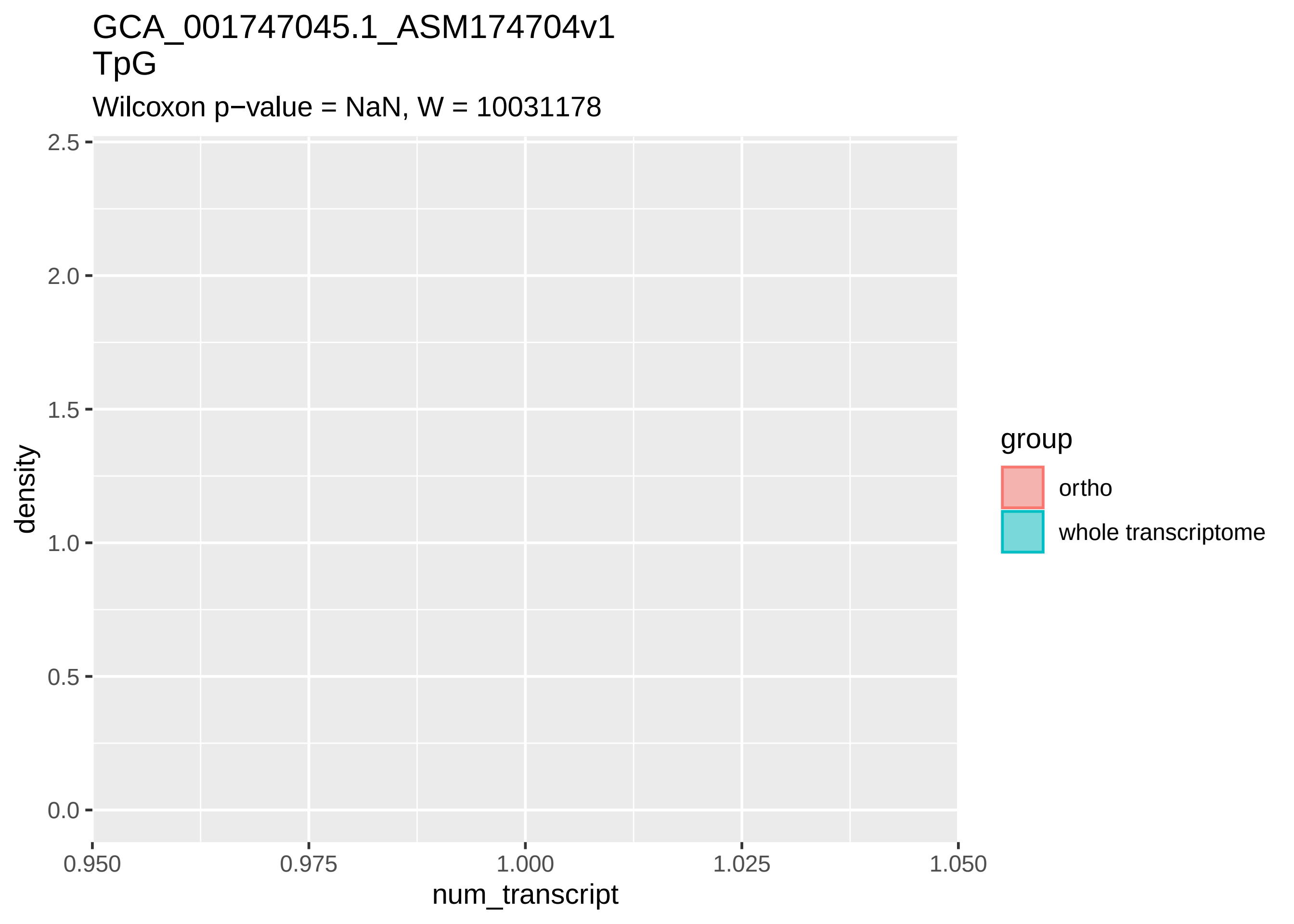

GCA_001747045.1_ASM174704v1
TpG
Wilcoxon p−value = NaN, W = 10031178
density
2.5
2.0
1.5
1.0
0.5
0.0
0.950
0.975
1.000
1.025
1.050
num_transcript
group
ortho
whole transcriptome

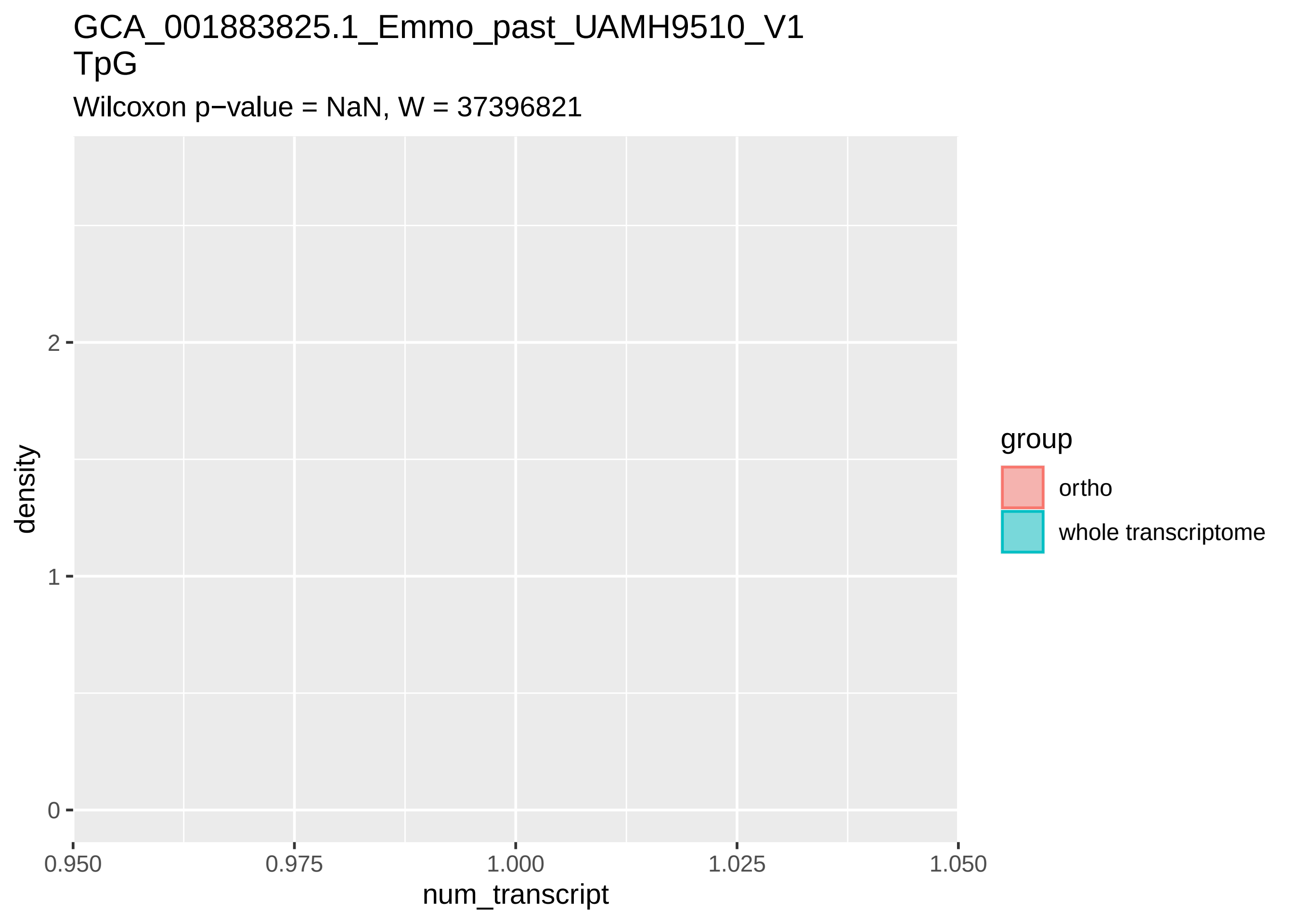
GCA_001883825.1_Emmo_past_UAMH9510_V1
TpG
Wilcoxon p−value = NaN, W = 37396821
density
2
1
0
0.950
0.975
1.000
1.025
1.050
num_transcript
group
ortho
whole transcriptome

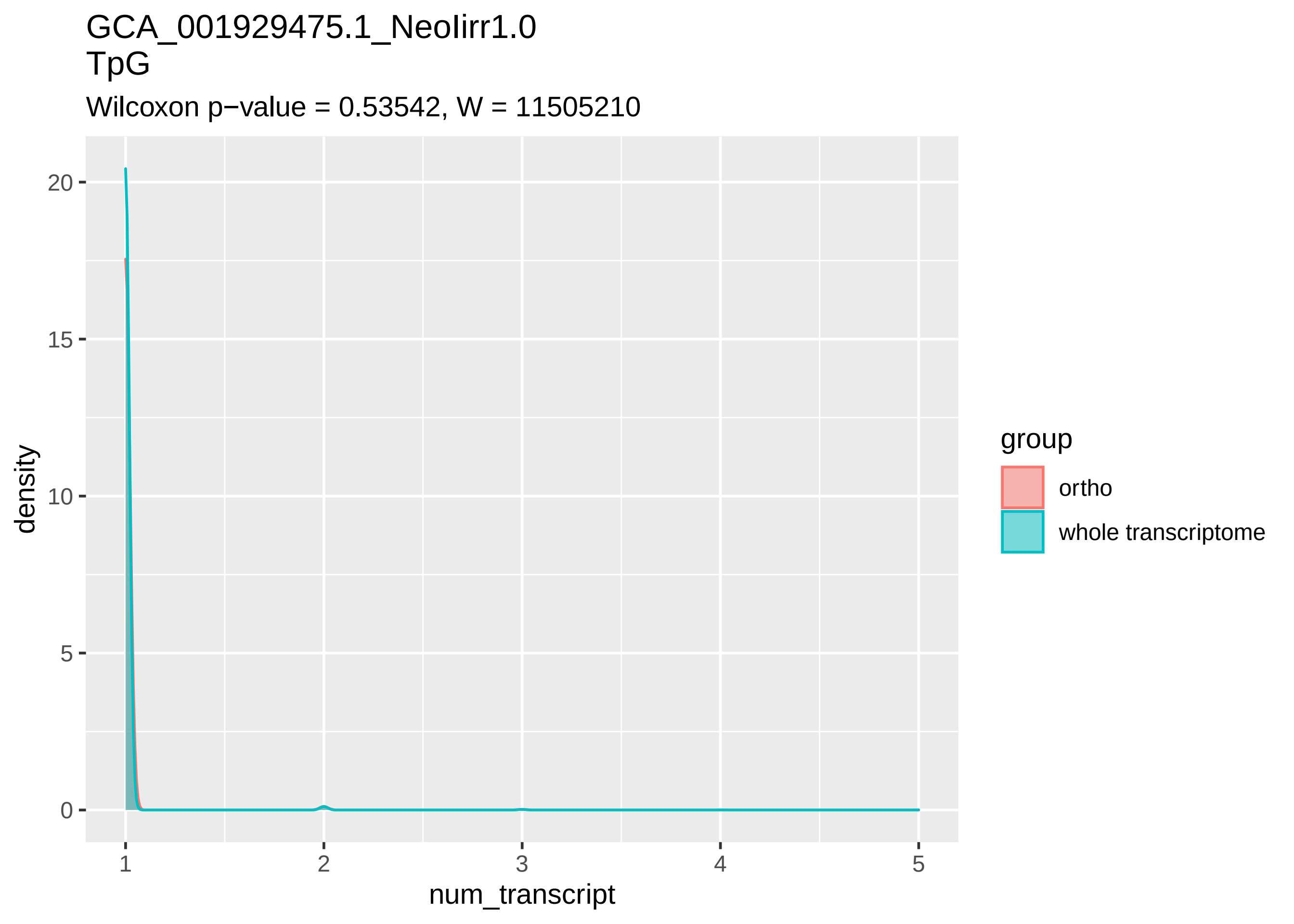
GCA_001929475.1_NeoIirr1.0
TpG
Wilcoxon p−value = 0.53542, W = 11505210
density
20
15
10
5
0
1
2
3
4
5
num_transcript
group
ortho
whole transcriptome

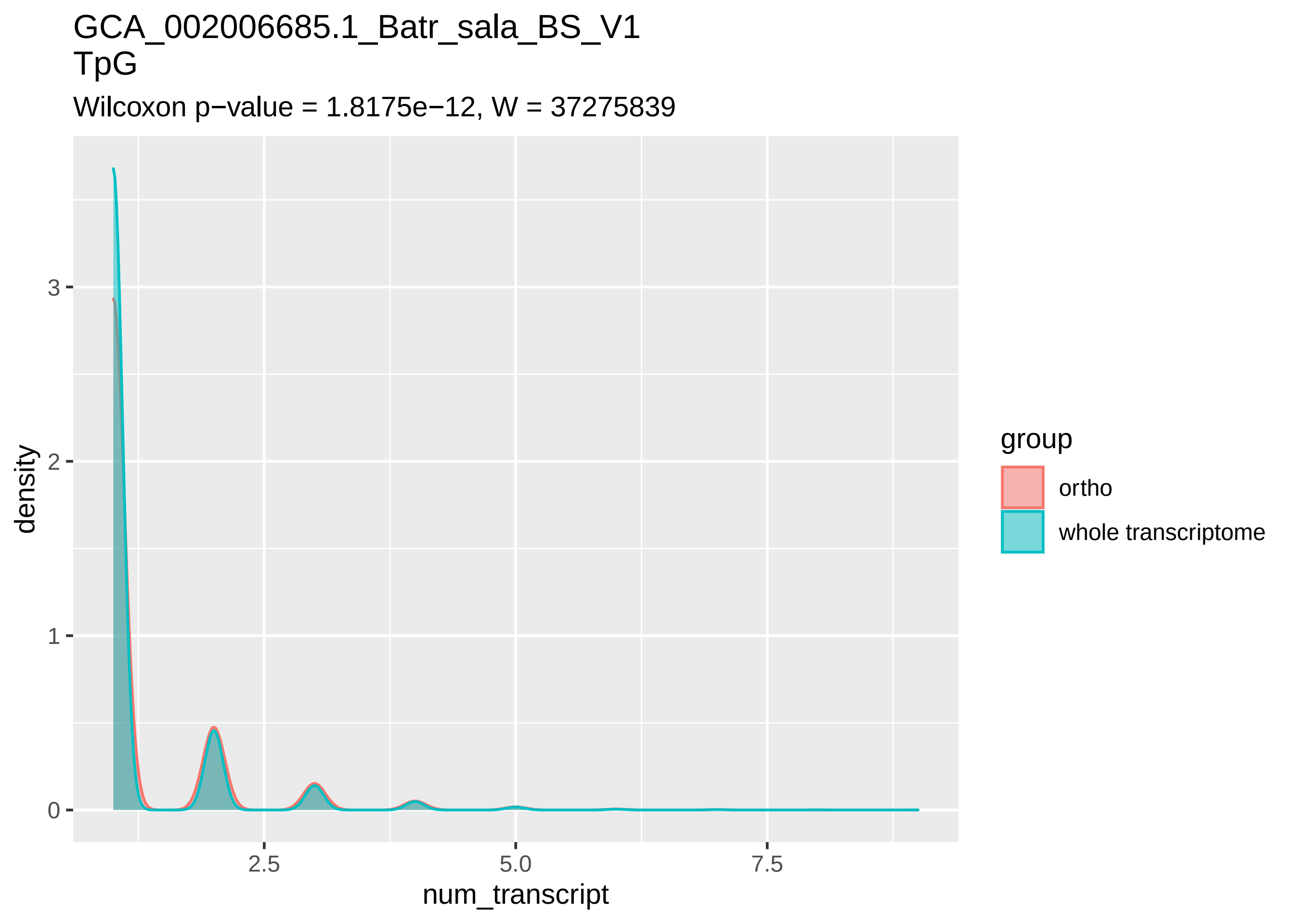

GCA_002006685.1_Batr_sala_BS_V1
TpG
Wilcoxon p−value = 1.8175e−12, W = 37275839
density
3
2
1
0
2.5
5.0
7.5
num_transcript
group
ortho
whole transcriptome

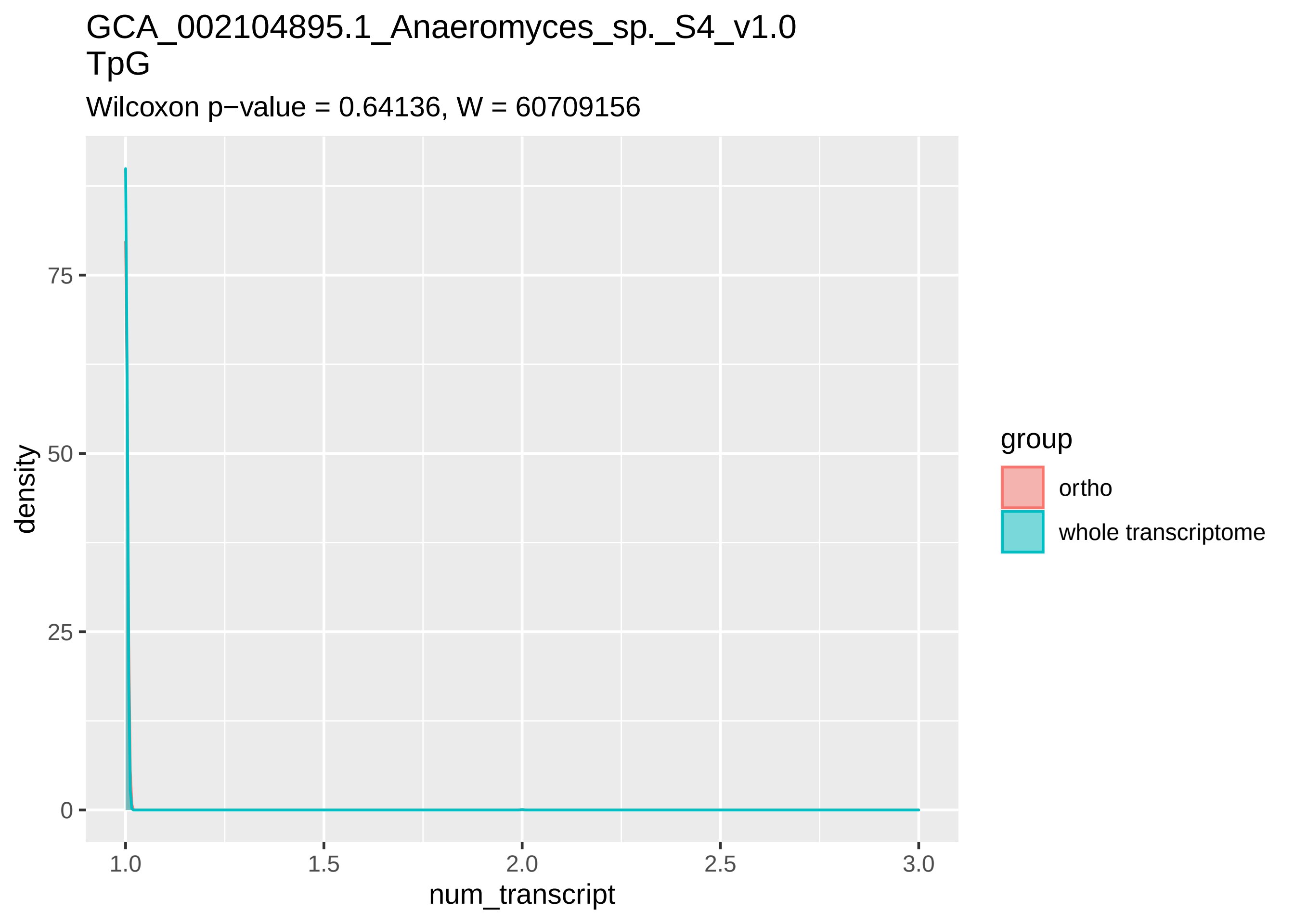

GCA_002104895.1_Anaeromyces_sp._S4_v1.0
TpG
Wilcoxon p−value = 0.64136, W = 60709156
density
num_transcript
0
25
50
75
1.0
1.5
2.0
2.5
3.0
group
ortho
whole transcriptome

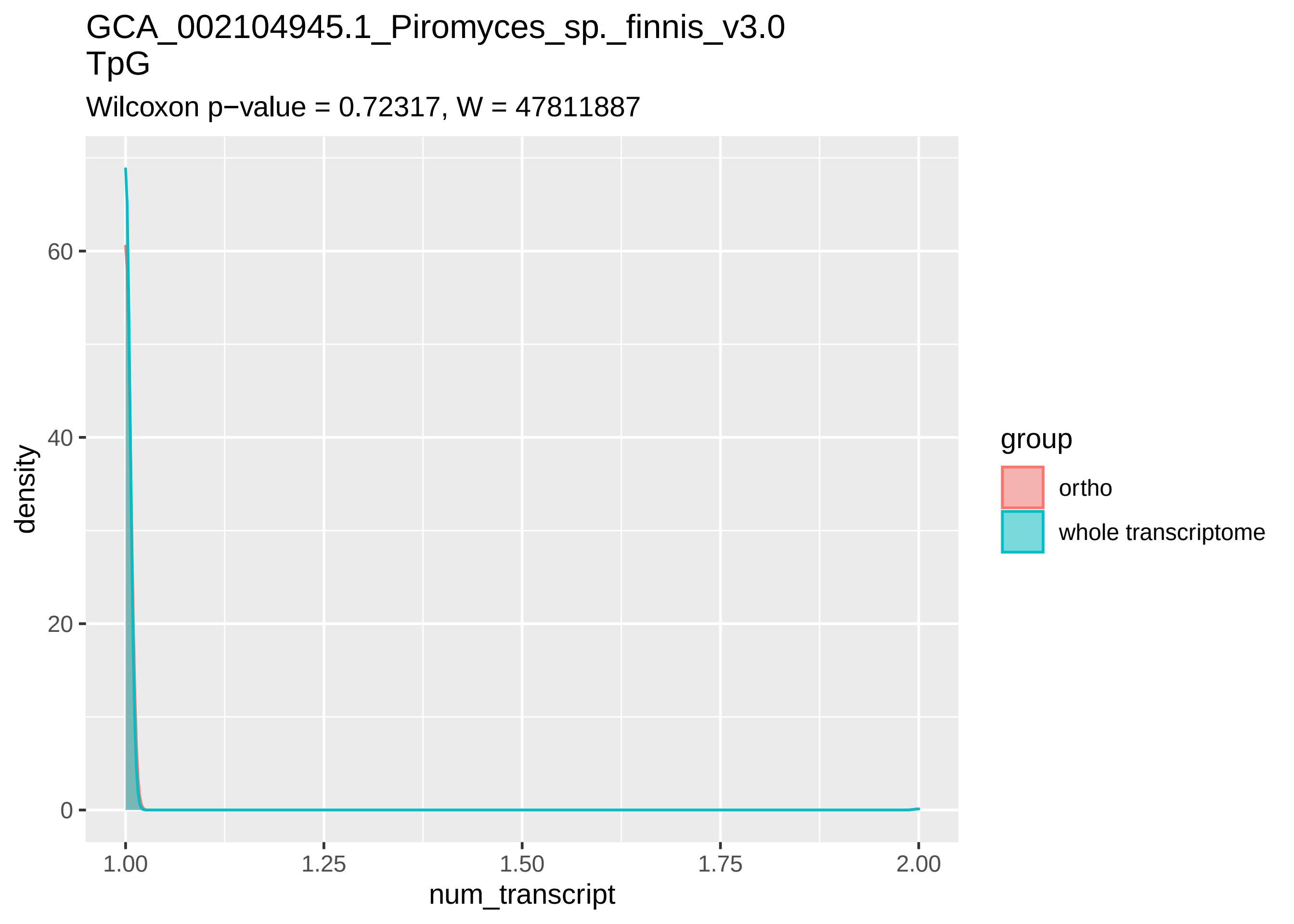

GCA_002104945.1_Piromyces_sp._finnis_v3.0
TpG
Wilcoxon p−value = 0.72317, W = 47811887
density
0
20
40
60
1.00
1.25
1.50
1.75
2.00
num_transcript
group
ortho
whole transcriptome

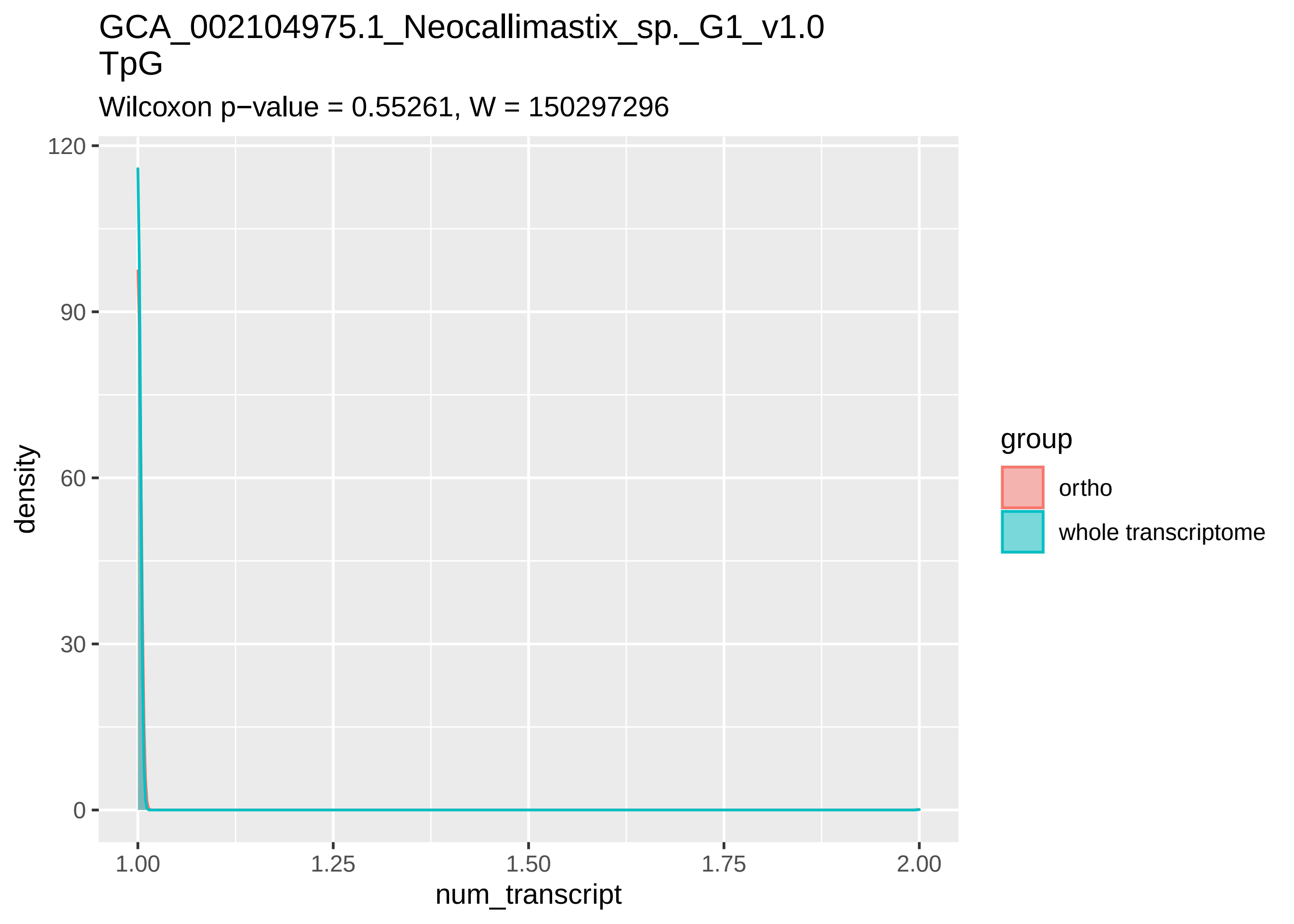

GCA_002104975.1_Neocallimastix_sp._G1_v1.0
TpG
Wilcoxon p−value = 0.55261, W = 150297296
density
120
90
60
30
0
1.00
1.25
1.50
1.75
2.00
num_transcript
group
ortho
whole transcriptome

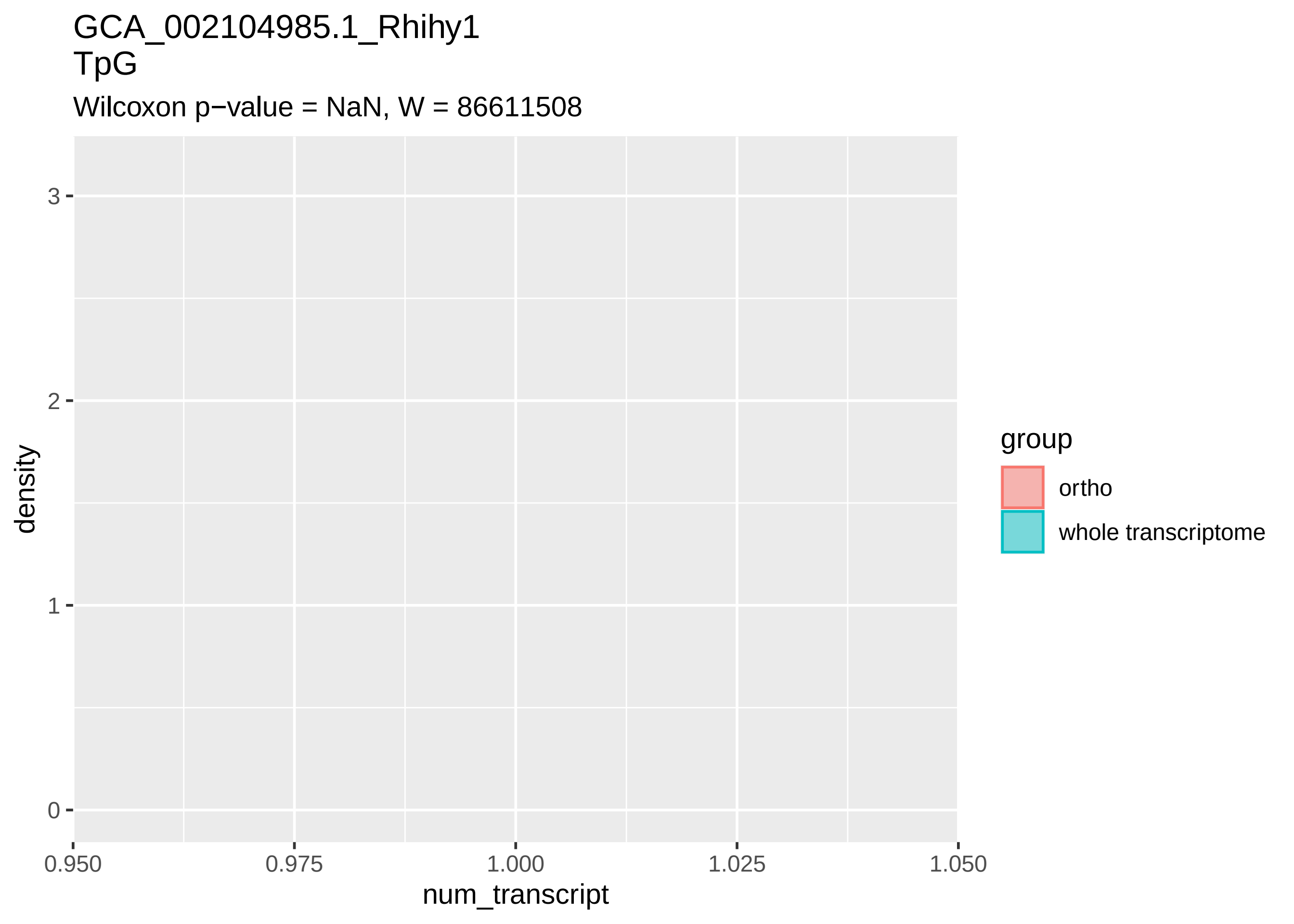

GCA_002104985.1_Rhihy1
TpG
Wilcoxon p−value = NaN, W = 86611508
density
3
2
1
0
0.950
0.975
1.000
1.025
1.050
num_transcript
group
ortho
whole transcriptome

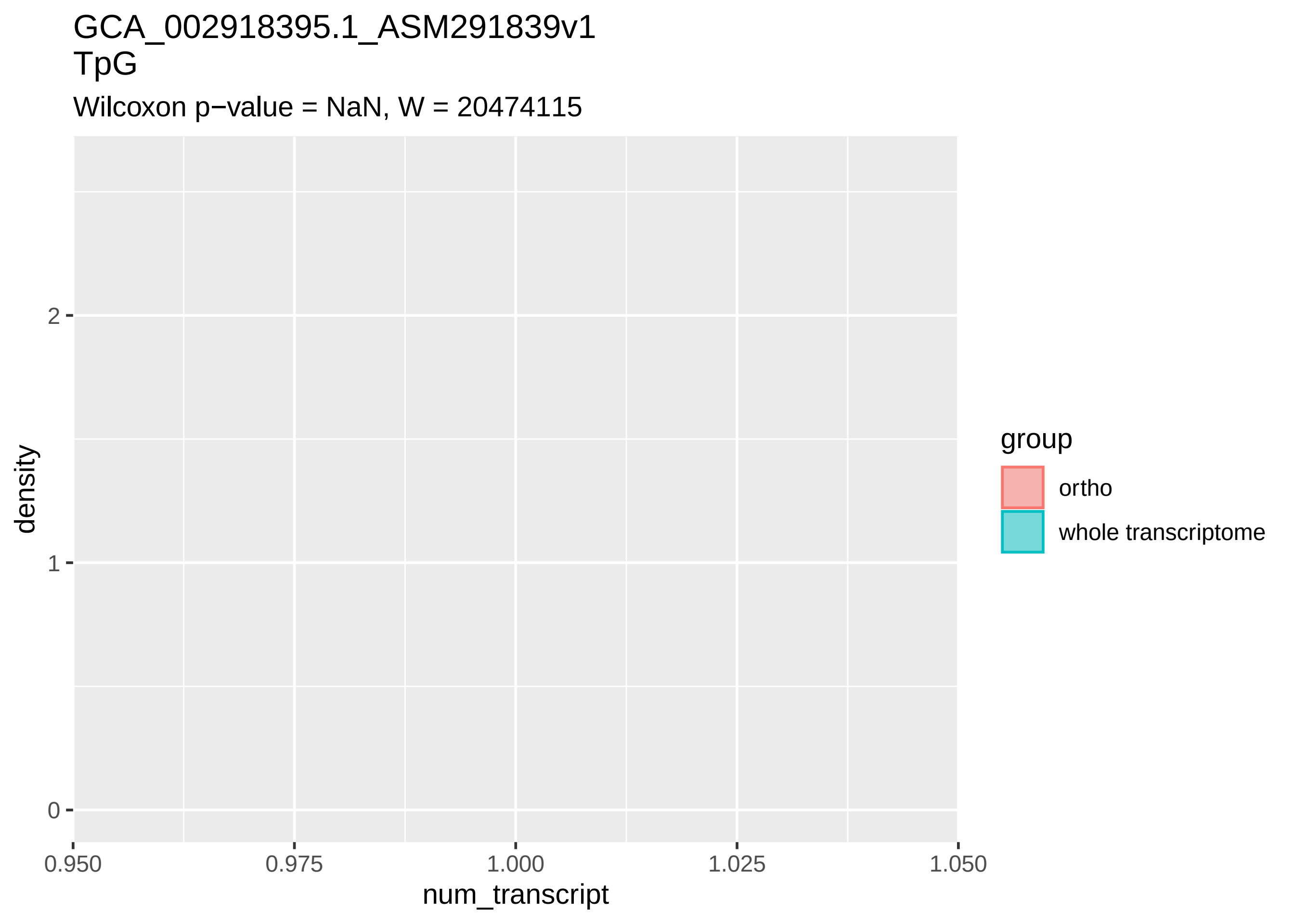
GCA_002918395.1_ASM291839v1
TpG
Wilcoxon p−value = NaN, W = 20474115
density
2
1
0
0.950
0.975
1.000
1.025
1.050
num_transcript
group
ortho
whole transcriptome

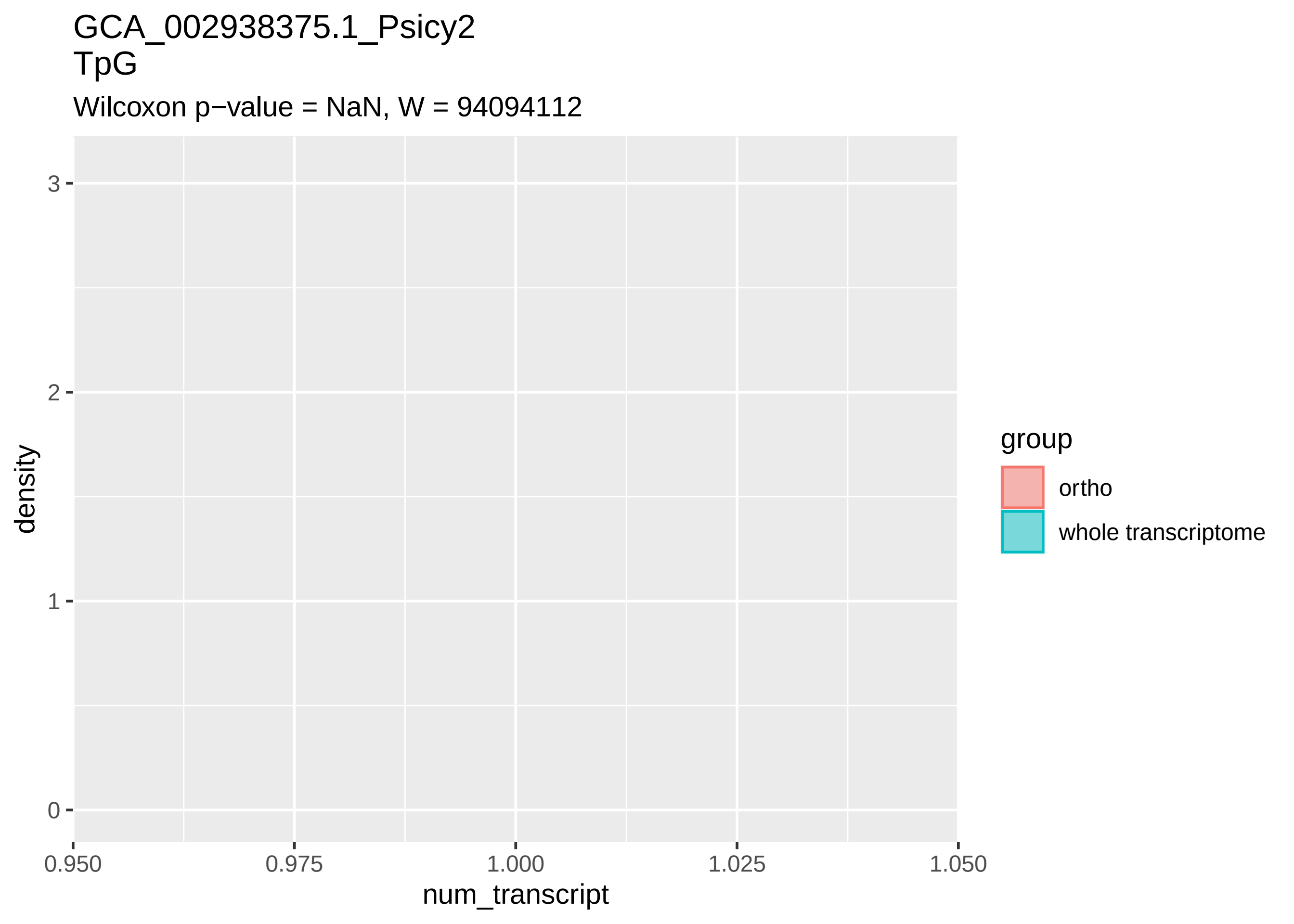
GCA_002938375.1_Psicy2
TpG
Wilcoxon p−value = NaN, W = 94094112
density
3
2
1
0
0.950
0.975
1.000
1.025
1.050
num_transcript
group
ortho
whole transcriptome

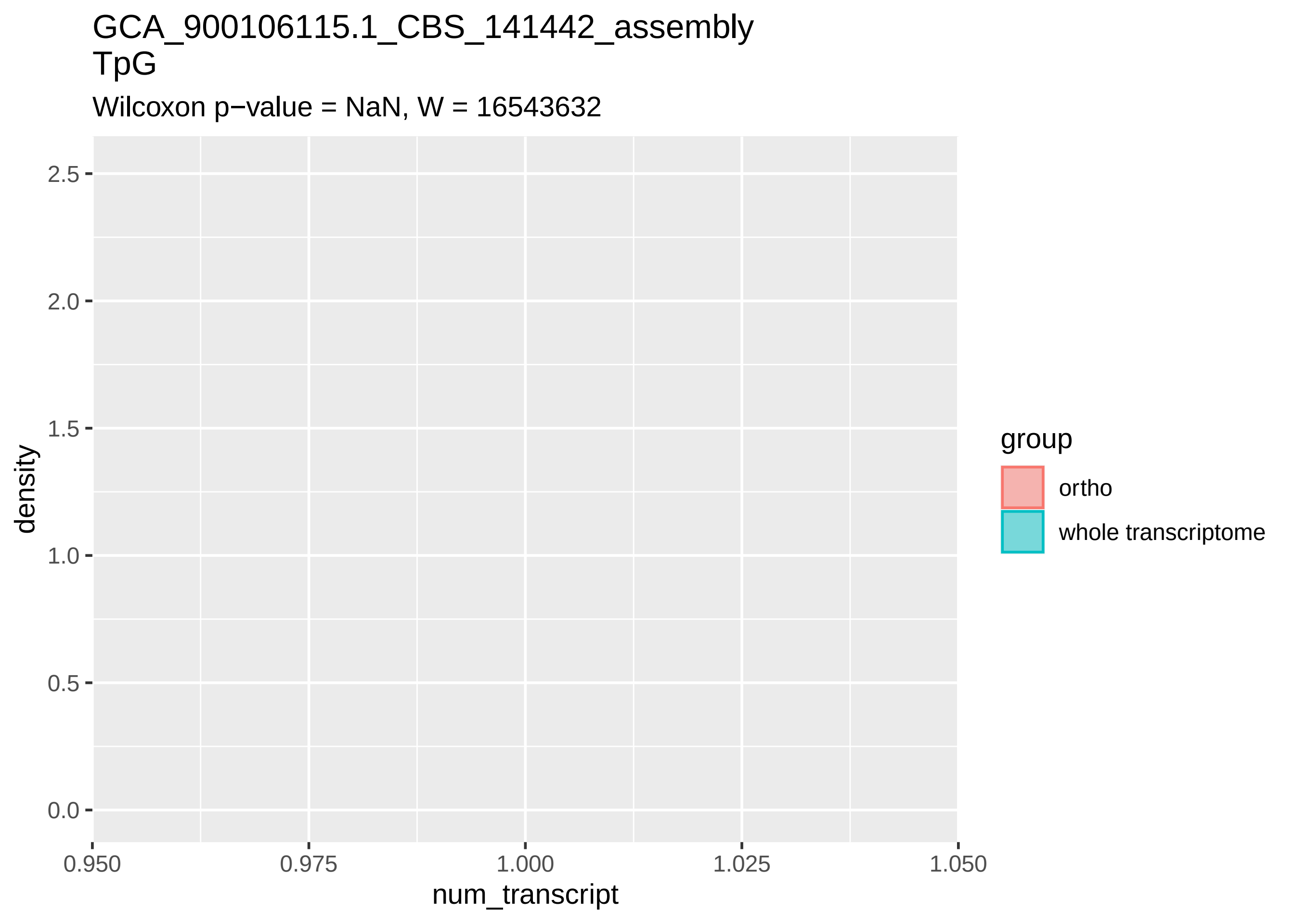
GCA_900106115.1_CBS_141442_assembly
TpG
Wilcoxon p−value = NaN, W = 16543632
density
2.5
2.0
1.5
1.0
0.5
0.0
0.950
0.975
1.000
1.025
1.050
num_transcript
group
ortho
whole transcriptome

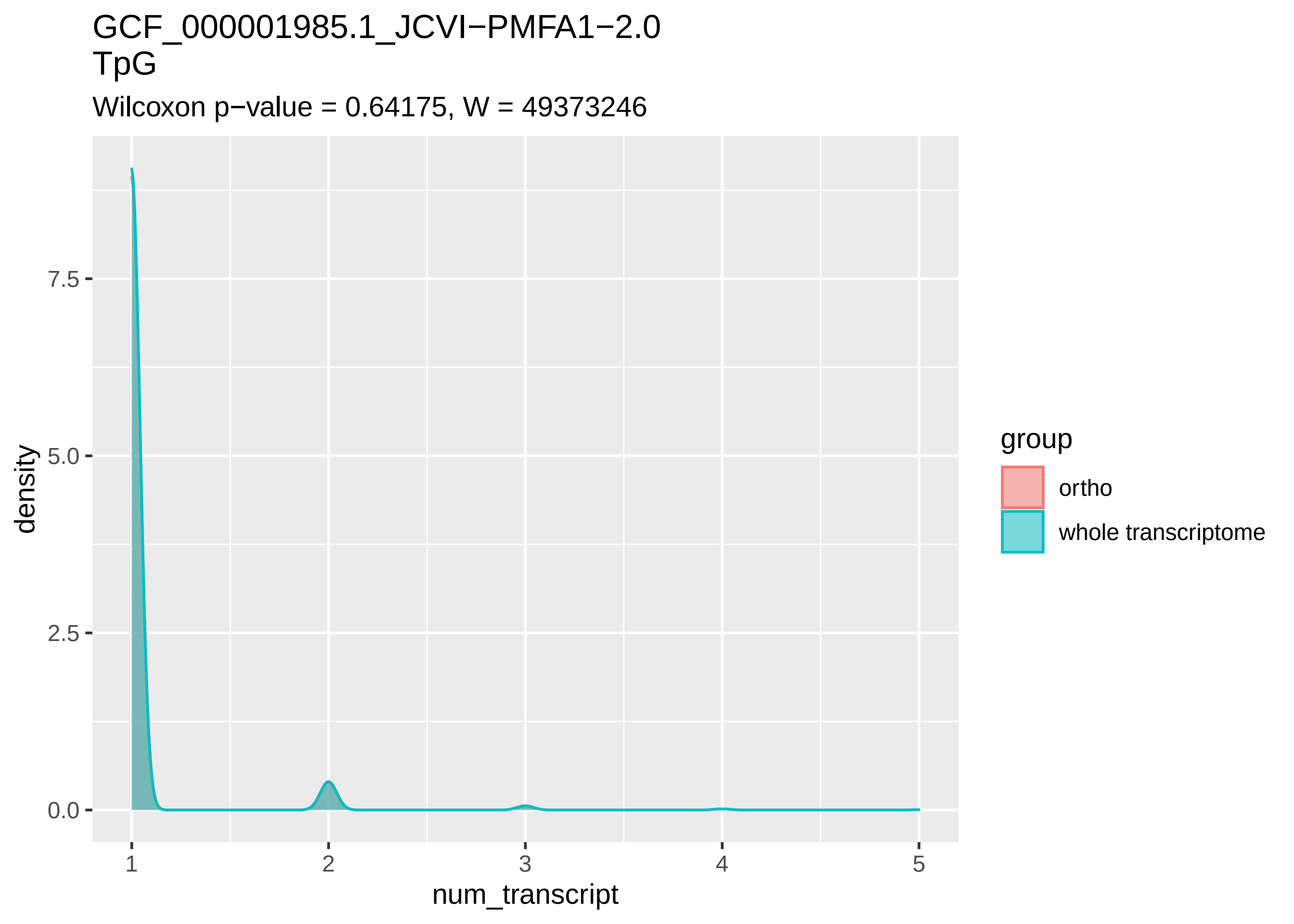
GCF_000001985.1_JCVI−PMFA1−2.0
TpG
Wilcoxon p−value = 0.64175, W = 49373246
density
7.5
5.0
2.5
0.0
1
2
3
4
5
num_transcript
group
ortho
whole transcriptome

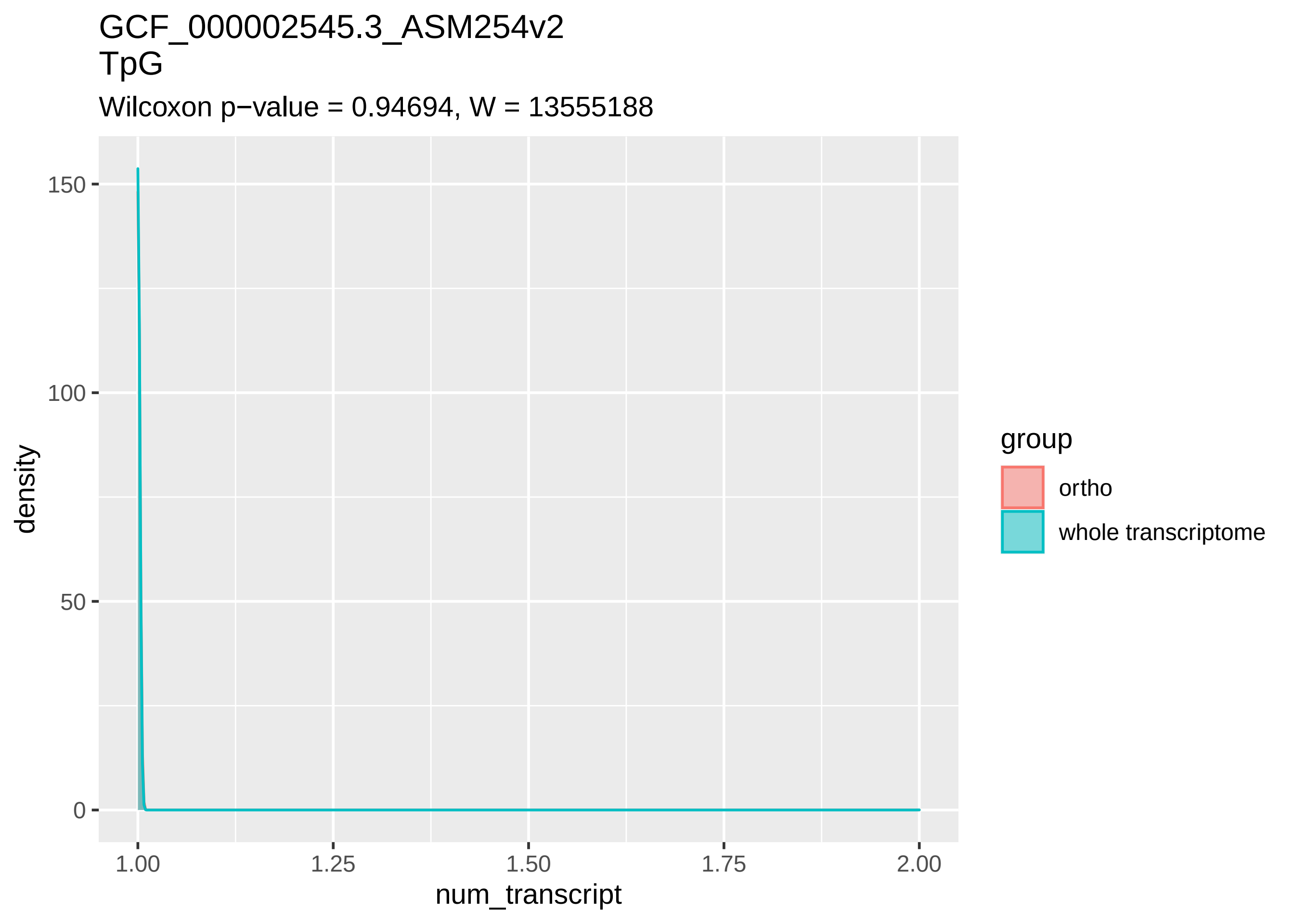

GCF_000002545.3_ASM254v2
TpG
Wilcoxon p−value = 0.94694, W = 13555188
150
100
50
0
1.00
1.25
1.50
1.75
2.00
num_transcript
density
group
ortho
whole transcriptome

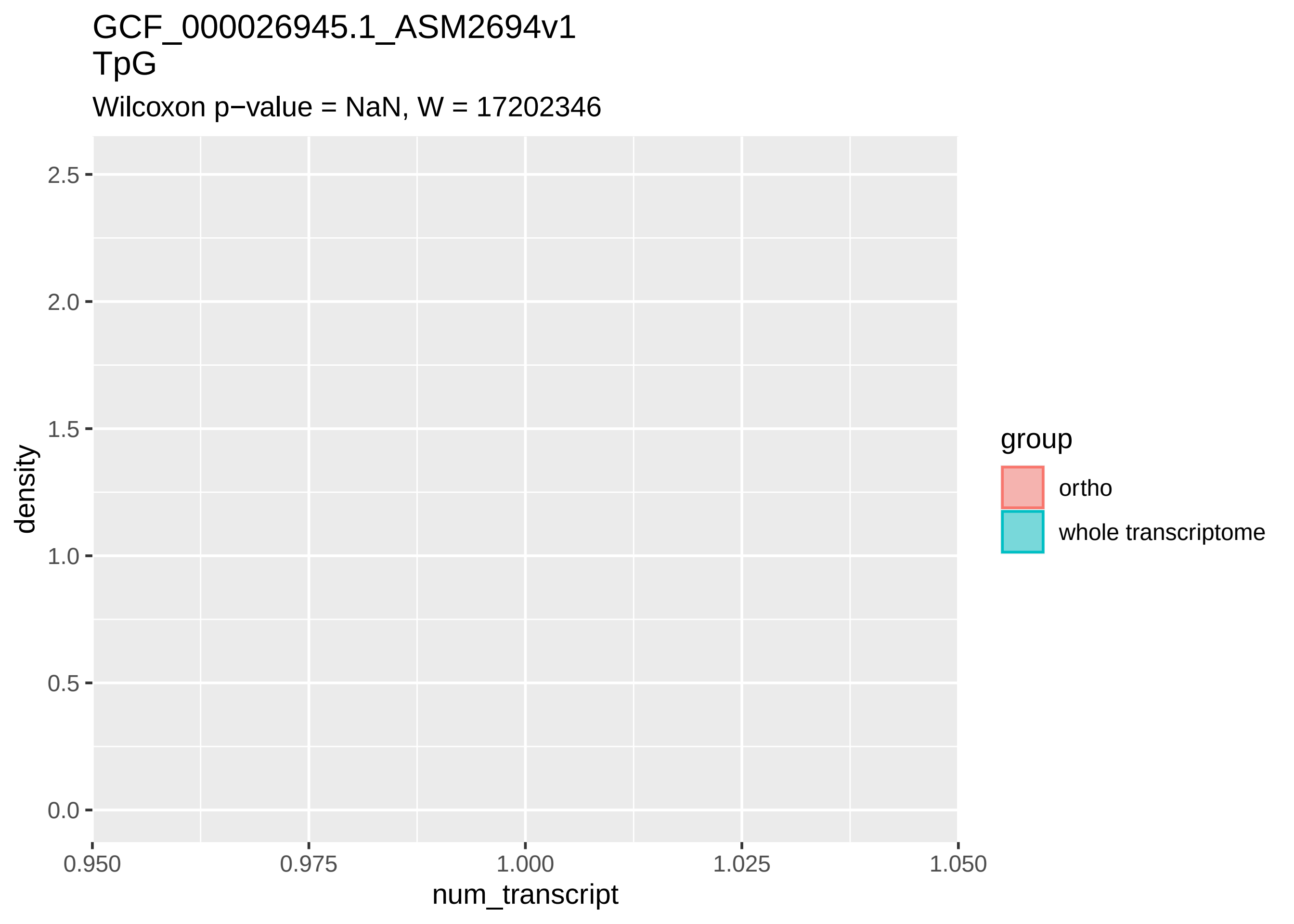
GCF_000026945.1_ASM2694v1
TpG
Wilcoxon p−value = NaN, W = 17202346
density
2.5
2.0
1.5
1.0
0.5
0.0
0.950
0.975
1.000
1.025
1.050
num_transcript
group
ortho
whole transcriptome

GCF_000091045.1_ASM9104v1
TpG

Wilcoxon p−value = 0.8188, W = 22024156

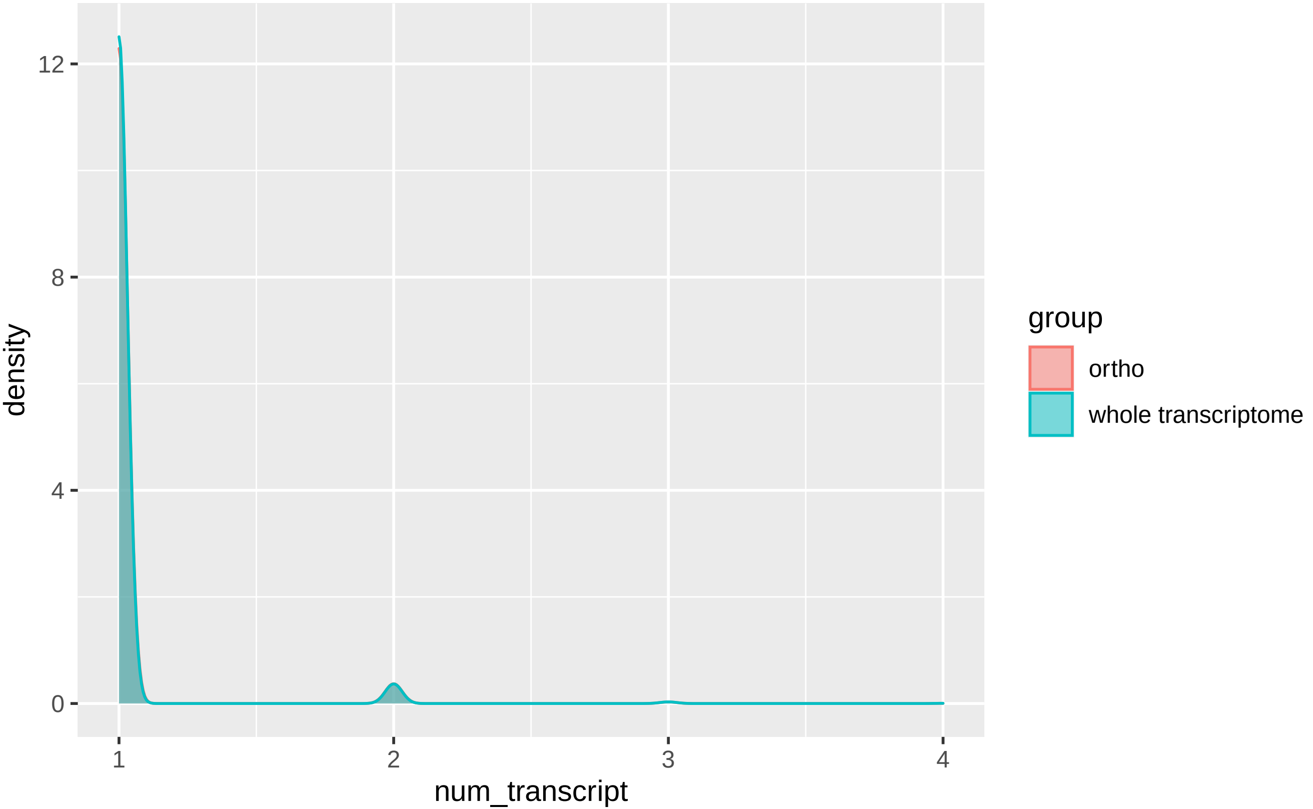

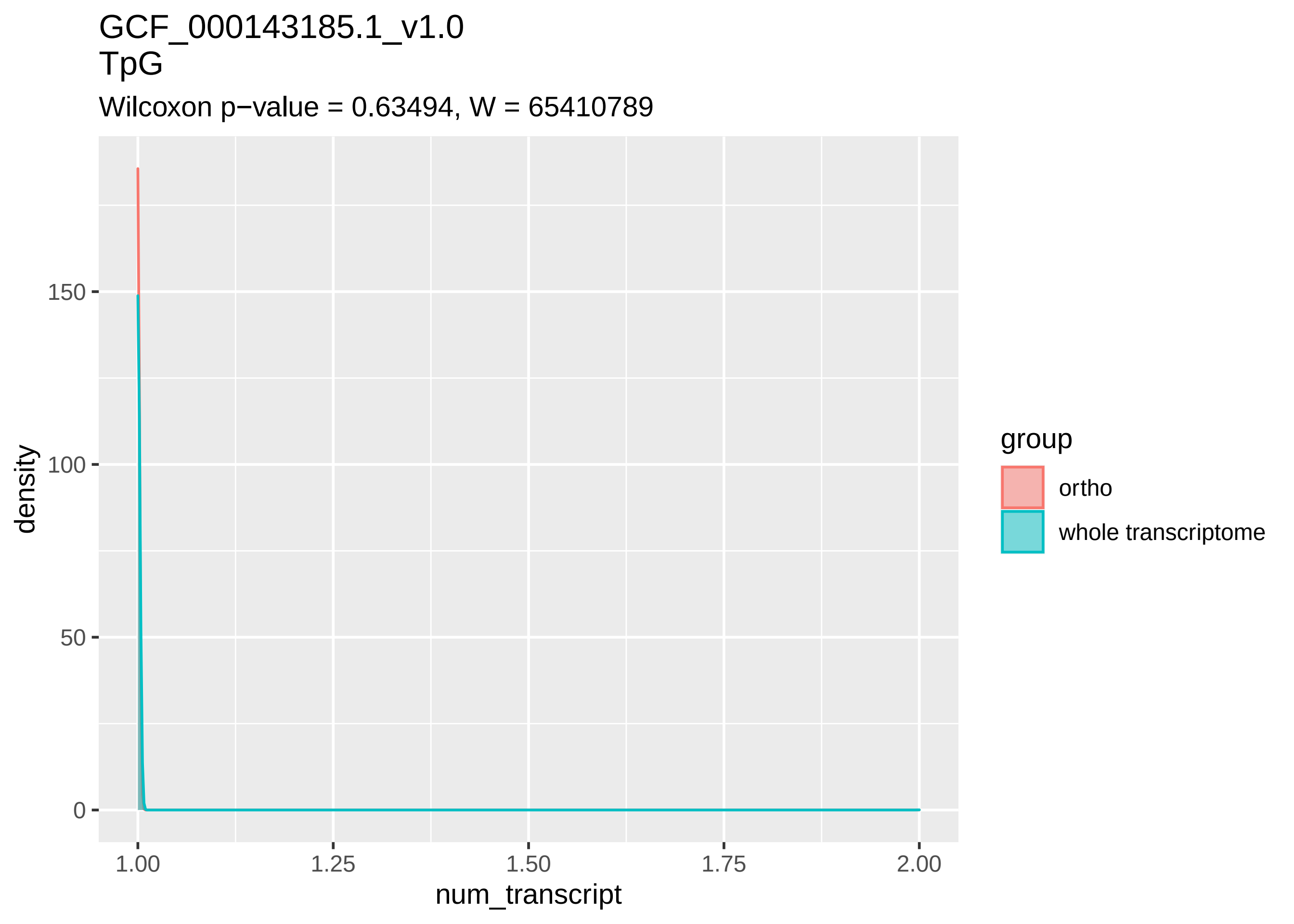
GCF_000143185.1_v1.0
TpG
Wilcoxon p−value = 0.63494, W = 65410789
density
150
100
50
0
1.00
1.25
1.50
1.75
2.00
num_transcript
group
ortho
whole transcriptome

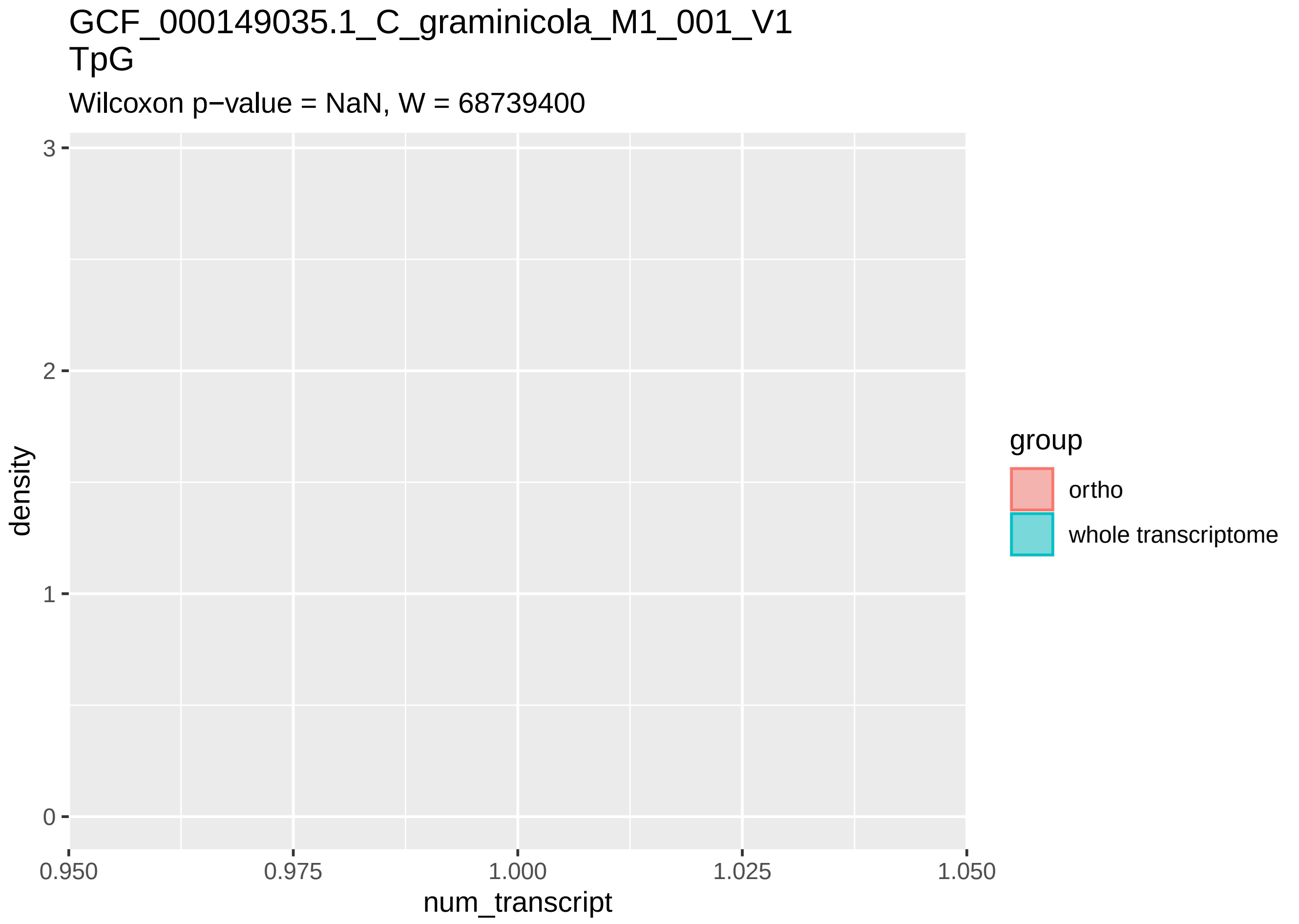
GCF_000149035.1_C_graminicola_M1_001_V1
TpG
Wilcoxon p−value = NaN, W = 68739400
density
num_transcript
0
1
2
3
0.950
0.975
1.000
1.025
1.050
group
ortho
whole transcriptome

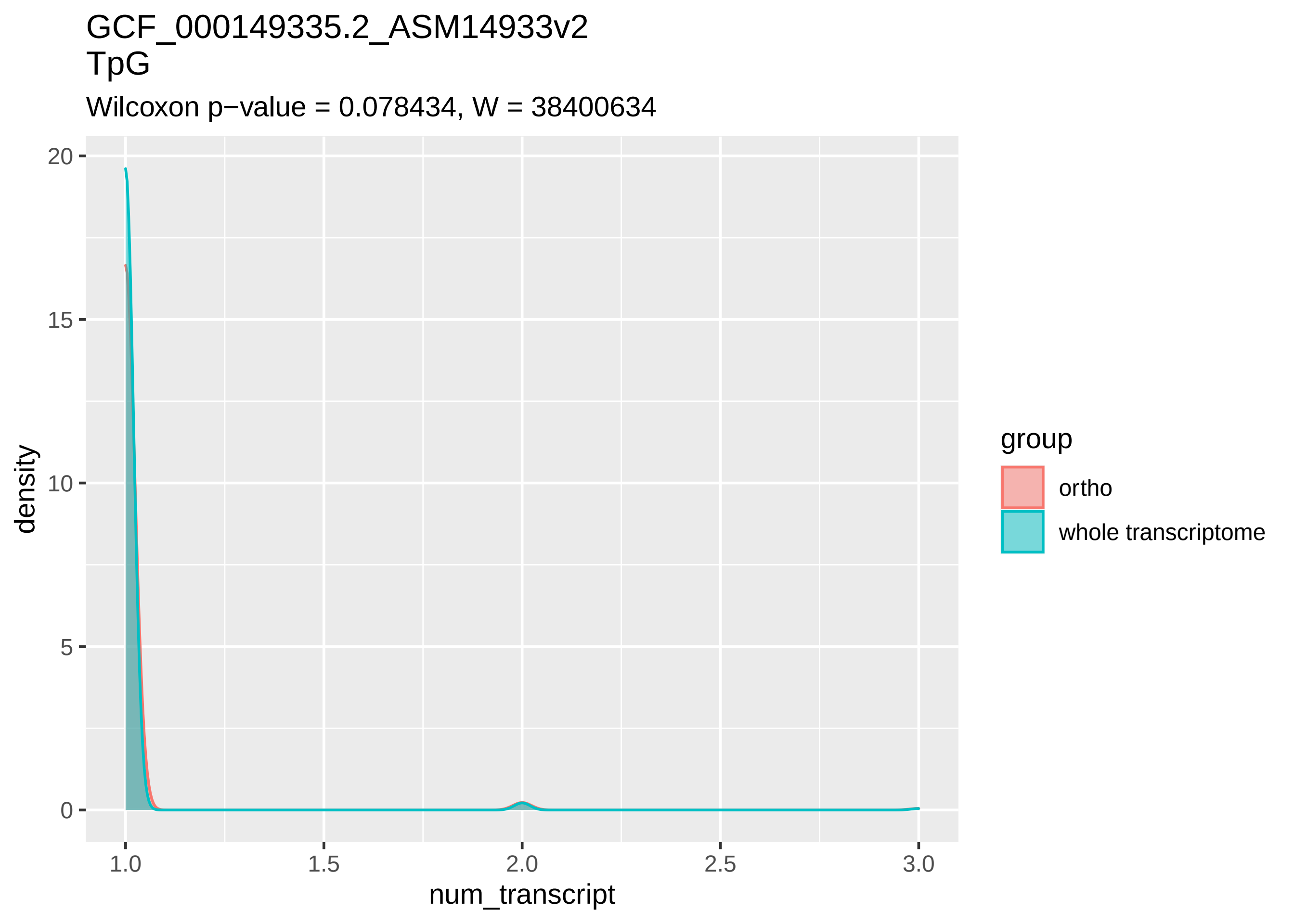
GCF_000149335.2_ASM14933v2
TpG
Wilcoxon p−value = 0.078434, W = 38400634
density
20
15
10
5
0
1.0
1.5
2.0
2.5
3.0
num_transcript
group
ortho
whole transcriptome

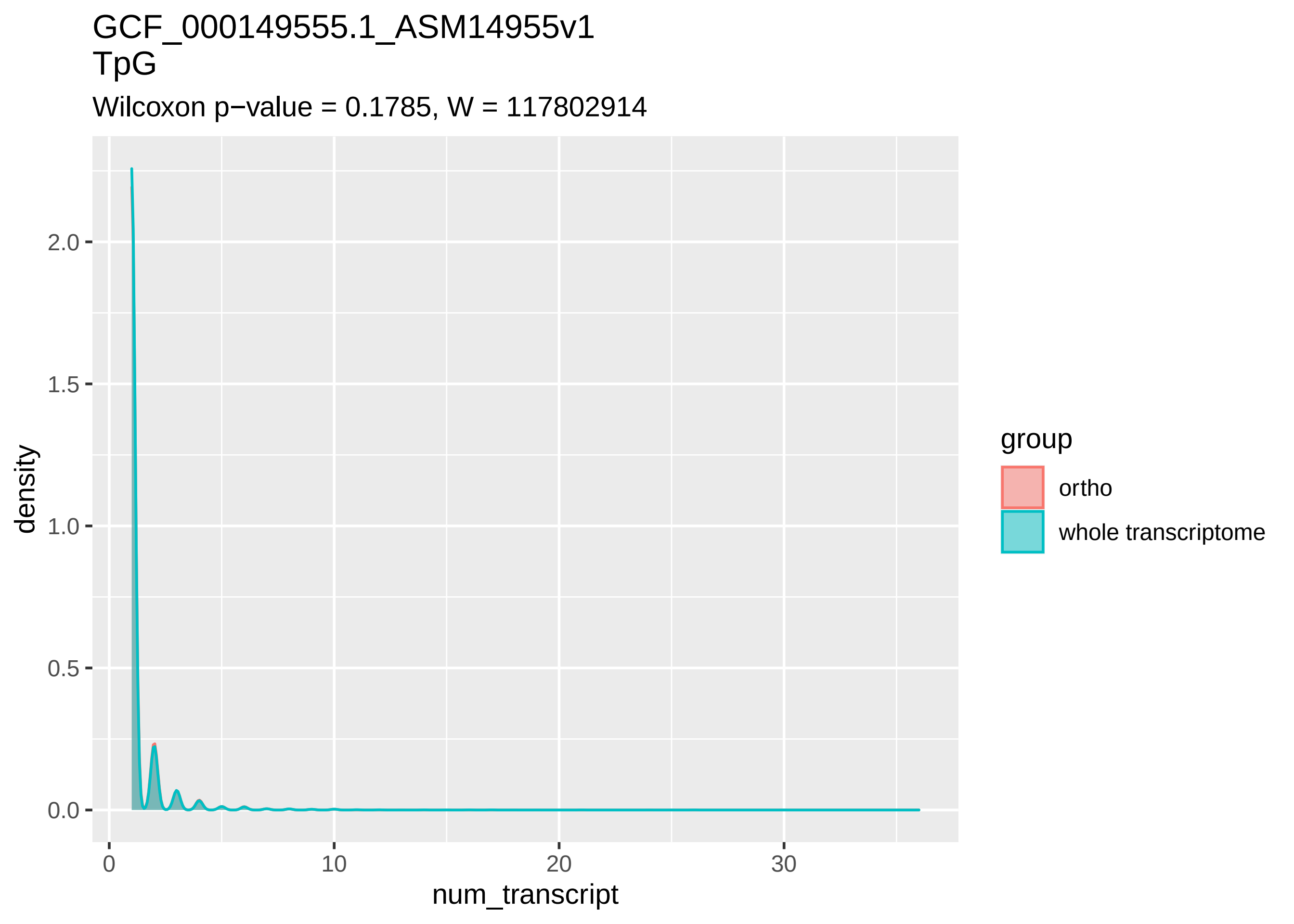
GCF_000149555.1_ASM14955v1
TpG
Wilcoxon p−value = 0.1785, W = 117802914
density
2.0
1.5
1.0
0.5
0.0
0
10
20
30
num_transcript
group
ortho
whole transcriptome

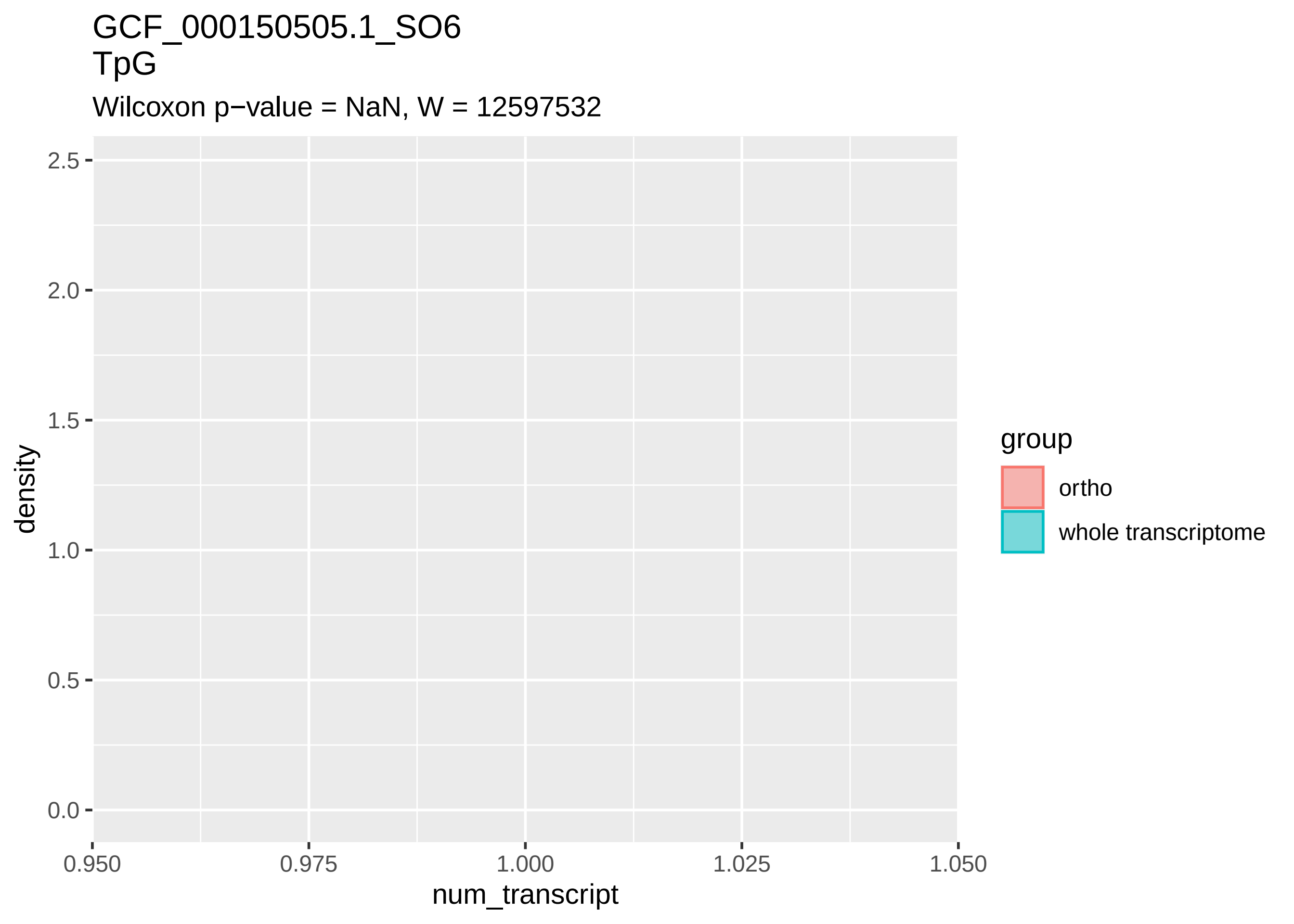

GCF_000150505.1_SO6
TpG
Wilcoxon p−value = NaN, W = 12597532
density
2.5
2.0
1.5
1.0
0.5
0.0
0.950
0.975
1.000
1.025
1.050
num_transcript
group
ortho
whole transcriptome

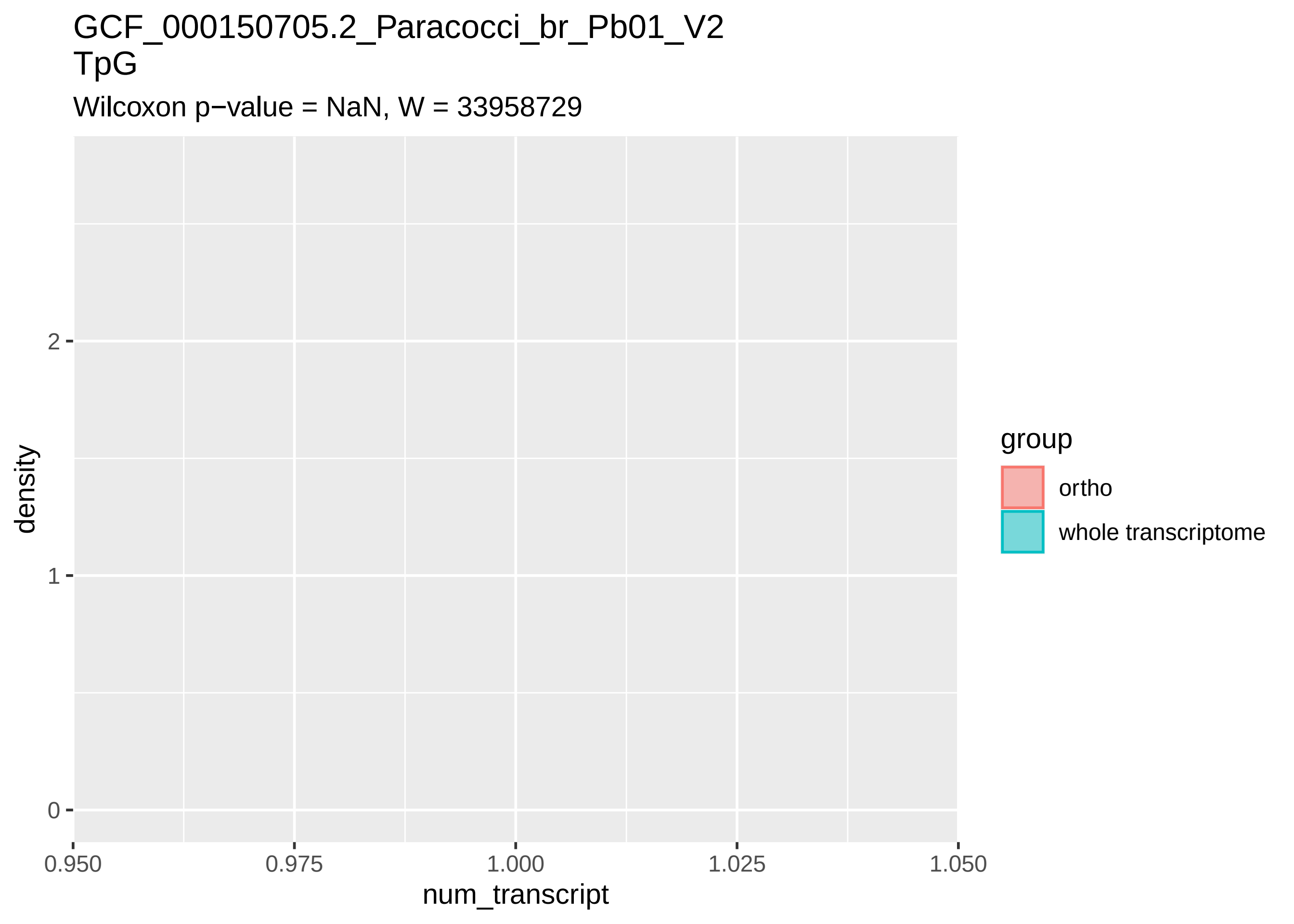
GCF_000150705.2_Paracocci_br_Pb01_V2
TpG
Wilcoxon p−value = NaN, W = 33958729
density
2
1
0
0.950
0.975
1.000
1.025
1.050
num_transcript
group
ortho
whole transcriptome

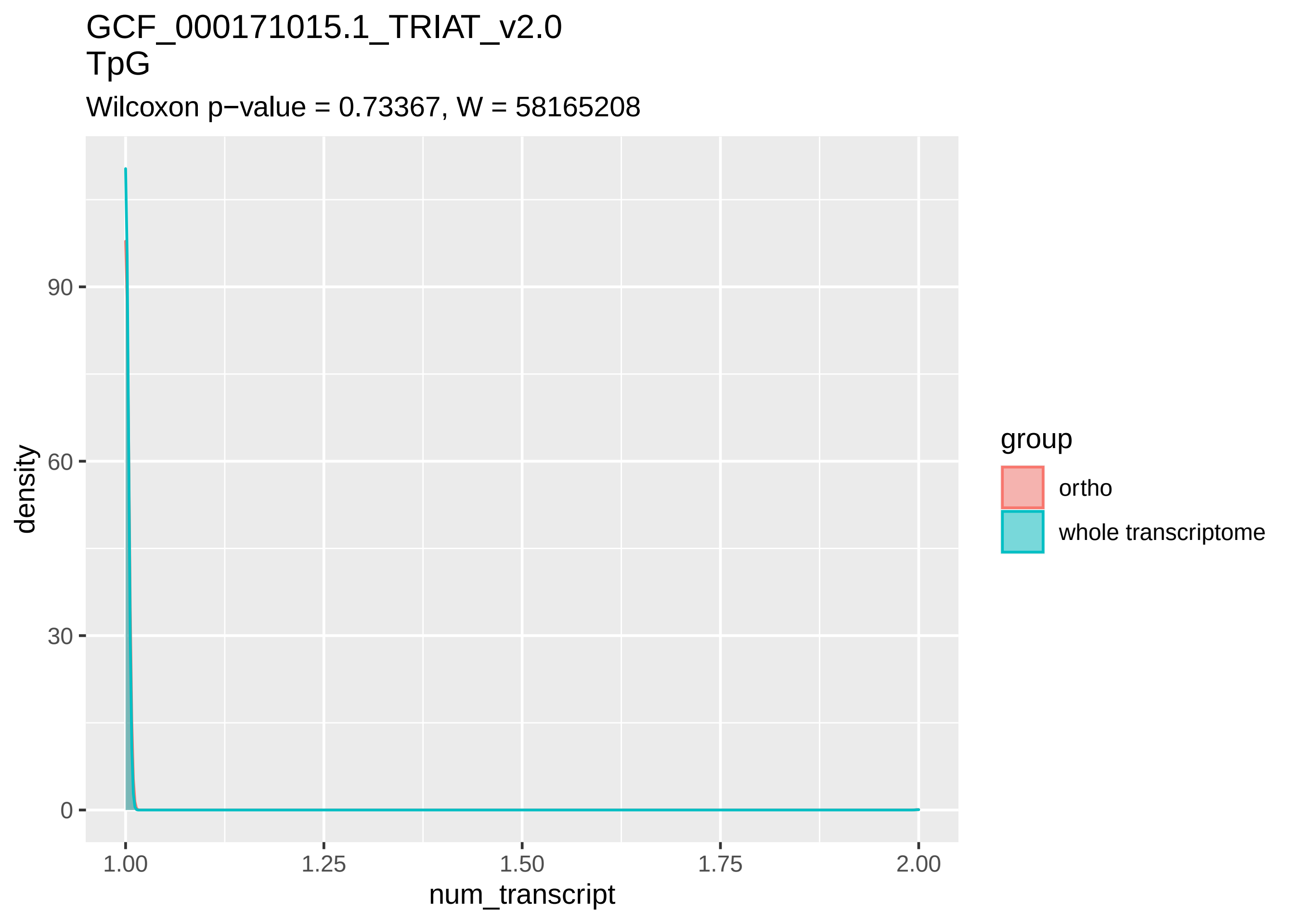

GCF_000171015.1_TRIAT_v2.0
TpG
Wilcoxon p−value = 0.73367, W = 58165208
density
90
60
30
0
1.00
1.25
1.50
1.75
2.00
num_transcript
group
ortho
whole transcriptome

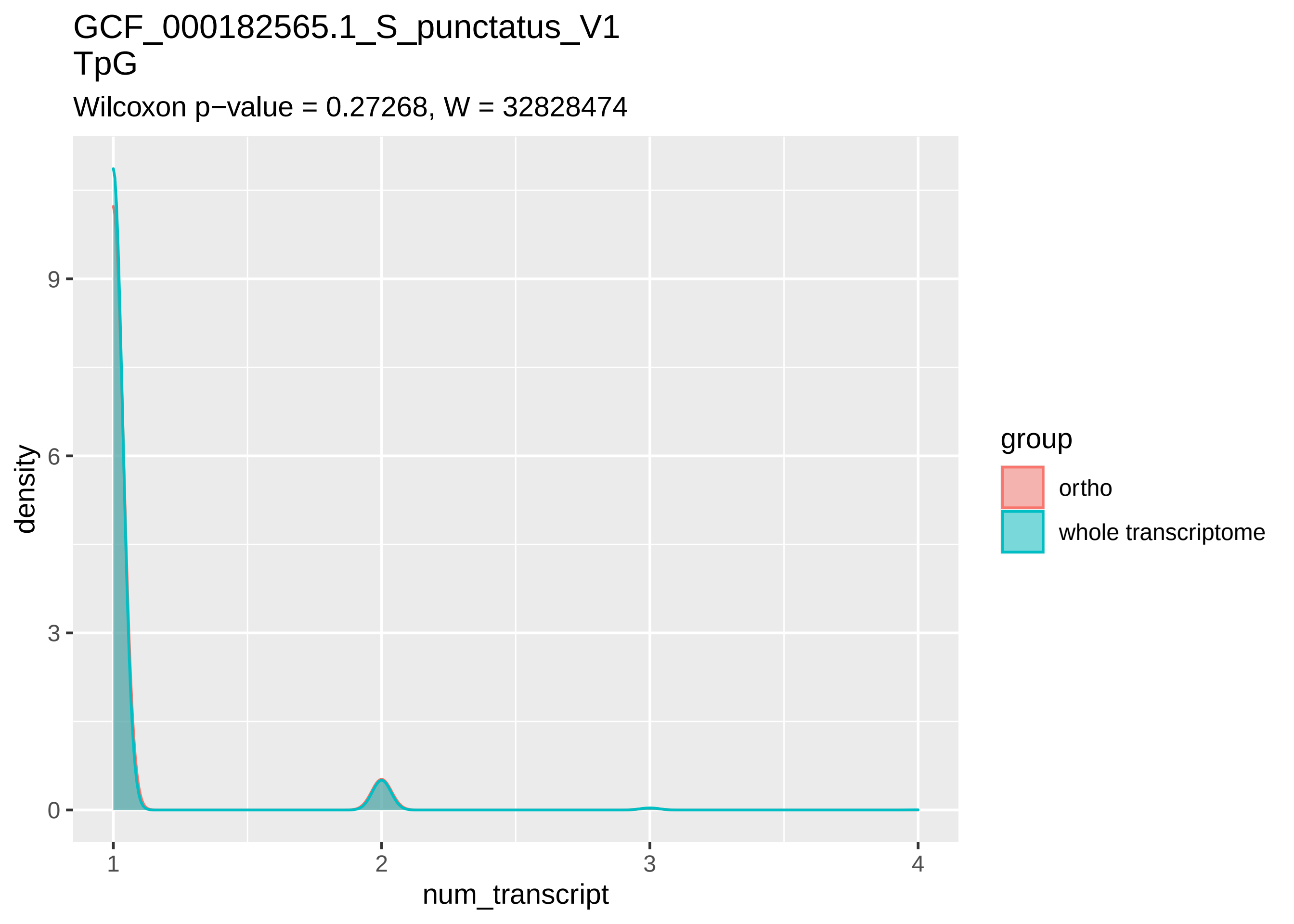
GCF_000182565.1_S_punctatus_V1
TpG
Wilcoxon p−value = 0.27268, W = 32828474
density
9
6
3
0
1
2
3
4
num_transcript
group
ortho
whole transcriptome

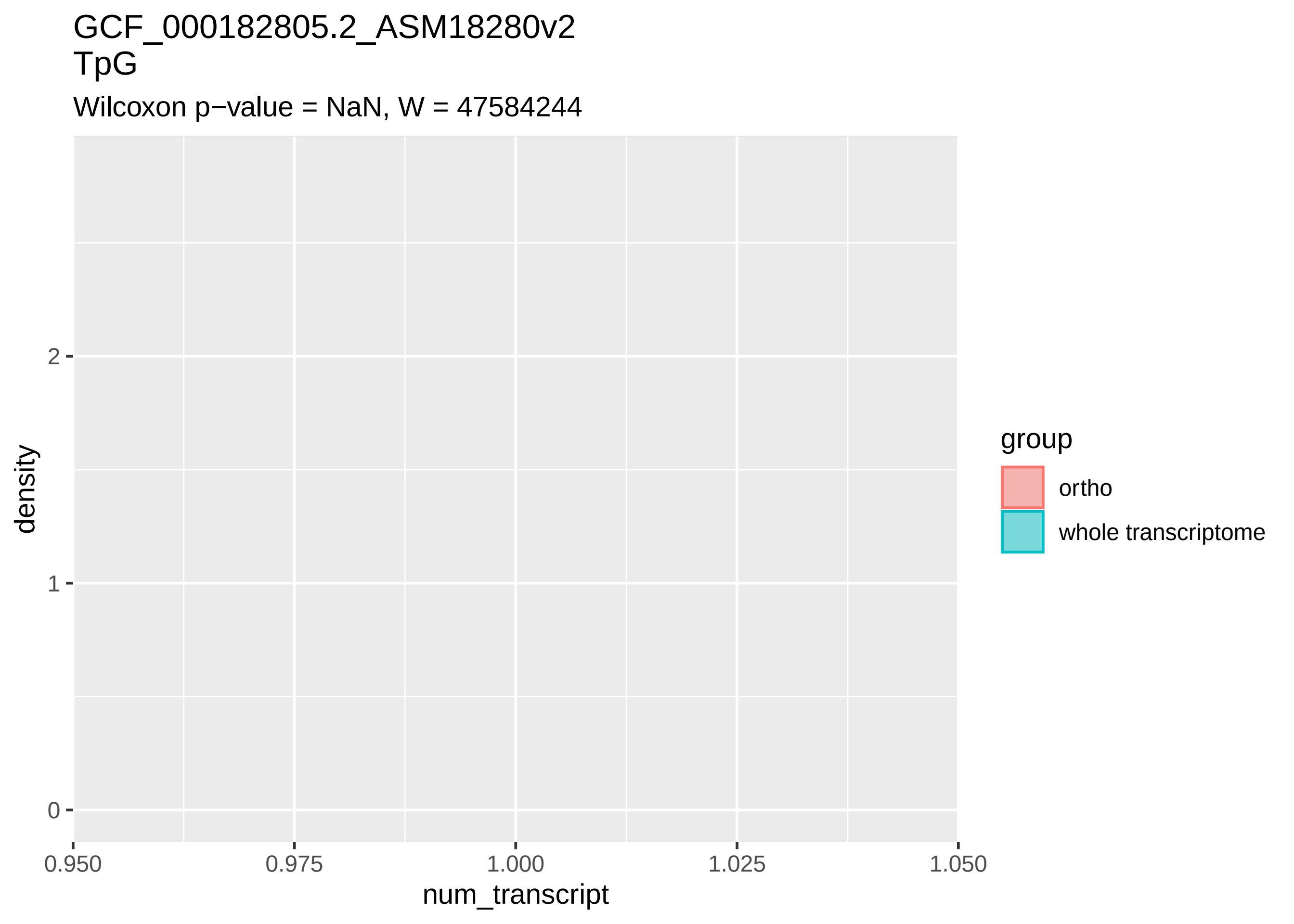

GCF_000182805.2_ASM18280v2
TpG
Wilcoxon p−value = NaN, W = 47584244
density
2
1
0
0.950
0.975
1.000
1.025
1.050
num_transcript
group
ortho
whole transcriptome

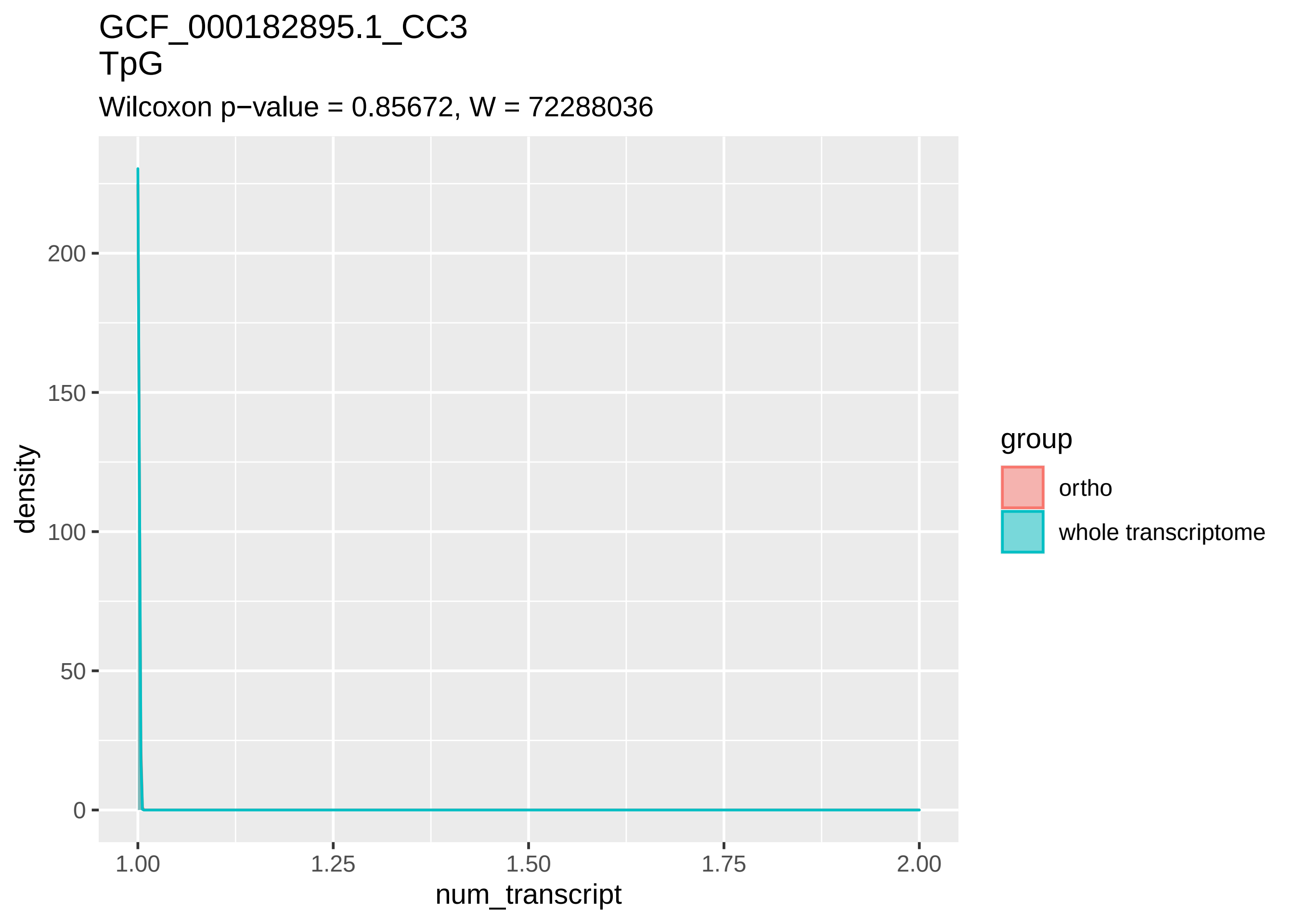
GCF_000182895.1_CC3
TpG
Wilcoxon p−value = 0.85672, W = 72288036
density
200
150
100
50
0
1.00
1.25
1.50
1.75
2.00
num_transcript
group
ortho
whole transcriptome

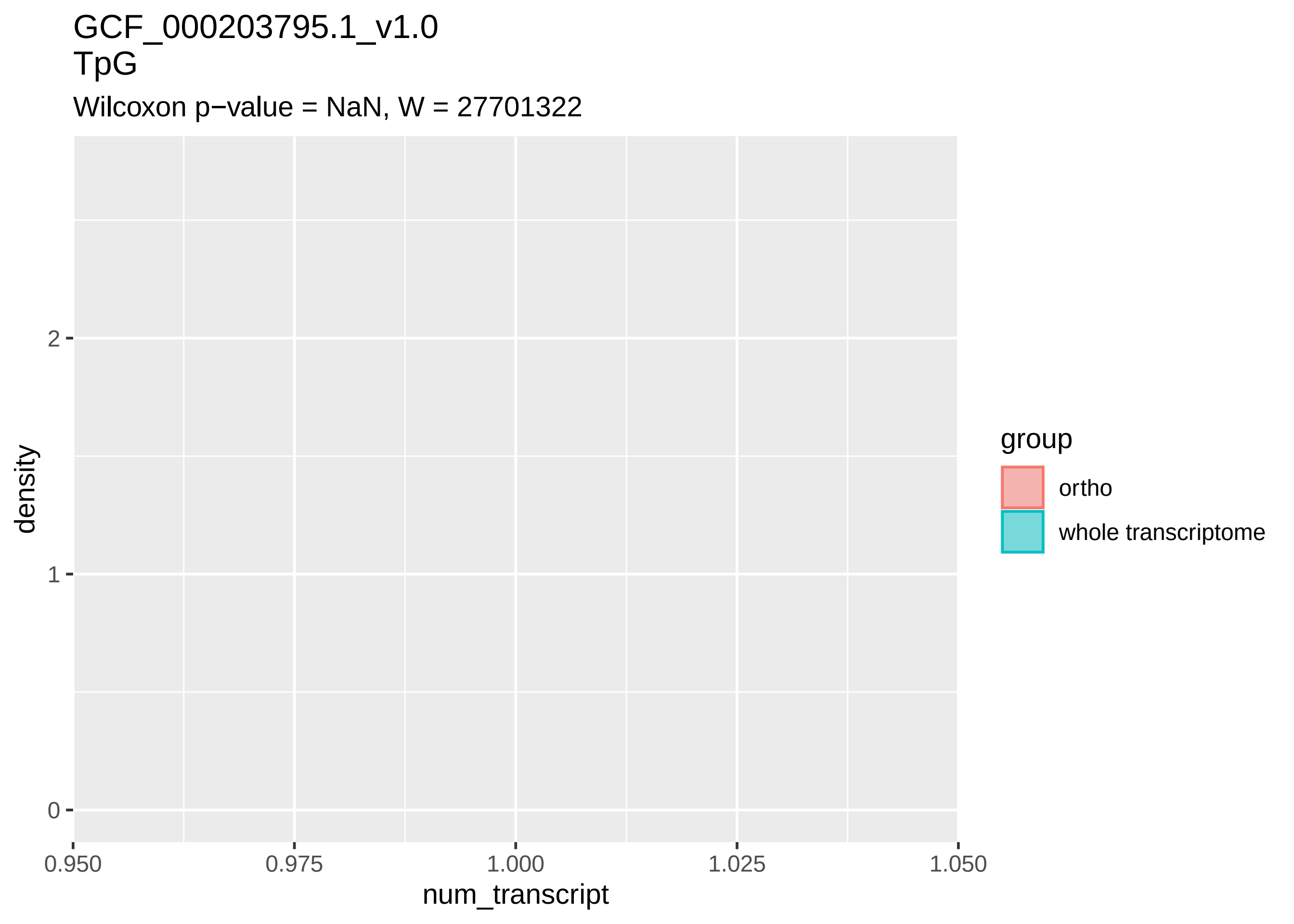

GCF_000203795.1_v1.0
TpG
Wilcoxon p−value = NaN, W = 27701322
density
2
1
0
0.950
0.975
1.000
1.025
1.050
num_transcript
group
ortho
whole transcriptome

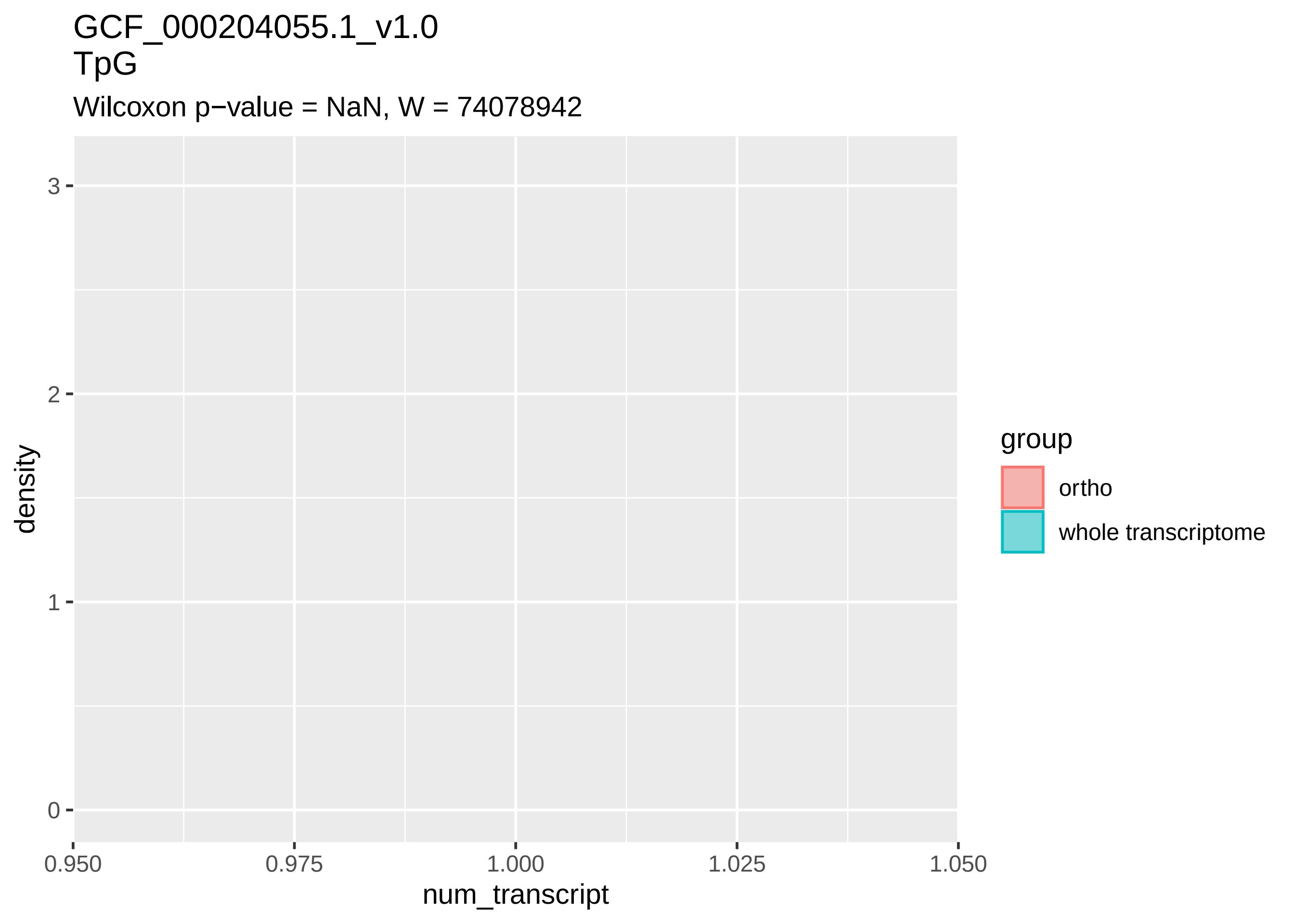
GCF_000204055.1_v1.0
TpG
Wilcoxon p−value = NaN, W = 74078942
density
3
2
1
0
0.950
0.975
1.000
1.025
1.050
num_transcript
group
ortho
whole transcriptome

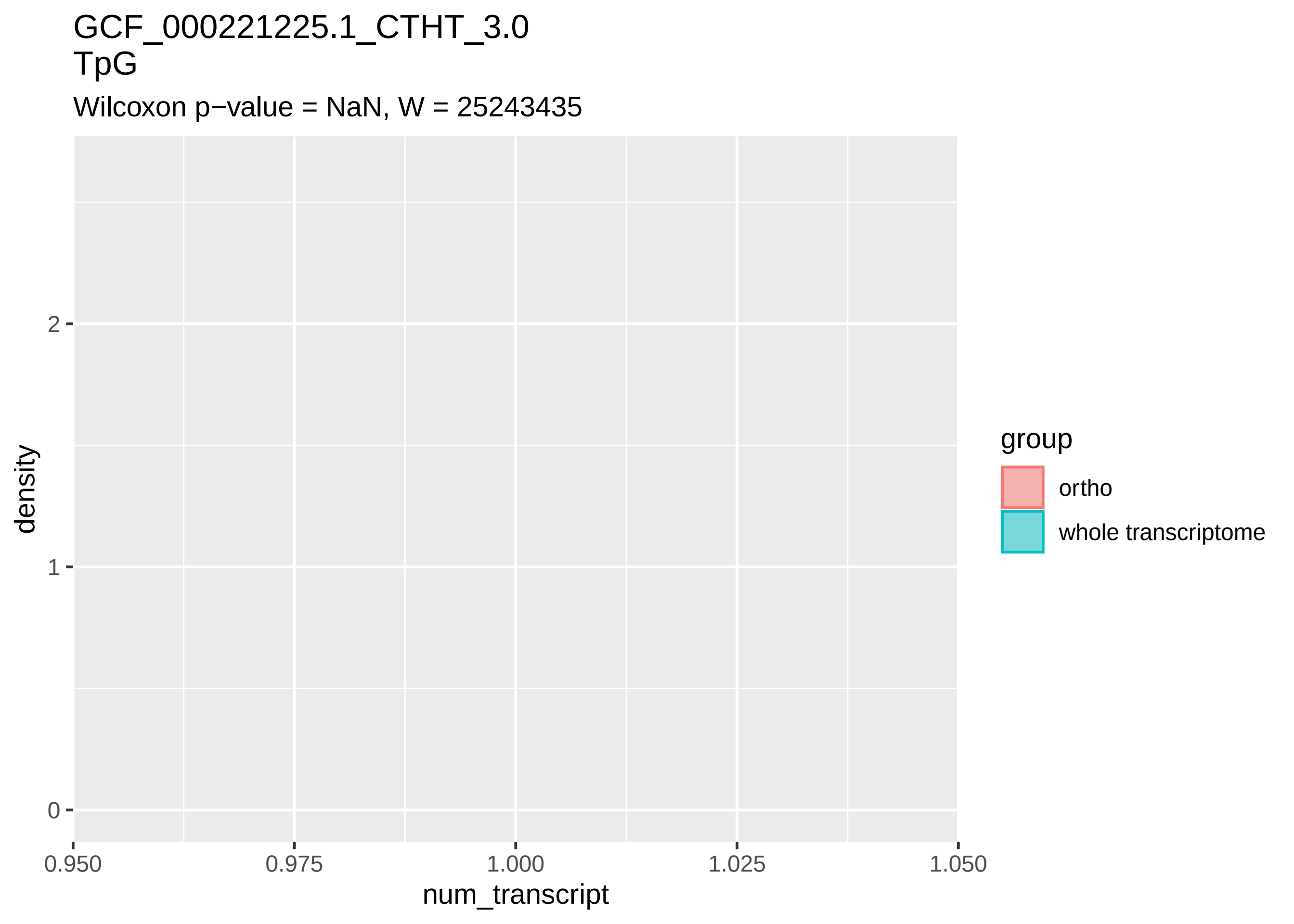
GCF_000221225.1_CTHT_3.0
TpG
Wilcoxon p−value = NaN, W = 25243435
density
2
1
0
0.950
0.975
1.000
1.025
1.050
num_transcript
group
ortho
whole transcriptome

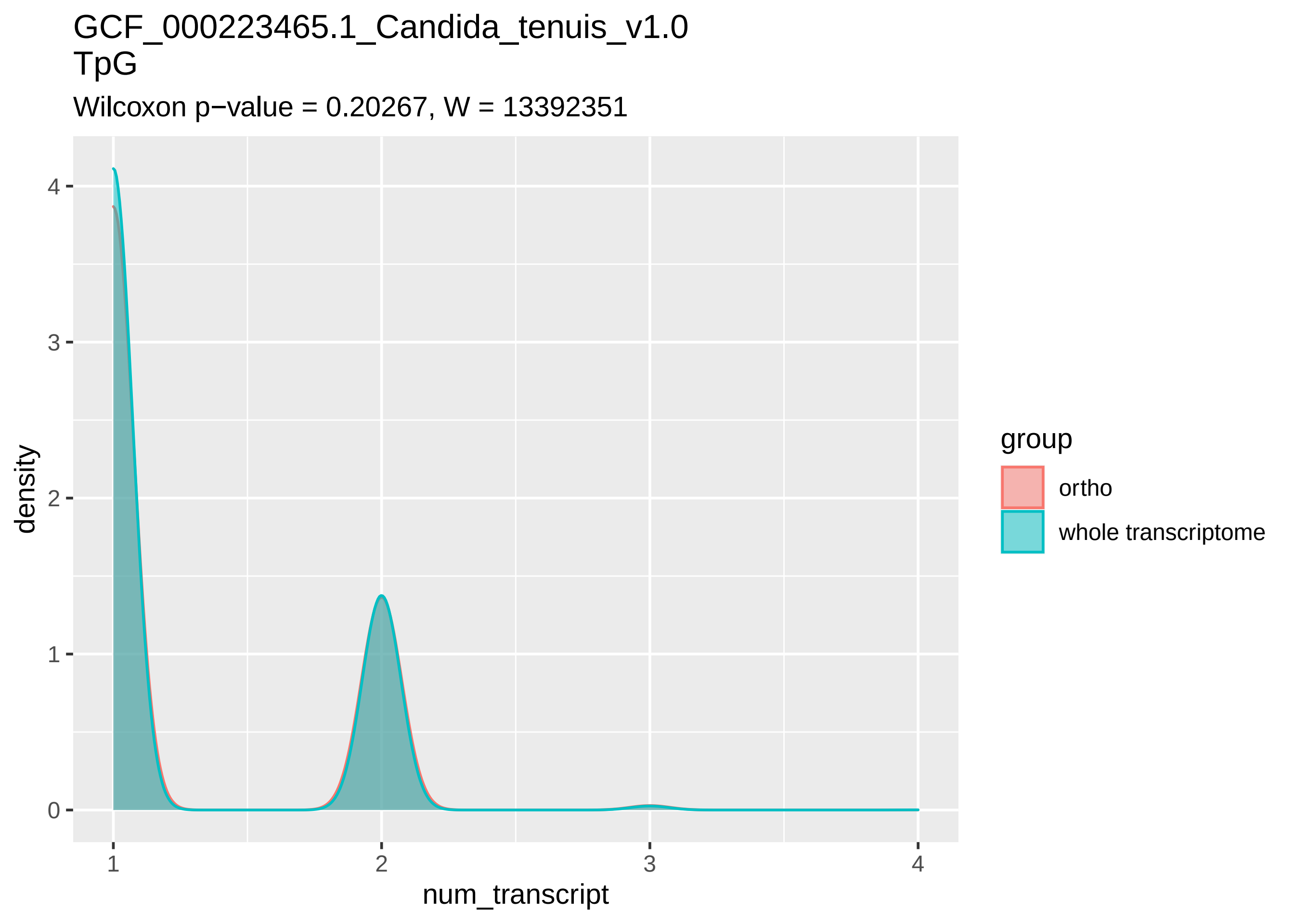
GCF_000223465.1_Candida_tenuis_v1.0
TpG
Wilcoxon p−value = 0.20267, W = 13392351
density
num_transcript
0
1
2
3
4
1
2
3
4
group
ortho
whole transcriptome

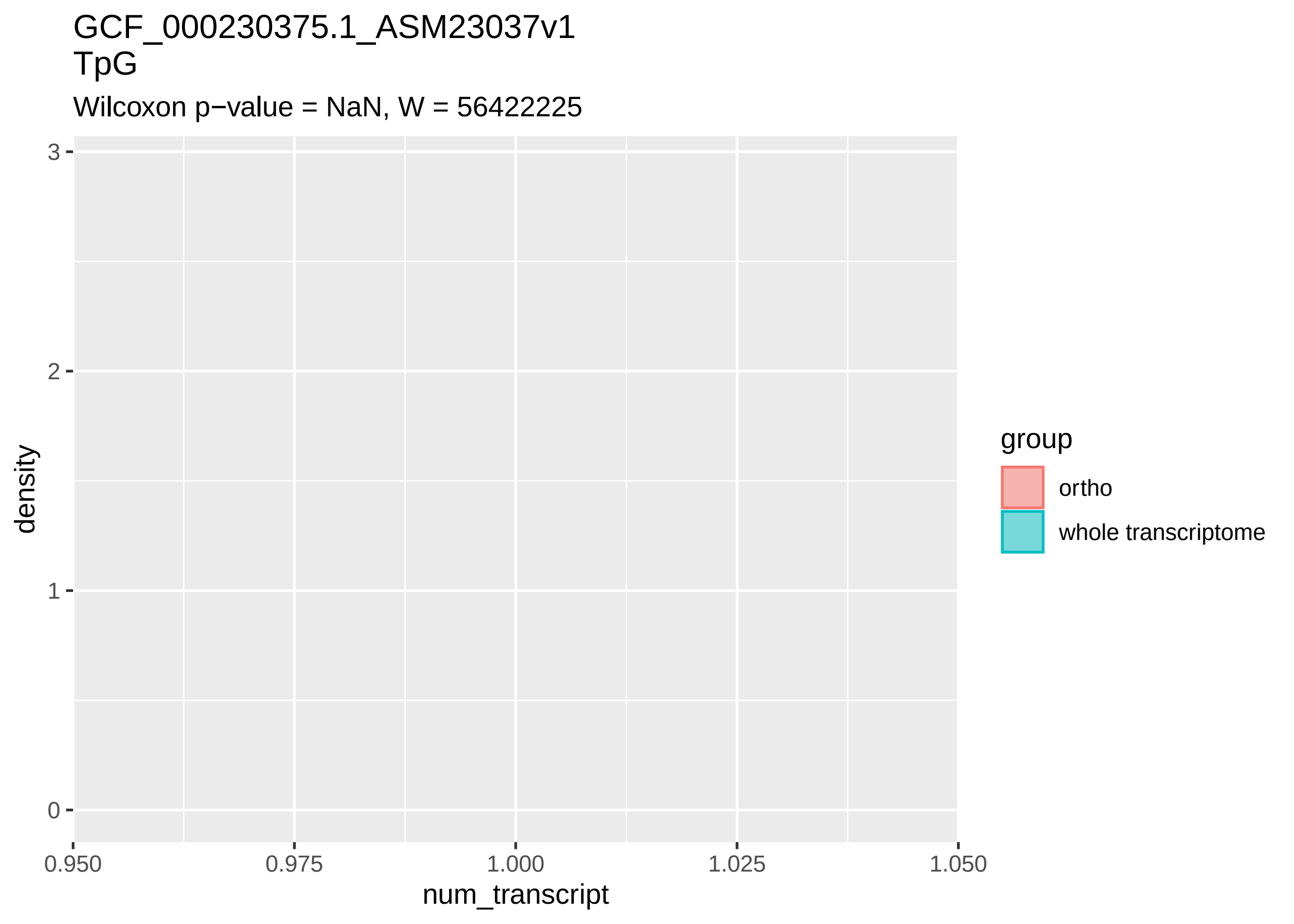
GCF_000230375.1_ASM23037v1
TpG
Wilcoxon p−value = NaN, W = 56422225
density
num_transcript
0
1
2
3
0.950
0.975
1.000
1.025
1.050
group
ortho
whole transcriptome

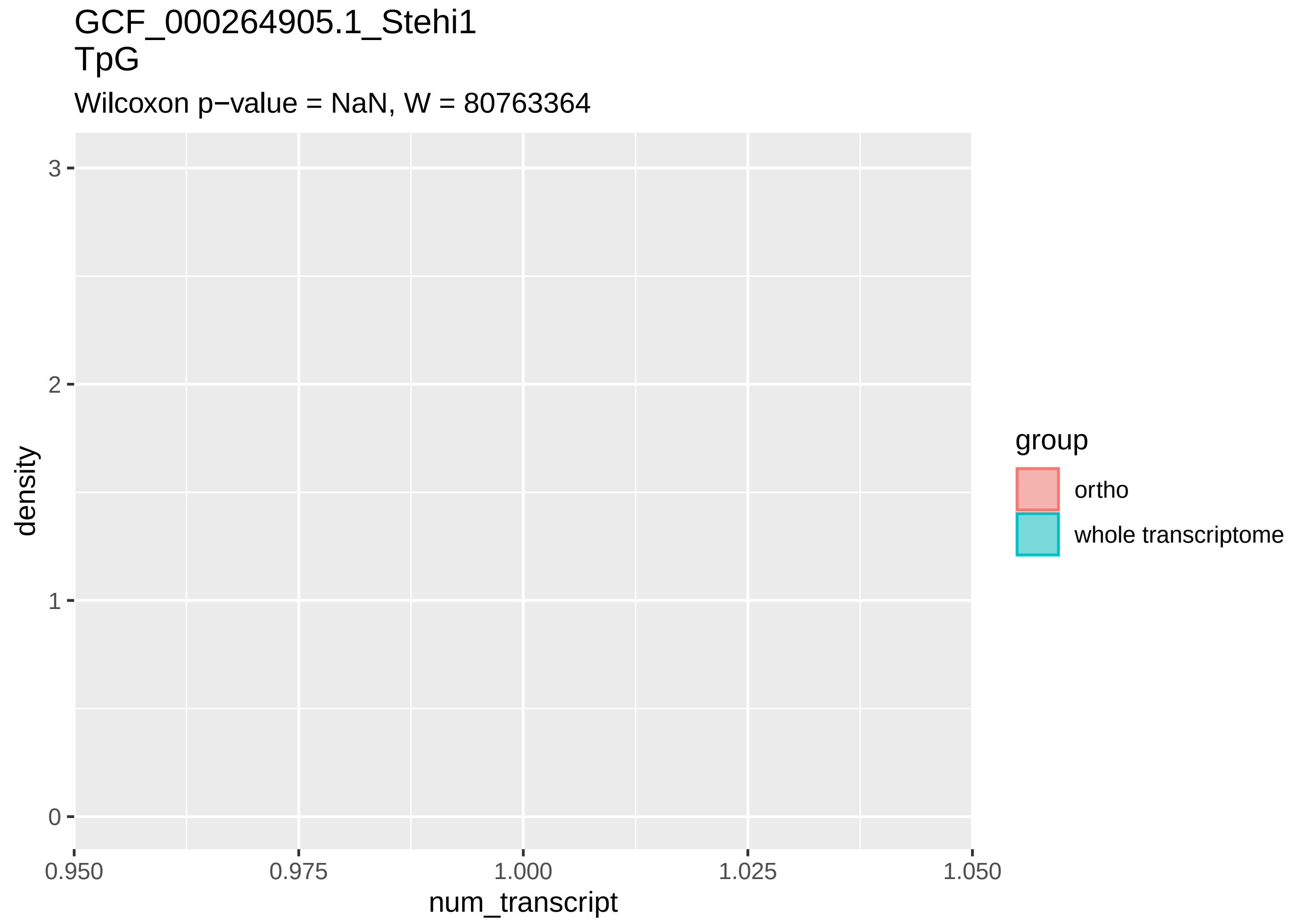
GCF_000264905.1_Stehi1
TpG
Wilcoxon p−value = NaN, W = 80763364
density
3
2
1
0
0.950
0.975
1.000
1.025
1.050
num_transcript
group
ortho
whole transcriptome

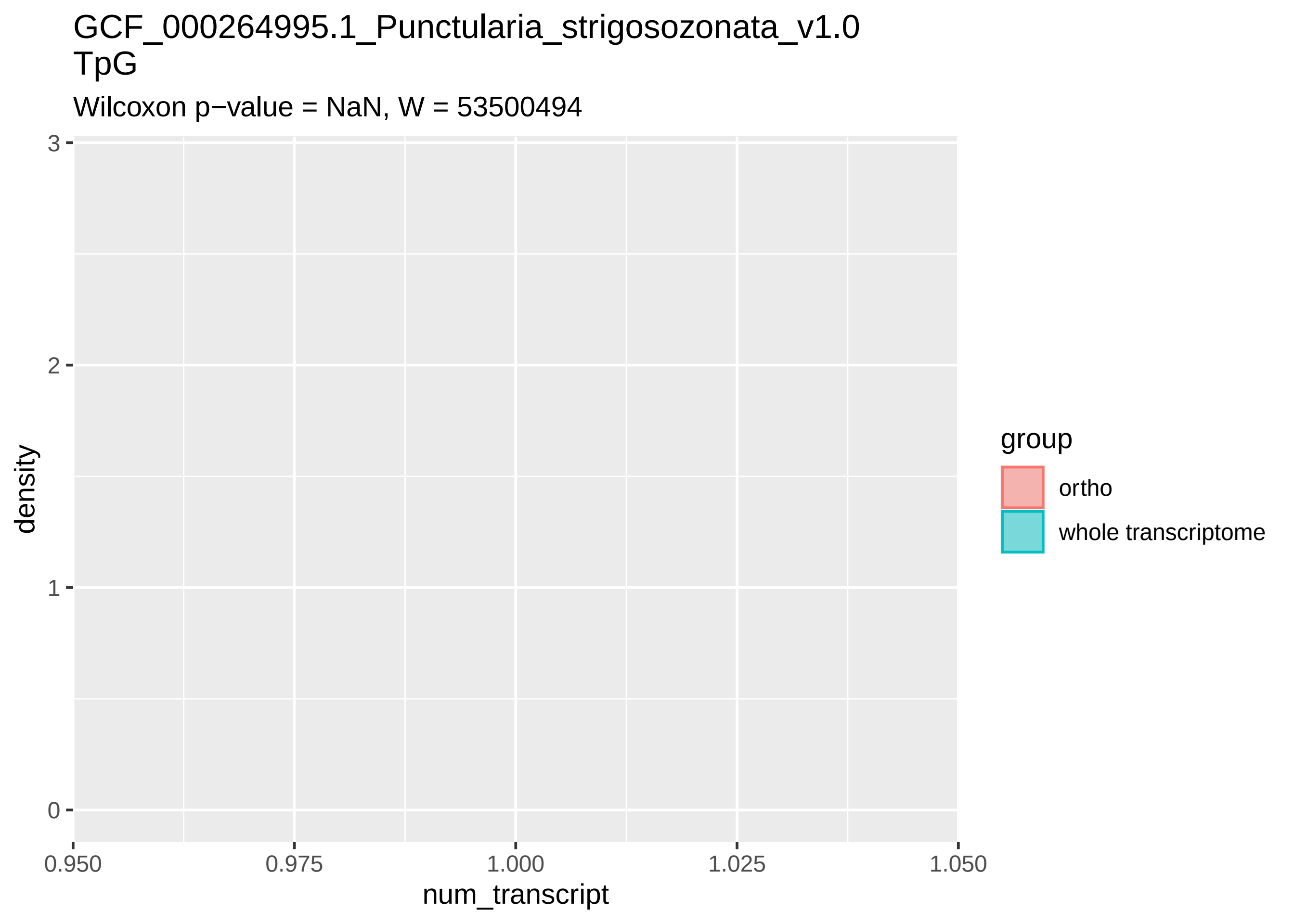

GCF_000264995.1_Punctularia_strigosozonata_v1.0
TpG
Wilcoxon p−value = NaN, W = 53500494
density
num_transcript
0
1
2
3
0.950
0.975
1.000
1.025
1.050
group
ortho
whole transcriptome

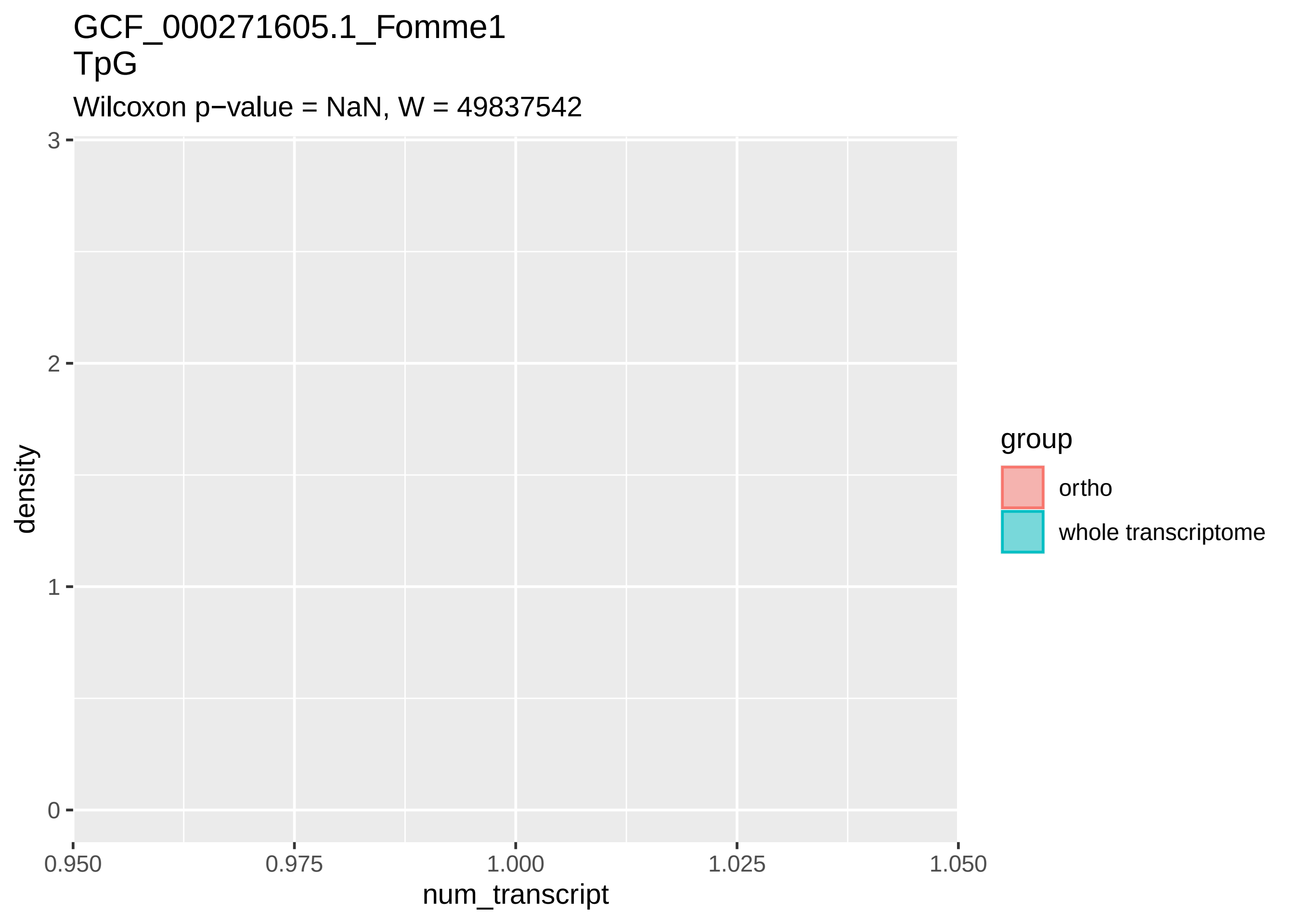
GCF_000271605.1_Fomme1
TpG
Wilcoxon p−value = NaN, W = 49837542
density
3
2
1
0
0.950
0.975
1.000
1.025
1.050
num_transcript
group
ortho
whole transcriptome

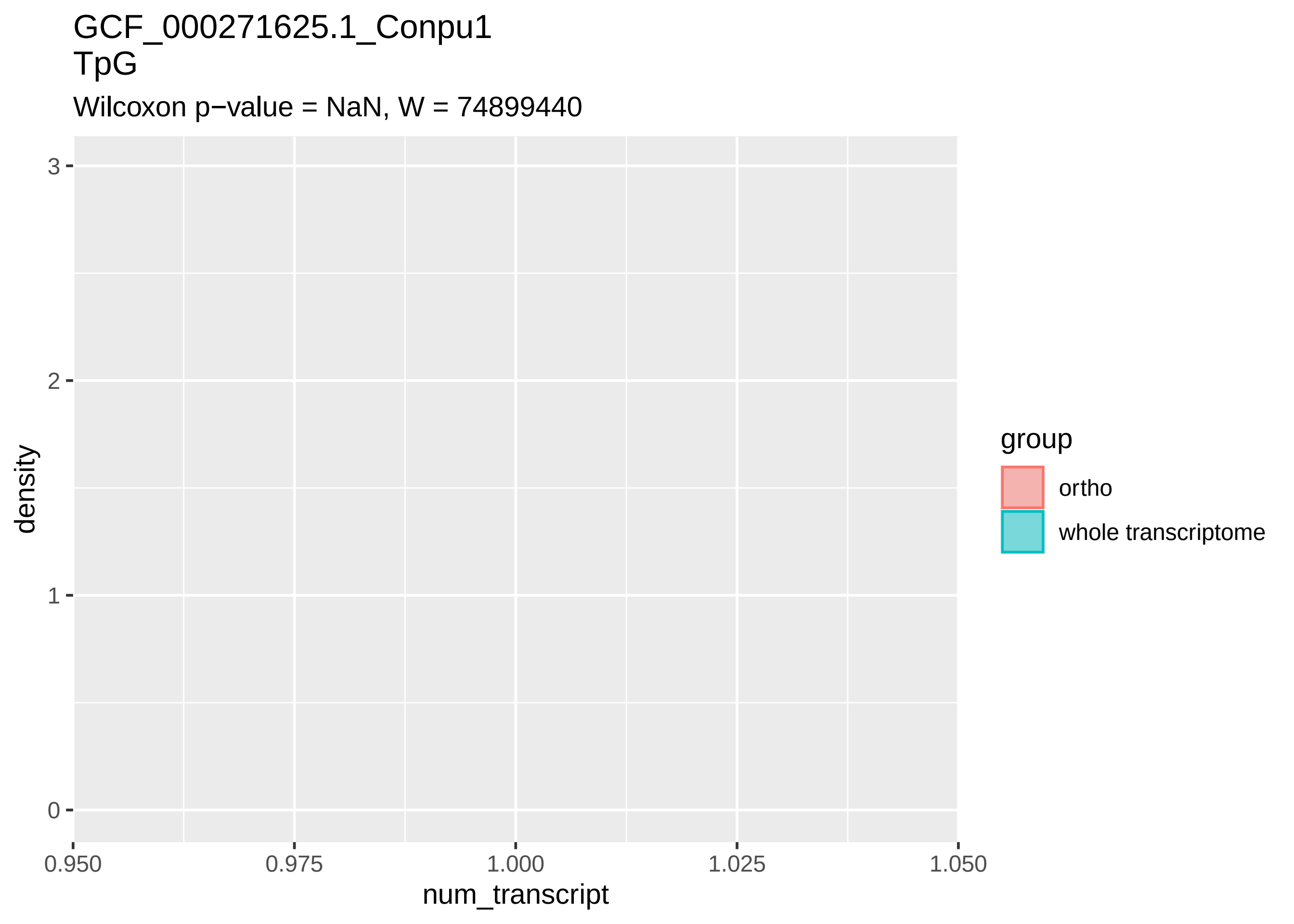
GCF_000271625.1_Conpu1
TpG
Wilcoxon p−value = NaN, W = 74899440
density
3
2
1
0
0.950
0.975
1.000
1.025
1.050
num_transcript
group
ortho
whole transcriptome

GCF_000271645.1_Treme1
TpG

Wilcoxon p−value = NaN, W = 23234184

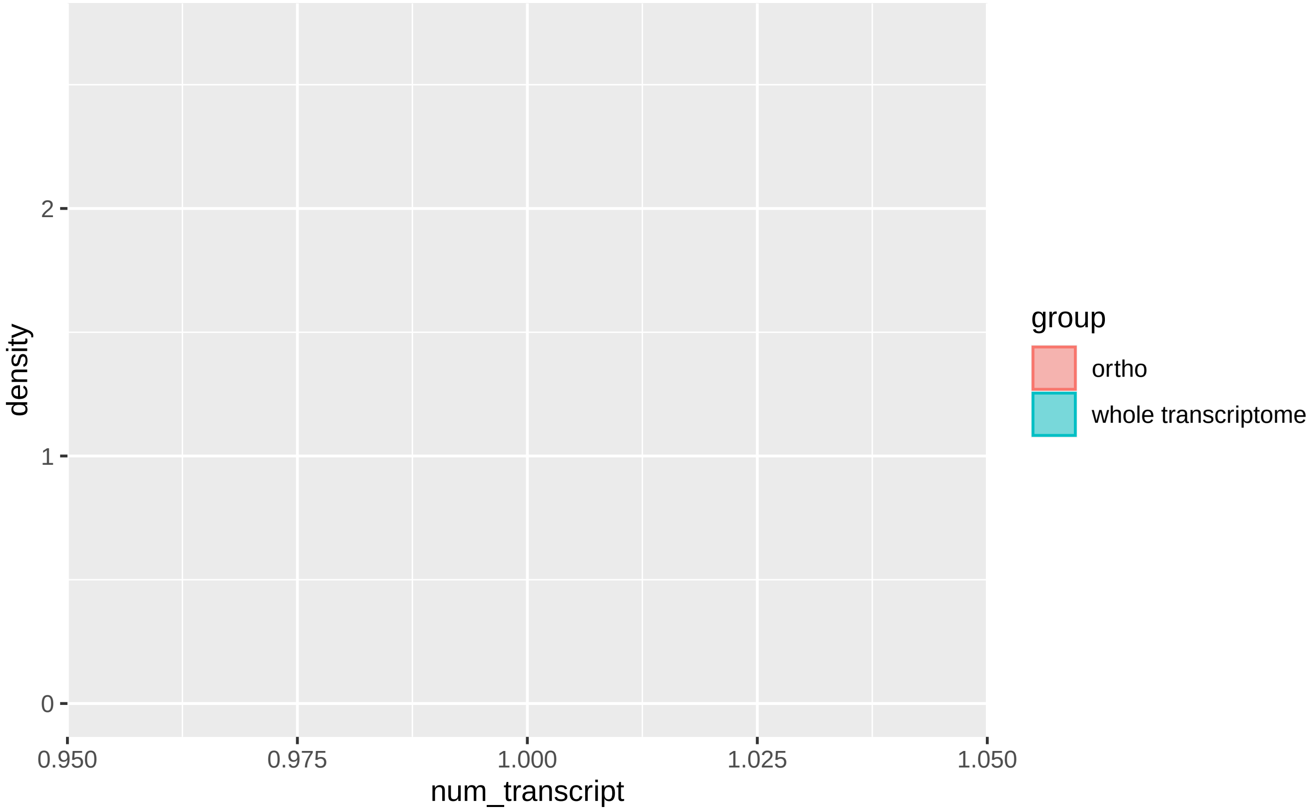

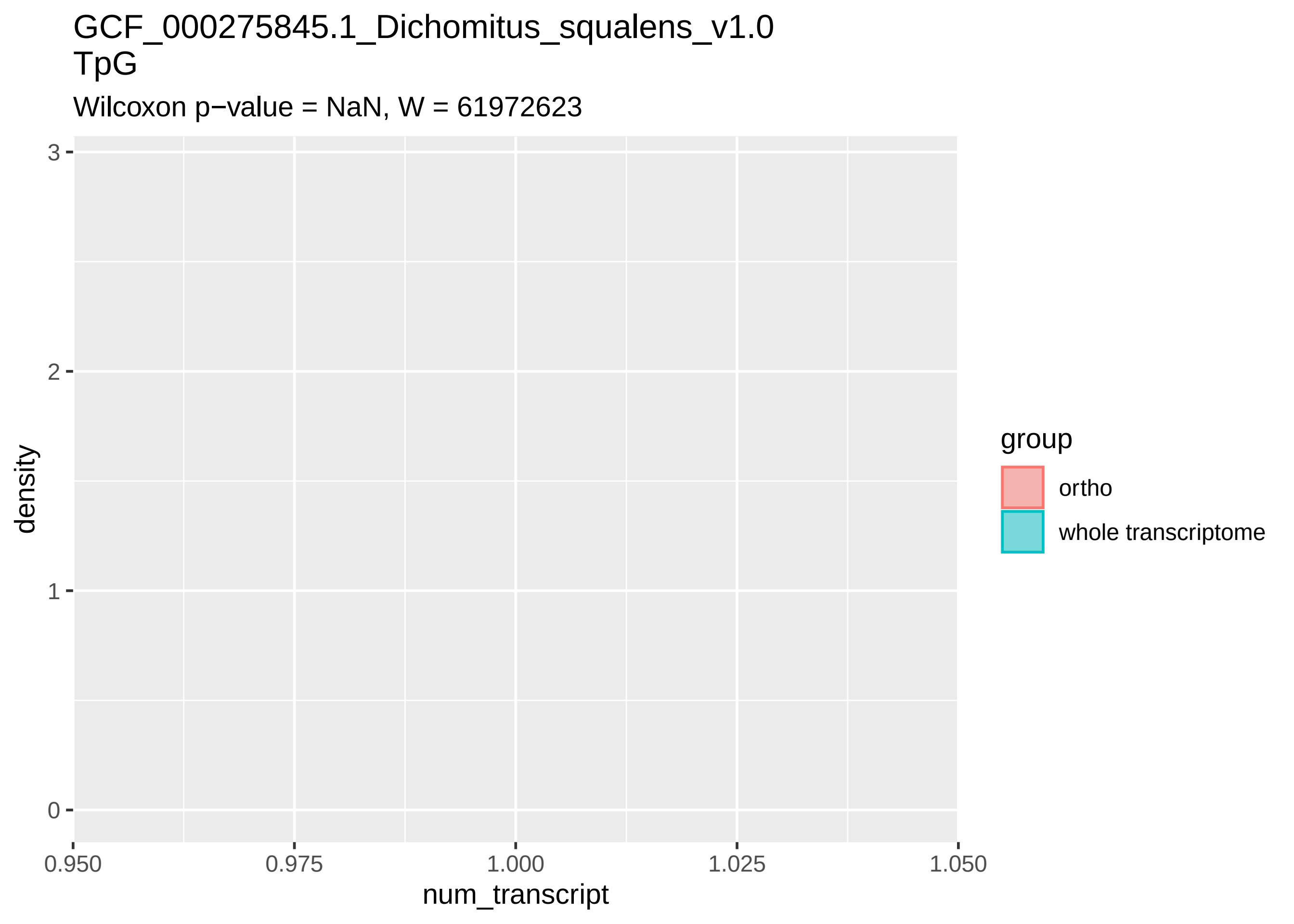

GCF_000275845.1_Dichomitus_squalens_v1.0
TpG
Wilcoxon p−value = NaN, W = 61972623
density
3
2
1
0
0.950
0.975
1.000
1.025
1.050
num_transcript
group
ortho
whole transcriptome

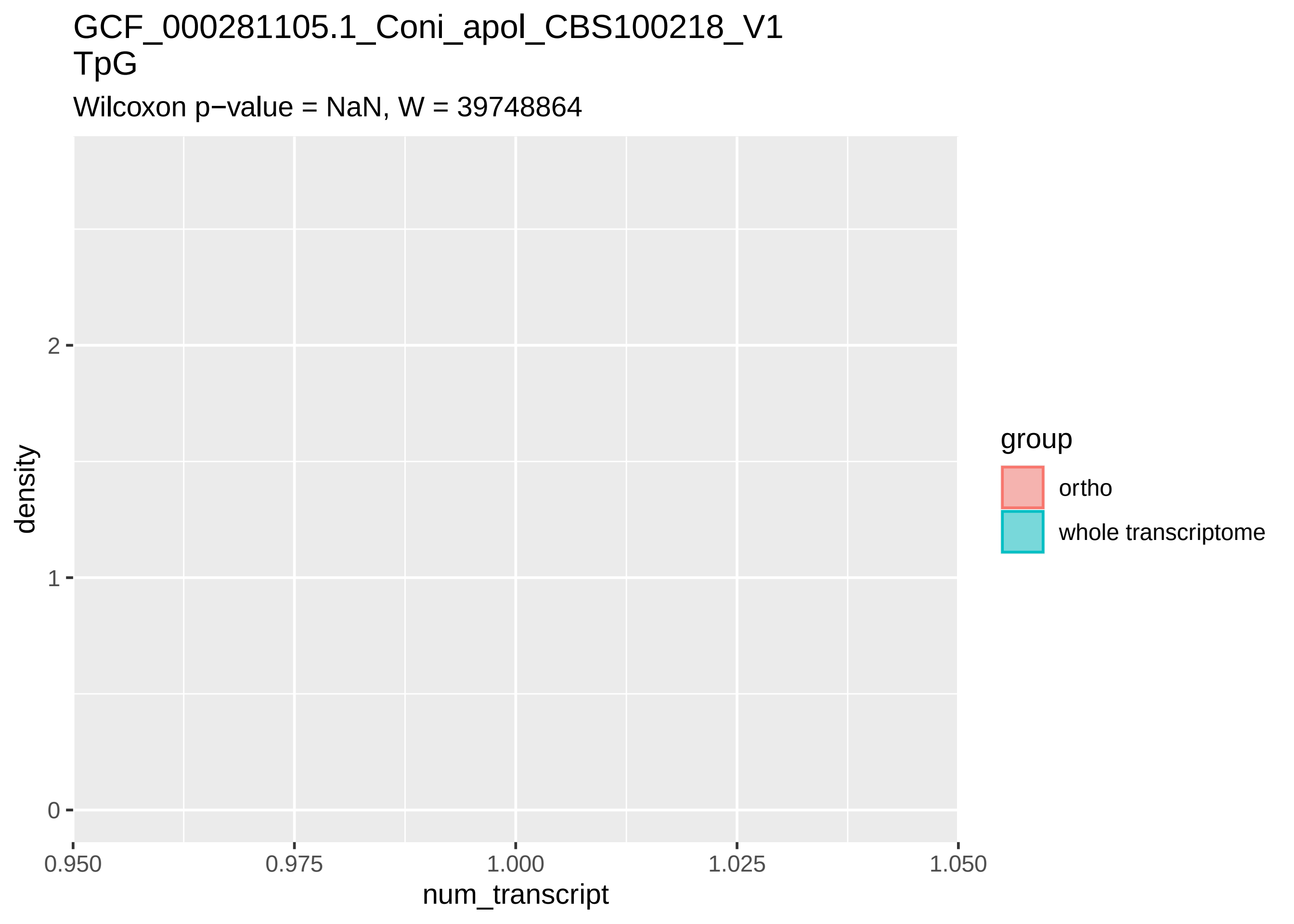

GCF_000281105.1_Coni_apol_CBS100218_V1
TpG
Wilcoxon p−value = NaN, W = 39748864
density
0
1
2
0.950
0.975
1.000
1.025
1.050
num_transcript
group
ortho
whole transcriptome

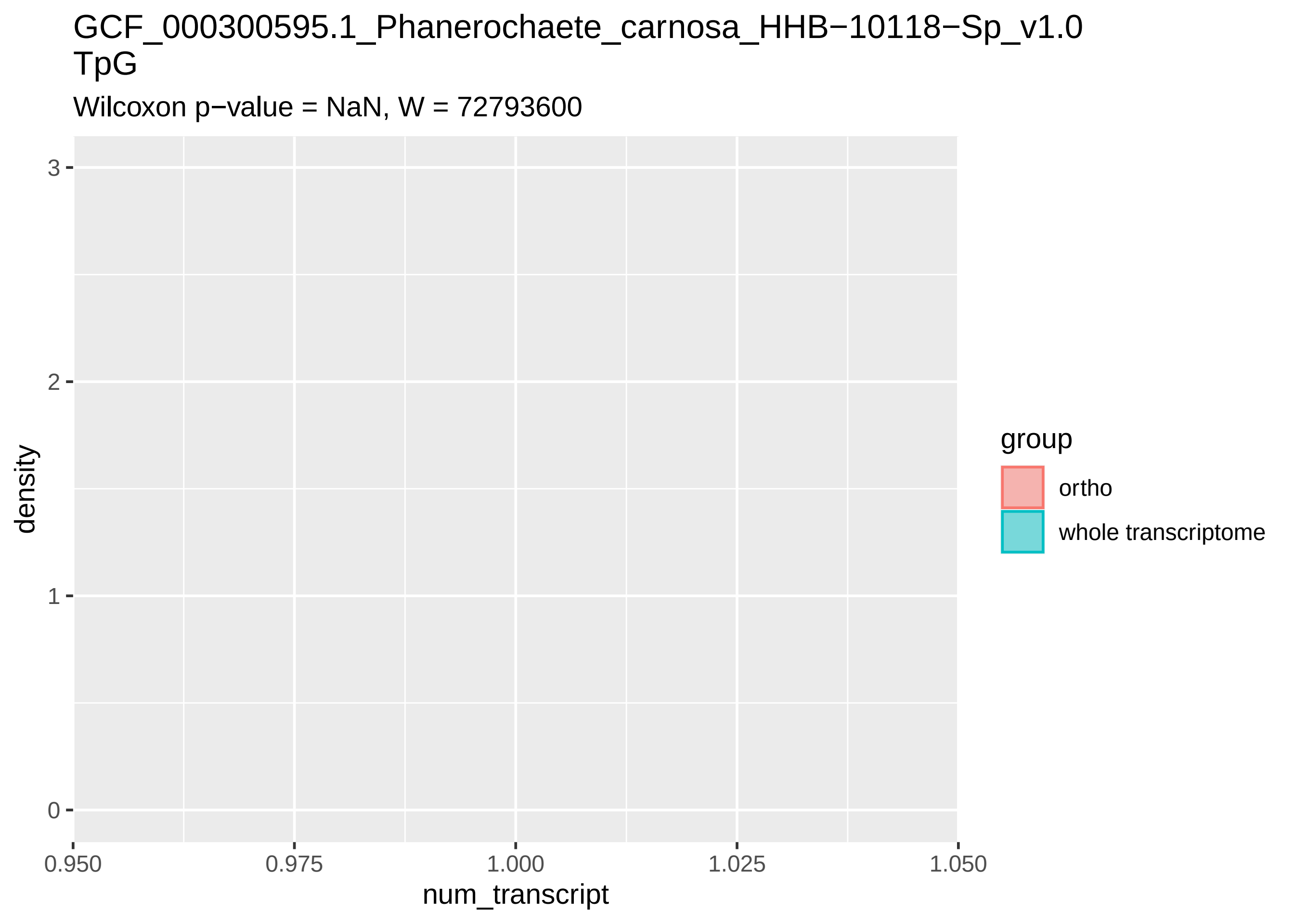
GCF_000300595.1_Phanerochaete_carnosa_HHB−10118−Sp_v1.0
TpG
Wilcoxon p−value = NaN, W = 72793600
density
3
2
1
0
0.950
0.975
1.000
1.025
1.050
num_transcript
group
ortho
whole transcriptome

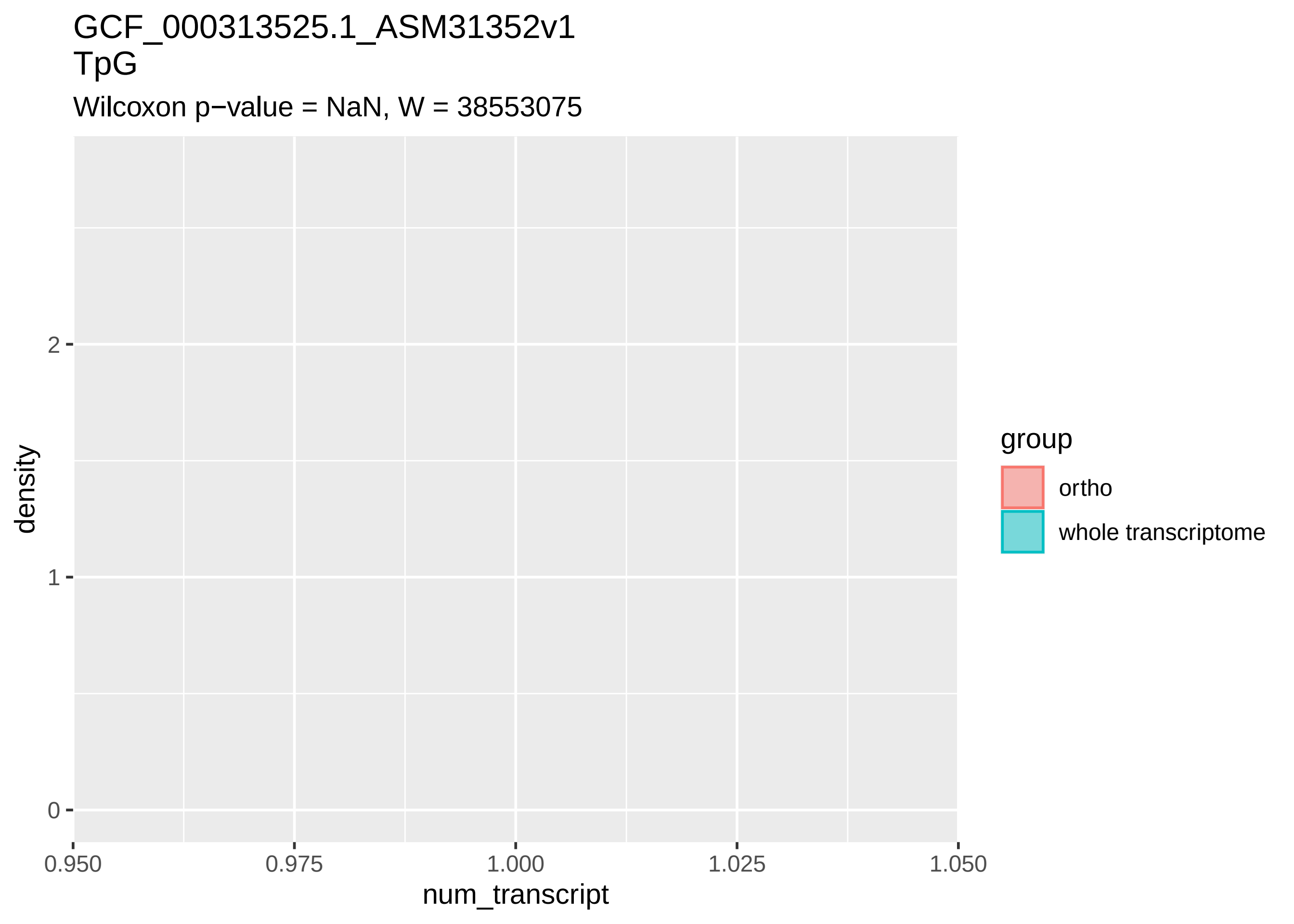
GCF_000313525.1_ASM31352v1
TpG
Wilcoxon p−value = NaN, W = 38553075
density
2
1
0
0.950
0.975
1.000
1.025
1.050
num_transcript
group
ortho
whole transcriptome

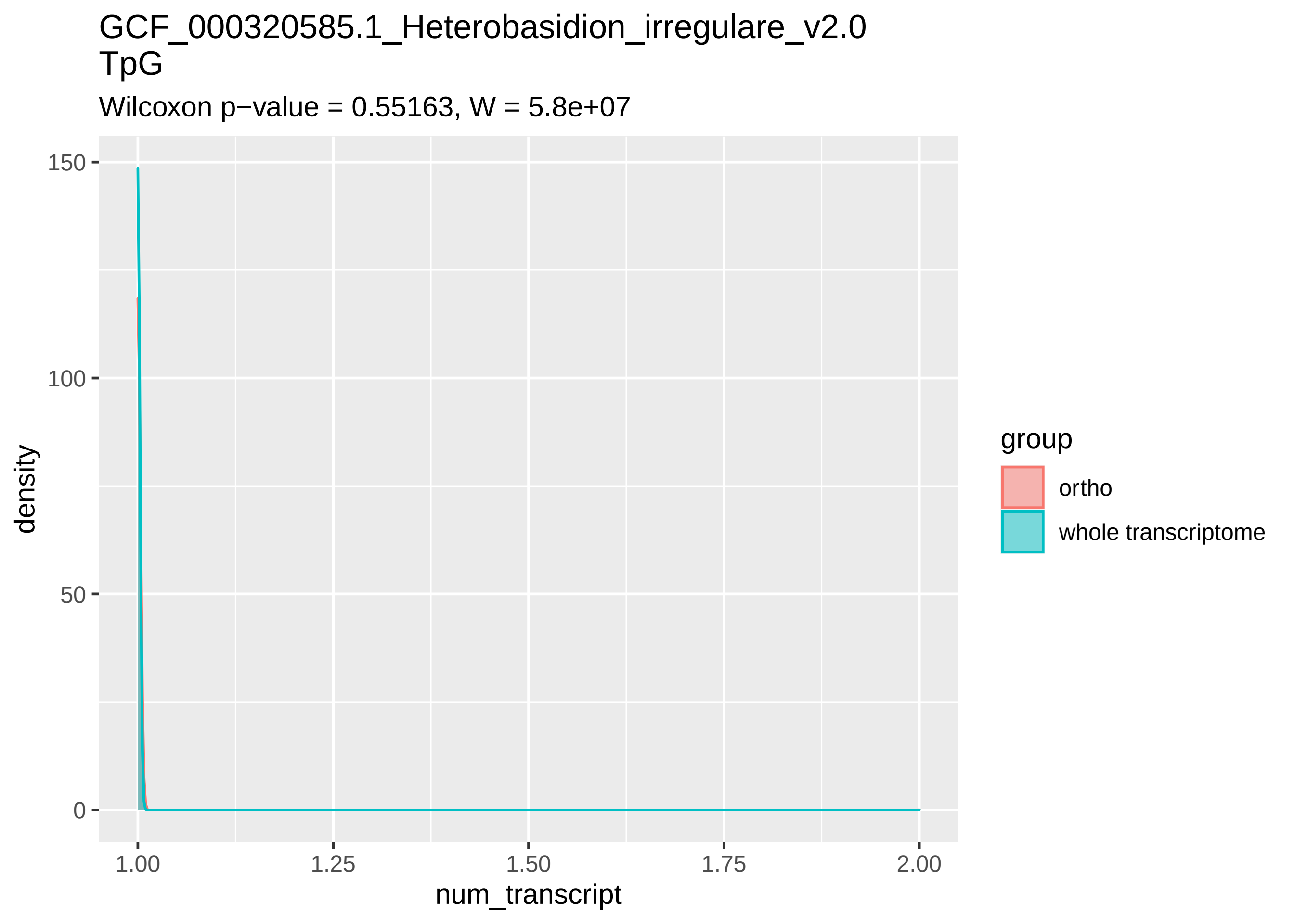
GCF_000320585.1_Heterobasidion_irregulare_v2.0
TpG
Wilcoxon p−value = 0.55163, W = 5.8e+07
150
100
50
0
density
1.00
1.25
1.50
1.75
2.00
num_transcript
group
ortho
whole transcriptome

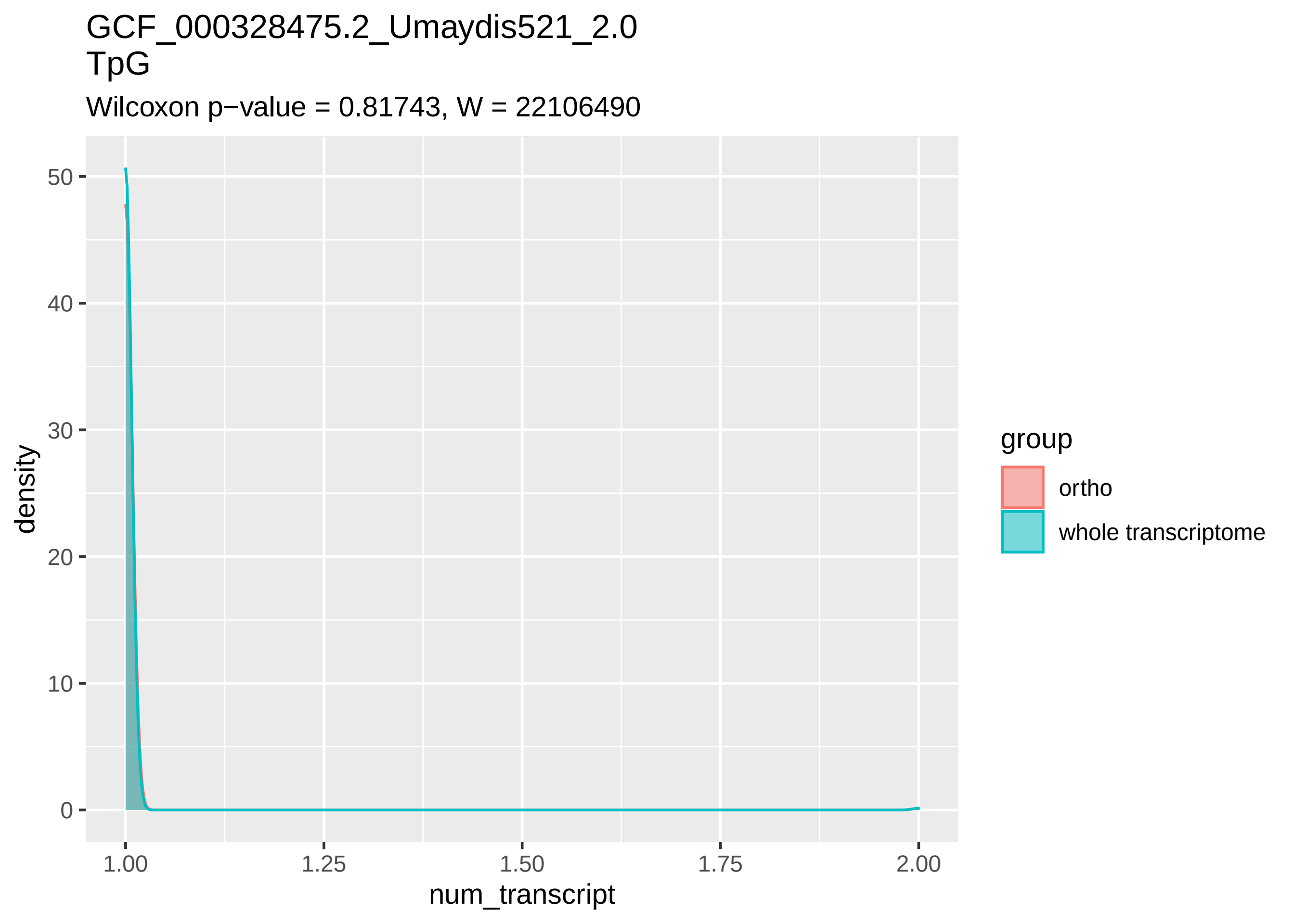

GCF_000328475.2_Umaydis521_2.0
TpG
Wilcoxon p−value = 0.81743, W = 22106490
density
num_transcript
0
10
20
30
40
50
1.00
1.25
1.50
1.75
2.00
group
ortho
whole transcriptome

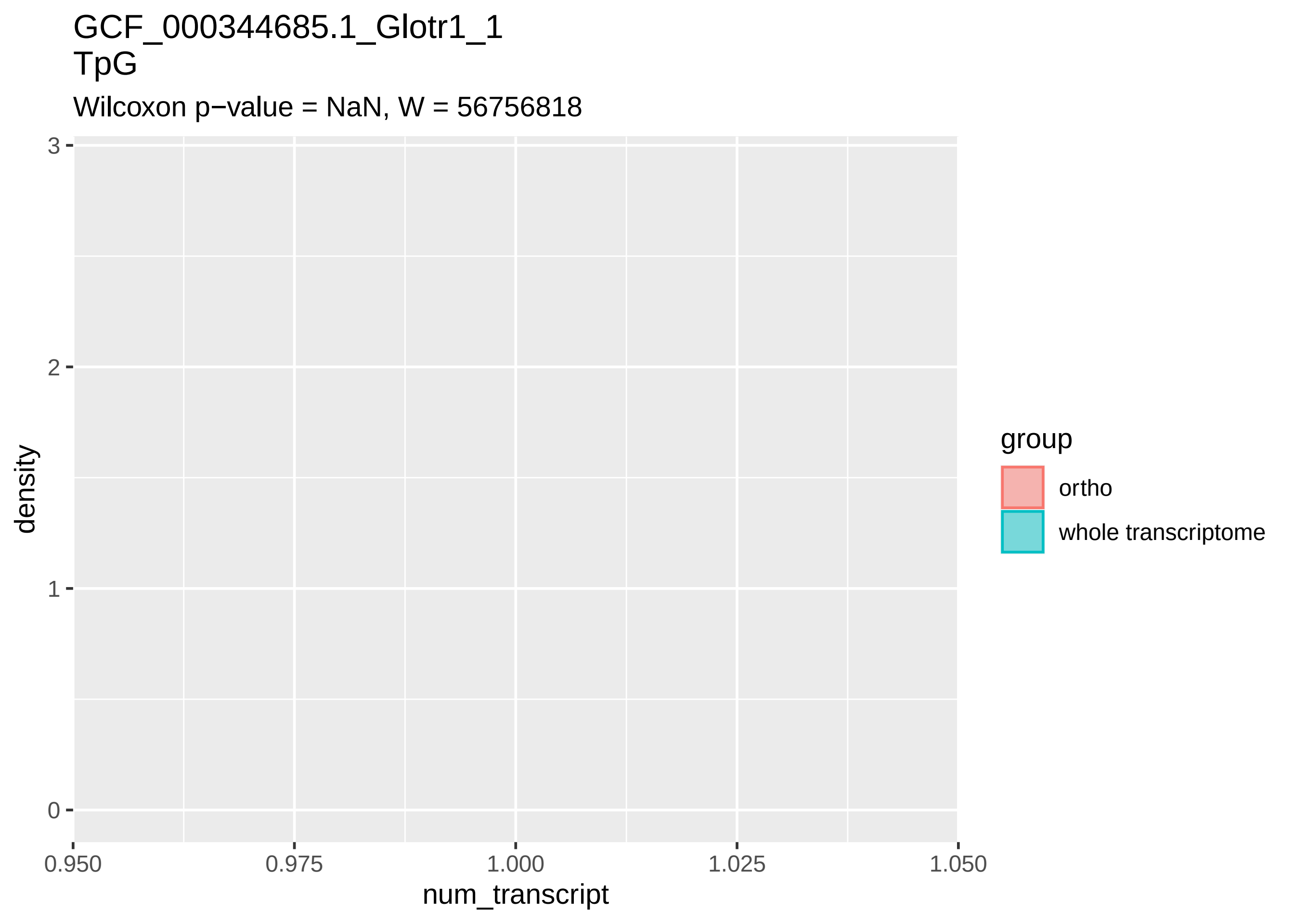
GCF_000344685.1_Glotr1_1
TpG
Wilcoxon p−value = NaN, W = 56756818
density
3
2
1
0
0.950
0.975
1.000
1.025
1.050
num_transcript
group
ortho
whole transcriptome

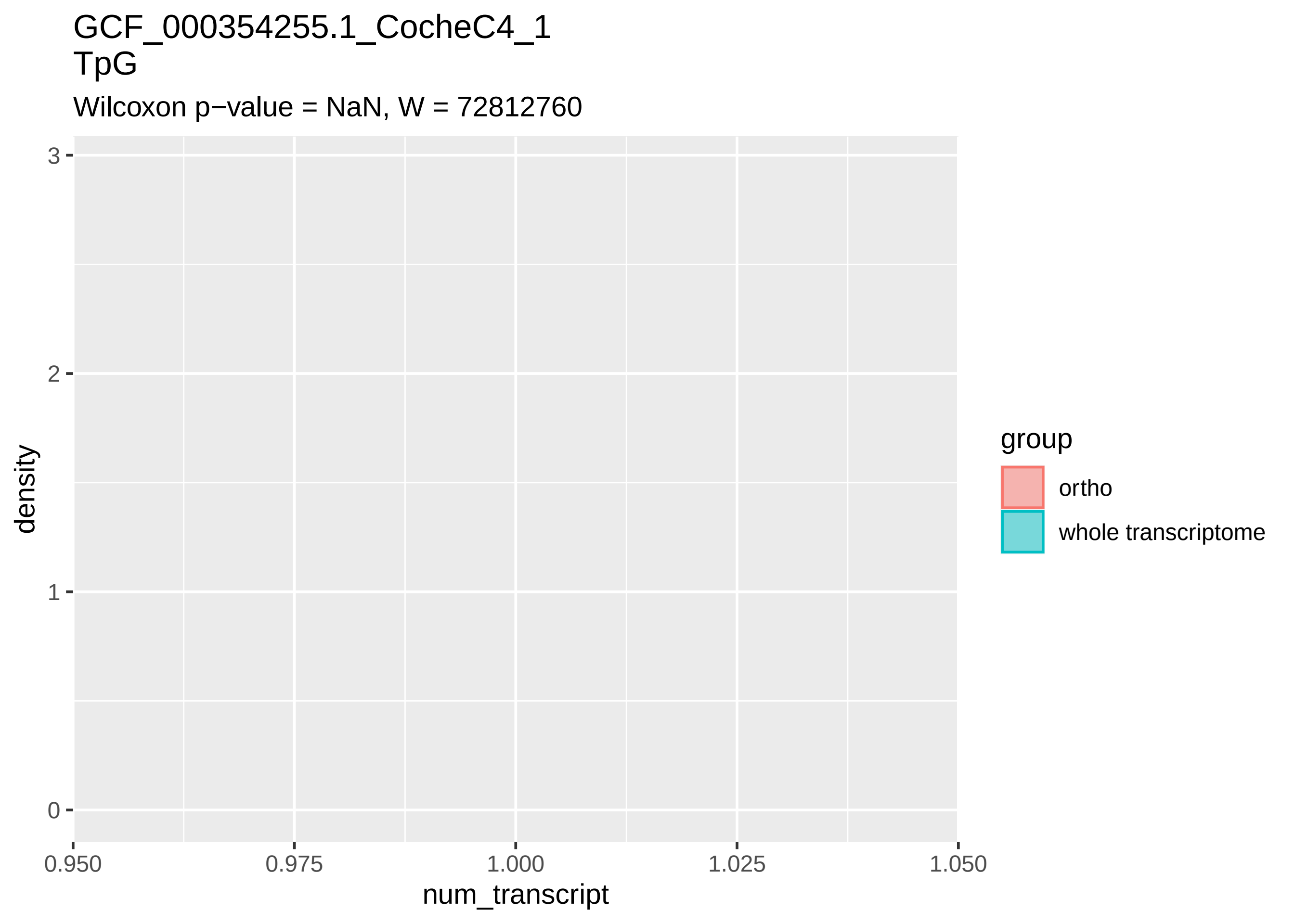

GCF_000354255.1_CocheC4_1
TpG
Wilcoxon p−value = NaN, W = 72812760
density
num_transcript
0
1
2
3
0.950
0.975
1.000
1.025
1.050
group
ortho
whole transcriptome

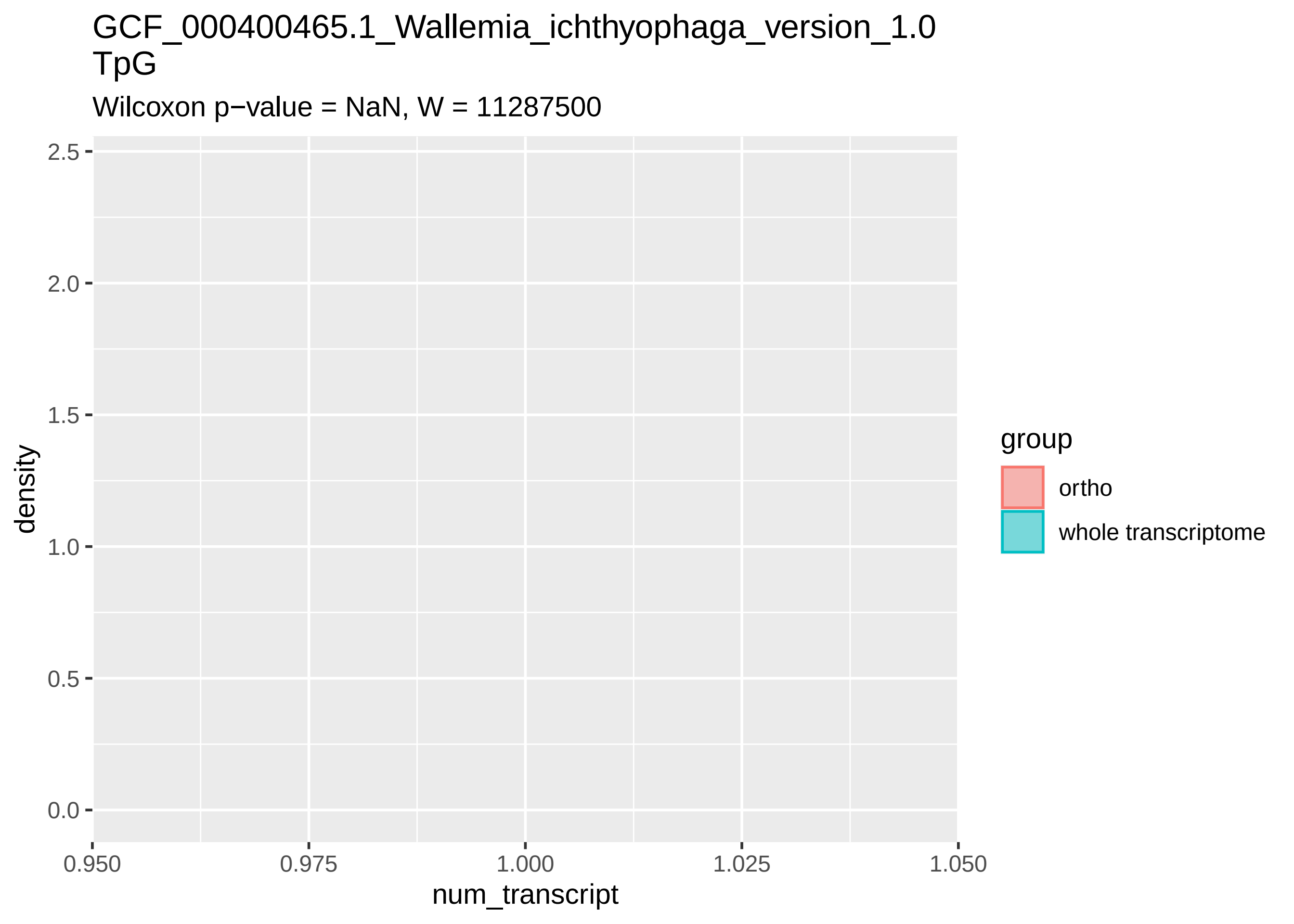
GCF_000400465.1_Wallemia_ichthyophaga_version_1.0
TpG
Wilcoxon p−value = NaN, W = 11287500
2.5
2.0
1.5
1.0
0.5
0.0
density
0.950
0.975
1.000
1.025
1.050
num_transcript
group
ortho
whole transcriptome

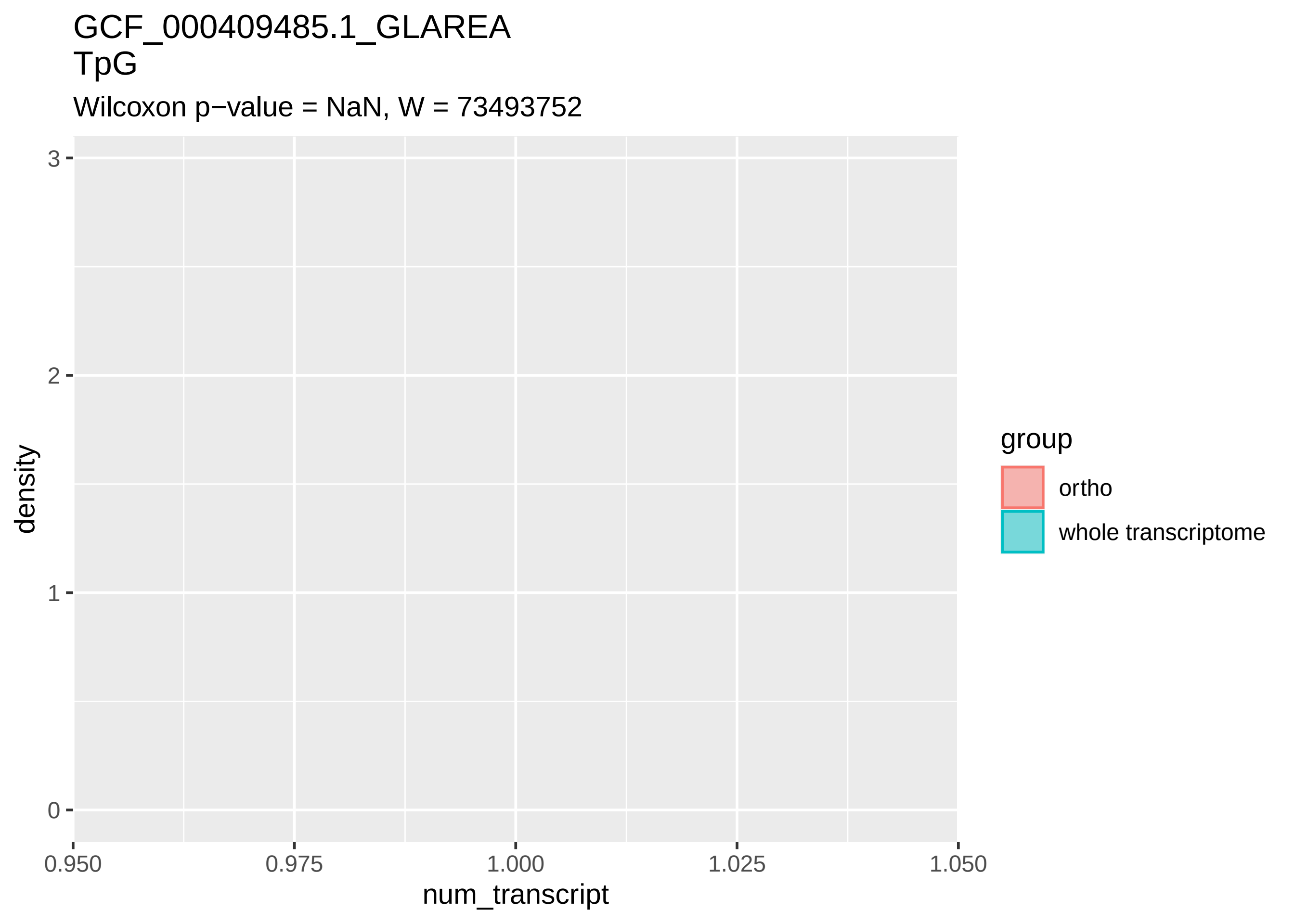

GCF_000409485.1_GLAREA
TpG
Wilcoxon p−value = NaN, W = 73493752
density
3
2
1
0
0.950
0.975
1.000
1.025
1.050
num_transcript
group
ortho
whole transcriptome

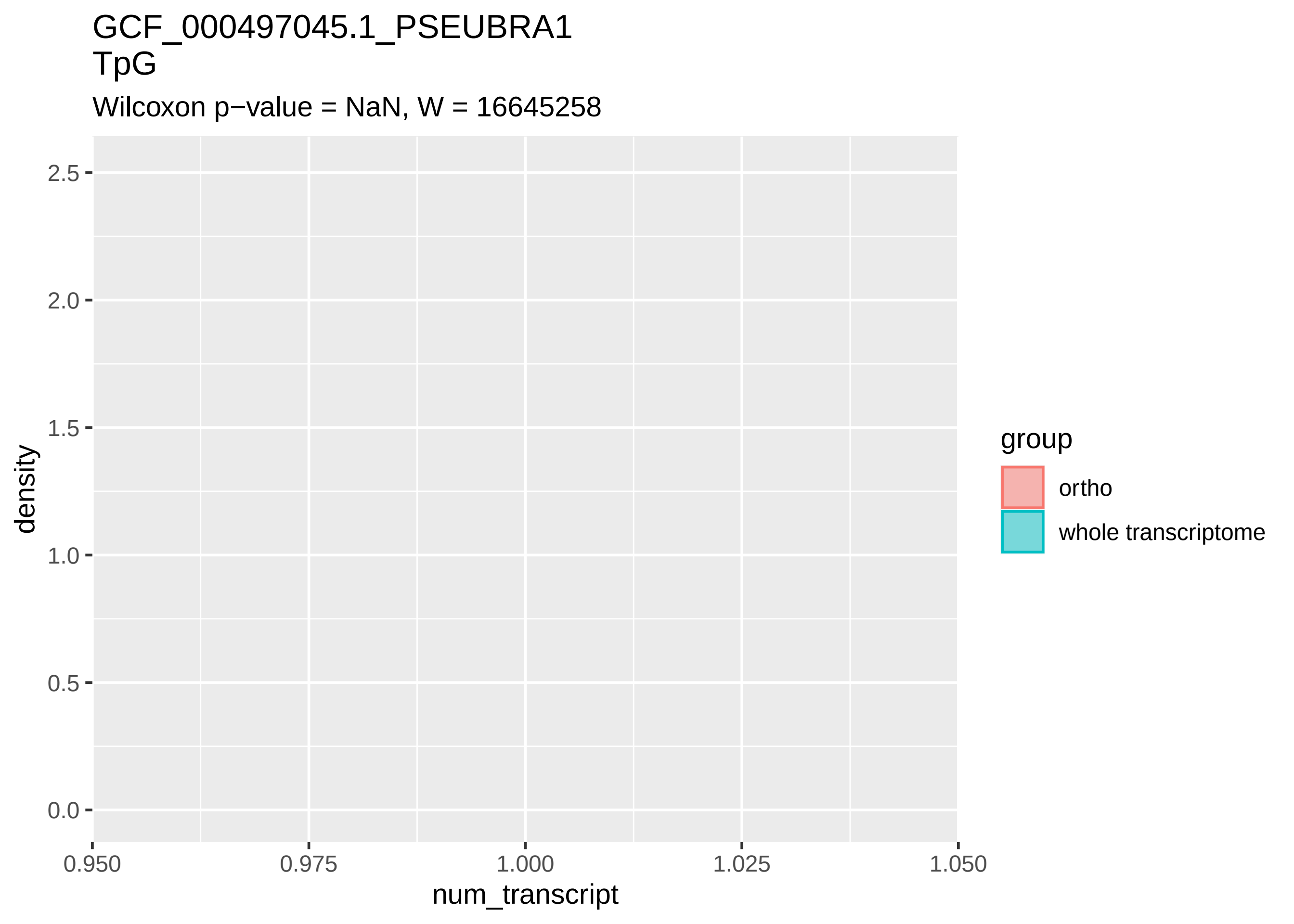
GCF_000497045.1_PSEUBRA1
TpG
Wilcoxon p−value = NaN, W = 16645258
density
2.5
2.0
1.5
1.0
0.5
0.0
0.950
0.975
1.000
1.025
1.050
num_transcript
group
ortho
whole transcriptome

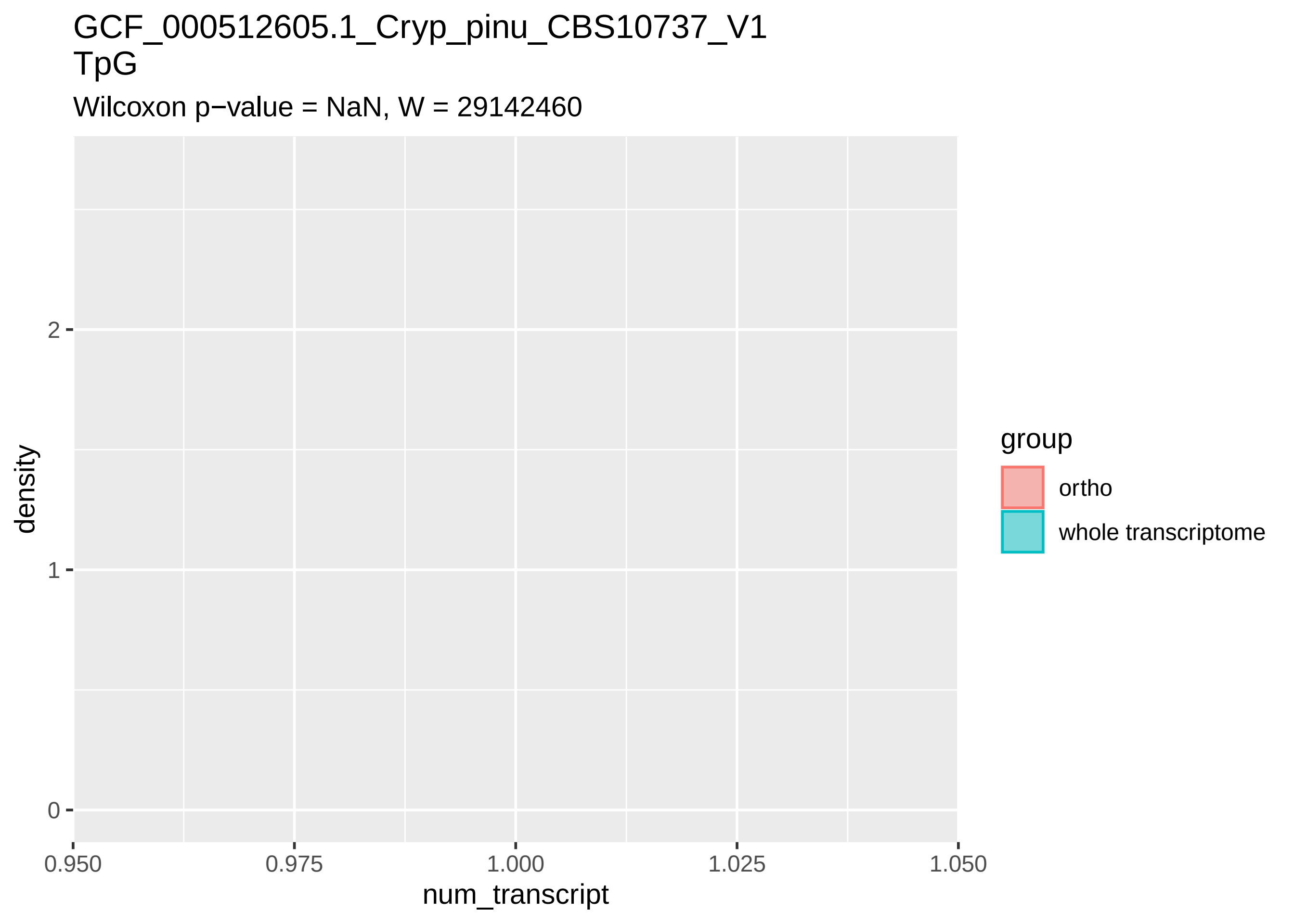

GCF_000512605.1_Cryp_pinu_CBS10737_V1
TpG
Wilcoxon p−value = NaN, W = 29142460
density
2
1
0
0.950
0.975
1.000
1.025
1.050
num_transcript
group
ortho
whole transcriptome

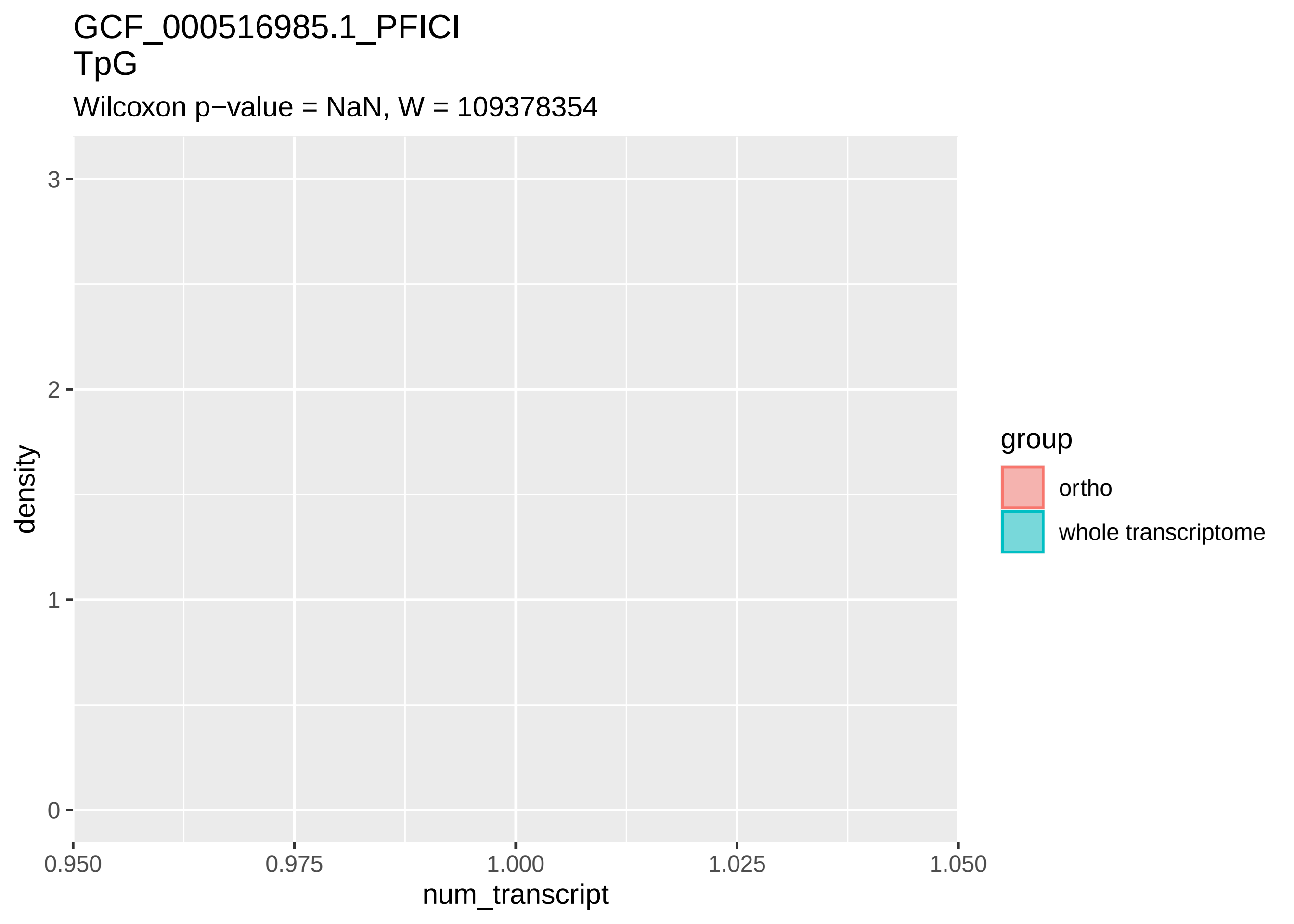

GCF_000516985.1_PFICI
TpG
Wilcoxon p−value = NaN, W = 109378354
density
num_transcript
0
1
2
3
0.950
0.975
1.000
1.025
1.050
group
ortho
whole transcriptome

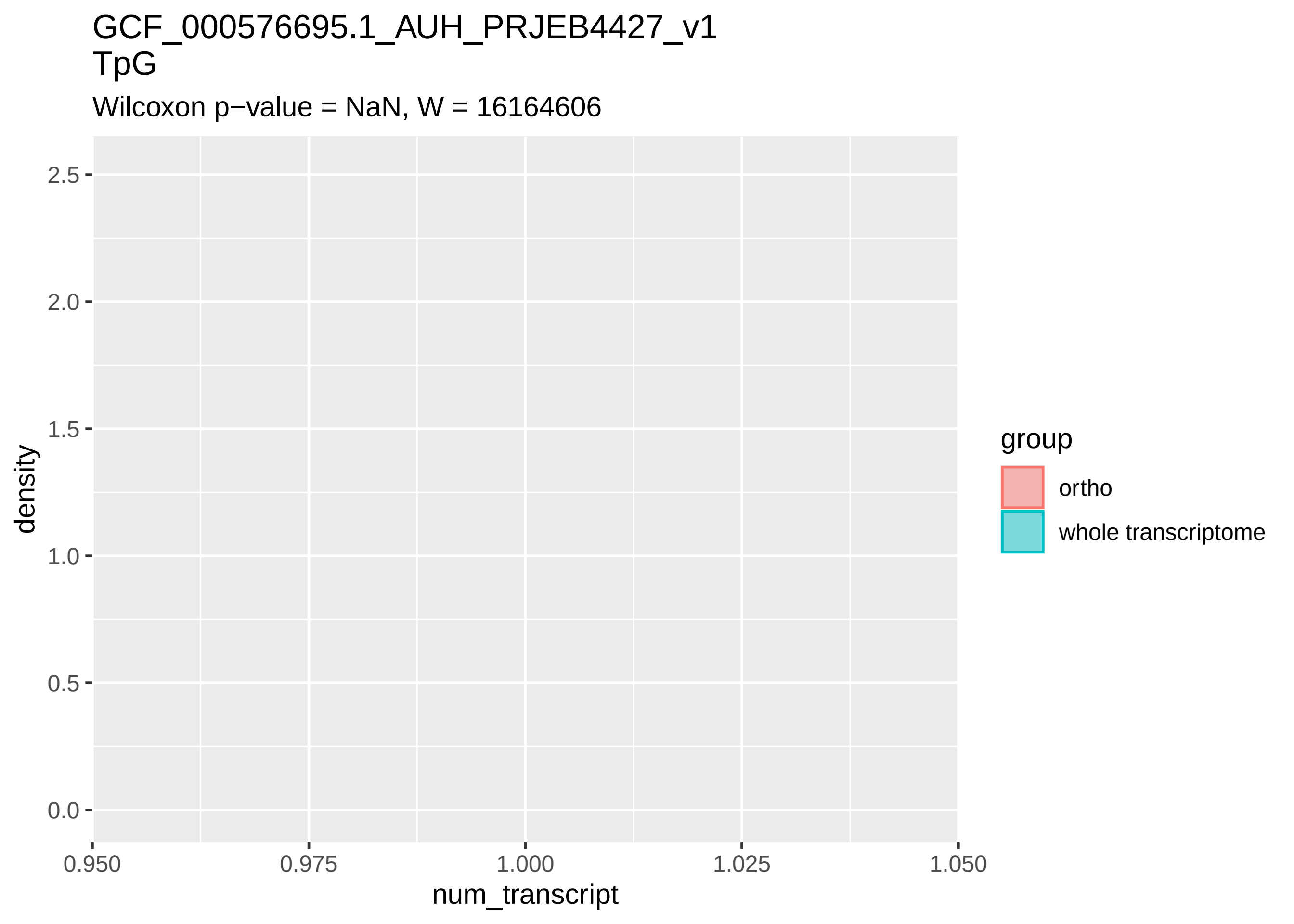
GCF_000576695.1_AUH_PRJEB4427_v1
TpG
Wilcoxon p−value = NaN, W = 16164606
density
2.5
2.0
1.5
1.0
0.5
0.0
0.950
0.975
1.000
1.025
1.050
num_transcript
group
ortho
whole transcriptome

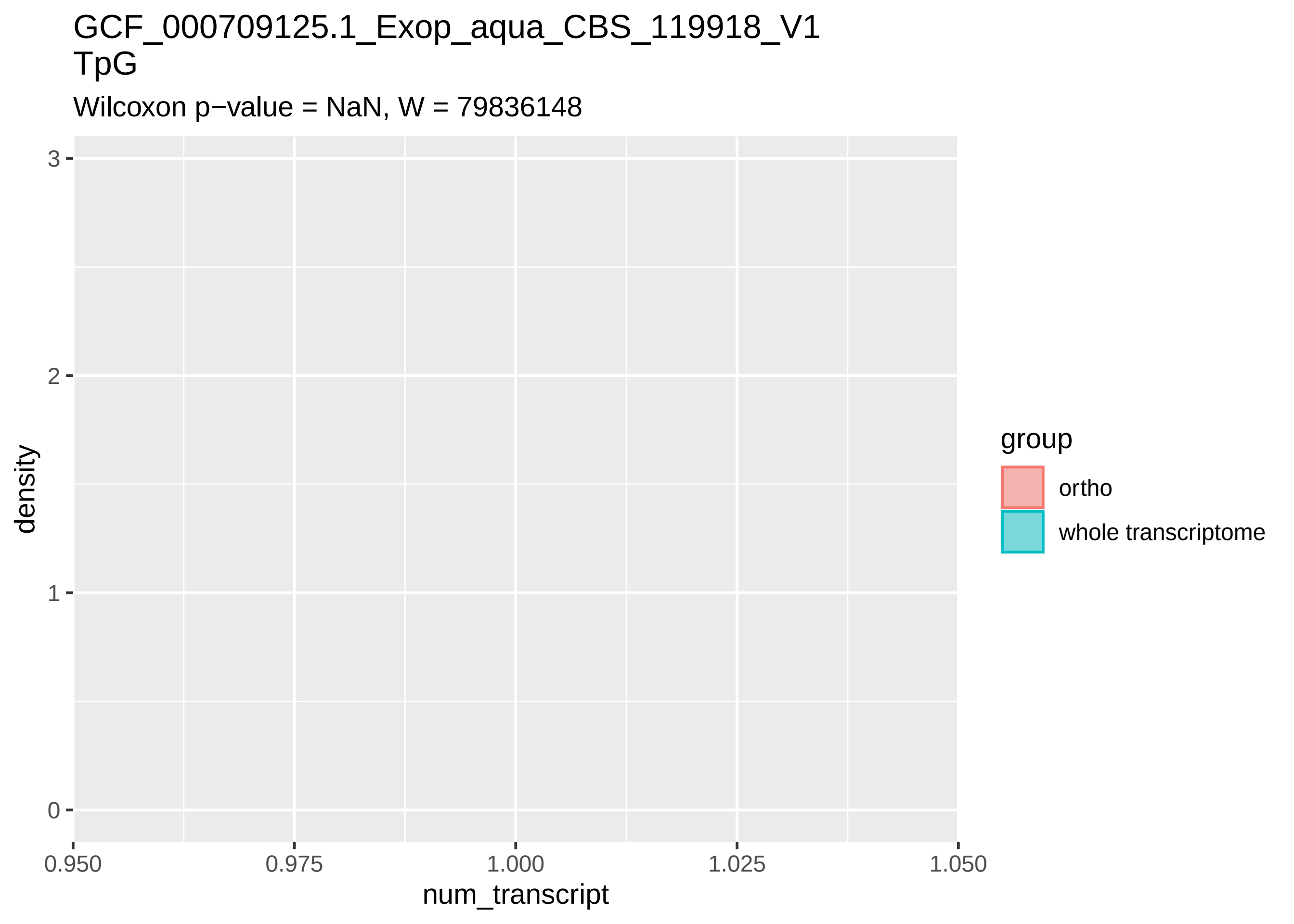
GCF_000709125.1_Exop_aqua_CBS_119918_V1
TpG
Wilcoxon p−value = NaN, W = 79836148
3
2
1
0
density
0.950
0.975
1.000
1.025
1.050
num_transcript
group
ortho
whole transcriptome

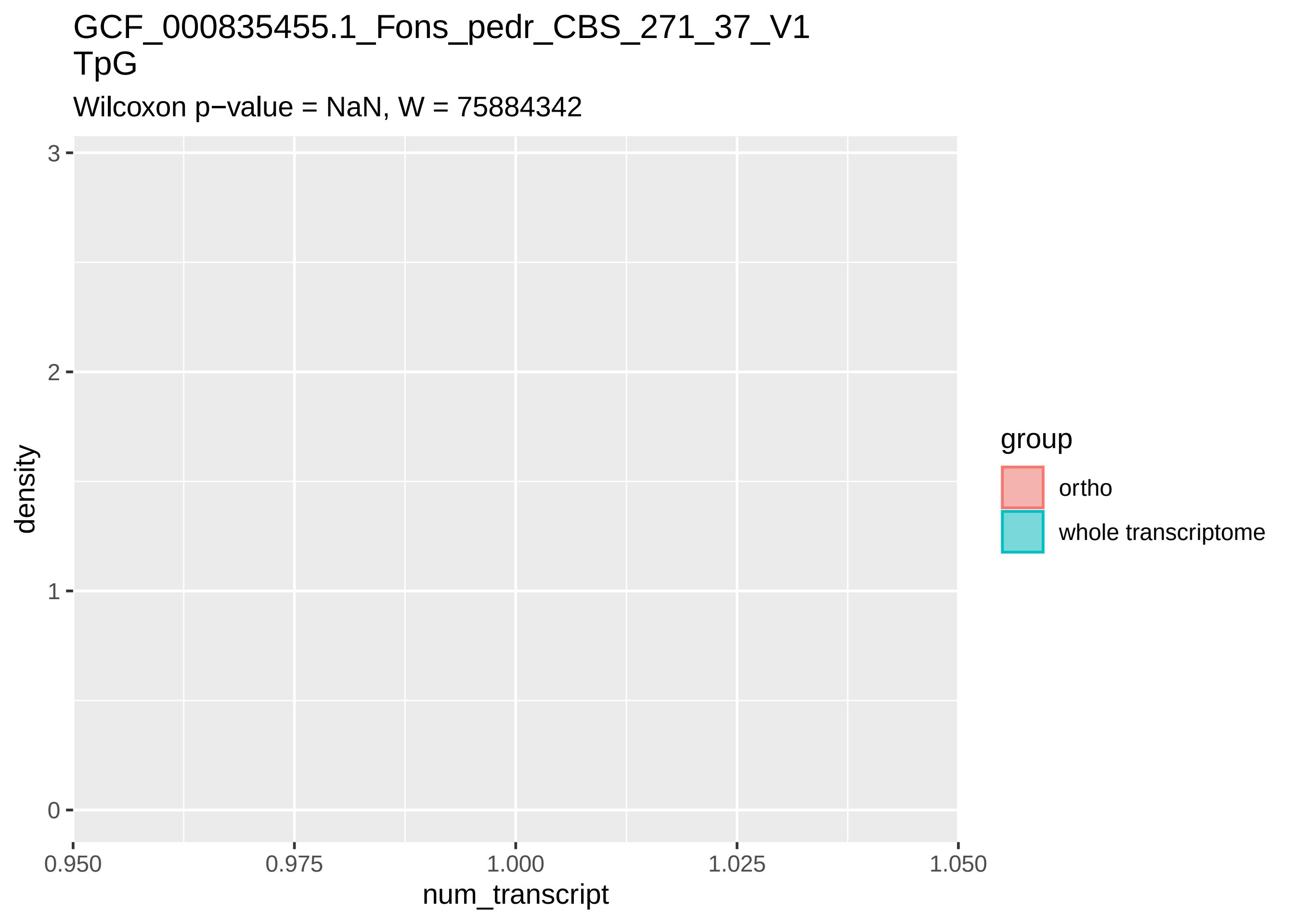
GCF_000835455.1_Fons_pedr_CBS_271_37_V1
TpG
Wilcoxon p−value = NaN, W = 75884342
density
3
2
1
0
0.950
0.975
1.000
1.025
1.050
num_transcript
group
ortho
whole transcriptome

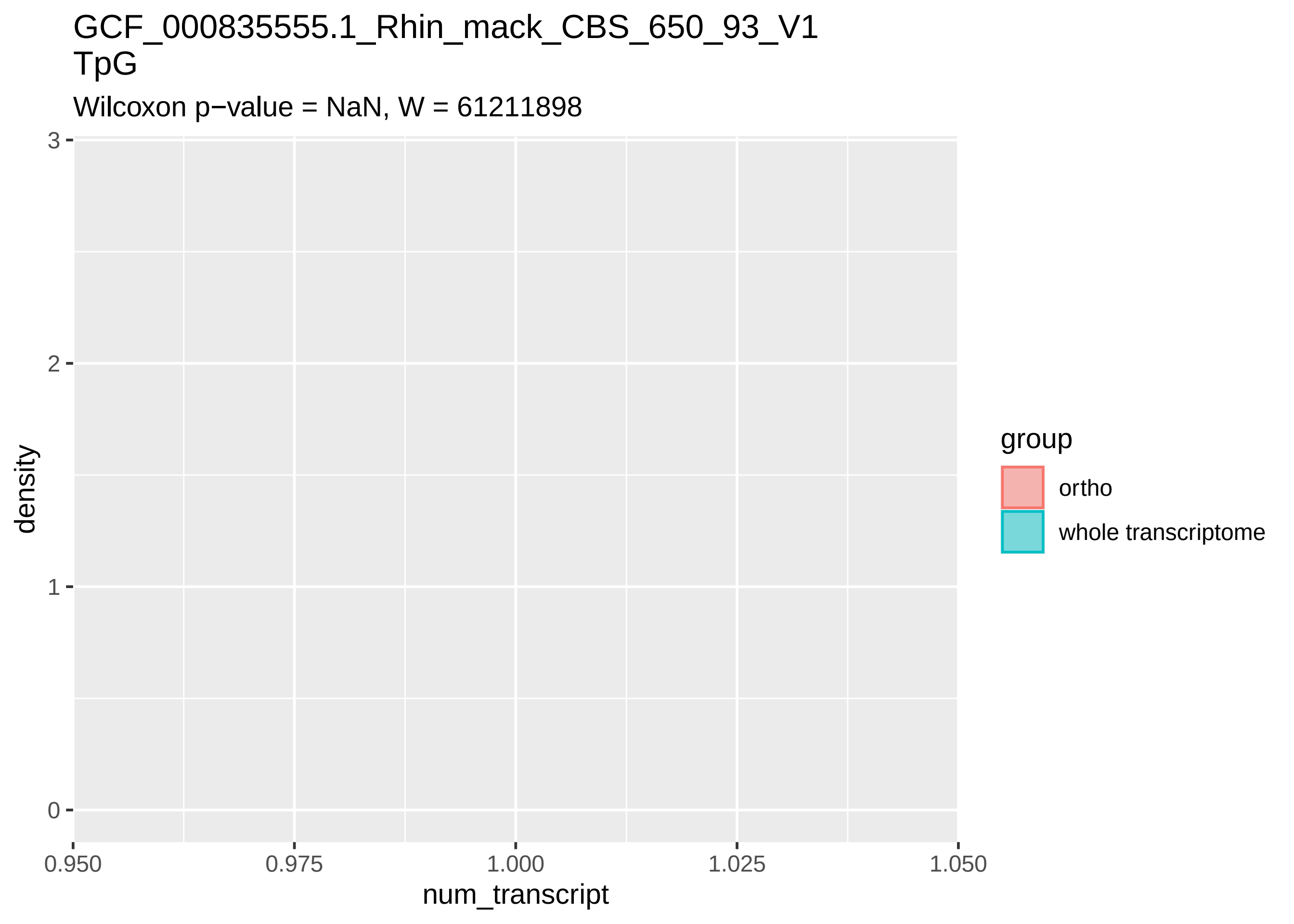
GCF_000835555.1_Rhin_mack_CBS_650_93_V1
TpG
Wilcoxon p−value = NaN, W = 61211898
density
num_transcript
0
1
2
3
0.950
0.975
1.000
1.025
1.050
group
ortho
whole transcriptome

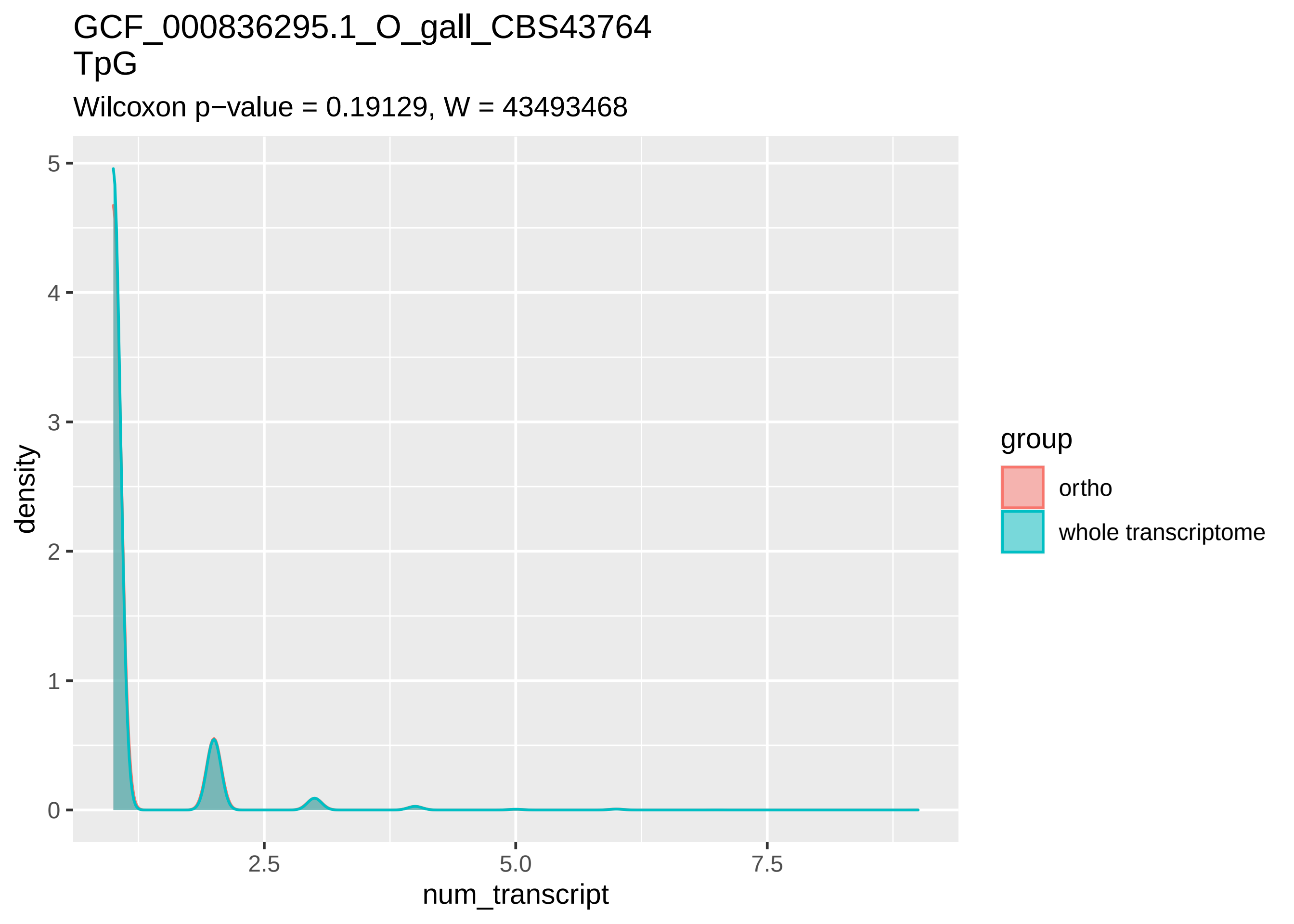
GCF_000836295.1_O_gall_CBS43764
TpG
Wilcoxon p−value = 0.19129, W = 43493468
density
num_transcript
0
1
2
3
4
5
2.5
5.0
7.5
group
ortho
whole transcriptome

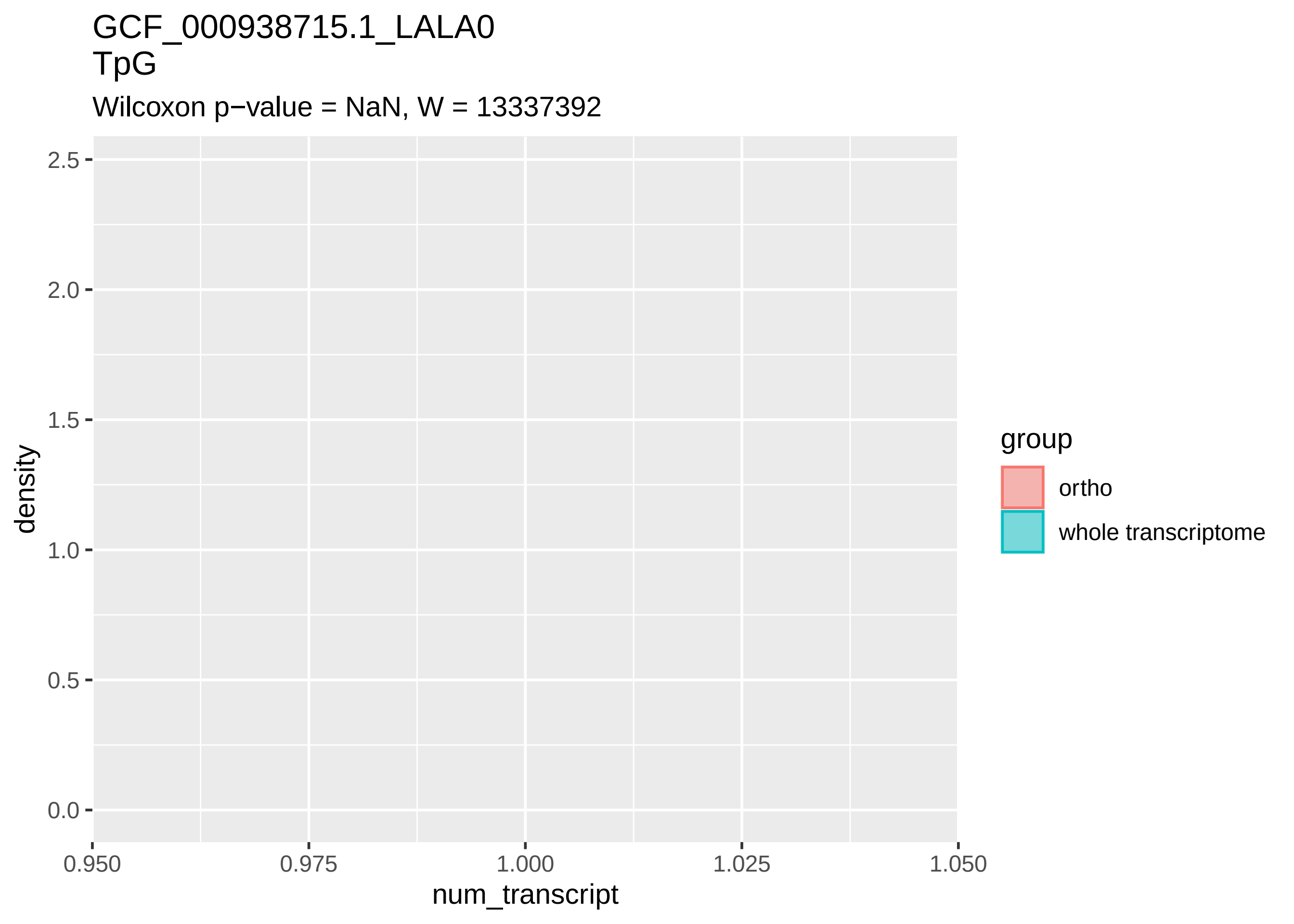
GCF_000938715.1_LALA0
TpG
Wilcoxon p−value = NaN, W = 13337392
2.5
2.0
1.5
1.0
0.5
0.0
density
0.950
0.975
1.000
1.025
1.050
num_transcript
group
ortho
whole transcriptome

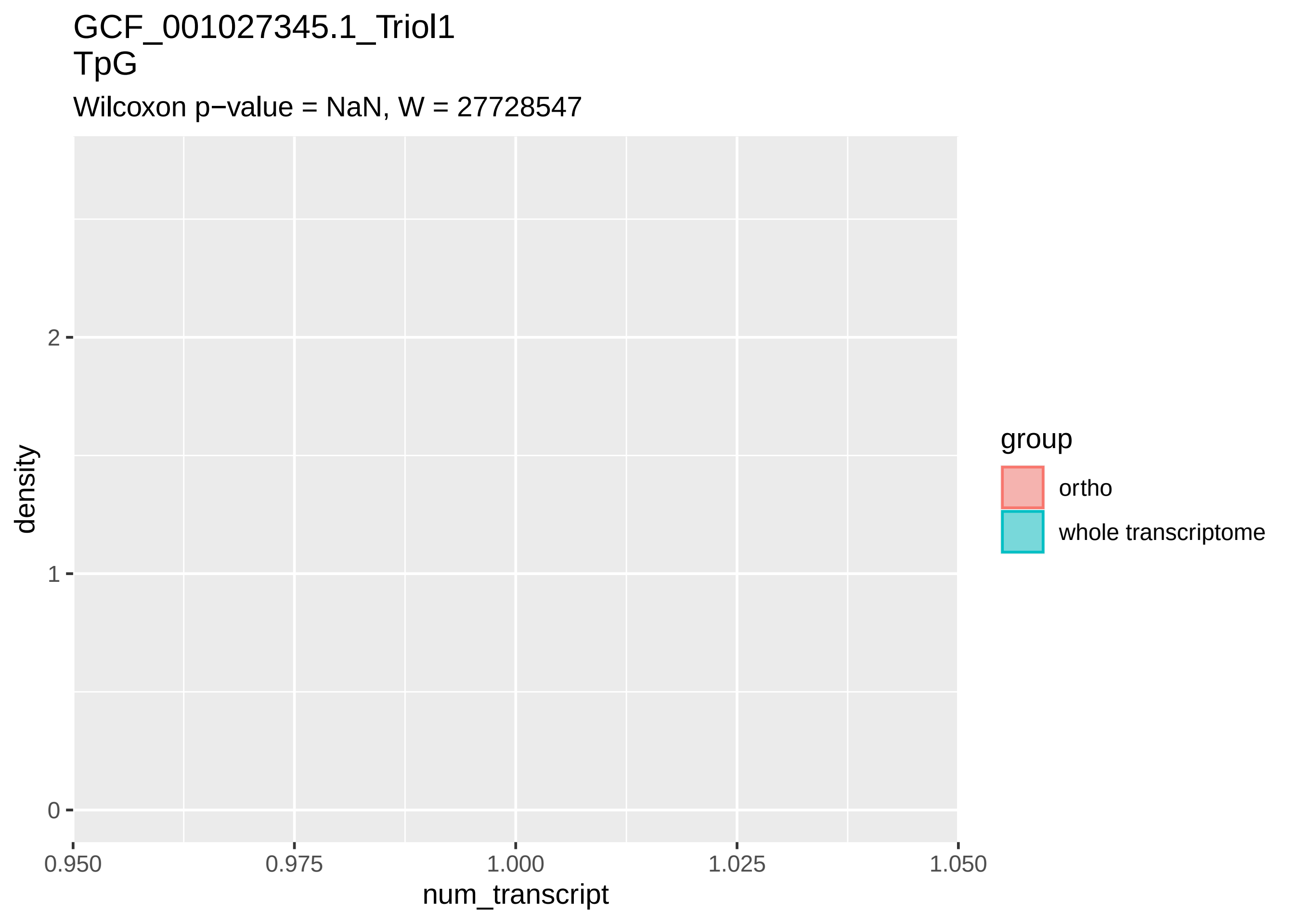
GCF_001027345.1_Triol1
TpG
Wilcoxon p−value = NaN, W = 27728547
density
2
1
0
0.950
0.975
1.000
1.025
1.050
num_transcript
group
ortho
whole transcriptome

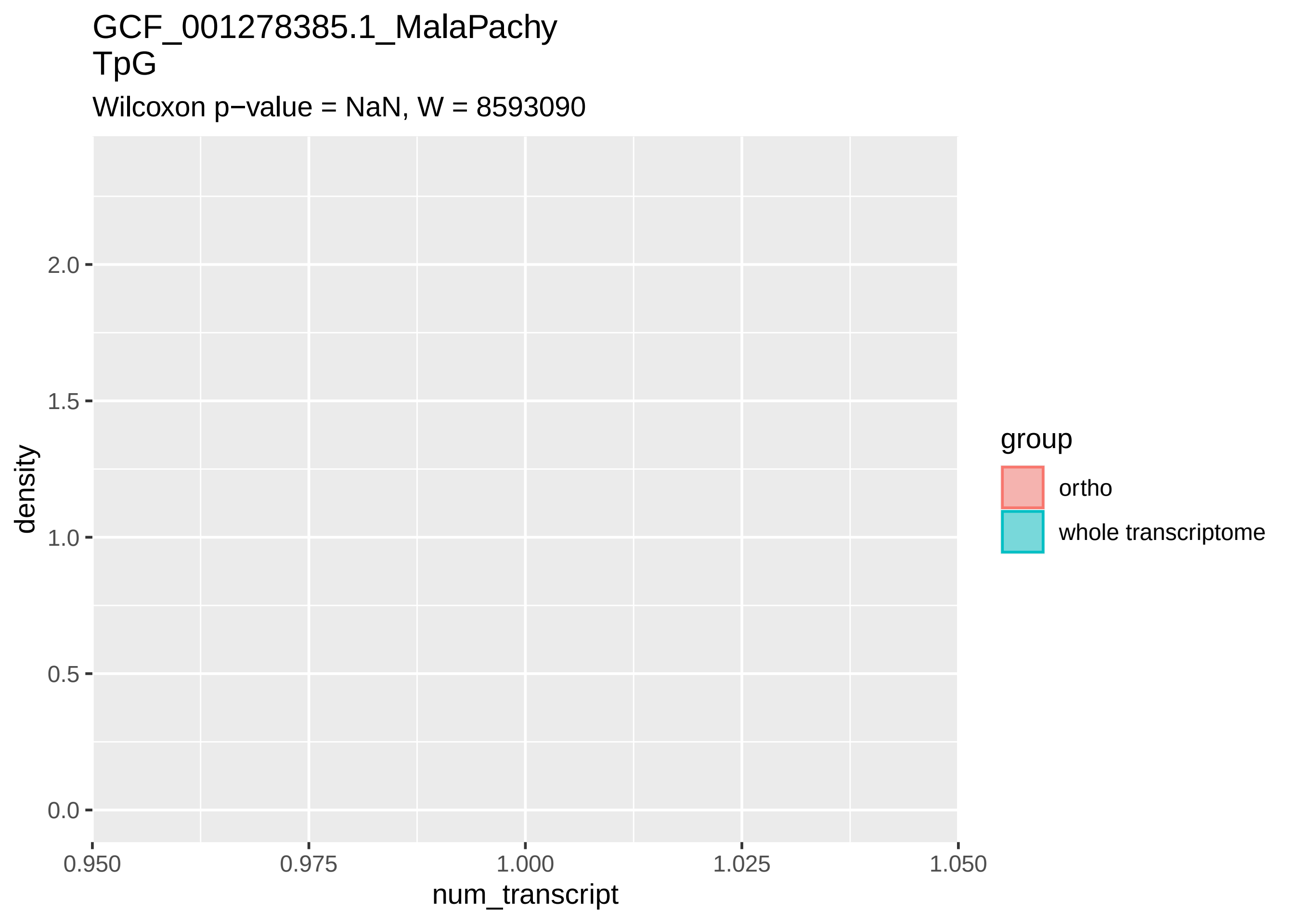
GCF_001278385.1_MalaPachy
TpG
Wilcoxon p−value = NaN, W = 8593090
density
2.0
1.5
1.0
0.5
0.0
0.950
0.975
1.000
1.025
1.050
num_transcript
group
ortho
whole transcriptome

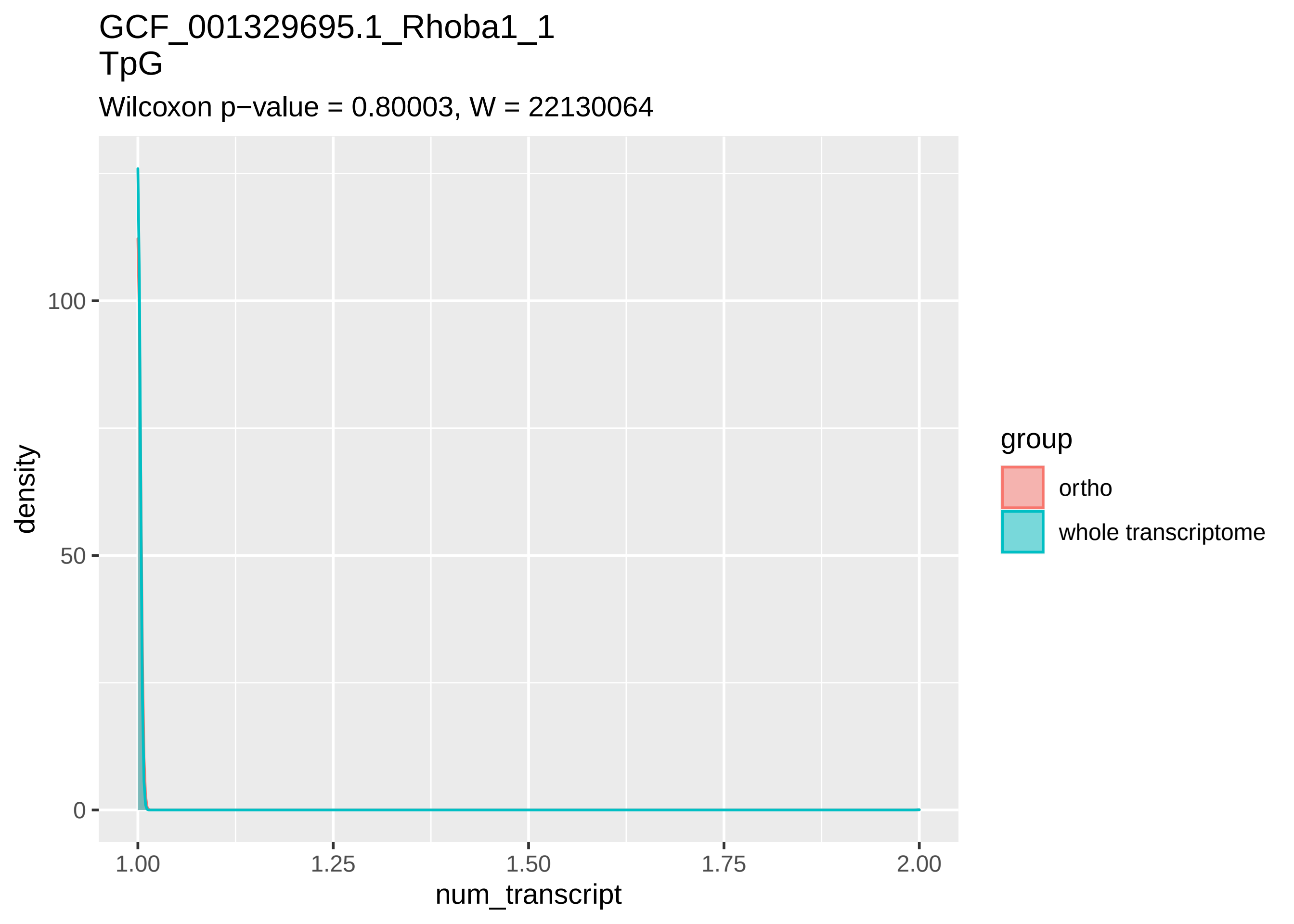
GCF_001329695.1_Rhoba1_1
TpG
Wilcoxon p−value = 0.80003, W = 22130064
density
100
50
0
1.00
1.25
1.50
1.75
2.00
num_transcript
group
ortho
whole transcriptome

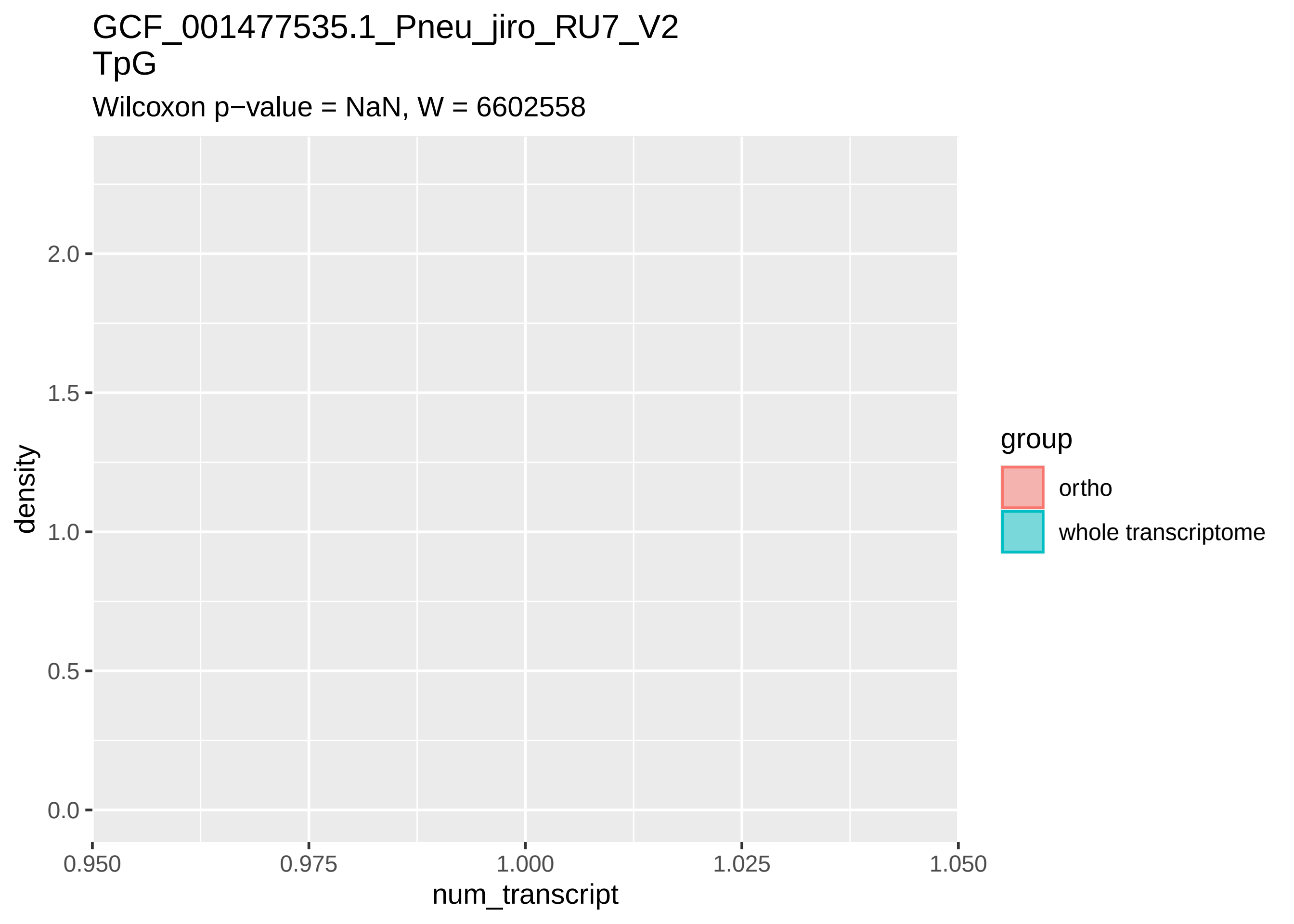
GCF_001477535.1_Pneu_jiro_RU7_V2
TpG
Wilcoxon p−value = NaN, W = 6602558
density
2.0
1.5
1.0
0.5
0.0
0.950
0.975
1.000
1.025
1.050
num_transcript
group
ortho
whole transcriptome

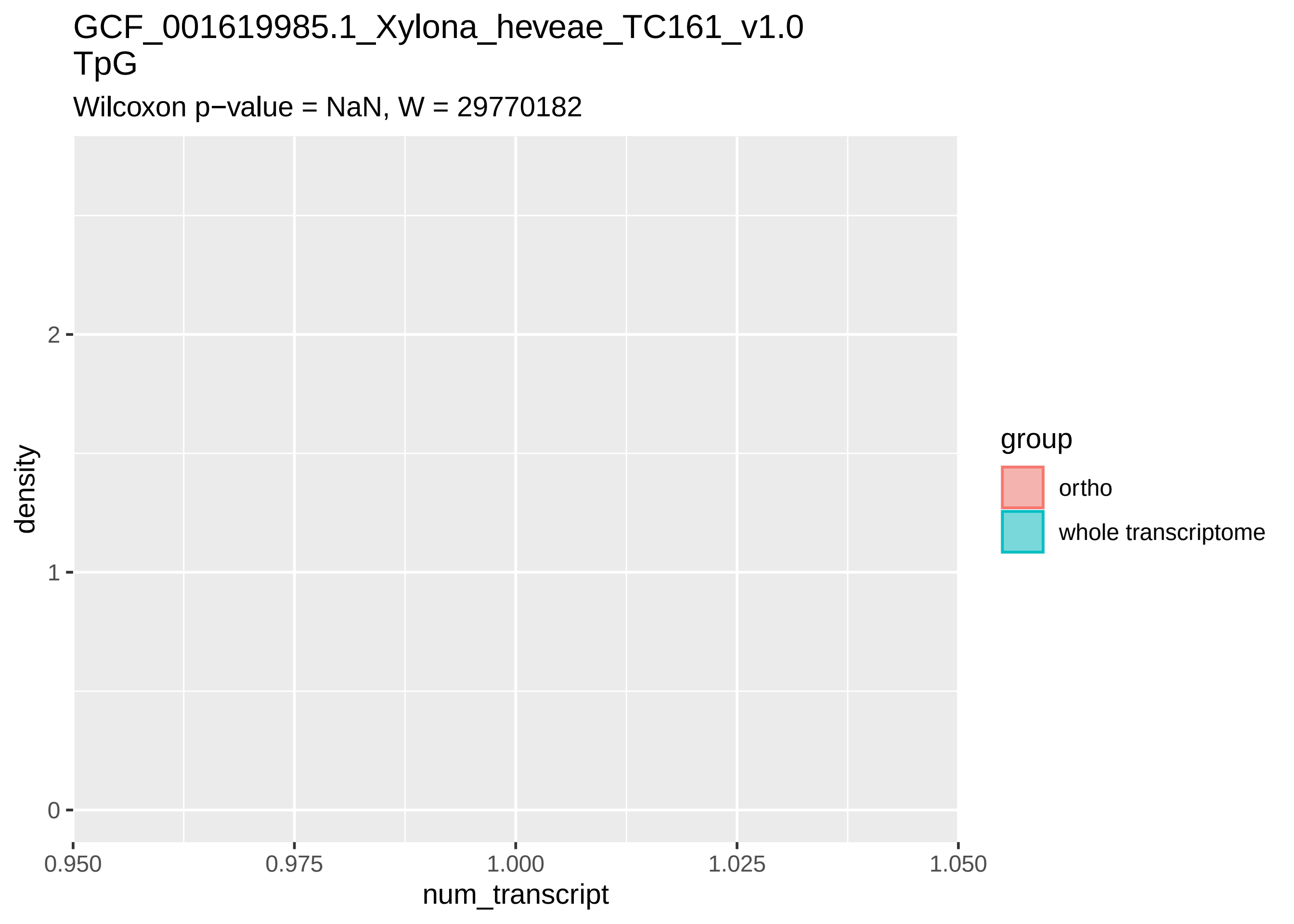
GCF_001619985.1_Xylona_heveae_TC161_v1.0
TpG
Wilcoxon p−value = NaN, W = 29770182
density
0
1
2
0.950
0.975
1.000
1.025
1.050
num_transcript
group
ortho
whole transcriptome

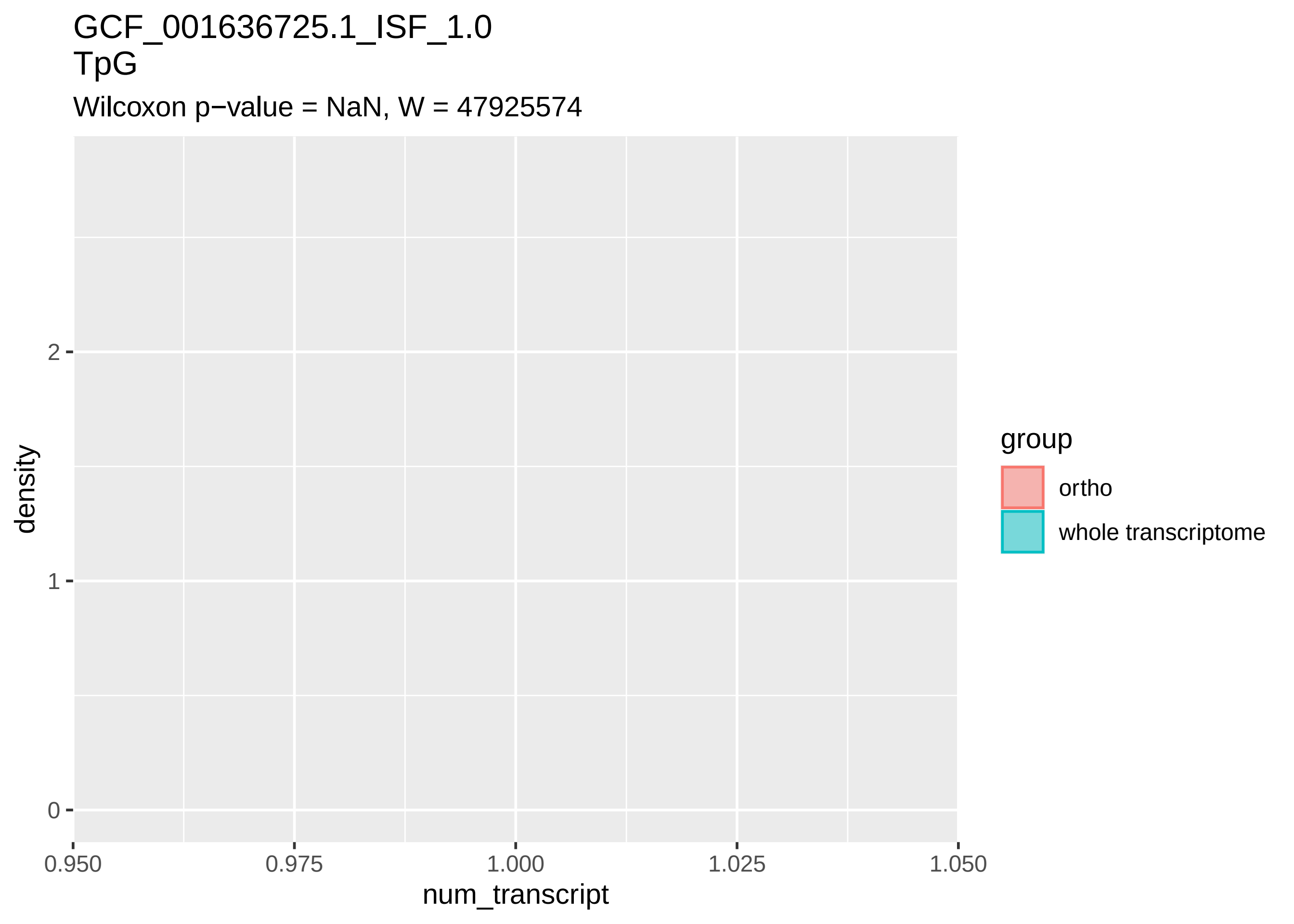
GCF_001636725.1_ISF_1.0
TpG
Wilcoxon p−value = NaN, W = 47925574
density
2
1
0
0.950
0.975
1.000
1.025
1.050
num_transcript
group
ortho
whole transcriptome

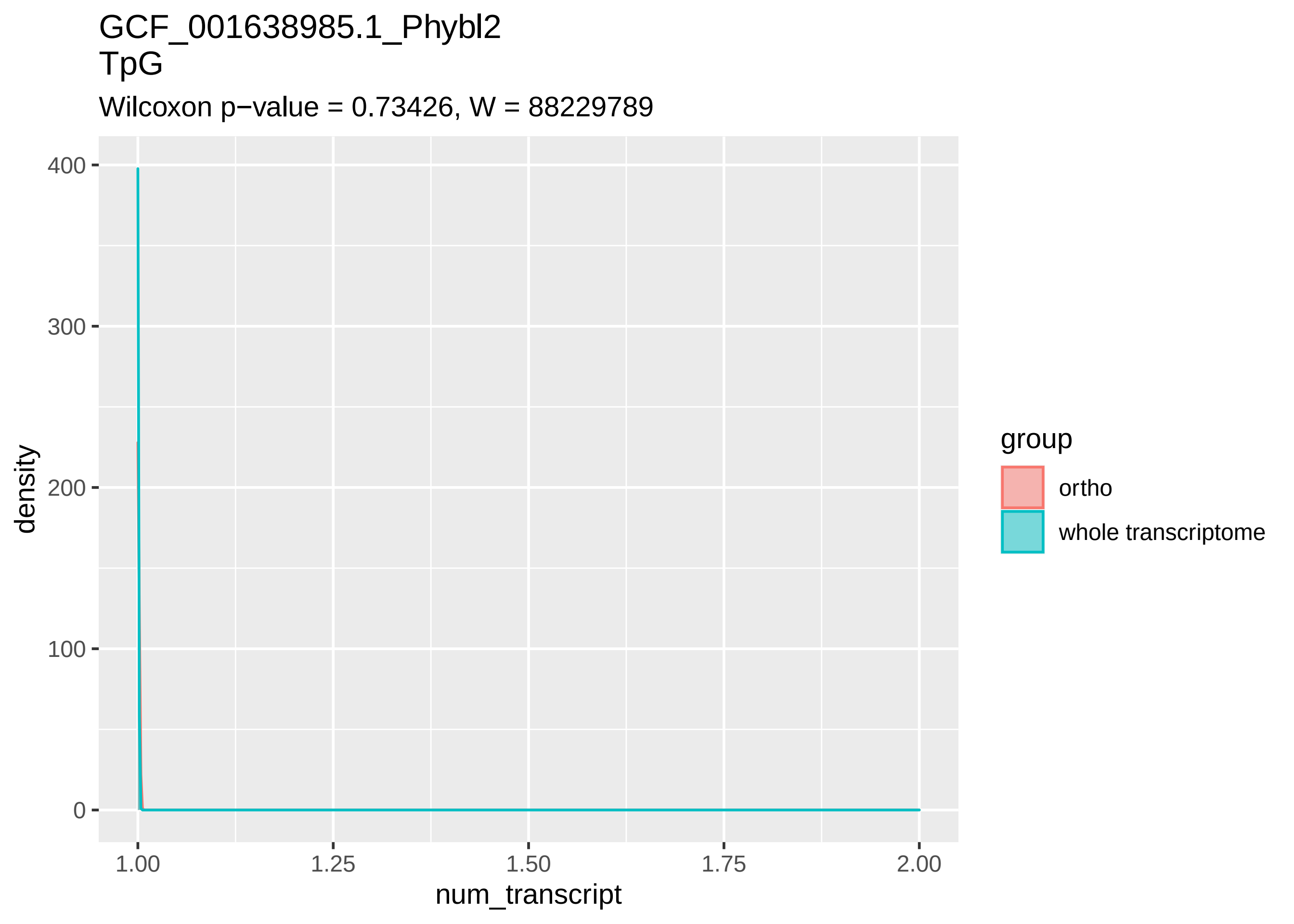

GCF_001638985.1_Phybl2
TpG
Wilcoxon p−value = 0.73426, W = 88229789
density
400
300
200
100
0
1.00
1.25
1.50
1.75
2.00
num_transcript
group
ortho
whole transcriptome

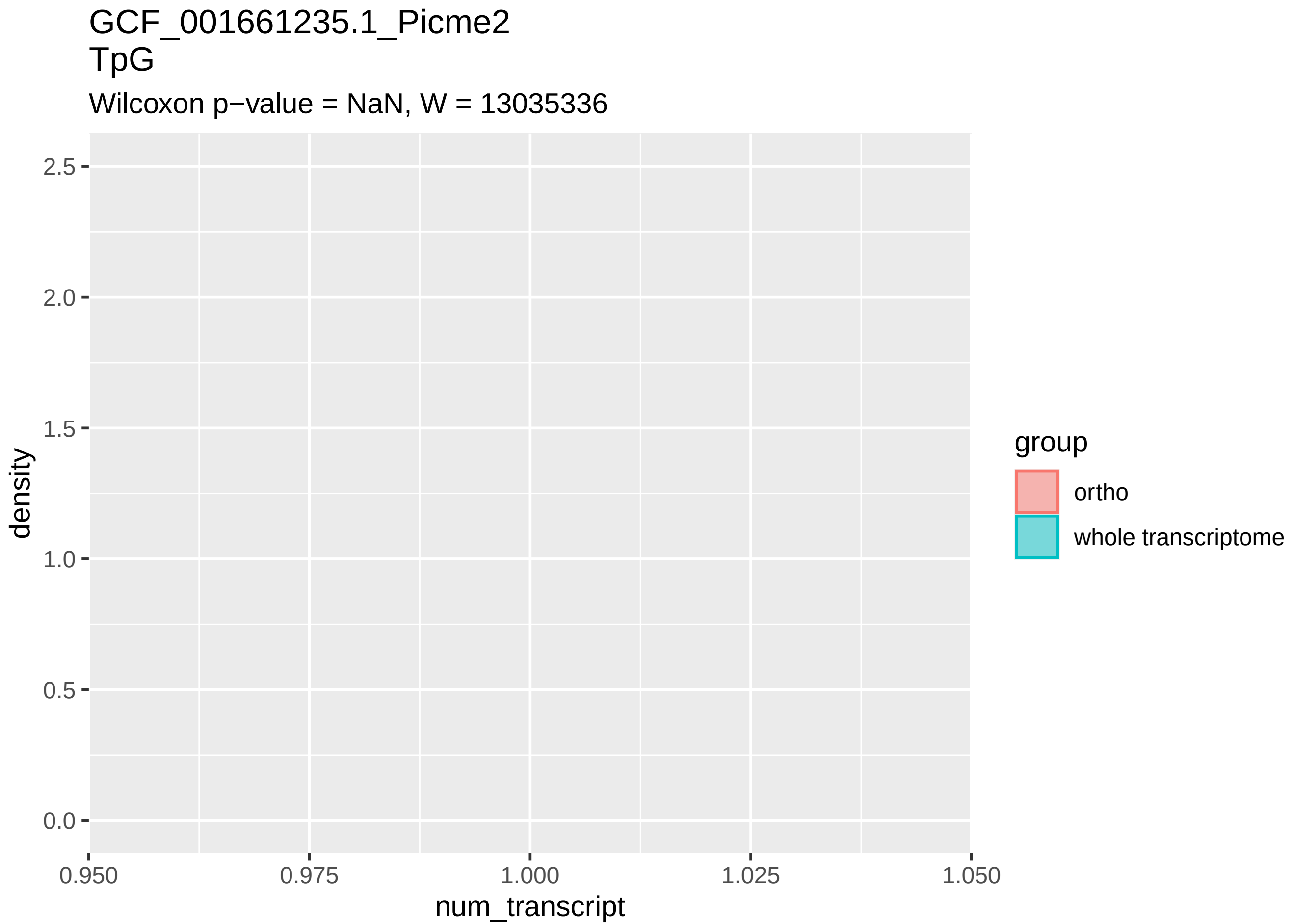
GCF_001661235.1_Picme2
TpG
Wilcoxon p−value = NaN, W = 13035336
density
2.5
2.0
1.5
1.0
0.5
0.0
0.950
0.975
1.000
1.025
1.050
num_transcript
group
ortho
whole transcriptome

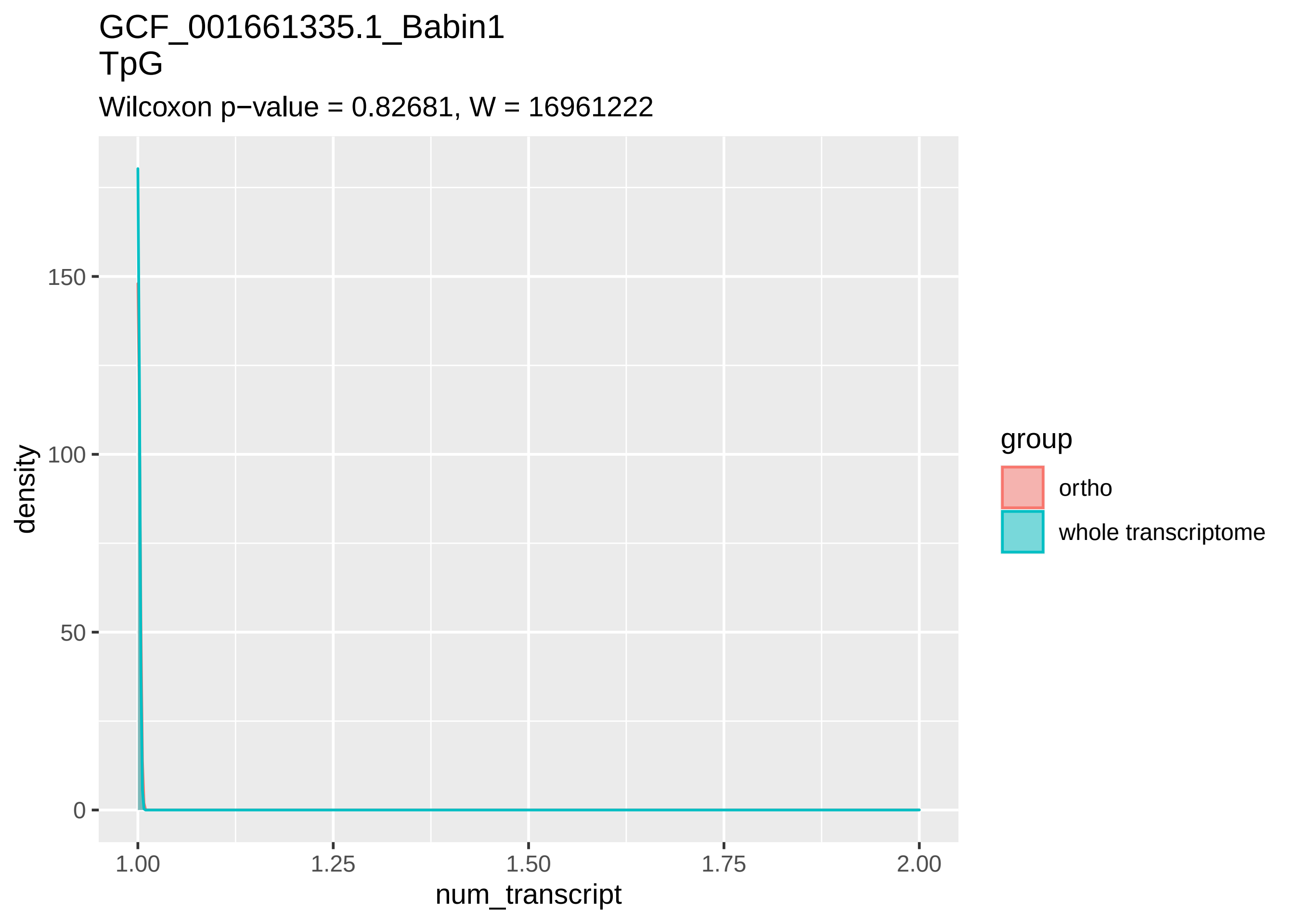
GCF_001661335.1_Babin1
TpG
Wilcoxon p−value = 0.82681, W = 16961222
density
150
100
50
0
1.00
1.25
1.50
1.75
2.00
num_transcript
group
ortho
whole transcriptome

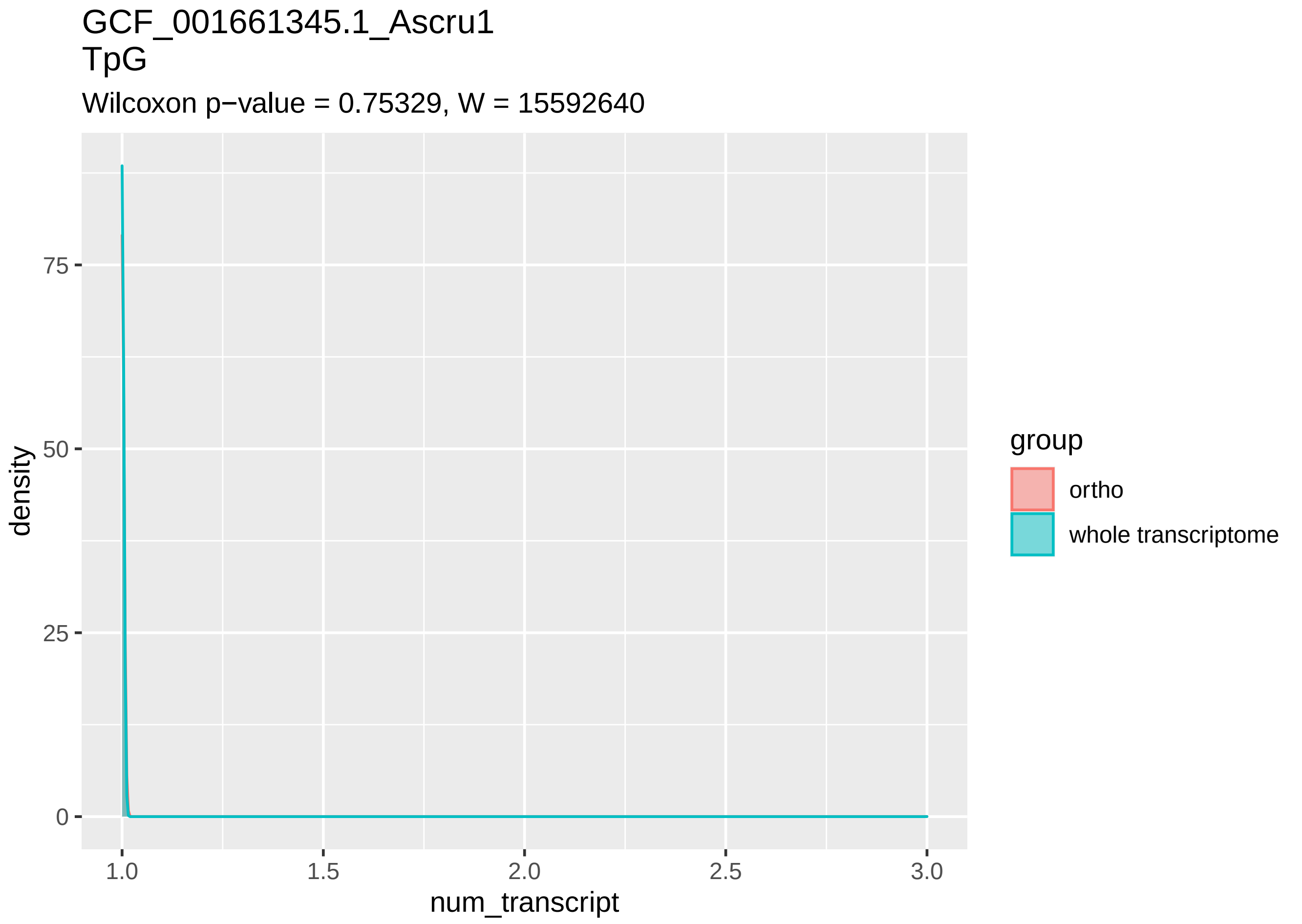
GCF_001661345.1_Ascru1
TpG
Wilcoxon p−value = 0.75329, W = 15592640
density
75
50
25
0
1.0
1.5
2.0
2.5
3.0
num_transcript
group
ortho
whole transcriptome

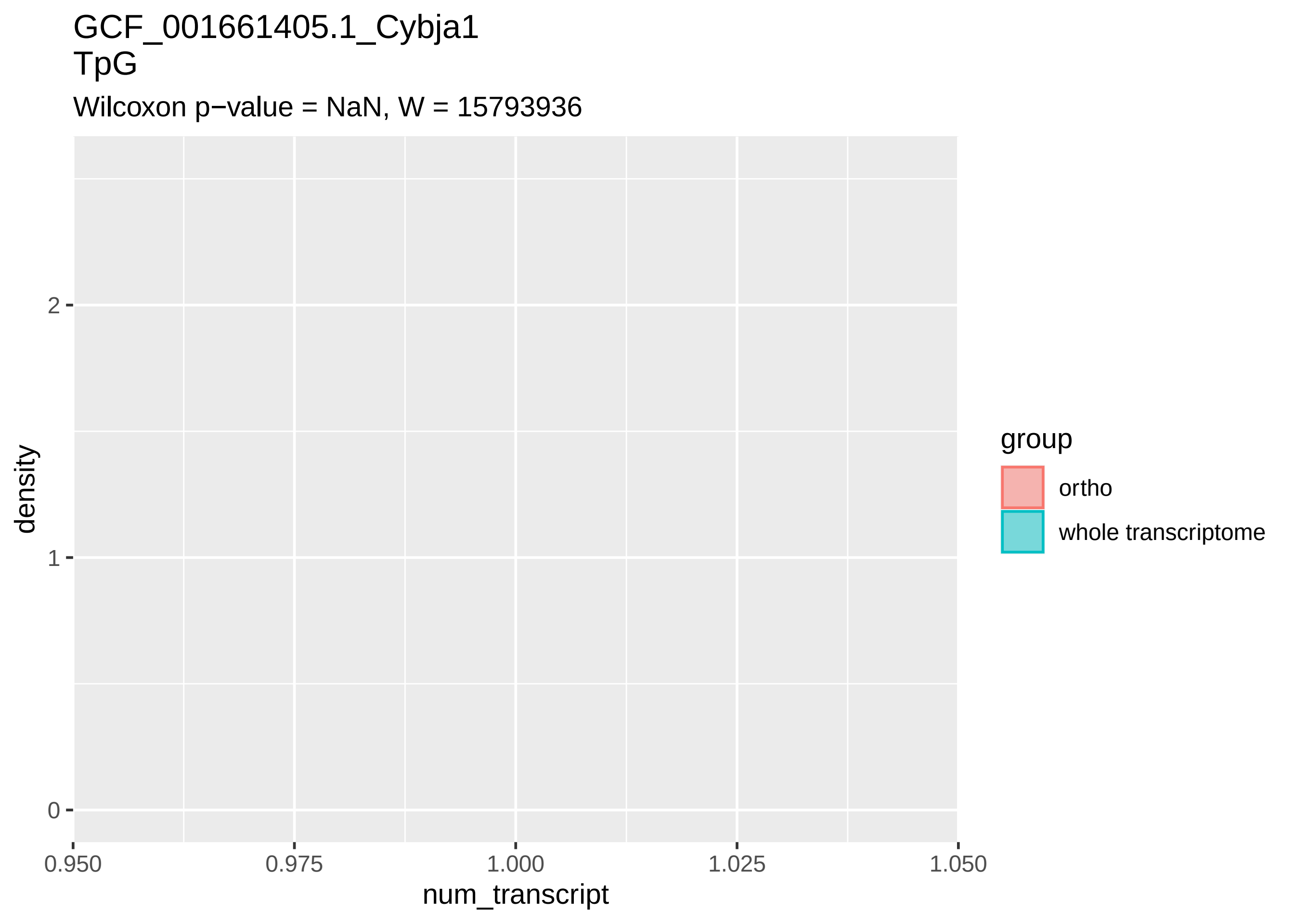

GCF_001661405.1_Cybja1
TpG
Wilcoxon p−value = NaN, W = 15793936
density
2
1
0
0.950
0.975
1.000
1.025
1.050
num_transcript
group
ortho
whole transcriptome

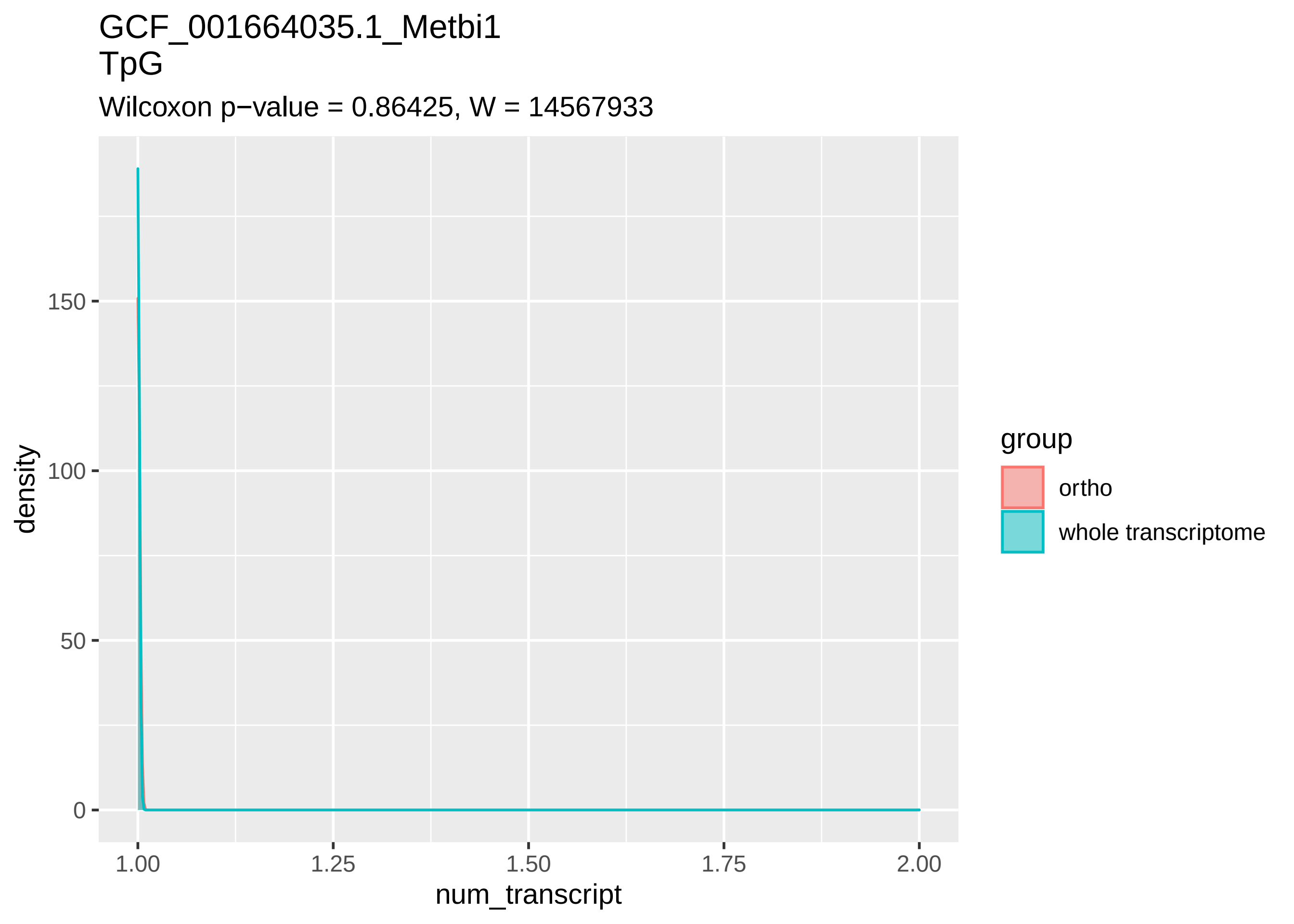
GCF_001664035.1_Metbi1
TpG
Wilcoxon p−value = 0.86425, W = 14567933
density
150
100
50
0
1.00
1.25
1.50
1.75
2.00
num_transcript
group
ortho
whole transcriptome

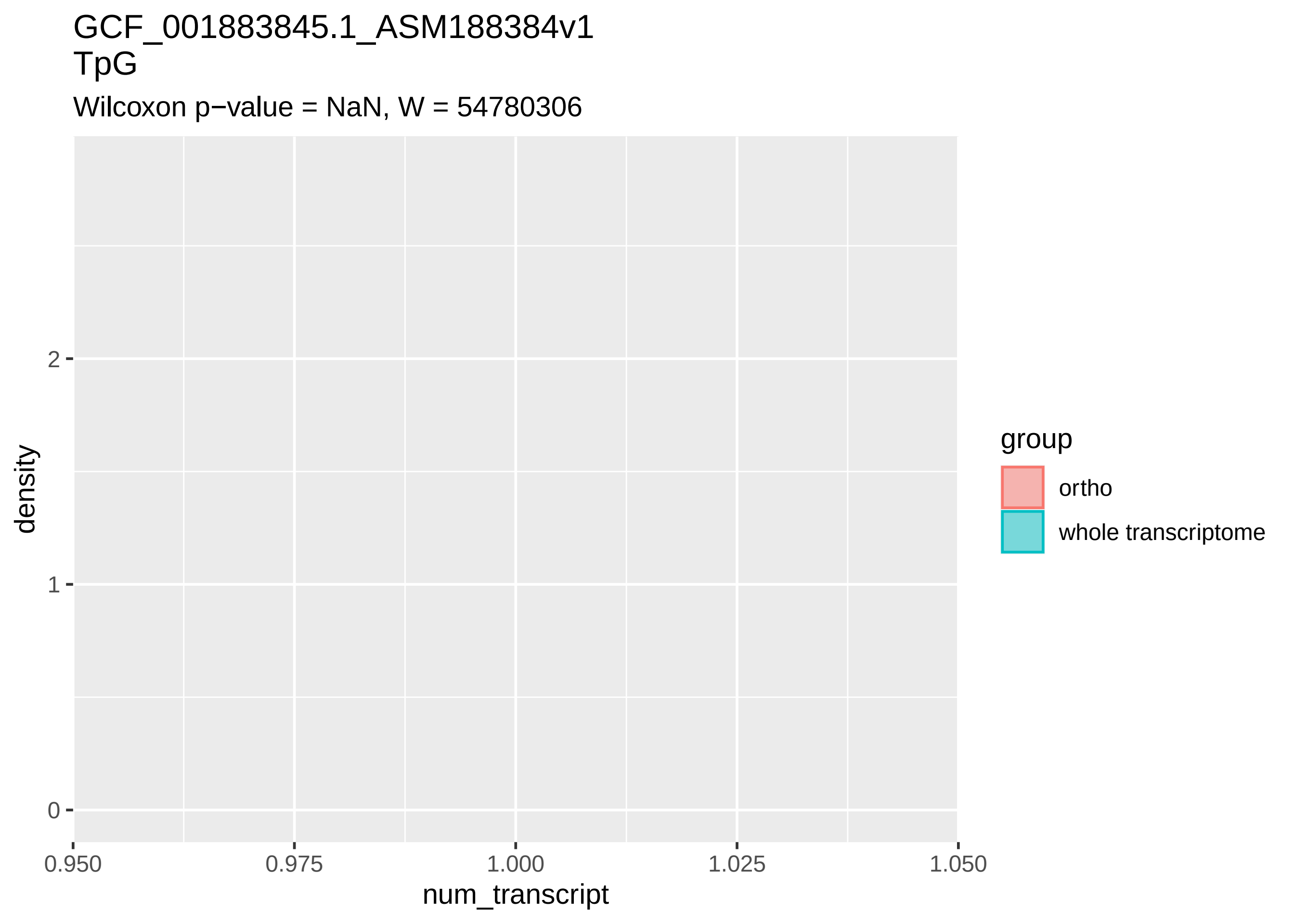

GCF_001883845.1_ASM188384v1
TpG
Wilcoxon p−value = NaN, W = 54780306
density
0
1
2
0.950
0.975
1.000
1.025
1.050
num_transcript
group
ortho
whole transcriptome

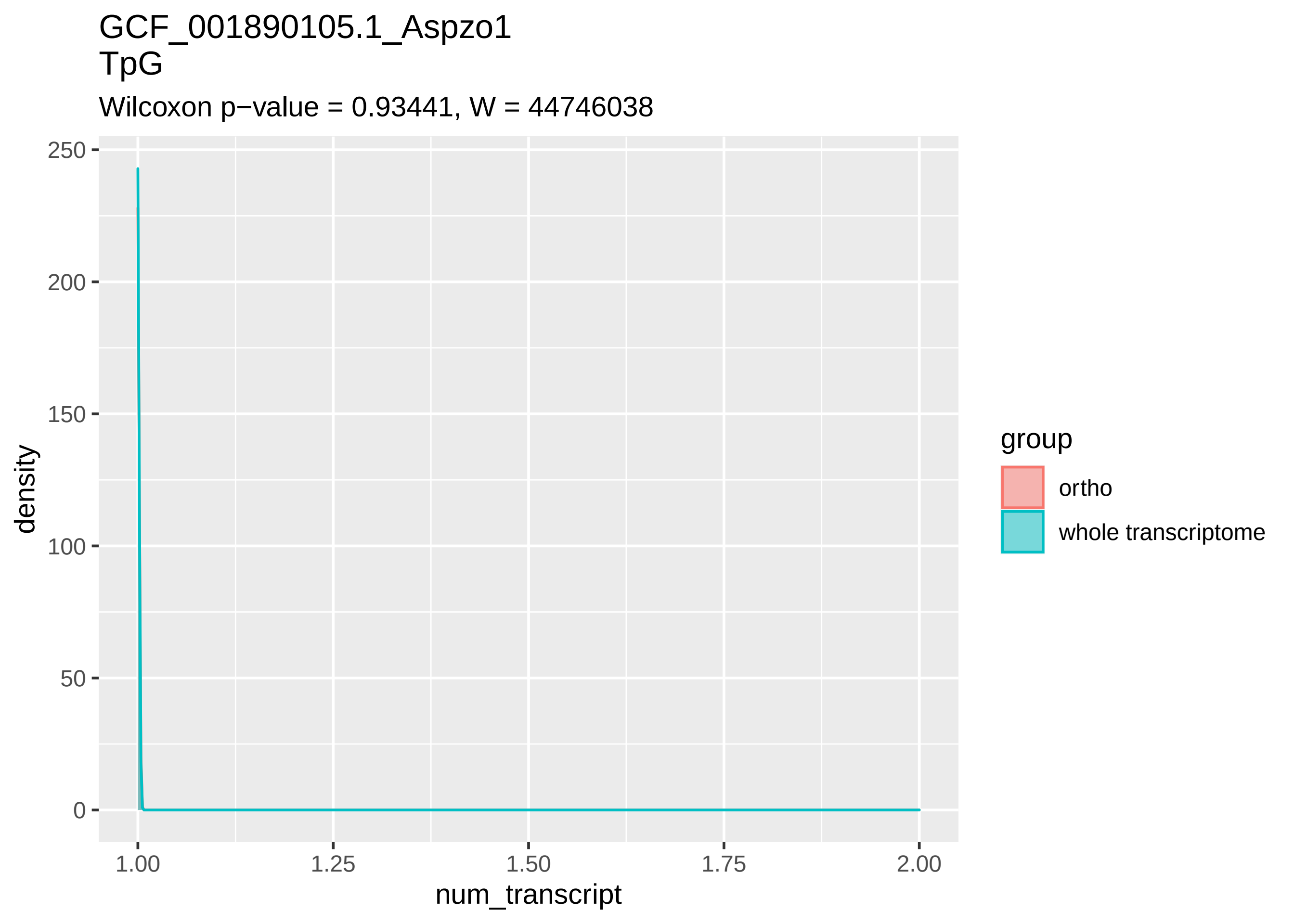

GCF_001890105.1_Aspzo1
TpG
Wilcoxon p−value = 0.93441, W = 44746038
density
250
200
150
100
50
0
1.00
1.25
1.50
1.75
2.00
num_transcript
group
ortho
whole transcriptome

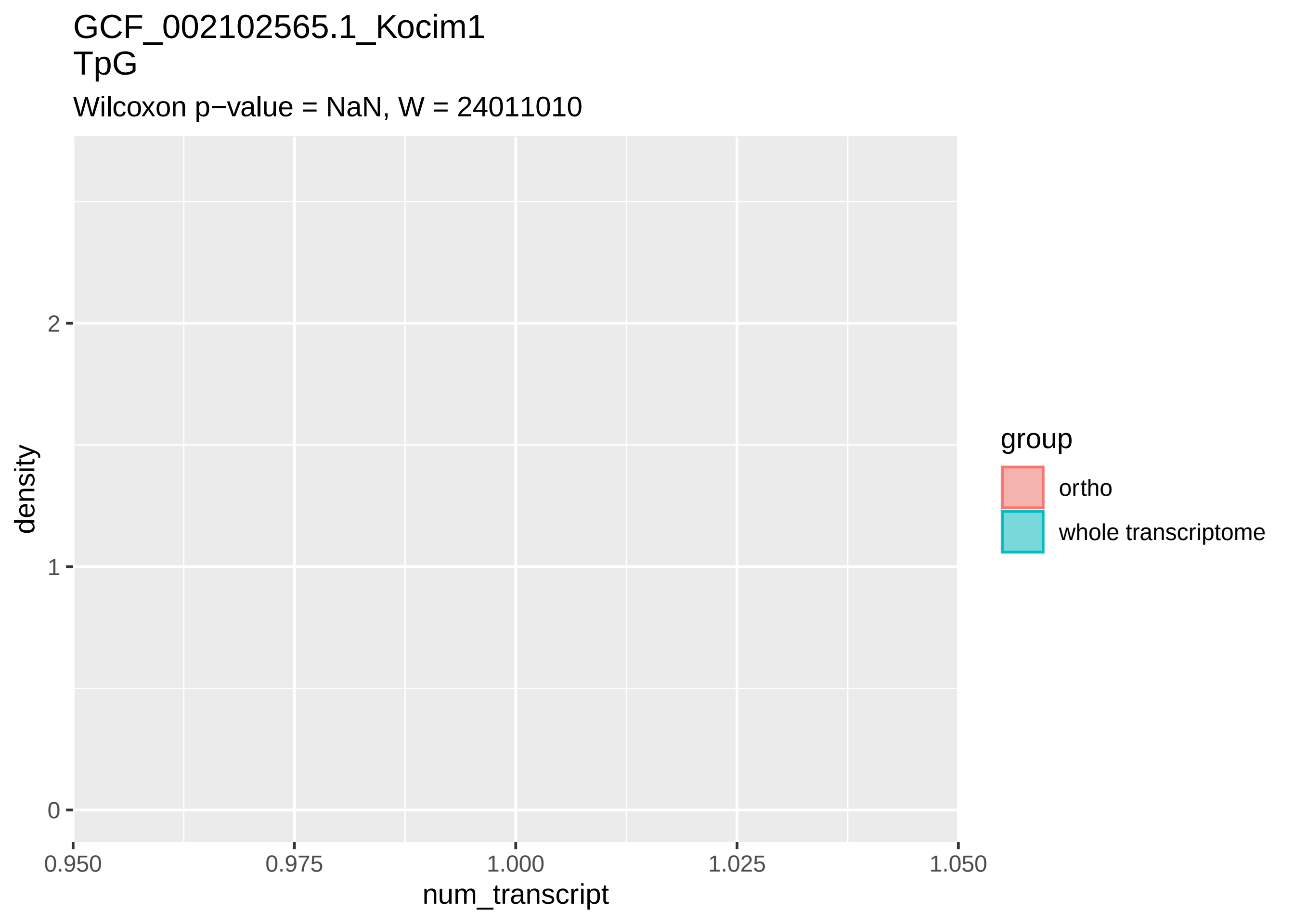
GCF_002102565.1_Kocim1
TpG
Wilcoxon p−value = NaN, W = 24011010
density
2
1
0
0.950
0.975
1.000
1.025
1.050
num_transcript
group
ortho
whole transcriptome

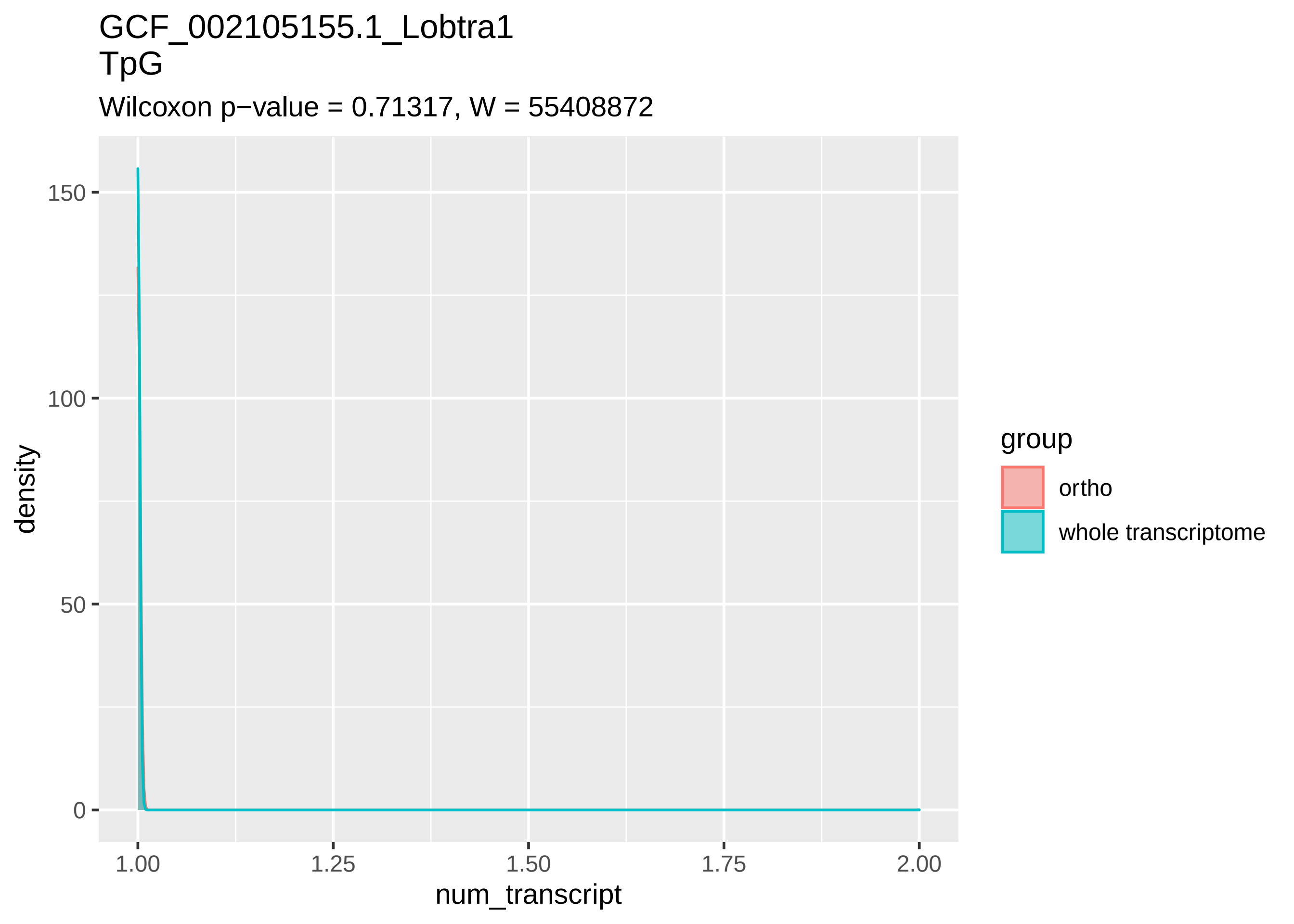
GCF_002105155.1_Lobtra1
TpG
Wilcoxon p−value = 0.71317, W = 55408872
150
100
50
0
density
1.00
1.25
1.50
1.75
2.00
num_transcript
group
ortho
whole transcriptome

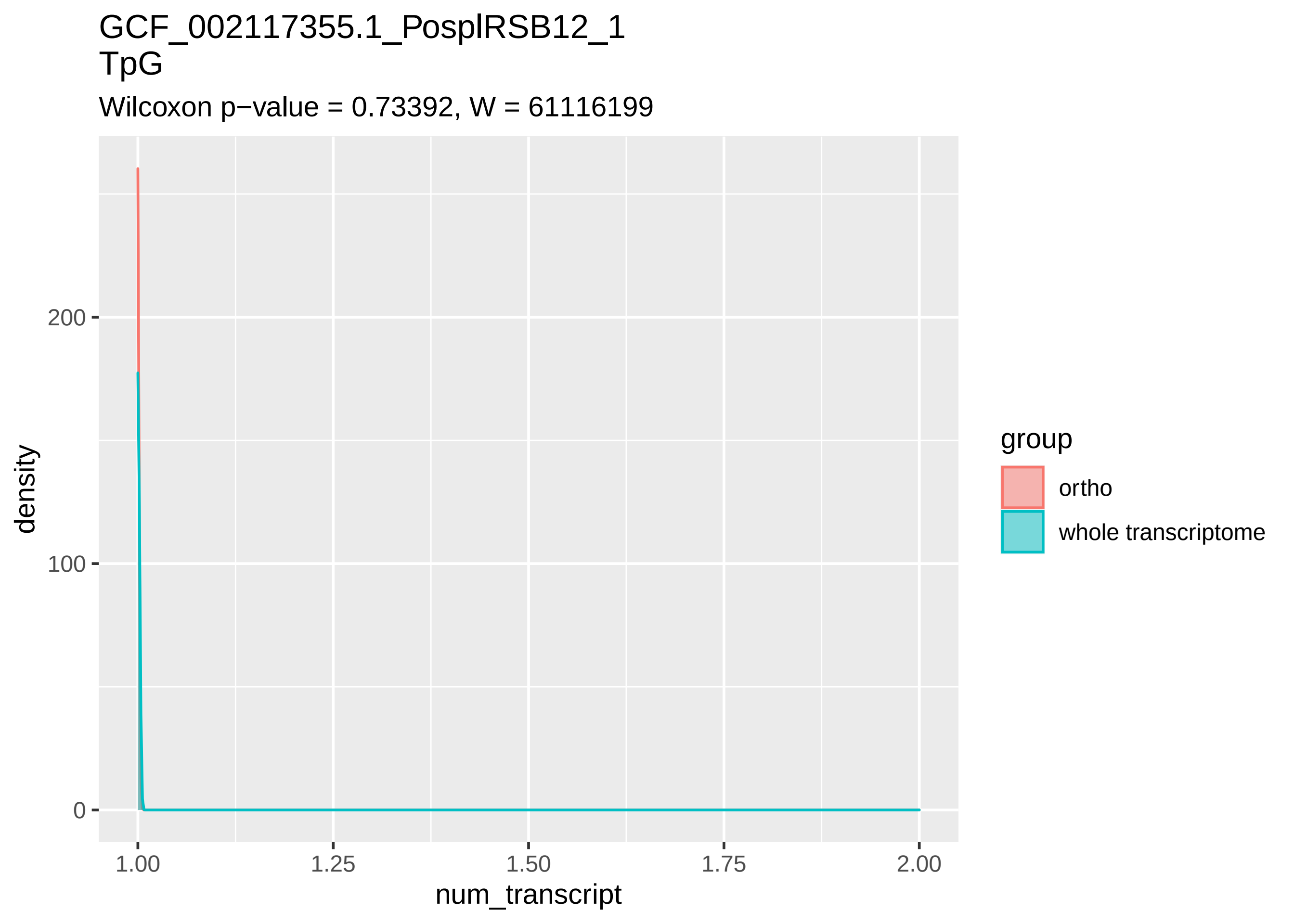
GCF_002117355.1_PosplRSB12_1
TpG
Wilcoxon p−value = 0.73392, W = 61116199
density
200
100
0
1.00
1.25
1.50
1.75
2.00
num_transcript
group
ortho
whole transcriptome

GCF_002847465.1_Aspnov1
TpG

Wilcoxon p−value = 0.9157, W = 60733097

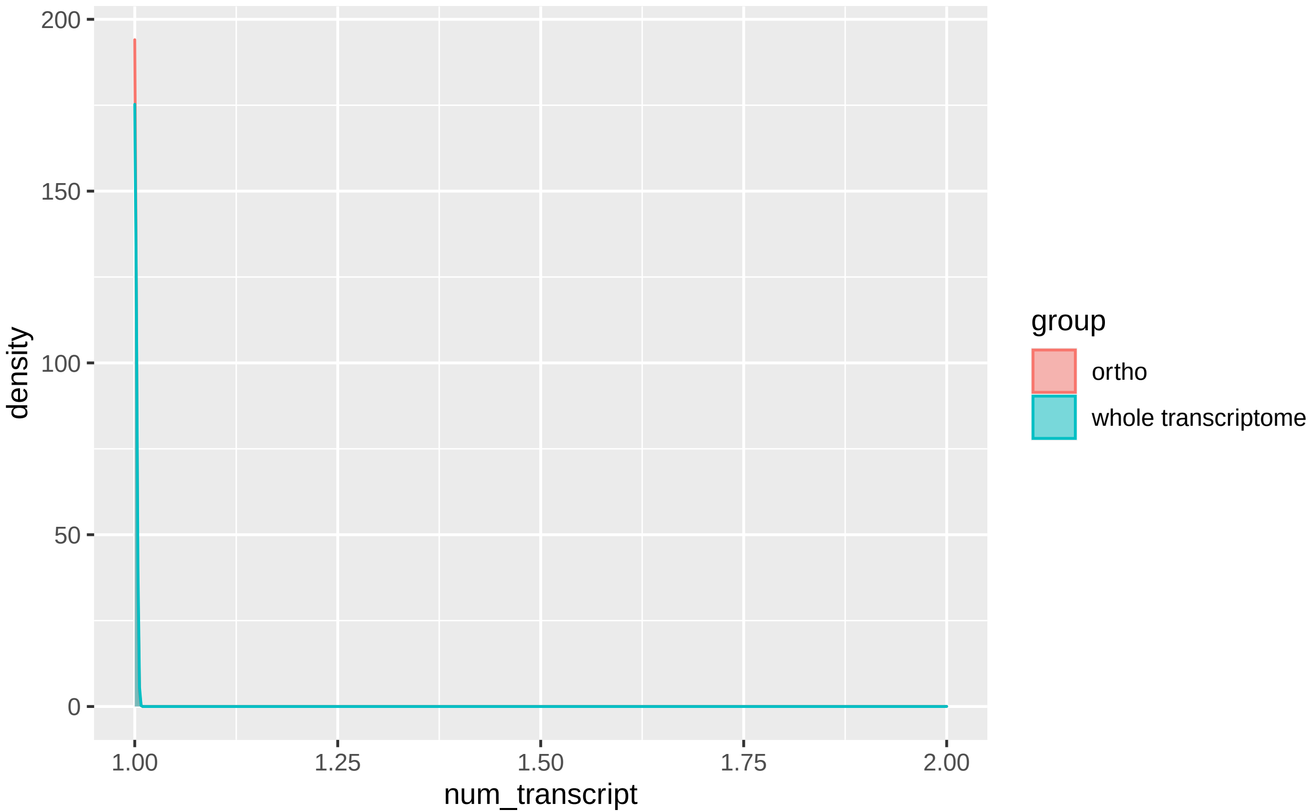

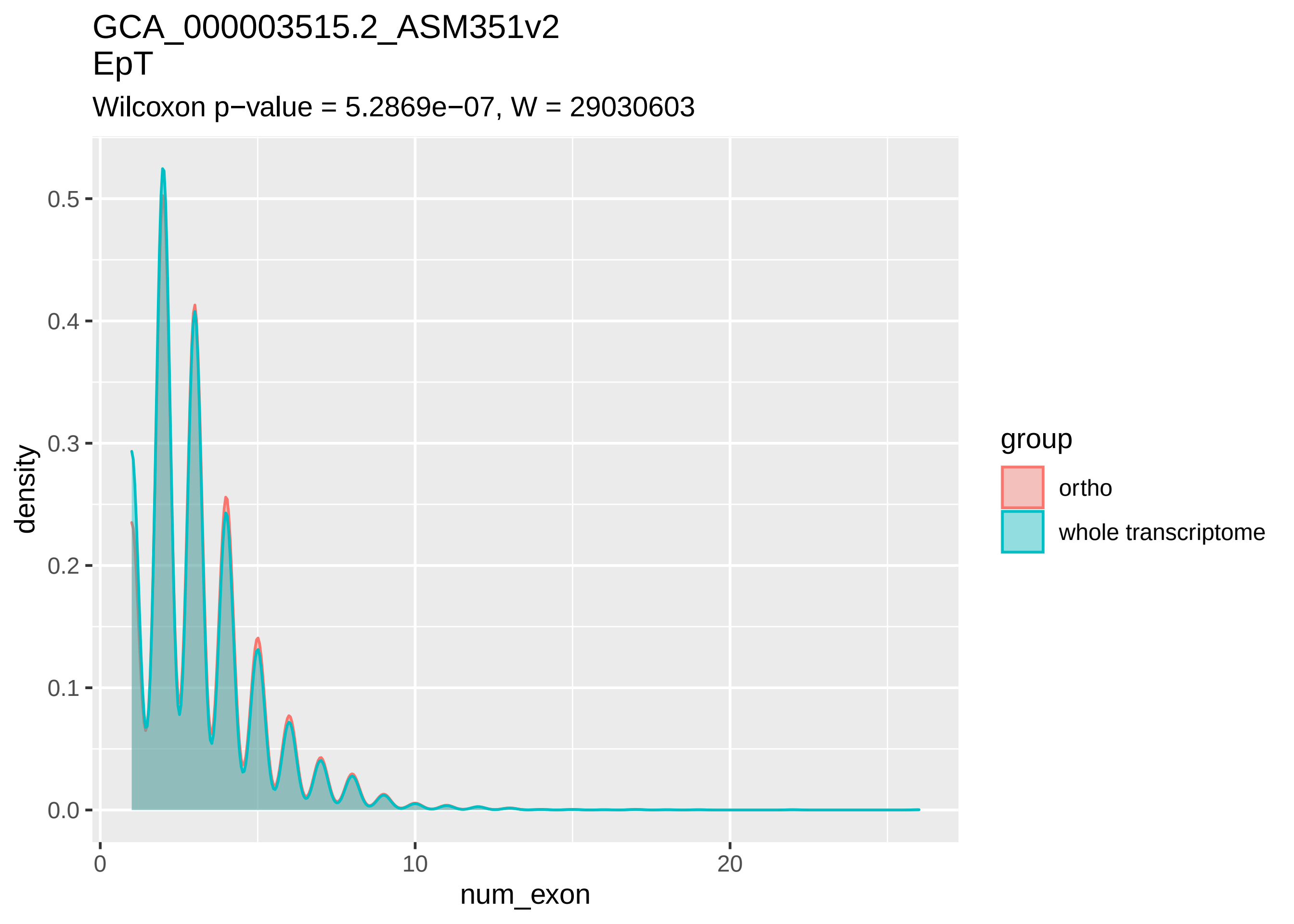

GCA_000003515.2_ASM351v2
EpT
Wilcoxon p−value = 5.2869e−07, W = 29030603
density
0.0
0.1
0.2
0.3
0.4
0.5
0
10
20
num_exon
group
ortho
whole transcriptome

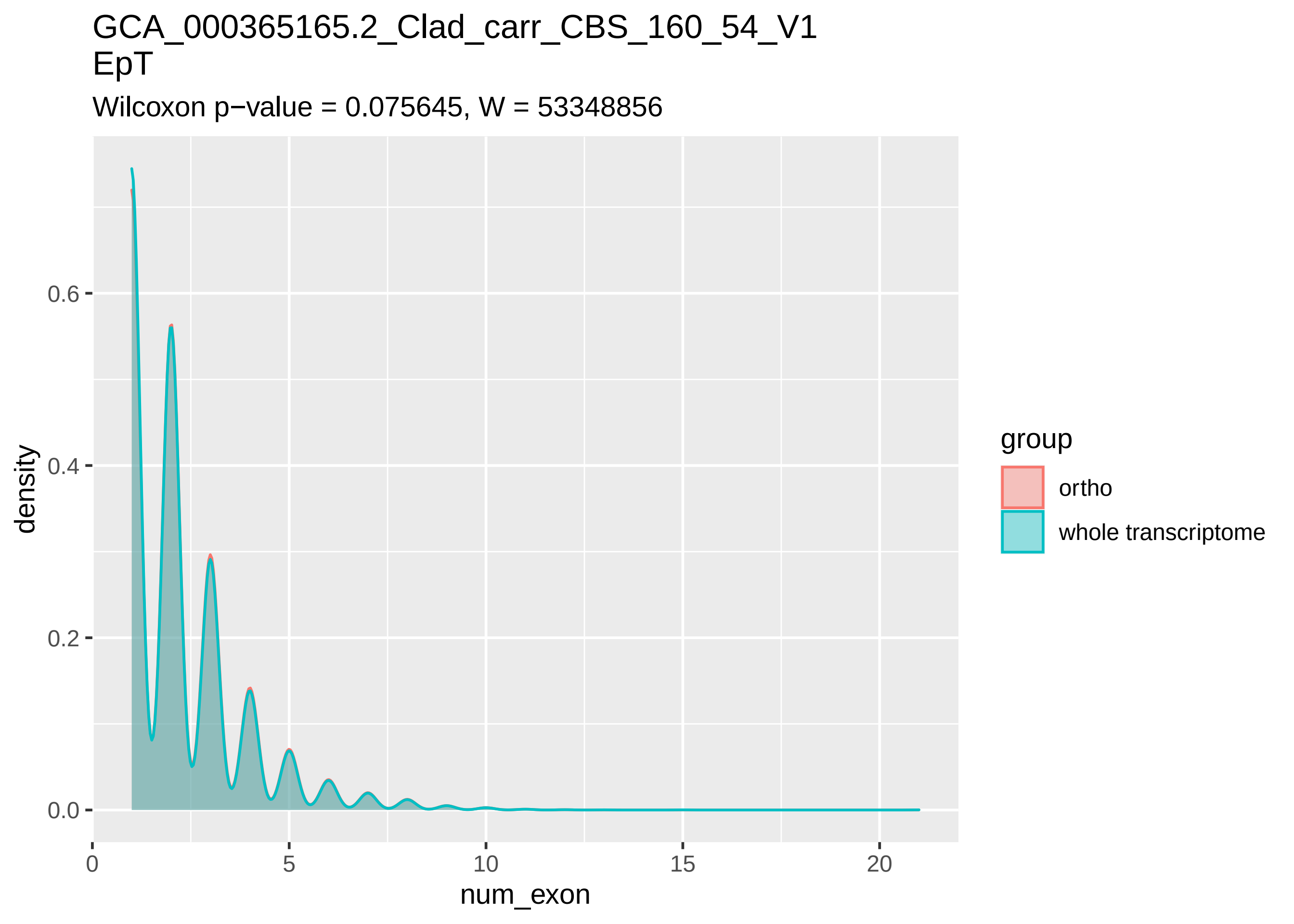

GCA_000365165.2_Clad_carr_CBS_160_54_V1
EpT
Wilcoxon p−value = 0.075645, W = 53348856
density
0.0
0.2
0.4
0.6
0
5
10
15
20
num_exon
group
ortho
whole transcriptome

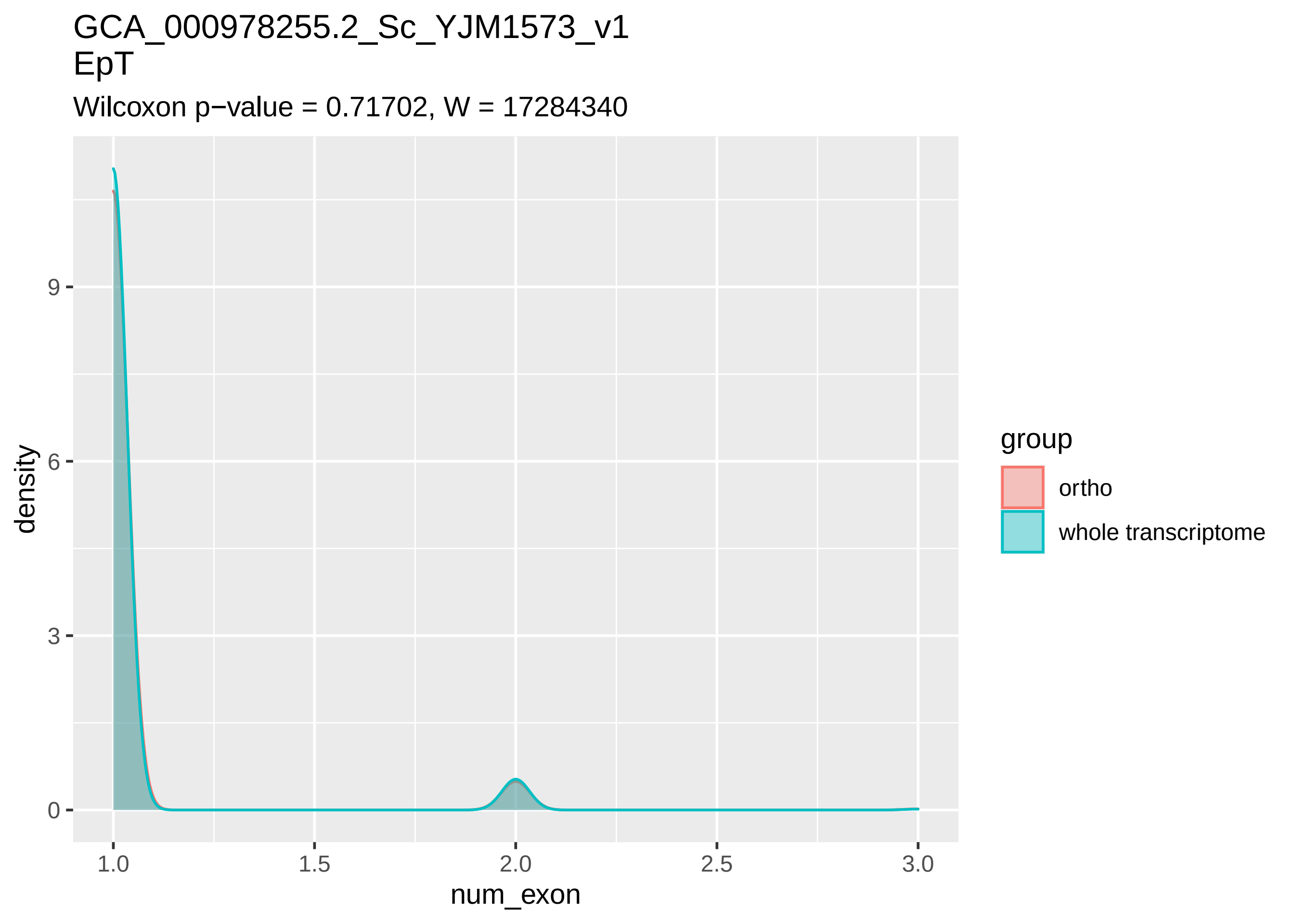
GCA_000978255.2_Sc_YJM1573_v1
EpT
Wilcoxon p−value = 0.71702, W = 17284340
density
num_exon
0
3
6
9
1.0
1.5
2.0
2.5
3.0
group
ortho
whole transcriptome

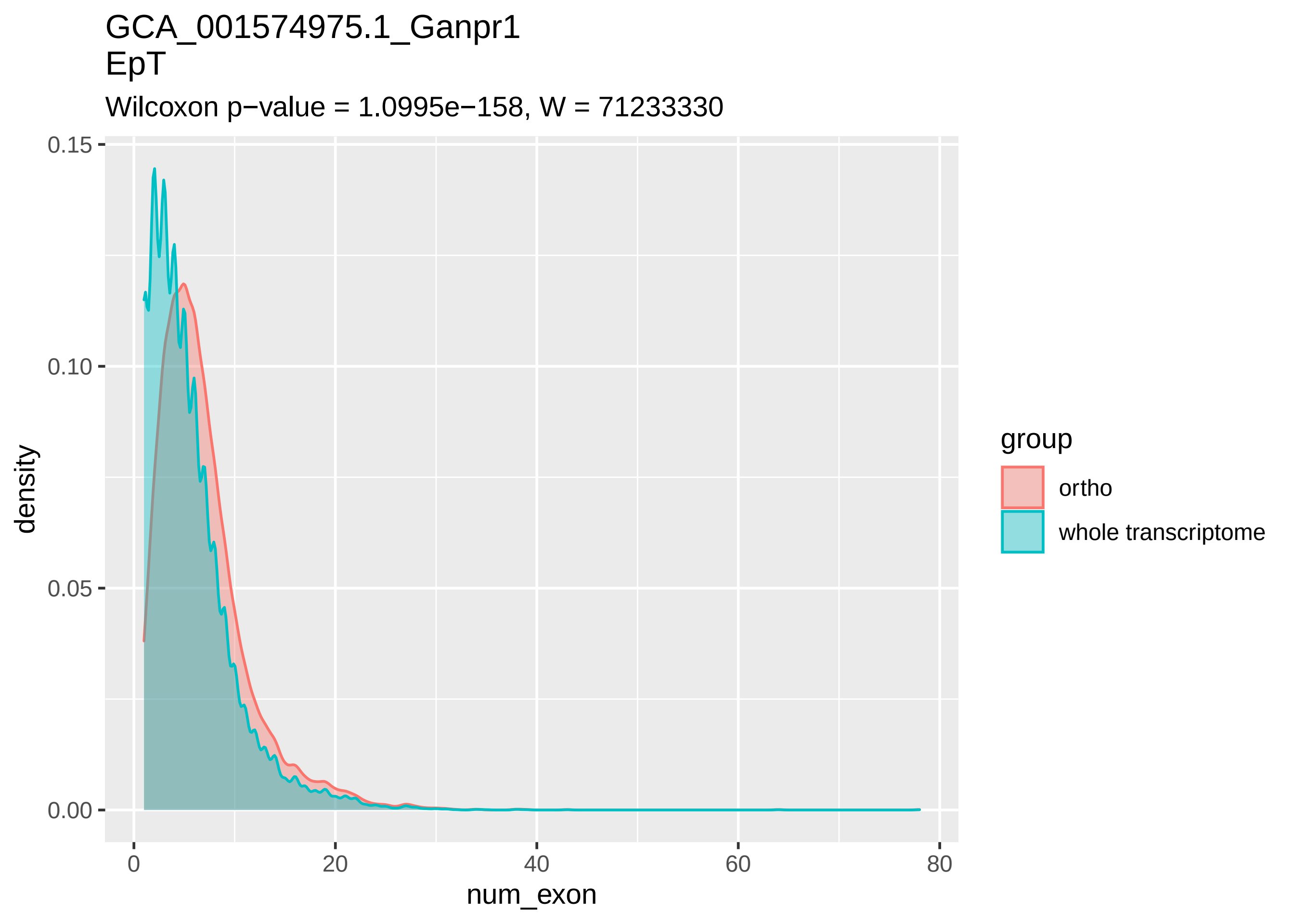

GCA_001574975.1_Ganpr1
EpT
Wilcoxon p−value = 1.0995e−158, W = 71233330
0.15
0.10
0.05
0.00
density
0
20
40
60
80
num_exon
group
ortho
whole transcriptome

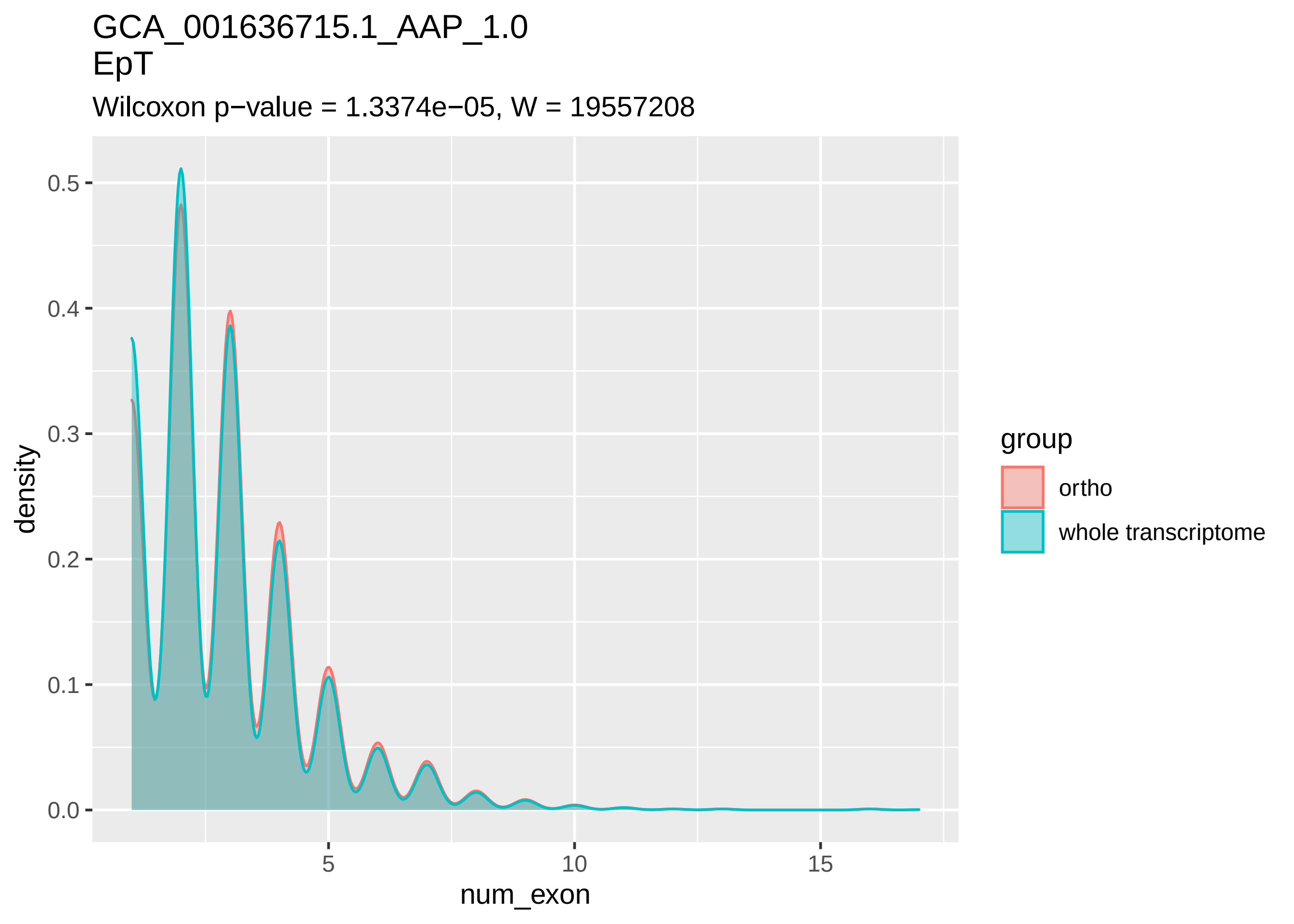
GCA_001636715.1_AAP_1.0
EpT
Wilcoxon p−value = 1.3374e−05, W = 19557208
density
0.5
0.4
0.3
0.2
0.1
0.0
5
10
15
num_exon
group
ortho
whole transcriptome

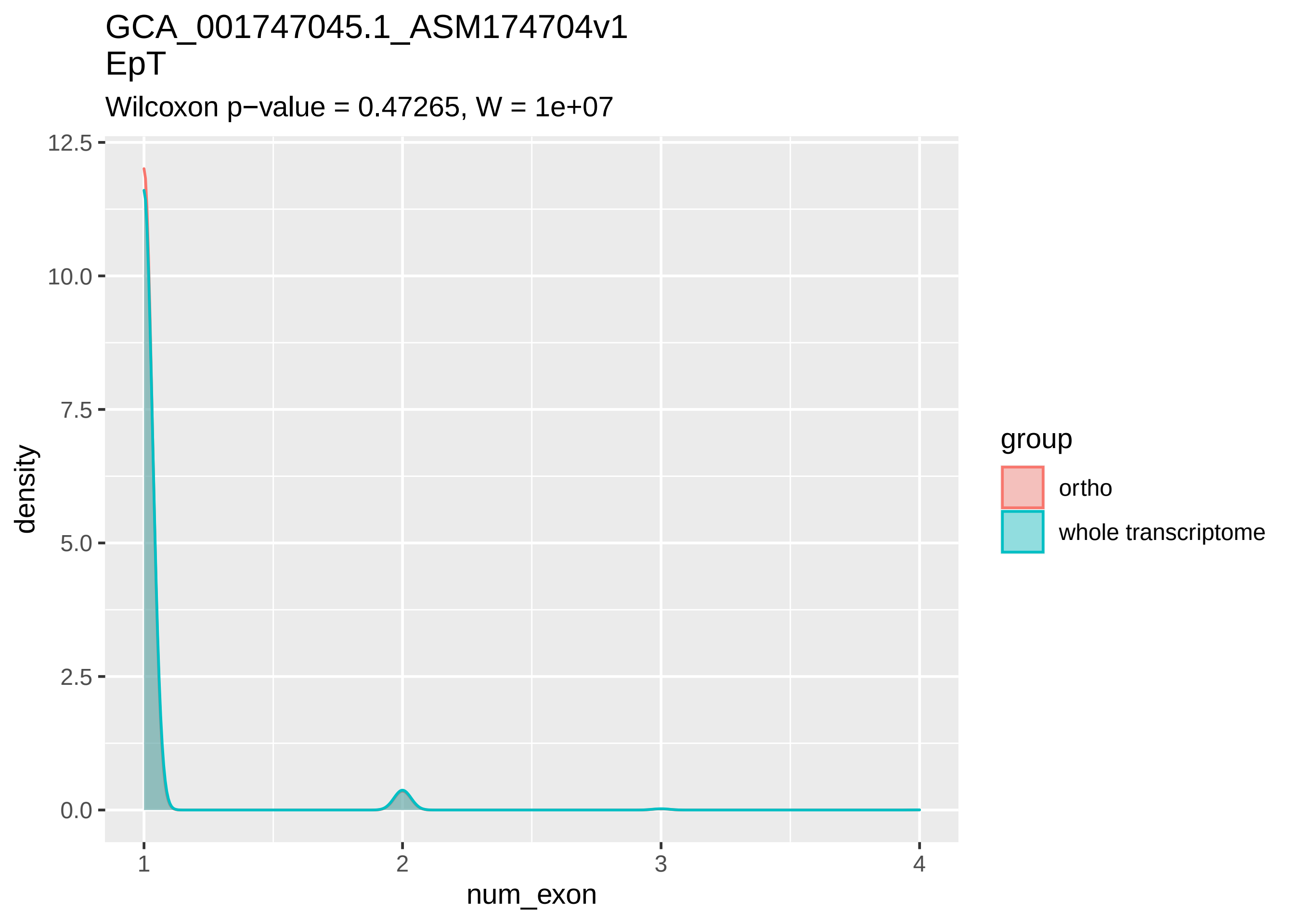

GCA_001747045.1_ASM174704v1
EpT
Wilcoxon p−value = 0.47265, W = 1e+07
12.5
10.0
7.5
5.0
2.5
0.0
density
1
2
3
4
num_exon
group
ortho
whole transcriptome

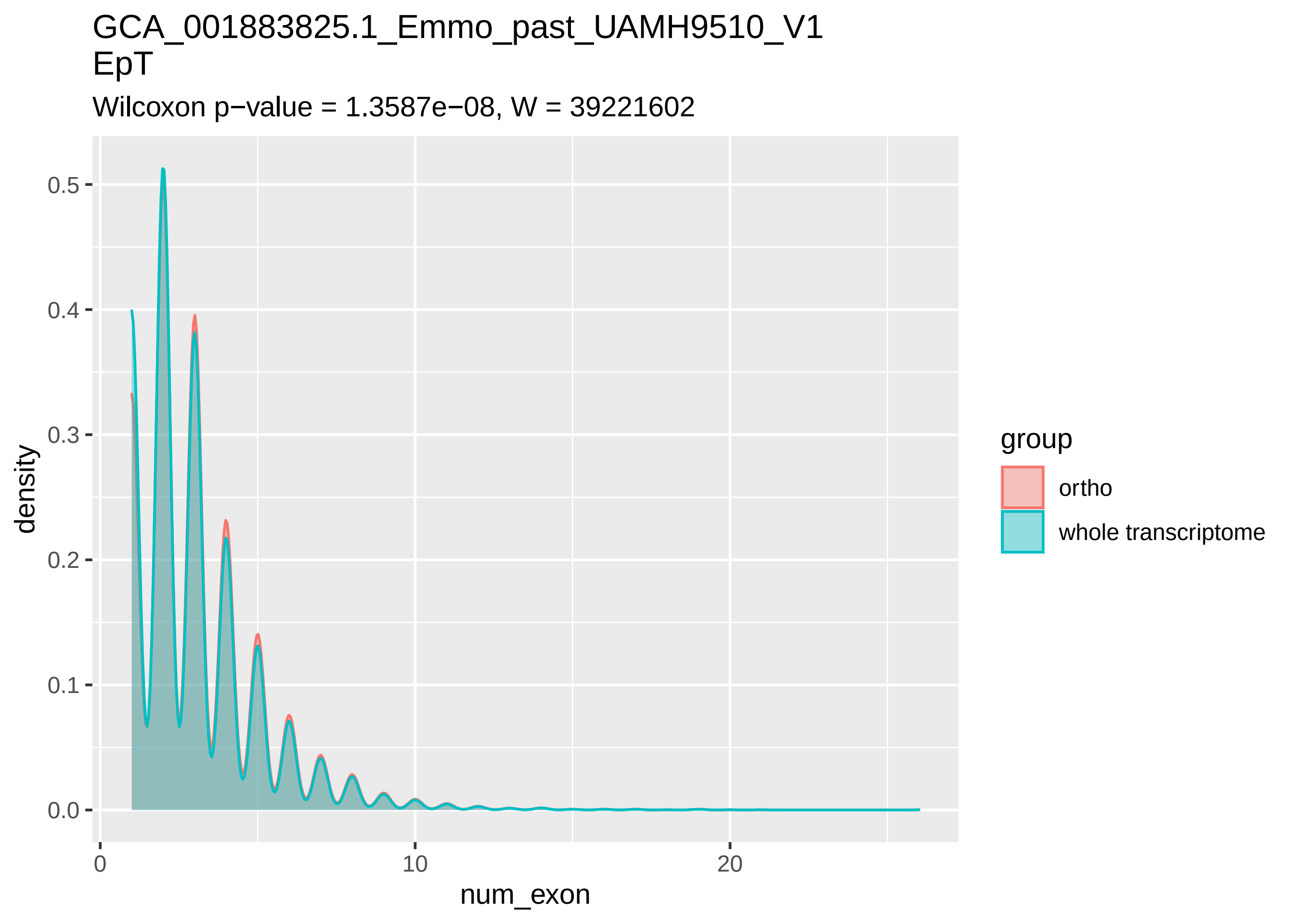

GCA_001883825.1_Emmo_past_UAMH9510_V1
EpT
Wilcoxon p−value = 1.3587e−08, W = 39221602
density
0.0
0.1
0.2
0.3
0.4
0.5
0
10
20
num_exon
group
ortho
whole transcriptome

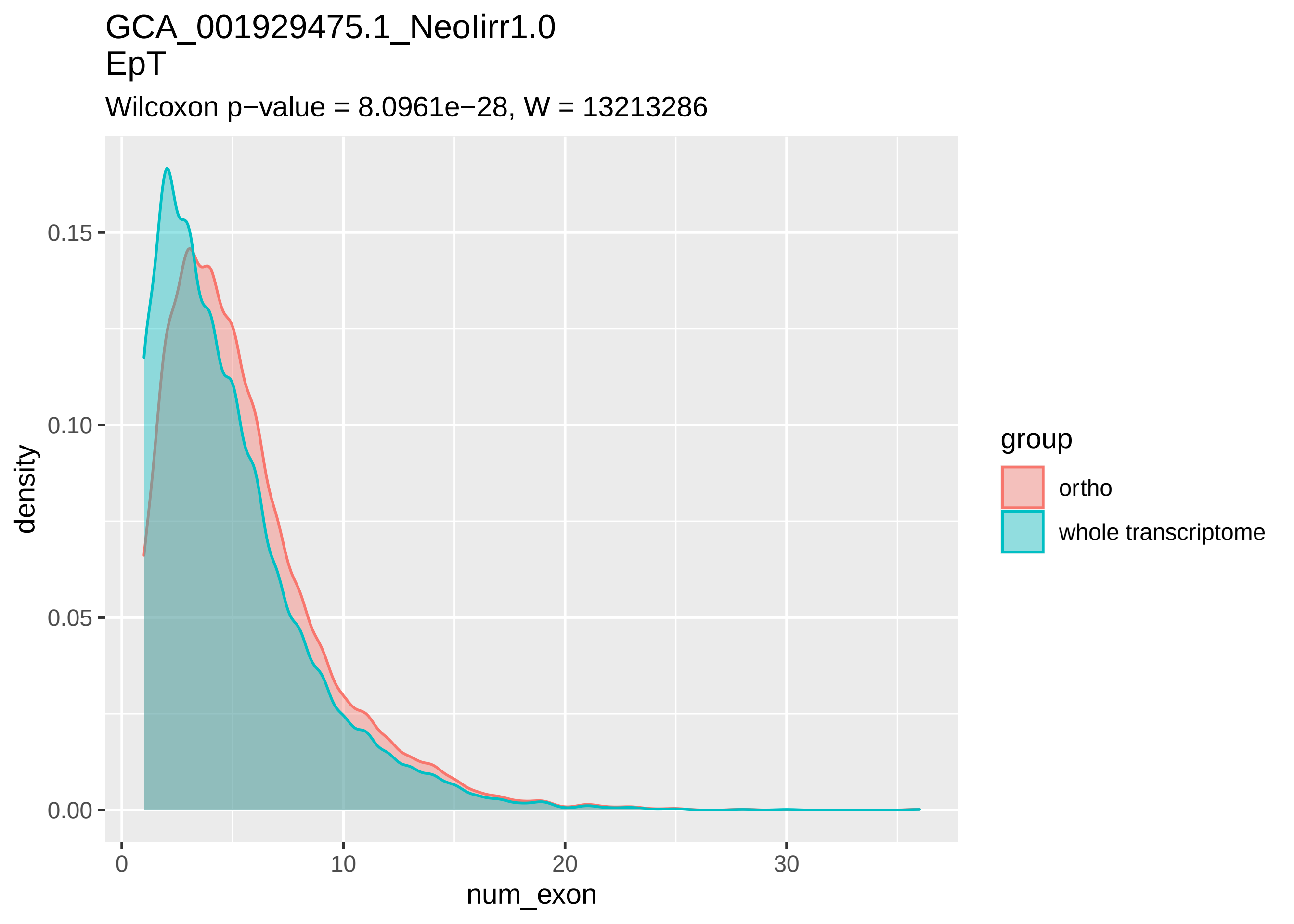

GCA_001929475.1_NeoIirr1.0
EpT
Wilcoxon p−value = 8.0961e−28, W = 13213286
density
0.15
0.10
0.05
0.00
0
10
20
30
num_exon
group
ortho
whole transcriptome

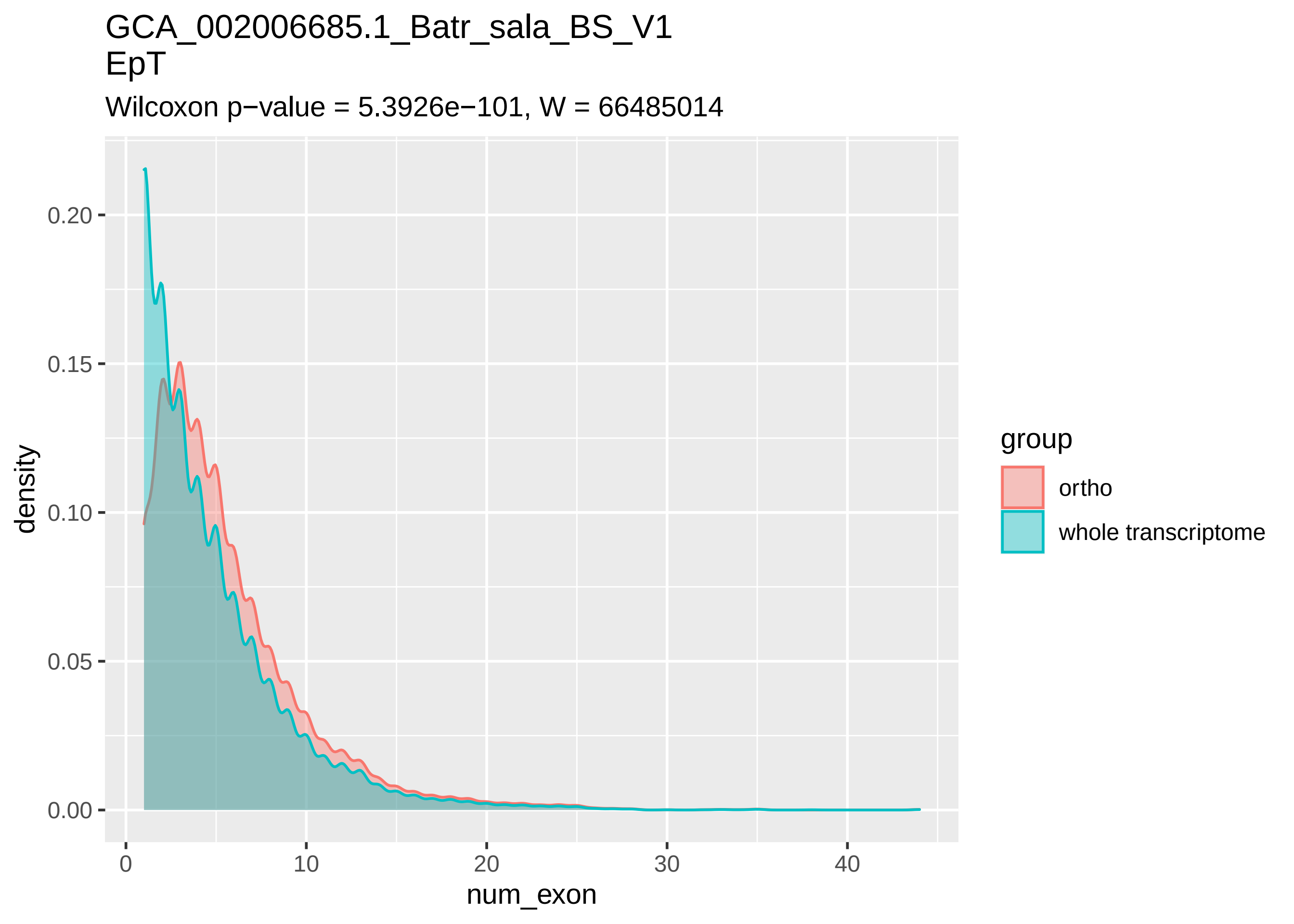
GCA_002006685.1_Batr_sala_BS_V1
EpT
Wilcoxon p−value = 5.3926e−101, W = 66485014
density
0.20
0.15
0.10
0.05
0.00
0
10
20
30
40
num_exon
group
ortho
whole transcriptome

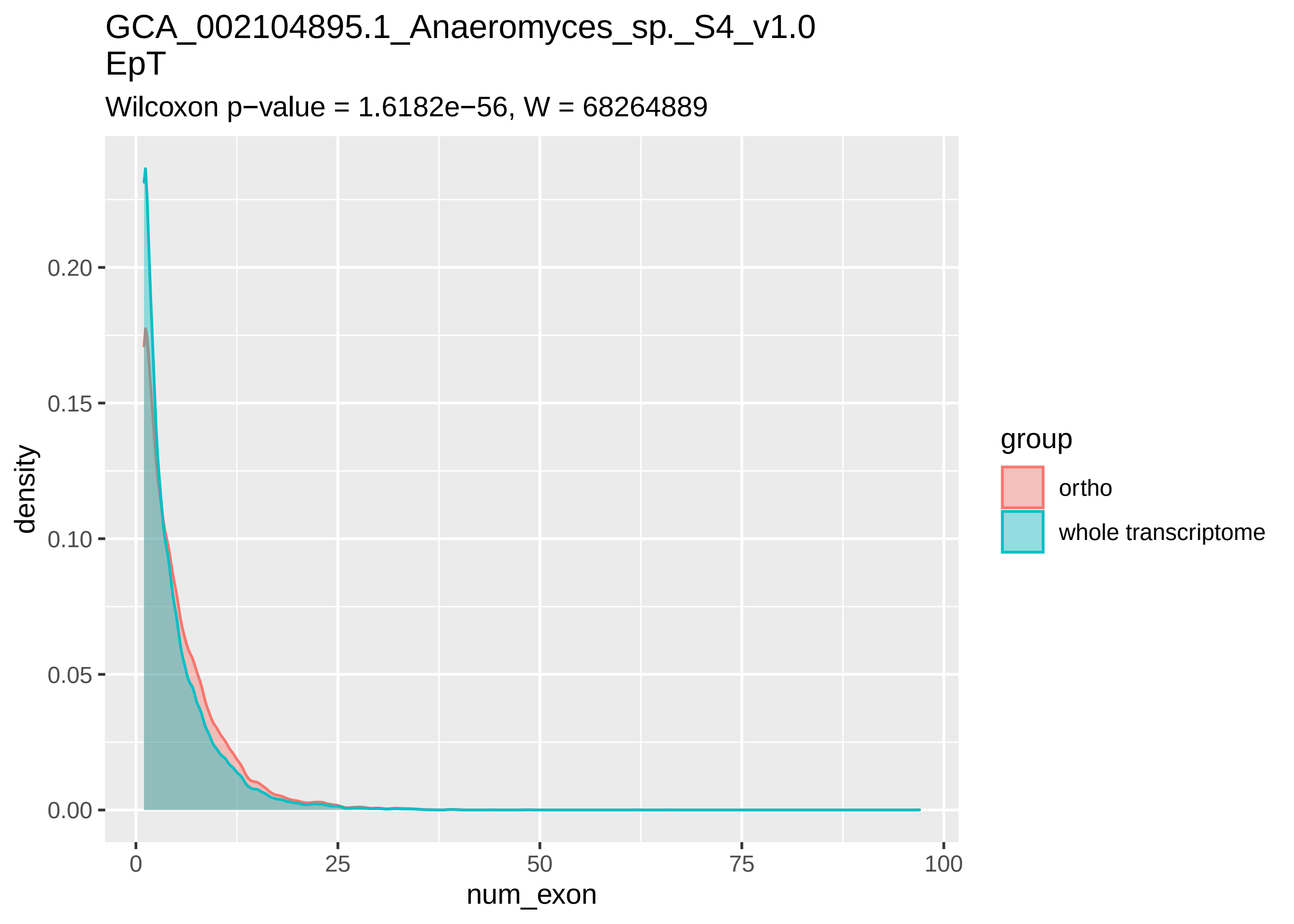
GCA_002104895.1_Anaeromyces_sp._S4_v1.0
EpT
Wilcoxon p−value = 1.6182e−56, W = 68264889
density
0.00
0.05
0.10
0.15
0.20
0
25
50
75
100
num_exon
group
ortho
whole transcriptome

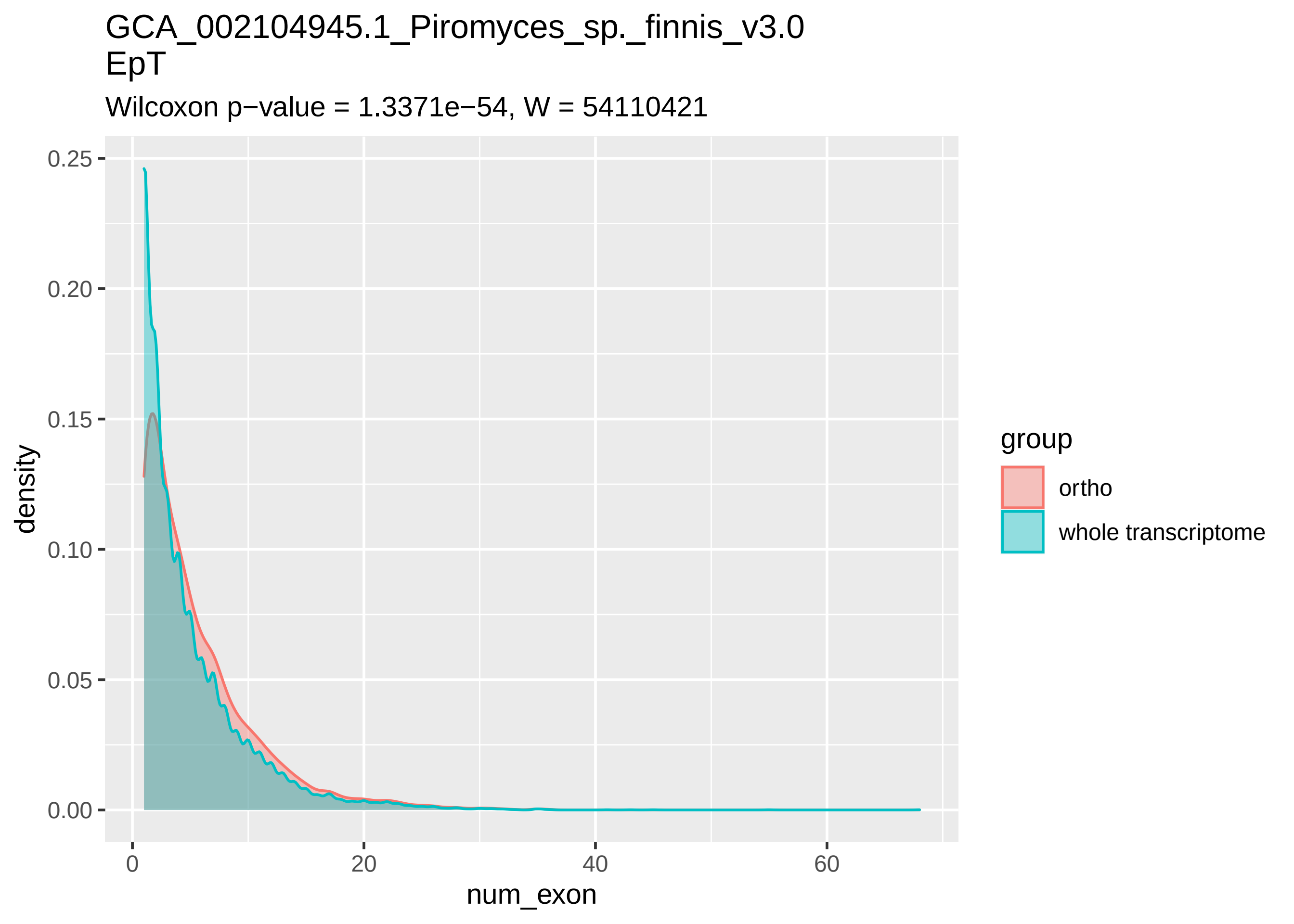
GCA_002104945.1_Piromyces_sp._finnis_v3.0
EpT
Wilcoxon p−value = 1.3371e−54, W = 54110421
density
0.25
0.20
0.15
0.10
0.05
0.00
0
20
40
60
num_exon
group
ortho
whole transcriptome

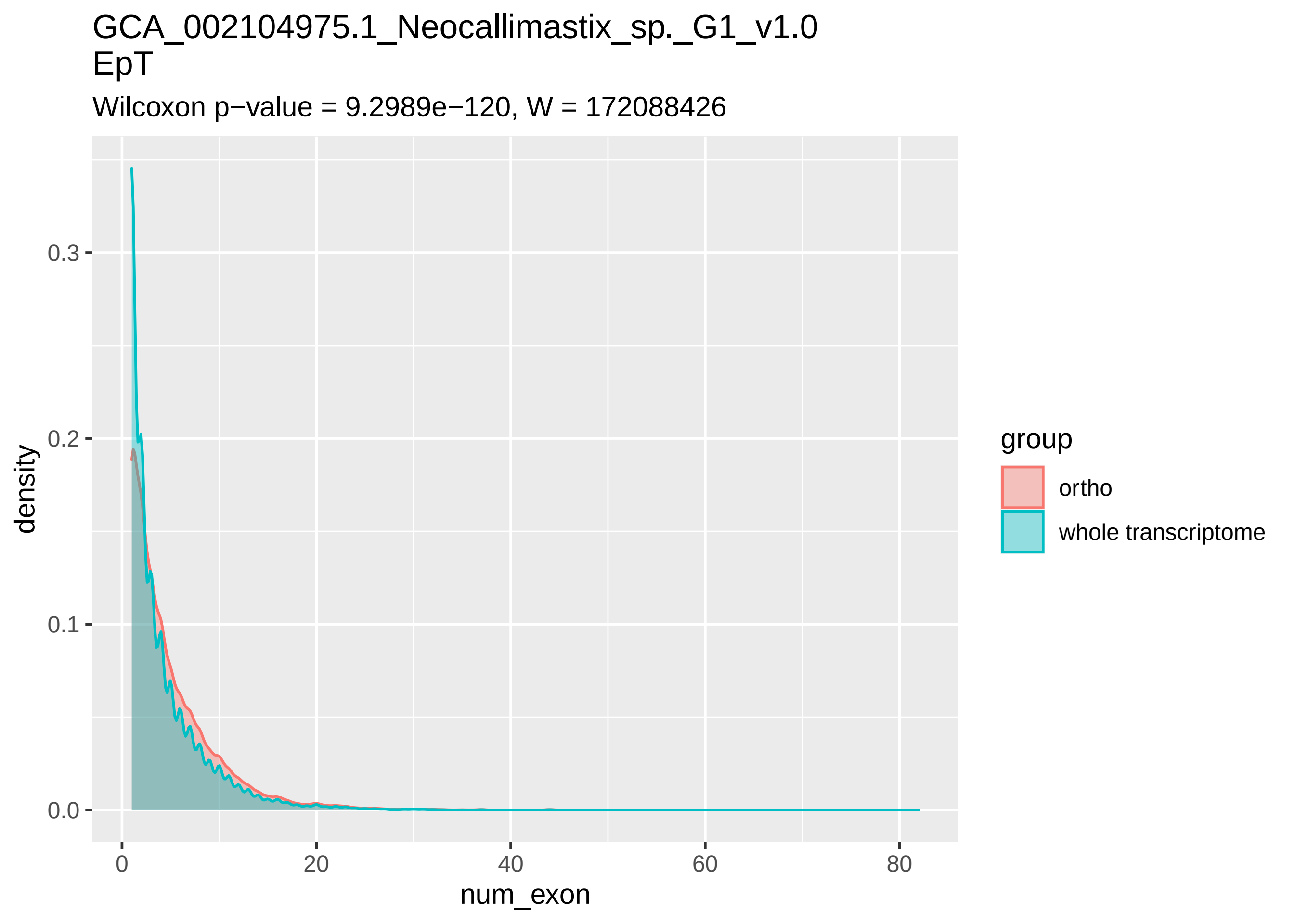
GCA_002104975.1_Neocallimastix_sp._G1_v1.0
EpT
Wilcoxon p−value = 9.2989e−120, W = 172088426
density
0.3
0.2
0.1
0.0
0
20
40
60
80
num_exon
group
ortho
whole transcriptome

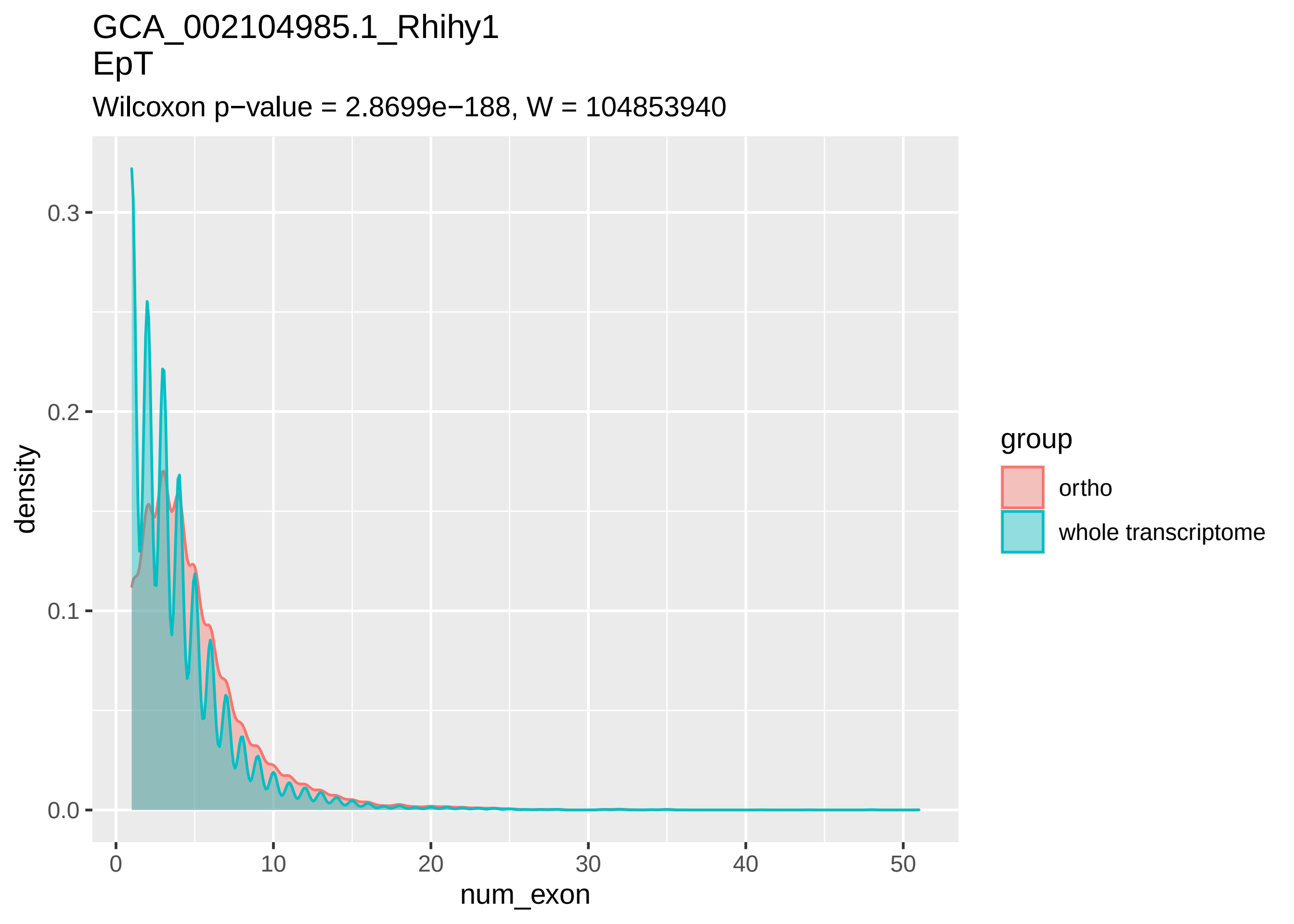

GCA_002104985.1_Rhihy1
EpT
Wilcoxon p−value = 2.8699e−188, W = 104853940
density
0.0
0.1
0.2
0.3
0
10
20
30
40
50
num_exon
group
ortho
whole transcriptome

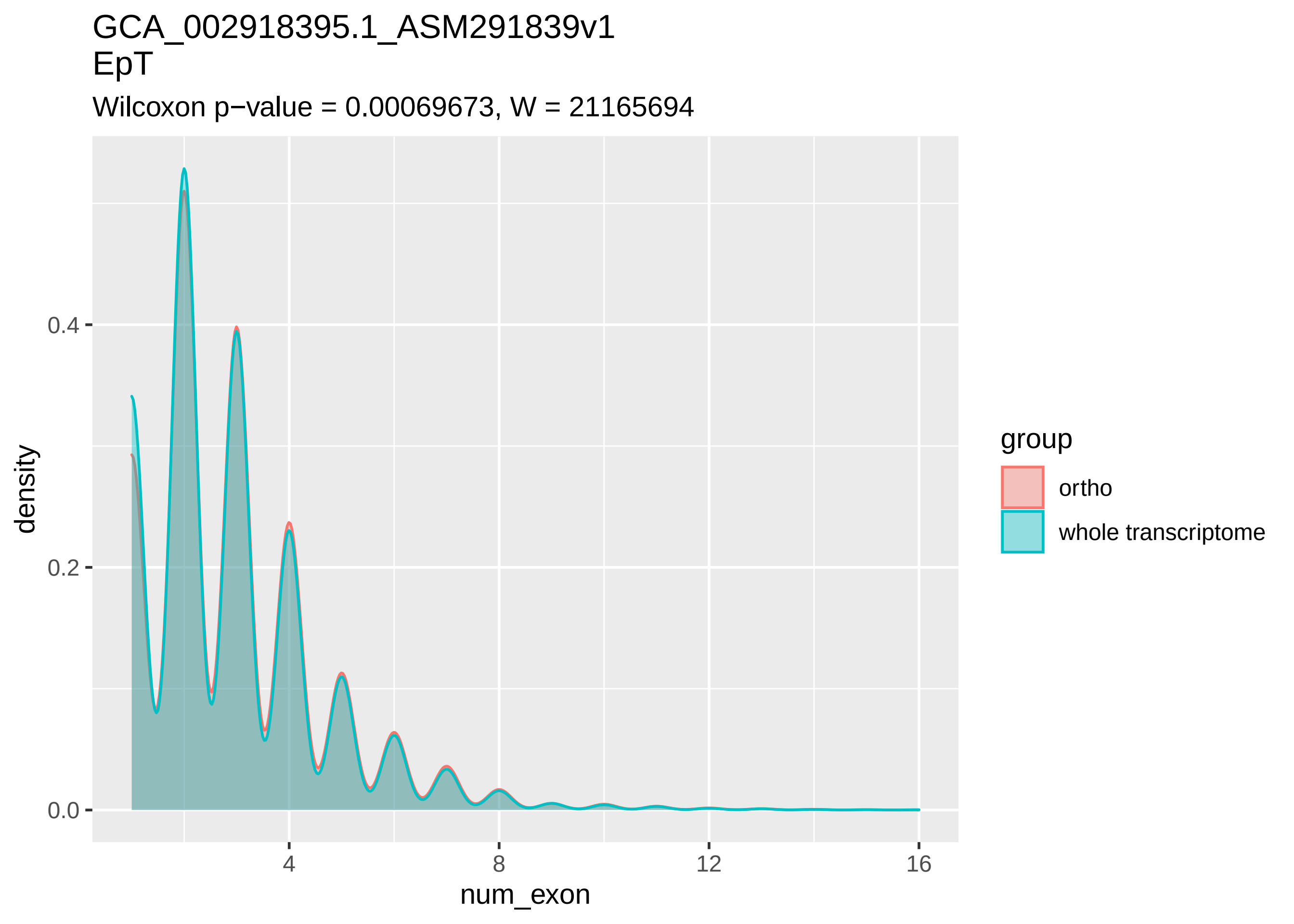
GCA_002918395.1_ASM291839v1
EpT
Wilcoxon p−value = 0.00069673, W = 21165694
density
0.0
0.2
0.4
4
8
12
16
num_exon
group
ortho
whole transcriptome

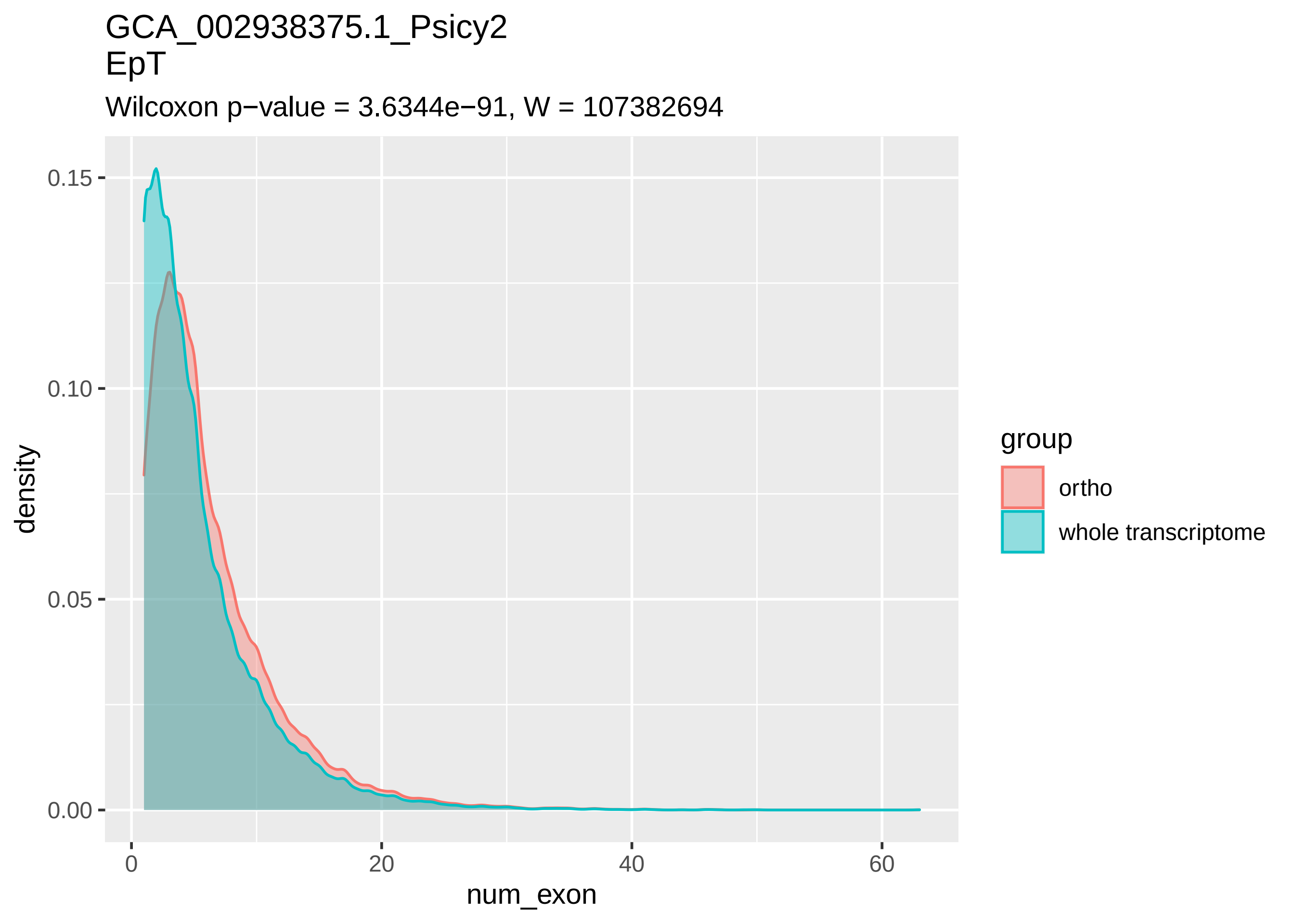

GCA_002938375.1_Psicy2
EpT
Wilcoxon p−value = 3.6344e−91, W = 107382694
0.15
0.10
0.05
0.00
density
0
20
40
60
num_exon
group
ortho
whole transcriptome

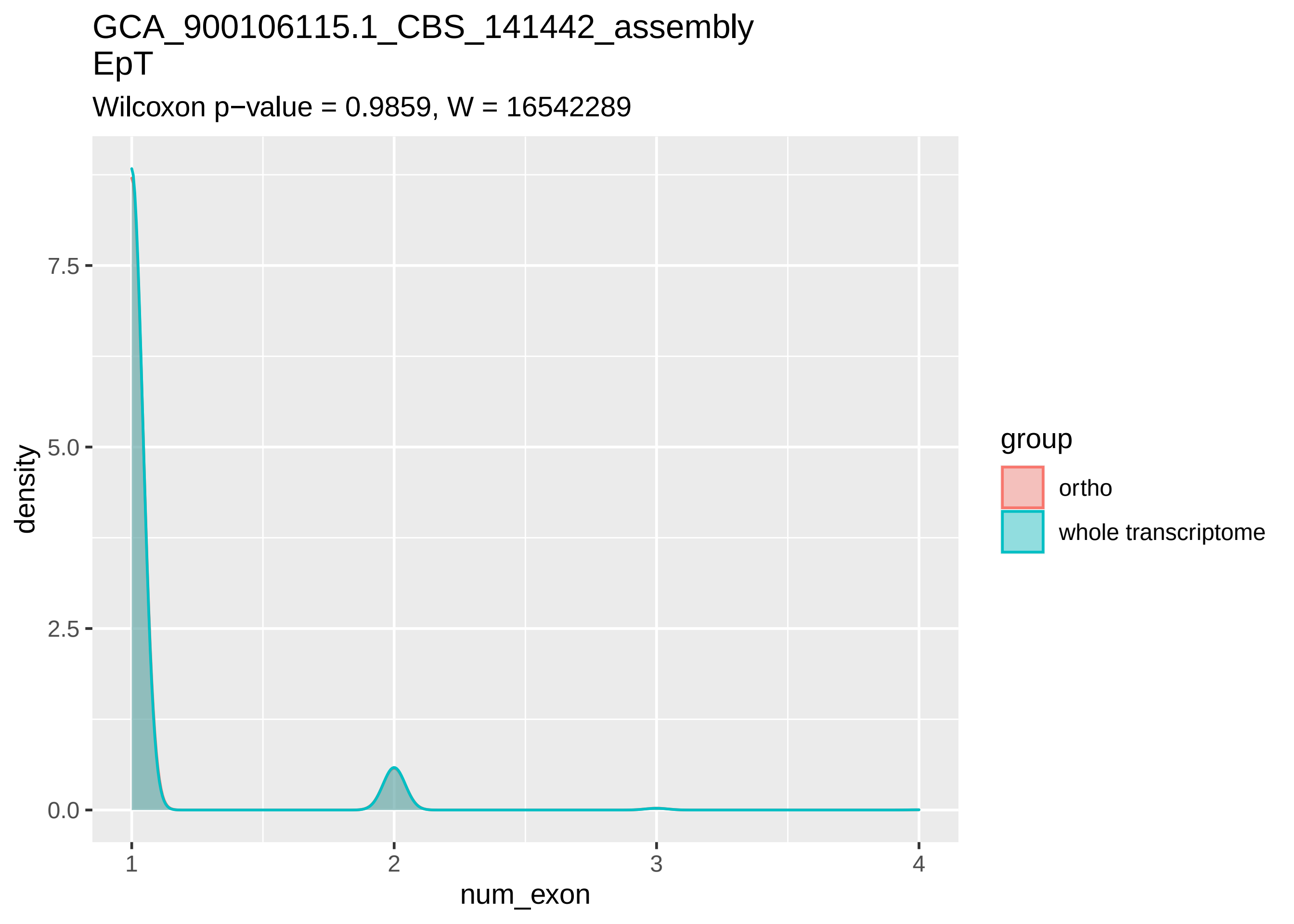
GCA_900106115.1_CBS_141442_assembly
EpT
Wilcoxon p−value = 0.9859, W = 16542289
density
7.5
5.0
2.5
0.0
1
2
3
4
num_exon
group
ortho
whole transcriptome

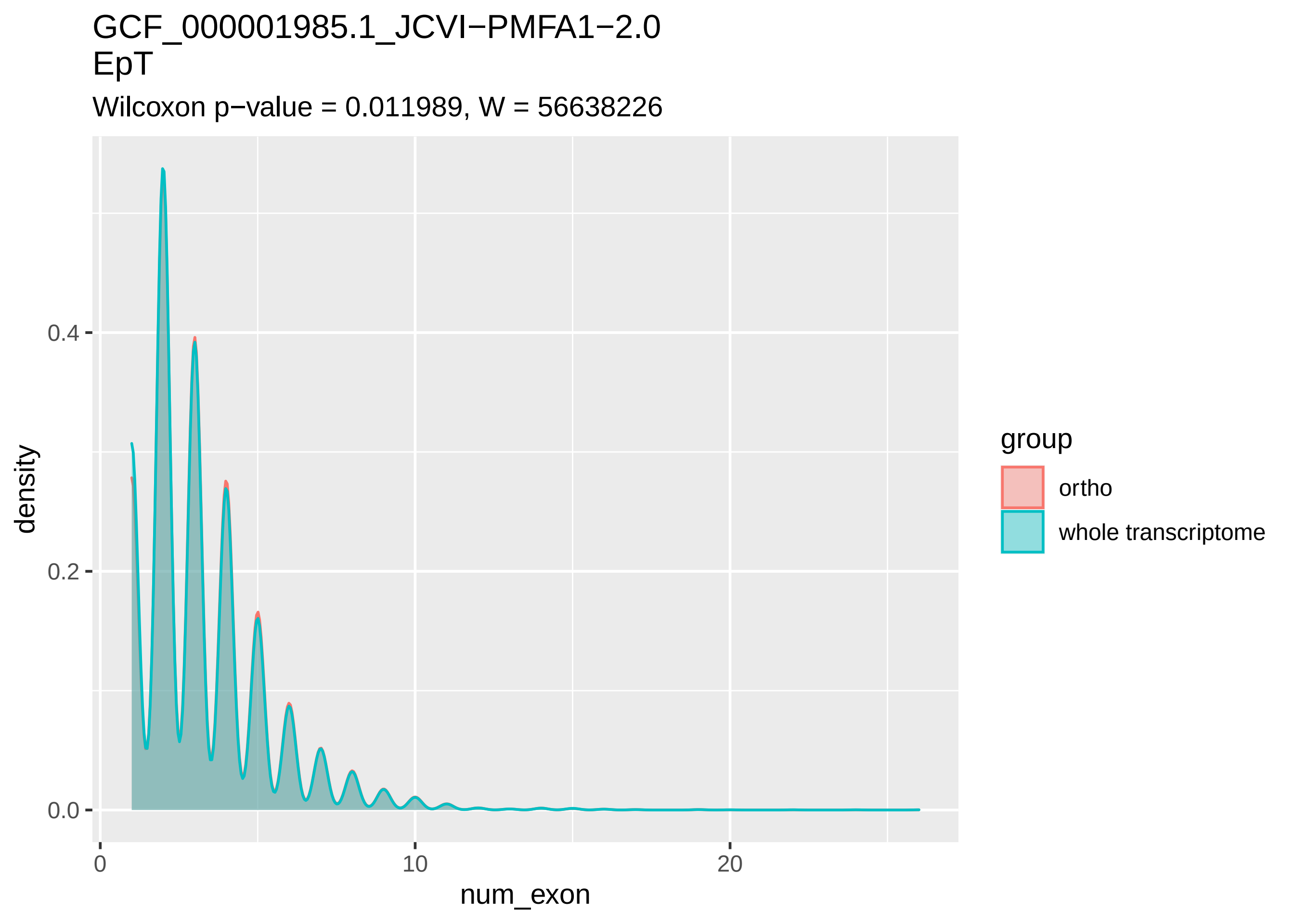
GCF_000001985.1_JCVI−PMFA1−2.0
EpT
Wilcoxon p−value = 0.011989, W = 56638226
density
0.0
0.2
0.4
0
10
20
num_exon
group
ortho
whole transcriptome

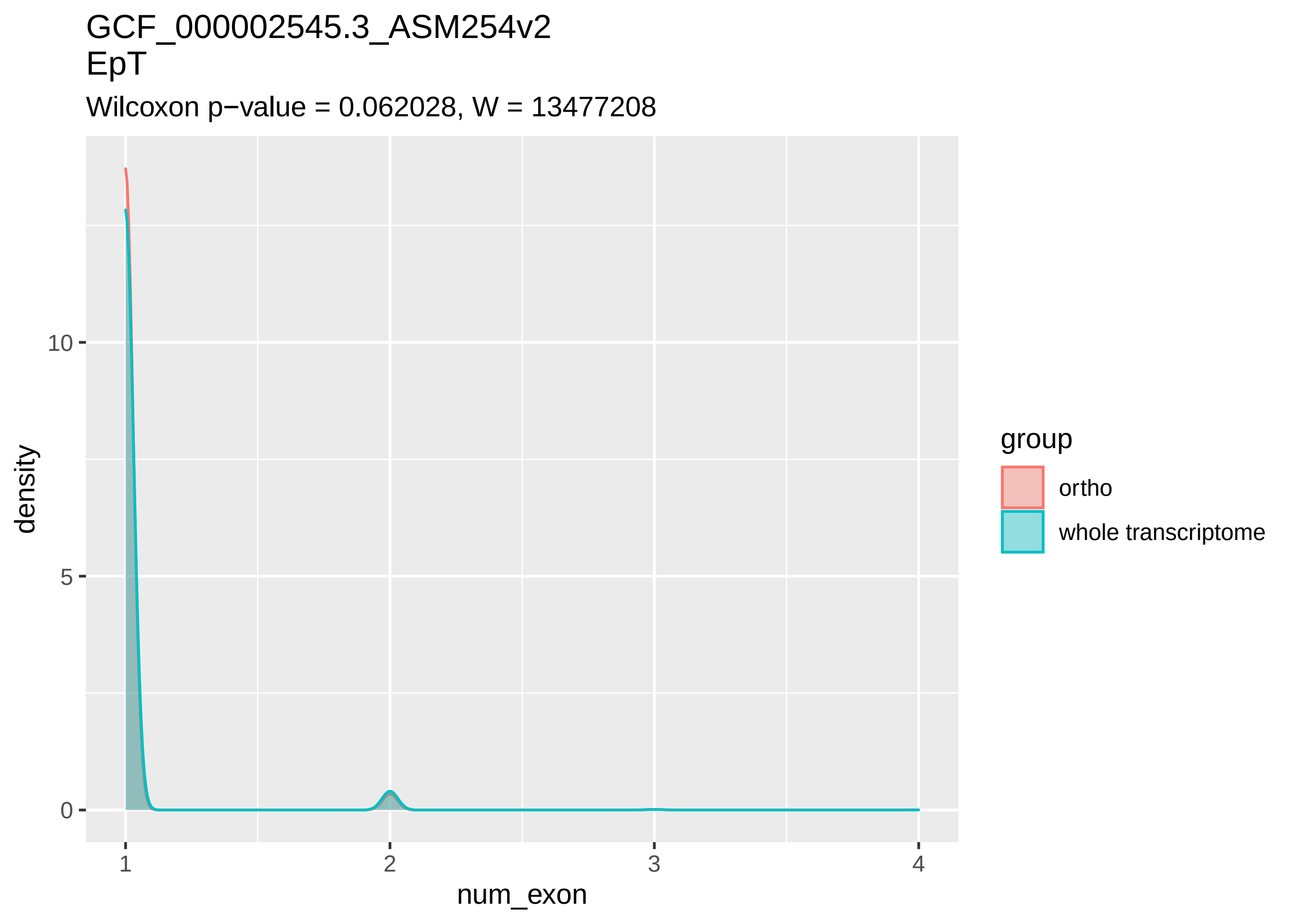
GCF_000002545.3_ASM254v2
EpT
Wilcoxon p−value = 0.062028, W = 13477208
density
10
5
0
1
2
3
4
num_exon
group
ortho
whole transcriptome

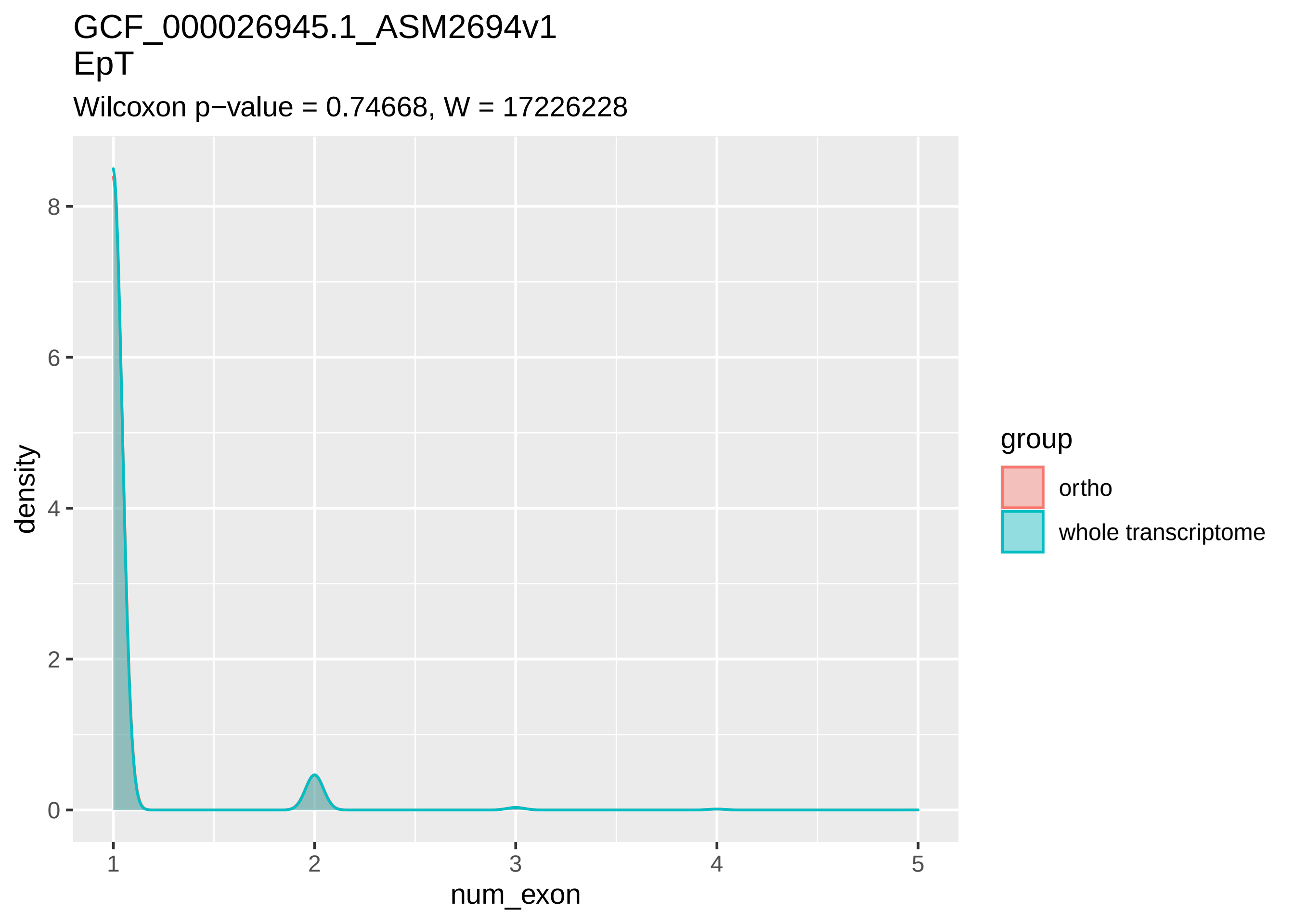

GCF_000026945.1_ASM2694v1
EpT
Wilcoxon p−value = 0.74668, W = 17226228
density
num_exon
0
2
4
6
8
1
2
3
4
5
group
ortho
whole transcriptome

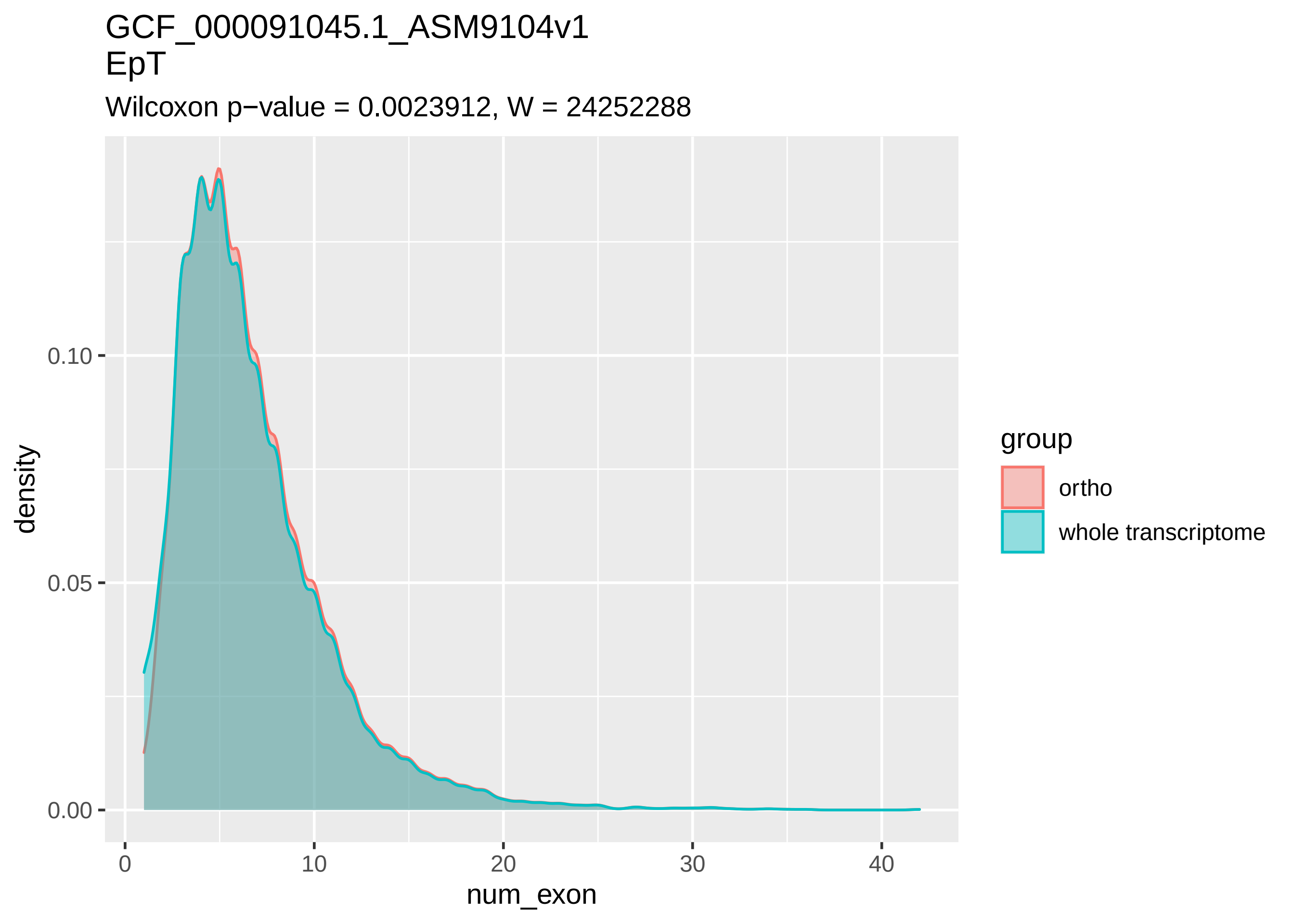

GCF_000091045.1_ASM9104v1
EpT
Wilcoxon p−value = 0.0023912, W = 24252288
density
0.10
0.05
0.00
0
10
20
30
40
num_exon
group
ortho
whole transcriptome

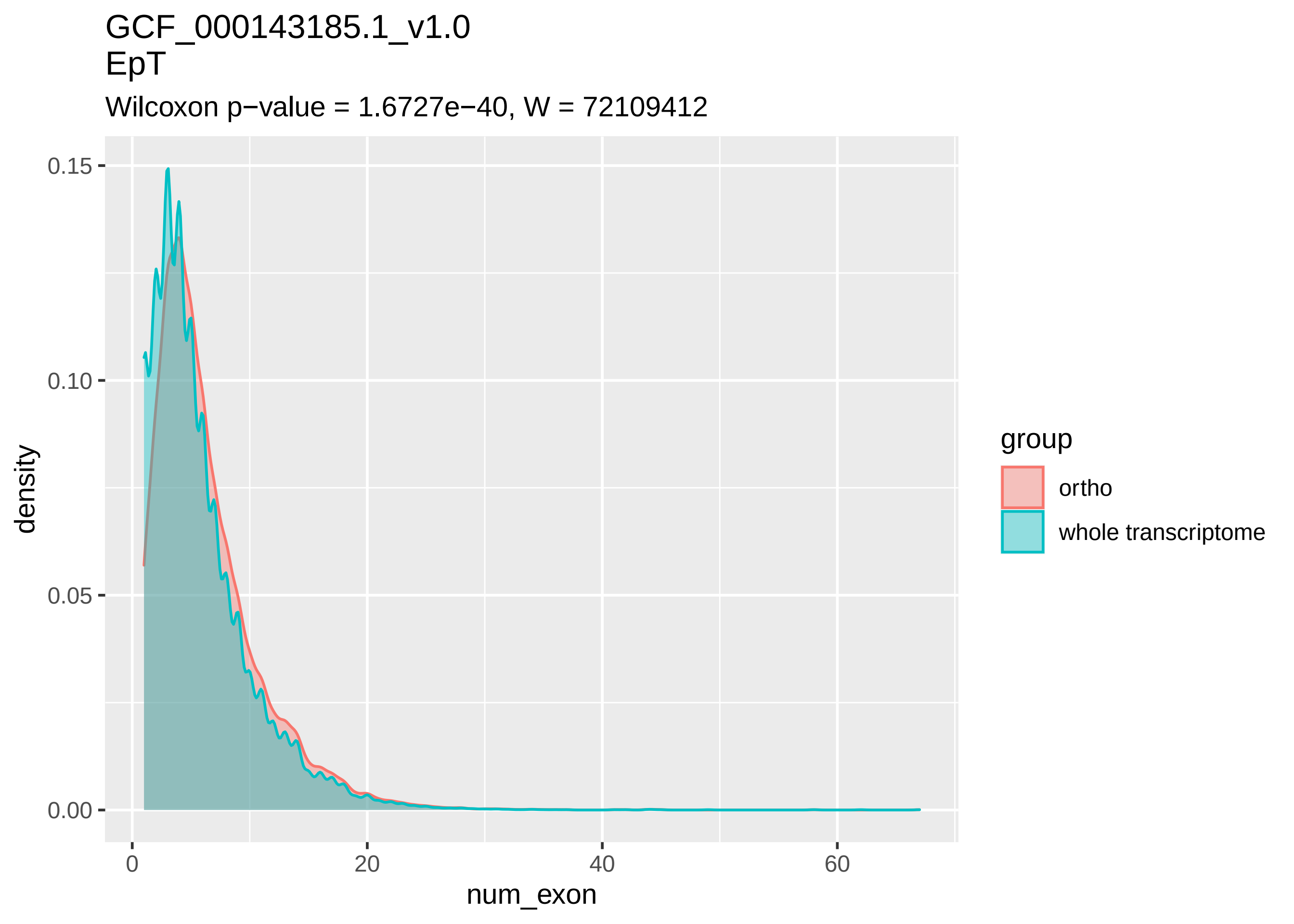
GCF_000143185.1_v1.0
EpT
Wilcoxon p−value = 1.6727e−40, W = 72109412
0.15
0.10
0.05
0.00
density
0
20
40
60
num_exon
group
ortho
whole transcriptome

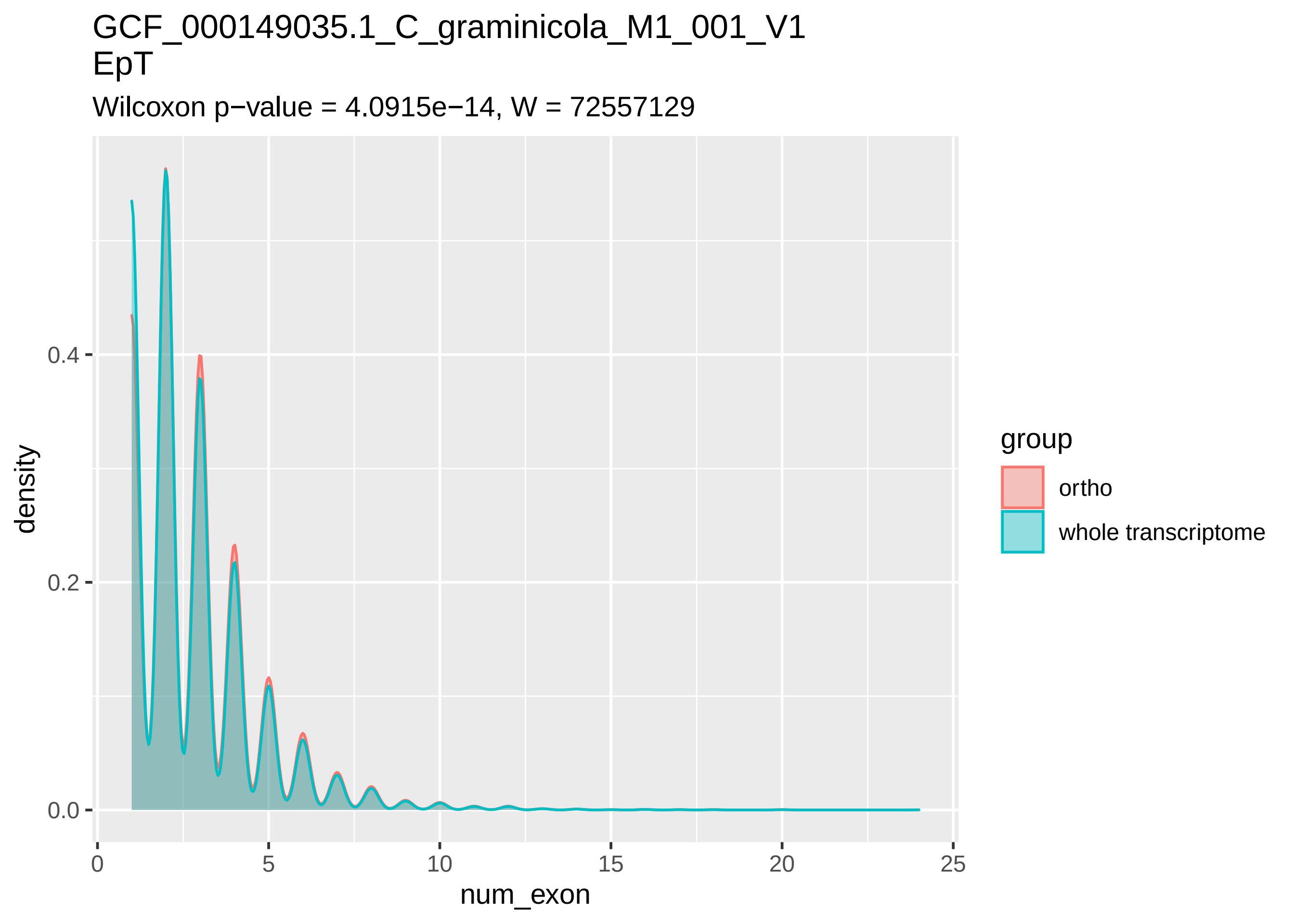
GCF_000149035.1_C_graminicola_M1_001_V1
EpT
Wilcoxon p−value = 4.0915e−14, W = 72557129
density
0.0
0.2
0.4
0
5
10
15
20
25
num_exon
group
ortho
whole transcriptome

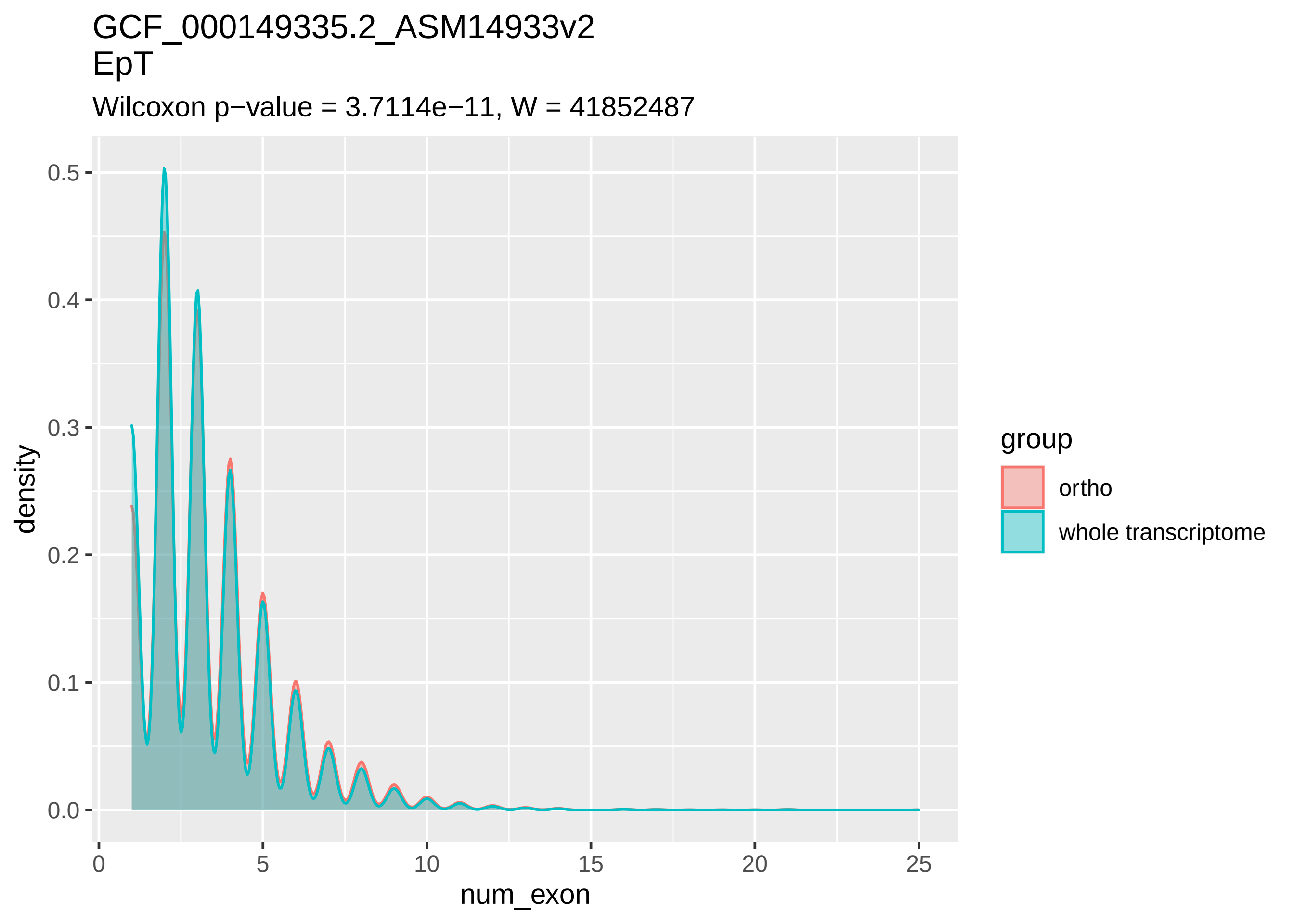
GCF_000149335.2_ASM14933v2
EpT
Wilcoxon p−value = 3.7114e−11, W = 41852487
density
0.0
0.1
0.2
0.3
0.4
0.5
0
5
10
15
20
25
num_exon
group
ortho
whole transcriptome

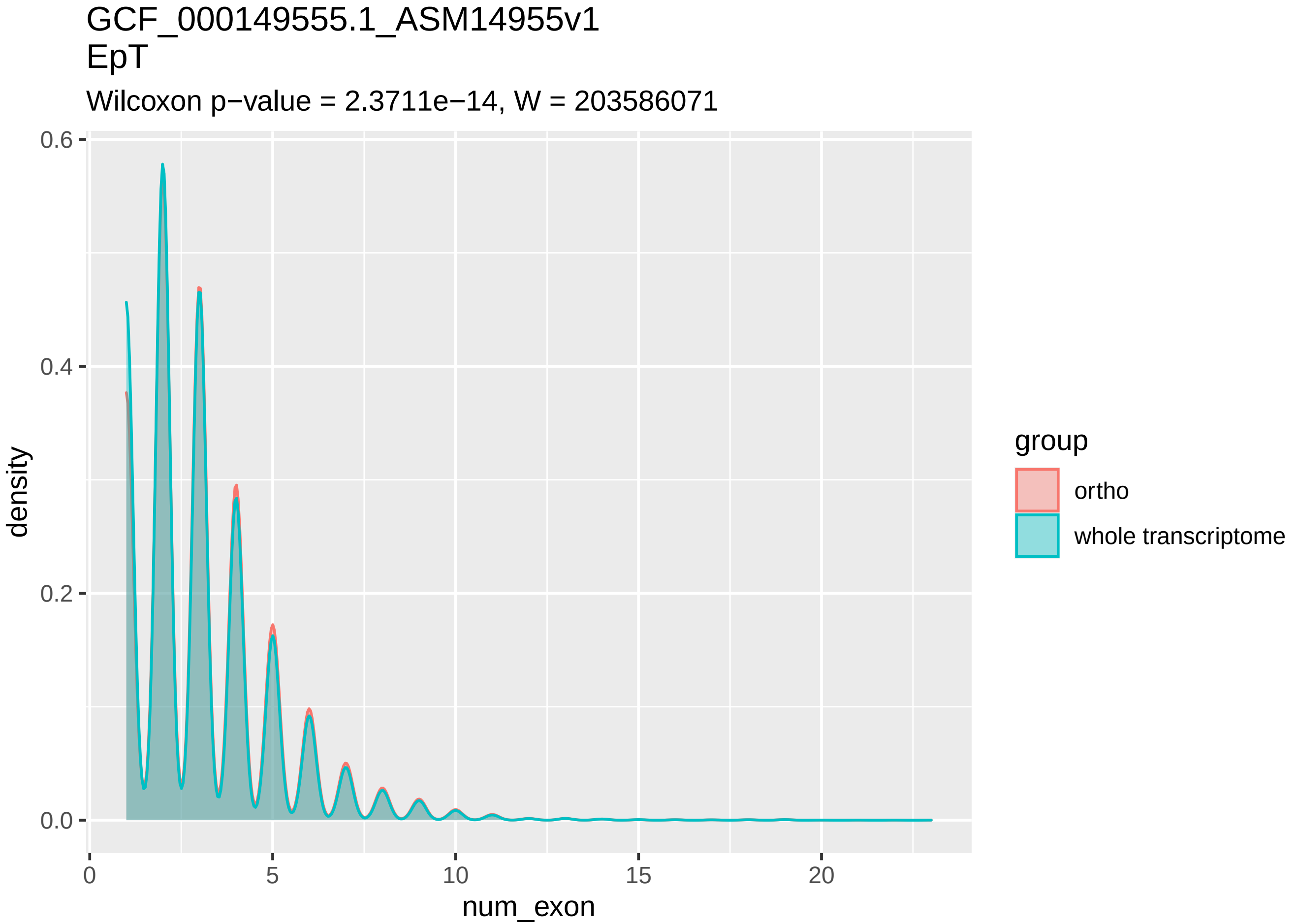
GCF_000149555.1_ASM14955v1
EpT
Wilcoxon p−value = 2.3711e−14, W = 203586071
0.6
0.4
0.2
0.0
density
0
5
10
15
20
num_exon
group
ortho
whole transcriptome

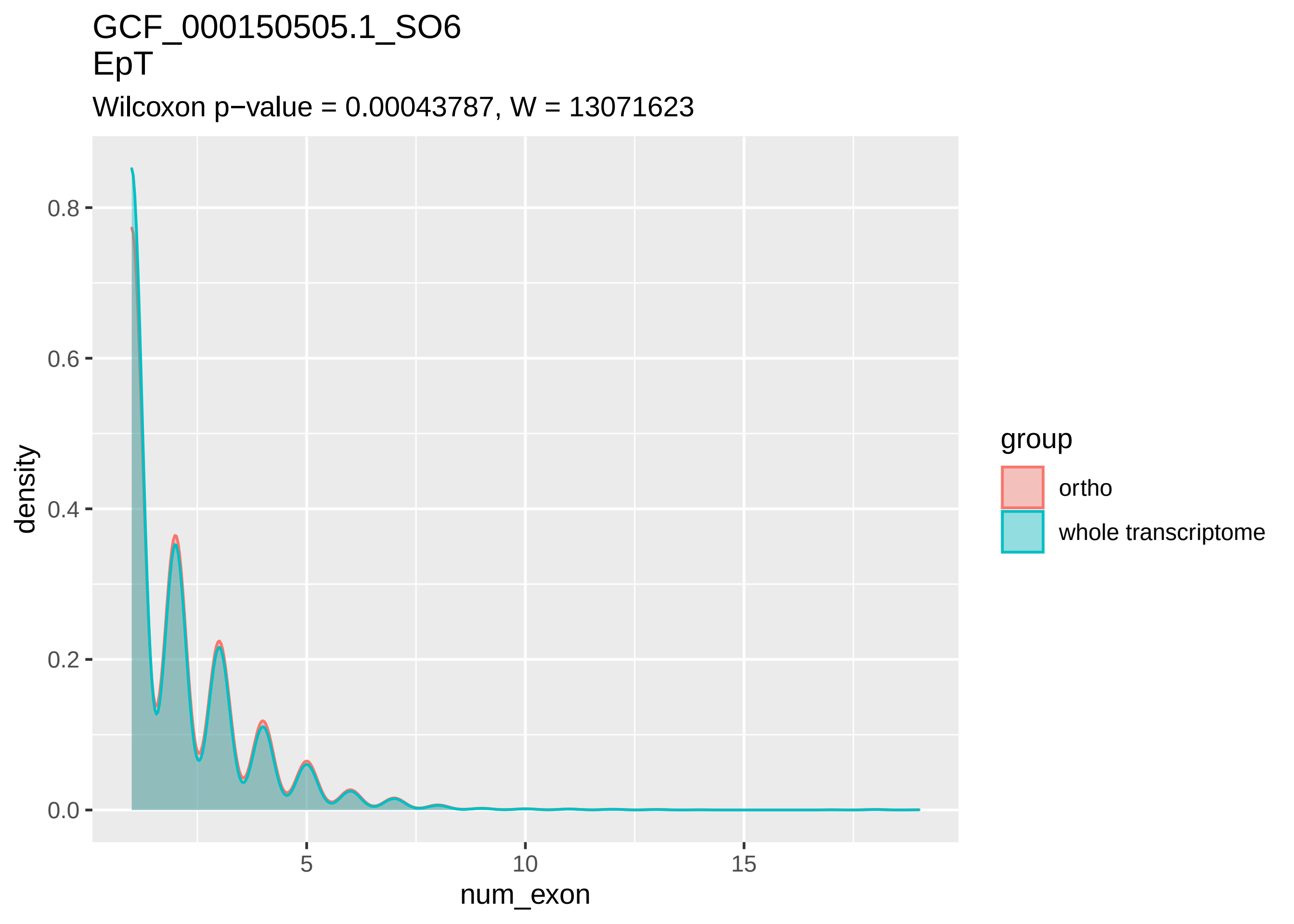
GCF_000150505.1_SO6
EpT
Wilcoxon p−value = 0.00043787, W = 13071623
0.8
0.6
0.4
0.2
0.0
density
5
10
15
num_exon
group
ortho
whole transcriptome

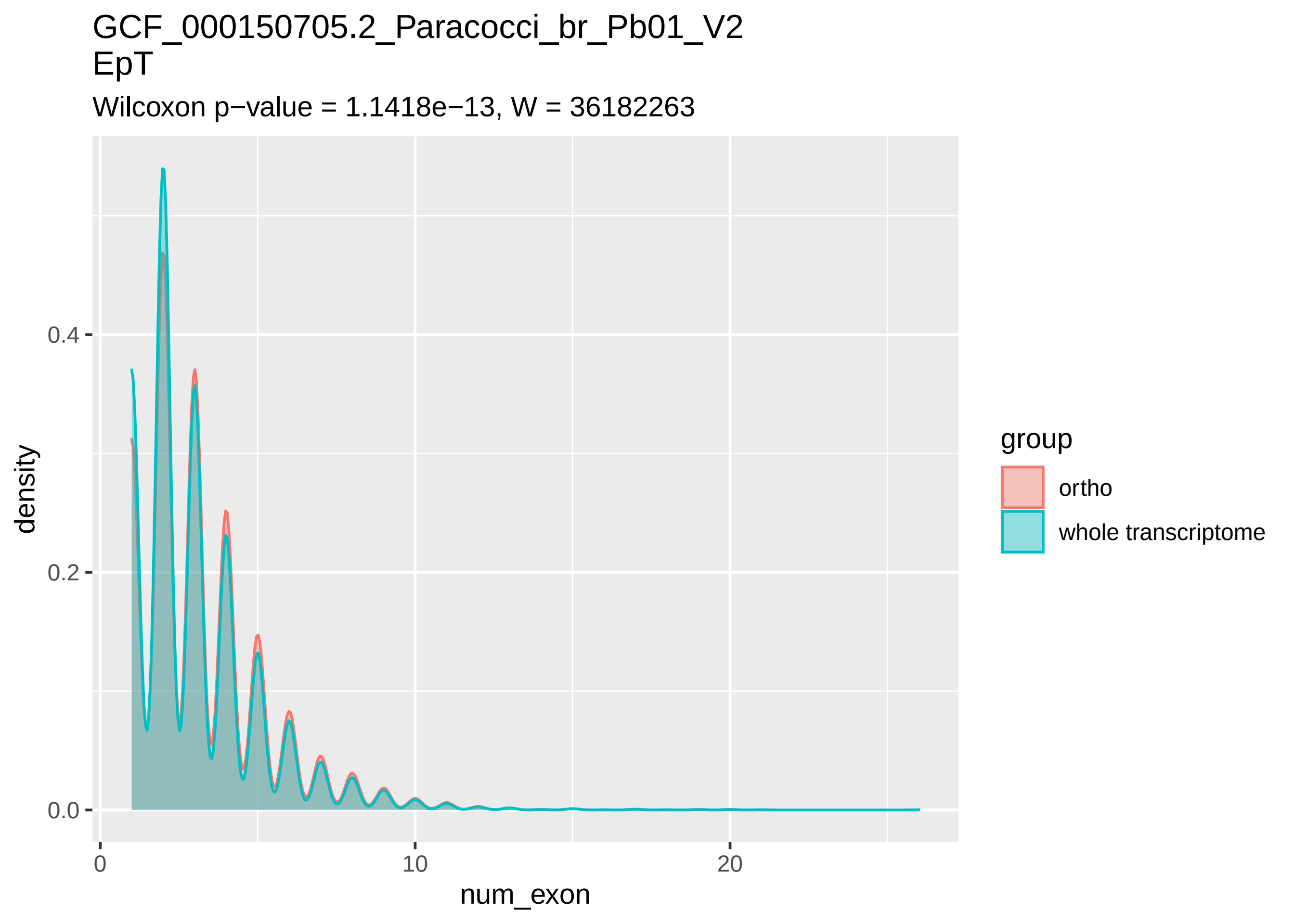
GCF_000150705.2_Paracocci_br_Pb01_V2
EpT
Wilcoxon p−value = 1.1418e−13, W = 36182263
density
0.0
0.2
0.4
0
10
20
num_exon
group
ortho
whole transcriptome

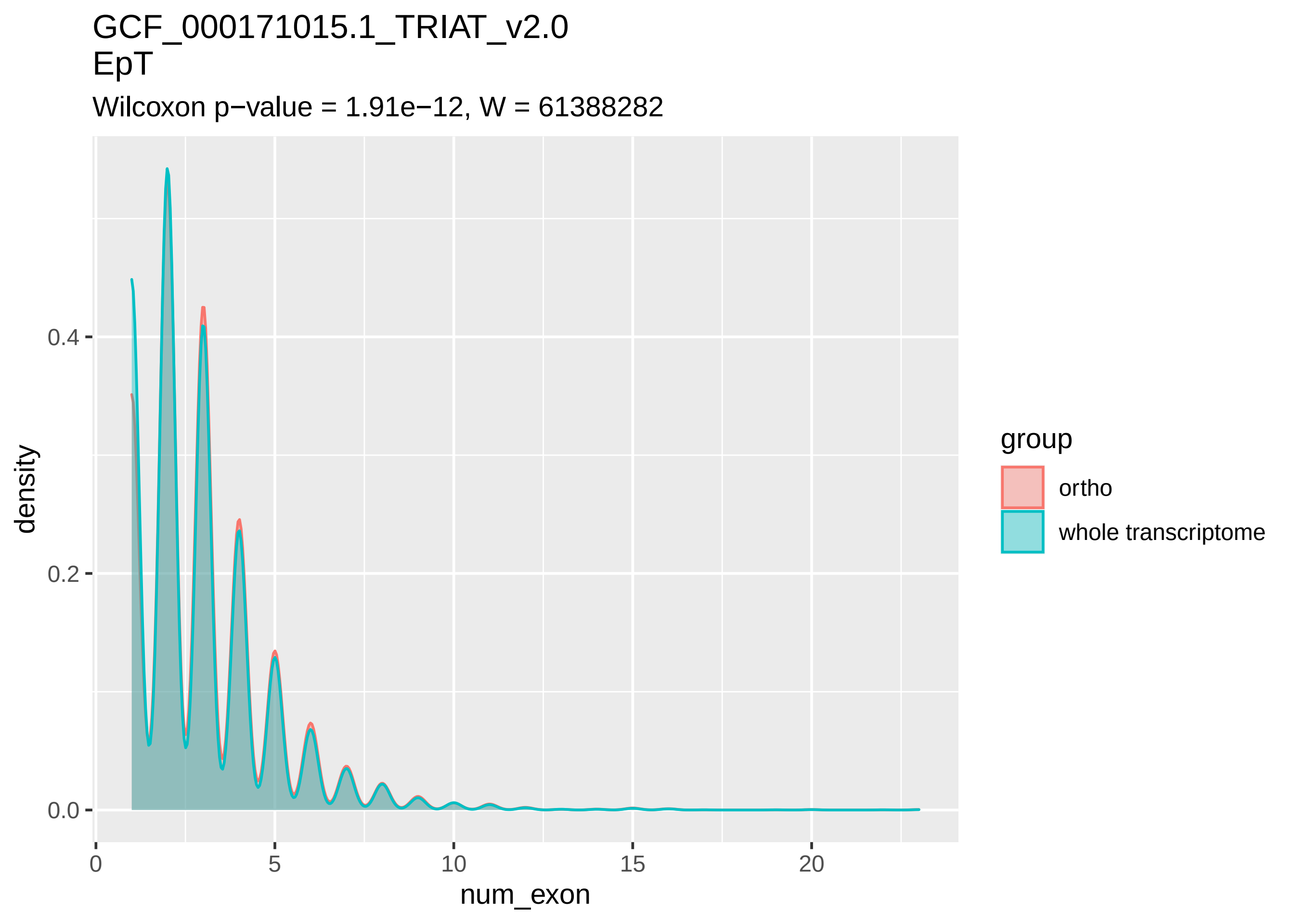

GCF_000171015.1_TRIAT_v2.0
EpT
Wilcoxon p−value = 1.91e−12, W = 61388282
density
0.0
0.2
0.4
0
5
10
15
20
num_exon
group
ortho
whole transcriptome

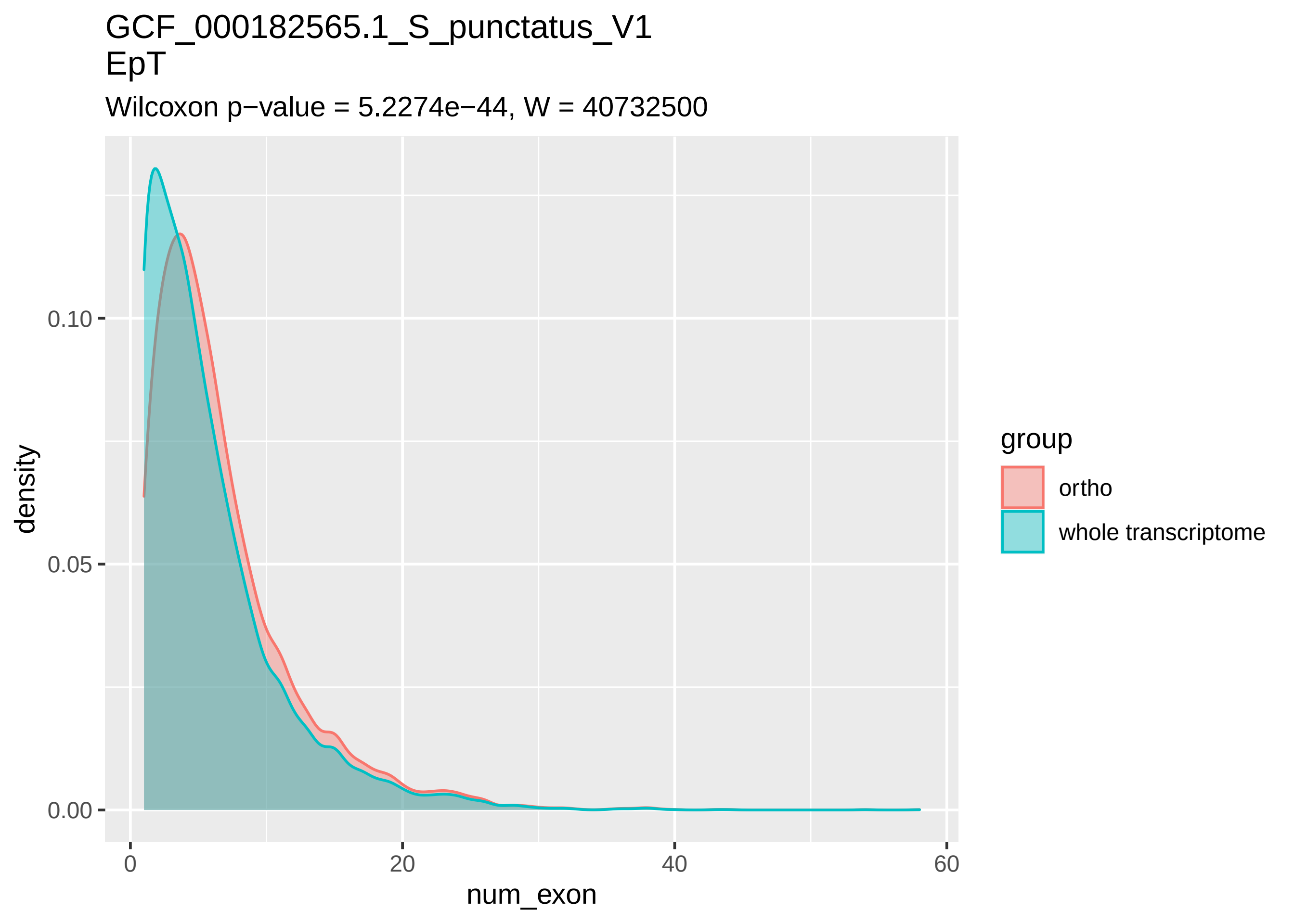
GCF_000182565.1_S_punctatus_V1
EpT
Wilcoxon p−value = 5.2274e−44, W = 40732500
density
0.10
0.05
0.00
0
20
40
60
num_exon
group
ortho
whole transcriptome

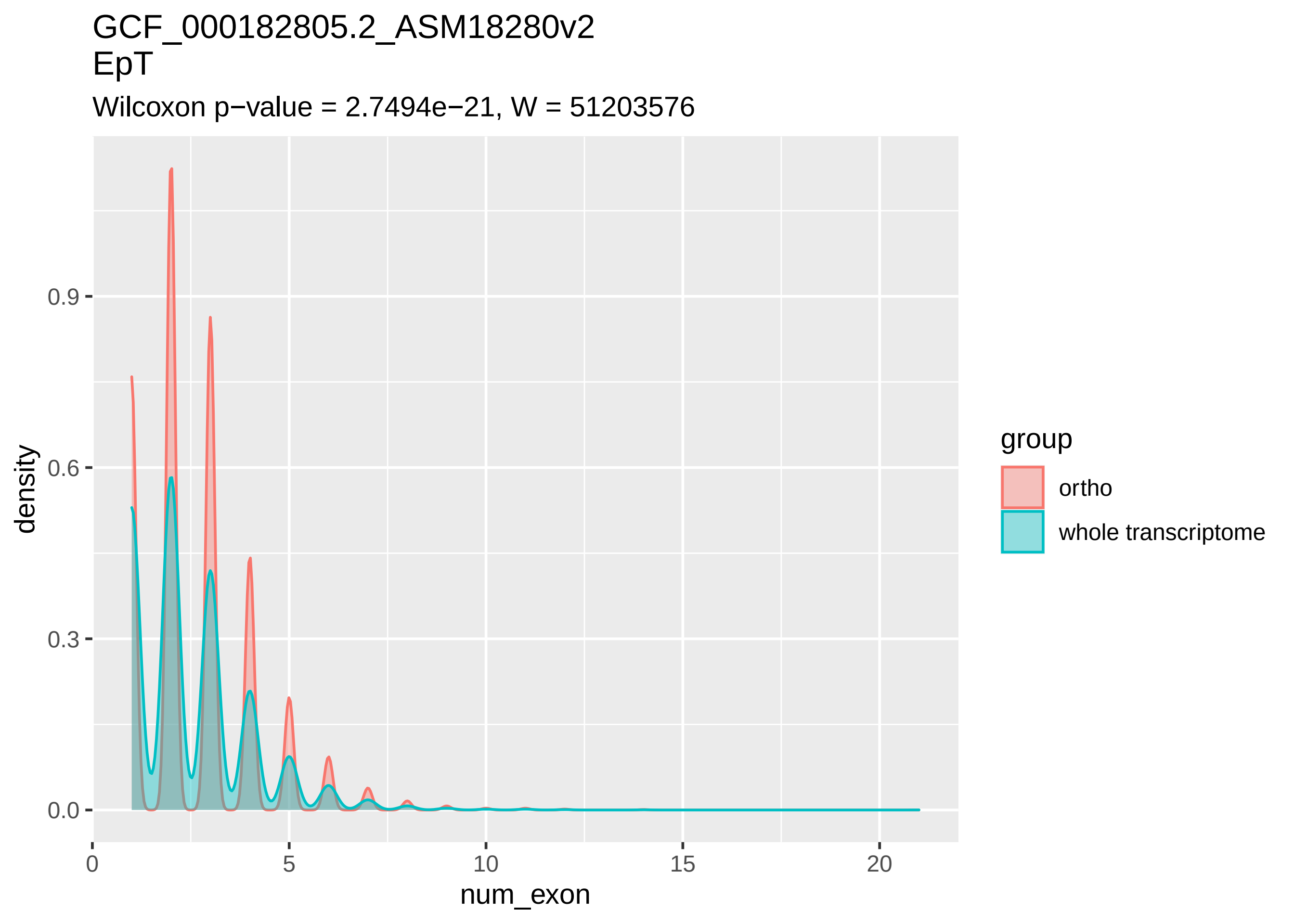
GCF_000182805.2_ASM18280v2
EpT
Wilcoxon p−value = 2.7494e−21, W = 51203576
density
0.0
0.3
0.6
0.9
0
5
10
15
20
num_exon
group
ortho
whole transcriptome

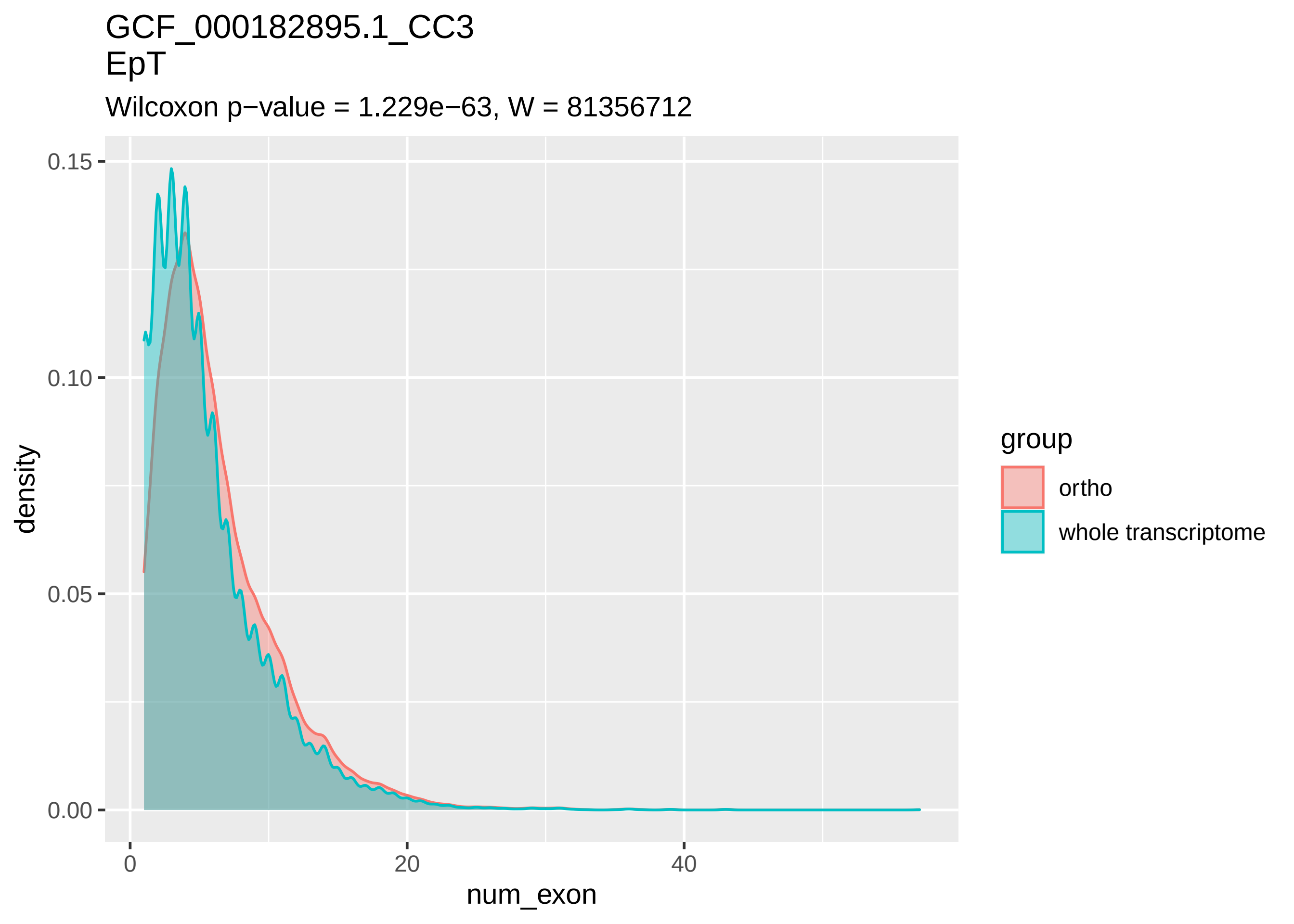
GCF_000182895.1_CC3
EpT
Wilcoxon p−value = 1.229e−63, W = 81356712
density
0.15
0.10
0.05
0.00
0
20
40
num_exon
group
ortho
whole transcriptome

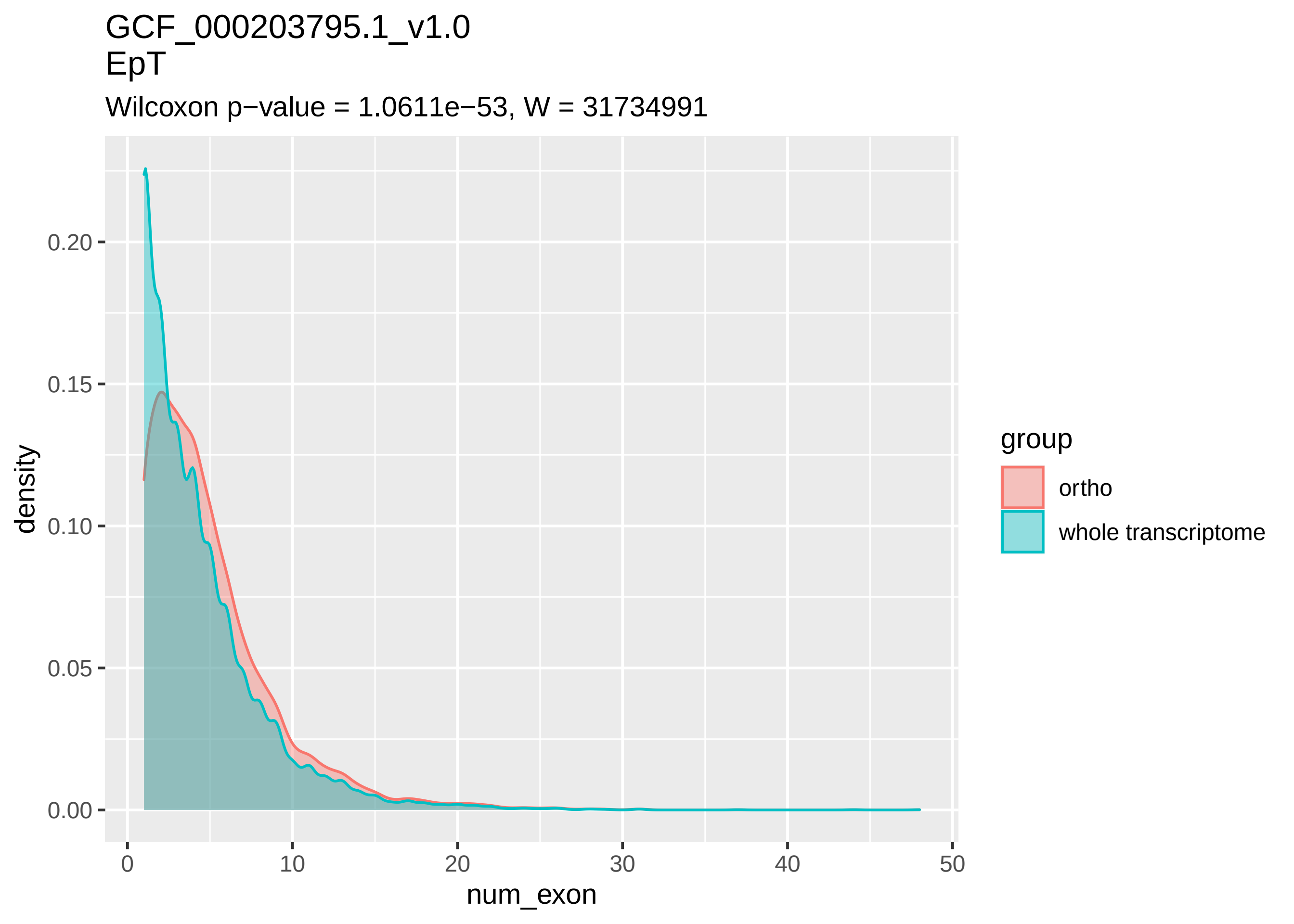
GCF_000203795.1_v1.0
EpT
Wilcoxon p−value = 1.0611e−53, W = 31734991
density
0.20
0.15
0.10
0.05
0.00
0
10
20
30
40
50
num_exon
group
ortho
whole transcriptome

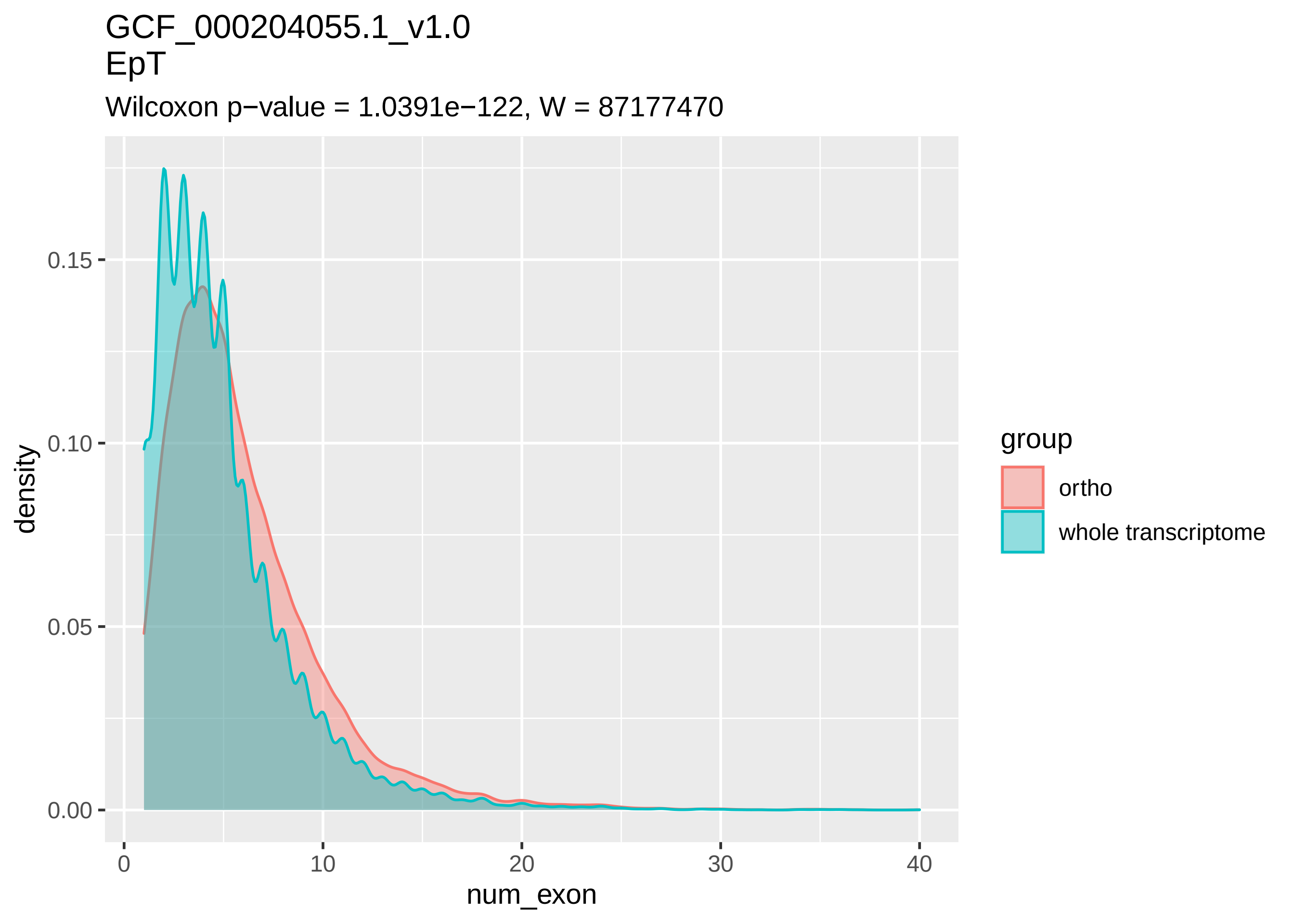

GCF_000204055.1_v1.0
EpT
Wilcoxon p−value = 1.0391e−122, W = 87177470
density
0.15
0.10
0.05
0.00
0
10
20
30
40
num_exon
group
ortho
whole transcriptome

GCF_000221225.1_CTHT_3.0
EpT
Wilcoxon p−value = 0.00092367, W = 26035496

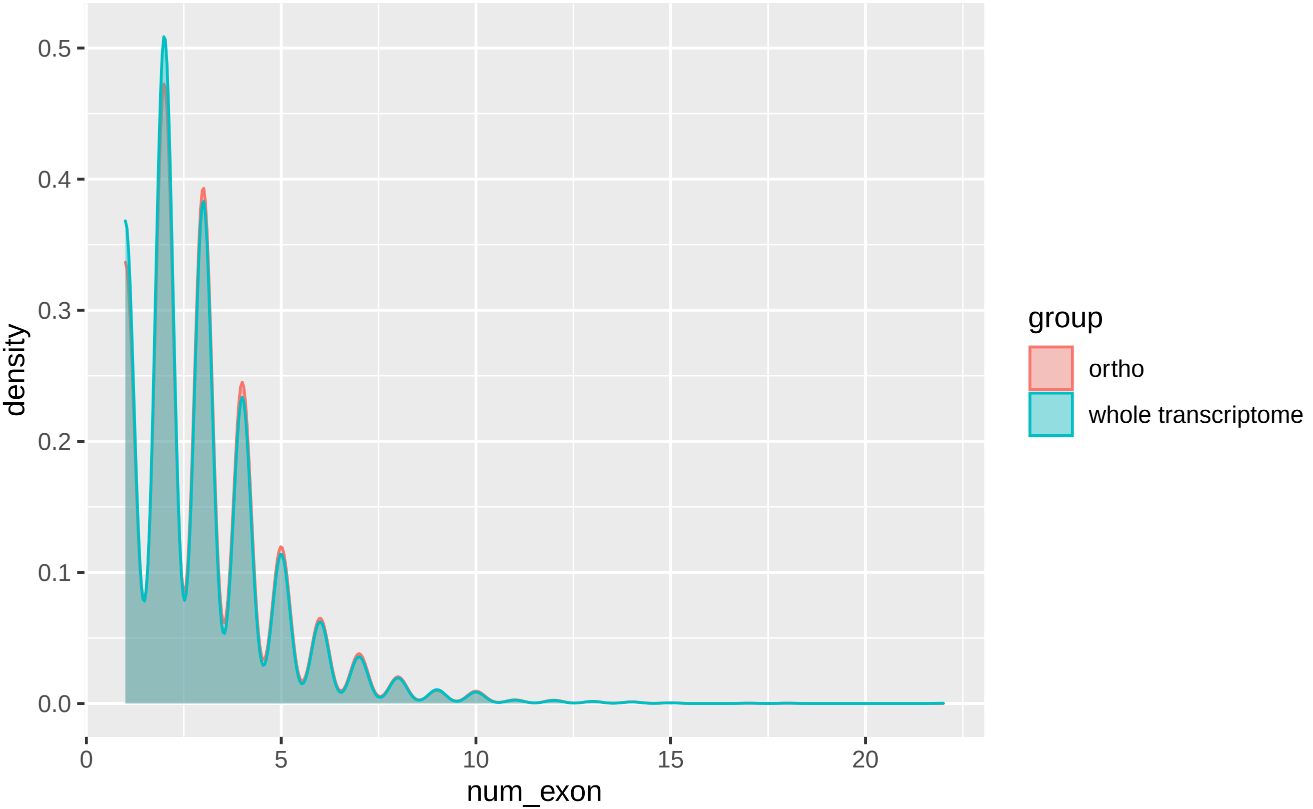

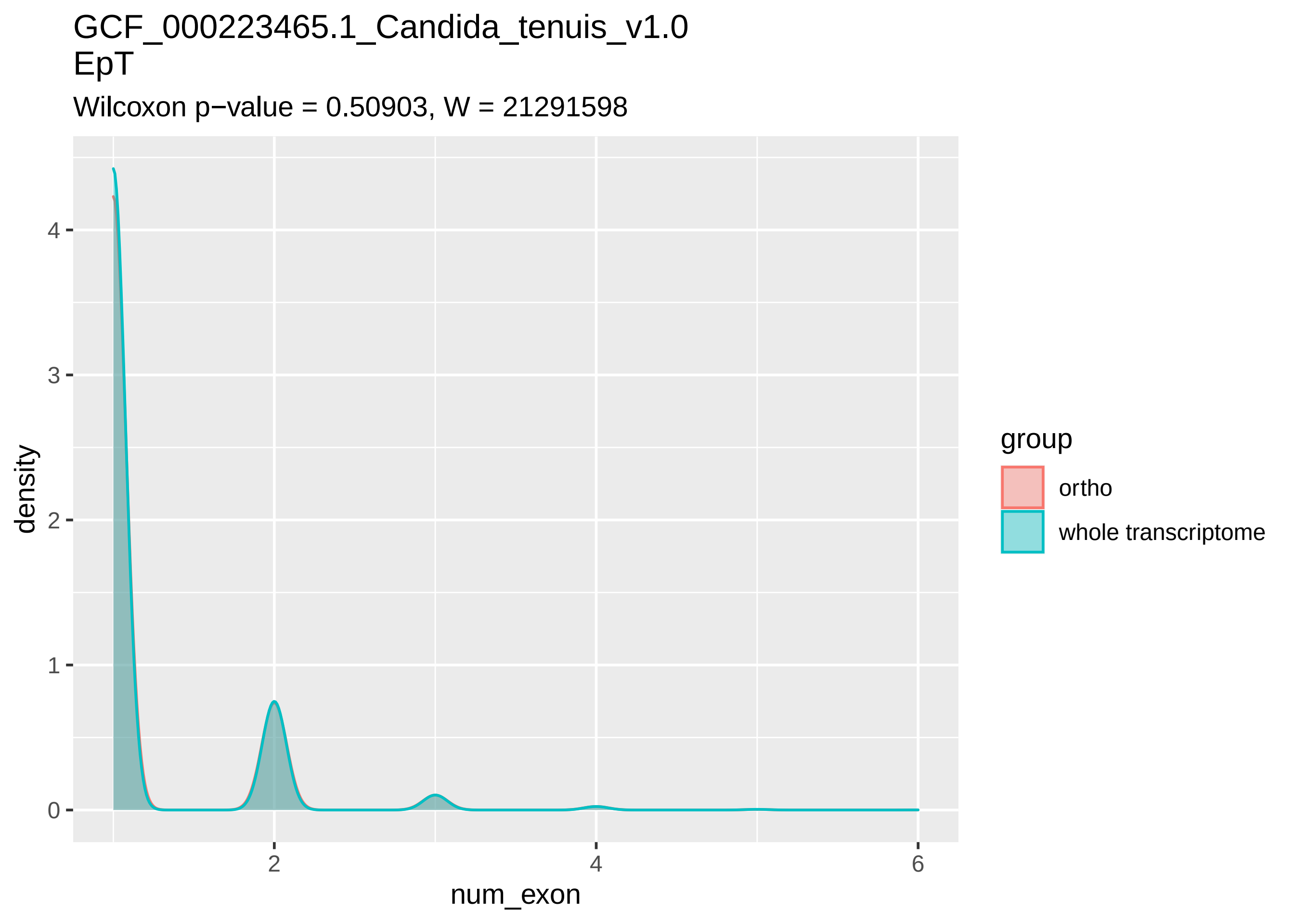

GCF_000223465.1_Candida_tenuis_v1.0
EpT
Wilcoxon p−value = 0.50903, W = 21291598
density
num_exon
0
1
2
3
4
2
4
6
group
ortho
whole transcriptome

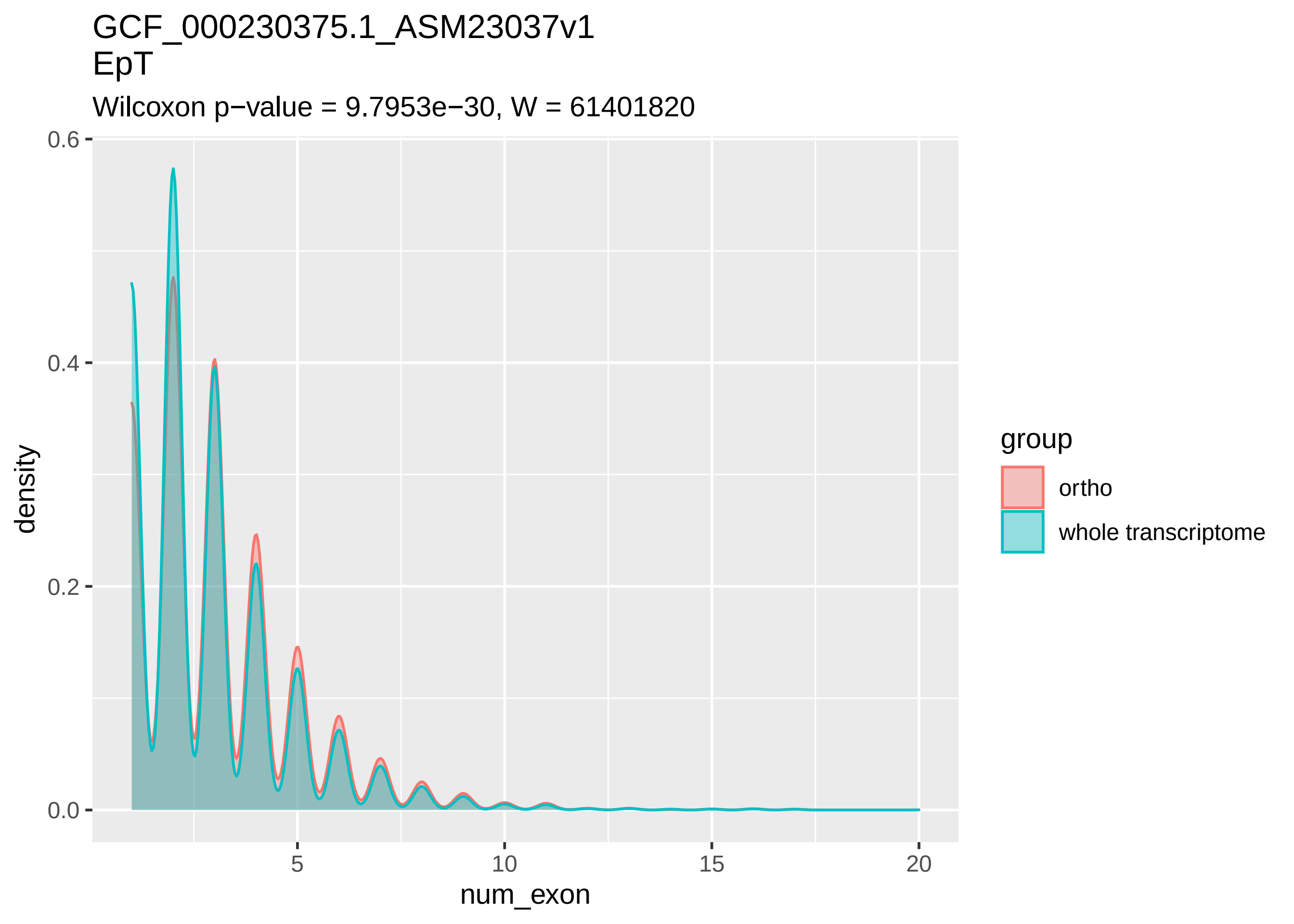
GCF_000230375.1_ASM23037v1
EpT
Wilcoxon p−value = 9.7953e−30, W = 61401820
density
0.0
0.2
0.4
0.6
num_exon
5
10
15
20
group
ortho
whole transcriptome

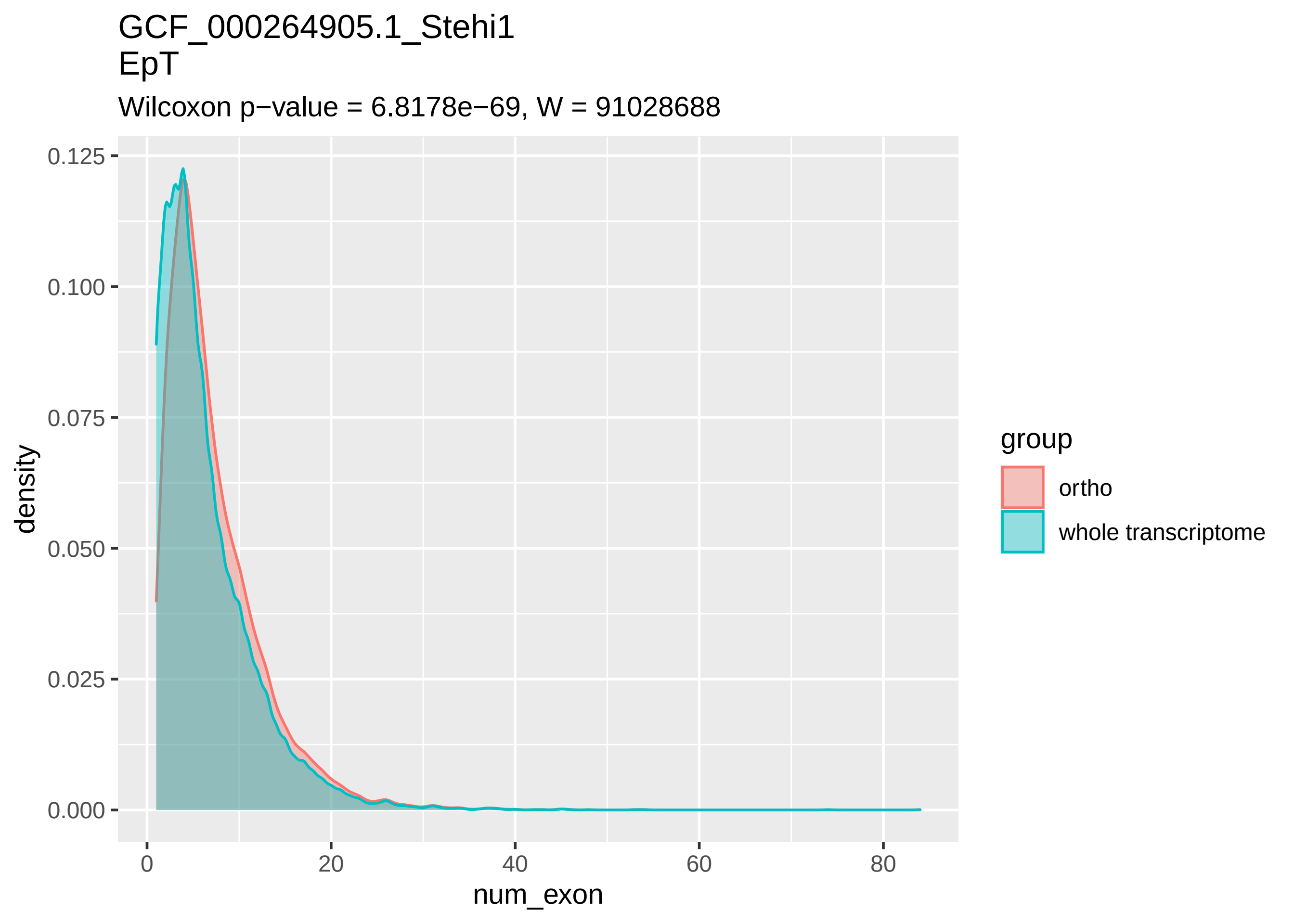

GCF_000264905.1_Stehi1
EpT
Wilcoxon p−value = 6.8178e−69, W = 91028688
0.125
0.100
0.075
0.050
0.025
0.000
density
0
20
40
60
80
num_exon
group
ortho
whole transcriptome

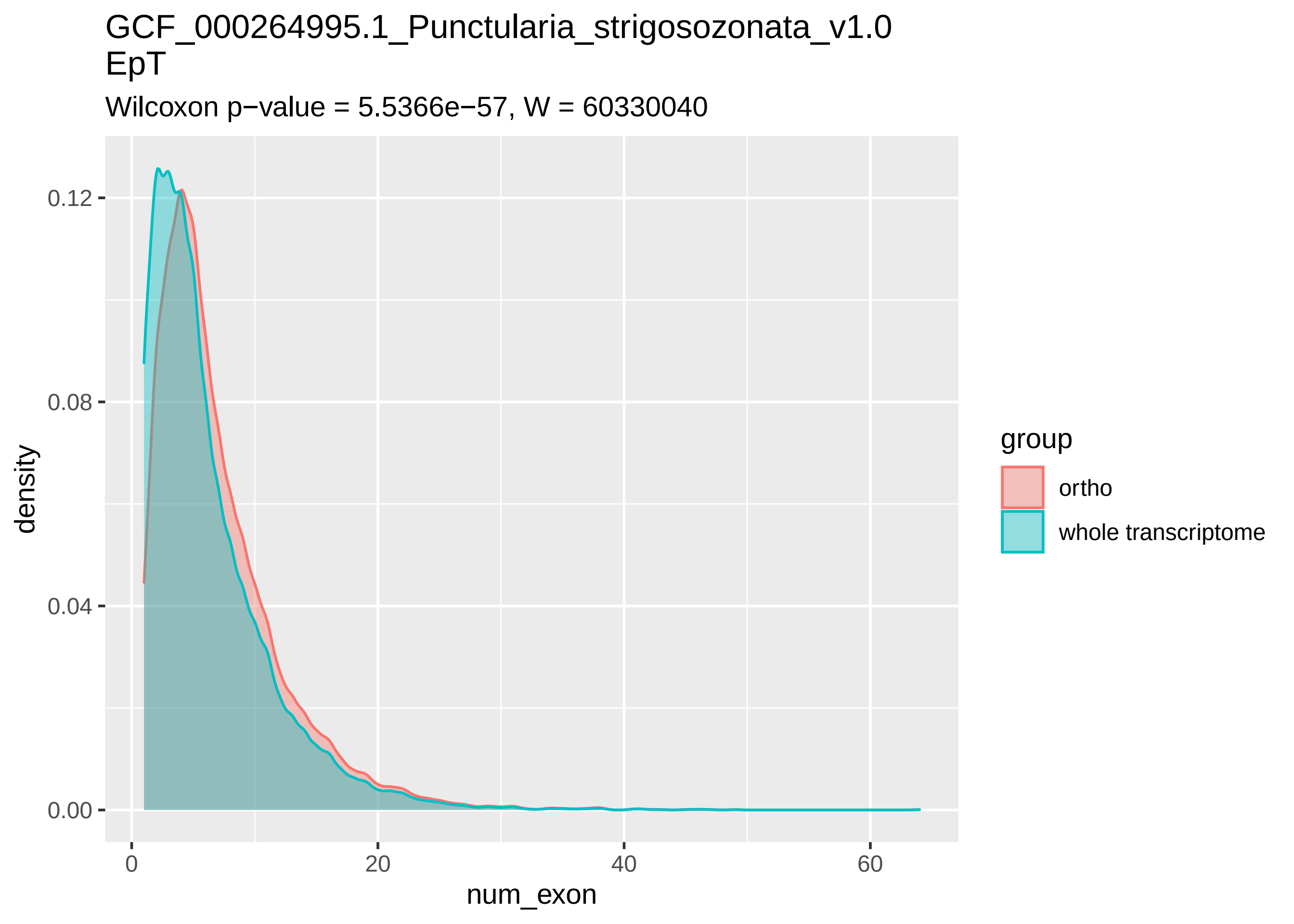
GCF_000264995.1_Punctularia_strigosozonata_v1.0
EpT
Wilcoxon p−value = 5.5366e−57, W = 60330040
density
0.12
0.08
0.04
0.00
0
20
40
60
num_exon
group
ortho
whole transcriptome

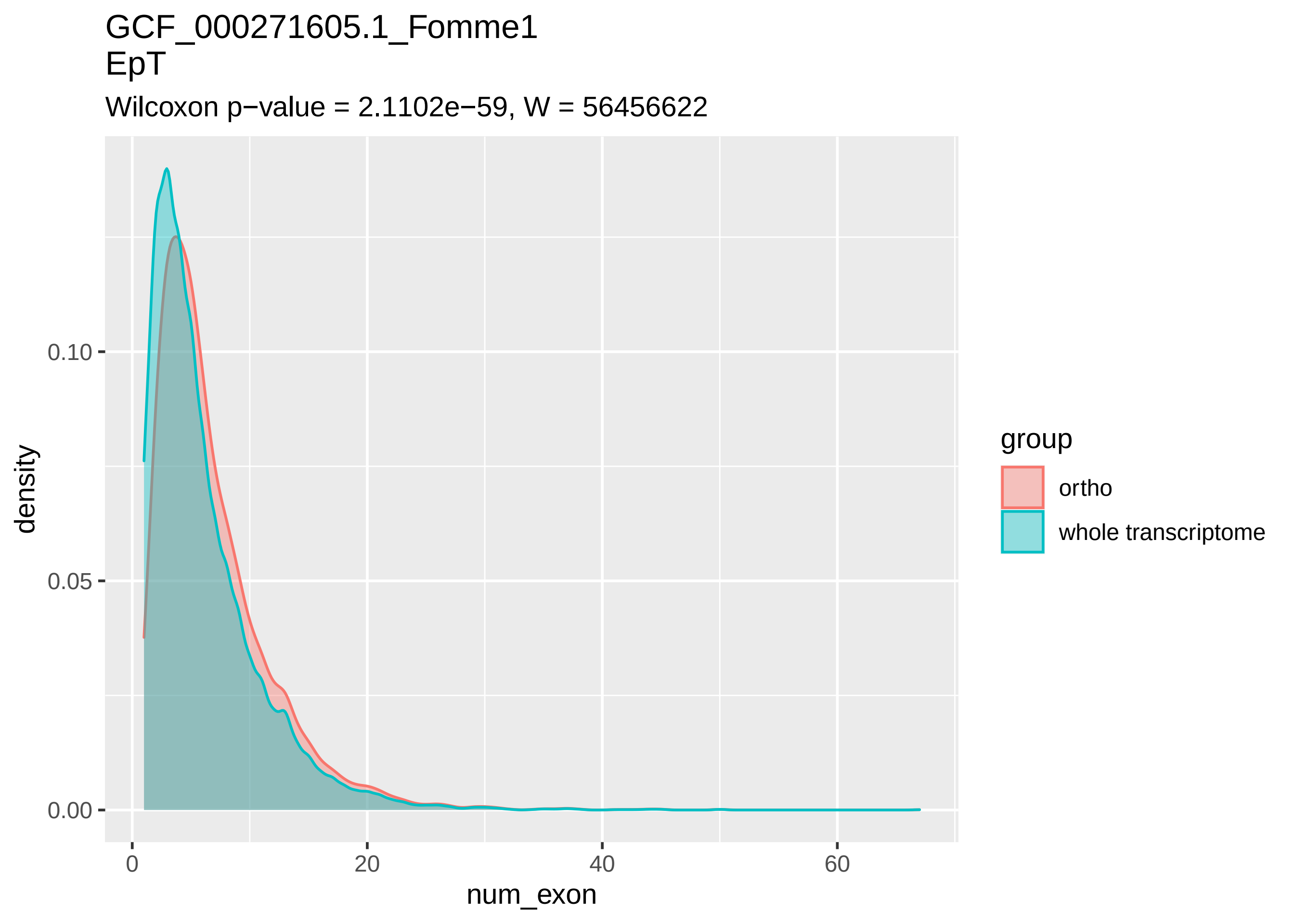

GCF_000271605.1_Fomme1
EpT
Wilcoxon p−value = 2.1102e−59, W = 56456622
0.10
0.05
0.00
density
0
20
40
60
num_exon
group
ortho
whole transcriptome

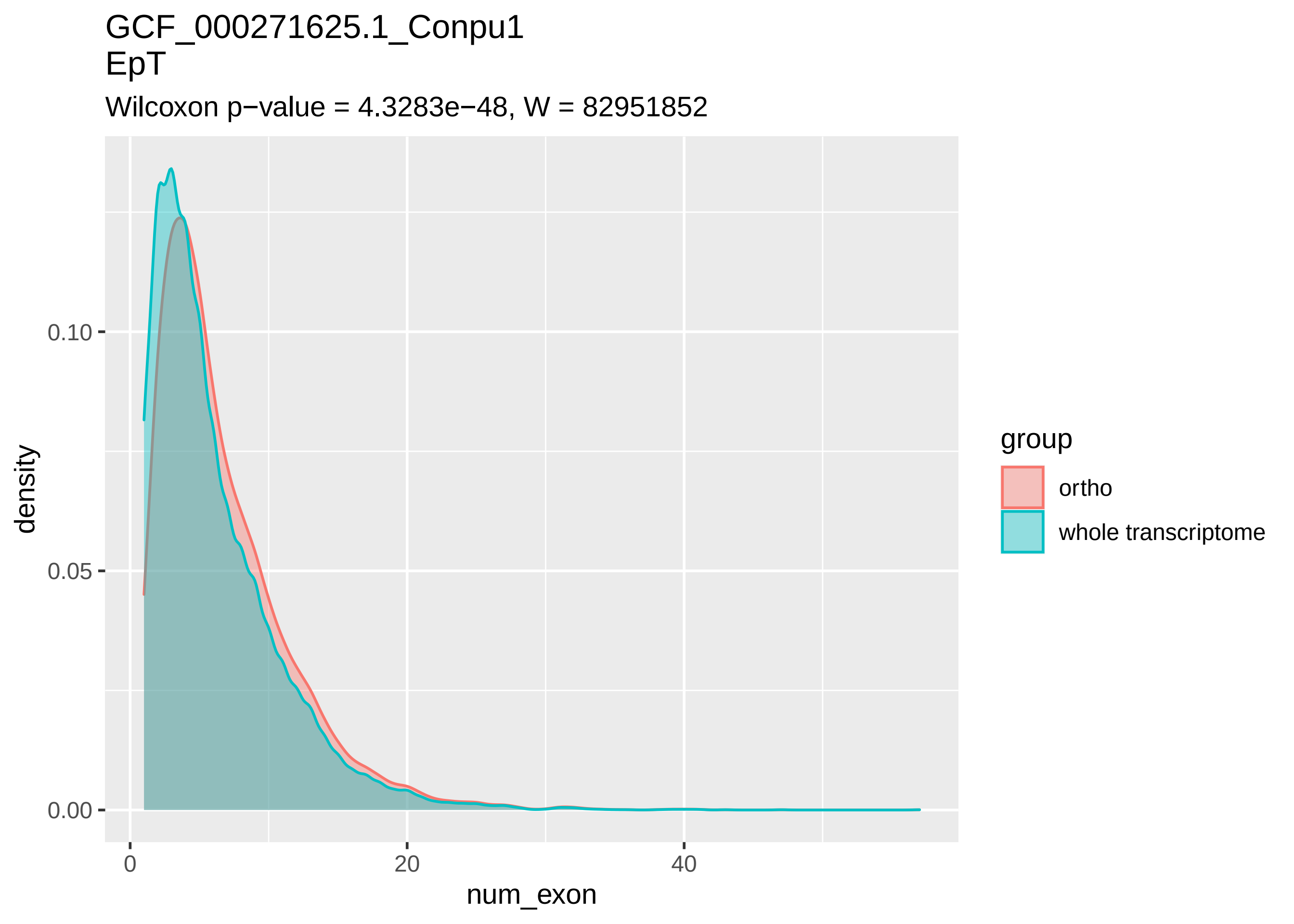
GCF_000271625.1_Conpu1
EpT
Wilcoxon p−value = 4.3283e−48, W = 82951852
density
0.10
0.05
0.00
0
20
40
num_exon
group
ortho
whole transcriptome

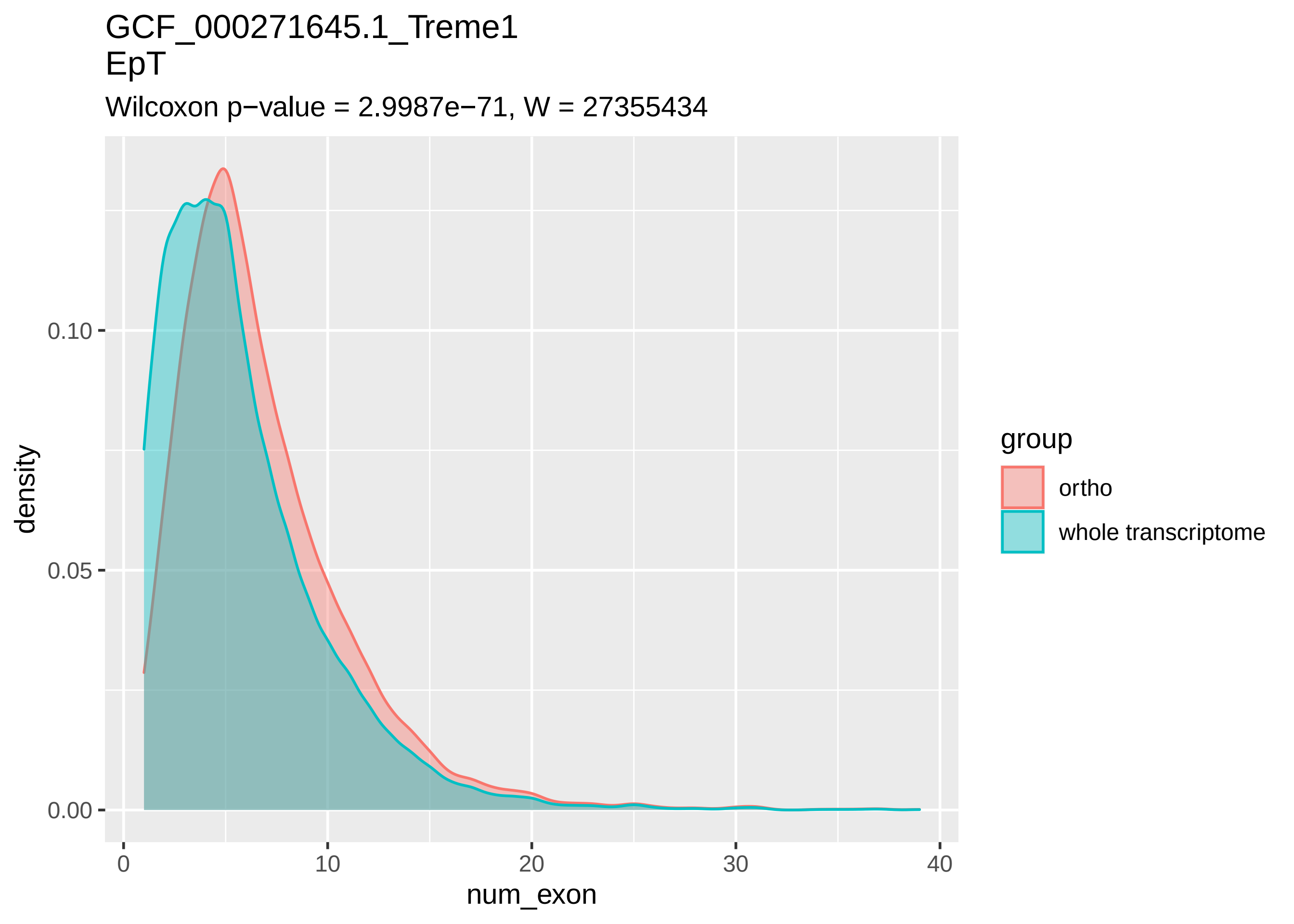
GCF_000271645.1_Treme1
EpT
Wilcoxon p−value = 2.9987e−71, W = 27355434
density
0.00
0.05
0.10
0
10
20
30
40
num_exon
group
ortho
whole transcriptome

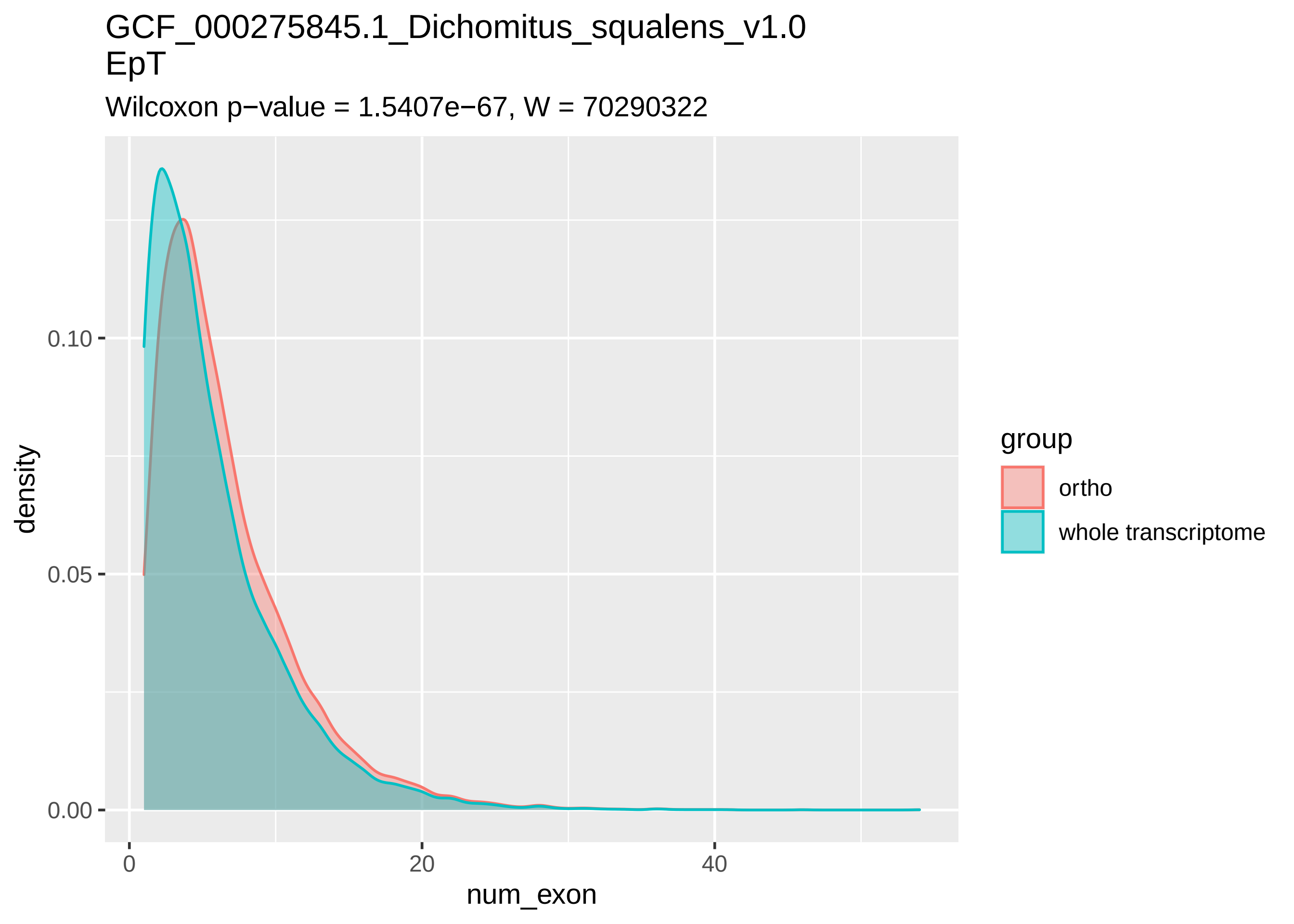
GCF_000275845.1_Dichomitus_squalens_v1.0
EpT
Wilcoxon p−value = 1.5407e−67, W = 70290322
density
0.10
0.05
0.00
0
20
40
num_exon
group
ortho
whole transcriptome

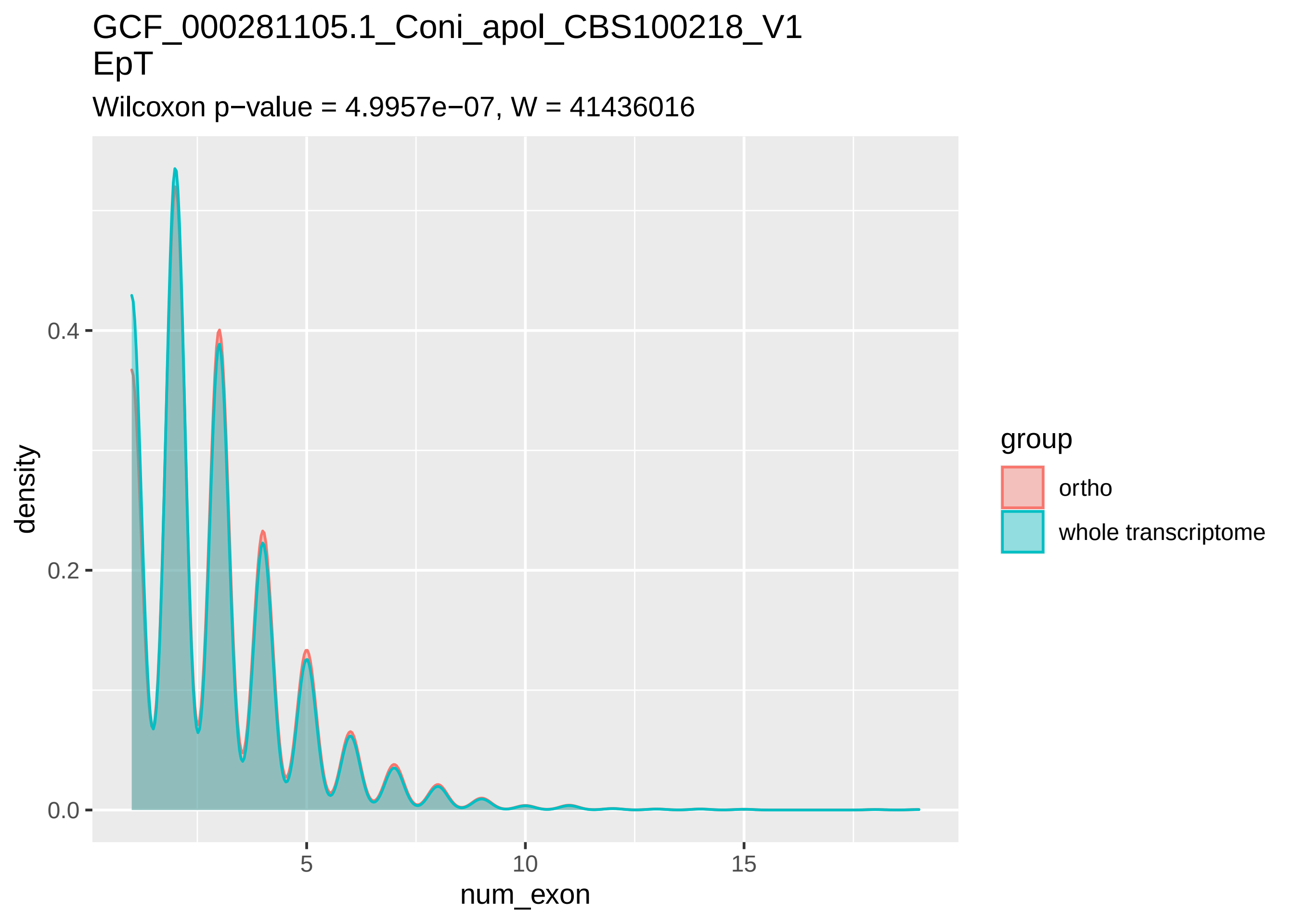
GCF_000281105.1_Coni_apol_CBS100218_V1
EpT
Wilcoxon p−value = 4.9957e−07, W = 41436016
density
0.4
0.2
0.0
5
10
15
num_exon
group
ortho
whole transcriptome

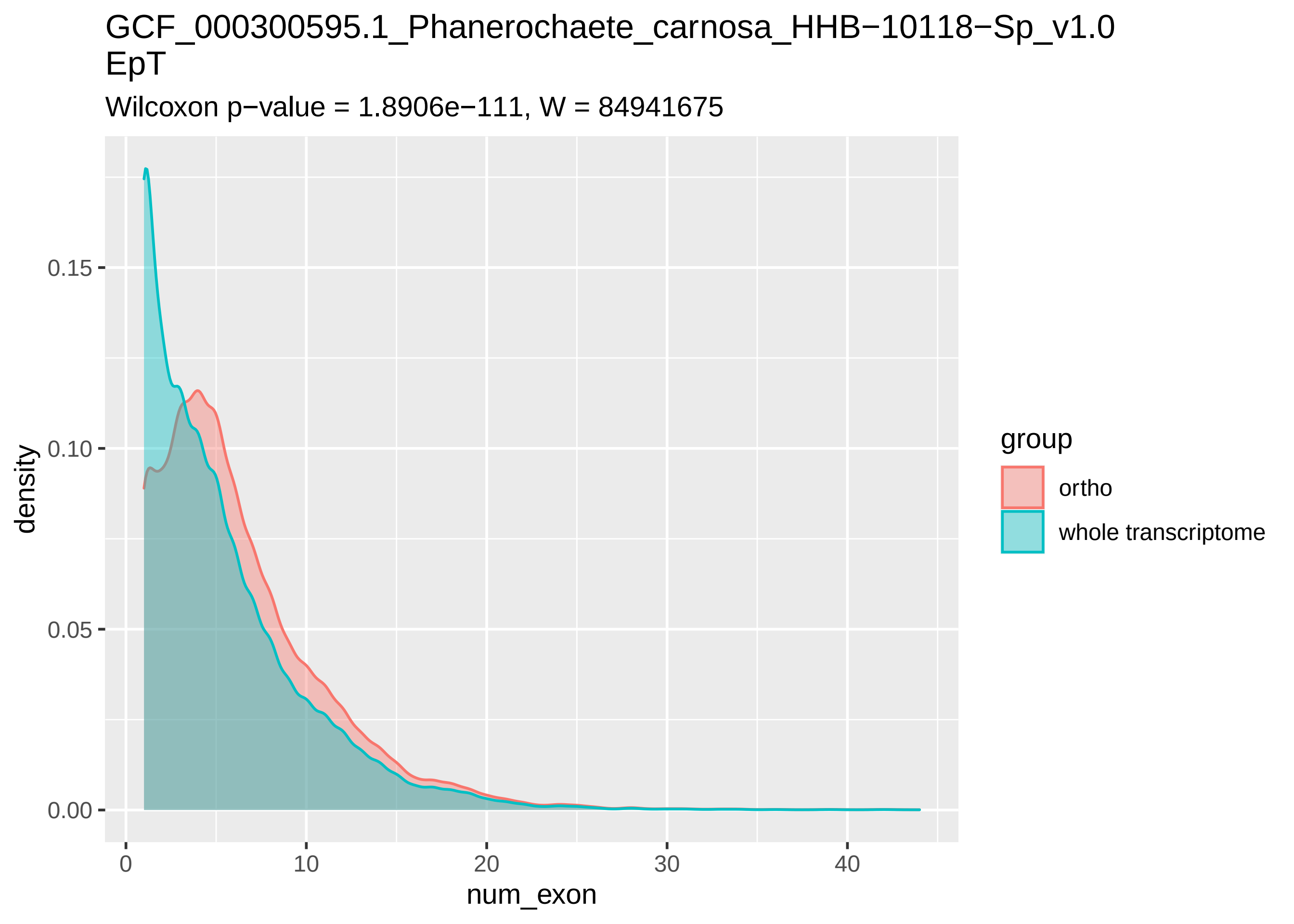

GCF_000300595.1_Phanerochaete_carnosa_HHB−10118−Sp_v1.0
EpT
Wilcoxon p−value = 1.8906e−111, W = 84941675
density
0.00
0.05
0.10
0.15
0
10
20
30
40
num_exon
group
ortho
whole transcriptome

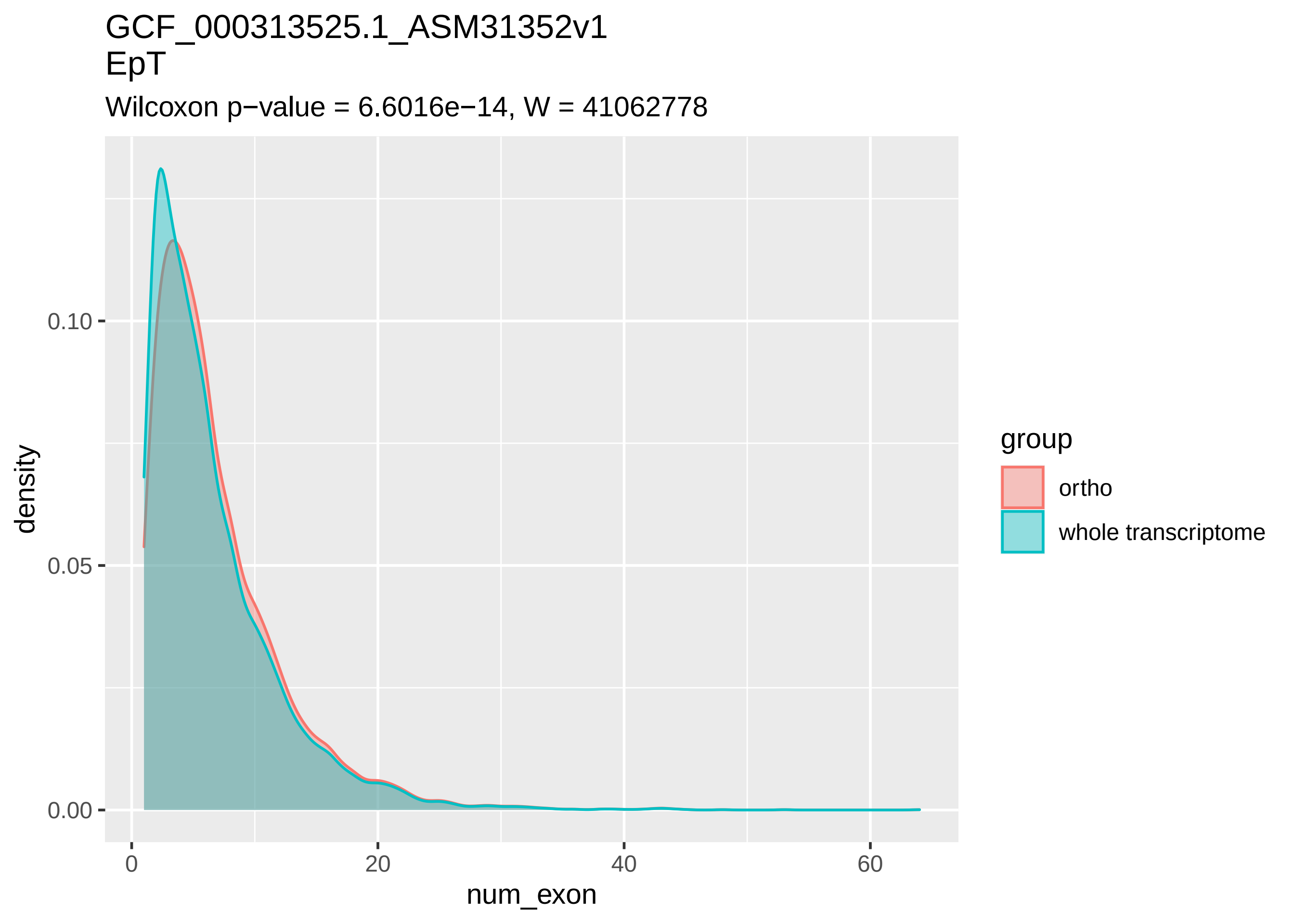
GCF_000313525.1_ASM31352v1
EpT
Wilcoxon p−value = 6.6016e−14, W = 41062778
density
0.10
0.05
0.00
0
20
40
60
num_exon
group
ortho
whole transcriptome

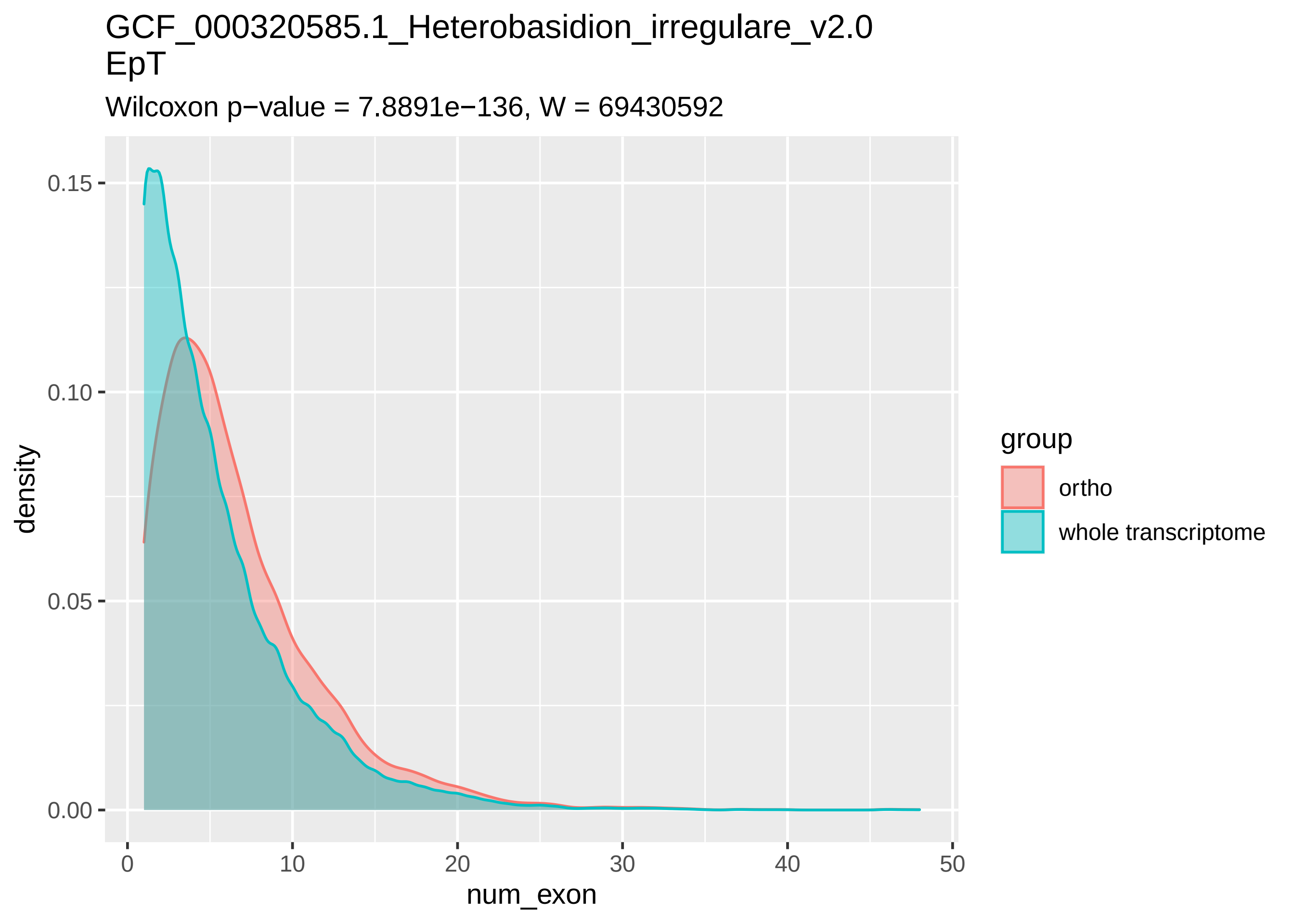
GCF_000320585.1_Heterobasidion_irregulare_v2.0
EpT
Wilcoxon p−value = 7.8891e−136, W = 69430592
density
0.15
0.10
0.05
0.00
0
10
20
30
40
50
num_exon
group
ortho
whole transcriptome

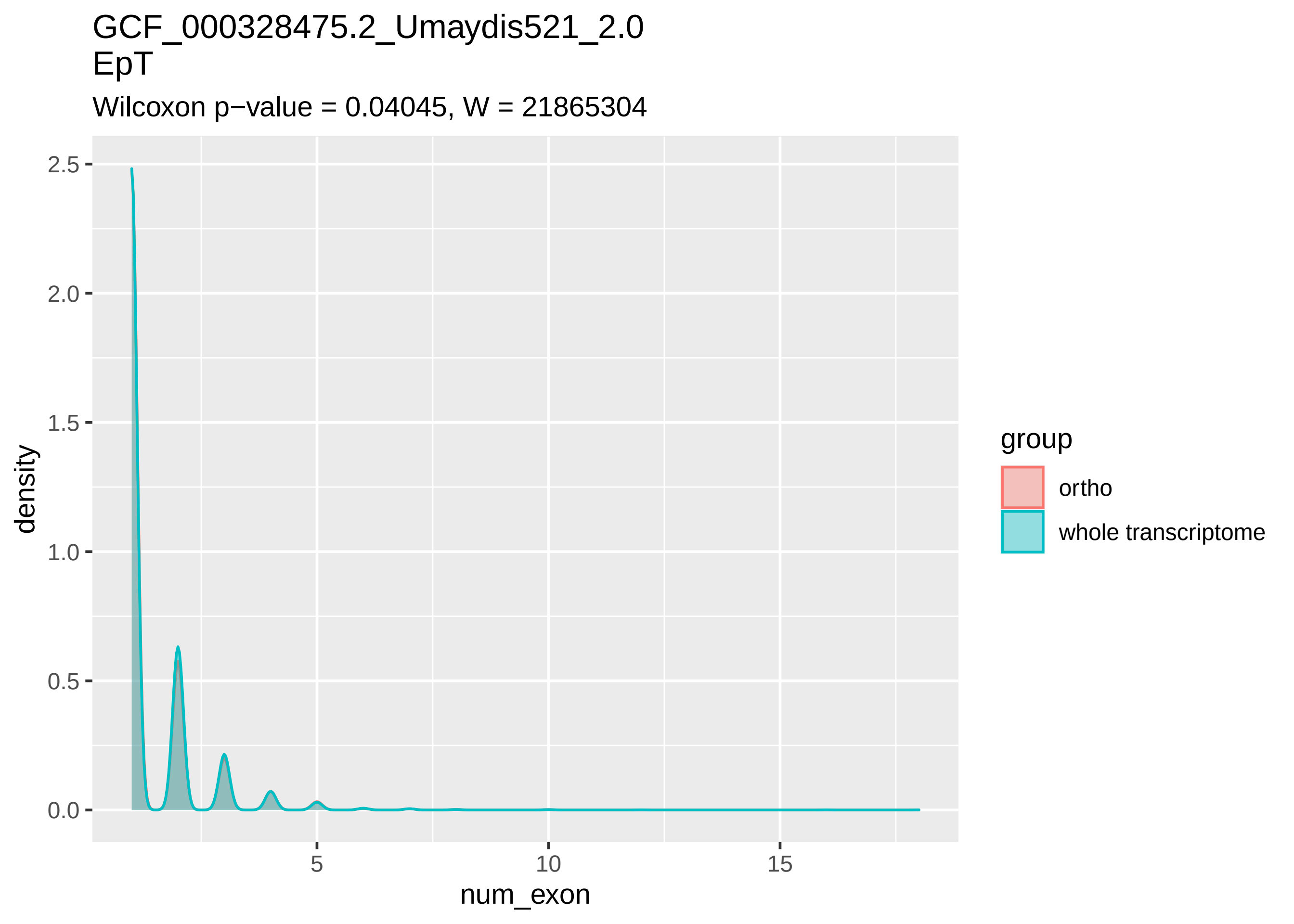
GCF_000328475.2_Umaydis521_2.0
EpT
Wilcoxon p−value = 0.04045, W = 21865304
2.5
2.0
1.5
1.0
0.5
0.0
density
5
10
15
num_exon
group
ortho
whole transcriptome

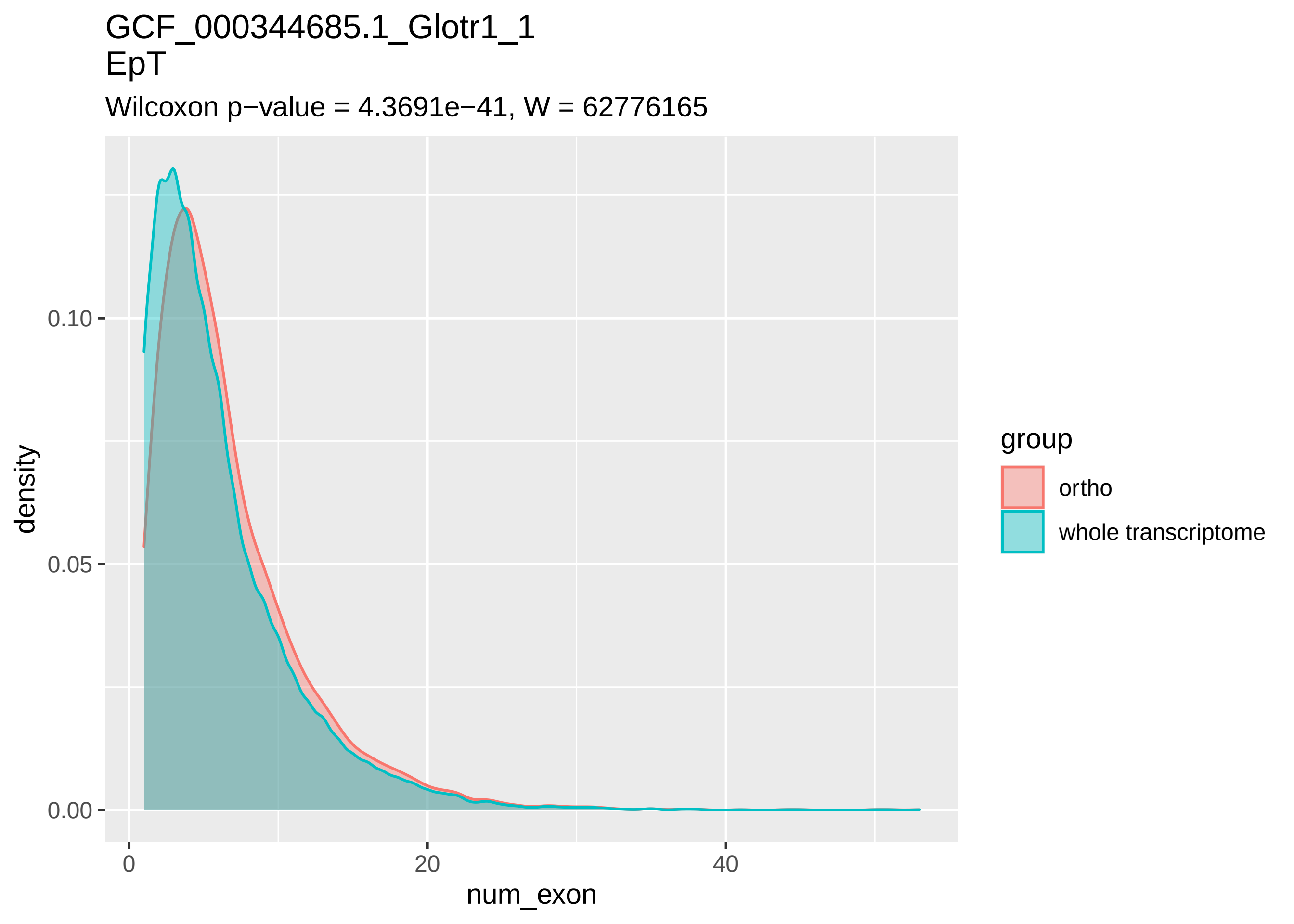
GCF_000344685.1_Glotr1_1
EpT
Wilcoxon p−value = 4.3691e−41, W = 62776165
density
0.10
0.05
0.00
0
20
40
num_exon
group
ortho
whole transcriptome

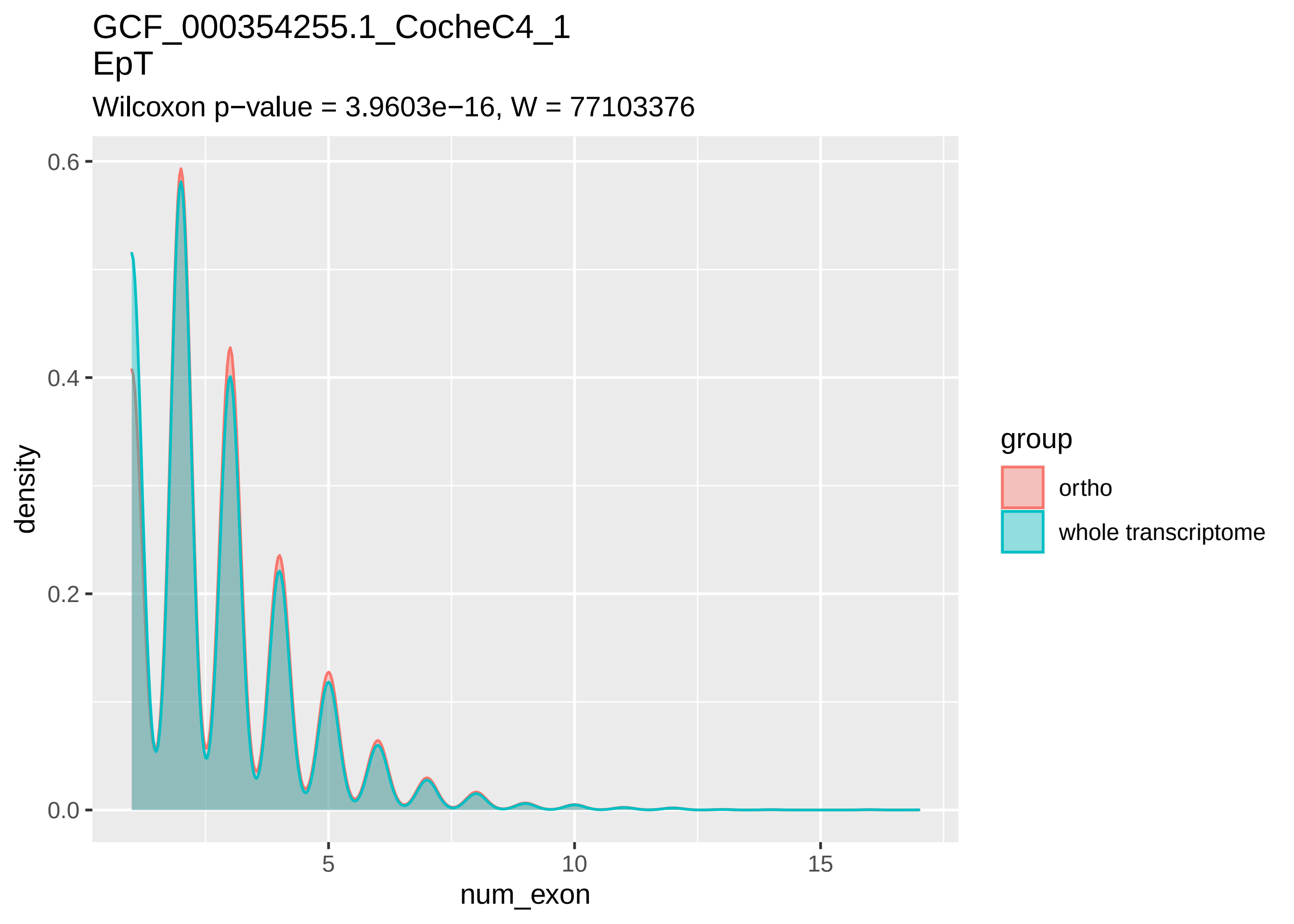

GCF_000354255.1_CocheC4_1
EpT
Wilcoxon p−value = 3.9603e−16, W = 77103376
0.6
0.4
0.2
0.0
density
5
10
15
num_exon
group
ortho
whole transcriptome

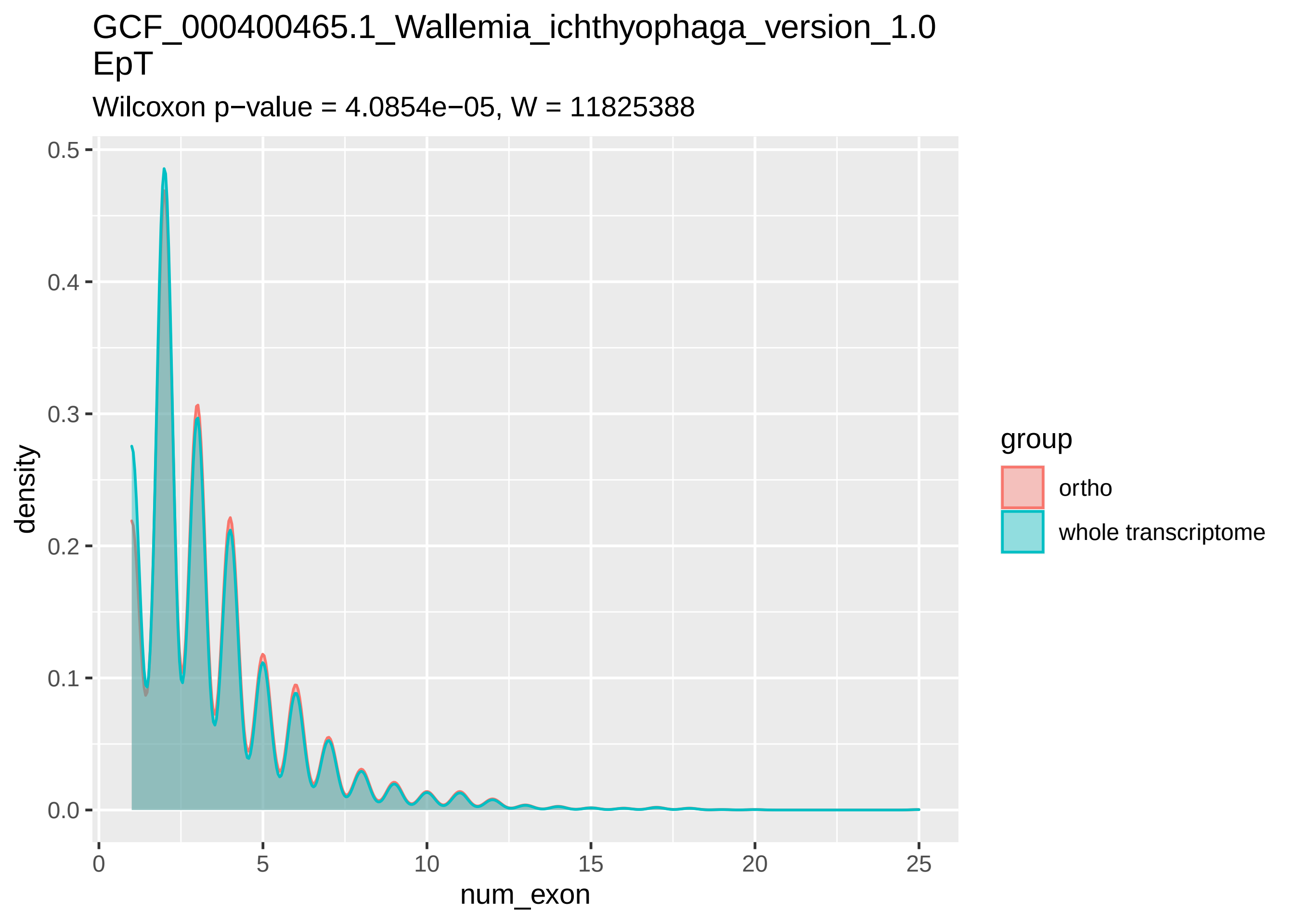
GCF_000400465.1_Wallemia_ichthyophaga_version_1.0
EpT
Wilcoxon p−value = 4.0854e−05, W = 11825388
density
0.5
0.4
0.3
0.2
0.1
0.0
0
5
10
15
20
25
num_exon
group
ortho
whole transcriptome

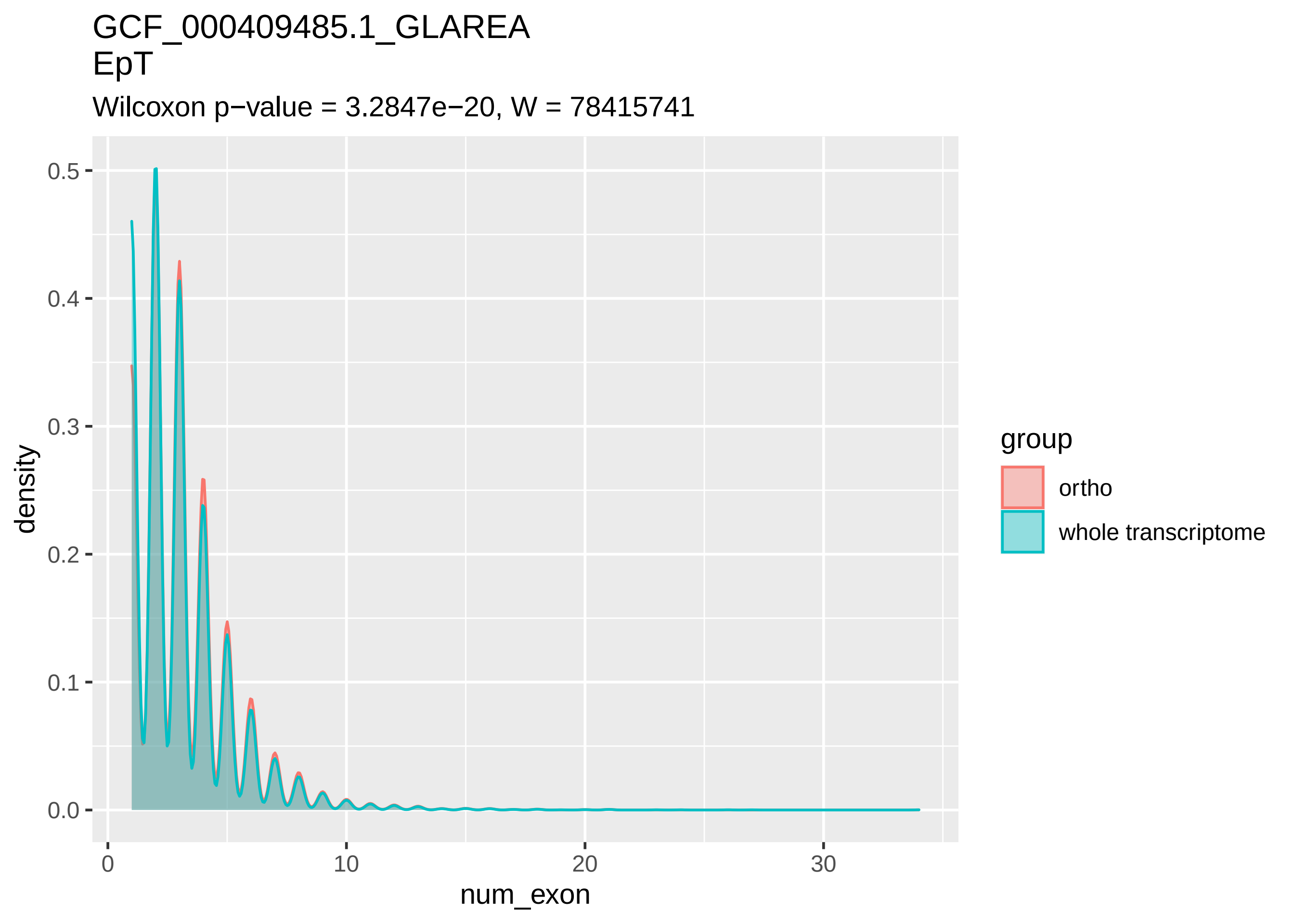
GCF_000409485.1_GLAREA
EpT
Wilcoxon p−value = 3.2847e−20, W = 78415741
density
0.0
0.1
0.2
0.3
0.4
0.5
0
10
20
30
num_exon
group
ortho
whole transcriptome

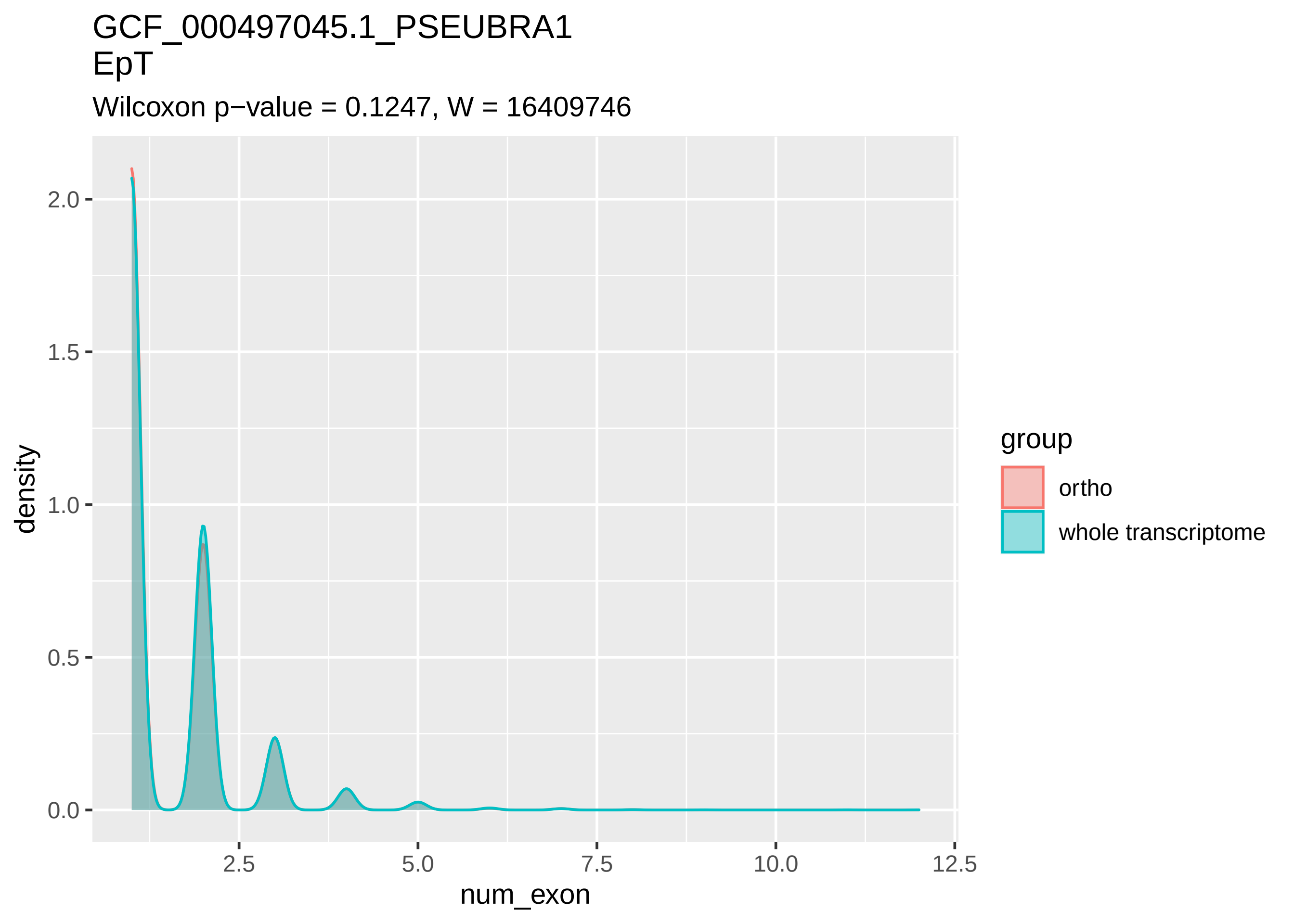

GCF_000497045.1_PSEUBRA1
EpT
Wilcoxon p−value = 0.1247, W = 16409746
density
2.0
1.5
1.0
0.5
0.0
2.5
5.0
7.5
10.0
12.5
num_exon
group
ortho
whole transcriptome

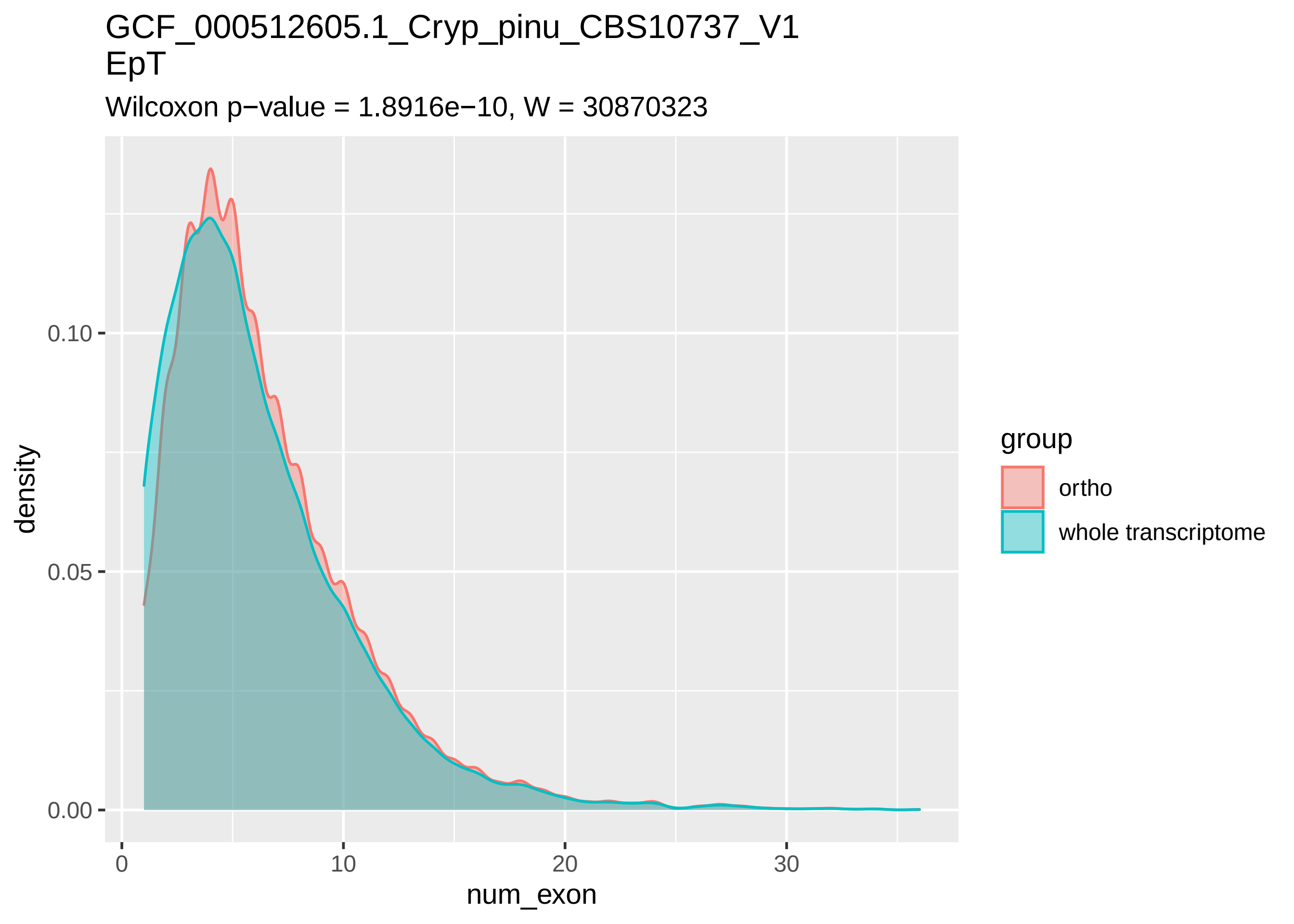
GCF_000512605.1_Cryp_pinu_CBS10737_V1
EpT
Wilcoxon p−value = 1.8916e−10, W = 30870323
density
0.10
0.05
0.00
0
10
20
30
num_exon
group
ortho
whole transcriptome

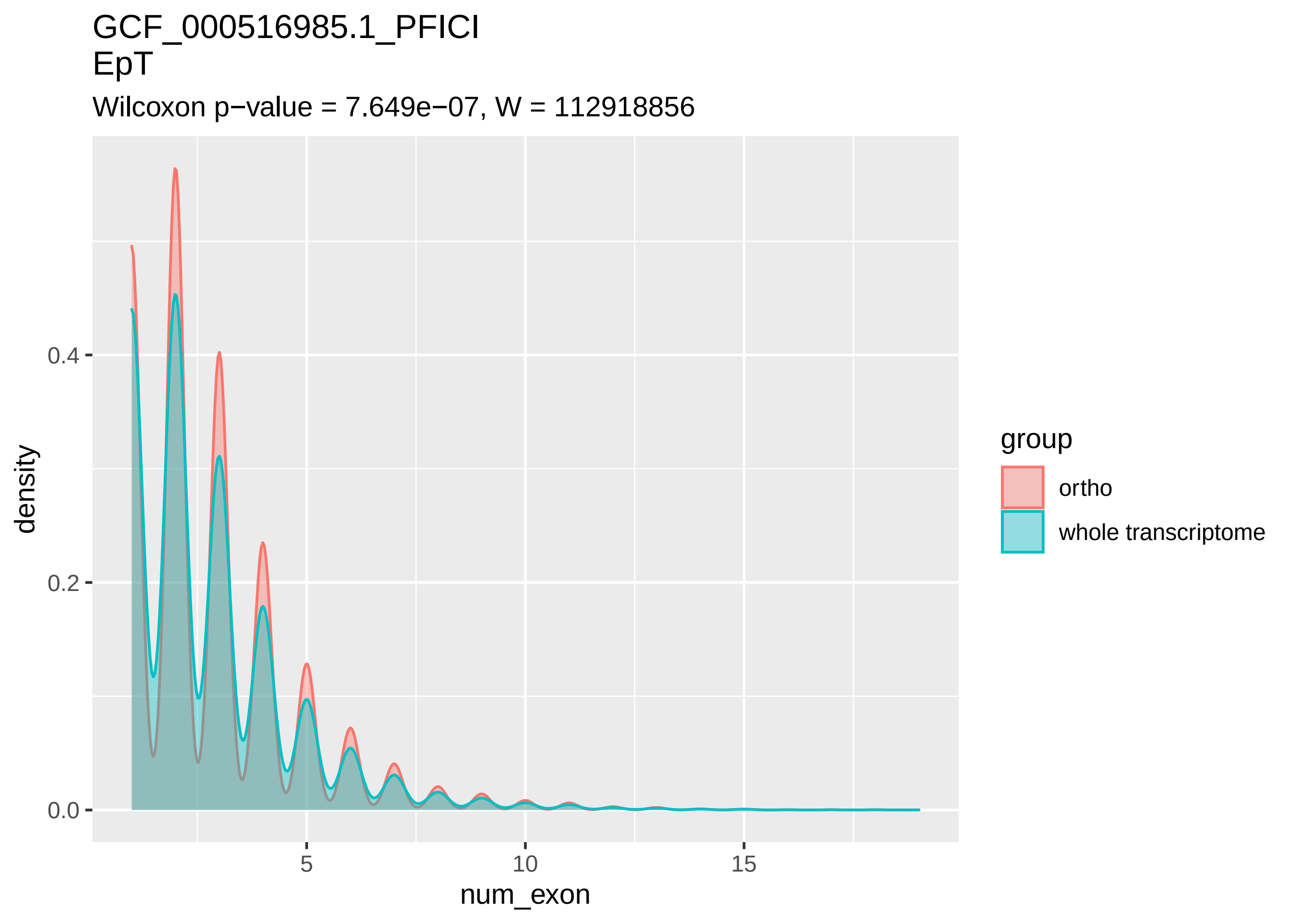
GCF_000516985.1_PFICI
EpT
Wilcoxon p−value = 7.649e−07, W = 112918856
density
0.0
0.2
0.4
5
10
15
num_exon
group
ortho
whole transcriptome

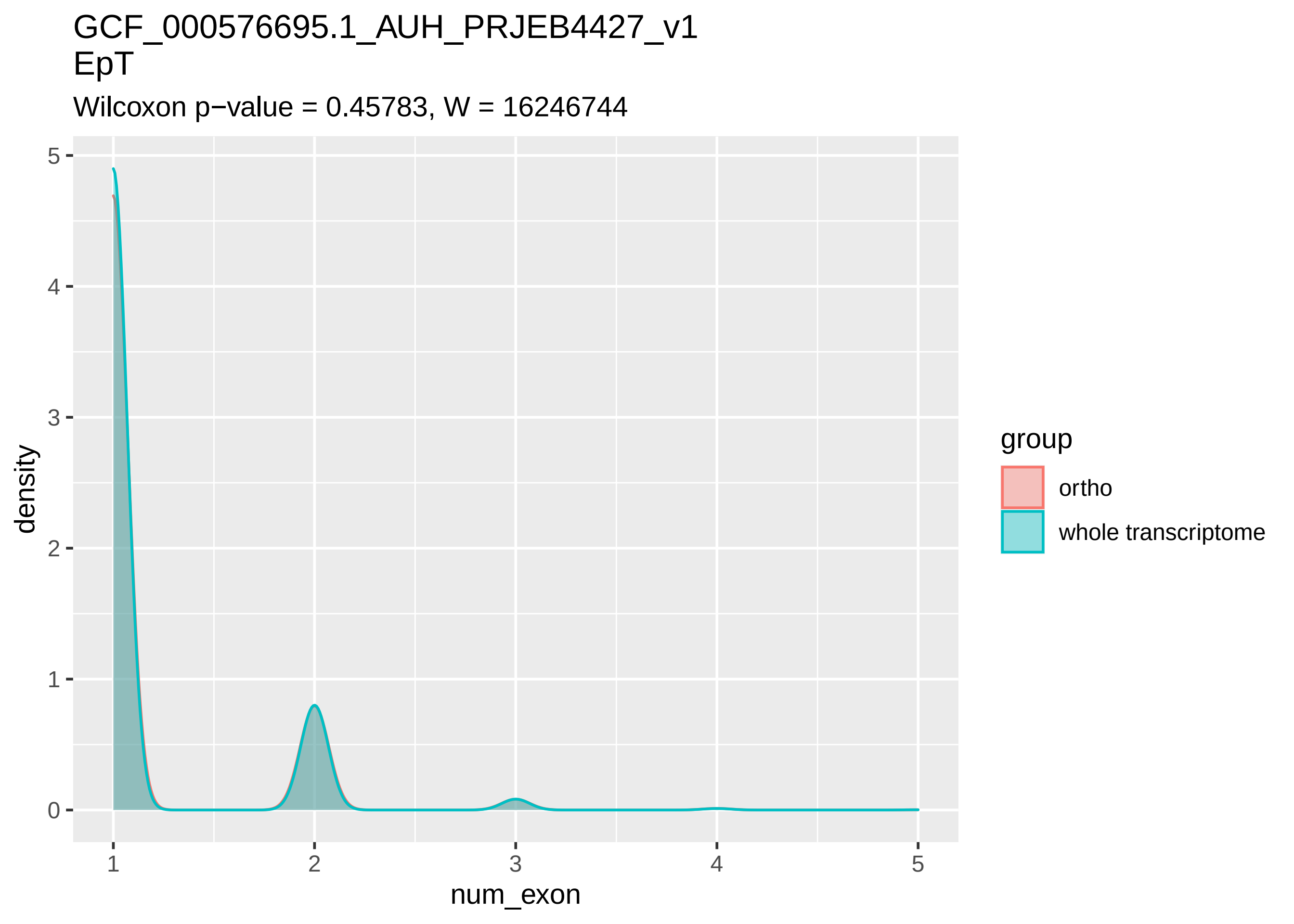
GCF_000576695.1_AUH_PRJEB4427_v1
EpT
Wilcoxon p−value = 0.45783, W = 16246744
density
num_exon
0
1
2
3
4
5
group
ortho
whole transcriptome

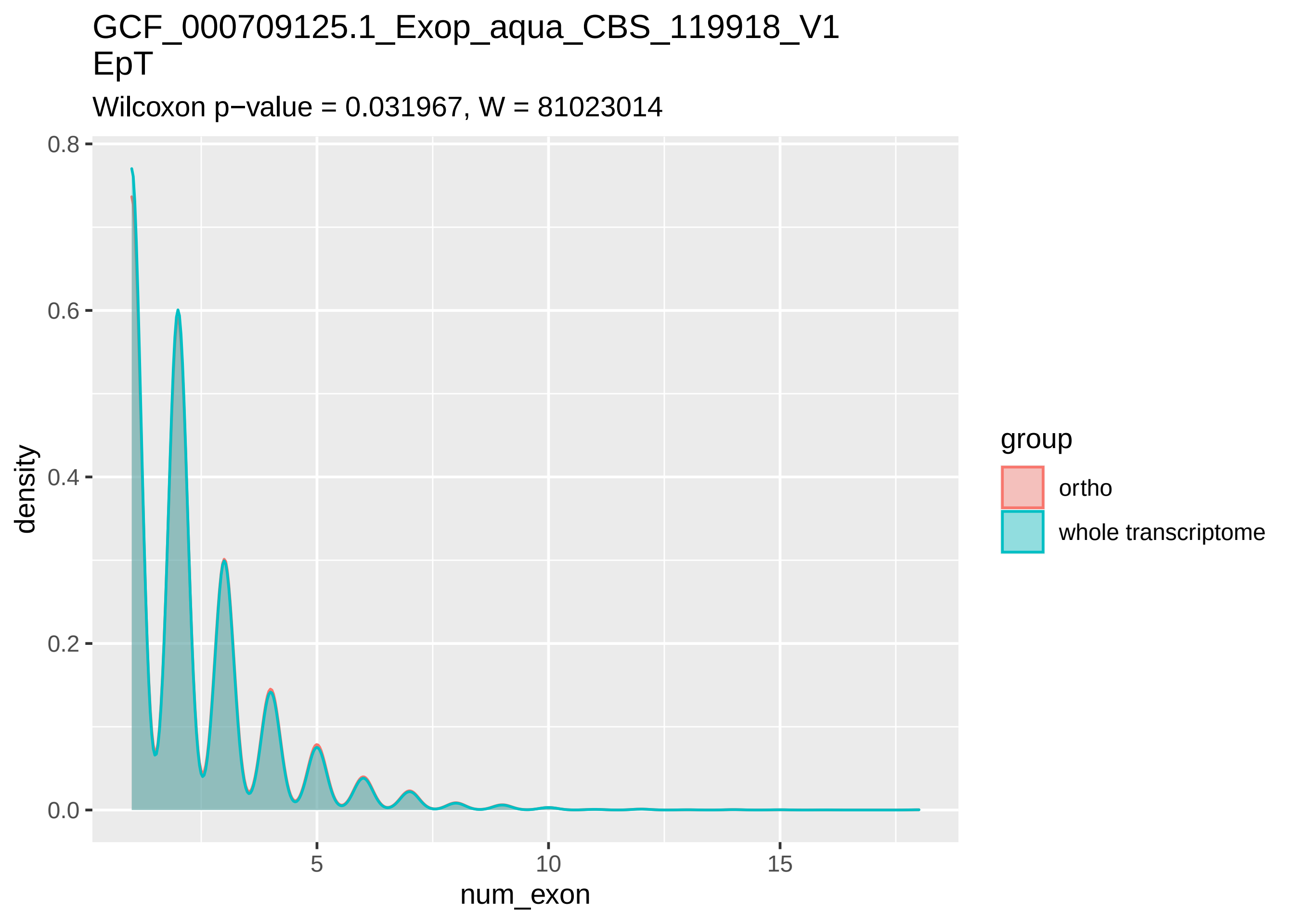
GCF_000709125.1_Exop_aqua_CBS_119918_V1
EpT
Wilcoxon p−value = 0.031967, W = 81023014
0.8
0.6
0.4
0.2
0.0
density
5
10
15
num_exon
group
ortho
whole transcriptome

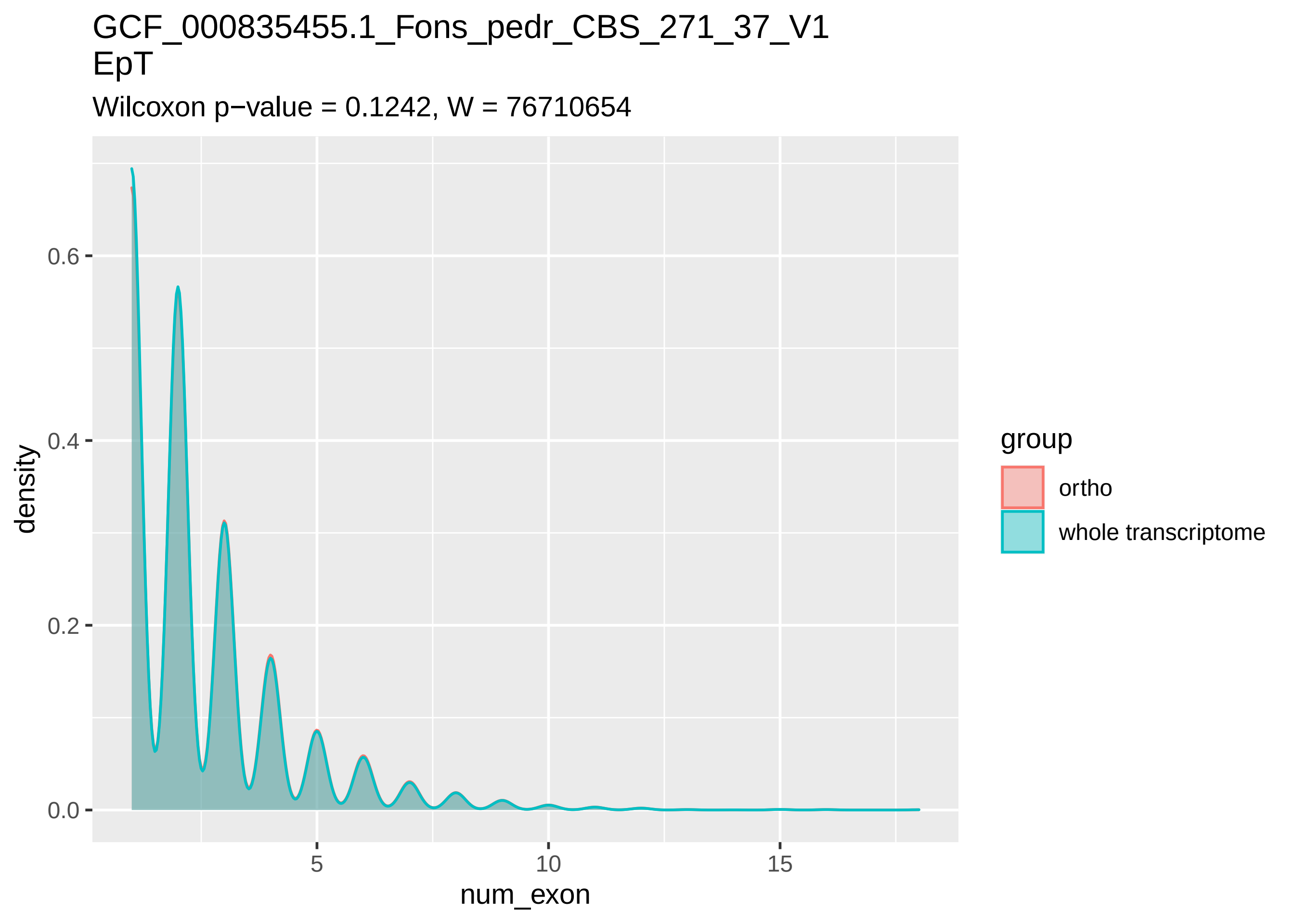
GCF_000835455.1_Fons_pedr_CBS_271_37_V1
EpT
Wilcoxon p−value = 0.1242, W = 76710654
density
0.0
0.2
0.4
0.6
5
10
15
num_exon
group
ortho
whole transcriptome

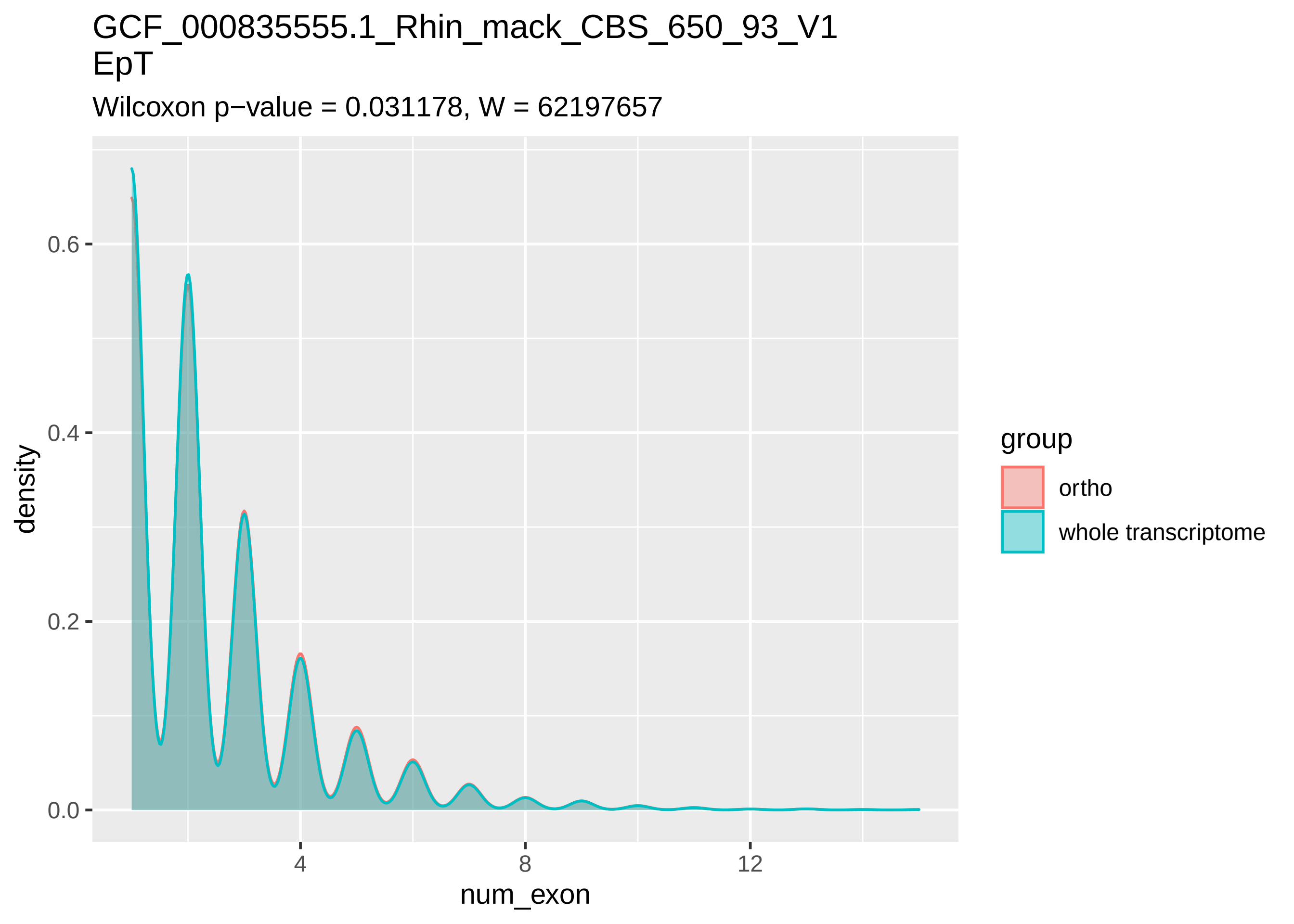
GCF_000835555.1_Rhin_mack_CBS_650_93_V1
EpT
Wilcoxon p−value = 0.031178, W = 62197657
density
0.0
0.2
0.4
0.6
4
8
12
num_exon
group
ortho
whole transcriptome

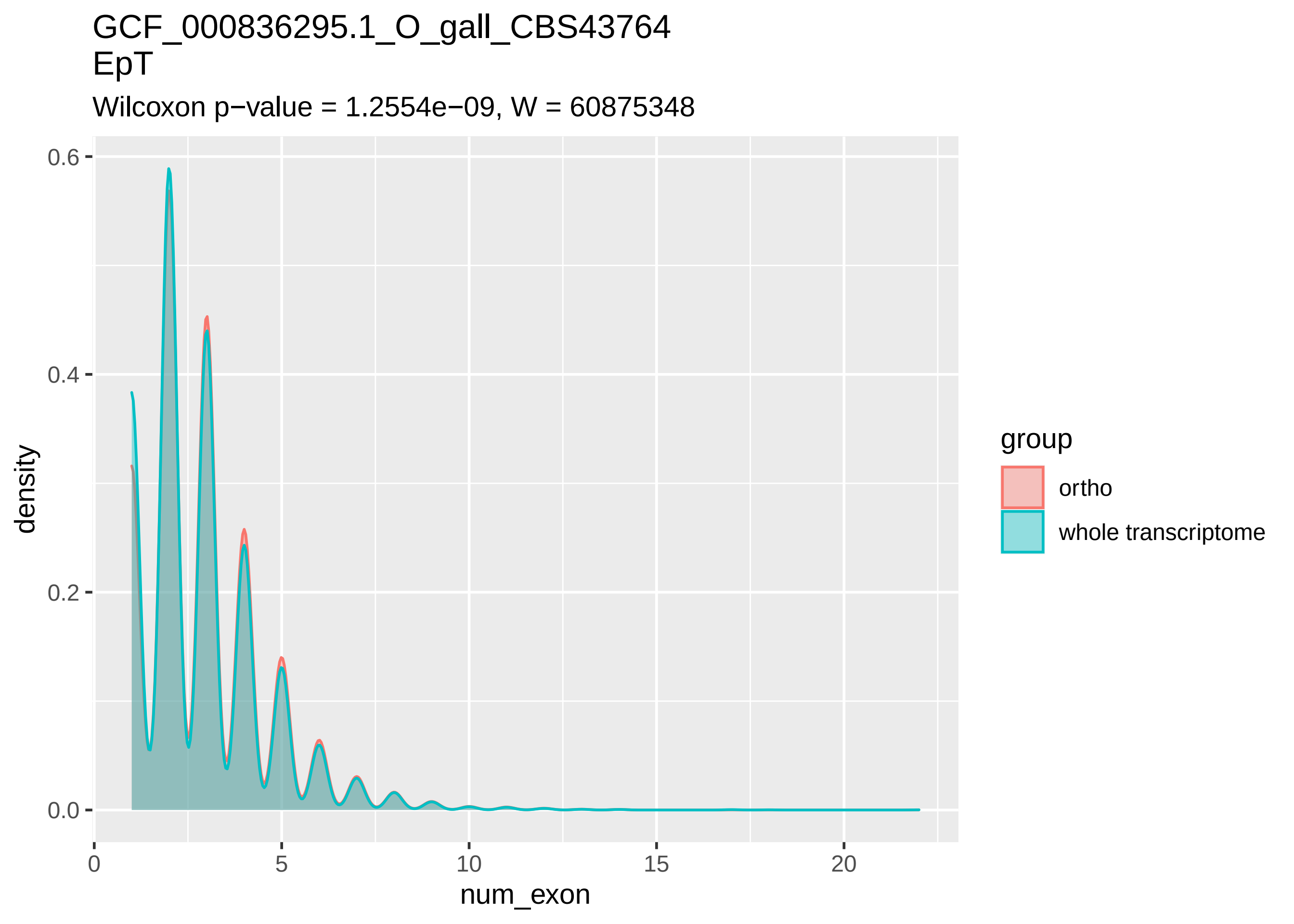
GCF_000836295.1_O_gall_CBS43764
EpT
Wilcoxon p−value = 1.2554e−09, W = 60875348
0.6
0.4
0.2
0.0
density
0
5
10
15
20
num_exon
group
ortho
whole transcriptome

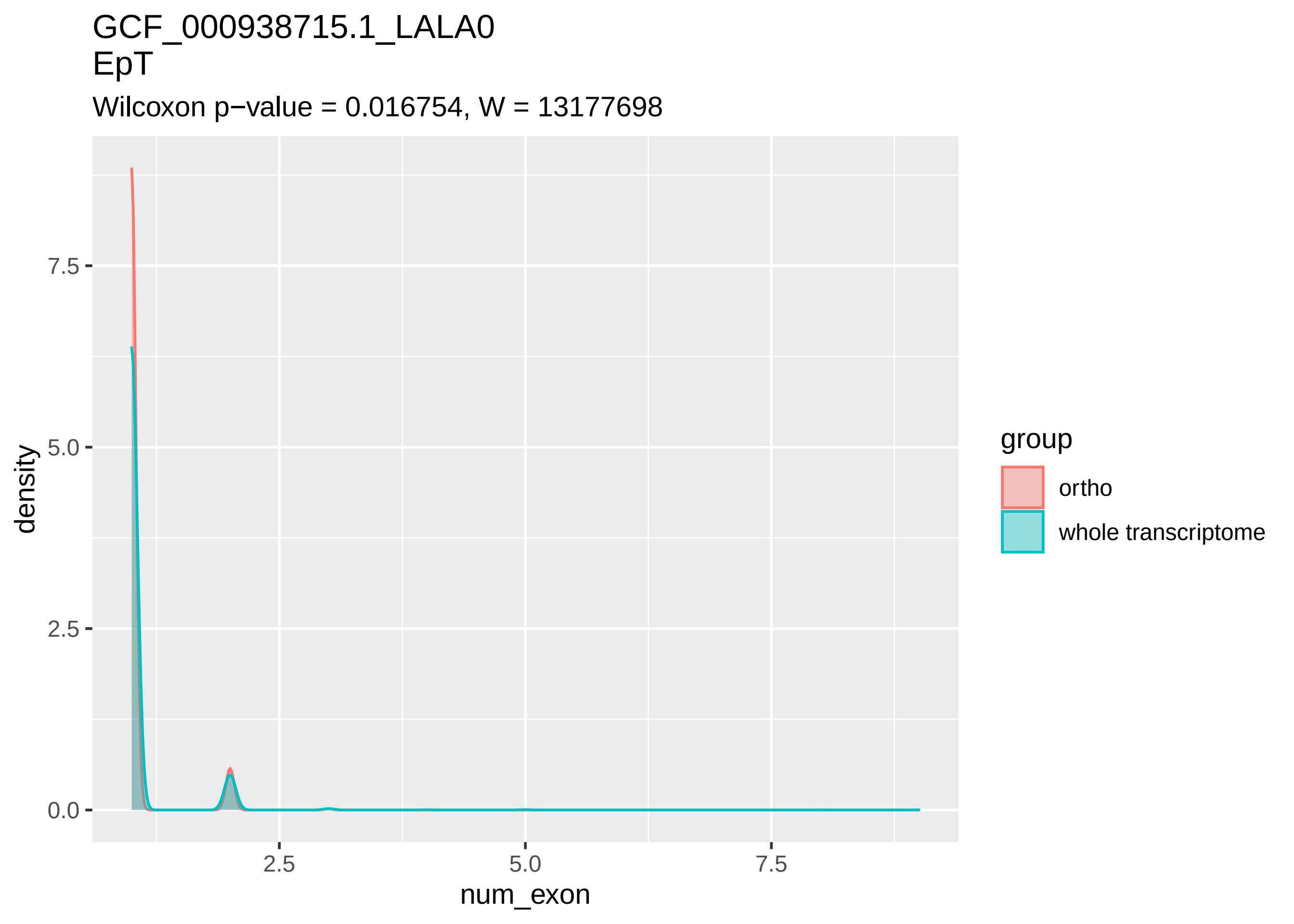

GCF_000938715.1_LALA0
EpT
Wilcoxon p−value = 0.016754, W = 13177698
density
7.5
5.0
2.5
0.0
2.5
5.0
7.5
num_exon
group
ortho
whole transcriptome

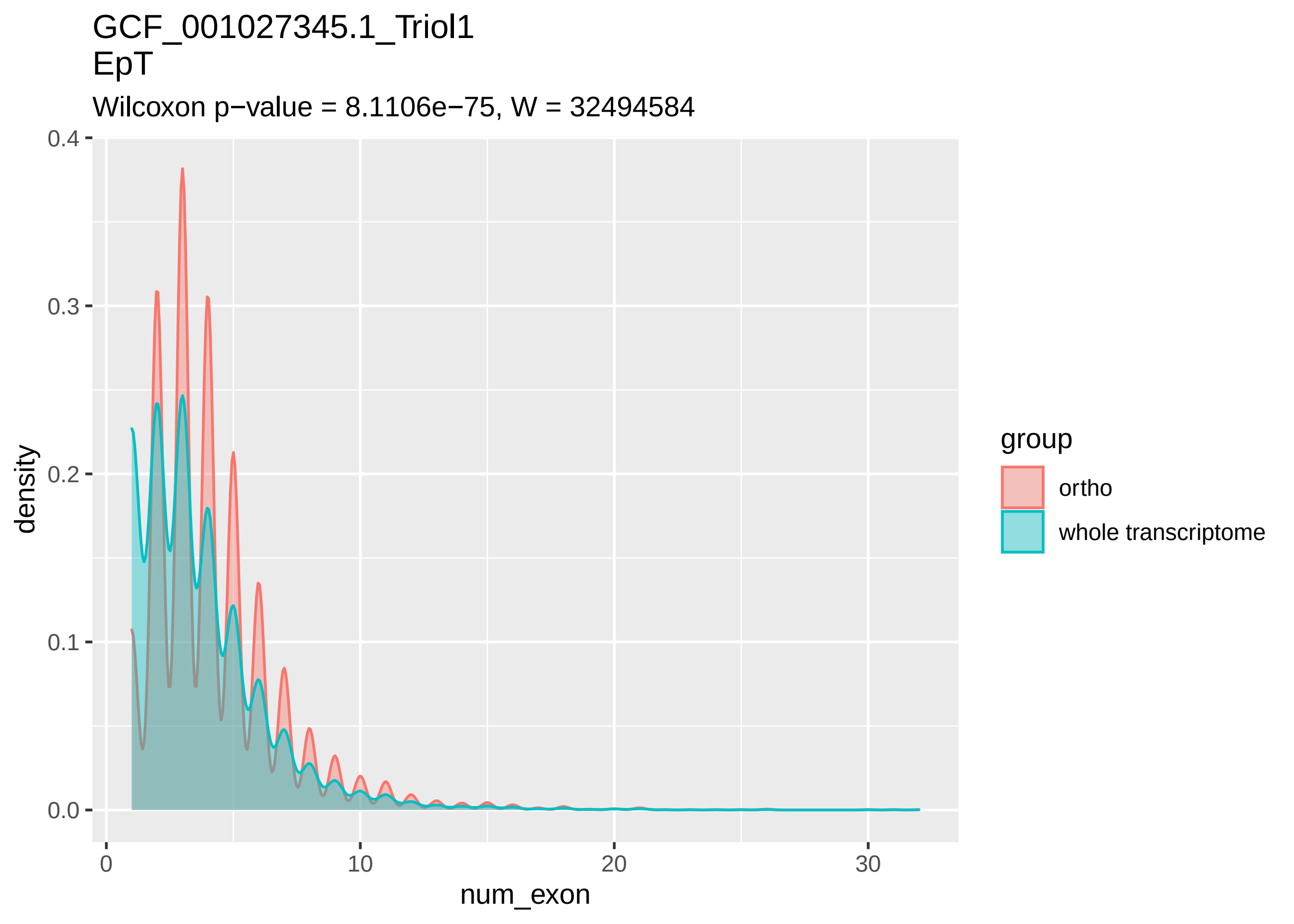
GCF_001027345.1_Triol1
EpT
Wilcoxon p−value = 8.1106e−75, W = 32494584
0.4
0.3
0.2
0.1
0.0
density
0
10
20
30
num_exon
group
ortho
whole transcriptome

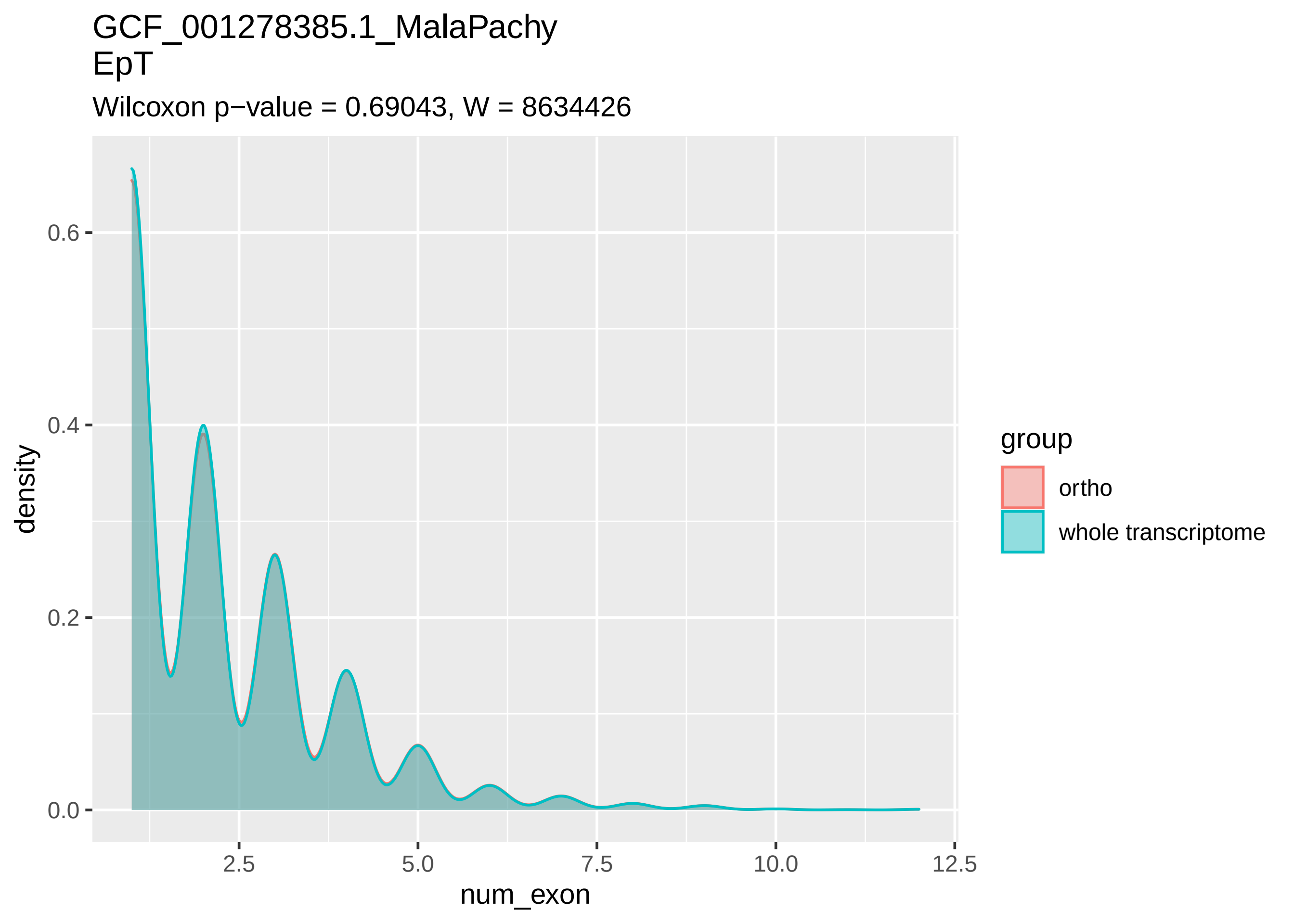
GCF_001278385.1_MalaPachy
EpT
Wilcoxon p−value = 0.69043, W = 8634426
density
0.0
0.2
0.4
0.6
2.5
5.0
7.5
10.0
12.5
num_exon
group
ortho
whole transcriptome

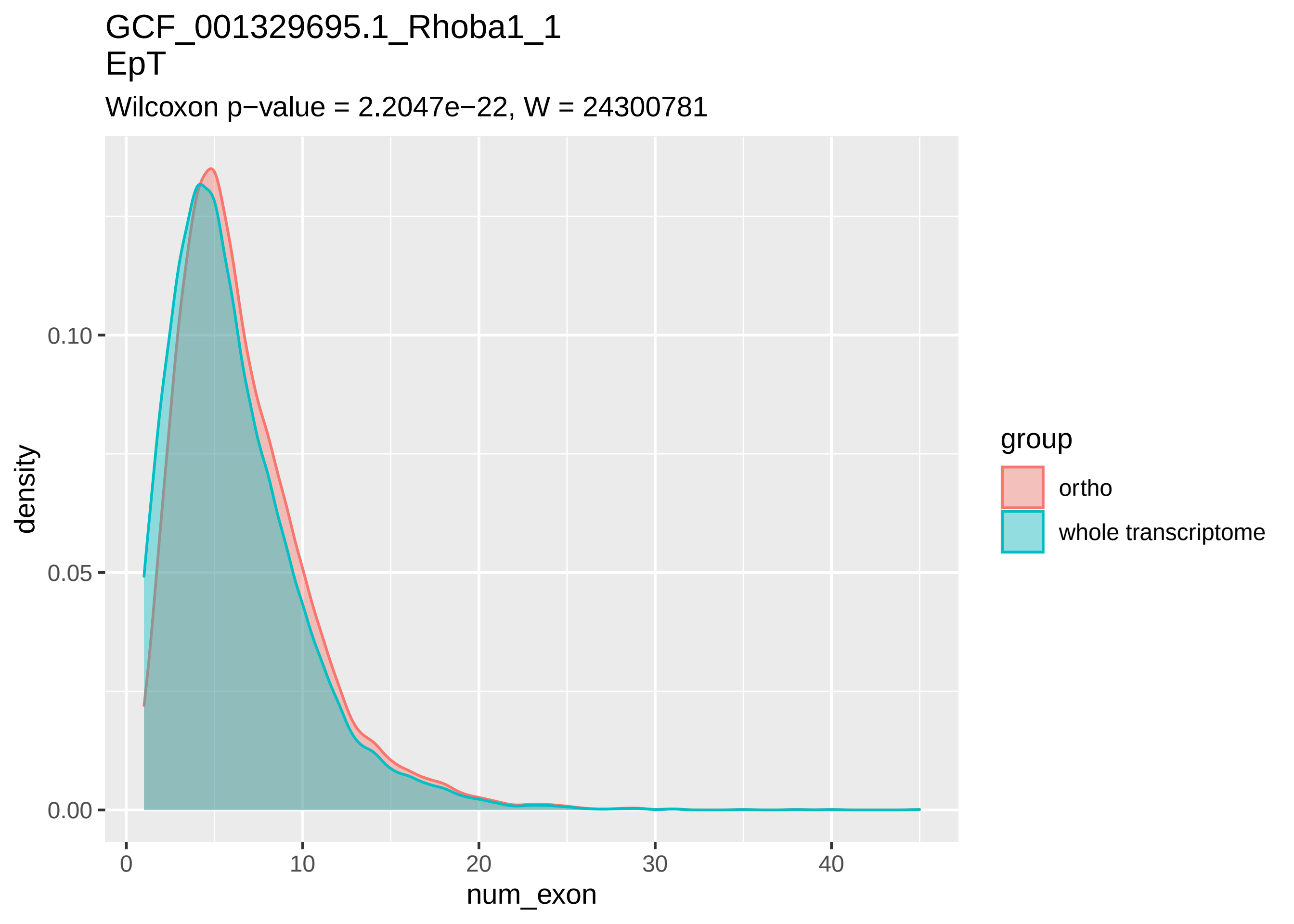
GCF_001329695.1_Rhoba1_1
EpT
Wilcoxon p−value = 2.2047e−22, W = 24300781
density
0.00
0.05
0.10
0
10
20
30
40
num_exon
group
ortho
whole transcriptome

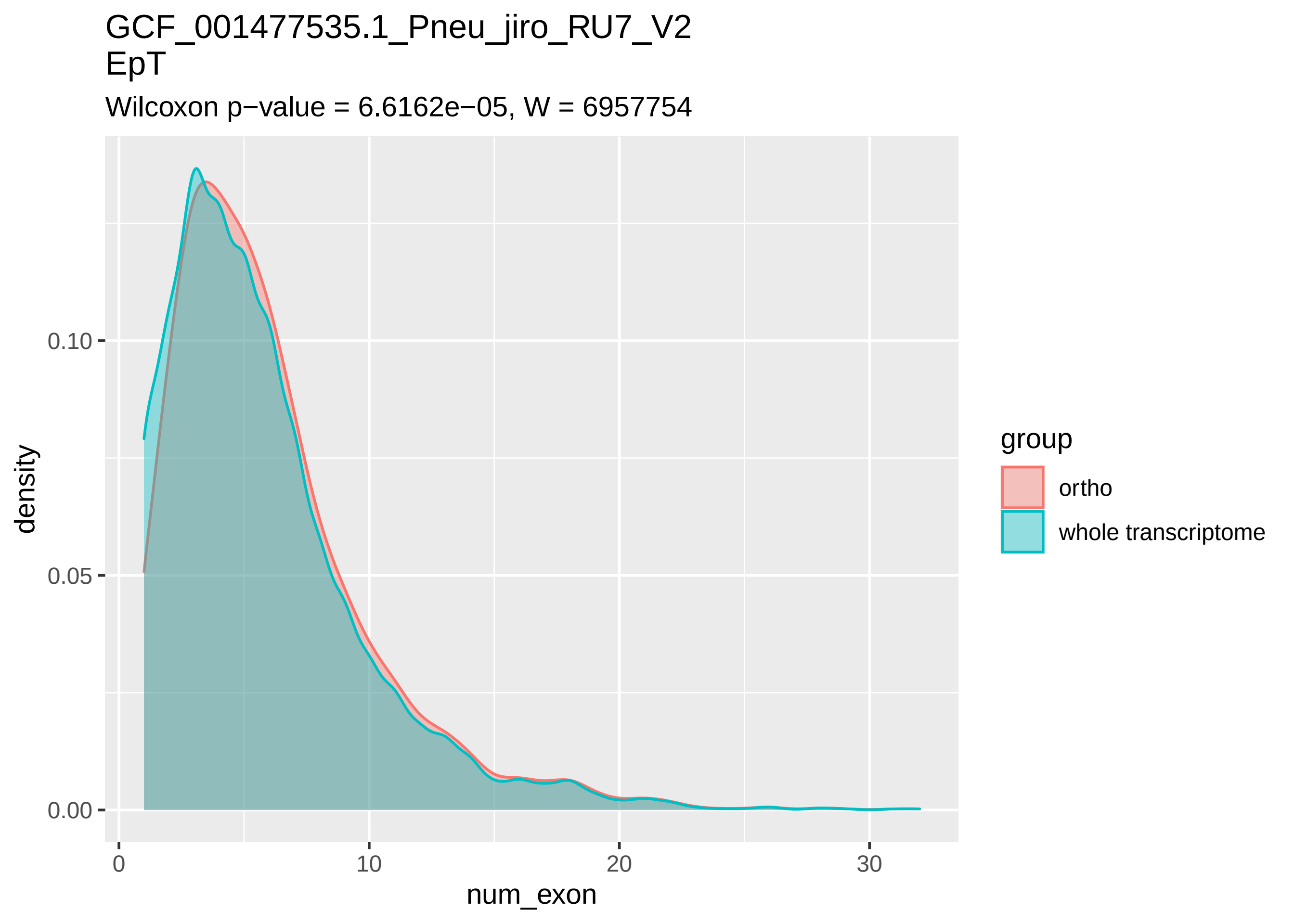

GCF_001477535.1_Pneu_jiro_RU7_V2
EpT
Wilcoxon p−value = 6.6162e−05, W = 6957754
density
0.00
0.05
0.10
0
10
20
30
num_exon
group
ortho
whole transcriptome

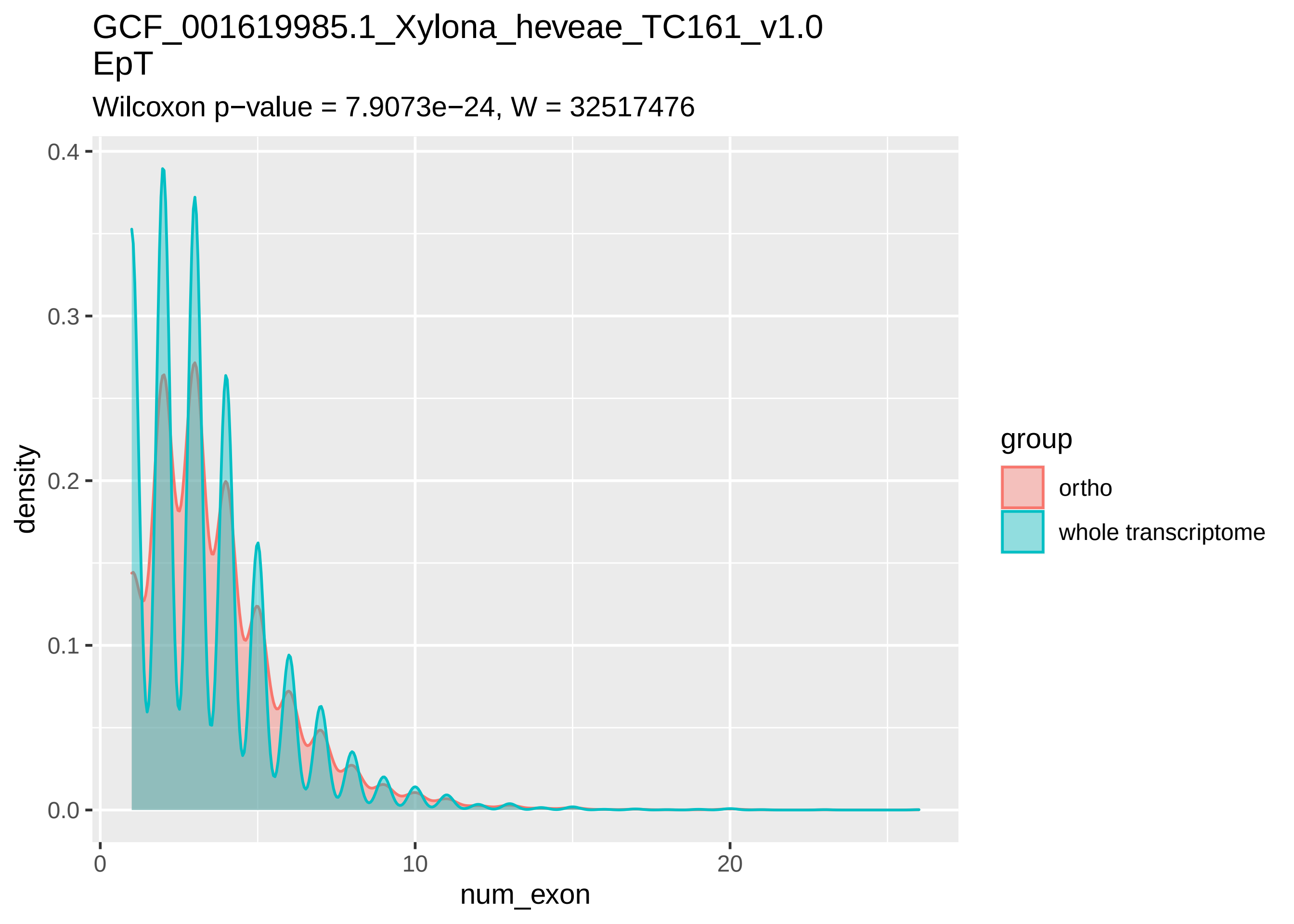

GCF_001619985.1_Xylona_heveae_TC161_v1.0
EpT
Wilcoxon p−value = 7.9073e−24, W = 32517476
density
num_exon
0.0
0.1
0.2
0.3
0.4
0
10
20
group
ortho
whole transcriptome

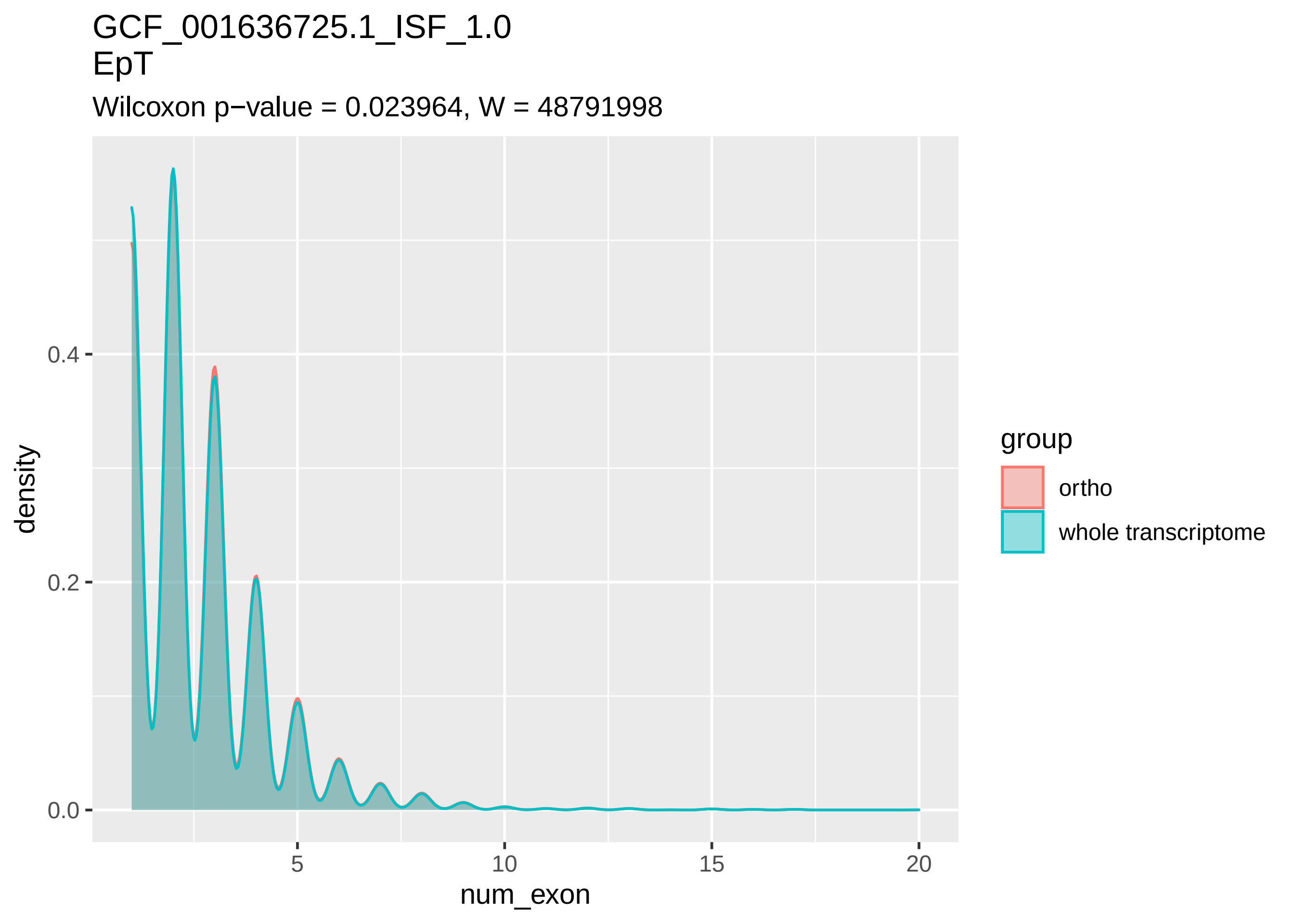
GCF_001636725.1_ISF_1.0
EpT
Wilcoxon p−value = 0.023964, W = 48791998
density
0.0
0.2
0.4
5
10
15
20
num_exon
group
ortho
whole transcriptome

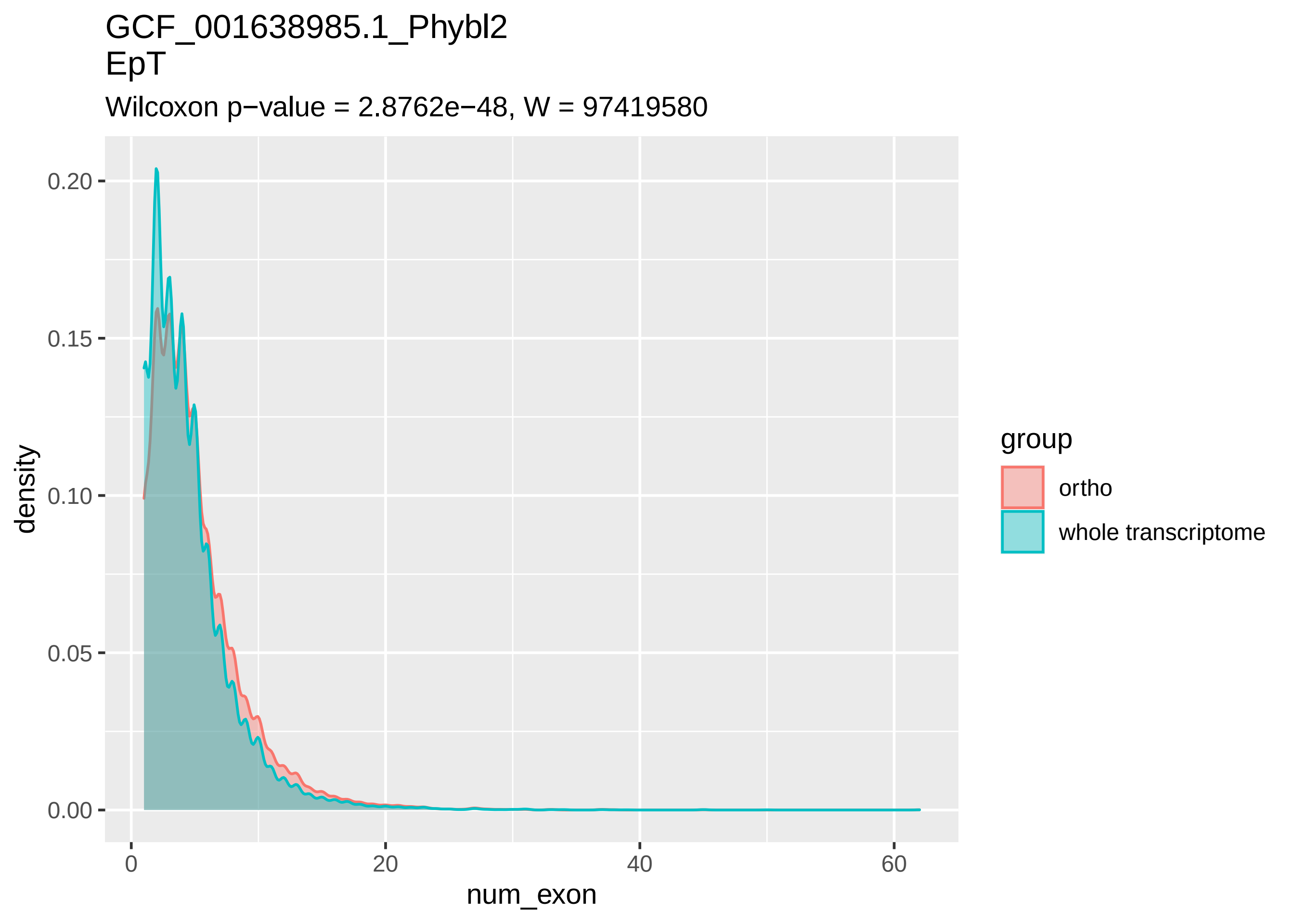
GCF_001638985.1_Phybl2
EpT
Wilcoxon p−value = 2.8762e−48, W = 97419580
density
0.20
0.15
0.10
0.05
0.00
0
20
40
60
num_exon
group
ortho
whole transcriptome

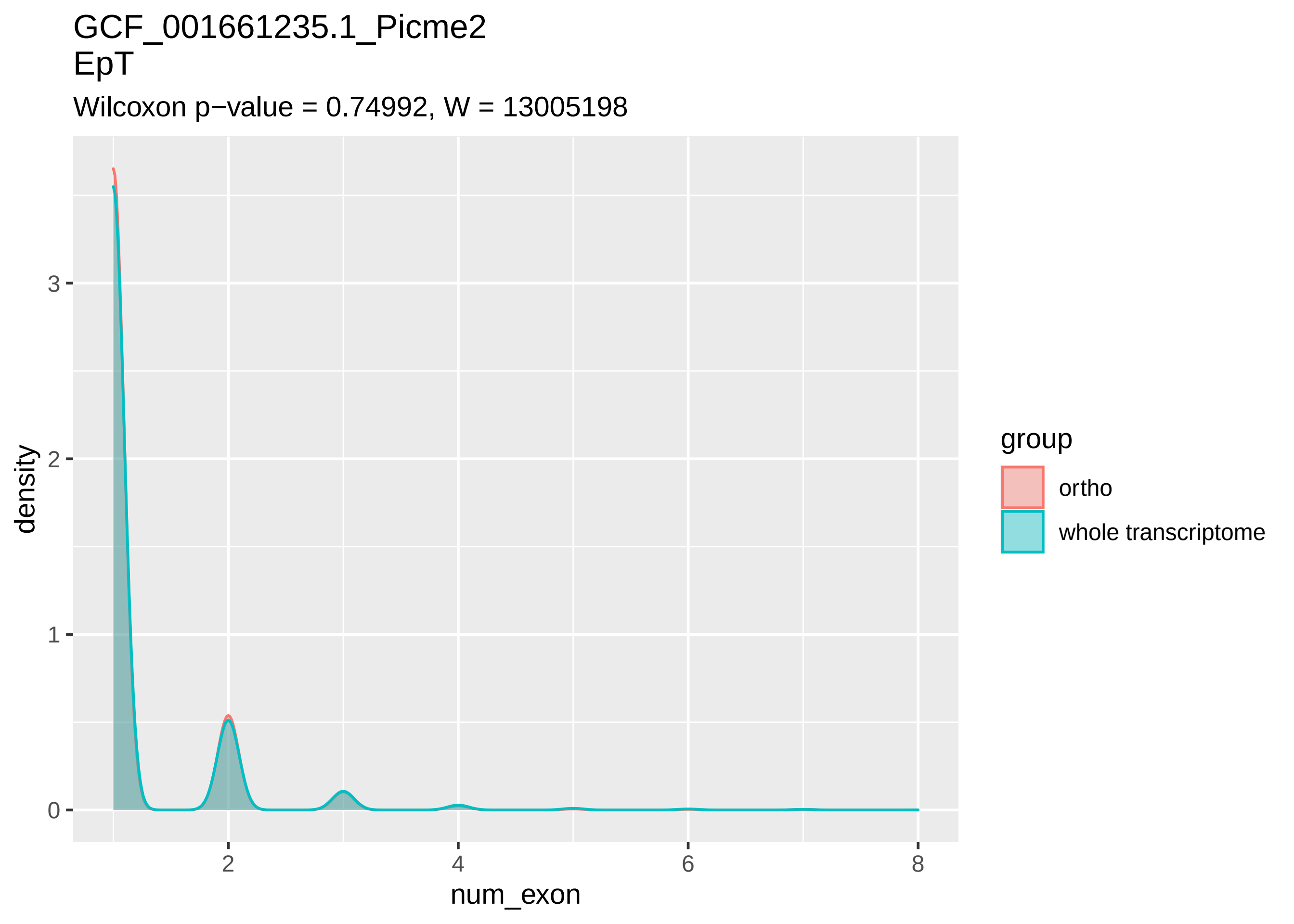
GCF_001661235.1_Picme2
EpT
Wilcoxon p−value = 0.74992, W = 13005198
density
3
2
1
0
2
4
6
8
num_exon
group
ortho
whole transcriptome

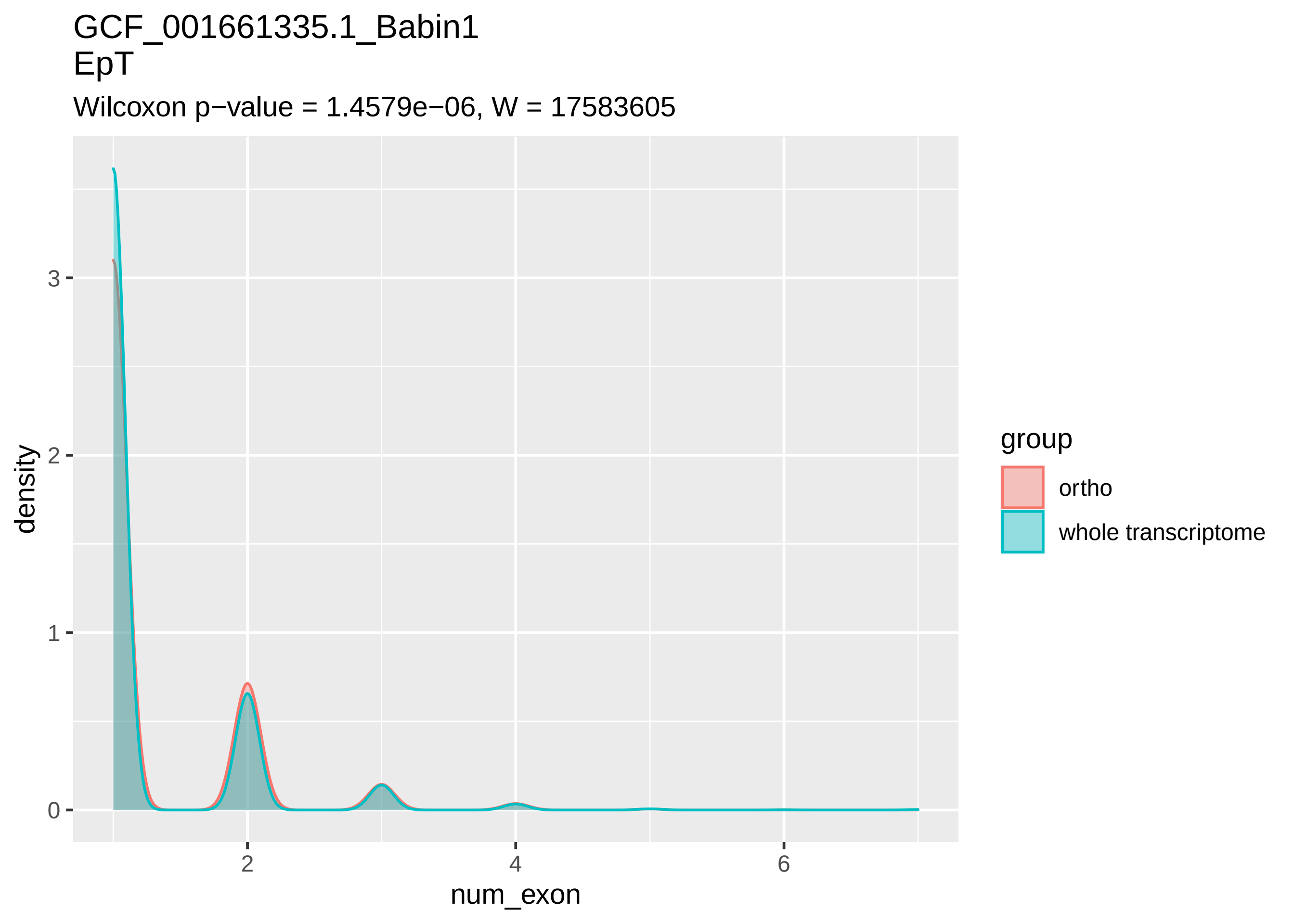
GCF_001661335.1_Babin1
EpT
Wilcoxon p−value = 1.4579e−06, W = 17583605
density
3
2
1
0
2
4
6
num_exon
group
ortho
whole transcriptome

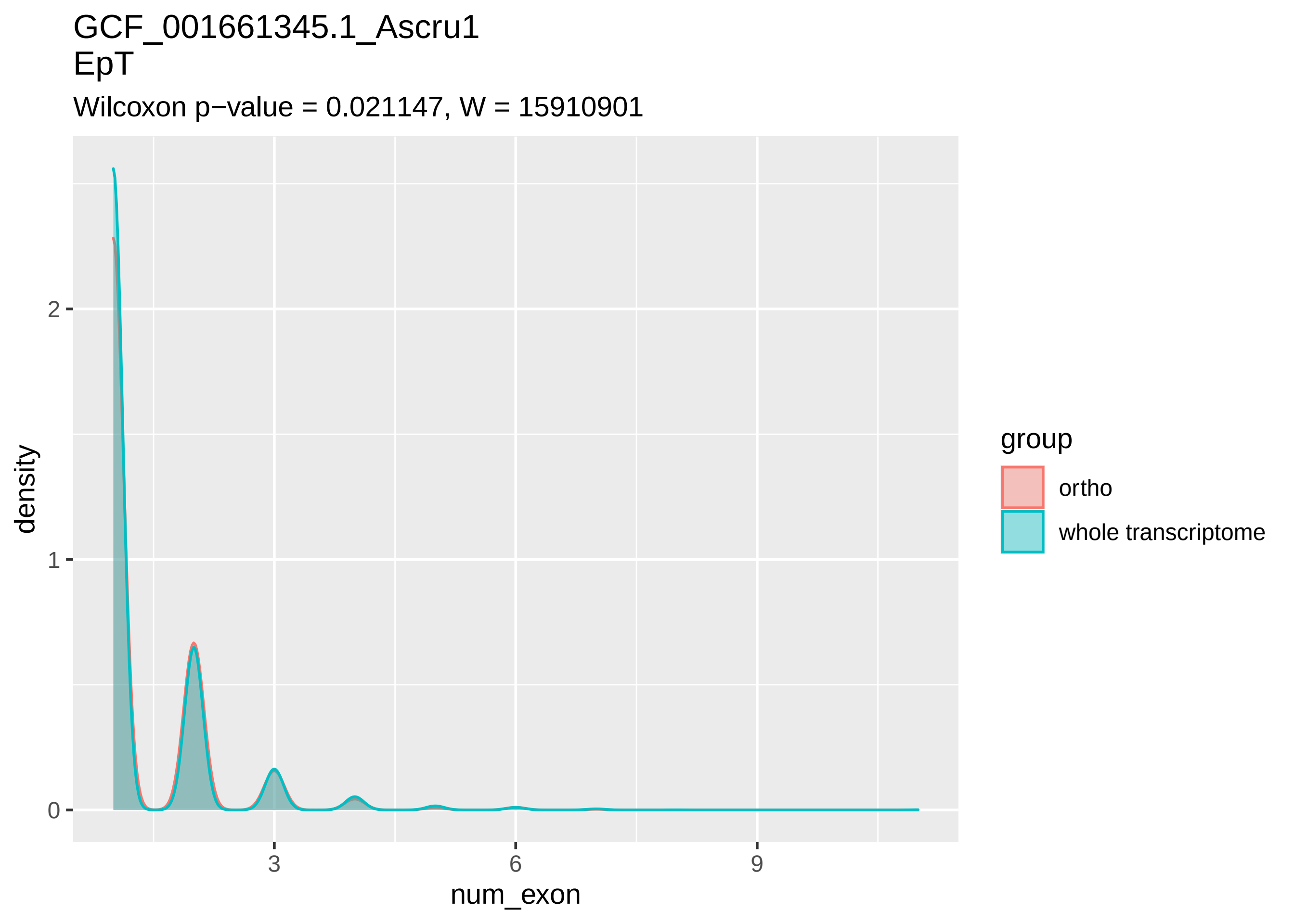
GCF_001661345.1_Ascru1
EpT
Wilcoxon p−value = 0.021147, W = 15910901
density
2
1
0
3
6
9
num_exon
group
ortho
whole transcriptome

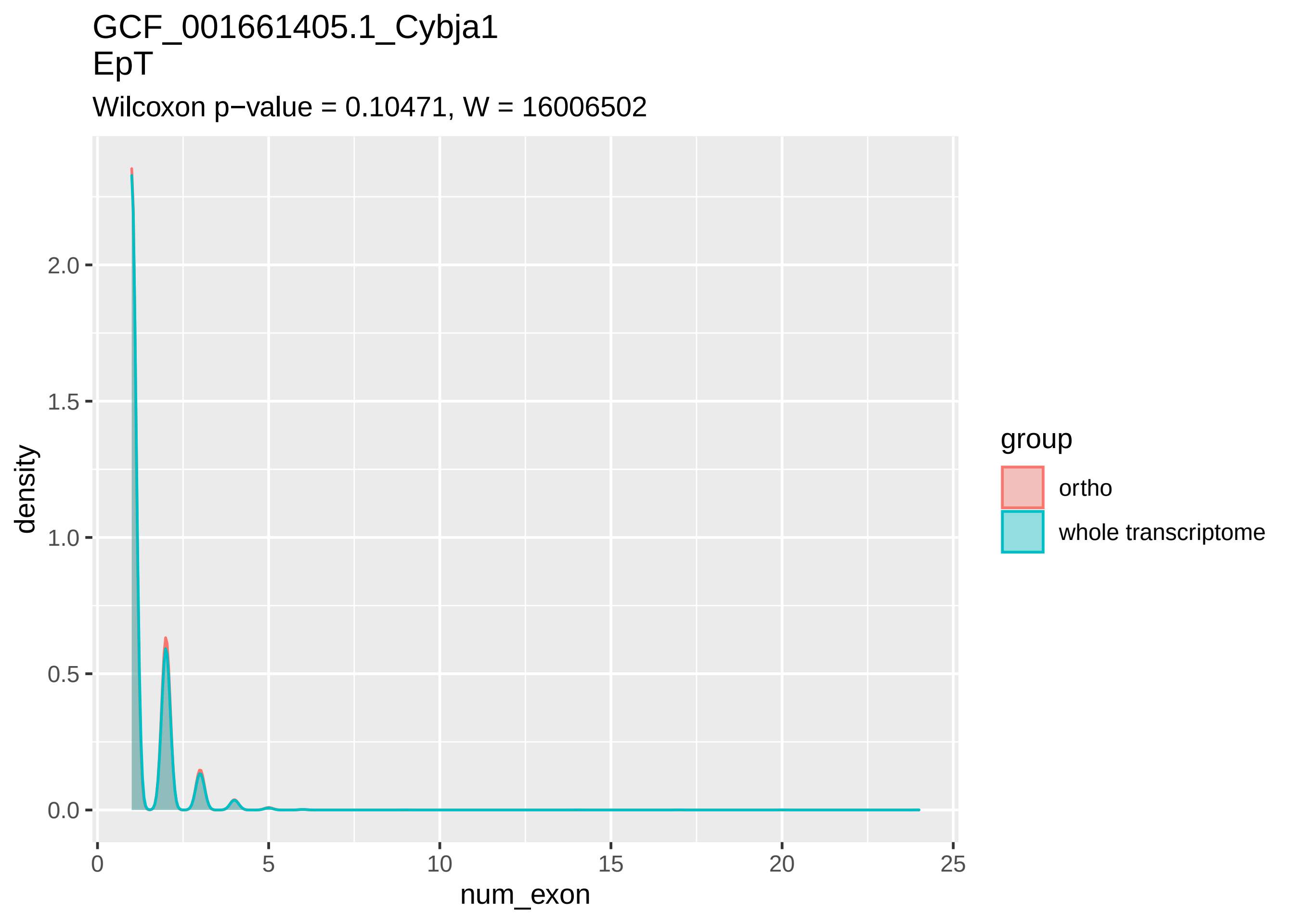
GCF_001661405.1_Cybja1
EpT
Wilcoxon p−value = 0.10471, W = 16006502
density
2.0
1.5
1.0
0.5
0.0
0
5
10
15
20
25
num_exon
group
ortho
whole transcriptome

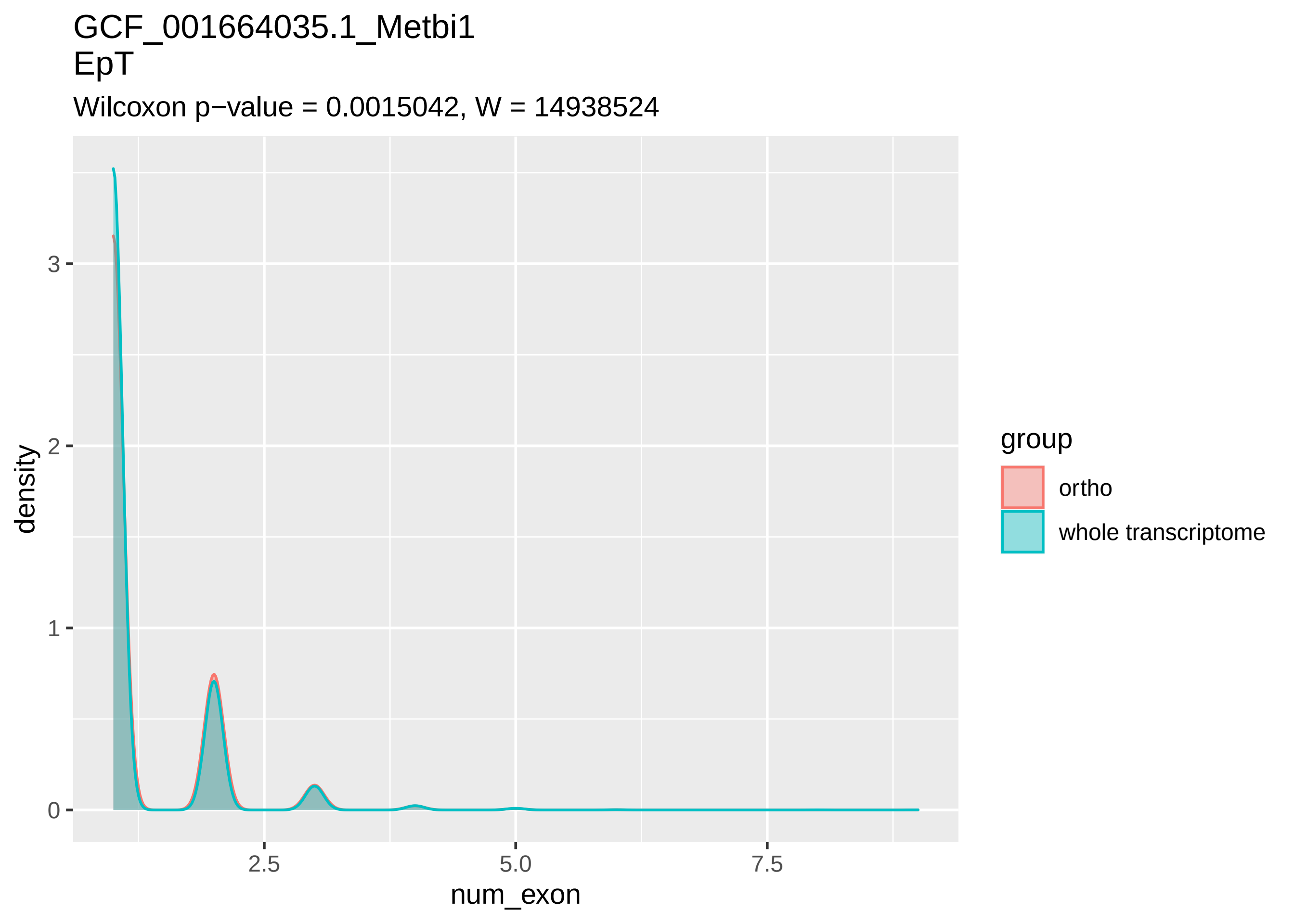
GCF_001664035.1_Metbi1
EpT
Wilcoxon p−value = 0.0015042, W = 14938524
density
num_exon
3
2
1
0
2.5
5.0
7.5
group
ortho
whole transcriptome

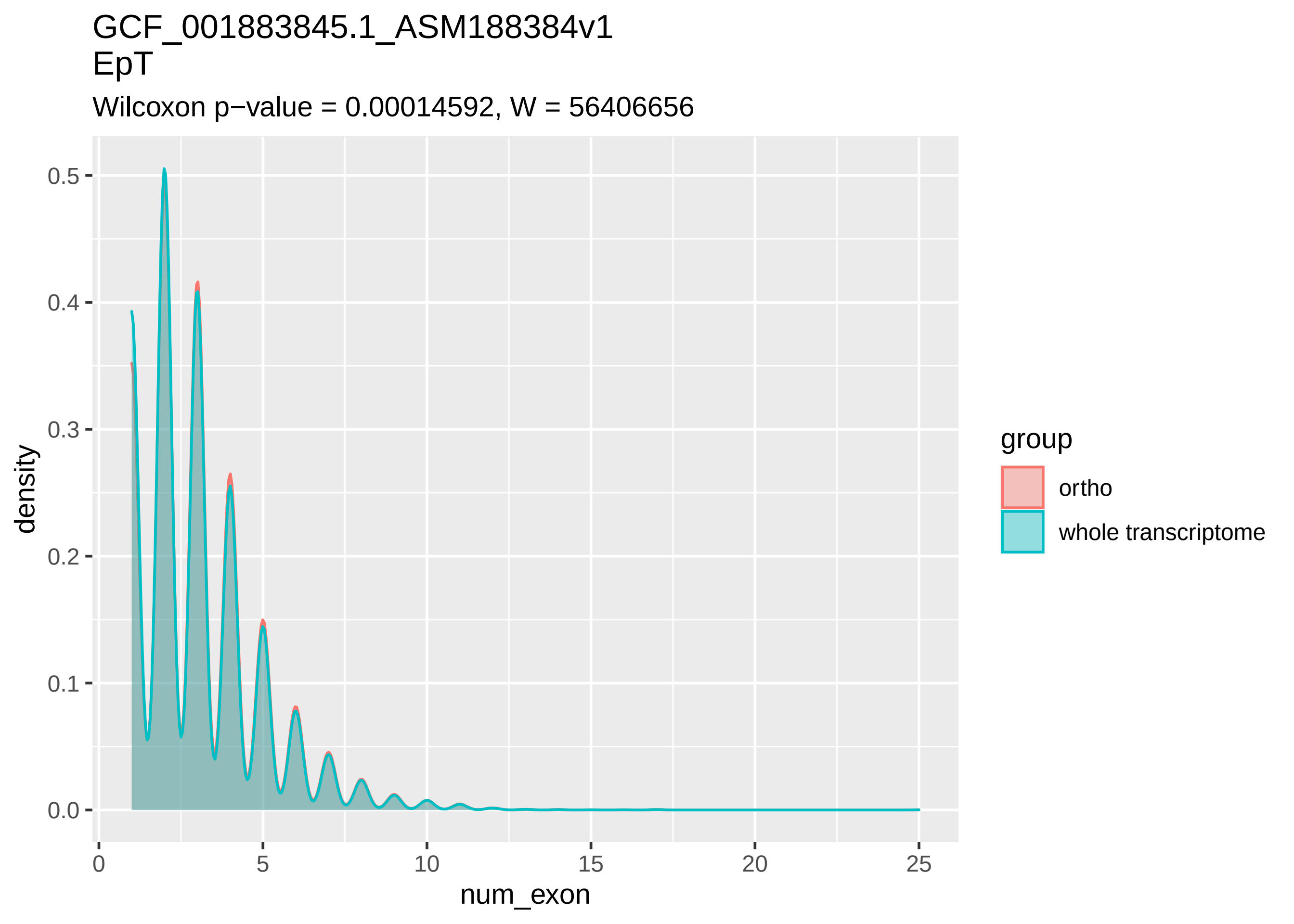
GCF_001883845.1_ASM188384v1
EpT
Wilcoxon p−value = 0.00014592, W = 56406656
density
0.0
0.1
0.2
0.3
0.4
0.5
0
5
10
15
20
25
num_exon
group
ortho
whole transcriptome

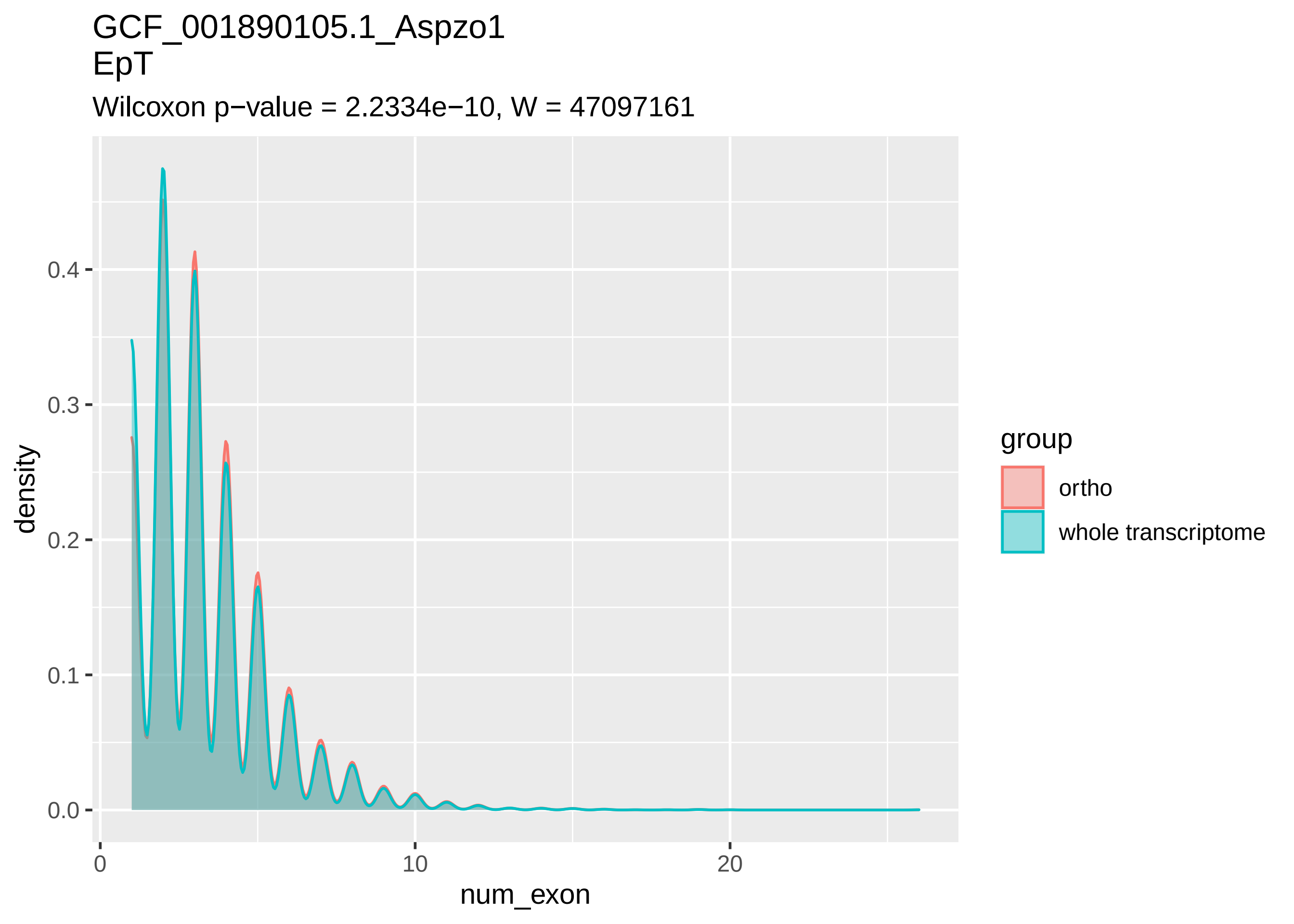
GCF_001890105.1_Aspzo1
EpT
Wilcoxon p−value = 2.2334e−10, W = 47097161
density
0.0
0.1
0.2
0.3
0.4
0
10
20
num_exon
group
ortho
whole transcriptome

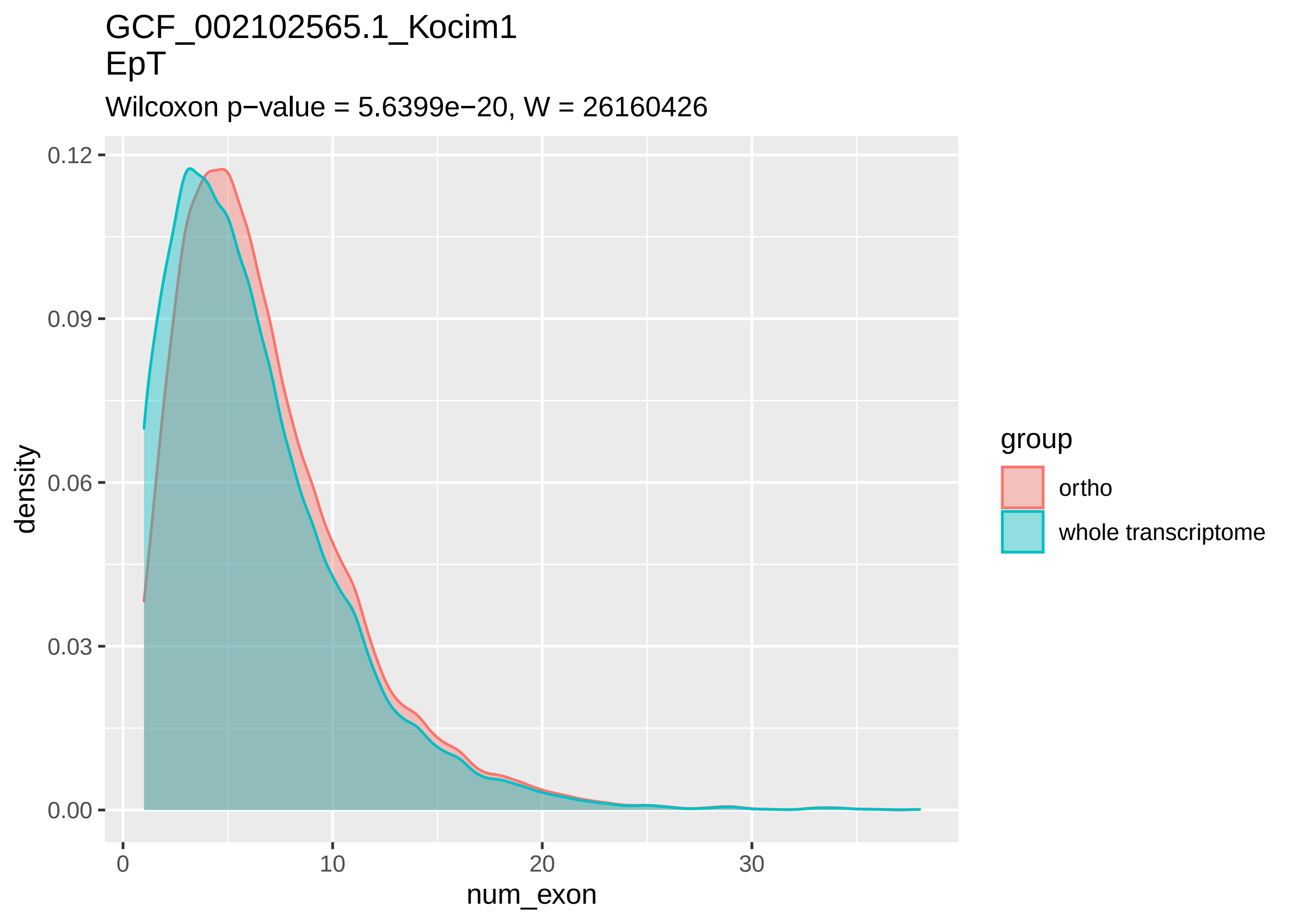
GCF_002102565.1_Kocim1
EpT
Wilcoxon p−value = 5.6399e−20, W = 26160426
0.12
0.09
0.06
0.03
0.00
density
0
10
20
30
num_exon
group
ortho
whole transcriptome

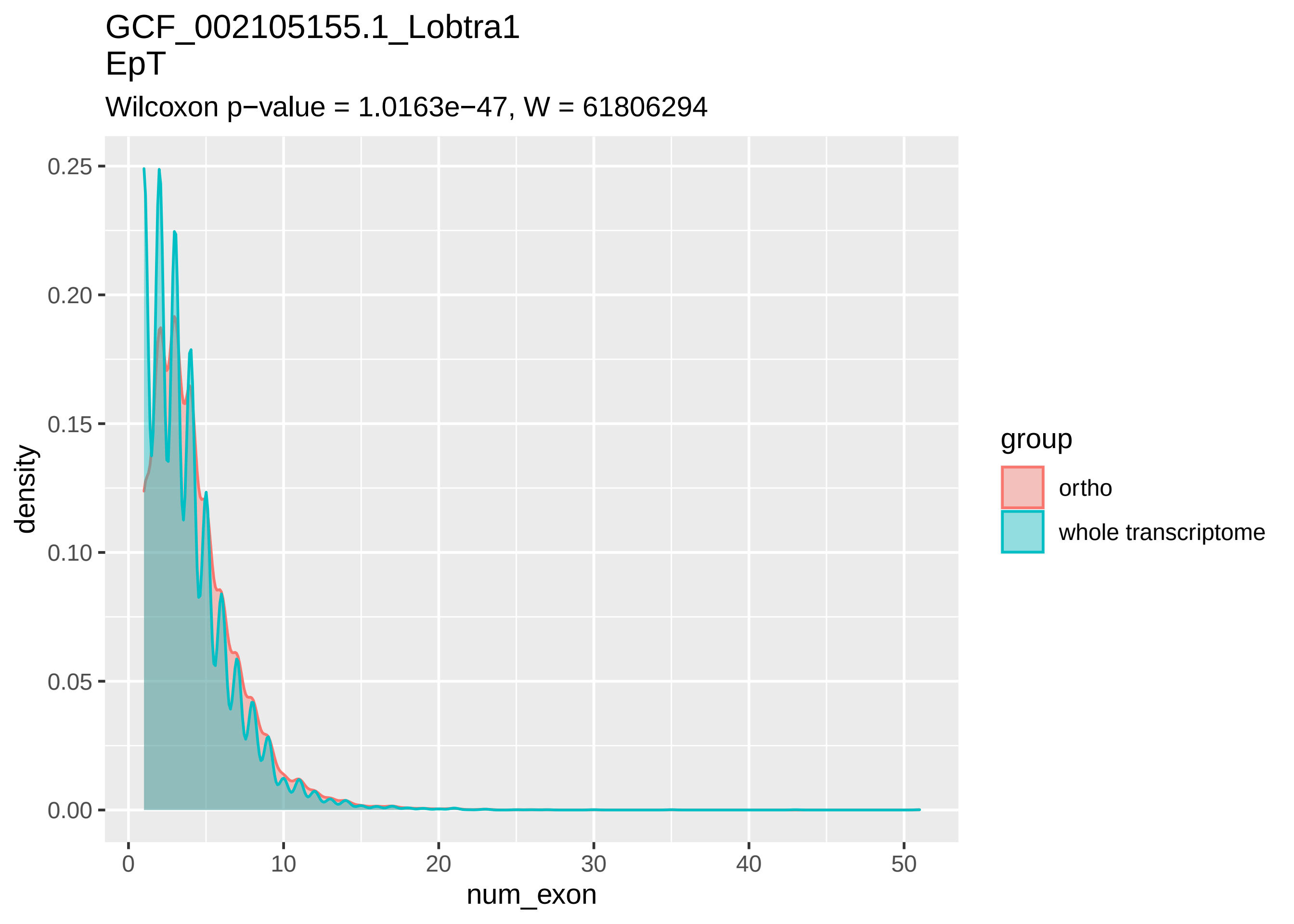

GCF_002105155.1_Lobtra1
EpT
Wilcoxon p−value = 1.0163e−47, W = 61806294
0.25
0.20
0.15
0.10
0.05
0.00
density
0
10
20
30
40
50
num_exon
group
ortho
whole transcriptome

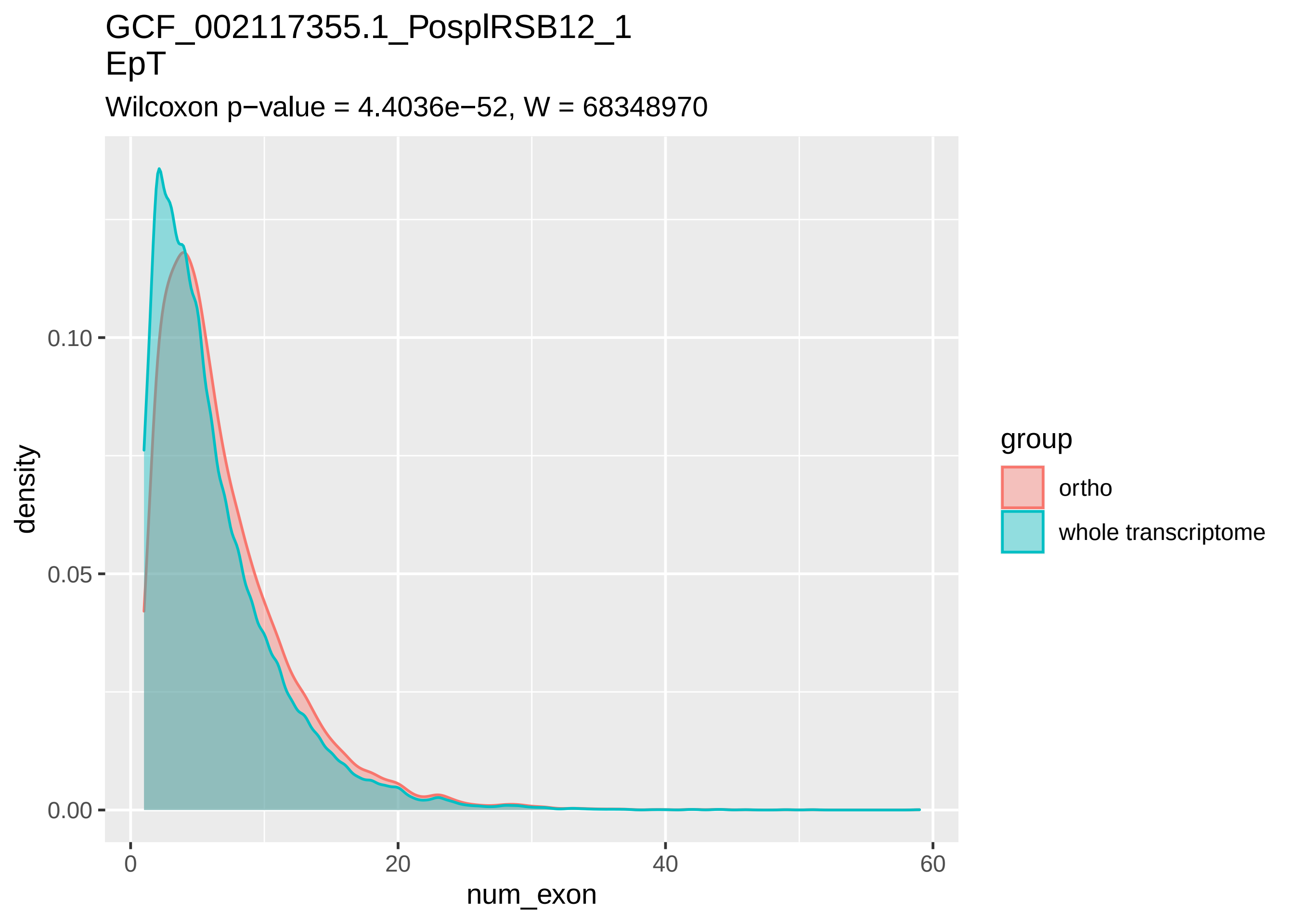

GCF_002117355.1_PosplRSB12_1
EpT
Wilcoxon p−value = 4.4036e−52, W = 68348970
density
0.10
0.05
0.00
0
20
40
60
num_exon
group
ortho
whole transcriptome

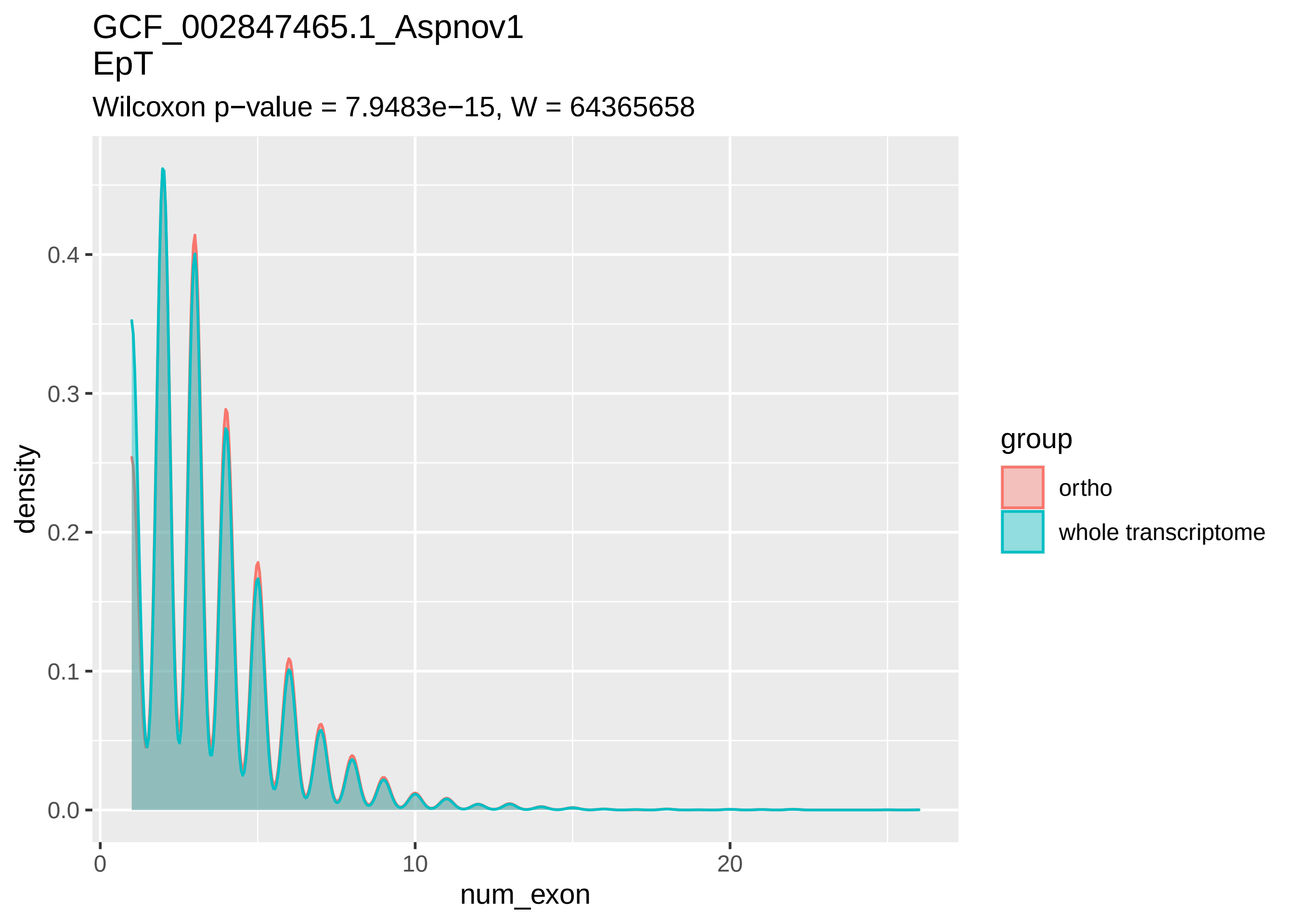

GCF_002847465.1_Aspnov1
EpT
Wilcoxon p−value = 7.9483e−15, W = 64365658
density
0.0
0.1
0.2
0.3
0.4
0
10
20
num_exon
group
ortho
whole transcriptome

GCA_000003515.2_ASM351v2
EpG
Wilcoxon p−value = 5.2869e−07, W = 29030603

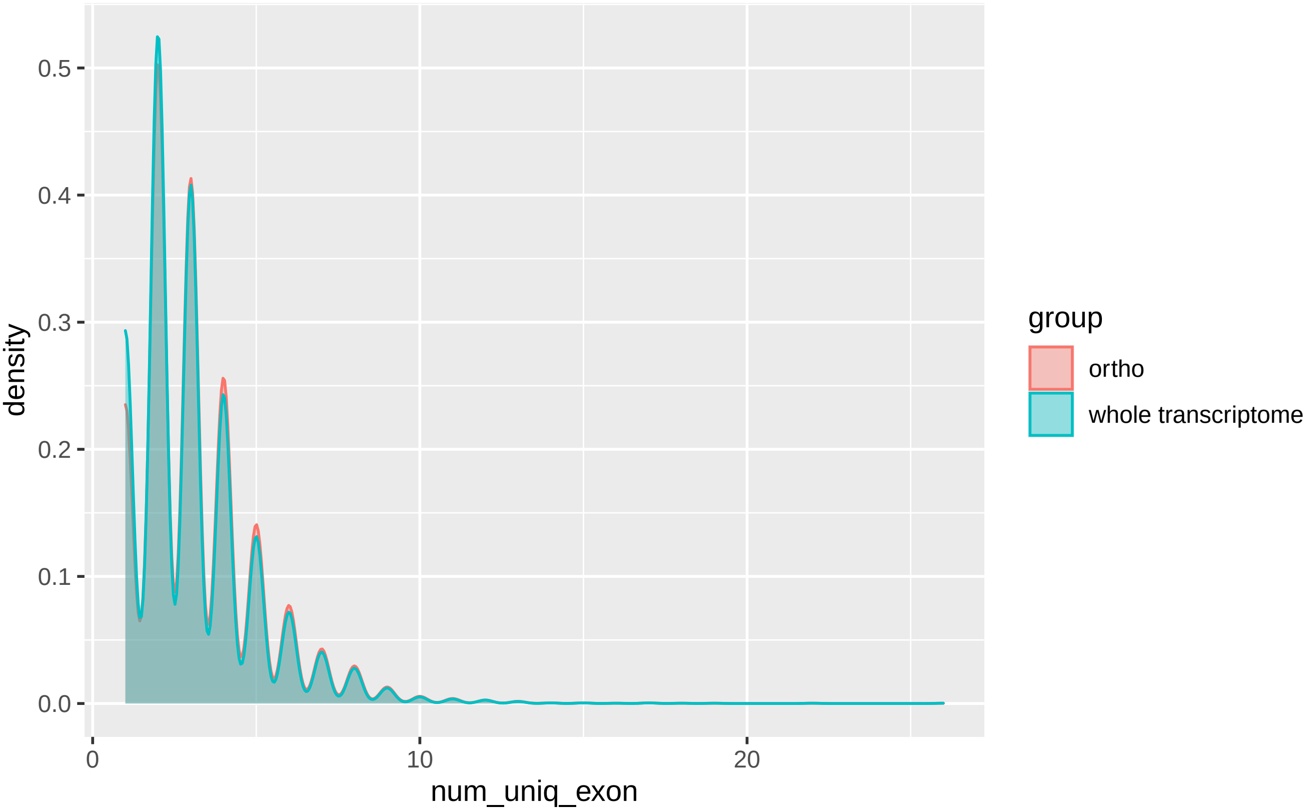

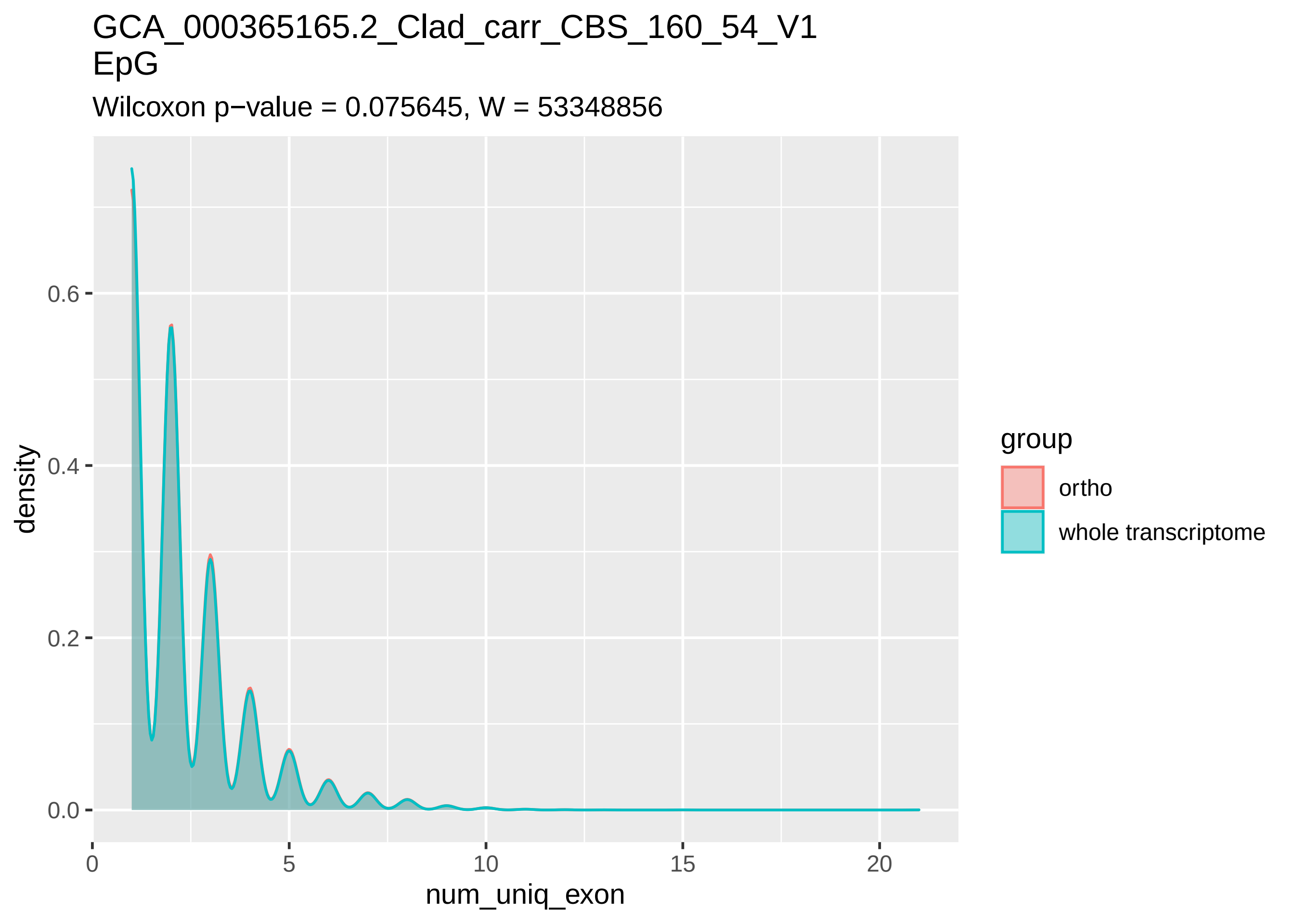
GCA_000365165.2_Clad_carr_CBS_160_54_V1
EpG
Wilcoxon p−value = 0.075645, W = 53348856
density
0.0
0.2
0.4
0.6
0
5
10
15
20
num_uniq_exon
group
ortho
whole transcriptome

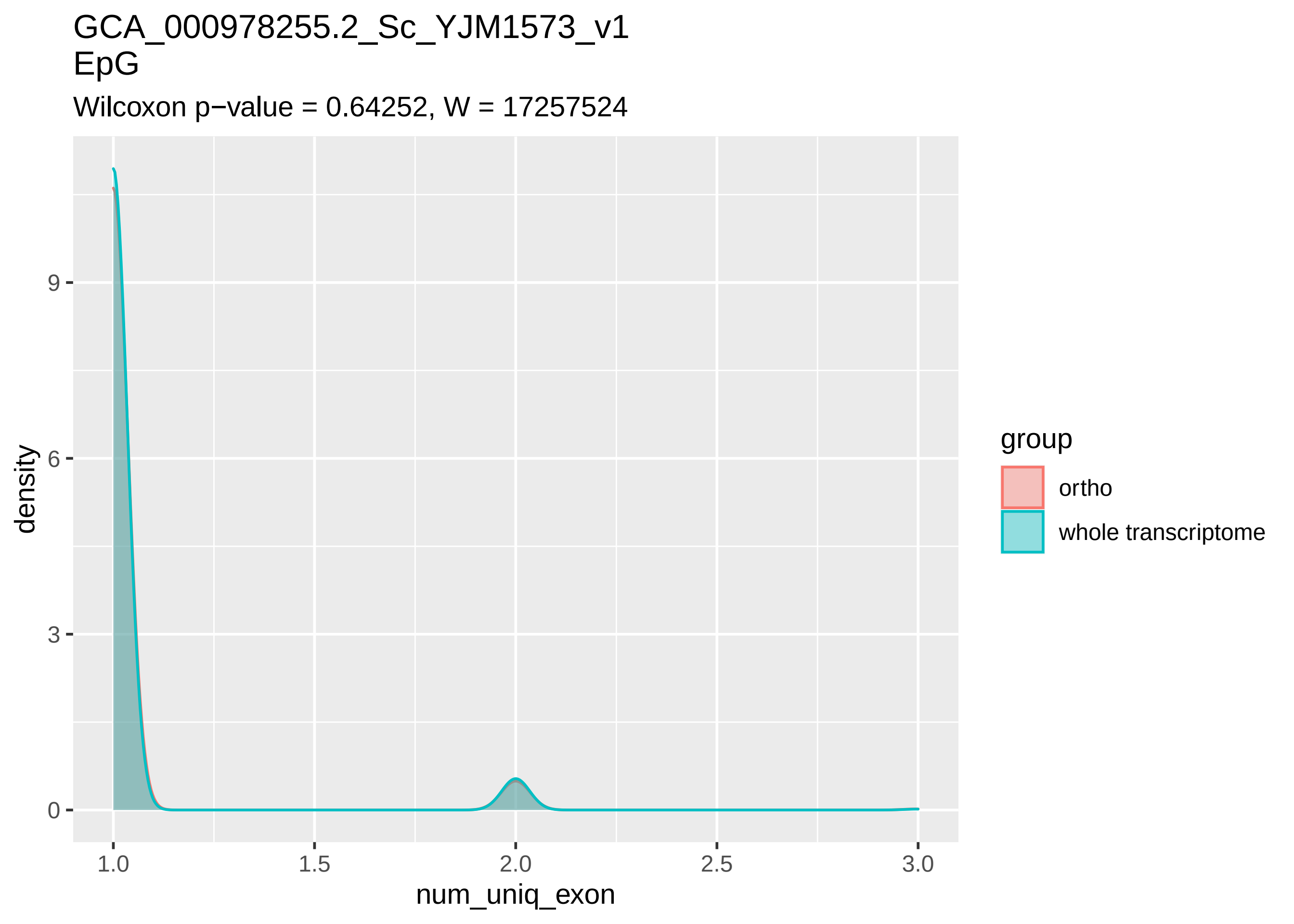

GCA_000978255.2_Sc_YJM1573_v1
EpG
Wilcoxon p−value = 0.64252, W = 17257524
density
num_uniq_exon
0
3
6
9
1.0
1.5
2.0
2.5
3.0
group
ortho
whole transcriptome

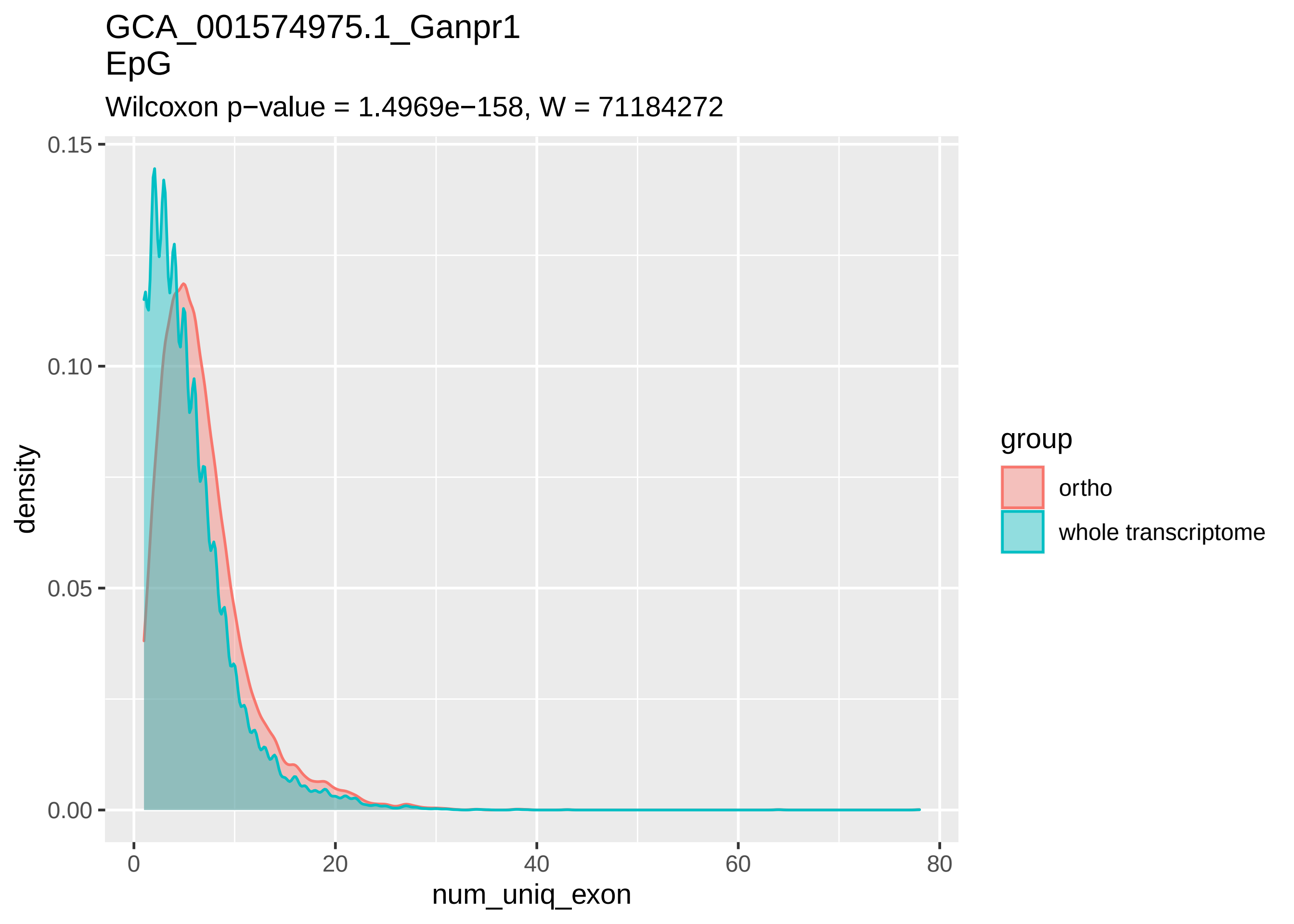

GCA_001574975.1_Ganpr1
EpG
Wilcoxon p−value = 1.4969e−158, W = 71184272
0.15
0.10
0.05
0.00
density
0
20
40
60
80
num_uniq_exon
group
ortho
whole transcriptome

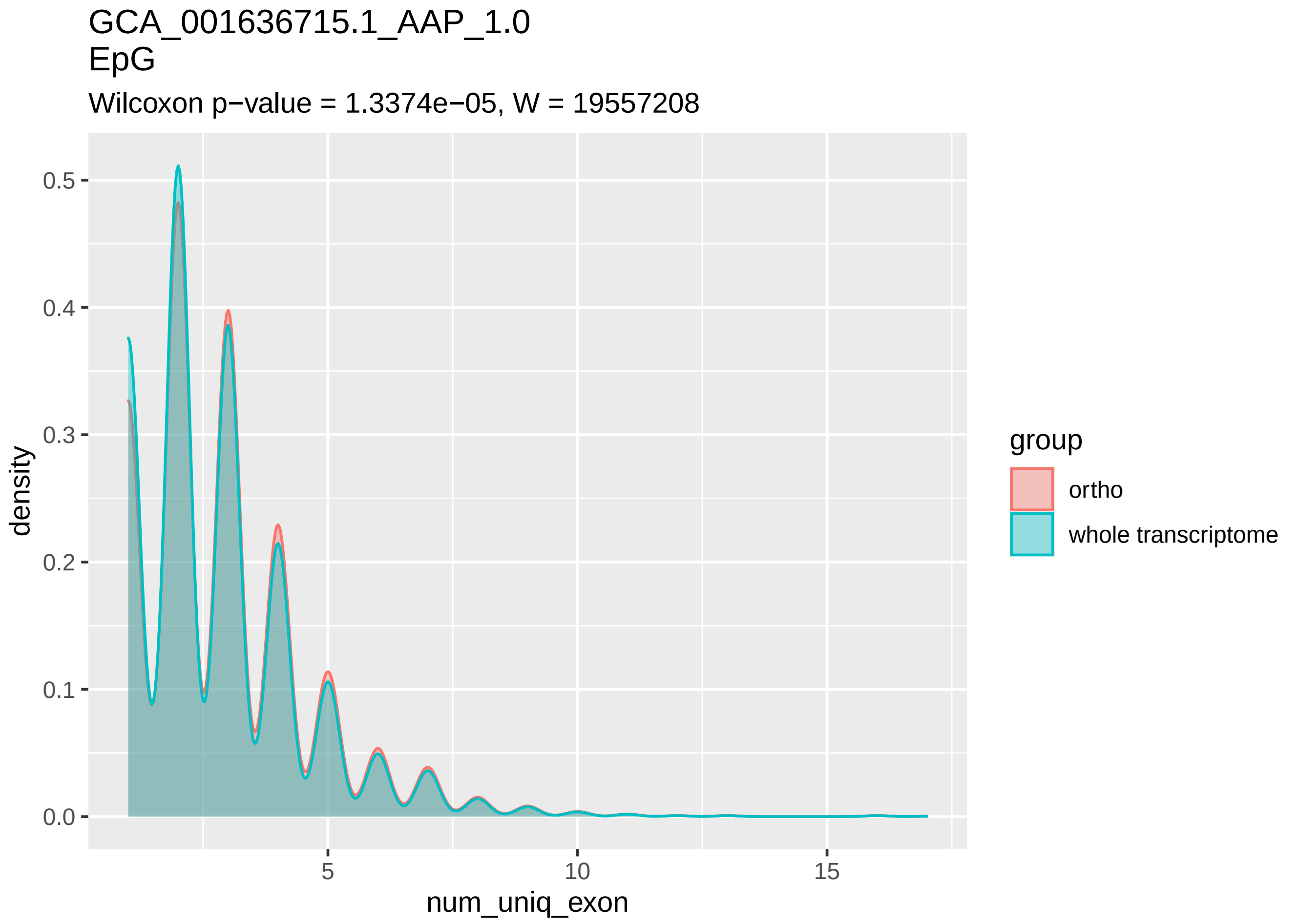
GCA_001636715.1_AAP_1.0
EpG
Wilcoxon p−value = 1.3374e−05, W = 19557208
density
0.0
0.1
0.2
0.3
0.4
0.5
5
10
15
num_uniq_exon
group
ortho
whole transcriptome

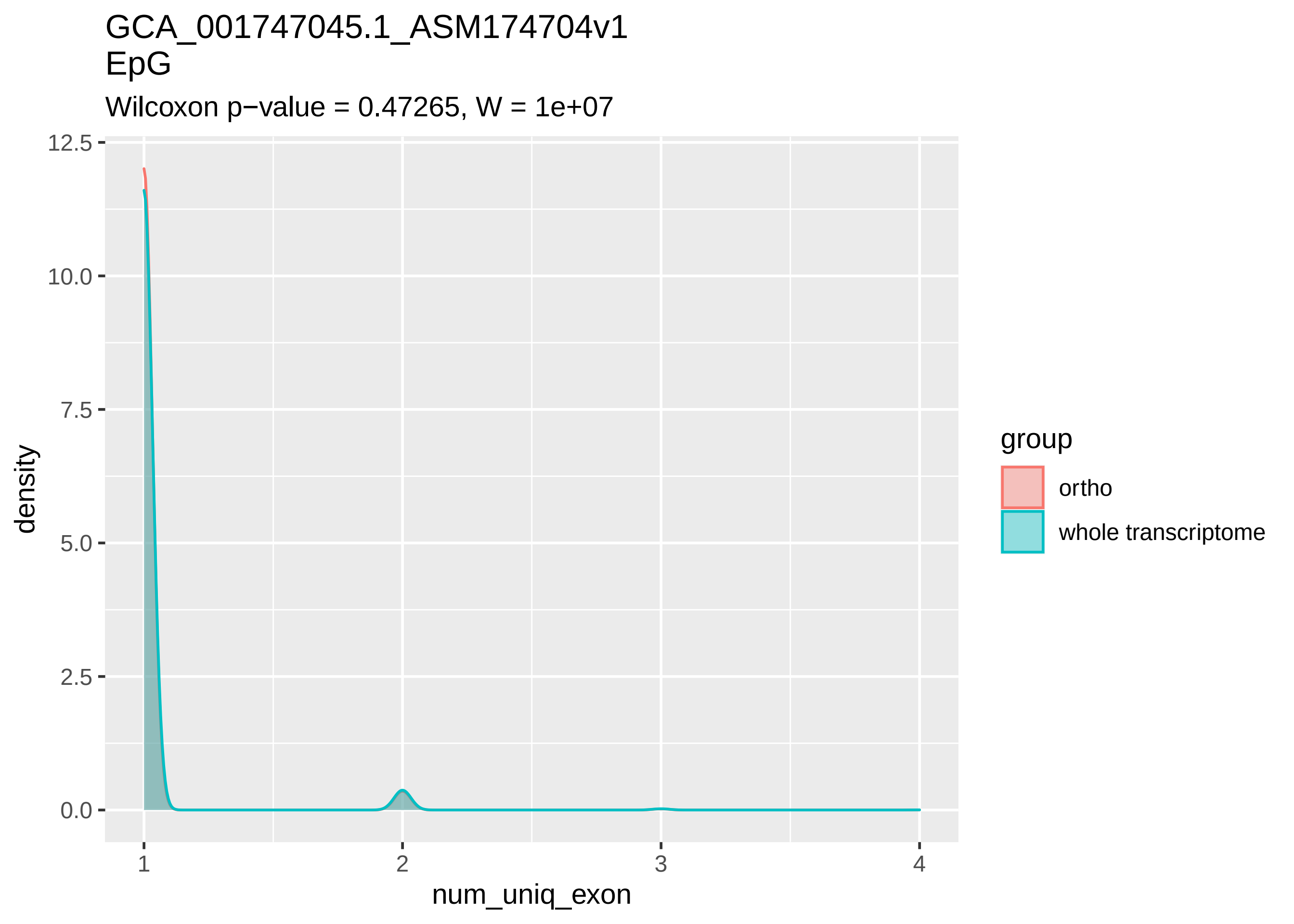

GCA_001747045.1_ASM174704v1
EpG
Wilcoxon p−value = 0.47265, W = 1e+07
density
12.5
10.0
7.5
5.0
2.5
0.0
1
2
3
4
num_uniq_exon
group
ortho
whole transcriptome

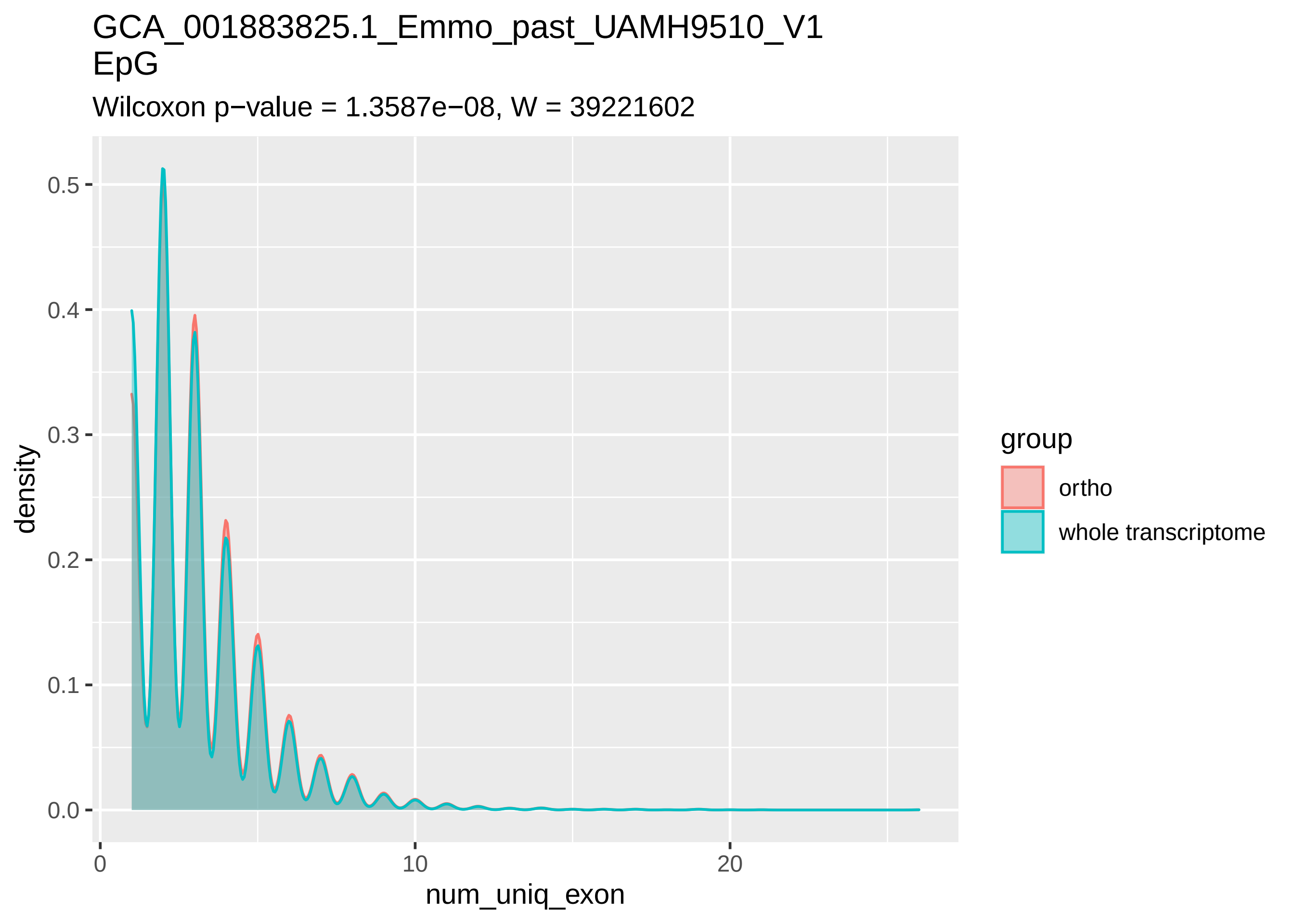
GCA_001883825.1_Emmo_past_UAMH9510_V1
EpG
Wilcoxon p−value = 1.3587e−08, W = 39221602
density
0.0
0.1
0.2
0.3
0.4
0.5
0
10
20
num_uniq_exon
group
ortho
whole transcriptome

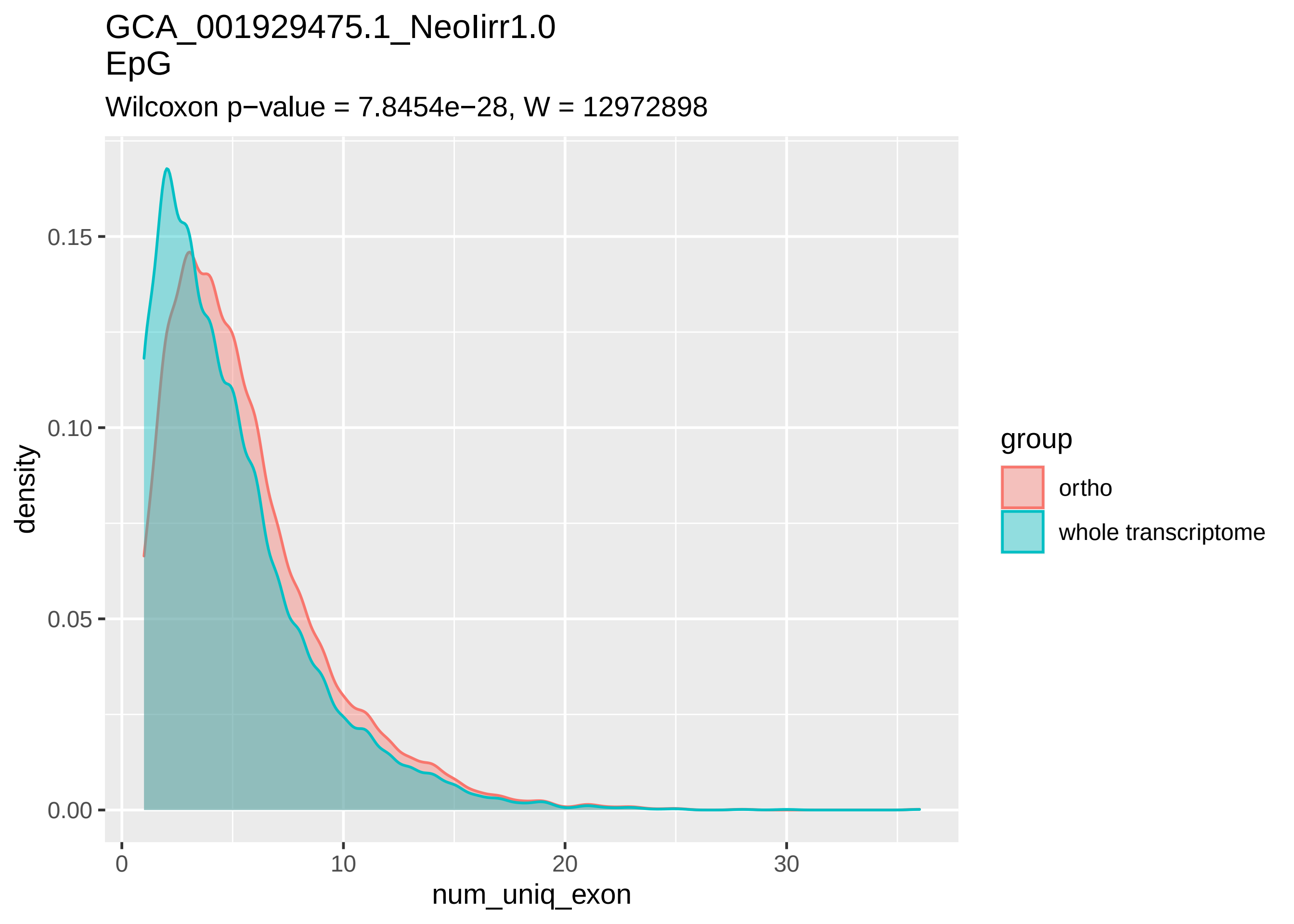

GCA_001929475.1_NeoIirr1.0
EpG
Wilcoxon p−value = 7.8454e−28, W = 12972898
density
0.00
0.05
0.10
0.15
0
10
20
30
num_uniq_exon
group
ortho
whole transcriptome

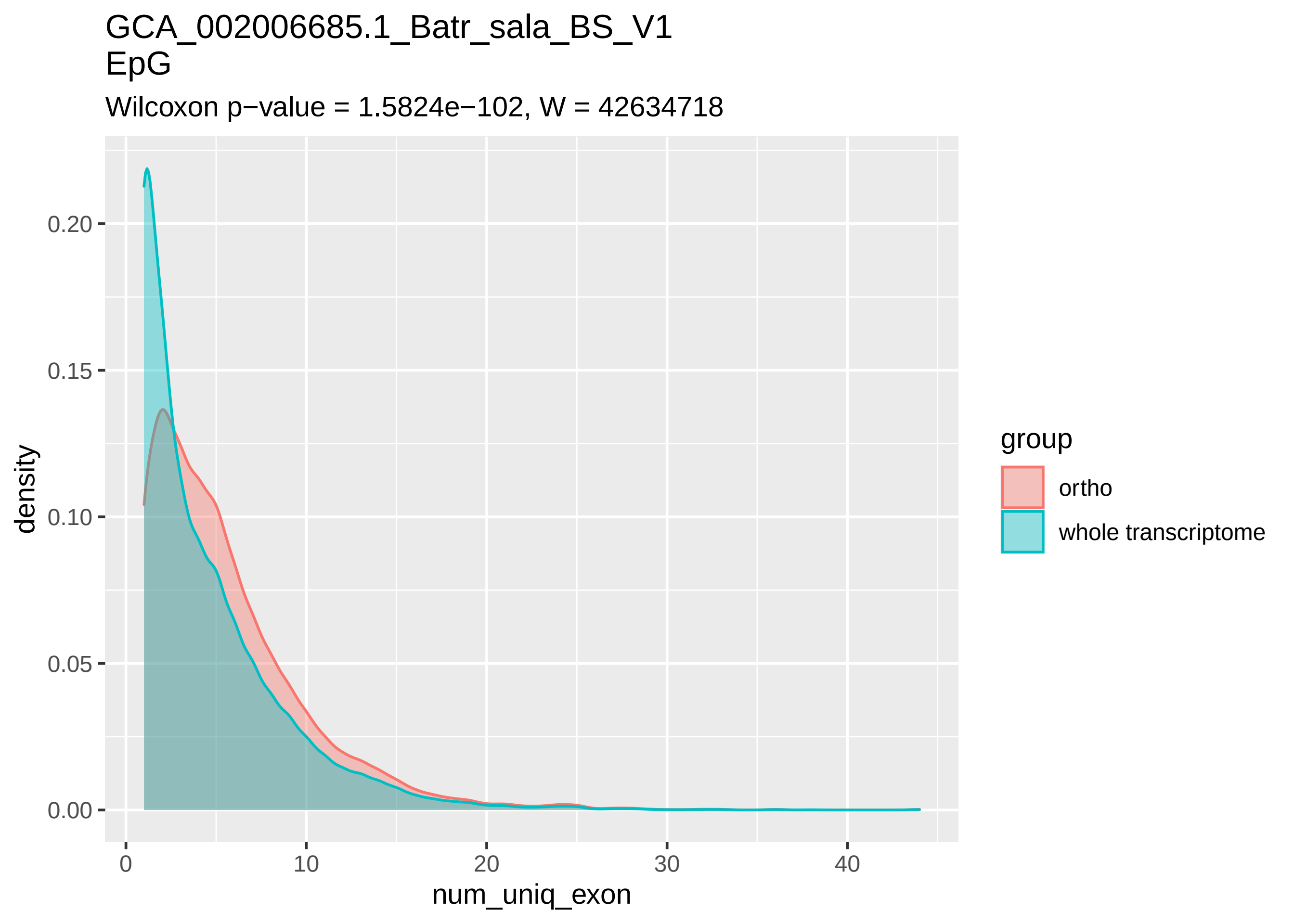
GCA_002006685.1_Batr_sala_BS_V1
EpG
Wilcoxon p−value = 1.5824e−102, W = 42634718
density
0.20
0.15
0.10
0.05
0.00
0
10
20
30
40
num_uniq_exon
group
ortho
whole transcriptome

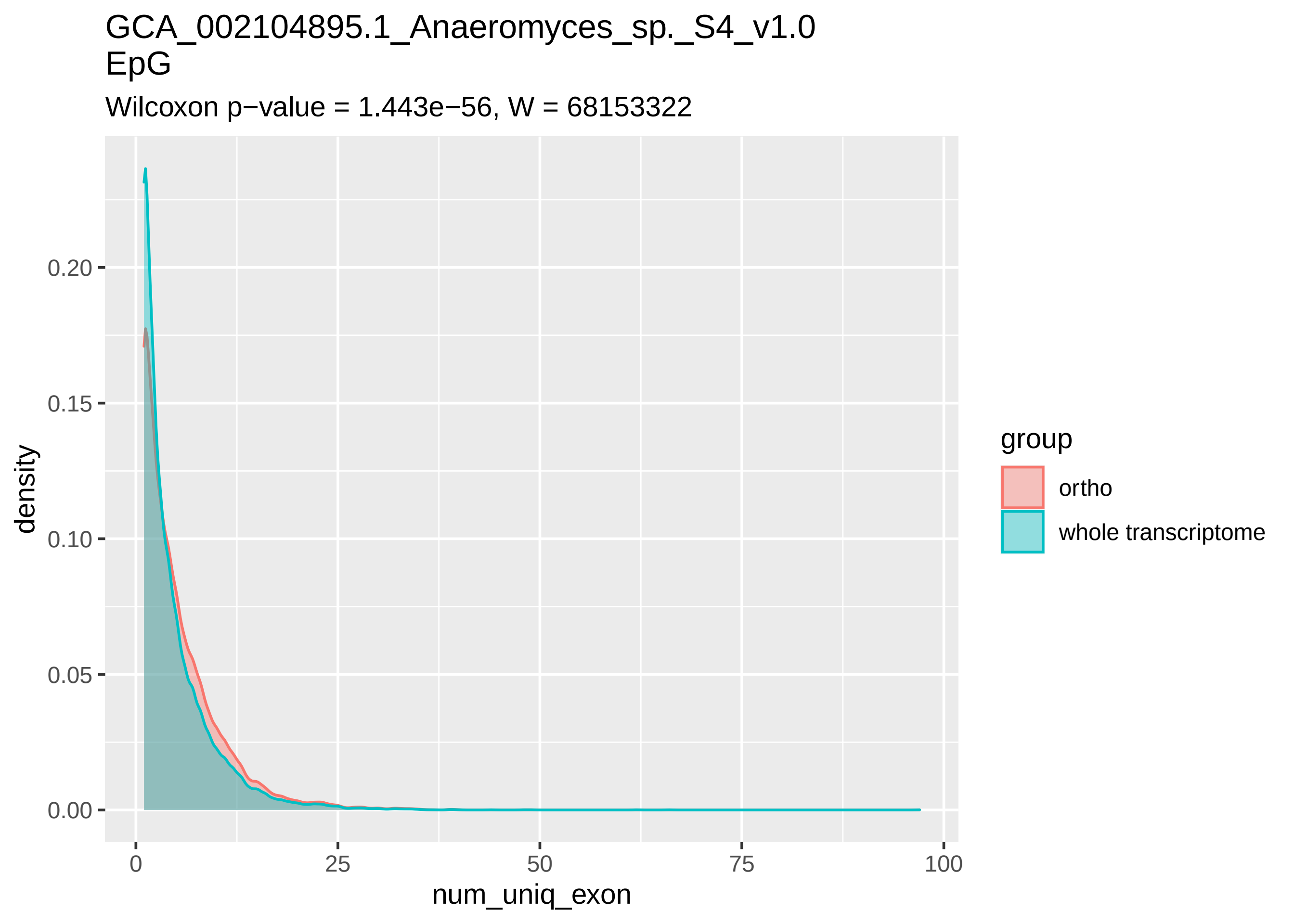

GCA_002104895.1_Anaeromyces_sp._S4_v1.0
EpG
Wilcoxon p−value = 1.443e−56, W = 68153322
density
0.20
0.15
0.10
0.05
0.00
0
25
50
75
100
num_uniq_exon
group
ortho
whole transcriptome

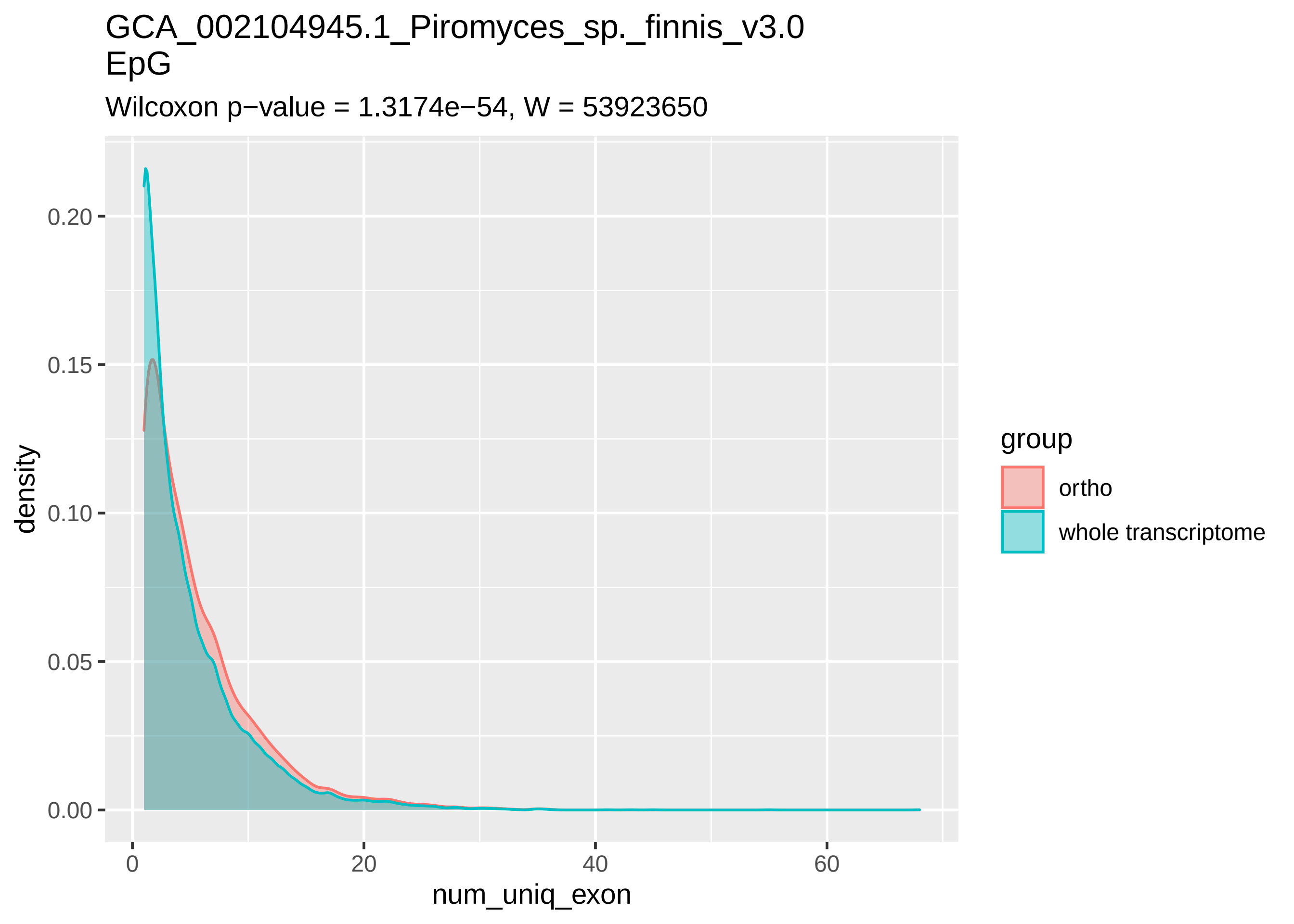
GCA_002104945.1_Piromyces_sp._finnis_v3.0
EpG
Wilcoxon p−value = 1.3174e−54, W = 53923650
density
0.20
0.15
0.10
0.05
0.00
0
20
40
60
num_uniq_exon
group
ortho
whole transcriptome

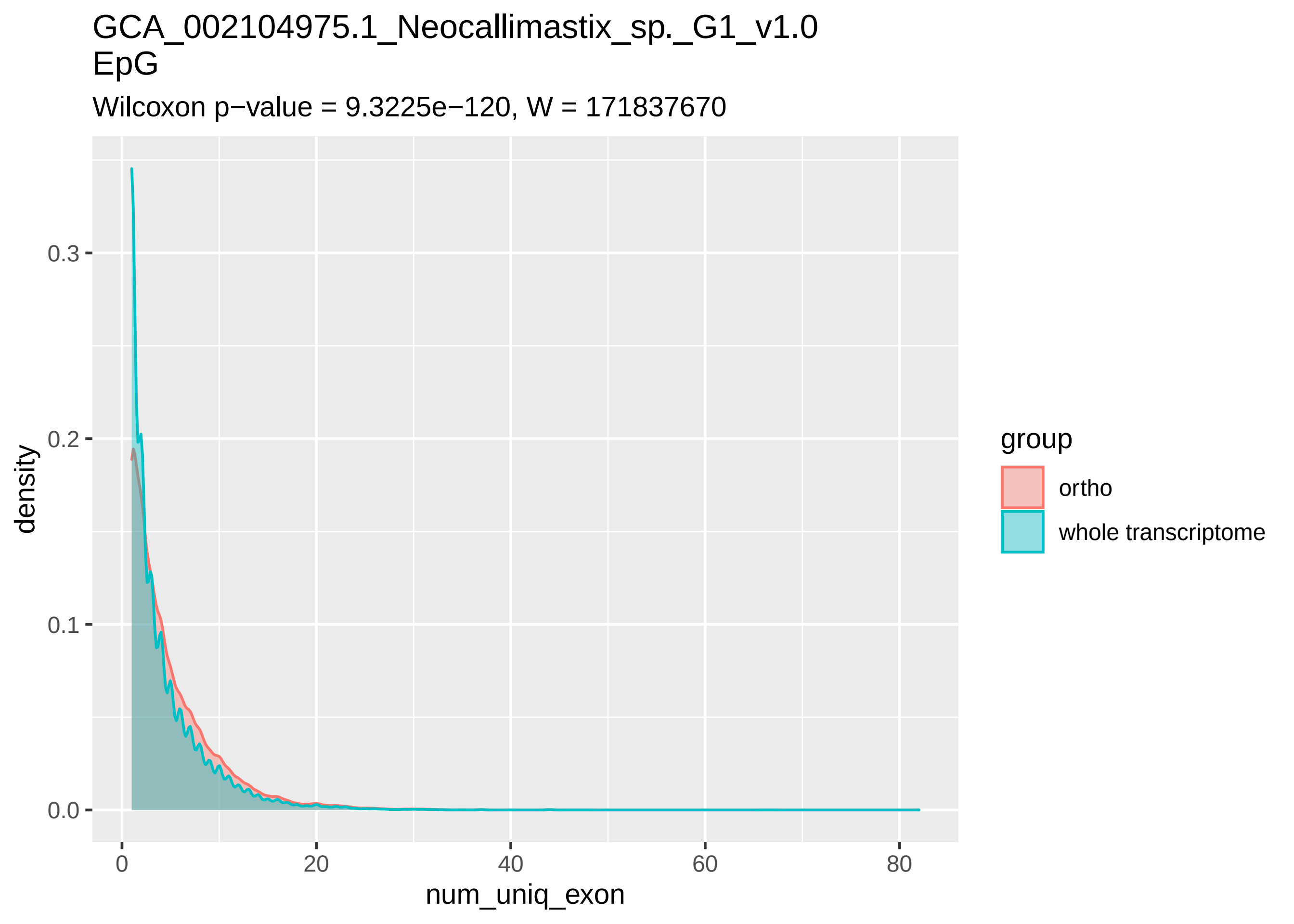
GCA_002104975.1_Neocallimastix_sp._G1_v1.0
EpG
Wilcoxon p−value = 9.3225e−120, W = 171837670
density
0.3
0.2
0.1
0.0
0
20
40
60
80
num_uniq_exon
group
ortho
whole transcriptome

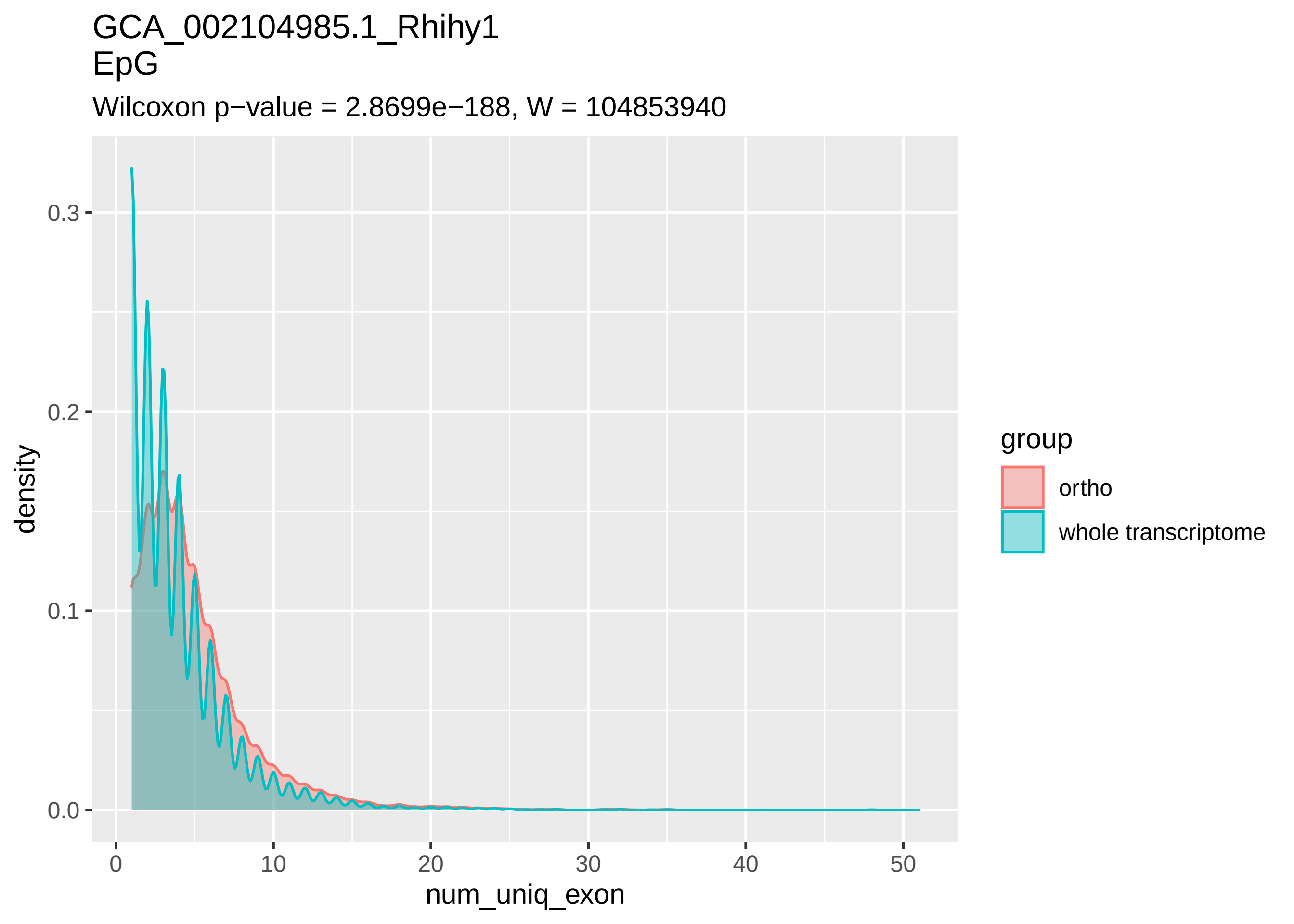

GCA_002104985.1_Rhihy1
EpG
Wilcoxon p−value = 2.8699e−188, W = 104853940
density
0.0
0.1
0.2
0.3
0
10
20
30
40
50
num_uniq_exon
group
ortho
whole transcriptome

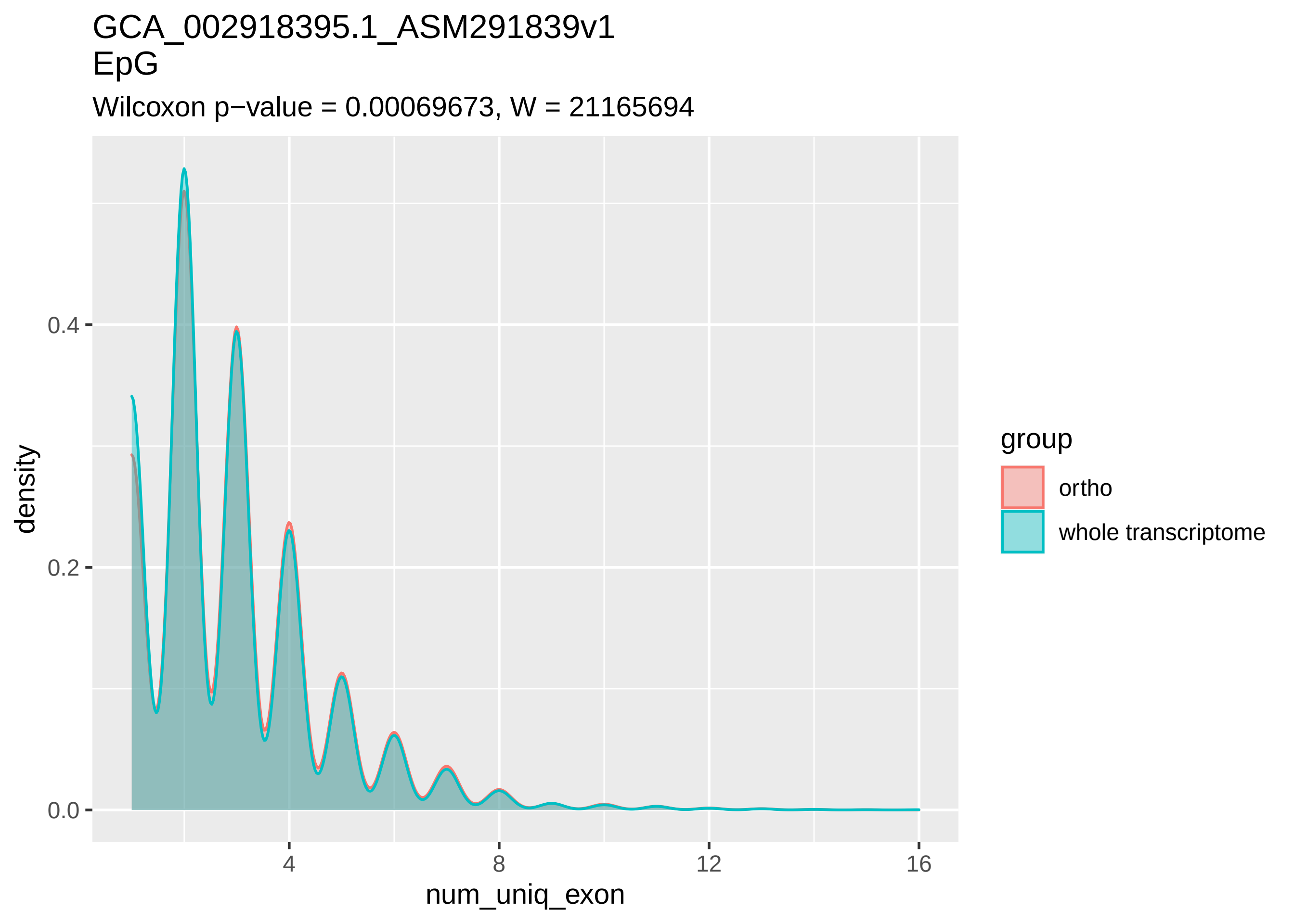

GCA_002918395.1_ASM291839v1
EpG
Wilcoxon p−value = 0.00069673, W = 21165694
density
0.0
0.2
0.4
4
8
12
16
num_uniq_exon
group
ortho
whole transcriptome

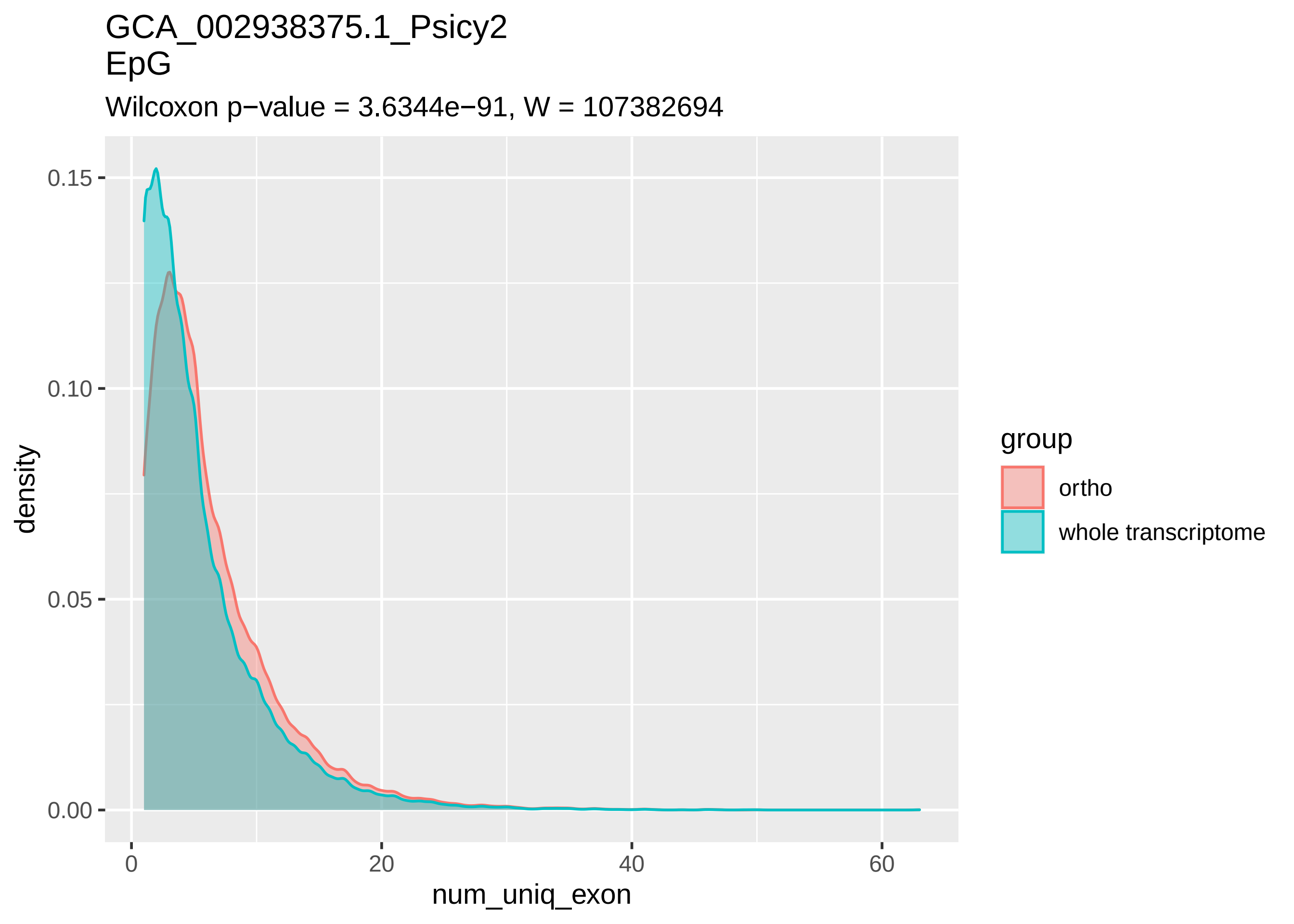

GCA_002938375.1_Psicy2
EpG
Wilcoxon p−value = 3.6344e−91, W = 107382694
0.15
0.10
0.05
0.00
density
0
20
40
60
num_uniq_exon
group
ortho
whole transcriptome

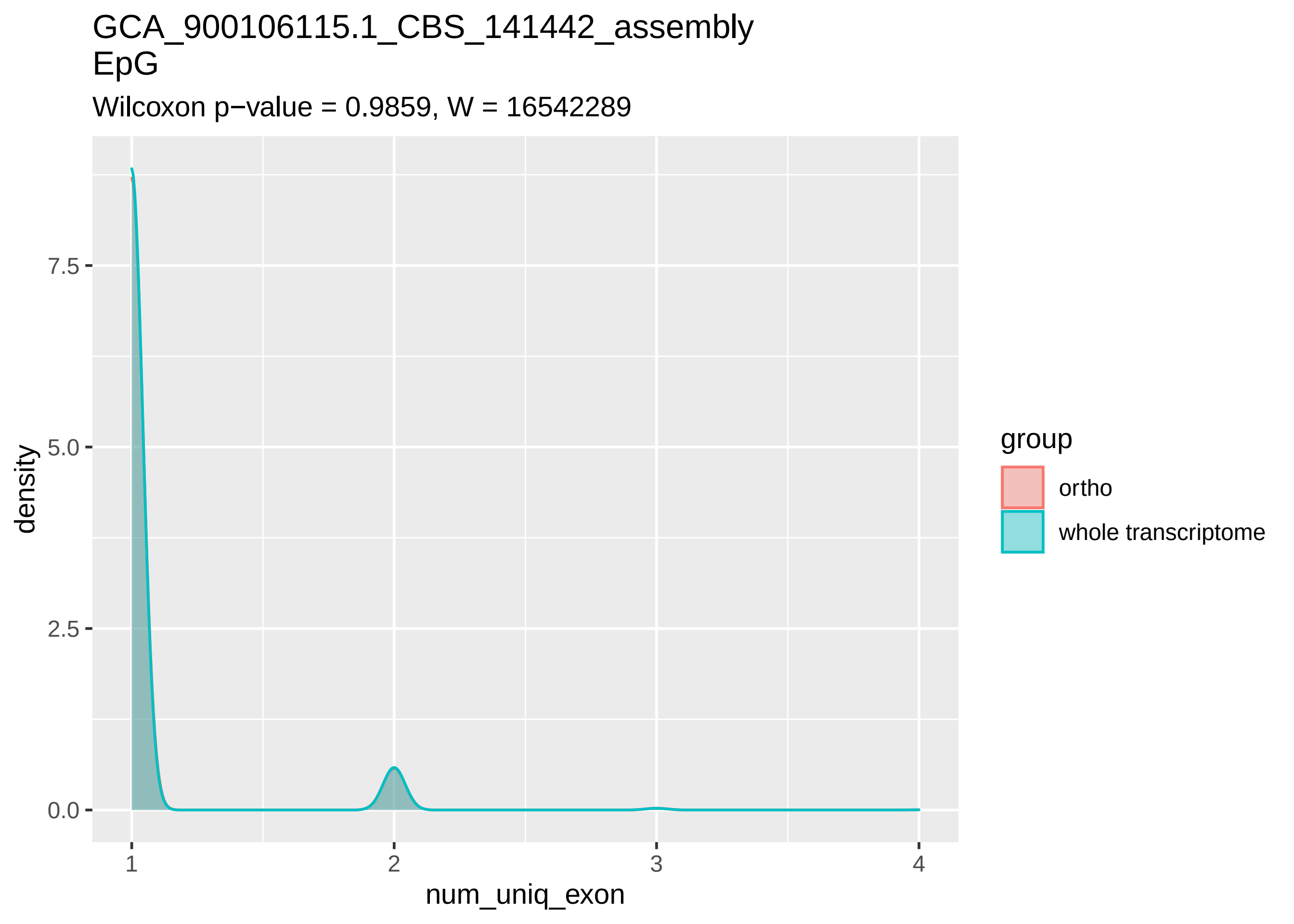
GCA_900106115.1_CBS_141442_assembly
EpG
Wilcoxon p−value = 0.9859, W = 16542289
density
7.5
5.0
2.5
0.0
1
2
3
4
num_uniq_exon
group
ortho
whole transcriptome

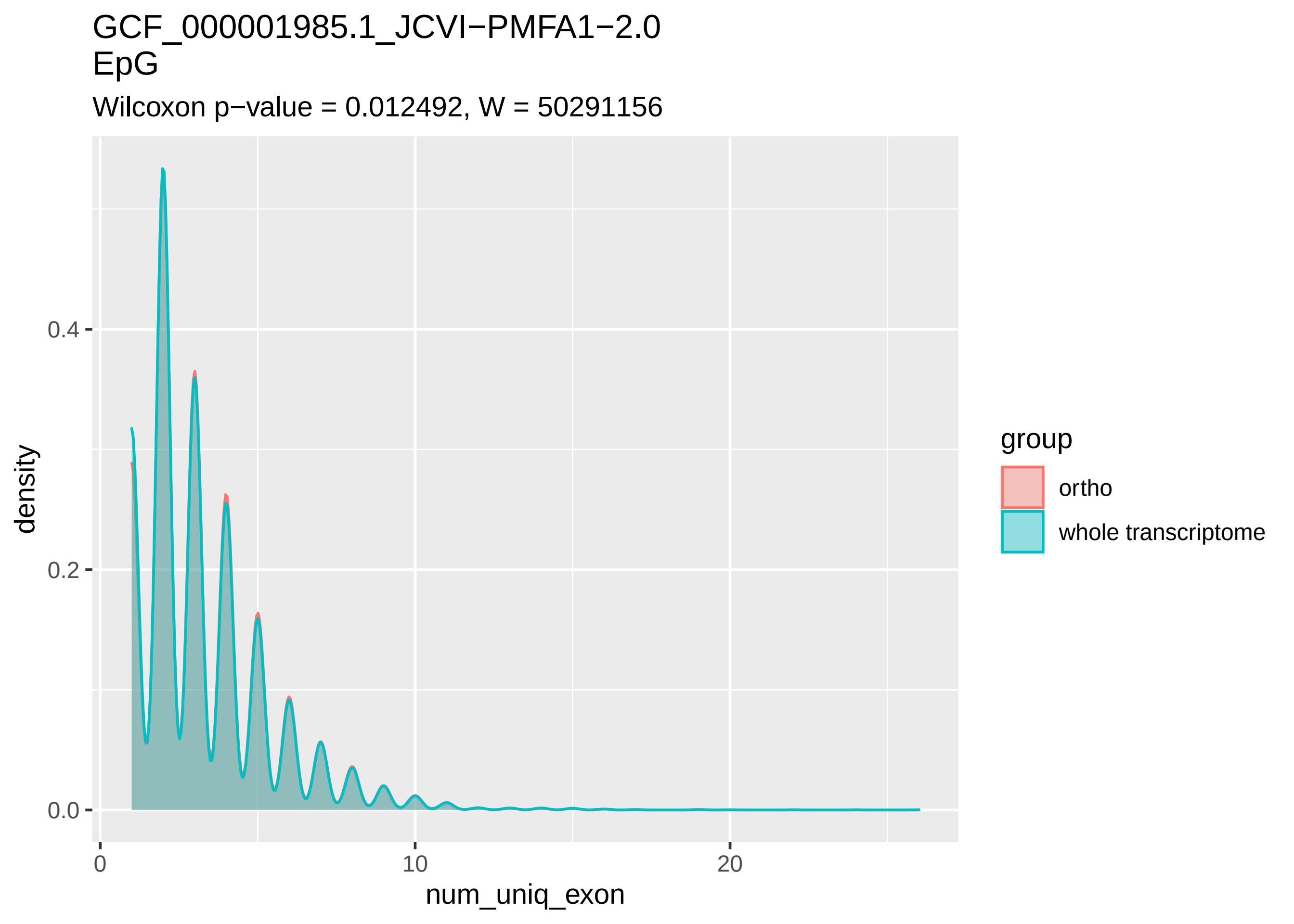
GCF_000001985.1_JCVI−PMFA1−2.0
EpG
Wilcoxon p−value = 0.012492, W = 50291156
density
0.4
0.2
0.0
0
10
20
num_uniq_exon
group
ortho
whole transcriptome

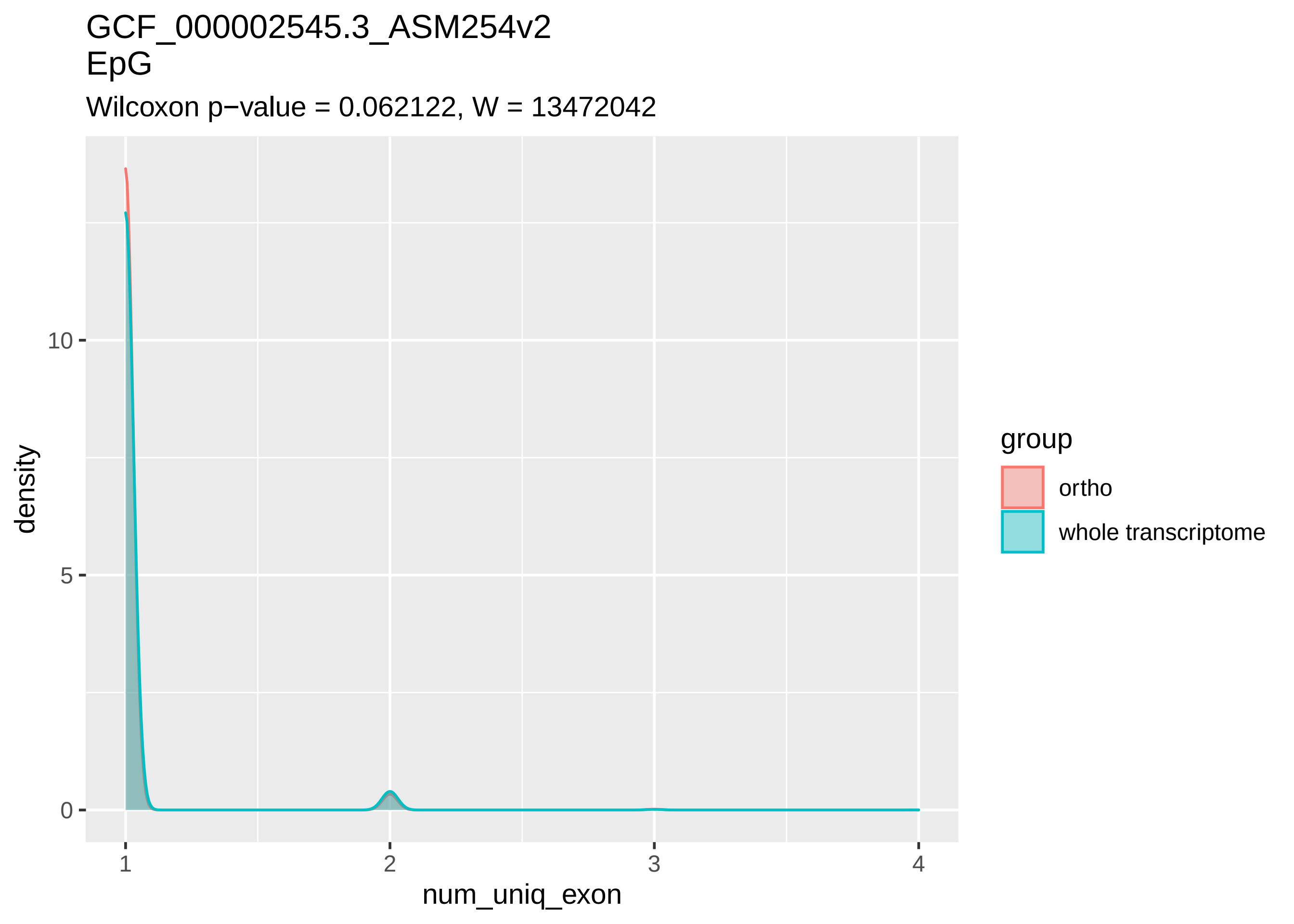
GCF_000002545.3_ASM254v2
EpG
Wilcoxon p−value = 0.062122, W = 13472042
density
10
5
0
1
2
3
4
num_uniq_exon
group
ortho
whole transcriptome

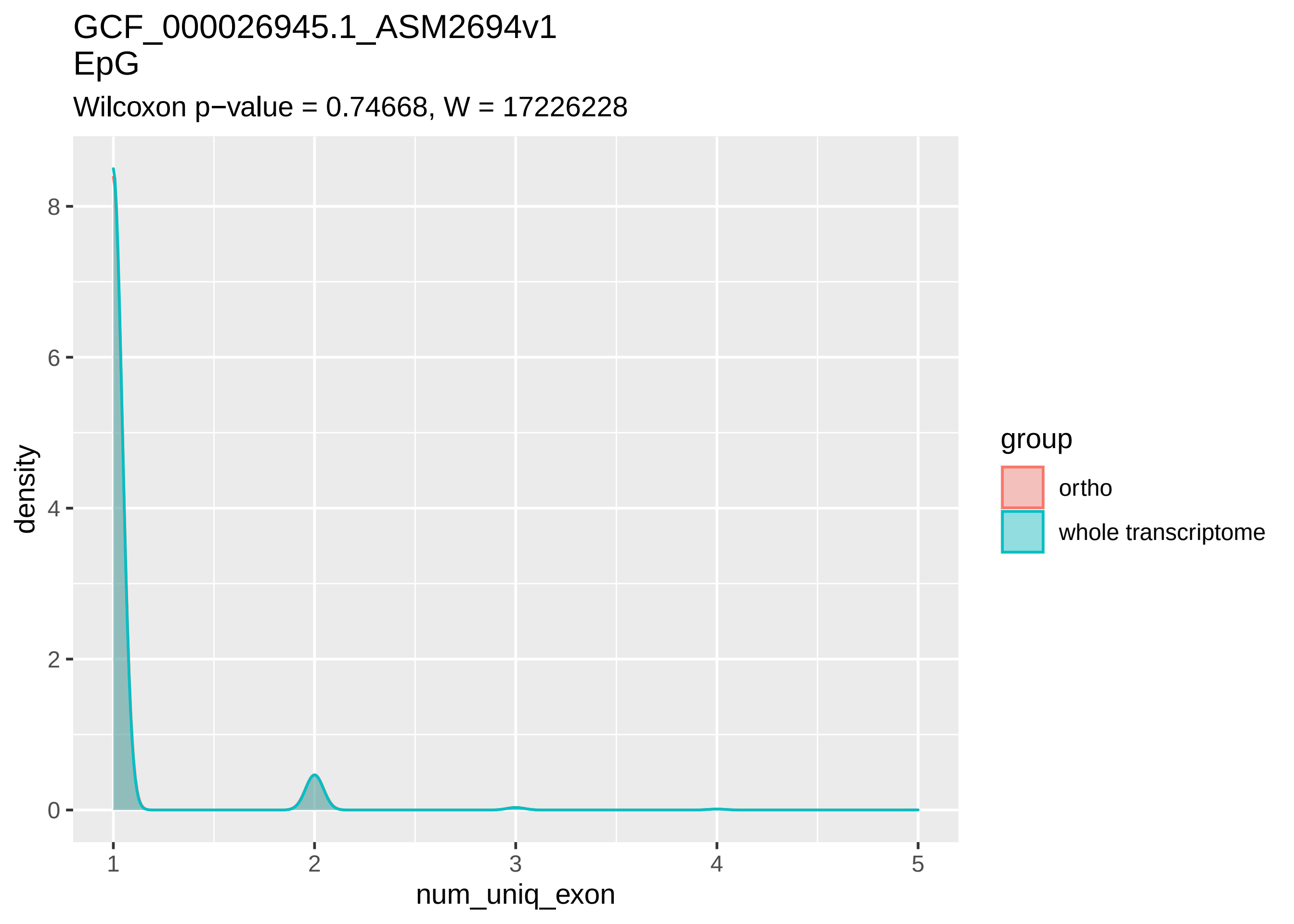

GCF_000026945.1_ASM2694v1
EpG
Wilcoxon p−value = 0.74668, W = 17226228
density
num_uniq_exon
0
2
4
6
8
1
2
3
4
5
group
ortho
whole transcriptome

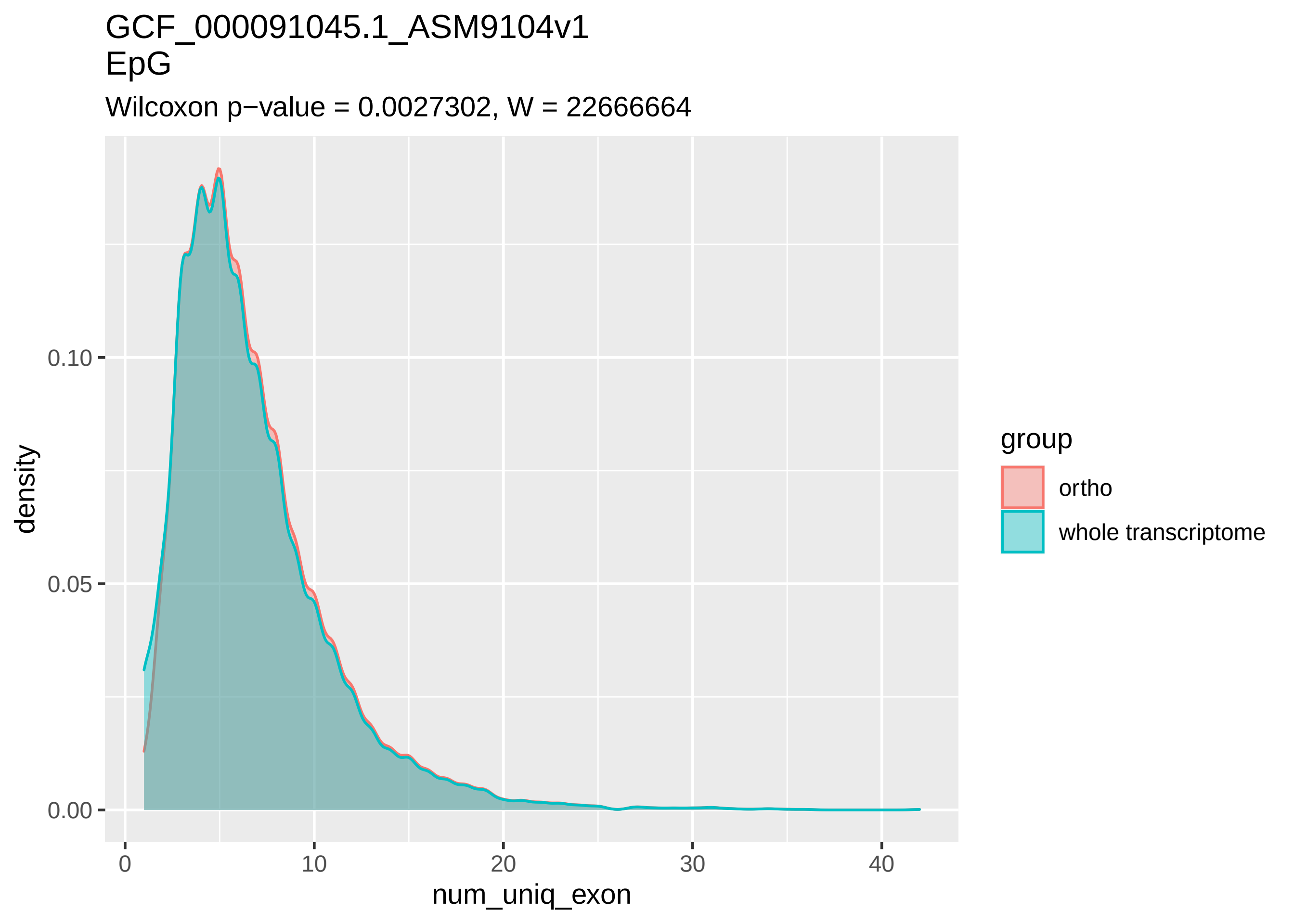
GCF_000091045.1_ASM9104v1
EpG
Wilcoxon p−value = 0.0027302, W = 22666664
density
0.00
0.05
0.10
0
10
20
30
40
num_uniq_exon
group
ortho
whole transcriptome

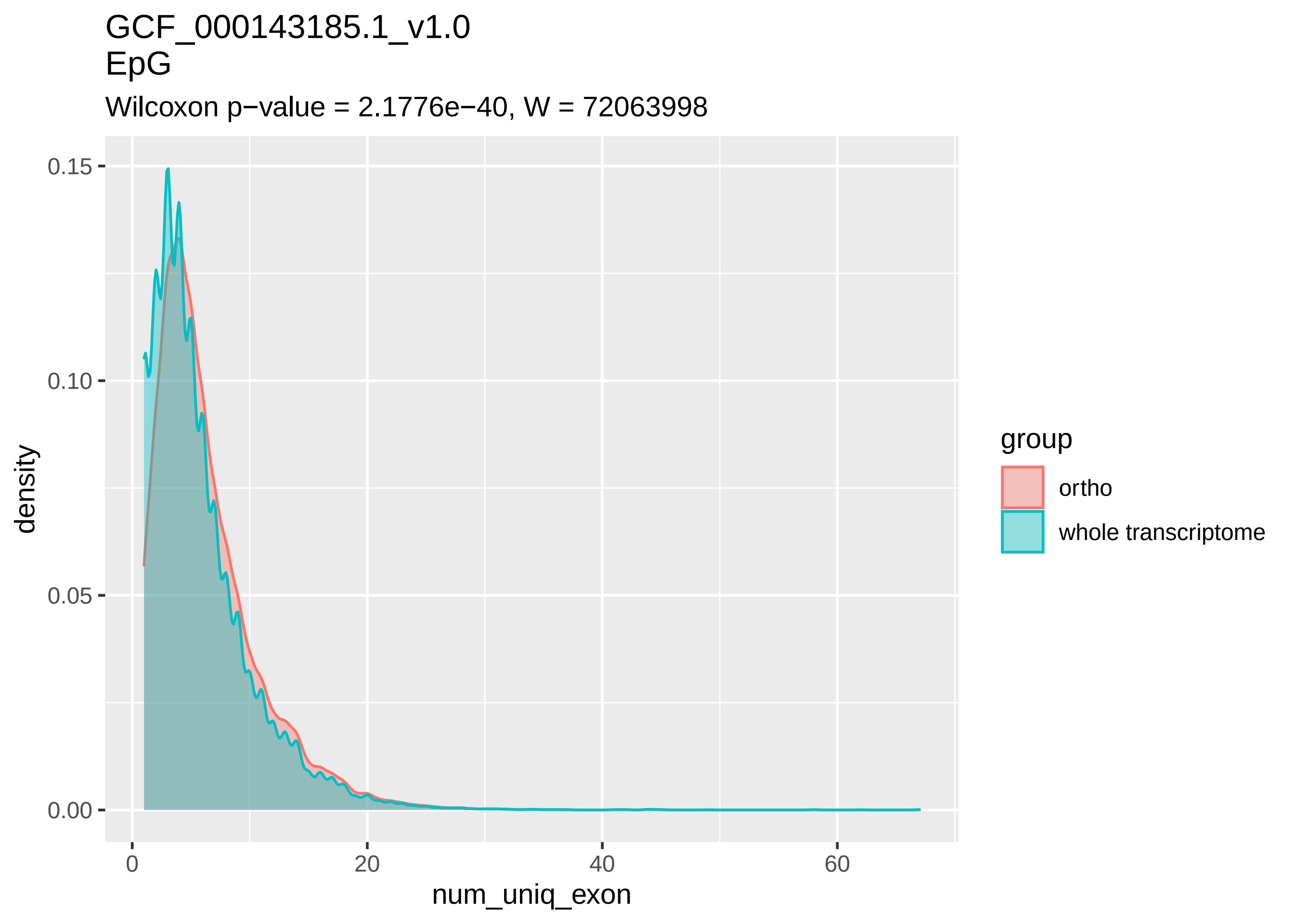
GCF_000143185.1_v1.0
EpG
Wilcoxon p−value = 2.1776e−40, W = 72063998
density
0.15
0.10
0.05
0.00
0
20
40
60
num_uniq_exon
group
ortho
whole transcriptome

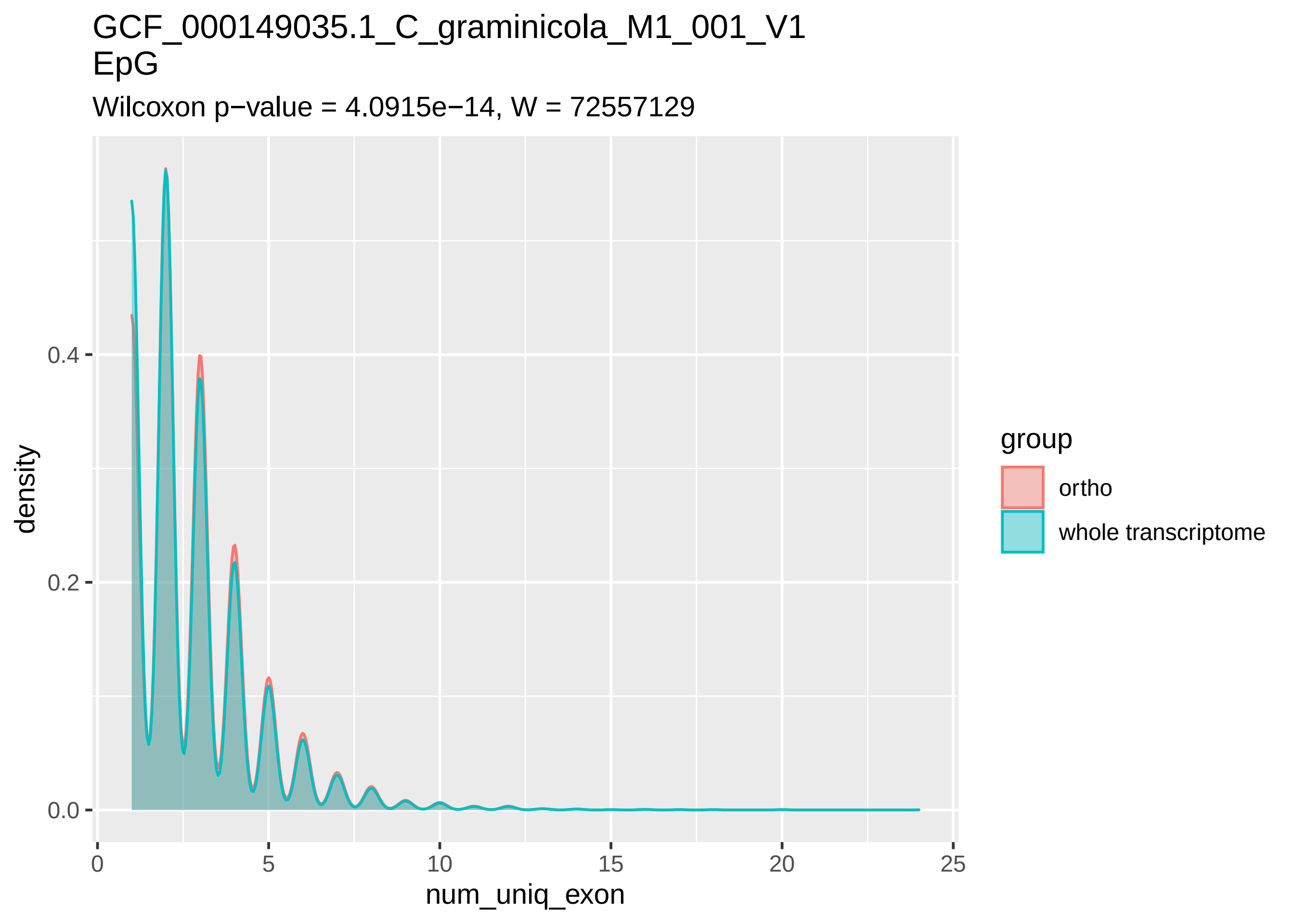

GCF_000149035.1_C_graminicola_M1_001_V1
EpG
Wilcoxon p−value = 4.0915e−14, W = 72557129
density
0.0
0.2
0.4
0
5
10
15
20
25
num_uniq_exon
group
ortho
whole transcriptome

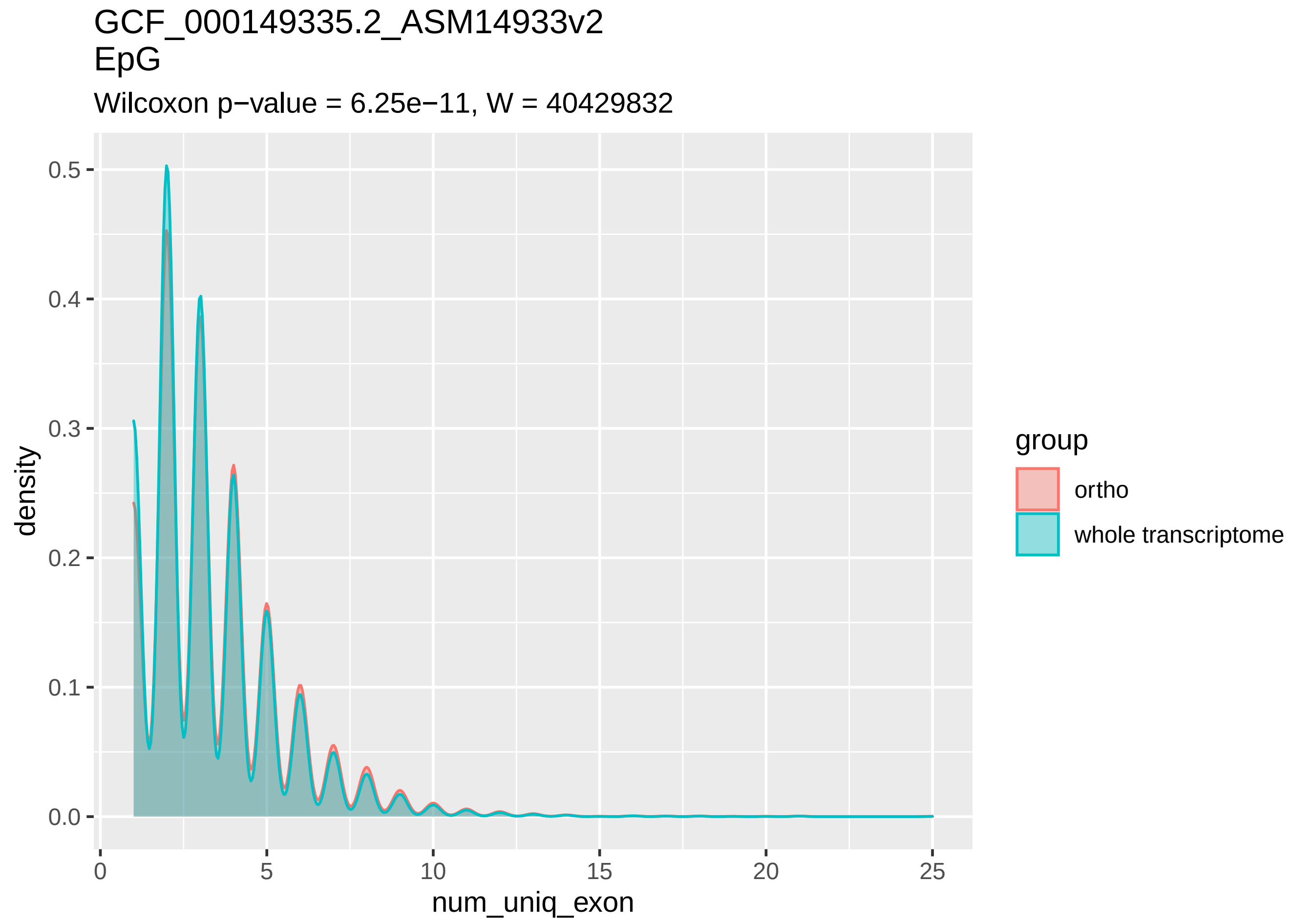

GCF_000149335.2_ASM14933v2
EpG
Wilcoxon p−value = 6.25e−11, W = 40429832
density
0.0
0.1
0.2
0.3
0.4
0.5
0
5
10
15
20
25
num_uniq_exon
group
ortho
whole transcriptome

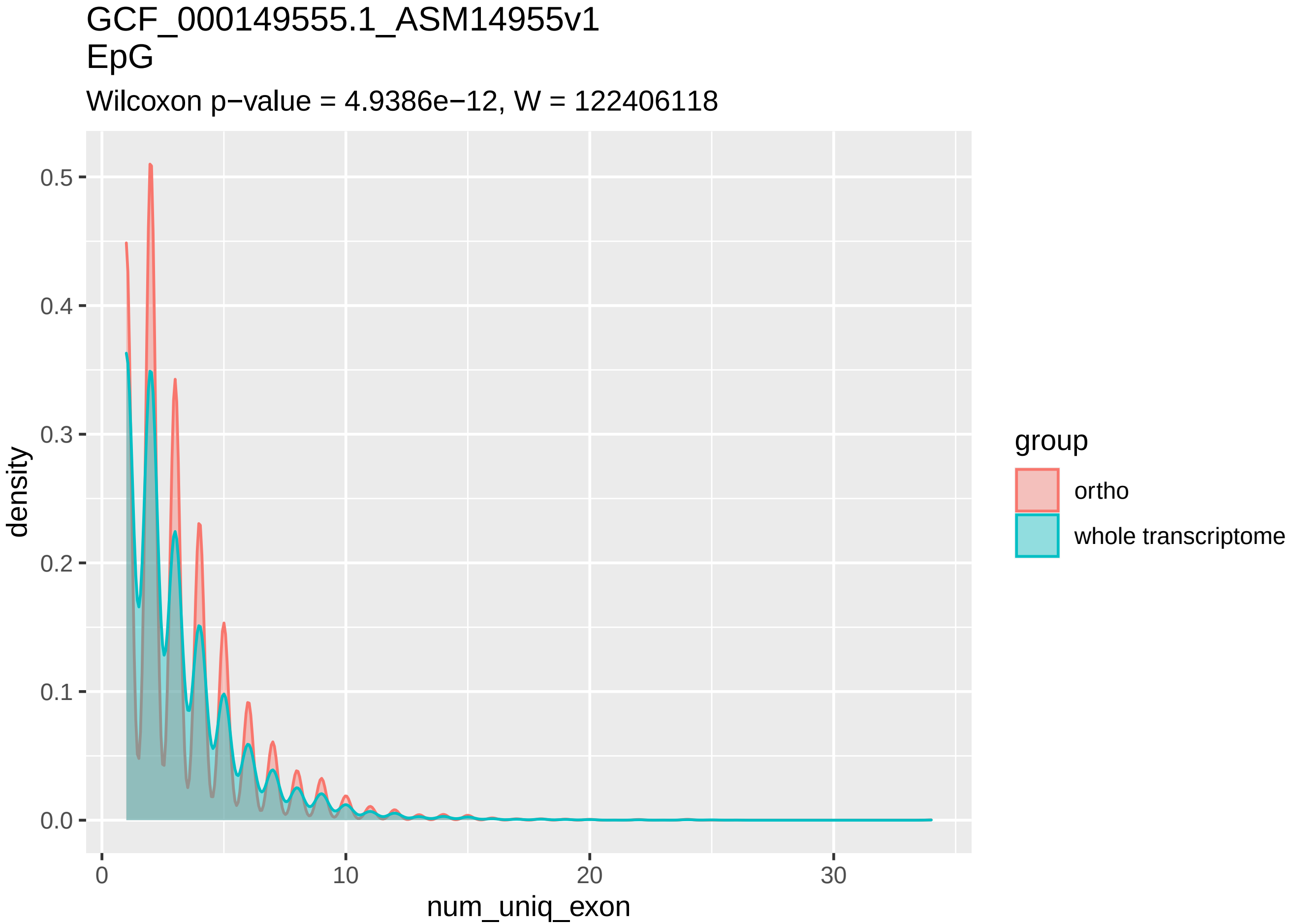

GCF_000149555.1_ASM14955v1
EpG
Wilcoxon p−value = 4.9386e−12, W = 122406118
density
0.0
0.1
0.2
0.3
0.4
0.5
0
10
20
30
num_uniq_exon
group
ortho
whole transcriptome

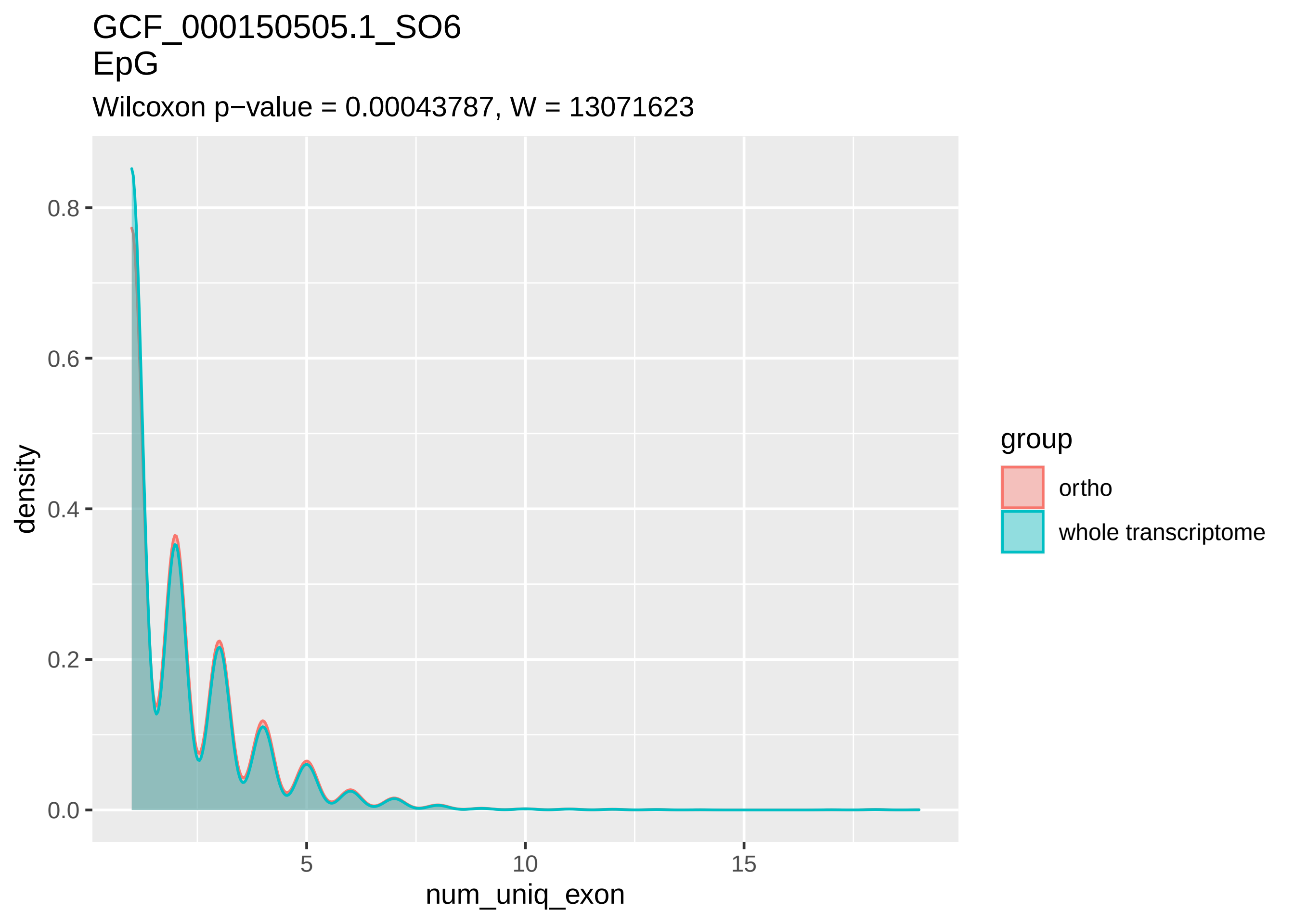

GCF_000150505.1_SO6
EpG
Wilcoxon p−value = 0.00043787, W = 13071623
density
0.0
0.2
0.4
0.6
0.8
5
10
15
num_uniq_exon
group
ortho
whole transcriptome

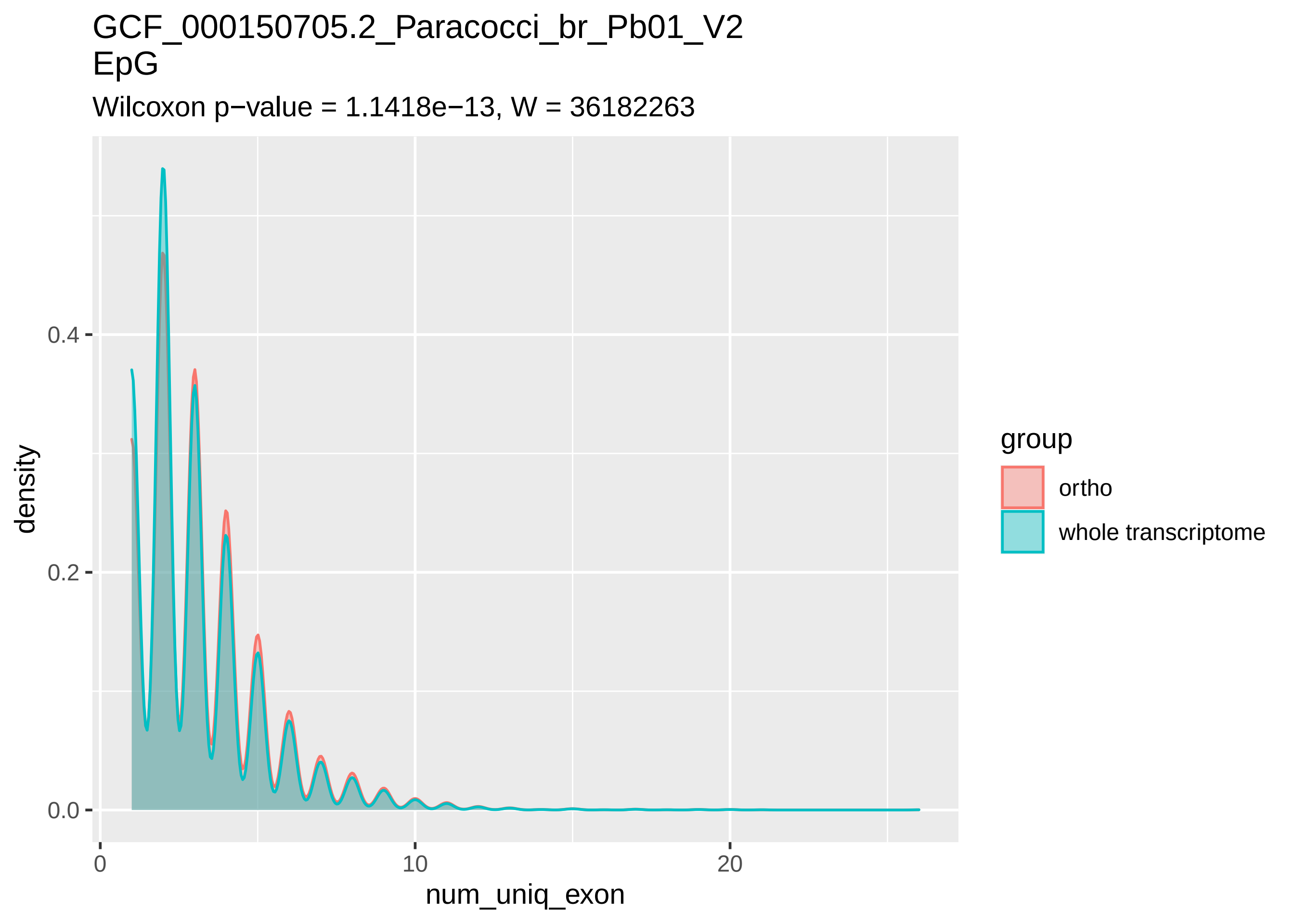
GCF_000150705.2_Paracocci_br_Pb01_V2
EpG
Wilcoxon p−value = 1.1418e−13, W = 36182263
density
0.0
0.2
0.4
0
10
20
num_uniq_exon
group
ortho
whole transcriptome

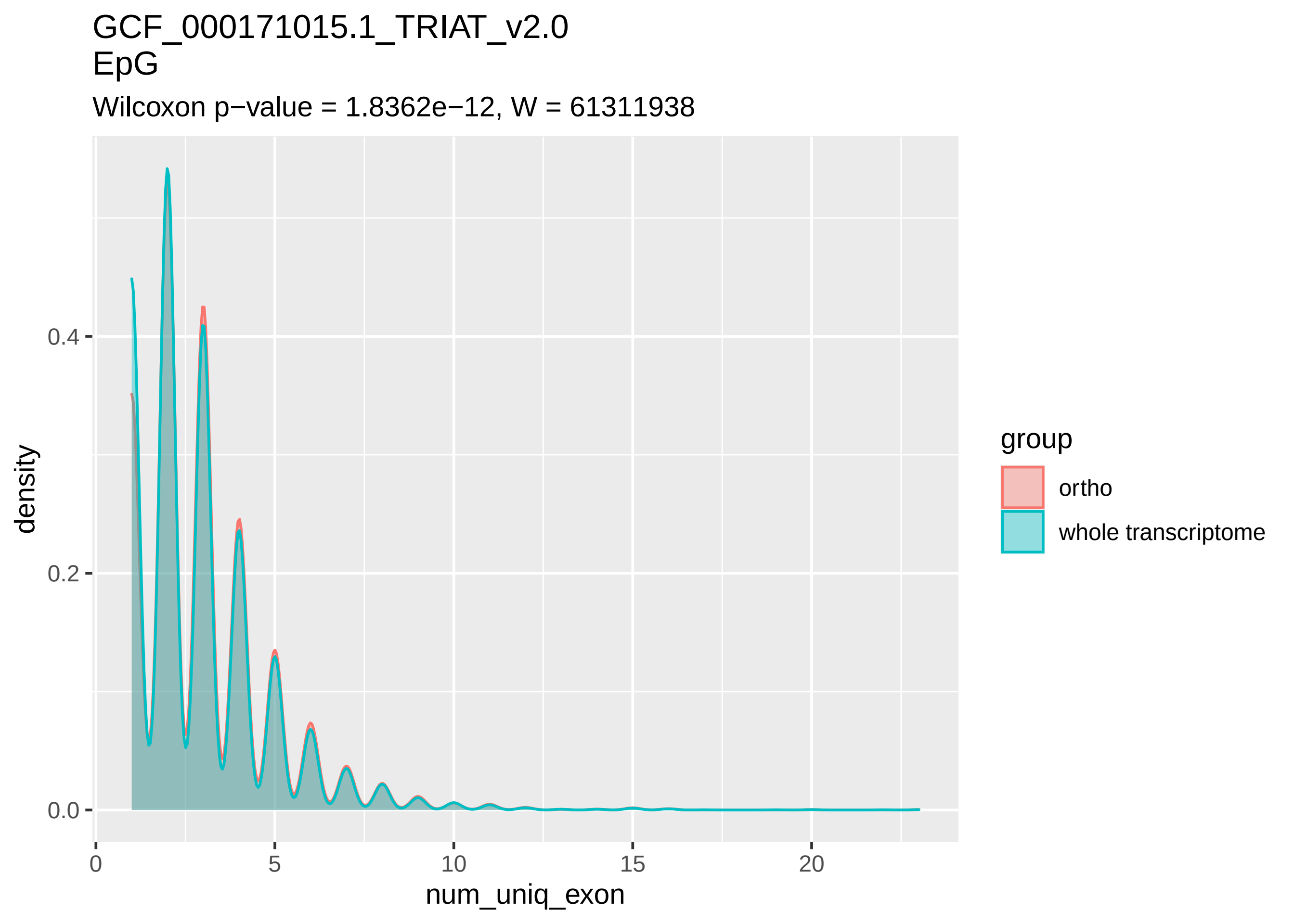
GCF_000171015.1_TRIAT_v2.0
EpG
Wilcoxon p−value = 1.8362e−12, W = 61311938
density
0.0
0.2
0.4
0
5
10
15
20
num_uniq_exon
group
ortho
whole transcriptome

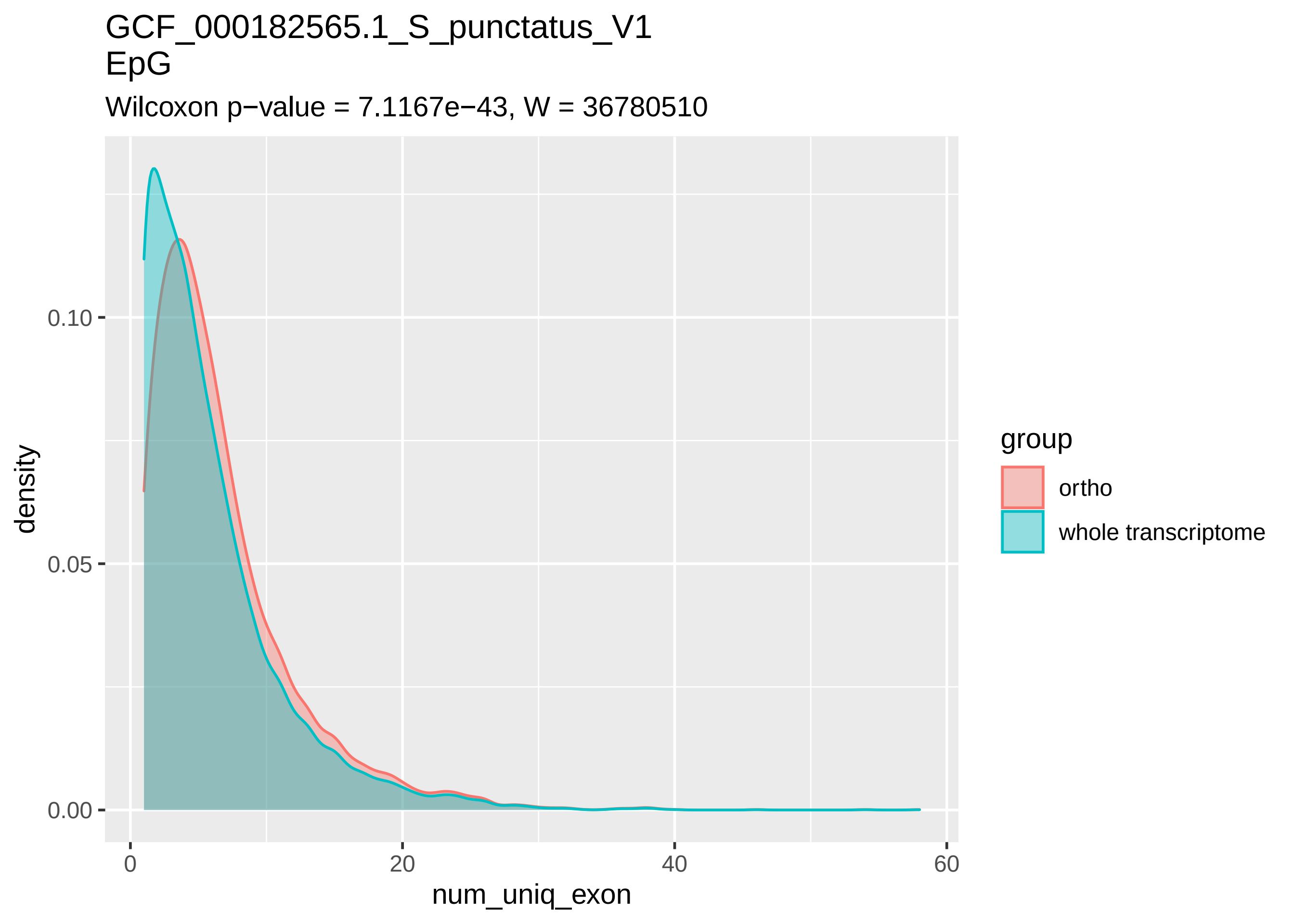
GCF_000182565.1_S_punctatus_V1
EpG
Wilcoxon p−value = 7.1167e−43, W = 36780510
density
0.10
0.05
0.00
0
20
40
60
num_uniq_exon
group
ortho
whole transcriptome

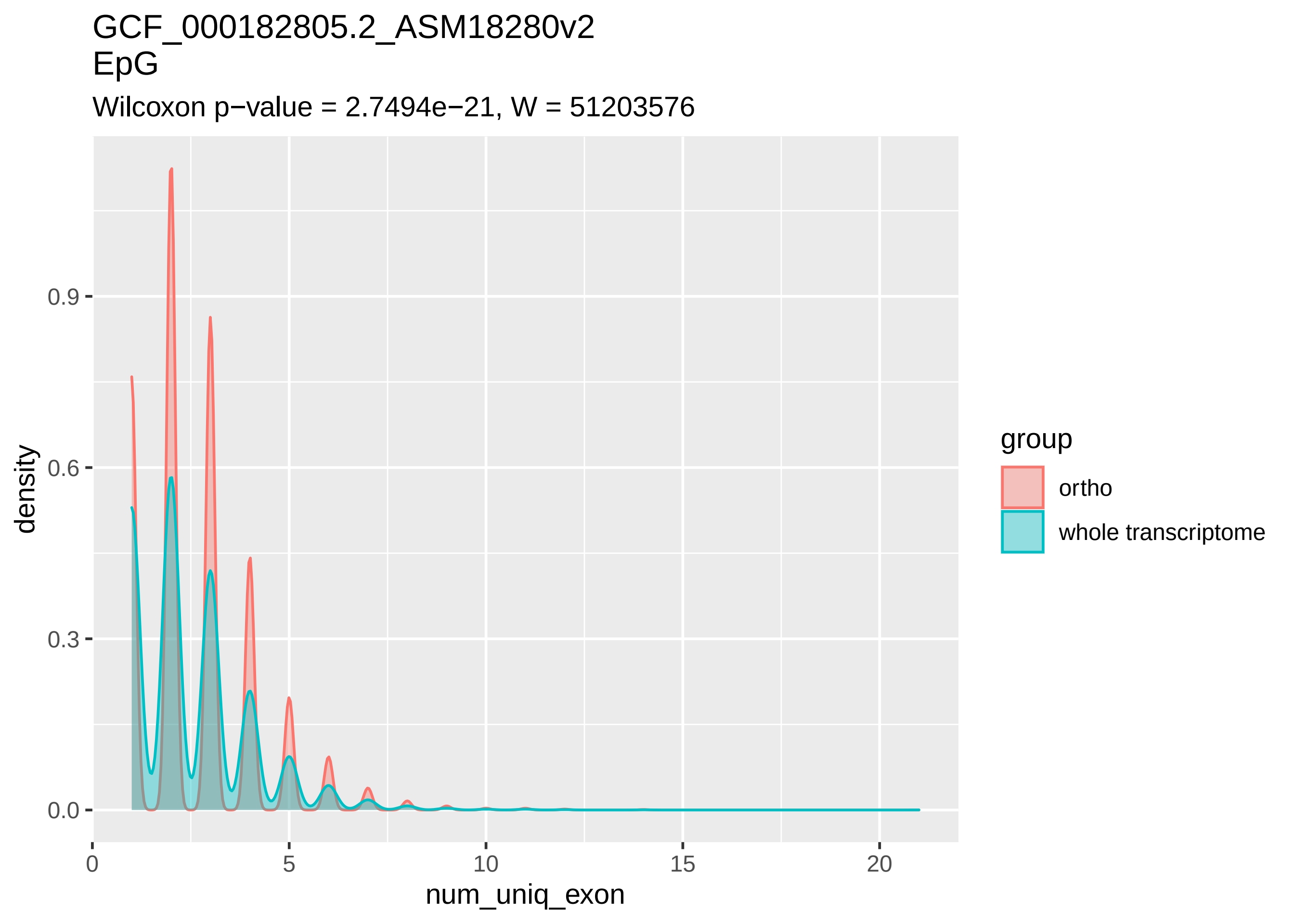
GCF_000182805.2_ASM18280v2
EpG
Wilcoxon p−value = 2.7494e−21, W = 51203576
density
0.9
0.6
0.3
0.0
0
5
10
15
20
num_uniq_exon
group
ortho
whole transcriptome

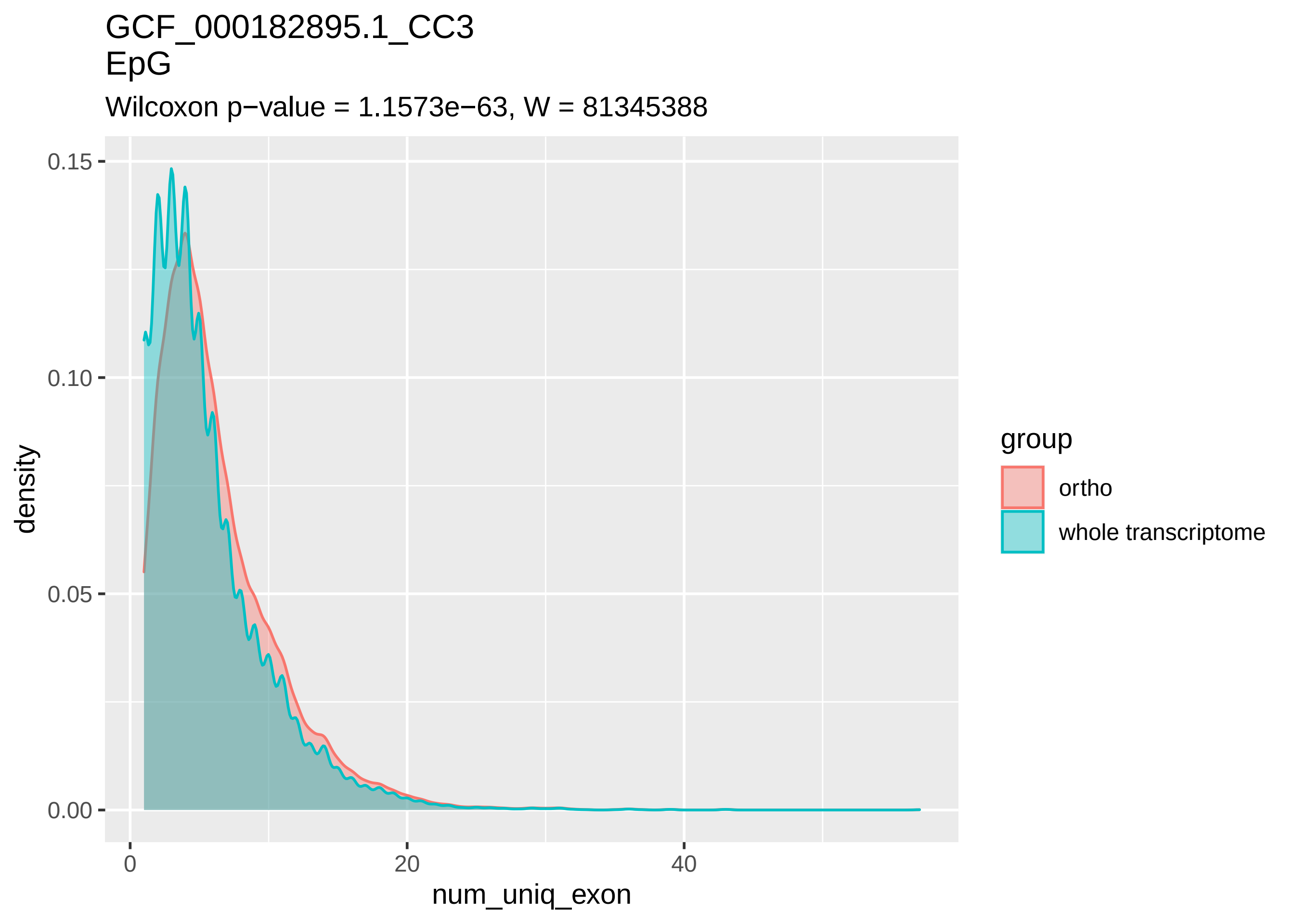

GCF_000182895.1_CC3
EpG
Wilcoxon p−value = 1.1573e−63, W = 81345388
0.15
0.10
0.05
0.00
density
0
20
40
num_uniq_exon
group
ortho
whole transcriptome

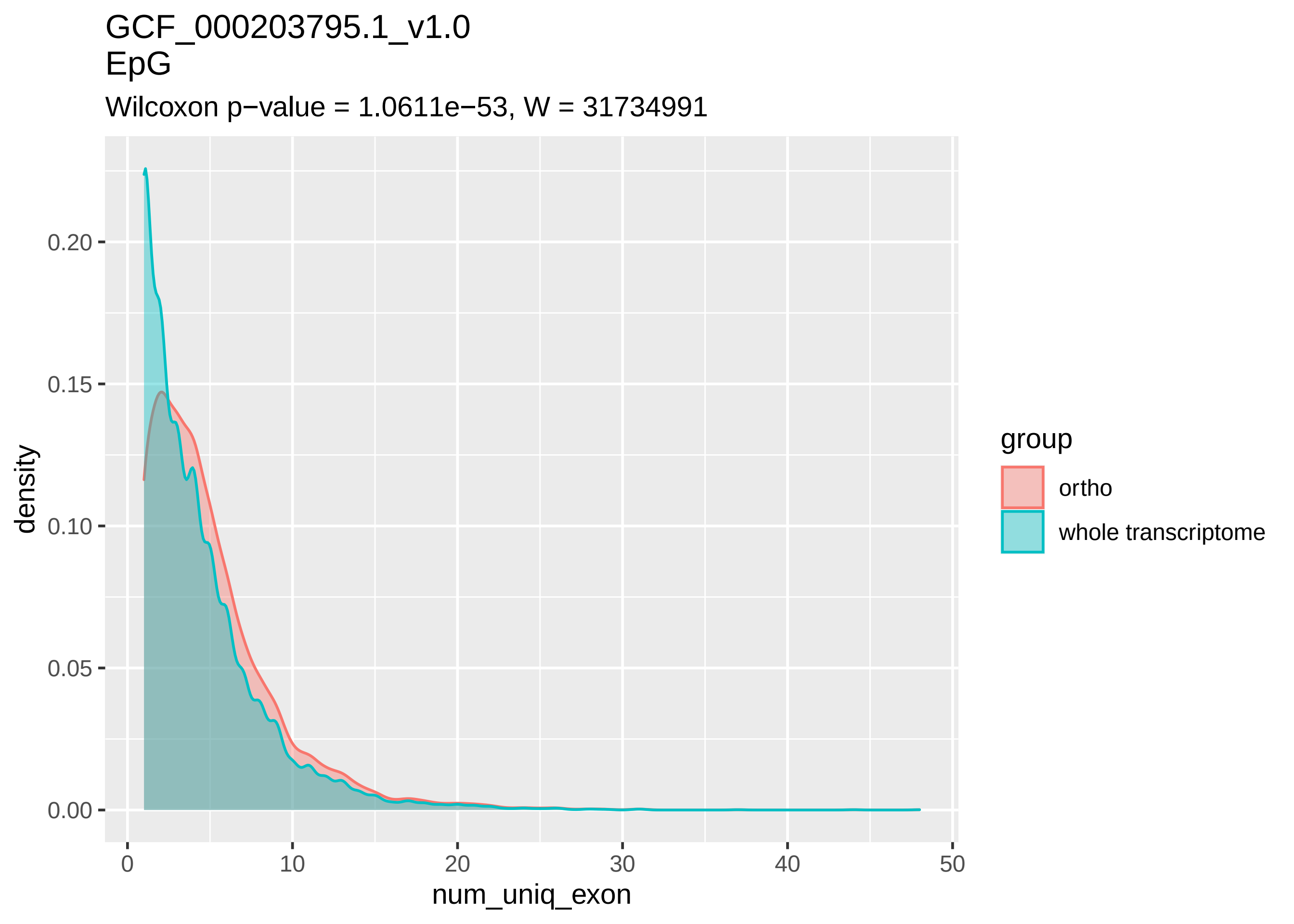
GCF_000203795.1_v1.0
EpG
Wilcoxon p−value = 1.0611e−53, W = 31734991
density
0.00
0.05
0.10
0.15
0.20
0
10
20
30
40
50
num_uniq_exon
group
ortho
whole transcriptome

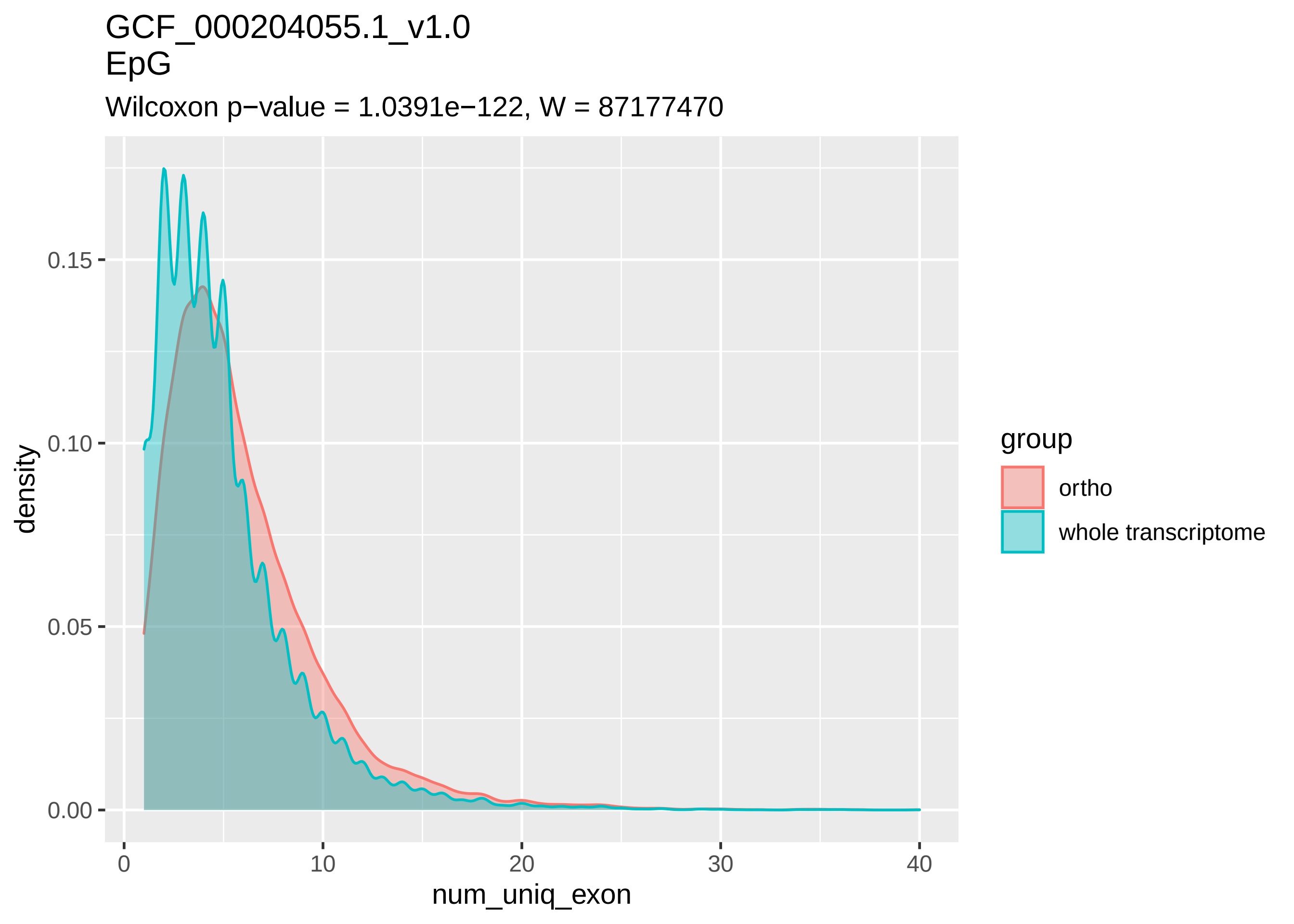
GCF_000204055.1_v1.0
EpG
Wilcoxon p−value = 1.0391e−122, W = 87177470
density
0.00
0.05
0.10
0.15
0
10
20
30
40
num_uniq_exon
group
ortho
whole transcriptome

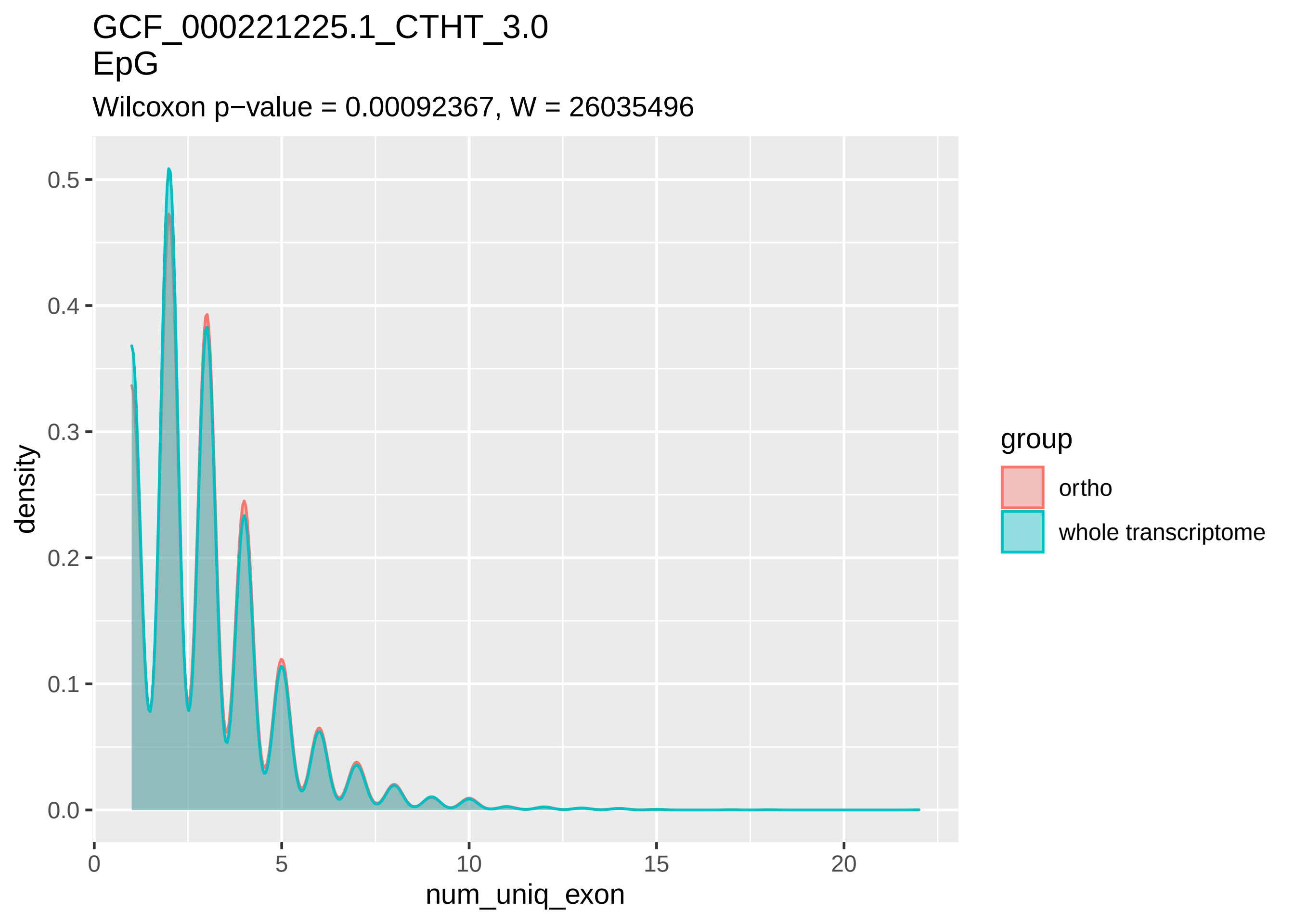
GCF_000221225.1_CTHT_3.0
EpG
Wilcoxon p−value = 0.00092367, W = 26035496
density
0.0
0.1
0.2
0.3
0.4
0.5
0
5
10
15
20
num_uniq_exon
group
ortho
whole transcriptome

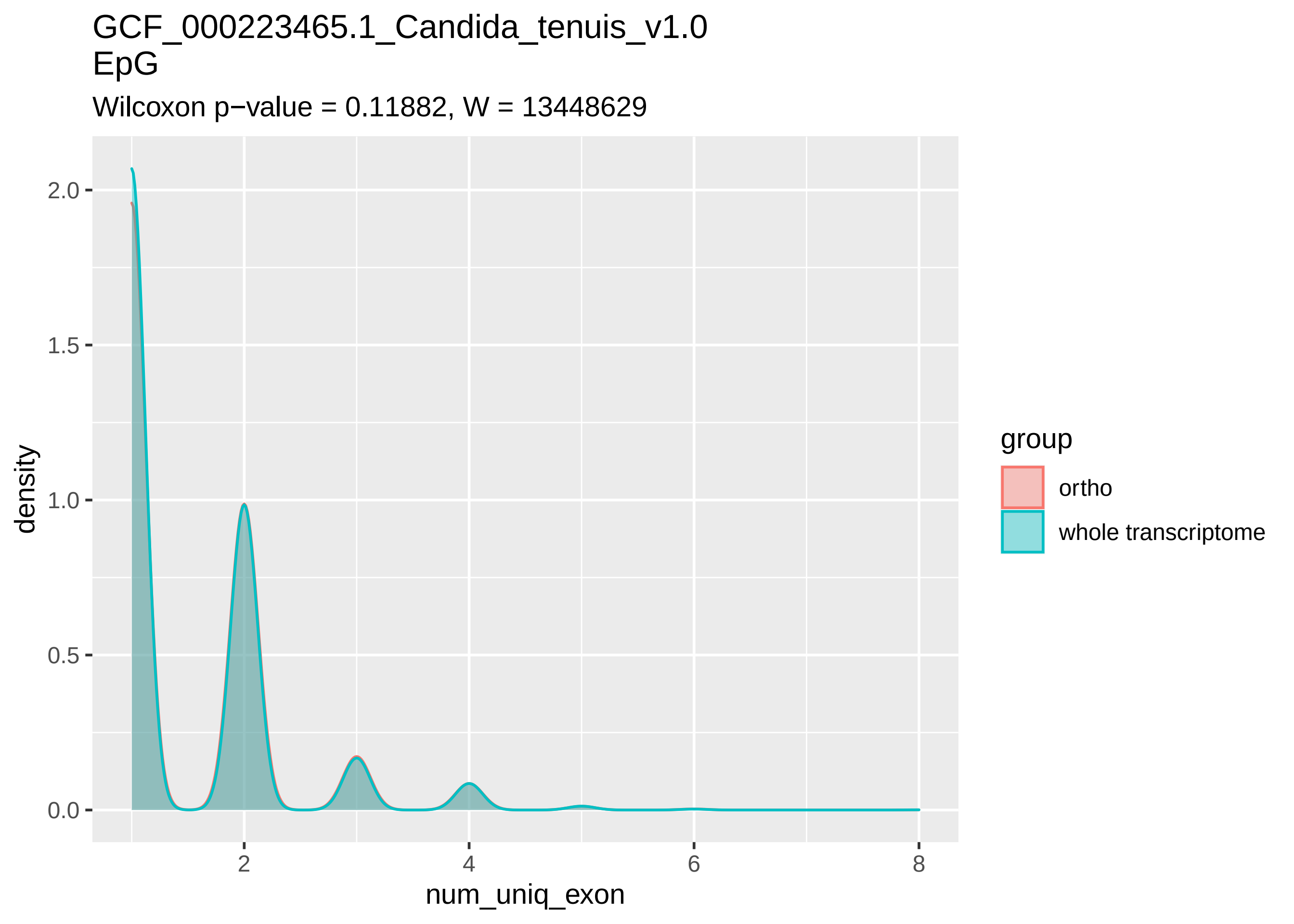
GCF_000223465.1_Candida_tenuis_v1.0
EpG
Wilcoxon p−value = 0.11882, W = 13448629
density
2.0
1.5
1.0
0.5
0.0
2
4
6
8
num_uniq_exon
group
ortho
whole transcriptome

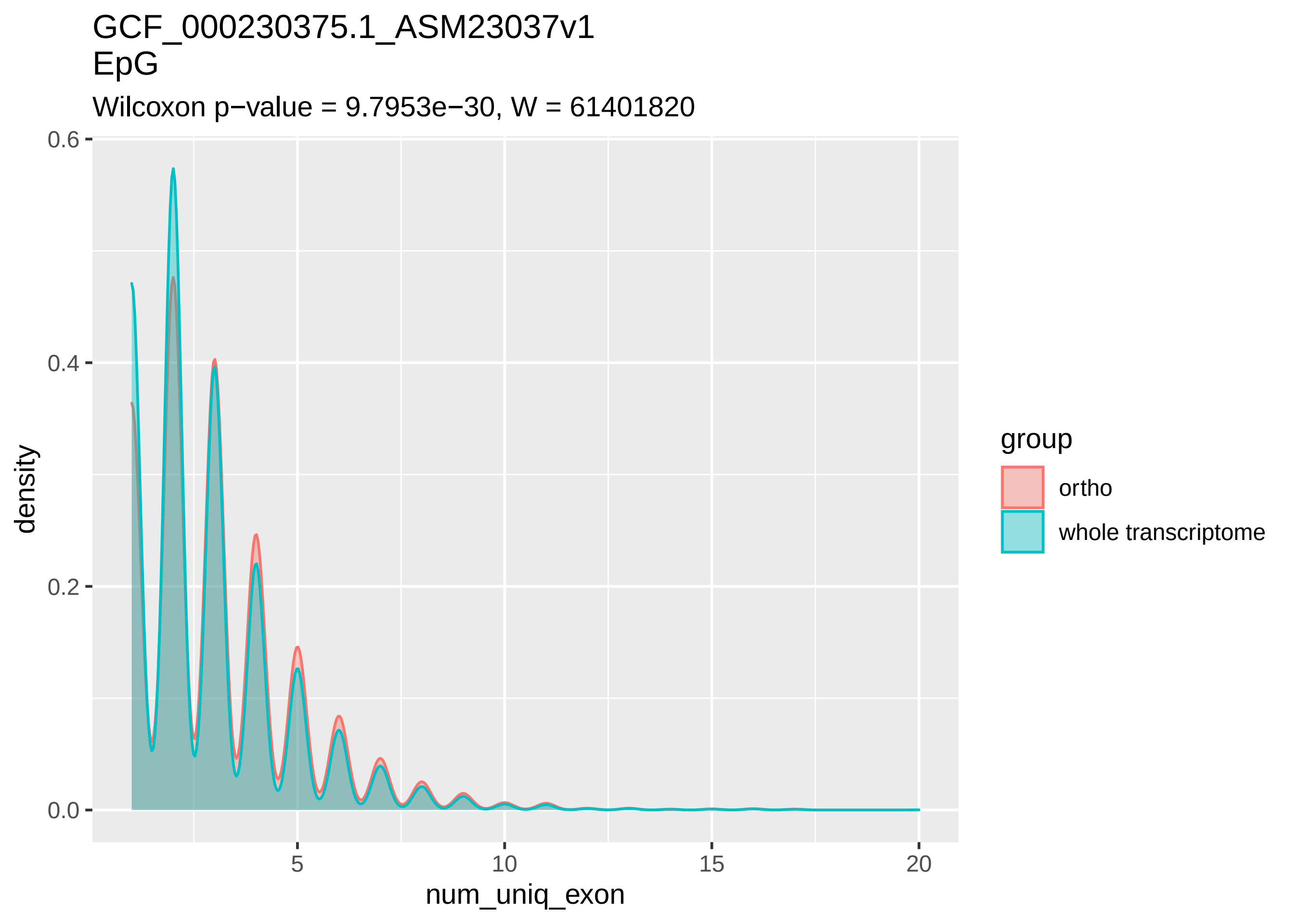

GCF_000230375.1_ASM23037v1
EpG
Wilcoxon p−value = 9.7953e−30, W = 61401820
density
0.0
0.2
0.4
0.6
5
10
15
20
num_uniq_exon
group
ortho
whole transcriptome

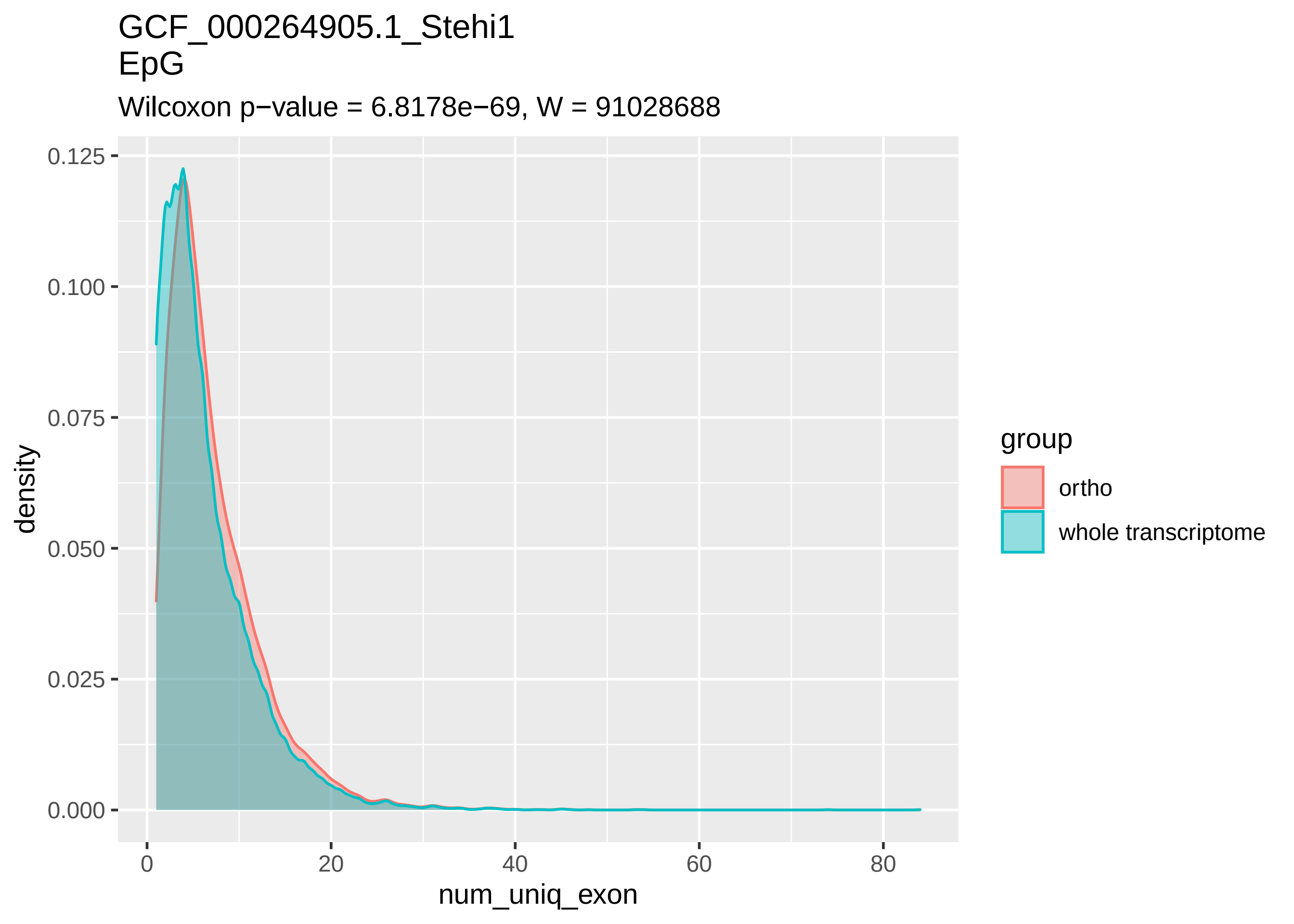

GCF_000264905.1_Stehi1
EpG
Wilcoxon p−value = 6.8178e−69, W = 91028688
0.125
0.100
0.075
0.050
0.025
0.000
density
0
20
40
60
80
num_uniq_exon
group
ortho
whole transcriptome

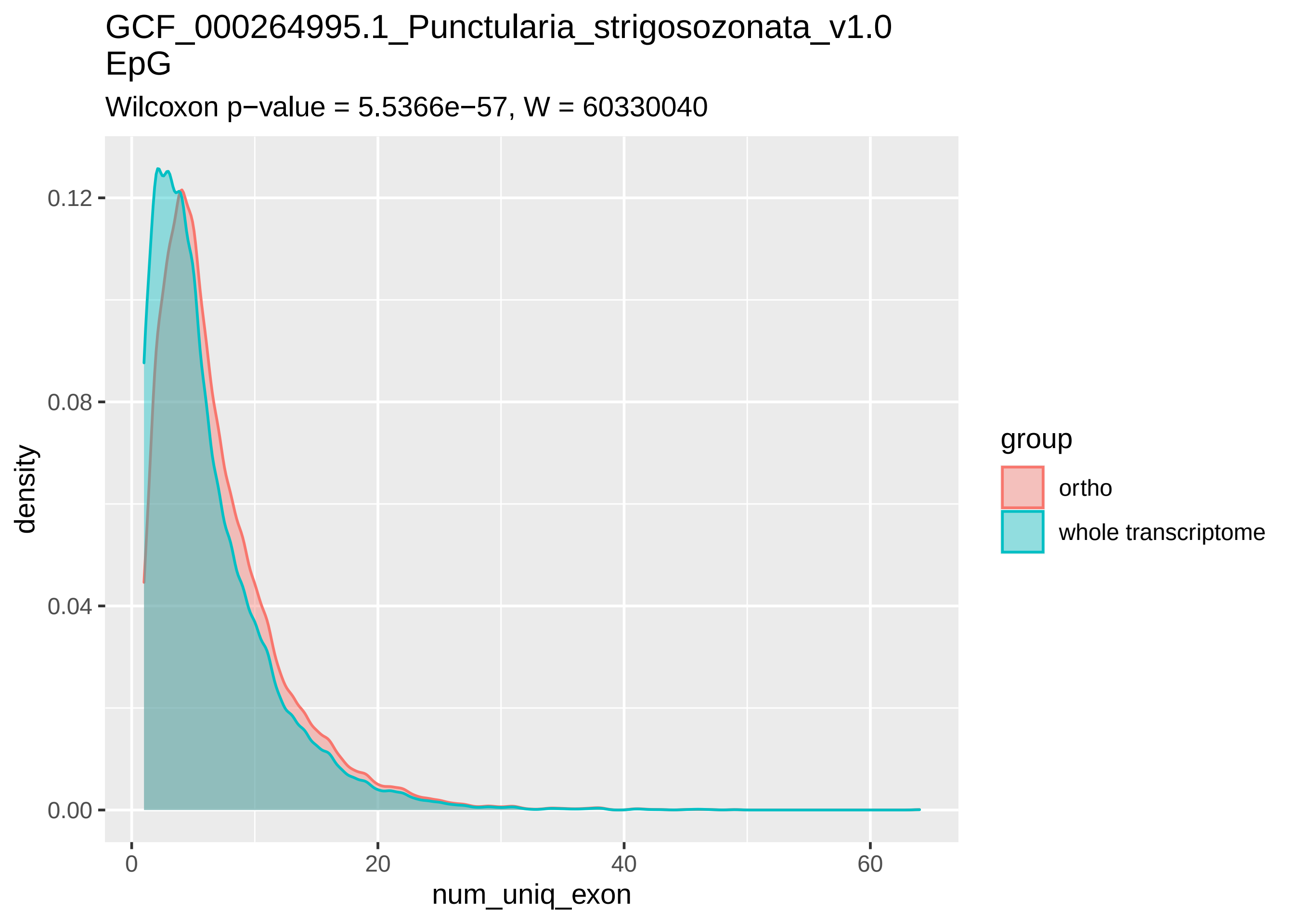
GCF_000264995.1_Punctularia_strigosozonata_v1.0
EpG
Wilcoxon p−value = 5.5366e−57, W = 60330040
0.12
0.08
0.04
0.00
density
0
20
40
60
num_uniq_exon
group
ortho
whole transcriptome

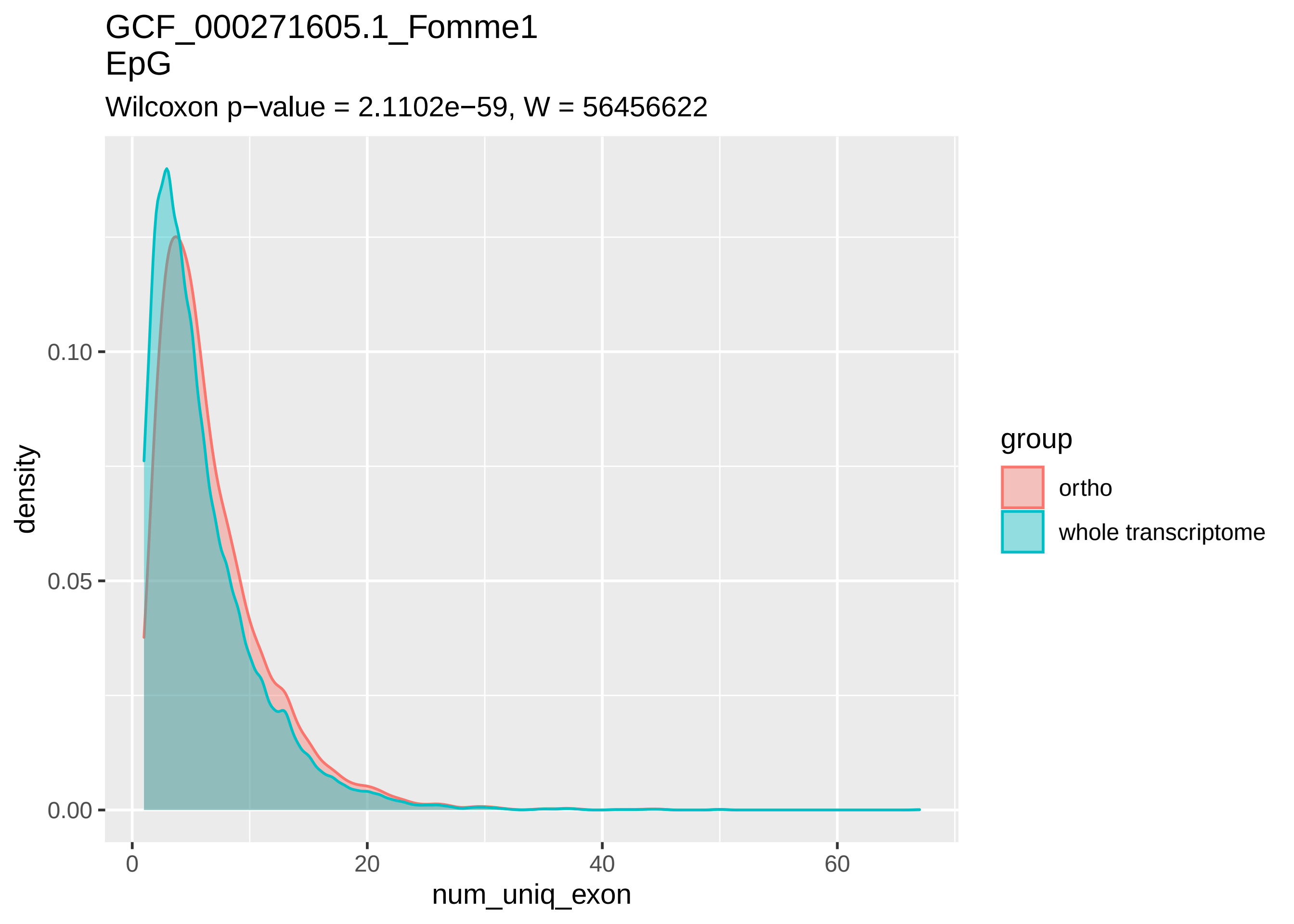
GCF_000271605.1_Fomme1
EpG
Wilcoxon p−value = 2.1102e−59, W = 56456622
density
0.10
0.05
0.00
0
20
40
60
num_uniq_exon
group
ortho
whole transcriptome

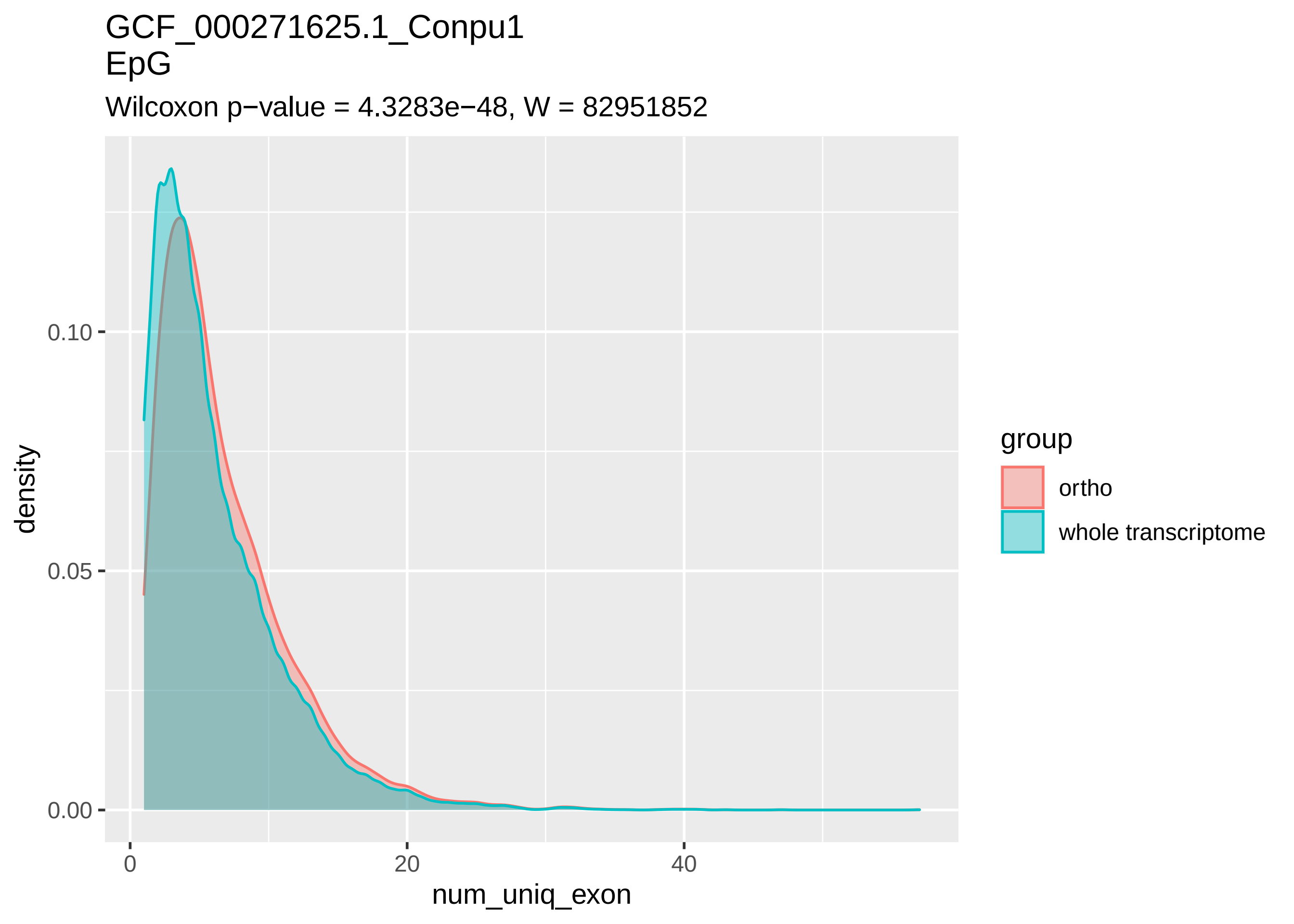
GCF_000271625.1_Conpu1
EpG
Wilcoxon p−value = 4.3283e−48, W = 82951852
0.10
0.05
0.00
density
0
20
40
num_uniq_exon
group
ortho
whole transcriptome

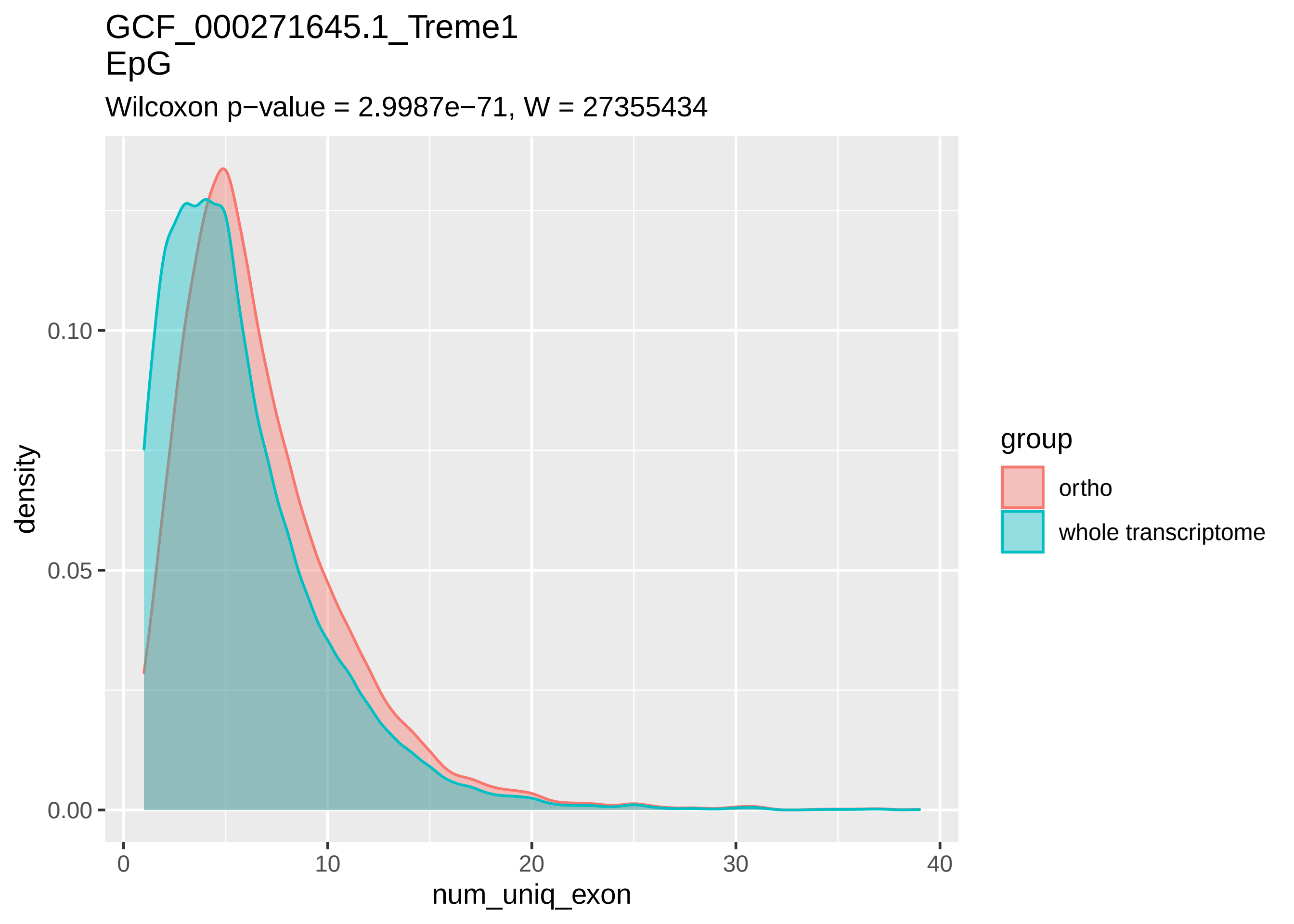

GCF_000271645.1_Treme1
EpG
Wilcoxon p−value = 2.9987e−71, W = 27355434
density
0.00
0.05
0.10
0
10
20
30
40
num_uniq_exon
group
ortho
whole transcriptome

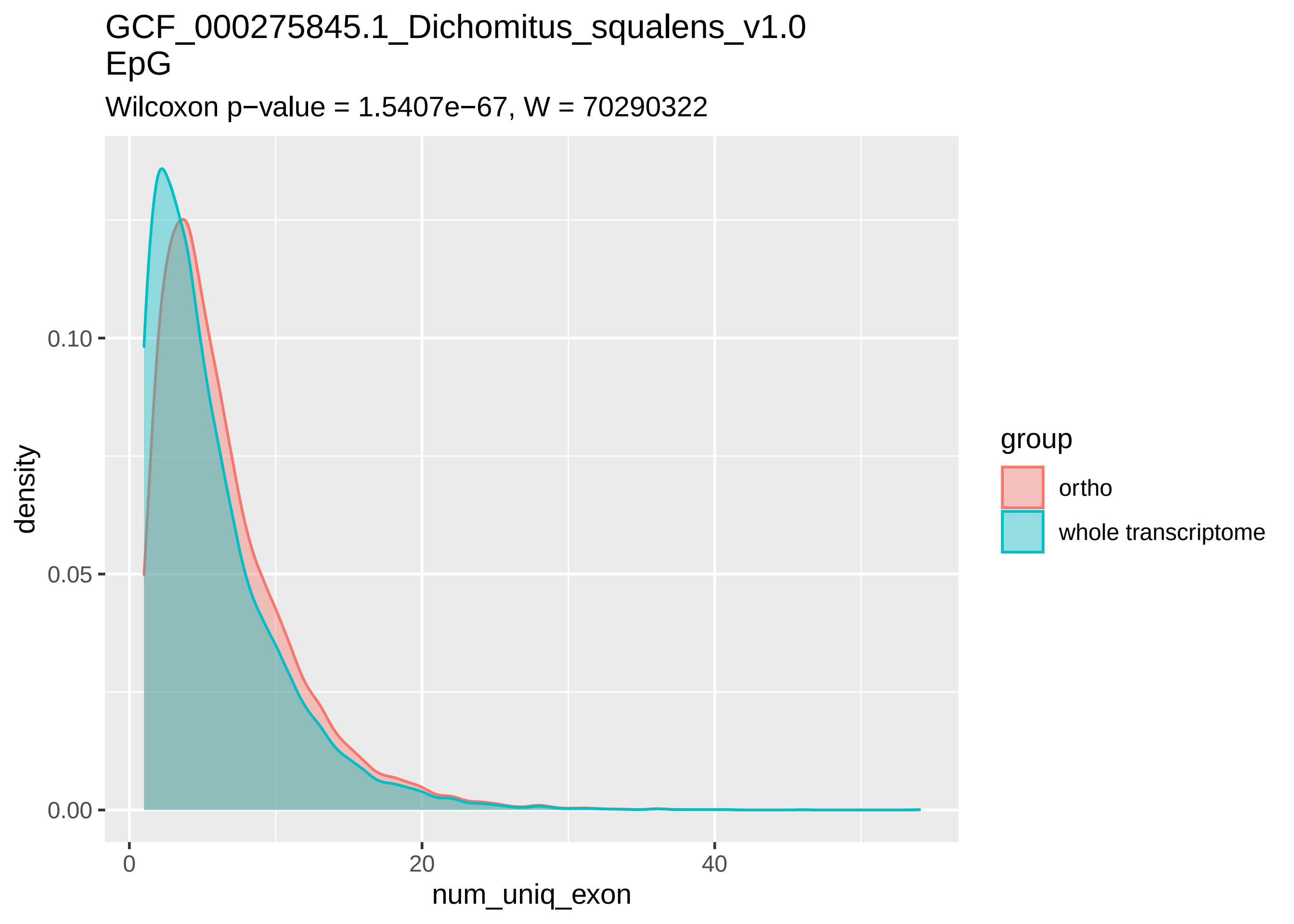
GCF_000275845.1_Dichomitus_squalens_v1.0
EpG
Wilcoxon p−value = 1.5407e−67, W = 70290322
density
0.10
0.05
0.00
0
20
40
num_uniq_exon
group
ortho
whole transcriptome

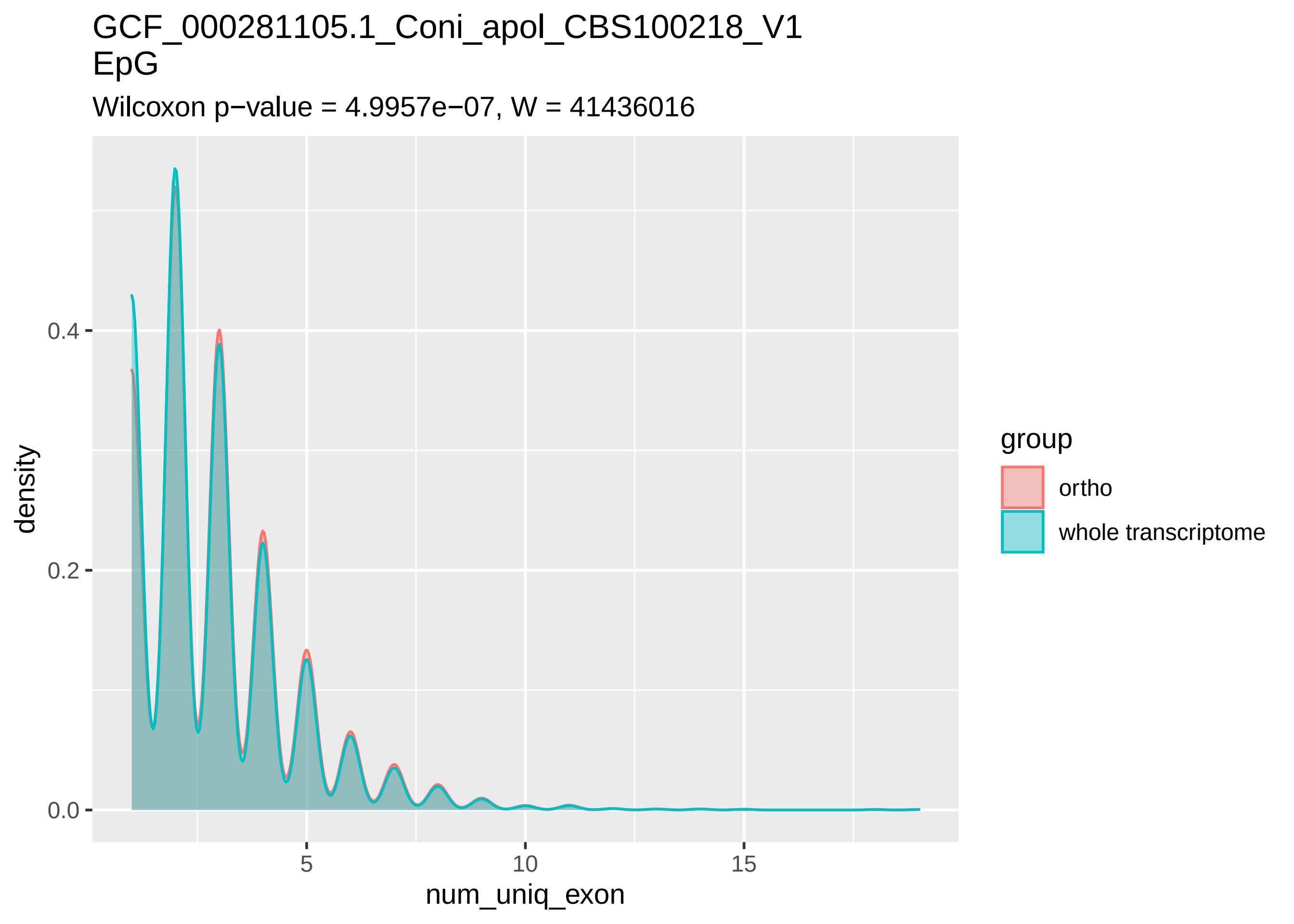
GCF_000281105.1_Coni_apol_CBS100218_V1
EpG
Wilcoxon p−value = 4.9957e−07, W = 41436016
density
0.0
0.2
0.4
5
10
15
num_uniq_exon
group
ortho
whole transcriptome

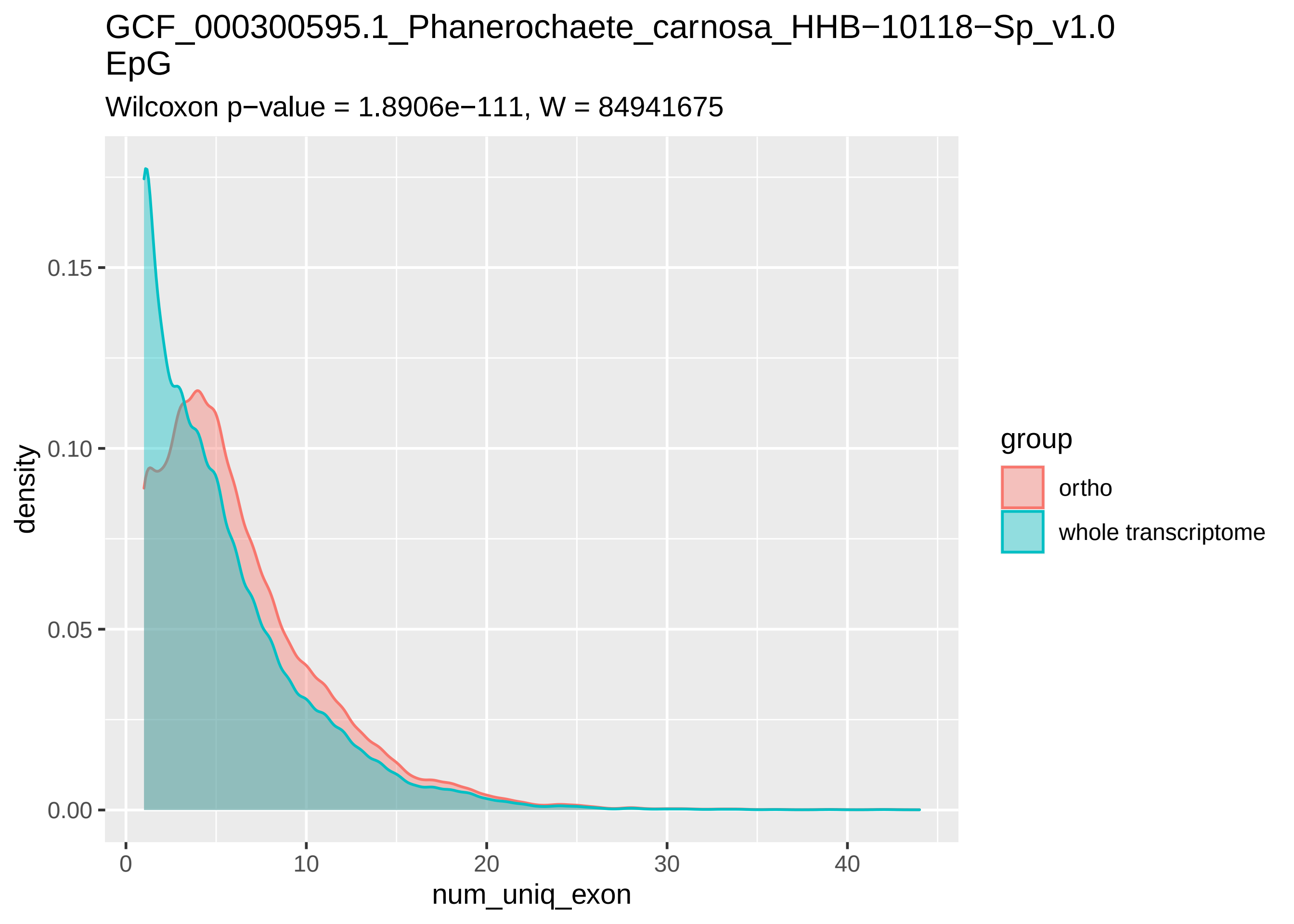
GCF_000300595.1_Phanerochaete_carnosa_HHB−10118−Sp_v1.0
EpG
Wilcoxon p−value = 1.8906e−111, W = 84941675
density
0.15
0.10
0.05
0.00
0
10
20
30
40
num_uniq_exon
group
ortho
whole transcriptome

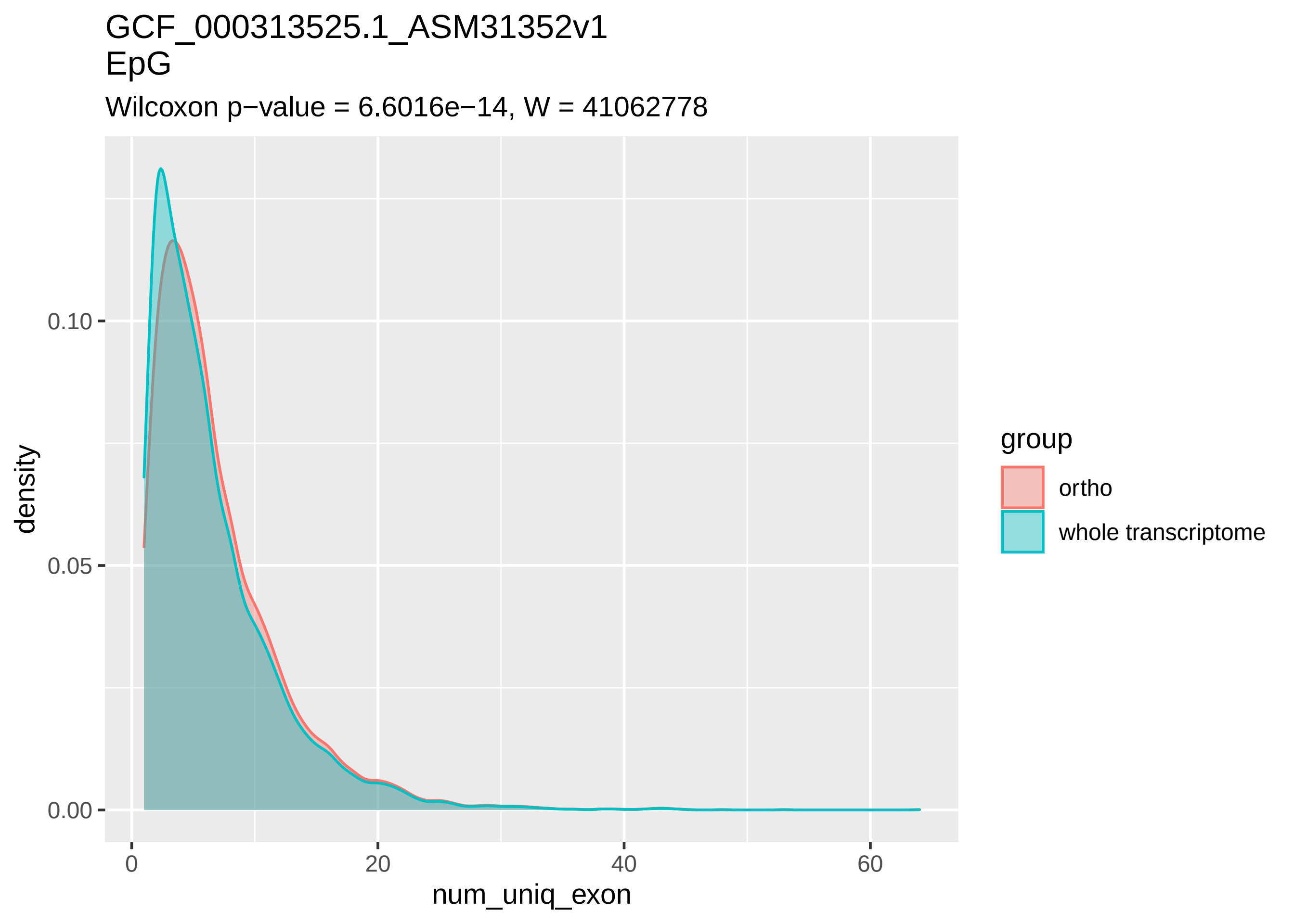
GCF_000313525.1_ASM31352v1
EpG
Wilcoxon p−value = 6.6016e−14, W = 41062778
density
0.10
0.05
0.00
0
20
40
60
num_uniq_exon
group
ortho
whole transcriptome

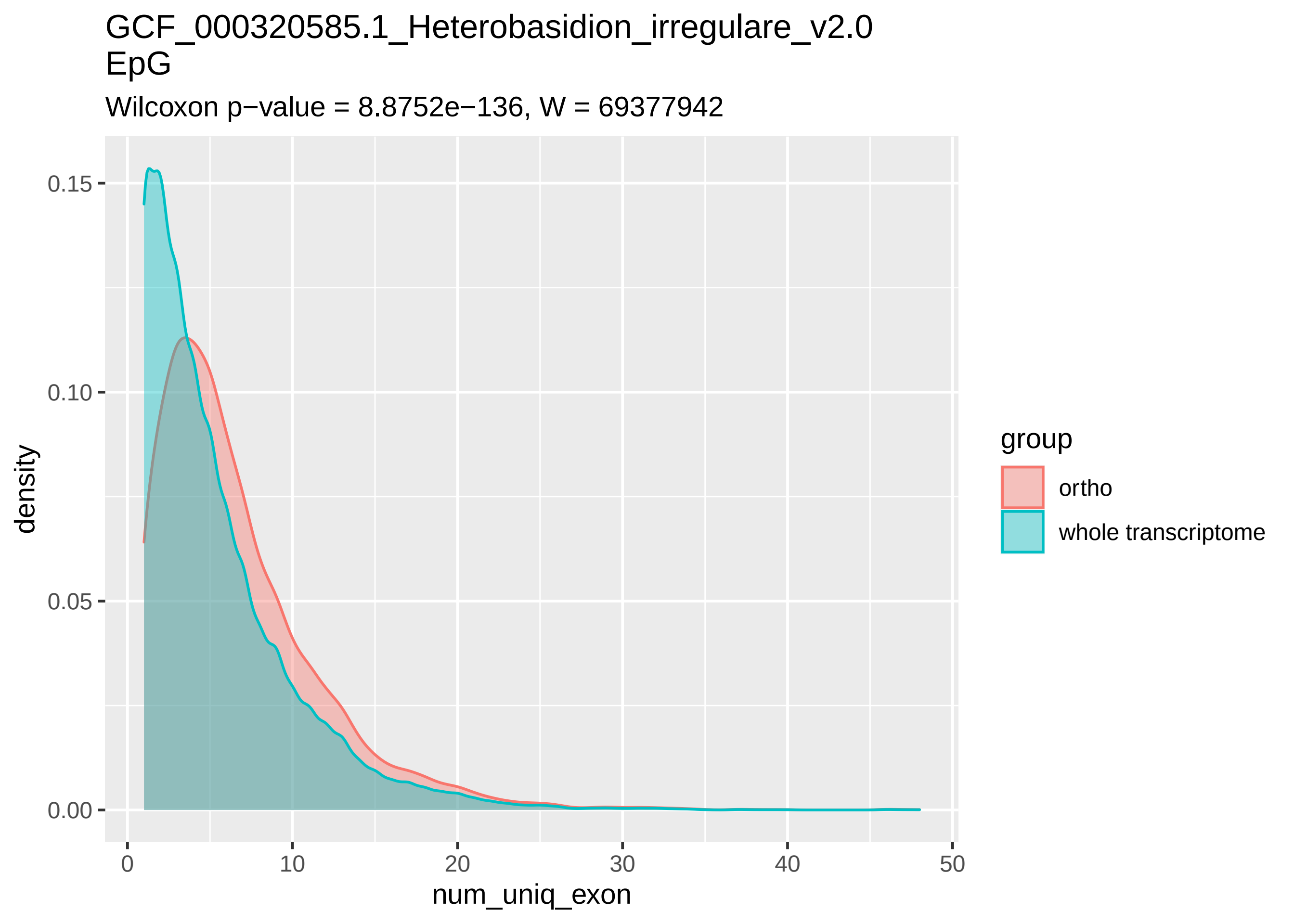
GCF_000320585.1_Heterobasidion_irregulare_v2.0
EpG
Wilcoxon p−value = 8.8752e−136, W = 69377942
density
0.15
0.10
0.05
0.00
0
10
20
30
40
50
num_uniq_exon
group
ortho
whole transcriptome

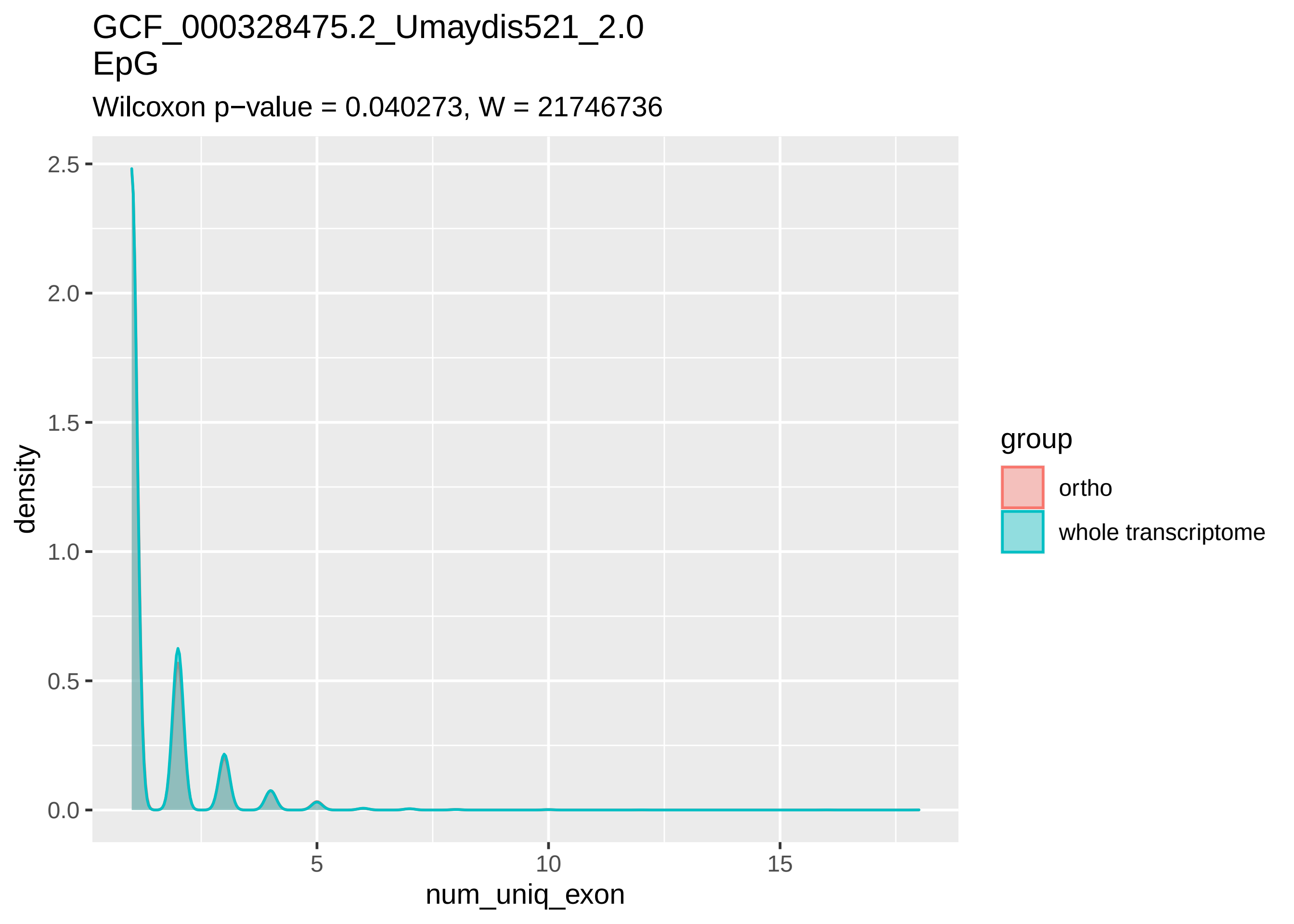
GCF_000328475.2_Umaydis521_2.0
EpG
Wilcoxon p−value = 0.040273, W = 21746736
density
2.5
2.0
1.5
1.0
0.5
0.0
5
10
15
num_uniq_exon
group
ortho
whole transcriptome

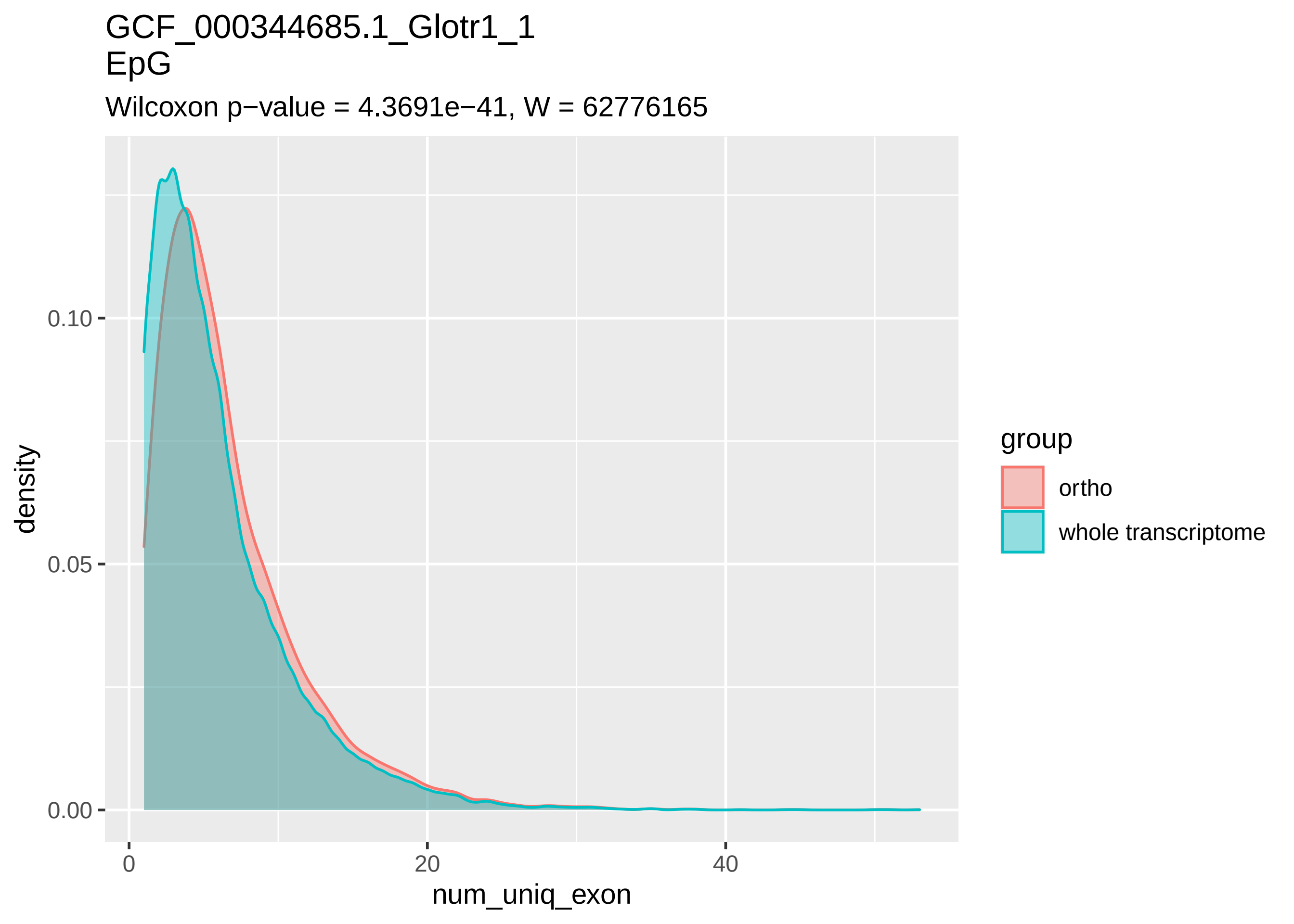
GCF_000344685.1_Glotr1_1
EpG
Wilcoxon p−value = 4.3691e−41, W = 62776165
density
0.10
0.05
0.00
0
20
40
num_uniq_exon
group
ortho
whole transcriptome

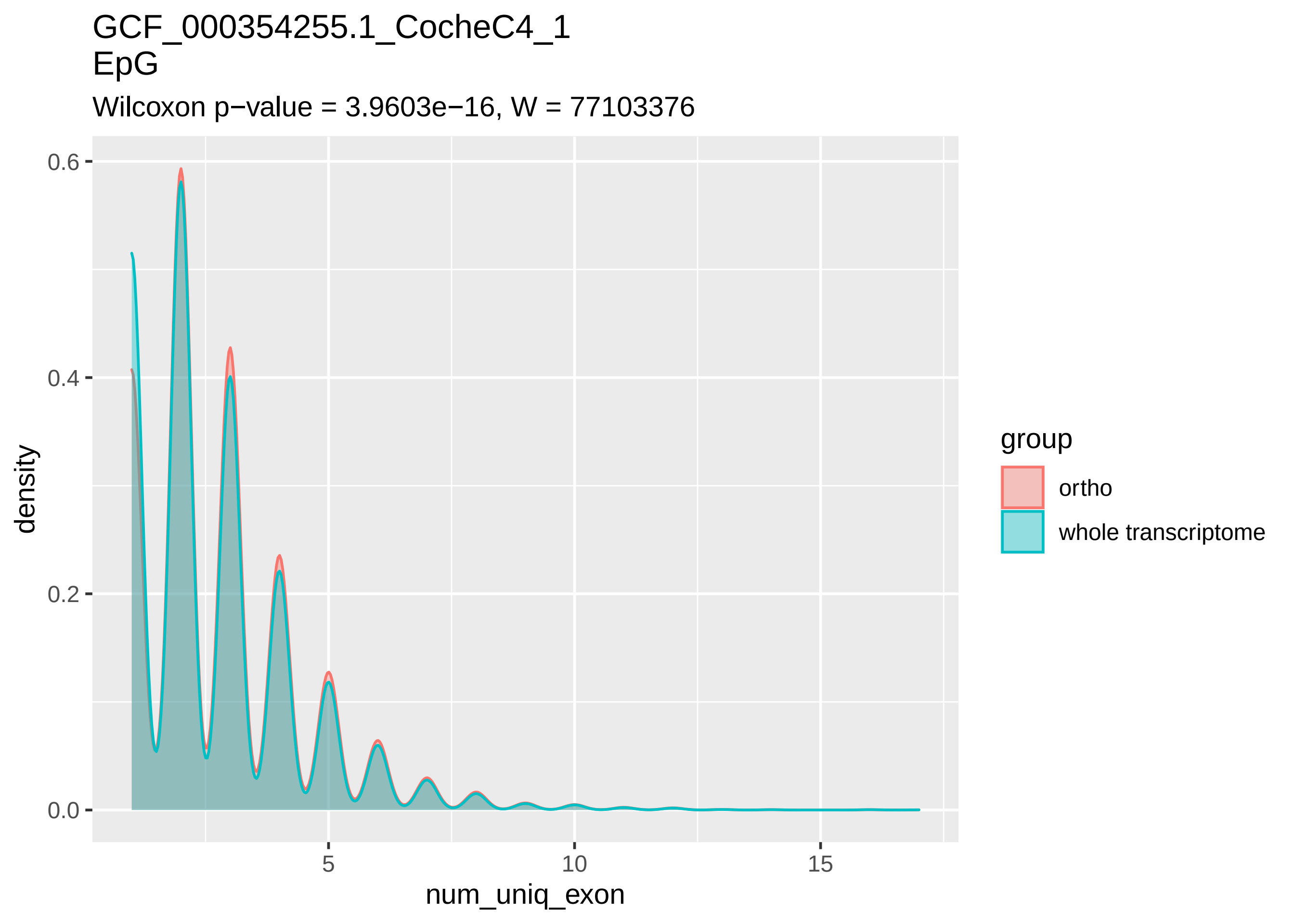
GCF_000354255.1_CocheC4_1
EpG
Wilcoxon p−value = 3.9603e−16, W = 77103376
0.6
0.4
0.2
0.0
density
5
10
15
num_uniq_exon
group
ortho
whole transcriptome

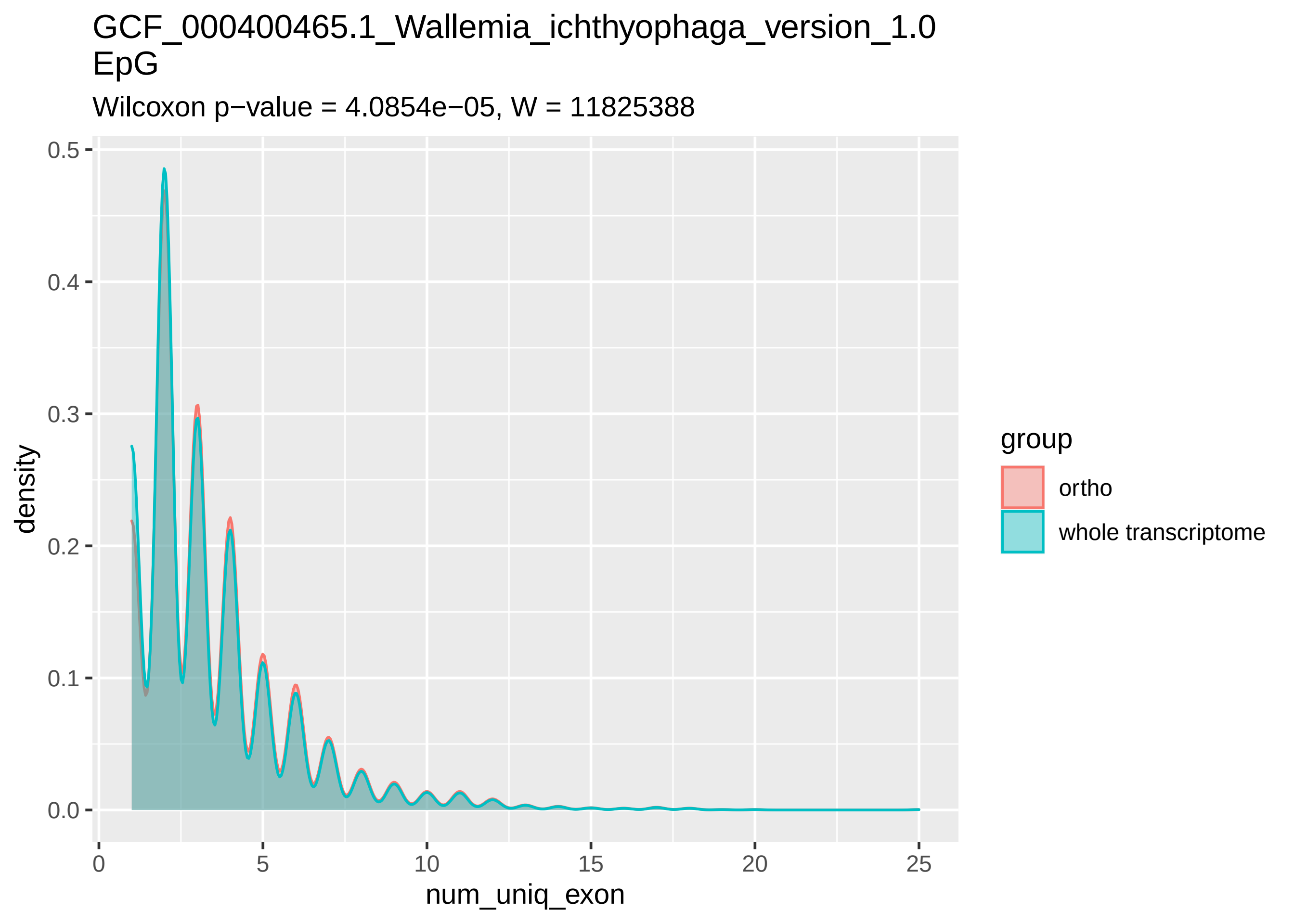
GCF_000400465.1_Wallemia_ichthyophaga_version_1.0
EpG
Wilcoxon p−value = 4.0854e−05, W = 11825388
density
0.0
0.1
0.2
0.3
0.4
0.5
0
5
10
15
20
25
num_uniq_exon
group
ortho
whole transcriptome

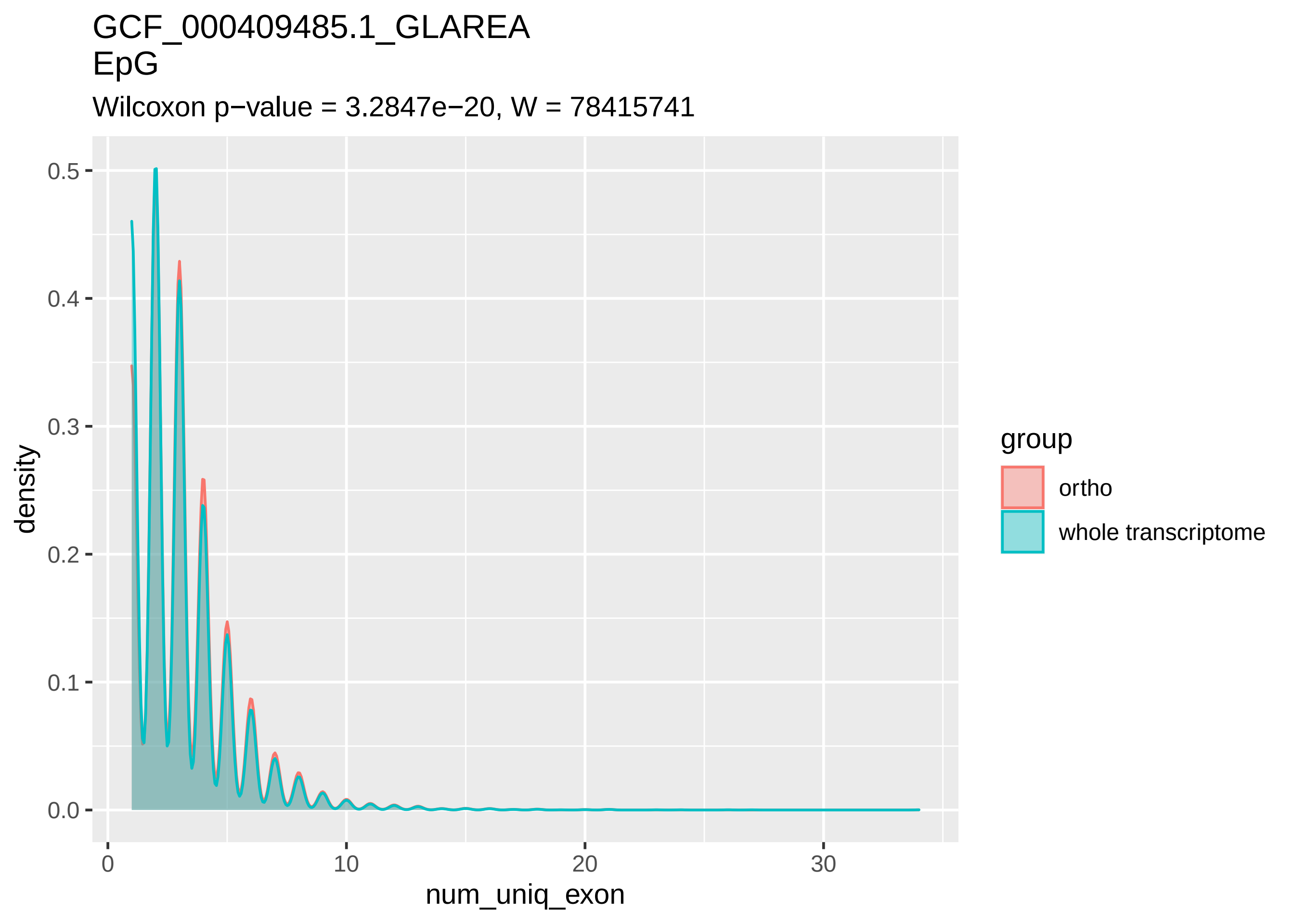
GCF_000409485.1_GLAREA
EpG
Wilcoxon p−value = 3.2847e−20, W = 78415741
density
0.5
0.4
0.3
0.2
0.1
0.0
0
10
20
30
num_uniq_exon
group
ortho
whole transcriptome

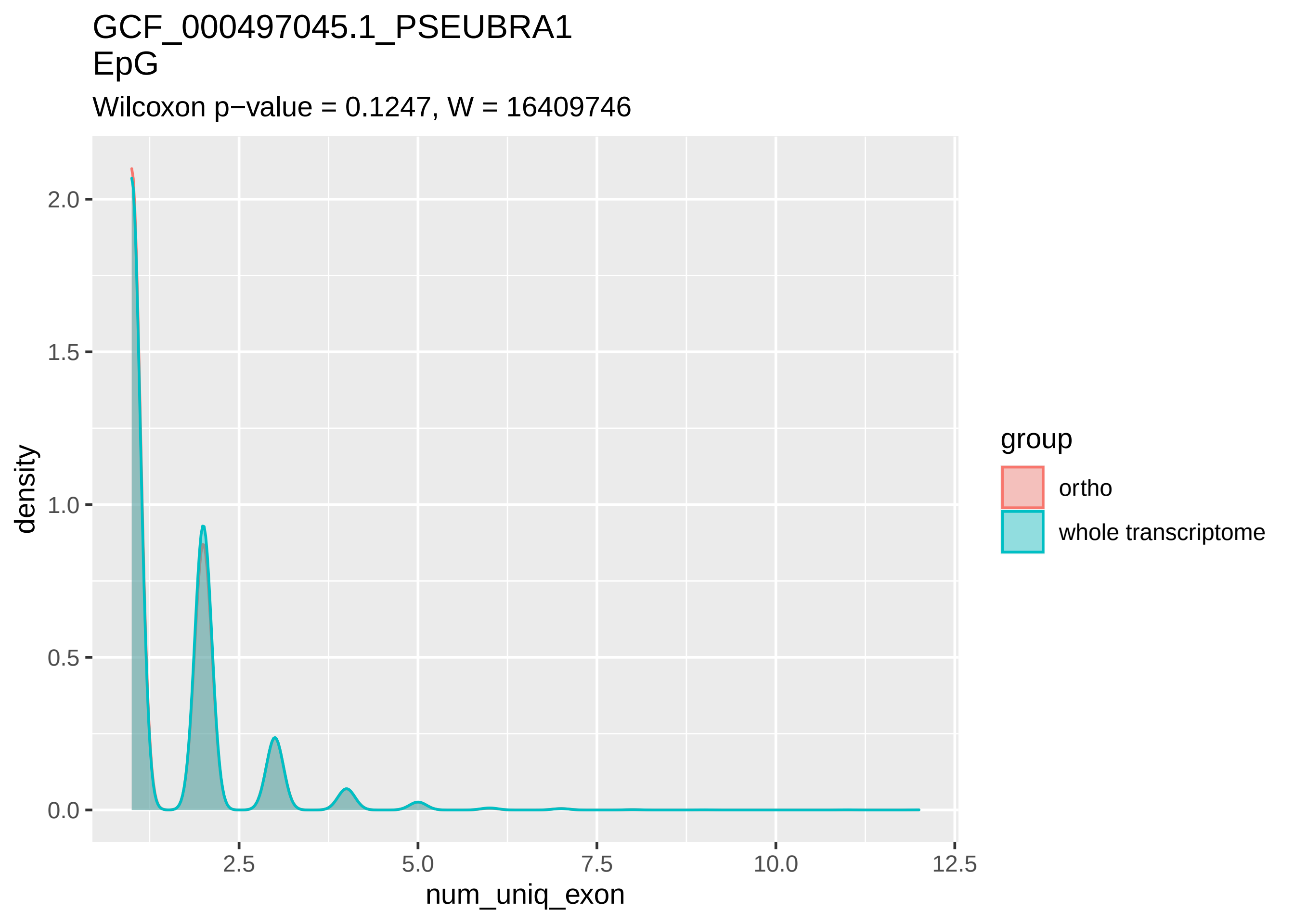

GCF_000497045.1_PSEUBRA1
EpG
Wilcoxon p−value = 0.1247, W = 16409746
density
2.0
1.5
1.0
0.5
0.0
2.5
5.0
7.5
10.0
12.5
num_uniq_exon
group
ortho
whole transcriptome

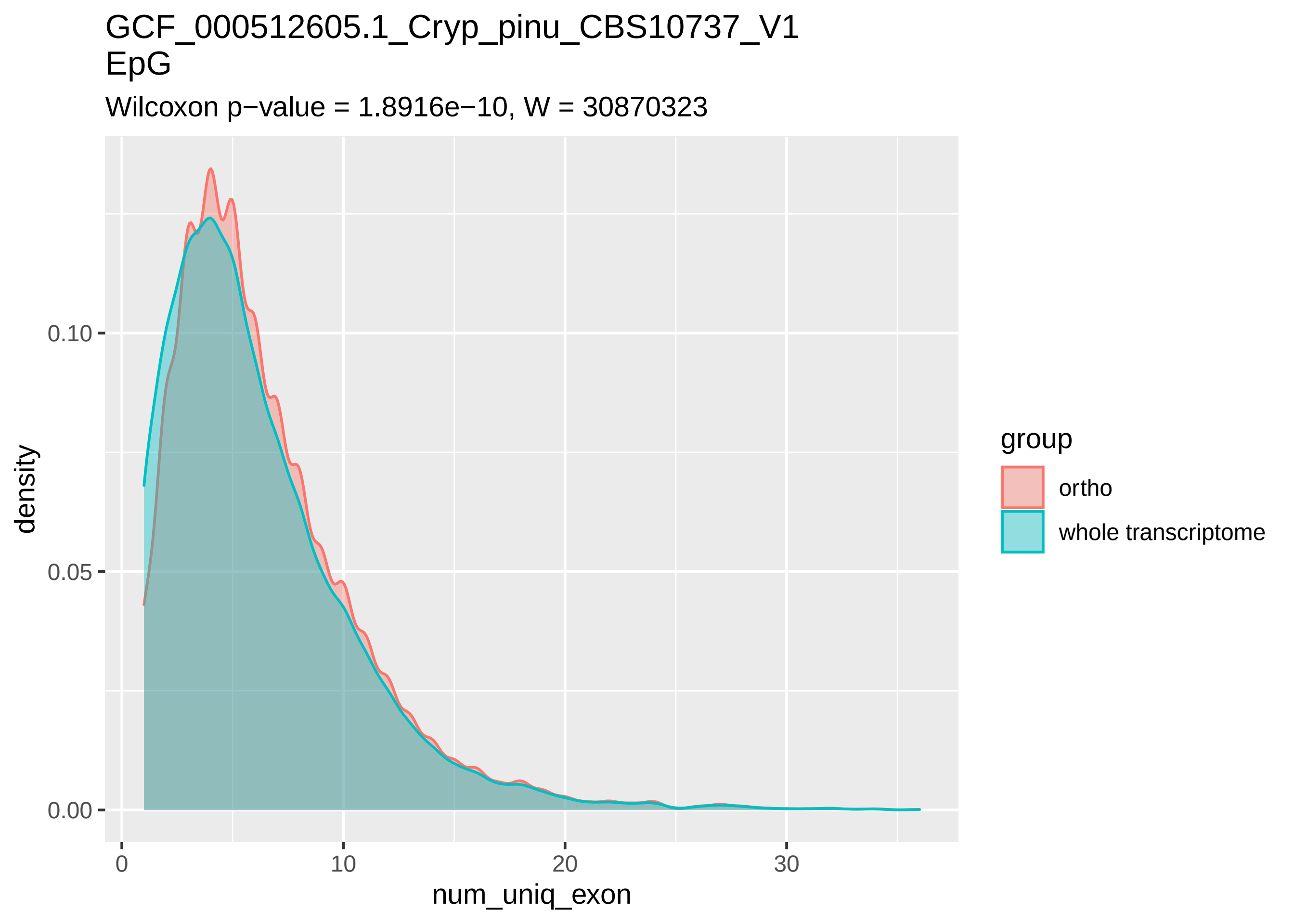
GCF_000512605.1_Cryp_pinu_CBS10737_V1
EpG
Wilcoxon p−value = 1.8916e−10, W = 30870323
density
0.10
0.05
0.00
0
10
20
30
num_uniq_exon
group
ortho
whole transcriptome

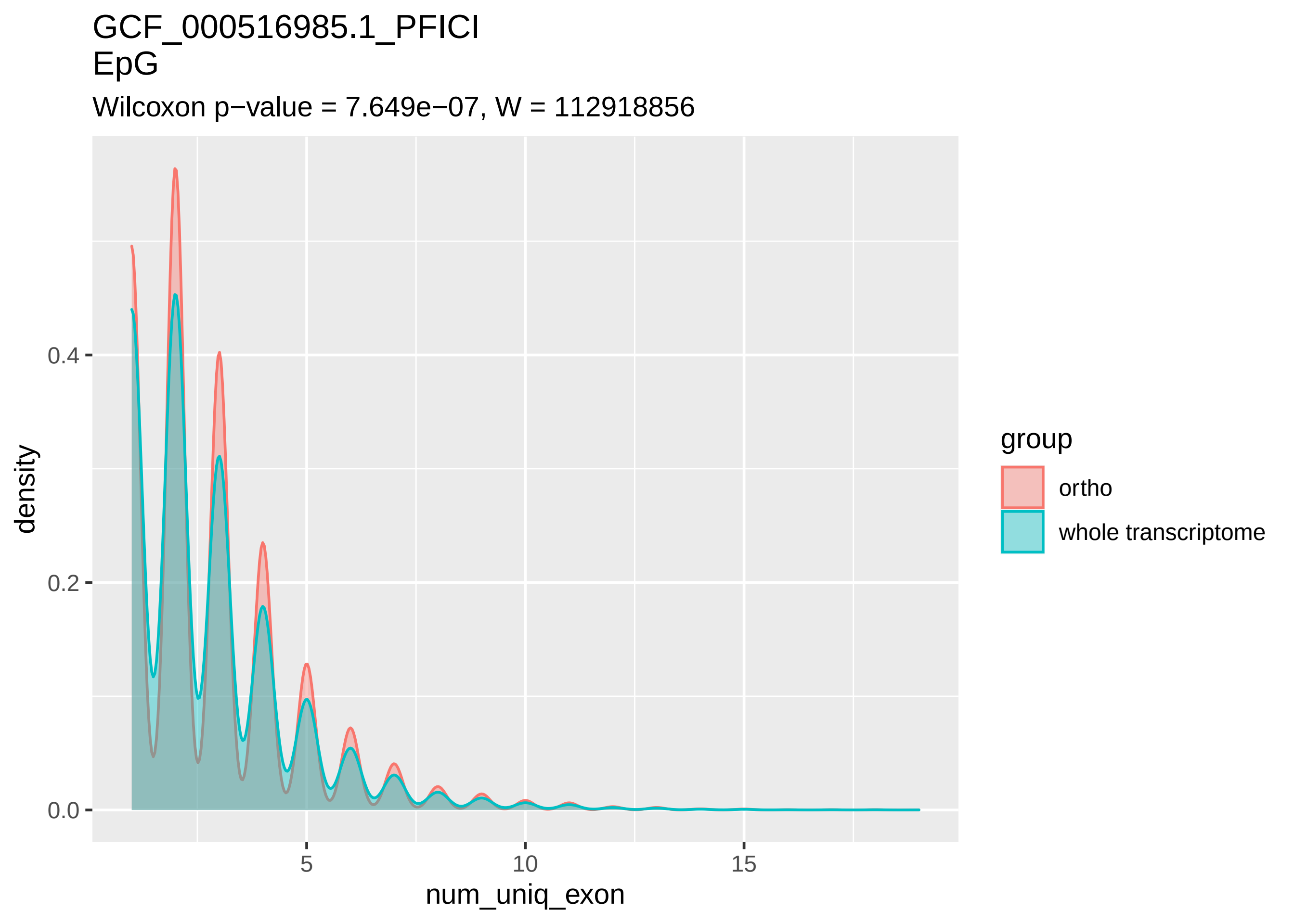

GCF_000516985.1_PFICI
EpG
Wilcoxon p−value = 7.649e−07, W = 112918856
density
0.0
0.2
0.4
5
10
15
num_uniq_exon
group
ortho
whole transcriptome

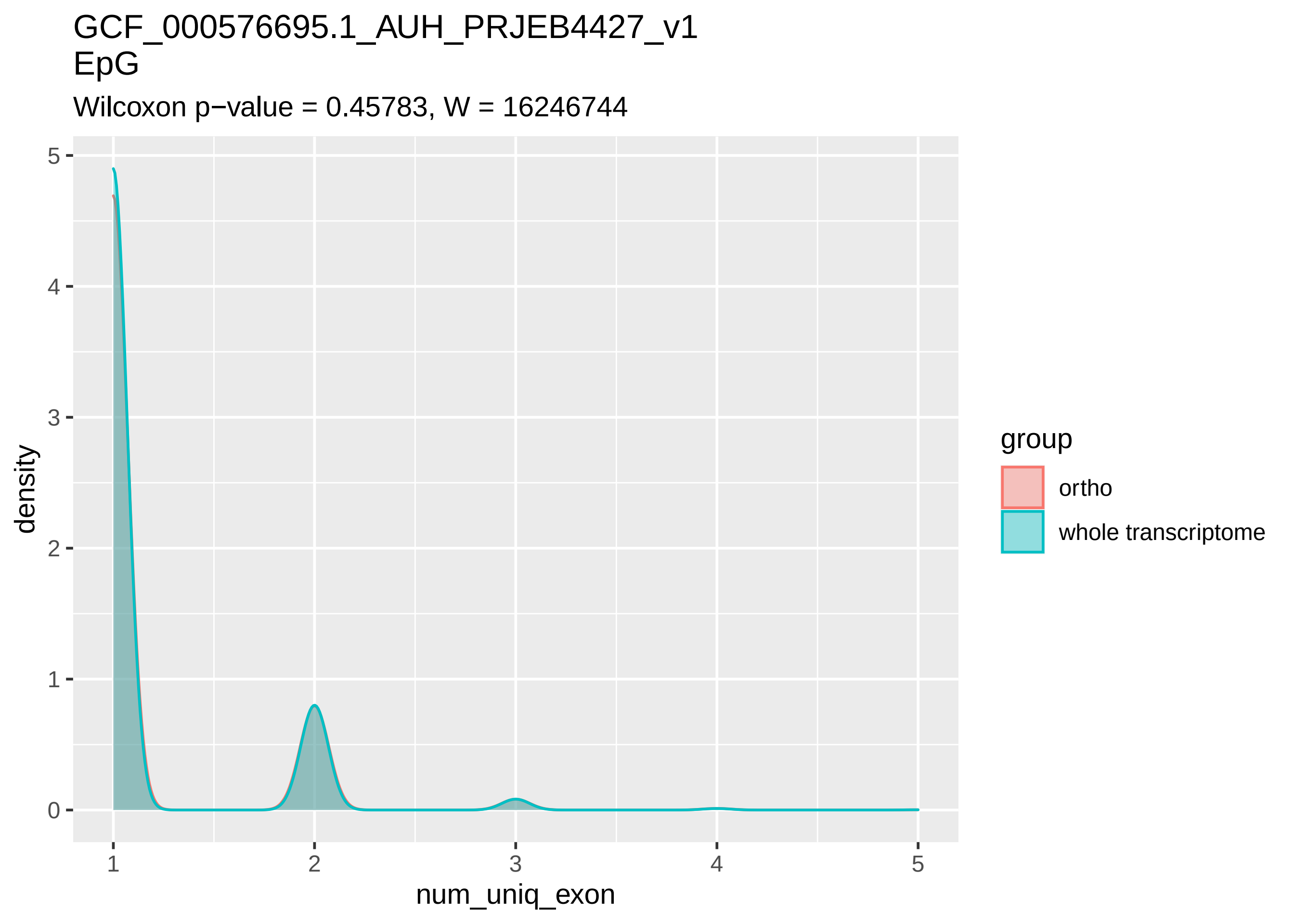
GCF_000576695.1_AUH_PRJEB4427_v1
EpG
Wilcoxon p−value = 0.45783, W = 16246744
density
num_uniq_exon
0
1
2
3
4
5
1
2
3
4
5
group
ortho
whole transcriptome

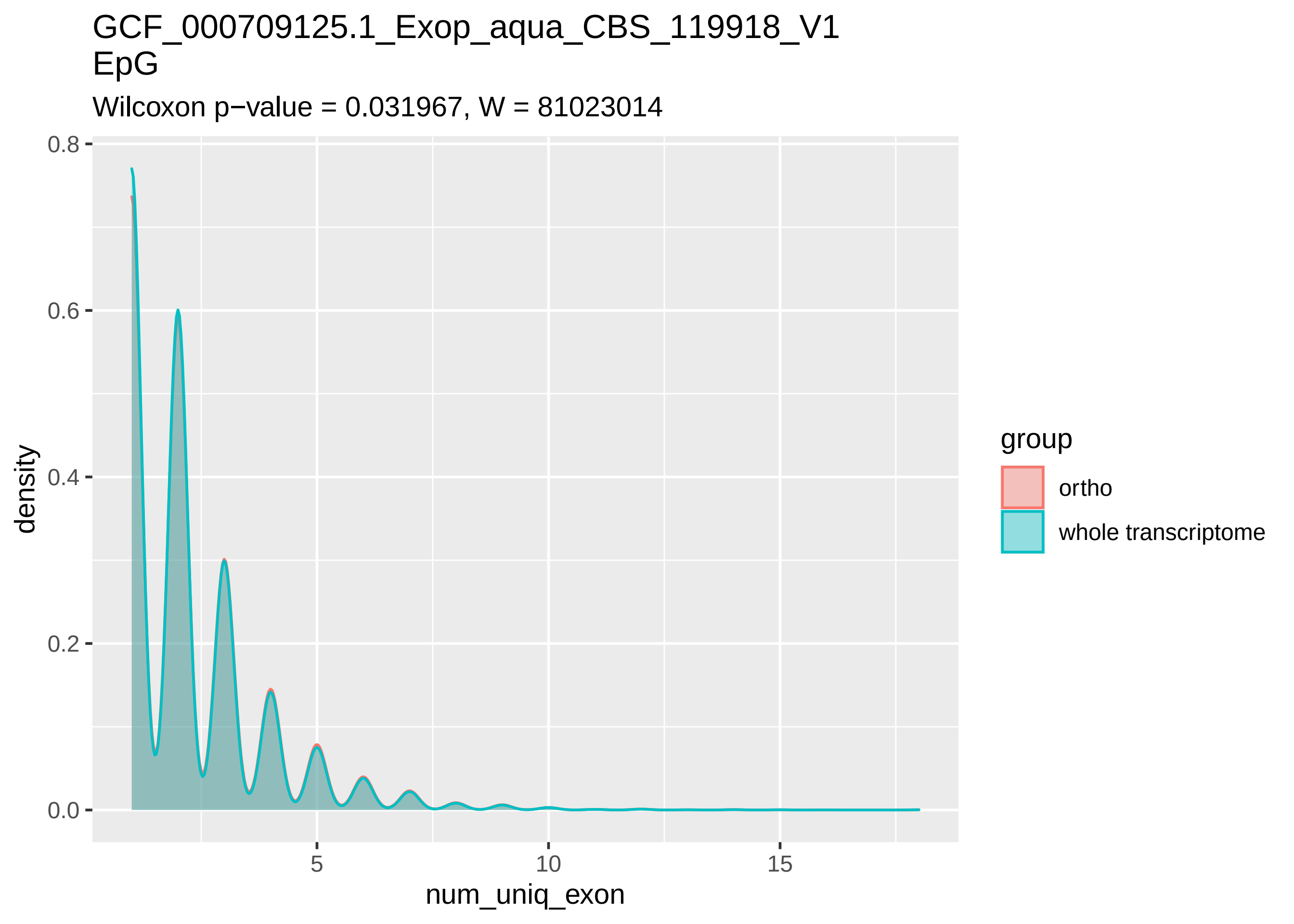

GCF_000709125.1_Exop_aqua_CBS_119918_V1
EpG
Wilcoxon p−value = 0.031967, W = 81023014
density
0.0
0.2
0.4
0.6
0.8
5
10
15
num_uniq_exon
group
ortho
whole transcriptome

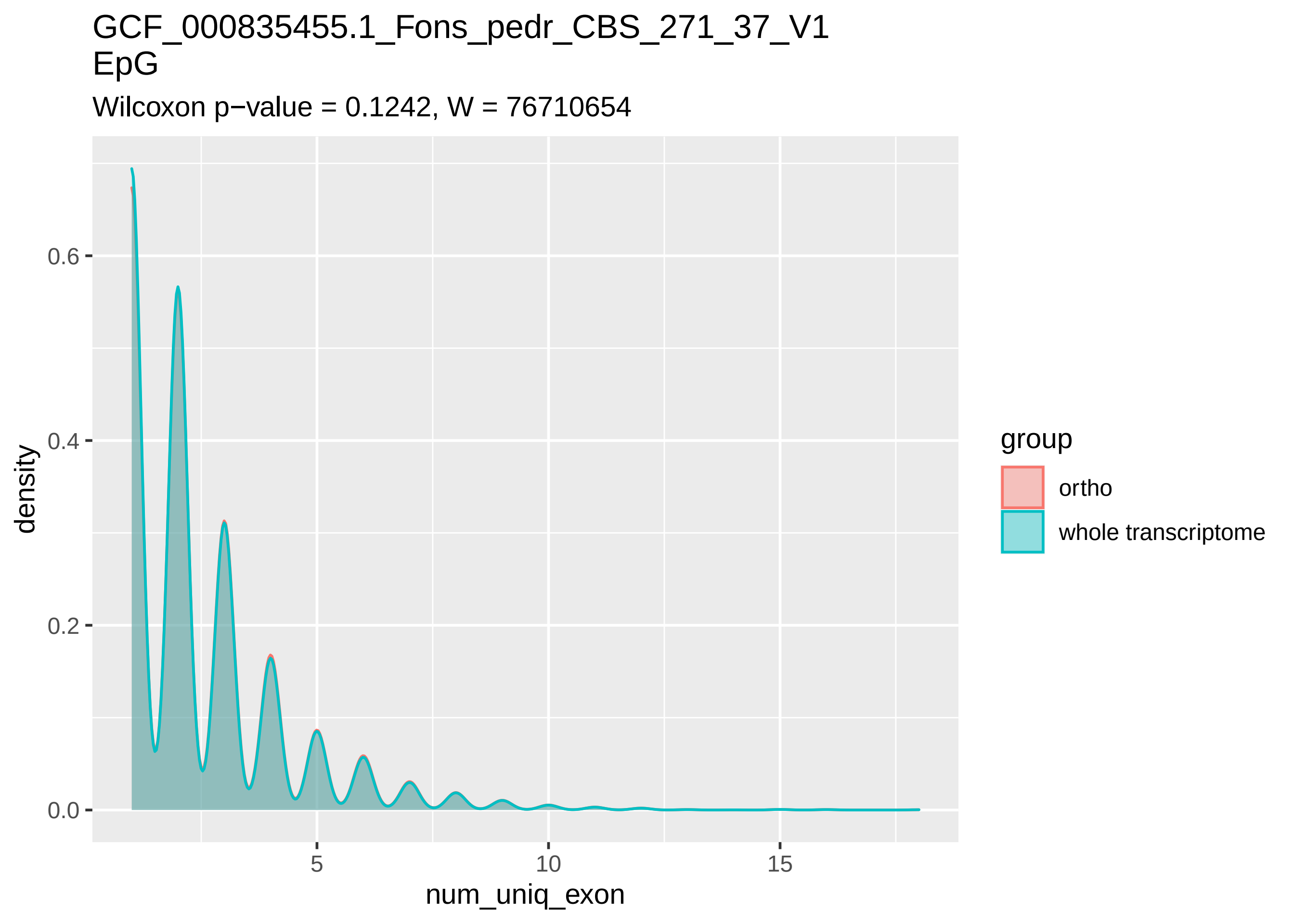
GCF_000835455.1_Fons_pedr_CBS_271_37_V1
EpG
Wilcoxon p−value = 0.1242, W = 76710654
density
0.0
0.2
0.4
0.6
5
10
15
num_uniq_exon
group
ortho
whole transcriptome

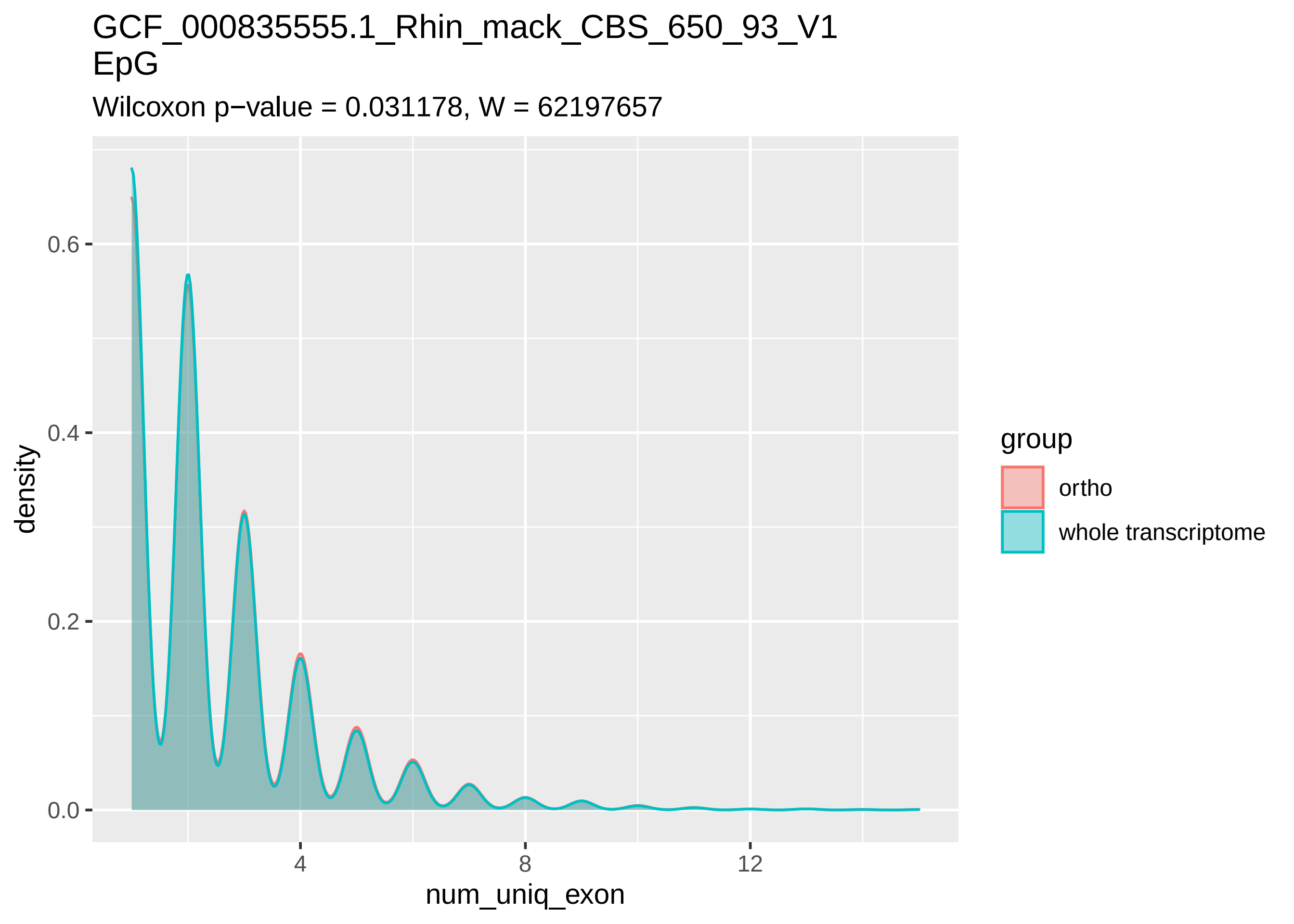

GCF_000835555.1_Rhin_mack_CBS_650_93_V1
EpG
Wilcoxon p−value = 0.031178, W = 62197657
density
0.0
0.2
0.4
0.6
4
8
12
num_uniq_exon
group
ortho
whole transcriptome

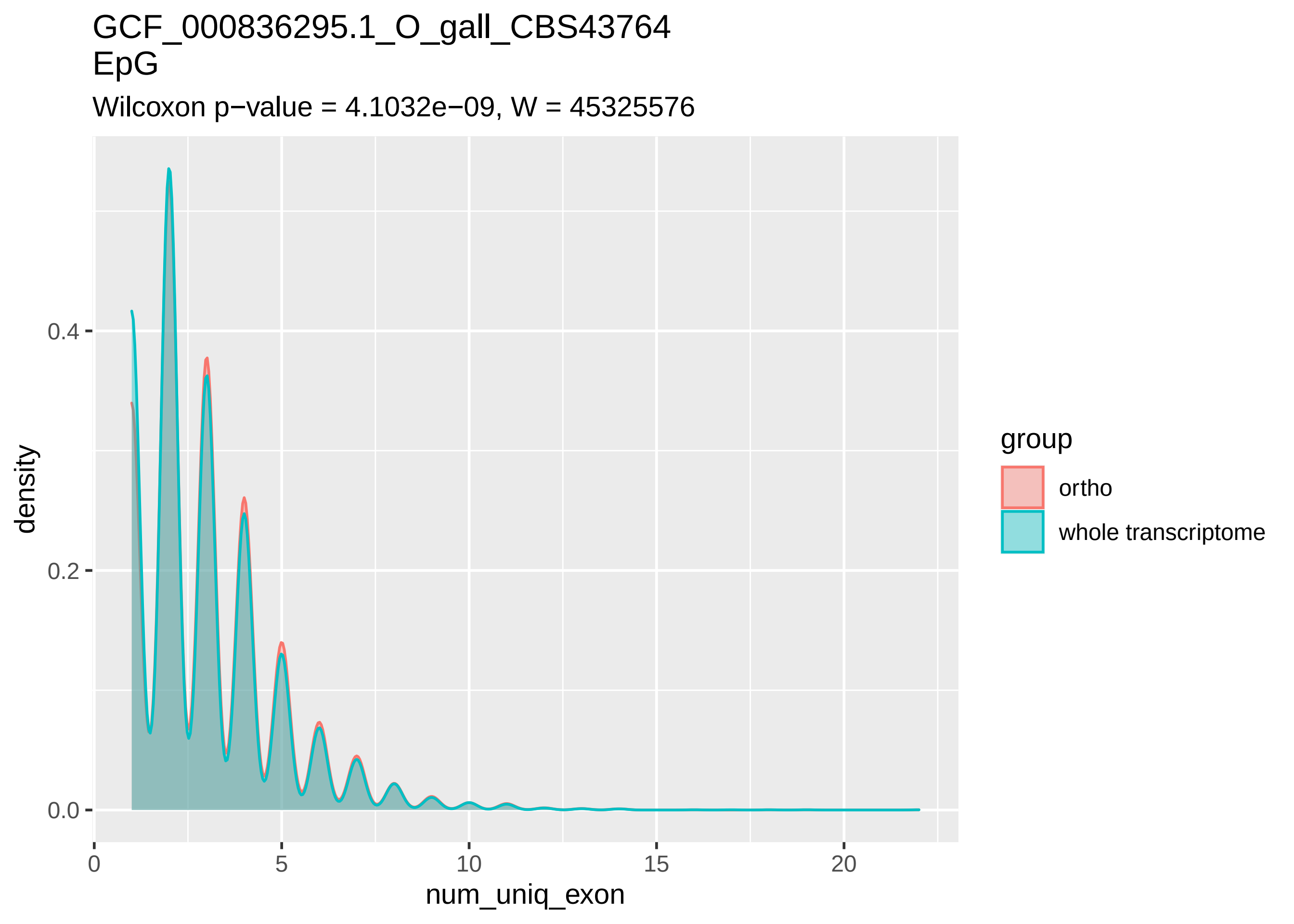
GCF_000836295.1_O_gall_CBS43764
EpG
Wilcoxon p−value = 4.1032e−09, W = 45325576
density
0.0
0.2
0.4
0
5
10
15
20
num_uniq_exon
group
ortho
whole transcriptome

GCF_000938715.1_LALA0
EpG

Wilcoxon p−value = 0.016754, W = 13177698

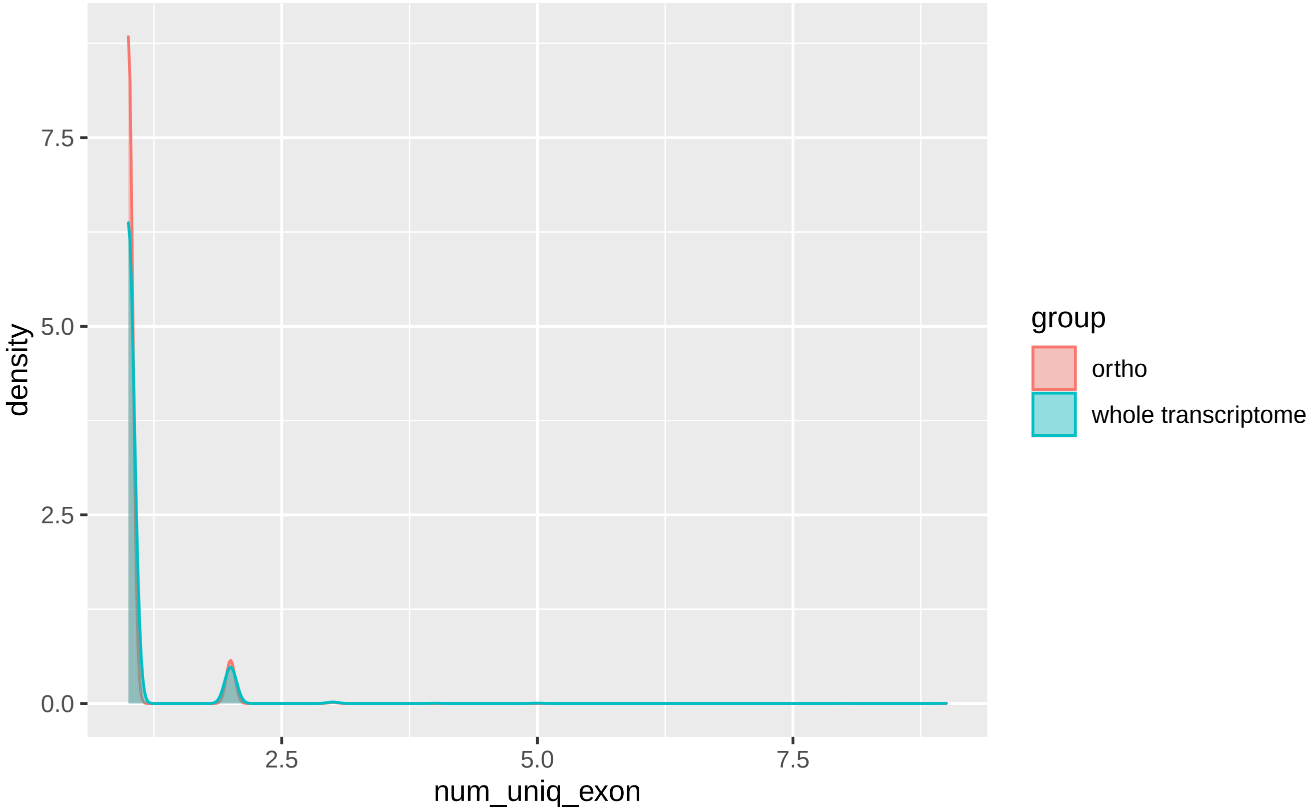

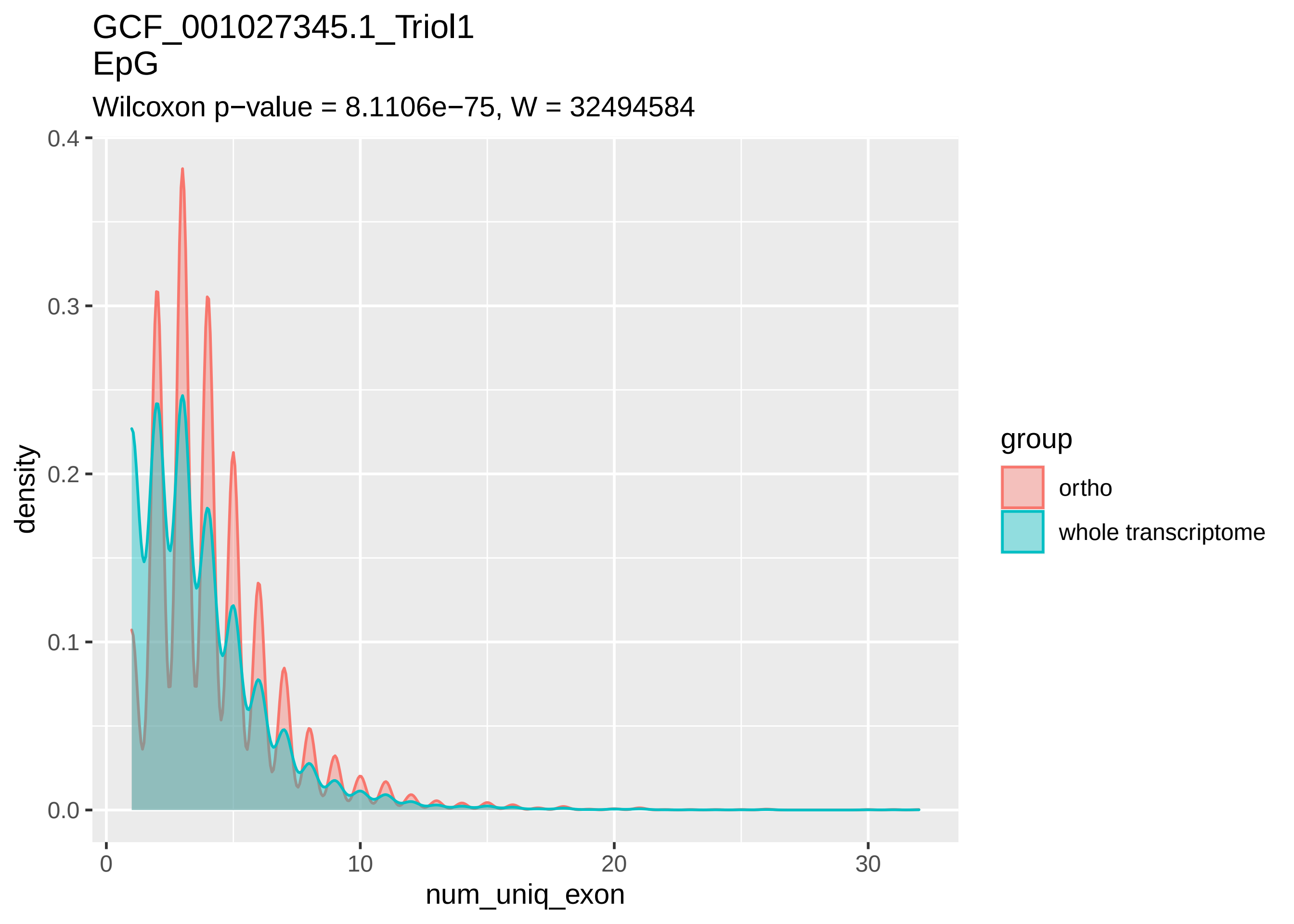

GCF_001027345.1_Triol1
EpG
Wilcoxon p−value = 8.1106e−75, W = 32494584
density
0.0
0.1
0.2
0.3
0.4
0
10
20
30
num_uniq_exon
group
ortho
whole transcriptome

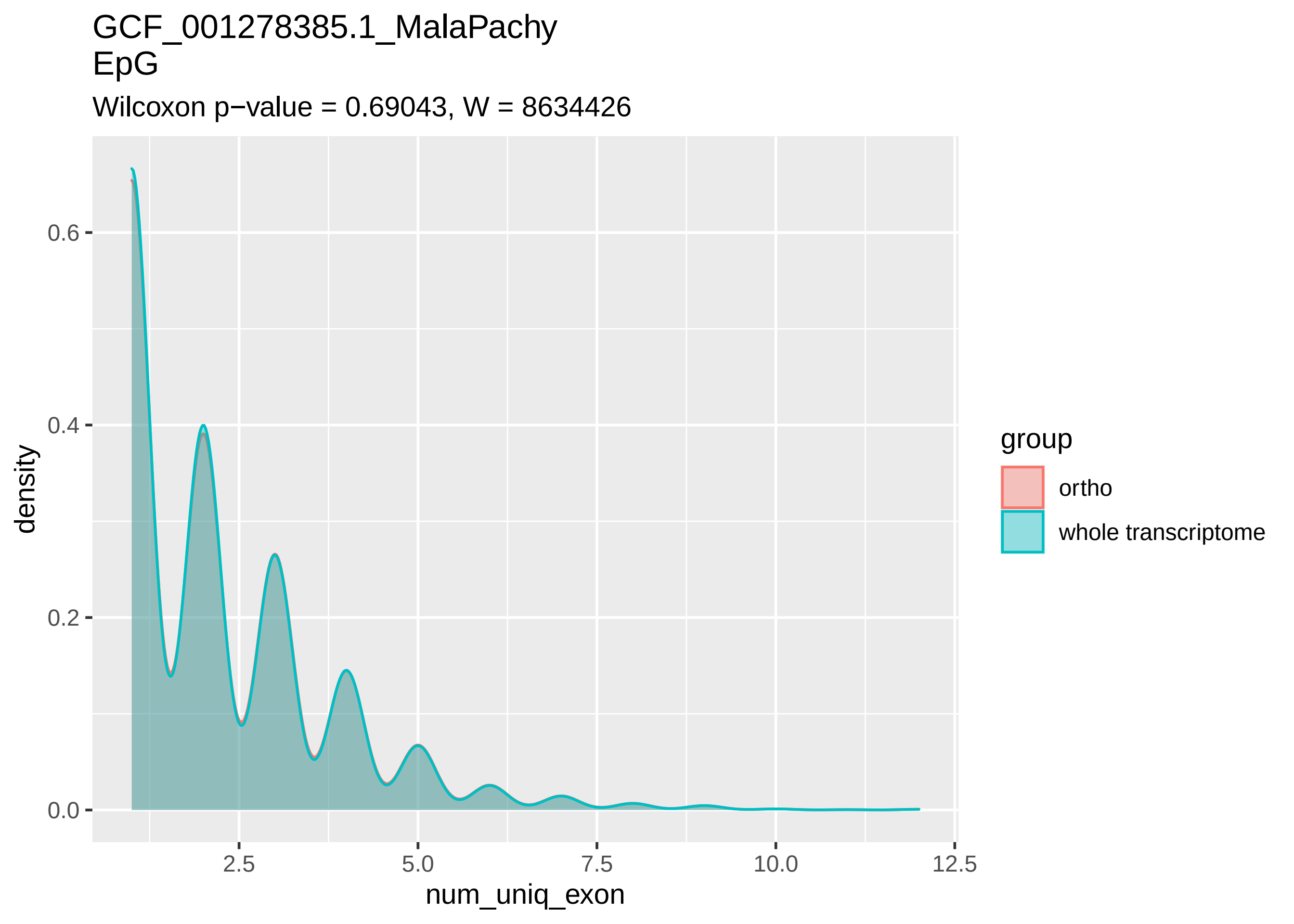
GCF_001278385.1_MalaPachy
EpG
Wilcoxon p−value = 0.69043, W = 8634426
0.6
0.4
0.2
0.0
density
2.5
5.0
7.5
10.0
12.5
num_uniq_exon
group
ortho
whole transcriptome

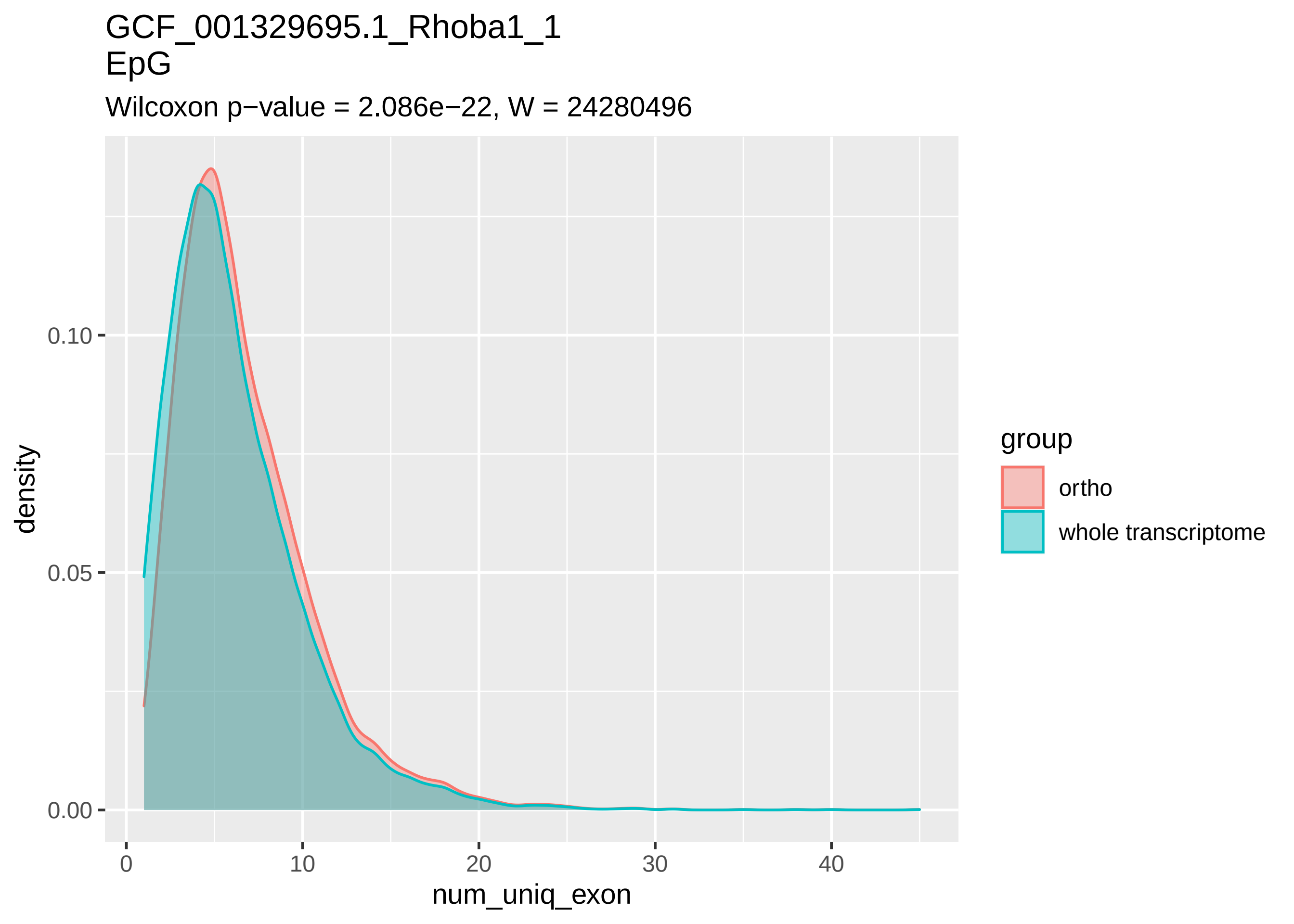

GCF_001329695.1_Rhoba1_1
EpG
Wilcoxon p−value = 2.086e−22, W = 24280496
density
0.00
0.05
0.10
0
10
20
30
40
num_uniq_exon
group
ortho
whole transcriptome

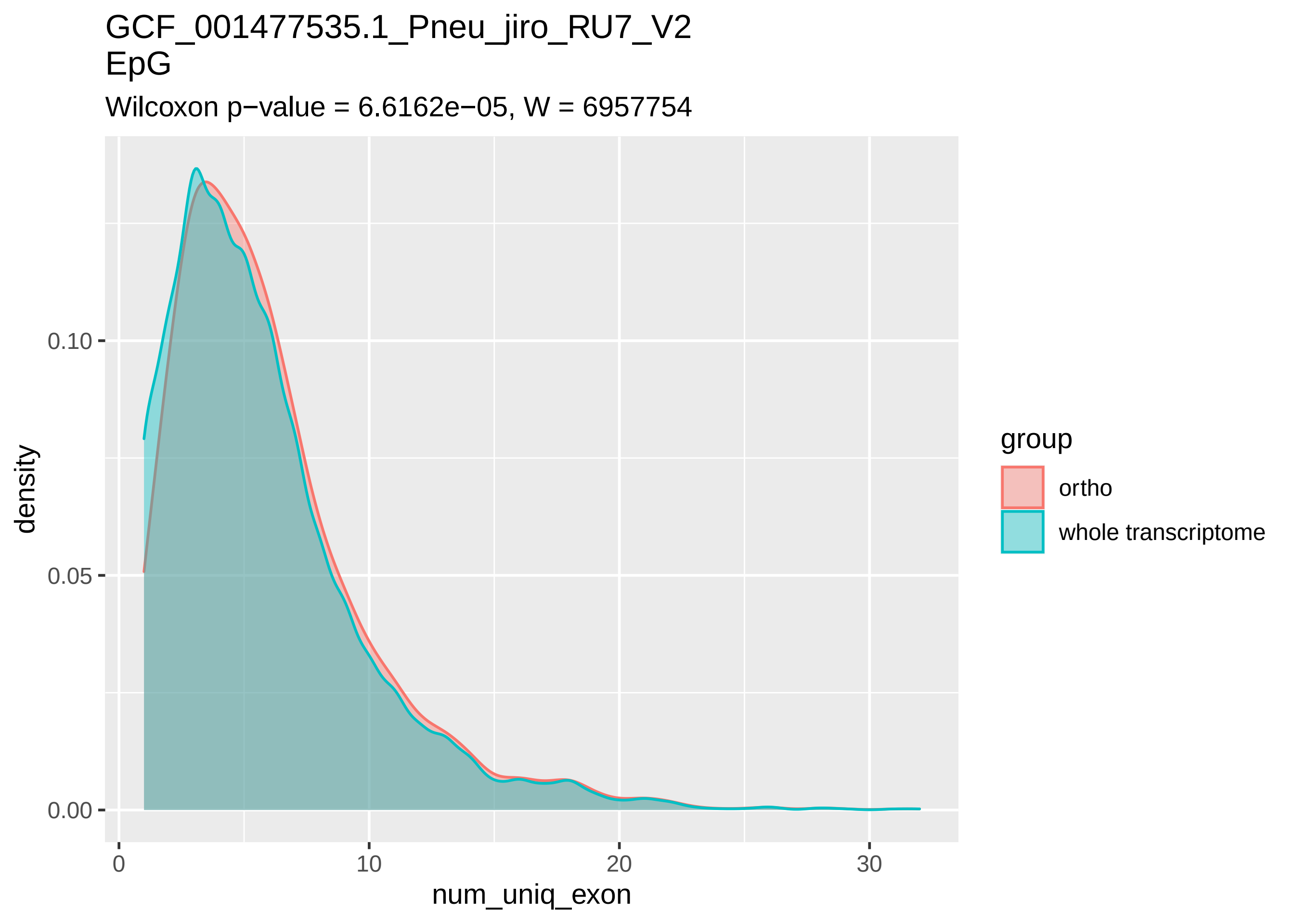
GCF_001477535.1_Pneu_jiro_RU7_V2
EpG
Wilcoxon p−value = 6.6162e−05, W = 6957754
density
0.10
0.05
0.00
0
10
20
30
num_uniq_exon
group
ortho
whole transcriptome

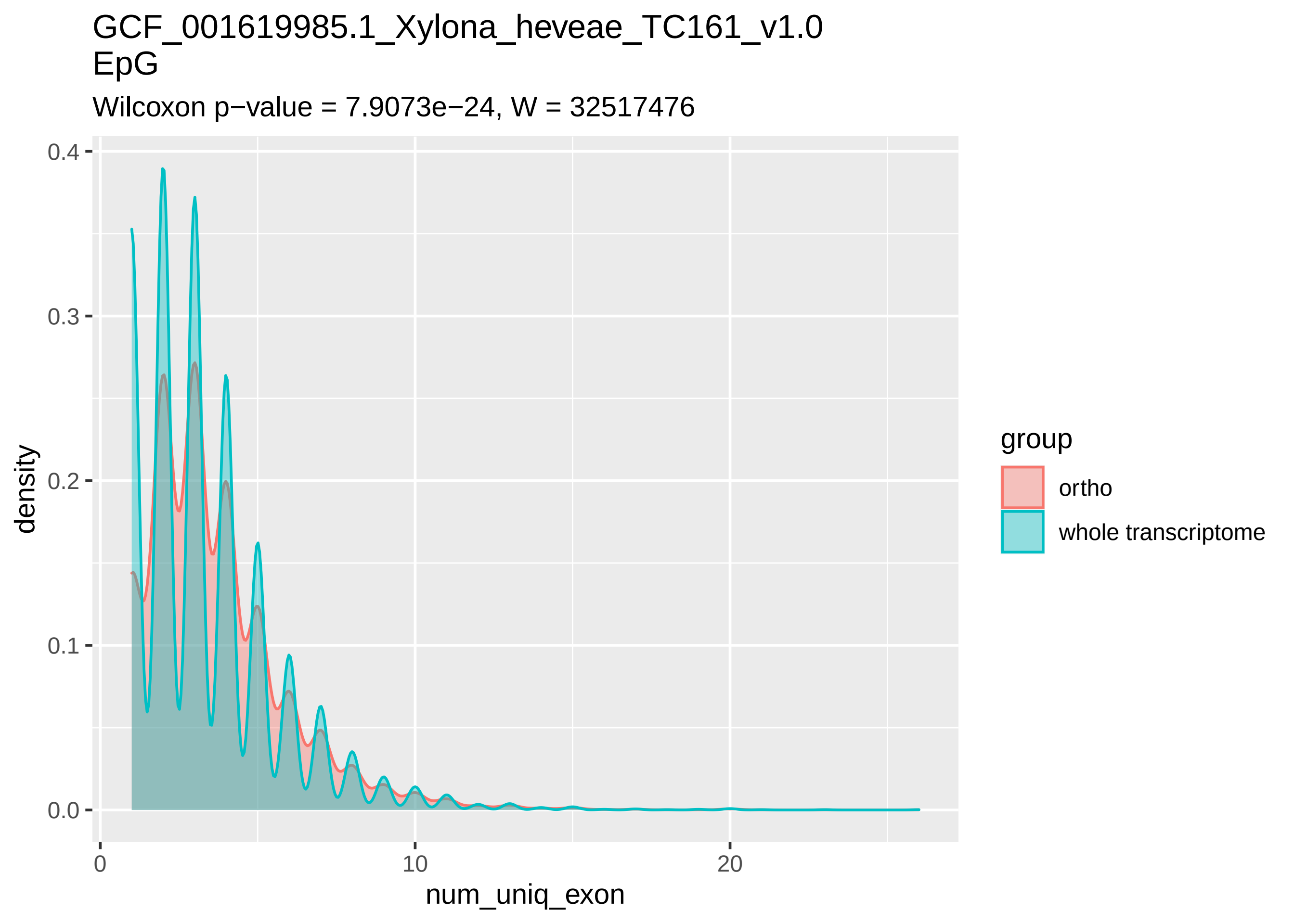
GCF_001619985.1_Xylona_heveae_TC161_v1.0
EpG
Wilcoxon p−value = 7.9073e−24, W = 32517476
density
0.0
0.1
0.2
0.3
0.4
0
10
20
num_uniq_exon
group
ortho
whole transcriptome

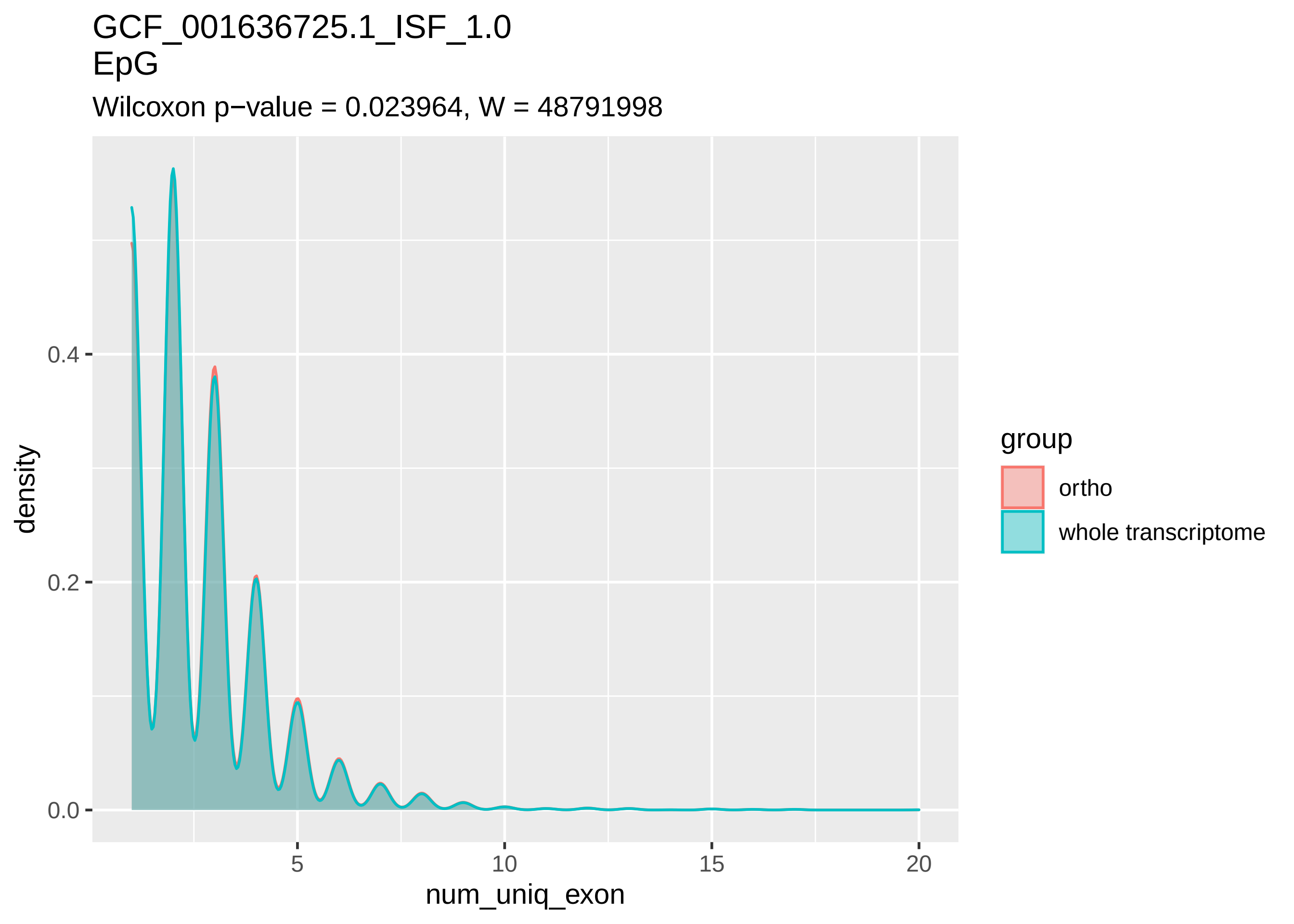
GCF_001636725.1_ISF_1.0
EpG
Wilcoxon p−value = 0.023964, W = 48791998
density
0.4
0.2
0.0
5
10
15
20
num_uniq_exon
group
ortho
whole transcriptome

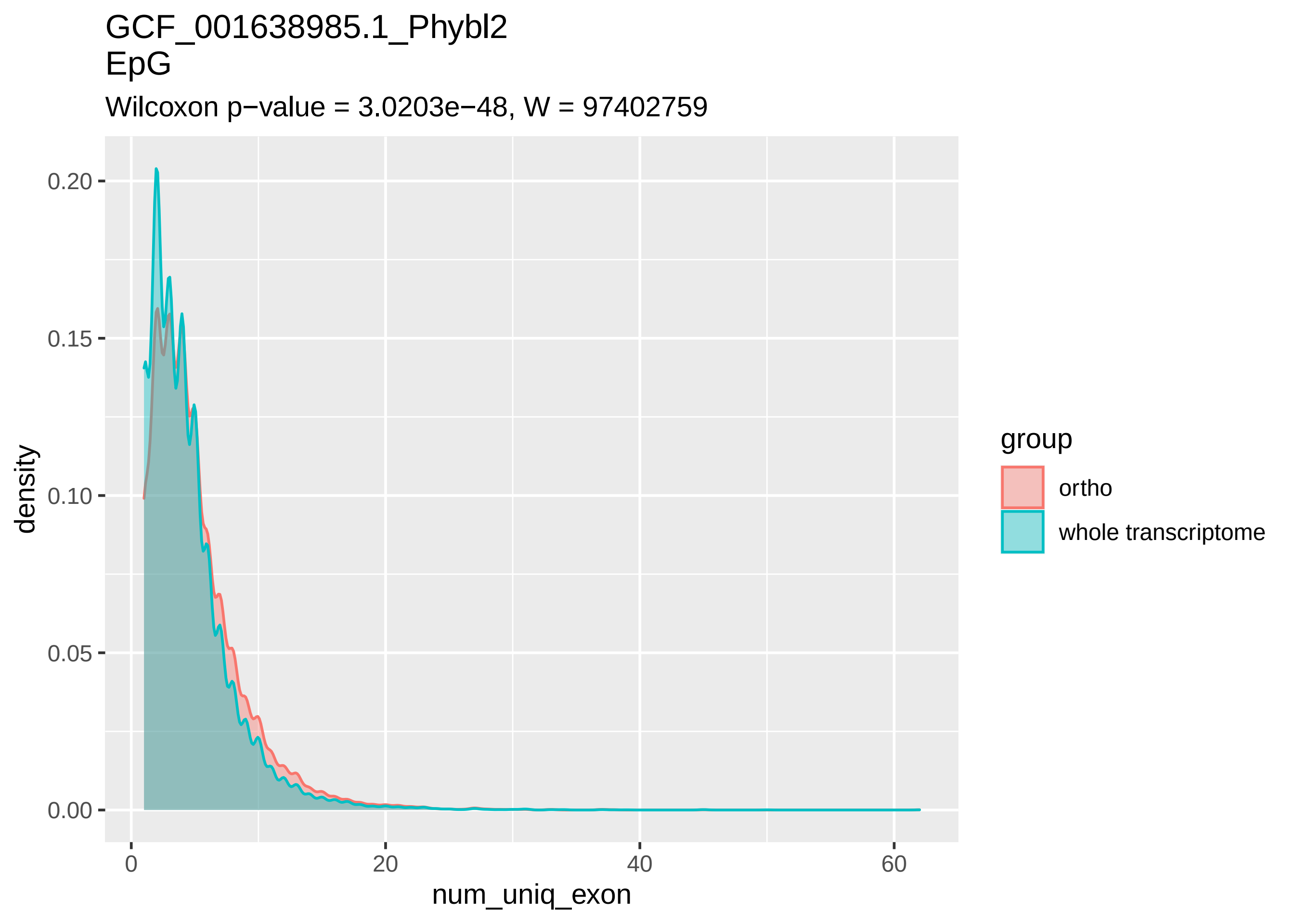
GCF_001638985.1_Phybl2
EpG
Wilcoxon p−value = 3.0203e−48, W = 97402759
density
0.20
0.15
0.10
0.05
0.00
0
20
40
60
num_uniq_exon
group
ortho
whole transcriptome

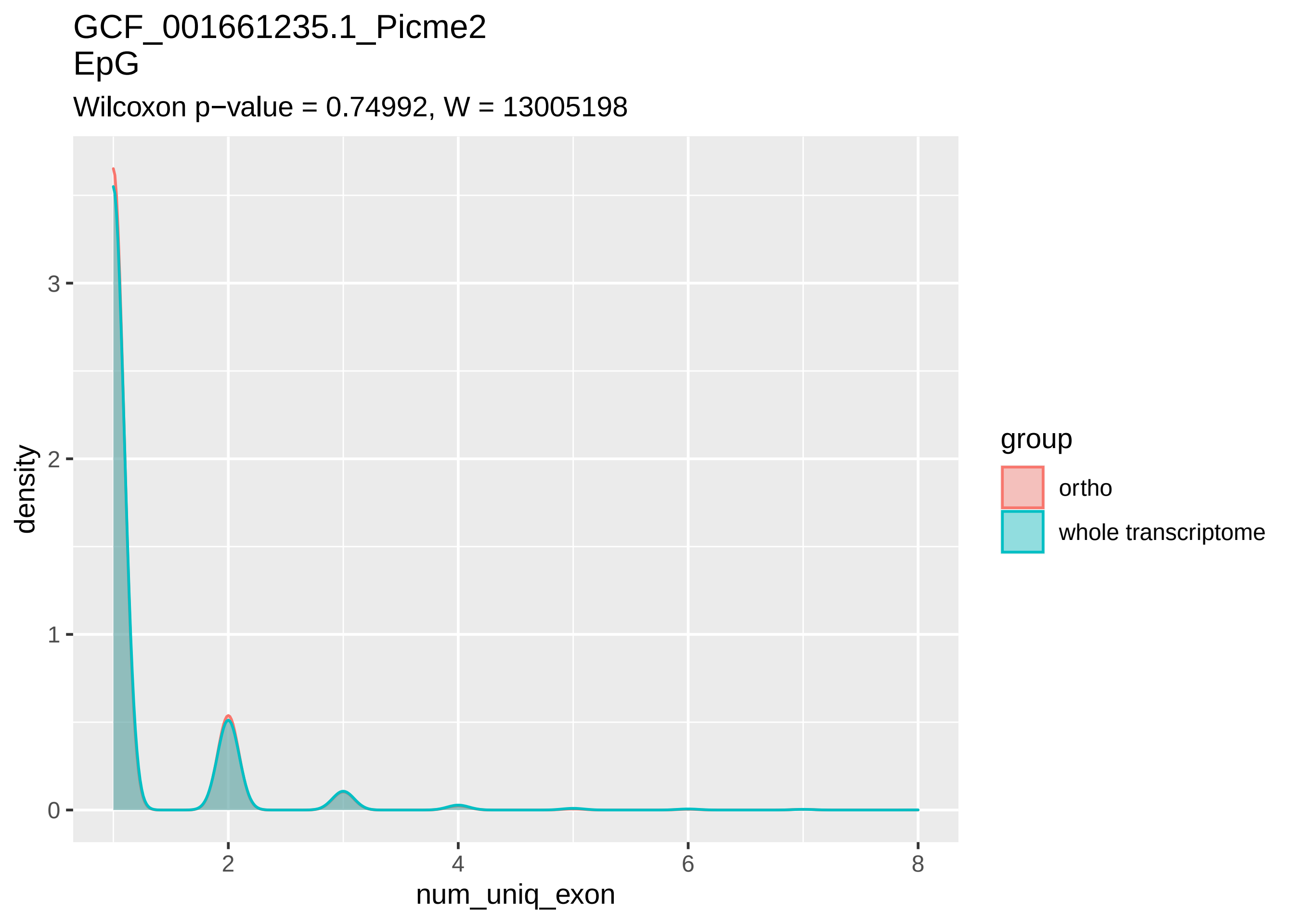
GCF_001661235.1_Picme2
EpG
Wilcoxon p−value = 0.74992, W = 13005198
density
num_uniq_exon
0
1
2
3
2
4
6
8
group
ortho
whole transcriptome

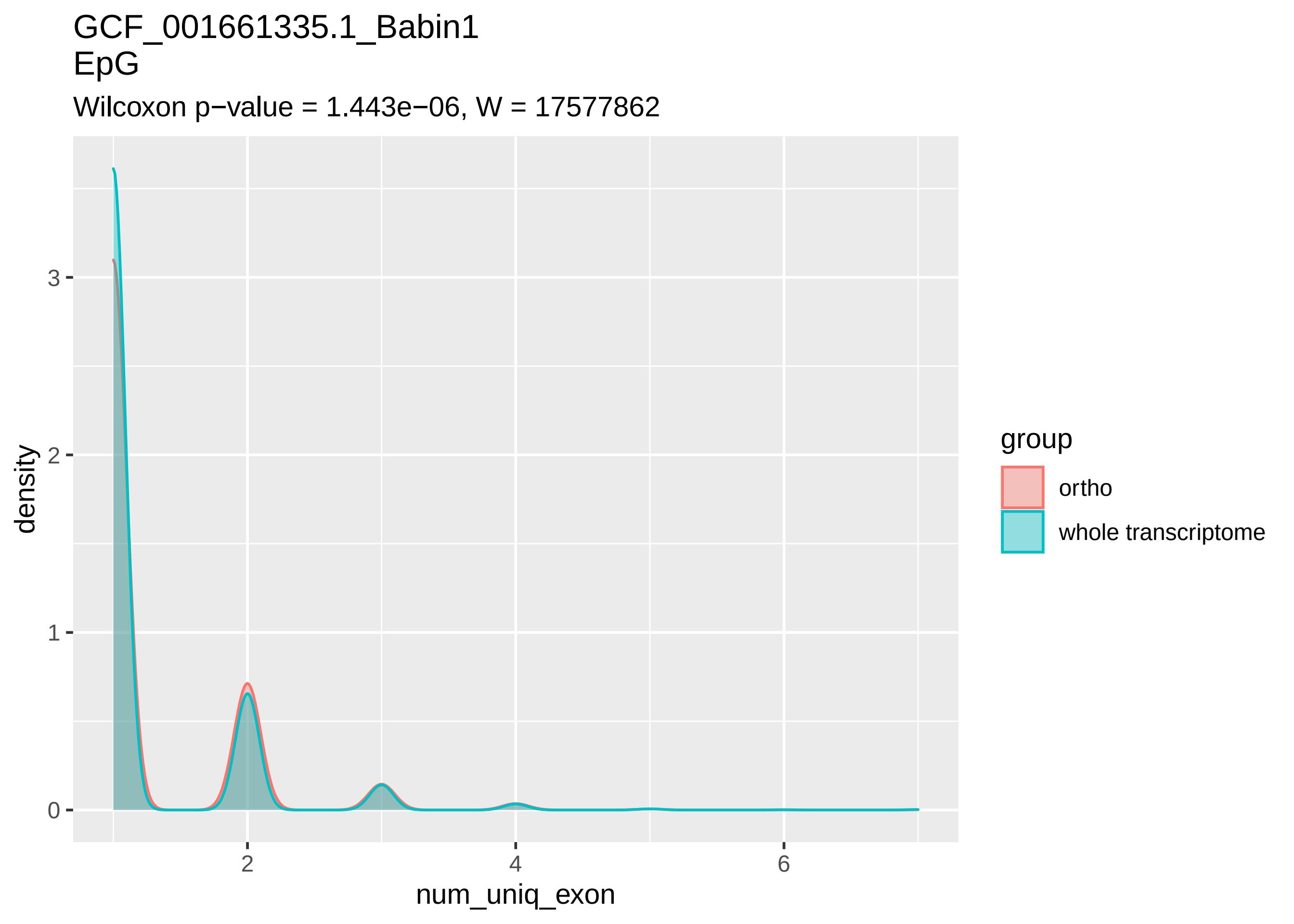

GCF_001661335.1_Babin1
EpG
Wilcoxon p−value = 1.443e−06, W = 17577862
density
0
1
2
3
2
4
6
num_uniq_exon
group
ortho
whole transcriptome

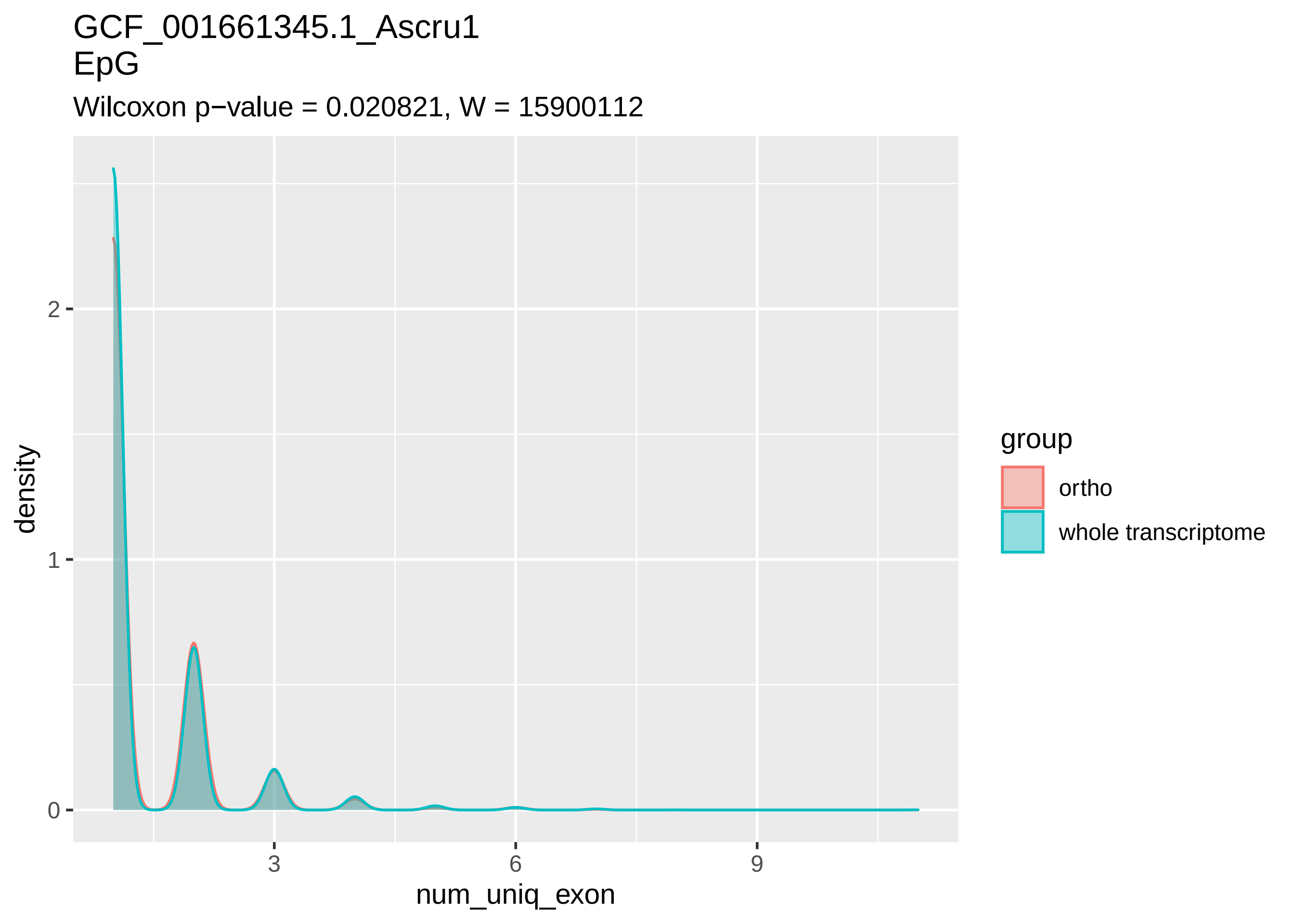

GCF_001661345.1_Ascru1
EpG
Wilcoxon p−value = 0.020821, W = 15900112
density
2
1
0
3
6
9
num_uniq_exon
group
ortho
whole transcriptome

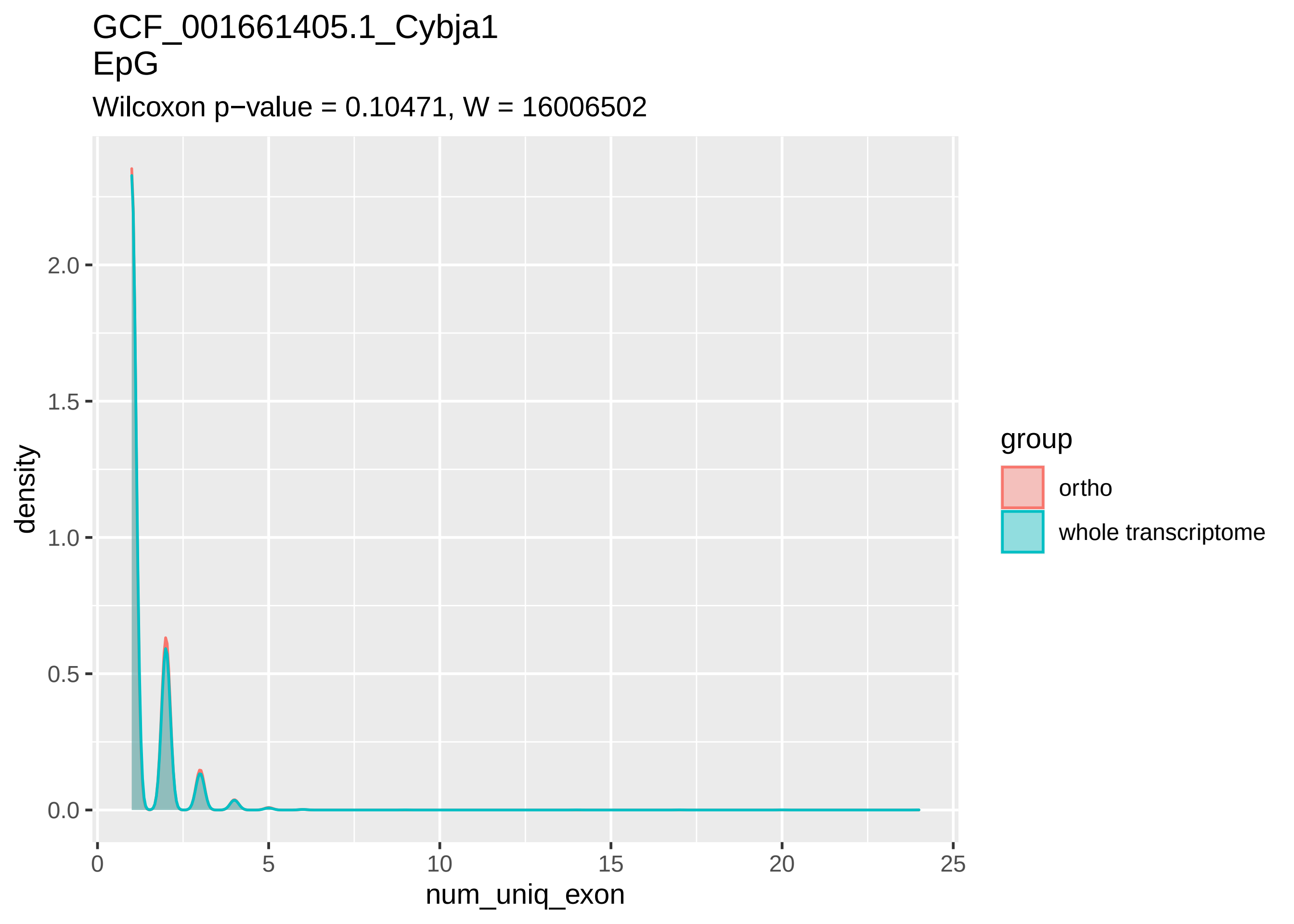
GCF_001661405.1_Cybja1
EpG
Wilcoxon p−value = 0.10471, W = 16006502
density
2.0
1.5
1.0
0.5
0.0
0
5
10
15
20
25
num_uniq_exon
group
ortho
whole transcriptome

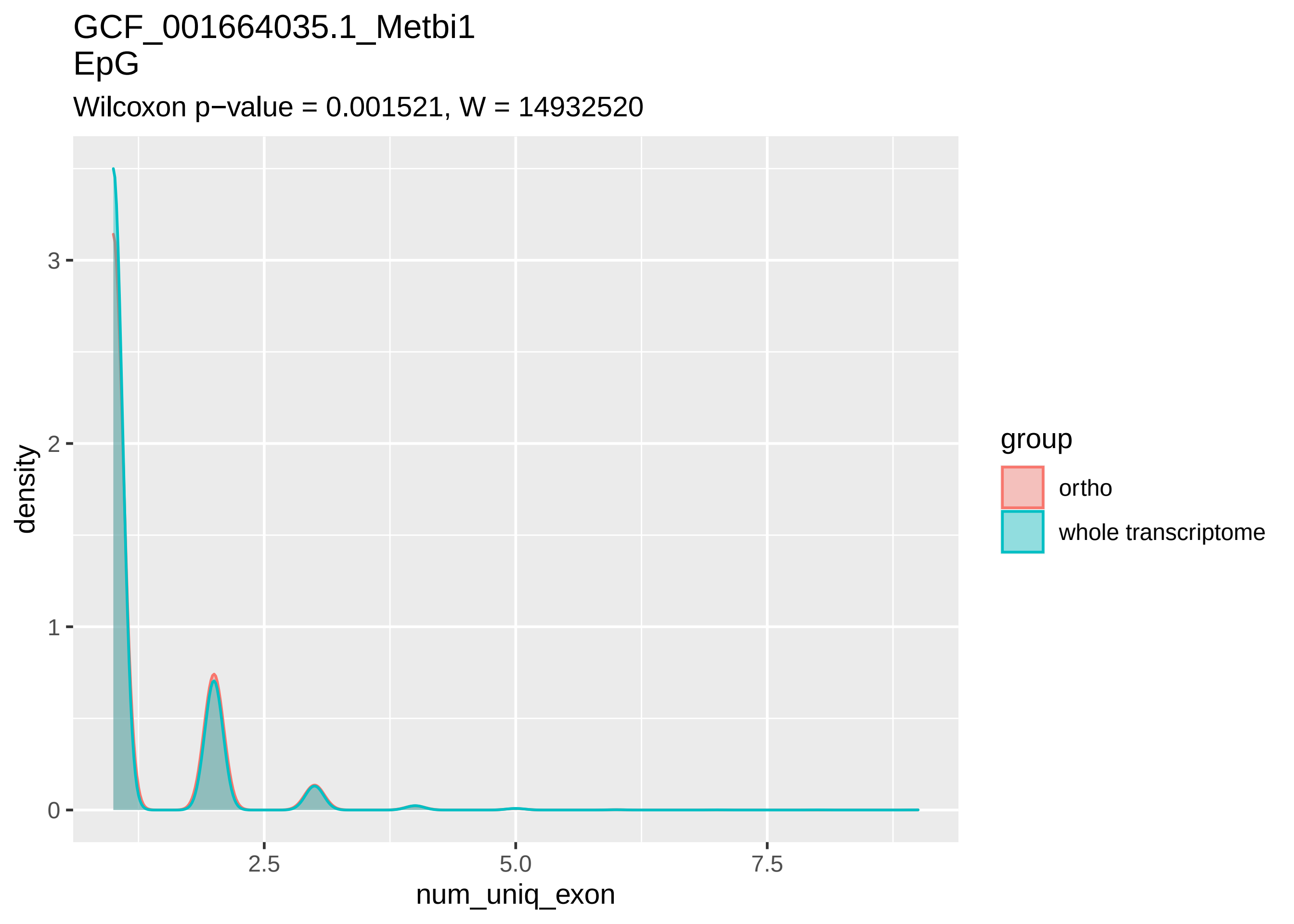
GCF_001664035.1_Metbi1
EpG
Wilcoxon p−value = 0.001521, W = 14932520
density
num_uniq_exon
0
1
2
3
2.5
5.0
7.5
group
ortho
whole transcriptome

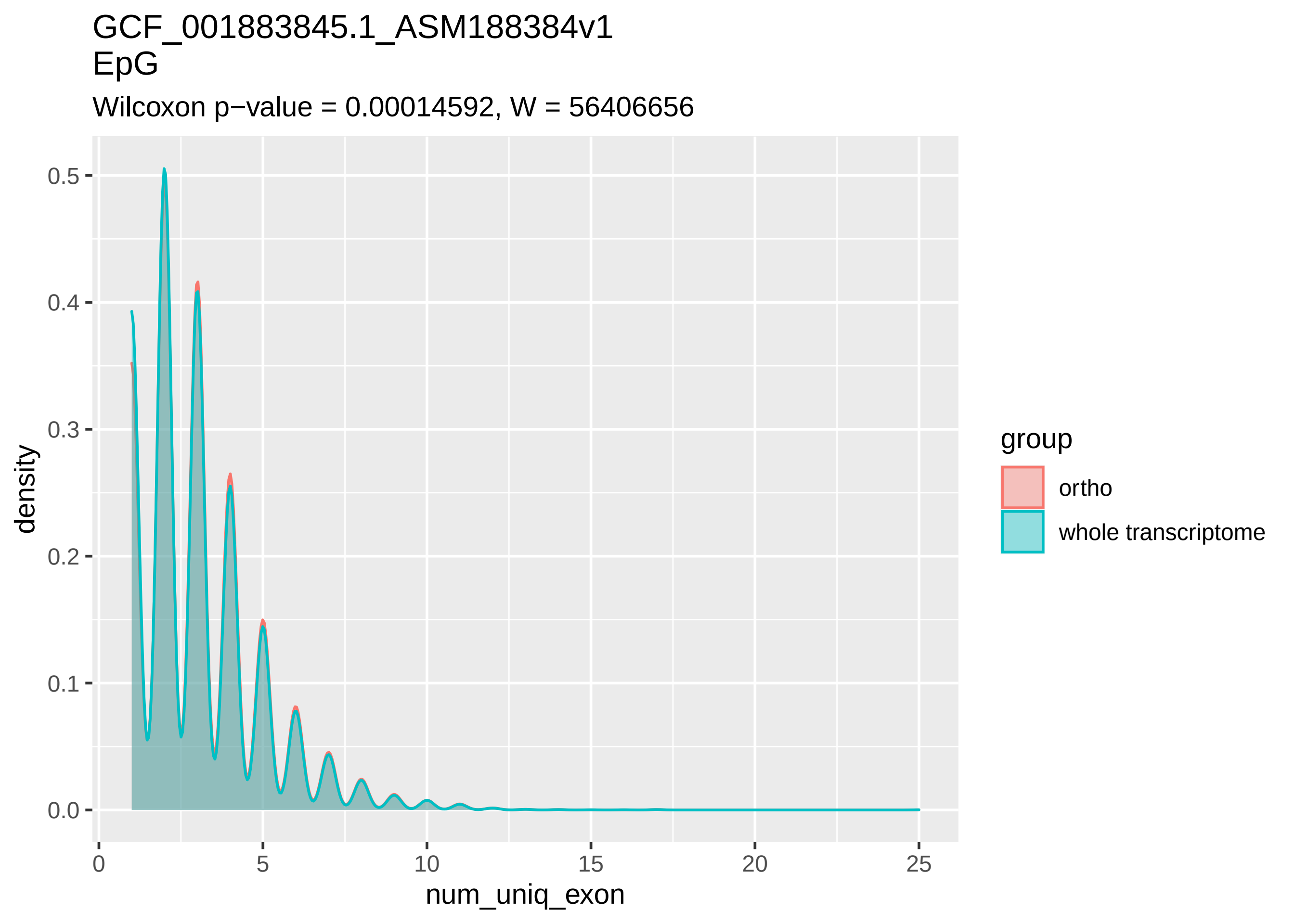
GCF_001883845.1_ASM188384v1
EpG
Wilcoxon p−value = 0.00014592, W = 56406656
density
0.0
0.1
0.2
0.3
0.4
0.5
0
5
10
15
20
25
num_uniq_exon
group
ortho
whole transcriptome

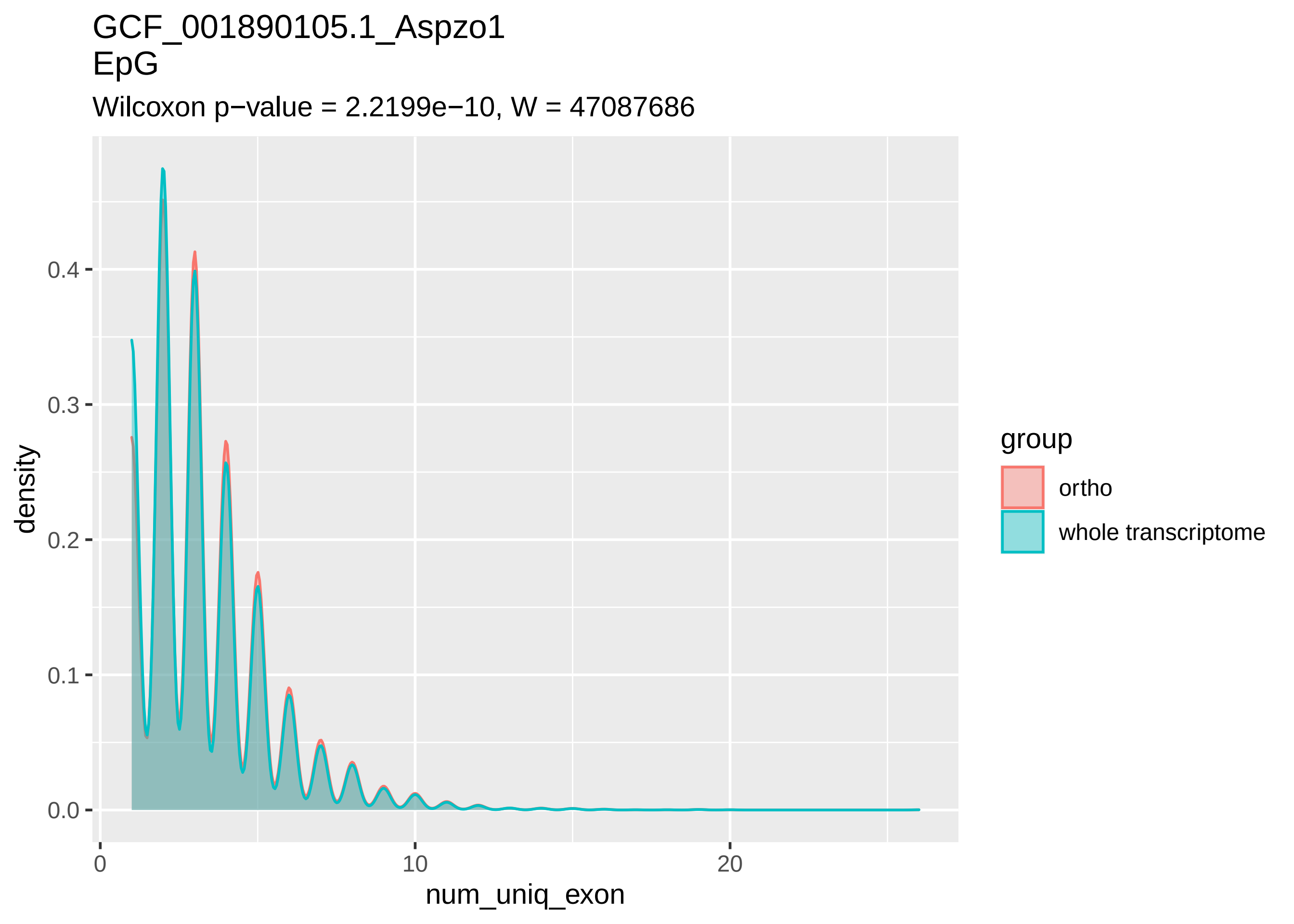
GCF_001890105.1_Aspzo1
EpG
Wilcoxon p−value = 2.2199e−10, W = 47087686
density
0.0
0.1
0.2
0.3
0.4
0
10
20
num_uniq_exon
group
ortho
whole transcriptome

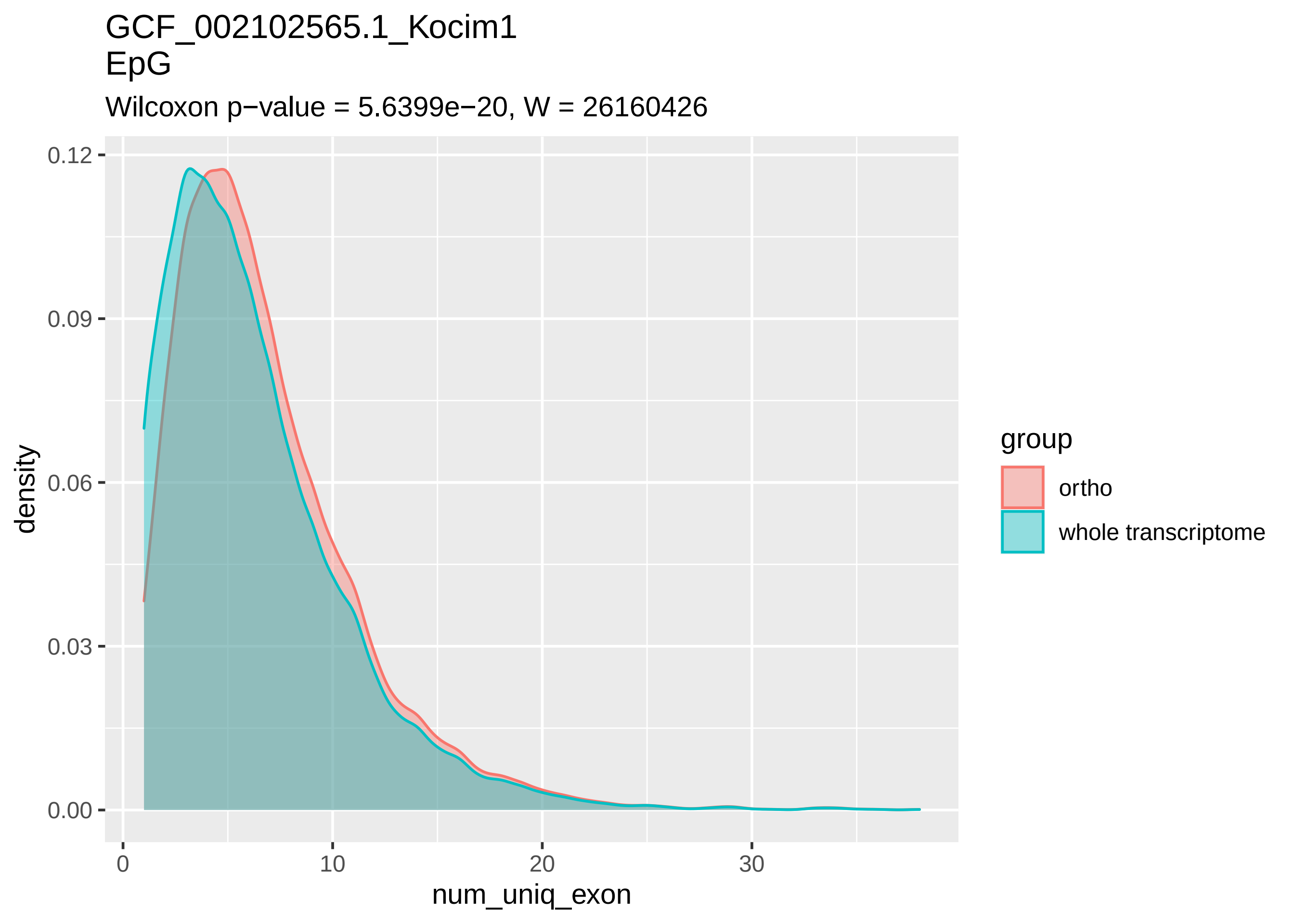

GCF_002102565.1_Kocim1
EpG
Wilcoxon p−value = 5.6399e−20, W = 26160426
0.12
0.09
0.06
0.03
0.00
density
0
10
20
30
num_uniq_exon
group
ortho
whole transcriptome

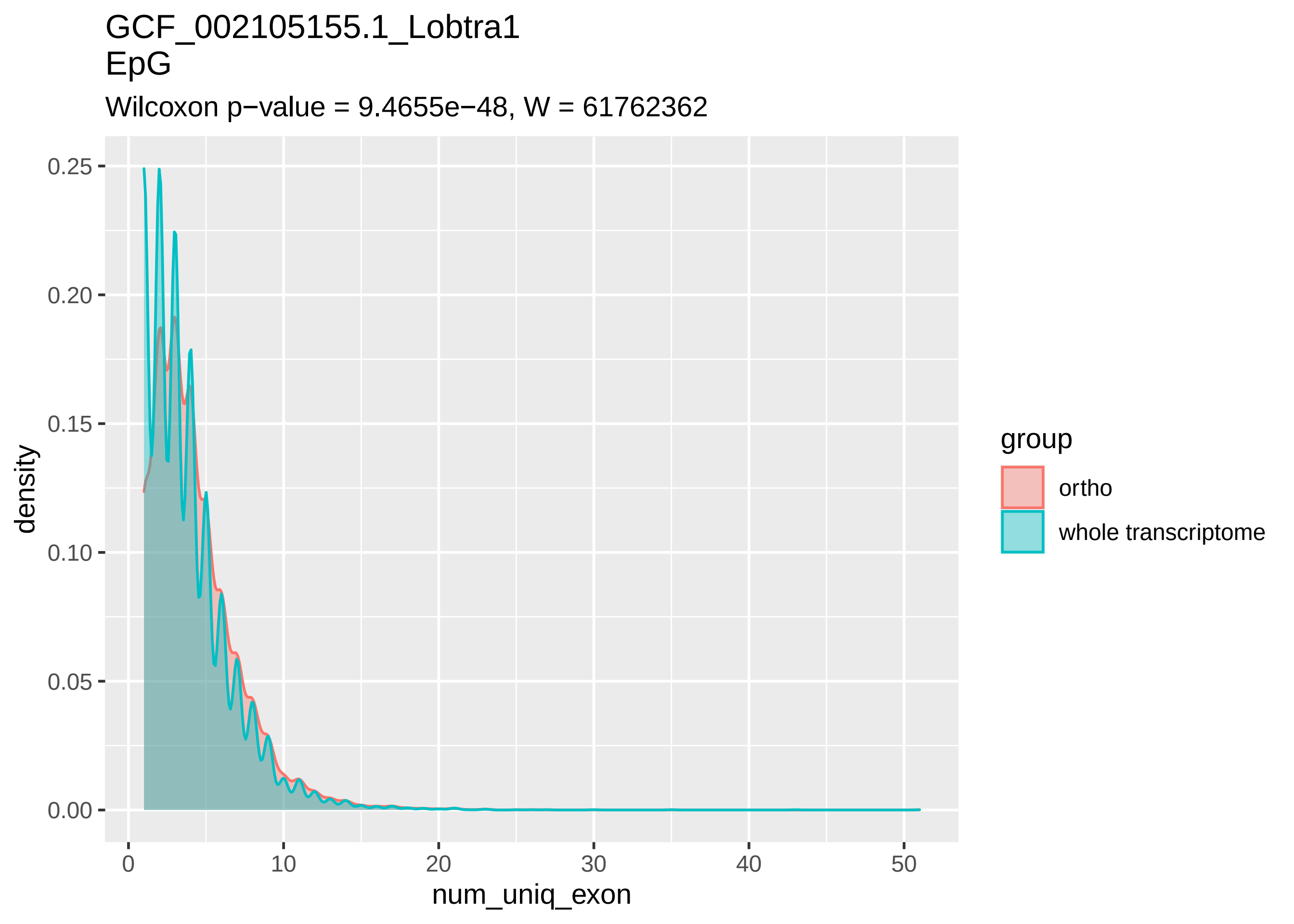
GCF_002105155.1_Lobtra1
EpG
Wilcoxon p−value = 9.4655e−48, W = 61762362
density
0.25
0.20
0.15
0.10
0.05
0.00
0
10
20
30
40
50
num_uniq_exon
group
ortho
whole transcriptome

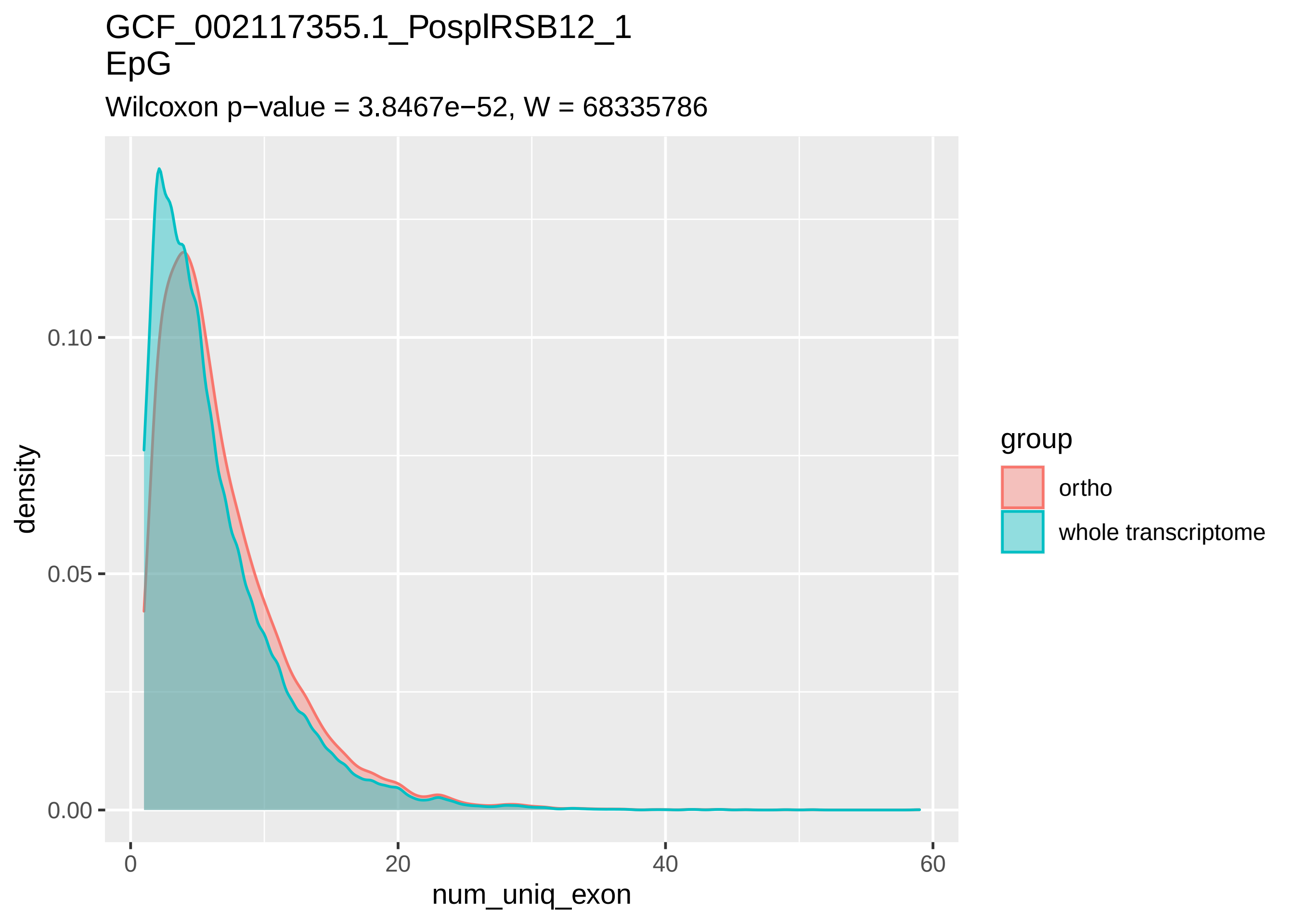

GCF_002117355.1_PosplRSB12_1
EpG
Wilcoxon p−value = 3.8467e−52, W = 68335786
density
0.10
0.05
0.00
0
20
40
60
num_uniq_exon
group
ortho
whole transcriptome

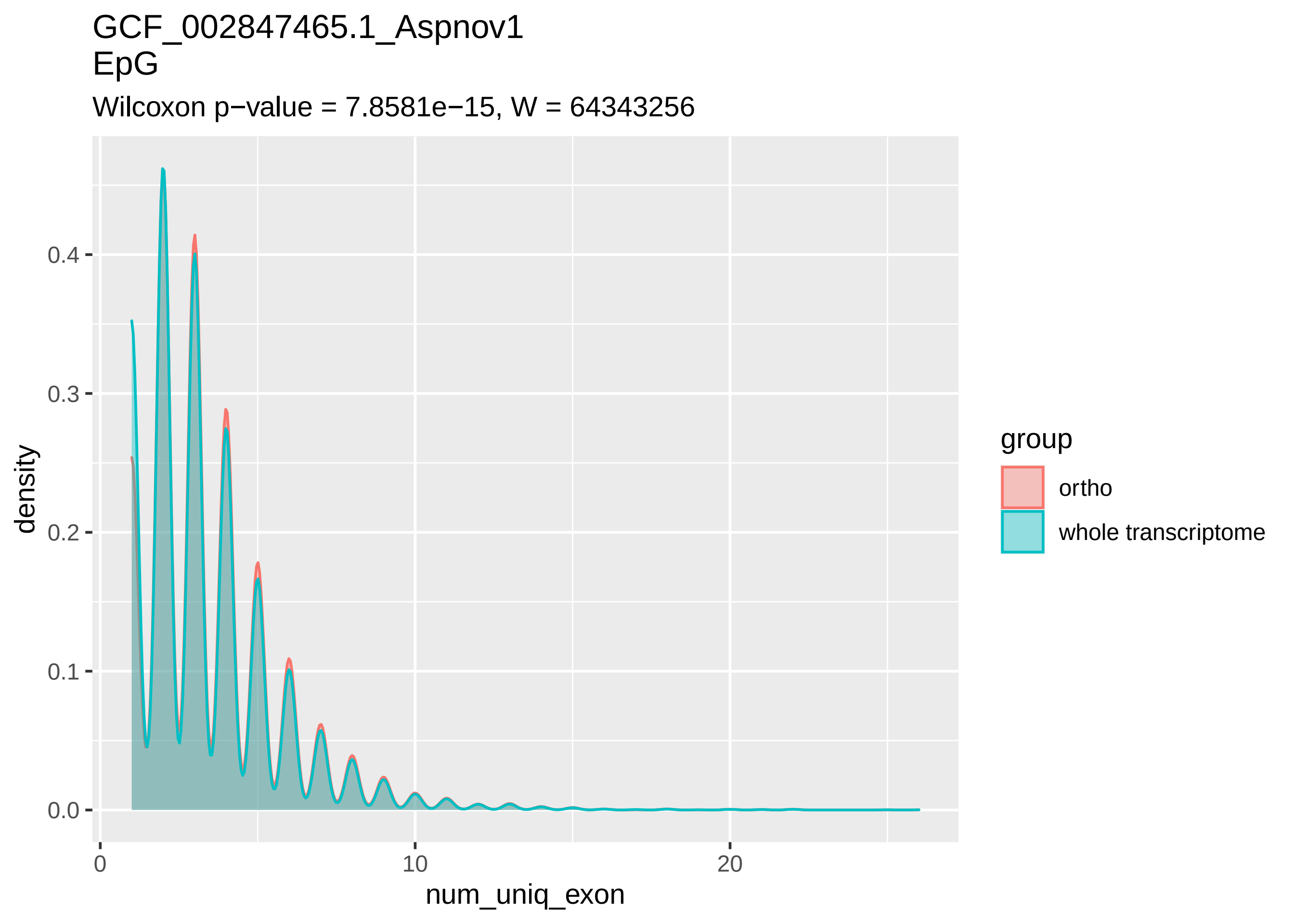
GCF_002847465.1_Aspnov1
EpG
Wilcoxon p−value = 7.8581e−15, W = 64343256
density
0.0
0.1
0.2
0.3
0.4
0
10
20
num_uniq_exon
group
ortho
whole transcriptome
